# Global, regional, and national burden of cardiovascular diseases in youths and young adults aged 15–39 years in 204 countries/territories, 1990–2019: a systematic analysis of Global Burden of Disease Study 2019

**DOI:** 10.1186/s12916-023-02925-4

**Published:** 2023-06-26

**Authors:** Jiahong Sun, Yanan Qiao, Min Zhao, Costan G. Magnussen, Bo Xi

**Affiliations:** 1 Department of Epidemiology, School of Public Health, Qilu Hospital, Cheeloo College of Medicine, Shandong University, 44 Wenhuaxi Road, Jinan, 250012 Shandong China; 2 Department of Nutrition and Food Hygiene, School of Public Health, Cheeloo College of Medicine, Shandong University, Jinan, Shandong China; 3 Baker Heart and Diabetes Institute, Melbourne, Australia; 4 Research Centre of Applied and Preventive Cardiovascular Medicine, University of Turku, Turku, Finland; 5 Centre for Population Health Research, University of Turku and Turku University Hospital, Turku, Finland

**Keywords:** Cardiovascular disease, Youths, Young adults, Global

## Abstract

**Background:**

Understanding the temporal trends in the burden of overall and type-specific cardiovascular diseases (CVDs) in youths and young adults and its attributable risk factors is important for effective and targeted prevention strategies and measures. We aimed to provide a standardized and comprehensive estimation of the prevalence, incidence, disability-adjusted life years (DALY), and mortality rate of CVDs and its associated risk factors in youths and young adults aged 15–39 years at global, regional, and national levels.

**Methods:**

We applied Global Burden of Disease, Injuries, and Risk Factors Study (GBD) 2019 analytical tools to calculate the age-standardized incidence, prevalence, DALY, and mortality rate of overall and type-specific CVDs (i.e., rheumatic heart disease, ischemic heart disease, stroke, hypertensive heart disease, non-rheumatic valvular heart disease, cardiomyopathy and myocarditis, atrial fibrillation and flutter, aortic aneurysm, and endocarditis) among youths and young adults aged 15–39 years by age, sex, region, sociodemographic index and across 204 countries/territories from 1990 to 2019, and proportional DALY of CVDs attributable to associated risk factors.

**Results:**

The global age-standardized DALY (per 100,000 population) for CVDs in youths and young adults significantly decreased from 1257.51 (95% confidence interval 1257.03, 1257.99) in 1990 to 990.64 (990.28, 990.99) in 2019 with an average annual percent change (AAPC) of − 0.81% (− 1.04%, − 0.58%, *P* < 0.001), and the age-standardized mortality rate also significantly decreased from 19.83 (19.77, 19.89) to 15.12 (15.08, 15.16) with an AAPC of − 0.93% (− 1.21%, − 0.66%, *P* < 0.001). However, the global age-standardized incidence rate (per 100,000 population) moderately increased from 126.80 (126.65, 126.95) in 1990 to 129.85 (129.72, 129.98) in 2019 with an AAPC of 0.08% (0.00%, 0.16%, *P* = 0.040), and the age-standardized prevalence rate significantly increased from 1477.54 (1477.03, 1478.06) to 1645.32 (1644.86, 1645.78) with an AAPC of 0.38% (0.35%, 0.40%,* P* < 0.001). In terms of type-specific CVDs, the age-standardized incidence and prevalence rate in rheumatic heart disease, prevalence rate in ischemic heart disease, and incidence rate in endocarditis increased from 1990 to 2019 (all *P* < 0.001). When stratified by sociodemographic index (SDI), the countries/territories with low and low-middle SDI had a higher burden of CVDs than the countries/territories with high and high-middle SDI. Women had a higher prevalence rate of CVDs than men, whereas men had a higher DALY and mortality rate than women. High systolic blood pressure, high body mass index, and low-density lipoprotein cholesterol were the main attributable risk factors for DALY of CVDs for all included countries and territories. Household air pollution from solid fuels was an additional attributable risk factor for DALY of CVDs in low and low-middle SDI countries compared with middle, high-middle, and high SDI countries. Compared with women, DALY for CVDs in men was more likely to be affected by almost all risk factors, especially for smoking.

**Conclusions:**

There is a substantial global burden of CVDs in youths and young adults in 2019. The burden of overall and type-specific CVDs varied by age, sex, SDI, region, and country. CVDs in young people are largely preventable, which deserve more attention in the targeted implementation of effective primary prevention strategies and expansion of young-people’s responsive healthcare systems.

**Supplementary Information:**

The online version contains supplementary material available at 10.1186/s12916-023-02925-4.

## Background

Globally, cardiovascular diseases (CVDs) are the leading cause of morbidity and mortality that inflict substantial social and economic costs [1, 2]. According to data from the Global Burden Disease, Injuries, and Risk Factors Study (GBD) 2019, the total number of CVD cases (272 million to 523 million), death (12.1 million to 18.6 million), and disability-adjusted life years (DALY, 279.8 million to 393.1 million) increased significantly in the overall population from 1990 to 2019 [3]. CVDs are not only severe diseases in high-income countries, over 80% of CVD cases and related deaths occur in low- and middle-income countries [4]. The total economic cost due to CVDs in low- and middle-income countries was approximately $ 3.7 trillion between 2011 and 2015 [5]. Although the economic and health burden of CVDs is high in low- and middle-income countries, healthcare resources are extremely scarce in these countries [6]. Therefore, there is an urgent need to develop and implement effective and targeted strategies for the primary prevention of CVDs, especially in low- and middle-income countries.

CVDs predominantly appear in middle-age and older adulthood. However, the occurrence of some CVDs tends to be trending younger in recent decades. For example, the prevalence of type-specific CVDs such as rheumatic heart disease reaches a peak between the age of 20 and 29 years old, and DALYs for alcoholic cardiomyopathy rises rapidly from 25 years old [3]. Moreover, adolescence is an important period for physical and mental development, which is commonly neglected for universal health coverage [7, 8]. It has been demonstrated that youths with CVDs experienced a substantial share in mortality burden [9]. For example, atherosclerosis can begin early in life and goes undetected for an extended time before developing into an advanced, clinically presentable, phase [10, 11]. Data from the Strong Heart Family Study showed that subclinical atherosclerosis in youths significantly increased the risk of all-cause mortality later in life [12]. Rheumatic heart disease is a major cause of CVD mortality in youths and young adults in developing countries, causing approximately 250,000 deaths every year [13]. In addition, infective endocarditis in youths without congenital heart disease was more similar to that in adults and is more often combined with acute heart failure which can cause significant short-term death, compared with those with congenital heart disease [14, 15].

In a prospective cohort study from the International Childhood Cardiovascular Cohort (i3C) Consortium, metabolic risk factors in youth aged 3–19 years such as high body mass index, high systolic blood pressure, and high cholesterol, and the increased change in these risk factors from childhood to adulthood can predict fatal or nonfatal CVD in midlife [16]. Therefore, an up-to-date understanding of the global burden of CVDs among young people is essential for informing policymakers to implement primary prevention and control.

The GBD data in the EU Member States from 1990 to 2019 found that the DALY and mortality rate due to CVDs in youths aged 10–24 years significantly decreased [17]. However, these findings cannot be generalized to other regions around the world. Although there have been reports on the global burden of all or type-specific CVDs in the overall population (aged 15–90 years) [3, 18–24], variations in the burden of overall and type-specific CVDs in young people (aged 15–39 years) across different age groups, by sex, region, country, and socioeconomic level remain unclear, and no studies have specifically reported the global burden of type-specific CVDs in young people. Also, evidence on potential risk factors for CVDs among young people globally is limited [25]. These gaps might preclude policymakers from establishing effective strategies to reduce the burden of CVDs in young people at the regional and national levels.

Therefore, based on data from the GBD 2019, we determined the epidemiological trends in age-standardized prevalence, incidence, DALY, and mortality rate of overall and type-specific CVDs and attributable risk factors in youths and young adults aged 15–39 years from 204 countries/territories at global, regional, and national levels from 1990 to 2019, stratified by age, sex, region, country, and sociodemographic index (SDI).

## Methods

### Study participants

The GBD 2019 provides a systematic scientific estimation of publicly available, published, and contributed data with enhanced method performance and standardization on prevalence, incidence, mortality, and DALY of 369 injuries and diseases for 204 countries and territories from 1990 to 2019 by age, sex, and country. Detail of the GBD 2019 has been reported prior [1]. We focused on CVDs with an age range of 15–39 years (i.e., 15–19 years as youths, 20–39 as young adults) as per earlier publications using GBD data [26, 27]. We extracted data on the prevalence, incidence, mortality, and DALY relating to overall and type-specific CVDs of rheumatic heart disease, ischemic heart disease, stroke (including ischemic stroke, intracerebral hemorrhage, and subarachnoid hemorrhage), hypertensive heart disease, non-rheumatic valvular heart disease, cardiomyopathy and myocarditis, atrial fibrillation and flutter, aortic aneurysm, endocarditis, and risk factors from the GBD 2019 dataset [28]. Ethical approval and informed consent were waived because the GBD is publicly available and no identifiable information was included in the analyses.

### Estimation framework of the disease burden of CVDs in young people aged 15–39 years in GBD 2019

Overall and type-specific CVDs and related deaths were identified based on standard definitions, which have been previously reported [3]. In GBD 2019, a Bayesian meta-regression disease modeling tool (i.e., DisMod-MR-2.1) was used to estimate the incidence and prevalence of overall and type-specific CVDs produced by population surveys, cohorts and registries, health system administrative data, and microdata from registry and cohort studies [29]. Data on mortality recorded from the vital registration sources defined by the International Classification of Diseases (ICD) codes including ICD-10, ICD-9, and ICD-8 were used to estimate the mortality rate for CVDs using the Cause of Death Ensemble model [29]. DALY due to overall and type-specific CVDs were calculated as the sum of years of life lost and years lived with disability after correction for comorbidity [3]. This study is performed according to the Guidelines for Accurate and Transparent Health Estimates Reporting guidelines [1].

### Estimated burden of CVDs in young people aged 15–39 years attributable to risk factors

The GBD 2019 yields 87 risk factors by counting all specific and aggregate risk factors at the global, regional, and national levels for 204 countries and territories [30]. The risk hierarchy in the GBD comparative risk assessment (CRA) includes four level risk factors (i.e., level 1: behavioral, environmental, metabolic, and occupational level; level 2: 20 risk factor or cluster of risk factors; level 3: 52 risk factors or cluster of risk factors; and level 4: 69 specific risk factors) [30]. In this study, attributable DALY of CVDs were calculated from the top 20 risk factors at level 3 and level 4 including ambient particulate matter pollution, diet high in red meat, diet high in sodium, diet high in trans fatty acids, diet low in fiber, diet low in fruits, diet low in legumes, diet low in nuts and seeds, diet low in polyunsaturated fatty acids, diet low in vegetables, diet low in whole grains, high body-mass index, high fasting plasma glucose, high low-density lipoprotein cholesterol, high systolic blood pressure, household air pollution from solid fuels, kidney dysfunction, low temperature, secondhand smoke, smoking in 2019 by multiplying total DALY by the population attributable fraction [23, 30]. The definitions of all included risk factors are on the Global Health Data Exchange website [31].

### Statistical analysis

Based on estimates downloaded from GBD 2019 analytical tools website (https://vizhub.healthdata.org/gbd-results/), we calculated age-standardized prevalence, incidence, DALY, and mortality rate per 100,000 people and their 95% confidence interval (CI) from 1990 to 2019 applying the world standard population in the GBD 2019 using “epitools” package in R version 4.1.0 [29]. We also calculated the average annual percent change (AAPC) and 95% CI for these metrics between 1990 and 2019 to reflect the magnitude and direction of temporal trends using the Joinpoint Regression Program software (version 4.9.0.0, National Cancer Institute, USA) [32]. A Monte Carlo permutation method was used to test the statistical significance. If AAPC > 0 and *P* value < 0.05, the age-standardized rate indicates an increasing trend during this study period; otherwise, if AAPC < 0 and *P* value < 0.05, the age-standardized rate indicates a decreasing trend; if *P* ≥ 0.05, the rate indicates an unchanged trend. We performed subgroup analyses stratified by age (15–19, 20–24, 25–29, 30–34, and 35–39 years), sex (men and women), SDI (high, high-middle, middle, low-middle, and low SDI categories) classified according to quintiles of the geometric mean of average years of education, total fertility rate with an age less than 25 years, and lag-distributed income per capita [29], 21 GBD regions (South Asia and East Asia, North Africa and Middle East, Western Sub-Saharan Africa, Southeast Asia, Eastern Sub-Saharan Africa, Central Latin America, Tropical Latin America, Central Sub-Saharan Africa, Western Europe, High-income North America, Central Asia, Eastern Europe, Southern Sub-Saharan Africa, High-income Asia Pacific, Central Europe, Andean Latin America, Caribbean, Southern Latin America, Oceania, Australasia), and 204 countries/territories. We examined the Pearson correlation of SDI with age-standardized rate and AAPC. We calculated the proportional contribution of each risk factor by dividing the age-standardized DALYs rate due to specific factors by the total age-standardized DALYs rate for CVD. Alcohol use as an influencing factor was excluded from our analysis because of its uncertain causality or its harmfulness being dependent on the amount of consumption [33–35]. We used R version 4.1.0 to perform data analyses. A two-sided *P* value < 0.05 indicates statistical significance.

### Role of the funding source

The funders had no role in the study design or implementation; data collection, management, analysis, or interpretation; manuscript preparation, review, or approval; or the decision to submit the manuscript for publication. The contents of the published material are solely the responsibility of the individual authors and do not reflect the views of the NHMRC.

## Results

Globally in 2019, the age-standardized incidence, prevalence, DALY, and mortality rate per 100,000 people of overall CVDs were 129.85 (95% CI 129.72, 129.98), 1645.32 (1644.86, 1645.78), 990.64 (990.28, 990.99), and 15.12 (15.08, 15.16), respectively (Additional file 1: Table S1).

**Fig. 1:**
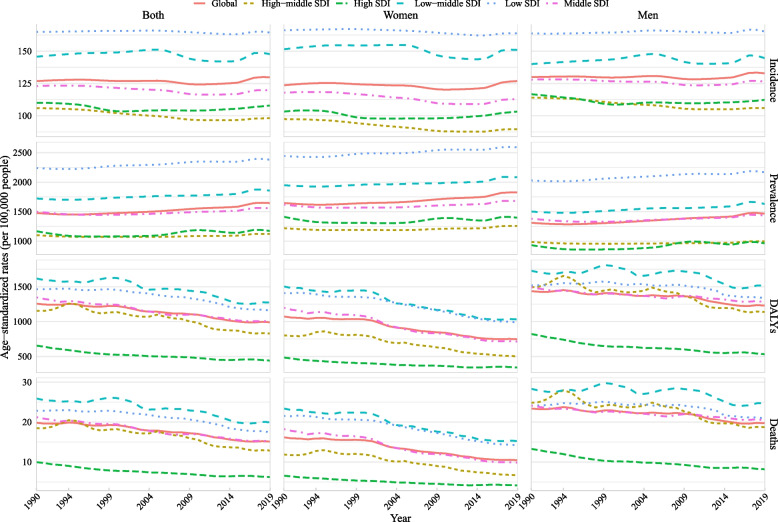
Temporal trends in age-standardized incidence, prevalence, DALYs, and mortality rate of cardiovascular diseases in youths and young adults overall and by sex (men and women) and sociodemographic index (high-income, high-middle income, middle income, low-middle income, and low-income categories) from 1990 to 2019. *Note:* DALY, disability-adjusted life years

**Fig. 2:**
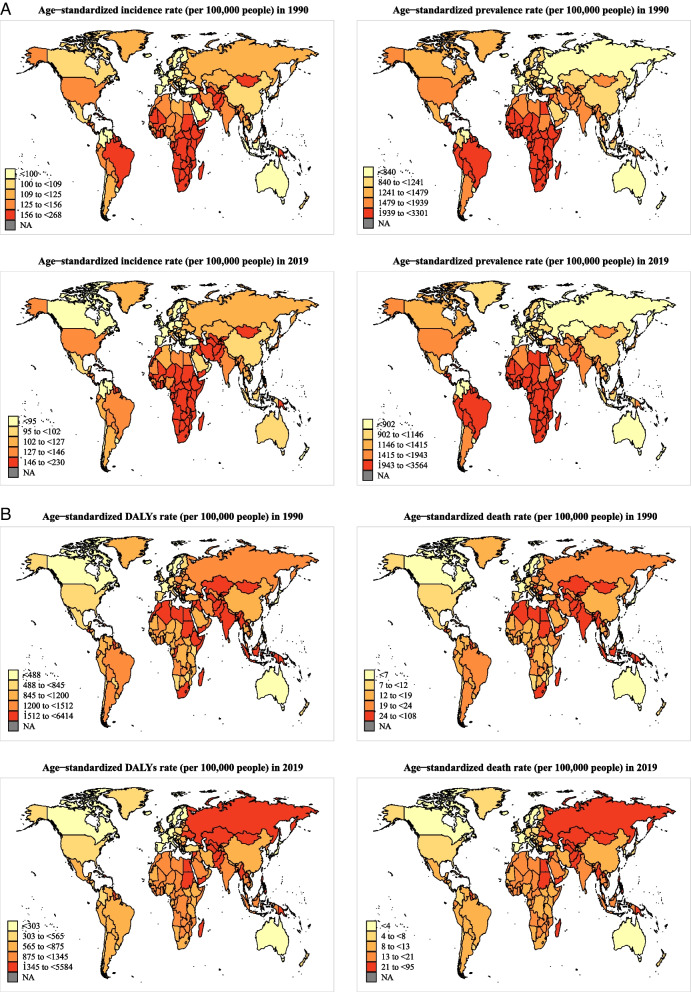
Age-standardized burden of cardiovascular disease across 204 countries/territories among youths and young adults overall, in 1990 and 2019. **A** Age-standardized incidence and prevalence in the total population; **B** age-standardized disability-adjusted life years (DALYs) and mortality rate in the total population

Globally in 2019, when stratified by SDI, low and low-middle SDI countries had the highest age-standardized prevalence, incidence, DALY, and mortality rate of CVDs, whereas high SDI countries had the lowest burden (Fig. 1 and Additional file 1: Table S1). At regional and national levels, countries/territories in regions of Eastern Sub-Saharan Africa, Southern Sub-Saharan Africa, Central Sub-Saharan Africa, and the Caribbean had the highest age-standardized incidence rate of CVDs, countries/territories in regions of Eastern Sub-Saharan Africa, Central Sub-Saharan Africa, Southern Sub-Saharan Africa, Western Sub-Saharan Africa had the highest prevalence rate, and countries/territories in regions of Eastern Europe and Oceania had the highest age-standardized DALY and mortality rate (Fig. 2 and Additional file 2: Figs. S1–2).

Globally in 2019, when stratified by sex, the age-standardized prevalence rate was higher in women than that in men, whereas the incidence, DALY, and mortality rate were higher in men than that in women (Fig. 1 and Additional file 1: Table S1). When stratified by age, the age-standardized rate of overall and type-specific CVDs increased with age (Additional files 1 and 2: Figs. S3–35, and Table S2). When stratified by age, sex, and SDI, women had a higher prevalence rate across all ages and SDI categories, whereas men aged 20–39 years had a higher DALY and mortality rate, regardless of SDI (Fig. 3 and Table S2). For different types of CVDs, age and sex differences in age-standardized incidence, prevalence, DALY, and mortality rate for these metrics varied across different types of CVDs (Additional files 2: Figs. S14–45 and Table S2). Men had a higher incidence, DALY, and mortality rate of ischemic heart disease and cardiomyopathy and myocarditis, and a higher DALY and mortality rate of stroke than women, whereas women had a higher incidence and prevalence rate of rheumatic heart disease than men (Additional file 2: Fig. S46). The sex and region differences in age-standardized rate also varied by different types of specific CVDs (Additional file 2: Figs. S47–56).

**Fig. 3:**
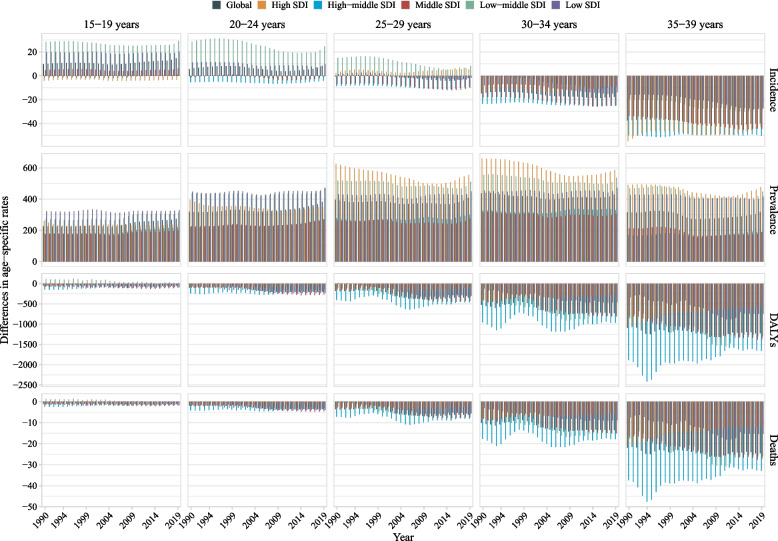
Difference in age-standardized incidence, prevalence, DALY, and mortality rate of cardiovascular diseases in youths and young adults between men and women by age and sociodemographic index (high-income, high-middle income, middle income, low-middle income, and low-income categories), from 1990 to 2019. *Note:* DALY, disability-adjusted life years. The difference indicates the age-standardized rate in women minus that in men. A difference > 0 suggests that women have a higher rate than men

**Fig. 4:**
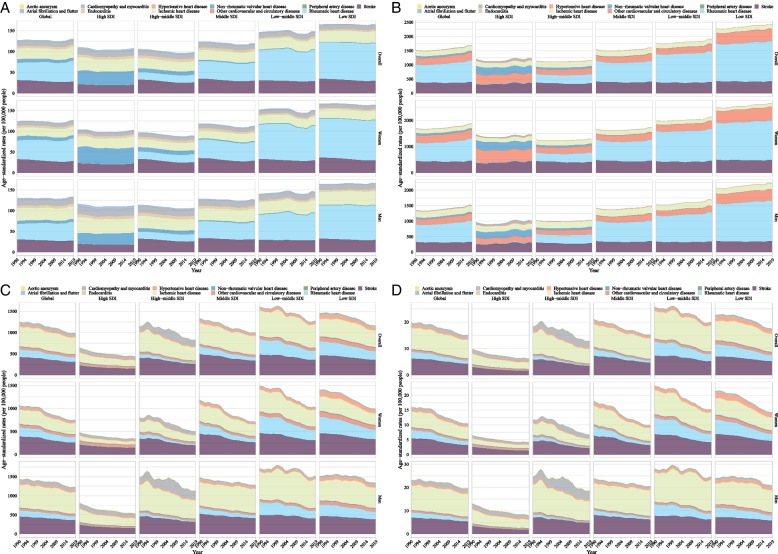
Temporal trends in age-standardized incidence, prevalence, DALY, and mortality rate of type-specific cardiovascular diseases in youths and young adults by sex and sociodemographic index from 1990 to 2019. **A** Incidence, **B** prevalence, **C** DALYs, and **D** deaths. *Note:* DALY, disability-adjusted life years

From 1990 to 2019, the age-standardized DALY rate of CVDs (per 100,000 population) among youths and young adults aged 15–39 years significantly decreased from 1257.51 (95% CI 1257.03, 1257.99) to 990.64 (990.28, 990.99) with an AAPC of − 0.81% (95% CI − 1.04%, − 0.58%, *P* < 0.0001), and the age-standardized mortality rate also significantly decreased from 19.83 (19.77, 19.89) to 15.12 (15.08, 15.16) with an AAPC of − 0.93% (− 1.21%, − 0.66%, *P* < 0.0001). However, the age-standardized incidence rate moderately increased from 126.80 (126.65, 126.95) in 1990 to 129.85 (129.72, 129.98) in 2019 with an AAPC of 0.08% (0.00%, 0.16%, *P* = 0.040), and age-standardized prevalence rate significantly increased from 1477.54 (1477.03, 1478.06) to 1645.32 (1644.86, 1645.78) with an AAPC of 0.38% (0.35%, 0.40%,* P* < 0.0001) (Figs. 1 and 4A–D; Additional file 1: Table S1).

**Fig. 5:**
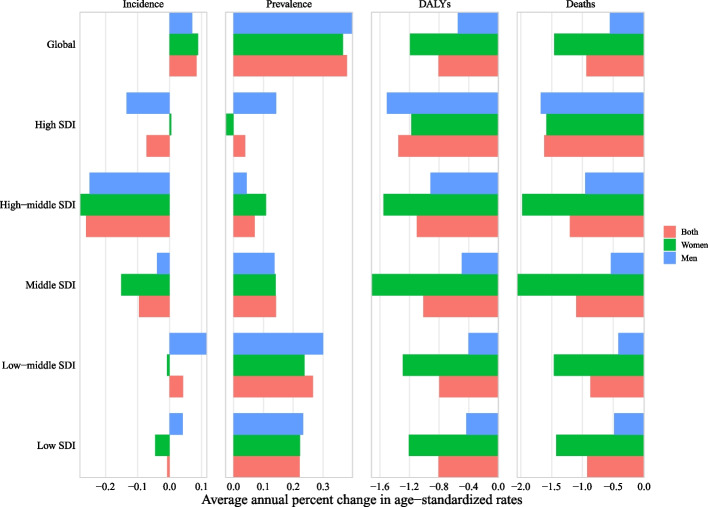
Average annual percent change in age-standardized incidence, prevalence, DALY, and mortality rate of cardiovascular diseases in youths and young adults by sex (men and women) and sociodemographic index (high-income, high-middle income, middle income, low-middle income, and low-income categories) from 1990 to 2019. *Note:* DALY, disability-adjusted life years

When stratified by SDI and GBD region, from 1990 to 2019, low and middle SDI countries/territories and 10 of 21 GBD regions had increased prevalence rate of CVDs, and 5 of 21 regions had increased incidence rate. Compared with countries/territories with high SDI (AAPC of prevalence: 0.04% (− 0.05%, 0.13%)) and high-middle SDI (0.07% (0.05%, 0.10%)), countries/territories with low SDI (0.22% (0.12%, 0.33%)), low-middle SDI (0.27% (0.22%, 0.31%)), and middle SDI (0.14% (0.11%, 0.18%)) increased faster. Only the Eastern European region had increased rates of incidence, prevalence, DALY, and mortality, and this region had the fastest increase in incidence and prevalence rate. Similar results were found among both sexes (Additional files 1 and 2: Table S1; Figs. 1, 4, and 5; and Additional file 2: Fig. S1). When stratified by age, prevalence rate increased from 1990 to 2019 with the largest AAPC value at 15–19 years (0.74 vs. 0.63 at 20–24 years, 0.42 at 25–29 years, 0.22 at 30–34 years, and 0.12 at 35–39 years) and DALY and mortality rate decreased across almost all ages with the largest AAPC value at 15–19 years (DALY: − 1.1 vs. − 0.71 at 20–24 years, − 0.82 at 25–29 years, − 0.79 at 30–34 years, and − 0.87 at 35–39 years; mortality: − 1.41 vs. − 0.88 at 20–24 years, − 0.94 at 25–29 years, − 0.85 at 30–34 years, and − 0.94 at 35–39 years). However, the incidence rate increased at nearly all ages of 15–29 years but decreased at 30–39 years (AAPC: 0.52 at 15–19 years, 0.31 at 20–24 years, 0.10 at 25–29 years, − 0.11 at 30–34 years, and − 0.21 at 35–39 years) (Fig. 3 and Fig. S57). When stratified by sex, both women and men had increased incidence and prevalence rates but decreased DALY and mortality rates with more decreased AAPC of global DALY (− 1.19% vs. − 0.55%) and mortality (− 1.46% vs. − 0.56%) rate in women compared with that in men (Fig. 5). When stratified by age, sex, and SDI, changes in these metrics are shown in Additional files 1 and 2: Table S2 and Fig. S57. The DALY and mortality rate of CVDs decreased across almost all groups by age, sex, and SDI or region (Additional files 1 and 2: Table S2, Fig. S57, and Fig. S58). The burden of CVDs from 1990 to 2019 stratified by country is shown in Additional files 1 and 2: Table S3 and Fig. S59.

From 1990 to 2019, for different types of specific CVDs, the age-standardized incidence and prevalence rate (per 100,000 population) in rheumatic heart disease (41.81 to 50.37 and 637.47 to 765.19, respectively), the prevalence rate in ischemic heart disease (177.62 to 180.42), and the incidence rate in endocarditis (5.53 to 5.73) significantly increased globally, whereas the incidence rate in stroke (30.99 to 27.48), the prevalence rate in hypertensive heart disease (9.47 to 9.15), and incidence and prevalence rate in non-rheumatic valvular heart disease (7.85 to 6.46 and 62.64 to 50.03, respectively), cardiomyopathy and myocarditis (11.79 to 11.12 and 6.19 to 4.59, respectively), and atrial fibrillation and flutter significantly decreased (2.48 to 2.36 and 8.24 to 7.85, respectively) (all *P* for AAPC < 0.05, Fig. 4A–D, and Additional file 1: Table S1). Almost all the type-specific DALY and mortality rate decreased, whereas there was an increase in atrial fibrillation and flutter and a non-significant decrease in cardiomyopathy and myocarditis and a non-significant increase in aortic aneurysm and endocarditis (Additional file 1: Table S1).

For different types of specific CVDs, when stratified by sex only men had an increased incidence, prevalence, DALY, and mortality rate of endocarditis, a decreased incidence rate but an increased prevalence rate of ischemic heart disease. Women had an increase in the incidence and prevalence rate of ischemic heart disease and an increase in the DALY rate of atrial fibrillation and flutter (Additional file 1: Table S1). When stratified by age, young people of any age had an increased incidence and prevalence rate of rheumatic heart disease (Additional file 1: Table S2). When stratified by age and sex, both men and women at the age of 15–30 years had an increased incidence rate of ischemic heart disease, whereas only women at the age of 30–39 years had an increased incidence rate. Men aged 25–39 years had increased incidence, prevalence, DALY, and mortality rate of endocarditis. Women aged 30–39 years had an increased DALY and mortality rate of atrial fibrillation and flutter (Additional file 1: Table S2).

For different types of specific CVDs, when stratified by sex and SDI or GBD region, low and middle SDI countries/territories and regions such as Central and South Asia, Sub-Saharan Africa, and Eastern Europe had increased burden of rheumatic heart disease, ischemic heart disease (most pronounced in women), aortic aneurysms, and endocarditis from 1990 to 2019. High SDI countries/territories and the regions such as high-income North America had an increased burden of atrial fibrillation and flutter (most pronounced in women) (Additional files 1 and 2: Table S1 and Figs. S47–56). When stratified by age, sex, and SDI or region, the change in age-standardized incidence, prevalence, DALY, and mortality rate varied by different types of CVDs (Additional files 1 and 2: Table S2, Figs. S60–79).

**Fig. 6:**
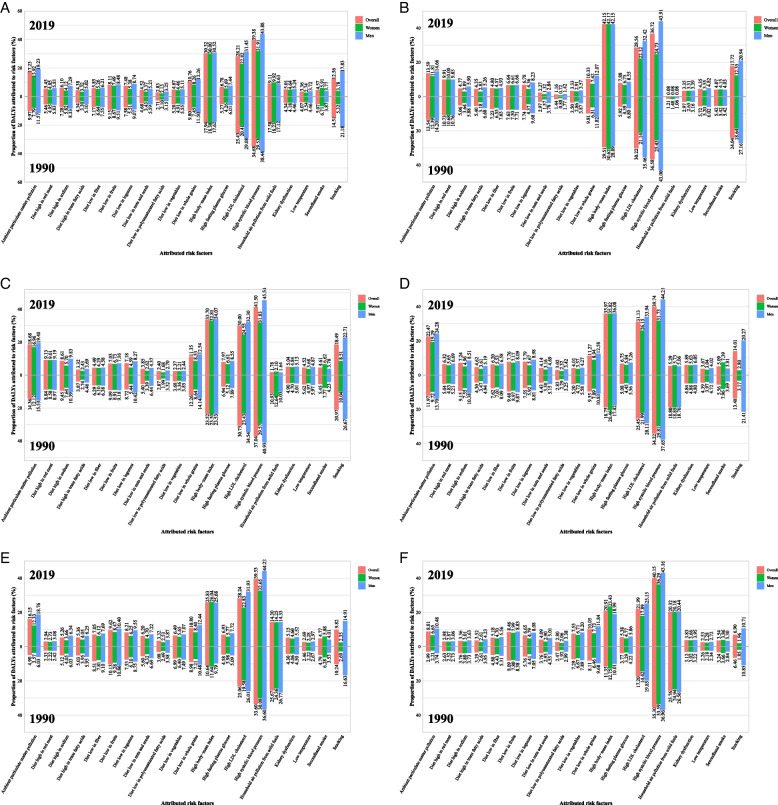
Proportion of DALY of cardiovascular disease attributed to 20 main risk factors in 1990 and 2019 overall and by sex and sociodemographic index. **A** Global, **B** high-income,** C** high-middle income, **D** middle-income, **E** low-middle income, and **F** low-income. *Note:* DALY, disability-adjusted life years

Among the top 20 risk factors for overall CVDs quantified in the GBD, high systolic blood pressure (39.38%), high body mass index (30.32%), high low-density lipoprotein cholesterol (28.21%), ambient particulate matter pollution (17.23%), smoking (12.56%), and diet low in whole grains (10.76%) mainly accounted for the age-standardized rate of CVDs DALY in 2019 (Fig. 6). High systolic blood pressure, high body mass index, and high low-density lipoprotein cholesterol were the three most important contributors across all categories of SDI, sex, and ages (Additional files 1 and 2: Tables S4–6 and Fig. S80). Men with CVDs were more likely to be affected by almost all risk factors especially for smoking compared with women (Fig. 6 and Additional file 1: Table S4). However, in low and low-middle SDI countries/territories, DALY for CVDs was additionally attributable to household air pollution from solid fuels in 2019 compared with that in middle, high-middle, and high SDI countries/territories (Additional files 1 and 2: Table S5 and Fig. 6A–F).

Proportions of DALYs of overall CVDs attributed to 20 risk factors in 2019 by age (i.e., 15–19, 20–24, 25–29, 30–34, and 35–39 years) are shown in Additional files 1 and 2: Table S6 and Fig. S80. Although the age-standardized rate of DALY and mortality attributed to high systolic blood pressure decreased slightly, the absolute numbers increased from 1990 to 2019 (Additional file 2: Fig. S81). High body mass index and high low-density lipoprotein cholesterol are the most important risk factor for stroke and ischemic heart disease, respectively. High systolic blood pressure is the second most important risk factor for stroke and ischemic heart disease and is the most important risk factor for hypertensive heart disease, rheumatic heart disease, aortic aneurysm, cardiomyopathy and myocarditis, atrial fibrillation and flutter, and endocarditis. (Additional file 2: Fig. S82).

The proportion of DALYs attributable to household air pollution from solid fuels in 1990 was near twice the proportion in 2019, whereas the proportion attributable to ambient particulate matter pollution and high body mass index in 2019 was nearly 1.7 times that in 1990, regardless of sex. The proportion attributable to high systolic blood pressure and high low-density lipoprotein cholesterol remained high in 2019 (39.38% and 28.21%) compared with that in 1990 (34.68% and 25.43%) (Additional files 1 and 2: Table S4 and Fig. 6).

In 2019, age-standardized prevalence, incidence, DALY, and mortality rate of CVDs were inversely associated with the SDI (i.e., the higher the SDI, the lower the burden), whereas the AAPC for these metrics fluctuated with the SDI (Additional file 2: Fig. S83).

## Discussion

Although the age-standardized DALY and mortality rate of overall CVDs significantly declined, the incidence and prevalence rate increased significantly from 1990 to 2019 among youths and young adults aged 15–39 years worldwide. In terms of type-specific CVDs, the age-standardized incidence and prevalence rate in rheumatic heart disease, prevalence rate in ischemic heart disease, and incidence rate in endocarditis increased from 1990 to 2019. We found the highest prevalence rate in countries/territories with low and low-middle SDI and the burden increased faster than that in countries/territories with high and high-middle SDI. Men aged 25–39 years had an increase in incidence, prevalence, DALY, and mortality rate of endocarditis, and women aged 30–39 years had an increase in DALY and mortality rate of atrial fibrillation and flutter. We also found that high systolic blood pressure, high body mass index, and high low-density lipoprotein cholesterol were the three most important contributors to DALY for CVDs. In low and low-middle SDI countries/territories, DALY for CVDs was additionally attributable to household air pollution from solid fuels compared with that in middle, high-middle, and high SDI countries/territories. Men were more likely to be affected by almost all risk factors especially for smoking compared with women. These findings provide a basic understanding of the global burden of CVDs and related risk factors in youths and young adults and call for urgent action, which can guide prioritization and allocation of resources for health care, research, and public health in young people from a global perspective.

Previous studies have reported the high global burden of overall or type-specific CVDs in the overall population (aged 15–90 years) [3, 18–22]. In contrast to the decreasing trend in overall CVDs among middle-aged and elderly adults worldwide [19, 36], we found that youths and young adults tended to show an increasing incidence and prevalence rate of CVDs globally. In addition, similar to a previous study, several type-specific CVDs such as rheumatic heart disease have become prevalent in young people [3]. Limited studies have focused on non-communicable diseases in young people [17], and the findings on ischemic stroke in young adults were inconsistent [23, 24]. A study in the EU Member States reported that the DALY and mortality rate of overall and type-specific CVDs among youths aged 10–24 years significantly decreased from 1990 to 2019, except for endocarditis [17]. However, countries in the EU Member States are mainly high SDI countries, with low and middle SDI countries not well represented. In this study, we included 204 countries and territories and found that the DALY and mortality rate of CVDs in youths and young adults aged 15–39 years decreased globally from 1990 to 2019. However, the incidence and prevalence rate of CVDs increased significantly worldwide. Our findings suggest that targeted strategies and measures are urgently needed for prevention and management of CVDs in youths and young adults. Several public health approaches such as health-promoting school-based programs, increments in health care services by implementing inter-professional teamwork, applying digital clinical decision-making tools, and setting up accessible and affordable rehabilitation facilities, especially for youths and young adults might be useful to address the challenges of CVDs [37–39].

The three main metabolic risk factors accounting for DALY of CVDs in young people are high systolic blood pressure, high body mass index, and high low-density lipoprotein cholesterol. Between 1990 and 2019, the global age-standardized rate of DALY due to high systolic blood pressure and high low-density lipoprotein cholesterol remain relatively static, whereas the rate of DALY due to high body mass index increased modestly in the overall population (aged 15–90 years) [3]. Similarly, we found that the age-standardized rate of DALY due to high body mass index significantly increased from 1990 to 2019 in youths and young adults. Although the DALY due to high systolic blood pressure and high low-density lipoprotein cholesterol slightly decreased, the proportion remained stable. In addition, our findings highlight that risk factors including ambient particulate matter pollution and lifestyle and behavioral risk factors (e.g., smoking and diet low in healthy ingredients) require attention. It has been demonstrated that CVDs can be largely preventable if effective and healthy lifestyle changes are made [40, 41]. Therefore, early warning to high-risk groups and effective measures to control the burden of CVDs should target addressing these risk factors through lifestyle changes such as smoking cessation, adopting healthy eating habits, and regular exercise in young people [42, 43].

Consistent with previous findings in the overall population (aged 15–90 years) [3, 19, 20, 44, 45] or young adults (regarding ischemic stroke) [23], we found that high SDI countries/territories had a lower burden of overall CVDs in young people compared with low and middle SDI countries/territories. We also found an inverse association of the SDI with age-standardized prevalence, incidence, DALY, and mortality rate. Low burden for CVDs in countries/territories with high SDI might be attributable to the better medical services available such as more comprehensive monitoring systems of CVD risk factors, which offer earlier detection and better management of CVD-related risk factors [46]. Increased burden of CVDs in low and middle SDI countries/territories might be due to changes in the socioeconomic status, dietary habits and lifestyle, living environment, and inequalities in access to curative and preventive services, which lead to an increased likelihood of exposure to CVD risk factors such as smoking, poor diet, obesity, and hypertension [1, 47]. Inadequate investment in health resources and unfavorable health awareness in young people additionally increase the CVD burden in this specific population in low- and middle-income countries/territories [48]. Besides, we found that household air pollution from solid fuels is an important risk factor in low and low-middle SDI countries/territories compared with middle, high-middle, and high SDI countries/territories, which might exacerbate the CVD burden in low SDI countries/territories. Therefore, effective measures to improve household conditions are recommended in low and middle SDI countries/territories. Some public interventions toward reducing household air pollution such as replacing cookstoves without chimneys implemented in low- and middle-income countries/territories have been shown to be effective [49]. A Cochrane systematic review showed feasibility and effectiveness of interventions targeting multiple risk factors for primary prevention of CVDs in low- and middle-income countries/territories [50]. We also found that countries/territories from regions of Africa, Eastern Europe, and Central Asia had faster increases in the incidence and prevalence rate of CVDs compared with countries/territories from other regions. Because of frequent military conflict and transitional recession, individuals in these regions have less access to healthcare [45, 51, 52]. Countries/territories from Sub-Saharan Africa have a low ability to respond to the rise in CVDs due to underfunding, insufficient resources, and weak healthcare systems [53]. Therefore, more targeted healthcare resources should be assigned to low and middle SDI countries/territories and there is a need to develop country-specific policies to reduce poverty, socioeconomic and health disparities for early prevention and management of CVDs in young people in different geographical locations. Interestingly, for several type-specific CVDs, we found that high SDI countries/territories had a higher burden of atrial fibrillation and flutter compared with low and middle SDI countries/territories. Young adults in high-income countries/territories reported more frequency of high stress and abdominal obesity, smoking, and alcohol use [54]. Thus, targeted interventions on these type-specific CVDs, especially in high SDI countries/territories, should also be considered in future.

The age-standardized DALY and mortality rate due to overall CVDs was higher in men than in women with the difference by sex becoming wider as age increased. In terms of type-specific CVDs, in line with previous findings on ischemic stroke in young and middle-aged adults aged 15–49 years [23], we found that age-standardized DALY and mortality rate due to stroke was higher in men than that in women in 2019. In addition, we found that men had a higher incidence, prevalence, DALY, and mortality rate of ischemic heart disease, cardiomyopathy, and myocarditis than that in women in 2019. We additionally found that only men aged 25–39 years had an increased burden due to endocarditis from 1990 to 2019. Experiments using gerbils showed that males have more histological damage and severe neurological sequelae than females after unilateral carotid artery occlusion [55]. Sex-specific pathophysiology in soluble epoxide hydrolase causing different responses to ischemic conditions might also contribute to this sex difference because of the protection in both preservations of blood flow and infarct volume in women [55]. In addition, estrogens in women may have a protective role in lipid homeostasis and endothelial function [56, 57]. However, consistent with one previous study [44], we found that women aged 30–39 years had an increased burden due to atrial fibrillation and flutter from 1990 to 2019. More atrial fibrosis in women with atrial fibrillation may predispose them to ischemic attack and death compared with men [58]. Another important explanation is related to the less treatment rate of catheter ablation as a rhythm control strategy and electrical cardioversion [59]. Also, young people aged 15–30 years had an increased incidence rate of ischemic heart disease from 1990 to 2019, regardless of sex, whereas only women aged 30–39 years had an increased incidence rate. Smoking contributed more to the burden of CVDs in men than that in women. Our findings highlight the need to mainstream age and sex in health services and establish targeted gender-sensitive health policies (such as screening) to deal with the challenges of CVDs in youths and young adults.

The main strength of this study is that we firstly made the systematic estimation of overall and type-specific CVDs burden among youths and young adults aged 15–39 years from 1990 to 2019 worldwide, across age groups, and groups of sexes, countries, regions, and socioeconomic levels. In addition, the AAPC between 1990 and 2019 provides temporal trends rather than the total percentage change, which offers more accurate information. However, several limitations related to estimates from the GBD data should be considered [1, 3, 30]. First, although more explicit corrections for bias have been implemented to improve data using higher-resource settings, estimates of CVD burden are surrounded by different data availability between countries/territories with considerable uncertainties [1, 3]. High-quality population-based data on the incidence or prevalence of CVDs in several low and middle SDI countries/territories or regions are limited, because they lack comprehensive surveillance systems and population-based registries [20, 21]. Although the GBD relies on predictive covariate values and statistical methods to estimate CVD burden in these locations, incidence, prevalence, DALY, and mortality rate might be underestimated in low and middle SDI countries. Second, although GBD adjusted alternative case definitions and differential access to health care, ICD codes for extracting CVD cases might lead to compositional bias due to that a proportion of patients are different from the confirmed cases defined by standard criteria. Third, clinical data in GBD might result in a skewed causal distribution in CVDs because of asymptomatic and mild CVDs in youths and young adults. Fourth, the association between DALY of CVDs and SDI among youths and young adults should be interpreted with caution because the effects of potential factors unavailable from the GBD on CVDs are not considered. Fifth, AAPC relies on the hypothesis that the age-standardized rate was constant over time. However, it might be influenced by possible sudden changes in national policies, the availability of preventive medicine services, medical innovations, and medications [19]. Sixth, the GBD database limits us to perform the longitudinal analysis on CVD burden. Seventh, comorbidity of CVDs and interactive effects of risk factors on CVDs among young people remain challenging issues, and future well-designed studies are needed. Eighth, the statistical power in this study was limited in some subgroup analyses for several type-specific CVDs because of the small number of annual recorded cases. Ninth, we did not consider alcohol use as a risk factor in our analysis because of its uncertain causality or its harmfulness being dependent on the amount of consumption [33–35]. Tenth, several type-specific CVDs such as data on pulmonary arterial hypertension, lower extremities peripheral arterial disease, and incidence estimate of hypertensive heart disease were unavailable in the GBD database. Eleventh, some main causes of CVDs such as HIV and syphilis in some specific regions (e.g., sub-Saharan Africa) were unavailable in the GBD database.

## Conclusions

Although the age-standardized DALY and mortality rate of CVDs in youths and young adults has significantly decreased from 1990 to 2019, the incidence and prevalence rate was still rising in young population, indicating that the CVDs burden in young people remains high globally. Specific regions, particularly low and middle SDI countries/territories present worrying increases in CVDs burden in young people. Targeted establishment and implementation of effective strategies and interventions are urgently needed to reduce the burden of CVDs in youths and young adults.

### Supplementary Information

**Additional file 1: Table S1.** Burden of the overall and type-specific cardiovascular disease from 1990 to 2019 by sex, sociodemographic index, and regions. **Table S2.** Burden of the overall and type-specific cardiovascular disease from 1990 to 2019 by age, sex, sociodemographic index, and regions. **Table S3.** Burden of the overall and type-specific cardiovascular disease from 1990 to 2019 by sex and countries/territories. **Table S4.** Age-standardized rate and proportion of disability-adjusted life years of overall cardiovascular disease attributed to 20 risk factors by sex, in 1990 and 2019. **Table S5. **Age-standardized rate and proportion of disability-adjusted life years of overall cardiovascular disease attributed to 20 risk factors in 2019 by sociodemographic index. **Table S6.** Age-specific rate and proportion of disability-adjusted life years of overall cardiovascular disease attributed to 20 risk factors in 2019 by age.

**Additional file 2: Fig S1.** Temporal trends in the burden of overall cardiovascular disease by sex and region from 1990 to 2019. **Fig S2.** Burden of overall cardiovascular disease across 204 countries/territories. **Fig S3-13.** Temporal trends in burden of overall and type-specific cardiovascular disease by age and sociodemographic index, from 1990 to 2019. **Fig S14-24.** Number and burden of overall and type-specific cardiovascular disease in 2019 by age and sex. **Fig S25-35.** Burden of overall and type-specific cardiovascular disease in 2019 by age, sex, and sociodemographic index. **Fig S36-45. **Difference in burden of type-specific cardiovascular disease by age and sociodemographic index, from 1990 to 2019. **Fig S46. **Burden of overall and type-specific cardiovascular diseases in 2019 by sex. **Fig S47-56.** Temporal trends in burden of type-specific cardiovascular disease by sex and regions from 1990 to 2019. **Fig S57-79.** Average annual percent change in burden of overall and type-specific cardiovascular disease by age sex, and sociodemographic index or region or countries/territories. **Fig S80.** Proportion of disability-adjusted life years attributed to 20 risk factors in 2019 by age.** Fig S81-82. **Burden of overall and type-specific cardiovascular disease attributed to systolic blood pressure. **Fig S83.** Burden of overall cardiovascular disease worldwide and in regions by sociodemographic index, 1990 to 2019.

## Data Availability

Data from the Global Health Data Exchange are publicly available online (https://www.healthdata.org/).

## Abbreviations

AAPC: Average annual percent change
CI: Confidence interval
CRA: Comparative risk assessment
CVD: Cardiovascular disease
DALY: Disability-adjusted life years
GBD: Global Burden Disease, Injuries, and Risk Factors Study
ICD: International Classification of Diseases
SDI: Sociodemographic index
