# Supplementary material for: Global, regional, and national burden of cardiovascular diseases in youths and young adults aged 15–39 years in 204 countries/territories, 1990–2019: a systematic analysis of Global Burden of Disease Study 2019
Source: BMC Med. 2023 Jun 26;21:222. doi: 10.1186/s12916-023-02925-4 (PMC10294522; doi:10.1186/s12916-023-02925-4)
Supplement: Supplementary file 1 — Additional file 1: Table S1. Burden of the overall and type-specific cardiovascular disease from 1990 to 2019 by sex, sociodemographic index, and regions. Table S2. Burden of the overall and type-specific cardiovascular disease from 1990 to 2019 by age, sex, sociodemographic index, and regions. Table S3. Burden of the overall and type-specific cardiovascular disease from 1990 to 2019 by sex and countries/territories. Table S4. Age-standardized rate and proportion of disability-adjusted life years of overall cardiovascular disease attributed to 20 risk factors by sex, in 1990 and 2019. Table S5. Age-standardized rate and proportion of disability-adjusted life years of overall cardiovascular disease attributed to 20 risk factors in 2019 by sociodemographic index. Table S6. Age-specific rate and proportion of disability-adjusted life years of overall cardiovascular disease attributed to 20 risk factors in 2019 by age. [file 12916_2023_2925_MOESM1_ESM.pdf]

**Table S1 Age-standardized incidence, prevalence, disability-adjusted life years, and death rate of the overall and type-specific cardiovascular disease in youths and young adults from 1990 to 2019 by sex, sociodemographic index, and 21 GBD regions**

| | Incidence | | | | Prevalence | | | | Disability-adjusted life years | | | | Death | | | |
|---|---|---|---|---|---|---|---|---|---|---|---|---|---|---|---|---|
| | Rate per 100,000 (95% CI) in 1990 | Rate per 100,000 (95% CI) in 2019 | Average annual percent change %, (95% CI) | *P*-value | Rate per 100,000 (95% CI) in 1990 | Rate per 100,000 (95% CI) in 2019 | Average annual percent change %, (95% CI) | *P*-value | Rate per 100,000 (95% CI) in 1990 | Rate per 100,000 (95% CI) in 2019 | Average annual percent change %, (95% CI) | *P*-value | Rate per 100,000 (95% CI) in 1990 | Rate per 100,000 (95% CI) in 2019 | Average annual percent change %, (95% CI) | *P*-value |
| **Cardiovascular diseases** | | | | | | | | | | | | | | | | |
| **Overall** | | | | | | | | | | | | | | | | |
| Global | 126.80 (126.65, 126.95) | 129.85 (129.72, 129.98) | 0.08 (0.00, 0.16) | 0.040 | 1477.54 (1477.03, 1478.06) | 1645.32 (1644.86, 1645.78) | 0.38 (0.35, 0.40) | <0.001 | 1257.51 (1257.03, 1257.99) | 990.64 (990.28, 990.99) | -0.81 (-1.03, -0.58) | <0.001 | 19.83 (19.77, 19.89) | 15.12 (15.08, 15.16) | -0.93 (-1.21, -0.66) | <0.001 |
| **SDI category** | | | | | | | | | | | | | | | | |
| High | 110.05 (109.69, 110.41) | 107.96 (107.61, 108.30) | -0.07 (-0.10, -0.05) | <0.001 | 1168.84 (1167.68, 1170.00) | 1173.12 (1171.99, 1174.25) | 0.04 (-0.05, 0.13) | 0.412 | 655.71 (654.84, 656.58) | 440.65 (439.96, 441.34) | -1.35 (-1.51, -1.20) | <0.001 | 9.96 (9.86, 10.07) | 6.22 (6.14, 6.30) | -1.62 (-1.79, -1.45) | <0.001 |
| High-middle | 105.89 (105.60, 106.18) | 98.05 (97.78, 98.31) | -0.26 (-0.31, -0.21) | <0.001 | 1101.55 (1100.62, 1102.49) | 1122.92 (1122.01, 1123.82) | 0.07 (0.05, 0.10) | <0.001 | 1153.93 (1152.97, 1154.89) | 830.49 (829.73, 831.24) | -1.10 (-1.60, -0.60) | <0.001 | 18.45 (18.33, 18.58) | 12.88 (12.78, 12.97) | -1.20 (-1.65, -0.75) | <0.001 |
| Middle | 122.99 (122.74, 123.25) | 119.64 (119.42, 119.86) | -0.10 (-0.13, -0.06) | <0.001 | 1498.89 (1497.99, 1499.79) | 1557.15 (1556.35, 1557.95) | 0.14 (0.11, 0.18) | <0.001 | 1347.68 (1346.81, 1348.55) | 998.02 (997.39, 998.66) | -1.01 (-1.24, -0.79) | <0.001 | 21.23 (21.12, 21.34) | 15.17 (15.09, 15.24) | -1.10 (-1.33, -0.87) | <0.001 |
| Low-middle | 145.70 (145.34, 146.07) | 147.68 (147.40, 147.96) | 0.04 (-0.04, 0.12) | 0.289 | 1722.74 (1721.48, 1724.00) | 1855.52 (1854.53, 1856.51) | 0.27 (0.22, 0.31) | <0.001 | 1617.87 (1616.63, 1619.11) | 1273.45 (1272.62, 1274.29) | -0.80 (-1.00, -0.59) | <0.001 | 25.90 (25.74, 26.05) | 19.98 (19.87, 20.08) | -0.87 (-1.11, -0.63) | <0.001 |
| Low | 164.85 (164.27, 165.44) | 164.40 (164.02, 164.78) | -0.01 (-0.02, 0.00) | 0.145 | 2238.91 (2236.74, 2241.08) | 2381.39 (2379.92, 2382.85) | 0.22 (0.12, 0.33) | <0.001 | 1463.49 (1461.69, 1465.30) | 1158.85 (1157.79, 1159.91) | -0.81 (-0.91, -0.71) | <0.001 | 22.83 (22.61, 23.06) | 17.49 (17.36, 17.62) | -0.92 (-1.03, -0.81) | <0.001 |
| **21 GBD region** | | | | | | | | | | | | | | | | |

| | | | | | | | | | | | | | | | | |
|---|---|---|---|---|---|---|---|---|---|---|---|---|---|---|---|---|
| South Asia | 145.75 (145.38, 146.11) | 145.79 (145.52, 146.06) | 0.00 (-0.13, 0.14) | 0.993 | 1609.07 (1607.84, 1610.29) | 1644.64 (1643.72, 1645.55) | 0.09 (0.07, 0.12) | <0.001 | 1680.14 (1678.87, 1681.41) | 1347.44 (1346.60, 1348.27) | -0.73 (-1.02, -0.45) | <0.001 | 27.08 (26.92, 27.24) | 21.53 (21.42, 21.63) | -0.76 (-1.09, -0.44) | <0.001 |
| East Asia | 108.76 (108.48, 109.04) | 96.11 (95.84, 96.38) | -0.43 (-0.48, -0.38) | <0.001 | 1234.04 (1233.11, 1234.97) | 1143.49 (1142.57, 1144.41) | -0.26 (-0.29, -0.22) | <0.001 | 1135.23 (1134.33, 1136.13) | 741.18 (740.45, 741.90) | -1.40 (-1.67, -1.13) | <0.001 | 17.68 (17.56, 17.79) | 11.02 (10.93, 11.11) | -1.56 (-1.85, -1.26) | <0.001 |
| North Africa and Middle East | 130.09 (129.46, 130.73) | 130.78 (130.35, 131.22) | 0.02 (0.00, 0.04) | 0.048 | 1720.04 (1717.74, 1722.34) | 1767.83 (1766.22, 1769.44) | 0.11 (0.02, 0.21) | 0.016 | 1847.86 (1845.45, 1850.28) | 1171.09 (1169.79, 1172.40) | -1.52 (-1.62, -1.42) | <0.001 | 29.68 (29.37, 29.99) | 17.83 (17.67, 17.99) | -1.70 (-1.80, -1.60) | <0.001 |
| Western Sub-Saharan Africa | 148.77 (147.85, 149.70) | 150.34 (149.76, 150.93) | 0.04 (0.03, 0.05) | <0.001 | 2548.75 (2544.95, 2552.55) | 2727.15 (2724.68, 2729.63) | 0.24 (0.20, 0.28) | <0.001 | 1053.82 (1051.30, 1056.35) | 866.58 (865.14, 868.03) | -0.68 (-0.82, -0.53) | <0.001 | 15.39 (15.08, 15.70) | 12.01 (11.84, 12.18) | -0.84 (-0.94, -0.75) | <0.001 |
| Southeast Asia | 109.68 (109.20, 110.16) | 107.95 (107.57, 108.34) | -0.05 (-0.07, -0.04) | <0.001 | 1157.37 (1155.82, 1158.92) | 1178.45 (1177.16, 1179.73) | 0.08 (-0.01, 0.17) | 0.096 | 1404.61 (1402.88, 1406.34) | 1182.42 (1181.14, 1183.70) | -0.54 (-0.67, -0.41) | <0.001 | 22.24 (22.02, 22.46) | 18.35 (18.19, 18.51) | -0.60 (-0.74, -0.45) | <0.001 |
| Eastern Sub-Saharan Africa | 186.31 (185.28, 187.35) | 179.93 (179.27, 180.58) | -0.12 (-0.13, -0.11) | <0.001 | 2825.83 (2821.78, 2829.90) | 2999.50 (2996.80, 3002.20) | 0.21 (0.13, 0.29) | <0.001 | 1351.55 (1348.64, 1354.46) | 958.27 (956.69, 959.84) | -1.18 (-1.27, -1.08) | <0.001 | 20.23 (19.87, 20.59) | 13.31 (13.12, 13.50) | -1.44 (-1.53, -1.34) | <0.001 |
| Central Latin America | 104.12 (103.33, 104.92) | 97.68 (97.07, 98.29) | -0.22 (-0.24, -0.19) | <0.001 | 1361.37 (1358.51, 1364.23) | 1380.09 (1377.80, 1382.39) | 0.05 (-0.01, 0.11) | 0.079 | 879.74 (877.40, 882.10) | 613.12 (611.59, 614.66) | -1.22 (-1.61, -0.82) | <0.001 | 13.74 (13.45, 14.04) | 9.12 (8.94, 9.31) | -1.38 (-1.82, -0.94) | <0.001 |
| Tropical Latin America | 162.75 (161.76, 163.76) | 140.39 (139.61, 141.18) | -0.51 (-0.55, -0.47) | <0.001 | 2285.52 (2281.77, 2289.27) | 2213.47 (2210.39, 2216.55) | -0.09 (-0.14, -0.04) | <0.001 | 1495.97 (1492.86, 1499.08) | 774.76 (772.96, 776.55) | -2.24 (-2.45, -2.04) | <0.001 | 24.07 (23.68, 24.47) | 11.30 (11.09, 11.52) | -2.58 (-2.83, -2.33) | <0.001 |
| Central Sub-Saharan Africa | 180.83 (178.96, 182.71) | 174.24 (173.09, 175.40) | -0.13 (-0.14, -0.12) | <0.001 | 2811.68 (2804.29, 2819.08) | 2829.22 (2824.55, 2833.90) | 0.03 (-0.02, 0.08) | 0.287 | 1205.66 (1200.64, 1210.69) | 922.14 (919.37, 924.91) | -0.95 (-1.01, -0.89) | <0.001 | 17.95 (17.33, 18.59) | 13.09 (12.76, 13.43) | -1.11 (-1.18, -1.04) | <0.001 |
| Western Europe | 85.23 (84.75, 85.70) | 78.00 (77.53, 78.47) | -0.31 (-0.32, -0.29) | <0.001 | 857.13 (855.63, 858.63) | 820.16 (818.64, 821.68) | -0.14 (-0.23, -0.05) | 0.003 | 584.53 (583.29, 585.76) | 275.54 (274.67, 276.41) | -2.62 (-2.79, -2.44) | <0.001 | 9.06 (8.91, 9.22) | 3.76 (3.66, 3.86) | -3.01 (-3.26, -2.76) | <0.001 |
| High-income North America | 138.38 (137.71, 139.05) | 137.53 (136.88, 138.18) | -0.04 (-0.09, 0.01) | 0.128 | 1625.82 (1623.53, 1628.12) | 1597.77 (1595.57, 1599.98) | -0.04 (-0.27, 0.20) | 0.743 | 651.65 (650.20, 653.09) | 539.12 (537.84, 540.40) | -0.63 (-0.90, -0.36) | <0.001 | 9.64 (9.46, 9.81) | 7.59 (7.44, 7.75) | -0.81 (-1.17, -0.46) | <0.001 |

| | | | | | | | | | | | | | | | | |
|---|---|---|---|---|---|---|---|---|---|---|---|---|---|---|---|---|
| Central Asia | 151.90 (150.45, 153.37) | 148.22 (147.00, 149.45) | -0.08 (-0.10, -0.06) | <0.001 | 1591.40 (1586.68, 1596.12) | 1636.99 (1632.93, 1641.06) | 0.11 (0.07, 0.15) | <0.001 | 1595.54 (1590.74, 1600.35) | 1401.24 (1397.52, 1404.97) | -0.43 (-0.77, -0.09) | 0.013 | 25.87 (25.26, 26.49) | 22.50 (22.03, 22.98) | -0.46 (-0.84, -0.09) | 0.017 |
| Eastern Europe | 116.67 (115.96, 117.37) | 125.11 (124.31, 125.92) | 0.24 (0.23, 0.26) | <0.001 | 829.71 (827.83, 831.58) | 903.76 (901.60, 905.93) | 0.31 (0.21, 0.42) | <0.001 | 1349.16 (1346.80, 1351.53) | 1784.53 (1781.60, 1787.46) | 1.09 (0.34, 1.84) | 0.004 | 22.52 (22.22, 22.83) | 30.24 (29.87, 30.62) | 1.15 (0.37, 1.94) | 0.004 |
| Southern Sub-Saharan Africa | 185.75 (183.91, 187.60) | 177.02 (175.60, 178.45) | -0.16 (-0.18, -0.15) | <0.001 | 2666.33 (2659.34, 2673.33) | 2651.97 (2646.48, 2657.48) | -0.01 (-0.04, 0.02) | 0.459 | 1398.63 (1393.36, 1403.92) | 857.44 (854.33, 860.57) | -1.61 (-2.62, -0.59) | 0.002 | 21.53 (20.87, 22.21) | 11.97 (11.61, 12.35) | -1.94 (-3.12, -0.75) | 0.001 |
| High-income Asia Pacific | 116.17 (115.36, 116.99) | 115.09 (114.20, 115.99) | -0.02 (-0.06, 0.01) | 0.217 | 983.49 (981.14, 985.86) | 1012.31 (1009.70, 1014.93) | 0.11 (-0.02, 0.23) | 0.100 | 687.08 (685.11, 689.06) | 275.18 (273.81, 276.55) | -3.12 (-3.39, -2.84) | <0.001 | 10.56 (10.32, 10.81) | 3.58 (3.43, 3.74) | -3.69 (-3.98, -3.40) | <0.001 |
| Central Europe | 96.64 (95.76, 97.53) | 92.68 (91.71, 93.66) | -0.14 (-0.16, -0.12) | <0.001 | 1091.07 (1088.09, 1094.05) | 1099.20 (1095.82, 1102.58) | 0.06 (-0.07, 0.19) | 0.392 | 1200.23 (1197.16, 1203.30) | 549.89 (547.57, 552.22) | -2.68 (-2.93, -2.44) | <0.001 | 19.56 (19.18, 19.96) | 7.98 (7.70, 8.26) | -3.08 (-3.36, -2.80) | <0.001 |
| Andean Latin America | 133.98 (132.14, 135.85) | 124.88 (123.51, 126.25) | -0.25 (-0.26, -0.23) | <0.001 | 2018.18 (2010.93, 2025.45) | 2039.06 (2033.54, 2044.60) | 0.04 (0.01, 0.07) | 0.010 | 1245.90 (1240.13, 1251.70) | 716.07 (712.79, 719.36) | -1.87 (-2.31, -1.43) | <0.001 | 19.09 (18.38, 19.83) | 10.09 (9.71, 10.49) | -2.15 (-2.65, -1.65) | <0.001 |
| Caribbean | 178.78 (176.58, 181.00) | 188.41 (186.41, 190.42) | 0.18 (0.17, 0.19) | <0.001 | 2053.59 (2046.12, 2061.09) | 2127.73 (2121.01, 2134.45) | 0.13 (0.08, 0.18) | <0.001 | 1373.90 (1367.66, 1380.16) | 1175.00 (1170.01, 1180.01) | -0.50 (-0.75, -0.26) | <0.001 | 21.97 (21.18, 22.78) | 18.47 (17.84, 19.10) | -0.57 (-0.84, -0.30) | <0.001 |
| Southern Latin America | 99.63 (98.21, 101.07) | 97.63 (96.42, 98.85) | -0.07 (-0.08, -0.06) | <0.001 | 1273.18 (1268.10, 1278.28) | 1386.00 (1381.44, 1390.57) | 0.29 (0.28, 0.31) | <0.001 | 1006.57 (1002.02, 1011.13) | 530.71 (527.91, 533.53) | -2.18 (-2.40, -1.96) | <0.001 | 15.85 (15.28, 16.43) | 7.50 (7.17, 7.84) | -2.54 (-2.80, -2.27) | <0.001 |
| Oceania | 160.13 (155.12, 165.28) | 165.28 (161.82, 168.80) | 0.11 (0.09, 0.13) | <0.001 | 1598.08 (1582.24, 1614.05) | 1650.65 (1639.70, 1661.66) | 0.12 (0.09, 0.15) | <0.001 | 2543.41 (2523.00, 2563.95) | 2561.34 (2547.55, 2575.19) | -0.02 (-0.14, 0.11) | 0.812 | 41.61 (39.01, 44.34) | 41.81 (40.06, 43.62) | -0.02 (-0.15, 0.10) | 0.709 |
| Australasia | 100.31 (98.18, 102.49) | 99.21 (97.29, 101.16) | -0.05 (-0.09, -0.01) | 0.017 | 729.55 (723.77, 735.36) | 753.84 (748.55, 759.16) | 0.11 (0.07, 0.15) | <0.001 | 501.61 (496.81, 506.43) | 278.09 (274.89, 281.33) | -2.02 (-2.26, -1.78) | <0.001 | 7.70 (7.12, 8.32) | 3.90 (3.53, 4.30) | -2.32 (-2.66, -1.98) | <0.001 |
| **Women** | | | | | | | | | | | | | | | | |
| Global | 123.58 (123.37, | 126.88 (126.70, | 0.09 (0.05, | <0.001 | 1647.49 (1646.72, | 1827.86 (1827.17, | 0.37 (0.32, | <0.001 | 1073.50 (1072.87, | 747.66 (747.22, | -1.19 (-1.37, | <0.001 | 16.18 (16.10, | 10.46 (10.41, | -1.46 (-1.64, | <0.001 |

| | | | | | | | | | | | | | | | | |
|---|---|---|---|---|---|---|---|---|---|---|---|---|---|---|---|---|
| | 123.79) | 127.07) | 0.13) | | 1648.27) | 1828.55) | 0.42) | | 1074.13) | 748.10) | -1.02) | | 16.25) | 10.51) | -1.28) | |
| **SDI category** | | | | | | | | | | | | | | | | |
| High | 103.22 (102.73, 103.72) | 103.24 (102.76, 103.73) | 0.01 (-0.04, 0.05) | 0.849 | 1412.96 (1411.14, 1414.79) | 1397.86 (1396.08, 1399.65) | -0.03 (-0.08, 0.02) | 0.312 | 484.49 (483.42, 485.56) | 342.08 (341.20, 342.97) | -1.18 (-1.41, -0.94) | <0.001 | 6.56 (6.44, 6.68) | 4.13 (4.03, 4.22) | -1.58 (-1.78, -1.38) | <0.001 |
| High-middle | 97.43 (97.03, 97.83) | 89.64 (89.27, 90.01) | -0.28 (-0.34, -0.22) | <0.001 | 1220.52 (1219.12, 1221.92) | 1258.04 (1256.66, 1259.42) | 0.11 (0.07, 0.15) | <0.001 | 803.89 (802.75, 805.03) | 502.64 (501.78, 503.50) | -1.55 (-2.22, -0.88) | <0.001 | 11.87 (11.73, 12.01) | 6.68 (6.58, 6.78) | -1.98 (-2.34, -1.61) | <0.001 |
| Middle | 117.81 (117.46, 118.17) | 112.88 (112.57, 113.18) | -0.15 (-0.19, -0.11) | <0.001 | 1618.93 (1617.60, 1620.27) | 1682.14 (1680.96, 1683.32) | 0.14 (0.12, 0.17) | <0.001 | 1198.50 (1197.34, 1199.66) | 713.80 (713.04, 714.56) | -1.72 (-2.04, -1.39) | <0.001 | 18.25 (18.10, 18.39) | 9.84 (9.75, 9.93) | -2.06 (-2.41, -1.70) | <0.001 |
| Low-middle | 151.49 (150.96, 152.01) | 150.94 (150.54, 151.34) | -0.01 (-0.04, 0.02) | 0.577 | 1948.11 (1946.21, 1950.01) | 2083.20 (2081.71, 2084.69) | 0.24 (0.17, 0.30) | <0.001 | 1504.04 (1502.34, 1505.73) | 1030.99 (1029.94, 1032.05) | -1.29 (-1.51, -1.07) | <0.001 | 23.39 (23.18, 23.61) | 15.25 (15.12, 15.38) | -1.46 (-1.74, -1.18) | <0.001 |
| Low | 166.13 (165.31, 166.96) | 163.79 (163.25, 164.33) | -0.05 (-0.07, -0.02) | <0.001 | 2443.27 (2440.09, 2446.45) | 2591.38 (2589.23, 2593.53) | 0.22 (0.13, 0.32) | <0.001 | 1409.16 (1406.67, 1411.65) | 987.40 (986.03, 988.76) | -1.21 (-1.32, -1.11) | <0.001 | 21.54 (21.23, 21.86) | 14.17 (14.01, 14.34) | -1.43 (-1.57, -1.29) | <0.001 |
| **21 GBD region** | | | | | | | | | | | | | | | | |
| South Asia | 156.31 (155.77, 156.85) | 152.78 (152.39, 153.18) | -0.07 (-0.14, -0.01) | 0.031 | 1866.99 (1865.09, 1868.89) | 1891.22 (1889.82, 1892.62) | 0.05 (0.01, 0.10) | 0.028 | 1536.91 (1535.18, 1538.65) | 1069.95 (1068.89, 1071.00) | -1.29 (-1.71, -0.86) | <0.001 | 23.94 (23.72, 24.16) | 16.11 (15.98, 16.25) | -1.42 (-1.90, -0.94) | <0.001 |
| East Asia | 101.30 (100.92, 101.68) | 87.09 (86.72, 87.46) | -0.54 (-0.62, -0.45) | <0.001 | 1301.25 (1299.88, 1302.63) | 1227.38 (1226.01, 1228.75) | -0.20 (-0.23, -0.17) | <0.001 | 979.67 (978.47, 980.88) | 441.69 (440.88, 442.50) | -2.81 (-3.48, -2.14) | <0.001 | 14.71 (14.56, 14.85) | 5.42 (5.33, 5.51) | -3.48 (-4.20, -2.76) | <0.001 |
| North Africa and Middle East | 127.04 (126.14, 127.94) | 125.34 (124.72, 125.96) | -0.05 (-0.06, -0.04) | <0.001 | 1828.66 (1825.26, 1832.06) | 1864.78 (1862.38, 1867.18) | 0.08 (0.00, 0.16) | 0.039 | 1688.07 (1684.78, 1691.38) | 1009.58 (1007.82, 1011.34) | -1.71 (-1.78, -1.63) | <0.001 | 26.44 (26.02, 26.86) | 14.77 (14.56, 14.98) | -1.98 (-2.16, -1.79) | <0.001 |
| Western Sub-Saharan Africa | 143.39 (142.12, 144.66) | 143.71 (142.93, 144.50) | 0.01 (0.00, 0.02) | 0.008 | 2606.85 (2601.47, 2612.24) | 2805.43 (2801.96, 2808.90) | 0.26 (0.24, 0.29) | <0.001 | 1032.71 (1029.19, 1036.24) | 759.51 (757.65, 761.38) | -1.06 (-1.17, -0.94) | <0.001 | 14.87 (14.44, 15.30) | 9.97 (9.75, 10.19) | -1.36 (-1.48, -1.25) | <0.001 |
| Southeast Asia | 99.99 (99.36, | 98.36 | -0.05 | <0.001 | 1233.67 | 1255.27 | 0.07 | 0.072 | 1264.31 | 871.35 | -1.26 | <0.001 | 19.43 | 12.60 | -1.46 | <0.001 |

| | | | | | | | | | | | | | | | | |
|---|---|---|---|---|---|---|---|---|---|---|---|---|---|---|---|---|
| | 100.63) | (97.83, 98.89) | (-0.07, -0.03) | | (1231.43, 1235.91) | (1253.38, 1257.16) | (-0.01, 0.15) | | (1262.01, 1266.61) | (869.79, 872.92) | (-1.37, -1.15) | | (19.14, 19.72) | (12.41, 12.79) | (-1.59, -1.34) | |
| Eastern Sub-Saharan Africa | 183.96 (182.54, 185.38) | 175.80 (174.90, 176.70) | -0.16 (-0.18, -0.15) | <0.001 | 3095.89 (3090.01, 3101.78) | 3293.90 (3289.97, 3297.85) | 0.23 (0.13, 0.32) | <0.001 | 1247.85 (1243.96, 1251.75) | 766.48 (764.52, 768.44) | -1.66 (-1.76, -1.56) | <0.001 | 18.11 (17.64, 18.60) | 9.61 (9.38, 9.83) | -2.16 (-2.26, -2.06) | <0.001 |
| Central Latin America | 108.30 (107.18, 109.43) | 96.77 (95.93, 97.63) | -0.39 (-0.46, -0.33) | <0.001 | 1634.31 (1629.94, 1638.69) | 1619.72 (1616.24, 1623.20) | -0.03 (-0.11, 0.06) | 0.583 | 815.37 (812.23, 818.52) | 479.55 (477.66, 481.45) | -1.82 (-2.37, -1.27) | <0.001 | 12.23 (11.84, 12.63) | 6.43 (6.21, 6.66) | -2.14 (-2.66, -1.61) | <0.001 |
| Tropical Latin America | 162.78 (161.38, 164.18) | 140.45 (139.34, 141.56) | -0.51 (-0.54, -0.48) | <0.001 | 2572.30 (2566.73, 2577.88) | 2520.45 (2515.81, 2525.09) | -0.06 (-0.14, 0.03) | 0.226 | 1283.03 (1279.01, 1287.06) | 634.92 (632.63, 637.22) | -2.40 (-2.53, -2.26) | <0.001 | 19.85 (19.35, 20.36) | 8.50 (8.24, 8.76) | -2.88 (-3.04, -2.72) | <0.001 |
| Central Sub-Saharan Africa | 176.77 (174.19, 179.39) | 169.91 (168.32, 171.52) | -0.13 (-0.15, -0.11) | <0.001 | 3016.07 (3005.32, 3026.86) | 3059.77 (3052.93, 3066.62) | 0.07 (0.01, 0.12) | 0.020 | 1100.56 (1093.82, 1107.33) | 781.24 (777.66, 784.83) | -1.17 (-1.28, -1.06) | <0.001 | 15.84 (15.03, 16.69) | 10.32 (9.90, 10.75) | -1.47 (-1.60, -1.33) | <0.001 |
| Western Europe | 74.45 (73.82, 75.08) | 69.52 (68.89, 70.16) | -0.23 (-0.26, -0.20) | <0.001 | 1071.60 (1069.21, 1073.98) | 1039.59 (1037.14, 1042.05) | -0.08 (-0.20, 0.04) | 0.199 | 399.89 (398.44, 401.35) | 207.95 (206.86, 209.04) | -2.24 (-2.41, -2.07) | <0.001 | 5.50 (5.33, 5.67) | 2.27 (2.16, 2.39) | -3.03 (-3.27, -2.80) | <0.001 |
| High-income North America | 141.01 (140.06, 141.97) | 143.54 (142.60, 144.48) | 0.06 (0.02, 0.10) | 0.003 | 1992.96 (1989.36, 1996.57) | 1921.78 (1918.36, 1925.22) | -0.11 (-0.28, 0.07) | 0.231 | 493.56 (491.77, 495.35) | 440.22 (438.58, 441.87) | -0.39 (-0.71, -0.08) | 0.014 | 6.37 (6.17, 6.57) | 5.48 (5.30, 5.66) | -0.48 (-0.80, -0.17) | 0.003 |
| Central Asia | 139.37 (137.42, 141.34) | 133.98 (132.32, 135.64) | -0.13 (-0.15, -0.11) | <0.001 | 1695.01 (1688.18, 1701.88) | 1721.40 (1715.48, 1727.33) | 0.06 (0.01, 0.12) | 0.021 | 1220.80 (1214.95, 1226.67) | 989.46 (985.01, 993.92) | -0.71 (-1.25, -0.16) | 0.011 | 18.57 (17.85, 19.31) | 14.73 (14.20, 15.29) | -0.78 (-1.39, -0.17) | 0.013 |
| Eastern Europe | 100.55 (99.63, 101.49) | 105.05 (103.99, 106.11) | 0.16 (0.14, 0.18) | <0.001 | 948.21 (945.36, 951.06) | 1029.89 (1026.57, 1033.22) | 0.32 (0.17, 0.47) | <0.001 | 654.03 (651.67, 656.40) | 838.91 (836.00, 841.82) | 1.00 (0.26, 1.75) | 0.008 | 9.53 (9.25, 9.82) | 12.73 (12.38, 13.08) | 1.03 (0.14, 1.92) | 0.023 |
| Southern Sub-Saharan Africa | 180.87 (178.36, 183.41) | 169.18 (167.21, 171.16) | -0.23 (-0.25, -0.21) | <0.001 | 2858.08 (2848.03, 2868.15) | 2813.09 (2805.09, 2821.11) | -0.05 (-0.11, 0.02) | 0.175 | 1376.41 (1369.19, 1383.66) | 686.64 (682.71, 690.60) | -2.27 (-3.66, -0.85) | 0.002 | 20.58 (19.69, 21.50) | 8.65 (8.21, 9.10) | -2.85 (-4.53, -1.14) | 0.001 |
| High-income Asia Pacific | 103.14 (102.05, 104.24) | 99.37 (98.18, 100.57) | -0.12 (-0.16, -0.09) | <0.001 | 1083.60 (1080.07, 1087.13) | 1090.37 (1086.44, 1094.30) | 0.05 (-0.09, 0.18) | 0.506 | 507.02 (504.60, 509.44) | 193.81 (192.14, 195.49) | -3.29 (-3.54, -3.04) | <0.001 | 7.19 (6.90, 7.48) | 1.93 (1.77, 2.10) | -4.51 (-4.78, -4.24) | <0.001 |
| Central Europe | 84.87 (83.69, | 81.06 | -0.15 | <0.001 | 1292.77 | 1311.89 | 0.08 | 0.310 | 737.38 | 370.28 | -2.36 | <0.001 | 10.64 | 4.20 (3.92, | -3.18 | <0.001 |

| | | | | | | | | | | | | | | | | |
|---|---|---|---|---|---|---|---|---|---|---|---|---|---|---|---|---|
| | 86.06) | (79.76, 82.38) | (-0.18, -0.13) | | (1288.13, 1297.42) | (1306.57, 1317.22) | (-0.08, 0.24) | | (733.93, 740.85) | (367.51, 373.08) | (-2.50, -2.23) | | (10.23, 11.06) | 4.51) | (-3.39, -2.96) | |
| Andean Latin America | 135.91 (133.32, 138.55) | 124.43 (122.50, 126.38) | -0.30 (-0.32, -0.29) | <0.001 | 2189.97 (2179.39, 2200.59) | 2214.39 (2206.24, 2222.57) | 0.04 (0.01, 0.06) | 0.005 | 1150.36 (1142.59, 1158.17) | 570.22 (566.09, 574.38) | -2.36 (-3.14, -1.57) | <0.001 | 17.27 (16.33, 18.27) | 7.40 (6.93, 7.88) | -2.85 (-3.80, -1.90) | <0.001 |
| Caribbean | 182.49 (179.39, 185.62) | 193.06 (190.22, 195.93) | 0.20 (0.18, 0.22) | <0.001 | 2223.42 (2212.55, 2234.33) | 2306.40 (2296.57, 2316.26) | 0.14 (0.07, 0.21) | <0.001 | 1393.96 (1385.23, 1402.74) | 1207.17 (1200.06, 1214.31) | -0.49 (-0.75, -0.22) | <0.001 | 21.94 (20.84, 23.07) | 18.60 (17.73, 19.51) | -0.56 (-0.86, -0.26) | <0.001 |
| Southern Latin America | 92.96 (91.04, 94.92) | 89.50 (87.87, 91.16) | -0.13 (-0.14, -0.11) | <0.001 | 1457.21 (1449.57, 1464.88) | 1587.17 (1580.29, 1594.08) | 0.30 (0.28, 0.32) | <0.001 | 833.16 (827.36, 838.99) | 421.36 (417.83, 424.91) | -2.30 (-2.56, -2.03) | <0.001 | 12.45 (11.75, 13.19) | 5.26 (4.87, 5.67) | -2.90 (-3.24, -2.55) | <0.001 |
| Oceania | 157.43 (150.36, 164.77) | 165.24 (160.34, 170.25) | 0.17 (0.15, 0.19) | <0.001 | 1681.20 (1657.97, 1704.69) | 1739.34 (1723.37, 1755.43) | 0.13 (0.09, 0.16) | <0.001 | 1884.19 (1859.26, 1909.39) | 1931.79 (1914.87, 1948.83) | 0.07 (-0.09, 0.22) | 0.388 | 29.41 (26.34, 32.75) | 30.18 (28.09, 32.39) | 0.08 (-0.08, 0.24) | 0.316 |
| Australasia | 65.88 (63.44, 68.40) | 65.19 (62.99, 67.46) | -0.04 (-0.06, -0.03) | <0.001 | 779.17 (770.71, 787.69) | 787.53 (779.86, 795.27) | 0.05 (0.00, 0.10) | 0.039 | 352.48 (346.80, 358.23) | 207.87 (203.94, 211.86) | -1.80 (-2.16, -1.43) | <0.001 | 4.78 (4.14, 5.49) | 2.37 (1.97, 2.83) | -2.38 (-2.95, -1.81) | <0.001 |
| **Men** | | | | | | | | | | | | | | | | |
| Global | 129.92 (129.71, 130.14) | 132.84 (132.66, 133.03) | 0.07 (0.00, 0.14) | 0.051 | 1311.41 (1310.73, 1312.09) | 1467.09 (1466.48, 1467.70) | 0.40 (0.37, 0.43) | <0.001 | 1436.68 (1435.96, 1437.41) | 1229.51 (1228.96, 1230.07) | -0.55 (-0.71, -0.39) | <0.001 | 23.39 (23.30, 23.48) | 19.70 (19.63, 19.77) | -0.56 (-0.81, -0.30) | <0.001 |
| **SDI category** | | | | | | | | | | | | | | | | |
| High | 116.70 (116.19, 117.22) | 112.36 (111.87, 112.85) | -0.13 (-0.15, -0.12) | <0.001 | 932.87 (931.42, 934.32) | 963.56 (962.14, 964.98) | 0.14 (0.01, 0.28) | 0.034 | 822.29 (820.93, 823.66) | 532.61 (531.56, 533.66) | -1.51 (-1.63, -1.38) | <0.001 | 13.28 (13.11, 13.45) | 8.17 (8.04, 8.30) | -1.68 (-1.87, -1.49) | <0.001 |
| High-middle | 114.08 (113.65, 114.50) | 106.00 (105.62, 106.39) | -0.25 (-0.28, -0.22) | <0.001 | 986.51 (985.27, 987.75) | 997.01 (995.83, 998.19) | 0.04 (0.02, 0.07) | <0.001 | 1492.62 (1491.09, 1494.15) | 1139.85 (1138.63, 1141.08) | -0.92 (-1.31, -0.52) | <0.001 | 24.83 (24.63, 25.02) | 18.73 (18.57, 18.88) | -0.96 (-1.38, -0.53) | <0.001 |
| Middle | 127.98 (127.61, 128.35) | 126.43 (126.11, 126.75) | -0.04 (-0.10, 0.02) | 0.214 | 1381.88 (1380.67, 1383.10) | 1434.55 (1433.48, 1435.63) | 0.14 (0.11, 0.17) | <0.001 | 1491.65 (1490.37, 1492.93) | 1280.09 (1279.08, 1281.10) | -0.50 (-0.70, -0.29) | <0.001 | 24.11 (23.95, 24.27) | 20.45 (20.33, 20.58) | -0.54 (-0.75, -0.33) | <0.001 |
| Low-middle | 140.00 (139.50, | 144.57 (144.18, | 0.12 (0.09, | <0.001 | 1501.80 (1500.15, | 1629.06 (1627.74, | 0.30 (0.24, | <0.001 | 1728.66 (1726.85, | 1516.40 (1515.12, | -0.40 (-0.75, | 0.024 | 28.33 (28.10, | 24.71 (24.55, | -0.42 (-0.81, | 0.038 |

| | | | | | | | | | | | | | | | | |
|---|---|---|---|---|---|---|---|---|---|---|---|---|---|---|---|---|
| | 140.50) | 144.96) | 0.14) | | 1503.45) | 1630.37) | 0.36) | | 1730.47) | 1517.69) | -0.05) | | 28.57) | 24.88) | -0.02) | |
| Low | 163.64 (162.81, 164.48) | 165.19 (164.64, 165.74) | 0.04 (0.02, 0.06) | <0.001 | 2029.51 (2026.57, 2032.45) | 2165.97 (2163.98, 2167.96) | 0.23 (0.18, 0.29) | <0.001 | 1518.81 (1516.19, 1521.43) | 1336.23 (1334.61, 1337.85) | -0.43 (-0.59, -0.27) | <0.001 | 24.15 (23.81, 24.48) | 20.93 (20.72, 21.14) | -0.48 (-0.66, -0.31) | <0.001 |
| **21 GBD region** | | | | | | | | | | | | | | | | |
| South Asia | 135.81 (135.32, 136.30) | 139.18 (138.81, 139.55) | 0.09 (0.05, 0.13) | <0.001 | 1367.36 (1365.79, 1368.93) | 1407.40 (1406.22, 1408.59) | 0.13 (0.06, 0.19) | <0.001 | 1813.20 (1811.36, 1815.04) | 1616.42 (1615.13, 1617.70) | -0.34 (-0.81, 0.12) | 0.148 | 30.00 (29.76, 30.24) | 26.77 (26.61, 26.94) | -0.33 (-0.84, 0.19) | 0.210 |
| East Asia | 115.75 (115.35, 116.15) | 104.75 (104.36, 105.14) | -0.35 (-0.37, -0.33) | <0.001 | 1170.58 (1169.32, 1171.85) | 1065.04 (1063.82, 1066.27) | -0.31 (-0.36, -0.26) | <0.001 | 1281.28 (1279.95, 1282.61) | 1027.65 (1026.47, 1028.84) | -0.74 (-1.34, -0.13) | 0.018 | 20.46 (20.30, 20.63) | 16.38 (16.23, 16.53) | -0.74 (-1.40, -0.08) | 0.029 |
| North Africa and Middle East | 132.97 (132.08, 133.87) | 135.69 (135.07, 136.31) | 0.07 (0.05, 0.10) | <0.001 | 1617.13 (1614.02, 1620.24) | 1679.47 (1677.30, 1681.65) | 0.15 (0.05, 0.26) | 0.005 | 1998.25 (1994.73, 2001.77) | 1317.86 (1315.94, 1319.78) | -1.39 (-1.57, -1.21) | <0.001 | 32.73 (32.28, 33.19) | 20.61 (20.37, 20.85) | -1.55 (-1.68, -1.41) | <0.001 |
| Western Sub-Saharan Africa | 154.38 (153.04, 155.73) | 157.79 (156.92, 158.66) | 0.08 (0.07, 0.09) | <0.001 | 2489.43 (2484.07, 2494.81) | 2639.52 (2635.99, 2643.05) | 0.21 (0.17, 0.25) | <0.001 | 1077.31 (1073.68, 1080.96) | 985.70 (983.46, 987.94) | -0.31 (-0.42, -0.19) | <0.001 | 15.95 (15.50, 16.40) | 14.28 (14.01, 14.56) | -0.38 (-0.51, -0.25) | <0.001 |
| Southeast Asia | 119.58 (118.87, 120.30) | 117.41 (116.85, 117.99) | -0.06 (-0.07, -0.05) | <0.001 | 1078.50 (1076.37, 1080.64) | 1103.34 (1101.59, 1105.09) | 0.10 (-0.00, 0.19) | 0.056 | 1548.52 (1545.93, 1551.11) | 1488.28 (1486.26, 1490.31) | -0.10 (-0.47, 0.28) | 0.605 | 25.12 (24.79, 25.46) | 24.00 (23.74, 24.25) | -0.15 (-0.52, 0.21) | 0.416 |
| Eastern Sub-Saharan Africa | 189.02 (187.51, 190.53) | 184.55 (183.60, 185.51) | -0.08 (-0.10, -0.05) | <0.001 | 2533.95 (2528.40, 2539.52) | 2689.46 (2685.78, 2693.13) | 0.21 (0.17, 0.25) | <0.001 | 1462.70 (1458.34, 1467.06) | 1162.01 (1159.52, 1164.51) | -0.78 (-0.94, -0.62) | <0.001 | 22.50 (21.96, 23.06) | 17.24 (16.93, 17.55) | -0.91 (-1.09, -0.74) | <0.001 |
| Central Latin America | 99.71 (98.60, 100.84) | 98.80 (97.93, 99.69) | -0.03 (-0.05, -0.01) | 0.003 | 1070.67 (1067.03, 1074.32) | 1129.59 (1126.63, 1132.56) | 0.19 (0.15, 0.23) | <0.001 | 948.48 (944.97, 952.00) | 755.12 (752.68, 757.57) | -0.77 (-1.18, -0.36) | <0.001 | 15.36 (14.91, 15.81) | 11.98 (11.68, 12.29) | -0.84 (-1.28, -0.40) | <0.001 |
| Tropical Latin America | 162.81 (161.39, 164.25) | 140.53 (139.42, 141.64) | -0.51 (-0.53, -0.49) | <0.001 | 1988.60 (1983.61, 1993.60) | 1902.61 (1898.56, 1906.66) | -0.14 (-0.17, -0.12) | <0.001 | 1718.11 (1713.34, 1722.89) | 918.38 (915.60, 921.16) | -2.15 (-2.34, -1.96) | <0.001 | 28.48 (27.86, 29.10) | 14.18 (13.84, 14.53) | -2.40 (-2.64, -2.17) | <0.001 |
| Central Sub-Saharan Africa | 185.09 (182.40, 187.81) | 178.71 (177.04, 180.38) | -0.12 (-0.15, -0.09) | <0.001 | 2603.88 (2593.76, 2614.03) | 2595.45 (2589.09, 2601.83) | -0.02 (-0.04, 0.00) | 0.073 | 1312.97 (1305.51, 1320.45) | 1065.48 (1061.26, 1069.72) | -0.77 (-0.88, -0.66) | <0.001 | 20.11 (19.18, 21.07) | 15.91 (15.39, 16.44) | -0.86 (-1.00, -0.72) | <0.001 |

| | | | | | | | | | | | | | | | | |
|---|---|---|---|---|---|---|---|---|---|---|---|---|---|---|---|---|
| Western Europe | 95.82 (95.12, 96.53) | 86.33 (85.64, 87.02) | -0.36 (-0.38, -0.34) | <0.001 | 647.97 (646.14, 649.81) | 608.72 (606.90, 610.55) | -0.22 (-0.26, -0.17) | <0.001 | 765.79 (763.80, 767.78) | 341.88 (340.52, 343.24) | -2.77 (-3.06, -2.47) | <0.001 | 12.56 (12.31, 12.82) | 5.21 (5.05, 5.38) | -3.02 (-3.33, -2.70) | <0.001 |
| High-income North America | 135.82 (134.89, 136.76) | 131.64 (130.75, 132.54) | -0.11 (-0.14, -0.09) | <0.001 | 1261.33 (1258.48, 1264.18) | 1277.30 (1274.52, 1280.08) | 0.09 (-0.22, 0.41) | 0.561 | 810.74 (808.47, 813.02) | 637.92 (635.96, 639.89) | -0.83 (-1.05, -0.60) | <0.001 | 12.92 (12.64, 13.21) | 9.71 (9.47, 9.95) | -0.98 (-1.36, -0.61) | <0.001 |
| Central Asia | 164.82 (162.67, 167.00) | 162.43 (160.63, 164.24) | -0.05 (-0.07, -0.03) | <0.001 | 1486.64 (1480.16, 1493.15) | 1554.81 (1549.24, 1560.40) | 0.16 (0.13, 0.18) | <0.001 | 1981.18 (1973.53, 1988.85) | 1811.17 (1805.20, 1817.16) | -0.31 (-0.79, 0.17) | 0.203 | 33.37 (32.38, 34.39) | 30.23 (29.46, 31.01) | -0.35 (-0.77, 0.07) | 0.102 |
| Eastern Europe | 132.86 (131.81, 133.92) | 145.23 (144.02, 146.45) | 0.31 (0.30, 0.32) | <0.001 | 713.01 (710.57, 715.45) | 781.60 (778.82, 784.39) | 0.33 (0.30, 0.35) | <0.001 | 2047.33 (2043.24, 2051.43) | 2729.57 (2724.49, 2734.67) | 1.11 (0.34, 1.88) | 0.004 | 35.58 (35.04, 36.12) | 47.77 (47.11, 48.45) | 1.14 (0.36, 1.93) | 0.004 |
| Southern Sub-Saharan Africa | 190.94 (188.25, 193.67) | 184.98 (182.93, 187.05) | -0.11 (-0.13, -0.09) | <0.001 | 2459.80 (2450.12, 2469.51) | 2490.28 (2482.73, 2497.83) | 0.05 (0.03, 0.07) | <0.001 | 1419.50 (1411.81, 1427.22) | 1028.90 (1024.06, 1033.75) | -1.21 (-1.84, -0.58) | <0.001 | 22.50 (21.53, 23.51) | 15.31 (14.72, 15.91) | -1.46 (-2.19, -0.72) | <0.001 |
| High-income Asia Pacific | 128.83 (127.63, 130.03) | 129.82 (128.51, 131.14) | 0.03 (0.01, 0.05) | 0.006 | 886.47 (883.33, 889.62) | 939.52 (936.04, 943.01) | 0.21 (0.15, 0.26) | <0.001 | 861.88 (858.79, 864.98) | 351.38 (349.25, 353.52) | -3.05 (-3.26, -2.83) | <0.001 | 13.84 (13.45, 14.24) | 5.13 (4.88, 5.39) | -3.38 (-3.62, -3.14) | <0.001 |
| Central Europe | 108.15 (106.85, 109.47) | 103.82 (102.40, 105.27) | -0.14 (-0.17, -0.11) | <0.001 | 895.64 (891.87, 899.43) | 896.45 (892.23, 900.69) | 0.02 (-0.06, 0.10) | 0.594 | 1653.27 (1648.23, 1658.32) | 722.39 (718.70, 726.10) | -2.85 (-3.15, -2.55) | <0.001 | 28.30 (27.65, 28.97) | 11.60 (11.14, 12.07) | -3.11 (-3.42, -2.80) | <0.001 |
| Andean Latin America | 132.00 (129.38, 134.67) | 125.53 (123.60, 127.48) | -0.18 (-0.19, -0.16) | <0.001 | 1838.64 (1828.76, 1848.55) | 1863.74 (1856.27, 1871.22) | 0.05 (0.03, 0.06) | <0.001 | 1345.42 (1336.85, 1354.03) | 862.26 (857.17, 867.37) | -1.52 (-1.84, -1.20) | <0.001 | 20.99 (19.92, 22.10) | 12.80 (12.19, 13.44) | -1.70 (-2.07, -1.34) | <0.001 |
| Caribbean | 175.04 (171.91, 178.20) | 183.85 (181.05, 186.68) | 0.17 (0.15, 0.19) | <0.001 | 1875.02 (1864.80, 1885.29) | 1945.46 (1936.34, 1954.61) | 0.13 (0.12, 0.15) | <0.001 | 1354.07 (1345.16, 1363.03) | 1143.95 (1136.93, 1150.99) | -0.53 (-0.71, -0.36) | <0.001 | 22.02 (20.89, 23.21) | 18.35 (17.47, 19.27) | -0.58 (-0.76, -0.39) | <0.001 |
| Southern Latin America | 106.50 (104.41, 108.62) | 105.92 (104.14, 107.72) | -0.02 (-0.03, -0.01) | 0.004 | 1084.84 (1078.17, 1091.54) | 1183.18 (1177.22, 1189.17) | 0.31 (0.27, 0.35) | <0.001 | 1185.14 (1178.11, 1192.20) | 642.45 (638.07, 646.85) | -2.09 (-2.41, -1.77) | <0.001 | 19.35 (18.46, 20.27) | 9.79 (9.25, 10.34) | -2.34 (-2.53, -2.15) | <0.001 |
| Oceania | 162.64 (155.58, 169.96) | 165.42 (160.54, 170.41) | 0.06 (0.05, 0.06) | <0.001 | 1518.76 (1497.20, 1540.56) | 1563.67 (1548.68, 1578.78) | 0.11 (0.08, 0.13) | <0.001 | 3168.92 (3137.00, 3201.10) | 3185.57 (3163.84, 3207.42) | -0.02 (-0.14, 0.09) | 0.713 | 53.18 (49.08, 57.54) | 53.36 (50.57, 56.27) | -0.03 (-0.14, 0.09) | 0.635 |

| | | | | | | | | | | | | | | | | |
|---|---|---|---|---|---|---|---|---|---|---|---|---|---|---|---|---|
| Australasia | 134.99 (131.49, 138.57) | 133.96 (130.80, 137.17) | -0.03 (-0.08, 0.02) | 0.228 | 680.88 (673.01, 688.83) | 721.75 (714.46, 729.11) | 0.20 (0.13, 0.27) | <0.001 | 651.78 (644.06, 659.56) | 350.21 (345.13, 355.36) | -2.12 (-2.47, -1.78) | <0.001 | 10.65 (9.68, 11.68) | 5.47 (4.86, 6.15) | -2.27 (-2.66, -1.89) | <0.001 |
| **Rheumatic heart disease** | | | | | | | | | | | | | | | | |
| **Overall** | | | | | | | | | | | | | | | | |
| Global | 41.81 (41.73, 41.90) | 50.37 (50.29, 50.45) | 0.65 (0.61, 0.68) | <0.001 | 637.47 (637.14, 637.81) | 765.19 (764.87, 765.50) | 0.64 (0.60, 0.68) | <0.001 | 190.36 (190.18, 190.54) | 116.18 (116.06, 116.31) | -1.69 (-1.85, -1.52) | <0.001 | 2.68 (2.66, 2.71) | 1.32 (1.31, 1.34) | -2.42 (-2.65, -2.19) | <0.001 |
| **SDI category** | | | | | | | | | | | | | | | | |
| High | 1.34 (1.30, 1.38) | 1.35 (1.31, 1.39) | 0.04 (-0.13, 0.22) | 0.636 | 20.67 (20.52, 20.83) | 24.52 (24.35, 24.68) | 0.60 (0.35, 0.86) | <0.001 | 17.05 (16.91, 17.20) | 6.96 (6.87, 7.05) | -2.97 (-3.34, -2.60) | <0.001 | 0.27 (0.25, 0.29) | 0.10 (0.09, 0.11) | -3.49 (-3.88, -3.11) | <0.001 |
| High-middle | 18.55 (18.43, 18.68) | 19.03 (18.90, 19.15) | 0.09 (0.05, 0.14) | <0.001 | 313.25 (312.75, 313.75) | 327.11 (326.61, 327.61) | 0.16 (0.11, 0.20) | <0.001 | 94.91 (94.64, 95.18) | 35.58 (35.42, 35.74) | -3.31 (-3.65, -2.96) | <0.001 | 1.36 (1.33, 1.40) | 0.33 (0.32, 0.35) | -4.68 (-5.21, -4.15) | <0.001 |
| Middle | 42.94 (42.79, 43.09) | 43.24 (43.10, 43.37) | 0.03 (-0.05, 0.11) | 0.520 | 735.18 (734.55, 735.80) | 727.91 (727.36, 728.45) | -0.03 (-0.05, -0.01) | 0.015 | 190.66 (190.34, 190.98) | 85.34 (85.15, 85.53) | -2.73 (-3.06, -2.40) | <0.001 | 2.64 (2.60, 2.68) | 0.84 (0.82, 0.86) | -3.84 (-4.30, -3.38) | <0.001 |
| Low-middle | 71.91 (71.66, 72.16) | 74.00 (73.80, 74.19) | 0.10 (0.02, 0.18) | 0.016 | 1004.28 (1003.33, 1005.23) | 1070.64 (1069.89, 1071.40) | 0.24 (0.18, 0.30) | <0.001 | 367.82 (367.24, 368.40) | 213.52 (213.19, 213.86) | -1.85 (-2.07, -1.63) | <0.001 | 5.37 (5.30, 5.44) | 2.73 (2.69, 2.77) | -2.31 (-2.58, -2.04) | <0.001 |
| Low | 87.55 (87.14, 87.97) | 90.01 (89.74, 90.28) | 0.11 (0.08, 0.13) | <0.001 | 1319.96 (1318.31, 1321.61) | 1403.41 (1402.29, 1404.53) | 0.22 (0.18, 0.25) | <0.001 | 339.17 (338.33, 340.02) | 204.51 (204.08, 204.94) | -1.74 (-1.90, -1.58) | <0.001 | 4.63 (4.53, 4.73) | 2.32 (2.27, 2.36) | -2.36 (-2.56, -2.16) | <0.001 |
| **21 GBD region** | | | | | | | | | | | | | | | | |
| South Asia | 76.62 (76.36, 76.88) | 75.07 (74.88, 75.26) | -0.08 (-0.22, 0.07) | 0.283 | 988.15 (987.19, 989.10) | 1011.88 (1011.16, 1012.59) | 0.11 (0.04, 0.18) | 0.001 | 475.23 (474.57, 475.90) | 275.71 (275.34, 276.09) | -1.89 (-2.17, -1.61) | <0.001 | 7.16 (7.07, 7.24) | 3.83 (3.78, 3.87) | -2.15 (-2.43, -1.87) | <0.001 |
| East Asia | 33.74 (33.59, 33.89) | 28.48 (28.32, 28.63) | -0.59 (-0.63, -0.54) | <0.001 | 615.21 (614.56, 615.86) | 507.75 (507.12, 508.38) | -0.66 (-0.69, -0.63) | <0.001 | 167.68 (167.34, 168.02) | 42.60 (42.42, 42.78) | -4.63 (-5.01, -4.25) | <0.001 | 2.38 (2.34, 2.42) | 0.31 (0.29, 0.32) | -6.77 (-7.35, -6.19) | <0.001 |

| | | | | | | | | | | | | | | | | |
|---|---|---|---|---|---|---|---|---|---|---|---|---|---|---|---|---|
| North Africa and Middle East | 33.12 (32.82, 33.43) | 35.58 (35.35, 35.81) | 0.25 (0.23, 0.26) | <0.001 | 521.68 (520.44, 522.93) | 555.06 (554.16, 555.97) | 0.22 (0.18, 0.27) | <0.001 | 114.76 (114.17, 115.34) | 54.42 (54.14, 54.71) | -2.52 (-2.62, -2.42) | <0.001 | 1.49 (1.42, 1.56) | 0.46 (0.44, 0.49) | -3.94 (-4.08, -3.79) | <0.001 |
| Western Sub-Saharan Africa | 75.09 (74.46, 75.73) | 77.85 (77.45, 78.26) | 0.12 (0.10, 0.14) | <0.001 | 1219.46 (1216.84, 1222.08) | 1285.81 (1284.12, 1287.51) | 0.18 (0.17, 0.20) | <0.001 | 139.08 (138.20, 139.96) | 91.96 (91.50, 92.41) | -1.42 (-1.49, -1.36) | <0.001 | 1.32 (1.23, 1.41) | 0.50 (0.47, 0.53) | -3.30 (-3.47, -3.12) | <0.001 |
| Southeast Asia | 23.77 (23.56, 23.99) | 23.21 (23.03, 23.39) | -0.09 (-0.13, -0.04) | <0.001 | 361.15 (360.30, 362.01) | 375.13 (374.41, 375.86) | 0.12 (0.07, 0.17) | <0.001 | 115.03 (114.55, 115.52) | 65.88 (65.58, 66.19) | -1.88 (-2.03, -1.73) | <0.001 | 1.64 (1.59, 1.70) | 0.79 (0.75, 0.82) | -2.45 (-2.66, -2.25) | <0.001 |
| Eastern Sub-Saharan Africa | 108.32 (107.56, 109.07) | 111.34 (110.84, 111.83) | 0.10 (0.09, 0.11) | <0.001 | 1752.35 (1749.18, 1755.52) | 1885.50 (1883.38, 1887.62) | 0.26 (0.22, 0.29) | <0.001 | 197.74 (196.69, 198.79) | 134.83 (134.26, 135.39) | -1.31 (-1.40, -1.22) | <0.001 | 1.84 (1.74, 1.95) | 0.72 (0.68, 0.76) | -3.18 (-3.38, -2.98) | <0.001 |
| Central Latin America | 28.33 (27.94, 28.73) | 29.61 (29.27, 29.94) | 0.15 (0.10, 0.20) | <0.001 | 494.83 (493.13, 496.53) | 515.22 (513.82, 516.62) | 0.14 (0.11, 0.18) | <0.001 | 83.06 (82.35, 83.77) | 34.64 (34.28, 35.01) | -2.94 (-3.14, -2.75) | <0.001 | 1.02 (0.94, 1.10) | 0.17 (0.15, 0.20) | -5.90 (-6.18, -5.62) | <0.001 |
| Tropical Latin America | 74.40 (73.74, 75.06) | 74.11 (73.53, 74.68) | -0.01 (-0.02, 0.00) | 0.039 | 1356.55 (1353.68, 1359.42) | 1374.14 (1371.71, 1376.58) | 0.04 (0.04, 0.05) | <0.001 | 151.71 (150.75, 152.68) | 90.13 (89.51, 90.76) | -1.78 (-1.89, -1.67) | <0.001 | 1.45 (1.36, 1.55) | 0.42 (0.38, 0.46) | -4.18 (-4.51, -3.86) | <0.001 |
| Central Sub-Saharan Africa | 102.91 (101.56, 104.28) | 104.93 (104.06, 105.80) | 0.07 (0.05, 0.08) | <0.001 | 1845.48 (1839.54, 1851.43) | 1872.85 (1869.07, 1876.63) | 0.05 (0.03, 0.07) | <0.001 | 193.04 (191.11, 194.98) | 135.23 (134.21, 136.25) | -1.25 (-1.29, -1.21) | <0.001 | 1.75 (1.56, 1.94) | 0.77 (0.69, 0.85) | -2.80 (-2.93, -2.68) | <0.001 |
| Western Europe | 0.64 (0.60, 0.68) | 0.39 (0.36, 0.43) | -1.71 (-1.94, -1.48) | <0.001 | 9.67 (9.51, 9.83) | 7.29 (7.14, 7.44) | -1.00 (-1.18, -0.81) | <0.001 | 13.98 (13.79, 14.17) | 4.70 (4.58, 4.81) | -3.75 (-4.00, -3.51) | <0.001 | 0.23 (0.21, 0.25) | 0.07 (0.06, 0.09) | -4.04 (-4.67, -3.40) | <0.001 |
| High-income North America | 1.91 (1.84, 1.99) | 1.32 (1.25, 1.38) | -1.26 (-1.34, -1.19) | <0.001 | 29.28 (28.97, 29.59) | 21.74 (21.48, 22.00) | -1.01 (-1.05, -0.96) | <0.001 | 16.34 (16.11, 16.57) | 6.45 (6.31, 6.59) | -3.13 (-3.55, -2.70) | <0.001 | 0.25 (0.23, 0.28) | 0.09 (0.07, 0.11) | -3.54 (-4.51, -2.57) | <0.001 |
| Central Asia | 48.82 (48.02, 49.63) | 51.62 (50.89, 52.36) | 0.19 (0.18, 0.21) | <0.001 | 833.56 (830.19, 836.94) | 905.61 (902.57, 908.65) | 0.29 (0.28, 0.29) | <0.001 | 313.67 (311.58, 315.77) | 140.78 (139.59, 141.97) | -2.69 (-3.36, -2.01) | <0.001 | 4.64 (4.38, 4.90) | 1.65 (1.52, 1.78) | -3.47 (-4.23, -2.70) | <0.001 |
| Eastern Europe | 5.77 (5.62, 5.93) | 3.18 (3.06, 3.32) | -2.03 (-2.22, -1.84) | <0.001 | 45.44 (45.00, 45.89) | 34.01 (33.58, 34.45) | -0.97 (-1.06, -0.87) | <0.001 | 76.30 (75.73, 76.87) | 16.60 (16.30, 16.90) | -4.98 (-6.11, -3.83) | <0.001 | 1.29 (1.22, 1.37) | 0.26 (0.22, 0.30) | -5.22 (-6.43, -3.99) | <0.001 |

| | | | | | | | | | | | | | | | | |
|---|---|---|---|---|---|---|---|---|---|---|---|---|---|---|---|---|
| Southern Sub-Saharan Africa | 98.39 (97.10, 99.69) | 99.25 (98.18, 100.32) | 0.03 (0.02, 0.04) | <0.001 | 1681.78 (1676.28, 1687.30) | 1707.54 (1703.13, 1711.96) | 0.05 (0.04, 0.07) | <0.001 | 243.16 (241.06, 245.29) | 159.77 (158.42, 161.12) | -1.43 (-1.89, -0.97) | <0.001 | 2.72 (2.49, 2.95) | 1.27 (1.15, 1.40) | -2.58 (-3.38, -1.76) | <0.001 |
| High-income Asia Pacific | 0.59 (0.53, 0.65) | 0.29 (0.25, 0.34) | -2.43 (-2.71, -2.16) | <0.001 | 13.16 (12.89, 13.44) | 9.57 (9.30, 9.84) | -1.11 (-1.20, -1.02) | <0.001 | 11.17 (10.92, 11.42) | 3.22 (3.07, 3.37) | -4.19 (-4.49, -3.90) | <0.001 | 0.18 (0.15, 0.21) | 0.04 (0.03, 0.07) | -4.86 (-5.17, -4.54) | <0.001 |
| Central Europe | 4.11 (3.93, 4.30) | 2.86 (2.68, 3.04) | -1.24 (-1.40, -1.09) | <0.001 | 49.28 (48.64, 49.92) | 41.19 (40.52, 41.88) | -0.63 (-0.75, -0.52) | <0.001 | 71.26 (70.50, 72.01) | 11.43 (11.09, 11.79) | -6.15 (-6.45, -5.85) | <0.001 | 1.21 (1.11, 1.31) | 0.16 (0.12, 0.21) | -6.78 (-7.07, -6.50) | <0.001 |
| Andean Latin America | 66.20 (64.95, 67.48) | 65.88 (64.90, 66.88) | -0.02 (-0.04, -0.01) | 0.002 | 1170.32 (1164.84, 1175.83) | 1187.99 (1183.77, 1192.21) | 0.05 (0.03, 0.07) | <0.001 | 117.11 (115.36, 118.87) | 73.96 (72.91, 75.02) | -1.58 (-1.73, -1.43) | <0.001 | 1.03 (0.87, 1.21) | 0.29 (0.23, 0.36) | -4.27 (-4.90, -3.63) | <0.001 |
| Caribbean | 62.97 (61.72, 64.25) | 68.30 (67.11, 69.52) | 0.28 (0.26, 0.31) | <0.001 | 1115.48 (1110.04, 1120.93) | 1175.23 (1170.25, 1180.23) | 0.18 (0.17, 0.19) | <0.001 | 246.92 (244.34, 249.53) | 163.39 (161.53, 165.26) | -1.37 (-1.55, -1.19) | <0.001 | 3.26 (2.96, 3.57) | 1.80 (1.61, 2.01) | -1.97 (-2.22, -1.72) | <0.001 |
| Southern Latin America | 33.49 (32.68, 34.32) | 35.67 (34.93, 36.42) | 0.22 (0.20, 0.24) | <0.001 | 634.17 (630.60, 637.77) | 690.28 (687.05, 693.51) | 0.29 (0.28, 0.31) | <0.001 | 79.42 (78.15, 80.70) | 43.86 (43.05, 44.68) | -2.03 (-2.13, -1.93) | <0.001 | 0.84 (0.72, 0.98) | 0.19 (0.14, 0.25) | -5.00 (-5.29, -4.72) | <0.001 |
| Oceania | 41.16 (38.71, 43.72) | 43.75 (42.00, 45.55) | 0.21 (0.20, 0.23) | <0.001 | 710.41 (700.13, 720.81) | 770.48 (763.10, 777.92) | 0.28 (0.24, 0.33) | <0.001 | 674.23 (664.04, 684.55) | 610.07 (603.46, 616.75) | -0.36 (-0.53, -0.20) | <0.001 | 10.74 (9.48, 12.14) | 9.56 (8.75, 10.44) | -0.42 (-0.59, -0.25) | <0.001 |
| Australasia | 1.20 (0.97, 1.46) | 0.94 (0.76, 1.15) | -0.84 (-0.98, -0.70) | <0.001 | 24.58 (23.51, 25.68) | 23.22 (22.28, 24.21) | -0.19 (-0.21, -0.17) | <0.001 | 28.66 (27.52, 29.84) | 12.47 (11.79, 13.19) | -2.79 (-3.12, -2.46) | <0.001 | 0.46 (0.32, 0.63) | 0.19 (0.12, 0.30) | -2.92 (-3.50, -2.33) | <0.001 |
| **Women** | | | | | | | | | | | | | | | | |
| Global | 45.76 (45.64, 45.89) | 55.68 (55.56, 55.80) | 0.68 (0.62, 0.73) | <0.001 | 704.89 (704.38, 705.39) | 854.09 (853.62, 854.56) | 0.67 (0.64, 0.70) | <0.001 | 208.13 (207.85, 208.40) | 119.84 (119.66, 120.02) | -1.89 (-2.10, -1.67) | <0.001 | 2.94 (2.91, 2.98) | 1.32 (1.31, 1.34) | -2.72 (-3.00, -2.44) | <0.001 |
| **SDI category** | | | | | | | | | | | | | | | | |
| High | 1.52 (1.46, 1.59) | 1.42 (1.36, 1.48) | -0.24 (-0.29, -0.19) | <0.001 | 22.44 (22.21, 22.67) | 23.54 (23.31, 23.78) | 0.17 (0.13, 0.22) | <0.001 | 18.09 (17.88, 18.29) | 6.93 (6.81, 7.06) | -3.22 (-3.53, -2.92) | <0.001 | 0.29 (0.26, 0.31) | 0.10 (0.08, 0.11) | -3.57 (-3.98, -3.15) | <0.001 |

| | | | | | | | | | | | | | | | | |
|---|---|---|---|---|---|---|---|---|---|---|---|---|---|---|---|---|
| High-middle | 19.10 (18.92, 19.27) | 20.28 (20.09, 20.47) | 0.21 (0.16, 0.26) | <0.001 | 338.83 (338.09, 339.57) | 366.42 (365.66, 367.19) | 0.28 (0.24, 0.31) | <0.001 | 99.41 (99.01, 99.81) | 35.23 (35.00, 35.47) | -3.51 (-3.85, -3.17) | <0.001 | 1.43 (1.38, 1.47) | 0.30 (0.28, 0.32) | -5.23 (-5.79, -4.68) | <0.001 |
| Middle | 44.87 (44.65, 45.08) | 45.78 (45.59, 45.98) | 0.06 (-0.04, 0.16) | 0.219 | 792.11 (791.19, 793.03) | 796.45 (795.63, 797.26) | 0.02 (0.00, 0.05) | 0.052 | 220.35 (219.86, 220.85) | 86.34 (86.07, 86.60) | -3.16 (-3.65, -2.66) | <0.001 | 3.12 (3.06, 3.18) | 0.81 (0.78, 0.83) | -4.51 (-5.03, -3.99) | <0.001 |
| Low-middle | 83.02 (82.64, 83.39) | 84.24 (83.94, 84.53) | 0.06 (0.03, 0.09) | <0.001 | 1160.57 (1159.12, 1162.03) | 1223.18 (1222.04, 1224.32) | 0.19 (0.18, 0.21) | <0.001 | 388.04 (387.19, 388.88) | 221.42 (220.93, 221.91) | -1.94 (-2.20, -1.68) | <0.001 | 5.60 (5.49, 5.70) | 2.76 (2.71, 2.81) | -2.41 (-2.70, -2.13) | <0.001 |
| Low | 94.39 (93.79, 94.99) | 96.98 (96.58, 97.38) | 0.10 (0.09, 0.11) | <0.001 | 1422.29 (1419.88, 1424.70) | 1510.56 (1508.93, 1512.19) | 0.21 (0.17, 0.25) | <0.001 | 366.56 (365.32, 367.80) | 210.62 (210.00, 211.24) | -1.92 (-2.17, -1.67) | <0.001 | 5.04 (4.90, 5.19) | 2.37 (2.30, 2.44) | -2.61 (-2.92, -2.29) | <0.001 |
| **21 GBD region** | | | | | | | | | | | | | | | | |
| South Asia | 91.34 (90.94, 91.75) | 87.77 (87.47, 88.07) | -0.13 (-0.19, -0.07) | <0.001 | 1171.89 (1170.39, 1173.39) | 1182.97 (1181.87, 1184.08) | 0.04 (0.02, 0.06) | <0.001 | 490.65 (489.68, 491.62) | 279.57 (279.03, 280.11) | -1.98 (-2.39, -1.56) | <0.001 | 7.28 (7.16, 7.40) | 3.78 (3.72, 3.84) | -2.30 (-2.77, -1.84) | <0.001 |
| East Asia | 34.01 (33.79, 34.22) | 29.08 (28.85, 29.31) | -0.55 (-0.59, -0.50) | <0.001 | 644.53 (643.58, 645.49) | 544.02 (543.08, 544.96) | -0.58 (-0.62, -0.55) | <0.001 | 210.94 (210.38, 211.49) | 43.57 (43.30, 43.83) | -5.30 (-5.97, -4.62) | <0.001 | 3.13 (3.06, 3.20) | 0.29 (0.27, 0.31) | -7.86 (-8.94, -6.76) | <0.001 |
| North Africa and Middle East | 34.08 (33.64, 34.53) | 36.74 (36.40, 37.08) | 0.26 (0.24, 0.28) | <0.001 | 555.73 (553.89, 557.58) | 595.68 (594.33, 597.05) | 0.24 (0.22, 0.27) | <0.001 | 144.64 (143.70, 145.58) | 62.34 (61.90, 62.78) | -2.85 (-2.94, -2.77) | <0.001 | 1.96 (1.85, 2.08) | 0.57 (0.53, 0.61) | -4.16 (-4.33, -3.99) | <0.001 |
| Western Sub-Saharan Africa | 76.90 (76.01, 77.79) | 80.43 (79.87, 81.00) | 0.16 (0.14, 0.17) | <0.001 | 1246.67 (1242.98, 1250.38) | 1328.86 (1326.48, 1331.24) | 0.22 (0.22, 0.23) | <0.001 | 158.81 (157.50, 160.13) | 95.91 (95.27, 96.55) | -1.72 (-1.77, -1.67) | <0.001 | 1.62 (1.49, 1.76) | 0.54 (0.49, 0.59) | -3.74 (-3.92, -3.56) | <0.001 |
| Southeast Asia | 27.10 (26.78, 27.42) | 26.36 (26.09, 26.64) | -0.10 (-0.14, -0.06) | <0.001 | 426.13 (424.83, 427.43) | 442.27 (441.15, 443.39) | 0.13 (0.10, 0.16) | <0.001 | 119.05 (118.36, 119.75) | 73.22 (72.76, 73.67) | -1.63 (-1.82, -1.44) | <0.001 | 1.66 (1.57, 1.74) | 0.86 (0.81, 0.91) | -2.20 (-2.48, -1.92) | <0.001 |
| Eastern Sub-Saharan Africa | 114.09 (113.02, 115.17) | 118.70 (117.98, 119.42) | 0.13 (0.13, 0.14) | <0.001 | 1861.25 (1856.73, 1865.79) | 2014.08 (2011.02, 2017.14) | 0.28 (0.24, 0.32) | <0.001 | 220.56 (219.01, 222.12) | 140.93 (140.13, 141.75) | -1.52 (-1.60, -1.45) | <0.001 | 2.17 (2.02, 2.33) | 0.74 (0.68, 0.80) | -3.62 (-3.76, -3.49) | <0.001 |
| Central Latin America | 30.43 (29.86, 31.01) | 31.78 (31.30, | 0.16 (0.14, | <0.001 | 559.75 (557.23, | 584.03 (581.94, | 0.15 (0.13, | <0.001 | 100.99 (99.89, 102.09) | 38.87 (38.33, 39.41) | -3.21 (-3.39, | <0.001 | 1.28 (1.16, 1.41) | 0.19 (0.15, 0.23) | -6.27 (-6.57, | <0.001 |

| | | | | | | | | | | | | | | | | |
|---|---|---|---|---|---|---|---|---|---|---|---|---|---|---|---|---|
| | | 32.28) | 0.17) | | 562.28) | 586.12) | 0.18) | | | | -3.04) | | | | -5.97) | |
| Tropical Latin America | 77.00 (76.06, 77.95) | 77.60 (76.77, 78.44) | 0.03 (-0.03, 0.09) | 0.285 | 1506.14 (1501.90, 1510.39) | 1549.94 (1546.29, 1553.59) | 0.10 (0.09, 0.11) | <0.001 | 167.82 (166.40, 169.25) | 100.37 (99.45, 101.30) | -1.75 (-1.88, -1.62) | <0.001 | 1.62 (1.48, 1.77) | 0.46 (0.40, 0.53) | -4.16 (-4.54, -3.78) | <0.001 |
| Central Sub-Saharan Africa | 105.89 (103.96, 107.85) | 108.75 (107.51, 110.00) | 0.10 (0.08, 0.12) | <0.001 | 1874.95 (1866.53, 1883.40) | 1923.92 (1918.53, 1929.33) | 0.09 (0.07, 0.12) | <0.001 | 228.42 (225.43, 231.44) | 142.09 (140.61, 143.58) | -1.64 (-1.72, -1.56) | <0.001 | 2.38 (2.08, 2.71) | 0.88 (0.76, 1.01) | -3.41 (-3.50, -3.32) | <0.001 |
| Western Europe | 0.64 (0.58, 0.70) | 0.37 (0.32, 0.42) | -1.90 (-1.97, -1.82) | <0.001 | 10.21 (9.98, 10.45) | 7.67 (7.46, 7.89) | -1.00 (-1.13, -0.88) | <0.001 | 13.07 (12.81, 13.33) | 4.29 (4.13, 4.45) | -3.82 (-3.98, -3.65) | <0.001 | 0.21 (0.18, 0.25) | 0.06 (0.05, 0.09) | -4.25 (-4.59, -3.90) | <0.001 |
| High-income North America | 2.29 (2.17, 2.42) | 1.59 (1.50, 1.70) | -1.25 (-1.31, -1.18) | <0.001 | 33.41 (32.94, 33.88) | 25.36 (24.96, 25.76) | -0.94 (-0.98, -0.89) | <0.001 | 17.84 (17.50, 18.18) | 6.98 (6.78, 7.19) | -3.15 (-3.51, -2.78) | <0.001 | 0.28 (0.23, 0.32) | 0.09 (0.07, 0.12) | -3.72 (-4.95, -2.48) | <0.001 |
| Central Asia | 49.28 (48.14, 50.44) | 51.70 (50.66, 52.77) | 0.17 (0.15, 0.20) | <0.001 | 894.70 (889.78, 899.64) | 966.57 (962.12, 971.04) | 0.27 (0.26, 0.28) | <0.001 | 351.03 (347.93, 354.16) | 151.22 (149.47, 152.98) | -2.82 (-3.42, -2.22) | <0.001 | 5.22 (4.84, 5.62) | 1.78 (1.60, 1.98) | -3.60 (-4.18, -3.01) | <0.001 |
| Eastern Europe | 5.03 (4.83, 5.24) | 3.09 (2.92, 3.28) | -1.62 (-1.71, -1.54) | <0.001 | 43.69 (43.08, 44.31) | 35.12 (34.49, 35.76) | -0.73 (-0.85, -0.62) | <0.001 | 56.96 (56.26, 57.66) | 12.06 (11.70, 12.43) | -5.05 (-6.13, -3.96) | <0.001 | 0.96 (0.87, 1.05) | 0.18 (0.14, 0.23) | -5.48 (-6.71, -4.24) | <0.001 |
| Southern Sub-Saharan Africa | 103.71 (101.87, 105.57) | 104.89 (103.34, 106.46) | 0.04 (0.02, 0.07) | <0.001 | 1788.66 (1780.78, 1796.57) | 1815.89 (1809.46, 1822.33) | 0.05 (0.04, 0.07) | <0.001 | 289.85 (286.64, 293.08) | 158.39 (156.49, 160.30) | -2.09 (-2.83, -1.35) | <0.001 | 3.43 (3.09, 3.81) | 1.18 (1.03, 1.36) | -3.58 (-4.81, -2.33) | <0.001 |
| High-income Asia Pacific | 0.63 (0.55, 0.72) | 0.31 (0.24, 0.38) | -2.46 (-2.75, -2.17) | <0.001 | 13.13 (12.74, 13.52) | 9.36 (8.99, 9.75) | -1.18 (-1.23, -1.12) | <0.001 | 11.92 (11.55, 12.29) | 3.17 (2.95, 3.39) | -4.48 (-4.86, -4.10) | <0.001 | 0.19 (0.14, 0.24) | 0.04 (0.02, 0.08) | -5.01 (-5.37, -4.66) | <0.001 |
| Central Europe | 3.67 (3.43, 3.93) | 2.64 (2.40, 2.91) | -1.13 (-1.29, -0.97) | <0.001 | 48.90 (47.99, 49.82) | 41.78 (40.80, 42.77) | -0.54 (-0.70, -0.38) | <0.001 | 61.00 (60.02, 62.00) | 9.82 (9.37, 10.29) | -6.12 (-6.42, -5.81) | <0.001 | 1.03 (0.91, 1.17) | 0.13 (0.09, 0.20) | -6.85 (-7.20, -6.50) | <0.001 |
| Andean Latin America | 69.29 (67.49, 71.13) | 69.40 (67.96, 70.86) | 0.00 (-0.01, 0.01) | 0.558 | 1270.45 (1262.45, 1278.49) | 1306.82 (1300.56, 1313.11) | 0.10 (0.09, 0.10) | <0.001 | 135.85 (133.22, 138.52) | 80.92 (79.37, 82.50) | -1.77 (-2.04, -1.50) | <0.001 | 1.28 (1.03, 1.57) | 0.32 (0.23, 0.43) | -4.68 (-5.41, -3.94) | <0.001 |
| Caribbean | 66.28 (64.49, 68.12) | 72.73 (70.99, | 0.33 (0.32, | <0.001 | 1213.37 (1205.45, | 1292.77 (1285.40, | 0.22 (0.21, | <0.001 | 328.40 (324.24, | 210.53 (207.57, | -1.48 (-1.74, | <0.001 | 4.57 (4.08, 5.10) | 2.51 (2.19, 2.85) | -2.01 (-2.34, | <0.001 |

| | | | | | | | | | | | | | | | | |
|---|---|---|---|---|---|---|---|---|---|---|---|---|---|---|---|---|
| | | 74.51) | 0.34) | | 1221.33) | 1300.16) | 0.23) | | 332.61) | 213.53) | -1.21) | | | | -1.68) | |
| Southern Latin America | 32.88 (31.75, 34.04) | 35.06 (34.02, 36.11) | 0.22 (0.20, 0.24) | <0.001 | 673.24 (668.06, 678.45) | 738.39 (733.69, 743.12) | 0.32 (0.29, 0.34) | <0.001 | 83.65 (81.82, 85.51) | 46.31 (45.13, 47.50) | -2.01 (-2.13, -1.89) | <0.001 | 0.89 (0.71, 1.10) | 0.19 (0.12, 0.28) | -5.17 (-5.54, -4.80) | <0.001 |
| Oceania | 43.62 (40.05, 47.45) | 46.66 (44.10, 49.33) | 0.24 (0.21, 0.26) | <0.001 | 774.06 (758.71, 789.66) | 840.99 (830.00, 852.09) | 0.30 (0.22, 0.38) | <0.001 | 672.52 (657.91, 687.39) | 620.19 (610.69, 629.80) | -0.28 (-0.47, -0.09) | 0.005 | 10.73 (8.94, 12.79) | 9.76 (8.60, 11.04) | -0.32 (-0.52, -0.13) | 0.001 |
| Australasia | 1.60 (1.24, 2.03) | 1.26 (0.97, 1.62) | -0.79 (-0.92, -0.65) | <0.001 | 30.62 (28.95, 32.37) | 28.96 (27.47, 30.52) | -0.19 (-0.23, -0.14) | <0.001 | 27.80 (26.22, 29.46) | 12.43 (11.48, 13.45) | -2.70 (-3.20, -2.20) | <0.001 | 0.44 (0.26, 0.70) | 0.19 (0.09, 0.36) | -2.80 (-2.91, -2.69) | <0.001 |
| **Men** | | | | | | | | | | | | | | | | |
| Global | 37.95 (37.84, 38.06) | 45.24 (45.13, 45.34) | 0.61 (0.56, 0.66) | <0.001 | 571.58 (571.13, 572.03) | 678.33 (677.92, 678.75) | 0.61 (0.56, 0.65) | <0.001 | 173.02 (172.78, 173.27) | 112.58 (112.41, 112.75) | -1.45 (-1.63, -1.27) | <0.001 | 2.43 (2.40, 2.46) | 1.32 (1.30, 1.34) | -2.06 (-2.29, -1.83) | <0.001 |
| **SDI category** | | | | | | | | | | | | | | | | |
| High | 1.17 (1.12, 1.22) | 1.28 (1.23, 1.33) | 0.29 (-0.01, 0.59) | 0.054 | 18.96 (18.75, 19.17) | 25.42 (25.19, 25.65) | 1.09 (0.88, 1.30) | <0.001 | 16.06 (15.87, 16.25) | 6.99 (6.87, 7.11) | -2.78 (-3.04, -2.51) | <0.001 | 0.26 (0.23, 0.28) | 0.10 (0.08, 0.11) | -3.18 (-3.57, -2.80) | <0.001 |
| High-middle | 18.03 (17.86, 18.20) | 17.87 (17.70, 18.04) | -0.02 (-0.07, 0.03) | 0.416 | 288.50 (287.83, 289.17) | 290.40 (289.74, 291.05) | 0.03 (-0.04, 0.10) | 0.364 | 90.55 (90.17, 90.93) | 35.90 (35.67, 36.13) | -3.12 (-3.41, -2.84) | <0.001 | 1.30 (1.26, 1.35) | 0.37 (0.34, 0.39) | -4.25 (-4.60, -3.90) | <0.001 |
| Middle | 41.05 (40.85, 41.25) | 40.79 (40.61, 40.97) | -0.04 (-0.15, 0.07) | 0.511 | 679.74 (678.90, 680.58) | 660.52 (659.78, 661.25) | -0.09 (-0.12, -0.05) | <0.001 | 161.87 (161.46, 162.29) | 84.36 (84.09, 84.62) | -2.21 (-2.50, -1.92) | <0.001 | 2.18 (2.13, 2.23) | 0.87 (0.85, 0.90) | -3.11 (-3.50, -2.72) | <0.001 |
| Low-middle | 61.00 (60.68, 61.32) | 63.91 (63.65, 64.16) | 0.19 (0.03, 0.35) | 0.018 | 851.09 (849.86, 852.32) | 918.79 (917.80, 919.77) | 0.29 (0.18, 0.40) | <0.001 | 348.12 (347.33, 348.92) | 205.64 (205.18, 206.11) | -1.78 (-2.05, -1.51) | <0.001 | 5.15 (5.05, 5.24) | 2.71 (2.65, 2.76) | -2.15 (-2.44, -1.85) | <0.001 |
| Low | 80.63 (80.07, 81.19) | 82.96 (82.59, 83.34) | 0.11 (0.09, 0.14) | <0.001 | 1214.92 (1212.67, 1217.17) | 1293.28 (1291.76, 1294.81) | 0.23 (0.18, 0.27) | <0.001 | 310.72 (309.57, 311.87) | 197.94 (197.34, 198.55) | -1.53 (-1.84, -1.22) | <0.001 | 4.21 (4.07, 4.34) | 2.26 (2.19, 2.33) | -2.12 (-2.54, -1.69) | <0.001 |
| **21 GBD region** | | | | | | | | | | | | | | | | |

| | | | | | | | | | | | | | | | | |
|---|---|---|---|---|---|---|---|---|---|---|---|---|---|---|---|---|
| South Asia | 62.80 (62.47, 63.12) | 62.94 (62.70, 63.19) | 0.06 (-0.24, 0.37) | 0.677 | 816.00 (814.79, 817.20) | 847.13 (846.21, 848.04) | 0.17 (0.03, 0.32) | 0.021 | 460.93 (460.02, 461.84) | 271.98 (271.46, 272.50) | -1.77 (-2.05, -1.50) | <0.001 | 7.04 (6.93, 7.16) | 3.87 (3.81, 3.93) | -2.02 (-2.32, -1.71) | <0.001 |
| East Asia | 33.48 (33.27, 33.69) | 27.94 (27.72, 28.15) | -0.63 (-0.69, -0.56) | <0.001 | 587.58 (586.70, 588.47) | 473.53 (472.68, 474.38) | -0.75 (-0.77, -0.72) | <0.001 | 127.16 (126.75, 127.58) | 41.66 (41.41, 41.91) | -3.81 (-4.05, -3.57) | <0.001 | 1.68 (1.63, 1.73) | 0.32 (0.30, 0.34) | -5.66 (-6.02, -5.30) | <0.001 |
| North Africa and Middle East | 32.20 (31.78, 32.62) | 34.50 (34.18, 34.82) | 0.24 (0.22, 0.27) | <0.001 | 489.42 (487.75, 491.11) | 518.03 (516.81, 519.24) | 0.20 (0.18, 0.23) | <0.001 | 86.41 (85.71, 87.12) | 47.20 (46.84, 47.57) | -2.03 (-2.15, -1.91) | <0.001 | 1.04 (0.96, 1.12) | 0.37 (0.34, 0.40) | -3.48 (-3.71, -3.26) | <0.001 |
| Western Sub-Saharan Africa | 73.11 (72.22, 74.02) | 75.05 (74.47, 75.62) | 0.09 (0.08, 0.10) | <0.001 | 1190.73 (1187.02, 1194.45) | 1238.08 (1235.66, 1240.49) | 0.14 (0.13, 0.15) | <0.001 | 117.86 (116.71, 119.03) | 87.54 (86.90, 88.18) | -1.02 (-1.11, -0.93) | <0.001 | 0.99 (0.88, 1.10) | 0.46 (0.41, 0.51) | -2.62 (-2.81, -2.44) | <0.001 |
| Southeast Asia | 20.35 (20.06, 20.63) | 20.13 (19.90, 20.37) | -0.03 (-0.08, 0.02) | 0.216 | 294.20 (293.11, 295.30) | 309.33 (308.40, 310.26) | 0.17 (0.13, 0.21) | <0.001 | 110.99 (110.31, 111.67) | 58.69 (58.29, 59.10) | -2.15 (-2.29, -2.02) | <0.001 | 1.63 (1.55, 1.72) | 0.71 (0.67, 0.76) | -2.76 (-3.03, -2.49) | <0.001 |
| Eastern Sub-Saharan Africa | 102.17 (101.11, 103.24) | 103.71 (103.03, 104.40) | 0.06 (0.03, 0.08) | <0.001 | 1634.38 (1629.97, 1638.81) | 1749.59 (1746.66, 1752.52) | 0.24 (0.20, 0.28) | <0.001 | 172.04 (170.65, 173.44) | 128.00 (127.22, 128.79) | -1.00 (-1.14, -0.85) | <0.001 | 1.47 (1.35, 1.61) | 0.69 (0.64, 0.75) | -2.54 (-2.78, -2.29) | <0.001 |
| Central Latin America | 26.12 (25.58, 26.67) | 27.41 (26.96, 27.88) | 0.18 (0.08, 0.28) | <0.001 | 425.77 (423.51, 428.04) | 443.44 (441.59, 445.30) | 0.14 (0.12, 0.17) | <0.001 | 63.94 (63.05, 64.83) | 30.21 (29.73, 30.70) | -2.52 (-2.72, -2.32) | <0.001 | 0.73 (0.64, 0.84) | 0.15 (0.12, 0.19) | -5.26 (-5.65, -4.86) | <0.001 |
| Tropical Latin America | 71.76 (70.84, 72.69) | 70.68 (69.89, 71.48) | -0.05 (-0.07, -0.03) | <0.001 | 1201.67 (1197.83, 1205.53) | 1195.90 (1192.68, 1199.13) | -0.02 (-0.03, -0.01) | <0.001 | 134.93 (133.64, 136.23) | 79.66 (78.84, 80.50) | -1.82 (-1.98, -1.65) | <0.001 | 1.27 (1.15, 1.41) | 0.37 (0.32, 0.43) | -4.16 (-4.46, -3.87) | <0.001 |
| Central Sub-Saharan Africa | 99.94 (98.05, 101.87) | 101.08 (99.88, 102.30) | 0.04 (0.01, 0.07) | 0.004 | 1815.12 (1806.76, 1823.51) | 1820.86 (1815.58, 1826.15) | 0.01 (-0.01, 0.04) | 0.352 | 156.24 (153.84, 158.67) | 128.11 (126.73, 129.51) | -0.70 (-0.75, -0.66) | <0.001 | 1.09 (0.90, 1.31) | 0.65 (0.55, 0.76) | -1.76 (-2.00, -1.53) | <0.001 |
| Western Europe | 0.64 (0.59, 0.70) | 0.41 (0.37, 0.46) | -1.56 (-1.71, -1.40) | <0.001 | 9.15 (8.93, 9.37) | 6.92 (6.72, 7.12) | -0.98 (-1.16, -0.80) | <0.001 | 14.87 (14.59, 15.15) | 5.10 (4.93, 5.27) | -3.66 (-4.25, -3.08) | <0.001 | 0.24 (0.21, 0.28) | 0.08 (0.06, 0.10) | -3.76 (-4.29, -3.23) | <0.001 |
| High-income North America | 1.54 (1.44, 1.65) | 1.04 (0.97, 1.13) | -1.34 (-1.45, -1.23) | <0.001 | 25.15 (24.74, 25.56) | 18.16 (17.82, 18.50) | -1.10 (-1.14, -1.07) | <0.001 | 14.83 (14.53, 15.15) | 5.92 (5.74, 6.12) | -3.09 (-3.68, -2.51) | <0.001 | 0.23 (0.20, 0.28) | 0.08 (0.06, 0.11) | -3.57 (-4.61, -2.52) | <0.001 |

| | | | | | | | | | | | | | | | | |
|---|---|---|---|---|---|---|---|---|---|---|---|---|---|---|---|---|
| Central Asia | 48.39 (47.26, 49.55) | 51.57 (50.54, 52.61) | 0.22 (0.19, 0.24) | <0.001 | 771.61 (767.02, 776.23) | 845.88 (841.76, 850.02) | 0.32 (0.30, 0.33) | <0.001 | 275.63 (272.86, 278.43) | 130.46 (128.85, 132.09) | -2.50 (-3.18, -1.82) | <0.001 | 4.04 (3.71, 4.40) | 1.51 (1.34, 1.70) | -3.28 (-4.19, -2.36) | <0.001 |
| Eastern Europe | 6.51 (6.28, 6.75) | 3.27 (3.09, 3.46) | -2.32 (-2.42, -2.22) | <0.001 | 47.20 (46.57, 47.84) | 32.92 (32.32, 33.53) | -1.22 (-1.38, -1.06) | <0.001 | 95.60 (94.70, 96.50) | 21.11 (20.64, 21.58) | -4.94 (-6.12, -3.74) | <0.001 | 1.63 (1.52, 1.75) | 0.34 (0.28, 0.41) | -5.14 (-6.37, -3.91) | <0.001 |
| Southern Sub-Saharan Africa | 92.67 (90.87, 94.50) | 93.61 (92.15, 95.09) | 0.04 (0.03, 0.05) | <0.001 | 1566.77 (1559.12, 1574.45) | 1598.63 (1592.59, 1604.68) | 0.07 (0.06, 0.08) | <0.001 | 192.66 (189.98, 195.38) | 160.98 (159.06, 162.92) | -0.60 (-0.83, -0.38) | <0.001 | 1.94 (1.68, 2.24) | 1.35 (1.18, 1.54) | -1.32 (-1.64, -1.00) | <0.001 |
| High-income Asia Pacific | 0.54 (0.47, 0.63) | 0.28 (0.22, 0.35) | -2.24 (-2.55, -1.92) | <0.001 | 13.19 (12.81, 13.58) | 9.76 (9.39, 10.14) | -1.03 (-1.10, -0.97) | <0.001 | 10.43 (10.09, 10.78) | 3.27 (3.06, 3.49) | -3.92 (-4.17, -3.66) | <0.001 | 0.16 (0.12, 0.21) | 0.05 (0.02, 0.08) | -4.25 (-4.59, -3.91) | <0.001 |
| Central Europe | 4.55 (4.28, 4.83) | 3.06 (2.80, 3.33) | -1.35 (-1.50, -1.20) | <0.001 | 49.67 (48.77, 50.58) | 40.64 (39.70, 41.59) | -0.71 (-0.85, -0.58) | <0.001 | 81.23 (80.10, 82.37) | 12.97 (12.46, 13.50) | -6.21 (-6.54, -5.89) | <0.001 | 1.38 (1.23, 1.53) | 0.19 (0.13, 0.26) | -6.67 (-7.13, -6.21) | <0.001 |
| Andean Latin America | 63.01 (61.26, 64.81) | 62.53 (61.18, 63.91) | -0.04 (-0.06, -0.01) | 0.001 | 1065.88 (1058.40, 1073.39) | 1069.81 (1064.17, 1075.48) | 0.00 (-0.02, 0.03) | 0.764 | 97.52 (95.26, 99.81) | 66.97 (65.56, 68.40) | -1.29 (-1.40, -1.18) | <0.001 | 0.77 (0.58, 1.00) | 0.26 (0.18, 0.36) | -3.64 (-4.11, -3.17) | <0.001 |
| Caribbean | 59.57 (57.83, 61.36) | 63.90 (62.26, 65.57) | 0.24 (0.22, 0.26) | <0.001 | 1013.02 (1005.62, 1020.46) | 1055.85 (1049.15, 1062.58) | 0.14 (0.10, 0.17) | <0.001 | 161.26 (158.29, 164.26) | 115.32 (113.11, 117.56) | -1.07 (-1.26, -0.88) | <0.001 | 1.88 (1.56, 2.23) | 1.08 (0.88, 1.32) | -1.77 (-2.08, -1.45) | <0.001 |
| Southern Latin America | 34.11 (32.95, 35.30) | 36.29 (35.25, 37.37) | 0.21 (0.19, 0.24) | <0.001 | 594.17 (589.25, 599.12) | 641.70 (637.30, 646.12) | 0.27 (0.25, 0.28) | <0.001 | 75.06 (73.31, 76.84) | 41.39 (40.28, 42.53) | -2.03 (-2.17, -1.89) | <0.001 | 0.79 (0.62, 1.00) | 0.18 (0.11, 0.27) | -5.01 (-5.37, -4.64) | <0.001 |
| Oceania | 38.82 (35.52, 42.36) | 40.99 (38.63, 43.47) | 0.19 (0.17, 0.21) | <0.001 | 649.91 (636.20, 663.86) | 702.31 (692.43, 712.30) | 0.27 (0.23, 0.31) | <0.001 | 675.52 (661.33, 689.95) | 598.05 (588.88, 607.33) | -0.46 (-0.60, -0.31) | <0.001 | 10.75 (9.01, 12.75) | 9.34 (8.21, 10.57) | -0.52 (-0.66, -0.38) | <0.001 |
| Australasia | 0.79 (0.55, 1.12) | 0.61 (0.42, 0.88) | -0.91 (-1.15, -0.67) | <0.001 | 18.51 (17.22, 19.88) | 17.41 (16.25, 18.64) | -0.20 (-0.28, -0.12) | <0.001 | 29.48 (27.84, 31.19) | 12.49 (11.53, 13.52) | -2.87 (-3.25, -2.48) | <0.001 | 0.47 (0.29, 0.74) | 0.20 (0.09, 0.37) | -2.87 (-3.23, -2.51) | <0.001 |
| **Ischemic heart disease** | | | | | | | | | | | | | | | | |
| **Overall** | | | | | | | | | | | | | | | | |

| | | | | | | | | | | | | | | | | |
|---|---|---|---|---|---|---|---|---|---|---|---|---|---|---|---|---|
| Global | 26.35 (26.28, 26.42) | 26.33 (26.27, 26.39) | -0.00 (-0.01, 0.01) | 0.842 | 177.62 (177.44, 177.81) | 180.42 (180.27, 180.57) | 0.05 (0.04, 0.07) | <0.001 | 459.57 (459.28, 459.86) | 402.58 (402.36, 402.81) | -0.46 (-0.74, -0.17) | 0.002 | 8.02 (7.98, 8.06) | 7.02 (6.99, 7.05) | -0.46 (-0.76, -0.16) | 0.003 |
| **SDI category** | | | | | | | | | | | | | | | | |
| High | 33.19 (32.99, 33.38) | 31.12 (30.94, 31.30) | -0.22 (-0.26, -0.18) | <0.001 | 177.53 (177.08, 177.98) | 186.09 (185.65, 186.53) | 0.17 (0.08, 0.26) | <0.001 | 233.21 (232.69, 233.72) | 141.17 (140.79, 141.56) | -1.71 (-1.92, -1.51) | <0.001 | 4.09 (4.03, 4.16) | 2.47 (2.42, 2.53) | -1.73 (-1.94, -1.51) | <0.001 |
| High-middle | 25.31 (25.16, 25.45) | 23.68 (23.56, 23.81) | -0.23 (-0.26, -0.20) | <0.001 | 192.94 (192.55, 193.33) | 193.38 (193.03, 193.74) | 0.01 (-0.01, 0.02) | 0.290 | 454.34 (453.74, 454.94) | 317.71 (317.25, 318.18) | -1.19 (-1.72, -0.66) | <0.001 | 8.00 (7.92, 8.08) | 5.53 (5.47, 5.59) | -1.22 (-1.77, -0.68) | <0.001 |
| Middle | 24.32 (24.20, 24.43) | 25.78 (25.68, 25.88) | 0.21 (0.19, 0.23) | <0.001 | 186.58 (186.25, 186.91) | 192.12 (191.84, 192.39) | 0.11 (0.08, 0.13) | <0.001 | 498.62 (498.09, 499.15) | 445.65 (445.23, 446.07) | -0.34 (-0.57, -0.10) | 0.005 | 8.65 (8.58, 8.72) | 7.72 (7.67, 7.78) | -0.34 (-0.57, -0.11) | 0.003 |
| Low-middle | 25.12 (24.96, 25.28) | 26.05 (25.93, 26.17) | 0.13 (0.11, 0.14) | <0.001 | 157.26 (156.86, 157.66) | 164.89 (164.59, 165.19) | 0.17 (0.15, 0.19) | <0.001 | 604.93 (604.16, 605.70) | 557.08 (556.53, 557.63) | -0.23 (-0.54, 0.09) | 0.154 | 10.59 (10.49, 10.69) | 9.80 (9.73, 9.88) | -0.21 (-0.54, 0.13) | 0.222 |
| Low | 26.55 (26.30, 26.80) | 27.85 (27.69, 28.02) | 0.16 (0.15, 0.18) | <0.001 | 144.95 (144.37, 145.54) | 153.84 (153.44, 154.23) | 0.21 (0.17, 0.26) | <0.001 | 413.41 (412.44, 414.39) | 389.40 (388.78, 390.02) | -0.19 (-0.33, -0.05) | 0.008 | 7.28 (7.15, 7.41) | 6.85 (6.77, 6.93) | -0.19 (-0.34, -0.04) | 0.014 |
| **21 GBD region** | | | | | | | | | | | | | | | | |
| South Asia | 25.73 (25.58, 25.89) | 26.42 (26.30, 26.54) | 0.09 (0.07, 0.11) | <0.001 | 152.78 (152.39, 153.17) | 161.75 (161.46, 162.05) | 0.20 (0.19, 0.21) | <0.001 | 726.51 (725.67, 727.35) | 682.58 (681.99, 683.18) | -0.18 (-0.59, 0.24) | 0.394 | 12.72 (12.60, 12.83) | 12.03 (11.95, 12.11) | -0.15 (-0.58, 0.28) | 0.501 |
| East Asia | 20.02 (19.90, 20.14) | 19.83 (19.71, 19.94) | -0.04 (-0.08, 0.00) | 0.050 | 212.36 (211.96, 212.75) | 223.15 (222.77, 223.53) | 0.16 (0.11, 0.21) | <0.001 | 354.92 (354.42, 355.43) | 300.63 (300.17, 301.09) | -0.45 (-0.75, -0.15) | 0.003 | 6.06 (5.99, 6.13) | 5.13 (5.07, 5.18) | -0.46 (-0.77, -0.14) | 0.005 |
| North Africa and Middle East | 38.74 (38.39, 39.10) | 39.01 (38.78, 39.25) | 0.03 (0.01, 0.04) | 0.002 | 255.31 (254.38, 256.24) | 260.00 (259.39, 260.61) | 0.09 (-0.02, 0.20) | 0.125 | 921.65 (919.91, 923.38) | 545.15 (544.26, 546.04) | -1.77 (-1.86, -1.68) | <0.001 | 16.12 (15.89, 16.35) | 9.42 (9.30, 9.54) | -1.81 (-1.90, -1.72) | <0.001 |
| Western Sub-Saharan Africa | 24.64 (24.25, 25.04) | 26.48 (26.22, 26.73) | 0.25 (0.23, 0.27) | <0.001 | 133.14 (132.20, 134.08) | 143.25 (142.63, 143.86) | 0.27 (0.21, 0.33) | <0.001 | 204.37 (203.23, 205.52) | 192.18 (191.48, 192.88) | -0.22 (-0.46, 0.02) | 0.076 | 3.59 (3.44, 3.75) | 3.36 (3.27, 3.45) | -0.24 (-0.48, 0.01) | 0.058 |
| Southeast Asia | 21.11 (20.90, | 22.04 | 0.15 | <0.001 | 146.72 | 156.60 | 0.23 | <0.001 | 460.20 | 459.83 | 0.04 | 0.667 | 8.04 (7.91, | 8.00 (7.89, | 0.03 | 0.789 |

| | | | | | | | | | | | | | | | | |
|---|---|---|---|---|---|---|---|---|---|---|---|---|---|---|---|---|
| | 21.33) | (21.86, 22.21) | (0.13, 0.16) | | (146.15, 147.30) | (156.14, 157.07) | (0.19, 0.28) | | (459.20, 461.20) | (459.03, 460.63) | (-0.16, 0.25) | | 8.18) | 8.10) | (-0.18, 0.23) | |
| Eastern Sub-Saharan Africa | 24.86 (24.46, 25.26) | 26.36 (26.10, 26.63) | 0.20 (0.19, 0.21) | <0.001 | 130.88 (129.93, 131.84) | 140.61 (139.98, 141.25) | 0.26 (0.23, 0.28) | <0.001 | 255.47 (254.18, 256.76) | 220.54 (219.77, 221.31) | -0.50 (-0.71, -0.29) | <0.001 | 4.43 (4.26, 4.61) | 3.80 (3.70, 3.91) | -0.52 (-0.74, -0.29) | <0.001 |
| Central Latin America | 25.79 (25.38, 26.19) | 23.60 (23.30, 23.90) | -0.30 (-0.31, -0.29) | <0.001 | 151.13 (150.12, 152.14) | 143.02 (142.27, 143.76) | -0.19 (-0.22, -0.16) | <0.001 | 339.84 (338.36, 341.32) | 270.17 (269.15, 271.19) | -0.77 (-1.30, -0.24) | 0.005 | 5.91 (5.72, 6.11) | 4.67 (4.54, 4.81) | -0.79 (-1.32, -0.26) | 0.004 |
| Tropical Latin America | 18.13 (17.79, 18.48) | 16.14 (15.88, 16.40) | -0.41 (-0.51, -0.31) | <0.001 | 134.02 (133.08, 134.97) | 132.84 (132.11, 133.58) | -0.04 (-0.06, -0.02) | 0.001 | 423.60 (421.92, 425.28) | 227.73 (226.77, 228.70) | -2.11 (-2.39, -1.82) | <0.001 | 7.57 (7.34, 7.79) | 3.97 (3.84, 4.09) | -2.20 (-2.50, -1.91) | <0.001 |
| Central Sub-Saharan Africa | 29.46 (28.67, 30.26) | 28.63 (28.14, 29.13) | -0.10 (-0.11, -0.09) | <0.001 | 133.47 (131.74, 135.23) | 131.04 (129.96, 132.13) | -0.06 (-0.08, -0.05) | <0.001 | 281.28 (278.79, 283.78) | 230.30 (228.89, 231.72) | -0.72 (-0.79, -0.66) | <0.001 | 4.95 (4.63, 5.30) | 4.05 (3.87, 4.25) | -0.73 (-0.80, -0.66) | <0.001 |
| Western Europe | 34.47 (34.18, 34.77) | 30.04 (29.76, 30.33) | -0.47 (-0.50, -0.44) | <0.001 | 187.42 (186.72, 188.12) | 181.32 (180.63, 182.01) | -0.10 (-0.17, -0.03) | 0.004 | 216.78 (216.03, 217.53) | 81.45 (80.99, 81.92) | -3.34 (-3.64, -3.04) | <0.001 | 3.84 (3.74, 3.94) | 1.43 (1.37, 1.49) | -3.37 (-3.68, -3.06) | <0.001 |
| High-income North America | 37.69 (37.35, 38.04) | 35.49 (35.17, 35.83) | -0.21 (-0.26, -0.16) | <0.001 | 173.25 (172.52, 173.98) | 185.59 (184.85, 186.33) | 0.24 (0.11, 0.36) | <0.001 | 217.15 (216.32, 217.97) | 159.16 (158.47, 159.85) | -1.02 (-1.41, -0.64) | <0.001 | 3.87 (3.76, 3.98) | 2.80 (2.71, 2.89) | -1.08 (-1.45, -0.70) | <0.001 |
| Central Asia | 36.15 (35.44, 36.88) | 34.86 (34.28, 35.45) | -0.12 (-0.14, -0.10) | <0.001 | 206.14 (204.40, 207.90) | 200.90 (199.50, 202.31) | -0.09 (-0.10, -0.08) | <0.001 | 666.28 (663.14, 669.43) | 617.05 (614.59, 619.53) | -0.27 (-0.70, 0.16) | 0.222 | 11.96 (11.54, 12.39) | 10.92 (10.59, 11.25) | -0.31 (-0.75, 0.13) | 0.164 |
| Eastern Europe | 36.14 (35.75, 36.53) | 36.26 (35.84, 36.69) | 0.01 (-0.03, 0.05) | 0.602 | 183.95 (183.09, 184.81) | 190.71 (189.78, 191.65) | 0.14 (0.10, 0.18) | <0.001 | 571.83 (570.31, 573.35) | 553.35 (551.74, 554.96) | 0.09 (-1.11, 1.30) | 0.885 | 10.37 (10.16, 10.57) | 9.92 (9.71, 10.14) | 0.06 (-1.14, 1.27) | 0.929 |
| Southern Sub-Saharan Africa | 35.50 (34.66, 36.36) | 34.15 (33.53, 34.77) | -0.13 (-0.15, -0.12) | <0.001 | 157.51 (155.69, 159.34) | 154.31 (152.99, 155.64) | -0.07 (-0.09, -0.04) | <0.001 | 297.46 (294.96, 299.98) | 184.08 (182.64, 185.53) | -1.57 (-2.87, -0.26) | 0.019 | 5.33 (5.00, 5.68) | 3.25 (3.06, 3.45) | -1.63 (-2.93, -0.32) | 0.015 |
| High-income Asia Pacific | 21.31 (20.96, 21.66) | 19.48 (19.12, 19.85) | -0.30 (-0.32, -0.28) | <0.001 | 123.30 (122.47, 124.14) | 117.47 (116.60, 118.34) | -0.16 (-0.21, -0.11) | <0.001 | 246.94 (245.75, 248.12) | 73.37 (72.68, 74.07) | -4.10 (-4.50, -3.70) | <0.001 | 4.19 (4.04, 4.35) | 1.27 (1.18, 1.36) | -4.08 (-4.46, -3.69) | <0.001 |
| Central Europe | 22.62 (22.20, | 20.64 | -0.32 | <0.001 | 187.74 | 174.50 | -0.24 | <0.001 | 531.71 | 173.40 | -3.84 | <0.001 | 9.58 (9.31, | 3.04 (2.88, | -3.93 | <0.001 |

| | | | | | | | | | | | | | | | | |
|---|---|---|---|---|---|---|---|---|---|---|---|---|---|---|---|---|
| | 23.05) | (20.19, 21.10) | (-0.34, -0.30) | | (186.54, 188.95) | (173.23, 175.78) | (-0.27, -0.22) | | (529.70, 533.73) | (172.14, 174.68) | (-4.22, -3.46) | | 9.85) | 3.21) | (-4.33, -3.53) | |
| Andean Latin America | 10.31 (9.78, 10.86) | 10.32 (9.92, 10.72) | 0.01 (-0.02, 0.04) | 0.593 | 83.72 (82.16, 85.31) | 87.14 (85.99, 88.30) | 0.14 (0.12, 0.17) | <0.001 | 380.24 (377.01, 383.49) | 222.19 (220.37, 224.03) | -1.81 (-2.38, -1.23) | <0.001 | 6.51 (6.09, 6.95) | 3.76 (3.53, 4.01) | -1.84 (-2.42, -1.26) | <0.001 |
| Caribbean | 59.63 (58.34, 60.95) | 61.73 (60.59, 62.88) | 0.13 (0.10, 0.16) | <0.001 | 295.98 (293.02, 298.97) | 295.79 (293.28, 298.31) | -0.00 (-0.01, 0.00) | 0.098 | 433.93 (430.37, 437.51) | 365.49 (362.71, 368.29) | -0.57 (-0.85, -0.30) | <0.001 | 7.65 (7.18, 8.14) | 6.42 (6.05, 6.80) | -0.59 (-0.89, -0.29) | <0.001 |
| Southern Latin America | 17.45 (16.86, 18.06) | 16.27 (15.78, 16.77) | -0.24 (-0.26, -0.22) | <0.001 | 95.72 (94.31, 97.14) | 93.11 (91.94, 94.28) | -0.10 (-0.11, -0.09) | <0.001 | 286.90 (284.46, 289.35) | 134.56 (133.16, 135.98) | -2.60 (-2.82, -2.37) | <0.001 | 5.06 (4.74, 5.39) | 2.35 (2.17, 2.55) | -2.59 (-2.77, -2.42) | <0.001 |
| Oceania | 26.02 (23.99, 28.19) | 26.44 (25.05, 27.88) | 0.06 (0.04, 0.07) | <0.001 | 196.59 (190.78, 202.53) | 201.22 (197.31, 205.19) | 0.08 (0.07, 0.09) | <0.001 | 758.08 (746.65, 769.66) | 824.73 (816.81, 832.71) | 0.26 (0.14, 0.37) | <0.001 | 13.65 (12.15, 15.30) | 14.80 (13.75, 15.90) | 0.25 (0.13, 0.36) | <0.001 |
| Australasia | 53.63 (52.07, 55.22) | 47.34 (46.03, 48.69) | -0.44 (-0.49, -0.39) | <0.001 | 243.83 (240.52, 247.18) | 229.94 (227.07, 232.83) | -0.24 (-0.43, -0.05) | 0.014 | 182.58 (179.72, 185.48) | 85.04 (83.31, 86.81) | -2.59 (-2.97, -2.21) | <0.001 | 3.26 (2.89, 3.67) | 1.52 (1.30, 1.78) | -2.59 (-3.00, -2.17) | <0.001 |
| **Women** | | | | | | | | | | | | | | | | |
| Global | 21.05 (20.96, 21.14) | 21.52 (21.44, 21.59) | 0.08 (0.06, 0.09) | <0.001 | 155.55 (155.31, 155.80) | 158.60 (158.40, 158.80) | 0.07 (0.03, 0.11) | <0.001 | 312.10 (311.76, 312.44) | 237.88 (237.63, 238.13) | -0.91 (-1.16, -0.66) | <0.001 | 5.34 (5.30, 5.39) | 4.07 (4.04, 4.10) | -0.91 (-1.16, -0.66) | <0.001 |
| **SDI category** | | | | | | | | | | | | | | | | |
| High | 26.42 (26.17, 26.67) | 26.69 (26.45, 26.94) | 0.04 (-0.02, 0.09) | 0.198 | 160.39 (159.78, 161.00) | 177.64 (177.03, 178.27) | 0.34 (0.22, 0.46) | <0.001 | 108.60 (108.09, 109.10) | 70.80 (70.41, 71.20) | -1.47 (-1.78, -1.15) | <0.001 | 1.84 (1.78, 1.91) | 1.21 (1.16, 1.27) | -1.44 (-1.74, -1.13) | <0.001 |
| High-middle | 19.92 (19.74, 20.10) | 19.58 (19.41, 19.74) | -0.06 (-0.08, -0.03) | <0.001 | 175.01 (174.48, 175.55) | 181.52 (181.03, 182.02) | 0.12 (0.09, 0.16) | <0.001 | 226.06 (225.46, 226.67) | 141.38 (140.93, 141.83) | -1.61 (-2.14, -1.09) | <0.001 | 3.87 (3.79, 3.95) | 2.38 (2.32, 2.44) | -1.68 (-2.20, -1.16) | <0.001 |
| Middle | 19.54 (19.39, 19.69) | 20.91 (20.78, 21.04) | 0.24 (0.23, 0.24) | <0.001 | 161.17 (160.73, 161.61) | 167.29 (166.93, 167.65) | 0.13 (0.11, 0.15) | <0.001 | 370.19 (369.54, 370.84) | 257.32 (256.87, 257.78) | -1.21 (-1.54, -0.87) | <0.001 | 6.34 (6.26, 6.43) | 4.37 (4.31, 4.43) | -1.23 (-1.55, -0.91) | <0.001 |
| Low-middle | 20.59 (20.39, 20.80) | 21.28 (21.13, | 0.11 (0.11, | <0.001 | 135.74 (135.21, | 140.39 (139.99, | 0.12 (0.10, | <0.001 | 479.85 (478.89, | 363.32 (362.69, | -0.94 (-1.23, | <0.001 | 8.26 (8.14, 8.39) | 6.29 (6.21, 6.37) | -0.93 (-1.22, | <0.001 |

| | | | | | | | | | | | | | | | | |
|---|---|---|---|---|---|---|---|---|---|---|---|---|---|---|---|---|
| | | 21.44) | 0.12) | | 136.27) | 140.78) | 0.14) | | 480.82) | 363.95) | -0.65) | | | | -0.63) | |
| Low | 21.60 (21.29, 21.92) | 22.36 (22.15, 22.57) | 0.12 (0.11, 0.13) | <0.001 | 115.73 (114.99, 116.47) | 119.67 (119.17, 120.16) | 0.12 (0.10, 0.14) | <0.001 | 319.32 (318.12, 320.52) | 250.14 (249.44, 250.83) | -0.83 (-1.00, -0.65) | <0.001 | 5.56 (5.40, 5.72) | 4.37 (4.28, 4.46) | -0.81 (-0.99, -0.63) | <0.001 |
| **21 GBD region** | | | | | | | | | | | | | | | | |
| South Asia | 21.53 (21.32, 21.74) | 22.01 (21.86, 22.16) | 0.07 (0.06, 0.09) | <0.001 | 137.18 (136.64, 137.71) | 143.79 (143.40, 144.18) | 0.16 (0.13, 0.19) | <0.001 | 562.94 (561.89, 564.00) | 434.26 (433.59, 434.94) | -0.92 (-1.42, -0.42) | <0.001 | 9.64 (9.50, 9.78) | 7.51 (7.42, 7.60) | -0.89 (-1.41, -0.37) | 0.001 |
| East Asia | 16.15 (16.00, 16.31) | 17.26 (17.10, 17.42) | 0.23 (0.22, 0.25) | <0.001 | 190.87 (190.34, 191.41) | 215.69 (215.15, 216.23) | 0.42 (0.40, 0.43) | <0.001 | 257.94 (257.32, 258.56) | 139.10 (138.66, 139.55) | -2.14 (-2.91, -1.37) | <0.001 | 4.37 (4.29, 4.45) | 2.27 (2.21, 2.33) | -2.29 (-3.05, -1.52) | <0.001 |
| North Africa and Middle East | 31.80 (31.34, 32.26) | 31.40 (31.09, 31.71) | -0.04 (-0.05, -0.04) | <0.001 | 209.14 (207.93, 210.35) | 206.14 (205.35, 206.93) | -0.04 (-0.11, 0.02) | 0.206 | 724.08 (721.90, 726.27) | 406.55 (405.44, 407.67) | -1.92 (-2.04, -1.80) | <0.001 | 12.50 (12.21, 12.79) | 6.98 (6.83, 7.12) | -1.95 (-2.06, -1.83) | <0.001 |
| Western Sub-Saharan Africa | 19.08 (18.60, 19.57) | 20.57 (20.26, 20.89) | 0.26 (0.25, 0.27) | <0.001 | 95.01 (93.89, 96.14) | 102.04 (101.33, 102.76) | 0.26 (0.21, 0.30) | <0.001 | 150.50 (149.12, 151.88) | 124.82 (124.05, 125.59) | -0.64 (-0.92, -0.36) | <0.001 | 2.62 (2.44, 2.81) | 2.16 (2.06, 2.26) | -0.66 (-0.93, -0.39) | <0.001 |
| Southeast Asia | 15.47 (15.21, 15.73) | 16.66 (16.44, 16.88) | 0.26 (0.24, 0.28) | <0.001 | 115.33 (114.62, 116.04) | 126.00 (125.41, 126.59) | 0.32 (0.24, 0.41) | <0.001 | 365.16 (363.91, 366.40) | 268.71 (267.84, 269.58) | -1.06 (-1.27, -0.85) | <0.001 | 6.34 (6.17, 6.50) | 4.63 (4.51, 4.74) | -1.09 (-1.30, -0.87) | <0.001 |
| Eastern Sub-Saharan Africa | 19.42 (18.93, 19.92) | 20.16 (19.84, 20.49) | 0.13 (0.11, 0.14) | <0.001 | 92.08 (90.98, 93.20) | 95.46 (94.74, 96.19) | 0.13 (0.12, 0.14) | <0.001 | 169.85 (168.41, 171.31) | 120.20 (119.42, 120.98) | -1.17 (-1.28, -1.06) | <0.001 | 2.91 (2.72, 3.11) | 2.04 (1.94, 2.15) | -1.21 (-1.32, -1.10) | <0.001 |
| Central Latin America | 21.77 (21.26, 22.29) | 18.85 (18.48, 19.23) | -0.48 (-0.52, -0.44) | <0.001 | 148.27 (146.89, 149.67) | 130.79 (129.80, 131.78) | -0.43 (-0.46, -0.40) | <0.001 | 234.68 (232.98, 236.39) | 144.50 (143.46, 145.54) | -1.59 (-2.21, -0.97) | <0.001 | 4.05 (3.82, 4.28) | 2.46 (2.33, 2.60) | -1.64 (-2.25, -1.02) | <0.001 |
| Tropical Latin America | 13.14 (12.74, 13.55) | 11.89 (11.57, 12.21) | -0.34 (-0.44, -0.25) | <0.001 | 92.16 (91.07, 93.26) | 90.33 (89.49, 91.18) | -0.07 (-0.09, -0.05) | <0.001 | 251.03 (249.24, 252.84) | 120.41 (119.42, 121.39) | -2.46 (-2.71, -2.21) | <0.001 | 4.46 (4.23, 4.71) | 2.10 (1.98, 2.24) | -2.52 (-2.77, -2.27) | <0.001 |
| Central Sub-Saharan Africa | 23.76 (22.77, 24.79) | 22.88 (22.27, 23.51) | -0.14 (-0.16, -0.11) | <0.001 | 104.19 (102.04, 106.37) | 100.82 (99.49, 102.17) | -0.11 (-0.12, -0.10) | <0.001 | 161.96 (159.32, 164.63) | 130.32 (128.83, 131.83) | -0.72 (-0.87, -0.56) | <0.001 | 2.84 (2.50, 3.21) | 2.28 (2.08, 2.49) | -0.73 (-0.88, -0.57) | <0.001 |

| | | | | | | | | | | | | | | | | |
|---|---|---|---|---|---|---|---|---|---|---|---|---|---|---|---|---|
| Western Europe | 28.39 (28.00, 28.78) | 26.62 (26.23, 27.01) | -0.21 (-0.26, -0.16) | <0.001 | 179.68 (178.72, 180.65) | 186.39 (185.39, 187.39) | 0.12 (0.09, 0.16) | <0.001 | 79.76 (79.11, 80.41) | 30.28 (29.88, 30.69) | -3.32 (-3.54, -3.10) | <0.001 | 1.37 (1.28, 1.45) | 0.51 (0.46, 0.56) | -3.37 (-3.52, -3.21) | <0.001 |
| High-income North America | 30.34 (29.90, 30.78) | 32.56 (32.12, 33.02) | 0.27 (0.18, 0.35) | <0.001 | 153.71 (152.73, 154.69) | 186.84 (185.79, 187.90) | 0.67 (0.59, 0.76) | <0.001 | 98.17 (97.39, 98.96) | 89.74 (89.01, 90.47) | -0.28 (-0.65, 0.09) | 0.142 | 1.70 (1.59, 1.80) | 1.56 (1.47, 1.66) | -0.27 (-0.64, 0.11) | 0.159 |
| Central Asia | 29.59 (28.69, 30.51) | 28.63 (27.88, 29.39) | -0.11 (-0.12, -0.11) | <0.001 | 190.93 (188.58, 193.30) | 182.44 (180.55, 184.35) | -0.14 (-0.19, -0.10) | <0.001 | 340.58 (337.47, 343.71) | 366.39 (363.70, 369.10) | 0.23 (-0.15, 0.62) | 0.237 | 5.93 (5.53, 6.36) | 6.35 (6.00, 6.72) | 0.22 (-0.18, 0.61) | 0.287 |
| Eastern Europe | 27.68 (27.20, 28.17) | 29.62 (29.07, 30.18) | 0.22 (0.19, 0.26) | <0.001 | 184.08 (182.86, 185.31) | 202.33 (200.95, 203.71) | 0.35 (0.21, 0.49) | <0.001 | 154.64 (153.51, 155.77) | 175.54 (174.24, 176.84) | 0.65 (-0.52, 1.82) | 0.277 | 2.72 (2.58, 2.88) | 3.09 (2.93, 3.27) | 0.66 (-0.52, 1.85) | 0.277 |
| Southern Sub-Saharan Africa | 28.81 (27.76, 29.89) | 26.11 (25.35, 26.89) | -0.35 (-0.40, -0.30) | <0.001 | 117.68 (115.49, 119.89) | 105.54 (104.00, 107.09) | -0.37 (-0.40, -0.34) | <0.001 | 223.29 (220.32, 226.30) | 103.70 (102.18, 105.24) | -2.47 (-4.47, -0.44) | 0.018 | 3.94 (3.55, 4.36) | 1.80 (1.60, 2.01) | -2.53 (-4.55, -0.47) | 0.016 |
| High-income Asia Pacific | 17.20 (16.76, 17.66) | 16.46 (15.97, 16.96) | -0.15 (-0.16, -0.14) | <0.001 | 119.22 (118.05, 120.39) | 121.37 (120.09, 122.67) | 0.09 (0.03, 0.14) | 0.005 | 139.15 (137.89, 140.43) | 25.22 (24.64, 25.82) | -5.78 (-6.23, -5.33) | <0.001 | 2.31 (2.15, 2.47) | 0.42 (0.35, 0.50) | -5.81 (-6.36, -5.26) | <0.001 |
| Central Europe | 16.82 (16.30, 17.36) | 15.76 (15.19, 16.34) | -0.23 (-0.28, -0.18) | <0.001 | 157.67 (156.10, 159.25) | 153.78 (152.07, 155.51) | -0.08 (-0.13, -0.02) | 0.010 | 182.53 (180.84, 184.24) | 67.47 (66.33, 68.63) | -3.43 (-3.77, -3.09) | <0.001 | 3.20 (2.98, 3.44) | 1.14 (1.00, 1.30) | -3.55 (-3.91, -3.19) | <0.001 |
| Andean Latin America | 7.61 (6.99, 8.28) | 7.50 (7.04, 7.99) | -0.05 (-0.08, -0.02) | 0.003 | 66.76 (64.82, 68.74) | 68.14 (66.71, 69.59) | 0.08 (0.02, 0.14) | 0.010 | 283.71 (279.83, 287.64) | 120.22 (118.32, 122.14) | -3.01 (-3.66, -2.36) | <0.001 | 4.84 (4.34, 5.38) | 2.02 (1.78, 2.28) | -3.07 (-3.73, -2.40) | <0.001 |
| Caribbean | 55.08 (53.36, 56.85) | 56.50 (54.98, 58.06) | 0.09 (0.08, 0.10) | <0.001 | 272.67 (268.72, 276.67) | 267.31 (263.98, 270.68) | -0.07 (-0.09, -0.04) | <0.001 | 321.05 (316.81, 325.34) | 284.02 (280.58, 287.49) | -0.38 (-0.61, -0.15) | 0.001 | 5.62 (5.07, 6.22) | 4.94 (4.49, 5.42) | -0.40 (-0.64, -0.16) | 0.001 |
| Southern Latin America | 13.70 (12.96, 14.46) | 12.57 (11.97, 13.19) | -0.29 (-0.31, -0.28) | <0.001 | 86.77 (84.90, 88.68) | 84.64 (83.08, 86.22) | -0.07 (-0.11, -0.04) | <0.001 | 157.73 (155.21, 160.28) | 62.69 (61.34, 64.07) | -3.15 (-3.60, -2.69) | <0.001 | 2.72 (2.40, 3.08) | 1.07 (0.90, 1.27) | -3.18 (-3.61, -2.74) | <0.001 |
| Oceania | 20.67 (18.11, 23.51) | 21.07 (19.33, 22.93) | 0.07 (0.05, 0.10) | <0.001 | 163.53 (155.99, 171.35) | 166.25 (161.24, 171.38) | 0.07 (0.01, 0.13) | 0.033 | 324.72 (314.17, 335.54) | 388.99 (381.35, 396.75) | 0.62 (0.49, 0.76) | <0.001 | 5.71 (4.38, 7.32) | 6.84 (5.85, 7.94) | 0.62 (0.49, 0.76) | <0.001 |

| | | | | | | | | | | | | | | | | |
|---|---|---|---|---|---|---|---|---|---|---|---|---|---|---|---|---|
| Australasia | 23.64 (22.19, 25.17) | 18.98 (17.81, 20.21) | -0.77 (-0.81, -0.73) | <0.001 | 143.41 (139.83, 147.06) | 123.50 (120.53, 126.52) | -0.50 (-0.56, -0.44) | <0.001 | 69.31 (66.83, 71.87) | 30.93 (29.46, 32.45) | -2.70 (-3.11, -2.28) | <0.001 | 1.20 (0.90, 1.58) | 0.54 (0.36, 0.78) | -2.68 (-3.14, -2.21) | <0.001 |
| **Men** | | | | | | | | | | | | | | | | |
| Global | 31.51 (31.40, 31.62) | 31.06 (30.97, 31.15) | -0.05 (-0.06, -0.04) | <0.001 | 199.09 (198.82, 199.37) | 201.90 (201.68, 202.13) | 0.05 (0.04, 0.06) | <0.001 | 603.18 (602.70, 603.65) | 564.60 (564.22, 564.98) | -0.18 (-0.55, 0.18) | 0.318 | 10.63 (10.57, 10.69) | 9.92 (9.87, 9.97) | -0.19 (-0.56, 0.18) | 0.309 |
| **SDI category** | | | | | | | | | | | | | | | | |
| High | 39.79 (39.49, 40.09) | 35.25 (34.98, 35.52) | -0.43 (-0.48, -0.37) | <0.001 | 194.29 (193.63, 194.95) | 194.00 (193.38, 194.62) | -0.00 (-0.05, 0.04) | 0.933 | 354.52 (353.63, 355.41) | 206.83 (206.19, 207.48) | -1.86 (-2.07, -1.64) | <0.001 | 6.29 (6.17, 6.41) | 3.65 (3.57, 3.74) | -1.88 (-2.11, -1.65) | <0.001 |
| High-middle | 30.52 (30.30, 30.74) | 27.58 (27.39, 27.77) | -0.36 (-0.39, -0.33) | <0.001 | 210.30 (209.72, 210.87) | 204.70 (204.20, 205.21) | -0.10 (-0.12, -0.08) | <0.001 | 675.22 (674.20, 676.25) | 484.09 (483.30, 484.89) | -1.10 (-1.67, -0.53) | <0.001 | 11.99 (11.85, 12.13) | 8.51 (8.40, 8.61) | -1.13 (-1.70, -0.56) | <0.001 |
| Middle | 28.93 (28.75, 29.11) | 30.63 (30.47, 30.78) | 0.20 (0.17, 0.23) | <0.001 | 211.04 (210.55, 211.53) | 216.86 (216.45, 217.27) | 0.10 (0.07, 0.13) | <0.001 | 622.54 (621.70, 623.37) | 632.82 (632.11, 633.52) | 0.11 (-0.10, 0.33) | 0.298 | 10.88 (10.76, 10.99) | 11.06 (10.96, 11.15) | 0.12 (-0.10, 0.33) | 0.284 |
| Low-middle | 29.54 (29.30, 29.78) | 30.82 (30.64, 31.01) | 0.15 (0.13, 0.16) | <0.001 | 178.22 (177.62, 178.82) | 189.43 (188.97, 189.89) | 0.21 (0.20, 0.22) | <0.001 | 726.78 (725.59, 727.97) | 751.24 (750.33, 752.15) | 0.18 (-0.31, 0.68) | 0.469 | 12.85 (12.70, 13.01) | 13.33 (13.20, 13.45) | 0.20 (-0.32, 0.72) | 0.453 |
| Low | 31.65 (31.27, 32.04) | 33.55 (33.29, 33.81) | 0.20 (0.19, 0.21) | <0.001 | 175.03 (174.11, 175.95) | 189.36 (188.72, 189.99) | 0.28 (0.21, 0.36) | <0.001 | 510.18 (508.62, 511.73) | 533.96 (532.91, 535.00) | 0.19 (-0.00, 0.38) | 0.055 | 9.04 (8.83, 9.25) | 9.43 (9.29, 9.57) | 0.18 (-0.01, 0.38) | 0.067 |
| **21 GBD region** | | | | | | | | | | | | | | | | |
| South Asia | 29.66 (29.43, 29.90) | 30.69 (30.51, 30.87) | 0.12 (0.10, 0.14) | <0.001 | 167.31 (166.74, 167.89) | 179.15 (178.72, 179.58) | 0.23 (0.20, 0.27) | <0.001 | 878.71 (877.41, 880.01) | 923.18 (922.21, 924.16) | 0.24 (-0.39, 0.87) | 0.452 | 15.58 (15.40, 15.75) | 16.41 (16.29, 16.55) | 0.26 (-0.39, 0.92) | 0.435 |
| East Asia | 23.63 (23.45, 23.81) | 22.30 (22.13, 22.47) | -0.19 (-0.27, -0.12) | <0.001 | 232.38 (231.80, 232.96) | 230.44 (229.90, 230.98) | -0.03 (-0.08, 0.03) | 0.338 | 445.83 (445.04, 446.62) | 455.19 (454.41, 455.97) | 0.14 (-0.59, 0.88) | 0.704 | 7.64 (7.54, 7.75) | 7.86 (7.76, 7.97) | 0.17 (-0.58, 0.92) | 0.657 |
| North Africa and Middle East | 45.30 (44.76, 45.84) | 45.88 (45.53, 46.24) | 0.05 (0.02, 0.07) | <0.001 | 298.79 (297.39, 300.20) | 308.35 (307.44, 309.27) | 0.13 (-0.01, 0.27) | 0.062 | 1107.62 (1104.96, 1110.29) | 670.23 (668.87, 671.59) | -1.69 (-1.81, -1.57) | <0.001 | 19.52 (19.17, 19.88) | 11.62 (11.44, 11.80) | -1.75 (-1.87, -1.64) | <0.001 |

| | | | | | | | | | | | | | | | | |
|---|---|---|---|---|---|---|---|---|---|---|---|---|---|---|---|---|
| Western Sub-Saharan Africa | 30.39 (29.78, 31.02) | 33.06 (32.64, 33.48) | 0.29 (0.27, 0.32) | <0.001 | 171.46 (169.95, 172.97) | 189.11 (188.09, 190.14) | 0.36 (0.28, 0.43) | <0.001 | 258.50 (256.67, 260.34) | 267.09 (265.89, 268.29) | 0.11 (-0.12, 0.33) | 0.350 | 4.56 (4.32, 4.81) | 4.69 (4.53, 4.86) | 0.09 (-0.14, 0.33) | 0.449 |
| Southeast Asia | 26.90 (26.56, 27.25) | 27.33 (27.06, 27.61) | 0.05 (0.03, 0.08) | <0.001 | 178.78 (177.88, 179.68) | 186.72 (186.01, 187.44) | 0.16 (0.13, 0.18) | <0.001 | 557.68 (556.11, 559.25) | 647.83 (646.50, 649.17) | 0.56 (0.21, 0.92) | 0.002 | 9.79 (9.59, 10.01) | 11.31 (11.13, 11.49) | 0.54 (0.19, 0.90) | 0.003 |
| Eastern Sub-Saharan Africa | 30.78 (30.14, 31.43) | 33.00 (32.57, 33.43) | 0.25 (0.22, 0.27) | <0.001 | 172.94 (171.36, 174.53) | 188.97 (187.92, 190.03) | 0.32 (0.25, 0.38) | <0.001 | 348.07 (345.88, 350.26) | 327.66 (326.31, 329.02) | -0.21 (-0.36, -0.05) | 0.008 | 6.07 (5.78, 6.37) | 5.68 (5.50, 5.87) | -0.23 (-0.38, -0.07) | 0.004 |
| Central Latin America | 30.07 (29.44, 30.71) | 28.66 (28.19, 29.14) | -0.17 (-0.20, -0.13) | <0.001 | 154.14 (152.67, 155.61) | 156.20 (155.08, 157.32) | 0.04 (0.02, 0.07) | 0.002 | 451.95 (449.49, 454.41) | 403.60 (401.81, 405.39) | -0.38 (-0.93, 0.16) | 0.170 | 7.90 (7.58, 8.24) | 7.03 (6.80, 7.27) | -0.40 (-0.94, 0.15) | 0.158 |
| Tropical Latin America | 23.34 (22.79, 23.90) | 20.51 (20.10, 20.93) | -0.44 (-0.49, -0.39) | <0.001 | 177.75 (176.20, 179.32) | 176.93 (175.73, 178.14) | -0.02 (-0.04, 0.01) | 0.122 | 603.65 (600.79, 606.52) | 338.10 (336.43, 339.78) | -1.98 (-2.23, -1.73) | <0.001 | 10.80 (10.42, 11.19) | 5.88 (5.66, 6.11) | -2.08 (-2.35, -1.82) | <0.001 |
| Central Sub-Saharan Africa | 35.32 (34.09, 36.59) | 34.49 (33.73, 35.27) | -0.08 (-0.09, -0.08) | <0.001 | 163.76 (161.02, 166.55) | 161.86 (160.16, 163.59) | -0.04 (-0.06, -0.02) | <0.001 | 404.12 (399.87, 408.40) | 332.12 (329.70, 334.54) | -0.70 (-0.78, -0.63) | <0.001 | 7.14 (6.58, 7.73) | 5.86 (5.54, 6.19) | -0.73 (-0.86, -0.60) | <0.001 |
| Western Europe | 40.48 (40.03, 40.94) | 33.47 (33.05, 33.90) | -0.66 (-0.68, -0.64) | <0.001 | 195.11 (194.11, 196.11) | 176.56 (175.60, 177.51) | -0.34 (-0.40, -0.28) | <0.001 | 351.45 (350.11, 352.79) | 131.67 (130.84, 132.49) | -3.39 (-3.67, -3.10) | <0.001 | 6.27 (6.09, 6.45) | 2.34 (2.23, 2.45) | -3.44 (-3.70, -3.18) | <0.001 |
| High-income North America | 45.13 (44.60, 45.67) | 38.49 (38.01, 38.97) | -0.55 (-0.60, -0.50) | <0.001 | 193.03 (191.93, 194.13) | 184.54 (183.50, 185.59) | -0.13 (-0.30, 0.03) | 0.120 | 337.00 (335.55, 338.45) | 228.55 (227.38, 229.72) | -1.28 (-1.75, -0.81) | <0.001 | 6.06 (5.87, 6.26) | 4.04 (3.89, 4.20) | -1.34 (-1.81, -0.87) | <0.001 |
| Central Asia | 42.91 (41.80, 44.05) | 41.08 (40.19, 41.99) | -0.14 (-0.17, -0.12) | <0.001 | 221.87 (219.29, 224.48) | 219.41 (217.34, 221.50) | -0.03 (-0.07, 0.00) | 0.058 | 1001.18 (995.68, 1006.71) | 866.74 (862.62, 870.88) | -0.52 (-0.98, -0.06) | 0.028 | 18.15 (17.41, 18.91) | 15.46 (14.92, 16.03) | -0.57 (-1.04, -0.10) | 0.018 |
| Eastern Europe | 44.66 (44.05, 45.27) | 42.99 (42.35, 43.64) | -0.12 (-0.17, -0.07) | <0.001 | 184.02 (182.80, 185.24) | 179.44 (178.16, 180.72) | -0.08 (-0.15, -0.01) | 0.037 | 991.53 (988.71, 994.36) | 931.31 (928.37, 934.25) | -0.01 (-1.23, 1.22) | 0.986 | 18.06 (17.69, 18.45) | 16.76 (16.37, 17.16) | -0.05 (-1.26, 1.17) | 0.935 |
| Southern Sub-Saharan Africa | 42.67 (41.35, 44.02) | 42.25 (41.28, 43.24) | -0.03 (-0.05, -0.02) | <0.001 | 199.88 (196.93, 202.86) | 203.41 (201.27, 205.57) | 0.07 (0.03, 0.10) | <0.001 | 375.51 (371.44, 379.61) | 264.89 (262.44, 267.35) | -1.34 (-2.48, -0.19) | 0.022 | 6.80 (6.26, 7.38) | 4.70 (4.38, 5.04) | -1.42 (-2.61, -0.22) | 0.021 |

| | | | | | | | | | | | | | | | | |
|---|---|---|---|---|---|---|---|---|---|---|---|---|---|---|---|---|
| High-income Asia Pacific | 25.30 (24.78, 25.84) | 22.32 (21.79, 22.86) | -0.43 (-0.46, -0.40) | <0.001 | 127.31 (126.13, 128.50) | 113.85 (112.67, 115.04) | -0.38 (-0.41, -0.34) | <0.001 | 351.57 (349.60, 353.56) | 118.44 (117.22, 119.67) | -3.71 (-3.95, -3.47) | <0.001 | 6.03 (5.77, 6.29) | 2.06 (1.91, 2.23) | -3.63 (-4.03, -3.22) | <0.001 |
| Central Europe | 28.31 (27.64, 28.98) | 25.34 (24.65, 26.04) | -0.38 (-0.40, -0.36) | <0.001 | 217.24 (215.43, 219.06) | 194.47 (192.61, 196.34) | -0.38 (-0.40, -0.36) | <0.001 | 873.57 (869.94, 877.21) | 275.13 (272.90, 277.37) | -4.01 (-4.42, -3.61) | <0.001 | 15.82 (15.34, 16.32) | 4.87 (4.58, 5.18) | -4.09 (-4.51, -3.68) | <0.001 |
| Andean Latin America | 13.13 (12.28, 14.02) | 13.18 (12.55, 13.82) | 0.01 (-0.06, 0.08) | 0.840 | 101.49 (99.02, 104.00) | 106.71 (104.90, 108.54) | 0.17 (0.16, 0.18) | <0.001 | 481.15 (475.94, 486.39) | 325.12 (321.98, 328.27) | -1.32 (-1.75, -0.87) | <0.001 | 8.26 (7.59, 8.98) | 5.53 (5.13, 5.96) | -1.35 (-1.79, -0.90) | <0.001 |
| Caribbean | 64.47 (62.55, 66.45) | 67.15 (65.46, 68.87) | 0.14 (0.12, 0.16) | <0.001 | 320.80 (316.36, 325.28) | 325.40 (321.66, 329.18) | 0.04 (0.01, 0.07) | 0.004 | 553.78 (548.00, 559.61) | 450.09 (445.69, 454.53) | -0.68 (-0.90, -0.46) | <0.001 | 9.80 (9.04, 10.61) | 7.96 (7.38, 8.57) | -0.69 (-0.90, -0.47) | <0.001 |
| Southern Latin America | 21.34 (20.40, 22.31) | 20.08 (19.32, 20.87) | -0.21 (-0.22, -0.20) | <0.001 | 104.99 (102.89, 107.12) | 101.91 (100.18, 103.66) | -0.11 (-0.13, -0.08) | <0.001 | 420.05 (415.85, 424.28) | 208.07 (205.59, 210.57) | -2.40 (-2.67, -2.14) | <0.001 | 7.46 (6.91, 8.05) | 3.66 (3.34, 4.01) | -2.43 (-2.79, -2.07) | <0.001 |
| Oceania | 31.12 (28.03, 34.48) | 31.78 (29.64, 34.04) | 0.07 (0.05, 0.09) | <0.001 | 227.99 (219.27, 236.98) | 236.30 (230.30, 242.41) | 0.12 (0.11, 0.13) | <0.001 | 1169.62 (1149.74, 1189.77) | 1260.89 (1247.02, 1274.89) | 0.23 (0.11, 0.36) | <0.001 | 21.19 (18.58, 24.08) | 22.77 (20.93, 24.72) | 0.22 (0.09, 0.35) | 0.001 |
| Australasia | 83.85 (81.10, 86.67) | 76.34 (73.98, 78.77) | -0.32 (-0.35, -0.29) | <0.001 | 345.30 (339.72, 350.95) | 339.42 (334.47, 344.43) | -0.12 (-0.42, 0.18) | 0.448 | 296.91 (291.74, 302.15) | 140.67 (137.50, 143.90) | -2.54 (-2.99, -2.09) | <0.001 | 5.34 (4.67, 6.09) | 2.53 (2.12, 3.00) | -2.55 (-3.01, -2.07) | <0.001 |
| **Stroke** | | | | | | | | | | | | | | | | |
| **Overall** | | | | | | | | | | | | | | | | |
| Global | 30.99 (30.91, 31.07) | 27.48 (27.42, 27.54) | -0.41 (-0.47, -0.35) | <0.001 | 386.75 (386.48, 387.01) | 388.58 (388.36, 388.80) | 0.08 (-0.28, 0.44) | 0.678 | 424.25 (423.97, 424.53) | 303.96 (303.76, 304.15) | -1.11 (-1.29, -0.92) | <0.001 | 6.25 (6.21, 6.28) | 4.20 (4.17, 4.22) | -1.35 (-1.62, -1.08) | <0.001 |
| **SDI category** | | | | | | | | | | | | | | | | |
| High | 21.03 (20.87, 21.19) | 19.74 (19.60, 19.89) | -0.22 (-0.26, -0.17) | <0.001 | 316.65 (316.05, 317.26) | 357.70 (357.07, 358.33) | 0.58 (-0.21, 1.39) | 0.150 | 225.92 (225.41, 226.43) | 144.01 (143.62, 144.41) | -1.52 (-1.70, -1.33) | <0.001 | 3.04 (2.98, 3.10) | 1.55 (1.50, 1.59) | -2.29 (-2.50, -2.09) | <0.001 |
| High-middle | 32.12 (31.96, 32.28) | 25.49 (25.35, 25.62) | -0.80 (-0.88, -0.72) | <0.001 | 375.75 (375.21, 376.30) | 345.62 (345.13, 346.12) | -0.24 (-0.45, -0.03) | 0.022 | 402.46 (401.89, 403.02) | 257.90 (257.48, 258.32) | -1.49 (-1.92, -1.05) | <0.001 | 5.81 (5.74, 5.87) | 3.41 (3.36, 3.46) | -1.81 (-2.34, -1.27) | <0.001 |

| | | | | | | | | | | | | | | | | |
|---|---|---|---|---|---|---|---|---|---|---|---|---|---|---|---|---|
| Middle | 34.20 (34.07, 34.34) | 29.68 (29.57, 29.79) | -0.48 (-0.56, -0.41) | <0.001 | 414.93 (414.45, 415.41) | 403.29 (402.88, 403.69) | -0.04 (-0.24, 0.17) | 0.731 | 492.96 (492.44, 493.49) | 331.71 (331.35, 332.08) | -1.35 (-1.64, -1.06) | <0.001 | 7.31 (7.25, 7.38) | 4.57 (4.53, 4.61) | -1.60 (-1.95, -1.25) | <0.001 |
| Low-middle | 30.89 (30.72, 31.06) | 29.03 (28.91, 29.16) | -0.21 (-0.26, -0.17) | <0.001 | 387.73 (387.13, 388.34) | 394.68 (394.22, 395.15) | 0.12 (-0.24, 0.48) | 0.515 | 481.96 (481.28, 482.64) | 353.41 (352.97, 353.85) | -1.03 (-1.21, -0.84) | <0.001 | 7.35 (7.27, 7.43) | 5.13 (5.08, 5.19) | -1.22 (-1.46, -0.98) | <0.001 |
| Low | 34.48 (34.21, 34.77) | 29.48 (29.31, 29.65) | -0.53 (-0.58, -0.48) | <0.001 | 427.73 (426.77, 428.70) | 419.12 (418.49, 419.75) | -0.03 (-0.30, 0.24) | 0.818 | 457.76 (456.75, 458.77) | 352.18 (351.60, 352.77) | -0.90 (-1.00, -0.78) | <0.001 | 6.93 (6.81, 7.06) | 5.10 (5.03, 5.17) | -1.05 (-1.15, -0.95) | <0.001 |
| **21 GBD region** | | | | | | | | | | | | | | | | |
| South Asia | 26.46 (26.31, 26.62) | 25.96 (25.85, 26.08) | -0.06 (-0.13, 0.01) | 0.088 | 348.23 (347.65, 348.80) | 357.76 (357.33, 358.18) | 0.15 (-0.24, 0.54) | 0.443 | 357.23 (356.65, 357.82) | 281.27 (280.89, 281.65) | -0.79 (-1.03, -0.53) | <0.001 | 5.29 (5.21, 5.36) | 3.96 (3.92, 4.01) | -0.96 (-1.27, -0.65) | <0.001 |
| East Asia | 28.92 (28.78, 29.06) | 23.92 (23.79, 24.05) | -0.68 (-0.73, -0.64) | <0.001 | 352.72 (352.22, 353.23) | 317.03 (316.56, 317.51) | -0.35 (-0.47, -0.24) | <0.001 | 490.29 (489.70, 490.88) | 321.63 (321.16, 322.11) | -1.39 (-1.94, -0.84) | <0.001 | 7.25 (7.18, 7.33) | 4.42 (4.37, 4.48) | -1.61 (-1.90, -1.30) | <0.001 |
| North Africa and Middle East | 39.78 (39.42, 40.14) | 36.01 (35.78, 36.24) | -0.33 (-0.35, -0.31) | <0.001 | 478.30 (477.07, 479.52) | 473.92 (473.09, 474.75) | 0.02 (-0.25, 0.29) | 0.889 | 556.93 (555.62, 558.24) | 376.36 (375.62, 377.10) | -1.33 (-1.51, -1.15) | <0.001 | 8.26 (8.10, 8.42) | 5.17 (5.08, 5.25) | -1.55 (-1.69, -1.41) | <0.001 |
| Western Sub-Saharan Africa | 32.42 (31.98, 32.87) | 28.90 (28.64, 29.17) | -0.38 (-0.41, -0.34) | <0.001 | 445.47 (443.83, 447.11) | 444.00 (442.97, 445.03) | 0.02 (-0.20, 0.24) | 0.860 | 396.04 (394.48, 397.61) | 316.40 (315.52, 317.28) | -0.77 (-0.88, -0.66) | <0.001 | 5.87 (5.68, 6.06) | 4.47 (4.36, 4.57) | -0.92 (-1.01, -0.84) | <0.001 |
| Southeast Asia | 44.99 (44.68, 45.30) | 40.99 (40.75, 41.23) | -0.31 (-0.33, -0.30) | <0.001 | 490.81 (489.79, 491.83) | 474.94 (474.13, 475.76) | -0.08 (-0.30, 0.15) | 0.507 | 646.91 (645.74, 648.09) | 490.43 (489.60, 491.25) | -0.93 (-1.05, -0.80) | <0.001 | 9.61 (9.46, 9.75) | 6.92 (6.82, 7.02) | -1.09 (-1.20, -0.98) | <0.001 |
| Eastern Sub-Saharan Africa | 37.17 (36.68, 37.67) | 25.90 (25.64, 26.16) | -1.23 (-1.29, -1.18) | <0.001 | 503.72 (501.96, 505.49) | 471.57 (470.48, 472.67) | -0.19 (-0.47, 0.09) | 0.184 | 557.22 (555.34, 559.10) | 362.29 (361.32, 363.26) | -1.47 (-1.59, -1.34) | <0.001 | 8.50 (8.27, 8.74) | 5.16 (5.05, 5.28) | -1.70 (-1.81, -1.60) | <0.001 |
| Central Latin America | 33.82 (33.36, 34.28) | 25.88 (25.57, 26.20) | -0.92 (-0.96, -0.88) | <0.001 | 483.29 (481.56, 485.03) | 438.07 (436.77, 439.36) | -0.29 (-0.58, 0.00) | 0.052 | 327.70 (326.27, 329.13) | 200.03 (199.16, 200.91) | -1.67 (-1.99, -1.35) | <0.001 | 4.86 (4.69, 5.04) | 2.73 (2.63, 2.84) | -1.94 (-2.25, -1.63) | <0.001 |
| Tropical Latin America | 52.04 (51.46, 52.62) | 29.47 (29.12, | -1.92 (-2.08, | <0.001 | 571.92 (570.02, | 451.30 (449.92, | -0.80 (-0.91, | <0.001 | 621.10 (619.10, | 235.22 (234.23, | -3.27 (-3.41, | <0.001 | 10.12 (9.86, 10.38) | 3.39 (3.27, 3.51) | -3.68 (-3.86, | <0.001 |

| | | | | | | | | | | | | | | | | |
|---|---|---|---|---|---|---|---|---|---|---|---|---|---|---|---|---|
| | | 29.82) | -1.76) | | 573.83) | 452.68) | -0.69) | | 623.12) | 236.21) | -3.12) | | | | -3.50) | |
| Central Sub-Saharan Africa | 33.26 (32.42, 34.11) | 24.98 (24.52, 25.44) | -0.98 (-1.01, -0.94) | <0.001 | 417.75 (414.81, 420.70) | 376.96 (375.21, 378.72) | -0.32 (-0.52, -0.11) | 0.003 | 442.44 (439.40, 445.50) | 310.50 (308.89, 312.11) | -1.25 (-1.31, -1.18) | <0.001 | 6.69 (6.31, 7.08) | 4.49 (4.30, 4.69) | -1.39 (-1.47, -1.32) | <0.001 |
| Western Europe | 18.64 (18.42, 18.86) | 14.58 (14.38, 14.78) | -0.84 (-0.92, -0.75) | <0.001 | 266.42 (265.59, 267.26) | 247.73 (246.89, 248.56) | -0.20 (-0.53, 0.14) | 0.252 | 211.69 (210.94, 212.43) | 92.28 (91.78, 92.79) | -2.84 (-3.06, -2.62) | <0.001 | 2.97 (2.88, 3.06) | 0.95 (0.90, 1.01) | -3.92 (-4.15, -3.69) | <0.001 |
| High-income North America | 18.18 (17.93, 18.42) | 17.64 (17.41, 17.88) | -0.09 (-0.17, -0.01) | 0.038 | 357.52 (356.44, 358.61) | 432.18 (431.03, 433.34) | 0.93 (-0.33, 2.20) | 0.149 | 181.80 (181.03, 182.57) | 143.12 (142.46, 143.78) | -0.78 (-1.12, -0.44) | <0.001 | 2.23 (2.14, 2.31) | 1.39 (1.32, 1.45) | -1.58 (-2.08, -1.07) | <0.001 |
| Central Asia | 40.00 (39.25, 40.77) | 32.41 (31.84, 32.98) | -0.71 (-0.75, -0.66) | <0.001 | 385.07 (382.73, 387.43) | 353.41 (351.54, 355.29) | -0.27 (-0.34, -0.20) | <0.001 | 404.39 (401.98, 406.81) | 343.21 (341.37, 345.06) | -0.57 (-0.87, -0.28) | <0.001 | 5.78 (5.50, 6.08) | 4.84 (4.62, 5.07) | -0.62 (-1.00, -0.25) | 0.001 |
| Eastern Europe | 35.28 (34.90, 35.67) | 33.58 (33.16, 33.99) | -0.16 (-0.22, -0.10) | <0.001 | 379.83 (378.56, 381.10) | 402.23 (400.78, 403.68) | 0.23 (0.08, 0.38) | 0.003 | 307.63 (306.49, 308.78) | 364.97 (363.63, 366.32) | 0.79 (-0.24, 1.83) | 0.133 | 4.09 (3.96, 4.22) | 5.09 (4.94, 5.25) | 1.02 (-0.27, 2.32) | 0.121 |
| Southern Sub-Saharan Africa | 32.33 (31.54, 33.15) | 23.04 (22.54, 23.56) | -1.16 (-1.22, -1.10) | <0.001 | 539.47 (536.27, 542.70) | 477.82 (475.49, 480.16) | -0.40 (-0.45, -0.35) | <0.001 | 493.73 (490.59, 496.89) | 272.05 (270.30, 273.81) | -2.30 (-2.99, -1.60) | <0.001 | 7.46 (7.07, 7.86) | 3.64 (3.44, 3.84) | -2.38 (-3.67, -1.07) | <0.001 |
| High-income Asia Pacific | 25.47 (25.10, 25.86) | 22.14 (21.75, 22.52) | -0.48 (-0.61, -0.35) | <0.001 | 269.63 (268.40, 270.87) | 272.91 (271.55, 274.28) | 0.06 (-0.28, 0.40) | 0.737 | 288.39 (287.11, 289.67) | 121.40 (120.50, 122.31) | -2.94 (-3.14, -2.74) | <0.001 | 4.16 (4.01, 4.31) | 1.27 (1.18, 1.36) | -4.02 (-4.30, -3.74) | <0.001 |
| Central Europe | 33.65 (33.14, 34.18) | 24.42 (23.92, 24.92) | -1.10 (-1.14, -1.05) | <0.001 | 372.92 (371.19, 374.65) | 335.46 (333.62, 337.30) | -0.31 (-0.61, -0.01) | 0.041 | 380.52 (378.78, 382.26) | 197.50 (196.09, 198.91) | -2.29 (-2.51, -2.07) | <0.001 | 5.42 (5.21, 5.63) | 2.27 (2.13, 2.43) | -3.00 (-3.33, -2.66) | <0.001 |
| Andean Latin America | 43.70 (42.62, 44.81) | 31.39 (30.71, 32.09) | -1.14 (-1.17, -1.11) | <0.001 | 487.98 (484.32, 491.66) | 424.45 (421.92, 426.99) | -0.46 (-0.52, -0.40) | <0.001 | 557.33 (553.48, 561.20) | 289.89 (287.81, 291.99) | -2.20 (-2.67, -1.73) | <0.001 | 8.64 (8.16, 9.14) | 4.21 (3.97, 4.47) | -2.42 (-2.94, -1.89) | <0.001 |
| Caribbean | 39.28 (38.23, 40.36) | 37.17 (36.28, 38.07) | -0.18 (-0.22, -0.14) | <0.001 | 461.09 (457.49, 464.70) | 447.85 (444.77, 450.94) | -0.06 (-0.30, 0.18) | 0.633 | 451.49 (447.92, 455.09) | 384.71 (381.86, 387.58) | -0.50 (-0.77, -0.23) | <0.001 | 7.14 (6.69, 7.61) | 5.99 (5.64, 6.36) | -0.56 (-0.84, -0.27) | <0.001 |
| Southern Latin America | 29.56 (28.78, 30.35) | 21.06 (20.50, | -1.15 (-1.22, | <0.001 | 300.04 (297.56, | 253.45 (251.52, | -0.56 (-0.72, | <0.001 | 450.34 (447.30, | 198.17 (196.46, | -2.79 (-3.06, | <0.001 | 6.97 (6.60, 7.36) | 2.68 (2.49, 2.89) | -3.23 (-3.50, | <0.001 |

| | | | | | | | | | | | | | | | | |
|---|---|---|---|---|---|---|---|---|---|---|---|---|---|---|---|---|
| | | 21.62) | -1.09) | | 302.54) | 255.40) | -0.40) | | 453.39) | 199.90) | -2.52) | | | | -2.97) | |
| Oceania | 72.51 (69.11, 76.05) | 73.53 (71.21, 75.91) | 0.05 (0.03, 0.08) | <0.001 | 591.61 (581.80, 601.55) | 587.62 (581.02, 594.28) | -0.01 (-0.06, 0.03) | 0.560 | 845.39 (833.67, 857.24) | 851.77 (843.84, 859.76) | -0.02 (-0.14, 0.11) | 0.777 | 12.74 (11.33, 14.29) | 12.83 (11.87, 13.85) | -0.03 (-0.16, 0.10) | 0.691 |
| Australasia | 18.42 (17.51, 19.37) | 16.21 (15.43, 17.02) | -0.44 (-0.47, -0.41) | <0.001 | 252.35 (248.94, 255.79) | 241.33 (238.31, 244.39) | -0.13 (-0.30, 0.04) | 0.133 | 142.57 (140.01, 145.16) | 79.59 (77.87, 81.35) | -2.00 (-2.26, -1.74) | <0.001 | 1.75 (1.48, 2.06) | 0.69 (0.54, 0.88) | -3.19 (-3.54, -2.84) | <0.001 |
| **Women** | | | | | | | | | | | | | | | | |
| Global | 32.02 (31.91, 32.13) | 27.40 (27.32, 27.48) | -0.53 (-0.60, -0.45) | <0.001 | 447.78 (447.37, 448.19) | 447.41 (447.07, 447.75) | 0.06 (-0.31, 0.42) | 0.760 | 393.26 (392.88, 393.64) | 254.26 (254.00, 254.52) | -1.43 (-1.67, -1.20) | <0.001 | 5.47 (5.43, 5.52) | 3.10 (3.07, 3.13) | -1.94 (-2.16, -1.71) | <0.001 |
| **SDI category** | | | | | | | | | | | | | | | | |
| High | 22.64 (22.41, 22.87) | 21.47 (21.25, 21.69) | -0.18 (-0.22, -0.14) | <0.001 | 377.72 (376.78, 378.67) | 423.86 (422.87, 424.85) | 0.55 (-0.16, 1.26) | 0.130 | 215.66 (214.95, 216.38) | 142.41 (141.84, 142.98) | -1.41 (-1.63, -1.19) | <0.001 | 2.65 (2.57, 2.73) | 1.28 (1.23, 1.33) | -2.47 (-2.65, -2.30) | <0.001 |
| High-middle | 32.50 (32.28, 32.73) | 25.16 (24.96, 25.35) | -0.87 (-0.92, -0.81) | <0.001 | 441.33 (440.49, 442.18) | 411.74 (410.96, 412.52) | -0.19 (-0.42, 0.04) | 0.102 | 343.90 (343.16, 344.65) | 197.80 (197.27, 198.34) | -1.89 (-2.27, -1.50) | <0.001 | 4.51 (4.42, 4.59) | 2.06 (2.01, 2.11) | -2.69 (-3.22, -2.14) | <0.001 |
| Middle | 34.89 (34.69, 35.08) | 28.57 (28.42, 28.73) | -0.67 (-0.75, -0.59) | <0.001 | 478.26 (477.53, 479.00) | 456.42 (455.81, 457.03) | -0.10 (-0.29, 0.09) | 0.311 | 454.82 (454.10, 455.54) | 258.64 (258.18, 259.10) | -1.91 (-2.10, -1.71) | <0.001 | 6.39 (6.31, 6.48) | 3.07 (3.02, 3.12) | -2.48 (-2.68, -2.28) | <0.001 |
| Low-middle | 32.54 (32.29, 32.79) | 29.41 (29.23, 29.59) | -0.33 (-0.42, -0.25) | <0.001 | 435.91 (435.00, 436.82) | 446.45 (445.75, 447.14) | 0.14 (-0.25, 0.54) | 0.483 | 466.52 (465.57, 467.47) | 305.67 (305.10, 306.25) | -1.43 (-1.62, -1.25) | <0.001 | 6.88 (6.77, 7.00) | 4.10 (4.03, 4.17) | -1.72 (-1.95, -1.48) | <0.001 |
| Low | 36.28 (35.88, 36.69) | 29.96 (29.73, 30.20) | -0.65 (-0.71, -0.59) | <0.001 | 494.73 (493.28, 496.20) | 482.84 (481.90, 483.79) | -0.05 (-0.31, 0.21) | 0.710 | 456.59 (455.17, 458.02) | 326.95 (326.16, 327.75) | -1.14 (-1.20, -1.07) | <0.001 | 6.76 (6.59, 6.94) | 4.50 (4.40, 4.59) | -1.39 (-1.49, -1.28) | <0.001 |
| **21 GBD region** | | | | | | | | | | | | | | | | |
| South Asia | 28.82 (28.58, 29.06) | 27.05 (26.88, 27.22) | -0.21 (-0.31, -0.12) | <0.001 | 389.38 (388.51, 390.26) | 404.66 (404.01, 405.31) | 0.20 (-0.24, 0.63) | 0.377 | 349.46 (348.63, 350.29) | 248.78 (248.27, 249.29) | -1.17 (-1.52, -0.83) | <0.001 | 4.94 (4.85, 5.05) | 3.19 (3.13, 3.24) | -1.54 (-1.98, -1.09) | <0.001 |

| | | | | | | | | | | | | | | | | |
|---|---|---|---|---|---|---|---|---|---|---|---|---|---|---|---|---|
| East Asia | 28.15 (27.95, 28.36) | 20.41 (20.23, 20.58) | -1.13 (-1.21, -1.05) | <0.001 | 408.42 (407.64, 409.19) | 367.33 (366.59, 368.07) | -0.35 (-0.49, -0.21) | <0.001 | 411.06 (410.29, 411.84) | 207.94 (207.38, 208.50) | -2.31 (-2.84, -1.78) | <0.001 | 5.61 (5.52, 5.70) | 2.13 (2.08, 2.19) | -3.43 (-4.09, -2.77) | <0.001 |
| North Africa and Middle East | 46.00 (45.45, 46.55) | 40.60 (40.25, 40.96) | -0.42 (-0.44, -0.40) | <0.001 | 552.95 (551.06, 554.85) | 540.51 (539.22, 541.80) | -0.03 (-0.27, 0.21) | 0.787 | 571.84 (569.93, 573.76) | 360.63 (359.58, 361.68) | -1.53 (-1.61, -1.45) | <0.001 | 8.30 (8.07, 8.54) | 4.71 (4.59, 4.84) | -1.88 (-2.11, -1.66) | <0.001 |
| Western Sub-Saharan Africa | 33.20 (32.57, 33.85) | 28.11 (27.75, 28.48) | -0.55 (-0.59, -0.52) | <0.001 | 502.21 (499.78, 504.65) | 492.63 (491.15, 494.12) | -0.03 (-0.24, 0.18) | 0.800 | 396.65 (394.43, 398.87) | 290.97 (289.80, 292.13) | -1.06 (-1.13, -0.99) | <0.001 | 5.75 (5.48, 6.03) | 3.89 (3.75, 4.03) | -1.33 (-1.46, -1.21) | <0.001 |
| Southeast Asia | 40.78 (40.37, 41.19) | 36.89 (36.57, 37.22) | -0.33 (-0.38, -0.28) | <0.001 | 521.31 (519.84, 522.78) | 508.68 (507.49, 509.88) | -0.05 (-0.26, 0.16) | 0.629 | 610.09 (608.49, 611.70) | 394.82 (393.77, 395.87) | -1.47 (-1.58, -1.37) | <0.001 | 8.72 (8.53, 8.91) | 5.02 (4.90, 5.14) | -1.87 (-2.05, -1.69) | <0.001 |
| Eastern Sub-Saharan Africa | 36.99 (36.31, 37.67) | 23.33 (22.98, 23.68) | -1.59 (-1.67, -1.51) | <0.001 | 595.84 (593.20, 598.49) | 551.14 (549.50, 552.78) | -0.23 (-0.49, 0.03) | 0.083 | 508.14 (505.62, 510.67) | 293.54 (292.31, 294.77) | -1.87 (-1.99, -1.76) | <0.001 | 7.53 (7.22, 7.85) | 3.83 (3.68, 3.97) | -2.30 (-2.42, -2.17) | <0.001 |
| Central Latin America | 41.67 (40.96, 42.39) | 30.45 (29.97, 30.93) | -1.08 (-1.12, -1.03) | <0.001 | 600.51 (597.82, 603.20) | 525.87 (523.89, 527.86) | -0.41 (-0.70, -0.12) | 0.005 | 355.13 (353.07, 357.21) | 196.61 (195.40, 197.83) | -2.01 (-2.38, -1.64) | <0.001 | 5.12 (4.87, 5.38) | 2.49 (2.35, 2.63) | -2.48 (-3.01, -1.95) | <0.001 |
| Tropical Latin America | 56.62 (55.78, 57.47) | 33.04 (32.52, 33.57) | -1.84 (-1.95, -1.74) | <0.001 | 651.17 (648.32, 654.03) | 521.95 (519.87, 524.04) | -0.75 (-0.86, -0.64) | <0.001 | 614.27 (611.48, 617.07) | 236.92 (235.53, 238.32) | -3.18 (-3.36, -3.00) | <0.001 | 9.79 (9.44, 10.15) | 3.27 (3.11, 3.43) | -3.68 (-3.90, -3.46) | <0.001 |
| Central Sub-Saharan Africa | 34.16 (32.97, 35.38) | 24.92 (24.28, 25.57) | -1.08 (-1.11, -1.05) | <0.001 | 494.12 (489.66, 498.62) | 446.34 (443.67, 449.02) | -0.32 (-0.52, -0.11) | 0.002 | 428.27 (424.05, 432.53) | 283.37 (281.20, 285.55) | -1.42 (-1.54, -1.30) | <0.001 | 6.29 (5.78, 6.83) | 3.86 (3.61, 4.13) | -1.75 (-1.84, -1.66) | <0.001 |
| Western Europe | 20.18 (19.86, 20.51) | 16.39 (16.09, 16.70) | -0.71 (-0.77, -0.65) | <0.001 | 321.06 (319.76, 322.37) | 307.10 (305.77, 308.44) | -0.10 (-0.44, 0.25) | 0.588 | 196.70 (195.68, 197.72) | 93.45 (92.72, 94.18) | -2.53 (-2.78, -2.29) | <0.001 | 2.56 (2.45, 2.68) | 0.81 (0.75, 0.88) | -3.93 (-4.15, -3.71) | <0.001 |
| High-income North America | 20.90 (20.53, 21.27) | 20.52 (20.17, 20.88) | -0.04 (-0.12, 0.03) | 0.230 | 418.79 (417.12, 420.45) | 505.40 (503.63, 507.18) | 0.89 (-0.22, 2.02) | 0.116 | 190.09 (188.98, 191.21) | 154.04 (153.07, 155.02) | -0.71 (-1.21, -0.20) | 0.007 | 2.15 (2.04, 2.27) | 1.32 (1.24, 1.42) | -1.62 (-2.08, -1.17) | <0.001 |
| Central Asia | 39.90 (38.85, 40.98) | 31.53 (30.74, 32.34) | -0.79 (-0.84, -0.74) | <0.001 | 455.60 (452.02, 459.20) | 420.73 (417.82, 423.64) | -0.24 (-0.34, -0.13) | <0.001 | 377.98 (374.71, 381.26) | 286.58 (284.19, 288.98) | -0.93 (-1.22, -0.65) | <0.001 | 4.99 (4.61, 5.38) | 3.53 (3.27, 3.81) | -1.25 (-1.66, -0.85) | <0.001 |

| | | | | | | | | | | | | | | | | |
|---|---|---|---|---|---|---|---|---|---|---|---|---|---|---|---|---|
| Eastern Europe | 34.34 (33.80, 34.89) | 33.30 (32.71, 33.90) | -0.07 (-0.16, 0.02) | 0.107 | 477.10 (475.08, 479.13) | 539.38 (536.96, 541.82) | 0.47 (0.25, 0.70) | <0.001 | 252.08 (250.60, 253.56) | 271.74 (270.06, 273.44) | 0.34 (-0.39, 1.06) | 0.364 | 2.69 (2.54, 2.85) | 2.90 (2.73, 3.07) | 0.40 (-0.65, 1.47) | 0.454 |
| Southern Sub-Saharan Africa | 30.79 (29.71, 31.89) | 21.80 (21.10, 22.52) | -1.18 (-1.26, -1.11) | <0.001 | 632.98 (628.17, 637.82) | 549.97 (546.44, 553.51) | -0.48 (-0.51, -0.44) | <0.001 | 482.42 (478.12, 486.75) | 223.79 (221.55, 226.05) | -2.52 (-3.92, -1.09) | 0.001 | 6.95 (6.43, 7.49) | 2.58 (2.34, 2.83) | -3.27 (-5.09, -1.42) | 0.001 |
| High-income Asia Pacific | 24.89 (24.36, 25.43) | 21.27 (20.73, 21.83) | -0.54 (-0.64, -0.44) | <0.001 | 330.38 (328.43, 332.34) | 326.58 (324.41, 328.75) | 0.06 (-0.41, 0.53) | 0.802 | 250.30 (248.60, 252.00) | 108.99 (107.75, 110.25) | -2.83 (-3.12, -2.55) | <0.001 | 3.27 (3.08, 3.47) | 0.84 (0.74, 0.96) | -4.63 (-5.01, -4.25) | <0.001 |
| Central Europe | 35.66 (34.90, 36.43) | 27.90 (27.14, 28.68) | -0.82 (-0.88, -0.77) | <0.001 | 487.25 (484.43, 490.08) | 466.12 (463.00, 469.26) | -0.10 (-0.41, 0.22) | 0.544 | 342.81 (340.45, 345.17) | 189.25 (187.27, 191.25) | -2.02 (-2.16, -1.87) | <0.001 | 4.30 (4.04, 4.57) | 1.66 (1.48, 1.85) | -3.28 (-3.53, -3.02) | <0.001 |
| Andean Latin America | 47.18 (45.60, 48.80) | 32.85 (31.86, 33.86) | -1.25 (-1.29, -1.22) | <0.001 | 526.06 (520.74, 531.43) | 453.23 (449.54, 456.94) | -0.49 (-0.63, -0.35) | <0.001 | 549.17 (543.80, 554.58) | 257.07 (254.29, 259.87) | -2.55 (-3.35, -1.74) | <0.001 | 8.44 (7.78, 9.14) | 3.58 (3.26, 3.92) | -2.88 (-3.81, -1.95) | <0.001 |
| Caribbean | 46.63 (45.04, 48.27) | 45.54 (44.17, 46.94) | -0.08 (-0.12, -0.03) | 0.001 | 525.39 (520.03, 530.79) | 516.01 (511.37, 520.68) | -0.02 (-0.27, 0.24) | 0.898 | 509.82 (504.53, 515.16) | 456.32 (451.95, 460.72) | -0.35 (-0.61, -0.09) | 0.009 | 7.98 (7.33, 8.68) | 7.05 (6.51, 7.61) | -0.41 (-0.71, -0.11) | 0.008 |
| Southern Latin America | 32.28 (31.15, 33.45) | 23.52 (22.70, 24.37) | -1.08 (-1.13, -1.03) | <0.001 | 360.19 (356.37, 364.04) | 307.51 (304.51, 310.53) | -0.53 (-0.70, -0.36) | <0.001 | 429.77 (425.61, 433.97) | 189.27 (186.91, 191.65) | -2.75 (-3.07, -2.44) | <0.001 | 6.44 (5.94, 6.97) | 2.36 (2.10, 2.64) | -3.38 (-3.79, -2.96) | <0.001 |
| Oceania | 74.48 (69.57, 79.65) | 77.43 (74.07, 80.91) | 0.14 (0.11, 0.17) | <0.001 | 644.18 (629.55, 659.09) | 644.47 (634.68, 654.37) | 0.01 (-0.04, 0.06) | 0.680 | 657.38 (642.63, 672.41) | 687.96 (677.86, 698.17) | 0.13 (-0.01, 0.27) | 0.078 | 9.12 (7.44, 11.07) | 9.64 (8.47, 10.92) | 0.17 (0.01, 0.32) | 0.041 |
| Australasia | 20.74 (19.37, 22.17) | 19.21 (18.01, 20.46) | -0.27 (-0.35, -0.18) | <0.001 | 324.36 (318.90, 329.89) | 319.71 (314.79, 324.69) | -0.02 (-0.24, 0.21) | 0.888 | 148.03 (144.36, 151.78) | 90.86 (88.27, 93.52) | -1.65 (-1.96, -1.35) | <0.001 | 1.64 (1.28, 2.08) | 0.67 (0.46, 0.93) | -3.07 (-3.69, -2.43) | <0.001 |
| **Men** | | | | | | | | | | | | | | | | |
| Global | 29.98 (29.88, 30.09) | 27.57 (27.49, 27.66) | -0.29 (-0.32, -0.27) | <0.001 | 327.15 (326.81, 327.50) | 331.13 (330.84, 331.42) | 0.10 (-0.25, 0.46) | 0.564 | 454.44 (454.03, 454.85) | 352.77 (352.47, 353.07) | -0.87 (-1.13, -0.61) | <0.001 | 7.00 (6.95, 7.05) | 5.27 (5.23, 5.31) | -0.98 (-1.25, -0.71) | <0.001 |
| **SDI category** | | | | | | | | | | | | | | | | |

| | | | | | | | | | | | | | | | | |
|---|---|---|---|---|---|---|---|---|---|---|---|---|---|---|---|---|
| High | 19.48 (19.27, 19.69) | 18.14 (17.94, 18.33) | -0.24 (-0.27, -0.21) | <0.001 | 257.59 (256.83, 258.36) | 295.99 (295.19, 296.79) | 0.67 (-0.25, 1.59) | 0.153 | 235.92 (235.18, 236.65) | 145.50 (144.95, 146.06) | -1.64 (-1.79, -1.49) | <0.001 | 3.42 (3.33, 3.51) | 1.79 (1.73, 1.86) | -2.23 (-2.40, -2.06) | <0.001 |
| High-middle | 31.75 (31.52, 31.97) | 25.83 (25.64, 26.01) | -0.71 (-0.75, -0.67) | <0.001 | 312.32 (311.62, 313.01) | 283.84 (283.22, 284.46) | -0.28 (-0.48, -0.09) | 0.005 | 459.12 (458.27, 459.97) | 314.69 (314.05, 315.34) | -1.28 (-1.65, -0.91) | <0.001 | 7.06 (6.96, 7.17) | 4.69 (4.61, 4.77) | -1.38 (-1.86, -0.89) | <0.001 |
| Middle | 33.53 (33.34, 33.72) | 30.81 (30.65, 30.97) | -0.29 (-0.32, -0.27) | <0.001 | 353.35 (352.73, 353.97) | 351.16 (350.63, 351.69) | 0.04 (-0.16, 0.24) | 0.700 | 529.89 (529.13, 530.65) | 404.06 (403.49, 404.62) | -0.90 (-1.16, -0.65) | <0.001 | 8.20 (8.11, 8.30) | 6.06 (5.99, 6.13) | -1.03 (-1.36, -0.71) | <0.001 |
| Low-middle | 29.28 (29.05, 29.52) | 28.66 (28.49, 28.84) | -0.08 (-0.10, -0.06) | <0.001 | 340.57 (339.77, 341.37) | 343.23 (342.62, 343.84) | 0.08 (-0.23, 0.39) | 0.611 | 496.99 (496.02, 497.96) | 401.21 (400.55, 401.87) | -0.70 (-0.92, -0.48) | <0.001 | 7.81 (7.68, 7.93) | 6.17 (6.09, 6.25) | -0.77 (-1.01, -0.53) | <0.001 |
| Low | 32.64 (32.26, 33.03) | 28.97 (28.74, 29.21) | -0.40 (-0.43, -0.37) | <0.001 | 359.18 (357.91, 360.45) | 353.86 (353.03, 354.69) | -0.01 (-0.30, 0.28) | 0.952 | 458.85 (457.42, 460.29) | 378.09 (377.23, 378.95) | -0.67 (-0.85, -0.50) | <0.001 | 7.10 (6.92, 7.28) | 5.72 (5.61, 5.83) | -0.73 (-0.92, -0.55) | <0.001 |
| **21 GBD region** | | | | | | | | | | | | | | | | |
| South Asia | 24.26 (24.05, 24.48) | 24.93 (24.77, 25.08) | 0.09 (0.03, 0.14) | 0.002 | 309.73 (308.97, 310.48) | 312.64 (312.08, 313.20) | 0.09 (-0.24, 0.42) | 0.607 | 364.34 (363.52, 365.17) | 312.80 (312.24, 313.37) | -0.47 (-0.82, -0.11) | 0.011 | 5.60 (5.50, 5.71) | 4.72 (4.65, 4.79) | -0.53 (-0.95, -0.09) | 0.017 |
| East Asia | 29.63 (29.43, 29.84) | 27.33 (27.14, 27.53) | -0.29 (-0.36, -0.23) | <0.001 | 300.28 (299.64, 300.93) | 270.09 (269.48, 270.70) | -0.35 (-0.44, -0.25) | <0.001 | 564.68 (563.80, 565.57) | 430.44 (429.68, 431.21) | -0.84 (-1.28, -0.39) | <0.001 | 8.80 (8.69, 8.91) | 6.61 (6.52, 6.71) | -0.89 (-1.56, -0.22) | 0.009 |
| North Africa and Middle East | 33.90 (33.44, 34.36) | 31.84 (31.55, 32.14) | -0.20 (-0.23, -0.18) | <0.001 | 407.66 (406.08, 409.25) | 413.40 (412.33, 414.48) | 0.10 (-0.20, 0.41) | 0.505 | 543.00 (541.20, 544.80) | 391.34 (390.29, 392.39) | -1.12 (-1.42, -0.82) | <0.001 | 8.22 (8.00, 8.45) | 5.59 (5.46, 5.72) | -1.33 (-1.62, -1.04) | <0.001 |
| Western Sub-Saharan Africa | 31.73 (31.10, 32.36) | 29.79 (29.40, 30.18) | -0.21 (-0.26, -0.16) | <0.001 | 385.22 (383.04, 387.40) | 390.52 (389.11, 391.93) | 0.06 (-0.17, 0.28) | 0.625 | 397.76 (395.55, 399.99) | 344.92 (343.60, 346.25) | -0.50 (-0.60, -0.39) | <0.001 | 6.03 (5.76, 6.31) | 5.11 (4.95, 5.27) | -0.57 (-0.69, -0.46) | <0.001 |
| Southeast Asia | 49.28 (48.81, 49.74) | 45.03 (44.68, 45.38) | -0.30 (-0.33, -0.27) | <0.001 | 459.32 (457.92, 460.73) | 441.94 (440.84, 443.05) | -0.09 (-0.34, 0.16) | 0.467 | 684.56 (682.84, 686.29) | 584.36 (583.09, 585.63) | -0.50 (-0.71, -0.29) | <0.001 | 10.52 (10.30, 10.74) | 8.79 (8.64, 8.95) | -0.56 (-0.78, -0.34) | <0.001 |
| Eastern Sub-Saharan | 37.38 (36.68, 38.10) | 28.64 (28.25, | -0.91 (-0.95, | <0.001 | 404.19 (401.90, | 387.99 (386.55, | -0.09 (-0.40, | 0.574 | 610.61 (607.81, | 435.05 (433.54, | -1.15 (-1.30, | <0.001 | 9.56 (9.21, 9.92) | 6.58 (6.40, 6.77) | -1.27 (-1.43, | <0.001 |

| | | | | | | | | | | | | | | | | |
|---|---|---|---|---|---|---|---|---|---|---|---|---|---|---|---|---|
| Africa | | 29.04) | -0.86) | | 406.50) | 389.43) | 0.22) | | 613.43) | 436.58) | -1.00) | | | | -1.12) | |
| Central Latin America | 25.46 (24.89, 26.05) | 21.11 (20.70, 21.52) | -0.65 (-0.69, -0.60) | <0.001 | 358.46 (356.33, 360.60) | 345.67 (344.02, 347.32) | -0.12 (-0.30, 0.06) | 0.203 | 298.57 (296.62, 300.54) | 203.78 (202.52, 205.05) | -1.30 (-1.66, -0.93) | <0.001 | 4.60 (4.35, 4.85) | 3.00 (2.85, 3.16) | -1.45 (-1.88, -1.02) | <0.001 |
| Tropical Latin America | 47.29 (46.50, 48.08) | 25.84 (25.37, 26.31) | -2.08 (-2.15, -2.01) | <0.001 | 489.53 (487.02, 492.06) | 379.04 (377.25, 380.83) | -0.86 (-0.98, -0.75) | <0.001 | 628.29 (625.40, 631.19) | 233.40 (232.00, 234.80) | -3.32 (-3.56, -3.08) | <0.001 | 10.46 (10.09, 10.84) | 3.51 (3.34, 3.69) | -3.66 (-3.93, -3.38) | <0.001 |
| Central Sub-Saharan Africa | 32.34 (31.16, 33.55) | 25.04 (24.39, 25.70) | -0.88 (-0.92, -0.84) | <0.001 | 339.85 (336.06, 343.68) | 306.53 (304.28, 308.80) | -0.32 (-0.60, -0.05) | 0.022 | 456.55 (452.18, 460.96) | 338.08 (335.72, 340.46) | -1.06 (-1.21, -0.92) | <0.001 | 7.09 (6.54, 7.66) | 5.13 (4.84, 5.43) | -1.16 (-1.31, -1.00) | <0.001 |
| Western Europe | 17.12 (16.83, 17.43) | 12.82 (12.56, 13.09) | -0.99 (-1.07, -0.91) | <0.001 | 213.08 (212.03, 214.13) | 190.36 (189.34, 191.39) | -0.34 (-0.65, -0.02) | 0.039 | 226.35 (225.27, 227.44) | 91.22 (90.52, 91.93) | -3.13 (-3.35, -2.92) | <0.001 | 3.36 (3.23, 3.49) | 1.09 (1.01, 1.17) | -3.88 (-4.22, -3.54) | <0.001 |
| High-income North America | 15.47 (15.15, 15.79) | 14.80 (14.50, 15.10) | -0.16 (-0.21, -0.11) | <0.001 | 296.59 (295.20, 297.99) | 359.85 (358.37, 361.34) | 0.98 (-0.48, 2.47) | 0.188 | 173.63 (172.57, 174.69) | 132.38 (131.49, 133.28) | -0.90 (-1.23, -0.58) | <0.001 | 2.30 (2.19, 2.43) | 1.45 (1.36, 1.55) | -1.55 (-2.01, -1.09) | <0.001 |
| Central Asia | 40.15 (39.08, 41.25) | 33.33 (32.52, 34.15) | -0.62 (-0.67, -0.58) | <0.001 | 313.62 (310.60, 316.67) | 287.52 (285.14, 289.91) | -0.29 (-0.33, -0.26) | <0.001 | 431.77 (428.22, 435.35) | 399.74 (396.93, 402.56) | -0.28 (-0.64, 0.09) | 0.143 | 6.60 (6.17, 7.06) | 6.14 (5.80, 6.50) | -0.26 (-0.67, 0.16) | 0.222 |
| Eastern Europe | 36.28 (35.73, 36.83) | 33.97 (33.39, 34.55) | -0.22 (-0.31, -0.13) | <0.001 | 283.66 (282.12, 285.20) | 268.00 (266.37, 269.63) | -0.19 (-0.23, -0.15) | <0.001 | 363.64 (361.90, 365.39) | 458.76 (456.66, 460.87) | 1.03 (-0.17, 2.25) | 0.093 | 5.49 (5.27, 5.70) | 7.30 (7.04, 7.56) | 1.10 (-0.49, 2.71) | 0.175 |
| Southern Sub-Saharan Africa | 33.98 (32.81, 35.19) | 24.30 (23.56, 25.06) | -1.13 (-1.27, -0.99) | <0.001 | 438.91 (434.72, 443.13) | 405.39 (402.36, 408.44) | -0.25 (-0.40, -0.10) | 0.001 | 505.17 (500.58, 509.79) | 320.53 (317.83, 323.24) | -1.65 (-2.43, -0.87) | <0.001 | 7.99 (7.42, 8.60) | 4.70 (4.37, 5.04) | -1.93 (-2.86, -0.99) | <0.001 |
| High-income Asia Pacific | 26.06 (25.52, 26.60) | 22.95 (22.41, 23.50) | -0.44 (-0.53, -0.35) | <0.001 | 210.82 (209.30, 212.36) | 222.77 (221.07, 224.48) | 0.21 (-0.03, 0.45) | 0.088 | 325.43 (323.53, 327.33) | 133.05 (131.75, 134.36) | -3.05 (-3.36, -2.75) | <0.001 | 5.02 (4.79, 5.26) | 1.66 (1.52, 1.81) | -3.75 (-4.17, -3.32) | <0.001 |
| Central Europe | 31.73 (31.03, 32.44) | 21.10 (20.47, 21.76) | -1.40 (-1.43, -1.38) | <0.001 | 261.84 (259.82, 263.88) | 210.65 (208.65, 212.67) | -0.70 (-0.96, -0.43) | <0.001 | 417.54 (414.99, 420.10) | 205.52 (203.54, 207.52) | -2.45 (-2.72, -2.18) | <0.001 | 6.51 (6.20, 6.83) | 2.87 (2.64, 3.11) | -2.83 (-3.22, -2.44) | <0.001 |
| Andean Latin America | 40.07 (38.59, 41.59) | 29.92 (28.97, | -1.01 (-1.04, | <0.001 | 448.10 (443.10, | 395.18 (391.73, | -0.42 (-0.46, | <0.001 | 565.54 (560.03, | 322.34 (319.23, | -1.90 (-2.24, | <0.001 | 8.85 (8.16, 9.58) | 4.85 (4.47, 5.25) | -2.01 (-2.51, | <0.001 |

| | | | | | | | | | | | | | | | | |
|---|---|---|---|---|---|---|---|---|---|---|---|---|---|---|---|---|
| | | 30.88) | -0.98) | | 453.14) | 398.66) | -0.39) | | 571.10) | 325.47) | -1.56) | | | | -1.51) | |
| Caribbean | 31.55 (30.20, 32.96) | 28.60 (27.50, 29.73) | -0.34 (-0.37, -0.31) | <0.001 | 393.01 (388.28, 397.79) | 377.64 (373.62, 381.69) | -0.09 (-0.33, 0.14) | 0.427 | 390.13 (385.36, 394.94) | 311.55 (307.90, 315.24) | -0.75 (-0.93, -0.57) | <0.001 | 6.25 (5.65, 6.89) | 4.91 (4.46, 5.40) | -0.81 (-1.00, -0.62) | <0.001 |
| Southern Latin America | 26.76 (25.72, 27.84) | 18.55 (17.81, 19.31) | -1.25 (-1.33, -1.17) | <0.001 | 238.33 (235.18, 241.51) | 198.67 (196.24, 201.12) | -0.62 (-0.68, -0.55) | <0.001 | 471.41 (466.99, 475.87) | 207.24 (204.76, 209.74) | -2.78 (-3.24, -2.33) | <0.001 | 7.52 (6.97, 8.11) | 3.02 (2.72, 3.33) | -3.10 (-3.41, -2.80) | <0.001 |
| Oceania | 70.58 (65.89, 75.53) | 69.66 (66.48, 72.97) | -0.04 (-0.05, -0.04) | <0.001 | 541.54 (528.45, 554.89) | 531.27 (522.43, 540.22) | -0.05 (-0.09, -0.01) | 0.015 | 1023.90 (1005.89, 1042.17) | 1012.80 (1000.60, 1025.10) | -0.09 (-0.21, 0.03) | 0.156 | 16.18 (13.97, 18.66) | 15.98 (14.48, 17.61) | -0.10 (-0.22, 0.03) | 0.128 |
| Australasia | 16.11 (14.91, 17.38) | 13.18 (12.19, 14.23) | -0.69 (-0.71, -0.67) | <0.001 | 180.38 (176.32, 184.51) | 162.18 (158.69, 165.74) | -0.33 (-0.52, -0.13) | 0.001 | 137.08 (133.54, 140.69) | 68.22 (65.97, 70.54) | -2.39 (-2.66, -2.13) | <0.001 | 1.86 (1.47, 2.33) | 0.72 (0.51, 1.00) | -3.26 (-3.69, -2.83) | <0.001 |
| **Hypertensive heart disease** | | | | | | | | | | | | | | | | |
| **Overall** | | | | | | | | | | | | | | | | |
| Global | — | — | — | — | 9.47 (9.43, 9.51) | 9.15 (9.11, 9.18) | -0.10 (-0.16, -0.05) | 0.001 | 40.53 (40.44, 40.62) | 29.68 (29.62, 29.75) | -1.07 (-1.20, -0.95) | <0.001 | 0.70 (0.69, 0.71) | 0.51 (0.50, 0.52) | -1.04 (-1.19, -0.89) | <0.001 |
| **SDI category** | | | | | | | | | | | | | | | | |
| High | — | — | — | — | 23.06 (22.90, 23.23) | 23.89 (23.74, 24.05) | 0.15 (0.01, 0.30) | 0.043 | 18.02 (17.88, 18.17) | 21.80 (21.65, 21.95) | 0.67 (0.34, 1.00) | <0.001 | 0.28 (0.26, 0.30) | 0.35 (0.34, 0.37) | 0.82 (0.45, 1.19) | <0.001 |
| High-middle | — | — | — | — | 5.96 (5.89, 6.03) | 6.88 (6.82, 6.95) | 0.48 (0.40, 0.56) | <0.001 | 24.93 (24.79, 25.07) | 13.61 (13.52, 13.71) | -2.08 (-2.30, -1.86) | <0.001 | 0.43 (0.41, 0.45) | 0.23 (0.22, 0.24) | -2.28 (-2.60, -1.95) | <0.001 |
| Middle | — | — | — | — | 9.19 (9.12, 9.27) | 9.65 (9.58, 9.71) | 0.16 (0.13, 0.19) | <0.001 | 52.06 (51.89, 52.23) | 29.91 (29.80, 30.02) | -1.90 (-1.96, -1.83) | <0.001 | 0.90 (0.88, 0.93) | 0.51 (0.50, 0.53) | -1.94 (-2.05, -1.84) | <0.001 |
| Low-middle | — | — | — | — | 3.93 (3.87, 3.99) | 4.30 (4.25, 4.35) | 0.31 (0.26, 0.36) | <0.001 | 44.16 (43.95, 44.37) | 31.65 (31.52, 31.78) | -1.13 (-1.28, -0.97) | <0.001 | 0.78 (0.75, 0.81) | 0.56 (0.54, 0.57) | -1.13 (-1.30, -0.95) | <0.001 |

| | | | | | | | | | | | | | | | | |
|---|---|---|---|---|---|---|---|---|---|---|---|---|---|---|---|---|
| Low | — | — | — | — | 5.10 (4.99, 5.21) | 5.79 (5.71, 5.87) | 0.44 (0.41, 0.47) | <0.001 | 76.56 (76.14, 76.99) | 57.12 (56.88, 57.36) | -1.00 (-1.11, -0.88) | <0.001 | 1.36 (1.30, 1.42) | 1.01 (0.98, 1.04) | -1.01 (-1.14, -0.89) | <0.001 |
| **21 GBD region** | | | | | | | | | | | | | | | | |
| South Asia | — | — | — | — | 1.71 (1.67, 1.75) | 1.77 (1.74, 1.80) | 0.13 (0.08, 0.17) | <0.001 | 28.50 (28.33, 28.67) | 20.46 (20.36, 20.57) | -1.13 (-1.39, -0.87) | <0.001 | 0.50 (0.48, 0.53) | 0.36 (0.35, 0.38) | -1.17 (-1.64, -0.70) | <0.001 |
| East Asia | — | — | — | — | 10.81 (10.72, 10.90) | 12.33 (12.24, 12.42) | 0.44 (0.39, 0.49) | <0.001 | 50.71 (50.52, 50.90) | 19.74 (19.62, 19.86) | -3.21 (-3.86, -2.57) | <0.001 | 0.87 (0.85, 0.90) | 0.33 (0.31, 0.34) | -3.23 (-4.11, -2.34) | <0.001 |
| North Africa and Middle East | — | — | — | — | 15.18 (14.95, 15.41) | 15.66 (15.51, 15.81) | 0.10 (0.08, 0.12) | <0.001 | 63.87 (63.42, 64.32) | 45.28 (45.02, 45.54) | -1.17 (-1.31, -1.02) | <0.001 | 1.09 (1.03, 1.15) | 0.77 (0.73, 0.80) | -1.19 (-1.24, -1.14) | <0.001 |
| Western Sub-Saharan Africa | — | — | — | — | 7.66 (7.44, 7.89) | 7.80 (7.66, 7.94) | 0.07 (0.02, 0.12) | 0.006 | 62.14 (61.51, 62.77) | 64.78 (64.37, 65.19) | 0.16 (0.03, 0.28) | 0.014 | 1.09 (1.01, 1.18) | 1.14 (1.08, 1.19) | 0.17 (0.02, 0.32) | 0.029 |
| Southeast Asia | — | — | — | — | 9.14 (9.00, 9.29) | 9.73 (9.62, 9.85) | 0.22 (0.20, 0.23) | <0.001 | 50.33 (49.99, 50.66) | 41.05 (40.81, 41.29) | -0.69 (-0.81, -0.57) | <0.001 | 0.89 (0.84, 0.93) | 0.72 (0.69, 0.75) | -0.72 (-0.91, -0.54) | <0.001 |
| Eastern Sub-Saharan Africa | — | — | — | — | 7.54 (7.31, 7.77) | 7.87 (7.72, 8.02) | 0.14 (0.13, 0.16) | <0.001 | 123.76 (122.84, 124.68) | 64.77 (64.35, 65.20) | -2.20 (-2.31, -2.10) | <0.001 | 2.21 (2.09, 2.33) | 1.15 (1.09, 1.20) | -2.23 (-2.36, -2.10) | <0.001 |
| Central Latin America | — | — | — | — | 1.80 (1.70, 1.91) | 2.26 (2.17, 2.35) | 0.79 (0.67, 0.91) | <0.001 | 22.24 (21.87, 22.63) | 12.71 (12.49, 12.94) | -1.89 (-2.14, -1.63) | <0.001 | 0.39 (0.34, 0.44) | 0.22 (0.19, 0.25) | -1.96 (-2.40, -1.53) | <0.001 |
| Tropical Latin America | — | — | — | — | 1.72 (1.62, 1.83) | 2.44 (2.34, 2.54) | 1.23 (1.08, 1.37) | <0.001 | 48.40 (47.84, 48.97) | 24.39 (24.07, 24.71) | -2.31 (-2.51, -2.12) | <0.001 | 0.87 (0.80, 0.95) | 0.43 (0.39, 0.48) | -2.37 (-2.57, -2.17) | <0.001 |
| Central Sub-Saharan Africa | — | — | — | — | 4.23 (3.92, 4.55) | 4.23 (4.04, 4.43) | 0.01 (-0.03, 0.04) | 0.770 | 86.72 (85.33, 88.13) | 62.90 (62.15, 63.65) | -1.14 (-1.20, -1.07) | <0.001 | 1.55 (1.37, 1.75) | 1.13 (1.03, 1.23) | -1.13 (-1.20, -1.06) | <0.001 |
| Western Europe | — | — | — | — | 2.85 (2.76, 2.93) | 2.30 (2.22, 2.38) | -0.73 (-0.82, | <0.001 | 6.31 (6.19, 6.44) | 4.08 (3.97, 4.18) | -1.49 (-1.67, | <0.001 | 0.11 (0.09, 0.13) | 0.07 (0.06, 0.08) | -1.45 (-2.69, | 0.023 |

| | | | | | | | | | | | | | | | | |
|---|---|---|---|---|---|---|---|---|---|---|---|---|---|---|---|---|
| | | | | | | | -0.64) | | | | -1.30) | | | | -0.20) | |
| High-income North America | — | — | — | — | 55.19 (54.78, 55.61) | 59.42 (59.00, 59.85) | 0.30 (0.16, 0.43) | <0.001 | 26.58 (26.29, 26.87) | 47.80 (47.42, 48.17) | 2.06 (1.82, 2.30) | <0.001 | 0.39 (0.36, 0.43) | 0.77 (0.72, 0.82) | 2.41 (2.12, 2.70) | <0.001 |
| Central Asia | — | — | — | — | 4.48 (4.23, 4.75) | 5.01 (4.80, 5.24) | 0.39 (0.35, 0.42) | <0.001 | 27.98 (27.35, 28.63) | 25.91 (25.41, 26.43) | -0.26 (-0.81, 0.29) | 0.354 | 0.49 (0.41, 0.59) | 0.45 (0.39, 0.53) | -0.25 (-0.83, 0.34) | 0.404 |
| Eastern Europe | — | — | — | — | 0.49 (0.44, 0.53) | 0.49 (0.45, 0.54) | 0.01 (-0.13, 0.15) | 0.871 | 10.26 (10.06, 10.47) | 13.26 (13.01, 13.51) | 1.03 (-0.14, 2.21) | 0.085 | 0.19 (0.16, 0.22) | 0.24 (0.21, 0.28) | 0.83 (-0.26, 1.93) | 0.136 |
| Southern Sub-Saharan Africa | — | — | — | — | 3.34 (3.08, 3.62) | 3.06 (2.88, 3.26) | -0.30 (-0.34, -0.26) | <0.001 | 107.03 (105.53, 108.54) | 54.99 (54.21, 55.79) | -2.51 (-3.63, -1.39) | <0.001 | 1.93 (1.73, 2.14) | 0.98 (0.88, 1.09) | -2.56 (-3.70, -1.40) | <0.001 |
| High-income Asia Pacific | — | — | — | — | 8.05 (7.84, 8.27) | 7.11 (6.90, 7.33) | -0.44 (-0.51, -0.38) | <0.001 | 21.26 (20.91, 21.61) | 4.37 (4.20, 4.55) | -5.36 (-5.68, -5.03) | <0.001 | 0.35 (0.30, 0.40) | 0.07 (0.05, 0.09) | -5.48 (-6.58, -4.37) | <0.001 |
| Central Europe | — | — | — | — | 3.23 (3.08, 3.40) | 2.87 (2.72, 3.04) | -0.41 (-0.47, -0.35) | <0.001 | 16.71 (16.35, 17.07) | 15.23 (14.86, 15.61) | -0.35 (-0.52, -0.19) | <0.001 | 0.30 (0.25, 0.35) | 0.27 (0.22, 0.33) | -0.37 (-0.64, -0.10) | 0.008 |
| Andean Latin America | — | — | — | — | 1.67 (1.47, 1.90) | 3.35 (3.13, 3.58) | 2.41 (2.31, 2.52) | <0.001 | 41.08 (40.02, 42.16) | 25.58 (24.96, 26.20) | -1.64 (-2.02, -1.25) | <0.001 | 0.71 (0.57, 0.86) | 0.43 (0.36, 0.52) | -1.71 (-2.15, -1.26) | <0.001 |
| Caribbean | — | — | — | — | 5.94 (5.53, 6.38) | 5.85 (5.51, 6.22) | -0.05 (-0.07, -0.03) | <0.001 | 52.01 (50.78, 53.26) | 54.36 (53.29, 55.45) | 0.19 (-0.02, 0.40) | 0.070 | 0.92 (0.76, 1.10) | 0.96 (0.83, 1.12) | 0.17 (0.00, 0.34) | 0.049 |
| Southern Latin America | — | — | — | — | 1.79 (1.61, 1.99) | 2.94 (2.74, 3.16) | 1.72 (1.61, 1.83) | <0.001 | 18.50 (17.89, 19.13) | 12.22 (11.80, 12.65) | -1.42 (-1.72, -1.12) | <0.001 | 0.32 (0.25, 0.42) | 0.21 (0.16, 0.27) | -1.46 (-1.63, -1.29) | <0.001 |
| Oceania | — | — | — | — | 7.45 (6.35, 8.69) | 7.10 (6.38, 7.88) | -0.17 (-0.19, -0.14) | <0.001 | 86.38 (82.56, 90.34) | 85.59 (83.05, 88.18) | -0.06 (-0.17, 0.05) | 0.276 | 1.55 (1.08, 2.17) | 1.54 (1.21, 1.92) | -0.05 (-0.18, 0.08) | 0.471 |
| Australasia | — | — | — | — | 2.34 (2.03, 2.69) | 1.67 (1.43, 1.93) | -1.16 (-1.22, | <0.001 | 3.72 (3.32, 4.16) | 2.66 (2.36, 2.99) | -1.12 (-1.42, | <0.001 | 0.06 (0.02, 0.15) | 0.05 (0.01, 0.11) | -0.61 (-0.61, | <0.001 |

| | | | | | | | | | | | | | | | | |
|---|---|---|---|---|---|---|---|---|---|---|---|---|---|---|---|---|
| | | | | | | | -1.11) | | | | -0.82) | | | | -0.61) | |
| **Women** | | | | | | | | | | | | | | | | |
| Global | — | — | — | — | 8.31 (8.25, 8.36) | 7.90 (7.85, 7.94) | -0.16 (-0.22, -0.10) | <0.001 | 40.84 (40.71, 40.96) | 27.17 (27.09, 27.26) | -1.37 (-1.50, -1.24) | <0.001 | 0.71 (0.69, 0.72) | 0.47 (0.46, 0.48) | -1.39 (-1.57, -1.21) | <0.001 |
| **SDI category** | | | | | | | | | | | | | | | | |
| High | — | — | — | — | 19.80 (19.58, 20.01) | 19.42 (19.21, 19.62) | -0.03 (-0.16, 0.11) | 0.709 | 12.30 (12.13, 12.47) | 13.67 (13.50, 13.84) | 0.38 (0.15, 0.62) | 0.001 | 0.19 (0.17, 0.21) | 0.22 (0.20, 0.24) | 0.55 (0.12, 0.98) | 0.012 |
| High-middle | — | — | — | — | 5.45 (5.36, 5.55) | 6.33 (6.24, 6.42) | 0.51 (0.44, 0.57) | <0.001 | 19.54 (19.36, 19.72) | 9.14 (9.03, 9.26) | -2.66 (-2.98, -2.35) | <0.001 | 0.34 (0.31, 0.36) | 0.15 (0.14, 0.17) | -2.85 (-3.42, -2.28) | <0.001 |
| Middle | — | — | — | — | 8.13 (8.03, 8.22) | 8.57 (8.49, 8.65) | 0.17 (0.11, 0.24) | <0.001 | 50.62 (50.38, 50.86) | 23.74 (23.60, 23.87) | -2.62 (-2.78, -2.45) | <0.001 | 0.88 (0.85, 0.91) | 0.41 (0.39, 0.42) | -2.64 (-2.81, -2.48) | <0.001 |
| Low-middle | — | — | — | — | 3.46 (3.38, 3.55) | 3.86 (3.80, 3.93) | 0.37 (0.34, 0.41) | <0.001 | 50.35 (50.03, 50.66) | 32.49 (32.30, 32.67) | -1.48 (-1.67, -1.30) | <0.001 | 0.88 (0.84, 0.93) | 0.57 (0.54, 0.60) | -1.46 (-1.67, -1.24) | <0.001 |
| Low | — | — | — | — | 4.48 (4.34, 4.63) | 4.99 (4.89, 5.09) | 0.37 (0.35, 0.40) | <0.001 | 96.49 (95.82, 97.15) | 63.42 (63.07, 63.77) | -1.44 (-1.51, -1.36) | <0.001 | 1.71 (1.62, 1.80) | 1.12 (1.07, 1.17) | -1.46 (-1.56, -1.35) | <0.001 |
| **21 GBD region** | | | | | | | | | | | | | | | | |
| South Asia | — | — | — | — | 1.68 (1.63, 1.75) | 1.75 (1.71, 1.79) | 0.12 (0.02, 0.23) | 0.019 | 34.87 (34.61, 35.14) | 21.33 (21.18, 21.48) | -1.74 (-2.20, -1.27) | <0.001 | 0.61 (0.58, 0.65) | 0.38 (0.36, 0.40) | -1.67 (-2.30, -1.05) | <0.001 |
| East Asia | — | — | — | — | 9.70 (9.58, 9.82) | 11.07 (10.95, 11.19) | 0.43 (0.36, 0.50) | <0.001 | 43.48 (43.23, 43.74) | 12.11 (11.98, 12.24) | -4.25 (-4.95, -3.54) | <0.001 | 0.75 (0.71, 0.78) | 0.19 (0.18, 0.21) | -4.55 (-5.47, -3.62) | <0.001 |
| North Africa and Middle East | — | — | — | — | 13.83 (13.52, 14.15) | 14.37 (14.16, 14.58) | 0.13 (0.12, 0.14) | <0.001 | 70.57 (69.89, 71.26) | 43.06 (42.70, 43.42) | -1.68 (-1.90, -1.46) | <0.001 | 1.21 (1.12, 1.30) | 0.73 (0.68, 0.78) | -1.71 (-1.95, -1.47) | <0.001 |

| | | | | | | | | | | | | | | | | |
|---|---|---|---|---|---|---|---|---|---|---|---|---|---|---|---|---|
| Western Sub-Saharan Africa | — | — | — | — | 6.65 (6.36, 6.96) | 6.92 (6.73, 7.11) | 0.14 (0.12, 0.16) | <0.001 | 84.09 (83.05, 85.13) | 76.76 (76.15, 77.37) | -0.30 (-0.42, -0.18) | <0.001 | 1.48 (1.35, 1.63) | 1.35 (1.27, 1.43) | -0.30 (-0.44, -0.16) | <0.001 |
| Southeast Asia | — | — | — | — | 7.58 (7.40, 7.77) | 7.96 (7.81, 8.11) | 0.16 (0.14, 0.19) | <0.001 | 51.44 (50.97, 51.92) | 33.59 (33.29, 33.90) | -1.45 (-1.59, -1.30) | <0.001 | 0.91 (0.85, 0.97) | 0.59 (0.55, 0.63) | -1.43 (-1.54, -1.33) | <0.001 |
| Eastern Sub-Saharan Africa | — | — | — | — | 6.19 (5.91, 6.49) | 6.39 (6.21, 6.58) | 0.11 (0.10, 0.12) | <0.001 | 151.01 (149.62, 152.42) | 66.77 (66.18, 67.37) | -2.77 (-2.88, -2.65) | <0.001 | 2.69 (2.51, 2.89) | 1.18 (1.10, 1.26) | -2.81 (-2.99, -2.63) | <0.001 |
| Central Latin America | — | — | — | — | 2.46 (2.28, 2.64) | 3.02 (2.87, 3.17) | 0.72 (0.60, 0.84) | <0.001 | 23.12 (22.59, 23.67) | 11.73 (11.44, 12.03) | -2.29 (-2.71, -1.87) | <0.001 | 0.40 (0.34, 0.48) | 0.20 (0.16, 0.24) | -2.34 (-2.74, -1.95) | <0.001 |
| Tropical Latin America | — | — | — | — | 2.48 (2.30, 2.66) | 3.42 (3.25, 3.59) | 1.11 (1.03, 1.19) | <0.001 | 45.75 (44.98, 46.53) | 21.34 (20.92, 21.75) | -2.57 (-2.86, -2.27) | <0.001 | 0.82 (0.72, 0.93) | 0.38 (0.33, 0.44) | -2.59 (-3.02, -2.16) | <0.001 |
| Central Sub-Saharan Africa | — | — | — | — | 3.81 (3.40, 4.25) | 3.82 (3.56, 4.09) | 0.01 (-0.02, 0.04) | 0.424 | 100.88 (98.78, 103.01) | 69.42 (68.32, 70.53) | -1.33 (-1.40, -1.25) | <0.001 | 1.80 (1.53, 2.11) | 1.24 (1.10, 1.40) | -1.34 (-1.42, -1.26) | <0.001 |
| Western Europe | — | — | — | — | 2.40 (2.29, 2.52) | 1.83 (1.73, 1.93) | -0.95 (-1.07, -0.84) | <0.001 | 3.78 (3.64, 3.92) | 2.52 (2.40, 2.64) | -1.40 (-1.54, -1.26) | <0.001 | 0.06 (0.05, 0.08) | 0.04 (0.03, 0.06) | -1.48 (-2.44, -0.50) | 0.003 |
| High-income North America | — | — | — | — | 46.82 (46.28, 47.36) | 46.93 (46.41, 47.46) | 0.06 (-0.11, 0.22) | 0.478 | 17.65 (17.32, 17.98) | 29.15 (28.74, 29.57) | 1.78 (1.49, 2.08) | <0.001 | 0.25 (0.21, 0.29) | 0.46 (0.41, 0.51) | 2.22 (1.78, 2.65) | <0.001 |
| Central Asia | — | — | — | — | 2.58 (2.31, 2.87) | 2.68 (2.46, 2.93) | 0.13 (0.08, 0.17) | <0.001 | 19.64 (18.89, 20.40) | 16.96 (16.38, 17.55) | -0.48 (-0.77, -0.18) | 0.001 | 0.34 (0.25, 0.46) | 0.30 (0.22, 0.39) | -0.43 (-0.76, -0.09) | 0.013 |
| Eastern Europe | — | — | — | — | 0.32 (0.27, 0.38) | 0.33 (0.28, 0.40) | 0.09 (-0.21, 0.39) | 0.561 | 4.14 (3.96, 4.33) | 5.79 (5.55, 6.03) | 1.28 (0.16, 2.42) | 0.025 | 0.07 (0.05, 0.10) | 0.10 (0.08, 0.14) | 1.17 (-0.02, 2.37) | 0.054 |
| Southern Sub-Saharan Africa | — | — | — | — | 3.17 (2.82, 3.56) | 2.84 (2.60, 3.11) | -0.38 (-0.41, -0.35) | <0.001 | 130.39 (128.10, 132.71) | 53.36 (52.27, 54.47) | -2.91 (-4.98, -0.80) | 0.007 | 2.34 (2.04, 2.67) | 0.95 (0.81, 1.11) | -2.95 (-5.03, -0.82) | 0.007 |

| | | | | | | | | | | | | | | | | |
|---|---|---|---|---|---|---|---|---|---|---|---|---|---|---|---|---|
| High-income Asia Pacific | — | — | — | — | 6.60 (6.33, 6.88) | 5.43 (5.16, 5.71) | -0.68 (-0.74, -0.62) | <0.001 | 15.22 (14.80, 15.65) | 2.83 (2.64, 3.04) | -5.67 (-6.08, -5.26) | <0.001 | 0.25 (0.20, 0.31) | 0.04 (0.02, 0.07) | -6.13 (-7.77, -4.45) | <0.001 |
| Central Europe | — | — | — | — | 2.33 (2.14, 2.53) | 2.00 (1.81, 2.20) | -0.53 (-0.59, -0.46) | <0.001 | 11.20 (10.78, 11.63) | 8.57 (8.17, 8.99) | -0.93 (-1.23, -0.63) | <0.001 | 0.20 (0.15, 0.26) | 0.15 (0.10, 0.22) | -1.03 (-1.31, -0.74) | <0.001 |
| Andean Latin America | — | — | — | — | 1.83 (1.53, 2.16) | 4.02 (3.68, 4.39) | 2.73 (2.67, 2.80) | <0.001 | 43.51 (41.99, 45.07) | 20.74 (19.95, 21.54) | -2.52 (-3.15, -1.88) | <0.001 | 0.75 (0.56, 0.99) | 0.35 (0.26, 0.47) | -2.60 (-3.26, -1.94) | <0.001 |
| Caribbean | — | — | — | — | 6.04 (5.46, 6.67) | 5.86 (5.38, 6.38) | -0.11 (-0.14, -0.08) | <0.001 | 58.65 (56.83, 60.50) | 59.09 (57.53, 60.69) | 0.07 (-0.21, 0.35) | 0.630 | 1.03 (0.81, 1.31) | 1.04 (0.84, 1.27) | 0.06 (-0.22, 0.35) | 0.660 |
| Southern Latin America | — | — | — | — | 1.89 (1.62, 2.18) | 3.08 (2.79, 3.40) | 1.65 (1.42, 1.88) | <0.001 | 15.06 (14.28, 15.86) | 9.01 (8.50, 9.54) | -1.76 (-2.04, -1.47) | <0.001 | 0.26 (0.17, 0.39) | 0.15 (0.09, 0.24) | -1.97 (-2.46, -1.49) | <0.001 |
| Oceania | — | — | — | — | 6.45 (5.02, 8.18) | 5.83 (4.92, 6.86) | -0.35 (-0.38, -0.33) | <0.001 | 83.50 (78.18, 89.10) | 78.15 (74.74, 81.68) | -0.25 (-0.38, -0.12) | <0.001 | 1.49 (0.85, 2.43) | 1.39 (0.97, 1.94) | -0.25 (-0.38, -0.12) | <0.001 |
| Australasia | — | — | — | — | 2.11 (1.70, 2.59) | 1.44 (1.14, 1.81) | -1.30 (-1.35, -1.24) | <0.001 | 2.53 (2.08, 3.06) | 1.69 (1.36, 2.09) | -1.33 (-1.49, -1.16) | <0.001 | 0.04 (0.00, 0.17) | 0.03 (0.00, 0.14) | -0.96 (-0.96, -0.96) | <0.001 |
| **Men** | | | | | | | | | | | | | | | | |
| Global | — | — | — | — | 10.60 (10.54, 10.66) | 10.38 (10.33, 10.43) | -0.06 (-0.13, 0.01) | 0.074 | 40.23 (40.10, 40.35) | 32.17 (32.08, 32.26) | -0.76 (-0.89, -0.63) | <0.001 | 0.70 (0.68, 0.71) | 0.56 (0.54, 0.57) | -0.78 (-1.00, -0.57) | <0.001 |
| **SDI category** | | | | | | | | | | | | | | | | |
| High | — | — | — | — | 26.25 (26.01, 26.49) | 28.07 (27.83, 28.30) | 0.26 (0.07, 0.44) | 0.006 | 23.58 (23.35, 23.81) | 29.39 (29.15, 29.63) | 0.79 (0.55, 1.03) | <0.001 | 0.38 (0.35, 0.41) | 0.48 (0.45, 0.52) | 0.81 (0.43, 1.20) | <0.001 |
| High-middle | — | — | — | — | 6.45 (6.35, 6.55) | 7.40 (7.31, 7.50) | 0.45 (0.36, 0.55) | <0.001 | 30.14 (29.93, 30.36) | 17.83 (17.68, 17.98) | -1.82 (-2.02, -1.62) | <0.001 | 0.52 (0.49, 0.55) | 0.31 (0.29, 0.33) | -1.88 (-2.17, -1.58) | <0.001 |

| | | | | | | | | | | | | | | | | |
|---|---|---|---|---|---|---|---|---|---|---|---|---|---|---|---|---|
| Middle | — | — | — | — | 10.22 (10.12, 10.33) | 10.72 (10.63, 10.81) | 0.16 (0.11, 0.21) | <0.001 | 53.44 (53.20, 53.69) | 36.04 (35.87, 36.21) | -1.35 (-1.48, -1.22) | <0.001 | 0.93 (0.90, 0.96) | 0.62 (0.60, 0.64) | -1.38 (-1.61, -1.14) | <0.001 |
| Low-middle | — | — | — | — | 4.39 (4.29, 4.48) | 4.75 (4.67, 4.82) | 0.27 (0.20, 0.34) | <0.001 | 38.11 (37.84, 38.38) | 30.83 (30.65, 31.02) | -0.72 (-0.91, -0.53) | <0.001 | 0.67 (0.64, 0.71) | 0.54 (0.52, 0.57) | -0.73 (-1.00, -0.47) | <0.001 |
| Low | — | — | — | — | 5.74 (5.57, 5.91) | 6.62 (6.50, 6.74) | 0.50 (0.47, 0.53) | <0.001 | 56.09 (55.58, 56.61) | 50.66 (50.34, 50.99) | -0.36 (-0.45, -0.27) | <0.001 | 1.00 (0.93, 1.07) | 0.90 (0.85, 0.94) | -0.38 (-0.46, -0.30) | <0.001 |
| **21 GBD region** | | | | | | | | | | | | | | | | |
| South Asia | — | — | — | — | 1.73 (1.68, 1.79) | 1.79 (1.74, 1.83) | 0.11 (0.08, 0.15) | <0.001 | 22.54 (22.33, 22.75) | 19.64 (19.50, 19.78) | -0.43 (-0.86, 0.00) | 0.052 | 0.40 (0.38, 0.43) | 0.35 (0.33, 0.37) | -0.44 (-0.97, 0.09) | 0.103 |
| East Asia | — | — | — | — | 11.84 (11.71, 11.97) | 13.54 (13.41, 13.67) | 0.44 (0.37, 0.51) | <0.001 | 57.50 (57.21, 57.78) | 27.05 (26.86, 27.24) | -2.58 (-2.96, -2.19) | <0.001 | 0.99 (0.95, 1.03) | 0.46 (0.43, 0.48) | -2.61 (-3.12, -2.09) | <0.001 |
| North Africa and Middle East | — | — | — | — | 16.44 (16.11, 16.78) | 16.81 (16.60, 17.03) | 0.07 (0.04, 0.11) | <0.001 | 57.53 (56.93, 58.13) | 47.29 (46.93, 47.65) | -0.67 (-0.84, -0.49) | <0.001 | 0.98 (0.90, 1.06) | 0.80 (0.75, 0.85) | -0.68 (-0.87, -0.50) | <0.001 |
| Western Sub-Saharan Africa | — | — | — | — | 8.67 (8.34, 9.02) | 8.78 (8.56, 9.00) | 0.06 (-0.03, 0.15) | 0.174 | 39.88 (39.16, 40.61) | 51.68 (51.15, 52.21) | 0.91 (0.71, 1.11) | <0.001 | 0.70 (0.61, 0.80) | 0.91 (0.84, 0.98) | 0.94 (0.66, 1.22) | <0.001 |
| Southeast Asia | — | — | — | — | 10.74 (10.52, 10.96) | 11.48 (11.30, 11.65) | 0.24 (0.21, 0.28) | <0.001 | 49.18 (48.71, 49.65) | 48.38 (48.02, 48.75) | -0.03 (-0.18, 0.13) | 0.755 | 0.86 (0.80, 0.93) | 0.85 (0.80, 0.90) | -0.02 (-0.25, 0.21) | 0.862 |
| Eastern Sub-Saharan Africa | — | — | — | — | 8.99 (8.63, 9.36) | 9.45 (9.22, 9.69) | 0.17 (0.14, 0.19) | <0.001 | 94.14 (92.99, 95.31) | 62.68 (62.08, 63.28) | -1.39 (-1.51, -1.27) | <0.001 | 1.68 (1.53, 1.85) | 1.11 (1.03, 1.19) | -1.42 (-1.59, -1.25) | <0.001 |
| Central Latin America | — | — | — | — | 1.11 (0.99, 1.23) | 1.44 (1.33, 1.55) | 0.87 (0.77, 0.97) | <0.001 | 21.32 (20.79, 21.86) | 13.76 (13.44, 14.10) | -1.47 (-1.84, -1.10) | <0.001 | 0.37 (0.31, 0.45) | 0.24 (0.20, 0.29) | -1.47 (-1.94, -1.00) | <0.001 |
| Tropical Latin America | — | — | — | — | 0.94 (0.83, 1.05) | 1.43 (1.32, 1.54) | 1.46 (1.33, | <0.001 | 51.17 (50.34, 52.01) | 27.52 (27.04, 28.00) | -2.13 (-2.30, | <0.001 | 0.92 (0.81, 1.04) | 0.49 (0.43, 0.56) | -2.13 (-2.33, | <0.001 |

| | | | | | | | | | | | | | | | | |
|---|---|---|---|---|---|---|---|---|---|---|---|---|---|---|---|---|
| | | | | | | | 1.59) | | | | -1.95) | | | | -1.93) | |
| Central Sub-Saharan Africa | — | — | — | — | 4.66 (4.20, 5.16) | 4.65 (4.36, 4.95) | -0.01 (-0.03, 0.01) | 0.402 | 72.16 (70.35, 74.01) | 56.28 (55.28, 57.29) | -0.89 (-1.03, -0.75) | <0.001 | 1.30 (1.06, 1.57) | 1.01 (0.88, 1.15) | -0.90 (-1.04, -0.76) | <0.001 |
| Western Europe | — | — | — | — | 3.28 (3.15, 3.41) | 2.76 (2.64, 2.89) | -0.60 (-0.66, -0.54) | <0.001 | 8.80 (8.59, 9.02) | 5.60 (5.43, 5.78) | -1.54 (-1.80, -1.29) | <0.001 | 0.15 (0.12, 0.18) | 0.10 (0.07, 0.12) | -1.58 (-2.12, -1.04) | <0.001 |
| High-income North America | — | — | — | — | 63.60 (62.98, 64.23) | 71.87 (71.22, 72.52) | 0.45 (0.26, 0.64) | <0.001 | 35.55 (35.08, 36.03) | 66.40 (65.77, 67.03) | 2.19 (1.95, 2.44) | <0.001 | 0.54 (0.49, 0.60) | 1.08 (1.01, 1.17) | 2.45 (2.20, 2.71) | <0.001 |
| Central Asia | — | — | — | — | 6.43 (6.00, 6.89) | 7.34 (6.97, 7.73) | 0.46 (0.39, 0.53) | <0.001 | 36.56 (35.52, 37.62) | 34.82 (33.99, 35.66) | -0.13 (-0.50, 0.24) | 0.493 | 0.65 (0.51, 0.80) | 0.61 (0.51, 0.73) | -0.16 (-0.55, 0.23) | 0.413 |
| Eastern Europe | — | — | — | — | 0.65 (0.58, 0.73) | 0.65 (0.58, 0.74) | -0.02 (-0.09, 0.05) | 0.612 | 16.41 (16.05, 16.78) | 20.75 (20.31, 21.19) | 0.95 (-0.26, 2.18) | 0.124 | 0.30 (0.25, 0.35) | 0.38 (0.32, 0.44) | 0.97 (-0.37, 2.33) | 0.155 |
| Southern Sub-Saharan Africa | — | — | — | — | 3.53 (3.14, 3.95) | 3.28 (3.01, 3.57) | -0.26 (-0.29, -0.22) | <0.001 | 81.97 (80.08, 83.89) | 56.61 (55.48, 57.75) | -1.43 (-2.37, -0.48) | 0.003 | 1.49 (1.24, 1.77) | 1.02 (0.87, 1.18) | -1.43 (-2.58, -0.26) | 0.017 |
| High-income Asia Pacific | — | — | — | — | 9.47 (9.15, 9.80) | 8.69 (8.37, 9.02) | -0.30 (-0.34, -0.26) | <0.001 | 27.10 (26.55, 27.66) | 5.82 (5.55, 6.09) | -5.19 (-5.41, -4.97) | <0.001 | 0.44 (0.38, 0.52) | 0.09 (0.06, 0.13) | -5.31 (-5.83, -4.79) | <0.001 |
| Central Europe | — | — | — | — | 4.12 (3.88, 4.38) | 3.72 (3.47, 3.99) | -0.35 (-0.38, -0.32) | <0.001 | 22.10 (21.52, 22.69) | 21.63 (21.01, 22.27) | -0.12 (-0.35, 0.12) | 0.334 | 0.39 (0.32, 0.48) | 0.38 (0.31, 0.48) | -0.17 (-0.64, 0.30) | 0.468 |
| Andean Latin America | — | — | — | — | 1.51 (1.25, 1.82) | 2.65 (2.37, 2.94) | 1.96 (1.88, 2.05) | <0.001 | 38.54 (37.08, 40.03) | 30.40 (29.45, 31.38) | -0.82 (-1.11, -0.54) | <0.001 | 0.66 (0.48, 0.88) | 0.51 (0.40, 0.66) | -0.85 (-1.15, -0.56) | <0.001 |
| Caribbean | — | — | — | — | 5.83 (5.25, 6.47) | 5.84 (5.35, 6.37) | 0.02 (-0.01, 0.05) | 0.243 | 45.01 (43.37, 46.69) | 49.54 (48.08, 51.03) | 0.38 (0.13, 0.62) | 0.002 | 0.80 (0.59, 1.05) | 0.88 (0.70, 1.10) | 0.38 (0.07, 0.69) | 0.017 |
| Southern Latin America | — | — | — | — | 1.69 (1.44, 1.98) | 2.79 (2.51, 3.10) | 1.79 (1.52, | <0.001 | 22.04 (21.08, 23.02) | 15.49 (14.82, 16.19) | -1.22 (-1.55, | <0.001 | 0.39 (0.27, 0.54) | 0.27 (0.19, 0.37) | -1.23 (-1.76, | <0.001 |

| | | | | | | | | | | | | | | | | |
|---|---|---|---|---|---|---|---|---|---|---|---|---|---|---|---|---|
| | | | | | | | 2.05) | | | | -0.88) | | | | -0.70) | |
| Oceania | — | — | — | — | 8.39 (6.78, 10.28) | 8.37 (7.27, 9.60) | -0.01 (-0.03, 0.01) | 0.335 | 89.02 (83.60, 94.71) | 92.98 (89.25, 96.84) | 0.11 (0.01, 0.22) | 0.038 | 1.61 (0.95, 2.56) | 1.68 (1.21, 2.27) | 0.11 (-0.01, 0.22) | 0.066 |
| Australasia | — | — | — | — | 2.58 (2.12, 3.12) | 1.90 (1.55, 2.31) | -1.06 (-1.11, -1.01) | <0.001 | 4.92 (4.27, 5.64) | 3.65 (3.15, 4.21) | -1.01 (-1.43, -0.60) | <0.001 | 0.08 (0.02, 0.23) | 0.06 (0.01, 0.19) | -1.07 (-2.82, 0.71) | 0.237 |
| **Non-rheumatic valvular heart disease** | | | | | | | | | | | | | | | | |
| **Overall** | | | | | | | | | | | | | | | | |
| Global | 7.85 (7.81, 7.89) | 6.46 (6.43, 6.48) | -0.67 (-0.72, -0.62) | <0.001 | 62.64 (62.53, 62.75) | 50.03 (49.95, 50.11) | -0.77 (-0.84, -0.70) | <0.001 | 10.82 (10.77, 10.86) | 8.85 (8.81, 8.88) | -0.69 (-0.80, -0.59) | <0.001 | 0.18 (0.17, 0.19) | 0.15 (0.14, 0.15) | — | — |
| **SDI category** | | | | | | | | | | | | | | | | |
| High | 30.68 (30.50, 30.87) | 32.21 (32.02, 32.39) | 0.15 (0.09, 0.21) | <0.001 | 247.02 (246.49, 247.54) | 255.83 (255.32, 256.35) | 0.11 (0.04, 0.18) | 0.002 | 11.98 (11.86, 12.10) | 7.91 (7.81, 8.00) | -1.44 (-1.67, -1.20) | <0.001 | 0.20 (0.19, 0.22) | 0.13 (0.12, 0.14) | -1.51 (-2.33, -0.68) | <0.001 |
| High-middle | 8.65 (8.57, 8.73) | 8.76 (8.68, 8.83) | 0.05 (0.00, 0.10) | 0.037 | 67.85 (67.61, 68.08) | 67.38 (67.17, 67.59) | -0.01 (-0.10, 0.09) | 0.926 | 9.76 (9.67, 9.85) | 6.89 (6.82, 6.96) | -1.21 (-1.44, -0.98) | <0.001 | 0.16 (0.15, 0.17) | 0.12 (0.11, 0.12) | -0.96 (-1.44, -0.47) | <0.001 |
| Middle | 1.86 (1.83, 1.89) | 1.94 (1.91, 1.97) | 0.14 (0.05, 0.24) | 0.002 | 12.13 (12.04, 12.21) | 11.61 (11.54, 11.67) | -0.18 (-0.23, -0.13) | <0.001 | 10.25 (10.18, 10.33) | 8.08 (8.02, 8.14) | -0.81 (-0.90, -0.72) | <0.001 | 0.17 (0.16, 0.18) | 0.13 (0.13, 0.14) | -1.11 (-1.22, -1.00) | <0.001 |
| Low-middle | 0.94 (0.91, 0.97) | 1.00 (0.98, 1.02) | 0.22 (0.08, 0.36) | 0.002 | 3.98 (3.92, 4.04) | 4.41 (4.36, 4.46) | 0.32 (0.17, 0.47) | <0.001 | 10.32 (10.22, 10.42) | 9.76 (9.69, 9.83) | -0.19 (-0.36, -0.02) | 0.027 | 0.17 (0.16, 0.19) | 0.16 (0.15, 0.17) | -0.22 (-0.59, 0.16) | 0.258 |
| Low | 0.65 (0.62, 0.69) | 0.71 (0.68, 0.74) | 0.28 (0.08, 0.48) | 0.006 | 1.42 (1.36, 1.48) | 2.01 (1.96, 2.06) | 1.11 (0.64, 1.59) | <0.001 | 13.90 (13.73, 14.08) | 11.77 (11.67, 11.88) | -0.56 (-0.72, -0.41) | <0.001 | 0.23 (0.21, 0.26) | 0.20 (0.18, 0.21) | -0.48 (-0.81, -0.14) | 0.006 |
| **21 GBD region** | | | | | | | | | | | | | | | | |

| | | | | | | | | | | | | | | | | |
|---|---|---|---|---|---|---|---|---|---|---|---|---|---|---|---|---|
| South Asia | 0.49 (0.46, 0.51) | 0.62 (0.60, 0.64) | 0.79 (0.55, 1.02) | <0.001 | — | 1.36 (1.33, 1.39) | — | — | 8.90 (8.81, 8.99) | 8.45 (8.38, 8.51) | -0.20 (-0.48, 0.09) | 0.175 | 0.15 (0.14, 0.16) | 0.14 (0.13, 0.15) | -0.11 (-0.69, 0.48) | 0.715 |
| East Asia | 3.30 (3.25, 3.35) | 4.04 (3.99, 4.09) | 0.69 (0.59, 0.79) | <0.001 | 24.77 (24.64, 24.91) | 28.71 (28.58, 28.85) | 0.50 (0.30, 0.70) | <0.001 | 5.25 (5.19, 5.31) | 3.03 (2.98, 3.08) | -1.87 (-2.16, -1.57) | <0.001 | 0.09 (0.08, 0.10) | 0.05 (0.05, 0.06) | -2.48 (-2.77, -2.19) | <0.001 |
| North Africa and Middle East | 2.17 (2.09, 2.26) | 2.43 (2.37, 2.49) | 0.39 (0.31, 0.48) | <0.001 | 14.22 (14.00, 14.45) | 15.88 (15.73, 16.03) | 0.37 (0.20, 0.53) | <0.001 | 29.11 (28.81, 29.41) | 19.43 (19.26, 19.60) | -1.36 (-1.43, -1.29) | <0.001 | 0.48 (0.45, 0.52) | 0.32 (0.30, 0.35) | -1.42 (-1.59, -1.25) | <0.001 |
| Western Sub-Saharan Africa | 0.77 (0.70, 0.84) | 0.84 (0.79, 0.89) | 0.29 (0.14, 0.45) | <0.001 | 2.53 (2.40, 2.67) | 3.48 (3.38, 3.58) | 1.02 (0.73, 1.31) | <0.001 | 13.21 (12.94, 13.49) | 12.48 (12.32, 12.65) | -0.21 (-0.37, -0.05) | 0.009 | 0.22 (0.18, 0.26) | 0.20 (0.18, 0.23) | -0.30 (-0.64, 0.03) | 0.073 |
| Southeast Asia | 1.06 (1.01, 1.11) | 1.25 (1.21, 1.29) | 0.56 (0.51, 0.63) | <0.001 | 6.10 (5.99, 6.22) | 7.47 (7.37, 7.57) | 0.69 (0.59, 0.79) | <0.001 | 4.40 (4.31, 4.50) | 3.87 (3.80, 3.95) | -0.39 (-0.47, -0.31) | <0.001 | 0.07 (0.06, 0.09) | 0.07 (0.06, 0.08) | 0.00 (0.00, 0.00)* | 0.008 |
| Eastern Sub-Saharan Africa | 0.62 (0.56, 0.69) | 0.60 (0.56, 0.65) | -0.17 (-0.29, -0.04) | 0.008 | 1.16 (1.07, 1.26) | 1.16 (1.10, 1.22) | 0.10 (-0.44, 0.63) | 0.720 | 18.87 (18.54, 19.21) | 12.16 (11.99, 12.34) | -1.51 (-1.71, -1.31) | <0.001 | 0.32 (0.27, 0.36) | 0.20 (0.18, 0.23) | -1.60 (-1.89, -1.31) | <0.001 |
| Central Latin America | 0.79 (0.72, 0.87) | 1.30 (1.23, 1.38) | 1.68 (1.50, 1.87) | <0.001 | 1.88 (1.76, 2.00) | 4.35 (4.23, 4.49) | 2.97 (2.18, 3.76) | <0.001 | 11.95 (11.68, 12.22) | 9.36 (9.17, 9.55) | -0.80 (-1.20, -0.41) | <0.001 | 0.20 (0.17, 0.24) | 0.16 (0.14, 0.19) | -0.57 (-1.26, 0.13) | 0.109 |
| Tropical Latin America | 1.64 (1.54, 1.75) | 1.65 (1.56, 1.73) | -0.01 (-0.30, 0.28) | 0.925 | 7.60 (7.38, 7.83) | 6.63 (6.47, 6.80) | -0.58 (-0.89, -0.26) | <0.001 | 33.66 (33.20, 34.12) | 15.25 (14.99, 15.50) | -2.72 (-3.02, -2.41) | <0.001 | 0.57 (0.51, 0.63) | 0.26 (0.23, 0.29) | -2.69 (-2.97, -2.40) | <0.001 |
| Central Sub-Saharan Africa | 0.62 (0.51, 0.75) | 0.59 (0.52, 0.66) | -0.21 (-0.41, 0.00) | 0.053 | 1.32 (1.14, 1.51) | 0.73 (0.65, 0.82) | -1.94 (-2.55, -1.32) | <0.001 | 12.82 (12.32, 13.34) | 9.80 (9.52, 10.08) | -0.99 (-1.07, -0.91) | <0.001 | 0.21 (0.15, 0.29) | 0.16 (0.13, 0.21) | -0.95 (-1.12, -0.78) | <0.001 |
| Western Europe | 8.59 (8.44, 8.74) | 9.86 (9.69, 10.02) | 0.47 (0.36, 0.59) | <0.001 | 65.34 (64.93, 65.75) | 75.79 (75.34, 76.24) | 0.51 (0.40, 0.62) | <0.001 | 12.65 (12.47, 12.84) | 7.79 (7.64, 7.94) | -1.71 (-1.89, -1.54) | <0.001 | 0.21 (0.19, 0.24) | 0.13 (0.11, 0.15) | -1.68 (-2.10, -1.26) | <0.001 |
| High-income North America | 55.73 (55.31, 56.16) | 57.85 (57.43, 58.27) | 0.10 (-0.02, 0.22) | 0.088 | 449.17 (447.99, 450.36) | 467.22 (466.04, 468.40) | 0.11 (-0.02, 0.23) | 0.089 | 15.35 (15.13, 15.58) | 8.94 (8.77, 9.10) | -1.83 (-2.44, -1.22) | <0.001 | 0.26 (0.23, 0.29) | 0.15 (0.13, 0.17) | -1.88 (-2.68, -1.08) | <0.001 |

| | | | | | | | | | | | | | | | | |
|---|---|---|---|---|---|---|---|---|---|---|---|---|---|---|---|---|
| Central Asia | 9.63 (9.27, 10.01) | 11.16 (10.83, 11.49) | 0.51 (0.48, 0.54) | <0.001 | 75.66 (74.61, 76.72) | 86.81 (85.89, 87.74) | 0.48 (0.44, 0.51) | <0.001 | 4.08 (3.85, 4.33) | 6.52 (6.26, 6.78) | 1.62 (0.99, 2.26) | <0.001 | 0.07 (0.04, 0.10) | 0.11 (0.08, 0.14) | 1.41 (0.16, 2.68) | 0.027 |
| Eastern Europe | 17.09 (16.82, 17.36) | 23.58 (23.23, 23.93) | 1.12 (1.09, 1.14) | <0.001 | 134.45 (133.71, 135.18) | 186.08 (185.15, 187.01) | 1.13 (1.09, 1.16) | <0.001 | 3.95 (3.82, 4.09) | 8.33 (8.11, 8.54) | 2.79 (1.61, 3.99) | <0.001 | 0.06 (0.05, 0.08) | 0.14 (0.11, 0.17) | 3.11 (1.53, 4.71) | <0.001 |
| Southern Sub-Saharan Africa | 1.25 (1.09, 1.41) | 2.67 (2.50, 2.85) | 2.64 (2.55, 2.74) | <0.001 | 5.48 (5.13, 5.84) | 15.41 (15.00, 15.83) | 3.66 (3.42, 3.91) | <0.001 | 29.42 (28.68, 30.19) | 15.52 (15.10, 15.95) | -2.15 (-3.01, -1.28) | <0.001 | 0.50 (0.41, 0.61) | 0.26 (0.21, 0.32) | -2.19 (-3.18, -1.20) | <0.001 |
| High-income Asia Pacific | 43.38 (42.89, 43.88) | 48.78 (48.21, 49.36) | 0.41 (0.37, 0.46) | <0.001 | 350.24 (348.84, 351.65) | 393.38 (391.79, 394.97) | 0.41 (0.34, 0.48) | <0.001 | 8.16 (7.95, 8.38) | 3.94 (3.77, 4.11) | -2.47 (-2.72, -2.23) | <0.001 | 0.13 (0.11, 0.16) | 0.06 (0.04, 0.09) | -2.76 (-3.07, -2.43) | <0.001 |
| Central Europe | 14.63 (14.29, 14.98) | 21.93 (21.46, 22.40) | 1.40 (1.36, 1.45) | <0.001 | 114.12 (113.18, 115.06) | 173.38 (172.12, 174.65) | 1.45 (1.41, 1.50) | <0.001 | 9.27 (9.00, 9.55) | 10.14 (9.81, 10.47) | 0.33 (0.17, 0.50) | <0.001 | 0.16 (0.12, 0.20) | 0.17 (0.13, 0.22) | 0.24 (-0.14, 0.62) | 0.211 |
| Andean Latin America | 0.71 (0.58, 0.87) | 1.53 (1.38, 1.69) | 2.71 (2.52, 2.89) | <0.001 | 1.62 (1.40, 1.86) | 6.36 (6.05, 6.68) | 4.91 (3.58, 6.26) | <0.001 | 12.62 (12.06, 13.20) | 9.33 (8.96, 9.71) | -1.04 (-1.52, -0.56) | <0.001 | 0.21 (0.14, 0.30) | 0.15 (0.11, 0.21) | -1.10 (-1.42, -0.78) | <0.001 |
| Caribbean | 1.40 (1.20, 1.61) | 1.91 (1.71, 2.12) | 1.04 (0.97, 1.12) | <0.001 | 7.05 (6.59, 7.53) | 10.20 (9.73, 10.67) | 1.24 (1.00, 1.48) | <0.001 | 20.80 (20.05, 21.57) | 17.51 (16.91, 18.13) | -0.57 (-0.73, -0.42) | <0.001 | 0.35 (0.26, 0.46) | 0.29 (0.22, 0.38) | -0.59 (-0.79, -0.38) | <0.001 |
| Southern Latin America | 2.78 (2.55, 3.04) | 4.64 (4.39, 4.91) | 1.78 (1.74, 1.82) | <0.001 | 19.31 (18.68, 19.96) | 33.61 (32.92, 34.32) | 1.97 (1.92, 2.02) | <0.001 | 11.67 (11.19, 12.17) | 8.98 (8.62, 9.35) | -0.89 (-1.17, -0.60) | <0.001 | 0.20 (0.14, 0.27) | 0.15 (0.11, 0.21) | -0.86 (-1.22, -0.49) | <0.001 |
| Oceania | 2.10 (1.55, 2.78) | 2.13 (1.75, 2.56) | 0.03 (-0.07, 0.13) | 0.531 | 13.70 (12.19, 15.35) | 13.67 (12.66, 14.74) | -0.04 (-0.21, 0.13) | 0.636 | 17.63 (16.05, 19.34) | 18.13 (17.01, 19.30) | 0.10 (-0.20, 0.40) | 0.531 | 0.29 (0.12, 0.60) | 0.29 (0.17, 0.48) | 0.06 (-0.46, 0.59) | 0.814 |
| Australasia | 4.40 (3.96, 4.87) | 11.58 (10.93, 12.26) | 3.39 (3.29, 3.49) | <0.001 | 31.53 (30.35, 32.75) | 89.92 (88.13, 91.73) | 3.68 (3.34, 4.01) | <0.001 | 10.44 (9.75, 11.16) | 6.88 (6.38, 7.41) | -1.44 (-1.72, -1.15) | <0.001 | 0.18 (0.10, 0.29) | 0.12 (0.06, 0.21) | -1.29 (-2.02, -0.56) | 0.001 |
| **Women** | | | | | | | | | | | | | | | | |
| Global | 9.01 (8.96, 9.07) | 6.91 (6.87, | -0.92 (-0.97, | <0.001 | 73.11 (72.94, 73.28) | 54.20 (54.09, | -1.03 (-1.12, | <0.001 | 10.22 (10.16, 10.28) | 8.33 (8.29, 8.38) | -0.71 (-0.87, | <0.001 | 0.17 (0.16, 0.18) | 0.14 (0.13, 0.14) | -0.72 (-0.98, | <0.001 |

| | | | | | | | | | | | | | | | | |
|---|---|---|---|---|---|---|---|---|---|---|---|---|---|---|---|---|
| | | 6.95) | -0.86) | | | 54.32) | -0.94) | | | | -0.55) | | | | -0.47) | |
| **SDI category** | | | | | | | | | | | | | | | | |
| High | 36.53 (36.24, 36.83) | 37.94 (37.65, 38.24) | 0.10 (0.00, 0.21) | 0.044 | 295.91 (295.09, 296.73) | 302.74 (301.94, 303.55) | 0.05 (-0.06, 0.17) | 0.364 | 8.61 (8.46, 8.75) | 6.58 (6.45, 6.70) | -0.92 (-1.15, -0.69) | <0.001 | 0.14 (0.12, 0.16) | 0.11 (0.09, 0.12) | -0.83 (-1.11, -0.55) | <0.001 |
| High-middle | 9.57 (9.45, 9.70) | 8.60 (8.49, 8.71) | -0.37 (-0.47, -0.26) | <0.001 | 75.75 (75.40, 76.10) | 66.13 (65.84, 66.43) | -0.46 (-0.57, -0.35) | <0.001 | 7.86 (7.75, 7.97) | 5.32 (5.23, 5.41) | -1.34 (-1.66, -1.03) | <0.001 | 0.13 (0.12, 0.15) | 0.09 (0.08, 0.10) | -1.26 (-1.90, -0.61) | <0.001 |
| Middle | 1.96 (1.91, 2.00) | 1.86 (1.82, 1.90) | -0.21 (-0.26, -0.15) | <0.001 | 13.29 (13.16, 13.41) | 11.55 (11.45, 11.64) | -0.49 (-0.64, -0.34) | <0.001 | 10.19 (10.09, 10.30) | 7.29 (7.21, 7.36) | -1.13 (-1.26, -1.00) | <0.001 | 0.17 (0.16, 0.18) | 0.12 (0.11, 0.13) | -1.19 (-1.49, -0.89) | <0.001 |
| Low-middle | 0.93 (0.89, 0.97) | 0.93 (0.90, 0.97) | -0.01 (-0.18, 0.16) | 0.903 | 4.45 (4.35, 4.55) | 4.20 (4.14, 4.27) | -0.43 (-0.69, -0.17) | 0.001 | 11.33 (11.19, 11.48) | 9.86 (9.76, 9.97) | -0.48 (-0.72, -0.25) | <0.001 | 0.19 (0.17, 0.21) | 0.16 (0.15, 0.18) | -0.62 (-0.98, -0.26) | 0.001 |
| Low | 0.59 (0.54, 0.64) | 0.63 (0.59, 0.66) | 0.20 (0.03, 0.38) | 0.020 | 1.31 (1.23, 1.40) | 1.50 (1.45, 1.56) | 0.61 (0.08, 1.14) | 0.024 | 15.75 (15.49, 16.00) | 12.35 (12.20, 12.50) | -0.83 (-0.92, -0.74) | <0.001 | 0.26 (0.23, 0.30) | 0.20 (0.19, 0.22) | -0.97 (-1.15, -0.78) | <0.001 |
| **21 GBD region** | | | | | | | | | | | | | | | | |
| South Asia | 0.41 (0.38, 0.44) | 0.51 (0.49, 0.53) | 0.74 (0.49, 0.99) | <0.001 | — | 0.71 (0.68, 0.74) | — | — | 10.14 (10.00, 10.28) | 8.46 (8.37, 8.56) | -0.66 (-1.11, -0.21) | 0.004 | 0.17 (0.15, 0.19) | 0.14 (0.13, 0.15) | -0.60 (-1.07, -0.12) | 0.014 |
| East Asia | 3.79 (3.71, 3.86) | 4.56 (4.48, 4.64) | 0.63 (0.55, 0.71) | <0.001 | 28.74 (28.53, 28.95) | 33.11 (32.91, 33.32) | 0.47 (0.34, 0.60) | <0.001 | 4.40 (4.32, 4.48) | 2.04 (1.99, 2.10) | -2.63 (-2.98, -2.29) | <0.001 | 0.07 (0.06, 0.08) | 0.03 (0.03, 0.04) | -2.95 (-3.93, -1.96) | <0.001 |
| North Africa and Middle East | 2.48 (2.35, 2.61) | 2.65 (2.57, 2.75) | 0.23 (0.15, 0.31) | <0.001 | 17.68 (17.33, 18.04) | 18.81 (18.57, 19.05) | 0.21 (0.07, 0.35) | 0.004 | 31.23 (30.79, 31.67) | 19.59 (19.34, 19.84) | -1.57 (-1.67, -1.47) | <0.001 | 0.52 (0.47, 0.59) | 0.33 (0.30, 0.36) | -1.57 (-1.70, -1.43) | <0.001 |
| Western Sub-Saharan Africa | 0.59 (0.51, 0.68) | 0.63 (0.58, 0.69) | 0.23 (0.08, 0.38) | 0.002 | 1.76 (1.60, 1.93) | 2.08 (1.97, 2.19) | 0.53 (0.36, 0.70) | <0.001 | 17.35 (16.92, 17.79) | 15.74 (15.49, 16.00) | -0.32 (-0.41, -0.24) | <0.001 | 0.28 (0.23, 0.34) | 0.25 (0.22, 0.29) | -0.37 (-0.58, -0.16) | 0.001 |
| Southeast Asia | 1.13 (1.06, | 1.32 | 0.52 | <0.001 | 6.63 (6.45, | 8.08 (7.93, | 0.65 | <0.001 | 4.40 (4.26, | 3.25 (3.15, | -1.03 | <0.001 | 0.07 (0.06, | 0.05 (0.04, | -0.99 | <0.001 |

| | | | | | | | | | | | | | | | | |
|---|---|---|---|---|---|---|---|---|---|---|---|---|---|---|---|---|
| | 1.20) | (1.26, 1.39) | (0.46, 0.58) | | 6.80) | 8.23) | (0.47, 0.83) | | 4.53) | 3.34) | (-1.16, -0.90) | | 0.09) | 0.07) | (-0.99, -0.99) | |
| Eastern Sub-Saharan Africa | 0.57 (0.49, 0.66) | 0.52 (0.47, 0.58) | -0.34 (-0.55, -0.13) | 0.002 | 0.98 (0.87, 1.11) | 0.87 (0.80, 0.94) | -0.22 (-1.43, 1.00) | 0.723 | 19.44 (18.97, 19.91) | 10.81 (10.58, 11.04) | -2.01 (-2.17, -1.84) | <0.001 | 0.32 (0.27, 0.39) | 0.18 (0.15, 0.21) | -2.03 (-2.38, -1.68) | <0.001 |
| Central Latin America | 0.88 (0.78, 0.99) | 1.11 (1.02, 1.20) | 0.80 (0.70, 0.90) | <0.001 | 3.62 (3.40, 3.85) | 3.77 (3.60, 3.94) | 0.16 (-0.02, 0.33) | 0.077 | 10.55 (10.20, 10.91) | 8.21 (7.97, 8.47) | -0.83 (-1.36, -0.30) | 0.002 | 0.18 (0.14, 0.23) | 0.14 (0.11, 0.18) | -0.87 (-1.70, -0.04) | 0.041 |
| Tropical Latin America | 1.93 (1.77, 2.09) | 1.81 (1.69, 1.94) | -0.26 (-0.38, -0.14) | <0.001 | 12.26 (11.86, 12.67) | 8.91 (8.65, 9.18) | -1.17 (-1.42, -0.93) | <0.001 | 30.01 (29.41, 30.63) | 13.64 (13.31, 13.98) | -2.73 (-2.84, -2.63) | <0.001 | 0.51 (0.43, 0.60) | 0.23 (0.19, 0.28) | -2.76 (-2.87, -2.64) | <0.001 |
| Central Sub-Saharan Africa | 0.57 (0.42, 0.75) | 0.52 (0.43, 0.63) | -0.34 (-0.48, -0.21) | <0.001 | 0.66 (0.49, 0.86) | 0.73 (0.61, 0.85) | 0.42 (0.19, 0.65) | <0.001 | 13.08 (12.38, 13.82) | 9.21 (8.83, 9.60) | -1.24 (-1.36, -1.11) | <0.001 | 0.22 (0.13, 0.34) | 0.15 (0.11, 0.21) | -1.32 (-1.41, -1.23) | <0.001 |
| Western Europe | 10.38 (10.15, 10.62) | 11.20 (10.95, 11.46) | 0.26 (0.17, 0.36) | <0.001 | 79.77 (79.13, 80.42) | 86.85 (86.17, 87.53) | 0.29 (0.20, 0.38) | <0.001 | 7.92 (7.71, 8.12) | 5.39 (5.21, 5.57) | -1.32 (-1.55, -1.10) | <0.001 | 0.13 (0.11, 0.16) | 0.09 (0.07, 0.12) | -1.26 (-1.68, -0.85) | <0.001 |
| High-income North America | 70.12 (69.45, 70.79) | 72.11 (71.45, 72.77) | 0.06 (-0.08, 0.20) | 0.388 | 566.86 (564.98, 568.74) | 583.45 (581.58, 585.31) | 0.06 (-0.09, 0.21) | 0.419 | 10.99 (10.73, 11.26) | 7.81 (7.59, 8.03) | -1.15 (-1.58, -0.72) | <0.001 | 0.18 (0.15, 0.22) | 0.12 (0.10, 0.16) | -1.35 (-2.04, -0.66) | <0.001 |
| Central Asia | 7.77 (7.31, 8.25) | 8.61 (8.20, 9.03) | 0.35 (0.33, 0.38) | <0.001 | 61.02 (59.70, 62.37) | 66.25 (65.11, 67.39) | 0.29 (0.22, 0.36) | <0.001 | 3.84 (3.52, 4.18) | 5.87 (5.52, 6.23) | 1.46 (0.84, 2.09) | <0.001 | 0.06 (0.03, 0.12) | 0.09 (0.06, 0.15) | 1.46 (0.45, 2.47) | 0.004 |
| Eastern Europe | 17.12 (16.74, 17.50) | 18.22 (17.79, 18.66) | 0.21 (0.18, 0.24) | <0.001 | 135.41 (134.37, 136.46) | 142.55 (141.40, 143.70) | 0.17 (0.14, 0.21) | <0.001 | 2.59 (2.44, 2.75) | 5.02 (4.78, 5.27) | 2.39 (1.50, 3.29) | <0.001 | 0.04 (0.02, 0.06) | 0.08 (0.05, 0.12) | 2.69 (2.15, 3.24) | <0.001 |
| Southern Sub-Saharan Africa | 1.06 (0.87, 1.29) | 0.90 (0.76, 1.06) | -0.58 (-0.67, -0.50) | <0.001 | 5.45 (4.98, 5.96) | 4.74 (4.42, 5.08) | -0.48 (-0.67, -0.29) | <0.001 | 30.87 (29.82, 31.96) | 13.31 (12.77, 13.87) | -2.75 (-4.01, -1.48) | <0.001 | 0.52 (0.39, 0.68) | 0.22 (0.16, 0.31) | -2.93 (-4.15, -1.70) | <0.001 |
| High-income Asia Pacific | 44.13 (43.42, 44.85) | 46.54 (45.74, 47.36) | 0.18 (0.14, 0.22) | <0.001 | 357.61 (355.59, 359.64) | 376.63 (374.40, 378.88) | 0.18 (0.14, 0.22) | <0.001 | 6.05 (5.79, 6.32) | 2.96 (2.75, 3.17) | -2.45 (-2.73, -2.17) | <0.001 | 0.10 (0.07, 0.14) | 0.04 (0.02, 0.08) | -2.74 (-3.22, -2.25) | <0.001 |
| Central Europe | 15.29 (14.79, | 20.65 | 1.03 | <0.001 | 119.27 | 163.31 | 1.09 | <0.001 | 6.81 (6.48, | 7.03 (6.64, | 0.11 | 0.198 | 0.11 (0.07, | 0.12 (0.07, | 0.22 | 0.008 |

| | | | | | | | | | | | | | | | | |
|---|---|---|---|---|---|---|---|---|---|---|---|---|---|---|---|---|
| | 15.79) | (20.01, 21.31) | (0.98, 1.09) | | (117.91, 120.65) | (161.56, 165.07) | (1.01, 1.17) | | 7.15) | 7.43) | (-0.06, 0.27) | | 0.17) | 0.18) | (0.06, 0.38) | |
| Andean Latin America | 0.81 (0.62, 1.06) | 1.10 (0.93, 1.30) | 1.06 (0.93, 1.19) | <0.001 | 3.17 (2.74, 3.64) | 3.26 (2.96, 3.59) | 0.27 (-1.18, 1.74) | 0.718 | 13.32 (12.52, 14.17) | 8.02 (7.54, 8.53) | -1.80 (-2.62, -0.97) | <0.001 | 0.22 (0.13, 0.36) | 0.13 (0.08, 0.21) | -2.04 (-2.35, -1.73) | <0.001 |
| Caribbean | 1.16 (0.92, 1.44) | 1.30 (1.08, 1.55) | 0.38 (0.29, 0.46) | <0.001 | 6.27 (5.67, 6.92) | 6.26 (5.76, 6.80) | -0.03 (-0.30, 0.23) | 0.801 | 16.36 (15.45, 17.32) | 15.22 (14.43, 16.05) | -0.22 (-0.40, -0.03) | 0.025 | 0.27 (0.16, 0.42) | 0.25 (0.16, 0.38) | -0.16 (-0.42, 0.10) | 0.224 |
| Southern Latin America | 3.00 (2.66, 3.37) | 4.03 (3.69, 4.39) | 1.00 (0.95, 1.05) | <0.001 | 21.45 (20.52, 22.41) | 27.77 (26.88, 28.67) | 0.88 (0.63, 1.13) | <0.001 | 9.33 (8.73, 9.96) | 6.42 (5.99, 6.87) | -1.25 (-1.57, -0.93) | <0.001 | 0.16 (0.09, 0.26) | 0.11 (0.06, 0.18) | -1.17 (-1.41, -0.93) | <0.001 |
| Oceania | 2.67 (1.81, 3.82) | 2.68 (2.08, 3.39) | -0.05 (-0.22, 0.12) | 0.546 | 19.08 (16.56, 21.89) | 19.04 (17.37, 20.83) | -0.06 (-0.19, 0.07) | 0.359 | 15.27 (13.16, 17.64) | 17.17 (15.63, 18.82) | 0.44 (0.37, 0.50) | <0.001 | 0.25 (0.05, 0.77) | 0.28 (0.12, 0.58) | 0.39 (-0.17, 0.94) | 0.173 |
| Australasia | 4.70 (4.07, 5.41) | 10.13 (9.28, 11.04) | 2.68 (2.63, 2.74) | <0.001 | 34.23 (32.50, 36.03) | 78.70 (76.37, 81.10) | 2.88 (2.71, 3.04) | <0.001 | 7.56 (6.75, 8.45) | 4.99 (4.40, 5.65) | -1.42 (-1.82, -1.02) | <0.001 | 0.13 (0.04, 0.30) | 0.08 (0.02, 0.22) | -1.41 (-2.29, -0.52) | 0.002 |
| **Men** | | | | | | | | | | | | | | | | |
| Global | 6.71 (6.66, 6.76) | 6.01 (5.97, 6.05) | -0.37 (-0.43, -0.31) | <0.001 | 52.43 (52.29, 52.57) | 45.92 (45.82, 46.03) | -0.45 (-0.52, -0.38) | <0.001 | 11.40 (11.33, 11.46) | 9.36 (9.31, 9.41) | -0.66 (-0.78, -0.55) | <0.001 | 0.19 (0.18, 0.20) | 0.16 (0.15, 0.16) | -0.66 (-1.05, -0.26) | 0.001 |
| **SDI category** | | | | | | | | | | | | | | | | |
| High | 25.01 (24.77, 25.25) | 26.85 (26.62, 27.09) | 0.23 (0.19, 0.28) | <0.001 | 199.43 (198.77, 200.10) | 212.09 (211.44, 212.74) | 0.20 (0.16, 0.24) | <0.001 | 15.25 (15.06, 15.43) | 9.15 (9.01, 9.29) | -1.75 (-2.00, -1.51) | <0.001 | 0.26 (0.24, 0.28) | 0.15 (0.14, 0.17) | -1.91 (-2.53, -1.28) | <0.001 |
| High-middle | 7.75 (7.65, 7.87) | 8.90 (8.80, 9.01) | 0.49 (0.43, 0.54) | <0.001 | 60.20 (59.90, 60.51) | 68.56 (68.27, 68.86) | 0.46 (0.39, 0.54) | <0.001 | 11.60 (11.47, 11.74) | 8.36 (8.26, 8.47) | -1.16 (-1.47, -0.84) | <0.001 | 0.19 (0.18, 0.21) | 0.14 (0.13, 0.16) | -1.03 (-1.35, -0.70) | <0.001 |
| Middle | 1.77 (1.73, 1.82) | 2.02 (1.98, 2.06) | 0.47 (0.35, 0.60) | <0.001 | 11.01 (10.90, 11.13) | 11.67 (11.57, 11.76) | 0.20 (0.14, 0.26) | <0.001 | 10.31 (10.21, 10.41) | 8.87 (8.78, 8.95) | -0.50 (-0.56, -0.44) | <0.001 | 0.17 (0.16, 0.19) | 0.15 (0.14, 0.16) | -0.43 (-0.43, -0.43) | <0.001 |
| Low-middle | 0.95 (0.91, 0.99) | 1.06 (1.03, | 0.38 (0.26, | <0.001 | 3.52 (3.44, 3.61) | 4.62 (4.55, 4.69) | 0.90 (0.54, | <0.001 | 9.32 (9.19, 9.46) | 9.67 (9.56, 9.77) | 0.18 (-0.10, | 0.206 | 0.16 (0.14, 0.18) | 0.16 (0.15, 0.18) | -0.00 (-0.52, | 0.991 |

| | | | | | | | | | | | | | | | | |
|---|---|---|---|---|---|---|---|---|---|---|---|---|---|---|---|---|
| | | 1.10) | 0.50) | | | | 1.26) | | | | 0.45) | | | | 0.52) | |
| Low | 0.72 (0.67, 0.78) | 0.80 (0.76, 0.84) | 0.31 (0.14, 0.47) | <0.001 | 1.52 (1.44, 1.61) | 2.54 (2.46, 2.61) | 1.72 (0.80, 2.65) | <0.001 | 12.02 (11.79, 12.25) | 11.20 (11.05, 11.34) | -0.23 (-0.45, -0.02) | 0.030 | 0.20 (0.17, 0.23) | 0.19 (0.17, 0.21) | -0.14 (-0.34, 0.05) | 0.155 |
| **21 GBD region** | | | | | | | | | | | | | | | | |
| South Asia | 0.56 (0.53, 0.59) | 0.72 (0.69, 0.75) | 0.87 (0.70, 1.03) | <0.001 | — | 1.99 (1.94, 2.03) | — | — | 7.73 (7.61, 7.85) | 8.44 (8.35, 8.53) | 0.34 (-0.04, 0.73) | 0.079 | 0.13 (0.12, 0.15) | 0.14 (0.13, 0.16) | 0.32 (-0.42, 1.07) | 0.396 |
| East Asia | 2.85 (2.79, 2.91) | 3.55 (3.48, 3.62) | 0.75 (0.59, 0.91) | <0.001 | 21.07 (20.89, 21.24) | 24.48 (24.30, 24.66) | 0.51 (0.38, 0.65) | <0.001 | 6.04 (5.95, 6.13) | 3.98 (3.90, 4.05) | -1.47 (-1.91, -1.02) | <0.001 | 0.10 (0.09, 0.11) | 0.07 (0.06, 0.08) | -1.16 (-1.72, -0.60) | <0.001 |
| North Africa and Middle East | 1.88 (1.78, 2.00) | 2.22 (2.14, 2.30) | 0.58 (0.48, 0.68) | <0.001 | 10.97 (10.69, 11.24) | 13.23 (13.04, 13.42) | 0.63 (0.41, 0.85) | <0.001 | 27.13 (26.73, 27.52) | 19.32 (19.09, 19.56) | -1.13 (-1.29, -0.97) | <0.001 | 0.45 (0.40, 0.50) | 0.32 (0.29, 0.35) | -1.22 (-1.56, -0.88) | <0.001 |
| Western Sub-Saharan Africa | 0.96 (0.85, 1.08) | 1.07 (0.99, 1.14) | 0.36 (0.26, 0.46) | <0.001 | 3.28 (3.07, 3.51) | 5.04 (4.88, 5.22) | 1.38 (0.97, 1.80) | <0.001 | 8.72 (8.40, 9.05) | 8.93 (8.72, 9.15) | 0.10 (-0.03, 0.23) | 0.129 | 0.15 (0.11, 0.20) | 0.15 (0.12, 0.18) | 0.00 (-0.27, 0.27) | 0.999 |
| Southeast Asia | 0.98 (0.92, 1.05) | 1.17 (1.11, 1.23) | 0.62 (0.56, 0.67) | <0.001 | 5.57 (5.41, 5.74) | 6.87 (6.74, 7.01) | 0.75 (0.63, 0.87) | <0.001 | 4.40 (4.27, 4.54) | 4.49 (4.38, 4.60) | 0.08 (-0.14, 0.30) | 0.469 | 0.08 (0.06, 0.10) | 0.08 (0.06, 0.09) | 0.44 (-0.35, 1.24) | 0.276 |
| Eastern Sub-Saharan Africa | 0.69 (0.59, 0.79) | 0.69 (0.63, 0.75) | -0.02 (-0.20, 0.16) | 0.820 | 1.36 (1.22, 1.51) | 1.47 (1.37, 1.57) | 0.19 (-0.27, 0.64) | 0.418 | 18.23 (17.75, 18.71) | 13.59 (13.32, 13.85) | -1.01 (-1.20, -0.82) | <0.001 | 0.31 (0.25, 0.38) | 0.23 (0.19, 0.26) | -1.07 (-1.41, -0.74) | <0.001 |
| Central Latin America | 0.70 (0.60, 0.80) | 1.51 (1.40, 1.62) | 2.73 (2.60, 2.86) | <0.001 | 0.03 (0.01, 0.06) | 4.98 (4.78, 5.18) | 8.59 (5.81, 11.45) | <0.001 | 13.44 (13.03, 13.86) | 10.58 (10.30, 10.87) | -0.84 (-1.13, -0.55) | <0.001 | 0.23 (0.18, 0.29) | 0.18 (0.14, 0.22) | -0.79 (-1.03, -0.55) | <0.001 |
| Tropical Latin America | 1.35 (1.22, 1.49) | 1.48 (1.37, 1.59) | 0.30 (0.11, 0.48) | 0.001 | 2.74 (2.54, 2.94) | 4.26 (4.08, 4.45) | 1.26 (0.89, 1.62) | <0.001 | 37.43 (36.74, 38.13) | 16.87 (16.49, 17.25) | -2.68 (-3.10, -2.26) | <0.001 | 0.63 (0.55, 0.73) | 0.28 (0.24, 0.34) | -2.76 (-3.27, -2.25) | <0.001 |
| Central Sub-Saharan Africa | 0.68 (0.52, 0.88) | 0.65 (0.55, 0.77) | -0.21 (-0.47, 0.05) | 0.112 | 2.00 (1.69, 2.34) | 0.74 (0.62, 0.87) | -3.04 (-4.18, -1.89) | <0.001 | 12.56 (11.86, 13.30) | 10.40 (9.99, 10.82) | -0.70 (-1.00, -0.41) | <0.001 | 0.21 (0.13, 0.33) | 0.18 (0.13, 0.24) | -0.61 (-0.80, -0.42) | <0.001 |
| Western Europe | 6.84 (6.65, | 8.55 | 0.77 | <0.001 | 51.17 (50.66, | 64.95 | 0.82 | <0.001 | 17.28 (16.98, | 10.11 (9.88, | -1.90 | <0.001 | 0.29 (0.26, | 0.17 (0.14, | -2.00 | <0.001 |

| | | | | | | | | | | | | | | | | |
|---|---|---|---|---|---|---|---|---|---|---|---|---|---|---|---|---|
| | 7.03) | (8.33, 8.76) | (0.65, 0.88) | | 51.69) | (64.37, 65.53) | (0.66, 0.97) | | 17.59) | 10.35) | (-2.14, -1.65) | | 0.33) | 0.21) | (-2.29, -1.71) | |
| High-income North America | 41.38 (40.87, 41.90) | 43.71 (43.20, 44.23) | 0.15 (0.05, 0.25) | 0.004 | 331.00 (329.56, 332.44) | 351.16 (349.71, 352.60) | 0.16 (0.06, 0.26) | 0.002 | 19.71 (19.36, 20.07) | 10.05 (9.80, 10.30) | -2.30 (-3.06, -1.53) | <0.001 | 0.34 (0.29, 0.39) | 0.17 (0.14, 0.20) | -2.41 (-3.21, -1.61) | <0.001 |
| Central Asia | 11.54 (10.97, 12.13) | 13.68 (13.16, 14.20) | 0.59 (0.57, 0.61) | <0.001 | 90.69 (89.05, 92.35) | 107.28 (105.84, 108.74) | 0.59 (0.53, 0.64) | <0.001 | 4.35 (4.00, 4.71) | 7.17 (6.80, 7.56) | 1.71 (0.88, 2.55) | <0.001 | 0.07 (0.03, 0.13) | 0.12 (0.07, 0.18) | 1.43 (1.03, 1.83) | <0.001 |
| Eastern Europe | 17.06 (16.68, 17.44) | 28.89 (28.35, 29.44) | 1.84 (1.80, 1.89) | <0.001 | 133.50 (132.46, 134.54) | 229.56 (228.11, 231.03) | 1.89 (1.83, 1.96) | <0.001 | 5.30 (5.09, 5.52) | 11.60 (11.25, 11.96) | 2.84 (1.42, 4.28) | <0.001 | 0.09 (0.06, 0.12) | 0.19 (0.15, 0.25) | 2.86 (1.17, 4.59) | 0.001 |
| Southern Sub-Saharan Africa | 1.44 (1.21, 1.71) | 4.45 (4.14, 4.78) | 3.95 (3.72, 4.18) | <0.001 | 5.50 (5.01, 6.03) | 26.17 (25.41, 26.95) | 5.56 (5.13, 5.99) | <0.001 | 27.82 (26.77, 28.91) | 17.73 (17.10, 18.38) | -1.61 (-2.08, -1.14) | <0.001 | 0.48 (0.35, 0.64) | 0.30 (0.22, 0.39) | -1.58 (-2.28, -0.87) | <0.001 |
| High-income Asia Pacific | 42.66 (41.97, 43.36) | 50.87 (50.06, 51.70) | 0.62 (0.57, 0.66) | <0.001 | 343.08 (341.13, 345.04) | 409.06 (406.81, 411.32) | 0.63 (0.56, 0.71) | <0.001 | 10.21 (9.88, 10.56) | 4.85 (4.60, 5.12) | -2.52 (-2.81, -2.23) | <0.001 | 0.17 (0.13, 0.22) | 0.08 (0.05, 0.12) | -2.79 (-3.09, -2.50) | <0.001 |
| Central Europe | 13.99 (13.53, 14.47) | 23.15 (22.49, 23.83) | 1.76 (1.72, 1.80) | <0.001 | 109.08 (107.79, 110.38) | 183.04 (181.23, 184.87) | 1.82 (1.78, 1.85) | <0.001 | 11.66 (11.24, 12.10) | 13.11 (12.61, 13.64) | 0.36 (0.24, 0.48) | <0.001 | 0.20 (0.15, 0.26) | 0.22 (0.16, 0.30) | 0.36 (0.02, 0.70) | 0.039 |
| Andean Latin America | 0.61 (0.44, 0.83) | 1.97 (1.73, 2.23) | 4.22 (3.89, 4.56) | <0.001 | — | 9.54 (9.00, 10.10) | — | — | 11.89 (11.11, 12.70) | 10.61 (10.06, 11.19) | -0.36 (-0.68, -0.04) | 0.029 | 0.19 (0.11, 0.33) | 0.17 (0.11, 0.26) | -0.39 (-0.72, -0.05) | 0.027 |
| Caribbean | 1.65 (1.35, 1.99) | 2.54 (2.22, 2.89) | 1.47 (1.40, 1.54) | <0.001 | 7.88 (7.18, 8.62) | 14.28 (13.50, 15.09) | 2.08 (1.72, 2.44) | <0.001 | 25.51 (24.32, 26.74) | 19.90 (18.99, 20.84) | -0.83 (-0.97, -0.70) | <0.001 | 0.43 (0.29, 0.62) | 0.34 (0.23, 0.48) | -0.80 (-1.03, -0.57) | <0.001 |
| Southern Latin America | 2.56 (2.25, 2.91) | 5.27 (4.88, 5.68) | 2.51 (2.44, 2.58) | <0.001 | 17.10 (16.26, 17.98) | 39.59 (38.52, 40.69) | 3.02 (2.83, 3.21) | <0.001 | 14.08 (13.33, 14.87) | 11.59 (11.01, 12.19) | -0.64 (-0.89, -0.38) | <0.001 | 0.24 (0.15, 0.37) | 0.20 (0.13, 0.29) | -0.65 (-0.97, -0.33) | <0.001 |
| Oceania | 1.55 (0.93, 2.45) | 1.58 (1.13, 2.15) | 0.02 (-0.12, 0.16) | 0.798 | 8.56 (6.91, 10.49) | 8.30 (7.19, 9.52) | -0.16 (-0.40, 0.07) | 0.177 | 19.84 (17.53, 22.39) | 18.91 (17.33, 20.60) | -0.18 (-0.47, 0.11) | 0.232 | 0.32 (0.09, 0.84) | 0.30 (0.13, 0.60) | -0.22 (-0.43, -0.01) | 0.040 |
| Australasia | 4.09 (3.50, | 13.05 | 4.10 | <0.001 | 28.81 (27.21, | 101.42 | 4.39 | <0.001 | 13.31 (12.22, | 8.79 (7.99, | -1.38 | <0.001 | 0.23 (0.11, | 0.15 (0.06, | -1.46 | <0.001 |

| | | | | | | | | | | | | | | | | |
|---|---|---|---|---|---|---|---|---|---|---|---|---|---|---|---|---|
| | 4.75) | (12.08, 14.09) | (4.01, 4.20) | | 30.47) | (98.73, 104.18) | (4.04, 4.75) | | 14.47) | 9.65) | (-1.78, -0.97) | | 0.43) | 0.31) | (-2.17, -0.75) | |
| **Cardiomyopathy and myocarditis** | | | | | | | | | | | | | | | | |
| **Overall** | | | | | | | | | | | | | | | | |
| Global | 11.79 (11.74, 11.83) | 11.12 (11.09, 11.16) | -0.20 (-0.22, -0.19) | <0.001 | 6.19 (6.16, 6.22) | 4.59 (4.56, 4.61) | -0.98 (-1.23, -0.72) | <0.001 | 55.45 (55.35, 55.55) | 53.87 (53.79, 53.96) | -0.11 (-0.68, 0.46) | 0.701 | 0.93 (0.91, 0.94) | 0.91 (0.90, 0.92) | -0.08 (-0.70, 0.55) | 0.812 |
| **SDI category** | | | | | | | | | | | | | | | | |
| High | 13.71 (13.58, 13.84) | 13.24 (13.11, 13.36) | -0.12 (-0.14, -0.10) | <0.001 | 38.06 (37.85, 38.27) | 39.21 (39.00, 39.42) | 0.15 (-0.06, 0.36) | 0.157 | 77.03 (76.73, 77.33) | 54.67 (54.42, 54.92) | -1.18 (-1.31, -1.05) | <0.001 | 1.20 (1.16, 1.24) | 0.82 (0.79, 0.86) | -1.33 (-1.54, -1.13) | <0.001 |
| High-middle | 12.07 (11.97, 12.16) | 11.53 (11.44, 11.63) | -0.15 (-0.17, -0.14) | <0.001 | 0.69 (0.67, 0.71) | 0.49 (0.47, 0.51) | -1.17 (-1.85, -0.48) | 0.001 | 98.56 (98.28, 98.84) | 134.57 (134.27, 134.87) | 1.10 (0.31, 1.90) | 0.006 | 1.70 (1.66, 1.74) | 2.37 (2.33, 2.41) | 1.20 (0.38, 2.02) | 0.004 |
| Middle | 11.60 (11.52, 11.68) | 10.89 (10.82, 10.95) | -0.22 (-0.24, -0.20) | <0.001 | 0.01 (0.01, 0.01) | 0.01 (0.01, 0.01) | — | — | 34.96 (34.82, 35.09) | 30.74 (30.63, 30.85) | -0.43 (-0.50, -0.36) | <0.001 | 0.58 (0.56, 0.59) | 0.50 (0.49, 0.52) | -0.53 (-0.61, -0.44) | <0.001 |
| Low-middle | 10.95 (10.85, 11.05) | 10.74 (10.66, 10.81) | -0.07 (-0.08, -0.06) | <0.001 | — | — | — | — | 25.98 (25.83, 26.14) | 25.82 (25.71, 25.94) | 0.02 (-0.13, 0.16) | 0.834 | 0.43 (0.41, 0.45) | 0.43 (0.42, 0.45) | -0.02 (-0.12, 0.08) | 0.732 |
| Low | 10.49 (10.34, 10.64) | 10.43 (10.33, 10.52) | -0.02 (-0.02, -0.02) | <0.001 | — | — | — | — | 42.70 (42.39, 43.00) | 38.99 (38.80, 39.18) | -0.31 (-0.44, -0.18) | <0.001 | 0.72 (0.68, 0.76) | 0.65 (0.63, 0.68) | -0.35 (-0.46, -0.24) | <0.001 |
| **21 GBD region** | | | | | | | | | | | | | | | | |
| South Asia | 11.05 (10.95, 11.15) | 11.04 (10.97, 11.12) | -0.00 (-0.01, 0.00) | 0.126 | — | — | — | — | 9.21 (9.12, 9.30) | 9.15 (9.08, 9.22) | -0.04 (-0.33, 0.26) | 0.794 | 0.15 (0.14, 0.16) | 0.15 (0.14, 0.16) | 0.04 (-0.47, 0.55) | 0.878 |
| East Asia | 12.82 (12.72, 12.91) | 11.96 (11.86, 12.05) | -0.25 (-0.28, -0.21) | <0.001 | — | — | — | — | 29.16 (29.02, 29.31) | 25.80 (25.66, 25.94) | -0.42 (-0.91, 0.08) | 0.100 | 0.47 (0.45, 0.49) | 0.41 (0.40, 0.43) | -0.43 (-0.86, 0.00) | 0.050 |
| North Africa and Middle East | 8.90 (8.74, 9.06) | 8.92 (8.80, | 0.01 (0.00, | 0.019 | 0.25 (0.22, 0.28) | 0.07 (0.06, 0.08) | -3.65 (-5.36, | <0.001 | 43.40 (43.05, 43.76) | 31.41 (31.20, 31.63) | -1.10 (-1.25, | <0.001 | 0.69 (0.65, 0.74) | 0.49 (0.47, 0.52) | -1.18 (-1.31, | <0.001 |

| | | | | | | | | | | | | | | | | |
|---|---|---|---|---|---|---|---|---|---|---|---|---|---|---|---|---|
| | | 9.03) | 0.02) | | | | -1.90) | | | | -0.94) | | | | -1.05) | |
| Western Sub-Saharan Africa | 10.53 (10.28, 10.78) | 10.47 (10.32, 10.63) | -0.02 (-0.02, -0.02) | <0.001 | — | — | — | — | 72.92 (72.27, 73.58) | 56.31 (55.95, 56.67) | -0.89 (-1.05, -0.72) | <0.001 | 1.21 (1.13, 1.30) | 0.93 (0.88, 0.98) | -0.90 (-1.06, -0.75) | <0.001 |
| Southeast Asia | 10.73 (10.59, 10.88) | 10.82 (10.70, 10.95) | 0.03 (0.03, 0.03) | <0.001 | — | 0.03 (0.02, 0.03) | — | — | 36.51 (36.23, 36.78) | 37.07 (36.84, 37.29) | 0.06 (-0.07, 0.19) | 0.367 | 0.61 (0.58, 0.65) | 0.62 (0.59, 0.65) | 0.13 (-0.07, 0.33) | 0.212 |
| Eastern Sub-Saharan Africa | 10.41 (10.17, 10.66) | 10.44 (10.28, 10.60) | 0.01 (0.01, 0.01) | <0.001 | — | — | — | — | 48.31 (47.76, 48.87) | 44.51 (44.17, 44.86) | -0.29 (-0.44, -0.14) | <0.001 | 0.83 (0.76, 0.91) | 0.76 (0.72, 0.81) | -0.30 (-0.51, -0.09) | 0.004 |
| Central Latin America | 9.72 (9.48, 9.97) | 9.72 (9.53, 9.91) | 0.00 (-0.01, 0.01) | 0.947 | — | — | — | — | 24.00 (23.62, 24.39) | 21.92 (21.63, 22.21) | -0.30 (-0.52, -0.09) | 0.006 | 0.40 (0.35, 0.45) | 0.36 (0.32, 0.40) | -0.28 (-0.67, 0.11) | 0.163 |
| Tropical Latin America | 10.22 (9.97, 10.47) | 10.23 (10.02, 10.44) | 0.00 (0.00, 0.00)* | <0.001 | — | — | — | — | 114.55 (113.70, 115.41) | 82.87 (82.29, 83.47) | -1.13 (-1.37, -0.89) | <0.001 | 1.97 (1.86, 2.09) | 1.41 (1.33, 1.49) | -1.16 (-1.33, -0.99) | <0.001 |
| Central Sub-Saharan Africa | 10.06 (9.62, 10.51) | 10.06 (9.78, 10.35) | 0.00 (0.00, 0.00)* | <0.001 | — | — | — | — | 80.62 (79.34, 81.92) | 77.32 (76.53, 78.12) | -0.20 (-0.32, -0.07) | 0.002 | 1.36 (1.20, 1.54) | 1.31 (1.20, 1.42) | -0.18 (-0.38, 0.01) | 0.065 |
| Western Europe | 12.78 (12.60, 12.97) | 12.80 (12.61, 13.00) | 0.00 (-0.02, 0.02) | 0.873 | 1.34 (1.28, 1.40) | 0.30 (0.27, 0.33) | -4.51 (-6.18, -2.82) | <0.001 | 52.90 (52.53, 53.28) | 29.79 (29.50, 30.08) | -1.98 (-2.22, -1.74) | <0.001 | 0.85 (0.80, 0.90) | 0.47 (0.44, 0.51) | -2.10 (-2.22, -1.99) | <0.001 |
| High-income North America | 14.04 (13.82, 14.26) | 14.00 (13.79, 14.21) | -0.00 (-0.05, 0.05) | 0.910 | 98.79 (98.23, 99.36) | 104.56 (103.99, 105.13) | 0.25 (0.02, 0.48) | 0.037 | 110.14 (109.54, 110.75) | 88.28 (87.76, 88.81) | -0.77 (-0.96, -0.57) | <0.001 | 1.67 (1.60, 1.75) | 1.29 (1.23, 1.35) | -0.91 (-1.16, -0.67) | <0.001 |
| Central Asia | 11.52 (11.12, 11.92) | 11.56 (11.22, 11.91) | 0.01 (0.01, 0.02) | <0.001 | 0.75 (0.64, 0.87) | 0.66 (0.58, 0.75) | -0.47 (-0.72, -0.22) | <0.001 | 104.61 (103.40, 105.84) | 201.60 (200.20, 203.02) | 2.15 (1.28, 3.03) | <0.001 | 1.79 (1.63, 1.96) | 3.53 (3.34, 3.72) | 2.24 (1.42, 3.07) | <0.001 |
| Eastern Europe | 13.50 (13.25, 13.75) | 13.53 (13.25, 13.81) | 0.01 (0.00, 0.01) | <0.001 | 1.47 (1.39, 1.54) | 1.31 (1.24, 1.38) | -0.40 (-0.75, -0.05) | 0.025 | 311.85 (310.71, 312.99) | 704.57 (702.74, 706.40) | 2.90 (1.96, 3.86) | <0.001 | 5.49 (5.34, 5.64) | 12.56 (12.32, 12.81) | 2.95 (2.02, 3.90) | <0.001 |
| Southern Sub-Saharan | 10.78 (10.33, 11.23) | 10.82 (10.47, | 0.02 (0.01, | <0.001 | — | — | — | — | 89.76 (88.45, 91.08) | 69.34 (68.45, 70.23) | -1.00 (-1.58, | 0.001 | 1.52 (1.35, 1.71) | 1.16 (1.04, 1.28) | -0.90 (-1.62, | 0.014 |

| | | | | | | | | | | | | | | | | |
|---|---|---|---|---|---|---|---|---|---|---|---|---|---|---|---|---|
| Africa | | 11.18) | 0.02) | | | | | | | | -0.43) | | | | -0.18) | |
| High-income Asia Pacific | 15.78 (15.48, 16.08) | 15.50 (15.16, 15.85) | -0.06 (-0.09, -0.04) | <0.001 | 12.43 (12.17, 12.70) | 9.86 (9.60, 10.14) | -0.80 (-0.95, -0.65) | <0.001 | 58.78 (58.20, 59.36) | 31.27 (30.80, 31.75) | -2.15 (-2.41, -1.88) | <0.001 | 0.91 (0.84, 0.99) | 0.46 (0.41, 0.53) | -2.33 (-2.54, -2.13) | <0.001 |
| Central Europe | 13.66 (13.32, 14.00) | 13.85 (13.46, 14.24) | 0.05 (0.03, 0.06) | <0.001 | 0.09 (0.07, 0.12) | 0.18 (0.14, 0.23) | 2.68 (1.24, 4.13) | <0.001 | 86.94 (86.11, 87.78) | 68.80 (67.97, 69.63) | -0.80 (-0.93, -0.67) | <0.001 | 1.49 (1.38, 1.60) | 1.18 (1.07, 1.29) | -0.81 (-0.94, -0.68) | <0.001 |
| Andean Latin America | 9.05 (8.57, 9.56) | 9.11 (8.74, 9.49) | 0.02 (0.01, 0.04) | <0.001 | — | — | — | — | 26.08 (25.27, 26.92) | 21.69 (21.12, 22.26) | -0.64 (-0.82, -0.46) | <0.001 | 0.43 (0.33, 0.55) | 0.35 (0.28, 0.43) | -0.67 (-0.98, -0.36) | <0.001 |
| Caribbean | 9.20 (8.71, 9.71) | 9.22 (8.78, 9.67) | 0.01 (0.00, 0.01) | 0.003 | — | — | — | — | 64.79 (63.45, 66.14) | 71.77 (70.54, 73.02) | 0.40 (0.24, 0.56) | <0.001 | 1.09 (0.92, 1.28) | 1.21 (1.06, 1.39) | 0.40 (0.22, 0.57) | <0.001 |
| Southern Latin America | 10.32 (9.87, 10.79) | 10.36 (9.96, 10.76) | 0.01 (0.01, 0.02) | <0.001 | 2.70 (2.48, 2.95) | 1.02 (0.91, 1.16) | -3.33 (-4.45, -2.19) | <0.001 | 70.72 (69.53, 71.94) | 53.66 (52.77, 54.56) | -0.92 (-1.09, -0.75) | <0.001 | 1.17 (1.02, 1.34) | 0.88 (0.77, 1.01) | -0.95 (-1.14, -0.76) | <0.001 |
| Oceania | 10.95 (9.70, 12.33) | 10.93 (10.06, 11.85) | -0.01 (-0.01, -0.00) | 0.001 | — | — | — | — | 51.44 (48.61, 54.40) | 58.40 (56.35, 60.50) | 0.40 (0.25, 0.54) | <0.001 | 0.88 (0.54, 1.35) | 0.99 (0.74, 1.30) | 0.39 (0.23, 0.55) | <0.001 |
| Australasia | 12.18 (11.44, 12.97) | 12.24 (11.55, 12.95) | 0.02 (0.01, 0.03) | 0.002 | 0.16 (0.09, 0.27) | 0.09 (0.04, 0.17) | -1.96 (-3.86, -0.02) | 0.048 | 86.44 (84.44, 88.47) | 47.96 (46.62, 49.33) | -1.99 (-2.50, -1.47) | <0.001 | 1.39 (1.15, 1.67) | 0.78 (0.62, 0.97) | -2.00 (-2.63, -1.36) | <0.001 |
| **Women** | | | | | | | | | | | | | | | | |
| Global | 8.78 (8.72, 8.83) | 8.49 (8.44, 8.54) | -0.12 (-0.13, -0.11) | <0.001 | 4.77 (4.73, 4.81) | 3.24 (3.21, 3.27) | -1.24 (-1.45, -1.03) | <0.001 | 34.06 (33.95, 34.17) | 29.07 (28.98, 29.16) | -0.52 (-0.95, -0.09) | 0.017 | 0.56 (0.54, 0.57) | 0.49 (0.47, 0.50) | -0.46 (-1.17, 0.25) | 0.203 |
| **SDI category** | | | | | | | | | | | | | | | | |
| High | 8.55 (8.41, 8.70) | 8.19 (8.05, 8.33) | -0.15 (-0.18, -0.13) | <0.001 | 29.97 (29.71, 30.24) | 28.81 (28.55, 29.07) | -0.03 (-0.28, 0.23) | 0.845 | 45.85 (45.52, 46.18) | 33.41 (33.13, 33.69) | -1.08 (-1.23, -0.92) | <0.001 | 0.68 (0.64, 0.72) | 0.48 (0.45, 0.52) | -1.20 (-1.44, -0.96) | <0.001 |
| High-middle | 8.49 (8.38, 8.61) | 8.09 (7.97, 8.20) | -0.18 (-0.21, -0.14) | <0.001 | 0.18 (0.16, 0.20) | 0.16 (0.14, 0.17) | -0.37 (-1.20, 0.47) | 0.384 | 49.91 (49.63, 50.19) | 61.26 (60.97, 61.55) | 0.79 (0.03, 1.56) | 0.043 | 0.85 (0.81, 0.88) | 1.07 (1.03, 1.10) | 0.88 (0.05, 1.71) | 0.037 |

| | | | | | | | | | | | | | | | | |
|---|---|---|---|---|---|---|---|---|---|---|---|---|---|---|---|---|
| Middle | 8.91 (8.81, 9.01) | 8.41 (8.32, 8.49) | -0.21 (-0.22, -0.19) | <0.001 | — | — | — | — | 26.30 (26.13, 26.47) | 19.47 (19.34, 19.59) | -0.98 (-1.15, -0.82) | <0.001 | 0.43 (0.41, 0.46) | 0.32 (0.30, 0.33) | -1.06 (-1.19, -0.92) | <0.001 |
| Low-middle | 9.01 (8.89, 9.14) | 8.87 (8.78, 8.97) | -0.06 (-0.07, -0.05) | <0.001 | — | — | — | — | 20.42 (20.23, 20.62) | 17.25 (17.12, 17.39) | -0.55 (-0.65, -0.45) | <0.001 | 0.34 (0.32, 0.37) | 0.29 (0.27, 0.31) | -0.53 (-0.69, -0.38) | <0.001 |
| Low | 8.81 (8.62, 9.01) | 8.77 (8.64, 8.90) | -0.02 (-0.02, -0.01) | <0.001 | — | — | — | — | 31.03 (30.66, 31.40) | 24.03 (23.82, 24.24) | -0.87 (-0.96, -0.79) | <0.001 | 0.53 (0.48, 0.58) | 0.40 (0.38, 0.43) | -0.98 (-1.14, -0.81) | <0.001 |
| **21 GBD region** | | | | | | | | | | | | | | | | |
| South Asia | 9.27 (9.14, 9.40) | 9.27 (9.18, 9.37) | 0.00 (0.00, 0.00)* | <0.001 | — | — | — | — | 7.11 (6.99, 7.23) | 6.09 (6.01, 6.17) | -0.54 (-0.88, -0.20) | 0.002 | 0.12 (0.10, 0.13) | 0.10 (0.09, 0.11) | -0.68 (-1.00, -0.36) | <0.001 |
| East Asia | 9.47 (9.36, 9.59) | 8.42 (8.30, 8.54) | -0.42 (-0.44, -0.39) | <0.001 | — | — | — | — | 21.68 (21.51, 21.86) | 16.12 (15.96, 16.29) | -1.10 (-1.65, -0.54) | <0.001 | 0.35 (0.32, 0.37) | 0.25 (0.23, 0.27) | -1.19 (-1.93, -0.45) | 0.002 |
| North Africa and Middle East | 7.08 (6.88, 7.29) | 7.07 (6.92, 7.22) | -0.00 (-0.01, 0.00) | 0.236 | — | — | — | — | 32.43 (31.99, 32.87) | 21.20 (20.94, 21.45) | -1.46 (-1.60, -1.33) | <0.001 | 0.52 (0.47, 0.58) | 0.34 (0.31, 0.37) | -1.50 (-1.61, -1.39) | <0.001 |
| Western Sub-Saharan Africa | 8.90 (8.59, 9.23) | 8.91 (8.71, 9.11) | 0.01 (0.01, 0.01) | <0.001 | — | — | — | — | 62.43 (61.57, 63.30) | 39.88 (39.46, 40.31) | -1.55 (-1.68, -1.42) | <0.001 | 1.07 (0.96, 1.19) | 0.68 (0.62, 0.73) | -1.56 (-1.72, -1.41) | <0.001 |
| Southeast Asia | 7.95 (7.78, 8.13) | 7.95 (7.80, 8.11) | 0.00 (-0.01, 0.01) | 0.853 | — | — | — | — | 27.15 (26.81, 27.48) | 24.15 (23.89, 24.41) | -0.40 (-0.51, -0.29) | <0.001 | 0.45 (0.41, 0.50) | 0.40 (0.37, 0.44) | -0.38 (-0.50, -0.25) | <0.001 |
| Eastern Sub-Saharan Africa | 8.84 (8.53, 9.16) | 8.84 (8.64, 9.05) | 0.0006 (-0.003, 0.004) | 0.752 | — | — | — | — | 24.69 (24.14, 25.24) | 19.13 (18.82, 19.45) | -0.87 (-0.98, -0.76) | <0.001 | 0.42 (0.35, 0.50) | 0.32 (0.28, 0.37) | -0.88 (-1.09, -0.67) | <0.001 |
| Central Latin America | 8.15 (7.85, 8.47) | 8.13 (7.89, 8.38) | -0.01 (-0.02, -0.00) | 0.018 | — | — | — | — | 16.50 (16.06, 16.95) | 14.02 (13.69, 14.34) | -0.53 (-0.91, -0.15) | 0.006 | 0.27 (0.22, 0.33) | 0.23 (0.19, 0.27) | -0.52 (-0.83, -0.20) | 0.001 |
| Tropical Latin America | 8.58 (8.26, 8.91) | 8.57 (8.31, | -0.00 (-0.00, | <0.001 | — | — | — | — | 73.85 (72.89, 74.81) | 43.97 (43.37, 44.59) | -1.78 (-2.00, | <0.001 | 1.26 (1.14, 1.39) | 0.74 (0.66, 0.82) | -1.83 (-2.12, | <0.001 |

| | | | | | | | | | | | | | | | | |
|---|---|---|---|---|---|---|---|---|---|---|---|---|---|---|---|---|
| | | 8.85) | -0.00)* | | | | | | | | -1.57) | | | | -1.55) | |
| Central Sub-Saharan Africa | 8.50 (7.93, 9.10) | 8.50 (8.14, 8.87) | 0.00 (0.00, 0.00) | 1.000 | — | — | — | — | 61.82 (60.24, 63.43) | 53.67 (52.75, 54.61) | -0.52 (-0.64, -0.40) | <0.001 | 1.05 (0.85, 1.28) | 0.90 (0.79, 1.04) | -0.53 (-0.68, -0.38) | <0.001 |
| Western Europe | 7.76 (7.55, 7.96) | 7.66 (7.45, 7.88) | -0.05 (-0.07, -0.02) | <0.001 | 0.47 (0.42, 0.52) | 0.01 (0.00, 0.02) | -13.71 (-20.13, -6.77) | <0.001 | 28.70 (28.31, 29.10) | 14.25 (13.96, 14.55) | -2.39 (-2.65, -2.13) | <0.001 | 0.45 (0.40, 0.50) | 0.22 (0.18, 0.25) | -2.53 (-2.80, -2.26) | <0.001 |
| High-income North America | 8.86 (8.62, 9.11) | 8.55 (8.32, 8.79) | -0.11 (-0.25, 0.02) | 0.094 | 82.61 (81.87, 83.35) | 78.13 (77.44, 78.84) | -0.07 (-0.33, 0.18) | 0.577 | 67.35 (66.68, 68.03) | 59.18 (58.57, 59.79) | -0.43 (-0.65, -0.21) | <0.001 | 0.96 (0.88, 1.04) | 0.83 (0.76, 0.91) | -0.47 (-0.80, -0.13) | 0.007 |
| Central Asia | 8.83 (8.35, 9.34) | 8.83 (8.41, 9.27) | 0.00 (0.00, 0.00)* | <0.001 | — | — | — | — | 66.11 (64.76, 67.48) | 109.52 (108.04, 111.01) | 1.72 (1.29, 2.15) | <0.001 | 1.12 (0.95, 1.31) | 1.89 (1.70, 2.10) | 1.80 (1.37, 2.24) | <0.001 |
| Eastern Europe | 9.57 (9.27, 9.86) | 9.58 (9.24, 9.92) | 0.00 (-0.00, 0.01) | 0.505 | — | — | — | — | 135.44 (134.38, 136.51) | 294.14 (292.45, 295.83) | 2.83 (1.82, 3.86) | <0.001 | 2.36 (2.22, 2.50) | 5.22 (5.00, 5.45) | 2.91 (1.89, 3.94) | <0.001 |
| Southern Sub-Saharan Africa | 9.13 (8.57, 9.73) | 9.12 (8.67, 9.58) | -0.00 (-0.01, 0.00) | 0.234 | — | — | — | — | 75.01 (73.36, 76.69) | 45.32 (44.31, 46.35) | -1.65 (-2.61, -0.69) | 0.001 | 1.26 (1.05, 1.50) | 0.75 (0.63, 0.90) | -1.70 (-2.71, -0.67) | 0.001 |
| High-income Asia Pacific | 9.47 (9.14, 9.81) | 8.89 (8.53, 9.27) | -0.22 (-0.24, -0.19) | <0.001 | 1.11 (1.00, 1.23) | 0.17 (0.12, 0.24) | -6.67 (-9.22, -4.05) | <0.001 | 34.23 (33.60, 34.87) | 16.91 (16.41, 17.43) | -2.40 (-2.67, -2.13) | <0.001 | 0.52 (0.44, 0.60) | 0.24 (0.18, 0.31) | -2.64 (-2.98, -2.29) | <0.001 |
| Central Europe | 7.66 (7.31, 8.03) | 7.68 (7.27, 8.11) | 0.00 (-0.05, 0.05) | 0.980 | — | — | — | — | 44.51 (43.65, 45.37) | 26.23 (25.49, 27.00) | -1.80 (-1.90, -1.70) | <0.001 | 0.75 (0.64, 0.87) | 0.44 (0.34, 0.54) | -1.84 (-1.98, -1.70) | <0.001 |
| Andean Latin America | 7.51 (6.90, 8.16) | 7.54 (7.08, 8.04) | 0.01 (0.00, 0.03) | 0.027 | — | — | — | — | 20.40 (19.40, 21.45) | 13.89 (13.25, 14.56) | -1.31 (-1.65, -0.97) | <0.001 | 0.33 (0.21, 0.50) | 0.22 (0.15, 0.32) | -1.38 (-1.87, -0.88) | <0.001 |
| Caribbean | 7.67 (7.05, 8.33) | 7.67 (7.11, 8.26) | 0.00 (0.00, 0.00)* | <0.001 | — | — | — | — | 48.97 (47.37, 50.62) | 54.42 (52.92, 55.95) | 0.39 (0.25, 0.54) | <0.001 | 0.81 (0.62, 1.05) | 0.91 (0.72, 1.12) | 0.40 (0.25, 0.55) | <0.001 |
| Southern Latin America | 6.27 (5.78, 6.79) | 6.27 (5.85, | 0.00 (0.00, | <0.001 | — | — | — | — | 44.28 (42.96, 45.64) | 29.34 (28.41, 30.29) | -1.39 (-1.72, | <0.001 | 0.72 (0.56, 0.92) | 0.47 (0.36, 0.61) | -1.50 (-2.07, | <0.001 |

| | | | | | | | | | | | | | | | | |
|---|---|---|---|---|---|---|---|---|---|---|---|---|---|---|---|---|
| | | 6.72) | 0.00)* | | | | | | | | -1.06) | | | | -0.93) | |
| Oceania | 8.12 (6.61, 9.90) | 8.12 (7.07, 9.28) | 0.00 (0.00, 0.00)* | <0.001 | — | — | — | — | 38.88 (35.36, 42.66) | 41.11 (38.67, 43.66) | 0.22 (0.12, 0.31) | <0.001 | 0.67 (0.28, 1.37) | 0.70 (0.42, 1.11) | 0.14 (0.01, 0.26) | 0.032 |
| Australasia | 7.21 (6.41, 8.08) | 7.16 (6.43, 7.96) | -0.02 (-0.04, -0.01) | 0.002 | — | — | — | — | 51.47 (49.30, 53.72) | 22.49 (21.20, 23.85) | -2.82 (-3.30, -2.34) | <0.001 | 0.81 (0.56, 1.14) | 0.35 (0.21, 0.57) | -2.89 (-3.39, -2.38) | <0.001 |
| **Men** | | | | | | | | | | | | | | | | |
| Global | 14.73 (14.66, 14.80) | 13.69 (13.63, 13.75) | -0.25 (-0.26, -0.24) | <0.001 | 7.58 (7.52, 7.63) | 5.91 (5.87, 5.95) | -0.82 (-1.04, -0.60) | <0.001 | 76.30 (76.14, 76.47) | 78.20 (78.06, 78.34) | 0.10 (-0.39, 0.59) | 0.690 | 1.28 (1.26, 1.31) | 1.34 (1.32, 1.35) | 0.17 (-0.30, 0.64) | 0.473 |
| **SDI category** | | | | | | | | | | | | | | | | |
| High | 18.68 (18.47, 18.89) | 17.95 (17.75, 18.15) | -0.14 (-0.16, -0.11) | <0.001 | 45.89 (45.57, 46.21) | 48.90 (48.57, 49.22) | 0.25 (0.05, 0.45) | 0.013 | 107.22 (106.72, 107.72) | 74.50 (74.10, 74.91) | -1.25 (-1.41, -1.09) | <0.001 | 1.70 (1.64, 1.77) | 1.14 (1.10, 1.19) | -1.40 (-1.55, -1.24) | <0.001 |
| High-middle | 15.52 (15.37, 15.68) | 14.75 (14.60, 14.90) | -0.18 (-0.18, -0.17) | <0.001 | 1.18 (1.14, 1.23) | 0.81 (0.78, 0.84) | -1.45 (-2.32, -0.57) | 0.001 | 145.61 (145.14, 146.09) | 203.68 (203.17, 204.20) | 1.17 (0.35, 1.99) | 0.005 | 2.52 (2.46, 2.59) | 3.60 (3.53, 3.67) | 1.25 (0.44, 2.07) | 0.002 |
| Middle | 14.22 (14.10, 14.34) | 13.31 (13.21, 13.42) | -0.23 (-0.25, -0.21) | <0.001 | 0.01 (0.01, 0.02) | 0.02 (0.01, 0.02) | — | — | 43.37 (43.15, 43.58) | 41.83 (41.64, 42.01) | -0.12 (-0.23, -0.01) | 0.036 | 0.71 (0.69, 0.74) | 0.69 (0.67, 0.71) | -0.16 (-0.36, 0.05) | 0.136 |
| Low-middle | 12.84 (12.69, 12.99) | 12.59 (12.48, 12.71) | -0.07 (-0.08, -0.06) | <0.001 | — | 0.01 (0.01, 0.01) | — | — | 31.42 (31.18, 31.66) | 34.34 (34.15, 34.53) | 0.35 (0.26, 0.44) | <0.001 | 0.52 (0.49, 0.55) | 0.57 (0.55, 0.60) | 0.36 (0.23, 0.48) | <0.001 |
| Low | 12.20 (11.98, 12.43) | 12.13 (11.98, 12.28) | -0.02 (-0.02, -0.02) | <0.001 | — | — | — | — | 54.59 (54.10, 55.08) | 54.36 (54.04, 54.69) | -0.01 (-0.18, 0.16) | 0.942 | 0.92 (0.85, 0.98) | 0.91 (0.87, 0.95) | -0.06 (-0.14, 0.02) | 0.141 |
| **21 GBD region** | | | | | | | | | | | | | | | | |
| South Asia | 12.72 (12.57, 12.88) | 12.74 (12.63, 12.85) | 0.01 (0.01, 0.01) | <0.001 | — | — | — | — | 11.18 (11.04, 11.32) | 12.09 (11.98, 12.20) | 0.33 (-0.05, 0.71) | 0.090 | 0.18 (0.17, 0.20) | 0.20 (0.18, 0.21) | 0.43 (-0.11, 0.97) | 0.121 |
| East Asia | 15.98 (15.83, 16.12) | 15.25 (15.09, | -0.16 (-0.17, | <0.001 | — | — | — | — | 36.22 (36.00, 36.44) | 34.94 (34.72, 35.17) | -0.08 (-0.39, | 0.602 | 0.58 (0.55, 0.61) | 0.57 (0.54, 0.60) | -0.04 (-0.23, | 0.716 |

| | | | | | | | | | | | | | | | | |
|---|---|---|---|---|---|---|---|---|---|---|---|---|---|---|---|---|
| | | 15.40) | -0.15) | | | | | | | | 0.23) | | | | 0.16) | |
| North Africa and Middle East | 10.63 (10.38, 10.88) | 10.62 (10.44, 10.79) | -0.00 (-0.01, 0.00) | 0.255 | 0.48 (0.43, 0.54) | 0.13 (0.11, 0.15) | -3.99 (-5.53, -2.43) | <0.001 | 53.83 (53.28, 54.39) | 40.82 (40.48, 41.16) | -0.96 (-1.18, -0.73) | <0.001 | 0.85 (0.78, 0.92) | 0.64 (0.60, 0.68) | -0.95 (-1.20, -0.69) | <0.001 |
| Western Sub-Saharan Africa | 12.25 (11.87, 12.63) | 12.20 (11.96, 12.45) | -0.01 (-0.02, -0.01) | <0.001 | — | — | — | — | 84.54 (83.55, 85.53) | 74.26 (73.67, 74.86) | -0.43 (-0.60, -0.25) | <0.001 | 1.38 (1.25, 1.51) | 1.21 (1.13, 1.29) | -0.45 (-0.70, -0.20) | <0.001 |
| Southeast Asia | 13.60 (13.37, 13.84) | 13.63 (13.44, 13.83) | 0.01 (0.01, 0.01) | <0.001 | — | 0.05 (0.04, 0.07) | — | — | 46.11 (45.67, 46.56) | 49.75 (49.38, 50.12) | 0.30 (0.13, 0.46) | <0.001 | 0.78 (0.72, 0.84) | 0.83 (0.79, 0.88) | 0.25 (0.08, 0.42) | 0.004 |
| Eastern Sub-Saharan Africa | 12.11 (11.73, 12.51) | 12.13 (11.88, 12.38) | 0.01 (0.00, 0.01) | 0.003 | — | — | — | — | 73.95 (72.96, 74.95) | 71.54 (70.92, 72.17) | -0.12 (-0.27, 0.04) | 0.138 | 1.27 (1.15, 1.41) | 1.23 (1.15, 1.31) | -0.10 (-0.30, 0.09) | 0.308 |
| Central Latin America | 11.39 (11.02, 11.77) | 11.37 (11.07, 11.67) | -0.01 (-0.01, -0.00) | <0.001 | — | — | — | — | 31.98 (31.35, 32.62) | 30.20 (29.71, 30.69) | -0.17 (-0.34, -0.00) | 0.049 | 0.53 (0.45, 0.62) | 0.50 (0.44, 0.57) | -0.17 (-0.52, 0.17) | 0.326 |
| Tropical Latin America | 11.92 (11.53, 12.31) | 11.91 (11.59, 12.23) | -0.00 (-0.00, -0.00)[*] | <0.001 | — | — | — | — | 156.91 (155.48, 158.35) | 122.69 (121.67, 123.71) | -0.87 (-1.05, -0.68) | <0.001 | 2.72 (2.53, 2.91) | 2.09 (1.96, 2.23) | -0.94 (-1.13, -0.75) | <0.001 |
| Central Sub-Saharan Africa | 11.64 (10.97, 12.35) | 11.64 (11.22, 12.08) | 0.00 (0.00, 0.00)[*] | <0.001 | — | — | — | — | 99.84 (97.82, 101.90) | 101.39 (100.10, 102.69) | 0.03 (-0.09, 0.16) | 0.580 | 1.69 (1.43, 1.98) | 1.72 (1.55, 1.90) | 0.04 (-0.08, 0.15) | 0.520 |
| Western Europe | 17.68 (17.38, 17.99) | 17.74 (17.42, 18.07) | 0.01 (-0.01, 0.02) | 0.394 | 2.19 (2.08, 2.30) | 0.58 (0.53, 0.64) | -4.17 (-6.03, -2.28) | <0.001 | 76.54 (75.91, 77.18) | 44.84 (44.34, 45.35) | -1.86 (-2.13, -1.59) | <0.001 | 1.24 (1.16, 1.32) | 0.72 (0.66, 0.79) | -1.88 (-2.17, -1.58) | <0.001 |
| High-income North America | 19.14 (18.78, 19.50) | 19.33 (18.98, 19.68) | 0.04 (-0.00, 0.09) | 0.061 | 114.89 (114.03, 115.76) | 130.60 (129.71, 131.50) | 0.47 (0.25, 0.70) | <0.001 | 152.68 (151.68, 153.68) | 116.98 (116.13, 117.83) | -0.95 (-1.11, -0.78) | <0.001 | 2.38 (2.26, 2.51) | 1.74 (1.64, 1.85) | -1.10 (-1.41, -0.78) | <0.001 |
| Central Asia | 14.23 (13.61, 14.87) | 14.23 (13.70, 14.78) | 0.00 (-0.00, 0.00) | 1.000 | 1.52 (1.30, 1.76) | 1.33 (1.17, 1.50) | -0.50 (-0.78, -0.21) | 0.001 | 143.99 (141.96, 146.04) | 293.01 (290.61, 295.42) | 2.31 (1.25, 3.37) | <0.001 | 2.48 (2.22, 2.77) | 5.16 (4.84, 5.48) | 2.40 (1.42, 3.39) | <0.001 |
| Eastern Europe | 17.37 (16.98, 17.77) | 17.38 (16.93, | 0.00 (-0.01, | 0.703 | 2.96 (2.81, 3.11) | 2.64 (2.50, 2.80) | -0.39 (-0.74, | 0.026 | 488.37 (486.37, | 1113.97 (1110.72, | 2.92 (1.96, | <0.001 | 8.63 (8.37, 8.90) | 19.90 (19.47, | 2.96 (2.01, | <0.001 |

| | | | | | | | | | | | | | | | | |
|---|---|---|---|---|---|---|---|---|---|---|---|---|---|---|---|---|
| | | 17.84) | 0.01) | | | | -0.05) | | 490.39) | 1117.22) | 3.89) | | | 20.34) | 3.92) | |
| Southern Sub-Saharan Africa | 12.54 (11.86, 13.26) | 12.53 (12.00, 13.08) | -0.00 (-0.01, 0.00) | 0.309 | — | — | — | — | 105.48 (103.43, 107.56) | 93.46 (92.01, 94.93) | -0.48 (-1.09, 0.13) | 0.119 | 1.80 (1.54, 2.09) | 1.56 (1.38, 1.76) | -0.59 (-1.17, -0.01) | 0.048 |
| High-income Asia Pacific | 21.87 (21.38, 22.37) | 21.68 (21.11, 22.25) | -0.03 (-0.05, -0.01) | 0.001 | 23.39 (22.88, 23.90) | 18.92 (18.41, 19.45) | -0.73 (-0.86, -0.59) | <0.001 | 82.56 (81.60, 83.52) | 44.70 (43.92, 45.50) | -2.10 (-2.39, -1.81) | <0.001 | 1.30 (1.18, 1.42) | 0.68 (0.58, 0.78) | -2.21 (-2.49, -1.93) | <0.001 |
| Central Europe | 19.48 (18.92, 20.06) | 19.72 (19.08, 20.39) | 0.04 (0.03, 0.06) | <0.001 | 0.19 (0.14, 0.25) | 0.36 (0.28, 0.44) | 2.65 (1.09, 4.23) | 0.001 | 128.37 (126.95, 129.79) | 109.61 (108.16, 111.07) | -0.55 (-0.69, -0.40) | <0.001 | 2.22 (2.03, 2.41) | 1.89 (1.71, 2.09) | -0.55 (-0.71, -0.40) | <0.001 |
| Andean Latin America | 10.66 (9.92, 11.45) | 10.67 (10.11, 11.25) | 0.00 (0.00, 0.00)* | <0.001 | — | — | — | — | 32.02 (30.73, 33.35) | 29.43 (28.50, 30.39) | -0.27 (-0.43, -0.11) | 0.001 | 0.52 (0.37, 0.72) | 0.48 (0.37, 0.61) | -0.28 (-0.45, -0.12) | 0.001 |
| Caribbean | 10.79 (10.04, 11.60) | 10.79 (10.13, 11.50) | 0.00 (0.00, 0.00)* | <0.001 | — | — | — | — | 81.49 (79.34, 83.69) | 89.70 (87.74, 91.68) | 0.38 (0.20, 0.55) | <0.001 | 1.38 (1.11, 1.70) | 1.53 (1.29, 1.81) | 0.39 (0.21, 0.56) | <0.001 |
| Southern Latin America | 14.47 (13.71, 15.26) | 14.47 (13.81, 15.14) | -0.00 (-0.01, 0.00) | 0.758 | 5.47 (5.01, 5.97) | 2.05 (1.82, 2.32) | -3.36 (-4.48, -2.21) | <0.001 | 97.86 (95.85, 99.89) | 78.33 (76.81, 79.88) | -0.74 (-0.99, -0.49) | <0.001 | 1.63 (1.38, 1.92) | 1.30 (1.11, 1.51) | -0.74 (-1.01, -0.47) | <0.001 |
| Oceania | 13.64 (11.71, 15.82) | 13.64 (12.29, 15.10) | 0.00 (0.00, 0.00)* | <0.001 | — | — | — | — | 63.43 (59.06, 68.04) | 75.40 (72.13, 78.79) | 0.56 (0.44, 0.69) | <0.001 | 1.07 (0.57, 1.86) | 1.27 (0.88, 1.79) | 0.55 (0.42, 0.69) | <0.001 |
| Australasia | 17.15 (15.91, 18.47) | 17.34 (16.20, 18.56) | 0.04 (0.03, 0.05) | <0.001 | 0.32 (0.17, 0.55) | 0.18 (0.09, 0.34) | -2.27 (-3.53, -0.99) | 0.001 | 121.40 (118.06, 124.82) | 73.80 (71.45, 76.22) | -1.68 (-2.25, -1.11) | <0.001 | 1.97 (1.57, 2.45) | 1.22 (0.93, 1.57) | -1.64 (-2.24, -1.04) | <0.001 |
| **Atrial fibrillation and flutter** | | | | | | | | | | | | | | | | |
| **Overall** | | | | | | | | | | | | | | | | |
| Global | 2.48 (2.46, 2.50) | 2.36 (2.35, 2.38) | -0.17 (-0.21, -0.13) | <0.001 | 8.24 (8.20, 8.28) | 7.85 (7.82, 7.88) | -0.17 (-0.20, -0.14) | <0.001 | 1.07 (1.05, 1.08) | 1.08 (1.07, 1.09) | 0.03 (0.03, 0.03) | <0.001 | 0.01 (0.01, 0.01) | 0.01 (0.01, 0.01) | — | — |
| **SDI category** | | | | | | | | | | | | | | | | |

| | | | | | | | | | | | | | | | | |
|---|---|---|---|---|---|---|---|---|---|---|---|---|---|---|---|---|
| High | 3.75 (3.69, 3.81) | 3.87 (3.81, 3.93) | 0.11 (0.08, 0.14) | <0.001 | 12.43 (12.32, 12.55) | 12.82 (12.71, 12.93) | 0.11 (0.08, 0.14) | <0.001 | 1.47 (1.43, 1.51) | 1.47 (1.44, 1.51) | 0.00 (-0.09, 0.10) | 0.936 | 0.01 (0.00, 0.01) | 0.01 (0.00, 0.01) | — | — |
| High-middle | 2.63 (2.59, 2.68) | 2.57 (2.53, 2.61) | -0.08 (-0.11, -0.05) | <0.001 | 8.74 (8.66, 8.82) | 8.53 (8.46, 8.60) | -0.09 (-0.10, -0.07) | <0.001 | 1.09 (1.06, 1.12) | 1.06 (1.04, 1.09) | -0.10 (-0.17, -0.03) | 0.007 | 0.01 (0.00, 0.01) | 0.01 (0.00, 0.01) | 0.00 (0.00, 0.00) | 1.000 |
| Middle | 2.14 (2.10, 2.17) | 2.15 (2.12, 2.18) | 0.01 (-0.03, 0.04) | 0.740 | 7.10 (7.03, 7.16) | 7.12 (7.07, 7.17) | 0.01 (-0.02, 0.03) | 0.731 | 1.01 (0.98, 1.03) | 1.08 (1.06, 1.11) | 0.22 (0.15, 0.29) | <0.001 | 0.01 (0.01, 0.01) | 0.01 (0.01, 0.01) | — | — |
| Low-middle | 2.04 (1.99, 2.09) | 2.02 (1.98, 2.05) | -0.03 (-0.07, 0.01) | 0.147 | 6.78 (6.69, 6.86) | 6.69 (6.63, 6.76) | -0.04 (-0.06, -0.03) | <0.001 | 0.86 (0.83, 0.89) | 0.95 (0.93, 0.97) | 0.36 (0.32, 0.40) | <0.001 | 0.01 (0.00, 0.01) | 0.01 (0.01, 0.01) | — | — |
| Low | 1.60 (1.53, 1.66) | 1.58 (1.54, 1.62) | -0.03 (-0.10, 0.05) | 0.450 | 5.30 (5.19, 5.42) | 5.25 (5.18, 5.33) | -0.03 (-0.05, -0.01) | 0.010 | 0.83 (0.79, 0.88) | 0.88 (0.85, 0.91) | 0.19 (0.14, 0.23) | <0.001 | 0.01 (0.00, 0.01) | 0.01 (0.01, 0.01) | — | — |
| **21 GBD region** | | | | | | | | | | | | | | | | |
| South Asia | 2.19 (2.14, 2.24) | 2.21 (2.18, 2.25) | 0.03 (-0.01, 0.06) | 0.114 | 7.26 (7.17, 7.34) | 7.34 (7.27, 7.40) | 0.03 (-0.01, 0.07) | 0.124 | 0.83 (0.80, 0.86) | 0.93 (0.91, 0.95) | 0.42 (0.31, 0.53) | <0.001 | — | 0.01 (0.00, 0.01) | — | — |
| East Asia | 2.38 (2.34, 2.42) | 2.51 (2.47, 2.55) | 0.18 (0.13, 0.24) | <0.001 | 7.90 (7.82, 7.97) | 8.33 (8.25, 8.40) | 0.17 (0.14, 0.21) | <0.001 | 0.91 (0.89, 0.94) | 0.93 (0.91, 0.96) | 0.05 (-0.04, 0.14) | 0.299 | — | — | — | — |
| North Africa and Middle East | 1.74 (1.66, 1.82) | 1.78 (1.73, 1.83) | 0.07 (0.02, 0.13) | 0.013 | 5.78 (5.64, 5.93) | 5.91 (5.82, 6.00) | 0.07 (0.04, 0.10) | <0.001 | 0.88 (0.82, 0.94) | 0.94 (0.90, 0.97) | 0.20 (0.17, 0.24) | <0.001 | 0.01 (0.00, 0.01) | 0.01 (0.00, 0.01) | 0.00 (0.00, 0.00) | 1.000 |
| Western Sub-Saharan Africa | 1.52 (1.42, 1.63) | 1.55 (1.49, 1.62) | 0.07 (0.01, 0.14) | 0.033 | 5.05 (4.87, 5.25) | 5.14 (5.03, 5.27) | 0.06 (0.03, 0.10) | <0.001 | 0.61 (0.55, 0.68) | 0.65 (0.60, 0.69) | 0.23 (0.17, 0.29) | <0.001 | — | — | — | — |
| Southeast Asia | 2.43 (2.35, 2.51) | 2.52 (2.46, 2.58) | 0.12 (0.07, 0.16) | <0.001 | 8.06 (7.92, 8.20) | 8.36 (8.25, 8.46) | 0.12 (0.09, 0.14) | <0.001 | 1.16 (1.10, 1.21) | 1.43 (1.39, 1.48) | 0.73 (0.67, 0.79) | <0.001 | 0.01 (0.00, 0.01) | 0.01 (0.01, 0.02) | — | — |
| Eastern Sub-Saharan | 0.96 (0.88, 1.05) | 0.99 (0.93, | 0.12 (0.01, | 0.035 | 3.19 (3.04, 3.35) | 3.27 (3.17, 3.37) | 0.09 (0.03, | 0.001 | 0.87 (0.80, 0.96) | 0.87 (0.82, 0.92) | -0.02 (-0.17, | 0.824 | 0.01 (0.00, 0.02) | 0.01 (0.01, 0.02) | 0.00 (0.00, | <0.001 |

| | | | | | | | | | | | | | | | | |
|---|---|---|---|---|---|---|---|---|---|---|---|---|---|---|---|---|
| Africa | | 1.04) | 0.23) | | | | 0.14) | | | | 0.14) | | | | 0.00)* | |
| Central Latin America | 1.03 (0.95, 1.12) | 1.03 (0.97, 1.10) | -0.00 (-0.06, 0.06) | 0.936 | 3.43 (3.27, 3.59) | 3.43 (3.32, 3.55) | 0.00 (-0.02, 0.03) | 0.699 | 0.85 (0.78, 0.93) | 0.81 (0.76, 0.87) | -0.15 (-0.31, 0.01) | 0.072 | 0.01 (0.00, 0.02) | 0.01 (0.00, 0.02) | 0.00 (0.00, 0.00)* | <0.001 |
| Tropical Latin America | 1.50 (1.40, 1.61) | 1.51 (1.43, 1.59) | 0.11 (0.04, 0.19) | 0.005 | 4.98 (4.80, 5.17) | 5.01 (4.87, 5.15) | 0.12 (0.06, 0.18) | <0.001 | 1.26 (1.17, 1.36) | 1.30 (1.23, 1.37) | 0.15 (-0.15, 0.44) | 0.334 | 0.02 (0.01, 0.03) | 0.02 (0.01, 0.03) | -0.25 (-3.55, 3.15) | 0.882 |
| Central Sub-Saharan Africa | 1.41 (1.23, 1.61) | 1.42 (1.30, 1.54) | 0.02 (-0.01, 0.04) | 0.255 | 4.68 (4.35, 5.03) | 4.70 (4.48, 4.91) | 0.02 (0.00, 0.03) | 0.016 | 0.88 (0.74, 1.04) | 0.89 (0.80, 0.99) | 0.06 (-0.00, 0.11) | 0.053 | 0.01 (0.00, 0.04) | 0.01 (0.00, 0.02) | 0.00 (0.00, 0.00)* | <0.001 |
| Western Europe | 3.63 (3.53, 3.73) | 3.32 (3.23, 3.41) | -0.29 (-0.33, -0.26) | <0.001 | 12.05 (11.87, 12.22) | 11.00 (10.84, 11.17) | -0.31 (-0.34, -0.28) | <0.001 | 1.62 (1.56, 1.69) | 1.40 (1.34, 1.46) | -0.50 (-0.56, -0.44) | <0.001 | 0.01 (0.01, 0.02) | 0.01 (0.00, 0.02) | 0.00 (0.00, 0.00)* | <0.001 |
| High-income North America | 4.56 (4.44, 4.68) | 5.56 (5.43, 5.69) | 0.68 (0.62, 0.75) | <0.001 | 15.14 (14.92, 15.35) | 18.44 (18.21, 18.67) | 0.68 (0.61, 0.74) | <0.001 | 1.57 (1.50, 1.64) | 1.92 (1.84, 1.99) | 0.70 (0.55, 0.85) | <0.001 | 0.01 (0.00, 0.01) | 0.01 (0.00, 0.01) | 0.00 (0.00, 0.00) | 1.000 |
| Central Asia | 2.64 (2.44, 2.85) | 2.77 (2.60, 2.94) | 0.16 (0.13, 0.20) | <0.001 | 8.77 (8.40, 9.15) | 9.18 (8.88, 9.49) | 0.16 (0.13, 0.18) | <0.001 | 1.10 (0.97, 1.24) | 1.19 (1.08, 1.30) | 0.27 (0.13, 0.42) | <0.001 | 0.01 (0.00, 0.03) | 0.01 (0.00, 0.02) | 0.00 (0.00, 0.00)* | <0.001 |
| Eastern Europe | 3.28 (3.17, 3.39) | 3.50 (3.38, 3.62) | 0.22 (0.19, 0.26) | <0.001 | 10.87 (10.67, 11.08) | 11.60 (11.39, 11.83) | 0.23 (0.20, 0.25) | <0.001 | 1.13 (1.06, 1.19) | 1.25 (1.18, 1.33) | 0.37 (0.27, 0.47) | <0.001 | — | — | — | — |
| Southern Sub-Saharan Africa | 1.60 (1.41, 1.80) | 1.59 (1.45, 1.73) | -0.01 (-0.07, 0.05) | 0.640 | 5.29 (4.95, 5.65) | 5.26 (5.02, 5.52) | -0.02 (-0.04, 0.01) | 0.262 | 2.24 (2.03, 2.48) | 1.52 (1.39, 1.66) | -1.48 (-2.20, -0.75) | <0.001 | 0.03 (0.01, 0.07) | 0.02 (0.01, 0.04) | -1.79 (-3.03, -0.53) | 0.005 |
| High-income Asia Pacific | 2.88 (2.75, 3.01) | 2.87 (2.74, 3.00) | -0.03 (-0.09, 0.03) | 0.367 | 9.53 (9.31, 9.76) | 9.51 (9.28, 9.75) | -0.02 (-0.08, 0.04) | 0.422 | 1.23 (1.15, 1.32) | 1.15 (1.07, 1.23) | -0.23 (-0.35, -0.11) | <0.001 | 0.01 (0.00, 0.02) | 0.01 (0.00, 0.02) | 0.00 (0.00, 0.00)* | <0.001 |
| Central Europe | 3.12 (2.97, 3.28) | 3.10 (2.95, 3.27) | -0.03 (-0.08, 0.03) | 0.368 | 10.37 (10.09, 10.64) | 10.31 (10.02, 10.60) | -0.02 (-0.04, 0.01) | 0.293 | 1.30 (1.21, 1.40) | 1.26 (1.16, 1.37) | -0.12 (-0.22, -0.01) | 0.031 | 0.01 (0.00, 0.02) | 0.01 (0.00, 0.02) | 0.00 (0.00, 0.00)* | <0.001 |
| Andean Latin America | 0.33 (0.24, 0.45) | 0.38 (0.31, | 0.52 (0.28, | <0.001 | 1.09 (0.91, 1.29) | 1.26 (1.12, 1.40) | 0.51 (0.33, | <0.001 | 1.03 (0.86, 1.22) | 0.96 (0.84, 1.09) | -0.28 (-0.54, | 0.044 | 0.02 (0.00, 0.06) | 0.02 (0.00, 0.04) | 0.00 (0.00, | <0.001 |

| | | | | | | | | | | | | | | | | |
|---|---|---|---|---|---|---|---|---|---|---|---|---|---|---|---|---|
| | | 0.46) | 0.76) | | | | 0.70) | | | | -0.01) | | | | 0.00)* | |
| Caribbean | 0.95 (0.79, 1.14) | 0.98 (0.84, 1.13) | 0.11 (0.06, 0.16) | <0.001 | 3.16 (2.85, 3.49) | 3.24 (2.98, 3.51) | 0.09 (0.05, 0.13) | <0.001 | 0.97 (0.80, 1.16) | 1.02 (0.87, 1.17) | 0.17 (-0.06, 0.39) | 0.152 | 0.01 (0.00, 0.05) | 0.01 (0.00, 0.04) | 0.00 (0.00, 0.00)* | <0.001 |
| Southern Latin America | 1.96 (1.76, 2.17) | 2.03 (1.86, 2.21) | 0.11 (0.05, 0.17) | 0.001 | 6.49 (6.13, 6.88) | 6.72 (6.41, 7.04) | 0.11 (0.08, 0.15) | <0.001 | 1.08 (0.94, 1.25) | 1.05 (0.93, 1.18) | -0.13 (-0.27, 0.02) | 0.088 | 0.01 (0.00, 0.04) | 0.01 (0.00, 0.03) | 0.00 (0.00, 0.00)* | <0.001 |
| Oceania | 2.34 (1.73, 3.10) | 2.37 (1.96, 2.85) | 0.04 (-0.03, 0.10) | 0.309 | 7.76 (6.60, 9.07) | 7.87 (7.10, 8.70) | 0.04 (-0.03, 0.11) | 0.283 | 4.64 (3.77, 5.66) | 5.56 (4.92, 6.27) | 0.61 (0.49, 0.73) | <0.001 | 0.07 (0.01, 0.32) | 0.09 (0.03, 0.23) | 0.75 (0.75, 0.75) | <0.001 |
| Australasia | 4.73 (4.28, 5.21) | 4.49 (4.11, 4.91) | -0.17 (-0.21, -0.13) | <0.001 | 15.69 (14.87, 16.55) | 14.92 (14.21, 15.66) | -0.17 (-0.20, -0.13) | <0.001 | 1.63 (1.37, 1.92) | 1.55 (1.33, 1.81) | -0.16 (-0.39, 0.08) | 0.195 | 0.01 (0.00, 0.06) | 0.01 (0.00, 0.06) | 0.00 (0.00, 0.00)* | <0.001 |
| **Women** | | | | | | | | | | | | | | | | |
| Global | 1.80 (1.78, 1.83) | 1.83 (1.81, 1.85) | 0.05 (-0.06, 0.16) | 0.359 | 5.99 (5.94, 6.04) | 6.06 (6.02, 6.10) | 0.03 (-0.03, 0.10) | 0.337 | 0.83 (0.81, 0.84) | 0.87 (0.85, 0.88) | 0.15 (0.05, 0.25) | 0.003 | 0.01 (0.00, 0.01) | 0.01 (0.01, 0.01) | — | — |
| **SDI category** | | | | | | | | | | | | | | | | |
| High | 1.66 (1.60, 1.72) | 1.86 (1.80, 1.92) | 0.39 (0.30, 0.49) | <0.001 | 5.50 (5.39, 5.61) | 6.16 (6.05, 6.27) | 0.39 (0.36, 0.42) | <0.001 | 0.77 (0.73, 0.81) | 0.80 (0.76, 0.84) | 0.13 (0.05, 0.21) | 0.001 | 0.01 (0.00, 0.01) | 0.01 (0.00, 0.01) | 0.00 (0.00, 0.00) | 1.000 |
| High-middle | 1.81 (1.76, 1.87) | 1.90 (1.85, 1.95) | 0.17 (0.08, 0.26) | <0.001 | 6.02 (5.92, 6.12) | 6.27 (6.18, 6.36) | 0.14 (0.11, 0.17) | <0.001 | 0.81 (0.77, 0.84) | 0.82 (0.79, 0.86) | 0.04 (-0.05, 0.14) | 0.392 | 0.01 (0.00, 0.01) | 0.01 (0.00, 0.01) | 0.00 (0.00, 0.00) | 1.000 |
| Middle | 1.95 (1.90, 2.00) | 1.94 (1.90, 1.98) | -0.04 (-0.15, 0.07) | 0.447 | 6.48 (6.39, 6.57) | 6.42 (6.34, 6.49) | -0.07 (-0.18, 0.05) | 0.247 | 0.92 (0.89, 0.96) | 0.96 (0.93, 0.99) | 0.09 (-0.05, 0.24) | 0.216 | 0.01 (0.00, 0.01) | 0.01 (0.01, 0.01) | — | — |
| Low-middle | 1.85 (1.79, 1.92) | 1.84 (1.79, 1.88) | -0.04 (-0.10, 0.02) | 0.199 | 6.16 (6.04, 6.27) | 6.09 (6.01, 6.18) | -0.05 (-0.13, 0.03) | 0.181 | 0.76 (0.72, 0.80) | 0.83 (0.80, 0.86) | 0.30 (0.21, 0.39) | <0.001 | — | 0.01 (0.00, 0.01) | — | — |
| Low | 1.40 (1.32, 1.49) | 1.38 (1.33, 1.44) | -0.05 (-0.10, 0.01) | 0.082 | 4.66 (4.51, 4.82) | 4.59 (4.50, 4.69) | -0.05 (-0.08, -0.02) | 0.002 | 0.77 (0.71, 0.83) | 0.81 (0.76, 0.85) | 0.17 (0.13, 0.21) | <0.001 | 0.01 (0.00, 0.02) | 0.01 (0.00, 0.01) | 0.00 (0.00, 0.00)* | <0.001 |

**21 GBD region**

| | | | | | | | | | | | | | | | | |
|---|---|---|---|---|---|---|---|---|---|---|---|---|---|---|---|---|
| South Asia | 2.04 (1.98, 2.11) | 2.10 (2.06, 2.15) | 0.10 (0.02, 0.19) | 0.015 | 6.78 (6.66, 6.90) | 6.98 (6.90, 7.07) | 0.11 (0.01, 0.20) | 0.024 | 0.74 (0.70, 0.78) | 0.82 (0.79, 0.85) | 0.32 (0.04, 0.60) | 0.025 | — | — | — | — |
| East Asia | 2.30 (2.24, 2.36) | 2.44 (2.38, 2.50) | 0.18 (0.09, 0.27) | <0.001 | 7.64 (7.53, 7.75) | 8.09 (7.99, 8.20) | 0.18 (0.13, 0.23) | <0.001 | 0.87 (0.84, 0.91) | 0.89 (0.86, 0.93) | 0.09 (-0.05, 0.23) | 0.209 | — | — | — | — |
| North Africa and Middle East | 1.28 (1.18, 1.38) | 1.29 (1.23, 1.36) | 0.02 (-0.04, 0.08) | 0.526 | 4.25 (4.07, 4.43) | 4.29 (4.18, 4.41) | 0.02 (-0.02, 0.06) | 0.265 | 0.73 (0.66, 0.81) | 0.81 (0.77, 0.86) | 0.36 (0.28, 0.44) | <0.001 | 0.01 (0.00, 0.02) | 0.01 (0.00, 0.01) | 0.00 (0.00, 0.00)* | <0.001 |
| Western Sub-Saharan Africa | 1.32 (1.18, 1.46) | 1.34 (1.25, 1.42) | 0.06 (0.00, 0.12) | 0.047 | 4.37 (4.12, 4.63) | 4.44 (4.28, 4.59) | 0.06 (0.03, 0.08) | <0.001 | 0.54 (0.46, 0.64) | 0.57 (0.52, 0.63) | 0.16 (0.09, 0.22) | <0.001 | — | — | — | — |
| Southeast Asia | 2.23 (2.12, 2.33) | 2.30 (2.22, 2.38) | 0.10 (0.05, 0.16) | <0.001 | 7.38 (7.20, 7.57) | 7.62 (7.47, 7.76) | 0.10 (0.07, 0.13) | <0.001 | 0.93 (0.87, 1.00) | 1.14 (1.09, 1.20) | 0.69 (0.60, 0.78) | <0.001 | 0.01 (0.00, 0.01) | 0.01 (0.00, 0.02) | 0.00 (0.00, 0.00)* | <0.001 |
| Eastern Sub-Saharan Africa | 0.80 (0.70, 0.92) | 0.81 (0.74, 0.88) | 0.05 (-0.01, 0.10) | 0.124 | 2.66 (2.47, 2.86) | 2.68 (2.55, 2.80) | 0.03 (-0.02, 0.08) | 0.197 | 0.85 (0.74, 0.97) | 0.81 (0.75, 0.88) | -0.17 (-0.38, 0.05) | 0.126 | 0.01 (0.00, 0.03) | 0.01 (0.00, 0.02) | 0.00 (0.00, 0.00)* | <0.001 |
| Central Latin America | 0.83 (0.73, 0.95) | 0.82 (0.74, 0.90) | -0.05 (-0.12, 0.02) | 0.164 | 2.76 (2.56, 2.96) | 2.71 (2.57, 2.86) | -0.07 (-0.12, -0.02) | 0.011 | 0.84 (0.74, 0.96) | 0.77 (0.69, 0.85) | -0.26 (-0.40, -0.13) | <0.001 | 0.01 (0.00, 0.03) | 0.01 (0.00, 0.02) | 0.00 (0.00, 0.00)* | <0.001 |
| Tropical Latin America | 1.13 (1.01, 1.26) | 1.14 (1.05, 1.24) | 0.13 (-0.02, 0.27) | 0.093 | 3.73 (3.51, 3.97) | 3.77 (3.61, 3.95) | 0.12 (0.02, 0.22) | 0.019 | 1.08 (0.97, 1.21) | 1.17 (1.08, 1.27) | 0.32 (0.06, 0.58) | 0.017 | 0.01 (0.00, 0.04) | 0.02 (0.01, 0.03) | 2.37 (-1.88, 6.79) | 0.279 |
| Central Sub-Saharan Africa | 1.22 (0.99, 1.49) | 1.20 (1.06, 1.36) | -0.06 (-0.11, -0.02) | 0.008 | 4.06 (3.63, 4.53) | 3.99 (3.71, 4.27) | -0.05 (-0.07, -0.03) | <0.001 | 0.81 (0.63, 1.03) | 0.81 (0.70, 0.95) | -0.02 (-0.07, 0.03) | 0.435 | 0.01 (0.00, 0.06) | 0.01 (0.00, 0.03) | 0.00 (-0.00, 0.00) | 1.000 |
| Western Europe | 1.13 (1.06, 1.21) | 1.01 (0.94, 1.08) | -0.39 (-0.56, -0.23) | <0.001 | 3.76 (3.62, 3.90) | 3.35 (3.22, 3.48) | -0.39 (-0.44, -0.35) | <0.001 | 0.73 (0.67, 0.79) | 0.63 (0.57, 0.69) | -0.49 (-0.63, -0.35) | <0.001 | 0.01 (0.00, 0.02) | 0.01 (0.00, 0.02) | 0.00 (0.00, 0.00)* | <0.001 |
| High-income North America | 2.43 (2.31, 2.56) | 3.11 (2.97, | 0.87 (0.77, | <0.001 | 8.07 (7.85, 8.29) | 10.31 (10.07, | 0.85 (0.80, | <0.001 | 0.90 (0.82, 0.97) | 1.11 (1.03, 1.20) | 0.78 (0.61, | <0.001 | — | — | — | — |

| | | | | | | | | | | | | | | | | |
|---|---|---|---|---|---|---|---|---|---|---|---|---|---|---|---|---|
| | | 3.24) | 0.96) | | | 10.56) | 0.91) | | | | 0.94) | | | | | |
| Central Asia | 1.43 (1.23, 1.66) | 1.46 (1.30, 1.65) | 0.08 (0.03, 0.13) | 0.004 | 4.75 (4.37, 5.16) | 4.86 (4.55, 5.19) | 0.08 (0.05, 0.11) | <0.001 | 0.71 (0.57, 0.88) | 0.75 (0.63, 0.88) | 0.20 (0.10, 0.29) | <0.001 | 0.01 (0.00, 0.04) | 0.01 (0.00, 0.03) | 0.00 (0.00, 0.00)* | <0.001 |
| Eastern Europe | 1.81 (1.69, 1.93) | 1.95 (1.83, 2.09) | 0.27 (0.17, 0.36) | <0.001 | 6.00 (5.78, 6.22) | 6.48 (6.25, 6.72) | 0.27 (0.23, 0.31) | <0.001 | 0.69 (0.62, 0.77) | 0.77 (0.69, 0.86) | 0.40 (0.22, 0.59) | <0.001 | — | — | — | — |
| Southern Sub-Saharan Africa | 1.37 (1.14, 1.64) | 1.33 (1.16, 1.52) | -0.11 (-0.21, -0.00) | 0.042 | 4.55 (4.11, 5.02) | 4.42 (4.10, 4.75) | -0.09 (-0.14, -0.03) | 0.002 | 2.51 (2.20, 2.86) | 1.37 (1.20, 1.56) | -1.95 (-3.00, -0.90) | <0.001 | 0.04 (0.01, 0.11) | 0.02 (0.00, 0.05) | -2.42 (-5.49, 0.75) | 0.133 |
| High-income Asia Pacific | 0.90 (0.80, 1.01) | 0.71 (0.62, 0.81) | -0.86 (-1.00, -0.72) | <0.001 | 2.99 (2.82, 3.18) | 2.35 (2.19, 2.52) | -0.85 (-0.92, -0.78) | <0.001 | 0.60 (0.52, 0.68) | 0.47 (0.40, 0.55) | -0.85 (-1.06, -0.65) | <0.001 | 0.01 (0.00, 0.02) | — | — | — |
| Central Europe | 1.30 (1.17, 1.45) | 1.28 (1.14, 1.44) | -0.05 (-0.13, 0.02) | 0.166 | 4.32 (4.07, 4.58) | 4.26 (4.00, 4.54) | -0.05 (-0.09, -0.02) | 0.003 | 0.71 (0.61, 0.82) | 0.67 (0.57, 0.79) | -0.20 (-0.33, -0.07) | 0.002 | 0.01 (0.00, 0.03) | 0.01 (0.00, 0.04) | 0.00 (0.00, 0.00)* | <0.001 |
| Andean Latin America | 0.25 (0.14, 0.41) | 0.29 (0.20, 0.40) | 0.60 (0.41, 0.80) | <0.001 | 0.82 (0.61, 1.08) | 0.96 (0.80, 1.15) | 0.54 (0.41, 0.66) | <0.001 | 0.95 (0.72, 1.22) | 0.85 (0.70, 1.03) | -0.43 (-0.94, 0.09) | 0.104 | 0.02 (0.00, 0.09) | 0.01 (0.00, 0.06) | -2.40 (-7.81, 3.33) | 0.404 |
| Caribbean | 0.71 (0.52, 0.95) | 0.72 (0.55, 0.91) | 0.03 (-0.02, 0.08) | 0.292 | 2.36 (2.00, 2.78) | 2.38 (2.07, 2.72) | 0.03 (0.00, 0.05) | 0.035 | 0.76 (0.56, 1.01) | 0.77 (0.60, 0.97) | 0.02 (-0.20, 0.24) | 0.842 | 0.01 (0.00, 0.08) | 0.01 (0.00, 0.06) | 0.00 (0.00, 0.00)* | <0.001 |
| Southern Latin America | 1.01 (0.82, 1.24) | 1.05 (0.88, 1.24) | 0.11 (0.01, 0.21) | 0.035 | 3.36 (3.00, 3.76) | 3.48 (3.18, 3.81) | 0.11 (0.07, 0.14) | <0.001 | 0.75 (0.59, 0.95) | 0.69 (0.56, 0.85) | -0.34 (-0.46, -0.22) | <0.001 | 0.01 (0.00, 0.06) | 0.01 (0.00, 0.04) | 0.00 (0.00, 0.00)* | 0.004 |
| Oceania | 2.11 (1.32, 3.22) | 2.16 (1.62, 2.83) | 0.06 (-0.02, 0.13) | 0.129 | 7.01 (5.47, 8.86) | 7.18 (6.15, 8.33) | 0.07 (0.02, 0.12) | 0.011 | 5.85 (4.45, 7.55) | 7.03 (6.01, 8.16) | 0.61 (0.48, 0.73) | <0.001 | 0.10 (0.00, 0.56) | 0.12 (0.02, 0.36) | 0.62 (0.62, 0.62)* | <0.001 |
| Australasia | 2.52 (2.06, 3.04) | 2.39 (2.00, 2.84) | -0.18 (-0.24, -0.12) | <0.001 | 8.35 (7.51, 9.26) | 7.93 (7.21, 8.71) | -0.18 (-0.20, -0.16) | <0.001 | 0.92 (0.65, 1.26) | 0.85 (0.63, 1.14) | -0.22 (-0.30, -0.15) | <0.001 | — | — | — | — |
| **Men** | | | | | | | | | | | | | | | | |
| Global | 3.14 (3.10, | 2.89 | -0.28 | <0.001 | 10.43 (10.37, | 9.61 (9.56, | -0.28 | <0.001 | 1.30 (1.28, | 1.29 (1.27, | -0.03 | 0.193 | 0.01 (0.01, | 0.01 (0.01, | — | — |

| | | | | | | | | | | | | | | | | |
|---|---|---|---|---|---|---|---|---|---|---|---|---|---|---|---|---|
| | 3.17) | (2.86, 2.91) | (-0.32, -0.24) | | 10.49) | 9.66) | (-0.32, -0.24) | | 1.33) | 1.31) | (-0.07, 0.01) | | 0.01) | 0.01) | | |
| **SDI category** | | | | | | | | | | | | | | | | |
| High | 5.79 (5.68, 5.90) | 5.74 (5.64, 5.84) | -0.02 (-0.08, 0.04) | 0.528 | 19.21 (19.00, 19.42) | 19.04 (18.85, 19.23) | -0.02 (-0.09, 0.05) | 0.548 | 2.15 (2.08, 2.22) | 2.10 (2.04, 2.17) | -0.07 (-0.19, 0.04) | 0.204 | 0.01 (0.01, 0.02) | 0.01 (0.01, 0.01) | — | — |
| High-middle | 3.43 (3.35, 3.50) | 3.21 (3.15, 3.27) | -0.23 (-0.28, -0.18) | <0.001 | 11.38 (11.24, 11.51) | 10.68 (10.57, 10.80) | -0.21 (-0.25, -0.18) | <0.001 | 1.37 (1.32, 1.41) | 1.30 (1.26, 1.34) | -0.19 (-0.27, -0.12) | <0.001 | 0.01 (0.00, 0.01) | 0.01 (0.00, 0.01) | 0.00 (0.00, 0.00) | 1.000 |
| Middle | 2.32 (2.26, 2.37) | 2.36 (2.32, 2.40) | 0.06 (0.04, 0.09) | <0.001 | 7.69 (7.59, 7.79) | 7.82 (7.74, 7.90) | 0.06 (0.03, 0.08) | <0.001 | 1.09 (1.05, 1.12) | 1.21 (1.18, 1.24) | 0.37 (0.27, 0.47) | <0.001 | 0.01 (0.01, 0.01) | 0.01 (0.01, 0.01) | — | — |
| Low-middle | 2.22 (2.16, 2.29) | 2.20 (2.15, 2.25) | -0.03 (-0.05, -0.00) | 0.037 | 7.38 (7.25, 7.50) | 7.29 (7.20, 7.39) | -0.04 (-0.07, -0.01) | 0.003 | 0.96 (0.91, 1.00) | 1.07 (1.03, 1.10) | 0.41 (0.28, 0.53) | <0.001 | 0.01 (0.00, 0.01) | 0.01 (0.01, 0.01) | — | — |
| Low | 1.79 (1.70, 1.89) | 1.79 (1.73, 1.85) | 0.01 (-0.02, 0.05) | 0.440 | 5.96 (5.78, 6.14) | 5.94 (5.82, 6.05) | -0.01 (-0.03, 0.01) | 0.346 | 0.90 (0.83, 0.97) | 0.96 (0.91, 1.00) | 0.21 (0.16, 0.26) | <0.001 | 0.01 (0.00, 0.02) | 0.01 (0.00, 0.01) | 0.00 (0.00, 0.00)* | <0.001 |
| **21 GBD region** | | | | | | | | | | | | | | | | |
| South Asia | 2.32 (2.25, 2.39) | 2.31 (2.27, 2.36) | -0.01 (-0.05, 0.02) | 0.492 | 7.70 (7.57, 7.82) | 7.68 (7.59, 7.77) | -0.01 (-0.03, 0.01) | 0.258 | 0.92 (0.88, 0.97) | 1.04 (1.00, 1.07) | 0.44 (0.32, 0.56) | <0.001 | 0.01 (0.00, 0.01) | 0.01 (0.00, 0.01) | — | — |
| East Asia | 2.45 (2.39, 2.51) | 2.58 (2.52, 2.63) | 0.18 (0.10, 0.25) | <0.001 | 8.14 (8.03, 8.25) | 8.55 (8.45, 8.66) | 0.17 (0.12, 0.21) | <0.001 | 0.95 (0.92, 0.99) | 0.97 (0.93, 1.00) | 0.04 (-0.03, 0.11) | 0.300 | — | — | — | — |
| North Africa and Middle East | 2.18 (2.05, 2.30) | 2.22 (2.14, 2.29) | 0.07 (0.02, 0.12) | 0.011 | 7.22 (7.00, 7.45) | 7.35 (7.21, 7.49) | 0.06 (0.03, 0.08) | <0.001 | 1.02 (0.94, 1.11) | 1.04 (0.99, 1.10) | 0.09 (0.01, 0.17) | 0.025 | 0.01 (0.00, 0.02) | 0.01 (0.00, 0.01) | 0.00 (0.00, 0.00)* | 0.020 |
| Western Sub-Saharan Africa | 1.72 (1.57, 1.88) | 1.79 (1.69, 1.89) | 0.15 (0.10, 0.19) | <0.001 | 5.71 (5.43, 6.00) | 5.93 (5.74, 6.12) | 0.14 (0.10, 0.17) | <0.001 | 0.68 (0.59, 0.79) | 0.73 (0.67, 0.80) | 0.28 (0.23, 0.33) | <0.001 | — | — | — | — |
| Southeast Asia | 2.64 (2.53, 2.75) | 2.74 (2.65, | 0.12 (0.08, | <0.001 | 8.75 (8.54, 8.96) | 9.08 (8.93, 9.24) | 0.13 (0.11, | <0.001 | 1.38 (1.30, 1.47) | 1.72 (1.65, 1.79) | 0.77 (0.68, | <0.001 | 0.01 (0.01, 0.02) | 0.02 (0.01, 0.03) | 2.34 (2.34, | <0.001 |

| | | | | | | | | | | | | | | | | |
|---|---|---|---|---|---|---|---|---|---|---|---|---|---|---|---|---|
| | | 2.82) | 0.16) | | | | 0.15) | | | | 0.86) | | | | 2.34) | |
| Eastern Sub-Saharan Africa | 1.13 (1.01, 1.27) | 1.18 (1.09, 1.26) | 0.15 (0.09, 0.21) | <0.001 | 3.76 (3.52, 4.01) | 3.90 (3.75, 4.06) | 0.12 (0.08, 0.16) | <0.001 | 0.90 (0.79, 1.03) | 0.93 (0.86, 1.01) | 0.07 (0.01, 0.13) | 0.027 | 0.01 (0.00, 0.03) | 0.01 (0.00, 0.02) | 0.00 (-0.00, 0.00) | 1.000 |
| Central Latin America | 1.25 (1.12, 1.39) | 1.27 (1.17, 1.37) | 0.06 (-0.00, 0.12) | 0.067 | 4.14 (3.90, 4.40) | 4.21 (4.03, 4.40) | 0.07 (0.03, 0.10) | <0.001 | 0.86 (0.75, 0.98) | 0.86 (0.78, 0.95) | 0.03 (-0.24, 0.30) | 0.836 | 0.01 (0.00, 0.03) | 0.01 (0.00, 0.02) | 0.00 (0.00, 0.00)* | <0.001 |
| Tropical Latin America | 1.89 (1.73, 2.07) | 1.90 (1.78, 2.02) | 0.11 (0.02, 0.19) | 0.013 | 6.29 (5.99, 6.59) | 6.30 (6.08, 6.52) | 0.10 (0.02, 0.19) | 0.016 | 1.45 (1.31, 1.60) | 1.43 (1.33, 1.54) | 0.01 (-0.24, 0.26) | 0.936 | 0.02 (0.01, 0.04) | 0.02 (0.01, 0.03) | 0.04 (-8.30, 9.14) | 0.993 |
| Central Sub-Saharan Africa | 1.60 (1.34, 1.91) | 1.63 (1.46, 1.82) | 0.06 (0.03, 0.09) | <0.001 | 5.32 (4.82, 5.87) | 5.42 (5.10, 5.75) | 0.07 (0.05, 0.08) | <0.001 | 0.95 (0.75, 1.20) | 0.97 (0.84, 1.12) | 0.09 (-0.12, 0.30) | 0.405 | 0.01 (0.00, 0.07) | 0.01 (0.00, 0.04) | 0.00 (0.00, 0.00)* | <0.001 |
| Western Europe | 6.09 (5.92, 6.27) | 5.60 (5.44, 5.77) | -0.28 (-0.32, -0.25) | <0.001 | 20.22 (19.90, 20.55) | 18.58 (18.28, 18.88) | -0.28 (-0.31, -0.26) | <0.001 | 2.51 (2.40, 2.62) | 2.17 (2.07, 2.27) | -0.51 (-0.61, -0.40) | <0.001 | 0.01 (0.01, 0.03) | 0.01 (0.00, 0.02) | 0.00 (0.00, 0.00)* | <0.001 |
| High-income North America | 6.71 (6.51, 6.92) | 8.03 (7.82, 8.25) | 0.62 (0.59, 0.66) | <0.001 | 22.29 (21.92, 22.66) | 26.67 (26.28, 27.06) | 0.63 (0.57, 0.69) | <0.001 | 2.25 (2.14, 2.37) | 2.73 (2.61, 2.86) | 0.69 (0.62, 0.76) | <0.001 | 0.01 (0.00, 0.02) | 0.01 (0.00, 0.02) | 0.00 (0.00, 0.00)* | <0.001 |
| Central Asia | 3.89 (3.55, 4.26) | 4.07 (3.79, 4.37) | 0.16 (0.14, 0.19) | <0.001 | 12.91 (12.27, 13.58) | 13.51 (12.99, 14.05) | 0.16 (0.14, 0.18) | <0.001 | 1.50 (1.29, 1.74) | 1.63 (1.46, 1.83) | 0.29 (0.11, 0.48) | 0.002 | 0.01 (0.00, 0.05) | 0.01 (0.00, 0.04) | 0.00 (0.00, 0.00)* | <0.001 |
| Eastern Europe | 4.77 (4.58, 4.96) | 5.07 (4.87, 5.28) | 0.22 (0.20, 0.25) | <0.001 | 15.82 (15.47, 16.18) | 16.82 (16.45, 17.20) | 0.21 (0.19, 0.24) | <0.001 | 1.57 (1.46, 1.68) | 1.75 (1.63, 1.87) | 0.38 (0.28, 0.48) | <0.001 | — | 0.01 (0.00, 0.02) | — | — |
| Southern Sub-Saharan Africa | 1.83 (1.55, 2.15) | 1.84 (1.64, 2.06) | 0.02 (-0.05, 0.08) | 0.574 | 6.08 (5.56, 6.63) | 6.11 (5.74, 6.50) | 0.02 (0.01, 0.04) | 0.012 | 1.95 (1.67, 2.28) | 1.67 (1.48, 1.87) | -0.68 (-1.20, -0.16) | 0.010 | 0.03 (0.00, 0.09) | 0.02 (0.01, 0.06) | -1.42 (-1.42, -1.42) | <0.001 |
| High-income Asia Pacific | 4.80 (4.58, 5.04) | 4.89 (4.66, 5.14) | 0.06 (0.02, 0.09) | 0.006 | 15.90 (15.49, 16.32) | 16.24 (15.82, 16.67) | 0.06 (0.04, 0.09) | <0.001 | 1.86 (1.72, 2.00) | 1.78 (1.64, 1.93) | -0.14 (-0.23, -0.06) | <0.001 | 0.01 (0.00, 0.03) | 0.01 (0.00, 0.03) | 0.00 (0.00, 0.00)* | <0.001 |
| Central Europe | 4.91 (4.65, 5.19) | 4.86 (4.58, | -0.03 (-0.07, | 0.158 | 16.31 (15.83, 16.80) | 16.13 (15.62, | -0.03 (-0.09, | 0.307 | 1.89 (1.73, 2.06) | 1.83 (1.67, 2.02) | -0.12 (-0.17, | <0.001 | 0.01 (0.00, 0.03) | 0.01 (0.00, 0.04) | 0.00 (-0.00, | 1.000 |

| | | | | | | | | | | | | | | | | |
|---|---|---|---|---|---|---|---|---|---|---|---|---|---|---|---|---|
| | | 5.15) | 0.01) | | | 16.65) | 0.03) | | | | -0.06) | | | | 0.00) | |
| Andean Latin America | 0.41 (0.27, 0.61) | 0.47 (0.36, 0.61) | 0.46 (0.25, 0.67) | <0.001 | 1.37 (1.09, 1.70) | 1.56 (1.35, 1.80) | 0.45 (0.39, 0.52) | <0.001 | 1.11 (0.86, 1.41) | 1.08 (0.90, 1.28) | -0.11 (-0.41, 0.18) | 0.453 | 0.02 (0.00, 0.10) | 0.02 (0.00, 0.06) | 0.00 (0.00, 0.00)* | <0.001 |
| Caribbean | 1.21 (0.94, 1.52) | 1.25 (1.02, 1.50) | 0.11 (0.07, 0.15) | <0.001 | 4.01 (3.51, 4.56) | 4.13 (3.72, 4.58) | 0.10 (0.06, 0.14) | <0.001 | 1.20 (0.93, 1.51) | 1.27 (1.04, 1.53) | 0.20 (0.01, 0.39) | 0.042 | 0.02 (0.00, 0.09) | 0.02 (0.00, 0.07) | -0.11 (-0.11, -0.11) | <0.001 |
| Southern Latin America | 2.93 (2.59, 3.31) | 3.03 (2.74, 3.35) | 0.11 (0.05, 0.16) | <0.001 | 9.73 (9.09, 10.40) | 10.07 (9.53, 10.63) | 0.11 (0.07, 0.15) | <0.001 | 1.42 (1.19, 1.69) | 1.42 (1.22, 1.64) | -0.01 (-0.26, 0.24) | 0.927 | 0.01 (0.00, 0.06) | 0.01 (0.00, 0.05) | 0.00 (0.00, 0.00)* | <0.001 |
| Oceania | 2.55 (1.69, 3.71) | 2.58 (1.98, 3.31) | 0.03 (-0.02, 0.09) | 0.263 | 8.47 (6.81, 10.42) | 8.56 (7.43, 9.82) | 0.03 (0.01, 0.05) | 0.002 | 3.51 (2.48, 4.81) | 4.10 (3.33, 4.98) | 0.52 (0.40, 0.65) | <0.001 | 0.05 (0.00, 0.45) | 0.06 (0.00, 0.27) | 0.62 (0.62, 0.62) | <0.001 |
| Australasia | 6.97 (6.20, 7.81) | 6.67 (6.00, 7.40) | -0.14 (-0.18, -0.10) | <0.001 | 23.13 (21.71, 24.62) | 22.13 (20.90, 23.42) | -0.15 (-0.18, -0.11) | <0.001 | 2.35 (1.91, 2.86) | 2.27 (1.89, 2.72) | -0.07 (-0.40, 0.26) | 0.669 | 0.01 (0.00, 0.11) | 0.01 (0.00, 0.10) | 0.00 (0.00, 0.00)* | <0.001 |
| **Aortic aneurysm** | | | | | | | | | | | | | | | | |
| **Overall** | | | | | | | | | | | | | | | | |
| Global | — | — | — | — | — | — | — | — | 8.04 (8.00, 8.08) | 8.07 (8.04, 8.10) | 0.01 (-0.19, 0.21) | 0.933 | 0.14 (0.14, 0.15) | 0.14 (0.14, 0.15) | — | — |
| **SDI category** | | | | | | | | | | | | | | | | |
| High | — | — | — | — | — | — | — | — | 10.82 (10.71, 10.94) | 8.92 (8.82, 9.02) | -0.70 (-0.95, -0.45) | <0.001 | 0.19 (0.17, 0.20) | 0.16 (0.14, 0.17) | -0.60 (-0.77, -0.43) | <0.001 |
| High-middle | — | — | — | — | — | — | — | — | 10.05 (9.96, 10.14) | 10.79 (10.71, 10.88) | 0.25 (-0.07, 0.57) | 0.126 | 0.18 (0.16, 0.19) | 0.19 (0.18, 0.20) | 0.23 (-0.05, 0.51) | 0.103 |
| Middle | — | — | — | — | — | — | — | — | 6.60 (6.54, 6.66) | 7.13 (7.08, 7.18) | 0.28 (0.06, 0.51) | 0.013 | 0.11 (0.11, 0.12) | 0.12 (0.12, 0.13) | 0.23 (0.23, 0.23) | <0.001 |

| | | | | | | | | | | | | | | | | |
|---|---|---|---|---|---|---|---|---|---|---|---|---|---|---|---|---|
| Low-middle | — | — | — | — | — | — | — | — | 5.83 (5.75, 5.90) | 7.21 (7.15, 7.28) | 0.74 (0.48, 0.99) | <0.001 | 0.10 (0.09, 0.11) | 0.13 (0.12, 0.14) | 1.06 (0.12, 2.01) | 0.028 |
| Low | — | — | — | — | — | — | — | — | 7.93 (7.80, 8.07) | 7.19 (7.10, 7.27) | -0.33 (-0.60, -0.07) | 0.015 | 0.14 (0.12, 0.16) | 0.13 (0.11, 0.14) | -0.23 (-0.56, 0.11) | 0.185 |
| **21 GBD region** | | | | | | | | | | | | | | | | |
| South Asia | — | — | — | — | — | — | — | — | 4.95 (4.88, 5.02) | 6.16 (6.10, 6.22) | 0.83 (0.37, 1.28) | <0.001 | 0.09 (0.08, 0.10) | 0.11 (0.10, 0.12) | 0.87 (-0.14, 1.90) | 0.091 |
| East Asia | — | — | — | — | — | — | — | — | 6.24 (6.18, 6.31) | 6.81 (6.74, 6.88) | 0.31 (-0.09, 0.70) | 0.125 | 0.11 (0.10, 0.12) | 0.12 (0.11, 0.13) | 0.31 (-0.13, 0.75) | 0.166 |
| North Africa and Middle East | — | — | — | — | — | — | — | — | 8.87 (8.70, 9.04) | 7.16 (7.06, 7.26) | -0.75 (-0.87, -0.62) | <0.001 | 0.15 (0.13, 0.18) | 0.12 (0.11, 0.14) | -0.80 (-1.11, -0.48) | <0.001 |
| Western Sub-Saharan Africa | — | — | — | — | — | — | — | — | 7.87 (7.65, 8.10) | 6.26 (6.14, 6.39) | -0.77 (-0.91, -0.63) | <0.001 | 0.14 (0.11, 0.18) | 0.11 (0.09, 0.13) | -0.82 (-1.28, -0.36) | <0.001 |
| Southeast Asia | — | — | — | — | — | — | — | — | 5.31 (5.20, 5.42) | 7.09 (6.99, 7.19) | 1.00 (0.78, 1.22) | <0.001 | 0.09 (0.08, 0.11) | 0.12 (0.11, 0.14) | 1.00 (0.20, 1.80) | 0.014 |
| Eastern Sub-Saharan Africa | — | — | — | — | — | — | — | — | 12.27 (11.99, 12.55) | 9.47 (9.31, 9.63) | -0.90 (-1.11, -0.68) | <0.001 | 0.21 (0.18, 0.25) | 0.16 (0.14, 0.19) | -0.94 (-1.54, -0.33) | 0.003 |
| Central Latin America | — | — | — | — | — | — | — | — | 7.42 (7.21, 7.64) | 7.11 (6.95, 7.28) | -0.09 (-0.28, 0.10) | 0.342 | 0.13 (0.10, 0.16) | 0.12 (0.10, 0.15) | -0.29 (-1.01, 0.43) | 0.423 |
| Tropical Latin America | — | — | — | — | — | — | — | — | 15.37 (15.06, 15.69) | 18.43 (18.16, 18.71) | 0.67 (0.48, 0.87) | <0.001 | 0.27 (0.23, 0.32) | 0.32 (0.29, 0.36) | 0.59 (0.31, 0.88) | <0.001 |
| Central Sub-Saharan | — | — | — | — | — | — | — | — | 9.91 (9.45, 10.38) | 8.20 (7.94, 8.47) | -0.70 (-0.94, | <0.001 | 0.17 (0.12, 0.25) | 0.14 (0.11, 0.18) | -0.74 (-1.20, | 0.002 |

| | | | | | | | | | | | | | | | | |
|---|---|---|---|---|---|---|---|---|---|---|---|---|---|---|---|---|
| Africa | | | | | | | | | | | -0.45) | | | | -0.27) | |
| Western Europe | — | — | — | — | — | — | — | — | 11.56 (11.38, 11.73) | 8.00 (7.85, 8.15) | -1.28 (-1.45, -1.12) | <0.001 | 0.20 (0.18, 0.22) | 0.14 (0.12, 0.16) | -1.22 (-1.59, -0.85) | <0.001 |
| High-income North America | — | — | — | — | — | — | — | — | 13.17 (12.96, 13.37) | 10.76 (10.59, 10.95) | -0.74 (-1.15, -0.34) | <0.001 | 0.23 (0.20, 0.26) | 0.19 (0.17, 0.22) | -0.64 (-1.25, -0.02) | 0.043 |
| Central Asia | — | — | — | — | — | — | — | — | 5.74 (5.46, 6.04) | 8.43 (8.14, 8.72) | 1.34 (0.91, 1.77) | <0.001 | 0.10 (0.07, 0.15) | 0.15 (0.11, 0.19) | 1.20 (0.58, 1.83) | <0.001 |
| Eastern Europe | — | — | — | — | — | — | — | — | 12.18 (11.96, 12.41) | 20.90 (20.58, 21.23) | 1.99 (0.87, 3.13) | <0.001 | 0.21 (0.18, 0.24) | 0.37 (0.33, 0.41) | 2.09 (1.14, 3.05) | <0.001 |
| Southern Sub-Saharan Africa | — | — | — | — | — | — | — | — | 19.89 (19.26, 20.54) | 11.73 (11.37, 12.10) | -1.94 (-2.68, -1.19) | <0.001 | 0.35 (0.27, 0.45) | 0.21 (0.16, 0.26) | -1.93 (-2.84, -1.00) | <0.001 |
| High-income Asia Pacific | — | — | — | — | — | — | — | — | 6.24 (6.05, 6.43) | 7.98 (7.76, 8.22) | 0.86 (0.69, 1.03) | <0.001 | 0.11 (0.09, 0.14) | 0.14 (0.11, 0.18) | 0.86 (0.62, 1.09) | <0.001 |
| Central Europe | — | — | — | — | — | — | — | — | 14.21 (13.87, 14.55) | 15.57 (15.18, 15.97) | 0.28 (0.10, 0.46) | 0.002 | 0.25 (0.21, 0.30) | 0.27 (0.22, 0.33) | 0.29 (0.05, 0.53) | 0.019 |
| Andean Latin America | — | — | — | — | — | — | — | — | 7.97 (7.52, 8.46) | 7.25 (6.92, 7.59) | -0.31 (-0.77, 0.14) | 0.177 | 0.14 (0.08, 0.21) | 0.12 (0.08, 0.18) | -0.48 (-0.87, -0.09) | 0.016 |
| Caribbean | — | — | — | — | — | — | — | — | 7.67 (7.21, 8.16) | 7.69 (7.29, 8.11) | 0.01 (-0.15, 0.17) | 0.866 | 0.13 (0.08, 0.21) | 0.13 (0.09, 0.20) | 0.03 (-0.32, 0.37) | 0.885 |
| Southern Latin America | — | — | — | — | — | — | — | — | 10.10 (9.65, 10.56) | 10.12 (9.74, 10.52) | 0.04 (-0.19, 0.27) | 0.732 | 0.17 (0.12, 0.25) | 0.18 (0.13, 0.24) | 0.02 (-0.33, 0.38) | 0.894 |
| Oceania | — | — | — | — | — | — | — | — | 13.64 (12.17, 15.25) | 12.50 (11.55, 13.51) | -0.31 (-0.41, | <0.001 | 0.24 (0.08, 0.56) | 0.22 (0.11, 0.40) | -0.30 (-0.42, | <0.001 |

| | | | | | | | | | | | | | | | | |
|---|---|---|---|---|---|---|---|---|---|---|---|---|---|---|---|---|
| | | | | | | | | | | | -0.20) | | | | -0.19) | |
| Australasia | — | — | — | — | — | — | — | — | 10.78 (10.08, 11.51) | 5.56 (5.11, 6.03) | -2.30 (-2.70, -1.89) | <0.001 | 0.19 (0.11, 0.31) | 0.10 (0.05, 0.18) | -2.11 (-3.09, -1.12) | <0.001 |
| **Women** | | | | | | | | | | | | | | | | |
| Global | — | — | — | — | — | — | — | — | 4.39 (4.35, 4.43) | 4.25 (4.21, 4.28) | -0.10 (-0.25, 0.04) | 0.164 | 0.08 (0.07, 0.08) | 0.07 (0.07, 0.08) | -0.45 (-0.45, -0.45) | <0.001 |
| **SDI category** | | | | | | | | | | | | | | | | |
| High | — | — | — | — | — | — | — | — | 5.35 (5.24, 5.46) | 4.38 (4.28, 4.48) | -0.68 (-0.83, -0.54) | <0.001 | 0.09 (0.08, 0.11) | 0.08 (0.06, 0.09) | -0.35 (-0.87, 0.17) | 0.191 |
| High-middle | — | — | — | — | — | — | — | — | 4.87 (4.78, 4.96) | 4.99 (4.91, 5.08) | 0.13 (-0.14, 0.39) | 0.352 | 0.08 (0.07, 0.10) | 0.09 (0.08, 0.10) | 0.05 (-0.06, 0.16) | 0.368 |
| Middle | — | — | — | — | — | — | — | — | 4.04 (3.98, 4.11) | 4.01 (3.96, 4.07) | -0.03 (-0.34, 0.27) | 0.842 | 0.07 (0.06, 0.08) | 0.07 (0.06, 0.08) | 0.01 (0.01, 0.01) | <0.001 |
| Low-middle | — | — | — | — | — | — | — | — | 3.50 (3.42, 3.58) | 4.16 (4.10, 4.23) | 0.62 (0.44, 0.79) | <0.001 | 0.06 (0.05, 0.07) | 0.07 (0.06, 0.08) | 0.53 (0.53, 0.53) | <0.001 |
| Low | — | — | — | — | — | — | — | — | 4.75 (4.61, 4.90) | 3.96 (3.87, 4.05) | -0.63 (-0.80, -0.46) | <0.001 | 0.08 (0.07, 0.11) | 0.07 (0.06, 0.08) | -0.62 (-1.03, -0.22) | 0.003 |
| **21 GBD region** | | | | | | | | | | | | | | | | |
| South Asia | — | — | — | — | — | — | — | — | 2.64 (2.56, 2.71) | 3.37 (3.31, 3.43) | 0.90 (0.57, 1.23) | <0.001 | 0.05 (0.04, 0.06) | 0.06 (0.05, 0.07) | 0.82 (-0.86, 2.54) | 0.341 |
| East Asia | — | — | — | — | — | — | — | — | 3.34 (3.27, 3.41) | 2.92 (2.86, 2.99) | -0.56 (-1.26, 0.14) | 0.115 | 0.06 (0.05, 0.07) | 0.05 (0.04, 0.06) | -0.41 (-1.43, 0.61) | 0.428 |

| | | | | | | | | | | | | | | | | |
|---|---|---|---|---|---|---|---|---|---|---|---|---|---|---|---|---|
| North Africa and Middle East | — | — | — | — | — | — | — | — | 3.62 (3.48, 3.78) | 3.72 (3.61, 3.83) | 0.11 (0.03, 0.18) | 0.004 | 0.06 (0.04, 0.08) | 0.06 (0.05, 0.08) | -0.00 (-0.00, -0.00)* | <0.001 |
| Western Sub-Saharan Africa | — | — | — | — | — | — | — | — | 6.61 (6.32, 6.92) | 3.57 (3.44, 3.70) | -2.10 (-2.28, -1.93) | <0.001 | 0.12 (0.08, 0.17) | 0.06 (0.05, 0.09) | -2.43 (-2.86, -2.01) | <0.001 |
| Southeast Asia | — | — | — | — | — | — | — | — | 3.43 (3.31, 3.55) | 4.59 (4.48, 4.71) | 1.01 (0.91, 1.12) | <0.001 | 0.06 (0.05, 0.08) | 0.08 (0.07, 0.10) | 1.00 (0.61, 1.39) | <0.001 |
| Eastern Sub-Saharan Africa | — | — | — | — | — | — | — | — | 7.32 (7.02, 7.63) | 5.48 (5.31, 5.65) | -1.00 (-1.16, -0.84) | <0.001 | 0.13 (0.09, 0.18) | 0.10 (0.07, 0.12) | -0.90 (-1.53, -0.26) | 0.006 |
| Central Latin America | — | — | — | — | — | — | — | — | 4.61 (4.37, 4.85) | 4.13 (3.95, 4.31) | -0.36 (-0.67, -0.05) | 0.023 | 0.08 (0.05, 0.12) | 0.07 (0.05, 0.10) | -0.43 (-1.28, 0.43) | 0.324 |
| Tropical Latin America | — | — | — | — | — | — | — | — | 9.79 (9.44, 10.15) | 11.30 (11.00, 11.61) | 0.55 (0.31, 0.79) | <0.001 | 0.18 (0.13, 0.23) | 0.20 (0.16, 0.24) | 0.43 (-0.14, 1.00) | 0.137 |
| Central Sub-Saharan Africa | — | — | — | — | — | — | — | — | 5.32 (4.85, 5.82) | 4.35 (4.09, 4.64) | -0.73 (-0.90, -0.55) | <0.001 | 0.09 (0.04, 0.18) | 0.08 (0.04, 0.12) | -0.35 (-0.90, 0.20) | 0.208 |
| Western Europe | — | — | — | — | — | — | — | — | 5.45 (5.28, 5.62) | 4.25 (4.09, 4.41) | -0.87 (-1.12, -0.61) | <0.001 | 0.09 (0.07, 0.12) | 0.07 (0.05, 0.10) | -1.15 (-1.67, -0.63) | <0.001 |
| High-income North America | — | — | — | — | — | — | — | — | 6.59 (6.39, 6.80) | 5.39 (5.21, 5.57) | -0.71 (-0.97, -0.45) | <0.001 | 0.11 (0.09, 0.15) | 0.09 (0.07, 0.12) | -0.67 (-0.67, -0.67) | <0.001 |
| Central Asia | — | — | — | — | — | — | — | — | 3.05 (2.76, 3.36) | 4.22 (3.93, 4.52) | 1.11 (0.88, 1.34) | <0.001 | 0.05 (0.02, 0.11) | 0.07 (0.04, 0.13) | 1.00 (1.00, 1.00) | <0.001 |
| Eastern Europe | — | — | — | — | — | — | — | — | 5.95 (5.73, 6.19) | 8.83 (8.53, 9.14) | 1.47 (0.61, 2.34) | 0.001 | 0.10 (0.08, 0.14) | 0.15 (0.12, 0.20) | 1.48 (0.37, 2.61) | 0.009 |

| | | | | | | | | | | | | | | | | |
|---|---|---|---|---|---|---|---|---|---|---|---|---|---|---|---|---|
| Southern Sub-Saharan Africa | — | — | — | — | — | — | — | — | 16.26 (15.48, 17.07) | 7.08 (6.69, 7.49) | -2.82 (-3.68, -1.96) | <0.001 | 0.28 (0.19, 0.41) | 0.12 (0.08, 0.19) | -2.80 (-3.89, -1.70) | <0.001 |
| High-income Asia Pacific | — | — | — | — | — | — | — | — | 2.81 (2.63, 3.00) | 3.59 (3.37, 3.83) | 0.87 (0.75, 0.99) | <0.001 | 0.05 (0.03, 0.08) | 0.06 (0.04, 0.10) | 0.62 (-0.35, 1.60) | 0.210 |
| Central Europe | — | — | — | — | — | — | — | — | 6.95 (6.62, 7.30) | 7.64 (7.25, 8.05) | 0.32 (0.15, 0.49) | <0.001 | 0.12 (0.08, 0.18) | 0.13 (0.09, 0.20) | 0.34 (-0.22, 0.89) | 0.235 |
| Andean Latin America | — | — | — | — | — | — | — | — | 5.39 (4.87, 5.95) | 5.03 (4.65, 5.44) | -0.25 (-0.92, 0.43) | 0.478 | 0.09 (0.04, 0.20) | 0.08 (0.04, 0.15) | -0.39 (-0.39, -0.39) | <0.001 |
| Caribbean | — | — | — | — | — | — | — | — | 4.19 (3.73, 4.71) | 4.55 (4.12, 5.00) | 0.28 (0.12, 0.43) | <0.001 | 0.07 (0.02, 0.17) | 0.08 (0.03, 0.16) | 0.64 (-0.26, 1.55) | 0.163 |
| Southern Latin America | — | — | — | — | — | — | — | — | 6.05 (5.57, 6.57) | 5.68 (5.27, 6.10) | -0.24 (-0.50, 0.03) | 0.079 | 0.10 (0.05, 0.19) | 0.10 (0.05, 0.17) | -0.31 (-0.83, 0.21) | 0.243 |
| Oceania | — | — | — | — | — | — | — | — | 3.73 (2.66, 5.10) | 3.30 (2.63, 4.10) | -0.41 (-0.56, -0.26) | <0.001 | 0.07 (0.00, 0.50) | 0.06 (0.00, 0.26) | -0.52 (-0.52, -0.52) | <0.001 |
| Australasia | — | — | — | — | — | — | — | — | 5.45 (4.76, 6.20) | 2.98 (2.53, 3.50) | -2.03 (-2.20, -1.86) | <0.001 | 0.09 (0.03, 0.25) | 0.05 (0.01, 0.18) | -2.00 (-2.58, -1.41) | <0.001 |
| **Men** | | | | | | | | | | | | | | | | |
| Global | — | — | — | — | — | — | — | — | 11.60 (11.53, 11.66) | 11.82 (11.77, 11.88) | 0.06 (-0.13, 0.24) | 0.564 | 0.20 (0.19, 0.21) | 0.21 (0.20, 0.22) | 0.20 (-0.38, 0.78) | 0.507 |
| **SDI category** | | | | | | | | | | | | | | | | |
| High | — | — | — | — | — | — | — | — | 16.13 (15.94, 16.33) | 13.15 (12.99, 13.32) | -0.75 (-0.99, -0.52) | <0.001 | 0.28 (0.26, 0.31) | 0.23 (0.21, 0.26) | -0.63 (-0.80, -0.45) | <0.001 |
| High-middle | — | — | — | — | — | — | — | — | 15.07 (14.91, | 16.26 (16.11, | 0.22 | 0.078 | 0.26 (0.24, | 0.29 (0.27, | 0.28 | 0.078 |

| | | | | | | | | | | | | | | | | |
|---|---|---|---|---|---|---|---|---|---|---|---|---|---|---|---|---|
| | | | | | | | | | 15.22) | 16.41) | (-0.03, 0.46) | | 0.28) | 0.31) | (-0.03, 0.59) | |
| Middle | — | — | — | — | — | — | — | — | 9.08 (8.98, 9.18) | 10.21 (10.13, 10.31) | 0.43 (0.14, 0.71) | 0.003 | 0.16 (0.15, 0.17) | 0.18 (0.17, 0.19) | 0.42 (0.42, 0.42) | <0.001 |
| Low-middle | — | — | — | — | — | — | — | — | 8.10 (7.98, 8.23) | 10.26 (10.16, 10.37) | 0.88 (0.60, 1.17) | <0.001 | 0.14 (0.13, 0.16) | 0.18 (0.17, 0.20) | 0.97 (0.63, 1.32) | <0.001 |
| Low | — | — | — | — | — | — | — | — | 11.20 (10.98, 11.43) | 10.52 (10.38, 10.67) | -0.24 (-0.48, 0.01) | 0.058 | 0.20 (0.17, 0.23) | 0.18 (0.17, 0.20) | -0.36 (-0.36, -0.36) | <0.001 |
| **21 GBD region** | | | | | | | | | | | | | | | | |
| South Asia | — | — | — | — | — | — | — | — | 7.10 (6.99, 7.22) | 8.85 (8.76, 8.95) | 0.85 (0.25, 1.45) | 0.006 | 0.13 (0.11, 0.14) | 0.16 (0.14, 0.17) | 0.68 (-0.06, 1.44) | 0.073 |
| East Asia | — | — | — | — | — | — | — | — | 8.96 (8.85, 9.07) | 10.54 (10.42, 10.66) | 0.57 (0.15, 1.01) | 0.009 | 0.16 (0.14, 0.17) | 0.19 (0.17, 0.20) | 0.60 (-0.31, 1.51) | 0.196 |
| North Africa and Middle East | — | — | — | — | — | — | — | — | 13.83 (13.53, 14.12) | 10.27 (10.11, 10.45) | -1.03 (-1.20, -0.86) | <0.001 | 0.24 (0.20, 0.28) | 0.18 (0.16, 0.20) | -0.99 (-1.44, -0.53) | <0.001 |
| Western Sub-Saharan Africa | — | — | — | — | — | — | — | — | 9.30 (8.96, 9.65) | 9.26 (9.04, 9.48) | 0.00 (-0.17, 0.17) | 0.987 | 0.16 (0.12, 0.22) | 0.16 (0.13, 0.19) | -0.06 (-0.43, 0.30) | 0.727 |
| Southeast Asia | — | — | — | — | — | — | — | — | 7.24 (7.07, 7.42) | 9.53 (9.37, 9.69) | 1.01 (0.64, 1.38) | <0.001 | 0.12 (0.10, 0.15) | 0.16 (0.14, 0.19) | 0.95 (0.66, 1.23) | <0.001 |
| Eastern Sub-Saharan Africa | — | — | — | — | — | — | — | — | 17.63 (17.15, 18.12) | 13.72 (13.44, 13.99) | -0.86 (-1.05, -0.66) | <0.001 | 0.31 (0.24, 0.38) | 0.24 (0.20, 0.28) | -0.93 (-1.27, -0.59) | <0.001 |
| Central Latin America | — | — | — | — | — | — | — | — | 10.41 (10.05, 10.79) | 10.27 (9.99, 10.56) | -0.03 (-0.28, 0.22) | 0.827 | 0.18 (0.13, 0.24) | 0.18 (0.14, 0.22) | -0.01 (-0.63, 0.61) | 0.964 |

| | | | | | | | | | | | | | | | | |
|---|---|---|---|---|---|---|---|---|---|---|---|---|---|---|---|---|
| Tropical Latin America | — | — | — | — | — | — | — | — | 21.18 (20.65, 21.72) | 25.74 (25.28, 26.21) | 0.68 (0.43, 0.94) | <0.001 | 0.38 (0.31, 0.46) | 0.45 (0.39, 0.52) | 0.62 (0.22, 1.03) | 0.003 |
| Central Sub-Saharan Africa | — | — | — | — | — | — | — | — | 14.62 (13.84, 15.44) | 12.12 (11.67, 12.58) | -0.69 (-1.00, -0.39) | <0.001 | 0.25 (0.16, 0.39) | 0.21 (0.16, 0.28) | -0.65 (-0.98, -0.32) | <0.001 |
| Western Europe | — | — | — | — | — | — | — | — | 17.53 (17.23, 17.84) | 11.66 (11.41, 11.91) | -1.42 (-1.66, -1.19) | <0.001 | 0.30 (0.26, 0.35) | 0.20 (0.17, 0.24) | -1.48 (-1.86, -1.11) | <0.001 |
| High-income North America | — | — | — | — | — | — | — | — | 19.73 (19.37, 20.09) | 16.12 (15.81, 16.43) | -0.76 (-1.15, -0.37) | <0.001 | 0.34 (0.30, 0.39) | 0.29 (0.25, 0.33) | -0.57 (-1.06, -0.08) | 0.023 |
| Central Asia | — | — | — | — | — | — | — | — | 8.51 (8.02, 9.02) | 12.59 (12.10, 13.10) | 1.37 (0.73, 2.01) | <0.001 | 0.15 (0.09, 0.23) | 0.22 (0.16, 0.30) | 1.32 (0.72, 1.92) | <0.001 |
| Eastern Europe | — | — | — | — | — | — | — | — | 18.40 (18.01, 18.80) | 32.93 (32.36, 33.51) | 2.14 (1.33, 2.97) | <0.001 | 0.32 (0.27, 0.38) | 0.58 (0.51, 0.66) | 2.22 (0.94, 3.51) | 0.001 |
| Southern Sub-Saharan Africa | — | — | — | — | — | — | — | — | 23.75 (22.76, 24.77) | 16.41 (15.81, 17.04) | -1.36 (-2.08, -0.63) | <0.001 | 0.42 (0.30, 0.58) | 0.29 (0.21, 0.38) | -1.40 (-2.29, -0.50) | 0.002 |
| High-income Asia Pacific | — | — | — | — | — | — | — | — | 9.57 (9.24, 9.90) | 12.10 (11.71, 12.50) | 0.82 (0.54, 1.11) | <0.001 | 0.17 (0.13, 0.22) | 0.22 (0.17, 0.28) | 0.92 (0.45, 1.38) | <0.001 |
| Central Europe | — | — | — | — | — | — | — | — | 21.28 (20.71, 21.87) | 23.16 (22.50, 23.84) | 0.27 (-0.07, 0.60) | 0.115 | 0.37 (0.30, 0.46) | 0.41 (0.32, 0.51) | 0.31 (0.04, 0.58) | 0.024 |
| Andean Latin America | — | — | — | — | — | — | — | — | 10.69 (9.92, 11.49) | 9.51 (8.98, 10.07) | -0.38 (-0.77, 0.02) | 0.063 | 0.18 (0.10, 0.32) | 0.16 (0.10, 0.25) | -0.42 (-0.81, -0.02) | 0.039 |
| Caribbean | — | — | — | — | — | — | — | — | 11.34 (10.54, 12.19) | 10.93 (10.25, 11.64) | -0.08 (-0.30, 0.14) | 0.482 | 0.20 (0.10, 0.34) | 0.19 (0.11, 0.31) | -0.13 (-0.38, 0.12) | 0.315 |

| | | | | | | | | | | | | | | | | |
|---|---|---|---|---|---|---|---|---|---|---|---|---|---|---|---|---|
| Southern Latin America | — | — | — | — | — | — | — | — | 14.25 (13.49, 15.04) | 14.65 (14.00, 15.33) | 0.14 (-0.14, 0.43) | 0.323 | 0.25 (0.16, 0.37) | 0.25 (0.18, 0.36) | 0.10 (-0.26, 0.46) | 0.599 |
| Oceania | — | — | — | — | — | — | — | — | 23.08 (20.42, 26.01) | 21.64 (19.88, 23.52) | -0.23 (-0.34, -0.13) | <0.001 | 0.41 (0.13, 0.99) | 0.38 (0.18, 0.71) | -0.26 (-0.40, -0.11) | 0.001 |
| Australasia | — | — | — | — | — | — | — | — | 16.14 (14.94, 17.41) | 8.20 (7.44, 9.02) | -2.35 (-2.73, -1.96) | <0.001 | 0.28 (0.14, 0.50) | 0.15 (0.06, 0.30) | -2.27 (-2.96, -1.57) | <0.001 |
| **Endocarditis** | | | | | | | | | | | | | | | | |
| **Overall** | | | | | | | | | | | | | | | | |
| Global | 5.53 (5.50, 5.56) | 5.73 (5.70, 5.76) | 0.13 (0.08, 0.18) | <0.001 | 0.97 (0.95, 0.98) | 0.96 (0.95, 0.97) | -0.02 (-0.09, 0.06) | 0.672 | 13.11 (13.06, 13.15) | 13.50 (13.46, 13.54) | 0.09 (-0.12, 0.30) | 0.390 | 0.22 (0.21, 0.23) | 0.23 (0.22, 0.23) | 0.15 (-0.11, 0.40) | 0.255 |
| **SDI category** | | | | | | | | | | | | | | | | |
| High | 6.35 (6.26, 6.43) | 6.43 (6.35, 6.52) | 0.04 (0.01, 0.07) | 0.024 | 1.98 (1.93, 2.03) | 1.87 (1.82, 1.91) | -0.19 (-0.29, -0.09) | <0.001 | 7.49 (7.39, 7.58) | 8.94 (8.84, 9.04) | 0.60 (0.44, 0.76) | <0.001 | 0.12 (0.11, 0.14) | 0.15 (0.14, 0.17) | 0.84 (0.38, 1.31) | <0.001 |
| High-middle | 6.56 (6.49, 6.63) | 6.98 (6.91, 7.06) | 0.21 (0.12, 0.30) | <0.001 | 0.92 (0.89, 0.94) | 1.05 (1.02, 1.07) | 0.46 (0.38, 0.54) | <0.001 | 12.54 (12.44, 12.64) | 14.04 (13.94, 14.14) | 0.39 (-0.25, 1.04) | 0.234 | 0.21 (0.20, 0.22) | 0.24 (0.23, 0.25) | 0.53 (0.08, 0.97) | 0.020 |
| Middle | 5.94 (5.88, 5.99) | 5.96 (5.91, 6.01) | 0.03 (-0.02, 0.08) | 0.256 | 0.91 (0.89, 0.93) | 0.98 (0.96, 1.00) | 0.26 (0.17, 0.34) | <0.001 | 14.99 (14.90, 15.08) | 14.21 (14.14, 14.29) | -0.18 (-0.36, 0.01) | 0.067 | 0.25 (0.24, 0.26) | 0.24 (0.23, 0.25) | -0.13 (-0.47, 0.22) | 0.467 |
| Low-middle | 3.86 (3.80, 3.92) | 4.85 (4.79, 4.90) | 0.80 (0.74, 0.86) | <0.001 | 0.49 (0.47, 0.51) | 0.62 (0.60, 0.63) | 0.82 (0.72, 0.92) | <0.001 | 13.62 (13.51, 13.73) | 13.72 (13.64, 13.81) | 0.02 (-0.18, 0.23) | 0.836 | 0.23 (0.21, 0.24) | 0.23 (0.22, 0.24) | 0.08 (-0.18, 0.34) | 0.567 |
| Low | 3.52 (3.44, 3.61) | 4.34 (4.28, 4.41) | 0.76 (0.64, 0.89) | <0.001 | 0.55 (0.52, 0.59) | 0.67 (0.64, 0.69) | 0.70 (0.61, 0.79) | <0.001 | 16.28 (16.09, 16.47) | 14.21 (14.09, 14.32) | -0.47 (-0.62, -0.32) | <0.001 | 0.27 (0.25, 0.30) | 0.24 (0.22, 0.26) | -0.41 (-0.41, -0.41) | <0.001 |
| **21 GBD region** | | | | | | | | | | | | | | | | |
| South Asia | 3.20 (3.15, 3.26) | 4.47 (4.42, | 1.19 (1.09, | <0.001 | 0.35 (0.33, 0.37) | 0.46 (0.45, 0.48) | 0.99 (0.84, | <0.001 | 10.90 (10.80, 11.00) | 11.56 (11.49, 11.64) | 0.19 (-0.15, | 0.271 | 0.18 (0.17, 0.20) | 0.20 (0.19, 0.21) | 0.28 (-0.40, | 0.421 |

| | | | | | | | | | | | | | | | | |
|---|---|---|---|---|---|---|---|---|---|---|---|---|---|---|---|---|
| | | 4.51) | 1.28) | | | | 1.14) | | | | 0.52) | | | | 0.97) | |
| East Asia | 7.58 (7.51, 7.65) | 5.38 (5.32, 5.44) | -1.14 (-1.41, -0.86) | <0.001 | 0.86 (0.84, 0.89) | 0.80 (0.78, 0.83) | -0.23 (-0.33, -0.14) | <0.001 | 8.11 (8.04, 8.19) | 4.09 (4.04, 4.15) | -2.31 (-2.80, -1.82) | <0.001 | 0.13 (0.12, 0.14) | 0.07 (0.06, 0.08) | -1.93 (-2.65, -1.20) | <0.001 |
| North Africa and Middle East | 5.64 (5.51, 5.77) | 7.06 (6.96, 7.16) | 0.79 (0.74, 0.84) | <0.001 | 0.92 (0.87, 0.97) | 1.04 (1.00, 1.08) | 0.41 (0.36, 0.45) | <0.001 | 11.14 (10.96, 11.33) | 8.74 (8.63, 8.85) | -0.81 (-0.92, -0.71) | <0.001 | 0.19 (0.16, 0.21) | 0.15 (0.13, 0.16) | -0.70 (-1.10, -0.30) | 0.001 |
| Western Sub-Saharan Africa | 3.79 (3.65, 3.94) | 4.25 (4.15, 4.35) | 0.46 (0.33, 0.58) | <0.001 | 0.79 (0.73, 0.86) | 0.88 (0.84, 0.93) | 0.38 (0.24, 0.52) | <0.001 | 17.99 (17.66, 18.32) | 12.44 (12.27, 12.61) | -1.25 (-1.37, -1.14) | <0.001 | 0.31 (0.26, 0.35) | 0.21 (0.19, 0.23) | -1.33 (-1.58, -1.08) | <0.001 |
| Southeast Asia | 5.59 (5.48, 5.69) | 7.13 (7.03, 7.23) | 0.84 (0.82, 0.86) | <0.001 | 1.55 (1.49, 1.60) | 1.66 (1.61, 1.71) | 0.23 (0.20, 0.25) | <0.001 | 34.40 (34.13, 34.67) | 34.14 (33.92, 34.35) | -0.03 (-0.25, 0.20) | 0.814 | 0.58 (0.55, 0.62) | 0.57 (0.55, 0.60) | -0.08 (-0.55, 0.40) | 0.752 |
| Eastern Sub-Saharan Africa | 3.97 (3.82, 4.13) | 4.29 (4.19, 4.40) | 0.32 (0.20, 0.43) | <0.001 | 0.83 (0.77, 0.91) | 0.93 (0.88, 0.98) | 0.41 (0.34, 0.47) | <0.001 | 24.96 (24.57, 25.35) | 19.07 (18.85, 19.29) | -0.93 (-1.11, -0.75) | <0.001 | 0.42 (0.37, 0.47) | 0.32 (0.29, 0.35) | -0.97 (-1.22, -0.73) | <0.001 |
| Central Latin America | 4.64 (4.48, 4.82) | 6.53 (6.37, 6.69) | 1.18 (1.13, 1.22) | <0.001 | 0.64 (0.58, 0.70) | 0.84 (0.78, 0.89) | 0.94 (0.84, 1.05) | <0.001 | 9.11 (8.88, 9.35) | 11.13 (10.92, 11.33) | 0.73 (0.47, 0.99) | <0.001 | 0.15 (0.12, 0.19) | 0.19 (0.16, 0.21) | 0.80 (0.19, 1.41) | 0.010 |
| Tropical Latin America | 4.82 (4.65, 5.00) | 7.29 (7.12, 7.47) | 1.42 (1.34, 1.49) | <0.001 | 0.71 (0.64, 0.77) | 0.98 (0.92, 1.05) | 1.11 (1.00, 1.23) | <0.001 | 23.89 (23.51, 24.27) | 20.99 (20.69, 21.29) | -0.44 (-0.67, -0.20) | <0.001 | 0.40 (0.35, 0.45) | 0.35 (0.31, 0.39) | -0.42 (-0.74, -0.10) | 0.011 |
| Central Sub-Saharan Africa | 3.11 (2.87, 3.37) | 3.64 (3.48, 3.82) | 0.59 (0.47, 0.72) | <0.001 | 0.71 (0.59, 0.84) | 0.77 (0.69, 0.85) | 0.30 (0.25, 0.34) | <0.001 | 17.54 (16.95, 18.15) | 15.22 (14.88, 15.58) | -0.53 (-0.62, -0.45) | <0.001 | 0.30 (0.22, 0.39) | 0.26 (0.21, 0.31) | -0.51 (-0.67, -0.35) | <0.001 |
| Western Europe | 6.47 (6.34, 6.60) | 7.01 (6.87, 7.15) | 0.27 (0.22, 0.33) | <0.001 | 1.20 (1.15, 1.26) | 1.07 (1.01, 1.12) | -0.42 (-0.53, -0.31) | <0.001 | 5.23 (5.11, 5.34) | 6.67 (6.53, 6.81) | 0.80 (0.66, 0.93) | <0.001 | 0.09 (0.07, 0.10) | 0.11 (0.10, 0.13) | 0.72 (0.01, 1.43) | 0.046 |
| High-income North America | 6.26 (6.11, 6.41) | 5.67 (5.54, 5.80) | -0.34 (-0.39, -0.28) | <0.001 | 3.09 (2.99, 3.19) | 3.01 (2.91, 3.11) | -0.05 (-0.22, 0.13) | 0.623 | 7.24 (7.09, 7.40) | 12.33 (12.13, 12.52) | 1.88 (1.56, 2.20) | <0.001 | 0.12 (0.10, 0.14) | 0.21 (0.19, 0.24) | 1.94 (1.42, 2.45) | <0.001 |
| Central Asia | 3.13 (2.93, 3.35) | 3.85 (3.65, | 0.73 (0.67, | <0.001 | 0.30 (0.24, 0.37) | 0.35 (0.30, 0.42) | 0.56 (0.38, | <0.001 | 5.30 (5.04, 5.58) | 7.98 (7.70, 8.27) | 1.42 (1.10, | <0.001 | 0.09 (0.06, 0.13) | 0.13 (0.10, 0.18) | 1.02 (0.22, | 0.013 |

| | | | | | | | | | | | | | | | | |
|---|---|---|---|---|---|---|---|---|---|---|---|---|---|---|---|---|
| | | 4.05) | 0.79) | | | | 0.74) | | | | 1.75) | | | | 1.84) | |
| Eastern Europe | 5.62 (5.46, 5.78) | 11.48 (11.23, 11.73) | 2.50 (2.28, 2.73) | <0.001 | 0.60 (0.55, 0.65) | 1.06 (0.98, 1.14) | 1.98 (1.77, 2.19) | <0.001 | 14.85 (14.60, 15.11) | 42.86 (42.39, 43.33) | 3.79 (2.61, 4.99) | <0.001 | 0.25 (0.22, 0.28) | 0.74 (0.68, 0.80) | 3.91 (2.58, 5.25) | <0.001 |
| Southern Sub-Saharan Africa | 5.91 (5.58, 6.25) | 5.51 (5.26, 5.76) | -0.23 (-0.32, -0.15) | <0.001 | 1.42 (1.27, 1.59) | 1.31 (1.19, 1.44) | -0.29 (-0.41, -0.17) | <0.001 | 45.22 (44.29, 46.16) | 25.49 (24.95, 26.03) | -1.92 (-3.12, -0.71) | 0.002 | 0.77 (0.65, 0.90) | 0.43 (0.36, 0.50) | -1.97 (-3.18, -0.75) | 0.002 |
| High-income Asia Pacific | 6.77 (6.57, 6.97) | 6.03 (5.83, 6.25) | -0.40 (-0.44, -0.36) | <0.001 | 1.72 (1.62, 1.82) | 1.49 (1.39, 1.60) | -0.50 (-0.54, -0.46) | <0.001 | 9.26 (9.03, 9.50) | 4.78 (4.59, 4.96) | -2.28 (-2.61, -1.95) | <0.001 | 0.15 (0.12, 0.18) | 0.08 (0.06, 0.11) | -2.28 (-2.67, -1.88) | <0.001 |
| Central Europe | 4.84 (4.64, 5.04) | 5.89 (5.64, 6.14) | 0.68 (0.52, 0.83) | <0.001 | 0.61 (0.54, 0.68) | 0.70 (0.61, 0.79) | 0.48 (0.34, 0.62) | <0.001 | 7.13 (6.89, 7.38) | 9.04 (8.74, 9.35) | 0.81 (0.48, 1.14) | <0.001 | 0.12 (0.09, 0.16) | 0.15 (0.12, 0.20) | 0.74 (0.25, 1.24) | 0.003 |
| Andean Latin America | 3.67 (3.37, 4.00) | 6.26 (5.96, 6.58) | 1.86 (1.82, 1.91) | <0.001 | 0.47 (0.37, 0.60) | 0.77 (0.67, 0.89) | 1.71 (1.56, 1.86) | <0.001 | 16.23 (15.59, 16.90) | 12.03 (11.61, 12.46) | -0.99 (-1.42, -0.56) | <0.001 | 0.27 (0.19, 0.37) | 0.20 (0.15, 0.26) | -1.01 (-1.74, -0.27) | 0.007 |
| Caribbean | 5.35 (4.98, 5.75) | 9.10 (8.67, 9.55) | 1.85 (1.82, 1.89) | <0.001 | 0.75 (0.61, 0.91) | 1.03 (0.89, 1.19) | 1.11 (1.06, 1.16) | <0.001 | 18.44 (17.74, 19.17) | 23.68 (22.98, 24.40) | 0.91 (0.64, 1.18) | <0.001 | 0.31 (0.22, 0.42) | 0.40 (0.31, 0.50) | 0.92 (0.52, 1.31) | <0.001 |
| Southern Latin America | 4.07 (3.78, 4.36) | 7.61 (7.27, 7.95) | 2.18 (2.09, 2.27) | <0.001 | 0.83 (0.70, 0.96) | 1.16 (1.03, 1.30) | 1.17 (1.10, 1.25) | <0.001 | 11.29 (10.82, 11.78) | 14.63 (14.17, 15.11) | 0.92 (0.67, 1.16) | <0.001 | 0.19 (0.13, 0.27) | 0.25 (0.19, 0.32) | 1.04 (0.75, 1.34) | <0.001 |
| Oceania | 5.05 (4.21, 6.03) | 6.14 (5.49, 6.85) | 0.69 (0.61, 0.77) | <0.001 | 0.64 (0.37, 1.06) | 0.68 (0.48, 0.94) | 0.19 (0.03, 0.34) | 0.019 | 22.84 (20.98, 24.83) | 31.05 (29.56, 32.59) | 1.03 (0.90, 1.16) | <0.001 | 0.39 (0.18, 0.74) | 0.53 (0.35, 0.77) | 1.02 (0.85, 1.20) | <0.001 |
| Australasia | 5.76 (5.25, 6.30) | 6.41 (5.92, 6.93) | 0.37 (0.29, 0.45) | <0.001 | 1.27 (1.04, 1.54) | 1.08 (0.88, 1.31) | -0.55 (-0.66, -0.44) | <0.001 | 5.93 (5.42, 6.48) | 7.63 (7.10, 8.19) | 0.96 (0.49, 1.43) | <0.001 | 0.10 (0.04, 0.19) | 0.13 (0.07, 0.22) | 0.99 (-0.04, 2.03) | 0.059 |
| **Women** | | | | | | | | | | | | | | | | |
| Global | 5.15 (5.11, 5.19) | 5.06 (5.02, 5.10) | -0.04 (-0.09, 0.02) | 0.167 | 1.01 (0.99, 1.03) | 0.95 (0.94, 0.97) | -0.19 (-0.30, -0.08) | 0.001 | 11.10 (11.04, 11.17) | 10.57 (10.51, 10.62) | -0.18 (-0.31, -0.04) | 0.013 | 0.18 (0.18, 0.19) | 0.18 (0.17, 0.18) | -0.06 (-0.37, 0.26) | 0.732 |

| | | | | | | | | | | | | | | | | |
|---|---|---|---|---|---|---|---|---|---|---|---|---|---|---|---|---|
| **SDI category** | | | | | | | | | | | | | | | | |
| High | 5.89 (5.78, 6.02) | 5.67 (5.55, 5.79) | -0.13 (-0.17, -0.10) | <0.001 | 2.28 (2.21, 2.35) | 2.05 (1.98, 2.12) | -0.34 (-0.43, -0.25) | <0.001 | 5.73 (5.61, 5.85) | 6.87 (6.75, 7.00) | 0.62 (0.42, 0.82) | <0.001 | 0.09 (0.08, 0.11) | 0.11 (0.10, 0.13) | 0.82 (0.10, 1.54) | 0.025 |
| High-middle | 6.03 (5.93, 6.13) | 6.05 (5.95, 6.14) | 0.01 (-0.06, 0.08) | 0.822 | 0.90 (0.86, 0.94) | 0.99 (0.95, 1.03) | 0.35 (0.29, 0.41) | <0.001 | 8.35 (8.23, 8.46) | 8.51 (8.40, 8.63) | 0.05 (-0.43, 0.53) | 0.830 | 0.14 (0.12, 0.15) | 0.14 (0.13, 0.16) | 0.21 (-0.54, 0.96) | 0.585 |
| Middle | 5.70 (5.63, 5.78) | 5.41 (5.34, 5.47) | -0.17 (-0.21, -0.13) | <0.001 | 0.95 (0.92, 0.98) | 0.97 (0.94, 1.00) | 0.07 (-0.04, 0.17) | 0.199 | 13.41 (13.29, 13.53) | 11.59 (11.49, 11.69) | -0.49 (-0.86, -0.12) | 0.010 | 0.22 (0.21, 0.24) | 0.19 (0.18, 0.21) | -0.51 (-1.11, 0.09) | 0.096 |
| Low-middle | 3.55 (3.47, 3.63) | 4.36 (4.30, 4.43) | 0.73 (0.63, 0.84) | <0.001 | 0.48 (0.45, 0.51) | 0.60 (0.57, 0.62) | 0.77 (0.68, 0.86) | <0.001 | 12.95 (12.79, 13.10) | 12.02 (11.91, 12.14) | -0.22 (-0.51, 0.08) | 0.156 | 0.21 (0.20, 0.24) | 0.20 (0.19, 0.22) | -0.16 (-0.45, 0.13) | 0.288 |
| Low | 3.05 (2.94, 3.17) | 3.72 (3.63, 3.80) | 0.76 (0.66, 0.86) | <0.001 | 0.54 (0.49, 0.59) | 0.64 (0.61, 0.67) | 0.61 (0.47, 0.76) | <0.001 | 14.77 (14.52, 15.02) | 11.16 (11.02, 11.30) | -0.97 (-1.11, -0.84) | <0.001 | 0.25 (0.22, 0.28) | 0.19 (0.17, 0.21) | -0.95 (-1.41, -0.49) | <0.001 |
| **21 GBD region** | | | | | | | | | | | | | | | | |
| South Asia | 2.90 (2.83, 2.98) | 4.07 (4.00, 4.13) | 1.20 (1.12, 1.29) | <0.001 | 0.34 (0.31, 0.36) | 0.44 (0.42, 0.46) | 0.98 (0.78, 1.18) | <0.001 | 10.18 (10.05, 10.32) | 10.15 (10.04, 10.25) | -0.06 (-0.56, 0.45) | 0.821 | 0.17 (0.15, 0.19) | 0.17 (0.16, 0.18) | -0.08 (-0.89, 0.75) | 0.855 |
| East Asia | 7.42 (7.32, 7.53) | 4.93 (4.84, 5.02) | -1.36 (-1.68, -1.03) | <0.001 | 0.84 (0.81, 0.88) | 0.75 (0.71, 0.78) | -0.37 (-0.48, -0.26) | <0.001 | 5.39 (5.30, 5.48) | 2.19 (2.13, 2.25) | -3.25 (-4.06, -2.43) | <0.001 | 0.09 (0.08, 0.10) | 0.04 (0.03, 0.04) | -2.72 (-3.73, -1.71) | <0.001 |
| North Africa and Middle East | 4.32 (4.16, 4.49) | 5.58 (5.45, 5.71) | 0.88 (0.85, 0.92) | <0.001 | 0.76 (0.69, 0.83) | 0.84 (0.79, 0.89) | 0.31 (0.22, 0.40) | <0.001 | 8.30 (8.07, 8.53) | 6.45 (6.31, 6.59) | -0.85 (-0.96, -0.73) | <0.001 | 0.14 (0.11, 0.17) | 0.11 (0.09, 0.13) | -0.89 (-1.43, -0.34) | 0.001 |
| Western Sub-Saharan Africa | 3.40 (3.21, 3.60) | 3.71 (3.59, 3.84) | 0.36 (0.23, 0.48) | <0.001 | 0.84 (0.74, 0.94) | 0.93 (0.87, 1.00) | 0.42 (0.34, 0.50) | <0.001 | 18.52 (18.06, 19.00) | 10.29 (10.07, 10.51) | -2.01 (-2.15, -1.88) | <0.001 | 0.32 (0.26, 0.39) | 0.17 (0.15, 0.20) | -2.11 (-2.55, -1.66) | <0.001 |
| Southeast Asia | 5.34 (5.20, 5.49) | 6.87 (6.73, 7.01) | 0.86 (0.84, 0.89) | <0.001 | 1.68 (1.60, 1.76) | 1.74 (1.67, 1.82) | 0.11 (0.06, 0.17) | <0.001 | 31.50 (31.15, 31.86) | 28.88 (28.59, 29.17) | -0.30 (-0.69, 0.09) | 0.128 | 0.53 (0.48, 0.58) | 0.48 (0.45, 0.52) | -0.34 (-0.78, 0.10) | 0.134 |

| | | | | | | | | | | | | | | | | |
|---|---|---|---|---|---|---|---|---|---|---|---|---|---|---|---|---|
| Eastern Sub-Saharan Africa | 3.25 (3.06, 3.45) | 3.44 (3.31, 3.57) | 0.25 (0.13, 0.36) | <0.001 | 0.79 (0.70, 0.89) | 0.88 (0.82, 0.95) | 0.36 (0.20, 0.51) | <0.001 | 21.82 (21.32, 22.33) | 13.83 (13.57, 14.09) | -1.56 (-1.75, -1.36) | <0.001 | 0.37 (0.30, 0.44) | 0.23 (0.20, 0.27) | -1.63 (-2.04, -1.22) | <0.001 |
| Central Latin America | 4.56 (4.33, 4.80) | 5.64 (5.43, 5.84) | 0.74 (0.63, 0.86) | <0.001 | 0.71 (0.62, 0.81) | 0.83 (0.76, 0.92) | 0.54 (0.46, 0.63) | <0.001 | 7.55 (7.26, 7.86) | 8.89 (8.63, 9.15) | 0.63 (0.07, 1.20) | 0.028 | 0.12 (0.09, 0.17) | 0.15 (0.12, 0.18) | 0.82 (0.47, 1.16) | <0.001 |
| Tropical Latin America | 4.39 (4.16, 4.63) | 6.39 (6.17, 6.63) | 1.29 (1.22, 1.36) | <0.001 | 0.77 (0.68, 0.87) | 1.02 (0.93, 1.12) | 0.96 (0.79, 1.12) | <0.001 | 20.19 (19.70, 20.69) | 18.15 (17.76, 18.55) | -0.32 (-0.62, -0.02) | 0.039 | 0.33 (0.27, 0.41) | 0.30 (0.25, 0.36) | -0.37 (-0.60, -0.14) | 0.002 |
| Central Sub-Saharan Africa | 2.67 (2.36, 3.01) | 3.14 (2.92, 3.36) | 0.60 (0.49, 0.72) | <0.001 | 0.74 (0.58, 0.93) | 0.79 (0.69, 0.91) | 0.22 (0.07, 0.37) | 0.005 | 14.79 (14.04, 15.58) | 11.86 (11.43, 12.31) | -0.80 (-1.00, -0.59) | <0.001 | 0.25 (0.16, 0.37) | 0.20 (0.15, 0.26) | -0.77 (-1.15, -0.39) | <0.001 |
| Western Europe | 5.97 (5.79, 6.15) | 6.27 (6.07, 6.46) | 0.16 (0.10, 0.22) | <0.001 | 1.25 (1.17, 1.34) | 1.05 (0.97, 1.13) | -0.65 (-0.82, -0.48) | <0.001 | 3.66 (3.52, 3.80) | 4.35 (4.19, 4.51) | 0.60 (0.45, 0.74) | <0.001 | 0.06 (0.04, 0.08) | 0.07 (0.05, 0.10) | 0.53 (-0.19, 1.26) | 0.149 |
| High-income North America | 6.07 (5.87, 6.28) | 5.10 (4.92, 5.28) | -0.59 (-0.64, -0.54) | <0.001 | 3.75 (3.60, 3.92) | 3.49 (3.34, 3.64) | -0.21 (-0.35, -0.08) | 0.002 | 5.21 (5.03, 5.40) | 10.36 (10.11, 10.62) | 2.48 (2.09, 2.88) | <0.001 | 0.08 (0.06, 0.11) | 0.17 (0.14, 0.21) | 2.73 (1.80, 3.67) | <0.001 |
| Central Asia | 2.57 (2.31, 2.85) | 3.21 (2.96, 3.48) | 0.78 (0.73, 0.84) | <0.001 | 0.25 (0.17, 0.34) | 0.29 (0.22, 0.38) | 0.64 (0.46, 0.82) | <0.001 | 3.51 (3.21, 3.83) | 5.50 (5.17, 5.84) | 1.49 (1.14, 1.84) | <0.001 | 0.06 (0.03, 0.12) | 0.09 (0.05, 0.15) | 1.27 (0.92, 1.62) | <0.001 |
| Eastern Europe | 5.01 (4.80, 5.23) | 9.28 (8.96, 9.61) | 2.13 (1.92, 2.35) | <0.001 | 0.56 (0.49, 0.64) | 0.91 (0.81, 1.02) | 1.68 (1.51, 1.84) | <0.001 | 7.58 (7.32, 7.85) | 20.45 (19.97, 20.94) | 3.54 (2.51, 4.59) | <0.001 | 0.12 (0.09, 0.16) | 0.34 (0.28, 0.41) | 3.69 (2.93, 4.47) | <0.001 |
| Southern Sub-Saharan Africa | 6.00 (5.55, 6.48) | 5.02 (4.69, 5.37) | -0.62 (-0.80, -0.43) | <0.001 | 1.58 (1.35, 1.83) | 1.40 (1.22, 1.59) | -0.40 (-0.52, -0.28) | <0.001 | 51.96 (50.59, 53.36) | 20.33 (19.66, 21.01) | -3.08 (-4.67, -1.46) | <0.001 | 0.88 (0.71, 1.08) | 0.34 (0.26, 0.44) | -3.12 (-4.81, -1.41) | <0.001 |
| High-income Asia Pacific | 5.92 (5.66, 6.18) | 5.18 (4.90, 5.47) | -0.46 (-0.49, -0.43) | <0.001 | 1.85 (1.71, 2.00) | 1.56 (1.40, 1.72) | -0.60 (-0.69, -0.51) | <0.001 | 8.77 (8.45, 9.10) | 3.60 (3.37, 3.84) | -3.08 (-3.57, -2.58) | <0.001 | 0.14 (0.10, 0.19) | 0.06 (0.03, 0.10) | -3.06 (-3.63, -2.48) | <0.001 |
| Central Europe | 4.47 (4.20, 4.75) | 5.15 (4.82, 5.50) | 0.49 (0.44, 0.53) | <0.001 | 0.56 (0.47, 0.67) | 0.61 (0.50, 0.74) | 0.29 (0.11, 0.47) | 0.002 | 4.37 (4.10, 4.65) | 5.05 (4.72, 5.39) | 0.50 (0.30, 0.71) | <0.001 | 0.07 (0.04, 0.12) | 0.08 (0.05, 0.14) | 0.49 (-0.04, 1.01) | 0.069 |

| | | | | | | | | | | | | | | | | |
|---|---|---|---|---|---|---|---|---|---|---|---|---|---|---|---|---|
| Andean Latin America | 3.26 (2.86, 3.70) | 5.73 (5.33, 6.16) | 1.97 (1.94, 2.00) | <0.001 | 0.45 (0.31, 0.63) | 0.76 (0.62, 0.93) | 1.81 (1.78, 1.84) | <0.001 | 15.07 (14.20, 15.98) | 9.83 (9.29, 10.39) | -1.44 (-2.40, -0.47) | 0.004 | 0.25 (0.15, 0.40) | 0.16 (0.10, 0.25) | -1.46 (-2.06, -0.85) | <0.001 |
| Caribbean | 4.94 (4.45, 5.49) | 8.60 (8.01, 9.22) | 1.94 (1.86, 2.02) | <0.001 | 0.76 (0.58, 0.99) | 1.02 (0.82, 1.25) | 1.03 (0.95, 1.11) | <0.001 | 17.62 (16.67, 18.62) | 22.99 (22.02, 23.99) | 0.96 (0.67, 1.25) | <0.001 | 0.29 (0.18, 0.45) | 0.38 (0.27, 0.53) | 0.94 (0.51, 1.37) | <0.001 |
| Southern Latin America | 3.82 (3.44, 4.23) | 7.00 (6.55, 7.48) | 2.13 (2.07, 2.19) | <0.001 | 0.89 (0.71, 1.10) | 1.19 (1.01, 1.39) | 1.00 (0.89, 1.10) | <0.001 | 9.72 (9.10, 10.37) | 10.68 (10.13, 11.26) | 0.37 (0.21, 0.53) | <0.001 | 0.16 (0.09, 0.27) | 0.18 (0.11, 0.27) | 0.39 (-0.28, 1.05) | 0.254 |
| Oceania | 5.76 (4.49, 7.30) | 7.12 (6.13, 8.22) | 0.75 (0.67, 0.82) | <0.001 | 0.76 (0.35, 1.48) | 0.80 (0.49, 1.22) | 0.16 (0.07, 0.26) | <0.001 | 25.91 (23.08, 29.01) | 35.79 (33.53, 38.17) | 1.08 (0.94, 1.22) | <0.001 | 0.44 (0.15, 1.05) | 0.61 (0.35, 1.00) | 1.09 (0.96, 1.23) | <0.001 |
| Australasia | 5.48 (4.79, 6.24) | 6.07 (5.40, 6.80) | 0.35 (0.26, 0.43) | <0.001 | 1.45 (1.10, 1.87) | 1.18 (0.90, 1.53) | -0.71 (-0.81, -0.62) | <0.001 | 4.63 (3.99, 5.34) | 5.77 (5.13, 6.48) | 0.87 (0.36, 1.39) | 0.001 | 0.08 (0.02, 0.22) | 0.10 (0.03, 0.24) | 0.87 (-0.13, 1.89) | 0.089 |
| **Men** | | | | | | | | | | | | | | | | |
| Global | 5.90 (5.85, 5.94) | 6.38 (6.34, 6.42) | 0.28 (0.21, 0.35) | <0.001 | 0.92 (0.90, 0.94) | 0.97 (0.95, 0.98) | 0.19 (0.09, 0.28) | <0.001 | 15.06 (14.98, 15.13) | 16.38 (16.31, 16.44) | 0.30 (0.00, 0.59) | 0.050 | 0.25 (0.24, 0.26) | 0.28 (0.27, 0.29) | 0.31 (0.11, 0.50) | 0.002 |
| **SDI category** | | | | | | | | | | | | | | | | |
| High | 6.79 (6.66, 6.91) | 7.15 (7.02, 7.27) | 0.18 (0.13, 0.23) | <0.001 | 1.69 (1.63, 1.76) | 1.69 (1.63, 1.75) | 0.00 (-0.10, 0.11) | 0.935 | 9.19 (9.05, 9.34) | 10.86 (10.71, 11.01) | 0.59 (0.38, 0.79) | <0.001 | 0.16 (0.14, 0.18) | 0.19 (0.17, 0.21) | 0.53 (-0.06, 1.12) | 0.080 |
| High-middle | 7.08 (6.97, 7.18) | 7.87 (7.76, 7.97) | 0.36 (0.27, 0.45) | <0.001 | 0.93 (0.90, 0.97) | 1.10 (1.06, 1.14) | 0.58 (0.52, 0.64) | <0.001 | 16.59 (16.43, 16.75) | 19.23 (19.06, 19.39) | 0.54 (-0.08, 1.15) | 0.087 | 0.28 (0.26, 0.30) | 0.33 (0.31, 0.35) | 0.65 (0.42, 0.88) | <0.001 |
| Middle | 6.17 (6.09, 6.25) | 6.51 (6.44, 6.58) | 0.19 (0.13, 0.26) | <0.001 | 0.88 (0.85, 0.91) | 0.99 (0.96, 1.02) | 0.40 (0.30, 0.49) | <0.001 | 16.53 (16.40, 16.66) | 16.82 (16.70, 16.93) | 0.06 (-0.13, 0.25) | 0.527 | 0.28 (0.26, 0.30) | 0.28 (0.27, 0.30) | -0.01 (-0.40, 0.39) | 0.966 |
| Low-middle | 4.16 (4.08, 4.25) | 5.32 (5.25, 5.40) | 0.87 (0.82, 0.92) | <0.001 | 0.50 (0.47, 0.53) | 0.63 (0.61, 0.66) | 0.83 (0.71, 0.95) | <0.001 | 14.27 (14.10, 14.43) | 15.43 (15.30, 15.56) | 0.28 (0.03, 0.52) | 0.027 | 0.24 (0.22, 0.26) | 0.26 (0.25, 0.28) | 0.34 (-0.07, 0.76) | 0.103 |
| Low | 4.01 (3.88, | 4.98 | 0.79 | <0.001 | 0.57 (0.52, | 0.69 (0.66, | 0.72 | <0.001 | 17.82 (17.54, | 17.35 (17.17, | -0.11 | 0.287 | 0.30 (0.27, | 0.29 (0.27, | -0.20 | 0.101 |

| | | | | | | | | | | | | | | | | |
|---|---|---|---|---|---|---|---|---|---|---|---|---|---|---|---|---|
| | 4.14) | (4.89, 5.08) | (0.69, 0.90) | | 0.62) | 0.73) | (0.59, 0.85) | | 18.10) | 17.54) | (-0.31, 0.09) | | 0.34) | 0.32) | (-0.43, 0.04) | |
| **21 GBD region** | | | | | | | | | | | | | | | | |
| South Asia | 3.48 (3.40, 3.56) | 4.85 (4.78, 4.92) | 1.16 (1.06, 1.26) | <0.001 | 0.36 (0.34, 0.39) | 0.48 (0.46, 0.51) | 1.05 (0.91, 1.18) | <0.001 | 11.56 (11.42, 11.71) | 12.94 (12.83, 13.06) | 0.42 (0.04, 0.81) | 0.031 | 0.20 (0.18, 0.22) | 0.22 (0.21, 0.24) | 0.42 (-0.07, 0.91) | 0.090 |
| East Asia | 7.73 (7.63, 7.83) | 5.81 (5.72, 5.91) | -0.94 (-1.18, -0.71) | <0.001 | 0.88 (0.85, 0.92) | 0.86 (0.82, 0.89) | -0.06 (-0.22, 0.09) | 0.426 | 10.68 (10.56, 10.80) | 5.90 (5.81, 5.99) | -2.01 (-2.43, -1.59) | <0.001 | 0.18 (0.16, 0.19) | 0.10 (0.09, 0.11) | -1.88 (-2.96, -0.78) | 0.001 |
| North Africa and Middle East | 6.89 (6.69, 7.09) | 8.41 (8.26, 8.56) | 0.70 (0.64, 0.76) | <0.001 | 1.07 (0.99, 1.15) | 1.21 (1.15, 1.27) | 0.42 (0.38, 0.47) | <0.001 | 13.84 (13.55, 14.13) | 10.83 (10.65, 11.00) | -0.82 (-0.96, -0.69) | <0.001 | 0.23 (0.20, 0.27) | 0.18 (0.16, 0.20) | -0.84 (-1.20, -0.48) | <0.001 |
| Western Sub-Saharan Africa | 4.22 (4.00, 4.45) | 4.84 (4.69, 4.99) | 0.53 (0.40, 0.66) | <0.001 | 0.74 (0.65, 0.84) | 0.83 (0.77, 0.89) | 0.42 (0.33, 0.52) | <0.001 | 17.43 (16.97, 17.90) | 14.82 (14.55, 15.10) | -0.55 (-0.67, -0.43) | <0.001 | 0.30 (0.24, 0.36) | 0.25 (0.22, 0.29) | -0.64 (-0.83, -0.45) | <0.001 |
| Southeast Asia | 5.84 (5.68, 5.99) | 7.38 (7.24, 7.53) | 0.82 (0.79, 0.84) | <0.001 | 1.41 (1.33, 1.49) | 1.58 (1.51, 1.65) | 0.41 (0.33, 0.49) | <0.001 | 37.39 (36.99, 37.79) | 39.30 (38.98, 39.64) | 0.25 (-0.14, 0.64) | 0.218 | 0.63 (0.58, 0.69) | 0.66 (0.62, 0.71) | 0.24 (-0.04, 0.52) | 0.087 |
| Eastern Sub-Saharan Africa | 4.75 (4.52, 5.00) | 5.20 (5.04, 5.36) | 0.33 (0.27, 0.39) | <0.001 | 0.88 (0.78, 0.99) | 0.98 (0.91, 1.06) | 0.42 (0.32, 0.51) | <0.001 | 28.34 (27.74, 28.95) | 24.65 (24.29, 25.01) | -0.48 (-0.71, -0.25) | <0.001 | 0.48 (0.40, 0.57) | 0.42 (0.37, 0.47) | -0.50 (-0.74, -0.26) | <0.001 |
| Central Latin America | 4.73 (4.49, 4.98) | 7.47 (7.23, 7.72) | 1.57 (1.50, 1.64) | <0.001 | 0.56 (0.48, 0.65) | 0.84 (0.76, 0.92) | 1.40 (1.24, 1.57) | <0.001 | 10.77 (10.41, 11.15) | 13.48 (13.16, 13.81) | 0.86 (0.36, 1.35) | 0.001 | 0.18 (0.14, 0.24) | 0.23 (0.19, 0.27) | 0.96 (0.79, 1.14) | <0.001 |
| Tropical Latin America | 5.27 (5.02, 5.54) | 8.21 (7.95, 8.48) | 1.51 (1.48, 1.55) | <0.001 | 0.64 (0.55, 0.73) | 0.95 (0.86, 1.05) | 1.36 (1.22, 1.51) | <0.001 | 27.72 (27.13, 28.31) | 23.87 (23.42, 24.32) | -0.50 (-0.69, -0.31) | <0.001 | 0.46 (0.39, 0.54) | 0.40 (0.34, 0.46) | -0.47 (-0.84, -0.11) | 0.012 |
| Central Sub-Saharan Africa | 3.56 (3.19, 3.96) | 4.16 (3.91, 4.43) | 0.59 (0.47, 0.71) | <0.001 | 0.68 (0.52, 0.86) | 0.74 (0.64, 0.86) | 0.37 (0.31, 0.42) | <0.001 | 20.36 (19.45, 21.31) | 18.65 (18.10, 19.22) | -0.37 (-0.50, -0.24) | <0.001 | 0.34 (0.23, 0.49) | 0.32 (0.25, 0.40) | -0.32 (-0.43, -0.20) | <0.001 |
| Western Europe | 6.96 (6.77, 7.15) | 7.73 (7.52, 7.94) | 0.35 (0.28, 0.43) | <0.001 | 1.16 (1.08, 1.24) | 1.09 (1.01, 1.17) | -0.22 (-0.34, -0.10) | <0.001 | 6.77 (6.58, 6.96) | 8.93 (8.71, 9.15) | 0.90 (0.71, 1.08) | <0.001 | 0.11 (0.09, 0.14) | 0.15 (0.12, 0.18) | 0.81 (0.36, 1.27) | <0.001 |

| | | | | | | | | | | | | | | | | |
|---|---|---|---|---|---|---|---|---|---|---|---|---|---|---|---|---|
| High-income North America | 6.44 (6.24, 6.66) | 6.24 (6.04, 6.44) | -0.07 (-0.14, -0.01) | 0.033 | 2.42 (2.30, 2.55) | 2.54 (2.42, 2.67) | 0.20 (0.03, 0.37) | 0.022 | 9.29 (9.05, 9.53) | 14.29 (14.00, 14.59) | 1.51 (1.06, 1.96) | <0.001 | 0.16 (0.13, 0.20) | 0.25 (0.21, 0.29) | 1.52 (1.04, 2.00) | <0.001 |
| Central Asia | 3.70 (3.39, 4.04) | 4.48 (4.18, 4.79) | 0.69 (0.62, 0.77) | <0.001 | 0.35 (0.26, 0.47) | 0.42 (0.33, 0.52) | 0.56 (0.40, 0.71) | <0.001 | 7.14 (6.70, 7.60) | 10.42 (9.97, 10.89) | 1.30 (0.89, 1.72) | <0.001 | 0.12 (0.07, 0.20) | 0.18 (0.12, 0.25) | 1.35 (0.81, 1.90) | <0.001 |
| Eastern Europe | 6.22 (5.99, 6.46) | 13.66 (13.28, 14.05) | 2.76 (2.62, 2.91) | <0.001 | 0.63 (0.56, 0.71) | 1.20 (1.09, 1.33) | 2.26 (2.03, 2.49) | <0.001 | 22.09 (21.66, 22.53) | 65.11 (64.30, 65.93) | 3.98 (2.66, 5.31) | <0.001 | 0.37 (0.32, 0.44) | 1.13 (1.03, 1.24) | 4.10 (2.74, 5.49) | <0.001 |
| Southern Sub-Saharan Africa | 5.81 (5.34, 6.30) | 5.99 (5.63, 6.38) | 0.10 (0.04, 0.15) | <0.001 | 1.25 (1.05, 1.49) | 1.23 (1.06, 1.41) | -0.07 (-0.13, 0.00) | 0.053 | 37.92 (36.69, 39.17) | 30.67 (29.85, 31.52) | -0.77 (-1.28, -0.25) | 0.004 | 0.65 (0.50, 0.84) | 0.52 (0.41, 0.64) | -0.82 (-1.42, -0.21) | 0.009 |
| High-income Asia Pacific | 7.59 (7.30, 7.89) | 6.83 (6.53, 7.15) | -0.36 (-0.40, -0.32) | <0.001 | 1.59 (1.46, 1.73) | 1.43 (1.29, 1.58) | -0.39 (-0.47, -0.30) | <0.001 | 9.74 (9.41, 10.08) | 5.87 (5.59, 6.16) | -1.73 (-1.90, -1.56) | <0.001 | 0.16 (0.12, 0.21) | 0.10 (0.07, 0.14) | -1.57 (-2.23, -0.90) | <0.001 |
| Central Europe | 5.19 (4.90, 5.49) | 6.59 (6.23, 6.97) | 0.85 (0.74, 0.95) | <0.001 | 0.65 (0.55, 0.76) | 0.79 (0.66, 0.93) | 0.67 (0.51, 0.84) | <0.001 | 9.83 (9.44, 10.24) | 12.86 (12.36, 13.38) | 0.91 (0.54, 1.27) | <0.001 | 0.17 (0.12, 0.23) | 0.22 (0.16, 0.30) | 0.90 (0.19, 1.61) | 0.013 |
| Andean Latin America | 4.11 (3.65, 4.61) | 6.79 (6.35, 7.26) | 1.76 (1.72, 1.80) | <0.001 | 0.50 (0.35, 0.69) | 0.78 (0.64, 0.95) | 1.58 (1.46, 1.70) | <0.001 | 17.43 (16.50, 18.42) | 14.15 (13.51, 14.81) | -0.68 (-0.94, -0.42) | <0.001 | 0.28 (0.17, 0.44) | 0.23 (0.15, 0.33) | -0.66 (-1.13, -0.20) | 0.006 |
| Caribbean | 5.78 (5.23, 6.39) | 9.63 (9.00, 10.29) | 1.78 (1.74, 1.83) | <0.001 | 0.73 (0.55, 0.97) | 1.04 (0.84, 1.27) | 1.22 (1.13, 1.31) | <0.001 | 19.33 (18.30, 20.41) | 24.43 (23.41, 25.47) | 0.85 (0.46, 1.24) | <0.001 | 0.33 (0.20, 0.50) | 0.42 (0.29, 0.57) | 0.85 (0.39, 1.30) | <0.001 |
| Southern Latin America | 4.32 (3.91, 4.77) | 8.22 (7.73, 8.73) | 2.27 (2.11, 2.44) | <0.001 | 0.76 (0.60, 0.96) | 1.13 (0.95, 1.33) | 1.37 (1.25, 1.49) | <0.001 | 12.91 (12.19, 13.67) | 18.66 (17.92, 19.43) | 1.32 (1.05, 1.59) | <0.001 | 0.22 (0.14, 0.34) | 0.32 (0.23, 0.43) | 1.38 (1.08, 1.69) | <0.001 |
| Oceania | 4.38 (3.31, 5.71) | 5.18 (4.36, 6.12) | 0.59 (0.51, 0.67) | <0.001 | 0.52 (0.21, 1.12) | 0.56 (0.32, 0.93) | 0.28 (0.17, 0.39) | <0.001 | 19.91 (17.52, 22.55) | 26.33 (24.42, 28.35) | 0.96 (0.82, 1.09) | <0.001 | 0.34 (0.09, 0.88) | 0.44 (0.23, 0.78) | 0.86 (0.69, 1.04) | <0.001 |
| Australasia | 6.04 (5.31, 6.84) | 6.76 (6.06, 7.53) | 0.39 (0.34, 0.44) | <0.001 | 1.09 (0.79, 1.46) | 0.98 (0.72, 1.30) | -0.36 (-0.48, -0.23) | <0.001 | 7.24 (6.44, 8.11) | 9.52 (8.69, 10.42) | 1.02 (0.47, 1.56) | <0.001 | 0.12 (0.04, 0.28) | 0.16 (0.07, 0.33) | 1.14 (-0.03, 2.34) | 0.057 |

| | | | | | | | | | | | | | | | | |
|---|---|---|---|---|---|---|---|---|---|---|---|---|---|---|---|---|
| **Other cardiovascular and circulatory diseases** | | | | | | | | | | | | | | | | |
| **Overall** | | | | | | | | | | | | | | | | |
| Global | — | — | — | — | 232.41 (232.21, 232.61) | 282.72 (282.53, 282.91) | 0.68 (0.66, 0.70) | <0.001 | 54.33 (54.23, 54.43) | 52.86 (52.78, 52.94) | -0.08 (-0.19, 0.04) | 0.181 | 0.70 (0.69, 0.72) | 0.63 (0.62, 0.64) | -0.38 (-0.52, -0.23) | <0.001 |
| **SDI category** | | | | | | | | | | | | | | | | |
| High | — | — | — | — | 377.89 (377.23, 378.56) | 318.09 (317.48, 318.69) | -0.58 (-0.59, -0.56) | <0.001 | 52.72 (52.47, 52.97) | 44.80 (44.57, 45.02) | -0.55 (-0.69, -0.41) | <0.001 | 0.55 (0.53, 0.58) | 0.48 (0.45, 0.50) | -0.48 (-0.76, -0.21) | 0.001 |
| High-middle | — | — | — | — | 176.12 (175.74, 176.49) | 212.31 (211.90, 212.73) | 0.64 (0.62, 0.67) | <0.001 | 45.30 (45.11, 45.49) | 38.33 (38.16, 38.50) | -0.58 (-0.90, -0.27) | <0.001 | 0.60 (0.58, 0.63) | 0.45 (0.44, 0.47) | -0.97 (-1.37, -0.56) | <0.001 |
| Middle | — | — | — | — | 180.21 (179.90, 180.51) | 249.94 (249.61, 250.26) | 1.14 (1.10, 1.19) | <0.001 | 45.57 (45.41, 45.73) | 44.17 (44.03, 44.30) | -0.09 (-0.18, -0.01) | 0.039 | 0.60 (0.58, 0.62) | 0.51 (0.50, 0.53) | -0.55 (-0.76, -0.35) | <0.001 |
| Low-middle | — | — | — | — | 198.86 (198.45, 199.27) | 251.24 (250.87, 251.60) | 0.81 (0.80, 0.82) | <0.001 | 62.39 (62.16, 62.63) | 60.32 (60.14, 60.50) | -0.11 (-0.28, 0.06) | 0.195 | 0.87 (0.84, 0.90) | 0.79 (0.77, 0.81) | -0.32 (-0.51, -0.12) | 0.001 |
| Low | — | — | — | — | 378.60 (377.73, 379.46) | 438.79 (438.18, 439.41) | 0.51 (0.50, 0.52) | <0.001 | 94.94 (94.49, 95.39) | 82.60 (82.32, 82.87) | -0.48 (-0.55, -0.40) | <0.001 | 1.26 (1.21, 1.32) | 0.99 (0.96, 1.03) | -0.85 (-0.90, -0.79) | <0.001 |
| **21 GBD region** | | | | | | | | | | | | | | | | |
| South Asia | — | — | — | — | 148.83 (148.46, 149.19) | 141.65 (141.38, 141.91) | -0.18 (-0.20, -0.16) | <0.001 | 57.88 (57.65, 58.11) | 51.16 (51.00, 51.32) | -0.42 (-0.70, -0.15) | 0.003 | 0.84 (0.81, 0.87) | 0.74 (0.72, 0.76) | -0.47 (-0.83, -0.10) | 0.012 |
| East Asia | — | — | — | — | 61.81 (61.61, 62.02) | 93.38 (93.11, 93.65) | 1.39 (1.23, 1.56) | <0.001 | 21.95 (21.83, 22.08) | 15.92 (15.81, 16.03) | -1.07 (-1.34, -0.81) | <0.001 | 0.31 (0.29, 0.32) | 0.18 (0.17, 0.19) | -1.78 (-2.26, -1.30) | <0.001 |
| North Africa and Middle East | — | — | — | — | 478.25 (477.07, | 492.43 (491.57, | 0.11 (0.06, | <0.001 | 97.26 (96.72, 97.80) | 82.21 (81.86, 82.56) | -0.57 (-0.68, | <0.001 | 1.20 (1.14, 1.26) | 0.93 (0.89, 0.96) | -0.86 (-0.99, | <0.001 |

| | | | | | | | | | | | | | | | | |
|---|---|---|---|---|---|---|---|---|---|---|---|---|---|---|---|---|
| | | | | | 479.43) | 493.29) | 0.15) | | | | -0.46) | | | | -0.74) | |
| Western Sub-Saharan Africa | — | — | — | — | 786.44 (784.39, 788.49) | 893.23 (891.85, 894.61) | 0.45 (0.42, 0.48) | <0.001 | 139.58 (138.69, 140.48) | 113.13 (112.62, 113.63) | -0.73 (-0.79, -0.68) | <0.001 | 1.64 (1.54, 1.74) | 1.09 (1.04, 1.14) | -1.42 (-1.52, -1.31) | <0.001 |
| Southeast Asia | — | — | — | — | 174.26 (173.68, 174.84) | 185.40 (184.88, 185.91) | 0.21 (0.19, 0.23) | <0.001 | 50.37 (50.05, 50.69) | 41.64 (41.39, 41.88) | -0.65 (-0.76, -0.53) | <0.001 | 0.69 (0.65, 0.73) | 0.53 (0.50, 0.56) | -0.89 (-1.05, -0.74) | <0.001 |
| Eastern Sub-Saharan Africa | — | — | — | — | 476.98 (475.35, 478.62) | 542.86 (541.73, 543.99) | 0.44 (0.42, 0.47) | <0.001 | 112.08 (111.26, 112.90) | 89.75 (89.29, 90.23) | -0.77 (-0.85, -0.68) | <0.001 | 1.46 (1.36, 1.56) | 1.01 (0.96, 1.07) | -1.26 (-1.40, -1.13) | <0.001 |
| Central Latin America | — | — | — | — | 254.17 (252.98, 255.37) | 302.98 (301.90, 304.05) | 0.60 (0.58, 0.63) | <0.001 | 53.57 (53.00, 54.13) | 45.23 (44.82, 45.65) | -0.56 (-0.78, -0.34) | <0.001 | 0.67 (0.61, 0.74) | 0.49 (0.44, 0.53) | -1.06 (-1.39, -0.72) | <0.001 |
| Tropical Latin America | — | — | — | — | 244.32 (243.13, 245.52) | 275.16 (274.05, 276.26) | 0.41 (0.38, 0.44) | <0.001 | 62.42 (61.80, 63.04) | 58.44 (57.94, 58.94) | -0.23 (-0.48, 0.02) | 0.072 | 0.84 (0.77, 0.92) | 0.74 (0.69, 0.80) | -0.35 (-0.64, -0.05) | 0.021 |
| Central Sub-Saharan Africa | — | — | — | — | 449.80 (446.91, 452.71) | 483.17 (481.27, 485.07) | 0.27 (0.25, 0.29) | <0.001 | 80.41 (79.15, 81.69) | 71.77 (71.02, 72.52) | -0.41 (-0.46, -0.35) | <0.001 | 0.96 (0.82, 1.11) | 0.78 (0.70, 0.86) | -0.74 (-0.87, -0.61) | <0.001 |
| Western Europe | — | — | — | — | 348.70 (347.74, 349.66) | 328.99 (328.00, 329.98) | -0.20 (-0.24, -0.17) | <0.001 | 51.81 (51.44, 52.18) | 39.38 (39.05, 39.72) | -0.98 (-1.13, -0.83) | <0.001 | 0.56 (0.53, 0.60) | 0.37 (0.34, 0.41) | -1.47 (-1.81, -1.13) | <0.001 |
| High-income North America | — | — | — | — | 501.92 (500.62, 503.23) | 366.29 (365.22, 367.36) | -1.06 (-1.09, -1.03) | <0.001 | 62.31 (61.86, 62.77) | 60.37 (59.94, 60.80) | -0.08 (-0.25, 0.08) | 0.324 | 0.61 (0.56, 0.65) | 0.70 (0.66, 0.75) | 0.54 (0.26, 0.82) | <0.001 |
| Central Asia | — | — | — | — | 116.71 (115.47, 117.96) | 115.05 (113.95, 116.15) | -0.05 (-0.06, -0.03) | <0.001 | 62.37 (61.44, 63.31) | 48.57 (47.88, 49.28) | -0.92 (-1.46, -0.38) | 0.001 | 0.94 (0.83, 1.07) | 0.72 (0.63, 0.81) | -1.01 (-1.68, -0.33) | 0.004 |
| Eastern Europe | — | — | — | — | 99.32 (98.63, 100.02) | 104.11 (103.27, 104.96) | 0.17 (0.15, 0.18) | <0.001 | 39.18 (38.76, 39.60) | 58.44 (57.89, 59.01) | 1.55 (0.39, 2.71) | 0.009 | 0.57 (0.52, 0.62) | 0.92 (0.85, 0.99) | 1.85 (0.53, 3.19) | 0.006 |
| Southern Sub-Saharan | — | — | — | — | 318.59 (316.25, | 332.38 (330.43, | 0.14 (0.11, | <0.001 | 70.71 (69.55, 71.88) | 62.96 (62.12, 63.82) | -0.53 (-1.03, | 0.040 | 0.92 (0.79, 1.07) | 0.77 (0.68, 0.87) | -0.74 (-1.51, | 0.062 |

| | | | | | | | | | | | | | | | | |
|---|---|---|---|---|---|---|---|---|---|---|---|---|---|---|---|---|
| Africa | | | | | 320.95) | 334.35) | 0.18) | | | | -0.02) | | | | 0.04) | |
| High-income Asia Pacific | — | — | — | — | 241.71 (240.53, 242.88) | 236.01 (234.69, 237.34) | -0.08 (-0.09, -0.07) | <0.001 | 35.66 (35.21, 36.12) | 23.70 (23.29, 24.12) | -1.40 (-1.51, -1.30) | <0.001 | 0.38 (0.33, 0.43) | 0.18 (0.15, 0.22) | -2.55 (-2.90, -2.19) | <0.001 |
| Central Europe | — | — | — | — | 386.02 (384.20, 387.85) | 394.19 (392.05, 396.35) | 0.08 (0.04, 0.12) | <0.001 | 81.17 (80.36, 81.99) | 47.52 (46.80, 48.24) | -1.85 (-2.04, -1.66) | <0.001 | 1.05 (0.96, 1.14) | 0.45 (0.38, 0.52) | -2.92 (-3.21, -2.63) | <0.001 |
| Andean Latin America | — | — | — | — | 305.24 (302.54, 307.97) | 365.49 (363.16, 367.83) | 0.62 (0.60, 0.64) | <0.001 | 86.21 (84.73, 87.70) | 53.19 (52.30, 54.09) | -1.62 (-1.98, -1.27) | <0.001 | 1.15 (0.98, 1.34) | 0.55 (0.46, 0.65) | -2.53 (-3.09, -1.96) | <0.001 |
| Caribbean | — | — | — | — | 209.62 (207.30, 211.97) | 234.01 (231.79, 236.25) | 0.38 (0.37, 0.39) | <0.001 | 76.88 (75.43, 78.34) | 85.38 (84.04, 86.73) | 0.39 (0.17, 0.61) | <0.001 | 1.11 (0.94, 1.30) | 1.23 (1.08, 1.41) | 0.38 (0.16, 0.60) | 0.001 |
| Southern Latin America | — | — | — | — | 243.50 (241.31, 245.72) | 334.59 (332.34, 336.85) | 1.10 (1.09, 1.12) | <0.001 | 66.54 (65.38, 67.71) | 53.45 (52.56, 54.36) | -0.76 (-0.99, -0.54) | <0.001 | 0.91 (0.77, 1.05) | 0.60 (0.51, 0.70) | -1.44 (-1.86, -1.02) | <0.001 |
| Oceania | — | — | — | — | 121.36 (117.23, 125.61) | 110.85 (108.09, 113.66) | -0.31 (-0.32, -0.30) | <0.001 | 69.12 (65.89, 72.48) | 63.55 (61.43, 65.73) | -0.30 (-0.41, -0.19) | <0.001 | 1.05 (0.68, 1.56) | 0.96 (0.72, 1.27) | -0.35 (-0.48, -0.22) | <0.001 |
| Australasia | — | — | — | — | 194.11 (191.12, 197.14) | 184.22 (181.57, 186.90) | -0.19 (-0.23, -0.15) | <0.001 | 28.86 (27.71, 30.05) | 28.76 (27.72, 29.83) | -0.03 (-0.33, 0.26) | 0.821 | 0.31 (0.20, 0.46) | 0.32 (0.22, 0.46) | 0.17 (-0.39, 0.72) | 0.556 |
| **Women** | | | | | | | | | | | | | | | | |
| Global | — | — | — | — | 291.83 (291.50, 292.15) | 341.21 (340.91, 341.51) | 0.54 (0.53, 0.56) | <0.001 | 58.58 (58.43, 58.72) | 55.43 (55.31, 55.55) | -0.17 (-0.29, -0.04) | 0.009 | 0.72 (0.70, 0.74) | 0.62 (0.61, 0.63) | -0.50 (-0.70, -0.30) | <0.001 |
| **SDI category** | | | | | | | | | | | | | | | | |
| High | — | — | — | — | 550.52 (549.37, 551.67) | 463.55 (462.50, 464.61) | -0.58 (-0.60, -0.56) | <0.001 | 63.53 (63.14, 63.92) | 56.23 (55.86, 56.59) | -0.42 (-0.55, -0.28) | <0.001 | 0.58 (0.54, 0.61) | 0.54 (0.50, 0.57) | -0.22 (-0.42, -0.01) | 0.039 |
| High-middle | — | — | — | — | 217.87 (217.28, 218.47) | 258.69 (258.04, 259.34) | 0.59 (0.56, 0.62) | <0.001 | 43.17 (42.91, 43.44) | 38.17 (37.93, 38.42) | -0.39 (-0.66, -0.12) | 0.004 | 0.53 (0.50, 0.56) | 0.41 (0.38, 0.43) | -0.82 (-1.17, -0.47) | <0.001 |

| | | | | | | | | | | | | | | | | |
|---|---|---|---|---|---|---|---|---|---|---|---|---|---|---|---|---|
| Middle | — | — | — | — | 206.69 (206.22, 207.16) | 281.40 (280.91, 281.88) | 1.08 (1.05, 1.10) | <0.001 | 47.64 (47.42, 47.87) | 44.45 (44.26, 44.64) | -0.21 (-0.39, -0.03) | 0.021 | 0.61 (0.58, 0.64) | 0.49 (0.47, 0.51) | -0.72 (-1.03, -0.42) | <0.001 |
| Low-middle | — | — | — | — | 242.66 (242.01, 243.31) | 301.34 (300.78, 301.90) | 0.75 (0.73, 0.76) | <0.001 | 70.33 (69.97, 70.69) | 63.96 (63.70, 64.22) | -0.33 (-0.54, -0.13) | 0.001 | 0.96 (0.92, 1.00) | 0.80 (0.77, 0.83) | -0.61 (-0.92, -0.30) | <0.001 |
| Low | — | — | — | — | 446.48 (445.15, 447.82) | 517.17 (516.23, 518.12) | 0.51 (0.49, 0.53) | <0.001 | 103.14 (102.48, 103.80) | 83.96 (83.57, 84.35) | -0.70 (-0.79, -0.62) | <0.001 | 1.34 (1.27, 1.42) | 0.95 (0.91, 0.99) | -1.20 (-1.36, -1.03) | <0.001 |
| **21 GBD region** | | | | | | | | | | | | | | | | |
| South Asia | — | — | — | — | 197.80 (197.19, 198.40) | 188.32 (187.88, 188.76) | -0.18 (-0.21, -0.16) | <0.001 | 68.19 (67.83, 68.55) | 57.12 (56.87, 57.36) | -0.65 (-1.03, -0.26) | 0.001 | 0.96 (0.92, 1.01) | 0.79 (0.76, 0.82) | -0.73 (-1.20, -0.25) | 0.003 |
| East Asia | — | — | — | — | 64.03 (63.73, 64.33) | 96.60 (96.20, 96.99) | 1.35 (1.08, 1.63) | <0.001 | 20.56 (20.39, 20.73) | 14.80 (14.65, 14.96) | -1.25 (-1.78, -0.71) | <0.001 | 0.28 (0.26, 0.30) | 0.16 (0.14, 0.17) | -1.93 (-2.87, -0.99) | <0.001 |
| North Africa and Middle East | — | — | — | — | 527.02 (525.23, 528.80) | 538.50 (537.21, 539.80) | 0.07 (0.05, 0.10) | <0.001 | 100.64 (99.85, 101.43) | 85.23 (84.72, 85.74) | -0.56 (-0.67, -0.44) | <0.001 | 1.21 (1.12, 1.30) | 0.94 (0.89, 1.00) | -0.85 (-1.00, -0.70) | <0.001 |
| Western Sub-Saharan Africa | — | — | — | — | 803.56 (800.64, 806.50) | 927.66 (925.69, 929.63) | 0.50 (0.46, 0.53) | <0.001 | 137.21 (135.96, 138.46) | 101.02 (100.36, 101.68) | -1.06 (-1.14, -0.99) | <0.001 | 1.60 (1.46, 1.74) | 0.87 (0.80, 0.93) | -2.08 (-2.26, -1.90) | <0.001 |
| Southeast Asia | — | — | — | — | 187.25 (186.40, 188.10) | 193.56 (192.82, 194.31) | 0.12 (0.10, 0.14) | <0.001 | 51.16 (50.71, 51.62) | 39.01 (38.68, 39.34) | -0.93 (-1.02, -0.83) | <0.001 | 0.69 (0.64, 0.75) | 0.48 (0.44, 0.52) | -1.26 (-1.42, -1.10) | <0.001 |
| Eastern Sub-Saharan Africa | — | — | — | — | 592.75 (590.21, 595.31) | 683.29 (681.51, 685.07) | 0.49 (0.44, 0.53) | <0.001 | 124.18 (122.99, 125.37) | 94.98 (94.31, 95.66) | -0.93 (-1.01, -0.84) | <0.001 | 1.56 (1.43, 1.70) | 0.98 (0.91, 1.05) | -1.62 (-1.78, -1.46) | <0.001 |
| Central Latin America | — | — | — | — | 349.39 (347.43, 351.36) | 402.98 (401.25, 404.72) | 0.49 (0.46, 0.52) | <0.001 | 61.39 (60.55, 62.24) | 51.83 (51.21, 52.46) | -0.59 (-0.82, -0.36) | <0.001 | 0.72 (0.63, 0.82) | 0.51 (0.45, 0.57) | -1.18 (-1.53, -0.82) | <0.001 |
| Tropical Latin America | — | — | — | — | 345.61 (343.61, | 384.20 (382.37, | 0.37 (0.34, | <0.001 | 69.22 (68.31, 70.14) | 67.64 (66.89, 68.40) | -0.03 (-0.19, | 0.680 | 0.86 (0.76, 0.97) | 0.80 (0.72, 0.88) | -0.18 (-0.41, | 0.116 |

| | | | | | | | | | | | | | | | | |
|---|---|---|---|---|---|---|---|---|---|---|---|---|---|---|---|---|
| | | | | | 347.62) | 386.04) | 0.40) | | | | 0.12) | | | | 0.05) | |
| Central Sub-Saharan Africa | — | — | — | — | 584.01 (579.36, 588.69) | 632.17 (629.10, 635.25) | 0.29 (0.21, 0.38) | <0.001 | 85.21 (83.39, 87.06) | 76.13 (75.05, 77.23) | -0.41 (-0.48, -0.33) | <0.001 | 0.92 (0.74, 1.14) | 0.72 (0.62, 0.84) | -0.91 (-1.05, -0.76) | <0.001 |
| Western Europe | — | — | — | — | 511.02 (509.37, 512.68) | 480.34 (478.64, 482.05) | -0.22 (-0.25, -0.19) | <0.001 | 60.14 (59.58, 60.71) | 48.55 (48.02, 49.09) | -0.76 (-0.88, -0.65) | <0.001 | 0.56 (0.50, 0.61) | 0.39 (0.34, 0.44) | -1.26 (-1.48, -1.04) | <0.001 |
| High-income North America | — | — | — | — | 748.78 (746.52, 751.04) | 551.98 (550.12, 553.85) | -1.02 (-1.06, -0.98) | <0.001 | 78.76 (78.04, 79.49) | 76.46 (75.78, 77.16) | -0.07 (-0.41, 0.26) | 0.665 | 0.66 (0.59, 0.73) | 0.81 (0.74, 0.88) | 0.78 (0.42, 1.14) | <0.001 |
| Central Asia | — | — | — | — | 122.99 (121.19, 124.81) | 115.17 (113.62, 116.73) | -0.22 (-0.24, -0.19) | <0.001 | 54.36 (53.15, 55.59) | 42.47 (41.54, 43.41) | -0.89 (-1.23, -0.54) | <0.001 | 0.80 (0.65, 0.96) | 0.61 (0.50, 0.73) | -1.00 (-1.41, -0.58) | <0.001 |
| Eastern Europe | — | — | — | — | 123.80 (122.71, 124.90) | 126.91 (125.60, 128.23) | 0.09 (0.08, 0.11) | <0.001 | 33.96 (33.40, 34.52) | 44.56 (43.86, 45.28) | 1.13 (0.23, 2.03) | 0.014 | 0.46 (0.40, 0.52) | 0.65 (0.57, 0.74) | 1.44 (0.23, 2.66) | 0.019 |
| Southern Sub-Saharan Africa | — | — | — | — | 355.86 (352.40, 359.35) | 378.38 (375.43, 381.34) | 0.22 (0.19, 0.24) | <0.001 | 73.84 (72.21, 75.50) | 59.99 (58.83, 61.17) | -0.70 (-1.56, 0.17) | 0.116 | 0.94 (0.76, 1.15) | 0.67 (0.56, 0.81) | -1.06 (-2.35, 0.25) | 0.113 |
| High-income Asia Pacific | — | — | — | — | 297.91 (296.05, 299.78) | 289.69 (287.59, 291.80) | -0.09 (-0.11, -0.08) | <0.001 | 37.97 (37.31, 38.64) | 26.06 (25.44, 26.70) | -1.31 (-1.49, -1.12) | <0.001 | 0.37 (0.30, 0.44) | 0.17 (0.13, 0.23) | -2.66 (-3.17, -2.13) | <0.001 |
| Central Europe | — | — | — | — | 505.20 (502.24, 508.18) | 513.44 (509.96, 516.94) | 0.07 (0.03, 0.11) | 0.002 | 76.50 (75.37, 77.64) | 48.55 (47.51, 49.61) | -1.55 (-1.70, -1.39) | <0.001 | 0.85 (0.73, 0.97) | 0.35 (0.27, 0.45) | -3.04 (-3.31, -2.77) | <0.001 |
| Andean Latin America | — | — | — | — | 357.74 (353.60, 361.93) | 418.11 (414.57, 421.68) | 0.53 (0.48, 0.59) | <0.001 | 82.99 (80.96, 85.06) | 53.65 (52.39, 54.94) | -1.51 (-1.87, -1.14) | <0.001 | 1.06 (0.84, 1.32) | 0.51 (0.40, 0.66) | -2.46 (-3.34, -1.56) | <0.001 |
| Caribbean | — | — | — | — | 245.10 (241.56, 248.69) | 262.61 (259.30, 265.96) | 0.23 (0.22, 0.25) | <0.001 | 88.13 (85.98, 90.33) | 99.25 (97.22, 101.31) | 0.43 (0.19, 0.67) | <0.001 | 1.27 (1.02, 1.56) | 1.44 (1.21, 1.71) | 0.47 (0.20, 0.74) | 0.001 |
| Southern Latin America | — | — | — | — | 341.14 (337.48, | 454.98 (451.29, | 1.00 (0.98, | <0.001 | 76.81 (75.06, 78.58) | 61.27 (59.92, 62.64) | -0.75 (-1.03, | <0.001 | 0.98 (0.80, 1.21) | 0.62 (0.49, 0.77) | -1.58 (-2.06, | <0.001 |

| | | | | | | | | | | | | | | | | |
|---|---|---|---|---|---|---|---|---|---|---|---|---|---|---|---|---|
| | | | | | 344.84) | 458.70) | 1.01) | | | | -0.47) | | | | -1.10) | |
| Oceania | — | — | — | — | 115.66 (109.86, 121.71) | 101.30 (97.53, 105.19) | -0.46 (-0.49, -0.42) | <0.001 | 56.44 (52.30, 60.84) | 52.11 (49.39, 54.94) | -0.31 (-0.45, -0.16) | <0.001 | 0.84 (0.40, 1.58) | 0.78 (0.48, 1.20) | -0.21 (-0.31, -0.12) | <0.001 |
| Australasia | — | — | — | — | 270.37 (265.39, 275.43) | 256.03 (251.64, 260.49) | -0.21 (-0.30, -0.11) | <0.001 | 34.77 (32.99, 36.62) | 34.86 (33.25, 36.53) | -0.01 (-0.43, 0.42) | 0.977 | 0.34 (0.19, 0.57) | 0.36 (0.22, 0.58) | 0.26 (-0.46, 0.98) | 0.481 |
| **Men** | | | | | | | | | | | | | | | | |
| Global | — | — | — | — | 174.36 (174.12, 174.61) | 225.48 (225.24, 225.72) | 0.89 (0.87, 0.91) | <0.001 | 50.16 (50.03, 50.30) | 50.35 (50.24, 50.46) | 0.00 (-0.12, 0.13) | 0.960 | 0.69 (0.67, 0.70) | 0.64 (0.63, 0.66) | -0.28 (-0.50, -0.06) | 0.013 |
| **SDI category** | | | | | | | | | | | | | | | | |
| High | — | — | — | — | 211.05 (210.35, 211.75) | 182.43 (181.79, 183.07) | -0.48 (-0.52, -0.45) | <0.001 | 42.28 (41.97, 42.59) | 34.14 (33.86, 34.41) | -0.72 (-0.88, -0.57) | <0.001 | 0.53 (0.49, 0.56) | 0.42 (0.39, 0.45) | -0.83 (-1.18, -0.48) | <0.001 |
| High-middle | — | — | — | — | 135.74 (135.28, 136.20) | 168.99 (168.48, 169.51) | 0.75 (0.72, 0.78) | <0.001 | 47.35 (47.08, 47.62) | 38.51 (38.27, 38.74) | -0.76 (-0.98, -0.54) | <0.001 | 0.68 (0.65, 0.71) | 0.50 (0.48, 0.53) | -1.11 (-1.37, -0.85) | <0.001 |
| Middle | — | — | — | — | 154.51 (154.12, 154.91) | 218.81 (218.38, 219.24) | 1.21 (1.18, 1.24) | <0.001 | 43.54 (43.32, 43.75) | 43.90 (43.71, 44.08) | 0.04 (-0.04, 0.11) | 0.329 | 0.59 (0.57, 0.62) | 0.54 (0.51, 0.56) | -0.29 (-0.46, -0.13) | 0.001 |
| Low-middle | — | — | — | — | 155.96 (155.45, 156.47) | 201.33 (200.87, 201.78) | 0.88 (0.87, 0.89) | <0.001 | 54.59 (54.28, 54.91) | 56.71 (56.46, 56.96) | 0.15 (-0.03, 0.32) | 0.095 | 0.79 (0.75, 0.82) | 0.78 (0.75, 0.81) | 0.01 (-0.30, 0.32) | 0.971 |
| Low | — | — | — | — | 309.00 (307.90, 310.10) | 358.01 (357.23, 358.80) | 0.51 (0.49, 0.53) | <0.001 | 86.44 (85.83, 87.05) | 81.18 (80.79, 81.57) | -0.21 (-0.31, -0.11) | <0.001 | 1.18 (1.11, 1.25) | 1.04 (1.00, 1.09) | -0.43 (-0.54, -0.33) | <0.001 |
| **21 GBD region** | | | | | | | | | | | | | | | | |
| South Asia | — | — | — | — | 102.93 (102.52, 103.35) | 96.76 (96.45, 97.07) | -0.23 (-0.27, -0.19) | <0.001 | 48.19 (47.89, 48.49) | 45.46 (45.24, 45.67) | -0.19 (-0.54, 0.17) | 0.310 | 0.73 (0.70, 0.77) | 0.69 (0.66, 0.72) | -0.20 (-0.64, 0.25) | 0.390 |

| | | | | | | | | | | | | | | | | |
|---|---|---|---|---|---|---|---|---|---|---|---|---|---|---|---|---|
| East Asia | — | — | — | — | 59.76 (59.48, 60.05) | 90.27 (89.90, 90.64) | 1.43 (1.34, 1.51) | <0.001 | 23.26 (23.08, 23.44) | 16.99 (16.83, 17.15) | -1.10 (-1.46, -0.75) | <0.001 | 0.33 (0.31, 0.36) | 0.20 (0.19, 0.22) | -1.73 (-2.44, -1.01) | <0.001 |
| North Africa and Middle East | — | — | — | — | 432.27 (430.72, 433.82) | 451.16 (450.02, 452.31) | 0.16 (0.09, 0.22) | <0.001 | 94.04 (93.30, 94.78) | 79.52 (79.04, 79.99) | -0.56 (-0.71, -0.40) | <0.001 | 1.18 (1.10, 1.27) | 0.91 (0.86, 0.96) | -0.87 (-1.07, -0.68) | <0.001 |
| Western Sub-Saharan Africa | — | — | — | — | 773.12 (770.23, 776.02) | 853.58 (851.65, 855.51) | 0.34 (0.32, 0.37) | <0.001 | 142.62 (141.34, 143.92) | 126.45 (125.68, 127.22) | -0.42 (-0.49, -0.36) | <0.001 | 1.68 (1.54, 1.83) | 1.34 (1.26, 1.42) | -0.78 (-0.91, -0.64) | <0.001 |
| Southeast Asia | — | — | — | — | 161.00 (160.21, 161.80) | 177.38 (176.68, 178.09) | 0.33 (0.32, 0.35) | <0.001 | 49.59 (49.13, 50.04) | 44.22 (43.87, 44.57) | -0.40 (-0.60, -0.19) | <0.001 | 0.69 (0.64, 0.75) | 0.58 (0.54, 0.62) | -0.62 (-0.89, -0.34) | <0.001 |
| Eastern Sub-Saharan Africa | — | — | — | — | 351.98 (349.99, 353.98) | 394.41 (393.05, 395.78) | 0.39 (0.37, 0.41) | <0.001 | 98.78 (97.68, 99.90) | 84.20 (83.55, 84.85) | -0.55 (-0.68, -0.42) | <0.001 | 1.34 (1.21, 1.48) | 1.05 (0.98, 1.13) | -0.84 (-1.01, -0.66) | <0.001 |
| Central Latin America | — | — | — | — | 152.68 (151.39, 153.98) | 198.37 (197.14, 199.61) | 0.90 (0.88, 0.93) | <0.001 | 45.23 (44.49, 45.98) | 38.37 (37.83, 38.92) | -0.56 (-0.88, -0.23) | 0.001 | 0.62 (0.54, 0.72) | 0.47 (0.41, 0.53) | -0.92 (-1.38, -0.46) | <0.001 |
| Tropical Latin America | — | — | — | — | 139.43 (138.17, 140.71) | 164.59 (163.38, 165.81) | 0.57 (0.56, 0.58) | <0.001 | 55.38 (54.55, 56.23) | 49.10 (48.45, 49.76) | -0.37 (-0.56, -0.17) | <0.001 | 0.82 (0.72, 0.93) | 0.68 (0.61, 0.76) | -0.59 (-0.86, -0.32) | <0.001 |
| Central Sub-Saharan Africa | — | — | — | — | 313.41 (310.02, 316.84) | 332.17 (329.96, 334.40) | 0.21 (0.19, 0.23) | <0.001 | 75.55 (73.81, 77.32) | 67.36 (66.33, 68.40) | -0.42 (-0.50, -0.33) | <0.001 | 1.00 (0.80, 1.23) | 0.84 (0.72, 0.96) | -0.63 (-0.76, -0.50) | <0.001 |
| Western Europe | — | — | — | — | 190.30 (189.30, 191.31) | 183.18 (182.14, 184.22) | -0.13 (-0.21, -0.06) | 0.001 | 43.69 (43.21, 44.17) | 30.58 (30.17, 31.00) | -1.28 (-1.53, -1.03) | <0.001 | 0.57 (0.52, 0.63) | 0.36 (0.31, 0.40) | -1.60 (-2.13, -1.07) | <0.001 |
| High-income North America | — | — | — | — | 257.56 (256.24, 258.89) | 183.15 (182.09, 184.23) | -1.15 (-1.21, -1.09) | <0.001 | 46.07 (45.52, 46.63) | 44.50 (43.98, 45.02) | -0.10 (-0.47, 0.27) | 0.597 | 0.55 (0.50, 0.62) | 0.60 (0.54, 0.66) | 0.28 (-0.09, 0.66) | 0.141 |
| Central Asia | — | — | — | — | 109.99 (108.30, 111.71) | 114.55 (113.01, 116.11) | 0.14 (0.13, 0.16) | <0.001 | 70.55 (69.15, 71.98) | 54.60 (53.56, 55.65) | -0.85 (-1.30, -0.41) | <0.001 | 1.10 (0.93, 1.29) | 0.82 (0.70, 0.96) | -1.04 (-1.46, -0.61) | <0.001 |

| | | | | | | | | | | | | | | | | |
|---|---|---|---|---|---|---|---|---|---|---|---|---|---|---|---|---|
| Eastern Europe | — | — | — | — | 75.22 (74.37, 76.08) | 81.90 (80.84, 82.97) | 0.30 (0.28, 0.32) | <0.001 | 44.42 (43.80, 45.05) | 72.31 (71.45, 73.18) | 1.82 (0.41, 3.26) | 0.012 | 0.69 (0.61, 0.77) | 1.19 (1.08, 1.30) | 2.05 (0.53, 3.60) | 0.008 |
| Southern Sub-Saharan Africa | — | — | — | — | 278.79 (275.67, 281.95) | 286.23 (283.66, 288.82) | 0.09 (0.07, 0.11) | <0.001 | 67.26 (65.64, 68.92) | 65.95 (64.73, 67.19) | -0.09 (-0.55, 0.37) | 0.696 | 0.91 (0.72, 1.13) | 0.86 (0.73, 1.01) | -0.24 (-0.85, 0.37) | 0.443 |
| High-income Asia Pacific | — | — | — | — | 187.10 (185.66, 188.56) | 185.88 (184.24, 187.54) | -0.03 (-0.13, 0.07) | 0.577 | 33.42 (32.81, 34.04) | 21.49 (20.95, 22.05) | -1.52 (-1.67, -1.36) | <0.001 | 0.39 (0.33, 0.46) | 0.19 (0.14, 0.25) | -2.49 (-2.65, -2.34) | <0.001 |
| Central Europe | — | — | — | — | 270.44 (268.29, 272.59) | 280.43 (277.88, 283.01) | 0.13 (0.11, 0.16) | <0.001 | 85.79 (84.62, 86.97) | 46.56 (45.58, 47.56) | -2.13 (-2.33, -1.94) | <0.001 | 1.24 (1.10, 1.39) | 0.54 (0.44, 0.66) | -2.89 (-3.17, -2.62) | <0.001 |
| Andean Latin America | — | — | — | — | 250.21 (246.78, 253.68) | 311.98 (308.96, 315.02) | 0.76 (0.74, 0.78) | <0.001 | 89.54 (87.40, 91.73) | 52.64 (51.40, 53.91) | -1.75 (-2.04, -1.46) | <0.001 | 1.25 (1.00, 1.54) | 0.59 (0.46, 0.74) | -2.49 (-2.94, -2.03) | <0.001 |
| Caribbean | — | — | — | — | 171.95 (168.99, 174.96) | 204.31 (201.37, 207.28) | 0.60 (0.58, 0.62) | <0.001 | 65.04 (63.15, 66.98) | 71.23 (69.49, 73.00) | 0.36 (0.23, 0.48) | <0.001 | 0.95 (0.73, 1.21) | 1.02 (0.83, 1.26) | 0.31 (0.14, 0.48) | <0.001 |
| Southern Latin America | — | — | — | — | 143.61 (141.23, 146.02) | 213.03 (210.49, 215.60) | 1.36 (1.33, 1.40) | <0.001 | 56.05 (54.54, 57.60) | 45.60 (44.44, 46.79) | -0.72 (-1.03, -0.41) | <0.001 | 0.83 (0.65, 1.03) | 0.58 (0.46, 0.73) | -1.22 (-1.58, -0.87) | <0.001 |
| Oceania | — | — | — | — | 126.58 (120.75, 132.65) | 119.27 (115.29, 123.35) | -0.20 (-0.21, -0.19) | <0.001 | 81.10 (76.23, 86.23) | 74.47 (71.25, 77.80) | -0.32 (-0.42, -0.22) | <0.001 | 1.25 (0.71, 2.07) | 1.14 (0.77, 1.63) | -0.34 (-0.46, -0.21) | <0.001 |
| Australasia | — | — | — | — | 117.65 (114.36, 121.01) | 111.34 (108.42, 114.32) | -0.19 (-0.21, -0.17) | <0.001 | 22.95 (21.51, 24.46) | 22.60 (21.30, 23.95) | -0.07 (-0.33, 0.19) | 0.597 | 0.28 (0.14, 0.49) | 0.29 (0.16, 0.48) | 0.08 (-0.38, 0.54) | 0.731 |

CI, confidence interval; GBD, global burden disease

"—" indicates that data are unavailable; * significantly different from zero (*P*<0.05).

**Table S2 Age-specific incidence, prevalence, disability-adjusted life years, and death rate of the overall and type-specific cardiovascular disease in youths and young adults from 1990 to 2019 by age, sex, sociodemographic index, and 21 GBD regions**

| | Incidence | | | | Prevalence | | | | Disability-adjusted life years | | | | Death | | | |
|---|---|---|---|---|---|---|---|---|---|---|---|---|---|---|---|---|
| | Rate per 100,000 (95% CI) in 1990 | Rate per 100,000 (95% CI) in 2019 | Average annual percent change %, (95% CI) | *P*-value | Rate per 100,000 (95% CI) in 1990 | Rate per 100,000 (95% CI) in 2019 | Average annual percent change %, (95% CI) | *P*-value | Rate per 100,000 (95% CI) in 1990 | Rate per 100,000 (95% CI) in 2019 | Average annual percent change %, (95% CI) | *P*-value | Rate per 100,000 (95% CI) in 1990 | Rate per 100,000 (95% CI) in 2019 | Average annual percent change %, (95% CI) | *P*-value |
| **Cardiovascular diseases** | | | | | | | | | | | | | | | | |
| **15-19 years** | | | | | | | | | | | | | | | | |
| **Women** | | | | | | | | | | | | | | | | |
| Global | 100.65 (68.81, 134.99) | 118.36 (77.85, 159.99) | 0.56 (0.54, 0.59) | <0.001 | 1142.56 (857.28, 1486.42) | 1408.64 (1038.03, 1859.13) | 0.73 (0.67, 0.79) | <0.001 | 548.20 (499.42, 601.93) | 394.75 (345.22, 448.86) | -1.15 (-1.45, -0.85) | <0.001 | 6.39 (5.83, 6.94) | 4.07 (3.58, 4.57) | -1.55 (-1.86, -1.23) | <0.001 |
| **SDI category** | | | | | | | | | | | | | | | | |
| High | 36.19 (27.72, 46.31) | 37.96 (29.61, 47.80) | 0.17 (0.14, 0.20) | <0.001 | 606.01 (436.28, 837.16) | 588.65 (442.99, 772.30) | -0.01 (-0.43, 0.41) | 0.960 | 234.53 (211.46, 260.65) | 142.53 (121.66, 166.82) | -1.67 (-1.97, -1.37) | <0.001 | 2.42 (2.26, 2.57) | 1.11 (1.03, 1.20) | -2.54 (-2.83, -2.24) | <0.001 |
| High-middle | 59.86 (44.98, 77.35) | 61.19 (45.38, 79.92) | 0.08 (0.02, 0.14) | 0.011 | 747.32 (580.70, 937.37) | 819.70 (630.44, 1043.74) | 0.32 (0.29, 0.35) | <0.001 | 368.07 (328.66, 408.20) | 237.26 (207.41, 268.96) | -1.48 (-1.69, -1.28) | <0.001 | 4.10 (3.72, 4.57) | 2.23 (2.00, 2.46) | -2.07 (-2.31, -1.82) | <0.001 |
| Middle | 98.71 (67.11, 133.90) | 98.07 (66.28, 132.24) | -0.01 (-0.09, 0.06) | 0.696 | 1144.92 (855.78, 1500.08) | 1236.98 (914.22, 1616.53) | 0.27 (0.23, 0.32) | <0.001 | 573.73 (522.52, 632.63) | 384.25 (337.24, 427.38) | -1.36 (-1.59, -1.13) | <0.001 | 6.70 (6.16, 7.28) | 4.01 (3.56, 4.49) | -1.74 (-2.03, -1.44) | <0.001 |
| Low-middle | 140.93 (92.64, 191.13) | 149.70 (96.46, 205.57) | 0.23 (0.12, 0.34) | <0.001 | 1357.18 (988.54, 1803.38) | 1553.25 (1119.53, 2103.19) | 0.48 (0.42, 0.53) | <0.001 | 770.66 (672.87, 869.66) | 503.65 (433.95, 583.50) | -1.49 (-1.89, -1.08) | <0.001 | 9.43 (8.09, 10.71) | 5.53 (4.67, 6.44) | -1.87 (-2.36, -1.37) | <0.001 |
| Low | 169.50 (104.69, 240.36) | 177.82 (106.09, 253.34) | 0.17 (0.10, 0.24) | <0.001 | 2020.87 (1424.13, 2727.23) | 2208.03 (1568.09, 3019.65) | 0.32 (0.25, 0.40) | <0.001 | 668.91 (558.67, 789.47) | 476.00 (397.11, 568.98) | -1.16 (-1.44, -0.87) | <0.001 | 7.53 (6.19, 8.93) | 4.67 (3.79, 5.68) | -1.64 (-1.84, -1.44) | <0.001 |

**21 GBD region**

| | | | | | | | | | | | | | | | | |
|---|---|---|---|---|---|---|---|---|---|---|---|---|---|---|---|---|
| South Asia | 145.54 (100.29, 194.27) | 148.50 (100.45, 201.88) | 0.08 (0.05, 0.12) | <0.001 | 1171.50 (839.93, 1569.21) | 1245.21 (895.75, 1715.62) | 0.23 (0.17, 0.30) | <0.001 | 851.04 (732.90, 978.58) | 515.85 (441.19, 598.52) | -1.80 (-2.46, -1.14) | <0.001 | 10.72 (8.97, 12.37) | 5.95 (4.99, 7.03) | -2.12 (-2.90, -1.33) | <0.001 |
| East Asia | 85.69 (58.07, 117.30) | 71.26 (48.31, 97.52) | -0.64 (-0.77, -0.50) | <0.001 | 889.82 (652.22, 1190.00) | 786.19 (585.35, 1035.65) | -0.38 (-0.53, -0.24) | <0.001 | 416.37 (354.69, 485.40) | 215.18 (180.49, 253.06) | -2.27 (-2.50, -2.03) | <0.001 | 4.59 (3.86, 5.55) | 1.85 (1.55, 2.14) | -3.14 (-3.38, -2.90) | <0.001 |
| North Africa and Middle East | 79.32 (56.70, 104.59) | 85.12 (59.40, 112.80) | 0.24 (0.22, 0.26) | <0.001 | 1236.93 (923.74, 1628.26) | 1328.51 (971.81, 1760.30) | 0.26 (0.21, 0.31) | <0.001 | 977.44 (854.95, 1116.94) | 569.34 (464.23, 693.15) | -1.79 (-2.00, -1.58) | <0.001 | 12.33 (10.69, 14.23) | 6.55 (5.23, 8.20) | -2.08 (-2.35, -1.81) | <0.001 |
| Western Sub-Saharan Africa | 140.28 (84.59, 202.19) | 147.97 (87.13, 212.89) | 0.19 (0.16, 0.23) | <0.001 | 2361.78 (1604.34, 3276.51) | 2545.40 (1721.46, 3546.21) | 0.27 (0.24, 0.29) | <0.001 | 541.37 (427.29, 675.70) | 414.31 (319.48, 520.99) | -0.92 (-1.00, -0.84) | <0.001 | 5.47 (4.13, 7.15) | 3.54 (2.62, 4.74) | -1.48 (-1.65, -1.31) | <0.001 |
| Southeast Asia | 68.89 (51.62, 90.65) | 70.67 (52.19, 92.42) | 0.10 (0.06, 0.14) | <0.001 | 783.12 (609.14, 993.92) | 842.06 (650.06, 1063.53) | 0.25 (0.17, 0.34) | <0.001 | 530.23 (449.18, 614.35) | 399.55 (344.20, 451.78) | -0.95 (-1.09, -0.80) | <0.001 | 6.24 (5.14, 7.30) | 4.35 (3.70, 4.99) | -1.21 (-1.38, -1.03) | <0.001 |
| Eastern Sub-Saharan Africa | 216.89 (121.55, 314.40) | 230.57 (127.11, 337.90) | 0.22 (0.17, 0.27) | <0.001 | 2784.93 (1966.85, 3770.29) | 3044.98 (2161.43, 4146.96) | 0.31 (0.19, 0.43) | <0.001 | 624.03 (500.03, 749.29) | 460.50 (364.33, 564.01) | -1.04 (-1.19, -0.88) | <0.001 | 6.27 (4.83, 7.78) | 3.78 (2.89, 4.79) | -1.73 (-1.90, -1.56) | <0.001 |
| Central Latin America | 83.15 (59.61, 108.70) | 82.38 (57.55, 109.58) | -0.02 (-0.08, 0.03) | 0.404 | 1126.23 (871.74, 1422.05) | 1154.00 (890.42, 1467.25) | 0.09 (-0.01, 0.20) | 0.083 | 407.68 (376.93, 442.40) | 262.96 (225.82, 304.94) | -1.46 (-1.77, -1.15) | <0.001 | 4.67 (4.40, 4.93) | 2.63 (2.25, 3.06) | -1.93 (-2.38, -1.49) | <0.001 |
| Tropical Latin America | 159.24 (97.24, 226.91) | 157.97 (94.85, 226.93) | -0.02 (-0.04, -0.00) | 0.017 | 1956.41 (1434.11, 2612.35) | 2012.05 (1449.71, 2698.55) | 0.10 (0.08, 0.12) | <0.001 | 467.57 (421.62, 526.51) | 317.89 (272.89, 377.25) | -1.31 (-1.61, -1.01) | <0.001 | 4.95 (4.65, 5.26) | 2.83 (2.61, 3.04) | -1.89 (-2.25, -1.54) | <0.001 |
| Central Sub-Saharan Africa | 200.65 (113.00, 306.50) | 207.27 (112.04, 317.60) | 0.12 (0.08, 0.15) | <0.001 | 2836.42 (1917.30, 3889.80) | 2918.42 (2026.30, 4031.81) | 0.11 (0.07, 0.14) | <0.001 | 585.16 (409.02, 772.21) | 438.28 (331.07, 567.51) | -1.04 (-1.15, -0.94) | <0.001 | 5.81 (3.65, 8.27) | 3.70 (2.54, 5.32) | -1.61 (-1.72, -1.49) | <0.001 |
| Western Europe | 30.04 (21.81, 39.71) | 29.20 (20.99, 39.11) | -0.10 (-0.15, -0.06) | <0.001 | 542.82 (386.17, 758.33) | 532.39 (374.79, 737.58) | -0.02 (-0.30, 0.27) | 0.916 | 190.69 (171.95, 213.07) | 102.90 (86.26, 124.42) | -2.16 (-2.40, -1.93) | <0.001 | 1.96 (1.87, 2.06) | 0.74 (0.68, 0.81) | -3.39 (-3.75, -3.03) | <0.001 |
| High-income North America | 42.80 (34.24, | 45.87 (37.22, | 0.24 (0.20, 0.27) | <0.001 | 825.91 (564.70, | 679.81 (519.27, | -0.55 (-1.04, | 0.030 | 197.93 (171.30, | 165.03 (139.02, | -0.55 (-0.85, | <0.001 | 1.71 (1.64, | 1.29 (1.20, | -0.90 (-2.04, | 0.124 |

| | | | | | | | | | | | | | | | | |
|---|---|---|---|---|---|---|---|---|---|---|---|---|---|---|---|---|
| | 53.61) | 56.65) | | | 1180.02) | 891.26) | -0.05) | | 230.60) | 192.76) | -0.24) | | 1.78) | 1.39) | 0.25) | |
| Central Asia | 106.83 (69.64, 146.94) | 109.89 (68.91, 153.86) | 0.10 (0.08, 0.12) | <0.001 | 1130.57 (825.42, 1501.60) | 1178.78 (852.05, 1587.35) | 0.15 (0.10, 0.20) | <0.001 | 517.32 (479.16, 561.88) | 380.56 (329.03, 438.30) | -1.00 (-1.34, -0.65) | <0.001 | 5.99 (5.62, 6.36) | 4.04 (3.49, 4.68) | -1.34 (-1.84, -0.83) | <0.001 |
| Eastern Europe | 38.27 (29.35, 49.59) | 41.88 (32.15, 54.15) | 0.34 (0.25, 0.42) | <0.001 | 460.87 (354.17, 593.39) | 526.82 (411.86, 663.98) | 0.50 (0.30, 0.71) | <0.001 | 269.08 (245.79, 296.17) | 244.36 (212.51, 280.73) | -0.32 (-1.32, 0.69) | 0.536 | 2.84 (2.74, 2.97) | 2.35 (2.09, 2.62) | -0.75 (-2.19, 0.71) | 0.315 |
| Southern Sub-Saharan Africa | 197.08 (112.10, 287.35) | 197.57 (110.68, 287.56) | 0.02 (-0.04, 0.08) | 0.489 | 2565.73 (1843.49, 3457.83) | 2584.74 (1840.44, 3485.57) | 0.03 (0.01, 0.05) | 0.010 | 541.89 (446.02, 639.41) | 396.64 (307.32, 513.18) | -1.01 (-1.39, -0.64) | <0.001 | 5.26 (4.30, 6.17) | 3.23 (2.27, 4.45) | -1.55 (-2.13, -0.97) | <0.001 |
| High-income Asia Pacific | 37.81 (29.94, 47.51) | 36.68 (28.77, 46.02) | -0.10 (-0.13, -0.07) | <0.001 | 343.30 (245.30, 471.09) | 368.37 (269.76, 490.11) | 0.28 (0.06, 0.51) | 0.014 | 293.77 (259.75, 326.69) | 96.53 (79.91, 114.78) | -3.78 (-4.25, -3.32) | <0.001 | 3.44 (3.05, 3.85) | 0.66 (0.60, 0.73) | -5.58 (-6.51, -4.63) | <0.001 |
| Central Europe | 35.33 (27.32, 45.62) | 35.50 (27.63, 45.88) | 0.02 (-0.03, 0.07) | 0.435 | 808.25 (574.84, 1106.55) | 808.74 (580.49, 1096.37) | 0.04 (-0.14, 0.22) | 0.641 | 280.64 (249.44, 315.45) | 174.54 (143.53, 209.85) | -1.62 (-1.85, -1.39) | <0.001 | 2.74 (2.59, 2.91) | 1.25 (1.08, 1.44) | -2.68 (-3.06, -2.29) | <0.001 |
| Andean Latin America | 138.58 (86.63, 193.88) | 136.69 (84.54, 195.26) | -0.04 (-0.07, -0.01) | 0.018 | 1712.40 (1259.42, 2292.10) | 1780.22 (1262.38, 2374.92) | 0.14 (0.08, 0.20) | <0.001 | 699.56 (579.23, 829.91) | 397.42 (311.20, 499.27) | -1.99 (-2.56, -1.41) | <0.001 | 8.39 (6.82, 10.13) | 4.12 (3.13, 5.38) | -2.49 (-3.17, -1.80) | <0.001 |
| Caribbean | 140.88 (91.17, 194.62) | 151.51 (98.08, 210.46) | 0.25 (0.22, 0.29) | <0.001 | 1521.39 (1128.45, 2030.70) | 1600.19 (1154.35, 2146.60) | 0.18 (0.15, 0.22) | <0.001 | 658.13 (529.40, 808.45) | 564.60 (397.21, 798.28) | -0.30 (-0.55, -0.05) | 0.017 | 7.97 (6.22, 10.10) | 6.61 (4.30, 9.97) | -0.38 (-0.68, -0.08) | 0.013 |
| Southern Latin America | 74.32 (48.87, 101.57) | 78.58 (52.71, 109.14) | 0.20 (0.14, 0.26) | <0.001 | 1084.22 (792.48, 1446.95) | 1179.68 (855.15, 1573.93) | 0.29 (0.21, 0.38) | <0.001 | 423.45 (377.87, 473.48) | 248.86 (209.40, 292.14) | -1.84 (-2.14, -1.54) | <0.001 | 4.82 (4.31, 5.34) | 2.33 (1.98, 2.71) | -2.50 (-2.90, -2.11) | <0.001 |
| Oceania | 86.14 (60.19, 123.69) | 90.22 (61.37, 129.36) | 0.17 (0.12, 0.22) | <0.001 | 1166.25 (871.80, 1555.12) | 1218.52 (905.20, 1621.43) | 0.17 (0.10, 0.24) | <0.001 | 773.66 (521.01, 1096.17) | 821.47 (561.84, 1152.76) | 0.30 (0.20, 0.39) | <0.001 | 9.41 (5.98, 14.11) | 10.05 (6.41, 14.71) | 0.32 (0.21, 0.43) | <0.001 |
| Australasia | 26.86 (18.81, 36.14) | 27.40 (19.87, 37.07) | 0.08 (0.02, 0.13) | 0.003 | 346.75 (247.08, 467.66) | 339.66 (243.15, 449.07) | -0.04 (-0.19, 0.12) | 0.630 | 155.89 (133.51, 181.67) | 86.61 (68.03, 107.92) | -1.92 (-2.87, -0.95) | <0.001 | 1.59 (1.40, 1.81) | 0.63 (0.52, 0.77) | -3.03 (-4.91, -1.11) | 0.002 |
| **Men** | | | | | | | | | | | | | | | | |
| Global | 90.98 | 103.67 | 0.47 (0.36, | <0.001 | 916.12 | 1135.66 | 0.75 (0.71, | <0.001 | 591.19 | 447.00 | -1.00 | <0.001 | 7.34 | 5.18 | -1.24 | <0.001 |

| | | | | | | | | | | | | | | | | |
|---|---|---|---|---|---|---|---|---|---|---|---|---|---|---|---|---|
| | (64.55, 120.95) | (71.03, 140.08) | 0.58) | | (664.17, 1203.77) | (819.87, 1515.89) | 0.80) | | (531.75, 649.50) | (389.94, 506.53) | (-1.50, -0.49) | | (6.54, 8.05) | (4.54, 5.93) | (-1.83, -0.65) | |
| **SDI category** | | | | | | | | | | | | | | | | |
| High | 40.28 (30.17, 53.36) | 41.41 (31.89, 53.62) | 0.09 (0.06, 0.12) | <0.001 | 344.97 (258.52, 469.81) | 352.30 (275.26, 449.94) | 0.20 (-0.33, 0.74) | 0.458 | 309.71 (261.09, 335.45) | 151.44 (136.91, 168.31) | -2.41 (-2.59, -2.23) | <0.001 | 3.81 (3.17, 4.09) | 1.58 (1.48, 1.69) | -3.01 (-3.25, -2.78) | <0.001 |
| High-middle | 61.18 (47.20, 78.61) | 60.21 (45.74, 77.43) | -0.04 (-0.11, 0.02) | 0.146 | 568.71 (429.57, 733.84) | 601.78 (452.63, 790.62) | 0.20 (0.16, 0.25) | <0.001 | 518.20 (458.31, 575.78) | 305.65 (272.37, 341.82) | -1.88 (-2.44, -1.31) | <0.001 | 6.54 (5.73, 7.38) | 3.56 (3.16, 4.03) | -2.17 (-2.83, -1.51) | <0.001 |
| Middle | 93.90 (65.34, 127.49) | 91.89 (64.39, 123.63) | -0.06 (-0.14, 0.03) | 0.188 | 968.99 (698.18, 1288.72) | 1038.56 (754.77, 1376.08) | 0.24 (0.16, 0.32) | <0.001 | 648.02 (575.57, 720.26) | 490.82 (424.68, 563.08) | -0.98 (-1.27, -0.69) | <0.001 | 8.07 (7.11, 9.06) | 5.84 (4.97, 6.86) | -1.16 (-1.49, -0.82) | <0.001 |
| Low-middle | 112.54 (76.44, 152.18) | 120.44 (80.54, 164.69) | 0.26 (0.10, 0.42) | 0.001 | 1082.78 (781.44, 1443.63) | 1238.18 (878.53, 1670.88) | 0.48 (0.43, 0.53) | <0.001 | 651.85 (560.65, 744.01) | 485.33 (410.59, 566.66) | -1.12 (-2.01, -0.23) | 0.014 | 8.09 (6.88, 9.36) | 5.66 (4.79, 6.74) | -1.35 (-2.37, -0.32) | 0.011 |
| Low | 149.79 (95.12, 208.40) | 157.53 (97.23, 222.85) | 0.19 (0.12, 0.26) | <0.001 | 1698.30 (1185.29, 2335.01) | 1878.26 (1309.08, 2573.72) | 0.35 (0.30, 0.41) | <0.001 | 728.39 (618.17, 845.85) | 564.31 (468.84, 668.65) | -0.94 (-1.20, -0.68) | <0.001 | 8.75 (7.21, 10.36) | 6.33 (5.17, 7.60) | -1.19 (-1.51, -0.87) | <0.001 |
| **21 GBD region** | | | | | | | | | | | | | | | | |
| South Asia | 109.26 (76.11, 146.27) | 113.64 (78.66, 153.80) | 0.19 (-0.04, 0.42) | 0.110 | 901.77 (649.70, 1217.23) | 939.24 (671.73, 1294.06) | 0.16 (0.07, 0.24) | <0.001 | 641.70 (535.20, 750.00) | 446.96 (369.45, 532.94) | -1.47 (-3.43, 0.54) | 0.150 | 8.09 (6.63, 9.60) | 5.35 (4.36, 6.55) | -1.95 (-3.92, 0.05) | 0.056 |
| East Asia | 88.62 (61.28, 121.09) | 74.28 (51.77, 100.16) | -0.61 (-0.70, -0.52) | <0.001 | 757.22 (533.41, 1049.31) | 627.88 (435.02, 865.94) | -0.64 (-0.75, -0.52) | <0.001 | 636.07 (510.08, 751.21) | 338.75 (273.85, 405.14) | -2.17 (-2.31, -2.04) | <0.001 | 8.02 (6.26, 9.59) | 3.97 (3.19, 4.89) | -2.39 (-2.60, -2.18) | <0.001 |
| North Africa and Middle East | 79.28 (58.10, 103.55) | 86.56 (62.92, 115.73) | 0.32 (0.28, 0.35) | <0.001 | 1146.60 (838.46, 1562.01) | 1254.46 (912.00, 1692.70) | 0.33 (0.26, 0.40) | <0.001 | 996.50 (850.65, 1171.06) | 739.40 (572.50, 919.75) | -0.99 (-1.26, -0.73) | <0.001 | 12.81 (10.84, 15.31) | 9.13 (6.92, 11.60) | -1.12 (-1.40, -0.84) | <0.001 |
| Western Sub-Saharan Africa | 139.47 (87.24, 197.06) | 144.16 (89.28, 204.76) | 0.12 (0.10, 0.13) | <0.001 | 2470.32 (1646.05, 3580.77) | 2613.22 (1735.33, 3795.15) | 0.20 (0.18, 0.22) | <0.001 | 588.00 (454.67, 749.33) | 484.71 (366.10, 619.38) | -0.67 (-0.79, -0.56) | <0.001 | 6.20 (4.66, 8.14) | 4.64 (3.38, 6.13) | -1.02 (-1.20, -0.84) | <0.001 |
| Southeast Asia | 62.70 (48.33, | 64.59 (49.51, | 0.11 (0.08, 0.13) | <0.001 | 610.45 (473.89, | 651.55 (498.68, | 0.23 (0.16, 0.30) | <0.001 | 490.78 (413.22, | 503.56 (429.02, | 0.12 (-0.07, | 0.218 | 6.01 (4.98, | 6.17 (5.09, | 0.13 (-0.11, 0.37) | 0.297 |

| | | | | | | | | | | | | | | | | |
|---|---|---|---|---|---|---|---|---|---|---|---|---|---|---|---|---|
| | 80.37) | 82.90) | | | 773.77) | 833.71) | | | 596.20) | 583.79) | 0.32) | | 7.56) | 7.31) | | |
| Eastern Sub-Saharan Africa | 197.01 (115.12, 279.73) | 207.22 (119.80, 302.74) | 0.19 (0.14, 0.25) | <0.001 | 2196.97 (1536.88, 3029.49) | 2424.14 (1696.33, 3310.72) | 0.36 (0.29, 0.42) | <0.001 | 881.56 (713.31, 1049.80) | 654.17 (536.39, 779.25) | -1.01 (-1.11, -0.91) | <0.001 | 10.55 (8.32, 12.76) | 7.20 (5.81, 8.81) | -1.30 (-1.39, -1.20) | <0.001 |
| Central Latin America | 69.40 (49.79, 91.64) | 71.31 (51.31, 95.14) | 0.09 (0.08, 0.11) | <0.001 | 826.65 (628.71, 1067.26) | 873.64 (663.05, 1130.57) | 0.20 (0.18, 0.21) | <0.001 | 406.27 (378.31, 439.39) | 304.02 (256.25, 356.86) | -0.86 (-1.18, -0.54) | <0.001 | 5.00 (4.67, 5.32) | 3.53 (2.91, 4.21) | -1.04 (-1.36, -0.72) | <0.001 |
| Tropical Latin America | 140.05 (88.42, 198.00) | 136.47 (85.08, 191.99) | -0.09 (-0.10, -0.08) | <0.001 | 1535.40 (1094.71, 2100.27) | 1519.63 (1077.80, 2067.14) | -0.03 (-0.05, -0.01) | 0.001 | 536.09 (490.61, 590.39) | 406.38 (358.90, 461.36) | -0.97 (-1.35, -0.58) | <0.001 | 6.31 (5.88, 6.79) | 4.51 (4.04, 4.98) | -1.17 (-1.68, -0.66) | <0.001 |
| Central Sub-Saharan Africa | 187.37 (105.01, 276.71) | 188.95 (105.71, 290.15) | 0.03 (0.00, 0.06) | 0.025 | 2373.57 (1637.61, 3236.98) | 2394.86 (1628.85, 3300.99) | 0.03 (-0.00, 0.06) | 0.093 | 775.56 (555.30, 1047.55) | 566.87 (411.67, 750.35) | -1.10 (-1.27, -0.93) | <0.001 | 8.99 (6.14, 12.73) | 6.06 (4.05, 8.63) | -1.43 (-1.58, -1.27) | <0.001 |
| Western Europe | 35.05 (25.10, 47.05) | 34.11 (24.36, 46.16) | -0.10 (-0.13, -0.07) | <0.001 | 288.42 (204.17, 404.43) | 276.29 (195.89, 380.12) | -0.14 (-0.31, 0.04) | 0.125 | 267.75 (251.67, 287.02) | 108.11 (96.97, 121.50) | -3.09 (-3.62, -2.56) | <0.001 | 3.33 (3.16, 3.52) | 1.11 (1.03, 1.20) | -3.73 (-4.29, -3.16) | <0.001 |
| High-income North America | 42.25 (31.79, 55.90) | 45.38 (35.58, 57.44) | 0.24 (0.19, 0.29) | <0.001 | 445.02 (334.12, 600.71) | 418.50 (325.82, 521.57) | 0.01 (-0.82, 0.85) | 0.981 | 236.32 (217.35, 257.07) | 186.10 (167.22, 206.38) | -0.72 (-1.14, -0.30) | 0.001 | 2.67 (2.54, 2.80) | 1.96 (1.84, 2.13) | -0.99 (-1.45, -0.52) | <0.001 |
| Central Asia | 102.50 (69.86, 139.72) | 107.39 (71.81, 149.95) | 0.16 (0.15, 0.17) | <0.001 | 966.33 (684.69, 1321.92) | 1036.85 (722.32, 1427.83) | 0.25 (0.20, 0.29) | <0.001 | 518.77 (479.62, 563.64) | 450.24 (380.67, 527.50) | -0.29 (-0.66, 0.08) | 0.120 | 6.30 (5.85, 6.78) | 5.30 (4.44, 6.30) | -0.36 (-0.78, 0.06) | 0.090 |
| Eastern Europe | 41.19 (30.81, 54.18) | 43.59 (33.17, 56.65) | 0.21 (0.17, 0.26) | <0.001 | 263.88 (186.56, 372.29) | 276.37 (188.83, 399.82) | 0.19 (0.10, 0.27) | <0.001 | 376.38 (358.87, 395.40) | 348.58 (300.15, 400.33) | 0.05 (-1.35, 1.48) | 0.941 | 4.79 (4.61, 4.96) | 4.40 (3.77, 5.10) | 0.06 (-1.37, 1.51) | 0.933 |
| Southern Sub-Saharan Africa | 178.18 (105.59, 257.28) | 182.22 (108.40, 261.05) | 0.08 (0.07, 0.09) | <0.001 | 2137.94 (1506.27, 2916.74) | 2200.60 (1530.97, 3001.95) | 0.10 (0.09, 0.12) | <0.001 | 535.30 (430.45, 648.87) | 538.55 (411.91, 697.65) | 0.18 (-0.06, 0.43) | 0.143 | 5.72 (4.54, 7.19) | 5.73 (4.14, 7.95) | 0.21 (-0.14, 0.57) | 0.245 |
| High-income Asia Pacific | 46.46 (35.72, 61.22) | 47.23 (36.50, 62.34) | 0.05 (-0.01, 0.11) | 0.078 | 288.19 (195.78, 417.50) | 301.91 (205.69, 431.93) | 0.17 (0.11, 0.24) | <0.001 | 474.90 (278.28, 550.65) | 113.04 (99.37, 128.19) | -4.82 (-5.20, -4.44) | <0.001 | 6.17 (3.49, 7.19) | 1.09 (1.01, 1.19) | -5.53 (-5.87, -5.19) | <0.001 |
| Central Europe | 40.05 (30.49, | 40.21 (30.76, | 0.02 (-0.05, 0.09) | 0.602 | 481.31 (323.84, | 479.20 (316.18, | 0.02 (-0.09, 0.12) | 0.754 | 350.99 (327.06, | 199.04 (169.59, | -1.95 (-2.38, | <0.001 | 4.27 (4.03, | 2.16 (1.81, | -2.35 (-2.79, | <0.001 |

| | | | | | | | | | | | | | | | | |
|---|---|---|---|---|---|---|---|---|---|---|---|---|---|---|---|---|
| | 53.02) | 52.73) | | | 711.09) | 708.67) | | | 379.81) | 232.30) | -1.52) | | 4.51) | 2.57) | -1.92) | |
| Andean Latin America | 127.63 (85.02, 179.55) | 122.77 (78.70, 171.92) | -0.14 (-0.16, -0.12) | <0.001 | 1549.57 (1112.52, 2112.98) | 1598.06 (1140.27, 2132.39) | 0.11 (0.07, 0.15) | <0.001 | 861.52 (698.44, 1057.91) | 515.21 (402.95, 653.68) | -1.73 (-2.13, -1.33) | <0.001 | 10.85 (8.69, 13.57) | 5.97 (4.49, 7.81) | -2.01 (-2.60, -1.41) | <0.001 |
| Caribbean | 120.59 (78.40, 168.87) | 129.35 (87.03, 180.84) | 0.24 (0.22, 0.27) | <0.001 | 1358.72 (997.77, 1821.17) | 1427.97 (1036.69, 1918.44) | 0.16 (0.14, 0.19) | <0.001 | 511.06 (434.56, 601.93) | 411.10 (302.87, 542.28) | -0.63 (-0.86, -0.41) | <0.001 | 6.10 (5.11, 7.33) | 4.65 (3.32, 6.42) | -0.78 (-1.06, -0.51) | <0.001 |
| Southern Latin America | 78.38 (54.44, 106.02) | 83.45 (58.47, 115.12) | 0.21 (0.17, 0.25) | <0.001 | 820.20 (572.80, 1118.65) | 897.25 (622.29, 1247.40) | 0.30 (0.21, 0.40) | <0.001 | 508.89 (453.59, 572.21) | 306.76 (260.48, 359.78) | -1.77 (-2.12, -1.41) | <0.001 | 6.33 (5.60, 7.13) | 3.45 (2.88, 4.08) | -2.11 (-2.52, -1.69) | <0.001 |
| Oceania | 86.67 (63.54, 117.71) | 88.01 (63.57, 122.41) | 0.06 (0.04, 0.08) | <0.001 | 1043.02 (763.46, 1383.20) | 1089.06 (790.95, 1447.29) | 0.16 (0.11, 0.21) | <0.001 | 1090.01 (761.18, 1560.39) | 1144.18 (750.09, 1709.50) | 0.10 (-0.08, 0.28) | 0.297 | 14.10 (9.38, 20.64) | 14.84 (9.49, 22.58) | 0.10 (-0.10, 0.30) | 0.329 |
| Australasia | 37.05 (25.78, 50.38) | 38.45 (27.54, 51.98) | 0.13 (0.12, 0.14) | <0.001 | 177.36 (120.26, 243.40) | 167.64 (111.44, 230.37) | -0.18 (-0.28, -0.09) | <0.001 | 220.40 (195.60, 248.57) | 90.91 (76.70, 107.30) | -3.03 (-3.73, -2.32) | <0.001 | 2.77 (2.44, 3.14) | 0.98 (0.81, 1.19) | -3.57 (-4.55, -2.58) | <0.001 |
| **20-24 years** | | | | | | | | | | | | | | | | |
| **Women** | | | | | | | | | | | | | | | | |
| Global | 105.24 (78.17, 134.88) | 116.25 (84.13, 152.68) | 0.34 (0.28, 0.40) | <0.001 | 1320.64 (1061.06, 1620.29) | 1580.08 (1238.95, 1955.56) | 0.63 (0.58, 0.68) | <0.001 | 732.41 (667.00, 799.83) | 537.20 (475.67, 600.00) | -1.06 (-1.22, -0.90) | <0.001 | 9.37 (8.43, 10.29) | 6.27 (5.49, 7.09) | -1.38 (-1.56, -1.19) | <0.001 |
| **SDI category** | | | | | | | | | | | | | | | | |
| High | 67.72 (57.45, 79.03) | 71.82 (61.50, 83.21) | 0.20 (0.16, 0.24) | <0.001 | 873.09 (676.41, 1137.83) | 857.32 (698.28, 1066.65) | 0.00 (-0.34, 0.34) | 0.993 | 311.34 (283.31, 344.25) | 208.53 (181.14, 239.32) | -1.37 (-1.63, -1.10) | <0.001 | 3.48 (3.29, 3.67) | 1.91 (1.76, 2.10) | -2.07 (-2.39, -1.75) | <0.001 |
| High-middle | 71.11 (57.44, 87.68) | 70.36 (56.12, 87.84) | -0.03 (-0.05, -0.02) | <0.001 | 866.48 (718.21, 1039.56) | 945.97 (776.27, 1147.03) | 0.31 (0.26, 0.36) | <0.001 | 503.61 (448.89, 566.59) | 329.99 (292.03, 371.78) | -1.43 (-1.92, -0.95) | <0.001 | 6.19 (5.50, 7.01) | 3.55 (3.16, 3.96) | -1.84 (-2.33, -1.33) | <0.001 |
| Middle | 98.98 (72.11, 129.32) | 99.61 (72.42, 131.14) | 0.03 (-0.08, 0.15) | 0.596 | 1310.23 (1044.39, 1607.65) | 1416.98 (1121.48, 1759.26) | 0.28 (0.21, 0.35) | <0.001 | 773.63 (692.07, 855.33) | 526.86 (465.80, 586.22) | -1.26 (-1.55, -0.96) | <0.001 | 9.91 (8.82, 11.09) | 6.17 (5.40, 6.99) | -1.58 (-1.89, -1.26) | <0.001 |
| Low-middle | 150.51 (104.53, | 150.96 (104.01, | 0.01 (-0.04, 0.06) | 0.577 | 1654.81 (1302.12, | 1827.92 (1404.89, | 0.36 (0.32, 0.40) | <0.001 | 1060.86 (918.63, | 713.62 (612.40, | -1.37 (-1.83, | <0.001 | 14.15 (11.97, | 8.80 (7.45, | -1.65 (-2.21, | <0.001 |

| | | | | | | | | | | | | | | | | |
|---|---|---|---|---|---|---|---|---|---|---|---|---|---|---|---|---|
| | 201.47) | 203.68) | | | 2060.29) | 2278.75) | | | 1205.34) | 826.26) | -0.91) | | 16.23) | 10.47) | -1.09) | |
| Low | 154.64 (103.56, 213.45) | 154.64 (101.06, 216.98) | 0.00 (-0.01, 0.01) | 0.786 | 2236.42 (1691.50, 2853.16) | 2415.73 (1807.87, 3099.54) | 0.29 (0.19, 0.39) | <0.001 | 921.16 (788.50, 1068.98) | 641.18 (539.44, 761.52) | -1.23 (-1.41, -1.05) | <0.001 | 11.65 (9.71, 13.68) | 7.28 (5.95, 8.79) | -1.60 (-1.80, -1.40) | <0.001 |
| **21 GBD region** | | | | | | | | | | | | | | | | |
| South Asia | 165.70 (114.07, 220.63) | 159.59 (110.31, 212.58) | -0.13 (-0.19, -0.06) | <0.001 | 1566.28 (1208.96, 1943.16) | 1609.02 (1239.44, 2015.07) | 0.11 (0.06, 0.16) | <0.001 | 1186.28 (1004.94, 1371.20) | 756.24 (632.21, 902.50) | -1.63 (-2.22, -1.04) | <0.001 | 16.16 (13.39, 18.86) | 9.64 (7.84, 11.78) | -1.89 (-2.56, -1.21) | <0.001 |
| East Asia | 81.35 (59.00, 107.35) | 71.20 (51.78, 93.03) | -0.47 (-0.53, -0.40) | <0.001 | 1001.36 (783.19, 1253.92) | 916.73 (724.41, 1129.80) | -0.26 (-0.45, -0.07) | 0.006 | 593.46 (484.57, 729.43) | 326.65 (269.47, 389.00) | -1.86 (-2.29, -1.44) | <0.001 | 7.31 (5.76, 9.37) | 3.39 (2.65, 4.19) | -2.38 (-2.89, -1.87) | <0.001 |
| North Africa and Middle East | 94.82 (72.65, 119.97) | 97.29 (73.35, 124.01) | 0.09 (0.08, 0.11) | <0.001 | 1404.96 (1112.79, 1776.59) | 1490.40 (1171.59, 1885.43) | 0.22 (0.14, 0.31) | <0.001 | 1124.33 (986.01, 1280.13) | 716.87 (584.75, 874.54) | -1.51 (-1.73, -1.28) | <0.001 | 15.19 (13.19, 17.43) | 8.98 (7.04, 11.35) | -1.77 (-2.02, -1.51) | <0.001 |
| Western Sub-Saharan Africa | 127.60 (83.60, 179.70) | 130.88 (85.44, 185.15) | 0.10 (0.06, 0.14) | <0.001 | 2453.10 (1773.96, 3295.29) | 2671.63 (1929.59, 3610.09) | 0.30 (0.28, 0.33) | <0.001 | 686.02 (541.71, 857.09) | 533.79 (412.28, 677.99) | -0.86 (-0.97, -0.75) | <0.001 | 7.93 (5.96, 10.25) | 5.45 (3.97, 7.43) | -1.27 (-1.42, -1.13) | <0.001 |
| Southeast Asia | 77.85 (59.48, 99.82) | 77.41 (59.17, 98.03) | -0.02 (-0.07, 0.03) | 0.525 | 926.92 (764.13, 1118.44) | 975.16 (798.74, 1181.03) | 0.19 (0.11, 0.27) | <0.001 | 760.00 (630.24, 890.36) | 558.13 (467.63, 643.50) | -1.06 (-1.21, -0.91) | <0.001 | 9.81 (7.82, 11.63) | 6.74 (5.55, 8.03) | -1.28 (-1.44, -1.11) | <0.001 |
| Eastern Sub-Saharan Africa | 165.13 (104.59, 239.97) | 165.05 (101.08, 240.42) | -0.01 (-0.02, 0.01) | 0.428 | 2920.92 (2170.77, 3728.62) | 3174.21 (2362.07, 4083.06) | 0.31 (0.19, 0.43) | <0.001 | 819.64 (672.51, 999.72) | 549.45 (439.72, 665.73) | -1.36 (-1.48, -1.23) | <0.001 | 9.53 (7.46, 11.85) | 5.25 (4.03, 6.59) | -2.02 (-2.15, -1.88) | <0.001 |
| Central Latin America | 87.33 (67.10, 109.65) | 83.92 (62.53, 107.48) | -0.13 (-0.16, -0.10) | <0.001 | 1351.14 (1105.17, 1639.83) | 1380.46 (1124.16, 1683.11) | 0.08 (-0.04, 0.20) | 0.172 | 538.73 (500.32, 580.47) | 357.23 (300.05, 424.49) | -1.44 (-2.12, -0.75) | <0.001 | 6.75 (6.40, 7.11) | 4.02 (3.27, 4.88) | -1.48 (-1.99, -0.96) | <0.001 |
| Tropical Latin America | 141.26 (96.28, 191.44) | 133.31 (89.13, 186.27) | -0.20 (-0.26, -0.13) | <0.001 | 2243.35 (1748.60, 2786.87) | 2272.32 (1746.21, 2831.38) | 0.05 (0.01, 0.09) | 0.005 | 687.08 (631.62, 746.88) | 415.43 (364.59, 478.44) | -1.66 (-2.14, -1.17) | <0.001 | 8.33 (7.81, 8.87) | 4.25 (3.92, 4.58) | -2.21 (-2.75, -1.66) | <0.001 |
| Central Sub-Saharan Africa | 159.42 (98.54, 232.54) | 158.19 (95.18, 232.45) | -0.01 (-0.05, 0.02) | 0.409 | 2889.06 (2087.93, 3815.86) | 2967.08 (2175.29, 3864.57) | 0.11 (0.05, 0.16) | <0.001 | 692.64 (496.25, 928.11) | 515.72 (389.02, 668.17) | -1.04 (-1.11, -0.96) | <0.001 | 7.79 (5.03, 10.96) | 5.05 (3.39, 7.12) | -1.51 (-1.60, -1.41) | <0.001 |

| | | | | | | | | | | | | | | | | |
|---|---|---|---|---|---|---|---|---|---|---|---|---|---|---|---|---|
| Western Europe | 54.14 (43.90, 65.79) | 52.50 (42.08, 63.89) | -0.10 (-0.13, -0.07) | <0.001 | 750.00 (560.53, 983.87) | 725.92 (554.78, 946.36) | -0.11 (-0.24, 0.03) | 0.125 | 267.03 (242.58, 296.11) | 144.77 (124.37, 170.38) | -2.08 (-2.39, -1.77) | <0.001 | 3.03 (2.88, 3.19) | 1.23 (1.14, 1.33) | -3.05 (-3.39, -2.70) | <0.001 |
| High-income North America | 89.52 (78.30, 102.37) | 97.37 (85.82, 110.00) | 0.29 (0.25, 0.34) | <0.001 | 1177.61 (879.15, 1597.62) | 1066.73 (863.00, 1334.34) | -0.29 (-0.56, -0.03) | 0.031 | 288.51 (255.54, 329.99) | 247.22 (215.43, 283.29) | -0.48 (-0.68, -0.28) | <0.001 | 2.88 (2.76, 2.99) | 2.27 (2.11, 2.43) | -0.93 (-1.44, -0.42) | <0.001 |
| Central Asia | 115.29 (86.45, 150.49) | 114.81 (83.85, 150.77) | -0.01 (-0.03, 0.00) | 0.186 | 1351.39 (1069.62, 1658.47) | 1402.25 (1103.55, 1763.69) | 0.15 (0.05, 0.24) | 0.003 | 884.11 (822.32, 946.21) | 666.21 (576.76, 767.07) | -1.06 (-1.43, -0.70) | <0.001 | 11.61 (10.93, 12.35) | 8.33 (7.04, 9.69) | -1.28 (-1.69, -0.86) | <0.001 |
| Eastern Europe | 63.17 (51.73, 76.10) | 68.27 (56.04, 82.13) | 0.28 (0.22, 0.34) | <0.001 | 544.76 (447.38, 662.42) | 617.69 (511.31, 746.42) | 0.44 (0.26, 0.62) | <0.001 | 380.77 (349.08, 414.21) | 406.23 (345.56, 476.89) | 0.10 (-0.79, 0.99) | 0.835 | 4.52 (4.24, 4.75) | 4.73 (3.93, 5.71) | -0.02 (-1.10, 1.07) | 0.965 |
| Southern Sub-Saharan Africa | 164.78 (105.29, 235.71) | 161.10 (101.64, 234.38) | -0.08 (-0.09, -0.06) | <0.001 | 2699.79 (2067.37, 3420.01) | 2695.18 (2071.36, 3405.34) | 0.00 (-0.07, 0.07) | 0.926 | 791.22 (666.98, 915.28) | 465.68 (347.27, 601.55) | -1.73 (-2.70, -0.76) | 0.001 | 9.25 (7.64, 10.85) | 4.39 (2.94, 6.14) | -2.45 (-3.87, -1.01) | 0.001 |
| High-income Asia Pacific | 69.31 (59.45, 80.02) | 66.95 (57.59, 77.34) | -0.11 (-0.17, -0.06) | <0.001 | 615.97 (485.90, 792.13) | 621.78 (494.75, 787.73) | 0.06 (-0.14, 0.25) | 0.566 | 360.22 (319.30, 401.88) | 131.28 (109.83, 155.89) | -3.46 (-3.72, -3.19) | <0.001 | 4.47 (3.95, 5.00) | 1.03 (0.93, 1.14) | -5.06 (-5.50, -4.62) | <0.001 |
| Central Europe | 55.78 (46.09, 67.28) | 56.73 (46.73, 68.30) | 0.07 (0.00, 0.14) | 0.041 | 938.38 (721.90, 1202.72) | 968.41 (745.75, 1254.76) | 0.13 (-0.04, 0.30) | 0.136 | 417.97 (382.05, 456.76) | 248.95 (205.41, 294.88) | -1.83 (-2.00, -1.66) | <0.001 | 4.76 (4.53, 4.99) | 2.19 (1.80, 2.65) | -2.81 (-3.27, -2.34) | <0.001 |
| Andean Latin America | 130.34 (89.84, 176.68) | 123.59 (83.39, 171.16) | -0.18 (-0.19, -0.17) | <0.001 | 1929.08 (1486.90, 2423.41) | 1982.99 (1525.83, 2516.37) | 0.09 (0.04, 0.14) | <0.001 | 900.30 (752.24, 1069.05) | 459.52 (356.27, 572.54) | -2.27 (-2.98, -1.56) | <0.001 | 11.80 (9.76, 14.35) | 5.15 (3.81, 6.72) | -2.80 (-3.69, -1.90) | <0.001 |
| Caribbean | 154.22 (113.22, 200.42) | 164.44 (117.03, 218.85) | 0.23 (0.21, 0.25) | <0.001 | 1824.93 (1436.53, 2281.85) | 1918.51 (1508.88, 2406.91) | 0.19 (0.14, 0.23) | <0.001 | 896.01 (708.65, 1088.83) | 824.03 (573.98, 1136.10) | -0.26 (-0.52, -0.00) | 0.048 | 11.87 (9.25, 14.72) | 10.72 (7.14, 15.33) | -0.32 (-0.62, -0.03) | 0.032 |
| Southern Latin America | 76.26 (56.39, 99.79) | 78.55 (57.75, 102.85) | 0.10 (0.09, 0.11) | <0.001 | 1234.20 (945.98, 1594.74) | 1332.74 (1027.82, 1694.51) | 0.25 (0.19, 0.32) | <0.001 | 565.91 (507.39, 630.77) | 314.46 (268.26, 365.65) | -1.97 (-2.42, -1.52) | <0.001 | 7.09 (6.32, 7.86) | 3.27 (2.82, 3.80) | -2.59 (-3.14, -2.04) | <0.001 |
| Oceania | 112.38 (85.22, 146.70) | 117.70 (87.69, 156.45) | 0.16 (0.14, 0.18) | <0.001 | 1155.97 (895.99, 1466.04) | 1219.06 (933.38, 1559.55) | 0.20 (0.08, 0.33) | 0.002 | 1138.05 (787.87, 1597.05) | 1171.45 (804.73, 1644.35) | 0.06 (-0.12, 0.23) | 0.550 | 15.30 (10.06, 22.43) | 15.77 (10.35, 22.92) | 0.05 (-0.15, 0.25) | 0.601 |

| | | | | | | | | | | | | | | | | |
|---|---|---|---|---|---|---|---|---|---|---|---|---|---|---|---|---|
| Australasia | 44.15 (34.43, 55.06) | 44.71 (35.20, 55.29) | 0.04 (0.01, 0.06) | 0.003 | 453.21 (336.84, 588.42) | 447.85 (340.30, 585.32) | -0.06 (-0.20, 0.08) | 0.435 | 223.19 (193.17, 256.41) | 123.22 (98.66, 151.18) | -2.01 (-3.03, -0.98) | <0.001 | 2.54 (2.20, 2.92) | 1.05 (0.85, 1.26) | -3.04 (-4.28, -1.79) | <0.001 |
| **Men** | | | | | | | | | | | | | | | | |
| Global | 99.54 (76.46, 126.86) | 107.85 (80.55, 139.49) | 0.28 (0.21, 0.36) | <0.001 | 1002.68 (795.92, 1240.91) | 1197.80 (937.44, 1497.27) | 0.62 (0.58, 0.67) | <0.001 | 833.09 (761.19, 903.54) | 734.24 (654.48, 821.20) | -0.43 (-0.56, -0.30) | <0.001 | 11.36 (10.36, 12.27) | 9.75 (8.71, 11.04) | -0.50 (-0.63, -0.36) | <0.001 |
| **SDI category** | | | | | | | | | | | | | | | | |
| High | 69.38 (56.86, 84.53) | 71.82 (60.06, 85.59) | 0.11 (0.07, 0.15) | <0.001 | 478.95 (385.88, 600.28) | 507.56 (423.06, 611.71) | 0.30 (-0.08, 0.69) | 0.121 | 398.18 (371.82, 423.33) | 257.61 (235.63, 280.97) | -1.50 (-1.75, -1.25) | <0.001 | 5.26 (4.94, 5.53) | 3.11 (2.90, 3.37) | -1.75 (-1.88, -1.62) | <0.001 |
| High-middle | 76.49 (61.85, 94.21) | 74.50 (59.99, 91.82) | -0.09 (-0.13, -0.04) | <0.001 | 646.21 (521.39, 785.97) | 676.23 (545.36, 831.32) | 0.17 (0.12, 0.22) | <0.001 | 747.08 (662.83, 839.82) | 547.46 (489.10, 609.58) | -1.21 (-1.64, -0.78) | <0.001 | 10.29 (9.04, 11.60) | 7.32 (6.47, 8.21) | -1.35 (-1.84, -0.86) | <0.001 |
| Middle | 100.13 (75.01, 129.87) | 100.30 (75.14, 128.69) | 0.01 (-0.04, 0.06) | 0.593 | 1084.20 (844.84, 1355.33) | 1146.63 (899.96, 1441.23) | 0.20 (0.17, 0.24) | <0.001 | 875.04 (779.60, 968.92) | 800.59 (699.32, 904.80) | -0.28 (-0.45, -0.11) | 0.001 | 11.88 (10.52, 13.15) | 10.75 (9.30, 12.28) | -0.32 (-0.50, -0.14) | 0.001 |
| Low-middle | 121.97 (88.11, 162.03) | 126.40 (90.21, 168.79) | 0.15 (-0.03, 0.33) | 0.103 | 1217.81 (943.52, 1537.45) | 1354.88 (1032.77, 1717.05) | 0.39 (0.33, 0.46) | <0.001 | 1055.43 (930.18, 1184.89) | 868.78 (744.17, 1010.44) | -0.65 (-1.08, -0.21) | 0.004 | 14.58 (12.76, 16.45) | 11.69 (9.94, 13.82) | -0.74 (-1.23, -0.26) | 0.003 |
| Low | 143.45 (98.66, 197.24) | 144.93 (97.41, 200.12) | 0.05 (0.01, 0.10) | 0.026 | 1789.36 (1348.37, 2278.80) | 1946.77 (1458.59, 2502.73) | 0.30 (0.23, 0.37) | <0.001 | 992.67 (842.15, 1137.89) | 868.30 (730.05, 1021.03) | -0.46 (-0.66, -0.27) | <0.001 | 13.25 (11.11, 15.45) | 11.26 (9.40, 13.53) | -0.57 (-0.80, -0.35) | <0.001 |
| **21 GBD region** | | | | | | | | | | | | | | | | |
| South Asia | 124.41 (88.51, 165.39) | 125.20 (89.51, 166.43) | 0.04 (-0.02, 0.10) | 0.172 | 1083.75 (826.81, 1369.45) | 1121.55 (848.94, 1426.95) | 0.15 (-0.01, 0.31) | 0.068 | 1141.42 (990.05, 1295.06) | 916.60 (769.01, 1088.78) | -0.76 (-1.36, -0.14) | 0.015 | 16.00 (13.75, 18.25) | 12.60 (10.53, 15.20) | -0.83 (-1.49, -0.17) | 0.014 |
| East Asia | 89.77 (67.03, 116.83) | 80.08 (60.78, 103.57) | -0.39 (-0.45, -0.33) | <0.001 | 856.41 (657.83, 1086.12) | 731.81 (562.91, 917.36) | -0.53 (-0.63, -0.43) | <0.001 | 793.36 (630.88, 937.53) | 608.30 (493.49, 728.02) | -0.79 (-1.40, -0.18) | 0.011 | 10.76 (8.42, 12.86) | 8.12 (6.37, 9.88) | -0.85 (-1.50, -0.20) | 0.010 |
| North Africa and Middle East | 97.13 (76.05, 122.16) | 101.59 (78.66, 128.22) | 0.16 (0.10, 0.23) | <0.001 | 1190.72 (929.21, 1522.57) | 1271.18 (986.08, 1639.79) | 0.24 (0.18, 0.31) | <0.001 | 1131.21 (972.87, 1302.37) | 912.96 (726.44, 1137.71) | -0.70 (-0.91, -0.49) | <0.001 | 15.66 (13.29, 18.27) | 12.31 (9.45, 15.70) | -0.79 (-1.01, -0.56) | <0.001 |

| | | | | | | | | | | | | | | | | |
|---|---|---|---|---|---|---|---|---|---|---|---|---|---|---|---|---|
| Western Sub-Saharan Africa | 133.75 (91.08, 184.60) | 137.08 (93.20, 189.78) | 0.08 (0.08, 0.09) | <0.001 | 2256.01 (1597.33, 3064.96) | 2398.17 (1704.77, 3282.86) | 0.22 (0.19, 0.25) | <0.001 | 765.94 (606.55, 963.79) | 691.95 (538.69, 881.34) | -0.35 (-0.43, -0.27) | <0.001 | 9.46 (7.22, 12.39) | 8.22 (5.98, 10.92) | -0.49 (-0.59, -0.40) | <0.001 |
| Southeast Asia | 79.26 (64.23, 99.24) | 79.35 (64.15, 99.53) | 0.01 (-0.02, 0.03) | 0.537 | 802.95 (650.63, 988.58) | 842.43 (672.55, 1051.78) | 0.17 (0.13, 0.21) | <0.001 | 843.13 (724.34, 981.23) | 893.55 (730.19, 1031.37) | 0.26 (0.01, 0.51) | 0.045 | 11.43 (9.67, 13.42) | 12.16 (9.53, 14.30) | 0.28 (0.01, 0.55) | 0.045 |
| Eastern Sub-Saharan Africa | 165.48 (109.93, 233.56) | 162.87 (106.31, 232.76) | -0.05 (-0.06, -0.04) | <0.001 | 2336.75 (1737.25, 3002.47) | 2534.49 (1882.88, 3276.00) | 0.30 (0.24, 0.36) | <0.001 | 1049.30 (858.82, 1237.56) | 848.42 (694.75, 1022.05) | -0.72 (-0.88, -0.55) | <0.001 | 13.69 (10.95, 16.49) | 10.51 (8.37, 13.14) | -0.90 (-1.08, -0.72) | <0.001 |
| Central Latin America | 76.92 (59.30, 97.49) | 79.19 (60.43, 101.16) | 0.10 (0.07, 0.14) | <0.001 | 906.02 (737.13, 1105.49) | 968.00 (781.75, 1181.93) | 0.24 (0.19, 0.29) | <0.001 | 584.02 (544.19, 623.04) | 469.33 (406.17, 539.78) | -0.72 (-1.21, -0.23) | 0.004 | 7.94 (7.46, 8.45) | 6.17 (5.26, 7.23) | -0.70 (-1.04, -0.35) | <0.001 |
| Tropical Latin America | 133.57 (93.47, 180.40) | 127.76 (87.84, 175.56) | -0.15 (-0.18, -0.13) | <0.001 | 1710.20 (1311.98, 2161.69) | 1679.75 (1293.60, 2123.85) | -0.06 (-0.08, -0.03) | <0.001 | 776.78 (719.81, 838.78) | 552.63 (501.07, 611.02) | -1.19 (-1.50, -0.87) | <0.001 | 10.22 (9.46, 10.92) | 6.88 (6.35, 7.52) | -1.38 (-1.81, -0.95) | <0.001 |
| Central Sub-Saharan Africa | 159.81 (103.04, 231.35) | 158.49 (99.72, 232.82) | -0.03 (-0.05, -0.01) | 0.004 | 2474.49 (1817.37, 3229.86) | 2500.16 (1834.44, 3279.96) | 0.04 (0.01, 0.07) | 0.009 | 862.87 (614.94, 1181.38) | 715.57 (520.14, 973.95) | -0.71 (-0.84, -0.58) | <0.001 | 10.86 (7.31, 15.84) | 8.63 (5.81, 12.32) | -0.87 (-1.02, -0.72) | <0.001 |
| Western Europe | 58.70 (46.55, 72.83) | 56.01 (44.26, 70.10) | -0.17 (-0.19, -0.14) | <0.001 | 364.78 (276.89, 473.96) | 353.46 (268.65, 465.16) | -0.11 (-0.16, -0.06) | <0.001 | 413.36 (388.32, 438.34) | 176.48 (161.26, 193.85) | -3.00 (-3.27, -2.74) | <0.001 | 5.64 (5.35, 5.95) | 2.12 (1.98, 2.27) | -3.46 (-3.75, -3.18) | <0.001 |
| High-income North America | 78.43 (64.89, 94.60) | 85.37 (73.37, 99.00) | 0.30 (0.23, 0.36) | <0.001 | 674.47 (528.40, 875.80) | 676.79 (563.46, 815.35) | 0.08 (-0.23, 0.39) | 0.628 | 349.14 (325.11, 374.48) | 327.24 (297.82, 355.46) | -0.20 (-0.62, 0.22) | 0.352 | 4.33 (4.16, 4.53) | 3.96 (3.66, 4.23) | -0.30 (-0.87, 0.27) | 0.298 |
| Central Asia | 119.18 (90.74, 152.84) | 121.36 (90.16, 157.32) | 0.06 (0.05, 0.08) | <0.001 | 1092.79 (838.63, 1388.48) | 1169.73 (878.70, 1497.69) | 0.23 (0.20, 0.27) | <0.001 | 844.66 (787.45, 907.14) | 856.18 (749.42, 975.93) | -0.03 (-0.73, 0.67) | 0.923 | 11.50 (10.76, 12.38) | 11.64 (10.15, 13.33) | -0.05 (-0.81, 0.71) | 0.889 |
| Eastern Europe | 71.57 (58.30, 87.13) | 79.47 (66.18, 94.67) | 0.37 (0.35, 0.39) | <0.001 | 290.37 (236.59, 352.02) | 309.76 (255.26, 374.77) | 0.25 (0.04, 0.46) | 0.018 | 719.63 (687.21, 750.66) | 926.35 (799.16, 1059.70) | 1.02 (-0.01, 2.06) | 0.052 | 10.22 (9.77, 10.61) | 13.36 (11.54, 15.38) | 1.07 (-0.00, 2.16) | 0.050 |
| Southern Sub-Saharan Africa | 162.13 (110.52, 221.43) | 162.98 (109.05, 227.01) | 0.03 (-0.01, 0.07) | 0.162 | 2245.98 (1695.62, 2883.25) | 2299.10 (1738.14, 2967.52) | 0.09 (0.06, 0.11) | <0.001 | 596.52 (494.14, 724.36) | 622.75 (499.88, 775.91) | 0.12 (-0.50, 0.74) | 0.712 | 6.94 (5.57, 8.78) | 7.29 (5.67, 9.61) | 0.13 (-0.65, 0.91) | 0.748 |

| | | | | | | | | | | | | | | | | |
|---|---|---|---|---|---|---|---|---|---|---|---|---|---|---|---|---|
| High-income Asia Pacific | 79.32 (66.13, 95.60) | 80.73 (68.23, 96.92) | 0.05 (-0.01, 0.12) | 0.116 | 384.39 (307.00, 486.96) | 403.53 (323.68, 505.45) | 0.19 (0.04, 0.34) | 0.015 | 460.86 (376.61, 511.15) | 175.15 (158.73, 194.34) | -3.26 (-3.64, -2.88) | <0.001 | 6.33 (5.05, 7.02) | 2.03 (1.89, 2.22) | -3.77 (-4.30, -3.24) | <0.001 |
| Central Europe | 64.57 (52.47, 78.68) | 66.79 (54.91, 81.04) | 0.12 (0.05, 0.19) | 0.001 | 521.24 (387.51, 691.85) | 530.45 (392.98, 716.06) | 0.08 (-0.10, 0.26) | 0.382 | 599.34 (567.73, 634.43) | 337.67 (292.62, 386.81) | -2.06 (-2.45, -1.68) | <0.001 | 8.21 (7.80, 8.64) | 4.31 (3.68, 5.00) | -2.36 (-2.77, -1.94) | <0.001 |
| Andean Latin America | 123.78 (87.23, 167.82) | 118.90 (81.49, 161.59) | -0.15 (-0.18, -0.12) | <0.001 | 1671.73 (1289.01, 2138.44) | 1688.54 (1300.59, 2148.31) | 0.04 (-0.00, 0.08) | 0.082 | 1118.72 (902.26, 1358.59) | 736.73 (558.34, 942.36) | -1.26 (-1.72, -0.80) | <0.001 | 15.38 (12.30, 18.88) | 9.63 (7.06, 12.71) | -1.42 (-1.94, -0.89) | <0.001 |
| Caribbean | 137.08 (100.02, 181.18) | 146.14 (107.24, 194.91) | 0.22 (0.20, 0.24) | <0.001 | 1537.18 (1203.34, 1937.72) | 1605.53 (1258.79, 2017.04) | 0.15 (0.12, 0.18) | <0.001 | 733.28 (638.19, 847.59) | 619.71 (462.01, 836.26) | -0.47 (-0.73, -0.20) | 0.001 | 9.74 (8.40, 11.32) | 7.98 (5.66, 11.23) | -0.65 (-1.03, -0.27) | 0.001 |
| Southern Latin America | 82.84 (62.49, 106.77) | 87.33 (65.15, 113.94) | 0.17 (0.13, 0.21) | <0.001 | 919.09 (692.54, 1179.27) | 981.81 (737.56, 1268.94) | 0.22 (0.10, 0.34) | <0.001 | 711.76 (637.92, 791.79) | 398.45 (340.05, 462.35) | -2.02 (-2.52, -1.51) | <0.001 | 9.70 (8.63, 10.85) | 4.96 (4.20, 5.81) | -2.32 (-2.89, -1.75) | <0.001 |
| Oceania | 109.20 (84.64, 139.68) | 110.76 (85.66, 142.57) | 0.05 (0.04, 0.06) | <0.001 | 1121.09 (883.54, 1408.12) | 1162.70 (899.95, 1461.83) | 0.14 (0.06, 0.21) | <0.001 | 1753.25 (1204.88, 2526.70) | 1745.30 (1130.37, 2479.87) | -0.07 (-0.32, 0.17) | 0.553 | 24.84 (16.55, 36.61) | 24.72 (15.43, 35.56) | -0.08 (-0.34, 0.18) | 0.538 |
| Australasia | 74.64 (58.86, 92.48) | 77.81 (61.51, 94.35) | 0.14 (0.12, 0.16) | <0.001 | 283.54 (204.96, 371.73) | 299.41 (222.05, 394.41) | 0.17 (0.13, 0.22) | <0.001 | 333.25 (294.88, 375.20) | 158.15 (132.06, 187.08) | -2.47 (-2.98, -1.97) | <0.001 | 4.53 (3.97, 5.13) | 1.94 (1.58, 2.35) | -2.86 (-3.55, -2.17) | <0.001 |
| **25-29 years** | | | | | | | | | | | | | | | | |
| **Women** | | | | | | | | | | | | | | | | |
| Global | 115.66 (93.32, 140.36) | 117.96 (94.36, 144.29) | 0.08 (0.04, 0.13) | <0.001 | 1629.15 (1372.31, 1974.41) | 1803.01 (1495.35, 2201.49) | 0.37 (0.30, 0.44) | <0.001 | 949.35 (868.82, 1036.07) | 648.68 (573.42, 723.60) | -1.22 (-1.71, -0.73) | <0.001 | 13.24 (12.02, 14.50) | 8.26 (7.21, 9.29) | -1.54 (-2.10, -0.99) | <0.001 |
| **SDI category** | | | | | | | | | | | | | | | | |
| High | 104.76 (87.97, 123.14) | 109.24 (92.61, 127.10) | 0.14 (0.10, 0.18) | <0.001 | 1441.24 (1156.42, 1837.35) | 1417.69 (1183.25, 1729.59) | -0.04 (-0.10, 0.02) | 0.173 | 437.84 (398.07, 484.50) | 306.45 (268.82, 352.12) | -1.21 (-1.50, -0.91) | <0.001 | 5.32 (5.11, 5.55) | 3.18 (2.95, 3.47) | -1.76 (-2.07, -1.46) | <0.001 |
| High-middle | 91.33 (75.44, 109.94) | 84.28 (68.60, 102.46) | -0.27 (-0.30, -0.24) | <0.001 | 1169.83 (1009.42, 1380.42) | 1204.03 (1027.98, 1426.25) | 0.12 (0.06, 0.18) | <0.001 | 682.49 (612.85, 760.03) | 412.92 (364.27, 465.52) | -1.65 (-2.44, -0.85) | <0.001 | 9.17 (8.18, 10.39) | 4.81 (4.24, 5.39) | -2.15 (-3.00, -1.29) | <0.001 |

| | | | | | | | | | | | | | | | | |
|---|---|---|---|---|---|---|---|---|---|---|---|---|---|---|---|---|
| Middle | 108.72 (85.79, 134.19) | 104.93 (82.59, 130.42) | -0.11 (-0.16, -0.06) | <0.001 | 1577.23 (1315.81, 1891.93) | 1644.40 (1365.52, 1989.97) | 0.16 (0.07, 0.26) | 0.001 | 1050.27 (944.85, 1162.13) | 623.54 (554.71, 698.67) | -1.62 (-2.44, -0.80) | <0.001 | 14.77 (13.27, 16.53) | 7.86 (6.84, 8.86) | -1.99 (-2.87, -1.09) | <0.001 |
| Low-middle | 146.08 (112.58, 184.13) | 142.96 (108.89, 179.68) | -0.05 (-0.19, 0.09) | 0.503 | 2004.08 (1618.26, 2443.41) | 2120.97 (1696.65, 2584.60) | 0.21 (0.15, 0.27) | <0.001 | 1343.66 (1170.07, 1506.67) | 891.41 (755.21, 1034.03) | -1.39 (-1.80, -0.97) | <0.001 | 19.46 (16.68, 22.03) | 12.02 (9.96, 14.17) | -1.63 (-2.11, -1.15) | <0.001 |
| Low | 154.20 (117.44, 195.83) | 151.67 (113.66, 194.65) | -0.06 (-0.07, -0.03) | <0.001 | 2471.44 (1955.65, 3111.07) | 2629.15 (2058.67, 3346.74) | 0.23 (0.15, 0.32) | <0.001 | 1233.68 (1058.64, 1425.19) | 832.72 (714.90, 973.29) | -1.34 (-1.49, -1.19) | <0.001 | 17.36 (14.54, 20.30) | 10.70 (8.87, 12.72) | -1.65 (-1.82, -1.48) | <0.001 |
| **21 GBD region** | | | | | | | | | | | | | | | | |
| South Asia | 153.95 (117.90, 193.92) | 147.55 (112.57, 186.37) | -0.15 (-0.21, -0.09) | <0.001 | 1967.34 (1564.37, 2410.01) | 1968.01 (1562.64, 2407.89) | 0.01 (-0.05, 0.07) | 0.750 | 1384.96 (1198.52, 1581.56) | 939.04 (779.42, 1125.01) | -1.34 (-2.13, -0.54) | 0.001 | 20.24 (17.13, 23.35) | 12.97 (10.35, 15.83) | -1.55 (-2.42, -0.66) | 0.001 |
| East Asia | 91.13 (71.98, 113.32) | 79.81 (62.76, 98.77) | -0.47 (-0.58, -0.37) | <0.001 | 1215.15 (1002.65, 1445.33) | 1156.54 (978.65, 1381.45) | -0.17 (-0.23, -0.12) | <0.001 | 839.98 (672.10, 1026.95) | 378.85 (307.69, 453.01) | -2.38 (-3.37, -1.38) | <0.001 | 11.50 (8.84, 14.56) | 4.12 (3.14, 5.21) | -3.38 (-4.60, -2.15) | <0.001 |
| North Africa and Middle East | 115.96 (94.40, 140.23) | 114.75 (93.02, 139.12) | -0.03 (-0.05, -0.01) | 0.001 | 1705.15 (1405.08, 2101.41) | 1757.93 (1450.79, 2169.56) | 0.12 (0.06, 0.19) | <0.001 | 1450.41 (1273.49, 1642.31) | 875.04 (706.33, 1088.62) | -1.69 (-2.01, -1.36) | <0.001 | 21.27 (18.61, 24.58) | 11.88 (9.31, 15.07) | -1.93 (-2.26, -1.61) | <0.001 |
| Western Sub-Saharan Africa | 130.95 (99.32, 167.70) | 131.81 (98.59, 170.58) | 0.03 (0.02, 0.04) | <0.001 | 2527.41 (1954.53, 3364.03) | 2753.75 (2099.88, 3723.75) | 0.30 (0.28, 0.33) | <0.001 | 865.68 (699.93, 1080.63) | 633.35 (500.85, 797.01) | -1.07 (-1.13, -1.02) | <0.001 | 11.33 (8.89, 14.69) | 7.36 (5.50, 9.87) | -1.47 (-1.57, -1.36) | <0.001 |
| Southeast Asia | 90.25 (72.89, 112.53) | 89.28 (71.53, 110.83) | -0.03 (-0.07, 0.00) | 0.080 | 1183.99 (1006.15, 1404.49) | 1204.38 (1023.27, 1418.05) | 0.08 (0.01, 0.14) | 0.016 | 1131.79 (947.71, 1320.41) | 750.63 (633.32, 877.51) | -1.37 (-1.51, -1.23) | <0.001 | 16.09 (13.22, 19.01) | 9.91 (8.13, 11.90) | -1.63 (-1.78, -1.48) | <0.001 |
| Eastern Sub-Saharan Africa | 163.06 (119.77, 214.99) | 156.10 (110.96, 208.21) | -0.16 (-0.18, -0.13) | <0.001 | 3135.26 (2447.77, 3993.87) | 3368.93 (2620.95, 4301.92) | 0.26 (0.19, 0.34) | <0.001 | 1088.08 (885.04, 1319.81) | 647.61 (532.72, 789.69) | -1.76 (-1.90, -1.63) | <0.001 | 14.38 (11.14, 17.79) | 7.04 (5.52, 8.91) | -2.42 (-2.58, -2.26) | <0.001 |
| Central Latin America | 101.91 (82.77, 125.96) | 93.04 (73.88, 114.77) | -0.31 (-0.36, -0.25) | <0.001 | 1678.02 (1409.95, 2016.26) | 1676.66 (1392.09, 2049.84) | 0.02 (-0.13, 0.18) | 0.771 | 732.52 (685.53, 783.65) | 446.19 (367.53, 535.97) | -1.62 (-2.14, -1.10) | <0.001 | 10.08 (9.61, 10.59) | 5.46 (4.33, 6.75) | -1.71 (-2.12, -1.29) | <0.001 |
| Tropical Latin America | 146.90 (112.70, | 130.82 (97.97, | -0.40 (-0.45, -0.35) | <0.001 | 2675.05 (2179.00, | 2631.91 (2113.67, | -0.04 (-0.14, | 0.468 | 1072.48 (1003.78, | 527.53 (468.80, | -2.45 (-2.63, | <0.001 | 14.82 (14.05, | 6.04 (5.54, | -3.11 (-3.52, | <0.001 |

| | | | | | | | | | | | | | | | | |
|---|---|---|---|---|---|---|---|---|---|---|---|---|---|---|---|---|
| | 187.68) | 170.08) | | | 3245.94) | 3215.63) | 0.06) | | 1145.84) | 599.74) | -2.26) | | 15.66) | 6.49) | -2.69) | |
| Central Sub-Saharan Africa | 159.97 (117.07, 211.50) | 153.29 (108.95, 203.72) | -0.14 (-0.16, -0.13) | <0.001 | 2979.15 (2288.42, 3824.21) | 3047.60 (2317.45, 3931.34) | 0.09 (0.01, 0.17) | 0.020 | 901.56 (638.68, 1222.66) | 612.20 (466.63, 796.97) | -1.32 (-1.44, -1.21) | <0.001 | 11.65 (7.38, 16.82) | 6.91 (4.80, 9.70) | -1.78 (-1.93, -1.64) | <0.001 |
| Western Europe | 82.98 (65.87, 100.98) | 79.94 (63.70, 97.64) | -0.12 (-0.15, -0.09) | <0.001 | 1218.59 (916.68, 1623.77) | 1179.71 (899.36, 1534.22) | -0.12 (-0.17, -0.06) | <0.001 | 367.93 (335.56, 408.45) | 196.95 (166.95, 233.73) | -2.17 (-2.56, -1.77) | <0.001 | 4.49 (4.31, 4.67) | 1.79 (1.68, 1.93) | -3.21 (-3.50, -2.93) | <0.001 |
| High-income North America | 141.14 (122.70, 161.87) | 153.30 (133.58, 174.01) | 0.29 (0.21, 0.38) | <0.001 | 1948.37 (1563.20, 2536.70) | 1851.82 (1568.04, 2228.85) | -0.15 (-0.22, -0.08) | <0.001 | 446.75 (400.24, 503.69) | 392.69 (346.15, 446.64) | -0.38 (-0.80, 0.04) | 0.076 | 5.08 (4.91, 5.25) | 4.23 (3.95, 4.61) | -0.56 (-1.24, 0.12) | 0.106 |
| Central Asia | 135.05 (110.09, 164.89) | 130.55 (105.03, 161.36) | -0.11 (-0.16, -0.06) | <0.001 | 1644.12 (1352.48, 1954.64) | 1684.81 (1389.15, 2015.04) | 0.09 (0.03, 0.15) | 0.002 | 1090.92 (1015.98, 1161.55) | 871.07 (746.92, 1023.41) | -0.80 (-1.21, -0.38) | <0.001 | 15.44 (14.39, 16.44) | 11.89 (9.98, 14.19) | -0.89 (-1.61, -0.17) | 0.016 |
| Eastern Europe | 94.87 (77.24, 115.65) | 102.34 (83.71, 123.82) | 0.27 (0.25, 0.29) | <0.001 | 817.38 (706.33, 939.38) | 905.71 (789.36, 1028.43) | 0.39 (0.22, 0.56) | <0.001 | 510.96 (462.74, 552.00) | 665.31 (543.20, 815.70) | 1.02 (0.10, 1.95) | 0.030 | 6.63 (6.05, 6.98) | 8.95 (7.09, 11.28) | 1.22 (0.01, 2.43) | 0.048 |
| Southern Sub-Saharan Africa | 167.40 (125.03, 218.53) | 156.51 (115.23, 206.61) | -0.23 (-0.27, -0.20) | <0.001 | 2869.13 (2275.72, 3523.94) | 2849.51 (2236.61, 3520.74) | -0.02 (-0.08, 0.05) | 0.607 | 1369.54 (1124.48, 1602.27) | 676.76 (451.54, 939.86) | -2.37 (-3.60, -1.12) | <0.001 | 19.14 (15.23, 22.65) | 7.99 (4.53, 12.26) | -2.80 (-4.57, -0.99) | 0.003 |
| High-income Asia Pacific | 102.53 (88.66, 117.86) | 99.98 (86.54, 114.86) | -0.08 (-0.12, -0.04) | <0.001 | 1056.64 (877.31, 1307.69) | 1061.54 (891.16, 1310.88) | 0.04 (-0.10, 0.18) | 0.607 | 483.69 (424.38, 533.96) | 175.92 (148.95, 207.79) | -3.48 (-3.75, -3.20) | <0.001 | 6.47 (5.83, 7.10) | 1.50 (1.35, 1.68) | -5.00 (-5.38, -4.61) | <0.001 |
| Central Europe | 81.40 (66.47, 98.77) | 81.50 (66.96, 98.48) | 0.01 (-0.04, 0.07) | 0.659 | 1309.10 (1058.63, 1649.90) | 1344.41 (1094.43, 1672.56) | 0.11 (-0.09, 0.32) | 0.270 | 589.85 (543.29, 641.58) | 333.80 (277.51, 391.39) | -1.92 (-2.08, -1.76) | <0.001 | 7.44 (7.13, 7.80) | 3.30 (2.67, 4.00) | -2.66 (-2.95, -2.36) | <0.001 |
| Andean Latin America | 130.95 (100.91, 166.02) | 120.09 (90.38, 154.49) | -0.30 (-0.32, -0.28) | <0.001 | 2297.33 (1857.37, 2846.68) | 2352.90 (1878.39, 2867.78) | 0.08 (0.05, 0.11) | <0.001 | 1107.50 (926.52, 1309.11) | 535.65 (415.62, 685.98) | -2.58 (-3.08, -2.08) | <0.001 | 15.68 (13.06, 18.99) | 6.40 (4.59, 8.73) | -3.15 (-3.75, -2.55) | <0.001 |
| Caribbean | 180.26 (146.01, 219.82) | 192.35 (153.54, 235.43) | 0.24 (0.21, 0.28) | <0.001 | 2195.67 (1802.05, 2650.62) | 2296.53 (1871.40, 2781.79) | 0.17 (0.07, 0.27) | 0.001 | 1194.82 (924.54, 1464.13) | 1075.69 (733.12, 1470.44) | -0.34 (-0.57, -0.12) | 0.003 | 17.34 (13.11, 21.73) | 15.33 (9.87, 21.86) | -0.41 (-0.66, -0.15) | 0.002 |
| Southern Latin America | 88.10 (70.04, | 86.76 (68.46, | -0.05 (-0.08, -0.02) | 0.002 | 1580.59 (1249.76, | 1675.63 (1317.80, | 0.20 (0.15, 0.26) | <0.001 | 736.11 (675.07, | 370.17 (316.28, | -2.37 (-2.99, | <0.001 | 10.01 (9.09, | 4.05 (3.40, | -3.08 (-3.77, | <0.001 |

| | | | | | | | | | | | | | | | | |
|---|---|---|---|---|---|---|---|---|---|---|---|---|---|---|---|---|
| | 108.34) | 107.39) | | | 1990.51) | 2102.18) | | | 808.23) | 429.85) | -1.73) | | 10.96) | 4.67) | -2.39) | |
| Oceania | 156.37 (131.38, 184.86) | 164.69 (136.44, 194.75) | 0.18 (0.13, 0.23) | <0.001 | 1627.81 (1346.99, 1970.35) | 1673.35 (1367.94, 2048.78) | 0.10 (0.04, 0.17) | 0.003 | 1720.28 (1223.51, 2276.63) | 1734.11 (1198.32, 2429.22) | 0.01 (-0.25, 0.28) | 0.910 | 25.10 (17.25, 33.85) | 25.31 (16.50, 36.60) | 0.01 (-0.28, 0.31) | 0.927 |
| Australasia | 66.66 (51.33, 83.37) | 66.06 (52.62, 82.06) | -0.04 (-0.09, 0.01) | 0.123 | 783.40 (583.37, 1008.45) | 799.30 (614.60, 1015.59) | 0.07 (0.01, 0.14) | 0.021 | 329.08 (290.00, 369.82) | 184.80 (152.93, 221.72) | -1.98 (-2.88, -1.07) | <0.001 | 4.09 (3.67, 4.57) | 1.80 (1.49, 2.15) | -2.77 (-3.95, -1.56) | <0.001 |
| **Men** | | | | | | | | | | | | | | | | |
| Global | 116.22 (95.62, 139.94) | 119.73 (97.40, 144.94) | 0.11 (0.04, 0.19) | 0.004 | 1234.26 (1033.85, 1482.32) | 1394.76 (1147.88, 1701.03) | 0.44 (0.36, 0.53) | <0.001 | 1133.13 (1056.61, 1211.44) | 995.08 (907.79, 1095.03) | -0.41 (-0.67, -0.16) | 0.002 | 16.86 (15.72, 18.01) | 14.51 (13.18, 16.11) | -0.48 (-0.76, -0.20) | 0.001 |
| **SDI category** | | | | | | | | | | | | | | | | |
| High | 102.26 (82.99, 122.66) | 103.83 (85.85, 122.84) | 0.05 (0.01, 0.09) | 0.011 | 815.55 (685.06, 986.35) | 864.20 (742.77, 1003.37) | 0.22 (0.12, 0.32) | <0.001 | 606.42 (573.52, 638.48) | 413.91 (379.16, 452.17) | -1.32 (-1.57, -1.07) | <0.001 | 8.76 (8.39, 9.11) | 5.58 (5.16, 6.09) | -1.54 (-1.79, -1.28) | <0.001 |
| High-middle | 99.94 (81.61, 120.67) | 93.65 (76.84, 113.01) | -0.22 (-0.24, -0.20) | <0.001 | 890.59 (759.72, 1053.83) | 905.21 (764.04, 1075.14) | 0.06 (0.03, 0.10) | <0.001 | 1086.81 (998.47, 1171.71) | 875.42 (795.72, 949.66) | -0.98 (-1.29, -0.66) | <0.001 | 16.33 (14.97, 17.72) | 12.94 (11.78, 14.06) | -1.05 (-1.38, -0.71) | <0.001 |
| Middle | 115.91 (93.32, 141.96) | 114.88 (92.20, 141.70) | -0.03 (-0.06, 0.01) | 0.137 | 1313.86 (1087.58, 1579.34) | 1370.02 (1127.93, 1663.75) | 0.16 (0.10, 0.21) | <0.001 | 1202.09 (1099.31, 1306.46) | 1046.49 (933.66, 1155.79) | -0.47 (-0.83, -0.11) | 0.011 | 17.83 (16.28, 19.48) | 15.29 (13.60, 17.07) | -0.52 (-0.93, -0.11) | 0.012 |
| Low-middle | 131.12 (104.41, 161.99) | 134.61 (106.55, 167.19) | 0.10 (0.07, 0.14) | <0.001 | 1486.02 (1191.82, 1820.39) | 1609.25 (1281.18, 1982.02) | 0.30 (0.19, 0.41) | <0.001 | 1445.21 (1289.50, 1604.07) | 1216.77 (1056.46, 1402.94) | -0.56 (-1.06, -0.05) | 0.032 | 21.78 (19.41, 24.36) | 17.99 (15.47, 20.93) | -0.63 (-1.16, -0.09) | 0.023 |
| Low | 152.82 (119.17, 192.83) | 153.37 (118.65, 194.42) | 0.02 (0.00, 0.03) | 0.010 | 2040.98 (1594.51, 2550.93) | 2180.42 (1688.89, 2759.37) | 0.24 (0.20, 0.28) | <0.001 | 1221.92 (1057.02, 1404.11) | 1132.80 (973.50, 1312.08) | -0.24 (-0.51, 0.03) | 0.078 | 17.77 (15.13, 20.66) | 16.20 (13.84, 19.07) | -0.30 (-0.60, 0.00) | 0.054 |
| **21 GBD region** | | | | | | | | | | | | | | | | |
| South Asia | 129.43 (101.92, 160.65) | 130.79 (103.38, 162.94) | 0.05 (-0.02, 0.11) | 0.149 | 1366.91 (1080.51, 1691.68) | 1388.60 (1096.46, 1720.30) | 0.09 (-0.02, 0.20) | 0.124 | 1496.02 (1319.63, 1677.92) | 1287.77 (1116.41, 1492.31) | -0.51 (-1.17, 0.16) | 0.137 | 22.75 (19.93, 25.63) | 19.36 (16.59, 22.66) | -0.55 (-1.25, 0.16) | 0.128 |

| | | | | | | | | | | | | | | | | |
|---|---|---|---|---|---|---|---|---|---|---|---|---|---|---|---|---|
| East Asia | 103.49 (83.17, 127.09) | 93.32 (75.19, 115.03) | -0.35 (-0.40, -0.30) | <0.001 | 1071.75 (888.83, 1286.45) | 974.43 (813.20, 1175.18) | -0.33 (-0.37, -0.29) | <0.001 | 982.61 (816.80, 1125.28) | 779.80 (649.51, 903.91) | -0.79 (-1.17, -0.40) | <0.001 | 14.37 (11.82, 16.73) | 11.20 (9.13, 13.29) | -0.84 (-1.28, -0.40) | <0.001 |
| North Africa and Middle East | 120.24 (97.93, 145.13) | 122.09 (98.73, 148.08) | 0.06 (0.03, 0.08) | <0.001 | 1553.45 (1249.29, 1957.33) | 1619.71 (1307.21, 2032.04) | 0.16 (0.05, 0.26) | 0.003 | 1628.81 (1416.02, 1921.40) | 1134.12 (920.86, 1399.97) | -1.19 (-1.49, -0.88) | <0.001 | 24.59 (21.19, 29.31) | 16.49 (13.08, 20.78) | -1.30 (-1.62, -0.99) | <0.001 |
| Western Sub-Saharan Africa | 145.86 (113.65, 183.55) | 148.57 (115.98, 187.91) | 0.06 (0.05, 0.07) | <0.001 | 2577.60 (1918.50, 3548.53) | 2778.56 (2051.04, 3823.01) | 0.27 (0.25, 0.30) | <0.001 | 948.92 (765.66, 1177.20) | 888.44 (704.95, 1093.20) | -0.23 (-0.32, -0.15) | <0.001 | 12.86 (10.06, 16.42) | 11.69 (9.08, 14.97) | -0.33 (-0.49, -0.17) | <0.001 |
| Southeast Asia | 102.09 (83.49, 123.74) | 101.42 (82.53, 123.69) | -0.02 (-0.03, -0.00) | 0.023 | 978.03 (833.73, 1148.15) | 1006.56 (848.68, 1192.61) | 0.12 (0.02, 0.21) | 0.017 | 1355.63 (1180.73, 1556.08) | 1292.17 (1023.04, 1493.57) | -0.15 (-0.38, 0.07) | 0.184 | 20.29 (17.38, 23.62) | 19.25 (15.08, 22.64) | -0.16 (-0.40, 0.07) | 0.170 |
| Eastern Sub-Saharan Africa | 172.91 (132.31, 221.49) | 168.18 (126.46, 218.32) | -0.09 (-0.12, -0.07) | <0.001 | 2530.93 (1975.44, 3167.49) | 2694.44 (2074.34, 3403.33) | 0.22 (0.18, 0.26) | <0.001 | 1175.82 (980.60, 1365.09) | 986.21 (813.99, 1179.38) | -0.63 (-0.81, -0.45) | <0.001 | 16.63 (13.62, 19.55) | 13.41 (10.80, 16.69) | -0.76 (-0.96, -0.57) | <0.001 |
| Central Latin America | 93.43 (74.38, 114.78) | 93.75 (74.62, 114.85) | 0.01 (-0.01, 0.04) | 0.222 | 1058.77 (884.67, 1266.33) | 1112.40 (913.47, 1350.07) | 0.18 (0.14, 0.23) | <0.001 | 843.81 (795.89, 892.58) | 672.55 (587.82, 766.48) | -0.76 (-1.29, -0.23) | 0.005 | 12.62 (11.98, 13.30) | 9.80 (8.49, 11.25) | -0.67 (-0.93, -0.42) | <0.001 |
| Tropical Latin America | 145.03 (113.06, 182.25) | 132.91 (102.03, 169.63) | -0.31 (-0.34, -0.28) | <0.001 | 1958.24 (1575.54, 2413.87) | 1892.64 (1519.94, 2326.21) | -0.11 (-0.14, -0.09) | <0.001 | 1278.57 (1198.17, 1365.66) | 757.42 (699.47, 823.29) | -1.79 (-2.00, -1.58) | <0.001 | 18.95 (17.80, 20.19) | 10.56 (9.84, 11.41) | -2.00 (-2.27, -1.73) | <0.001 |
| Central Sub-Saharan Africa | 169.77 (128.76, 221.53) | 164.25 (121.39, 219.52) | -0.11 (-0.13, -0.10) | <0.001 | 2578.58 (1977.56, 3343.99) | 2592.36 (1931.95, 3341.12) | 0.02 (-0.01, 0.05) | 0.250 | 991.34 (699.11, 1350.90) | 831.11 (602.80, 1118.66) | -0.64 (-0.74, -0.54) | <0.001 | 13.69 (9.17, 19.54) | 11.07 (7.41, 15.77) | -0.77 (-0.89, -0.66) | <0.001 |
| Western Europe | 85.79 (66.31, 106.14) | 79.67 (62.20, 98.65) | -0.25 (-0.29, -0.21) | <0.001 | 591.98 (463.20, 745.73) | 571.14 (452.75, 717.22) | -0.13 (-0.20, -0.06) | <0.001 | 591.73 (561.24, 623.52) | 280.87 (259.79, 305.08) | -2.60 (-2.97, -2.24) | <0.001 | 8.76 (8.34, 9.19) | 3.79 (3.56, 4.00) | -2.93 (-3.37, -2.48) | <0.001 |
| High-income North America | 119.08 (97.69, 142.53) | 127.14 (107.34, 147.55) | 0.22 (0.18, 0.26) | <0.001 | 1103.86 (926.79, 1336.40) | 1151.98 (996.96, 1334.16) | 0.22 (-0.03, 0.47) | 0.089 | 573.46 (541.06, 609.61) | 510.26 (454.01, 556.18) | -0.38 (-0.68, -0.09) | 0.011 | 7.95 (7.66, 8.25) | 6.82 (6.12, 7.31) | -0.50 (-0.95, -0.06) | 0.028 |
| Central Asia | 146.26 (120.48, 175.96) | 145.38 (117.90, 176.79) | -0.02 (-0.04, 0.00) | 0.051 | 1367.99 (1123.97, 1659.20) | 1442.60 (1155.30, 1767.86) | 0.18 (0.14, 0.22) | <0.001 | 1384.77 (1299.13, 1467.09) | 1373.42 (1218.52, 1540.16) | -0.04 (-0.75, 0.67) | 0.903 | 20.86 (19.61, 22.15) | 20.65 (18.29, 23.24) | 0.03 (-0.83, 0.89) | 0.954 |

| | | | | | | | | | | | | | | | | |
|---|---|---|---|---|---|---|---|---|---|---|---|---|---|---|---|---|
| Eastern Europe | 110.15 (89.23, 132.24) | 124.56 (104.04, 147.38) | 0.43 (0.41, 0.45) | <0.001 | 527.24 (460.51, 599.28) | 588.21 (515.20, 667.33) | 0.38 (0.34, 0.43) | <0.001 | 1369.93 (1319.97, 1416.53) | 2163.54 (1926.71, 2402.79) | 1.62 (0.37, 2.89) | 0.011 | 21.33 (20.49, 22.00) | 34.28 (30.49, 38.14) | 1.70 (0.43, 2.98) | 0.009 |
| Southern Sub-Saharan Africa | 177.70 (137.64, 223.91) | 174.57 (134.31, 221.69) | -0.06 (-0.08, -0.03) | <0.001 | 2429.56 (1895.17, 3029.36) | 2469.09 (1948.74, 3080.51) | 0.06 (0.04, 0.08) | <0.001 | 1023.61 (859.31, 1219.00) | 821.34 (675.90, 999.69) | -0.83 (-1.32, -0.34) | 0.001 | 14.23 (11.70, 17.33) | 10.94 (8.71, 13.80) | -0.93 (-1.50, -0.35) | 0.002 |
| High-income Asia Pacific | 113.27 (96.27, 131.12) | 114.13 (97.42, 131.30) | 0.04 (-0.02, 0.11) | 0.195 | 740.37 (619.48, 901.45) | 794.88 (676.19, 946.74) | 0.26 (0.19, 0.33) | <0.001 | 699.42 (615.43, 766.28) | 277.65 (251.93, 307.58) | -3.17 (-3.58, -2.75) | <0.001 | 10.44 (9.00, 11.50) | 3.61 (3.32, 4.01) | -3.51 (-3.85, -3.18) | <0.001 |
| Central Europe | 93.60 (76.00, 113.48) | 95.77 (78.48, 114.11) | 0.08 (0.06, 0.11) | <0.001 | 793.04 (642.04, 984.62) | 812.08 (657.92, 1003.33) | 0.09 (-0.01, 0.19) | 0.070 | 1072.47 (1026.22, 1122.72) | 539.78 (481.78, 604.21) | -2.37 (-2.73, -2.00) | <0.001 | 16.26 (15.65, 16.96) | 7.70 (6.83, 8.65) | -2.56 (-2.95, -2.18) | <0.001 |
| Andean Latin America | 128.25 (101.02, 161.17) | 123.02 (95.02, 155.57) | -0.15 (-0.17, -0.13) | <0.001 | 1910.34 (1498.17, 2382.31) | 1935.63 (1554.52, 2399.95) | 0.05 (0.03, 0.06) | <0.001 | 1223.86 (1003.83, 1453.36) | 845.19 (664.34, 1079.91) | -1.22 (-1.87, -0.56) | <0.001 | 18.05 (14.62, 21.79) | 11.91 (9.05, 15.60) | -1.33 (-1.92, -0.74) | <0.001 |
| Caribbean | 166.84 (131.58, 206.83) | 175.43 (138.95, 216.41) | 0.18 (0.17, 0.19) | <0.001 | 1807.83 (1462.53, 2197.91) | 1887.11 (1512.67, 2293.67) | 0.15 (0.12, 0.18) | <0.001 | 1096.48 (968.38, 1231.80) | 910.09 (700.90, 1202.84) | -0.63 (-0.84, -0.42) | <0.001 | 16.21 (14.13, 18.37) | 13.13 (9.76, 17.83) | -0.71 (-0.95, -0.47) | <0.001 |
| Southern Latin America | 94.24 (75.09, 114.25) | 96.89 (77.31, 119.72) | 0.09 (0.05, 0.13) | <0.001 | 1099.43 (874.58, 1383.34) | 1177.05 (915.13, 1471.33) | 0.23 (0.16, 0.30) | <0.001 | 927.73 (845.73, 1019.11) | 528.00 (453.17, 603.88) | -1.93 (-2.21, -1.64) | <0.001 | 13.73 (12.41, 15.12) | 7.24 (6.13, 8.27) | -2.18 (-2.50, -1.86) | <0.001 |
| Oceania | 141.35 (116.13, 170.59) | 145.83 (120.84, 174.29) | 0.11 (0.10, 0.12) | <0.001 | 1331.31 (1097.99, 1622.29) | 1369.11 (1100.22, 1673.64) | 0.10 (0.07, 0.14) | <0.001 | 2363.39 (1669.93, 3182.71) | 2439.18 (1650.15, 3486.78) | 0.04 (-0.07, 0.15) | 0.451 | 36.24 (25.02, 49.45) | 37.47 (24.66, 54.04) | 0.05 (-0.07, 0.16) | 0.429 |
| Australasia | 121.13 (88.48, 154.51) | 123.63 (93.86, 156.49) | 0.07 (0.04, 0.10) | <0.001 | 540.54 (423.09, 680.79) | 586.42 (458.43, 733.15) | 0.27 (0.20, 0.34) | <0.001 | 521.56 (471.14, 579.98) | 281.78 (240.21, 328.37) | -2.06 (-2.87, -1.24) | <0.001 | 7.73 (6.98, 8.62) | 3.91 (3.27, 4.63) | -2.29 (-3.18, -1.38) | <0.001 |
| **30-34 years** | | | | | | | | | | | | | | | | |
| **Women** | | | | | | | | | | | | | | | | |
| Global | 137.24 (119.17, 157.25) | 130.97 (112.03, 152.02) | -0.16 (-0.21, -0.10) | <0.001 | 1909.73 (1656.10, 2225.90) | 2012.52 (1715.63, 2367.07) | 0.18 (0.14, 0.23) | <0.001 | 1329.67 (1221.20, 1432.47) | 909.24 (811.73, 1007.44) | -1.30 (-1.67, -0.94) | <0.001 | 20.71 (18.92, 22.48) | 13.24 (11.77, 14.66) | -1.53 (-1.93, -1.13) | <0.001 |
| **SDI category** | | | | | | | | | | | | | | | | |

| | | | | | | | | | | | | | | | | |
|---|---|---|---|---|---|---|---|---|---|---|---|---|---|---|---|---|
| High | 143.32 (125.62, 161.97) | 141.56 (125.16, 159.53) | -0.04 (-0.11, 0.04) | 0.364 | 1862.91 (1566.28, 2280.57) | 1859.56 (1615.35, 2189.92) | 0.02 (-0.10, 0.14) | 0.710 | 618.12 (574.30, 669.85) | 454.25 (408.20, 507.45) | -1.04 (-1.29, -0.79) | <0.001 | 8.64 (8.35, 8.95) | 5.72 (5.29, 6.25) | -1.35 (-1.71, -0.98) | <0.001 |
| High-middle | 120.11 (104.30, 138.94) | 106.15 (90.70, 123.99) | -0.42 (-0.48, -0.37) | <0.001 | 1483.35 (1309.24, 1700.71) | 1490.48 (1310.63, 1712.97) | 0.02 (-0.01, 0.05) | 0.201 | 1002.59 (914.04, 1107.53) | 635.57 (566.73, 708.63) | -1.67 (-2.53, -0.80) | <0.001 | 15.15 (13.83, 16.91) | 8.73 (7.72, 9.76) | -2.02 (-3.15, -0.88) | 0.001 |
| Middle | 129.76 (110.93, 150.50) | 120.35 (101.85, 141.28) | -0.26 (-0.31, -0.20) | <0.001 | 1867.72 (1606.29, 2183.04) | 1894.77 (1613.60, 2240.10) | 0.05 (-0.04, 0.14) | 0.304 | 1507.30 (1363.29, 1654.08) | 854.27 (758.08, 948.82) | -1.85 (-2.58, -1.12) | <0.001 | 23.63 (21.26, 26.09) | 12.19 (10.67, 13.65) | -2.16 (-2.92, -1.38) | <0.001 |
| Low-middle | 153.28 (129.45, 179.22) | 147.91 (124.13, 174.79) | -0.12 (-0.15, -0.09) | <0.001 | 2256.48 (1873.83, 2683.35) | 2339.81 (1948.61, 2798.40) | 0.12 (0.06, 0.20) | <0.001 | 1900.55 (1647.22, 2140.00) | 1282.82 (1087.89, 1467.60) | -1.35 (-1.64, -1.05) | <0.001 | 30.63 (25.92, 34.78) | 19.66 (16.42, 22.77) | -1.53 (-1.86, -1.19) | <0.001 |
| Low | 165.74 (140.28, 194.35) | 158.82 (132.20, 187.84) | -0.15 (-0.16, -0.14) | <0.001 | 2604.43 (2122.15, 3197.44) | 2718.38 (2201.85, 3369.93) | 0.16 (0.09, 0.24) | <0.001 | 1771.13 (1515.23, 2034.26) | 1240.26 (1062.52, 1452.16) | -1.23 (-1.33, -1.14) | <0.001 | 28.09 (23.87, 32.51) | 18.63 (15.57, 21.95) | -1.44 (-1.54, -1.33) | <0.001 |
| **21 GBD region** | | | | | | | | | | | | | | | | |
| South Asia | 153.77 (128.75, 181.13) | 148.24 (124.13, 176.17) | -0.12 (-0.15, -0.10) | <0.001 | 2228.78 (1834.04, 2658.84) | 2214.53 (1817.74, 2659.62) | -0.00 (-0.09, 0.08) | 0.948 | 1920.00 (1654.70, 2181.49) | 1346.11 (1104.50, 1594.36) | -1.23 (-1.73, -0.72) | <0.001 | 31.10 (26.53, 35.90) | 20.96 (16.71, 25.22) | -1.38 (-1.94, -0.82) | <0.001 |
| East Asia | 111.90 (95.18, 130.81) | 97.04 (82.05, 114.59) | -0.49 (-0.52, -0.46) | <0.001 | 1503.34 (1309.86, 1753.37) | 1460.26 (1263.98, 1697.14) | -0.10 (-0.19, -0.00) | 0.044 | 1206.54 (972.57, 1472.22) | 538.95 (439.52, 648.26) | -2.86 (-4.34, -1.35) | <0.001 | 18.45 (14.64, 23.10) | 6.82 (5.26, 8.57) | -3.23 (-4.25, -2.20) | <0.001 |
| North Africa and Middle East | 152.98 (133.01, 175.24) | 147.18 (126.90, 169.64) | -0.14 (-0.15, -0.12) | <0.001 | 2146.64 (1810.89, 2572.58) | 2141.48 (1810.72, 2556.90) | 0.01 (-0.08, 0.10) | 0.820 | 2028.64 (1791.17, 2294.05) | 1231.32 (999.35, 1489.54) | -1.69 (-1.86, -1.53) | <0.001 | 32.72 (28.58, 37.38) | 18.68 (14.77, 22.97) | -1.88 (-2.03, -1.72) | <0.001 |
| Western Sub-Saharan Africa | 146.98 (123.30, 172.98) | 143.82 (119.64, 170.64) | -0.08 (-0.09, -0.07) | <0.001 | 2728.13 (2136.26, 3663.97) | 2946.43 (2276.99, 4089.83) | 0.27 (0.25, 0.30) | <0.001 | 1280.51 (1040.72, 1577.46) | 930.60 (746.15, 1157.99) | -1.09 (-1.20, -0.99) | <0.001 | 19.30 (15.09, 24.43) | 12.94 (9.77, 16.90) | -1.36 (-1.46, -1.25) | <0.001 |
| Southeast Asia | 116.66 (100.46, 136.51) | 113.52 (97.64, 133.09) | -0.09 (-0.10, -0.08) | <0.001 | 1488.41 (1298.41, 1712.42) | 1488.72 (1294.51, 1703.97) | 0.01 (-0.08, 0.10) | 0.818 | 1643.60 (1402.63, 1893.12) | 1085.64 (916.11, 1262.36) | -1.42 (-1.55, -1.28) | <0.001 | 25.83 (21.49, 30.17) | 16.05 (13.25, 19.21) | -1.63 (-1.77, -1.48) | <0.001 |
| Eastern Sub-Saharan Africa | 174.63 (143.92, | 155.83 (124.00, | -0.40 (-0.42, -0.38) | <0.001 | 3116.74 (2516.95, | 3257.93 (2556.45, | 0.17 (0.11, 0.22) | <0.001 | 1514.21 (1228.32, | 886.98 (723.06, | -1.82 (-1.93, | <0.001 | 23.01 (18.00, | 11.90 (9.40, | -2.25 (-2.37, | <0.001 |

| | | | | | | | | | | | | | | | | |
|---|---|---|---|---|---|---|---|---|---|---|---|---|---|---|---|---|
| | 207.65) | 189.43) | | | 3889.06) | 4076.18) | | | 1810.67) | 1064.85) | -1.71) | | 27.98) | 14.62) | -2.12) | |
| Central Latin America | 124.16 (105.91, 146.51) | 106.16 (89.32, 126.12) | -0.54 (-0.56, -0.52) | <0.001 | 1855.83 (1596.44, 2169.85) | 1831.76 (1562.98, 2153.78) | -0.04 (-0.12, 0.05) | 0.404 | 999.37 (941.34, 1056.16) | 568.72 (466.89, 685.54) | -1.75 (-2.13, -1.36) | <0.001 | 15.51 (14.78, 16.28) | 7.96 (6.31, 9.83) | -2.00 (-2.50, -1.50) | <0.001 |
| Tropical Latin America | 167.95 (140.93, 199.48) | 134.55 (110.63, 162.50) | -0.76 (-0.82, -0.71) | <0.001 | 2839.08 (2352.21, 3383.12) | 2740.07 (2264.28, 3301.59) | -0.12 (-0.16, -0.07) | <0.001 | 1716.12 (1624.00, 1810.03) | 773.18 (704.50, 849.21) | -2.67 (-2.82, -2.52) | <0.001 | 27.29 (26.09, 28.55) | 10.81 (10.11, 11.61) | -3.10 (-3.28, -2.92) | <0.001 |
| Central Sub-Saharan Africa | 171.04 (140.47, 204.06) | 157.27 (126.47, 190.27) | -0.29 (-0.30, -0.28) | <0.001 | 2996.05 (2353.76, 3767.56) | 3022.85 (2372.13, 3856.28) | 0.05 (-0.03, 0.12) | 0.209 | 1361.12 (949.87, 1820.47) | 947.79 (684.25, 1233.96) | -1.23 (-1.34, -1.11) | <0.001 | 20.61 (13.59, 28.93) | 13.36 (8.93, 18.43) | -1.47 (-1.61, -1.34) | <0.001 |
| Western Europe | 102.18 (85.30, 120.69) | 95.30 (78.85, 112.77) | -0.23 (-0.27, -0.20) | <0.001 | 1387.77 (1107.25, 1764.54) | 1354.80 (1090.21, 1704.90) | -0.06 (-0.13, 0.02) | 0.141 | 502.45 (465.76, 542.67) | 264.63 (233.40, 301.31) | -2.18 (-2.33, -2.02) | <0.001 | 7.13 (6.85, 7.39) | 3.04 (2.87, 3.24) | -2.87 (-3.07, -2.67) | <0.001 |
| High-income North America | 197.85 (177.73, 219.72) | 201.79 (182.10, 223.07) | 0.07 (0.02, 0.12) | 0.005 | 2615.54 (2192.69, 3255.39) | 2640.47 (2288.72, 3122.76) | 0.09 (-0.12, 0.30) | 0.412 | 657.83 (603.38, 721.95) | 609.45 (552.71, 674.29) | -0.16 (-0.59, 0.28) | 0.485 | 8.83 (8.56, 9.11) | 7.91 (7.48, 8.41) | -0.34 (-1.01, 0.34) | 0.332 |
| Central Asia | 159.93 (138.04, 185.34) | 149.89 (127.93, 175.63) | -0.22 (-0.24, -0.20) | <0.001 | 2005.39 (1734.53, 2302.75) | 2015.85 (1711.68, 2354.53) | 0.03 (-0.01, 0.06) | 0.150 | 1564.45 (1479.60, 1649.86) | 1313.39 (1155.19, 1517.60) | -0.61 (-1.01, -0.20) | 0.003 | 24.52 (23.22, 25.85) | 20.23 (17.53, 23.69) | -0.68 (-1.14, -0.21) | 0.005 |
| Eastern Europe | 136.41 (117.02, 158.63) | 142.25 (122.15, 164.98) | 0.14 (0.13, 0.16) | <0.001 | 1209.47 (1079.34, 1347.44) | 1304.58 (1165.22, 1456.92) | 0.29 (0.15, 0.43) | <0.001 | 816.62 (748.23, 871.88) | 1158.93 (950.17, 1404.87) | 1.16 (0.23, 2.10) | 0.014 | 12.09 (11.16, 12.64) | 17.92 (14.43, 22.30) | 1.47 (0.59, 2.36) | 0.001 |
| Southern Sub-Saharan Africa | 178.37 (147.27, 212.98) | 159.03 (129.81, 191.80) | -0.40 (-0.45, -0.35) | <0.001 | 2937.41 (2413.98, 3582.37) | 2857.39 (2321.77, 3493.36) | -0.09 (-0.12, -0.05) | <0.001 | 1943.12 (1575.44, 2242.53) | 877.89 (606.92, 1214.22) | -2.55 (-4.27, -0.81) | 0.004 | 30.69 (23.88, 35.86) | 12.15 (7.26, 18.02) | -2.98 (-5.05, -0.88) | 0.006 |
| High-income Asia Pacific | 139.13 (124.96, 155.67) | 137.05 (122.35, 153.17) | -0.05 (-0.13, 0.04) | 0.268 | 1456.59 (1251.48, 1717.88) | 1502.23 (1307.43, 1764.74) | 0.12 (0.00, 0.23) | 0.043 | 633.17 (576.51, 687.73) | 241.38 (209.18, 280.64) | -3.25 (-3.44, -3.06) | <0.001 | 9.37 (8.51, 10.18) | 2.51 (2.31, 2.76) | -4.44 (-4.73, -4.14) | <0.001 |
| Central Europe | 113.13 (97.03, 131.47) | 107.75 (92.34, 125.53) | -0.16 (-0.20, -0.12) | <0.001 | 1574.03 (1318.26, 1922.39) | 1604.24 (1342.41, 1952.94) | 0.07 (-0.10, 0.24) | 0.426 | 951.89 (896.84, 1014.30) | 462.28 (391.23, 538.99) | -2.41 (-2.59, -2.23) | <0.001 | 14.01 (13.53, 14.58) | 5.45 (4.57, 6.51) | -3.23 (-3.48, -2.97) | <0.001 |
| Andean Latin America | 135.80 (112.72, | 119.22 (97.66, | -0.45 (-0.46, -0.44) | <0.001 | 2416.86 (1995.15, | 2432.13 (1971.32, | 0.02 (-0.04, 0.08) | 0.492 | 1335.55 (1110.17, | 657.70 (509.77, | -2.39 (-3.41, | <0.001 | 20.96 (17.15, | 9.02 (6.51, | -2.88 (-4.37, | <0.001 |

| | | | | | | | | | | | | | | | | |
|---|---|---|---|---|---|---|---|---|---|---|---|---|---|---|---|---|
| | 162.10) | 144.22) | | | 2898.44) | 2928.28) | | | 1580.07) | 836.53) | -1.37) | | 25.15) | 12.01) | -1.37) | |
| Caribbean | 209.33 (178.28, 242.37) | 220.34 (187.08, 256.59) | 0.19 (0.15, 0.22) | <0.001 | 2605.64 (2219.22, 3024.36) | 2683.30 (2250.85, 3144.23) | 0.11 (0.02, 0.19) | 0.013 | 1794.01 (1449.29, 2154.19) | 1510.34 (1080.50, 2069.69) | -0.59 (-0.90, -0.27) | <0.001 | 29.10 (22.82, 35.08) | 24.02 (16.36, 33.96) | -0.66 (-1.02, -0.29) | <0.001 |
| Southern Latin America | 105.32 (90.01, 123.19) | 97.53 (82.12, 115.80) | -0.26 (-0.29, -0.23) | <0.001 | 1640.58 (1282.25, 2052.03) | 1849.45 (1508.61, 2269.84) | 0.43 (0.38, 0.48) | <0.001 | 1007.27 (924.05, 1093.20) | 498.58 (433.33, 564.29) | -2.30 (-2.72, -1.88) | <0.001 | 15.57 (14.28, 16.85) | 6.45 (5.64, 7.29) | -2.89 (-3.42, -2.35) | <0.001 |
| Oceania | 202.06 (178.88, 226.20) | 212.62 (188.84, 239.13) | 0.18 (0.16, 0.19) | <0.001 | 1962.93 (1706.86, 2290.15) | 2024.24 (1748.93, 2379.37) | 0.11 (0.07, 0.15) | <0.001 | 2735.21 (1978.62, 3628.51) | 2743.80 (1933.45, 3699.94) | -0.04 (-0.18, 0.10) | 0.574 | 44.21 (30.94, 59.90) | 44.28 (30.10, 61.42) | -0.05 (-0.20, 0.10) | 0.527 |
| Australasia | 89.43 (73.08, 106.82) | 87.74 (72.81, 103.97) | -0.07 (-0.10, -0.05) | <0.001 | 987.46 (790.02, 1237.10) | 1000.16 (793.64, 1233.55) | 0.05 (-0.05, 0.16) | 0.306 | 457.92 (409.95, 506.46) | 271.66 (231.89, 317.60) | -1.78 (-2.26, -1.30) | <0.001 | 6.44 (5.85, 7.05) | 3.24 (2.74, 3.81) | -2.32 (-2.91, -1.73) | <0.001 |
| **Men** | | | | | | | | | | | | | | | | |
| Global | 151.84 (131.60, 174.73) | 148.73 (128.19, 171.07) | -0.07 (-0.10, -0.04) | <0.001 | 1471.73 (1284.00, 1687.15) | 1583.69 (1370.77, 1828.81) | 0.27 (0.20, 0.35) | <0.001 | 1851.30 (1743.61, 1961.35) | 1634.11 (1504.97, 1780.73) | -0.42 (-0.56, -0.28) | <0.001 | 30.72 (28.96, 32.56) | 26.82 (24.64, 29.30) | -0.46 (-0.61, -0.31) | <0.001 |
| **SDI category** | | | | | | | | | | | | | | | | |
| High | 158.15 (136.10, 182.26) | 150.75 (131.86, 171.40) | -0.17 (-0.20, -0.15) | <0.001 | 1203.24 (1074.40, 1347.46) | 1273.74 (1152.15, 1402.62) | 0.23 (-0.03, 0.48) | 0.087 | 1055.93 (1014.64, 1098.99) | 729.68 (666.81, 805.93) | -1.29 (-1.61, -0.97) | <0.001 | 17.16 (16.62, 17.79) | 11.31 (10.29, 12.53) | -1.45 (-1.92, -0.97) | <0.001 |
| High-middle | 143.72 (124.93, 165.24) | 131.16 (113.63, 151.95) | -0.32 (-0.34, -0.30) | <0.001 | 1157.89 (1025.38, 1307.42) | 1160.55 (1030.14, 1310.90) | 0.01 (-0.02, 0.04) | 0.403 | 1960.69 (1836.07, 2092.67) | 1602.81 (1468.76, 1739.62) | -0.72 (-1.33, -0.12) | 0.020 | 32.84 (30.71, 35.01) | 26.58 (24.23, 28.91) | -0.76 (-1.39, -0.14) | 0.017 |
| Middle | 147.88 (126.84, 171.51) | 145.18 (124.37, 168.44) | -0.07 (-0.09, -0.04) | <0.001 | 1553.41 (1340.86, 1807.52) | 1587.87 (1371.72, 1860.07) | 0.09 (0.05, 0.14) | <0.001 | 1921.45 (1779.73, 2078.70) | 1656.30 (1497.21, 1824.96) | -0.54 (-0.77, -0.31) | <0.001 | 31.73 (29.36, 34.38) | 27.07 (24.35, 29.91) | -0.58 (-0.82, -0.34) | <0.001 |
| Low-middle | 154.05 (131.02, 177.38) | 156.88 (132.93, 181.47) | 0.05 (-0.03, 0.14) | 0.197 | 1701.17 (1431.28, 2018.27) | 1801.88 (1511.40, 2150.97) | 0.21 (0.13, 0.30) | <0.001 | 2300.78 (2082.02, 2527.07) | 2096.47 (1854.60, 2376.06) | -0.28 (-0.57, 0.01) | 0.060 | 38.53 (34.72, 42.46) | 34.87 (30.63, 39.78) | -0.30 (-0.60, -0.01) | 0.043 |
| Low | 173.74 (147.37, | 172.64 (144.59, | -0.02 (-0.04, 0.00) | 0.080 | 2149.79 (1781.54, | 2246.30 (1860.05, | 0.16 (0.11, 0.20) | <0.001 | 1895.61 (1662.77, | 1700.44 (1465.66, | -0.43 (-0.56, | <0.001 | 31.07 (27.01, | 27.55 (23.45, | -0.48 (-0.61, | <0.001 |

| | | | | | | | | | | | | | | | | |
|---|---|---|---|---|---|---|---|---|---|---|---|---|---|---|---|---|
| | 202.47) | 202.01) | | | 2587.25) | 2711.78) | | | 2151.97) | 1966.82) | -0.30) | | 35.60) | 32.05) | -0.34) | |
| **21 GBD region** | | | | | | | | | | | | | | | | |
| South Asia | 146.99 (123.71, 170.01) | 150.40 (126.09, 174.90) | 0.07 (-0.03, 0.17) | 0.158 | 1605.67 (1330.41, 1928.16) | 1645.81 (1364.05, 1970.56) | 0.11 (0.05, 0.17) | <0.001 | 2444.07 (2188.15, 2722.00) | 2274.34 (1970.43, 2612.20) | -0.26 (-0.64, 0.12) | 0.181 | 41.23 (36.69, 45.99) | 38.22 (32.94, 44.03) | -0.26 (-0.59, 0.07) | 0.117 |
| East Asia | 132.00 (112.68, 153.92) | 122.16 (104.55, 142.68) | -0.28 (-0.30, -0.25) | <0.001 | 1358.03 (1173.97, 1589.78) | 1268.20 (1096.03, 1484.68) | -0.22 (-0.30, -0.14) | <0.001 | 1558.06 (1316.34, 1792.33) | 1366.86 (1126.28, 1599.88) | -0.35 (-0.78, 0.09) | 0.120 | 25.36 (21.23, 29.41) | 22.10 (17.78, 26.29) | -0.36 (-0.83, 0.11) | 0.132 |
| North Africa and Middle East | 161.90 (139.38, 187.09) | 162.52 (139.32, 188.32) | 0.01 (-0.02, 0.04) | 0.469 | 1794.82 (1566.36, 2102.73) | 1837.37 (1597.42, 2134.98) | 0.10 (-0.01, 0.21) | 0.073 | 2442.82 (2159.74, 2852.00) | 1555.92 (1281.39, 1925.98) | -1.52 (-1.69, -1.34) | <0.001 | 40.86 (35.78, 48.13) | 25.16 (20.44, 31.75) | -1.64 (-1.82, -1.46) | <0.001 |
| Western Sub-Saharan Africa | 165.89 (140.39, 193.84) | 168.64 (142.33, 197.05) | 0.06 (0.05, 0.06) | <0.001 | 2382.71 (1937.61, 3020.53) | 2522.49 (2038.88, 3233.53) | 0.21 (0.15, 0.26) | <0.001 | 1294.96 (1034.50, 1639.21) | 1222.58 (982.04, 1504.59) | -0.21 (-0.29, -0.12) | <0.001 | 20.24 (15.76, 26.28) | 18.83 (14.70, 23.78) | -0.26 (-0.34, -0.17) | <0.001 |
| Southeast Asia | 149.91 (130.84, 172.20) | 146.10 (126.96, 168.77) | -0.09 (-0.10, -0.08) | <0.001 | 1239.85 (1093.43, 1406.76) | 1257.78 (1115.59, 1428.86) | 0.07 (-0.02, 0.15) | 0.115 | 2087.58 (1838.20, 2336.03) | 1939.74 (1634.86, 2231.04) | -0.26 (-0.59, 0.08) | 0.131 | 34.38 (29.92, 38.93) | 31.81 (26.50, 37.13) | -0.27 (-0.62, 0.08) | 0.130 |
| Eastern Sub-Saharan Africa | 192.05 (161.29, 224.69) | 181.55 (150.73, 215.13) | -0.19 (-0.21, -0.17) | <0.001 | 2625.28 (2165.54, 3197.98) | 2722.11 (2208.79, 3319.89) | 0.13 (0.07, 0.19) | <0.001 | 1673.34 (1414.16, 1942.34) | 1384.56 (1144.20, 1660.44) | -0.68 (-0.83, -0.52) | <0.001 | 26.72 (22.39, 31.41) | 21.56 (17.49, 26.49) | -0.76 (-0.93, -0.58) | <0.001 |
| Central Latin America | 118.12 (100.14, 138.53) | 115.00 (96.93, 135.98) | -0.09 (-0.10, -0.08) | <0.001 | 1125.81 (973.92, 1297.57) | 1206.47 (1047.91, 1388.48) | 0.24 (0.19, 0.29) | <0.001 | 1236.62 (1176.57, 1302.97) | 998.54 (868.50, 1151.69) | -0.58 (-1.08, -0.09) | 0.020 | 20.66 (19.73, 21.76) | 16.39 (14.17, 18.98) | -0.63 (-1.14, -0.12) | 0.015 |
| Tropical Latin America | 176.96 (151.56, 205.83) | 145.01 (121.87, 171.89) | -0.70 (-0.73, -0.66) | <0.001 | 2151.50 (1799.72, 2598.64) | 2049.90 (1708.58, 2462.30) | -0.16 (-0.19, -0.12) | <0.001 | 2345.56 (2222.16, 2477.41) | 1178.16 (1098.87, 1264.80) | -2.33 (-2.59, -2.07) | <0.001 | 39.28 (37.21, 41.52) | 18.80 (17.55, 20.11) | -2.49 (-2.79, -2.19) | <0.001 |
| Central Sub-Saharan Africa | 190.74 (159.42, 224.29) | 179.65 (146.84, 215.00) | -0.21 (-0.22, -0.19) | <0.001 | 2632.75 (2083.50, 3274.96) | 2602.71 (2089.05, 3222.61) | -0.03 (-0.06, -0.01) | 0.011 | 1575.35 (1135.37, 2119.88) | 1321.35 (940.07, 1803.73) | -0.64 (-0.76, -0.53) | <0.001 | 25.12 (17.17, 34.86) | 20.68 (14.01, 29.07) | -0.71 (-0.84, -0.58) | <0.001 |
| Western Europe | 128.92 (107.89, 150.99) | 114.94 (95.79, 134.31) | -0.39 (-0.43, -0.36) | <0.001 | 788.71 (669.63, 914.39) | 748.17 (645.94, 865.48) | -0.18 (-0.23, -0.14) | <0.001 | 977.81 (933.97, 1023.74) | 461.00 (434.74, 490.37) | -2.65 (-2.91, -2.40) | <0.001 | 16.18 (15.51, 16.86) | 7.18 (6.80, 7.57) | -2.87 (-3.12, -2.62) | <0.001 |

| | | | | | | | | | | | | | | | | |
|---|---|---|---|---|---|---|---|---|---|---|---|---|---|---|---|---|
| High-income North America | 186.57 (160.82, 215.26) | 179.95 (159.40, 202.50) | -0.13 (-0.15, -0.11) | <0.001 | 1652.98 (1462.56, 1872.55) | 1758.65 (1568.73, 1968.51) | 0.24 (-0.13, 0.61) | 0.203 | 1055.81 (1013.15, 1103.29) | 862.21 (768.93, 925.22) | -0.71 (-1.15, -0.26) | 0.002 | 16.78 (16.28, 17.33) | 13.18 (11.77, 14.10) | -0.81 (-1.41, -0.21) | 0.009 |
| Central Asia | 199.10 (174.52, 227.56) | 192.80 (167.10, 220.55) | -0.11 (-0.14, -0.08) | <0.001 | 1724.20 (1490.01, 2011.00) | 1788.58 (1522.39, 2098.48) | 0.13 (0.10, 0.15) | <0.001 | 2736.11 (2590.04, 2882.26) | 2515.63 (2262.64, 2789.71) | -0.32 (-0.96, 0.33) | 0.336 | 46.04 (43.60, 48.47) | 42.21 (37.78, 46.99) | -0.33 (-1.00, 0.35) | 0.345 |
| Eastern Europe | 182.32 (157.90, 208.64) | 201.04 (177.18, 227.61) | 0.34 (0.32, 0.35) | <0.001 | 901.42 (809.04, 1003.67) | 1006.17 (908.58, 1116.78) | 0.38 (0.36, 0.40) | <0.001 | 2875.85 (2761.76, 2954.80) | 4119.44 (3649.84, 4605.61) | 1.13 (0.13, 2.13) | 0.027 | 49.35 (47.42, 50.78) | 71.42 (63.12, 79.94) | 1.16 (0.16, 2.18) | 0.024 |
| Southern Sub-Saharan Africa | 204.02 (170.51, 238.00) | 192.64 (160.09, 227.03) | -0.19 (-0.21, -0.18) | <0.001 | 2558.49 (2128.25, 3108.46) | 2572.19 (2134.44, 3125.43) | 0.03 (-0.01, 0.06) | 0.119 | 1966.07 (1660.88, 2317.69) | 1315.17 (1046.64, 1617.13) | -1.52 (-3.04, 0.02) | 0.054 | 31.91 (26.69, 38.34) | 20.50 (15.84, 25.76) | -1.67 (-3.34, 0.04) | 0.056 |
| High-income Asia Pacific | 171.95 (153.53, 191.77) | 174.40 (156.15, 194.74) | 0.05 (0.03, 0.08) | <0.001 | 1143.05 (1024.04, 1286.87) | 1238.90 (1111.46, 1375.29) | 0.28 (0.16, 0.40) | <0.001 | 1110.79 (996.23, 1204.43) | 463.28 (423.81, 507.54) | -3.02 (-3.29, -2.75) | <0.001 | 18.35 (16.38, 19.90) | 6.94 (6.43, 7.58) | -3.34 (-3.66, -3.01) | <0.001 |
| Central Europe | 144.27 (125.35, 165.54) | 138.74 (120.52, 158.86) | -0.14 (-0.16, -0.12) | <0.001 | 1042.84 (915.15, 1191.23) | 1067.13 (933.17, 1211.82) | 0.09 (0.03, 0.15) | 0.003 | 2266.28 (2184.36, 2353.03) | 964.48 (855.77, 1069.45) | -2.96 (-3.31, -2.61) | <0.001 | 38.44 (37.18, 39.90) | 15.63 (13.86, 17.37) | -3.18 (-3.57, -2.79) | <0.001 |
| Andean Latin America | 135.94 (114.92, 160.85) | 128.72 (108.26, 153.21) | -0.19 (-0.20, -0.18) | <0.001 | 1915.01 (1574.55, 2330.73) | 1939.35 (1598.15, 2333.75) | 0.04 (0.01, 0.07) | 0.009 | 1571.03 (1322.45, 1850.39) | 1032.12 (811.07, 1291.19) | -1.45 (-1.82, -1.08) | <0.001 | 25.78 (21.43, 30.55) | 16.27 (12.34, 20.70) | -1.59 (-2.00, -1.18) | <0.001 |
| Caribbean | 207.76 (176.09, 241.75) | 216.05 (182.84, 251.33) | 0.14 (0.12, 0.15) | <0.001 | 2099.85 (1779.49, 2473.62) | 2171.61 (1832.88, 2550.01) | 0.12 (0.05, 0.18) | 0.001 | 1804.05 (1620.23, 2049.25) | 1520.91 (1183.24, 1900.16) | -0.51 (-0.82, -0.19) | 0.002 | 29.97 (26.73, 34.26) | 24.94 (18.97, 31.73) | -0.55 (-0.89, -0.20) | 0.002 |
| Southern Latin America | 121.98 (104.56, 140.48) | 118.60 (101.20, 138.03) | -0.10 (-0.12, -0.07) | <0.001 | 1122.67 (922.05, 1364.66) | 1296.61 (1054.61, 1566.50) | 0.49 (0.39, 0.58) | <0.001 | 1438.46 (1328.36, 1563.20) | 794.57 (693.37, 893.12) | -2.01 (-2.41, -1.61) | <0.001 | 23.94 (22.05, 26.06) | 12.48 (10.71, 14.16) | -2.21 (-2.64, -1.77) | <0.001 |
| Oceania | 203.75 (180.35, 231.24) | 208.55 (183.76, 234.82) | 0.08 (0.07, 0.09) | <0.001 | 1717.09 (1506.36, 1998.35) | 1763.92 (1509.52, 2066.19) | 0.09 (0.05, 0.14) | <0.001 | 4103.24 (2989.87, 5441.92) | 4179.80 (3077.46, 5578.79) | 0.02 (-0.13, 0.16) | 0.812 | 69.38 (49.68, 92.51) | 70.72 (50.90, 95.44) | 0.02 (-0.13, 0.17) | 0.813 |
| Australasia | 189.89 (155.48, 230.28) | 187.62 (153.94, 225.94) | -0.04 (-0.08, 0.00) | 0.052 | 873.62 (720.00, 1025.52) | 968.25 (821.82, 1142.51) | 0.28 (0.19, 0.38) | <0.001 | 817.06 (745.39, 889.25) | 472.91 (399.50, 550.53) | -1.88 (-2.25, -1.50) | <0.001 | 13.40 (12.25, 14.63) | 7.45 (6.23, 8.76) | -2.02 (-2.42, -1.60) | <0.001 |

| | | | | | | | | | | | | | | | | |
|---|---|---|---|---|---|---|---|---|---|---|---|---|---|---|---|---|
| **35-39 years** | | | | | | | | | | | | | | | | |
| **Women** | | | | | | | | | | | | | | | | |
| Global | 166.94 (143.63, 194.78) | 155.19 (132.90, 182.38) | -0.25 (-0.30, -0.20) | <0.001 | 2380.40 (2138.49, 2692.48) | 2455.45 (2187.32, 2807.78) | 0.11 (0.08, 0.14) | <0.001 | 1972.85 (1821.21, 2134.09) | 1359.07 (1225.77, 1497.68) | -1.27 (-1.44, -1.10) | <0.001 | 34.40 (31.70, 37.51) | 22.58 (20.13, 24.85) | -1.43 (-1.62, -1.24) | <0.001 |
| **SDI category** | | | | | | | | | | | | | | | | |
| High | 181.27 (159.76, 206.48) | 171.36 (152.90, 193.19) | -0.21 (-0.28, -0.13) | <0.001 | 2506.73 (2218.87, 2867.31) | 2491.97 (2252.95, 2758.60) | 0.01 (-0.16, 0.17) | 0.940 | 898.39 (847.66, 953.44) | 659.43 (598.58, 728.79) | -1.06 (-1.30, -0.82) | <0.001 | 14.32 (13.91, 14.78) | 9.72 (8.92, 10.63) | -1.28 (-1.49, -1.07) | <0.001 |
| High-middle | 156.16 (134.77, 182.40) | 134.92 (115.48, 158.81) | -0.50 (-0.53, -0.47) | <0.001 | 1981.65 (1788.01, 2200.86) | 1964.53 (1758.26, 2192.62) | -0.03 (-0.05, 0.00) | 0.055 | 1605.50 (1464.47, 1763.55) | 983.84 (887.86, 1089.22) | -1.59 (-1.88, -1.30) | <0.001 | 27.43 (24.95, 30.37) | 15.62 (13.98, 17.37) | -1.81 (-2.16, -1.47) | <0.001 |
| Middle | 160.26 (135.91, 190.53) | 147.16 (123.72, 175.79) | -0.29 (-0.33, -0.26) | <0.001 | 2333.73 (2080.02, 2649.49) | 2345.88 (2075.81, 2683.30) | 0.02 (0.00, 0.04) | 0.052 | 2286.51 (2055.76, 2548.76) | 1282.42 (1141.22, 1426.87) | -1.98 (-2.13, -1.82) | <0.001 | 40.10 (35.74, 44.97) | 20.91 (18.38, 23.39) | -2.23 (-2.36, -2.10) | <0.001 |
| Low-middle | 169.64 (143.12, 200.38) | 164.64 (138.11, 195.33) | -0.10 (-0.13, -0.08) | <0.001 | 2614.43 (2284.66, 3011.42) | 2707.17 (2352.75, 3140.76) | 0.13 (0.04, 0.22) | 0.006 | 2665.12 (2356.35, 2992.11) | 1927.39 (1647.97, 2216.04) | -1.11 (-1.43, -0.79) | <0.001 | 47.75 (42.08, 54.05) | 33.44 (28.12, 38.76) | -1.22 (-1.57, -0.87) | <0.001 |
| Low | 189.07 (159.40, 223.31) | 176.14 (147.28, 209.85) | -0.24 (-0.26, -0.23) | <0.001 | 2993.37 (2547.85, 3613.26) | 3083.73 (2606.70, 3749.30) | 0.12 (0.04, 0.19) | 0.002 | 2684.52 (2318.49, 3091.71) | 1913.43 (1638.04, 2223.55) | -1.19 (-1.36, -1.01) | <0.001 | 47.76 (40.83, 55.43) | 32.84 (27.47, 38.69) | -1.32 (-1.52, -1.12) | <0.001 |
| **21 GBD region** | | | | | | | | | | | | | | | | |
| South Asia | 164.00 (136.71, 195.39) | 160.90 (133.98, 192.60) | -0.07 (-0.10, -0.04) | <0.001 | 2562.41 (2221.87, 2968.71) | 2572.59 (2228.95, 2977.18) | 0.04 (-0.09, 0.16) | 0.587 | 2538.07 (2227.10, 2910.43) | 1958.90 (1618.94, 2331.62) | -1.03 (-1.62, -0.43) | 0.001 | 45.53 (39.43, 52.49) | 34.32 (27.76, 41.65) | -1.11 (-1.75, -0.47) | 0.001 |
| East Asia | 143.41 (120.92, 171.39) | 122.24 (102.78, 146.20) | -0.57 (-0.62, -0.52) | <0.001 | 2029.43 (1789.88, 2296.79) | 1954.08 (1725.51, 2216.44) | -0.14 (-0.20, -0.07) | <0.001 | 2026.55 (1650.47, 2442.48) | 817.11 (665.86, 988.31) | -3.08 (-3.26, -2.89) | <0.001 | 35.17 (27.88, 43.29) | 12.08 (9.40, 15.42) | -3.62 (-3.84, -3.40) | <0.001 |
| North Africa and Middle East | 207.08 (176.39, 244.63) | 195.26 (165.97, 232.88) | -0.20 (-0.22, -0.18) | <0.001 | 2837.33 (2481.51, 3324.59) | 2774.46 (2442.11, 3206.20) | -0.07 (-0.13, -0.01) | 0.022 | 3107.45 (2751.34, 3483.05) | 1797.78 (1445.56, 2143.89) | -1.87 (-2.02, -1.72) | <0.001 | 55.68 (49.01, 62.67) | 30.52 (23.77, 37.23) | -2.05 (-2.21, -1.89) | <0.001 |

| | | | | | | | | | | | | | | | | |
|---|---|---|---|---|---|---|---|---|---|---|---|---|---|---|---|---|
| Western Sub-Saharan Africa | 175.49 (147.59, 207.67) | 166.54 (139.21, 198.23) | -0.18 (-0.20, -0.16) | <0.001 | 3042.42 (2518.66, 3865.74) | 3183.89 (2585.90, 4131.86) | 0.17 (0.12, 0.22) | <0.001 | 1954.05 (1579.22, 2411.82) | 1399.35 (1116.47, 1740.23) | -1.15 (-1.27, -1.03) | <0.001 | 33.56 (26.24, 42.13) | 22.75 (17.43, 29.16) | -1.33 (-1.44, -1.22) | <0.001 |
| Southeast Asia | 156.57 (133.31, 187.03) | 150.23 (127.37, 179.82) | -0.14 (-0.16, -0.13) | <0.001 | 1920.07 (1733.63, 2143.98) | 1889.55 (1698.98, 2108.60) | -0.04 (-0.10, 0.02) | 0.207 | 2484.17 (2166.02, 2850.69) | 1714.36 (1445.79, 2021.11) | -1.27 (-1.37, -1.16) | <0.001 | 43.50 (37.65, 50.31) | 28.76 (23.72, 34.59) | -1.41 (-1.54, -1.28) | <0.001 |
| Eastern Sub-Saharan Africa | 198.91 (166.19, 234.85) | 164.85 (134.60, 198.68) | -0.65 (-0.68, -0.62) | <0.001 | 3611.45 (2987.50, 4525.89) | 3691.02 (3021.98, 4664.91) | 0.08 (0.06, 0.10) | <0.001 | 2397.15 (1981.12, 2890.23) | 1394.58 (1155.59, 1671.07) | -1.85 (-1.92, -1.78) | <0.001 | 41.42 (33.09, 50.88) | 22.16 (17.68, 27.24) | -2.13 (-2.23, -2.03) | <0.001 |
| Central Latin America | 153.34 (129.65, 184.14) | 123.29 (101.87, 149.43) | -0.75 (-0.80, -0.71) | <0.001 | 2294.88 (2030.75, 2619.11) | 2173.17 (1906.87, 2496.15) | -0.17 (-0.28, -0.06) | 0.002 | 1527.91 (1457.78, 1601.03) | 826.95 (689.89, 994.07) | -1.94 (-2.28, -1.59) | <0.001 | 26.67 (25.59, 27.75) | 13.31 (10.76, 16.40) | -2.15 (-2.55, -1.75) | <0.001 |
| Tropical Latin America | 204.21 (170.28, 244.47) | 144.41 (120.72, 172.09) | -1.19 (-1.29, -1.09) | <0.001 | 3302.66 (2864.35, 3817.25) | 3067.06 (2632.35, 3588.89) | -0.25 (-0.27, -0.24) | <0.001 | 2737.74 (2616.84, 2855.73) | 1247.41 (1149.55, 1340.23) | -2.64 (-2.85, -2.43) | <0.001 | 49.00 (46.97, 51.07) | 20.59 (19.24, 21.99) | -2.90 (-3.13, -2.66) | <0.001 |
| Central Sub-Saharan Africa | 192.64 (161.57, 229.26) | 170.03 (139.33, 204.90) | -0.43 (-0.44, -0.42) | <0.001 | 3445.27 (2821.54, 4330.99) | 3392.96 (2729.46, 4344.00) | -0.03 (-0.10, 0.03) | 0.336 | 2144.12 (1547.29, 2942.01) | 1517.20 (1086.64, 2085.84) | -1.21 (-1.32, -1.09) | <0.001 | 36.98 (25.30, 52.44) | 25.04 (16.89, 35.90) | -1.36 (-1.49, -1.23) | <0.001 |
| Western Europe | 112.77 (95.76, 133.58) | 99.05 (83.58, 117.13) | -0.45 (-0.47, -0.42) | <0.001 | 1584.21 (1352.13, 1868.99) | 1525.46 (1325.97, 1763.11) | -0.11 (-0.20, -0.02) | 0.016 | 734.45 (696.41, 780.22) | 360.62 (327.78, 396.64) | -2.44 (-2.55, -2.34) | <0.001 | 12.07 (11.66, 12.47) | 5.06 (4.78, 5.40) | -2.98 (-3.08, -2.89) | <0.001 |
| High-income North America | 259.19 (230.75, 294.86) | 242.58 (221.37, 267.03) | -0.26 (-0.34, -0.19) | <0.001 | 3740.83 (3298.93, 4415.99) | 3732.07 (3362.79, 4174.89) | -0.00 (-0.09, 0.09) | 0.990 | 967.31 (902.90, 1043.22) | 870.56 (804.41, 942.61) | -0.36 (-0.66, -0.07) | 0.016 | 14.88 (14.49, 15.29) | 13.07 (12.49, 13.61) | -0.49 (-0.84, -0.14) | 0.007 |
| Central Asia | 189.65 (164.70, 221.01) | 172.25 (147.94, 203.62) | -0.33 (-0.35, -0.31) | <0.001 | 2504.69 (2246.01, 2810.31) | 2477.22 (2209.52, 2794.38) | -0.03 (-0.07, 0.01) | 0.103 | 2245.09 (2115.65, 2363.77) | 1890.97 (1674.53, 2166.30) | -0.69 (-1.01, -0.37) | <0.001 | 39.10 (36.83, 41.09) | 32.48 (28.31, 37.58) | -0.75 (-1.09, -0.40) | <0.001 |
| Eastern Europe | 187.64 (160.89, 219.91) | 187.71 (161.82, 219.57) | -0.00 (-0.02, 0.01) | 0.838 | 1875.19 (1706.43, 2073.14) | 1964.31 (1780.47, 2175.70) | 0.18 (0.07, 0.30) | 0.002 | 1425.44 (1325.00, 1507.13) | 1915.35 (1589.91, 2299.86) | 1.44 (0.52, 2.37) | 0.002 | 24.00 (22.36, 25.11) | 33.27 (27.03, 40.86) | 1.63 (0.57, 2.70) | 0.003 |
| Southern Sub-Saharan Africa | 197.43 (162.32, 236.13) | 168.92 (137.58, 203.87) | -0.55 (-0.61, -0.48) | <0.001 | 3300.40 (2831.46, 3907.87) | 3139.88 (2630.10, 3764.21) | -0.17 (-0.18, -0.16) | <0.001 | 2469.91 (2053.14, 2831.98) | 1101.50 (793.90, 1450.17) | -2.63 (-3.99, -1.24) | <0.001 | 43.12 (34.89, 49.96) | 17.14 (11.57, 23.51) | -3.00 (-4.61, -1.36) | <0.001 |

| | | | | | | | | | | | | | | | | |
|---|---|---|---|---|---|---|---|---|---|---|---|---|---|---|---|---|
| High-income Asia Pacific | 183.99 (165.39, 208.26) | 172.17 (153.04, 195.05) | -0.22 (-0.26, -0.19) | <0.001 | 2157.18 (1945.06, 2394.18) | 2102.95 (1919.09, 2309.23) | -0.06 (-0.14, 0.01) | 0.094 | 827.52 (758.98, 889.68) | 353.76 (309.64, 399.90) | -2.90 (-3.24, -2.56) | <0.001 | 13.37 (12.49, 14.23) | 4.35 (4.02, 4.78) | -3.85 (-4.22, -3.47) | <0.001 |
| Central Europe | 152.49 (131.53, 177.58) | 135.66 (116.72, 159.26) | -0.41 (-0.43, -0.39) | <0.001 | 1973.16 (1752.21, 2227.74) | 1972.39 (1743.52, 2217.92) | 0.02 (-0.10, 0.15) | 0.704 | 1598.96 (1520.30, 1680.69) | 691.50 (586.13, 800.98) | -2.91 (-3.22, -2.60) | <0.001 | 27.03 (26.16, 27.99) | 9.81 (8.17, 11.61) | -3.51 (-3.91, -3.10) | <0.001 |
| Andean Latin America | 144.79 (121.73, 170.74) | 120.87 (99.19, 146.14) | -0.62 (-0.63, -0.61) | <0.001 | 2710.42 (2314.30, 3175.61) | 2622.70 (2215.10, 3058.59) | -0.10 (-0.13, -0.07) | <0.001 | 1838.81 (1545.02, 2154.30) | 853.86 (660.69, 1085.50) | -2.57 (-3.54, -1.58) | <0.001 | 32.27 (26.74, 38.16) | 13.37 (9.82, 17.73) | -2.95 (-4.09, -1.80) | <0.001 |
| Caribbean | 239.58 (203.15, 283.84) | 248.30 (209.97, 293.80) | 0.13 (0.10, 0.15) | <0.001 | 3161.32 (2785.90, 3603.62) | 3222.87 (2826.74, 3664.38) | 0.07 (0.01, 0.13) | 0.028 | 2660.95 (2218.25, 3135.28) | 2256.01 (1651.89, 3033.51) | -0.55 (-0.88, -0.21) | 0.002 | 48.08 (39.61, 57.31) | 40.21 (28.57, 55.08) | -0.59 (-0.97, -0.22) | 0.002 |
| Southern Latin America | 127.20 (108.28, 149.33) | 109.93 (91.89, 130.51) | -0.51 (-0.53, -0.48) | <0.001 | 1835.01 (1545.00, 2172.92) | 1997.62 (1685.01, 2362.07) | 0.28 (0.23, 0.33) | <0.001 | 1563.91 (1453.36, 1685.95) | 729.87 (640.36, 824.96) | -2.66 (-3.12, -2.20) | <0.001 | 27.36 (25.38, 29.36) | 11.19 (9.78, 12.66) | -3.11 (-3.63, -2.60) | <0.001 |
| Oceania | 249.74 (219.02, 286.79) | 261.43 (228.43, 299.73) | 0.16 (0.12, 0.20) | <0.001 | 2673.75 (2382.34, 3020.14) | 2744.07 (2430.81, 3086.31) | 0.09 (0.06, 0.13) | <0.001 | 3370.87 (2452.38, 4526.69) | 3514.43 (2470.68, 5009.28) | 0.15 (0.04, 0.27) | 0.010 | 59.06 (41.63, 81.11) | 61.73 (41.58, 91.26) | 0.16 (0.04, 0.29) | 0.012 |
| Australasia | 112.45 (92.16, 137.22) | 109.80 (90.38, 132.21) | -0.08 (-0.11, -0.06) | <0.001 | 1455.17 (1201.00, 1782.91) | 1485.18 (1226.02, 1790.52) | 0.07 (-0.01, 0.15) | 0.086 | 654.80 (593.73, 719.79) | 411.02 (354.87, 473.30) | -1.69 (-1.82, -1.57) | <0.001 | 10.26 (9.36, 11.20) | 5.71 (4.87, 6.64) | -2.09 (-2.24, -1.94) | <0.001 |
| **Men** | | | | | | | | | | | | | | | | |
| Global | 204.37 (172.80, 242.30) | 194.96 (164.79, 230.15) | -0.16 (-0.18, -0.15) | <0.001 | 2064.98 (1863.10, 2307.38) | 2138.71 (1921.82, 2411.16) | 0.13 (0.09, 0.18) | <0.001 | 3061.04 (2889.66, 3230.47) | 2585.11 (2377.12, 2813.68) | -0.54 (-0.96, -0.12) | 0.012 | 56.33 (53.20, 59.66) | 47.10 (43.03, 51.23) | -0.57 (-1.01, -0.13) | 0.011 |
| **SDI category** | | | | | | | | | | | | | | | | |
| High | 236.34 (198.64, 284.46) | 214.12 (184.12, 250.44) | -0.34 (-0.35, -0.33) | <0.001 | 2016.07 (1827.63, 2223.88) | 2014.75 (1847.38, 2195.02) | 0.04 (-0.15, 0.23) | 0.702 | 1929.03 (1872.02, 1991.73) | 1236.97 (1139.50, 1379.02) | -1.47 (-1.62, -1.32) | <0.001 | 34.99 (34.13, 35.94) | 21.60 (19.93, 24.34) | -1.59 (-1.85, -1.32) | <0.001 |
| High-middle | 206.05 (175.64, 243.45) | 185.11 (157.36, 219.85) | -0.37 (-0.41, -0.34) | <0.001 | 1813.18 (1641.02, 2008.44) | 1777.37 (1607.92, 1983.89) | -0.06 (-0.11, -0.00) | 0.032 | 3495.52 (3279.23, 3734.09) | 2641.57 (2409.28, 2882.06) | -0.86 (-1.29, -0.43) | <0.001 | 64.84 (60.82, 69.26) | 48.47 (44.09, 53.06) | -0.90 (-1.35, -0.44) | <0.001 |

| | | | | | | | | | | | | | | | | |
|---|---|---|---|---|---|---|---|---|---|---|---|---|---|---|---|---|
| Middle | 193.92 (162.26, 232.86) | 191.60 (160.81, 228.18) | -0.04 (-0.06, -0.03) | <0.001 | 2121.91 (1894.29, 2410.65) | 2158.37 (1926.39, 2450.80) | 0.06 (0.02, 0.10) | 0.003 | 3096.71 (2843.68, 3356.53) | 2655.36 (2400.38, 2919.40) | -0.49 (-0.60, -0.38) | <0.001 | 56.68 (51.94, 61.67) | 48.19 (43.24, 53.20) | -0.57 (-0.62, -0.53) | <0.001 |
| Low-middle | 189.21 (158.39, 225.30) | 192.94 (161.21, 229.58) | 0.06 (0.04, 0.08) | <0.001 | 2145.00 (1897.69, 2451.04) | 2259.59 (1995.91, 2585.02) | 0.19 (0.11, 0.27) | <0.001 | 3524.08 (3193.81, 3911.72) | 3235.95 (2846.46, 3708.30) | -0.18 (-0.85, 0.50) | 0.607 | 65.33 (59.03, 72.45) | 59.64 (52.19, 68.73) | -0.20 (-0.91, 0.52) | 0.589 |
| Low | 205.16 (172.21, 242.51) | 203.16 (169.00, 240.94) | -0.03 (-0.04, -0.03) | <0.001 | 2569.34 (2231.02, 2983.50) | 2668.10 (2310.66, 3121.03) | 0.14 (0.00, 0.26) | 0.042 | 3019.61 (2624.29, 3410.89) | 2656.02 (2288.01, 3070.18) | -0.39 (-0.63, -0.14) | 0.002 | 55.25 (47.81, 62.88) | 48.13 (41.07, 56.20) | -0.42 (-0.68, -0.16) | 0.001 |
| **21 GBD region** | | | | | | | | | | | | | | | | |
| South Asia | 176.51 (145.72, 211.98) | 183.86 (152.28, 220.38) | 0.13 (0.11, 0.15) | <0.001 | 2007.94 (1765.89, 2314.39) | 2074.19 (1817.29, 2381.01) | 0.13 (0.09, 0.17) | <0.001 | 3697.20 (3282.96, 4146.99) | 3514.07 (2997.18, 4073.77) | -0.05 (-0.99, 0.90) | 0.922 | 68.98 (61.19, 77.59) | 65.33 (55.38, 76.01) | -0.06 (-1.04, 0.94) | 0.911 |
| East Asia | 175.16 (146.30, 212.56) | 164.55 (138.71, 197.42) | -0.23 (-0.26, -0.19) | <0.001 | 1947.63 (1720.54, 2228.68) | 1867.07 (1642.81, 2133.48) | -0.14 (-0.17, -0.12) | <0.001 | 2670.15 (2242.98, 3096.46) | 2266.58 (1837.72, 2703.40) | -0.44 (-0.90, 0.03) | 0.065 | 48.44 (40.24, 56.73) | 40.72 (32.41, 48.96) | -0.47 (-0.96, 0.03) | 0.065 |
| North Africa and Middle East | 223.13 (183.90, 270.70) | 221.48 (182.41, 269.42) | -0.02 (-0.04, 0.01) | 0.186 | 2564.77 (2289.66, 2941.73) | 2567.48 (2298.53, 2937.82) | 0.02 (-0.07, 0.12) | 0.636 | 4159.43 (3718.94, 4763.94) | 2442.13 (2033.71, 3013.82) | -1.82 (-1.94, -1.70) | <0.001 | 77.15 (68.48, 89.48) | 43.94 (36.13, 54.94) | -1.93 (-2.05, -1.80) | <0.001 |
| Western Sub-Saharan Africa | 193.60 (161.22, 229.71) | 197.00 (163.73, 233.95) | 0.06 (0.05, 0.07) | <0.001 | 2798.46 (2400.93, 3370.93) | 2920.56 (2492.19, 3522.83) | 0.16 (0.11, 0.20) | <0.001 | 1944.57 (1544.58, 2508.82) | 1791.26 (1434.34, 2217.51) | -0.28 (-0.41, -0.15) | <0.001 | 34.19 (26.31, 45.06) | 31.10 (24.18, 39.20) | -0.33 (-0.47, -0.19) | <0.001 |
| Southeast Asia | 222.62 (188.32, 265.43) | 212.99 (181.62, 254.33) | -0.15 (-0.16, -0.15) | <0.001 | 1907.71 (1721.05, 2115.69) | 1899.07 (1714.56, 2105.84) | 0.01 (-0.09, 0.11) | 0.908 | 3291.87 (2925.93, 3669.05) | 3112.50 (2622.52, 3636.03) | -0.14 (-0.41, 0.12) | 0.297 | 59.75 (52.65, 66.93) | 56.36 (46.76, 66.40) | -0.15 (-0.44, 0.14) | 0.321 |
| Eastern Sub-Saharan Africa | 221.22 (184.12, 261.68) | 203.46 (166.75, 242.39) | -0.28 (-0.30, -0.26) | <0.001 | 3078.67 (2641.96, 3649.29) | 3151.96 (2689.30, 3760.03) | 0.09 (0.02, 0.15) | 0.008 | 2745.15 (2311.99, 3215.07) | 2102.35 (1764.05, 2526.56) | -0.94 (-1.06, -0.81) | <0.001 | 49.33 (40.93, 57.66) | 36.91 (30.31, 45.09) | -1.01 (-1.13, -0.90) | <0.001 |
| Central Latin America | 150.32 (124.08, 181.24) | 143.29 (117.67, 174.37) | -0.17 (-0.20, -0.13) | <0.001 | 1513.05 (1349.17, 1700.73) | 1565.55 (1397.96, 1771.11) | 0.12 (0.06, 0.17) | <0.001 | 1839.25 (1762.55, 1921.97) | 1467.04 (1248.87, 1708.18) | -0.64 (-1.00, -0.28) | <0.001 | 33.94 (32.52, 35.48) | 26.72 (22.55, 31.32) | -0.67 (-1.03, -0.31) | <0.001 |
| Tropical Latin America | 229.10 (192.21, | 163.95 (136.89, | -1.16 (-1.21, -1.12) | <0.001 | 2722.71 (2376.76, | 2480.70 (2166.07, | -0.32 (-0.34, | <0.001 | 4067.12 (3862.17, | 1867.29 (1746.12, | -2.62 (-2.88, | <0.001 | 75.72 (71.79, | 33.51 (31.41, | -2.74 (-3.02, | <0.001 |

| | | | | | | | | | | | | | | | | |
|---|---|---|---|---|---|---|---|---|---|---|---|---|---|---|---|---|
| | 274.60) | 195.04) | | | 3143.94) | 2888.48) | -0.29) | | 4264.15) | 1991.19) | -2.36) | | 79.55) | 35.75) | -2.46) | |
| Central Sub-Saharan Africa | 222.57 (184.16, 264.80) | 204.72 (167.06, 246.05) | -0.29 (-0.30, -0.28) | <0.001 | 3032.08 (2562.82, 3649.81) | 2946.34 (2456.98, 3587.26) | -0.10 (-0.12, -0.07) | <0.001 | 2567.62 (1892.47, 3417.24) | 2066.25 (1503.03, 2850.05) | -0.77 (-0.90, -0.64) | <0.001 | 46.18 (33.59, 62.42) | 36.63 (25.88, 51.68) | -0.82 (-0.97, -0.68) | <0.001 |
| Western Europe | 188.59 (152.28, 232.35) | 161.83 (132.47, 197.71) | -0.53 (-0.56, -0.50) | <0.001 | 1325.73 (1176.26, 1494.14) | 1201.87 (1075.67, 1333.01) | -0.32 (-0.42, -0.21) | <0.001 | 1748.34 (1689.73, 1809.57) | 758.76 (718.63, 800.14) | -2.86 (-3.07, -2.65) | <0.001 | 32.19 (31.16, 33.28) | 13.29 (12.65, 13.96) | -3.03 (-3.26, -2.80) | <0.001 |
| High-income North America | 280.58 (233.06, 341.36) | 243.55 (212.28, 279.86) | -0.48 (-0.54, -0.43) | <0.001 | 2691.68 (2420.97, 3021.27) | 2642.77 (2407.86, 2897.06) | -0.04 (-0.18, 0.10) | 0.572 | 2048.02 (1981.01, 2117.65) | 1450.61 (1341.13, 1532.04) | -1.14 (-1.52, -0.76) | <0.001 | 36.79 (35.90, 37.81) | 25.30 (23.42, 26.63) | -1.25 (-1.68, -0.82) | <0.001 |
| Central Asia | 277.61 (237.90, 326.52) | 263.57 (225.31, 313.57) | -0.17 (-0.19, -0.16) | <0.001 | 2454.69 (2201.25, 2750.98) | 2506.73 (2226.10, 2832.65) | 0.07 (0.05, 0.10) | <0.001 | 4936.88 (4729.35, 5164.54) | 4309.75 (3873.71, 4791.57) | -0.66 (-1.28, -0.04) | 0.038 | 92.11 (88.09, 96.46) | 80.01 (71.52, 89.16) | -0.68 (-1.32, -0.04) | 0.037 |
| Eastern Europe | 287.91 (244.83, 339.67) | 308.49 (264.55, 362.55) | 0.24 (0.22, 0.25) | <0.001 | 1755.44 (1595.76, 1932.23) | 1919.04 (1750.27, 2117.67) | 0.30 (0.29, 0.32) | <0.001 | 5490.27 (5303.41, 5629.25) | 6860.50 (5963.55, 7811.09) | 1.11 (0.02, 2.21) | 0.046 | 103.65 (99.98, 106.20) | 130.19 (112.88, 148.55) | 1.13 (0.01, 2.26) | 0.047 |
| Southern Sub-Saharan Africa | 240.59 (198.44, 288.96) | 217.18 (177.17, 262.51) | -0.35 (-0.37, -0.33) | <0.001 | 3027.78 (2630.95, 3529.82) | 3000.67 (2593.56, 3504.95) | -0.02 (-0.06, 0.02) | 0.297 | 3305.95 (2796.89, 3808.15) | 2021.74 (1562.75, 2565.38) | -1.69 (-2.37, -1.01) | <0.001 | 60.13 (50.30, 69.75) | 35.58 (26.79, 45.83) | -1.81 (-2.54, -1.07) | <0.001 |
| High-income Asia Pacific | 257.68 (227.51, 296.10) | 257.04 (226.82, 293.42) | -0.01 (-0.03, 0.02) | 0.536 | 2085.11 (1895.02, 2289.05) | 2177.68 (1987.52, 2385.30) | 0.16 (0.06, 0.27) | 0.002 | 1712.14 (1587.82, 1817.79) | 808.39 (743.65, 885.53) | -2.58 (-2.77, -2.40) | <0.001 | 30.86 (28.59, 32.74) | 13.39 (12.42, 14.66) | -2.88 (-3.09, -2.67) | <0.001 |
| Central Europe | 219.16 (187.63, 257.61) | 195.78 (168.27, 228.69) | -0.40 (-0.42, -0.37) | <0.001 | 1790.98 (1588.42, 2013.89) | 1739.95 (1557.88, 1955.60) | -0.08 (-0.15, -0.01) | 0.018 | 4453.16 (4326.30, 4589.56) | 1750.42 (1546.06, 1966.53) | -3.27 (-3.55, -2.99) | <0.001 | 83.46 (81.28, 85.95) | 31.59 (27.62, 35.67) | -3.39 (-3.69, -3.10) | <0.001 |
| Andean Latin America | 146.81 (122.96, 172.71) | 135.97 (113.25, 161.58) | -0.27 (-0.30, -0.24) | <0.001 | 2222.13 (1917.29, 2587.04) | 2228.82 (1919.12, 2597.02) | 0.01 (-0.01, 0.03) | 0.539 | 2092.04 (1784.64, 2455.03) | 1267.70 (992.81, 1641.72) | -1.66 (-2.14, -1.18) | <0.001 | 37.98 (32.15, 45.10) | 22.09 (16.88, 29.25) | -1.80 (-2.31, -1.28) | <0.001 |
| Caribbean | 259.38 (215.82, 313.82) | 268.77 (221.46, 323.96) | 0.12 (0.11, 0.14) | <0.001 | 2729.53 (2419.50, 3109.00) | 2792.47 (2488.42, 3177.87) | 0.09 (0.04, 0.13) | <0.001 | 2905.69 (2580.66, 3283.31) | 2501.39 (1955.88, 3201.13) | -0.48 (-0.76, -0.21) | 0.001 | 53.65 (47.39, 60.82) | 45.85 (35.34, 59.49) | -0.51 (-0.80, -0.22) | 0.001 |
| Southern Latin America | 165.26 (139.64, | 151.33 (128.23, | -0.31 (-0.33, -0.28) | <0.001 | 1542.63 (1335.03, | 1649.99 (1401.53, | 0.24 (0.19, 0.29) | <0.001 | 2574.16 (2387.70, | 1299.04 (1146.56, | -2.28 (-2.52, | <0.001 | 47.76 (44.23, | 23.06 (20.28, | -2.43 (-2.69, | <0.001 |

| | | | | | | | | | | | | | | | | |
|---|---|---|---|---|---|---|---|---|---|---|---|---|---|---|---|---|
| | 194.47) | 179.73) | | | 1799.47) | 1945.95) | | | 2768.30) | 1434.09) | -2.04) | | 51.27) | 25.76) | -2.17) | |
| Oceania | 296.84 (258.00, 341.04) | 298.68 (257.95, 344.71) | 0.02 (0.01, 0.02) | <0.001 | 2556.08 (2257.87, 2877.26) | 2609.35 (2309.76, 2956.89) | 0.07 (0.05, 0.09) | <0.001 | 7241.79 (5509.20, 9186.72) | 7110.21 (5258.00, 9471.64) | -0.09 (-0.16, -0.01) | 0.026 | 135.20 (101.53, 172.77) | 132.66 (96.57, 177.95) | -0.09 (-0.17, -0.01) | 0.025 |
| Australasia | 280.85 (219.00, 361.99) | 269.35 (214.44, 341.86) | -0.15 (-0.21, -0.09) | <0.001 | 1705.02 (1475.40, 1940.61) | 1773.10 (1545.12, 2013.64) | 0.14 (-0.01, 0.29) | 0.065 | 1514.88 (1411.60, 1622.07) | 833.59 (721.20, 951.20) | -2.03 (-2.45, -1.60) | <0.001 | 27.63 (25.67, 29.53) | 14.67 (12.55, 16.95) | -2.14 (-2.59, -1.69) | <0.001 |
| **Rheumatic heart disease** | | | | | | | | | | | | | | | | |
| **15-19 years** | | | | | | | | | | | | | | | | |
| **Women** | | | | | | | | | | | | | | | | |
| Global | 67.98 (36.69, 101.12) | 87.35 (47.03, 130.26) | 0.87 (0.79, 0.94) | <0.001 | 610.90 (392.36, 905.00) | 761.15 (482.49, 1143.20) | 0.77 (0.72, 0.82) | <0.001 | 146.37 (122.60, 173.41) | 95.59 (75.07, 123.70) | -1.52 (-1.60, -1.45) | <0.001 | 1.64 (1.38, 1.88) | 0.83 (0.71, 0.97) | -2.35 (-2.83, -1.88) | <0.001 |
| **SDI category** | | | | | | | | | | | | | | | | |
| High | 0.73 (0.38, 1.19) | 0.85 (0.55, 1.21) | 0.55 (0.47, 0.62) | <0.001 | 13.39 (10.00, 17.63) | 14.15 (11.42, 17.28) | 0.19 (0.04, 0.33) | 0.010 | 9.67 (8.19, 11.67) | 3.26 (2.74, 3.91) | -3.62 (-3.83, -3.41) | <0.001 | 0.13 (0.11, 0.15) | 0.04 (0.03, 0.04) | -4.23 (-4.48, -3.97) | <0.001 |
| High-middle | 26.06 (12.93, 40.00) | 29.76 (15.02, 45.95) | 0.47 (0.43, 0.51) | <0.001 | 296.20 (190.41, 426.76) | 318.20 (204.19, 468.29) | 0.26 (0.18, 0.34) | <0.001 | 58.49 (48.52, 70.58) | 28.08 (19.94, 39.54) | -2.55 (-2.77, -2.33) | <0.001 | 0.62 (0.53, 0.73) | 0.18 (0.14, 0.23) | -4.19 (-4.58, -3.81) | <0.001 |
| Middle | 64.65 (32.49, 100.13) | 66.38 (34.40, 101.64) | 0.10 (0.04, 0.15) | <0.001 | 670.99 (428.18, 981.63) | 649.68 (407.38, 952.79) | -0.09 (-0.24, 0.05) | 0.204 | 130.08 (108.32, 155.67) | 69.18 (52.85, 93.25) | -2.19 (-2.68, -1.71) | <0.001 | 1.37 (1.19, 1.56) | 0.53 (0.44, 0.62) | -3.29 (-3.83, -2.74) | <0.001 |
| Low-middle | 111.16 (65.97, 160.01) | 119.80 (68.33, 176.03) | 0.27 (0.15, 0.39) | <0.001 | 828.84 (524.23, 1243.56) | 932.28 (576.12, 1423.34) | 0.42 (0.34, 0.50) | <0.001 | 272.70 (221.97, 326.73) | 152.31 (120.39, 191.82) | -2.04 (-2.64, -1.44) | <0.001 | 3.26 (2.61, 3.96) | 1.51 (1.22, 1.83) | -2.70 (-3.39, -2.00) | <0.001 |
| Low | 140.63 (73.72, 210.52) | 149.32 (76.04, 224.88) | 0.22 (0.17, 0.27) | <0.001 | 1198.40 (777.18, 1800.14) | 1310.34 (851.97, 1963.22) | 0.31 (0.27, 0.35) | <0.001 | 247.36 (186.16, 315.90) | 140.96 (103.72, 188.43) | -1.94 (-2.14, -1.74) | <0.001 | 2.67 (1.97, 3.44) | 1.10 (0.84, 1.41) | -3.06 (-3.30, -2.81) | <0.001 |
| **21 GBD region** | | | | | | | | | | | | | | | | |
| South Asia | 117.38 (72.62, | 118.92 (71.78, | 0.05 (-0.05, 0.16) | 0.329 | 725.90 (456.77, | 791.22 (486.31, | 0.33 (0.23, 0.44) | <0.001 | 357.46 (290.28, | 182.99 (148.97, | -2.39 (-3.25, | <0.001 | 4.52 (3.58, | 2.03 (1.68, | -2.83 (-3.54, | <0.001 |

| | | | | | | | | | | | | | | | | |
|---|---|---|---|---|---|---|---|---|---|---|---|---|---|---|---|---|
| | 164.72) | 170.64) | | | 1114.13) | 1252.74) | | | 428.41) | 221.98) | -1.51) | | 5.47) | 2.46) | -2.11) | |
| East Asia | 51.25 (23.39, 82.56) | 42.67 (19.90, 68.54) | -0.62 (-0.76, -0.47) | <0.001 | 623.99 (398.74, 910.98) | 497.55 (316.61, 731.35) | -0.77 (-0.96, -0.58) | <0.001 | 99.99 (78.87, 125.94) | 32.63 (21.02, 50.12) | -3.86 (-4.29, -3.42) | <0.001 | 0.98 (0.79, 1.21) | 0.12 (0.09, 0.15) | -7.01 (-7.24, -6.79) | <0.001 |
| North Africa and Middle East | 44.75 (23.29, 67.92) | 50.65 (26.14, 77.71) | 0.43 (0.40, 0.46) | <0.001 | 436.06 (282.16, 638.08) | 485.71 (299.73, 726.81) | 0.37 (0.32, 0.42) | <0.001 | 107.91 (73.14, 141.97) | 47.10 (31.50, 65.26) | -2.80 (-2.90, -2.70) | <0.001 | 1.22 (0.76, 1.67) | 0.33 (0.22, 0.49) | -4.34 (-4.60, -4.08) | <0.001 |
| Western Sub-Saharan Africa | 111.67 (54.50, 172.00) | 119.56 (58.54, 184.47) | 0.24 (0.22, 0.25) | <0.001 | 1097.12 (687.40, 1654.57) | 1176.85 (744.09, 1771.15) | 0.24 (0.23, 0.26) | <0.001 | 142.39 (100.00, 192.42) | 84.84 (55.26, 128.71) | -1.78 (-1.87, -1.69) | <0.001 | 1.26 (0.83, 1.72) | 0.40 (0.27, 0.60) | -3.88 (-4.00, -3.75) | <0.001 |
| Southeast Asia | 35.09 (19.33, 53.28) | 36.69 (18.70, 56.43) | 0.15 (0.11, 0.19) | <0.001 | 342.65 (220.40, 503.84) | 380.87 (239.52, 560.11) | 0.37 (0.33, 0.40) | <0.001 | 89.42 (70.84, 109.19) | 63.45 (51.02, 78.83) | -1.17 (-1.34, -1.00) | <0.001 | 1.02 (0.79, 1.25) | 0.63 (0.51, 0.74) | -1.61 (-1.78, -1.43) | <0.001 |
| Eastern Sub-Saharan Africa | 189.24 (93.08, 284.34) | 204.18 (99.66, 312.62) | 0.27 (0.22, 0.31) | <0.001 | 1704.41 (1121.23, 2542.57) | 1896.76 (1237.19, 2807.80) | 0.37 (0.29, 0.46) | <0.001 | 191.85 (137.85, 261.74) | 132.97 (87.57, 198.30) | -1.24 (-1.39, -1.09) | <0.001 | 1.55 (1.10, 2.08) | 0.59 (0.44, 0.81) | -3.24 (-3.41, -3.08) | <0.001 |
| Central Latin America | 47.31 (24.65, 71.22) | 49.90 (26.05, 75.70) | 0.19 (0.14, 0.23) | <0.001 | 477.07 (310.07, 686.98) | 496.18 (321.14, 725.85) | 0.14 (0.10, 0.17) | <0.001 | 55.82 (44.88, 71.81) | 29.73 (18.48, 46.26) | -2.11 (-2.23, -1.99) | <0.001 | 0.46 (0.42, 0.51) | 0.08 (0.07, 0.10) | -5.66 (-5.94, -5.38) | <0.001 |
| Tropical Latin America | 123.70 (62.25, 189.33) | 126.17 (63.04, 194.85) | 0.07 (0.05, 0.08) | <0.001 | 1285.61 (839.78, 1854.15) | 1328.49 (865.73, 1896.22) | 0.11 (0.08, 0.14) | <0.001 | 127.32 (98.48, 171.36) | 78.28 (48.38, 122.96) | -1.67 (-1.83, -1.51) | <0.001 | 0.93 (0.85, 1.01) | 0.21 (0.19, 0.23) | -4.99 (-5.41, -4.56) | <0.001 |
| Central Sub-Saharan Africa | 172.23 (83.67, 280.90) | 180.55 (85.26, 293.32) | 0.16 (0.14, 0.19) | <0.001 | 1778.50 (1120.26, 2685.70) | 1838.25 (1171.84, 2726.72) | 0.11 (0.09, 0.14) | <0.001 | 172.79 (105.13, 253.39) | 120.34 (72.24, 181.86) | -1.25 (-1.41, -1.09) | <0.001 | 1.24 (0.59, 2.09) | 0.46 (0.24, 0.85) | -3.40 (-3.60, -3.19) | <0.001 |
| Western Europe | 0.29 (0.10, 0.56) | 0.21 (0.05, 0.43) | -1.23 (-1.38, -1.09) | <0.001 | 7.93 (5.86, 10.54) | 6.36 (4.58, 8.51) | -0.80 (-0.91, -0.68) | <0.001 | 6.67 (6.02, 7.46) | 2.56 (2.19, 2.96) | -3.35 (-3.94, -2.75) | <0.001 | 0.09 (0.08, 0.10) | 0.03 (0.03, 0.04) | -3.60 (-4.21, -3.00) | <0.001 |
| High-income North America | 1.11 (0.46, 1.95) | 0.85 (0.45, 1.26) | -0.92 (-0.97, -0.88) | <0.001 | 17.09 (11.71, 24.09) | 13.00 (10.60, 15.85) | -0.93 (-0.97, -0.89) | <0.001 | 6.48 (5.81, 7.21) | 2.51 (2.16, 2.94) | -3.11 (-3.79, -2.43) | <0.001 | 0.08 (0.07, 0.08) | 0.03 (0.02, 0.03) | -3.72 (-4.92, -2.51) | <0.001 |
| Central Asia | 72.68 (37.13, | 77.99 (39.30, | 0.26 (0.24, 0.27) | <0.001 | 770.27 (493.16, | 835.50 (530.38, | 0.28 (0.26, 0.30) | <0.001 | 211.43 (187.85, | 102.98 (79.79, 135.94) | -2.33 (-2.60, | <0.001 | 2.45 (2.23, | 0.88 (0.70, | -3.28 (-3.67, | <0.001 |

| | | | | | | | | | | | | | | | | |
|---|---|---|---|---|---|---|---|---|---|---|---|---|---|---|---|---|
| | 113.25) | 121.77) | | | 1131.42) | 1228.62) | | | 239.62) | | -2.06) | | 2.69) | 1.09) | -2.89) | |
| Eastern Europe | 1.74 (1.04, 2.52) | 1.68 (1.01, 2.45) | -0.13 (-0.19, -0.07) | <0.001 | 24.56 (18.78, 31.37) | 23.75 (18.17, 30.31) | -0.12 (-0.15, -0.08) | <0.001 | 25.42 (23.16, 27.46) | 6.92 (5.77, 8.19) | -4.36 (-5.99, -2.70) | <0.001 | 0.34 (0.31, 0.36) | 0.08 (0.07, 0.10) | -4.81 (-6.53, -3.06) | <0.001 |
| Southern Sub-Saharan Africa | 164.83 (77.33, 256.77) | 167.26 (81.25, 259.21) | 0.05 (0.01, 0.08) | 0.015 | 1647.98 (1051.58, 2454.18) | 1671.64 (1067.41, 2480.96) | 0.06 (0.01, 0.11) | 0.015 | 200.60 (147.11, 266.11) | 156.86 (103.38, 226.73) | -0.89 (-1.30, -0.47) | <0.001 | 1.71 (1.24, 2.17) | 1.08 (0.64, 1.67) | -1.39 (-2.37, -0.41) | 0.005 |
| High-income Asia Pacific | 0.35 (0.13, 0.66) | 0.19 (0.05, 0.38) | -2.09 (-2.15, -2.03) | <0.001 | 10.04 (7.38, 13.33) | 7.75 (5.94, 9.97) | -0.90 (-0.93, -0.87) | <0.001 | 7.90 (6.29, 9.86) | 2.06 (1.66, 2.52) | -4.54 (-5.45, -3.64) | <0.001 | 0.10 (0.08, 0.13) | 0.02 (0.02, 0.03) | -5.14 (-6.23, -4.05) | <0.001 |
| Central Europe | 3.33 (1.89, 4.90) | 2.97 (1.62, 4.50) | -0.39 (-0.58, -0.20) | <0.001 | 39.40 (27.29, 54.95) | 37.10 (25.83, 51.25) | -0.18 (-0.36, -0.00) | 0.048 | 19.89 (17.87, 22.22) | 5.10 (4.08, 6.51) | -4.60 (-5.17, -4.03) | <0.001 | 0.25 (0.23, 0.28) | 0.05 (0.04, 0.06) | -5.68 (-6.46, -4.90) | <0.001 |
| Andean Latin America | 103.86 (52.23, 159.04) | 105.86 (52.50, 165.26) | 0.06 (0.04, 0.08) | <0.001 | 1073.13 (688.20, 1571.99) | 1101.14 (702.64, 1633.72) | 0.09 (0.08, 0.10) | <0.001 | 101.83 (71.82, 141.86) | 66.42 (40.51, 106.35) | -1.48 (-1.69, -1.27) | <0.001 | 0.71 (0.50, 1.00) | 0.19 (0.13, 0.28) | -4.54 (-5.20, -3.88) | <0.001 |
| Caribbean | 100.25 (51.92, 152.58) | 109.61 (53.71, 167.69) | 0.31 (0.27, 0.35) | <0.001 | 1027.68 (668.98, 1485.66) | 1094.35 (703.12, 1620.25) | 0.21 (0.20, 0.23) | <0.001 | 226.87 (160.89, 309.13) | 155.99 (94.59, 249.42) | -1.11 (-1.36, -0.86) | <0.001 | 2.49 (1.67, 3.58) | 1.45 (0.73, 2.64) | -1.62 (-1.98, -1.26) | <0.001 |
| Southern Latin America | 48.41 (24.06, 75.24) | 53.17 (26.21, 83.91) | 0.34 (0.28, 0.40) | <0.001 | 575.11 (367.79, 841.61) | 632.95 (406.53, 909.99) | 0.33 (0.27, 0.38) | <0.001 | 51.94 (37.53, 72.71) | 36.95 (22.12, 58.56) | -1.16 (-1.33, -0.98) | <0.001 | 0.34 (0.28, 0.41) | 0.09 (0.07, 0.11) | -4.36 (-4.78, -3.93) | <0.001 |
| Oceania | 46.91 (21.27, 81.06) | 50.45 (21.86, 88.96) | 0.26 (0.23, 0.29) | <0.001 | 770.04 (510.85, 1140.57) | 841.46 (565.52, 1226.28) | 0.31 (0.24, 0.39) | <0.001 | 424.10 (259.64, 652.56) | 426.40 (272.75, 654.84) | 0.17 (0.06, 0.29) | 0.003 | 5.42 (3.10, 8.65) | 5.40 (3.22, 8.64) | 0.15 (0.03, 0.28) | 0.014 |
| Australasia | 0.92 (0.31, 1.76) | 0.78 (0.23, 1.58) | -0.52 (-0.64, -0.40) | <0.001 | 23.43 (15.13, 33.44) | 22.91 (15.05, 32.67) | -0.06 (-0.12, 0.01) | 0.082 | 13.58 (11.27, 16.29) | 5.29 (3.93, 6.98) | -3.16 (-4.70, -1.60) | <0.001 | 0.17 (0.14, 0.21) | 0.06 (0.05, 0.07) | -3.56 (-5.47, -1.62) | <0.001 |
| **Men** | | | | | | | | | | | | | | | | |
| Global | 54.81 (28.30, 83.84) | 69.11 (35.55, 105.10) | 0.82 (0.69, 0.94) | <0.001 | 523.41 (334.77, 777.60) | 639.62 (407.29, 963.19) | 0.71 (0.65, 0.76) | <0.001 | 135.24 (112.90, 160.39) | 96.50 (77.55, 121.15) | -1.23 (-2.03, -0.42) | 0.003 | 1.54 (1.29, 1.82) | 0.92 (0.77, 1.09) | -1.85 (-2.99, -0.70) | 0.002 |
| **SDI category** | | | | | | | | | | | | | | | | |

| | | | | | | | | | | | | | | | | |
|---|---|---|---|---|---|---|---|---|---|---|---|---|---|---|---|---|
| High | 0.56 (0.30, 0.95) | 0.64 (0.41, 0.92) | 0.50 (0.06, 0.95) | 0.027 | 12.23 (9.28, 15.92) | 12.60 (10.12, 15.44) | 0.08 (-0.00, 0.17) | 0.055 | 8.61 (7.37, 10.22) | 2.86 (2.43, 3.37) | -3.72 (-3.92, -3.52) | <0.001 | 0.11 (0.10, 0.13) | 0.03 (0.03, 0.04) | -4.30 (-4.52, -4.08) | <0.001 |
| High-middle | 23.27 (11.50, 36.21) | 25.18 (13.00, 39.08) | 0.29 (0.23, 0.34) | <0.001 | 259.33 (164.99, 380.67) | 264.07 (167.93, 389.64) | 0.10 (0.02, 0.18) | 0.014 | 61.32 (50.67, 72.86) | 29.02 (21.14, 38.79) | -2.64 (-2.83, -2.44) | <0.001 | 0.68 (0.58, 0.80) | 0.23 (0.17, 0.30) | -3.71 (-4.77, -2.63) | <0.001 |
| Middle | 56.45 (27.04, 88.38) | 56.52 (28.58, 86.95) | 0.03 (-0.04, 0.11) | 0.398 | 595.75 (380.21, 881.64) | 559.22 (349.50, 836.81) | -0.20 (-0.29, -0.12) | <0.001 | 116.93 (95.77, 142.31) | 66.79 (51.40, 87.59) | -2.04 (-2.54, -1.55) | <0.001 | 1.23 (1.04, 1.50) | 0.56 (0.46, 0.67) | -2.78 (-3.52, -2.03) | <0.001 |
| Low-middle | 80.26 (44.11, 119.35) | 87.99 (47.99, 132.95) | 0.35 (0.16, 0.55) | <0.001 | 666.95 (420.19, 1007.14) | 747.13 (465.08, 1129.42) | 0.41 (0.35, 0.46) | <0.001 | 237.12 (193.11, 289.33) | 143.60 (113.56, 182.34) | -1.88 (-3.17, -0.58) | 0.005 | 2.87 (2.31, 3.57) | 1.51 (1.17, 1.93) | -2.33 (-3.67, -0.97) | 0.001 |
| Low | 117.01 (60.46, 175.34) | 124.49 (61.01, 189.93) | 0.23 (0.20, 0.26) | <0.001 | 1058.95 (685.09, 1591.19) | 1166.88 (754.79, 1754.59) | 0.34 (0.26, 0.42) | <0.001 | 264.53 (204.35, 338.07) | 166.30 (127.64, 213.22) | -1.64 (-2.31, -0.96) | <0.001 | 2.99 (2.22, 3.84) | 1.54 (1.18, 1.95) | -2.35 (-3.17, -1.52) | <0.001 |
| **21 GBD region** | | | | | | | | | | | | | | | | |
| South Asia | 79.43 (46.47, 116.26) | 83.02 (48.04, 123.07) | 0.21 (-0.07, 0.50) | 0.144 | 562.95 (352.25, 854.65) | 609.40 (378.35, 936.78) | 0.32 (0.16, 0.47) | <0.001 | 312.74 (254.07, 379.08) | 178.91 (141.41, 219.71) | -2.06 (-3.88, -0.21) | 0.029 | 4.00 (3.23, 4.88) | 2.09 (1.64, 2.59) | -2.23 (-3.40, -1.04) | <0.001 |
| East Asia | 48.35 (21.42, 77.92) | 39.84 (18.76, 64.07) | -0.65 (-0.79, -0.51) | <0.001 | 580.82 (366.56, 859.29) | 451.29 (281.54, 674.45) | -0.88 (-0.96, -0.80) | <0.001 | 95.32 (72.56, 122.98) | 32.91 (21.62, 49.25) | -3.63 (-3.79, -3.46) | <0.001 | 0.94 (0.71, 1.22) | 0.15 (0.11, 0.20) | -6.15 (-6.47, -5.82) | <0.001 |
| North Africa and Middle East | 42.21 (21.94, 63.73) | 47.76 (24.78, 73.28) | 0.43 (0.41, 0.44) | <0.001 | 404.60 (261.64, 598.82) | 447.04 (275.95, 680.66) | 0.35 (0.29, 0.41) | <0.001 | 75.98 (49.64, 126.82) | 41.84 (26.46, 64.13) | -2.02 (-2.17, -1.88) | <0.001 | 0.79 (0.46, 1.46) | 0.28 (0.16, 0.50) | -3.41 (-3.67, -3.15) | <0.001 |
| Western Sub-Saharan Africa | 104.80 (50.96, 162.11) | 108.87 (52.47, 169.45) | 0.13 (0.11, 0.15) | <0.001 | 1070.71 (671.93, 1621.68) | 1123.89 (702.22, 1698.40) | 0.17 (0.14, 0.20) | <0.001 | 125.51 (85.42, 176.69) | 84.59 (54.05, 129.26) | -1.35 (-1.43, -1.26) | <0.001 | 1.04 (0.64, 1.56) | 0.43 (0.28, 0.66) | -3.00 (-3.17, -2.83) | <0.001 |
| Southeast Asia | 24.35 (12.29, 37.70) | 26.12 (12.90, 40.66) | 0.24 (0.19, 0.29) | <0.001 | 248.92 (158.14, 370.24) | 279.63 (175.87, 414.66) | 0.40 (0.37, 0.43) | <0.001 | 67.77 (52.01, 89.22) | 54.23 (40.66, 68.01) | -0.73 (-0.90, -0.57) | <0.001 | 0.78 (0.58, 1.06) | 0.57 (0.41, 0.73) | -1.05 (-1.26, -0.85) | <0.001 |
| Eastern Sub-Saharan Africa | 162.22 (77.24, | 173.06 (82.52, | 0.24 (0.18, 0.29) | <0.001 | 1503.70 (985.95, | 1685.02 (1089.66, | 0.41 (0.33, 0.49) | <0.001 | 265.26 (197.48, | 169.76 (121.72, | -1.52 (-1.63, | <0.001 | 2.71 (1.93, | 1.24 (0.94, | -2.63 (-2.78, | <0.001 |

| | | | | | | | | | | | | | | | | |
|---|---|---|---|---|---|---|---|---|---|---|---|---|---|---|---|---|
| | 246.30) | 266.35) | | | 2265.10) | 2500.37) | | | 354.96) | 231.61) | -1.41) | | 3.75) | 1.64) | -2.48) | |
| Central Latin America | 38.55 (19.43, 58.43) | 40.37 (20.60, 61.97) | 0.16 (0.14, 0.19) | <0.001 | 384.90 (247.28, 571.85) | 403.27 (255.00, 605.46) | 0.16 (0.14, 0.19) | <0.001 | 48.70 (39.45, 62.79) | 26.02 (16.78, 40.85) | -2.11 (-2.27, -1.96) | <0.001 | 0.42 (0.38, 0.47) | 0.09 (0.07, 0.11) | -4.97 (-5.28, -4.66) | <0.001 |
| Tropical Latin America | 106.64 (53.04, 164.09) | 104.92 (52.08, 160.53) | -0.06 (-0.07, -0.05) | <0.001 | 1059.70 (668.81, 1576.15) | 1054.91 (673.14, 1556.94) | -0.02 (-0.06, 0.02) | 0.318 | 134.29 (108.64, 173.83) | 71.25 (46.63, 108.73) | -2.13 (-2.27, -2.00) | <0.001 | 1.17 (1.06, 1.31) | 0.29 (0.24, 0.32) | -4.74 (-5.14, -4.34) | <0.001 |
| Central Sub-Saharan Africa | 156.45 (71.38, 246.88) | 158.90 (74.74, 262.80) | 0.06 (0.04, 0.08) | <0.001 | 1786.25 (1099.04, 2608.90) | 1798.49 (1124.73, 2660.26) | 0.02 (0.01, 0.03) | 0.001 | 233.48 (149.22, 331.07) | 164.53 (101.16, 266.98) | -1.23 (-1.31, -1.16) | <0.001 | 2.08 (1.12, 3.40) | 1.10 (0.53, 2.37) | -2.20 (-2.33, -2.07) | <0.001 |
| Western Europe | 0.30 (0.12, 0.57) | 0.21 (0.06, 0.44) | -1.28 (-1.54, -1.02) | <0.001 | 7.15 (5.18, 9.70) | 5.65 (4.00, 7.77) | -0.85 (-0.95, -0.74) | <0.001 | 7.93 (7.11, 8.76) | 2.43 (1.96, 2.80) | -4.15 (-4.83, -3.46) | <0.001 | 0.11 (0.09, 0.12) | 0.03 (0.02, 0.03) | -4.39 (-5.13, -3.65) | <0.001 |
| High-income North America | 0.70 (0.24, 1.32) | 0.41 (0.19, 0.68) | -1.76 (-1.82, -1.70) | <0.001 | 15.47 (11.08, 21.31) | 11.50 (9.64, 13.66) | -0.99 (-1.08, -0.90) | <0.001 | 6.26 (5.56, 7.03) | 2.40 (2.06, 2.84) | -3.10 (-3.64, -2.55) | <0.001 | 0.08 (0.07, 0.08) | 0.02 (0.02, 0.03) | -3.63 (-4.24, -3.02) | <0.001 |
| Central Asia | 67.10 (34.32, 103.27) | 73.48 (38.04, 115.08) | 0.32 (0.30, 0.34) | <0.001 | 674.23 (426.18, 1000.83) | 735.70 (457.47, 1093.16) | 0.30 (0.29, 0.32) | <0.001 | 181.35 (159.50, 209.16) | 99.57 (76.36, 132.14) | -2.00 (-2.37, -1.64) | <0.001 | 2.08 (1.89, 2.28) | 0.90 (0.72, 1.13) | -2.81 (-3.37, -2.25) | <0.001 |
| Eastern Europe | 2.00 (1.22, 2.87) | 1.65 (1.01, 2.39) | -0.61 (-0.71, -0.52) | <0.001 | 25.24 (19.28, 32.22) | 22.01 (16.73, 28.12) | -0.46 (-0.52, -0.41) | <0.001 | 35.43 (30.37, 38.86) | 8.45 (6.70, 10.30) | -4.51 (-5.31, -3.70) | <0.001 | 0.48 (0.41, 0.53) | 0.10 (0.08, 0.13) | -4.82 (-5.66, -3.97) | <0.001 |
| Southern Sub-Saharan Africa | 142.16 (68.14, 221.64) | 145.75 (70.24, 224.93) | 0.08 (0.07, 0.09) | <0.001 | 1455.89 (923.69, 2189.66) | 1498.61 (952.09, 2239.47) | 0.10 (0.09, 0.11) | <0.001 | 210.70 (150.17, 285.34) | 207.85 (137.38, 299.49) | -0.00 (-0.36, 0.36) | 0.987 | 1.97 (1.35, 2.78) | 1.90 (1.10, 3.03) | -0.06 (-0.56, 0.44) | 0.805 |
| High-income Asia Pacific | 0.36 (0.14, 0.68) | 0.20 (0.05, 0.40) | -2.13 (-2.26, -2.00) | <0.001 | 10.83 (7.99, 14.26) | 8.51 (6.45, 10.93) | -0.84 (-0.92, -0.77) | <0.001 | 7.53 (5.58, 9.21) | 1.96 (1.62, 2.29) | -4.58 (-4.78, -4.37) | <0.001 | 0.10 (0.07, 0.12) | 0.02 (0.02, 0.02) | -5.15 (-5.51, -4.78) | <0.001 |
| Central Europe | 3.44 (2.07, 5.10) | 3.13 (1.71, 4.84) | -0.28 (-0.49, -0.07) | 0.009 | 37.87 (25.88, 53.83) | 35.02 (23.90, 50.39) | -0.21 (-0.42, -0.00) | 0.047 | 30.18 (27.03, 33.35) | 6.75 (5.45, 8.28) | -4.95 (-5.72, -4.17) | <0.001 | 0.40 (0.36, 0.44) | 0.07 (0.06, 0.09) | -5.71 (-6.40, -5.02) | <0.001 |
| Andean Latin America | 91.03 (47.21, | 89.56 (44.88, | -0.06 (-0.08, -0.04) | <0.001 | 923.49 (582.32, | 923.99 (576.28, | -0.01 (-0.06, | 0.791 | 88.34 (61.18, 124.76) | 59.66 (37.56, 93.38) | -1.36 (-1.48, | <0.001 | 0.61 (0.43, | 0.21 (0.14, | -3.64 (-3.87, | <0.001 |

| | | | | | | | | | | | | | | | | |
|---|---|---|---|---|---|---|---|---|---|---|---|---|---|---|---|---|
| | 141.89) | 139.79) | | | 1380.31) | 1361.45) | 0.04) | | | | -1.25) | | 0.86) | 0.30) | -3.41) | |
| Caribbean | 85.31 (42.53, 132.32) | 91.61 (46.56, 143.05) | 0.24 (0.22, 0.26) | <0.001 | 878.32 (546.99, 1315.64) | 912.39 (574.18, 1365.91) | 0.13 (0.11, 0.15) | <0.001 | 140.61 (107.34, 186.28) | 96.38 (62.68, 147.68) | -1.21 (-1.48, -0.94) | <0.001 | 1.38 (1.03, 1.91) | 0.73 (0.39, 1.33) | -1.99 (-2.41, -1.56) | <0.001 |
| Southern Latin America | 48.69 (24.53, 75.17) | 53.06 (26.21, 82.14) | 0.29 (0.21, 0.38) | <0.001 | 518.50 (328.75, 757.34) | 567.74 (359.30, 854.47) | 0.31 (0.25, 0.38) | <0.001 | 50.72 (37.68, 70.32) | 33.98 (20.22, 54.42) | -1.38 (-1.53, -1.23) | <0.001 | 0.36 (0.31, 0.43) | 0.09 (0.07, 0.11) | -4.66 (-5.17, -4.14) | <0.001 |
| Oceania | 39.18 (17.45, 69.95) | 41.88 (18.67, 75.93) | 0.23 (0.22, 0.25) | <0.001 | 647.07 (423.64, 931.55) | 710.07 (460.98, 1035.63) | 0.32 (0.29, 0.34) | <0.001 | 504.46 (309.08, 825.82) | 506.45 (289.70, 821.74) | -0.02 (-0.20, 0.15) | 0.798 | 6.63 (3.91, 11.14) | 6.62 (3.60, 11.30) | -0.04 (-0.23, 0.14) | 0.640 |
| Australasia | 0.54 (0.16, 1.13) | 0.42 (0.09, 0.91) | -0.80 (-0.98, -0.62) | <0.001 | 18.50 (12.51, 26.10) | 17.32 (11.89, 24.77) | -0.21 (-0.26, -0.17) | <0.001 | 19.16 (15.84, 23.43) | 6.29 (4.93, 7.98) | -3.62 (-4.73, -2.50) | <0.001 | 0.26 (0.21, 0.31) | 0.08 (0.06, 0.10) | -3.95 (-5.10, -2.79) | <0.001 |
| **20-24 years** | | | | | | | | | | | | | | | | |
| **Women** | | | | | | | | | | | | | | | | |
| Global | 56.18 (30.36, 84.80) | 70.06 (37.80, 106.32) | 0.76 (0.67, 0.85) | <0.001 | 726.10 (524.75, 967.23) | 902.05 (645.91, 1212.37) | 0.76 (0.71, 0.81) | <0.001 | 181.28 (150.24, 213.94) | 113.27 (91.85, 141.89) | -1.61 (-1.86, -1.36) | <0.001 | 2.20 (1.82, 2.63) | 1.05 (0.90, 1.26) | -2.53 (-2.87, -2.20) | <0.001 |
| **SDI category** | | | | | | | | | | | | | | | | |
| High | 0.89 (0.44, 1.55) | 0.91 (0.58, 1.34) | 0.03 (-0.18, 0.24) | 0.777 | 16.50 (12.58, 21.37) | 16.93 (14.02, 20.15) | 0.13 (-0.24, 0.51) | 0.484 | 12.19 (10.60, 14.16) | 4.60 (3.88, 5.60) | -3.28 (-3.48, -3.08) | <0.001 | 0.17 (0.15, 0.20) | 0.06 (0.05, 0.07) | -3.81 (-4.12, -3.49) | <0.001 |
| High-middle | 20.59 (10.81, 32.66) | 23.54 (12.64, 36.88) | 0.47 (0.41, 0.52) | <0.001 | 340.27 (245.76, 455.12) | 371.41 (263.61, 502.04) | 0.32 (0.24, 0.40) | <0.001 | 74.09 (61.96, 88.89) | 31.23 (22.76, 42.91) | -2.95 (-3.23, -2.68) | <0.001 | 0.87 (0.73, 1.03) | 0.20 (0.16, 0.26) | -4.90 (-5.35, -4.44) | <0.001 |
| Middle | 51.87 (26.70, 80.81) | 55.23 (29.37, 84.64) | 0.23 (0.07, 0.40) | 0.004 | 784.98 (556.77, 1059.61) | 791.54 (560.01, 1070.47) | 0.04 (-0.01, 0.09) | 0.125 | 170.77 (142.58, 202.82) | 80.39 (61.72, 103.48) | -2.58 (-2.96, -2.19) | <0.001 | 2.00 (1.69, 2.34) | 0.64 (0.50, 0.80) | -3.92 (-4.44, -3.38) | <0.001 |
| Low-middle | 107.79 (60.90, 157.78) | 108.89 (61.24, 160.46) | 0.04 (-0.03, 0.11) | 0.297 | 1122.08 (810.47, 1483.03) | 1216.22 (865.78, 1629.87) | 0.29 (0.24, 0.34) | <0.001 | 340.82 (266.25, 422.18) | 188.59 (150.04, 234.34) | -2.09 (-2.62, -1.54) | <0.001 | 4.32 (3.22, 5.47) | 1.96 (1.56, 2.43) | -2.74 (-3.34, -2.13) | <0.001 |
| Low | 111.74 (59.04, | 113.65 (58.82, | 0.06 (0.04, 0.09) | <0.001 | 1445.74 (1031.26, | 1551.46 (1105.49, | 0.26 (0.20, 0.33) | <0.001 | 332.84 (248.28, | 183.25 (138.91, | -2.03 (-2.31, | <0.001 | 3.97 (2.84, | 1.65 (1.22, | -2.99 (-3.31, | <0.001 |

| | | | | | | | | | | | | | | | | |
|---|---|---|---|---|---|---|---|---|---|---|---|---|---|---|---|---|
| | 169.84) | 175.28) | | | 1955.36) | 2102.79) | | | 425.70) | 238.21) | -1.75) | | 5.26) | 2.18) | -2.68) | |
| **21 GBD region** | | | | | | | | | | | | | | | | |
| South Asia | 125.19 (74.92, 179.37) | 118.55 (68.99, 172.38) | -0.18 (-0.28, -0.10) | <0.001 | 1113.97 (783.67, 1462.33) | 1145.61 (808.45, 1523.36) | 0.10 (0.04, 0.17) | 0.001 | 444.97 (345.75, 553.25) | 229.83 (184.29, 281.98) | -2.34 (-2.94, -1.73) | <0.001 | 5.89 (4.46, 7.46) | 2.63 (2.12, 3.28) | -2.85 (-3.55, -2.15) | <0.001 |
| East Asia | 36.54 (17.00, 61.48) | 31.80 (15.36, 51.93) | -0.50 (-0.67, -0.33) | <0.001 | 657.39 (467.62, 884.70) | 536.59 (378.22, 731.31) | -0.69 (-0.75, -0.64) | <0.001 | 138.24 (107.50, 177.13) | 37.61 (25.71, 54.38) | -4.50 (-4.85, -4.14) | <0.001 | 1.60 (1.21, 2.12) | 0.17 (0.13, 0.23) | -7.35 (-7.92, -6.78) | <0.001 |
| North Africa and Middle East | 40.04 (20.48, 63.02) | 44.03 (21.84, 69.30) | 0.33 (0.30, 0.36) | <0.001 | 532.34 (379.44, 720.16) | 582.08 (405.00, 797.72) | 0.30 (0.26, 0.34) | <0.001 | 134.73 (87.02, 171.48) | 57.39 (40.42, 78.94) | -2.88 (-3.03, -2.73) | <0.001 | 1.64 (0.97, 2.17) | 0.44 (0.29, 0.64) | -4.40 (-4.56, -4.23) | <0.001 |
| Western Sub-Saharan Africa | 87.29 (43.00, 138.64) | 91.64 (45.72, 145.62) | 0.17 (0.15, 0.19) | <0.001 | 1265.79 (883.31, 1737.38) | 1353.06 (944.35, 1855.22) | 0.23 (0.22, 0.24) | <0.001 | 186.52 (133.58, 245.52) | 107.97 (74.79, 153.45) | -1.86 (-1.94, -1.79) | <0.001 | 1.90 (1.23, 2.65) | 0.65 (0.44, 0.97) | -3.63 (-3.73, -3.53) | <0.001 |
| Southeast Asia | 30.94 (15.54, 49.32) | 30.39 (15.49, 47.88) | -0.07 (-0.14, -0.01) | 0.029 | 412.68 (295.71, 555.98) | 446.18 (316.73, 606.14) | 0.26 (0.22, 0.30) | <0.001 | 100.75 (77.89, 123.08) | 68.80 (52.68, 85.02) | -1.30 (-1.46, -1.14) | <0.001 | 1.22 (0.91, 1.53) | 0.71 (0.53, 0.89) | -1.82 (-2.08, -1.57) | <0.001 |
| Eastern Sub-Saharan Africa | 124.70 (63.47, 202.10) | 128.97 (64.46, 203.17) | 0.11 (0.09, 0.13) | <0.001 | 1941.74 (1372.83, 2659.60) | 2126.22 (1500.30, 2909.64) | 0.32 (0.28, 0.35) | <0.001 | 251.47 (177.28, 333.23) | 157.09 (107.95, 225.20) | -1.60 (-1.70, -1.49) | <0.001 | 2.40 (1.48, 3.34) | 0.84 (0.61, 1.13) | -3.55 (-3.66, -3.43) | <0.001 |
| Central Latin America | 35.07 (19.03, 53.27) | 38.35 (20.45, 58.99) | 0.32 (0.30, 0.34) | <0.001 | 552.83 (395.71, 743.00) | 594.64 (427.55, 802.28) | 0.26 (0.23, 0.29) | <0.001 | 76.65 (64.75, 92.77) | 36.90 (24.34, 54.54) | -2.42 (-2.73, -2.11) | <0.001 | 0.75 (0.70, 0.82) | 0.13 (0.10, 0.16) | -5.86 (-6.43, -5.29) | <0.001 |
| Tropical Latin America | 89.58 (47.52, 140.59) | 90.03 (47.13, 141.34) | 0.02 (-0.04, 0.09) | 0.437 | 1502.32 (1072.82, 2014.06) | 1546.77 (1094.75, 2095.85) | 0.10 (0.08, 0.11) | <0.001 | 155.43 (123.48, 198.49) | 92.47 (60.57, 138.50) | -1.77 (-2.01, -1.54) | <0.001 | 1.26 (1.16, 1.39) | 0.28 (0.25, 0.32) | -4.98 (-5.43, -4.54) | <0.001 |
| Central Sub-Saharan Africa | 116.50 (56.93, 190.60) | 119.67 (56.33, 194.20) | 0.11 (0.07, 0.14) | <0.001 | 1961.09 (1356.15, 2732.37) | 2021.30 (1411.78, 2755.46) | 0.11 (0.09, 0.13) | <0.001 | 206.52 (122.57, 311.89) | 134.12 (84.01, 198.12) | -1.49 (-1.57, -1.40) | <0.001 | 1.72 (0.74, 3.01) | 0.58 (0.29, 1.07) | -3.68 (-3.79, -3.58) | <0.001 |
| Western Europe | 0.31 (0.11, 0.68) | 0.20 (0.05, 0.47) | -1.50 (-1.59, -1.40) | <0.001 | 8.63 (6.50, 11.28) | 6.87 (5.08, 9.13) | -0.82 (-0.92, -0.72) | <0.001 | 9.13 (8.36, 10.01) | 3.55 (3.12, 4.04) | -3.23 (-3.76, -2.70) | <0.001 | 0.13 (0.12, 0.14) | 0.05 (0.04, 0.05) | -3.43 (-4.02, -2.85) | <0.001 |
| High-income North | 1.50 (0.67, | 1.14 (0.64, | -0.95 (-1.01, | <0.001 | 23.12 (16.14, | 17.78 (14.54, | -0.88 | <0.001 | 10.51 (9.62, | 4.02 (3.53, | -3.27 | <0.001 | 0.14 | 0.04 | -3.87 | <0.001 |

| | | | | | | | | | | | | | | | | |
|---|---|---|---|---|---|---|---|---|---|---|---|---|---|---|---|---|
| America | 2.64) | 1.74) | -0.88) | | 31.40) | 21.24) | (-0.93, -0.84) | | 11.50) | 4.60) | (-4.05, -2.48) | | (0.13, 0.15) | (0.04, 0.05) | (-4.90, -2.83) | |
| Central Asia | 56.85 (29.91, 89.25) | 60.80 (31.79, 94.49) | 0.24 (0.18, 0.30) | <0.001 | 904.60 (645.17, 1213.57) | 973.18 (694.57, 1327.13) | 0.25 (0.23, 0.27) | <0.001 | 305.40 (275.96, 339.07) | 130.16 (99.85, 164.55) | -3.01 (-3.53, -2.49) | <0.001 | 3.94 (3.62, 4.28) | 1.26 (0.99, 1.58) | -4.01 (-4.66, -3.36) | <0.001 |
| Eastern Europe | 1.99 (1.20, 3.06) | 1.63 (0.97, 2.49) | -0.64 (-0.72, -0.56) | <0.001 | 29.57 (23.44, 36.50) | 26.90 (21.11, 32.89) | -0.34 (-0.41, -0.27) | <0.001 | 34.98 (31.72, 38.27) | 8.60 (6.92, 10.62) | -4.56 (-5.24, -3.88) | <0.001 | 0.50 (0.46, 0.55) | 0.11 (0.09, 0.14) | -4.85 (-5.63, -4.07) | <0.001 |
| Southern Sub-Saharan Africa | 117.01 (57.70, 186.74) | 118.94 (60.18, 187.63) | 0.05 (0.04, 0.06) | <0.001 | 1861.93 (1299.31, 2542.48) | 1887.83 (1320.85, 2575.70) | 0.05 (0.04, 0.06) | <0.001 | 264.07 (202.18, 336.53) | 159.83 (108.97, 221.70) | -1.67 (-2.35, -0.99) | <0.001 | 2.64 (1.88, 3.43) | 1.05 (0.66, 1.63) | -3.10 (-4.35, -1.83) | <0.001 |
| High-income Asia Pacific | 0.35 (0.11, 0.81) | 0.18 (0.05, 0.42) | -2.37 (-2.46, -2.27) | <0.001 | 11.50 (8.63, 15.15) | 8.54 (6.54, 10.88) | -1.04 (-1.08, -0.99) | <0.001 | 7.65 (6.10, 9.37) | 2.25 (1.86, 2.72) | -4.22 (-4.63, -3.81) | <0.001 | 0.11 (0.08, 0.13) | 0.03 (0.02, 0.03) | -4.74 (-5.21, -4.26) | <0.001 |
| Central Europe | 2.93 (1.72, 4.36) | 2.49 (1.39, 3.92) | -0.46 (-0.85, -0.07) | 0.020 | 47.41 (35.76, 61.26) | 45.40 (33.60, 58.77) | -0.13 (-0.33, 0.07) | 0.213 | 33.98 (30.48, 37.90) | 7.22 (5.84, 9.00) | -5.21 (-5.66, -4.76) | <0.001 | 0.48 (0.43, 0.53) | 0.08 (0.06, 0.09) | -6.30 (-6.98, -5.61) | <0.001 |
| Andean Latin America | 82.09 (42.47, 127.94) | 82.48 (43.17, 125.52) | 0.01 (0.01, 0.02) | <0.001 | 1258.03 (903.07, 1702.04) | 1292.96 (919.67, 1748.00) | 0.10 (0.09, 0.11) | <0.001 | 121.24 (88.64, 163.08) | 75.94 (47.77, 114.41) | -1.61 (-1.89, -1.32) | <0.001 | 0.92 (0.66, 1.24) | 0.21 (0.14, 0.31) | -4.93 (-5.61, -4.26) | <0.001 |
| Caribbean | 79.51 (41.06, 124.56) | 87.05 (43.30, 137.15) | 0.32 (0.28, 0.35) | <0.001 | 1221.85 (867.00, 1644.16) | 1295.34 (919.24, 1787.10) | 0.20 (0.18, 0.22) | <0.001 | 265.03 (182.16, 376.61) | 180.22 (109.08, 284.06) | -1.23 (-1.45, -1.00) | <0.001 | 3.11 (1.95, 4.72) | 1.78 (0.89, 3.26) | -1.84 (-2.19, -1.50) | <0.001 |
| Southern Latin America | 36.33 (18.89, 58.04) | 40.34 (21.04, 62.91) | 0.36 (0.32, 0.39) | <0.001 | 646.73 (458.07, 870.99) | 723.10 (520.17, 966.81) | 0.38 (0.26, 0.49) | <0.001 | 64.94 (49.15, 85.94) | 42.69 (26.38, 65.30) | -1.44 (-1.66, -1.22) | <0.001 | 0.51 (0.43, 0.62) | 0.12 (0.09, 0.15) | -4.80 (-5.35, -4.26) | <0.001 |
| Oceania | 43.62 (18.53, 77.32) | 46.88 (19.50, 83.76) | 0.25 (0.20, 0.31) | <0.001 | 772.94 (539.45, 1062.59) | 842.09 (588.86, 1149.29) | 0.31 (0.24, 0.39) | <0.001 | 532.96 (325.69, 832.51) | 486.30 (290.84, 761.35) | -0.38 (-0.59, -0.16) | 0.001 | 7.46 (4.35, 11.95) | 6.70 (3.82, 10.76) | -0.43 (-0.66, -0.20) | <0.001 |
| Australasia | 1.04 (0.33, 2.21) | 0.85 (0.27, 1.94) | -0.64 (-0.77, -0.52) | <0.001 | 26.40 (18.05, 35.79) | 25.47 (17.75, 34.89) | -0.09 (-0.15, -0.03) | 0.003 | 21.32 (17.49, 25.89) | 8.27 (6.42, 10.56) | -3.12 (-4.18, -2.05) | <0.001 | 0.30 (0.24, 0.36) | 0.10 (0.08, 0.14) | -3.47 (-4.60, -2.31) | <0.001 |
| **Men** | | | | | | | | | | | | | | | | |

| | | | | | | | | | | | | | | | | |
|---|---|---|---|---|---|---|---|---|---|---|---|---|---|---|---|---|
| Global | 44.22 (22.75, 69.13) | 54.20 (27.84, 84.50) | 0.70 (0.56, 0.84) | <0.001 | 594.84 (421.26, 807.35) | 720.27 (509.85, 979.94) | 0.68 (0.61, 0.75) | <0.001 | 161.26 (134.51, 191.39) | 114.66 (93.57, 140.62) | -1.18 (-1.49, -0.88) | <0.001 | 1.99 (1.66, 2.35) | 1.20 (0.99, 1.44) | -1.73 (-2.21, -1.25) | <0.001 |
| **SDI category** | | | | | | | | | | | | | | | | |
| High | 0.68 (0.34, 1.23) | 0.62 (0.38, 0.97) | -0.35 (-1.05, 0.36) | 0.336 | 14.66 (11.52, 18.54) | 14.00 (11.58, 16.78) | -0.15 (-0.72, 0.42) | 0.604 | 11.25 (10.02, 12.84) | 4.28 (3.71, 4.97) | -3.31 (-3.60, -3.02) | <0.001 | 0.16 (0.14, 0.18) | 0.05 (0.05, 0.06) | -3.72 (-3.88, -3.55) | <0.001 |
| High-middle | 18.91 (9.57, 29.98) | 20.23 (10.50, 31.97) | 0.24 (0.17, 0.30) | <0.001 | 294.27 (210.17, 402.09) | 296.43 (211.22, 403.50) | 0.04 (-0.04, 0.12) | 0.286 | 72.90 (61.55, 85.98) | 33.83 (25.04, 45.43) | -2.60 (-2.96, -2.25) | <0.001 | 0.88 (0.74, 1.04) | 0.29 (0.22, 0.40) | -3.68 (-4.23, -3.12) | <0.001 |
| Middle | 45.88 (22.81, 73.82) | 47.31 (24.12, 73.37) | 0.11 (0.06, 0.17) | <0.001 | 684.79 (482.92, 928.59) | 661.70 (464.17, 902.14) | -0.11 (-0.15, -0.07) | <0.001 | 140.11 (115.69, 168.88) | 81.35 (62.87, 104.82) | -1.87 (-2.27, -1.46) | <0.001 | 1.61 (1.31, 1.93) | 0.74 (0.59, 0.92) | -2.66 (-3.24, -2.09) | <0.001 |
| Low-middle | 73.67 (39.08, 112.02) | 77.39 (40.73, 119.01) | 0.20 (-0.01, 0.40) | 0.061 | 837.04 (585.52, 1122.26) | 920.67 (646.12, 1249.16) | 0.36 (0.26, 0.46) | <0.001 | 324.96 (261.99, 401.46) | 192.15 (149.50, 239.15) | -1.81 (-2.32, -1.29) | <0.001 | 4.28 (3.38, 5.39) | 2.22 (1.70, 2.86) | -2.24 (-2.97, -1.50) | <0.001 |
| Low | 92.73 (47.59, 145.06) | 94.28 (47.23, 148.60) | 0.08 (0.02, 0.15) | 0.010 | 1237.32 (873.44, 1691.11) | 1335.14 (946.26, 1826.64) | 0.28 (0.24, 0.31) | <0.001 | 291.68 (219.52, 382.73) | 194.48 (151.68, 251.82) | -1.38 (-1.70, -1.07) | <0.001 | 3.49 (2.45, 4.70) | 1.96 (1.47, 2.59) | -1.97 (-2.38, -1.55) | <0.001 |
| **21 GBD region** | | | | | | | | | | | | | | | | |
| South Asia | 79.90 (44.26, 119.22) | 79.40 (43.31, 119.94) | 0.02 (-0.34, 0.39) | 0.898 | 787.05 (540.19, 1050.47) | 826.43 (565.08, 1121.31) | 0.21 (0.00, 0.43) | 0.046 | 447.52 (366.28, 543.30) | 258.36 (207.58, 317.21) | -1.84 (-2.50, -1.17) | <0.001 | 6.15 (4.95, 7.56) | 3.28 (2.61, 4.09) | -2.13 (-2.86, -1.40) | <0.001 |
| East Asia | 35.34 (16.26, 60.26) | 30.00 (13.96, 50.54) | -0.56 (-0.73, -0.38) | <0.001 | 605.97 (425.30, 833.29) | 472.90 (329.39, 651.57) | -0.86 (-0.89, -0.82) | <0.001 | 104.29 (79.43, 132.34) | 37.44 (26.04, 52.57) | -3.55 (-3.80, -3.30) | <0.001 | 1.13 (0.83, 1.46) | 0.22 (0.16, 0.29) | -5.62 (-6.04, -5.19) | <0.001 |
| North Africa and Middle East | 37.43 (18.82, 58.83) | 40.89 (20.40, 64.12) | 0.31 (0.25, 0.36) | <0.001 | 480.30 (339.34, 650.11) | 516.69 (356.02, 704.70) | 0.24 (0.19, 0.30) | <0.001 | 87.74 (56.45, 140.48) | 46.55 (30.46, 68.31) | -2.14 (-2.40, -1.88) | <0.001 | 0.97 (0.53, 1.71) | 0.32 (0.16, 0.55) | -3.68 (-3.94, -3.42) | <0.001 |
| Western Sub-Saharan Africa | 81.73 (39.45, 131.45) | 84.38 (41.22, 136.43) | 0.11 (0.10, 0.12) | <0.001 | 1215.41 (848.21, 1672.17) | 1268.01 (889.98, 1746.40) | 0.15 (0.14, 0.16) | <0.001 | 125.13 (86.73, 173.62) | 92.31 (61.05, 134.11) | -1.04 (-1.14, -0.94) | <0.001 | 1.01 (0.61, 1.53) | 0.47 (0.27, 0.74) | -2.58 (-2.82, -2.33) | <0.001 |
| Southeast Asia | 21.89 | 21.94 | 0.01 (-0.11, | 0.824 | 294.08 | 316.52 | 0.24 (0.18, | <0.001 | 86.84 (66.12, | 57.74 (42.22, | -1.36 | <0.001 | 1.09 | 0.64 | -1.78 | <0.001 |

| | | | | | | | | | | | | | | | | |
|---|---|---|---|---|---|---|---|---|---|---|---|---|---|---|---|---|
| | (10.75, 35.33) | (10.80, 35.45) | 0.14) | | (205.90, 396.27) | (225.42, 435.93) | 0.31) | | 110.33) | 71.66) | (-1.57, -1.15) | | (0.80, 1.44) | (0.44, 0.80) | (-2.04, -1.53) | |
| Eastern Sub-Saharan Africa | 111.63 (55.96, 179.36) | 110.97 (53.15, 178.85) | -0.02 (-0.04, 0.01) | 0.162 | 1701.06 (1205.25, 2331.62) | 1850.78 (1297.67, 2540.15) | 0.30 (0.24, 0.35) | <0.001 | 169.61 (119.95, 236.96) | 131.95 (91.35, 188.35) | -0.84 (-0.97, -0.71) | <0.001 | 1.33 (0.89, 1.99) | 0.65 (0.45, 0.93) | -2.41 (-2.59, -2.23) | <0.001 |
| Central Latin America | 29.64 (15.26, 47.50) | 32.58 (16.62, 51.58) | 0.33 (0.29, 0.37) | <0.001 | 430.27 (304.21, 584.64) | 465.17 (330.28, 635.00) | 0.27 (0.25, 0.29) | <0.001 | 53.75 (43.81, 67.78) | 29.72 (19.62, 44.05) | -2.02 (-2.13, -1.90) | <0.001 | 0.50 (0.45, 0.55) | 0.11 (0.09, 0.13) | -5.00 (-5.38, -4.62) | <0.001 |
| Tropical Latin America | 82.56 (41.89, 129.43) | 81.87 (41.43, 127.69) | -0.03 (-0.04, -0.01) | 0.002 | 1221.00 (852.92, 1684.72) | 1213.51 (856.41, 1647.86) | -0.02 (-0.03, -0.02) | <0.001 | 125.10 (97.25, 163.34) | 75.85 (49.53, 114.21) | -1.72 (-1.93, -1.51) | <0.001 | 1.00 (0.90, 1.12) | 0.26 (0.23, 0.29) | -4.39 (-4.78, -4.01) | <0.001 |
| Central Sub-Saharan Africa | 109.07 (53.23, 177.85) | 110.82 (51.27, 183.77) | 0.05 (0.02, 0.09) | 0.003 | 1907.68 (1347.55, 2598.85) | 1919.27 (1332.95, 2640.80) | 0.02 (-0.00, 0.05) | 0.057 | 163.27 (104.30, 243.09) | 133.94 (83.20, 210.98) | -0.69 (-0.77, -0.61) | <0.001 | 1.09 (0.53, 1.99) | 0.63 (0.25, 1.51) | -1.90 (-2.02, -1.78) | <0.001 |
| Western Europe | 0.35 (0.12, 0.72) | 0.22 (0.06, 0.49) | -1.58 (-1.71, -1.46) | <0.001 | 7.73 (5.76, 10.37) | 6.05 (4.32, 8.27) | -0.88 (-0.98, -0.79) | <0.001 | 11.82 (10.78, 13.07) | 3.82 (3.30, 4.39) | -3.94 (-4.51, -3.35) | <0.001 | 0.17 (0.16, 0.19) | 0.05 (0.04, 0.06) | -4.12 (-4.71, -3.52) | <0.001 |
| High-income North America | 0.89 (0.34, 1.82) | 0.54 (0.27, 0.92) | -1.66 (-1.78, -1.55) | <0.001 | 18.92 (13.89, 25.00) | 13.68 (11.45, 16.19) | -1.10 (-1.13, -1.07) | <0.001 | 9.13 (8.22, 10.11) | 3.82 (3.35, 4.46) | -2.98 (-3.89, -2.06) | <0.001 | 0.12 (0.11, 0.13) | 0.05 (0.04, 0.05) | -3.34 (-4.53, -2.13) | <0.001 |
| Central Asia | 55.10 (28.57, 86.98) | 59.89 (30.09, 93.10) | 0.28 (0.26, 0.31) | <0.001 | 784.03 (550.74, 1065.80) | 860.78 (604.73, 1169.15) | 0.32 (0.30, 0.34) | <0.001 | 217.65 (192.54, 246.55) | 110.23 (83.41, 142.79) | -2.35 (-2.86, -1.83) | <0.001 | 2.71 (2.46, 2.99) | 1.03 (0.81, 1.31) | -3.29 (-4.09, -2.48) | <0.001 |
| Eastern Europe | 2.50 (1.61, 3.67) | 1.75 (1.07, 2.67) | -1.17 (-1.35, -0.99) | <0.001 | 30.51 (23.85, 37.42) | 24.78 (19.47, 30.40) | -0.71 (-0.84, -0.58) | <0.001 | 54.42 (48.27, 60.56) | 13.01 (10.48, 16.46) | -4.56 (-5.47, -3.64) | <0.001 | 0.80 (0.70, 0.89) | 0.18 (0.14, 0.23) | -4.78 (-5.75, -3.81) | <0.001 |
| Southern Sub-Saharan Africa | 103.42 (51.63, 164.69) | 104.75 (50.66, 167.84) | 0.05 (0.03, 0.06) | <0.001 | 1630.52 (1144.99, 2244.92) | 1669.39 (1174.42, 2306.60) | 0.08 (0.07, 0.09) | <0.001 | 159.19 (112.06, 219.73) | 155.95 (105.44, 222.32) | -0.04 (-0.26, 0.17) | 0.710 | 1.22 (0.82, 1.75) | 1.14 (0.67, 1.86) | -0.17 (-0.56, 0.23) | 0.407 |
| High-income Asia Pacific | 0.35 (0.11, 0.77) | 0.18 (0.05, 0.44) | -2.28 (-2.43, -2.13) | <0.001 | 11.88 (9.04, 15.42) | 9.09 (6.98, 11.64) | -0.91 (-1.05, -0.77) | <0.001 | 8.31 (7.07, 9.54) | 2.68 (2.23, 3.12) | -3.76 (-4.14, -3.38) | <0.001 | 0.12 (0.10, 0.13) | 0.03 (0.03, 0.04) | -4.18 (-4.60, -3.75) | <0.001 |
| Central Europe | 3.36 (2.04, | 2.79 (1.60, | -0.60 (-0.86, | <0.001 | 45.04 (34.09, | 42.74 (31.62, | -0.05 | 0.819 | 47.02 (42.43, | 9.62 (7.99, | -5.56 | <0.001 | 0.67 | 0.11 | -6.25 | <0.001 |

| | | | | | | | | | | | | | | | | |
|---|---|---|---|---|---|---|---|---|---|---|---|---|---|---|---|---|
| | 4.88) | 4.16) | -0.34) | | 58.57) | 55.83) | (-0.49, 0.39) | | 51.39) | 11.48) | (-6.19, -4.93) | | (0.61, 0.74) | (0.09, 0.13) | (-6.96, -5.52) | |
| Andean Latin America | 72.56 (36.23, 114.68) | 72.33 (36.49, 114.37) | -0.02 (-0.05, 0.01) | 0.166 | 1066.63 (741.25, 1467.71) | 1067.00 (753.10, 1452.37) | -0.01 (-0.04, 0.02) | 0.502 | 90.42 (63.30, 126.97) | 65.06 (41.04, 99.90) | -1.14 (-1.19, -1.10) | <0.001 | 0.59 (0.41, 0.85) | 0.21 (0.14, 0.31) | -3.47 (-3.82, -3.12) | <0.001 |
| Caribbean | 69.07 (34.18, 109.20) | 74.29 (37.66, 115.58) | 0.25 (0.21, 0.29) | <0.001 | 1025.52 (717.79, 1404.16) | 1061.87 (744.24, 1447.15) | 0.12 (0.10, 0.14) | <0.001 | 142.66 (108.24, 184.16) | 103.49 (67.85, 157.63) | -1.04 (-1.31, -0.78) | <0.001 | 1.40 (1.05, 1.92) | 0.79 (0.42, 1.49) | -1.87 (-2.38, -1.36) | <0.001 |
| Southern Latin America | 38.00 (19.07, 60.32) | 41.77 (21.33, 67.04) | 0.32 (0.23, 0.41) | <0.001 | 579.00 (405.36, 786.82) | 638.05 (451.65, 869.50) | 0.33 (0.22, 0.43) | <0.001 | 64.38 (49.56, 83.07) | 39.31 (25.05, 60.14) | -1.67 (-1.94, -1.41) | <0.001 | 0.55 (0.46, 0.66) | 0.13 (0.10, 0.16) | -4.82 (-5.60, -4.04) | <0.001 |
| Oceania | 36.96 (15.48, 66.88) | 38.99 (17.02, 69.03) | 0.19 (0.17, 0.20) | <0.001 | 644.83 (436.76, 882.36) | 701.05 (472.22, 980.80) | 0.29 (0.23, 0.34) | <0.001 | 659.65 (362.98, 1143.24) | 605.26 (320.88, 1007.81) | -0.35 (-0.66, -0.03) | 0.034 | 9.44 (5.07, 16.69) | 8.59 (4.34, 14.70) | -0.38 (-0.72, -0.04) | 0.027 |
| Australasia | 0.50 (0.14, 1.17) | 0.39 (0.09, 0.95) | -0.80 (-0.92, -0.67) | <0.001 | 19.07 (13.22, 25.93) | 17.89 (12.37, 24.64) | -0.20 (-0.30, -0.11) | <0.001 | 27.39 (22.47, 33.35) | 9.96 (7.70, 12.68) | -3.39 (-3.98, -2.79) | <0.001 | 0.40 (0.32, 0.48) | 0.14 (0.10, 0.18) | -3.59 (-4.22, -2.96) | <0.001 |
| **25-29 years** | | | | | | | | | | | | | | | | |
| **Women** | | | | | | | | | | | | | | | | |
| Global | 43.51 (26.87, 62.35) | 51.53 (31.59, 73.86) | 0.60 (0.53, 0.67) | <0.001 | 756.04 (548.79, 1000.05) | 912.03 (650.31, 1201.72) | 0.65 (0.61, 0.69) | <0.001 | 198.76 (164.50, 232.86) | 117.65 (94.35, 147.87) | -1.75 (-2.23, -1.28) | <0.001 | 2.64 (2.16, 3.12) | 1.21 (0.98, 1.47) | -2.63 (-3.20, -2.06) | <0.001 |
| **SDI category** | | | | | | | | | | | | | | | | |
| High | 1.23 (0.60, 2.07) | 1.07 (0.66, 1.61) | -0.51 (-0.65, -0.37) | <0.001 | 21.06 (16.16, 27.16) | 19.83 (16.58, 23.49) | -0.20 (-0.48, 0.08) | 0.166 | 17.44 (15.78, 19.34) | 6.74 (5.83, 7.86) | -3.23 (-3.73, -2.72) | <0.001 | 0.26 (0.24, 0.29) | 0.09 (0.08, 0.11) | -3.58 (-4.00, -3.16) | <0.001 |
| High-middle | 17.54 (11.04, 25.85) | 18.31 (11.31, 26.77) | 0.15 (0.10, 0.21) | <0.001 | 358.40 (259.93, 474.81) | 379.01 (272.31, 501.24) | 0.18 (0.10, 0.26) | <0.001 | 92.33 (78.61, 107.27) | 33.96 (25.46, 45.18) | -3.57 (-4.29, -2.84) | <0.001 | 1.22 (1.05, 1.43) | 0.26 (0.21, 0.32) | -5.14 (-6.03, -4.24) | <0.001 |
| Middle | 43.04 (25.87, 63.42) | 44.12 (26.61, 64.62) | 0.10 (0.01, 0.19) | 0.035 | 832.81 (593.18, 1099.58) | 853.20 (609.91, 1125.66) | 0.08 (0.05, 0.12) | <0.001 | 211.08 (179.69, 246.93) | 86.84 (66.60, 112.08) | -3.21 (-3.88, -2.54) | <0.001 | 2.78 (2.37, 3.25) | 0.75 (0.60, 0.93) | -4.36 (-5.42, -3.30) | <0.001 |
| Low-middle | 85.05 | 83.53 | -0.03 (-0.21, | 0.784 | 1289.27 | 1343.54 | 0.16 (0.10, | <0.001 | 366.25 | 216.01 | -1.79 | <0.001 | 4.95 | 2.47 | -2.34 | <0.001 |

| | | | | | | | | | | | | | | | | |
|---|---|---|---|---|---|---|---|---|---|---|---|---|---|---|---|---|
| | (53.01, 119.87) | (51.48, 118.03) | 0.16) | | (923.69, 1708.52) | (951.72, 1780.80) | 0.22) | | (277.38, 453.32) | (165.79, 274.08) | (-2.19, -1.40) | | (3.58, 6.32) | (1.82, 3.24) | (-2.85, -1.83) | |
| Low | 90.75 (54.80, 129.79) | 91.76 (54.88, 133.58) | 0.04 (0.01, 0.07) | 0.015 | 1539.50 (1096.13, 2045.10) | 1624.32 (1156.20, 2136.22) | 0.19 (0.16, 0.22) | <0.001 | 357.33 (260.06, 464.79) | 203.53 (152.09, 265.08) | -1.94 (-2.12, -1.76) | <0.001 | 4.62 (3.07, 6.27) | 2.06 (1.47, 2.78) | -2.73 (-2.94, -2.53) | <0.001 |
| **21 GBD region** | | | | | | | | | | | | | | | | |
| South Asia | 96.07 (61.02, 134.09) | 90.08 (56.19, 126.72) | -0.23 (-0.32, -0.14) | <0.001 | 1340.25 (944.72, 1763.34) | 1334.81 (940.13, 1753.69) | -0.02 (-0.13, 0.09) | 0.698 | 458.20 (342.74, 570.16) | 270.77 (210.26, 346.60) | -1.81 (-2.42, -1.20) | <0.001 | 6.40 (4.62, 8.19) | 3.37 (2.55, 4.36) | -2.22 (-2.92, -1.52) | <0.001 |
| East Asia | 30.69 (17.24, 47.33) | 26.72 (15.59, 40.22) | -0.49 (-0.57, -0.42) | <0.001 | 651.56 (460.67, 866.56) | 560.57 (395.43, 742.89) | -0.52 (-0.54, -0.50) | <0.001 | 196.28 (153.37, 245.33) | 42.20 (29.68, 58.43) | -5.43 (-6.37, -4.48) | <0.001 | 2.68 (2.04, 3.44) | 0.24 (0.17, 0.33) | -8.21 (-9.37, -7.04) | <0.001 |
| North Africa and Middle East | 33.50 (20.28, 48.64) | 35.73 (21.47, 51.73) | 0.22 (0.20, 0.24) | <0.001 | 587.74 (415.63, 781.30) | 628.87 (440.20, 848.11) | 0.23 (0.19, 0.28) | <0.001 | 157.30 (109.47, 196.94) | 68.00 (49.62, 89.45) | -2.81 (-2.95, -2.67) | <0.001 | 2.10 (1.37, 2.69) | 0.62 (0.42, 0.86) | -4.10 (-4.27, -3.94) | <0.001 |
| Western Sub-Saharan Africa | 73.26 (43.37, 108.10) | 76.11 (45.32, 113.18) | 0.13 (0.13, 0.14) | <0.001 | 1322.44 (931.23, 1778.54) | 1408.94 (983.94, 1883.96) | 0.22 (0.21, 0.23) | <0.001 | 155.38 (109.68, 207.51) | 97.94 (65.99, 139.62) | -1.58 (-1.62, -1.53) | <0.001 | 1.51 (0.92, 2.17) | 0.51 (0.34, 0.75) | -3.67 (-3.74, -3.60) | <0.001 |
| Southeast Asia | 26.14 (15.64, 37.93) | 25.05 (15.18, 36.92) | -0.16 (-0.20, -0.11) | <0.001 | 447.29 (321.92, 595.72) | 465.83 (333.02, 622.60) | 0.14 (0.11, 0.17) | <0.001 | 118.09 (91.68, 141.83) | 76.39 (60.18, 95.06) | -1.46 (-1.74, -1.17) | <0.001 | 1.57 (1.18, 1.92) | 0.88 (0.67, 1.10) | -1.90 (-2.31, -1.50) | <0.001 |
| Eastern Sub-Saharan Africa | 102.62 (59.81, 152.39) | 105.13 (61.07, 158.19) | 0.08 (0.06, 0.09) | <0.001 | 1974.76 (1406.27, 2641.89) | 2130.36 (1528.41, 2823.49) | 0.27 (0.21, 0.32) | <0.001 | 229.88 (151.00, 311.34) | 144.70 (98.81, 206.70) | -1.57 (-1.65, -1.50) | <0.001 | 2.23 (1.13, 3.35) | 0.71 (0.52, 0.97) | -3.81 (-4.00, -3.63) | <0.001 |
| Central Latin America | 27.96 (17.36, 40.59) | 29.89 (17.94, 43.79) | 0.26 (0.23, 0.30) | <0.001 | 584.60 (429.19, 749.21) | 625.66 (455.78, 808.46) | 0.26 (0.22, 0.29) | <0.001 | 100.24 (87.05, 116.33) | 41.22 (28.28, 58.69) | -2.94 (-3.20, -2.67) | <0.001 | 1.18 (1.09, 1.27) | 0.19 (0.14, 0.24) | -5.90 (-6.37, -5.43) | <0.001 |
| Tropical Latin America | 71.62 (42.02, 106.63) | 71.88 (42.04, 108.00) | 0.02 (0.00, 0.04) | 0.052 | 1594.51 (1163.08, 2054.87) | 1637.85 (1181.94, 2113.51) | 0.10 (0.08, 0.11) | <0.001 | 175.86 (141.53, 218.21) | 102.23 (68.75, 146.18) | -1.82 (-2.14, -1.50) | <0.001 | 1.63 (1.49, 1.79) | 0.40 (0.36, 0.45) | -4.69 (-5.10, -4.29) | <0.001 |
| Central Sub-Saharan Africa | 96.15 (53.70, 146.92) | 97.98 (53.62, 146.66) | 0.07 (0.05, 0.10) | <0.001 | 1968.92 (1376.35, 2632.27) | 2019.91 (1440.71, 2675.68) | 0.09 (0.07, 0.11) | <0.001 | 218.07 (143.89, 318.39) | 135.66 (88.64, 195.14) | -1.63 (-1.72, -1.54) | <0.001 | 2.04 (1.06, 3.42) | 0.66 (0.35, 1.23) | -3.82 (-4.04, -3.60) | <0.001 |

| | | | | | | | | | | | | | | | | |
|---|---|---|---|---|---|---|---|---|---|---|---|---|---|---|---|---|
| Western Europe | 0.38 (0.12, 0.81) | 0.24 (0.07, 0.56) | -1.68 (-1.80, -1.55) | <0.001 | 9.39 (7.05, 12.52) | 7.30 (5.37, 9.94) | -0.89 (-1.00, -0.77) | <0.001 | 12.00 (10.99, 13.04) | 3.95 (3.51, 4.50) | -3.82 (-4.30, -3.33) | <0.001 | 0.19 (0.17, 0.20) | 0.06 (0.05, 0.07) | -4.06 (-4.70, -3.41) | <0.001 |
| High-income North America | 2.05 (0.91, 3.47) | 1.51 (0.87, 2.24) | -1.06 (-1.17, -0.94) | <0.001 | 31.28 (22.30, 42.35) | 24.12 (20.30, 28.58) | -0.88 (-0.92, -0.85) | <0.001 | 17.66 (16.16, 19.13) | 7.54 (6.61, 8.67) | -2.88 (-3.40, -2.36) | <0.001 | 0.26 (0.24, 0.27) | 0.10 (0.09, 0.11) | -3.28 (-4.05, -2.50) | <0.001 |
| Central Asia | 46.01 (28.15, 67.74) | 48.60 (28.91, 71.94) | 0.19 (0.16, 0.22) | <0.001 | 945.27 (678.09, 1239.30) | 1015.17 (727.43, 1330.79) | 0.25 (0.23, 0.27) | <0.001 | 351.38 (317.41, 384.86) | 149.30 (117.52, 190.82) | -2.85 (-3.62, -2.07) | <0.001 | 4.98 (4.51, 5.41) | 1.64 (1.28, 2.11) | -3.70 (-4.55, -2.85) | <0.001 |
| Eastern Europe | 2.72 (1.45, 4.23) | 1.99 (1.04, 3.15) | -1.03 (-1.11, -0.95) | <0.001 | 35.71 (27.95, 43.65) | 30.76 (24.36, 38.33) | -0.50 (-0.57, -0.44) | <0.001 | 44.04 (38.48, 48.31) | 9.78 (7.57, 12.46) | -4.97 (-6.15, -3.77) | <0.001 | 0.69 (0.60, 0.75) | 0.13 (0.10, 0.18) | -5.37 (-6.66, -4.06) | <0.001 |
| Southern Sub-Saharan Africa | 95.81 (55.41, 144.58) | 96.87 (56.28, 146.39) | 0.03 (0.02, 0.05) | <0.001 | 1896.92 (1361.26, 2548.87) | 1927.74 (1367.65, 2546.04) | 0.06 (0.04, 0.07) | <0.001 | 329.87 (248.66, 418.17) | 165.77 (114.05, 227.75) | -2.35 (-3.18, -1.50) | <0.001 | 3.91 (2.63, 5.12) | 1.22 (0.73, 1.90) | -3.90 (-5.29, -2.48) | <0.001 |
| High-income Asia Pacific | 0.41 (0.12, 0.88) | 0.20 (0.05, 0.48) | -2.50 (-2.67, -2.33) | <0.001 | 12.51 (9.33, 16.69) | 9.17 (6.94, 11.87) | -1.08 (-1.16, -1.01) | <0.001 | 12.67 (10.49, 15.16) | 3.33 (2.77, 3.98) | -4.54 (-5.02, -4.07) | <0.001 | 0.19 (0.16, 0.24) | 0.05 (0.04, 0.05) | -4.96 (-5.49, -4.42) | <0.001 |
| Central Europe | 2.94 (1.95, 4.17) | 2.03 (1.28, 3.03) | -1.32 (-1.61, -1.03) | <0.001 | 54.21 (41.39, 69.44) | 43.50 (33.08, 54.53) | -0.82 (-1.08, -0.57) | <0.001 | 48.07 (43.95, 52.77) | 9.47 (7.73, 11.43) | -5.33 (-5.86, -4.80) | <0.001 | 0.74 (0.67, 0.81) | 0.12 (0.09, 0.15) | -6.07 (-6.61, -5.52) | <0.001 |
| Andean Latin America | 66.43 (39.37, 98.89) | 66.25 (38.59, 98.15) | -0.01 (-0.02, -0.01) | <0.001 | 1345.60 (977.72, 1774.27) | 1384.00 (1000.07, 1792.60) | 0.09 (0.08, 0.11) | <0.001 | 131.64 (95.82, 174.62) | 81.83 (51.46, 121.02) | -1.68 (-1.85, -1.50) | <0.001 | 1.10 (0.79, 1.48) | 0.26 (0.16, 0.39) | -4.95 (-5.71, -4.17) | <0.001 |
| Caribbean | 63.53 (37.79, 93.29) | 69.90 (40.75, 103.68) | 0.34 (0.31, 0.36) | <0.001 | 1303.48 (929.94, 1707.86) | 1384.19 (995.71, 1801.74) | 0.21 (0.19, 0.23) | <0.001 | 332.11 (213.62, 469.98) | 219.57 (137.40, 343.34) | -1.37 (-1.55, -1.18) | <0.001 | 4.39 (2.55, 6.61) | 2.50 (1.29, 4.48) | -1.86 (-2.11, -1.60) | <0.001 |
| Southern Latin America | 30.30 (17.82, 45.45) | 32.56 (19.79, 48.17) | 0.25 (0.18, 0.32) | <0.001 | 686.16 (494.62, 887.97) | 762.94 (562.09, 979.23) | 0.36 (0.24, 0.48) | <0.001 | 85.37 (69.01, 108.02) | 47.11 (31.29, 68.65) | -2.08 (-2.49, -1.66) | <0.001 | 0.85 (0.72, 1.01) | 0.18 (0.14, 0.23) | -5.34 (-6.26, -4.41) | <0.001 |
| Oceania | 42.93 (25.16, 64.08) | 46.03 (26.61, 69.53) | 0.25 (0.23, 0.27) | <0.001 | 771.33 (545.80, 1049.77) | 839.41 (592.27, 1149.14) | 0.30 (0.23, 0.38) | <0.001 | 568.42 (313.09, 919.15) | 522.64 (272.67, 866.07) | -0.29 (-0.60, 0.01) | 0.061 | 8.63 (4.52, 14.36) | 7.83 (3.79, 13.52) | -0.36 (-0.64, -0.08) | 0.011 |

| | | | | | | | | | | | | | | | | |
|---|---|---|---|---|---|---|---|---|---|---|---|---|---|---|---|---|
| Australasia | 1.35 (0.44, 2.59) | 1.08 (0.32, 2.12) | -0.76 (-0.93, -0.58) | <0.001 | 29.71 (20.78, 40.40) | 28.24 (19.89, 38.57) | -0.15 (-0.25, -0.04) | 0.007 | 31.19 (25.63, 37.46) | 12.94 (10.17, 16.26) | -2.83 (-4.80, -0.82) | 0.006 | 0.48 (0.39, 0.58) | 0.19 (0.15, 0.24) | -3.04 (-5.14, -0.88) | 0.006 |
| **Men** | | | | | | | | | | | | | | | | |
| Global | 35.63 (21.77, 51.33) | 41.64 (25.10, 60.12) | 0.55 (0.41, 0.69) | <0.001 | 602.04 (427.79, 805.48) | 711.36 (501.74, 954.64) | 0.58 (0.53, 0.64) | <0.001 | 167.66 (139.98, 196.92) | 114.36 (93.74, 139.65) | -1.33 (-1.60, -1.06) | <0.001 | 2.25 (1.84, 2.65) | 1.30 (1.10, 1.52) | -1.86 (-2.30, -1.43) | <0.001 |
| **SDI category** | | | | | | | | | | | | | | | | |
| High | 0.91 (0.46, 1.58) | 0.82 (0.52, 1.24) | -0.45 (-0.79, -0.10) | 0.011 | 18.12 (14.22, 23.01) | 17.52 (14.55, 20.85) | -0.04 (-0.54, 0.46) | 0.871 | 14.84 (13.59, 16.41) | 6.21 (5.40, 7.18) | -2.97 (-3.24, -2.70) | <0.001 | 0.22 (0.21, 0.25) | 0.08 (0.07, 0.10) | -3.32 (-3.52, -3.12) | <0.001 |
| High-middle | 16.57 (10.06, 24.46) | 16.15 (9.86, 23.84) | -0.10 (-0.21, 0.01) | 0.090 | 302.57 (216.94, 407.86) | 295.16 (209.95, 397.78) | -0.08 (-0.12, -0.05) | <0.001 | 80.53 (69.47, 92.51) | 35.96 (27.50, 46.69) | -2.67 (-3.13, -2.21) | <0.001 | 1.07 (0.94, 1.23) | 0.35 (0.27, 0.46) | -3.66 (-4.29, -3.03) | <0.001 |
| Middle | 39.37 (23.22, 58.07) | 39.35 (23.64, 57.63) | -0.01 (-0.16, 0.14) | 0.920 | 711.51 (500.83, 950.38) | 702.44 (493.25, 943.61) | -0.04 (-0.10, 0.01) | 0.147 | 152.71 (129.64, 180.91) | 86.57 (68.39, 108.92) | -1.95 (-2.23, -1.66) | <0.001 | 1.92 (1.65, 2.25) | 0.86 (0.70, 1.05) | -2.79 (-3.29, -2.30) | <0.001 |
| Low-middle | 61.02 (37.69, 86.90) | 62.86 (38.22, 89.94) | 0.14 (-0.07, 0.36) | 0.199 | 925.25 (649.65, 1242.00) | 994.92 (697.72, 1338.15) | 0.29 (0.14, 0.43) | <0.001 | 361.57 (287.62, 452.25) | 214.71 (168.82, 264.29) | -1.73 (-2.20, -1.26) | <0.001 | 5.15 (3.94, 6.57) | 2.71 (2.08, 3.46) | -2.15 (-2.69, -1.60) | <0.001 |
| Low | 77.35 (46.62, 111.10) | 78.29 (46.03, 113.86) | 0.05 (0.01, 0.10) | 0.018 | 1305.34 (921.45, 1751.70) | 1376.70 (974.99, 1838.34) | 0.20 (0.16, 0.23) | <0.001 | 291.73 (213.60, 392.43) | 199.20 (154.42, 252.87) | -1.27 (-1.84, -0.68) | <0.001 | 3.73 (2.52, 5.25) | 2.16 (1.63, 2.77) | -1.82 (-2.38, -1.25) | <0.001 |
| **21 GBD region** | | | | | | | | | | | | | | | | |
| South Asia | 64.53 (40.21, 91.31) | 63.71 (39.19, 90.83) | -0.01 (-0.27, 0.25) | 0.915 | 911.87 (636.39, 1234.80) | 939.84 (654.49, 1274.34) | 0.15 (-0.03, 0.32) | 0.100 | 482.20 (382.99, 593.89) | 291.37 (237.85, 350.43) | -1.74 (-2.15, -1.34) | <0.001 | 7.11 (5.51, 8.90) | 4.00 (3.25, 4.86) | -1.96 (-2.39, -1.52) | <0.001 |
| East Asia | 30.57 (17.11, 46.57) | 25.55 (14.52, 38.61) | -0.61 (-0.71, -0.52) | <0.001 | 593.59 (416.84, 794.89) | 478.20 (339.50, 650.67) | -0.74 (-0.77, -0.71) | <0.001 | 102.49 (81.32, 127.43) | 37.91 (26.60, 52.50) | -3.45 (-3.71, -3.19) | <0.001 | 1.20 (0.93, 1.49) | 0.24 (0.18, 0.30) | -5.49 (-5.82, -5.16) | <0.001 |
| North Africa and Middle East | 31.35 (19.05, 45.17) | 33.08 (19.55, 47.86) | 0.19 (0.15, 0.24) | <0.001 | 515.87 (364.00, 697.88) | 541.36 (376.09, 733.32) | 0.16 (0.14, 0.19) | <0.001 | 77.35 (53.47, 111.96) | 45.24 (31.67, 63.47) | -1.81 (-2.00, -1.63) | <0.001 | 0.85 (0.52, 1.36) | 0.31 (0.19, 0.48) | -3.42 (-3.66, -3.18) | <0.001 |

| | | | | | | | | | | | | | | | | |
|---|---|---|---|---|---|---|---|---|---|---|---|---|---|---|---|---|
| Western Sub-Saharan Africa | 69.58 (40.92, 103.19) | 71.22 (41.70, 106.16) | 0.08 (0.06, 0.09) | <0.001 | 1257.59 (884.29, 1682.41) | 1305.91 (928.15, 1755.87) | 0.13 (0.12, 0.14) | <0.001 | 102.17 (67.53, 142.07) | 83.69 (54.04, 123.72) | -0.67 (-0.73, -0.62) | <0.001 | 0.69 (0.41, 1.04) | 0.34 (0.22, 0.51) | -2.36 (-2.58, -2.13) | <0.001 |
| Southeast Asia | 19.49 (12.01, 28.79) | 19.00 (11.47, 27.66) | -0.09 (-0.14, -0.05) | <0.001 | 306.30 (217.34, 419.40) | 321.17 (229.91, 438.61) | 0.15 (0.05, 0.25) | 0.002 | 129.61 (98.83, 166.81) | 66.06 (49.26, 81.25) | -2.26 (-2.48, -2.04) | <0.001 | 1.86 (1.36, 2.46) | 0.82 (0.60, 1.03) | -2.75 (-3.00, -2.49) | <0.001 |
| Eastern Sub-Saharan Africa | 93.30 (54.51, 136.98) | 92.39 (53.33, 139.00) | -0.03 (-0.04, -0.02) | <0.001 | 1731.77 (1233.65, 2315.11) | 1837.60 (1315.17, 2448.68) | 0.21 (0.18, 0.24) | <0.001 | 139.71 (96.98, 194.27) | 116.51 (78.70, 168.59) | -0.62 (-0.75, -0.49) | <0.001 | 0.93 (0.64, 1.28) | 0.47 (0.35, 0.63) | -2.32 (-2.53, -2.12) | <0.001 |
| Central Latin America | 24.29 (14.77, 36.29) | 26.24 (15.56, 38.82) | 0.28 (0.20, 0.35) | <0.001 | 443.24 (315.91, 590.19) | 474.58 (337.17, 629.92) | 0.24 (0.22, 0.27) | <0.001 | 62.75 (52.59, 76.36) | 31.54 (21.24, 44.71) | -2.33 (-2.55, -2.11) | <0.001 | 0.67 (0.61, 0.75) | 0.14 (0.12, 0.17) | -5.09 (-5.34, -4.84) | <0.001 |
| Tropical Latin America | 68.19 (40.66, 100.58) | 67.37 (39.73, 99.59) | -0.04 (-0.07, -0.00) | 0.041 | 1274.87 (897.70, 1695.53) | 1267.62 (908.09, 1671.78) | -0.02 (-0.03, -0.01) | <0.001 | 133.06 (104.45, 168.84) | 83.47 (56.12, 119.74) | -1.59 (-1.73, -1.45) | <0.001 | 1.17 (1.05, 1.31) | 0.37 (0.33, 0.41) | -3.87 (-4.23, -3.50) | <0.001 |
| Central Sub-Saharan Africa | 91.68 (51.07, 136.37) | 92.75 (50.90, 142.10) | 0.04 (0.02, 0.07) | 0.001 | 1886.45 (1378.06, 2543.25) | 1894.25 (1336.77, 2540.52) | 0.01 (-0.01, 0.04) | 0.263 | 127.62 (83.00, 187.77) | 116.15 (72.26, 182.16) | -0.34 (-0.37, -0.30) | <0.001 | 0.62 (0.33, 1.10) | 0.42 (0.16, 1.04) | -1.31 (-1.40, -1.22) | <0.001 |
| Western Europe | 0.44 (0.15, 0.89) | 0.28 (0.07, 0.63) | -1.68 (-1.79, -1.56) | <0.001 | 8.40 (6.12, 11.45) | 6.42 (4.59, 8.96) | -0.94 (-1.13, -0.76) | <0.001 | 14.17 (12.79, 15.61) | 4.86 (4.28, 5.45) | -3.74 (-4.21, -3.28) | <0.001 | 0.22 (0.20, 0.24) | 0.07 (0.06, 0.08) | -3.88 (-4.37, -3.40) | <0.001 |
| High-income North America | 1.22 (0.48, 2.34) | 0.79 (0.40, 1.29) | -1.47 (-1.52, -1.42) | <0.001 | 23.45 (17.23, 31.53) | 16.71 (14.17, 19.84) | -1.15 (-1.18, -1.12) | <0.001 | 13.19 (12.00, 14.53) | 6.18 (5.26, 7.16) | -2.53 (-3.14, -1.92) | <0.001 | 0.19 (0.18, 0.21) | 0.08 (0.07, 0.10) | -2.80 (-3.54, -2.06) | <0.001 |
| Central Asia | 46.07 (28.07, 67.79) | 49.22 (29.14, 71.80) | 0.23 (0.20, 0.26) | <0.001 | 817.80 (577.35, 1097.01) | 894.50 (632.45, 1194.06) | 0.31 (0.29, 0.33) | <0.001 | 273.08 (244.11, 304.88) | 128.88 (101.56, 163.28) | -2.50 (-3.36, -1.62) | <0.001 | 3.80 (3.44, 4.16) | 1.39 (1.10, 1.75) | -3.34 (-4.27, -2.40) | <0.001 |
| Eastern Europe | 3.66 (2.06, 5.44) | 2.29 (1.26, 3.52) | -1.56 (-1.87, -1.25) | <0.001 | 37.63 (29.51, 46.80) | 28.79 (22.69, 35.94) | -0.89 (-1.02, -0.76) | <0.001 | 78.12 (66.86, 89.31) | 18.88 (15.17, 23.33) | -4.70 (-6.16, -3.22) | <0.001 | 1.24 (1.05, 1.42) | 0.28 (0.23, 0.36) | -4.87 (-6.39, -3.33) | <0.001 |
| Southern Sub-Saharan Africa | 86.24 (50.77, 127.64) | 86.72 (50.44, 128.65) | 0.02 (0.01, 0.03) | 0.002 | 1656.58 (1186.80, 2209.49) | 1688.98 (1208.81, 2248.98) | 0.07 (0.06, 0.07) | <0.001 | 177.16 (127.66, 237.60) | 142.82 (100.14, 195.75) | -0.75 (-0.90, -0.61) | <0.001 | 1.60 (1.15, 2.24) | 1.01 (0.71, 1.44) | -1.61 (-2.07, -1.14) | <0.001 |

| | | | | | | | | | | | | | | | | |
|---|---|---|---|---|---|---|---|---|---|---|---|---|---|---|---|---|
| High-income Asia Pacific | 0.40 (0.12, 0.86) | 0.20 (0.05, 0.48) | -2.34 (-2.48, -2.21) | <0.001 | 12.83 (9.76, 16.74) | 9.58 (7.33, 12.42) | -1.04 (-1.18, -0.90) | <0.001 | 10.61 (9.11, 12.22) | 3.44 (2.82, 4.04) | -3.82 (-4.18, -3.46) | <0.001 | 0.16 (0.14, 0.19) | 0.05 (0.04, 0.05) | -4.15 (-4.68, -3.61) | <0.001 |
| Central Europe | 3.74 (2.49, 5.14) | 2.50 (1.59, 3.56) | -1.42 (-1.78, -1.07) | <0.001 | 51.79 (39.83, 65.09) | 41.98 (32.00, 53.11) | -0.79 (-1.12, -0.47) | <0.001 | 67.72 (62.48, 73.49) | 12.55 (10.75, 14.48) | -5.64 (-6.18, -5.09) | <0.001 | 1.06 (0.97, 1.15) | 0.17 (0.14, 0.20) | -6.09 (-6.66, -5.52) | <0.001 |
| Andean Latin America | 60.46 (33.66, 90.79) | 60.25 (35.92, 89.82) | -0.02 (-0.05, 0.00) | 0.079 | 1125.99 (786.75, 1530.02) | 1129.22 (809.79, 1520.97) | 0.00 (-0.02, 0.03) | 0.746 | 104.32 (74.64, 143.49) | 71.77 (47.05, 105.34) | -1.28 (-1.51, -1.06) | <0.001 | 0.82 (0.57, 1.20) | 0.29 (0.19, 0.42) | -3.50 (-3.98, -3.02) | <0.001 |
| Caribbean | 57.42 (33.75, 84.88) | 61.67 (36.10, 89.84) | 0.25 (0.22, 0.28) | <0.001 | 1082.10 (763.62, 1455.48) | 1123.99 (789.35, 1499.31) | 0.13 (0.12, 0.14) | <0.001 | 157.53 (118.67, 204.73) | 115.92 (76.52, 173.43) | -1.04 (-1.21, -0.86) | <0.001 | 1.72 (1.30, 2.28) | 1.01 (0.56, 1.83) | -1.80 (-2.10, -1.50) | <0.001 |
| Southern Latin America | 31.82 (18.27, 46.93) | 34.00 (19.87, 51.29) | 0.22 (0.14, 0.31) | <0.001 | 606.43 (428.00, 809.47) | 660.29 (464.07, 882.56) | 0.29 (0.22, 0.36) | <0.001 | 72.11 (57.15, 91.92) | 41.10 (26.21, 61.38) | -1.92 (-2.06, -1.78) | <0.001 | 0.70 (0.59, 0.84) | 0.15 (0.12, 0.20) | -5.07 (-5.55, -4.58) | <0.001 |
| Oceania | 38.13 (23.60, 55.64) | 40.24 (25.03, 58.98) | 0.19 (0.18, 0.20) | <0.001 | 642.96 (454.03, 890.83) | 694.80 (485.07, 970.19) | 0.28 (0.24, 0.31) | <0.001 | 503.09 (241.40, 963.84) | 448.43 (198.60, 862.91) | -0.44 (-0.54, -0.34) | <0.001 | 7.66 (3.52, 15.13) | 6.73 (2.80, 13.51) | -0.49 (-0.60, -0.39) | <0.001 |
| Australasia | 0.57 (0.14, 1.25) | 0.43 (0.08, 1.05) | -0.92 (-1.12, -0.71) | <0.001 | 18.34 (12.78, 25.54) | 17.32 (11.77, 23.98) | -0.16 (-0.29, -0.03) | 0.018 | 33.93 (27.94, 41.65) | 14.64 (11.26, 18.74) | -2.73 (-3.60, -1.84) | <0.001 | 0.53 (0.44, 0.65) | 0.22 (0.17, 0.28) | -2.84 (-3.74, -1.94) | <0.001 |
| **30-34 years** | | | | | | | | | | | | | | | | |
| **Women** | | | | | | | | | | | | | | | | |
| Global | 32.50 (22.42, 43.21) | 36.96 (25.01, 49.60) | 0.45 (0.38, 0.52) | <0.001 | 736.08 (556.22, 963.60) | 875.46 (652.45, 1150.49) | 0.61 (0.56, 0.66) | <0.001 | 231.45 (192.39, 269.27) | 128.57 (101.90, 158.66) | -2.01 (-2.40, -1.62) | <0.001 | 3.47 (2.85, 4.09) | 1.54 (1.16, 1.95) | -2.77 (-3.23, -2.30) | <0.001 |
| **SDI category** | | | | | | | | | | | | | | | | |
| High | 1.99 (1.26, 2.82) | 1.77 (1.26, 2.31) | -0.40 (-0.51, -0.29) | <0.001 | 27.35 (20.52, 35.75) | 27.95 (23.60, 33.28) | 0.06 (-0.04, 0.15) | 0.254 | 22.84 (20.94, 25.01) | 9.11 (7.85, 10.94) | -3.08 (-3.50, -2.66) | <0.001 | 0.37 (0.34, 0.41) | 0.13 (0.11, 0.16) | -3.50 (-3.95, -3.05) | <0.001 |
| High-middle | 15.22 (11.03, 20.16) | 15.16 (10.63, 20.41) | 0.00 (-0.09, 0.10) | 0.919 | 351.02 (267.72, 463.55) | 387.88 (289.09, 515.51) | 0.36 (0.30, 0.43) | <0.001 | 115.16 (98.70, 133.43) | 38.56 (30.18, 49.88) | -3.72 (-4.13, -3.31) | <0.001 | 1.74 (1.50, 2.03) | 0.35 (0.29, 0.43) | -5.38 (-6.30, -4.46) | <0.001 |

| | | | | | | | | | | | | | | | | |
|---|---|---|---|---|---|---|---|---|---|---|---|---|---|---|---|---|
| Middle | 34.27 (22.88, 46.72) | 33.95 (22.93, 46.46) | -0.03 (-0.14, 0.09) | 0.641 | 845.75 (631.11, 1110.99) | 865.25 (644.54, 1138.33) | 0.08 (0.00, 0.16) | 0.043 | 253.82 (217.48, 295.48) | 92.48 (73.72, 116.72) | -3.43 (-3.99, -2.86) | <0.001 | 3.77 (3.24, 4.40) | 0.90 (0.74, 1.09) | -4.77 (-5.82, -3.71) | <0.001 |
| Low-middle | 62.80 (42.77, 82.20) | 60.51 (40.64, 80.64) | -0.10 (-0.25, 0.05) | 0.182 | 1338.04 (990.27, 1733.75) | 1360.63 (1001.57, 1782.10) | 0.04 (-0.08, 0.17) | 0.514 | 446.23 (329.49, 553.71) | 256.68 (186.64, 332.68) | -1.87 (-2.22, -1.52) | <0.001 | 6.76 (4.74, 8.68) | 3.40 (2.27, 4.71) | -2.34 (-2.65, -2.02) | <0.001 |
| Low | 69.76 (47.28, 93.31) | 70.16 (46.48, 93.65) | 0.02 (0.01, 0.03) | <0.001 | 1526.26 (1124.75, 2006.45) | 1595.03 (1186.05, 2089.04) | 0.15 (0.14, 0.17) | <0.001 | 424.72 (302.86, 555.61) | 248.59 (184.44, 325.16) | -1.85 (-2.23, -1.47) | <0.001 | 6.23 (4.21, 8.53) | 3.06 (2.06, 4.25) | -2.45 (-2.92, -1.98) | <0.001 |
| **21 GBD region** | | | | | | | | | | | | | | | | |
| South Asia | 68.07 (47.41, 89.78) | 63.41 (43.10, 83.89) | -0.25 (-0.34, -0.16) | <0.001 | 1405.26 (1027.59, 1827.34) | 1379.70 (1002.42, 1810.73) | -0.07 (-0.21, 0.07) | 0.327 | 560.25 (411.48, 702.18) | 330.83 (244.53, 434.97) | -1.80 (-2.27, -1.33) | <0.001 | 8.72 (6.15, 11.21) | 4.69 (3.24, 6.42) | -2.14 (-2.69, -1.58) | <0.001 |
| East Asia | 25.74 (16.95, 35.46) | 22.65 (14.94, 31.12) | -0.42 (-0.55, -0.29) | <0.001 | 635.27 (469.76, 833.52) | 573.43 (421.58, 755.95) | -0.35 (-0.43, -0.28) | <0.001 | 253.23 (201.00, 319.85) | 48.09 (35.48, 64.49) | -5.61 (-6.43, -4.79) | <0.001 | 3.93 (3.05, 5.01) | 0.35 (0.26, 0.47) | -8.38 (-10.04, -6.68) | <0.001 |
| North Africa and Middle East | 27.41 (18.35, 36.62) | 28.25 (18.96, 37.79) | 0.09 (0.05, 0.14) | <0.001 | 615.21 (454.04, 813.86) | 649.60 (472.98, 862.07) | 0.20 (0.17, 0.23) | <0.001 | 158.92 (112.23, 199.89) | 69.77 (52.26, 90.79) | -2.80 (-2.94, -2.66) | <0.001 | 2.29 (1.53, 2.97) | 0.69 (0.47, 0.95) | -4.03 (-4.19, -3.87) | <0.001 |
| Western Sub-Saharan Africa | 59.31 (39.15, 80.86) | 60.83 (39.59, 82.87) | 0.08 (0.05, 0.11) | <0.001 | 1312.41 (968.88, 1753.46) | 1394.39 (1018.05, 1847.40) | 0.21 (0.20, 0.22) | <0.001 | 159.02 (110.52, 212.33) | 97.91 (66.83, 136.71) | -1.66 (-1.72, -1.60) | <0.001 | 1.72 (1.07, 2.40) | 0.57 (0.38, 0.83) | -3.73 (-3.81, -3.66) | <0.001 |
| Southeast Asia | 22.36 (15.43, 29.91) | 20.65 (14.10, 27.85) | -0.27 (-0.31, -0.23) | <0.001 | 466.98 (347.62, 613.07) | 468.84 (350.69, 621.01) | 0.01 (-0.02, 0.05) | 0.455 | 127.74 (102.53, 152.28) | 75.80 (59.90, 92.95) | -1.76 (-1.91, -1.62) | <0.001 | 1.86 (1.45, 2.30) | 0.94 (0.74, 1.17) | -2.28 (-2.53, -2.03) | <0.001 |
| Eastern Sub-Saharan Africa | 80.71 (52.55, 111.30) | 81.32 (52.31, 112.69) | 0.02 (-0.01, 0.04) | 0.255 | 1913.64 (1414.11, 2537.19) | 2036.79 (1502.10, 2672.08) | 0.22 (0.20, 0.23) | <0.001 | 226.26 (147.91, 306.15) | 141.13 (97.76, 197.87) | -1.61 (-1.66, -1.56) | <0.001 | 2.42 (1.23, 3.53) | 0.80 (0.56, 1.08) | -3.70 (-3.92, -3.48) | <0.001 |
| Central Latin America | 21.93 (15.02, 29.65) | 21.95 (14.75, 29.98) | 0.01 (-0.02, 0.04) | 0.575 | 599.04 (455.24, 770.21) | 620.98 (468.65, 801.18) | 0.14 (0.09, 0.19) | <0.001 | 120.32 (106.25, 136.46) | 41.57 (28.99, 58.10) | -3.57 (-3.78, -3.36) | <0.001 | 1.63 (1.51, 1.74) | 0.21 (0.17, 0.27) | -6.69 (-7.05, -6.33) | <0.001 |
| Tropical Latin America | 53.91 (35.14, | 53.86 (35.19, | -0.00 (-0.03, 0.02) | 0.863 | 1611.77 (1204.09, | 1655.52 (1225.67, | 0.10 (0.08, 0.11) | <0.001 | 186.94 (151.81, | 111.53 (79.10, 155.37) | -1.76 (-1.95, | <0.001 | 1.97 (1.81, | 0.59 (0.53, | -4.08 (-4.58, | <0.001 |

| | | | | | | | | | | | | | | | | |
|---|---|---|---|---|---|---|---|---|---|---|---|---|---|---|---|---|
| | 74.48) | 75.26) | | | 2092.92) | 2164.25) | | | 228.34) | | -1.57) | | 2.12) | 0.66) | -3.58) | |
| Central Sub-Saharan Africa | 75.95 (48.21, 105.47) | 76.53 (47.11, 104.91) | 0.03 (0.01, 0.05) | 0.001 | 1897.52 (1387.81, 2517.08) | 1938.08 (1429.32, 2524.10) | 0.08 (0.06, 0.10) | <0.001 | 261.88 (168.70, 389.85) | 155.09 (104.11, 222.27) | -1.79 (-1.88, -1.71) | <0.001 | 3.06 (1.54, 5.21) | 1.14 (0.61, 2.11) | -3.37 (-3.45, -3.29) | <0.001 |
| Western Europe | 0.80 (0.45, 1.25) | 0.44 (0.20, 0.79) | -2.02 (-2.38, -1.66) | <0.001 | 10.80 (7.92, 14.88) | 8.02 (5.72, 11.28) | -1.04 (-1.17, -0.91) | <0.001 | 16.40 (15.10, 17.75) | 5.29 (4.74, 5.94) | -3.83 (-4.10, -3.55) | <0.001 | 0.28 (0.26, 0.30) | 0.08 (0.08, 0.09) | -4.05 (-4.37, -3.73) | <0.001 |
| High-income North America | 2.95 (1.73, 4.35) | 2.02 (1.36, 2.69) | -1.27 (-1.37, -1.17) | <0.001 | 42.22 (29.67, 57.55) | 32.31 (26.79, 38.34) | -0.93 (-0.99, -0.87) | <0.001 | 23.88 (22.12, 25.92) | 10.15 (8.98, 11.60) | -2.90 (-3.49, -2.29) | <0.001 | 0.38 (0.35, 0.40) | 0.14 (0.13, 0.16) | -3.32 (-4.03, -2.60) | <0.001 |
| Central Asia | 36.75 (25.15, 49.81) | 37.72 (25.06, 51.34) | 0.09 (0.06, 0.12) | <0.001 | 947.47 (704.01, 1236.20) | 1021.84 (766.81, 1337.68) | 0.27 (0.24, 0.29) | <0.001 | 415.56 (380.67, 451.81) | 174.47 (139.87, 215.23) | -2.91 (-3.39, -2.43) | <0.001 | 6.54 (6.03, 7.05) | 2.22 (1.77, 2.78) | -3.62 (-4.15, -3.09) | <0.001 |
| Eastern Europe | 6.57 (4.79, 8.50) | 3.81 (2.66, 5.12) | -1.81 (-1.93, -1.70) | <0.001 | 48.68 (37.71, 61.20) | 39.05 (30.53, 49.12) | -0.73 (-0.86, -0.60) | <0.001 | 67.65 (57.75, 74.15) | 13.71 (10.68, 17.30) | -5.33 (-6.32, -4.32) | <0.001 | 1.15 (0.98, 1.27) | 0.21 (0.16, 0.27) | -5.64 (-7.16, -4.10) | <0.001 |
| Southern Sub-Saharan Africa | 74.72 (48.22, 102.03) | 75.11 (48.83, 103.33) | 0.02 (0.00, 0.03) | 0.009 | 1836.93 (1361.41, 2430.13) | 1867.02 (1353.54, 2457.69) | 0.06 (0.04, 0.07) | <0.001 | 353.43 (254.18, 456.24) | 161.78 (114.59, 217.66) | -2.59 (-3.66, -1.49) | <0.001 | 4.71 (2.97, 6.28) | 1.32 (0.77, 1.99) | -4.30 (-5.91, -2.67) | <0.001 |
| High-income Asia Pacific | 0.76 (0.39, 1.27) | 0.36 (0.16, 0.64) | -2.59 (-2.68, -2.50) | <0.001 | 14.02 (10.31, 18.89) | 9.90 (7.55, 13.16) | -1.21 (-1.28, -1.15) | <0.001 | 15.13 (12.96, 17.73) | 4.00 (3.45, 4.64) | -4.48 (-4.80, -4.16) | <0.001 | 0.25 (0.21, 0.30) | 0.06 (0.05, 0.07) | -4.86 (-5.23, -4.48) | <0.001 |
| Central Europe | 3.72 (2.87, 4.70) | 2.30 (1.63, 3.12) | -1.68 (-1.80, -1.56) | <0.001 | 51.46 (41.07, 63.86) | 39.41 (30.97, 48.86) | -0.97 (-1.23, -0.71) | <0.001 | 78.62 (72.67, 85.04) | 12.00 (9.96, 14.39) | -6.30 (-6.58, -6.01) | <0.001 | 1.34 (1.24, 1.45) | 0.18 (0.14, 0.22) | -6.77 (-7.08, -6.46) | <0.001 |
| Andean Latin America | 50.87 (33.18, 70.25) | 50.11 (32.31, 69.77) | -0.05 (-0.06, -0.05) | <0.001 | 1367.79 (1012.32, 1787.13) | 1408.25 (1032.36, 1834.69) | 0.10 (0.07, 0.12) | <0.001 | 154.55 (116.86, 202.38) | 89.12 (59.08, 126.77) | -1.89 (-2.25, -1.52) | <0.001 | 1.59 (1.18, 2.17) | 0.40 (0.26, 0.59) | -4.69 (-5.73, -3.65) | <0.001 |
| Caribbean | 47.79 (31.43, 65.88) | 52.89 (34.17, 72.25) | 0.36 (0.32, 0.39) | <0.001 | 1293.42 (946.94, 1692.27) | 1392.25 (1025.56, 1815.91) | 0.26 (0.24, 0.27) | <0.001 | 393.26 (261.40, 563.63) | 238.50 (149.24, 383.29) | -1.67 (-1.76, -1.58) | <0.001 | 5.86 (3.47, 8.83) | 3.05 (1.56, 5.46) | -2.20 (-2.31, -2.08) | <0.001 |
| Southern Latin America | 25.46 (17.25, | 25.94 (17.32, | 0.07 (-0.01, 0.14) | 0.093 | 724.04 (542.16, | 792.16 (596.73, | 0.32 (0.26, 0.38) | <0.001 | 98.09 (81.02, 119.90) | 50.60 (34.72, 72.18) | -2.23 (-2.48, | <0.001 | 1.13 (0.97, | 0.23 (0.19, | -5.23 (-5.74, | <0.001 |

| | | | | | | | | | | | | | | | | |
|---|---|---|---|---|---|---|---|---|---|---|---|---|---|---|---|---|
| | 35.61) | 35.28) | | | 936.20) | 1010.32) | | | | | -1.99) | | 1.30) | 0.29) | -4.72) | |
| Oceania | 42.30 (28.83, 56.63) | 45.11 (30.27, 60.80) | 0.22 (0.18, 0.26) | <0.001 | 774.37 (579.60, 1044.82) | 839.43 (624.77, 1135.56) | 0.29 (0.23, 0.35) | <0.001 | 1072.28 (627.75, 1674.81) | 966.84 (547.58, 1525.63) | -0.40 (-0.56, -0.24) | <0.001 | 18.28 (10.46, 29.03) | 16.36 (8.85, 26.17) | -0.43 (-0.60, -0.26) | <0.001 |
| Australasia | 1.98 (1.00, 3.25) | 1.54 (0.70, 2.61) | -0.86 (-0.98, -0.75) | <0.001 | 33.95 (23.33, 47.11) | 31.88 (21.79, 43.95) | -0.18 (-0.26, -0.10) | <0.001 | 36.29 (30.58, 43.21) | 16.56 (13.27, 20.28) | -2.58 (-3.08, -2.09) | <0.001 | 0.61 (0.51, 0.73) | 0.26 (0.20, 0.33) | -2.75 (-3.28, -2.21) | <0.001 |
| **Men** | | | | | | | | | | | | | | | | |
| Global | 28.43 (19.56, 37.68) | 31.94 (21.48, 43.01) | 0.42 (0.37, 0.47) | <0.001 | 580.57 (430.73, 773.74) | 679.81 (500.60, 907.95) | 0.56 (0.49, 0.63) | <0.001 | 178.98 (149.55, 212.54) | 110.41 (90.87, 133.02) | -1.65 (-1.81, -1.50) | <0.001 | 2.67 (2.15, 3.19) | 1.37 (1.14, 1.65) | -2.28 (-2.47, -2.09) | <0.001 |
| **SDI category** | | | | | | | | | | | | | | | | |
| High | 1.51 (0.97, 2.18) | 2.06 (1.57, 2.64) | 1.15 (0.95, 1.35) | <0.001 | 22.59 (17.71, 28.90) | 41.14 (32.99, 50.64) | 2.11 (1.69, 2.53) | <0.001 | 20.17 (18.60, 22.15) | 9.90 (8.24, 12.24) | -2.19 (-2.93, -1.45) | <0.001 | 0.33 (0.31, 0.37) | 0.14 (0.11, 0.17) | -2.83 (-3.34, -2.31) | <0.001 |
| High-middle | 15.13 (10.93, 20.02) | 14.08 (9.77, 19.06) | -0.22 (-0.36, -0.08) | 0.002 | 293.73 (220.96, 390.03) | 302.74 (224.78, 403.97) | 0.12 (0.06, 0.17) | <0.001 | 102.14 (90.35, 116.04) | 36.93 (29.39, 46.45) | -3.45 (-3.68, -3.21) | <0.001 | 1.55 (1.38, 1.76) | 0.39 (0.32, 0.49) | -4.64 (-4.94, -4.33) | <0.001 |
| Middle | 32.89 (21.84, 44.91) | 32.14 (21.12, 43.67) | -0.07 (-0.20, 0.06) | 0.299 | 710.30 (523.71, 950.57) | 709.26 (520.28, 948.85) | 0.02 (-0.08, 0.12) | 0.710 | 177.20 (150.59, 208.81) | 87.61 (69.21, 109.32) | -2.40 (-2.70, -2.10) | <0.001 | 2.53 (2.14, 2.94) | 0.94 (0.78, 1.17) | -3.44 (-3.75, -3.13) | <0.001 |
| Low-middle | 48.74 (33.63, 64.31) | 49.15 (33.21, 65.31) | 0.04 (-0.13, 0.21) | 0.676 | 946.97 (695.01, 1264.07) | 999.23 (734.21, 1327.80) | 0.20 (0.12, 0.28) | <0.001 | 368.65 (281.43, 480.84) | 222.92 (174.02, 279.95) | -1.69 (-1.92, -1.46) | <0.001 | 5.71 (4.18, 7.65) | 3.09 (2.27, 4.01) | -2.08 (-2.31, -1.85) | <0.001 |
| Low | 61.86 (41.28, 82.45) | 62.39 (41.23, 84.76) | 0.04 (0.01, 0.06) | 0.001 | 1287.99 (949.15, 1709.36) | 1342.62 (986.47, 1781.68) | 0.16 (0.10, 0.21) | <0.001 | 317.81 (217.18, 449.05) | 198.71 (153.01, 253.00) | -1.62 (-1.75, -1.49) | <0.001 | 4.53 (2.90, 6.80) | 2.38 (1.75, 3.15) | -2.27 (-2.46, -2.08) | <0.001 |
| **21 GBD region** | | | | | | | | | | | | | | | | |
| South Asia | 49.67 (34.31, 65.51) | 48.61 (33.27, 64.48) | -0.06 (-0.23, 0.11) | 0.496 | 946.49 (682.26, 1265.91) | 965.99 (698.57, 1285.89) | 0.12 (-0.01, 0.25) | 0.070 | 481.67 (367.00, 621.30) | 289.80 (230.99, 355.31) | -1.73 (-1.98, -1.48) | <0.001 | 7.71 (5.73, 10.14) | 4.30 (3.32, 5.42) | -2.02 (-2.29, -1.74) | <0.001 |
| East Asia | 26.46 (17.27, | 22.53 (14.52, | -0.53 (-0.65, -0.41) | <0.001 | 574.27 (421.64, | 488.70 (358.79, | -0.55 (-0.62, | <0.001 | 137.93 (109.55, | 45.57 (33.37, 60.57) | -3.77 (-4.21, | <0.001 | 1.95 (1.54, | 0.39 (0.30, | -5.49 (-6.12, | <0.001 |

| | | | | | | | | | | | | | | | | |
|---|---|---|---|---|---|---|---|---|---|---|---|---|---|---|---|---|
| | 36.48) | 30.85) | | | 768.08) | 660.21) | -0.48) | | 169.42) | | -3.33) | | 2.42) | 0.49) | -4.86) | |
| North Africa and Middle East | 25.97 (17.55, 35.14) | 26.63 (17.66, 35.48) | 0.10 (0.02, 0.17) | 0.009 | 527.13 (386.81, 705.38) | 552.09 (405.87, 740.84) | 0.17 (0.12, 0.22) | <0.001 | 84.14 (58.91, 119.80) | 48.14 (34.36, 66.33) | -1.90 (-2.00, -1.79) | <0.001 | 1.04 (0.64, 1.60) | 0.38 (0.24, 0.56) | -3.39 (-3.51, -3.26) | <0.001 |
| Western Sub-Saharan Africa | 57.43 (37.89, 78.10) | 58.23 (38.54, 80.04) | 0.05 (0.03, 0.06) | <0.001 | 1240.75 (915.20, 1658.93) | 1284.11 (944.87, 1715.75) | 0.12 (0.11, 0.13) | <0.001 | 119.41 (81.67, 165.14) | 91.15 (60.51, 132.56) | -0.91 (-0.99, -0.83) | <0.001 | 1.07 (0.65, 1.61) | 0.53 (0.35, 0.80) | -2.40 (-2.57, -2.23) | <0.001 |
| Southeast Asia | 17.99 (12.61, 23.83) | 16.95 (11.63, 22.47) | -0.19 (-0.26, -0.12) | <0.001 | 311.98 (231.29, 425.24) | 319.53 (237.33, 428.78) | 0.08 (0.05, 0.11) | <0.001 | 126.06 (96.12, 162.11) | 57.87 (45.55, 71.82) | -2.63 (-2.82, -2.45) | <0.001 | 1.96 (1.47, 2.57) | 0.75 (0.57, 0.94) | -3.25 (-3.45, -3.05) | <0.001 |
| Eastern Sub-Saharan Africa | 75.15 (49.10, 102.31) | 73.83 (47.41, 102.16) | -0.06 (-0.08, -0.04) | <0.001 | 1679.62 (1242.18, 2210.47) | 1751.40 (1291.26, 2325.19) | 0.15 (0.13, 0.17) | <0.001 | 125.37 (85.81, 175.29) | 105.70 (68.99, 157.81) | -0.58 (-0.68, -0.48) | <0.001 | 0.81 (0.55, 1.15) | 0.40 (0.30, 0.54) | -2.42 (-2.62, -2.21) | <0.001 |
| Central Latin America | 19.84 (13.44, 27.24) | 20.14 (13.41, 27.22) | 0.06 (0.01, 0.11) | 0.024 | 443.89 (328.36, 586.67) | 454.75 (336.99, 602.06) | 0.09 (0.04, 0.15) | 0.001 | 74.99 (63.76, 87.91) | 32.22 (22.37, 45.18) | -2.85 (-3.05, -2.63) | <0.001 | 0.95 (0.86, 1.05) | 0.19 (0.15, 0.22) | -5.40 (-5.84, -4.95) | <0.001 |
| Tropical Latin America | 53.96 (35.54, 74.09) | 53.01 (34.67, 72.03) | -0.06 (-0.08, -0.04) | <0.001 | 1264.28 (925.39, 1673.49) | 1258.62 (928.20, 1670.06) | -0.02 (-0.02, -0.01) | <0.001 | 138.08 (110.27, 174.63) | 84.50 (57.84, 119.90) | -1.69 (-1.83, -1.54) | <0.001 | 1.38 (1.25, 1.52) | 0.43 (0.38, 0.50) | -3.88 (-4.34, -3.42) | <0.001 |
| Central Sub-Saharan Africa | 74.40 (47.45, 102.22) | 74.77 (46.59, 105.19) | 0.02 (0.01, 0.02) | <0.001 | 1804.37 (1336.43, 2403.32) | 1805.78 (1331.23, 2406.06) | 0.01 (-0.02, 0.04) | 0.658 | 117.54 (76.11, 170.14) | 107.84 (66.32, 164.72) | -0.30 (-0.33, -0.26) | <0.001 | 0.57 (0.30, 1.01) | 0.39 (0.16, 0.96) | -1.29 (-1.52, -1.06) | <0.001 |
| Western Europe | 0.81 (0.47, 1.28) | 0.51 (0.25, 0.85) | -1.61 (-1.72, -1.51) | <0.001 | 9.69 (6.88, 13.64) | 7.22 (4.99, 10.65) | -1.03 (-1.18, -0.88) | <0.001 | 18.36 (16.71, 20.28) | 6.43 (5.62, 7.33) | -3.59 (-4.22, -2.96) | <0.001 | 0.31 (0.29, 0.35) | 0.11 (0.09, 0.12) | -3.71 (-4.37, -3.06) | <0.001 |
| High-income North America | 1.99 (1.10, 3.06) | 1.38 (0.95, 1.83) | -1.23 (-1.29, -1.17) | <0.001 | 29.84 (21.41, 40.73) | 21.30 (17.70, 25.74) | -1.14 (-1.17, -1.10) | <0.001 | 20.31 (18.71, 21.97) | 8.31 (6.92, 9.58) | -3.01 (-3.98, -2.03) | <0.001 | 0.33 (0.30, 0.36) | 0.12 (0.10, 0.14) | -3.30 (-4.46, -2.13) | <0.001 |
| Central Asia | 38.13 (26.36, 51.32) | 39.61 (26.71, 54.10) | 0.13 (0.09, 0.17) | <0.001 | 811.78 (595.87, 1076.46) | 890.31 (654.39, 1174.01) | 0.32 (0.30, 0.34) | <0.001 | 327.32 (292.48, 364.81) | 147.63 (117.28, 187.31) | -2.71 (-3.19, -2.22) | <0.001 | 5.09 (4.59, 5.58) | 1.85 (1.44, 2.30) | -3.46 (-3.99, -2.92) | <0.001 |
| Eastern Europe | 8.69 (6.57, 10.92) | 4.14 (3.01, 5.43) | -2.53 (-2.65, -2.41) | <0.001 | 52.59 (40.80, 65.84) | 36.88 (28.64, 46.70) | -1.21 (-1.40, | <0.001 | 125.00 (104.75, | 26.31 (21.30, 33.05) | -5.17 (-6.10, | <0.001 | 2.16 (1.82, | 0.43 (0.35, | -5.39 (-6.48, | <0.001 |

| | | | | | | | | | | | | | | | | |
|---|---|---|---|---|---|---|---|---|---|---|---|---|---|---|---|---|
| | | | | | | | -1.03) | | 146.45) | | -4.24) | | 2.55) | 0.55) | -4.30) | |
| Southern Sub-Saharan Africa | 69.19 (44.80, 94.66) | 68.92 (44.57, 95.30) | -0.01 (-0.03, 0.00) | 0.065 | 1603.19 (1175.34, 2122.75) | 1628.24 (1198.43, 2155.36) | 0.05 (0.05, 0.06) | <0.001 | 205.14 (155.31, 266.43) | 147.16 (107.30, 195.49) | -1.22 (-1.66, -0.77) | <0.001 | 2.28 (1.67, 3.08) | 1.24 (0.83, 1.81) | -2.24 (-3.01, -1.48) | <0.001 |
| High-income Asia Pacific | 0.62 (0.30, 1.07) | 0.32 (0.14, 0.59) | -2.27 (-2.34, -2.20) | <0.001 | 13.93 (10.47, 18.70) | 10.21 (7.73, 13.37) | -1.06 (-1.14, -0.97) | <0.001 | 11.45 (10.11, 13.13) | 3.89 (3.34, 4.50) | -3.65 (-4.07, -3.22) | <0.001 | 0.19 (0.16, 0.22) | 0.06 (0.05, 0.07) | -3.98 (-4.43, -3.52) | <0.001 |
| Central Europe | 5.05 (3.92, 6.28) | 2.89 (2.05, 3.82) | -1.92 (-2.13, -1.70) | <0.001 | 53.14 (42.16, 65.12) | 40.22 (31.55, 50.47) | -0.98 (-1.17, -0.80) | <0.001 | 108.22 (99.33, 117.42) | 15.84 (13.62, 18.29) | -6.46 (-6.85, -6.06) | <0.001 | 1.87 (1.71, 2.03) | 0.24 (0.21, 0.29) | -6.84 (-7.32, -6.36) | <0.001 |
| Andean Latin America | 48.40 (31.44, 66.80) | 48.20 (31.80, 65.97) | -0.02 (-0.04, -0.00) | 0.038 | 1133.17 (829.94, 1513.03) | 1139.62 (851.35, 1511.48) | 0.01 (-0.00, 0.03) | 0.112 | 95.57 (68.16, 130.91) | 68.44 (44.15, 100.86) | -1.15 (-1.33, -0.96) | <0.001 | 0.73 (0.53, 1.02) | 0.25 (0.16, 0.36) | -3.70 (-4.15, -3.25) | <0.001 |
| Caribbean | 45.76 (30.05, 61.93) | 49.11 (32.32, 65.13) | 0.24 (0.23, 0.26) | <0.001 | 1067.99 (788.21, 1416.51) | 1126.06 (818.09, 1506.10) | 0.18 (0.16, 0.20) | <0.001 | 175.12 (133.30, 232.23) | 127.06 (84.28, 189.59) | -1.02 (-1.23, -0.82) | <0.001 | 2.19 (1.57, 3.00) | 1.29 (0.70, 2.34) | -1.71 (-2.02, -1.40) | <0.001 |
| Southern Latin America | 26.89 (17.66, 36.62) | 27.59 (18.20, 38.80) | 0.11 (0.06, 0.15) | <0.001 | 631.69 (470.14, 839.81) | 676.23 (487.80, 900.99) | 0.24 (0.17, 0.31) | <0.001 | 87.46 (70.68, 109.63) | 44.92 (30.38, 64.38) | -2.27 (-2.54, -2.01) | <0.001 | 1.02 (0.84, 1.20) | 0.22 (0.17, 0.29) | -5.11 (-5.41, -4.80) | <0.001 |
| Oceania | 39.36 (27.37, 52.47) | 41.42 (28.85, 54.41) | 0.18 (0.16, 0.19) | <0.001 | 650.49 (486.77, 880.98) | 697.49 (514.82, 947.32) | 0.25 (0.21, 0.28) | <0.001 | 930.69 (435.97, 1711.76) | 805.74 (380.17, 1469.70) | -0.51 (-0.59, -0.43) | <0.001 | 15.87 (7.14, 29.72) | 13.62 (6.01, 25.36) | -0.54 (-0.62, -0.45) | <0.001 |
| Australasia | 0.92 (0.43, 1.61) | 0.71 (0.29, 1.37) | -0.91 (-1.04, -0.78) | <0.001 | 17.41 (11.82, 24.53) | 16.54 (11.24, 23.46) | -0.15 (-0.30, -0.00) | 0.046 | 32.09 (26.04, 38.86) | 15.14 (11.70, 19.45) | -2.51 (-3.22, -1.79) | <0.001 | 0.55 (0.44, 0.67) | 0.25 (0.19, 0.33) | -2.61 (-3.34, -1.87) | <0.001 |
| **35-39 years** | | | | | | | | | | | | | | | | |
| **Women** | | | | | | | | | | | | | | | | |
| Global | 23.39 (15.98, 33.23) | 25.17 (16.86, 36.37) | 0.25 (0.22, 0.28) | <0.001 | 704.35 (549.37, 899.97) | 824.34 (636.43, 1058.92) | 0.55 (0.50, 0.59) | <0.001 | 300.02 (255.53, 349.26) | 150.14 (116.23, 189.84) | -2.35 (-2.56, -2.14) | <0.001 | 5.16 (4.33, 6.02) | 2.15 (1.55, 2.84) | -2.96 (-3.20, -2.72) | <0.001 |
| **SDI category** | | | | | | | | | | | | | | | | |
| High | 3.05 (1.79, 4.59) | 2.71 (1.76, 3.88) | -0.40 (-0.47, -0.34) | <0.001 | 36.62 (27.69, 46.27) | 42.06 (36.00, 49.13) | 0.45 (0.31, 0.59) | <0.001 | 30.78 (28.50, 33.48) | 11.99 (10.18, 14.53) | -3.18 (-3.46, | <0.001 | 0.55 (0.51, | 0.19 (0.15, | -3.65 (-4.10, | <0.001 |

| | | | | | | | | | | | | | | | | |
|---|---|---|---|---|---|---|---|---|---|---|---|---|---|---|---|---|
| | | | | | | | | | | | -2.90) | | 0.60) | 0.23) | -3.20) | |
| High-middle | 14.78 (10.69, 19.62) | 12.64 (8.75, 17.46) | -0.54 (-0.62, -0.47) | <0.001 | 354.23 (277.55, 451.60) | 382.40 (295.12, 497.03) | 0.26 (0.16, 0.37) | <0.001 | 169.67 (147.08, 195.66) | 46.44 (37.68, 57.70) | -4.40 (-4.69, -4.11) | <0.001 | 2.95 (2.55, 3.44) | 0.54 (0.45, 0.65) | -5.73 (-6.19, -5.27) | <0.001 |
| Middle | 26.05 (16.71, 38.35) | 24.31 (15.77, 35.21) | -0.24 (-0.27, -0.21) | <0.001 | 845.04 (653.78, 1082.71) | 843.82 (651.36, 1081.49) | 0.01 (-0.09, 0.10) | 0.897 | 362.32 (304.77, 429.28) | 107.00 (88.17, 132.99) | -4.11 (-4.43, -3.79) | <0.001 | 6.23 (5.18, 7.47) | 1.29 (1.09, 1.54) | -5.27 (-5.79, -4.74) | <0.001 |
| Low-middle | 39.50 (26.44, 56.67) | 38.63 (25.37, 56.13) | -0.07 (-0.10, -0.03) | <0.001 | 1275.20 (984.21, 1630.78) | 1303.91 (1005.62, 1677.79) | 0.09 (-0.06, 0.24) | 0.244 | 545.31 (416.47, 685.07) | 311.91 (216.58, 430.75) | -2.01 (-2.41, -1.60) | <0.001 | 9.39 (6.96, 12.06) | 4.85 (2.94, 7.09) | -2.34 (-2.75, -1.94) | <0.001 |
| Low | 48.42 (31.80, 70.80) | 48.46 (31.54, 71.01) | -0.01 (-0.06, 0.04) | 0.778 | 1425.60 (1099.74, 1855.78) | 1489.59 (1136.51, 1914.17) | 0.15 (0.14, 0.16) | <0.001 | 498.92 (351.42, 662.25) | 294.45 (214.18, 399.23) | -1.82 (-2.07, -1.57) | <0.001 | 8.36 (5.70, 11.37) | 4.35 (2.87, 6.26) | -2.25 (-2.53, -1.97) | <0.001 |
| **21 GBD region** | | | | | | | | | | | | | | | | |
| South Asia | 40.19 (27.08, 56.94) | 37.78 (25.39, 54.38) | -0.21 (-0.26, -0.15) | <0.001 | 1344.00 (1021.80, 1723.04) | 1322.99 (1005.24, 1705.59) | -0.05 (-0.12, 0.01) | 0.134 | 667.61 (500.32, 853.50) | 409.72 (287.84, 566.25) | -1.69 (-2.14, -1.23) | <0.001 | 11.69 (8.54, 15.38) | 6.72 (4.35, 9.68) | -1.92 (-2.41, -1.43) | <0.001 |
| East Asia | 22.61 (14.43, 33.36) | 18.89 (12.19, 27.41) | -0.63 (-0.85, -0.40) | <0.001 | 656.89 (504.56, 841.70) | 559.19 (433.84, 713.14) | -0.56 (-0.77, -0.35) | <0.001 | 401.38 (311.53, 503.01) | 60.46 (47.05, 77.50) | -6.46 (-6.86, -6.07) | <0.001 | 7.15 (5.45, 9.06) | 0.63 (0.46, 0.83) | -8.13 (-8.53, -7.72) | <0.001 |
| North Africa and Middle East | 22.01 (13.71, 33.09) | 21.60 (13.68, 31.76) | -0.06 (-0.10, -0.03) | 0.001 | 629.12 (486.47, 808.54) | 650.59 (497.69, 835.81) | 0.12 (0.08, 0.15) | <0.001 | 171.40 (124.40, 216.13) | 72.35 (52.93, 94.92) | -2.93 (-3.09, -2.77) | <0.001 | 2.74 (1.86, 3.55) | 0.80 (0.54, 1.13) | -4.14 (-4.33, -3.95) | <0.001 |
| Western Sub-Saharan Africa | 45.39 (29.14, 66.15) | 45.62 (28.90, 66.11) | 0.02 (-0.01, 0.05) | 0.296 | 1251.70 (961.73, 1626.67) | 1326.40 (1011.60, 1728.06) | 0.20 (0.19, 0.21) | <0.001 | 150.49 (108.27, 195.54) | 91.07 (63.01, 127.25) | -1.72 (-1.77, -1.67) | <0.001 | 1.78 (1.12, 2.51) | 0.56 (0.38, 0.77) | -3.95 (-4.08, -3.81) | <0.001 |
| Southeast Asia | 19.07 (13.06, 26.66) | 16.73 (11.15, 23.69) | -0.46 (-0.49, -0.42) | <0.001 | 475.99 (372.50, 607.23) | 457.79 (353.18, 584.00) | -0.14 (-0.16, -0.12) | <0.001 | 168.13 (135.13, 199.64) | 83.91 (67.65, 103.07) | -2.33 (-2.49, -2.16) | <0.001 | 2.81 (2.20, 3.44) | 1.20 (0.93, 1.50) | -2.83 (-3.08, -2.58) | <0.001 |
| Eastern Sub-Saharan Africa | 58.79 (37.55, 85.77) | 57.80 (36.96, 84.85) | -0.07 (-0.11, -0.03) | <0.001 | 1776.39 (1352.06, 2309.19) | 1873.18 (1419.97, 2416.69) | 0.18 (0.17, 0.19) | <0.001 | 203.37 (136.90, 271.48) | 127.58 (89.31, 176.61) | -1.60 (-1.65, -1.55) | <0.001 | 2.34 (1.26, 3.35) | 0.78 (0.55, 1.07) | -3.71 (-3.90, -3.52) | <0.001 |
| Central Latin | 16.37 | 14.76 | -0.35 (-0.41, | <0.001 | 598.66 | 592.72 | -0.03 | 0.158 | 164.32 | 46.75 (34.78, | -4.19 | <0.001 | 2.63 | 0.36 | -6.49 | <0.001 |

| | | | | | | | | | | | | | | | | |
|---|---|---|---|---|---|---|---|---|---|---|---|---|---|---|---|---|
| America | (11.43, 22.79) | (10.01, 21.31) | -0.28) | | (469.01, 756.12) | (462.48, 747.55) | (-0.08, 0.01) | | (149.34, 181.98) | 61.78) | (-4.48, -3.89) | | (2.45, 2.83) | (0.29, 0.45) | (-6.82, -6.15) | |
| Tropical Latin America | 36.29 (23.05, 53.59) | 35.87 (22.54, 53.32) | -0.04 (-0.10, 0.02) | 0.232 | 1567.34 (1212.02, 2000.18) | 1612.07 (1243.65, 2063.16) | 0.10 (0.09, 0.11) | <0.001 | 202.02 (170.50, 242.52) | 122.39 (92.06, 163.43) | -1.71 (-1.80, -1.63) | <0.001 | 2.50 (2.29, 2.70) | 0.91 (0.81, 1.01) | -3.36 (-3.85, -2.86) | <0.001 |
| Central Sub-Saharan Africa | 55.70 (35.42, 81.75) | 55.14 (34.31, 82.00) | -0.04 (-0.06, -0.01) | 0.001 | 1763.72 (1346.12, 2302.99) | 1793.37 (1348.22, 2302.41) | 0.06 (0.04, 0.08) | <0.001 | 297.29 (182.63, 437.51) | 171.08 (111.93, 251.84) | -1.93 (-1.99, -1.87) | <0.001 | 4.17 (2.05, 6.87) | 1.70 (0.87, 3.16) | -3.08 (-3.15, -3.00) | <0.001 |
| Western Europe | 1.54 (0.78, 2.57) | 0.83 (0.31, 1.62) | -2.13 (-2.39, -1.86) | <0.001 | 15.12 (10.89, 20.13) | 10.25 (7.28, 13.90) | -1.36 (-1.48, -1.24) | <0.001 | 23.05 (21.24, 24.91) | 6.54 (5.88, 7.33) | -4.25 (-4.50, -4.01) | <0.001 | 0.43 (0.39, 0.46) | 0.11 (0.10, 0.13) | -4.45 (-4.72, -4.19) | <0.001 |
| High-income North America | 4.20 (2.32, 6.31) | 2.66 (1.65, 3.83) | -1.55 (-1.63, -1.48) | <0.001 | 58.14 (42.72, 75.58) | 43.11 (36.53, 50.05) | -1.02 (-1.10, -0.93) | <0.001 | 33.89 (31.41, 36.43) | 11.82 (10.55, 13.36) | -3.63 (-4.14, -3.11) | <0.001 | 0.59 (0.55, 0.63) | 0.18 (0.16, 0.20) | -4.14 (-4.68, -3.59) | <0.001 |
| Central Asia | 29.06 (19.85, 41.52) | 27.59 (17.86, 40.22) | -0.18 (-0.22, -0.14) | <0.001 | 921.87 (712.79, 1171.52) | 1005.18 (780.46, 1283.40) | 0.31 (0.29, 0.32) | <0.001 | 504.51 (460.08, 547.72) | 211.61 (168.58, 258.23) | -2.92 (-3.50, -2.34) | <0.001 | 8.93 (8.19, 9.72) | 3.18 (2.48, 3.97) | -3.44 (-4.04, -2.84) | <0.001 |
| Eastern Europe | 13.49 (8.36, 18.38) | 6.98 (4.17, 9.93) | -2.19 (-2.33, -2.06) | <0.001 | 86.95 (66.68, 108.32) | 59.16 (45.97, 73.91) | -1.28 (-1.40, -1.16) | <0.001 | 123.87 (106.36, 133.94) | 23.12 (18.16, 29.30) | -5.53 (-6.37, -4.68) | <0.001 | 2.32 (1.97, 2.52) | 0.39 (0.30, 0.50) | -5.84 (-6.72, -4.95) | <0.001 |
| Southern Sub-Saharan Africa | 53.68 (34.61, 78.61) | 53.49 (33.84, 79.06) | -0.01 (-0.03, -0.00) | 0.009 | 1702.70 (1298.00, 2202.22) | 1728.71 (1323.58, 2230.31) | 0.06 (0.02, 0.10) | 0.004 | 315.40 (230.72, 397.74) | 146.65 (103.13, 196.69) | -2.68 (-3.52, -1.84) | <0.001 | 4.55 (3.03, 6.01) | 1.28 (0.82, 1.87) | -4.15 (-5.43, -2.85) | <0.001 |
| High-income Asia Pacific | 1.42 (0.59, 2.46) | 0.67 (0.25, 1.27) | -2.54 (-2.75, -2.32) | <0.001 | 18.51 (13.62, 24.65) | 11.91 (8.97, 15.68) | -1.53 (-1.61, -1.45) | <0.001 | 17.45 (15.63, 19.55) | 4.49 (3.86, 5.23) | -4.61 (-4.80, -4.42) | <0.001 | 0.31 (0.28, 0.35) | 0.07 (0.06, 0.09) | -5.02 (-5.30, -4.74) | <0.001 |
| Central Europe | 5.71 (3.88, 7.66) | 3.49 (2.17, 5.16) | -1.67 (-1.79, -1.56) | <0.001 | 53.49 (43.80, 64.81) | 43.94 (35.30, 53.70) | -0.69 (-0.90, -0.47) | <0.001 | 138.00 (128.46, 148.64) | 16.67 (13.62, 20.03) | -7.21 (-7.55, -6.86) | <0.001 | 2.62 (2.43, 2.83) | 0.28 (0.22, 0.34) | -7.60 (-7.97, -7.24) | <0.001 |
| Andean Latin America | 35.31 (22.40, 52.00) | 34.02 (21.41, 50.31) | -0.12 (-0.14, -0.11) | <0.001 | 1336.97 (1018.38, 1722.68) | 1378.54 (1049.28, 1762.40) | 0.10 (0.09, 0.11) | <0.001 | 178.88 (137.42, 228.75) | 94.55 (64.83, 133.48) | -2.22 (-2.41, -2.02) | <0.001 | 2.25 (1.68, 3.00) | 0.57 (0.38, 0.84) | -4.61 (-5.36, -3.86) | <0.001 |
| Caribbean | 32.50 | 35.66 | 0.33 (0.30, | <0.001 | 1243.34 | 1322.75 | 0.22 (0.19, | <0.001 | 451.70 | 271.98 | -1.70 | <0.001 | 7.60 | 4.06 | -2.10 | <0.001 |

| | | | | | | | | | | | | | | | | |
|---|---|---|---|---|---|---|---|---|---|---|---|---|---|---|---|---|
| | (21.03, 47.95) | (22.97, 53.05) | 0.36) | | (952.59, 1593.24) | (1005.59, 1690.83) | 0.25) | | (304.20, 631.68) | (169.22, 450.69) | (-1.96, -1.43) | | (4.70, 11.01) | (2.13, 7.17) | (-2.43, -1.77) | |
| Southern Latin America | 20.81 (13.58, 30.87) | 19.43 (12.37, 29.06) | -0.23 (-0.29, -0.18) | <0.001 | 754.65 (583.81, 963.52) | 799.55 (624.25, 1008.08) | 0.20 (0.15, 0.24) | <0.001 | 126.55 (107.09, 150.87) | 56.38 (40.19, 78.59) | -2.73 (-2.96, -2.49) | <0.001 | 1.77 (1.52, 2.06) | 0.36 (0.28, 0.47) | -5.27 (-5.61, -4.93) | <0.001 |
| Oceania | 41.81 (25.17, 59.62) | 44.11 (26.45, 63.10) | 0.16 (0.06, 0.27) | 0.001 | 782.99 (614.58, 1012.80) | 842.59 (653.68, 1096.32) | 0.26 (0.22, 0.30) | <0.001 | 823.69 (465.88, 1398.31) | 747.85 (391.65, 1312.64) | -0.35 (-0.54, -0.16) | <0.001 | 15.21 (8.25, 26.40) | 13.68 (6.77, 24.42) | -0.39 (-0.59, -0.19) | <0.001 |
| Australasia | 2.94 (1.54, 4.78) | 2.24 (1.03, 3.68) | -0.93 (-1.03, -0.83) | <0.001 | 41.73 (29.38, 55.45) | 38.06 (26.47, 51.03) | -0.27 (-0.37, -0.17) | <0.001 | 39.74 (32.84, 48.04) | 20.96 (16.56, 26.38) | -2.14 (-2.64, -1.64) | <0.001 | 0.72 (0.59, 0.88) | 0.36 (0.28, 0.46) | -2.27 (-2.81, -1.73) | <0.001 |
| **Men** | | | | | | | | | | | | | | | | |
| Global | 22.95 (15.07, 32.74) | 24.04 (15.47, 35.03) | 0.17 (0.12, 0.22) | <0.001 | 559.78 (436.21, 720.87) | 638.59 (492.31, 826.82) | 0.46 (0.39, 0.53) | <0.001 | 232.39 (192.65, 278.53) | 130.15 (105.57, 159.02) | -1.95 (-2.30, -1.59) | <0.001 | 3.98 (3.22, 4.86) | 1.92 (1.55, 2.37) | -2.45 (-2.85, -2.06) | <0.001 |
| **SDI category** | | | | | | | | | | | | | | | | |
| High | 2.39 (1.45, 3.63) | 2.50 (1.69, 3.43) | 0.14 (0.01, 0.28) | 0.038 | 29.18 (22.73, 36.70) | 46.19 (38.54, 55.46) | 1.56 (1.37, 1.75) | <0.001 | 27.64 (25.63, 30.09) | 12.90 (10.60, 16.38) | -2.52 (-2.69, -2.36) | <0.001 | 0.50 (0.47, 0.55) | 0.20 (0.17, 0.27) | -3.07 (-3.25, -2.90) | <0.001 |
| High-middle | 15.35 (11.02, 20.34) | 12.22 (8.26, 17.10) | -0.79 (-0.81, -0.77) | <0.001 | 296.10 (233.60, 376.36) | 296.97 (230.56, 383.43) | 0.00 (-0.09, 0.09) | 0.988 | 145.42 (128.74, 165.46) | 45.51 (37.01, 56.37) | -3.85 (-4.21, -3.49) | <0.001 | 2.54 (2.25, 2.87) | 0.60 (0.49, 0.74) | -4.81 (-5.29, -4.32) | <0.001 |
| Middle | 27.31 (17.06, 39.88) | 24.96 (15.73, 36.41) | -0.31 (-0.45, -0.16) | <0.001 | 708.05 (547.35, 917.61) | 683.50 (528.96, 885.51) | -0.12 (-0.15, -0.07) | <0.001 | 235.70 (199.90, 273.22) | 103.32 (82.49, 126.36) | -2.82 (-3.14, -2.49) | <0.001 | 3.90 (3.28, 4.58) | 1.36 (1.10, 1.68) | -3.61 (-4.04, -3.18) | <0.001 |
| Low-middle | 36.01 (23.13, 52.50) | 35.95 (23.32, 52.64) | -0.00 (-0.04, 0.03) | 0.880 | 906.05 (694.41, 1183.56) | 954.49 (733.41, 1244.90) | 0.20 (0.05, 0.36) | 0.011 | 473.37 (349.87, 640.73) | 268.31 (202.67, 351.60) | -1.79 (-2.52, -1.05) | <0.001 | 8.33 (5.91, 11.51) | 4.31 (3.10, 5.83) | -2.10 (-2.91, -1.28) | <0.001 |
| Low | 46.06 (29.67, 66.89) | 46.53 (29.71, 67.88) | 0.04 (0.02, 0.05) | <0.001 | 1199.68 (916.30, 1551.11) | 1252.83 (956.20, 1623.76) | 0.16 (0.13, 0.19) | <0.001 | 402.82 (270.98, 590.04) | 238.39 (178.31, 310.97) | -1.70 (-2.16, -1.22) | <0.001 | 6.70 (4.15, 10.17) | 3.46 (2.43, 4.66) | -2.15 (-2.71, -1.59) | <0.001 |
| **21 GBD region** | | | | | | | | | | | | | | | | |

| | | | | | | | | | | | | | | | | |
|---|---|---|---|---|---|---|---|---|---|---|---|---|---|---|---|---|
| South Asia | 34.90 (22.99, 51.24) | 33.97 (22.30, 49.68) | -0.09 (-0.14, -0.04) | <0.001 | 910.79 (695.05, 1191.25) | 929.73 (711.22, 1211.63) | 0.11 (0.02, 0.20) | 0.018 | 611.36 (453.87, 816.54) | 360.55 (279.36, 463.92) | -1.66 (-2.40, -0.91) | <0.001 | 10.99 (7.96, 15.04) | 6.11 (4.61, 8.00) | -1.87 (-2.67, -1.06) | <0.001 |
| East Asia | 23.95 (14.59, 34.91) | 19.50 (12.21, 28.48) | -0.76 (-1.06, -0.47) | <0.001 | 582.22 (445.88, 750.68) | 479.86 (371.65, 621.74) | -0.70 (-0.88, -0.52) | <0.001 | 208.58 (166.08, 256.19) | 57.20 (43.18, 73.65) | -4.47 (-4.80, -4.14) | <0.001 | 3.49 (2.72, 4.34) | 0.65 (0.49, 0.85) | -5.69 (-6.17, -5.20) | <0.001 |
| North Africa and Middle East | 21.60 (13.45, 31.59) | 20.99 (13.01, 30.86) | -0.09 (-0.16, -0.02) | 0.011 | 533.41 (410.80, 690.81) | 543.49 (418.84, 702.47) | 0.06 (0.03, 0.10) | <0.001 | 110.23 (77.66, 153.27) | 55.71 (40.51, 75.86) | -2.33 (-2.42, -2.25) | <0.001 | 1.64 (1.04, 2.44) | 0.57 (0.38, 0.84) | -3.57 (-3.68, -3.46) | <0.001 |
| Western Sub-Saharan Africa | 45.29 (28.66, 65.28) | 45.32 (28.72, 64.81) | 0.01 (-0.05, 0.06) | 0.814 | 1180.13 (903.50, 1530.34) | 1217.49 (928.38, 1587.25) | 0.11 (0.10, 0.12) | <0.001 | 116.05 (78.24, 161.49) | 86.05 (57.10, 121.78) | -1.02 (-1.10, -0.94) | <0.001 | 1.16 (0.68, 1.83) | 0.54 (0.35, 0.85) | -2.61 (-2.84, -2.38) | <0.001 |
| Southeast Asia | 17.16 (11.39, 23.78) | 15.41 (10.03, 21.43) | -0.37 (-0.40, -0.33) | <0.001 | 317.00 (247.55, 412.24) | 313.07 (243.45, 407.32) | -0.05 (-0.06, -0.03) | <0.001 | 154.59 (117.93, 199.36) | 57.89 (45.48, 71.67) | -3.31 (-3.46, -3.16) | <0.001 | 2.70 (2.00, 3.54) | 0.83 (0.63, 1.06) | -3.96 (-4.15, -3.78) | <0.001 |
| Eastern Sub-Saharan Africa | 56.88 (36.43, 82.48) | 55.43 (35.28, 81.09) | -0.08 (-0.13, -0.03) | 0.001 | 1559.26 (1181.62, 2013.68) | 1611.06 (1230.49, 2094.29) | 0.12 (0.10, 0.15) | <0.001 | 147.40 (102.96, 196.57) | 109.30 (75.77, 153.25) | -1.01 (-1.08, -0.93) | <0.001 | 1.43 (0.94, 2.07) | 0.64 (0.48, 0.89) | -2.73 (-2.83, -2.63) | <0.001 |
| Central Latin America | 15.68 (10.51, 22.30) | 14.75 (9.69, 21.34) | -0.22 (-0.24, -0.20) | <0.001 | 431.43 (333.50, 553.09) | 420.38 (324.42, 541.15) | -0.08 (-0.11, -0.06) | <0.001 | 83.77 (73.53, 95.79) | 32.23 (23.37, 43.54) | -3.24 (-3.43, -3.05) | <0.001 | 1.22 (1.12, 1.35) | 0.24 (0.19, 0.29) | -5.49 (-5.93, -5.04) | <0.001 |
| Tropical Latin America | 39.79 (25.37, 58.25) | 38.66 (24.45, 57.38) | -0.09 (-0.12, -0.06) | <0.001 | 1203.46 (915.87, 1548.60) | 1200.01 (913.42, 1564.43) | -0.01 (-0.01, 0.00) | 0.129 | 145.79 (119.97, 177.49) | 84.88 (60.63, 116.96) | -1.83 (-2.09, -1.57) | <0.001 | 1.72 (1.57, 1.87) | 0.54 (0.49, 0.61) | -3.84 (-4.19, -3.48) | <0.001 |
| Central Sub-Saharan Africa | 57.09 (36.02, 85.40) | 56.83 (35.83, 86.12) | -0.02 (-0.03, -0.00) | 0.021 | 1675.16 (1276.60, 2154.17) | 1668.53 (1264.63, 2172.63) | -0.01 (-0.03, 0.00) | 0.160 | 127.45 (86.17, 181.18) | 112.11 (71.18, 173.46) | -0.45 (-0.49, -0.40) | <0.001 | 0.95 (0.51, 1.62) | 0.64 (0.28, 1.59) | -1.31 (-1.50, -1.12) | <0.001 |
| Western Europe | 1.44 (0.75, 2.40) | 0.93 (0.42, 1.69) | -1.51 (-1.61, -1.41) | <0.001 | 13.48 (9.50, 18.25) | 9.70 (6.67, 13.50) | -1.16 (-1.31, -1.02) | <0.001 | 23.90 (22.03, 25.89) | 8.65 (7.64, 9.79) | -3.48 (-3.78, -3.18) | <0.001 | 0.45 (0.41, 0.49) | 0.16 (0.14, 0.18) | -3.60 (-3.91, -3.28) | <0.001 |
| High-income North America | 3.20 (1.79, 4.92) | 2.31 (1.38, 3.30) | -1.12 (-1.21, -1.03) | <0.001 | 41.01 (30.29, 53.67) | 29.70 (25.23, 34.81) | -1.09 (-1.12, -1.07) | <0.001 | 27.86 (25.83, 30.09) | 9.80 (8.70, 11.09) | -3.54 (-4.02, -3.06) | <0.001 | 0.49 (0.46, 0.53) | 0.16 (0.14, 0.18) | -3.90 (-4.45, -3.36) | <0.001 |

| | | | | | | | | | | | | | | | | |
|---|---|---|---|---|---|---|---|---|---|---|---|---|---|---|---|---|
| Central Asia | 31.43 (21.66, 43.74) | 30.69 (19.69, 45.33) | -0.08 (-0.11, -0.04) | <0.001 | 781.30 (603.92, 1007.81) | 861.04 (664.21, 1101.27) | 0.34 (0.32, 0.35) | <0.001 | 405.06 (364.08, 449.81) | 174.88 (141.64, 216.82) | -2.83 (-3.45, -2.20) | <0.001 | 7.12 (6.41, 7.89) | 2.59 (2.05, 3.25) | -3.35 (-4.06, -2.65) | <0.001 |
| Eastern Europe | 17.50 (11.45, 23.32) | 7.19 (4.46, 9.96) | -3.04 (-3.12, -2.96) | <0.001 | 98.25 (76.91, 121.23) | 56.03 (44.01, 69.37) | -1.93 (-2.02, -1.83) | <0.001 | 204.65 (169.90, 240.28) | 42.83 (35.10, 52.32) | -5.05 (-6.14, -3.94) | <0.001 | 3.87 (3.21, 4.56) | 0.78 (0.63, 0.96) | -5.19 (-6.30, -4.05) | <0.001 |
| Southern Sub-Saharan Africa | 52.24 (33.02, 76.70) | 51.34 (32.58, 75.75) | -0.06 (-0.09, -0.04) | <0.001 | 1488.80 (1133.80, 1921.19) | 1506.00 (1150.37, 1944.27) | 0.04 (0.03, 0.05) | <0.001 | 213.22 (161.30, 279.61) | 144.74 (104.27, 197.56) | -1.33 (-1.69, -0.97) | <0.001 | 2.76 (1.94, 3.77) | 1.43 (0.94, 2.21) | -2.25 (-2.84, -1.65) | <0.001 |
| High-income Asia Pacific | 1.06 (0.45, 1.98) | 0.55 (0.19, 1.11) | -2.19 (-2.34, -2.04) | <0.001 | 17.20 (12.94, 22.53) | 11.75 (8.82, 15.27) | -1.32 (-1.41, -1.24) | <0.001 | 15.14 (13.69, 16.82) | 4.67 (4.05, 5.37) | -3.95 (-4.29, -3.61) | <0.001 | 0.27 (0.25, 0.30) | 0.08 (0.07, 0.09) | -4.29 (-4.72, -3.86) | <0.001 |
| Central Europe | 7.63 (5.30, 10.02) | 4.07 (2.57, 5.87) | -2.15 (-2.29, -2.01) | <0.001 | 63.31 (51.69, 76.25) | 44.01 (34.94, 54.88) | -1.23 (-1.46, -1.00) | <0.001 | 169.23 (157.46, 180.99) | 21.85 (18.82, 25.34) | -6.89 (-7.12, -6.66) | <0.001 | 3.21 (2.98, 3.45) | 0.38 (0.32, 0.44) | -7.18 (-7.43, -6.93) | <0.001 |
| Andean Latin America | 36.34 (22.76, 54.28) | 36.19 (22.49, 53.30) | -0.02 (-0.04, -0.00) | 0.019 | 1099.22 (836.84, 1429.72) | 1109.56 (847.83, 1418.17) | 0.03 (0.01, 0.04) | <0.001 | 111.39 (83.02, 147.68) | 71.15 (47.18, 103.85) | -1.51 (-1.68, -1.33) | <0.001 | 1.14 (0.83, 1.57) | 0.35 (0.23, 0.52) | -3.91 (-4.23, -3.60) | <0.001 |
| Caribbean | 34.45 (21.76, 49.18) | 36.44 (22.85, 53.23) | 0.20 (0.18, 0.21) | <0.001 | 1026.60 (793.65, 1325.54) | 1072.29 (822.14, 1381.94) | 0.15 (0.14, 0.16) | <0.001 | 197.22 (141.80, 262.46) | 138.80 (92.19, 212.16) | -1.12 (-1.35, -0.88) | <0.001 | 2.87 (1.95, 4.06) | 1.70 (0.94, 3.00) | -1.65 (-2.02, -1.29) | <0.001 |
| Southern Latin America | 22.14 (14.18, 32.29) | 21.42 (13.70, 30.86) | -0.11 (-0.26, 0.04) | 0.141 | 650.06 (494.50, 837.59) | 678.29 (519.92, 877.71) | 0.14 (0.10, 0.18) | <0.001 | 107.06 (89.63, 128.03) | 49.38 (34.82, 69.20) | -2.63 (-2.77, -2.49) | <0.001 | 1.48 (1.24, 1.76) | 0.33 (0.25, 0.43) | -4.98 (-5.26, -4.69) | <0.001 |
| Oceania | 40.70 (25.84, 58.26) | 42.59 (25.82, 61.02) | 0.16 (0.10, 0.21) | <0.001 | 666.41 (518.90, 869.55) | 707.89 (553.27, 919.81) | 0.21 (0.19, 0.24) | <0.001 | 821.48 (363.84, 1689.52) | 645.80 (281.74, 1307.16) | -0.84 (-1.02, -0.65) | <0.001 | 15.27 (6.45, 32.04) | 11.83 (4.77, 24.64) | -0.89 (-1.08, -0.69) | <0.001 |
| Australasia | 1.55 (0.65, 2.68) | 1.21 (0.47, 2.26) | -0.83 (-0.95, -0.72) | <0.001 | 19.28 (13.16, 26.61) | 18.01 (12.28, 25.04) | -0.24 (-0.34, -0.14) | <0.001 | 36.68 (29.68, 44.28) | 17.75 (13.83, 22.67) | -2.53 (-3.26, -1.79) | <0.001 | 0.69 (0.55, 0.83) | 0.32 (0.25, 0.41) | -2.62 (-3.39, -1.85) | <0.001 |
| **Ischemic heart disease** | | | | | | | | | | | | | | | | |
| **15-19 years** | | | | | | | | | | | | | | | | |

| | | | | | | | | | | | | | | | | |
|---|---|---|---|---|---|---|---|---|---|---|---|---|---|---|---|---|
| **Women** | | | | | | | | | | | | | | | | |
| Global | 4.11 (1.10, 8.63) | 4.29 (1.14, 8.97) | 0.15 (0.13, 0.17) | <0.001 | 14.72 (10.59, 19.89) | 14.53 (10.44, 19.46) | -0.03 (-0.11, 0.05) | 0.485 | 130.37 (113.79, 151.40) | 92.92 (80.55, 106.06) | -1.16 (-1.58, -0.74) | <0.001 | 1.82 (1.59, 2.11) | 1.30 (1.13, 1.48) | -1.16 (-1.58, -0.74) | <0.001 |
| **SDI category** | | | | | | | | | | | | | | | | |
| High | 5.46 (1.55, 11.06) | 6.48 (2.20, 12.45) | 0.60 (0.54, 0.66) | <0.001 | 19.36 (13.87, 26.06) | 22.95 (16.62, 30.74) | 0.62 (0.50, 0.73) | <0.001 | 41.98 (36.73, 47.86) | 13.27 (11.25, 15.46) | -3.80 (-4.16, -3.44) | <0.001 | 0.59 (0.51, 0.67) | 0.18 (0.16, 0.21) | -3.83 (-4.19, -3.46) | <0.001 |
| High-middle | 4.07 (1.13, 8.57) | 4.14 (1.09, 8.66) | 0.05 (0.01, 0.09) | 0.018 | 18.27 (13.33, 24.32) | 18.96 (13.84, 25.01) | 0.13 (0.10, 0.16) | <0.001 | 76.56 (64.74, 91.78) | 47.81 (40.13, 55.49) | -1.62 (-2.03, -1.21) | <0.001 | 1.07 (0.90, 1.28) | 0.67 (0.56, 0.77) | -1.63 (-2.04, -1.22) | <0.001 |
| Middle | 3.84 (1.02, 8.09) | 4.06 (1.08, 8.51) | 0.19 (0.16, 0.23) | <0.001 | 14.65 (10.32, 19.75) | 15.43 (10.98, 20.84) | 0.19 (0.14, 0.24) | <0.001 | 149.44 (130.41, 169.49) | 107.26 (91.58, 124.39) | -1.10 (-1.39, -0.81) | <0.001 | 2.09 (1.82, 2.37) | 1.50 (1.28, 1.74) | -1.10 (-1.39, -0.81) | <0.001 |
| Low-middle | 3.89 (0.87, 8.34) | 4.04 (0.86, 8.64) | 0.12 (0.09, 0.15) | <0.001 | 11.67 (8.24, 15.90) | 12.12 (8.52, 16.57) | 0.15 (0.04, 0.27) | 0.008 | 198.31 (159.41, 244.63) | 129.23 (106.58, 153.60) | -1.46 (-1.94, -0.98) | <0.001 | 2.77 (2.23, 3.42) | 1.81 (1.49, 2.15) | -1.46 (-1.94, -0.98) | <0.001 |
| Low | 4.05 (0.94, 8.59) | 4.10 (0.96, 8.79) | 0.04 (0.01, 0.07) | 0.010 | 9.61 (6.71, 13.21) | 9.85 (6.87, 13.61) | 0.10 (-0.00, 0.21) | 0.062 | 117.73 (89.36, 156.88) | 87.77 (70.52, 109.61) | -1.00 (-1.22, -0.79) | <0.001 | 1.65 (1.25, 2.19) | 1.23 (0.99, 1.53) | -1.00 (-1.22, -0.79) | <0.001 |
| **21 GBD region** | | | | | | | | | | | | | | | | |
| South Asia | 3.95 (0.81, 8.51) | 4.05 (0.80, 8.64) | 0.08 (0.04, 0.12) | <0.001 | 10.79 (7.41, 14.94) | 11.62 (8.07, 16.17) | 0.28 (0.16, 0.41) | <0.001 | 250.53 (194.35, 312.34) | 155.34 (127.91, 183.56) | -1.70 (-2.38, -1.02) | <0.001 | 3.51 (2.72, 4.37) | 2.17 (1.79, 2.57) | -1.70 (-2.38, -1.03) | <0.001 |
| East Asia | 3.15 (0.64, 7.13) | 3.31 (0.61, 7.36) | 0.18 (0.10, 0.25) | <0.001 | 15.02 (10.26, 21.07) | 18.32 (12.66, 25.08) | 0.68 (0.65, 0.72) | <0.001 | 82.54 (66.87, 100.59) | 43.75 (35.67, 51.89) | -2.27 (-2.65, -1.88) | <0.001 | 1.15 (0.93, 1.40) | 0.61 (0.49, 0.72) | -2.28 (-2.66, -1.89) | <0.001 |
| North Africa and Middle East | 5.20 (1.44, 10.44) | 5.14 (1.45, 10.18) | -0.04 (-0.06, -0.02) | <0.001 | 21.98 (15.75, 29.16) | 21.83 (15.82, 28.83) | -0.02 (-0.05, 0.00) | 0.068 | 358.79 (297.94, 426.96) | 187.19 (141.56, 241.84) | -2.16 (-2.48, -1.83) | <0.001 | 5.01 (4.16, 5.97) | 2.62 (1.98, 3.38) | -2.16 (-2.48, -1.83) | <0.001 |
| Western Sub-Saharan Africa | 3.65 (0.75, 8.14) | 3.78 (0.75, 8.45) | 0.12 (0.10, 0.13) | <0.001 | 7.81 (5.26, 11.01) | 7.90 (5.29, 11.17) | 0.06 (-0.04, 0.16) | 0.229 | 73.93 (51.83, 101.13) | 58.86 (42.13, 81.05) | -0.79 (-0.97, -0.60) | <0.001 | 1.03 (0.72, 1.41) | 0.82 (0.59, 1.13) | -0.79 (-0.97, -0.60) | <0.001 |

| | | | | | | | | | | | | | | | | |
|---|---|---|---|---|---|---|---|---|---|---|---|---|---|---|---|---|
| Southeast Asia | 3.25 (0.81, 7.14) | 3.64 (1.01, 7.69) | 0.40 (0.34, 0.46) | <0.001 | 13.16 (9.30, 17.54) | 14.52 (10.35, 19.08) | 0.36 (0.25, 0.48) | <0.001 | 117.98 (93.28, 156.53) | 97.51 (81.27, 113.44) | -0.65 (-0.84, -0.46) | <0.001 | 1.65 (1.30, 2.19) | 1.36 (1.14, 1.58) | -0.65 (-0.84, -0.46) | <0.001 |
| Eastern Sub-Saharan Africa | 3.68 (0.76, 8.05) | 3.76 (0.81, 8.37) | 0.06 (0.03, 0.10) | 0.001 | 7.11 (4.80, 10.16) | 7.18 (4.78, 10.16) | 0.05 (-0.04, 0.14) | 0.274 | 90.69 (63.24, 134.95) | 71.97 (52.46, 93.69) | -0.79 (-0.93, -0.65) | <0.001 | 1.27 (0.88, 1.89) | 1.01 (0.73, 1.31) | -0.79 (-0.93, -0.65) | <0.001 |
| Central Latin America | 4.87 (1.26, 10.01) | 4.14 (0.85, 8.84) | -0.55 (-0.60, -0.50) | <0.001 | 15.63 (11.11, 21.14) | 13.64 (9.64, 18.78) | -0.46 (-0.49, -0.44) | <0.001 | 89.83 (82.72, 96.90) | 53.78 (45.43, 63.70) | -1.76 (-2.50, -1.02) | <0.001 | 1.26 (1.16, 1.35) | 0.75 (0.63, 0.89) | -1.76 (-2.51, -1.02) | <0.001 |
| Tropical Latin America | 3.43 (0.76, 7.75) | 3.42 (0.81, 7.72) | -0.02 (-0.11, 0.08) | 0.731 | 10.41 (7.07, 14.47) | 10.66 (7.32, 14.78) | 0.10 (-0.00, 0.20) | 0.060 | 45.78 (41.88, 49.78) | 30.94 (27.64, 34.31) | -1.21 (-1.68, -0.75) | <0.001 | 0.64 (0.58, 0.70) | 0.43 (0.39, 0.48) | -1.22 (-1.68, -0.75) | <0.001 |
| Central Sub-Saharan Africa | 4.46 (0.95, 9.57) | 4.30 (0.87, 9.29) | -0.13 (-0.15, -0.11) | <0.001 | 8.98 (6.14, 12.44) | 8.55 (5.69, 12.14) | -0.15 (-0.20, -0.11) | <0.001 | 50.03 (29.56, 83.97) | 41.98 (26.28, 65.41) | -0.63 (-0.76, -0.51) | <0.001 | 0.70 (0.41, 1.17) | 0.59 (0.37, 0.91) | -0.63 (-0.76, -0.51) | <0.001 |
| Western Europe | 6.71 (2.22, 12.79) | 6.92 (2.35, 13.23) | 0.12 (0.09, 0.15) | <0.001 | 21.29 (15.06, 28.91) | 24.39 (17.53, 32.60) | 0.49 (0.44, 0.53) | <0.001 | 20.60 (18.75, 22.54) | 5.87 (5.07, 6.57) | -4.30 (-4.65, -3.95) | <0.001 | 0.29 (0.26, 0.31) | 0.08 (0.07, 0.09) | -4.34 (-4.70, -3.99) | <0.001 |
| High-income North America | 5.23 (1.10, 11.29) | 7.52 (2.71, 14.25) | 1.26 (1.21, 1.32) | <0.001 | 17.65 (12.53, 24.28) | 22.99 (16.14, 30.83) | 0.93 (0.84, 1.01) | <0.001 | 15.96 (14.61, 17.21) | 12.72 (11.17, 14.14) | -0.49 (-1.24, 0.26) | 0.200 | 0.22 (0.20, 0.24) | 0.18 (0.15, 0.20) | -0.74 (-1.92, 0.46) | 0.227 |
| Central Asia | 6.09 (1.80, 11.92) | 5.93 (1.69, 11.65) | -0.09 (-0.11, -0.06) | <0.001 | 22.23 (16.24, 29.59) | 21.27 (15.19, 28.69) | -0.14 (-0.19, -0.10) | <0.001 | 83.82 (74.80, 93.17) | 86.33 (72.26, 102.35) | 0.20 (-0.24, 0.65) | 0.364 | 1.17 (1.05, 1.30) | 1.21 (1.01, 1.43) | 0.20 (-0.24, 0.65) | 0.363 |
| Eastern Europe | 5.60 (1.46, 11.14) | 5.98 (1.71, 11.71) | 0.22 (0.19, 0.25) | <0.001 | 24.57 (17.61, 33.03) | 27.38 (19.59, 36.09) | 0.39 (0.34, 0.44) | <0.001 | 35.23 (31.77, 38.38) | 29.22 (24.26, 34.03) | -0.67 (-2.11, 0.79) | 0.367 | 0.49 (0.44, 0.53) | 0.41 (0.34, 0.48) | -0.67 (-2.12, 0.80) | 0.367 |
| Southern Sub-Saharan Africa | 5.01 (1.19, 10.40) | 4.60 (0.94, 9.75) | -0.29 (-0.33, -0.26) | <0.001 | 11.01 (7.63, 15.31) | 9.78 (6.78, 13.72) | -0.40 (-0.43, -0.36) | <0.001 | 36.10 (28.54, 44.41) | 24.51 (16.61, 35.57) | -1.15 (-1.81, -0.49) | 0.001 | 0.50 (0.40, 0.62) | 0.34 (0.23, 0.50) | -1.16 (-1.82, -0.50) | 0.001 |
| High-income Asia Pacific | 4.34 (1.05, 9.02) | 4.42 (1.09, 9.17) | 0.06 (0.03, 0.09) | <0.001 | 17.64 (12.60, 24.06) | 19.60 (14.05, 26.96) | 0.39 (0.33, 0.45) | <0.001 | 94.42 (74.84, 113.62) | 7.20 (5.77, 8.57) | -8.55 (-9.52, -7.58) | <0.001 | 1.32 (1.05, 1.59) | 0.10 (0.08, 0.12) | -8.59 (-9.55, -7.62) | <0.001 |

| | | | | | | | | | | | | | | | | |
|---|---|---|---|---|---|---|---|---|---|---|---|---|---|---|---|---|
| Central Europe | 4.04 (1.09, 8.63) | 4.10 (1.08, 8.57) | 0.05 (-0.01, 0.11) | 0.108 | 24.53 (18.41, 32.22) | 25.86 (19.15, 33.88) | 0.21 (0.15, 0.28) | <0.001 | 31.85 (28.78, 34.94) | 13.56 (11.26, 16.03) | -2.92 (-3.33, -2.51) | <0.001 | 0.44 (0.40, 0.49) | 0.19 (0.16, 0.22) | -2.94 (-3.36, -2.53) | <0.001 |
| Andean Latin America | 2.24 (0.28, 5.77) | 2.25 (0.29, 5.61) | 0.02 (-0.03, 0.06) | 0.439 | 7.70 (5.14, 10.77) | 8.15 (5.59, 11.26) | 0.21 (0.18, 0.23) | <0.001 | 138.60 (106.34, 177.82) | 69.44 (50.46, 94.47) | -2.45 (-3.25, -1.64) | <0.001 | 1.94 (1.49, 2.49) | 0.97 (0.70, 1.32) | -2.46 (-3.26, -1.64) | <0.001 |
| Caribbean | 12.11 (5.17, 21.25) | 12.06 (5.33, 21.70) | -0.01 (-0.04, 0.01) | 0.288 | 28.23 (20.35, 36.89) | 27.90 (20.14, 36.79) | -0.04 (-0.05, -0.03) | <0.001 | 93.80 (71.88, 119.54) | 82.32 (49.95, 126.72) | -0.41 (-1.11, 0.30) | 0.261 | 1.31 (1.00, 1.67) | 1.15 (0.70, 1.77) | -0.40 (-1.11, 0.30) | 0.261 |
| Southern Latin America | 3.31 (0.56, 7.34) | 3.22 (0.62, 7.37) | -0.07 (-0.14, 0.00) | 0.061 | 13.06 (9.22, 17.95) | 15.19 (10.92, 20.68) | 0.55 (0.47, 0.62) | <0.001 | 67.73 (55.84, 81.33) | 24.37 (19.76, 29.69) | -3.65 (-4.22, -3.08) | <0.001 | 0.95 (0.78, 1.14) | 0.34 (0.28, 0.41) | -3.65 (-4.22, -3.08) | <0.001 |
| Oceania | 3.92 (0.69, 8.37) | 3.96 (0.53, 8.66) | 0.04 (0.01, 0.08) | 0.011 | 15.41 (10.69, 20.84) | 15.73 (11.10, 21.44) | 0.09 (0.04, 0.14) | <0.001 | 58.66 (31.80, 101.24) | 75.48 (42.26, 125.98) | 0.81 (0.28, 1.34) | 0.003 | 0.82 (0.44, 1.42) | 1.05 (0.59, 1.76) | 0.81 (0.28, 1.35) | 0.003 |
| Australasia | 4.97 (1.09, 10.42) | 4.32 (0.79, 9.17) | -0.49 (-0.55, -0.43) | <0.001 | 21.42 (15.55, 29.33) | 21.75 (15.35, 29.32) | 0.06 (0.01, 0.12) | 0.021 | 11.10 (9.03, 13.77) | 2.78 (2.14, 3.55) | -4.51 (-6.14, -2.85) | <0.001 | 0.15 (0.12, 0.19) | 0.04 (0.03, 0.05) | -4.57 (-6.23, -2.88) | <0.001 |
| **Men** | | | | | | | | | | | | | | | | |
| Global | 4.75 (1.22, 9.82) | 5.04 (1.41, 10.31) | 0.21 (0.19, 0.22) | <0.001 | 14.23 (10.05, 19.56) | 14.69 (10.42, 19.72) | 0.12 (0.05, 0.19) | <0.001 | 141.65 (123.69, 157.75) | 113.12 (96.00, 133.94) | -0.88 (-1.53, -0.22) | 0.009 | 1.98 (1.73, 2.21) | 1.58 (1.34, 1.87) | -0.88 (-1.53, -0.22) | 0.009 |
| **SDI category** | | | | | | | | | | | | | | | | |
| High | 5.13 (1.44, 10.67) | 5.76 (1.76, 11.34) | 0.40 (0.39, 0.42) | <0.001 | 16.41 (11.73, 22.37) | 17.89 (12.85, 24.06) | 0.31 (0.28, 0.33) | <0.001 | 83.40 (57.69, 95.79) | 25.41 (22.68, 28.46) | -3.98 (-4.26, -3.70) | <0.001 | 1.16 (0.81, 1.34) | 0.35 (0.32, 0.40) | -3.98 (-4.27, -3.70) | <0.001 |
| High-middle | 4.46 (1.02, 9.42) | 4.29 (1.04, 9.08) | -0.14 (-0.16, -0.11) | <0.001 | 15.41 (10.93, 20.97) | 15.12 (10.77, 20.49) | -0.06 (-0.10, -0.03) | 0.001 | 135.12 (111.24, 157.86) | 84.24 (72.63, 97.95) | -1.75 (-2.41, -1.09) | <0.001 | 1.89 (1.55, 2.21) | 1.18 (1.02, 1.37) | -1.75 (-2.41, -1.09) | <0.001 |
| Middle | 4.60 (1.24, 9.51) | 4.82 (1.30, 9.89) | 0.16 (0.14, 0.17) | <0.001 | 14.49 (10.20, 19.87) | 15.47 (11.05, 21.13) | 0.24 (0.19, 0.28) | <0.001 | 175.14 (151.38, 198.38) | 150.62 (125.77, 182.81) | -0.54 (-1.10, 0.03) | 0.065 | 2.45 (2.12, 2.77) | 2.11 (1.76, 2.56) | -0.53 (-1.09, 0.04) | 0.067 |
| Low-middle | 4.76 (1.16, | 5.02 (1.24, | 0.19 (0.18, | <0.001 | 12.71 (8.84, | 13.90 (9.77, | 0.32 (0.22, | <0.001 | 135.24 | 115.83 (93.48, | -0.74 | 0.379 | 1.89 | 1.62 | -0.74 | 0.381 |

| | | | | | | | | | | | | | | | | |
|---|---|---|---|---|---|---|---|---|---|---|---|---|---|---|---|---|
| | 9.92) | 10.34) | 0.20) | | 17.57) | 19.02) | 0.41) | | (112.66, 162.20) | 143.20) | (-2.37, 0.92) | | (1.58, 2.27) | (1.31, 2.00) | (-2.37, 0.92) | |
| Low | 5.32 (1.33, 10.87) | 5.58 (1.56, 11.16) | 0.16 (0.14, 0.19) | <0.001 | 11.87 (8.32, 16.38) | 12.75 (8.89, 17.69) | 0.27 (0.14, 0.40) | <0.001 | 119.78 (93.11, 151.36) | 114.16 (88.66, 141.63) | -0.15 (-0.81, 0.51) | 0.657 | 1.67 (1.30, 2.12) | 1.60 (1.24, 1.98) | -0.15 (-0.80, 0.51) | 0.662 |
| **21 GBD region** | | | | | | | | | | | | | | | | |
| South Asia | 4.73 (1.07, 9.92) | 4.84 (1.11, 10.11) | 0.09 (0.08, 0.10) | <0.001 | 12.20 (8.37, 17.09) | 13.61 (9.35, 18.90) | 0.38 (0.35, 0.42) | <0.001 | 143.13 (111.98, 178.34) | 121.08 (91.96, 153.83) | -1.13 (-3.63, 1.43) | 0.383 | 2.00 (1.57, 2.50) | 1.70 (1.29, 2.15) | -1.13 (-3.62, 1.43) | 0.384 |
| East Asia | 3.73 (0.68, 8.38) | 3.52 (0.67, 8.04) | -0.20 (-0.30, -0.10) | <0.001 | 13.36 (8.95, 18.75) | 13.48 (9.07, 18.98) | 0.04 (-0.01, 0.07) | 0.093 | 165.89 (122.60, 202.74) | 105.02 (85.08, 129.29) | -1.54 (-1.93, -1.16) | <0.001 | 2.32 (1.71, 2.83) | 1.47 (1.19, 1.81) | -1.54 (-1.93, -1.15) | <0.001 |
| North Africa and Middle East | 6.65 (2.22, 12.95) | 6.65 (2.15, 12.87) | 0.01 (-0.07, 0.10) | 0.784 | 25.17 (18.07, 33.52) | 26.01 (18.75, 34.20) | 0.14 (0.08, 0.19) | <0.001 | 356.58 (293.58, 432.94) | 252.31 (182.27, 338.12) | -1.16 (-1.42, -0.90) | <0.001 | 4.98 (4.10, 6.05) | 3.53 (2.55, 4.73) | -1.16 (-1.41, -0.90) | <0.001 |
| Western Sub-Saharan Africa | 5.24 (1.31, 10.78) | 5.67 (1.49, 11.30) | 0.27 (0.26, 0.28) | <0.001 | 10.97 (7.64, 15.24) | 11.62 (8.05, 16.28) | 0.24 (0.07, 0.40) | 0.004 | 70.46 (50.29, 97.36) | 65.57 (46.86, 91.11) | -0.23 (-0.42, -0.04) | 0.017 | 0.98 (0.70, 1.36) | 0.92 (0.65, 1.27) | -0.23 (-0.41, -0.04) | 0.018 |
| Southeast Asia | 4.22 (1.11, 8.88) | 4.31 (1.18, 9.14) | 0.07 (0.03, 0.11) | <0.001 | 14.09 (9.96, 18.92) | 14.38 (10.17, 19.41) | 0.09 (0.02, 0.16) | 0.009 | 102.42 (82.93, 128.55) | 138.05 (112.07, 164.82) | 1.07 (0.93, 1.22) | <0.001 | 1.43 (1.16, 1.80) | 1.93 (1.57, 2.31) | 1.08 (0.93, 1.22) | <0.001 |
| Eastern Sub-Saharan Africa | 5.28 (1.24, 10.91) | 5.60 (1.48, 11.34) | 0.21 (0.19, 0.22) | <0.001 | 10.07 (6.95, 14.07) | 10.94 (7.58, 15.10) | 0.32 (0.16, 0.47) | <0.001 | 133.98 (95.79, 179.61) | 124.10 (92.09, 159.96) | -0.24 (-0.38, -0.10) | 0.001 | 1.87 (1.34, 2.51) | 1.74 (1.29, 2.24) | -0.24 (-0.38, -0.10) | 0.001 |
| Central Latin America | 5.21 (1.11, 10.74) | 5.04 (1.18, 10.47) | -0.12 (-0.13, -0.10) | <0.001 | 12.23 (8.37, 16.88) | 12.51 (8.68, 17.42) | 0.09 (0.06, 0.11) | <0.001 | 118.88 (109.71, 128.98) | 99.22 (81.90, 117.80) | -0.56 (-1.06, -0.05) | 0.030 | 1.67 (1.54, 1.81) | 1.39 (1.15, 1.65) | -0.55 (-1.06, -0.05) | 0.032 |
| Tropical Latin America | 4.23 (0.98, 9.34) | 4.22 (0.92, 9.19) | -0.02 (-0.06, 0.02) | 0.429 | 11.44 (7.79, 16.07) | 11.99 (8.21, 17.02) | 0.18 (0.14, 0.21) | <0.001 | 79.63 (71.60, 87.28) | 87.27 (77.21, 97.51) | 0.26 (-0.33, 0.86) | 0.389 | 1.11 (1.00, 1.22) | 1.22 (1.08, 1.37) | 0.26 (-0.33, 0.86) | 0.388 |
| Central Sub-Saharan Africa | 6.11 (1.36, 11.89) | 5.94 (1.40, 11.87) | -0.07 (-0.23, 0.09) | 0.390 | 11.18 (7.66, 15.57) | 11.09 (7.76, 15.52) | -0.01 (-0.07, 0.05) | 0.748 | 153.96 (98.76, 240.80) | 112.33 (68.64, 174.12) | -1.17 (-1.24, -1.09) | <0.001 | 2.15 (1.38, 3.37) | 1.57 (0.96, 2.44) | -1.16 (-1.24, -1.08) | <0.001 |

| | | | | | | | | | | | | | | | | |
|---|---|---|---|---|---|---|---|---|---|---|---|---|---|---|---|---|
| Western Europe | 5.84 (1.71, 11.57) | 5.65 (1.68, 11.18) | -0.12 (-0.13, -0.10) | <0.001 | 16.10 (11.25, 22.02) | 17.25 (12.38, 23.42) | 0.23 (0.22, 0.25) | <0.001 | 52.79 (47.39, 58.01) | 13.58 (11.45, 15.28) | -4.54 (-5.22, -3.86) | <0.001 | 0.74 (0.66, 0.81) | 0.19 (0.16, 0.21) | -4.56 (-5.24, -3.88) | <0.001 |
| High-income North America | 4.75 (0.90, 10.53) | 6.29 (1.84, 12.41) | 0.97 (0.90, 1.04) | <0.001 | 16.87 (11.61, 23.23) | 18.10 (12.66, 24.41) | 0.29 (0.16, 0.42) | <0.001 | 33.24 (29.40, 36.54) | 29.45 (26.74, 33.51) | -0.42 (-1.30, 0.47) | 0.357 | 0.46 (0.41, 0.51) | 0.41 (0.37, 0.47) | -0.41 (-1.30, 0.49) | 0.370 |
| Central Asia | 6.02 (1.56, 11.97) | 5.60 (1.38, 11.39) | -0.23 (-0.28, -0.18) | <0.001 | 17.03 (12.05, 23.31) | 16.87 (11.93, 23.07) | -0.03 (-0.04, -0.02) | <0.001 | 102.52 (92.60, 113.97) | 118.32 (95.95, 144.20) | 0.64 (-0.31, 1.60) | 0.190 | 1.43 (1.30, 1.60) | 1.66 (1.34, 2.02) | 0.64 (-0.31, 1.59) | 0.189 |
| Eastern Europe | 5.67 (1.37, 11.54) | 5.28 (1.21, 10.79) | -0.25 (-0.27, -0.23) | <0.001 | 15.92 (11.07, 22.03) | 14.61 (10.39, 20.09) | -0.29 (-0.34, -0.25) | <0.001 | 78.40 (68.00, 84.63) | 74.48 (61.50, 89.65) | -0.23 (-2.08, 1.66) | 0.813 | 1.10 (0.95, 1.18) | 1.04 (0.86, 1.26) | -0.22 (-2.08, 1.67) | 0.817 |
| Southern Sub-Saharan Africa | 6.64 (1.96, 12.81) | 6.71 (2.03, 13.08) | 0.04 (0.03, 0.06) | <0.001 | 13.63 (9.48, 18.60) | 14.25 (9.86, 19.50) | 0.17 (0.13, 0.20) | <0.001 | 29.81 (21.93, 40.21) | 35.48 (23.57, 53.27) | 0.91 (0.66, 1.15) | <0.001 | 0.42 (0.31, 0.56) | 0.50 (0.33, 0.74) | 0.91 (0.66, 1.15) | <0.001 |
| High-income Asia Pacific | 3.61 (0.76, 8.00) | 3.50 (0.72, 7.76) | -0.11 (-0.14, -0.08) | <0.001 | 11.85 (8.12, 16.31) | 11.63 (8.13, 16.08) | -0.06 (-0.10, -0.02) | 0.002 | 198.16 (96.75, 245.89) | 19.91 (16.32, 22.93) | -7.64 (-8.11, -7.16) | <0.001 | 2.77 (1.35, 3.44) | 0.28 (0.23, 0.32) | -7.64 (-8.12, -7.17) | <0.001 |
| Central Europe | 4.33 (1.06, 9.20) | 4.17 (1.02, 8.86) | -0.13 (-0.17, -0.09) | <0.001 | 19.37 (14.23, 25.47) | 18.17 (13.25, 24.15) | -0.22 (-0.25, -0.19) | <0.001 | 73.66 (67.57, 80.53) | 32.39 (25.71, 40.78) | -2.85 (-3.68, -2.01) | <0.001 | 1.03 (0.95, 1.13) | 0.45 (0.36, 0.57) | -2.85 (-3.68, -2.02) | <0.001 |
| Andean Latin America | 2.85 (0.48, 6.84) | 2.90 (0.56, 6.84) | 0.06 (0.02, 0.11) | 0.004 | 7.66 (4.98, 10.83) | 8.40 (5.54, 11.68) | 0.32 (0.30, 0.34) | <0.001 | 202.78 (151.81, 266.29) | 130.58 (91.17, 179.91) | -1.45 (-1.95, -0.94) | <0.001 | 2.84 (2.12, 3.73) | 1.83 (1.28, 2.52) | -1.44 (-1.95, -0.94) | <0.001 |
| Caribbean | 11.80 (5.19, 20.79) | 12.67 (5.66, 22.16) | 0.26 (0.21, 0.30) | <0.001 | 25.74 (18.70, 34.32) | 26.29 (19.08, 34.33) | 0.07 (0.05, 0.09) | <0.001 | 91.19 (73.98, 111.20) | 72.90 (47.70, 109.26) | -0.70 (-1.07, -0.32) | <0.001 | 1.28 (1.03, 1.55) | 1.02 (0.67, 1.53) | -0.69 (-1.07, -0.31) | <0.001 |
| Southern Latin America | 3.14 (0.64, 7.28) | 3.24 (0.49, 7.48) | 0.11 (0.07, 0.15) | <0.001 | 10.24 (7.02, 13.83) | 11.75 (8.30, 16.04) | 0.49 (0.44, 0.55) | <0.001 | 105.70 (87.92, 125.38) | 54.54 (43.49, 67.10) | -2.31 (-2.68, -1.94) | <0.001 | 1.48 (1.23, 1.76) | 0.76 (0.61, 0.94) | -2.31 (-2.68, -1.94) | <0.001 |
| Oceania | 4.90 (1.03, 10.36) | 4.96 (0.85, 10.39) | 0.04 (0.01, 0.06) | 0.003 | 15.46 (10.94, 21.37) | 15.77 (11.00, 22.11) | 0.08 (0.06, 0.10) | <0.001 | 69.83 (43.07, 115.38) | 86.37 (51.59, 154.34) | 0.69 (0.36, 1.03) | <0.001 | 0.98 (0.60, 1.61) | 1.21 (0.72, 2.16) | 0.70 (0.36, 1.03) | <0.001 |

| | | | | | | | | | | | | | | | | |
|---|---|---|---|---|---|---|---|---|---|---|---|---|---|---|---|---|
| Australasia | 11.44 (4.95, 20.68) | 11.60 (5.36, 20.64) | 0.03 (-0.06, 0.11) | 0.554 | 29.15 (20.39, 39.46) | 28.97 (20.27, 39.09) | -0.10 (-0.44, 0.24) | 0.575 | 33.37 (26.79, 40.82) | 8.40 (6.25, 10.81) | -4.74 (-5.98, -3.49) | <0.001 | 0.47 (0.37, 0.57) | 0.12 (0.09, 0.15) | -4.78 (-6.02, -3.52) | <0.001 |
| **20-24 years** | | | | | | | | | | | | | | | | |
| **Women** | | | | | | | | | | | | | | | | |
| Global | 12.83 (7.91, 19.34) | 13.46 (8.40, 20.18) | 0.16 (0.14, 0.18) | <0.001 | 39.04 (29.98, 50.36) | 38.13 (29.38, 48.36) | -0.08 (-0.11, -0.05) | <0.001 | 184.89 (161.41, 211.25) | 149.86 (129.37, 172.14) | -0.74 (-0.98, -0.49) | <0.001 | 2.76 (2.41, 3.16) | 2.24 (1.93, 2.57) | -0.74 (-0.98, -0.49) | <0.001 |
| **SDI category** | | | | | | | | | | | | | | | | |
| High | 18.30 (11.98, 26.79) | 20.66 (13.97, 29.23) | 0.43 (0.35, 0.52) | <0.001 | 44.28 (34.28, 55.76) | 51.69 (40.54, 64.85) | 0.55 (0.43, 0.68) | <0.001 | 54.32 (48.57, 60.74) | 28.22 (24.10, 33.57) | -2.27 (-2.71, -1.84) | <0.001 | 0.80 (0.72, 0.90) | 0.41 (0.35, 0.49) | -2.32 (-2.76, -1.87) | <0.001 |
| High-middle | 12.44 (7.63, 18.96) | 12.51 (7.60, 18.84) | 0.02 (-0.03, 0.07) | 0.456 | 45.75 (34.73, 58.90) | 46.58 (35.33, 59.82) | 0.06 (0.03, 0.08) | <0.001 | 123.08 (103.59, 145.90) | 85.64 (73.14, 100.10) | -1.16 (-1.61, -0.70) | <0.001 | 1.83 (1.54, 2.17) | 1.27 (1.08, 1.49) | -1.17 (-1.64, -0.70) | <0.001 |
| Middle | 11.54 (6.93, 17.80) | 12.48 (7.76, 18.79) | 0.27 (0.25, 0.29) | <0.001 | 40.53 (30.67, 53.28) | 40.64 (30.97, 52.40) | 0.01 (-0.02, 0.04) | 0.666 | 212.11 (184.71, 242.32) | 175.93 (149.17, 204.78) | -0.60 (-0.94, -0.25) | 0.001 | 3.17 (2.75, 3.62) | 2.63 (2.22, 3.06) | -0.60 (-0.95, -0.25) | 0.001 |
| Low-middle | 12.10 (7.24, 18.46) | 12.64 (7.68, 19.36) | 0.15 (0.14, 0.16) | <0.001 | 32.17 (24.84, 41.13) | 32.77 (25.13, 41.97) | 0.07 (0.01, 0.13) | 0.015 | 289.70 (233.18, 358.57) | 219.47 (179.04, 263.47) | -0.99 (-1.67, -0.30) | 0.005 | 4.34 (3.49, 5.37) | 3.29 (2.68, 3.95) | -0.99 (-1.67, -0.30) | 0.005 |
| Low | 12.68 (7.58, 19.33) | 13.05 (8.01, 19.86) | 0.10 (0.08, 0.12) | <0.001 | 26.60 (20.47, 33.68) | 27.33 (21.00, 34.65) | 0.10 (0.04, 0.15) | 0.001 | 160.84 (119.50, 217.90) | 120.21 (94.47, 149.60) | -0.99 (-1.37, -0.62) | <0.001 | 2.41 (1.78, 3.27) | 1.80 (1.41, 2.24) | -1.00 (-1.37, -0.62) | <0.001 |
| **21 GBD region** | | | | | | | | | | | | | | | | |
| South Asia | 12.49 (7.50, 18.85) | 12.80 (7.76, 19.46) | 0.08 (0.06, 0.10) | <0.001 | 30.23 (23.16, 38.76) | 31.84 (24.29, 40.88) | 0.18 (0.17, 0.20) | <0.001 | 382.35 (304.82, 476.01) | 272.43 (216.77, 338.30) | -1.30 (-2.03, -0.56) | 0.001 | 5.73 (4.57, 7.14) | 4.08 (3.24, 5.07) | -1.30 (-2.03, -0.56) | 0.001 |
| East Asia | 9.31 (4.95, 15.11) | 9.93 (5.30, 15.79) | 0.22 (0.21, 0.24) | <0.001 | 47.54 (33.94, 65.04) | 55.37 (39.66, 75.32) | 0.53 (0.50, 0.57) | <0.001 | 147.26 (114.06, 187.53) | 97.27 (75.72, 120.55) | -1.07 (-1.61, -0.52) | <0.001 | 2.18 (1.68, 2.79) | 1.43 (1.11, 1.78) | -1.09 (-1.65, -0.53) | <0.001 |
| North Africa and Middle East | 16.82 (10.85, | 16.40 (10.42, | -0.09 (-0.11, -0.07) | <0.001 | 51.77 (40.33, 64.73) | 51.08 (39.69, 63.97) | -0.04 (-0.07, | 0.004 | 372.95 (294.65, | 243.61 (186.28, | -1.40 (-1.66, | <0.001 | 5.59 (4.42, | 3.65 (2.79, | -1.40 (-1.66, | <0.001 |

| | | | | | | | | | | | | | | | | |
|---|---|---|---|---|---|---|---|---|---|---|---|---|---|---|---|---|
| | 24.69) | 24.28) | | | | | -0.01) | | 465.28) | 319.26) | -1.13) | | 6.97) | 4.79) | -1.14) | |
| Western Sub-Saharan Africa | 11.29 (6.48, 17.56) | 12.07 (7.02, 18.59) | 0.23 (0.21, 0.24) | <0.001 | 22.56 (17.19, 29.09) | 23.43 (17.86, 30.22) | 0.14 (0.10, 0.19) | <0.001 | 66.00 (47.05, 90.73) | 65.25 (45.93, 90.36) | -0.03 (-0.36, 0.29) | 0.843 | 0.99 (0.70, 1.36) | 0.97 (0.68, 1.35) | -0.04 (-0.37, 0.30) | 0.833 |
| Southeast Asia | 9.28 (5.41, 15.05) | 10.56 (6.35, 16.52) | 0.45 (0.37, 0.53) | <0.001 | 32.86 (24.14, 43.62) | 36.27 (26.52, 48.16) | 0.36 (0.26, 0.46) | <0.001 | 179.58 (137.12, 238.39) | 156.09 (125.69, 187.71) | -0.47 (-0.78, -0.16) | 0.003 | 2.68 (2.05, 3.56) | 2.33 (1.87, 2.80) | -0.48 (-0.79, -0.16) | 0.003 |
| Eastern Sub-Saharan Africa | 11.47 (6.54, 17.86) | 11.89 (7.02, 18.66) | 0.12 (0.10, 0.14) | <0.001 | 21.27 (16.17, 27.34) | 21.65 (16.48, 27.90) | 0.06 (0.05, 0.08) | <0.001 | 99.53 (69.80, 154.63) | 72.92 (53.48, 96.10) | -1.06 (-1.18, -0.94) | <0.001 | 1.49 (1.04, 2.32) | 1.09 (0.80, 1.44) | -1.07 (-1.19, -0.95) | <0.001 |
| Central Latin America | 14.81 (9.13, 22.38) | 12.71 (7.65, 19.81) | -0.51 (-0.56, -0.46) | <0.001 | 37.53 (28.73, 47.61) | 33.05 (25.10, 42.66) | -0.44 (-0.45, -0.42) | <0.001 | 126.02 (116.39, 136.30) | 93.11 (76.26, 114.86) | -0.86 (-1.39, -0.33) | 0.001 | 1.88 (1.74, 2.04) | 1.39 (1.14, 1.72) | -0.87 (-1.40, -0.33) | 0.002 |
| Tropical Latin America | 9.81 (5.34, 15.98) | 9.59 (5.13, 15.72) | -0.07 (-0.17, 0.03) | 0.192 | 24.67 (18.31, 32.55) | 25.06 (18.54, 33.17) | 0.06 (0.01, 0.10) | 0.011 | 83.26 (75.67, 90.63) | 56.01 (49.14, 62.85) | -1.30 (-1.92, -0.68) | <0.001 | 1.24 (1.13, 1.36) | 0.83 (0.73, 0.94) | -1.31 (-1.94, -0.68) | <0.001 |
| Central Sub-Saharan Africa | 14.18 (8.40, 21.63) | 13.65 (8.40, 20.88) | -0.14 (-0.16, -0.11) | <0.001 | 25.02 (18.82, 32.36) | 23.97 (18.17, 30.72) | -0.14 (-0.16, -0.12) | <0.001 | 62.12 (34.59, 103.32) | 54.93 (31.53, 92.07) | -0.40 (-0.51, -0.30) | <0.001 | 0.93 (0.51, 1.55) | 0.82 (0.46, 1.38) | -0.41 (-0.51, -0.30) | <0.001 |
| Western Europe | 22.40 (14.96, 31.94) | 22.27 (15.25, 31.40) | -0.01 (-0.04, 0.03) | 0.713 | 50.54 (39.28, 63.42) | 55.22 (43.27, 69.22) | 0.32 (0.25, 0.38) | <0.001 | 37.55 (34.16, 41.31) | 12.27 (10.87, 13.71) | -3.82 (-4.20, -3.44) | <0.001 | 0.55 (0.50, 0.61) | 0.18 (0.16, 0.20) | -3.92 (-4.30, -3.52) | <0.001 |
| High-income North America | 18.03 (10.93, 26.88) | 24.97 (17.12, 34.84) | 1.17 (0.94, 1.40) | <0.001 | 38.41 (29.21, 49.53) | 51.74 (40.11, 65.02) | 1.04 (0.92, 1.17) | <0.001 | 29.40 (26.60, 32.68) | 28.61 (25.92, 31.27) | -0.24 (-0.82, 0.33) | 0.405 | 0.43 (0.39, 0.48) | 0.42 (0.38, 0.46) | -0.23 (-0.82, 0.37) | 0.449 |
| Central Asia | 19.65 (12.87, 28.27) | 19.01 (12.20, 27.43) | -0.11 (-0.13, -0.10) | <0.001 | 49.36 (39.21, 61.89) | 47.55 (37.16, 59.70) | -0.12 (-0.15, -0.08) | <0.001 | 197.95 (178.39, 217.12) | 242.35 (197.33, 296.30) | 0.76 (0.23, 1.28) | 0.005 | 2.96 (2.67, 3.25) | 3.63 (2.96, 4.44) | 0.76 (0.24, 1.28) | 0.004 |
| Eastern Europe | 18.17 (11.82, 26.77) | 19.41 (12.51, 28.21) | 0.22 (0.19, 0.25) | <0.001 | 49.08 (38.16, 62.49) | 54.07 (41.98, 68.80) | 0.36 (0.29, 0.42) | <0.001 | 59.64 (54.42, 64.47) | 69.09 (55.31, 85.16) | 0.65 (-0.45, 1.75) | 0.248 | 0.89 (0.81, 0.96) | 1.03 (0.82, 1.27) | 0.66 (-0.45, 1.77) | 0.244 |
| Southern Sub-Saharan Africa | 16.59 (10.32, | 15.10 (9.36, 23.11) | -0.33 (-0.38, -0.29) | <0.001 | 27.67 (21.08, 35.47) | 24.92 (18.96, 32.14) | -0.35 (-0.40, | <0.001 | 68.18 (52.42, 87.26) | 41.02 (25.85, 60.08) | -1.62 (-3.35, | 0.073 | 1.02 (0.78, | 0.61 (0.38, | -1.64 (-3.40, | 0.073 |

| | | | | | | | | | | | | | | | | |
|---|---|---|---|---|---|---|---|---|---|---|---|---|---|---|---|---|
| | 24.96) | | | | | | -0.31) | | | | 0.15) | | 1.31) | 0.89) | 0.16) | |
| High-income Asia Pacific | 13.63 (8.40, 20.46) | 13.55 (8.48, 20.63) | -0.01 (-0.05, 0.02) | 0.478 | 36.88 (27.50, 48.01) | 39.48 (30.08, 51.59) | 0.26 (0.19, 0.34) | <0.001 | 106.30 (85.65, 129.40) | 13.40 (11.14, 15.68) | -6.91 (-7.56, -6.25) | <0.001 | 1.59 (1.28, 1.94) | 0.19 (0.16, 0.23) | -7.02 (-7.67, -6.36) | <0.001 |
| Central Europe | 12.05 (7.31, 18.58) | 11.78 (7.07, 17.97) | -0.08 (-0.13, -0.03) | 0.001 | 45.66 (35.57, 57.66) | 47.77 (36.99, 60.34) | 0.18 (0.06, 0.30) | 0.004 | 71.29 (65.01, 77.69) | 31.51 (25.29, 39.53) | -3.00 (-3.73, -2.27) | <0.001 | 1.06 (0.96, 1.15) | 0.46 (0.37, 0.58) | -3.05 (-3.80, -2.30) | <0.001 |
| Andean Latin America | 6.10 (2.76, 11.25) | 6.09 (2.73, 11.04) | 0.01 (-0.02, 0.03) | 0.506 | 18.94 (13.64, 25.61) | 19.59 (13.99, 26.35) | 0.12 (0.11, 0.14) | <0.001 | 193.94 (148.49, 251.62) | 88.41 (61.25, 120.97) | -2.64 (-3.90, -1.36) | <0.001 | 2.91 (2.22, 3.77) | 1.32 (0.91, 1.81) | -2.65 (-3.91, -1.38) | <0.001 |
| Caribbean | 37.76 (25.92, 50.32) | 38.57 (26.95, 51.29) | 0.07 (0.05, 0.10) | <0.001 | 69.95 (56.35, 85.38) | 68.95 (55.62, 84.32) | -0.05 (-0.07, -0.02) | <0.001 | 144.10 (112.68, 186.50) | 156.94 (96.21, 228.91) | 0.27 (-0.21, 0.75) | 0.270 | 2.15 (1.68, 2.79) | 2.35 (1.44, 3.44) | 0.27 (-0.21, 0.76) | 0.267 |
| Southern Latin America | 10.44 (5.82, 16.47) | 9.98 (5.68, 16.11) | -0.15 (-0.19, -0.11) | <0.001 | 26.85 (20.14, 35.25) | 27.68 (20.78, 37.13) | 0.14 (0.06, 0.21) | <0.001 | 95.36 (80.32, 111.49) | 42.18 (34.46, 51.75) | -2.70 (-3.26, -2.14) | <0.001 | 1.43 (1.20, 1.67) | 0.63 (0.51, 0.77) | -2.71 (-3.28, -2.14) | <0.001 |
| Oceania | 11.87 (6.68, 18.80) | 12.07 (6.63, 18.82) | 0.07 (0.02, 0.11) | 0.003 | 40.19 (29.69, 53.22) | 40.79 (30.53, 53.86) | 0.07 (0.02, 0.13) | 0.006 | 93.34 (54.18, 153.75) | 126.13 (75.59, 201.30) | 0.97 (0.70, 1.25) | <0.001 | 1.38 (0.80, 2.30) | 1.88 (1.11, 3.00) | 0.98 (0.70, 1.26) | <0.001 |
| Australasia | 16.05 (9.67, 23.78) | 13.32 (7.69, 20.77) | -0.65 (-0.67, -0.64) | <0.001 | 41.48 (31.47, 53.59) | 39.06 (29.35, 51.03) | -0.19 (-0.24, -0.14) | <0.001 | 23.34 (18.60, 29.20) | 8.23 (6.29, 10.55) | -3.50 (-4.75, -2.24) | <0.001 | 0.34 (0.27, 0.43) | 0.12 (0.09, 0.15) | -3.57 (-4.86, -2.27) | <0.001 |
| **Men** | | | | | | | | | | | | | | | | |
| Global | 15.92 (10.25, 23.54) | 16.75 (10.96, 24.70) | 0.18 (0.17, 0.19) | <0.001 | 41.69 (32.24, 52.69) | 42.17 (32.91, 53.21) | 0.05 (0.03, 0.06) | <0.001 | 254.79 (224.84, 282.25) | 263.67 (233.63, 307.58) | 0.14 (-0.07, 0.35) | 0.197 | 3.81 (3.36, 4.22) | 3.95 (3.50, 4.60) | 0.14 (-0.07, 0.36) | 0.181 |
| **SDI category** | | | | | | | | | | | | | | | | |
| High | 18.43 (11.91, 27.27) | 19.45 (13.01, 28.07) | 0.18 (0.16, 0.20) | <0.001 | 40.22 (30.90, 51.19) | 43.27 (33.95, 54.11) | 0.26 (0.19, 0.33) | <0.001 | 106.75 (93.56, 115.64) | 63.35 (57.02, 71.65) | -1.77 (-2.14, -1.40) | <0.001 | 1.59 (1.38, 1.72) | 0.94 (0.84, 1.06) | -1.79 (-2.17, -1.40) | <0.001 |
| High-middle | 14.94 (9.48, 22.35) | 14.14 (8.88, 21.24) | -0.19 (-0.22, -0.16) | <0.001 | 44.29 (33.62, 57.22) | 42.93 (32.73, 55.01) | -0.11 (-0.13, -0.10) | <0.001 | 247.77 (208.28, 283.68) | 210.13 (179.90, 246.39) | -0.66 (-1.02, -0.30) | <0.001 | 3.70 (3.10, 4.24) | 3.14 (2.68, 3.69) | -0.66 (-1.03, -0.30) | <0.001 |

| | | | | | | | | | | | | | | | | |
|---|---|---|---|---|---|---|---|---|---|---|---|---|---|---|---|---|
| Middle | 15.14 (9.63, 22.55) | 16.09 (10.45, 23.66) | 0.22 (0.19, 0.24) | <0.001 | 44.39 (33.97, 56.91) | 45.03 (34.75, 57.00) | 0.05 (0.04, 0.06) | <0.001 | 288.83 (252.58, 323.97) | 332.10 (288.11, 384.62) | 0.52 (0.31, 0.72) | <0.001 | 4.32 (3.77, 4.83) | 4.97 (4.31, 5.76) | 0.52 (0.32, 0.73) | <0.001 |
| Low-middle | 15.84 (10.15, 23.74) | 16.72 (10.67, 24.81) | 0.19 (0.17, 0.21) | <0.001 | 37.72 (29.29, 47.52) | 40.05 (31.19, 50.64) | 0.21 (0.18, 0.24) | <0.001 | 321.07 (269.91, 384.40) | 318.31 (266.62, 389.32) | -0.01 (-0.58, 0.57) | 0.983 | 4.81 (4.04, 5.75) | 4.77 (3.99, 5.84) | -0.00 (-0.58, 0.58) | 0.993 |
| Low | 17.72 (11.41, 26.20) | 18.69 (12.00, 27.57) | 0.19 (0.18, 0.20) | <0.001 | 36.21 (28.55, 45.34) | 38.96 (30.62, 48.49) | 0.25 (0.19, 0.31) | <0.001 | 200.13 (157.73, 253.26) | 227.45 (180.68, 282.54) | 0.40 (0.04, 0.77) | 0.031 | 2.99 (2.36, 3.79) | 3.41 (2.70, 4.23) | 0.41 (0.04, 0.77) | 0.029 |
| **21 GBD region** | | | | | | | | | | | | | | | | |
| South Asia | 15.90 (9.98, 23.97) | 16.33 (10.30, 24.46) | 0.09 (0.06, 0.12) | <0.001 | 35.10 (26.90, 44.57) | 37.97 (29.23, 48.37) | 0.27 (0.25, 0.29) | <0.001 | 409.48 (337.29, 486.54) | 405.79 (334.39, 502.02) | -0.04 (-0.83, 0.76) | 0.928 | 6.14 (5.05, 7.30) | 6.09 (5.02, 7.53) | -0.03 (-0.82, 0.76) | 0.935 |
| East Asia | 12.22 (7.23, 18.75) | 11.45 (6.69, 17.78) | -0.21 (-0.31, -0.12) | <0.001 | 47.70 (35.16, 63.35) | 48.14 (35.35, 65.15) | 0.03 (-0.02, 0.08) | 0.259 | 249.88 (187.57, 302.57) | 250.43 (199.79, 307.13) | 0.20 (-0.48, 0.89) | 0.564 | 3.72 (2.79, 4.51) | 3.73 (2.97, 4.57) | 0.20 (-0.48, 0.90) | 0.563 |
| North Africa and Middle East | 22.69 (15.05, 31.98) | 22.41 (14.86, 31.49) | -0.04 (-0.07, -0.01) | 0.019 | 65.01 (52.08, 80.30) | 66.78 (53.70, 82.44) | 0.12 (0.06, 0.17) | <0.001 | 419.34 (339.06, 506.86) | 346.58 (253.13, 460.31) | -0.59 (-0.89, -0.29) | <0.001 | 6.28 (5.07, 7.59) | 5.18 (3.79, 6.90) | -0.59 (-0.89, -0.29) | <0.001 |
| Western Sub-Saharan Africa | 17.41 (10.93, 25.87) | 18.93 (12.18, 28.01) | 0.29 (0.27, 0.30) | <0.001 | 35.41 (28.00, 44.39) | 38.23 (30.26, 47.62) | 0.29 (0.19, 0.39) | <0.001 | 111.90 (81.51, 152.78) | 121.89 (86.82, 167.02) | 0.30 (0.02, 0.58) | 0.035 | 1.67 (1.21, 2.29) | 1.82 (1.29, 2.49) | 0.30 (0.02, 0.58) | 0.036 |
| Southeast Asia | 13.66 (8.57, 20.80) | 14.02 (8.85, 21.27) | 0.09 (0.06, 0.12) | <0.001 | 39.50 (29.89, 51.03) | 40.92 (31.07, 53.02) | 0.12 (0.07, 0.18) | <0.001 | 219.93 (182.84, 268.24) | 323.74 (247.83, 382.66) | 1.39 (1.23, 1.55) | <0.001 | 3.29 (2.73, 4.01) | 4.84 (3.70, 5.73) | 1.40 (1.24, 1.56) | <0.001 |
| Eastern Sub-Saharan Africa | 17.56 (10.94, 26.01) | 18.84 (11.92, 27.80) | 0.24 (0.22, 0.27) | <0.001 | 34.39 (27.18, 42.90) | 37.22 (29.60, 46.29) | 0.29 (0.22, 0.36) | <0.001 | 141.02 (105.67, 184.56) | 139.06 (102.89, 183.97) | -0.03 (-0.25, 0.19) | 0.784 | 2.10 (1.57, 2.76) | 2.07 (1.53, 2.75) | -0.03 (-0.25, 0.19) | 0.781 |
| Central Latin America | 17.35 (10.71, 25.70) | 16.53 (10.11, 24.79) | -0.16 (-0.18, -0.15) | <0.001 | 32.71 (25.37, 41.54) | 33.17 (25.38, 42.44) | 0.05 (0.02, 0.07) | <0.001 | 218.48 (201.90, 233.43) | 213.89 (181.71, 248.51) | 0.19 (-0.20, 0.59) | 0.344 | 3.27 (3.03, 3.50) | 3.20 (2.72, 3.73) | 0.19 (-0.21, 0.59) | 0.347 |
| Tropical Latin America | 13.85 (8.47, 21.35) | 13.53 (8.38, 20.87) | -0.08 (-0.12, -0.04) | <0.001 | 34.45 (26.25, 44.31) | 35.66 (27.10, 45.63) | 0.11 (0.07, 0.15) | <0.001 | 167.93 (152.10, | 158.09 (142.88, | -0.23 (-0.59, | 0.198 | 2.51 (2.27, | 2.36 (2.13, | -0.24 (-0.59, | 0.193 |

| | | | | | | | | | | | | | | | | |
|---|---|---|---|---|---|---|---|---|---|---|---|---|---|---|---|---|
| | | | | | | | | | 182.23) | 175.59) | 0.12) | | 2.72) | 2.62) | 0.12) | |
| Central Sub-Saharan Africa | 20.47 (12.88, 29.76) | 19.90 (12.48, 29.23) | -0.09 (-0.12, -0.07) | <0.001 | 34.14 (26.78, 42.64) | 33.93 (26.45, 42.25) | -0.01 (-0.04, 0.01) | 0.266 | 125.26 (77.82, 192.68) | 107.01 (64.86, 165.90) | -0.58 (-0.68, -0.48) | <0.001 | 1.87 (1.16, 2.89) | 1.60 (0.97, 2.48) | -0.58 (-0.68, -0.48) | <0.001 |
| Western Europe | 20.61 (13.24, 29.55) | 18.66 (12.40, 27.13) | -0.34 (-0.38, -0.30) | <0.001 | 41.33 (31.68, 52.71) | 41.01 (31.85, 51.62) | -0.03 (-0.09, 0.02) | 0.244 | 113.51 (104.42, 122.84) | 36.11 (32.38, 40.57) | -4.01 (-4.37, -3.65) | <0.001 | 1.69 (1.56, 1.83) | 0.53 (0.48, 0.60) | -4.05 (-4.41, -3.68) | <0.001 |
| High-income North America | 18.21 (10.44, 27.73) | 22.24 (14.59, 31.41) | 0.69 (0.63, 0.74) | <0.001 | 36.94 (28.42, 47.79) | 41.26 (31.70, 52.40) | 0.38 (0.24, 0.52) | <0.001 | 64.76 (58.55, 69.79) | 74.46 (68.10, 86.40) | 0.54 (-0.33, 1.43) | 0.225 | 0.95 (0.86, 1.03) | 1.10 (1.01, 1.28) | 0.57 (-0.32, 1.46) | 0.211 |
| Central Asia | 20.85 (13.47, 29.93) | 19.73 (12.68, 28.40) | -0.18 (-0.21, -0.14) | <0.001 | 44.61 (35.20, 54.89) | 44.31 (34.95, 55.24) | -0.03 (-0.04, -0.02) | <0.001 | 250.13 (227.87, 273.05) | 348.76 (292.32, 409.35) | 1.16 (0.23, 2.09) | 0.014 | 3.75 (3.42, 4.09) | 5.23 (4.39, 6.15) | 1.16 (0.23, 2.10) | 0.014 |
| Eastern Europe | 20.48 (13.12, 29.57) | 19.22 (12.10, 28.05) | -0.22 (-0.25, -0.20) | <0.001 | 36.92 (28.58, 46.99) | 34.82 (26.94, 44.83) | -0.21 (-0.24, -0.17) | <0.001 | 211.76 (193.89, 228.45) | 281.73 (232.83, 355.40) | 1.42 (-0.23, 3.10) | 0.091 | 3.17 (2.91, 3.43) | 4.23 (3.50, 5.34) | 1.43 (-0.22, 3.10) | 0.090 |
| Southern Sub-Saharan Africa | 23.53 (15.13, 33.25) | 23.51 (15.47, 33.21) | 0.00 (-0.01, 0.02) | 0.576 | 40.34 (31.95, 50.64) | 41.60 (32.81, 52.02) | 0.12 (0.07, 0.15) | <0.001 | 57.61 (42.88, 78.22) | 74.04 (55.89, 100.05) | 0.82 (-0.07, 1.72) | 0.073 | 0.85 (0.63, 1.16) | 1.10 (0.83, 1.49) | 0.83 (-0.08, 1.74) | 0.073 |
| High-income Asia Pacific | 12.22 (7.36, 18.63) | 11.35 (6.70, 17.32) | -0.26 (-0.28, -0.24) | <0.001 | 28.18 (21.14, 36.98) | 26.61 (19.68, 35.11) | -0.20 (-0.22, -0.17) | <0.001 | 167.45 (121.97, 197.10) | 48.07 (40.13, 53.82) | -4.22 (-4.93, -3.50) | <0.001 | 2.50 (1.81, 2.95) | 0.71 (0.59, 0.80) | -4.25 (-4.97, -3.52) | <0.001 |
| Central Europe | 14.34 (9.00, 21.43) | 13.47 (8.50, 20.28) | -0.21 (-0.24, -0.17) | <0.001 | 45.33 (35.47, 56.80) | 42.37 (32.62, 53.61) | -0.23 (-0.25, -0.21) | <0.001 | 199.59 (186.30, 212.64) | 92.51 (76.66, 111.02) | -2.78 (-3.38, -2.17) | <0.001 | 2.98 (2.78, 3.18) | 1.37 (1.14, 1.65) | -2.79 (-3.40, -2.19) | <0.001 |
| Andean Latin America | 8.61 (4.20, 14.40) | 8.76 (4.55, 14.55) | 0.07 (0.05, 0.08) | <0.001 | 22.35 (16.61, 29.42) | 23.89 (17.77, 31.22) | 0.23 (0.21, 0.25) | <0.001 | 320.58 (242.71, 422.45) | 239.90 (169.98, 328.71) | -0.82 (-1.32, -0.33) | 0.001 | 4.81 (3.64, 6.34) | 3.59 (2.54, 4.93) | -0.83 (-1.32, -0.33) | 0.001 |
| Caribbean | 38.87 (26.85, 52.10) | 40.90 (28.61, 54.88) | 0.18 (0.17, 0.20) | <0.001 | 70.47 (57.24, 85.62) | 71.94 (58.55, 86.78) | 0.06 (-0.00, 0.12) | 0.057 | 201.81 (168.79, 239.03) | 173.21 (113.69, 255.37) | -0.51 (-0.97, -0.05) | 0.030 | 3.02 (2.51, 3.58) | 2.59 (1.70, 3.82) | -0.51 (-0.98, -0.05) | 0.030 |
| Southern Latin America | 10.52 (6.06, 16.62) | 10.53 (6.15, 16.75) | 0.01 (-0.01, 0.02) | 0.490 | 23.48 (17.57, 30.96) | 23.97 (18.07, 31.80) | 0.08 (0.04, 0.11) | <0.001 | 181.23 (149.62, | 92.90 (75.44, 113.71) | -2.31 (-2.65, | <0.001 | 2.71 (2.23, | 1.39 (1.12, | -2.32 (-2.66, | <0.001 |

| | | | | | | | | | | | | | | | | |
|---|---|---|---|---|---|---|---|---|---|---|---|---|---|---|---|---|
| | | | | | | | | | 216.07) | | -1.98) | | 3.23) | 1.70) | -1.98) | |
| Oceania | 16.32 (9.67, 24.82) | 16.57 (10.01, 24.54) | 0.04 (-0.01, 0.09) | 0.158 | 47.09 (35.70, 61.66) | 48.19 (36.16, 62.78) | 0.08 (0.06, 0.09) | <0.001 | 288.44 (184.29, 465.82) | 338.93 (205.26, 547.23) | 0.46 (0.16, 0.75) | 0.003 | 4.31 (2.75, 6.97) | 5.07 (3.07, 8.20) | 0.46 (0.16, 0.76) | 0.003 |
| Australasia | 41.24 (28.20, 55.74) | 39.81 (27.52, 53.59) | -0.11 (-0.16, -0.07) | <0.001 | 69.57 (55.83, 85.41) | 70.03 (56.53, 86.03) | -0.05 (-0.39, 0.30) | 0.789 | 74.29 (60.82, 89.10) | 26.78 (19.93, 34.68) | -3.36 (-4.22, -2.50) | <0.001 | 1.10 (0.90, 1.33) | 0.39 (0.29, 0.51) | -3.40 (-4.27, -2.53) | <0.001 |
| **25-29 years** | | | | | | | | | | | | | | | | |
| **Women** | | | | | | | | | | | | | | | | |
| Global | 22.53 (13.76, 32.98) | 23.13 (14.26, 33.31) | 0.10 (0.06, 0.13) | <0.001 | 109.16 (89.93, 130.93) | 110.97 (91.52, 131.75) | 0.05 (0.03, 0.08) | <0.001 | 261.33 (231.07, 297.86) | 186.72 (161.96, 212.96) | -1.03 (-1.75, -0.31) | 0.005 | 4.19 (3.71, 4.78) | 2.98 (2.58, 3.41) | -1.04 (-1.77, -0.30) | 0.006 |
| **SDI category** | | | | | | | | | | | | | | | | |
| High | 32.73 (20.08, 46.32) | 36.08 (22.98, 49.44) | 0.34 (0.27, 0.40) | <0.001 | 121.39 (101.22, 142.28) | 139.91 (118.38, 162.71) | 0.49 (0.42, 0.55) | <0.001 | 83.19 (76.10, 89.59) | 48.87 (42.90, 56.52) | -1.73 (-2.13, -1.33) | <0.001 | 1.31 (1.20, 1.41) | 0.76 (0.66, 0.88) | -1.78 (-2.19, -1.37) | <0.001 |
| High-middle | 21.86 (13.48, 32.00) | 21.20 (13.00, 31.08) | -0.09 (-0.13, -0.06) | <0.001 | 125.47 (102.65, 151.27) | 130.30 (106.76, 156.84) | 0.14 (0.12, 0.15) | <0.001 | 181.40 (155.88, 212.56) | 103.82 (89.32, 119.37) | -2.12 (-3.29, -0.92) | 0.001 | 2.88 (2.47, 3.39) | 1.62 (1.39, 1.88) | -2.19 (-3.42, -0.95) | 0.001 |
| Middle | 19.51 (11.78, 29.13) | 20.97 (12.74, 30.98) | 0.25 (0.22, 0.28) | <0.001 | 111.22 (90.41, 135.95) | 116.22 (95.11, 140.26) | 0.15 (0.13, 0.17) | <0.001 | 317.40 (278.68, 360.09) | 210.19 (179.59, 241.33) | -1.22 (-2.13, -0.31) | 0.009 | 5.09 (4.46, 5.77) | 3.35 (2.85, 3.86) | -1.24 (-2.16, -0.31) | 0.009 |
| Low-middle | 20.83 (12.47, 30.42) | 21.74 (12.97, 31.67) | 0.15 (0.14, 0.16) | <0.001 | 91.87 (75.37, 110.24) | 95.36 (78.26, 115.05) | 0.13 (0.10, 0.15) | <0.001 | 402.10 (330.03, 494.92) | 285.52 (232.92, 346.93) | -1.16 (-1.82, -0.50) | 0.001 | 6.48 (5.29, 7.99) | 4.60 (3.74, 5.59) | -1.17 (-1.83, -0.50) | 0.001 |
| Low | 21.92 (13.27, 31.91) | 22.86 (13.75, 32.98) | 0.14 (0.13, 0.15) | <0.001 | 77.47 (63.90, 92.70) | 80.55 (66.74, 96.37) | 0.14 (0.12, 0.15) | <0.001 | 239.77 (179.55, 323.43) | 169.69 (134.99, 212.07) | -1.15 (-1.41, -0.90) | <0.001 | 3.86 (2.88, 5.21) | 2.73 (2.16, 3.42) | -1.16 (-1.42, -0.90) | <0.001 |
| **21 GBD region** | | | | | | | | | | | | | | | | |
| South Asia | 21.67 (12.98, 31.51) | 22.21 (13.35, 32.23) | 0.09 (0.04, 0.14) | <0.001 | 91.14 (74.19, 109.91) | 95.73 (77.85, 115.80) | 0.17 (0.13, 0.20) | <0.001 | 486.78 (393.01, 594.50) | 348.84 (275.70, 435.66) | -1.07 (-2.11, -0.03) | 0.044 | 7.86 (6.34, 9.60) | 5.62 (4.44, 7.03) | -1.08 (-2.12, -0.02) | 0.045 |
| East Asia | 15.33 (8.75, | 16.59 (9.63, | 0.27 (0.26, | <0.001 | 132.37 | 151.52 | 0.46 (0.45, | <0.001 | 228.27 | 113.64 (86.82, | -2.15 | <0.001 | 3.62 | 1.74 | -2.26 | <0.001 |

| | | | | | | | | | | | | | | | | |
|---|---|---|---|---|---|---|---|---|---|---|---|---|---|---|---|---|
| | 23.81) | 25.49) | 0.28) | | (102.01, 167.40) | (117.92, 188.77) | 0.48) | | (175.76, 295.06) | 142.56) | (-3.34, -0.95) | | (2.77, 4.71) | (1.32, 2.21) | (-3.50, -1.00) | |
| North Africa and Middle East | 29.45 (17.59, 42.19) | 28.85 (17.27, 41.68) | -0.07 (-0.09, -0.05) | <0.001 | 143.00 (119.86, 167.07) | 140.39 (118.00, 163.66) | -0.05 (-0.08, -0.01) | 0.007 | 581.86 (489.71, 680.75) | 322.76 (248.44, 414.51) | -1.98 (-2.34, -1.62) | <0.001 | 9.39 (7.90, 11.00) | 5.19 (3.99, 6.68) | -1.99 (-2.35, -1.62) | <0.001 |
| Western Sub-Saharan Africa | 19.33 (11.49, 28.84) | 21.10 (12.57, 31.14) | 0.30 (0.28, 0.32) | <0.001 | 65.04 (53.77, 77.65) | 69.34 (57.48, 83.14) | 0.23 (0.21, 0.25) | <0.001 | 66.20 (47.22, 90.88) | 53.71 (38.47, 72.02) | -0.71 (-0.89, -0.53) | <0.001 | 1.06 (0.75, 1.46) | 0.85 (0.60, 1.15) | -0.73 (-0.92, -0.54) | <0.001 |
| Southeast Asia | 14.78 (8.52, 23.10) | 17.06 (10.15, 25.78) | 0.50 (0.46, 0.55) | <0.001 | 81.77 (66.25, 98.94) | 92.05 (74.72, 110.55) | 0.43 (0.34, 0.52) | <0.001 | 312.08 (248.94, 404.50) | 217.41 (176.35, 265.12) | -1.21 (-1.41, -1.01) | <0.001 | 5.02 (4.01, 6.52) | 3.49 (2.81, 4.27) | -1.22 (-1.42, -1.02) | <0.001 |
| Eastern Sub-Saharan Africa | 19.73 (11.78, 29.41) | 20.63 (12.38, 30.52) | 0.15 (0.13, 0.16) | <0.001 | 62.28 (51.44, 75.27) | 64.24 (53.16, 77.33) | 0.11 (0.07, 0.14) | <0.001 | 122.09 (90.26, 197.17) | 77.07 (56.43, 102.75) | -1.56 (-1.74, -1.38) | <0.001 | 1.96 (1.44, 3.18) | 1.23 (0.89, 1.65) | -1.59 (-1.76, -1.41) | <0.001 |
| Central Latin America | 24.92 (15.24, 35.78) | 21.58 (13.26, 31.36) | -0.49 (-0.50, -0.47) | <0.001 | 105.74 (87.52, 125.79) | 93.08 (76.68, 110.65) | -0.44 (-0.47, -0.41) | <0.001 | 188.77 (175.72, 201.92) | 122.45 (97.91, 151.66) | -1.07 (-1.54, -0.60) | <0.001 | 3.02 (2.82, 3.23) | 1.95 (1.55, 2.42) | -1.08 (-1.56, -0.60) | <0.001 |
| Tropical Latin America | 15.76 (9.09, 24.38) | 15.11 (8.79, 23.18) | -0.14 (-0.21, -0.07) | <0.001 | 66.73 (53.79, 82.00) | 66.92 (54.53, 81.35) | 0.00 (-0.02, 0.02) | 0.700 | 167.65 (154.70, 180.10) | 77.79 (69.58, 85.80) | -2.52 (-3.02, -2.02) | <0.001 | 2.70 (2.49, 2.90) | 1.24 (1.10, 1.37) | -2.56 (-3.06, -2.05) | <0.001 |
| Central Sub-Saharan Africa | 24.69 (14.60, 35.73) | 23.72 (14.16, 34.77) | -0.14 (-0.16, -0.11) | <0.001 | 71.82 (58.96, 85.36) | 69.35 (57.61, 83.01) | -0.12 (-0.13, -0.11) | <0.001 | 116.65 (69.28, 185.30) | 87.93 (53.05, 141.47) | -0.97 (-1.16, -0.78) | <0.001 | 1.87 (1.11, 2.99) | 1.41 (0.84, 2.28) | -0.98 (-1.17, -0.78) | <0.001 |
| Western Europe | 39.99 (25.44, 55.54) | 39.11 (25.25, 52.62) | -0.06 (-0.14, 0.01) | 0.072 | 142.30 (121.02, 165.04) | 152.69 (130.76, 176.24) | 0.25 (0.19, 0.32) | <0.001 | 58.79 (54.82, 62.69) | 19.84 (17.83, 21.90) | -3.71 (-4.18, -3.24) | <0.001 | 0.92 (0.86, 0.98) | 0.30 (0.27, 0.33) | -3.84 (-4.37, -3.31) | <0.001 |
| High-income North America | 33.19 (19.10, 49.48) | 44.83 (28.72, 61.27) | 1.09 (0.83, 1.36) | <0.001 | 108.45 (88.24, 130.57) | 145.28 (120.59, 174.18) | 1.02 (0.87, 1.16) | <0.001 | 64.65 (60.17, 68.42) | 61.09 (55.49, 68.47) | -0.13 (-0.61, 0.35) | 0.592 | 1.00 (0.93, 1.05) | 0.95 (0.87, 1.07) | -0.11 (-0.67, 0.46) | 0.709 |
| Central Asia | 34.62 (21.16, 48.64) | 33.33 (19.93, 47.42) | -0.13 (-0.15, -0.11) | <0.001 | 138.16 (117.18, 159.66) | 132.44 (112.58, 153.62) | -0.14 (-0.17, -0.11) | <0.001 | 270.81 (246.69, 293.90) | 314.76 (254.43, 384.94) | 0.60 (-0.07, 1.29) | 0.080 | 4.36 (3.97, 4.74) | 5.08 (4.10, 6.23) | 0.61 (-0.07, 1.30) | 0.078 |
| Eastern Europe | 32.12 | 34.32 | 0.22 (0.19, | <0.001 | 133.17 | 146.68 | 0.35 (0.25, | <0.001 | 95.41 (82.63, | 121.28 (92.65, | 0.92 | 0.037 | 1.52 | 1.94 | 0.94 (0.06, | 0.036 |

| | | | | | | | | | | | | | | | | |
|---|---|---|---|---|---|---|---|---|---|---|---|---|---|---|---|---|
| | (19.52, 44.83) | (21.12, 48.65) | 0.25) | | (110.34, 158.46) | (121.40, 173.47) | 0.45) | | 105.77) | 158.12) | (0.05, 1.80) | | (1.30, 1.69) | (1.47, 2.54) | 1.83) | |
| Southern Sub-Saharan Africa | 29.73 (17.70, 42.89) | 26.86 (15.99, 38.93) | -0.34 (-0.39, -0.30) | <0.001 | 79.35 (64.30, 95.77) | 70.97 (57.68, 85.79) | -0.38 (-0.42, -0.34) | <0.001 | 191.11 (149.14, 239.84) | 85.13 (44.99, 136.43) | -2.55 (-4.53, -0.53) | 0.014 | 3.08 (2.39, 3.87) | 1.36 (0.71, 2.19) | -2.58 (-4.58, -0.53) | 0.014 |
| High-income Asia Pacific | 23.33 (14.65, 33.20) | 23.28 (14.74, 32.73) | -0.01 (-0.04, 0.03) | 0.665 | 94.35 (78.25, 111.34) | 99.89 (82.87, 117.92) | 0.21 (0.16, 0.27) | <0.001 | 137.63 (113.10, 161.32) | 19.41 (16.03, 22.72) | -6.60 (-7.29, -5.91) | <0.001 | 2.21 (1.81, 2.59) | 0.29 (0.24, 0.35) | -6.78 (-7.50, -6.05) | <0.001 |
| Central Europe | 19.91 (11.95, 29.54) | 19.11 (11.72, 28.34) | -0.14 (-0.19, -0.09) | <0.001 | 115.50 (95.67, 137.96) | 116.38 (97.22, 138.78) | 0.03 (-0.03, 0.10) | 0.300 | 120.61 (111.56, 129.80) | 49.19 (39.76, 59.92) | -2.93 (-3.39, -2.46) | <0.001 | 1.91 (1.77, 2.06) | 0.76 (0.61, 0.94) | -3.00 (-3.49, -2.52) | <0.001 |
| Andean Latin America | 9.35 (4.51, 15.56) | 9.27 (4.44, 15.58) | -0.03 (-0.09, 0.03) | 0.324 | 49.07 (39.82, 59.94) | 50.40 (40.19, 62.23) | 0.09 (0.06, 0.12) | <0.001 | 270.00 (210.38, 345.28) | 104.94 (73.17, 146.94) | -3.35 (-4.06, -2.64) | <0.001 | 4.36 (3.40, 5.57) | 1.68 (1.16, 2.37) | -3.39 (-4.10, -2.66) | <0.001 |
| Caribbean | 65.00 (41.76, 89.79) | 67.42 (43.99, 93.68) | 0.14 (0.12, 0.16) | <0.001 | 200.47 (173.94, 229.26) | 196.45 (169.75, 224.32) | -0.07 (-0.07, -0.06) | <0.001 | 229.91 (182.52, 303.09) | 223.54 (135.01, 335.22) | -0.05 (-0.65, 0.56) | 0.880 | 3.69 (2.92, 4.89) | 3.59 (2.15, 5.40) | -0.04 (-0.66, 0.57) | 0.884 |
| Southern Latin America | 18.06 (10.92, 26.41) | 17.07 (10.25, 25.09) | -0.19 (-0.21, -0.17) | <0.001 | 69.04 (57.59, 81.64) | 68.51 (57.27, 81.84) | -0.02 (-0.07, 0.03) | 0.453 | 128.21 (109.17, 150.66) | 46.02 (36.22, 56.67) | -3.52 (-4.52, -2.50) | <0.001 | 2.06 (1.75, 2.43) | 0.73 (0.57, 0.90) | -3.55 (-4.57, -2.52) | <0.001 |
| Oceania | 19.94 (11.33, 29.66) | 20.36 (11.69, 30.75) | 0.08 (0.04, 0.11) | <0.001 | 109.20 (87.12, 134.53) | 110.67 (87.24, 137.02) | 0.06 (-0.00, 0.12) | 0.053 | 245.20 (140.38, 407.27) | 292.93 (167.57, 497.64) | 0.61 (0.39, 0.83) | <0.001 | 3.92 (2.23, 6.56) | 4.70 (2.68, 7.98) | 0.62 (0.40, 0.84) | <0.001 |
| Australasia | 28.27 (17.00, 40.19) | 22.63 (13.52, 33.47) | -0.78 (-0.80, -0.75) | <0.001 | 107.94 (89.89, 127.15) | 95.12 (78.71, 113.44) | -0.41 (-0.49, -0.34) | <0.001 | 49.92 (41.71, 59.35) | 17.28 (13.84, 21.29) | -3.54 (-5.11, -1.94) | <0.001 | 0.78 (0.65, 0.94) | 0.26 (0.21, 0.33) | -3.61 (-5.25, -1.95) | <0.001 |
| **Men** | | | | | | | | | | | | | | | | |
| Global | 29.21 (17.49, 42.29) | 29.83 (18.06, 42.85) | 0.07 (0.06, 0.09) | <0.001 | 129.11 (105.71, 157.43) | 131.85 (108.17, 158.96) | 0.07 (0.06, 0.09) | <0.001 | 417.97 (381.47, 455.22) | 417.19 (374.27, 476.21) | 0.03 (-0.42, 0.49) | 0.883 | 6.71 (6.13, 7.31) | 6.71 (6.01, 7.66) | 0.04 (-0.42, 0.50) | 0.874 |
| **SDI category** | | | | | | | | | | | | | | | | |
| High | 34.80 (20.32, | 35.26 (21.57, | 0.04 (0.01, 0.06) | 0.003 | 122.77 (100.41, | 127.30 (106.05, | 0.13 (0.09, 0.16) | <0.001 | 204.68 (188.49, | 128.28 (116.81, | -1.61 (-1.90, | <0.001 | 3.26 (2.99, | 2.03 (1.84, | -1.64 (-1.94, | <0.001 |

| | | | | | | | | | | | | | | | | |
|---|---|---|---|---|---|---|---|---|---|---|---|---|---|---|---|---|
| | 50.55) | 49.59) | | | 146.89) | 148.49) | | | 216.64) | 145.07) | -1.32) | | 3.46) | 2.30) | -1.34) | |
| High-middle | 27.67 (16.62, 39.70) | 25.17 (15.17, 36.33) | -0.33 (-0.35, -0.30) | <0.001 | 136.65 (110.54, 169.07) | 134.46 (108.52, 164.19) | -0.06 (-0.10, -0.03) | <0.001 | 426.01 (377.30, 470.94) | 352.11 (313.91, 398.21) | -0.66 (-1.26, -0.06) | 0.032 | 6.83 (6.05, 7.55) | 5.64 (5.01, 6.37) | -0.67 (-1.27, -0.06) | 0.032 |
| Middle | 27.32 (16.42, 39.68) | 28.66 (17.33, 41.32) | 0.17 (0.14, 0.19) | <0.001 | 137.82 (111.77, 169.30) | 141.11 (115.50, 171.95) | 0.08 (0.07, 0.10) | <0.001 | 470.59 (426.68, 515.75) | 487.72 (429.03, 551.22) | 0.12 (-0.39, 0.65) | 0.636 | 7.56 (6.83, 8.31) | 7.84 (6.89, 8.88) | 0.13 (-0.40, 0.66) | 0.634 |
| Low-middle | 28.62 (17.12, 41.15) | 30.02 (17.93, 42.88) | 0.17 (0.15, 0.19) | <0.001 | 116.59 (95.55, 140.66) | 123.88 (101.64, 149.15) | 0.20 (0.18, 0.22) | <0.001 | 524.54 (446.16, 615.38) | 532.36 (449.70, 639.10) | 0.08 (-0.60, 0.76) | 0.823 | 8.46 (7.20, 9.94) | 8.59 (7.24, 10.33) | 0.08 (-0.60, 0.77) | 0.819 |
| Low | 31.91 (19.30, 45.53) | 33.89 (20.63, 48.19) | 0.21 (0.19, 0.22) | <0.001 | 114.49 (94.26, 136.48) | 124.32 (102.81, 148.12) | 0.29 (0.26, 0.32) | <0.001 | 326.58 (261.61, 404.00) | 379.93 (307.01, 461.83) | 0.55 (0.22, 0.88) | 0.001 | 5.26 (4.20, 6.52) | 6.12 (4.93, 7.44) | 0.55 (0.22, 0.88) | 0.001 |
| **21 GBD region** | | | | | | | | | | | | | | | | |
| South Asia | 28.80 (17.10, 41.32) | 29.60 (17.54, 41.98) | 0.10 (0.08, 0.12) | <0.001 | 108.91 (88.32, 131.88) | 116.63 (94.87, 140.84) | 0.23 (0.19, 0.28) | <0.001 | 635.23 (531.01, 755.27) | 662.08 (559.00, 789.82) | 0.13 (-0.74, 1.01) | 0.769 | 10.27 (8.58, 12.20) | 10.71 (9.05, 12.78) | 0.13 (-0.74, 1.01) | 0.765 |
| East Asia | 21.73 (12.63, 32.14) | 20.28 (11.80, 30.02) | -0.23 (-0.31, -0.15) | <0.001 | 152.59 (117.95, 193.04) | 152.18 (116.04, 194.29) | -0.00 (-0.08, 0.07) | 0.899 | 333.15 (264.81, 390.14) | 335.10 (278.66, 398.72) | 0.04 (-0.52, 0.61) | 0.877 | 5.30 (4.19, 6.24) | 5.32 (4.38, 6.35) | 0.04 (-0.54, 0.62) | 0.888 |
| North Africa and Middle East | 41.17 (24.85, 58.15) | 41.11 (24.97, 58.00) | 0.00 (-0.03, 0.03) | 0.983 | 196.18 (165.16, 229.03) | 201.67 (171.38, 234.85) | 0.11 (0.02, 0.20) | 0.013 | 779.14 (647.04, 942.34) | 504.91 (382.91, 679.42) | -1.44 (-1.78, -1.10) | <0.001 | 12.56 (10.45, 15.22) | 8.11 (6.15, 10.95) | -1.45 (-1.79, -1.11) | <0.001 |
| Western Sub-Saharan Africa | 31.29 (19.05, 44.44) | 34.13 (20.41, 48.48) | 0.30 (0.28, 0.31) | <0.001 | 113.62 (94.08, 135.89) | 124.61 (103.57, 149.55) | 0.34 (0.28, 0.39) | <0.001 | 136.15 (99.58, 182.58) | 149.40 (108.33, 198.02) | 0.31 (0.04, 0.59) | 0.027 | 2.17 (1.58, 2.91) | 2.38 (1.72, 3.17) | 0.31 (0.03, 0.59) | 0.031 |
| Southeast Asia | 23.88 (14.03, 35.09) | 24.76 (14.63, 36.39) | 0.13 (0.10, 0.15) | <0.001 | 116.05 (94.11, 143.02) | 122.41 (98.68, 150.76) | 0.19 (0.16, 0.22) | <0.001 | 485.80 (407.14, 567.88) | 579.31 (431.32, 685.67) | 0.63 (0.32, 0.95) | <0.001 | 7.81 (6.54, 9.15) | 9.33 (6.93, 11.05) | 0.63 (0.32, 0.95) | <0.001 |
| Eastern Sub-Saharan Africa | 31.66 (19.29, 45.37) | 34.19 (20.70, 48.56) | 0.27 (0.23, 0.31) | <0.001 | 113.21 (91.94, 135.90) | 123.13 (100.46, 147.20) | 0.30 (0.27, 0.33) | <0.001 | 234.19 (187.77, 283.72) | 238.59 (176.99, 310.33) | 0.05 (-0.18, 0.29) | 0.661 | 3.75 (3.00, 4.57) | 3.82 (2.83, 4.99) | 0.05 (-0.19, 0.29) | 0.680 |
| Central Latin | 31.16 | 29.46 | -0.19 (-0.20, | <0.001 | 100.43 (82.90, | 101.63 (83.49, | 0.04 (0.02, | 0.001 | 368.35 | 340.88 | -0.09 | 0.599 | 5.93 | 5.48 | -0.10 | 0.595 |

| | | | | | | | | | | | | | | | | |
|---|---|---|---|---|---|---|---|---|---|---|---|---|---|---|---|---|
| America | (18.70, 44.61) | (17.77, 42.65) | -0.17) | | 121.05) | 122.23) | 0.06) | | (347.35, 390.49) | (296.41, 392.70) | (-0.44, 0.26) | | (5.59, 6.30) | (4.77, 6.32) | (-0.45, 0.26) | |
| Tropical Latin America | 24.65 (14.60, 35.79) | 23.71 (14.26, 34.63) | -0.13 (-0.20, -0.06) | <0.001 | 113.48 (89.21, 143.55) | 116.32 (92.24, 146.36) | 0.08 (0.04, 0.12) | <0.001 | 372.06 (342.27, 402.46) | 241.73 (221.43, 264.59) | -1.45 (-1.83, -1.05) | <0.001 | 5.97 (5.49, 6.46) | 3.86 (3.54, 4.22) | -1.47 (-1.86, -1.07) | <0.001 |
| Central Sub-Saharan Africa | 37.00 (22.39, 52.62) | 35.94 (21.76, 50.97) | -0.10 (-0.12, -0.07) | <0.001 | 108.63 (90.63, 128.43) | 107.56 (90.61, 128.38) | -0.04 (-0.04, -0.03) | <0.001 | 236.51 (150.48, 366.28) | 199.19 (123.86, 300.16) | -0.61 (-0.68, -0.54) | <0.001 | 3.80 (2.41, 5.91) | 3.20 (1.98, 4.83) | -0.61 (-0.68, -0.54) | <0.001 |
| Western Europe | 38.22 (23.15, 54.64) | 33.35 (20.08, 47.07) | -0.48 (-0.52, -0.44) | <0.001 | 126.61 (103.44, 149.83) | 119.35 (99.35, 139.19) | -0.20 (-0.25, -0.14) | <0.001 | 210.88 (197.75, 222.49) | 82.83 (77.55, 89.52) | -3.24 (-3.52, -2.95) | <0.001 | 3.37 (3.16, 3.56) | 1.31 (1.23, 1.42) | -3.27 (-3.57, -2.98) | <0.001 |
| High-income North America | 35.69 (19.29, 55.34) | 41.56 (25.80, 59.18) | 0.52 (0.48, 0.56) | <0.001 | 113.00 (90.31, 137.77) | 121.02 (99.25, 147.52) | 0.27 (0.07, 0.47) | 0.009 | 165.79 (153.41, 175.95) | 144.86 (132.24, 163.51) | -0.46 (-0.97, 0.05) | 0.077 | 2.62 (2.42, 2.79) | 2.29 (2.09, 2.59) | -0.46 (-0.98, 0.06) | 0.083 |
| Central Asia | 38.55 (23.14, 54.73) | 36.85 (21.69, 52.32) | -0.14 (-0.17, -0.12) | <0.001 | 139.58 (118.10, 163.59) | 138.20 (116.55, 163.23) | -0.04 (-0.05, -0.03) | <0.001 | 519.11 (478.37, 560.08) | 591.35 (510.71, 677.12) | 0.46 (-0.15, 1.06) | 0.139 | 8.38 (7.72, 9.05) | 9.56 (8.26, 10.95) | 0.46 (-0.15, 1.07) | 0.137 |
| Eastern Europe | 38.77 (23.05, 55.54) | 36.57 (21.54, 52.95) | -0.20 (-0.24, -0.16) | <0.001 | 112.68 (91.47, 135.90) | 108.60 (87.87, 131.33) | -0.13 (-0.17, -0.09) | <0.001 | 517.16 (455.47, 559.38) | 660.58 (560.34, 839.09) | 1.03 (-0.60, 2.69) | 0.217 | 8.36 (7.37, 9.05) | 10.70 (9.07, 13.61) | 1.04 (-0.59, 2.70) | 0.214 |
| Southern Sub-Saharan Africa | 44.14 (26.84, 62.28) | 43.66 (26.85, 61.71) | -0.03 (-0.05, -0.02) | <0.001 | 130.30 (105.40, 158.05) | 132.92 (108.70, 159.72) | 0.07 (0.03, 0.12) | 0.001 | 163.10 (123.32, 213.88) | 147.70 (114.18, 189.72) | -0.39 (-1.11, 0.34) | 0.296 | 2.60 (1.95, 3.42) | 2.35 (1.82, 3.03) | -0.40 (-1.13, 0.34) | 0.287 |
| High-income Asia Pacific | 22.20 (13.15, 32.64) | 20.11 (11.82, 29.84) | -0.34 (-0.37, -0.31) | <0.001 | 81.91 (66.17, 98.97) | 75.03 (61.12, 90.04) | -0.29 (-0.34, -0.25) | <0.001 | 269.51 (214.96, 309.90) | 85.55 (72.80, 96.48) | -3.89 (-4.23, -3.54) | <0.001 | 4.33 (3.44, 4.99) | 1.35 (1.15, 1.53) | -3.94 (-4.29, -3.59) | <0.001 |
| Central Europe | 25.69 (14.94, 37.55) | 23.92 (14.49, 34.48) | -0.26 (-0.29, -0.22) | <0.001 | 139.44 (112.40, 172.78) | 127.31 (104.25, 154.73) | -0.32 (-0.35, -0.28) | <0.001 | 469.17 (446.98, 492.27) | 172.08 (148.86, 197.29) | -3.42 (-4.00, -2.84) | <0.001 | 7.54 (7.19, 7.90) | 2.73 (2.35, 3.14) | -3.46 (-4.04, -2.87) | <0.001 |
| Andean Latin America | 14.47 (7.95, 22.89) | 14.64 (8.14, 22.81) | 0.05 (0.02, 0.08) | 0.002 | 67.73 (53.63, 83.56) | 71.88 (57.22, 88.61) | 0.20 (0.18, 0.21) | <0.001 | 398.73 (307.46, 496.10) | 284.86 (210.89, 381.58) | -1.17 (-1.76, -0.57) | <0.001 | 6.42 (4.94, 8.01) | 4.58 (3.38, 6.16) | -1.18 (-1.78, -0.58) | <0.001 |
| Caribbean | 69.58 | 71.88 | 0.13 (0.10, | <0.001 | 218.31 | 221.20 | 0.03 (-0.03, | 0.297 | 408.75 | 320.26 | -0.81 | <0.001 | 6.57 | 5.14 | -0.82 | <0.001 |

| | | | | | | | | | | | | | | | | |
|---|---|---|---|---|---|---|---|---|---|---|---|---|---|---|---|---|
| | (44.02, 96.61) | (45.76, 100.58) | 0.15) | | (187.15, 252.72) | (189.85, 253.15) | 0.10) | | (351.90, 469.61) | (229.51, 446.95) | (-1.24, -0.39) | | (5.65, 7.57) | (3.66, 7.21) | (-1.25, -0.39) | |
| Southern Latin America | 18.96 (10.82, 28.32) | 18.66 (10.86, 28.28) | -0.05 (-0.07, -0.04) | <0.001 | 67.43 (55.85, 80.79) | 66.67 (55.11, 80.07) | -0.05 (-0.10, -0.00) | 0.039 | 262.96 (228.66, 299.78) | 147.37 (121.62, 176.34) | -2.05 (-2.57, -1.52) | <0.001 | 4.23 (3.68, 4.84) | 2.36 (1.95, 2.84) | -2.06 (-2.60, -1.53) | <0.001 |
| Oceania | 29.39 (17.14, 42.44) | 29.87 (17.45, 43.49) | 0.06 (-0.01, 0.12) | 0.075 | 145.95 (114.35, 183.24) | 150.25 (116.88, 187.35) | 0.10 (0.07, 0.12) | <0.001 | 644.82 (436.66, 990.11) | 744.32 (467.32, 1156.94) | 0.45 (0.21, 0.69) | <0.001 | 10.37 (6.99, 15.91) | 11.99 (7.51, 18.70) | 0.46 (0.21, 0.70) | <0.001 |
| Australasia | 77.89 (48.27, 110.32) | 73.03 (45.00, 104.33) | -0.20 (-0.28, -0.12) | <0.001 | 220.13 (184.71, 257.43) | 221.12 (187.27, 258.74) | -0.06 (-0.38, 0.27) | 0.736 | 163.99 (141.45, 190.85) | 77.29 (61.95, 93.52) | -2.50 (-3.63, -1.36) | <0.001 | 2.61 (2.25, 3.05) | 1.23 (0.98, 1.49) | -2.52 (-3.66, -1.36) | <0.001 |
| **30-34 years** | | | | | | | | | | | | | | | | |
| **Women** | | | | | | | | | | | | | | | | |
| Global | 31.40 (21.64, 41.71) | 31.75 (21.97, 41.78) | 0.04 (0.02, 0.06) | <0.001 | 232.06 (190.28, 280.57) | 242.08 (199.26, 292.26) | 0.15 (0.13, 0.17) | <0.001 | 420.10 (377.28, 471.10) | 314.35 (276.41, 354.53) | -0.94 (-1.41, -0.46) | <0.001 | 7.31 (6.56, 8.19) | 5.44 (4.76, 6.13) | -0.96 (-1.43, -0.48) | <0.001 |
| **SDI category** | | | | | | | | | | | | | | | | |
| High | 39.92 (27.35, 53.38) | 40.00 (26.97, 52.57) | 0.02 (-0.04, 0.07) | 0.568 | 249.75 (209.87, 295.23) | 283.29 (240.78, 332.57) | 0.44 (0.35, 0.53) | <0.001 | 144.37 (135.76, 153.40) | 107.32 (94.77, 122.55) | -0.97 (-1.36, -0.58) | <0.001 | 2.46 (2.31, 2.61) | 1.81 (1.59, 2.09) | -1.00 (-1.42, -0.58) | <0.001 |
| High-middle | 29.71 (20.56, 39.47) | 28.94 (19.87, 38.53) | -0.10 (-0.12, -0.08) | <0.001 | 262.19 (214.61, 318.78) | 277.75 (225.09, 338.24) | 0.20 (0.17, 0.24) | <0.001 | 301.23 (267.65, 347.10) | 190.88 (166.24, 218.30) | -1.62 (-2.50, -0.74) | <0.001 | 5.19 (4.60, 5.99) | 3.23 (2.79, 3.71) | -1.68 (-2.61, -0.75) | <0.001 |
| Middle | 28.63 (19.82, 38.15) | 30.43 (21.04, 40.30) | 0.21 (0.18, 0.24) | <0.001 | 235.02 (190.33, 286.52) | 252.78 (206.07, 307.71) | 0.26 (0.23, 0.28) | <0.001 | 514.34 (457.73, 575.53) | 335.53 (289.45, 380.75) | -1.50 (-2.12, -0.87) | <0.001 | 8.96 (7.96, 10.03) | 5.80 (4.99, 6.59) | -1.53 (-2.16, -0.89) | <0.001 |
| Low-middle | 30.25 (20.77, 40.46) | 31.23 (21.61, 41.36) | 0.11 (0.10, 0.12) | <0.001 | 199.64 (162.97, 241.12) | 208.86 (171.44, 252.47) | 0.16 (0.13, 0.18) | <0.001 | 658.60 (553.16, 801.86) | 480.91 (393.14, 570.49) | -1.09 (-1.54, -0.63) | <0.001 | 11.54 (9.69, 14.07) | 8.41 (6.82, 9.98) | -1.09 (-1.55, -0.64) | <0.001 |
| Low | 31.68 (21.89, 41.97) | 32.98 (22.99, 43.87) | 0.14 (0.13, 0.14) | <0.001 | 170.69 (142.23, 203.85) | 177.54 (147.68, 211.21) | 0.14 (0.12, 0.16) | <0.001 | 434.33 (341.07, 559.94) | 345.73 (279.89, 425.73) | -0.76 (-0.91, -0.60) | <0.001 | 7.61 (5.96, 9.82) | 6.04 (4.87, 7.46) | -0.76 (-0.92, -0.61) | <0.001 |
| **21 GBD region** | | | | | | | | | | | | | | | | |

| | | | | | | | | | | | | | | | | |
|---|---|---|---|---|---|---|---|---|---|---|---|---|---|---|---|---|
| South Asia | 31.64 (21.80, 42.15) | 32.35 (22.33, 42.95) | 0.08 (0.06, 0.10) | <0.001 | 203.45 (165.42, 246.63) | 213.29 (174.09, 258.92) | 0.16 (0.12, 0.20) | <0.001 | 760.13 (628.43, 917.62) | 577.78 (458.98, 705.09) | -0.97 (-1.69, -0.25) | 0.009 | 13.33 (10.99, 16.11) | 10.12 (8.04, 12.37) | -0.98 (-1.70, -0.25) | 0.008 |
| East Asia | 23.28 (15.83, 31.55) | 25.05 (17.17, 33.51) | 0.25 (0.24, 0.26) | <0.001 | 281.68 (215.25, 356.62) | 320.39 (245.52, 400.26) | 0.44 (0.42, 0.46) | <0.001 | 346.34 (268.52, 439.66) | 185.40 (142.89, 231.17) | -2.03 (-3.73, -0.30) | 0.022 | 5.92 (4.60, 7.57) | 3.05 (2.33, 3.84) | -2.17 (-3.93, -0.38) | 0.017 |
| North Africa and Middle East | 46.62 (33.49, 60.89) | 46.24 (33.05, 60.95) | -0.03 (-0.05, -0.01) | <0.001 | 308.76 (261.63, 362.48) | 304.69 (258.80, 358.30) | -0.03 (-0.09, 0.02) | 0.237 | 963.70 (834.87, 1104.30) | 558.25 (437.07, 695.43) | -1.82 (-2.00, -1.65) | <0.001 | 16.91 (14.61, 19.40) | 9.76 (7.64, 12.18) | -1.84 (-2.01, -1.66) | <0.001 |
| Western Sub-Saharan Africa | 27.94 (19.18, 37.21) | 30.36 (20.93, 40.63) | 0.29 (0.27, 0.30) | <0.001 | 141.73 (117.96, 169.35) | 152.53 (126.67, 182.11) | 0.26 (0.24, 0.28) | <0.001 | 221.36 (160.74, 295.06) | 184.26 (133.10, 246.81) | -0.62 (-0.90, -0.35) | <0.001 | 3.87 (2.80, 5.19) | 3.21 (2.31, 4.31) | -0.64 (-0.91, -0.36) | <0.001 |
| Southeast Asia | 22.06 (15.06, 30.37) | 23.89 (16.36, 32.45) | 0.28 (0.26, 0.31) | <0.001 | 167.97 (137.69, 201.54) | 186.23 (152.02, 224.46) | 0.37 (0.33, 0.42) | <0.001 | 534.46 (426.11, 681.07) | 364.10 (300.10, 439.98) | -1.29 (-1.49, -1.09) | <0.001 | 9.35 (7.46, 11.94) | 6.34 (5.20, 7.69) | -1.31 (-1.51, -1.10) | <0.001 |
| Eastern Sub-Saharan Africa | 28.51 (19.38, 37.85) | 29.65 (20.31, 39.52) | 0.13 (0.12, 0.14) | <0.001 | 136.90 (112.92, 164.60) | 141.76 (117.08, 170.13) | 0.12 (0.10, 0.15) | <0.001 | 197.85 (145.91, 295.36) | 139.01 (103.55, 182.44) | -1.20 (-1.32, -1.08) | <0.001 | 3.44 (2.53, 5.15) | 2.40 (1.77, 3.17) | -1.23 (-1.35, -1.10) | <0.001 |
| Central Latin America | 31.98 (22.14, 42.37) | 27.78 (18.88, 37.36) | -0.48 (-0.49, -0.46) | <0.001 | 224.23 (186.29, 269.68) | 197.99 (163.63, 238.05) | -0.43 (-0.46, -0.40) | <0.001 | 308.47 (288.89, 326.90) | 186.98 (150.50, 232.40) | -1.71 (-2.63, -0.78) | <0.001 | 5.37 (5.01, 5.70) | 3.23 (2.59, 4.02) | -1.74 (-2.65, -0.81) | <0.001 |
| Tropical Latin America | 19.03 (12.15, 26.82) | 17.11 (10.61, 24.00) | -0.36 (-0.48, -0.25) | <0.001 | 138.52 (111.88, 169.74) | 137.01 (110.72, 166.92) | -0.04 (-0.07, -0.02) | 0.001 | 368.54 (345.16, 392.53) | 159.54 (145.01, 173.94) | -2.85 (-3.20, -2.50) | <0.001 | 6.45 (6.05, 6.87) | 2.76 (2.51, 3.02) | -2.89 (-3.24, -2.53) | <0.001 |
| Central Sub-Saharan Africa | 35.04 (24.58, 46.81) | 33.72 (23.36, 45.86) | -0.13 (-0.14, -0.12) | <0.001 | 156.17 (130.61, 184.71) | 151.42 (126.56, 179.69) | -0.11 (-0.12, -0.10) | <0.001 | 238.21 (143.18, 381.63) | 189.06 (115.76, 296.38) | -0.80 (-1.00, -0.59) | <0.001 | 4.16 (2.48, 6.69) | 3.30 (2.01, 5.21) | -0.80 (-1.01, -0.60) | <0.001 |
| Western Europe | 43.31 (28.98, 58.15) | 40.58 (26.84, 54.40) | -0.21 (-0.25, -0.17) | <0.001 | 289.33 (246.19, 340.03) | 305.43 (262.43, 353.91) | 0.20 (0.12, 0.28) | <0.001 | 105.89 (98.29, 113.38) | 42.12 (38.67, 46.16) | -3.17 (-3.32, -3.03) | <0.001 | 1.80 (1.67, 1.92) | 0.69 (0.64, 0.76) | -3.27 (-3.42, -3.13) | <0.001 |
| High-income North America | 45.67 (30.96, 62.50) | 49.71 (33.43, 65.61) | 0.32 (0.24, 0.39) | <0.001 | 233.41 (190.83, 281.78) | 299.26 (244.80, 361.25) | 0.87 (0.70, 1.03) | <0.001 | 140.60 (132.01, 148.07) | 136.74 (126.12, 152.03) | -0.04 (-0.65, 0.58) | 0.907 | 2.37 (2.23, 2.48) | 2.33 (2.15, 2.59) | 0.01 (-0.54, 0.57) | 0.958 |

| | | | | | | | | | | | | | | | | |
|---|---|---|---|---|---|---|---|---|---|---|---|---|---|---|---|---|
| Central Asia | 44.25 (31.18, 58.39) | 42.70 (29.57, 56.26) | -0.12 (-0.13, -0.12) | <0.001 | 291.56 (250.17, 336.27) | 278.36 (239.76, 321.46) | -0.15 (-0.18, -0.13) | <0.001 | 490.58 (455.54, 526.04) | 527.22 (443.08, 633.29) | 0.07 (-0.38, 0.51) | 0.772 | 8.58 (7.96, 9.22) | 9.24 (7.78, 11.11) | 0.07 (-0.37, 0.52) | 0.751 |
| Eastern Europe | 41.38 (28.60, 54.64) | 44.31 (30.64, 58.13) | 0.23 (0.20, 0.26) | <0.001 | 278.36 (229.65, 333.38) | 306.43 (252.51, 368.35) | 0.35 (0.24, 0.46) | <0.001 | 202.47 (169.38, 224.29) | 236.45 (182.89, 306.26) | 0.66 (-0.04, 1.36) | 0.067 | 3.51 (2.93, 3.88) | 4.10 (3.15, 5.34) | 0.66 (-0.05, 1.38) | 0.068 |
| Southern Sub-Saharan Africa | 42.83 (30.20, 56.77) | 38.72 (27.24, 51.66) | -0.36 (-0.40, -0.31) | <0.001 | 174.11 (141.98, 210.06) | 155.77 (127.59, 188.83) | -0.38 (-0.42, -0.34) | <0.001 | 358.80 (285.45, 440.80) | 158.21 (94.00, 239.85) | -3.08 (-6.50, 0.47) | 0.088 | 6.27 (4.99, 7.72) | 2.74 (1.62, 4.19) | -3.11 (-6.58, 0.49) | 0.090 |
| High-income Asia Pacific | 25.65 (16.73, 34.63) | 24.87 (16.06, 33.86) | -0.09 (-0.14, -0.05) | <0.001 | 186.62 (155.83, 221.47) | 194.73 (160.63, 232.12) | 0.16 (0.08, 0.24) | <0.001 | 174.06 (146.25, 201.83) | 34.69 (30.28, 39.16) | -5.46 (-5.67, -5.24) | <0.001 | 3.02 (2.53, 3.51) | 0.57 (0.50, 0.65) | -5.62 (-5.85, -5.40) | <0.001 |
| Central Europe | 24.36 (16.01, 33.03) | 22.85 (15.13, 31.34) | -0.22 (-0.25, -0.20) | <0.001 | 232.76 (191.79, 282.10) | 229.52 (190.73, 275.48) | -0.03 (-0.07, 0.01) | 0.101 | 247.68 (232.98, 263.48) | 92.33 (76.10, 112.54) | -3.38 (-3.73, -3.04) | <0.001 | 4.27 (4.02, 4.52) | 1.54 (1.26, 1.87) | -3.49 (-3.85, -3.14) | <0.001 |
| Andean Latin America | 10.82 (5.84, 16.56) | 10.64 (5.57, 16.56) | -0.06 (-0.08, -0.03) | <0.001 | 99.94 (80.22, 122.80) | 102.26 (81.70, 125.48) | 0.08 (0.04, 0.13) | <0.001 | 344.71 (268.56, 437.93) | 142.06 (101.73, 194.62) | -3.12 (-4.03, -2.21) | <0.001 | 6.04 (4.71, 7.67) | 2.46 (1.72, 3.40) | -3.17 (-4.07, -2.26) | <0.001 |
| Caribbean | 81.86 (57.83, 105.83) | 84.27 (59.62, 109.69) | 0.11 (0.09, 0.12) | <0.001 | 422.51 (364.92, 483.30) | 413.56 (358.84, 472.78) | -0.07 (-0.08, -0.06) | <0.001 | 453.98 (368.80, 561.03) | 381.86 (242.22, 557.53) | -0.61 (-1.21, -0.02) | 0.044 | 7.93 (6.43, 9.81) | 6.66 (4.22, 9.78) | -0.62 (-1.22, -0.01) | 0.045 |
| Southern Latin America | 20.53 (13.32, 28.27) | 18.80 (11.98, 26.17) | -0.30 (-0.32, -0.29) | <0.001 | 136.71 (114.80, 159.95) | 133.23 (113.18, 156.76) | -0.09 (-0.12, -0.06) | <0.001 | 203.36 (170.71, 238.54) | 80.51 (65.31, 97.19) | -3.06 (-3.48, -2.64) | <0.001 | 3.57 (2.99, 4.18) | 1.40 (1.13, 1.70) | -3.09 (-3.52, -2.66) | <0.001 |
| Oceania | 30.00 (20.38, 40.66) | 30.63 (20.74, 41.83) | 0.08 (0.06, 0.10) | <0.001 | 235.42 (187.52, 292.11) | 238.64 (188.85, 297.11) | 0.06 (0.00, 0.12) | 0.035 | 542.53 (322.21, 883.81) | 641.44 (400.61, 1048.27) | 0.54 (0.38, 0.69) | <0.001 | 9.45 (5.57, 15.38) | 11.19 (6.87, 18.34) | 0.55 (0.39, 0.70) | <0.001 |
| Australasia | 35.39 (23.52, 47.02) | 28.04 (18.78, 38.67) | -0.82 (-0.85, -0.79) | <0.001 | 220.35 (187.21, 258.70) | 188.38 (158.48, 222.32) | -0.52 (-0.61, -0.44) | <0.001 | 102.57 (87.98, 118.50) | 44.60 (36.10, 54.29) | -2.83 (-3.65, -1.99) | <0.001 | 1.75 (1.49, 2.03) | 0.75 (0.61, 0.92) | -2.84 (-3.70, -1.97) | <0.001 |
| **Men** | | | | | | | | | | | | | | | | |
| Global | 46.90 (33.38, | 45.69 (32.68, | -0.09 (-0.12, -0.07) | <0.001 | 291.29 (237.27, | 300.50 (246.34, | 0.11 (0.09, 0.13) | <0.001 | 848.97 (793.02, | 818.23 (741.26, | -0.13 (-0.39, | 0.324 | 14.84 (13.85, | 14.30 (12.97, | -0.13 (-0.40, | 0.325 |

| | | | | | | | | | | | | | | | | |
|---|---|---|---|---|---|---|---|---|---|---|---|---|---|---|---|---|
| | 61.50) | 59.55) | | | 350.72) | 361.92) | | | 909.35) | 911.34) | 0.13) | | 15.90) | 15.96) | 0.13) | |
| **SDI category** | | | | | | | | | | | | | | | | |
| High | 59.21 (42.20, 78.46) | 52.43 (37.52, 68.18) | -0.42 (-0.48, -0.36) | <0.001 | 283.13 (236.51, 338.48) | 290.83 (247.18, 340.29) | 0.10 (0.02, 0.19) | 0.017 | 469.19 (447.17, 490.06) | 302.01 (271.41, 344.04) | -1.54 (-1.96, -1.13) | <0.001 | 8.14 (7.77, 8.49) | 5.20 (4.68, 5.96) | -1.57 (-2.00, -1.13) | <0.001 |
| High-middle | 45.46 (32.43, 59.62) | 40.47 (28.80, 53.11) | -0.41 (-0.44, -0.37) | <0.001 | 307.17 (247.25, 377.42) | 305.02 (244.26, 374.15) | -0.02 (-0.05, -0.00) | 0.019 | 940.34 (850.01, 1013.50) | 702.66 (634.35, 797.60) | -1.08 (-1.40, -0.76) | <0.001 | 16.43 (14.85, 17.71) | 12.22 (11.00, 13.92) | -1.10 (-1.42, -0.77) | <0.001 |
| Middle | 43.05 (30.64, 56.26) | 45.03 (32.14, 58.81) | 0.16 (0.12, 0.20) | <0.001 | 308.48 (248.26, 376.31) | 322.12 (260.89, 390.75) | 0.15 (0.13, 0.17) | <0.001 | 876.40 (808.17, 953.96) | 883.86 (794.81, 989.31) | 0.01 (-0.23, 0.26) | 0.936 | 15.30 (14.06, 16.69) | 15.43 (13.88, 17.29) | 0.01 (-0.24, 0.26) | 0.945 |
| Low-middle | 43.88 (31.01, 57.45) | 45.64 (32.36, 59.69) | 0.13 (0.12, 0.15) | <0.001 | 262.02 (216.35, 315.40) | 279.67 (230.62, 336.57) | 0.23 (0.21, 0.24) | <0.001 | 1082.82 (942.58, 1237.84) | 1144.24 (992.03, 1334.15) | 0.23 (-0.28, 0.74) | 0.372 | 19.01 (16.50, 21.77) | 20.10 (17.41, 23.47) | 0.23 (-0.28, 0.75) | 0.370 |
| Low | 47.00 (33.26, 61.72) | 49.91 (35.40, 65.60) | 0.20 (0.19, 0.22) | <0.001 | 259.74 (215.16, 309.47) | 281.02 (233.30, 336.19) | 0.28 (0.25, 0.31) | <0.001 | 727.74 (601.20, 876.23) | 766.40 (638.11, 916.52) | 0.10 (-0.06, 0.27) | 0.225 | 12.75 (10.53, 15.37) | 13.42 (11.16, 16.06) | 0.10 (-0.07, 0.27) | 0.236 |
| **21 GBD region** | | | | | | | | | | | | | | | | |
| South Asia | 44.02 (30.94, 57.58) | 45.52 (32.06, 59.46) | 0.11 (0.09, 0.13) | <0.001 | 247.23 (203.88, 297.65) | 264.19 (216.68, 317.92) | 0.23 (0.19, 0.27) | <0.001 | 1323.46 (1139.66, 1520.52) | 1418.31 (1205.77, 1667.65) | 0.22 (-0.28, 0.73) | 0.378 | 23.29 (20.04, 26.77) | 24.96 (21.23, 29.38) | 0.23 (-0.27, 0.73) | 0.374 |
| East Asia | 34.74 (24.14, 46.05) | 32.77 (22.61, 43.68) | -0.19 (-0.26, -0.12) | <0.001 | 343.70 (261.66, 435.80) | 342.59 (258.82, 434.80) | -0.01 (-0.06, 0.04) | 0.722 | 576.65 (474.80, 678.22) | 635.80 (523.06, 754.50) | 0.37 (-0.24, 0.98) | 0.235 | 9.94 (8.11, 11.66) | 10.96 (9.01, 13.07) | 0.37 (-0.26, 1.00) | 0.249 |
| North Africa and Middle East | 66.91 (48.81, 85.79) | 68.16 (49.73, 87.51) | 0.06 (0.03, 0.09) | <0.001 | 440.39 (372.91, 514.99) | 456.24 (387.83, 535.69) | 0.15 (0.04, 0.25) | 0.007 | 1494.23 (1294.23, 1767.05) | 886.85 (717.73, 1115.55) | -1.76 (-1.90, -1.63) | <0.001 | 26.18 (22.71, 31.01) | 15.45 (12.50, 19.51) | -1.78 (-1.92, -1.65) | <0.001 |
| Western Sub-Saharan Africa | 45.21 (31.93, 59.41) | 49.24 (34.69, 64.92) | 0.29 (0.26, 0.32) | <0.001 | 256.77 (213.03, 306.47) | 282.95 (235.43, 339.29) | 0.35 (0.30, 0.40) | <0.001 | 338.42 (253.33, 454.95) | 363.04 (268.67, 471.72) | 0.24 (-0.01, 0.49) | 0.065 | 5.88 (4.35, 7.92) | 6.30 (4.64, 8.24) | 0.23 (-0.02, 0.49) | 0.076 |
| Southeast Asia | 39.22 (27.65, | 40.10 (28.49, | 0.08 (0.06, 0.10) | <0.001 | 258.62 (207.04, | 272.83 (218.63, | 0.19 (0.15, 0.23) | <0.001 | 810.92 (701.73, | 906.64 (736.60, | 0.46 (0.09, | 0.016 | 14.16 (12.24, | 15.84 (12.81, | 0.47 (0.09, 0.85) | 0.016 |

| | | | | | | | | | | | | | | | | |
|---|---|---|---|---|---|---|---|---|---|---|---|---|---|---|---|---|
| | 52.04) | 52.92) | | | 317.35) | 334.88) | | | 930.90) | 1071.46) | 0.84) | | 16.25) | 18.73) | | |
| Eastern Sub-Saharan Africa | 45.89 (32.23, 60.79) | 49.23 (34.84, 64.62) | 0.24 (0.20, 0.28) | <0.001 | 258.36 (212.59, 310.07) | 281.39 (232.23, 339.12) | 0.30 (0.26, 0.34) | <0.001 | 445.74 (352.43, 536.31) | 438.87 (333.59, 564.06) | -0.07 (-0.28, 0.14) | 0.490 | 7.75 (6.10, 9.35) | 7.62 (5.76, 9.83) | -0.08 (-0.29, 0.13) | 0.463 |
| Central Latin America | 44.75 (31.41, 59.36) | 42.55 (29.51, 56.70) | -0.17 (-0.21, -0.14) | <0.001 | 227.04 (187.15, 273.90) | 230.15 (189.49, 277.63) | 0.04 (0.01, 0.08) | 0.012 | 635.31 (603.82, 670.36) | 573.18 (496.07, 659.56) | -0.12 (-0.69, 0.45) | 0.680 | 11.12 (10.57, 11.75) | 10.02 (8.64, 11.56) | -0.12 (-0.69, 0.45) | 0.680 |
| Tropical Latin America | 34.72 (24.01, 46.44) | 30.56 (20.57, 41.04) | -0.44 (-0.52, -0.37) | <0.001 | 260.14 (201.22, 328.92) | 262.38 (204.85, 331.45) | 0.02 (-0.02, 0.06) | 0.289 | 886.92 (826.09, 952.69) | 469.93 (435.36, 508.53) | -2.18 (-2.53, -1.83) | <0.001 | 15.51 (14.44, 16.63) | 8.14 (7.56, 8.84) | -2.21 (-2.57, -1.86) | <0.001 |
| Central Sub-Saharan Africa | 52.71 (37.44, 69.77) | 51.43 (35.56, 68.26) | -0.09 (-0.10, -0.08) | <0.001 | 246.19 (207.45, 288.93) | 243.43 (205.37, 289.20) | -0.04 (-0.05, -0.03) | <0.001 | 566.11 (367.79, 798.10) | 490.82 (306.48, 745.85) | -0.52 (-0.64, -0.39) | <0.001 | 9.92 (6.44, 14.03) | 8.59 (5.34, 13.06) | -0.52 (-0.64, -0.39) | <0.001 |
| Western Europe | 60.29 (43.16, 78.70) | 49.64 (35.47, 64.65) | -0.68 (-0.71, -0.65) | <0.001 | 288.77 (241.00, 342.44) | 264.74 (225.57, 309.06) | -0.30 (-0.35, -0.25) | <0.001 | 470.67 (448.90, 493.50) | 190.29 (179.56, 203.97) | -3.13 (-3.55, -2.71) | <0.001 | 8.19 (7.81, 8.59) | 3.28 (3.10, 3.53) | -3.16 (-3.59, -2.73) | <0.001 |
| High-income North America | 67.11 (46.66, 91.34) | 58.12 (41.67, 75.47) | -0.51 (-0.56, -0.45) | <0.001 | 272.16 (223.35, 329.68) | 273.44 (225.88, 328.44) | 0.05 (-0.14, 0.23) | 0.630 | 436.96 (416.91, 459.26) | 319.19 (288.79, 353.14) | -1.07 (-1.56, -0.58) | <0.001 | 7.54 (7.21, 7.92) | 5.49 (4.97, 6.11) | -1.09 (-1.59, -0.58) | <0.001 |
| Central Asia | 63.69 (46.45, 83.14) | 61.06 (43.94, 79.90) | -0.14 (-0.16, -0.12) | <0.001 | 322.66 (273.43, 376.40) | 318.63 (270.53, 373.71) | -0.04 (-0.06, -0.03) | <0.001 | 1475.26 (1384.83, 1568.65) | 1267.11 (1106.43, 1431.82) | -0.64 (-1.35, 0.07) | 0.078 | 25.93 (24.34, 27.57) | 22.27 (19.43, 25.12) | -0.64 (-1.35, 0.07) | 0.079 |
| Eastern Europe | 66.41 (48.39, 86.37) | 63.92 (46.91, 83.84) | -0.12 (-0.17, -0.08) | <0.001 | 263.02 (217.36, 316.39) | 255.86 (213.17, 307.25) | -0.10 (-0.14, -0.06) | <0.001 | 1423.22 (1216.96, 1527.00) | 1383.79 (1176.39, 1754.18) | -0.29 (-1.05, 0.48) | 0.460 | 25.05 (21.42, 26.90) | 24.37 (20.71, 30.94) | -0.29 (-1.05, 0.48) | 0.464 |
| Southern Sub-Saharan Africa | 64.30 (45.74, 83.63) | 63.47 (45.75, 82.93) | -0.04 (-0.06, -0.03) | <0.001 | 296.11 (240.65, 360.08) | 301.41 (246.55, 362.35) | 0.07 (0.04, 0.09) | <0.001 | 540.36 (431.72, 676.40) | 394.48 (293.22, 501.29) | -1.25 (-2.89, 0.41) | 0.138 | 9.41 (7.51, 11.80) | 6.84 (5.05, 8.74) | -1.27 (-2.94, 0.42) | 0.140 |
| High-income Asia Pacific | 37.06 (25.98, 48.92) | 32.83 (22.88, 43.68) | -0.41 (-0.43, -0.39) | <0.001 | 184.43 (152.55, 221.41) | 167.11 (138.72, 200.55) | -0.34 (-0.36, -0.31) | <0.001 | 486.94 (411.33, 547.70) | 172.02 (153.58, 190.25) | -3.62 (-3.92, -3.32) | <0.001 | 8.50 (7.17, 9.58) | 2.96 (2.65, 3.28) | -3.67 (-3.97, -3.36) | <0.001 |
| Central Europe | 41.26 (29.02, | 37.20 (26.11, | -0.36 (-0.39, -0.33) | <0.001 | 313.48 (250.51, | 283.59 (230.18, | -0.34 (-0.37, | <0.001 | 1245.22 (1195.56, | 381.71 (332.82, | -4.09 (-4.61, | <0.001 | 21.83 (20.97, | 6.59 (5.73, | -4.14 (-4.67, | <0.001 |

| | | | | | | | | | | | | | | | | |
|---|---|---|---|---|---|---|---|---|---|---|---|---|---|---|---|---|
| | 54.54) | 49.22) | | | 386.71) | 346.28) | -0.30) | | 1297.17) | 434.33) | -3.57) | | 22.74) | 7.54) | -3.61) | |
| Andean Latin America | 19.22 (12.46, 26.79) | 19.26 (12.35, 26.96) | 0.01 (-0.07, 0.08) | 0.853 | 149.74 (118.35, 185.41) | 157.95 (125.16, 196.63) | 0.18 (0.16, 0.20) | <0.001 | 651.92 (521.17, 806.00) | 447.45 (334.77, 589.76) | -1.29 (-1.76, -0.82) | <0.001 | 11.43 (9.14, 14.16) | 7.81 (5.82, 10.29) | -1.30 (-1.78, -0.83) | <0.001 |
| Caribbean | 96.34 (68.97, 124.54) | 99.39 (72.38, 128.02) | 0.11 (0.08, 0.14) | <0.001 | 483.76 (414.25, 560.74) | 490.28 (418.56, 565.81) | 0.04 (-0.00, 0.08) | 0.061 | 795.52 (698.28, 905.19) | 642.43 (468.48, 846.27) | -0.64 (-1.01, -0.26) | 0.001 | 13.90 (12.18, 15.82) | 11.20 (8.13, 14.82) | -0.64 (-1.02, -0.26) | 0.001 |
| Southern Latin America | 31.44 (21.67, 41.96) | 29.53 (20.23, 39.41) | -0.21 (-0.23, -0.20) | <0.001 | 152.40 (126.84, 181.37) | 148.41 (125.16, 174.36) | -0.10 (-0.14, -0.05) | <0.001 | 549.98 (487.82, 621.43) | 277.31 (234.13, 322.74) | -2.37 (-2.79, -1.95) | <0.001 | 9.67 (8.56, 10.92) | 4.85 (4.10, 5.65) | -2.38 (-2.80, -1.96) | <0.001 |
| Oceania | 46.00 (32.10, 60.78) | 46.98 (32.88, 62.81) | 0.07 (0.05, 0.09) | <0.001 | 330.58 (257.87, 416.20) | 341.78 (266.42, 430.59) | 0.11 (0.09, 0.13) | <0.001 | 1687.22 (1193.49, 2434.94) | 1873.97 (1293.95, 2736.14) | 0.35 (0.17, 0.53) | <0.001 | 29.59 (20.85, 42.75) | 32.88 (22.66, 48.07) | 0.35 (0.17, 0.54) | <0.001 |
| Australasia | 125.35 (93.78, 163.32) | 113.65 (82.82, 147.20) | -0.33 (-0.37, -0.30) | <0.001 | 513.32 (437.09, 594.22) | 511.09 (439.79, 591.50) | -0.08 (-0.37, 0.21) | 0.597 | 372.03 (336.52, 407.41) | 200.15 (166.94, 235.14) | -2.10 (-2.58, -1.63) | <0.001 | 6.46 (5.82, 7.07) | 3.47 (2.88, 4.08) | -2.11 (-2.59, -1.63) | <0.001 |
| **35-39 years** | | | | | | | | | | | | | | | | |
| **Women** | | | | | | | | | | | | | | | | |
| Global | 38.44 (24.85, 57.45) | 39.05 (25.62, 57.41) | 0.05 (0.05, 0.06) | <0.001 | 431.89 (355.38, 524.68) | 437.33 (364.54, 526.80) | 0.05 (0.00, 0.09) | 0.048 | 621.74 (560.05, 691.92) | 491.71 (434.42, 550.46) | -0.79 (-1.11, -0.47) | <0.001 | 11.80 (10.63, 13.18) | 9.31 (8.16, 10.40) | -0.80 (-1.12, -0.48) | <0.001 |
| **SDI category** | | | | | | | | | | | | | | | | |
| High | 39.82 (25.45, 60.20) | 33.52 (22.39, 48.79) | -0.59 (-0.63, -0.55) | <0.001 | 414.38 (353.90, 485.42) | 440.76 (381.59, 513.27) | 0.21 (0.14, 0.27) | <0.001 | 242.66 (231.57, 253.63) | 175.58 (157.90, 198.65) | -1.14 (-1.53, -0.74) | <0.001 | 4.52 (4.33, 4.72) | 3.26 (2.93, 3.69) | -1.15 (-1.58, -0.72) | <0.001 |
| High-middle | 35.20 (22.76, 52.48) | 34.71 (22.85, 51.28) | -0.05 (-0.07, -0.03) | <0.001 | 477.56 (386.24, 587.89) | 489.87 (398.46, 602.66) | 0.09 (0.06, 0.12) | <0.001 | 496.86 (443.19, 554.46) | 308.94 (275.28, 346.66) | -1.55 (-2.00, -1.10) | <0.001 | 9.35 (8.34, 10.48) | 5.70 (5.06, 6.43) | -1.61 (-2.07, -1.14) | <0.001 |
| Middle | 38.25 (24.59, 57.26) | 40.94 (27.04, 60.19) | 0.24 (0.20, 0.29) | <0.001 | 456.07 (367.28, 568.81) | 464.38 (380.06, 570.57) | 0.06 (0.03, 0.10) | 0.001 | 726.59 (645.85, 817.59) | 503.36 (441.11, 567.26) | -1.20 (-1.43, -0.96) | <0.001 | 13.79 (12.25, 15.58) | 9.49 (8.30, 10.71) | -1.22 (-1.45, -0.98) | <0.001 |
| Low-middle | 40.16 (26.11, | 41.13 (26.90, | 0.08 (0.06, 0.09) | <0.001 | 387.39 (322.39, | 398.21 (331.97, | 0.10 (0.07, 0.12) | <0.001 | 938.28 (794.37, | 776.19 (636.17, | -0.59 (-0.97, | 0.002 | 17.97 (15.23, | 14.85 (12.15, | -0.59 (-0.97, | 0.002 |

| | | | | | | | | | | | | | | | | |
|---|---|---|---|---|---|---|---|---|---|---|---|---|---|---|---|---|
| | 59.65) | 60.62) | | | 470.79) | 482.97) | | | 1119.16) | 941.18) | -0.21) | | 21.50) | 17.98) | -0.22) | |
| Low | 42.17 (27.62, 62.35) | 43.42 (28.66, 64.12) | 0.10 (0.08, 0.12) | <0.001 | 332.08 (280.84, 396.75) | 342.06 (289.21, 410.69) | 0.11 (0.08, 0.14) | <0.001 | 714.09 (573.09, 910.98) | 585.90 (470.72, 713.13) | -0.71 (-0.86, -0.56) | <0.001 | 13.68 (11.00, 17.48) | 11.21 (8.98, 13.66) | -0.72 (-0.87, -0.56) | <0.001 |
| **21 GBD region** | | | | | | | | | | | | | | | | |
| South Asia | 42.40 (27.76, 61.91) | 43.25 (28.36, 63.62) | 0.06 (0.03, 0.09) | <0.001 | 395.42 (328.35, 480.41) | 413.65 (341.11, 505.35) | 0.15 (0.12, 0.18) | <0.001 | 1026.45 (858.12, 1237.72) | 903.30 (725.20, 1114.04) | -0.58 (-1.35, 0.20) | 0.144 | 19.68 (16.45, 23.72) | 17.31 (13.88, 21.37) | -0.58 (-1.35, 0.20) | 0.143 |
| East Asia | 33.20 (20.84, 50.71) | 35.13 (22.46, 52.25) | 0.19 (0.18, 0.20) | <0.001 | 539.18 (407.48, 702.34) | 601.30 (455.94, 778.92) | 0.37 (0.34, 0.39) | <0.001 | 538.25 (424.34, 676.58) | 282.66 (220.50, 350.69) | -2.19 (-2.51, -1.87) | <0.001 | 10.01 (7.74, 12.73) | 5.01 (3.82, 6.37) | -2.36 (-2.70, -2.02) | <0.001 |
| North Africa and Middle East | 68.28 (45.40, 101.70) | 67.69 (44.92, 101.43) | -0.02 (-0.04, 0.00) | 0.057 | 586.51 (505.71, 688.39) | 578.09 (496.70, 678.42) | -0.04 (-0.11, 0.04) | 0.319 | 1477.52 (1287.28, 1723.36) | 793.46 (616.86, 991.75) | -2.12 (-2.32, -1.92) | <0.001 | 28.36 (24.69, 33.14) | 15.15 (11.75, 19.02) | -2.14 (-2.34, -1.94) | <0.001 |
| Western Sub-Saharan Africa | 37.12 (23.81, 55.98) | 39.79 (25.99, 58.68) | 0.24 (0.20, 0.28) | <0.001 | 268.56 (227.55, 320.94) | 290.19 (245.70, 348.71) | 0.28 (0.24, 0.32) | <0.001 | 359.86 (268.75, 485.00) | 289.92 (215.82, 396.75) | -0.74 (-0.95, -0.53) | <0.001 | 6.87 (5.10, 9.30) | 5.51 (4.07, 7.57) | -0.76 (-0.97, -0.55) | <0.001 |
| Southeast Asia | 31.22 (19.81, 47.39) | 31.37 (20.26, 46.73) | 0.02 (-0.02, 0.06) | 0.346 | 316.40 (259.45, 385.06) | 339.07 (279.03, 412.51) | 0.26 (0.17, 0.34) | <0.001 | 758.83 (621.45, 955.39) | 562.82 (461.58, 692.26) | -1.01 (-1.12, -0.91) | <0.001 | 14.51 (11.88, 18.35) | 10.71 (8.71, 13.20) | -1.03 (-1.13, -0.93) | <0.001 |
| Eastern Sub-Saharan Africa | 37.70 (24.23, 56.25) | 39.02 (24.93, 57.97) | 0.12 (0.10, 0.14) | <0.001 | 262.88 (220.33, 318.09) | 273.75 (227.98, 330.02) | 0.14 (0.12, 0.16) | <0.001 | 371.22 (278.93, 546.27) | 261.83 (196.10, 340.70) | -1.20 (-1.30, -1.10) | <0.001 | 7.06 (5.27, 10.43) | 4.94 (3.67, 6.49) | -1.23 (-1.33, -1.12) | <0.001 |
| Central Latin America | 35.94 (22.92, 53.90) | 31.23 (19.17, 47.46) | -0.48 (-0.49, -0.46) | <0.001 | 404.19 (337.68, 486.52) | 356.78 (298.55, 428.97) | -0.43 (-0.46, -0.39) | <0.001 | 509.24 (481.36, 536.07) | 293.72 (240.18, 361.87) | -1.69 (-2.04, -1.34) | <0.001 | 9.68 (9.15, 10.18) | 5.53 (4.50, 6.81) | -1.73 (-2.09, -1.37) | <0.001 |
| Tropical Latin America | 19.57 (11.06, 32.12) | 15.65 (8.76, 25.80) | -0.76 (-0.78, -0.72) | <0.001 | 248.63 (198.61, 305.80) | 239.09 (191.95, 293.87) | -0.13 (-0.15, -0.11) | <0.001 | 662.62 (625.14, 699.75) | 309.75 (284.27, 336.50) | -2.50 (-2.72, -2.28) | <0.001 | 12.70 (11.97, 13.40) | 5.88 (5.39, 6.38) | -2.53 (-2.76, -2.31) | <0.001 |
| Central Sub-Saharan Africa | 45.24 (29.47, 67.64) | 43.65 (28.46, 65.05) | -0.13 (-0.14, -0.11) | <0.001 | 292.30 (252.08, 346.01) | 283.15 (241.24, 335.73) | -0.10 (-0.12, -0.09) | <0.001 | 382.77 (233.16, 636.41) | 309.66 (189.61, 489.31) | -0.74 (-0.88, -0.59) | <0.001 | 7.31 (4.41, 12.24) | 5.91 (3.58, 9.39) | -0.74 (-0.89, -0.59) | <0.001 |

| | | | | | | | | | | | | | | | | |
|---|---|---|---|---|---|---|---|---|---|---|---|---|---|---|---|---|
| Western Europe | 32.73 (20.31, 48.27) | 26.69 (16.78, 39.16) | -0.69 (-0.73, -0.65) | <0.001 | 446.30 (384.17, 522.47) | 445.47 (386.78, 513.53) | 0.02 (-0.06, 0.10) | 0.593 | 196.19 (185.29, 207.38) | 79.87 (74.32, 86.31) | -3.04 (-3.16, -2.92) | <0.001 | 3.66 (3.47, 3.85) | 1.46 (1.36, 1.57) | -3.10 (-3.37, -2.83) | <0.001 |
| High-income North America | 55.53 (34.69, 85.53) | 39.74 (27.36, 55.29) | -1.14 (-1.18, -1.10) | <0.001 | 418.06 (350.61, 498.41) | 468.70 (398.35, 554.76) | 0.40 (0.33, 0.47) | <0.001 | 269.96 (256.97, 282.44) | 235.98 (223.11, 256.07) | -0.54 (-0.90, -0.17) | 0.005 | 5.02 (4.80, 5.22) | 4.44 (4.20, 4.82) | -0.48 (-0.82, -0.13) | 0.007 |
| Central Asia | 48.36 (32.26, 70.77) | 47.06 (31.53, 68.38) | -0.09 (-0.11, -0.08) | <0.001 | 511.41 (440.41, 596.81) | 488.01 (423.62, 569.48) | -0.15 (-0.20, -0.09) | <0.001 | 735.84 (686.52, 782.59) | 736.68 (630.26, 859.45) | -0.06 (-0.43, 0.30) | 0.733 | 14.09 (13.12, 14.97) | 14.11 (12.05, 16.48) | -0.06 (-0.43, 0.30) | 0.728 |
| Eastern Europe | 45.93 (30.63, 66.91) | 49.23 (32.98, 72.10) | 0.23 (0.20, 0.26) | <0.001 | 490.53 (410.67, 584.17) | 537.69 (449.83, 643.42) | 0.35 (0.14, 0.55) | 0.001 | 425.17 (365.52, 464.71) | 472.55 (375.27, 604.59) | 0.46 (-0.46, 1.40) | 0.327 | 8.08 (6.95, 8.83) | 8.97 (7.06, 11.47) | 0.46 (-0.47, 1.41) | 0.334 |
| Southern Sub-Saharan Africa | 55.88 (37.04, 80.89) | 50.70 (33.31, 74.41) | -0.33 (-0.38, -0.28) | <0.001 | 334.21 (281.57, 401.75) | 300.34 (253.14, 360.14) | -0.36 (-0.41, -0.32) | <0.001 | 521.45 (421.44, 621.88) | 235.02 (155.66, 331.18) | -2.57 (-4.27, -0.83) | 0.004 | 9.94 (8.04, 11.92) | 4.43 (2.90, 6.27) | -2.60 (-4.33, -0.84) | 0.004 |
| High-income Asia Pacific | 21.09 (12.75, 32.40) | 17.86 (10.78, 27.36) | -0.57 (-0.63, -0.52) | <0.001 | 293.77 (248.62, 349.38) | 285.47 (240.90, 337.28) | -0.07 (-0.13, -0.01) | 0.019 | 195.89 (171.57, 221.63) | 57.23 (51.28, 63.61) | -4.12 (-4.56, -3.68) | <0.001 | 3.68 (3.23, 4.17) | 1.04 (0.93, 1.16) | -4.24 (-4.72, -3.77) | <0.001 |
| Central Europe | 26.39 (15.39, 41.65) | 23.20 (14.11, 35.85) | -0.46 (-0.54, -0.38) | <0.001 | 416.18 (337.26, 516.36) | 392.95 (323.00, 477.20) | -0.18 (-0.22, -0.14) | <0.001 | 494.43 (472.08, 519.10) | 168.61 (140.60, 202.28) | -3.69 (-4.15, -3.23) | <0.001 | 9.35 (8.95, 9.80) | 3.07 (2.55, 3.75) | -3.81 (-4.28, -3.34) | <0.001 |
| Andean Latin America | 10.52 (4.99, 17.73) | 10.21 (4.88, 17.33) | -0.11 (-0.14, -0.07) | <0.001 | 178.27 (140.53, 224.14) | 180.70 (141.66, 227.75) | 0.06 (-0.01, 0.12) | 0.085 | 514.44 (404.10, 639.49) | 212.61 (148.84, 288.76) | -3.10 (-3.83, -2.37) | <0.001 | 9.85 (7.71, 12.25) | 4.01 (2.79, 5.48) | -3.16 (-3.89, -2.42) | <0.001 |
| Caribbean | 87.68 (59.48, 126.79) | 89.41 (60.67, 130.82) | 0.06 (0.04, 0.08) | <0.001 | 725.18 (637.38, 831.62) | 711.00 (625.35, 810.30) | -0.06 (-0.10, -0.02) | 0.001 | 762.22 (628.69, 939.50) | 639.76 (427.76, 919.13) | -0.61 (-0.99, -0.23) | 0.002 | 14.57 (11.99, 18.06) | 12.21 (8.12, 17.65) | -0.62 (-1.01, -0.23) | 0.002 |
| Southern Latin America | 17.91 (10.50, 28.02) | 15.23 (8.57, 24.63) | -0.57 (-0.59, -0.55) | <0.001 | 212.19 (182.23, 249.21) | 201.07 (174.30, 236.52) | -0.17 (-0.21, -0.12) | <0.001 | 323.72 (288.53, 366.11) | 132.73 (110.85, 159.22) | -3.03 (-3.19, -2.87) | <0.001 | 6.21 (5.52, 7.01) | 2.52 (2.10, 3.04) | -3.06 (-3.22, -2.90) | <0.001 |
| Oceania | 42.07 (27.02, 62.44) | 42.88 (27.69, 65.11) | 0.07 (0.05, 0.09) | <0.001 | 470.52 (369.49, 599.07) | 479.51 (372.70, 607.74) | 0.08 (0.02, 0.13) | 0.004 | 771.76 (472.77, 1241.42) | 911.66 (579.25, 1501.78) | 0.59 (0.47, 0.71) | <0.001 | 14.64 (8.83, 23.82) | 17.34 (10.85, 28.77) | 0.60 (0.48, 0.73) | <0.001 |

| | | | | | | | | | | | | | | | | |
|---|---|---|---|---|---|---|---|---|---|---|---|---|---|---|---|---|
| Australasia | 37.42 (24.09, 56.39) | 29.58 (17.52, 45.84) | -0.81 (-0.87, -0.76) | <0.001 | 367.16 (316.56, 430.17) | 307.32 (266.06, 361.19) | -0.58 (-0.71, -0.45) | <0.001 | 179.50 (157.27, 204.00) | 92.11 (78.76, 105.73) | -2.24 (-2.51, -1.96) | <0.001 | 3.36 (2.92, 3.82) | 1.72 (1.46, 1.98) | -2.37 (-2.61, -2.12) | <0.001 |
| **Men** | | | | | | | | | | | | | | | | |
| Global | 68.24 (45.33, 101.45) | 65.01 (43.27, 95.57) | -0.16 (-0.19, -0.14) | <0.001 | 586.01 (483.12, 715.24) | 587.58 (489.23, 712.43) | 0.01 (-0.01, 0.03) | 0.524 | 1512.59 (1420.91, 1614.09) | 1356.12 (1235.38, 1507.79) | -0.35 (-0.69, 0.00) | 0.050 | 28.94 (27.15, 30.91) | 25.92 (23.58, 28.83) | -0.35 (-0.71, 0.01) | 0.055 |
| **SDI category** | | | | | | | | | | | | | | | | |
| High | 91.52 (60.97, 136.09) | 71.04 (48.54, 102.81) | -0.87 (-0.89, -0.85) | <0.001 | 574.15 (489.39, 687.34) | 553.85 (478.55, 649.61) | -0.13 (-0.16, -0.09) | <0.001 | 1016.53 (983.99, 1051.55) | 579.85 (526.94, 664.46) | -1.87 (-2.06, -1.67) | <0.001 | 19.38 (18.78, 20.03) | 10.98 (9.95, 12.62) | -1.91 (-2.10, -1.72) | <0.001 |
| High-middle | 67.47 (44.77, 99.83) | 60.37 (39.73, 88.65) | -0.38 (-0.41, -0.36) | <0.001 | 618.47 (499.63, 766.49) | 593.99 (478.31, 735.07) | -0.15 (-0.19, -0.11) | <0.001 | 1822.91 (1661.06, 1947.65) | 1201.85 (1087.23, 1340.56) | -1.33 (-1.86, -0.80) | <0.001 | 34.91 (31.77, 37.32) | 22.88 (20.72, 25.52) | -1.35 (-1.88, -0.81) | <0.001 |
| Middle | 61.20 (40.61, 90.86) | 65.68 (44.01, 95.43) | 0.26 (0.18, 0.34) | <0.001 | 620.89 (497.78, 777.21) | 633.05 (518.64, 774.09) | 0.07 (0.03, 0.12) | <0.001 | 1451.79 (1334.62, 1583.97) | 1461.85 (1317.70, 1623.60) | 0.07 (-0.09, 0.24) | 0.395 | 27.70 (25.46, 30.27) | 27.92 (25.13, 31.06) | 0.08 (-0.09, 0.25) | 0.378 |
| Low-middle | 61.24 (40.35, 91.72) | 63.59 (41.96, 94.63) | 0.13 (0.11, 0.14) | <0.001 | 521.65 (436.49, 631.18) | 552.79 (462.72, 671.08) | 0.20 (0.17, 0.22) | <0.001 | 1761.70 (1555.03, 2020.48) | 1850.13 (1592.51, 2160.42) | 0.16 (-0.71, 1.03) | 0.724 | 33.84 (29.85, 38.79) | 35.53 (30.49, 41.58) | 0.16 (-0.71, 1.03) | 0.725 |
| Low | 63.11 (42.00, 94.66) | 66.92 (44.40, 98.32) | 0.20 (0.17, 0.23) | <0.001 | 511.43 (430.74, 616.21) | 553.10 (465.79, 665.04) | 0.28 (0.22, 0.35) | <0.001 | 1316.98 (1113.93, 1565.82) | 1323.43 (1110.54, 1574.13) | 0.10 (-0.26, 0.46) | 0.578 | 25.26 (21.38, 30.03) | 25.36 (21.20, 30.20) | 0.10 (-0.26, 0.46) | 0.594 |
| **21 GBD region** | | | | | | | | | | | | | | | | |
| South Asia | 61.55 (40.33, 91.81) | 64.11 (42.07, 96.12) | 0.13 (0.11, 0.16) | <0.001 | 489.03 (408.88, 591.31) | 523.07 (435.78, 635.72) | 0.24 (0.19, 0.29) | <0.001 | 2113.79 (1820.41, 2423.79) | 2260.72 (1899.96, 2658.62) | 0.39 (-0.66, 1.45) | 0.471 | 40.71 (35.04, 46.73) | 43.52 (36.58, 51.27) | 0.39 (-0.67, 1.46) | 0.474 |
| East Asia | 51.31 (33.64, 75.93) | 48.78 (32.11, 72.19) | -0.18 (-0.22, -0.13) | <0.001 | 682.94 (518.94, 881.79) | 673.12 (511.22, 870.34) | -0.04 (-0.09, 0.00) | 0.062 | 999.72 (818.29, 1180.17) | 1059.81 (862.87, 1270.10) | 0.31 (-0.02, 0.64) | 0.062 | 18.83 (15.29, 22.29) | 19.96 (16.17, 24.02) | 0.32 (-0.02, 0.66) | 0.068 |
| North Africa and Middle East | 100.02 (67.28, | 102.30 (69.01, | 0.09 (0.04, 0.13) | <0.001 | 865.80 (744.23, | 892.81 (769.72, | 0.13 (0.00, 0.26) | 0.044 | 2772.42 (2449.26, | 1506.88 (1233.60, | -2.08 (-2.23, | <0.001 | 53.24 (46.96, | 28.75 (23.51, | -2.10 (-2.25, | <0.001 |

| | | | | | | | | | | | | | | | | |
|---|---|---|---|---|---|---|---|---|---|---|---|---|---|---|---|---|
| | 146.23) | 147.69) | | | 1029.51) | 1063.63) | | | 3159.61) | 1891.19) | -1.93) | | 60.71) | 36.15) | -1.95) | |
| Western Sub-Saharan Africa | 59.17 (39.65, 86.22) | 64.25 (43.02, 92.66) | 0.28 (0.24, 0.32) | <0.001 | 497.71 (419.14, 600.43) | 551.57 (461.20, 666.75) | 0.37 (0.30, 0.44) | <0.001 | 708.74 (532.80, 963.20) | 709.69 (535.03, 913.90) | 0.02 (-0.24, 0.27) | 0.893 | 13.51 (10.12, 18.50) | 13.48 (10.04, 17.44) | 0.01 (-0.25, 0.26) | 0.961 |
| Southeast Asia | 60.05 (39.92, 89.89) | 59.97 (40.28, 87.83) | -0.01 (-0.02, 0.01) | 0.376 | 525.30 (426.12, 653.64) | 545.15 (440.56, 680.73) | 0.13 (0.10, 0.16) | <0.001 | 1312.14 (1139.26, 1487.06) | 1443.40 (1192.35, 1714.55) | 0.36 (0.05, 0.68) | 0.023 | 25.06 (21.74, 28.48) | 27.58 (22.59, 32.74) | 0.37 (0.05, 0.69) | 0.024 |
| Eastern Sub-Saharan Africa | 59.96 (39.83, 87.29) | 64.02 (42.70, 92.71) | 0.23 (0.21, 0.24) | <0.001 | 506.98 (417.03, 620.58) | 556.14 (460.60, 679.97) | 0.33 (0.27, 0.39) | <0.001 | 871.34 (711.71, 1051.76) | 774.40 (587.87, 1007.18) | -0.43 (-0.60, -0.26) | <0.001 | 16.58 (13.50, 20.05) | 14.68 (11.11, 19.14) | -0.45 (-0.61, -0.28) | <0.001 |
| Central Latin America | 58.11 (38.77, 84.66) | 55.70 (36.80, 81.36) | -0.14 (-0.18, -0.11) | <0.001 | 449.52 (377.06, 544.00) | 455.45 (382.50, 552.94) | 0.04 (0.02, 0.07) | 0.001 | 1024.98 (977.08, 1077.42) | 882.43 (749.15, 1028.21) | -0.34 (-0.74, 0.06) | 0.099 | 19.62 (18.70, 20.62) | 16.86 (14.26, 19.71) | -0.34 (-0.74, 0.06) | 0.096 |
| Tropical Latin America | 43.93 (27.90, 65.13) | 34.10 (21.44, 51.62) | -0.88 (-0.91, -0.85) | <0.001 | 529.87 (411.48, 683.39) | 517.62 (401.79, 666.34) | -0.08 (-0.10, -0.06) | <0.001 | 1701.95 (1606.34, 1799.22) | 818.67 (760.70, 882.73) | -2.45 (-2.75, -2.16) | <0.001 | 32.57 (30.75, 34.41) | 15.48 (14.40, 16.74) | -2.49 (-2.79, -2.19) | <0.001 |
| Central Sub-Saharan Africa | 67.57 (45.82, 100.90) | 66.38 (44.75, 97.82) | -0.07 (-0.09, -0.05) | <0.001 | 473.02 (403.15, 560.08) | 466.96 (399.16, 554.82) | -0.04 (-0.07, -0.01) | 0.010 | 1045.39 (709.16, 1486.56) | 838.85 (561.72, 1223.65) | -0.76 (-0.89, -0.63) | <0.001 | 20.06 (13.49, 28.55) | 16.06 (10.68, 23.48) | -0.77 (-0.90, -0.63) | <0.001 |
| Western Europe | 86.99 (58.41, 126.53) | 67.32 (44.95, 100.06) | -0.89 (-0.93, -0.84) | <0.001 | 567.43 (484.54, 675.49) | 497.00 (431.36, 579.91) | -0.45 (-0.50, -0.39) | <0.001 | 1020.47 (982.75, 1057.81) | 377.97 (359.04, 400.57) | -3.39 (-3.65, -3.14) | <0.001 | 19.52 (18.83, 20.18) | 7.17 (6.81, 7.63) | -3.42 (-3.68, -3.16) | <0.001 |
| High-income North America | 112.50 (72.44, 167.56) | 72.03 (50.63, 99.97) | -1.52 (-1.58, -1.45) | <0.001 | 593.11 (495.58, 724.53) | 528.95 (451.63, 620.97) | -0.38 (-0.51, -0.24) | <0.001 | 1106.95 (1068.82, 1156.52) | 646.06 (602.48, 710.92) | -1.82 (-2.26, -1.38) | <0.001 | 21.06 (20.32, 22.00) | 12.27 (11.46, 13.54) | -1.83 (-2.27, -1.38) | <0.001 |
| Central Asia | 96.01 (64.74, 140.30) | 92.34 (61.81, 137.16) | -0.13 (-0.14, -0.12) | <0.001 | 660.70 (566.57, 777.91) | 653.41 (563.42, 772.92) | -0.04 (-0.07, -0.02) | 0.001 | 2996.89 (2839.62, 3144.95) | 2257.35 (1975.48, 2551.13) | -0.99 (-1.69, -0.29) | 0.006 | 57.80 (54.76, 60.68) | 43.44 (38.01, 49.06) | -1.00 (-1.70, -0.29) | 0.006 |
| Eastern Europe | 103.41 (70.66, 150.39) | 101.21 (68.01, 149.71) | -0.07 (-0.13, -0.01) | 0.024 | 554.27 (466.67, 667.89) | 545.03 (458.60, 658.10) | -0.07 (-0.11, -0.02) | 0.002 | 3075.06 (2623.33, 3277.01) | 2545.07 (2144.17, 3178.34) | -0.34 (-1.64, 0.98) | 0.612 | 59.34 (50.61, 63.23) | 49.04 (41.31, 61.21) | -0.34 (-1.64, 0.98) | 0.610 |
| Southern Sub-Saharan Africa | 83.90 (56.91, | 82.92 (56.76, | -0.04 (-0.05, -0.02) | <0.001 | 586.15 (483.40, | 594.98 (495.65, | 0.06 (0.02, 0.10) | 0.005 | 1225.77 (1009.60, | 757.51 (570.94, | -1.66 (-2.50, | <0.001 | 23.37 (19.22, | 14.34 (10.70, | -1.66 (-2.58, | <0.001 |

| | | | | | | | | | | | | | | | | |
|---|---|---|---|---|---|---|---|---|---|---|---|---|---|---|---|---|
| | 120.34) | 118.09) | | | 711.11) | 717.22) | | | 1459.82) | 981.69) | -0.82) | | 27.83) | 18.58) | -0.74) | |
| High-income Asia Pacific | 57.75 (38.14, 84.20) | 49.15 (33.01, 71.69) | -0.55 (-0.57, -0.53) | <0.001 | 372.31 (314.27, 445.39) | 325.71 (278.03, 384.44) | -0.45 (-0.49, -0.42) | <0.001 | 697.99 (613.59, 765.10) | 299.35 (271.94, 331.43) | -2.92 (-3.24, -2.60) | <0.001 | 13.31 (11.69, 14.57) | 5.64 (5.13, 6.27) | -2.96 (-3.29, -2.63) | <0.001 |
| Central Europe | 62.73 (40.54, 95.00) | 53.75 (35.88, 79.76) | -0.53 (-0.55, -0.51) | <0.001 | 641.20 (512.08, 808.98) | 564.92 (459.47, 698.63) | -0.43 (-0.45, -0.41) | <0.001 | 2682.78 (2598.87, 2768.28) | 783.49 (675.58, 904.58) | -4.28 (-4.73, -3.82) | <0.001 | 51.54 (49.97, 53.22) | 14.84 (12.79, 17.20) | -4.33 (-4.79, -3.86) | <0.001 |
| Andean Latin America | 22.86 (12.92, 36.07) | 22.63 (12.81, 35.93) | -0.03 (-0.05, -0.02) | <0.001 | 293.44 (230.33, 372.19) | 306.38 (240.30, 388.02) | 0.15 (0.13, 0.16) | <0.001 | 915.35 (749.40, 1102.54) | 574.74 (435.03, 788.10) | -1.54 (-2.12, -0.95) | <0.001 | 17.52 (14.33, 21.15) | 10.91 (8.24, 15.07) | -1.56 (-2.15, -0.97) | <0.001 |
| Caribbean | 118.39 (81.98, 170.41) | 124.04 (84.47, 177.23) | 0.16 (0.12, 0.20) | <0.001 | 909.96 (785.20, 1065.87) | 923.01 (799.92, 1082.20) | 0.05 (0.04, 0.06) | <0.001 | 1427.97 (1265.68, 1599.31) | 1169.35 (883.08, 1494.93) | -0.65 (-0.99, -0.31) | <0.001 | 27.28 (24.16, 30.58) | 22.30 (16.80, 28.60) | -0.66 (-1.01, -0.31) | <0.001 |
| Southern Latin America | 47.85 (31.31, 70.72) | 43.12 (27.93, 63.36) | -0.36 (-0.38, -0.34) | <0.001 | 305.98 (262.38, 362.01) | 291.56 (251.11, 339.96) | -0.17 (-0.19, -0.14) | <0.001 | 1116.27 (1007.68, 1235.71) | 522.23 (446.94, 596.57) | -2.54 (-2.79, -2.28) | <0.001 | 21.52 (19.43, 23.84) | 10.01 (8.57, 11.45) | -2.56 (-2.81, -2.30) | <0.001 |
| Oceania | 66.18 (43.68, 98.09) | 67.89 (45.41, 98.85) | 0.09 (0.06, 0.12) | <0.001 | 678.20 (526.29, 860.35) | 705.95 (549.44, 907.93) | 0.14 (0.13, 0.14) | <0.001 | 3562.46 (2686.79, 4901.35) | 3680.20 (2647.20, 5158.66) | 0.09 (-0.07, 0.26) | 0.261 | 68.50 (51.49, 94.38) | 70.75 (50.86, 99.47) | 0.09 (-0.07, 0.26) | 0.267 |
| Australasia | 183.56 (125.60, 263.15) | 161.21 (109.84, 227.71) | -0.47 (-0.53, -0.41) | <0.001 | 1009.57 (873.21, 1155.42) | 977.76 (850.15, 1121.43) | -0.16 (-0.39, 0.07) | 0.168 | 944.22 (868.00, 1024.15) | 440.86 (380.44, 500.27) | -2.58 (-3.09, -2.06) | <0.001 | 18.04 (16.61, 19.59) | 8.39 (7.21, 9.53) | -2.59 (-3.11, -2.07) | <0.001 |
| **Stroke** | | | | | | | | | | | | | | | | |
| **15-19 years** | | | | | | | | | | | | | | | | |
| **Women** | | | | | | | | | | | | | | | | |
| Global | 15.55 (9.90, 22.78) | 14.86 (9.30, 22.11) | -0.13 (-0.20, -0.05) | 0.001 | 232.87 (180.93, 291.83) | 255.33 (202.14, 317.15) | 0.38 (0.04, 0.72) | 0.028 | 179.01 (159.20, 200.86) | 122.84 (105.12, 141.73) | -1.28 (-1.51, -1.04) | <0.001 | 1.88 (1.70, 2.06) | 1.09 (0.95, 1.23) | -1.92 (-2.13, -1.71) | <0.001 |
| **SDI category** | | | | | | | | | | | | | | | | |
| High | 11.15 (6.38, 17.28) | 11.41 (6.47, 18.13) | 0.09 (0.07, 0.11) | <0.001 | 184.36 (141.43, 232.22) | 230.65 (175.78, 289.24) | 0.97 (0.11, 1.84) | 0.027 | 99.14 (86.21, 114.47) | 65.06 (50.61, 81.58) | -1.41 (-1.73, -1.10) | <0.001 | 0.87 (0.80, 0.94) | 0.32 (0.28, 0.36) | -3.26 (-3.56, -2.96) | <0.001 |
| High-middle | 15.56 (9.55, | 14.63 (8.87, | -0.22 (-0.35, | 0.001 | 213.65 | 215.96 | 0.09 (-0.18, | 0.503 | 155.32 | 95.60 (79.04, | -1.62 | <0.001 | 1.52 | 0.69 | -2.63 | <0.001 |

| | | | | | | | | | | | | | | | | |
|---|---|---|---|---|---|---|---|---|---|---|---|---|---|---|---|---|
| | 23.18) | 22.42) | -0.09) | | (160.65, 273.75) | (164.85, 274.05) | 0.37) | | (134.97, 177.87) | 114.49) | (-1.81, -1.42) | | (1.36, 1.71) | (0.62, 0.77) | (-2.87, -2.38) | |
| Middle | 17.22 (10.89, 25.17) | 16.28 (10.21, 24.13) | -0.21 (-0.35, -0.06) | 0.006 | 241.26 (183.78, 304.02) | 249.15 (193.96, 309.85) | 0.17 (-0.15, 0.49) | 0.304 | 204.42 (179.55, 229.72) | 130.84 (111.99, 151.64) | -1.53 (-1.69, -1.37) | <0.001 | 2.18 (1.94, 2.44) | 1.16 (1.02, 1.33) | -2.11 (-2.29, -1.94) | <0.001 |
| Low-middle | 15.38 (10.01, 22.35) | 15.18 (9.66, 22.19) | 0.01 (-0.11, 0.12) | 0.922 | 233.88 (182.83, 291.89) | 252.57 (198.68, 313.58) | 0.34 (-0.02, 0.72) | 0.067 | 202.22 (173.39, 232.68) | 137.27 (114.34, 161.43) | -1.34 (-1.61, -1.06) | <0.001 | 2.26 (1.90, 2.65) | 1.32 (1.08, 1.59) | -1.86 (-2.16, -1.57) | <0.001 |
| Low | 15.07 (9.86, 21.94) | 14.10 (8.87, 20.75) | -0.19 (-0.31, -0.07) | 0.002 | 292.97 (235.13, 361.63) | 304.59 (241.70, 372.58) | 0.19 (-0.03, 0.40) | 0.090 | 175.35 (148.38, 206.52) | 135.14 (112.58, 160.22) | -0.88 (-1.07, -0.68) | <0.001 | 1.83 (1.50, 2.22) | 1.24 (0.99, 1.54) | -1.32 (-1.50, -1.13) | <0.001 |
| **21 GBD region** | | | | | | | | | | | | | | | | |
| South Asia | 14.45 (9.41, 21.35) | 14.85 (9.36, 21.84) | 0.11 (0.07, 0.15) | <0.001 | 209.28 (158.95, 263.87) | 226.29 (172.52, 285.38) | 0.35 (-0.03, 0.72) | 0.070 | 163.51 (135.00, 194.98) | 114.97 (93.19, 137.28) | -1.29 (-1.79, -0.78) | <0.001 | 1.77 (1.42, 2.18) | 1.05 (0.84, 1.30) | -1.89 (-2.62, -1.16) | <0.001 |
| East Asia | 14.71 (8.78, 22.89) | 13.37 (7.55, 21.56) | -0.30 (-0.48, -0.12) | 0.001 | 212.13 (149.36, 284.32) | 198.69 (137.99, 265.99) | -0.21 (-0.31, -0.10) | <0.001 | 173.59 (144.42, 206.41) | 103.10 (81.78, 127.56) | -1.75 (-2.02, -1.48) | <0.001 | 1.68 (1.39, 2.06) | 0.70 (0.58, 0.83) | -2.98 (-3.25, -2.71) | <0.001 |
| North Africa and Middle East | 19.79 (13.44, 27.58) | 18.92 (12.25, 26.94) | -0.12 (-0.16, -0.08) | <0.001 | 263.35 (211.11, 320.79) | 269.30 (217.96, 322.44) | 0.15 (-0.17, 0.46) | 0.361 | 337.06 (279.75, 406.48) | 203.49 (163.65, 254.01) | -1.69 (-1.95, -1.44) | <0.001 | 4.08 (3.34, 4.98) | 2.20 (1.68, 2.92) | -2.08 (-2.40, -1.77) | <0.001 |
| Western Sub-Saharan Africa | 14.68 (9.50, 21.84) | 13.94 (8.51, 21.02) | -0.12 (-0.28, 0.03) | 0.121 | 302.45 (238.27, 376.33) | 313.99 (246.20, 388.41) | 0.17 (0.00, 0.34) | 0.043 | 154.95 (122.25, 194.96) | 123.03 (97.07, 158.77) | -0.79 (-0.92, -0.65) | <0.001 | 1.55 (1.15, 2.06) | 1.08 (0.78, 1.56) | -1.22 (-1.39, -1.04) | <0.001 |
| Southeast Asia | 19.49 (12.80, 27.97) | 18.30 (11.76, 26.65) | -0.21 (-0.27, -0.15) | <0.001 | 245.21 (194.68, 302.08) | 255.51 (200.64, 315.85) | 0.19 (-0.03, 0.41) | 0.096 | 241.62 (194.87, 290.63) | 166.06 (138.21, 193.79) | -1.27 (-1.40, -1.15) | <0.001 | 2.59 (2.05, 3.17) | 1.51 (1.24, 1.79) | -1.84 (-1.99, -1.68) | <0.001 |
| Eastern Sub-Saharan Africa | 13.98 (8.89, 20.85) | 12.37 (7.33, 19.15) | -0.41 (-0.56, -0.27) | <0.001 | 396.97 (317.03, 488.97) | 404.09 (320.92, 497.13) | 0.11 (-0.09, 0.31) | 0.277 | 184.53 (147.63, 225.71) | 130.75 (103.05, 160.37) | -1.19 (-1.36, -1.01) | <0.001 | 1.80 (1.37, 2.31) | 1.03 (0.76, 1.35) | -1.89 (-2.12, -1.66) | <0.001 |
| Central Latin America | 20.99 (13.64, 30.15) | 17.91 (11.49, 26.30) | -0.53 (-0.63, -0.43) | <0.001 | 301.61 (241.79, 368.85) | 281.64 (225.60, 340.93) | -0.20 (-0.47, 0.08) | 0.161 | 184.27 (168.98, 200.51) | 110.58 (93.90, 129.54) | -1.64 (-1.75, -1.52) | <0.001 | 2.13 (1.97, 2.30) | 1.13 (0.93, 1.34) | -2.04 (-2.20, -1.88) | <0.001 |

| | | | | | | | | | | | | | | | | |
|---|---|---|---|---|---|---|---|---|---|---|---|---|---|---|---|---|
| Tropical Latin America | 21.56 (14.39, 30.67) | 17.89 (11.59, 25.94) | -0.56 (-0.68, -0.43) | <0.001 | 292.34 (226.02, 367.57) | 272.31 (215.26, 336.36) | -0.23 (-0.32, -0.13) | <0.001 | 172.86 (155.85, 191.57) | 103.72 (90.30, 118.64) | -1.68 (-2.09, -1.26) | <0.001 | 1.99 (1.81, 2.19) | 1.05 (0.94, 1.18) | -2.08 (-2.51, -1.65) | <0.001 |
| Central Sub-Saharan Africa | 14.62 (9.53, 21.52) | 12.68 (7.83, 19.40) | -0.48 (-0.50, -0.45) | <0.001 | 296.03 (219.97, 377.71) | 288.26 (219.99, 372.24) | -0.06 (-0.24, 0.13) | 0.561 | 203.59 (136.97, 286.26) | 137.47 (99.38, 190.76) | -1.39 (-1.51, -1.27) | <0.001 | 2.25 (1.35, 3.41) | 1.33 (0.84, 2.02) | -1.83 (-1.95, -1.71) | <0.001 |
| Western Europe | 10.27 (5.67, 16.17) | 9.38 (5.05, 15.26) | -0.28 (-0.36, -0.20) | <0.001 | 162.41 (119.87, 202.11) | 166.13 (123.87, 210.88) | 0.24 (-0.20, 0.68) | 0.279 | 92.52 (81.70, 105.23) | 45.22 (35.37, 56.89) | -2.47 (-2.77, -2.16) | <0.001 | 0.89 (0.83, 0.96) | 0.23 (0.20, 0.26) | -4.62 (-5.47, -3.77) | <0.001 |
| High-income North America | 10.32 (5.43, 16.92) | 11.91 (6.56, 19.44) | 0.50 (0.45, 0.55) | <0.001 | 212.95 (154.03, 279.09) | 282.78 (210.28, 362.33) | 1.23 (0.06, 2.42) | 0.039 | 79.43 (65.46, 96.51) | 73.53 (56.23, 93.52) | -0.18 (-0.79, 0.43) | 0.553 | 0.56 (0.51, 0.60) | 0.35 (0.31, 0.39) | -1.58 (-2.33, -0.83) | <0.001 |
| Central Asia | 17.37 (11.42, 25.00) | 14.58 (8.89, 22.10) | -0.58 (-0.70, -0.45) | <0.001 | 189.10 (146.81, 234.72) | 187.65 (140.59, 237.71) | 0.03 (-0.18, 0.25) | 0.752 | 138.67 (119.57, 160.00) | 102.05 (82.65, 123.31) | -1.05 (-1.38, -0.71) | <0.001 | 1.33 (1.17, 1.49) | 0.82 (0.65, 1.01) | -1.66 (-2.21, -1.11) | <0.001 |
| Eastern Europe | 15.57 (9.54, 23.65) | 16.73 (10.19, 25.52) | 0.28 (0.18, 0.38) | <0.001 | 223.56 (166.60, 288.79) | 280.15 (213.88, 355.84) | 0.84 (0.54, 1.15) | <0.001 | 126.12 (106.69, 148.06) | 110.61 (87.80, 137.41) | -0.36 (-0.95, 0.23) | 0.229 | 1.02 (0.94, 1.09) | 0.66 (0.55, 0.77) | -1.54 (-2.82, -0.25) | 0.019 |
| Southern Sub-Saharan Africa | 15.27 (9.36, 23.38) | 13.99 (8.51, 21.61) | -0.25 (-0.38, -0.12) | <0.001 | 411.64 (318.02, 515.66) | 381.06 (290.46, 482.03) | -0.24 (-0.30, -0.19) | <0.001 | 156.15 (127.73, 188.69) | 103.55 (77.65, 133.03) | -1.37 (-1.78, -0.95) | <0.001 | 1.36 (1.06, 1.64) | 0.68 (0.46, 0.97) | -2.20 (-2.89, -1.51) | <0.001 |
| High-income Asia Pacific | 11.06 (6.56, 17.08) | 9.79 (5.50, 15.60) | -0.41 (-0.58, -0.23) | <0.001 | 125.66 (90.71, 164.09) | 155.22 (112.09, 203.33) | 0.84 (0.47, 1.22) | <0.001 | 116.32 (97.86, 134.76) | 50.18 (37.96, 64.95) | -2.85 (-3.29, -2.39) | <0.001 | 1.16 (0.96, 1.35) | 0.20 (0.16, 0.24) | -5.85 (-6.56, -5.14) | <0.001 |
| Central Europe | 15.07 (9.13, 22.65) | 14.10 (8.43, 21.90) | -0.22 (-0.28, -0.16) | <0.001 | 220.85 (172.40, 278.23) | 227.40 (176.02, 282.66) | 0.18 (-0.17, 0.52) | 0.315 | 137.50 (117.65, 161.11) | 86.85 (67.98, 109.52) | -1.52 (-1.88, -1.15) | <0.001 | 1.19 (1.09, 1.29) | 0.47 (0.39, 0.54) | -3.20 (-3.61, -2.79) | <0.001 |
| Andean Latin America | 24.40 (18.04, 32.57) | 19.01 (12.81, 26.50) | -0.84 (-0.88, -0.81) | <0.001 | 212.85 (163.93, 270.30) | 208.85 (158.95, 262.78) | -0.03 (-0.19, 0.12) | 0.687 | 325.43 (258.93, 402.09) | 169.09 (130.16, 218.21) | -2.32 (-3.23, -1.40) | <0.001 | 4.22 (3.28, 5.29) | 2.04 (1.50, 2.73) | -2.57 (-3.52, -1.62) | <0.001 |
| Caribbean | 18.58 (12.36, 26.45) | 17.70 (11.78, 25.35) | -0.16 (-0.22, -0.10) | <0.001 | 236.16 (185.56, 290.43) | 232.43 (183.15, 284.35) | -0.01 (-0.30, 0.29) | 0.965 | 212.10 (161.76, 278.51) | 192.12 (130.44, 288.85) | -0.14 (-0.40, 0.14) | 0.326 | 2.61 (1.94, 3.52) | 2.33 (1.48, 3.67) | -0.17 (-0.49, 0.15) | 0.285 |

| | | | | | | | | | | | | | | | | |
|---|---|---|---|---|---|---|---|---|---|---|---|---|---|---|---|---|
| Southern Latin America | 14.44 (9.12, 21.57) | 11.81 (7.09, 17.89) | -0.65 (-0.70, -0.60) | <0.001 | 143.73 (100.80, 195.14) | 128.03 (85.09, 171.22) | -0.41 (-0.78, -0.05) | 0.027 | 198.45 (174.27, 223.53) | 101.94 (80.71, 123.59) | -2.27 (-2.73, -1.81) | <0.001 | 2.36 (2.07, 2.66) | 1.03 (0.78, 1.30) | -2.80 (-3.38, -2.22) | <0.001 |
| Oceania | 23.20 (16.18, 32.21) | 22.87 (15.96, 31.60) | -0.02 (-0.06, 0.02) | 0.397 | 220.21 (167.25, 281.85) | 215.88 (156.32, 280.82) | -0.03 (-0.20, 0.15) | 0.752 | 206.02 (142.76, 303.10) | 222.64 (152.10, 326.27) | 0.24 (-0.30, 0.78) | 0.385 | 2.12 (1.28, 3.43) | 2.36 (1.42, 3.77) | 0.38 (0.27, 0.49) | <0.001 |
| Australasia | 10.87 (5.94, 17.70) | 11.03 (5.97, 17.82) | 0.06 (-0.03, 0.14) | 0.199 | 164.88 (111.67, 229.03) | 170.14 (110.79, 239.62) | 0.17 (-0.14, 0.47) | 0.282 | 64.56 (48.98, 84.64) | 42.24 (27.89, 59.01) | -1.36 (-2.12, -0.59) | 0.001 | 0.47 (0.35, 0.60) | 0.15 (0.10, 0.21) | -3.69 (-6.02, -1.29) | 0.003 |
| **Men** | | | | | | | | | | | | | | | | |
| Global | 13.06 (8.41, 19.10) | 12.48 (7.93, 18.58) | -0.14 (-0.20, -0.07) | <0.001 | 151.28 (115.46, 191.20) | 163.07 (126.99, 204.88) | 0.34 (-0.03, 0.71) | 0.072 | 205.96 (182.39, 230.70) | 139.40 (120.61, 160.25) | -1.38 (-1.62, -1.14) | <0.001 | 2.50 (2.21, 2.82) | 1.57 (1.35, 1.84) | -1.63 (-1.94, -1.31) | <0.001 |
| **SDI category** | | | | | | | | | | | | | | | | |
| High | 8.38 (4.92, 13.17) | 8.33 (4.71, 13.37) | 0.01 (-0.08, 0.10) | 0.839 | 113.07 (83.56, 148.64) | 151.89 (111.19, 197.09) | 1.24 (0.20, 2.29) | 0.019 | 102.79 (86.22, 115.85) | 49.73 (40.84, 60.35) | -2.37 (-2.70, -2.03) | <0.001 | 1.13 (0.90, 1.25) | 0.34 (0.31, 0.40) | -4.00 (-4.18, -3.82) | <0.001 |
| High-middle | 12.73 (8.04, 18.90) | 11.13 (6.76, 17.09) | -0.46 (-0.50, -0.41) | <0.001 | 125.27 (93.35, 162.79) | 116.19 (85.69, 149.78) | -0.17 (-0.42, 0.08) | 0.175 | 201.05 (172.75, 230.51) | 100.48 (86.57, 115.73) | -2.38 (-2.60, -2.17) | <0.001 | 2.44 (2.05, 2.85) | 1.05 (0.90, 1.22) | -2.83 (-3.22, -2.44) | <0.001 |
| Middle | 14.51 (9.48, 21.08) | 13.76 (8.76, 20.15) | -0.17 (-0.22, -0.11) | <0.001 | 158.28 (118.43, 199.64) | 163.69 (126.11, 206.76) | 0.18 (-0.13, 0.48) | 0.255 | 251.04 (219.70, 285.41) | 176.84 (145.35, 208.86) | -1.16 (-1.33, -0.99) | <0.001 | 3.10 (2.70, 3.57) | 2.07 (1.67, 2.49) | -1.34 (-1.52, -1.16) | <0.001 |
| Low-middle | 13.12 (8.54, 19.16) | 12.70 (8.05, 18.84) | -0.10 (-0.19, -0.01) | 0.037 | 170.54 (131.54, 215.42) | 172.82 (133.68, 217.71) | 0.11 (-0.19, 0.41) | 0.479 | 194.24 (166.00, 226.02) | 137.61 (115.86, 162.79) | -1.25 (-1.85, -0.65) | <0.001 | 2.34 (1.94, 2.77) | 1.55 (1.31, 1.87) | -1.51 (-2.19, -0.82) | <0.001 |
| Low | 14.00 (9.21, 20.30) | 13.16 (8.27, 19.47) | -0.20 (-0.29, -0.10) | <0.001 | 178.80 (141.80, 220.85) | 185.95 (146.49, 230.14) | 0.21 (0.01, 0.41) | 0.038 | 204.42 (167.06, 245.83) | 154.45 (124.37, 190.26) | -0.97 (-1.28, -0.66) | <0.001 | 2.49 (1.97, 3.06) | 1.79 (1.40, 2.25) | -1.16 (-1.51, -0.80) | <0.001 |
| **21 GBD region** | | | | | | | | | | | | | | | | |
| South Asia | 11.97 (7.65, 17.99) | 11.64 (7.20, 17.62) | -0.08 (-0.14, -0.03) | 0.003 | 164.29 (123.96, 210.80) | 161.92 (120.63, 210.44) | 0.00 (-0.26, 0.26) | 0.989 | 135.35 (108.87, 164.11) | 97.81 (78.67, 123.01) | -1.29 (-2.69, 0.14) | 0.077 | 1.52 (1.18, 1.90) | 1.00 (0.77, 1.32) | -1.67 (-3.45, 0.14) | 0.071 |

| | | | | | | | | | | | | | | | | |
|---|---|---|---|---|---|---|---|---|---|---|---|---|---|---|---|---|
| East Asia | 13.56 (8.53, 20.04) | 11.68 (7.05, 18.25) | -0.51 (-0.59, -0.43) | <0.001 | 125.30 (85.93, 168.68) | 96.18 (65.29, 131.91) | -0.89 (-1.14, -0.64) | <0.001 | 278.65 (223.38, 336.34) | 146.77 (116.14, 176.46) | -2.20 (-2.44, -1.96) | <0.001 | 3.48 (2.70, 4.26) | 1.68 (1.30, 2.09) | -2.50 (-2.77, -2.22) | <0.001 |
| North Africa and Middle East | 15.18 (10.08, 21.80) | 15.61 (10.25, 22.56) | 0.12 (-0.01, 0.25) | 0.061 | 194.86 (151.63, 239.59) | 204.33 (163.28, 248.55) | 0.29 (-0.02, 0.59) | 0.068 | 365.71 (282.88, 453.26) | 273.98 (191.84, 364.34) | -0.94 (-1.17, -0.71) | <0.001 | 4.69 (3.58, 5.94) | 3.39 (2.30, 4.64) | -1.09 (-1.31, -0.87) | <0.001 |
| Western Sub-Saharan Africa | 14.94 (9.17, 22.17) | 14.42 (8.65, 21.54) | -0.11 (-0.15, -0.07) | <0.001 | 190.98 (149.79, 236.13) | 201.94 (159.72, 246.48) | 0.24 (-0.01, 0.49) | 0.062 | 145.94 (111.96, 186.18) | 118.89 (90.24, 151.23) | -0.70 (-0.84, -0.56) | <0.001 | 1.68 (1.21, 2.24) | 1.28 (0.90, 1.74) | -0.94 (-1.07, -0.80) | <0.001 |
| Southeast Asia | 17.38 (11.85, 24.74) | 16.40 (10.92, 23.71) | -0.16 (-0.28, -0.04) | 0.010 | 180.98 (139.73, 224.15) | 176.77 (136.45, 221.93) | -0.02 (-0.33, 0.29) | 0.906 | 242.74 (199.46, 305.96) | 222.09 (184.49, 260.96) | -0.28 (-0.50, -0.06) | 0.011 | 2.86 (2.29, 3.71) | 2.57 (2.11, 3.12) | -0.34 (-0.57, -0.10) | 0.006 |
| Eastern Sub-Saharan Africa | 15.80 (10.61, 22.45) | 14.21 (9.09, 20.88) | -0.36 (-0.38, -0.34) | <0.001 | 204.41 (162.52, 254.39) | 214.98 (168.93, 263.80) | 0.23 (-0.07, 0.53) | 0.130 | 313.37 (241.38, 389.11) | 214.75 (169.06, 270.45) | -1.28 (-1.37, -1.19) | <0.001 | 4.00 (2.99, 5.04) | 2.60 (1.97, 3.36) | -1.46 (-1.55, -1.36) | <0.001 |
| Central Latin America | 13.11 (8.47, 19.23) | 11.91 (7.29, 17.87) | -0.32 (-0.37, -0.27) | <0.001 | 205.26 (159.07, 253.61) | 206.85 (162.40, 256.11) | 0.03 (-0.10, 0.17) | 0.633 | 155.63 (141.63, 169.86) | 105.18 (87.30, 125.13) | -1.25 (-1.63, -0.88) | <0.001 | 1.93 (1.75, 2.11) | 1.22 (0.99, 1.51) | -1.46 (-1.85, -1.07) | <0.001 |
| Tropical Latin America | 15.78 (10.20, 23.01) | 13.29 (8.40, 19.70) | -0.52 (-0.61, -0.44) | <0.001 | 236.40 (181.16, 304.89) | 206.87 (158.05, 263.81) | -0.43 (-0.60, -0.27) | <0.001 | 171.92 (155.98, 189.68) | 107.47 (93.77, 122.69) | -1.58 (-1.96, -1.19) | <0.001 | 2.13 (1.92, 2.33) | 1.25 (1.08, 1.43) | -1.77 (-2.25, -1.29) | <0.001 |
| Central Sub-Saharan Africa | 12.23 (7.84, 18.20) | 10.94 (6.76, 16.74) | -0.34 (-0.47, -0.21) | <0.001 | 167.99 (119.64, 223.79) | 163.28 (115.16, 216.71) | -0.04 (-0.27, 0.19) | 0.729 | 222.29 (149.38, 322.50) | 147.65 (98.37, 215.79) | -1.43 (-1.59, -1.27) | <0.001 | 2.78 (1.77, 4.17) | 1.74 (1.06, 2.67) | -1.63 (-1.80, -1.46) | <0.001 |
| Western Europe | 8.20 (4.72, 12.72) | 6.92 (3.77, 11.16) | -0.58 (-0.67, -0.49) | <0.001 | 93.65 (65.49, 123.95) | 90.10 (64.66, 119.56) | -0.01 (-0.43, 0.40) | 0.946 | 104.37 (94.05, 115.87) | 36.10 (29.70, 43.40) | -3.66 (-4.09, -3.22) | <0.001 | 1.22 (1.10, 1.34) | 0.27 (0.23, 0.31) | -5.07 (-5.85, -4.28) | <0.001 |
| High-income North America | 7.95 (4.32, 13.15) | 8.82 (4.80, 14.34) | 0.39 (0.29, 0.49) | <0.001 | 153.78 (107.55, 209.51) | 213.05 (149.69, 286.02) | 1.39 (0.12, 2.67) | 0.031 | 68.09 (58.11, 80.63) | 57.15 (44.88, 71.20) | -0.57 (-1.03, -0.10) | 0.018 | 0.59 (0.53, 0.66) | 0.37 (0.32, 0.43) | -1.64 (-2.18, -1.11) | <0.001 |
| Central Asia | 12.84 (8.22, 19.03) | 10.76 (6.48, 16.91) | -0.59 (-0.68, -0.51) | <0.001 | 77.43 (54.05, 102.46) | 79.42 (54.34, 108.40) | 0.19 (-0.28, 0.66) | 0.422 | 124.43 (108.71, 139.72) | 100.54 (82.20, 122.35) | -0.57 (-1.00, -0.15) | 0.009 | 1.40 (1.24, 1.60) | 1.07 (0.83, 1.36) | -0.68 (-1.22, -0.14) | 0.014 |

| | | | | | | | | | | | | | | | | |
|---|---|---|---|---|---|---|---|---|---|---|---|---|---|---|---|---|
| Eastern Europe | 10.57 (6.22, 16.48) | 9.33 (5.37, 14.82) | -0.42 (-0.60, -0.23) | <0.001 | 70.43 (47.42, 97.09) | 68.01 (45.08, 92.29) | -0.03 (-0.47, 0.41) | 0.899 | 108.04 (94.59, 120.66) | 85.86 (70.93, 103.65) | -0.59 (-1.82, 0.66) | 0.354 | 1.18 (1.03, 1.28) | 0.88 (0.70, 1.09) | -0.76 (-2.30, 0.80) | 0.338 |
| Southern Sub-Saharan Africa | 14.14 (9.03, 20.78) | 13.46 (8.27, 20.44) | -0.15 (-0.34, 0.04) | 0.111 | 230.87 (175.41, 295.44) | 235.90 (175.78, 300.49) | 0.12 (-0.04, 0.28) | 0.157 | 151.50 (116.08, 192.99) | 140.77 (104.16, 200.01) | -0.18 (-0.72, 0.37) | 0.523 | 1.68 (1.22, 2.23) | 1.53 (1.03, 2.29) | -0.20 (-0.80, 0.41) | 0.527 |
| High-income Asia Pacific | 7.94 (4.59, 12.23) | 6.81 (3.62, 11.32) | -0.51 (-0.80, -0.21) | 0.001 | 52.43 (33.38, 73.53) | 74.06 (48.97, 103.30) | 1.26 (0.97, 1.56) | <0.001 | 147.77 (79.02, 181.00) | 38.44 (30.64, 46.78) | -4.53 (-5.15, -3.90) | <0.001 | 1.81 (0.86, 2.24) | 0.26 (0.21, 0.30) | -6.25 (-7.09, -5.40) | <0.001 |
| Central Europe | 9.78 (5.90, 15.11) | 8.08 (4.59, 12.82) | -0.63 (-0.70, -0.56) | <0.001 | 63.58 (43.16, 84.91) | 54.33 (36.19, 74.65) | -0.43 (-1.23, 0.37) | 0.287 | 124.68 (111.00, 138.53) | 63.77 (52.14, 75.08) | -2.27 (-2.64, -1.91) | <0.001 | 1.43 (1.28, 1.57) | 0.60 (0.48, 0.73) | -3.04 (-3.53, -2.54) | <0.001 |
| Andean Latin America | 22.65 (17.01, 29.79) | 17.49 (11.95, 24.41) | -0.89 (-0.93, -0.85) | <0.001 | 207.66 (158.71, 263.38) | 203.31 (154.76, 257.11) | -0.06 (-0.13, 0.01) | 0.073 | 415.27 (326.26, 526.65) | 210.36 (154.91, 282.66) | -2.31 (-2.88, -1.73) | <0.001 | 5.57 (4.32, 7.12) | 2.71 (1.94, 3.74) | -2.45 (-3.06, -1.83) | <0.001 |
| Caribbean | 10.84 (6.74, 16.46) | 10.24 (6.20, 15.86) | -0.18 (-0.22, -0.15) | <0.001 | 216.82 (165.40, 268.78) | 212.75 (161.36, 264.57) | -0.03 (-0.23, 0.17) | 0.761 | 147.90 (119.91, 185.04) | 111.30 (77.58, 160.59) | -0.86 (-1.10, -0.63) | <0.001 | 1.80 (1.41, 2.31) | 1.30 (0.83, 1.96) | -0.97 (-1.25, -0.70) | <0.001 |
| Southern Latin America | 11.70 (7.44, 17.03) | 9.53 (5.71, 14.43) | -0.70 (-0.73, -0.68) | <0.001 | 76.47 (46.77, 108.54) | 66.53 (40.09, 97.66) | -0.43 (-0.65, -0.22) | <0.001 | 223.52 (187.86, 263.75) | 107.32 (80.87, 138.53) | -2.52 (-2.99, -2.04) | <0.001 | 2.89 (2.41, 3.45) | 1.27 (0.91, 1.71) | -2.86 (-3.38, -2.33) | <0.001 |
| Oceania | 25.91 (19.87, 33.68) | 23.89 (17.84, 31.73) | -0.27 (-0.29, -0.24) | <0.001 | 179.93 (131.17, 233.72) | 176.82 (126.65, 233.65) | -0.04 (-0.12, 0.03) | 0.281 | 369.81 (225.50, 590.34) | 385.19 (230.70, 637.37) | 0.15 (-0.00, 0.30) | 0.055 | 4.62 (2.59, 7.67) | 4.85 (2.67, 8.36) | 0.08 (0.01, 0.16) | 0.021 |
| Australasia | 7.59 (3.97, 12.36) | 7.29 (3.82, 12.20) | -0.14 (-0.20, -0.08) | <0.001 | 74.92 (39.09, 113.10) | 74.84 (39.63, 118.76) | 0.04 (-0.31, 0.38) | 0.842 | 59.41 (48.38, 72.91) | 26.10 (18.90, 34.44) | -2.81 (-3.68, -1.93) | <0.001 | 0.61 (0.49, 0.77) | 0.15 (0.10, 0.23) | -4.75 (-6.00, -3.49) | <0.001 |
| **20-24 years** | | | | | | | | | | | | | | | | |
| **Women** | | | | | | | | | | | | | | | | |
| Global | 19.14 (13.69, 26.69) | 17.10 (11.79, 24.53) | -0.38 (-0.41, -0.35) | <0.001 | 305.76 (242.07, 369.69) | 326.65 (263.64, 393.21) | 0.30 (-0.07, 0.68) | 0.117 | 249.00 (221.63, 278.67) | 172.70 (149.57, 196.16) | -1.25 (-1.45, -1.05) | <0.001 | 2.87 (2.59, 3.20) | 1.74 (1.51, 1.97) | -1.66 (-1.89, -1.43) | <0.001 |
| **SDI category** | | | | | | | | | | | | | | | | |

| | | | | | | | | | | | | | | | | |
|---|---|---|---|---|---|---|---|---|---|---|---|---|---|---|---|---|
| High | 13.72 (9.13, 20.08) | 13.46 (8.69, 20.34) | -0.06 (-0.12, 0.00) | 0.066 | 257.50 (202.70, 310.98) | 305.24 (240.70, 369.07) | 0.76 (0.06, 1.46) | 0.034 | 136.53 (120.72, 155.20) | 90.07 (72.41, 110.45) | -1.36 (-1.75, -0.97) | <0.001 | 1.35 (1.25, 1.45) | 0.56 (0.49, 0.64) | -3.06 (-3.34, -2.77) | <0.001 |
| High-middle | 19.51 (13.86, 27.49) | 16.90 (11.29, 25.29) | -0.46 (-0.61, -0.32) | <0.001 | 285.30 (219.70, 350.01) | 287.23 (223.78, 350.69) | 0.09 (-0.18, 0.36) | 0.525 | 209.38 (181.72, 238.74) | 131.43 (108.03, 156.89) | -1.52 (-2.03, -1.02) | <0.001 | 2.22 (1.96, 2.55) | 1.08 (0.95, 1.23) | -2.39 (-2.92, -1.85) | <0.001 |
| Middle | 21.08 (15.16, 29.21) | 18.68 (12.91, 26.72) | -0.41 (-0.46, -0.36) | <0.001 | 321.81 (253.39, 392.13) | 331.95 (268.19, 398.92) | 0.17 (-0.08, 0.42) | 0.179 | 278.42 (242.52, 313.64) | 181.04 (155.28, 208.62) | -1.37 (-1.71, -1.04) | <0.001 | 3.22 (2.82, 3.65) | 1.80 (1.56, 2.08) | -1.91 (-2.23, -1.59) | <0.001 |
| Low-middle | 18.75 (13.59, 25.94) | 17.21 (12.01, 24.46) | -0.27 (-0.39, -0.16) | <0.001 | 304.16 (242.96, 366.33) | 324.27 (258.49, 391.89) | 0.32 (-0.07, 0.72) | 0.109 | 302.09 (260.61, 350.41) | 198.96 (168.65, 236.37) | -1.40 (-1.73, -1.07) | <0.001 | 3.75 (3.14, 4.46) | 2.19 (1.80, 2.64) | -1.85 (-2.28, -1.41) | <0.001 |
| Low | 19.23 (14.26, 25.96) | 16.42 (11.61, 23.13) | -0.51 (-0.58, -0.44) | <0.001 | 360.25 (296.81, 429.81) | 366.09 (296.10, 438.53) | 0.12 (-0.04, 0.28) | 0.128 | 259.03 (217.12, 305.73) | 198.86 (165.04, 234.48) | -0.90 (-1.02, -0.77) | <0.001 | 3.09 (2.49, 3.77) | 2.16 (1.72, 2.62) | -1.22 (-1.33, -1.11) | <0.001 |
| **21 GBD region** | | | | | | | | | | | | | | | | |
| South Asia | 16.96 (11.95, 23.85) | 16.15 (10.93, 23.22) | -0.14 (-0.28, 0.00) | 0.055 | 276.28 (217.76, 336.33) | 296.57 (230.17, 363.94) | 0.34 (-0.06, 0.75) | 0.094 | 250.74 (209.73, 301.40) | 170.13 (138.90, 204.69) | -1.38 (-1.87, -0.89) | <0.001 | 3.06 (2.46, 3.76) | 1.80 (1.43, 2.27) | -1.92 (-2.60, -1.24) | <0.001 |
| East Asia | 17.66 (11.97, 25.34) | 14.80 (9.37, 22.92) | -0.60 (-0.66, -0.54) | <0.001 | 272.94 (198.29, 351.69) | 268.68 (199.02, 340.68) | -0.03 (-0.10, 0.05) | 0.474 | 228.69 (183.83, 279.16) | 144.55 (113.12, 177.93) | -1.67 (-2.46, -0.88) | <0.001 | 2.40 (1.86, 3.11) | 1.16 (0.91, 1.46) | -2.16 (-2.78, -1.53) | <0.001 |
| North Africa and Middle East | 26.62 (20.40, 34.84) | 24.69 (18.33, 32.96) | -0.25 (-0.28, -0.21) | <0.001 | 362.98 (301.34, 425.33) | 365.60 (301.21, 426.76) | 0.10 (-0.21, 0.41) | 0.528 | 414.78 (339.56, 505.09) | 270.36 (214.66, 337.09) | -1.44 (-1.71, -1.17) | <0.001 | 5.33 (4.22, 6.68) | 3.14 (2.39, 4.12) | -1.77 (-2.10, -1.45) | <0.001 |
| Western Sub-Saharan Africa | 17.67 (12.78, 24.79) | 15.45 (10.72, 22.64) | -0.42 (-0.54, -0.30) | <0.001 | 369.53 (297.96, 443.32) | 373.51 (301.85, 447.88) | 0.07 (-0.13, 0.28) | 0.480 | 221.25 (176.38, 279.43) | 184.78 (146.51, 237.29) | -0.61 (-0.72, -0.50) | <0.001 | 2.53 (1.90, 3.36) | 1.96 (1.42, 2.69) | -0.86 (-0.99, -0.72) | <0.001 |
| Southeast Asia | 24.96 (18.31, 34.05) | 22.41 (16.01, 31.22) | -0.36 (-0.41, -0.32) | <0.001 | 343.96 (277.22, 409.80) | 348.13 (281.49, 418.85) | 0.07 (-0.12, 0.27) | 0.453 | 374.62 (299.10, 450.96) | 244.65 (203.18, 285.62) | -1.44 (-1.57, -1.31) | <0.001 | 4.48 (3.45, 5.54) | 2.52 (2.02, 3.06) | -1.95 (-2.11, -1.78) | <0.001 |
| Eastern Sub-Saharan Africa | 17.68 (12.89, | 12.76 (8.30, 19.33) | -1.06 (-1.21, -0.92) | <0.001 | 457.20 (372.13, | 454.16 (368.03, | 0.02 (-0.20, 0.23) | 0.868 | 269.13 (217.74, | 176.93 (141.69, | -1.44 (-1.53, | <0.001 | 3.08 (2.37, | 1.69 (1.25, | -2.04 (-2.15, | <0.001 |

| | | | | | | | | | | | | | | | | |
|---|---|---|---|---|---|---|---|---|---|---|---|---|---|---|---|---|
| | 24.42) | | | | 551.61) | 552.57) | | | 336.52) | 215.79) | -1.35) | | 4.01) | 2.17) | -1.93) | |
| Central Latin America | 25.89 (18.82, 35.65) | 20.56 (14.34, 29.49) | -0.80 (-0.96, -0.64) | <0.001 | 419.40 (341.18, 499.29) | 385.80 (317.12, 455.84) | -0.24 (-0.55, 0.07) | 0.134 | 239.10 (218.96, 260.50) | 144.47 (118.25, 175.98) | -1.60 (-1.94, -1.26) | <0.001 | 2.97 (2.76, 3.20) | 1.59 (1.25, 2.02) | -1.95 (-2.43, -1.46) | <0.001 |
| Tropical Latin America | 29.22 (21.70, 39.06) | 20.41 (14.12, 29.04) | -1.17 (-1.36, -0.97) | <0.001 | 400.71 (315.81, 489.83) | 361.82 (295.28, 435.96) | -0.33 (-0.44, -0.23) | <0.001 | 282.09 (255.88, 311.04) | 140.22 (121.93, 162.41) | -2.32 (-2.80, -1.83) | <0.001 | 3.62 (3.29, 3.95) | 1.55 (1.35, 1.78) | -2.83 (-3.39, -2.26) | <0.001 |
| Central Sub-Saharan Africa | 18.31 (13.43, 25.20) | 14.04 (9.48, 20.96) | -0.88 (-0.94, -0.82) | <0.001 | 364.63 (280.35, 450.91) | 341.02 (258.24, 416.78) | -0.20 (-0.42, 0.01) | 0.057 | 244.69 (169.64, 340.10) | 171.52 (119.39, 233.61) | -1.24 (-1.33, -1.16) | <0.001 | 2.91 (1.77, 4.28) | 1.83 (1.09, 2.72) | -1.62 (-1.71, -1.53) | <0.001 |
| Western Europe | 12.55 (8.24, 18.55) | 10.91 (6.72, 16.83) | -0.46 (-0.56, -0.36) | <0.001 | 221.80 (168.49, 274.45) | 220.96 (170.57, 271.59) | 0.08 (-0.30, 0.46) | 0.680 | 129.31 (116.43, 145.32) | 63.07 (50.87, 77.70) | -2.42 (-2.80, -2.04) | <0.001 | 1.39 (1.30, 1.49) | 0.40 (0.35, 0.46) | -4.20 (-4.49, -3.91) | <0.001 |
| High-income North America | 12.75 (7.94, 19.78) | 13.64 (8.51, 21.13) | 0.24 (0.19, 0.29) | <0.001 | 287.46 (218.83, 359.81) | 372.32 (287.28, 461.16) | 1.16 (0.15, 2.17) | 0.024 | 115.01 (96.99, 135.09) | 98.63 (77.59, 122.10) | -0.51 (-1.21, 0.19) | 0.151 | 0.97 (0.89, 1.05) | 0.56 (0.50, 0.63) | -1.85 (-2.26, -1.44) | <0.001 |
| Central Asia | 23.95 (18.20, 31.87) | 19.11 (13.30, 26.70) | -0.75 (-0.91, -0.58) | <0.001 | 284.46 (231.57, 338.46) | 274.95 (219.23, 333.72) | -0.08 (-0.34, 0.19) | 0.575 | 259.21 (230.36, 288.98) | 170.28 (137.71, 204.55) | -1.51 (-1.92, -1.11) | <0.001 | 2.99 (2.67, 3.32) | 1.68 (1.33, 2.10) | -2.19 (-2.88, -1.49) | <0.001 |
| Eastern Europe | 19.47 (13.34, 28.05) | 19.87 (13.15, 29.28) | 0.09 (0.00, 0.17) | 0.048 | 299.79 (234.09, 373.87) | 366.93 (287.81, 447.98) | 0.76 (0.43, 1.10) | <0.001 | 169.77 (144.07, 197.65) | 159.28 (127.27, 196.04) | -0.14 (-0.94, 0.66) | 0.727 | 1.51 (1.36, 1.65) | 1.18 (0.93, 1.49) | -0.82 (-1.75, 0.11) | 0.085 |
| Southern Sub-Saharan Africa | 17.13 (11.61, 25.46) | 13.79 (8.79, 21.35) | -0.71 (-0.80, -0.61) | <0.001 | 501.24 (391.43, 614.46) | 449.85 (350.90, 550.52) | -0.36 (-0.45, -0.27) | <0.001 | 245.46 (200.39, 292.16) | 139.36 (102.85, 179.41) | -1.85 (-2.95, -0.73) | 0.001 | 2.65 (2.10, 3.25) | 1.14 (0.72, 1.60) | -2.77 (-4.53, -0.98) | 0.003 |
| High-income Asia Pacific | 13.79 (9.22, 20.16) | 11.30 (6.93, 17.64) | -0.68 (-0.74, -0.61) | <0.001 | 217.09 (172.15, 261.96) | 220.68 (170.11, 270.70) | 0.13 (-0.33, 0.59) | 0.578 | 157.22 (131.92, 182.27) | 68.34 (52.49, 85.62) | -2.83 (-3.07, -2.59) | <0.001 | 1.70 (1.44, 1.99) | 0.36 (0.30, 0.42) | -5.33 (-5.76, -4.91) | <0.001 |
| Central Europe | 20.76 (14.83, 29.02) | 18.44 (12.56, 26.64) | -0.39 (-0.47, -0.32) | <0.001 | 305.62 (241.40, 363.87) | 309.15 (243.68, 375.76) | 0.13 (-0.35, 0.62) | 0.596 | 197.56 (174.14, 223.89) | 125.35 (98.75, 153.54) | -1.53 (-1.81, -1.25) | <0.001 | 1.94 (1.80, 2.09) | 0.84 (0.67, 1.03) | -3.04 (-3.47, -2.61) | <0.001 |
| Andean Latin America | 32.54 (25.76, | 23.31 (17.13, | -1.14 (-1.17, -1.11) | <0.001 | 328.67 (265.75, | 303.57 (241.58, | -0.22 (-0.35, | <0.001 | 423.93 (343.18, | 199.34 (150.53, | -2.51 (-3.27, | <0.001 | 5.85 (4.62, | 2.51 (1.79, | -2.81 (-3.67, | <0.001 |

| | | | | | | | | | | | | | | | | |
|---|---|---|---|---|---|---|---|---|---|---|---|---|---|---|---|---|
| | 41.16) | 31.44) | | | 402.14) | 377.50) | -0.10) | | 519.88) | 256.51) | -1.75) | | 7.28) | 3.36) | -1.95) | |
| Caribbean | 25.29 (19.11, 33.59) | 24.31 (18.04, 32.75) | -0.10 (-0.18, -0.03) | 0.009 | 332.77 (272.08, 395.63) | 330.68 (270.66, 396.54) | 0.06 (-0.12, 0.24) | 0.518 | 303.03 (238.63, 387.10) | 278.99 (187.33, 410.55) | -0.25 (-0.56, 0.06) | 0.116 | 4.04 (3.10, 5.35) | 3.68 (2.31, 5.67) | -0.29 (-0.59, 0.01) | 0.062 |
| Southern Latin America | 19.03 (13.72, 26.55) | 14.68 (10.03, 21.20) | -0.86 (-0.93, -0.78) | <0.001 | 215.28 (158.28, 274.91) | 191.90 (142.32, 250.19) | -0.39 (-0.65, -0.12) | 0.005 | 273.90 (232.10, 316.74) | 128.50 (103.44, 159.41) | -2.50 (-3.06, -1.94) | <0.001 | 3.50 (2.93, 4.11) | 1.36 (1.05, 1.75) | -3.11 (-3.77, -2.45) | <0.001 |
| Oceania | 42.60 (35.09, 51.93) | 43.32 (35.61, 53.31) | 0.06 (0.04, 0.08) | <0.001 | 352.71 (279.89, 426.44) | 346.82 (266.65, 432.89) | -0.03 (-0.20, 0.15) | 0.773 | 376.79 (251.95, 554.03) | 408.49 (269.57, 610.43) | 0.24 (0.06, 0.41) | 0.007 | 4.47 (2.71, 7.02) | 4.96 (2.97, 8.08) | 0.31 (0.11, 0.50) | 0.002 |
| Australasia | 13.47 (8.41, 20.40) | 13.32 (8.30, 20.71) | -0.05 (-0.12, 0.03) | 0.215 | 225.05 (157.03, 303.68) | 220.14 (151.60, 288.48) | -0.06 (-0.58, 0.46) | 0.817 | 92.06 (72.77, 116.55) | 56.58 (39.47, 77.43) | -1.68 (-2.51, -0.84) | <0.001 | 0.78 (0.62, 0.97) | 0.24 (0.16, 0.33) | -4.06 (-5.53, -2.56) | <0.001 |
| **Men** | | | | | | | | | | | | | | | | |
| Global | 16.75 (12.19, 23.00) | 15.47 (10.99, 21.74) | -0.26 (-0.36, -0.15) | <0.001 | 207.92 (161.05, 252.55) | 219.65 (172.03, 265.72) | 0.27 (-0.12, 0.65) | 0.174 | 285.00 (255.07, 315.62) | 231.25 (202.64, 263.07) | -0.69 (-0.86, -0.53) | <0.001 | 3.74 (3.32, 4.15) | 2.94 (2.55, 3.39) | -0.78 (-0.95, -0.60) | <0.001 |
| **SDI category** | | | | | | | | | | | | | | | | |
| High | 10.66 (7.10, 15.52) | 9.92 (6.47, 15.08) | -0.22 (-0.36, -0.08) | 0.003 | 163.84 (125.06, 202.90) | 200.08 (153.25, 247.42) | 0.91 (-0.06, 1.89) | 0.067 | 136.21 (121.88, 150.43) | 77.42 (65.20, 91.24) | -1.85 (-2.16, -1.54) | <0.001 | 1.62 (1.45, 1.76) | 0.68 (0.60, 0.78) | -2.94 (-3.08, -2.81) | <0.001 |
| High-middle | 17.09 (12.36, 23.55) | 14.34 (9.87, 20.49) | -0.59 (-0.69, -0.49) | <0.001 | 186.08 (142.43, 228.05) | 175.14 (133.20, 215.05) | -0.19 (-0.37, -0.01) | 0.040 | 272.07 (236.04, 311.88) | 163.79 (140.67, 188.67) | -1.77 (-2.15, -1.39) | <0.001 | 3.53 (3.01, 4.07) | 1.95 (1.66, 2.28) | -2.08 (-2.55, -1.61) | <0.001 |
| Middle | 18.75 (13.75, 25.41) | 17.45 (12.54, 23.97) | -0.25 (-0.31, -0.19) | <0.001 | 221.25 (171.96, 270.91) | 231.01 (182.52, 279.08) | 0.16 (0.05, 0.28) | 0.005 | 320.75 (281.59, 366.50) | 265.71 (216.50, 308.43) | -0.60 (-0.85, -0.35) | <0.001 | 4.23 (3.66, 4.81) | 3.42 (2.73, 3.99) | -0.73 (-0.90, -0.56) | <0.001 |
| Low-middle | 16.32 (11.81, 22.48) | 15.62 (11.13, 21.81) | -0.12 (-0.22, -0.02) | 0.015 | 224.22 (174.63, 273.67) | 227.82 (178.23, 279.83) | 0.12 (-0.19, 0.42) | 0.448 | 304.40 (257.10, 354.16) | 251.85 (212.18, 293.98) | -0.63 (-0.81, -0.45) | <0.001 | 4.04 (3.39, 4.75) | 3.26 (2.71, 3.87) | -0.72 (-0.90, -0.53) | <0.001 |
| Low | 18.04 (13.48, 24.29) | 16.13 (11.60, 22.57) | -0.38 (-0.41, -0.34) | <0.001 | 233.06 (187.45, 283.56) | 238.88 (189.81, 287.56) | 0.14 (-0.16, 0.43) | 0.358 | 348.73 (292.56, 408.05) | 293.50 (238.84, 359.26) | -0.59 (-0.72, -0.45) | <0.001 | 4.73 (3.90, 5.63) | 3.90 (3.10, 4.89) | -0.69 (-0.79, -0.59) | <0.001 |

**21 GBD region**

| | | | | | | | | | | | | | | | | |
|---|---|---|---|---|---|---|---|---|---|---|---|---|---|---|---|---|
| South Asia | 13.90 (9.61, 19.85) | 13.55 (9.24, 19.65) | -0.07 (-0.21, 0.07) | 0.347 | 211.24 (162.13, 262.10) | 212.88 (162.23, 268.39) | 0.09 (-0.21, 0.38) | 0.570 | 216.22 (174.71, 261.47) | 184.01 (152.76, 221.69) | -0.53 (-1.25, 0.19) | 0.146 | 2.75 (2.18, 3.40) | 2.26 (1.81, 2.81) | -0.56 (-1.49, 0.37) | 0.235 |
| East Asia | 17.57 (12.56, 24.21) | 15.69 (10.96, 22.32) | -0.38 (-0.48, -0.27) | <0.001 | 182.69 (135.16, 229.57) | 158.47 (115.64, 200.43) | -0.48 (-0.62, -0.34) | <0.001 | 334.52 (267.65, 399.95) | 247.06 (189.19, 296.26) | -0.95 (-1.46, -0.43) | <0.001 | 4.41 (3.41, 5.35) | 3.16 (2.32, 3.87) | -1.05 (-1.63, -0.47) | <0.001 |
| North Africa and Middle East | 19.70 (14.83, 26.30) | 19.66 (14.65, 26.53) | 0.01 (-0.07, 0.09) | 0.777 | 271.97 (223.02, 322.10) | 282.94 (233.00, 336.93) | 0.21 (-0.14, 0.55) | 0.235 | 408.40 (323.51, 502.58) | 342.20 (248.09, 442.66) | -0.61 (-0.95, -0.26) | 0.001 | 5.54 (4.27, 6.95) | 4.52 (3.17, 6.02) | -0.69 (-1.13, -0.24) | 0.003 |
| Western Sub-Saharan Africa | 19.16 (14.21, 26.34) | 17.68 (12.60, 25.05) | -0.24 (-0.32, -0.17) | <0.001 | 250.12 (199.50, 303.54) | 258.87 (206.85, 310.96) | 0.17 (-0.05, 0.39) | 0.133 | 315.12 (245.34, 402.78) | 276.39 (211.54, 365.52) | -0.45 (-0.59, -0.30) | <0.001 | 4.22 (3.20, 5.48) | 3.62 (2.66, 4.92) | -0.53 (-0.68, -0.38) | <0.001 |
| Southeast Asia | 24.78 (19.02, 32.34) | 23.02 (17.38, 30.44) | -0.23 (-0.37, -0.09) | 0.001 | 269.08 (217.02, 324.27) | 262.49 (212.59, 315.32) | -0.02 (-0.31, 0.27) | 0.871 | 400.46 (329.28, 475.27) | 366.28 (293.63, 432.49) | -0.32 (-0.50, -0.15) | <0.001 | 5.22 (4.19, 6.38) | 4.71 (3.65, 5.67) | -0.33 (-0.59, -0.08) | 0.011 |
| Eastern Sub-Saharan Africa | 20.80 (15.78, 27.82) | 17.01 (12.37, 23.89) | -0.68 (-0.72, -0.65) | <0.001 | 263.78 (214.46, 315.33) | 269.74 (216.28, 323.59) | 0.14 (-0.15, 0.43) | 0.335 | 537.64 (429.90, 647.42) | 397.06 (313.61, 499.62) | -1.03 (-1.20, -0.85) | <0.001 | 7.54 (5.92, 9.16) | 5.42 (4.14, 6.97) | -1.12 (-1.29, -0.95) | <0.001 |
| Central Latin America | 15.47 (11.05, 21.94) | 13.40 (9.00, 19.44) | -0.49 (-0.53, -0.45) | <0.001 | 261.57 (211.50, 314.01) | 262.31 (210.61, 313.12) | 0.05 (-0.14, 0.23) | 0.599 | 208.17 (187.15, 227.93) | 137.43 (115.37, 161.77) | -1.29 (-1.56, -1.01) | <0.001 | 2.79 (2.51, 3.08) | 1.73 (1.44, 2.08) | -1.51 (-1.80, -1.22) | <0.001 |
| Tropical Latin America | 21.37 (15.47, 29.12) | 15.16 (10.32, 22.02) | -1.17 (-1.30, -1.05) | <0.001 | 308.05 (240.38, 378.99) | 270.37 (215.05, 326.95) | -0.43 (-0.61, -0.26) | <0.001 | 273.85 (249.86, 299.75) | 142.29 (125.38, 160.66) | -2.18 (-2.58, -1.77) | <0.001 | 3.72 (3.36, 4.06) | 1.79 (1.58, 2.03) | -2.42 (-2.92, -1.91) | <0.001 |
| Central Sub-Saharan Africa | 16.23 (11.93, 22.09) | 13.16 (9.00, 18.95) | -0.70 (-0.77, -0.63) | <0.001 | 218.51 (158.01, 280.82) | 209.24 (153.49, 272.88) | -0.12 (-0.31, 0.07) | 0.216 | 379.59 (262.25, 544.73) | 288.52 (198.03, 415.57) | -1.01 (-1.19, -0.84) | <0.001 | 5.25 (3.48, 7.80) | 3.90 (2.51, 5.82) | -1.11 (-1.28, -0.93) | <0.001 |
| Western Europe | 10.46 (7.02, 15.03) | 8.27 (5.13, 12.80) | -0.80 (-0.90, -0.70) | <0.001 | 133.00 (99.45, 166.56) | 125.57 (94.35, 157.92) | -0.13 (-0.48, 0.23) | 0.480 | 158.67 (143.80, 173.98) | 57.29 (48.49, 67.12) | -3.54 (-3.92, -3.16) | <0.001 | 2.04 (1.86, 2.25) | 0.53 (0.47, 0.61) | -4.70 (-5.05, -4.35) | <0.001 |
| High-income North America | 9.28 (5.69, 14.54) | 9.70 (5.99, 15.26) | 0.20 (0.06, 0.34) | 0.005 | 211.89 (153.15, | 272.52 (198.82, | 1.19 (-0.16, 2.56) | 0.083 | 98.08 (85.44, 113.36) | 84.00 (69.03, 100.83) | -0.49 (-1.22, | 0.186 | 0.99 (0.90, | 0.68 (0.60, | -1.27 (-1.88, | <0.001 |

| | | | | | | | | | | | | | | | | |
|---|---|---|---|---|---|---|---|---|---|---|---|---|---|---|---|---|
| | | | | | 271.16) | 349.85) | | | | | 0.24) | | 1.08) | 0.79) | -0.65) | |
| Central Asia | 19.48 (14.98, 25.55) | 16.09 (11.59, 22.33) | -0.65 (-0.69, -0.62) | <0.001 | 153.33 (117.68, 192.02) | 144.28 (105.26, 185.80) | -0.20 (-0.34, -0.05) | 0.009 | 206.49 (185.63, 228.05) | 181.21 (150.54, 217.68) | -0.54 (-1.03, -0.05) | 0.030 | 2.59 (2.33, 2.86) | 2.23 (1.81, 2.70) | -0.67 (-1.37, 0.04) | 0.063 |
| Eastern Europe | 15.87 (11.45, 21.89) | 14.16 (9.98, 19.77) | -0.37 (-0.44, -0.30) | <0.001 | 133.11 (96.85, 170.84) | 121.68 (88.73, 157.41) | -0.25 (-0.65, 0.16) | 0.231 | 183.49 (164.47, 200.67) | 181.86 (148.42, 234.49) | -0.11 (-1.53, 1.32) | 0.876 | 2.27 (2.07, 2.45) | 2.26 (1.79, 3.01) | -0.11 (-1.71, 1.51) | 0.893 |
| Southern Sub-Saharan Africa | 17.89 (12.97, 24.79) | 15.29 (10.62, 22.01) | -0.52 (-0.63, -0.41) | <0.001 | 299.70 (235.46, 369.87) | 296.24 (227.64, 363.17) | 0.02 (-0.13, 0.17) | 0.806 | 208.13 (157.55, 269.71) | 195.53 (154.76, 253.38) | -0.08 (-1.11, 0.96) | 0.882 | 2.54 (1.81, 3.43) | 2.36 (1.78, 3.21) | -0.07 (-1.30, 1.17) | 0.907 |
| High-income Asia Pacific | 11.00 (7.43, 15.61) | 8.62 (5.25, 13.54) | -0.83 (-0.95, -0.70) | <0.001 | 99.06 (73.11, 127.74) | 104.46 (71.97, 136.53) | 0.20 (-0.17, 0.56) | 0.292 | 153.50 (118.85, 177.66) | 56.29 (45.77, 67.43) | -3.38 (-3.85, -2.92) | <0.001 | 1.94 (1.39, 2.27) | 0.46 (0.38, 0.54) | -4.82 (-5.47, -4.16) | <0.001 |
| Central Europe | 14.79 (10.73, 20.11) | 11.55 (7.85, 16.67) | -0.83 (-0.92, -0.75) | <0.001 | 122.40 (93.25, 154.68) | 97.74 (72.79, 124.01) | -0.66 (-1.09, -0.22) | 0.003 | 195.24 (177.40, 212.26) | 107.63 (90.05, 125.37) | -2.13 (-2.56, -1.69) | <0.001 | 2.46 (2.25, 2.66) | 1.18 (0.97, 1.41) | -2.67 (-3.28, -2.05) | <0.001 |
| Andean Latin America | 29.45 (23.52, 36.85) | 21.82 (16.41, 28.80) | -1.04 (-1.09, -0.99) | <0.001 | 295.71 (234.44, 360.57) | 265.71 (212.20, 323.64) | -0.34 (-0.46, -0.21) | <0.001 | 505.95 (395.86, 627.40) | 287.63 (205.85, 382.14) | -1.76 (-2.30, -1.21) | <0.001 | 7.22 (5.60, 9.06) | 3.97 (2.78, 5.37) | -1.82 (-2.40, -1.24) | <0.001 |
| Caribbean | 14.14 (10.00, 19.87) | 12.83 (8.71, 18.52) | -0.33 (-0.38, -0.28) | <0.001 | 276.11 (220.40, 336.40) | 269.33 (212.96, 327.95) | -0.03 (-0.26, 0.20) | 0.804 | 230.38 (194.24, 275.49) | 180.40 (129.90, 261.96) | -0.73 (-1.10, -0.37) | <0.001 | 3.11 (2.58, 3.77) | 2.37 (1.59, 3.63) | -0.81 (-1.22, -0.40) | <0.001 |
| Southern Latin America | 15.56 (11.26, 21.21) | 11.81 (7.90, 16.94) | -0.94 (-1.03, -0.84) | <0.001 | 138.97 (93.73, 186.64) | 113.71 (77.60, 155.13) | -0.67 (-0.83, -0.51) | <0.001 | 311.39 (266.29, 363.57) | 138.53 (107.24, 176.11) | -2.83 (-3.38, -2.28) | <0.001 | 4.32 (3.66, 5.07) | 1.74 (1.30, 2.26) | -3.17 (-3.80, -2.53) | <0.001 |
| Oceania | 37.48 (30.99, 45.33) | 36.01 (29.43, 44.13) | -0.13 (-0.15, -0.11) | <0.001 | 315.52 (249.31, 388.23) | 305.59 (234.39, 389.50) | -0.08 (-0.21, 0.05) | 0.226 | 607.07 (381.18, 982.54) | 601.91 (357.14, 937.41) | -0.16 (-0.42, 0.09) | 0.206 | 8.25 (4.98, 13.88) | 8.19 (4.61, 13.13) | -0.15 (-0.40, 0.10) | 0.249 |
| Australasia | 9.95 (6.28, 15.09) | 8.84 (5.27, 13.89) | -0.40 (-0.44, -0.35) | <0.001 | 108.54 (66.87, 156.19) | 105.40 (63.68, 159.01) | -0.04 (-0.35, 0.26) | 0.780 | 87.29 (71.54, 105.52) | 40.12 (29.44, 53.08) | -2.58 (-2.92, -2.23) | <0.001 | 1.00 (0.79, 1.25) | 0.30 (0.20, 0.43) | -3.92 (-4.48, -3.35) | <0.001 |
| **25-29 years** | | | | | | | | | | | | | | | | |
| **Women** | | | | | | | | | | | | | | | | |

| | | | | | | | | | | | | | | | | |
|---|---|---|---|---|---|---|---|---|---|---|---|---|---|---|---|---|
| Global | 27.10 (19.22, 38.22) | 22.99 (15.52, 33.51) | -0.56 (-0.66, -0.45) | <0.001 | 413.51 (339.85, 487.54) | 417.00 (344.57, 487.39) | 0.09 (-0.26, 0.45) | 0.610 | 334.43 (300.00, 370.63) | 214.16 (185.52, 243.67) | -1.42 (-1.90, -0.94) | <0.001 | 4.22 (3.82, 4.64) | 2.30 (1.99, 2.62) | -2.14 (-2.57, -1.70) | <0.001 |
| **SDI category** | | | | | | | | | | | | | | | | |
| High | 19.21 (12.75, 28.93) | 18.25 (11.72, 28.40) | -0.18 (-0.20, -0.16) | <0.001 | 356.97 (293.57, 418.50) | 402.08 (329.88, 471.89) | 0.57 (-0.10, 1.25) | 0.095 | 188.84 (166.94, 210.73) | 124.70 (102.35, 150.83) | -1.39 (-1.67, -1.12) | <0.001 | 2.09 (1.97, 2.22) | 0.93 (0.84, 1.06) | -2.67 (-2.96, -2.38) | <0.001 |
| High-middle | 27.94 (19.08, 39.90) | 22.12 (14.64, 33.86) | -0.80 (-0.91, -0.68) | <0.001 | 401.84 (325.28, 479.34) | 384.31 (312.07, 457.33) | -0.09 (-0.44, 0.27) | 0.630 | 285.42 (248.29, 324.21) | 164.32 (137.55, 194.33) | -1.97 (-2.95, -0.98) | <0.001 | 3.32 (2.95, 3.78) | 1.42 (1.24, 1.60) | -2.96 (-3.76, -2.16) | <0.001 |
| Middle | 29.81 (21.02, 42.03) | 24.20 (16.26, 35.76) | -0.69 (-0.78, -0.60) | <0.001 | 440.32 (360.60, 524.14) | 423.46 (347.71, 496.89) | -0.08 (-0.40, 0.24) | 0.628 | 378.33 (332.96, 422.60) | 218.51 (188.47, 251.23) | -1.86 (-2.70, -1.01) | <0.001 | 4.78 (4.25, 5.38) | 2.27 (1.98, 2.62) | -2.34 (-3.26, -1.42) | <0.001 |
| Low-middle | 26.71 (19.31, 37.79) | 23.52 (16.23, 34.32) | -0.43 (-0.49, -0.38) | <0.001 | 403.98 (335.56, 474.68) | 416.81 (345.00, 487.15) | 0.20 (-0.17, 0.58) | 0.296 | 410.66 (348.89, 473.70) | 252.91 (213.06, 292.83) | -1.61 (-1.93, -1.29) | <0.001 | 5.59 (4.60, 6.53) | 3.01 (2.47, 3.59) | -2.07 (-2.59, -1.55) | <0.001 |
| Low | 29.02 (21.47, 39.91) | 23.94 (17.11, 34.00) | -0.65 (-0.70, -0.61) | <0.001 | 459.16 (386.61, 534.88) | 453.20 (381.27, 525.10) | 0.000027 (-0.241829, 0.242469) | 1.000 | 369.61 (313.55, 433.20) | 265.33 (221.94, 320.26) | -1.12 (-1.30, -0.95) | <0.001 | 4.92 (4.09, 5.87) | 3.22 (2.60, 3.98) | -1.44 (-1.57, -1.30) | <0.001 |
| **21 GBD region** | | | | | | | | | | | | | | | | |
| South Asia | 23.61 (16.53, 34.44) | 21.44 (14.48, 32.01) | -0.33 (-0.46, -0.19) | <0.001 | 364.46 (297.52, 431.24) | 382.12 (308.64, 452.86) | 0.26 (-0.13, 0.65) | 0.193 | 310.85 (258.48, 370.39) | 213.45 (174.44, 257.49) | -1.26 (-2.07, -0.44) | 0.002 | 4.07 (3.26, 4.93) | 2.44 (1.91, 3.08) | -1.76 (-2.82, -0.69) | 0.001 |
| East Asia | 24.85 (17.07, 36.21) | 18.79 (11.99, 29.78) | -0.99 (-1.07, -0.91) | <0.001 | 372.73 (288.60, 460.34) | 343.43 (266.02, 424.06) | -0.27 (-0.41, -0.14) | <0.001 | 328.06 (265.98, 400.06) | 175.87 (138.86, 216.12) | -1.82 (-2.75, -0.89) | <0.001 | 3.88 (3.00, 4.97) | 1.49 (1.14, 1.90) | -3.11 (-4.43, -1.78) | <0.001 |
| North Africa and Middle East | 39.14 (29.89, 51.82) | 35.11 (25.75, 47.74) | -0.37 (-0.40, -0.34) | <0.001 | 505.04 (432.50, 578.35) | 504.49 (429.73, 578.14) | 0.05 (-0.19, 0.29) | 0.687 | 485.62 (415.06, 565.96) | 313.85 (251.17, 390.51) | -1.44 (-1.74, -1.13) | <0.001 | 6.58 (5.48, 7.79) | 3.79 (2.88, 4.93) | -1.87 (-2.15, -1.60) | <0.001 |
| Western Sub-Saharan Africa | 25.44 (17.97, 35.88) | 21.34 (14.32, 31.22) | -0.54 (-0.60, -0.48) | <0.001 | 467.52 (388.04, 549.13) | 463.11 (383.43, 542.17) | 0.00 (-0.19, 0.19) | 0.984 | 299.00 (235.74, 384.05) | 223.06 (175.17, 289.38) | -1.00 (-1.10, -0.91) | <0.001 | 3.80 (2.86, 5.15) | 2.56 (1.88, 3.67) | -1.34 (-1.44, -1.24) | <0.001 |
| Southeast Asia | 34.76 | 30.80 | -0.40 (-0.48, | <0.001 | 477.96 | 461.74 | -0.08 | 0.541 | 535.90 | 325.37 | -1.67 | <0.001 | 7.01 | 3.63 | -2.22 | <0.001 |

| | | | | | | | | | | | | | | | | |
|---|---|---|---|---|---|---|---|---|---|---|---|---|---|---|---|---|
| | (24.67, 48.72) | (21.14, 44.24) | -0.31) | | (398.16, 561.01) | (381.77, 542.53) | (-0.33, 0.17) | | (426.27, 639.53) | (270.78, 381.98) | (-1.78, -1.56) | | (5.36, 8.49) | (2.91, 4.42) | (-2.35, -2.10) | |
| Eastern Sub-Saharan Africa | 27.94 (20.40, 38.81) | 17.51 (11.30, 27.05) | -1.59 (-1.69, -1.50) | <0.001 | 556.67 (462.00, 651.91) | 523.97 (431.25, 620.79) | -0.16 (-0.41, 0.09) | 0.210 | 382.48 (307.15, 494.42) | 215.58 (175.44, 263.52) | -1.95 (-2.11, -1.78) | <0.001 | 4.98 (3.85, 6.72) | 2.31 (1.72, 3.02) | -2.60 (-2.75, -2.46) | <0.001 |
| Central Latin America | 35.42 (24.59, 50.97) | 26.79 (17.68, 39.59) | -0.96 (-1.05, -0.87) | <0.001 | 555.42 (462.62, 649.53) | 495.67 (417.51, 576.61) | -0.34 (-0.62, -0.07) | 0.015 | 317.67 (293.01, 343.24) | 178.68 (146.62, 218.61) | -1.77 (-2.04, -1.49) | <0.001 | 4.27 (4.00, 4.57) | 2.09 (1.62, 2.66) | -2.14 (-2.52, -1.76) | <0.001 |
| Tropical Latin America | 44.53 (32.15, 61.43) | 26.88 (18.40, 38.67) | -1.73 (-1.92, -1.53) | <0.001 | 577.97 (466.37, 681.46) | 476.91 (394.20, 557.31) | -0.63 (-0.77, -0.49) | <0.001 | 495.85 (462.17, 533.29) | 184.74 (162.81, 207.65) | -3.31 (-3.58, -3.03) | <0.001 | 7.14 (6.65, 7.65) | 2.21 (1.97, 2.43) | -3.95 (-4.50, -3.39) | <0.001 |
| Central Sub-Saharan Africa | 27.38 (19.95, 37.72) | 19.43 (12.74, 29.57) | -1.18 (-1.21, -1.14) | <0.001 | 462.65 (370.27, 568.05) | 421.68 (338.47, 513.21) | -0.25 (-0.49, -0.01) | 0.040 | 341.67 (227.73, 485.92) | 212.95 (155.19, 293.58) | -1.62 (-1.75, -1.48) | <0.001 | 4.53 (2.75, 6.77) | 2.50 (1.59, 3.80) | -2.09 (-2.20, -1.99) | <0.001 |
| Western Europe | 17.85 (11.96, 26.69) | 14.89 (9.26, 23.52) | -0.61 (-0.70, -0.52) | <0.001 | 302.03 (243.51, 357.66) | 291.65 (235.11, 350.63) | -0.05 (-0.45, 0.35) | 0.791 | 178.47 (160.44, 196.86) | 84.26 (68.52, 102.14) | -2.59 (-3.02, -2.15) | <0.001 | 2.14 (2.02, 2.28) | 0.63 (0.56, 0.70) | -4.17 (-4.75, -3.59) | <0.001 |
| High-income North America | 17.81 (11.09, 28.16) | 18.01 (11.08, 28.91) | 0.04 (-0.01, 0.09) | 0.155 | 398.19 (316.47, 478.23) | 488.39 (396.94, 584.29) | 0.97 (-0.04, 1.98) | 0.059 | 169.93 (146.23, 196.95) | 138.70 (113.07, 167.07) | -0.60 (-1.22, 0.01) | 0.054 | 1.72 (1.61, 1.82) | 1.01 (0.90, 1.14) | -1.79 (-2.50, -1.08) | <0.001 |
| Central Asia | 35.06 (24.71, 49.22) | 27.76 (18.23, 41.01) | -0.77 (-0.84, -0.70) | <0.001 | 408.25 (342.28, 477.92) | 384.41 (311.44, 459.34) | -0.17 (-0.30, -0.05) | 0.007 | 320.17 (284.86, 356.51) | 240.29 (195.81, 293.06) | -0.92 (-1.36, -0.48) | <0.001 | 3.83 (3.47, 4.19) | 2.60 (2.03, 3.26) | -1.27 (-1.89, -0.65) | <0.001 |
| Eastern Europe | 27.76 (18.18, 41.54) | 27.70 (18.06, 42.12) | 0.05 (-0.04, 0.14) | 0.306 | 436.55 (350.26, 522.45) | 513.50 (421.53, 605.33) | 0.62 (0.35, 0.89) | <0.001 | 203.81 (169.78, 240.45) | 215.23 (169.86, 268.64) | 0.30 (-0.38, 1.00) | 0.388 | 1.83 (1.60, 2.03) | 1.82 (1.35, 2.45) | -0.09 (-0.83, 0.64) | 0.801 |
| Southern Sub-Saharan Africa | 25.33 (16.82, 37.37) | 17.70 (11.06, 27.96) | -1.25 (-1.34, -1.15) | <0.001 | 606.27 (489.71, 726.09) | 533.35 (429.63, 638.01) | -0.42 (-0.49, -0.35) | <0.001 | 466.53 (376.97, 561.75) | 219.52 (148.94, 306.16) | -2.54 (-3.81, -1.26) | <0.001 | 6.25 (4.84, 7.69) | 2.38 (1.30, 3.70) | -3.09 (-5.00, -1.14) | 0.002 |
| High-income Asia Pacific | 19.11 (12.67, 29.44) | 15.02 (8.88, 25.25) | -0.83 (-0.92, -0.73) | <0.001 | 309.29 (252.98, 369.86) | 290.46 (234.70, 351.08) | -0.12 (-0.52, 0.27) | 0.540 | 217.65 (184.62, 246.27) | 92.19 (72.18, 114.94) | -2.92 (-3.34, -2.50) | <0.001 | 2.60 (2.26, 2.97) | 0.57 (0.46, 0.67) | -5.14 (-5.71, -4.56) | <0.001 |
| Central Europe | 30.96 | 26.50 | -0.53 (-0.59, | <0.001 | 448.51 | 447.62 | 0.06 (-0.31, | 0.739 | 281.64 | 171.08 | -1.71 | <0.001 | 3.08 | 1.30 | -2.87 | <0.001 |

| | | | | | | | | | | | | | | | | |
|---|---|---|---|---|---|---|---|---|---|---|---|---|---|---|---|---|
| | (20.83, 44.69) | (17.35, 39.88) | -0.46) | | (376.13, 522.09) | (371.64, 526.77) | 0.44) | | (251.05, 316.90) | (137.62, 207.31) | (-1.87, -1.56) | | (2.90, 3.29) | (1.05, 1.60) | (-3.12, -2.62) | |
| Andean Latin America | 43.52 (33.64, 56.44) | 30.08 (20.90, 42.36) | -1.27 (-1.31, -1.22) | <0.001 | 476.44 (395.65, 560.35) | 417.83 (343.57, 497.49) | -0.44 (-0.58, -0.30) | <0.001 | 526.94 (425.89, 640.15) | 237.59 (177.35, 313.43) | -2.74 (-3.70, -1.76) | <0.001 | 7.75 (6.19, 9.55) | 3.15 (2.18, 4.35) | -3.10 (-4.22, -1.98) | <0.001 |
| Caribbean | 37.81 (27.44, 51.91) | 37.34 (26.97, 51.30) | -0.03 (-0.06, 0.00) | 0.071 | 465.72 (391.38, 537.39) | 463.19 (384.69, 542.21) | 0.09 (-0.16, 0.34) | 0.482 | 423.64 (323.62, 539.22) | 394.63 (258.43, 572.03) | -0.22 (-0.46, 0.02) | 0.068 | 6.12 (4.48, 8.02) | 5.65 (3.45, 8.54) | -0.25 (-0.51, 0.01) | 0.057 |
| Southern Latin America | 26.64 (19.05, 37.42) | 19.86 (13.06, 30.38) | -0.98 (-1.04, -0.92) | <0.001 | 320.22 (252.09, 391.47) | 278.95 (212.42, 352.18) | -0.45 (-0.63, -0.27) | <0.001 | 351.37 (310.49, 400.62) | 156.82 (124.54, 187.89) | -2.75 (-3.33, -2.16) | <0.001 | 4.82 (4.21, 5.51) | 1.73 (1.33, 2.15) | -3.46 (-4.16, -2.75) | <0.001 |
| Oceania | 76.85 (64.17, 93.99) | 80.30 (67.13, 99.33) | 0.15 (0.08, 0.22) | <0.001 | 576.53 (482.43, 675.39) | 573.25 (469.11, 681.36) | -0.01 (-0.10, 0.09) | 0.884 | 681.26 (476.19, 967.17) | 701.08 (478.47, 1032.57) | 0.06 (-0.08, 0.19) | 0.413 | 9.07 (5.77, 13.52) | 9.40 (5.75, 14.79) | 0.07 (-0.09, 0.23) | 0.371 |
| Australasia | 19.49 (12.09, 30.85) | 18.59 (11.45, 29.70) | -0.17 (-0.31, -0.03) | 0.016 | 306.33 (226.20, 393.89) | 309.89 (230.61, 399.16) | 0.08 (-0.17, 0.34) | 0.527 | 136.16 (110.19, 164.47) | 78.66 (56.94, 103.12) | -1.89 (-2.62, -1.16) | <0.001 | 1.37 (1.12, 1.65) | 0.44 (0.31, 0.60) | -3.88 (-5.10, -2.65) | <0.001 |
| **Men** | | | | | | | | | | | | | | | | |
| Global | 24.07 (17.47, 33.91) | 22.12 (15.63, 31.88) | -0.29 (-0.32, -0.26) | <0.001 | 293.78 (239.67, 345.68) | 298.73 (244.84, 350.57) | 0.12 (-0.20, 0.45) | 0.467 | 366.64 (333.09, 398.77) | 285.48 (254.62, 322.01) | -0.85 (-1.07, -0.62) | <0.001 | 5.18 (4.68, 5.65) | 3.88 (3.42, 4.39) | -0.99 (-1.27, -0.71) | <0.001 |
| **SDI category** | | | | | | | | | | | | | | | | |
| High | 15.11 (10.14, 22.33) | 13.75 (8.85, 21.49) | -0.31 (-0.36, -0.26) | <0.001 | 229.85 (183.79, 274.63) | 268.02 (214.87, 319.16) | 0.72 (-0.18, 1.62) | 0.116 | 192.86 (174.18, 210.74) | 117.03 (100.72, 136.20) | -1.69 (-1.96, -1.43) | <0.001 | 2.52 (2.31, 2.70) | 1.23 (1.09, 1.40) | -2.44 (-2.73, -2.15) | <0.001 |
| High-middle | 25.15 (17.91, 35.42) | 20.78 (13.97, 31.03) | -0.67 (-0.70, -0.64) | <0.001 | 274.52 (220.76, 327.73) | 253.05 (201.56, 302.17) | -0.22 (-0.42, -0.03) | 0.025 | 352.65 (314.30, 393.38) | 234.71 (205.18, 267.22) | -1.43 (-1.76, -1.10) | <0.001 | 4.91 (4.34, 5.54) | 3.06 (2.66, 3.54) | -1.65 (-2.04, -1.26) | <0.001 |
| Middle | 27.19 (19.49, 37.79) | 24.89 (17.44, 35.57) | -0.30 (-0.35, -0.25) | <0.001 | 317.73 (260.56, 373.18) | 316.44 (260.29, 373.31) | 0.05 (-0.23, 0.32) | 0.734 | 421.54 (377.57, 471.29) | 324.94 (279.13, 368.37) | -0.86 (-1.12, -0.60) | <0.001 | 5.98 (5.27, 6.67) | 4.45 (3.69, 5.12) | -0.97 (-1.26, -0.68) | <0.001 |
| Low-middle | 23.51 (17.22, | 22.78 (16.51, | -0.12 (-0.14, -0.10) | <0.001 | 312.60 (254.95, | 312.38 (255.72, | 0.06 (-0.23, 0.36) | 0.662 | 418.20 (352.13, | 325.24 (278.39, | -0.77 (-1.29, | 0.005 | 6.04 (5.06, | 4.54 (3.79, | -0.89 (-1.45, | 0.002 |

| | | | | | | | | | | | | | | | | |
|---|---|---|---|---|---|---|---|---|---|---|---|---|---|---|---|---|
| | 33.35) | 32.69) | | | 367.99) | 366.90) | | | 480.03) | 380.16) | -0.23) | | 6.99) | 5.39) | -0.33) | |
| Low | 26.61 (20.01, 36.75) | 23.31 (17.06, 32.97) | -0.44 (-0.49, -0.40) | <0.001 | 323.87 (267.71, 378.72) | 320.57 (265.94, 375.42) | 0.03 (-0.24, 0.29) | 0.851 | 383.98 (324.27, 447.87) | 332.59 (273.88, 402.37) | -0.48 (-0.61, -0.34) | <0.001 | 5.53 (4.55, 6.50) | 4.69 (3.73, 5.83) | -0.56 (-0.70, -0.41) | <0.001 |
| **21 GBD category** | | | | | | | | | | | | | | | | |
| South Asia | 19.36 (13.37, 28.50) | 19.24 (13.30, 28.29) | -0.03 (-0.08, 0.01) | 0.166 | 288.23 (229.36, 345.91) | 286.60 (229.42, 343.99) | 0.05 (-0.24, 0.33) | 0.746 | 286.24 (233.39, 339.77) | 239.25 (202.02, 282.60) | -0.56 (-1.14, 0.03) | 0.063 | 3.96 (3.11, 4.82) | 3.19 (2.61, 3.90) | -0.54 (-1.18, 0.10) | 0.099 |
| East Asia | 24.94 (17.43, 35.05) | 22.90 (15.42, 33.70) | -0.28 (-0.41, -0.15) | <0.001 | 266.96 (211.06, 323.20) | 238.08 (184.96, 291.44) | -0.37 (-0.50, -0.25) | <0.001 | 425.93 (355.05, 498.69) | 317.34 (253.07, 369.56) | -1.00 (-1.42, -0.59) | <0.001 | 6.01 (4.92, 7.23) | 4.33 (3.35, 5.21) | -1.14 (-1.62, -0.66) | <0.001 |
| North Africa and Middle East | 28.52 (21.28, 38.68) | 27.05 (19.59, 37.03) | -0.17 (-0.22, -0.11) | <0.001 | 375.00 (317.24, 434.15) | 384.81 (327.76, 443.53) | 0.18 (-0.01, 0.37) | 0.068 | 532.34 (436.24, 645.41) | 386.32 (297.89, 488.09) | -1.11 (-1.49, -0.74) | <0.001 | 7.79 (6.26, 9.69) | 5.39 (4.00, 7.07) | -1.27 (-1.64, -0.90) | <0.001 |
| Western Sub-Saharan Africa | 27.68 (20.30, 38.20) | 25.26 (18.04, 35.77) | -0.30 (-0.32, -0.27) | <0.001 | 346.62 (287.49, 408.13) | 355.28 (294.17, 415.51) | 0.15 (-0.08, 0.38) | 0.206 | 376.41 (302.85, 469.28) | 329.20 (257.08, 417.62) | -0.46 (-0.56, -0.36) | <0.001 | 5.38 (4.18, 6.92) | 4.60 (3.52, 6.00) | -0.54 (-0.67, -0.42) | <0.001 |
| Southeast Asia | 37.97 (28.37, 51.73) | 35.07 (25.54, 48.32) | -0.25 (-0.34, -0.17) | <0.001 | 409.87 (342.10, 478.83) | 394.75 (325.97, 463.98) | -0.07 (-0.33, 0.19) | 0.587 | 556.08 (462.72, 660.45) | 459.76 (361.18, 544.63) | -0.63 (-1.02, -0.24) | 0.002 | 7.81 (6.33, 9.42) | 6.28 (4.67, 7.57) | -0.67 (-1.09, -0.25) | 0.002 |
| Eastern Sub-Saharan Africa | 30.23 (22.87, 41.14) | 23.46 (16.98, 33.46) | -0.87 (-0.92, -0.81) | <0.001 | 363.65 (302.24, 424.96) | 354.46 (294.18, 414.81) | -0.02 (-0.32, 0.29) | 0.913 | 511.73 (406.89, 612.89) | 379.84 (296.27, 486.83) | -1.03 (-1.17, -0.88) | <0.001 | 7.56 (5.86, 9.21) | 5.43 (4.11, 7.15) | -1.12 (-1.28, -0.97) | <0.001 |
| Central Latin America | 21.11 (14.48, 30.69) | 17.85 (11.81, 27.09) | -0.58 (-0.61, -0.55) | <0.001 | 328.91 (268.24, 387.32) | 321.33 (262.88, 379.86) | -0.04 (-0.32, 0.24) | 0.772 | 283.30 (258.59, 307.60) | 188.33 (160.74, 218.70) | -1.26 (-1.54, -0.99) | <0.001 | 4.13 (3.74, 4.50) | 2.60 (2.19, 3.09) | -1.42 (-1.73, -1.10) | <0.001 |
| Tropical Latin America | 33.64 (23.38, 48.20) | 20.55 (13.49, 31.24) | -1.71 (-1.79, -1.63) | <0.001 | 428.50 (345.14, 510.46) | 351.25 (285.92, 417.68) | -0.67 (-0.82, -0.53) | <0.001 | 466.73 (430.32, 505.05) | 188.07 (165.84, 210.41) | -3.12 (-3.43, -2.81) | <0.001 | 7.00 (6.45, 7.63) | 2.57 (2.28, 2.92) | -3.46 (-3.79, -3.12) | <0.001 |
| Central Sub-Saharan Africa | 25.19 (18.76, 34.48) | 19.12 (13.32, 28.33) | -0.94 (-0.96, -0.91) | <0.001 | 294.15 (227.79, 362.94) | 265.99 (203.84, 336.93) | -0.32 (-0.55, -0.07) | 0.010 | 378.08 (250.68, 547.93) | 283.05 (190.97, 410.19) | -1.05 (-1.17, -0.93) | <0.001 | 5.51 (3.44, 8.28) | 4.00 (2.49, 6.11) | -1.16 (-1.30, -1.02) | <0.001 |
| Western Europe | 14.80 | 11.31 (7.13, | -0.92 (-1.00, | <0.001 | 189.14 | 175.02 | -0.22 | 0.163 | 205.69 | 82.44 (70.32, | -3.19 | <0.001 | 2.85 | 0.88 | -4.16 | <0.001 |

| | | | | | | | | | | | | | | | | |
|---|---|---|---|---|---|---|---|---|---|---|---|---|---|---|---|---|
| | (10.16, 21.73) | 17.75) | -0.84) | | (149.39, 230.17) | (139.00, 213.14) | (-0.52, 0.09) | | (187.23, 226.32) | 95.01) | (-3.65, -2.73) | | (2.61, 3.13) | (0.79, 1.00) | (-4.65, -3.66) | |
| High-income North America | 12.51 (7.73, 20.21) | 12.56 (7.73, 20.32) | 0.06 (-0.01, 0.12) | 0.105 | 273.85 (210.44, 340.36) | 338.18 (257.99, 417.74) | 1.03 (-0.28, 2.36) | 0.124 | 143.95 (127.15, 162.60) | 114.79 (96.39, 136.58) | -0.74 (-1.21, -0.27) | 0.002 | 1.68 (1.53, 1.82) | 1.08 (0.94, 1.27) | -1.44 (-2.00, -0.87) | <0.001 |
| Central Asia | 31.00 (23.45, 41.50) | 25.76 (18.05, 36.59) | -0.65 (-0.70, -0.59) | <0.001 | 257.18 (208.32, 308.51) | 234.28 (183.99, 289.12) | -0.30 (-0.45, -0.16) | <0.001 | 350.30 (314.81, 382.84) | 308.52 (253.62, 371.41) | -0.55 (-1.24, 0.15) | 0.122 | 4.87 (4.35, 5.34) | 4.23 (3.43, 5.19) | -0.62 (-1.40, 0.17) | 0.123 |
| Eastern Europe | 25.79 (18.04, 36.93) | 23.44 (16.05, 33.56) | -0.32 (-0.38, -0.25) | <0.001 | 231.66 (181.01, 283.46) | 221.45 (173.01, 269.85) | -0.14 (-0.20, -0.07) | <0.001 | 268.77 (237.69, 296.07) | 333.94 (278.01, 437.22) | 0.82 (-0.52, 2.17) | 0.230 | 3.61 (3.15, 3.91) | 4.70 (3.81, 6.29) | 1.00 (-0.57, 2.59) | 0.215 |
| Southern Sub-Saharan Africa | 27.58 (19.07, 39.27) | 21.05 (14.28, 31.13) | -0.92 (-0.98, -0.86) | <0.001 | 400.85 (320.79, 480.06) | 378.79 (304.01, 457.68) | -0.16 (-0.30, -0.02) | 0.028 | 387.37 (298.15, 496.32) | 270.09 (216.20, 341.41) | -1.30 (-1.83, -0.77) | <0.001 | 5.48 (4.02, 7.19) | 3.61 (2.78, 4.68) | -1.52 (-2.13, -0.91) | <0.001 |
| High-income Asia Pacific | 16.53 (11.41, 24.11) | 12.60 (7.74, 20.97) | -0.93 (-1.05, -0.81) | <0.001 | 166.24 (130.93, 204.23) | 178.89 (135.89, 223.87) | 0.32 (-0.10, 0.73) | 0.132 | 246.29 (204.09, 280.73) | 96.03 (80.70, 113.32) | -3.19 (-3.62, -2.76) | <0.001 | 3.45 (2.81, 3.96) | 1.00 (0.82, 1.17) | -4.22 (-4.83, -3.62) | <0.001 |
| Central Europe | 23.46 (16.33, 33.13) | 17.58 (11.54, 26.82) | -0.98 (-1.07, -0.88) | <0.001 | 221.36 (177.44, 264.72) | 192.39 (154.84, 233.54) | -0.40 (-0.78, -0.03) | 0.036 | 311.75 (287.26, 336.99) | 164.82 (140.98, 189.81) | -2.22 (-2.61, -1.82) | <0.001 | 4.34 (4.02, 4.65) | 2.03 (1.73, 2.38) | -2.63 (-3.12, -2.14) | <0.001 |
| Andean Latin America | 37.82 (29.15, 48.70) | 28.49 (20.58, 39.58) | -0.98 (-1.03, -0.94) | <0.001 | 423.45 (345.08, 501.03) | 379.11 (311.11, 449.88) | -0.37 (-0.43, -0.31) | <0.001 | 511.36 (410.07, 631.52) | 327.72 (239.12, 441.21) | -1.46 (-2.19, -0.72) | <0.001 | 7.72 (6.06, 9.76) | 4.80 (3.36, 6.66) | -1.56 (-2.35, -0.76) | <0.001 |
| Caribbean | 21.72 (14.67, 31.84) | 19.35 (12.36, 29.31) | -0.38 (-0.46, -0.30) | <0.001 | 344.08 (280.75, 408.20) | 333.11 (275.18, 398.41) | -0.09 (-0.27, 0.09) | 0.333 | 312.62 (265.51, 367.51) | 241.10 (173.46, 344.56) | -0.87 (-1.05, -0.70) | <0.001 | 4.59 (3.83, 5.47) | 3.44 (2.36, 5.20) | -0.95 (-1.15, -0.76) | <0.001 |
| Southern Latin America | 21.29 (15.72, 29.15) | 15.52 (10.45, 22.52) | -1.06 (-1.17, -0.96) | <0.001 | 204.09 (156.34, 255.86) | 186.81 (132.85, 246.94) | -0.28 (-0.34, -0.22) | <0.001 | 399.34 (341.86, 469.39) | 173.59 (132.57, 215.28) | -2.86 (-3.28, -2.44) | <0.001 | 5.93 (4.97, 7.04) | 2.32 (1.68, 2.96) | -3.22 (-3.69, -2.74) | <0.001 |
| Oceania | 54.10 (44.03, 68.64) | 55.13 (44.25, 70.55) | 0.06 (0.05, 0.08) | <0.001 | 473.36 (390.28, 559.13) | 462.29 (371.96, 555.18) | -0.06 (-0.13, 0.01) | 0.113 | 961.45 (615.59, 1415.66) | 970.13 (615.91, 1511.16) | 0.02 (-0.25, 0.29) | 0.868 | 14.21 (8.62, 21.65) | 14.38 (8.72, 23.35) | 0.03 (-0.26, 0.33) | 0.824 |
| Australasia | 14.78 (9.10, | 12.09 (7.12, | -0.69 (-0.73, | <0.001 | 152.07 | 143.26 (93.05, | -0.14 | 0.219 | 129.57 | 59.80 (44.83, | -2.58 | <0.001 | 1.65 | 0.54 | -3.73 | <0.001 |

| | | | | | | | | | | | | | | | | |
|---|---|---|---|---|---|---|---|---|---|---|---|---|---|---|---|---|
| | 23.25) | 19.88) | -0.64) | | (102.27, 210.47) | 199.99) | (-0.36, 0.08) | | (108.16, 154.03) | 78.08) | (-3.30, -1.86) | | (1.34, 2.02) | (0.36, 0.80) | (-4.77, -2.69) | |
| **30-34 years** | | | | | | | | | | | | | | | | |
| **Women** | | | | | | | | | | | | | | | | |
| Global | 41.77 (32.68, 53.04) | 34.38 (25.98, 45.46) | -0.67 (-0.72, -0.62) | <0.001 | 567.27 (478.19, 658.97) | 548.09 (460.70, 633.43) | -0.05 (-0.37, 0.28) | 0.774 | 483.22 (435.64, 532.20) | 307.93 (270.02, 349.06) | -1.53 (-1.86, -1.20) | <0.001 | 6.82 (6.23, 7.44) | 3.80 (3.36, 4.29) | -2.08 (-2.50, -1.66) | <0.001 |
| **SDI category** | | | | | | | | | | | | | | | | |
| High | 29.29 (22.13, 39.26) | 27.16 (19.88, 37.60) | -0.25 (-0.34, -0.15) | <0.001 | 482.14 (407.71, 563.31) | 525.78 (442.37, 612.19) | 0.44 (-0.13, 1.02) | 0.128 | 276.51 (249.38, 304.10) | 181.45 (153.44, 213.34) | -1.43 (-1.65, -1.20) | <0.001 | 3.51 (3.32, 3.67) | 1.70 (1.53, 1.91) | -2.45 (-2.72, -2.19) | <0.001 |
| High-middle | 42.59 (33.01, 55.12) | 31.06 (22.58, 43.15) | -1.08 (-1.18, -0.97) | <0.001 | 572.80 (477.03, 672.06) | 519.16 (433.37, 606.81) | -0.27 (-0.51, -0.03) | 0.027 | 421.70 (374.18, 471.89) | 243.00 (207.24, 281.20) | -1.95 (-2.51, -1.40) | <0.001 | 5.56 (5.01, 6.25) | 2.54 (2.23, 2.90) | -2.71 (-3.61, -1.80) | <0.001 |
| Middle | 45.72 (35.80, 58.12) | 35.05 (26.36, 46.63) | -0.90 (-0.97, -0.84) | <0.001 | 614.50 (514.22, 715.29) | 563.84 (469.60, 654.02) | -0.24 (-0.57, 0.09) | 0.146 | 550.90 (489.51, 615.41) | 302.24 (261.31, 347.64) | -2.00 (-2.40, -1.60) | <0.001 | 7.80 (6.98, 8.72) | 3.56 (3.09, 4.06) | -2.64 (-3.13, -2.14) | <0.001 |
| Low-middle | 42.55 (33.95, 52.99) | 37.39 (29.04, 47.96) | -0.44 (-0.52, -0.37) | <0.001 | 545.15 (461.71, 630.72) | 548.73 (461.83, 638.30) | 0.10 (-0.23, 0.42) | 0.560 | 589.27 (506.15, 682.30) | 379.67 (321.82, 441.57) | -1.49 (-1.64, -1.33) | <0.001 | 8.90 (7.46, 10.40) | 5.20 (4.35, 6.19) | -1.77 (-2.00, -1.54) | <0.001 |
| Low | 48.34 (39.61, 58.99) | 39.01 (31.24, 49.00) | -0.74 (-0.78, -0.70) | <0.001 | 597.30 (514.83, 685.95) | 571.38 (487.43, 656.73) | -0.11 (-0.32, 0.09) | 0.285 | 589.06 (499.79, 683.43) | 414.16 (349.92, 487.29) | -1.22 (-1.33, -1.10) | <0.001 | 8.90 (7.41, 10.49) | 5.85 (4.80, 7.05) | -1.45 (-1.57, -1.32) | <0.001 |
| **21 GBD region** | | | | | | | | | | | | | | | | |
| South Asia | 37.04 (28.72, 47.23) | 33.89 (25.87, 44.17) | -0.29 (-0.41, -0.16) | <0.001 | 484.22 (401.08, 569.70) | 496.92 (411.89, 587.33) | 0.15 (-0.20, 0.51) | 0.401 | 437.00 (365.74, 514.13) | 307.28 (252.94, 367.31) | -1.20 (-1.56, -0.83) | <0.001 | 6.37 (5.18, 7.70) | 4.03 (3.17, 5.05) | -1.55 (-1.97, -1.14) | <0.001 |
| East Asia | 36.37 (27.51, 47.51) | 24.73 (17.27, 35.42) | -1.35 (-1.47, -1.23) | <0.001 | 523.76 (423.35, 626.72) | 465.50 (374.10, 556.50) | -0.38 (-0.57, -0.19) | <0.001 | 491.37 (401.14, 598.52) | 250.39 (199.66, 302.88) | -2.44 (-3.71, -1.16) | <0.001 | 6.66 (5.29, 8.44) | 2.59 (1.99, 3.25) | -3.00 (-4.10, -1.88) | <0.001 |
| North Africa and Middle East | 60.67 (50.73, 73.65) | 52.58 (42.83, 65.48) | -0.49 (-0.50, -0.47) | <0.001 | 710.62 (618.42, 810.25) | 683.73 (595.32, 777.84) | -0.07 (-0.20, 0.06) | 0.272 | 643.38 (547.23, 747.59) | 409.40 (325.72, 500.58) | -1.53 (-1.69, -1.38) | <0.001 | 9.44 (7.83, 11.16) | 5.36 (4.12, 6.87) | -1.89 (-2.05, -1.73) | <0.001 |

| | | | | | | | | | | | | | | | | |
|---|---|---|---|---|---|---|---|---|---|---|---|---|---|---|---|---|
| Western Sub-Saharan Africa | 43.43 (35.01, 53.86) | 35.96 (27.94, 45.92) | -0.64 (-0.66, -0.61) | <0.001 | 596.12 (510.09, 690.27) | 579.20 (486.50, 673.61) | -0.06 (-0.23, 0.10) | 0.469 | 506.36 (401.96, 634.59) | 365.48 (289.29, 462.11) | -1.12 (-1.22, -1.01) | <0.001 | 7.48 (5.70, 9.65) | 5.03 (3.71, 6.69) | -1.35 (-1.47, -1.23) | <0.001 |
| Southeast Asia | 52.77 (41.53, 66.52) | 47.28 (36.44, 60.90) | -0.37 (-0.41, -0.32) | <0.001 | 667.87 (561.58, 785.73) | 640.79 (537.47, 749.15) | -0.10 (-0.30, 0.09) | 0.284 | 753.65 (619.92, 886.69) | 472.76 (397.27, 551.08) | -1.59 (-1.68, -1.49) | <0.001 | 10.84 (8.68, 12.96) | 5.96 (4.77, 7.23) | -2.06 (-2.32, -1.80) | <0.001 |
| Eastern Sub-Saharan Africa | 50.07 (41.20, 60.90) | 29.44 (22.45, 38.90) | -1.81 (-1.93, -1.69) | <0.001 | 692.42 (596.17, 794.45) | 621.84 (520.56, 723.90) | -0.33 (-0.54, -0.12) | 0.002 | 676.68 (539.38, 832.66) | 372.30 (297.44, 450.74) | -2.04 (-2.16, -1.92) | <0.001 | 10.28 (7.97, 12.94) | 5.06 (3.84, 6.34) | -2.41 (-2.54, -2.28) | <0.001 |
| Central Latin America | 53.34 (41.50, 68.82) | 37.77 (27.72, 51.42) | -1.18 (-1.24, -1.13) | <0.001 | 765.98 (647.02, 885.89) | 660.69 (562.11, 765.63) | -0.46 (-0.73, -0.19) | 0.001 | 431.85 (398.77, 469.04) | 233.25 (189.55, 286.22) | -1.97 (-2.49, -1.45) | <0.001 | 6.36 (5.95, 6.78) | 3.00 (2.32, 3.84) | -2.37 (-2.75, -1.99) | <0.001 |
| Tropical Latin America | 76.04 (61.17, 93.87) | 41.43 (31.72, 53.62) | -2.08 (-2.22, -1.94) | <0.001 | 851.60 (707.43, 1005.66) | 662.46 (554.97, 772.94) | -0.84 (-0.99, -0.70) | <0.001 | 844.43 (791.51, 904.12) | 295.47 (266.26, 328.43) | -3.52 (-3.73, -3.31) | <0.001 | 13.55 (12.71, 14.51) | 4.12 (3.71, 4.52) | -4.08 (-4.27, -3.90) | <0.001 |
| Central Sub-Saharan Africa | 45.22 (36.94, 55.15) | 31.79 (24.53, 41.67) | -1.22 (-1.24, -1.20) | <0.001 | 586.47 (487.69, 693.92) | 523.35 (431.45, 625.13) | -0.36 (-0.54, -0.17) | <0.001 | 527.22 (347.72, 757.37) | 345.29 (241.68, 472.31) | -1.44 (-1.56, -1.32) | <0.001 | 7.90 (4.82, 11.91) | 4.82 (3.03, 7.11) | -1.69 (-1.83, -1.55) | <0.001 |
| Western Europe | 26.12 (19.67, 34.77) | 20.73 (14.68, 29.64) | -0.79 (-0.85, -0.73) | <0.001 | 412.45 (348.85, 485.82) | 386.84 (320.41, 456.48) | -0.17 (-0.44, 0.10) | 0.206 | 249.43 (226.13, 272.48) | 119.94 (100.65, 142.39) | -2.50 (-2.68, -2.32) | <0.001 | 3.35 (3.16, 3.54) | 1.11 (1.01, 1.22) | -3.72 (-3.96, -3.48) | <0.001 |
| High-income North America | 26.96 (19.46, 37.54) | 25.57 (18.08, 36.62) | -0.17 (-0.22, -0.12) | <0.001 | 527.91 (432.09, 627.15) | 623.54 (516.97, 734.41) | 0.82 (-0.10, 1.75) | 0.081 | 256.28 (227.48, 287.26) | 202.59 (170.90, 236.69) | -0.76 (-1.13, -0.39) | <0.001 | 3.07 (2.89, 3.25) | 1.89 (1.73, 2.09) | -1.61 (-2.27, -0.94) | <0.001 |
| Central Asia | 52.87 (42.46, 67.21) | 41.43 (31.43, 55.79) | -0.83 (-0.87, -0.78) | <0.001 | 598.69 (510.40, 689.95) | 545.68 (451.95, 648.69) | -0.29 (-0.37, -0.21) | <0.001 | 473.57 (427.51, 524.51) | 370.25 (309.69, 441.84) | -0.84 (-1.13, -0.55) | <0.001 | 6.27 (5.77, 6.79) | 4.62 (3.78, 5.65) | -1.05 (-1.45, -0.64) | <0.001 |
| Eastern Europe | 44.82 (33.79, 60.17) | 42.72 (32.16, 58.16) | -0.14 (-0.20, -0.09) | <0.001 | 616.61 (508.02, 736.03) | 681.68 (572.05, 808.57) | 0.39 (0.17, 0.61) | <0.001 | 297.80 (252.37, 348.29) | 345.99 (275.45, 430.43) | 0.66 (-0.03, 1.35) | 0.061 | 3.15 (2.71, 3.46) | 3.78 (2.89, 5.05) | 0.88 (0.18, 1.58) | 0.013 |
| Southern Sub-Saharan Africa | 40.18 (30.50, 52.99) | 26.40 (19.09, 36.89) | -1.46 (-1.56, -1.36) | <0.001 | 741.55 (614.88, 885.09) | 635.09 (523.94, 757.30) | -0.53 (-0.60, -0.47) | <0.001 | 702.03 (562.82, 855.73) | 300.16 (210.54, 408.41) | -2.73 (-4.47, -0.96) | 0.003 | 10.60 (8.15, 13.05) | 3.78 (2.26, 5.66) | -3.32 (-5.51, -1.07) | 0.004 |

| | | | | | | | | | | | | | | | | |
|---|---|---|---|---|---|---|---|---|---|---|---|---|---|---|---|---|
| High-income Asia Pacific | 32.78 (25.06, 43.99) | 27.19 (19.56, 37.87) | -0.64 (-0.79, -0.48) | <0.001 | 424.04 (357.14, 502.13) | 402.84 (334.69, 481.98) | -0.15 (-0.39, 0.08) | 0.205 | 320.61 (279.65, 357.85) | 135.73 (111.37, 164.59) | -2.89 (-3.12, -2.66) | <0.001 | 4.33 (3.79, 4.81) | 1.09 (0.94, 1.25) | -4.64 (-4.98, -4.29) | <0.001 |
| Central Europe | 47.50 (37.02, 61.97) | 36.37 (26.49, 50.45) | -0.90 (-1.00, -0.80) | <0.001 | 640.51 (543.76, 741.77) | 604.45 (508.84, 704.02) | -0.14 (-0.43, 0.16) | 0.365 | 448.18 (404.13, 493.01) | 240.89 (196.51, 289.62) | -2.06 (-2.23, -1.89) | <0.001 | 5.75 (5.43, 6.07) | 2.18 (1.80, 2.64) | -3.26 (-3.52, -2.99) | <0.001 |
| Andean Latin America | 60.06 (49.51, 72.48) | 40.78 (31.15, 53.17) | -1.33 (-1.37, -1.29) | <0.001 | 707.82 (604.54, 811.37) | 599.32 (506.46, 701.33) | -0.55 (-0.69, -0.40) | <0.001 | 637.84 (516.71, 781.42) | 304.97 (230.41, 391.46) | -2.53 (-3.46, -1.59) | <0.001 | 10.08 (8.04, 12.51) | 4.37 (3.07, 5.96) | -2.86 (-3.97, -1.74) | <0.001 |
| Caribbean | 62.61 (51.57, 78.48) | 61.37 (50.30, 78.31) | -0.07 (-0.11, -0.02) | 0.004 | 676.51 (573.96, 785.17) | 656.19 (552.07, 771.39) | -0.06 (-0.26, 0.13) | 0.525 | 664.62 (525.70, 833.13) | 590.14 (412.42, 829.54) | -0.39 (-0.68, -0.10) | 0.009 | 10.59 (8.15, 13.49) | 9.29 (6.14, 13.41) | -0.44 (-0.77, -0.10) | 0.011 |
| Southern Latin America | 41.98 (33.22, 52.80) | 30.00 (22.51, 40.74) | -1.14 (-1.18, -1.09) | <0.001 | 482.81 (397.92, 590.02) | 410.51 (329.61, 502.53) | -0.54 (-0.78, -0.30) | <0.001 | 521.32 (468.24, 575.11) | 225.16 (184.98, 267.84) | -2.75 (-3.27, -2.22) | <0.001 | 7.90 (7.12, 8.81) | 2.82 (2.26, 3.39) | -3.37 (-4.05, -2.68) | <0.001 |
| Oceania | 107.72 (94.00, 123.66) | 113.17 (98.88, 131.13) | 0.17 (0.15, 0.20) | <0.001 | 902.71 (777.96, 1036.68) | 907.62 (770.66, 1050.52) | 0.02 (-0.06, 0.10) | 0.695 | 838.11 (602.61, 1175.04) | 853.84 (571.79, 1238.04) | 0.04 (-0.17, 0.25) | 0.725 | 11.59 (7.44, 17.68) | 11.84 (7.31, 18.32) | 0.04 (-0.22, 0.32) | 0.745 |
| Australasia | 26.99 (19.20, 38.38) | 24.50 (16.77, 35.81) | -0.34 (-0.40, -0.29) | <0.001 | 411.15 (317.96, 512.30) | 402.58 (307.78, 510.72) | -0.04 (-0.19, 0.10) | 0.561 | 191.94 (160.07, 227.12) | 118.06 (90.81, 148.08) | -1.59 (-2.23, -0.94) | <0.001 | 2.20 (1.84, 2.58) | 0.92 (0.68, 1.21) | -3.02 (-3.91, -2.12) | <0.001 |
| **Men** | | | | | | | | | | | | | | | | |
| Global | 39.55 (31.62, 49.21) | 36.02 (28.36, 45.94) | -0.33 (-0.36, -0.30) | <0.001 | 420.68 (353.48, 489.28) | 419.16 (351.30, 488.22) | 0.06 (-0.25, 0.37) | 0.699 | 565.51 (520.43, 615.53) | 450.84 (403.85, 501.89) | -0.77 (-1.12, -0.42) | <0.001 | 8.85 (8.10, 9.60) | 6.86 (6.13, 7.65) | -0.88 (-1.32, -0.45) | <0.001 |
| **SDI category** | | | | | | | | | | | | | | | | |
| High | 25.49 (19.98, 33.53) | 23.53 (17.88, 32.18) | -0.28 (-0.38, -0.19) | <0.001 | 330.60 (274.50, 389.00) | 372.48 (309.11, 437.86) | 0.61 (-0.21, 1.44) | 0.143 | 297.40 (275.48, 319.63) | 193.71 (169.83, 219.67) | -1.46 (-1.73, -1.20) | <0.001 | 4.38 (4.12, 4.65) | 2.45 (2.17, 2.82) | -1.92 (-2.08, -1.75) | <0.001 |
| High-middle | 42.31 (33.66, 52.84) | 34.19 (26.32, 44.38) | -0.75 (-0.79, -0.70) | <0.001 | 407.57 (337.92, 479.59) | 368.62 (304.12, 439.39) | -0.29 (-0.49, -0.08) | 0.006 | 566.59 (508.19, 636.01) | 428.53 (374.70, 488.68) | -0.94 (-1.47, -0.40) | 0.001 | 8.80 (7.83, 9.87) | 6.46 (5.54, 7.46) | -1.04 (-1.64, -0.43) | 0.001 |
| Middle | 44.71 | 40.11 | -0.39 (-0.42, | <0.001 | 462.90 | 445.99 | -0.08 | 0.519 | 657.62 | 502.21 | -0.93 | <0.001 | 10.33 | 7.65 | -1.04 | <0.001 |

| | | | | | | | | | | | | | | | | |
|---|---|---|---|---|---|---|---|---|---|---|---|---|---|---|---|---|
| | (35.57, 55.82) | (31.49, 50.72) | -0.35) | | (388.66, 542.06) | (373.23, 521.86) | (-0.33, 0.17) | | (594.86, 733.53) | (430.74, 565.88) | (-1.25, -0.60) | | (9.26, 11.62) | (6.49, 8.68) | (-1.43, -0.65) | |
| Low-middle | 38.47 (31.17, 47.86) | 37.64 (29.95, 47.21) | -0.08 (-0.12, -0.05) | <0.001 | 431.35 (364.38, 500.17) | 437.10 (368.90, 510.79) | 0.11 (-0.15, 0.37) | 0.409 | 656.68 (575.49, 745.45) | 533.75 (461.51, 611.49) | -0.64 (-0.86, -0.43) | <0.001 | 10.53 (9.16, 11.99) | 8.37 (7.19, 9.76) | -0.73 (-0.99, -0.46) | <0.001 |
| Low | 43.43 (35.48, 53.06) | 37.84 (30.47, 47.11) | -0.47 (-0.51, -0.44) | <0.001 | 461.27 (396.37, 532.04) | 449.73 (386.64, 517.90) | -0.05 (-0.27, 0.17) | 0.669 | 557.30 (476.07, 648.82) | 459.46 (381.86, 548.69) | -0.68 (-0.77, -0.58) | <0.001 | 8.82 (7.42, 10.41) | 7.11 (5.76, 8.72) | -0.74 (-0.84, -0.64) | <0.001 |
| **21 GBD region** | | | | | | | | | | | | | | | | |
| South Asia | 31.22 (24.27, 40.78) | 32.32 (25.28, 41.81) | 0.11 (0.07, 0.15) | <0.001 | 384.68 (318.33, 451.69) | 394.39 (327.00, 470.97) | 0.14 (-0.14, 0.41) | 0.327 | 498.95 (415.01, 582.32) | 426.52 (363.85, 493.35) | -0.51 (-0.72, -0.30) | <0.001 | 7.86 (6.45, 9.28) | 6.57 (5.44, 7.71) | -0.60 (-0.82, -0.38) | <0.001 |
| East Asia | 38.90 (29.62, 50.39) | 36.27 (27.33, 47.38) | -0.27 (-0.38, -0.16) | <0.001 | 402.78 (330.19, 479.69) | 366.71 (296.28, 442.14) | -0.30 (-0.46, -0.15) | <0.001 | 685.90 (577.03, 813.86) | 564.50 (436.55, 663.08) | -0.56 (-1.02, -0.10) | 0.018 | 10.80 (8.95, 13.07) | 8.75 (6.48, 10.45) | -0.61 (-1.10, -0.11) | 0.018 |
| North Africa and Middle East | 44.37 (36.56, 54.67) | 40.73 (32.73, 51.33) | -0.28 (-0.30, -0.26) | <0.001 | 518.76 (445.53, 593.66) | 525.24 (450.52, 601.25) | 0.12 (-0.05, 0.29) | 0.168 | 594.99 (482.82, 731.07) | 409.97 (328.83, 524.61) | -1.24 (-1.39, -1.10) | <0.001 | 9.27 (7.32, 11.79) | 5.98 (4.61, 7.92) | -1.47 (-1.66, -1.28) | <0.001 |
| Western Sub-Saharan Africa | 41.61 (33.22, 52.06) | 38.75 (30.46, 48.95) | -0.24 (-0.27, -0.21) | <0.001 | 502.35 (428.69, 580.01) | 503.45 (427.99, 576.03) | 0.04 (-0.15, 0.23) | 0.657 | 469.69 (371.22, 590.45) | 416.14 (327.37, 520.09) | -0.41 (-0.50, -0.31) | <0.001 | 7.24 (5.52, 9.43) | 6.28 (4.74, 8.13) | -0.47 (-0.57, -0.37) | <0.001 |
| Southeast Asia | 66.63 (55.03, 80.68) | 60.64 (49.32, 74.22) | -0.32 (-0.34, -0.30) | <0.001 | 598.73 (505.99, 701.29) | 576.50 (484.77, 677.92) | -0.09 (-0.28, 0.10) | 0.353 | 902.13 (772.92, 1043.74) | 736.02 (608.56, 865.96) | -0.69 (-1.01, -0.38) | <0.001 | 14.04 (11.73, 16.53) | 11.17 (9.05, 13.35) | -0.78 (-1.12, -0.44) | <0.001 |
| Eastern Sub-Saharan Africa | 49.58 (40.30, 61.19) | 36.66 (29.06, 46.28) | -1.03 (-1.08, -0.99) | <0.001 | 519.36 (446.04, 596.80) | 490.61 (418.25, 566.91) | -0.14 (-0.39, 0.11) | 0.265 | 702.97 (575.51, 838.05) | 512.50 (398.33, 650.84) | -1.10 (-1.21, -0.98) | <0.001 | 11.33 (9.07, 13.76) | 8.02 (6.09, 10.43) | -1.18 (-1.30, -1.07) | <0.001 |
| Central Latin America | 32.45 (25.24, 42.10) | 26.43 (19.69, 36.18) | -0.71 (-0.77, -0.65) | <0.001 | 435.14 (369.09, 507.11) | 414.80 (350.32, 480.71) | -0.15 (-0.40, 0.10) | 0.247 | 370.57 (340.81, 399.95) | 252.28 (212.92, 296.96) | -1.25 (-1.75, -0.75) | <0.001 | 5.89 (5.41, 6.36) | 3.82 (3.18, 4.61) | -1.40 (-1.92, -0.88) | <0.001 |
| Tropical Latin America | 64.15 (50.86, 80.14) | 32.81 (24.72, 43.41) | -2.31 (-2.42, -2.21) | <0.001 | 609.10 (491.11, 728.99) | 462.23 (380.92, 545.23) | -0.93 (-1.02, -0.84) | <0.001 | 851.04 (778.59, 935.94) | 290.26 (259.59, 323.84) | -3.54 (-3.77, -3.32) | <0.001 | 14.16 (12.97, 15.63) | 4.44 (3.94, 4.98) | -3.84 (-4.10, -3.58) | <0.001 |

| | | | | | | | | | | | | | | | | |
|---|---|---|---|---|---|---|---|---|---|---|---|---|---|---|---|---|
| Central Sub-Saharan Africa | 43.59 (35.97, 52.79) | 32.80 (26.10, 42.03) | -0.98 (-1.00, -0.95) | <0.001 | 434.76 (351.32, 518.35) | 385.03 (306.15, 472.73) | -0.37 (-0.62, -0.11) | 0.005 | 540.87 (367.53, 776.60) | 403.61 (271.86, 591.05) | -1.03 (-1.16, -0.89) | <0.001 | 8.63 (5.62, 12.78) | 6.28 (3.99, 9.65) | -1.12 (-1.27, -0.96) | <0.001 |
| Western Europe | 22.26 (17.45, 29.01) | 16.31 (11.76, 23.03) | -1.06 (-1.13, -1.00) | <0.001 | 271.57 (222.34, 320.93) | 240.45 (199.18, 283.51) | -0.37 (-0.65, -0.08) | 0.012 | 284.36 (260.59, 310.24) | 121.38 (107.70, 138.05) | -2.98 (-3.31, -2.66) | <0.001 | 4.33 (3.98, 4.70) | 1.51 (1.35, 1.67) | -3.65 (-4.18, -3.11) | <0.001 |
| High-income North America | 19.63 (14.16, 28.11) | 18.25 (12.96, 26.58) | -0.24 (-0.31, -0.16) | <0.001 | 368.34 (297.25, 445.63) | 436.46 (345.40, 531.65) | 0.92 (-0.44, 2.29) | 0.188 | 225.68 (205.08, 248.19) | 169.09 (145.58, 194.11) | -0.97 (-1.43, -0.51) | <0.001 | 3.07 (2.91, 3.27) | 1.93 (1.70, 2.24) | -1.53 (-2.17, -0.89) | <0.001 |
| Central Asia | 55.05 (46.58, 65.95) | 45.63 (37.88, 55.89) | -0.63 (-0.70, -0.56) | <0.001 | 437.88 (366.95, 508.10) | 397.87 (324.23, 481.04) | -0.33 (-0.38, -0.29) | <0.001 | 570.71 (516.87, 625.32) | 524.41 (439.44, 617.93) | -0.37 (-0.75, 0.01) | 0.055 | 8.74 (7.96, 9.58) | 8.02 (6.57, 9.71) | -0.32 (-0.84, 0.20) | 0.231 |
| Eastern Europe | 49.96 (39.57, 61.89) | 46.85 (37.45, 58.23) | -0.21 (-0.29, -0.14) | <0.001 | 379.42 (308.31, 473.74) | 355.90 (290.04, 432.28) | -0.22 (-0.26, -0.18) | <0.001 | 473.31 (408.67, 518.78) | 643.57 (534.94, 855.03) | 1.04 (0.47, 1.60) | <0.001 | 7.14 (6.09, 7.76) | 10.21 (8.35, 13.82) | 1.21 (0.58, 1.84) | <0.001 |
| Southern Sub-Saharan Africa | 45.57 (35.44, 58.08) | 30.74 (23.10, 40.68) | -1.36 (-1.43, -1.30) | <0.001 | 552.38 (458.04, 661.15) | 498.12 (406.78, 592.06) | -0.33 (-0.49, -0.17) | <0.001 | 747.52 (596.01, 927.00) | 425.86 (339.34, 532.47) | -2.05 (-3.44, -0.65) | 0.004 | 12.03 (9.37, 15.19) | 6.45 (4.90, 8.23) | -2.27 (-3.87, -0.64) | 0.006 |
| High-income Asia Pacific | 35.87 (28.86, 43.98) | 30.80 (23.16, 40.05) | -0.52 (-0.77, -0.27) | <0.001 | 287.56 (232.99, 342.55) | 295.79 (240.74, 357.63) | 0.13 (-0.04, 0.30) | 0.144 | 415.89 (365.43, 462.99) | 174.09 (150.91, 199.50) | -2.98 (-3.42, -2.55) | <0.001 | 6.49 (5.60, 7.28) | 2.21 (1.94, 2.52) | -3.69 (-4.26, -3.12) | <0.001 |
| Central Europe | 43.38 (35.53, 52.79) | 28.45 (22.01, 37.61) | -1.45 (-1.48, -1.43) | <0.001 | 356.12 (296.33, 423.32) | 294.23 (236.18, 355.92) | -0.61 (-0.83, -0.40) | <0.001 | 551.87 (514.63, 590.07) | 272.38 (236.30, 310.32) | -2.46 (-2.80, -2.12) | <0.001 | 8.61 (8.09, 9.16) | 3.84 (3.29, 4.41) | -2.86 (-3.30, -2.41) | <0.001 |
| Andean Latin America | 49.86 (40.91, 60.86) | 37.28 (29.19, 48.25) | -1.01 (-1.05, -0.96) | <0.001 | 580.94 (483.08, 674.11) | 499.77 (411.77, 588.29) | -0.51 (-0.59, -0.43) | <0.001 | 620.99 (506.64, 756.30) | 366.45 (273.58, 475.91) | -1.85 (-2.24, -1.47) | <0.001 | 10.12 (8.08, 12.52) | 5.73 (4.11, 7.68) | -2.00 (-2.43, -1.57) | <0.001 |
| Caribbean | 42.46 (34.15, 53.57) | 38.15 (29.98, 48.92) | -0.36 (-0.42, -0.29) | <0.001 | 466.87 (392.32, 543.10) | 444.29 (367.97, 522.69) | -0.12 (-0.43, 0.20) | 0.467 | 518.22 (440.86, 614.48) | 416.01 (301.14, 561.32) | -0.61 (-0.97, -0.25) | 0.001 | 8.46 (7.15, 10.12) | 6.68 (4.70, 9.28) | -0.64 (-1.03, -0.25) | 0.001 |
| Southern Latin America | 34.60 (28.73, 42.51) | 23.41 (17.86, 30.83) | -1.33 (-1.40, -1.26) | <0.001 | 311.86 (248.99, 379.05) | 263.04 (204.88, 330.23) | -0.56 (-0.64, -0.48) | <0.001 | 549.42 (481.67, 624.72) | 247.46 (193.31, 303.41) | -2.71 (-3.13, -2.28) | <0.001 | 8.87 (7.75, 10.19) | 3.64 (2.73, 4.62) | -3.02 (-3.52, -2.52) | <0.001 |

| | | | | | | | | | | | | | | | | |
|---|---|---|---|---|---|---|---|---|---|---|---|---|---|---|---|---|
| Oceania | 93.77 (81.29, 107.20) | 94.54 (80.94, 109.26) | 0.03 (0.01, 0.05) | 0.003 | 728.49 (620.47, 849.67) | 717.02 (592.66, 841.85) | -0.05 (-0.14, 0.04) | 0.317 | 1176.34 (778.61, 1769.00) | 1168.98 (755.31, 1752.26) | -0.06 (-0.11, 0.00) | 0.065 | 18.56 (11.43, 28.78) | 18.46 (11.19, 28.65) | -0.06 (-0.12, 0.01) | 0.082 |
| Australasia | 21.31 (15.52, 29.75) | 16.73 (11.27, 24.72) | -0.83 (-0.86, -0.79) | <0.001 | 229.99 (162.48, 302.85) | 207.15 (142.28, 276.62) | -0.29 (-0.76, 0.18) | 0.222 | 182.21 (149.45, 218.66) | 89.49 (66.04, 116.86) | -2.50 (-2.98, -2.01) | <0.001 | 2.56 (2.07, 3.12) | 0.98 (0.67, 1.37) | -3.37 (-4.04, -2.69) | <0.001 |
| **35-39 years** | | | | | | | | | | | | | | | | |
| **Women** | | | | | | | | | | | | | | | | |
| Global | 62.04 (48.35, 78.83) | 52.05 (40.02, 67.18) | -0.60 (-0.64, -0.56) | <0.001 | 784.25 (682.45, 902.21) | 747.32 (650.49, 859.55) | -0.11 (-0.39, 0.18) | 0.463 | 790.82 (720.43, 866.39) | 496.23 (441.64, 556.94) | -1.59 (-1.77, -1.42) | <0.001 | 12.81 (11.71, 14.07) | 7.28 (6.44, 8.12) | -1.95 (-2.12, -1.77) | <0.001 |
| **SDI category** | | | | | | | | | | | | | | | | |
| High | 43.65 (32.23, 58.15) | 40.51 (28.96, 54.27) | -0.24 (-0.31, -0.18) | <0.001 | 663.76 (573.69, 769.01) | 711.70 (611.49, 825.76) | 0.38 (-0.16, 0.92) | 0.173 | 414.04 (380.22, 449.33) | 275.24 (237.28, 316.83) | -1.36 (-1.61, -1.12) | <0.001 | 6.03 (5.76, 6.31) | 3.21 (2.84, 3.68) | -2.11 (-2.25, -1.98) | <0.001 |
| High-middle | 62.49 (48.29, 79.44) | 44.60 (33.24, 58.95) | -1.15 (-1.21, -1.08) | <0.001 | 802.57 (691.78, 932.45) | 709.95 (611.56, 829.77) | -0.38 (-0.55, -0.21) | <0.001 | 711.56 (637.16, 802.27) | 388.25 (339.52, 442.10) | -2.02 (-2.36, -1.68) | <0.001 | 11.00 (9.81, 12.44) | 5.06 (4.48, 5.72) | -2.59 (-3.08, -2.11) | <0.001 |
| Middle | 66.45 (50.99, 85.14) | 52.96 (40.37, 69.03) | -0.77 (-0.83, -0.70) | <0.001 | 844.51 (729.86, 979.94) | 774.79 (670.59, 894.21) | -0.28 (-0.37, -0.20) | <0.001 | 946.90 (841.43, 1074.38) | 502.51 (437.50, 570.31) | -2.14 (-2.26, -2.02) | <0.001 | 15.51 (13.62, 17.87) | 7.21 (6.25, 8.27) | -2.58 (-2.70, -2.46) | <0.001 |
| Low-middle | 65.19 (51.26, 81.92) | 58.91 (45.75, 75.48) | -0.34 (-0.41, -0.28) | <0.001 | 753.22 (656.65, 859.47) | 747.61 (650.39, 862.94) | 0.03 (-0.25, 0.31) | 0.816 | 909.92 (797.65, 1032.69) | 614.21 (523.51, 710.59) | -1.33 (-1.50, -1.15) | <0.001 | 15.42 (13.33, 17.73) | 9.74 (8.16, 11.47) | -1.58 (-1.74, -1.42) | <0.001 |
| Low | 77.06 (62.56, 94.35) | 62.01 (49.77, 77.74) | -0.75 (-0.80, -0.71) | <0.001 | 826.12 (730.75, 934.01) | 773.45 (681.33, 883.56) | -0.19 (-0.36, -0.03) | 0.020 | 983.40 (835.42, 1137.68) | 684.51 (580.38, 811.25) | -1.24 (-1.34, -1.14) | <0.001 | 16.80 (13.94, 19.72) | 11.14 (9.19, 13.51) | -1.42 (-1.50, -1.33) | <0.001 |
| **21 GBD region** | | | | | | | | | | | | | | | | |
| South Asia | 57.05 (43.16, 73.69) | 53.47 (40.13, 70.25) | -0.22 (-0.28, -0.16) | <0.001 | 665.98 (570.82, 778.53) | 673.46 (572.97, 789.92) | 0.10 (-0.22, 0.43) | 0.532 | 639.79 (539.93, 754.76) | 479.79 (392.27, 579.64) | -1.07 (-1.61, -0.54) | <0.001 | 10.46 (8.68, 12.68) | 7.33 (5.74, 9.18) | -1.31 (-1.92, -0.69) | <0.001 |
| East Asia | 51.56 (37.96, | 32.64 (23.74, | -1.58 (-1.65, -1.51) | <0.001 | 720.63 (605.92, | 608.49 (504.30, | -0.57 (-0.78, | <0.001 | 918.51 (754.20, | 399.52 (322.93, | -2.75 (-3.04, | <0.001 | 14.93 (11.85, | 5.23 (4.05, | -3.53 (-3.75, | <0.001 |

| | | | | | | | | | | | | | | | | |
|---|---|---|---|---|---|---|---|---|---|---|---|---|---|---|---|---|
| | 68.84) | 45.00) | | | 848.98) | 722.52) | -0.36) | | 1118.63) | 480.72) | -2.46) | | 18.75) | 6.69) | -3.30) | |
| North Africa and Middle East | 92.30 (76.55, 111.84) | 78.75 (63.38, 99.15) | -0.54 (-0.58, -0.51) | <0.001 | 1010.28 (901.62, 1138.71) | 960.18 (851.99, 1080.69) | -0.15 (-0.26, -0.04) | 0.010 | 1059.82 (902.48, 1231.45) | 656.86 (531.90, 791.90) | -1.63 (-1.80, -1.46) | <0.001 | 17.59 (14.64, 20.61) | 9.94 (7.70, 12.47) | -1.94 (-2.15, -1.72) | <0.001 |
| Western Sub-Saharan Africa | 71.48 (57.92, 88.46) | 59.19 (47.15, 74.76) | -0.66 (-0.69, -0.62) | <0.001 | 837.40 (734.99, 951.31) | 788.44 (686.79, 900.61) | -0.16 (-0.25, -0.08) | <0.001 | 885.97 (700.35, 1114.86) | 614.73 (485.26, 777.85) | -1.26 (-1.33, -1.19) | <0.001 | 14.95 (11.45, 19.49) | 9.82 (7.42, 12.89) | -1.44 (-1.58, -1.30) | <0.001 |
| Southeast Asia | 78.92 (61.12, 100.15) | 72.00 (55.16, 93.46) | -0.32 (-0.35, -0.29) | <0.001 | 954.39 (827.21, 1100.11) | 913.95 (787.74, 1057.10) | -0.11 (-0.23, 0.00) | 0.055 | 1261.01 (1077.13, 1459.16) | 841.77 (701.86, 985.09) | -1.36 (-1.45, -1.27) | <0.001 | 20.72 (17.46, 24.46) | 12.75 (10.40, 15.40) | -1.63 (-1.73, -1.53) | <0.001 |
| Eastern Sub-Saharan Africa | 83.46 (67.39, 102.15) | 48.87 (38.08, 63.59) | -1.85 (-1.92, -1.77) | <0.001 | 938.94 (826.66, 1063.40) | 797.20 (689.66, 916.33) | -0.54 (-0.73, -0.34) | <0.001 | 1139.07 (931.11, 1400.52) | 629.74 (506.81, 769.15) | -2.03 (-2.10, -1.95) | <0.001 | 19.58 (15.49, 24.41) | 10.09 (7.73, 12.78) | -2.25 (-2.37, -2.14) | <0.001 |
| Central Latin America | 79.59 (62.10, 102.99) | 53.41 (38.38, 72.61) | -1.38 (-1.49, -1.26) | <0.001 | 1047.40 (915.53, 1199.34) | 874.87 (755.31, 1012.51) | -0.56 (-0.73, -0.40) | <0.001 | 657.38 (616.54, 701.80) | 342.64 (281.36, 416.80) | -2.12 (-2.38, -1.85) | <0.001 | 10.86 (10.27, 11.53) | 5.06 (3.96, 6.37) | -2.43 (-2.81, -2.06) | <0.001 |
| Tropical Latin America | 123.75 (96.53, 157.49) | 64.02 (48.36, 82.85) | -2.25 (-2.39, -2.12) | <0.001 | 1245.28 (1059.83, 1452.00) | 911.22 (780.88, 1059.98) | -1.06 (-1.18, -0.94) | <0.001 | 1422.38 (1327.99, 1518.25) | 506.86 (460.48, 556.51) | -3.45 (-3.64, -3.25) | <0.001 | 25.38 (23.70, 27.13) | 8.22 (7.39, 9.00) | -3.77 (-4.00, -3.54) | <0.001 |
| Central Sub-Saharan Africa | 72.04 (59.30, 89.37) | 51.20 (40.01, 66.47) | -1.18 (-1.20, -1.17) | <0.001 | 821.66 (705.04, 944.46) | 705.92 (595.26, 824.27) | -0.49 (-0.59, -0.39) | <0.001 | 905.45 (620.37, 1290.55) | 603.12 (417.73, 845.74) | -1.41 (-1.53, -1.29) | <0.001 | 15.38 (9.76, 22.87) | 9.80 (6.25, 14.41) | -1.56 (-1.72, -1.41) | <0.001 |
| Western Europe | 37.35 (27.82, 48.81) | 28.33 (19.49, 39.27) | -0.95 (-1.00, -0.90) | <0.001 | 552.56 (473.11, 638.07) | 510.38 (433.47, 595.15) | -0.23 (-0.47, 0.01) | 0.056 | 365.51 (336.60, 397.58) | 169.24 (146.36, 194.18) | -2.61 (-2.73, -2.49) | <0.001 | 5.58 (5.26, 5.91) | 1.89 (1.73, 2.08) | -3.67 (-3.88, -3.45) | <0.001 |
| High-income North America | 40.19 (27.79, 54.72) | 36.36 (24.32, 50.42) | -0.35 (-0.38, -0.32) | <0.001 | 727.63 (612.64, 861.85) | 823.11 (695.54, 972.81) | 0.67 (-0.22, 1.56) | 0.141 | 363.41 (327.63, 403.54) | 281.37 (242.81, 321.28) | -0.88 (-1.28, -0.47) | <0.001 | 4.95 (4.69, 5.18) | 3.13 (2.90, 3.44) | -1.60 (-1.99, -1.22) | <0.001 |
| Central Asia | 77.32 (63.11, 96.73) | 60.15 (46.79, 77.18) | -0.85 (-0.90, -0.80) | <0.001 | 878.01 (762.32, 990.68) | 780.15 (665.11, 911.85) | -0.39 (-0.46, -0.32) | <0.001 | 769.51 (696.08, 841.53) | 608.24 (514.07, 719.80) | -0.88 (-1.15, -0.60) | <0.001 | 11.68 (10.72, 12.58) | 8.88 (7.33, 10.75) | -1.03 (-1.34, -0.71) | <0.001 |
| Eastern Europe | 70.57 (52.28, | 65.21 (48.63, | -0.27 (-0.31, -0.24) | <0.001 | 887.04 (746.21, | 931.21 (791.91, | 0.21 (0.06, 0.35) | 0.005 | 505.97 (434.50, | 581.98 (471.04, | 0.72 (0.07, | 0.030 | 6.59 (5.72, | 7.88 (6.16, | 0.94 (0.11, 1.77) | 0.026 |

| | | | | | | | | | | | | | | | | |
|---|---|---|---|---|---|---|---|---|---|---|---|---|---|---|---|---|
| | 93.65) | 84.61) | | | 1056.76) | 1109.18) | | | 570.77) | 720.29) | 1.37) | | 7.16) | 10.32) | | |
| Southern Sub-Saharan Africa | 61.54 (46.08, 81.11) | 40.26 (29.77, 54.01) | -1.47 (-1.56, -1.38) | <0.001 | 968.70 (817.23, 1136.73) | 799.02 (672.28, 941.69) | -0.66 (-0.70, -0.62) | <0.001 | 936.28 (769.46, 1098.52) | 390.83 (285.34, 512.26) | -2.85 (-4.08, -1.59) | <0.001 | 15.58 (12.33, 18.51) | 5.49 (3.64, 7.76) | -3.39 (-4.99, -1.76) | <0.001 |
| High-income Asia Pacific | 52.60 (38.82, 72.02) | 47.48 (32.50, 65.41) | -0.35 (-0.46, -0.25) | <0.001 | 634.24 (543.07, 734.07) | 616.69 (516.96, 723.68) | -0.09 (-0.21, 0.04) | 0.162 | 482.44 (431.66, 526.74) | 217.88 (181.99, 255.24) | -2.69 (-3.02, -2.36) | <0.001 | 7.28 (6.60, 7.90) | 2.20 (1.94, 2.49) | -4.07 (-4.58, -3.56) | <0.001 |
| Central Europe | 70.56 (54.93, 89.09) | 48.13 (34.40, 64.84) | -1.30 (-1.39, -1.21) | <0.001 | 901.05 (786.00, 1034.44) | 811.01 (698.43, 945.03) | -0.32 (-0.56, -0.09) | 0.007 | 716.60 (653.18, 780.66) | 352.97 (289.63, 417.07) | -2.45 (-2.72, -2.19) | <0.001 | 10.62 (10.08, 11.18) | 3.89 (3.20, 4.64) | -3.50 (-3.90, -3.09) | <0.001 |
| Andean Latin America | 82.16 (67.12, 100.75) | 55.36 (41.22, 73.21) | -1.35 (-1.38, -1.33) | <0.001 | 996.80 (870.49, 1145.60) | 807.18 (698.06, 932.80) | -0.69 (-0.83, -0.56) | <0.001 | 896.75 (745.63, 1068.72) | 401.55 (304.36, 528.00) | -2.70 (-3.65, -1.73) | <0.001 | 15.60 (12.78, 18.84) | 6.31 (4.47, 8.79) | -3.04 (-4.17, -1.90) | <0.001 |
| Caribbean | 98.26 (79.39, 121.15) | 96.23 (77.89, 119.29) | -0.07 (-0.14, 0.00) | 0.065 | 1005.57 (883.78, 1144.18) | 984.94 (860.55, 1124.73) | -0.06 (-0.16, 0.04) | 0.206 | 1042.48 (847.52, 1280.41) | 909.11 (636.35, 1279.05) | -0.44 (-0.78, -0.11) | 0.009 | 18.38 (14.71, 22.93) | 15.84 (10.58, 22.92) | -0.49 (-0.86, -0.10) | 0.012 |
| Southern Latin America | 65.28 (51.71, 82.36) | 45.17 (32.91, 60.97) | -1.26 (-1.30, -1.22) | <0.001 | 704.92 (598.12, 823.01) | 581.54 (485.60, 685.83) | -0.64 (-0.80, -0.48) | <0.001 | 881.84 (789.42, 972.58) | 363.94 (301.64, 427.04) | -2.98 (-3.25, -2.71) | <0.001 | 15.07 (13.34, 16.81) | 5.33 (4.34, 6.38) | -3.49 (-3.82, -3.16) | <0.001 |
| Oceania | 135.51 (114.64, 160.19) | 141.82 (121.16, 165.96) | 0.16 (0.12, 0.19) | <0.001 | 1296.54 (1142.34, 1452.55) | 1308.61 (1143.56, 1491.18) | 0.04 (-0.01, 0.08) | 0.106 | 1311.82 (961.72, 1778.70) | 1386.19 (982.02, 1964.28) | 0.20 (0.08, 0.31) | 0.001 | 20.43 (14.11, 29.54) | 21.82 (13.94, 33.14) | 0.23 (0.09, 0.36) | 0.001 |
| Australasia | 35.85 (24.29, 49.50) | 31.00 (20.72, 44.61) | -0.47 (-0.56, -0.38) | <0.001 | 560.74 (444.79, 678.37) | 539.30 (421.22, 660.81) | -0.10 (-0.26, 0.05) | 0.184 | 280.75 (241.16, 321.01) | 174.28 (135.69, 216.79) | -1.67 (-1.93, -1.40) | <0.001 | 3.77 (3.28, 4.26) | 1.77 (1.33, 2.28) | -2.59 (-2.92, -2.26) | <0.001 |
| **Men** | | | | | | | | | | | | | | | | |
| Global | 62.28 (48.33, 79.08) | 57.01 (44.13, 72.71) | -0.31 (-0.36, -0.26) | <0.001 | 616.55 (532.57, 709.34) | 606.85 (524.03, 699.37) | 0.00 (-0.26, 0.26) | 0.978 | 932.67 (857.80, 1018.23) | 723.75 (657.06, 799.00) | -0.84 (-1.17, -0.51) | <0.001 | 16.32 (14.88, 17.95) | 12.33 (11.14, 13.70) | -0.93 (-1.26, -0.60) | <0.001 |
| **SDI category** | | | | | | | | | | | | | | | | |
| High | 41.66 (32.31, 53.40) | 38.72 (29.13, 51.21) | -0.26 (-0.32, -0.19) | <0.001 | 495.00 (422.67, 574.93) | 531.89 (457.01, 622.19) | 0.39 (-0.24, 1.03) | 0.229 | 495.84 (466.61, 526.32) | 321.21 (283.01, 365.94) | -1.48 (-1.67, -1.28) | <0.001 | 8.27 (7.84, 8.71) | 4.78 (4.27, 5.51) | -1.88 (-2.04, -1.71) | <0.001 |

| | | | | | | | | | | | | | | | | |
|---|---|---|---|---|---|---|---|---|---|---|---|---|---|---|---|---|
| High-middle | 67.95 (52.58, 86.31) | 53.70 (40.92, 70.04) | -0.81 (-0.87, -0.75) | <0.001 | 626.54 (532.84, 735.02) | 557.52 (475.09, 654.66) | -0.36 (-0.46, -0.26) | <0.001 | 994.17 (889.14, 1118.42) | 717.89 (633.37, 809.50) | -1.05 (-1.43, -0.66) | <0.001 | 17.35 (15.44, 19.69) | 12.19 (10.58, 13.86) | -1.13 (-1.57, -0.68) | <0.001 |
| Middle | 68.92 (52.98, 89.19) | 63.72 (49.51, 80.89) | -0.28 (-0.32, -0.24) | <0.001 | 666.32 (570.71, 769.57) | 655.87 (564.32, 760.07) | -0.05 (-0.14, 0.04) | 0.287 | 1096.17 (979.52, 1248.90) | 824.55 (723.62, 925.79) | -0.93 (-1.21, -0.65) | <0.001 | 19.25 (17.02, 22.30) | 14.07 (12.25, 15.90) | -1.03 (-1.36, -0.71) | <0.001 |
| Low-middle | 60.59 (47.82, 75.77) | 60.18 (47.16, 75.90) | -0.04 (-0.09, 0.02) | 0.222 | 616.37 (536.52, 703.31) | 618.33 (533.96, 709.13) | 0.06 (-0.16, 0.28) | 0.584 | 1005.85 (885.25, 1134.26) | 838.34 (733.48, 962.23) | -0.56 (-0.87, -0.26) | <0.001 | 17.89 (15.51, 20.37) | 14.64 (12.67, 17.01) | -0.62 (-0.95, -0.29) | <0.001 |
| Low | 67.45 (53.88, 83.83) | 59.94 (47.27, 74.97) | -0.42 (-0.45, -0.39) | <0.001 | 654.99 (572.33, 742.68) | 625.97 (546.93, 705.77) | -0.12 (-0.30, 0.06) | 0.196 | 874.93 (744.84, 1013.86) | 712.53 (596.00, 856.49) | -0.69 (-0.88, -0.51) | <0.001 | 15.38 (13.01, 18.04) | 12.29 (10.00, 15.03) | -0.77 (-1.06, -0.48) | <0.001 |
| **21 GBD region** | | | | | | | | | | | | | | | | |
| South Asia | 49.27 (37.12, 63.74) | 52.72 (39.95, 68.23) | 0.22 (0.15, 0.29) | <0.001 | 544.63 (466.93, 632.01) | 553.23 (471.26, 647.29) | 0.11 (-0.15, 0.38) | 0.407 | 757.92 (632.21, 888.13) | 684.34 (582.50, 801.04) | -0.24 (-0.82, 0.35) | 0.424 | 13.30 (10.90, 15.72) | 11.83 (9.89, 14.03) | -0.30 (-1.04, 0.44) | 0.421 |
| East Asia | 58.49 (42.98, 80.31) | 55.26 (41.36, 73.23) | -0.22 (-0.28, -0.15) | <0.001 | 576.95 (481.22, 679.70) | 543.42 (451.45, 650.07) | -0.20 (-0.32, -0.07) | 0.002 | 1205.13 (1002.24, 1438.72) | 971.38 (756.33, 1152.16) | -0.61 (-1.17, -0.04) | 0.035 | 21.35 (17.44, 26.10) | 16.91 (12.82, 20.39) | -0.67 (-1.16, -0.17) | 0.008 |
| North Africa and Middle East | 67.93 (55.42, 83.98) | 61.56 (48.72, 78.79) | -0.34 (-0.37, -0.31) | <0.001 | 741.48 (655.03, 840.60) | 731.25 (645.08, 823.85) | 0.01 (-0.10, 0.12) | 0.843 | 871.11 (730.34, 1093.96) | 577.07 (473.93, 726.56) | -1.39 (-1.54, -1.25) | <0.001 | 14.99 (12.37, 19.43) | 9.30 (7.37, 11.98) | -1.61 (-1.73, -1.50) | <0.001 |
| Western Sub-Saharan Africa | 60.66 (47.62, 76.99) | 57.99 (45.84, 73.54) | -0.15 (-0.18, -0.12) | <0.001 | 695.79 (609.22, 791.88) | 690.95 (602.84, 790.24) | 0.00 (-0.12, 0.12) | 0.994 | 747.50 (594.61, 944.79) | 641.28 (507.38, 802.95) | -0.53 (-0.63, -0.43) | <0.001 | 12.86 (10.03, 16.74) | 10.81 (8.27, 13.91) | -0.60 (-0.71, -0.49) | <0.001 |
| Southeast Asia | 110.53 (89.11, 138.81) | 99.78 (79.56, 125.16) | -0.36 (-0.37, -0.34) | <0.001 | 924.58 (799.58, 1059.34) | 881.35 (760.91, 1017.95) | -0.12 (-0.31, 0.07) | 0.205 | 1462.47 (1257.30, 1681.20) | 1255.37 (1035.31, 1474.04) | -0.48 (-0.66, -0.31) | <0.001 | 25.25 (21.27, 29.37) | 21.36 (17.32, 25.61) | -0.52 (-0.75, -0.30) | <0.001 |
| Eastern Sub-Saharan Africa | 77.80 (62.37, 96.65) | 56.90 (44.42, 71.74) | -1.07 (-1.12, -1.03) | <0.001 | 732.01 (636.86, 832.41) | 663.12 (576.05, 753.54) | -0.31 (-0.47, -0.15) | <0.001 | 1069.59 (870.10, 1285.21) | 726.70 (566.94, 914.40) | -1.33 (-1.43, -1.23) | <0.001 | 19.03 (15.28, 23.17) | 12.54 (9.47, 16.28) | -1.43 (-1.52, -1.35) | <0.001 |
| Central Latin America | 49.47 (38.48, | 39.17 (29.08, | -0.81 (-0.92, -0.70) | <0.001 | 608.17 (527.98, | 564.46 (485.10, | -0.21 (-0.40, | 0.030 | 517.31 (480.63, | 366.05 (304.01, | -1.11 (-1.44, | <0.001 | 9.07 (8.46, | 6.20 (5.05, | -1.22 (-1.56, | <0.001 |

| | | | | | | | | | | | | | | | | |
|---|---|---|---|---|---|---|---|---|---|---|---|---|---|---|---|---|
| | 63.86) | 52.11) | | | 698.24) | 654.88) | -0.02) | | 556.91) | 430.46) | -0.79) | | 9.76) | 7.39) | -0.87) | |
| Tropical Latin America | 112.86 (85.49, 143.50) | 51.91 (37.92, 69.09) | -2.65 (-2.72, -2.59) | <0.001 | 948.21 (803.80, 1119.95) | 656.55 (554.49, 769.34) | -1.17 (-1.30, -1.04) | <0.001 | 1536.69 (1405.55, 1674.22) | 482.11 (434.73, 532.91) | -3.89 (-4.15, -3.63) | <0.001 | 28.33 (25.85, 30.98) | 8.33 (7.46, 9.30) | -4.11 (-4.39, -3.82) | <0.001 |
| Central Sub-Saharan Africa | 71.43 (57.74, 88.34) | 54.29 (43.24, 68.21) | -0.95 (-0.96, -0.93) | <0.001 | 639.10 (539.99, 748.42) | 554.91 (461.82, 654.31) | -0.45 (-0.55, -0.34) | <0.001 | 828.93 (589.12, 1153.22) | 619.22 (420.23, 904.32) | -1.03 (-1.16, -0.90) | <0.001 | 14.58 (9.93, 20.73) | 10.69 (6.78, 16.30) | -1.10 (-1.25, -0.95) | <0.001 |
| Western Europe | 32.82 (25.22, 42.12) | 23.22 (16.43, 32.11) | -1.18 (-1.26, -1.11) | <0.001 | 415.40 (355.64, 480.44) | 351.00 (295.57, 411.49) | -0.51 (-0.73, -0.30) | <0.001 | 414.31 (386.35, 444.77) | 175.14 (156.73, 195.93) | -2.96 (-3.15, -2.76) | <0.001 | 7.02 (6.55, 7.51) | 2.50 (2.27, 2.76) | -3.52 (-3.88, -3.15) | <0.001 |
| High-income North America | 30.63 (22.26, 41.40) | 26.78 (18.94, 36.89) | -0.47 (-0.50, -0.44) | <0.001 | 517.22 (423.83, 629.11) | 581.96 (478.13, 701.80) | 0.70 (-0.53, 1.95) | 0.266 | 367.08 (341.10, 395.73) | 260.42 (228.21, 295.29) | -1.18 (-1.65, -0.70) | <0.001 | 5.79 (5.53, 6.14) | 3.57 (3.23, 4.04) | -1.64 (-2.15, -1.14) | <0.001 |
| Central Asia | 91.64 (78.01, 108.04) | 76.06 (63.70, 90.45) | -0.63 (-0.69, -0.56) | <0.001 | 716.90 (625.10, 816.87) | 648.14 (551.54, 756.41) | -0.34 (-0.39, -0.29) | <0.001 | 1009.35 (929.98, 1090.81) | 985.81 (831.73, 1150.83) | -0.16 (-0.45, 0.14) | 0.296 | 17.25 (15.89, 18.64) | 16.97 (14.11, 20.33) | -0.13 (-0.44, 0.17) | 0.392 |
| Eastern Europe | 88.31 (68.11, 112.97) | 84.88 (65.50, 108.23) | -0.11 (-0.20, -0.03) | 0.007 | 673.34 (560.51, 798.90) | 638.96 (536.36, 757.56) | -0.17 (-0.20, -0.14) | <0.001 | 872.36 (766.02, 941.35) | 1175.26 (978.41, 1494.20) | 1.28 (0.11, 2.46) | 0.031 | 14.79 (12.78, 15.91) | 20.73 (16.97, 26.84) | 1.45 (0.48, 2.43) | 0.003 |
| Southern Sub-Saharan Africa | 71.51 (53.95, 92.91) | 44.66 (33.57, 59.22) | -1.62 (-1.71, -1.53) | <0.001 | 774.49 (654.40, 910.05) | 668.61 (564.74, 784.61) | -0.50 (-0.56, -0.44) | <0.001 | 1151.61 (922.68, 1377.98) | 627.71 (477.51, 802.94) | -2.09 (-2.90, -1.28) | <0.001 | 20.47 (16.14, 24.80) | 10.60 (7.71, 13.82) | -2.25 (-3.12, -1.38) | <0.001 |
| High-income Asia Pacific | 65.70 (51.26, 83.56) | 62.38 (45.51, 82.42) | -0.19 (-0.28, -0.11) | <0.001 | 500.86 (425.61, 583.79) | 511.74 (430.77, 606.46) | 0.10 (-0.09, 0.29) | 0.296 | 732.44 (666.79, 798.23) | 334.65 (295.32, 377.39) | -2.65 (-2.96, -2.34) | <0.001 | 12.67 (11.50, 13.85) | 4.92 (4.37, 5.58) | -3.22 (-3.53, -2.90) | <0.001 |
| Central Europe | 74.81 (62.44, 90.38) | 44.09 (34.32, 55.99) | -1.81 (-1.86, -1.77) | <0.001 | 609.09 (524.36, 709.53) | 462.61 (387.30, 550.84) | -0.92 (-1.13, -0.70) | <0.001 | 1006.18 (946.67, 1065.84) | 465.48 (400.44, 530.15) | -2.72 (-2.99, -2.46) | <0.001 | 17.57 (16.62, 18.60) | 7.48 (6.35, 8.63) | -3.02 (-3.33, -2.71) | <0.001 |
| Andean Latin America | 65.59 (53.00, 81.42) | 48.15 (36.94, 62.33) | -1.06 (-1.09, -1.04) | <0.001 | 802.59 (694.49, 915.65) | 684.65 (580.43, 793.40) | -0.55 (-0.60, -0.50) | <0.001 | 819.07 (671.18, 981.90) | 445.76 (337.03, 593.02) | -2.02 (-2.50, -1.54) | <0.001 | 14.62 (11.78, 17.75) | 7.58 (5.43, 10.47) | -2.18 (-2.72, -1.65) | <0.001 |
| Caribbean | 76.25 (61.82, | 69.32 (54.73, | -0.33 (-0.36, -0.30) | <0.001 | 718.72 (623.92, | 682.41 (587.83, | -0.15 (-0.31, | 0.074 | 819.65 (695.90, | 674.26 (490.76, | -0.62 (-0.90, | <0.001 | 14.78 (12.39, | 12.02 (8.56, | -0.66 (-0.95, | <0.001 |

| | | | | | | | | | | | | | | | | |
|---|---|---|---|---|---|---|---|---|---|---|---|---|---|---|---|---|
| | 95.28) | 86.64) | | | 819.14) | 786.51) | 0.01) | | 970.80) | 924.49) | -0.34) | | 17.75) | 16.78) | -0.36) | |
| Southern Latin America | 55.82 (45.51, 68.86) | 35.52 (26.43, 46.38) | -1.55 (-1.62, -1.48) | <0.001 | 510.11 (419.69, 611.16) | 402.28 (320.68, 490.80) | -0.82 (-0.86, -0.77) | <0.001 | 956.01 (840.56, 1084.09) | 403.13 (322.59, 491.87) | -2.90 (-3.40, -2.41) | <0.001 | 17.22 (14.98, 19.58) | 6.72 (5.18, 8.39) | -3.16 (-3.72, -2.60) | <0.001 |
| Oceania | 156.89 (133.41, 185.09) | 153.99 (129.59, 182.46) | -0.06 (-0.08, -0.05) | <0.001 | 1119.74 (979.98, 1266.39) | 1102.48 (960.06, 1271.19) | -0.04 (-0.09, 0.00) | 0.054 | 2210.64 (1456.75, 3144.97) | 2134.10 (1402.55, 3072.78) | -0.15 (-0.22, -0.08) | <0.001 | 39.13 (24.68, 57.27) | 37.72 (23.80, 56.13) | -0.16 (-0.23, -0.09) | <0.001 |
| Australasia | 29.51 (20.90, 40.99) | 22.77 (15.04, 33.46) | -0.89 (-0.93, -0.85) | <0.001 | 370.56 (284.81, 460.72) | 307.31 (226.33, 399.83) | -0.65 (-0.88, -0.42) | <0.001 | 249.35 (211.87, 288.83) | 138.73 (104.92, 182.63) | -2.05 (-2.52, -1.57) | <0.001 | 3.87 (3.26, 4.54) | 1.82 (1.26, 2.51) | -2.62 (-3.27, -1.97) | <0.001 |
| **Hypertensive heart disease** | | | | | | | | | | | | | | | | |
| **15-19 years** | | | | | | | | | | | | | | | | |
| **Women** | | | | | | | | | | | | | | | | |
| Global | — | — | — | — | 0.18 (0.07, 0.33) | 0.19 (0.07, 0.37) | 0.28 (0.24, 0.32) | <0.001 | 12.78 (7.29, 18.21) | 9.22 (6.09, 11.96) | -1.12 (-1.28, -0.96) | <0.001 | 0.18 (0.10, 0.25) | 0.13 (0.09, 0.17) | -1.12 (-1.28, -0.96) | <0.001 |
| **SDI category** | | | | | | | | | | | | | | | | |
| High | — | — | — | — | 0.30 (0.13, 0.55) | 0.29 (0.13, 0.55) | -0.01 (-0.07, 0.05) | 0.789 | 3.85 (2.02, 4.90) | 1.62 (1.16, 1.90) | -2.89 (-3.23, -2.56) | <0.001 | 0.05 (0.03, 0.07) | 0.02 (0.02, 0.03) | -2.92 (-3.25, -2.59) | <0.001 |
| High-middle | — | — | — | — | 0.15 (0.06, 0.29) | 0.15 (0.06, 0.28) | -0.06 (-0.12, -0.01) | 0.019 | 6.07 (4.62, 7.62) | 2.77 (2.31, 3.25) | -2.66 (-2.91, -2.41) | <0.001 | 0.08 (0.06, 0.11) | 0.04 (0.03, 0.05) | -2.66 (-2.91, -2.42) | <0.001 |
| Middle | — | — | — | — | 0.17 (0.07, 0.33) | 0.17 (0.07, 0.34) | 0.11 (0.07, 0.15) | <0.001 | 15.00 (9.62, 19.80) | 7.53 (5.67, 9.36) | -2.36 (-2.51, -2.21) | <0.001 | 0.21 (0.13, 0.28) | 0.11 (0.08, 0.13) | -2.36 (-2.51, -2.21) | <0.001 |
| Low-middle | — | — | — | — | 0.11 (0.04, 0.22) | 0.14 (0.05, 0.28) | 0.69 (0.66, 0.71) | <0.001 | 14.62 (5.93, 24.06) | 9.58 (5.81, 13.01) | -1.49 (-1.75, -1.23) | <0.001 | 0.20 (0.08, 0.34) | 0.13 (0.08, 0.18) | -1.49 (-1.75, -1.24) | <0.001 |
| Low | — | — | — | — | 0.25 (0.09, 0.52) | 0.27 (0.09, 0.55) | 0.23 (0.21, 0.26) | <0.001 | 23.91 (8.62, 41.73) | 18.77 (9.05, 27.29) | -0.82 (-0.97, -0.67) | <0.001 | 0.33 (0.12, 0.58) | 0.26 (0.13, 0.38) | -0.82 (-0.98, -0.67) | <0.001 |

**21 GBD region**

| | | | | | | | | | | | | | | | | |
|---|---|---|---|---|---|---|---|---|---|---|---|---|---|---|---|---|
| South Asia | — | — | — | — | 0.06 (0.02, 0.12) | 0.06 (0.02, 0.13) | 0.12 (0.08, 0.15) | <0.001 | 10.17 (1.94, 19.82) | 5.34 (2.37, 8.23) | -2.32 (-3.01, -1.62) | <0.001 | 0.14 (0.03, 0.28) | 0.07 (0.03, 0.12) | -2.32 (-3.01, -1.62) | <0.001 |
| East Asia | — | — | — | — | 0.21 (0.09, 0.40) | 0.23 (0.10, 0.45) | 0.38 (0.36, 0.40) | <0.001 | 13.87 (9.03, 17.71) | 3.91 (2.63, 5.18) | -4.32 (-4.61, -4.03) | <0.001 | 0.19 (0.13, 0.25) | 0.05 (0.04, 0.07) | -4.33 (-4.62, -4.04) | <0.001 |
| North Africa and Middle East | — | — | — | — | 0.12 (0.05, 0.24) | 0.13 (0.05, 0.25) | 0.15 (0.09, 0.21) | <0.001 | 31.11 (14.34, 48.53) | 18.29 (11.82, 26.71) | -1.74 (-2.10, -1.36) | <0.001 | 0.43 (0.20, 0.68) | 0.26 (0.17, 0.37) | -1.73 (-2.10, -1.36) | <0.001 |
| Western Sub-Saharan Africa | — | — | — | — | 0.50 (0.18, 1.03) | 0.51 (0.18, 1.04) | 0.04 (-0.04, 0.11) | 0.337 | 33.94 (15.87, 50.07) | 31.99 (15.02, 47.31) | -0.20 (-0.32, -0.07) | 0.002 | 0.47 (0.22, 0.70) | 0.45 (0.21, 0.66) | -0.20 (-0.32, -0.07) | 0.002 |
| Southeast Asia | — | — | — | — | 0.11 (0.04, 0.21) | 0.11 (0.05, 0.22) | 0.25 (0.21, 0.28) | <0.001 | 9.09 (4.21, 14.42) | 6.40 (4.05, 8.19) | -1.19 (-1.37, -1.01) | <0.001 | 0.13 (0.06, 0.20) | 0.09 (0.06, 0.11) | -1.17 (-1.35, -0.99) | <0.001 |
| Eastern Sub-Saharan Africa | — | — | — | — | 0.43 (0.16, 0.88) | 0.43 (0.15, 0.91) | 0.06 (0.03, 0.10) | 0.001 | 27.81 (10.37, 53.23) | 15.20 (7.60, 23.78) | -2.05 (-2.18, -1.92) | <0.001 | 0.39 (0.14, 0.74) | 0.21 (0.11, 0.33) | -2.05 (-2.19, -1.92) | <0.001 |
| Central Latin America | — | — | — | — | 0.11 (0.04, 0.22) | 0.10 (0.04, 0.21) | -0.13 (-0.16, -0.10) | <0.001 | 7.15 (4.44, 8.35) | 4.44 (3.40, 5.79) | -1.57 (-2.00, -1.14) | <0.001 | 0.10 (0.06, 0.12) | 0.06 (0.05, 0.08) | -1.57 (-2.00, -1.14) | <0.001 |
| Tropical Latin America | — | — | — | — | 0.11 (0.04, 0.23) | 0.11 (0.04, 0.22) | -0.14 (-0.16, -0.11) | <0.001 | 7.39 (5.15, 8.62) | 4.34 (3.58, 6.69) | -1.82 (-2.43, -1.21) | <0.001 | 0.10 (0.07, 0.12) | 0.06 (0.05, 0.09) | -1.82 (-2.43, -1.21) | <0.001 |
| Central Sub-Saharan Africa | — | — | — | — | 0.19 (0.06, 0.41) | 0.18 (0.06, 0.41) | 0.01 (-0.08, 0.10) | 0.843 | 22.45 (6.68, 42.54) | 16.29 (7.59, 27.79) | -1.11 (-1.23, -1.00) | <0.001 | 0.31 (0.09, 0.60) | 0.23 (0.11, 0.39) | -1.12 (-1.23, -1.00) | <0.001 |
| Western Europe | — | — | — | — | 0.18 (0.08, 0.35) | 0.15 (0.07, 0.28) | -0.71 (-0.83, -0.59) | <0.001 | 1.33 (1.05, 1.60) | 1.05 (0.82, 1.28) | -0.81 (-1.04, -0.58) | <0.001 | 0.02 (0.01, 0.02) | 0.01 (0.01, 0.02) | -0.81 (-1.04, -0.58) | <0.001 |
| High-income North America | — | — | — | — | 0.47 (0.21, 0.85) | 0.45 (0.20, 0.84) | -0.10 (-0.16, | 0.005 | 1.38 (1.17, 1.69) | 1.55 (0.94, 1.81) | 0.50 (-0.29, | 0.218 | 0.02 (0.02, | 0.02 (0.01, | 0.52 (-0.27, 1.31) | 0.197 |

| | | | | | | | | | | | | | | | | |
|---|---|---|---|---|---|---|---|---|---|---|---|---|---|---|---|---|
| | | | | | | | -0.03) | | | | 1.29) | | 0.02) | 0.02) | | |
| Central Asia | — | — | — | — | 0.06 (0.02, 0.14) | 0.06 (0.02, 0.14) | -0.13 (-0.20, -0.07) | <0.001 | 5.87 (4.06, 7.32) | 5.22 (3.77, 6.91) | -0.36 (-0.61, -0.11) | 0.005 | 0.08 (0.06, 0.10) | 0.07 (0.05, 0.10) | -0.36 (-0.61, -0.12) | 0.004 |
| Eastern Europe | — | — | — | — | 0.04 (0.01, 0.09) | 0.04 (0.01, 0.09) | 0.01 (-0.08, 0.10) | 0.821 | 1.06 (0.74, 1.17) | 1.53 (0.85, 1.84) | 1.14 (0.02, 2.28) | 0.046 | 0.01 (0.01, 0.02) | 0.02 (0.01, 0.03) | 1.15 (0.02, 2.29) | 0.047 |
| Southern Sub-Saharan Africa | — | — | — | — | 0.27 (0.10, 0.57) | 0.23 (0.08, 0.49) | -0.52 (-0.59, -0.46) | <0.001 | 11.77 (8.24, 15.40) | 7.67 (4.93, 11.76) | -1.26 (-2.02, -0.49) | 0.001 | 0.16 (0.12, 0.22) | 0.11 (0.07, 0.16) | -1.27 (-2.03, -0.50) | 0.001 |
| High-income Asia Pacific | — | — | — | — | 0.30 (0.13, 0.56) | 0.30 (0.13, 0.55) | -0.01 (-0.05, 0.03) | 0.517 | 9.65 (2.72, 13.63) | 1.27 (0.88, 1.80) | -6.84 (-7.61, -6.07) | <0.001 | 0.13 (0.04, 0.19) | 0.02 (0.01, 0.02) | -6.90 (-7.67, -6.12) | <0.001 |
| Central Europe | — | — | — | — | 0.06 (0.02, 0.12) | 0.05 (0.02, 0.09) | -0.83 (-0.87, -0.80) | <0.001 | 2.32 (1.86, 2.76) | 2.06 (1.23, 2.61) | -0.43 (-1.05, 0.18) | 0.169 | 0.03 (0.03, 0.04) | 0.03 (0.02, 0.04) | -0.43 (-1.05, 0.19) | 0.172 |
| Andean Latin America | — | — | — | — | 0.08 (0.03, 0.17) | 0.09 (0.03, 0.19) | 0.28 (0.24, 0.32) | <0.001 | 19.68 (13.56, 27.08) | 10.80 (6.91, 16.24) | -2.11 (-2.84, -1.37) | <0.001 | 0.28 (0.19, 0.38) | 0.15 (0.10, 0.23) | -2.11 (-2.85, -1.37) | <0.001 |
| Caribbean | — | — | — | — | 0.08 (0.03, 0.16) | 0.08 (0.03, 0.16) | -0.17 (-0.18, -0.15) | <0.001 | 12.36 (7.42, 19.73) | 11.91 (6.49, 22.00) | 0.14 (-0.16, 0.44) | 0.375 | 0.17 (0.10, 0.28) | 0.17 (0.09, 0.31) | 0.14 (-0.17, 0.44) | 0.376 |
| Southern Latin America | — | — | — | — | 0.15 (0.06, 0.27) | 0.13 (0.05, 0.25) | -0.45 (-0.51, -0.39) | <0.001 | 4.09 (1.63, 5.83) | 2.96 (1.15, 4.25) | -1.14 (-1.54, -0.75) | <0.001 | 0.06 (0.02, 0.08) | 0.04 (0.02, 0.06) | -1.14 (-1.54, -0.75) | <0.001 |
| Oceania | — | — | — | — | 0.09 (0.03, 0.18) | 0.09 (0.03, 0.17) | -0.11 (-0.12, -0.10) | <0.001 | 11.69 (5.11, 22.89) | 12.51 (4.98, 25.52) | 0.16 (-0.10, 0.43) | 0.229 | 0.16 (0.07, 0.32) | 0.17 (0.07, 0.36) | 0.16 (-0.10, 0.43) | 0.228 |
| Australasia | — | — | — | — | 0.11 (0.05, 0.21) | 0.09 (0.04, 0.17) | -0.71 (-0.81, -0.61) | <0.001 | 0.73 (0.64, 0.83) | 0.60 (0.48, 0.72) | -0.64 (-1.52, 0.25) | 0.157 | 0.01 (0.01, 0.01) | 0.01 (0.01, 0.01) | -0.64 (-1.53, 0.26) | 0.163 |
| **Men** | | | | | | | | | | | | | | | | |
| Global | — | — | — | — | 0.20 (0.08, | 0.22 (0.09, | 0.41 (0.35, | <0.001 | 11.30 (6.57, | 6.30 (4.53, | -2.02 | <0.001 | 0.16 | 0.09 | -2.02 | <0.001 |

| | | | | | | | | | | | | | | | | |
|---|---|---|---|---|---|---|---|---|---|---|---|---|---|---|---|---|
| | | | | | 0.38) | 0.43) | 0.48) | | 14.81) | 8.17) | (-2.17, -1.87) | | (0.09, 0.21) | (0.06, 0.11) | (-2.18, -1.87) | |
| **SDI category** | | | | | | | | | | | | | | | | |
| High | — | — | — | — | 0.31 (0.14, 0.58) | 0.33 (0.15, 0.61) | 0.18 (0.13, 0.23) | <0.001 | 7.46 (2.36, 9.96) | 2.50 (1.60, 2.97) | -3.65 (-4.12, -3.18) | <0.001 | 0.10 (0.03, 0.14) | 0.03 (0.02, 0.04) | -3.56 (-4.02, -3.09) | <0.001 |
| High-middle | — | — | — | — | 0.16 (0.07, 0.31) | 0.16 (0.07, 0.31) | -0.05 (-0.08, -0.01) | 0.006 | 9.17 (6.69, 11.95) | 3.67 (2.98, 4.52) | -3.18 (-3.38, -2.98) | <0.001 | 0.13 (0.09, 0.17) | 0.05 (0.04, 0.06) | -3.18 (-3.38, -2.98) | <0.001 |
| Middle | — | — | — | — | 0.19 (0.08, 0.38) | 0.20 (0.08, 0.39) | 0.13 (0.08, 0.17) | <0.001 | 16.71 (8.98, 23.02) | 9.35 (6.19, 13.42) | -1.99 (-2.17, -1.81) | <0.001 | 0.23 (0.13, 0.32) | 0.13 (0.09, 0.19) | -1.99 (-2.17, -1.81) | <0.001 |
| Low-middle | — | — | — | — | 0.13 (0.05, 0.26) | 0.17 (0.07, 0.33) | 0.83 (0.80, 0.86) | <0.001 | 8.00 (4.86, 10.82) | 5.35 (3.89, 6.98) | -1.36 (-1.78, -0.93) | <0.001 | 0.11 (0.07, 0.15) | 0.07 (0.05, 0.10) | -1.36 (-1.79, -0.93) | <0.001 |
| Low | — | — | — | — | 0.30 (0.11, 0.60) | 0.33 (0.12, 0.67) | 0.36 (0.31, 0.40) | <0.001 | 8.24 (3.99, 12.78) | 6.62 (4.03, 9.26) | -0.77 (-0.94, -0.59) | <0.001 | 0.11 (0.05, 0.18) | 0.09 (0.06, 0.13) | -0.77 (-0.94, -0.59) | <0.001 |
| **21 GBD region** | | | | | | | | | | | | | | | | |
| South Asia | — | — | — | — | 0.06 (0.02, 0.11) | 0.06 (0.02, 0.12) | 0.12 (0.10, 0.15) | <0.001 | 2.83 (1.57, 4.30) | 2.44 (1.75, 3.46) | -0.70 (-2.23, 0.86) | 0.377 | 0.04 (0.02, 0.06) | 0.03 (0.02, 0.05) | -0.70 (-2.23, 0.86) | 0.378 |
| East Asia | — | — | — | — | 0.23 (0.09, 0.45) | 0.25 (0.10, 0.48) | 0.25 (0.19, 0.30) | <0.001 | 22.53 (10.32, 29.91) | 7.46 (4.70, 9.80) | -3.75 (-4.03, -3.48) | <0.001 | 0.31 (0.14, 0.42) | 0.10 (0.07, 0.14) | -3.75 (-4.03, -3.47) | <0.001 |
| North Africa and Middle East | — | — | — | — | 0.18 (0.08, 0.34) | 0.18 (0.08, 0.35) | 0.12 (0.09, 0.16) | <0.001 | 25.04 (13.29, 46.59) | 20.94 (10.50, 38.18) | -0.57 (-0.76, -0.39) | <0.001 | 0.35 (0.19, 0.65) | 0.29 (0.15, 0.53) | -0.57 (-0.75, -0.39) | <0.001 |
| Western Sub-Saharan Africa | — | — | — | — | 0.64 (0.24, 1.30) | 0.64 (0.24, 1.30) | 0.04 (-0.01, 0.08) | 0.141 | 3.10 (1.54, 4.93) | 3.75 (1.91, 6.35) | 0.66 (0.47, 0.86) | <0.001 | 0.04 (0.02, 0.07) | 0.05 (0.03, 0.09) | 0.68 (0.48, 0.88) | <0.001 |
| Southeast Asia | — | — | — | — | 0.13 (0.05, 0.25) | 0.13 (0.05, 0.26) | 0.21 (0.20, 0.23) | <0.001 | 6.68 (3.42, 10.27) | 7.09 (5.23, 9.25) | 0.24 (0.07, | 0.006 | 0.09 (0.05, | 0.10 (0.07, | 0.24 (0.07, 0.41) | 0.005 |

| | | | | | | | | | | | | | | | | |
|---|---|---|---|---|---|---|---|---|---|---|---|---|---|---|---|---|
| | | | | | | | | | | | 0.41) | | 0.14) | 0.13) | | |
| Eastern Sub-Saharan Africa | — | — | — | — | 0.60 (0.22, 1.19) | 0.62 (0.23, 1.26) | 0.15 (0.11, 0.18) | <0.001 | 14.47 (7.05, 22.96) | 9.91 (5.98, 16.39) | -1.29 (-1.36, -1.21) | <0.001 | 0.20 (0.10, 0.32) | 0.14 (0.08, 0.23) | -1.29 (-1.37, -1.22) | <0.001 |
| Central Latin America | — | — | — | — | 0.10 (0.04, 0.20) | 0.10 (0.04, 0.20) | 0.08 (0.04, 0.12) | <0.001 | 5.82 (3.86, 6.88) | 3.73 (2.78, 4.87) | -1.45 (-1.75, -1.14) | <0.001 | 0.08 (0.05, 0.10) | 0.05 (0.04, 0.07) | -1.45 (-1.75, -1.14) | <0.001 |
| Tropical Latin America | — | — | — | — | 0.11 (0.04, 0.21) | 0.11 (0.04, 0.22) | -0.01 (-0.05, 0.02) | 0.415 | 6.69 (5.45, 8.04) | 5.10 (4.28, 7.20) | -0.97 (-1.34, -0.61) | <0.001 | 0.09 (0.08, 0.11) | 0.07 (0.06, 0.10) | -0.97 (-1.34, -0.61) | <0.001 |
| Central Sub-Saharan Africa | — | — | — | — | 0.22 (0.07, 0.48) | 0.22 (0.07, 0.47) | 0.08 (-0.05, 0.22) | 0.230 | 10.36 (3.93, 18.88) | 7.33 (3.70, 12.83) | -1.21 (-1.36, -1.06) | <0.001 | 0.14 (0.05, 0.26) | 0.10 (0.05, 0.18) | -1.21 (-1.36, -1.06) | <0.001 |
| Western Europe | — | — | — | — | 0.17 (0.07, 0.31) | 0.14 (0.06, 0.27) | -0.70 (-0.75, -0.66) | <0.001 | 1.93 (1.24, 2.34) | 1.15 (0.91, 1.40) | -1.78 (-2.22, -1.35) | <0.001 | 0.03 (0.02, 0.03) | 0.02 (0.01, 0.02) | -1.79 (-2.23, -1.35) | <0.001 |
| High-income North America | — | — | — | — | 0.53 (0.24, 0.97) | 0.55 (0.24, 1.03) | 0.17 (0.10, 0.25) | <0.001 | 2.58 (2.19, 3.23) | 3.71 (2.02, 4.41) | 1.29 (0.50, 2.09) | 0.001 | 0.04 (0.03, 0.04) | 0.05 (0.03, 0.06) | 1.32 (0.52, 2.12) | 0.001 |
| Central Asia | — | — | — | — | 0.10 (0.04, 0.19) | 0.11 (0.04, 0.21) | 0.23 (0.19, 0.27) | <0.001 | 8.08 (6.46, 10.57) | 9.89 (7.38, 12.96) | 0.84 (0.05, 1.64) | 0.037 | 0.11 (0.09, 0.15) | 0.14 (0.10, 0.18) | 0.84 (0.05, 1.64) | 0.036 |
| Eastern Europe | — | — | — | — | 0.04 (0.02, 0.09) | 0.05 (0.02, 0.10) | 0.14 (0.12, 0.17) | <0.001 | 1.30 (0.63, 1.50) | 1.60 (0.81, 2.02) | 0.77 (-0.26, 1.80) | 0.145 | 0.02 (0.01, 0.02) | 0.02 (0.01, 0.03) | 0.77 (-0.26, 1.81) | 0.141 |
| Southern Sub-Saharan Africa | — | — | — | — | 0.30 (0.11, 0.62) | 0.27 (0.10, 0.55) | -0.36 (-0.46, -0.26) | <0.001 | 7.59 (4.97, 11.11) | 8.75 (5.34, 14.19) | 0.71 (0.30, 1.12) | 0.001 | 0.11 (0.07, 0.16) | 0.12 (0.07, 0.20) | 0.71 (0.30, 1.12) | 0.001 |
| High-income Asia Pacific | — | — | — | — | 0.32 (0.15, 0.60) | 0.31 (0.14, 0.59) | -0.14 (-0.20, -0.08) | <0.001 | 22.40 (2.44, 32.33) | 1.42 (0.98, 2.21) | -9.22 (-9.71, -8.73) | <0.001 | 0.31 (0.03, 0.45) | 0.02 (0.01, 0.03) | -9.28 (-9.77, -8.78) | <0.001 |
| Central Europe | — | — | — | — | 0.07 (0.03, 0.13) | 0.06 (0.02, 0.11) | -0.61 (-0.65, | <0.001 | 2.95 (2.15, 3.48) | 2.75 (1.50, 3.66) | -0.28 (-0.87, | 0.358 | 0.04 (0.03, | 0.04 (0.02, | -0.28 (-0.87, | 0.363 |

| | | | | | | | | | | | | | | | | |
|---|---|---|---|---|---|---|---|---|---|---|---|---|---|---|---|---|
| | | | | | | | -0.57) | | | | 0.32) | | 0.05) | 0.05) | 0.32) | |
| Andean Latin America | — | — | — | — | 0.10 (0.04, 0.20) | 0.11 (0.04, 0.23) | 0.28 (0.25, 0.30) | <0.001 | 18.65 (12.42, 26.84) | 13.72 (8.57, 21.49) | -1.02 (-1.44, -0.60) | <0.001 | 0.26 (0.17, 0.38) | 0.19 (0.12, 0.30) | -1.02 (-1.44, -0.60) | <0.001 |
| Caribbean | — | — | — | — | 0.09 (0.04, 0.18) | 0.09 (0.04, 0.18) | -0.06 (-0.08, -0.04) | <0.001 | 8.99 (6.58, 12.48) | 8.59 (5.31, 13.44) | -0.08 (-0.52, 0.36) | 0.718 | 0.13 (0.09, 0.17) | 0.12 (0.07, 0.19) | -0.08 (-0.52, 0.36) | 0.727 |
| Southern Latin America | — | — | — | — | 0.21 (0.09, 0.39) | 0.19 (0.08, 0.35) | -0.42 (-0.51, -0.33) | <0.001 | 7.05 (3.95, 9.81) | 5.19 (3.08, 7.17) | -1.06 (-1.33, -0.79) | <0.001 | 0.10 (0.05, 0.14) | 0.07 (0.04, 0.10) | -1.06 (-1.33, -0.79) | <0.001 |
| Oceania | — | — | — | — | 0.11 (0.04, 0.21) | 0.11 (0.04, 0.21) | -0.05 (-0.07, -0.03) | <0.001 | 10.75 (5.43, 20.18) | 12.39 (5.37, 25.59) | 0.44 (0.16, 0.72) | 0.002 | 0.15 (0.08, 0.28) | 0.17 (0.08, 0.36) | 0.44 (0.16, 0.73) | 0.002 |
| Australasia | — | — | — | — | 0.11 (0.05, 0.21) | 0.09 (0.04, 0.17) | -0.88 (-0.92, -0.84) | <0.001 | 0.87 (0.71, 1.06) | 0.63 (0.48, 0.79) | -1.12 (-1.59, -0.64) | <0.001 | 0.01 (0.01, 0.01) | 0.01 (0.01, 0.01) | -1.12 (-1.59, -0.64) | <0.001 |
| **20-24 years** | | | | | | | | | | | | | | | | |
| **Women** | | | | | | | | | | | | | | | | |
| Global | — | — | — | — | 1.69 (0.86, 2.95) | 1.54 (0.78, 2.68) | -0.32 (-0.43, -0.22) | <0.001 | 19.65 (11.75, 28.23) | 12.13 (8.37, 15.49) | -1.63 (-1.78, -1.48) | <0.001 | 0.29 (0.17, 0.42) | 0.18 (0.12, 0.23) | -1.64 (-1.79, -1.49) | <0.001 |
| **SDI category** | | | | | | | | | | | | | | | | |
| High | — | — | — | — | 3.26 (1.82, 5.34) | 3.70 (2.09, 5.98) | 0.47 (0.25, 0.69) | <0.001 | 4.99 (3.60, 6.00) | 3.48 (2.34, 4.04) | -1.17 (-1.46, -0.87) | <0.001 | 0.07 (0.05, 0.09) | 0.05 (0.03, 0.05) | -1.32 (-1.63, -1.01) | <0.001 |
| High-middle | — | — | — | — | 1.50 (0.73, 2.61) | 1.47 (0.72, 2.52) | -0.07 (-0.15, 0.00) | 0.051 | 10.35 (7.87, 13.37) | 4.37 (3.61, 5.21) | -2.97 (-3.37, -2.57) | <0.001 | 0.15 (0.12, 0.20) | 0.06 (0.05, 0.08) | -3.04 (-3.45, -2.63) | <0.001 |
| Middle | — | — | — | — | 1.92 (0.91, 3.41) | 1.74 (0.81, 3.08) | -0.35 (-0.42, -0.29) | <0.001 | 25.06 (16.17, 32.75) | 10.91 (8.44, 13.18) | -2.83 (-3.18, -2.48) | <0.001 | 0.37 (0.24, 0.49) | 0.16 (0.12, 0.20) | -2.85 (-3.21, -2.50) | <0.001 |
| Low-middle | — | — | — | — | 0.86 (0.41, 1.54) | 0.86 (0.41, 1.56) | 0.02 (-0.02, 0.05) | 0.314 | 22.61 (9.15, 38.01) | 13.44 (8.16, 18.46) | -1.78 (-2.06, | <0.001 | 0.34 (0.14, | 0.20 (0.12, | -1.79 (-2.07, | <0.001 |

| | | | | | | | | | | | | | | | | |
|---|---|---|---|---|---|---|---|---|---|---|---|---|---|---|---|---|
| | | | | | | | | | | | -1.50) | | 0.57) | 0.28) | -1.50) | |
| Low | — | — | — | — | 0.98 (0.44, 1.83) | 1.06 (0.48, 1.99) | 0.28 (0.25, 0.32) | <0.001 | 33.19 (10.35, 63.82) | 23.55 (11.05, 34.63) | -1.17 (-1.25, -1.09) | <0.001 | 0.50 (0.15, 0.96) | 0.35 (0.16, 0.52) | -1.17 (-1.25, -1.09) | <0.001 |
| **21 GBD region** | | | | | | | | | | | | | | | | |
| South Asia | — | — | — | — | 0.38 (0.17, 0.68) | 0.39 (0.18, 0.71) | 0.12 (0.10, 0.14) | <0.001 | 16.22 (3.08, 31.37) | 9.01 (3.91, 14.08) | -2.11 (-2.78, -1.43) | <0.001 | 0.24 (0.05, 0.47) | 0.13 (0.06, 0.21) | -2.11 (-2.79, -1.43) | <0.001 |
| East Asia | — | — | — | — | 2.45 (1.17, 4.33) | 2.60 (1.25, 4.58) | 0.21 (0.18, 0.24) | <0.001 | 26.13 (16.98, 35.16) | 7.51 (5.07, 10.47) | -4.14 (-5.12, -3.15) | <0.001 | 0.39 (0.25, 0.53) | 0.11 (0.07, 0.15) | -4.22 (-5.23, -3.20) | <0.001 |
| North Africa and Middle East | — | — | — | — | 2.45 (1.17, 4.37) | 2.51 (1.22, 4.43) | 0.08 (0.04, 0.13) | <0.001 | 35.78 (16.78, 54.88) | 22.17 (13.58, 32.87) | -1.65 (-1.99, -1.31) | <0.001 | 0.53 (0.25, 0.82) | 0.33 (0.20, 0.49) | -1.66 (-2.01, -1.32) | <0.001 |
| Western Sub-Saharan Africa | — | — | — | — | 1.56 (0.69, 2.91) | 1.58 (0.69, 2.90) | 0.05 (0.01, 0.10) | 0.023 | 26.34 (11.96, 38.08) | 27.46 (13.12, 40.41) | 0.16 (0.04, 0.28) | 0.007 | 0.39 (0.18, 0.57) | 0.41 (0.20, 0.61) | 0.16 (0.04, 0.28) | 0.007 |
| Southeast Asia | — | — | — | — | 1.49 (0.68, 2.68) | 1.59 (0.73, 2.83) | 0.22 (0.19, 0.26) | <0.001 | 17.72 (7.52, 30.38) | 11.82 (7.17, 15.24) | -1.38 (-1.61, -1.15) | <0.001 | 0.26 (0.11, 0.45) | 0.18 (0.11, 0.23) | -1.40 (-1.63, -1.16) | <0.001 |
| Eastern Sub-Saharan Africa | — | — | — | — | 1.36 (0.61, 2.56) | 1.39 (0.61, 2.59) | 0.06 (0.04, 0.09) | <0.001 | 47.40 (15.32, 96.58) | 23.38 (11.88, 34.50) | -2.39 (-2.55, -2.24) | <0.001 | 0.71 (0.23, 1.45) | 0.35 (0.18, 0.52) | -2.40 (-2.56, -2.25) | <0.001 |
| Central Latin America | — | — | — | — | 0.99 (0.47, 1.79) | 0.96 (0.44, 1.74) | -0.08 (-0.10, -0.06) | <0.001 | 10.81 (7.11, 12.51) | 6.29 (4.60, 8.90) | -1.92 (-2.63, -1.19) | <0.001 | 0.16 (0.11, 0.19) | 0.09 (0.07, 0.13) | -1.94 (-2.67, -1.20) | <0.001 |
| Tropical Latin America | — | — | — | — | 1.14 (0.52, 2.04) | 1.14 (0.51, 2.10) | 0.01 (-0.02, 0.03) | 0.667 | 13.60 (9.53, 15.46) | 7.43 (6.25, 11.34) | -2.04 (-2.45, -1.62) | <0.001 | 0.20 (0.14, 0.23) | 0.11 (0.09, 0.17) | -2.06 (-2.48, -1.64) | <0.001 |
| Central Sub-Saharan Africa | — | — | — | — | 0.67 (0.27, 1.29) | 0.66 (0.27, 1.31) | -0.03 (-0.11, 0.06) | 0.540 | 33.96 (11.21, 67.57) | 24.19 (10.74, 41.93) | -1.14 (-1.25, -1.03) | <0.001 | 0.51 (0.17, 1.02) | 0.36 (0.16, 0.63) | -1.14 (-1.25, -1.03) | <0.001 |
| Western Europe | — | — | — | — | 0.76 (0.42, | 0.62 (0.33, | -0.72 | <0.001 | 1.86 (1.43, | 1.29 (0.99, | -1.25 | <0.001 | 0.03 | 0.02 | -1.28 | <0.001 |

| | | | | | | | | | | | | | | | | |
|---|---|---|---|---|---|---|---|---|---|---|---|---|---|---|---|---|
| | | | | | 1.24) | 1.04) | (-0.81, -0.64) | | 2.27) | 1.56) | (-1.66, -0.84) | | (0.02, 0.03) | (0.01, 0.02) | (-1.70, -0.85) | |
| High-income North America | — | — | — | — | 7.52 (4.24, 12.45) | 7.87 (4.40, 12.88) | 0.20 (0.06, 0.35) | 0.007 | 4.20 (3.59, 5.64) | 5.65 (3.32, 6.57) | 0.96 (0.56, 1.36) | <0.001 | 0.05 (0.05, 0.07) | 0.07 (0.04, 0.09) | 1.09 (0.64, 1.54) | <0.001 |
| Central Asia | — | — | — | — | 0.53 (0.21, 1.00) | 0.55 (0.22, 1.08) | 0.12 (0.07, 0.17) | <0.001 | 9.44 (6.77, 11.66) | 8.00 (5.63, 10.82) | -0.61 (-0.92, -0.29) | <0.001 | 0.14 (0.10, 0.17) | 0.12 (0.08, 0.16) | -0.61 (-0.93, -0.29) | <0.001 |
| Eastern Europe | — | — | — | — | 0.08 (0.04, 0.15) | 0.08 (0.04, 0.16) | 0.04 (-0.01, 0.09) | 0.119 | 1.02 (0.70, 1.12) | 1.69 (0.94, 2.19) | 1.85 (0.76, 2.94) | 0.001 | 0.02 (0.01, 0.02) | 0.03 (0.01, 0.03) | 1.86 (0.77, 2.96) | 0.001 |
| Southern Sub-Saharan Africa | — | — | — | — | 0.71 (0.32, 1.32) | 0.62 (0.27, 1.17) | -0.47 (-0.56, -0.39) | <0.001 | 35.68 (26.55, 46.02) | 16.35 (10.16, 24.34) | -2.54 (-4.15, -0.90) | 0.003 | 0.54 (0.40, 0.69) | 0.24 (0.15, 0.37) | -2.55 (-4.17, -0.90) | 0.003 |
| High-income Asia Pacific | — | — | — | — | 1.80 (0.90, 3.13) | 1.66 (0.85, 2.87) | -0.31 (-0.41, -0.20) | <0.001 | 10.70 (4.33, 15.06) | 1.76 (1.27, 2.45) | -6.05 (-6.42, -5.68) | <0.001 | 0.16 (0.06, 0.22) | 0.02 (0.02, 0.03) | -6.29 (-6.68, -5.90) | <0.001 |
| Central Europe | — | — | — | — | 0.46 (0.21, 0.85) | 0.38 (0.17, 0.67) | -0.71 (-0.74, -0.69) | <0.001 | 4.97 (3.49, 5.94) | 3.88 (2.06, 5.14) | -0.94 (-1.23, -0.65) | <0.001 | 0.07 (0.05, 0.09) | 0.06 (0.03, 0.08) | -0.94 (-1.23, -0.64) | <0.001 |
| Andean Latin America | — | — | — | — | 1.43 (0.62, 2.65) | 1.57 (0.67, 2.92) | 0.32 (0.29, 0.34) | <0.001 | 27.21 (18.72, 37.08) | 13.28 (8.80, 19.63) | -2.39 (-3.14, -1.64) | <0.001 | 0.41 (0.28, 0.56) | 0.20 (0.13, 0.29) | -2.41 (-3.16, -1.65) | <0.001 |
| Caribbean | — | — | — | — | 1.31 (0.60, 2.36) | 1.31 (0.59, 2.36) | 0.00 (-0.05, 0.06) | 0.887 | 28.10 (16.38, 45.40) | 29.02 (15.66, 52.32) | 0.19 (-0.15, 0.53) | 0.283 | 0.42 (0.24, 0.68) | 0.43 (0.23, 0.79) | 0.19 (-0.16, 0.54) | 0.284 |
| Southern Latin America | — | — | — | — | 1.39 (0.70, 2.46) | 1.14 (0.56, 2.08) | -0.69 (-0.71, -0.67) | <0.001 | 6.10 (3.76, 8.04) | 4.55 (2.61, 6.25) | -0.99 (-1.52, -0.46) | <0.001 | 0.09 (0.05, 0.12) | 0.07 (0.04, 0.09) | -1.00 (-1.54, -0.45) | <0.001 |
| Oceania | — | — | — | — | 1.20 (0.56, 2.19) | 1.10 (0.51, 2.04) | -0.29 (-0.32, -0.26) | <0.001 | 23.65 (10.13, 46.32) | 23.31 (8.78, 46.93) | -0.10 (-0.38, 0.19) | 0.508 | 0.35 (0.15, 0.69) | 0.35 (0.13, 0.70) | -0.09 (-0.38, 0.19) | 0.520 |
| Australasia | — | — | — | — | 0.34 (0.18, | 0.25 (0.13, | -1.01 | <0.001 | 0.72 (0.62, | 0.59 (0.49, | -0.70 | 0.002 | 0.01 | 0.01 | -0.68 | 0.003 |

| | | | | | | | | | | | | | | | | |
|---|---|---|---|---|---|---|---|---|---|---|---|---|---|---|---|---|
| | | | | | 0.56) | 0.42) | (-1.04, -0.99) | | 0.84) | 0.69) | (-1.13, -0.27) | | (0.01, 0.01) | (0.01, 0.01) | (-1.13, -0.24) | |
| **Men** | | | | | | | | | | | | | | | | |
| Global | — | — | — | — | 2.06 (1.04, 3.53) | 1.93 (1.01, 3.31) | -0.22 (-0.32, -0.12) | <0.001 | 17.75 (11.21, 22.66) | 13.26 (9.45, 15.93) | -1.00 (-1.17, -0.84) | <0.001 | 0.26 (0.17, 0.34) | 0.20 (0.14, 0.24) | -1.01 (-1.18, -0.85) | <0.001 |
| **SDI category** | | | | | | | | | | | | | | | | |
| High | — | — | — | — | 4.47 (2.48, 7.32) | 5.60 (3.21, 9.18) | 0.82 (0.61, 1.03) | <0.001 | 8.63 (5.91, 10.14) | 9.16 (5.13, 10.68) | 0.21 (-0.07, 0.48) | 0.145 | 0.12 (0.08, 0.15) | 0.13 (0.07, 0.15) | 0.18 (-0.11, 0.47) | 0.228 |
| High-middle | — | — | — | — | 1.65 (0.81, 2.84) | 1.63 (0.83, 2.83) | -0.04 (-0.13, 0.04) | 0.331 | 14.61 (10.35, 19.02) | 7.29 (5.90, 9.02) | -2.47 (-2.73, -2.20) | <0.001 | 0.22 (0.15, 0.28) | 0.11 (0.09, 0.13) | -2.50 (-2.77, -2.23) | <0.001 |
| Middle | — | — | — | — | 2.27 (1.09, 3.96) | 2.01 (0.99, 3.54) | -0.43 (-0.49, -0.38) | <0.001 | 24.50 (13.58, 32.74) | 16.31 (10.67, 20.72) | -1.43 (-1.63, -1.23) | <0.001 | 0.36 (0.20, 0.49) | 0.24 (0.16, 0.31) | -1.44 (-1.64, -1.23) | <0.001 |
| Low-middle | — | — | — | — | 0.98 (0.48, 1.73) | 0.99 (0.48, 1.75) | 0.02 (-0.01, 0.04) | 0.173 | 14.87 (9.47, 20.34) | 11.76 (9.04, 15.00) | -0.84 (-1.09, -0.59) | <0.001 | 0.22 (0.14, 0.30) | 0.18 (0.13, 0.22) | -0.84 (-1.09, -0.59) | <0.001 |
| Low | — | — | — | — | 1.14 (0.53, 2.09) | 1.30 (0.62, 2.35) | 0.45 (0.39, 0.50) | <0.001 | 18.50 (9.61, 27.77) | 18.11 (11.56, 25.11) | -0.06 (-0.15, 0.03) | 0.175 | 0.28 (0.14, 0.42) | 0.27 (0.17, 0.38) | -0.06 (-0.15, 0.02) | 0.166 |
| **21 GBD region** | | | | | | | | | | | | | | | | |
| South Asia | — | — | — | — | 0.33 (0.15, 0.59) | 0.34 (0.16, 0.61) | 0.11 (0.10, 0.13) | <0.001 | 6.80 (4.38, 9.67) | 6.35 (4.57, 8.98) | -0.21 (-0.91, 0.50) | 0.564 | 0.10 (0.07, 0.14) | 0.09 (0.07, 0.13) | -0.21 (-0.91, 0.50) | 0.567 |
| East Asia | — | — | — | — | 2.91 (1.40, 5.07) | 3.07 (1.51, 5.43) | 0.18 (0.14, 0.22) | <0.001 | 30.06 (14.30, 39.65) | 13.07 (7.71, 16.86) | -2.91 (-3.35, -2.46) | <0.001 | 0.45 (0.21, 0.59) | 0.19 (0.11, 0.25) | -2.95 (-3.40, -2.49) | <0.001 |
| North Africa and Middle East | — | — | — | — | 2.71 (1.38, 4.71) | 2.75 (1.39, 4.77) | 0.05 (0.01, 0.09) | 0.020 | 28.27 (16.03, 52.50) | 24.16 (13.23, 43.36) | -0.52 (-0.76, -0.28) | <0.001 | 0.42 (0.24, 0.78) | 0.36 (0.20, 0.65) | -0.53 (-0.77, -0.28) | <0.001 |
| Western | — | — | — | — | 1.97 (0.92, | 1.96 (0.89, | 0.00 (-0.06, | 0.973 | 16.23 (8.59, | 21.99 (11.14, | 1.07 | <0.001 | 0.24 | 0.33 | 1.08 (0.84, | <0.001 |

| | | | | | | | | | | | | | | | | |
|---|---|---|---|---|---|---|---|---|---|---|---|---|---|---|---|---|
| Sub-Saharan Africa | | | | | 3.54) | 3.56) | 0.06) | | 25.41) | 36.99) | (0.83, 1.31) | | (0.13, 0.38) | (0.16, 0.55) | 1.33) | |
| Southeast Asia | — | — | — | — | 1.78 (0.84, 3.18) | 1.94 (0.93, 3.52) | 0.30 (0.27, 0.32) | <0.001 | 18.21 (9.86, 28.34) | 19.74 (10.57, 25.11) | 0.29 (0.10, 0.48) | 0.003 | 0.27 (0.14, 0.42) | 0.29 (0.16, 0.37) | 0.29 (0.10, 0.48) | 0.003 |
| Eastern Sub-Saharan Africa | — | — | — | — | 1.87 (0.85, 3.45) | 1.93 (0.90, 3.58) | 0.11 (0.06, 0.17) | <0.001 | 29.18 (14.46, 45.24) | 21.69 (12.91, 35.02) | -1.01 (-1.15, -0.87) | <0.001 | 0.44 (0.21, 0.67) | 0.32 (0.19, 0.52) | -1.02 (-1.16, -0.88) | <0.001 |
| Central Latin America | — | — | — | — | 0.82 (0.39, 1.47) | 0.83 (0.40, 1.49) | 0.05 (0.01, 0.10) | 0.016 | 11.20 (8.97, 13.74) | 7.35 (5.55, 10.03) | -1.40 (-1.74, -1.06) | <0.001 | 0.17 (0.13, 0.20) | 0.11 (0.08, 0.15) | -1.41 (-1.75, -1.07) | <0.001 |
| Tropical Latin America | — | — | — | — | 0.92 (0.44, 1.62) | 0.92 (0.42, 1.66) | 0.03 (-0.02, 0.07) | 0.204 | 15.46 (12.24, 18.51) | 11.80 (9.91, 15.30) | -0.90 (-1.28, -0.53) | <0.001 | 0.23 (0.18, 0.28) | 0.18 (0.15, 0.23) | -0.92 (-1.29, -0.54) | <0.001 |
| Central Sub-Saharan Africa | — | — | — | — | 0.77 (0.33, 1.52) | 0.78 (0.33, 1.55) | 0.09 (-0.04, 0.22) | 0.192 | 23.00 (8.67, 43.43) | 18.54 (9.35, 32.90) | -0.75 (-0.98, -0.52) | <0.001 | 0.34 (0.13, 0.65) | 0.28 (0.14, 0.49) | -0.76 (-0.99, -0.52) | <0.001 |
| Western Europe | — | — | — | — | 0.83 (0.46, 1.36) | 0.71 (0.38, 1.16) | -0.54 (-0.64, -0.44) | <0.001 | 3.95 (2.51, 5.05) | 2.11 (1.56, 3.09) | -2.25 (-2.79, -1.71) | <0.001 | 0.06 (0.04, 0.07) | 0.03 (0.02, 0.05) | -2.29 (-2.87, -1.70) | <0.001 |
| High-income North America | — | — | — | — | 11.14 (6.14, 18.31) | 13.04 (7.33, 21.12) | 0.58 (0.43, 0.73) | <0.001 | 9.87 (8.51, 13.66) | 19.60 (9.62, 23.13) | 2.37 (1.93, 2.81) | <0.001 | 0.13 (0.11, 0.19) | 0.28 (0.12, 0.33) | 2.54 (2.05, 3.02) | <0.001 |
| Central Asia | — | — | — | — | 1.05 (0.48, 1.89) | 1.20 (0.56, 2.20) | 0.45 (0.39, 0.51) | <0.001 | 10.03 (7.38, 13.17) | 10.11 (7.45, 13.85) | 0.10 (-0.88, 1.08) | 0.849 | 0.15 (0.11, 0.20) | 0.15 (0.11, 0.21) | 0.09 (-0.89, 1.08) | 0.857 |
| Eastern Europe | — | — | — | — | 0.12 (0.06, 0.21) | 0.13 (0.06, 0.23) | 0.14 (0.11, 0.18) | <0.001 | 4.71 (3.33, 5.42) | 5.74 (4.28, 7.46) | 0.78 (-0.31, 1.90) | 0.162 | 0.07 (0.05, 0.08) | 0.09 (0.06, 0.11) | 0.79 (-0.31, 1.90) | 0.160 |
| Southern Sub-Saharan Africa | — | — | — | — | 0.77 (0.35, 1.43) | 0.70 (0.32, 1.27) | -0.36 (-0.47, -0.26) | <0.001 | 12.81 (8.34, 20.12) | 14.91 (10.66, 21.11) | 0.56 (-0.07, 1.19) | 0.082 | 0.19 (0.12, 0.30) | 0.22 (0.16, 0.32) | 0.56 (-0.08, 1.20) | 0.085 |
| High-income Asia | — | — | — | — | 1.96 (1.04, | 1.79 (0.94, | -0.34 | <0.001 | 14.29 (3.81, | 1.93 (1.32, | -6.70 | <0.001 | 0.21 | 0.03 | -6.90 | <0.001 |

| | | | | | | | | | | | | | | | | |
|---|---|---|---|---|---|---|---|---|---|---|---|---|---|---|---|---|
| Pacific | | | | | 3.30) | 3.02) | (-0.41, -0.27) | | 20.03) | 3.12) | (-7.08, -6.31) | | (0.05, 0.30) | (0.02, 0.04) | (-7.34, -6.45) | |
| Central Europe | — | — | — | — | 0.65 (0.31, 1.14) | 0.55 (0.28, 0.94) | -0.53 (-0.57, -0.48) | <0.001 | 7.11 (5.89, 8.56) | 6.77 (4.26, 9.04) | -0.32 (-0.64, -0.00) | 0.047 | 0.11 (0.09, 0.13) | 0.10 (0.06, 0.14) | -0.32 (-0.64, -0.00) | 0.048 |
| Andean Latin America | — | — | — | — | 1.55 (0.69, 2.80) | 1.71 (0.76, 3.07) | 0.34 (0.32, 0.36) | <0.001 | 31.24 (21.70, 43.03) | 25.99 (15.78, 39.95) | -0.59 (-0.89, -0.30) | <0.001 | 0.47 (0.32, 0.64) | 0.39 (0.24, 0.60) | -0.60 (-0.89, -0.30) | <0.001 |
| Caribbean | — | — | — | — | 1.21 (0.56, 2.21) | 1.22 (0.56, 2.23) | 0.02 (0.01, 0.04) | 0.001 | 15.69 (11.82, 20.93) | 16.94 (10.80, 24.79) | 0.33 (0.01, 0.65) | 0.043 | 0.23 (0.18, 0.31) | 0.25 (0.16, 0.37) | 0.33 (0.01, 0.65) | 0.043 |
| Southern Latin America | — | — | — | — | 1.63 (0.84, 2.74) | 1.40 (0.69, 2.40) | -0.51 (-0.57, -0.45) | <0.001 | 12.32 (6.44, 17.42) | 8.55 (4.94, 12.46) | -1.24 (-1.60, -0.88) | <0.001 | 0.18 (0.09, 0.26) | 0.13 (0.07, 0.19) | -1.25 (-1.61, -0.88) | <0.001 |
| Oceania | — | — | — | — | 1.36 (0.64, 2.44) | 1.33 (0.63, 2.36) | -0.10 (-0.11, -0.09) | <0.001 | 29.25 (15.84, 53.22) | 31.55 (14.90, 58.34) | 0.18 (-0.02, 0.39) | 0.078 | 0.44 (0.24, 0.80) | 0.47 (0.22, 0.88) | 0.18 (-0.02, 0.39) | 0.074 |
| Australasia | — | — | — | — | 0.42 (0.24, 0.67) | 0.32 (0.17, 0.52) | -0.98 (-1.08, -0.88) | <0.001 | 1.59 (1.14, 2.15) | 0.87 (0.66, 1.40) | -2.04 (-2.46, -1.63) | <0.001 | 0.02 (0.02, 0.03) | 0.01 (0.01, 0.02) | -2.07 (-2.49, -1.65) | <0.001 |
| **25-29 years** | | | | | | | | | | | | | | | | |
| **Women** | | | | | | | | | | | | | | | | |
| Global | — | — | — | — | 6.10 (2.93, 10.66) | 5.86 (2.83, 10.39) | -0.12 (-0.18, -0.06) | <0.001 | 31.30 (18.66, 43.16) | 20.66 (15.17, 25.66) | -1.38 (-1.86, -0.89) | <0.001 | 0.50 (0.30, 0.69) | 0.33 (0.24, 0.41) | -1.40 (-1.90, -0.91) | <0.001 |
| **SDI category** | | | | | | | | | | | | | | | | |
| High | — | — | — | — | 13.63 (7.07, 23.28) | 15.63 (8.44, 26.09) | 0.53 (0.42, 0.64) | <0.001 | 8.77 (7.02, 10.21) | 9.10 (5.71, 10.68) | 0.18 (-0.13, 0.49) | 0.246 | 0.12 (0.10, 0.14) | 0.13 (0.07, 0.15) | 0.25 (-0.00, 0.50) | 0.052 |
| High-middle | — | — | — | — | 4.69 (2.16, 8.31) | 5.19 (2.41, 9.39) | 0.36 (0.27, 0.45) | <0.001 | 14.03 (11.16, 17.42) | 6.42 (5.38, 7.67) | -2.61 (-3.42, -1.80) | <0.001 | 0.22 (0.17, 0.27) | 0.10 (0.08, 0.12) | -2.91 (-3.69, -2.13) | <0.001 |
| Middle | — | — | — | — | 6.42 (2.89, | 6.38 (2.87, | -0.02 | 0.345 | 37.37 (25.18, | 17.70 (12.89, | -2.48 | <0.001 | 0.60 | 0.28 | -2.54 | <0.001 |

| | | | | | | | | | | | | | | | | |
|---|---|---|---|---|---|---|---|---|---|---|---|---|---|---|---|---|
| | | | | | 11.52) | 11.48) | (-0.06, 0.02) | | 47.87) | 21.38) | (-3.26, -1.70) | | (0.39, 0.77) | (0.20, 0.34) | (-3.33, -1.75) | |
| Low-middle | — | — | — | — | 2.78 (1.22, 5.10) | 2.88 (1.26, 5.36) | 0.12 (0.10, 0.14) | <0.001 | 38.06 (16.74, 59.28) | 23.54 (15.48, 32.39) | -1.62 (-1.93, -1.30) | <0.001 | 0.61 (0.27, 0.96) | 0.38 (0.25, 0.52) | -1.63 (-1.95, -1.31) | <0.001 |
| Low | — | — | — | — | 2.86 (1.15, 5.72) | 3.20 (1.29, 6.44) | 0.40 (0.36, 0.44) | <0.001 | 75.47 (29.62, 128.24) | 48.21 (26.77, 66.74) | -1.54 (-1.62, -1.46) | <0.001 | 1.22 (0.48, 2.08) | 0.78 (0.43, 1.08) | -1.55 (-1.63, -1.47) | <0.001 |
| **21 GBD region** | | | | | | | | | | | | | | | | |
| South Asia | — | — | — | — | 1.14 (0.46, 2.21) | 1.19 (0.48, 2.29) | 0.17 (0.15, 0.20) | <0.001 | 25.86 (6.55, 46.49) | 15.36 (8.92, 23.69) | -1.79 (-2.52, -1.04) | <0.001 | 0.42 (0.10, 0.75) | 0.25 (0.14, 0.38) | -1.79 (-2.53, -1.05) | <0.001 |
| East Asia | — | — | — | — | 8.07 (3.63, 14.49) | 8.74 (3.97, 15.70) | 0.27 (0.24, 0.29) | <0.001 | 32.00 (21.14, 42.44) | 8.70 (5.96, 11.66) | -4.39 (-6.32, -2.41) | <0.001 | 0.51 (0.33, 0.68) | 0.13 (0.08, 0.18) | -4.64 (-6.71, -2.52) | <0.001 |
| North Africa and Middle East | — | — | — | — | 9.94 (4.58, 17.71) | 10.46 (4.84, 18.62) | 0.17 (0.14, 0.20) | <0.001 | 55.46 (27.02, 82.50) | 35.28 (22.32, 50.23) | -1.46 (-1.80, -1.13) | <0.001 | 0.89 (0.43, 1.33) | 0.56 (0.35, 0.80) | -1.50 (-1.84, -1.15) | <0.001 |
| Western Sub-Saharan Africa | — | — | — | — | 4.22 (1.64, 8.66) | 4.31 (1.61, 9.04) | 0.09 (0.04, 0.14) | 0.001 | 61.94 (33.07, 89.29) | 58.89 (31.34, 83.04) | -0.15 (-0.28, -0.03) | 0.014 | 1.00 (0.53, 1.44) | 0.95 (0.50, 1.34) | -0.15 (-0.28, -0.03) | 0.014 |
| Southeast Asia | — | — | — | — | 5.19 (2.26, 9.35) | 5.47 (2.38, 9.85) | 0.18 (0.15, 0.21) | <0.001 | 38.78 (18.79, 58.22) | 23.64 (14.46, 30.18) | -1.65 (-1.84, -1.46) | <0.001 | 0.62 (0.30, 0.94) | 0.38 (0.22, 0.48) | -1.68 (-1.88, -1.48) | <0.001 |
| Eastern Sub-Saharan Africa | — | — | — | — | 3.66 (1.34, 7.62) | 3.73 (1.39, 7.85) | 0.07 (0.05, 0.09) | <0.001 | 123.58 (49.74, 221.92) | 52.57 (29.59, 74.58) | -2.89 (-3.05, -2.73) | <0.001 | 2.00 (0.80, 3.60) | 0.85 (0.48, 1.21) | -2.90 (-3.07, -2.74) | <0.001 |
| Central Latin America | — | — | — | — | 3.34 (1.46, 6.30) | 3.25 (1.38, 6.05) | -0.10 (-0.13, -0.06) | <0.001 | 16.49 (12.30, 18.94) | 9.22 (6.59, 13.69) | -1.97 (-2.54, -1.40) | <0.001 | 0.26 (0.19, 0.30) | 0.14 (0.10, 0.22) | -2.02 (-2.62, -1.42) | <0.001 |
| Tropical Latin America | — | — | — | — | 3.73 (1.59, 6.83) | 3.69 (1.50, 7.11) | -0.04 (-0.06, -0.02) | 0.001 | 27.21 (18.54, 30.92) | 12.12 (9.98, 19.55) | -2.76 (-3.10, -2.42) | <0.001 | 0.44 (0.30, 0.50) | 0.19 (0.16, 0.31) | -2.82 (-3.17, -2.47) | <0.001 |

| | | | | | | | | | | | | | | | | |
|---|---|---|---|---|---|---|---|---|---|---|---|---|---|---|---|---|
| Central Sub-Saharan Africa | — | — | — | — | 1.96 (0.68, 4.17) | 1.93 (0.68, 4.19) | -0.04 (-0.10, 0.03) | 0.282 | 60.35 (24.32, 104.83) | 39.18 (19.48, 63.09) | -1.50 (-1.60, -1.41) | <0.001 | 0.98 (0.39, 1.70) | 0.63 (0.31, 1.02) | -1.51 (-1.60, -1.42) | <0.001 |
| Western Europe | — | — | — | — | 2.04 (1.02, 3.57) | 1.62 (0.78, 2.85) | -0.80 (-0.85, -0.75) | <0.001 | 2.64 (1.67, 3.13) | 1.68 (1.15, 2.08) | -1.64 (-1.75, -1.53) | <0.001 | 0.04 (0.02, 0.05) | 0.02 (0.02, 0.03) | -1.69 (-1.81, -1.57) | <0.001 |
| High-income North America | — | — | — | — | 32.21 (16.88, 55.26) | 36.10 (19.47, 60.72) | 0.46 (0.25, 0.66) | <0.001 | 11.91 (9.77, 16.05) | 18.62 (10.70, 22.13) | 1.60 (1.17, 2.03) | <0.001 | 0.15 (0.13, 0.21) | 0.25 (0.12, 0.30) | 1.92 (1.42, 2.43) | <0.001 |
| Central Asia | — | — | — | — | 1.77 (0.64, 3.48) | 1.83 (0.66, 3.79) | 0.11 (0.05, 0.18) | 0.001 | 12.73 (9.25, 15.65) | 10.69 (7.68, 14.40) | -0.60 (-0.89, -0.31) | <0.001 | 0.20 (0.15, 0.25) | 0.17 (0.12, 0.23) | -0.61 (-0.90, -0.32) | <0.001 |
| Eastern Europe | — | — | — | — | 0.17 (0.06, 0.34) | 0.17 (0.07, 0.34) | 0.02 (-0.05, 0.09) | 0.566 | 2.03 (1.21, 2.39) | 3.37 (1.72, 4.58) | 1.73 (0.58, 2.89) | 0.003 | 0.03 (0.02, 0.04) | 0.05 (0.03, 0.07) | 1.75 (0.59, 2.92) | 0.003 |
| Southern Sub-Saharan Africa | — | — | — | — | 1.69 (0.65, 3.46) | 1.52 (0.58, 3.13) | -0.38 (-0.42, -0.33) | <0.001 | 97.45 (68.56, 124.68) | 43.36 (22.55, 69.95) | -2.59 (-4.55, -0.60) | 0.011 | 1.58 (1.11, 2.02) | 0.70 (0.36, 1.13) | -2.60 (-4.57, -0.60) | 0.011 |
| High-income Asia Pacific | — | — | — | — | 5.48 (2.63, 9.97) | 4.71 (2.27, 8.45) | -0.54 (-0.62, -0.45) | <0.001 | 13.00 (4.95, 17.45) | 2.04 (1.49, 2.73) | -6.30 (-6.79, -5.81) | <0.001 | 0.20 (0.07, 0.27) | 0.03 (0.02, 0.04) | -6.95 (-7.48, -6.43) | <0.001 |
| Central Europe | — | — | — | — | 1.56 (0.60, 3.08) | 1.35 (0.61, 2.49) | -0.49 (-0.58, -0.40) | <0.001 | 7.05 (5.75, 8.65) | 5.69 (3.86, 7.61) | -0.63 (-1.50, 0.24) | 0.155 | 0.11 (0.09, 0.14) | 0.09 (0.06, 0.12) | -0.64 (-1.52, 0.25) | 0.160 |
| Andean Latin America | — | — | — | — | 5.20 (2.13, 9.86) | 5.62 (2.25, 10.58) | 0.27 (0.24, 0.30) | <0.001 | 33.03 (22.95, 46.66) | 15.79 (10.37, 23.02) | -2.63 (-3.39, -1.86) | <0.001 | 0.53 (0.36, 0.75) | 0.25 (0.16, 0.37) | -2.70 (-3.48, -1.91) | <0.001 |
| Caribbean | — | — | — | — | 4.43 (1.85, 8.12) | 4.41 (1.90, 8.07) | -0.01 (-0.05, 0.03) | 0.577 | 37.27 (21.82, 61.29) | 39.05 (21.07, 67.82) | 0.25 (-0.15, 0.66) | 0.216 | 0.60 (0.35, 0.99) | 0.63 (0.34, 1.10) | 0.26 (-0.15, 0.67) | 0.216 |
| Southern Latin America | — | — | — | — | 5.10 (2.41, 9.13) | 3.98 (1.88, 7.20) | -0.85 (-0.87, -0.84) | <0.001 | 11.80 (8.35, 15.63) | 7.37 (5.20, 10.06) | -1.65 (-2.10, -1.20) | <0.001 | 0.18 (0.13, 0.25) | 0.11 (0.08, 0.16) | -1.69 (-2.15, -1.22) | <0.001 |

| | | | | | | | | | | | | | | | | |
|---|---|---|---|---|---|---|---|---|---|---|---|---|---|---|---|---|
| Oceania | — | — | — | — | 4.37 (1.87, 8.27) | 3.90 (1.73, 7.16) | -0.39 (-0.42, -0.36) | <0.001 | 64.35 (30.08, 117.69) | 59.72 (25.08, 121.38) | -0.27 (-0.49, -0.05) | 0.015 | 1.04 (0.48, 1.91) | 0.96 (0.40, 1.96) | -0.27 (-0.49, -0.05) | 0.016 |
| Australasia | — | — | — | — | 0.91 (0.42, 1.66) | 0.64 (0.29, 1.16) | -1.22 (-1.25, -1.20) | <0.001 | 0.88 (0.73, 1.03) | 0.64 (0.53, 0.76) | -1.04 (-1.60, -0.47) | <0.001 | 0.01 (0.01, 0.01) | 0.01 (0.01, 0.01) | -1.01 (-1.62, -0.40) | 0.001 |
| **Men** | | | | | | | | | | | | | | | | |
| Global | — | — | — | — | 7.77 (3.87, 13.38) | 7.78 (3.94, 13.35) | 0.02 (-0.03, 0.07) | 0.432 | 28.95 (22.04, 33.94) | 23.40 (16.24, 27.32) | -0.72 (-0.87, -0.56) | <0.001 | 0.46 (0.34, 0.54) | 0.37 (0.25, 0.43) | -0.74 (-0.91, -0.57) | <0.001 |
| **SDI category** | | | | | | | | | | | | | | | | |
| High | — | — | — | — | 19.77 (10.32, 33.43) | 25.21 (13.69, 41.81) | 0.88 (0.78, 0.98) | <0.001 | 16.56 (13.84, 19.41) | 21.32 (12.17, 25.18) | 0.88 (0.51, 1.26) | <0.001 | 0.24 (0.19, 0.28) | 0.31 (0.16, 0.37) | 0.88 (0.51, 1.25) | <0.001 |
| High-middle | — | — | — | — | 5.24 (2.44, 9.24) | 5.91 (2.82, 10.42) | 0.40 (0.31, 0.49) | <0.001 | 21.51 (17.11, 26.36) | 12.27 (9.87, 14.58) | -1.96 (-2.38, -1.55) | <0.001 | 0.34 (0.27, 0.42) | 0.19 (0.15, 0.23) | -2.04 (-2.47, -1.61) | <0.001 |
| Middle | — | — | — | — | 7.70 (3.50, 13.70) | 7.58 (3.53, 13.84) | -0.05 (-0.10, 0.00) | 0.049 | 38.74 (26.38, 47.91) | 25.70 (15.86, 31.10) | -1.39 (-1.57, -1.20) | <0.001 | 0.62 (0.42, 0.76) | 0.41 (0.25, 0.49) | -1.42 (-1.60, -1.23) | <0.001 |
| Low-middle | — | — | — | — | 3.23 (1.44, 5.75) | 3.33 (1.52, 6.10) | 0.10 (0.05, 0.14) | <0.001 | 26.67 (18.99, 33.78) | 21.43 (16.30, 26.27) | -0.76 (-1.05, -0.46) | <0.001 | 0.43 (0.30, 0.54) | 0.34 (0.26, 0.42) | -0.77 (-1.07, -0.47) | <0.001 |
| Low | — | — | — | — | 3.37 (1.40, 6.66) | 4.00 (1.74, 7.82) | 0.60 (0.53, 0.67) | <0.001 | 38.30 (24.23, 52.15) | 38.26 (23.21, 53.32) | 0.02 (-0.18, 0.21) | 0.870 | 0.62 (0.39, 0.84) | 0.61 (0.36, 0.86) | 0.01 (-0.19, 0.21) | 0.911 |
| **21 GBD region** | | | | | | | | | | | | | | | | |
| South Asia | — | — | — | — | 0.97 (0.40, 1.88) | 1.01 (0.42, 1.94) | 0.10 (0.08, 0.12) | <0.001 | 13.76 (9.32, 20.79) | 12.29 (8.29, 17.98) | -0.37 (-0.83, 0.10) | 0.119 | 0.22 (0.15, 0.34) | 0.20 (0.13, 0.29) | -0.37 (-0.83, 0.10) | 0.119 |
| East Asia | — | — | — | — | 9.52 (4.26, 16.93) | 10.40 (4.78, 18.24) | 0.29 (0.19, 0.39) | <0.001 | 44.39 (26.59, 55.95) | 20.17 (11.07, 25.48) | -2.65 (-3.12, -2.18) | <0.001 | 0.71 (0.42, 0.88) | 0.31 (0.17, 0.39) | -2.75 (-3.24, -2.26) | <0.001 |

| | | | | | | | | | | | | | | | | |
|---|---|---|---|---|---|---|---|---|---|---|---|---|---|---|---|---|
| North Africa and Middle East | — | — | — | — | 11.31 (5.51, 19.95) | 11.63 (5.62, 20.54) | 0.09 (0.07, 0.12) | <0.001 | 43.41 (24.90, 72.76) | 36.75 (20.66, 60.06) | -0.55 (-0.72, -0.38) | <0.001 | 0.69 (0.39, 1.16) | 0.58 (0.32, 0.96) | -0.57 (-0.75, -0.40) | <0.001 |
| Western Sub-Saharan Africa | — | — | — | — | 5.33 (2.10, 10.73) | 5.29 (2.12, 10.61) | -0.01 (-0.06, 0.04) | 0.650 | 34.90 (21.54, 50.52) | 47.27 (23.71, 72.08) | 1.06 (0.84, 1.28) | <0.001 | 0.56 (0.34, 0.80) | 0.76 (0.38, 1.16) | 1.08 (0.86, 1.30) | <0.001 |
| Southeast Asia | — | — | — | — | 6.60 (3.06, 11.87) | 7.14 (3.31, 13.06) | 0.28 (0.22, 0.33) | <0.001 | 32.83 (18.62, 46.02) | 31.57 (14.70, 39.33) | -0.09 (-0.27, 0.09) | 0.329 | 0.52 (0.29, 0.74) | 0.50 (0.23, 0.63) | -0.10 (-0.28, 0.09) | 0.297 |
| Eastern Sub-Saharan Africa | — | — | — | — | 5.01 (1.96, 10.30) | 5.18 (2.05, 10.69) | 0.10 (0.07, 0.14) | <0.001 | 62.26 (35.26, 91.70) | 44.19 (24.67, 74.32) | -1.17 (-1.40, -0.95) | <0.001 | 1.00 (0.56, 1.48) | 0.71 (0.39, 1.20) | -1.18 (-1.41, -0.96) | <0.001 |
| Central Latin America | — | — | — | — | 2.75 (1.19, 5.09) | 2.74 (1.20, 5.00) | -0.02 (-0.06, 0.02) | 0.344 | 17.10 (13.94, 21.05) | 11.13 (8.63, 16.31) | -1.44 (-1.77, -1.10) | <0.001 | 0.27 (0.22, 0.34) | 0.18 (0.14, 0.26) | -1.47 (-1.81, -1.12) | <0.001 |
| Tropical Latin America | — | — | — | — | 2.97 (1.33, 5.39) | 2.95 (1.25, 5.62) | -0.02 (-0.05, -0.00) | 0.041 | 31.93 (23.63, 37.24) | 19.95 (16.84, 29.51) | -1.58 (-2.02, -1.14) | <0.001 | 0.51 (0.38, 0.60) | 0.32 (0.27, 0.47) | -1.60 (-2.05, -1.16) | <0.001 |
| Central Sub-Saharan Africa | — | — | — | — | 2.25 (0.83, 4.90) | 2.29 (0.87, 4.97) | 0.08 (0.02, 0.14) | 0.010 | 44.46 (19.29, 73.88) | 35.02 (19.48, 63.08) | -0.89 (-1.03, -0.75) | <0.001 | 0.72 (0.31, 1.20) | 0.56 (0.31, 1.02) | -0.90 (-1.04, -0.76) | <0.001 |
| Western Europe | — | — | — | — | 2.51 (1.28, 4.32) | 2.17 (1.07, 3.83) | -0.50 (-0.58, -0.42) | <0.001 | 6.85 (4.48, 8.47) | 4.36 (3.17, 5.89) | -1.51 (-2.15, -0.88) | <0.001 | 0.11 (0.07, 0.13) | 0.07 (0.05, 0.09) | -1.55 (-2.22, -0.88) | <0.001 |
| High-income North America | — | — | — | — | 49.35 (26.03, 83.17) | 62.98 (34.25, 104.38) | 0.89 (0.66, 1.11) | <0.001 | 23.59 (19.81, 34.49) | 48.03 (24.73, 58.23) | 2.59 (2.03, 3.15) | <0.001 | 0.31 (0.27, 0.48) | 0.69 (0.31, 0.84) | 2.85 (2.33, 3.36) | <0.001 |
| Central Asia | — | — | — | — | 4.06 (1.71, 7.74) | 4.55 (1.88, 8.54) | 0.40 (0.35, 0.44) | <0.001 | 24.89 (18.42, 33.48) | 22.73 (17.13, 32.12) | -0.34 (-0.81, 0.15) | 0.172 | 0.40 (0.29, 0.54) | 0.36 (0.27, 0.51) | -0.35 (-0.83, 0.14) | 0.158 |
| Eastern Europe | — | — | — | — | 0.31 (0.13, 0.60) | 0.32 (0.13, 0.60) | 0.04 (0.00, 0.08) | 0.039 | 7.73 (4.23, 9.17) | 10.26 (6.95, 13.24) | 1.09 (-0.37, 2.57) | 0.143 | 0.13 (0.07, 0.15) | 0.17 (0.11, 0.21) | 1.10 (-0.36, 2.57) | 0.140 |

| | | | | | | | | | | | | | | | | |
|---|---|---|---|---|---|---|---|---|---|---|---|---|---|---|---|---|
| Southern Sub-Saharan Africa | — | — | — | — | 1.83 (0.72, 3.77) | 1.68 (0.67, 3.43) | -0.34 (-0.42, -0.25) | <0.001 | 45.34 (31.03, 65.95) | 36.55 (26.01, 51.37) | -0.83 (-1.58, -0.07) | 0.031 | 0.73 (0.50, 1.07) | 0.59 (0.42, 0.83) | -0.84 (-1.59, -0.08) | 0.031 |
| High-income Asia Pacific | — | — | — | — | 6.48 (3.25, 11.60) | 5.88 (2.99, 10.64) | -0.33 (-0.48, -0.17) | <0.001 | 22.42 (8.75, 31.01) | 4.35 (3.11, 7.02) | -5.52 (-5.79, -5.25) | <0.001 | 0.35 (0.13, 0.49) | 0.06 (0.04, 0.11) | -5.88 (-6.19, -5.58) | <0.001 |
| Central Europe | — | — | — | — | 2.49 (1.15, 4.61) | 2.22 (1.06, 3.97) | -0.39 (-0.43, -0.35) | <0.001 | 13.80 (11.69, 17.53) | 14.06 (9.45, 18.11) | 0.09 (-0.14, 0.32) | 0.435 | 0.22 (0.19, 0.28) | 0.22 (0.15, 0.29) | 0.10 (-0.14, 0.34) | 0.415 |
| Andean Latin America | — | — | — | — | 5.65 (2.34, 10.75) | 6.14 (2.58, 11.39) | 0.30 (0.25, 0.35) | <0.001 | 31.77 (22.81, 42.37) | 26.49 (17.30, 41.29) | -0.64 (-0.99, -0.28) | <0.001 | 0.51 (0.36, 0.67) | 0.42 (0.27, 0.66) | -0.65 (-1.02, -0.29) | <0.001 |
| Caribbean | — | — | — | — | 4.14 (1.77, 7.66) | 4.14 (1.83, 7.74) | 0.01 (-0.03, 0.05) | 0.619 | 27.35 (20.73, 35.80) | 30.27 (20.67, 44.58) | 0.40 (0.12, 0.67) | 0.004 | 0.44 (0.33, 0.57) | 0.49 (0.33, 0.72) | 0.39 (0.07, 0.72) | 0.018 |
| Southern Latin America | — | — | — | — | 5.90 (2.84, 10.52) | 5.01 (2.37, 8.78) | -0.56 (-0.57, -0.54) | <0.001 | 15.51 (9.02, 21.59) | 11.29 (6.87, 16.24) | -1.06 (-1.47, -0.65) | <0.001 | 0.24 (0.14, 0.34) | 0.18 (0.10, 0.26) | -1.08 (-1.50, -0.66) | <0.001 |
| Oceania | — | — | — | — | 5.05 (2.29, 9.50) | 4.92 (2.21, 8.97) | -0.09 (-0.10, -0.08) | <0.001 | 44.38 (23.23, 80.28) | 47.52 (21.87, 90.28) | 0.22 (0.11, 0.33) | <0.001 | 0.71 (0.37, 1.29) | 0.76 (0.34, 1.46) | 0.22 (0.11, 0.34) | <0.001 |
| Australasia | — | — | — | — | 1.27 (0.61, 2.25) | 0.94 (0.44, 1.66) | -1.05 (-1.11, -0.98) | <0.001 | 3.58 (2.07, 5.17) | 2.45 (1.38, 3.81) | -1.34 (-1.94, -0.73) | <0.001 | 0.06 (0.03, 0.08) | 0.04 (0.02, 0.06) | -1.34 (-1.96, -0.72) | <0.001 |
| **30-34 years** | | | | | | | | | | | | | | | | |
| **Women** | | | | | | | | | | | | | | | | |
| Global | — | — | — | — | 12.79 (7.03, 21.14) | 12.45 (6.78, 20.75) | -0.08 (-0.15, -0.02) | 0.012 | 55.86 (35.83, 75.34) | 35.99 (26.96, 43.32) | -1.47 (-1.84, -1.09) | <0.001 | 0.97 (0.61, 1.31) | 0.62 (0.46, 0.75) | -1.51 (-1.89, -1.13) | <0.001 |
| **SDI category** | | | | | | | | | | | | | | | | |
| High | — | — | — | — | 33.24 (19.78, 51.94) | 35.03 (21.24, 54.42) | 0.22 (0.09, 0.35) | 0.001 | 17.79 (15.30, 21.33) | 21.43 (13.57, 24.65) | 0.66 (0.24, 1.09) | 0.002 | 0.26 (0.23, 0.31) | 0.32 (0.18, 0.37) | 0.81 (0.44, 1.17) | <0.001 |

| | | | | | | | | | | | | | | | | |
|---|---|---|---|---|---|---|---|---|---|---|---|---|---|---|---|---|
| High-middle | — | — | — | — | 7.90 (3.88, 13.79) | 9.73 (4.91, 16.88) | 0.72 (0.64, 0.81) | <0.001 | 26.23 (20.97, 32.05) | 12.26 (10.16, 14.73) | -2.57 (-3.41, -1.72) | <0.001 | 0.45 (0.36, 0.56) | 0.20 (0.17, 0.24) | -2.72 (-3.66, -1.77) | <0.001 |
| Middle | — | — | — | — | 11.85 (5.81, 20.65) | 13.00 (6.58, 22.37) | 0.32 (0.28, 0.37) | <0.001 | 70.64 (47.75, 88.40) | 32.03 (23.41, 38.55) | -2.69 (-2.98, -2.40) | <0.001 | 1.23 (0.82, 1.54) | 0.54 (0.40, 0.66) | -2.81 (-3.15, -2.47) | <0.001 |
| Low-middle | — | — | — | — | 4.87 (2.31, 8.64) | 5.54 (2.60, 9.91) | 0.46 (0.36, 0.56) | <0.001 | 70.91 (32.49, 110.35) | 44.34 (29.68, 59.17) | -1.54 (-1.72, -1.37) | <0.001 | 1.24 (0.56, 1.94) | 0.77 (0.52, 1.04) | -1.56 (-1.74, -1.39) | <0.001 |
| Low | — | — | — | — | 6.19 (2.71, 11.43) | 6.92 (3.02, 12.73) | 0.39 (0.38, 0.40) | <0.001 | 129.16 (54.63, 211.93) | 83.18 (47.95, 112.58) | -1.53 (-1.63, -1.42) | <0.001 | 2.27 (0.95, 3.73) | 1.46 (0.84, 1.98) | -1.53 (-1.64, -1.43) | <0.001 |
| **21 GBD region** | | | | | | | | | | | | | | | | |
| South Asia | — | — | — | — | 2.35 (1.08, 4.22) | 2.47 (1.13, 4.50) | 0.17 (0.11, 0.23) | <0.001 | 50.30 (15.17, 88.60) | 30.69 (18.29, 45.45) | -1.70 (-2.30, -1.09) | <0.001 | 0.88 (0.26, 1.56) | 0.54 (0.32, 0.80) | -1.71 (-2.31, -1.10) | <0.001 |
| East Asia | — | — | — | — | 14.73 (7.25, 25.35) | 16.57 (8.55, 28.61) | 0.40 (0.32, 0.48) | <0.001 | 59.61 (40.71, 79.47) | 16.53 (11.35, 22.35) | -4.14 (-5.83, -2.42) | <0.001 | 1.03 (0.70, 1.38) | 0.27 (0.17, 0.36) | -4.60 (-6.16, -3.03) | <0.001 |
| North Africa and Middle East | — | — | — | — | 21.58 (11.08, 36.18) | 22.78 (11.85, 38.24) | 0.18 (0.16, 0.21) | <0.001 | 89.92 (44.18, 129.15) | 57.24 (36.14, 79.33) | -1.49 (-1.63, -1.34) | <0.001 | 1.55 (0.75, 2.24) | 0.97 (0.61, 1.36) | -1.54 (-1.69, -1.39) | <0.001 |
| Western Sub-Saharan Africa | — | — | — | — | 9.06 (3.97, 16.92) | 9.34 (4.01, 17.75) | 0.10 (0.07, 0.14) | <0.001 | 97.37 (56.24, 134.82) | 89.77 (48.50, 122.35) | -0.26 (-0.38, -0.15) | <0.001 | 1.70 (0.97, 2.36) | 1.57 (0.85, 2.14) | -0.26 (-0.38, -0.15) | <0.001 |
| Southeast Asia | — | — | — | — | 10.94 (5.28, 18.82) | 11.51 (5.68, 19.70) | 0.17 (0.16, 0.18) | <0.001 | 74.24 (38.20, 112.09) | 46.58 (28.47, 59.45) | -1.55 (-1.63, -1.47) | <0.001 | 1.29 (0.66, 1.96) | 0.80 (0.49, 1.03) | -1.58 (-1.66, -1.50) | <0.001 |
| Eastern Sub-Saharan Africa | — | — | — | — | 8.12 (3.33, 15.44) | 8.34 (3.47, 15.66) | 0.09 (0.07, 0.10) | <0.001 | 204.66 (89.57, 360.14) | 89.17 (49.60, 126.14) | -2.82 (-2.96, -2.68) | <0.001 | 3.60 (1.57, 6.33) | 1.56 (0.87, 2.22) | -2.83 (-2.97, -2.69) | <0.001 |
| Central Latin America | — | — | — | — | 2.46 (0.33, 6.64) | 3.55 (0.64, 8.31) | 1.26 (1.10, 1.43) | <0.001 | 31.21 (22.10, 35.45) | 15.42 (11.22, 24.83) | -2.31 (-2.72, | <0.001 | 0.55 (0.39, | 0.27 (0.19, | -2.36 (-2.77, | <0.001 |

| | | | | | | | | | | | | | | | | |
|---|---|---|---|---|---|---|---|---|---|---|---|---|---|---|---|---|
| | | | | | | | | | | | -1.89) | | 0.62) | 0.43) | -1.94) | |
| Tropical Latin America | — | — | — | — | 2.16 (0.00, 7.03) | 3.80 (0.54, 9.31) | 1.98 (1.85, 2.11) | <0.001 | 69.44 (45.24, 77.10) | 29.94 (25.31, 47.62) | -2.79 (-3.21, -2.37) | <0.001 | 1.22 (0.79, 1.36) | 0.52 (0.44, 0.84) | -2.82 (-3.25, -2.40) | <0.001 |
| Central Sub-Saharan Africa | — | — | — | — | 4.94 (1.98, 9.94) | 4.91 (1.92, 9.74) | -0.01 (-0.03, 0.00) | 0.059 | 139.10 (58.75, 225.30) | 94.68 (47.91, 154.20) | -1.34 (-1.41, -1.26) | <0.001 | 2.45 (1.03, 3.97) | 1.66 (0.84, 2.72) | -1.34 (-1.41, -1.26) | <0.001 |
| Western Europe | — | — | — | — | 3.75 (1.97, 6.32) | 2.94 (1.43, 5.14) | -0.84 (-0.90, -0.77) | <0.001 | 5.43 (4.13, 6.57) | 3.66 (2.68, 4.45) | -1.40 (-1.73, -1.06) | <0.001 | 0.09 (0.07, 0.11) | 0.06 (0.04, 0.07) | -1.44 (-1.77, -1.12) | <0.001 |
| High-income North America | — | — | — | — | 77.80 (46.40, 123.11) | 87.42 (52.83, 134.29) | 0.46 (0.32, 0.60) | <0.001 | 26.87 (22.48, 37.17) | 47.34 (26.13, 55.65) | 2.03 (1.54, 2.52) | <0.001 | 0.36 (0.32, 0.54) | 0.70 (0.32, 0.81) | 2.45 (1.98, 2.93) | <0.001 |
| Central Asia | — | — | — | — | 3.69 (1.47, 7.11) | 3.84 (1.38, 7.49) | 0.14 (0.11, 0.17) | <0.001 | 25.59 (17.83, 30.88) | 22.29 (16.66, 28.47) | -0.49 (-0.88, -0.11) | 0.012 | 0.45 (0.31, 0.54) | 0.39 (0.29, 0.50) | -0.50 (-0.90, -0.11) | 0.013 |
| Eastern Europe | — | — | — | — | 0.39 (0.14, 0.75) | 0.40 (0.16, 0.77) | 0.10 (-0.00, 0.19) | 0.052 | 4.31 (2.63, 5.02) | 6.80 (4.04, 9.18) | 1.53 (0.36, 2.72) | 0.010 | 0.08 (0.05, 0.09) | 0.12 (0.07, 0.16) | 1.54 (0.37, 2.73) | 0.010 |
| Southern Sub-Saharan Africa | — | — | — | — | 3.85 (1.65, 7.20) | 3.47 (1.48, 6.49) | -0.36 (-0.40, -0.32) | <0.001 | 203.49 (140.43, 262.41) | 82.59 (47.04, 125.55) | -3.37 (-6.12, -0.54) | 0.020 | 3.58 (2.47, 4.62) | 1.45 (0.82, 2.21) | -3.38 (-6.13, -0.53) | 0.020 |
| High-income Asia Pacific | — | — | — | — | 10.34 (5.24, 17.44) | 8.32 (4.44, 13.71) | -0.77 (-0.88, -0.65) | <0.001 | 20.59 (11.45, 25.78) | 3.96 (3.08, 5.66) | -5.56 (-6.00, -5.12) | <0.001 | 0.35 (0.18, 0.44) | 0.06 (0.04, 0.09) | -6.13 (-6.61, -5.66) | <0.001 |
| Central Europe | — | — | — | — | 3.36 (1.32, 6.30) | 3.00 (1.41, 5.32) | -0.38 (-0.42, -0.33) | <0.001 | 13.81 (10.85, 16.05) | 10.89 (6.57, 14.10) | -0.77 (-1.07, -0.47) | <0.001 | 0.24 (0.19, 0.28) | 0.19 (0.11, 0.24) | -0.78 (-1.10, -0.47) | <0.001 |
| Andean Latin America | — | — | — | — | 0.70 (0.00, 5.26) | 4.69 (0.00, 11.71) | 6.74 (6.59, 6.88) | <0.001 | 60.39 (42.73, 81.47) | 27.11 (17.79, 39.59) | -2.73 (-3.61, -1.84) | <0.001 | 1.07 (0.75, 1.44) | 0.47 (0.31, 0.70) | -2.78 (-3.66, -1.89) | <0.001 |
| Caribbean | — | — | — | — | 8.94 (4.01, 15.52) | 8.51 (3.82, 14.84) | -0.17 (-0.20, | <0.001 | 82.36 (51.76, 133.01) | 81.28 (46.95, 139.32) | -0.04 (-0.19, | 0.617 | 1.44 (0.90, | 1.42 (0.82, | -0.04 (-0.19, | 0.637 |

| | | | | | | | | | | | | | | | |
|---|---|---|---|---|---|---|---|---|---|---|---|---|---|---|---|
| | | | | | | | -0.15) | | | | 0.11) | | 2.33) | 2.44) | 0.12) | |
| Southern Latin America | — | — | — | — | 1.06 (0.05, 5.08) | 4.15 (0.85, 9.95) | 4.80 (4.59, 5.02) | <0.001 | 22.18 (15.56, 28.26) | 13.05 (9.34, 17.61) | -1.78 (-2.12, -1.45) | <0.001 | 0.39 (0.27, 0.50) | 0.22 (0.16, 0.31) | -1.85 (-2.17, -1.53) | <0.001 |
| Oceania | — | — | — | — | 9.41 (4.49, 16.51) | 8.37 (4.00, 14.80) | -0.40 (-0.42, -0.39) | <0.001 | 127.68 (63.41, 233.85) | 116.08 (50.13, 223.65) | -0.37 (-0.57, -0.16) | <0.001 | 2.24 (1.11, 4.11) | 2.04 (0.87, 3.93) | -0.37 (-0.58, -0.16) | <0.001 |
| Australasia | — | — | — | — | 2.75 (1.41, 4.50) | 1.87 (0.93, 3.21) | -1.32 (-1.34, -1.29) | <0.001 | 3.21 (2.42, 4.02) | 1.92 (1.42, 2.67) | -1.72 (-2.02, -1.41) | <0.001 | 0.05 (0.04, 0.07) | 0.03 (0.02, 0.04) | -1.75 (-2.09, -1.42) | <0.001 |
| **Men** | | | | | | | | | | | | | | | | |
| Global | — | — | — | — | 16.42 (9.02, 27.01) | 16.56 (9.19, 27.21) | 0.04 (-0.02, 0.10) | 0.172 | 53.21 (40.87, 61.14) | 45.65 (31.97, 52.17) | -0.52 (-0.64, -0.41) | <0.001 | 0.91 (0.70, 1.06) | 0.78 (0.54, 0.90) | -0.54 (-0.66, -0.43) | <0.001 |
| **SDI category** | | | | | | | | | | | | | | | | |
| High | — | — | — | — | 45.09 (26.52, 71.10) | 52.44 (31.49, 82.40) | 0.54 (0.29, 0.79) | <0.001 | 33.30 (29.19, 38.80) | 45.38 (27.48, 55.52) | 1.12 (0.80, 1.43) | <0.001 | 0.52 (0.45, 0.60) | 0.72 (0.39, 0.89) | 1.17 (0.84, 1.50) | <0.001 |
| High-middle | — | — | — | — | 9.19 (4.85, 15.60) | 11.26 (5.92, 19.36) | 0.69 (0.48, 0.89) | <0.001 | 38.49 (32.16, 45.63) | 25.32 (21.80, 29.54) | -1.49 (-1.83, -1.15) | <0.001 | 0.67 (0.55, 0.79) | 0.43 (0.37, 0.50) | -1.55 (-1.91, -1.20) | <0.001 |
| Middle | — | — | — | — | 14.70 (7.52, 24.87) | 15.93 (8.28, 27.10) | 0.28 (0.14, 0.41) | <0.001 | 68.78 (47.42, 82.06) | 49.85 (31.34, 58.40) | -1.14 (-1.41, -0.87) | <0.001 | 1.19 (0.82, 1.42) | 0.85 (0.52, 1.01) | -1.18 (-1.46, -0.90) | <0.001 |
| Low-middle | — | — | — | — | 6.08 (3.10, 10.50) | 6.64 (3.39, 11.48) | 0.32 (0.15, 0.49) | <0.001 | 53.27 (37.93, 66.30) | 44.40 (34.36, 54.57) | -0.64 (-0.76, -0.53) | <0.001 | 0.93 (0.66, 1.16) | 0.77 (0.60, 0.96) | -0.66 (-0.78, -0.54) | <0.001 |
| Low | — | — | — | — | 7.66 (3.54, 13.80) | 8.96 (4.30, 15.99) | 0.55 (0.50, 0.60) | <0.001 | 79.02 (49.37, 108.45) | 73.12 (46.62, 105.33) | -0.28 (-0.37, -0.19) | <0.001 | 1.38 (0.86, 1.90) | 1.28 (0.81, 1.85) | -0.29 (-0.38, -0.20) | <0.001 |
| **21 GBD region** | | | | | | | | | | | | | | | | |
| South Asia | — | — | — | — | 2.28 (1.08, 4.05) | 2.34 (1.09, 4.20) | 0.10 (0.09, 0.11) | <0.001 | 33.38 (23.06, 49.02) | 29.18 (20.48, 40.83) | -0.49 (-0.63, | <0.001 | 0.59 (0.40, | 0.51 (0.36, | -0.49 (-0.63, | <0.001 |

| | | | | | | | | | | | | | | | | |
|---|---|---|---|---|---|---|---|---|---|---|---|---|---|---|---|---|
| | | | | | | | | | | | -0.35) | | 0.86) | 0.72) | -0.35) | |
| East Asia | — | — | — | — | 17.55 (8.99, 30.19) | 20.05 (10.43, 34.50) | 0.47 (0.37, 0.58) | <0.001 | 67.63 (42.62, 83.22) | 36.31 (20.01, 45.12) | -2.09 (-2.52, -1.65) | <0.001 | 1.17 (0.73, 1.44) | 0.61 (0.32, 0.77) | -2.20 (-2.69, -1.71) | <0.001 |
| North Africa and Middle East | — | — | — | — | 25.44 (13.98, 42.54) | 26.64 (14.37, 44.84) | 0.16 (0.12, 0.19) | <0.001 | 70.46 (39.41, 116.99) | 60.03 (34.25, 96.89) | -0.50 (-0.66, -0.33) | <0.001 | 1.20 (0.66, 2.03) | 1.02 (0.57, 1.65) | -0.51 (-0.69, -0.34) | <0.001 |
| Western Sub-Saharan Africa | — | — | — | — | 11.59 (5.26, 20.91) | 11.64 (5.30, 21.16) | 0.03 (-0.04, 0.10) | 0.428 | 59.01 (37.42, 84.40) | 77.41 (38.76, 115.53) | 0.95 (0.81, 1.10) | <0.001 | 1.02 (0.64, 1.47) | 1.35 (0.66, 2.02) | 0.97 (0.83, 1.11) | <0.001 |
| Southeast Asia | — | — | — | — | 15.00 (7.80, 25.16) | 16.11 (8.39, 27.03) | 0.25 (0.22, 0.28) | <0.001 | 75.54 (45.30, 101.11) | 71.12 (35.51, 87.46) | -0.19 (-0.37, -0.02) | 0.033 | 1.31 (0.77, 1.75) | 1.23 (0.60, 1.52) | -0.20 (-0.38, -0.02) | 0.029 |
| Eastern Sub-Saharan Africa | — | — | — | — | 11.41 (5.09, 20.90) | 11.91 (5.42, 21.69) | 0.14 (0.12, 0.16) | <0.001 | 129.86 (71.21, 194.01) | 89.41 (49.95, 149.69) | -1.27 (-1.48, -1.06) | <0.001 | 2.27 (1.24, 3.41) | 1.56 (0.86, 2.63) | -1.28 (-1.49, -1.08) | <0.001 |
| Central Latin America | — | — | — | — | 0.49 (0.00, 1.98) | 1.07 (0.09, 3.19) | 2.62 (2.31, 2.93) | <0.001 | 30.59 (23.79, 35.45) | 19.00 (14.87, 26.88) | -1.61 (-2.10, -1.13) | <0.001 | 0.54 (0.42, 0.62) | 0.33 (0.26, 0.47) | -1.62 (-2.11, -1.14) | <0.001 |
| Tropical Latin America | — | — | — | — | 0.13 (0.00, 1.00) | 0.79 (0.00, 3.27) | 6.42 (6.09, 6.75) | <0.001 | 78.30 (51.54, 88.88) | 39.64 (33.96, 57.34) | -2.32 (-2.60, -2.04) | <0.001 | 1.38 (0.91, 1.57) | 0.70 (0.60, 1.01) | -2.33 (-2.61, -2.05) | <0.001 |
| Central Sub-Saharan Africa | — | — | — | — | 5.84 (2.42, 11.53) | 5.87 (2.50, 11.35) | 0.02 (-0.03, 0.07) | 0.462 | 99.59 (41.07, 167.26) | 79.32 (41.21, 142.08) | -0.81 (-0.95, -0.66) | <0.001 | 1.75 (0.72, 2.95) | 1.39 (0.72, 2.50) | -0.81 (-0.96, -0.67) | <0.001 |
| Western Europe | — | — | — | — | 5.02 (2.68, 8.28) | 4.45 (2.38, 7.39) | -0.41 (-0.50, -0.32) | <0.001 | 12.17 (8.21, 14.33) | 8.12 (6.03, 9.88) | -1.42 (-1.77, -1.06) | <0.001 | 0.21 (0.14, 0.24) | 0.14 (0.10, 0.17) | -1.46 (-1.82, -1.10) | <0.001 |
| High-income North America | — | — | — | — | 109.28 (64.25, 172.04) | 140.41 (84.27, 218.65) | 0.91 (0.58, 1.24) | <0.001 | 53.22 (46.25, 74.20) | 104.13 (55.25, 122.13) | 2.43 (2.07, 2.79) | <0.001 | 0.77 (0.69, 1.15) | 1.62 (0.75, 1.92) | 2.67 (2.38, 2.96) | <0.001 |
| Central Asia | — | — | — | — | 9.29 (4.44, 16.37) | 10.57 (4.97, 18.41) | 0.44 (0.38, 0.51) | <0.001 | 49.72 (37.00, 64.37) | 46.72 (34.40, 62.29) | -0.21 (-0.64, | 0.316 | 0.86 (0.64, | 0.81 (0.59, | -0.23 (-0.65, | 0.295 |

| | | | | | | | | | | | | | | | | |
|---|---|---|---|---|---|---|---|---|---|---|---|---|---|---|---|---|
| | | | | | | | | | | | 0.21) | | 1.12) | 1.09) | 0.20) | |
| Eastern Europe | — | — | — | — | 0.80 (0.37, 1.41) | 0.79 (0.37, 1.41) | -0.01 (-0.07, 0.06) | 0.793 | 25.51 (18.47, 30.21) | 34.91 (27.96, 47.27) | 1.22 (0.06, 2.39) | 0.039 | 0.45 (0.32, 0.53) | 0.62 (0.49, 0.83) | 1.22 (0.06, 2.40) | 0.040 |
| Southern Sub-Saharan Africa | — | — | — | — | 4.20 (1.86, 7.95) | 3.89 (1.76, 7.45) | -0.28 (-0.32, -0.23) | <0.001 | 120.69 (87.62, 162.40) | 82.04 (59.72, 108.95) | -1.48 (-2.84, -0.09) | 0.037 | 2.12 (1.54, 2.86) | 1.44 (1.04, 1.91) | -1.48 (-2.85, -0.09) | 0.037 |
| High-income Asia Pacific | — | — | — | — | 14.11 (7.69, 23.25) | 12.36 (6.82, 20.05) | -0.46 (-0.54, -0.39) | <0.001 | 34.03 (16.31, 43.93) | 7.86 (6.02, 13.03) | -4.97 (-5.43, -4.52) | <0.001 | 0.58 (0.26, 0.75) | 0.12 (0.09, 0.21) | -5.40 (-5.84, -4.97) | <0.001 |
| Central Europe | — | — | — | — | 5.80 (2.92, 9.89) | 5.39 (2.88, 8.82) | -0.25 (-0.28, -0.22) | <0.001 | 29.83 (25.33, 35.41) | 29.72 (19.87, 37.10) | -0.07 (-0.34, 0.20) | 0.618 | 0.52 (0.44, 0.61) | 0.52 (0.34, 0.64) | -0.06 (-0.34, 0.21) | 0.647 |
| Andean Latin America | — | — | — | — | 0.04 (0.00, 0.57) | 1.63 (0.00, 7.57) | 13.16 (12.55, 13.78) | <0.001 | 49.96 (35.66, 66.48) | 39.58 (26.99, 56.94) | -0.92 (-1.30, -0.55) | <0.001 | 0.88 (0.63, 1.17) | 0.70 (0.47, 1.00) | -0.94 (-1.31, -0.56) | <0.001 |
| Caribbean | — | — | — | — | 8.48 (3.86, 14.87) | 8.38 (4.02, 14.64) | -0.04 (-0.06, -0.01) | 0.001 | 65.44 (50.74, 85.42) | 68.75 (46.26, 100.89) | 0.20 (0.02, 0.37) | 0.027 | 1.14 (0.88, 1.49) | 1.20 (0.80, 1.76) | 0.20 (0.02, 0.37) | 0.026 |
| Southern Latin America | — | — | — | — | 0.18 (0.00, 0.63) | 3.05 (0.50, 9.20) | 10.26 (9.61, 10.91) | <0.001 | 27.45 (19.87, 35.99) | 20.97 (15.31, 29.30) | -0.92 (-1.35, -0.49) | <0.001 | 0.48 (0.35, 0.64) | 0.37 (0.26, 0.51) | -0.97 (-1.40, -0.54) | <0.001 |
| Oceania | — | — | — | — | 11.75 (5.83, 20.35) | 11.65 (5.82, 20.08) | -0.03 (-0.04, -0.01) | <0.001 | 98.39 (51.40, 177.83) | 106.66 (49.88, 201.83) | 0.26 (0.15, 0.36) | <0.001 | 1.72 (0.88, 3.12) | 1.86 (0.86, 3.54) | 0.26 (0.15, 0.37) | <0.001 |
| Australasia | — | — | — | — | 3.41 (1.90, 5.44) | 2.49 (1.33, 4.06) | -1.08 (-1.11, -1.05) | <0.001 | 6.53 (4.61, 8.50) | 4.87 (3.25, 7.11) | -1.02 (-1.52, -0.52) | <0.001 | 0.11 (0.08, 0.14) | 0.08 (0.05, 0.12) | -1.01 (-1.54, -0.48) | <0.001 |
| **35-39 years** | | | | | | | | | | | | | | | | |
| **Women** | | | | | | | | | | | | | | | | |
| Global | — | — | — | — | 23.53 (11.86, 41.95) | 22.04 (11.09, 38.74) | -0.20 (-0.29, -0.10) | <0.001 | 94.13 (62.91, 124.04) | 64.29 (48.84, 77.57) | -1.32 (-1.47, -1.17) | <0.001 | 1.78 (1.16, 2.37) | 1.21 (0.91, 1.45) | -1.35 (-1.50, -1.20) | <0.001 |

| | | | | | | | | | | | | | | | | |
|---|---|---|---|---|---|---|---|---|---|---|---|---|---|---|---|---|
| **SDI category** | | | | | | | | | | | | | | | | |
| High | — | — | — | — | 55.16 (29.28, 95.64) | 48.36 (27.32, 81.54) | -0.40 (-0.83, 0.04) | 0.076 | 29.13 (24.74, 34.99) | 36.93 (22.35, 42.74) | 0.79 (0.50, 1.08) | <0.001 | 0.47 (0.40, 0.56) | 0.63 (0.35, 0.72) | 1.02 (0.72, 1.33) | <0.001 |
| High-middle | — | — | — | — | 14.71 (7.19, 26.98) | 17.13 (8.49, 30.95) | 0.48 (0.31, 0.65) | <0.001 | 45.60 (36.62, 55.21) | 22.13 (18.75, 26.57) | -2.50 (-2.78, -2.23) | <0.001 | 0.86 (0.69, 1.03) | 0.40 (0.34, 0.48) | -2.65 (-2.94, -2.36) | <0.001 |
| Middle | — | — | — | — | 22.92 (10.84, 42.00) | 24.42 (11.85, 43.57) | 0.20 (0.11, 0.29) | <0.001 | 116.98 (80.36, 147.46) | 56.20 (40.79, 66.52) | -2.56 (-2.89, -2.22) | <0.001 | 2.22 (1.50, 2.81) | 1.05 (0.76, 1.25) | -2.63 (-2.98, -2.28) | <0.001 |
| Low-middle | — | — | — | — | 9.80 (4.43, 18.86) | 11.17 (5.10, 21.32) | 0.46 (0.40, 0.52) | <0.001 | 117.68 (58.84, 176.58) | 79.74 (54.84, 105.16) | -1.33 (-1.59, -1.08) | <0.001 | 2.26 (1.12, 3.40) | 1.52 (1.03, 2.01) | -1.35 (-1.61, -1.09) | <0.001 |
| Low | — | — | — | — | 13.68 (5.77, 26.74) | 15.24 (6.47, 29.74) | 0.37 (0.36, 0.38) | <0.001 | 246.67 (114.97, 387.07) | 159.80 (93.24, 216.38) | -1.48 (-1.59, -1.38) | <0.001 | 4.75 (2.20, 7.46) | 3.07 (1.77, 4.15) | -1.49 (-1.60, -1.39) | <0.001 |
| **21 GBD region** | | | | | | | | | | | | | | | | |
| South Asia | — | — | — | — | 5.08 (2.22, 9.89) | 5.24 (2.34, 10.05) | 0.11 (0.09, 0.13) | <0.001 | 80.10 (30.15, 138.08) | 51.73 (30.76, 76.84) | -1.62 (-2.11, -1.12) | <0.001 | 1.54 (0.58, 2.66) | 0.99 (0.59, 1.47) | -1.62 (-2.12, -1.12) | <0.001 |
| East Asia | — | — | — | — | 26.09 (12.69, 47.70) | 30.81 (15.03, 55.64) | 0.55 (0.46, 0.65) | <0.001 | 95.20 (66.72, 124.90) | 26.49 (17.80, 35.47) | -4.36 (-4.66, -4.05) | <0.001 | 1.80 (1.25, 2.36) | 0.46 (0.30, 0.62) | -4.64 (-4.96, -4.32) | <0.001 |
| North Africa and Middle East | — | — | — | — | 39.75 (19.28, 72.68) | 40.81 (20.05, 74.27) | 0.09 (0.08, 0.10) | <0.001 | 155.08 (71.30, 229.60) | 90.91 (55.19, 124.13) | -1.83 (-1.98, -1.69) | <0.001 | 2.93 (1.30, 4.35) | 1.69 (0.99, 2.33) | -1.89 (-2.04, -1.74) | <0.001 |
| Western Sub-Saharan Africa | — | — | — | — | 20.19 (8.98, 37.99) | 21.23 (9.15, 39.79) | 0.17 (0.16, 0.19) | <0.001 | 222.60 (131.68, 307.92) | 194.43 (104.32, 268.63) | -0.46 (-0.57, -0.34) | <0.001 | 4.27 (2.52, 5.93) | 3.72 (1.97, 5.16) | -0.46 (-0.57, -0.35) | <0.001 |
| Southeast Asia | — | — | — | — | 22.82 (10.85, 41.39) | 23.88 (11.46, 42.99) | 0.15 (0.11, 0.19) | <0.001 | 131.79 (69.64, 190.66) | 89.15 (51.76, 114.63) | -1.33 (-1.49, -1.17) | <0.001 | 2.51 (1.31, 3.66) | 1.68 (0.97, 2.18) | -1.35 (-1.52, -1.19) | <0.001 |
| Eastern Sub-Saharan | — | — | — | — | 19.59 (8.13, | 20.36 (8.42, | 0.13 (0.12, | <0.001 | 394.25 | 171.71 (95.93, | -2.82 | <0.001 | 7.59 | 3.29 | -2.84 | <0.001 |

| | | | | | | | | | | | | | | | | |
|---|---|---|---|---|---|---|---|---|---|---|---|---|---|---|---|---|
| Africa | | | | | 38.31) | 39.58) | 0.14) | | (183.64, 653.36) | 242.39) | (-2.92, -2.72) | | (3.52, 12.58) | (1.81, 4.65) | (-2.94, -2.74) | |
| Central Latin America | — | — | — | — | 6.02 (1.14, 14.71) | 8.13 (2.10, 18.07) | 1.04 (0.90, 1.19) | <0.001 | 55.59 (36.16, 63.00) | 25.78 (19.12, 38.12) | -2.62 (-3.06, -2.18) | <0.001 | 1.07 (0.69, 1.21) | 0.48 (0.36, 0.73) | -2.68 (-3.14, -2.23) | <0.001 |
| Tropical Latin America | — | — | — | — | 5.84 (0.36, 15.66) | 9.37 (2.17, 21.10) | 1.64 (1.58, 1.71) | <0.001 | 125.05 (80.82, 138.14) | 59.24 (49.74, 86.26) | -2.50 (-2.77, -2.24) | <0.001 | 2.41 (1.56, 2.66) | 1.13 (0.95, 1.65) | -2.54 (-2.81, -2.27) | <0.001 |
| Central Sub-Saharan Africa | — | — | — | — | 12.73 (4.75, 26.87) | 12.86 (4.75, 27.23) | 0.04 (0.03, 0.04) | <0.001 | 278.29 (127.81, 448.56) | 193.27 (97.03, 309.77) | -1.33 (-1.41, -1.25) | <0.001 | 5.36 (2.45, 8.66) | 3.72 (1.86, 5.98) | -1.33 (-1.41, -1.25) | <0.001 |
| Western Europe | — | — | — | — | 5.98 (2.77, 11.07) | 4.30 (1.94, 8.16) | -1.14 (-1.24, -1.03) | <0.001 | 8.48 (6.33, 10.00) | 5.46 (4.17, 6.53) | -1.49 (-1.64, -1.33) | <0.001 | 0.15 (0.11, 0.18) | 0.10 (0.07, 0.12) | -1.52 (-1.64, -1.40) | <0.001 |
| High-income North America | — | — | — | — | 131.86 (71.30, 227.77) | 117.43 (64.72, 198.64) | -0.33 (-0.62, -0.04) | 0.024 | 49.58 (41.47, 63.94) | 82.24 (44.12, 95.32) | 1.72 (1.50, 1.94) | <0.001 | 0.74 (0.67, 1.01) | 1.40 (0.67, 1.58) | 2.18 (1.87, 2.49) | <0.001 |
| Central Asia | — | — | — | — | 7.75 (2.93, 15.70) | 8.07 (2.93, 16.51) | 0.14 (0.08, 0.20) | <0.001 | 49.57 (36.13, 59.10) | 42.94 (32.45, 55.16) | -0.42 (-1.02, 0.18) | 0.167 | 0.95 (0.68, 1.13) | 0.82 (0.61, 1.05) | -0.44 (-1.04, 0.17) | 0.157 |
| Eastern Europe | — | — | — | — | 1.04 (0.38, 2.13) | 1.10 (0.42, 2.24) | 0.21 (0.14, 0.28) | <0.001 | 13.69 (8.43, 15.90) | 17.34 (12.47, 22.72) | 0.74 (-0.43, 1.93) | 0.215 | 0.26 (0.16, 0.31) | 0.33 (0.24, 0.44) | 0.74 (-0.44, 1.94) | 0.220 |
| Southern Sub-Saharan Africa | — | — | — | — | 10.49 (4.40, 21.23) | 9.42 (3.88, 19.74) | -0.36 (-0.44, -0.29) | <0.001 | 342.97 (243.74, 429.35) | 131.69 (87.87, 192.55) | -3.09 (-4.71, -1.44) | <0.001 | 6.60 (4.69, 8.27) | 2.53 (1.68, 3.70) | -3.10 (-4.73, -1.44) | <0.001 |
| High-income Asia Pacific | — | — | — | — | 17.06 (7.57, 31.25) | 13.77 (6.91, 24.44) | -0.74 (-0.87, -0.62) | <0.001 | 23.96 (15.16, 29.20) | 5.67 (4.37, 8.95) | -4.91 (-5.18, -4.63) | <0.001 | 0.43 (0.27, 0.53) | 0.09 (0.07, 0.15) | -5.54 (-5.89, -5.18) | <0.001 |
| Central Europe | — | — | — | — | 7.00 (2.56, 14.27) | 5.89 (2.54, 11.22) | -0.59 (-0.63, -0.55) | <0.001 | 31.08 (25.73, 36.58) | 22.69 (15.42, 29.24) | -1.10 (-1.31, -0.89) | <0.001 | 0.59 (0.49, 0.69) | 0.43 (0.29, 0.56) | -1.11 (-1.32, -0.89) | <0.001 |
| Andean Latin | — | — | — | — | 1.84 (0.00, | 9.15 (1.15, | 5.67 (5.40, | <0.001 | 85.02 (60.48, | 40.18 (26.59, | -2.54 | <0.001 | 1.64 | 0.76 | -2.61 | <0.001 |

| | | | | | | | | | | | | | | | | |
|---|---|---|---|---|---|---|---|---|---|---|---|---|---|---|---|---|
| America | | | | | 9.34) | 19.94) | 5.95) | | 115.03) | 57.28) | (-3.22, -1.87) | | (1.16, 2.22) | (0.50, 1.09) | (-3.28, -1.93) | |
| Caribbean | — | — | — | — | 17.49 (7.75, 32.78) | 16.98 (7.43, 31.68) | -0.12 (-0.16, -0.07) | <0.001 | 148.92 (92.03, 235.42) | 150.08 (90.15, 248.26) | 0.05 (-0.21, 0.31) | 0.689 | 2.85 (1.75, 4.52) | 2.87 (1.73, 4.78) | 0.06 (-0.21, 0.32) | 0.680 |
| Southern Latin America | — | — | — | — | 1.87 (0.12, 8.02) | 6.78 (1.26, 17.33) | 4.52 (4.21, 4.83) | <0.001 | 34.80 (23.69, 44.57) | 19.05 (13.91, 27.66) | -2.09 (-2.49, -1.69) | <0.001 | 0.67 (0.45, 0.86) | 0.36 (0.26, 0.52) | -2.10 (-2.42, -1.78) | <0.001 |
| Oceania | — | — | — | — | 19.47 (8.49, 36.71) | 17.75 (7.88, 33.99) | -0.33 (-0.37, -0.29) | <0.001 | 214.29 (107.42, 373.49) | 201.53 (91.39, 381.18) | -0.23 (-0.34, -0.11) | <0.001 | 4.11 (2.05, 7.20) | 3.87 (1.73, 7.34) | -0.23 (-0.34, -0.11) | <0.001 |
| Australasia | — | — | — | — | 7.24 (3.48, 13.42) | 4.91 (2.29, 9.03) | -1.31 (-1.35, -1.28) | <0.001 | 7.95 (5.70, 10.12) | 5.23 (3.69, 7.08) | -1.46 (-1.82, -1.10) | <0.001 | 0.14 (0.10, 0.18) | 0.09 (0.06, 0.13) | -1.47 (-1.86, -1.08) | <0.001 |
| **Men** | | | | | | | | | | | | | | | | |
| Global | — | — | — | — | 30.07 (15.30, 52.77) | 28.82 (15.10, 50.20) | -0.12 (-0.21, -0.04) | 0.006 | 100.20 (73.69, 116.20) | 80.92 (56.07, 92.39) | -0.70 (-0.94, -0.46) | <0.001 | 1.89 (1.36, 2.19) | 1.52 (1.05, 1.73) | -0.73 (-0.96, -0.50) | <0.001 |
| **SDI category** | | | | | | | | | | | | | | | | |
| High | — | — | — | — | 70.06 (38.10, 119.09) | 64.88 (36.83, 109.11) | -0.22 (-0.49, 0.05) | 0.104 | 57.95 (49.99, 67.29) | 77.44 (45.60, 95.20) | 1.01 (0.72, 1.30) | <0.001 | 1.00 (0.86, 1.17) | 1.39 (0.78, 1.73) | 1.12 (0.87, 1.38) | <0.001 |
| High-middle | — | — | — | — | 18.07 (8.98, 33.39) | 20.44 (10.34, 37.28) | 0.37 (0.17, 0.57) | <0.001 | 74.40 (60.04, 88.81) | 45.48 (37.96, 52.79) | -1.69 (-2.01, -1.38) | <0.001 | 1.41 (1.13, 1.69) | 0.84 (0.71, 0.98) | -1.76 (-2.07, -1.44) | <0.001 |
| Middle | — | — | — | — | 29.70 (14.52, 53.08) | 31.53 (15.70, 56.60) | 0.19 (0.07, 0.30) | 0.001 | 131.73 (86.46, 159.04) | 88.15 (54.79, 102.58) | -1.38 (-1.62, -1.14) | <0.001 | 2.50 (1.62, 3.02) | 1.65 (1.01, 1.93) | -1.42 (-1.68, -1.17) | <0.001 |
| Low-middle | — | — | — | — | 13.00 (6.22, 24.00) | 14.24 (6.80, 26.22) | 0.31 (0.26, 0.36) | <0.001 | 98.24 (69.78, 125.39) | 79.95 (61.77, 99.18) | -0.68 (-0.91, -0.46) | <0.001 | 1.88 (1.34, 2.40) | 1.52 (1.18, 1.89) | -0.70 (-0.93, -0.47) | <0.001 |
| Low | — | — | — | — | 18.28 (8.08, 34.68) | 20.86 (9.38, 39.86) | 0.46 (0.43, 0.50) | <0.001 | 153.26 (92.24, | 131.82 (81.43, 189.03) | -0.53 (-0.65, | <0.001 | 2.93 (1.77, | 2.51 (1.53, | -0.54 (-0.66, | <0.001 |

| | | | | | | | | | | | | | | | | |
|---|---|---|---|---|---|---|---|---|---|---|---|---|---|---|---|---|
| | | | | | | | | | 209.59) | | -0.41) | | 4.03) | 3.62) | -0.42) | |
| **21 GBD region** | | | | | | | | | | | | | | | | |
| South Asia | — | — | — | — | 5.68 (2.52, 10.89) | 5.85 (2.60, 11.26) | 0.10 (0.09, 0.12) | <0.001 | 62.96 (43.85, 91.82) | 53.96 (37.36, 74.29) | -0.42 (-1.09, 0.26) | 0.226 | 1.21 (0.84, 1.77) | 1.03 (0.71, 1.42) | -0.42 (-1.10, 0.26) | 0.226 |
| East Asia | — | — | — | — | 32.81 (16.01, 58.82) | 38.37 (19.35, 68.97) | 0.53 (0.38, 0.68) | <0.001 | 135.70 (84.64, 170.57) | 64.86 (36.53, 81.94) | -2.48 (-3.00, -1.96) | <0.001 | 2.57 (1.60, 3.25) | 1.19 (0.64, 1.51) | -2.59 (-3.14, -2.03) | <0.001 |
| North Africa and Middle East | — | — | — | — | 48.24 (24.14, 87.82) | 48.62 (24.47, 86.76) | 0.02 (-0.02, 0.06) | 0.347 | 132.95 (69.76, 227.02) | 104.31 (62.03, 165.25) | -0.84 (-0.97, -0.71) | <0.001 | 2.49 (1.27, 4.31) | 1.93 (1.12, 3.13) | -0.87 (-0.97, -0.77) | <0.001 |
| Western Sub-Saharan Africa | — | — | — | — | 26.86 (11.98, 49.39) | 27.44 (12.21, 51.12) | 0.10 (0.01, 0.18) | 0.023 | 97.13 (64.38, 139.37) | 121.80 (63.10, 172.86) | 0.80 (0.56, 1.04) | <0.001 | 1.83 (1.20, 2.64) | 2.31 (1.16, 3.30) | 0.81 (0.57, 1.06) | <0.001 |
| Southeast Asia | — | — | — | — | 34.09 (16.66, 62.21) | 36.23 (17.88, 65.13) | 0.21 (0.19, 0.23) | <0.001 | 126.82 (75.86, 165.58) | 126.29 (60.40, 158.57) | -0.00 (-0.20, 0.20) | 0.985 | 2.40 (1.42, 3.17) | 2.38 (1.11, 3.01) | -0.01 (-0.21, 0.19) | 0.942 |
| Eastern Sub-Saharan Africa | — | — | — | — | 29.34 (12.77, 54.91) | 31.09 (13.73, 57.72) | 0.20 (0.18, 0.21) | <0.001 | 263.82 (138.36, 383.64) | 166.45 (95.43, 287.39) | -1.58 (-1.69, -1.47) | <0.001 | 5.05 (2.64, 7.37) | 3.17 (1.81, 5.53) | -1.60 (-1.72, -1.49) | <0.001 |
| Central Latin America | — | — | — | — | 1.50 (0.00, 4.79) | 2.71 (0.49, 7.21) | 2.04 (1.94, 2.14) | <0.001 | 46.72 (36.25, 54.18) | 30.75 (23.65, 43.20) | -1.41 (-1.65, -1.18) | <0.001 | 0.90 (0.70, 1.04) | 0.59 (0.45, 0.83) | -1.43 (-1.67, -1.19) | <0.001 |
| Tropical Latin America | — | — | — | — | 0.56 (0.00, 3.40) | 2.60 (0.00, 8.43) | 5.38 (5.03, 5.72) | <0.001 | 139.14 (91.90, 156.61) | 68.51 (58.80, 102.48) | -2.40 (-2.64, -2.17) | <0.001 | 2.69 (1.78, 3.03) | 1.32 (1.13, 1.97) | -2.41 (-2.65, -2.18) | <0.001 |
| Central Sub-Saharan Africa | — | — | — | — | 16.01 (6.39, 34.18) | 15.86 (6.28, 34.17) | -0.03 (-0.04, -0.02) | <0.001 | 206.01 (89.06, 340.44) | 158.71 (84.80, 276.77) | -0.93 (-1.08, -0.79) | <0.001 | 3.96 (1.70, 6.56) | 3.04 (1.62, 5.33) | -0.94 (-1.08, -0.79) | <0.001 |
| Western Europe | — | — | — | — | 8.92 (4.55, 15.77) | 7.20 (3.56, 12.73) | -0.74 (-0.78, -0.70) | <0.001 | 21.38 (14.85, 24.98) | 13.77 (10.63, 17.09) | -1.52 (-1.86, -1.19) | <0.001 | 0.40 (0.27, 0.47) | 0.25 (0.19, 0.31) | -1.56 (-1.91, -1.21) | <0.001 |
| High-income North | — | — | — | — | 168.10 (91.80, | 163.15 (90.33, | -0.04 | 0.799 | 99.89 (87.92, | 177.05 (88.81, | 2.00 | <0.001 | 1.65 | 3.15 | 2.29 (1.89, | <0.001 |

| | | | | | | | | | | | | | | | | |
|---|---|---|---|---|---|---|---|---|---|---|---|---|---|---|---|---|
| America | | | | | 287.59) | 274.58) | (-0.34, 0.26) | | 132.12) | 206.03) | (1.71, 2.29) | | (1.49, 2.24) | (1.44, 3.63) | 2.69) | |
| Central Asia | — | — | — | — | 19.98 (8.69, 37.58) | 22.92 (9.76, 42.91) | 0.48 (0.42, 0.54) | <0.001 | 100.90 (70.24, 131.39) | 94.55 (71.62, 122.22) | -0.17 (-0.55, 0.20) | 0.357 | 1.92 (1.33, 2.50) | 1.79 (1.35, 2.31) | -0.19 (-0.57, 0.19) | 0.327 |
| Eastern Europe | — | — | — | — | 2.25 (0.98, 4.37) | 2.23 (0.96, 4.36) | -0.04 (-0.06, -0.02) | 0.001 | 48.32 (30.81, 57.85) | 57.98 (44.20, 75.41) | 0.65 (-0.68, 1.99) | 0.342 | 0.93 (0.59, 1.12) | 1.12 (0.85, 1.46) | 0.65 (-0.68, 2.00) | 0.341 |
| Southern Sub-Saharan Africa | — | — | — | — | 11.85 (4.91, 24.04) | 11.09 (4.56, 22.82) | -0.23 (-0.28, -0.18) | <0.001 | 252.11 (196.63, 318.60) | 158.35 (116.71, 212.15) | -1.58 (-2.46, -0.69) | <0.001 | 4.85 (3.77, 6.14) | 3.04 (2.24, 4.09) | -1.59 (-2.47, -0.70) | 0.001 |
| High-income Asia Pacific | — | — | — | — | 27.67 (14.16, 48.73) | 26.09 (13.90, 44.24) | -0.20 (-0.26, -0.14) | <0.001 | 45.42 (26.76, 56.24) | 15.12 (11.48, 25.67) | -3.71 (-3.89, -3.52) | <0.001 | 0.83 (0.47, 1.03) | 0.25 (0.18, 0.46) | -4.08 (-4.29, -3.87) | <0.001 |
| Central Europe | — | — | — | — | 13.12 (6.05, 24.95) | 11.73 (5.92, 21.01) | -0.38 (-0.40, -0.36) | <0.001 | 63.79 (54.99, 76.30) | 61.65 (39.27, 77.15) | -0.15 (-0.60, 0.31) | 0.528 | 1.21 (1.05, 1.47) | 1.17 (0.74, 1.47) | -0.14 (-0.60, 0.32) | 0.549 |
| Andean Latin America | — | — | — | — | 0.15 (0.00, 2.10) | 4.00 (0.00, 13.55) | 11.88 (11.32, 12.44) | <0.001 | 66.55 (47.53, 89.75) | 50.39 (33.34, 72.49) | -0.92 (-1.27, -0.57) | <0.001 | 1.29 (0.92, 1.74) | 0.97 (0.64, 1.39) | -0.95 (-1.30, -0.59) | <0.001 |
| Caribbean | — | — | — | — | 17.23 (7.48, 31.82) | 17.39 (7.67, 32.40) | 0.04 (-0.01, 0.08) | 0.137 | 120.68 (91.86, 166.32) | 138.14 (96.40, 205.23) | 0.47 (0.19, 0.76) | 0.001 | 2.30 (1.74, 3.20) | 2.64 (1.84, 3.93) | 0.48 (0.19, 0.77) | 0.001 |
| Southern Latin America | — | — | — | — | 0.48 (0.00, 1.58) | 4.83 (0.28, 15.82) | 8.29 (7.60, 8.98) | <0.001 | 53.06 (38.81, 68.14) | 34.92 (25.54, 49.02) | -1.40 (-1.82, -0.98) | <0.001 | 1.03 (0.75, 1.32) | 0.67 (0.49, 0.94) | -1.45 (-1.86, -1.03) | <0.001 |
| Oceania | — | — | — | — | 26.76 (12.77, 47.96) | 26.97 (12.77, 48.64) | 0.02 (0.01, 0.04) | <0.001 | 293.64 (154.93, 496.97) | 298.68 (146.48, 520.93) | 0.04 (-0.06, 0.13) | 0.448 | 5.64 (2.97, 9.58) | 5.73 (2.77, 10.06) | 0.04 (-0.06, 0.13) | 0.453 |
| Australasia | — | — | — | — | 8.70 (4.36, 15.55) | 6.38 (3.12, 11.47) | -1.06 (-1.10, -1.02) | <0.001 | 13.47 (9.66, 17.90) | 10.57 (7.15, 15.13) | -0.77 (-1.16, -0.37) | <0.001 | 0.24 (0.17, 0.33) | 0.19 (0.13, 0.28) | -0.74 (-1.16, -0.32) | 0.001 |
| **Non-rheumatic** | | | | | | | | | | | | | | | | |

| | | | | | | | | | | | | | | | | |
|---|---|---|---|---|---|---|---|---|---|---|---|---|---|---|---|---|
| **valvular heart disease** | | | | | | | | | | | | | | | | |
| **15-19 years** | | | | | | | | | | | | | | | | |
| **Women** | | | | | | | | | | | | | | | | |
| Global | 1.45 (1.35, 1.57) | 1.19 (1.11, 1.28) | -0.70 (-0.74, -0.65) | <0.001 | 1.41 (1.19, 1.65) | 1.15 (0.97, 1.34) | -0.70 (-0.85, -0.56) | <0.001 | 8.00 (4.88, 11.63) | 7.12 (4.98, 9.51) | -0.38 (-0.48, -0.29) | <0.001 | 0.11 (0.07, 0.16) | 0.10 (0.07, 0.13) | -0.38 (-0.48, -0.29) | <0.001 |
| **SDI category** | | | | | | | | | | | | | | | | |
| High | 7.54 (7.08, 8.02) | 8.43 (7.96, 8.94) | 0.36 (0.22, 0.49) | <0.001 | 10.55 (8.81, 12.54) | 11.45 (9.44, 13.40) | 0.25 (0.02, 0.47) | 0.030 | 4.33 (3.92, 4.78) | 2.99 (2.55, 3.47) | -1.14 (-1.46, -0.81) | <0.001 | 0.06 (0.05, 0.07) | 0.04 (0.04, 0.05) | -1.14 (-1.46, -0.81) | <0.001 |
| High-middle | 1.89 (1.73, 2.07) | 1.75 (1.59, 1.92) | -0.26 (-0.32, -0.20) | <0.001 | 0.88 (0.62, 1.19) | 0.71 (0.51, 0.93) | -0.73 (-1.14, -0.33) | <0.001 | 5.37 (4.16, 6.52) | 3.95 (3.45, 4.52) | -1.06 (-1.35, -0.76) | <0.001 | 0.08 (0.06, 0.09) | 0.06 (0.05, 0.06) | -1.06 (-1.35, -0.76) | <0.001 |
| Middle | 0.40 (0.35, 0.46) | 0.37 (0.31, 0.43) | -0.31 (-0.36, -0.26) | <0.001 | — | — | — | — | 8.16 (5.45, 11.09) | 6.15 (4.90, 7.57) | -0.98 (-1.19, -0.78) | <0.001 | 0.11 (0.08, 0.16) | 0.09 (0.07, 0.11) | -0.99 (-1.19, -0.78) | <0.001 |
| Low-middle | 0.20 (0.16, 0.23) | 0.18 (0.15, 0.22) | -0.31 (-0.44, -0.18) | <0.001 | — | — | — | — | 9.34 (4.08, 16.59) | 7.98 (4.89, 11.77) | -0.57 (-0.71, -0.42) | <0.001 | 0.13 (0.06, 0.23) | 0.11 (0.07, 0.16) | -0.57 (-0.71, -0.42) | <0.001 |
| Low | 0.12 (0.09, 0.16) | 0.13 (0.10, 0.16) | 0.09 (0.05, 0.14) | <0.001 | — | — | — | — | 13.61 (5.57, 25.25) | 11.25 (6.15, 17.69) | -0.64 (-0.77, -0.52) | <0.001 | 0.19 (0.08, 0.35) | 0.16 (0.09, 0.25) | -0.65 (-0.77, -0.52) | <0.001 |
| **21 GBD region** | | | | | | | | | | | | | | | | |
| South Asia | 0.08 (0.07, 0.10) | 0.11 (0.09, 0.13) | 0.74 (0.59, 0.88) | <0.001 | — | — | — | — | 8.14 (2.26, 16.37) | 5.99 (2.88, 9.76) | -1.08 (-1.42, -0.74) | <0.001 | 0.11 (0.03, 0.23) | 0.08 (0.04, 0.14) | -1.08 (-1.42, -0.75) | <0.001 |
| East Asia | 0.74 (0.67, 0.81) | 0.83 (0.76, 0.91) | 0.41 (0.27, 0.55) | <0.001 | 0.08 (0.00, 0.18) | 0.13 (0.00, 0.30) | 1.84 (1.15, 2.53) | <0.001 | 3.36 (1.96, 4.76) | 1.45 (1.17, 1.75) | -2.96 (-3.07, -2.84) | <0.001 | 0.05 (0.03, 0.07) | 0.02 (0.02, 0.02) | -2.96 (-3.08, -2.85) | <0.001 |
| North Africa and Middle East | 0.51 (0.38, 0.66) | 0.52 (0.39, 0.68) | 0.09 (0.03, 0.14) | 0.002 | — | — | — | — | 24.55 (14.79, 35.47) | 15.44 (11.19, 21.09) | -1.54 (-1.84, -1.24) | <0.001 | 0.34 (0.21, 0.50) | 0.22 (0.16, 0.29) | -1.54 (-1.84, -1.24) | <0.001 |

| | | | | | | | | | | | | | | | | |
|---|---|---|---|---|---|---|---|---|---|---|---|---|---|---|---|---|
| Western Sub-Saharan Africa | 0.12 (0.10, 0.15) | 0.13 (0.10, 0.16) | 0.22 (0.14, 0.31) | <0.001 | — | — | — | — | 22.29 (8.85, 43.14) | 20.60 (11.19, 33.23) | -0.28 (-0.39, -0.17) | <0.001 | 0.31 (0.12, 0.60) | 0.29 (0.16, 0.46) | -0.28 (-0.39, -0.17) | <0.001 |
| Southeast Asia | 0.23 (0.18, 0.29) | 0.27 (0.22, 0.34) | 0.53 (0.50, 0.56) | <0.001 | — | — | — | — | 2.87 (1.56, 4.57) | 2.16 (1.70, 2.65) | -0.95 (-1.05, -0.84) | <0.001 | 0.04 (0.02, 0.06) | 0.03 (0.02, 0.04) | -0.95 (-1.06, -0.84) | <0.001 |
| Eastern Sub-Saharan Africa | 0.12 (0.09, 0.15) | 0.11 (0.08, 0.14) | -0.33 (-0.49, -0.17) | <0.001 | — | — | — | — | 14.40 (6.48, 25.78) | 8.23 (5.02, 12.29) | -1.91 (-2.12, -1.71) | <0.001 | 0.20 (0.09, 0.36) | 0.11 (0.07, 0.17) | -1.92 (-2.12, -1.72) | <0.001 |
| Central Latin America | 0.18 (0.15, 0.22) | 0.23 (0.18, 0.28) | 0.79 (0.73, 0.85) | <0.001 | — | — | — | — | 6.61 (5.73, 7.52) | 5.48 (4.42, 6.75) | -0.58 (-1.31, 0.16) | 0.124 | 0.09 (0.08, 0.11) | 0.08 (0.06, 0.09) | -0.58 (-1.31, 0.16) | 0.125 |
| Tropical Latin America | 0.40 (0.35, 0.44) | 0.38 (0.33, 0.42) | -0.25 (-0.46, -0.03) | 0.026 | — | — | — | — | 16.13 (14.51, 17.86) | 8.37 (7.43, 9.39) | -2.19 (-2.67, -1.71) | <0.001 | 0.23 (0.20, 0.25) | 0.12 (0.10, 0.13) | -2.19 (-2.67, -1.71) | <0.001 |
| Central Sub-Saharan Africa | 0.12 (0.08, 0.17) | 0.11 (0.07, 0.16) | -0.35 (-0.45, -0.24) | <0.001 | — | — | — | — | 11.01 (4.38, 23.30) | 7.47 (3.76, 14.24) | -1.33 (-1.63, -1.03) | <0.001 | 0.15 (0.06, 0.33) | 0.10 (0.05, 0.20) | -1.33 (-1.63, -1.03) | <0.001 |
| Western Europe | 2.46 (2.31, 2.63) | 2.31 (2.14, 2.49) | -0.19 (-0.36, -0.02) | 0.027 | 3.14 (2.15, 4.25) | 2.34 (1.62, 3.05) | -1.00 (-1.52, -0.47) | <0.001 | 4.57 (4.11, 5.03) | 3.13 (2.69, 3.58) | -1.35 (-1.83, -0.86) | <0.001 | 0.06 (0.06, 0.07) | 0.04 (0.04, 0.05) | -1.39 (-1.86, -0.92) | <0.001 |
| High-income North America | 14.79 (13.86, 15.67) | 15.14 (14.33, 15.91) | 0.05 (-0.09, 0.19) | 0.507 | 23.27 (18.53, 28.32) | 23.01 (18.60, 27.47) | -0.10 (-0.35, 0.16) | 0.454 | 4.07 (3.68, 4.42) | 2.58 (2.31, 2.88) | -1.49 (-2.33, -0.64) | 0.001 | 0.06 (0.05, 0.06) | 0.04 (0.03, 0.04) | -1.50 (-2.34, -0.65) | 0.001 |
| Central Asia | 1.60 (1.16, 2.11) | 1.77 (1.30, 2.31) | 0.34 (0.32, 0.36) | <0.001 | — | — | — | — | 4.16 (3.50, 4.85) | 6.47 (5.03, 8.35) | 1.66 (0.86, 2.45) | <0.001 | 0.06 (0.05, 0.07) | 0.09 (0.07, 0.12) | 1.66 (0.87, 2.45) | <0.001 |
| Eastern Europe | 3.56 (3.22, 3.94) | 3.80 (3.45, 4.16) | 0.22 (0.20, 0.24) | <0.001 | — | — | — | — | 2.55 (2.29, 2.80) | 4.47 (3.79, 5.59) | 2.21 (1.22, 3.22) | <0.001 | 0.04 (0.03, 0.04) | 0.06 (0.05, 0.08) | 2.22 (1.22, 3.23) | <0.001 |
| Southern Sub-Saharan Africa | 0.22 (0.19, 0.25) | 0.19 (0.16, 0.22) | -0.54 (-0.60, -0.48) | <0.001 | — | — | — | — | 16.59 (12.44, 20.93) | 8.04 (5.27, 11.77) | -2.59 (-3.26, -1.91) | <0.001 | 0.23 (0.17, 0.29) | 0.11 (0.07, 0.16) | -2.60 (-3.27, -1.91) | <0.001 |

| | | | | | | | | | | | | | | | | |
|---|---|---|---|---|---|---|---|---|---|---|---|---|---|---|---|---|
| High-income Asia Pacific | 9.29 (8.64, 10.01) | 9.82 (9.13, 10.59) | 0.19 (0.15, 0.23) | <0.001 | 11.59 (8.63, 14.89) | 11.46 (8.79, 14.45) | -0.02 (-0.14, 0.10) | 0.718 | 3.68 (3.16, 4.26) | 1.88 (1.42, 2.33) | -2.30 (-3.29, -1.30) | <0.001 | 0.05 (0.04, 0.06) | 0.03 (0.02, 0.03) | -2.31 (-3.31, -1.31) | <0.001 |
| Central Europe | 3.16 (2.59, 3.77) | 4.31 (3.62, 5.03) | 1.09 (1.05, 1.13) | <0.001 | — | — | — | — | 3.83 (3.22, 4.46) | 4.27 (3.50, 5.05) | 0.44 (-0.04, 0.92) | 0.070 | 0.05 (0.04, 0.06) | 0.06 (0.05, 0.07) | 0.44 (-0.03, 0.92) | 0.068 |
| Andean Latin America | 0.17 (0.12, 0.22) | 0.23 (0.17, 0.30) | 1.11 (1.02, 1.19) | <0.001 | — | — | — | — | 10.99 (6.86, 16.05) | 6.66 (4.38, 9.63) | -1.80 (-2.33, -1.26) | <0.001 | 0.15 (0.10, 0.22) | 0.09 (0.06, 0.13) | -1.80 (-2.34, -1.26) | <0.001 |
| Caribbean | 0.24 (0.17, 0.30) | 0.26 (0.20, 0.33) | 0.36 (0.30, 0.43) | <0.001 | — | — | — | — | 16.42 (9.96, 26.09) | 15.73 (8.69, 28.70) | 0.02 (-0.37, 0.42) | 0.900 | 0.23 (0.14, 0.37) | 0.22 (0.12, 0.40) | 0.03 (-0.37, 0.42) | 0.897 |
| Southern Latin America | 0.62 (0.45, 0.84) | 0.83 (0.62, 1.09) | 0.97 (0.92, 1.01) | <0.001 | — | — | — | — | 5.71 (4.28, 7.44) | 3.93 (3.07, 4.94) | -1.30 (-1.76, -0.84) | <0.001 | 0.08 (0.06, 0.10) | 0.05 (0.04, 0.07) | -1.30 (-1.76, -0.84) | <0.001 |
| Oceania | 0.55 (0.41, 0.73) | 0.56 (0.40, 0.73) | -0.01 (-0.17, 0.15) | 0.899 | — | — | — | — | 9.08 (4.30, 17.73) | 11.49 (5.72, 21.31) | 0.74 (-0.11, 1.61) | 0.088 | 0.13 (0.06, 0.25) | 0.16 (0.08, 0.30) | 0.74 (-0.11, 1.60) | 0.088 |
| Australasia | 1.00 (0.83, 1.21) | 2.13 (1.72, 2.62) | 2.65 (2.61, 2.69) | <0.001 | — | — | — | — | 2.72 (2.15, 3.46) | 1.45 (1.06, 1.94) | -2.01 (-3.53, -0.46) | 0.011 | 0.04 (0.03, 0.05) | 0.02 (0.01, 0.03) | -2.01 (-3.53, -0.46) | 0.011 |
| **Men** | | | | | | | | | | | | | | | | |
| Global | 1.13 (1.03, 1.24) | 1.01 (0.92, 1.12) | -0.37 (-0.39, -0.34) | <0.001 | 0.82 (0.65, 1.01) | 0.63 (0.47, 0.78) | -0.88 (-1.07, -0.70) | <0.001 | 7.34 (5.85, 9.11) | 6.24 (5.16, 7.59) | -0.57 (-0.73, -0.40) | <0.001 | 0.10 (0.08, 0.13) | 0.09 (0.07, 0.11) | -0.57 (-0.73, -0.40) | <0.001 |
| **SDI category** | | | | | | | | | | | | | | | | |
| High | 5.36 (4.98, 5.77) | 5.96 (5.55, 6.39) | 0.36 (0.30, 0.41) | <0.001 | 6.23 (4.87, 7.81) | 6.10 (4.53, 7.66) | -0.08 (-0.24, 0.08) | 0.321 | 7.00 (6.18, 7.76) | 4.03 (3.55, 4.59) | -1.88 (-2.08, -1.67) | <0.001 | 0.10 (0.09, 0.11) | 0.06 (0.05, 0.06) | -1.87 (-2.08, -1.66) | <0.001 |
| High-middle | 1.50 (1.37, 1.67) | 1.80 (1.58, 2.04) | 0.60 (0.53, 0.67) | <0.001 | 0.38 (0.21, 0.57) | 0.42 (0.23, 0.60) | 0.32 (-0.24, 0.89) | 0.261 | 7.64 (6.45, 9.06) | 5.02 (4.39, 6.15) | -1.46 (-1.76, -1.15) | <0.001 | 0.11 (0.09, 0.13) | 0.07 (0.06, 0.09) | -1.46 (-1.76, -1.15) | <0.001 |
| Middle | 0.36 (0.31, | 0.41 (0.34, | 0.41 (0.31, | <0.001 | — | — | — | — | 7.44 (5.46, | 6.25 (4.83, | -0.57 | <0.001 | 0.10 | 0.09 | -0.57 | <0.001 |

| | | | | | | | | | | | | | | | | |
|---|---|---|---|---|---|---|---|---|---|---|---|---|---|---|---|---|
| | 0.42) | 0.48) | 0.50) | | | | | | 9.47) | 8.26) | (-0.74, -0.41) | | (0.08, 0.13) | (0.07, 0.12) | (-0.73, -0.41) | |
| Low-middle | 0.20 (0.16, 0.24) | 0.21 (0.17, 0.25) | 0.16 (0.02, 0.29) | 0.024 | — | — | — | — | 6.11 (4.20, 8.75) | 6.16 (4.65, 8.16) | -0.03 (-0.37, 0.30) | 0.844 | 0.09 (0.06, 0.12) | 0.09 (0.07, 0.11) | -0.03 (-0.37, 0.30) | 0.853 |
| Low | 0.15 (0.12, 0.19) | 0.17 (0.13, 0.20) | 0.28 (0.22, 0.34) | <0.001 | — | — | — | — | 9.46 (5.21, 14.81) | 8.19 (5.70, 11.07) | -0.51 (-0.76, -0.25) | <0.001 | 0.13 (0.07, 0.21) | 0.11 (0.08, 0.15) | -0.50 (-0.76, -0.25) | <0.001 |
| **21 GBD region** | | | | | | | | | | | | | | | | |
| South Asia | 0.12 (0.09, 0.14) | 0.15 (0.12, 0.18) | 0.84 (0.80, 0.87) | <0.001 | — | — | — | — | 4.28 (2.51, 7.04) | 4.41 (3.02, 6.29) | -0.04 (-1.23, 1.16) | 0.945 | 0.06 (0.04, 0.10) | 0.06 (0.04, 0.09) | -0.04 (-1.22, 1.16) | 0.951 |
| East Asia | 0.55 (0.50, 0.62) | 0.64 (0.58, 0.72) | 0.50 (0.36, 0.65) | <0.001 | — | — | — | — | 3.97 (2.63, 5.52) | 1.72 (1.33, 2.35) | -2.86 (-3.05, -2.67) | <0.001 | 0.06 (0.04, 0.08) | 0.02 (0.02, 0.03) | -2.86 (-3.05, -2.67) | <0.001 |
| North Africa and Middle East | 0.37 (0.28, 0.48) | 0.41 (0.31, 0.52) | 0.30 (0.26, 0.34) | <0.001 | — | — | — | — | 22.03 (15.23, 31.21) | 16.00 (10.55, 23.69) | -1.07 (-1.29, -0.85) | <0.001 | 0.31 (0.21, 0.44) | 0.22 (0.15, 0.33) | -1.07 (-1.29, -0.85) | <0.001 |
| Western Sub-Saharan Africa | 0.20 (0.16, 0.25) | 0.22 (0.18, 0.27) | 0.33 (0.25, 0.41) | <0.001 | — | — | — | — | 8.07 (4.12, 13.39) | 7.80 (4.91, 11.47) | -0.11 (-0.26, 0.03) | 0.123 | 0.11 (0.06, 0.19) | 0.11 (0.07, 0.16) | -0.11 (-0.26, 0.03) | 0.129 |
| Southeast Asia | 0.20 (0.16, 0.26) | 0.24 (0.19, 0.30) | 0.59 (0.58, 0.61) | <0.001 | — | — | — | — | 2.15 (1.45, 3.65) | 2.42 (1.75, 3.83) | 0.44 (0.05, 0.83) | 0.027 | 0.03 (0.02, 0.05) | 0.03 (0.02, 0.05) | 0.44 (0.05, 0.83) | 0.026 |
| Eastern Sub-Saharan Africa | 0.14 (0.11, 0.18) | 0.14 (0.11, 0.18) | -0.03 (-0.11, 0.05) | 0.474 | — | — | — | — | 15.15 (8.40, 23.82) | 11.13 (7.65, 15.65) | -1.05 (-1.21, -0.89) | <0.001 | 0.21 (0.12, 0.33) | 0.16 (0.11, 0.22) | -1.05 (-1.21, -0.89) | <0.001 |
| Central Latin America | 0.14 (0.11, 0.18) | 0.31 (0.24, 0.39) | 2.68 (2.54, 2.83) | <0.001 | — | — | — | — | 8.31 (7.18, 9.63) | 6.41 (5.07, 8.03) | -0.87 (-1.59, -0.15) | 0.018 | 0.12 (0.10, 0.13) | 0.09 (0.07, 0.11) | -0.87 (-1.58, -0.15) | 0.018 |
| Tropical Latin America | 0.27 (0.23, 0.33) | 0.30 (0.25, 0.37) | 0.35 (0.20, 0.49) | <0.001 | — | — | — | — | 23.65 (21.05, 26.64) | 11.74 (10.09, 13.54) | -2.42 (-2.85, -2.00) | <0.001 | 0.33 (0.29, 0.37) | 0.16 (0.14, 0.19) | -2.42 (-2.85, -2.00) | <0.001 |

| | | | | | | | | | | | | | | | | |
|---|---|---|---|---|---|---|---|---|---|---|---|---|---|---|---|---|
| Central Sub-Saharan Africa | 0.14 (0.10, 0.19) | 0.14 (0.09, 0.19) | -0.16 (-0.22, -0.10) | <0.001 | — | — | — | — | 10.11 (4.62, 18.98) | 7.32 (4.01, 12.69) | -1.15 (-1.33, -0.97) | <0.001 | 0.14 (0.06, 0.27) | 0.10 (0.06, 0.18) | -1.15 (-1.33, -0.97) | <0.001 |
| Western Europe | 1.59 (1.48, 1.72) | 1.75 (1.59, 1.93) | 0.34 (0.22, 0.47) | <0.001 | 1.32 (0.70, 2.01) | 1.05 (0.59, 1.56) | -0.71 (-1.64, 0.24) | 0.141 | 9.27 (7.96, 10.47) | 4.96 (4.07, 5.83) | -2.19 (-2.72, -1.66) | <0.001 | 0.13 (0.11, 0.15) | 0.07 (0.06, 0.08) | -2.19 (-2.72, -1.66) | <0.001 |
| High-income North America | 8.65 (8.11, 9.21) | 9.12 (8.56, 9.64) | 0.15 (0.06, 0.24) | 0.001 | 12.26 (8.91, 16.00) | 11.40 (7.80, 14.96) | -0.27 (-0.41, -0.12) | <0.001 | 7.79 (6.75, 8.70) | 4.37 (3.87, 5.15) | -1.97 (-2.57, -1.36) | <0.001 | 0.11 (0.09, 0.12) | 0.06 (0.05, 0.07) | -1.97 (-2.57, -1.36) | <0.001 |
| Central Asia | 2.39 (1.73, 3.16) | 2.80 (2.10, 3.65) | 0.55 (0.53, 0.57) | <0.001 | — | — | — | — | 2.46 (2.06, 2.94) | 4.55 (3.34, 6.01) | 2.24 (0.90, 3.60) | 0.001 | 0.03 (0.03, 0.04) | 0.06 (0.05, 0.08) | 2.25 (0.90, 3.60) | 0.001 |
| Eastern Europe | 3.54 (3.23, 3.89) | 5.99 (5.14, 6.99) | 1.83 (1.78, 1.88) | <0.001 | — | 0.91 (0.00, 2.00) | — | — | 3.85 (3.33, 4.31) | 5.10 (4.03, 6.34) | 1.38 (0.70, 2.06) | <0.001 | 0.05 (0.05, 0.06) | 0.07 (0.06, 0.09) | 1.38 (0.70, 2.07) | <0.001 |
| Southern Sub-Saharan Africa | 0.28 (0.23, 0.33) | 0.80 (0.59, 1.03) | 3.64 (3.35, 3.93) | <0.001 | — | — | — | — | 14.07 (10.15, 18.95) | 11.41 (7.88, 17.39) | -0.60 (-1.24, 0.04) | 0.067 | 0.20 (0.14, 0.27) | 0.16 (0.11, 0.24) | -0.60 (-1.24, 0.04) | 0.067 |
| High-income Asia Pacific | 8.86 (8.11, 9.71) | 10.61 (9.69, 11.68) | 0.63 (0.54, 0.72) | <0.001 | 9.30 (6.64, 12.34) | 9.42 (6.46, 12.62) | 0.16 (-0.12, 0.44) | 0.259 | 5.12 (4.04, 6.29) | 2.10 (1.66, 2.50) | -3.09 (-3.47, -2.71) | <0.001 | 0.07 (0.06, 0.09) | 0.03 (0.02, 0.03) | -3.10 (-3.47, -2.72) | <0.001 |
| Central Europe | 2.90 (2.38, 3.47) | 4.88 (4.08, 5.81) | 1.82 (1.75, 1.90) | <0.001 | — | — | — | — | 6.85 (5.83, 7.93) | 7.16 (5.72, 8.78) | 0.32 (-0.19, 0.83) | 0.224 | 0.10 (0.08, 0.11) | 0.10 (0.08, 0.12) | 0.32 (-0.19, 0.83) | 0.217 |
| Andean Latin America | 0.13 (0.09, 0.17) | 0.42 (0.31, 0.54) | 4.18 (3.97, 4.40) | <0.001 | — | — | — | — | 11.31 (7.39, 16.29) | 9.93 (6.48, 14.69) | -0.50 (-0.86, -0.15) | 0.006 | 0.16 (0.10, 0.23) | 0.14 (0.09, 0.21) | -0.50 (-0.85, -0.14) | 0.006 |
| Caribbean | 0.34 (0.26, 0.42) | 0.51 (0.41, 0.64) | 1.44 (1.34, 1.54) | <0.001 | — | — | — | — | 15.31 (10.70, 19.95) | 11.69 (7.46, 17.53) | -0.91 (-1.24, -0.57) | <0.001 | 0.21 (0.15, 0.28) | 0.16 (0.10, 0.25) | -0.91 (-1.24, -0.57) | <0.001 |
| Southern Latin America | 0.53 (0.38, 0.72) | 1.08 (0.84, 1.35) | 2.45 (2.40, 2.50) | <0.001 | — | — | — | — | 9.43 (7.16, 12.61) | 7.75 (5.75, 10.09) | -0.69 (-1.01, -0.37) | <0.001 | 0.13 (0.10, 0.18) | 0.11 (0.08, 0.14) | -0.69 (-1.01, -0.37) | <0.001 |

| | | | | | | | | | | | | | | | | |
|---|---|---|---|---|---|---|---|---|---|---|---|---|---|---|---|---|
| Oceania | 0.32 (0.23, 0.43) | 0.33 (0.23, 0.45) | 0.05 (-0.10, 0.19) | 0.515 | — | — | — | — | 27.55 (14.70, 48.32) | 27.20 (14.71, 45.82) | -0.05 (-0.33, 0.23) | 0.717 | 0.39 (0.21, 0.68) | 0.38 (0.21, 0.64) | -0.05 (-0.33, 0.23) | 0.717 |
| Australasia | 0.87 (0.71, 1.08) | 2.73 (2.13, 3.40) | 4.03 (3.91, 4.15) | <0.001 | — | — | — | — | 7.34 (5.57, 9.57) | 3.37 (2.36, 4.63) | -2.71 (-3.83, -1.57) | <0.001 | 0.10 (0.08, 0.13) | 0.05 (0.03, 0.06) | -2.71 (-3.83, -1.57) | <0.001 |
| **20-24 years** | | | | | | | | | | | | | | | | |
| **Women** | | | | | | | | | | | | | | | | |
| Global | 4.61 (4.28, 4.96) | 3.79 (3.52, 4.08) | -0.69 (-0.77, -0.61) | <0.001 | 14.72 (13.39, 16.08) | 11.69 (10.69, 12.71) | -0.80 (-0.90, -0.70) | <0.001 | 8.77 (5.45, 12.84) | 7.44 (5.18, 10.10) | -0.55 (-0.67, -0.44) | <0.001 | 0.13 (0.08, 0.19) | 0.11 (0.08, 0.15) | -0.55 (-0.67, -0.44) | <0.001 |
| **SDI category** | | | | | | | | | | | | | | | | |
| High | 22.17 (20.81, 23.57) | 24.52 (23.14, 26.04) | 0.33 (0.17, 0.49) | <0.001 | 82.76 (75.88, 90.28) | 90.61 (83.47, 98.53) | 0.28 (0.04, 0.53) | 0.024 | 5.86 (5.39, 6.40) | 4.47 (3.92, 5.17) | -0.92 (-1.23, -0.60) | <0.001 | 0.09 (0.08, 0.09) | 0.07 (0.06, 0.08) | -0.93 (-1.25, -0.61) | <0.001 |
| High-middle | 5.51 (5.06, 6.03) | 5.23 (4.77, 5.74) | -0.17 (-0.23, -0.12) | <0.001 | 17.84 (15.13, 20.86) | 15.76 (12.97, 18.86) | -0.37 (-0.54, -0.21) | <0.001 | 6.74 (5.12, 8.47) | 4.39 (3.85, 5.07) | -1.46 (-1.67, -1.25) | <0.001 | 0.10 (0.08, 0.13) | 0.07 (0.06, 0.08) | -1.46 (-1.68, -1.25) | <0.001 |
| Middle | 1.23 (1.07, 1.40) | 1.11 (0.94, 1.28) | -0.36 (-0.43, -0.30) | <0.001 | 0.77 (0.36, 1.31) | 0.72 (0.35, 1.17) | -0.21 (-0.33, -0.09) | 0.001 | 8.43 (5.60, 11.57) | 6.12 (4.97, 7.59) | -1.08 (-1.32, -0.84) | <0.001 | 0.13 (0.08, 0.17) | 0.09 (0.07, 0.11) | -1.08 (-1.32, -0.84) | <0.001 |
| Low-middle | 0.59 (0.49, 0.71) | 0.56 (0.46, 0.67) | -0.25 (-0.32, -0.17) | <0.001 | 0.18 (0.07, 0.34) | 0.18 (0.06, 0.32) | -0.16 (-0.46, 0.15) | 0.325 | 10.91 (4.53, 19.83) | 9.07 (5.19, 13.91) | -0.64 (-1.04, -0.24) | 0.002 | 0.16 (0.07, 0.30) | 0.14 (0.08, 0.21) | -0.64 (-1.04, -0.24) | 0.002 |
| Low | 0.37 (0.28, 0.47) | 0.39 (0.29, 0.49) | 0.14 (0.05, 0.22) | 0.002 | — | — | — | — | 13.75 (5.31, 25.87) | 11.40 (5.97, 18.53) | -0.64 (-0.74, -0.54) | <0.001 | 0.21 (0.08, 0.39) | 0.17 (0.09, 0.28) | -0.64 (-0.74, -0.54) | <0.001 |
| **21 GBD region** | | | | | | | | | | | | | | | | |
| South Asia | 0.25 (0.20, 0.31) | 0.32 (0.26, 0.38) | 0.75 (0.60, 0.89) | <0.001 | — | — | — | — | 10.55 (2.81, 21.44) | 8.09 (3.80, 13.86) | -0.98 (-1.55, -0.41) | 0.001 | 0.16 (0.04, 0.32) | 0.12 (0.06, 0.21) | -0.98 (-1.55, -0.41) | 0.001 |
| East Asia | 2.24 (2.05, 2.48) | 2.76 (2.53, 3.01) | 0.74 (0.60, 0.88) | <0.001 | 3.52 (2.18, 5.15) | 5.52 (3.08, 8.53) | 1.61 (0.97, 2.26) | <0.001 | 4.10 (2.18, 6.39) | 1.81 (1.40, 2.32) | -2.74 (-3.21, | <0.001 | 0.06 (0.03, | 0.03 (0.02, | -2.73 (-3.38, | <0.001 |

| | | | | | | | | | | | | | | | | |
|---|---|---|---|---|---|---|---|---|---|---|---|---|---|---|---|---|
| | | | | | | | | | | | -2.27) | | 0.10) | 0.03) | -2.07) | |
| North Africa and Middle East | 1.54 (1.15, 1.98) | 1.60 (1.19, 2.07) | 0.15 (0.10, 0.20) | <0.001 | — | — | — | — | 23.58 (14.33, 33.85) | 13.67 (9.67, 18.92) | -1.84 (-1.99, -1.70) | <0.001 | 0.35 (0.22, 0.51) | 0.20 (0.14, 0.28) | -1.85 (-1.99, -1.70) | <0.001 |
| Western Sub-Saharan Africa | 0.37 (0.29, 0.46) | 0.39 (0.31, 0.49) | 0.23 (0.14, 0.31) | <0.001 | — | — | — | — | 15.60 (6.30, 30.99) | 15.82 (8.67, 25.85) | 0.06 (-0.10, 0.22) | 0.456 | 0.23 (0.09, 0.47) | 0.24 (0.13, 0.39) | 0.06 (-0.10, 0.21) | 0.459 |
| Southeast Asia | 0.70 (0.55, 0.88) | 0.82 (0.66, 1.02) | 0.54 (0.52, 0.56) | <0.001 | — | — | — | — | 4.03 (2.15, 6.43) | 2.98 (2.35, 3.72) | -0.98 (-1.11, -0.85) | <0.001 | 0.06 (0.03, 0.10) | 0.04 (0.04, 0.06) | -0.99 (-1.11, -0.86) | <0.001 |
| Eastern Sub-Saharan Africa | 0.35 (0.27, 0.45) | 0.33 (0.24, 0.42) | -0.30 (-0.41, -0.19) | <0.001 | — | — | — | — | 16.51 (6.75, 31.39) | 9.82 (5.58, 15.33) | -1.77 (-1.95, -1.60) | <0.001 | 0.25 (0.10, 0.47) | 0.15 (0.08, 0.23) | -1.78 (-1.95, -1.60) | <0.001 |
| Central Latin America | 0.55 (0.44, 0.66) | 0.68 (0.55, 0.83) | 0.78 (0.73, 0.83) | <0.001 | — | — | — | — | 6.72 (5.89, 7.54) | 5.82 (4.54, 7.46) | -0.51 (-1.09, 0.07) | 0.085 | 0.10 (0.09, 0.11) | 0.09 (0.07, 0.11) | -0.51 (-1.09, 0.07) | 0.084 |
| Tropical Latin America | 1.20 (1.07, 1.33) | 1.13 (0.99, 1.27) | -0.25 (-0.47, -0.03) | 0.023 | — | — | — | — | 23.14 (20.71, 25.94) | 10.03 (8.75, 11.58) | -2.87 (-3.12, -2.62) | <0.001 | 0.35 (0.31, 0.39) | 0.15 (0.13, 0.17) | -2.87 (-3.12, -2.62) | <0.001 |
| Central Sub-Saharan Africa | 0.35 (0.23, 0.51) | 0.32 (0.21, 0.47) | -0.32 (-0.37, -0.26) | <0.001 | — | — | — | — | 11.69 (3.84, 25.87) | 8.65 (3.71, 16.76) | -1.03 (-1.18, -0.88) | <0.001 | 0.18 (0.06, 0.39) | 0.13 (0.06, 0.25) | -1.03 (-1.18, -0.88) | <0.001 |
| Western Europe | 6.89 (6.45, 7.38) | 6.95 (6.44, 7.51) | 0.03 (-0.10, 0.15) | 0.677 | 25.00 (22.23, 27.88) | 22.31 (19.71, 25.02) | -0.39 (-0.47, -0.31) | <0.001 | 6.13 (5.49, 6.84) | 4.34 (3.83, 5.00) | -1.18 (-1.66, -0.71) | <0.001 | 0.09 (0.08, 0.10) | 0.06 (0.06, 0.07) | -1.19 (-1.66, -0.71) | <0.001 |
| High-income North America | 44.30 (41.53, 46.93) | 45.36 (42.90, 47.67) | 0.04 (-0.11, 0.20) | 0.572 | 167.38 (151.72, 183.71) | 171.02 (155.12, 187.53) | 0.03 (-0.16, 0.23) | 0.740 | 6.45 (5.87, 7.01) | 4.70 (4.24, 5.24) | -1.17 (-2.21, -0.11) | 0.031 | 0.09 (0.09, 0.10) | 0.07 (0.06, 0.08) | -1.19 (-2.25, -0.12) | 0.029 |
| Central Asia | 4.81 (3.48, 6.34) | 5.31 (3.89, 6.95) | 0.34 (0.32, 0.36) | <0.001 | 18.28 (9.77, 28.89) | 19.54 (10.41, 31.06) | 0.22 (0.02, 0.42) | 0.035 | 3.66 (3.03, 4.48) | 4.92 (3.81, 6.34) | 0.95 (0.39, 1.53) | 0.001 | 0.05 (0.05, 0.07) | 0.07 (0.06, 0.09) | 0.96 (0.39, 1.53) | 0.001 |
| Eastern Europe | 10.68 (9.67, 11.82) | 11.37 (10.32, | 0.21 (0.18, 0.24) | <0.001 | 40.88 (30.96, 51.80) | 41.98 (33.65, 50.94) | 0.10 (0.00, 0.20) | 0.042 | 2.37 (2.15, 2.61) | 4.37 (3.39, 6.10) | 2.18 (1.15, | <0.001 | 0.04 (0.03, | 0.07 (0.05, | 2.21 (1.16, 3.27) | <0.001 |

| | | | | | | | | | | | | | | | | |
|---|---|---|---|---|---|---|---|---|---|---|---|---|---|---|---|---|
| | | 12.45) | | | | | | | | | 3.23) | | 0.04) | 0.09) | | |
| Southern Sub-Saharan Africa | 0.66 (0.58, 0.74) | 0.56 (0.47, 0.65) | -0.56 (-0.61, -0.51) | <0.001 | — | — | — | — | 21.12 (15.97, 26.83) | 9.59 (6.11, 14.45) | -2.62 (-3.82, -1.41) | <0.001 | 0.32 (0.24, 0.40) | 0.14 (0.09, 0.22) | -2.63 (-3.83, -1.41) | <0.001 |
| High-income Asia Pacific | 27.86 (25.90, 30.04) | 29.04 (26.96, 31.38) | 0.14 (0.10, 0.18) | <0.001 | 104.81 (90.18, 120.16) | 111.60 (95.13, 128.53) | 0.20 (0.09, 0.32) | <0.001 | 4.39 (3.78, 5.23) | 2.46 (2.00, 3.00) | -2.00 (-2.35, -1.65) | <0.001 | 0.07 (0.06, 0.08) | 0.04 (0.03, 0.04) | -2.03 (-2.39, -1.68) | <0.001 |
| Central Europe | 9.45 (7.72, 11.33) | 12.89 (10.85, 15.00) | 1.08 (1.04, 1.13) | <0.001 | 34.45 (24.89, 45.07) | 46.88 (36.51, 59.37) | 1.09 (0.71, 1.47) | <0.001 | 5.25 (4.35, 6.10) | 5.67 (4.54, 7.06) | 0.29 (0.02, 0.56) | 0.032 | 0.08 (0.06, 0.09) | 0.08 (0.07, 0.11) | 0.29 (0.02, 0.56) | 0.033 |
| Andean Latin America | 0.51 (0.36, 0.67) | 0.69 (0.52, 0.89) | 1.10 (1.02, 1.18) | <0.001 | — | — | — | — | 14.38 (7.87, 21.55) | 7.59 (5.07, 11.06) | -2.17 (-3.10, -1.24) | <0.001 | 0.22 (0.12, 0.32) | 0.11 (0.08, 0.17) | -2.17 (-3.10, -1.24) | <0.001 |
| Caribbean | 0.71 (0.53, 0.91) | 0.80 (0.60, 1.01) | 0.37 (0.29, 0.45) | <0.001 | — | — | — | — | 14.17 (8.47, 23.70) | 13.67 (7.07, 28.02) | -0.04 (-0.32, 0.24) | 0.801 | 0.21 (0.13, 0.36) | 0.21 (0.11, 0.42) | -0.03 (-0.31, 0.25) | 0.812 |
| Southern Latin America | 1.87 (1.34, 2.52) | 2.50 (1.86, 3.28) | 1.00 (0.96, 1.04) | <0.001 | — | — | — | — | 8.28 (6.27, 10.70) | 5.87 (4.63, 7.52) | -1.12 (-1.51, -0.73) | <0.001 | 0.12 (0.09, 0.16) | 0.09 (0.07, 0.11) | -1.12 (-1.51, -0.73) | <0.001 |
| Oceania | 1.66 (1.22, 2.20) | 1.67 (1.21, 2.21) | -0.04 (-0.17, 0.09) | 0.547 | — | — | — | — | 24.00 (10.63, 48.11) | 27.34 (11.68, 56.55) | 0.41 (0.05, 0.77) | 0.026 | 0.36 (0.16, 0.72) | 0.41 (0.18, 0.85) | 0.41 (0.05, 0.77) | 0.026 |
| Australasia | 2.96 (2.44, 3.59) | 6.35 (5.11, 7.85) | 2.67 (2.58, 2.75) | <0.001 | 4.84 (1.74, 8.47) | 17.24 (4.77, 33.63) | 4.53 (4.06, 5.00) | <0.001 | 7.11 (5.37, 9.15) | 3.86 (2.91, 5.03) | -2.00 (-2.90, -1.10) | <0.001 | 0.11 (0.08, 0.14) | 0.06 (0.04, 0.08) | -2.00 (-2.90, -1.10) | <0.001 |
| **Men** | | | | | | | | | | | | | | | | |
| Global | 3.55 (3.27, 3.89) | 3.27 (2.98, 3.60) | -0.30 (-0.37, -0.23) | <0.001 | 10.45 (9.34, 11.65) | 9.75 (8.71, 10.82) | -0.24 (-0.32, -0.15) | <0.001 | 8.87 (7.40, 10.76) | 7.63 (6.46, 9.15) | -0.50 (-0.67, -0.33) | <0.001 | 0.13 (0.11, 0.16) | 0.11 (0.10, 0.14) | -0.50 (-0.66, -0.33) | <0.001 |
| **SDI category** | | | | | | | | | | | | | | | | |
| High | 15.55 (14.46, 16.74) | 17.40 (16.17, 18.68) | 0.36 (0.24, 0.47) | <0.001 | 56.05 (49.91, 62.41) | 65.06 (58.24, 72.39) | 0.50 (0.40, 0.59) | <0.001 | 9.76 (8.86, 10.63) | 6.23 (5.54, 7.19) | -1.55 (-1.89, -1.21) | <0.001 | 0.15 (0.13, 0.16) | 0.09 (0.08, 0.11) | -1.59 (-1.96, -1.23) | <0.001 |

| | | | | | | | | | | | | | | | | |
|---|---|---|---|---|---|---|---|---|---|---|---|---|---|---|---|---|
| High-middle | 4.38 (3.98, 4.85) | 5.24 (4.61, 5.92) | 0.63 (0.57, 0.69) | <0.001 | 13.05 (10.87, 15.37) | 16.63 (13.68, 19.74) | 0.84 (0.70, 0.98) | <0.001 | 9.58 (8.02, 11.80) | 6.28 (5.43, 7.83) | -1.41 (-1.78, -1.04) | <0.001 | 0.14 (0.12, 0.18) | 0.09 (0.08, 0.12) | -1.42 (-1.78, -1.05) | <0.001 |
| Middle | 1.11 (0.96, 1.27) | 1.23 (1.04, 1.45) | 0.38 (0.32, 0.44) | <0.001 | 0.52 (0.23, 0.84) | 0.92 (0.50, 1.43) | 2.12 (1.84, 2.40) | <0.001 | 8.87 (7.01, 11.25) | 8.08 (6.15, 10.88) | -0.32 (-0.42, -0.22) | <0.001 | 0.13 (0.11, 0.17) | 0.12 (0.09, 0.16) | -0.32 (-0.42, -0.22) | <0.001 |
| Low-middle | 0.60 (0.50, 0.72) | 0.64 (0.53, 0.77) | 0.21 (0.16, 0.26) | <0.001 | 0.27 (0.13, 0.45) | 0.27 (0.12, 0.44) | -0.02 (-0.33, 0.28) | 0.877 | 7.31 (5.26, 10.14) | 7.27 (5.67, 9.34) | -0.01 (-0.33, 0.32) | 0.973 | 0.11 (0.08, 0.15) | 0.11 (0.09, 0.14) | -0.00 (-0.33, 0.32) | 0.981 |
| Low | 0.45 (0.35, 0.56) | 0.50 (0.39, 0.61) | 0.30 (0.24, 0.36) | <0.001 | — | — | — | — | 9.49 (5.78, 15.02) | 9.46 (6.62, 13.01) | 0.00 (-0.17, 0.18) | 0.979 | 0.14 (0.09, 0.23) | 0.14 (0.10, 0.20) | 0.00 (-0.17, 0.18) | 0.970 |
| **21 GBD region** | | | | | | | | | | | | | | | | |
| South Asia | 0.35 (0.28, 0.42) | 0.45 (0.37, 0.53) | 0.85 (0.80, 0.89) | <0.001 | — | — | — | — | 5.53 (3.60, 8.52) | 5.94 (4.45, 8.38) | 0.27 (-0.22, 0.76) | 0.289 | 0.08 (0.05, 0.13) | 0.09 (0.07, 0.13) | 0.27 (-0.22, 0.76) | 0.280 |
| East Asia | 1.70 (1.54, 1.89) | 2.10 (1.90, 2.35) | 0.73 (0.66, 0.80) | <0.001 | 1.51 (0.68, 2.53) | 3.06 (1.38, 5.64) | 2.48 (1.89, 3.07) | <0.001 | 4.39 (2.93, 6.13) | 2.56 (1.99, 3.29) | -1.92 (-2.37, -1.46) | <0.001 | 0.07 (0.04, 0.09) | 0.04 (0.03, 0.05) | -1.92 (-2.37, -1.46) | <0.001 |
| North Africa and Middle East | 1.14 (0.86, 1.45) | 1.27 (0.97, 1.61) | 0.41 (0.30, 0.52) | <0.001 | 0.02 (0.00, 0.05) | 0.09 (0.00, 0.27) | 5.38 (4.71, 6.05) | <0.001 | 30.95 (21.61, 42.69) | 22.04 (14.80, 32.28) | -1.13 (-1.32, -0.94) | <0.001 | 0.46 (0.32, 0.64) | 0.33 (0.22, 0.48) | -1.13 (-1.32, -0.94) | <0.001 |
| Western Sub-Saharan Africa | 0.60 (0.48, 0.74) | 0.66 (0.53, 0.81) | 0.33 (0.26, 0.40) | <0.001 | — | — | — | — | 6.56 (3.62, 10.67) | 6.92 (4.49, 10.25) | 0.21 (0.08, 0.34) | 0.001 | 0.10 (0.05, 0.16) | 0.10 (0.07, 0.15) | 0.21 (0.08, 0.34) | 0.001 |
| Southeast Asia | 0.61 (0.48, 0.78) | 0.73 (0.58, 0.91) | 0.60 (0.57, 0.62) | <0.001 | — | — | — | — | 3.54 (2.56, 5.39) | 3.73 (2.87, 5.19) | 0.18 (-0.06, 0.44) | 0.148 | 0.05 (0.04, 0.08) | 0.06 (0.04, 0.08) | 0.18 (-0.07, 0.44) | 0.150 |
| Eastern Sub-Saharan Africa | 0.43 (0.33, 0.54) | 0.43 (0.33, 0.54) | -0.03 (-0.11, 0.05) | 0.515 | — | — | — | — | 14.47 (8.76, 22.24) | 11.62 (7.91, 16.75) | -0.75 (-0.92, -0.58) | <0.001 | 0.22 (0.13, 0.33) | 0.17 (0.12, 0.25) | -0.75 (-0.91, -0.58) | <0.001 |
| Central Latin America | 0.43 (0.33, 0.55) | 0.92 (0.71, 1.17) | 2.68 (2.56, 2.80) | <0.001 | — | — | — | — | 8.60 (7.37, 10.05) | 5.87 (4.60, 7.33) | -1.25 (-1.56, | <0.001 | 0.13 (0.11, | 0.09 (0.07, | -1.25 (-1.56, | <0.001 |

| | | | | | | | | | | | | | | | | |
|---|---|---|---|---|---|---|---|---|---|---|---|---|---|---|---|---|
| | | | | | | | | | | | -0.94) | | 0.15) | 0.11) | -0.94) | |
| Tropical Latin America | 0.83 (0.69, 0.99) | 0.92 (0.75, 1.12) | 0.32 (0.18, 0.47) | <0.001 | — | — | — | — | 30.15 (26.57, 34.06) | 12.81 (11.24, 14.47) | -2.90 (-3.48, -2.32) | <0.001 | 0.45 (0.40, 0.51) | 0.19 (0.17, 0.22) | -2.91 (-3.48, -2.33) | <0.001 |
| Central Sub-Saharan Africa | 0.42 (0.29, 0.58) | 0.41 (0.27, 0.57) | -0.16 (-0.24, -0.08) | <0.001 | — | — | — | — | 10.25 (4.54, 20.36) | 8.68 (4.42, 15.25) | -0.69 (-0.86, -0.51) | <0.001 | 0.15 (0.07, 0.31) | 0.13 (0.07, 0.23) | -0.68 (-0.86, -0.51) | <0.001 |
| Western Europe | 4.49 (4.17, 4.84) | 5.29 (4.80, 5.83) | 0.57 (0.32, 0.84) | <0.001 | 15.26 (13.12, 17.72) | 17.72 (15.40, 20.15) | 0.53 (0.34, 0.73) | <0.001 | 13.47 (11.80, 15.31) | 7.36 (6.28, 8.70) | -2.18 (-2.57, -1.79) | <0.001 | 0.20 (0.18, 0.23) | 0.11 (0.09, 0.13) | -2.18 (-2.58, -1.79) | <0.001 |
| High-income North America | 26.01 (24.39, 27.68) | 27.33 (25.66, 28.90) | 0.13 (0.02, 0.24) | 0.022 | 93.68 (82.06, 105.50) | 104.95 (92.66, 117.96) | 0.38 (0.28, 0.47) | <0.001 | 10.39 (9.24, 11.43) | 6.14 (5.34, 7.19) | -1.81 (-2.25, -1.37) | <0.001 | 0.16 (0.14, 0.17) | 0.09 (0.08, 0.11) | -1.82 (-2.27, -1.38) | <0.001 |
| Central Asia | 7.17 (5.19, 9.48) | 8.40 (6.32, 10.93) | 0.55 (0.52, 0.59) | <0.001 | 27.32 (15.53, 40.91) | 29.16 (16.49, 43.94) | 0.20 (0.09, 0.30) | <0.001 | 3.92 (3.17, 4.84) | 6.08 (4.76, 7.72) | 1.47 (0.47, 2.49) | 0.004 | 0.06 (0.05, 0.07) | 0.09 (0.07, 0.12) | 1.48 (0.48, 2.49) | 0.004 |
| Eastern Europe | 10.64 (9.69, 11.69) | 17.96 (15.41, 20.94) | 1.83 (1.79, 1.87) | <0.001 | 38.00 (29.71, 46.75) | 69.27 (55.83, 85.22) | 2.09 (2.00, 2.19) | <0.001 | 4.86 (4.32, 5.52) | 8.65 (6.79, 11.72) | 1.99 (1.21, 2.79) | <0.001 | 0.07 (0.06, 0.08) | 0.13 (0.10, 0.18) | 2.00 (1.22, 2.80) | <0.001 |
| Southern Sub-Saharan Africa | 0.87 (0.73, 1.04) | 2.56 (1.89, 3.33) | 3.76 (3.50, 4.02) | <0.001 | — | 4.75 (1.11, 9.77) | — | — | 15.00 (10.26, 21.88) | 13.44 (9.88, 19.72) | -0.37 (-0.90, 0.16) | 0.174 | 0.23 (0.15, 0.33) | 0.20 (0.15, 0.30) | -0.37 (-0.91, 0.16) | 0.173 |
| High-income Asia Pacific | 26.97 (24.62, 29.54) | 31.79 (28.96, 35.10) | 0.59 (0.50, 0.68) | <0.001 | 102.55 (87.11, 119.59) | 122.84 (102.40, 143.79) | 0.64 (0.55, 0.74) | <0.001 | 7.68 (6.72, 8.81) | 3.73 (2.96, 4.44) | -2.43 (-3.12, -1.74) | <0.001 | 0.11 (0.10, 0.13) | 0.06 (0.04, 0.07) | -2.45 (-3.16, -1.73) | <0.001 |
| Central Europe | 8.67 (7.10, 10.42) | 14.48 (12.13, 17.16) | 1.80 (1.76, 1.83) | <0.001 | 28.87 (20.63, 39.53) | 55.71 (42.76, 69.98) | 2.27 (2.02, 2.51) | <0.001 | 6.78 (5.78, 7.94) | 7.16 (5.80, 8.59) | 0.13 (-0.20, 0.46) | 0.434 | 0.10 (0.09, 0.12) | 0.11 (0.09, 0.13) | 0.13 (-0.20, 0.46) | 0.440 |
| Andean Latin America | 0.38 (0.26, 0.52) | 1.24 (0.93, 1.60) | 4.18 (3.99, 4.38) | <0.001 | — | — | — | — | 12.41 (7.92, 18.40) | 10.63 (6.95, 15.51) | -0.44 (-0.82, -0.06) | 0.025 | 0.19 (0.12, 0.28) | 0.16 (0.10, 0.23) | -0.44 (-0.82, -0.05) | 0.025 |
| Caribbean | 1.01 (0.78, 1.25) | 1.55 (1.23, 1.93) | 1.48 (1.38, 1.58) | <0.001 | — | 1.40 (0.32, 3.13) | — | — | 18.13 (13.56, 23.00) | 12.93 (9.02, 18.87) | -1.13 (-1.39, | <0.001 | 0.27 (0.20, | 0.19 (0.14, | -1.13 (-1.39, | <0.001 |

| | | | | | | | | | | | | | | | | |
|---|---|---|---|---|---|---|---|---|---|---|---|---|---|---|---|---|
| | | | | | | | | | | | -0.88) | | 0.35) | 0.28) | -0.88) | |
| Southern Latin America | 1.60 (1.15, 2.16) | 3.27 (2.55, 4.06) | 2.48 (2.42, 2.54) | <0.001 | — | 2.95 (0.00, 7.94) | — | — | 8.03 (5.94, 10.80) | 6.60 (4.97, 8.50) | -0.68 (-1.09, -0.26) | 0.001 | 0.12 (0.09, 0.16) | 0.10 (0.07, 0.13) | -0.68 (-1.09, -0.26) | 0.001 |
| Oceania | 0.96 (0.68, 1.31) | 0.98 (0.69, 1.34) | 0.02 (-0.13, 0.17) | 0.786 | — | — | — | — | 23.85 (12.80, 44.19) | 21.99 (12.00, 40.92) | -0.37 (-0.56, -0.17) | <0.001 | 0.36 (0.19, 0.66) | 0.33 (0.18, 0.61) | -0.37 (-0.56, -0.17) | <0.001 |
| Australasia | 2.58 (2.10, 3.20) | 8.16 (6.35, 10.19) | 4.05 (3.90, 4.19) | <0.001 | 3.35 (1.00, 6.44) | 25.41 (8.86, 46.20) | 7.36 (6.70, 8.03) | <0.001 | 9.97 (7.47, 12.76) | 5.44 (3.92, 7.56) | -2.00 (-2.59, -1.41) | <0.001 | 0.15 (0.11, 0.19) | 0.08 (0.06, 0.11) | -2.01 (-2.60, -1.41) | <0.001 |
| **25-29 years** | | | | | | | | | | | | | | | | |
| **Women** | | | | | | | | | | | | | | | | |
| Global | 8.78 (8.15, 9.44) | 6.86 (6.37, 7.37) | -0.84 (-0.93, -0.74) | <0.001 | 52.41 (48.29, 56.63) | 38.93 (35.87, 42.14) | -1.01 (-1.11, -0.92) | <0.001 | 10.21 (6.79, 14.23) | 8.19 (6.02, 10.73) | -0.74 (-0.98, -0.51) | <0.001 | 0.16 (0.11, 0.23) | 0.13 (0.10, 0.17) | -0.74 (-0.98, -0.50) | <0.001 |
| **SDI category** | | | | | | | | | | | | | | | | |
| High | 37.28 (34.99, 39.65) | 40.17 (37.89, 42.68) | 0.22 (0.10, 0.35) | <0.001 | 232.09 (215.53, 249.17) | 249.79 (232.63, 267.78) | 0.21 (0.07, 0.36) | 0.004 | 8.43 (7.80, 9.14) | 6.56 (5.81, 7.76) | -0.86 (-1.19, -0.54) | <0.001 | 0.13 (0.12, 0.14) | 0.10 (0.09, 0.12) | -0.88 (-1.23, -0.54) | <0.001 |
| High-middle | 9.70 (8.92, 10.63) | 8.65 (7.91, 9.47) | -0.39 (-0.45, -0.33) | <0.001 | 59.13 (52.31, 66.85) | 49.89 (43.38, 56.99) | -0.57 (-0.72, -0.42) | <0.001 | 7.48 (5.77, 9.25) | 4.89 (4.25, 5.61) | -1.40 (-1.98, -0.81) | <0.001 | 0.12 (0.09, 0.15) | 0.08 (0.07, 0.09) | -1.40 (-1.99, -0.81) | <0.001 |
| Middle | 2.03 (1.76, 2.32) | 1.93 (1.66, 2.22) | -0.17 (-0.20, -0.13) | <0.001 | 9.23 (6.91, 11.83) | 7.14 (5.15, 9.38) | -0.90 (-1.03, -0.77) | <0.001 | 10.61 (7.19, 14.35) | 7.42 (5.96, 9.22) | -1.20 (-1.52, -0.88) | <0.001 | 0.17 (0.12, 0.23) | 0.12 (0.10, 0.15) | -1.20 (-1.52, -0.89) | <0.001 |
| Low-middle | 0.98 (0.80, 1.18) | 0.97 (0.80, 1.16) | -0.05 (-0.13, 0.02) | 0.179 | 3.45 (2.19, 4.96) | 2.25 (1.37, 3.23) | -1.46 (-1.69, -1.24) | <0.001 | 11.10 (5.15, 18.97) | 9.70 (5.92, 14.28) | -0.45 (-0.84, -0.05) | 0.026 | 0.18 (0.08, 0.31) | 0.16 (0.10, 0.23) | -0.45 (-0.84, -0.05) | 0.027 |
| Low | 0.61 (0.46, 0.78) | 0.65 (0.50, 0.83) | 0.20 (0.06, 0.34) | 0.006 | 0.50 (0.13, 0.98) | 0.48 (0.10, 0.98) | -0.24 (-0.94, 0.47) | 0.511 | 16.70 (6.31, 31.67) | 12.57 (6.39, 20.55) | -0.97 (-1.09, -0.85) | <0.001 | 0.27 (0.10, 0.51) | 0.20 (0.10, 0.33) | -0.97 (-1.09, -0.85) | <0.001 |
| **21 GBD region** | | | | | | | | | | | | | | | | |
| South Asia | 0.42 (0.34, | 0.53 (0.44, | 0.76 (0.61, | <0.001 | — | — | — | — | 9.49 (3.00, | 8.13 (4.06, | -0.44 | 0.169 | 0.15 | 0.13 | -0.44 | 0.171 |

| | | | | | | | | | | | | | | | | |
|---|---|---|---|---|---|---|---|---|---|---|---|---|---|---|---|---|
| | 0.51) | 0.63) | 0.90) | | | | | | 18.03) | 13.05) | (-1.07, 0.19) | | (0.05, 0.29) | (0.07, 0.21) | (-1.07, 0.19) | |
| East Asia | 3.94 (3.61, 4.34) | 4.59 (4.22, 5.02) | 0.54 (0.43, 0.65) | <0.001 | 23.65 (19.28, 28.76) | 24.95 (19.27, 31.13) | 0.17 (0.00, 0.33) | 0.050 | 4.27 (2.13, 7.02) | 1.96 (1.48, 2.56) | -2.47 (-3.94, -0.99) | 0.001 | 0.07 (0.03, 0.11) | 0.03 (0.02, 0.04) | -2.50 (-3.98, -1.00) | 0.001 |
| North Africa and Middle East | 2.58 (1.93, 3.32) | 2.72 (2.04, 3.50) | 0.19 (0.14, 0.25) | <0.001 | 15.37 (9.42, 22.73) | 15.47 (9.39, 22.84) | 0.02 (-0.17, 0.21) | 0.839 | 33.21 (19.98, 49.15) | 21.34 (15.26, 29.38) | -1.49 (-1.62, -1.36) | <0.001 | 0.54 (0.32, 0.80) | 0.35 (0.25, 0.48) | -1.49 (-1.62, -1.36) | <0.001 |
| Western Sub-Saharan Africa | 0.61 (0.47, 0.78) | 0.66 (0.51, 0.83) | 0.24 (0.16, 0.32) | <0.001 | — | — | — | — | 19.29 (7.15, 41.25) | 17.35 (9.01, 28.94) | -0.35 (-0.49, -0.21) | <0.001 | 0.31 (0.12, 0.67) | 0.28 (0.15, 0.47) | -0.35 (-0.49, -0.21) | <0.001 |
| Southeast Asia | 1.17 (0.91, 1.48) | 1.37 (1.09, 1.71) | 0.55 (0.53, 0.56) | <0.001 | 0.002 (0.000, 0.004) | 0.64 (0.02, 1.56) | 26.76 (20.60, 33.23) | <0.001 | 3.99 (2.22, 6.15) | 2.86 (2.20, 3.62) | -1.12 (-1.24, -1.01) | <0.001 | 0.06 (0.04, 0.10) | 0.05 (0.04, 0.06) | -1.13 (-1.24, -1.01) | <0.001 |
| Eastern Sub-Saharan Africa | 0.59 (0.45, 0.75) | 0.54 (0.41, 0.70) | -0.31 (-0.50, -0.13) | 0.001 | — | — | — | — | 21.88 (8.35, 40.96) | 11.40 (6.47, 17.60) | -2.23 (-2.45, -2.00) | <0.001 | 0.35 (0.14, 0.66) | 0.18 (0.10, 0.29) | -2.23 (-2.45, -2.00) | <0.001 |
| Central Latin America | 0.92 (0.74, 1.12) | 1.15 (0.92, 1.39) | 0.78 (0.72, 0.83) | <0.001 | — | — | — | — | 10.39 (9.07, 11.79) | 8.25 (6.13, 10.75) | -0.80 (-1.53, -0.06) | 0.034 | 0.17 (0.15, 0.19) | 0.13 (0.10, 0.17) | -0.80 (-1.54, -0.06) | 0.035 |
| Tropical Latin America | 2.00 (1.78, 2.22) | 1.88 (1.65, 2.13) | -0.26 (-0.48, -0.04) | 0.021 | 10.56 (6.35, 15.51) | — | — | — | 30.20 (27.21, 33.58) | 12.81 (11.39, 14.45) | -2.99 (-3.14, -2.83) | <0.001 | 0.49 (0.44, 0.55) | 0.21 (0.18, 0.23) | -2.99 (-3.15, -2.83) | <0.001 |
| Central Sub-Saharan Africa | 0.59 (0.38, 0.84) | 0.54 (0.34, 0.79) | -0.31 (-0.41, -0.22) | <0.001 | — | — | — | — | 11.05 (3.66, 24.37) | 7.25 (3.27, 13.94) | -1.46 (-1.60, -1.33) | <0.001 | 0.18 (0.06, 0.40) | 0.12 (0.05, 0.23) | -1.47 (-1.60, -1.33) | <0.001 |
| Western Europe | 11.03 (10.32, 11.82) | 11.75 (10.88, 12.71) | 0.25 (0.04, 0.46) | 0.020 | 65.29 (60.00, 70.95) | 68.53 (62.47, 74.95) | 0.16 (-0.08, 0.41) | 0.198 | 7.40 (6.64, 8.27) | 4.94 (4.44, 5.59) | -1.41 (-1.89, -0.93) | <0.001 | 0.12 (0.11, 0.13) | 0.08 (0.07, 0.09) | -1.42 (-1.90, -0.93) | <0.001 |
| High-income North America | 73.18 (68.63, 77.54) | 75.49 (71.42, 79.35) | 0.08 (-0.05, 0.21) | 0.245 | 458.53 (424.86, 494.48) | 473.51 (440.71, 506.86) | 0.08 (-0.06, 0.22) | 0.259 | 11.40 (10.50, 12.36) | 7.79 (6.83, 8.88) | -1.32 (-2.07, -0.57) | 0.001 | 0.18 (0.16, 0.19) | 0.12 (0.11, 0.14) | -1.37 (-2.18, -0.56) | 0.001 |
| Central Asia | 8.05 (5.83, | 8.92 (6.56, | 0.35 (0.31, | <0.001 | 49.70 (31.58, | 52.45 (33.36, | 0.17 (0.12, | <0.001 | 3.87 (3.31, | 6.50 (4.90, | 1.71 | <0.001 | 0.06 | 0.10 | 1.73 (1.05, | <0.001 |

| | | | | | | | | | | | | | | | | |
|---|---|---|---|---|---|---|---|---|---|---|---|---|---|---|---|---|
| | 10.61) | 11.67) | 0.39) | | 70.71) | 75.11) | 0.23) | | 4.53) | 8.59) | (1.03, 2.39) | | (0.05, 0.07) | (0.08, 0.14) | 2.41) | |
| Eastern Europe | 17.81 (16.12, 19.68) | 18.92 (17.17, 20.73) | 0.21 (0.19, 0.22) | <0.001 | 111.04 (93.72, 129.23) | 114.32 (99.25, 132.40) | 0.10 (0.07, 0.13) | <0.001 | 2.06 (1.77, 2.39) | 4.06 (2.97, 6.04) | 2.58 (2.02, 3.14) | <0.001 | 0.03 (0.03, 0.04) | 0.06 (0.05, 0.10) | 2.69 (2.09, 3.28) | <0.001 |
| Southern Sub-Saharan Africa | 1.10 (0.97, 1.25) | 0.94 (0.80, 1.09) | -0.56 (-0.61, -0.51) | <0.001 | — | — | — | — | 37.46 (27.81, 48.48) | 15.34 (7.81, 25.23) | -3.04 (-4.65, -1.39) | <0.001 | 0.61 (0.45, 0.79) | 0.25 (0.13, 0.41) | -3.04 (-4.66, -1.39) | <0.001 |
| High-income Asia Pacific | 44.73 (41.41, 48.29) | 47.90 (44.40, 51.79) | 0.23 (0.17, 0.29) | <0.001 | 279.99 (248.88, 310.54) | 300.92 (267.99, 335.48) | 0.23 (0.16, 0.31) | <0.001 | 5.18 (4.43, 6.16) | 2.68 (2.21, 3.27) | -2.42 (-2.76, -2.07) | <0.001 | 0.08 (0.07, 0.10) | 0.04 (0.03, 0.05) | -2.53 (-2.90, -2.16) | <0.001 |
| Central Europe | 15.83 (13.04, 18.85) | 21.37 (18.04, 24.80) | 1.05 (1.00, 1.09) | <0.001 | 94.27 (72.73, 118.95) | 134.08 (107.82, 159.51) | 1.24 (1.08, 1.40) | <0.001 | 5.91 (5.01, 6.86) | 6.63 (5.18, 8.28) | 0.37 (0.06, 0.68) | 0.018 | 0.09 (0.08, 0.11) | 0.11 (0.08, 0.13) | 0.38 (0.06, 0.70) | 0.020 |
| Andean Latin America | 0.84 (0.60, 1.11) | 1.14 (0.85, 1.47) | 1.05 (0.96, 1.14) | <0.001 | — | — | — | — | 14.98 (8.93, 22.16) | 8.75 (5.73, 12.63) | -1.98 (-2.41, -1.53) | <0.001 | 0.24 (0.14, 0.36) | 0.14 (0.09, 0.20) | -1.98 (-2.42, -1.53) | <0.001 |
| Caribbean | 1.20 (0.89, 1.54) | 1.33 (1.01, 1.69) | 0.35 (0.29, 0.40) | <0.001 | 0.05 (0.01, 0.10) | 0.86 (0.01, 2.21) | 10.30 (8.98, 11.63) | <0.001 | 13.77 (8.86, 21.20) | 13.48 (7.61, 24.37) | -0.05 (-0.38, 0.29) | 0.774 | 0.22 (0.14, 0.34) | 0.22 (0.12, 0.40) | -0.05 (-0.38, 0.29) | 0.780 |
| Southern Latin America | 3.12 (2.24, 4.19) | 4.20 (3.13, 5.50) | 1.02 (0.95, 1.09) | <0.001 | 18.72 (5.59, 35.31) | 22.79 (8.85, 41.20) | 0.76 (0.39, 1.12) | <0.001 | 10.39 (7.65, 13.33) | 6.78 (5.32, 8.55) | -1.45 (-2.04, -0.85) | <0.001 | 0.17 (0.12, 0.22) | 0.11 (0.09, 0.14) | -1.46 (-2.05, -0.86) | <0.001 |
| Oceania | 2.77 (2.04, 3.68) | 2.78 (2.02, 3.68) | -0.03 (-0.20, 0.13) | 0.698 | 16.88 (5.65, 32.74) | 16.75 (3.40, 32.91) | -0.06 (-0.28, 0.17) | 0.627 | 4.85 (2.19, 9.56) | 5.17 (2.18, 10.29) | 0.20 (-0.05, 0.44) | 0.112 | 0.08 (0.04, 0.16) | 0.08 (0.04, 0.17) | 0.20 (-0.04, 0.44) | 0.108 |
| Australasia | 4.94 (4.08, 5.99) | 10.52 (8.43, 13.05) | 2.64 (2.59, 2.69) | <0.001 | 29.12 (13.34, 48.07) | 66.51 (36.05, 103.22) | 2.79 (2.45, 3.13) | <0.001 | 7.69 (6.09, 9.48) | 4.81 (3.79, 6.02) | -1.57 (-2.93, -0.20) | 0.025 | 0.12 (0.10, 0.15) | 0.08 (0.06, 0.10) | -1.58 (-2.95, -0.20) | 0.025 |
| **Men** | | | | | | | | | | | | | | | | |
| Global | 6.63 (6.12, 7.24) | 5.96 (5.42, 6.57) | -0.35 (-0.44, -0.27) | <0.001 | 37.95 (34.61, 41.64) | 32.15 (29.05, 35.29) | -0.55 (-0.64, -0.46) | <0.001 | 10.16 (8.67, 12.15) | 8.38 (7.41, 9.78) | -0.64 (-0.83, -0.45) | <0.001 | 0.16 (0.14, 0.20) | 0.14 (0.12, 0.16) | -0.64 (-0.83, -0.45) | <0.001 |

| | | | | | | | | | | | | | | | | |
|---|---|---|---|---|---|---|---|---|---|---|---|---|---|---|---|---|
| **SDI category** | | | | | | | | | | | | | | | | |
| High | 25.49 (23.74, 27.43) | 28.42 (26.39, 30.52) | 0.38 (0.34, 0.41) | <0.001 | 158.19 (144.94, 172.22) | 175.56 (160.34, 190.95) | 0.35 (0.31, 0.40) | <0.001 | 13.46 (12.33, 14.63) | 8.84 (7.91, 9.98) | -1.46 (-1.89, -1.03) | <0.001 | 0.22 (0.20, 0.23) | 0.14 (0.13, 0.16) | -1.48 (-1.91, -1.04) | <0.001 |
| High-middle | 7.83 (7.15, 8.66) | 8.78 (7.72, 9.92) | 0.40 (0.33, 0.47) | <0.001 | 45.79 (40.14, 52.60) | 50.83 (43.57, 58.46) | 0.36 (0.23, 0.50) | <0.001 | 10.87 (9.25, 13.22) | 7.75 (6.75, 9.61) | -1.20 (-1.66, -0.74) | <0.001 | 0.18 (0.15, 0.21) | 0.13 (0.11, 0.16) | -1.20 (-1.67, -0.74) | <0.001 |
| Middle | 1.85 (1.61, 2.13) | 2.13 (1.80, 2.50) | 0.47 (0.39, 0.55) | <0.001 | 7.36 (5.16, 9.83) | 6.59 (4.92, 8.46) | -0.42 (-0.83, -0.01) | 0.042 | 9.48 (7.78, 11.30) | 8.27 (6.75, 10.49) | -0.47 (-0.58, -0.37) | <0.001 | 0.15 (0.13, 0.18) | 0.13 (0.11, 0.17) | -0.47 (-0.58, -0.37) | <0.001 |
| Low-middle | 1.01 (0.83, 1.21) | 1.11 (0.92, 1.32) | 0.30 (0.22, 0.38) | <0.001 | 2.44 (1.39, 3.69) | 1.76 (1.06, 2.59) | -1.13 (-1.35, -0.91) | <0.001 | 8.08 (5.81, 11.61) | 8.20 (6.44, 10.69) | 0.07 (-0.29, 0.44) | 0.697 | 0.13 (0.09, 0.19) | 0.13 (0.10, 0.17) | 0.07 (-0.29, 0.44) | 0.694 |
| Low | 0.75 (0.59, 0.94) | 0.83 (0.65, 1.03) | 0.32 (0.27, 0.38) | <0.001 | — | — | — | — | 9.70 (5.62, 16.30) | 9.36 (6.36, 13.47) | -0.13 (-0.42, 0.16) | 0.372 | 0.16 (0.09, 0.26) | 0.15 (0.10, 0.22) | -0.13 (-0.42, 0.16) | 0.369 |
| **21 GBD region** | | | | | | | | | | | | | | | | |
| South Asia | 0.58 (0.47, 0.70) | 0.75 (0.62, 0.88) | 0.85 (0.82, 0.88) | <0.001 | — | — | — | — | 6.29 (4.02, 9.78) | 7.18 (5.33, 9.70) | 0.47 (0.03, 0.91) | 0.037 | 0.10 (0.07, 0.16) | 0.12 (0.09, 0.16) | 0.47 (0.03, 0.91) | 0.036 |
| East Asia | 2.97 (2.70, 3.29) | 3.62 (3.28, 4.03) | 0.70 (0.61, 0.78) | <0.001 | 17.18 (13.39, 21.21) | 18.33 (13.69, 23.12) | 0.21 (0.06, 0.36) | 0.006 | 5.80 (4.12, 8.08) | 4.13 (3.26, 5.19) | -1.20 (-1.45, -0.94) | <0.001 | 0.09 (0.07, 0.13) | 0.07 (0.05, 0.08) | -1.20 (-1.46, -0.95) | <0.001 |
| North Africa and Middle East | 1.95 (1.47, 2.48) | 2.21 (1.69, 2.78) | 0.44 (0.36, 0.52) | <0.001 | 5.01 (1.94, 9.11) | 5.90 (2.73, 9.89) | 0.44 (-0.99, 1.89) | 0.550 | 25.33 (18.35, 35.60) | 18.64 (13.34, 25.92) | -1.05 (-1.26, -0.84) | <0.001 | 0.41 (0.30, 0.58) | 0.30 (0.22, 0.42) | -1.05 (-1.26, -0.84) | <0.001 |
| Western Sub-Saharan Africa | 1.00 (0.81, 1.23) | 1.11 (0.89, 1.37) | 0.34 (0.26, 0.41) | <0.001 | — | — | — | — | 4.56 (2.76, 7.15) | 4.90 (3.37, 6.79) | 0.27 (0.16, 0.38) | <0.001 | 0.07 (0.04, 0.12) | 0.08 (0.05, 0.11) | 0.27 (0.16, 0.38) | <0.001 |
| Southeast Asia | 1.02 (0.80, 1.30) | 1.21 (0.96, 1.52) | 0.60 (0.58, 0.61) | <0.001 | — | — | — | — | 3.62 (2.63, 5.13) | 3.74 (2.93, 5.17) | 0.15 (-0.17, 0.47) | 0.366 | 0.06 (0.04, 0.08) | 0.06 (0.05, 0.08) | 0.15 (-0.17, 0.47) | 0.366 |
| Eastern Sub-Saharan | 0.72 (0.55, | 0.71 (0.55, | -0.03 (-0.11, | 0.494 | — | — | — | — | 16.23 (9.30, | 12.10 (8.10, | -1.00 | <0.001 | 0.26 | 0.20 | -1.00 | <0.001 |

| | | | | | | | | | | | | | | | | |
|---|---|---|---|---|---|---|---|---|---|---|---|---|---|---|---|---|
| Africa | 0.90) | 0.90) | 0.05) | | | | | | 26.07) | 17.21) | (-1.18, -0.81) | | (0.15, 0.42) | (0.13, 0.28) | (-1.18, -0.81) | |
| Central Latin America | 0.72 (0.55, 0.92) | 1.56 (1.20, 1.97) | 2.70 (2.57, 2.82) | <0.001 | — | 2.05 (1.04, 3.32) | — | — | 12.75 (10.73, 14.95) | 10.33 (8.13, 12.77) | -0.72 (-1.14, -0.30) | 0.001 | 0.21 (0.17, 0.24) | 0.17 (0.13, 0.21) | -0.72 (-1.14, -0.30) | 0.001 |
| Tropical Latin America | 1.40 (1.16, 1.67) | 1.53 (1.25, 1.87) | 0.30 (0.16, 0.44) | <0.001 | — | 0.25 (0.00, 0.57) | — | — | 34.95 (30.59, 39.89) | 15.29 (13.27, 17.86) | -2.75 (-3.13, -2.37) | <0.001 | 0.57 (0.50, 0.65) | 0.25 (0.22, 0.29) | -2.76 (-3.14, -2.38) | <0.001 |
| Central Sub-Saharan Africa | 0.71 (0.48, 0.96) | 0.68 (0.46, 0.95) | -0.17 (-0.22, -0.12) | <0.001 | — | — | — | — | 10.40 (4.18, 20.86) | 8.68 (4.48, 15.66) | -0.67 (-0.95, -0.39) | <0.001 | 0.17 (0.07, 0.34) | 0.14 (0.07, 0.25) | -0.67 (-0.95, -0.39) | <0.001 |
| Western Europe | 7.20 (6.69, 7.77) | 8.93 (8.11, 9.84) | 0.75 (0.60, 0.90) | <0.001 | 41.02 (37.02, 45.40) | 50.64 (45.23, 56.20) | 0.71 (0.46, 0.96) | <0.001 | 16.79 (14.59, 19.10) | 9.89 (8.64, 11.40) | -1.92 (-2.32, -1.52) | <0.001 | 0.27 (0.24, 0.31) | 0.16 (0.14, 0.18) | -1.93 (-2.33, -1.52) | <0.001 |
| High-income North America | 43.15 (40.48, 45.90) | 45.65 (42.86, 48.25) | 0.16 (0.06, 0.27) | 0.002 | 270.28 (246.55, 295.75) | 285.78 (259.99, 309.86) | 0.16 (0.05, 0.27) | 0.004 | 16.37 (14.69, 17.92) | 9.41 (7.46, 11.22) | -1.96 (-2.84, -1.07) | <0.001 | 0.26 (0.24, 0.29) | 0.15 (0.12, 0.18) | -2.00 (-2.90, -1.09) | <0.001 |
| Central Asia | 11.97 (8.67, 15.84) | 14.16 (10.67, 18.37) | 0.59 (0.57, 0.61) | <0.001 | 74.19 (48.42, 105.38) | 85.93 (58.32, 116.81) | 0.52 (0.43, 0.61) | <0.001 | 4.24 (3.45, 5.26) | 7.45 (5.71, 9.37) | 1.92 (1.44, 2.40) | <0.001 | 0.07 (0.06, 0.08) | 0.12 (0.09, 0.15) | 1.94 (1.46, 2.42) | <0.001 |
| Eastern Europe | 17.74 (16.18, 19.47) | 29.80 (25.54, 34.69) | 1.81 (1.78, 1.83) | <0.001 | 106.89 (89.89, 124.94) | 186.35 (156.59, 222.53) | 1.94 (1.88, 1.99) | <0.001 | 4.64 (3.98, 5.42) | 11.20 (9.02, 15.74) | 3.20 (1.72, 4.70) | <0.001 | 0.07 (0.06, 0.09) | 0.18 (0.14, 0.25) | 3.23 (1.73, 4.74) | <0.001 |
| Southern Sub-Saharan Africa | 1.50 (1.25, 1.80) | 4.63 (3.41, 6.06) | 3.95 (3.67, 4.24) | <0.001 | — | 18.62 (9.61, 30.70) | — | — | 24.66 (17.03, 33.51) | 15.49 (11.09, 23.32) | -1.69 (-1.96, -1.42) | <0.001 | 0.40 (0.28, 0.54) | 0.25 (0.18, 0.38) | -1.70 (-1.97, -1.43) | <0.001 |
| High-income Asia Pacific | 44.15 (40.20, 48.49) | 52.51 (47.71, 57.95) | 0.59 (0.51, 0.68) | <0.001 | 277.17 (244.18, 316.18) | 328.84 (286.85, 375.71) | 0.60 (0.51, 0.69) | <0.001 | 8.87 (7.77, 10.22) | 4.65 (3.73, 5.64) | -2.27 (-2.52, -2.02) | <0.001 | 0.14 (0.12, 0.16) | 0.07 (0.06, 0.09) | -2.34 (-2.60, -2.09) | <0.001 |
| Central Europe | 14.48 (11.92, 17.38) | 23.94 (20.13, 28.32) | 1.75 (1.70, 1.81) | <0.001 | 87.68 (67.40, 110.84) | 149.79 (119.62, 181.49) | 1.88 (1.81, 1.95) | <0.001 | 9.47 (7.96, 11.30) | 12.10 (9.87, 14.50) | 0.94 (0.47, 1.40) | <0.001 | 0.15 (0.13, 0.18) | 0.19 (0.16, 0.23) | 0.87 (0.47, 1.28) | <0.001 |
| Andean Latin | 0.64 (0.43, | 2.04 (1.52, | 4.12 (3.92, | <0.001 | — | 5.18 (0.00, | — | — | 13.20 (8.71, | 12.45 (8.42, | -0.15 | 0.551 | 0.21 | 0.20 | -0.15 | 0.544 |

| | | | | | | | | | | | | | | | | |
|---|---|---|---|---|---|---|---|---|---|---|---|---|---|---|---|---|
| America | 0.87) | 2.63) | 4.33) | | | 10.95) | | | 19.06) | 18.01) | (-0.64, 0.35) | | (0.14, 0.31) | (0.14, 0.29) | (-0.65, 0.34) | |
| Caribbean | 1.69 (1.32, 2.09) | 2.59 (2.05, 3.22) | 1.47 (1.38, 1.55) | <0.001 | 1.44 (0.06, 3.26) | 3.76 (1.59, 6.50) | 2.61 (2.10, 3.12) | <0.001 | 26.72 (20.30, 33.78) | 20.74 (14.27, 30.04) | -0.86 (-1.10, -0.61) | <0.001 | 0.43 (0.33, 0.55) | 0.34 (0.23, 0.49) | -0.86 (-1.10, -0.61) | <0.001 |
| Southern Latin America | 2.67 (1.91, 3.59) | 5.51 (4.30, 6.83) | 2.52 (2.44, 2.60) | <0.001 | 14.95 (3.76, 29.37) | 34.62 (16.65, 56.76) | 3.12 (2.01, 4.24) | <0.001 | 11.92 (8.74, 15.76) | 10.20 (7.60, 13.42) | -0.50 (-0.86, -0.14) | 0.007 | 0.19 (0.14, 0.26) | 0.16 (0.12, 0.22) | -0.50 (-0.87, -0.14) | 0.006 |
| Oceania | 1.61 (1.15, 2.20) | 1.64 (1.15, 2.24) | 0.00 (-0.15, 0.15) | 0.978 | 0.88 (0.34, 1.61) | 0.28 (0.09, 0.57) | -3.97 (-5.93, -1.97) | <0.001 | 14.11 (7.17, 29.22) | 13.91 (6.64, 27.90) | -0.07 (-0.36, 0.21) | 0.619 | 0.23 (0.12, 0.47) | 0.23 (0.11, 0.45) | -0.07 (-0.36, 0.22) | 0.624 |
| Australasia | 4.27 (3.48, 5.31) | 13.57 (10.53, 16.99) | 4.06 (3.91, 4.21) | <0.001 | 23.60 (9.24, 43.64) | 84.43 (47.31, 126.84) | 4.54 (3.97, 5.12) | <0.001 | 12.49 (9.80, 15.80) | 8.71 (6.48, 11.15) | -1.19 (-2.14, -0.24) | 0.015 | 0.20 (0.16, 0.26) | 0.14 (0.10, 0.18) | -1.20 (-2.15, -0.25) | 0.014 |
| **30-34 years** | | | | | | | | | | | | | | | | |
| **Women** | | | | | | | | | | | | | | | | |
| Global | 13.85 (12.89, 14.87) | 10.09 (9.38, 10.85) | -1.12 (-1.21, -1.02) | <0.001 | 118.02 (109.56, 127.44) | 83.20 (77.20, 90.08) | -1.23 (-1.32, -1.13) | <0.001 | 10.67 (7.64, 14.17) | 7.94 (6.21, 9.87) | -1.03 (-1.28, -0.79) | <0.001 | 0.19 (0.13, 0.25) | 0.14 (0.11, 0.17) | -1.03 (-1.27, -0.78) | <0.001 |
| **SDI category** | | | | | | | | | | | | | | | | |
| High | 53.57 (50.31, 56.97) | 54.50 (51.39, 57.84) | 0.03 (-0.10, 0.15) | 0.681 | 466.71 (435.59, 499.20) | 474.91 (444.84, 508.53) | 0.03 (-0.10, 0.15) | 0.682 | 11.15 (10.31, 12.17) | 8.22 (7.36, 9.41) | -1.01 (-1.32, -0.70) | <0.001 | 0.19 (0.18, 0.20) | 0.14 (0.12, 0.16) | -1.04 (-1.43, -0.66) | <0.001 |
| High-middle | 14.31 (13.12, 15.70) | 12.25 (11.20, 13.41) | -0.53 (-0.61, -0.46) | <0.001 | 122.21 (110.36, 136.21) | 101.43 (91.12, 112.44) | -0.64 (-0.71, -0.57) | <0.001 | 8.46 (6.89, 10.06) | 5.52 (4.86, 6.21) | -1.42 (-1.88, -0.96) | <0.001 | 0.15 (0.12, 0.17) | 0.10 (0.08, 0.11) | -1.43 (-1.90, -0.95) | <0.001 |
| Middle | 2.80 (2.43, 3.22) | 2.78 (2.41, 3.18) | 0.01 (-0.10, 0.11) | 0.894 | 22.22 (18.04, 27.02) | 19.25 (15.44, 23.52) | -0.48 (-0.59, -0.37) | <0.001 | 10.55 (7.52, 13.78) | 6.87 (5.73, 8.23) | -1.49 (-1.83, -1.15) | <0.001 | 0.19 (0.13, 0.24) | 0.12 (0.10, 0.14) | -1.50 (-1.84, -1.15) | <0.001 |
| Low-middle | 1.32 (1.08, 1.60) | 1.39 (1.14, 1.67) | 0.16 (0.08, 0.23) | <0.001 | 6.82 (5.04, 8.90) | 6.51 (4.58, 8.47) | -0.23 (-0.46, 0.00) | 0.054 | 11.53 (5.54, 19.15) | 9.95 (6.40, 14.15) | -0.46 (-0.83, -0.08) | 0.017 | 0.20 (0.10, 0.34) | 0.18 (0.11, 0.25) | -0.45 (-0.83, -0.08) | 0.017 |
| Low | 0.85 (0.64, | 0.91 (0.69, | 0.24 (0.17, | <0.001 | 1.77 (0.96, | 1.67 (0.78, | -0.40 | 0.214 | 14.47 (6.02, | 10.76 (5.79, | -1.03 | <0.001 | 0.26 | 0.19 | -1.03 | <0.001 |

| | | | | | | | | | | | | | | | | |
|---|---|---|---|---|---|---|---|---|---|---|---|---|---|---|---|---|
| | 1.08) | 1.16) | 0.31) | | 2.76) | 2.69) | (-1.03, 0.23) | | 26.09) | 16.94) | (-1.16, -0.90) | | (0.11, 0.46) | (0.10, 0.30) | (-1.16, -0.90) | |
| **21 GBD region** | | | | | | | | | | | | | | | | |
| South Asia | 0.59 (0.48, 0.71) | 0.74 (0.61, 0.88) | 0.76 (0.62, 0.91) | <0.001 | — | — | — | — | 10.90 (3.94, 19.40) | 9.62 (5.37, 14.16) | -0.45 (-0.90, 0.01) | 0.055 | 0.19 (0.07, 0.34) | 0.17 (0.09, 0.25) | -0.45 (-0.90, 0.01) | 0.055 |
| East Asia | 5.76 (5.29, 6.33) | 6.60 (6.08, 7.22) | 0.43 (0.26, 0.61) | <0.001 | 49.25 (42.13, 57.11) | 51.59 (44.25, 59.57) | 0.13 (-0.07, 0.32) | 0.208 | 4.46 (2.34, 7.11) | 2.15 (1.64, 2.75) | -2.37 (-3.66, -1.06) | <0.001 | 0.08 (0.04, 0.12) | 0.04 (0.03, 0.05) | -2.41 (-3.71, -1.09) | <0.001 |
| North Africa and Middle East | 3.62 (2.71, 4.68) | 3.89 (2.97, 4.97) | 0.26 (0.19, 0.34) | <0.001 | 30.17 (19.51, 42.33) | 32.10 (21.20, 43.73) | 0.21 (0.11, 0.32) | <0.001 | 26.58 (16.18, 39.31) | 17.11 (12.44, 22.76) | -1.49 (-1.60, -1.39) | <0.001 | 0.47 (0.28, 0.69) | 0.30 (0.22, 0.40) | -1.50 (-1.60, -1.39) | <0.001 |
| Western Sub-Saharan Africa | 0.86 (0.66, 1.09) | 0.92 (0.71, 1.16) | 0.25 (0.16, 0.34) | <0.001 | — | 0.02 (0.00, 0.04) | — | — | 8.35 (3.49, 16.17) | 7.17 (4.06, 11.28) | -0.53 (-0.69, -0.38) | <0.001 | 0.15 (0.06, 0.29) | 0.13 (0.07, 0.20) | -0.53 (-0.69, -0.38) | <0.001 |
| Southeast Asia | 1.65 (1.28, 2.07) | 1.93 (1.53, 2.40) | 0.54 (0.53, 0.56) | <0.001 | 13.14 (8.36, 18.49) | 16.30 (10.93, 22.85) | 0.72 (0.56, 0.89) | <0.001 | 5.06 (2.98, 7.51) | 3.63 (2.83, 4.46) | -1.13 (-1.27, -0.99) | <0.001 | 0.09 (0.05, 0.13) | 0.06 (0.05, 0.08) | -1.14 (-1.27, -1.00) | <0.001 |
| Eastern Sub-Saharan Africa | 0.83 (0.62, 1.05) | 0.76 (0.57, 0.98) | -0.31 (-0.47, -0.16) | <0.001 | 1.01 (0.15, 2.27) | — | — | — | 20.10 (8.51, 36.13) | 10.98 (6.32, 16.46) | -2.07 (-2.25, -1.89) | <0.001 | 0.35 (0.15, 0.64) | 0.19 (0.11, 0.29) | -2.07 (-2.25, -1.89) | <0.001 |
| Central Latin America | 1.29 (1.03, 1.57) | 1.61 (1.30, 1.95) | 0.79 (0.75, 0.84) | <0.001 | 3.97 (0.69, 8.49) | 3.86 (0.90, 7.96) | -0.11 (-0.56, 0.35) | 0.648 | 11.93 (10.55, 13.53) | 9.26 (6.96, 12.08) | -0.81 (-1.75, 0.14) | 0.096 | 0.21 (0.19, 0.24) | 0.16 (0.12, 0.21) | -0.81 (-1.76, 0.14) | 0.095 |
| Tropical Latin America | 2.80 (2.50, 3.11) | 2.63 (2.32, 2.98) | -0.26 (-0.48, -0.04) | 0.021 | 20.90 (14.48, 27.85) | 18.39 (12.97, 25.03) | -0.50 (-0.60, -0.39) | <0.001 | 36.25 (32.84, 39.81) | 16.07 (14.22, 18.14) | -2.75 (-2.91, -2.60) | <0.001 | 0.64 (0.58, 0.70) | 0.28 (0.25, 0.32) | -2.76 (-2.91, -2.60) | <0.001 |
| Central Sub-Saharan Africa | 0.83 (0.54, 1.18) | 0.76 (0.48, 1.11) | -0.29 (-0.33, -0.24) | <0.001 | 0.18 (0.00, 0.51) | 0.12 (0.00, 0.28) | -1.27 (-3.76, 1.28) | 0.326 | 14.10 (5.22, 30.34) | 9.78 (4.53, 18.42) | -1.28 (-1.39, -1.17) | <0.001 | 0.25 (0.09, 0.54) | 0.17 (0.08, 0.32) | -1.28 (-1.39, -1.17) | <0.001 |
| Western Europe | 14.75 (13.80, 15.81) | 16.22 (15.03, 17.55) | 0.34 (0.21, 0.47) | <0.001 | 122.99 (114.32, 132.04) | 135.24 (124.68, 146.70) | 0.33 (0.10, 0.55) | 0.005 | 10.32 (9.27, 11.55) | 6.95 (6.28, 7.84) | -1.38 (-1.76, -1.01) | <0.001 | 0.18 (0.16, 0.20) | 0.12 (0.11, 0.14) | -1.40 (-1.78, -1.02) | <0.001 |

| | | | | | | | | | | | | | | | | |
|---|---|---|---|---|---|---|---|---|---|---|---|---|---|---|---|---|
| High-income North America | 101.89 (95.59, 107.91) | 104.99 (99.34, 110.38) | 0.06 (-0.10, 0.23) | 0.428 | 892.07 (831.22, 953.48) | 920.46 (863.86, 977.03) | 0.07 (-0.11, 0.25) | 0.452 | 15.22 (13.97, 16.86) | 11.12 (9.70, 12.84) | -1.01 (-1.70, -0.31) | 0.005 | 0.25 (0.23, 0.27) | 0.18 (0.16, 0.20) | -1.06 (-1.81, -0.31) | 0.006 |
| Central Asia | 11.32 (8.20, 14.89) | 12.55 (9.24, 16.39) | 0.36 (0.33, 0.38) | <0.001 | 96.72 (65.06, 136.38) | 104.77 (70.06, 145.98) | 0.30 (0.16, 0.44) | <0.001 | 3.81 (3.29, 4.43) | 5.64 (4.44, 7.17) | 1.32 (0.61, 2.03) | <0.001 | 0.07 (0.06, 0.08) | 0.10 (0.08, 0.12) | 1.36 (0.65, 2.08) | <0.001 |
| Eastern Europe | 24.91 (22.58, 27.50) | 26.53 (24.11, 29.02) | 0.21 (0.18, 0.24) | <0.001 | 214.84 (188.03, 243.67) | 226.28 (201.14, 253.01) | 0.17 (0.10, 0.24) | <0.001 | 2.68 (2.21, 3.18) | 5.57 (4.10, 8.13) | 2.63 (1.89, 3.37) | <0.001 | 0.04 (0.04, 0.05) | 0.09 (0.07, 0.14) | 3.12 (2.46, 3.79) | <0.001 |
| Southern Sub-Saharan Africa | 1.55 (1.36, 1.75) | 1.31 (1.12, 1.52) | -0.59 (-0.67, -0.51) | <0.001 | 10.18 (5.80, 14.90) | 8.93 (4.67, 13.94) | -0.44 (-0.65, -0.24) | <0.001 | 41.26 (29.80, 54.26) | 16.87 (9.94, 25.96) | -2.88 (-4.50, -1.24) | 0.001 | 0.73 (0.53, 0.96) | 0.30 (0.18, 0.46) | -2.88 (-4.50, -1.24) | 0.001 |
| High-income Asia Pacific | 61.90 (57.31, 66.80) | 68.69 (63.82, 74.13) | 0.37 (0.28, 0.46) | <0.001 | 541.41 (495.43, 596.59) | 601.87 (549.45, 662.01) | 0.38 (0.28, 0.48) | <0.001 | 6.77 (5.88, 7.92) | 3.20 (2.64, 4.06) | -2.56 (-2.98, -2.13) | <0.001 | 0.11 (0.10, 0.13) | 0.05 (0.04, 0.06) | -2.75 (-3.20, -2.30) | <0.001 |
| Central Europe | 22.31 (18.48, 26.48) | 30.07 (25.39, 34.86) | 1.03 (0.99, 1.08) | <0.001 | 189.74 (152.25, 231.59) | 260.34 (215.17, 310.33) | 1.10 (1.03, 1.18) | <0.001 | 7.51 (6.35, 8.71) | 7.56 (6.12, 9.28) | 0.05 (-0.28, 0.38) | 0.763 | 0.13 (0.11, 0.15) | 0.13 (0.10, 0.16) | 0.04 (-0.29, 0.38) | 0.802 |
| Andean Latin America | 1.18 (0.84, 1.56) | 1.60 (1.19, 2.05) | 1.05 (0.98, 1.12) | <0.001 | 2.82 (0.00, 8.05) | — | — | — | 11.91 (6.93, 17.61) | 7.96 (5.35, 11.42) | -1.44 (-2.10, -0.78) | <0.001 | 0.21 (0.12, 0.31) | 0.14 (0.09, 0.20) | -1.45 (-2.11, -0.78) | <0.001 |
| Caribbean | 1.68 (1.25, 2.16) | 1.88 (1.42, 2.39) | 0.35 (0.28, 0.42) | <0.001 | 12.33 (5.96, 19.82) | 10.65 (5.47, 17.45) | -0.57 (-1.13, -0.00) | 0.049 | 17.53 (11.24, 26.36) | 15.01 (8.91, 26.68) | -0.49 (-0.62, -0.37) | <0.001 | 0.31 (0.20, 0.47) | 0.26 (0.16, 0.47) | -0.49 (-0.62, -0.36) | <0.001 |
| Southern Latin America | 4.36 (3.13, 5.88) | 5.87 (4.37, 7.68) | 1.01 (0.95, 1.07) | <0.001 | 36.04 (16.98, 60.15) | 44.18 (23.03, 71.07) | 0.67 (0.41, 0.94) | <0.001 | 11.25 (8.46, 14.52) | 7.55 (5.90, 9.53) | -1.24 (-1.99, -0.49) | 0.001 | 0.20 (0.15, 0.26) | 0.13 (0.10, 0.17) | -1.25 (-1.99, -0.51) | 0.001 |
| Oceania | 3.89 (2.87, 5.17) | 3.89 (2.82, 5.15) | -0.06 (-0.18, 0.07) | 0.379 | 32.30 (16.11, 53.77) | 32.08 (13.73, 56.73) | -0.11 (-0.28, 0.06) | 0.212 | 24.94 (11.61, 45.53) | 26.88 (12.17, 52.24) | 0.21 (-0.10, 0.52) | 0.176 | 0.44 (0.20, 0.80) | 0.47 (0.21, 0.92) | 0.21 (-0.10, 0.52) | 0.176 |
| Australasia | 6.83 (5.62, 8.31) | 14.68 (11.73, 18.24) | 2.67 (2.64, 2.71) | <0.001 | 56.19 (33.90, 85.35) | 126.58 (80.30, 177.26) | 2.81 (2.45, 3.17) | <0.001 | 10.10 (8.00, 12.72) | 7.46 (5.60, 9.68) | -0.97 (-1.53, -0.40) | 0.001 | 0.18 (0.14, 0.22) | 0.13 (0.10, 0.17) | -0.99 (-1.55, -0.42) | 0.001 |

| | | | | | | | | | | | | | | | | |
|---|---|---|---|---|---|---|---|---|---|---|---|---|---|---|---|---|
| **Men** | | | | | | | | | | | | | | | | |
| Global | 10.22 (9.44, 11.18) | 8.85 (8.04, 9.78) | -0.51 (-0.58, -0.45) | <0.001 | 84.77 (77.41, 92.83) | 70.61 (64.21, 77.77) | -0.65 (-0.72, -0.57) | <0.001 | 13.36 (11.58, 15.76) | 10.50 (9.24, 12.25) | -0.85 (-0.97, -0.74) | <0.001 | 0.23 (0.20, 0.28) | 0.18 (0.16, 0.21) | -0.85 (-0.97, -0.74) | <0.001 |
| **SDI category** | | | | | | | | | | | | | | | | |
| High | 36.21 (33.74, 38.96) | 38.00 (35.22, 40.80) | 0.15 (0.06, 0.23) | 0.001 | 312.17 (287.65, 338.06) | 328.20 (301.57, 357.61) | 0.15 (0.06, 0.23) | 0.001 | 20.12 (18.52, 21.66) | 11.89 (10.64, 13.34) | -1.82 (-2.21, -1.43) | <0.001 | 0.35 (0.32, 0.38) | 0.20 (0.18, 0.23) | -1.85 (-2.26, -1.44) | <0.001 |
| High-middle | 11.68 (10.66, 12.93) | 12.89 (11.32, 14.60) | 0.38 (0.18, 0.57) | <0.001 | 98.48 (87.80, 109.46) | 105.29 (92.17, 119.23) | 0.26 (-0.01, 0.54) | 0.058 | 13.15 (11.37, 15.53) | 9.71 (8.66, 11.37) | -1.05 (-1.44, -0.67) | <0.001 | 0.23 (0.20, 0.27) | 0.17 (0.15, 0.20) | -1.05 (-1.34, -0.75) | <0.001 |
| Middle | 2.56 (2.22, 2.95) | 3.01 (2.56, 3.52) | 0.58 (0.47, 0.69) | <0.001 | 18.82 (14.69, 23.57) | 19.61 (15.89, 23.77) | 0.14 (-0.07, 0.36) | 0.190 | 11.45 (9.60, 13.37) | 9.26 (7.94, 10.89) | -0.80 (-0.98, -0.61) | <0.001 | 0.20 (0.17, 0.24) | 0.16 (0.14, 0.19) | -0.80 (-0.98, -0.62) | <0.001 |
| Low-middle | 1.37 (1.12, 1.65) | 1.57 (1.30, 1.87) | 0.47 (0.40, 0.55) | <0.001 | 5.33 (3.56, 7.22) | 5.89 (4.16, 7.80) | 0.40 (0.11, 0.69) | 0.007 | 10.69 (7.78, 15.32) | 11.37 (8.70, 14.89) | 0.20 (-0.01, 0.41) | 0.061 | 0.19 (0.14, 0.27) | 0.20 (0.15, 0.26) | 0.20 (-0.05, 0.44) | 0.113 |
| Low | 1.05 (0.82, 1.31) | 1.16 (0.91, 1.44) | 0.33 (0.27, 0.39) | <0.001 | 1.80 (0.87, 2.97) | 3.22 (1.91, 4.83) | 1.82 (0.90, 2.75) | <0.001 | 13.25 (8.02, 21.50) | 12.25 (8.45, 17.20) | -0.27 (-0.38, -0.17) | <0.001 | 0.23 (0.14, 0.38) | 0.22 (0.15, 0.30) | -0.27 (-0.38, -0.17) | <0.001 |
| **21 GBD region** | | | | | | | | | | | | | | | | |
| South Asia | 0.81 (0.66, 0.97) | 1.05 (0.87, 1.23) | 0.86 (0.82, 0.89) | <0.001 | — | 0.02 (0.00, 0.05) | — | — | 9.42 (6.28, 14.47) | 10.46 (7.62, 14.22) | 0.35 (0.08, 0.61) | 0.011 | 0.17 (0.11, 0.26) | 0.18 (0.13, 0.25) | 0.35 (0.08, 0.61) | 0.010 |
| East Asia | 4.30 (3.91, 4.75) | 5.20 (4.72, 5.77) | 0.64 (0.55, 0.74) | <0.001 | 36.48 (30.28, 43.18) | 37.99 (31.86, 44.22) | 0.06 (-0.18, 0.31) | 0.619 | 7.15 (5.31, 9.56) | 5.21 (4.16, 6.35) | -1.15 (-1.70, -0.60) | <0.001 | 0.13 (0.09, 0.17) | 0.09 (0.07, 0.11) | -1.16 (-1.70, -0.61) | <0.001 |
| North Africa and Middle East | 2.77 (2.10, 3.53) | 3.34 (2.59, 4.16) | 0.65 (0.52, 0.77) | <0.001 | 20.40 (12.97, 29.82) | 24.61 (16.85, 33.29) | 0.62 (0.42, 0.83) | <0.001 | 22.33 (15.18, 32.38) | 15.87 (11.96, 20.45) | -1.16 (-1.29, -1.03) | <0.001 | 0.39 (0.27, 0.57) | 0.28 (0.21, 0.36) | -1.16 (-1.30, -1.03) | <0.001 |
| Western Sub-Saharan Africa | 1.40 (1.13, 1.71) | 1.55 (1.25, 1.92) | 0.34 (0.27, 0.42) | <0.001 | 2.27 (0.81, 4.29) | 9.91 (6.70, 13.54) | 4.50 (3.17, 5.84) | <0.001 | 10.97 (6.82, 16.82) | 11.49 (8.18, 15.89) | 0.18 (0.07, 0.28) | 0.001 | 0.19 (0.12, 0.30) | 0.20 (0.14, 0.28) | 0.18 (0.07, 0.28) | 0.001 |

| | | | | | | | | | | | | | | | | |
|---|---|---|---|---|---|---|---|---|---|---|---|---|---|---|---|---|
| Southeast Asia | 1.43 (1.12, 1.83) | 1.70 (1.34, 2.14) | 0.60 (0.58, 0.62) | <0.001 | 10.90 (6.74, 15.73) | 14.05 (9.16, 19.81) | 0.85 (0.64, 1.06) | <0.001 | 5.09 (3.89, 7.20) | 5.04 (4.02, 6.82) | 0.00 (-0.33, 0.33) | 0.991 | 0.09 (0.07, 0.13) | 0.09 (0.07, 0.12) | 0.00 (-0.33, 0.33) | 0.990 |
| Eastern Sub-Saharan Africa | 1.00 (0.77, 1.26) | 1.01 (0.78, 1.27) | -0.01 (-0.14, 0.13) | 0.917 | 1.32 (0.22, 2.74) | 0.72 (0.00, 2.08) | -2.31 (-2.99, -1.62) | <0.001 | 19.02 (11.01, 30.01) | 13.94 (9.74, 19.20) | -1.07 (-1.32, -0.82) | <0.001 | 0.34 (0.19, 0.53) | 0.25 (0.17, 0.34) | -1.07 (-1.32, -0.82) | <0.001 |
| Central Latin America | 1.01 (0.78, 1.28) | 2.21 (1.70, 2.80) | 2.74 (2.59, 2.90) | <0.001 | — | 8.29 (4.92, 12.35) | — | — | 16.59 (14.24, 19.22) | 13.83 (10.96, 17.72) | -0.61 (-1.04, -0.18) | 0.005 | 0.29 (0.25, 0.34) | 0.24 (0.19, 0.31) | -0.62 (-1.04, -0.19) | 0.005 |
| Tropical Latin America | 1.97 (1.64, 2.35) | 2.15 (1.75, 2.63) | 0.29 (0.15, 0.43) | <0.001 | — | 0.48 (0.15, 0.94) | — | — | 44.80 (40.05, 49.81) | 19.80 (17.57, 22.35) | -2.72 (-3.02, -2.42) | <0.001 | 0.79 (0.71, 0.88) | 0.35 (0.31, 0.39) | -2.73 (-3.02, -2.43) | <0.001 |
| Central Sub-Saharan Africa | 0.99 (0.68, 1.35) | 0.95 (0.64, 1.33) | -0.17 (-0.23, -0.10) | <0.001 | 0.23 (0.00, 0.60) | 0.29 (0.07, 0.63) | 0.53 (-0.25, 1.32) | 0.183 | 13.21 (5.39, 25.90) | 11.12 (5.68, 20.20) | -0.62 (-0.87, -0.37) | <0.001 | 0.23 (0.09, 0.46) | 0.20 (0.10, 0.36) | -0.62 (-0.87, -0.37) | <0.001 |
| Western Europe | 9.74 (9.05, 10.52) | 12.37 (11.22, 13.64) | 0.83 (0.69, 0.97) | <0.001 | 79.76 (72.30, 87.33) | 101.48 (91.31, 112.46) | 0.82 (0.53, 1.11) | <0.001 | 21.87 (19.10, 24.88) | 13.23 (11.78, 14.92) | -1.77 (-2.15, -1.38) | <0.001 | 0.38 (0.34, 0.44) | 0.23 (0.21, 0.26) | -1.78 (-2.16, -1.39) | <0.001 |
| High-income North America | 60.20 (56.48, 64.03) | 63.71 (59.83, 67.34) | 0.15 (0.05, 0.26) | 0.003 | 521.69 (481.40, 563.92) | 553.51 (511.72, 597.01) | 0.16 (0.06, 0.27) | 0.003 | 27.43 (25.05, 29.76) | 13.34 (10.48, 15.56) | -2.49 (-3.26, -1.72) | <0.001 | 0.48 (0.43, 0.51) | 0.23 (0.18, 0.26) | -2.56 (-3.37, -1.75) | <0.001 |
| Central Asia | 16.81 (12.18, 22.25) | 19.95 (15.05, 25.85) | 0.60 (0.58, 0.62) | <0.001 | 144.12 (97.79, 195.78) | 172.07 (124.95, 230.45) | 0.63 (0.55, 0.71) | <0.001 | 4.85 (3.99, 6.16) | 7.66 (6.12, 9.55) | 1.45 (0.84, 2.06) | <0.001 | 0.08 (0.07, 0.10) | 0.13 (0.11, 0.16) | 1.49 (0.87, 2.11) | <0.001 |
| Eastern Europe | 24.82 (22.64, 27.24) | 42.07 (36.07, 49.08) | 1.82 (1.77, 1.88) | <0.001 | 213.55 (188.12, 239.56) | 363.78 (308.29, 423.72) | 1.85 (1.78, 1.92) | <0.001 | 5.91 (4.90, 7.13) | 15.25 (12.15, 20.92) | 3.33 (2.71, 3.97) | <0.001 | 0.10 (0.08, 0.12) | 0.26 (0.21, 0.36) | 3.41 (2.77, 4.06) | <0.001 |
| Southern Sub-Saharan Africa | 2.12 (1.77, 2.54) | 6.69 (4.92, 8.78) | 4.04 (3.75, 4.32) | <0.001 | 9.35 (5.14, 14.73) | 44.62 (28.41, 64.25) | 5.83 (4.94, 6.73) | <0.001 | 43.25 (31.41, 56.95) | 23.63 (16.99, 35.12) | -2.23 (-3.53, -0.91) | 0.001 | 0.76 (0.55, 1.00) | 0.42 (0.30, 0.62) | -2.24 (-3.54, -0.92) | 0.001 |
| High-income Asia Pacific | 60.68 (55.14, 66.80) | 74.53 (67.94, 82.20) | 0.74 (0.62, 0.86) | <0.001 | 527.52 (470.10, 591.45) | 649.12 (577.84, 728.18) | 0.75 (0.66, 0.84) | <0.001 | 12.05 (10.65, 13.92) | 5.94 (5.03, 7.29) | -2.42 (-2.76, -2.07) | <0.001 | 0.20 (0.18, 0.23) | 0.10 (0.08, 0.12) | -2.57 (-3.03, -2.10) | <0.001 |

| | | | | | | | | | | | | | | | | |
|---|---|---|---|---|---|---|---|---|---|---|---|---|---|---|---|---|
| Central Europe | 20.38 (16.84, 24.36) | 33.73 (28.37, 39.85) | 1.76 (1.68, 1.83) | <0.001 | 174.94 (140.98, 214.17) | 290.67 (236.42, 352.02) | 1.78 (1.73, 1.82) | <0.001 | 14.37 (12.07, 16.83) | 16.99 (14.32, 19.88) | 0.58 (0.39, 0.78) | <0.001 | 0.25 (0.21, 0.29) | 0.30 (0.25, 0.35) | 0.58 (0.38, 0.78) | <0.001 |
| Andean Latin America | 0.89 (0.61, 1.21) | 2.85 (2.13, 3.67) | 4.12 (3.94, 4.30) | <0.001 | — | 16.27 (5.42, 28.44) | — | — | 10.22 (7.00, 14.49) | 9.48 (6.34, 13.55) | -0.34 (-0.64, -0.03) | 0.032 | 0.18 (0.12, 0.26) | 0.17 (0.11, 0.24) | -0.34 (-0.65, -0.04) | 0.029 |
| Caribbean | 2.41 (1.88, 3.00) | 3.66 (2.90, 4.54) | 1.44 (1.39, 1.48) | <0.001 | 15.45 (7.89, 25.06) | 24.89 (15.30, 35.41) | 1.61 (1.21, 2.02) | <0.001 | 32.91 (25.92, 40.40) | 25.65 (18.38, 35.88) | -0.90 (-1.08, -0.71) | <0.001 | 0.58 (0.46, 0.71) | 0.45 (0.32, 0.63) | -0.90 (-1.08, -0.72) | <0.001 |
| Southern Latin America | 3.73 (2.68, 5.04) | 7.70 (6.00, 9.54) | 2.51 (2.39, 2.63) | <0.001 | 29.08 (12.71, 49.44) | 66.40 (38.58, 100.09) | 2.97 (2.41, 3.53) | <0.001 | 15.76 (12.10, 20.21) | 12.99 (10.00, 16.77) | -0.65 (-1.03, -0.26) | 0.001 | 0.28 (0.21, 0.36) | 0.23 (0.18, 0.29) | -0.66 (-1.04, -0.27) | 0.001 |
| Oceania | 2.26 (1.61, 3.08) | 2.29 (1.61, 3.14) | 0.01 (-0.15, 0.16) | 0.942 | 17.36 (5.15, 33.51) | 17.21 (3.53, 34.63) | -0.12 (-0.48, 0.23) | 0.493 | 8.31 (4.33, 16.03) | 8.09 (4.16, 15.29) | -0.13 (-0.22, -0.03) | 0.014 | 0.15 (0.08, 0.28) | 0.14 (0.07, 0.27) | -0.13 (-0.23, -0.03) | 0.014 |
| Australasia | 5.93 (4.81, 7.39) | 18.97 (14.69, 23.78) | 4.08 (3.95, 4.22) | <0.001 | 48.43 (25.08, 76.39) | 162.26 (106.22, 224.99) | 4.23 (3.90, 4.56) | <0.001 | 15.97 (12.09, 20.07) | 11.10 (7.76, 15.22) | -1.20 (-1.81, -0.58) | <0.001 | 0.28 (0.21, 0.35) | 0.19 (0.13, 0.27) | -1.23 (-1.84, -0.61) | <0.001 |
| **35-39 years** | | | | | | | | | | | | | | | | |
| **Women** | | | | | | | | | | | | | | | | |
| Global | 18.40 (17.15, 19.74) | 14.12 (13.12, 15.16) | -0.90 (-0.99, -0.82) | <0.001 | 203.03 (189.38, 218.62) | 154.06 (142.97, 165.62) | -0.95 (-1.06, -0.84) | <0.001 | 14.14 (10.24, 18.31) | 11.42 (9.06, 14.01) | -0.72 (-0.87, -0.56) | <0.001 | 0.27 (0.19, 0.35) | 0.22 (0.17, 0.27) | -0.70 (-0.86, -0.55) | <0.001 |
| **SDI category** | | | | | | | | | | | | | | | | |
| High | 69.39 (65.22, 73.71) | 69.20 (65.17, 73.55) | -0.03 (-0.15, 0.09) | 0.630 | 778.52 (731.61, 831.35) | 777.78 (731.26, 826.36) | -0.03 (-0.16, 0.11) | 0.708 | 14.48 (13.26, 16.16) | 11.63 (10.11, 13.77) | -0.77 (-1.06, -0.48) | <0.001 | 0.26 (0.24, 0.28) | 0.21 (0.18, 0.24) | -0.80 (-1.17, -0.43) | <0.001 |
| High-middle | 18.41 (16.88, 20.22) | 16.88 (15.47, 18.45) | -0.29 (-0.37, -0.21) | <0.001 | 202.74 (184.24, 225.16) | 184.47 (166.75, 204.00) | -0.32 (-0.41, -0.24) | <0.001 | 11.97 (9.51, 14.52) | 8.32 (7.29, 9.53) | -1.22 (-1.58, -0.87) | <0.001 | 0.23 (0.18, 0.28) | 0.16 (0.14, 0.18) | -1.23 (-1.59, -0.87) | <0.001 |
| Middle | 3.71 (3.25, 4.24) | 3.47 (2.99, 3.99) | -0.28 (-0.49, -0.06) | 0.013 | 38.90 (31.38, 46.64) | 34.80 (28.90, 41.88) | -0.44 (-0.74, -0.14) | 0.004 | 13.84 (9.99, 18.22) | 10.32 (8.63, 12.26) | -0.99 (-1.16, -0.82) | <0.001 | 0.27 (0.19, 0.35) | 0.20 (0.17, 0.24) | -0.99 (-1.16, -0.82) | <0.001 |

| | | | | | | | | | | | | | | | | |
|---|---|---|---|---|---|---|---|---|---|---|---|---|---|---|---|---|
| Low-middle | 1.72 (1.41, 2.05) | 1.76 (1.45, 2.11) | 0.06 (-0.14, 0.27) | 0.559 | 13.37 (9.95, 17.14) | 13.70 (10.46, 17.49) | -0.12 (-0.61, 0.36) | 0.617 | 14.31 (7.49, 22.66) | 13.16 (8.82, 18.11) | -0.27 (-0.50, -0.04) | 0.019 | 0.28 (0.14, 0.44) | 0.25 (0.17, 0.35) | -0.27 (-0.50, -0.04) | 0.020 |
| Low | 1.09 (0.83, 1.38) | 1.17 (0.89, 1.48) | 0.24 (0.17, 0.30) | <0.001 | 4.85 (3.21, 6.85) | 6.05 (3.92, 8.60) | 0.72 (0.05, 1.40) | 0.036 | 20.96 (8.94, 37.43) | 16.27 (9.06, 25.51) | -0.87 (-1.03, -0.72) | <0.001 | 0.40 (0.17, 0.72) | 0.31 (0.17, 0.49) | -0.87 (-1.02, -0.72) | <0.001 |
| **21 GBD region** | | | | | | | | | | | | | | | | |
| South Asia | 0.76 (0.61, 0.91) | 0.96 (0.79, 1.13) | 0.77 (0.64, 0.91) | <0.001 | — | 3.97 (2.63, 5.47) | — | — | 12.00 (4.98, 20.10) | 11.05 (6.71, 15.48) | -0.37 (-0.95, 0.22) | 0.219 | 0.23 (0.10, 0.39) | 0.21 (0.13, 0.30) | -0.36 (-0.94, 0.22) | 0.220 |
| East Asia | 7.02 (6.44, 7.73) | 8.95 (8.24, 9.78) | 0.86 (0.75, 0.98) | <0.001 | 76.55 (66.14, 87.84) | 94.54 (83.64, 106.32) | 0.74 (0.59, 0.90) | <0.001 | 6.09 (3.39, 8.97) | 3.02 (2.32, 3.89) | -2.39 (-2.63, -2.15) | <0.001 | 0.12 (0.06, 0.17) | 0.06 (0.04, 0.07) | -2.46 (-2.70, -2.22) | <0.001 |
| North Africa and Middle East | 4.63 (3.46, 5.99) | 5.06 (3.88, 6.46) | 0.32 (0.21, 0.42) | <0.001 | 48.85 (32.96, 66.76) | 52.95 (37.84, 71.27) | 0.26 (0.13, 0.40) | <0.001 | 51.00 (29.01, 75.71) | 32.20 (22.97, 43.60) | -1.56 (-1.66, -1.46) | <0.001 | 0.98 (0.56, 1.46) | 0.62 (0.44, 0.84) | -1.56 (-1.67, -1.46) | <0.001 |
| Western Sub-Saharan Africa | 1.10 (0.85, 1.40) | 1.19 (0.92, 1.49) | 0.24 (0.16, 0.33) | <0.001 | 9.82 (6.57, 13.56) | 11.56 (7.87, 15.78) | 0.53 (0.37, 0.69) | <0.001 | 20.85 (8.19, 43.31) | 17.16 (9.28, 28.47) | -0.68 (-0.84, -0.51) | <0.001 | 0.40 (0.16, 0.84) | 0.33 (0.18, 0.55) | -0.68 (-0.85, -0.51) | <0.001 |
| Southeast Asia | 2.12 (1.64, 2.66) | 2.48 (1.97, 3.08) | 0.55 (0.53, 0.57) | <0.001 | 22.79 (15.88, 30.90) | 26.78 (19.08, 36.61) | 0.55 (0.41, 0.69) | <0.001 | 6.43 (3.94, 9.14) | 4.91 (3.84, 6.06) | -0.92 (-1.05, -0.79) | <0.001 | 0.12 (0.08, 0.18) | 0.09 (0.07, 0.12) | -0.93 (-1.06, -0.79) | <0.001 |
| Eastern Sub-Saharan Africa | 1.05 (0.80, 1.34) | 0.98 (0.73, 1.26) | -0.25 (-0.35, -0.15) | <0.001 | 4.39 (2.58, 6.75) | 4.84 (2.47, 7.93) | 0.62 (-1.37, 2.65) | 0.542 | 25.51 (11.39, 45.32) | 14.24 (8.73, 21.32) | -1.99 (-2.08, -1.90) | <0.001 | 0.49 (0.22, 0.88) | 0.27 (0.17, 0.41) | -1.99 (-2.08, -1.90) | <0.001 |
| Central Latin America | 1.65 (1.33, 2.02) | 2.08 (1.68, 2.53) | 0.81 (0.75, 0.86) | <0.001 | 15.90 (10.08, 23.01) | 16.85 (11.43, 23.22) | 0.20 (0.03, 0.38) | 0.024 | 18.48 (16.30, 20.65) | 13.16 (9.98, 17.14) | -1.14 (-1.53, -0.74) | <0.001 | 0.36 (0.31, 0.40) | 0.25 (0.19, 0.33) | -1.14 (-1.53, -0.75) | <0.001 |
| Tropical Latin America | 3.61 (3.21, 4.00) | 3.39 (2.99, 3.84) | -0.26 (-0.47, -0.04) | 0.023 | 34.01 (25.53, 44.52) | 29.88 (21.96, 37.84) | -0.49 (-0.59, -0.39) | <0.001 | 47.99 (43.84, 52.81) | 22.58 (20.04, 25.40) | -2.60 (-2.94, -2.25) | <0.001 | 0.93 (0.85, 1.02) | 0.44 (0.39, 0.49) | -2.60 (-2.94, -2.26) | <0.001 |
| Central Sub-Saharan Africa | 1.06 (0.69, 1.52) | 0.98 (0.62, 1.43) | -0.30 (-0.41, -0.20) | <0.001 | 3.48 (0.65, 7.57) | 3.92 (0.75, 8.59) | 0.42 (0.01, 0.82) | 0.044 | 18.42 (7.33, 37.33) | 13.56 (6.70, 24.96) | -1.11 (-1.23, | <0.001 | 0.36 (0.14, | 0.26 (0.13, | -1.11 (-1.23, | <0.001 |

| | | | | | | | | | | | | | | | | |
|---|---|---|---|---|---|---|---|---|---|---|---|---|---|---|---|---|
| | | | | | | | | | | | -1.00) | | 0.72) | 0.48) | -1.00) | |
| Western Europe | 18.66 (17.46, 19.99) | 20.97 (19.44, 22.68) | 0.40 (0.29, 0.51) | <0.001 | 206.38 (191.98, 222.38) | 233.09 (213.94, 253.62) | 0.42 (0.28, 0.56) | <0.001 | 12.06 (10.71, 13.43) | 8.18 (7.43, 9.13) | -1.34 (-1.49, -1.20) | <0.001 | 0.23 (0.20, 0.25) | 0.15 (0.14, 0.17) | -1.38 (-1.53, -1.24) | <0.001 |
| High-income North America | 129.93 (121.96, 137.55) | 133.44 (126.29, 140.23) | 0.05 (-0.11, 0.21) | 0.547 | 1463.93 (1370.00, 1562.33) | 1505.17 (1419.39, 1588.76) | 0.06 (-0.11, 0.22) | 0.518 | 19.68 (17.72, 22.30) | 14.24 (12.55, 16.90) | -1.14 (-1.72, -0.56) | <0.001 | 0.34 (0.32, 0.37) | 0.24 (0.22, 0.27) | -1.25 (-1.84, -0.64) | <0.001 |
| Central Asia | 14.58 (10.58, 19.16) | 16.19 (11.93, 21.13) | 0.36 (0.34, 0.39) | <0.001 | 159.22 (110.46, 222.14) | 175.14 (123.67, 238.00) | 0.31 (0.19, 0.43) | <0.001 | 3.65 (3.06, 4.33) | 5.77 (4.46, 7.30) | 1.64 (1.03, 2.25) | <0.001 | 0.07 (0.06, 0.08) | 0.11 (0.08, 0.14) | 1.74 (1.09, 2.38) | <0.001 |
| Eastern Europe | 31.97 (29.00, 35.29) | 34.03 (30.91, 37.20) | 0.21 (0.19, 0.23) | <0.001 | 351.88 (311.78, 395.51) | 374.43 (334.37, 416.51) | 0.21 (0.18, 0.24) | <0.001 | 3.40 (2.78, 4.25) | 6.94 (5.17, 9.80) | 2.59 (1.70, 3.49) | <0.001 | 0.05 (0.05, 0.06) | 0.12 (0.09, 0.18) | 2.99 (1.97, 4.02) | <0.001 |
| Southern Sub-Saharan Africa | 1.99 (1.75, 2.24) | 1.69 (1.44, 1.96) | -0.59 (-0.67, -0.52) | <0.001 | 19.44 (12.90, 26.67) | 16.81 (10.66, 23.87) | -0.50 (-0.67, -0.33) | <0.001 | 41.03 (30.89, 52.78) | 17.97 (11.25, 26.48) | -2.76 (-3.84, -1.68) | <0.001 | 0.79 (0.60, 1.02) | 0.35 (0.22, 0.51) | -2.77 (-3.84, -1.68) | <0.001 |
| High-income Asia Pacific | 85.71 (80.11, 92.10) | 86.28 (80.12, 93.21) | 0.02 (-0.06, 0.10) | 0.605 | 961.63 (888.80, 1040.68) | 971.44 (890.77, 1061.29) | 0.03 (-0.04, 0.11) | 0.389 | 11.07 (9.77, 12.99) | 4.89 (3.90, 6.74) | -2.86 (-3.15, -2.58) | <0.001 | 0.19 (0.17, 0.21) | 0.07 (0.06, 0.09) | -3.32 (-3.50, -3.15) | <0.001 |
| Central Europe | 28.68 (23.78, 34.00) | 38.64 (32.68, 44.74) | 1.03 (0.99, 1.07) | <0.001 | 315.18 (258.08, 376.37) | 425.68 (353.94, 505.83) | 1.04 (0.98, 1.10) | <0.001 | 12.50 (10.74, 14.47) | 11.84 (9.50, 14.50) | -0.19 (-0.63, 0.26) | 0.408 | 0.23 (0.20, 0.27) | 0.22 (0.17, 0.27) | -0.21 (-0.68, 0.27) | 0.394 |
| Andean Latin America | 1.52 (1.09, 2.00) | 2.06 (1.52, 2.64) | 1.04 (0.94, 1.15) | <0.001 | 14.61 (5.00, 26.80) | 18.19 (7.22, 31.83) | 0.78 (-0.67, 2.25) | 0.292 | 14.59 (8.73, 21.23) | 9.45 (6.42, 13.34) | -1.51 (-2.24, -0.77) | <0.001 | 0.28 (0.17, 0.41) | 0.18 (0.12, 0.26) | -1.51 (-2.25, -0.77) | <0.001 |
| Caribbean | 2.19 (1.63, 2.80) | 2.47 (1.87, 3.12) | 0.39 (0.33, 0.46) | <0.001 | 21.65 (12.02, 33.96) | 22.49 (13.03, 34.20) | 0.11 (-0.00, 0.23) | 0.055 | 20.46 (13.22, 31.45) | 18.58 (11.22, 31.82) | -0.29 (-0.55, -0.04) | 0.024 | 0.40 (0.25, 0.61) | 0.36 (0.22, 0.62) | -0.29 (-0.55, -0.04) | 0.025 |
| Southern Latin America | 5.60 (4.02, 7.54) | 7.52 (5.60, 9.85) | 1.02 (0.99, 1.05) | <0.001 | 59.78 (32.00, 96.05) | 81.68 (48.36, 119.65) | 1.12 (0.71, 1.53) | <0.001 | 11.70 (8.87, 14.87) | 8.46 (6.72, 10.62) | -1.11 (-1.68, -0.54) | <0.001 | 0.22 (0.17, 0.29) | 0.16 (0.13, 0.20) | -1.13 (-1.71, -0.56) | <0.001 |
| Oceania | 5.01 (3.69, 6.66) | 5.02 (3.65, 6.65) | -0.01 (-0.06, 0.03) | 0.631 | 52.68 (27.66, 83.64) | 52.83 (25.95, 83.58) | -0.03 (-0.16, | 0.681 | 14.00 (6.68, 25.29) | 15.34 (6.90, 28.73) | 0.28 (0.08, | 0.005 | 0.27 (0.13, | 0.29 (0.13, | 0.28 (0.09, 0.48) | 0.005 |

| | | | | | | | | | | | | | | | | |
|---|---|---|---|---|---|---|---|---|---|---|---|---|---|---|---|---|
| | | | | | | | 0.11) | | | | 0.48) | | 0.49) | 0.55) | | |
| Australasia | 8.70 (7.14, 10.62) | 18.91 (15.13, 23.46) | 2.71 (2.65, 2.77) | <0.001 | 92.11 (56.98, 131.21) | 208.02 (145.04, 283.51) | 2.78 (2.60, 2.97) | <0.001 | 11.13 (8.45, 14.19) | 8.18 (6.11, 10.76) | -1.20 (-1.84, -0.55) | <0.001 | 0.21 (0.16, 0.27) | 0.15 (0.11, 0.20) | -1.25 (-1.92, -0.59) | <0.001 |
| **Men** | | | | | | | | | | | | | | | | |
| Global | 13.50 (12.49, 14.72) | 12.30 (11.20, 13.59) | -0.30 (-0.36, -0.23) | <0.001 | 145.43 (133.74, 157.68) | 131.93 (119.92, 145.48) | -0.32 (-0.39, -0.25) | <0.001 | 18.56 (16.13, 22.21) | 15.05 (13.21, 17.45) | -0.71 (-0.77, -0.65) | <0.001 | 0.35 (0.31, 0.42) | 0.29 (0.25, 0.34) | -0.71 (-0.77, -0.65) | <0.001 |
| **SDI category** | | | | | | | | | | | | | | | | |
| High | 47.35 (44.16, 50.85) | 49.55 (45.89, 53.29) | 0.14 (0.08, 0.21) | <0.001 | 526.02 (484.86, 565.70) | 549.61 (506.47, 595.46) | 0.14 (0.07, 0.21) | <0.001 | 28.43 (26.54, 30.53) | 16.17 (14.39, 18.50) | -1.86 (-2.11, -1.61) | <0.001 | 0.54 (0.50, 0.57) | 0.30 (0.27, 0.34) | -1.91 (-2.16, -1.65) | <0.001 |
| High-middle | 14.99 (13.67, 16.58) | 17.68 (15.53, 20.01) | 0.58 (0.54, 0.61) | <0.001 | 162.72 (146.01, 181.25) | 192.18 (166.98, 219.51) | 0.58 (0.52, 0.64) | <0.001 | 17.93 (15.13, 21.69) | 14.09 (12.62, 16.38) | -0.80 (-1.00, -0.60) | <0.001 | 0.34 (0.29, 0.41) | 0.27 (0.24, 0.31) | -0.81 (-1.01, -0.61) | <0.001 |
| Middle | 3.32 (2.89, 3.80) | 3.74 (3.17, 4.39) | 0.39 (0.20, 0.58) | <0.001 | 32.29 (26.30, 39.16) | 35.49 (29.11, 42.57) | 0.31 (0.15, 0.47) | <0.001 | 15.19 (12.76, 18.44) | 13.21 (11.46, 15.66) | -0.47 (-0.57, -0.37) | <0.001 | 0.29 (0.25, 0.36) | 0.25 (0.22, 0.30) | -0.48 (-0.58, -0.38) | <0.001 |
| Low-middle | 1.75 (1.44, 2.08) | 1.99 (1.64, 2.37) | 0.43 (0.21, 0.65) | <0.001 | 10.86 (7.93, 13.93) | 17.12 (12.82, 21.71) | 1.57 (0.98, 2.16) | <0.001 | 15.49 (11.36, 22.19) | 16.53 (12.69, 22.12) | 0.24 (-0.21, 0.70) | 0.296 | 0.30 (0.22, 0.43) | 0.32 (0.24, 0.43) | 0.24 (-0.21, 0.70) | 0.296 |
| Low | 1.34 (1.05, 1.66) | 1.49 (1.18, 1.85) | 0.36 (0.30, 0.41) | <0.001 | 6.55 (4.12, 9.28) | 10.67 (6.87, 14.90) | 1.64 (0.91, 2.38) | <0.001 | 19.34 (11.53, 31.31) | 17.81 (12.45, 25.26) | -0.27 (-0.47, -0.06) | 0.010 | 0.37 (0.22, 0.61) | 0.34 (0.24, 0.49) | -0.27 (-0.47, -0.06) | 0.010 |
| **21 GBD region** | | | | | | | | | | | | | | | | |
| South Asia | 1.04 (0.85, 1.25) | 1.35 (1.12, 1.59) | 0.87 (0.83, 0.90) | <0.001 | 0.004 (0.000, 0.010) | 11.05 (7.87, 14.52) | 25.55 (21.55, 29.68) | <0.001 | 14.29 (9.68, 21.64) | 15.49 (11.11, 21.70) | 0.29 (-0.45, 1.04) | 0.442 | 0.28 (0.19, 0.42) | 0.30 (0.21, 0.42) | 0.29 (-0.45, 1.04) | 0.444 |
| East Asia | 5.28 (4.80, 5.86) | 6.90 (6.26, 7.66) | 0.94 (0.80, 1.09) | <0.001 | 57.18 (48.04, 67.87) | 71.49 (61.03, 82.70) | 0.81 (0.67, 0.94) | <0.001 | 9.55 (6.93, 13.06) | 6.89 (5.37, 8.63) | -1.16 (-1.48, -0.84) | <0.001 | 0.18 (0.13, 0.25) | 0.13 (0.10, 0.17) | -1.14 (-1.52, -0.76) | <0.001 |
| North Africa and Middle East | 3.56 (2.69, 4.54) | 4.34 (3.37, 5.41) | 0.70 (0.64, 0.75) | <0.001 | 33.53 (22.40, 47.44) | 40.52 (29.13, 53.90) | 0.68 (0.35, 1.02) | <0.001 | 36.11 (22.48, 54.22) | 24.71 (19.23, 31.36) | -1.30 (-1.40, | <0.001 | 0.70 (0.43, | 0.48 (0.37, | -1.30 (-1.40, | <0.001 |

| | | | | | | | | | | | | | | | | |
|---|---|---|---|---|---|---|---|---|---|---|---|---|---|---|---|---|
| | | | | | | | | | | | -1.20) | | 1.05) | 0.60) | -1.20) | |
| Western Sub-Saharan Africa | 1.80 (1.46, 2.20) | 2.00 (1.60, 2.47) | 0.35 (0.27, 0.43) | <0.001 | 15.86 (11.15, 21.09) | 17.45 (12.45, 23.50) | 0.32 (0.16, 0.48) | <0.001 | 14.28 (9.10, 21.41) | 14.41 (10.22, 19.43) | 0.04 (-0.05, 0.12) | 0.422 | 0.28 (0.18, 0.41) | 0.28 (0.20, 0.38) | 0.03 (-0.05, 0.12) | 0.439 |
| Southeast Asia | 1.84 (1.44, 2.35) | 2.20 (1.74, 2.76) | 0.60 (0.58, 0.62) | <0.001 | 19.32 (13.36, 26.59) | 23.18 (16.15, 31.39) | 0.63 (0.57, 0.69) | <0.001 | 8.28 (6.49, 12.37) | 8.14 (6.30, 12.98) | -0.04 (-0.26, 0.19) | 0.759 | 0.16 (0.12, 0.24) | 0.16 (0.12, 0.25) | -0.04 (-0.26, 0.19) | 0.750 |
| Eastern Sub-Saharan Africa | 1.27 (0.99, 1.59) | 1.30 (1.00, 1.63) | 0.03 (-0.04, 0.10) | 0.477 | 6.15 (3.47, 9.67) | 7.41 (4.03, 11.39) | 0.59 (0.13, 1.05) | 0.011 | 27.71 (15.88, 43.28) | 20.14 (14.20, 28.30) | -1.10 (-1.29, -0.91) | <0.001 | 0.54 (0.31, 0.84) | 0.39 (0.27, 0.55) | -1.10 (-1.29, -0.91) | <0.001 |
| Central Latin America | 1.30 (0.99, 1.64) | 2.85 (2.19, 3.62) | 2.77 (2.62, 2.92) | <0.001 | 0.18 (0.00, 0.48) | 16.53 (10.28, 23.38) | 15.17 (8.53, 22.22) | <0.001 | 22.68 (19.74, 25.87) | 17.91 (14.05, 22.45) | -0.74 (-1.00, -0.48) | <0.001 | 0.44 (0.38, 0.50) | 0.35 (0.27, 0.43) | -0.74 (-1.01, -0.48) | <0.001 |
| Tropical Latin America | 2.53 (2.10, 3.02) | 2.77 (2.25, 3.38) | 0.30 (0.16, 0.43) | <0.001 | 15.26 (10.16, 21.60) | 22.97 (15.96, 31.58) | 1.31 (0.95, 1.68) | <0.001 | 57.52 (51.63, 63.99) | 26.45 (23.47, 30.16) | -2.63 (-2.99, -2.28) | <0.001 | 1.11 (1.00, 1.24) | 0.51 (0.45, 0.58) | -2.64 (-2.99, -2.28) | <0.001 |
| Central Sub-Saharan Africa | 1.28 (0.87, 1.74) | 1.22 (0.83, 1.70) | -0.18 (-0.24, -0.11) | <0.001 | 10.87 (1.90, 23.19) | 3.81 (0.75, 7.79) | -3.22 (-4.30, -2.13) | <0.001 | 19.97 (8.70, 38.03) | 17.30 (9.27, 30.64) | -0.53 (-0.78, -0.28) | <0.001 | 0.39 (0.17, 0.74) | 0.33 (0.18, 0.59) | -0.53 (-0.78, -0.28) | <0.001 |
| Western Europe | 12.42 (11.54, 13.42) | 16.05 (14.57, 17.67) | 0.88 (0.73, 1.03) | <0.001 | 134.20 (123.34, 146.14) | 174.31 (157.13, 193.20) | 0.90 (0.66, 1.14) | <0.001 | 27.10 (24.11, 30.58) | 16.49 (14.67, 18.87) | -1.74 (-2.04, -1.43) | <0.001 | 0.52 (0.46, 0.59) | 0.32 (0.28, 0.36) | -1.75 (-2.07, -1.44) | <0.001 |
| High-income North America | 76.89 (72.14, 81.76) | 81.21 (76.28, 85.84) | 0.14 (0.05, 0.24) | 0.004 | 857.42 (795.34, 922.85) | 906.20 (842.33, 970.17) | 0.14 (0.04, 0.24) | 0.006 | 40.48 (37.55, 43.78) | 18.69 (15.90, 21.33) | -2.68 (-3.22, -2.13) | <0.001 | 0.76 (0.71, 0.82) | 0.34 (0.29, 0.39) | -2.78 (-3.34, -2.22) | <0.001 |
| Central Asia | 21.63 (15.67, 28.61) | 25.77 (19.47, 33.38) | 0.61 (0.57, 0.65) | <0.001 | 235.70 (166.37, 318.98) | 282.83 (206.38, 370.91) | 0.64 (0.55, 0.73) | <0.001 | 6.71 (5.53, 8.42) | 10.79 (8.49, 13.38) | 1.61 (1.04, 2.17) | <0.001 | 0.12 (0.10, 0.15) | 0.20 (0.16, 0.25) | 1.68 (1.08, 2.27) | <0.001 |
| Eastern Europe | 31.87 (29.07, 34.95) | 54.28 (46.50, 63.34) | 1.85 (1.80, 1.90) | <0.001 | 350.59 (312.92, 391.30) | 598.13 (510.49, 705.00) | 1.87 (1.83, 1.91) | <0.001 | 7.66 (6.21, 9.34) | 19.46 (15.38, 25.57) | 3.71 (2.81, 4.61) | <0.001 | 0.14 (0.11, 0.17) | 0.36 (0.28, 0.48) | 3.86 (2.97, 4.76) | <0.001 |
| Southern Sub-Saharan Africa | 2.72 (2.26, 3.25) | 8.47 (6.24, 11.10) | 3.99 (3.69, 4.28) | <0.001 | 20.59 (13.65, 28.58) | 71.50 (49.10, 100.74) | 4.40 (4.14, 4.67) | <0.001 | 46.38 (34.32, 57.83) | 26.57 (18.69, 38.04) | -1.91 (-2.43, | <0.001 | 0.89 (0.66, | 0.51 (0.36, | -1.92 (-2.44, | <0.001 |

| | | | | | | | | | | | | | | | | |
|---|---|---|---|---|---|---|---|---|---|---|---|---|---|---|---|---|
| | | | | | | | | | | | -1.38) | | 1.12) | 0.73) | -1.39) | |
| High-income Asia Pacific | 81.02 (74.48, 88.24) | 94.82 (86.22, 104.60) | 0.55 (0.43, 0.67) | <0.001 | 904.14 (822.27, 994.24) | 1059.58 (952.94, 1181.11) | 0.55 (0.45, 0.66) | <0.001 | 18.88 (16.92, 21.34) | 8.58 (7.07, 10.83) | -2.68 (-3.08, -2.29) | <0.001 | 0.34 (0.31, 0.38) | 0.14 (0.12, 0.17) | -2.96 (-3.44, -2.48) | <0.001 |
| Central Europe | 26.27 (21.70, 31.43) | 43.23 (36.37, 50.97) | 1.74 (1.68, 1.80) | <0.001 | 288.14 (232.25, 349.53) | 475.21 (391.88, 563.76) | 1.76 (1.67, 1.85) | <0.001 | 22.73 (19.65, 26.43) | 24.23 (19.92, 28.80) | 0.20 (-0.19, 0.58) | 0.317 | 0.43 (0.37, 0.50) | 0.46 (0.37, 0.55) | 0.18 (-0.22, 0.58) | 0.383 |
| Andean Latin America | 1.14 (0.78, 1.56) | 3.67 (2.74, 4.73) | 4.13 (3.90, 4.36) | <0.001 | 0.00 (0.00, 0.00) | 29.88 (14.86, 47.68) | — | — | 12.30 (8.14, 17.66) | 10.59 (7.13, 15.37) | -0.47 (-0.82, -0.13) | 0.007 | 0.24 (0.16, 0.34) | 0.20 (0.14, 0.30) | -0.49 (-0.83, -0.14) | 0.006 |
| Caribbean | 3.14 (2.45, 3.89) | 4.87 (3.85, 6.07) | 1.51 (1.47, 1.55) | <0.001 | 25.65 (15.35, 38.15) | 46.95 (31.59, 64.46) | 2.10 (1.84, 2.35) | <0.001 | 37.16 (28.89, 47.19) | 30.83 (21.80, 42.64) | -0.65 (-0.85, -0.45) | <0.001 | 0.72 (0.56, 0.91) | 0.60 (0.42, 0.82) | -0.66 (-0.85, -0.45) | <0.001 |
| Southern Latin America | 4.78 (3.43, 6.49) | 9.83 (7.67, 12.20) | 2.50 (2.46, 2.55) | <0.001 | 47.26 (22.42, 75.60) | 107.02 (68.53, 146.05) | 2.88 (2.56, 3.19) | <0.001 | 27.39 (20.85, 35.18) | 22.09 (16.98, 28.32) | -0.63 (-1.14, -0.12) | 0.016 | 0.53 (0.40, 0.68) | 0.42 (0.33, 0.55) | -0.64 (-1.16, -0.13) | 0.014 |
| Oceania | 2.92 (2.08, 3.97) | 2.96 (2.07, 4.04) | -0.01 (-0.15, 0.13) | 0.904 | 28.05 (11.92, 49.68) | 27.40 (9.02, 49.79) | -0.12 (-0.31, 0.07) | 0.213 | 24.62 (12.86, 45.06) | 22.48 (11.62, 41.73) | -0.32 (-0.47, -0.16) | <0.001 | 0.48 (0.25, 0.87) | 0.43 (0.22, 0.81) | -0.32 (-0.47, -0.16) | <0.001 |
| Australasia | 7.58 (6.14, 9.45) | 24.37 (18.87, 30.53) | 4.11 (3.95, 4.28) | <0.001 | 78.13 (50.47, 116.41) | 266.72 (187.90, 359.95) | 4.29 (4.01, 4.58) | <0.001 | 22.51 (17.60, 28.52) | 16.90 (12.03, 23.04) | -0.94 (-1.69, -0.18) | 0.016 | 0.43 (0.34, 0.55) | 0.32 (0.23, 0.44) | -0.98 (-1.74, -0.21) | 0.013 |
| **Cardiomyopathy and myocarditis** | | | | | | | | | | | | | | | | |
| **15-19 years** | | | | | | | | | | | | | | | | |
| **Women** | | | | | | | | | | | | | | | | |
| Global | 7.33 (4.12, 11.85) | 6.90 (3.88, 11.19) | -0.21 (-0.23, -0.20) | <0.001 | 1.05 (0.58, 1.61) | 0.86 (0.46, 1.34) | -0.60 (-0.87, -0.33) | <0.001 | 19.14 (15.31, 27.43) | 13.42 (10.83, 16.09) | -1.17 (-1.26, -1.08) | <0.001 | 0.26 (0.20, 0.37) | 0.18 (0.14, 0.22) | -1.20 (-1.29, -1.10) | <0.001 |
| **SDI category** | | | | | | | | | | | | | | | | |
| High | 6.94 (3.84, 11.32) | 6.79 (4.00, 10.75) | -0.08 (-0.13, -0.03) | 0.003 | 8.81 (4.90, 13.64) | 9.23 (4.94, 14.43) | 0.34 (-0.02, 0.69) | 0.062 | 30.82 (27.26, 34.92) | 20.53 (17.74, 23.69) | -1.24 (-1.50, -0.98) | <0.001 | 0.38 (0.34, 0.43) | 0.24 (0.21, 0.27) | -1.50 (-1.86, -1.14) | <0.001 |

| | | | | | | | | | | | | | | | | |
|---|---|---|---|---|---|---|---|---|---|---|---|---|---|---|---|---|
| High-middle | 7.20 (3.99, 11.84) | 6.67 (3.80, 10.75) | -0.27 (-0.33, -0.20) | <0.001 | 0.07 (0.01, 0.14) | 0.08 (0.01, 0.16) | 0.52 (0.19, 0.84) | 0.002 | 24.43 (20.41, 33.72) | 19.73 (15.15, 22.52) | -0.76 (-0.91, -0.61) | <0.001 | 0.33 (0.28, 0.46) | 0.27 (0.20, 0.30) | -0.78 (-0.94, -0.62) | <0.001 |
| Middle | 7.69 (4.33, 12.47) | 6.90 (3.85, 11.23) | -0.38 (-0.39, -0.36) | <0.001 | — | — | — | — | 18.68 (13.79, 31.57) | 13.81 (10.90, 18.00) | -1.02 (-1.12, -0.92) | <0.001 | 0.26 (0.19, 0.44) | 0.19 (0.15, 0.25) | -1.04 (-1.14, -0.94) | <0.001 |
| Low-middle | 7.29 (4.06, 11.83) | 7.06 (3.91, 11.51) | -0.11 (-0.12, -0.09) | <0.001 | — | — | — | — | 12.22 (7.53, 18.38) | 9.28 (7.01, 12.14) | -0.95 (-1.05, -0.84) | <0.001 | 0.17 (0.10, 0.25) | 0.13 (0.09, 0.17) | -0.93 (-1.03, -0.83) | <0.001 |
| Low | 6.89 (3.83, 11.28) | 6.88 (3.82, 11.26) | -0.01 (-0.01, -0.01) | <0.001 | — | — | — | — | 12.66 (6.14, 19.48) | 11.22 (7.42, 15.83) | -0.44 (-0.53, -0.34) | <0.001 | 0.17 (0.08, 0.27) | 0.15 (0.10, 0.22) | -0.45 (-0.54, -0.35) | <0.001 |
| **21 GBD region** | | | | | | | | | | | | | | | | |
| South Asia | 7.26 (4.05, 11.94) | 7.27 (4.05, 11.96) | 0.01 (0.01, 0.01) | <0.001 | — | — | — | — | 4.17 (1.89, 7.19) | 3.27 (2.20, 4.88) | -0.85 (-1.13, -0.56) | <0.001 | 0.06 (0.02, 0.10) | 0.04 (0.03, 0.07) | -0.87 (-1.16, -0.59) | <0.001 |
| East Asia | 8.81 (4.93, 14.45) | 7.30 (4.24, 11.66) | -0.65 (-0.70, -0.61) | <0.001 | — | — | — | — | 20.39 (14.09, 39.73) | 15.08 (9.41, 19.18) | -0.95 (-1.20, -0.70) | <0.001 | 0.28 (0.19, 0.55) | 0.20 (0.13, 0.26) | -0.96 (-1.21, -0.72) | <0.001 |
| North Africa and Middle East | 5.36 (2.87, 9.12) | 5.32 (2.84, 9.05) | -0.02 (-0.03, -0.02) | <0.001 | — | — | — | — | 29.31 (16.97, 49.24) | 19.42 (13.17, 32.48) | -1.39 (-1.57, -1.21) | <0.001 | 0.40 (0.23, 0.68) | 0.26 (0.18, 0.45) | -1.42 (-1.60, -1.23) | <0.001 |
| Western Sub-Saharan Africa | 7.00 (3.90, 11.46) | 7.00 (3.90, 11.47) | 0.00 (0.00, 0.00)* | 0.032 | — | — | — | — | 13.02 (6.13, 22.13) | 9.51 (5.90, 13.96) | -1.11 (-1.25, -0.96) | <0.001 | 0.18 (0.08, 0.31) | 0.13 (0.08, 0.19) | -1.13 (-1.24, -1.01) | <0.001 |
| Southeast Asia | 7.15 (4.01, 11.62) | 7.18 (4.03, 11.62) | 0.01 (0.01, 0.02) | <0.001 | — | — | — | — | 14.64 (8.42, 24.92) | 13.33 (9.32, 18.08) | -0.28 (-0.47, -0.09) | 0.004 | 0.20 (0.11, 0.34) | 0.18 (0.12, 0.25) | -0.30 (-0.50, -0.11) | 0.003 |
| Eastern Sub-Saharan Africa | 6.93 (3.86, 11.37) | 6.93 (3.86, 11.37) | 0.00 (0.00, 0.00)* | <0.001 | — | — | — | — | 11.17 (6.40, 17.15) | 9.56 (6.13, 15.04) | -0.54 (-0.66, -0.41) | <0.001 | 0.15 (0.09, 0.24) | 0.13 (0.08, 0.21) | -0.55 (-0.67, -0.42) | <0.001 |
| Central Latin America | 6.36 (3.47, 10.40) | 6.33 (3.42, 10.36) | -0.02 (-0.02, -0.02) | <0.001 | — | — | — | — | 11.58 (9.68, 14.03) | 11.24 (8.46, 13.61) | 0.07 (-0.41, | 0.782 | 0.15 (0.13, | 0.15 (0.11, | 0.08 (-0.42, 0.58) | 0.749 |

| | | | | | | | | | | | | | | | | |
|---|---|---|---|---|---|---|---|---|---|---|---|---|---|---|---|---|
| | | | | | | | | | | | 0.55) | | 0.19) | 0.18) | | |
| Tropical Latin America | 6.69 (3.65, 10.91) | 6.68 (3.65, 10.90) | -0.0048 (-0.0050, -0.0047) | <0.001 | — | — | — | — | 33.11 (27.85, 38.61) | 26.56 (22.11, 30.48) | -0.66 (-1.07, -0.24) | 0.002 | 0.45 (0.38, 0.52) | 0.36 (0.30, 0.41) | -0.68 (-1.11, -0.24) | 0.002 |
| Central Sub-Saharan Africa | 6.67 (3.70, 11.26) | 6.67 (3.70, 11.26) | 0.00 (0.00, 0.00)* | <0.001 | — | — | — | — | 39.37 (12.25, 76.15) | 36.87 (16.85, 66.02) | -0.27 (-0.42, -0.13) | <0.001 | 0.55 (0.17, 1.06) | 0.51 (0.23, 0.92) | -0.27 (-0.42, -0.13) | <0.001 |
| Western Europe | 6.23 (3.41, 10.41) | 6.17 (3.40, 10.32) | -0.04 (-0.06, -0.01) | 0.006 | 0.42 (0.08, 0.93) | — | — | — | 22.25 (18.49, 24.98) | 10.33 (8.63, 13.32) | -2.64 (-3.06, -2.22) | <0.001 | 0.28 (0.23, 0.32) | 0.12 (0.10, 0.17) | -2.94 (-3.30, -2.58) | <0.001 |
| High-income North America | 6.63 (3.55, 11.03) | 6.86 (4.07, 10.72) | 0.11 (0.04, 0.17) | 0.001 | 26.78 (14.85, 41.88) | 22.49 (12.08, 35.10) | -0.57 (-0.97, -0.17) | 0.005 | 37.97 (33.42, 43.64) | 30.79 (25.69, 35.58) | -0.61 (-1.16, -0.06) | 0.030 | 0.45 (0.40, 0.50) | 0.36 (0.30, 0.40) | -0.69 (-1.25, -0.14) | 0.014 |
| Central Asia | 7.20 (3.98, 11.82) | 7.20 (3.98, 11.82) | 0.00 (0.00, 0.00)* | <0.001 | — | — | — | — | 29.95 (23.24, 40.09) | 41.70 (28.58, 52.77) | 1.14 (0.80, 1.48) | <0.001 | 0.41 (0.32, 0.56) | 0.58 (0.40, 0.73) | 1.16 (0.81, 1.50) | <0.001 |
| Eastern Europe | 8.00 (4.48, 13.10) | 8.01 (4.49, 13.13) | 0.00 (0.00, 0.01) | <0.001 | — | — | — | — | 39.69 (34.86, 54.36) | 51.34 (34.85, 60.32) | 0.72 (0.05, 1.40) | 0.036 | 0.55 (0.48, 0.75) | 0.71 (0.48, 0.83) | 0.74 (0.05, 1.43) | 0.034 |
| Southern Sub-Saharan Africa | 7.14 (3.96, 11.72) | 7.11 (3.94, 11.63) | -0.02 (-0.02, -0.01) | <0.001 | — | — | — | — | 44.74 (30.12, 59.54) | 31.33 (19.48, 49.07) | -1.17 (-1.71, -0.63) | <0.001 | 0.62 (0.42, 0.83) | 0.44 (0.27, 0.68) | -1.18 (-1.73, -0.64) | <0.001 |
| High-income Asia Pacific | 8.35 (4.74, 13.40) | 8.34 (4.81, 13.22) | -0.01 (-0.02, 0.00) | 0.210 | — | — | — | — | 30.01 (25.29, 38.12) | 13.92 (11.07, 17.37) | -2.66 (-3.03, -2.29) | <0.001 | 0.38 (0.32, 0.50) | 0.15 (0.12, 0.19) | -3.11 (-3.54, -2.67) | <0.001 |
| Central Europe | 6.48 (3.58, 10.60) | 6.47 (3.57, 10.59) | -0.01 (-0.05, 0.03) | 0.653 | — | — | — | — | 28.25 (24.45, 32.96) | 17.01 (14.16, 20.32) | -1.64 (-2.30, -0.96) | <0.001 | 0.39 (0.34, 0.45) | 0.23 (0.19, 0.28) | -1.66 (-2.33, -0.98) | <0.001 |
| Andean Latin America | 5.74 (3.13, 9.65) | 5.79 (3.15, 9.71) | 0.03 (0.03, 0.03) | <0.001 | — | — | — | — | 18.32 (10.99, 28.00) | 13.04 (7.90, 19.97) | -1.20 (-1.46, -0.94) | <0.001 | 0.25 (0.15, 0.39) | 0.18 (0.10, 0.27) | -1.24 (-1.51, -0.96) | <0.001 |
| Caribbean | 5.95 (3.20, 9.91) | 5.95 (3.20, 9.91) | 0.00 (0.00, 0.00)* | <0.001 | — | — | — | — | 31.00 (20.17, 50.39) | 34.74 (17.76, 69.61) | 0.59 (0.33, | <0.001 | 0.43 (0.27, | 0.48 (0.24, | 0.60 (0.33, 0.87) | <0.001 |

| | | | | | | | | | | | | | | | | |
|---|---|---|---|---|---|---|---|---|---|---|---|---|---|---|---|---|
| | | | | | | | | | | | 0.85) | | 0.70) | 0.97) | | |
| Southern Latin America | 4.78 (2.60, 8.08) | 4.78 (2.60, 8.08) | 0.00 (-0.00, 0.00) | 0.888 | — | — | — | — | 32.28 (23.52, 45.49) | 22.86 (17.12, 30.46) | -1.35 (-1.58, -1.11) | <0.001 | 0.43 (0.31, 0.62) | 0.30 (0.22, 0.41) | -1.40 (-1.64, -1.16) | <0.001 |
| Oceania | 7.25 (4.04, 11.84) | 7.25 (4.04, 11.84) | 0.00 (0.00, 0.00)* | <0.001 | — | — | — | — | 18.12 (8.02, 37.13) | 23.12 (9.68, 46.49) | 0.78 (0.26, 1.29) | 0.003 | 0.25 (0.11, 0.52) | 0.32 (0.13, 0.65) | 0.78 (0.27, 1.30) | 0.003 |
| Australasia | 5.48 (2.99, 9.12) | 5.46 (3.00, 8.92) | -0.02 (-0.03, -0.00) | 0.032 | — | — | — | — | 38.15 (29.19, 46.48) | 13.51 (10.18, 17.77) | -3.46 (-4.57, -2.33) | <0.001 | 0.51 (0.38, 0.62) | 0.17 (0.12, 0.23) | -3.60 (-4.91, -2.27) | <0.001 |
| **Men** | | | | | | | | | | | | | | | | |
| Global | 12.43 (6.93, 20.39) | 11.51 (6.48, 18.45) | -0.26 (-0.28, -0.25) | <0.001 | 2.01 (1.17, 2.95) | 1.82 (1.08, 2.66) | -0.26 (-0.52, -0.00) | 0.048 | 39.33 (32.75, 52.35) | 32.70 (26.42, 40.50) | -0.65 (-0.87, -0.43) | <0.001 | 0.54 (0.45, 0.72) | 0.45 (0.36, 0.56) | -0.63 (-0.81, -0.45) | <0.001 |
| **SDI category** | | | | | | | | | | | | | | | | |
| High | 15.72 (8.65, 25.58) | 16.19 (9.60, 25.16) | 0.10 (0.06, 0.13) | <0.001 | 16.65 (9.69, 24.49) | 19.24 (11.36, 28.18) | 0.57 (0.31, 0.83) | <0.001 | 64.34 (53.74, 74.10) | 42.64 (36.24, 48.16) | -1.42 (-1.64, -1.21) | <0.001 | 0.84 (0.69, 0.98) | 0.54 (0.45, 0.60) | -1.56 (-1.82, -1.30) | <0.001 |
| High-middle | 13.46 (7.49, 22.08) | 12.93 (7.17, 21.02) | -0.14 (-0.16, -0.12) | <0.001 | 0.08 (0.02, 0.17) | 0.12 (0.04, 0.23) | 1.77 (1.19, 2.35) | <0.001 | 54.41 (47.20, 71.71) | 44.37 (34.02, 51.35) | -0.76 (-1.45, -0.06) | 0.033 | 0.75 (0.65, 0.99) | 0.61 (0.46, 0.71) | -0.77 (-1.46, -0.07) | 0.032 |
| Middle | 12.68 (7.14, 20.83) | 11.68 (6.52, 19.03) | -0.29 (-0.30, -0.27) | <0.001 | — | — | — | — | 33.01 (26.25, 50.26) | 29.30 (23.42, 40.66) | -0.41 (-0.50, -0.32) | <0.001 | 0.45 (0.36, 0.69) | 0.40 (0.32, 0.56) | -0.41 (-0.50, -0.32) | <0.001 |
| Low-middle | 10.61 (5.94, 17.31) | 10.33 (5.79, 16.92) | -0.09 (-0.10, -0.08) | <0.001 | — | — | — | — | 23.10 (15.32, 32.62) | 24.23 (18.26, 31.33) | 0.16 (0.02, 0.29) | 0.025 | 0.32 (0.21, 0.45) | 0.34 (0.25, 0.43) | 0.16 (0.03, 0.30) | 0.019 |
| Low | 9.73 (5.46, 15.89) | 9.71 (5.46, 15.86) | -0.00 (-0.01, -0.00) | <0.001 | — | — | — | — | 39.00 (21.56, 55.84) | 36.94 (26.52, 50.81) | -0.16 (-0.33, -0.00) | 0.047 | 0.54 (0.30, 0.78) | 0.51 (0.37, 0.71) | -0.16 (-0.33, -0.00) | 0.048 |
| **21 GBD region** | | | | | | | | | | | | | | | | |
| South Asia | 10.07 (5.63, 16.51) | 10.09 (5.64, 16.55) | 0.0060 (0.0057, | <0.001 | — | — | — | — | 7.66 (3.75, 13.93) | 8.01 (5.19, 11.30) | 0.21 (-0.37, | 0.476 | 0.10 (0.05, | 0.11 (0.07, | 0.22 (-0.38, 0.82) | 0.472 |

| | | | | | | | | | | | | | | | | |
|---|---|---|---|---|---|---|---|---|---|---|---|---|---|---|---|---|
| | | | 0.0063) | | | | | | | | 0.80) | | 0.19) | 0.16) | | |
| East Asia | 15.14 (8.57, 24.87) | 14.89 (8.42, 23.77) | -0.06 (-0.07, -0.05) | <0.001 | — | — | — | — | 37.81 (28.37, 59.11) | 27.54 (18.61, 36.22) | -1.10 (-1.35, -0.86) | <0.001 | 0.52 (0.39, 0.81) | 0.37 (0.25, 0.49) | -1.14 (-1.39, -0.89) | <0.001 |
| North Africa and Middle East | 9.54 (5.33, 15.74) | 9.52 (5.29, 15.72) | -0.00 (-0.01, 0.01) | 0.514 | — | — | — | — | 58.68 (39.59, 96.98) | 44.96 (27.56, 76.67) | -0.90 (-1.13, -0.66) | <0.001 | 0.81 (0.54, 1.34) | 0.61 (0.37, 1.06) | -0.91 (-1.15, -0.68) | <0.001 |
| Western Sub-Saharan Africa | 9.70 (5.43, 15.83) | 9.70 (5.43, 15.83) | 0.00 (0.00, 0.00) | 0.085 | — | — | — | — | 92.17 (49.26, 136.91) | 77.05 (52.96, 106.03) | -0.65 (-0.79, -0.51) | <0.001 | 1.29 (0.69, 1.91) | 1.08 (0.74, 1.48) | -0.65 (-0.79, -0.51) | <0.001 |
| Southeast Asia | 12.70 (7.18, 21.00) | 12.84 (7.21, 21.11) | 0.04 (0.03, 0.04) | <0.001 | — | — | — | — | 21.21 (13.70, 38.42) | 24.37 (17.73, 34.71) | 0.50 (0.20, 0.80) | 0.001 | 0.29 (0.18, 0.53) | 0.33 (0.24, 0.48) | 0.50 (0.20, 0.81) | 0.001 |
| Eastern Sub-Saharan Africa | 9.63 (5.39, 15.68) | 9.64 (5.39, 15.70) | 0.00 (0.00, 0.00)* | <0.001 | — | — | — | — | 30.44 (18.52, 41.64) | 29.66 (17.04, 44.93) | -0.08 (-0.21, 0.04) | 0.189 | 0.42 (0.26, 0.58) | 0.41 (0.23, 0.63) | -0.08 (-0.21, 0.04) | 0.191 |
| Central Latin America | 8.88 (4.94, 14.67) | 8.83 (4.92, 14.66) | -0.02 (-0.02, -0.01) | <0.001 | — | — | — | — | 21.44 (17.67, 26.28) | 21.54 (14.38, 26.97) | 0.07 (-0.44, 0.58) | 0.799 | 0.29 (0.24, 0.36) | 0.29 (0.19, 0.37) | 0.07 (-0.45, 0.59) | 0.786 |
| Tropical Latin America | 9.24 (5.09, 15.31) | 9.23 (5.08, 15.29) | -0.0039 (-0.0041, -0.0038) | <0.001 | — | — | — | — | 58.44 (46.40, 68.13) | 60.45 (41.57, 70.68) | 0.14 (-0.14, 0.41) | 0.339 | 0.80 (0.63, 0.93) | 0.83 (0.57, 0.97) | 0.14 (-0.14, 0.42) | 0.340 |
| Central Sub-Saharan Africa | 9.28 (5.16, 15.07) | 9.28 (5.16, 15.07) | 0.00 (0.00, 0.00)* | <0.001 | — | — | — | — | 67.11 (30.85, 114.86) | 60.68 (34.14, 102.34) | -0.37 (-0.58, -0.15) | 0.001 | 0.94 (0.43, 1.60) | 0.85 (0.48, 1.43) | -0.37 (-0.58, -0.15) | 0.001 |
| Western Europe | 14.34 (7.81, 23.22) | 14.74 (7.93, 23.95) | 0.09 (0.08, 0.11) | <0.001 | 0.45 (0.09, 0.94) | — | — | — | 53.98 (41.98, 64.38) | 25.62 (21.79, 32.73) | -2.49 (-2.93, -2.05) | <0.001 | 0.72 (0.55, 0.87) | 0.33 (0.28, 0.43) | -2.63 (-3.16, -2.10) | <0.001 |
| High-income North America | 14.85 (7.92, 24.64) | 16.82 (10.49, 25.47) | 0.43 (0.34, 0.51) | <0.001 | 39.73 (23.34, 58.66) | 42.21 (24.53, 62.05) | 0.27 (-0.03, 0.56) | 0.078 | 84.03 (71.82, 93.74) | 63.51 (52.28, 71.33) | -0.99 (-1.24, -0.73) | <0.001 | 1.08 (0.90, 1.22) | 0.79 (0.64, 0.88) | -1.10 (-1.40, -0.81) | <0.001 |
| Central Asia | 11.31 (6.06, 18.33) | 11.31 (6.06, 18.33) | 0.00009 (0.00006, | <0.001 | — | — | — | — | 48.16 (38.09, 68.77) | 70.21 (48.54, 88.72) | 1.34 (0.43, | 0.004 | 0.67 (0.53, | 0.98 (0.67, | 1.36 (0.44, 2.29) | 0.004 |

| | | | | | | | | | | | | | | | | |
|---|---|---|---|---|---|---|---|---|---|---|---|---|---|---|---|---|
| | | | 0.00012) | | | | | | | | 2.27) | | 0.96) | 1.24) | | |
| Eastern Europe | 15.12 (8.39, 24.38) | 15.13 (8.38, 24.40) | 0.00 (-0.00, 0.00) | 0.231 | — | — | — | — | 102.20 (90.97, 132.07) | 126.36 (91.28, 150.14) | 0.87 (-0.52, 2.28) | 0.220 | 1.42 (1.26, 1.83) | 1.76 (1.27, 2.10) | 0.88 (-0.51, 2.30) | 0.216 |
| Southern Sub-Saharan Africa | 9.90 (5.54, 16.16) | 9.87 (5.52, 16.11) | -0.01 (-0.01, -0.01) | <0.001 | — | — | — | — | 57.67 (40.73, 80.78) | 64.49 (40.66, 96.36) | 0.37 (-0.09, 0.83) | 0.114 | 0.80 (0.57, 1.13) | 0.90 (0.57, 1.34) | 0.44 (-0.08, 0.96) | 0.096 |
| High-income Asia Pacific | 20.11 (11.31, 32.91) | 21.67 (12.55, 34.48) | 0.25 (0.24, 0.27) | <0.001 | 14.85 (7.82, 23.39) | 14.33 (7.53, 23.12) | -0.19 (-0.52, 0.14) | 0.258 | 54.79 (45.76, 71.78) | 26.43 (22.48, 34.58) | -2.52 (-2.78, -2.27) | <0.001 | 0.71 (0.60, 0.96) | 0.32 (0.27, 0.43) | -2.82 (-3.08, -2.56) | <0.001 |
| Central Europe | 15.53 (8.41, 25.58) | 15.62 (8.52, 25.66) | 0.03 (0.00, 0.05) | 0.015 | — | — | — | — | 52.50 (45.81, 66.31) | 40.41 (30.47, 50.28) | -0.88 (-1.28, -0.47) | <0.001 | 0.72 (0.63, 0.92) | 0.55 (0.41, 0.69) | -0.88 (-1.29, -0.47) | <0.001 |
| Andean Latin America | 8.10 (4.41, 13.38) | 8.11 (4.37, 13.35) | 0.00 (0.00, 0.00)* | <0.001 | — | — | — | — | 24.70 (15.89, 36.51) | 22.24 (13.96, 33.62) | -0.37 (-0.70, -0.04) | 0.026 | 0.34 (0.21, 0.50) | 0.30 (0.19, 0.46) | -0.39 (-0.72, -0.05) | 0.023 |
| Caribbean | 8.41 (4.65, 14.03) | 8.41 (4.65, 14.03) | 0.00 (0.00, 0.00)* | <0.001 | — | — | — | — | 43.82 (33.67, 61.04) | 44.27 (29.29, 67.10) | 0.14 (-0.06, 0.34) | 0.164 | 0.60 (0.46, 0.85) | 0.61 (0.40, 0.93) | 0.14 (-0.06, 0.34) | 0.162 |
| Southern Latin America | 11.20 (6.09, 18.46) | 11.20 (6.09, 18.46) | -0.00 (-0.00, 0.00) | 0.466 | — | — | — | — | 61.43 (46.21, 86.43) | 48.50 (37.56, 66.39) | -0.90 (-1.21, -0.59) | <0.001 | 0.83 (0.62, 1.18) | 0.65 (0.50, 0.91) | -0.92 (-1.24, -0.59) | <0.001 |
| Oceania | 12.56 (7.03, 20.54) | 12.56 (7.03, 20.54) | 0.00 (-0.00, 0.00) | 1.000 | — | — | — | — | 33.08 (17.55, 61.55) | 44.10 (22.83, 89.79) | 0.92 (0.78, 1.06) | <0.001 | 0.46 (0.24, 0.85) | 0.61 (0.31, 1.25) | 0.93 (0.79, 1.07) | <0.001 |
| Australasia | 12.52 (6.85, 20.87) | 12.56 (6.90, 20.73) | 0.01 (0.01, 0.02) | <0.001 | — | — | — | — | 78.36 (57.80, 94.77) | 31.88 (25.11, 40.63) | -3.10 (-3.74, -2.45) | <0.001 | 1.07 (0.78, 1.30) | 0.42 (0.33, 0.54) | -3.19 (-3.85, -2.53) | <0.001 |
| **20-24 years** | | | | | | | | | | | | | | | | |
| **Women** | | | | | | | | | | | | | | | | |
| Global | 8.00 (4.55, 12.76) | 7.67 (4.42, 12.06) | -0.14 (-0.16, -0.13) | <0.001 | 2.91 (1.89, 4.20) | 2.40 (1.52, 3.46) | -0.58 (-0.92, -0.24) | 0.001 | 24.16 (19.38, 32.87) | 18.19 (14.28, 21.30) | -0.96 (-1.32, -0.61) | <0.001 | 0.35 (0.27, 0.48) | 0.26 (0.20, 0.31) | -0.98 (-1.36, -0.60) | <0.001 |

| | | | | | | | | | | | | | | | | |
|---|---|---|---|---|---|---|---|---|---|---|---|---|---|---|---|---|
| **SDI category** | | | | | | | | | | | | | | | | |
| High | 7.56 (4.28, 12.18) | 7.44 (4.45, 11.41) | -0.06 (-0.11, -0.02) | 0.004 | 22.29 (14.45, 32.17) | 23.66 (14.96, 34.14) | 0.32 (0.01, 0.62) | 0.046 | 36.43 (32.35, 41.68) | 26.60 (23.05, 30.66) | -1.11 (-1.36, -0.85) | <0.001 | 0.48 (0.42, 0.53) | 0.33 (0.28, 0.36) | -1.27 (-1.41, -1.13) | <0.001 |
| High-middle | 7.73 (4.32, 12.50) | 7.27 (4.20, 11.43) | -0.22 (-0.25, -0.19) | <0.001 | 0.14 (0.06, 0.25) | 0.16 (0.07, 0.29) | 0.70 (0.16, 1.23) | 0.010 | 30.75 (26.28, 41.03) | 28.01 (20.15, 32.88) | -0.21 (-0.76, 0.34) | 0.457 | 0.45 (0.38, 0.60) | 0.41 (0.29, 0.48) | -0.21 (-0.78, 0.36) | 0.468 |
| Middle | 8.27 (4.67, 13.15) | 7.61 (4.34, 12.04) | -0.29 (-0.30, -0.27) | <0.001 | — | — | — | — | 22.29 (16.58, 35.81) | 16.75 (12.61, 20.82) | -0.95 (-1.09, -0.81) | <0.001 | 0.33 (0.24, 0.53) | 0.24 (0.18, 0.30) | -0.97 (-1.12, -0.83) | <0.001 |
| Low-middle | 8.13 (4.64, 12.82) | 7.95 (4.55, 12.50) | -0.08 (-0.08, -0.07) | <0.001 | — | — | — | — | 15.53 (9.70, 21.86) | 12.40 (8.77, 15.68) | -0.71 (-0.98, -0.45) | <0.001 | 0.23 (0.14, 0.32) | 0.18 (0.13, 0.23) | -0.78 (-1.07, -0.50) | <0.001 |
| Low | 7.86 (4.49, 12.44) | 7.82 (4.47, 12.40) | -0.02 (-0.02, -0.01) | <0.001 | — | — | — | — | 19.21 (8.22, 29.63) | 16.62 (10.52, 23.87) | -0.48 (-0.59, -0.37) | <0.001 | 0.29 (0.12, 0.44) | 0.25 (0.15, 0.36) | -0.49 (-0.60, -0.38) | <0.001 |
| **21 GBD region** | | | | | | | | | | | | | | | | |
| South Asia | 8.22 (4.73, 12.90) | 8.23 (4.74, 12.92) | 0.0038 (0.0035, 0.0041) | <0.001 | — | — | — | — | 4.63 (2.15, 7.83) | 3.78 (2.45, 5.40) | -0.73 (-1.00, -0.46) | <0.001 | 0.07 (0.03, 0.12) | 0.06 (0.03, 0.08) | -0.75 (-1.03, -0.47) | <0.001 |
| East Asia | 9.01 (4.96, 14.54) | 7.76 (4.45, 12.20) | -0.51 (-0.59, -0.44) | <0.001 | — | — | — | — | 22.31 (14.81, 42.23) | 18.65 (10.63, 25.44) | -0.70 (-1.45, 0.05) | 0.069 | 0.33 (0.21, 0.62) | 0.27 (0.15, 0.37) | -0.73 (-1.51, 0.05) | 0.067 |
| North Africa and Middle East | 6.29 (3.61, 9.97) | 6.25 (3.58, 9.86) | -0.02 (-0.02, -0.02) | <0.001 | — | — | — | — | 34.28 (19.40, 55.26) | 21.36 (14.46, 35.23) | -1.63 (-1.73, -1.53) | <0.001 | 0.51 (0.28, 0.82) | 0.31 (0.21, 0.52) | -1.68 (-1.78, -1.57) | <0.001 |
| Western Sub-Saharan Africa | 7.95 (4.54, 12.62) | 7.95 (4.54, 12.62) | 0.00 (0.00, 0.00) | 0.087 | — | — | — | — | 35.33 (14.80, 58.91) | 25.03 (15.56, 37.44) | -1.18 (-1.31, -1.05) | <0.001 | 0.53 (0.22, 0.88) | 0.37 (0.23, 0.56) | -1.20 (-1.33, -1.07) | <0.001 |
| Southeast Asia | 7.47 (4.17, 11.90) | 7.48 (4.17, 11.92) | 0.01 (0.00, 0.01) | <0.001 | — | — | — | — | 16.30 (8.31, 25.87) | 15.44 (8.82, 20.69) | -0.17 (-0.31, -0.02) | 0.025 | 0.24 (0.12, 0.38) | 0.22 (0.12, 0.30) | -0.18 (-0.33, -0.03) | 0.018 |
| Eastern Sub-Saharan | 7.89 (4.50, | 7.89 (4.50, | -0.00 (-0.00, | 0.006 | — | — | — | — | 13.09 (6.70, | 11.36 (7.01, | -0.48 | <0.001 | 0.19 | 0.17 | -0.50 | <0.001 |

| | | | | | | | | | | | | | | | | |
|---|---|---|---|---|---|---|---|---|---|---|---|---|---|---|---|---|
| Africa | 12.57) | 12.57) | -0.00)* | | | | | | 22.12) | 17.63) | (-0.62, -0.33) | | (0.10, 0.33) | (0.10, 0.26) | (-0.64, -0.35) | |
| Central Latin America | 7.23 (4.05, 11.40) | 7.20 (4.03, 11.39) | -0.01 (-0.02, -0.01) | <0.001 | — | — | — | — | 13.25 (11.25, 15.80) | 11.98 (9.01, 15.14) | -0.37 (-0.99, 0.25) | 0.242 | 0.19 (0.16, 0.23) | 0.17 (0.13, 0.22) | -0.38 (-1.04, 0.28) | 0.260 |
| Tropical Latin America | 7.59 (4.27, 11.91) | 7.58 (4.26, 11.89) | -0.0039 (-0.0041, -0.0037) | <0.001 | — | — | — | — | 48.83 (37.51, 56.41) | 31.78 (26.45, 37.18) | -1.42 (-1.61, -1.22) | <0.001 | 0.72 (0.55, 0.83) | 0.46 (0.38, 0.54) | -1.46 (-1.66, -1.26) | <0.001 |
| Central Sub-Saharan Africa | 7.63 (4.29, 12.26) | 7.63 (4.29, 12.26) | 0.00 (0.00, 0.00)* | <0.001 | — | — | — | — | 42.04 (12.12, 82.97) | 40.38 (17.69, 70.69) | -0.16 (-0.28, -0.04) | 0.010 | 0.63 (0.18, 1.25) | 0.60 (0.26, 1.06) | -0.16 (-0.28, -0.04) | 0.009 |
| Western Europe | 6.95 (3.97, 11.16) | 6.90 (3.97, 10.96) | -0.03 (-0.05, -0.01) | 0.001 | 0.54 (0.16, 1.02) | — | — | — | 24.30 (19.32, 27.72) | 12.25 (10.52, 15.10) | -2.31 (-2.45, -2.16) | <0.001 | 0.33 (0.26, 0.38) | 0.16 (0.14, 0.20) | -2.47 (-2.64, -2.30) | <0.001 |
| High-income North America | 7.58 (4.26, 12.28) | 7.79 (4.75, 11.41) | 0.09 (0.05, 0.13) | <0.001 | 65.71 (42.40, 94.96) | 60.64 (38.33, 87.31) | -0.18 (-0.45, 0.09) | 0.192 | 51.75 (45.07, 59.82) | 43.51 (35.55, 50.07) | -0.64 (-1.07, -0.20) | 0.004 | 0.64 (0.56, 0.71) | 0.52 (0.40, 0.57) | -0.71 (-1.19, -0.23) | 0.004 |
| Central Asia | 7.89 (4.52, 12.42) | 7.89 (4.52, 12.42) | 0.00 (0.00, 0.00)* | <0.001 | — | — | — | — | 50.22 (38.40, 70.39) | 66.33 (49.37, 84.31) | 0.96 (0.64, 1.28) | <0.001 | 0.75 (0.57, 1.05) | 0.99 (0.73, 1.26) | 0.97 (0.65, 1.29) | <0.001 |
| Eastern Europe | 8.57 (4.96, 13.68) | 8.58 (4.97, 13.69) | 0.00 (0.00, 0.00)* | <0.001 | — | — | — | — | 71.01 (63.66, 85.10) | 108.78 (66.94, 137.52) | 1.42 (0.62, 2.21) | <0.001 | 1.05 (0.95, 1.27) | 1.62 (0.99, 2.06) | 1.44 (0.64, 2.24) | <0.001 |
| Southern Sub-Saharan Africa | 8.11 (4.64, 12.79) | 8.08 (4.62, 12.74) | -0.01 (-0.01, -0.01) | <0.001 | — | — | — | — | 57.45 (36.39, 76.29) | 35.33 (20.28, 56.76) | -1.64 (-2.48, -0.78) | <0.001 | 0.86 (0.55, 1.15) | 0.53 (0.30, 0.85) | -1.65 (-2.50, -0.79) | <0.001 |
| High-income Asia Pacific | 8.57 (4.86, 13.75) | 8.22 (4.67, 13.15) | -0.15 (-0.16, -0.13) | <0.001 | 1.25 (0.37, 2.38) | 0.75 (0.17, 1.54) | -1.84 (-5.22, 1.66) | 0.298 | 30.75 (26.35, 38.58) | 15.03 (12.11, 19.12) | -2.49 (-2.72, -2.25) | <0.001 | 0.42 (0.36, 0.53) | 0.18 (0.15, 0.24) | -2.88 (-3.16, -2.60) | <0.001 |
| Central Europe | 6.91 (3.85, 11.04) | 6.93 (3.87, 11.08) | 0.00 (-0.04, 0.04) | 0.889 | — | — | — | — | 31.34 (26.38, 37.53) | 19.00 (15.33, 23.67) | -1.72 (-2.14, -1.31) | <0.001 | 0.46 (0.39, 0.55) | 0.28 (0.22, 0.35) | -1.76 (-2.19, -1.32) | <0.001 |
| Andean Latin | 6.57 (3.72, | 6.62 (3.73, | 0.03 (0.02, | <0.001 | — | — | — | — | 18.60 (10.46, | 11.94 (7.60, | -1.52 | <0.001 | 0.27 | 0.17 | -1.58 | <0.001 |

| | | | | | | | | | | | | | | | | |
|---|---|---|---|---|---|---|---|---|---|---|---|---|---|---|---|---|
| America | 10.45) | 10.52) | 0.03) | | | | | | 29.72) | 17.74) | (-1.93, -1.12) | | (0.15, 0.44) | (0.11, 0.26) | (-1.99, -1.16) | |
| Caribbean | 6.80 (3.80, 10.90) | 6.80 (3.80, 10.90) | 0.00 (-0.00, 0.00) | 1.000 | — | — | — | — | 42.90 (27.42, 71.80) | 49.77 (25.09, 96.61) | 0.58 (0.40, 0.77) | <0.001 | 0.63 (0.40, 1.07) | 0.74 (0.36, 1.43) | 0.60 (0.41, 0.79) | <0.001 |
| Southern Latin America | 5.35 (2.96, 8.53) | 5.35 (2.96, 8.53) | 0.00 (-0.00, 0.00) | 0.512 | — | — | — | — | 36.89 (27.58, 49.67) | 24.46 (18.51, 31.93) | -1.35 (-1.62, -1.08) | <0.001 | 0.52 (0.39, 0.72) | 0.34 (0.26, 0.45) | -1.39 (-1.70, -1.09) | <0.001 |
| Oceania | 7.66 (4.46, 12.19) | 7.66 (4.46, 12.19) | 0.00 (0.00, 0.00)* | <0.001 | — | — | — | — | 23.87 (10.02, 46.86) | 26.17 (9.72, 52.95) | 0.25 (0.09, 0.41) | 0.002 | 0.36 (0.15, 0.70) | 0.39 (0.14, 0.79) | 0.25 (0.10, 0.41) | 0.002 |
| Australasia | 6.17 (3.50, 9.97) | 6.15 (3.51, 9.80) | -0.01 (-0.02, -0.01) | <0.001 | — | — | — | — | 43.77 (32.42, 53.66) | 15.58 (11.94, 21.17) | -3.53 (-4.62, -2.42) | <0.001 | 0.62 (0.45, 0.77) | 0.21 (0.15, 0.29) | -3.75 (-4.93, -2.54) | <0.001 |
| **Men** | | | | | | | | | | | | | | | | |
| Global | 14.07 (8.11, 22.09) | 13.14 (7.76, 20.20) | -0.24 (-0.25, -0.22) | <0.001 | 4.76 (3.18, 6.61) | 4.39 (2.91, 6.24) | -0.23 (-0.60, 0.14) | 0.229 | 46.30 (40.16, 58.52) | 41.78 (33.53, 50.23) | -0.38 (-0.54, -0.22) | <0.001 | 0.67 (0.58, 0.85) | 0.61 (0.49, 0.74) | -0.38 (-0.56, -0.21) | <0.001 |
| **SDI category** | | | | | | | | | | | | | | | | |
| High | 18.28 (10.78, 28.52) | 18.76 (11.93, 27.84) | 0.09 (0.04, 0.13) | <0.001 | 34.93 (23.21, 48.58) | 41.24 (27.34, 58.53) | 0.67 (0.08, 1.26) | 0.027 | 79.57 (69.21, 91.59) | 59.56 (51.94, 67.83) | -1.00 (-1.12, -0.88) | <0.001 | 1.10 (0.95, 1.29) | 0.80 (0.68, 0.92) | -1.13 (-1.30, -0.96) | <0.001 |
| High-middle | 15.11 (8.66, 23.78) | 14.69 (8.63, 22.83) | -0.10 (-0.11, -0.08) | <0.001 | 0.51 (0.21, 0.90) | 0.38 (0.16, 0.71) | -0.68 (-1.40, 0.05) | 0.066 | 69.73 (62.17, 86.98) | 75.00 (51.47, 86.84) | 0.13 (-0.34, 0.59) | 0.590 | 1.03 (0.91, 1.28) | 1.11 (0.75, 1.28) | 0.13 (-0.34, 0.61) | 0.580 |
| Middle | 13.95 (7.99, 21.99) | 12.98 (7.53, 20.12) | -0.26 (-0.28, -0.23) | <0.001 | — | — | — | — | 35.48 (28.81, 51.84) | 35.26 (28.05, 47.11) | -0.01 (-0.18, 0.15) | 0.881 | 0.52 (0.42, 0.77) | 0.52 (0.41, 0.70) | -0.01 (-0.18, 0.15) | 0.884 |
| Low-middle | 11.89 (6.81, 18.40) | 11.62 (6.73, 17.92) | -0.08 (-0.09, -0.08) | <0.001 | — | — | — | — | 24.84 (17.64, 33.63) | 25.90 (19.16, 34.11) | 0.18 (0.03, 0.33) | 0.020 | 0.37 (0.26, 0.50) | 0.38 (0.28, 0.51) | 0.19 (0.03, 0.34) | 0.018 |
| Low | 10.98 (6.32, 17.00) | 10.95 (6.29, 16.96) | -0.01 (-0.01, -0.01) | <0.001 | — | — | — | — | 35.34 (20.75, 49.69) | 37.24 (26.59, 52.12) | 0.19 (0.04, | 0.013 | 0.53 (0.31, | 0.56 (0.40, | 0.19 (0.04, 0.34) | 0.013 |

| | | | | | | | | | | | | | | | | |
|---|---|---|---|---|---|---|---|---|---|---|---|---|---|---|---|---|
| | | | | | | | | | | | 0.34) | | 0.74) | 0.78) | | |
| **21 GBD region** | | | | | | | | | | | | | | | | |
| South Asia | 11.33 (6.53, 17.34) | 11.35 (6.54, 17.37) | 0.00 (0.00, 0.00)* | <0.001 | — | — | — | — | 10.48 (6.24, 15.26) | 11.43 (7.76, 16.37) | 0.30 (-0.07, 0.68) | 0.109 | 0.15 (0.09, 0.23) | 0.17 (0.11, 0.24) | 0.31 (-0.06, 0.69) | 0.103 |
| East Asia | 16.21 (9.14, 25.72) | 16.49 (9.84, 25.20) | 0.06 (0.03, 0.08) | <0.001 | — | — | — | — | 33.91 (25.37, 53.23) | 31.84 (20.35, 41.97) | -0.23 (-0.52, 0.06) | 0.124 | 0.50 (0.36, 0.79) | 0.46 (0.29, 0.62) | -0.24 (-0.55, 0.06) | 0.113 |
| North Africa and Middle East | 10.61 (6.01, 16.70) | 10.59 (5.99, 16.63) | -0.00 (-0.01, 0.01) | 0.481 | 0.02 (0.00, 0.04) | — | — | — | 54.68 (37.56, 89.89) | 41.17 (25.60, 71.94) | -0.95 (-1.17, -0.73) | <0.001 | 0.80 (0.54, 1.33) | 0.59 (0.36, 1.06) | -0.99 (-1.21, -0.76) | <0.001 |
| Western Sub-Saharan Africa | 10.96 (6.31, 16.99) | 10.94 (6.29, 16.97) | -0.0070 (-0.0074, -0.0066) | <0.001 | — | — | — | — | 52.69 (27.77, 79.64) | 47.00 (31.76, 67.72) | -0.38 (-0.60, -0.16) | 0.001 | 0.79 (0.41, 1.19) | 0.70 (0.47, 1.01) | -0.38 (-0.60, -0.16) | 0.001 |
| Southeast Asia | 13.66 (7.74, 21.45) | 13.76 (7.79, 21.57) | 0.02 (0.02, 0.03) | <0.001 | — | — | — | — | 32.23 (22.07, 51.52) | 37.28 (26.24, 49.78) | 0.51 (0.34, 0.68) | <0.001 | 0.47 (0.32, 0.77) | 0.55 (0.38, 0.74) | 0.52 (0.34, 0.69) | <0.001 |
| Eastern Sub-Saharan Africa | 10.88 (6.23, 16.88) | 10.89 (6.24, 16.91) | 0.00 (0.00, 0.00)* | <0.001 | — | — | — | — | 42.38 (27.12, 58.18) | 43.64 (25.29, 67.13) | 0.11 (-0.07, 0.28) | 0.226 | 0.63 (0.40, 0.87) | 0.65 (0.38, 1.00) | 0.11 (-0.07, 0.28) | 0.228 |
| Central Latin America | 10.09 (5.70, 15.89) | 10.06 (5.66, 15.84) | -0.01 (-0.01, -0.01) | <0.001 | — | — | — | — | 27.02 (22.79, 33.82) | 23.33 (17.51, 28.97) | -0.40 (-0.57, -0.23) | <0.001 | 0.39 (0.33, 0.49) | 0.34 (0.25, 0.42) | -0.41 (-0.59, -0.24) | <0.001 |
| Tropical Latin America | 10.49 (5.98, 16.45) | 10.48 (5.98, 16.44) | -0.0033 (-0.0034, -0.0032) | <0.001 | — | — | — | — | 86.70 (67.17, 99.98) | 77.19 (56.34, 89.93) | -0.42 (-0.75, -0.09) | 0.014 | 1.28 (0.99, 1.48) | 1.14 (0.83, 1.33) | -0.43 (-0.77, -0.09) | 0.014 |
| Central Sub-Saharan Africa | 10.51 (5.94, 16.43) | 10.51 (5.94, 16.43) | 0.00 (0.00, 0.00)* | <0.001 | — | — | — | — | 73.00 (33.69, 127.80) | 77.04 (41.03, 128.83) | 0.13 (-0.02, 0.28) | 0.080 | 1.09 (0.50, 1.92) | 1.15 (0.61, 1.93) | 0.14 (-0.02, 0.29) | 0.079 |
| Western Europe | 17.01 (10.22, 26.12) | 17.45 (10.34, 27.02) | 0.09 (0.07, 0.10) | <0.001 | 2.11 (0.37, 4.71) | 0.15 (0.08, 0.24) | -8.44 (-10.62, -6.21) | <0.001 | 62.04 (49.26, 74.07) | 35.32 (29.12, 44.75) | -1.97 (-2.32, -1.62) | <0.001 | 0.88 (0.69, 1.07) | 0.49 (0.39, 0.62) | -2.05 (-2.42, -1.68) | <0.001 |
| High-income North | 18.24 | 20.45 | 0.41 (0.36, | <0.001 | 91.80 (59.43, | 102.52 (67.42, | 0.44 (-0.07, | 0.091 | 108.67 | 93.55 (76.98, | -0.51 | <0.001 | 1.46 | 1.22 | -0.63 | <0.001 |

| | | | | | | | | | | | | | | | | |
|---|---|---|---|---|---|---|---|---|---|---|---|---|---|---|---|---|
| America | (10.64, 28.31) | (13.83, 28.40) | 0.46) | | 128.51) | 146.07) | 0.94) | | (93.83, 122.03) | 106.93) | (-0.69, -0.33) | | (1.24, 1.65) | (0.96, 1.39) | (-0.81, -0.46) | |
| Central Asia | 13.48 (7.73, 20.71) | 13.48 (7.73, 20.71) | 0.000069 (0.000063, 0.000076) | <0.001 | — | — | — | — | 91.48 (72.97, 134.24) | 141.23 (103.22, 175.62) | 1.54 (0.13, 2.97) | 0.032 | 1.36 (1.09, 2.01) | 2.11 (1.54, 2.63) | 1.55 (0.13, 2.99) | 0.032 |
| Eastern Europe | 16.98 (9.83, 26.59) | 16.99 (9.83, 26.61) | 0.00 (-0.00, 0.00) | 0.458 | — | — | — | — | 200.37 (169.64, 229.07) | 346.62 (215.55, 420.47) | 1.88 (1.12, 2.65) | <0.001 | 2.99 (2.53, 3.43) | 5.20 (3.22, 6.31) | 1.89 (1.13, 2.66) | <0.001 |
| Southern Sub-Saharan Africa | 11.18 (6.46, 17.17) | 11.15 (6.44, 17.16) | -0.01 (-0.01, -0.01) | <0.001 | — | — | — | — | 67.80 (45.34, 99.88) | 82.35 (54.95, 121.78) | 0.68 (0.33, 1.04) | <0.001 | 1.02 (0.68, 1.50) | 1.23 (0.82, 1.82) | 0.68 (0.33, 1.04) | <0.001 |
| High-income Asia Pacific | 22.46 (12.99, 35.26) | 23.39 (13.82, 36.96) | 0.14 (0.12, 0.16) | <0.001 | 18.16 (10.74, 27.58) | 16.12 (9.41, 24.15) | -0.42 (-0.61, -0.24) | <0.001 | 71.77 (63.30, 93.05) | 36.77 (31.02, 51.08) | -2.27 (-2.57, -1.97) | <0.001 | 1.01 (0.89, 1.33) | 0.49 (0.41, 0.70) | -2.46 (-2.80, -2.13) | <0.001 |
| Central Europe | 18.84 (11.08, 28.71) | 19.18 (11.42, 29.16) | 0.08 (0.05, 0.11) | <0.001 | — | — | — | — | 68.80 (60.25, 89.45) | 56.52 (42.98, 69.14) | -0.67 (-0.94, -0.40) | <0.001 | 1.01 (0.88, 1.33) | 0.83 (0.63, 1.02) | -0.69 (-0.97, -0.41) | <0.001 |
| Andean Latin America | 9.47 (5.37, 14.89) | 9.45 (5.38, 14.90) | -0.01 (-0.01, -0.00) | <0.001 | — | — | — | — | 31.69 (20.41, 49.69) | 29.02 (18.25, 42.69) | -0.16 (-0.52, 0.20) | 0.379 | 0.47 (0.30, 0.73) | 0.43 (0.26, 0.63) | -0.17 (-0.54, 0.20) | 0.365 |
| Caribbean | 9.63 (5.41, 15.33) | 9.63 (5.41, 15.33) | 0.00 (0.00, 0.00)* | <0.001 | — | — | — | — | 51.67 (40.19, 68.21) | 52.61 (36.03, 77.94) | 0.15 (-0.12, 0.42) | 0.269 | 0.76 (0.59, 1.01) | 0.78 (0.53, 1.15) | 0.15 (-0.12, 0.43) | 0.278 |
| Southern Latin America | 13.51 (7.72, 20.77) | 13.51 (7.72, 20.77) | 0.00 (-0.00, 0.00) | 0.836 | 8.71 (2.05, 17.89) | 2.73 (0.00, 7.94) | -3.98 (-5.73, -2.19) | <0.001 | 71.77 (54.61, 97.96) | 54.82 (42.43, 73.69) | -0.93 (-1.32, -0.54) | <0.001 | 1.03 (0.77, 1.42) | 0.78 (0.60, 1.07) | -0.95 (-1.36, -0.55) | <0.001 |
| Oceania | 13.67 (7.74, 21.75) | 13.67 (7.74, 21.75) | 0.00 (0.00, 0.00)* | <0.001 | — | — | — | — | 38.63 (20.47, 71.21) | 46.65 (22.85, 88.79) | 0.52 (0.29, 0.74) | <0.001 | 0.57 (0.30, 1.06) | 0.69 (0.34, 1.32) | 0.53 (0.30, 0.75) | <0.001 |
| Australasia | 15.41 (8.96, 23.76) | 15.60 (9.25, 23.83) | 0.04 (0.03, 0.06) | <0.001 | — | — | — | — | 99.04 (77.12, 118.77) | 50.05 (37.95, 64.08) | -2.30 (-2.88, -1.71) | <0.001 | 1.44 (1.11, 1.74) | 0.71 (0.54, 0.93) | -2.36 (-2.97, -1.75) | <0.001 |
| **25-29 years** | | | | | | | | | | | | | | | | |

| | | | | | | | | | | | | | | | | |
|---|---|---|---|---|---|---|---|---|---|---|---|---|---|---|---|---|
| **Women** | | | | | | | | | | | | | | | | |
| Global | 8.67 (4.66, 14.31) | 8.45 (4.60, 13.79) | -0.09 (-0.10, -0.08) | <0.001 | 6.10 (3.67, 9.13) | 4.82 (2.91, 7.29) | -0.69 (-1.04, -0.33) | <0.001 | 33.51 (26.56, 45.42) | 27.65 (22.21, 31.93) | -0.59 (-1.05, -0.14) | 0.011 | 0.52 (0.41, 0.71) | 0.43 (0.34, 0.50) | -0.60 (-1.06, -0.14) | 0.011 |
| **SDI category** | | | | | | | | | | | | | | | | |
| High | 8.30 (4.43, 13.67) | 7.92 (4.45, 12.66) | -0.16 (-0.19, -0.14) | <0.001 | 39.65 (23.83, 59.49) | 43.81 (26.44, 66.12) | 0.43 (0.20, 0.66) | <0.001 | 45.76 (40.78, 52.27) | 34.16 (29.64, 39.84) | -0.98 (-1.21, -0.76) | <0.001 | 0.64 (0.57, 0.72) | 0.45 (0.40, 0.51) | -1.17 (-1.40, -0.93) | <0.001 |
| High-middle | 8.37 (4.47, 13.83) | 7.94 (4.33, 12.96) | -0.18 (-0.21, -0.15) | <0.001 | 0.23 (0.10, 0.42) | 0.22 (0.11, 0.39) | 0.07 (-1.03, 1.19) | 0.897 | 43.41 (38.00, 58.41) | 46.58 (34.03, 56.18) | 0.15 (-1.48, 1.81) | 0.857 | 0.69 (0.60, 0.93) | 0.74 (0.54, 0.90) | 0.16 (-1.48, 1.84) | 0.846 |
| Middle | 8.79 (4.76, 14.44) | 8.38 (4.54, 13.72) | -0.16 (-0.17, -0.15) | <0.001 | — | — | — | — | 26.26 (19.51, 41.17) | 20.73 (15.50, 25.65) | -0.67 (-1.23, -0.10) | 0.022 | 0.42 (0.31, 0.66) | 0.33 (0.25, 0.41) | -0.68 (-1.26, -0.10) | 0.021 |
| Low-middle | 9.01 (4.85, 14.77) | 8.91 (4.80, 14.64) | -0.04 (-0.04, -0.04) | <0.001 | — | — | — | — | 22.47 (13.49, 31.72) | 19.38 (13.95, 24.54) | -0.45 (-0.79, -0.11) | 0.010 | 0.36 (0.21, 0.51) | 0.31 (0.22, 0.39) | -0.45 (-0.80, -0.11) | 0.010 |
| Low | 8.90 (4.76, 14.72) | 8.84 (4.72, 14.62) | -0.02 (-0.02, -0.02) | <0.001 | — | — | — | — | 39.91 (15.47, 62.78) | 29.52 (18.23, 41.49) | -1.04 (-1.12, -0.95) | <0.001 | 0.64 (0.25, 1.02) | 0.48 (0.29, 0.67) | -1.05 (-1.13, -0.96) | <0.001 |
| **21 GBD region** | | | | | | | | | | | | | | | | |
| South Asia | 9.32 (5.00, 15.36) | 9.32 (5.00, 15.37) | 0.00 (0.00, 0.00)* | 0.012 | — | — | — | — | 7.13 (3.28, 12.03) | 6.32 (3.92, 9.12) | -0.40 (-0.81, 0.01) | 0.057 | 0.11 (0.05, 0.19) | 0.10 (0.06, 0.15) | -0.41 (-0.83, 0.01) | 0.059 |
| East Asia | 9.21 (4.96, 15.12) | 8.17 (4.53, 13.16) | -0.43 (-0.47, -0.38) | <0.001 | — | — | — | — | 20.92 (13.71, 41.21) | 16.07 (9.65, 22.10) | -0.68 (-1.84, 0.50) | 0.257 | 0.33 (0.21, 0.66) | 0.25 (0.14, 0.35) | -0.71 (-1.90, 0.50) | 0.247 |
| North Africa and Middle East | 7.36 (3.86, 12.44) | 7.34 (3.85, 12.43) | -0.01 (-0.01, -0.00) | <0.001 | — | — | — | — | 27.97 (16.53, 48.34) | 19.18 (12.97, 32.09) | -1.28 (-1.36, -1.21) | <0.001 | 0.44 (0.25, 0.78) | 0.30 (0.20, 0.51) | -1.32 (-1.40, -1.24) | <0.001 |
| Western Sub-Saharan Africa | 8.98 (4.81, 14.87) | 8.99 (4.81, 14.88) | 0.00 (0.00, 0.00)* | <0.001 | — | — | — | — | 104.71 (42.18, 169.39) | 67.73 (41.59, 96.08) | -1.50 (-1.62, -1.38) | <0.001 | 1.69 (0.68, 2.74) | 1.09 (0.67, 1.55) | -1.50 (-1.62, -1.39) | <0.001 |

| | | | | | | | | | | | | | | | | |
|---|---|---|---|---|---|---|---|---|---|---|---|---|---|---|---|---|
| Southeast Asia | 7.79 (4.25, 12.72) | 7.79 (4.20, 12.71) | 0.00 (-0.00, 0.00) | 0.830 | — | — | — | — | 28.35 (15.13, 46.90) | 26.45 (16.31, 35.51) | -0.18 (-0.28, -0.09) | <0.001 | 0.45 (0.23, 0.76) | 0.42 (0.25, 0.57) | -0.19 (-0.28, -0.10) | <0.001 |
| Eastern Sub-Saharan Africa | 8.93 (4.77, 14.77) | 8.93 (4.77, 14.77) | -0.00 (-0.00, 0.00) | 0.236 | — | — | — | — | 24.17 (11.62, 46.59) | 18.49 (11.72, 27.65) | -0.91 (-1.12, -0.69) | <0.001 | 0.39 (0.19, 0.75) | 0.30 (0.19, 0.44) | -0.92 (-1.14, -0.71) | <0.001 |
| Central Latin America | 8.19 (4.32, 13.63) | 8.17 (4.31, 13.59) | -0.01 (-0.01, -0.00) | <0.001 | — | — | — | — | 16.00 (13.63, 19.96) | 13.71 (10.09, 17.49) | -0.55 (-1.04, -0.06) | 0.029 | 0.25 (0.21, 0.31) | 0.21 (0.15, 0.27) | -0.57 (-1.07, -0.06) | 0.028 |
| Tropical Latin America | 8.59 (4.53, 14.48) | 8.59 (4.53, 14.46) | -0.0037 (-0.0038, -0.0035) | <0.001 | — | — | — | — | 65.77 (52.15, 74.27) | 36.89 (31.61, 43.27) | -1.95 (-2.22, -1.68) | <0.001 | 1.05 (0.83, 1.19) | 0.58 (0.49, 0.68) | -1.99 (-2.27, -1.72) | <0.001 |
| Central Sub-Saharan Africa | 8.61 (4.47, 14.50) | 8.61 (4.47, 14.50) | 0.00 (-0.00, 0.00) | 1.000 | — | — | — | — | 56.43 (17.61, 104.64) | 46.68 (19.88, 82.16) | -0.68 (-0.82, -0.55) | <0.001 | 0.91 (0.28, 1.69) | 0.75 (0.32, 1.33) | -0.69 (-0.82, -0.55) | <0.001 |
| Western Europe | 7.61 (4.06, 12.46) | 7.53 (4.04, 12.27) | -0.04 (-0.07, -0.01) | 0.006 | 0.35 (0.07, 0.75) | 0.01 (0.00, 0.03) | — | — | 27.33 (23.17, 30.70) | 13.92 (12.11, 17.19) | -2.30 (-2.56, -2.04) | <0.001 | 0.41 (0.34, 0.46) | 0.20 (0.17, 0.25) | -2.42 (-2.70, -2.15) | <0.001 |
| High-income North America | 8.65 (4.55, 14.39) | 8.16 (4.80, 12.75) | -0.21 (-0.25, -0.16) | <0.001 | 110.10 (65.96, 164.32) | 115.83 (69.92, 175.04) | 0.30 (-0.00, 0.60) | 0.051 | 69.45 (60.23, 80.27) | 60.66 (48.54, 72.94) | -0.42 (-0.78, -0.06) | 0.021 | 0.92 (0.80, 1.00) | 0.77 (0.61, 0.88) | -0.57 (-0.97, -0.18) | 0.004 |
| Central Asia | 8.74 (4.60, 14.62) | 8.74 (4.60, 14.62) | 0.00 (0.00, 0.00)* | <0.001 | — | — | — | — | 70.63 (54.48, 104.21) | 99.56 (75.71, 134.03) | 1.11 (0.60, 1.62) | <0.001 | 1.14 (0.88, 1.68) | 1.61 (1.22, 2.17) | 1.13 (0.61, 1.64) | <0.001 |
| Eastern Europe | 9.42 (5.08, 15.60) | 9.43 (5.08, 15.61) | 0.00 (0.00, 0.00)* | 0.012 | — | — | — | — | 116.48 (101.20, 151.58) | 230.55 (153.08, 297.27) | 2.46 (1.65, 3.28) | <0.001 | 1.88 (1.63, 2.44) | 3.73 (2.47, 4.82) | 2.48 (1.67, 3.31) | <0.001 |
| Southern Sub-Saharan Africa | 9.20 (4.94, 15.18) | 9.19 (4.94, 15.15) | -0.00 (-0.00, -0.00)* | <0.001 | — | — | — | — | 79.04 (47.50, 108.34) | 48.89 (23.60, 84.27) | -1.57 (-2.91, -0.20) | 0.024 | 1.28 (0.77, 1.76) | 0.79 (0.38, 1.36) | -1.57 (-2.93, -0.20) | 0.025 |
| High-income Asia Pacific | 9.05 (4.83, 14.96) | 8.44 (4.64, 13.64) | -0.24 (-0.26, -0.23) | <0.001 | 1.30 (0.40, 2.63) | 0.09 (0.00, 0.21) | — | — | 36.55 (30.74, 46.57) | 17.09 (13.97, 22.73) | -2.60 (-2.93, -2.27) | <0.001 | 0.55 (0.46, 0.71) | 0.24 (0.19, 0.32) | -2.86 (-3.28, -2.44) | <0.001 |

| | | | | | | | | | | | | | | | | |
|---|---|---|---|---|---|---|---|---|---|---|---|---|---|---|---|---|
| Central Europe | 7.36 (3.93, 12.22) | 7.38 (3.95, 12.18) | -0.01 (-0.06, 0.05) | 0.850 | — | — | — | — | 40.10 (34.22, 47.84) | 24.40 (19.28, 30.96) | -1.64 (-2.03, -1.25) | <0.001 | 0.64 (0.54, 0.76) | 0.39 (0.30, 0.49) | -1.68 (-2.07, -1.28) | <0.001 |
| Andean Latin America | 7.58 (4.02, 12.63) | 7.62 (4.05, 12.92) | 0.02 (0.01, 0.02) | <0.001 | — | — | — | — | 18.08 (10.58, 28.41) | 12.23 (7.63, 19.17) | -1.42 (-1.77, -1.07) | <0.001 | 0.29 (0.17, 0.45) | 0.19 (0.12, 0.30) | -1.48 (-1.83, -1.11) | <0.001 |
| Caribbean | 7.75 (4.08, 12.93) | 7.75 (4.08, 12.93) | 0.00 (0.00, 0.00) | 0.188 | — | — | — | — | 45.43 (30.04, 72.54) | 49.93 (27.42, 87.32) | 0.44 (-0.04, 0.93) | 0.074 | 0.73 (0.48, 1.17) | 0.80 (0.44, 1.41) | 0.46 (-0.04, 0.95) | 0.070 |
| Southern Latin America | 6.11 (3.23, 9.94) | 6.11 (3.23, 9.94) | 0.00 (-0.00, 0.00) | 0.835 | — | — | — | — | 43.01 (31.17, 59.61) | 26.42 (20.28, 34.26) | -1.65 (-2.01, -1.29) | <0.001 | 0.66 (0.47, 0.94) | 0.40 (0.30, 0.52) | -1.70 (-2.08, -1.31) | <0.001 |
| Oceania | 7.99 (4.29, 13.19) | 7.99 (4.29, 13.19) | 0.00 (0.00, 0.00)* | <0.001 | — | — | — | — | 51.83 (22.94, 95.23) | 52.92 (18.89, 107.80) | 0.06 (-0.12, 0.24) | 0.534 | 0.84 (0.37, 1.54) | 0.86 (0.30, 1.75) | 0.06 (-0.12, 0.24) | 0.514 |
| Australasia | 7.04 (3.73, 11.67) | 7.02 (3.78, 11.59) | -0.01 (-0.01, -0.00) | <0.001 | — | — | — | — | 50.63 (39.84, 61.53) | 21.77 (16.39, 30.90) | -2.85 (-3.93, -1.75) | <0.001 | 0.77 (0.60, 0.95) | 0.32 (0.24, 0.47) | -2.97 (-4.14, -1.78) | <0.001 |
| **Men** | | | | | | | | | | | | | | | | |
| Global | 14.98 (8.51, 24.10) | 14.03 (8.01, 22.38) | -0.23 (-0.24, -0.21) | <0.001 | 8.48 (5.18, 12.62) | 7.86 (4.93, 11.67) | -0.12 (-0.50, 0.26) | 0.535 | 66.24 (59.15, 82.52) | 67.05 (52.65, 75.67) | 0.03 (-0.32, 0.39) | 0.851 | 1.04 (0.93, 1.31) | 1.06 (0.83, 1.20) | 0.04 (-0.33, 0.42) | 0.818 |
| **SDI category** | | | | | | | | | | | | | | | | |
| High | 19.24 (11.02, 30.92) | 18.47 (11.23, 28.04) | -0.14 (-0.17, -0.10) | <0.001 | 53.21 (32.50, 79.23) | 67.35 (42.25, 99.83) | 0.89 (0.60, 1.19) | <0.001 | 98.77 (86.24, 114.37) | 74.62 (63.76, 85.55) | -0.97 (-1.12, -0.82) | <0.001 | 1.47 (1.27, 1.73) | 1.07 (0.89, 1.22) | -1.11 (-1.34, -0.88) | <0.001 |
| High-middle | 15.83 (8.96, 25.50) | 15.14 (8.68, 24.16) | -0.15 (-0.17, -0.14) | <0.001 | 0.93 (0.39, 1.64) | 0.65 (0.28, 1.11) | -1.04 (-3.96, 1.97) | 0.493 | 118.82 (107.18, 151.16) | 159.68 (110.12, 184.10) | 1.00 (0.35, 1.66) | 0.003 | 1.90 (1.72, 2.42) | 2.57 (1.77, 2.97) | 1.02 (0.36, 1.68) | 0.002 |
| Middle | 14.30 (8.06, 23.11) | 13.58 (7.75, 21.75) | -0.18 (-0.20, -0.16) | <0.001 | — | — | — | — | 40.03 (33.54, 58.19) | 40.76 (31.82, 52.16) | 0.08 (-0.05, 0.21) | 0.229 | 0.64 (0.53, 0.93) | 0.65 (0.51, 0.83) | 0.07 (-0.06, 0.20) | 0.259 |
| Low-middle | 12.93 (7.10, | 12.69 (6.96, | -0.06 (-0.07, | <0.001 | — | — | — | — | 30.30 (22.55, | 33.31 (24.69, | 0.34 | <0.001 | 0.48 | 0.53 | 0.34 (0.22, | <0.001 |

| | | | | | | | | | | | | | | | | |
|---|---|---|---|---|---|---|---|---|---|---|---|---|---|---|---|---|
| | 20.88) | 20.50) | -0.05) | | | | | | 41.20) | 41.82) | (0.22, 0.46) | | (0.36, 0.66) | (0.40, 0.67) | 0.47) | |
| Low | 12.30 (6.64, 20.01) | 12.21 (6.59, 19.86) | -0.02 (-0.03, -0.02) | <0.001 | — | — | — | — | 53.63 (33.08, 71.63) | 56.25 (39.60, 74.97) | 0.17 (0.03, 0.32) | 0.021 | 0.87 (0.53, 1.16) | 0.91 (0.64, 1.21) | 0.17 (0.02, 0.32) | 0.022 |
| **21 GBD region** | | | | | | | | | | | | | | | | |
| South Asia | 12.79 (6.80, 20.86) | 12.81 (6.81, 20.89) | 0.00 (0.00, 0.00)* | <0.001 | — | — | — | — | 11.56 (7.44, 15.81) | 13.15 (8.96, 18.05) | 0.45 (0.03, 0.87) | 0.034 | 0.18 (0.12, 0.25) | 0.21 (0.14, 0.29) | 0.46 (0.04, 0.89) | 0.034 |
| East Asia | 16.02 (8.89, 25.77) | 15.40 (8.94, 23.85) | -0.14 (-0.15, -0.13) | <0.001 | — | — | — | — | 31.74 (24.12, 50.67) | 32.98 (21.38, 42.15) | 0.17 (-0.10, 0.44) | 0.223 | 0.50 (0.38, 0.81) | 0.52 (0.33, 0.66) | 0.16 (-0.12, 0.45) | 0.269 |
| North Africa and Middle East | 10.77 (6.02, 17.49) | 10.73 (6.00, 17.43) | -0.01 (-0.02, -0.01) | <0.001 | 0.07 (0.02, 0.15) | — | — | — | 50.64 (35.85, 90.09) | 38.70 (26.18, 65.41) | -0.92 (-1.12, -0.70) | <0.001 | 0.79 (0.55, 1.44) | 0.60 (0.40, 1.04) | -0.95 (-1.17, -0.74) | <0.001 |
| Western Sub-Saharan Africa | 12.35 (6.66, 20.12) | 12.30 (6.63, 20.05) | -0.01 (-0.01, -0.01) | <0.001 | — | — | — | — | 91.65 (54.75, 130.83) | 86.23 (58.74, 118.25) | -0.20 (-0.35, -0.04) | 0.011 | 1.48 (0.88, 2.12) | 1.40 (0.95, 1.92) | -0.20 (-0.35, -0.05) | 0.010 |
| Southeast Asia | 13.76 (7.74, 21.84) | 13.79 (7.81, 22.15) | 0.01 (0.00, 0.01) | <0.001 | — | — | — | — | 42.40 (31.12, 70.78) | 47.11 (33.17, 63.99) | 0.38 (0.08, 0.69) | 0.012 | 0.67 (0.49, 1.14) | 0.75 (0.52, 1.02) | 0.37 (0.08, 0.66) | 0.012 |
| Eastern Sub-Saharan Africa | 12.23 (6.61, 19.95) | 12.25 (6.62, 19.97) | 0.00 (0.00, 0.01) | <0.001 | — | — | — | — | 68.69 (44.24, 93.43) | 68.93 (37.69, 101.85) | -0.02 (-0.21, 0.18) | 0.867 | 1.11 (0.71, 1.51) | 1.11 (0.61, 1.65) | -0.02 (-0.21, 0.18) | 0.859 |
| Central Latin America | 11.47 (6.25, 18.62) | 11.45 (6.25, 18.61) | -0.00 (-0.01, -0.00) | 0.001 | — | — | — | — | 30.94 (26.08, 39.56) | 28.40 (21.06, 35.21) | -0.22 (-0.46, 0.01) | 0.061 | 0.49 (0.41, 0.63) | 0.45 (0.33, 0.56) | -0.23 (-0.46, 0.01) | 0.062 |
| Tropical Latin America | 11.97 (6.43, 19.37) | 11.96 (6.42, 19.35) | -0.00 (-0.00, -0.00)* | <0.001 | — | — | — | — | 140.74 (108.37, 160.97) | 113.18 (83.99, 130.69) | -0.75 (-0.96, -0.54) | <0.001 | 2.26 (1.74, 2.59) | 1.81 (1.33, 2.09) | -0.76 (-0.97, -0.55) | <0.001 |
| Central Sub-Saharan Africa | 11.79 (6.37, 19.28) | 11.79 (6.37, 19.28) | 0.00 (0.00, 0.00)* | <0.001 | — | — | — | — | 89.08 (37.83, 161.76) | 93.44 (48.82, 155.64) | 0.16 (-0.07, 0.38) | 0.177 | 1.44 (0.61, 2.62) | 1.51 (0.79, 2.52) | 0.16 (-0.07, 0.38) | 0.180 |

| | | | | | | | | | | | | | | | | |
|---|---|---|---|---|---|---|---|---|---|---|---|---|---|---|---|---|
| Western Europe | 18.17 (10.30, 28.69) | 18.14 (10.24, 28.53) | -0.01 (-0.02, 0.00) | 0.227 | 3.65 (0.95, 7.40) | 1.10 (0.58, 1.75) | -3.57 (-7.74, 0.79) | 0.107 | 69.31 (55.92, 84.25) | 43.63 (34.39, 52.01) | -1.62 (-1.89, -1.35) | <0.001 | 1.07 (0.85, 1.30) | 0.66 (0.52, 0.81) | -1.68 (-1.97, -1.38) | <0.001 |
| High-income North America | 20.15 (11.39, 32.74) | 20.09 (13.00, 28.90) | -0.00 (-0.04, 0.03) | 0.813 | 136.59 (81.47, 202.87) | 177.09 (110.43, 263.81) | 0.97 (0.64, 1.30) | <0.001 | 140.54 (117.07, 159.35) | 116.44 (90.64, 139.15) | -0.64 (-0.91, -0.36) | <0.001 | 2.02 (1.64, 2.31) | 1.58 (1.19, 1.89) | -0.86 (-1.22, -0.51) | <0.001 |
| Central Asia | 15.11 (8.54, 24.79) | 15.11 (8.53, 24.77) | -0.000 (-0.00030, -0.00022) | <0.001 | — | — | — | — | 126.93 (98.77, 197.67) | 238.14 (158.23, 295.21) | 2.23 (1.58, 2.88) | <0.001 | 2.04 (1.59, 3.19) | 3.85 (2.56, 4.78) | 2.24 (1.59, 2.90) | <0.001 |
| Eastern Europe | 17.91 (10.22, 29.05) | 17.91 (10.22, 29.07) | 0.00 (-0.00, 0.00) | 0.897 | — | — | — | — | 409.19 (355.31, 512.36) | 949.76 (631.17, 1122.11) | 3.08 (2.10, 4.08) | <0.001 | 6.62 (5.74, 8.30) | 15.41 (10.23, 18.21) | 3.10 (2.11, 4.09) | <0.001 |
| Southern Sub-Saharan Africa | 12.64 (6.82, 20.54) | 12.63 (6.81, 20.53) | -0.00 (-0.00, 0.00) | 0.043 | — | — | — | — | 95.07 (64.58, 137.00) | 88.64 (58.60, 128.92) | -0.36 (-0.67, -0.04) | 0.027 | 1.54 (1.04, 2.22) | 1.43 (0.94, 2.09) | -0.36 (-0.68, -0.04) | 0.026 |
| High-income Asia Pacific | 22.54 (13.05, 35.50) | 22.02 (13.16, 33.61) | -0.09 (-0.10, -0.07) | <0.001 | 20.60 (10.95, 32.87) | 17.62 (9.55, 27.63) | -0.58 (-0.78, -0.38) | <0.001 | 86.55 (76.87, 112.93) | 46.20 (38.62, 65.93) | -2.16 (-2.49, -1.82) | <0.001 | 1.32 (1.17, 1.76) | 0.67 (0.56, 1.00) | -2.30 (-2.62, -1.97) | <0.001 |
| Central Europe | 21.08 (12.07, 33.58) | 21.33 (12.20, 34.00) | 0.04 (-0.03, 0.12) | 0.240 | — | — | — | — | 95.95 (83.03, 123.69) | 84.34 (63.17, 101.68) | -0.44 (-0.65, -0.23) | <0.001 | 1.53 (1.32, 1.98) | 1.34 (1.00, 1.62) | -0.45 (-0.67, -0.24) | <0.001 |
| Andean Latin America | 10.88 (5.95, 18.03) | 10.88 (5.94, 18.05) | 0.00 (-0.00, 0.00) | 0.692 | — | — | — | — | 34.07 (22.15, 50.70) | 33.80 (21.69, 49.25) | -0.00 (-0.21, 0.20) | 0.963 | 0.54 (0.35, 0.81) | 0.54 (0.34, 0.79) | -0.01 (-0.22, 0.20) | 0.904 |
| Caribbean | 10.92 (6.04, 17.87) | 10.92 (6.04, 17.87) | 0.00 (0.00, 0.00)* | <0.001 | — | — | — | — | 73.69 (58.06, 101.27) | 79.68 (56.26, 116.73) | 0.34 (0.01, 0.67) | 0.046 | 1.18 (0.93, 1.62) | 1.28 (0.89, 1.88) | 0.34 (0.00, 0.67) | 0.048 |
| Southern Latin America | 15.20 (8.37, 25.37) | 15.20 (8.37, 25.37) | 0.00 (-0.00, 0.00) | 0.907 | 18.25 (6.19, 35.46) | 7.41 (1.66, 15.40) | -2.99 (-3.37, -2.62) | <0.001 | 86.93 (65.98, 114.00) | 70.50 (54.74, 89.85) | -0.68 (-0.99, -0.37) | <0.001 | 1.35 (1.01, 1.79) | 1.09 (0.84, 1.41) | -0.68 (-1.00, -0.36) | <0.001 |
| Oceania | 13.81 (7.54, 22.11) | 13.81 (7.54, 22.11) | 0.00 (0.00, 0.00)* | <0.001 | — | — | — | — | 70.41 (38.98, 130.84) | 86.10 (43.25, 163.31) | 0.69 (0.55, 0.82) | <0.001 | 1.13 (0.62, 2.11) | 1.39 (0.69, 2.64) | 0.70 (0.56, 0.83) | <0.001 |

| | | | | | | | | | | | | | | | | |
|---|---|---|---|---|---|---|---|---|---|---|---|---|---|---|---|---|
| Australasia | 17.68 (9.77, 29.04) | 17.97 (10.29, 28.95) | 0.06 (0.05, 0.07) | <0.001 | 0.26 (0.00, 0.63) | — | — | — | 127.49 (99.15, 154.58) | 76.90 (58.23, 100.20) | -1.62 (-2.53, -0.70) | 0.001 | 2.00 (1.54, 2.46) | 1.20 (0.89, 1.57) | -1.64 (-2.59, -0.69) | 0.001 |
| **30-34 years** | | | | | | | | | | | | | | | | |
| **Women** | | | | | | | | | | | | | | | | |
| Global | 9.56 (5.78, 15.06) | 9.37 (5.71, 14.67) | -0.07 (-0.09, -0.04) | <0.001 | 8.24 (4.58, 12.85) | 5.34 (2.95, 8.53) | -1.44 (-1.84, -1.03) | <0.001 | 42.67 (35.18, 57.15) | 37.82 (30.49, 43.56) | -0.40 (-1.21, 0.42) | 0.338 | 0.72 (0.59, 0.98) | 0.65 (0.52, 0.75) | -0.37 (-1.22, 0.48) | 0.389 |
| **SDI category** | | | | | | | | | | | | | | | | |
| High | 9.44 (5.79, 14.73) | 8.89 (5.61, 13.53) | -0.21 (-0.23, -0.19) | <0.001 | 47.51 (26.44, 74.25) | 45.97 (25.34, 73.48) | -0.03 (-0.26, 0.20) | 0.773 | 55.11 (48.93, 62.72) | 41.48 (35.00, 48.19) | -0.96 (-1.36, -0.56) | <0.001 | 0.85 (0.77, 0.95) | 0.62 (0.53, 0.69) | -1.07 (-1.40, -0.73) | <0.001 |
| High-middle | 9.23 (5.55, 14.52) | 8.90 (5.43, 13.74) | -0.13 (-0.15, -0.10) | <0.001 | 0.28 (0.11, 0.49) | 0.21 (0.09, 0.37) | -1.17 (-2.15, -0.19) | 0.020 | 66.32 (58.20, 91.72) | 88.43 (64.04, 107.16) | 0.99 (0.10, 1.88) | 0.028 | 1.16 (1.01, 1.60) | 1.55 (1.12, 1.88) | 1.01 (0.11, 1.91) | 0.027 |
| Middle | 9.51 (5.76, 14.90) | 9.26 (5.63, 14.56) | -0.10 (-0.12, -0.08) | <0.001 | — | — | — | — | 30.78 (23.19, 49.72) | 21.94 (16.98, 28.70) | -1.12 (-1.33, -0.91) | <0.001 | 0.54 (0.40, 0.87) | 0.38 (0.29, 0.50) | -1.14 (-1.35, -0.93) | <0.001 |
| Low-middle | 9.98 (6.00, 15.77) | 9.92 (5.99, 15.65) | -0.02 (-0.02, -0.02) | <0.001 | — | — | — | — | 24.36 (14.82, 35.53) | 20.70 (14.42, 26.40) | -0.52 (-0.79, -0.24) | <0.001 | 0.43 (0.26, 0.62) | 0.36 (0.25, 0.46) | -0.52 (-0.81, -0.24) | <0.001 |
| Low | 9.94 (5.92, 15.85) | 9.87 (5.89, 15.76) | -0.02 (-0.02, -0.02) | <0.001 | — | — | — | — | 35.40 (14.74, 57.27) | 26.64 (16.67, 38.42) | -1.00 (-1.08, -0.92) | <0.001 | 0.62 (0.26, 1.01) | 0.46 (0.29, 0.67) | -1.01 (-1.09, -0.94) | <0.001 |
| **21 GBD region** | | | | | | | | | | | | | | | | |
| South Asia | 10.45 (6.27, 16.39) | 10.45 (6.27, 16.39) | 0.00 (-0.00, 0.00) | 0.256 | — | — | — | — | 9.59 (4.46, 15.80) | 8.39 (4.83, 12.05) | -0.44 (-0.83, -0.06) | 0.025 | 0.17 (0.08, 0.28) | 0.15 (0.08, 0.21) | -0.45 (-0.84, -0.06) | 0.026 |
| East Asia | 9.75 (5.87, 15.36) | 9.00 (5.61, 13.68) | -0.27 (-0.31, -0.23) | <0.001 | — | — | — | — | 20.20 (13.50, 40.96) | 14.69 (8.65, 20.54) | -0.96 (-2.04, 0.13) | 0.083 | 0.35 (0.23, 0.71) | 0.25 (0.14, 0.35) | -0.93 (-1.99, 0.14) | 0.088 |
| North Africa and Middle East | 8.15 (4.85, 12.91) | 8.16 (4.85, 12.92) | 0.00 (0.00, 0.01) | 0.018 | — | — | — | — | 34.00 (20.00, 60.01) | 22.08 (14.78, 38.76) | -1.47 (-1.59, | <0.001 | 0.59 (0.34, | 0.38 (0.25, | -1.52 (-1.64, | <0.001 |

| | | | | | | | | | | | | | | | | |
|---|---|---|---|---|---|---|---|---|---|---|---|---|---|---|---|---|
| | | | | | | | | | | | -1.35) | | 1.05) | 0.67) | -1.39) | |
| Western Sub-Saharan Africa | 10.01 (5.97, 15.97) | 10.02 (5.98, 15.98) | 0.00 (0.00, 0.00)* | <0.001 | — | — | — | — | 66.31 (26.09, 110.16) | 41.25 (25.65, 60.34) | -1.63 (-1.77, -1.50) | <0.001 | 1.16 (0.45, 1.94) | 0.72 (0.45, 1.06) | -1.64 (-1.77, -1.51) | <0.001 |
| Southeast Asia | 8.37 (5.05, 13.18) | 8.35 (5.05, 13.14) | -0.01 (-0.01, -0.00) | <0.001 | — | — | — | — | 38.10 (22.68, 65.28) | 33.04 (22.28, 45.20) | -0.48 (-0.56, -0.41) | <0.001 | 0.66 (0.39, 1.14) | 0.57 (0.38, 0.79) | -0.50 (-0.57, -0.42) | <0.001 |
| Eastern Sub-Saharan Africa | 9.95 (5.93, 15.91) | 9.96 (5.94, 15.92) | 0.00 (0.00, 0.00)* | <0.001 | — | — | — | — | 29.67 (13.01, 57.64) | 22.55 (13.51, 33.21) | -0.94 (-1.08, -0.79) | <0.001 | 0.52 (0.22, 1.01) | 0.39 (0.23, 0.58) | -0.95 (-1.10, -0.80) | <0.001 |
| Central Latin America | 9.20 (5.52, 14.57) | 9.19 (5.52, 14.56) | -0.00 (-0.00, 0.00) | 0.306 | — | — | — | — | 18.22 (15.28, 21.94) | 14.59 (10.90, 18.98) | -0.63 (-0.95, -0.31) | <0.001 | 0.32 (0.26, 0.38) | 0.25 (0.19, 0.33) | -0.66 (-0.98, -0.33) | <0.001 |
| Tropical Latin America | 9.68 (5.79, 15.33) | 9.67 (5.78, 15.32) | -0.0027 (-0.0028, -0.0025) | <0.001 | — | — | — | — | 99.23 (72.66, 111.91) | 51.65 (43.78, 61.23) | -2.26 (-2.48, -2.04) | <0.001 | 1.75 (1.28, 1.96) | 0.90 (0.76, 1.07) | -2.29 (-2.51, -2.07) | <0.001 |
| Central Sub-Saharan Africa | 9.56 (5.75, 15.39) | 9.56 (5.75, 15.39) | 0.00 (-0.00, 0.00) | 1.000 | — | — | — | — | 70.83 (22.28, 136.60) | 58.66 (25.29, 103.33) | -0.68 (-0.87, -0.49) | <0.001 | 1.24 (0.39, 2.40) | 1.03 (0.44, 1.82) | -0.69 (-0.88, -0.50) | <0.001 |
| Western Europe | 8.57 (5.20, 13.44) | 8.46 (5.16, 13.13) | -0.05 (-0.07, -0.03) | <0.001 | 0.53 (0.08, 1.17) | 0.03 (0.01, 0.06) | — | — | 31.16 (25.00, 35.75) | 15.98 (14.07, 19.57) | -2.28 (-2.44, -2.12) | <0.001 | 0.51 (0.41, 0.59) | 0.26 (0.22, 0.32) | -2.39 (-2.58, -2.21) | <0.001 |
| High-income North America | 10.12 (6.12, 15.62) | 9.28 (6.16, 13.58) | -0.30 (-0.35, -0.25) | <0.001 | 125.68 (69.81, 196.04) | 130.30 (72.02, 208.08) | 0.22 (-0.05, 0.49) | 0.105 | 85.55 (74.83, 99.47) | 78.88 (62.52, 95.21) | -0.24 (-0.70, 0.22) | 0.311 | 1.24 (1.10, 1.38) | 1.12 (0.87, 1.28) | -0.33 (-0.79, 0.14) | 0.165 |
| Central Asia | 9.78 (5.93, 15.50) | 9.78 (5.93, 15.50) | 0.00 (0.00, 0.00)* | <0.001 | — | — | — | — | 86.67 (65.73, 138.86) | 149.66 (103.63, 188.64) | 1.86 (1.46, 2.26) | <0.001 | 1.52 (1.15, 2.44) | 2.63 (1.81, 3.32) | 1.88 (1.48, 2.28) | <0.001 |
| Eastern Europe | 10.50 (6.37, 16.15) | 10.51 (6.38, 16.17) | 0.00 (0.00, 0.01) | <0.001 | — | — | — | — | 191.16 (161.26, 282.18) | 458.98 (317.95, 583.62) | 3.13 (1.65, 4.63) | <0.001 | 3.36 (2.83, 4.96) | 8.09 (5.60, 10.30) | 3.15 (1.67, 4.65) | <0.001 |
| Southern Sub-Saharan Africa | 10.30 (6.19, 16.32) | 10.30 (6.18, 16.31) | -0.00 (-0.00, 0.00) | 0.140 | — | — | — | — | 94.32 (53.35, 134.36) | 53.87 (29.23, 88.35) | -1.81 (-3.33, | 0.023 | 1.66 (0.94, | 0.94 (0.51, | -1.81 (-3.35, | 0.023 |

| | | | | | | | | | | | | | | | | |
|---|---|---|---|---|---|---|---|---|---|---|---|---|---|---|---|---|
| | | | | | | | | | | | -0.25) | | 2.36) | 1.55) | -0.25) | |
| High-income Asia Pacific | 10.06 (6.17, 15.54) | 9.21 (5.66, 14.25) | -0.30 (-0.33, -0.27) | <0.001 | — | — | — | — | 37.10 (31.57, 50.66) | 19.26 (15.72, 26.71) | -2.23 (-2.46, -1.99) | <0.001 | 0.60 (0.51, 0.84) | 0.30 (0.24, 0.43) | -2.40 (-2.67, -2.13) | <0.001 |
| Central Europe | 8.27 (5.08, 12.65) | 8.30 (5.09, 12.71) | 0.01 (-0.04, 0.06) | 0.771 | — | — | — | — | 52.14 (43.80, 61.13) | 30.27 (24.34, 37.83) | -1.81 (-2.05, -1.56) | <0.001 | 0.91 (0.76, 1.06) | 0.52 (0.42, 0.65) | -1.84 (-2.08, -1.60) | <0.001 |
| Andean Latin America | 8.57 (5.21, 13.52) | 8.60 (5.24, 13.65) | 0.01 (0.01, 0.01) | <0.001 | — | — | — | — | 22.08 (12.53, 34.47) | 15.17 (9.36, 24.16) | -1.31 (-1.70, -0.93) | <0.001 | 0.39 (0.22, 0.61) | 0.26 (0.16, 0.42) | -1.36 (-1.75, -0.96) | <0.001 |
| Caribbean | 8.68 (5.22, 13.85) | 8.68 (5.22, 13.85) | 0.00 (0.00, 0.00)* | 0.029 | — | — | — | — | 59.93 (39.96, 94.85) | 65.04 (37.54, 118.06) | 0.28 (0.05, 0.52) | 0.019 | 1.04 (0.70, 1.66) | 1.14 (0.65, 2.07) | 0.29 (0.05, 0.53) | 0.018 |
| Southern Latin America | 7.13 (4.31, 10.88) | 7.13 (4.31, 10.88) | 0.00 (-0.00, 0.00) | 0.805 | — | — | — | — | 46.74 (34.15, 64.41) | 29.69 (22.51, 40.15) | -1.48 (-1.92, -1.03) | <0.001 | 0.82 (0.60, 1.14) | 0.51 (0.38, 0.68) | -1.57 (-2.02, -1.11) | <0.001 |
| Oceania | 8.56 (5.17, 13.62) | 8.56 (5.17, 13.62) | 0.00 (0.00, 0.00)* | <0.001 | — | — | — | — | 13.73 (6.47, 26.44) | 13.75 (5.35, 26.20) | -0.03 (-0.18, 0.11) | 0.646 | 0.24 (0.11, 0.46) | 0.24 (0.09, 0.46) | -0.02 (-0.17, 0.12) | 0.738 |
| Australasia | 8.19 (4.99, 12.51) | 8.14 (4.97, 12.33) | -0.02 (-0.03, -0.01) | <0.001 | — | — | — | — | 59.02 (43.93, 71.53) | 26.76 (19.88, 36.35) | -2.70 (-3.19, -2.20) | <0.001 | 0.98 (0.72, 1.20) | 0.43 (0.32, 0.61) | -2.79 (-3.33, -2.25) | <0.001 |
| **Men** | | | | | | | | | | | | | | | | |
| Global | 15.89 (9.74, 24.38) | 14.74 (9.12, 22.44) | -0.26 (-0.28, -0.24) | <0.001 | 12.42 (6.77, 19.23) | 9.25 (5.24, 14.45) | -1.02 (-1.70, -0.33) | 0.004 | 100.41 (89.28, 127.80) | 108.44 (81.62, 123.35) | 0.28 (-0.34, 0.89) | 0.376 | 1.73 (1.53, 2.21) | 1.88 (1.42, 2.14) | 0.30 (-0.33, 0.94) | 0.355 |
| **SDI category** | | | | | | | | | | | | | | | | |
| High | 20.06 (12.37, 30.86) | 18.15 (11.61, 27.22) | -0.34 (-0.37, -0.32) | <0.001 | 70.87 (38.70, 109.88) | 74.70 (42.35, 116.53) | 0.21 (-0.30, 0.73) | 0.426 | 129.57 (104.12, 146.81) | 89.30 (73.83, 104.81) | -1.30 (-1.50, -1.09) | <0.001 | 2.12 (1.68, 2.44) | 1.41 (1.13, 1.68) | -1.44 (-1.67, -1.21) | <0.001 |
| High-middle | 16.56 (10.12, 25.77) | 15.45 (9.38, 23.91) | -0.24 (-0.26, -0.22) | <0.001 | 0.87 (0.22, 1.81) | 0.31 (0.14, 0.54) | -3.61 (-5.44, -1.75) | <0.001 | 205.82 (179.27, 281.32) | 307.42 (209.95, 358.54) | 1.47 (0.66, 2.28) | <0.001 | 3.61 (3.14, 4.94) | 5.41 (3.70, 6.31) | 1.48 (0.67, 2.30) | <0.001 |

| | | | | | | | | | | | | | | | | |
|---|---|---|---|---|---|---|---|---|---|---|---|---|---|---|---|---|
| Middle | 14.79 (9.04, 22.79) | 14.07 (8.61, 21.62) | -0.17 (-0.19, -0.15) | <0.001 | — | — | — | — | 49.66 (41.84, 71.95) | 46.98 (37.03, 61.78) | -0.24 (-0.44, -0.04) | 0.021 | 0.86 (0.73, 1.26) | 0.81 (0.64, 1.08) | -0.25 (-0.46, -0.04) | 0.017 |
| Low-middle | 14.03 (8.50, 21.58) | 13.82 (8.41, 21.29) | -0.05 (-0.05, -0.04) | <0.001 | 0.02 (0.00, 0.04) | 0.03 (0.00, 0.08) | 1.77 (1.45, 2.09) | <0.001 | 36.38 (28.06, 49.72) | 41.21 (30.75, 51.06) | 0.46 (0.29, 0.63) | <0.001 | 0.64 (0.49, 0.87) | 0.72 (0.54, 0.90) | 0.46 (0.30, 0.63) | <0.001 |
| Low | 13.63 (8.23, 21.11) | 13.52 (8.15, 20.86) | -0.03 (-0.03, -0.03) | <0.001 | — | — | — | — | 70.21 (41.42, 93.91) | 69.68 (46.81, 93.04) | -0.02 (-0.11, 0.06) | 0.561 | 1.23 (0.72, 1.65) | 1.22 (0.82, 1.64) | -0.03 (-0.11, 0.06) | 0.530 |
| **21 GBD region** | | | | | | | | | | | | | | | | |
| South Asia | 14.28 (8.67, 22.18) | 14.30 (8.69, 22.20) | 0.0040 (0.0037, 0.0043) | <0.001 | — | — | — | — | 11.71 (7.98, 16.67) | 12.72 (9.09, 17.88) | 0.29 (0.13, 0.44) | <0.001 | 0.20 (0.14, 0.29) | 0.22 (0.16, 0.31) | 0.28 (0.06, 0.51) | 0.013 |
| East Asia | 16.07 (9.81, 24.94) | 14.78 (9.10, 22.80) | -0.29 (-0.34, -0.23) | <0.001 | — | — | — | — | 34.42 (25.90, 56.72) | 37.51 (23.21, 48.40) | 0.32 (-0.18, 0.83) | 0.213 | 0.59 (0.45, 0.99) | 0.65 (0.39, 0.84) | 0.32 (-0.20, 0.84) | 0.235 |
| North Africa and Middle East | 11.03 (6.64, 17.11) | 11.03 (6.64, 17.11) | 0.00 (-0.01, 0.01) | 0.991 | 2.29 (0.10, 5.93) | 0.39 (0.07, 0.87) | -5.79 (-6.55, -5.03) | <0.001 | 48.00 (32.79, 88.00) | 36.09 (25.85, 61.48) | -0.97 (-1.10, -0.85) | <0.001 | 0.82 (0.55, 1.53) | 0.61 (0.42, 1.06) | -1.02 (-1.15, -0.89) | <0.001 |
| Western Sub-Saharan Africa | 13.73 (8.30, 21.26) | 13.66 (8.26, 21.14) | -0.02 (-0.02, -0.02) | <0.001 | — | — | — | — | 117.44 (65.79, 168.12) | 105.72 (69.60, 145.45) | -0.35 (-0.52, -0.18) | <0.001 | 2.07 (1.15, 2.96) | 1.86 (1.22, 2.56) | -0.35 (-0.52, -0.18) | <0.001 |
| Southeast Asia | 13.92 (8.51, 21.69) | 13.88 (8.47, 21.52) | -0.01 (-0.01, -0.01) | <0.001 | — | — | — | — | 56.46 (41.87, 93.07) | 58.75 (42.55, 84.10) | 0.15 (-0.02, 0.32) | 0.082 | 0.98 (0.72, 1.61) | 1.01 (0.73, 1.47) | 0.15 (-0.02, 0.32) | 0.094 |
| Eastern Sub-Saharan Africa | 13.56 (8.18, 20.98) | 13.59 (8.21, 21.02) | 0.01 (0.01, 0.01) | <0.001 | — | — | — | — | 95.95 (60.62, 129.40) | 94.17 (53.30, 137.59) | -0.06 (-0.22, 0.10) | 0.468 | 1.68 (1.06, 2.28) | 1.65 (0.93, 2.42) | -0.06 (-0.22, 0.10) | 0.464 |
| Central Latin America | 12.86 (7.81, 19.78) | 12.86 (7.81, 19.78) | 0.00 (-0.00, 0.00) | 0.577 | — | — | — | — | 35.49 (30.02, 45.32) | 35.33 (24.25, 43.84) | 0.00 (-0.20, 0.21) | 0.996 | 0.62 (0.53, 0.80) | 0.62 (0.42, 0.77) | 0.00 (-0.21, 0.22) | 0.991 |
| Tropical Latin America | 13.50 (8.22, 20.61) | 13.49 (8.21, 20.59) | -0.0028 (-0.0029, | <0.001 | — | — | — | — | 219.34 (158.31, | 159.26 (113.59, | -1.09 (-1.36, | <0.001 | 3.87 (2.79, | 2.80 (2.00, | -1.10 (-1.37, | <0.001 |

| | | | | | | | | | | | | | | | | |
|---|---|---|---|---|---|---|---|---|---|---|---|---|---|---|---|---|
| | | | -0.0027) | | | | | | 250.99) | 186.95) | -0.82) | | 4.43) | 3.29) | -0.82) | |
| Central Sub-Saharan Africa | 13.02 (7.80, 20.20) | 13.02 (7.80, 20.20) | 0.00 (0.00, 0.00)* | 0.001 | — | — | — | — | 117.18 (46.72, 216.83) | 121.48 (62.06, 205.45) | 0.12 (-0.01, 0.25) | 0.080 | 2.06 (0.81, 3.82) | 2.14 (1.09, 3.62) | 0.12 (-0.01, 0.25) | 0.082 |
| Western Europe | 19.26 (11.90, 29.10) | 18.97 (11.58, 28.84) | -0.06 (-0.08, -0.03) | <0.001 | 4.05 (1.04, 8.33) | 1.27 (0.63, 2.07) | -3.70 (-7.22, -0.04) | 0.048 | 84.08 (65.25, 100.88) | 53.21 (41.73, 64.16) | -1.59 (-1.85, -1.33) | <0.001 | 1.42 (1.09, 1.72) | 0.89 (0.69, 1.09) | -1.64 (-1.91, -1.36) | <0.001 |
| High-income North America | 21.37 (13.06, 33.03) | 19.83 (13.25, 28.46) | -0.25 (-0.29, -0.21) | <0.001 | 177.37 (96.78, 277.57) | 216.65 (122.00, 338.76) | 0.73 (0.39, 1.08) | <0.001 | 193.00 (148.12, 218.11) | 144.34 (109.16, 177.02) | -1.01 (-1.41, -0.61) | <0.001 | 3.08 (2.35, 3.42) | 2.15 (1.55, 2.63) | -1.26 (-1.71, -0.81) | <0.001 |
| Central Asia | 15.91 (9.61, 24.48) | 15.91 (9.61, 24.50) | -0.00046 (-0.00047, -0.00046) | <0.001 | — | — | — | — | 205.79 (153.60, 353.23) | 430.32 (274.88, 532.44) | 2.48 (1.86, 3.11) | <0.001 | 3.61 (2.68, 6.22) | 7.58 (4.82, 9.37) | 2.50 (1.88, 3.12) | <0.001 |
| Eastern Europe | 18.50 (11.50, 28.33) | 18.52 (11.51, 28.37) | 0.00 (0.00, 0.00)* | 0.002 | — | — | — | — | 719.22 (597.53, 1017.09) | 1768.86 (1201.79, 2100.99) | 3.30 (2.54, 4.06) | <0.001 | 12.66 (10.52, 17.93) | 31.22 (21.20, 37.09) | 3.31 (2.55, 4.07) | <0.001 |
| Southern Sub-Saharan Africa | 14.10 (8.56, 21.85) | 14.11 (8.56, 21.85) | 0.00 (0.00, 0.00)* | <0.001 | — | — | — | — | 142.29 (101.90, 200.66) | 110.96 (74.27, 166.90) | -0.81 (-1.56, -0.06) | 0.035 | 2.50 (1.79, 3.53) | 1.95 (1.30, 2.94) | -0.81 (-1.56, -0.06) | 0.034 |
| High-income Asia Pacific | 22.32 (13.63, 34.31) | 20.95 (13.06, 31.85) | -0.22 (-0.26, -0.19) | <0.001 | 24.41 (12.38, 39.64) | 20.19 (10.03, 32.94) | -0.63 (-0.86, -0.40) | <0.001 | 90.62 (79.47, 125.12) | 52.79 (43.33, 80.47) | -1.84 (-2.08, -1.59) | <0.001 | 1.50 (1.32, 2.09) | 0.84 (0.69, 1.34) | -1.97 (-2.26, -1.68) | <0.001 |
| Central Europe | 21.65 (13.33, 33.31) | 21.91 (13.49, 33.73) | 0.03 (0.01, 0.06) | 0.004 | — | — | — | — | 168.60 (134.04, 207.37) | 145.74 (102.88, 176.24) | -0.55 (-0.79, -0.30) | <0.001 | 2.94 (2.33, 3.63) | 2.53 (1.79, 3.07) | -0.56 (-0.81, -0.31) | <0.001 |
| Andean Latin America | 12.16 (7.29, 19.18) | 12.18 (7.30, 19.20) | 0.00 (0.00, 0.00)* | <0.001 | — | — | — | — | 32.02 (21.05, 46.98) | 29.34 (17.77, 43.89) | -0.33 (-0.54, -0.12) | 0.002 | 0.56 (0.37, 0.83) | 0.51 (0.31, 0.77) | -0.35 (-0.56, -0.14) | 0.001 |
| Caribbean | 12.18 (7.31, 18.95) | 12.18 (7.31, 18.95) | 0.00 (0.00, 0.00)* | <0.001 | — | — | — | — | 101.52 (78.31, 143.92) | 112.64 (81.08, 161.52) | 0.43 (0.28, 0.57) | <0.001 | 1.77 (1.36, 2.52) | 1.97 (1.41, 2.83) | 0.43 (0.28, 0.57) | <0.001 |
| Southern Latin America | 16.29 (9.81, 25.51) | 16.29 (9.81, 25.51) | -0.00 (-0.00, 0.00) | 0.709 | — | — | — | — | 113.10 (86.22, | 96.89 (73.68, 124.38) | -0.51 (-0.81, | 0.001 | 1.99 (1.51, | 1.69 (1.28, | -0.55 (-0.85, | <0.001 |

| | | | | | | | | | | | | | | | | |
|---|---|---|---|---|---|---|---|---|---|---|---|---|---|---|---|---|
| | | | | | | | | | 150.79) | | -0.21) | | 2.66) | 2.17) | -0.25) | |
| Oceania | 14.03 (8.30, 22.10) | 14.03 (8.30, 22.10) | 0.00 (0.00, 0.00)* | <0.001 | — | — | — | — | 80.72 (44.34, 152.08) | 94.51 (47.54, 179.76) | 0.52 (0.42, 0.62) | <0.001 | 1.41 (0.77, 2.67) | 1.66 (0.83, 3.16) | 0.52 (0.43, 0.62) | <0.001 |
| Australasia | 19.70 (12.11, 29.54) | 19.97 (12.46, 29.64) | 0.05 (0.02, 0.07) | <0.001 | 0.50 (0.11, 1.07) | 0.17 (0.00, 0.42) | -3.74 (-5.05, -2.42) | <0.001 | 144.87 (111.18, 172.69) | 93.69 (69.10, 127.94) | -1.60 (-2.48, -0.71) | <0.001 | 2.47 (1.87, 2.98) | 1.59 (1.15, 2.17) | -1.62 (-2.54, -0.70) | 0.001 |
| **35-39 years** | | | | | | | | | | | | | | | | |
| **Women** | | | | | | | | | | | | | | | | |
| Global | 10.72 (5.75, 17.47) | 10.46 (5.73, 16.89) | -0.09 (-0.09, -0.08) | <0.001 | 6.25 (3.67, 9.98) | 3.08 (1.86, 4.90) | -2.34 (-2.64, -2.04) | <0.001 | 55.09 (46.06, 73.78) | 52.92 (43.45, 60.02) | -0.03 (-0.53, 0.47) | 0.898 | 1.04 (0.86, 1.40) | 1.00 (0.82, 1.14) | -0.01 (-0.51, 0.50) | 0.976 |
| **SDI category** | | | | | | | | | | | | | | | | |
| High | 10.98 (5.89, 18.05) | 10.30 (5.81, 16.36) | -0.22 (-0.25, -0.20) | <0.001 | 34.99 (20.57, 55.95) | 23.41 (14.17, 37.12) | -1.25 (-1.91, -0.60) | <0.001 | 65.25 (58.39, 73.02) | 47.48 (41.63, 53.20) | -1.10 (-1.37, -0.82) | <0.001 | 1.15 (1.03, 1.28) | 0.84 (0.74, 0.93) | -1.10 (-1.40, -0.81) | <0.001 |
| High-middle | 10.31 (5.57, 16.87) | 10.05 (5.48, 16.31) | -0.09 (-0.10, -0.07) | <0.001 | 0.19 (0.07, 0.35) | 0.12 (0.05, 0.24) | -1.32 (-2.75, 0.13) | 0.075 | 92.80 (80.65, 135.45) | 137.63 (95.76, 167.50) | 1.47 (0.42, 2.53) | 0.006 | 1.78 (1.54, 2.61) | 2.64 (1.84, 3.22) | 1.48 (0.43, 2.55) | 0.006 |
| Middle | 10.63 (5.78, 17.31) | 10.27 (5.59, 16.64) | -0.12 (-0.15, -0.10) | <0.001 | — | — | — | — | 35.46 (27.43, 52.28) | 25.43 (20.51, 32.88) | -1.12 (-1.30, -0.93) | <0.001 | 0.68 (0.52, 1.00) | 0.48 (0.39, 0.63) | -1.14 (-1.32, -0.96) | <0.001 |
| Low-middle | 11.10 (6.06, 18.03) | 10.99 (6.03, 17.80) | -0.03 (-0.04, -0.03) | <0.001 | — | — | — | — | 29.57 (18.70, 40.03) | 26.50 (19.03, 33.10) | -0.38 (-0.46, -0.31) | <0.001 | 0.57 (0.36, 0.77) | 0.51 (0.36, 0.63) | -0.40 (-0.47, -0.32) | <0.001 |
| Low | 10.97 (5.93, 17.80) | 10.90 (5.88, 17.68) | -0.02 (-0.02, -0.02) | <0.001 | — | — | — | — | 52.46 (22.09, 81.59) | 39.28 (24.28, 55.30) | -1.00 (-1.05, -0.94) | <0.001 | 1.00 (0.42, 1.57) | 0.75 (0.46, 1.07) | -1.01 (-1.07, -0.95) | <0.001 |
| **21 GBD region** | | | | | | | | | | | | | | | | |
| South Asia | 11.62 (6.33, 19.09) | 11.61 (6.32, 19.07) | -0.00 (-0.00, -0.00)* | <0.001 | — | — | — | — | 10.88 (5.28, 18.27) | 9.45 (5.88, 13.65) | -0.58 (-0.93, -0.24) | 0.001 | 0.21 (0.10, 0.35) | 0.18 (0.11, 0.26) | -0.59 (-0.94, -0.24) | 0.001 |
| East Asia | 10.82 (5.85, | 10.21 (5.84, | -0.21 (-0.26, | <0.001 | — | — | — | — | 24.98 (17.03, | 16.07 (10.22, | -1.52 | <0.001 | 0.47 | 0.30 | -1.57 | <0.001 |

| | | | | | | | | | | | | | | | | |
|---|---|---|---|---|---|---|---|---|---|---|---|---|---|---|---|---|
| | 17.77) | 16.44) | -0.16) | | | | | | 46.46) | 21.70) | (-1.87, -1.16) | | (0.32, 0.89) | (0.19, 0.41) | (-1.93, -1.22) | |
| North Africa and Middle East | 8.64 (4.70, 14.07) | 8.67 (4.71, 14.08) | 0.01 (0.01, 0.01) | <0.001 | — | — | — | — | 37.40 (21.95, 63.97) | 24.48 (16.79, 38.72) | -1.43 (-1.57, -1.29) | <0.001 | 0.71 (0.41, 1.23) | 0.46 (0.31, 0.73) | -1.46 (-1.61, -1.31) | <0.001 |
| Western Sub-Saharan Africa | 11.04 (5.94, 17.88) | 11.06 (5.96, 17.93) | 0.01 (0.00, 0.01) | <0.001 | — | — | — | — | 102.53 (41.34, 169.06) | 61.47 (38.13, 87.45) | -1.77 (-1.94, -1.60) | <0.001 | 1.97 (0.79, 3.26) | 1.17 (0.73, 1.67) | -1.79 (-1.96, -1.62) | <0.001 |
| Southeast Asia | 9.22 (4.98, 15.04) | 9.19 (4.97, 14.97) | -0.01 (-0.01, -0.01) | <0.001 | — | — | — | — | 41.81 (25.41, 66.82) | 35.30 (25.29, 50.17) | -0.57 (-0.66, -0.48) | <0.001 | 0.80 (0.48, 1.28) | 0.67 (0.48, 0.96) | -0.58 (-0.67, -0.49) | <0.001 |
| Eastern Sub-Saharan Africa | 10.97 (5.88, 17.83) | 10.98 (5.88, 17.83) | 0.00 (-0.00, 0.00) | 0.141 | — | — | — | — | 49.81 (21.55, 94.81) | 36.86 (22.07, 52.52) | -1.04 (-1.14, -0.93) | <0.001 | 0.95 (0.41, 1.81) | 0.70 (0.41, 1.00) | -1.06 (-1.16, -0.95) | <0.001 |
| Central Latin America | 10.25 (5.51, 16.71) | 10.24 (5.51, 16.70) | -0.00 (-0.00, 0.00) | 0.056 | — | — | — | — | 24.96 (21.18, 29.60) | 19.49 (14.71, 25.30) | -0.77 (-1.02, -0.52) | <0.001 | 0.48 (0.40, 0.57) | 0.37 (0.28, 0.48) | -0.79 (-1.05, -0.53) | <0.001 |
| Tropical Latin America | 10.84 (5.93, 17.74) | 10.84 (5.93, 17.73) | -0.00 (-0.00, -0.00)* | <0.001 | — | — | — | — | 134.27 (101.56, 149.54) | 79.03 (67.24, 91.63) | -1.82 (-2.07, -1.57) | <0.001 | 2.59 (1.96, 2.88) | 1.51 (1.29, 1.76) | -1.84 (-2.09, -1.59) | <0.001 |
| Central Sub-Saharan Africa | 10.47 (5.60, 17.00) | 10.47 (5.60, 17.00) | 0.00 (0.00, 0.00)* | <0.001 | — | — | — | — | 108.42 (35.90, 212.31) | 92.02 (40.94, 164.37) | -0.60 (-0.77, -0.43) | <0.001 | 2.08 (0.68, 4.10) | 1.77 (0.78, 3.17) | -0.60 (-0.77, -0.43) | <0.001 |
| Western Europe | 9.85 (5.33, 16.08) | 9.67 (5.30, 15.76) | -0.07 (-0.08, -0.06) | <0.001 | 0.51 (0.09, 1.12) | — | — | — | 40.57 (31.78, 46.58) | 19.87 (17.49, 23.72) | -2.41 (-2.58, -2.25) | <0.001 | 0.75 (0.58, 0.87) | 0.36 (0.32, 0.44) | -2.45 (-2.63, -2.28) | <0.001 |
| High-income North America | 11.96 (6.38, 19.81) | 11.15 (6.73, 17.24) | -0.24 (-0.31, -0.17) | <0.001 | 93.18 (54.77, 148.43) | 67.94 (41.09, 107.95) | -0.99 (-1.64, -0.33) | 0.003 | 99.34 (87.10, 113.24) | 89.10 (72.73, 101.24) | -0.38 (-0.74, -0.03) | 0.035 | 1.70 (1.48, 1.90) | 1.54 (1.24, 1.72) | -0.35 (-0.68, -0.02) | 0.038 |
| Central Asia | 11.00 (6.06, 17.72) | 11.00 (6.06, 17.72) | 0.00 (0.00, 0.00)* | <0.001 | — | — | — | — | 101.45 (78.46, 172.41) | 210.25 (139.01, 265.53) | 2.58 (2.10, 3.07) | <0.001 | 1.95 (1.50, 3.32) | 4.06 (2.67, 5.13) | 2.60 (2.12, 3.08) | <0.001 |
| Eastern Europe | 11.79 (6.29, | 11.81 (6.30, | 0.00 (0.00, | 0.002 | — | — | — | — | 288.16 | 698.80 | 3.36 | <0.001 | 5.55 | 13.48 | 3.37 (2.09, | <0.001 |

| | | | | | | | | | | | | | | | | |
|---|---|---|---|---|---|---|---|---|---|---|---|---|---|---|---|---|
| | 19.08) | 19.10) | 0.01) | | | | | | (239.45, 458.75) | (464.82, 886.88) | (2.08, 4.65) | | (4.60, 8.85) | (8.96, 17.14) | 4.67) | |
| Southern Sub-Saharan Africa | 11.42 (6.28, 18.55) | 11.41 (6.27, 18.53) | -0.00 (-0.01, -0.00) | 0.018 | — | — | — | — | 106.98 (65.84, 143.94) | 60.78 (36.74, 95.64) | -1.84 (-2.71, -0.96) | <0.001 | 2.06 (1.26, 2.76) | 1.16 (0.70, 1.83) | -1.85 (-2.72, -0.97) | <0.001 |
| High-income Asia Pacific | 11.72 (6.35, 19.29) | 10.50 (5.79, 17.03) | -0.37 (-0.39, -0.35) | <0.001 | 3.29 (1.33, 6.05) | — | — | — | 37.71 (32.77, 50.46) | 20.00 (16.74, 27.13) | -2.21 (-2.61, -1.81) | <0.001 | 0.67 (0.59, 0.92) | 0.35 (0.29, 0.48) | -2.40 (-2.60, -2.19) | <0.001 |
| Central Europe | 9.67 (5.15, 15.87) | 9.70 (5.16, 15.89) | 0.00 (-0.04, 0.04) | 0.985 | — | — | — | — | 76.30 (64.70, 88.72) | 43.56 (34.93, 53.65) | -1.90 (-2.15, -1.66) | <0.001 | 1.46 (1.24, 1.70) | 0.83 (0.66, 1.02) | -1.92 (-2.17, -1.67) | <0.001 |
| Andean Latin America | 9.53 (5.12, 15.56) | 9.55 (5.15, 15.58) | 0.01 (0.00, 0.01) | <0.001 | — | — | — | — | 25.84 (15.52, 40.03) | 17.68 (10.94, 26.67) | -1.35 (-1.88, -0.82) | <0.001 | 0.50 (0.30, 0.77) | 0.34 (0.21, 0.51) | -1.39 (-1.93, -0.85) | <0.001 |
| Caribbean | 9.61 (5.13, 15.84) | 9.61 (5.13, 15.84) | 0.00 (0.00, 0.00)* | <0.001 | — | — | — | — | 70.14 (47.20, 109.08) | 77.44 (45.91, 134.74) | 0.35 (0.11, 0.60) | 0.005 | 1.34 (0.90, 2.10) | 1.49 (0.87, 2.59) | 0.36 (0.11, 0.61) | 0.004 |
| Southern Latin America | 8.41 (4.52, 14.44) | 8.41 (4.52, 14.44) | 0.00 (0.00, 0.00) | 0.283 | — | — | — | — | 66.27 (47.99, 92.93) | 45.79 (35.14, 60.14) | -1.31 (-1.73, -0.89) | <0.001 | 1.28 (0.92, 1.80) | 0.87 (0.66, 1.14) | -1.36 (-1.78, -0.93) | <0.001 |
| Oceania | 9.38 (4.98, 15.23) | 9.38 (4.98, 15.23) | 0.00 (0.00, 0.00) | 0.104 | — | — | — | — | 94.13 (42.15, 174.35) | 96.57 (40.01, 193.97) | 0.08 (-0.10, 0.26) | 0.394 | 1.81 (0.81, 3.37) | 1.86 (0.77, 3.75) | 0.05 (-0.07, 0.17) | 0.442 |
| Australasia | 9.64 (5.16, 15.93) | 9.51 (5.23, 15.68) | -0.05 (-0.06, -0.03) | <0.001 | — | — | — | — | 69.48 (55.61, 84.12) | 37.69 (28.76, 49.78) | -2.07 (-2.42, -1.72) | <0.001 | 1.28 (1.01, 1.55) | 0.68 (0.51, 0.91) | -2.15 (-2.70, -1.60) | <0.001 |
| **Men** | | | | | | | | | | | | | | | | |
| Global | 16.77 (9.48, 26.68) | 15.49 (8.78, 24.55) | -0.28 (-0.29, -0.27) | <0.001 | 11.41 (6.74, 18.27) | 6.89 (4.24, 10.67) | -1.71 (-2.14, -1.28) | <0.001 | 141.46 (125.77, 186.76) | 155.82 (116.11, 178.31) | 0.34 (-0.30, 0.98) | 0.304 | 2.69 (2.38, 3.58) | 2.98 (2.21, 3.41) | 0.35 (-0.29, 1.00) | 0.281 |
| **SDI category** | | | | | | | | | | | | | | | | |
| High | 20.65 (11.84, | 18.37 (11.05, | -0.40 (-0.44, -0.36) | <0.001 | 59.17 (35.03, 95.57) | 45.53 (27.59, 71.92) | -0.88 (-1.31, | <0.001 | 176.97 (136.71, | 114.65 (94.90, 130.63) | -1.50 (-1.66, | <0.001 | 3.26 (2.50, | 2.09 (1.71, | -1.55 (-1.72, | <0.001 |

| | | | | | | | | | | | | | | | | |
|---|---|---|---|---|---|---|---|---|---|---|---|---|---|---|---|---|
| | 32.94) | 28.41) | | | | | -0.45) | | 197.02) | | -1.34) | | 3.68) | 2.40) | -1.38) | |
| High-middle | 17.05 (9.69, 26.99) | 15.84 (9.03, 25.24) | -0.25 (-0.29, -0.20) | <0.001 | 3.93 (1.91, 6.82) | 2.87 (1.38, 4.84) | -1.10 (-1.48, -0.73) | <0.001 | 309.93 (261.66, 453.77) | 484.67 (328.56, 571.24) | 1.58 (0.68, 2.49) | 0.001 | 5.96 (5.04, 8.74) | 9.34 (6.32, 11.02) | 1.58 (0.68, 2.50) | 0.001 |
| Middle | 15.70 (8.78, 25.15) | 14.57 (8.18, 23.25) | -0.26 (-0.31, -0.22) | <0.001 | 0.08 (0.02, 0.18) | 0.10 (0.02, 0.24) | 0.81 (0.11, 1.51) | 0.023 | 62.11 (51.90, 90.93) | 60.36 (48.44, 83.96) | -0.09 (-0.26, 0.07) | 0.273 | 1.19 (0.99, 1.74) | 1.15 (0.91, 1.61) | -0.10 (-0.27, 0.07) | 0.234 |
| Low-middle | 15.28 (8.46, 24.60) | 15.03 (8.32, 24.13) | -0.06 (-0.06, -0.06) | <0.001 | — | 0.02 (0.00, 0.07) | — | — | 45.09 (34.74, 63.63) | 50.19 (37.99, 62.28) | 0.39 (0.27, 0.51) | <0.001 | 0.86 (0.66, 1.22) | 0.96 (0.72, 1.20) | 0.39 (0.26, 0.51) | <0.001 |
| Low | 14.99 (8.17, 24.33) | 14.84 (8.10, 24.13) | -0.03 (-0.04, -0.03) | <0.001 | — | — | — | — | 80.14 (48.11, 109.13) | 76.83 (50.68, 105.19) | -0.15 (-0.25, -0.05) | 0.003 | 1.54 (0.92, 2.10) | 1.47 (0.96, 2.02) | -0.16 (-0.26, -0.06) | 0.002 |
| **21 GBD region** | | | | | | | | | | | | | | | | |
| South Asia | 15.82 (8.64, 25.40) | 15.83 (8.66, 25.42) | 0.0028 (0.0025, 0.0031) | <0.001 | — | — | — | — | 15.27 (9.76, 22.86) | 15.97 (10.93, 23.48) | 0.16 (-0.65, 0.98) | 0.697 | 0.29 (0.18, 0.44) | 0.30 (0.21, 0.45) | 0.16 (-0.66, 1.00) | 0.701 |
| East Asia | 16.58 (9.35, 26.43) | 14.57 (8.26, 23.08) | -0.46 (-0.52, -0.40) | <0.001 | — | — | — | — | 43.88 (32.90, 71.31) | 47.01 (28.45, 61.89) | 0.28 (-0.04, 0.59) | 0.085 | 0.83 (0.62, 1.36) | 0.89 (0.53, 1.18) | 0.26 (-0.05, 0.58) | 0.103 |
| North Africa and Middle East | 11.39 (6.22, 18.31) | 11.42 (6.23, 18.35) | 0.01 (0.00, 0.01) | 0.001 | 0.14 (0.04, 0.29) | 0.29 (0.05, 0.64) | 2.32 (0.62, 4.05) | 0.007 | 56.79 (36.00, 104.11) | 42.82 (30.82, 70.60) | -0.98 (-1.08, -0.87) | <0.001 | 1.07 (0.67, 2.00) | 0.80 (0.57, 1.33) | -1.01 (-1.12, -0.90) | <0.001 |
| Western Sub-Saharan Africa | 15.13 (8.17, 24.54) | 15.02 (8.11, 24.44) | -0.02 (-0.02, -0.02) | <0.001 | — | — | — | — | 68.61 (38.22, 103.54) | 55.08 (36.17, 75.83) | -0.75 (-0.88, -0.63) | <0.001 | 1.32 (0.73, 1.99) | 1.05 (0.69, 1.46) | -0.76 (-0.89, -0.64) | <0.001 |
| Southeast Asia | 14.12 (7.81, 22.58) | 14.04 (7.74, 22.59) | -0.02 (-0.02, -0.02) | <0.001 | — | 0.30 (0.00, 0.76) | — | — | 85.59 (63.33, 141.22) | 88.41 (62.66, 138.84) | 0.16 (-0.04, 0.36) | 0.112 | 1.63 (1.21, 2.71) | 1.68 (1.19, 2.66) | 0.16 (-0.04, 0.36) | 0.124 |
| Eastern Sub-Saharan Africa | 14.90 (8.05, 24.27) | 14.92 (8.06, 24.32) | 0.00 (0.00, 0.01) | <0.001 | — | — | — | — | 145.82 (90.71, 194.85) | 133.51 (72.35, 197.74) | -0.35 (-0.46, -0.24) | <0.001 | 2.80 (1.74, 3.75) | 2.56 (1.38, 3.81) | -0.35 (-0.46, -0.24) | <0.001 |
| Central Latin | 14.27 (7.89, | 14.27 (7.89, | 0.00 (-0.00, | 0.943 | — | — | — | — | 47.97 (40.06, | 45.19 (31.74, | -0.15 | 0.225 | 0.92 | 0.87 | -0.16 | 0.211 |

| | | | | | | | | | | | | | | | | |
|---|---|---|---|---|---|---|---|---|---|---|---|---|---|---|---|---|
| America | 23.23) | 23.23) | 0.00) | | | | | | 60.72) | 57.61) | (-0.41, 0.10) | | (0.77, 1.17) | (0.61, 1.11) | (-0.42, 0.09) | |
| Tropical Latin America | 15.08 (8.52, 24.91) | 15.07 (8.52, 24.89) | -0.00 (-0.00, -0.00)* | <0.001 | — | — | — | — | 309.25 (225.42, 352.64) | 222.58 (151.11, 260.29) | -1.10 (-1.42, -0.77) | <0.001 | 5.98 (4.36, 6.81) | 4.29 (2.92, 5.02) | -1.11 (-1.43, -0.78) | <0.001 |
| Central Sub-Saharan Africa | 14.21 (7.67, 23.18) | 14.21 (7.67, 23.18) | 0.00 (0.00, 0.00)* | <0.001 | — | — | — | — | 164.23 (68.08, 302.49) | 166.55 (85.73, 284.61) | 0.01 (-0.09, 0.12) | 0.828 | 3.16 (1.31, 5.84) | 3.21 (1.65, 5.49) | 0.01 (-0.09, 0.12) | 0.812 |
| Western Europe | 20.30 (11.68, 32.58) | 19.98 (11.44, 32.22) | -0.06 (-0.09, -0.03) | <0.001 | 0.76 (0.23, 1.47) | 0.46 (0.11, 0.95) | -1.26 (-2.85, 0.36) | 0.127 | 120.85 (94.12, 146.41) | 71.67 (54.55, 86.39) | -1.81 (-2.02, -1.61) | <0.001 | 2.28 (1.76, 2.75) | 1.34 (1.01, 1.61) | -1.83 (-2.04, -1.62) | <0.001 |
| High-income North America | 21.90 (12.27, 35.23) | 19.69 (12.64, 28.83) | -0.36 (-0.42, -0.31) | <0.001 | 141.85 (84.12, 228.07) | 126.36 (75.55, 199.88) | -0.40 (-0.88, 0.08) | 0.106 | 257.60 (189.64, 284.98) | 180.52 (136.89, 213.53) | -1.26 (-1.48, -1.05) | <0.001 | 4.69 (3.44, 5.18) | 3.23 (2.45, 3.84) | -1.30 (-1.73, -0.88) | <0.001 |
| Central Asia | 15.88 (8.70, 24.71) | 15.88 (8.74, 24.66) | -0.000567 (-0.000573, -0.000562) | <0.001 | 8.46 (2.38, 17.51) | 7.40 (1.98, 15.20) | -0.51 (-0.78, -0.25) | <0.001 | 274.33 (201.45, 482.73) | 654.29 (380.38, 826.66) | 2.99 (2.49, 3.48) | <0.001 | 5.27 (3.84, 9.32) | 12.63 (7.33, 15.97) | 3.00 (2.51, 3.49) | <0.001 |
| Eastern Europe | 18.76 (10.63, 30.26) | 18.77 (10.63, 30.28) | 0.00 (0.00, 0.00)* | 0.007 | 16.48 (7.54, 28.92) | 14.74 (6.45, 25.57) | -0.38 (-0.73, -0.04) | 0.029 | 1133.24 (906.43, 1770.57) | 2686.34 (1836.16, 3233.14) | 3.15 (2.18, 4.13) | <0.001 | 21.86 (17.48, 34.22) | 51.89 (35.48, 62.49) | 3.16 (2.18, 4.14) | <0.001 |
| Southern Sub-Saharan Africa | 15.56 (8.47, 25.02) | 15.56 (8.46, 25.01) | -0.00 (-0.00, 0.00) | 0.057 | — | — | — | — | 179.46 (119.48, 247.60) | 128.27 (85.80, 190.55) | -1.10 (-1.64, -0.56) | <0.001 | 3.45 (2.29, 4.75) | 2.46 (1.65, 3.67) | -1.11 (-1.65, -0.57) | <0.001 |
| High-income Asia Pacific | 22.12 (12.61, 34.70) | 20.10 (11.68, 30.99) | -0.33 (-0.40, -0.26) | <0.001 | 41.89 (22.77, 69.44) | 27.87 (15.24, 45.33) | -1.46 (-1.75, -1.17) | <0.001 | 115.78 (102.81, 154.98) | 65.82 (54.40, 100.16) | -1.92 (-2.14, -1.70) | <0.001 | 2.11 (1.87, 2.86) | 1.16 (0.94, 1.81) | -2.03 (-2.29, -1.76) | <0.001 |
| Central Europe | 20.92 (12.19, 32.83) | 21.22 (12.47, 33.12) | 0.04 (0.01, 0.07) | 0.019 | 1.03 (0.37, 2.10) | 1.98 (0.78, 3.59) | 2.42 (0.70, 4.17) | 0.006 | 282.89 (221.57, 340.38) | 244.90 (163.43, 299.40) | -0.49 (-0.66, -0.32) | <0.001 | 5.42 (4.26, 6.55) | 4.68 (3.11, 5.72) | -0.50 (-0.67, -0.33) | <0.001 |
| Andean Latin America | 13.31 (7.22, 21.99) | 13.34 (7.24, 21.77) | 0.01 (0.00, 0.01) | <0.001 | — | — | — | — | 39.05 (25.40, 59.83) | 33.91 (21.47, 52.39) | -0.46 (-0.67, -0.25) | <0.001 | 0.75 (0.49, 1.16) | 0.65 (0.41, 1.01) | -0.48 (-0.69, -0.27) | <0.001 |
| Caribbean | 13.42 (7.32, | 13.42 (7.32, | 0.00 (0.00, | 0.027 | — | — | — | — | 149.15 | 174.57 | 0.56 | <0.001 | 2.86 | 3.35 | 0.56 (0.26, | <0.001 |

| | | | | | | | | | | | | | | | | |
|---|---|---|---|---|---|---|---|---|---|---|---|---|---|---|---|---|
| | 21.87) | 21.87) | 0.00)* | | | | | | (112.76, 219.01) | (125.16, 249.75) | (0.26, 0.85) | | (2.16, 4.22) | (2.40, 4.80) | 0.86) | |
| Southern Latin America | 16.79 (9.31, 26.40) | 16.79 (9.31, 26.40) | 0.00 (-0.00, 0.00) | 0.646 | — | — | — | — | 168.35 (124.78, 226.50) | 130.74 (100.02, 172.13) | -0.78 (-0.96, -0.58) | <0.001 | 3.25 (2.41, 4.38) | 2.51 (1.92, 3.31) | -0.80 (-0.99, -0.61) | <0.001 |
| Oceania | 14.32 (7.98, 23.35) | 14.32 (7.98, 23.35) | 0.00 (0.00, 0.00)* | <0.001 | — | — | — | — | 102.71 (57.21, 182.46) | 114.31 (60.88, 209.68) | 0.36 (0.26, 0.45) | <0.001 | 1.97 (1.09, 3.51) | 2.20 (1.17, 4.05) | 0.37 (0.31, 0.42) | <0.001 |
| Australasia | 21.49 (12.05, 34.23) | 21.67 (12.47, 34.04) | 0.03 (0.01, 0.05) | <0.001 | 0.97 (0.28, 1.94) | 0.81 (0.32, 1.59) | -0.74 (-1.87, 0.40) | 0.202 | 167.76 (135.51, 209.03) | 127.58 (95.30, 173.93) | -0.97 (-1.22, -0.73) | <0.001 | 3.13 (2.51, 3.95) | 2.38 (1.77, 3.28) | -0.97 (-1.23, -0.72) | <0.001 |
| **Atrial fibrillation and flutter** | | | | | | | | | | | | | | | | |
| **30-34 years** | | | | | | | | | | | | | | | | |
| **Women** | | | | | | | | | | | | | | | | |
| Global | 2.45 (1.10, 4.40) | 2.53 (1.16, 4.58) | 0.11 (0.06, 0.15) | <0.001 | 4.16 (1.87, 7.48) | 4.29 (1.97, 7.79) | 0.11 (0.06, 0.15) | <0.001 | 1.12 (0.87, 1.49) | 1.22 (0.95, 1.59) | 0.27 (0.22, 0.33) | <0.001 | 0.01 (0.01, 0.01) | 0.01 (0.01, 0.02) | 0.37 (0.28, 0.46) | <0.001 |
| **SDI category** | | | | | | | | | | | | | | | | |
| High | 2.29 (1.04, 4.15) | 2.58 (1.30, 4.44) | 0.41 (0.35, 0.47) | <0.001 | 3.90 (1.77, 7.05) | 4.39 (2.21, 7.55) | 0.41 (0.35, 0.47) | <0.001 | 1.06 (0.82, 1.39) | 1.08 (0.82, 1.44) | 0.06 (-0.02, 0.13) | 0.124 | 0.01 (0.01, 0.01) | 0.01 (0.01, 0.01) | -0.12 (-0.34, 0.10) | 0.278 |
| High-middle | 2.47 (1.10, 4.43) | 2.66 (1.22, 4.83) | 0.26 (0.22, 0.29) | <0.001 | 4.20 (1.87, 7.53) | 4.52 (2.07, 8.20) | 0.26 (0.22, 0.29) | <0.001 | 1.09 (0.85, 1.44) | 1.09 (0.82, 1.44) | -0.04 (-0.16, 0.08) | 0.532 | 0.01 (0.01, 0.01) | 0.01 (0.01, 0.01) | -0.18 (-0.56, 0.21) | 0.366 |
| Middle | 2.62 (1.18, 4.69) | 2.68 (1.23, 4.79) | 0.08 (0.04, 0.13) | <0.001 | 4.45 (2.01, 7.97) | 4.55 (2.09, 8.14) | 0.08 (0.04, 0.13) | <0.001 | 1.30 (1.00, 1.69) | 1.41 (1.05, 1.83) | 0.29 (-0.02, 0.60) | 0.072 | 0.02 (0.01, 0.02) | 0.02 (0.01, 0.02) | 0.30 (-0.19, 0.79) | 0.231 |
| Low-middle | 2.51 (1.15, 4.55) | 2.51 (1.14, 4.54) | -0.00 (-0.04, 0.03) | 0.804 | 4.27 (1.95, 7.73) | 4.27 (1.94, 7.72) | -0.00 (-0.04, 0.03) | 0.808 | 0.92 (0.67, 1.28) | 1.12 (0.84, 1.50) | 0.67 (0.55, 0.79) | <0.001 | 0.01 (0.01, 0.01) | 0.01 (0.01, 0.02) | 1.14 (0.87, 1.40) | <0.001 |
| Low | 1.89 (0.82, 3.42) | 1.87 (0.80, 3.40) | -0.04 (-0.05, -0.03) | <0.001 | 3.22 (1.40, 5.82) | 3.18 (1.35, 5.77) | -0.04 (-0.05, | <0.001 | 1.13 (0.74, 1.59) | 1.24 (0.88, 1.63) | 0.33 (0.18, | <0.001 | 0.01 (0.01, | 0.02 (0.01, | 0.46 (0.24, 0.68) | <0.001 |

| | | | | | | | | | | | | | | | | |
|---|---|---|---|---|---|---|---|---|---|---|---|---|---|---|---|---|
| | | | | | | | -0.03) | | | | 0.48) | | 0.02) | 0.02) | | |
| **21 GBD region** | | | | | | | | | | | | | | | | |
| South Asia | 2.79 (1.29, 5.00) | 2.88 (1.34, 5.10) | 0.10 (0.02, 0.17) | 0.008 | 4.75 (2.19, 8.50) | 4.89 (2.27, 8.67) | 0.10 (0.02, 0.17) | 0.008 | 0.86 (0.59, 1.27) | 1.05 (0.74, 1.46) | 0.73 (0.52, 0.93) | <0.001 | 0.01 (0.01, 0.01) | 0.01 (0.01, 0.01) | 1.31 (0.93, 1.70) | <0.001 |
| East Asia | 3.15 (1.48, 5.57) | 3.35 (1.55, 5.94) | 0.20 (0.14, 0.25) | <0.001 | 5.35 (2.51, 9.47) | 5.69 (2.63, 10.09) | 0.20 (0.14, 0.25) | <0.001 | 1.05 (0.73, 1.49) | 1.06 (0.72, 1.54) | -0.03 (-0.37, 0.30) | 0.844 | 0.01 (0.01, 0.01) | 0.01 (0.01, 0.01) | -0.25 (-0.77, 0.27) | 0.346 |
| North Africa and Middle East | 1.75 (0.73, 3.19) | 1.77 (0.73, 3.26) | 0.02 (-0.00, 0.04) | 0.063 | 2.98 (1.25, 5.43) | 3.00 (1.25, 5.55) | 0.02 (-0.00, 0.04) | 0.060 | 1.28 (0.89, 1.83) | 1.55 (0.99, 2.30) | 0.66 (0.51, 0.82) | <0.001 | 0.02 (0.01, 0.03) | 0.02 (0.01, 0.04) | 0.82 (0.62, 1.03) | <0.001 |
| Western Sub-Saharan Africa | 1.80 (0.75, 3.32) | 1.83 (0.76, 3.39) | 0.05 (0.04, 0.06) | <0.001 | 3.06 (1.28, 5.65) | 3.11 (1.30, 5.76) | 0.05 (0.04, 0.06) | <0.001 | 0.66 (0.44, 0.94) | 0.73 (0.52, 1.04) | 0.37 (0.28, 0.45) | <0.001 | 0.01 (0.00, 0.01) | 0.01 (0.01, 0.01) | 0.60 (0.49, 0.71) | <0.001 |
| Southeast Asia | 3.04 (1.41, 5.46) | 3.14 (1.45, 5.59) | 0.10 (0.07, 0.13) | <0.001 | 5.17 (2.40, 9.29) | 5.34 (2.47, 9.50) | 0.10 (0.07, 0.13) | <0.001 | 1.14 (0.79, 1.60) | 1.66 (1.13, 2.20) | 1.31 (1.19, 1.44) | <0.001 | 0.01 (0.01, 0.02) | 0.02 (0.01, 0.03) | 1.96 (1.79, 2.13) | <0.001 |
| Eastern Sub-Saharan Africa | 1.10 (0.40, 2.08) | 1.10 (0.41, 2.08) | 0.03 (0.01, 0.04) | 0.001 | 1.86 (0.69, 3.53) | 1.88 (0.70, 3.53) | 0.03 (0.01, 0.04) | 0.001 | 1.69 (0.70, 2.62) | 1.73 (0.95, 2.52) | 0.07 (-0.06, 0.21) | 0.292 | 0.03 (0.01, 0.04) | 0.03 (0.01, 0.04) | 0.08 (-0.07, 0.23) | 0.303 |
| Central Latin America | 1.14 (0.42, 2.15) | 1.12 (0.40, 2.13) | -0.07 (-0.12, -0.01) | 0.017 | 1.93 (0.71, 3.66) | 1.90 (0.69, 3.62) | -0.07 (-0.12, -0.01) | 0.017 | 1.20 (0.67, 1.47) | 1.07 (0.66, 1.41) | -0.35 (-0.45, -0.26) | <0.001 | 0.02 (0.01, 0.02) | 0.02 (0.01, 0.02) | -0.40 (-0.52, -0.29) | <0.001 |
| Tropical Latin America | 1.54 (0.62, 2.83) | 1.55 (0.63, 2.85) | 0.11 (0.01, 0.21) | 0.023 | 2.62 (1.05, 4.81) | 2.64 (1.07, 4.85) | 0.11 (0.01, 0.21) | 0.023 | 1.55 (1.27, 2.03) | 1.54 (1.18, 1.84) | -0.02 (-0.35, 0.31) | 0.882 | 0.02 (0.02, 0.03) | 0.02 (0.02, 0.03) | 0.06 (-0.14, 0.26) | 0.569 |
| Central Sub-Saharan Africa | 1.67 (0.69, 2.97) | 1.64 (0.68, 2.95) | -0.06 (-0.07, -0.06) | <0.001 | 2.85 (1.18, 5.05) | 2.79 (1.16, 5.01) | -0.06 (-0.07, -0.06) | <0.001 | 1.34 (0.58, 2.60) | 1.45 (0.71, 2.52) | 0.24 (-0.00, 0.49) | 0.055 | 0.02 (0.01, 0.04) | 0.02 (0.01, 0.04) | 0.32 (0.02, 0.61) | 0.034 |
| Western Europe | 1.55 (0.62, 2.84) | 1.39 (0.55, 2.57) | -0.38 (-0.40, -0.35) | <0.001 | 2.63 (1.06, 4.83) | 2.36 (0.93, 4.36) | -0.38 (-0.40, -0.35) | <0.001 | 1.14 (0.76, 1.45) | 1.06 (0.75, 1.34) | -0.25 (-0.32, -0.17) | <0.001 | 0.02 (0.01, 0.02) | 0.01 (0.01, 0.02) | -0.24 (-0.32, -0.16) | <0.001 |
| High-income North | 3.33 (1.63, | 4.25 (2.24, | 0.86 (0.81, | <0.001 | 5.65 (2.76, | 7.23 (3.80, | 0.86 (0.81, | <0.001 | 1.09 (0.79, | 1.18 (0.83, | 0.32 | <0.001 | 0.01 | 0.01 | -0.27 | 0.259 |

| | | | | | | | | | | | | | | | | |
|---|---|---|---|---|---|---|---|---|---|---|---|---|---|---|---|---|
| America | 5.94) | 7.18) | 0.90) | | 10.09) | 12.20) | 0.90) | | 1.52) | 1.70) | (0.19, 0.44) | | (0.01, 0.01) | (0.01, 0.01) | (-0.74, 0.20) | |
| Central Asia | 1.96 (0.80, 3.60) | 2.00 (0.82, 3.68) | 0.08 (0.06, 0.10) | <0.001 | 3.33 (1.36, 6.13) | 3.40 (1.39, 6.25) | 0.08 (0.06, 0.10) | <0.001 | 1.13 (0.70, 1.49) | 1.19 (0.87, 1.52) | 0.15 (0.02, 0.29) | 0.028 | 0.01 (0.01, 0.02) | 0.02 (0.01, 0.02) | 0.15 (-0.05, 0.34) | 0.147 |
| Eastern Europe | 2.47 (1.08, 4.49) | 2.67 (1.14, 4.82) | 0.27 (0.23, 0.31) | <0.001 | 4.20 (1.83, 7.64) | 4.54 (1.94, 8.19) | 0.27 (0.23, 0.31) | <0.001 | 0.90 (0.65, 1.24) | 1.05 (0.73, 1.44) | 0.53 (0.36, 0.70) | <0.001 | 0.01 (0.01, 0.01) | 0.01 (0.01, 0.02) | 0.70 (0.44, 0.95) | <0.001 |
| Southern Sub-Saharan Africa | 1.88 (0.79, 3.45) | 1.82 (0.76, 3.38) | -0.09 (-0.14, -0.05) | <0.001 | 3.19 (1.34, 5.87) | 3.10 (1.30, 5.75) | -0.09 (-0.14, -0.05) | <0.001 | 5.31 (3.54, 7.21) | 2.63 (1.50, 4.15) | -2.28 (-3.95, -0.57) | 0.009 | 0.09 (0.06, 0.12) | 0.04 (0.02, 0.07) | -2.46 (-4.27, -0.62) | 0.009 |
| High-income Asia Pacific | 1.22 (0.46, 2.30) | 0.97 (0.32, 1.87) | -0.84 (-0.92, -0.75) | <0.001 | 2.08 (0.78, 3.91) | 1.65 (0.55, 3.17) | -0.84 (-0.92, -0.75) | <0.001 | 0.89 (0.72, 1.09) | 0.74 (0.59, 0.92) | -0.61 (-0.81, -0.41) | <0.001 | 0.01 (0.01, 0.01) | 0.01 (0.01, 0.01) | -0.58 (-0.72, -0.44) | <0.001 |
| Central Europe | 1.78 (0.70, 3.34) | 1.75 (0.70, 3.33) | -0.06 (-0.08, -0.03) | <0.001 | 3.02 (1.19, 5.67) | 2.98 (1.19, 5.66) | -0.06 (-0.08, -0.03) | <0.001 | 1.20 (0.86, 1.52) | 1.12 (0.81, 1.48) | -0.24 (-0.31, -0.17) | <0.001 | 0.02 (0.01, 0.02) | 0.02 (0.01, 0.02) | -0.28 (-0.37, -0.18) | <0.001 |
| Andean Latin America | 0.34 (0.05, 0.78) | 0.40 (0.06, 0.90) | 0.53 (0.44, 0.62) | <0.001 | 0.58 (0.09, 1.33) | 0.67 (0.11, 1.52) | 0.53 (0.44, 0.62) | <0.001 | 1.43 (0.72, 2.77) | 1.24 (0.75, 2.02) | -0.54 (-1.07, -0.01) | 0.045 | 0.02 (0.01, 0.05) | 0.02 (0.01, 0.03) | -0.59 (-1.15, -0.03) | 0.040 |
| Caribbean | 0.97 (0.33, 1.94) | 0.98 (0.33, 1.94) | 0.02 (0.01, 0.03) | 0.001 | 1.66 (0.56, 3.30) | 1.67 (0.56, 3.30) | 0.02 (0.01, 0.03) | 0.001 | 1.59 (1.05, 2.47) | 1.61 (0.99, 2.74) | 0.10 (-0.15, 0.35) | 0.446 | 0.03 (0.02, 0.04) | 0.03 (0.02, 0.04) | 0.10 (-0.17, 0.37) | 0.462 |
| Southern Latin America | 1.39 (0.53, 2.56) | 1.44 (0.58, 2.69) | 0.11 (0.06, 0.16) | <0.001 | 2.36 (0.89, 4.35) | 2.44 (0.98, 4.57) | 0.11 (0.06, 0.16) | <0.001 | 1.21 (0.76, 1.63) | 1.07 (0.70, 1.42) | -0.44 (-0.60, -0.28) | <0.001 | 0.02 (0.01, 0.02) | 0.02 (0.01, 0.02) | -0.56 (-0.77, -0.34) | <0.001 |
| Oceania | 2.89 (1.36, 5.11) | 2.96 (1.39, 5.22) | 0.07 (0.01, 0.12) | 0.027 | 4.91 (2.31, 8.68) | 5.03 (2.37, 8.87) | 0.07 (0.01, 0.12) | 0.026 | 6.79 (3.63, 14.28) | 7.79 (3.86, 16.69) | 0.49 (0.46, 0.53) | <0.001 | 0.11 (0.06, 0.24) | 0.13 (0.06, 0.28) | 0.52 (0.49, 0.56) | <0.001 |
| Australasia | 3.44 (1.61, 5.99) | 3.27 (1.51, 5.74) | -0.18 (-0.20, -0.16) | <0.001 | 5.85 (2.74, 10.18) | 5.56 (2.56, 9.76) | -0.18 (-0.20, -0.16) | <0.001 | 1.17 (0.84, 1.65) | 1.05 (0.73, 1.51) | -0.34 (-0.40, -0.28) | <0.001 | 0.01 (0.01, 0.01) | 0.01 (0.01, 0.01) | -0.50 (-0.64, -0.37) | <0.001 |
| **Men** | | | | | | | | | | | | | | | | |

| | | | | | | | | | | | | | | | | |
|---|---|---|---|---|---|---|---|---|---|---|---|---|---|---|---|---|
| Global | 4.27 (2.17, 7.51) | 3.89 (1.99, 6.81) | -0.32 (-0.36, -0.28) | <0.001 | 7.26 (3.68, 12.77) | 6.61 (3.39, 11.57) | -0.32 (-0.36, -0.28) | <0.001 | 1.50 (1.09, 2.07) | 1.51 (1.12, 2.02) | 0.01 (-0.10, 0.13) | 0.793 | 0.01 (0.01, 0.02) | 0.02 (0.01, 0.02) | 0.26 (0.02, 0.49) | 0.035 |
| **SDI category** | | | | | | | | | | | | | | | | |
| High | 7.93 (4.26, 13.57) | 7.88 (4.34, 13.13) | -0.02 (-0.06, 0.00) | 0.108 | 13.49 (7.23, 23.06) | 13.40 (7.37, 22.31) | -0.02 (-0.06, 0.00) | 0.109 | 2.26 (1.53, 3.27) | 2.22 (1.49, 3.19) | -0.09 (-0.20, 0.03) | 0.140 | 0.02 (0.01, 0.02) | 0.02 (0.01, 0.02) | -0.17 (-0.41, 0.06) | 0.150 |
| High-middle | 4.68 (2.39, 8.19) | 4.31 (2.18, 7.63) | -0.29 (-0.31, -0.27) | <0.001 | 7.96 (4.06, 13.92) | 7.33 (3.71, 12.98) | -0.29 (-0.31, -0.27) | <0.001 | 1.55 (1.08, 2.19) | 1.45 (1.05, 2.00) | -0.27 (-0.43, -0.11) | 0.001 | 0.01 (0.01, 0.02) | 0.01 (0.01, 0.02) | -0.21 (-0.38, -0.04) | 0.017 |
| Middle | 3.16 (1.52, 5.59) | 3.24 (1.55, 5.73) | 0.08 (0.07, 0.10) | <0.001 | 5.37 (2.59, 9.50) | 5.50 (2.64, 9.75) | 0.08 (0.07, 0.10) | <0.001 | 1.35 (1.04, 1.79) | 1.48 (1.08, 1.94) | 0.29 (0.02, 0.56) | 0.035 | 0.02 (0.01, 0.02) | 0.02 (0.01, 0.02) | 0.39 (0.04, 0.74) | 0.031 |
| Low-middle | 3.03 (1.42, 5.45) | 3.01 (1.40, 5.31) | -0.02 (-0.04, -0.01) | <0.001 | 5.16 (2.41, 9.27) | 5.11 (2.37, 9.02) | -0.02 (-0.04, -0.01) | <0.001 | 1.08 (0.78, 1.49) | 1.28 (0.95, 1.71) | 0.59 (0.50, 0.68) | <0.001 | 0.01 (0.01, 0.01) | 0.01 (0.01, 0.02) | 0.98 (0.83, 1.14) | <0.001 |
| Low | 2.43 (1.09, 4.34) | 2.43 (1.08, 4.40) | 0.00 (-0.01, 0.01) | 0.838 | 4.14 (1.85, 7.38) | 4.14 (1.83, 7.47) | 0.00 (-0.01, 0.01) | 0.828 | 1.20 (0.81, 1.70) | 1.38 (0.99, 1.88) | 0.46 (0.40, 0.51) | <0.001 | 0.01 (0.01, 0.02) | 0.02 (0.01, 0.03) | 0.65 (0.51, 0.79) | <0.001 |
| **21 GBD region** | | | | | | | | | | | | | | | | |
| South Asia | 3.17 (1.47, 5.67) | 3.16 (1.46, 5.62) | -0.01 (-0.02, 0.00) | 0.038 | 5.39 (2.49, 9.63) | 5.38 (2.48, 9.56) | -0.01 (-0.02, 0.00) | 0.038 | 0.99 (0.68, 1.42) | 1.17 (0.84, 1.64) | 0.58 (0.48, 0.68) | <0.001 | 0.01 (0.01, 0.01) | 0.01 (0.01, 0.02) | 1.06 (0.87, 1.24) | <0.001 |
| East Asia | 3.36 (1.64, 5.88) | 3.52 (1.73, 6.24) | 0.16 (0.11, 0.22) | <0.001 | 5.71 (2.79, 9.99) | 5.99 (2.94, 10.61) | 0.16 (0.11, 0.22) | <0.001 | 1.19 (0.85, 1.66) | 1.18 (0.85, 1.65) | -0.05 (-0.31, 0.20) | 0.684 | 0.01 (0.01, 0.01) | 0.01 (0.01, 0.01) | -0.24 (-0.71, 0.23) | 0.314 |
| North Africa and Middle East | 2.98 (1.45, 5.26) | 3.03 (1.49, 5.39) | 0.07 (0.03, 0.11) | 0.001 | 5.06 (2.46, 8.94) | 5.15 (2.53, 9.16) | 0.07 (0.03, 0.11) | 0.001 | 1.36 (1.03, 1.84) | 1.42 (0.98, 1.97) | 0.12 (0.05, 0.20) | 0.001 | 0.02 (0.01, 0.02) | 0.02 (0.01, 0.02) | 0.20 (0.03, 0.36) | 0.018 |
| Western Sub-Saharan Africa | 2.35 (1.03, 4.24) | 2.44 (1.09, 4.43) | 0.13 (0.11, 0.15) | <0.001 | 4.00 (1.75, 7.20) | 4.15 (1.85, 7.53) | 0.13 (0.11, 0.15) | <0.001 | 0.86 (0.60, 1.22) | 0.97 (0.70, 1.34) | 0.43 (0.36, 0.51) | <0.001 | 0.01 (0.01, 0.01) | 0.01 (0.01, 0.01) | 0.63 (0.53, 0.72) | <0.001 |
| Southeast Asia | 3.60 (1.75, | 3.74 (1.81, | 0.13 (0.11, | <0.001 | 6.13 (2.97, | 6.36 (3.08, | 0.13 (0.11, | <0.001 | 1.21 (0.73, | 1.68 (0.80, | 1.12 | <0.001 | 0.01 | 0.02 | 1.78 (1.66, | <0.001 |

| | | | | | | | | | | | | | | | | |
|---|---|---|---|---|---|---|---|---|---|---|---|---|---|---|---|---|
| | 6.39) | 6.58) | 0.15) | | 10.86) | 11.18) | 0.15) | | 1.74) | 2.37) | (1.03, 1.21) | | (0.01, 0.02) | (0.01, 0.03) | 1.91) | |
| Eastern Sub-Saharan Africa | 1.55 (0.64, 2.87) | 1.61 (0.67, 2.98) | 0.12 (0.09, 0.15) | <0.001 | 2.63 (1.08, 4.87) | 2.73 (1.14, 5.06) | 0.12 (0.09, 0.15) | <0.001 | 1.42 (0.80, 2.09) | 1.59 (0.96, 2.39) | 0.40 (0.28, 0.53) | <0.001 | 0.02 (0.01, 0.03) | 0.02 (0.01, 0.04) | 0.46 (0.31, 0.61) | <0.001 |
| Central Latin America | 1.71 (0.71, 3.08) | 1.73 (0.72, 3.14) | 0.06 (0.04, 0.09) | <0.001 | 2.90 (1.21, 5.23) | 2.95 (1.23, 5.33) | 0.06 (0.04, 0.09) | <0.001 | 1.36 (0.85, 1.74) | 1.35 (0.86, 1.76) | 0.01 (-0.15, 0.18) | 0.863 | 0.02 (0.01, 0.02) | 0.02 (0.01, 0.02) | -0.00 (-0.20, 0.20) | 0.982 |
| Tropical Latin America | 2.59 (1.15, 4.71) | 2.59 (1.16, 4.69) | 0.09 (-0.01, 0.19) | 0.068 | 4.41 (1.96, 8.01) | 4.41 (1.97, 7.97) | 0.09 (-0.01, 0.19) | 0.068 | 2.15 (1.57, 2.90) | 2.13 (1.46, 2.69) | 0.10 (-0.32, 0.51) | 0.644 | 0.03 (0.02, 0.04) | 0.03 (0.02, 0.04) | -0.01 (-0.22, 0.21) | 0.951 |
| Central Sub-Saharan Africa | 2.19 (0.96, 3.96) | 2.23 (0.96, 4.01) | 0.06 (0.05, 0.07) | <0.001 | 3.73 (1.63, 6.74) | 3.80 (1.64, 6.82) | 0.06 (0.05, 0.07) | <0.001 | 1.26 (0.64, 2.84) | 1.36 (0.77, 2.47) | 0.22 (0.14, 0.31) | <0.001 | 0.02 (0.01, 0.04) | 0.02 (0.01, 0.04) | 0.28 (0.16, 0.40) | <0.001 |
| Western Europe | 8.34 (4.47, 14.20) | 7.71 (4.13, 13.11) | -0.27 (-0.29, -0.24) | <0.001 | 14.18 (7.59, 24.13) | 13.11 (7.01, 22.29) | -0.26 (-0.29, -0.24) | <0.001 | 2.67 (1.58, 3.85) | 2.29 (1.37, 3.38) | -0.54 (-0.68, -0.38) | <0.001 | 0.02 (0.01, 0.03) | 0.02 (0.01, 0.03) | -0.80 (-1.13, -0.47) | <0.001 |
| High-income North America | 9.19 (4.86, 15.68) | 11.00 (6.14, 18.71) | 0.64 (0.55, 0.72) | <0.001 | 15.62 (8.26, 26.66) | 18.70 (10.45, 31.81) | 0.64 (0.55, 0.72) | <0.001 | 2.17 (1.40, 3.27) | 2.63 (1.71, 3.92) | 0.66 (0.50, 0.83) | <0.001 | 0.01 (0.01, 0.02) | 0.02 (0.01, 0.02) | 0.72 (0.40, 1.05) | <0.001 |
| Central Asia | 5.32 (2.68, 9.17) | 5.57 (2.78, 9.50) | 0.16 (0.15, 0.17) | <0.001 | 9.04 (4.55, 15.58) | 9.46 (4.72, 16.15) | 0.16 (0.15, 0.17) | <0.001 | 1.55 (0.92, 2.29) | 1.68 (1.09, 2.46) | 0.28 (0.22, 0.33) | <0.001 | 0.01 (0.01, 0.02) | 0.01 (0.01, 0.02) | 0.40 (0.30, 0.52) | <0.001 |
| Eastern Europe | 6.52 (3.36, 11.33) | 6.93 (3.58, 11.89) | 0.21 (0.19, 0.23) | <0.001 | 11.09 (5.71, 19.27) | 11.79 (6.09, 20.21) | 0.21 (0.19, 0.23) | <0.001 | 1.66 (1.03, 2.53) | 1.93 (1.30, 2.83) | 0.53 (0.35, 0.71) | <0.001 | 0.01 (0.01, 0.01) | 0.02 (0.01, 0.02) | 0.92 (0.36, 1.47) | 0.001 |
| Southern Sub-Saharan Africa | 2.51 (1.13, 4.53) | 2.52 (1.12, 4.51) | 0.03 (0.01, 0.04) | <0.001 | 4.26 (1.92, 7.70) | 4.29 (1.90, 7.67) | 0.03 (0.01, 0.04) | <0.001 | 3.68 (2.75, 4.72) | 3.17 (2.27, 4.23) | -0.68 (-2.07, 0.73) | 0.344 | 0.06 (0.04, 0.08) | 0.05 (0.03, 0.07) | -0.76 (-2.36, 0.85) | 0.352 |
| High-income Asia Pacific | 6.67 (3.54, 11.30) | 6.69 (3.54, 11.32) | 0.00 (-0.03, 0.04) | 0.741 | 11.33 (6.02, 19.21) | 11.37 (6.03, 19.24) | 0.01 (-0.03, 0.04) | 0.738 | 2.30 (1.63, 3.19) | 1.99 (1.39, 2.84) | -0.52 (-0.65, -0.38) | <0.001 | 0.02 (0.02, 0.03) | 0.02 (0.01, 0.02) | -0.97 (-1.30, -0.65) | <0.001 |
| Central Europe | 6.71 (3.42, | 6.64 (3.43, | -0.02 (-0.07, | 0.479 | 11.40 (5.81, | 11.29 (5.83, | -0.02 | 0.480 | 1.77 (1.16, | 1.81 (1.22, | 0.07 | <0.001 | 0.01 | 0.01 | 0.18 (0.10, | <0.001 |

| | | | | | | | | | | | | | | | | |
|---|---|---|---|---|---|---|---|---|---|---|---|---|---|---|---|---|
| | 11.58) | 11.54) | 0.03) | | 19.68) | 19.61) | (-0.07, 0.04) | | 2.68) | 2.67) | (0.04, 0.11) | | (0.01, 0.02) | (0.01, 0.02) | 0.27) | |
| Andean Latin America | 0.57 (0.11, 1.12) | 0.64 (0.16, 1.29) | 0.47 (0.38, 0.57) | <0.001 | 0.96 (0.19, 1.91) | 1.09 (0.27, 2.19) | 0.47 (0.38, 0.57) | <0.001 | 1.95 (1.41, 2.70) | 2.06 (1.38, 2.89) | 0.14 (-0.09, 0.38) | 0.236 | 0.03 (0.02, 0.05) | 0.03 (0.02, 0.05) | 0.13 (-0.12, 0.38) | 0.317 |
| Caribbean | 1.65 (0.69, 2.96) | 1.70 (0.71, 3.09) | 0.10 (0.07, 0.12) | <0.001 | 2.81 (1.17, 5.04) | 2.89 (1.21, 5.26) | 0.10 (0.07, 0.12) | <0.001 | 2.57 (1.88, 3.52) | 2.60 (1.87, 3.71) | 0.03 (-0.23, 0.30) | 0.804 | 0.04 (0.03, 0.06) | 0.04 (0.03, 0.06) | 0.03 (-0.27, 0.33) | 0.844 |
| Southern Latin America | 4.02 (2.02, 6.84) | 4.15 (2.13, 7.18) | 0.10 (0.07, 0.14) | <0.001 | 6.83 (3.43, 11.62) | 7.06 (3.62, 12.21) | 0.10 (0.07, 0.14) | <0.001 | 1.60 (1.00, 2.26) | 1.57 (0.96, 2.20) | -0.06 (-0.22, 0.09) | 0.418 | 0.02 (0.01, 0.02) | 0.02 (0.01, 0.02) | -0.17 (-0.41, 0.07) | 0.172 |
| Oceania | 3.49 (1.68, 6.25) | 3.53 (1.72, 6.25) | 0.03 (0.02, 0.04) | <0.001 | 5.94 (2.86, 10.63) | 6.00 (2.92, 10.63) | 0.03 (0.02, 0.04) | <0.001 | 8.00 (4.90, 16.16) | 9.93 (5.65, 19.11) | 0.72 (0.53, 0.92) | <0.001 | 0.13 (0.08, 0.28) | 0.17 (0.09, 0.33) | 0.77 (0.56, 0.97) | <0.001 |
| Australasia | 9.53 (5.16, 15.94) | 9.12 (4.87, 15.97) | -0.15 (-0.18, -0.11) | <0.001 | 16.20 (8.77, 27.10) | 15.50 (8.28, 27.15) | -0.14 (-0.18, -0.11) | <0.001 | 2.22 (1.41, 3.44) | 2.12 (1.34, 3.28) | -0.15 (-0.25, -0.05) | 0.004 | 0.01 (0.01, 0.02) | 0.01 (0.01, 0.02) | -0.17 (-0.41, 0.07) | 0.170 |
| **35-39 years** | | | | | | | | | | | | | | | | |
| **Women** | | | | | | | | | | | | | | | | |
| Global | 7.42 (3.35, 13.32) | 7.48 (3.44, 13.56) | 0.02 (-0.03, 0.07) | 0.425 | 28.92 (13.05, 51.95) | 29.18 (13.39, 52.88) | 0.02 (-0.03, 0.07) | 0.424 | 3.39 (1.89, 5.57) | 3.51 (2.01, 5.73) | 0.11 (0.02, 0.20) | 0.014 | 0.02 (0.02, 0.02) | 0.02 (0.02, 0.02) | 0.29 (0.23, 0.35) | <0.001 |
| **SDI category** | | | | | | | | | | | | | | | | |
| High | 6.79 (3.07, 12.32) | 7.59 (3.80, 13.02) | 0.38 (0.36, 0.41) | <0.001 | 26.48 (11.97, 48.05) | 29.61 (14.83, 50.79) | 0.38 (0.36, 0.41) | <0.001 | 3.13 (1.75, 5.15) | 3.30 (1.87, 5.47) | 0.17 (0.08, 0.26) | <0.001 | 0.02 (0.01, 0.02) | 0.02 (0.01, 0.02) | -0.32 (-0.52, -0.12) | 0.002 |
| High-middle | 7.45 (3.33, 13.36) | 7.72 (3.49, 14.00) | 0.12 (0.09, 0.15) | <0.001 | 29.03 (12.98, 52.10) | 30.09 (13.60, 54.59) | 0.12 (0.09, 0.15) | <0.001 | 3.32 (1.76, 5.60) | 3.42 (1.81, 5.80) | 0.08 (-0.04, 0.20) | 0.178 | 0.02 (0.01, 0.02) | 0.02 (0.01, 0.02) | -0.04 (-0.15, 0.07) | 0.432 |
| Middle | 8.03 (3.65, 14.36) | 7.91 (3.62, 14.15) | -0.07 (-0.15, 0.01) | 0.090 | 31.31 (14.25, 55.98) | 30.86 (14.12, 55.19) | -0.07 (-0.15, 0.01) | 0.090 | 3.76 (2.10, 6.20) | 3.85 (2.22, 6.29) | 0.08 (-0.05, 0.20) | 0.230 | 0.02 (0.02, 0.03) | 0.02 (0.02, 0.03) | 0.32 (0.29, 0.34) | <0.001 |
| Low-middle | 7.62 (3.49, | 7.53 (3.42, | -0.06 (-0.11, | 0.041 | 29.72 (13.60, | 29.37 (13.33, | -0.06 | 0.042 | 3.22 (1.60, | 3.42 (1.85, | 0.22 | 0.001 | 0.01 | 0.02 | 0.79 (0.59, | <0.001 |

| | | | | | | | | | | | | | | | | |
|---|---|---|---|---|---|---|---|---|---|---|---|---|---|---|---|---|
| | 13.76) | 13.62) | -0.00) | | 53.65) | 53.10) | (-0.11, -0.00) | | 5.47) | 5.63) | (0.08, 0.35) | | (0.01, 0.02) | (0.01, 0.02) | 0.99) | |
| Low | 5.78 (2.51, 10.48) | 5.69 (2.43, 10.30) | -0.05 (-0.08, -0.02) | <0.001 | 22.53 (9.80, 40.86) | 22.18 (9.49, 40.16) | -0.05 (-0.08, -0.02) | <0.001 | 3.07 (1.70, 5.08) | 3.15 (1.81, 5.15) | 0.09 (-0.01, 0.18) | 0.072 | 0.02 (0.01, 0.04) | 0.02 (0.02, 0.04) | 0.26 (0.17, 0.34) | <0.001 |
| **21 GBD region** | | | | | | | | | | | | | | | | |
| South Asia | 8.39 (3.87, 15.01) | 8.63 (4.01, 15.32) | 0.10 (0.02, 0.18) | 0.018 | 32.69 (15.10, 58.53) | 33.66 (15.63, 59.71) | 0.10 (0.02, 0.18) | 0.018 | 3.18 (1.41, 5.78) | 3.43 (1.72, 5.95) | 0.23 (0.05, 0.41) | 0.013 | 0.01 (0.01, 0.02) | 0.01 (0.01, 0.02) | 0.75 (0.24, 1.25) | 0.004 |
| East Asia | 9.44 (4.44, 16.73) | 10.00 (4.64, 17.70) | 0.18 (0.12, 0.24) | <0.001 | 36.82 (17.29, 65.23) | 38.98 (18.09, 69.04) | 0.18 (0.12, 0.24) | <0.001 | 3.73 (1.80, 6.60) | 3.83 (1.80, 6.97) | 0.07 (-0.13, 0.28) | 0.486 | 0.01 (0.01, 0.01) | 0.01 (0.01, 0.01) | -0.62 (-0.82, -0.42) | <0.001 |
| North Africa and Middle East | 5.25 (2.20, 9.55) | 5.31 (2.20, 9.79) | 0.03 (-0.01, 0.06) | 0.141 | 20.46 (8.57, 37.22) | 20.68 (8.58, 38.18) | 0.03 (-0.01, 0.06) | 0.132 | 2.69 (1.47, 4.45) | 2.87 (1.62, 4.74) | 0.23 (0.15, 0.31) | <0.001 | 0.02 (0.01, 0.02) | 0.02 (0.01, 0.03) | 0.62 (0.41, 0.84) | <0.001 |
| Western Sub-Saharan Africa | 5.41 (2.26, 9.96) | 5.49 (2.29, 10.16) | 0.05 (0.04, 0.06) | <0.001 | 21.08 (8.80, 38.84) | 21.38 (8.93, 39.62) | 0.05 (0.04, 0.06) | <0.001 | 2.33 (1.11, 4.22) | 2.39 (1.17, 4.29) | 0.08 (0.04, 0.12) | <0.001 | 0.01 (0.01, 0.01) | 0.01 (0.01, 0.01) | 0.24 (0.14, 0.35) | <0.001 |
| Southeast Asia | 9.13 (4.23, 16.37) | 9.42 (4.36, 16.75) | 0.10 (0.07, 0.13) | <0.001 | 35.58 (16.49, 63.84) | 36.72 (17.01, 65.30) | 0.10 (0.07, 0.13) | <0.001 | 3.97 (2.05, 6.73) | 4.57 (2.52, 7.46) | 0.46 (0.36, 0.56) | <0.001 | 0.02 (0.01, 0.03) | 0.03 (0.02, 0.04) | 1.26 (1.14, 1.38) | <0.001 |
| Eastern Sub-Saharan Africa | 3.29 (1.21, 6.23) | 3.31 (1.23, 6.23) | 0.03 (0.01, 0.04) | 0.001 | 12.81 (4.71, 24.28) | 12.90 (4.80, 24.29) | 0.03 (0.01, 0.04) | 0.001 | 2.91 (1.68, 4.61) | 2.67 (1.65, 4.05) | -0.31 (-0.42, -0.19) | <0.001 | 0.03 (0.02, 0.06) | 0.03 (0.02, 0.04) | -0.54 (-0.73, -0.34) | <0.001 |
| Central Latin America | 3.41 (1.25, 6.45) | 3.35 (1.21, 6.38) | -0.07 (-0.11, -0.03) | 0.001 | 13.29 (4.87, 25.14) | 13.06 (4.73, 24.88) | -0.07 (-0.11, -0.03) | 0.001 | 3.40 (2.49, 4.70) | 3.13 (2.12, 4.58) | -0.21 (-0.35, -0.08) | 0.002 | 0.04 (0.04, 0.06) | 0.04 (0.03, 0.05) | -0.33 (-0.55, -0.10) | 0.004 |
| Tropical Latin America | 4.62 (1.85, 8.50) | 4.67 (1.88, 8.56) | 0.12 (0.01, 0.22) | 0.030 | 18.00 (7.22, 33.12) | 18.20 (7.34, 33.38) | 0.12 (0.01, 0.22) | 0.030 | 4.38 (3.20, 6.09) | 4.89 (3.51, 6.57) | 0.40 (0.08, 0.73) | 0.015 | 0.05 (0.05, 0.07) | 0.06 (0.04, 0.07) | 0.54 (0.21, 0.86) | 0.001 |
| Central Sub-Saharan Africa | 5.02 (2.08, 8.91) | 4.93 (2.04, 8.85) | -0.06 (-0.07, -0.06) | <0.001 | 19.57 (8.12, 34.73) | 19.21 (7.95, 34.49) | -0.06 (-0.07, -0.06) | <0.001 | 3.08 (1.62, 5.12) | 2.98 (1.63, 4.86) | -0.14 (-0.21, -0.06) | <0.001 | 0.03 (0.01, 0.05) | 0.02 (0.01, 0.05) | -0.23 (-0.41, -0.05) | 0.013 |

| | | | | | | | | | | | | | | | | |
|---|---|---|---|---|---|---|---|---|---|---|---|---|---|---|---|---|
| Western Europe | 4.65 (1.87, 8.51) | 4.14 (1.64, 7.67) | -0.39 (-0.41, -0.37) | <0.001 | 18.12 (7.30, 33.19) | 16.14 (6.39, 29.92) | -0.39 (-0.41, -0.37) | <0.001 | 2.82 (1.69, 4.52) | 2.37 (1.38, 3.90) | -0.60 (-0.65, -0.55) | <0.001 | 0.02 (0.01, 0.03) | 0.02 (0.01, 0.02) | -0.92 (-1.05, -0.80) | <0.001 |
| High-income North America | 9.97 (4.88, 17.81) | 12.74 (6.70, 21.49) | 0.85 (0.80, 0.90) | <0.001 | 38.89 (19.02, 69.45) | 49.69 (26.13, 83.81) | 0.85 (0.80, 0.90) | <0.001 | 3.82 (1.78, 6.67) | 4.93 (2.70, 8.29) | 0.89 (0.71, 1.08) | <0.001 | 0.01 (0.01, 0.02) | 0.02 (0.01, 0.02) | 1.11 (0.56, 1.66) | <0.001 |
| Central Asia | 5.88 (2.41, 10.83) | 6.01 (2.46, 11.03) | 0.08 (0.06, 0.10) | <0.001 | 22.92 (9.39, 42.22) | 23.43 (9.59, 43.00) | 0.08 (0.06, 0.10) | <0.001 | 2.75 (1.43, 4.71) | 2.88 (1.54, 4.89) | 0.15 (0.12, 0.19) | <0.001 | 0.01 (0.01, 0.02) | 0.02 (0.01, 0.02) | 0.38 (0.24, 0.52) | <0.001 |
| Eastern Europe | 7.41 (3.23, 13.48) | 8.01 (3.43, 14.45) | 0.27 (0.22, 0.32) | <0.001 | 28.91 (12.61, 52.58) | 31.23 (13.37, 56.37) | 0.27 (0.22, 0.32) | <0.001 | 2.88 (1.29, 5.23) | 3.17 (1.42, 5.73) | 0.33 (0.20, 0.47) | <0.001 | 0.01 (0.01, 0.01) | 0.01 (0.01, 0.01) | 0.66 (0.30, 1.02) | <0.001 |
| Southern Sub-Saharan Africa | 5.63 (2.36, 10.35) | 5.46 (2.29, 10.15) | -0.10 (-0.14, -0.06) | <0.001 | 21.93 (9.21, 40.35) | 21.29 (8.91, 39.56) | -0.10 (-0.14, -0.06) | <0.001 | 8.30 (5.79, 11.00) | 4.80 (2.91, 7.33) | -1.84 (-2.58, -1.09) | <0.001 | 0.12 (0.09, 0.16) | 0.06 (0.03, 0.09) | -2.56 (-3.59, -1.51) | <0.001 |
| High-income Asia Pacific | 3.71 (1.40, 6.92) | 2.91 (0.97, 5.60) | -0.85 (-0.97, -0.74) | <0.001 | 14.45 (5.47, 27.00) | 11.33 (3.79, 21.85) | -0.85 (-0.97, -0.74) | <0.001 | 2.37 (1.45, 3.72) | 1.82 (1.06, 2.99) | -0.92 (-1.02, -0.81) | <0.001 | 0.02 (0.02, 0.03) | 0.02 (0.01, 0.02) | -1.00 (-1.28, -0.71) | <0.001 |
| Central Europe | 5.34 (2.10, 10.03) | 5.27 (2.11, 10.01) | -0.05 (-0.08, -0.02) | 0.002 | 20.83 (8.19, 39.12) | 20.55 (8.22, 39.05) | -0.05 (-0.08, -0.02) | 0.002 | 2.66 (1.45, 4.47) | 2.54 (1.35, 4.30) | -0.16 (-0.22, -0.10) | <0.001 | 0.02 (0.01, 0.02) | 0.01 (0.01, 0.02) | -0.45 (-0.54, -0.36) | <0.001 |
| Andean Latin America | 1.02 (0.16, 2.34) | 1.19 (0.19, 2.69) | 0.53 (0.47, 0.59) | <0.001 | 3.97 (0.62, 9.12) | 4.64 (0.75, 10.47) | 0.53 (0.47, 0.59) | <0.001 | 3.74 (2.16, 6.68) | 3.40 (2.10, 5.27) | -0.46 (-0.93, 0.00) | 0.051 | 0.07 (0.04, 0.12) | 0.06 (0.03, 0.09) | -0.61 (-1.14, -0.09) | 0.022 |
| Caribbean | 2.92 (0.98, 5.83) | 2.94 (0.99, 5.83) | 0.02 (0.01, 0.04) | <0.001 | 11.39 (3.84, 22.72) | 11.48 (3.85, 22.74) | 0.02 (0.01, 0.04) | <0.001 | 2.53 (1.60, 3.97) | 2.57 (1.52, 4.20) | 0.04 (-0.18, 0.27) | 0.711 | 0.03 (0.02, 0.05) | 0.03 (0.02, 0.05) | 0.12 (-0.19, 0.43) | 0.442 |
| Southern Latin America | 4.15 (1.57, 7.68) | 4.31 (1.73, 8.05) | 0.11 (0.08, 0.13) | <0.001 | 16.20 (6.13, 29.92) | 16.79 (6.74, 31.38) | 0.11 (0.08, 0.13) | <0.001 | 2.90 (1.67, 4.47) | 2.72 (1.60, 4.30) | -0.22 (-0.32, -0.12) | <0.001 | 0.03 (0.01, 0.04) | 0.02 (0.01, 0.03) | -0.60 (-0.85, -0.34) | <0.001 |
| Oceania | 8.67 (4.08, 15.32) | 8.87 (4.19, 15.65) | 0.07 (0.01, 0.12) | 0.021 | 33.80 (15.91, 59.74) | 34.59 (16.32, 61.00) | 0.07 (0.01, 0.12) | 0.021 | 25.31 (13.58, 50.66) | 30.78 (15.50, 65.26) | 0.64 (0.53, 0.74) | <0.001 | 0.44 (0.22, 0.94) | 0.54 (0.25, 1.23) | 0.70 (0.58, 0.82) | <0.001 |

| | | | | | | | | | | | | | | | | |
|---|---|---|---|---|---|---|---|---|---|---|---|---|---|---|---|---|
| Australasia | 10.32 (4.84, 17.96) | 9.80 (4.52, 17.21) | -0.18 (-0.20, -0.16) | <0.001 | 40.23 (18.87, 70.06) | 38.23 (17.64, 67.11) | -0.18 (-0.20, -0.16) | <0.001 | 3.85 (0.87, 8.79) | 3.63 (0.76, 8.44) | -0.16 (-0.21, -0.11) | <0.001 | 0.01 (0.01, 0.01) | 0.01 (0.01, 0.01) | -0.26 (-0.59, 0.08) | 0.133 |
| **Men** | | | | | | | | | | | | | | | | |
| Global | 12.90 (6.55, 22.69) | 11.91 (6.10, 20.82) | -0.26 (-0.33, -0.20) | <0.001 | 50.32 (25.52, 88.49) | 46.45 (23.77, 81.19) | -0.26 (-0.33, -0.20) | <0.001 | 5.66 (3.26, 9.27) | 5.56 (3.35, 8.80) | -0.05 (-0.09, -0.00) | 0.032 | 0.03 (0.02, 0.03) | 0.03 (0.02, 0.04) | 0.51 (0.39, 0.63) | <0.001 |
| **SDI category** | | | | | | | | | | | | | | | | |
| High | 23.73 (12.73, 40.52) | 23.51 (12.94, 39.13) | -0.01 (-0.08, 0.05) | 0.666 | 92.56 (49.64, 158.13) | 91.72 (50.49, 152.70) | -0.01 (-0.08, 0.05) | 0.671 | 9.54 (5.20, 16.40) | 9.33 (5.22, 15.08) | -0.07 (-0.17, 0.02) | 0.147 | 0.03 (0.02, 0.04) | 0.03 (0.02, 0.04) | -0.30 (-0.56, -0.04) | 0.021 |
| High-middle | 14.06 (7.17, 24.57) | 13.25 (6.77, 23.35) | -0.20 (-0.24, -0.17) | <0.001 | 54.85 (27.98, 95.85) | 51.67 (26.40, 91.07) | -0.20 (-0.23, -0.17) | <0.001 | 5.95 (3.28, 10.01) | 5.67 (3.11, 9.52) | -0.17 (-0.22, -0.11) | <0.001 | 0.03 (0.02, 0.03) | 0.02 (0.02, 0.03) | -0.10 (-0.20, 0.00) | 0.052 |
| Middle | 9.51 (4.60, 16.79) | 9.66 (4.62, 17.13) | 0.05 (0.04, 0.07) | <0.001 | 37.08 (17.95, 65.49) | 37.68 (18.00, 66.80) | 0.05 (0.04, 0.07) | <0.001 | 4.60 (2.69, 7.36) | 5.13 (3.14, 8.05) | 0.39 (0.34, 0.45) | <0.001 | 0.03 (0.02, 0.03) | 0.04 (0.03, 0.04) | 1.08 (0.91, 1.26) | <0.001 |
| Low-middle | 9.13 (4.27, 16.40) | 9.02 (4.18, 15.89) | -0.04 (-0.05, -0.03) | <0.001 | 35.58 (16.63, 63.96) | 35.16 (16.30, 61.98) | -0.04 (-0.05, -0.03) | <0.001 | 4.16 (2.29, 7.08) | 4.58 (2.67, 7.25) | 0.34 (0.18, 0.50) | <0.001 | 0.02 (0.02, 0.03) | 0.03 (0.02, 0.04) | 1.15 (0.74, 1.56) | <0.001 |
| Low | 7.38 (3.31, 13.15) | 7.35 (3.26, 13.27) | -0.01 (-0.03, 0.00) | 0.133 | 28.76 (12.91, 51.27) | 28.64 (12.69, 51.74) | -0.01 (-0.03, 0.00) | 0.141 | 3.72 (2.02, 6.18) | 3.85 (2.24, 6.26) | 0.12 (0.04, 0.20) | 0.005 | 0.02 (0.01, 0.04) | 0.03 (0.02, 0.04) | 0.32 (0.12, 0.52) | 0.002 |
| **21 GBD region** | | | | | | | | | | | | | | | | |
| South Asia | 9.51 (4.40, 17.01) | 9.49 (4.38, 16.86) | -0.01 (-0.01, 0.00) | 0.076 | 37.09 (17.16, 66.30) | 37.01 (17.08, 65.76) | -0.01 (-0.01, 0.00) | 0.082 | 4.07 (2.17, 6.92) | 4.51 (2.47, 7.27) | 0.36 (0.11, 0.61) | 0.005 | 0.02 (0.01, 0.03) | 0.03 (0.02, 0.04) | 1.29 (0.32, 2.26) | 0.009 |
| East Asia | 10.06 (4.91, 17.61) | 10.57 (5.19, 18.71) | 0.17 (0.11, 0.22) | <0.001 | 39.23 (19.16, 68.67) | 41.23 (20.24, 72.98) | 0.17 (0.11, 0.22) | <0.001 | 4.03 (1.97, 6.99) | 4.12 (2.01, 7.26) | 0.06 (0.02, 0.11) | 0.008 | 0.01 (0.01, 0.01) | 0.01 (0.01, 0.01) | -0.37 (-0.68, -0.05) | 0.022 |
| North Africa and Middle East | 8.93 (4.35, 15.78) | 9.08 (4.45, 16.14) | 0.06 (0.04, 0.07) | <0.001 | 34.81 (16.94, 61.52) | 35.43 (17.36, 62.96) | 0.06 (0.04, 0.08) | <0.001 | 4.22 (2.35, 7.02) | 4.29 (2.37, 7.19) | 0.07 (0.02, 0.12) | 0.005 | 0.02 (0.02, 0.04) | 0.02 (0.02, 0.04) | 0.08 (-0.09, 0.25) | 0.372 |

| | | | | | | | | | | | | | | | | |
|---|---|---|---|---|---|---|---|---|---|---|---|---|---|---|---|---|
| Western Sub-Saharan Africa | 7.06 (3.09, 12.72) | 7.33 (3.27, 13.28) | 0.13 (0.11, 0.15) | <0.001 | 27.54 (12.06, 49.57) | 28.57 (12.75, 51.80) | 0.13 (0.11, 0.15) | <0.001 | 2.88 (1.41, 5.12) | 3.04 (1.51, 5.48) | 0.23 (0.19, 0.26) | <0.001 | 0.01 (0.01, 0.01) | 0.01 (0.01, 0.02) | 0.36 (0.27, 0.46) | <0.001 |
| Southeast Asia | 10.82 (5.24, 19.16) | 11.23 (5.43, 19.69) | 0.13 (0.11, 0.14) | <0.001 | 42.17 (20.45, 74.70) | 43.78 (21.17, 76.78) | 0.13 (0.11, 0.14) | <0.001 | 6.40 (4.17, 9.79) | 7.78 (4.97, 11.55) | 0.69 (0.61, 0.77) | <0.001 | 0.05 (0.04, 0.07) | 0.08 (0.05, 0.10) | 1.33 (1.18, 1.49) | <0.001 |
| Eastern Sub-Saharan Africa | 4.65 (1.91, 8.59) | 4.82 (2.01, 8.92) | 0.12 (0.09, 0.16) | <0.001 | 18.11 (7.44, 33.48) | 18.80 (7.82, 34.78) | 0.12 (0.09, 0.16) | <0.001 | 3.50 (2.00, 5.70) | 3.47 (2.05, 5.49) | -0.03 (-0.15, 0.09) | 0.581 | 0.04 (0.02, 0.07) | 0.03 (0.02, 0.05) | -0.18 (-0.30, -0.06) | 0.003 |
| Central Latin America | 5.12 (2.14, 9.23) | 5.21 (2.16, 9.40) | 0.06 (0.04, 0.08) | <0.001 | 19.96 (8.33, 35.99) | 20.30 (8.43, 36.66) | 0.06 (0.04, 0.08) | <0.001 | 3.35 (2.05, 5.13) | 3.37 (2.03, 5.21) | 0.02 (-0.21, 0.26) | 0.843 | 0.03 (0.02, 0.04) | 0.03 (0.02, 0.04) | -0.00 (-0.33, 0.32) | 0.983 |
| Tropical Latin America | 7.77 (3.46, 14.12) | 7.79 (3.48, 14.07) | 0.10 (0.01, 0.19) | 0.029 | 30.30 (13.47, 55.07) | 30.36 (13.57, 54.86) | 0.10 (0.01, 0.19) | 0.029 | 5.78 (3.74, 8.56) | 5.70 (3.74, 8.19) | -0.01 (-0.24, 0.21) | 0.894 | 0.06 (0.04, 0.09) | 0.06 (0.04, 0.08) | -0.14 (-0.35, 0.07) | 0.187 |
| Central Sub-Saharan Africa | 6.58 (2.88, 11.90) | 6.70 (2.89, 12.04) | 0.06 (0.05, 0.07) | <0.001 | 25.65 (11.23, 46.39) | 26.11 (11.26, 46.92) | 0.06 (0.05, 0.07) | <0.001 | 3.95 (1.47, 8.79) | 3.96 (1.56, 7.74) | 0.08 (0.00, 0.15) | 0.038 | 0.03 (0.01, 0.11) | 0.03 (0.02, 0.08) | -0.05 (-0.17, 0.07) | 0.431 |
| Western Europe | 24.98 (13.40, 42.51) | 22.93 (12.25, 38.97) | -0.29 (-0.31, -0.26) | <0.001 | 97.47 (52.27, 165.85) | 89.44 (47.77, 152.08) | -0.29 (-0.31, -0.26) | <0.001 | 11.11 (6.31, 18.44) | 9.62 (5.21, 15.84) | -0.48 (-0.57, -0.40) | <0.001 | 0.05 (0.03, 0.07) | 0.04 (0.02, 0.05) | -1.13 (-1.40, -0.85) | <0.001 |
| High-income North America | 27.54 (14.58, 46.99) | 32.94 (18.42, 55.97) | 0.63 (0.57, 0.68) | <0.001 | 107.43 (56.87, 183.38) | 128.51 (71.85, 218.45) | 0.63 (0.57, 0.68) | <0.001 | 10.22 (5.22, 17.18) | 12.39 (6.80, 20.95) | 0.66 (0.55, 0.78) | <0.001 | 0.02 (0.02, 0.04) | 0.03 (0.02, 0.04) | 1.13 (0.69, 1.57) | <0.001 |
| Central Asia | 15.96 (8.04, 27.50) | 16.70 (8.34, 28.50) | 0.16 (0.15, 0.18) | <0.001 | 62.24 (31.36, 107.24) | 65.14 (32.51, 111.16) | 0.16 (0.15, 0.18) | <0.001 | 6.69 (3.30, 11.71) | 7.29 (3.75, 12.86) | 0.30 (0.07, 0.54) | 0.012 | 0.03 (0.01, 0.04) | 0.03 (0.02, 0.04) | 0.57 (-0.28, 1.43) | 0.190 |
| Eastern Europe | 19.55 (10.06, 33.96) | 20.79 (10.74, 35.64) | 0.21 (0.19, 0.24) | <0.001 | 76.25 (39.24, 132.50) | 81.09 (41.87, 139.04) | 0.21 (0.19, 0.24) | <0.001 | 6.95 (3.29, 12.70) | 7.65 (3.86, 13.35) | 0.34 (0.26, 0.43) | <0.001 | 0.01 (0.01, 0.01) | 0.01 (0.01, 0.02) | 1.14 (0.47, 1.82) | 0.001 |
| Southern Sub-Saharan Africa | 7.51 (3.38, 13.57) | 7.56 (3.35, 13.53) | 0.03 (0.01, 0.04) | <0.001 | 29.29 (13.18, 52.91) | 29.46 (13.07, 52.75) | 0.03 (0.01, 0.04) | <0.001 | 6.93 (4.58, 9.74) | 5.88 (3.70, 8.74) | -0.57 (-0.96, -0.18) | 0.004 | 0.09 (0.06, 0.12) | 0.07 (0.04, 0.09) | -0.92 (-1.60, -0.23) | 0.009 |

| | | | | | | | | | | | | | | | | |
|---|---|---|---|---|---|---|---|---|---|---|---|---|---|---|---|---|
| High-income Asia Pacific | 19.59 (10.41, 33.01) | 20.07 (10.66, 33.95) | 0.07 (0.04, 0.11) | <0.001 | 76.42 (40.60, 128.77) | 78.30 (41.56, 132.47) | 0.07 (0.04, 0.11) | <0.001 | 7.86 (3.97, 13.80) | 7.79 (3.85, 14.08) | -0.03 (-0.12, 0.06) | 0.487 | 0.03 (0.01, 0.04) | 0.02 (0.01, 0.03) | -0.80 (-1.00, -0.61) | <0.001 |
| Central Europe | 20.16 (10.28, 34.74) | 19.93 (10.31, 34.61) | -0.02 (-0.09, 0.04) | 0.489 | 78.62 (40.08, 135.51) | 77.75 (40.19, 135.02) | -0.02 (-0.09, 0.04) | 0.494 | 8.61 (4.80, 14.34) | 8.28 (4.47, 14.33) | -0.15 (-0.23, -0.06) | 0.001 | 0.04 (0.03, 0.05) | 0.03 (0.02, 0.04) | -0.72 (-0.98, -0.45) | <0.001 |
| Andean Latin America | 1.70 (0.34, 3.37) | 1.93 (0.47, 3.87) | 0.47 (0.34, 0.61) | <0.001 | 6.61 (1.32, 13.15) | 7.53 (1.83, 15.08) | 0.47 (0.34, 0.61) | <0.001 | 4.10 (2.80, 5.80) | 3.80 (2.50, 5.41) | -0.24 (-0.50, 0.02) | 0.075 | 0.07 (0.05, 0.10) | 0.06 (0.04, 0.09) | -0.39 (-0.77, -0.01) | 0.044 |
| Caribbean | 4.96 (2.05, 8.89) | 5.11 (2.14, 9.29) | 0.10 (0.07, 0.13) | <0.001 | 19.33 (8.00, 34.66) | 19.92 (8.34, 36.22) | 0.10 (0.07, 0.13) | <0.001 | 3.90 (2.57, 5.69) | 4.27 (2.85, 6.17) | 0.32 (0.16, 0.47) | <0.001 | 0.04 (0.03, 0.06) | 0.05 (0.03, 0.07) | 0.53 (0.23, 0.83) | 0.001 |
| Southern Latin America | 12.03 (6.04, 20.43) | 12.44 (6.38, 21.50) | 0.11 (0.07, 0.15) | <0.001 | 46.90 (23.56, 79.66) | 48.53 (24.88, 83.86) | 0.11 (0.07, 0.15) | <0.001 | 6.21 (2.68, 11.73) | 6.20 (2.77, 11.57) | -0.04 (-0.31, 0.23) | 0.763 | 0.04 (0.02, 0.07) | 0.04 (0.02, 0.06) | -0.17 (-0.72, 0.38) | 0.531 |
| Oceania | 10.47 (5.06, 18.75) | 10.59 (5.16, 18.76) | 0.03 (0.02, 0.04) | <0.001 | 40.84 (19.72, 73.09) | 41.28 (20.10, 73.14) | 0.03 (0.02, 0.05) | <0.001 | 10.93 (6.39, 20.00) | 12.14 (7.28, 20.25) | 0.39 (0.37, 0.42) | <0.001 | 0.14 (0.09, 0.30) | 0.17 (0.10, 0.30) | 0.52 (0.43, 0.61) | <0.001 |
| Australasia | 28.58 (15.47, 47.73) | 27.34 (14.62, 47.86) | -0.15 (-0.18, -0.11) | <0.001 | 111.49 (60.33, 186.26) | 106.66 (57.01, 186.76) | -0.15 (-0.18, -0.11) | <0.001 | 10.69 (4.43, 21.29) | 10.38 (4.25, 20.14) | -0.04 (-0.34, 0.26) | 0.770 | 0.03 (0.02, 0.04) | 0.03 (0.02, 0.05) | 0.30 (-0.28, 0.89) | 0.306 |
| **Aortic aneurysm** | | | | | | | | | | | | | | | | |
| **15-19 years** | | | | | | | | | | | | | | | | |
| **Women** | | | | | | | | | | | | | | | | |
| Global | — | — | — | — | — | — | — | — | 1.52 (1.32, 2.44) | 1.51 (1.33, 1.83) | -0.04 (-0.14, 0.07) | 0.528 | 0.02 (0.02, 0.03) | 0.02 (0.02, 0.03) | -0.03 (-0.14, 0.07) | 0.533 |
| **SDI category** | | | | | | | | | | | | | | | | |
| High | — | — | — | — | — | — | — | — | 1.81 (1.67, 1.98) | 1.29 (1.14, 1.41) | -1.13 (-1.30, -0.96) | <0.001 | 0.03 (0.02, 0.03) | 0.02 (0.02, 0.02) | -1.13 (-1.30, -0.96) | <0.001 |
| High-middle | — | — | — | — | — | — | — | — | 1.94 (1.74, 2.29) | 2.09 (1.84, 2.33) | 0.25 (-0.10, | 0.165 | 0.03 (0.02, | 0.03 (0.03, | 0.25 (-0.10, 0.61) | 0.163 |

| | | | | | | | | | | | | | | | | |
|---|---|---|---|---|---|---|---|---|---|---|---|---|---|---|---|---|
| | | | | | | | | | | | 0.61) | | 0.03) | 0.03) | | |
| Middle | — | — | — | — | — | — | — | — | 1.50 (1.27, 2.33) | 1.69 (1.40, 2.06) | 0.43 (0.25, 0.62) | <0.001 | 0.02 (0.02, 0.03) | 0.02 (0.02, 0.03) | 0.43 (0.25, 0.62) | <0.001 |
| Low-middle | — | — | — | — | — | — | — | — | 1.13 (0.81, 2.80) | 1.30 (1.02, 2.01) | 0.45 (0.36, 0.55) | <0.001 | 0.02 (0.01, 0.04) | 0.02 (0.01, 0.03) | 0.45 (0.36, 0.54) | <0.001 |
| Low | — | — | — | — | — | — | — | — | 1.39 (0.78, 3.03) | 1.26 (0.90, 1.79) | -0.34 (-0.55, -0.14) | 0.001 | 0.02 (0.01, 0.04) | 0.02 (0.01, 0.03) | -0.35 (-0.55, -0.14) | 0.001 |
| **21 GBD region** | | | | | | | | | | | | | | | | |
| South Asia | — | — | — | — | — | — | — | — | 0.82 (0.49, 2.67) | 0.97 (0.70, 1.82) | 0.58 (0.36, 0.81) | <0.001 | 0.01 (0.01, 0.04) | 0.01 (0.01, 0.03) | 0.58 (0.36, 0.81) | <0.001 |
| East Asia | — | — | — | — | — | — | — | — | 1.20 (0.96, 1.82) | 0.90 (0.74, 1.08) | -0.98 (-1.20, -0.76) | <0.001 | 0.02 (0.01, 0.03) | 0.01 (0.01, 0.02) | -0.98 (-1.19, -0.76) | <0.001 |
| North Africa and Middle East | — | — | — | — | — | — | — | — | 2.53 (1.83, 4.24) | 2.77 (2.04, 3.79) | 0.31 (0.14, 0.49) | <0.001 | 0.04 (0.03, 0.06) | 0.04 (0.03, 0.05) | 0.32 (0.15, 0.48) | <0.001 |
| Western Sub-Saharan Africa | — | — | — | — | — | — | — | — | 1.11 (0.73, 1.79) | 0.82 (0.58, 1.20) | -1.05 (-1.21, -0.88) | <0.001 | 0.02 (0.01, 0.03) | 0.01 (0.01, 0.02) | -1.05 (-1.21, -0.88) | <0.001 |
| Southeast Asia | — | — | — | — | — | — | — | — | 1.20 (0.84, 2.41) | 1.76 (1.39, 2.34) | 1.35 (1.22, 1.49) | <0.001 | 0.02 (0.01, 0.03) | 0.02 (0.02, 0.03) | 1.35 (1.22, 1.49) | <0.001 |
| Eastern Sub-Saharan Africa | — | — | — | — | — | — | — | — | 2.45 (1.21, 4.82) | 1.97 (1.31, 2.76) | -0.75 (-1.01, -0.49) | <0.001 | 0.03 (0.02, 0.07) | 0.03 (0.02, 0.04) | -0.75 (-1.01, -0.50) | <0.001 |
| Central Latin America | — | — | — | — | — | — | — | — | 1.75 (1.54, 1.98) | 1.64 (1.34, 2.02) | -0.09 (-0.44, 0.25) | 0.600 | 0.02 (0.02, 0.03) | 0.02 (0.02, 0.03) | -0.09 (-0.44, 0.26) | 0.606 |
| Tropical Latin | — | — | — | — | — | — | — | — | 2.03 (1.82, | 3.19 (2.78, | 1.68 | <0.001 | 0.03 | 0.04 | 1.68 (1.56, | <0.001 |

| | | | | | | | | | | | | | | | | |
|---|---|---|---|---|---|---|---|---|---|---|---|---|---|---|---|---|
| America | | | | | | | | | 2.27) | 3.62) | (1.56, 1.79) | | (0.03, 0.03) | (0.04, 0.05) | 1.79) | |
| Central Sub-Saharan Africa | — | — | — | — | — | — | — | — | 1.84 (0.83, 4.01) | 1.63 (0.91, 2.72) | -0.44 (-0.54, -0.33) | <0.001 | 0.03 (0.01, 0.06) | 0.02 (0.01, 0.04) | -0.44 (-0.55, -0.33) | <0.001 |
| Western Europe | — | — | — | — | — | — | — | — | 1.92 (1.72, 2.17) | 1.46 (1.23, 1.66) | -1.01 (-1.20, -0.81) | <0.001 | 0.03 (0.02, 0.03) | 0.02 (0.02, 0.02) | -1.00 (-1.20, -0.81) | <0.001 |
| High-income North America | — | — | — | — | — | — | — | — | 2.61 (2.38, 2.88) | 1.41 (1.23, 1.58) | -2.00 (-2.67, -1.34) | <0.001 | 0.04 (0.03, 0.04) | 0.02 (0.02, 0.02) | -2.00 (-2.66, -1.34) | <0.001 |
| Central Asia | — | — | — | — | — | — | — | — | 1.01 (0.82, 1.25) | 1.22 (0.96, 1.54) | 0.61 (0.43, 0.80) | <0.001 | 0.01 (0.01, 0.02) | 0.02 (0.01, 0.02) | 0.61 (0.43, 0.79) | <0.001 |
| Eastern Europe | — | — | — | — | — | — | — | — | 2.73 (2.44, 3.00) | 3.94 (3.24, 4.65) | 1.32 (0.32, 2.33) | 0.009 | 0.04 (0.03, 0.04) | 0.06 (0.05, 0.07) | 1.32 (0.33, 2.33) | 0.009 |
| Southern Sub-Saharan Africa | — | — | — | — | — | — | — | — | 3.09 (2.40, 3.88) | 1.65 (1.10, 2.38) | -2.13 (-2.62, -1.63) | <0.001 | 0.04 (0.03, 0.05) | 0.02 (0.02, 0.03) | -2.13 (-2.63, -1.63) | <0.001 |
| High-income Asia Pacific | — | — | — | — | — | — | — | — | 0.89 (0.78, 1.09) | 0.99 (0.77, 1.15) | 0.36 (0.06, 0.66) | 0.019 | 0.01 (0.01, 0.02) | 0.01 (0.01, 0.02) | 0.36 (0.07, 0.66) | 0.016 |
| Central Europe | — | — | — | — | — | — | — | — | 2.15 (1.83, 2.55) | 2.61 (2.09, 3.19) | 0.71 (0.40, 1.03) | <0.001 | 0.03 (0.03, 0.04) | 0.04 (0.03, 0.04) | 0.71 (0.40, 1.03) | <0.001 |
| Andean Latin America | — | — | — | — | — | — | — | — | 4.65 (3.05, 7.01) | 4.31 (2.73, 6.16) | -0.34 (-0.91, 0.24) | 0.249 | 0.07 (0.04, 0.10) | 0.06 (0.04, 0.09) | -0.34 (-0.91, 0.24) | 0.248 |
| Caribbean | — | — | — | — | — | — | — | — | 1.01 (0.81, 1.57) | 1.03 (0.71, 1.79) | 0.12 (-0.09, 0.34) | 0.266 | 0.01 (0.01, 0.02) | 0.01 (0.01, 0.03) | 0.12 (-0.09, 0.34) | 0.266 |
| Southern Latin | — | — | — | — | — | — | — | — | 3.19 (2.53, | 2.88 (2.28, | -0.36 | 0.002 | 0.04 | 0.04 | -0.36 | 0.002 |

| | | | | | | | | | | | | | | | | |
|---|---|---|---|---|---|---|---|---|---|---|---|---|---|---|---|---|
| America | | | | | | | | | 4.04) | 3.58) | (-0.59, -0.14) | | (0.04, 0.06) | (0.03, 0.05) | (-0.59, -0.14) | |
| Oceania | — | — | — | — | — | — | — | — | 0.48 (0.30, 0.77) | 0.63 (0.38, 1.02) | 0.91 (0.50, 1.33) | <0.001 | 0.01 (0.00, 0.01) | 0.01 (0.01, 0.01) | 0.91 (0.50, 1.33) | <0.001 |
| Australasia | — | — | — | — | — | — | — | — | 1.85 (1.43, 2.41) | 0.86 (0.62, 1.19) | -2.53 (-3.35, -1.70) | <0.001 | 0.03 (0.02, 0.03) | 0.01 (0.01, 0.02) | -2.53 (-3.35, -1.71) | <0.001 |
| **Men** | | | | | | | | | | | | | | | | |
| Global | — | — | — | — | — | — | — | — | 4.27 (3.32, 5.48) | 3.73 (3.19, 4.28) | -0.51 (-0.82, -0.19) | 0.002 | 0.06 (0.05, 0.08) | 0.05 (0.04, 0.06) | -0.50 (-0.82, -0.19) | 0.002 |
| **SDI category** | | | | | | | | | | | | | | | | |
| High | — | — | — | — | — | — | — | — | 6.04 (5.40, 6.71) | 3.08 (2.75, 3.52) | -2.33 (-2.81, -1.85) | <0.001 | 0.08 (0.08, 0.09) | 0.04 (0.04, 0.05) | -2.33 (-2.80, -1.85) | <0.001 |
| High-middle | — | — | — | — | — | — | — | — | 5.87 (4.91, 7.09) | 4.86 (4.21, 5.73) | -0.67 (-1.23, -0.11) | 0.019 | 0.08 (0.07, 0.10) | 0.07 (0.06, 0.08) | -0.67 (-1.23, -0.11) | 0.020 |
| Middle | — | — | — | — | — | — | — | — | 3.71 (2.82, 4.80) | 4.15 (3.35, 5.23) | 0.39 (0.26, 0.52) | <0.001 | 0.05 (0.04, 0.07) | 0.06 (0.05, 0.07) | 0.40 (0.27, 0.52) | <0.001 |
| Low-middle | — | — | — | — | — | — | — | — | 2.84 (1.65, 4.58) | 3.10 (2.39, 3.90) | 0.35 (0.02, 0.69) | 0.039 | 0.04 (0.02, 0.06) | 0.04 (0.03, 0.05) | 0.36 (0.02, 0.69) | 0.036 |
| Low | — | — | — | — | — | — | — | — | 4.23 (1.93, 7.51) | 3.48 (2.12, 5.10) | -0.69 (-1.04, -0.35) | <0.001 | 0.06 (0.03, 0.11) | 0.05 (0.03, 0.07) | -0.69 (-1.04, -0.34) | <0.001 |
| **21 GBD region** | | | | | | | | | | | | | | | | |
| South Asia | — | — | — | — | — | — | — | — | 2.06 (0.96, 3.79) | 2.18 (1.49, 2.93) | 0.31 (-0.55, 1.17) | 0.481 | 0.03 (0.01, 0.05) | 0.03 (0.02, 0.04) | 0.31 (-0.54, 1.17) | 0.475 |
| East Asia | — | — | — | — | — | — | — | — | 3.46 (2.28, | 2.23 (1.70, | -1.57 | <0.001 | 0.05 | 0.03 | -1.56 | <0.001 |

| | | | | | | | | | | | | | | | | |
|---|---|---|---|---|---|---|---|---|---|---|---|---|---|---|---|---|
| | | | | | | | | | 4.92) | 2.87) | (-1.96, -1.19) | | (0.03, 0.07) | (0.02, 0.04) | (-1.95, -1.18) | |
| North Africa and Middle East | — | — | — | — | — | — | — | — | 6.28 (3.91, 9.25) | 5.96 (4.05, 8.90) | -0.14 (-0.44, 0.17) | 0.379 | 0.09 (0.05, 0.13) | 0.08 (0.06, 0.12) | -0.13 (-0.43, 0.17) | 0.389 |
| Western Sub-Saharan Africa | — | — | — | — | — | — | — | — | 2.11 (1.09, 3.47) | 2.06 (1.33, 2.88) | -0.06 (-0.27, 0.14) | 0.529 | 0.03 (0.02, 0.05) | 0.03 (0.02, 0.04) | -0.06 (-0.26, 0.14) | 0.540 |
| Southeast Asia | — | — | — | — | — | — | — | — | 4.25 (2.41, 6.44) | 5.99 (4.30, 8.36) | 1.28 (1.01, 1.54) | <0.001 | 0.06 (0.03, 0.09) | 0.08 (0.06, 0.12) | 1.28 (1.01, 1.54) | <0.001 |
| Eastern Sub-Saharan Africa | — | — | — | — | — | — | — | — | 7.91 (3.53, 14.63) | 5.81 (3.52, 8.81) | -1.05 (-1.13, -0.97) | <0.001 | 0.11 (0.05, 0.20) | 0.08 (0.05, 0.12) | -1.05 (-1.13, -0.97) | <0.001 |
| Central Latin America | — | — | — | — | — | — | — | — | 4.54 (4.00, 5.24) | 3.99 (3.14, 5.11) | -0.34 (-0.77, 0.09) | 0.119 | 0.06 (0.06, 0.07) | 0.06 (0.04, 0.07) | -0.34 (-0.77, 0.09) | 0.122 |
| Tropical Latin America | — | — | — | — | — | — | — | — | 4.97 (4.38, 5.66) | 8.66 (7.57, 9.98) | 2.02 (1.46, 2.60) | <0.001 | 0.07 (0.06, 0.08) | 0.12 (0.11, 0.14) | 2.02 (1.46, 2.59) | <0.001 |
| Central Sub-Saharan Africa | — | — | — | — | — | — | — | — | 6.15 (2.53, 12.57) | 4.23 (2.12, 7.38) | -1.33 (-1.47, -1.19) | <0.001 | 0.09 (0.04, 0.18) | 0.06 (0.03, 0.10) | -1.33 (-1.46, -1.19) | <0.001 |
| Western Europe | — | — | — | — | — | — | — | — | 7.75 (6.82, 8.74) | 3.47 (2.97, 4.03) | -2.73 (-3.34, -2.13) | <0.001 | 0.11 (0.10, 0.12) | 0.05 (0.04, 0.06) | -2.73 (-3.33, -2.13) | <0.001 |
| High-income North America | — | — | — | — | — | — | — | — | 7.33 (6.53, 8.16) | 3.44 (3.05, 3.98) | -2.61 (-3.22, -1.99) | <0.001 | 0.10 (0.09, 0.11) | 0.05 (0.04, 0.06) | -2.60 (-3.21, -1.98) | <0.001 |
| Central Asia | — | — | — | — | — | — | — | — | 1.62 (1.24, 2.01) | 3.19 (2.48, 4.09) | 2.39 (1.80, 2.98) | <0.001 | 0.02 (0.02, 0.03) | 0.04 (0.03, 0.06) | 2.39 (1.80, 2.98) | <0.001 |
| Eastern Europe | — | — | — | — | — | — | — | — | 6.98 (5.89, | 6.99 (5.63, | 0.13 | 0.869 | 0.10 | 0.10 | 0.14 (-1.44, | 0.865 |

| | | | | | | | | | | | | | | | | |
|---|---|---|---|---|---|---|---|---|---|---|---|---|---|---|---|---|
| | | | | | | | | | 7.78) | 8.74) | (-1.44, 1.73) | | (0.08, 0.11) | (0.08, 0.12) | 1.74) | |
| Southern Sub-Saharan Africa | — | — | — | — | — | — | — | — | 3.47 (2.57, 4.63) | 3.14 (2.08, 4.73) | -0.19 (-1.20, 0.83) | 0.718 | 0.05 (0.04, 0.06) | 0.04 (0.03, 0.07) | -0.19 (-1.20, 0.83) | 0.716 |
| High-income Asia Pacific | — | — | — | — | — | — | — | — | 3.70 (2.85, 4.62) | 2.52 (1.95, 2.98) | -1.29 (-1.60, -0.98) | <0.001 | 0.05 (0.04, 0.06) | 0.04 (0.03, 0.04) | -1.28 (-1.59, -0.97) | <0.001 |
| Central Europe | — | — | — | — | — | — | — | — | 7.74 (6.70, 8.70) | 7.69 (6.28, 9.41) | -0.09 (-0.59, 0.41) | 0.717 | 0.11 (0.09, 0.12) | 0.11 (0.09, 0.13) | -0.09 (-0.59, 0.41) | 0.721 |
| Andean Latin America | — | — | — | — | — | — | — | — | 4.45 (2.67, 7.08) | 3.70 (2.40, 5.54) | -0.58 (-1.00, -0.16) | 0.007 | 0.06 (0.04, 0.10) | 0.05 (0.03, 0.08) | -0.57 (-0.99, -0.15) | 0.008 |
| Caribbean | — | — | — | — | — | — | — | — | 5.59 (4.21, 7.49) | 5.09 (3.43, 7.69) | -0.31 (-0.53, -0.09) | 0.006 | 0.08 (0.06, 0.10) | 0.07 (0.05, 0.11) | -0.30 (-0.52, -0.08) | 0.007 |
| Southern Latin America | — | — | — | — | — | — | — | — | 6.46 (5.05, 8.54) | 6.00 (4.76, 7.64) | -0.40 (-0.91, 0.12) | 0.131 | 0.09 (0.07, 0.12) | 0.08 (0.07, 0.11) | -0.40 (-0.91, 0.12) | 0.133 |
| Oceania | — | — | — | — | — | — | — | — | 5.41 (2.55, 10.74) | 5.43 (2.45, 11.38) | -0.07 (-0.45, 0.30) | 0.698 | 0.08 (0.04, 0.15) | 0.08 (0.03, 0.16) | -0.07 (-0.45, 0.30) | 0.700 |
| Australasia | — | — | — | — | — | — | — | — | 4.54 (3.59, 5.62) | 1.37 (1.02, 1.82) | -4.08 (-5.38, -2.76) | <0.001 | 0.06 (0.05, 0.08) | 0.02 (0.01, 0.03) | -4.08 (-5.37, -2.76) | <0.001 |
| **20-24 years** | | | | | | | | | | | | | | | | |
| **Women** | | | | | | | | | | | | | | | | |
| Global | — | — | — | — | — | — | — | — | 2.62 (2.22, 4.51) | 2.47 (2.16, 3.07) | -0.20 (-0.32, -0.09) | 0.001 | 0.04 (0.03, 0.07) | 0.04 (0.03, 0.05) | -0.20 (-0.32, -0.09) | 0.001 |
| **SDI category** | | | | | | | | | | | | | | | | |
| High | — | — | — | — | — | — | — | — | 3.12 (2.86, | 2.51 (2.26, | -0.75 | <0.001 | 0.05 | 0.04 | -0.74 | <0.001 |

| | | | | | | | | | | | | | | | | |
|---|---|---|---|---|---|---|---|---|---|---|---|---|---|---|---|---|
| | | | | | | | | | 3.45) | 2.78) | (-0.89, -0.61) | | (0.04, 0.05) | (0.03, 0.04) | (-0.88, -0.61) | |
| High-middle | — | — | — | — | — | — | — | — | 2.91 (2.49, 3.91) | 2.98 (2.58, 3.41) | 0.11 (-0.20, 0.42) | 0.480 | 0.04 (0.04, 0.06) | 0.04 (0.04, 0.05) | 0.11 (-0.20, 0.43) | 0.478 |
| Middle | — | — | — | — | — | — | — | — | 2.75 (2.23, 4.76) | 2.64 (2.23, 3.22) | -0.15 (-0.41, 0.11) | 0.256 | 0.04 (0.03, 0.07) | 0.04 (0.03, 0.05) | -0.15 (-0.41, 0.11) | 0.254 |
| Low-middle | — | — | — | — | — | — | — | — | 2.03 (1.43, 5.27) | 2.27 (1.77, 3.66) | 0.39 (0.07, 0.70) | 0.016 | 0.03 (0.02, 0.08) | 0.03 (0.03, 0.05) | 0.39 (0.07, 0.70) | 0.016 |
| Low | — | — | — | — | — | — | — | — | 2.15 (1.10, 5.36) | 2.06 (1.42, 3.22) | -0.18 (-0.29, -0.06) | 0.003 | 0.03 (0.02, 0.08) | 0.03 (0.02, 0.05) | -0.18 (-0.29, -0.06) | 0.002 |
| **21 GBD region** | | | | | | | | | | | | | | | | |
| South Asia | — | — | — | — | — | — | — | — | 1.46 (0.88, 5.05) | 1.78 (1.24, 3.39) | 0.70 (0.51, 0.90) | <0.001 | 0.02 (0.01, 0.08) | 0.03 (0.02, 0.05) | 0.70 (0.51, 0.90) | <0.001 |
| East Asia | — | — | — | — | — | — | — | — | 2.80 (1.98, 5.16) | 2.43 (1.85, 3.09) | -0.33 (-0.80, 0.14) | 0.164 | 0.04 (0.03, 0.08) | 0.04 (0.03, 0.05) | -0.33 (-0.80, 0.13) | 0.163 |
| North Africa and Middle East | — | — | — | — | — | — | — | — | 2.68 (1.93, 4.55) | 2.64 (1.93, 3.72) | -0.05 (-0.12, 0.03) | 0.221 | 0.04 (0.03, 0.07) | 0.04 (0.03, 0.06) | -0.05 (-0.12, 0.03) | 0.209 |
| Western Sub-Saharan Africa | — | — | — | — | — | — | — | — | 1.58 (1.02, 2.55) | 1.37 (0.93, 2.00) | -0.53 (-0.68, -0.37) | <0.001 | 0.02 (0.02, 0.04) | 0.02 (0.01, 0.03) | -0.53 (-0.68, -0.37) | <0.001 |
| Southeast Asia | — | — | — | — | — | — | — | — | 1.85 (1.33, 3.84) | 2.64 (2.02, 3.55) | 1.25 (1.06, 1.45) | <0.001 | 0.03 (0.02, 0.06) | 0.04 (0.03, 0.05) | 1.25 (1.06, 1.45) | <0.001 |
| Eastern Sub-Saharan Africa | — | — | — | — | — | — | — | — | 3.79 (1.75, 8.21) | 3.14 (2.01, 4.38) | -0.66 (-0.91, -0.40) | <0.001 | 0.06 (0.03, 0.12) | 0.05 (0.03, 0.07) | -0.66 (-0.91, -0.41) | <0.001 |

| | | | | | | | | | | | | | | | | |
|---|---|---|---|---|---|---|---|---|---|---|---|---|---|---|---|---|
| Central Latin America | — | — | — | — | — | — | — | — | 3.25 (2.81, 3.75) | 3.14 (2.40, 3.98) | -0.08 (-0.73, 0.57) | 0.801 | 0.05 (0.04, 0.06) | 0.05 (0.04, 0.06) | -0.08 (-0.73, 0.57) | 0.802 |
| Tropical Latin America | — | — | — | — | — | — | — | — | 3.41 (2.99, 3.90) | 5.11 (4.42, 5.96) | 1.43 (1.01, 1.86) | <0.001 | 0.05 (0.04, 0.06) | 0.08 (0.07, 0.09) | 1.43 (1.01, 1.85) | <0.001 |
| Central Sub-Saharan Africa | — | — | — | — | — | — | — | — | 2.81 (1.28, 6.11) | 2.50 (1.34, 4.28) | -0.42 (-0.56, -0.29) | <0.001 | 0.04 (0.02, 0.09) | 0.04 (0.02, 0.06) | -0.43 (-0.56, -0.29) | <0.001 |
| Western Europe | — | — | — | — | — | — | — | — | 3.41 (3.00, 3.91) | 2.63 (2.25, 3.07) | -0.91 (-1.12, -0.69) | <0.001 | 0.05 (0.05, 0.06) | 0.04 (0.03, 0.05) | -0.91 (-1.12, -0.69) | <0.001 |
| High-income North America | — | — | — | — | — | — | — | — | 3.80 (3.44, 4.18) | 2.90 (2.61, 3.24) | -0.94 (-1.30, -0.58) | <0.001 | 0.06 (0.05, 0.06) | 0.04 (0.04, 0.05) | -0.94 (-1.29, -0.57) | <0.001 |
| Central Asia | — | — | — | — | — | — | — | — | 1.23 (0.99, 1.60) | 1.70 (1.32, 2.19) | 1.14 (0.88, 1.40) | <0.001 | 0.02 (0.01, 0.02) | 0.03 (0.02, 0.03) | 1.12 (0.86, 1.38) | <0.001 |
| Eastern Europe | — | — | — | — | — | — | — | — | 2.96 (2.54, 3.32) | 4.31 (3.28, 5.55) | 1.31 (0.17, 2.48) | 0.024 | 0.04 (0.04, 0.05) | 0.06 (0.05, 0.08) | 1.32 (0.17, 2.48) | 0.024 |
| Southern Sub-Saharan Africa | — | — | — | — | — | — | — | — | 10.96 (8.35, 13.73) | 4.77 (2.88, 7.15) | -2.82 (-3.34, -2.30) | <0.001 | 0.17 (0.13, 0.21) | 0.07 (0.04, 0.11) | -2.83 (-3.35, -2.30) | <0.001 |
| High-income Asia Pacific | — | — | — | — | — | — | — | — | 1.48 (1.23, 1.86) | 1.98 (1.57, 2.27) | 1.02 (0.69, 1.36) | <0.001 | 0.02 (0.02, 0.03) | 0.03 (0.02, 0.03) | 1.02 (0.69, 1.36) | <0.001 |
| Central Europe | — | — | — | — | — | — | — | — | 4.32 (3.67, 5.14) | 4.96 (3.87, 6.16) | 0.44 (0.20, 0.68) | <0.001 | 0.06 (0.06, 0.08) | 0.07 (0.06, 0.09) | 0.44 (0.20, 0.68) | <0.001 |
| Andean Latin America | — | — | — | — | — | — | — | — | 3.99 (2.62, 6.08) | 3.60 (2.35, 5.17) | -0.36 (-0.90, 0.18) | 0.193 | 0.06 (0.04, 0.09) | 0.05 (0.04, 0.08) | -0.36 (-0.90, 0.18) | 0.191 |

| | | | | | | | | | | | | | | | | |
|---|---|---|---|---|---|---|---|---|---|---|---|---|---|---|---|---|
| Caribbean | — | — | — | — | — | — | — | — | 2.11 (1.60, 3.72) | 2.15 (1.39, 3.88) | 0.04 (-0.11, 0.20) | 0.576 | 0.03 (0.02, 0.06) | 0.03 (0.02, 0.06) | 0.05 (-0.11, 0.20) | 0.557 |
| Southern Latin America | — | — | — | — | — | — | — | — | 3.74 (2.82, 4.89) | 3.54 (2.69, 4.65) | -0.20 (-0.48, 0.09) | 0.179 | 0.06 (0.04, 0.07) | 0.05 (0.04, 0.07) | -0.20 (-0.48, 0.09) | 0.175 |
| Oceania | — | — | — | — | — | — | — | — | 0.60 (0.36, 1.03) | 0.61 (0.36, 1.04) | -0.03 (-0.28, 0.23) | 0.830 | 0.01 (0.01, 0.02) | 0.01 (0.01, 0.02) | -0.03 (-0.28, 0.23) | 0.833 |
| Australasia | — | — | — | — | — | — | — | — | 3.87 (2.71, 5.28) | 1.85 (1.26, 2.70) | -2.52 (-3.00, -2.05) | <0.001 | 0.06 (0.04, 0.08) | 0.03 (0.02, 0.04) | -2.52 (-3.00, -2.04) | <0.001 |
| **Men** | | | | | | | | | | | | | | | | |
| Global | — | — | — | — | — | — | — | — | 6.07 (4.88, 7.64) | 5.84 (5.14, 6.61) | -0.13 (-0.30, 0.03) | 0.114 | 0.09 (0.07, 0.11) | 0.09 (0.08, 0.10) | -0.13 (-0.30, 0.04) | 0.122 |
| **SDI category** | | | | | | | | | | | | | | | | |
| High | — | — | — | — | — | — | — | — | 9.10 (8.25, 9.93) | 5.81 (5.26, 6.49) | -1.56 (-1.71, -1.40) | <0.001 | 0.14 (0.12, 0.15) | 0.09 (0.08, 0.10) | -1.55 (-1.71, -1.40) | <0.001 |
| High-middle | — | — | — | — | — | — | — | — | 8.26 (6.91, 10.10) | 8.20 (7.05, 9.64) | -0.11 (-0.72, 0.52) | 0.735 | 0.12 (0.10, 0.15) | 0.12 (0.11, 0.14) | -0.10 (-0.72, 0.52) | 0.740 |
| Middle | — | — | — | — | — | — | — | — | 4.90 (3.79, 6.30) | 5.71 (4.83, 6.89) | 0.58 (0.39, 0.76) | <0.001 | 0.07 (0.06, 0.09) | 0.09 (0.07, 0.10) | 0.58 (0.40, 0.76) | <0.001 |
| Low-middle | — | — | — | — | — | — | — | — | 4.08 (2.48, 6.52) | 4.81 (3.79, 5.96) | 0.64 (0.20, 1.07) | 0.004 | 0.06 (0.04, 0.10) | 0.07 (0.06, 0.09) | 0.64 (0.20, 1.07) | 0.004 |
| Low | — | — | — | — | — | — | — | — | 5.73 (2.78, 10.14) | 5.53 (3.42, 8.14) | -0.14 (-0.34, 0.06) | 0.176 | 0.09 (0.04, 0.15) | 0.08 (0.05, 0.12) | -0.14 (-0.34, 0.06) | 0.181 |
| **21 GBD region** | | | | | | | | | | | | | | | | |

| | | | | | | | | | | | | | | | | |
|---|---|---|---|---|---|---|---|---|---|---|---|---|---|---|---|---|
| South Asia | — | — | — | — | — | — | — | — | 3.21 (1.62, 5.65) | 3.74 (2.67, 4.92) | 0.65 (-0.15, 1.46) | 0.114 | 0.05 (0.02, 0.08) | 0.06 (0.04, 0.07) | 0.65 (-0.15, 1.46) | 0.111 |
| East Asia | — | — | — | — | — | — | — | — | 5.14 (3.35, 7.46) | 4.88 (3.75, 6.23) | -0.12 (-0.60, 0.36) | 0.625 | 0.08 (0.05, 0.11) | 0.07 (0.06, 0.09) | -0.12 (-0.59, 0.37) | 0.639 |
| North Africa and Middle East | — | — | — | — | — | — | — | — | 6.55 (4.02, 10.13) | 5.72 (4.13, 8.17) | -0.43 (-0.69, -0.17) | 0.001 | 0.10 (0.06, 0.15) | 0.09 (0.06, 0.12) | -0.43 (-0.69, -0.17) | 0.001 |
| Western Sub-Saharan Africa | — | — | — | — | — | — | — | — | 4.47 (2.36, 7.04) | 4.49 (2.98, 6.33) | 0.04 (-0.16, 0.24) | 0.702 | 0.07 (0.04, 0.11) | 0.07 (0.04, 0.10) | 0.04 (-0.16, 0.24) | 0.693 |
| Southeast Asia | — | — | — | — | — | — | — | — | 4.41 (2.59, 6.78) | 6.26 (4.50, 8.38) | 1.29 (0.95, 1.62) | <0.001 | 0.07 (0.04, 0.10) | 0.09 (0.07, 0.13) | 1.29 (0.95, 1.62) | <0.001 |
| Eastern Sub-Saharan Africa | — | — | — | — | — | — | — | — | 9.76 (4.72, 17.39) | 8.06 (4.80, 12.15) | -0.65 (-0.83, -0.48) | <0.001 | 0.15 (0.07, 0.26) | 0.12 (0.07, 0.18) | -0.65 (-0.82, -0.48) | <0.001 |
| Central Latin America | — | — | — | — | — | — | — | — | 6.33 (5.44, 7.34) | 6.22 (4.97, 7.76) | -0.02 (-0.29, 0.26) | 0.906 | 0.10 (0.08, 0.11) | 0.09 (0.07, 0.12) | -0.02 (-0.29, 0.26) | 0.910 |
| Tropical Latin America | — | — | — | — | — | — | — | — | 8.33 (7.26, 9.58) | 14.03 (12.17, 16.21) | 1.83 (1.31, 2.35) | <0.001 | 0.13 (0.11, 0.14) | 0.21 (0.18, 0.24) | 1.83 (1.31, 2.35) | <0.001 |
| Central Sub-Saharan Africa | — | — | — | — | — | — | — | — | 8.26 (3.67, 17.14) | 6.80 (3.64, 12.15) | -0.70 (-0.88, -0.51) | <0.001 | 0.12 (0.06, 0.26) | 0.10 (0.05, 0.18) | -0.70 (-0.88, -0.51) | <0.001 |
| Western Europe | — | — | — | — | — | — | — | — | 10.99 (9.55, 12.39) | 5.87 (5.17, 6.84) | -2.18 (-2.70, -1.66) | <0.001 | 0.17 (0.14, 0.19) | 0.09 (0.08, 0.10) | -2.18 (-2.70, -1.66) | <0.001 |
| High-income North America | — | — | — | — | — | — | — | — | 10.67 (9.55, 11.77) | 6.99 (6.05, 8.11) | -1.51 (-2.05, -0.97) | <0.001 | 0.16 (0.14, 0.18) | 0.11 (0.09, 0.12) | -1.51 (-2.04, -0.97) | <0.001 |

| | | | | | | | | | | | | | | | | |
|---|---|---|---|---|---|---|---|---|---|---|---|---|---|---|---|---|
| Central Asia | — | — | — | — | — | — | — | — | 4.81 (3.71, 6.02) | 7.42 (5.88, 9.18) | 1.43 (0.40, 2.46) | 0.006 | 0.07 (0.06, 0.09) | 0.11 (0.09, 0.14) | 1.43 (0.40, 2.47) | 0.006 |
| Eastern Europe | — | — | — | — | — | — | — | — | 11.54 (10.10, 13.35) | 18.17 (14.56, 23.83) | 1.55 (1.11, 1.98) | <0.001 | 0.17 (0.15, 0.20) | 0.27 (0.22, 0.36) | 1.55 (1.12, 1.99) | <0.001 |
| Southern Sub-Saharan Africa | — | — | — | — | — | — | — | — | 10.41 (7.60, 14.63) | 10.16 (7.51, 13.76) | -0.13 (-1.13, 0.88) | 0.800 | 0.16 (0.11, 0.22) | 0.15 (0.11, 0.21) | -0.13 (-1.14, 0.88) | 0.796 |
| High-income Asia Pacific | — | — | — | — | — | — | — | — | 5.11 (4.12, 6.49) | 4.64 (3.74, 5.51) | -0.27 (-0.72, 0.18) | 0.233 | 0.08 (0.06, 0.10) | 0.07 (0.06, 0.08) | -0.27 (-0.72, 0.18) | 0.242 |
| Central Europe | — | — | — | — | — | — | — | — | 11.26 (9.75, 12.89) | 11.45 (9.49, 13.75) | 0.04 (-0.50, 0.57) | 0.892 | 0.17 (0.15, 0.19) | 0.17 (0.14, 0.21) | 0.04 (-0.49, 0.57) | 0.890 |
| Andean Latin America | — | — | — | — | — | — | — | — | 7.05 (4.29, 10.93) | 6.17 (3.91, 9.55) | -0.24 (-0.78, 0.30) | 0.385 | 0.11 (0.06, 0.16) | 0.09 (0.06, 0.14) | -0.24 (-0.77, 0.30) | 0.389 |
| Caribbean | — | — | — | — | — | — | — | — | 4.88 (3.60, 6.45) | 4.11 (2.80, 5.94) | -0.62 (-0.87, -0.38) | <0.001 | 0.07 (0.05, 0.10) | 0.06 (0.04, 0.09) | -0.62 (-0.87, -0.37) | <0.001 |
| Southern Latin America | — | — | — | — | — | — | — | — | 9.00 (6.61, 12.22) | 7.95 (5.96, 10.55) | -0.32 (-0.98, 0.35) | 0.351 | 0.14 (0.10, 0.18) | 0.12 (0.09, 0.16) | -0.32 (-0.97, 0.35) | 0.351 |
| Oceania | — | — | — | — | — | — | — | — | 7.03 (3.33, 14.38) | 6.54 (2.97, 13.11) | -0.34 (-0.71, 0.04) | 0.083 | 0.11 (0.05, 0.22) | 0.10 (0.04, 0.20) | -0.33 (-0.71, 0.04) | 0.083 |
| Australasia | — | — | — | — | — | — | — | — | 8.55 (6.36, 11.32) | 3.09 (2.07, 4.42) | -3.42 (-3.78, -3.06) | <0.001 | 0.13 (0.10, 0.17) | 0.05 (0.03, 0.07) | -3.42 (-3.77, -3.06) | <0.001 |
| **25-29 years** | | | | | | | | | | | | | | | | |
| **Women** | | | | | | | | | | | | | | | | |
| Global | — | — | — | — | — | — | — | — | 3.82 (3.34, | 3.69 (3.25, | -0.08 | 0.580 | 0.06 | 0.06 | -0.08 | 0.583 |

| | | | | | | | | | | | | | | | | |
|---|---|---|---|---|---|---|---|---|---|---|---|---|---|---|---|---|
| | | | | | | | | | 6.28) | 4.47) | (-0.39, 0.22) | | (0.05, 0.10) | (0.05, 0.07) | (-0.39, 0.22) | |
| **SDI category** | | | | | | | | | | | | | | | | |
| High | — | — | — | — | — | — | — | — | 5.38 (4.99, 5.81) | 4.10 (3.75, 4.52) | -0.91 (-1.06, -0.76) | <0.001 | 0.09 (0.08, 0.09) | 0.07 (0.06, 0.07) | -0.91 (-1.06, -0.76) | <0.001 |
| High-middle | — | — | — | — | — | — | — | — | 4.27 (3.75, 5.46) | 4.40 (3.81, 5.07) | 0.14 (-0.23, 0.50) | 0.466 | 0.07 (0.06, 0.09) | 0.07 (0.06, 0.08) | 0.14 (-0.23, 0.50) | 0.464 |
| Middle | — | — | — | — | — | — | — | — | 3.39 (2.79, 5.94) | 3.47 (2.92, 4.16) | 0.14 (-0.25, 0.54) | 0.480 | 0.06 (0.05, 0.10) | 0.06 (0.05, 0.07) | 0.14 (-0.26, 0.54) | 0.483 |
| Low-middle | — | — | — | — | — | — | — | — | 2.87 (2.07, 7.21) | 3.47 (2.76, 5.30) | 0.70 (0.34, 1.07) | <0.001 | 0.05 (0.03, 0.12) | 0.06 (0.04, 0.09) | 0.70 (0.34, 1.07) | <0.001 |
| Low | — | — | — | — | — | — | — | — | 3.82 (2.06, 9.51) | 3.38 (2.39, 5.42) | -0.42 (-0.55, -0.29) | <0.001 | 0.06 (0.03, 0.15) | 0.05 (0.04, 0.09) | -0.42 (-0.55, -0.28) | <0.001 |
| **21 GBD region** | | | | | | | | | | | | | | | | |
| South Asia | — | — | — | — | — | — | — | — | 2.20 (1.37, 6.53) | 2.90 (2.04, 4.83) | 0.98 (0.63, 1.33) | <0.001 | 0.04 (0.02, 0.11) | 0.05 (0.03, 0.08) | 0.98 (0.63, 1.33) | <0.001 |
| East Asia | — | — | — | — | — | — | — | — | 2.90 (1.99, 6.02) | 2.59 (1.94, 3.38) | -0.24 (-1.25, 0.77) | 0.639 | 0.05 (0.03, 0.10) | 0.04 (0.03, 0.05) | -0.24 (-1.25, 0.78) | 0.639 |
| North Africa and Middle East | — | — | — | — | — | — | — | — | 3.98 (2.85, 7.71) | 4.12 (3.02, 5.84) | 0.12 (0.02, 0.21) | 0.016 | 0.06 (0.05, 0.13) | 0.07 (0.05, 0.09) | 0.12 (0.02, 0.21) | 0.014 |
| Western Sub-Saharan Africa | — | — | — | — | — | — | — | — | 2.75 (1.81, 4.90) | 2.26 (1.52, 3.32) | -0.68 (-0.83, -0.53) | <0.001 | 0.04 (0.03, 0.08) | 0.04 (0.02, 0.05) | -0.68 (-0.83, -0.53) | <0.001 |
| Southeast Asia | — | — | — | — | — | — | — | — | 2.31 (1.67, 4.74) | 3.14 (2.40, 4.17) | 1.09 (0.90, | <0.001 | 0.04 (0.03, | 0.05 (0.04, | 1.09 (0.90, 1.29) | <0.001 |

| | | | | | | | | | | | | | | | | |
|---|---|---|---|---|---|---|---|---|---|---|---|---|---|---|---|---|
| | | | | | | | | | | | 1.29) | | 0.08) | 0.07) | | |
| Eastern Sub-Saharan Africa | — | — | — | — | — | — | — | — | 7.04 (3.32, 16.02) | 5.32 (3.48, 7.41) | -1.00 (-1.15, -0.84) | <0.001 | 0.11 (0.05, 0.26) | 0.09 (0.06, 0.12) | -0.99 (-1.14, -0.84) | <0.001 |
| Central Latin America | — | — | — | — | — | — | — | — | 4.05 (3.52, 4.73) | 3.70 (2.78, 4.82) | -0.22 (-0.74, 0.30) | 0.401 | 0.07 (0.06, 0.08) | 0.06 (0.05, 0.08) | -0.22 (-0.74, 0.30) | 0.398 |
| Tropical Latin America | — | — | — | — | — | — | — | — | 6.98 (6.17, 7.89) | 9.32 (7.89, 10.85) | 1.05 (0.47, 1.63) | <0.001 | 0.11 (0.10, 0.13) | 0.15 (0.13, 0.18) | 1.05 (0.47, 1.63) | <0.001 |
| Central Sub-Saharan Africa | — | — | — | — | — | — | — | — | 3.82 (1.93, 8.23) | 3.05 (1.74, 5.23) | -0.78 (-1.00, -0.56) | <0.001 | 0.06 (0.03, 0.13) | 0.05 (0.03, 0.08) | -0.78 (-1.00, -0.56) | <0.001 |
| Western Europe | — | — | — | — | — | — | — | — | 5.57 (4.91, 6.36) | 4.21 (3.69, 4.88) | -0.98 (-1.15, -0.81) | <0.001 | 0.09 (0.08, 0.10) | 0.07 (0.06, 0.08) | -0.98 (-1.15, -0.81) | <0.001 |
| High-income North America | — | — | — | — | — | — | — | — | 6.77 (6.20, 7.31) | 4.88 (4.36, 5.69) | -1.03 (-1.40, -0.67) | <0.001 | 0.11 (0.10, 0.12) | 0.08 (0.07, 0.09) | -1.03 (-1.39, -0.66) | <0.001 |
| Central Asia | — | — | — | — | — | — | — | — | 2.57 (2.07, 3.27) | 3.43 (2.61, 4.49) | 1.00 (0.79, 1.21) | <0.001 | 0.04 (0.03, 0.05) | 0.06 (0.04, 0.07) | 1.00 (0.79, 1.21) | <0.001 |
| Eastern Europe | — | — | — | — | — | — | — | — | 6.14 (4.67, 7.10) | 8.92 (6.33, 11.98) | 1.29 (0.74, 1.84) | <0.001 | 0.10 (0.08, 0.12) | 0.15 (0.10, 0.19) | 1.30 (0.75, 1.85) | <0.001 |
| Southern Sub-Saharan Africa | — | — | — | — | — | — | — | — | 15.38 (12.10, 19.84) | 7.05 (3.59, 11.19) | -2.71 (-3.76, -1.65) | <0.001 | 0.25 (0.20, 0.32) | 0.11 (0.06, 0.18) | -2.71 (-3.76, -1.65) | <0.001 |
| High-income Asia Pacific | — | — | — | — | — | — | — | — | 2.57 (2.16, 3.25) | 3.03 (2.39, 3.57) | 0.58 (0.27, 0.88) | <0.001 | 0.04 (0.04, 0.05) | 0.05 (0.04, 0.06) | 0.58 (0.27, 0.88) | <0.001 |
| Central Europe | — | — | — | — | — | — | — | — | 6.64 (5.82, 7.87) | 7.78 (6.11, 9.57) | 0.53 (0.33, | <0.001 | 0.11 (0.09, | 0.13 (0.10, | 0.53 (0.33, 0.74) | <0.001 |

| | | | | | | | | | | | | | | | | |
|---|---|---|---|---|---|---|---|---|---|---|---|---|---|---|---|---|
| | | | | | | | | | | | 0.74) | | 0.13) | 0.16) | | |
| Andean Latin America | — | — | — | — | — | — | — | — | 3.87 (2.56, 5.99) | 3.56 (2.31, 5.41) | -0.42 (-1.07, 0.22) | 0.197 | 0.06 (0.04, 0.10) | 0.06 (0.04, 0.09) | -0.42 (-1.07, 0.22) | 0.199 |
| Caribbean | — | — | — | — | — | — | — | — | 4.88 (3.74, 8.00) | 5.23 (3.36, 9.13) | 0.24 (0.01, 0.47) | 0.040 | 0.08 (0.06, 0.13) | 0.08 (0.05, 0.15) | 0.24 (0.01, 0.47) | 0.039 |
| Southern Latin America | — | — | — | — | — | — | — | — | 3.97 (2.92, 5.38) | 3.65 (2.75, 4.71) | -0.30 (-0.57, -0.03) | 0.028 | 0.06 (0.05, 0.09) | 0.06 (0.04, 0.08) | -0.30 (-0.57, -0.04) | 0.027 |
| Oceania | — | — | — | — | — | — | — | — | 0.92 (0.51, 1.80) | 0.83 (0.43, 1.73) | -0.39 (-0.58, -0.20) | <0.001 | 0.01 (0.01, 0.03) | 0.01 (0.01, 0.03) | -0.39 (-0.57, -0.20) | <0.001 |
| Australasia | — | — | — | — | — | — | — | — | 4.40 (3.20, 5.97) | 2.08 (1.40, 3.00) | -2.56 (-3.11, -2.00) | <0.001 | 0.07 (0.05, 0.10) | 0.03 (0.02, 0.05) | -2.55 (-3.10, -2.00) | <0.001 |
| **Men** | | | | | | | | | | | | | | | | |
| Global | — | — | — | — | — | — | — | — | 10.16 (8.39, 12.62) | 10.25 (9.16, 11.49) | 0.05 (-0.03, 0.14) | 0.243 | 0.16 (0.14, 0.20) | 0.17 (0.15, 0.19) | 0.05 (-0.03, 0.14) | 0.238 |
| **SDI category** | | | | | | | | | | | | | | | | |
| High | — | — | — | — | — | — | — | — | 14.44 (13.22, 15.69) | 10.74 (9.73, 11.89) | -1.07 (-1.32, -0.81) | <0.001 | 0.23 (0.21, 0.25) | 0.17 (0.16, 0.19) | -1.07 (-1.33, -0.81) | <0.001 |
| High-middle | — | — | — | — | — | — | — | — | 12.83 (10.88, 15.43) | 13.53 (11.88, 15.77) | 0.15 (-0.40, 0.71) | 0.588 | 0.21 (0.18, 0.25) | 0.22 (0.19, 0.26) | 0.15 (-0.40, 0.71) | 0.586 |
| Middle | — | — | — | — | — | — | — | — | 7.93 (6.37, 9.97) | 8.96 (7.63, 10.48) | 0.43 (0.29, 0.57) | <0.001 | 0.13 (0.10, 0.16) | 0.15 (0.12, 0.17) | 0.43 (0.29, 0.57) | <0.001 |
| Low-middle | — | — | — | — | — | — | — | — | 7.23 (4.39, 11.27) | 8.95 (7.10, 11.11) | 0.76 (0.34, 1.18) | <0.001 | 0.12 (0.07, 0.18) | 0.15 (0.12, 0.18) | 0.76 (0.34, 1.18) | <0.001 |

| | | | | | | | | | | | | | | | | |
|---|---|---|---|---|---|---|---|---|---|---|---|---|---|---|---|---|
| Low | — | — | — | — | — | — | — | — | 10.91 (5.54, 18.47) | 10.67 (6.95, 15.27) | -0.09 (-0.32, 0.15) | 0.482 | 0.18 (0.09, 0.30) | 0.17 (0.11, 0.25) | -0.09 (-0.32, 0.15) | 0.481 |
| **21 GBD region** | | | | | | | | | | | | | | | | |
| South Asia | — | — | — | — | — | — | — | — | 5.75 (2.98, 9.75) | 7.29 (5.22, 9.51) | 0.82 (0.12, 1.53) | 0.022 | 0.09 (0.05, 0.16) | 0.12 (0.08, 0.15) | 0.82 (0.12, 1.53) | 0.021 |
| East Asia | — | — | — | — | — | — | — | — | 7.89 (5.38, 11.13) | 8.53 (6.86, 10.63) | 0.25 (0.01, 0.49) | 0.039 | 0.13 (0.09, 0.18) | 0.14 (0.11, 0.17) | 0.25 (0.01, 0.49) | 0.039 |
| North Africa and Middle East | — | — | — | — | — | — | — | — | 12.00 (7.35, 18.44) | 9.31 (7.00, 12.17) | -0.94 (-1.26, -0.62) | <0.001 | 0.19 (0.12, 0.30) | 0.15 (0.11, 0.20) | -0.94 (-1.26, -0.62) | <0.001 |
| Western Sub-Saharan Africa | — | — | — | — | — | — | — | — | 13.26 (7.44, 20.04) | 13.14 (9.29, 17.67) | -0.01 (-0.20, 0.18) | 0.918 | 0.22 (0.12, 0.33) | 0.21 (0.15, 0.29) | -0.01 (-0.20, 0.18) | 0.921 |
| Southeast Asia | — | — | — | — | — | — | — | — | 6.84 (4.20, 10.00) | 8.84 (6.43, 11.36) | 0.85 (0.31, 1.40) | 0.002 | 0.11 (0.07, 0.16) | 0.14 (0.10, 0.18) | 0.85 (0.31, 1.40) | 0.002 |
| Eastern Sub-Saharan Africa | — | — | — | — | — | — | — | — | 16.48 (8.72, 28.92) | 13.14 (8.11, 19.27) | -0.77 (-0.97, -0.57) | <0.001 | 0.27 (0.14, 0.47) | 0.21 (0.13, 0.31) | -0.77 (-0.97, -0.57) | <0.001 |
| Central Latin America | — | — | — | — | — | — | — | — | 9.24 (8.02, 10.69) | 9.40 (7.63, 11.43) | 0.17 (-0.07, 0.41) | 0.167 | 0.15 (0.13, 0.17) | 0.15 (0.12, 0.19) | 0.17 (-0.07, 0.41) | 0.168 |
| Tropical Latin America | — | — | — | — | — | — | — | — | 14.96 (13.07, 17.33) | 21.28 (18.63, 24.59) | 1.31 (0.98, 1.63) | <0.001 | 0.24 (0.21, 0.28) | 0.34 (0.30, 0.40) | 1.30 (0.98, 1.62) | <0.001 |
| Central Sub-Saharan Africa | — | — | — | — | — | — | — | — | 12.69 (5.77, 24.71) | 10.61 (6.09, 17.55) | -0.65 (-0.98, -0.32) | <0.001 | 0.21 (0.09, 0.40) | 0.17 (0.10, 0.28) | -0.65 (-0.98, -0.32) | <0.001 |
| Western Europe | — | — | — | — | — | — | — | — | 16.87 (14.78, 18.94) | 10.66 (9.48, 12.05) | -1.63 (-2.10, | <0.001 | 0.27 (0.24, | 0.17 (0.15, | -1.63 (-2.10, | <0.001 |

| | | | | | | | | | | | | | | | | |
|---|---|---|---|---|---|---|---|---|---|---|---|---|---|---|---|---|
| | | | | | | | | | | | -1.16) | | 0.31) | 0.20) | -1.16) | |
| High-income North America | — | — | — | — | — | — | — | — | 17.13 (15.64, 18.68) | 12.97 (10.54, 15.07) | -1.03 (-1.57, -0.48) | <0.001 | 0.28 (0.25, 0.30) | 0.21 (0.17, 0.24) | -1.03 (-1.58, -0.48) | <0.001 |
| Central Asia | — | — | — | — | — | — | — | — | 8.00 (6.67, 9.79) | 10.90 (8.85, 13.53) | 1.06 (0.10, 2.02) | 0.029 | 0.13 (0.11, 0.16) | 0.18 (0.14, 0.22) | 1.06 (0.10, 2.02) | 0.030 |
| Eastern Europe | — | — | — | — | — | — | — | — | 14.12 (11.38, 17.00) | 28.25 (23.14, 37.72) | 2.50 (0.81, 4.22) | 0.004 | 0.23 (0.18, 0.28) | 0.46 (0.38, 0.61) | 2.51 (0.35, 4.72) | 0.023 |
| Southern Sub-Saharan Africa | — | — | — | — | — | — | — | — | 21.30 (16.20, 28.83) | 15.90 (11.52, 21.36) | -1.07 (-1.48, -0.67) | <0.001 | 0.35 (0.26, 0.47) | 0.26 (0.19, 0.35) | -1.08 (-1.49, -0.67) | <0.001 |
| High-income Asia Pacific | — | — | — | — | — | — | — | — | 7.73 (6.28, 9.77) | 7.44 (6.03, 9.01) | -0.15 (-0.71, 0.41) | 0.589 | 0.13 (0.10, 0.16) | 0.12 (0.10, 0.15) | -0.15 (-0.71, 0.41) | 0.590 |
| Central Europe | — | — | — | — | — | — | — | — | 18.95 (16.25, 21.52) | 20.79 (17.71, 23.99) | 0.31 (-0.14, 0.77) | 0.180 | 0.31 (0.26, 0.35) | 0.34 (0.29, 0.39) | 0.31 (-0.14, 0.77) | 0.179 |
| Andean Latin America | — | — | — | — | — | — | — | — | 9.22 (6.19, 13.41) | 8.87 (6.03, 12.70) | -0.09 (-0.62, 0.44) | 0.741 | 0.15 (0.10, 0.22) | 0.14 (0.10, 0.21) | -0.09 (-0.62, 0.44) | 0.741 |
| Caribbean | — | — | — | — | — | — | — | — | 9.95 (7.76, 12.68) | 8.64 (6.32, 11.64) | -0.49 (-0.77, -0.21) | 0.001 | 0.16 (0.13, 0.21) | 0.14 (0.10, 0.19) | -0.49 (-0.77, -0.21) | 0.001 |
| Southern Latin America | — | — | — | — | — | — | — | — | 12.66 (9.65, 16.58) | 13.03 (10.06, 16.88) | 0.09 (-0.56, 0.75) | 0.781 | 0.21 (0.16, 0.27) | 0.21 (0.16, 0.27) | 0.09 (-0.56, 0.75) | 0.783 |
| Oceania | — | — | — | — | — | — | — | — | 20.62 (9.28, 40.89) | 19.86 (8.94, 37.20) | -0.15 (-0.49, 0.20) | 0.403 | 0.33 (0.15, 0.66) | 0.32 (0.15, 0.60) | -0.14 (-0.48, 0.20) | 0.408 |
| Australasia | — | — | — | — | — | — | — | — | 17.59 (13.42, 22.38) | 8.04 (5.54, 11.38) | -2.64 (-3.47, | <0.001 | 0.29 (0.22, | 0.13 (0.09, | -2.64 (-3.46, | <0.001 |

| | | | | | | | | | | | | | | | |
|---|---|---|---|---|---|---|---|---|---|---|---|---|---|---|---|
| | | | | | | | | | | | -1.81) | | 0.36) | 0.18) | -1.81) |
| **30-34 years** | | | | | | | | | | | | | | | |
| **Women** | | | | | | | | | | | | | | | |
| Global | — | — | — | — | — | — | — | — | 5.93 (5.27, 9.40) | 5.69 (5.06, 6.81) | -0.14 (-0.36, 0.09) | 0.247 | 0.10 (0.09, 0.17) | 0.10 (0.09, 0.12) | -0.14 (-0.36, 0.09) | 0.246 |
| **SDI category** | | | | | | | | | | | | | | | |
| High | — | — | — | — | — | — | — | — | 7.20 (6.69, 7.78) | 6.03 (5.55, 6.69) | -0.58 (-0.81, -0.35) | <0.001 | 0.13 (0.12, 0.14) | 0.11 (0.10, 0.12) | -0.58 (-0.81, -0.36) | <0.001 |
| High-middle | — | — | — | — | — | — | — | — | 6.60 (5.89, 8.10) | 6.42 (5.57, 7.41) | -0.07 (-0.38, 0.23) | 0.629 | 0.12 (0.10, 0.14) | 0.11 (0.10, 0.13) | -0.07 (-0.38, 0.23) | 0.633 |
| Middle | — | — | — | — | — | — | — | — | 5.49 (4.57, 9.24) | 5.32 (4.58, 6.41) | -0.08 (-0.34, 0.18) | 0.551 | 0.10 (0.08, 0.16) | 0.09 (0.08, 0.11) | -0.08 (-0.34, 0.18) | 0.555 |
| Low-middle | — | — | — | — | — | — | — | — | 4.68 (3.48, 11.15) | 5.73 (4.62, 8.58) | 0.74 (0.47, 1.00) | <0.001 | 0.08 (0.06, 0.20) | 0.10 (0.08, 0.15) | 0.74 (0.48, 1.00) | <0.001 |
| Low | — | — | — | — | — | — | — | — | 5.91 (3.26, 13.80) | 5.09 (3.61, 7.94) | -0.56 (-0.68, -0.44) | <0.001 | 0.10 (0.06, 0.24) | 0.09 (0.06, 0.14) | -0.55 (-0.67, -0.43) | <0.001 |
| **21 GBD region** | | | | | | | | | | | | | | | |
| South Asia | — | — | — | — | — | — | — | — | 3.78 (2.54, 9.89) | 4.96 (3.59, 7.86) | 1.04 (0.74, 1.33) | <0.001 | 0.07 (0.04, 0.17) | 0.09 (0.06, 0.14) | 1.04 (0.75, 1.33) | <0.001 |
| East Asia | — | — | — | — | — | — | — | — | 4.35 (3.04, 9.04) | 3.92 (2.95, 5.02) | -0.27 (-1.20, 0.66) | 0.566 | 0.08 (0.05, 0.16) | 0.07 (0.05, 0.09) | -0.27 (-1.19, 0.65) | 0.563 |
| North Africa and Middle East | — | — | — | — | — | — | — | — | 4.34 (3.08, 8.23) | 4.46 (3.37, 6.13) | 0.07 (-0.07, 0.22) | 0.311 | 0.08 (0.05, 0.15) | 0.08 (0.06, 0.11) | 0.07 (-0.07, 0.22) | 0.319 |
| Western | — | — | — | — | — | — | — | — | 5.74 (3.76, | 3.53 (2.43, | -1.67 | <0.001 | 0.10 | 0.06 | -1.67 | <0.001 |

| | | | | | | | | | | | | | | | | |
|---|---|---|---|---|---|---|---|---|---|---|---|---|---|---|---|---|
| Sub-Saharan Africa | | | | | | | | | 9.33) | 5.09) | (-1.87, -1.48) | | (0.07, 0.16) | (0.04, 0.09) | (-1.87, -1.48) | |
| Southeast Asia | — | — | — | — | — | — | — | — | 5.29 (3.88, 10.18) | 6.93 (5.46, 9.23) | 0.95 (0.89, 1.01) | <0.001 | 0.09 (0.07, 0.18) | 0.12 (0.10, 0.16) | 0.95 (0.90, 1.01) | <0.001 |
| Eastern Sub-Saharan Africa | — | — | — | — | — | — | — | — | 9.47 (4.43, 21.12) | 7.02 (4.56, 9.62) | -1.09 (-1.22, -0.95) | <0.001 | 0.17 (0.08, 0.37) | 0.12 (0.08, 0.17) | -1.08 (-1.22, -0.95) | <0.001 |
| Central Latin America | — | — | — | — | — | — | — | — | 5.35 (4.56, 6.18) | 4.74 (3.58, 6.45) | -0.31 (-0.52, -0.10) | 0.004 | 0.09 (0.08, 0.11) | 0.08 (0.06, 0.11) | -0.31 (-0.52, -0.10) | 0.004 |
| Tropical Latin America | — | — | — | — | — | — | — | — | 14.07 (12.35, 16.00) | 14.06 (12.19, 16.30) | 0.10 (-0.45, 0.66) | 0.713 | 0.25 (0.22, 0.28) | 0.25 (0.22, 0.29) | 0.10 (-0.45, 0.66) | 0.711 |
| Central Sub-Saharan Africa | — | — | — | — | — | — | — | — | 7.21 (3.50, 15.78) | 5.72 (3.09, 10.07) | -0.85 (-1.07, -0.64) | <0.001 | 0.13 (0.06, 0.28) | 0.10 (0.05, 0.18) | -0.85 (-1.07, -0.64) | <0.001 |
| Western Europe | — | — | — | — | — | — | — | — | 7.00 (6.17, 8.03) | 5.62 (4.92, 6.49) | -0.78 (-0.99, -0.56) | <0.001 | 0.12 (0.11, 0.14) | 0.10 (0.09, 0.11) | -0.78 (-0.99, -0.56) | <0.001 |
| High-income North America | — | — | — | — | — | — | — | — | 8.60 (7.87, 9.33) | 7.41 (6.54, 8.81) | -0.52 (-1.02, -0.01) | 0.044 | 0.15 (0.14, 0.16) | 0.13 (0.12, 0.16) | -0.52 (-1.02, -0.02) | 0.043 |
| Central Asia | — | — | — | — | — | — | — | — | 3.78 (3.06, 4.58) | 5.06 (3.92, 6.46) | 1.03 (0.80, 1.26) | <0.001 | 0.07 (0.05, 0.08) | 0.09 (0.07, 0.11) | 1.03 (0.80, 1.26) | <0.001 |
| Eastern Europe | — | — | — | — | — | — | — | — | 7.94 (5.81, 9.10) | 12.06 (8.73, 16.93) | 1.50 (0.93, 2.08) | <0.001 | 0.14 (0.10, 0.16) | 0.21 (0.15, 0.30) | 1.51 (0.93, 2.08) | <0.001 |
| Southern Sub-Saharan Africa | — | — | — | — | — | — | — | — | 24.17 (18.43, 31.71) | 9.97 (5.78, 15.67) | -2.97 (-4.27, -1.66) | <0.001 | 0.43 (0.33, 0.56) | 0.18 (0.10, 0.28) | -2.97 (-4.27, -1.66) | <0.001 |
| High-income Asia | — | — | — | — | — | — | — | — | 4.44 (3.59, | 5.55 (4.53, | 0.78 | <0.001 | 0.08 | 0.10 | 0.78 (0.41, | <0.001 |

| | | | | | | | | | | | | | | | | |
|---|---|---|---|---|---|---|---|---|---|---|---|---|---|---|---|---|
| Pacific | | | | | | | | | 5.96) | 6.64) | (0.41, 1.15) | | (0.06, 0.11) | (0.08, 0.12) | 1.15) | |
| Central Europe | — | — | — | — | — | — | — | — | 9.18 (7.96, 10.61) | 9.69 (7.76, 11.91) | 0.21 (0.04, 0.38) | 0.013 | 0.16 (0.14, 0.19) | 0.17 (0.14, 0.21) | 0.21 (0.04, 0.38) | 0.013 |
| Andean Latin America | — | — | — | — | — | — | — | — | 7.20 (4.70, 11.71) | 6.90 (4.28, 10.55) | -0.20 (-0.75, 0.36) | 0.493 | 0.13 (0.08, 0.21) | 0.12 (0.08, 0.19) | -0.20 (-0.75, 0.37) | 0.495 |
| Caribbean | — | — | — | — | — | — | — | — | 4.71 (3.64, 7.54) | 5.38 (3.56, 9.01) | 0.47 (0.19, 0.74) | 0.001 | 0.08 (0.06, 0.13) | 0.10 (0.06, 0.16) | 0.47 (0.19, 0.74) | 0.001 |
| Southern Latin America | — | — | — | — | — | — | — | — | 8.90 (6.62, 11.70) | 8.33 (6.13, 10.75) | -0.21 (-0.52, 0.10) | 0.190 | 0.16 (0.12, 0.21) | 0.15 (0.11, 0.19) | -0.21 (-0.52, 0.10) | 0.189 |
| Oceania | — | — | — | — | — | — | — | — | 7.47 (4.31, 13.07) | 6.36 (3.36, 12.38) | -0.58 (-0.83, -0.32) | <0.001 | 0.13 (0.08, 0.23) | 0.11 (0.06, 0.22) | -0.58 (-0.83, -0.32) | <0.001 |
| Australasia | — | — | — | — | — | — | — | — | 7.34 (5.44, 9.80) | 4.22 (2.87, 6.19) | -1.88 (-2.09, -1.66) | <0.001 | 0.13 (0.10, 0.17) | 0.07 (0.05, 0.11) | -1.88 (-2.09, -1.66) | <0.001 |
| **Men** | | | | | | | | | | | | | | | | |
| Global | — | — | — | — | — | — | — | — | 15.13 (12.72, 18.60) | 15.49 (14.01, 17.19) | 0.04 (-0.16, 0.25) | 0.663 | 0.27 (0.22, 0.33) | 0.27 (0.25, 0.30) | 0.05 (-0.16, 0.25) | 0.660 |
| **SDI category** | | | | | | | | | | | | | | | | |
| High | — | — | — | — | — | — | — | — | 21.36 (19.95, 22.77) | 18.35 (16.32, 20.27) | -0.59 (-1.05, -0.12) | 0.013 | 0.38 (0.35, 0.40) | 0.32 (0.29, 0.36) | -0.59 (-1.05, -0.13) | 0.013 |
| High-middle | — | — | — | — | — | — | — | — | 19.64 (16.83, 23.44) | 21.24 (18.77, 24.62) | 0.21 (-0.22, 0.66) | 0.338 | 0.35 (0.30, 0.41) | 0.38 (0.33, 0.43) | 0.22 (-0.22, 0.66) | 0.336 |
| Middle | — | — | — | — | — | — | — | — | 11.62 (9.36, 14.57) | 13.10 (11.48, 15.18) | 0.36 (-0.08, | 0.106 | 0.21 (0.17, | 0.23 (0.20, | 0.36 (-0.08, 0.81) | 0.108 |

| | | | | | | | | | | | | | | | | |
|---|---|---|---|---|---|---|---|---|---|---|---|---|---|---|---|---|
| | | | | | | | | | | | 0.81) | | 0.26) | 0.27) | | |
| Low-middle | — | — | — | — | — | — | — | — | 10.33 (6.07, 16.49) | 13.52 (9.65, 17.25) | 0.96 (0.72, 1.20) | <0.001 | 0.18 (0.11, 0.29) | 0.24 (0.17, 0.30) | 0.96 (0.72, 1.20) | <0.001 |
| Low | — | — | — | — | — | — | — | — | 13.30 (6.63, 22.69) | 12.56 (8.16, 17.98) | -0.24 (-0.39, -0.08) | 0.003 | 0.23 (0.12, 0.40) | 0.22 (0.14, 0.32) | -0.24 (-0.39, -0.08) | 0.003 |
| **21 GBD region** | | | | | | | | | | | | | | | | |
| South Asia | — | — | — | — | — | — | — | — | 9.61 (4.84, 16.77) | 12.25 (7.69, 16.41) | 0.86 (0.54, 1.18) | <0.001 | 0.17 (0.09, 0.30) | 0.22 (0.14, 0.29) | 0.86 (0.55, 1.18) | <0.001 |
| East Asia | — | — | — | — | — | — | — | — | 11.67 (8.34, 16.31) | 15.27 (12.46, 18.73) | 0.93 (0.59, 1.27) | <0.001 | 0.21 (0.15, 0.29) | 0.27 (0.22, 0.33) | 0.93 (0.59, 1.27) | <0.001 |
| North Africa and Middle East | — | — | — | — | — | — | — | — | 18.08 (11.24, 29.12) | 12.60 (9.69, 16.75) | -1.26 (-1.55, -0.96) | <0.001 | 0.32 (0.20, 0.51) | 0.22 (0.17, 0.30) | -1.26 (-1.56, -0.96) | <0.001 |
| Western Sub-Saharan Africa | — | — | — | — | — | — | — | — | 6.52 (3.75, 9.71) | 6.75 (4.82, 9.08) | 0.15 (0.02, 0.28) | 0.025 | 0.12 (0.07, 0.17) | 0.12 (0.09, 0.16) | 0.15 (0.02, 0.28) | 0.025 |
| Southeast Asia | — | — | — | — | — | — | — | — | 8.09 (5.47, 11.35) | 10.35 (7.89, 13.21) | 0.94 (0.47, 1.41) | <0.001 | 0.14 (0.10, 0.20) | 0.18 (0.14, 0.23) | 0.94 (0.47, 1.41) | <0.001 |
| Eastern Sub-Saharan Africa | — | — | — | — | — | — | — | — | 21.52 (10.86, 38.13) | 17.09 (10.75, 24.70) | -0.80 (-1.03, -0.56) | <0.001 | 0.38 (0.19, 0.67) | 0.30 (0.19, 0.44) | -0.80 (-1.03, -0.56) | <0.001 |
| Central Latin America | — | — | — | — | — | — | — | — | 13.34 (11.65, 15.24) | 13.03 (10.56, 16.04) | -0.05 (-0.34, 0.24) | 0.737 | 0.24 (0.21, 0.27) | 0.23 (0.19, 0.28) | -0.05 (-0.34, 0.24) | 0.733 |
| Tropical Latin America | — | — | — | — | — | — | — | — | 29.51 (26.46, 33.06) | 34.16 (30.34, 38.58) | 0.52 (0.12, 0.94) | 0.012 | 0.52 (0.47, 0.58) | 0.60 (0.54, 0.68) | 0.52 (0.11, 0.93) | 0.012 |
| Central Sub-Saharan | — | — | — | — | — | — | — | — | 18.06 (8.42, | 15.27 (8.66, | -0.61 | <0.001 | 0.32 | 0.27 | -0.61 | <0.001 |

| | | | | | | | | | | | | | | | | |
|---|---|---|---|---|---|---|---|---|---|---|---|---|---|---|---|---|
| Africa | | | | | | | | | 33.81) | 25.02) | (-0.89, -0.32) | | (0.15, 0.60) | (0.15, 0.44) | (-0.89, -0.32) | |
| Western Europe | — | — | — | — | — | — | — | — | 21.62 (19.45, 23.85) | 15.59 (14.16, 17.39) | -1.14 (-1.50, -0.79) | <0.001 | 0.38 (0.34, 0.42) | 0.28 (0.25, 0.31) | -1.14 (-1.50, -0.79) | <0.001 |
| High-income North America | — | — | — | — | — | — | — | — | 26.87 (24.96, 28.89) | 23.09 (17.91, 26.66) | -0.54 (-1.16, 0.09) | 0.092 | 0.47 (0.44, 0.51) | 0.41 (0.32, 0.47) | -0.54 (-1.16, 0.08) | 0.089 |
| Central Asia | — | — | — | — | — | — | — | — | 10.98 (9.01, 14.46) | 15.85 (12.89, 19.55) | 1.18 (0.70, 1.66) | <0.001 | 0.19 (0.16, 0.26) | 0.28 (0.23, 0.35) | 1.18 (0.71, 1.66) | <0.001 |
| Eastern Europe | — | — | — | — | — | — | — | — | 22.78 (17.59, 28.45) | 40.78 (33.33, 54.52) | 2.01 (1.34, 2.68) | <0.001 | 0.40 (0.31, 0.50) | 0.72 (0.59, 0.96) | 2.01 (1.34, 2.68) | <0.001 |
| Southern Sub-Saharan Africa | — | — | — | — | — | — | — | — | 34.43 (27.27, 44.05) | 22.00 (16.46, 29.11) | -1.68 (-3.28, -0.06) | 0.043 | 0.61 (0.48, 0.78) | 0.39 (0.29, 0.51) | -1.68 (-3.29, -0.06) | 0.043 |
| High-income Asia Pacific | — | — | — | — | — | — | — | — | 12.15 (10.25, 14.93) | 15.69 (13.75, 18.04) | 0.81 (0.56, 1.06) | <0.001 | 0.21 (0.18, 0.26) | 0.28 (0.24, 0.32) | 0.81 (0.56, 1.06) | <0.001 |
| Central Europe | — | — | — | — | — | — | — | — | 29.72 (25.60, 33.80) | 31.83 (27.64, 36.48) | 0.15 (-0.13, 0.44) | 0.295 | 0.53 (0.45, 0.60) | 0.56 (0.49, 0.64) | 0.15 (-0.13, 0.44) | 0.295 |
| Andean Latin America | — | — | — | — | — | — | — | — | 15.25 (10.67, 21.61) | 13.75 (9.43, 19.55) | -0.43 (-0.78, -0.07) | 0.018 | 0.27 (0.19, 0.38) | 0.24 (0.17, 0.34) | -0.43 (-0.79, -0.07) | 0.018 |
| Caribbean | — | — | — | — | — | — | — | — | 14.35 (11.46, 18.12) | 14.10 (9.96, 18.80) | 0.00 (-0.29, 0.30) | 0.991 | 0.25 (0.20, 0.32) | 0.25 (0.18, 0.33) | 0.00 (-0.29, 0.30) | 0.992 |
| Southern Latin America | — | — | — | — | — | — | — | — | 17.40 (13.92, 21.81) | 19.63 (15.54, 24.33) | 0.48 (0.10, 0.86) | 0.014 | 0.31 (0.25, 0.39) | 0.35 (0.27, 0.43) | 0.48 (0.10, 0.86) | 0.014 |
| Oceania | — | — | — | — | — | — | — | — | 39.42 (18.10, | 37.54 (16.99, | -0.21 | <0.001 | 0.70 | 0.66 | -0.21 | <0.001 |

| | | | | | | | | | | | | | | | | |
|---|---|---|---|---|---|---|---|---|---|---|---|---|---|---|---|---|
| | | | | | | | | | 76.53) | 69.58) | (-0.32, -0.11) | | (0.32, 1.35) | (0.30, 1.23) | (-0.32, -0.11) | |
| Australasia | — | — | — | — | — | — | — | — | 23.00 (18.62, 29.05) | 11.96 (8.52, 16.11) | -2.24 (-2.69, -1.80) | <0.001 | 0.41 (0.33, 0.51) | 0.21 (0.15, 0.28) | -2.24 (-2.68, -1.80) | <0.001 |
| **35-39 years** | | | | | | | | | | | | | | | | |
| **Women** | | | | | | | | | | | | | | | | |
| Global | — | — | — | — | — | — | — | — | 8.92 (8.00, 13.23) | 8.71 (7.84, 10.15) | -0.08 (-0.19, 0.04) | 0.181 | 0.17 (0.15, 0.26) | 0.17 (0.15, 0.20) | -0.07 (-0.19, 0.04) | 0.191 |
| **SDI category** | | | | | | | | | | | | | | | | |
| High | — | — | — | — | — | — | — | — | 10.22 (9.60, 10.95) | 8.85 (8.23, 9.65) | -0.50 (-0.65, -0.34) | <0.001 | 0.20 (0.19, 0.21) | 0.17 (0.16, 0.19) | -0.50 (-0.65, -0.34) | <0.001 |
| High-middle | — | — | — | — | — | — | — | — | 9.54 (8.68, 10.99) | 9.99 (8.75, 11.25) | 0.16 (0.05, 0.28) | 0.007 | 0.18 (0.17, 0.21) | 0.19 (0.17, 0.22) | 0.16 (0.04, 0.28) | 0.007 |
| Middle | — | — | — | — | — | — | — | — | 7.83 (6.61, 12.46) | 7.63 (6.62, 8.82) | -0.08 (-0.19, 0.03) | 0.145 | 0.15 (0.13, 0.24) | 0.15 (0.13, 0.17) | -0.08 (-0.19, 0.03) | 0.151 |
| Low-middle | — | — | — | — | — | — | — | — | 7.54 (5.76, 15.19) | 8.96 (7.35, 12.30) | 0.58 (0.30, 0.85) | <0.001 | 0.15 (0.11, 0.29) | 0.17 (0.14, 0.24) | 0.58 (0.31, 0.86) | <0.001 |
| Low | — | — | — | — | — | — | — | — | 11.68 (6.80, 24.80) | 8.87 (6.36, 13.30) | -0.95 (-1.14, -0.77) | <0.001 | 0.23 (0.13, 0.48) | 0.17 (0.12, 0.26) | -0.95 (-1.13, -0.77) | <0.001 |
| **21 GBD region** | | | | | | | | | | | | | | | | |
| South Asia | — | — | — | — | — | — | — | — | 5.48 (3.86, 12.34) | 6.98 (5.19, 9.93) | 0.82 (0.46, 1.20) | <0.001 | 0.11 (0.07, 0.24) | 0.14 (0.10, 0.19) | 0.83 (0.46, 1.20) | <0.001 |
| East Asia | — | — | — | — | — | — | — | — | 6.01 (4.35, 11.01) | 5.25 (3.99, 6.66) | -0.47 (-0.77, -0.17) | 0.002 | 0.12 (0.08, 0.21) | 0.10 (0.08, 0.13) | -0.48 (-0.80, -0.16) | 0.003 |

| | | | | | | | | | | | | | | | | |
|---|---|---|---|---|---|---|---|---|---|---|---|---|---|---|---|---|
| North Africa and Middle East | — | — | — | — | — | — | — | — | 4.88 (3.43, 9.84) | 4.90 (3.67, 6.81) | 0.05 (-0.03, 0.13) | 0.225 | 0.09 (0.07, 0.19) | 0.09 (0.07, 0.13) | 0.05 (-0.03, 0.13) | 0.188 |
| Western Sub-Saharan Africa | — | — | — | — | — | — | — | — | 24.41 (16.24, 39.16) | 10.98 (7.69, 15.42) | -2.72 (-2.92, -2.52) | <0.001 | 0.47 (0.31, 0.76) | 0.21 (0.15, 0.30) | -2.72 (-2.92, -2.52) | <0.001 |
| Southeast Asia | — | — | — | — | — | — | — | — | 7.20 (5.34, 13.39) | 9.43 (7.25, 12.47) | 0.94 (0.82, 1.07) | <0.001 | 0.14 (0.10, 0.26) | 0.18 (0.14, 0.24) | 0.95 (0.82, 1.07) | <0.001 |
| Eastern Sub-Saharan Africa | — | — | — | — | — | — | — | — | 15.36 (7.23, 31.86) | 10.98 (7.02, 15.22) | -1.16 (-1.33, -0.98) | <0.001 | 0.30 (0.14, 0.62) | 0.21 (0.14, 0.29) | -1.15 (-1.33, -0.98) | <0.001 |
| Central Latin America | — | — | — | — | — | — | — | — | 9.48 (8.25, 10.96) | 8.13 (6.08, 10.78) | -0.46 (-0.91, 0.00) | 0.053 | 0.18 (0.16, 0.21) | 0.16 (0.12, 0.21) | -0.46 (-0.91, 0.00) | 0.053 |
| Tropical Latin America | — | — | — | — | — | — | — | — | 25.20 (22.70, 28.16) | 27.60 (24.43, 31.44) | 0.32 (0.08, 0.56) | 0.010 | 0.49 (0.44, 0.55) | 0.53 (0.47, 0.61) | 0.32 (0.08, 0.56) | 0.010 |
| Central Sub-Saharan Africa | — | — | — | — | — | — | — | — | 12.09 (6.32, 24.36) | 9.81 (5.53, 17.32) | -0.76 (-1.10, -0.42) | <0.001 | 0.23 (0.12, 0.47) | 0.19 (0.11, 0.34) | -0.76 (-1.09, -0.41) | <0.001 |
| Western Europe | — | — | — | — | — | — | — | — | 10.32 (9.18, 11.67) | 8.09 (7.24, 9.08) | -0.85 (-1.07, -0.64) | <0.001 | 0.20 (0.18, 0.23) | 0.16 (0.14, 0.18) | -0.85 (-1.07, -0.63) | <0.001 |
| High-income North America | — | — | — | — | — | — | — | — | 12.32 (11.34, 13.25) | 11.53 (10.57, 13.13) | -0.24 (-0.59, 0.11) | 0.175 | 0.24 (0.22, 0.26) | 0.22 (0.20, 0.25) | -0.24 (-0.59, 0.11) | 0.178 |
| Central Asia | — | — | — | — | — | — | — | — | 7.38 (5.98, 8.79) | 10.76 (8.43, 13.61) | 1.32 (1.19, 1.45) | <0.001 | 0.14 (0.12, 0.17) | 0.21 (0.16, 0.26) | 1.32 (1.19, 1.45) | <0.001 |
| Eastern Europe | — | — | — | — | — | — | — | — | 11.02 (7.90, 12.59) | 16.47 (12.46, 22.11) | 1.42 (0.86, 1.97) | <0.001 | 0.21 (0.15, 0.24) | 0.32 (0.24, 0.43) | 1.41 (0.86, 1.97) | <0.001 |

| | | | | | | | | | | | | | | | | |
|---|---|---|---|---|---|---|---|---|---|---|---|---|---|---|---|---|
| Southern Sub-Saharan Africa | — | — | — | — | — | — | — | — | 30.95 (24.44, 39.78) | 13.33 (8.28, 19.21) | -2.80 (-3.51, -2.08) | <0.001 | 0.60 (0.47, 0.77) | 0.26 (0.16, 0.37) | -2.80 (-3.51, -2.08) | <0.001 |
| High-income Asia Pacific | — | — | — | — | — | — | — | — | 5.21 (4.55, 6.24) | 7.17 (6.05, 8.29) | 1.07 (0.79, 1.35) | <0.001 | 0.10 (0.09, 0.12) | 0.14 (0.12, 0.16) | 1.07 (0.79, 1.35) | <0.001 |
| Central Europe | — | — | — | — | — | — | — | — | 13.82 (12.30, 15.67) | 14.52 (11.71, 17.69) | 0.17 (-0.02, 0.36) | 0.076 | 0.27 (0.24, 0.30) | 0.28 (0.23, 0.34) | 0.15 (-0.02, 0.32) | 0.083 |
| Andean Latin America | — | — | — | — | — | — | — | — | 7.67 (5.19, 12.05) | 7.21 (4.58, 10.57) | -0.29 (-0.86, 0.28) | 0.321 | 0.15 (0.10, 0.23) | 0.14 (0.09, 0.20) | -0.29 (-0.86, 0.29) | 0.325 |
| Caribbean | — | — | — | — | — | — | — | — | 9.17 (7.35, 14.41) | 9.95 (6.74, 16.10) | 0.28 (0.17, 0.39) | <0.001 | 0.18 (0.14, 0.28) | 0.19 (0.13, 0.31) | 0.28 (0.17, 0.39) | <0.001 |
| Southern Latin America | — | — | — | — | — | — | — | — | 11.50 (8.91, 14.51) | 10.97 (8.50, 13.84) | -0.15 (-0.38, 0.08) | 0.205 | 0.22 (0.17, 0.28) | 0.21 (0.16, 0.27) | -0.15 (-0.38, 0.08) | 0.197 |
| Oceania | — | — | — | — | — | — | — | — | 10.43 (5.99, 18.22) | 9.14 (5.00, 16.90) | -0.46 (-0.76, -0.16) | 0.003 | 0.20 (0.12, 0.35) | 0.18 (0.10, 0.33) | -0.46 (-0.76, -0.16) | 0.003 |
| Australasia | — | — | — | — | — | — | — | — | 10.79 (8.46, 13.77) | 6.55 (4.82, 8.74) | -1.76 (-1.97, -1.56) | <0.001 | 0.21 (0.16, 0.27) | 0.13 (0.09, 0.17) | -1.76 (-1.97, -1.56) | <0.001 |
| **Men** | | | | | | | | | | | | | | | | |
| Global | — | — | — | — | — | — | — | — | 24.73 (20.68, 30.47) | 26.46 (23.82, 29.46) | 0.26 (0.07, 0.44) | 0.006 | 0.48 (0.40, 0.59) | 0.51 (0.46, 0.57) | 0.26 (0.07, 0.44) | 0.006 |
| **SDI category** | | | | | | | | | | | | | | | | |
| High | — | — | — | — | — | — | — | — | 32.86 (30.88, 34.97) | 31.03 (28.52, 33.83) | -0.24 (-0.59, 0.10) | 0.169 | 0.64 (0.60, 0.68) | 0.60 (0.55, 0.65) | -0.24 (-0.59, 0.11) | 0.174 |
| High-middle | — | — | — | — | — | — | — | — | 31.76 (27.16, | 37.19 (33.29, | 0.60 | <0.001 | 0.61 | 0.72 | 0.60 (0.30, | <0.001 |

| | | | | | | | | | | | | | | | | |
|---|---|---|---|---|---|---|---|---|---|---|---|---|---|---|---|---|
| | | | | | | | | | 37.78) | 42.38) | (0.30, 0.90) | | (0.53, 0.73) | (0.64, 0.82) | 0.90) | |
| Middle | — | — | — | — | — | — | — | — | 19.00 (15.01, 24.37) | 21.13 (18.55, 24.31) | 0.39 (0.12, 0.66) | 0.005 | 0.37 (0.29, 0.47) | 0.41 (0.36, 0.47) | 0.39 (0.11, 0.66) | 0.006 |
| Low-middle | — | — | — | — | — | — | — | — | 17.75 (10.47, 28.31) | 23.28 (16.24, 29.93) | 0.97 (0.42, 1.52) | 0.001 | 0.34 (0.20, 0.55) | 0.45 (0.31, 0.58) | 0.97 (0.42, 1.52) | <0.001 |
| Low | — | — | — | — | — | — | — | — | 24.13 (12.15, 41.45) | 22.54 (14.56, 32.26) | -0.24 (-0.47, -0.00) | 0.047 | 0.47 (0.24, 0.80) | 0.44 (0.28, 0.62) | -0.24 (-0.47, -0.00) | 0.046 |
| **21 GBD region** | | | | | | | | | | | | | | | | |
| South Asia | — | — | — | — | — | — | — | — | 16.56 (8.21, 28.78) | 21.02 (12.10, 29.16) | 0.85 (-0.06, 1.77) | 0.067 | 0.32 (0.16, 0.56) | 0.41 (0.23, 0.56) | 0.85 (-0.06, 1.77) | 0.066 |
| East Asia | — | — | — | — | — | — | — | — | 18.37 (12.97, 26.05) | 24.39 (19.40, 29.96) | 1.01 (0.56, 1.46) | <0.001 | 0.36 (0.25, 0.50) | 0.47 (0.38, 0.58) | 1.01 (0.56, 1.46) | <0.001 |
| North Africa and Middle East | — | — | — | — | — | — | — | — | 28.91 (17.43, 45.38) | 19.39 (15.10, 24.56) | -1.37 (-1.55, -1.19) | <0.001 | 0.56 (0.34, 0.88) | 0.38 (0.29, 0.48) | -1.37 (-1.55, -1.19) | <0.001 |
| Western Sub-Saharan Africa | — | — | — | — | — | — | — | — | 22.24 (12.49, 34.36) | 21.91 (15.28, 30.17) | -0.04 (-0.18, 0.10) | 0.592 | 0.43 (0.24, 0.67) | 0.42 (0.30, 0.58) | -0.04 (-0.18, 0.10) | 0.588 |
| Southeast Asia | — | — | — | — | — | — | — | — | 13.73 (9.88, 18.94) | 17.52 (13.37, 22.73) | 0.84 (0.55, 1.13) | <0.001 | 0.27 (0.19, 0.37) | 0.34 (0.26, 0.44) | 0.84 (0.56, 1.13) | <0.001 |
| Eastern Sub-Saharan Africa | — | — | — | — | — | — | — | — | 35.69 (18.21, 64.27) | 26.92 (17.05, 38.87) | -0.97 (-1.07, -0.86) | <0.001 | 0.69 (0.35, 1.24) | 0.52 (0.33, 0.75) | -0.97 (-1.07, -0.86) | <0.001 |
| Central Latin America | — | — | — | — | — | — | — | — | 20.47 (18.26, 23.37) | 20.62 (15.99, 26.01) | 0.01 (-0.26, 0.28) | 0.961 | 0.40 (0.35, 0.45) | 0.40 (0.31, 0.50) | 0.01 (-0.26, 0.28) | 0.959 |

| | | | | | | | | | | | | | | | | |
|---|---|---|---|---|---|---|---|---|---|---|---|---|---|---|---|---|
| Tropical Latin America | — | — | — | — | — | — | — | — | 53.84 (49.17, 59.52) | 56.09 (49.79, 62.94) | 0.17 (-0.20, 0.53) | 0.372 | 1.04 (0.95, 1.15) | 1.08 (0.96, 1.22) | 0.17 (-0.20, 0.53) | 0.372 |
| Central Sub-Saharan Africa | — | — | — | — | — | — | — | — | 30.80 (15.35, 55.94) | 26.19 (15.13, 43.61) | -0.59 (-0.84, -0.33) | <0.001 | 0.60 (0.30, 1.08) | 0.51 (0.29, 0.84) | -0.59 (-0.85, -0.33) | <0.001 |
| Western Europe | — | — | — | — | — | — | — | — | 33.39 (30.59, 36.42) | 25.22 (22.90, 27.82) | -1.07 (-1.29, -0.85) | <0.001 | 0.65 (0.59, 0.70) | 0.49 (0.44, 0.54) | -1.07 (-1.29, -0.85) | <0.001 |
| High-income North America | — | — | — | — | — | — | — | — | 40.56 (38.04, 43.60) | 38.18 (32.39, 43.01) | -0.19 (-0.79, 0.41) | 0.532 | 0.78 (0.74, 0.84) | 0.74 (0.63, 0.83) | -0.19 (-0.79, 0.41) | 0.531 |
| Central Asia | — | — | — | — | — | — | — | — | 19.10 (15.57, 25.33) | 28.43 (23.83, 34.67) | 1.21 (0.69, 1.73) | <0.001 | 0.37 (0.30, 0.49) | 0.55 (0.46, 0.67) | 1.21 (0.69, 1.73) | <0.001 |
| Eastern Europe | — | — | — | — | — | — | — | — | 40.34 (31.49, 50.72) | 78.46 (63.30, 101.28) | 2.46 (1.12, 3.82) | <0.001 | 0.78 (0.61, 0.98) | 1.52 (1.22, 1.96) | 2.46 (1.12, 3.82) | <0.001 |
| Southern Sub-Saharan Africa | — | — | — | — | — | — | — | — | 55.20 (42.97, 71.26) | 34.41 (24.77, 45.92) | -1.62 (-2.36, -0.88) | <0.001 | 1.07 (0.83, 1.38) | 0.67 (0.48, 0.89) | -1.62 (-2.36, -0.88) | <0.001 |
| High-income Asia Pacific | — | — | — | — | — | — | — | — | 21.15 (18.59, 24.40) | 33.77 (30.14, 38.71) | 1.65 (1.29, 2.00) | <0.001 | 0.41 (0.36, 0.47) | 0.65 (0.58, 0.75) | 1.64 (1.29, 2.00) | <0.001 |
| Central Europe | — | — | — | — | — | — | — | — | 42.96 (37.61, 48.19) | 48.92 (41.81, 56.63) | 0.38 (0.05, 0.72) | 0.025 | 0.83 (0.73, 0.93) | 0.95 (0.81, 1.10) | 0.38 (0.05, 0.72) | 0.025 |
| Andean Latin America | — | — | — | — | — | — | — | — | 19.24 (13.18, 26.30) | 16.65 (11.46, 23.78) | -0.45 (-0.89, -0.00) | 0.049 | 0.37 (0.25, 0.51) | 0.32 (0.22, 0.46) | -0.45 (-0.89, -0.00) | 0.048 |
| Caribbean | — | — | — | — | — | — | — | — | 24.13 (18.96, 30.94) | 25.08 (17.90, 34.16) | 0.17 (-0.02, 0.37) | 0.074 | 0.47 (0.37, 0.60) | 0.49 (0.35, 0.66) | 0.18 (-0.01, 0.37) | 0.070 |

| | | | | | | | | | | | | | | | | |
|---|---|---|---|---|---|---|---|---|---|---|---|---|---|---|---|---|
| Southern Latin America | — | — | — | — | — | — | — | — | 28.22 (22.45, 35.52) | 29.43 (23.19, 36.78) | 0.20 (-0.29, 0.69) | 0.418 | 0.55 (0.43, 0.69) | 0.57 (0.45, 0.71) | 0.20 (-0.29, 0.68) | 0.424 |
| Oceania | — | — | — | — | — | — | — | — | 48.38 (23.00, 93.27) | 43.78 (20.96, 81.65) | -0.34 (-0.43, -0.25) | <0.001 | 0.94 (0.45, 1.81) | 0.85 (0.41, 1.58) | -0.34 (-0.43, -0.25) | <0.001 |
| Australasia | — | — | — | — | — | — | — | — | 30.07 (23.74, 37.63) | 18.57 (12.94, 24.77) | -1.66 (-2.27, -1.05) | <0.001 | 0.58 (0.46, 0.73) | 0.36 (0.25, 0.48) | -1.66 (-2.27, -1.05) | <0.001 |
| **Endocarditis** | | | | | | | | | | | | | | | | |
| **15-19 years** | | | | | | | | | | | | | | | | |
| **Women** | | | | | | | | | | | | | | | | |
| Global | 4.23 (2.23, 6.88) | 3.76 (1.89, 6.16) | -0.39 (-0.50, -0.28) | <0.001 | 0.71 (0.44, 1.04) | 0.67 (0.41, 1.00) | -0.21 (-0.29, -0.13) | <0.001 | 7.27 (4.33, 9.34) | 6.22 (4.28, 8.13) | -0.52 (-0.70, -0.35) | <0.001 | 0.10 (0.06, 0.13) | 0.09 (0.06, 0.11) | -0.53 (-0.70, -0.35) | <0.001 |
| **SDI category** | | | | | | | | | | | | | | | | |
| High | 4.36 (2.14, 7.18) | 4.01 (2.05, 6.64) | -0.29 (-0.34, -0.24) | <0.001 | 1.09 (0.71, 1.52) | 0.95 (0.62, 1.33) | -0.44 (-0.48, -0.41) | <0.001 | 3.26 (2.27, 4.56) | 2.40 (1.40, 2.86) | -1.03 (-1.47, -0.59) | <0.001 | 0.04 (0.03, 0.06) | 0.03 (0.02, 0.04) | -1.04 (-1.50, -0.59) | <0.001 |
| High-middle | 5.10 (2.72, 8.33) | 4.25 (2.08, 7.11) | -0.61 (-0.72, -0.50) | <0.001 | 0.78 (0.49, 1.12) | 0.78 (0.47, 1.14) | -0.00 (-0.04, 0.04) | 0.929 | 6.35 (4.28, 7.86) | 5.29 (4.22, 6.91) | -0.57 (-1.06, -0.07) | 0.024 | 0.09 (0.06, 0.11) | 0.07 (0.06, 0.10) | -0.57 (-1.07, -0.07) | 0.024 |
| Middle | 4.90 (2.63, 7.86) | 4.08 (2.05, 6.70) | -0.62 (-0.69, -0.54) | <0.001 | 0.81 (0.50, 1.19) | 0.79 (0.47, 1.21) | -0.07 (-0.10, -0.05) | <0.001 | 8.61 (5.35, 11.32) | 7.65 (5.67, 10.24) | -0.40 (-0.58, -0.22) | <0.001 | 0.12 (0.07, 0.16) | 0.11 (0.08, 0.14) | -0.40 (-0.58, -0.22) | <0.001 |
| Low-middle | 3.02 (1.56, 4.95) | 3.44 (1.73, 5.66) | 0.51 (0.40, 0.61) | <0.001 | 0.43 (0.25, 0.64) | 0.49 (0.30, 0.75) | 0.52 (0.41, 0.64) | <0.001 | 8.09 (3.88, 13.01) | 6.90 (4.31, 9.98) | -0.56 (-0.74, -0.37) | <0.001 | 0.11 (0.05, 0.18) | 0.10 (0.06, 0.14) | -0.56 (-0.74, -0.38) | <0.001 |
| Low | 2.74 (1.41, 4.48) | 3.29 (1.69, 5.41) | 0.68 (0.53, 0.82) | <0.001 | 0.46 (0.26, 0.72) | 0.53 (0.30, 0.82) | 0.49 (0.44, 0.54) | <0.001 | 7.11 (2.76, 11.23) | 5.56 (3.00, 7.81) | -0.87 (-1.19, -0.54) | <0.001 | 0.10 (0.04, 0.16) | 0.08 (0.04, 0.11) | -0.87 (-1.21, -0.53) | <0.001 |
| **21 GBD region** | | | | | | | | | | | | | | | | |
| South Asia | 2.43 (1.24, | 3.30 (1.66, | 1.14 (0.99, | <0.001 | 0.30 (0.18, | 0.38 (0.23, | 0.84 (0.74, | <0.001 | 6.69 (2.52, | 5.82 (2.99, | -0.51 | 0.010 | 0.09 | 0.08 | -0.52 | 0.009 |

| | | | | | | | | | | | | | | | |
|---|---|---|---|---|---|---|---|---|---|---|---|---|---|---|---|
| | 4.04) | 5.37) | 1.29) | | 0.46) | 0.58) | 0.93) | | 12.69) | 9.59) | (-0.90, -0.12) | | (0.03, 0.18) | (0.04, 0.13) | (-0.91, -0.13) | |
| East Asia | 7.03 (3.91, 11.16) | 3.79 (1.75, 6.45) | -2.11 (-2.36, -1.86) | <0.001 | 0.80 (0.51, 1.15) | 0.62 (0.37, 0.92) | -0.84 (-0.95, -0.72) | <0.001 | 4.83 (1.94, 6.98) | 1.63 (1.18, 2.54) | -3.69 (-4.04, -3.34) | <0.001 | 0.07 (0.03, 0.10) | 0.02 (0.02, 0.04) | -3.75 (-4.11, -3.39) | <0.001 |
| North Africa and Middle East | 3.70 (1.88, 6.04) | 4.57 (2.34, 7.50) | 0.72 (0.69, 0.75) | <0.001 | 0.52 (0.32, 0.77) | 0.61 (0.37, 0.90) | 0.54 (0.52, 0.57) | <0.001 | 5.94 (3.45, 8.89) | 4.37 (2.72, 6.27) | -1.00 (-1.29, -0.72) | <0.001 | 0.08 (0.05, 0.12) | 0.06 (0.04, 0.09) | -1.02 (-1.30, -0.73) | <0.001 |
| Western Sub-Saharan Africa | 3.16 (1.64, 5.14) | 3.56 (1.80, 5.82) | 0.47 (0.26, 0.68) | <0.001 | 0.52 (0.30, 0.82) | 0.57 (0.33, 0.88) | 0.32 (0.24, 0.41) | <0.001 | 4.52 (1.71, 7.38) | 2.58 (1.58, 3.91) | -1.94 (-2.08, -1.81) | <0.001 | 0.06 (0.02, 0.10) | 0.04 (0.02, 0.05) | -1.97 (-2.11, -1.83) | <0.001 |
| Southeast Asia | 3.68 (1.92, 5.98) | 4.59 (2.35, 7.36) | 0.77 (0.74, 0.80) | <0.001 | 1.16 (0.67, 1.81) | 1.21 (0.73, 1.86) | 0.14 (0.08, 0.19) | <0.001 | 18.00 (10.53, 28.76) | 17.21 (11.73, 24.09) | -0.18 (-0.37, -0.00) | 0.048 | 0.25 (0.15, 0.40) | 0.24 (0.16, 0.34) | -0.18 (-0.37, -0.00) | 0.048 |
| Eastern Sub-Saharan Africa | 2.94 (1.57, 4.77) | 3.22 (1.67, 5.28) | 0.37 (0.26, 0.49) | <0.001 | 0.74 (0.39, 1.23) | 0.80 (0.42, 1.34) | 0.27 (0.18, 0.35) | <0.001 | 11.86 (4.00, 21.29) | 8.40 (3.90, 14.30) | -1.18 (-1.42, -0.94) | <0.001 | 0.17 (0.06, 0.30) | 0.12 (0.05, 0.20) | -1.19 (-1.43, -0.95) | <0.001 |
| Central Latin America | 3.43 (1.70, 5.65) | 3.87 (1.75, 6.56) | 0.40 (0.29, 0.52) | <0.001 | 0.62 (0.37, 0.93) | 0.66 (0.39, 1.02) | 0.25 (0.22, 0.28) | <0.001 | 5.70 (4.55, 8.45) | 6.72 (4.43, 8.82) | 0.72 (0.30, 1.14) | 0.001 | 0.08 (0.06, 0.12) | 0.09 (0.06, 0.12) | 0.72 (0.31, 1.14) | 0.001 |
| Tropical Latin America | 3.46 (1.74, 5.74) | 3.44 (1.49, 5.99) | -0.02 (-0.24, 0.20) | 0.841 | 0.89 (0.49, 1.43) | 0.95 (0.52, 1.54) | 0.23 (0.21, 0.25) | <0.001 | 15.37 (11.70, 21.69) | 13.01 (10.24, 22.14) | -0.46 (-0.62, -0.31) | <0.001 | 0.21 (0.16, 0.30) | 0.18 (0.14, 0.31) | -0.47 (-0.62, -0.31) | <0.001 |
| Central Sub-Saharan Africa | 2.55 (1.34, 4.12) | 2.95 (1.51, 4.86) | 0.55 (0.37, 0.73) | <0.001 | 0.69 (0.35, 1.21) | 0.72 (0.37, 1.25) | 0.20 (0.13, 0.26) | <0.001 | 9.40 (2.99, 18.90) | 7.61 (3.23, 14.59) | -0.73 (-1.02, -0.44) | <0.001 | 0.13 (0.04, 0.26) | 0.11 (0.04, 0.20) | -0.74 (-1.03, -0.44) | <0.001 |
| Western Europe | 4.08 (1.95, 6.78) | 4.22 (1.97, 7.03) | 0.11 (0.03, 0.20) | 0.009 | 0.90 (0.58, 1.29) | 0.79 (0.50, 1.16) | -0.48 (-0.61, -0.35) | <0.001 | 2.23 (1.69, 3.65) | 2.41 (1.22, 3.06) | 0.27 (0.01, 0.53) | 0.043 | 0.03 (0.02, 0.05) | 0.03 (0.02, 0.04) | 0.29 (0.03, 0.55) | 0.027 |
| High-income North America | 4.72 (2.34, 7.85) | 3.59 (1.92, 5.78) | -0.93 (-0.99, -0.87) | <0.001 | 1.22 (0.80, 1.76) | 1.03 (0.68, 1.50) | -0.54 (-0.66, -0.41) | <0.001 | 1.40 (1.11, 2.43) | 2.18 (1.13, 2.66) | 1.68 (0.81, 2.56) | <0.001 | 0.02 (0.01, 0.03) | 0.03 (0.01, 0.04) | 1.80 (0.89, 2.71) | <0.001 |
| Central Asia | 1.89 (0.89, | 2.42 (1.18, | 0.86 (0.81, | <0.001 | 0.19 (0.10, | 0.23 (0.12, | 0.69 (0.66, | <0.001 | 1.69 (1.22, | 3.18 (2.23, | 2.22 | <0.001 | 0.02 | 0.04 | 2.23 (1.67, | <0.001 |

| | | | | | | | | | | | | | | | | |
|---|---|---|---|---|---|---|---|---|---|---|---|---|---|---|---|---|
| | 3.37) | 4.07) | 0.91) | | 0.31) | 0.37) | 0.71) | | 2.78) | 4.68) | (1.66, 2.79) | | (0.02, 0.04) | (0.03, 0.07) | 2.80) | |
| Eastern Europe | 3.79 (1.87, 6.24) | 5.69 (2.91, 9.27) | 1.40 (1.27, 1.53) | <0.001 | 0.52 (0.31, 0.80) | 0.68 (0.41, 1.03) | 0.88 (0.85, 0.92) | <0.001 | 6.25 (5.09, 10.20) | 9.13 (5.60, 11.59) | 1.30 (0.29, 2.33) | 0.012 | 0.09 (0.07, 0.14) | 0.13 (0.08, 0.16) | 1.31 (0.29, 2.34) | 0.012 |
| Southern Sub-Saharan Africa | 4.61 (2.45, 7.38) | 4.43 (2.32, 7.16) | -0.11 (-0.31, 0.10) | 0.296 | 1.86 (0.96, 3.26) | 1.65 (0.85, 2.89) | -0.41 (-0.46, -0.36) | <0.001 | 22.73 (11.72, 32.07) | 9.59 (5.85, 15.85) | -2.80 (-3.42, -2.18) | <0.001 | 0.32 (0.16, 0.45) | 0.13 (0.08, 0.22) | -2.83 (-3.45, -2.21) | <0.001 |
| High-income Asia Pacific | 4.43 (2.17, 7.27) | 4.13 (2.00, 6.87) | -0.26 (-0.28, -0.23) | <0.001 | 1.31 (0.80, 1.91) | 1.22 (0.75, 1.77) | -0.25 (-0.30, -0.21) | <0.001 | 6.25 (3.48, 9.74) | 2.10 (1.59, 3.35) | -3.87 (-5.18, -2.53) | <0.001 | 0.09 (0.05, 0.13) | 0.03 (0.02, 0.05) | -3.97 (-5.30, -2.62) | <0.001 |
| Central Europe | 3.25 (1.52, 5.46) | 3.54 (1.68, 6.01) | 0.30 (0.24, 0.35) | <0.001 | 0.37 (0.22, 0.57) | 0.37 (0.21, 0.58) | 0.01 (-0.04, 0.06) | 0.665 | 2.26 (1.80, 3.54) | 2.56 (1.29, 3.39) | 0.49 (0.11, 0.87) | 0.011 | 0.03 (0.02, 0.05) | 0.04 (0.02, 0.05) | 0.50 (0.12, 0.88) | 0.010 |
| Andean Latin America | 2.17 (1.08, 3.66) | 3.55 (1.75, 5.90) | 1.73 (1.66, 1.79) | <0.001 | 0.46 (0.26, 0.73) | 0.61 (0.36, 0.96) | 1.02 (0.99, 1.05) | <0.001 | 11.28 (6.96, 17.64) | 8.02 (4.73, 12.84) | -1.21 (-1.87, -0.54) | <0.001 | 0.16 (0.10, 0.25) | 0.11 (0.07, 0.18) | -1.22 (-1.89, -0.54) | <0.001 |
| Caribbean | 3.75 (1.91, 6.14) | 5.94 (3.14, 9.43) | 1.60 (1.55, 1.66) | <0.001 | 0.59 (0.36, 0.88) | 0.73 (0.46, 1.08) | 0.78 (0.74, 0.82) | <0.001 | 10.26 (6.96, 14.75) | 12.16 (7.54, 19.89) | 0.78 (0.52, 1.04) | <0.001 | 0.14 (0.10, 0.21) | 0.17 (0.10, 0.28) | 0.78 (0.52, 1.04) | <0.001 |
| Southern Latin America | 2.76 (1.34, 4.68) | 4.77 (2.40, 7.94) | 1.92 (1.87, 1.97) | <0.001 | 0.77 (0.46, 1.18) | 0.91 (0.57, 1.38) | 0.56 (0.51, 0.61) | <0.001 | 5.65 (3.36, 8.75) | 6.54 (4.52, 9.66) | 0.46 (0.19, 0.73) | 0.001 | 0.08 (0.05, 0.12) | 0.09 (0.06, 0.13) | 0.46 (0.19, 0.73) | 0.001 |
| Oceania | 4.30 (2.23, 6.94) | 5.12 (2.83, 8.11) | 0.62 (0.56, 0.68) | <0.001 | 0.53 (0.33, 0.78) | 0.56 (0.35, 0.83) | 0.20 (0.18, 0.22) | <0.001 | 6.80 (3.64, 12.66) | 10.64 (5.11, 19.73) | 1.48 (0.94, 2.02) | <0.001 | 0.09 (0.05, 0.18) | 0.15 (0.07, 0.28) | 1.48 (0.94, 2.02) | <0.001 |
| Australasia | 3.62 (1.74, 6.05) | 3.68 (1.68, 6.32) | 0.05 (-0.01, 0.11) | 0.108 | 0.71 (0.45, 1.02) | 0.62 (0.37, 0.91) | -0.45 (-0.49, -0.40) | <0.001 | 2.32 (1.60, 3.91) | 2.35 (1.03, 3.54) | -0.08 (-1.20, 1.05) | 0.888 | 0.03 (0.02, 0.05) | 0.03 (0.01, 0.05) | -0.07 (-1.21, 1.08) | 0.902 |
| **Men** | | | | | | | | | | | | | | | | |
| Global | 4.80 (2.57, 7.77) | 4.51 (2.32, 7.31) | -0.20 (-0.33, -0.07) | 0.003 | 0.66 (0.42, 0.96) | 0.65 (0.40, 0.95) | -0.07 (-0.12, -0.01) | 0.024 | 8.07 (5.25, 10.12) | 7.13 (5.01, 9.13) | -0.41 (-0.59, -0.23) | <0.001 | 0.11 (0.07, 0.14) | 0.10 (0.07, 0.13) | -0.41 (-0.59, -0.23) | <0.001 |

| | | | | | | | | | | | | | | | | |
|---|---|---|---|---|---|---|---|---|---|---|---|---|---|---|---|---|
| **SDI category** | | | | | | | | | | | | | | | | |
| High | 5.13 (2.57, 8.41) | 4.53 (2.34, 7.32) | -0.43 (-0.47, -0.40) | <0.001 | 0.86 (0.56, 1.21) | 0.76 (0.51, 1.07) | -0.42 (-0.44, -0.40) | <0.001 | 3.81 (2.42, 5.30) | 2.75 (1.42, 3.46) | -1.13 (-1.36, -0.90) | <0.001 | 0.05 (0.03, 0.07) | 0.04 (0.02, 0.05) | -1.14 (-1.38, -0.90) | <0.001 |
| High-middle | 5.75 (3.13, 9.20) | 4.88 (2.44, 8.00) | -0.57 (-0.67, -0.47) | <0.001 | 0.76 (0.49, 1.09) | 0.76 (0.47, 1.10) | 0.00 (-0.03, 0.04) | 0.932 | 9.53 (6.05, 12.59) | 6.72 (5.33, 8.90) | -1.12 (-1.62, -0.61) | <0.001 | 0.13 (0.08, 0.18) | 0.09 (0.07, 0.12) | -1.12 (-1.63, -0.61) | <0.001 |
| Middle | 5.29 (2.87, 8.53) | 4.70 (2.39, 7.64) | -0.40 (-0.46, -0.33) | <0.001 | 0.74 (0.47, 1.08) | 0.75 (0.46, 1.10) | 0.03 (-0.01, 0.08) | 0.128 | 9.09 (6.14, 11.85) | 8.70 (6.18, 11.30) | -0.15 (-0.32, 0.03) | 0.100 | 0.13 (0.09, 0.16) | 0.12 (0.09, 0.16) | -0.15 (-0.32, 0.03) | 0.110 |
| Low-middle | 3.59 (1.88, 5.84) | 4.18 (2.16, 6.78) | 0.55 (0.41, 0.69) | <0.001 | 0.44 (0.26, 0.66) | 0.51 (0.31, 0.77) | 0.59 (0.46, 0.72) | <0.001 | 7.17 (4.45, 9.62) | 6.80 (4.69, 9.41) | -0.19 (-0.57, 0.19) | 0.330 | 0.10 (0.06, 0.13) | 0.09 (0.07, 0.13) | -0.19 (-0.57, 0.19) | 0.330 |
| Low | 3.59 (1.90, 5.76) | 4.42 (2.33, 7.21) | 0.74 (0.68, 0.80) | <0.001 | 0.45 (0.27, 0.68) | 0.54 (0.33, 0.82) | 0.69 (0.57, 0.80) | <0.001 | 8.60 (4.32, 15.05) | 7.57 (4.23, 12.03) | -0.44 (-0.81, -0.07) | 0.020 | 0.12 (0.06, 0.21) | 0.11 (0.06, 0.17) | -0.44 (-0.81, -0.07) | 0.020 |
| **21 GBD region** | | | | | | | | | | | | | | | | |
| South Asia | 2.94 (1.49, 4.85) | 3.91 (1.99, 6.45) | 1.00 (0.90, 1.10) | <0.001 | 0.33 (0.20, 0.51) | 0.42 (0.25, 0.64) | 0.85 (0.80, 0.90) | <0.001 | 4.62 (2.86, 6.72) | 4.65 (3.01, 6.95) | 0.06 (-1.51, 1.64) | 0.945 | 0.06 (0.04, 0.09) | 0.06 (0.04, 0.10) | 0.06 (-1.50, 1.64) | 0.945 |
| East Asia | 7.28 (4.08, 11.43) | 3.71 (1.75, 6.19) | -2.23 (-2.61, -1.83) | <0.001 | 0.79 (0.51, 1.14) | 0.59 (0.36, 0.89) | -0.96 (-1.15, -0.77) | <0.001 | 8.38 (3.25, 12.80) | 2.36 (1.67, 3.81) | -4.19 (-5.01, -3.36) | <0.001 | 0.12 (0.04, 0.18) | 0.03 (0.02, 0.05) | -4.22 (-5.05, -3.39) | <0.001 |
| North Africa and Middle East | 5.33 (2.79, 8.59) | 6.61 (3.57, 10.61) | 0.75 (0.70, 0.80) | <0.001 | 0.70 (0.44, 1.00) | 0.83 (0.53, 1.19) | 0.61 (0.57, 0.65) | <0.001 | 8.09 (5.07, 12.68) | 6.54 (3.88, 10.59) | -0.71 (-0.86, -0.56) | <0.001 | 0.11 (0.07, 0.18) | 0.09 (0.05, 0.15) | -0.71 (-0.86, -0.56) | <0.001 |
| Western Sub-Saharan Africa | 4.60 (2.56, 7.42) | 5.28 (2.92, 8.57) | 0.55 (0.32, 0.78) | <0.001 | 0.52 (0.32, 0.78) | 0.59 (0.36, 0.87) | 0.46 (0.30, 0.61) | <0.001 | 4.82 (2.50, 8.07) | 3.80 (2.37, 5.83) | -0.81 (-1.00, -0.62) | <0.001 | 0.07 (0.03, 0.11) | 0.05 (0.03, 0.08) | -0.82 (-1.01, -0.63) | <0.001 |
| Southeast Asia | 3.85 (1.99, 6.24) | 4.68 (2.46, 7.51) | 0.71 (0.63, 0.79) | <0.001 | 0.92 (0.55, 1.36) | 0.99 (0.60, 1.46) | 0.27 (0.23, 0.30) | <0.001 | 15.38 (10.21, 24.30) | 18.82 (12.25, 26.00) | 0.80 (0.42, 1.18) | <0.001 | 0.21 (0.14, 0.34) | 0.26 (0.17, 0.36) | 0.80 (0.42, 1.19) | <0.001 |
| Eastern Sub-Saharan | 3.95 (2.10, | 4.57 (2.42, | 0.54 (0.40, | <0.001 | 0.67 (0.39, | 0.76 (0.44, | 0.44 (0.36, | <0.001 | 16.14 (7.30, | 13.32 (6.86, | -0.66 | <0.001 | 0.23 | 0.19 | -0.66 | <0.001 |

| | | | | | | | | | | | | | | | | |
|---|---|---|---|---|---|---|---|---|---|---|---|---|---|---|---|---|
| Africa | 6.20) | 7.26) | 0.68) | | 1.05) | 1.17) | 0.51) | | 30.86) | 23.06) | (-0.78, -0.54) | | (0.10, 0.43) | (0.09, 0.32) | (-0.79, -0.54) | |
| Central Latin America | 3.51 (1.74, 5.88) | 4.85 (2.24, 8.18) | 1.08 (1.02, 1.15) | <0.001 | 0.51 (0.31, 0.78) | 0.66 (0.40, 1.02) | 0.85 (0.81, 0.89) | <0.001 | 7.04 (5.41, 10.05) | 7.91 (5.15, 10.56) | 0.44 (0.16, 0.73) | 0.002 | 0.10 (0.08, 0.14) | 0.11 (0.07, 0.15) | 0.44 (0.16, 0.73) | 0.002 |
| Tropical Latin America | 3.89 (2.08, 6.25) | 4.50 (2.05, 7.66) | 0.50 (0.36, 0.64) | <0.001 | 0.75 (0.43, 1.14) | 0.87 (0.50, 1.34) | 0.52 (0.45, 0.60) | <0.001 | 19.90 (14.09, 26.83) | 16.71 (12.75, 29.04) | -0.61 (-1.06, -0.16) | 0.007 | 0.28 (0.20, 0.38) | 0.23 (0.18, 0.41) | -0.61 (-1.06, -0.16) | 0.007 |
| Central Sub-Saharan Africa | 3.17 (1.66, 5.17) | 3.76 (1.92, 6.16) | 0.61 (0.56, 0.66) | <0.001 | 0.51 (0.29, 0.81) | 0.56 (0.32, 0.88) | 0.41 (0.33, 0.48) | <0.001 | 10.95 (5.02, 24.96) | 8.66 (3.90, 17.08) | -0.86 (-1.17, -0.54) | <0.001 | 0.15 (0.07, 0.35) | 0.12 (0.05, 0.24) | -0.86 (-1.18, -0.54) | <0.001 |
| Western Europe | 4.76 (2.31, 7.85) | 4.82 (2.32, 7.96) | 0.04 (-0.05, 0.13) | 0.392 | 0.75 (0.48, 1.08) | 0.68 (0.43, 1.01) | -0.35 (-0.41, -0.30) | <0.001 | 2.78 (2.05, 4.81) | 3.18 (1.28, 4.37) | 0.43 (0.15, 0.72) | 0.003 | 0.04 (0.03, 0.07) | 0.04 (0.02, 0.06) | 0.45 (0.16, 0.74) | 0.002 |
| High-income North America | 5.35 (2.71, 8.76) | 3.92 (2.15, 6.25) | -1.00 (-1.09, -0.91) | <0.001 | 0.88 (0.57, 1.23) | 0.75 (0.51, 1.04) | -0.50 (-0.56, -0.44) | <0.001 | 1.67 (1.29, 3.03) | 2.45 (1.22, 3.10) | 1.32 (0.63, 2.01) | <0.001 | 0.02 (0.02, 0.04) | 0.03 (0.02, 0.04) | 1.38 (0.66, 2.10) | <0.001 |
| Central Asia | 2.84 (1.40, 4.80) | 3.44 (1.69, 5.68) | 0.68 (0.61, 0.74) | <0.001 | 0.28 (0.16, 0.45) | 0.34 (0.19, 0.53) | 0.64 (0.56, 0.71) | <0.001 | 3.03 (1.87, 4.58) | 4.81 (3.02, 6.86) | 1.58 (1.04, 2.13) | <0.001 | 0.04 (0.03, 0.06) | 0.07 (0.04, 0.10) | 1.58 (1.04, 2.13) | <0.001 |
| Eastern Europe | 4.29 (2.15, 6.98) | 6.21 (3.17, 10.16) | 1.29 (1.20, 1.38) | <0.001 | 0.50 (0.31, 0.75) | 0.67 (0.41, 1.01) | 1.00 (0.91, 1.09) | <0.001 | 8.43 (6.25, 11.75) | 10.34 (6.59, 14.10) | 0.76 (-0.06, 1.59) | 0.068 | 0.12 (0.09, 0.16) | 0.14 (0.09, 0.20) | 0.77 (-0.06, 1.59) | 0.067 |
| Southern Sub-Saharan Africa | 5.06 (2.73, 7.89) | 5.64 (3.05, 8.81) | 0.36 (0.31, 0.42) | <0.001 | 1.51 (0.81, 2.59) | 1.47 (0.80, 2.44) | -0.09 (-0.15, -0.02) | 0.008 | 16.12 (10.23, 23.88) | 14.72 (8.86, 21.98) | -0.34 (-1.04, 0.37) | 0.345 | 0.22 (0.14, 0.33) | 0.20 (0.12, 0.31) | -0.34 (-1.05, 0.38) | 0.350 |
| High-income Asia Pacific | 5.57 (2.83, 8.93) | 4.45 (2.18, 7.31) | -0.78 (-0.83, -0.72) | <0.001 | 1.06 (0.68, 1.48) | 0.93 (0.59, 1.32) | -0.46 (-0.51, -0.42) | <0.001 | 6.33 (2.76, 11.65) | 2.19 (1.09, 2.92) | -3.56 (-4.07, -3.04) | <0.001 | 0.09 (0.04, 0.16) | 0.03 (0.01, 0.04) | -3.62 (-4.14, -3.09) | <0.001 |
| Central Europe | 4.06 (1.99, 6.68) | 4.33 (2.17, 7.02) | 0.21 (0.18, 0.24) | <0.001 | 0.43 (0.26, 0.65) | 0.44 (0.27, 0.67) | 0.08 (0.06, 0.11) | <0.001 | 3.74 (2.92, 5.64) | 4.10 (2.06, 5.61) | 0.39 (0.03, 0.74) | 0.032 | 0.05 (0.04, 0.08) | 0.06 (0.03, 0.08) | 0.39 (0.04, 0.75) | 0.031 |
| Andean Latin | 2.86 (1.47, | 4.29 (2.18, | 1.41 (1.36, | <0.001 | 0.55 (0.33, | 0.73 (0.43, | 0.97 (0.95, | <0.001 | 16.56 (9.26, | 12.78 (7.48, | -0.87 | <0.001 | 0.23 | 0.18 | -0.87 | <0.001 |

| | | | | | | | | | | | | | | | | |
|---|---|---|---|---|---|---|---|---|---|---|---|---|---|---|---|---|
| America | 4.71) | 6.99) | 1.47) | | 0.84) | 1.11) | 1.00) | | 28.15) | 21.20) | (-1.22, -0.51) | | (0.13, 0.39) | (0.10, 0.30) | (-1.22, -0.52) | |
| Caribbean | 3.89 (1.97, 6.35) | 5.90 (3.10, 9.41) | 1.46 (1.38, 1.54) | <0.001 | 0.57 (0.35, 0.84) | 0.74 (0.47, 1.07) | 0.91 (0.87, 0.96) | <0.001 | 10.78 (7.63, 15.39) | 12.93 (8.04, 21.75) | 0.63 (0.10, 1.16) | 0.020 | 0.15 (0.11, 0.22) | 0.18 (0.11, 0.30) | 0.63 (0.10, 1.17) | 0.019 |
| Southern Latin America | 3.12 (1.55, 5.35) | 5.34 (2.71, 8.75) | 1.85 (1.73, 1.97) | <0.001 | 0.69 (0.44, 1.00) | 0.86 (0.54, 1.24) | 0.75 (0.71, 0.78) | <0.001 | 6.25 (3.57, 9.70) | 8.75 (5.43, 12.90) | 1.13 (0.89, 1.37) | <0.001 | 0.09 (0.05, 0.14) | 0.12 (0.08, 0.18) | 1.14 (0.90, 1.38) | <0.001 |
| Oceania | 3.80 (1.96, 6.18) | 4.39 (2.32, 6.96) | 0.53 (0.47, 0.58) | <0.001 | 0.60 (0.38, 0.89) | 0.61 (0.38, 0.89) | 0.05 (-0.01, 0.11) | 0.095 | 16.90 (9.14, 31.34) | 24.43 (11.94, 44.30) | 1.25 (0.96, 1.55) | <0.001 | 0.24 (0.13, 0.44) | 0.34 (0.17, 0.62) | 1.26 (0.96, 1.55) | <0.001 |
| Australasia | 4.09 (1.97, 6.85) | 3.84 (1.87, 6.41) | -0.22 (-0.27, -0.17) | <0.001 | 0.65 (0.42, 0.93) | 0.55 (0.34, 0.79) | -0.58 (-0.63, -0.54) | <0.001 | 3.98 (2.52, 7.11) | 3.18 (1.59, 5.02) | -0.78 (-1.38, -0.17) | 0.012 | 0.06 (0.03, 0.10) | 0.04 (0.02, 0.07) | -0.77 (-1.38, -0.16) | 0.013 |
| **20-24 years** | | | | | | | | | | | | | | | | |
| **Women** | | | | | | | | | | | | | | | | |
| Global | 4.48 (2.39, 7.19) | 4.17 (2.22, 6.64) | -0.23 (-0.28, -0.18) | <0.001 | 0.92 (0.59, 1.33) | 0.89 (0.56, 1.31) | -0.14 (-0.18, -0.10) | <0.001 | 10.03 (5.91, 13.24) | 9.61 (6.71, 12.19) | -0.15 (-0.35, 0.05) | 0.140 | 0.15 (0.09, 0.20) | 0.14 (0.10, 0.18) | -0.15 (-0.35, 0.05) | 0.142 |
| **SDI category** | | | | | | | | | | | | | | | | |
| High | 5.08 (2.69, 8.05) | 4.83 (2.73, 7.54) | -0.16 (-0.20, -0.13) | <0.001 | 2.10 (1.37, 3.03) | 1.96 (1.29, 2.82) | -0.21 (-0.29, -0.13) | <0.001 | 4.97 (3.67, 7.24) | 5.22 (2.92, 6.28) | 0.20 (-0.08, 0.49) | 0.170 | 0.07 (0.05, 0.11) | 0.08 (0.04, 0.09) | 0.21 (-0.08, 0.51) | 0.158 |
| High-middle | 5.34 (2.81, 8.59) | 4.90 (2.59, 7.78) | -0.30 (-0.35, -0.26) | <0.001 | 0.89 (0.56, 1.29) | 0.95 (0.60, 1.37) | 0.19 (0.15, 0.23) | <0.001 | 7.89 (5.38, 9.99) | 7.33 (5.19, 9.08) | -0.29 (-0.78, 0.20) | 0.245 | 0.12 (0.08, 0.15) | 0.11 (0.08, 0.14) | -0.30 (-0.79, 0.20) | 0.240 |
| Middle | 4.99 (2.67, 7.97) | 4.50 (2.39, 7.20) | -0.34 (-0.40, -0.28) | <0.001 | 0.88 (0.54, 1.30) | 0.91 (0.56, 1.35) | 0.14 (0.02, 0.27) | 0.024 | 10.90 (7.23, 15.05) | 10.25 (7.59, 13.76) | -0.21 (-0.44, 0.02) | 0.080 | 0.16 (0.11, 0.22) | 0.15 (0.11, 0.21) | -0.21 (-0.44, 0.02) | 0.076 |
| Low-middle | 3.16 (1.68, 5.08) | 3.71 (1.95, 5.95) | 0.59 (0.53, 0.66) | <0.001 | 0.48 (0.29, 0.71) | 0.58 (0.35, 0.86) | 0.68 (0.63, 0.73) | <0.001 | 12.47 (5.65, 20.07) | 11.25 (6.92, 16.78) | -0.38 (-0.82, 0.07) | 0.097 | 0.19 (0.08, 0.30) | 0.17 (0.10, 0.25) | -0.38 (-0.82, 0.07) | 0.096 |
| Low | 2.77 (1.46, | 3.31 (1.72, | 0.68 (0.54, | <0.001 | 0.57 (0.30, | 0.66 (0.36, | 0.52 (0.49, | <0.001 | 12.87 (4.53, | 10.32 (6.01, | -0.76 | <0.001 | 0.19 | 0.15 | -0.77 | <0.001 |

| | | | | | | | | | | | | | | | | |
|---|---|---|---|---|---|---|---|---|---|---|---|---|---|---|---|---|
| | 4.45) | 5.42) | 0.81) | | 0.94) | 1.08) | 0.55) | | 21.04) | 14.49) | (-0.94, -0.58) | | (0.07, 0.32) | (0.09, 0.22) | (-0.95, -0.59) | |
| **21 GBD region** | | | | | | | | | | | | | | | | |
| South Asia | 2.59 (1.32, 4.24) | 3.55 (1.84, 5.72) | 1.15 (1.03, 1.27) | <0.001 | 0.32 (0.20, 0.50) | 0.41 (0.25, 0.62) | 0.86 (0.77, 0.95) | <0.001 | 10.89 (4.17, 19.99) | 10.02 (5.08, 16.55) | -0.37 (-1.02, 0.29) | 0.272 | 0.16 (0.06, 0.30) | 0.15 (0.08, 0.25) | -0.37 (-1.03, 0.29) | 0.273 |
| East Asia | 6.58 (3.46, 10.60) | 4.16 (2.06, 6.85) | -1.51 (-1.83, -1.19) | <0.001 | 0.80 (0.50, 1.21) | 0.72 (0.43, 1.08) | -0.36 (-0.45, -0.27) | <0.001 | 5.39 (2.52, 7.98) | 2.22 (1.51, 3.34) | -3.04 (-4.09, -1.98) | <0.001 | 0.08 (0.04, 0.12) | 0.03 (0.02, 0.05) | -3.09 (-4.15, -2.02) | <0.001 |
| North Africa and Middle East | 3.51 (1.78, 5.83) | 4.30 (2.19, 7.02) | 0.69 (0.65, 0.73) | <0.001 | 0.60 (0.37, 0.87) | 0.67 (0.41, 0.97) | 0.38 (0.35, 0.40) | <0.001 | 7.57 (4.83, 11.94) | 5.15 (3.56, 7.63) | -1.31 (-1.47, -1.15) | <0.001 | 0.11 (0.07, 0.18) | 0.08 (0.05, 0.11) | -1.32 (-1.48, -1.16) | <0.001 |
| Western Sub-Saharan Africa | 3.03 (1.58, 4.89) | 3.36 (1.72, 5.52) | 0.43 (0.32, 0.53) | <0.001 | 0.87 (0.43, 1.51) | 0.96 (0.48, 1.62) | 0.35 (0.24, 0.45) | <0.001 | 14.34 (5.63, 23.50) | 8.77 (5.45, 13.64) | -1.68 (-1.80, -1.56) | <0.001 | 0.21 (0.08, 0.35) | 0.13 (0.08, 0.20) | -1.70 (-1.82, -1.58) | <0.001 |
| Southeast Asia | 4.50 (2.60, 6.88) | 5.74 (3.34, 8.73) | 0.84 (0.81, 0.88) | <0.001 | 1.44 (0.88, 2.15) | 1.53 (0.95, 2.26) | 0.20 (0.12, 0.27) | <0.001 | 23.65 (14.64, 37.94) | 22.67 (15.37, 31.50) | -0.15 (-0.46, 0.17) | 0.370 | 0.35 (0.22, 0.57) | 0.34 (0.23, 0.47) | -0.15 (-0.47, 0.17) | 0.367 |
| Eastern Sub-Saharan Africa | 3.03 (1.66, 4.78) | 3.22 (1.69, 5.26) | 0.26 (0.17, 0.34) | <0.001 | 0.86 (0.42, 1.47) | 0.94 (0.47, 1.61) | 0.31 (0.26, 0.36) | <0.001 | 18.50 (5.47, 33.07) | 12.71 (5.99, 21.15) | -1.32 (-1.57, -1.07) | <0.001 | 0.28 (0.08, 0.50) | 0.19 (0.09, 0.32) | -1.33 (-1.58, -1.08) | <0.001 |
| Central Latin America | 3.78 (1.96, 6.10) | 4.42 (2.22, 7.32) | 0.54 (0.45, 0.64) | <0.001 | 0.75 (0.46, 1.11) | 0.81 (0.49, 1.20) | 0.24 (0.21, 0.27) | <0.001 | 6.78 (5.30, 10.36) | 8.26 (5.34, 11.21) | 0.77 (0.26, 1.28) | 0.003 | 0.10 (0.08, 0.15) | 0.12 (0.08, 0.17) | 0.77 (0.26, 1.29) | 0.003 |
| Tropical Latin America | 3.86 (2.06, 6.16) | 4.57 (2.30, 7.45) | 0.58 (0.42, 0.74) | <0.001 | 0.91 (0.52, 1.43) | 1.00 (0.59, 1.54) | 0.34 (0.31, 0.36) | <0.001 | 18.31 (12.88, 27.79) | 15.71 (12.25, 26.02) | -0.52 (-0.89, -0.14) | 0.007 | 0.27 (0.19, 0.42) | 0.23 (0.18, 0.39) | -0.52 (-0.90, -0.14) | 0.007 |
| Central Sub-Saharan Africa | 2.46 (1.29, 3.98) | 2.88 (1.47, 4.69) | 0.59 (0.46, 0.72) | <0.001 | 0.82 (0.38, 1.45) | 0.86 (0.41, 1.57) | 0.17 (0.09, 0.25) | <0.001 | 13.74 (3.97, 28.92) | 11.42 (4.83, 22.74) | -0.71 (-0.89, -0.54) | <0.001 | 0.21 (0.06, 0.43) | 0.17 (0.07, 0.34) | -0.72 (-0.90, -0.54) | <0.001 |
| Western Europe | 5.04 (2.69, 7.99) | 5.27 (2.81, 8.36) | 0.14 (0.10, 0.19) | <0.001 | 1.31 (0.88, 1.78) | 1.08 (0.72, 1.50) | -0.67 (-0.80, -0.55) | <0.001 | 3.52 (2.54, 6.16) | 3.96 (2.02, 4.94) | 0.40 (0.03, 0.78) | 0.036 | 0.05 (0.04, 0.09) | 0.06 (0.03, 0.07) | 0.43 (0.05, 0.82) | 0.028 |

| | | | | | | | | | | | | | | | | |
|---|---|---|---|---|---|---|---|---|---|---|---|---|---|---|---|---|
| High-income North America | 5.36 (2.82, 8.54) | 4.48 (2.77, 6.64) | -0.59 (-0.68, -0.50) | <0.001 | 3.31 (2.01, 5.07) | 3.11 (1.87, 4.81) | -0.18 (-0.27, -0.09) | <0.001 | 3.70 (2.82, 6.88) | 6.93 (3.17, 8.70) | 2.14 (1.54, 2.74) | <0.001 | 0.05 (0.04, 0.10) | 0.10 (0.04, 0.13) | 2.33 (1.62, 3.05) | <0.001 |
| Central Asia | 2.13 (1.03, 3.58) | 2.70 (1.33, 4.44) | 0.83 (0.79, 0.88) | <0.001 | 0.21 (0.12, 0.34) | 0.25 (0.14, 0.40) | 0.57 (0.54, 0.59) | <0.001 | 3.63 (2.56, 5.31) | 5.13 (3.69, 7.88) | 1.17 (0.78, 1.56) | <0.001 | 0.05 (0.04, 0.08) | 0.08 (0.06, 0.12) | 1.17 (0.78, 1.56) | <0.001 |
| Eastern Europe | 4.30 (2.27, 6.91) | 7.41 (4.27, 11.05) | 1.93 (1.79, 2.06) | <0.001 | 0.53 (0.32, 0.81) | 0.78 (0.48, 1.15) | 1.31 (1.25, 1.37) | <0.001 | 7.06 (4.84, 9.66) | 15.57 (5.96, 22.36) | 2.89 (1.74, 4.05) | <0.001 | 0.11 (0.07, 0.14) | 0.23 (0.09, 0.34) | 2.90 (1.75, 4.07) | <0.001 |
| Southern Sub-Saharan Africa | 5.27 (3.06, 8.04) | 4.62 (2.46, 7.41) | -0.46 (-0.62, -0.29) | <0.001 | 1.65 (0.84, 2.78) | 1.45 (0.72, 2.44) | -0.43 (-0.49, -0.37) | <0.001 | 35.12 (18.06, 50.31) | 13.73 (7.08, 24.30) | -3.10 (-4.45, -1.73) | <0.001 | 0.53 (0.27, 0.76) | 0.20 (0.10, 0.36) | -3.12 (-4.48, -1.74) | <0.001 |
| High-income Asia Pacific | 5.11 (2.74, 8.15) | 4.66 (2.46, 7.44) | -0.33 (-0.35, -0.31) | <0.001 | 1.98 (1.21, 2.95) | 1.73 (1.08, 2.55) | -0.48 (-0.51, -0.45) | <0.001 | 8.34 (4.91, 12.56) | 3.24 (2.47, 4.93) | -3.25 (-3.84, -2.65) | <0.001 | 0.12 (0.07, 0.19) | 0.05 (0.03, 0.07) | -3.33 (-3.94, -2.72) | <0.001 |
| Central Europe | 3.69 (1.90, 6.05) | 4.19 (2.21, 6.81) | 0.44 (0.40, 0.49) | <0.001 | 0.52 (0.32, 0.76) | 0.54 (0.33, 0.81) | 0.14 (0.10, 0.17) | <0.001 | 3.88 (3.04, 5.94) | 4.60 (2.35, 6.15) | 0.52 (0.15, 0.90) | 0.005 | 0.06 (0.05, 0.09) | 0.07 (0.03, 0.09) | 0.53 (0.15, 0.90) | 0.005 |
| Andean Latin America | 2.54 (1.32, 4.11) | 4.39 (2.35, 6.95) | 1.91 (1.89, 1.94) | <0.001 | 0.45 (0.28, 0.67) | 0.64 (0.39, 0.93) | 1.18 (1.15, 1.20) | <0.001 | 12.00 (6.59, 19.86) | 7.62 (4.57, 12.25) | -1.68 (-2.60, -0.74) | <0.001 | 0.18 (0.10, 0.30) | 0.11 (0.07, 0.18) | -1.69 (-2.62, -0.75) | <0.001 |
| Caribbean | 4.15 (2.26, 6.58) | 6.91 (4.01, 10.37) | 1.78 (1.75, 1.80) | <0.001 | 0.77 (0.48, 1.12) | 0.96 (0.62, 1.39) | 0.80 (0.74, 0.85) | <0.001 | 18.93 (12.46, 30.11) | 25.24 (14.82, 45.59) | 1.02 (0.71, 1.33) | <0.001 | 0.28 (0.19, 0.45) | 0.38 (0.22, 0.68) | 1.02 (0.71, 1.33) | <0.001 |
| Southern Latin America | 3.24 (1.67, 5.26) | 5.70 (3.00, 8.97) | 1.98 (1.94, 2.02) | <0.001 | 1.21 (0.77, 1.75) | 1.29 (0.82, 1.88) | 0.22 (0.17, 0.26) | <0.001 | 8.78 (5.44, 14.25) | 9.82 (6.44, 15.90) | 0.47 (0.14, 0.79) | 0.005 | 0.13 (0.08, 0.21) | 0.15 (0.10, 0.24) | 0.47 (0.14, 0.80) | 0.005 |
| Oceania | 4.98 (2.86, 7.54) | 6.10 (3.76, 9.17) | 0.70 (0.68, 0.73) | <0.001 | 0.77 (0.51, 1.11) | 0.79 (0.52, 1.16) | 0.08 (0.03, 0.13) | 0.002 | 32.64 (18.14, 57.30) | 44.84 (22.16, 79.98) | 1.12 (0.75, 1.49) | <0.001 | 0.49 (0.27, 0.86) | 0.67 (0.33, 1.20) | 1.12 (0.76, 1.49) | <0.001 |
| Australasia | 4.46 (2.35, 7.18) | 4.73 (2.42, 7.56) | 0.20 (0.08, 0.31) | 0.001 | 1.36 (0.94, 1.88) | 1.10 (0.73, 1.53) | -0.75 (-0.78, -0.72) | <0.001 | 4.49 (2.92, 7.21) | 4.75 (2.07, 7.38) | 0.24 (-0.68, 1.18) | 0.610 | 0.07 (0.04, 0.11) | 0.07 (0.03, 0.11) | 0.27 (-0.67, 1.22) | 0.576 |

| | | | | | | | | | | | | | | | | |
|---|---|---|---|---|---|---|---|---|---|---|---|---|---|---|---|---|
| **Men** | | | | | | | | | | | | | | | | |
| Global | 5.03 (2.68, 7.97) | 5.02 (2.74, 7.86) | 0.01 (-0.04, 0.05) | 0.724 | 0.85 (0.54, 1.22) | 0.86 (0.55, 1.25) | 0.07 (0.03, 0.11) | <0.001 | 12.76 (9.52, 15.89) | 12.99 (9.96, 16.84) | 0.05 (-0.16, 0.25) | 0.657 | 0.19 (0.14, 0.24) | 0.19 (0.15, 0.25) | 0.05 (-0.15, 0.25) | 0.645 |
| **SDI category** | | | | | | | | | | | | | | | | |
| High | 5.79 (3.10, 9.26) | 5.67 (3.27, 8.68) | -0.08 (-0.12, -0.04) | <0.001 | 1.45 (0.97, 2.03) | 1.44 (0.96, 1.99) | -0.03 (-0.08, 0.02) | 0.245 | 6.60 (5.00, 9.44) | 7.19 (3.87, 8.86) | 0.34 (-0.09, 0.78) | 0.117 | 0.10 (0.07, 0.14) | 0.11 (0.06, 0.13) | 0.35 (-0.08, 0.79) | 0.112 |
| High-middle | 6.07 (3.36, 9.59) | 5.87 (3.26, 8.98) | -0.12 (-0.16, -0.07) | <0.001 | 0.93 (0.60, 1.34) | 1.01 (0.66, 1.43) | 0.30 (0.27, 0.33) | <0.001 | 14.98 (10.69, 18.70) | 12.91 (10.11, 15.89) | -0.49 (-1.21, 0.23) | 0.185 | 0.22 (0.16, 0.28) | 0.19 (0.15, 0.24) | -0.49 (-1.21, 0.24) | 0.185 |
| Middle | 5.31 (2.80, 8.49) | 5.24 (2.83, 8.22) | -0.04 (-0.08, -0.01) | 0.010 | 0.86 (0.53, 1.25) | 0.93 (0.58, 1.37) | 0.29 (0.23, 0.34) | <0.001 | 13.81 (10.70, 19.11) | 14.61 (11.48, 19.74) | 0.22 (-0.02, 0.46) | 0.071 | 0.21 (0.16, 0.29) | 0.22 (0.17, 0.30) | 0.22 (-0.02, 0.46) | 0.069 |
| Low-middle | 3.66 (1.92, 5.94) | 4.42 (2.35, 7.05) | 0.67 (0.58, 0.76) | <0.001 | 0.48 (0.30, 0.72) | 0.59 (0.36, 0.88) | 0.72 (0.64, 0.80) | <0.001 | 11.78 (7.99, 15.29) | 12.30 (8.91, 16.79) | 0.17 (-0.11, 0.46) | 0.235 | 0.18 (0.12, 0.23) | 0.18 (0.13, 0.25) | 0.17 (-0.11, 0.46) | 0.235 |
| Low | 3.54 (1.91, 5.62) | 4.39 (2.34, 6.90) | 0.78 (0.69, 0.87) | <0.001 | 0.56 (0.32, 0.88) | 0.67 (0.39, 1.06) | 0.69 (0.64, 0.74) | <0.001 | 14.83 (8.22, 24.28) | 14.85 (9.64, 21.96) | 0.01 (-0.14, 0.16) | 0.889 | 0.22 (0.12, 0.36) | 0.22 (0.14, 0.33) | 0.01 (-0.14, 0.16) | 0.894 |
| **21 GBD region** | | | | | | | | | | | | | | | | |
| South Asia | 3.04 (1.55, 5.00) | 4.12 (2.14, 6.70) | 1.07 (0.94, 1.20) | <0.001 | 0.31 (0.19, 0.49) | 0.41 (0.24, 0.64) | 0.94 (0.86, 1.01) | <0.001 | 8.35 (5.41, 11.44) | 9.03 (6.01, 12.84) | 0.28 (-0.33, 0.89) | 0.366 | 0.13 (0.08, 0.17) | 0.14 (0.09, 0.19) | 0.28 (-0.32, 0.89) | 0.360 |
| East Asia | 6.72 (3.51, 10.76) | 4.35 (2.23, 7.08) | -1.45 (-1.71, -1.20) | <0.001 | 0.89 (0.55, 1.32) | 0.84 (0.51, 1.26) | -0.17 (-0.27, -0.07) | 0.001 | 11.82 (5.71, 16.51) | 5.97 (4.28, 8.18) | -2.23 (-2.54, -1.91) | <0.001 | 0.18 (0.08, 0.25) | 0.09 (0.06, 0.12) | -2.24 (-2.56, -1.92) | <0.001 |
| North Africa and Middle East | 5.56 (2.98, 8.86) | 6.77 (3.57, 10.71) | 0.68 (0.66, 0.70) | <0.001 | 0.93 (0.60, 1.32) | 1.07 (0.69, 1.53) | 0.48 (0.45, 0.52) | <0.001 | 13.52 (8.70, 21.95) | 10.84 (6.92, 16.67) | -0.72 (-0.88, -0.55) | <0.001 | 0.20 (0.13, 0.33) | 0.16 (0.10, 0.25) | -0.72 (-0.89, -0.56) | <0.001 |
| Western Sub-Saharan Africa | 3.89 (2.07, 6.25) | 4.49 (2.34, 7.18) | 0.55 (0.42, 0.68) | <0.001 | 0.92 (0.48, 1.54) | 1.01 (0.54, 1.69) | 0.33 (0.25, 0.41) | <0.001 | 19.01 (10.29, 30.73) | 16.44 (10.63, 24.74) | -0.51 (-0.66, -0.36) | <0.001 | 0.28 (0.15, 0.46) | 0.25 (0.16, 0.37) | -0.52 (-0.67, -0.36) | <0.001 |

| | | | | | | | | | | | | | | | | |
|---|---|---|---|---|---|---|---|---|---|---|---|---|---|---|---|---|
| Southeast Asia | 4.66 (2.72, 7.11) | 5.87 (3.45, 8.80) | 0.80 (0.77, 0.83) | <0.001 | 1.23 (0.77, 1.77) | 1.36 (0.88, 1.93) | 0.35 (0.31, 0.40) | <0.001 | 26.11 (17.91, 43.98) | 29.82 (21.70, 42.32) | 0.51 (0.35, 0.67) | <0.001 | 0.39 (0.27, 0.66) | 0.45 (0.32, 0.63) | 0.51 (0.35, 0.67) | <0.001 |
| Eastern Sub-Saharan Africa | 4.18 (2.40, 6.43) | 4.72 (2.63, 7.30) | 0.47 (0.39, 0.55) | <0.001 | 0.83 (0.45, 1.34) | 0.92 (0.50, 1.49) | 0.39 (0.35, 0.42) | <0.001 | 22.47 (11.21, 41.62) | 20.38 (11.51, 33.59) | -0.34 (-0.49, -0.18) | <0.001 | 0.34 (0.17, 0.62) | 0.31 (0.17, 0.50) | -0.34 (-0.49, -0.18) | <0.001 |
| Central Latin America | 3.94 (2.04, 6.38) | 5.70 (2.88, 9.36) | 1.25 (1.20, 1.30) | <0.001 | 0.65 (0.41, 0.97) | 0.83 (0.51, 1.23) | 0.80 (0.77, 0.84) | <0.001 | 9.77 (7.64, 14.21) | 12.25 (7.34, 16.07) | 0.80 (0.54, 1.07) | <0.001 | 0.15 (0.11, 0.21) | 0.18 (0.11, 0.24) | 0.80 (0.54, 1.07) | <0.001 |
| Tropical Latin America | 4.47 (2.53, 6.91) | 5.81 (2.99, 9.35) | 0.89 (0.81, 0.97) | <0.001 | 0.75 (0.45, 1.14) | 0.91 (0.55, 1.35) | 0.67 (0.64, 0.69) | <0.001 | 25.69 (18.43, 33.52) | 21.16 (16.68, 34.02) | -0.67 (-1.02, -0.31) | <0.001 | 0.39 (0.28, 0.50) | 0.32 (0.25, 0.51) | -0.67 (-1.03, -0.32) | <0.001 |
| Central Sub-Saharan Africa | 3.11 (1.63, 5.01) | 3.69 (1.94, 6.00) | 0.64 (0.45, 0.82) | <0.001 | 0.65 (0.35, 1.09) | 0.72 (0.38, 1.19) | 0.34 (0.26, 0.41) | <0.001 | 16.59 (8.00, 33.55) | 15.26 (7.75, 28.83) | -0.38 (-0.56, -0.20) | <0.001 | 0.25 (0.12, 0.50) | 0.23 (0.12, 0.43) | -0.38 (-0.56, -0.20) | <0.001 |
| Western Europe | 5.78 (3.12, 9.17) | 6.12 (3.34, 9.61) | 0.20 (0.12, 0.27) | <0.001 | 1.12 (0.76, 1.52) | 1.00 (0.67, 1.40) | -0.39 (-0.42, -0.36) | <0.001 | 5.51 (4.07, 9.67) | 6.63 (2.97, 8.66) | 0.58 (0.28, 0.89) | <0.001 | 0.08 (0.06, 0.14) | 0.10 (0.04, 0.13) | 0.60 (0.29, 0.91) | <0.001 |
| High-income North America | 5.80 (3.05, 9.34) | 5.10 (3.16, 7.48) | -0.41 (-0.47, -0.35) | <0.001 | 1.89 (1.20, 2.81) | 1.94 (1.21, 2.93) | 0.12 (-0.01, 0.25) | 0.073 | 4.20 (3.28, 7.59) | 7.93 (3.60, 10.02) | 2.32 (1.80, 2.83) | <0.001 | 0.06 (0.05, 0.11) | 0.12 (0.05, 0.15) | 2.38 (1.86, 2.90) | <0.001 |
| Central Asia | 3.10 (1.59, 5.15) | 3.76 (1.97, 6.11) | 0.69 (0.62, 0.76) | <0.001 | 0.32 (0.19, 0.50) | 0.38 (0.22, 0.59) | 0.61 (0.55, 0.68) | <0.001 | 6.25 (4.43, 9.23) | 9.38 (6.58, 14.62) | 1.42 (0.94, 1.91) | <0.001 | 0.09 (0.07, 0.14) | 0.14 (0.10, 0.22) | 1.43 (0.94, 1.92) | <0.001 |
| Eastern Europe | 5.10 (2.86, 7.91) | 9.40 (5.76, 13.79) | 2.13 (1.95, 2.31) | <0.001 | 0.56 (0.35, 0.83) | 0.89 (0.58, 1.30) | 1.61 (1.48, 1.73) | <0.001 | 16.88 (13.59, 27.83) | 32.77 (20.59, 43.38) | 2.42 (0.73, 4.14) | 0.005 | 0.25 (0.20, 0.42) | 0.49 (0.31, 0.65) | 2.43 (0.74, 4.14) | 0.005 |
| Southern Sub-Saharan Africa | 5.24 (2.97, 8.21) | 5.72 (3.23, 8.96) | 0.32 (0.25, 0.39) | <0.001 | 1.47 (0.75, 2.50) | 1.44 (0.76, 2.41) | -0.07 (-0.10, -0.03) | <0.001 | 23.30 (15.34, 35.54) | 25.48 (17.65, 35.28) | 0.31 (-0.07, 0.70) | 0.114 | 0.35 (0.23, 0.53) | 0.38 (0.26, 0.53) | 0.31 (-0.08, 0.70) | 0.118 |
| High-income Asia Pacific | 6.32 (3.42, 9.94) | 5.40 (2.97, 8.41) | -0.54 (-0.58, -0.51) | <0.001 | 1.53 (0.99, 2.17) | 1.35 (0.88, 1.93) | -0.45 (-0.48, -0.42) | <0.001 | 8.74 (5.82, 12.93) | 5.10 (3.07, 6.80) | -1.83 (-2.38, -1.28) | <0.001 | 0.13 (0.09, 0.19) | 0.08 (0.04, 0.10) | -1.85 (-2.42, -1.28) | <0.001 |

| | | | | | | | | | | | | | | | | |
|---|---|---|---|---|---|---|---|---|---|---|---|---|---|---|---|---|
| Central Europe | 4.57 (2.41, 7.37) | 5.33 (3.01, 8.23) | 0.54 (0.49, 0.58) | <0.001 | 0.63 (0.41, 0.92) | 0.70 (0.47, 0.99) | 0.33 (0.29, 0.38) | <0.001 | 7.76 (6.07, 11.35) | 9.75 (4.97, 12.98) | 0.72 (0.38, 1.06) | <0.001 | 0.12 (0.09, 0.17) | 0.15 (0.07, 0.19) | 0.72 (0.38, 1.07) | <0.001 |
| Andean Latin America | 3.33 (1.81, 5.26) | 5.30 (2.94, 8.30) | 1.63 (1.59, 1.66) | <0.001 | 0.56 (0.35, 0.82) | 0.77 (0.49, 1.13) | 1.11 (1.08, 1.14) | <0.001 | 19.99 (11.73, 31.21) | 16.00 (9.77, 25.31) | -0.58 (-1.01, -0.15) | 0.008 | 0.30 (0.18, 0.47) | 0.24 (0.15, 0.38) | -0.59 (-1.01, -0.15) | 0.008 |
| Caribbean | 4.38 (2.37, 6.87) | 6.95 (3.96, 10.72) | 1.62 (1.56, 1.68) | <0.001 | 0.63 (0.40, 0.92) | 0.83 (0.54, 1.19) | 0.93 (0.90, 0.96) | <0.001 | 14.27 (10.31, 19.39) | 18.01 (11.94, 26.72) | 0.85 (0.37, 1.34) | 0.001 | 0.21 (0.15, 0.29) | 0.27 (0.18, 0.40) | 0.85 (0.37, 1.34) | 0.001 |
| Southern Latin America | 3.65 (1.95, 5.95) | 6.43 (3.55, 10.35) | 1.97 (1.89, 2.05) | <0.001 | 0.97 (0.64, 1.33) | 1.14 (0.75, 1.59) | 0.57 (0.53, 0.61) | <0.001 | 9.84 (6.08, 15.30) | 13.57 (8.99, 19.49) | 1.17 (0.83, 1.52) | <0.001 | 0.15 (0.09, 0.23) | 0.20 (0.13, 0.29) | 1.18 (0.83, 1.53) | <0.001 |
| Oceania | 3.80 (2.05, 6.15) | 4.53 (2.48, 7.04) | 0.62 (0.58, 0.67) | <0.001 | 0.33 (0.19, 0.52) | 0.38 (0.22, 0.59) | 0.50 (0.46, 0.55) | <0.001 | 5.28 (2.94, 10.00) | 6.96 (3.71, 12.16) | 0.91 (0.63, 1.18) | <0.001 | 0.08 (0.04, 0.15) | 0.10 (0.06, 0.18) | 0.91 (0.64, 1.18) | <0.001 |
| Australasia | 4.96 (2.64, 7.90) | 5.01 (2.74, 7.83) | 0.02 (-0.03, 0.08) | 0.420 | 0.93 (0.63, 1.25) | 0.78 (0.53, 1.06) | -0.59 (-0.62, -0.56) | <0.001 | 5.91 (3.84, 8.91) | 6.31 (2.58, 10.12) | 0.28 (-0.22, 0.78) | 0.267 | 0.09 (0.06, 0.13) | 0.09 (0.04, 0.15) | 0.29 (-0.21, 0.80) | 0.251 |
| **25-29 years** | | | | | | | | | | | | | | | | |
| **Women** | | | | | | | | | | | | | | | | |
| Global | 5.07 (2.55, 8.25) | 5.00 (2.48, 8.26) | -0.04 (-0.08, 0.01) | 0.094 | 1.20 (0.83, 1.69) | 1.11 (0.76, 1.59) | -0.23 (-0.30, -0.17) | <0.001 | 13.70 (8.56, 18.51) | 12.58 (9.17, 16.73) | -0.29 (-0.56, -0.02) | 0.034 | 0.22 (0.14, 0.30) | 0.20 (0.15, 0.27) | -0.29 (-0.57, -0.02) | 0.036 |
| **SDI category** | | | | | | | | | | | | | | | | |
| High | 6.01 (2.90, 9.83) | 5.74 (2.99, 9.19) | -0.16 (-0.19, -0.13) | <0.001 | 3.01 (2.08, 4.22) | 2.84 (1.93, 4.05) | -0.16 (-0.25, -0.07) | <0.001 | 6.69 (4.98, 10.20) | 8.27 (4.20, 10.31) | 0.78 (0.43, 1.13) | <0.001 | 0.10 (0.08, 0.16) | 0.13 (0.06, 0.16) | 0.80 (0.36, 1.24) | <0.001 |
| High-middle | 5.91 (2.92, 9.71) | 6.06 (2.99, 9.93) | 0.07 (0.01, 0.13) | 0.016 | 1.02 (0.70, 1.41) | 1.13 (0.76, 1.58) | 0.34 (0.32, 0.36) | <0.001 | 10.34 (7.55, 13.32) | 10.25 (6.92, 12.63) | -0.16 (-1.10, 0.79) | 0.736 | 0.17 (0.12, 0.21) | 0.17 (0.11, 0.20) | -0.16 (-1.10, 0.79) | 0.735 |
| Middle | 5.54 (2.81, 8.98) | 5.33 (2.65, 8.76) | -0.12 (-0.17, -0.07) | <0.001 | 1.05 (0.69, 1.49) | 1.06 (0.70, 1.51) | 0.04 (0.00, 0.07) | 0.035 | 16.34 (11.27, 23.98) | 13.66 (10.39, 20.36) | -0.66 (-1.00, -0.32) | <0.001 | 0.26 (0.18, 0.39) | 0.22 (0.17, 0.33) | -0.66 (-1.00, -0.32) | <0.001 |

| | | | | | | | | | | | | | | | | |
|---|---|---|---|---|---|---|---|---|---|---|---|---|---|---|---|---|
| Low-middle | 3.50 (1.75, 5.77) | 4.29 (2.09, 7.14) | 0.70 (0.61, 0.79) | <0.001 | 0.52 (0.33, 0.76) | 0.65 (0.41, 0.95) | 0.76 (0.71, 0.81) | <0.001 | 15.87 (7.25, 24.71) | 14.15 (9.02, 20.85) | -0.39 (-0.77, -0.01) | 0.041 | 0.26 (0.12, 0.40) | 0.23 (0.15, 0.34) | -0.39 (-0.77, -0.02) | 0.040 |
| Low | 3.00 (1.51, 4.94) | 3.61 (1.77, 5.99) | 0.68 (0.59, 0.78) | <0.001 | 0.59 (0.34, 0.97) | 0.70 (0.40, 1.15) | 0.57 (0.53, 0.60) | <0.001 | 19.22 (6.44, 31.40) | 13.50 (8.04, 19.16) | -1.23 (-1.41, -1.04) | <0.001 | 0.31 (0.10, 0.51) | 0.22 (0.13, 0.31) | -1.23 (-1.42, -1.04) | <0.001 |
| **21 GBD region** | | | | | | | | | | | | | | | | |
| South Asia | 2.85 (1.37, 4.81) | 3.97 (1.92, 6.64) | 1.20 (1.09, 1.32) | <0.001 | 0.35 (0.21, 0.50) | 0.45 (0.28, 0.65) | 0.96 (0.87, 1.04) | <0.001 | 11.55 (5.04, 20.82) | 11.73 (6.67, 18.16) | 0.13 (-0.60, 0.87) | 0.725 | 0.19 (0.08, 0.34) | 0.19 (0.11, 0.29) | 0.13 (-0.60, 0.88) | 0.724 |
| East Asia | 7.11 (3.52, 11.53) | 4.94 (2.28, 8.35) | -1.24 (-1.35, -1.13) | <0.001 | 0.92 (0.59, 1.30) | 0.86 (0.56, 1.23) | -0.20 (-0.31, -0.08) | 0.001 | 6.78 (3.24, 9.70) | 2.71 (1.82, 4.17) | -3.04 (-4.24, -1.82) | <0.001 | 0.11 (0.05, 0.16) | 0.04 (0.03, 0.07) | -3.09 (-4.30, -1.86) | <0.001 |
| North Africa and Middle East | 3.93 (1.87, 6.64) | 4.99 (2.36, 8.18) | 0.82 (0.81, 0.83) | <0.001 | 0.74 (0.49, 1.05) | 0.83 (0.55, 1.16) | 0.34 (0.28, 0.41) | <0.001 | 9.30 (5.45, 15.42) | 7.56 (4.76, 11.31) | -0.71 (-0.84, -0.58) | <0.001 | 0.15 (0.09, 0.25) | 0.12 (0.08, 0.18) | -0.71 (-0.84, -0.58) | <0.001 |
| Western Sub-Saharan Africa | 3.33 (1.69, 5.44) | 3.61 (1.78, 6.01) | 0.33 (0.23, 0.43) | <0.001 | 1.07 (0.56, 1.88) | 1.19 (0.62, 2.13) | 0.40 (0.33, 0.46) | <0.001 | 27.57 (10.73, 44.83) | 15.62 (9.91, 24.18) | -1.94 (-2.06, -1.82) | <0.001 | 0.45 (0.17, 0.73) | 0.25 (0.16, 0.39) | -1.95 (-2.07, -1.83) | <0.001 |
| Southeast Asia | 5.61 (3.10, 8.66) | 7.21 (3.88, 11.18) | 0.87 (0.85, 0.88) | <0.001 | 1.97 (1.19, 2.92) | 2.04 (1.31, 2.95) | 0.13 (0.10, 0.15) | <0.001 | 39.18 (25.89, 64.42) | 35.08 (25.41, 54.60) | -0.39 (-0.76, -0.01) | 0.044 | 0.63 (0.42, 1.04) | 0.57 (0.41, 0.88) | -0.39 (-0.76, -0.01) | 0.044 |
| Eastern Sub-Saharan Africa | 3.25 (1.77, 5.10) | 3.36 (1.69, 5.58) | 0.16 (0.08, 0.25) | <0.001 | 0.83 (0.45, 1.44) | 0.92 (0.50, 1.61) | 0.36 (0.33, 0.39) | <0.001 | 28.68 (7.99, 51.66) | 17.01 (8.27, 26.81) | -1.78 (-2.02, -1.55) | <0.001 | 0.46 (0.13, 0.84) | 0.27 (0.13, 0.43) | -1.79 (-2.02, -1.56) | <0.001 |
| Central Latin America | 4.50 (2.16, 7.52) | 5.46 (2.54, 9.14) | 0.68 (0.60, 0.76) | <0.001 | 0.88 (0.57, 1.27) | 0.95 (0.61, 1.38) | 0.28 (0.26, 0.31) | <0.001 | 8.76 (6.85, 13.72) | 10.41 (6.67, 14.08) | 0.74 (-0.08, 1.57) | 0.076 | 0.14 (0.11, 0.22) | 0.17 (0.11, 0.23) | 0.75 (-0.08, 1.58) | 0.076 |
| Tropical Latin America | 4.39 (2.16, 7.20) | 6.49 (2.82, 11.17) | 1.31 (1.19, 1.44) | <0.001 | 0.92 (0.58, 1.39) | 1.12 (0.70, 1.67) | 0.64 (0.55, 0.73) | <0.001 | 21.91 (15.67, 34.94) | 17.66 (13.76, 28.97) | -0.71 (-1.04, -0.38) | <0.001 | 0.35 (0.25, 0.57) | 0.29 (0.22, 0.47) | -0.72 (-1.05, -0.38) | <0.001 |
| Central Sub-Saharan Africa | 2.54 (1.25, 4.06) | 3.00 (1.45, 4.99) | 0.62 (0.53, 0.72) | <0.001 | 0.71 (0.35, 1.25) | 0.76 (0.40, 1.33) | 0.25 (0.21, 0.29) | <0.001 | 14.47 (4.17, 29.07) | 11.04 (4.58, 21.04) | -0.96 (-1.20, | <0.001 | 0.23 (0.07, | 0.18 (0.07, | -0.97 (-1.20, | <0.001 |

| | | | | | | | | | | | | | | | | |
|---|---|---|---|---|---|---|---|---|---|---|---|---|---|---|---|---|
| | | | | | | | | | | | -0.73) | | 0.47) | 0.34) | -0.73) | |
| Western Europe | 6.13 (2.95, 10.10) | 6.43 (3.07, 10.61) | 0.16 (0.11, 0.21) | <0.001 | 1.43 (1.00, 1.94) | 1.18 (0.80, 1.62) | -0.69 (-0.77, -0.61) | <0.001 | 3.92 (2.81, 7.20) | 4.55 (2.40, 5.58) | 0.45 (0.14, 0.75) | 0.004 | 0.06 (0.04, 0.12) | 0.07 (0.04, 0.09) | 0.48 (0.18, 0.79) | 0.002 |
| High-income North America | 6.25 (3.06, 10.24) | 5.30 (2.98, 8.03) | -0.53 (-0.67, -0.40) | <0.001 | 5.32 (3.51, 7.82) | 5.17 (3.35, 7.78) | -0.06 (-0.21, 0.10) | 0.491 | 5.73 (4.46, 10.42) | 12.22 (5.11, 15.92) | 2.72 (2.13, 3.31) | <0.001 | 0.09 (0.07, 0.16) | 0.19 (0.08, 0.25) | 2.90 (2.34, 3.46) | <0.001 |
| Central Asia | 2.55 (1.17, 4.40) | 3.19 (1.50, 5.60) | 0.78 (0.73, 0.83) | <0.001 | 0.25 (0.14, 0.38) | 0.29 (0.16, 0.46) | 0.54 (0.50, 0.58) | <0.001 | 3.75 (2.64, 5.16) | 5.71 (3.94, 8.25) | 1.50 (1.02, 1.97) | <0.001 | 0.06 (0.04, 0.08) | 0.09 (0.06, 0.13) | 1.50 (1.03, 1.98) | <0.001 |
| Eastern Europe | 5.04 (2.47, 8.30) | 9.97 (5.47, 15.54) | 2.35 (2.04, 2.65) | <0.001 | 0.61 (0.38, 0.91) | 1.00 (0.61, 1.46) | 1.71 (1.59, 1.83) | <0.001 | 10.70 (8.15, 18.80) | 31.08 (13.55, 44.64) | 3.92 (3.23, 4.61) | <0.001 | 0.17 (0.13, 0.30) | 0.50 (0.22, 0.73) | 3.93 (3.24, 4.62) | <0.001 |
| Southern Sub-Saharan Africa | 6.23 (3.62, 9.31) | 4.96 (2.48, 8.14) | -0.76 (-0.87, -0.66) | <0.001 | 1.55 (0.89, 2.65) | 1.37 (0.76, 2.37) | -0.41 (-0.56, -0.25) | <0.001 | 70.01 (32.37, 100.81) | 28.60 (12.61, 52.47) | -2.86 (-4.41, -1.29) | <0.001 | 1.14 (0.52, 1.64) | 0.46 (0.20, 0.85) | -2.87 (-4.43, -1.29) | <0.001 |
| High-income Asia Pacific | 5.91 (2.87, 9.62) | 5.15 (2.51, 8.46) | -0.48 (-0.51, -0.46) | <0.001 | 2.32 (1.52, 3.45) | 1.92 (1.27, 2.74) | -0.67 (-0.73, -0.60) | <0.001 | 11.51 (6.68, 17.66) | 4.39 (3.34, 6.74) | -3.33 (-3.99, -2.68) | <0.001 | 0.18 (0.11, 0.28) | 0.07 (0.05, 0.11) | -3.40 (-4.07, -2.73) | <0.001 |
| Central Europe | 4.40 (2.08, 7.42) | 5.10 (2.41, 8.54) | 0.51 (0.45, 0.57) | <0.001 | 0.66 (0.41, 0.97) | 0.71 (0.46, 1.02) | 0.21 (0.16, 0.26) | <0.001 | 5.86 (4.59, 9.02) | 6.68 (3.43, 9.06) | 0.40 (0.06, 0.74) | 0.019 | 0.09 (0.07, 0.15) | 0.11 (0.05, 0.15) | 0.40 (0.06, 0.75) | 0.020 |
| Andean Latin America | 3.22 (1.54, 5.49) | 5.74 (2.72, 9.54) | 2.02 (1.96, 2.07) | <0.001 | 0.58 (0.35, 0.87) | 0.82 (0.50, 1.21) | 1.25 (1.23, 1.27) | <0.001 | 13.23 (7.16, 22.14) | 8.26 (4.75, 13.72) | -1.58 (-2.28, -0.89) | <0.001 | 0.21 (0.12, 0.36) | 0.13 (0.08, 0.22) | -1.60 (-2.29, -0.89) | <0.001 |
| Caribbean | 4.98 (2.49, 8.14) | 8.61 (4.49, 13.64) | 1.90 (1.85, 1.94) | <0.001 | 0.79 (0.51, 1.13) | 1.04 (0.67, 1.48) | 0.95 (0.92, 0.99) | <0.001 | 18.80 (12.55, 29.46) | 25.77 (15.19, 43.31) | 1.16 (0.88, 1.45) | <0.001 | 0.30 (0.20, 0.48) | 0.42 (0.25, 0.70) | 1.16 (0.88, 1.46) | <0.001 |
| Southern Latin America | 3.87 (1.88, 6.56) | 6.96 (3.30, 11.31) | 2.06 (2.02, 2.09) | <0.001 | 1.44 (0.95, 2.07) | 1.50 (0.99, 2.11) | 0.13 (0.12, 0.14) | <0.001 | 10.67 (6.26, 16.86) | 10.88 (7.35, 16.03) | 0.08 (-0.26, 0.42) | 0.632 | 0.17 (0.10, 0.27) | 0.17 (0.12, 0.26) | 0.08 (-0.26, 0.43) | 0.630 |
| Oceania | 5.90 (3.14, 9.10) | 7.23 (4.13, 10.94) | 0.72 (0.68, 0.76) | <0.001 | 0.59 (0.39, 0.83) | 0.65 (0.42, 0.91) | 0.31 (0.28, 0.34) | <0.001 | 11.25 (6.14, 20.01) | 15.24 (7.72, 28.28) | 1.02 (0.82, | <0.001 | 0.18 (0.10, | 0.25 (0.12, | 1.02 (0.82, 1.22) | <0.001 |

| | | | | | | | | | | | | | | | | |
|---|---|---|---|---|---|---|---|---|---|---|---|---|---|---|---|---|
| | | | | | | | | | | | 1.22) | | 0.32) | 0.46) | | |
| Australasia | 5.58 (2.69, 9.23) | 6.22 (2.89, 10.41) | 0.38 (0.35, 0.40) | <0.001 | 1.80 (1.22, 2.50) | 1.42 (0.94, 2.02) | -0.81 (-0.85, -0.78) | <0.001 | 6.01 (4.05, 9.77) | 6.91 (3.47, 10.45) | 0.51 (-0.25, 1.27) | 0.191 | 0.10 (0.06, 0.16) | 0.11 (0.06, 0.17) | 0.54 (-0.23, 1.32) | 0.168 |
| **Men** | | | | | | | | | | | | | | | | |
| Global | 5.71 (2.92, 9.24) | 6.15 (3.12, 9.93) | 0.26 (0.24, 0.29) | <0.001 | 0.99 (0.68, 1.36) | 1.03 (0.71, 1.43) | 0.17 (0.07, 0.27) | 0.001 | 15.96 (13.15, 21.87) | 17.68 (13.53, 23.05) | 0.33 (0.02, 0.65) | 0.036 | 0.26 (0.21, 0.35) | 0.29 (0.22, 0.37) | 0.34 (0.02, 0.65) | 0.036 |
| **SDI category** | | | | | | | | | | | | | | | | |
| High | 6.69 (3.30, 10.90) | 7.11 (3.71, 11.27) | 0.20 (0.17, 0.24) | <0.001 | 1.97 (1.38, 2.69) | 2.09 (1.47, 2.86) | 0.24 (0.14, 0.33) | <0.001 | 8.83 (7.00, 13.89) | 10.95 (5.91, 13.19) | 0.76 (0.46, 1.05) | <0.001 | 0.14 (0.11, 0.22) | 0.17 (0.09, 0.21) | 0.77 (0.47, 1.06) | <0.001 |
| High-middle | 6.89 (3.55, 11.00) | 7.63 (3.96, 12.15) | 0.35 (0.30, 0.40) | <0.001 | 0.99 (0.67, 1.35) | 1.16 (0.79, 1.59) | 0.56 (0.54, 0.59) | <0.001 | 18.20 (14.32, 24.44) | 21.31 (15.11, 25.98) | 0.63 (-0.19, 1.45) | 0.131 | 0.29 (0.23, 0.40) | 0.34 (0.24, 0.42) | 0.63 (-0.18, 1.46) | 0.130 |
| Middle | 5.88 (3.00, 9.47) | 6.28 (3.17, 10.16) | 0.22 (0.20, 0.25) | <0.001 | 0.91 (0.61, 1.28) | 1.02 (0.68, 1.42) | 0.38 (0.29, 0.46) | <0.001 | 17.76 (13.41, 27.88) | 18.97 (14.88, 28.13) | 0.21 (-0.28, 0.69) | 0.401 | 0.29 (0.22, 0.45) | 0.31 (0.24, 0.46) | 0.21 (-0.28, 0.70) | 0.406 |
| Low-middle | 4.03 (2.01, 6.68) | 5.14 (2.55, 8.43) | 0.85 (0.78, 0.93) | <0.001 | 0.51 (0.33, 0.72) | 0.64 (0.41, 0.91) | 0.79 (0.75, 0.84) | <0.001 | 15.06 (10.52, 20.14) | 16.00 (11.76, 21.81) | 0.22 (-0.09, 0.54) | 0.162 | 0.24 (0.17, 0.33) | 0.26 (0.19, 0.35) | 0.22 (-0.09, 0.54) | 0.164 |
| Low | 3.90 (2.06, 6.21) | 4.83 (2.48, 7.76) | 0.79 (0.72, 0.86) | <0.001 | 0.56 (0.36, 0.86) | 0.68 (0.43, 1.02) | 0.69 (0.64, 0.74) | <0.001 | 17.98 (10.12, 28.41) | 18.34 (11.46, 26.63) | 0.05 (-0.12, 0.22) | 0.556 | 0.29 (0.16, 0.46) | 0.30 (0.19, 0.43) | 0.05 (-0.12, 0.22) | 0.566 |
| **21 GBD region** | | | | | | | | | | | | | | | | |
| South Asia | 3.36 (1.61, 5.69) | 4.68 (2.30, 7.66) | 1.19 (1.11, 1.26) | <0.001 | 0.36 (0.22, 0.52) | 0.48 (0.28, 0.70) | 1.00 (0.93, 1.06) | <0.001 | 11.50 (7.32, 15.82) | 13.13 (9.11, 18.19) | 0.46 (0.02, 0.90) | 0.040 | 0.19 (0.12, 0.26) | 0.21 (0.15, 0.29) | 0.46 (0.02, 0.90) | 0.040 |
| East Asia | 7.26 (3.59, 11.67) | 5.57 (2.55, 9.22) | -0.88 (-1.08, -0.68) | <0.001 | 0.90 (0.59, 1.28) | 0.90 (0.59, 1.28) | 0.02 (-0.05, 0.09) | 0.633 | 10.06 (5.55, 13.58) | 6.09 (4.35, 8.16) | -1.73 (-2.24, -1.22) | <0.001 | 0.16 (0.09, 0.22) | 0.10 (0.07, 0.13) | -1.75 (-2.26, -1.23) | <0.001 |
| North Africa and Middle East | 6.49 (3.24, 10.58) | 7.91 (3.87, 13.15) | 0.68 (0.65, 0.72) | <0.001 | 0.98 (0.66, 1.36) | 1.12 (0.75, 1.55) | 0.45 (0.42, 0.47) | <0.001 | 11.29 (7.63, 18.43) | 9.20 (6.10, 13.32) | -0.69 (-0.90, | <0.001 | 0.18 (0.12, | 0.15 (0.10, | -0.70 (-0.91, | <0.001 |

| | | | | | | | | | | | | | | | | |
|---|---|---|---|---|---|---|---|---|---|---|---|---|---|---|---|---|
| | | | | | | | | | | | -0.49) | | 0.30) | 0.21) | -0.49) | |
| Western Sub-Saharan Africa | 3.97 (2.04, 6.45) | 4.56 (2.32, 7.38) | 0.54 (0.46, 0.63) | <0.001 | 0.68 (0.41, 1.06) | 0.76 (0.47, 1.16) | 0.42 (0.37, 0.47) | <0.001 | 17.04 (9.44, 27.79) | 15.17 (9.66, 22.77) | -0.40 (-0.52, -0.28) | <0.001 | 0.28 (0.15, 0.45) | 0.25 (0.16, 0.37) | -0.40 (-0.52, -0.28) | <0.001 |
| Southeast Asia | 5.97 (3.36, 9.05) | 7.59 (4.29, 11.43) | 0.83 (0.81, 0.86) | <0.001 | 1.58 (1.02, 2.26) | 1.79 (1.20, 2.54) | 0.42 (0.38, 0.46) | <0.001 | 46.87 (30.87, 84.53) | 49.69 (36.19, 78.66) | 0.29 (0.06, 0.53) | 0.015 | 0.76 (0.50, 1.37) | 0.80 (0.58, 1.27) | 0.29 (0.06, 0.53) | 0.016 |
| Eastern Sub-Saharan Africa | 4.78 (2.68, 7.19) | 5.18 (2.81, 8.05) | 0.31 (0.24, 0.38) | <0.001 | 0.88 (0.54, 1.45) | 0.98 (0.59, 1.58) | 0.37 (0.34, 0.41) | <0.001 | 29.80 (14.85, 52.75) | 26.73 (14.73, 44.05) | -0.39 (-0.62, -0.15) | 0.001 | 0.48 (0.24, 0.85) | 0.43 (0.24, 0.71) | -0.39 (-0.62, -0.16) | 0.001 |
| Central Latin America | 4.69 (2.25, 7.84) | 7.18 (3.34, 12.10) | 1.47 (1.37, 1.57) | <0.001 | 0.66 (0.43, 0.95) | 0.90 (0.55, 1.32) | 1.03 (1.00, 1.06) | <0.001 | 10.21 (7.86, 15.26) | 12.57 (7.56, 16.61) | 0.77 (0.46, 1.08) | <0.001 | 0.16 (0.13, 0.25) | 0.20 (0.12, 0.27) | 0.77 (0.46, 1.08) | <0.001 |
| Tropical Latin America | 5.18 (2.69, 8.33) | 7.78 (3.49, 13.13) | 1.38 (1.33, 1.42) | <0.001 | 0.73 (0.48, 1.07) | 1.00 (0.62, 1.45) | 1.04 (1.00, 1.08) | <0.001 | 29.70 (21.64, 42.77) | 25.22 (19.78, 41.98) | -0.56 (-0.75, -0.37) | <0.001 | 0.48 (0.35, 0.69) | 0.41 (0.32, 0.68) | -0.57 (-0.76, -0.38) | <0.001 |
| Central Sub-Saharan Africa | 3.40 (1.78, 5.44) | 3.97 (2.04, 6.53) | 0.57 (0.48, 0.66) | <0.001 | 0.68 (0.38, 1.16) | 0.75 (0.43, 1.25) | 0.34 (0.30, 0.38) | <0.001 | 20.07 (9.46, 39.50) | 18.82 (9.33, 36.95) | -0.25 (-0.34, -0.15) | <0.001 | 0.32 (0.15, 0.64) | 0.30 (0.15, 0.60) | -0.29 (-0.52, -0.06) | 0.014 |
| Western Europe | 6.96 (3.40, 11.36) | 7.66 (3.73, 12.58) | 0.32 (0.24, 0.41) | <0.001 | 1.25 (0.87, 1.68) | 1.17 (0.80, 1.59) | -0.25 (-0.26, -0.23) | <0.001 | 6.96 (5.18, 12.12) | 9.09 (3.92, 11.56) | 0.89 (0.66, 1.11) | <0.001 | 0.11 (0.08, 0.19) | 0.15 (0.06, 0.19) | 0.90 (0.68, 1.13) | <0.001 |
| High-income North America | 6.37 (3.16, 10.32) | 6.48 (3.67, 9.75) | 0.09 (0.03, 0.15) | 0.002 | 3.11 (2.10, 4.49) | 3.50 (2.32, 5.20) | 0.45 (0.26, 0.64) | <0.001 | 8.32 (6.66, 14.78) | 14.19 (6.72, 18.77) | 1.88 (1.41, 2.35) | <0.001 | 0.13 (0.10, 0.24) | 0.23 (0.10, 0.30) | 1.91 (1.43, 2.40) | <0.001 |
| Central Asia | 3.55 (1.69, 6.13) | 4.29 (2.08, 7.14) | 0.67 (0.53, 0.82) | <0.001 | 0.35 (0.20, 0.53) | 0.41 (0.24, 0.62) | 0.60 (0.52, 0.69) | <0.001 | 7.93 (5.73, 11.71) | 11.40 (7.90, 19.60) | 1.26 (0.77, 1.76) | <0.001 | 0.13 (0.09, 0.19) | 0.18 (0.13, 0.32) | 1.27 (0.77, 1.76) | <0.001 |
| Eastern Europe | 6.29 (3.39, 9.97) | 14.55 (8.75, 21.35) | 2.92 (2.72, 3.13) | <0.001 | 0.67 (0.43, 0.95) | 1.30 (0.85, 1.82) | 2.30 (2.11, 2.49) | <0.001 | 28.73 (20.52, 58.38) | 82.75 (48.76, 107.75) | 3.77 (2.37, 5.19) | <0.001 | 0.47 (0.33, 0.95) | 1.34 (0.79, 1.75) | 3.78 (2.38, 5.20) | <0.001 |
| Southern Sub-Saharan Africa | 5.60 (3.11, 8.64) | 5.87 (3.17, 9.25) | 0.17 (0.12, 0.22) | <0.001 | 1.19 (0.70, 1.98) | 1.18 (0.70, 1.95) | -0.03 (-0.06, | 0.006 | 44.49 (29.84, 65.34) | 38.27 (25.93, 55.43) | -0.59 (-0.91, | <0.001 | 0.72 (0.48, | 0.62 (0.42, | -0.60 (-0.92, | <0.001 |

| | | | | | | | | | | | | | | | | |
|---|---|---|---|---|---|---|---|---|---|---|---|---|---|---|---|---|
| | | | | | | | -0.01) | | | | -0.28) | | 1.06) | 0.90) | -0.28) | |
| High-income Asia Pacific | 7.46 (3.71, 12.18) | 6.71 (3.36, 10.73) | -0.38 (-0.42, -0.33) | <0.001 | 1.63 (1.14, 2.23) | 1.45 (1.01, 1.99) | -0.41 (-0.47, -0.35) | <0.001 | 9.86 (6.36, 14.91) | 5.90 (3.99, 8.21) | -1.73 (-2.02, -1.44) | <0.001 | 0.16 (0.10, 0.24) | 0.09 (0.06, 0.13) | -1.75 (-2.05, -1.45) | <0.001 |
| Central Europe | 5.15 (2.54, 8.37) | 6.51 (3.41, 10.30) | 0.83 (0.73, 0.93) | <0.001 | 0.69 (0.45, 0.97) | 0.83 (0.56, 1.15) | 0.65 (0.58, 0.71) | <0.001 | 10.32 (8.19, 15.35) | 13.77 (6.32, 18.20) | 1.03 (0.60, 1.46) | <0.001 | 0.17 (0.13, 0.25) | 0.22 (0.10, 0.29) | 1.03 (0.60, 1.46) | <0.001 |
| Andean Latin America | 3.99 (1.93, 6.52) | 6.72 (3.30, 11.04) | 1.82 (1.79, 1.85) | <0.001 | 0.58 (0.36, 0.83) | 0.84 (0.53, 1.21) | 1.33 (1.28, 1.37) | <0.001 | 17.45 (10.46, 27.46) | 15.01 (9.01, 24.54) | -0.50 (-1.02, 0.04) | 0.067 | 0.28 (0.17, 0.44) | 0.24 (0.15, 0.40) | -0.50 (-1.03, 0.03) | 0.065 |
| Caribbean | 5.50 (2.75, 8.98) | 9.02 (4.60, 14.38) | 1.73 (1.66, 1.79) | <0.001 | 0.74 (0.48, 1.06) | 1.01 (0.64, 1.45) | 1.05 (0.99, 1.10) | <0.001 | 18.08 (13.55, 24.94) | 22.80 (15.96, 32.77) | 0.85 (0.50, 1.20) | <0.001 | 0.29 (0.22, 0.40) | 0.37 (0.26, 0.53) | 0.85 (0.50, 1.20) | <0.001 |
| Southern Latin America | 4.29 (2.11, 7.10) | 8.01 (3.99, 13.02) | 2.17 (2.10, 2.24) | <0.001 | 1.23 (0.83, 1.71) | 1.45 (0.99, 1.96) | 0.56 (0.52, 0.61) | <0.001 | 12.61 (7.43, 19.37) | 17.69 (11.62, 25.19) | 1.22 (0.81, 1.64) | <0.001 | 0.20 (0.12, 0.31) | 0.28 (0.19, 0.41) | 1.23 (0.81, 1.65) | <0.001 |
| Oceania | 4.31 (2.29, 6.83) | 5.14 (2.82, 8.08) | 0.64 (0.55, 0.72) | <0.001 | 0.59 (0.40, 0.82) | 0.62 (0.42, 0.87) | 0.18 (0.14, 0.23) | <0.001 | 30.58 (17.84, 55.45) | 40.33 (22.36, 68.68) | 0.92 (0.73, 1.11) | <0.001 | 0.50 (0.29, 0.90) | 0.65 (0.36, 1.11) | 0.92 (0.73, 1.11) | <0.001 |
| Australasia | 5.95 (2.83, 9.67) | 6.53 (3.14, 10.87) | 0.32 (0.30, 0.34) | <0.001 | 1.15 (0.79, 1.56) | 1.00 (0.66, 1.42) | -0.48 (-0.52, -0.45) | <0.001 | 7.05 (4.64, 10.70) | 9.54 (3.70, 15.07) | 0.96 (0.03, 1.90) | 0.043 | 0.11 (0.07, 0.17) | 0.15 (0.06, 0.24) | 0.98 (0.04, 1.93) | 0.040 |
| **30-34 years** | | | | | | | | | | | | | | | | |
| **Women** | | | | | | | | | | | | | | | | |
| Global | 5.72 (3.15, 9.09) | 5.90 (3.19, 9.32) | 0.13 (0.08, 0.17) | <0.001 | 1.19 (0.80, 1.67) | 1.07 (0.70, 1.53) | -0.35 (-0.45, -0.25) | <0.001 | 12.23 (8.05, 16.57) | 11.44 (8.20, 14.62) | -0.22 (-0.48, 0.04) | 0.098 | 0.21 (0.14, 0.29) | 0.20 (0.14, 0.26) | -0.22 (-0.48, 0.04) | 0.100 |
| **SDI category** | | | | | | | | | | | | | | | | |
| High | 6.83 (3.65, 10.84) | 6.65 (3.75, 10.34) | -0.09 (-0.13, -0.04) | <0.001 | 2.96 (1.86, 4.46) | 2.67 (1.69, 4.11) | -0.33 (-0.47, -0.19) | <0.001 | 6.79 (5.19, 10.45) | 8.93 (4.71, 10.90) | 1.01 (0.67, 1.35) | <0.001 | 0.12 (0.09, 0.18) | 0.15 (0.08, 0.19) | 1.08 (0.75, 1.40) | <0.001 |
| High-middle | 6.58 (3.57, 10.45) | 7.19 (3.90, 11.31) | 0.30 (0.27, 0.34) | <0.001 | 0.91 (0.59, 1.32) | 1.06 (0.70, 1.52) | 0.52 (0.42, 0.61) | <0.001 | 8.32 (6.05, 10.64) | 9.22 (5.60, 11.58) | 0.36 (-0.30, | 0.282 | 0.15 (0.11, | 0.16 (0.10, | 0.36 (-0.30, 1.03) | 0.282 |

| | | | | | | | | | | | | | | | | |
|---|---|---|---|---|---|---|---|---|---|---|---|---|---|---|---|---|
| | | | | | | | | | | | 1.03) | | 0.19) | 0.20) | | |
| Middle | 6.21 (3.49, 9.72) | 6.20 (3.34, 9.92) | 0.02 (-0.03, 0.07) | 0.502 | 1.04 (0.68, 1.54) | 1.02 (0.65, 1.48) | -0.06 (-0.10, -0.01) | 0.010 | 16.21 (11.63, 24.37) | 12.32 (9.36, 17.81) | -0.97 (-1.56, -0.37) | 0.001 | 0.28 (0.20, 0.43) | 0.22 (0.16, 0.31) | -0.97 (-1.57, -0.37) | 0.001 |
| Low-middle | 3.86 (2.10, 6.14) | 4.96 (2.69, 7.92) | 0.88 (0.82, 0.94) | <0.001 | 0.47 (0.29, 0.70) | 0.61 (0.38, 0.91) | 0.94 (0.90, 0.98) | <0.001 | 14.06 (7.00, 21.17) | 13.20 (8.70, 19.01) | -0.21 (-0.47, 0.04) | 0.099 | 0.25 (0.12, 0.37) | 0.23 (0.15, 0.33) | -0.21 (-0.47, 0.04) | 0.097 |
| Low | 3.28 (1.80, 5.26) | 4.01 (2.15, 6.54) | 0.75 (0.65, 0.85) | <0.001 | 0.48 (0.28, 0.80) | 0.58 (0.34, 0.94) | 0.67 (0.56, 0.77) | <0.001 | 15.38 (5.95, 23.69) | 11.75 (7.09, 16.04) | -0.94 (-1.11, -0.76) | <0.001 | 0.27 (0.10, 0.42) | 0.21 (0.12, 0.28) | -0.94 (-1.12, -0.76) | <0.001 |
| **21 GBD region** | | | | | | | | | | | | | | | | |
| South Asia | 3.19 (1.66, 5.24) | 4.53 (2.41, 7.31) | 1.24 (1.14, 1.34) | <0.001 | 0.36 (0.22, 0.53) | 0.48 (0.29, 0.72) | 1.03 (0.95, 1.12) | <0.001 | 10.83 (5.49, 18.56) | 11.32 (6.80, 17.38) | 0.16 (-0.42, 0.74) | 0.596 | 0.19 (0.10, 0.33) | 0.20 (0.12, 0.31) | 0.16 (-0.42, 0.74) | 0.596 |
| East Asia | 7.87 (4.26, 12.51) | 5.66 (2.76, 9.22) | -1.12 (-1.32, -0.92) | <0.001 | 0.87 (0.54, 1.29) | 0.79 (0.48, 1.17) | -0.36 (-0.46, -0.26) | <0.001 | 5.09 (2.43, 7.12) | 2.12 (1.45, 3.20) | -2.99 (-4.78, -1.17) | 0.001 | 0.09 (0.04, 0.12) | 0.04 (0.02, 0.06) | -3.04 (-4.85, -1.20) | 0.001 |
| North Africa and Middle East | 4.76 (2.53, 7.54) | 6.30 (3.41, 9.83) | 0.97 (0.95, 0.99) | <0.001 | 0.95 (0.63, 1.37) | 1.02 (0.67, 1.47) | 0.26 (0.23, 0.29) | <0.001 | 8.18 (4.52, 12.17) | 6.75 (4.02, 9.40) | -0.65 (-0.89, -0.42) | <0.001 | 0.14 (0.08, 0.21) | 0.12 (0.07, 0.16) | -0.65 (-0.86, -0.45) | <0.001 |
| Western Sub-Saharan Africa | 3.63 (2.03, 5.66) | 3.90 (2.08, 6.39) | 0.30 (0.21, 0.40) | <0.001 | 0.65 (0.37, 1.17) | 0.73 (0.41, 1.30) | 0.41 (0.35, 0.48) | <0.001 | 17.49 (6.57, 28.85) | 9.56 (6.14, 15.04) | -2.07 (-2.23, -1.90) | <0.001 | 0.31 (0.11, 0.51) | 0.17 (0.11, 0.26) | -2.08 (-2.24, -1.91) | <0.001 |
| Southeast Asia | 6.42 (4.00, 9.62) | 8.29 (5.03, 12.63) | 0.87 (0.83, 0.91) | <0.001 | 2.09 (1.28, 3.22) | 2.13 (1.38, 3.11) | 0.06 (0.01, 0.11) | 0.011 | 38.75 (26.45, 65.45) | 34.26 (25.09, 52.77) | -0.42 (-0.85, 0.02) | 0.061 | 0.68 (0.46, 1.15) | 0.60 (0.44, 0.93) | -0.41 (-0.85, 0.02) | 0.062 |
| Eastern Sub-Saharan Africa | 3.47 (2.01, 5.35) | 3.58 (1.93, 5.85) | 0.16 (0.07, 0.26) | 0.001 | 0.69 (0.38, 1.27) | 0.78 (0.42, 1.43) | 0.43 (0.41, 0.46) | <0.001 | 22.44 (6.77, 39.64) | 13.75 (6.72, 22.17) | -1.68 (-1.86, -1.49) | <0.001 | 0.39 (0.12, 0.70) | 0.24 (0.12, 0.39) | -1.68 (-1.87, -1.49) | <0.001 |
| Central Latin America | 5.29 (2.78, 8.44) | 6.73 (3.40, 10.71) | 0.82 (0.70, 0.94) | <0.001 | 0.60 (0.31, 0.97) | 0.80 (0.44, 1.24) | 1.01 (0.98, 1.03) | <0.001 | 7.33 (5.60, 10.62) | 8.20 (5.35, 11.06) | 0.47 (-0.06, 1.01) | 0.083 | 0.13 (0.10, 0.19) | 0.14 (0.09, 0.19) | 0.47 (-0.07, 1.01) | 0.088 |
| Tropical Latin | 4.95 (2.75, | 8.29 (4.33, | 1.74 (1.45, | <0.001 | 0.50 (0.24, | 0.93 (0.50, | 2.11 (2.00, | <0.001 | 22.15 (16.47, | 21.04 (15.93, | -0.16 | 0.502 | 0.39 | 0.37 | -0.16 | 0.492 |

| | | | | | | | | | | | | | | | | |
|---|---|---|---|---|---|---|---|---|---|---|---|---|---|---|---|---|
| America | 7.76) | 13.51) | 2.04) | | 0.89) | 1.46) | 2.22) | | 36.96) | 31.32) | (-0.61, 0.30) | | (0.29, 0.65) | (0.28, 0.55) | (-0.61, 0.30) | |
| Central Sub-Saharan Africa | 2.76 (1.51, 4.38) | 3.27 (1.71, 5.37) | 0.64 (0.56, 0.72) | <0.001 | 0.66 (0.34, 1.28) | 0.72 (0.38, 1.38) | 0.34 (0.29, 0.39) | <0.001 | 15.93 (4.77, 31.93) | 12.45 (5.30, 22.44) | -0.88 (-1.11, -0.65) | <0.001 | 0.28 (0.08, 0.56) | 0.22 (0.09, 0.40) | -0.88 (-1.11, -0.65) | <0.001 |
| Western Europe | 7.09 (3.68, 11.19) | 7.49 (3.97, 11.95) | 0.20 (0.13, 0.27) | <0.001 | 1.39 (0.89, 1.99) | 1.15 (0.75, 1.65) | -0.66 (-0.78, -0.55) | <0.001 | 4.09 (2.94, 7.84) | 5.21 (2.83, 6.39) | 0.81 (0.55, 1.08) | <0.001 | 0.07 (0.05, 0.14) | 0.09 (0.05, 0.11) | 0.85 (0.58, 1.12) | <0.001 |
| High-income North America | 6.93 (3.69, 10.99) | 5.97 (3.60, 8.94) | -0.49 (-0.54, -0.45) | <0.001 | 5.22 (3.05, 8.41) | 5.12 (2.99, 8.67) | -0.02 (-0.16, 0.12) | 0.772 | 7.49 (5.91, 13.35) | 14.85 (6.73, 18.83) | 2.50 (1.75, 3.26) | <0.001 | 0.12 (0.10, 0.23) | 0.25 (0.11, 0.32) | 2.61 (1.85, 3.38) | <0.001 |
| Central Asia | 3.00 (1.49, 4.87) | 3.71 (1.90, 6.21) | 0.74 (0.69, 0.79) | <0.001 | 0.29 (0.16, 0.45) | 0.33 (0.19, 0.53) | 0.47 (0.44, 0.51) | <0.001 | 4.75 (3.48, 6.47) | 7.28 (5.31, 10.78) | 1.48 (1.15, 1.80) | <0.001 | 0.08 (0.06, 0.11) | 0.13 (0.09, 0.19) | 1.48 (1.16, 1.81) | <0.001 |
| Eastern Europe | 5.76 (3.06, 9.10) | 11.71 (7.08, 17.59) | 2.46 (2.25, 2.67) | <0.001 | 0.55 (0.32, 0.83) | 1.04 (0.65, 1.53) | 2.21 (2.04, 2.39) | <0.001 | 6.47 (4.77, 10.40) | 24.09 (7.62, 36.55) | 4.78 (3.87, 5.70) | <0.001 | 0.11 (0.08, 0.18) | 0.42 (0.13, 0.64) | 4.79 (3.88, 5.71) | <0.001 |
| Southern Sub-Saharan Africa | 6.91 (4.37, 10.13) | 5.38 (2.98, 8.59) | -0.83 (-0.93, -0.74) | <0.001 | 1.27 (0.74, 2.21) | 1.11 (0.62, 1.99) | -0.46 (-0.54, -0.37) | <0.001 | 71.25 (30.44, 103.07) | 27.90 (15.00, 48.99) | -3.00 (-5.12, -0.83) | 0.007 | 1.25 (0.53, 1.82) | 0.49 (0.26, 0.86) | -3.00 (-5.12, -0.84) | 0.007 |
| High-income Asia Pacific | 6.76 (3.64, 10.71) | 5.76 (3.02, 9.26) | -0.55 (-0.60, -0.51) | <0.001 | 1.93 (1.20, 2.93) | 1.53 (0.99, 2.23) | -0.84 (-0.99, -0.69) | <0.001 | 9.18 (5.26, 13.90) | 3.83 (2.94, 5.93) | -3.03 (-3.30, -2.75) | <0.001 | 0.16 (0.09, 0.24) | 0.07 (0.05, 0.10) | -3.08 (-3.36, -2.80) | <0.001 |
| Central Europe | 5.21 (2.63, 8.44) | 6.11 (3.21, 9.71) | 0.55 (0.51, 0.60) | <0.001 | 0.62 (0.37, 0.93) | 0.70 (0.43, 1.03) | 0.41 (0.35, 0.47) | <0.001 | 4.70 (3.66, 6.88) | 5.36 (2.73, 7.31) | 0.50 (-0.05, 1.05) | 0.074 | 0.08 (0.06, 0.12) | 0.09 (0.05, 0.13) | 0.50 (-0.05, 1.05) | 0.075 |
| Andean Latin America | 3.95 (2.23, 6.23) | 7.10 (3.90, 10.98) | 2.05 (2.00, 2.10) | <0.001 | 0.34 (0.17, 0.64) | 0.82 (0.41, 1.33) | 3.12 (3.06, 3.18) | <0.001 | 17.23 (9.25, 28.21) | 11.33 (6.87, 18.34) | -1.39 (-2.41, -0.36) | 0.008 | 0.30 (0.16, 0.50) | 0.20 (0.12, 0.32) | -1.40 (-2.42, -0.37) | 0.008 |
| Caribbean | 5.73 (3.25, 8.81) | 10.26 (6.10, 15.53) | 2.03 (1.96, 2.10) | <0.001 | 0.83 (0.53, 1.20) | 1.14 (0.74, 1.60) | 1.11 (1.07, 1.15) | <0.001 | 17.16 (11.95, 25.69) | 21.39 (12.67, 33.97) | 0.73 (0.36, 1.10) | <0.001 | 0.30 (0.21, 0.45) | 0.38 (0.22, 0.60) | 0.73 (0.35, 1.11) | <0.001 |
| Southern Latin | 4.46 (2.39, | 8.34 (4.57, | 2.19 (2.16, | <0.001 | 0.43 (0.22, | 1.02 (0.55, | 3.02 (2.93, | <0.001 | 8.18 (5.10, | 9.11 (6.24, | 0.47 | 0.003 | 0.14 | 0.16 | 0.46 (0.14, | 0.005 |

| | | | | | | | | | | | | | | | | |
|---|---|---|---|---|---|---|---|---|---|---|---|---|---|---|---|---|
| America | 7.19) | 12.94) | 2.22) | | 0.78) | 1.60) | 3.10) | | 13.10) | 13.71) | (0.15, 0.79) | | (0.09, 0.23) | (0.11, 0.24) | 0.78) | |
| Oceania | 6.68 (4.11, 9.92) | 8.30 (5.43, 12.12) | 0.77 (0.71, 0.82) | <0.001 | 0.91 (0.60, 1.31) | 0.92 (0.64, 1.29) | 0.04 (0.02, 0.06) | <0.001 | 42.21 (23.51, 73.91) | 57.38 (30.45, 101.83) | 1.04 (0.94, 1.13) | <0.001 | 0.74 (0.41, 1.30) | 1.01 (0.54, 1.80) | 1.04 (0.94, 1.14) | <0.001 |
| Australasia | 6.61 (3.56, 10.29) | 7.57 (4.08, 11.98) | 0.47 (0.39, 0.54) | <0.001 | 1.89 (1.25, 2.71) | 1.50 (0.98, 2.16) | -0.80 (-0.86, -0.74) | <0.001 | 5.85 (4.08, 9.61) | 8.04 (3.94, 12.28) | 1.11 (0.61, 1.61) | <0.001 | 0.10 (0.07, 0.17) | 0.14 (0.07, 0.22) | 1.16 (0.65, 1.67) | <0.001 |
| **Men** | | | | | | | | | | | | | | | | |
| Global | 6.58 (3.74, 10.18) | 7.59 (4.42, 11.56) | 0.50 (0.46, 0.53) | <0.001 | 1.07 (0.73, 1.52) | 1.14 (0.76, 1.62) | 0.21 (0.14, 0.29) | <0.001 | 18.42 (15.27, 25.14) | 20.81 (15.55, 25.69) | 0.44 (0.18, 0.70) | 0.001 | 0.32 (0.27, 0.44) | 0.37 (0.27, 0.45) | 0.44 (0.18, 0.71) | 0.001 |
| **SDI category** | | | | | | | | | | | | | | | | |
| High | 7.74 (4.29, 12.19) | 8.71 (5.19, 13.12) | 0.40 (0.36, 0.45) | <0.001 | 2.28 (1.52, 3.28) | 2.35 (1.59, 3.38) | 0.13 (0.03, 0.23) | 0.013 | 12.46 (10.00, 19.66) | 15.72 (8.57, 19.16) | 0.79 (0.56, 1.01) | <0.001 | 0.22 (0.17, 0.34) | 0.27 (0.15, 0.33) | 0.79 (0.57, 1.02) | <0.001 |
| High-middle | 7.89 (4.59, 11.96) | 9.76 (5.86, 14.49) | 0.73 (0.69, 0.77) | <0.001 | 0.99 (0.66, 1.38) | 1.26 (0.85, 1.74) | 0.83 (0.81, 0.86) | <0.001 | 19.59 (15.68, 26.26) | 26.82 (15.88, 33.37) | 1.09 (0.72, 1.46) | <0.001 | 0.34 (0.28, 0.46) | 0.47 (0.28, 0.59) | 1.09 (0.72, 1.46) | <0.001 |
| Middle | 6.72 (3.87, 10.31) | 7.59 (4.29, 11.68) | 0.43 (0.36, 0.49) | <0.001 | 0.92 (0.61, 1.32) | 1.06 (0.70, 1.51) | 0.47 (0.44, 0.49) | <0.001 | 20.44 (15.21, 33.39) | 19.50 (15.58, 29.39) | -0.17 (-0.40, 0.07) | 0.163 | 0.36 (0.27, 0.59) | 0.34 (0.27, 0.52) | -0.17 (-0.40, 0.07) | 0.159 |
| Low-middle | 4.52 (2.50, 7.16) | 6.06 (3.42, 9.36) | 1.03 (0.92, 1.14) | <0.001 | 0.52 (0.34, 0.75) | 0.69 (0.44, 0.99) | 0.99 (0.94, 1.04) | <0.001 | 17.60 (12.93, 23.89) | 19.59 (14.56, 26.73) | 0.36 (0.21, 0.52) | <0.001 | 0.31 (0.23, 0.42) | 0.35 (0.26, 0.47) | 0.36 (0.21, 0.52) | <0.001 |
| Low | 4.33 (2.58, 6.63) | 5.39 (3.12, 8.34) | 0.80 (0.73, 0.87) | <0.001 | 0.59 (0.36, 0.95) | 0.72 (0.45, 1.13) | 0.73 (0.69, 0.77) | <0.001 | 21.61 (12.23, 33.93) | 21.24 (13.55, 31.94) | -0.11 (-0.22, 0.01) | 0.067 | 0.38 (0.22, 0.60) | 0.37 (0.24, 0.56) | -0.11 (-0.22, 0.01) | 0.064 |
| **21 GBD region** | | | | | | | | | | | | | | | | |
| South Asia | 3.82 (2.08, 6.19) | 5.44 (3.04, 8.57) | 1.25 (1.13, 1.37) | <0.001 | 0.41 (0.26, 0.60) | 0.55 (0.34, 0.82) | 1.04 (1.01, 1.08) | <0.001 | 15.44 (10.56, 21.17) | 17.62 (12.70, 23.90) | 0.44 (0.18, 0.69) | 0.001 | 0.27 (0.19, 0.37) | 0.31 (0.22, 0.42) | 0.44 (0.19, 0.69) | 0.001 |
| East Asia | 8.18 (4.54, | 7.10 (3.73, | -0.45 (-0.65, | <0.001 | 0.90 (0.57, | 0.94 (0.59, | 0.17 (0.09, | <0.001 | 11.17 (6.63, | 7.49 (5.27, | -1.27 | <0.001 | 0.20 | 0.13 | -1.29 | <0.001 |

| | | | | | | | | | | | | | | | | |
|---|---|---|---|---|---|---|---|---|---|---|---|---|---|---|---|---|
| | 12.82) | 11.07) | -0.25) | | 1.31) | 1.38) | 0.25) | | 14.97) | 9.96) | (-1.59, -0.95) | | (0.12, 0.26) | (0.09, 0.17) | (-1.61, -0.96) | |
| North Africa and Middle East | 7.88 (4.55, 11.97) | 9.59 (5.41, 14.79) | 0.68 (0.64, 0.72) | <0.001 | 1.33 (0.92, 1.86) | 1.49 (1.01, 2.08) | 0.39 (0.33, 0.46) | <0.001 | 16.00 (10.99, 26.76) | 12.66 (8.21, 18.64) | -0.80 (-0.95, -0.64) | <0.001 | 0.28 (0.19, 0.47) | 0.22 (0.14, 0.33) | -0.80 (-0.96, -0.65) | <0.001 |
| Western Sub-Saharan Africa | 4.16 (2.33, 6.55) | 4.76 (2.62, 7.53) | 0.52 (0.44, 0.60) | <0.001 | 0.69 (0.40, 1.19) | 0.78 (0.46, 1.31) | 0.45 (0.39, 0.51) | <0.001 | 20.34 (11.60, 33.62) | 17.42 (11.27, 26.25) | -0.53 (-0.65, -0.40) | <0.001 | 0.36 (0.20, 0.59) | 0.31 (0.20, 0.46) | -0.53 (-0.65, -0.41) | <0.001 |
| Southeast Asia | 7.13 (4.56, 10.40) | 9.09 (5.86, 13.21) | 0.84 (0.79, 0.89) | <0.001 | 1.66 (1.10, 2.40) | 1.87 (1.29, 2.63) | 0.42 (0.35, 0.48) | <0.001 | 47.65 (29.65, 88.28) | 47.64 (33.78, 79.43) | 0.09 (-0.42, 0.61) | 0.718 | 0.84 (0.52, 1.56) | 0.84 (0.60, 1.40) | 0.10 (-0.41, 0.61) | 0.716 |
| Eastern Sub-Saharan Africa | 5.33 (3.35, 7.76) | 5.64 (3.43, 8.57) | 0.22 (0.12, 0.32) | <0.001 | 0.91 (0.55, 1.65) | 1.02 (0.60, 1.81) | 0.40 (0.38, 0.42) | <0.001 | 33.15 (16.97, 60.39) | 29.44 (16.29, 51.13) | -0.44 (-0.59, -0.29) | <0.001 | 0.58 (0.30, 1.06) | 0.52 (0.29, 0.90) | -0.44 (-0.59, -0.29) | <0.001 |
| Central Latin America | 5.49 (2.91, 8.66) | 9.07 (4.77, 14.37) | 1.73 (1.66, 1.79) | <0.001 | 0.45 (0.23, 0.73) | 0.82 (0.45, 1.29) | 2.09 (2.06, 2.12) | <0.001 | 13.36 (10.52, 20.83) | 17.27 (10.58, 23.33) | 0.93 (0.60, 1.27) | <0.001 | 0.24 (0.19, 0.37) | 0.30 (0.19, 0.41) | 0.93 (0.60, 1.26) | <0.001 |
| Tropical Latin America | 6.06 (3.61, 9.21) | 10.40 (5.77, 16.21) | 1.84 (1.76, 1.93) | <0.001 | 0.42 (0.24, 0.66) | 0.86 (0.47, 1.34) | 2.41 (2.34, 2.48) | <0.001 | 29.35 (20.94, 45.21) | 25.12 (19.32, 39.64) | -0.51 (-0.88, -0.14) | 0.007 | 0.52 (0.37, 0.80) | 0.44 (0.34, 0.70) | -0.52 (-0.89, -0.15) | 0.006 |
| Central Sub-Saharan Africa | 3.84 (2.27, 5.78) | 4.45 (2.57, 6.87) | 0.57 (0.50, 0.63) | <0.001 | 0.71 (0.41, 1.35) | 0.78 (0.44, 1.43) | 0.34 (0.30, 0.37) | <0.001 | 24.22 (11.45, 48.84) | 22.81 (11.50, 42.93) | -0.23 (-0.32, -0.15) | <0.001 | 0.43 (0.20, 0.86) | 0.40 (0.20, 0.76) | -0.23 (-0.32, -0.15) | <0.001 |
| Western Europe | 8.22 (4.47, 13.00) | 9.43 (5.37, 14.57) | 0.46 (0.42, 0.50) | <0.001 | 1.39 (0.93, 1.92) | 1.33 (0.90, 1.86) | -0.15 (-0.18, -0.13) | <0.001 | 8.96 (6.44, 16.87) | 12.22 (5.91, 15.78) | 1.01 (0.83, 1.20) | <0.001 | 0.16 (0.11, 0.30) | 0.21 (0.10, 0.28) | 1.03 (0.84, 1.21) | <0.001 |
| High-income North America | 7.08 (3.90, 11.20) | 7.65 (4.83, 11.35) | 0.27 (0.17, 0.37) | <0.001 | 3.55 (2.15, 5.65) | 4.06 (2.38, 6.62) | 0.50 (0.27, 0.73) | <0.001 | 14.68 (11.98, 24.51) | 22.10 (10.37, 28.97) | 1.41 (0.94, 1.87) | <0.001 | 0.25 (0.21, 0.43) | 0.38 (0.18, 0.51) | 1.42 (0.95, 1.89) | <0.001 |
| Central Asia | 4.21 (2.21, 6.76) | 5.07 (2.78, 8.09) | 0.68 (0.61, 0.76) | <0.001 | 0.37 (0.22, 0.58) | 0.45 (0.26, 0.68) | 0.64 (0.56, 0.73) | <0.001 | 8.14 (5.11, 11.96) | 11.76 (8.62, 17.18) | 1.26 (0.84, 1.68) | <0.001 | 0.14 (0.09, 0.21) | 0.21 (0.15, 0.30) | 1.26 (0.85, 1.68) | <0.001 |
| Eastern Europe | 7.42 (4.50, | 18.60 | 3.24 (3.06, | <0.001 | 0.68 (0.45, | 1.53 (1.06, | 2.87 (2.61, | <0.001 | 27.80 (19.72, | 103.16 (46.14, | 4.64 | <0.001 | 0.49 | 1.82 | 4.65 (3.88, | <0.001 |

| | | | | | | | | | | | | | | | | |
|---|---|---|---|---|---|---|---|---|---|---|---|---|---|---|---|---|
| | 11.09) | (12.60, 25.73) | 3.41) | | 0.97) | 2.12) | 3.14) | | 54.94) | 137.93) | (3.87, 5.42) | | (0.35, 0.97) | (0.81, 2.44) | 5.42) | |
| Southern Sub-Saharan Africa | 6.23 (3.90, 9.18) | 6.18 (3.67, 9.47) | -0.03 (-0.08, 0.02) | 0.262 | 0.91 (0.57, 1.50) | 0.91 (0.55, 1.50) | -0.02 (-0.07, 0.02) | 0.342 | 48.98 (30.95, 67.43) | 36.26 (24.43, 51.47) | -1.20 (-2.58, 0.19) | 0.091 | 0.86 (0.54, 1.19) | 0.64 (0.43, 0.91) | -1.20 (-2.58, 0.20) | 0.091 |
| High-income Asia Pacific | 8.73 (4.90, 13.62) | 8.28 (4.65, 12.68) | -0.17 (-0.22, -0.13) | <0.001 | 1.90 (1.29, 2.65) | 1.70 (1.15, 2.36) | -0.39 (-0.44, -0.35) | <0.001 | 11.37 (7.27, 17.12) | 7.66 (5.37, 9.98) | -1.39 (-1.68, -1.09) | <0.001 | 0.20 (0.13, 0.30) | 0.13 (0.09, 0.17) | -1.40 (-1.70, -1.10) | <0.001 |
| Central Europe | 5.84 (3.17, 9.22) | 7.92 (4.68, 11.96) | 1.08 (0.95, 1.22) | <0.001 | 0.73 (0.48, 1.05) | 0.96 (0.65, 1.33) | 0.90 (0.80, 1.00) | <0.001 | 13.09 (10.32, 20.90) | 17.60 (8.39, 23.43) | 1.02 (0.61, 1.43) | <0.001 | 0.23 (0.18, 0.37) | 0.31 (0.15, 0.41) | 1.02 (0.61, 1.43) | <0.001 |
| Andean Latin America | 4.84 (2.62, 7.61) | 8.30 (4.58, 12.82) | 1.89 (1.83, 1.95) | <0.001 | 0.36 (0.19, 0.58) | 0.71 (0.38, 1.10) | 2.36 (2.27, 2.46) | <0.001 | 15.16 (9.05, 23.11) | 12.60 (7.45, 20.67) | -0.70 (-0.98, -0.41) | <0.001 | 0.27 (0.16, 0.41) | 0.22 (0.13, 0.36) | -0.70 (-0.99, -0.41) | <0.001 |
| Caribbean | 6.95 (4.10, 10.43) | 11.85 (7.27, 17.50) | 1.86 (1.82, 1.91) | <0.001 | 0.84 (0.55, 1.19) | 1.22 (0.82, 1.70) | 1.29 (1.26, 1.33) | <0.001 | 26.24 (19.48, 36.38) | 32.27 (22.02, 45.89) | 0.75 (0.48, 1.02) | <0.001 | 0.46 (0.34, 0.64) | 0.57 (0.39, 0.81) | 0.75 (0.48, 1.02) | <0.001 |
| Southern Latin America | 5.02 (2.80, 7.90) | 9.93 (5.89, 14.99) | 2.38 (2.29, 2.47) | <0.001 | 0.41 (0.23, 0.64) | 1.04 (0.60, 1.60) | 3.27 (3.22, 3.33) | <0.001 | 14.60 (8.93, 22.76) | 22.22 (13.99, 32.38) | 1.52 (1.24, 1.80) | <0.001 | 0.26 (0.16, 0.40) | 0.39 (0.25, 0.57) | 1.51 (1.24, 1.79) | <0.001 |
| Oceania | 4.83 (2.83, 7.37) | 5.76 (3.50, 8.57) | 0.62 (0.49, 0.75) | <0.001 | 0.50 (0.33, 0.71) | 0.55 (0.37, 0.78) | 0.35 (0.24, 0.46) | <0.001 | 17.77 (10.02, 31.75) | 23.43 (14.27, 36.73) | 0.97 (0.76, 1.18) | <0.001 | 0.31 (0.18, 0.56) | 0.41 (0.25, 0.65) | 0.97 (0.77, 1.18) | <0.001 |
| Australasia | 7.15 (3.86, 11.14) | 8.47 (4.76, 13.01) | 0.58 (0.52, 0.64) | <0.001 | 1.43 (0.98, 1.97) | 1.28 (0.86, 1.78) | -0.37 (-0.39, -0.35) | <0.001 | 10.44 (6.99, 17.56) | 14.93 (6.93, 24.62) | 1.32 (0.67, 1.98) | <0.001 | 0.18 (0.12, 0.31) | 0.26 (0.12, 0.43) | 1.34 (0.68, 2.00) | <0.001 |
| **35-39 years** | | | | | | | | | | | | | | | | |
| **Women** | | | | | | | | | | | | | | | | |
| Global | 6.53 (3.16, 10.55) | 6.85 (3.36, 10.98) | 0.18 (0.12, 0.24) | <0.001 | 1.09 (0.72, 1.51) | 1.06 (0.69, 1.48) | -0.07 (-0.15, 0.02) | 0.115 | 12.92 (9.05, 17.61) | 13.78 (9.95, 17.69) | 0.23 (0.08, 0.39) | 0.003 | 0.25 (0.17, 0.34) | 0.27 (0.19, 0.34) | 0.24 (0.08, 0.40) | 0.003 |
| **SDI category** | | | | | | | | | | | | | | | | |
| High | 7.58 (3.52, | 7.52 (3.78, | -0.02 (-0.08, | 0.413 | 2.39 (1.64, | 1.94 (1.37, | -0.68 | <0.001 | 7.42 (5.78, | 10.48 (5.81, | 1.18 | <0.001 | 0.14 | 0.20 | 1.22 (0.98, | <0.001 |

| | | | | | | | | | | | | | | | | |
|---|---|---|---|---|---|---|---|---|---|---|---|---|---|---|---|---|
| | 12.50) | 11.87) | 0.03) | | 3.30) | 2.66) | (-0.74, -0.63) | | 12.12) | 12.82) | (0.95, 1.42) | | (0.11, 0.23) | (0.11, 0.25) | 1.46) | |
| High-middle | 7.51 (3.57, 12.16) | 8.32 (4.01, 13.28) | 0.37 (0.26, 0.48) | <0.001 | 0.89 (0.56, 1.26) | 1.05 (0.67, 1.49) | 0.58 (0.54, 0.62) | <0.001 | 9.10 (6.78, 12.52) | 11.11 (7.46, 13.51) | 0.75 (0.34, 1.15) | <0.001 | 0.18 (0.13, 0.24) | 0.21 (0.14, 0.26) | 0.75 (0.34, 1.15) | <0.001 |
| Middle | 7.14 (3.53, 11.40) | 7.30 (3.57, 11.62) | 0.10 (0.04, 0.16) | 0.001 | 0.97 (0.63, 1.37) | 1.08 (0.71, 1.52) | 0.38 (0.31, 0.45) | <0.001 | 15.87 (11.66, 23.44) | 14.83 (11.24, 21.78) | -0.19 (-0.30, -0.08) | 0.001 | 0.31 (0.22, 0.45) | 0.29 (0.22, 0.42) | -0.19 (-0.30, -0.08) | 0.001 |
| Low-middle | 4.35 (2.12, 7.12) | 5.68 (2.79, 9.31) | 0.96 (0.84, 1.08) | <0.001 | 0.49 (0.30, 0.70) | 0.68 (0.42, 0.98) | 1.15 (1.13, 1.17) | <0.001 | 14.96 (8.48, 22.14) | 15.51 (10.38, 21.62) | 0.13 (-0.07, 0.34) | 0.198 | 0.29 (0.16, 0.43) | 0.30 (0.20, 0.42) | 0.14 (-0.07, 0.34) | 0.194 |
| Low | 3.59 (1.84, 5.76) | 4.50 (2.22, 7.37) | 0.85 (0.73, 0.96) | <0.001 | 0.61 (0.35, 0.98) | 0.76 (0.44, 1.20) | 0.76 (0.71, 0.80) | <0.001 | 20.65 (8.29, 31.87) | 15.72 (9.01, 21.91) | -0.93 (-1.05, -0.82) | <0.001 | 0.40 (0.16, 0.62) | 0.30 (0.17, 0.42) | -0.94 (-1.05, -0.82) | <0.001 |
| **21 GBD region** | | | | | | | | | | | | | | | | |
| South Asia | 3.60 (1.68, 6.02) | 5.20 (2.50, 8.59) | 1.30 (1.16, 1.44) | <0.001 | 0.36 (0.21, 0.53) | 0.50 (0.30, 0.75) | 1.16 (1.06, 1.25) | <0.001 | 11.41 (6.65, 18.81) | 12.55 (7.90, 18.63) | 0.24 (-0.20, 0.68) | 0.280 | 0.22 (0.13, 0.36) | 0.24 (0.15, 0.36) | 0.25 (-0.19, 0.69) | 0.270 |
| East Asia | 8.76 (4.08, 14.11) | 6.42 (2.72, 11.05) | -1.02 (-1.29, -0.75) | <0.001 | 0.81 (0.46, 1.19) | 0.74 (0.43, 1.12) | -0.28 (-0.41, -0.15) | <0.001 | 4.85 (2.69, 6.65) | 2.35 (1.52, 3.32) | -2.48 (-2.81, -2.15) | <0.001 | 0.09 (0.05, 0.13) | 0.04 (0.03, 0.06) | -2.52 (-2.85, -2.19) | <0.001 |
| North Africa and Middle East | 5.97 (2.86, 9.85) | 8.18 (3.98, 13.16) | 1.09 (1.07, 1.11) | <0.001 | 1.07 (0.70, 1.50) | 1.15 (0.76, 1.60) | 0.25 (0.18, 0.31) | <0.001 | 11.02 (5.85, 16.42) | 8.92 (5.10, 12.82) | -0.71 (-0.85, -0.57) | <0.001 | 0.21 (0.11, 0.32) | 0.17 (0.10, 0.25) | -0.71 (-0.85, -0.57) | <0.001 |
| Western Sub-Saharan Africa | 3.94 (2.17, 6.13) | 4.21 (2.06, 6.84) | 0.30 (0.20, 0.39) | <0.001 | 1.13 (0.64, 1.95) | 1.28 (0.71, 2.20) | 0.44 (0.39, 0.49) | <0.001 | 31.43 (11.87, 51.93) | 16.25 (10.27, 25.16) | -2.26 (-2.37, -2.15) | <0.001 | 0.61 (0.23, 1.00) | 0.31 (0.20, 0.48) | -2.27 (-2.39, -2.16) | <0.001 |
| Southeast Asia | 6.90 (4.06, 10.24) | 9.05 (5.18, 13.66) | 0.93 (0.90, 0.96) | <0.001 | 1.80 (1.17, 2.54) | 1.89 (1.30, 2.60) | 0.16 (0.12, 0.19) | <0.001 | 40.68 (28.14, 64.69) | 37.57 (26.38, 57.97) | -0.27 (-0.51, -0.03) | 0.028 | 0.78 (0.54, 1.25) | 0.72 (0.51, 1.12) | -0.27 (-0.51, -0.03) | 0.028 |
| Eastern Sub-Saharan Africa | 3.65 (2.06, 5.62) | 3.89 (1.95, 6.34) | 0.27 (0.17, 0.37) | <0.001 | 0.86 (0.49, 1.47) | 1.00 (0.57, 1.73) | 0.53 (0.47, 0.59) | <0.001 | 29.46 (9.35, 51.60) | 18.26 (8.45, 30.53) | -1.64 (-1.76, -1.51) | <0.001 | 0.57 (0.18, 1.00) | 0.35 (0.16, 0.59) | -1.64 (-1.77, -1.51) | <0.001 |

| | | | | | | | | | | | | | | | | |
|---|---|---|---|---|---|---|---|---|---|---|---|---|---|---|---|---|
| Central Latin America | 6.14 (2.83, 10.06) | 8.22 (3.59, 13.88) | 1.01 (0.92, 1.10) | <0.001 | 0.71 (0.40, 1.09) | 0.98 (0.55, 1.48) | 1.09 (1.05, 1.13) | <0.001 | 9.61 (7.46, 14.85) | 11.30 (7.31, 15.51) | 0.61 (0.30, 0.93) | <0.001 | 0.19 (0.14, 0.29) | 0.22 (0.14, 0.30) | 0.61 (0.29, 0.93) | <0.001 |
| Tropical Latin America | 5.54 (2.99, 8.59) | 9.97 (4.82, 16.04) | 1.99 (1.78, 2.21) | <0.001 | 0.58 (0.31, 0.92) | 1.09 (0.61, 1.63) | 2.18 (2.09, 2.28) | <0.001 | 24.22 (18.91, 42.37) | 24.71 (18.56, 36.88) | 0.09 (-0.07, 0.26) | 0.275 | 0.47 (0.37, 0.82) | 0.48 (0.36, 0.71) | 0.09 (-0.08, 0.26) | 0.300 |
| Central Sub-Saharan Africa | 3.10 (1.59, 5.02) | 3.67 (1.84, 6.16) | 0.63 (0.56, 0.71) | <0.001 | 0.83 (0.45, 1.48) | 0.92 (0.50, 1.60) | 0.34 (0.31, 0.37) | <0.001 | 21.73 (6.85, 42.35) | 17.86 (7.40, 34.45) | -0.69 (-0.93, -0.46) | <0.001 | 0.42 (0.13, 0.82) | 0.34 (0.14, 0.66) | -0.70 (-0.93, -0.46) | <0.001 |
| Western Europe | 7.98 (3.63, 13.24) | 8.42 (3.87, 13.86) | 0.16 (0.09, 0.24) | <0.001 | 1.26 (0.83, 1.80) | 1.06 (0.66, 1.54) | -0.60 (-0.68, -0.52) | <0.001 | 4.80 (3.36, 9.52) | 6.02 (3.45, 7.39) | 0.81 (0.66, 0.96) | <0.001 | 0.09 (0.06, 0.18) | 0.12 (0.07, 0.14) | 0.84 (0.65, 1.03) | <0.001 |
| High-income North America | 7.41 (3.48, 12.13) | 6.48 (3.64, 9.88) | -0.45 (-0.49, -0.41) | <0.001 | 4.03 (2.71, 5.80) | 3.28 (2.25, 4.68) | -0.67 (-0.88, -0.46) | <0.001 | 8.59 (6.67, 16.25) | 17.40 (8.46, 21.39) | 2.44 (2.07, 2.81) | <0.001 | 0.16 (0.12, 0.31) | 0.33 (0.16, 0.41) | 2.52 (2.14, 2.90) | <0.001 |
| Central Asia | 3.46 (1.50, 5.93) | 4.25 (1.92, 7.12) | 0.73 (0.69, 0.77) | <0.001 | 0.31 (0.16, 0.49) | 0.36 (0.18, 0.58) | 0.53 (0.49, 0.57) | <0.001 | 3.99 (2.82, 5.98) | 6.60 (4.71, 10.23) | 1.76 (1.41, 2.11) | <0.001 | 0.08 (0.05, 0.12) | 0.13 (0.09, 0.20) | 1.76 (1.41, 2.11) | <0.001 |
| Eastern Europe | 6.48 (3.07, 10.70) | 12.44 (6.76, 19.16) | 2.30 (2.18, 2.42) | <0.001 | 0.60 (0.34, 0.93) | 1.10 (0.65, 1.60) | 2.12 (2.02, 2.21) | <0.001 | 7.53 (5.57, 14.44) | 24.09 (10.54, 34.04) | 4.21 (3.38, 5.04) | <0.001 | 0.15 (0.11, 0.28) | 0.47 (0.20, 0.66) | 4.21 (3.38, 5.04) | <0.001 |
| Southern Sub-Saharan Africa | 7.29 (4.39, 10.74) | 5.91 (3.05, 9.42) | -0.70 (-0.84, -0.57) | <0.001 | 1.52 (0.92, 2.45) | 1.34 (0.79, 2.17) | -0.41 (-0.47, -0.36) | <0.001 | 66.14 (29.23, 96.73) | 23.62 (12.90, 41.94) | -3.45 (-4.86, -2.02) | <0.001 | 1.27 (0.56, 1.87) | 0.45 (0.25, 0.81) | -3.46 (-4.87, -2.02) | <0.001 |
| High-income Asia Pacific | 7.76 (3.66, 12.73) | 6.49 (2.98, 10.74) | -0.61 (-0.66, -0.57) | <0.001 | 1.75 (1.12, 2.52) | 1.39 (0.94, 1.95) | -0.80 (-0.89, -0.71) | <0.001 | 8.85 (5.68, 13.08) | 4.72 (3.54, 7.44) | -2.19 (-2.44, -1.94) | <0.001 | 0.17 (0.11, 0.25) | 0.09 (0.07, 0.14) | -2.22 (-2.47, -1.97) | <0.001 |
| Central Europe | 6.14 (2.73, 10.43) | 7.24 (3.30, 11.84) | 0.57 (0.50, 0.64) | <0.001 | 0.68 (0.39, 1.00) | 0.75 (0.44, 1.14) | 0.37 (0.29, 0.45) | <0.001 | 5.49 (4.41, 8.92) | 6.43 (3.31, 8.70) | 0.63 (0.27, 0.98) | 0.001 | 0.11 (0.08, 0.17) | 0.12 (0.06, 0.17) | 0.63 (0.27, 0.99) | 0.001 |
| Andean Latin America | 4.72 (2.42, 7.57) | 8.48 (4.08, 14.02) | 2.04 (2.01, 2.07) | <0.001 | 0.41 (0.20, 0.74) | 0.97 (0.54, 1.45) | 2.97 (2.92, 3.02) | <0.001 | 22.98 (12.83, 36.89) | 14.75 (8.98, 23.56) | -1.44 (-2.36, -0.51) | 0.002 | 0.44 (0.25, 0.71) | 0.28 (0.17, 0.45) | -1.44 (-2.37, -0.51) | 0.002 |

| | | | | | | | | | | | | | | | | |
|---|---|---|---|---|---|---|---|---|---|---|---|---|---|---|---|---|
| Caribbean | 6.43 (3.34, 10.22) | 11.99 (6.65, 18.25) | 2.17 (2.12, 2.22) | <0.001 | 0.88 (0.59, 1.22) | 1.29 (0.84, 1.77) | 1.33 (1.31, 1.35) | <0.001 | 24.29 (17.54, 37.58) | 32.24 (20.75, 51.21) | 1.01 (0.69, 1.32) | <0.001 | 0.47 (0.34, 0.73) | 0.62 (0.40, 0.99) | 1.01 (0.69, 1.32) | <0.001 |
| Southern Latin America | 5.03 (2.42, 8.21) | 9.86 (4.94, 16.00) | 2.35 (2.29, 2.41) | <0.001 | 0.52 (0.25, 0.97) | 1.23 (0.67, 1.97) | 3.03 (2.96, 3.10) | <0.001 | 16.37 (10.55, 26.93) | 18.22 (12.03, 31.01) | 0.36 (0.04, 0.68) | 0.029 | 0.32 (0.20, 0.52) | 0.35 (0.23, 0.60) | 0.35 (0.03, 0.67) | 0.033 |
| Oceania | 7.29 (4.51, 10.69) | 9.34 (6.00, 13.32) | 0.87 (0.80, 0.93) | <0.001 | 1.09 (0.73, 1.52) | 1.13 (0.81, 1.57) | 0.15 (0.13, 0.16) | <0.001 | 40.35 (22.22, 73.21) | 55.89 (29.65, 99.82) | 1.11 (0.81, 1.41) | <0.001 | 0.78 (0.43, 1.42) | 1.08 (0.57, 1.93) | 1.11 (0.81, 1.41) | <0.001 |
| Australasia | 7.59 (3.42, 12.63) | 8.76 (4.11, 14.24) | 0.50 (0.41, 0.59) | <0.001 | 1.56 (1.05, 2.17) | 1.34 (0.87, 1.86) | -0.54 (-0.62, -0.47) | <0.001 | 4.73 (3.18, 7.58) | 7.42 (3.46, 11.76) | 1.52 (1.05, 2.00) | <0.001 | 0.09 (0.06, 0.14) | 0.14 (0.07, 0.23) | 1.57 (1.09, 2.05) | <0.001 |
| **Men** | | | | | | | | | | | | | | | | |
| Global | 7.72 (4.00, 12.09) | 9.20 (4.89, 14.33) | 0.61 (0.56, 0.66) | <0.001 | 1.09 (0.73, 1.50) | 1.23 (0.82, 1.68) | 0.42 (0.37, 0.48) | <0.001 | 21.62 (17.89, 30.43) | 25.36 (19.73, 32.83) | 0.59 (0.29, 0.89) | <0.001 | 0.42 (0.34, 0.59) | 0.49 (0.38, 0.63) | 0.59 (0.29, 0.89) | <0.001 |
| **SDI category** | | | | | | | | | | | | | | | | |
| High | 9.04 (4.48, 14.37) | 10.42 (5.67, 16.13) | 0.49 (0.44, 0.54) | <0.001 | 2.05 (1.42, 2.84) | 1.97 (1.41, 2.64) | -0.11 (-0.17, -0.06) | <0.001 | 15.66 (12.98, 24.69) | 19.67 (11.41, 23.54) | 0.78 (0.63, 0.92) | <0.001 | 0.30 (0.25, 0.48) | 0.38 (0.22, 0.45) | 0.78 (0.64, 0.93) | <0.001 |
| High-middle | 9.19 (4.92, 14.13) | 12.05 (6.53, 18.58) | 0.94 (0.89, 0.98) | <0.001 | 1.04 (0.68, 1.43) | 1.39 (0.91, 1.92) | 1.00 (0.97, 1.03) | <0.001 | 22.05 (17.72, 31.29) | 31.30 (19.88, 38.22) | 1.25 (0.69, 1.81) | <0.001 | 0.43 (0.34, 0.60) | 0.60 (0.38, 0.74) | 1.25 (0.69, 1.81) | <0.001 |
| Middle | 7.95 (4.07, 12.41) | 9.28 (4.77, 14.76) | 0.54 (0.47, 0.61) | <0.001 | 0.98 (0.64, 1.36) | 1.23 (0.80, 1.72) | 0.78 (0.72, 0.84) | <0.001 | 23.17 (17.33, 38.13) | 23.99 (18.47, 38.22) | 0.14 (-0.09, 0.38) | 0.224 | 0.45 (0.33, 0.74) | 0.46 (0.36, 0.74) | 0.14 (-0.09, 0.37) | 0.234 |
| Low-middle | 5.21 (2.68, 8.35) | 7.18 (3.73, 11.22) | 1.14 (1.05, 1.22) | <0.001 | 0.55 (0.35, 0.78) | 0.78 (0.49, 1.10) | 1.18 (1.13, 1.24) | <0.001 | 21.33 (15.39, 29.14) | 24.47 (17.96, 33.44) | 0.49 (0.05, 0.94) | 0.030 | 0.41 (0.30, 0.56) | 0.47 (0.35, 0.65) | 0.49 (0.05, 0.93) | 0.030 |
| Low | 4.82 (2.72, 7.39) | 6.09 (3.28, 9.43) | 0.85 (0.79, 0.92) | <0.001 | 0.70 (0.43, 1.07) | 0.87 (0.55, 1.33) | 0.79 (0.76, 0.81) | <0.001 | 28.27 (15.95, 44.42) | 26.92 (17.60, 40.00) | -0.18 (-0.40, 0.04) | 0.113 | 0.55 (0.31, 0.86) | 0.52 (0.34, 0.77) | -0.18 (-0.40, 0.04) | 0.108 |
| **21 GBD region** | | | | | | | | | | | | | | | | |

| | | | | | | | | | | | | | | | | |
|---|---|---|---|---|---|---|---|---|---|---|---|---|---|---|---|---|
| South Asia | 4.42 (2.23, 7.17) | 6.40 (3.30, 10.10) | 1.30 (1.20, 1.40) | <0.001 | 0.42 (0.26, 0.61) | 0.59 (0.36, 0.86) | 1.18 (1.11, 1.26) | <0.001 | 19.65 (13.23, 27.00) | 22.34 (15.39, 30.91) | 0.45 (-0.44, 1.35) | 0.319 | 0.38 (0.26, 0.52) | 0.43 (0.30, 0.60) | 0.45 (-0.43, 1.35) | 0.319 |
| East Asia | 9.49 (4.63, 15.13) | 8.97 (4.10, 14.90) | -0.14 (-0.36, 0.08) | 0.206 | 0.93 (0.56, 1.33) | 1.06 (0.63, 1.55) | 0.51 (0.35, 0.67) | <0.001 | 12.32 (7.40, 16.75) | 8.22 (5.80, 10.76) | -1.33 (-1.86, -0.80) | <0.001 | 0.24 (0.14, 0.32) | 0.16 (0.11, 0.21) | -1.34 (-1.87, -0.81) | <0.001 |
| North Africa and Middle East | 9.70 (5.24, 15.01) | 11.79 (5.98, 19.03) | 0.68 (0.61, 0.76) | <0.001 | 1.49 (1.02, 2.05) | 1.64 (1.09, 2.25) | 0.33 (0.24, 0.42) | <0.001 | 21.77 (14.60, 37.74) | 15.89 (10.93, 23.64) | -1.10 (-1.23, -0.97) | <0.001 | 0.42 (0.28, 0.73) | 0.31 (0.21, 0.46) | -1.11 (-1.24, -0.98) | <0.001 |
| Western Sub-Saharan Africa | 4.48 (2.35, 7.13) | 5.10 (2.58, 8.22) | 0.53 (0.44, 0.63) | <0.001 | 0.93 (0.55, 1.50) | 1.04 (0.61, 1.67) | 0.41 (0.32, 0.51) | <0.001 | 28.33 (15.21, 51.23) | 23.26 (14.01, 36.64) | -0.69 (-0.81, -0.56) | <0.001 | 0.55 (0.29, 0.99) | 0.45 (0.27, 0.71) | -0.69 (-0.82, -0.56) | <0.001 |
| Southeast Asia | 8.09 (4.94, 11.83) | 10.35 (6.26, 15.01) | 0.85 (0.81, 0.89) | <0.001 | 1.76 (1.21, 2.44) | 2.01 (1.40, 2.75) | 0.45 (0.42, 0.47) | <0.001 | 55.63 (33.39, 108.88) | 54.70 (36.09, 100.04) | -0.01 (-0.31, 0.29) | 0.945 | 1.07 (0.64, 2.10) | 1.06 (0.70, 1.93) | -0.01 (-0.32, 0.29) | 0.939 |
| Eastern Sub-Saharan Africa | 5.76 (3.54, 8.46) | 6.08 (3.52, 9.16) | 0.20 (0.11, 0.29) | <0.001 | 1.12 (0.67, 1.86) | 1.28 (0.76, 2.13) | 0.46 (0.44, 0.47) | <0.001 | 43.20 (20.96, 77.01) | 35.95 (19.47, 61.68) | -0.63 (-0.83, -0.43) | <0.001 | 0.83 (0.40, 1.49) | 0.69 (0.38, 1.19) | -0.63 (-0.83, -0.43) | <0.001 |
| Central Latin America | 6.37 (3.05, 10.40) | 11.34 (5.29, 18.61) | 1.99 (1.88, 2.11) | <0.001 | 0.53 (0.27, 0.83) | 1.02 (0.52, 1.63) | 2.30 (2.27, 2.33) | <0.001 | 14.34 (11.23, 22.25) | 18.67 (11.21, 24.87) | 0.96 (0.69, 1.24) | <0.001 | 0.28 (0.22, 0.43) | 0.36 (0.22, 0.48) | 0.96 (0.68, 1.24) | <0.001 |
| Tropical Latin America | 7.13 (4.00, 10.87) | 13.65 (6.94, 21.78) | 2.22 (2.13, 2.31) | <0.001 | 0.51 (0.30, 0.76) | 1.14 (0.61, 1.79) | 2.80 (2.71, 2.88) | <0.001 | 35.61 (25.54, 55.65) | 32.88 (23.65, 49.84) | -0.26 (-0.59, 0.07) | 0.126 | 0.69 (0.49, 1.08) | 0.63 (0.46, 0.96) | -0.27 (-0.59, 0.06) | 0.107 |
| Central Sub-Saharan Africa | 4.42 (2.48, 6.71) | 5.10 (2.78, 8.01) | 0.56 (0.50, 0.61) | <0.001 | 0.86 (0.51, 1.44) | 0.94 (0.55, 1.55) | 0.32 (0.28, 0.35) | <0.001 | 32.41 (14.95, 65.56) | 30.10 (15.54, 58.77) | -0.31 (-0.43, -0.18) | <0.001 | 0.63 (0.29, 1.27) | 0.58 (0.30, 1.14) | -0.31 (-0.43, -0.18) | <0.001 |
| Western Europe | 9.63 (4.61, 15.57) | 11.40 (5.84, 18.26) | 0.57 (0.55, 0.59) | <0.001 | 1.35 (0.88, 1.88) | 1.34 (0.85, 1.88) | -0.03 (-0.07, 0.01) | 0.180 | 10.51 (7.42, 20.64) | 14.90 (7.14, 19.04) | 1.13 (0.84, 1.42) | <0.001 | 0.20 (0.14, 0.40) | 0.29 (0.14, 0.37) | 1.14 (0.85, 1.44) | <0.001 |
| High-income North America | 7.93 (4.05, 12.60) | 8.60 (5.20, 12.63) | 0.29 (0.20, 0.38) | <0.001 | 2.93 (2.00, 4.20) | 2.72 (1.91, 3.87) | -0.21 (-0.43, -0.00) | 0.047 | 19.76 (16.51, 32.34) | 27.83 (14.59, 34.25) | 1.24 (0.99, 1.48) | <0.001 | 0.38 (0.31, 0.62) | 0.53 (0.28, 0.66) | 1.25 (1.01, 1.50) | <0.001 |

| | | | | | | | | | | | | | | | | |
|---|---|---|---|---|---|---|---|---|---|---|---|---|---|---|---|---|
| Central Asia | 5.06 (2.35, 8.46) | 6.13 (2.98, 9.81) | 0.70 (0.59, 0.82) | <0.001 | 0.45 (0.24, 0.69) | 0.53 (0.30, 0.81) | 0.65 (0.58, 0.72) | <0.001 | 11.21 (7.32, 16.57) | 15.95 (11.57, 25.62) | 1.19 (0.75, 1.63) | <0.001 | 0.22 (0.14, 0.32) | 0.31 (0.22, 0.50) | 1.18 (0.75, 1.62) | <0.001 |
| Eastern Europe | 8.50 (4.86, 12.98) | 21.37 (13.40, 30.91) | 3.29 (3.03, 3.54) | <0.001 | 0.78 (0.50, 1.09) | 1.76 (1.17, 2.47) | 2.91 (2.67, 3.15) | <0.001 | 31.21 (21.50, 66.95) | 108.67 (50.90, 145.65) | 4.49 (3.50, 5.48) | <0.001 | 0.60 (0.42, 1.30) | 2.10 (0.98, 2.82) | 4.49 (3.50, 5.48) | <0.001 |
| Southern Sub-Saharan Africa | 7.14 (4.37, 10.63) | 6.68 (3.80, 10.26) | -0.22 (-0.30, -0.14) | <0.001 | 1.11 (0.71, 1.71) | 1.08 (0.67, 1.67) | -0.11 (-0.14, -0.08) | <0.001 | 62.17 (34.89, 86.37) | 41.66 (26.62, 58.78) | -1.37 (-2.15, -0.59) | 0.001 | 1.20 (0.67, 1.67) | 0.80 (0.51, 1.13) | -1.37 (-2.15, -0.59) | 0.001 |
| High-income Asia Pacific | 10.44 (5.18, 16.85) | 9.97 (4.98, 16.25) | -0.16 (-0.24, -0.09) | <0.001 | 1.94 (1.31, 2.68) | 1.83 (1.28, 2.49) | -0.21 (-0.24, -0.17) | <0.001 | 13.16 (8.85, 19.53) | 9.32 (6.98, 13.41) | -1.21 (-1.50, -0.92) | <0.001 | 0.25 (0.17, 0.38) | 0.18 (0.13, 0.26) | -1.22 (-1.51, -0.93) | <0.001 |
| Central Europe | 6.64 (3.24, 10.78) | 9.49 (5.22, 14.78) | 1.24 (1.12, 1.36) | <0.001 | 0.80 (0.51, 1.12) | 1.07 (0.71, 1.49) | 0.99 (0.91, 1.07) | <0.001 | 15.60 (12.37, 24.41) | 21.05 (9.91, 27.68) | 1.05 (0.78, 1.32) | <0.001 | 0.30 (0.24, 0.47) | 0.41 (0.19, 0.53) | 1.05 (0.78, 1.32) | <0.001 |
| Andean Latin America | 5.86 (2.85, 9.43) | 10.05 (4.98, 16.18) | 1.89 (1.85, 1.93) | <0.001 | 0.44 (0.22, 0.71) | 0.87 (0.45, 1.37) | 2.40 (2.30, 2.51) | <0.001 | 17.97 (11.70, 26.37) | 14.39 (8.49, 22.37) | -0.69 (-1.09, -0.29) | 0.001 | 0.35 (0.23, 0.51) | 0.28 (0.16, 0.43) | -0.70 (-1.10, -0.30) | 0.001 |
| Caribbean | 8.78 (4.82, 13.52) | 15.57 (9.09, 23.23) | 1.99 (1.96, 2.02) | <0.001 | 0.93 (0.61, 1.30) | 1.48 (0.97, 2.06) | 1.62 (1.59, 1.65) | <0.001 | 29.59 (22.02, 43.33) | 39.27 (27.62, 55.20) | 0.98 (0.52, 1.44) | <0.001 | 0.57 (0.42, 0.84) | 0.76 (0.53, 1.07) | 0.98 (0.52, 1.44) | <0.001 |
| Southern Latin America | 5.84 (3.05, 9.45) | 12.20 (6.57, 19.14) | 2.56 (2.53, 2.59) | <0.001 | 0.47 (0.25, 0.73) | 1.19 (0.66, 1.80) | 3.23 (3.15, 3.32) | <0.001 | 23.14 (15.66, 36.99) | 33.93 (23.21, 50.58) | 1.37 (0.85, 1.90) | <0.001 | 0.45 (0.30, 0.72) | 0.65 (0.45, 0.98) | 1.36 (0.84, 1.89) | <0.001 |
| Oceania | 5.36 (2.95, 8.18) | 6.34 (3.58, 9.65) | 0.60 (0.49, 0.71) | <0.001 | 0.60 (0.40, 0.83) | 0.66 (0.44, 0.91) | 0.36 (0.26, 0.46) | <0.001 | 30.83 (18.11, 55.69) | 38.43 (23.51, 61.60) | 0.74 (0.62, 0.85) | <0.001 | 0.60 (0.35, 1.08) | 0.74 (0.45, 1.19) | 0.74 (0.62, 0.85) | <0.001 |
| Australasia | 8.58 (4.00, 14.09) | 10.77 (5.58, 17.64) | 0.79 (0.76, 0.83) | <0.001 | 1.38 (0.93, 1.92) | 1.37 (0.92, 1.95) | -0.03 (-0.06, 0.00) | 0.085 | 9.53 (6.40, 13.80) | 15.13 (5.70, 25.31) | 1.60 (1.06, 2.14) | <0.001 | 0.18 (0.12, 0.26) | 0.29 (0.11, 0.49) | 1.62 (1.08, 2.16) | <0.001 |
| **Other cardiovascular and circulatory diseases** | | | | | | | | | | | | | | | | |

| | | | | | | | | | | | | | | | | |
|---|---|---|---|---|---|---|---|---|---|---|---|---|---|---|---|---|
| **15-19 years** | | | | | | | | | | | | | | | | |
| **Women** | | | | | | | | | | | | | | | | |
| Global | — | — | — | — | 299.83 (165.10, 498.41) | 394.48 (212.90, 669.72) | 0.95 (0.93, 0.98) | <0.001 | 43.74 (34.33, 56.63) | 45.90 (33.57, 63.66) | 0.17 (0.04, 0.30) | 0.011 | 0.38 (0.32, 0.44) | 0.34 (0.29, 0.41) | -0.42 (-0.59, -0.25) | <0.001 |
| **SDI category** | | | | | | | | | | | | | | | | |
| High | — | — | — | — | 380.77 (216.67, 604.07) | 312.54 (186.75, 487.44) | -0.64 (-0.69, -0.58) | <0.001 | 39.67 (28.87, 54.98) | 32.10 (23.39, 44.29) | -0.69 (-0.81, -0.58) | <0.001 | 0.27 (0.24, 0.33) | 0.21 (0.18, 0.29) | -0.71 (-1.10, -0.32) | <0.001 |
| High-middle | — | — | — | — | 232.74 (134.72, 371.97) | 279.48 (161.12, 443.33) | 0.65 (0.53, 0.76) | <0.001 | 33.54 (26.50, 43.93) | 31.95 (22.76, 44.76) | -0.18 (-0.45, 0.10) | 0.205 | 0.29 (0.25, 0.35) | 0.23 (0.18, 0.35) | -0.74 (-1.07, -0.41) | <0.001 |
| Middle | — | — | — | — | 237.76 (129.05, 395.39) | 340.09 (178.21, 581.24) | 1.24 (1.21, 1.27) | <0.001 | 37.86 (29.84, 48.57) | 40.13 (29.41, 55.32) | 0.21 (0.13, 0.29) | <0.001 | 0.35 (0.29, 0.40) | 0.30 (0.25, 0.37) | -0.48 (-0.64, -0.32) | <0.001 |
| Low-middle | — | — | — | — | 302.39 (168.32, 506.15) | 374.26 (199.07, 639.81) | 0.73 (0.71, 0.76) | <0.001 | 52.05 (38.13, 68.85) | 49.81 (37.11, 67.31) | -0.18 (-0.38, 0.02) | 0.071 | 0.50 (0.33, 0.65) | 0.41 (0.33, 0.51) | -0.64 (-0.85, -0.43) | <0.001 |
| Low | — | — | — | — | 544.72 (260.33, 981.29) | 611.73 (290.67, 1103.29) | 0.40 (0.39, 0.41) | <0.001 | 69.78 (50.67, 100.11) | 64.06 (43.03, 95.87) | -0.30 (-0.38, -0.21) | <0.001 | 0.57 (0.43, 0.72) | 0.43 (0.33, 0.56) | -0.93 (-1.11, -0.74) | <0.001 |
| **21 GBD region** | | | | | | | | | | | | | | | | |
| South Asia | — | — | — | — | 241.71 (134.29, 407.16) | 229.73 (122.45, 392.43) | -0.18 (-0.20, -0.15) | <0.001 | 49.56 (32.02, 65.96) | 41.17 (30.68, 53.88) | -0.63 (-0.90, -0.36) | <0.001 | 0.51 (0.28, 0.69) | 0.40 (0.29, 0.51) | -0.93 (-1.50, -0.35) | 0.002 |
| East Asia | — | — | — | — | 62.42 (32.02, 105.45) | 89.44 (46.42, 149.80) | 1.24 (1.15, 1.33) | <0.001 | 16.58 (11.99, 21.32) | 12.72 (9.11, 17.77) | -0.95 (-1.49, -0.40) | 0.001 | 0.18 (0.13, 0.23) | 0.11 (0.08, 0.16) | -1.88 (-2.51, -1.24) | <0.001 |
| North Africa and Middle East | — | — | — | — | 528.17 (300.03, 865.89) | 566.02 (311.35, 940.04) | 0.24 (0.22, 0.25) | <0.001 | 80.23 (60.61, 105.73) | 71.27 (51.45, 98.51) | -0.36 (-0.53, -0.18) | <0.001 | 0.72 (0.55, 0.94) | 0.57 (0.42, 0.75) | -0.75 (-0.93, -0.57) | <0.001 |
| Western Sub-Saharan Africa | — | — | — | — | 986.99 (455.45, | 1084.10 (505.27, | 0.33 (0.31, 0.35) | <0.001 | 95.21 (61.08, 145.84) | 82.07 (46.49, 137.59) | -0.50 (-0.56, | <0.001 | 0.58 (0.42, | 0.33 (0.24, | -1.99 (-2.11, | <0.001 |

| | | | | | | | | | | | | | | | |
|---|---|---|---|---|---|---|---|---|---|---|---|---|---|---|---|
| | | | | | 1770.84) | 1931.62) | | | | | -0.45) | | 0.79) | 0.46) | -1.87) | |
| Southeast Asia | — | — | — | — | 196.30 (110.52, 313.90) | 206.54 (116.77, 334.50) | 0.17 (0.16, 0.19) | <0.001 | 35.41 (27.05, 45.87) | 31.67 (23.87, 41.88) | -0.36 (-0.52, -0.21) | <0.001 | 0.35 (0.26, 0.43) | 0.29 (0.21, 0.37) | -0.62 (-0.84, -0.40) | <0.001 |
| Eastern Sub-Saharan Africa | — | — | — | — | 709.93 (328.80, 1303.64) | 776.02 (353.46, 1426.39) | 0.30 (0.28, 0.33) | <0.001 | 89.26 (61.67, 129.44) | 81.45 (52.29, 125.03) | -0.32 (-0.40, -0.24) | <0.001 | 0.71 (0.50, 0.97) | 0.55 (0.35, 0.77) | -0.88 (-1.04, -0.71) | <0.001 |
| Central Latin America | — | — | — | — | 341.60 (194.56, 541.35) | 372.64 (212.61, 592.20) | 0.31 (0.29, 0.33) | <0.001 | 44.98 (34.94, 59.44) | 39.34 (27.66, 55.35) | -0.44 (-0.69, -0.18) | 0.001 | 0.37 (0.33, 0.41) | 0.27 (0.21, 0.37) | -0.94 (-1.28, -0.59) | <0.001 |
| Tropical Latin America | — | — | — | — | 392.82 (208.62, 663.26) | 423.17 (219.65, 709.08) | 0.26 (0.22, 0.31) | <0.001 | 47.57 (35.82, 66.98) | 49.49 (35.80, 70.34) | 0.17 (-0.08, 0.43) | 0.185 | 0.37 (0.33, 0.42) | 0.37 (0.31, 0.46) | 0.09 (-0.35, 0.53) | 0.683 |
| Central Sub-Saharan Africa | — | — | — | — | 788.02 (348.96, 1491.70) | 815.02 (367.09, 1563.27) | 0.12 (0.10, 0.14) | <0.001 | 74.67 (42.36, 123.93) | 68.62 (38.71, 116.97) | -0.32 (-0.38, -0.26) | <0.001 | 0.46 (0.26, 0.74) | 0.35 (0.20, 0.56) | -0.98 (-1.13, -0.83) | <0.001 |
| Western Europe | — | — | — | — | 359.26 (212.10, 568.90) | 343.86 (201.34, 539.71) | -0.15 (-0.17, -0.12) | <0.001 | 38.59 (28.58, 53.26) | 30.88 (21.22, 45.04) | -0.78 (-0.91, -0.64) | <0.001 | 0.27 (0.24, 0.33) | 0.17 (0.14, 0.24) | -1.60 (-1.86, -1.34) | <0.001 |
| High-income North America | — | — | — | — | 539.43 (293.05, 896.19) | 329.67 (186.45, 520.14) | -1.64 (-1.72, -1.56) | <0.001 | 48.63 (32.50, 72.86) | 37.76 (28.15, 51.78) | -0.80 (-1.08, -0.52) | <0.001 | 0.27 (0.24, 0.33) | 0.28 (0.24, 0.36) | 0.30 (-0.57, 1.18) | 0.497 |
| Central Asia | — | — | — | — | 160.95 (90.86, 255.96) | 146.79 (84.00, 237.26) | -0.29 (-0.38, -0.21) | <0.001 | 40.73 (24.99, 50.98) | 31.42 (20.24, 57.15) | -0.89 (-1.26, -0.52) | <0.001 | 0.45 (0.25, 0.54) | 0.33 (0.18, 0.70) | -1.12 (-1.61, -0.64) | <0.001 |
| Eastern Europe | — | — | — | — | 192.87 (107.94, 315.31) | 200.56 (112.34, 325.59) | 0.14 (0.11, 0.17) | <0.001 | 30.04 (21.12, 39.48) | 27.20 (18.32, 47.21) | -0.35 (-0.63, -0.08) | 0.012 | 0.27 (0.17, 0.35) | 0.23 (0.15, 0.50) | -0.61 (-1.62, 0.40) | 0.232 |
| Southern Sub-Saharan Africa | — | — | — | — | 520.28 (237.93, 945.61) | 554.67 (257.14, 1017.31) | 0.22 (0.18, 0.25) | <0.001 | 50.13 (31.97, 77.88) | 53.44 (32.47, 85.06) | 0.26 (-0.13, 0.65) | 0.195 | 0.31 (0.23, 0.41) | 0.33 (0.19, 0.52) | 0.28 (-0.52, 1.08) | 0.495 |
| High-income Asia Pacific | — | — | — | — | 188.49 (102.25, | 185.48 (102.47, | -0.05 (-0.10, | 0.028 | 24.64 (18.04, 33.95) | 16.94 (10.79, 25.32) | -1.28 (-1.64, | <0.001 | 0.20 (0.16, | 0.10 (0.07, | -2.53 (-3.16, | <0.001 |

| | | | | | | | | | | | | | | | | |
|---|---|---|---|---|---|---|---|---|---|---|---|---|---|---|---|---|
| | | | | | 309.90) | 301.49) | -0.01) | | | | -0.92) | | 0.28) | 0.17) | -1.90) | |
| Central Europe | — | — | — | — | 536.47 (315.75, 829.91) | 531.52 (310.50, 811.78) | -0.03 (-0.19, 0.14) | 0.743 | 52.59 (37.54, 73.48) | 40.52 (26.00, 61.97) | -0.86 (-1.08, -0.64) | <0.001 | 0.32 (0.30, 0.36) | 0.16 (0.13, 0.21) | -2.38 (-3.02, -1.72) | <0.001 |
| Andean Latin America | — | — | — | — | 438.87 (253.80, 708.11) | 485.20 (285.29, 785.75) | 0.34 (0.32, 0.36) | <0.001 | 68.78 (50.83, 91.11) | 49.65 (33.57, 70.70) | -1.12 (-1.47, -0.77) | <0.001 | 0.63 (0.47, 0.82) | 0.32 (0.22, 0.46) | -2.33 (-3.22, -1.43) | <0.001 |
| Caribbean | — | — | — | — | 240.78 (141.35, 386.72) | 259.10 (152.12, 411.23) | 0.24 (0.20, 0.28) | <0.001 | 54.31 (41.72, 72.36) | 58.60 (39.27, 85.60) | 0.43 (0.23, 0.63) | <0.001 | 0.58 (0.43, 0.80) | 0.62 (0.39, 0.98) | 0.52 (0.24, 0.80) | <0.001 |
| Southern Latin America | — | — | — | — | 365.05 (215.53, 575.99) | 411.22 (235.64, 653.55) | 0.42 (0.39, 0.44) | <0.001 | 54.41 (39.55, 72.72) | 46.43 (32.48, 64.22) | -0.54 (-0.88, -0.19) | 0.002 | 0.48 (0.36, 0.63) | 0.34 (0.25, 0.43) | -1.23 (-1.79, -0.67) | <0.001 |
| Oceania | — | — | — | — | 175.32 (99.93, 290.35) | 157.62 (88.74, 261.04) | -0.37 (-0.39, -0.35) | <0.001 | 38.71 (24.98, 57.97) | 38.57 (24.67, 58.28) | -0.08 (-0.54, 0.37) | 0.713 | 0.41 (0.24, 0.66) | 0.42 (0.25, 0.68) | 0.00 (-0.58, 0.59) | 0.991 |
| Australasia | — | — | — | — | 144.24 (81.72, 225.97) | 131.09 (74.91, 213.14) | -0.32 (-0.37, -0.28) | <0.001 | 20.88 (14.59, 29.37) | 17.52 (11.84, 25.53) | -0.65 (-1.44, 0.15) | 0.112 | 0.18 (0.14, 0.28) | 0.15 (0.10, 0.21) | -0.66 (-2.17, 0.88) | 0.402 |
| **Men** | | | | | | | | | | | | | | | | |
| Global | — | — | — | — | 240.08 (132.64, 400.48) | 331.75 (175.89, 561.24) | 1.12 (1.09, 1.14) | <0.001 | 38.04 (29.30, 49.92) | 41.88 (30.32, 57.88) | 0.32 (0.16, 0.48) | <0.001 | 0.35 (0.29, 0.43) | 0.33 (0.27, 0.41) | -0.22 (-0.41, -0.04) | 0.018 |
| **SDI category** | | | | | | | | | | | | | | | | |
| High | — | — | — | — | 190.40 (111.42, 308.14) | 156.81 (95.83, 248.13) | -0.66 (-0.69, -0.63) | <0.001 | 26.26 (20.71, 34.83) | 18.43 (13.85, 24.84) | -1.21 (-1.34, -1.08) | <0.001 | 0.22 (0.19, 0.26) | 0.14 (0.12, 0.18) | -1.60 (-1.84, -1.36) | <0.001 |
| High-middle | — | — | — | — | 182.93 (106.17, 295.50) | 219.54 (130.02, 358.71) | 0.63 (0.59, 0.68) | <0.001 | 34.10 (26.90, 42.83) | 27.26 (20.02, 37.99) | -0.78 (-1.04, -0.52) | <0.001 | 0.34 (0.27, 0.41) | 0.21 (0.17, 0.31) | -1.59 (-2.03, -1.15) | <0.001 |
| Middle | — | — | — | — | 218.19 (119.85, 366.84) | 316.10 (169.16, 536.28) | 1.28 (1.26, 1.31) | <0.001 | 34.95 (25.68, 46.83) | 38.83 (28.24, 55.08) | 0.36 (0.21, 0.51) | <0.001 | 0.32 (0.25, 0.42) | 0.30 (0.24, 0.40) | -0.22 (-0.43, -0.00) | 0.049 |

| | | | | | | | | | | | | | | | | |
|---|---|---|---|---|---|---|---|---|---|---|---|---|---|---|---|---|
| Low-middle | — | — | — | — | 246.79 (133.39, 409.66) | 319.35 (168.75, 543.66) | 0.88 (0.86, 0.90) | <0.001 | 38.03 (28.03, 51.48) | 42.65 (31.08, 59.35) | 0.33 (0.09, 0.56) | 0.006 | 0.34 (0.25, 0.46) | 0.35 (0.26, 0.45) | -0.09 (-0.30, 0.12) | 0.398 |
| Low | — | — | — | — | 469.34 (226.60, 838.15) | 533.17 (256.17, 954.89) | 0.44 (0.42, 0.46) | <0.001 | 70.14 (50.83, 95.78) | 66.61 (45.32, 95.67) | -0.18 (-0.34, -0.04) | 0.016 | 0.62 (0.48, 0.83) | 0.52 (0.38, 0.71) | -0.60 (-0.82, -0.38) | <0.001 |
| **21 GBD region** | | | | | | | | | | | | | | | | |
| South Asia | — | — | — | — | 172.72 (91.45, 292.99) | 164.13 (85.17, 281.00) | -0.20 (-0.25, -0.15) | <0.001 | 29.04 (19.91, 40.49) | 27.47 (19.14, 37.69) | -0.15 (-0.80, 0.50) | 0.646 | 0.27 (0.18, 0.40) | 0.26 (0.17, 0.35) | -0.14 (-1.06, 0.79) | 0.766 |
| East Asia | — | — | — | — | 61.63 (32.86, 104.41) | 89.25 (48.49, 151.08) | 1.25 (1.07, 1.43) | <0.001 | 20.08 (12.20, 27.56) | 12.75 (9.12, 18.60) | -1.61 (-1.82, -1.40) | <0.001 | 0.23 (0.13, 0.33) | 0.11 (0.08, 0.17) | -2.60 (-2.86, -2.35) | <0.001 |
| North Africa and Middle East | — | — | — | — | 534.69 (306.07, 901.00) | 593.55 (334.44, 1001.60) | 0.36 (0.33, 0.40) | <0.001 | 78.11 (53.83, 113.77) | 76.87 (52.51, 113.67) | -0.06 (-0.18, 0.07) | 0.390 | 0.68 (0.48, 1.06) | 0.62 (0.41, 0.98) | -0.32 (-0.50, -0.15) | <0.001 |
| Western Sub-Saharan Africa | — | — | — | — | 1232.30 (575.05, 2194.92) | 1311.89 (615.17, 2344.41) | 0.21 (0.19, 0.24) | <0.001 | 135.82 (88.66, 206.74) | 121.20 (74.74, 193.91) | -0.40 (-0.46, -0.34) | <0.001 | 0.96 (0.68, 1.35) | 0.69 (0.50, 0.99) | -1.12 (-1.29, -0.96) | <0.001 |
| Southeast Asia | — | — | — | — | 179.62 (104.94, 290.31) | 193.43 (112.85, 313.03) | 0.26 (0.24, 0.27) | <0.001 | 28.18 (20.56, 37.32) | 30.48 (23.00, 40.77) | 0.27 (0.11, 0.43) | 0.001 | 0.26 (0.19, 0.34) | 0.28 (0.21, 0.35) | 0.32 (0.13, 0.50) | 0.001 |
| Eastern Sub-Saharan Africa | — | — | — | — | 501.54 (231.39, 913.08) | 537.65 (244.24, 981.67) | 0.24 (0.23, 0.26) | <0.001 | 84.85 (60.97, 117.03) | 75.72 (50.42, 110.15) | -0.39 (-0.45, -0.32) | <0.001 | 0.81 (0.58, 1.09) | 0.65 (0.42, 0.91) | -0.73 (-0.83, -0.63) | <0.001 |
| Central Latin America | — | — | — | — | 234.06 (135.84, 378.91) | 261.33 (151.43, 422.63) | 0.38 (0.36, 0.40) | <0.001 | 35.92 (28.88, 46.52) | 30.02 (21.45, 41.78) | -0.55 (-0.70, -0.40) | <0.001 | 0.32 (0.29, 0.37) | 0.22 (0.17, 0.29) | -1.20 (-1.43, -0.96) | <0.001 |
| Tropical Latin America | — | — | — | — | 243.01 (130.77, 416.41) | 257.79 (138.22, 435.64) | 0.20 (0.18, 0.22) | <0.001 | 36.59 (28.48, 48.66) | 37.73 (28.98, 50.78) | 0.11 (-0.30, 0.51) | 0.609 | 0.33 (0.29, 0.38) | 0.33 (0.27, 0.40) | 0.06 (-0.43, 0.56) | 0.810 |
| Central Sub-Saharan Africa | — | — | — | — | 436.28 (194.00, | 442.72 (192.57, | 0.05 (0.04, 0.07) | <0.001 | 61.16 (38.33, 95.15) | 54.13 (32.82, 85.70) | -0.46 (-0.52, | <0.001 | 0.53 (0.32, | 0.42 (0.24, | -0.84 (-0.98, | <0.001 |

| | | | | | | | | | | | | | | | | |
|---|---|---|---|---|---|---|---|---|---|---|---|---|---|---|---|---|
| | | | | | 822.71) | 838.61) | | | | | -0.41) | | 0.85) | 0.70) | -0.69) | |
| Western Europe | — | — | — | — | 179.35 (105.96, 288.46) | 170.78 (102.24, 276.73) | -0.15 (-0.25, -0.06) | 0.002 | 26.95 (21.52, 35.08) | 17.62 (12.17, 24.89) | -1.48 (-1.69, -1.27) | <0.001 | 0.24 (0.21, 0.30) | 0.11 (0.10, 0.16) | -2.53 (-3.18, -1.89) | <0.001 |
| High-income North America | — | — | — | — | 214.68 (120.25, 357.11) | 135.49 (77.64, 219.41) | -1.55 (-1.60, -1.50) | <0.001 | 25.33 (18.94, 35.57) | 19.63 (15.37, 25.70) | -0.87 (-1.20, -0.54) | <0.001 | 0.19 (0.17, 0.23) | 0.17 (0.14, 0.22) | -0.29 (-0.61, 0.03) | 0.080 |
| Central Asia | — | — | — | — | 204.81 (117.91, 334.80) | 212.90 (120.52, 345.08) | 0.13 (0.12, 0.15) | <0.001 | 47.12 (36.29, 58.60) | 39.16 (27.79, 56.56) | -0.50 (-1.27, 0.27) | 0.205 | 0.50 (0.38, 0.59) | 0.38 (0.28, 0.63) | -0.74 (-1.31, -0.18) | 0.010 |
| Eastern Europe | — | — | — | — | 156.66 (84.25, 263.18) | 174.29 (94.17, 294.78) | 0.37 (0.32, 0.41) | <0.001 | 31.74 (24.62, 41.28) | 29.38 (20.86, 49.32) | -0.16 (-0.59, 0.27) | 0.465 | 0.32 (0.24, 0.41) | 0.28 (0.20, 0.57) | -0.57 (-1.08, -0.06) | 0.028 |
| Southern Sub-Saharan Africa | — | — | — | — | 456.03 (208.61, 825.88) | 468.01 (219.21, 862.12) | 0.09 (0.06, 0.12) | <0.001 | 44.38 (28.01, 69.41) | 51.91 (31.69, 80.31) | 0.57 (0.41, 0.74) | <0.001 | 0.27 (0.20, 0.37) | 0.37 (0.23, 0.57) | 1.09 (0.79, 1.40) | <0.001 |
| High-income Asia Pacific | — | — | — | — | 200.92 (113.40, 330.67) | 199.07 (111.58, 324.62) | -0.04 (-0.07, -0.00) | 0.039 | 29.10 (22.37, 39.45) | 18.07 (12.17, 27.24) | -1.66 (-1.86, -1.45) | <0.001 | 0.25 (0.18, 0.31) | 0.10 (0.08, 0.13) | -3.15 (-3.62, -2.67) | <0.001 |
| Central Europe | — | — | — | — | 372.21 (219.87, 590.86) | 382.30 (222.80, 608.13) | 0.10 (0.07, 0.13) | <0.001 | 48.68 (37.82, 64.11) | 34.03 (23.18, 50.37) | -1.21 (-1.57, -0.86) | <0.001 | 0.39 (0.35, 0.44) | 0.18 (0.14, 0.23) | -2.68 (-3.36, -2.00) | <0.001 |
| Andean Latin America | — | — | — | — | 424.71 (245.95, 697.66) | 480.60 (269.13, 803.08) | 0.43 (0.40, 0.46) | <0.001 | 79.47 (57.12, 108.51) | 52.24 (34.91, 75.90) | -1.42 (-1.71, -1.14) | <0.001 | 0.79 (0.55, 1.09) | 0.36 (0.24, 0.54) | -2.60 (-3.07, -2.12) | <0.001 |
| Caribbean | — | — | — | — | 247.63 (144.76, 397.73) | 286.70 (166.15, 463.87) | 0.50 (0.48, 0.53) | <0.001 | 46.88 (35.21, 62.15) | 47.94 (32.76, 66.93) | 0.15 (-0.05, 0.35) | 0.132 | 0.47 (0.35, 0.61) | 0.45 (0.30, 0.67) | 0.00 (-0.28, 0.29) | 0.982 |
| Southern Latin America | — | — | — | — | 227.99 (130.10, 373.08) | 262.11 (152.18, 435.92) | 0.47 (0.41, 0.53) | <0.001 | 38.33 (29.20, 50.87) | 34.72 (25.41, 48.21) | -0.37 (-0.63, -0.11) | 0.005 | 0.36 (0.28, 0.46) | 0.28 (0.22, 0.35) | -0.84 (-1.27, -0.41) | <0.001 |
| Oceania | — | — | — | — | 212.39 (119.11, | 201.09 (111.67, | -0.17 (-0.20, | <0.001 | 52.21 (34.58, 75.24) | 52.64 (33.59, 81.35) | -0.01 (-0.25, | 0.964 | 0.57 (0.36, | 0.58 (0.34, | 0.05 (-0.24, 0.34) | 0.722 |

| | | | | | | | | | | | | | | | | |
|---|---|---|---|---|---|---|---|---|---|---|---|---|---|---|---|---|
| | | | | | 351.71) | 336.78) | -0.15) | | | | 0.24) | | 0.89) | 0.97) | | |
| Australasia | — | — | — | — | 66.51 (38.92, 109.68) | 58.57 (35.30, 94.10) | -0.44 (-0.46, -0.42) | <0.001 | 13.36 (9.57, 19.85) | 9.69 (6.46, 14.19) | -1.08 (-1.76, -0.40) | 0.002 | 0.14 (0.10, 0.22) | 0.09 (0.06, 0.13) | -1.32 (-2.27, -0.36) | 0.007 |
| **20-24 years** | | | | | | | | | | | | | | | | |
| **Women** | | | | | | | | | | | | | | | | |
| Global | — | — | — | — | 260.95 (143.01, 432.85) | 327.10 (171.31, 554.00) | 0.80 (0.74, 0.85) | <0.001 | 52.02 (42.07, 65.27) | 51.52 (40.55, 67.31) | -0.04 (-0.12, 0.04) | 0.321 | 0.57 (0.45, 0.66) | 0.51 (0.43, 0.60) | -0.38 (-0.62, -0.12) | 0.003 |
| **SDI category** | | | | | | | | | | | | | | | | |
| High | — | — | — | — | 475.62 (286.03, 736.99) | 393.37 (243.73, 598.56) | -0.62 (-0.67, -0.57) | <0.001 | 52.92 (40.13, 72.67) | 43.36 (32.57, 58.79) | -0.69 (-0.93, -0.45) | <0.001 | 0.41 (0.38, 0.48) | 0.33 (0.29, 0.42) | -0.71 (-0.92, -0.49) | <0.001 |
| High-middle | — | — | — | — | 205.95 (121.77, 320.68) | 251.27 (147.58, 392.21) | 0.69 (0.65, 0.73) | <0.001 | 38.43 (31.71, 47.72) | 34.61 (25.97, 47.19) | -0.35 (-0.61, -0.08) | 0.010 | 0.41 (0.36, 0.47) | 0.31 (0.25, 0.46) | -0.90 (-1.19, -0.61) | <0.001 |
| Middle | — | — | — | — | 194.61 (103.88, 327.84) | 281.93 (145.84, 483.81) | 1.30 (1.24, 1.37) | <0.001 | 42.91 (35.12, 52.97) | 42.81 (33.20, 56.59) | -0.00 (-0.12, 0.11) | 0.959 | 0.49 (0.39, 0.56) | 0.41 (0.33, 0.50) | -0.52 (-0.78, -0.25) | <0.001 |
| Low-middle | — | — | — | — | 221.34 (115.37, 376.00) | 280.33 (141.64, 480.74) | 0.81 (0.79, 0.84) | <0.001 | 64.71 (45.55, 82.70) | 58.16 (44.29, 74.11) | -0.38 (-0.75, -0.01) | 0.044 | 0.79 (0.51, 1.02) | 0.65 (0.50, 0.80) | -0.72 (-1.20, -0.25) | 0.003 |
| Low | — | — | — | — | 431.48 (186.08, 805.32) | 502.23 (216.50, 922.45) | 0.53 (0.50, 0.55) | <0.001 | 87.29 (64.33, 115.38) | 74.89 (55.14, 104.15) | -0.52 (-0.59, -0.46) | <0.001 | 0.96 (0.69, 1.24) | 0.72 (0.56, 0.89) | -1.00 (-1.10, -0.91) | <0.001 |
| **21 GBD region** | | | | | | | | | | | | | | | | |
| South Asia | — | — | — | — | 167.22 (85.59, 284.19) | 157.51 (79.77, 270.57) | -0.21 (-0.25, -0.18) | <0.001 | 64.46 (38.53, 86.50) | 51.17 (36.42, 66.43) | -0.86 (-1.36, -0.35) | 0.001 | 0.83 (0.45, 1.14) | 0.64 (0.44, 0.84) | -0.98 (-1.56, -0.39) | 0.001 |
| East Asia | — | — | — | — | 58.83 (29.91, 99.05) | 86.70 (43.55, 146.64) | 1.34 (1.25, 1.43) | <0.001 | 18.54 (13.82, 24.47) | 14.60 (10.48, 20.44) | -0.65 (-1.02, -0.27) | 0.001 | 0.23 (0.16, 0.31) | 0.15 (0.10, 0.21) | -1.30 (-1.72, -0.87) | <0.001 |
| North Africa and | — | — | — | — | 485.34 | 519.62 | 0.24 (0.20, | <0.001 | 97.98 (75.43, | 80.52 (59.96, | -0.67 | <0.001 | 1.08 | 0.79 | -1.08 | <0.001 |

| | | | | | | | | | | | | | | | | |
|---|---|---|---|---|---|---|---|---|---|---|---|---|---|---|---|---|
| Middle East | | | | | (271.48, 796.59) | (284.60, 867.29) | 0.27) | | 126.25) | 107.74) | (-0.80, -0.53) | | (0.84, 1.36) | (0.59, 1.05) | (-1.27, -0.88) | |
| Western Sub-Saharan Africa | — | — | — | — | 826.90 (346.98, 1546.03) | 958.93 (401.91, 1757.42) | 0.52 (0.49, 0.54) | <0.001 | 119.06 (84.40, 168.84) | 97.34 (62.19, 148.42) | -0.68 (-0.77, -0.60) | <0.001 | 1.12 (0.82, 1.54) | 0.69 (0.49, 0.97) | -1.68 (-1.78, -1.59) | <0.001 |
| Southeast Asia | — | — | — | — | 165.68 (95.16, 264.97) | 172.43 (100.04, 272.86) | 0.14 (0.12, 0.16) | <0.001 | 41.50 (30.69, 52.83) | 33.04 (25.84, 41.93) | -0.78 (-0.94, -0.61) | <0.001 | 0.49 (0.35, 0.62) | 0.35 (0.27, 0.46) | -1.09 (-1.29, -0.89) | <0.001 |
| Eastern Sub-Saharan Africa | — | — | — | — | 533.31 (220.41, 1014.92) | 610.88 (251.24, 1143.86) | 0.47 (0.44, 0.50) | <0.001 | 100.22 (70.18, 137.02) | 82.10 (55.67, 118.56) | -0.69 (-0.84, -0.55) | <0.001 | 1.08 (0.75, 1.49) | 0.74 (0.50, 1.05) | -1.31 (-1.49, -1.12) | <0.001 |
| Central Latin America | — | — | — | — | 363.34 (214.31, 562.68) | 389.20 (227.15, 606.19) | 0.25 (0.21, 0.30) | <0.001 | 56.14 (45.52, 71.49) | 47.27 (34.31, 64.82) | -0.50 (-0.67, -0.33) | <0.001 | 0.55 (0.50, 0.61) | 0.39 (0.30, 0.52) | -0.98 (-1.34, -0.61) | <0.001 |
| Tropical Latin America | — | — | — | — | 342.35 (172.21, 586.03) | 365.62 (182.80, 624.43) | 0.23 (0.21, 0.26) | <0.001 | 58.99 (47.87, 75.01) | 56.67 (44.36, 75.65) | -0.11 (-0.41, 0.20) | 0.498 | 0.61 (0.55, 0.68) | 0.56 (0.47, 0.68) | -0.20 (-0.67, 0.28) | 0.411 |
| Central Sub-Saharan Africa | — | — | — | — | 566.59 (224.32, 1071.38) | 610.67 (245.84, 1206.27) | 0.28 (0.18, 0.37) | <0.001 | 75.07 (45.37, 113.63) | 68.00 (40.51, 107.74) | -0.35 (-0.44, -0.26) | <0.001 | 0.67 (0.39, 1.09) | 0.53 (0.31, 0.86) | -0.89 (-1.00, -0.79) | <0.001 |
| Western Europe | — | — | — | — | 467.12 (282.63, 707.52) | 440.17 (268.87, 662.42) | -0.21 (-0.24, -0.17) | <0.001 | 51.84 (39.32, 69.06) | 41.41 (29.61, 58.44) | -0.78 (-0.89, -0.67) | <0.001 | 0.40 (0.36, 0.48) | 0.27 (0.23, 0.35) | -1.40 (-1.70, -1.11) | <0.001 |
| High-income North America | — | — | — | — | 626.87 (332.55, 1046.12) | 423.74 (236.75, 682.67) | -1.31 (-1.36, -1.25) | <0.001 | 63.69 (45.35, 91.73) | 52.29 (39.67, 70.72) | -0.71 (-0.94, -0.47) | <0.001 | 0.45 (0.41, 0.52) | 0.44 (0.39, 0.55) | -0.16 (-0.62, 0.31) | 0.507 |
| Central Asia | — | — | — | — | 119.16 (71.48, 186.80) | 111.06 (67.93, 172.96) | -0.23 (-0.28, -0.19) | <0.001 | 53.36 (39.52, 63.05) | 37.35 (26.05, 59.53) | -1.30 (-1.66, -0.94) | <0.001 | 0.70 (0.50, 0.83) | 0.47 (0.32, 0.80) | -1.44 (-1.82, -1.05) | <0.001 |
| Eastern Europe | — | — | — | — | 140.41 (71.84, 239.40) | 142.74 (72.32, 242.84) | 0.06 (0.04, 0.08) | <0.001 | 31.97 (24.67, 39.28) | 34.55 (24.46, 60.37) | 0.26 (-0.42, 0.94) | 0.458 | 0.36 (0.27, 0.44) | 0.40 (0.28, 0.79) | 0.32 (-0.55, 1.20) | 0.474 |
| Southern | — | — | — | — | 337.47 | 360.85 | 0.23 (0.20, | <0.001 | 53.17 (38.78, | 45.71 (29.08, | -0.50 | 0.131 | 0.53 | 0.39 | -0.95 | 0.114 |

| | | | | | | | | | | | | | | | | |
|---|---|---|---|---|---|---|---|---|---|---|---|---|---|---|---|---|
| Sub-Saharan Africa | | | | | (141.17, 631.33) | (150.61, 658.45) | 0.26) | | 72.90) | 69.35) | (-1.14, 0.15) | | (0.41, 0.67) | (0.25, 0.61) | (-2.13, 0.23) | |
| High-income Asia Pacific | — | — | — | — | 264.74 (147.23, 427.90) | 259.63 (147.21, 412.12) | -0.07 (-0.10, -0.03) | <0.001 | 33.40 (25.13, 44.75) | 22.81 (14.97, 34.13) | -1.33 (-1.55, -1.11) | <0.001 | 0.29 (0.23, 0.38) | 0.13 (0.10, 0.22) | -2.71 (-3.13, -2.29) | <0.001 |
| Central Europe | — | — | — | — | 531.12 (326.71, 796.24) | 545.60 (335.14, 831.46) | 0.11 (-0.03, 0.24) | 0.115 | 65.39 (51.14, 86.66) | 46.76 (32.57, 68.39) | -1.12 (-1.32, -0.92) | <0.001 | 0.55 (0.51, 0.60) | 0.26 (0.19, 0.33) | -2.75 (-2.94, -2.55) | <0.001 |
| Andean Latin America | — | — | — | — | 353.58 (205.65, 558.05) | 393.87 (236.05, 625.24) | 0.37 (0.33, 0.40) | <0.001 | 85.01 (65.21, 109.44) | 51.81 (36.34, 71.14) | -1.66 (-2.17, -1.14) | <0.001 | 0.99 (0.74, 1.29) | 0.46 (0.31, 0.65) | -2.59 (-3.37, -1.80) | <0.001 |
| Caribbean | — | — | — | — | 227.83 (138.21, 352.11) | 250.55 (151.30, 384.90) | 0.32 (0.28, 0.37) | <0.001 | 77.64 (56.70, 104.21) | 88.03 (56.57, 132.32) | 0.47 (0.17, 0.76) | 0.002 | 0.98 (0.67, 1.37) | 1.12 (0.67, 1.79) | 0.50 (0.04, 0.97) | 0.034 |
| Southern Latin America | — | — | — | — | 363.78 (216.56, 572.19) | 403.43 (238.38, 632.02) | 0.36 (0.32, 0.40) | <0.001 | 67.93 (54.40, 86.93) | 52.84 (39.35, 72.35) | -0.80 (-1.18, -0.41) | <0.001 | 0.72 (0.60, 0.86) | 0.46 (0.36, 0.61) | -1.42 (-1.96, -0.88) | <0.001 |
| Oceania | — | — | — | — | 42.16 (23.87, 67.77) | 37.59 (21.34, 60.34) | -0.40 (-0.44, -0.36) | <0.001 | 30.18 (19.80, 45.30) | 28.26 (16.97, 44.88) | -0.24 (-0.50, 0.01) | 0.060 | 0.42 (0.26, 0.64) | 0.39 (0.22, 0.64) | -0.23 (-0.51, 0.04) | 0.097 |
| Australasia | — | — | — | — | 172.09 (100.97, 270.29) | 157.31 (93.16, 240.99) | -0.31 (-0.34, -0.28) | <0.001 | 26.50 (18.94, 37.30) | 23.51 (16.40, 33.27) | -0.38 (-1.30, 0.55) | 0.422 | 0.26 (0.19, 0.38) | 0.22 (0.16, 0.30) | -0.53 (-1.85, 0.80) | 0.433 |
| **Men** | | | | | | | | | | | | | | | | |
| Global | — | — | — | — | 172.46 (88.48, 299.23) | 230.35 (114.57, 407.62) | 1.01 (0.97, 1.05) | <0.001 | 40.30 (32.17, 50.92) | 43.16 (33.51, 56.15) | 0.24 (0.09, 0.40) | 0.002 | 0.46 (0.38, 0.58) | 0.46 (0.38, 0.56) | -0.03 (-0.23, 0.17) | 0.780 |
| **SDI category** | | | | | | | | | | | | | | | | |
| High | — | — | — | — | 193.39 (108.26, 311.90) | 167.89 (96.63, 261.94) | -0.46 (-0.50, -0.42) | <0.001 | 30.31 (24.37, 39.08) | 24.61 (19.35, 32.23) | -0.73 (-0.95, -0.51) | <0.001 | 0.30 (0.27, 0.34) | 0.23 (0.21, 0.29) | -0.86 (-1.14, -0.58) | <0.001 |
| High-middle | — | — | — | — | 138.12 (76.70, 222.79) | 173.09 (96.98, 279.08) | 0.80 (0.70, 0.90) | <0.001 | 37.18 (30.61, 44.88) | 30.03 (23.42, 40.60) | -0.73 (-0.91, | <0.001 | 0.44 (0.37, | 0.31 (0.26, | -1.27 (-1.65, | <0.001 |

| | | | | | | | | | | | | | | | | |
|---|---|---|---|---|---|---|---|---|---|---|---|---|---|---|---|---|
| | | | | | | | | | | | -0.56) | | 0.52) | 0.44) | -0.88) | |
| Middle | — | — | — | — | 165.79 (84.52, 291.03) | 238.03 (120.79, 423.17) | 1.27 (1.18, 1.36) | <0.001 | 37.79 (29.42, 49.47) | 41.45 (32.10, 54.76) | 0.33 (0.26, 0.40) | <0.001 | 0.43 (0.34, 0.56) | 0.43 (0.35, 0.53) | -0.03 (-0.20, 0.14) | 0.727 |
| Low-middle | — | — | — | — | 146.04 (73.13, 256.39) | 194.40 (95.06, 348.64) | 0.99 (0.96, 1.02) | <0.001 | 42.11 (32.00, 57.78) | 44.42 (33.72, 58.13) | 0.19 (-0.09, 0.47) | 0.179 | 0.51 (0.39, 0.72) | 0.51 (0.39, 0.65) | -0.02 (-0.40, 0.36) | 0.898 |
| Low | — | — | — | — | 310.74 (130.12, 591.88) | 364.37 (154.43, 694.43) | 0.55 (0.52, 0.58) | <0.001 | 68.24 (51.64, 92.32) | 67.68 (48.66, 93.24) | -0.02 (-0.13, 0.09) | 0.719 | 0.77 (0.60, 1.04) | 0.72 (0.53, 0.97) | -0.24 (-0.39, -0.08) | 0.003 |
| **21 GBD region** | | | | | | | | | | | | | | | | |
| South Asia | — | — | — | — | 76.86 (36.86, 135.49) | 72.13 (34.19, 128.63) | -0.23 (-0.28, -0.19) | <0.001 | 33.84 (23.73, 51.22) | 31.96 (22.48, 44.16) | -0.18 (-0.77, 0.40) | 0.537 | 0.44 (0.30, 0.70) | 0.42 (0.29, 0.59) | -0.17 (-0.83, 0.49) | 0.606 |
| East Asia | — | — | — | — | 55.59 (26.25, 98.36) | 82.82 (38.33, 146.36) | 1.38 (1.33, 1.44) | <0.001 | 19.34 (12.39, 26.59) | 15.04 (11.00, 21.36) | -0.82 (-1.25, -0.39) | <0.001 | 0.24 (0.15, 0.34) | 0.16 (0.12, 0.23) | -1.35 (-2.28, -0.42) | 0.005 |
| North Africa and Middle East | — | — | — | — | 407.28 (217.41, 691.77) | 439.11 (234.78, 738.78) | 0.26 (0.22, 0.30) | <0.001 | 81.75 (58.44, 115.54) | 73.70 (53.06, 103.28) | -0.34 (-0.54, -0.14) | 0.001 | 0.89 (0.64, 1.34) | 0.75 (0.52, 1.10) | -0.57 (-0.83, -0.31) | <0.001 |
| Western Sub-Saharan Africa | — | — | — | — | 788.28 (312.95, 1527.88) | 869.41 (346.09, 1661.40) | 0.34 (0.31, 0.37) | <0.001 | 114.83 (77.42, 168.40) | 104.52 (68.40, 159.80) | -0.33 (-0.41, -0.25) | <0.001 | 1.08 (0.78, 1.48) | 0.86 (0.61, 1.19) | -0.79 (-0.92, -0.65) | <0.001 |
| Southeast Asia | — | — | — | — | 228.21 (126.09, 370.16) | 251.53 (137.12, 414.46) | 0.34 (0.33, 0.35) | <0.001 | 51.41 (39.69, 65.01) | 48.96 (38.96, 63.19) | -0.16 (-0.32, -0.00) | 0.047 | 0.58 (0.45, 0.75) | 0.53 (0.43, 0.66) | -0.34 (-0.63, -0.06) | 0.017 |
| Eastern Sub-Saharan Africa | — | — | — | — | 365.17 (139.16, 707.57) | 408.20 (159.34, 791.27) | 0.38 (0.35, 0.41) | <0.001 | 82.77 (59.88, 115.08) | 74.95 (52.37, 108.24) | -0.34 (-0.48, -0.20) | <0.001 | 0.95 (0.71, 1.31) | 0.79 (0.54, 1.13) | -0.61 (-0.77, -0.44) | <0.001 |
| Central Latin America | — | — | — | — | 200.91 (106.94, 328.49) | 226.58 (125.83, 369.68) | 0.41 (0.39, 0.43) | <0.001 | 40.69 (33.76, 50.22) | 33.26 (24.83, 44.18) | -0.68 (-0.97, -0.39) | <0.001 | 0.45 (0.40, 0.51) | 0.31 (0.25, 0.39) | -1.10 (-1.47, -0.73) | <0.001 |
| Tropical Latin | — | — | — | — | 172.73 (78.93, | 185.56 (86.44, | 0.25 (0.24, | <0.001 | 43.57 (36.40, | 39.42 (31.84, | -0.31 | 0.033 | 0.51 | 0.44 | -0.49 | 0.011 |

| | | | | | | | | | | | | | | | | |
|---|---|---|---|---|---|---|---|---|---|---|---|---|---|---|---|---|
| America | | | | | 308.08) | 325.54) | 0.26) | | 53.91) | 49.79) | (-0.60, -0.02) | | (0.45, 0.59) | (0.38, 0.51) | (-0.87, -0.11) | |
| Central Sub-Saharan Africa | — | — | — | — | 342.93 (131.26, 669.90) | 362.54 (137.57, 742.15) | 0.20 (0.16, 0.24) | <0.001 | 63.65 (39.32, 100.11) | 59.78 (36.04, 93.60) | -0.24 (-0.30, -0.18) | <0.001 | 0.68 (0.40, 1.14) | 0.60 (0.34, 1.02) | -0.46 (-0.62, -0.31) | <0.001 |
| Western Europe | — | — | — | — | 188.98 (111.44, 297.79) | 181.59 (107.08, 285.90) | -0.15 (-0.21, -0.09) | <0.001 | 33.40 (27.41, 42.28) | 21.96 (16.37, 30.42) | -1.48 (-1.71, -1.25) | <0.001 | 0.35 (0.31, 0.41) | 0.18 (0.16, 0.24) | -2.29 (-2.65, -1.94) | <0.001 |
| High-income North America | — | — | — | — | 246.94 (119.85, 427.44) | 169.77 (86.57, 281.61) | -1.25 (-1.36, -1.14) | <0.001 | 33.36 (25.20, 45.15) | 30.74 (24.87, 39.07) | -0.29 (-0.79, 0.20) | 0.248 | 0.30 (0.26, 0.34) | 0.32 (0.28, 0.38) | 0.29 (-0.37, 0.96) | 0.387 |
| Central Asia | — | — | — | — | 109.32 (63.22, 175.37) | 111.73 (63.39, 183.84) | 0.07 (0.06, 0.09) | <0.001 | 53.91 (42.55, 63.57) | 41.76 (32.27, 58.38) | -0.92 (-1.26, -0.58) | <0.001 | 0.72 (0.57, 0.86) | 0.54 (0.40, 0.80) | -1.10 (-1.48, -0.72) | <0.001 |
| Eastern Europe | — | — | — | — | 71.73 (34.08, 123.90) | 78.41 (37.61, 137.28) | 0.31 (0.27, 0.34) | <0.001 | 31.58 (25.37, 39.47) | 37.79 (27.73, 70.88) | 0.34 (-0.14, 0.82) | 0.165 | 0.41 (0.32, 0.53) | 0.50 (0.36, 0.99) | 0.35 (-0.18, 0.88) | 0.197 |
| Southern Sub-Saharan Africa | — | — | — | — | 303.35 (118.57, 596.65) | 313.71 (123.54, 608.99) | 0.11 (0.09, 0.13) | <0.001 | 42.27 (28.38, 61.20) | 50.89 (33.84, 76.83) | 0.70 (0.30, 1.10) | 0.001 | 0.39 (0.28, 0.53) | 0.51 (0.33, 0.75) | 1.03 (0.44, 1.62) | 0.001 |
| High-income Asia Pacific | — | — | — | — | 144.84 (77.01, 243.24) | 143.90 (76.34, 240.22) | -0.03 (-0.07, 0.01) | 0.152 | 24.02 (18.30, 31.51) | 15.93 (11.17, 23.69) | -1.39 (-1.62, -1.17) | <0.001 | 0.24 (0.19, 0.29) | 0.12 (0.10, 0.15) | -2.35 (-2.66, -2.03) | <0.001 |
| Central Europe | — | — | — | — | 306.12 (180.59, 486.87) | 319.12 (185.39, 507.51) | 0.15 (0.10, 0.19) | <0.001 | 55.79 (46.33, 69.19) | 36.27 (26.48, 51.07) | -1.50 (-1.76, -1.24) | <0.001 | 0.59 (0.53, 0.65) | 0.28 (0.22, 0.36) | -2.58 (-3.01, -2.13) | <0.001 |
| Andean Latin America | — | — | — | — | 316.85 (181.78, 515.30) | 360.70 (200.75, 606.54) | 0.45 (0.42, 0.48) | <0.001 | 99.39 (72.31, 131.12) | 56.33 (39.74, 78.49) | -1.75 (-2.14, -1.36) | <0.001 | 1.23 (0.86, 1.68) | 0.55 (0.36, 0.80) | -2.53 (-3.01, -2.06) | <0.001 |
| Caribbean | — | — | — | — | 192.80 (108.96, 304.10) | 228.77 (130.69, 374.39) | 0.59 (0.58, 0.61) | <0.001 | 53.78 (41.91, 69.14) | 58.01 (40.65, 81.61) | 0.29 (-0.05, 0.64) | 0.093 | 0.65 (0.50, 0.85) | 0.68 (0.46, 1.02) | 0.22 (-0.24, 0.68) | 0.347 |
| Southern Latin | — | — | — | — | 184.45 | 214.20 | 0.50 (0.46, | <0.001 | 43.80 (34.97, | 36.20 (27.52, | -0.67 | <0.001 | 0.51 | 0.37 | -1.10 | <0.001 |

| | | | | | | | | | | | | | | | | |
|---|---|---|---|---|---|---|---|---|---|---|---|---|---|---|---|---|
| America | | | | | (105.57, 301.51) | (121.92, 358.70) | 0.55) | | 55.77) | 47.80) | (-1.04, -0.30) | | (0.40, 0.63) | (0.29, 0.46) | (-1.62, -0.58) | |
| Oceania | — | — | — | — | 150.59 (78.83, 260.33) | 137.94 (73.21, 239.17) | -0.30 (-0.33, -0.28) | <0.001 | 94.06 (61.94, 138.96) | 85.50 (53.94, 136.07) | -0.41 (-0.62, -0.20) | <0.001 | 1.29 (0.80, 1.97) | 1.17 (0.71, 1.93) | -0.42 (-0.66, -0.19) | 0.001 |
| Australasia | — | — | — | — | 100.12 (57.65, 163.73) | 93.94 (54.54, 151.95) | -0.21 (-0.24, -0.19) | <0.001 | 19.23 (13.94, 26.28) | 15.52 (10.46, 22.12) | -0.68 (-0.97, -0.39) | <0.001 | 0.21 (0.16, 0.27) | 0.16 (0.11, 0.22) | -0.84 (-1.24, -0.44) | <0.001 |
| **25-29 years** | | | | | | | | | | | | | | | | |
| **Women** | | | | | | | | | | | | | | | | |
| Global | — | — | — | — | 333.51 (187.57, 539.34) | 361.10 (194.62, 597.71) | 0.27 (0.22, 0.32) | <0.001 | 62.28 (50.36, 77.39) | 57.37 (45.63, 72.71) | -0.27 (-0.49, -0.05) | 0.018 | 0.72 (0.58, 0.84) | 0.62 (0.52, 0.72) | -0.54 (-0.92, -0.14) | 0.007 |
| **SDI category** | | | | | | | | | | | | | | | | |
| High | — | — | — | — | 716.47 (434.41, 1101.34) | 600.64 (379.00, 883.70) | -0.60 (-0.63, -0.56) | <0.001 | 73.35 (54.53, 99.43) | 63.96 (48.12, 84.76) | -0.46 (-0.61, -0.31) | <0.001 | 0.57 (0.52, 0.66) | 0.52 (0.45, 0.66) | -0.31 (-0.52, -0.10) | 0.004 |
| High-middle | — | — | — | — | 260.68 (153.59, 405.32) | 295.16 (175.31, 452.78) | 0.43 (0.36, 0.50) | <0.001 | 43.82 (36.18, 53.85) | 38.28 (28.78, 51.31) | -0.45 (-0.94, 0.04) | 0.073 | 0.48 (0.42, 0.54) | 0.36 (0.30, 0.51) | -0.96 (-1.88, -0.03) | 0.043 |
| Middle | — | — | — | — | 226.25 (121.12, 364.17) | 286.42 (153.07, 468.69) | 0.82 (0.76, 0.87) | <0.001 | 49.49 (40.33, 60.16) | 45.04 (35.90, 57.15) | -0.29 (-0.57, -0.00) | 0.049 | 0.61 (0.48, 0.69) | 0.48 (0.40, 0.57) | -0.81 (-1.20, -0.42) | <0.001 |
| Low-middle | — | — | — | — | 257.01 (130.70, 426.87) | 307.57 (153.01, 521.59) | 0.62 (0.59, 0.65) | <0.001 | 74.28 (54.34, 96.80) | 66.73 (51.69, 82.84) | -0.35 (-0.77, 0.06) | 0.093 | 0.98 (0.68, 1.29) | 0.81 (0.64, 1.00) | -0.62 (-1.19, -0.06) | 0.031 |
| Low | — | — | — | — | 438.77 (193.48, 836.98) | 518.36 (229.70, 978.63) | 0.58 (0.55, 0.60) | <0.001 | 111.84 (83.78, 145.42) | 86.99 (65.51, 117.05) | -0.86 (-0.95, -0.77) | <0.001 | 1.44 (1.01, 1.84) | 0.96 (0.76, 1.20) | -1.39 (-1.48, -1.31) | <0.001 |
| **21 GBD region** | | | | | | | | | | | | | | | | |
| South Asia | — | — | — | — | 212.17 (107.22, 353.58) | 197.15 (100.66, 331.25) | -0.26 (-0.31, -0.22) | <0.001 | 72.90 (47.70, 97.28) | 61.54 (46.33, 78.62) | -0.50 (-1.14, 0.13) | 0.119 | 1.00 (0.61, 1.38) | 0.83 (0.61, 1.08) | -0.55 (-1.29, 0.19) | 0.143 |

| | | | | | | | | | | | | | | | | |
|---|---|---|---|---|---|---|---|---|---|---|---|---|---|---|---|---|
| East Asia | — | — | — | — | 76.21 (42.52, 123.04) | 113.08 (62.09, 182.43) | 1.35 (1.30, 1.40) | <0.001 | 20.50 (15.00, 26.82) | 15.11 (10.92, 21.27) | -1.17 (-2.68, 0.35) | 0.132 | 0.26 (0.18, 0.35) | 0.14 (0.10, 0.20) | -2.02 (-3.75, -0.25) | 0.026 |
| North Africa and Middle East | — | — | — | — | 501.09 (266.06, 803.91) | 517.55 (278.11, 825.09) | 0.11 (0.09, 0.13) | <0.001 | 95.71 (74.02, 119.15) | 82.95 (62.66, 110.11) | -0.46 (-0.63, -0.30) | <0.001 | 1.12 (0.83, 1.37) | 0.90 (0.67, 1.18) | -0.74 (-0.97, -0.51) | <0.001 |
| Western Sub-Saharan Africa | — | — | — | — | 718.86 (318.05, 1352.45) | 866.31 (386.39, 1621.83) | 0.65 (0.61, 0.68) | <0.001 | 128.84 (90.32, 178.17) | 96.80 (63.74, 145.89) | -0.97 (-1.01, -0.94) | <0.001 | 1.47 (1.03, 2.01) | 0.82 (0.59, 1.13) | -2.02 (-2.13, -1.91) | <0.001 |
| Southeast Asia | — | — | — | — | 211.04 (117.13, 324.32) | 219.20 (125.66, 336.85) | 0.13 (0.12, 0.15) | <0.001 | 53.09 (39.68, 65.66) | 40.29 (31.15, 50.77) | -0.94 (-1.11, -0.76) | <0.001 | 0.68 (0.47, 0.85) | 0.46 (0.35, 0.59) | -1.30 (-1.53, -1.07) | <0.001 |
| Eastern Sub-Saharan Africa | — | — | — | — | 593.93 (248.19, 1140.82) | 707.64 (307.00, 1345.56) | 0.61 (0.56, 0.66) | <0.001 | 148.29 (106.34, 199.81) | 105.48 (73.75, 150.39) | -1.17 (-1.34, -0.99) | <0.001 | 1.89 (1.31, 2.55) | 1.10 (0.79, 1.57) | -1.86 (-2.02, -1.70) | <0.001 |
| Central Latin America | — | — | — | — | 471.73 (269.88, 737.00) | 501.47 (283.12, 785.77) | 0.23 (0.20, 0.26) | <0.001 | 70.15 (56.57, 88.66) | 58.56 (41.98, 80.07) | -0.52 (-0.67, -0.36) | <0.001 | 0.73 (0.67, 0.80) | 0.51 (0.38, 0.67) | -1.01 (-1.32, -0.70) | <0.001 |
| Tropical Latin America | — | — | — | — | 470.12 (244.08, 788.60) | 496.85 (261.84, 824.54) | 0.19 (0.18, 0.20) | <0.001 | 81.05 (66.73, 100.97) | 73.97 (57.88, 95.25) | -0.29 (-0.50, -0.08) | 0.006 | 0.91 (0.82, 1.02) | 0.77 (0.66, 0.91) | -0.56 (-0.89, -0.22) | 0.001 |
| Central Sub-Saharan Africa | — | — | — | — | 517.22 (217.30, 993.75) | 587.58 (238.59, 1186.10) | 0.47 (0.37, 0.57) | <0.001 | 79.06 (48.54, 120.54) | 68.47 (41.47, 108.33) | -0.50 (-0.60, -0.41) | <0.001 | 0.84 (0.49, 1.35) | 0.61 (0.36, 0.98) | -1.19 (-1.34, -1.04) | <0.001 |
| Western Europe | — | — | — | — | 740.63 (441.70, 1132.31) | 698.21 (419.15, 1042.64) | -0.21 (-0.26, -0.16) | <0.001 | 71.82 (52.29, 101.36) | 59.59 (40.27, 86.69) | -0.65 (-0.77, -0.52) | <0.001 | 0.52 (0.47, 0.66) | 0.36 (0.31, 0.49) | -1.35 (-1.90, -0.80) | <0.001 |
| High-income North America | — | — | — | — | 899.91 (501.16, 1474.90) | 646.73 (393.35, 1004.38) | -1.09 (-1.18, -1.01) | <0.001 | 89.25 (64.51, 125.29) | 81.19 (63.17, 106.77) | -0.29 (-0.60, 0.03) | 0.073 | 0.67 (0.59, 0.79) | 0.76 (0.66, 0.97) | 0.51 (0.02, 1.01) | 0.042 |
| Central Asia | — | — | — | — | 139.27 (78.85, 209.60) | 135.09 (76.55, 206.92) | -0.10 (-0.14, -0.05) | <0.001 | 55.01 (42.04, 65.84) | 40.84 (30.43, 63.50) | -1.02 (-1.56, -0.48) | <0.001 | 0.77 (0.56, 0.91) | 0.54 (0.39, 0.90) | -1.20 (-1.84, -0.56) | <0.001 |

| | | | | | | | | | | | | | | | | |
|---|---|---|---|---|---|---|---|---|---|---|---|---|---|---|---|---|
| Eastern Europe | — | — | — | — | 127.04 (70.87, 203.24) | 128.59 (71.91, 208.23) | 0.05 (0.01, 0.10) | 0.021 | 30.29 (24.47, 36.95) | 41.04 (28.98, 70.50) | 1.09 (0.43, 1.75) | 0.001 | 0.38 (0.30, 0.44) | 0.55 (0.38, 1.05) | 1.35 (0.55, 2.15) | 0.001 |
| Southern Sub-Saharan Africa | — | — | — | — | 337.80 (149.14, 637.39) | 362.42 (162.15, 699.26) | 0.24 (0.22, 0.26) | <0.001 | 82.69 (61.43, 106.67) | 63.10 (39.06, 93.61) | -0.93 (-2.06, 0.22) | 0.112 | 1.05 (0.77, 1.33) | 0.71 (0.40, 1.09) | -1.22 (-3.16, 0.76) | 0.226 |
| High-income Asia Pacific | — | — | — | — | 398.72 (235.05, 630.99) | 395.05 (239.79, 618.48) | -0.03 (-0.06, -0.00) | 0.025 | 46.93 (34.75, 63.60) | 31.76 (20.61, 47.74) | -1.35 (-1.52, -1.18) | <0.001 | 0.41 (0.34, 0.53) | 0.17 (0.13, 0.29) | -3.06 (-3.44, -2.68) | <0.001 |
| Central Europe | — | — | — | — | 628.75 (388.82, 938.05) | 638.29 (396.69, 947.55) | 0.06 (-0.06, 0.17) | 0.346 | 73.96 (57.73, 97.64) | 52.89 (36.02, 75.05) | -1.21 (-1.29, -1.12) | <0.001 | 0.65 (0.60, 0.71) | 0.30 (0.23, 0.39) | -2.63 (-2.93, -2.34) | <0.001 |
| Andean Latin America | — | — | — | — | 475.67 (242.78, 764.98) | 544.11 (296.41, 851.54) | 0.46 (0.44, 0.49) | <0.001 | 95.72 (72.18, 122.07) | 62.72 (42.95, 86.88) | -1.50 (-1.80, -1.19) | <0.001 | 1.14 (0.87, 1.47) | 0.54 (0.37, 0.79) | -2.55 (-3.46, -1.63) | <0.001 |
| Caribbean | — | — | — | — | 272.39 (146.72, 424.74) | 292.94 (158.95, 473.93) | 0.25 (0.21, 0.28) | <0.001 | 89.00 (65.32, 117.34) | 104.49 (67.66, 149.89) | 0.62 (0.34, 0.91) | <0.001 | 1.21 (0.83, 1.64) | 1.44 (0.88, 2.18) | 0.70 (0.35, 1.05) | <0.001 |
| Southern Latin America | — | — | — | — | 521.44 (288.66, 830.33) | 572.05 (316.57, 880.50) | 0.33 (0.30, 0.35) | <0.001 | 91.33 (73.51, 115.52) | 65.13 (45.97, 90.51) | -1.18 (-1.69, -0.68) | <0.001 | 1.03 (0.86, 1.24) | 0.56 (0.41, 0.76) | -2.14 (-2.86, -1.43) | <0.001 |
| Oceania | — | — | — | — | 194.86 (98.20, 327.56) | 167.08 (84.60, 279.34) | -0.54 (-0.61, -0.47) | <0.001 | 92.19 (60.73, 139.15) | 83.58 (50.17, 134.33) | -0.36 (-0.59, -0.12) | 0.003 | 1.33 (0.81, 2.07) | 1.21 (0.67, 2.06) | -0.28 (-0.70, 0.14) | 0.188 |
| Australasia | — | — | — | — | 346.25 (196.78, 542.11) | 325.05 (180.97, 503.67) | -0.22 (-0.26, -0.19) | <0.001 | 42.21 (30.24, 59.63) | 39.72 (27.67, 56.29) | -0.19 (-0.86, 0.48) | 0.579 | 0.38 (0.31, 0.50) | 0.36 (0.26, 0.50) | -0.13 (-1.15, 0.91) | 0.808 |
| **Men** | | | | | | | | | | | | | | | | |
| Global | — | — | — | — | 199.21 (102.10, 334.94) | 249.94 (124.54, 434.05) | 0.78 (0.72, 0.84) | <0.001 | 49.39 (40.53, 62.29) | 51.29 (40.70, 65.31) | 0.12 (-0.10, 0.34) | 0.283 | 0.63 (0.54, 0.79) | 0.61 (0.52, 0.73) | -0.05 (-0.30, 0.20) | 0.709 |
| SDI category | | | | | | | | | | | | | | | | |
| High | — | — | — | — | 262.41 | 233.68 | -0.38 | <0.001 | 41.98 (34.11, | 35.92 (28.58, | -0.54 | <0.001 | 0.45 | 0.38 | -0.60 | <0.001 |

| | | | | | | | | | | | | | | | |
|---|---|---|---|---|---|---|---|---|---|---|---|---|---|---|---|
| | | | | | (146.23, 412.59) | (136.23, 359.05) | (-0.45, -0.32) | | 52.68) | 44.82) | (-0.72, -0.35) | | (0.41, 0.51) | (0.33, 0.45) | (-0.83, -0.38) | |
| High-middle | — | — | — | — | 164.06 (89.28, 256.65) | 205.33 (112.58, 322.70) | 0.77 (0.69, 0.84) | <0.001 | 45.39 (38.42, 53.98) | 38.11 (30.48, 50.09) | -0.63 (-1.07, -0.20) | 0.004 | 0.59 (0.51, 0.69) | 0.44 (0.37, 0.60) | -1.01 (-1.63, -0.38) | 0.002 |
| Middle | — | — | — | — | 179.39 (90.12, 304.08) | 241.80 (123.10, 415.43) | 1.03 (0.95, 1.12) | <0.001 | 43.33 (34.89, 56.74) | 44.61 (35.19, 58.06) | 0.09 (-0.01, 0.20) | 0.084 | 0.54 (0.46, 0.70) | 0.51 (0.43, 0.62) | -0.24 (-0.40, -0.08) | 0.004 |
| Low-middle | — | — | — | — | 166.57 (79.55, 291.10) | 217.14 (103.74, 386.17) | 0.92 (0.88, 0.96) | <0.001 | 53.55 (41.73, 73.82) | 56.57 (43.98, 74.23) | 0.23 (-0.01, 0.48) | 0.057 | 0.72 (0.57, 1.05) | 0.73 (0.58, 0.93) | 0.07 (-0.24, 0.38) | 0.641 |
| Low | — | — | — | — | 336.38 (137.64, 651.43) | 400.33 (166.68, 771.13) | 0.60 (0.58, 0.63) | <0.001 | 89.12 (69.56, 120.12) | 88.20 (66.20, 120.13) | -0.02 (-0.11, 0.07) | 0.663 | 1.15 (0.93, 1.58) | 1.08 (0.83, 1.48) | -0.22 (-0.40, -0.05) | 0.014 |
| **21 GBD region** | | | | | | | | | | | | | | | | |
| South Asia | — | — | — | — | 97.36 (47.23, 170.69) | 89.35 (43.62, 155.73) | -0.32 (-0.39, -0.25) | <0.001 | 43.49 (30.24, 70.01) | 42.03 (31.37, 58.06) | -0.10 (-0.60, 0.41) | 0.713 | 0.62 (0.42, 1.02) | 0.60 (0.44, 0.86) | -0.07 (-0.62, 0.49) | 0.808 |
| East Asia | — | — | — | — | 78.50 (39.69, 133.59) | 119.55 (62.52, 203.17) | 1.48 (1.42, 1.53) | <0.001 | 21.15 (15.36, 28.44) | 17.55 (12.99, 23.75) | -0.64 (-0.95, -0.33) | <0.001 | 0.27 (0.19, 0.37) | 0.18 (0.14, 0.24) | -1.46 (-1.63, -1.29) | <0.001 |
| North Africa and Middle East | — | — | — | — | 510.91 (259.35, 850.89) | 538.48 (278.09, 882.78) | 0.19 (0.12, 0.26) | <0.001 | 97.30 (72.78, 135.07) | 85.04 (60.84, 118.93) | -0.44 (-0.67, -0.21) | <0.001 | 1.13 (0.86, 1.58) | 0.90 (0.65, 1.25) | -0.73 (-0.92, -0.55) | <0.001 |
| Western Sub-Saharan Africa | — | — | — | — | 909.20 (384.79, 1793.79) | 1041.19 (444.72, 2061.24) | 0.47 (0.44, 0.51) | <0.001 | 172.78 (123.16, 236.84) | 159.44 (112.98, 225.08) | -0.27 (-0.39, -0.15) | <0.001 | 2.01 (1.44, 2.79) | 1.67 (1.24, 2.31) | -0.62 (-0.73, -0.52) | <0.001 |
| Southeast Asia | — | — | — | — | 179.44 (96.28, 283.01) | 200.51 (107.45, 313.99) | 0.39 (0.37, 0.40) | <0.001 | 51.57 (41.32, 64.35) | 46.10 (36.56, 57.66) | -0.39 (-0.64, -0.14) | 0.002 | 0.68 (0.54, 0.85) | 0.57 (0.46, 0.70) | -0.61 (-1.01, -0.21) | 0.003 |
| Eastern Sub-Saharan Africa | — | — | — | — | 359.06 (145.52, 708.04) | 416.67 (170.73, 829.67) | 0.51 (0.48, 0.54) | <0.001 | 96.73 (73.34, 135.96) | 86.18 (61.36, 124.01) | -0.42 (-0.60, -0.24) | <0.001 | 1.26 (0.96, 1.80) | 1.03 (0.74, 1.58) | -0.68 (-0.83, -0.52) | <0.001 |

| | | | | | | | | | | | | | | | | |
|---|---|---|---|---|---|---|---|---|---|---|---|---|---|---|---|---|
| Central Latin America | — | — | — | — | 218.99 (115.62, 360.64) | 246.42 (131.35, 401.78) | 0.40 (0.38, 0.42) | <0.001 | 49.16 (41.46, 59.39) | 39.97 (30.69, 51.29) | -0.64 (-0.86, -0.42) | <0.001 | 0.60 (0.54, 0.68) | 0.43 (0.34, 0.52) | -1.00 (-1.35, -0.66) | <0.001 |
| Tropical Latin America | — | — | — | — | 167.62 (83.33, 291.92) | 182.13 (91.30, 315.26) | 0.29 (0.28, 0.30) | <0.001 | 54.44 (47.15, 63.81) | 49.23 (41.83, 58.84) | -0.34 (-0.64, -0.05) | 0.023 | 0.74 (0.66, 0.83) | 0.64 (0.56, 0.74) | -0.48 (-0.82, -0.14) | 0.005 |
| Central Sub-Saharan Africa | — | — | — | — | 325.32 (124.92, 652.82) | 360.46 (136.49, 755.57) | 0.36 (0.32, 0.41) | <0.001 | 72.42 (44.25, 109.13) | 66.13 (42.15, 104.21) | -0.35 (-0.51, -0.18) | <0.001 | 0.89 (0.52, 1.40) | 0.76 (0.45, 1.30) | -0.62 (-0.84, -0.39) | <0.001 |
| Western Europe | — | — | — | — | 261.14 (146.40, 409.52) | 254.60 (145.09, 397.68) | -0.09 (-0.13, -0.06) | <0.001 | 44.22 (35.81, 55.40) | 33.12 (25.29, 43.62) | -1.01 (-1.29, -0.73) | <0.001 | 0.49 (0.44, 0.56) | 0.31 (0.27, 0.39) | -1.56 (-1.95, -1.17) | <0.001 |
| High-income North America | — | — | — | — | 298.11 (158.16, 510.38) | 214.76 (121.94, 339.62) | -1.10 (-1.17, -1.03) | <0.001 | 44.58 (35.47, 57.48) | 43.38 (34.99, 52.87) | -0.09 (-0.45, 0.27) | 0.613 | 0.46 (0.42, 0.52) | 0.51 (0.42, 0.61) | 0.37 (-0.17, 0.92) | 0.179 |
| Central Asia | — | — | — | — | 114.72 (58.72, 184.66) | 122.99 (64.86, 200.80) | 0.24 (0.22, 0.25) | <0.001 | 70.28 (56.59, 83.33) | 54.05 (42.78, 72.43) | -0.86 (-1.53, -0.19) | 0.012 | 1.04 (0.84, 1.23) | 0.77 (0.60, 1.07) | -1.00 (-1.74, -0.25) | 0.009 |
| Eastern Europe | — | — | — | — | 69.08 (35.71, 116.85) | 74.04 (37.90, 124.89) | 0.24 (0.22, 0.26) | <0.001 | 41.47 (35.57, 49.79) | 67.92 (52.49, 112.82) | 1.80 (0.23, 3.39) | 0.024 | 0.61 (0.52, 0.74) | 1.04 (0.79, 1.77) | 1.93 (0.24, 3.65) | 0.025 |
| Southern Sub-Saharan Africa | — | — | — | — | 277.53 (121.08, 541.01) | 285.95 (123.28, 560.54) | 0.10 (0.07, 0.14) | <0.001 | 65.11 (48.13, 86.45) | 65.88 (47.18, 94.52) | 0.00 (-0.29, 0.29) | 0.993 | 0.81 (0.62, 1.07) | 0.82 (0.57, 1.22) | -0.03 (-0.41, 0.35) | 0.868 |
| High-income Asia Pacific | — | — | — | — | 227.48 (125.40, 364.82) | 226.36 (128.40, 356.81) | -0.03 (-0.10, 0.04) | 0.424 | 37.57 (28.41, 49.54) | 24.11 (17.25, 33.68) | -1.50 (-1.76, -1.24) | <0.001 | 0.41 (0.32, 0.51) | 0.19 (0.16, 0.24) | -2.59 (-2.94, -2.24) | <0.001 |
| Central Europe | — | — | — | — | 316.35 (179.78, 489.95) | 328.73 (187.97, 509.66) | 0.12 (0.02, 0.22) | 0.020 | 75.33 (64.97, 89.38) | 45.26 (34.44, 58.67) | -1.78 (-2.08, -1.48) | <0.001 | 0.94 (0.86, 1.04) | 0.44 (0.36, 0.55) | -2.60 (-2.91, -2.28) | <0.001 |
| Andean Latin America | — | — | — | — | 332.66 (162.20, 560.35) | 391.76 (195.11, 643.13) | 0.57 (0.56, 0.58) | <0.001 | 103.72 (72.80, 139.06) | 64.21 (44.28, 90.06) | -1.60 (-1.95, -1.25) | <0.001 | 1.39 (0.95, 1.89) | 0.70 (0.46, 1.04) | -2.34 (-2.83, -1.85) | <0.001 |

| | | | | | | | | | | | | | | | | |
|---|---|---|---|---|---|---|---|---|---|---|---|---|---|---|---|---|
| Caribbean | — | — | — | — | 199.62 (98.44, 329.62) | 242.56 (115.67, 402.23) | 0.68 (0.64, 0.73) | <0.001 | 61.80 (48.08, 76.87) | 70.68 (50.08, 96.19) | 0.49 (0.30, 0.69) | <0.001 | 0.83 (0.65, 1.05) | 0.93 (0.64, 1.33) | 0.46 (0.18, 0.74) | 0.001 |
| Southern Latin America | — | — | — | — | 225.24 (118.13, 369.80) | 255.42 (136.97, 413.29) | 0.43 (0.38, 0.49) | <0.001 | 53.69 (41.95, 68.42) | 43.24 (32.18, 57.36) | -0.76 (-1.15, -0.36) | <0.001 | 0.67 (0.54, 0.84) | 0.48 (0.37, 0.61) | -1.24 (-1.79, -0.70) | <0.001 |
| Oceania | — | — | — | — | 110.32 (53.16, 189.33) | 103.54 (50.09, 181.68) | -0.21 (-0.24, -0.19) | <0.001 | 73.92 (49.63, 109.28) | 68.60 (42.27, 101.26) | -0.28 (-0.40, -0.17) | <0.001 | 1.10 (0.71, 1.64) | 1.02 (0.61, 1.56) | -0.29 (-0.41, -0.16) | <0.001 |
| Australasia | — | — | — | — | 160.72 (83.55, 260.64) | 151.07 (80.97, 241.62) | -0.21 (-0.23, -0.19) | <0.001 | 25.87 (18.91, 36.09) | 24.43 (16.71, 34.56) | -0.25 (-0.67, 0.18) | 0.252 | 0.28 (0.21, 0.37) | 0.26 (0.19, 0.38) | -0.25 (-0.83, 0.33) | 0.391 |
| **30-34 years** | | | | | | | | | | | | | | | | |
| **Women** | | | | | | | | | | | | | | | | |
| Global | — | — | — | — | 291.11 (150.89, 503.79) | 302.64 (151.23, 541.45) | 0.13 (0.06, 0.20) | <0.001 | 66.43 (55.18, 80.80) | 58.28 (47.58, 74.20) | -0.47 (-0.68, -0.26) | <0.001 | 0.90 (0.76, 1.01) | 0.74 (0.64, 0.85) | -0.68 (-0.97, -0.39) | <0.001 |
| **SDI category** | | | | | | | | | | | | | | | | |
| High | — | — | — | — | 621.83 (353.89, 1040.60) | 532.08 (315.33, 859.61) | -0.52 (-0.56, -0.48) | <0.001 | 75.30 (58.05, 101.61) | 69.21 (53.60, 91.07) | -0.24 (-0.55, 0.06) | 0.121 | 0.75 (0.69, 0.87) | 0.72 (0.62, 0.91) | -0.08 (-0.60, 0.45) | 0.775 |
| High-middle | — | — | — | — | 216.63 (115.61, 363.31) | 244.95 (134.28, 413.40) | 0.41 (0.35, 0.47) | <0.001 | 47.48 (40.53, 57.44) | 40.19 (31.51, 52.80) | -0.53 (-0.82, -0.25) | <0.001 | 0.63 (0.58, 0.70) | 0.48 (0.40, 0.67) | -0.97 (-1.33, -0.62) | <0.001 |
| Middle | — | — | — | — | 197.44 (97.57, 352.32) | 241.39 (120.63, 433.98) | 0.71 (0.56, 0.85) | <0.001 | 53.26 (44.28, 63.56) | 44.13 (35.24, 57.15) | -0.72 (-1.07, -0.37) | <0.001 | 0.75 (0.63, 0.84) | 0.55 (0.46, 0.64) | -1.10 (-1.55, -0.65) | <0.001 |
| Low-middle | — | — | — | — | 211.92 (100.99, 384.58) | 261.39 (125.40, 484.08) | 0.73 (0.67, 0.80) | <0.001 | 79.99 (61.28, 101.17) | 70.51 (56.71, 86.99) | -0.43 (-0.65, -0.21) | <0.001 | 1.21 (0.89, 1.54) | 1.00 (0.79, 1.22) | -0.66 (-0.95, -0.38) | <0.001 |
| Low | — | — | — | — | 356.70 (150.02, 775.40) | 422.51 (173.28, 932.88) | 0.59 (0.56, 0.62) | <0.001 | 121.58 (92.34, 152.61) | 93.13 (72.10, 123.64) | -0.92 (-1.05, -0.79) | <0.001 | 1.81 (1.36, 2.25) | 1.25 (1.00, 1.54) | -1.29 (-1.46, -1.12) | <0.001 |

**21 GBD region**

| | | | | | | | | | | | | | | | | |
|---|---|---|---|---|---|---|---|---|---|---|---|---|---|---|---|---|
| South Asia | — | — | — | — | 181.64 (88.66, 328.00) | 170.74 (83.31, 308.03) | -0.22 (-0.28, -0.16) | <0.001 | 76.37 (56.64, 99.26) | 64.19 (47.98, 81.64) | -0.61 (-1.00, -0.22) | 0.002 | 1.18 (0.83, 1.54) | 0.97 (0.71, 1.26) | -0.65 (-1.18, -0.11) | 0.019 |
| East Asia | — | — | — | — | 64.54 (32.43, 118.59) | 96.29 (47.11, 178.66) | 1.40 (1.16, 1.63) | <0.001 | 20.83 (15.32, 27.12) | 14.59 (10.64, 20.76) | -1.39 (-2.42, -0.34) | 0.010 | 0.30 (0.21, 0.40) | 0.16 (0.12, 0.23) | -2.02 (-2.90, -1.14) | <0.001 |
| North Africa and Middle East | — | — | — | — | 537.95 (285.43, 895.80) | 524.84 (281.20, 881.03) | -0.08 (-0.12, -0.05) | <0.001 | 98.35 (76.80, 124.20) | 84.72 (63.75, 110.86) | -0.51 (-0.60, -0.41) | <0.001 | 1.23 (0.93, 1.51) | 1.00 (0.75, 1.29) | -0.69 (-0.88, -0.50) | <0.001 |
| Western Sub-Saharan Africa | — | — | — | — | 732.22 (292.39, 1657.30) | 878.69 (347.02, 1983.20) | 0.63 (0.59, 0.67) | <0.001 | 197.86 (144.62, 272.50) | 130.94 (90.40, 194.68) | -1.42 (-1.49, -1.36) | <0.001 | 2.81 (2.05, 3.69) | 1.49 (1.11, 2.00) | -2.17 (-2.29, -2.04) | <0.001 |
| Southeast Asia | — | — | — | — | 208.57 (112.78, 339.46) | 214.29 (113.32, 354.69) | 0.10 (0.06, 0.13) | <0.001 | 65.18 (51.18, 79.78) | 46.89 (36.22, 58.52) | -1.12 (-1.23, -1.01) | <0.001 | 0.95 (0.72, 1.17) | 0.62 (0.48, 0.78) | -1.44 (-1.61, -1.27) | <0.001 |
| Eastern Sub-Saharan Africa | — | — | — | — | 422.98 (167.38, 964.18) | 510.16 (203.10, 1159.57) | 0.66 (0.63, 0.68) | <0.001 | 125.39 (91.06, 171.60) | 89.35 (63.03, 132.99) | -1.16 (-1.26, -1.06) | <0.001 | 1.82 (1.32, 2.51) | 1.10 (0.80, 1.56) | -1.73 (-1.85, -1.62) | <0.001 |
| Central Latin America | — | — | — | — | 294.23 (126.56, 521.69) | 380.43 (202.27, 635.70) | 0.89 (0.84, 0.94) | <0.001 | 63.48 (52.39, 78.42) | 53.66 (39.50, 72.51) | -0.48 (-1.00, 0.04) | 0.069 | 0.84 (0.77, 0.94) | 0.59 (0.43, 0.79) | -1.07 (-1.79, -0.34) | 0.004 |
| Tropical Latin America | — | — | — | — | 258.60 (109.75, 501.51) | 312.21 (149.10, 580.33) | 0.65 (0.63, 0.66) | <0.001 | 73.53 (63.04, 88.86) | 72.34 (58.89, 91.99) | 0.04 (-0.20, 0.28) | 0.734 | 1.06 (0.97, 1.17) | 0.98 (0.85, 1.16) | -0.15 (-0.45, 0.15) | 0.318 |
| Central Sub-Saharan Africa | — | — | — | — | 407.73 (156.86, 948.30) | 466.37 (174.18, 1070.54) | 0.49 (0.35, 0.62) | <0.001 | 85.31 (54.53, 125.97) | 75.62 (46.50, 116.92) | -0.42 (-0.52, -0.32) | <0.001 | 1.13 (0.70, 1.76) | 0.90 (0.55, 1.40) | -0.84 (-0.94, -0.74) | <0.001 |
| Western Europe | — | — | — | — | 596.09 (325.75, 955.78) | 561.46 (311.71, 892.96) | -0.22 (-0.31, -0.14) | <0.001 | 71.59 (54.57, 97.44) | 58.80 (42.58, 81.51) | -0.68 (-0.79, -0.56) | <0.001 | 0.71 (0.64, 0.87) | 0.51 (0.45, 0.68) | -1.10 (-1.40, -0.81) | <0.001 |
| High-income North America | — | — | — | — | 805.54 (421.77, | 640.65 (351.47, | -0.75 (-0.81, | <0.001 | 92.25 (67.92, 130.57) | 99.18 (79.32, 129.78) | 0.34 (-0.02, | 0.067 | 0.87 (0.78, | 1.15 (0.99, | 1.04 (0.49, 1.58) | <0.001 |

| | | | | | | | | | | | | | | | | |
|---|---|---|---|---|---|---|---|---|---|---|---|---|---|---|---|---|
| | | | | | 1429.20) | 1105.75) | -0.69) | | | | 0.70) | | 1.03) | 1.46) | | |
| Central Asia | — | — | — | — | 112.00 (59.70, 185.04) | 106.28 (54.76, 176.84) | -0.17 (-0.20, -0.14) | <0.001 | 59.00 (46.82, 70.12) | 50.33 (39.10, 73.89) | -0.64 (-0.92, -0.35) | <0.001 | 0.93 (0.73, 1.11) | 0.79 (0.60, 1.23) | -0.69 (-1.00, -0.38) | <0.001 |
| Eastern Europe | — | — | — | — | 79.06 (39.65, 142.37) | 79.91 (40.23, 143.37) | 0.04 (0.01, 0.07) | 0.003 | 35.25 (29.15, 42.44) | 54.22 (39.11, 92.04) | 1.76 (0.92, 2.61) | <0.001 | 0.55 (0.45, 0.65) | 0.88 (0.62, 1.55) | 1.96 (1.02, 2.90) | <0.001 |
| Southern Sub-Saharan Africa | — | — | — | — | 225.67 (91.63, 498.20) | 240.93 (97.61, 537.87) | 0.23 (0.21, 0.24) | <0.001 | 89.06 (65.17, 113.69) | 63.89 (39.05, 91.73) | -1.06 (-2.58, 0.47) | 0.174 | 1.36 (0.95, 1.70) | 0.90 (0.53, 1.36) | -1.32 (-3.35, 0.75) | 0.210 |
| High-income Asia Pacific | — | — | — | — | 342.07 (178.37, 606.13) | 341.63 (184.78, 590.53) | 0.01 (-0.05, 0.07) | 0.751 | 44.40 (32.75, 62.22) | 30.41 (20.05, 47.60) | -1.30 (-1.41, -1.18) | <0.001 | 0.46 (0.39, 0.60) | 0.21 (0.17, 0.39) | -2.64 (-2.88, -2.40) | <0.001 |
| Central Europe | — | — | — | — | 490.78 (266.17, 813.11) | 507.36 (281.57, 848.42) | 0.15 (-0.01, 0.30) | 0.069 | 88.86 (75.01, 110.54) | 52.17 (36.91, 74.23) | -1.76 (-1.97, -1.54) | <0.001 | 1.11 (1.03, 1.19) | 0.44 (0.35, 0.56) | -3.03 (-3.39, -2.67) | <0.001 |
| Andean Latin America | — | — | — | — | 272.54 (108.30, 482.79) | 362.23 (179.31, 613.99) | 0.98 (0.91, 1.04) | <0.001 | 78.20 (58.80, 99.19) | 51.84 (35.71, 72.85) | -1.38 (-2.09, -0.67) | <0.001 | 1.12 (0.85, 1.45) | 0.57 (0.39, 0.84) | -2.38 (-3.53, -1.22) | <0.001 |
| Caribbean | — | — | — | — | 251.93 (136.79, 407.23) | 262.91 (136.64, 434.37) | 0.15 (0.13, 0.17) | <0.001 | 98.87 (73.88, 129.60) | 110.12 (73.38, 159.38) | 0.38 (0.18, 0.59) | <0.001 | 1.51 (1.08, 2.02) | 1.70 (1.06, 2.55) | 0.40 (0.13, 0.66) | 0.003 |
| Southern Latin America | — | — | — | — | 298.16 (52.22, 621.60) | 510.67 (260.52, 849.65) | 1.88 (1.81, 1.94) | <0.001 | 86.03 (66.62, 108.46) | 73.51 (54.13, 96.87) | -0.48 (-0.79, -0.16) | 0.003 | 1.24 (1.02, 1.50) | 0.82 (0.63, 1.05) | -1.34 (-1.74, -0.94) | <0.001 |
| Oceania | — | — | — | — | 67.32 (34.07, 110.38) | 56.46 (28.42, 93.67) | -0.61 (-0.66, -0.57) | <0.001 | 59.48 (38.07, 88.82) | 53.45 (32.48, 84.91) | -0.41 (-0.51, -0.31) | <0.001 | 0.98 (0.61, 1.49) | 0.89 (0.51, 1.44) | -0.40 (-0.50, -0.29) | <0.001 |
| Australasia | — | — | — | — | 307.00 (169.50, 483.67) | 294.07 (159.54, 465.50) | -0.16 (-0.21, -0.11) | <0.001 | 40.43 (29.33, 55.24) | 42.98 (30.03, 58.93) | 0.28 (-0.08, 0.63) | 0.126 | 0.42 (0.34, 0.59) | 0.48 (0.35, 0.67) | 0.57 (-0.01, 1.15) | 0.053 |
| **Men** | | | | | | | | | | | | | | | | |
| Global | — | — | — | — | 117.23 (61.25, | 138.48 (72.37, | 0.56 (0.51, | <0.001 | 55.81 (48.17, | 52.23 (44.58, | -0.26 | <0.001 | 0.87 | 0.79 | -0.39 | <0.001 |

| | | | | | | | | | | | | | | | | |
|---|---|---|---|---|---|---|---|---|---|---|---|---|---|---|---|---|
| | | | | | 208.97) | 253.61) | 0.61) | | 68.43) | 63.09) | (-0.36, -0.17) | | (0.77, 1.09) | (0.69, 0.91) | (-0.52, -0.25) | |
| **SDI category** | | | | | | | | | | | | | | | | |
| High | — | — | — | — | 186.69 (107.54, 300.58) | 163.64 (100.02, 257.28) | -0.44 (-0.49, -0.40) | <0.001 | 50.10 (44.06, 58.28) | 41.20 (34.85, 48.81) | -0.68 (-0.94, -0.43) | <0.001 | 0.71 (0.66, 0.77) | 0.57 (0.49, 0.68) | -0.76 (-1.11, -0.40) | <0.001 |
| High-middle | — | — | — | — | 91.00 (49.20, 151.41) | 115.04 (63.91, 189.12) | 0.80 (0.74, 0.86) | <0.001 | 53.37 (47.10, 60.99) | 42.74 (36.58, 54.87) | -0.78 (-1.16, -0.40) | <0.001 | 0.85 (0.76, 0.98) | 0.64 (0.56, 0.84) | -0.99 (-1.43, -0.54) | <0.001 |
| Middle | — | — | — | — | 95.99 (47.91, 177.83) | 130.43 (66.20, 239.84) | 1.07 (0.97, 1.17) | <0.001 | 46.93 (39.27, 59.73) | 42.44 (35.61, 53.15) | -0.37 (-0.57, -0.18) | <0.001 | 0.74 (0.62, 0.95) | 0.62 (0.54, 0.74) | -0.60 (-0.86, -0.34) | <0.001 |
| Low-middle | — | — | — | — | 97.60 (48.05, 180.07) | 121.47 (59.67, 229.66) | 0.74 (0.68, 0.80) | <0.001 | 63.27 (50.38, 90.18) | 64.19 (52.27, 79.85) | 0.04 (-0.17, 0.26) | 0.703 | 1.02 (0.80, 1.49) | 1.02 (0.83, 1.27) | -0.03 (-0.26, 0.20) | 0.811 |
| Low | — | — | — | — | 179.30 (74.39, 388.47) | 209.10 (88.48, 443.81) | 0.54 (0.52, 0.56) | <0.001 | 94.18 (74.44, 130.08) | 85.65 (65.72, 118.19) | -0.32 (-0.40, -0.24) | <0.001 | 1.49 (1.19, 2.11) | 1.31 (1.02, 1.85) | -0.44 (-0.53, -0.34) | <0.001 |
| **21 GBD region** | | | | | | | | | | | | | | | | |
| South Asia | — | — | — | — | 73.80 (36.43, 133.75) | 67.61 (33.56, 123.35) | -0.32 (-0.35, -0.28) | <0.001 | 59.43 (41.71, 94.27) | 56.31 (43.56, 75.10) | -0.21 (-0.38, -0.04) | 0.015 | 0.98 (0.68, 1.59) | 0.93 (0.71, 1.26) | -0.21 (-0.39, -0.02) | 0.033 |
| East Asia | — | — | — | — | 47.96 (23.92, 86.56) | 71.78 (35.76, 130.21) | 1.39 (1.34, 1.44) | <0.001 | 24.34 (18.02, 32.66) | 18.03 (13.98, 23.55) | -1.05 (-1.30, -0.80) | <0.001 | 0.38 (0.28, 0.53) | 0.25 (0.20, 0.33) | -1.49 (-1.77, -1.21) | <0.001 |
| North Africa and Middle East | — | — | — | — | 316.78 (170.09, 528.17) | 311.23 (168.11, 531.22) | -0.07 (-0.12, -0.01) | 0.014 | 93.22 (72.39, 122.55) | 72.29 (55.02, 95.02) | -0.86 (-0.98, -0.74) | <0.001 | 1.34 (1.03, 1.79) | 0.98 (0.74, 1.29) | -1.08 (-1.21, -0.94) | <0.001 |
| Western Sub-Saharan Africa | — | — | — | — | 420.77 (168.91, 932.04) | 486.18 (193.28, 1077.24) | 0.51 (0.46, 0.55) | <0.001 | 152.29 (111.21, 212.93) | 132.48 (96.27, 187.13) | -0.47 (-0.56, -0.38) | <0.001 | 2.29 (1.65, 3.31) | 1.87 (1.38, 2.66) | -0.68 (-0.82, -0.54) | <0.001 |
| Southeast Asia | — | — | — | — | 100.89 (55.43, 162.37) | 112.44 (61.80, 183.58) | 0.37 (0.35, 0.39) | <0.001 | 54.42 (44.23, 68.17) | 44.63 (35.73, 55.57) | -0.67 (-0.90, | <0.001 | 0.86 (0.69, | 0.68 (0.54, | -0.81 (-1.09, | <0.001 |

| | | | | | | | | | | | | | | | | |
|---|---|---|---|---|---|---|---|---|---|---|---|---|---|---|---|---|
| | | | | | | | | | | | -0.43) | | 1.09) | 0.86) | -0.52) | |
| Eastern Sub-Saharan Africa | — | — | — | — | 202.07 (79.31, 461.95) | 237.31 (93.36, 530.74) | 0.55 (0.52, 0.59) | <0.001 | 98.34 (75.34, 146.66) | 81.87 (59.71, 125.09) | -0.66 (-0.76, -0.55) | <0.001 | 1.54 (1.18, 2.36) | 1.22 (0.88, 1.93) | -0.83 (-0.95, -0.72) | <0.001 |
| Central Latin America | — | — | — | — | 46.58 (5.43, 109.27) | 119.66 (58.35, 208.07) | 3.29 (3.19, 3.39) | <0.001 | 45.02 (40.44, 51.09) | 41.06 (32.96, 50.16) | -0.23 (-0.74, 0.28) | 0.382 | 0.75 (0.68, 0.83) | 0.61 (0.48, 0.74) | -0.61 (-1.12, -0.10) | 0.020 |
| Tropical Latin America | — | — | — | — | 47.42 (11.51, 101.30) | 88.19 (41.39, 161.98) | 2.16 (2.10, 2.23) | <0.001 | 66.07 (59.90, 73.56) | 53.38 (47.18, 61.83) | -0.80 (-1.21, -0.40) | <0.001 | 1.12 (1.02, 1.24) | 0.86 (0.77, 0.97) | -0.83 (-1.34, -0.32) | 0.002 |
| Central Sub-Saharan Africa | — | — | — | — | 179.90 (68.06, 413.78) | 200.75 (75.96, 458.91) | 0.38 (0.36, 0.41) | <0.001 | 77.32 (47.33, 122.32) | 67.73 (42.13, 110.44) | -0.47 (-0.57, -0.38) | <0.001 | 1.20 (0.72, 1.98) | 1.00 (0.61, 1.70) | -0.62 (-0.73, -0.51) | <0.001 |
| Western Europe | — | — | — | — | 177.90 (98.85, 277.93) | 172.66 (97.95, 266.94) | -0.11 (-0.18, -0.03) | 0.004 | 53.07 (46.50, 61.32) | 38.24 (32.20, 46.21) | -1.16 (-1.37, -0.96) | <0.001 | 0.77 (0.70, 0.85) | 0.51 (0.46, 0.59) | -1.46 (-1.75, -1.16) | <0.001 |
| High-income North America | — | — | — | — | 217.59 (113.04, 382.99) | 165.25 (89.86, 280.16) | -0.92 (-0.98, -0.86) | <0.001 | 55.48 (47.91, 66.52) | 56.00 (45.50, 66.96) | 0.06 (-0.38, 0.49) | 0.801 | 0.77 (0.71, 0.86) | 0.83 (0.66, 1.00) | 0.25 (-0.34, 0.85) | 0.405 |
| Central Asia | — | — | — | — | 56.04 (29.41, 90.62) | 59.76 (30.48, 98.07) | 0.21 (0.20, 0.23) | <0.001 | 81.79 (68.24, 95.91) | 62.50 (50.90, 84.89) | -0.95 (-1.38, -0.52) | <0.001 | 1.39 (1.15, 1.64) | 1.04 (0.85, 1.43) | -1.01 (-1.46, -0.55) | <0.001 |
| Eastern Europe | — | — | — | — | 30.68 (15.87, 56.00) | 32.11 (16.69, 58.78) | 0.16 (0.14, 0.17) | <0.001 | 51.44 (44.32, 62.89) | 100.88 (80.66, 154.44) | 2.50 (1.36, 3.65) | <0.001 | 0.88 (0.75, 1.08) | 1.75 (1.39, 2.69) | 2.57 (1.41, 3.75) | <0.001 |
| Southern Sub-Saharan Africa | — | — | — | — | 134.28 (54.09, 298.44) | 137.76 (55.38, 305.99) | 0.09 (0.05, 0.12) | <0.001 | 79.73 (63.82, 99.10) | 69.62 (49.92, 95.28) | -0.59 (-1.31, 0.14) | 0.110 | 1.28 (1.02, 1.60) | 1.10 (0.78, 1.53) | -0.66 (-1.47, 0.16) | 0.113 |
| High-income Asia Pacific | — | — | — | — | 152.18 (86.04, 252.10) | 152.15 (86.04, 248.72) | -0.01 (-0.08, 0.06) | 0.812 | 33.98 (27.16, 42.35) | 21.35 (16.28, 28.30) | -1.66 (-1.91, -1.42) | <0.001 | 0.45 (0.37, 0.55) | 0.23 (0.20, 0.29) | -2.43 (-2.78, -2.08) | <0.001 |
| Central Europe | — | — | — | — | 174.57 (96.24, 283.22) | 185.06 (103.19, | 0.22 (0.17, 0.26) | <0.001 | 103.59 (95.05, | 50.87 (42.20, 61.48) | -2.45 (-2.67, | <0.001 | 1.66 (1.54, | 0.72 (0.59, | -2.88 (-3.14, | <0.001 |

| | | | | | | | | | | | | | | | | |
|---|---|---|---|---|---|---|---|---|---|---|---|---|---|---|---|---|
| | | | | | | 303.29) | | | 113.29) | | -2.23) | | 1.80) | 0.86) | -2.62) | |
| Andean Latin America | — | — | — | — | 78.80 (3.18, 175.23) | 155.33 (71.05, 270.26) | 2.35 (2.24, 2.46) | <0.001 | 77.99 (55.93, 101.36) | 42.97 (30.57, 59.90) | -2.02 (-2.32, -1.72) | <0.001 | 1.30 (0.92, 1.70) | 0.61 (0.40, 0.90) | -2.59 (-2.95, -2.22) | <0.001 |
| Caribbean | — | — | — | — | 104.20 (55.31, 166.93) | 125.82 (65.84, 206.21) | 0.65 (0.61, 0.69) | <0.001 | 72.15 (59.64, 88.81) | 79.37 (57.24, 109.23) | 0.33 (0.10, 0.55) | 0.004 | 1.17 (0.95, 1.47) | 1.28 (0.89, 1.80) | 0.33 (0.06, 0.60) | 0.015 |
| Southern Latin America | — | — | — | — | 37.97 (1.49, 134.22) | 168.69 (85.07, 275.36) | 5.22 (5.07, 5.37) | <0.001 | 61.67 (50.55, 74.41) | 50.63 (39.79, 62.63) | -0.66 (-1.13, -0.18) | 0.007 | 1.05 (0.87, 1.27) | 0.73 (0.58, 0.91) | -1.22 (-1.75, -0.68) | <0.001 |
| Oceania | — | — | — | — | 50.08 (25.14, 84.01) | 47.43 (24.27, 79.38) | -0.19 (-0.21, -0.16) | <0.001 | 56.39 (37.24, 83.24) | 50.94 (32.81, 74.34) | -0.38 (-0.58, -0.18) | <0.001 | 0.95 (0.60, 1.42) | 0.85 (0.53, 1.27) | -0.39 (-0.58, -0.20) | <0.001 |
| Australasia | — | — | — | — | 119.45 (68.11, 186.91) | 115.31 (65.84, 177.21) | -0.12 (-0.14, -0.11) | <0.001 | 27.69 (21.35, 36.30) | 29.46 (21.43, 38.97) | 0.21 (-0.48, 0.91) | 0.552 | 0.37 (0.30, 0.51) | 0.41 (0.30, 0.55) | 0.31 (-0.58, 1.21) | 0.497 |
| **35-39 years** | | | | | | | | | | | | | | | | |
| **Women** | | | | | | | | | | | | | | | | |
| Global | — | — | — | — | 271.76 (148.44, 468.97) | 311.64 (172.92, 544.14) | 0.48 (0.38, 0.58) | <0.001 | 71.69 (61.73, 84.03) | 66.35 (55.96, 81.74) | -0.25 (-0.40, -0.10) | 0.001 | 1.11 (0.98, 1.23) | 0.96 (0.84, 1.08) | -0.46 (-0.62, -0.31) | <0.001 |
| **SDI category** | | | | | | | | | | | | | | | | |
| High | — | — | — | — | 581.76 (340.92, 960.49) | 501.79 (312.27, 766.07) | -0.52 (-0.59, -0.45) | <0.001 | 81.28 (65.54, 103.71) | 77.93 (63.75, 96.77) | -0.16 (-0.42, 0.09) | 0.213 | 0.98 (0.91, 1.15) | 1.00 (0.86, 1.24) | 0.02 (-0.29, 0.34) | 0.893 |
| High-middle | — | — | — | — | 166.83 (89.84, 285.39) | 215.74 (127.34, 341.16) | 0.88 (0.85, 0.92) | <0.001 | 55.07 (48.83, 62.93) | 47.61 (39.49, 60.06) | -0.49 (-0.82, -0.16) | 0.004 | 0.89 (0.81, 0.99) | 0.70 (0.60, 0.91) | -0.64 (-0.87, -0.40) | <0.001 |
| Middle | — | — | — | — | 170.72 (90.26, 298.53) | 246.47 (135.31, 428.38) | 1.33 (1.14, 1.52) | <0.001 | 56.96 (48.96, 66.56) | 51.30 (41.98, 64.05) | -0.32 (-0.49, -0.15) | <0.001 | 0.92 (0.79, 1.05) | 0.74 (0.63, 0.86) | -0.74 (-0.88, -0.59) | <0.001 |
| Low-middle | — | — | — | — | 211.30 (110.10, | 272.52 (141.84, | 0.89 (0.80, 0.97) | <0.001 | 84.34 (67.84, 103.70) | 77.80 (62.56, 96.50) | -0.24 (-0.50, | 0.073 | 1.42 (1.16, | 1.23 (0.99, | -0.48 (-0.86, | 0.011 |

| | | | | | | | | | | | | | | | | |
|---|---|---|---|---|---|---|---|---|---|---|---|---|---|---|---|---|
| | | | | | 380.79) | 489.22) | | | | | 0.02) | | 1.72) | 1.50) | -0.11) | |
| Low | — | — | — | — | 449.03 (201.99, 951.96) | 519.44 (233.49, 1111.47) | 0.50 (0.46, 0.54) | <0.001 | 132.62 (103.47, 166.34) | 105.47 (81.21, 142.55) | -0.79 (-0.90, -0.68) | <0.001 | 2.11 (1.65, 2.65) | 1.51 (1.21, 1.91) | -1.16 (-1.34, -0.97) | <0.001 |
| **21 GBD region** | | | | | | | | | | | | | | | | |
| South Asia | — | — | — | — | 180.72 (98.87, 310.86) | 182.34 (100.52, 312.07) | 0.03 (0.01, 0.04) | <0.001 | 81.16 (63.44, 102.31) | 70.91 (53.25, 89.81) | -0.56 (-0.97, -0.15) | 0.007 | 1.39 (1.07, 1.76) | 1.19 (0.87, 1.54) | -0.64 (-1.11, -0.17) | 0.008 |
| East Asia | — | — | — | — | 57.79 (31.30, 98.63) | 98.65 (54.45, 170.32) | 1.84 (1.71, 1.98) | <0.001 | 27.54 (20.76, 34.52) | 17.46 (12.86, 23.12) | -1.58 (-1.77, -1.40) | <0.001 | 0.47 (0.35, 0.60) | 0.23 (0.17, 0.31) | -2.50 (-2.84, -2.15) | <0.001 |
| North Africa and Middle East | — | — | — | — | 591.02 (311.25, 1008.26) | 564.88 (317.12, 955.12) | -0.16 (-0.23, -0.10) | <0.001 | 136.64 (105.02, 169.48) | 110.83 (82.94, 143.01) | -0.72 (-0.83, -0.60) | <0.001 | 2.04 (1.44, 2.47) | 1.57 (1.12, 2.01) | -0.90 (-1.02, -0.77) | <0.001 |
| Western Sub-Saharan Africa | — | — | — | — | 724.14 (321.08, 1516.76) | 821.81 (367.72, 1737.14) | 0.44 (0.41, 0.48) | <0.001 | 153.59 (111.15, 207.28) | 100.95 (70.66, 153.14) | -1.44 (-1.53, -1.35) | <0.001 | 2.23 (1.62, 2.95) | 1.11 (0.83, 1.50) | -2.38 (-2.52, -2.24) | <0.001 |
| Southeast Asia | — | — | — | — | 151.48 (82.77, 256.17) | 151.08 (82.87, 248.66) | -0.01 (-0.03, 0.01) | 0.254 | 64.31 (51.86, 78.00) | 44.95 (35.91, 54.91) | -1.22 (-1.28, -1.16) | <0.001 | 1.09 (0.86, 1.32) | 0.71 (0.55, 0.88) | -1.43 (-1.52, -1.33) | <0.001 |
| Eastern Sub-Saharan Africa | — | — | — | — | 699.65 (301.34, 1528.75) | 812.66 (354.26, 1740.88) | 0.52 (0.50, 0.54) | <0.001 | 166.18 (122.20, 227.36) | 120.70 (83.37, 183.32) | -1.10 (-1.17, -1.03) | <0.001 | 2.50 (1.86, 3.46) | 1.51 (1.11, 2.21) | -1.75 (-1.84, -1.65) | <0.001 |
| Central Latin America | — | — | — | — | 265.08 (104.29, 515.53) | 369.88 (200.18, 620.80) | 1.16 (1.10, 1.21) | <0.001 | 75.45 (64.89, 89.90) | 62.86 (47.89, 82.60) | -0.65 (-1.07, -0.22) | 0.003 | 1.19 (1.09, 1.30) | 0.84 (0.63, 1.06) | -1.13 (-1.39, -0.86) | <0.001 |
| Tropical Latin America | — | — | — | — | 245.81 (109.22, 467.20) | 309.29 (157.70, 548.27) | 0.80 (0.78, 0.81) | <0.001 | 89.61 (78.42, 103.79) | 90.35 (76.86, 110.19) | 0.04 (-0.14, 0.22) | 0.676 | 1.48 (1.36, 1.65) | 1.43 (1.25, 1.69) | -0.11 (-0.34, 0.12) | 0.334 |
| Central Sub-Saharan Africa | — | — | — | — | 619.39 (255.02, 1351.68) | 661.96 (270.41, 1496.50) | 0.24 (0.17, 0.31) | <0.001 | 116.58 (74.53, 182.41) | 103.84 (63.66, 164.44) | -0.42 (-0.56, -0.29) | <0.001 | 1.63 (1.02, 2.64) | 1.33 (0.83, 2.21) | -0.78 (-0.93, -0.64) | <0.001 |
| Western Europe | — | — | — | — | 398.32 | 362.07 | -0.34 | <0.001 | 70.67 (58.22, | 54.98 (43.78, | -0.86 | <0.001 | 0.96 | 0.69 | -1.07 | <0.001 |

| | | | | | | | | | | | | | | | | |
|---|---|---|---|---|---|---|---|---|---|---|---|---|---|---|---|---|
| | | | | | (219.69, 667.80) | (202.49, 575.16) | (-0.40, -0.28) | | 89.02) | 70.01) | (-1.06, -0.66) | | (0.87, 1.17) | (0.61, 0.89) | (-1.45, -0.70) | |
| High-income North America | — | — | — | — | 914.96 (514.25, 1504.91) | 770.20 (486.92, 1182.44) | -0.59 (-0.69, -0.49) | <0.001 | 106.72 (82.02, 144.13) | 121.94 (99.65, 151.99) | 0.49 (0.25, 0.73) | <0.001 | 1.13 (1.02, 1.40) | 1.58 (1.39, 2.01) | 1.06 (0.58, 1.54) | <0.001 |
| Central Asia | — | — | — | — | 74.61 (39.83, 123.47) | 68.50 (36.30, 111.56) | -0.30 (-0.31, -0.28) | <0.001 | 66.45 (54.54, 78.53) | 55.25 (42.95, 80.85) | -0.57 (-1.01, -0.13) | 0.012 | 1.21 (0.98, 1.43) | 0.99 (0.76, 1.49) | -0.60 (-1.07, -0.12) | 0.015 |
| Eastern Europe | — | — | — | — | 64.72 (34.03, 120.12) | 67.31 (36.20, 121.28) | 0.14 (0.11, 0.17) | <0.001 | 43.76 (36.22, 52.62) | 70.89 (53.63, 109.23) | 2.16 (1.24, 3.09) | <0.001 | 0.78 (0.64, 0.93) | 1.30 (0.97, 2.01) | 2.30 (1.33, 3.29) | <0.001 |
| Southern Sub-Saharan Africa | — | — | — | — | 336.53 (154.66, 699.55) | 348.99 (157.27, 730.26) | 0.12 (0.10, 0.15) | <0.001 | 100.40 (75.91, 126.92) | 76.81 (50.91, 107.69) | -0.84 (-1.65, -0.01) | 0.046 | 1.60 (1.20, 1.99) | 1.13 (0.73, 1.61) | -1.07 (-2.19, 0.05) | 0.062 |
| High-income Asia Pacific | — | — | — | — | 309.46 (168.11, 509.40) | 277.77 (162.80, 431.85) | -0.38 (-0.42, -0.34) | <0.001 | 42.59 (32.49, 57.15) | 29.91 (21.60, 42.77) | -1.20 (-1.38, -1.02) | <0.001 | 0.50 (0.43, 0.66) | 0.29 (0.24, 0.48) | -1.90 (-2.23, -1.56) | <0.001 |
| Central Europe | — | — | — | — | 314.14 (166.62, 516.42) | 320.80 (175.72, 508.86) | 0.11 (0.01, 0.20) | 0.027 | 108.08 (97.77, 121.51) | 51.67 (40.17, 66.59) | -2.55 (-2.86, -2.24) | <0.001 | 1.77 (1.65, 1.89) | 0.67 (0.52, 0.84) | -3.37 (-3.97, -2.77) | <0.001 |
| Andean Latin America | — | — | — | — | 222.96 (86.55, 438.95) | 283.07 (143.08, 499.34) | 0.82 (0.76, 0.88) | <0.001 | 88.91 (68.79, 113.61) | 52.47 (36.67, 72.40) | -1.90 (-2.14, -1.67) | <0.001 | 1.49 (1.15, 1.90) | 0.72 (0.49, 1.03) | -2.41 (-3.60, -1.22) | <0.001 |
| Caribbean | — | — | — | — | 232.42 (124.47, 392.12) | 246.54 (129.64, 418.39) | 0.21 (0.19, 0.23) | <0.001 | 129.04 (97.29, 171.79) | 144.31 (97.44, 216.86) | 0.39 (0.14, 0.64) | 0.002 | 2.26 (1.68, 3.06) | 2.54 (1.64, 3.91) | 0.41 (0.12, 0.70) | 0.005 |
| Southern Latin America | — | — | — | — | 129.98 (4.67, 377.36) | 376.87 (186.93, 651.55) | 3.70 (3.41, 3.99) | <0.001 | 88.25 (71.08, 109.69) | 71.62 (54.59, 92.65) | -0.66 (-0.93, -0.39) | <0.001 | 1.57 (1.31, 1.90) | 1.00 (0.77, 1.26) | -1.46 (-2.10, -0.81) | <0.001 |
| Oceania | — | — | — | — | 90.88 (47.71, 160.19) | 80.61 (41.16, 139.43) | -0.41 (-0.45, -0.38) | <0.001 | 65.08 (42.30, 92.40) | 59.48 (36.73, 90.17) | -0.35 (-0.48, -0.22) | <0.001 | 1.16 (0.74, 1.68) | 1.07 (0.63, 1.65) | -0.34 (-0.48, -0.20) | <0.001 |
| Australasia | — | — | — | — | 413.58 | 404.49 | -0.09 | <0.001 | 46.86 (33.12, | 54.96 (39.36, | 0.55 | 0.003 | 0.48 | 0.65 | 1.09 (0.55, | <0.001 |

| | | | | | | | | | | | | | | | |
|---|---|---|---|---|---|---|---|---|---|---|---|---|---|---|---|
| | | | | | (225.65, 698.69) | (225.12, 651.90) | (-0.13, -0.05) | | 65.99) | 74.31) | (0.19, 0.92) | | (0.38, 0.66) | (0.46, 0.89) | 1.64) | |
| **Men** | | | | | | | | | | | | | | | |
| Global | — | — | — | — | 129.88 (70.85, 232.71) | 156.23 (85.73, 276.82) | 0.66 (0.54, 0.79) | <0.001 | 71.15 (62.06, 88.06) | 65.93 (57.60, 77.28) | -0.22 (-0.33, -0.11) | <0.001 | 1.24 (1.09, 1.56) | 1.11 (0.98, 1.29) | -0.34 (-0.47, -0.20) | <0.001 |
| **SDI category** | | | | | | | | | | | | | | | |
| High | — | — | — | — | 225.34 (127.85, 372.42) | 193.33 (121.10, 294.36) | -0.51 (-0.57, -0.44) | <0.001 | 67.60 (60.39, 76.95) | 54.73 (48.05, 64.26) | -0.73 (-0.94, -0.52) | <0.001 | 1.07 (1.01, 1.18) | 0.86 (0.77, 1.00) | -0.80 (-1.18, -0.41) | <0.001 |
| High-middle | — | — | — | — | 91.72 (50.71, 155.45) | 119.83 (69.98, 195.28) | 0.92 (0.87, 0.98) | <0.001 | 71.00 (63.84, 81.83) | 57.93 (50.33, 70.24) | -0.65 (-0.91, -0.38) | <0.001 | 1.28 (1.15, 1.47) | 0.99 (0.87, 1.23) | -0.81 (-1.11, -0.50) | <0.001 |
| Middle | — | — | — | — | 98.78 (51.25, 179.83) | 147.09 (78.78, 264.82) | 1.42 (1.28, 1.55) | <0.001 | 57.26 (47.78, 73.59) | 53.67 (45.96, 64.23) | -0.22 (-0.40, -0.05) | 0.011 | 1.00 (0.84, 1.31) | 0.88 (0.77, 1.04) | -0.42 (-0.68, -0.16) | 0.002 |
| Low-middle | — | — | — | — | 107.32 (56.26, 194.75) | 133.34 (69.32, 247.89) | 0.75 (0.71, 0.80) | <0.001 | 81.09 (62.89, 115.22) | 80.17 (66.52, 98.26) | 0.06 (-0.39, 0.52) | 0.788 | 1.46 (1.12, 2.11) | 1.41 (1.16, 1.75) | 0.00 (-0.50, 0.51) | 0.987 |
| Low | — | — | — | — | 219.95 (101.51, 448.44) | 249.48 (115.57, 508.24) | 0.43 (0.41, 0.45) | <0.001 | 116.03 (90.97, 162.67) | 101.91 (78.13, 139.32) | -0.44 (-0.69, -0.20) | <0.001 | 2.02 (1.58, 2.89) | 1.71 (1.32, 2.38) | -0.54 (-0.74, -0.34) | <0.001 |
| **21 GBD region** | | | | | | | | | | | | | | | |
| South Asia | — | — | — | — | 85.36 (45.59, 150.43) | 82.54 (44.08, 145.88) | -0.13 (-0.15, -0.10) | <0.001 | 81.34 (57.42, 127.17) | 75.18 (58.69, 98.67) | -0.20 (-0.86, 0.46) | 0.553 | 1.48 (1.03, 2.36) | 1.37 (1.04, 1.82) | -0.21 (-0.91, 0.50) | 0.562 |
| East Asia | — | — | — | — | 54.02 (28.90, 91.84) | 87.21 (47.33, 146.92) | 1.66 (1.55, 1.76) | <0.001 | 32.87 (24.08, 45.64) | 22.70 (17.65, 29.11) | -1.31 (-1.55, -1.07) | <0.001 | 0.58 (0.41, 0.82) | 0.35 (0.27, 0.44) | -1.78 (-2.11, -1.44) | <0.001 |
| North Africa and Middle East | — | — | — | — | 372.13 (201.01, 628.11) | 343.83 (194.37, 583.11) | -0.27 (-0.31, -0.22) | <0.001 | 124.92 (99.17, 156.52) | 91.05 (71.50, 115.70) | -1.08 (-1.20, -0.95) | <0.001 | 2.03 (1.60, 2.56) | 1.40 (1.11, 1.79) | -1.26 (-1.39, -1.13) | <0.001 |
| Western | — | — | — | — | 421.16 | 460.39 | 0.31 (0.28, | <0.001 | 138.82 | 114.74 (82.52, | -0.64 | <0.001 | 2.25 | 1.74 | -0.86 | <0.001 |

| | | | | | | | | | | | | | | | | |
|---|---|---|---|---|---|---|---|---|---|---|---|---|---|---|---|---|
| Sub-Saharan Africa | | | | | (191.03, 874.89) | (205.76, 963.62) | 0.33) | | (102.92, 200.46) | 163.88) | (-0.74, -0.54) | | (1.65, 3.50) | (1.25, 2.56) | (-0.98, -0.73) | |
| Southeast Asia | — | — | — | — | 104.67 (58.15, 178.38) | 116.12 (64.15, 195.41) | 0.35 (0.33, 0.37) | <0.001 | 66.22 (53.54, 85.58) | 53.00 (42.25, 68.33) | -0.75 (-0.96, -0.53) | <0.001 | 1.17 (0.93, 1.51) | 0.90 (0.72, 1.18) | -0.87 (-1.12, -0.62) | <0.001 |
| Eastern Sub-Saharan Africa | — | — | — | — | 307.38 (134.91, 661.59) | 347.56 (150.65, 743.04) | 0.42 (0.40, 0.44) | <0.001 | 137.08 (104.81, 206.60) | 105.53 (76.61, 160.74) | -0.90 (-1.00, -0.80) | <0.001 | 2.34 (1.78, 3.53) | 1.68 (1.21, 2.77) | -1.14 (-1.29, -1.00) | <0.001 |
| Central Latin America | — | — | — | — | 37.41 (2.37, 111.22) | 119.72 (56.34, 217.68) | 4.08 (4.00, 4.15) | <0.001 | 57.66 (52.54, 64.01) | 49.82 (38.68, 60.40) | -0.45 (-0.72, -0.17) | 0.001 | 1.07 (0.99, 1.18) | 0.84 (0.66, 1.02) | -0.77 (-1.39, -0.15) | 0.015 |
| Tropical Latin America | — | — | — | — | 41.84 (5.88, 101.14) | 89.05 (43.02, 162.68) | 2.63 (2.52, 2.75) | <0.001 | 81.54 (74.49, 89.32) | 69.42 (61.52, 78.68) | -0.52 (-0.85, -0.19) | 0.002 | 1.53 (1.40, 1.67) | 1.25 (1.11, 1.42) | -0.67 (-1.02, -0.31) | <0.001 |
| Central Sub-Saharan Africa | — | — | — | — | 259.52 (107.50, 581.00) | 271.74 (113.37, 619.29) | 0.16 (0.14, 0.17) | <0.001 | 108.48 (69.62, 175.39) | 93.25 (58.54, 155.10) | -0.54 (-0.64, -0.45) | <0.001 | 1.83 (1.13, 3.06) | 1.52 (0.93, 2.71) | -0.66 (-0.76, -0.55) | <0.001 |
| Western Europe | — | — | — | — | 139.17 (76.89, 235.25) | 131.46 (74.72, 211.94) | -0.21 (-0.29, -0.13) | <0.001 | 65.32 (58.80, 73.67) | 45.31 (39.61, 52.61) | -1.29 (-1.60, -0.98) | <0.001 | 1.12 (1.02, 1.24) | 0.74 (0.67, 0.84) | -1.46 (-1.80, -1.12) | <0.001 |
| High-income North America | — | — | — | — | 319.83 (178.74, 535.11) | 240.73 (149.88, 374.42) | -0.97 (-1.04, -0.90) | <0.001 | 77.64 (67.32, 92.54) | 79.68 (68.63, 93.41) | 0.14 (-0.19, 0.46) | 0.412 | 1.17 (1.09, 1.32) | 1.29 (1.12, 1.52) | 0.34 (-0.11, 0.79) | 0.138 |
| Central Asia | — | — | — | — | 47.68 (24.53, 80.98) | 47.12 (24.27, 80.29) | -0.04 (-0.05, -0.03) | <0.001 | 106.63 (89.62, 128.77) | 80.41 (64.82, 106.23) | -0.99 (-1.30, -0.68) | <0.001 | 2.01 (1.69, 2.44) | 1.50 (1.21, 2.00) | -1.02 (-1.34, -0.70) | <0.001 |
| Eastern Europe | — | — | — | — | 34.56 (18.75, 63.46) | 35.41 (18.94, 64.85) | 0.08 (0.07, 0.09) | <0.001 | 70.48 (60.96, 89.00) | 138.79 (112.71, 195.45) | 2.88 (1.91, 3.85) | <0.001 | 1.33 (1.14, 1.68) | 2.64 (2.14, 3.75) | 2.93 (1.95, 3.91) | <0.001 |
| Southern Sub-Saharan Africa | — | — | — | — | 190.80 (86.18, 403.35) | 192.46 (87.55, 403.42) | 0.02 (0.01, 0.04) | 0.002 | 113.09 (89.52, 141.45) | 96.63 (69.38, 134.08) | -0.55 (-1.32, 0.23) | 0.169 | 1.99 (1.56, 2.52) | 1.67 (1.19, 2.37) | -0.60 (-1.47, 0.27) | 0.174 |
| High-income Asia | — | — | — | — | 211.38 | 209.21 | -0.04 | 0.421 | 44.30 (36.29, | 29.33 (22.70, | -1.42 | <0.001 | 0.63 | 0.35 | -2.07 | <0.001 |

| | | | | | | | | | | | | | | | | |
|---|---|---|---|---|---|---|---|---|---|---|---|---|---|---|---|---|
| Pacific | | | | | (119.19, 347.78) | (125.52, 329.06) | (-0.14, 0.06) | | 54.72) | 38.28) | (-1.69, -1.16) | | (0.54, 0.75) | (0.30, 0.44) | (-2.44, -1.70) | |
| Central Europe | — | — | — | — | 156.57 (84.62, 259.01) | 159.78 (90.33, 259.69) | 0.07 (-0.02, 0.15) | 0.124 | 158.36 (147.25, 170.53) | 70.59 (58.99, 83.47) | -2.79 (-3.14, -2.44) | <0.001 | 2.90 (2.71, 3.11) | 1.20 (0.99, 1.42) | -3.06 (-3.43, -2.69) | <0.001 |
| Andean Latin America | — | — | — | — | 52.09 (1.59, 155.92) | 128.84 (58.84, 229.74) | 3.16 (2.89, 3.43) | <0.001 | 87.01 (64.25, 111.12) | 46.32 (32.28, 68.27) | -2.09 (-2.58, -1.58) | <0.001 | 1.63 (1.22, 2.08) | 0.76 (0.51, 1.14) | -2.53 (-3.09, -1.96) | <0.001 |
| Caribbean | — | — | — | — | 97.31 (50.42, 165.70) | 117.01 (60.00, 198.64) | 0.64 (0.61, 0.68) | <0.001 | 96.24 (76.86, 120.08) | 106.82 (77.37, 149.22) | 0.36 (0.12, 0.61) | 0.003 | 1.76 (1.38, 2.22) | 1.94 (1.39, 2.75) | 0.35 (0.09, 0.61) | 0.008 |
| Southern Latin America | — | — | — | — | 15.55 (1.60, 106.71) | 151.91 (69.82, 275.24) | 8.05 (7.75, 8.34) | <0.001 | 88.47 (72.92, 107.63) | 66.98 (53.43, 83.12) | -0.92 (-1.42, -0.42) | <0.001 | 1.70 (1.39, 2.08) | 1.14 (0.90, 1.42) | -1.34 (-1.88, -0.80) | <0.001 |
| Oceania | — | — | — | — | 94.57 (49.00, 171.99) | 92.65 (46.48, 162.24) | -0.07 (-0.11, -0.04) | <0.001 | 136.10 (90.22, 193.86) | 120.30 (77.81, 178.74) | -0.42 (-0.48, -0.36) | <0.001 | 2.54 (1.63, 3.67) | 2.23 (1.41, 3.33) | -0.43 (-0.50, -0.37) | <0.001 |
| Australasia | — | — | — | — | 150.20 (80.55, 237.96) | 147.12 (80.51, 243.06) | -0.08 (-0.12, -0.04) | <0.001 | 30.60 (23.55, 41.24) | 37.11 (26.84, 50.02) | 0.67 (0.24, 1.10) | 0.002 | 0.43 (0.35, 0.55) | 0.56 (0.40, 0.76) | 0.91 (0.18, 1.65) | 0.014 |

CI, confidence interval; GBD, global burden disease

"—" indicates that data are unavailable; * significantly different from zero (*P*<0.05). The estimates on atrial fibrillation burden for people aged 15-29 years in the GBD database were all treated as "0". Therefore, we used "0" to calculate the age-standardized burden of atrial fibrillation.

**Table S3 Age-standardized incidence, prevalence, disability-adjusted life years, and death rate of the overall and type-specific cardiovascular disease in youths and young adults from 1990 to 2019 by sex and countries/territories**

| | Incidence | | | | Prevalence | | | | Disability-adjusted life years | | | | Death | | | |
|---|---|---|---|---|---|---|---|---|---|---|---|---|---|---|---|---|
| | Rate per 100,000 (95% CI) in 1990 | Rate per 100,000 (95% CI) in 2019 | Average annual percent change % , (95% CI) | *P*-value | Rate per 100,000 (95% CI) in 1990 | Rate per 100,000 (95% CI) in 2019 | Average annual percent change % , (95% CI) | *P*-value | Rate per 100,000 (95% CI) in 1990 | Rate per 100,000 (95% CI) in 2019 | Average annual percent change % , (95% CI) | *P*-value | Rate per 100,000 (95% CI) in 1990 | Rate per 100,000 (95% CI) in 2019 | Average annual percent change % , (95% CI) | *P*-value |
| **Cardiovascular diseases** | | | | | | | | | | | | | | | | |
| **Overall** | | | | | | | | | | | | | | | | |
| Afghanistan | 170.49 (165.77, 175.32) | 174.49 (172.17, 176.82) | 0.08 (0.07, 0.09) | <0.001 | 1723.11 (1708.15 , 1738.20) | 1789.73 (1782.35 , 1797.13) | 0.15 (0.10, 0.20) | <0.001 | 3411.05 (3388.96 , 3433.26) | 2255.35 (2246.60 , 2264.12) | -1.33 (-1.47, -1.20) | <0.001 | 57.82 (54.95, 60.82) | 37.61 (36.47, 38.77) | -1.39 (-1.53, -1.25) | <0.001 |
| Albania | 136.22 (130.14, 142.53) | 136.62 (129.33, 144.23) | 0.02 (-0.01, 0.05) | 0.192 | 2216.46 (2191.74 , 2241.41) | 2248.05 (2218.17 , 2278.24) | 0.05 (-0.01, 0.11) | 0.072 | 1023.31 (1006.39 , 1040.45) | 1091.89 (1071.16 , 1112.93) | 0.08 (-0.72, 0.90) | 0.841 | 14.73 (12.75, 16.94) | 15.93 (13.51, 18.67) | 0.10 (-0.85, 1.05) | 0.843 |
| Algeria | 131.24 (128.87, 133.64) | 120.65 (119.03, 122.29) | -0.29 (-0.31, -0.27) | <0.001 | 1578.30 (1570.08 , 1586.56) | 1608.50 (1602.55 , 1614.46) | 0.09 (-0.01, 0.18) | 0.063 | 2057.11 (2047.50 , 2066.76) | 987.40 (982.80, 992.00) | -2.47 (-2.73, -2.21) | <0.001 | 33.65 (32.42, 34.92) | 15.06 (14.50, 15.64) | -2.64 (-2.89, -2.39) | <0.001 |
| American Samoa | 141.48 (92.99, 207.73) | 145.87 (96.61, 212.88) | 0.11 (0.09, 0.13) | <0.001 | 1859.58 (1671.01 , 2064.79) | 1886.23 (1697.15 , 2091.89) | 0.05 (0.03, 0.07) | <0.001 | 1775.84 (1587.47 , 1981.33) | 1779.92 (1591.28 , 1985.70) | 0.01 (-0.15, 0.16) | 0.937 | 27.98 (9.08, 66.71) | 28.03 (9.12, 66.83) | 0.00 (-0.17, 0.18) | 0.986 |
| Andorra | 68.85 (40.75, 111.65) | 66.07 (38.83, 107.40) | -0.14 (-0.18, -0.10) | <0.001 | 594.25 (504.17, 697.81) | 596.65 (507.69, 698.50) | 0.03 (-0.04, 0.10) | 0.359 | 350.11 (282.06, 431.80) | 237.22 (183.38, 304.05) | -1.32 (-1.41, -1.23) | <0.001 | 5.19 (0.31, 28.43) | 3.27 (0.07, 24.26) | -1.57 (-1.66, -1.47) | <0.001 |
| Angola | 182.49 (178.22, | 167.49 (165.07, | -0.29 (-0.30, | <0.001 | 2805.50 (2788.65 | 2880.98 (2870.83 | 0.10 (0.04, | 0.001 | 1304.11 (1292.31 | 931.91 (925.95, | -1.13 (-1.82, | 0.001 | 19.54 (18.11, | 13.09 (12.39, | -1.34 (-2.14, | 0.001 |

| | | | | | | | | | | | | | | | | |
|---|---|---|---|---|---|---|---|---|---|---|---|---|---|---|---|---|
| | 186.85) | 169.95) | -0.28) | | , 2822.42) | , 2891.16) | 0.16) | | , 1316.00) | 937.90) | -0.44) | | 21.07) | 13.84) | -0.53) | |
| Antigua and Barbuda | 174.13 (126.53, 234.45) | 172.02 (131.25, 221.86) | -0.04 (-0.06, -0.03) | <0.001 | 2542.45 (2349.81, 2747.26) | 2521.67 (2357.67, 2694.42) | -0.02 (-0.04, -0.01) | 0.003 | 997.51 (876.86, 1130.63) | 621.65 (542.15, 709.94) | -1.60 (-2.45, -0.75) | <0.001 | 15.01 (3.78, 40.79) | 8.41 (1.73, 25.47) | -1.95 (-3.02, -0.88) | <0.001 |
| Argentina | 117.76 (115.84, 119.71) | 110.76 (109.21, 112.34) | -0.21 (-0.22, -0.21) | <0.001 | 1617.04 (1609.89, 1624.21) | 1702.00 (1695.90, 1708.13) | 0.18 (0.15, 0.20) | <0.001 | 1226.31 (1220.07, 1232.58) | 601.06 (597.44, 604.68) | -2.43 (-2.64, -2.21) | <0.001 | 19.36 (18.58, 20.16) | 8.44 (8.02, 8.88) | -2.81 (-3.03, -2.58) | <0.001 |
| Armenia | 159.30 (152.82, 165.99) | 148.66 (141.53, 156.09) | -0.23 (-0.26, -0.19) | <0.001 | 1960.24 (1937.37, 1983.31) | 1860.01 (1834.85, 1885.46) | -0.18 (-0.19, -0.17) | <0.001 | 1005.90 (989.52, 1022.50) | 617.74 (603.77, 631.99) | -1.67 (-2.52, -0.81) | <0.001 | 15.33 (13.36, 17.52) | 8.63 (7.08, 10.49) | -1.98 (-2.95, -0.99) | <0.001 |
| Australia | 100.17 (97.83, 102.55) | 100.32 (98.24, 102.44) | -0.01 (-0.08, 0.06) | 0.818 | 748.53 (742.12, 754.98) | 771.44 (765.67, 777.25) | 0.10 (0.06, 0.14) | <0.001 | 472.51 (467.41, 477.64) | 270.93 (267.52, 274.38) | -1.91 (-2.27, -1.54) | <0.001 | 7.17 (6.55, 7.82) | 3.77 (3.38, 4.20) | -2.19 (-2.62, -1.77) | <0.001 |
| Austria | 88.75 (85.43, 92.18) | 83.62 (80.36, 87.00) | -0.20 (-0.24, -0.17) | <0.001 | 846.71 (836.34, 857.20) | 803.56 (793.30, 813.93) | -0.18 (-0.25, -0.10) | <0.001 | 542.28 (533.97, 550.70) | 228.59 (223.19, 234.12) | -2.95 (-3.41, -2.49) | <0.001 | 8.29 (7.29, 9.39) | 2.92 (2.35, 3.62) | -3.53 (-4.16, -2.89) | <0.001 |
| Azerbaijan | 160.70 (156.22, 165.28) | 151.67 (147.98, 155.43) | -0.20 (-0.21, -0.18) | <0.001 | 1850.11 (1834.82, 1865.51) | 1798.00 (1785.34, 1810.73) | -0.10 (-0.13, -0.07) | <0.001 | 1654.59 (1639.84, 1669.46) | 1201.70 (1191.49, 1211.99) | -1.26 (-1.98, -0.54) | 0.001 | 26.62 (24.76, 28.59) | 18.58 (17.34, 19.90) | -1.23 (-2.21, -0.24) | 0.015 |
| Bahamas | 180.42 (156.61, 207.05) | 184.35 (163.29, 207.43) | 0.08 (0.06, 0.10) | <0.001 | 2245.37 (2159.20, 2334.29) | 2232.18 (2157.34, 2309.02) | -0.02 (-0.04, 0.01) | 0.197 | 1370.08 (1301.34, 1441.64) | 1228.72 (1173.73, 1285.69) | -0.52 (-1.18, 0.15) | 0.131 | 21.98 (14.01, 33.03) | 19.60 (13.23, 28.06) | -0.45 (-1.30, 0.42) | 0.311 |
| Bahrain | 94.24 (82.89, 106.96) | 89.92 (82.67, 97.76) | -0.15 (-0.20, -0.11) | <0.001 | 1064.13 (1024.95, 1104.64) | 1019.71 (994.80, 1045.19) | -0.13 (-0.17, -0.08) | <0.001 | 1371.89 (1326.67, 1418.47) | 540.59 (522.16, 559.61) | -3.16 (-3.61, -2.71) | <0.001 | 21.82 (16.49, 28.60) | 7.71 (5.71, 10.34) | -3.53 (-4.02, -3.04) | <0.001 |
| Bangladesh | 133.26 | 133.52 | 0.01 | 0.333 | 1560.03 | 1626.25 | 0.17 | 0.044 | 1573.66 | 1012.46 | -1.38 | <0.001 | 25.20 | 15.55 | -1.54 | <0.001 |

| | | | | | | | | | | | | | | | | |
|---|---|---|---|---|---|---|---|---|---|---|---|---|---|---|---|---|
| | (132.13, 134.39) | (132.64, 134.39) | (-0.01, 0.02) | | (1556.10 , 1563.97) | (1623.20 , 1629.31) | (0.00, 0.33) | | (1569.64 , 1577.68) | (1010.04 , 1014.89) | (-1.77, -0.99) | | (24.69, 25.72) | (15.25, 15.85) | (-1.92, -1.15) | |
| Barbados | 178.63 (154.45, 205.59) | 182.30 (156.78, 210.90) | 0.07 (0.05, 0.09) | <0.001 | 2352.64 (2262.59 , 2445.42) | 2375.35 (2280.83 , 2472.89) | 0.03 (0.02, 0.05) | <0.001 | 919.39 (863.40, 978.11) | 634.17 (586.23, 685.11) | -1.47 (-2.21, -0.73) | <0.001 | 13.77 (7.71, 22.78) | 8.79 (4.04, 16.87) | -1.75 (-2.59, -0.89) | <0.001 |
| Belarus | 112.98 (109.76, 116.27) | 113.74 (110.13, 117.45) | 0.02 (0.00, 0.05) | 0.028 | 790.47 (781.93, 799.08) | 816.24 (806.62, 825.97) | 0.11 (0.07, 0.16) | <0.001 | 1257.82 (1247.11 , 1268.60) | 1106.45 (1095.62 , 1117.39) | -0.42 (-1.36, 0.54) | 0.393 | 21.04 (19.68, 22.48) | 18.42 (17.06, 19.88) | -0.43 (-1.44, 0.60) | 0.411 |
| Belgium | 69.94 (67.33, 72.63) | 66.78 (64.15, 69.48) | -0.16 (-0.18, -0.13) | <0.001 | 758.25 (749.57, 767.03) | 808.94 (799.72, 818.25) | 0.24 (0.16, 0.32) | <0.001 | 572.47 (564.94, 580.09) | 227.74 (222.90, 232.67) | -3.12 (-3.55, -2.70) | <0.001 | 8.85 (7.94, 9.85) | 2.86 (2.35, 3.47) | -3.80 (-4.31, -3.29) | <0.001 |
| Belize | 178.47 (147.90, 214.09) | 187.32 (167.53, 208.89) | 0.17 (0.16, 0.19) | <0.001 | 2184.57 (2074.19 , 2299.84) | 2339.39 (2268.04 , 2412.48) | 0.24 (0.21, 0.27) | <0.001 | 786.71 (719.36, 859.14) | 830.00 (787.12, 874.65) | 0.16 (-0.39, 0.71) | 0.564 | 11.49 (4.65, 24.28) | 12.21 (7.49, 18.86) | 0.10 (-0.55, 0.75) | 0.766 |
| Benin | 150.35 (144.44, 156.46) | 149.95 (146.43, 153.54) | -0.01 (-0.02, -0.00) | 0.001 | 2501.66 (2477.46 , 2526.05) | 2610.15 (2595.45 , 2624.91) | 0.16 (0.09, 0.23) | <0.001 | 1011.19 (995.37, 1027.22) | 906.82 (897.86, 915.87) | -0.38 (-0.59, -0.17) | 0.001 | 14.67 (12.79, 16.76) | 12.80 (11.74, 13.94) | -0.48 (-0.71, -0.24) | <0.001 |
| Bermuda | 109.35 (73.66, 158.99) | 109.49 (67.84, 170.19) | 0.02 (-0.04, 0.07) | 0.583 | 1254.32 (1122.82 , 1398.92) | 1219.74 (1066.74 , 1390.57) | -0.10 (-0.19, -0.02) | 0.020 | 766.71 (665.59, 880.91) | 355.82 (276.28, 453.56) | -2.82 (-3.14, -2.49) | <0.001 | 11.94 (2.70, 37.84) | 4.89 (0.11, 33.97) | -3.30 (-3.72, -2.87) | <0.001 |
| Bhutan | 137.18 (122.68, 153.09) | 126.76 (115.24, 139.15) | -0.27 (-0.30, -0.24) | <0.001 | 1470.61 (1421.20 , 1521.44) | 1444.12 (1404.60 , 1484.50) | -0.04 (-0.17, 0.09) | 0.574 | 1340.89 (1292.56 , 1390.69) | 847.98 (817.71, 879.11) | -1.58 (-1.71, -1.44) | <0.001 | 21.50 (15.71, 28.87) | 12.93 (9.44, 17.34) | -1.75 (-1.89, -1.60) | <0.001 |
| Bolivia (Plurinational State of) | 149.83 (144.97, 154.83) | 133.43 (130.19, 136.73) | -0.40 (-0.42, -0.37) | <0.001 | 1987.20 (1969.26 , | 1966.75 (1954.23 , | -0.03 (-0.07, 0.01) | 0.193 | 1430.32 (1414.90 , | 738.19 (730.48, 745.97) | -2.24 (-2.38, -2.09) | <0.001 | 22.33 (20.43, 24.37) | 10.58 (9.67, 11.56) | -2.53 (-2.68, -2.37) | <0.001 |

| | | | | | | | | | | | | | | | | |
|---|---|---|---|---|---|---|---|---|---|---|---|---|---|---|---|---|
| | | | | | 2005.26) | 1979.33) | | | 1445.87) | | | | | | | |
| Bosnia and Herzegovina | 93.30 (89.07, 97.69) | 99.40 (93.57, 105.53) | 0.22 (0.18, 0.26) | <0.001 | 949.97 (936.32, 963.78) | 1015.98 (997.03, 1035.23) | 0.24 (0.18, 0.31) | <0.001 | 867.10 (854.09, 880.26) | 574.73 (560.63, 589.12) | -1.56 (-1.99, -1.14) | <0.001 | 13.60 (12.01, 15.34) | 8.26 (6.66, 10.16) | -1.89 (-2.38, -1.40) | <0.001 |
| Botswana | 178.60 (166.86, 191.02) | 169.19 (161.42, 177.24) | -0.19 (-0.21, -0.17) | <0.001 | 2476.60 (2432.18 , 2521.70) | 2469.90 (2440.01 , 2500.08) | -0.01 (-0.04, 0.03) | 0.739 | 1096.05 (1065.37 , 1127.45) | 902.89 (885.01, 921.05) | -0.63 (-0.81, -0.45) | <0.001 | 16.24 (12.64, 20.60) | 12.99 (10.93, 15.33) | -0.73 (-0.93, -0.53) | <0.001 |
| Brazil | 163.41 (162.39, 164.42) | 140.67 (139.87, 141.46) | -0.52 (-0.56, -0.48) | <0.001 | 2290.36 (2286.57 , 2294.17) | 2216.35 (2213.21 , 2219.49) | -0.09 (-0.15, -0.04) | <0.001 | 1511.98 (1508.82 , 1515.15) | 780.03 (778.20, 781.86) | -2.26 (-2.46, -2.06) | <0.001 | 24.35 (23.95, 24.76) | 11.39 (11.17, 11.61) | -2.59 (-2.84, -2.35) | <0.001 |
| Brunei Darussalam | 115.78 (97.52, 136.56) | 103.87 (90.58, 118.72) | -0.37 (-0.40, -0.34) | <0.001 | 815.08 (765.24, 867.40) | 785.63 (748.58, 824.19) | -0.10 (-0.33, 0.13) | 0.385 | 1503.22 (1435.32 , 1573.59) | 768.36 (731.53, 806.72) | -2.30 (-2.75, -1.85) | <0.001 | 24.52 (16.54, 35.13) | 12.28 (8.05, 18.15) | -2.41 (-2.79, -2.04) | <0.001 |
| Bulgaria | 99.22 (95.73, 102.81) | 98.45 (94.27, 102.78) | -0.03 (-0.04, -0.01) | <0.001 | 1492.20 (1478.39 , 1506.10) | 1520.31 (1502.90 , 1537.89) | 0.08 (-0.02, 0.17) | 0.106 | 1645.45 (1631.20 , 1659.79) | 1215.22 (1200.59 , 1230.00) | -1.00 (-1.39, -0.60) | <0.001 | 26.62 (24.84, 28.49) | 19.18 (17.42, 21.11) | -1.07 (-1.51, -0.63) | <0.001 |
| Burkina Faso | 142.99 (138.74, 147.35) | 143.19 (140.61, 145.81) | 0.01 (-0.02, 0.03) | 0.561 | 2240.02 (2223.07 , 2257.08) | 2281.71 (2271.35 , 2292.11) | 0.07 (0.03, 0.12) | 0.002 | 924.49 (913.16, 935.93) | 912.84 (906.09, 919.64) | -0.01 (-0.23, 0.22) | 0.934 | 13.59 (12.23, 15.07) | 13.32 (12.51, 14.18) | -0.03 (-0.30, 0.24) | 0.830 |
| Burundi | 211.74 (205.41, 218.24) | 190.26 (186.27, 194.31) | -0.38 (-0.40, -0.35) | <0.001 | 2980.15 (2956.21 , 3004.26) | 3022.03 (3005.91 , 3038.22) | 0.05 (0.01, 0.09) | 0.009 | 1793.21 (1774.16 , 1812.42) | 1108.12 (1098.14 , 1118.18) | -1.69 (-1.82, -1.55) | <0.001 | 27.49 (25.15, 30.00) | 15.83 (14.64, 17.09) | -1.93 (-2.08, -1.78) | <0.001 |
| Cabo Verde | 146.44 (125.08, 170.95) | 140.61 (126.23, 156.22) | -0.14 (-0.15, -0.13) | <0.001 | 3123.01 (3022.99 , 3226.00) | 3209.90 (3139.74 , 3281.25) | 0.10 (0.09, 0.11) | <0.001 | 1281.81 (1213.79 , 1353.05) | 726.45 (693.32, 760.80) | -2.01 (-2.22, -1.80) | <0.001 | 18.83 (11.27, 30.06) | 9.01 (5.67, 13.64) | -2.59 (-2.88, -2.31) | <0.001 |
| Cambodia | 124.36 | 112.93 | -0.33 | <0.001 | 1233.23 | 1203.06 | -0.08 | 0.066 | 1493.53 | 887.69 | -1.76 | <0.001 | 24.06 | 13.55 | -1.93 | <0.001 |

| | | | | | | | | | | | | | | | | |
|---|---|---|---|---|---|---|---|---|---|---|---|---|---|---|---|---|
| | (120.71, 128.11) | (110.46, 115.44) | (-0.34, -0.32) | | (1221.67, 1244.87) | (1194.99, 1211.18) | (-0.15, 0.01) | | (1480.49, 1506.67) | (880.72, 894.69) | (-1.90, -1.62) | | (22.41, 25.80) | (12.70, 14.45) | (-2.11, -1.76) | |
| Cameroon | 143.14 (139.24, 147.13) | 151.66 (149.43, 153.92) | 0.20 (0.17, 0.23) | <0.001 | 2279.93 (2264.33, 2295.61) | 2504.35 (2495.28, 2513.45) | 0.32 (0.31, 0.34) | <0.001 | 1036.51 (1025.62, 1047.49) | 1017.95 (1012.00, 1023.94) | -0.06 (-0.21, 0.09) | 0.418 | 15.36 (14.04, 16.78) | 14.84 (14.12, 15.59) | -0.12 (-0.23, -0.01) | 0.035 |
| Canada | 104.98 (103.13, 106.85) | 95.01 (93.28, 96.77) | -0.33 (-0.37, -0.29) | <0.001 | 1385.06 (1378.33, 1391.83) | 1257.90 (1251.59, 1264.24) | -0.29 (-0.52, -0.06) | 0.013 | 468.32 (464.42, 472.25) | 302.94 (299.85, 306.05) | -1.47 (-1.69, -1.26) | <0.001 | 6.32 (5.88, 6.79) | 3.65 (3.32, 4.01) | -1.86 (-2.16, -1.55) | <0.001 |
| Central African Republic | 199.71 (191.01, 208.73) | 203.71 (197.52, 210.05) | 0.07 (0.06, 0.08) | <0.001 | 2807.79 (2774.98, 2840.92) | 2870.41 (2847.04, 2893.94) | 0.09 (0.03, 0.14) | 0.002 | 1487.24 (1462.39, 1512.42) | 1344.70 (1328.13, 1361.44) | -0.34 (-0.59, -0.09) | 0.008 | 22.74 (19.71, 26.12) | 20.32 (18.30, 22.51) | -0.38 (-0.66, -0.09) | 0.009 |
| Chad | 150.17 (144.82, 155.68) | 152.79 (149.47, 156.18) | 0.06 (0.05, 0.07) | <0.001 | 2281.62 (2260.60, 2302.79) | 2366.05 (2352.92, 2379.25) | 0.14 (0.09, 0.18) | <0.001 | 1051.38 (1036.73, 1066.19) | 1023.39 (1014.43, 1032.42) | 0.02 (-0.10, 0.14) | 0.712 | 15.55 (13.79, 17.48) | 15.01 (13.93, 16.17) | 0.01 (-0.13, 0.14) | 0.907 |
| Chile | 67.02 (64.87, 69.22) | 68.54 (66.61, 70.51) | 0.08 (0.06, 0.10) | <0.001 | 656.41 (649.69, 663.18) | 693.67 (687.50, 699.89) | 0.21 (0.14, 0.27) | <0.001 | 555.43 (549.20, 561.70) | 370.28 (365.79, 374.83) | -1.38 (-1.79, -0.98) | <0.001 | 8.52 (7.76, 9.33) | 5.34 (4.81, 5.91) | -1.60 (-2.06, -1.13) | <0.001 |
| China | 109.24 (108.96, 109.53) | 96.00 (95.72, 96.27) | -0.45 (-0.51, -0.40) | <0.001 | 1241.01 (1240.06, 1241.95) | 1145.62 (1144.69, 1146.56) | -0.27 (-0.31, -0.24) | <0.001 | 1140.57 (1139.65, 1141.49) | 734.19 (733.45, 734.92) | -1.45 (-1.73, -1.17) | <0.001 | 17.77 (17.65, 17.88) | 10.90 (10.81, 10.99) | -1.61 (-1.90, -1.31) | <0.001 |
| Colombia | 75.98 (74.49, 77.49) | 65.69 (64.55, 66.85) | -0.49 (-0.53, -0.45) | <0.001 | 887.53 (882.47, 892.61) | 852.55 (848.41, 856.70) | -0.12 (-0.24, -0.00) | 0.043 | 899.89 (894.74, 905.06) | 469.66 (466.60, 472.74) | -2.40 (-2.59, -2.21) | <0.001 | 14.49 (13.84, 15.17) | 7.07 (6.70, 7.46) | -2.64 (-2.85, -2.42) | <0.001 |
| Comoros | 197.00 (175.75, 220.36) | 179.14 (164.17, 195.16) | -0.33 (-0.35, -0.31) | <0.001 | 3096.46 (3009.95, 3185.06) | 3083.25 (3019.79, 3147.75) | -0.01 (-0.03, 0.00) | 0.113 | 1219.67 (1163.49, 1278.05) | 930.77 (895.51, 967.09) | -0.90 (-2.91, 1.15) | 0.387 | 17.58 (11.33, 26.25) | 12.76 (8.91, 17.76) | -1.14 (-3.55, 1.33) | 0.362 |

| | | | | | | | | | | | | | | | | |
|---|---|---|---|---|---|---|---|---|---|---|---|---|---|---|---|---|
| Congo | 199.71 (190.46, 209.33) | 177.58 (171.93, 183.38) | -0.41 (-0.42, -0.39) | <0.001 | 2976.49 (2940.63, 3012.71) | 2941.85 (2918.67, 2965.17) | -0.04 (-0.10, 0.03) | 0.257 | 1602.19 (1574.57, 1630.21) | 955.56 (942.26, 969.01) | -1.82 (-2.58, -1.05) | <0.001 | 24.69 (21.31, 28.47) | 13.56 (12.01, 15.25) | -2.10 (-2.95, -1.25) | <0.001 |
| Cook Islands | 105.69 (44.21, 216.80) | 106.62 (39.73, 231.29) | 0.03 (-0.01, 0.07) | 0.088 | 1085.17 (858.28, 1356.85) | 1109.39 (854.92, 1416.87) | 0.09 (0.02, 0.15) | 0.009 | 1879.21 (1572.03, 2231.43) | 1354.84 (1070.57, 1692.19) | -1.11 (-1.27, -0.95) | <0.001 | 30.61 (4.06, 111.82) | 21.50 (0.98, 105.76) | -1.20 (-1.39, -1.02) | <0.001 |
| Costa Rica | 135.07 (128.73, 141.66) | 133.24 (128.11, 138.53) | -0.04 (-0.06, -0.02) | <0.001 | 2223.52 (2197.50, 2249.80) | 2244.48 (2223.38, 2265.74) | 0.04 (0.02, 0.06) | <0.001 | 730.55 (715.38, 745.97) | 560.57 (550.13, 571.17) | -0.73 (-1.16, -0.29) | 0.001 | 10.53 (8.76, 12.56) | 7.51 (6.35, 8.84) | -0.92 (-1.47, -0.36) | 0.001 |
| Cote d'Ivoire | 164.24 (160.48, 168.07) | 160.58 (158.17, 163.01) | -0.08 (-0.09, -0.06) | <0.001 | 2529.48 (2514.78, 2544.25) | 2569.30 (2559.67, 2578.96) | 0.06 (0.03, 0.10) | 0.001 | 1218.28 (1207.75, 1228.89) | 995.04 (988.96, 1001.15) | -0.67 (-0.92, -0.43) | <0.001 | 18.12 (16.84, 19.48) | 14.31 (13.58, 15.07) | -0.79 (-0.98, -0.60) | <0.001 |
| Croatia | 87.49 (83.34, 91.81) | 87.39 (82.51, 92.52) | 0.00 (-0.06, 0.06) | 0.991 | 858.04 (844.99, 871.25) | 846.30 (831.08, 861.76) | -0.03 (-0.19, 0.14) | 0.750 | 819.22 (806.57, 832.03) | 339.72 (330.19, 349.50) | -2.92 (-3.76, -2.08) | <0.001 | 13.05 (11.51, 14.76) | 4.69 (3.65, 5.99) | -3.63 (-4.40, -2.85) | <0.001 |
| Cuba | 172.37 (168.61, 176.21) | 167.95 (163.76, 172.21) | -0.09 (-0.12, -0.06) | <0.001 | 2123.74 (2110.43, 2137.11) | 2074.17 (2059.43, 2089.00) | -0.08 (-0.10, -0.05) | <0.001 | 971.69 (962.46, 980.98) | 523.58 (516.25, 530.99) | -2.09 (-2.49, -1.70) | <0.001 | 15.07 (13.93, 16.28) | 7.25 (6.41, 8.17) | -2.48 (-2.94, -2.01) | <0.001 |
| Cyprus | 56.79 (48.73, 65.85) | 56.17 (49.92, 63.15) | -0.04 (-0.05, -0.02) | <0.001 | 574.05 (547.77, 601.30) | 629.76 (608.58, 651.64) | 0.40 (0.11, 0.69) | 0.006 | 546.98 (521.27, 573.66) | 333.46 (318.31, 349.30) | -1.70 (-2.33, -1.07) | <0.001 | 8.67 (5.71, 12.67) | 4.87 (3.23, 7.30) | -1.87 (-2.41, -1.33) | <0.001 |
| Czechia | 106.44 (103.18, 109.78) | 106.52 (103.01, 110.15) | 0.01 (-0.00, 0.03) | 0.111 | 1379.96 (1368.11, 1391.89) | 1352.64 (1339.85, 1365.53) | -0.06 (-0.10, -0.02) | 0.003 | 848.22 (839.11, 857.41) | 361.15 (354.71, 367.69) | -2.94 (-3.29, -2.58) | <0.001 | 13.02 (11.93, 14.20) | 4.47 (3.80, 5.25) | -3.66 (-4.08, -3.24) | <0.001 |
| Democratic People's Republic of Korea | 116.44 (114.07, 118.84) | 119.00 (116.89, 121.14) | 0.08 (0.06, 0.09) | <0.001 | 1222.42 (1214.73 , | 1236.52 (1229.71 , | 0.05 (0.01, 0.10) | 0.013 | 1369.00 (1360.78 , | 1331.85 (1324.83 , | -0.08 (-0.17, 0.00) | 0.058 | 21.66 (20.63, 22.73) | 20.94 (20.07, 21.84) | -0.12 (-0.18, -0.07) | <0.001 |

| | | | | | | | | | | | | | | | | |
|---|---|---|---|---|---|---|---|---|---|---|---|---|---|---|---|---|
| | | | | | 1230.16) | 1243.35) | | | 1377.25) | 1338.90) | | | | | | |
| Democratic Republic of the Congo | 178.03 (175.80, 180.28) | 175.17 (173.76, 176.58) | -0.06 (-0.07, -0.04) | <0.001 | 2802.80 (2793.90, 2811.72) | 2804.80 (2799.14, 2810.48) | 0.01 (-0.05, 0.06) | 0.749 | 1130.76 (1124.88, 1136.66) | 899.14 (895.81, 902.49) | -0.82 (-0.87, -0.77) | <0.001 | 16.70 (15.98, 17.44) | 12.76 (12.36, 13.17) | -0.95 (-1.01, -0.90) | <0.001 |
| Denmark | 84.15 (80.13, 88.33) | 71.07 (67.26, 75.05) | -0.58 (-0.60, -0.56) | <0.001 | 867.03 (853.98, 880.23) | 776.04 (763.29, 788.95) | -0.38 (-0.45, -0.30) | <0.001 | 472.16 (462.62, 481.86) | 185.45 (179.27, 191.81) | -3.23 (-3.64, -2.83) | <0.001 | 7.19 (6.06, 8.48) | 2.21 (1.59, 3.02) | -4.09 (-4.67, -3.50) | <0.001 |
| Djibouti | 176.52 (158.04, 196.76) | 173.17 (161.85, 185.08) | -0.07 (-0.08, -0.06) | <0.001 | 2851.37 (2775.01, 2929.47) | 3038.05 (2990.18, 3086.50) | 0.22 (0.18, 0.27) | <0.001 | 1105.19 (1056.05, 1156.16) | 909.89 (883.93, 936.44) | -0.63 (-1.02, -0.25) | 0.001 | 15.98 (10.49, 23.48) | 12.42 (9.56, 15.88) | -0.82 (-1.27, -0.38) | <0.001 |
| Dominica | 183.37 (136.15, 242.75) | 184.06 (135.43, 244.60) | 0.01 (0.00, 0.03) | 0.041 | 2234.13 (2059.82, 2420.11) | 2251.95 (2072.28, 2443.11) | 0.03 (0.01, 0.05) | 0.002 | 1223.23 (1092.23, 1366.30) | 938.91 (824.09, 1065.32) | -0.93 (-1.11, -0.75) | <0.001 | 19.16 (6.09, 46.31) | 14.20 (3.56, 38.06) | -1.02 (-1.24, -0.79) | <0.001 |
| Dominican Republic | 175.34 (170.51, 180.27) | 184.22 (180.26, 188.25) | 0.17 (0.16, 0.19) | <0.001 | 2151.18 (2134.19, 2168.27) | 2219.88 (2206.08, 2233.74) | 0.11 (0.08, 0.15) | <0.001 | 1358.46 (1344.63, 1372.42) | 1390.07 (1379.10, 1401.11) | 0.13 (-0.13, 0.39) | 0.321 | 21.46 (19.73, 23.31) | 22.20 (20.83, 23.64) | 0.17 (-0.12, 0.45) | 0.244 |
| Ecuador | 134.80 (131.22, 138.46) | 127.93 (125.33, 130.58) | -0.18 (-0.19, -0.18) | <0.001 | 1946.63 (1932.83, 1960.51) | 1942.75 (1932.53, 1953.01) | 0.00 (-0.02, 0.03) | 0.913 | 1278.35 (1267.01, 1289.77) | 871.36 (864.49, 878.27) | -1.38 (-1.90, -0.86) | <0.001 | 19.81 (18.41, 21.29) | 12.87 (12.04, 13.74) | -1.55 (-2.10, -1.00) | <0.001 |
| Egypt | 140.48 (138.87, 142.11) | 143.19 (142.03, 144.36) | 0.07 (0.05, 0.09) | <0.001 | 2305.26 (2298.77, 2311.76) | 2461.74 (2456.93, 2466.56) | 0.25 (0.18, 0.32) | <0.001 | 3112.53 (3104.93, 3120.15) | 2037.27 (2032.89, 2041.66) | -1.43 (-1.82, -1.03) | <0.001 | 49.91 (48.94, 50.89) | 31.50 (30.96, 32.06) | -1.56 (-1.99, -1.12) | <0.001 |
| El Salvador | 141.60 (136.39, 146.96) | 135.45 (130.95, 140.06) | -0.16 (-0.19, -0.12) | <0.001 | 1995.56 (1975.72, 2015.57) | 2074.36 (2056.59, 2092.26) | 0.14 (0.09, 0.20) | <0.001 | 1577.47 (1559.49, 1595.61) | 695.36 (684.91, 705.92) | -2.73 (-3.38, -2.07) | <0.001 | 24.77 (22.54, 27.18) | 9.96 (8.74, 11.31) | -3.02 (-3.75, -2.28) | <0.001 |
| Equatorial Guinea | 195.05 | 152.37 | -0.86 | <0.001 | 2910.66 | 2773.79 | -0.17 | <0.001 | 1428.82 | 647.25 | -2.66 | <0.001 | 21.63 | 8.28 | -3.21 | <0.001 |

| | | | | | | | | | | | | | | | | |
|---|---|---|---|---|---|---|---|---|---|---|---|---|---|---|---|---|
| | (172.88, 219.45) | (142.78, 162.52) | (-0.90, -0.81) | | (2823.00, 3000.51) | (2732.10, 2816.02) | (-0.19, -0.14) | | (1365.93, 1493.97) | (626.37, 668.71) | (-2.95, -2.36) | | (14.46, 31.25) | (6.02, 11.16) | (-3.57, -2.85) | |
| Eritrea | 217.15 (208.57, 226.03) | 197.54 (192.41, 202.79) | -0.33 (-0.38, -0.28) | <0.001 | 3189.94 (3156.51, 3223.66) | 3205.53 (3184.49, 3226.68) | 0.02 (-0.05, 0.09) | 0.520 | 1466.28 (1442.75, 1490.11) | 1185.93 (1172.71, 1199.27) | -0.73 (-1.14, -0.33) | <0.001 | 22.03 (19.19, 25.20) | 17.28 (15.69, 18.98) | -0.81 (-1.31, -0.31) | 0.001 |
| Estonia | 130.58 (121.57, 140.13) | 130.27 (119.76, 141.61) | 0.01 (-0.04, 0.06) | 0.742 | 942.37 (917.83, 967.44) | 971.64 (942.40, 1001.68) | 0.11 (0.07, 0.14) | <0.001 | 1117.64 (1091.20, 1144.59) | 428.86 (409.98, 448.54) | -3.19 (-3.90, -2.48) | <0.001 | 18.51 (15.26, 22.29) | 6.21 (4.16, 9.13) | -3.60 (-4.46, -2.73) | <0.001 |
| Eswatini | 172.55 (157.48, 188.82) | 176.40 (164.90, 188.53) | 0.08 (0.05, 0.11) | <0.001 | 2397.06 (2339.51, 2455.80) | 2418.30 (2375.14, 2462.08) | 0.03 (0.01, 0.05) | 0.003 | 917.24 (880.28, 955.46) | 974.25 (946.33, 1002.81) | 0.12 (-0.42, 0.65) | 0.673 | 13.21 (9.04, 18.75) | 14.29 (11.06, 18.20) | 0.17 (-0.43, 0.77) | 0.579 |
| Ethiopia | 175.27 (173.33, 177.23) | 164.85 (163.64, 166.07) | -0.21 (-0.22, -0.20) | <0.001 | 2498.85 (2491.45, 2506.27) | 2725.29 (2720.29, 2730.30) | 0.31 (0.27, 0.35) | <0.001 | 1537.91 (1531.93, 1543.91) | 730.84 (728.17, 733.51) | -2.53 (-2.63, -2.44) | <0.001 | 23.76 (23.01, 24.52) | 9.66 (9.35, 9.98) | -3.06 (-3.19, -2.94) | <0.001 |
| Fiji | 128.90 (116.53, 142.28) | 132.24 (120.64, 144.66) | 0.09 (0.05, 0.13) | <0.001 | 1515.87 (1472.78, 1559.95) | 1731.69 (1688.98, 1775.22) | 0.48 (0.42, 0.54) | <0.001 | 2963.01 (2901.98, 3025.04) | 2293.82 (2244.81, 2343.65) | -0.83 (-1.05, -0.62) | <0.001 | 48.78 (41.18, 57.41) | 37.01 (31.02, 43.82) | -0.92 (-1.49, -0.35) | 0.002 |
| Finland | 91.05 (86.83, 95.44) | 82.75 (78.54, 87.14) | -0.32 (-0.36, -0.28) | <0.001 | 843.64 (830.62, 856.84) | 790.04 (776.89, 803.36) | -0.19 (-0.32, -0.07) | 0.003 | 714.10 (702.42, 725.95) | 291.61 (283.74, 299.65) | -3.07 (-3.55, -2.59) | <0.001 | 11.48 (10.06, 13.08) | 4.01 (3.14, 5.06) | -3.61 (-4.12, -3.10) | <0.001 |
| France | 67.79 (66.72, 68.88) | 63.80 (62.71, 64.91) | -0.20 (-0.22, -0.19) | <0.001 | 792.67 (788.99, 796.36) | 746.76 (743.01, 750.53) | -0.20 (-0.29, -0.12) | <0.001 | 461.69 (458.90, 464.49) | 241.42 (239.31, 243.55) | -2.27 (-2.62, -1.92) | <0.001 | 6.95 (6.61, 7.30) | 3.18 (2.95, 3.44) | -2.74 (-3.20, -2.28) | <0.001 |
| Gabon | 162.50 (149.71, 176.15) | 156.48 (147.57, 165.82) | -0.12 (-0.14, -0.11) | <0.001 | 2757.34 (2703.93, 2811.61) | 2807.27 (2769.06, 2845.90) | 0.07 (0.05, 0.09) | <0.001 | 1159.45 (1123.67, 1196.12) | 778.65 (758.21, 799.52) | -1.41 (-1.59, -1.22) | <0.001 | 17.22 (13.06, 22.34) | 10.54 (8.26, 13.27) | -1.81 (-2.00, -1.63) | <0.001 |

| | | | | | | | | | | | | | | | | |
|---|---|---|---|---|---|---|---|---|---|---|---|---|---|---|---|---|
| Gambia | 156.27 (143.47, 170.02) | 162.58 (154.30, 171.22) | 0.14 (0.13, 0.14) | <0.001 | 2606.12 (2553.26, 2659.91) | 2742.28 (2708.05, 2776.86) | 0.18 (0.14, 0.23) | <0.001 | 974.37 (941.02, 1008.70) | 992.97 (971.77, 1014.54) | 0.14 (-0.81, 1.11) | 0.770 | 13.88 (10.09, 18.71) | 14.10 (11.64, 16.95) | 0.14 (-0.98, 1.29) | 0.802 |
| Georgia | 168.74 (163.27, 174.34) | 164.93 (157.72, 172.41) | -0.07 (-0.14, -0.00) | 0.046 | 2066.62 (2047.38, 2085.99) | 2104.22 (2078.26, 2130.46) | 0.06 (0.03, 0.09) | <0.001 | 2101.38 (2082.08, 2120.83) | 1328.99 (1309.01, 1349.22) | -1.62 (-2.85, -0.37) | 0.011 | 34.11 (31.70, 36.67) | 20.77 (18.37, 23.45) | -1.70 (-3.29, -0.09) | 0.038 |
| Germany | 85.10 (84.08, 86.14) | 75.64 (74.61, 76.69) | -0.41 (-0.46, -0.36) | <0.001 | 941.61 (938.16, 945.08) | 839.29 (835.80, 842.79) | -0.39 (-0.51, -0.26) | <0.001 | 729.70 (726.68, 732.74) | 292.01 (289.97, 294.05) | -3.14 (-3.33, -2.94) | <0.001 | 11.48 (11.11, 11.87) | 3.97 (3.74, 4.21) | -3.63 (-3.86, -3.40) | <0.001 |
| Ghana | 158.75 (155.42, 162.14) | 156.83 (154.71, 158.98) | -0.04 (-0.06, -0.02) | 0.001 | 2566.33 (2552.97, 2579.75) | 2609.97 (2601.31, 2618.65) | 0.06 (0.05, 0.07) | <0.001 | 1259.91 (1250.24, 1269.65) | 1032.70 (1027.14, 1038.28) | -0.70 (-0.92, -0.48) | <0.001 | 18.96 (17.77, 20.20) | 14.96 (14.29, 15.66) | -0.83 (-1.08, -0.58) | <0.001 |
| Greece | 69.31 (66.68, 72.01) | 65.55 (62.70, 68.51) | -0.19 (-0.20, -0.18) | <0.001 | 582.44 (574.79, 590.16) | 576.34 (567.87, 584.92) | 0.00 (-0.21, 0.21) | 0.999 | 686.36 (678.07, 694.74) | 484.40 (476.81, 492.08) | -1.25 (-1.54, -0.95) | <0.001 | 10.95 (9.93, 12.06) | 7.64 (6.72, 8.65) | -1.30 (-1.65, -0.96) | <0.001 |
| Greenland | 123.40 (84.72, 176.92) | 103.57 (64.72, 159.20) | -0.60 (-0.63, -0.56) | <0.001 | 1259.37 (1127.56, 1404.85) | 1123.03 (984.31, 1277.37) | -0.36 (-0.58, -0.13) | 0.002 | 1020.05 (901.30, 1152.59) | 503.21 (412.12, 610.14) | -2.39 (-2.62, -2.16) | <0.001 | 16.41 (4.73, 45.65) | 7.37 (0.58, 34.49) | -2.72 (-2.90, -2.55) | <0.001 |
| Grenada | 194.45 (148.90, 250.37) | 191.73 (151.06, 240.47) | -0.05 (-0.07, -0.03) | <0.001 | 2345.11 (2179.08, 2521.13) | 2339.37 (2191.17, 2495.38) | -0.01 (-0.05, 0.04) | 0.811 | 1634.78 (1493.15, 1786.75) | 861.95 (771.54, 960.40) | -2.15 (-2.46, -1.84) | <0.001 | 26.52 (11.31, 53.41) | 12.90 (4.11, 30.96) | -2.41 (-3.09, -1.73) | <0.001 |
| Guam | 118.34 (92.90, 148.80) | 137.07 (108.83, 170.55) | 0.51 (0.46, 0.57) | <0.001 | 1598.67 (1500.95, 1701.27) | 1753.35 (1648.22, 1863.55) | 0.34 (0.24, 0.43) | <0.001 | 1255.28 (1168.63, 1346.82) | 1805.36 (1697.45, 1918.42) | 1.23 (0.88, 1.58) | <0.001 | 19.02 (9.74, 33.66) | 28.93 (16.73, 46.68) | 1.42 (1.03, 1.81) | <0.001 |
| Guatemala | 159.19 (154.54, 163.96) | 142.48 (139.84, 145.17) | -0.38 (-0.43, -0.32) | <0.001 | 2051.78 (2034.91, | 2164.30 (2153.91, | 0.19 (0.17, 0.21) | <0.001 | 1724.67 (1708.91, | 892.72 (885.94, 899.54) | -2.29 (-3.01, -1.57) | <0.001 | 27.31 (25.34, 29.40) | 13.20 (12.38, 14.06) | -2.53 (-3.32, -1.74) | <0.001 |

| | | | | | | | | | | | | | | | | |
|---|---|---|---|---|---|---|---|---|---|---|---|---|---|---|---|---|
| | | | | | 2068.77) | 2174.73) | | | 1740.54) | | | | | | | |
| Guinea | 130.82 (125.93, 135.85) | 143.87 (140.39, 147.41) | 0.32 (0.28, 0.36) | <0.001 | 2019.54 (2000.30, 2038.92) | 2314.15 (2300.26, 2328.11) | 0.47 (0.44, 0.49) | <0.001 | 1090.90 (1076.48, 1105.48) | 1114.60 (1104.66, 1124.61) | 0.10 (-0.12, 0.33) | 0.362 | 16.43 (14.69, 18.33) | 16.61 (15.40, 17.90) | 0.07 (-0.17, 0.32) | 0.558 |
| Guinea-Bissau | 175.69 (162.01, 190.29) | 178.93 (169.56, 188.71) | 0.05 (0.00, 0.10) | 0.036 | 2164.00 (2115.36, 2213.55) | 2266.46 (2232.85, 2300.49) | 0.17 (0.14, 0.20) | <0.001 | 1692.48 (1648.32, 1737.59) | 1472.44 (1444.60, 1500.70) | -0.47 (-0.58, -0.35) | <0.001 | 26.66 (21.32, 33.00) | 22.95 (19.56, 26.79) | -0.51 (-0.66, -0.36) | <0.001 |
| Guyana | 218.26 (202.36, 235.16) | 208.51 (192.73, 225.30) | -0.16 (-0.18, -0.13) | <0.001 | 2296.77 (2244.48, 2350.04) | 2248.75 (2196.18, 2302.31) | -0.06 (-0.14, 0.02) | 0.123 | 2262.36 (2209.16, 2316.58) | 1738.53 (1691.41, 1786.68) | -0.96 (-1.68, -0.23) | 0.010 | 37.17 (30.57, 44.83) | 28.29 (22.52, 35.12) | -1.00 (-1.77, -0.22) | 0.012 |
| Haiti | 229.73 (223.61, 235.98) | 220.55 (216.55, 224.60) | -0.14 (-0.17, -0.12) | <0.001 | 2215.46 (2196.34, 2234.72) | 2212.31 (2199.61, 2225.08) | 0.01 (-0.07, 0.08) | 0.848 | 2748.50 (2726.88, 2770.25) | 1784.54 (1773.05, 1796.08) | -1.44 (-1.59, -1.29) | <0.001 | 45.59 (42.83, 48.49) | 28.97 (27.52, 30.48) | -1.52 (-1.67, -1.36) | <0.001 |
| Honduras | 148.50 (142.66, 154.53) | 142.73 (139.10, 146.43) | -0.14 (-0.17, -0.11) | <0.001 | 1978.57 (1956.92, 2000.43) | 2043.25 (2029.36, 2057.21) | 0.12 (0.07, 0.16) | <0.001 | 1429.66 (1410.68, 1448.83) | 678.14 (669.94, 686.43) | -2.50 (-3.12, -1.87) | <0.001 | 22.79 (20.42, 25.36) | 9.83 (8.85, 10.89) | -2.83 (-3.50, -2.15) | <0.001 |
| Hungary | 115.53 (112.17, 118.97) | 102.49 (98.91, 106.18) | -0.42 (-0.44, -0.40) | <0.001 | 1096.80 (1086.36, 1107.33) | 1030.74 (1019.29, 1042.30) | -0.21 (-0.25, -0.17) | <0.001 | 1607.13 (1594.83, 1619.50) | 532.84 (524.80, 541.00) | -3.74 (-4.21, -3.27) | <0.001 | 26.97 (25.42, 28.61) | 7.82 (6.88, 8.86) | -4.19 (-4.74, -3.65) | <0.001 |
| Iceland | 75.93 (60.19, 94.63) | 71.68 (57.56, 88.48) | -0.19 (-0.28, -0.10) | <0.001 | 625.08 (578.18, 674.86) | 619.19 (576.15, 664.82) | -0.01 (-0.11, 0.10) | 0.907 | 487.80 (446.43, 532.06) | 222.76 (197.35, 250.78) | -2.68 (-2.93, -2.44) | <0.001 | 7.49 (3.21, 15.00) | 3.00 (0.78, 8.37) | -3.15 (-3.50, -2.80) | <0.001 |
| India | 144.64 (144.23, 145.05) | 142.95 (142.64, 143.25) | -0.04 (-0.22, 0.14) | 0.652 | 1594.88 (1593.51, 1596.24) | 1609.96 (1608.93, 1610.98) | 0.06 (-0.02, 0.14) | 0.136 | 1695.85 (1694.43, 1697.27) | 1279.87 (1278.95, 1280.78) | -0.95 (-1.35, -0.56) | <0.001 | 27.35 (27.17, 27.53) | 20.46 (20.34, 20.58) | -0.99 (-1.50, -0.47) | <0.001 |
| Indonesia | 97.48 | 98.86 | 0.05 | <0.001 | 1020.78 | 1053.90 | 0.12 | 0.003 | 1650.74 | 1234.21 | -0.99 | <0.001 | 26.36 | 19.16 | -1.08 | <0.001 |

| | | | | | | | | | | | | | | | | |
|---|---|---|---|---|---|---|---|---|---|---|---|---|---|---|---|---|
| | (96.76, 98.21) | (98.27, 99.46) | (0.04, 0.06) | | (1018.47, 1023.10) | (1051.96, 1055.85) | (0.04, 0.20) | | (1647.76, 1653.73) | (1232.11, 1236.31) | (-1.10, -0.88) | | (25.98, 26.75) | (18.90, 19.43) | (-1.20, -0.97) | |
| Iran (Islamic Republic of) | 154.18 (152.46, 155.91) | 148.50 (147.25, 149.76) | -0.13 (-0.16, -0.10) | <0.001 | 1900.82 (1894.82, 1906.82) | 1922.80 (1918.25, 1927.36) | 0.04 (0.03, 0.06) | <0.001 | 1311.63 (1306.55, 1316.72) | 837.73 (834.76, 840.71) | -1.53 (-1.64, -1.42) | <0.001 | 20.45 (19.81, 21.10) | 12.07 (11.72, 12.43) | -1.81 (-1.95, -1.67) | <0.001 |
| Iraq | 159.77 (156.56, 163.02) | 154.18 (152.34, 156.02) | -0.12 (-0.14, -0.11) | <0.001 | 1922.06 (1910.88, 1933.30) | 1910.54 (1904.07, 1917.03) | -0.01 (-0.05, 0.04) | 0.839 | 1723.64 (1712.86, 1734.47) | 1062.76 (1057.88, 1067.67) | -1.69 (-1.90, -1.49) | <0.001 | 27.38 (26.03, 28.80) | 15.88 (15.28, 16.50) | -1.91 (-2.14, -1.67) | <0.001 |
| Ireland | 77.06 (72.43, 81.92) | 68.76 (64.82, 72.88) | -0.39 (-0.41, -0.37) | <0.001 | 763.29 (748.54, 778.25) | 733.11 (720.14, 746.27) | -0.13 (-0.23, -0.03) | 0.009 | 540.75 (528.36, 553.36) | 220.64 (213.65, 227.82) | -3.02 (-3.65, -2.38) | <0.001 | 8.43 (6.95, 10.15) | 2.85 (2.12, 3.77) | -3.67 (-4.42, -2.92) | <0.001 |
| Israel | 65.50 (61.88, 69.27) | 62.54 (59.85, 65.32) | -0.16 (-0.18, -0.13) | <0.001 | 594.22 (583.25, 605.34) | 598.60 (590.23, 607.06) | 0.05 (-0.06, 0.15) | 0.378 | 504.70 (494.60, 514.97) | 185.50 (180.86, 190.23) | -3.39 (-4.11, -2.67) | <0.001 | 7.81 (6.60, 9.18) | 2.31 (1.82, 2.90) | -4.17 (-4.67, -3.67) | <0.001 |
| Italy | 123.93 (122.45, 125.43) | 117.28 (115.65, 118.92) | -0.19 (-0.21, -0.16) | <0.001 | 1073.78 (1069.39, 1078.19) | 1079.63 (1074.71, 1084.57) | 0.02 (-0.11, 0.14) | 0.763 | 519.56 (516.51, 522.63) | 254.65 (252.25, 257.06) | -2.48 (-2.64, -2.32) | <0.001 | 7.98 (7.60, 8.37) | 3.43 (3.17, 3.72) | -2.89 (-3.12, -2.67) | <0.001 |
| Jamaica | 166.76 (158.50, 175.37) | 185.68 (177.98, 193.63) | 0.38 (0.35, 0.41) | <0.001 | 2166.80 (2136.61, 2197.34) | 2344.70 (2317.15, 2372.50) | 0.28 (0.26, 0.29) | <0.001 | 790.60 (772.14, 809.41) | 721.43 (706.11, 737.02) | -0.14 (-0.96, 0.69) | 0.738 | 11.55 (9.38, 14.09) | 10.40 (8.62, 12.43) | -0.14 (-1.15, 0.88) | 0.786 |
| Japan | 125.37 (124.34, 126.41) | 130.00 (128.81, 131.20) | 0.13 (0.10, 0.17) | <0.001 | 1079.56 (1076.54, 1082.59) | 1163.56 (1160.00, 1167.12) | 0.26 (0.24, 0.29) | <0.001 | 444.86 (442.93, 446.81) | 294.88 (293.09, 296.68) | -1.42 (-1.65, -1.20) | <0.001 | 6.52 (6.29, 6.76) | 3.82 (3.62, 4.03) | -1.83 (-2.10, -1.57) | <0.001 |
| Jordan | 105.31 (99.53, 111.37) | 95.22 (92.48, 98.02) | -0.34 (-0.38, -0.31) | <0.001 | 1301.02 (1280.76, 1321.54) | 1253.34 (1243.39, 1263.35) | -0.11 (-0.25, 0.02) | 0.096 | 905.31 (887.96, 922.93) | 483.14 (476.93, 489.41) | -2.15 (-2.48, -1.81) | <0.001 | 13.88 (11.77, 16.28) | 6.54 (5.84, 7.32) | -2.52 (-3.03, -2.00) | <0.001 |

| | | | | | | | | | | | | | | | | |
|---|---|---|---|---|---|---|---|---|---|---|---|---|---|---|---|---|
| Kazakhstan | 120.67 (118.07, 123.32) | 103.19 (100.88, 105.56) | -0.54 (-0.57, -0.51) | <0.001 | 949.11 (941.77, 956.49) | 880.24 (873.39, 887.15) | -0.26 (-0.29, -0.22) | <0.001 | 1599.75 (1590.22, 1609.34) | 1358.72 (1350.36, 1367.12) | -0.55 (-1.43, 0.33) | 0.218 | 26.46 (25.25, 27.72) | 22.65 (21.58, 23.76) | -0.52 (-1.46, 0.42) | 0.278 |
| Kenya | 172.18 (169.33, 175.07) | 170.96 (169.21, 172.73) | -0.02 (-0.03, -0.01) | 0.004 | 2884.93 (2873.11, 2896.79) | 3061.49 (3053.99, 3069.01) | 0.21 (0.19, 0.24) | <0.001 | 776.54 (770.17, 782.96) | 805.50 (801.56, 809.46) | 0.11 (0.02, 0.20) | 0.018 | 10.16 (9.42, 10.94) | 10.52 (10.07, 10.99) | 0.09 (-0.03, 0.21) | 0.145 |
| Kiribati | 267.96 (210.82, 336.57) | 230.45 (189.27, 278.15) | -0.52 (-0.54, -0.50) | <0.001 | 2063.60 (1899.59, 2238.68) | 1967.51 (1843.40, 2097.96) | -0.16 (-0.19, -0.14) | <0.001 | 6414.36 (6118.88, 6720.99) | 5583.80 (5371.90, 5802.08) | -0.49 (-0.59, -0.40) | <0.001 | 108.26 (72.75, 155.74) | 93.98 (68.22, 126.44) | -0.50 (-0.58, -0.41) | <0.001 |
| Kuwait | 91.94 (85.78, 98.48) | 94.41 (90.40, 98.61) | 0.10 (0.03, 0.17) | 0.005 | 1085.20 (1063.47, 1107.31) | 1147.90 (1133.25, 1162.74) | 0.20 (0.16, 0.24) | <0.001 | 974.47 (954.01, 995.32) | 679.85 (668.97, 690.91) | -1.12 (-2.30, 0.08) | 0.067 | 15.14 (12.69, 17.97) | 10.10 (8.85, 11.54) | -1.24 (-2.58, 0.11) | 0.072 |
| Kyrgyzstan | 168.55 (162.50, 174.78) | 150.45 (145.80, 155.23) | -0.39 (-0.41, -0.38) | <0.001 | 1871.71 (1851.42, 1892.17) | 1779.44 (1763.36, 1795.64) | -0.18 (-0.20, -0.16) | <0.001 | 1780.53 (1760.36, 1800.88) | 1031.92 (1019.55, 1044.40) | -1.83 (-2.75, -0.89) | <0.001 | 28.85 (26.32, 31.57) | 16.18 (14.66, 17.82) | -1.92 (-2.97, -0.86) | <0.001 |
| Lao People's Democratic Republic | 172.33 (165.59, 179.30) | 144.30 (140.09, 148.61) | -0.62 (-0.67, -0.56) | <0.001 | 1653.37 (1632.32, 1674.63) | 1530.84 (1517.06, 1544.71) | -0.25 (-0.30, -0.19) | <0.001 | 1660.46 (1638.89, 1682.25) | 1452.01 (1438.47, 1465.66) | -0.44 (-0.62, -0.25) | <0.001 | 26.24 (23.56, 29.15) | 22.81 (21.13, 24.59) | -0.45 (-0.65, -0.25) | <0.001 |
| Latvia | 104.94 (98.65, 111.54) | 108.11 (99.97, 116.83) | 0.11 (0.09, 0.13) | <0.001 | 699.61 (683.29, 716.25) | 758.54 (737.10, 780.54) | 0.33 (0.15, 0.50) | <0.001 | 1434.42 (1411.05, 1458.10) | 983.28 (959.25, 1007.86) | -1.23 (-2.73, 0.29) | 0.112 | 24.33 (21.38, 27.60) | 16.26 (13.33, 19.77) | -1.35 (-2.97, 0.31) | 0.109 |
| Lebanon | 89.23 (83.71, 95.04) | 90.37 (86.39, 94.50) | 0.05 (0.01, 0.09) | 0.018 | 1278.27 (1257.40, 1299.42) | 1358.64 (1342.83, 1374.61) | 0.22 (0.16, 0.27) | <0.001 | 1668.19 (1643.99, 1692.68) | 1124.30 (1110.05, 1138.71) | -1.34 (-1.45, -1.23) | <0.001 | 27.12 (24.09, 30.44) | 17.40 (15.68, 19.28) | -1.51 (-1.62, -1.39) | <0.001 |
| Lesotho | 173.10 | 189.92 | 0.33 | <0.001 | 2325.47 | 2405.57 | 0.13 | <0.001 | 767.86 | 1083.52 | 1.22 | <0.001 | 10.70 | 16.26 | 1.49 | <0.001 |

| | | | | | | | | | | | | | | | | |
|---|---|---|---|---|---|---|---|---|---|---|---|---|---|---|---|---|
| | (163.20, 183.50) | (181.20, 198.95) | (0.30, 0.35) | | (2288.49, 2362.95) | (2374.32, 2437.15) | (0.08, 0.18) | | (746.02, 790.22) | (1062.27, 1105.10) | (1.05, 1.40) | | (8.22, 13.73) | (13.73, 19.12) | (1.27, 1.71) | |
| Liberia | 155.91 (146.43, 165.87) | 160.17 (154.56, 165.94) | 0.09 (0.08, 0.10) | <0.001 | 2348.83 (2311.51, 2386.61) | 2519.71 (2497.29, 2542.28) | 0.25 (0.21, 0.30) | <0.001 | 1122.06 (1096.09, 1148.49) | 928.38 (914.51, 942.42) | -0.69 (-0.95, -0.43) | <0.001 | 16.68 (13.64, 20.21) | 13.27 (11.64, 15.07) | -0.83 (-1.13, -0.53) | <0.001 |
| Libya | 125.07 (119.40, 130.95) | 137.64 (133.53, 141.85) | 0.33 (0.29, 0.36) | <0.001 | 1729.47 (1708.28, 1750.88) | 1965.18 (1949.53, 1980.92) | 0.44 (0.36, 0.53) | <0.001 | 1650.15 (1629.19, 1671.33) | 1344.24 (1331.41, 1357.17) | -0.78 (-1.36, -0.20) | 0.009 | 26.01 (23.41, 28.84) | 20.59 (19.04, 22.24) | -0.90 (-1.57, -0.23) | 0.009 |
| Lithuania | 116.47 (110.90, 122.25) | 116.09 (109.09, 123.47) | -0.00 (-0.04, 0.04) | 0.854 | 950.06 (934.02, 966.32) | 960.09 (939.69, 980.86) | 0.06 (-0.03, 0.15) | 0.181 | 1155.27 (1137.58, 1173.17) | 713.43 (696.11, 731.12) | -1.67 (-2.91, -0.42) | 0.009 | 19.14 (16.92, 21.58) | 11.30 (9.23, 13.77) | -1.81 (-3.18, -0.42) | 0.011 |
| Luxembourg | 66.38 (54.15, 80.96) | 62.59 (52.66, 74.10) | -0.19 (-0.24, -0.14) | <0.001 | 931.04 (882.91, 981.47) | 933.31 (893.18, 974.98) | 0.03 (-0.05, 0.12) | 0.434 | 651.03 (611.24, 693.08) | 261.70 (241.00, 283.94) | -3.15 (-3.65, -2.65) | <0.001 | 10.02 (5.69, 16.94) | 3.31 (1.41, 7.03) | -3.82 (-4.45, -3.18) | <0.001 |
| Madagascar | 219.46 (215.02, 223.98) | 207.30 (204.55, 210.08) | -0.21 (-0.23, -0.18) | <0.001 | 3301.29 (3284.04, 3318.62) | 3457.53 (3446.27, 3468.82) | 0.17 (0.14, 0.19) | <0.001 | 3039.25 (3022.16, 3056.42) | 2014.90 (2006.04, 2023.80) | -1.36 (-1.72, -1.00) | <0.001 | 48.50 (46.34, 50.75) | 31.03 (29.93, 32.17) | -1.47 (-1.87, -1.07) | <0.001 |
| Malawi | 193.92 (189.32, 198.62) | 194.56 (191.39, 197.77) | 0.01 (0.00, 0.02) | 0.047 | 2965.77 (2947.48, 2984.15) | 3104.92 (3092.01, 3117.87) | 0.17 (0.09, 0.25) | <0.001 | 1200.14 (1188.11, 1212.26) | 963.18 (955.79, 970.62) | -0.71 (-0.83, -0.59) | <0.001 | 17.45 (16.00, 19.00) | 13.25 (12.38, 14.17) | -0.90 (-1.04, -0.77) | <0.001 |
| Malaysia | 134.07 (131.41, 136.77) | 124.30 (122.45, 126.18) | -0.26 (-0.28, -0.24) | <0.001 | 1893.89 (1883.85, 1903.97) | 1860.94 (1853.76, 1868.15) | -0.06 (-0.09, -0.03) | <0.001 | 1244.63 (1236.43, 1252.88) | 997.96 (992.71, 1003.24) | -0.79 (-1.50, -0.07) | 0.032 | 18.46 (17.46, 19.49) | 14.69 (14.06, 15.34) | -0.82 (-1.64, 0.01) | 0.052 |
| Maldives | 128.75 (103.34, 159.27) | 108.93 (96.08, 123.37) | -0.58 (-0.63, -0.53) | <0.001 | 1622.50 (1529.07, | 1541.36 (1492.21, | -0.17 (-0.20, -0.14) | <0.001 | 1822.58 (1718.86, | 667.46 (636.49, 699.91) | -3.40 (-3.80, -3.00) | <0.001 | 29.36 (17.39, 47.05) | 9.31 (6.05, 14.28) | -3.87 (-4.51, -3.22) | <0.001 |

| | | | | | | | | | | | | | | | | |
|---|---|---|---|---|---|---|---|---|---|---|---|---|---|---|---|---|
| | | | | | 1720.79) | 1592.03) | | | 1931.41) | | | | | | | |
| Mali | 172.70 (167.90, 177.61) | 163.73 (160.84, 166.65) | -0.18 (-0.19, -0.18) | <0.001 | 2654.61 (2635.73, 2673.60) | 2696.66 (2684.94, 2708.43) | 0.06 (0.03, 0.09) | <0.001 | 1494.00 (1479.44, 1508.67) | 1081.64 (1073.90, 1089.42) | -1.09 (-1.30, -0.87) | <0.001 | 22.79 (21.01, 24.69) | 15.71 (14.77, 16.69) | -1.25 (-1.49, -1.01) | <0.001 |
| Malta | 66.51 (53.82, 81.48) | 61.98 (49.83, 76.68) | -0.24 (-0.26, -0.23) | <0.001 | 633.29 (592.66, 676.13) | 594.15 (555.07, 635.66) | -0.19 (-0.36, -0.03) | 0.022 | 537.18 (499.87, 576.71) | 331.69 (302.86, 362.95) | -1.69 (-2.17, -1.21) | <0.001 | 8.24 (4.25, 14.70) | 4.82 (1.98, 10.61) | -1.87 (-2.18, -1.56) | <0.001 |
| Marshall Islands | 176.53 (116.84, 257.36) | 178.80 (129.08, 241.56) | 0.05 (0.01, 0.08) | 0.006 | 1718.01 (1520.15, 1935.72) | 1746.10 (1582.05, 1922.71) | 0.06 (0.04, 0.08) | <0.001 | 3414.64 (3128.59, 3720.78) | 3976.74 (3726.81, 4239.19) | 0.53 (0.46, 0.60) | <0.001 | 55.93 (24.85, 109.61) | 66.91 (38.14, 109.13) | 0.64 (0.55, 0.73) | <0.001 |
| Mauritania | 158.97 (149.92, 168.44) | 145.95 (139.94, 152.17) | -0.30 (-0.31, -0.28) | <0.001 | 2602.11 (2565.25, 2639.39) | 2614.95 (2589.33, 2640.78) | 0.01 (-0.00, 0.03) | 0.133 | 1193.43 (1167.86, 1219.43) | 675.34 (661.98, 688.91) | -1.91 (-2.05, -1.78) | <0.001 | 17.60 (14.58, 21.08) | 8.75 (7.26, 10.46) | -2.34 (-2.51, -2.18) | <0.001 |
| Mauritius | 128.13 (118.36, 138.52) | 117.68 (108.11, 127.89) | -0.29 (-0.30, -0.28) | <0.001 | 1711.60 (1675.42, 1748.38) | 1629.41 (1593.26, 1666.20) | -0.16 (-0.19, -0.13) | <0.001 | 1704.31 (1667.99, 1741.23) | 1022.90 (994.65, 1051.78) | -1.64 (-1.82, -1.47) | <0.001 | 27.48 (23.03, 32.55) | 15.49 (12.20, 19.43) | -1.87 (-2.07, -1.68) | <0.001 |
| Mexico | 108.22 (107.09, 109.35) | 97.18 (96.32, 98.05) | -0.37 (-0.41, -0.34) | <0.001 | 1479.08 (1474.92, 1483.24) | 1402.02 (1398.75, 1405.31) | -0.18 (-0.23, -0.13) | <0.001 | 708.11 (705.16, 711.07) | 607.69 (605.53, 609.86) | -0.46 (-0.92, 0.01) | 0.055 | 10.68 (10.32, 11.05) | 9.01 (8.75, 9.28) | -0.51 (-1.05, 0.02) | 0.061 |
| Micronesia (Federated States of) | 194.32 (151.63, 245.78) | 167.90 (129.85, 214.05) | -0.51 (-0.52, -0.49) | <0.001 | 1777.75 (1644.52, 1919.38) | 1679.94 (1555.14, 1812.50) | -0.18 (-0.23, -0.12) | <0.001 | 4255.70 (4043.07, 4476.93) | 3873.14 (3678.38, 4075.82) | -0.33 (-0.38, -0.27) | <0.001 | 71.80 (46.47, 106.36) | 64.74 (41.68, 96.29) | -0.36 (-0.42, -0.30) | <0.001 |
| Monaco | 70.73 (28.02, 159.82) | 70.64 (27.87, 150.19) | -0.01 (-0.02, 0.01) | 0.400 | 565.55 (425.86, 745.07) | 572.18 (431.74, 745.97) | 0.07 (-0.03, 0.18) | 0.181 | 545.31 (408.77, 721.63) | 349.68 (242.35, 490.79) | -1.52 (-1.57, -1.47) | <0.001 | 8.59 (0.13, 73.93) | 5.23 (0.01, 53.60) | -1.71 (-1.76, -1.65) | <0.001 |
| Mongolia | 187.73 (178.30, | 176.38 (169.39, | -0.21 (-0.23, | <0.001 | 1938.73 (1908.18 | 1929.83 (1906.58 | -0.00 (-0.04, | 0.861 | 2010.50 (1977.96 | 1765.74 (1744.25 | -0.40 (-0.84, | 0.077 | 32.87 (28.77, | 28.89 (26.21, | -0.41 (-0.89, | 0.099 |

| | | | | | | | | | | | | | | | | |
|---|---|---|---|---|---|---|---|---|---|---|---|---|---|---|---|---|
| | 197.58) | 183.62) | -0.20) | | , 1969.71) | , 1953.31) | 0.03) | | , 2043.48) | , 1787.46) | 0.04) | | 37.43) | 31.80) | 0.08) | |
| Montenegro | 104.24 (92.04, 117.62) | 98.10 (85.45, 112.19) | -0.21 (-0.24, -0.17) | <0.001 | 926.21 (889.10, 964.49) | 917.31 (877.81, 958.22) | -0.02 (-0.11, 0.07) | 0.714 | 1192.30 (1150.23 , 1235.55) | 682.58 (648.85, 717.71) | -2.15 (-3.51, -0.77) | 0.002 | 19.36 (14.33, 25.60) | 10.48 (6.71, 15.75) | -2.36 (-3.78, -0.92) | 0.001 |
| Morocco | 148.03 (145.62, 150.48) | 146.35 (144.40, 148.32) | -0.04 (-0.05, -0.03) | <0.001 | 1882.55 (1873.93 , 1891.21) | 1919.43 (1912.35 , 1926.53) | 0.08 (0.01, 0.16) | 0.028 | 1622.12 (1613.95 , 1630.32) | 1008.05 (1002.93 , 1013.19) | -1.58 (-1.81, -1.35) | <0.001 | 25.89 (24.86, 26.96) | 15.05 (14.43, 15.69) | -1.81 (-2.07, -1.54) | <0.001 |
| Mozambique | 201.36 (197.22, 205.57) | 205.69 (202.98, 208.43) | 0.07 (0.05, 0.09) | <0.001 | 2981.07 (2964.90 , 2997.32) | 3139.40 (3128.64 , 3150.19) | 0.19 (0.11, 0.28) | <0.001 | 887.04 (878.03, 896.12) | 1054.63 (1048.14 , 1061.14) | 0.62 (0.43, 0.81) | <0.001 | 12.14 (11.09, 13.26) | 14.89 (14.12, 15.70) | 0.72 (0.61, 0.84) | <0.001 |
| Myanmar | 149.04 (147.16, 150.93) | 129.74 (128.24, 131.26) | -0.48 (-0.51, -0.46) | <0.001 | 1574.67 (1568.55 , 1580.80) | 1492.64 (1487.54 , 1497.75) | -0.17 (-0.29, -0.04) | 0.011 | 2791.62 (2783.35 , 2799.92) | 1464.24 (1459.18 , 1469.31) | -2.22 (-2.36, -2.08) | <0.001 | 45.94 (44.88, 47.02) | 22.87 (22.24, 23.51) | -2.40 (-2.61, -2.20) | <0.001 |
| Namibia | 169.74 (158.74, 181.38) | 159.04 (151.35, 167.04) | -0.23 (-0.24, -0.21) | <0.001 | 2443.87 (2401.37 , 2486.99) | 2433.37 (2402.93 , 2464.13) | -0.02 (-0.03, -0.00) | 0.025 | 844.86 (818.97, 871.41) | 664.05 (647.90, 680.52) | -0.77 (-1.08, -0.47) | <0.001 | 11.93 (8.98, 15.59) | 8.84 (7.05, 10.97) | -0.95 (-1.34, -0.56) | <0.001 |
| Nauru | 154.17 (56.13, 341.08) | 147.35 (55.98, 318.02) | -0.14 (-0.30, 0.02) | 0.093 | 1167.69 (851.80, 1565.22) | 1130.34 (836.04, 1497.46) | -0.11 (-0.19, -0.03) | 0.004 | 4750.29 (4083.52 , 5497.04) | 4989.01 (4341.68 , 5707.75) | 0.17 (0.00, 0.33) | 0.046 | 80.88 (17.05, 237.79) | 84.97 (21.08, 231.46) | 0.17 (0.00, 0.33) | 0.050 |
| Nepal | 130.73 (128.07, 133.43) | 122.52 (120.60, 124.46) | -0.21 (-0.26, -0.16) | <0.001 | 1305.87 (1297.30 , 1314.49) | 1302.02 (1295.67 , 1308.41) | 0.01 (-0.04, 0.06) | 0.743 | 1360.77 (1351.89 , 1369.70) | 813.89 (808.81, 819.00) | -1.74 (-1.88, -1.60) | <0.001 | 22.11 (20.98, 23.29) | 12.51 (11.88, 13.17) | -1.93 (-2.09, -1.77) | <0.001 |
| Netherlands | 92.69 (90.31, 95.12) | 83.97 (81.54, 86.46) | -0.34 (-0.36, -0.32) | <0.001 | 786.96 (779.98, 793.99) | 807.40 (799.82, 815.03) | 0.09 (0.04, 0.13) | <0.001 | 479.11 (473.69, 484.59) | 213.24 (209.36, 217.16) | -2.76 (-3.00, -2.51) | <0.001 | 7.37 (6.72, 8.08) | 2.67 (2.25, 3.14) | -3.47 (-3.78, -3.17) | <0.001 |

| | | | | | | | | | | | | | | | | |
|---|---|---|---|---|---|---|---|---|---|---|---|---|---|---|---|---|
| New Zealand | 101.05 (95.83, 106.48) | 92.04 (87.14, 97.15) | -0.31 (-0.37, -0.24) | <0.001 | 634.89 (621.71, 648.28) | 645.00 (631.94, 658.26) | 0.07 (-0.04, 0.17) | 0.200 | 647.75 (634.44, 661.28) | 322.27 (313.06, 331.69) | -2.42 (-2.65, -2.19) | <0.001 | 10.41 (8.78, 12.25) | 4.70 (3.65, 5.97) | -2.68 (-3.09, -2.28) | <0.001 |
| Nicaragua | 143.35 (137.10, 149.84) | 140.06 (135.70, 144.53) | -0.08 (-0.10, -0.06) | <0.001 | 2090.27 (2065.89, 2114.89) | 2134.10 (2116.94, 2151.36) | 0.08 (0.05, 0.10) | <0.001 | 707.75 (693.12, 722.62) | 472.14 (464.05, 480.33) | -1.43 (-2.09, -0.76) | <0.001 | 10.23 (8.51, 12.21) | 5.98 (5.10, 6.98) | -1.89 (-2.87, -0.89) | <0.001 |
| Niger | 152.54 (147.82, 157.39) | 151.65 (148.84, 154.51) | -0.02 (-0.04, 0.00) | 0.077 | 2436.65 (2417.75, 2455.67) | 2576.80 (2565.22, 2588.44) | 0.21 (0.12, 0.29) | <0.001 | 1156.18 (1142.73, 1169.76) | 943.44 (936.10, 950.84) | -0.69 (-0.99, -0.40) | <0.001 | 17.16 (15.53, 18.93) | 13.48 (12.60, 14.41) | -0.82 (-1.20, -0.45) | <0.001 |
| Nigeria | 142.15 (140.85, 143.45) | 144.89 (144.06, 145.73) | 0.08 (0.04, 0.11) | <0.001 | 2658.64 (2653.05, 2664.24) | 2934.69 (2930.93, 2938.45) | 0.35 (0.32, 0.39) | <0.001 | 944.64 (941.19, 948.11) | 713.42 (711.50, 715.35) | -0.98 (-1.19, -0.77) | <0.001 | 13.44 (13.03, 13.87) | 9.19 (8.97, 9.42) | -1.31 (-1.54, -1.08) | <0.001 |
| Niue | 113.42 (1.67, 727.88) | 107.43 (0.29, 931.99) | -0.19 (-0.19, -0.18) | <0.001 | 1039.41 (445.49, 2072.84) | 1043.85 (373.66, 2338.98) | 0.02 (-0.02, 0.07) | 0.349 | 2615.29 (1586.21, 4070.42) | 1924.01 (945.02, 3509.01) | -1.07 (-1.30, -0.84) | <0.001 | 43.55 (0.00, 603.26) | 31.07 (0.00, 792.18) | -1.18 (-1.41, -0.94) | <0.001 |
| North Macedonia | 97.37 (90.73, 104.37) | 92.39 (85.94, 99.26) | -0.19 (-0.20, -0.17) | <0.001 | 850.38 (830.54, 870.58) | 850.18 (830.38, 870.39) | 0.02 (-0.12, 0.16) | 0.752 | 1214.93 (1191.20, 1239.02) | 763.75 (745.03, 782.88) | -1.53 (-2.09, -0.97) | <0.001 | 19.48 (16.58, 22.75) | 11.73 (9.54, 14.36) | -1.65 (-2.27, -1.03) | <0.001 |
| Northern Mariana Islands | 135.74 (93.05, 192.98) | 131.38 (73.33, 221.99) | -0.11 (-0.15, -0.07) | <0.001 | 1780.75 (1614.07, 1961.23) | 1822.90 (1586.75, 2088.08) | 0.09 (0.01, 0.17) | 0.020 | 1749.92 (1587.67, 1925.70) | 1354.84 (1141.43, 1599.27) | -0.76 (-1.05, -0.48) | <0.001 | 27.83 (11.02, 60.42) | 20.90 (2.78, 77.84) | -0.84 (-1.20, -0.47) | <0.001 |
| Norway | 108.02 (103.04, 113.18) | 101.83 (97.26, 106.57) | -0.20 (-0.22, -0.19) | <0.001 | 925.35 (910.68, 940.22) | 945.96 (931.93, 960.17) | 0.09 (0.01, 0.16) | 0.022 | 487.99 (477.38, 498.79) | 186.30 (180.12, 192.66) | -3.26 (-3.62, -2.89) | <0.001 | 7.45 (6.19, 8.90) | 2.11 (1.51, 2.90) | -4.22 (-4.71, -3.72) | <0.001 |
| Oman | 91.86 (85.38, 98.72) | 92.58 (88.92, 96.40) | 0.03 (-0.04, 0.11) | 0.355 | 1149.01 (1125.65 , | 1280.36 (1265.75 , | 0.38 (0.30, 0.47) | <0.001 | 1365.60 (1340.16 , | 765.40 (754.15, 776.82) | -1.94 (-2.41, -1.46) | <0.001 | 21.92 (18.80, 25.42) | 11.18 (9.88, 12.65) | -2.27 (-2.78, -1.76) | <0.001 |

| | | | | | | | | | | | | | | | | |
|---|---|---|---|---|---|---|---|---|---|---|---|---|---|---|---|---|
| | | | | | 1172.75) | 1295.14) | | | 1391.41) | | | | | | | |
| Pakistan | 171.95 (170.64, 173.28) | 177.44 (176.56, 178.33) | 0.11 (0.08, 0.15) | <0.001 | 1852.43 (1848.03, 1856.83) | 1952.01 (1949.05, 1954.98) | 0.20 (0.10, 0.31) | <0.001 | 1727.87 (1723.51, 1732.24) | 2158.13 (2154.96, 2161.31) | 0.79 (0.70, 0.87) | <0.001 | 27.84 (27.29, 28.41) | 35.11 (34.71, 35.53) | 0.82 (0.73, 0.91) | <0.001 |
| Palau | 113.59 (48.02, 228.92) | 114.91 (47.58, 238.23) | 0.04 (0.00, 0.08) | 0.032 | 1239.10 (989.22, 1534.52) | 1269.42 (1005.69, 1584.49) | 0.10 (0.03, 0.18) | 0.009 | 2624.02 (2250.61, 3042.90) | 2592.37 (2217.34, 3016.67) | -0.10 (-0.39, 0.20) | 0.513 | 43.57 (8.66, 131.55) | 43.11 (8.48, 137.20) | -0.11 (-0.44, 0.23) | 0.528 |
| Palestine | 131.18 (122.54, 140.33) | 127.43 (122.45, 132.57) | -0.10 (-0.13, -0.07) | <0.001 | 1784.74 (1752.31, 1817.68) | 1824.37 (1805.34, 1843.56) | 0.09 (0.01, 0.17) | 0.025 | 1023.86 (998.52, 1049.73) | 651.03 (639.46, 662.76) | -1.57 (-1.83, -1.30) | <0.001 | 15.62 (12.58, 19.22) | 9.08 (7.75, 10.60) | -1.88 (-2.19, -1.57) | <0.001 |
| Panama | 132.14 (125.05, 139.56) | 127.07 (121.60, 132.73) | -0.13 (-0.14, -0.12) | <0.001 | 1969.45 (1941.59, 1997.62) | 1971.49 (1949.74, 1993.44) | 0.01 (-0.06, 0.07) | 0.859 | 766.16 (748.54, 784.10) | 518.05 (506.90, 529.39) | -1.27 (-1.87, -0.67) | <0.001 | 11.23 (9.16, 13.63) | 6.98 (5.74, 8.41) | -1.54 (-2.28, -0.80) | <0.001 |
| Papua New Guinea | 163.66 (157.26, 170.27) | 167.76 (163.74, 171.87) | 0.09 (0.08, 0.10) | <0.001 | 1566.91 (1547.08, 1586.95) | 1627.91 (1615.35, 1640.54) | 0.14 (0.10, 0.18) | <0.001 | 2212.80 (2188.76, 2237.05) | 2335.95 (2320.76, 2351.22) | 0.13 (-0.06, 0.31) | 0.186 | 35.79 (32.76, 39.03) | 37.77 (35.85, 39.77) | 0.12 (-0.07, 0.31) | 0.220 |
| Paraguay | 136.40 (130.61, 142.40) | 133.07 (128.94, 137.29) | -0.08 (-0.10, -0.06) | <0.001 | 2089.36 (2066.39, 2112.52) | 2130.33 (2113.72, 2147.03) | 0.07 (0.04, 0.10) | <0.001 | 844.40 (829.48, 859.53) | 613.56 (604.61, 622.61) | -0.97 (-1.56, -0.38) | 0.001 | 12.68 (10.89, 14.68) | 8.58 (7.54, 9.71) | -1.21 (-2.07, -0.35) | 0.006 |
| Peru | 129.15 (126.76, 131.58) | 120.31 (118.48, 122.17) | -0.25 (-0.27, -0.23) | <0.001 | 2060.03 (2050.35, 2069.74) | 2116.18 (2108.46, 2123.91) | 0.09 (0.07, 0.11) | <0.001 | 1178.07 (1170.67, 1185.51) | 629.35 (625.15, 633.57) | -2.09 (-3.02, -1.15) | <0.001 | 17.83 (16.92, 18.78) | 8.52 (8.03, 9.02) | -2.49 (-3.58, -1.38) | <0.001 |
| Philippines | 142.31 (140.81, 143.84) | 139.59 (138.50, 140.69) | -0.07 (-0.08, -0.05) | <0.001 | 1309.44 (1304.85, 1314.04) | 1360.67 (1357.26, 1364.09) | 0.14 (0.10, 0.18) | <0.001 | 671.63 (668.29, 674.98) | 1695.99 (1692.15, 1699.84) | 3.35 (2.74, 3.96) | <0.001 | 9.47 (9.08, 9.88) | 27.32 (26.83, 27.81) | 3.82 (3.09, 4.55) | <0.001 |

| | | | | | | | | | | | | | | | | |
|---|---|---|---|---|---|---|---|---|---|---|---|---|---|---|---|---|
| Poland | 88.41 (86.92, 89.91) | 79.61 (78.10, 81.14) | -0.35 (-0.41, -0.30) | <0.001 | 1077.89 (1072.62, 1083.19) | 1067.07 (1061.42, 1072.75) | 0.00 (-0.19, 0.19) | 0.993 | 1289.85 (1284.29, 1295.42) | 435.14 (431.71, 438.61) | -3.81 (-4.45, -3.15) | <0.001 | 21.33 (20.63, 22.05) | 6.01 (5.63, 6.42) | -4.39 (-5.19, -3.59) | <0.001 |
| Portugal | 61.55 (59.07, 64.11) | 51.55 (49.07, 54.13) | -0.61 (-0.64, -0.59) | <0.001 | 739.22 (730.55, 747.96) | 702.06 (692.80, 711.43) | -0.12 (-0.33, 0.09) | 0.257 | 763.89 (755.07, 772.79) | 262.05 (256.53, 267.66) | -3.69 (-4.22, -3.15) | <0.001 | 12.09 (11.01, 13.26) | 3.55 (2.94, 4.25) | -4.23 (-4.88, -3.58) | <0.001 |
| Puerto Rico | 110.96 (105.48, 116.66) | 121.09 (114.80, 127.64) | 0.31 (0.28, 0.34) | <0.001 | 1004.34 (987.74, 1021.16) | 1051.81 (1033.11, 1070.77) | 0.16 (0.11, 0.21) | <0.001 | 710.42 (696.42, 724.64) | 399.07 (387.63, 410.76) | -1.90 (-2.67, -1.12) | <0.001 | 11.41 (9.69, 13.34) | 5.96 (4.64, 7.55) | -2.14 (-3.03, -1.24) | <0.001 |
| Qatar | 101.90 (89.75, 115.68) | 92.27 (87.62, 97.25) | -0.32 (-0.37, -0.27) | <0.001 | 1184.38 (1141.32, 1229.04) | 1085.20 (1068.44, 1102.29) | -0.30 (-0.33, -0.26) | <0.001 | 853.22 (816.24, 891.82) | 392.77 (382.62, 403.27) | -2.52 (-3.07, -1.96) | <0.001 | 12.83 (8.73, 18.76) | 5.15 (4.08, 6.62) | -3.00 (-3.80, -2.20) | <0.001 |
| Republic of Korea | 97.54 (96.19, 98.90) | 88.74 (87.38, 90.11) | -0.33 (-0.37, -0.28) | <0.001 | 784.47 (780.63, 788.32) | 759.88 (755.94, 763.84) | -0.08 (-0.16, 0.00) | 0.058 | 1214.16 (1209.39, 1218.95) | 233.31 (231.13, 235.52) | -5.54 (-5.95, -5.13) | <0.001 | 19.43 (18.83, 20.05) | 3.02 (2.78, 3.27) | -6.24 (-6.74, -5.74) | <0.001 |
| Republic of Moldova | 112.50 (107.69, 117.48) | 110.07 (104.67, 115.72) | -0.07 (-0.09, -0.06) | <0.001 | 775.32 (762.67, 788.14) | 778.53 (764.17, 793.14) | 0.01 (-0.03, 0.05) | 0.614 | 1014.95 (1000.44, 1029.64) | 715.55 (702.00, 729.34) | -1.20 (-2.14, -0.26) | 0.013 | 16.47 (14.68, 18.44) | 11.36 (9.73, 13.23) | -1.28 (-2.32, -0.23) | 0.018 |
| Romania | 90.73 (88.74, 92.76) | 96.32 (93.84, 98.85) | 0.19 (0.14, 0.25) | <0.001 | 803.93 (797.99, 809.91) | 887.39 (879.88, 894.94) | 0.38 (0.22, 0.54) | <0.001 | 1168.99 (1161.88, 1176.15) | 691.66 (685.09, 698.28) | -1.78 (-2.54, -1.01) | <0.001 | 19.27 (18.37, 20.21) | 10.73 (9.94, 11.58) | -1.99 (-2.86, -1.12) | <0.001 |
| Russian Federation | 118.82 (117.96, 119.68) | 126.02 (125.06, 126.99) | 0.20 (0.18, 0.22) | <0.001 | 828.93 (826.66, 831.21) | 901.98 (899.40, 904.56) | 0.31 (0.26, 0.36) | <0.001 | 1436.19 (1433.24, 1439.14) | 1764.00 (1760.52, 1767.48) | 0.64 (-0.60, 1.89) | 0.312 | 24.10 (23.72, 24.48) | 29.95 (29.51, 30.40) | 0.69 (-0.57, 1.97) | 0.285 |
| Rwanda | 210.76 (205.24, | 169.88 (166.42, | -0.75 (-0.77, | <0.001 | 2905.40 (2884.76 | 2832.07 (2817.74 | -0.07 (-0.14, | 0.059 | 1812.81 (1796.08 | 825.67 (817.81, | -2.69 (-2.97, | <0.001 | 28.01 (25.95, | 11.23 (10.32, | -3.12 (-3.40, | <0.001 |

| | | | | | | | | | | | | | | | | |
|---|---|---|---|---|---|---|---|---|---|---|---|---|---|---|---|---|
| | 216.40) | 173.40) | -0.72) | | , 2926.17) | , 2846.47) | 0.00) | | , 1829.67) | 833.59) | -2.41) | | 30.21) | 12.20) | -2.84) | |
| Saint Kitts and Nevis | 134.30 (83.91, 204.99) | 117.23 (77.60, 170.93) | -0.47 (-0.51, -0.43) | <0.001 | 1128.38 (971.19, 1304.72) | 1054.71 (927.14, 1195.69) | -0.22 (-0.32, -0.12) | <0.001 | 1925.90 (1716.16 , 2154.95) | 550.61 (459.44, 655.35) | -4.06 (-5.07, -3.04) | <0.001 | 33.00 (11.01, 77.09) | 8.34 (0.98, 32.17) | -4.49 (-5.63, -3.34) | <0.001 |
| Saint Lucia | 194.04 (157.81, 236.84) | 192.46 (160.89, 228.60) | -0.03 (-0.04, -0.02) | <0.001 | 2533.16 (2397.21 , 2675.44) | 2481.47 (2364.51 , 2602.89) | -0.07 (-0.10, -0.04) | <0.001 | 1261.67 (1162.45 , 1367.57) | 784.93 (720.22, 854.10) | -1.59 (-1.92, -1.25) | <0.001 | 19.79 (9.09, 38.10) | 11.36 (4.87, 22.82) | -1.86 (-2.27, -1.45) | <0.001 |
| Saint Vincent and the Grenadines | 184.61 (145.89, 231.51) | 186.69 (147.64, 233.02) | 0.04 (0.03, 0.05) | <0.001 | 2478.29 (2330.31 , 2634.11) | 2521.68 (2372.00 , 2678.43) | 0.06 (0.03, 0.10) | 0.001 | 1423.83 (1307.55 , 1548.35) | 951.00 (860.06, 1049.06) | -1.29 (-2.29, -0.28) | 0.012 | 22.60 (10.07, 44.63) | 14.21 (5.21, 31.17) | -1.48 (-2.43, -0.52) | 0.002 |
| Samoa | 156.06 (125.06, 193.05) | 151.61 (125.17, 182.25) | -0.10 (-0.11, -0.08) | <0.001 | 1792.39 (1684.51 , 1905.95) | 1800.87 (1707.38 , 1898.41) | 0.04 (-0.04, 0.11) | 0.376 | 2185.99 (2061.79 , 2316.15) | 2328.47 (2219.10 , 2442.01) | 0.23 (0.15, 0.30) | <0.001 | 35.25 (20.95, 56.16) | 37.77 (24.93, 55.11) | 0.25 (0.22, 0.29) | <0.001 |
| San Marino | 70.11 (26.37, 151.61) | 66.45 (26.70, 139.09) | -0.18 (-0.20, -0.16) | <0.001 | 1135.03 (925.33, 1378.93) | 1126.04 (931.66, 1350.31) | -0.03 (-0.05, -0.00) | 0.021 | 336.62 (226.82, 481.86) | 307.23 (210.95, 434.07) | -0.32 (-0.41, -0.22) | <0.001 | 4.42 (0.00, 52.39) | 3.96 (0.00, 46.58) | -0.38 (-0.54, -0.22) | <0.001 |
| Sao Tome and Principe | 168.52 (129.46, 217.03) | 172.31 (145.41, 202.92) | 0.07 (0.06, 0.09) | <0.001 | 3248.35 (3071.38 , 3434.07) | 3564.50 (3438.83 , 3693.76) | 0.33 (0.31, 0.34) | <0.001 | 1110.10 (1000.62 , 1229.13) | 1140.85 (1069.10 , 1216.29) | 0.13 (-0.39, 0.66) | 0.623 | 15.74 (5.16, 37.77) | 15.53 (8.17, 26.96) | 0.03 (-0.58, 0.65) | 0.925 |
| Saudi Arabia | 84.52 (82.23, 86.85) | 97.54 (96.17, 98.94) | 0.50 (0.47, 0.53) | <0.001 | 1147.69 (1139.31 , 1156.13) | 1243.98 (1238.98 , 1249.01) | 0.43 (0.03, 0.83) | 0.037 | 1116.90 (1108.52 , 1125.32) | 973.59 (969.29, 977.90) | -0.47 (-0.66, -0.28) | <0.001 | 17.57 (16.52, 18.66) | 15.06 (14.54, 15.60) | -0.53 (-0.84, -0.22) | 0.001 |
| Senegal | 154.99 (150.19, 159.92) | 151.86 (148.71, 155.07) | -0.07 (-0.09, -0.06) | <0.001 | 2499.59 (2480.23 , 2519.06) | 2540.57 (2527.63 , 2553.56) | 0.07 (0.02, 0.12) | 0.009 | 1024.90 (1012.11 , 1037.82) | 851.46 (843.78, 859.20) | -0.50 (-0.67, -0.33) | <0.001 | 14.81 (13.29, 16.48) | 11.83 (10.92, 12.79) | -0.61 (-0.81, -0.41) | <0.001 |

| | | | | | | | | | | | | | | | | |
|---|---|---|---|---|---|---|---|---|---|---|---|---|---|---|---|---|
| Serbia | 104.78 (101.45, 108.20) | 103.22 (99.60, 106.95) | -0.05 (-0.07, -0.03) | <0.001 | 990.04 (979.73, 1000.44) | 1035.15 (1023.59, 1046.83) | 0.20 (-0.01, 0.42) | 0.061 | 1078.59 (1067.91, 1089.36) | 505.81 (497.84, 513.88) | -2.56 (-2.89, -2.22) | <0.001 | 17.37 (16.04, 18.78) | 7.26 (6.35, 8.29) | -2.96 (-3.38, -2.54) | <0.001 |
| Seychelles | 124.64 (87.45, 173.63) | 119.32 (87.61, 159.58) | -0.14 (-0.17, -0.12) | <0.001 | 1668.52 (1524.20, 1823.91) | 1602.96 (1480.03, 1734.04) | -0.13 (-0.17, -0.09) | <0.001 | 1694.01 (1543.55, 1855.98) | 1104.73 (1005.10, 1212.35) | -1.38 (-1.97, -0.78) | <0.001 | 27.27 (11.24, 56.55) | 16.76 (6.71, 36.04) | -1.58 (-2.27, -0.88) | <0.001 |
| Sierra Leone | 166.54 (159.63, 173.69) | 172.30 (167.84, 176.85) | 0.12 (0.11, 0.13) | <0.001 | 2685.76 (2657.90, 2713.86) | 2838.32 (2820.26, 2856.48) | 0.20 (0.17, 0.23) | <0.001 | 1109.85 (1091.56, 1128.37) | 1143.57 (1131.74, 1155.49) | 0.14 (-0.01, 0.30) | 0.061 | 16.10 (13.94, 18.51) | 16.58 (15.16, 18.10) | 0.15 (-0.03, 0.33) | 0.110 |
| Singapore | 106.33 (101.24, 111.63) | 104.48 (100.29, 108.84) | -0.05 (-0.12, 0.01) | 0.121 | 803.47 (789.38, 817.76) | 781.91 (770.86, 793.12) | -0.08 (-0.15, -0.01) | 0.028 | 697.02 (683.88, 710.36) | 273.84 (267.05, 280.80) | -3.13 (-3.87, -2.38) | <0.001 | 11.03 (9.44, 12.84) | 3.76 (3.02, 4.68) | -3.61 (-4.53, -2.68) | <0.001 |
| Slovakia | 93.07 (89.01, 97.27) | 88.20 (84.04, 92.55) | -0.19 (-0.22, -0.16) | <0.001 | 1181.23 (1166.53, 1196.06) | 1141.13 (1125.67, 1156.78) | -0.10 (-0.14, -0.06) | <0.001 | 978.55 (965.47, 991.77) | 461.47 (452.02, 471.10) | -2.63 (-3.25, -2.01) | <0.001 | 15.54 (13.94, 17.28) | 6.39 (5.35, 7.62) | -3.14 (-3.91, -2.35) | <0.001 |
| Slovenia | 87.96 (81.59, 94.72) | 89.56 (82.36, 97.30) | 0.06 (0.04, 0.08) | <0.001 | 943.53 (922.31, 965.15) | 919.82 (896.25, 943.94) | -0.08 (-0.13, -0.04) | <0.001 | 600.06 (583.45, 617.06) | 215.56 (204.41, 227.26) | -3.52 (-3.79, -3.24) | <0.001 | 9.21 (7.27, 11.55) | 2.48 (1.45, 4.12) | -4.51 (-4.88, -4.13) | <0.001 |
| Solomon Islands | 186.20 (161.13, 214.32) | 173.70 (157.61, 191.02) | -0.24 (-0.25, -0.23) | <0.001 | 1603.58 (1529.74, 1680.34) | 1577.16 (1528.34, 1627.19) | -0.04 (-0.14, 0.06) | 0.419 | 5499.40 (5355.22, 5646.65) | 5516.29 (5422.58, 5611.23) | 0.01 (-0.12, 0.14) | 0.902 | 94.80 (76.55, 116.27) | 95.18 (83.17, 108.45) | 0.01 (-0.12, 0.14) | 0.883 |
| Somalia | 208.73 (203.05, 214.54) | 206.51 (203.26, 209.79) | -0.04 (-0.05, -0.03) | <0.001 | 2880.03 (2858.58, 2901.60) | 2993.59 (2981.05, 3006.17) | 0.13 (0.08, 0.19) | <0.001 | 1546.75 (1530.88, 1562.75) | 1291.05 (1282.54, 1299.60) | -0.60 (-0.72, -0.48) | <0.001 | 23.69 (21.75, 25.75) | 19.12 (18.09, 20.21) | -0.73 (-0.88, -0.57) | <0.001 |
| South Africa | 192.91 (190.72, 195.12) | 177.86 (176.16, 179.56) | -0.28 (-0.30, -0.26) | <0.001 | 2707.83 (2699.60 , | 2661.58 (2655.04 , | -0.05 (-0.09, -0.01) | 0.012 | 1579.57 (1573.04 , | 772.75 (769.29, 776.23) | -2.40 (-3.64, -1.14) | <0.001 | 24.78 (23.96, 25.63) | 10.66 (10.26, 11.07) | -2.78 (-4.18, -1.36) | <0.001 |

| | | | | | | | | | | | | | | | | |
|---|---|---|---|---|---|---|---|---|---|---|---|---|---|---|---|---|
| | | | | | 2716.08) | 2668.14) | | | 1586.12) | | | | | | | |
| South Sudan | 161.75 (156.43, 167.23) | 160.89 (156.53, 165.36) | -0.02 (-0.03, -0.00) | 0.025 | 2755.24 (2732.95, 2777.68) | 2911.89 (2892.89, 2931.00) | 0.20 (0.12, 0.27) | <0.001 | 968.87 (955.08, 982.82) | 752.58 (742.72, 762.53) | -0.81 (-1.15, -0.47) | <0.001 | 13.78 (12.14, 15.59) | 9.83 (8.71, 11.05) | -1.15 (-1.59, -0.72) | <0.001 |
| Spain | 75.64 (74.24, 77.07) | 68.01 (66.63, 69.42) | -0.35 (-0.45, -0.26) | <0.001 | 676.08 (671.86, 680.32) | 659.27 (654.93, 663.63) | -0.06 (-0.17, 0.04) | 0.238 | 686.43 (682.18, 690.70) | 262.93 (260.23, 265.65) | -3.38 (-3.65, -3.12) | <0.001 | 10.78 (10.25, 11.33) | 3.62 (3.32, 3.95) | -3.85 (-4.17, -3.53) | <0.001 |
| Sri Lanka | 73.85 (71.88, 75.87) | 71.80 (69.97, 73.66) | -0.09 (-0.10, -0.07) | <0.001 | 730.30 (724.06, 736.59) | 727.85 (722.01, 733.72) | 0.03 (-0.16, 0.22) | 0.771 | 1347.16 (1338.67, 1355.69) | 764.83 (758.85, 770.83) | -1.85 (-3.13, -0.55) | 0.006 | 21.67 (20.61, 22.79) | 11.60 (10.87, 12.35) | -2.04 (-3.43, -0.63) | 0.005 |
| Sudan | 157.57 (154.62, 160.56) | 158.53 (156.59, 160.48) | 0.02 (0.00, 0.05) | 0.022 | 1789.64 (1779.69, 1799.64) | 1899.79 (1893.08, 1906.51) | 0.23 (0.09, 0.36) | 0.001 | 2466.08 (2454.09, 2478.12) | 1549.74 (1543.59, 1555.92) | -1.58 (-1.67, -1.49) | <0.001 | 40.71 (39.16, 42.30) | 24.44 (23.67, 25.24) | -1.73 (-1.79, -1.68) | <0.001 |
| Suriname | 187.84 (166.48, 211.41) | 193.25 (175.11, 212.78) | 0.09 (0.05, 0.13) | <0.001 | 2128.87 (2055.23, 2204.69) | 2197.95 (2135.73, 2261.55) | 0.12 (0.06, 0.19) | <0.001 | 1462.86 (1399.70, 1528.29) | 1063.39 (1020.31, 1107.84) | -1.01 (-2.41, 0.40) | 0.160 | 23.63 (16.13, 33.60) | 16.57 (11.59, 22.99) | -1.13 (-2.69, 0.46) | 0.162 |
| Sweden | 79.32 (76.17, 82.56) | 87.14 (83.99, 90.39) | 0.33 (0.31, 0.34) | <0.001 | 1004.98 (993.64, 1016.43) | 961.24 (950.67, 971.89) | -0.09 (-0.24, 0.07) | 0.288 | 449.89 (442.37, 457.52) | 229.68 (224.53, 234.92) | -2.24 (-2.42, -2.06) | <0.001 | 6.32 (5.46, 7.28) | 2.40 (1.90, 2.99) | -3.26 (-3.50, -3.02) | <0.001 |
| Switzerland | 65.35 (62.36, 68.47) | 61.06 (58.26, 63.97) | -0.23 (-0.25, -0.21) | <0.001 | 624.98 (615.68, 634.39) | 590.04 (581.32, 598.87) | -0.15 (-0.36, 0.05) | 0.144 | 431.25 (423.53, 439.09) | 159.37 (154.88, 163.98) | -3.44 (-3.84, -3.04) | <0.001 | 6.57 (5.66, 7.62) | 1.94 (1.48, 2.52) | -4.22 (-4.76, -3.67) | <0.001 |
| Syrian Arab Republic | 160.56 (156.74, 164.46) | 142.96 (139.54, 146.45) | -0.40 (-0.44, -0.36) | <0.001 | 1973.35 (1959.83, 1986.95) | 1988.35 (1975.46, 2001.30) | 0.04 (0.01, 0.07) | 0.018 | 3543.66 (3525.45, 3561.93) | 1814.37 (1802.06, 1826.76) | -2.25 (-2.64, -1.85) | <0.001 | 57.64 (55.31, 60.05) | 28.32 (26.79, 29.92) | -2.38 (-2.82, -1.95) | <0.001 |
| Taiwan (Province of China) | 72.36 (70.65, 74.11) | 75.74 (73.88, 77.63) | 0.17 (0.12, 0.21) | <0.001 | 829.27 (823.45, 835.13) | 899.67 (893.31, 906.07) | 0.28 (0.21, 0.35) | <0.001 | 639.16 (634.04, 644.30) | 450.52 (446.09, 454.99) | -1.24 (-1.79, -0.69) | <0.001 | 9.41 (8.80, 10.05) | 6.25 (5.74, 6.79) | -1.45 (-2.11, -0.78) | <0.001 |

| | | | | | | | | | | | | | | | | |
|---|---|---|---|---|---|---|---|---|---|---|---|---|---|---|---|---|
| Tajikistan | 156.07 (150.57, 161.74) | 154.40 (150.56, 158.31) | -0.03 (-0.05, -0.02) | <0.001 | 1668.00 (1649.77, 1686.40) | 1669.76 (1657.07, 1682.54) | 0.00 (-0.03, 0.04) | 0.820 | 1304.56 (1287.75, 1321.55) | 980.41 (970.52, 990.38) | -0.89 (-1.32, -0.47) | <0.001 | 21.05 (18.94, 23.35) | 15.45 (14.22, 16.75) | -0.97 (-1.43, -0.51) | <0.001 |
| Thailand | 118.73 (117.39, 120.09) | 115.11 (113.78, 116.46) | -0.11 (-0.12, -0.10) | <0.001 | 1443.66 (1438.97, 1448.37) | 1414.69 (1409.99, 1419.39) | -0.06 (-0.09, -0.03) | <0.001 | 1003.55 (999.58, 1007.53) | 927.19 (923.46, 930.93) | -0.12 (-1.28, 1.05) | 0.838 | 15.31 (14.82, 15.81) | 13.96 (13.51, 14.42) | -0.16 (-1.46, 1.16) | 0.814 |
| Timor-Leste | 137.07 (124.18, 151.00) | 133.39 (123.11, 144.39) | -0.09 (-0.10, -0.09) | <0.001 | 1451.23 (1408.55, 1494.96) | 1488.72 (1453.73, 1524.43) | 0.10 (0.02, 0.18) | 0.013 | 945.04 (910.03, 981.11) | 1135.45 (1104.14, 1167.48) | 0.69 (0.07, 1.32) | 0.029 | 14.09 (10.07, 19.23) | 17.17 (13.46, 21.65) | 0.71 (0.10, 1.32) | 0.022 |
| Togo | 160.37 (153.47, 167.54) | 160.89 (156.46, 165.41) | 0.01 (0.00, 0.02) | 0.034 | 2617.51 (2589.58, 2645.70) | 2724.60 (2706.33, 2742.97) | 0.14 (0.09, 0.20) | <0.001 | 1204.64 (1184.99, 1224.56) | 1064.64 (1053.00, 1076.38) | -0.41 (-0.68, -0.14) | 0.003 | 17.80 (15.45, 20.44) | 15.39 (14.00, 16.87) | -0.48 (-0.77, -0.18) | 0.002 |
| Tokelau | 118.90 (0.47, 1030.81) | 109.81 (0.12, 1090.92) | -0.27 (-0.28, -0.27) | <0.001 | 1021.58 (354.93, 2369.07) | 1008.25 (310.30, 2470.64) | -0.04 (-0.09, 0.00) | 0.066 | 2360.51 (1237.11, 4136.90) | 2105.61 (993.22, 3953.16) | -0.39 (-0.42, -0.36) | <0.001 | 39.03 (0.00, 892.80) | 34.66 (0.00, 954.30) | -0.40 (-0.44, -0.37) | <0.001 |
| Tonga | 142.61 (103.87, 192.45) | 140.55 (104.67, 185.18) | -0.03 (-0.16, 0.10) | 0.602 | 2198.54 (2039.40, 2368.00) | 2235.71 (2085.46, 2394.30) | 0.07 (-0.02, 0.17) | 0.130 | 1117.49 (998.28, 1247.83) | 1144.27 (1035.01, 1262.18) | 0.11 (-0.06, 0.28) | 0.204 | 16.51 (5.00, 41.26) | 16.79 (6.02, 37.36) | 0.07 (-0.08, 0.22) | 0.383 |
| Trinidad and Tobago | 202.14 (189.79, 215.12) | 195.02 (183.13, 207.52) | -0.12 (-0.14, -0.11) | <0.001 | 2435.62 (2392.20, 2479.65) | 2422.95 (2380.76, 2465.76) | -0.01 (-0.03, 0.01) | 0.387 | 1325.18 (1292.89, 1358.09) | 878.53 (853.87, 903.78) | -1.44 (-2.17, -0.70) | <0.001 | 20.93 (17.03, 25.47) | 13.11 (10.30, 16.55) | -1.63 (-2.46, -0.80) | <0.001 |
| Tunisia | 80.91 (77.75, 84.16) | 83.75 (81.14, 86.41) | 0.13 (0.09, 0.18) | <0.001 | 940.57 (929.85, 951.39) | 1059.14 (1049.73, 1068.61) | 0.39 (0.32, 0.46) | <0.001 | 1007.22 (995.95, 1018.59) | 756.80 (748.97, 764.70) | -0.97 (-1.16, -0.78) | <0.001 | 16.24 (14.82, 17.76) | 11.66 (10.72, 12.67) | -1.13 (-1.31, -0.96) | <0.001 |
| Turkey | 83.76 | 71.63 | -0.54 | <0.001 | 1256.77 | 1123.25 | -0.38 | <0.001 | 1088.51 | 478.06 | -2.79 | <0.001 | 17.28 | 6.61 | -3.23 | <0.001 |

| | | | | | | | | | | | | | | | | |
|---|---|---|---|---|---|---|---|---|---|---|---|---|---|---|---|---|
| | (82.58, 84.96) | (70.75, 72.52) | (-0.61, -0.47) | | (1252.19, 1261.37) | (1119.73, 1126.78) | (-0.44, -0.33) | | (1084.17, 1092.85) | (475.78, 480.35) | (-3.11, -2.47) | | (16.74, 17.84) | (6.34, 6.88) | (-3.58, -2.89) | |
| Turkmenistan | 160.20 (153.75, 166.87) | 177.21 (171.48, 183.08) | 0.36 (0.33, 0.39) | <0.001 | 1747.83 (1726.32, 1769.57) | 1846.19 (1827.60, 1864.92) | 0.19 (0.14, 0.23) | <0.001 | 2069.95 (2045.94, 2094.20) | 2157.21 (2137.17, 2177.39) | 0.18 (-0.72, 1.09) | 0.701 | 33.96 (30.92, 37.24) | 34.99 (32.48, 37.65) | 0.14 (-0.81, 1.09) | 0.776 |
| Tuvalu | 139.17 (44.72, 328.97) | 119.37 (40.88, 274.65) | -0.53 (-0.54, -0.52) | <0.001 | 1028.27 (721.58, 1422.84) | 970.56 (705.19, 1305.94) | -0.18 (-0.22, -0.14) | <0.001 | 3463.11 (2876.54, 4135.45) | 3280.12 (2770.50, 3858.58) | -0.18 (-0.27, -0.10) | <0.001 | 58.20 (7.31, 209.92) | 55.54 (9.04, 183.41) | -0.16 (-0.25, -0.07) | 0.001 |
| Uganda | 190.44 (186.97, 193.96) | 180.26 (178.15, 182.39) | -0.19 (-0.21, -0.17) | <0.001 | 3012.35 (2998.33, 3026.42) | 3240.03 (3230.93, 3249.15) | 0.28 (0.17, 0.38) | <0.001 | 881.98 (874.03, 889.99) | 907.31 (902.28, 912.36) | 0.09 (-0.19, 0.38) | 0.522 | 12.05 (11.11, 13.05) | 12.22 (11.63, 12.84) | -0.01 (-0.37, 0.34) | 0.938 |
| Ukraine | 111.19 (109.73, 112.67) | 126.84 (125.09, 128.62) | 0.46 (0.36, 0.56) | <0.001 | 840.41 (836.39, 844.44) | 938.56 (933.77, 943.38) | 0.39 (0.34, 0.44) | <0.001 | 1143.19 (1138.55, 1147.86) | 2214.82 (2207.68, 2221.98) | 2.21 (-0.05, 4.53) | 0.056 | 18.72 (18.13, 19.32) | 37.54 (36.63, 38.47) | 2.47 (1.08, 3.87) | <0.001 |
| United Arab Emirates | 136.74 (129.39, 144.47) | 141.96 (137.73, 146.31) | 0.13 (0.10, 0.16) | <0.001 | 1590.56 (1565.46, 1616.05) | 1617.26 (1603.01, 1631.63) | 0.10 (-0.01, 0.21) | 0.067 | 1107.44 (1086.58, 1128.68) | 874.74 (865.09, 884.51) | -0.84 (-1.11, -0.56) | <0.001 | 16.98 (14.53, 19.84) | 13.11 (12.02, 14.32) | -0.92 (-1.22, -0.62) | <0.001 |
| United Kingdom | 82.91 (81.69, 84.15) | 78.35 (77.21, 79.51) | -0.19 (-0.26, -0.12) | <0.001 | 867.87 (863.91, 871.85) | 871.26 (867.43, 875.11) | 0.01 (-0.02, 0.04) | 0.511 | 539.39 (536.26, 542.53) | 360.96 (358.53, 363.40) | -1.32 (-1.64, -1.00) | <0.001 | 8.34 (7.96, 8.74) | 5.26 (4.97, 5.56) | -1.57 (-2.00, -1.14) | <0.001 |
| United Republic of Tanzania | 174.47 (171.79, 177.19) | 178.38 (176.61, 180.17) | 0.08 (0.06, 0.10) | <0.001 | 2820.17 (2809.21, 2831.16) | 3043.45 (3036.02, 3050.90) | 0.28 (0.20, 0.37) | <0.001 | 1085.33 (1078.28, 1092.41) | 868.64 (864.57, 872.72) | -0.73 (-0.80, -0.66) | <0.001 | 15.64 (14.79, 16.53) | 11.67 (11.19, 12.16) | -0.96 (-1.04, -0.88) | <0.001 |
| United States of America | 142.07 (141.35, 142.78) | 142.09 (141.40, 142.78) | -0.02 (-0.07, 0.04) | 0.525 | 1652.45 (1650.02 , | 1634.76 (1632.42 , | -0.01 (-0.29, 0.27) | 0.935 | 671.81 (670.26, 673.36) | 564.66 (563.28, 566.03) | -0.57 (-0.85, -0.30) | <0.001 | 10.00 (9.81, 10.19) | 8.02 (7.86, 8.19) | -0.76 (-1.11, -0.41) | <0.001 |

| | | | | | | | | | | | | | | | | |
|---|---|---|---|---|---|---|---|---|---|---|---|---|---|---|---|---|
| | | | | | 1654.89) | 1637.11) | | | | | | | | | | |
| United States Virgin Islands | 107.98 (77.91, 146.12) | 117.35 (82.41, 162.61) | 0.29 (0.26, 0.32) | <0.001 | 975.91 (880.88, 1078.68) | 1018.49 (909.51, 1137.36) | 0.15 (0.13, 0.18) | <0.001 | 1091.92 (991.36, 1200.19) | 578.68 (498.01, 669.20) | -2.13 (-2.68, -1.57) | <0.001 | 18.01 (7.32, 37.32) | 9.13 (1.84, 28.04) | -2.28 (-2.92, -1.64) | <0.001 |
| Uruguay | 69.10 (64.33, 74.13) | 67.50 (62.98, 72.28) | -0.07 (-0.11, -0.03) | 0.001 | 690.67 (675.43, 706.17) | 724.93 (709.88, 740.23) | 0.19 (0.03, 0.36) | 0.023 | 859.54 (842.50, 876.85) | 425.55 (414.09, 437.25) | -2.32 (-2.61, -2.03) | <0.001 | 13.84 (11.75, 16.20) | 6.23 (4.91, 7.79) | -2.63 (-2.96, -2.30) | <0.001 |
| Uzbekistan | 156.24 (153.54, 158.98) | 157.40 (155.34, 159.47) | 0.04 (0.01, 0.06) | 0.014 | 1683.33 (1674.41 , 1692.28) | 1804.79 (1797.81 , 1811.78) | 0.24 (0.19, 0.29) | <0.001 | 1434.86 (1426.43 , 1443.33) | 1596.79 (1590.24 , 1603.36) | 0.43 (-0.02, 0.89) | 0.058 | 22.98 (21.91, 24.08) | 25.65 (24.83, 26.50) | 0.45 (-0.05, 0.95) | 0.075 |
| Vanuatu | 199.60 (163.66, 241.43) | 184.35 (160.02, 211.48) | -0.28 (-0.28, -0.27) | <0.001 | 1966.90 (1851.10 , 2088.37) | 1900.98 (1821.34 , 1983.35) | -0.11 (-0.17, -0.05) | <0.001 | 3359.23 (3203.86 , 3520.42) | 4081.00 (3961.92 , 4202.86) | 0.65 (0.44, 0.86) | <0.001 | 55.53 (37.08, 80.22) | 68.28 (53.58, 85.90) | 0.69 (0.47, 0.90) | <0.001 |
| Venezuela (Bolivarian Republic of) | 79.23 (77.23, 81.26) | 71.57 (69.99, 73.18) | -0.35 (-0.36, -0.33) | <0.001 | 795.60 (789.26, 801.97) | 763.10 (757.91, 768.31) | -0.11 (-0.32, 0.10) | 0.319 | 1052.18 (1044.82 , 1059.58) | 706.52 (701.55, 711.51) | -1.34 (-2.26, -0.40) | 0.005 | 17.38 (16.44, 18.37) | 11.32 (10.70, 11.96) | -1.45 (-2.40, -0.48) | 0.004 |
| Viet Nam | 79.75 (78.66, 80.85) | 76.91 (76.06, 77.76) | -0.11 (-0.19, -0.03) | 0.008 | 752.30 (748.97, 755.65) | 796.72 (793.98, 799.47) | 0.24 (-0.02, 0.50) | 0.073 | 971.07 (967.22, 974.94) | 695.87 (693.34, 698.41) | -1.14 (-1.25, -1.03) | <0.001 | 15.49 (15.00, 15.99) | 10.42 (10.12, 10.74) | -1.36 (-1.48, -1.25) | <0.001 |
| Yemen | 164.75 (160.93, 168.64) | 162.92 (160.70, 165.17) | -0.04 (-0.06, -0.02) | <0.001 | 1868.27 (1855.34 , 1881.27) | 1951.54 (1943.83 , 1959.28) | 0.16 (-0.02, 0.33) | 0.076 | 1822.10 (1808.91 , 1835.37) | 1459.41 (1452.64 , 1466.21) | -0.73 (-0.94, -0.51) | <0.001 | 30.10 (28.41, 31.86) | 23.33 (22.47, 24.21) | -0.83 (-1.10, -0.56) | <0.001 |
| Zambia | 184.31 (179.29, 189.46) | 179.07 (176.04, 182.14) | -0.10 (-0.12, -0.09) | <0.001 | 2826.10 (2806.13 , 2846.19) | 2865.94 (2853.64 , 2878.29) | 0.05 (0.01, 0.08) | 0.014 | 1265.34 (1251.25 , 1279.57) | 1067.85 (1060.08 , 1075.67) | -0.57 (-0.81, -0.34) | <0.001 | 18.78 (17.06, 20.64) | 15.43 (14.49, 16.41) | -0.67 (-0.93, -0.40) | <0.001 |
| Zimbabwe | 161.73 (157.66, 165.89) | 177.68 (174.34, 181.07) | 0.33 (0.30, 0.36) | <0.001 | 2611.79 (2595.29 , | 2720.18 (2707.04 , | 0.14 (0.11, 0.18) | <0.001 | 826.90 (817.48, 836.42) | 1119.77 (1111.27 , | 1.06 (0.52, 1.60) | <0.001 | 11.13 (10.04, 12.31) | 15.97 (14.96, 17.03) | 1.26 (0.72, 1.81) | <0.001 |

| | | | | | | | | | | | | | | | | |
|---|---|---|---|---|---|---|---|---|---|---|---|---|---|---|---|---|
| | | | | | 2628.39) | 2733.37) | | | | 1128.32) | | | | | | |
| **Women** | | | | | | | | | | | | | | | | |
| Afghanistan | 179.23 (172.76, 185.90) | 182.98 (179.58, 186.43) | 0.07 (0.04, 0.09) | <0.001 | 1759.41 (1739.16, 1779.87) | 1814.87 (1804.18, 1825.62) | 0.12 (0.09, 0.15) | <0.001 | 4077.77 (4045.76, 4109.99) | 2557.39 (2544.06, 2570.78) | -1.54 (-1.73, -1.35) | <0.001 | 68.97 (64.82, 73.33) | 42.56 (40.83, 44.34) | -1.59 (-1.78, -1.40) | <0.001 |
| Albania | 126.52 (118.22, 135.29) | 123.35 (113.45, 133.90) | -0.08 (-0.11, -0.05) | <0.001 | 2482.15 (2444.89, 2519.87) | 2521.39 (2475.94, 2567.48) | 0.06 (0.00, 0.12) | 0.051 | 915.28 (892.50, 938.53) | 671.87 (648.55, 695.83) | -1.21 (-1.49, -0.93) | <0.001 | 12.42 (9.87, 15.45) | 8.30 (5.90, 11.39) | -1.58 (-1.94, -1.22) | <0.001 |
| Algeria | 128.13 (124.79, 131.55) | 115.57 (113.32, 117.86) | -0.36 (-0.38, -0.35) | <0.001 | 1651.16 (1639.15, 1663.23) | 1679.79 (1671.16, 1688.45) | 0.08 (-0.01, 0.18) | 0.093 | 1984.66 (1971.21, 1998.19) | 894.74 (888.53, 900.99) | -2.69 (-2.89, -2.49) | <0.001 | 32.05 (30.34, 33.84) | 13.20 (12.47, 13.98) | -2.99 (-3.20, -2.78) | <0.001 |
| American Samoa | 129.97 (67.77, 229.53) | 149.83 (82.65, 252.89) | 0.49 (0.48, 0.51) | <0.001 | 1942.81 (1672.63, 2247.02) | 2025.75 (1752.46, 2331.98) | 0.16 (0.09, 0.22) | <0.001 | 1133.20 (925.85, 1375.62) | 1307.45 (1085.34, 1563.58) | 0.53 (0.38, 0.68) | <0.001 | 16.25 (1.10, 75.50) | 19.12 (1.84, 78.30) | 0.59 (0.42, 0.76) | <0.001 |
| Andorra | 56.01 (21.94, 123.59) | 56.95 (23.24, 121.68) | 0.07 (0.02, 0.11) | 0.008 | 720.07 (575.73, 893.64) | 733.57 (592.57, 901.66) | 0.08 (0.01, 0.16) | 0.036 | 266.48 (181.91, 381.40) | 185.89 (119.29, 280.83) | -1.19 (-1.35, -1.02) | <0.001 | 3.48 (0.00, 48.42) | 2.14 (0.00, 42.97) | -1.62 (-1.84, -1.41) | <0.001 |
| Angola | 177.84 (171.88, 183.97) | 161.32 (158.05, 164.64) | -0.34 (-0.35, -0.32) | <0.001 | 2982.77 (2958.10, 3007.61) | 3075.52 (3061.08, 3090.03) | 0.12 (0.05, 0.18) | <0.001 | 1161.63 (1145.77, 1177.66) | 738.47 (731.18, 745.82) | -1.54 (-1.86, -1.23) | <0.001 | 16.83 (14.94, 18.89) | 9.45 (8.62, 10.33) | -1.95 (-2.37, -1.52) | <0.001 |
| Antigua and Barbuda | 173.19 (109.46, 261.74) | 170.49 (115.07, 244.54) | -0.05 (-0.07, -0.03) | <0.001 | 2774.84 (2497.03, 3076.05) | 2770.63 (2530.82, 3028.01) | -0.00 (-0.03, 0.03) | 0.884 | 869.48 (716.20, 1046.75) | 477.25 (381.52, 590.88) | -2.11 (-2.39, -1.82) | <0.001 | 12.42 (1.00, 52.99) | 5.57 (0.15, 33.72) | -2.79 (-3.65, -1.92) | <0.001 |
| Argentina | 110.23 (107.62, 112.89) | 102.11 (100.00, 104.25) | -0.26 (-0.27, -0.26) | <0.001 | 1810.60 (1799.97, 1821.29) | 1914.98 (1905.86, 1924.14) | 0.20 (0.18, 0.22) | <0.001 | 1018.39 (1010.39, 1026.43) | 484.96 (480.39, 489.56) | -2.52 (-2.88, -2.15) | <0.001 | 15.34 (14.37, 16.35) | 6.08 (5.58, 6.61) | -3.12 (-3.60, -2.64) | <0.001 |

| | | | | | | | | | | | | | | | | |
|---|---|---|---|---|---|---|---|---|---|---|---|---|---|---|---|---|
| Armenia | 144.86 (136.21, 153.93) | 137.32 (127.57, 147.72) | -0.17 (-0.21, -0.14) | <0.001 | 2120.31 (2086.96, 2154.08) | 2022.14 (1984.92, 2059.99) | -0.16 (-0.18, -0.14) | <0.001 | 666.62 (647.99, 685.68) | 342.07 (327.18, 357.58) | -2.24 (-2.89, -1.58) | <0.001 | 8.67 (6.67, 11.12) | 3.29 (2.02, 5.27) | -3.25 (-4.19, -2.30) | <0.001 |
| Australia | 63.05 (60.43, 65.76) | 64.18 (61.82, 66.61) | 0.06 (0.04, 0.07) | <0.001 | 796.18 (786.81, 805.64) | 801.03 (792.67, 809.47) | 0.02 (-0.04, 0.08) | 0.587 | 331.80 (325.76, 337.93) | 202.68 (198.49, 206.95) | -1.67 (-2.19, -1.16) | <0.001 | 4.38 (3.72, 5.14) | 2.28 (1.86, 2.77) | -2.21 (-2.89, -1.52) | <0.001 |
| Austria | 78.44 (74.01, 83.07) | 75.40 (70.97, 80.08) | -0.13 (-0.15, -0.10) | <0.001 | 1092.16 (1075.34, 1109.20) | 1045.20 (1028.29, 1062.34) | -0.14 (-0.22, -0.06) | <0.001 | 401.06 (390.90, 411.43) | 184.66 (177.63, 191.92) | -2.73 (-3.33, -2.13) | <0.001 | 5.45 (4.33, 6.80) | 1.83 (1.21, 2.72) | -3.80 (-4.32, -3.28) | <0.001 |
| Azerbaijan | 148.19 (142.22, 154.36) | 138.11 (133.07, 143.31) | -0.24 (-0.25, -0.23) | <0.001 | 1990.73 (1968.62, 2013.05) | 1942.24 (1923.42, 1961.22) | -0.08 (-0.10, -0.06) | <0.001 | 1246.07 (1228.47, 1263.87) | 829.74 (817.54, 842.10) | -1.43 (-2.05, -0.80) | <0.001 | 18.68 (16.56, 21.00) | 11.51 (10.13, 13.06) | -1.68 (-2.07, -1.29) | <0.001 |
| Bahamas | 179.42 (146.74, 217.62) | 180.26 (151.47, 213.08) | 0.03 (-0.01, 0.06) | 0.171 | 2454.75 (2329.15, 2585.71) | 2445.74 (2336.46, 2558.94) | -0.01 (-0.05, 0.04) | 0.715 | 1147.67 (1060.82, 1240.02) | 892.10 (827.07, 961.04) | -1.07 (-1.31, -0.83) | <0.001 | 17.70 (8.41, 33.16) | 13.25 (6.46, 24.33) | -1.21 (-1.54, -0.89) | <0.001 |
| Bahrain | 86.72 (69.10, 107.74) | 82.71 (71.84, 94.93) | -0.16 (-0.23, -0.08) | <0.001 | 1221.73 (1152.66, 1294.08) | 1180.43 (1138.31, 1223.88) | -0.09 (-0.18, 0.00) | 0.054 | 965.45 (903.82, 1030.40) | 388.49 (363.92, 414.42) | -3.10 (-3.92, -2.29) | <0.001 | 13.88 (7.40, 24.05) | 4.64 (2.35, 8.47) | -3.61 (-4.78, -2.42) | <0.001 |
| Bangladesh | 146.96 (145.28, 148.65) | 139.83 (138.59, 141.07) | -0.18 (-0.19, -0.16) | <0.001 | 1910.52 (1904.28, 1916.79) | 1912.01 (1907.41, 1916.62) | 0.03 (-0.18, 0.24) | 0.780 | 1535.63 (1529.94, 1541.34) | 800.60 (797.61, 803.60) | -2.14 (-2.92, -1.36) | <0.001 | 23.95 (23.24, 24.69) | 11.30 (10.95, 11.67) | -2.37 (-2.99, -1.76) | <0.001 |
| Barbados | 175.61 (142.46, 214.36) | 180.31 (145.12, 221.77) | 0.10 (0.04, 0.16) | 0.001 | 2560.75 (2429.44, 2697.51) | 2597.47 (2459.13, 2741.86) | 0.05 (0.03, 0.08) | <0.001 | 718.79 (650.02, 793.05) | 528.63 (467.72, 595.61) | -1.14 (-1.50, -0.77) | <0.001 | 9.85 (3.41, 22.51) | 6.61 (1.59, 18.78) | -1.48 (-1.95, -1.02) | <0.001 |
| Belarus | 95.59 (91.41, | 96.63 (91.86, | 0.04 (0.03, | <0.001 | 900.91 (887.99, | 928.16 (913.34, | 0.12 (0.05, | 0.001 | 587.87 (577.44, | 493.64 (483.17, | -0.64 (-1.81, | 0.291 | 8.50 (7.29, | 6.92 (5.76, | -0.77 (-2.20, | 0.299 |

| | | | | | | | | | | | | | | | | |
|---|---|---|---|---|---|---|---|---|---|---|---|---|---|---|---|---|
| | 99.92) | 101.62) | 0.05) | | 913.99) | 943.20) | 0.18) | | 598.44) | 504.33) | 0.55) | | 9.86) | 8.31) | 0.69) | |
| Belgium | 60.35 (56.90, 63.98) | 58.80 (55.31, 62.46) | -0.08 (-0.16, 0.00) | 0.058 | 966.46 (952.45, 980.64) | 1052.22 (1037.26, 1067.36) | 0.31 (0.22, 0.40) | <0.001 | 456.46 (446.85, 466.24) | 206.55 (199.98, 213.30) | -2.69 (-3.17, -2.20) | <0.001 | 6.51 (5.42, 7.78) | 2.16 (1.55, 2.97) | -3.72 (-4.31, -3.13) | <0.001 |
| Belize | 177.99 (135.63, 230.90) | 185.72 (158.50, 216.43) | 0.15 (0.12, 0.19) | <0.001 | 2399.17 (2235.31, 2573.07) | 2608.17 (2502.87, 2716.87) | 0.29 (0.24, 0.34) | <0.001 | 668.47 (580.86, 766.63) | 652.15 (599.55, 708.23) | -0.23 (-0.81, 0.35) | 0.430 | 9.22 (1.73, 29.91) | 8.72 (3.63, 17.72) | -0.41 (-1.16, 0.34) | 0.286 |
| Benin | 140.93 (133.25, 148.96) | 141.64 (136.93, 146.48) | 0.02 (0.01, 0.03) | <0.001 | 2510.27 (2477.55, 2543.35) | 2642.99 (2622.49, 2663.64) | 0.19 (0.15, 0.23) | <0.001 | 834.24 (814.92, 853.94) | 751.02 (739.79, 762.40) | -0.36 (-0.57, -0.14) | 0.001 | 11.50 (9.30, 14.09) | 9.95 (8.67, 11.37) | -0.49 (-0.74, -0.24) | <0.001 |
| Bermuda | 107.17 (59.55, 184.22) | 107.63 (52.60, 202.46) | 0.03 (-0.02, 0.08) | 0.279 | 1404.26 (1210.94, 1624.03) | 1362.53 (1138.86, 1621.92) | -0.11 (-0.20, -0.03) | 0.012 | 527.47 (411.72, 670.47) | 240.67 (152.59, 366.85) | -2.92 (-3.25, -2.59) | <0.001 | 7.53 (0.21, 52.53) | 2.65 (0.00, 56.91) | -3.90 (-4.46, -3.34) | <0.001 |
| Bhutan | 144.12 (122.66, 168.61) | 129.38 (112.61, 147.98) | -0.37 (-0.38, -0.35) | <0.001 | 1667.76 (1590.90, 1747.64) | 1629.19 (1568.16, 1692.04) | -0.06 (-0.18, 0.06) | 0.349 | 1329.26 (1259.04, 1402.59) | 685.95 (646.40, 727.33) | -2.28 (-2.55, -2.01) | <0.001 | 21.00 (12.94, 32.49) | 9.96 (5.72, 16.16) | -2.56 (-2.85, -2.27) | <0.001 |
| Bolivia (Plurinational State of) | 158.81 (151.84, 166.03) | 136.23 (131.60, 140.99) | -0.53 (-0.56, -0.49) | <0.001 | 2140.00 (2114.02, 2166.24) | 2118.93 (2100.53, 2137.45) | -0.02 (-0.05, 0.01) | 0.133 | 1555.35 (1532.94, 1578.02) | 722.43 (711.63, 733.35) | -2.60 (-2.70, -2.50) | <0.001 | 24.22 (21.47, 27.23) | 10.17 (8.92, 11.55) | -2.93 (-3.03, -2.82) | <0.001 |
| Bosnia and Herzegovina | 79.56 (73.96, 85.48) | 86.29 (78.61, 94.57) | 0.28 (0.24, 0.32) | <0.001 | 1101.13 (1079.96, 1122.62) | 1159.25 (1130.33, 1188.77) | 0.21 (0.08, 0.34) | 0.001 | 542.28 (527.50, 557.39) | 420.79 (403.62, 438.56) | -0.91 (-1.37, -0.44) | <0.001 | 7.28 (5.66, 9.23) | 5.10 (3.39, 7.45) | -1.25 (-2.50, 0.02) | 0.054 |
| Botswana | 163.72 (148.66, 180.05) | 162.95 (152.24, 174.23) | -0.01 (-0.03, 0.00) | 0.071 | 2578.20 (2516.79, 2640.85) | 2604.34 (2561.12, 2648.13) | 0.05 (-0.01, 0.11) | 0.089 | 880.20 (842.94, 918.78) | 764.92 (741.77, 788.63) | -0.46 (-0.92, 0.00) | 0.050 | 12.34 (8.22, 17.91) | 10.29 (7.78, 13.39) | -0.62 (-1.15, -0.08) | 0.025 |

| | | | | | | | | | | | | | | | | |
|---|---|---|---|---|---|---|---|---|---|---|---|---|---|---|---|---|
| Brazil | 163.41 (162.00, 164.84) | 140.77 (139.65, 141.90) | -0.51 (-0.55, -0.48) | <0.001 | 2579.38 (2573.73, 2585.03) | 2526.51 (2521.79, 2531.24) | -0.06 (-0.14, 0.03) | 0.220 | 1293.83 (1289.74, 1297.92) | 639.52 (637.18, 641.86) | -2.39 (-2.53, -2.26) | <0.001 | 20.03 (19.53, 20.55) | 8.57 (8.30, 8.84) | -2.87 (-3.03, -2.72) | <0.001 |
| Brunei Darussalam | 93.55 (70.00, 122.79) | 82.07 (65.04, 102.52) | -0.45 (-0.48, -0.43) | <0.001 | 851.02 (776.24, 931.28) | 826.54 (770.58, 885.79) | -0.05 (-0.22, 0.13) | 0.612 | 979.24 (898.75, 1065.23) | 486.72 (443.52, 533.27) | -2.38 (-2.55, -2.22) | <0.001 | 15.45 (6.89, 30.16) | 6.96 (2.74, 15.03) | -2.73 (-2.97, -2.50) | <0.001 |
| Bulgaria | 83.55 (79.01, 88.28) | 83.10 (77.59, 88.95) | -0.02 (-0.03, -0.01) | <0.001 | 1713.50 (1692.45, 1734.75) | 1728.99 (1702.15, 1756.19) | 0.06 (-0.07, 0.18) | 0.384 | 1077.48 (1061.06, 1094.10) | 783.85 (766.61, 801.42) | -1.09 (-1.42, -0.76) | <0.001 | 15.84 (13.92, 17.96) | 10.86 (8.96, 13.11) | -1.32 (-1.72, -0.92) | <0.001 |
| Burkina Faso | 137.68 (132.13, 143.42) | 136.02 (132.59, 139.52) | -0.04 (-0.05, -0.03) | <0.001 | 2346.31 (2323.13, 2369.68) | 2396.87 (2382.34, 2411.48) | 0.08 (0.05, 0.11) | <0.001 | 889.75 (874.99, 904.71) | 791.56 (782.99, 800.19) | -0.38 (-0.68, -0.07) | 0.015 | 12.80 (11.06, 14.74) | 10.98 (9.98, 12.06) | -0.49 (-0.86, -0.13) | 0.009 |
| Burundi | 208.91 (200.20, 217.93) | 187.41 (181.91, 193.04) | -0.38 (-0.41, -0.34) | <0.001 | 3266.55 (3231.76, 3301.66) | 3340.68 (3316.99, 3364.51) | 0.09 (0.02, 0.16) | 0.015 | 1614.59 (1589.38, 1640.13) | 924.45 (911.63, 937.40) | -1.92 (-2.11, -1.73) | <0.001 | 24.15 (21.11, 27.52) | 12.21 (10.76, 13.82) | -2.37 (-2.63, -2.11) | <0.001 |
| Cabo Verde | 132.12 (105.36, 164.46) | 126.12 (106.74, 148.08) | -0.15 (-0.18, -0.13) | <0.001 | 3231.07 (3093.64, 3373.76) | 3386.96 (3283.32, 3493.11) | 0.17 (0.15, 0.19) | <0.001 | 1056.70 (975.73, 1143.22) | 564.88 (522.92, 609.38) | -2.16 (-2.36, -1.97) | <0.001 | 14.55 (6.41, 29.23) | 5.99 (2.42, 12.40) | -3.12 (-3.87, -2.37) | <0.001 |
| Cambodia | 120.77 (115.96, 125.73) | 107.27 (103.88, 110.75) | -0.41 (-0.42, -0.39) | <0.001 | 1303.94 (1288.01, 1320.04) | 1279.37 (1267.59, 1291.23) | -0.06 (-0.15, 0.03) | 0.182 | 1506.45 (1488.98, 1524.07) | 772.96 (763.78, 782.22) | -2.25 (-2.40, -2.09) | <0.001 | 23.98 (21.80, 26.33) | 11.31 (10.22, 12.48) | -2.54 (-2.71, -2.36) | <0.001 |
| Cameroon | 136.33 (131.12, 141.71) | 143.36 (140.34, 146.43) | 0.18 (0.15, 0.20) | <0.001 | 2308.03 (2286.41, 2329.83) | 2541.55 (2528.77, 2554.39) | 0.33 (0.30, 0.36) | <0.001 | 989.52 (974.82, 1004.39) | 839.95 (832.40, 847.55) | -0.54 (-0.68, -0.41) | <0.001 | 14.46 (12.71, 16.41) | 11.62 (10.73, 12.56) | -0.73 (-0.87, -0.58) | <0.001 |
| Canada | 99.09 (96.56, | 91.23 (88.83, | -0.27 (-0.32, | <0.001 | 1699.34 (1688.78 | 1576.53 (1566.52 | -0.23 (-0.39, | 0.007 | 362.38 (357.50, | 253.26 (249.25, | -1.21 (-1.62, | <0.001 | 4.08 (3.59, | 2.44 (2.06, | -1.78 (-2.27, | <0.001 |

| | | | | | | | | | | | | | | | | |
|---|---|---|---|---|---|---|---|---|---|---|---|---|---|---|---|---|
| | 101.68) | 93.68) | -0.22) | | , 1709.95) | , 1586.59) | -0.06) | | 367.31) | 257.33) | -0.81) | | 4.64) | 2.87) | -1.29) | |
| Central African Republic | 191.27 (179.53, 203.65) | 199.81 (191.41, 208.51) | 0.15 (0.14, 0.16) | <0.001 | 2984.23 (2937.18 , 3031.92) | 3091.94 (3058.48 , 3125.70) | 0.13 (0.10, 0.16) | <0.001 | 1251.40 (1219.48 , 1283.98) | 1122.49 (1101.53 , 1143.78) | -0.29 (-0.66, 0.09) | 0.130 | 18.58 (14.82, 23.07) | 16.25 (13.77, 19.05) | -0.36 (-0.80, 0.08) | 0.106 |
| Chad | 143.25 (136.17, 150.63) | 144.87 (140.46, 149.40) | 0.04 (0.03, 0.04) | <0.001 | 2342.79 (2313.77 , 2372.11) | 2431.65 (2413.39 , 2450.02) | 0.15 (0.06, 0.24) | 0.001 | 978.90 (959.67, 998.44) | 873.56 (862.22, 885.01) | -0.34 (-0.58, -0.11) | 0.005 | 14.21 (11.95, 16.79) | 12.30 (10.96, 13.76) | -0.45 (-0.72, -0.17) | 0.002 |
| Chile | 61.82 (58.95, 64.79) | 61.11 (58.54, 63.78) | -0.03 (-0.09, 0.04) | 0.461 | 828.22 (817.68, 838.86) | 862.92 (853.15, 872.78) | 0.15 (0.08, 0.22) | <0.001 | 457.53 (449.64, 465.52) | 269.02 (263.58, 274.55) | -1.75 (-1.89, -1.62) | <0.001 | 6.50 (5.59, 7.53) | 3.25 (2.68, 3.91) | -2.27 (-2.44, -2.10) | <0.001 |
| China | 101.92 (101.53, 102.31) | 87.02 (86.65, 87.40) | -0.56 (-0.65, -0.48) | <0.001 | 1308.40 (1307.00 , 1309.80) | 1229.84 (1228.44 , 1231.24) | -0.21 (-0.24, -0.18) | <0.001 | 986.96 (985.73, 988.18) | 434.80 (433.98, 435.62) | -2.89 (-3.57, -2.21) | <0.001 | 14.83 (14.68, 14.98) | 5.30 (5.21, 5.39) | -3.63 (-4.29, -2.96) | <0.001 |
| Colombia | 77.54 (75.45, 79.67) | 63.63 (62.05, 65.25) | -0.68 (-0.73, -0.64) | <0.001 | 1108.31 (1100.43 , 1116.24) | 1052.06 (1045.59 , 1058.56) | -0.16 (-0.32, 0.01) | 0.060 | 809.65 (802.84, 816.50) | 387.32 (383.41, 391.27) | -2.46 (-2.81, -2.11) | <0.001 | 12.64 (11.79, 13.53) | 5.35 (4.90, 5.83) | -2.85 (-3.24, -2.47) | <0.001 |
| Comoros | 195.11 (166.18, 228.11) | 177.57 (156.64, 200.65) | -0.33 (-0.35, -0.31) | <0.001 | 3436.50 (3310.61 , 3566.37) | 3427.27 (3332.43 , 3524.23) | -0.01 (-0.03, 0.02) | 0.599 | 1191.81 (1115.43 , 1272.36) | 887.92 (839.25, 938.76) | -1.00 (-3.11, 1.17) | 0.364 | 16.52 (8.56, 29.22) | 11.46 (6.52, 18.79) | -1.23 (-3.94, 1.55) | 0.381 |
| Congo | 199.90 (187.06, 213.46) | 178.51 (170.65, 186.64) | -0.39 (-0.42, -0.37) | <0.001 | 3185.82 (3134.06 , 3238.30) | 3178.60 (3145.07 , 3212.40) | -0.01 (-0.05, 0.03) | 0.505 | 1299.41 (1264.69 , 1334.90) | 897.56 (879.64, 915.76) | -1.32 (-2.00, -0.64) | <0.001 | 19.14 (15.07, 24.02) | 12.24 (10.22, 14.54) | -1.58 (-2.34, -0.82) | <0.001 |
| Cook Islands | 97.98 (22.95, 278.66) | 100.76 (21.62, 291.92) | 0.12 (0.06, 0.18) | <0.001 | 1096.93 (777.43, 1509.35) | 1123.05 (780.90, 1565.74) | 0.10 (0.01, 0.19) | 0.026 | 1409.23 (1038.83 , 1873.22) | 755.61 (480.48, 1132.64) | -2.12 (-2.19, -2.04) | <0.001 | 21.98 (0.18, 161.09) | 10.52 (0.00, 144.57) | -2.50 (-2.58, -2.42) | <0.001 |

| | | | | | | | | | | | | | | | | |
|---|---|---|---|---|---|---|---|---|---|---|---|---|---|---|---|---|
| Costa Rica | 137.08 (128.15, 146.50) | 132.24 (125.12, 139.69) | -0.12 (-0.14, -0.09) | <0.001 | 2642.48 (2602.61, 2682.83) | 2640.77 (2608.88, 2672.96) | 0.00 (-0.02, 0.02) | 0.927 | 647.01 (627.03, 667.50) | 445.11 (432.18, 458.36) | -1.27 (-1.51, -1.03) | <0.001 | 8.56 (6.38, 11.28) | 5.05 (3.77, 6.66) | -1.61 (-2.15, -1.07) | <0.001 |
| Cote d'Ivoire | 150.77 (145.65, 156.05) | 152.22 (148.89, 155.60) | 0.04 (0.01, 0.06) | 0.002 | 2509.14 (2488.18, 2530.24) | 2590.87 (2577.11, 2604.70) | 0.12 (0.09, 0.16) | <0.001 | 984.20 (970.58, 997.98) | 813.59 (805.77, 821.47) | -0.65 (-0.85, -0.44) | <0.001 | 14.03 (12.41, 15.81) | 11.03 (10.13, 11.99) | -0.82 (-1.08, -0.56) | <0.001 |
| Croatia | 77.38 (71.83, 83.28) | 74.99 (68.55, 81.93) | -0.11 (-0.15, -0.07) | <0.001 | 1002.52 (982.29, 1023.08) | 956.90 (933.58, 980.72) | -0.13 (-0.30, 0.05) | 0.151 | 509.84 (495.50, 524.52) | 229.26 (217.95, 241.07) | -2.77 (-3.38, -2.15) | <0.001 | 7.01 (5.43, 8.94) | 2.30 (1.32, 3.84) | -3.73 (-4.61, -2.84) | <0.001 |
| Cuba | 173.17 (167.84, 178.62) | 167.79 (161.80, 173.96) | -0.10 (-0.13, -0.07) | <0.001 | 2284.33 (2264.79, 2303.99) | 2247.66 (2225.66, 2269.82) | -0.05 (-0.08, -0.03) | <0.001 | 823.91 (811.93, 836.02) | 398.38 (389.20, 407.72) | -2.42 (-2.60, -2.24) | <0.001 | 12.21 (10.78, 13.78) | 4.76 (3.82, 5.89) | -3.11 (-3.35, -2.86) | <0.001 |
| Cyprus | 46.45 (36.24, 58.76) | 46.95 (38.87, 56.59) | 0.04 (0.02, 0.06) | <0.001 | 746.80 (704.04, 791.58) | 829.27 (794.53, 865.46) | 0.44 (0.17, 0.72) | 0.002 | 393.03 (362.09, 426.00) | 177.30 (161.37, 194.71) | -2.71 (-3.46, -1.96) | <0.001 | 5.71 (2.56, 11.12) | 1.83 (0.59, 5.03) | -3.84 (-4.98, -2.68) | <0.001 |
| Czechia | 89.15 (84.90, 93.56) | 89.21 (84.59, 94.05) | 0.02 (-0.01, 0.04) | 0.175 | 1760.80 (1741.63, 1780.14) | 1707.29 (1686.45, 1728.35) | -0.10 (-0.17, -0.04) | 0.002 | 549.77 (539.23, 560.49) | 296.33 (287.80, 305.08) | -2.16 (-2.89, -1.42) | <0.001 | 6.89 (5.77, 8.18) | 2.63 (1.90, 3.59) | -3.32 (-4.35, -2.27) | <0.001 |
| Democratic People's Republic of Korea | 103.03 (99.95, 106.18) | 105.37 (102.48, 108.33) | 0.08 (0.06, 0.10) | <0.001 | 1258.40 (1247.56, 1269.33) | 1295.78 (1285.63, 1306.00) | 0.13 (0.02, 0.23) | 0.016 | 1081.50 (1071.32, 1091.76) | 949.04 (940.39, 957.76) | -0.46 (-0.52, -0.39) | <0.001 | 16.47 (15.23, 17.80) | 14.09 (13.06, 15.19) | -0.55 (-0.64, -0.45) | <0.001 |
| Democratic Republic of the Congo | 174.37 (171.28, 177.49) | 171.09 (169.13, 173.07) | -0.06 (-0.10, -0.02) | 0.007 | 3018.11 (3005.17, 3031.11) | 3047.18 (3038.80, 3055.58) | 0.05 (-0.01, 0.12) | 0.098 | 1065.45 (1057.47, 1073.48) | 775.00 (770.61, 779.41) | -1.17 (-1.42, -0.92) | <0.001 | 15.25 (14.30, 16.26) | 10.26 (9.75, 10.79) | -1.38 (-1.51, -1.25) | <0.001 |
| Denmark | 74.74 (69.35, 80.47) | 63.61 (58.50, 69.08) | -0.55 (-0.57, -0.52) | <0.001 | 1134.74 (1113.33, | 1034.30 (1013.27, | -0.32 (-0.39, -0.24) | <0.001 | 364.12 (352.13, 376.43) | 161.82 (153.58, 170.40) | -2.75 (-3.61, -1.89) | <0.001 | 4.85 (3.57, 6.48) | 1.45 (0.77, 2.51) | -4.05 (-5.30, -2.79) | <0.001 |

| | | | | | | | | | | | | | | | | |
|---|---|---|---|---|---|---|---|---|---|---|---|---|---|---|---|---|
| | | | | | 1156.48) | 1055.68) | | | | | | | | | | |
| Djibouti | 165.71 (139.63, 195.69) | 166.75 (150.69, 184.11) | 0.02 (0.01, 0.02) | <0.001 | 3118.37 (3000.23, 3240.31) | 3349.06 (3276.41, 3422.99) | 0.26 (0.19, 0.33) | <0.001 | 873.03 (808.74, 941.34) | 664.75 (633.08, 697.67) | -0.92 (-1.31, -0.52) | <0.001 | 11.59 (5.22, 22.57) | 7.75 (4.72, 12.15) | -1.35 (-1.86, -0.83) | <0.001 |
| Dominica | 184.96 (118.91, 277.20) | 184.44 (117.19, 276.50) | -0.01 (-0.06, 0.05) | 0.859 | 2505.79 (2240.62, 2795.65) | 2525.10 (2255.06, 2818.78) | 0.04 (-0.01, 0.09) | 0.107 | 1137.39 (958.10, 1342.22) | 828.70 (677.31, 1004.06) | -1.11 (-1.74, -0.48) | 0.001 | 17.16 (2.23, 63.51) | 11.78 (0.83, 51.23) | -1.25 (-1.66, -0.84) | <0.001 |
| Dominican Republic | 174.72 (168.10, 181.56) | 183.04 (177.44, 188.78) | 0.17 (0.15, 0.18) | <0.001 | 2302.22 (2277.87, 2326.78) | 2382.06 (2361.72, 2402.53) | 0.12 (0.09, 0.16) | <0.001 | 1245.28 (1227.00, 1263.79) | 1224.59 (1209.95, 1239.37) | -0.10 (-0.55, 0.36) | 0.684 | 19.25 (17.01, 21.72) | 18.97 (17.18, 20.89) | -0.10 (-0.62, 0.42) | 0.699 |
| Ecuador | 134.67 (129.68, 139.81) | 125.94 (122.30, 129.66) | -0.23 (-0.25, -0.22) | <0.001 | 2136.51 (2116.27, 2156.92) | 2132.24 (2117.13, 2147.43) | 0.01 (-0.05, 0.06) | 0.803 | 1134.41 (1119.47, 1149.51) | 630.70 (622.46, 639.01) | -2.03 (-2.67, -1.38) | <0.001 | 17.11 (15.30, 19.09) | 8.54 (7.60, 9.56) | -2.40 (-3.17, -1.62) | <0.001 |
| Egypt | 136.61 (134.34, 138.90) | 137.12 (135.49, 138.76) | 0.01 (-0.00, 0.03) | 0.081 | 2425.47 (2415.96, 2435.01) | 2579.32 (2572.26, 2586.39) | 0.23 (0.15, 0.30) | <0.001 | 2803.49 (2793.22, 2813.78) | 1552.38 (1546.89, 1557.87) | -1.96 (-2.61, -1.31) | <0.001 | 44.14 (42.85, 45.46) | 23.18 (22.51, 23.86) | -2.13 (-2.86, -1.40) | <0.001 |
| El Salvador | 142.11 (134.97, 149.56) | 134.30 (128.18, 140.65) | -0.19 (-0.23, -0.14) | <0.001 | 2298.87 (2269.50, 2328.55) | 2381.89 (2355.78, 2408.23) | 0.14 (0.06, 0.21) | <0.001 | 1191.59 (1170.12, 1213.38) | 498.18 (486.19, 510.39) | -3.01 (-3.60, -2.41) | <0.001 | 17.90 (15.33, 20.82) | 6.16 (4.89, 7.68) | -3.66 (-4.37, -2.95) | <0.001 |
| Equatorial Guinea | 189.20 (160.26, 222.12) | 148.29 (134.36, 163.39) | -0.85 (-0.91, -0.78) | <0.001 | 3055.12 (2934.86, 3179.27) | 3009.18 (2944.74, 3074.78) | -0.05 (-0.10, 0.01) | 0.084 | 1198.31 (1121.35, 1279.32) | 557.46 (529.06, 587.07) | -2.56 (-3.15, -1.97) | <0.001 | 17.54 (9.32, 30.22) | 6.40 (3.65, 10.51) | -3.35 (-3.88, -2.82) | <0.001 |
| Eritrea | 204.82 (193.19, 217.03) | 192.16 (184.97, 199.58) | -0.22 (-0.26, -0.19) | <0.001 | 3437.21 (3388.46, 3486.52) | 3518.68 (3487.22, 3550.38) | 0.09 (0.00, 0.18) | 0.047 | 1184.72 (1154.97, 1215.07) | 844.68 (828.75, 860.86) | -1.17 (-1.33, -1.01) | <0.001 | 16.99 (13.55, 21.08) | 10.97 (9.19, 13.01) | -1.52 (-1.67, -1.37) | <0.001 |

| | | | | | | | | | | | | | | | | |
|---|---|---|---|---|---|---|---|---|---|---|---|---|---|---|---|---|
| Estonia | 109.03 (97.45, 121.70) | 111.52 (97.59, 127.16) | 0.08 (0.06, 0.10) | <0.001 | 1100.68 (1062.95, 1139.48) | 1121.38 (1075.80, 1168.64) | 0.07 (0.02, 0.12) | 0.010 | 528.51 (502.62, 555.47) | 259.58 (238.01, 282.84) | -2.15 (-2.91, -1.39) | <0.001 | 7.38 (4.64, 11.27) | 2.70 (0.97, 6.50) | -3.26 (-5.24, -1.24) | 0.002 |
| Eswatini | 165.65 (146.15, 187.33) | 164.94 (149.64, 181.46) | -0.01 (-0.04, 0.03) | 0.629 | 2514.58 (2435.99, 2595.32) | 2522.14 (2461.08, 2584.40) | 0.02 (-0.01, 0.05) | 0.249 | 866.64 (818.79, 916.75) | 689.98 (657.70, 723.50) | -0.84 (-1.29, -0.39) | <0.001 | 12.10 (6.97, 19.78) | 9.06 (5.68, 13.79) | -1.07 (-1.66, -0.49) | <0.001 |
| Ethiopia | 178.40 (175.71, 181.12) | 162.59 (160.91, 164.28) | -0.32 (-0.34, -0.30) | <0.001 | 2794.70 (2783.95, 2805.48) | 3012.04 (3004.68, 3019.41) | 0.26 (0.24, 0.28) | <0.001 | 1531.00 (1522.78, 1539.25) | 612.10 (608.70, 615.52) | -3.10 (-3.26, -2.93) | <0.001 | 23.35 (22.33, 24.41) | 7.22 (6.85, 7.61) | -3.95 (-4.17, -3.74) | <0.001 |
| Fiji | 126.87 (109.63, 146.15) | 130.33 (114.02, 148.34) | 0.10 (0.09, 0.11) | <0.001 | 1573.49 (1511.25, 1637.73) | 1806.02 (1743.77, 1869.96) | 0.48 (0.41, 0.55) | <0.001 | 2155.95 (2082.79, 2231.11) | 1725.29 (1664.57, 1787.68) | -0.77 (-0.98, -0.55) | <0.001 | 33.80 (25.16, 44.56) | 26.43 (19.41, 35.20) | -0.86 (-1.11, -0.60) | <0.001 |
| Finland | 77.65 (72.07, 83.59) | 74.44 (68.72, 80.53) | -0.14 (-0.18, -0.09) | <0.001 | 1081.65 (1060.46, 1103.19) | 1035.87 (1014.20, 1057.92) | -0.12 (-0.24, -0.01) | 0.039 | 416.45 (403.53, 429.72) | 214.05 (204.30, 224.19) | -2.29 (-2.97, -1.60) | <0.001 | 5.70 (4.30, 7.48) | 2.24 (1.36, 3.54) | -3.44 (-3.91, -2.97) | <0.001 |
| France | 55.69 (54.31, 57.09) | 54.55 (53.12, 56.01) | -0.06 (-0.10, -0.03) | 0.001 | 952.74 (947.02, 958.49) | 919.98 (914.09, 925.90) | -0.12 (-0.19, -0.04) | 0.002 | 316.87 (313.58, 320.18) | 186.28 (183.65, 188.94) | -1.87 (-2.25, -1.49) | <0.001 | 4.08 (3.72, 4.47) | 1.97 (1.71, 2.25) | -2.55 (-2.88, -2.21) | <0.001 |
| Gabon | 151.67 (134.18, 171.01) | 149.96 (138.02, 162.70) | -0.04 (-0.05, -0.02) | <0.001 | 2911.27 (2832.46, 2991.91) | 2985.24 (2930.95, 3040.33) | 0.09 (0.07, 0.12) | <0.001 | 838.78 (794.70, 884.81) | 543.18 (519.71, 567.47) | -1.53 (-1.79, -1.26) | <0.001 | 11.44 (6.75, 18.31) | 6.20 (3.90, 9.40) | -2.13 (-2.48, -1.79) | <0.001 |
| Gambia | 145.59 (128.67, 164.38) | 151.85 (140.88, 163.52) | 0.15 (0.14, 0.16) | <0.001 | 2671.15 (2597.01, 2747.10) | 2821.47 (2773.47, 2870.15) | 0.19 (0.11, 0.27) | <0.001 | 806.46 (764.44, 850.37) | 790.05 (763.88, 816.93) | 0.04 (-0.76, 0.83) | 0.930 | 10.82 (6.38, 17.40) | 10.50 (7.63, 14.13) | 0.03 (-0.97, 1.03) | 0.959 |
| Georgia | 148.70 (141.53, | 142.27 (132.64, | -0.15 (-0.18, | <0.001 | 2162.45 (2134.90 | 2182.72 (2144.83 | 0.03 (0.00, | 0.037 | 1381.42 (1359.51 | 650.53 (630.13, | -2.53 (-3.54, | <0.001 | 20.56 (17.97, | 8.22 (6.10, | -3.09 (-4.21, | <0.001 |

| | | | | | | | | | | | | | | | | |
|---|---|---|---|---|---|---|---|---|---|---|---|---|---|---|---|---|
| | 156.16) | 152.49) | -0.12) | | , 2190.29) | , 2221.19) | 0.06) | | , 1403.60) | 671.50) | -1.51) | | 23.43) | 10.94) | -1.96) | |
| Germany | 73.18 (71.82, 74.57) | 66.57 (65.15, 68.01) | -0.32 (-0.35, -0.29) | <0.001 | 1249.92 (1244.20, 1255.66) | 1135.99 (1130.07, 1141.94) | -0.31 (-0.43, -0.19) | <0.001 | 506.11 (502.49, 509.75) | 238.79 (236.10, 241.51) | -2.60 (-2.97, -2.23) | <0.001 | 7.11 (6.69, 7.55) | 2.64 (2.37, 2.94) | -3.45 (-3.66, -3.24) | <0.001 |
| Ghana | 159.16 (154.54, 163.90) | 148.35 (145.50, 151.25) | -0.25 (-0.26, -0.23) | <0.001 | 2634.07 (2615.26, 2653.00) | 2651.55 (2639.48, 2663.66) | 0.03 (0.01, 0.05) | 0.016 | 1440.21 (1425.79, 1454.74) | 973.24 (965.80, 980.73) | -1.36 (-1.65, -1.07) | <0.001 | 21.99 (20.22, 23.88) | 13.85 (12.97, 14.78) | -1.56 (-1.91, -1.22) | <0.001 |
| Greece | 56.55 (53.21, 60.04) | 56.38 (52.61, 60.36) | 0.00 (-0.03, 0.04) | 0.933 | 686.93 (675.17, 698.85) | 703.56 (690.21, 717.13) | 0.13 (-0.12, 0.38) | 0.309 | 436.14 (426.79, 445.65) | 288.82 (280.43, 297.42) | -1.27 (-1.80, -0.74) | <0.001 | 6.33 (5.25, 7.57) | 3.92 (3.02, 5.05) | -1.52 (-2.12, -0.92) | <0.001 |
| Greenland | 119.91 (65.72, 207.96) | 101.83 (49.69, 189.22) | -0.56 (-0.57, -0.54) | <0.001 | 1459.75 (1249.50, 1700.26) | 1319.51 (1106.16, 1564.86) | -0.30 (-0.53, -0.06) | 0.014 | 967.43 (796.27, 1169.46) | 467.92 (344.96, 623.67) | -2.42 (-2.75, -2.08) | <0.001 | 15.12 (1.44, 70.06) | 6.48 (0.03, 55.95) | -2.85 (-3.16, -2.54) | <0.001 |
| Grenada | 194.91 (132.25, 278.80) | 189.85 (133.27, 263.41) | -0.09 (-0.11, -0.07) | <0.001 | 2512.97 (2270.57, 2775.55) | 2505.86 (2286.34, 2741.61) | -0.01 (-0.05, 0.03) | 0.659 | 1441.98 (1257.16, 1647.48) | 786.34 (664.00, 925.40) | -1.99 (-2.41, -1.56) | <0.001 | 22.48 (5.21, 64.29) | 11.27 (1.43, 41.31) | -2.25 (-2.74, -1.76) | <0.001 |
| Guam | 116.69 (80.39, 164.11) | 134.66 (95.22, 185.32) | 0.52 (0.41, 0.63) | <0.001 | 1753.66 (1602.93, 1915.01) | 1900.18 (1742.54, 2068.55) | 0.29 (0.22, 0.36) | <0.001 | 956.12 (845.74, 1077.18) | 1175.53 (1051.58, 1310.35) | 0.74 (0.17, 1.31) | 0.011 | 13.31 (3.44, 35.48) | 17.00 (5.28, 41.11) | 0.83 (0.08, 1.59) | 0.030 |
| Guatemala | 167.65 (161.05, 174.47) | 144.45 (140.76, 148.21) | -0.50 (-0.58, -0.43) | <0.001 | 2355.89 (2330.74, 2381.26) | 2448.24 (2432.88, 2463.67) | 0.14 (0.10, 0.19) | <0.001 | 1706.57 (1684.82, 1728.55) | 756.22 (747.62, 764.90) | -3.04 (-3.86, -2.21) | <0.001 | 26.64 (23.96, 29.55) | 10.40 (9.41, 11.48) | -3.51 (-4.45, -2.56) | <0.001 |
| Guinea | 126.01 (119.54, 132.75) | 137.49 (132.93, 142.18) | 0.30 (0.28, 0.32) | <0.001 | 2061.15 (2034.80, 2087.77) | 2355.52 (2336.60, 2374.56) | 0.46 (0.44, 0.49) | <0.001 | 1128.83 (1108.98, 1148.96) | 1026.74 (1013.92, 1039.70) | -0.30 (-0.49, -0.12) | 0.002 | 17.00 (14.62, 19.67) | 14.99 (13.45, 16.65) | -0.41 (-0.62, -0.19) | <0.001 |

| | | | | | | | | | | | | | | | | |
|---|---|---|---|---|---|---|---|---|---|---|---|---|---|---|---|---|
| Guinea-Bissau | 168.34 (150.36, 188.02) | 173.18 (160.60, 186.55) | 0.10 (0.06, 0.13) | <0.001 | 2256.84 (2189.71, 2325.65) | 2380.62 (2333.40, 2428.63) | 0.19 (0.16, 0.22) | <0.001 | 1614.81 (1556.41, 1674.94) | 1333.40 (1297.09, 1370.50) | -0.65 (-0.78, -0.52) | <0.001 | 25.27 (18.37, 34.02) | 20.43 (16.12, 25.59) | -0.72 (-0.87, -0.57) | <0.001 |
| Guyana | 213.24 (191.47, 236.99) | 206.33 (184.53, 230.10) | -0.11 (-0.13, -0.09) | <0.001 | 2450.69 (2375.07, 2528.26) | 2430.57 (2354.06, 2509.01) | -0.01 (-0.12, 0.11) | 0.912 | 1701.01 (1636.84, 1767.17) | 1443.50 (1383.92, 1505.04) | -0.56 (-1.54, 0.43) | 0.267 | 26.93 (19.33, 36.67) | 22.64 (15.69, 31.68) | -0.60 (-1.64, 0.46) | 0.268 |
| Haiti | 250.49 (241.73, 259.50) | 235.79 (230.10, 241.58) | -0.21 (-0.23, -0.20) | <0.001 | 2423.43 (2395.93, 2451.18) | 2397.29 (2379.07, 2415.61) | -0.02 (-0.12, 0.08) | 0.705 | 3513.41 (3479.89, 3547.18) | 2143.16 (2125.89, 2160.53) | -1.67 (-2.05, -1.28) | <0.001 | 58.23 (53.96, 62.76) | 34.63 (32.46, 36.90) | -1.76 (-2.12, -1.40) | <0.001 |
| Honduras | 157.28 (148.98, 165.96) | 147.19 (142.15, 152.38) | -0.23 (-0.24, -0.21) | <0.001 | 2269.48 (2237.23, 2302.11) | 2327.97 (2307.59, 2348.50) | 0.10 (0.00, 0.19) | 0.042 | 1590.29 (1562.58, 1618.39) | 763.31 (751.39, 775.38) | -2.46 (-2.86, -2.06) | <0.001 | 25.09 (21.68, 28.90) | 10.98 (9.58, 12.54) | -2.79 (-3.25, -2.32) | <0.001 |
| Hungary | 101.76 (97.28, 106.42) | 89.24 (84.47, 94.23) | -0.45 (-0.48, -0.42) | <0.001 | 1275.03 (1258.90, 1291.33) | 1193.78 (1176.04, 1211.76) | -0.21 (-0.26, -0.17) | <0.001 | 982.58 (968.83, 996.49) | 363.58 (353.99, 373.39) | -3.37 (-3.84, -2.89) | <0.001 | 15.02 (13.38, 16.82) | 4.23 (3.27, 5.42) | -4.22 (-4.84, -3.61) | <0.001 |
| Iceland | 60.59 (41.22, 86.18) | 57.33 (39.71, 80.62) | -0.18 (-0.21, -0.15) | <0.001 | 759.69 (686.20, 839.12) | 779.36 (709.96, 854.13) | 0.12 (-0.07, 0.31) | 0.218 | 331.23 (283.31, 385.15) | 164.68 (133.66, 201.19) | -2.36 (-2.53, -2.20) | <0.001 | 4.64 (0.72, 15.68) | 1.74 (0.05, 10.51) | -3.35 (-3.52, -3.19) | <0.001 |
| India | 154.82 (154.21, 155.42) | 149.48 (149.03, 149.92) | -0.11 (-0.18, -0.04) | 0.002 | 1845.42 (1843.31, 1847.54) | 1851.22 (1849.64, 1852.79) | 0.03 (-0.02, 0.07) | 0.250 | 1528.57 (1526.64, 1530.50) | 1009.14 (1007.97, 1010.30) | -1.52 (-2.06, -0.97) | <0.001 | 23.75 (23.51, 23.99) | 15.12 (14.97, 15.26) | -1.60 (-2.24, -0.95) | <0.001 |
| Indonesia | 92.59 (91.61, 93.59) | 88.92 (88.12, 89.73) | -0.14 (-0.16, -0.13) | <0.001 | 1095.04 (1091.66, 1098.42) | 1100.42 (1097.59, 1103.25) | 0.03 (-0.05, 0.11) | 0.460 | 1651.90 (1647.70, 1656.10) | 1027.33 (1024.61, 1030.06) | -1.60 (-1.72, -1.48) | <0.001 | 25.88 (25.35, 26.42) | 15.22 (14.90, 15.56) | -1.79 (-1.92, -1.66) | <0.001 |
| Iran (Islamic Republic of) | 141.17 (138.85, | 130.86 (129.17, | -0.25 (-0.29, | <0.001 | 1932.55 (1923.96 | 1910.37 (1903.89 | -0.04 (-0.05, | <0.001 | 1148.19 (1141.48 | 625.56 (621.87, | -2.07 (-2.23, | <0.001 | 17.26 (16.44, | 8.23 (7.82, | -2.53 (-2.69, | <0.001 |

| | | | | | | | | | | | | | | | | |
|---|---|---|---|---|---|---|---|---|---|---|---|---|---|---|---|---|
| | 143.52) | 132.56) | -0.22) | | , 1941.18) | , 1916.86) | -0.02) | | , 1154.93) | 629.27) | -1.90) | | 18.12) | 8.66) | -2.38) | |
| Iraq | 154.47 (149.97, 159.07) | 142.59 (140.07, 145.15) | -0.28 (-0.30, -0.25) | <0.001 | 2042.74 (2026.26, 2059.33) | 1990.77 (1981.31, 2000.27) | -0.07 (-0.12, -0.03) | 0.001 | 1601.69 (1586.89, 1616.59) | 923.72 (917.19, 930.29) | -1.92 (-2.33, -1.52) | <0.001 | 24.75 (22.92, 26.69) | 13.24 (12.46, 14.06) | -2.19 (-2.65, -1.72) | <0.001 |
| Ireland | 65.36 (59.37, 71.80) | 59.64 (54.47, 65.19) | -0.32 (-0.34, -0.29) | <0.001 | 956.88 (933.55, 980.66) | 937.46 (916.76, 958.53) | -0.06 (-0.17, 0.05) | 0.286 | 407.35 (392.19, 422.94) | 173.07 (164.32, 182.18) | -2.91 (-3.88, -1.93) | <0.001 | 5.74 (4.07, 7.88) | 1.74 (0.98, 2.90) | -4.07 (-5.44, -2.69) | <0.001 |
| Israel | 54.60 (49.99, 59.53) | 53.26 (49.75, 56.96) | -0.08 (-0.13, -0.02) | 0.006 | 707.32 (690.50, 724.45) | 728.82 (715.69, 742.14) | 0.13 (0.02, 0.23) | 0.018 | 331.65 (320.19, 343.43) | 137.00 (131.34, 142.83) | -3.02 (-3.26, -2.78) | <0.001 | 4.57 (3.32, 6.16) | 1.25 (0.77, 1.93) | -4.45 (-4.88, -4.01) | <0.001 |
| Italy | 120.33 (118.26, 122.44) | 114.81 (112.50, 117.15) | -0.15 (-0.19, -0.12) | <0.001 | 1311.86 (1304.98, 1318.77) | 1319.64 (1311.85, 1327.47) | 0.04 (-0.05, 0.12) | 0.413 | 354.66 (351.09, 358.27) | 180.75 (177.85, 183.70) | -2.31 (-2.47, -2.14) | <0.001 | 4.86 (4.45, 5.30) | 1.90 (1.62, 2.22) | -3.20 (-3.57, -2.84) | <0.001 |
| Jamaica | 168.69 (157.23, 180.84) | 189.24 (178.37, 200.61) | 0.41 (0.36, 0.46) | <0.001 | 2362.65 (2318.82, 2407.17) | 2568.24 (2527.80, 2609.17) | 0.29 (0.27, 0.32) | <0.001 | 875.60 (848.53, 903.38) | 785.72 (763.41, 808.54) | -0.17 (-1.52, 1.20) | 0.808 | 12.79 (9.67, 16.67) | 11.18 (8.66, 14.23) | -0.13 (-1.04, 0.80) | 0.791 |
| Japan | 115.18 (113.77, 116.60) | 116.03 (114.42, 117.67) | 0.03 (-0.01, 0.07) | 0.122 | 1204.94 (1200.38, 1209.50) | 1271.19 (1265.86, 1276.54) | 0.22 (0.08, 0.36) | 0.002 | 312.27 (309.95, 314.59) | 205.71 (203.56, 207.89) | -1.43 (-1.64, -1.22) | <0.001 | 3.96 (3.71, 4.23) | 2.04 (1.83, 2.26) | -2.26 (-2.78, -1.74) | <0.001 |
| Jordan | 102.43 (94.20, 111.25) | 88.66 (84.74, 92.72) | -0.48 (-0.59, -0.37) | <0.001 | 1478.86 (1447.57, 1510.72) | 1427.25 (1411.43, 1443.20) | -0.11 (-0.22, -0.00) | 0.042 | 786.85 (763.70, 810.57) | 355.48 (347.59, 363.51) | -2.64 (-3.21, -2.06) | <0.001 | 11.28 (8.61, 14.55) | 3.86 (3.07, 4.79) | -3.58 (-4.30, -2.86) | <0.001 |
| Kazakhstan | 106.15 (102.71, 109.67) | 88.86 (85.83, 91.99) | -0.62 (-0.67, -0.58) | <0.001 | 1033.77 (1022.97, 1044.67) | 947.45 (937.39, 957.61) | -0.30 (-0.34, -0.26) | <0.001 | 999.84 (989.21, 1010.57) | 800.90 (791.80, 810.08) | -0.72 (-1.26, -0.18) | 0.010 | 15.14 (13.86, 16.51) | 12.22 (11.13, 13.41) | -0.70 (-1.32, -0.07) | 0.029 |
| Kenya | 165.34 | 164.44 | -0.02 | <0.001 | 3145.79 | 3369.03 | 0.24 | <0.001 | 689.53 | 657.17 | -0.19 | <0.001 | 8.24 | 7.56 | -0.29 | <0.001 |

| | | | | | | | | | | | | | | | | |
|---|---|---|---|---|---|---|---|---|---|---|---|---|---|---|---|---|
| | (161.46, 169.30) | (162.04, 166.87) | (-0.03, -0.01) | | (3128.51 , 3163.14) | (3358.01 , 3380.08) | (0.21, 0.27) | | (681.10, 698.05) | (652.18, 662.19) | (-0.27, -0.12) | | (7.32, 9.26) | (7.03, 8.13) | (-0.43, -0.15) | |
| Kiribati | 237.93 (165.73, 332.56) | 214.61 (160.53, 281.42) | -0.36 (-0.39, -0.34) | <0.001 | 2033.88 (1809.36 , 2279.89) | 1938.78 (1768.19 , 2121.69) | -0.16 (-0.18, -0.13) | <0.001 | 4183.65 (3856.85 , 4531.91) | 3425.23 (3196.39 , 3666.41) | -0.69 (-0.77, -0.61) | <0.001 | 68.51 (32.56, 128.50) | 55.72 (30.05, 94.79) | -0.72 (-0.82, -0.61) | <0.001 |
| Kuwait | 85.03 (75.69, 95.25) | 86.14 (80.63, 92.05) | 0.06 (0.02, 0.09) | 0.002 | 1301.64 (1264.34 , 1339.81) | 1354.77 (1331.36 , 1378.58) | 0.13 (0.09, 0.18) | <0.001 | 710.46 (682.93, 738.85) | 342.71 (331.17, 354.65) | -2.34 (-3.12, -1.55) | <0.001 | 10.11 (7.07, 14.09) | 3.81 (2.71, 5.34) | -3.15 (-4.19, -2.10) | <0.001 |
| Kyrgyzstan | 154.48 (146.36, 162.94) | 136.95 (130.68, 143.45) | -0.41 (-0.44, -0.38) | <0.001 | 2001.40 (1971.85 , 2031.29) | 1914.05 (1890.46 , 1937.88) | -0.15 (-0.17, -0.14) | <0.001 | 1351.81 (1327.25 , 1376.74) | 631.23 (617.61, 645.09) | -2.56 (-3.00, -2.12) | <0.001 | 20.63 (17.68, 23.96) | 8.54 (7.01, 10.30) | -2.96 (-3.50, -2.42) | <0.001 |
| Lao People's Democratic Republic | 169.83 (160.62, 179.46) | 140.36 (134.50, 146.42) | -0.66 (-0.75, -0.57) | <0.001 | 1785.49 (1755.23 , 1816.17) | 1655.11 (1634.80 , 1675.63) | -0.25 (-0.29, -0.21) | <0.001 | 1529.71 (1500.99 , 1558.87) | 1241.72 (1223.96 , 1259.69) | -0.67 (-0.93, -0.42) | <0.001 | 23.60 (20.12, 27.54) | 18.84 (16.69, 21.18) | -0.73 (-1.02, -0.44) | <0.001 |
| Latvia | 88.50 (80.40, 97.24) | 88.90 (78.39, 100.61) | 0.02 (-0.01, 0.05) | 0.151 | 769.84 (745.64, 794.66) | 820.24 (788.03, 853.60) | 0.27 (0.10, 0.44) | 0.002 | 656.26 (633.93, 679.23) | 398.31 (376.08, 421.71) | -1.62 (-3.01, -0.22) | 0.024 | 9.95 (7.38, 13.18) | 5.29 (3.06, 8.83) | -2.07 (-3.73, -0.37) | 0.017 |
| Lebanon | 84.05 (76.68, 91.95) | 84.01 (78.60, 89.74) | 0.01 (-0.03, 0.05) | 0.676 | 1288.83 (1259.71 , 1318.47) | 1345.12 (1322.82 , 1367.75) | 0.16 (0.11, 0.20) | <0.001 | 1070.61 (1043.87 , 1097.87) | 637.41 (622.19, 652.95) | -1.75 (-1.85, -1.66) | <0.001 | 16.54 (13.35, 20.28) | 8.85 (7.15, 10.87) | -2.12 (-2.24, -2.00) | <0.001 |
| Lesotho | 166.71 (153.35, 181.04) | 176.70 (164.78, 189.28) | 0.21 (0.18, 0.23) | <0.001 | 2465.55 (2412.72 , 2519.34) | 2530.42 (2484.66 , 2576.84) | 0.10 (0.04, 0.17) | 0.003 | 668.54 (640.28, 697.82) | 834.69 (808.04, 862.01) | 0.66 (0.33, 1.00) | <0.001 | 8.75 (5.74, 12.87) | 11.65 (8.67, 15.35) | 0.84 (0.37, 1.31) | <0.001 |
| Liberia | 151.12 (138.57, 164.54) | 153.82 (146.19, 161.76) | 0.06 (0.05, 0.07) | <0.001 | 2413.53 (2362.39 , | 2590.60 (2558.91 , | 0.26 (0.20, 0.31) | <0.001 | 1105.19 (1070.21 , | 876.25 (857.50, 895.33) | -0.78 (-0.88, -0.67) | <0.001 | 16.22 (12.21, 21.15) | 12.18 (10.04, 14.65) | -0.96 (-1.09, -0.84) | <0.001 |

| | | | | | | | | | | | | | | | | |
|---|---|---|---|---|---|---|---|---|---|---|---|---|---|---|---|---|
| | | | | | 2465.52) | 2622.60) | | | 1141.05) | | | | | | | |
| Libya | 124.89 (116.39, 133.90) | 136.32 (130.45, 142.41) | 0.30 (0.26, 0.33) | <0.001 | 1876.95 (1843.77, 1910.63) | 2111.48 (2088.14, 2135.02) | 0.41 (0.33, 0.50) | <0.001 | 1534.33 (1503.83, 1565.34) | 1173.10 (1155.82, 1190.59) | -0.87 (-1.42, -0.33) | 0.002 | 23.70 (19.99, 27.95) | 17.22 (15.19, 19.45) | -1.01 (-1.72, -0.29) | 0.006 |
| Lithuania | 103.84 (96.46, 111.65) | 102.06 (92.74, 112.17) | -0.05 (-0.11, 0.01) | 0.085 | 1144.07 (1119.22, 1169.36) | 1140.08 (1108.26, 1172.67) | 0.02 (-0.09, 0.13) | 0.712 | 556.45 (539.16, 574.18) | 378.60 (360.52, 397.46) | -1.23 (-2.56, 0.11) | 0.072 | 7.70 (5.79, 10.07) | 4.67 (2.89, 7.32) | -1.57 (-3.41, 0.30) | 0.099 |
| Luxembourg | 54.28 (38.96, 74.64) | 53.72 (40.85, 69.92) | -0.04 (-0.06, -0.01) | 0.014 | 1244.54 (1165.21, 1328.58) | 1256.22 (1189.78, 1325.83) | 0.05 (-0.02, 0.12) | 0.157 | 444.45 (398.13, 495.48) | 215.37 (188.56, 245.42) | -2.45 (-2.86, -2.05) | <0.001 | 6.08 (1.88, 16.19) | 2.12 (0.33, 8.11) | -3.74 (-4.33, -3.15) | <0.001 |
| Madagascar | 210.56 (204.47, 216.81) | 202.11 (198.32, 205.97) | -0.16 (-0.20, -0.11) | <0.001 | 3618.25 (3592.90, 3643.74) | 3841.25 (3824.56, 3858.00) | 0.21 (0.18, 0.25) | <0.001 | 2602.80 (2580.52, 2625.24) | 1692.04 (1680.59, 1703.54) | -1.43 (-1.82, -1.04) | <0.001 | 40.66 (37.88, 43.60) | 25.06 (23.66, 26.51) | -1.60 (-2.04, -1.16) | <0.001 |
| Malawi | 193.62 (187.25, 200.18) | 190.71 (186.41, 195.09) | -0.06 (-0.07, -0.04) | <0.001 | 3205.89 (3179.46, 3232.50) | 3366.66 (3348.13, 3385.27) | 0.18 (0.11, 0.26) | <0.001 | 1161.80 (1145.29, 1178.50) | 686.33 (677.75, 695.00) | -1.75 (-1.90, -1.61) | <0.001 | 16.43 (14.49, 18.58) | 8.11 (7.18, 9.13) | -2.34 (-2.53, -2.14) | <0.001 |
| Malaysia | 121.40 (117.84, 125.05) | 113.71 (111.14, 116.33) | -0.22 (-0.26, -0.18) | <0.001 | 2079.96 (2065.09, 2094.91) | 2079.86 (2068.83, 2090.95) | 0.00 (-0.03, 0.04) | 0.810 | 937.40 (927.36, 947.54) | 579.98 (574.15, 585.85) | -1.64 (-1.88, -1.39) | <0.001 | 12.74 (11.59, 13.99) | 6.80 (6.18, 7.46) | -2.12 (-2.46, -1.78) | <0.001 |
| Maldives | 121.08 (87.44, 165.27) | 102.92 (82.70, 127.12) | -0.57 (-0.62, -0.51) | <0.001 | 1604.07 (1473.27, 1744.81) | 1524.62 (1444.76, 1608.22) | -0.15 (-0.20, -0.09) | <0.001 | 1479.24 (1349.70, 1619.09) | 376.00 (337.28, 418.49) | -4.59 (-4.84, -4.34) | <0.001 | 22.89 (9.32, 48.72) | 4.07 (1.07, 11.79) | -5.78 (-6.05, -5.51) | <0.001 |
| Mali | 179.24 (172.56, 186.12) | 165.39 (161.41, 169.44) | -0.28 (-0.29, -0.27) | <0.001 | 2821.45 (2794.86, 2848.24) | 2858.63 (2842.02, 2875.32) | 0.05 (0.03, 0.07) | <0.001 | 1935.59 (1912.92, 1958.47) | 1239.43 (1228.03, 1250.91) | -1.50 (-1.73, -1.26) | <0.001 | 30.23 (27.43, 33.26) | 18.23 (16.85, 19.70) | -1.73 (-2.03, -1.44) | <0.001 |

| | | | | | | | | | | | | | | | | |
|---|---|---|---|---|---|---|---|---|---|---|---|---|---|---|---|---|
| Malta | 53.46 (37.70, 74.05) | 51.01 (35.59, 71.88) | -0.16 (-0.21, -0.10) | <0.001 | 769.58 (705.70, 838.03) | 737.06 (674.47, 804.69) | -0.11 (-0.33, 0.11) | 0.325 | 421.86 (375.20, 473.10) | 243.67 (208.20, 284.30) | -1.84 (-2.21, -1.46) | <0.001 | 6.04 (1.75, 15.74) | 3.03 (0.41, 12.49) | -2.33 (-2.79, -1.86) | <0.001 |
| Marshall Islands | 172.64 (92.52, 297.72) | 181.34 (111.90, 278.36) | 0.17 (0.14, 0.21) | <0.001 | 1812.45 (1525.59, 2140.61) | 1864.85 (1623.43, 2132.48) | 0.10 (0.07, 0.12) | <0.001 | 2264.11 (1934.63, 2635.87) | 2870.36 (2567.96, 3198.93) | 0.81 (0.72, 0.90) | <0.001 | 36.08 (6.40, 116.46) | 46.50 (15.74, 106.60) | 0.87 (0.78, 0.95) | <0.001 |
| Mauritania | 151.39 (139.22, 164.40) | 140.44 (132.34, 148.94) | -0.26 (-0.28, -0.24) | <0.001 | 2655.50 (2603.78, 2708.04) | 2694.86 (2658.88, 2731.23) | 0.05 (0.01, 0.10) | 0.025 | 1138.54 (1103.90, 1174.04) | 646.24 (628.22, 664.66) | -1.90 (-2.01, -1.79) | <0.001 | 16.49 (12.52, 21.38) | 8.04 (6.11, 10.40) | -2.41 (-2.54, -2.27) | <0.001 |
| Mauritius | 119.72 (106.38, 134.30) | 109.87 (96.82, 124.23) | -0.30 (-0.31, -0.28) | <0.001 | 1885.09 (1831.06, 1940.33) | 1806.25 (1752.16, 1861.63) | -0.13 (-0.17, -0.10) | <0.001 | 1166.50 (1123.91, 1210.32) | 656.95 (624.74, 690.43) | -1.96 (-2.32, -1.60) | <0.001 | 17.07 (12.27, 23.17) | 8.53 (5.24, 13.20) | -2.38 (-2.84, -1.91) | <0.001 |
| Mexico | 114.03 (112.43, 115.64) | 95.43 (94.23, 96.63) | -0.61 (-0.65, -0.57) | <0.001 | 1785.34 (1778.98, 1791.71) | 1646.77 (1641.80, 1651.75) | -0.27 (-0.34, -0.21) | <0.001 | 672.81 (668.84, 676.81) | 412.49 (410.01, 414.99) | -1.59 (-2.15, -1.03) | <0.001 | 9.61 (9.14, 10.11) | 5.24 (4.97, 5.53) | -1.98 (-2.67, -1.29) | <0.001 |
| Micronesia (Federated States of) | 196.63 (136.94, 274.92) | 165.21 (112.77, 234.59) | -0.60 (-0.64, -0.56) | <0.001 | 1908.77 (1712.52, 2122.57) | 1774.29 (1591.61, 1973.03) | -0.24 (-0.28, -0.20) | <0.001 | 2730.73 (2490.33, 2989.02) | 2566.68 (2342.47, 2807.19) | -0.22 (-0.29, -0.15) | <0.001 | 43.84 (18.30, 89.70) | 41.10 (17.30, 83.23) | -0.23 (-0.31, -0.15) | <0.001 |
| Monaco | 57.98 (10.89, 209.52) | 61.17 (12.21, 189.85) | 0.19 (0.18, 0.21) | <0.001 | 685.47 (470.83, 983.83) | 719.90 (500.90, 1007.69) | 0.20 (0.01, 0.39) | 0.038 | 381.48 (226.75, 623.25) | 244.92 (125.90, 436.12) | -1.52 (-1.64, -1.39) | <0.001 | 5.45 (0.00, 133.90) | 3.14 (0.00, 96.76) | -1.88 (-1.98, -1.78) | <0.001 |
| Mongolia | 181.59 (168.62, 195.40) | 160.86 (151.38, 170.84) | -0.42 (-0.43, -0.41) | <0.001 | 2062.84 (2018.35, 2108.17) | 2013.76 (1980.16, 2047.85) | -0.07 (-0.13, -0.00) | 0.045 | 2116.36 (2069.39, 2164.21) | 1185.81 (1160.72, 1211.37) | -2.03 (-2.79, -1.26) | <0.001 | 34.24 (28.42, 40.99) | 18.04 (15.09, 21.48) | -2.25 (-3.08, -1.41) | <0.001 |
| Montenegro | 89.12 (73.17, 107.54) | 86.17 (69.51, 105.86) | -0.11 (-0.17, -0.06) | <0.001 | 1071.07 (1013.80 , | 1064.53 (1003.55 , | 0.00 (-0.12, 0.13) | 0.946 | 655.36 (610.79, 702.35) | 515.74 (473.93, 560.47) | -0.68 (-1.53, 0.18) | 0.123 | 9.28 (4.70, 16.50) | 6.96 (2.95, 14.28) | -0.80 (-1.90, 0.32) | 0.162 |

| | | | | | | | | | | | | | | | | |
|---|---|---|---|---|---|---|---|---|---|---|---|---|---|---|---|---|
| | | | | | 1130.76) | 1128.45) | | | | | | | | | | |
| Morocco | 145.27 (141.92, 148.69) | 145.10 (142.36, 147.88) | -0.01 (-0.02, 0.01) | 0.598 | 1923.61 (1911.37, 1935.93) | 1967.61 (1957.47, 1977.78) | 0.09 (0.02, 0.16) | 0.009 | 1476.30 (1465.42, 1487.25) | 989.02 (981.86, 996.23) | -1.34 (-1.57, -1.11) | <0.001 | 22.89 (21.54, 24.31) | 14.47 (13.62, 15.37) | -1.52 (-1.80, -1.25) | <0.001 |
| Mozambique | 195.34 (189.89, 200.91) | 194.54 (190.97, 198.17) | -0.02 (-0.05, 0.00) | 0.050 | 3153.36 (3131.06, 3175.79) | 3336.19 (3321.05, 3351.40) | 0.21 (0.12, 0.30) | <0.001 | 786.96 (775.57, 798.47) | 688.51 (681.38, 695.70) | -0.46 (-0.64, -0.28) | <0.001 | 10.10 (8.83, 11.50) | 8.19 (7.41, 9.04) | -0.73 (-0.95, -0.52) | <0.001 |
| Myanmar | 142.09 (139.55, 144.67) | 123.42 (121.39, 125.48) | -0.48 (-0.56, -0.41) | <0.001 | 1753.87 (1744.83, 1762.96) | 1630.42 (1623.01, 1637.86) | -0.24 (-0.37, -0.12) | <0.001 | 2829.55 (2817.85, 2841.29) | 1102.88 (1096.79, 1109.00) | -3.21 (-3.36, -3.06) | <0.001 | 46.40 (44.90, 47.94) | 16.60 (15.86, 17.37) | -3.50 (-3.66, -3.35) | <0.001 |
| Namibia | 162.75 (147.97, 178.76) | 152.58 (142.13, 163.63) | -0.22 (-0.24, -0.21) | <0.001 | 2593.68 (2533.13, 2655.45) | 2582.72 (2538.99, 2627.05) | -0.01 (-0.03, 0.01) | 0.146 | 804.68 (769.69, 840.96) | 490.52 (471.31, 510.35) | -1.67 (-1.99, -1.35) | <0.001 | 10.99 (7.18, 16.22) | 5.53 (3.65, 8.08) | -2.30 (-2.80, -1.80) | <0.001 |
| Nauru | 150.15 (29.54, 456.88) | 142.23 (29.90, 420.56) | -0.16 (-0.35, 0.02) | 0.085 | 1209.44 (768.49, 1817.29) | 1166.77 (756.09, 1726.26) | -0.11 (-0.26, 0.04) | 0.154 | 3022.44 (2293.43, 3914.50) | 3416.50 (2679.24, 4298.66) | 0.41 (0.16, 0.67) | 0.002 | 49.65 (1.03, 298.21) | 56.43 (2.42, 287.23) | 0.43 (0.16, 0.71) | 0.002 |
| Nepal | 127.96 (124.32, 131.68) | 121.56 (119.00, 124.16) | -0.17 (-0.24, -0.09) | <0.001 | 1395.98 (1383.66, 1408.39) | 1395.52 (1386.70, 1404.39) | 0.01 (-0.03, 0.05) | 0.593 | 1296.72 (1284.63, 1308.91) | 600.17 (594.31, 606.08) | -2.63 (-2.80, -2.46) | <0.001 | 20.88 (19.36, 22.50) | 8.78 (8.08, 9.53) | -2.94 (-3.13, -2.76) | <0.001 |
| Netherlands | 76.16 (73.09, 79.34) | 68.91 (65.79, 72.15) | -0.33 (-0.39, -0.27) | <0.001 | 1006.42 (995.11, 1017.83) | 1047.10 (1034.79, 1059.53) | 0.14 (0.08, 0.20) | <0.001 | 375.58 (368.71, 382.55) | 194.82 (189.56, 200.21) | -2.23 (-2.62, -1.85) | <0.001 | 5.17 (4.39, 6.05) | 2.00 (1.50, 2.62) | -3.12 (-3.91, -2.33) | <0.001 |
| New Zealand | 80.03 (73.54, 86.95) | 71.11 (65.13, 77.51) | -0.40 (-0.44, -0.35) | <0.001 | 696.20 (676.79, 716.04) | 707.40 (688.30, 726.91) | 0.06 (-0.07, 0.20) | 0.365 | 455.66 (439.98, 471.77) | 239.15 (228.11, 250.61) | -2.21 (-2.59, -1.84) | <0.001 | 6.78 (4.99, 9.01) | 2.94 (1.84, 4.49) | -2.95 (-3.43, -2.47) | <0.001 |
| Nicaragua | 146.86 | 141.38 | -0.13 | <0.001 | 2390.43 | 2437.84 | 0.10 | 0.029 | 750.32 | 449.39 | -1.82 | <0.001 | 10.57 | 5.30 | -2.44 | <0.001 |

| | | | | | | | | | | | | | | | | |
|---|---|---|---|---|---|---|---|---|---|---|---|---|---|---|---|---|
| | (138.19, 155.99) | (135.21, 147.75) | (-0.16, -0.10) | | (2354.42, 2426.89) | (2411.97, 2463.92) | (0.01, 0.19) | | (729.63, 771.48) | (438.27, 460.73) | (-2.52, -1.11) | | (8.21, 13.44) | (4.15, 6.67) | (-3.37, -1.50) | |
| Niger | 148.22 (141.80, 154.89) | 145.26 (141.42, 149.18) | -0.07 (-0.09, -0.06) | <0.001 | 2506.63 (2480.04, 2533.45) | 2671.24 (2654.68, 2687.89) | 0.24 (0.13, 0.35) | <0.001 | 1156.34 (1137.63, 1175.32) | 861.85 (852.02, 871.76) | -1.04 (-1.32, -0.77) | <0.001 | 17.02 (14.78, 19.52) | 11.87 (10.72, 13.12) | -1.26 (-1.65, -0.88) | <0.001 |
| Nigeria | 136.37 (134.57, 138.19) | 138.77 (137.65, 139.89) | 0.07 (0.04, 0.09) | <0.001 | 2721.64 (2713.56, 2729.74) | 3019.95 (3014.71, 3025.21) | 0.37 (0.34, 0.40) | <0.001 | 890.19 (885.36, 895.03) | 601.14 (598.72, 603.57) | -1.36 (-1.50, -1.22) | <0.001 | 12.29 (11.71, 12.88) | 7.02 (6.76, 7.29) | -1.92 (-2.16, -1.68) | <0.001 |
| Niue | 102.44 (0.01, 1240.07) | 101.87 (0.00, 1657.91) | -0.02 (-0.04, 0.01) | 0.196 | 1066.24 (286.43, 2772.19) | 1085.25 (220.43, 3258.83) | 0.07 (0.01, 0.14) | 0.026 | 1452.07 (492.47, 3324.51) | 1266.89 (300.96, 3530.04) | -0.47 (-0.69, -0.24) | <0.001 | 22.68 (0.00, 1085.97) | 19.32 (0.00, 1499.66) | -0.55 (-0.78, -0.31) | <0.001 |
| North Macedonia | 90.49 (81.41, 100.33) | 80.81 (72.16, 90.35) | -0.39 (-0.43, -0.36) | <0.001 | 957.10 (927.03, 987.92) | 940.63 (910.51, 971.62) | -0.03 (-0.20, 0.14) | 0.732 | 1061.58 (1029.88, 1094.02) | 588.45 (564.61, 613.15) | -2.00 (-2.90, -1.09) | <0.001 | 16.09 (12.40, 20.54) | 8.11 (5.56, 11.61) | -2.34 (-3.33, -1.34) | <0.001 |
| Northern Mariana Islands | 138.56 (79.37, 226.90) | 128.59 (49.97, 284.78) | -0.26 (-0.27, -0.24) | <0.001 | 1906.69 (1663.37, 2177.19) | 1922.71 (1566.87, 2344.92) | 0.03 (0.00, 0.06) | 0.038 | 1454.99 (1244.04, 1693.14) | 937.98 (682.81, 1264.87) | -1.48 (-1.67, -1.28) | <0.001 | 21.97 (3.74, 73.01) | 13.04 (0.04, 121.51) | -1.75 (-1.98, -1.52) | <0.001 |
| Norway | 92.82 (86.25, 99.78) | 90.15 (84.00, 96.66) | -0.10 (-0.18, -0.01) | 0.025 | 1009.86 (987.88, 1032.22) | 1090.63 (1068.91, 1112.70) | 0.27 (0.21, 0.33) | <0.001 | 321.62 (309.28, 334.35) | 146.19 (138.29, 154.45) | -2.71 (-3.02, -2.40) | <0.001 | 4.30 (2.98, 6.01) | 1.17 (0.57, 2.17) | -4.43 (-4.92, -3.94) | <0.001 |
| Oman | 89.58 (78.61, 101.73) | 86.04 (79.77, 92.74) | -0.13 (-0.20, -0.06) | <0.001 | 1295.68 (1253.33, 1339.15) | 1435.66 (1409.13, 1462.60) | 0.38 (0.31, 0.45) | <0.001 | 1175.69 (1135.11, 1217.40) | 710.19 (691.59, 729.20) | -1.56 (-1.77, -1.35) | <0.001 | 18.16 (13.40, 24.12) | 9.80 (7.74, 12.30) | -1.97 (-2.21, -1.73) | <0.001 |
| Pakistan | 186.39 (184.42, | 190.10 (188.81, | 0.08 (-0.05, | 0.232 | 2125.51 (2118.68 | 2232.13 (2227.62 | 0.18 (0.08, | <0.001 | 1711.10 (1704.81 | 1800.97 (1796.85 | 0.19 (0.10, | <0.001 | 27.13 (26.34, | 28.50 (27.98, | 0.18 (0.09, | <0.001 |

| | | | | | | | | | | | | | | | | |
|---|---|---|---|---|---|---|---|---|---|---|---|---|---|---|---|---|
| | 188.39) | 191.39) | 0.21) | | , 2132.36) | , 2236.64) | 0.28) | | , 1717.41) | , 1805.09) | 0.28) | | 27.95) | 29.03) | 0.28) | |
| Palau | 92.72 (19.29, 274.55) | 94.96 (15.31, 325.26) | 0.09 (0.06, 0.12) | <0.001 | 1128.59 (794.51, 1558.84) | 1162.94 (783.52, 1672.20) | 0.12 (0.01, 0.24) | 0.040 | 1288.20 (927.80, 1744.87) | 1145.40 (770.86, 1649.16) | -0.41 (-0.48, -0.34) | <0.001 | 19.88 (0.08, 158.25) | 17.28 (0.01, 200.76) | -0.49 (-0.56, -0.42) | <0.001 |
| Palestine | 126.10 (114.16, 139.05) | 121.85 (114.97, 129.07) | -0.12 (-0.15, -0.09) | <0.001 | 1997.70 (1948.99, 2047.42) | 2051.98 (2023.27, 2081.01) | 0.11 (0.02, 0.20) | 0.019 | 803.82 (772.42, 836.25) | 519.39 (504.88, 534.25) | -1.49 (-1.72, -1.25) | <0.001 | 11.30 (7.80, 15.93) | 6.30 (4.78, 8.18) | -1.98 (-2.15, -1.81) | <0.001 |
| Panama | 134.13 (124.09, 144.82) | 127.41 (119.65, 135.56) | -0.17 (-0.21, -0.13) | <0.001 | 2311.37 (2268.68, 2354.70) | 2307.07 (2273.61, 2340.90) | 0.01 (-0.10, 0.12) | 0.845 | 709.77 (685.91, 734.29) | 442.37 (427.77, 457.35) | -1.59 (-2.42, -0.75) | <0.001 | 9.80 (7.16, 13.15) | 5.23 (3.75, 7.10) | -2.09 (-3.25, -0.93) | <0.001 |
| Papua New Guinea | 159.63 (150.63, 169.05) | 167.05 (161.37, 172.89) | 0.16 (0.13, 0.19) | <0.001 | 1641.37 (1612.31, 1670.86) | 1711.64 (1693.35, 1730.08) | 0.16 (0.09, 0.22) | <0.001 | 1627.56 (1598.26, 1657.28) | 1756.86 (1738.25, 1775.62) | 0.22 (-0.03, 0.47) | 0.088 | 25.00 (21.45, 28.99) | 27.11 (24.83, 29.54) | 0.23 (-0.05, 0.51) | 0.101 |
| Paraguay | 136.48 (128.32, 145.06) | 131.30 (125.47, 137.33) | -0.13 (-0.16, -0.10) | <0.001 | 2277.70 (2243.76, 2312.06) | 2338.01 (2313.21, 2363.03) | 0.10 (0.04, 0.16) | 0.002 | 828.69 (807.78, 850.03) | 491.29 (479.88, 502.91) | -1.66 (-2.29, -1.03) | <0.001 | 12.08 (9.65, 14.95) | 6.18 (4.96, 7.62) | -2.12 (-3.09, -1.14) | <0.001 |
| Peru | 130.04 (126.69, 133.46) | 119.51 (116.91, 122.16) | -0.29 (-0.31, -0.28) | <0.001 | 2229.11 (2215.00, 2243.28) | 2292.89 (2281.50, 2304.33) | 0.10 (0.07, 0.12) | <0.001 | 1042.79 (1033.03, 1052.63) | 488.15 (482.91, 493.44) | -2.49 (-3.53, -1.45) | <0.001 | 15.37 (14.19, 16.62) | 5.88 (5.32, 6.49) | -3.12 (-4.39, -1.83) | <0.001 |
| Philippines | 126.93 (124.93, 128.96) | 132.78 (131.27, 134.32) | 0.15 (0.15, 0.16) | <0.001 | 1367.66 (1361.04, 1374.31) | 1489.10 (1484.00, 1494.21) | 0.30 (0.24, 0.37) | <0.001 | 495.39 (491.35, 499.45) | 1188.84 (1184.25, 1193.44) | 3.17 (2.71, 3.62) | <0.001 | 6.12 (5.68, 6.60) | 17.94 (17.37, 18.51) | 3.91 (3.32, 4.50) | <0.001 |
| Poland | 77.05 (75.08, 79.07) | 70.86 (68.82, 72.94) | -0.29 (-0.34, -0.24) | <0.001 | 1322.98 (1314.61, 1331.39) | 1352.28 (1343.16, 1361.46) | 0.09 (-0.08, 0.26) | 0.296 | 684.64 (678.81, 690.51) | 288.33 (284.21, 292.50) | -3.02 (-3.35, -2.68) | <0.001 | 9.80 (9.12, 10.52) | 2.74 (2.36, 3.16) | -4.52 (-4.92, -4.12) | <0.001 |

| | | | | | | | | | | | | | | | | |
|---|---|---|---|---|---|---|---|---|---|---|---|---|---|---|---|---|
| Portugal | 49.76 (46.63, 53.04) | 42.70 (39.52, 46.08) | -0.51 (-0.62, -0.40) | <0.001 | 887.19 (873.83, 900.71) | 856.54 (842.09, 871.18) | -0.10 (-0.23, 0.02) | 0.104 | 516.31 (506.13, 526.64) | 188.18 (181.56, 195.00) | -3.39 (-4.02, -2.76) | <0.001 | 7.52 (6.33, 8.86) | 1.98 (1.37, 2.79) | -4.55 (-5.26, -3.84) | <0.001 |
| Puerto Rico | 108.61 (101.15, 116.47) | 116.61 (108.04, 125.70) | 0.28 (0.22, 0.33) | <0.001 | 1137.60 (1113.18, 1162.42) | 1167.55 (1140.07, 1195.55) | 0.09 (0.07, 0.11) | <0.001 | 522.70 (506.18, 539.62) | 278.31 (265.03, 292.10) | -2.26 (-2.90, -1.61) | <0.001 | 7.74 (5.85, 10.05) | 3.60 (2.24, 5.51) | -2.80 (-3.66, -1.94) | <0.001 |
| Qatar | 97.83 (75.82, 124.83) | 84.60 (75.41, 94.89) | -0.49 (-0.55, -0.43) | <0.001 | 1338.61 (1252.62, 1429.41) | 1257.13 (1220.07, 1295.27) | -0.22 (-0.25, -0.19) | <0.001 | 913.56 (842.99, 988.93) | 366.76 (346.61, 388.00) | -2.93 (-4.16, -1.68) | <0.001 | 13.45 (6.23, 26.14) | 4.18 (2.33, 7.34) | -3.71 (-5.30, -2.10) | <0.001 |
| Republic of Korea | 78.47 (76.75, 80.22) | 69.26 (67.51, 71.06) | -0.42 (-0.50, -0.35) | <0.001 | 828.35 (822.74, 834.00) | 777.31 (771.46, 783.21) | -0.17 (-0.26, -0.09) | <0.001 | 934.78 (928.82, 940.77) | 171.97 (169.19, 174.78) | -5.72 (-6.16, -5.27) | <0.001 | 14.33 (13.60, 15.09) | 1.71 (1.45, 2.01) | -7.14 (-7.76, -6.51) | <0.001 |
| Republic of Moldova | 97.84 (91.58, 104.44) | 93.61 (86.50, 101.25) | -0.14 (-0.17, -0.11) | <0.001 | 857.43 (838.77, 876.43) | 862.51 (840.76, 884.77) | 0.02 (-0.02, 0.07) | 0.284 | 665.60 (649.08, 682.45) | 366.35 (352.23, 380.98) | -2.01 (-2.98, -1.03) | <0.001 | 9.93 (8.02, 12.20) | 4.72 (3.27, 6.72) | -2.46 (-3.62, -1.28) | <0.001 |
| Romania | 81.30 (78.63, 84.06) | 84.24 (80.91, 87.67) | 0.13 (0.10, 0.15) | <0.001 | 890.51 (881.62, 899.48) | 956.21 (944.99, 967.54) | 0.28 (0.08, 0.48) | 0.006 | 740.21 (732.13, 748.35) | 430.03 (422.53, 437.63) | -1.82 (-2.36, -1.28) | <0.001 | 11.07 (10.11, 12.11) | 5.60 (4.78, 6.52) | -2.31 (-2.88, -1.73) | <0.001 |
| Russian Federation | 102.20 (101.07, 103.35) | 106.20 (104.93, 107.48) | 0.12 (0.10, 0.15) | <0.001 | 943.01 (939.56, 946.47) | 1022.47 (1018.51, 1026.44) | 0.31 (0.17, 0.44) | <0.001 | 688.70 (685.76, 691.65) | 870.64 (867.11, 874.18) | 0.83 (0.06, 1.60) | 0.035 | 10.17 (9.82, 10.53) | 13.36 (12.93, 13.80) | 0.98 (0.13, 1.84) | 0.023 |
| Rwanda | 209.40 (201.73, 217.32) | 167.22 (162.43, 172.12) | -0.78 (-0.82, -0.74) | <0.001 | 3184.98 (3154.71, 3215.49) | 3140.56 (3119.40, 3161.84) | -0.04 (-0.08, 0.01) | 0.096 | 1793.59 (1770.14, 1817.30) | 677.05 (667.09, 687.11) | -3.35 (-3.69, -3.01) | <0.001 | 27.43 (24.56, 30.55) | 8.24 (7.16, 9.44) | -4.10 (-4.47, -3.73) | <0.001 |
| Saint Kitts and Nevis | 138.22 (69.94, 247.58) | 113.06 (60.59, 194.58) | -0.69 (-0.74, -0.64) | <0.001 | 1257.73 (1027.34, 1526.28) | 1128.43 (944.15, 1339.72) | -0.36 (-0.47, -0.25) | <0.001 | 1970.01 (1677.61, 2300.45) | 384.95 (280.23, 517.56) | -5.47 (-5.86, -5.08) | <0.001 | 33.08 (6.00, 105.33) | 5.25 (0.02, 45.81) | -6.17 (-6.57, -5.76) | <0.001 |
| Saint Lucia | 192.46 | 188.67 | -0.07 | <0.001 | 2795.74 | 2752.43 | -0.05 | 0.001 | 1100.38 | 610.22 | -1.98 | <0.001 | 16.45 | 7.95 | -2.45 | <0.001 |

| | | | | | | | | | | | | | | | | |
|---|---|---|---|---|---|---|---|---|---|---|---|---|---|---|---|---|
| | (143.47, 254.35) | (145.17, 241.55) | (-0.10, -0.04) | | (2597.50, 3006.31) | (2578.64, 2935.25) | (-0.08, -0.02) | | (973.83, 1239.83) | (530.46, 699.05) | (-2.41, -1.54) | | (4.45, 43.91) | (1.50, 25.20) | (-2.96, -1.94) | |
| Saint Vincent and the Grenadines | 187.04 (133.16, 257.80) | 186.77 (132.60, 255.86) | -0.00 (-0.05, 0.04) | 0.870 | 2667.57 (2449.80, 2901.32) | 2712.75 (2493.13, 2946.69) | 0.06 (0.00, 0.12) | 0.045 | 1263.70 (1111.42, 1432.59) | 873.46 (751.23, 1010.11) | -1.23 (-1.98, -0.48) | 0.001 | 19.25 (4.86, 53.85) | 12.50 (2.20, 39.54) | -1.45 (-2.29, -0.60) | 0.001 |
| Samoa | 150.82 (108.12, 206.07) | 155.21 (117.79, 201.32) | 0.10 (0.09, 0.12) | <0.001 | 1893.47 (1734.30, 2064.42) | 1927.73 (1790.18, 2073.58) | 0.09 (-0.03, 0.20) | 0.133 | 1675.46 (1520.77, 1842.45) | 1898.73 (1759.05, 2046.94) | 0.44 (0.40, 0.48) | <0.001 | 25.81 (9.91, 55.92) | 29.66 (14.53, 54.29) | 0.49 (0.44, 0.53) | <0.001 |
| San Marino | 58.27 (9.99, 190.19) | 58.26 (12.81, 172.40) | 0.01 (-0.03, 0.04) | 0.704 | 1517.26 (1176.07, 1929.94) | 1488.07 (1181.46, 1853.67) | -0.07 (-0.09, -0.04) | <0.001 | 270.11 (138.55, 478.15) | 246.54 (133.20, 422.41) | -0.31 (-0.43, -0.19) | <0.001 | 2.79 (0.00, 98.74) | 2.45 (0.00, 83.42) | -0.43 (-0.56, -0.30) | <0.001 |
| Sao Tome and Principe | 168.04 (115.49, 238.76) | 164.31 (127.81, 208.42) | -0.08 (-0.09, -0.06) | <0.001 | 3546.53 (3291.10, 3818.58) | 3888.97 (3703.33, 4081.89) | 0.32 (0.30, 0.35) | <0.001 | 1108.23 (960.43, 1273.91) | 1104.05 (1004.19, 1211.44) | 0.17 (-0.59, 0.94) | 0.654 | 15.08 (2.66, 49.74) | 14.61 (5.25, 32.60) | 0.11 (-0.77, 1.00) | 0.806 |
| Saudi Arabia | 80.11 (76.58, 83.76) | 96.79 (94.66, 98.97) | 0.65 (0.62, 0.69) | <0.001 | 1385.72 (1371.18, 1400.40) | 1487.30 (1478.76, 1495.89) | 0.36 (0.07, 0.64) | 0.013 | 1376.36 (1361.65, 1391.21) | 1019.93 (1012.99, 1026.92) | -0.98 (-1.25, -0.71) | <0.001 | 21.37 (19.55, 23.32) | 15.19 (14.36, 16.06) | -1.21 (-1.39, -1.03) | <0.001 |
| Senegal | 146.05 (139.69, 152.66) | 143.96 (139.68, 148.34) | -0.05 (-0.07, -0.04) | <0.001 | 2554.53 (2527.66, 2581.64) | 2617.69 (2599.30, 2636.20) | 0.11 (0.00, 0.21) | 0.044 | 903.34 (886.87, 920.06) | 682.32 (672.73, 692.01) | -0.95 (-1.62, -0.28) | 0.006 | 12.55 (10.65, 14.72) | 8.72 (7.65, 9.90) | -1.20 (-1.38, -1.01) | <0.001 |
| Serbia | 95.80 (91.27, 100.51) | 94.42 (89.47, 99.60) | -0.05 (-0.07, -0.03) | <0.001 | 1128.46 (1112.73, 1144.36) | 1192.15 (1174.33, 1210.18) | 0.25 (0.01, 0.49) | 0.040 | 790.35 (777.27, 803.61) | 372.31 (362.48, 382.36) | -2.52 (-2.83, -2.21) | <0.001 | 11.67 (10.14, 13.38) | 4.33 (3.34, 5.55) | -3.34 (-3.73, -2.94) | <0.001 |
| Seychelles | 111.48 (63.67, | 108.82 (65.43, | -0.08 (-0.10, | <0.001 | 1835.14 (1619.89 | 1800.12 (1606.52 | -0.06 (-0.09, | 0.003 | 1224.69 (1045.47 | 694.57 (577.43, | -1.93 (-2.38, | <0.001 | 18.29 (2.90, | 9.12 (0.78, | -2.36 (-2.97, | <0.001 |

| | | | | | | | | | | | | | | | | |
|---|---|---|---|---|---|---|---|---|---|---|---|---|---|---|---|---|
| | 185.44) | 171.18) | -0.05) | | , 2074.06) | , 2011.45) | -0.02) | | , 1428.46) | 829.49) | -1.48) | | 64.65) | 39.31) | -1.74) | |
| Sierra Leone | 156.44 (147.32, 166.02) | 167.49 (161.40, 173.77) | 0.24 (0.22, 0.26) | <0.001 | 2737.02 (2698.49, 2775.99) | 2929.30 (2903.74, 2955.04) | 0.24 (0.22, 0.26) | <0.001 | 1031.89 (1007.73, 1056.51) | 1122.26 (1105.96, 1138.75) | 0.33 (0.13, 0.52) | 0.001 | 14.61 (11.83, 17.87) | 15.98 (14.06, 18.10) | 0.33 (0.18, 0.48) | <0.001 |
| Singapore | 86.91 (80.40, 93.86) | 82.89 (77.53, 88.62) | -0.17 (-0.20, -0.13) | <0.001 | 852.52 (831.89, 873.57) | 801.92 (785.61, 818.60) | -0.19 (-0.26, -0.11) | <0.001 | 441.37 (426.48, 456.69) | 160.37 (152.71, 168.41) | -3.46 (-4.11, -2.79) | <0.001 | 6.27 (4.61, 8.39) | 1.56 (0.90, 2.67) | -4.81 (-5.75, -3.87) | <0.001 |
| Slovakia | 80.09 (74.74, 85.74) | 73.43 (68.00, 79.24) | -0.30 (-0.34, -0.27) | <0.001 | 1434.89 (1411.76, 1458.33) | 1365.53 (1341.12, 1390.33) | -0.16 (-0.20, -0.11) | <0.001 | 618.69 (603.77, 633.91) | 335.27 (323.48, 347.44) | -2.01 (-2.38, -1.64) | <0.001 | 8.38 (6.74, 10.33) | 3.55 (2.46, 5.05) | -2.80 (-3.37, -2.24) | <0.001 |
| Slovenia | 74.96 (66.63, 84.11) | 74.34 (64.96, 84.89) | -0.03 (-0.06, -0.00) | 0.035 | 1183.48 (1149.31, 1218.45) | 1122.01 (1083.76, 1161.42) | -0.16 (-0.24, -0.08) | <0.001 | 398.73 (379.26, 419.00) | 173.38 (158.60, 189.33) | -2.83 (-3.26, -2.40) | <0.001 | 5.05 (3.10, 7.87) | 1.25 (0.33, 3.64) | -4.95 (-5.71, -4.19) | <0.001 |
| Solomon Islands | 202.99 (166.21, 246.08) | 189.63 (166.20, 215.50) | -0.23 (-0.25, -0.22) | <0.001 | 1771.53 (1661.14, 1887.92) | 1726.72 (1654.94, 1800.88) | -0.07 (-0.14, -0.01) | 0.034 | 4771.58 (4581.73, 4967.69) | 4659.32 (4539.07, 4782.00) | -0.08 (-0.20, 0.04) | 0.186 | 80.61 (57.42, 110.51) | 78.69 (63.70, 96.20) | -0.09 (-0.21, 0.04) | 0.172 |
| Somalia | 207.97 (199.99, 216.20) | 204.96 (200.34, 209.66) | -0.05 (-0.08, -0.02) | 0.002 | 3159.11 (3127.43, 3191.04) | 3299.67 (3280.77, 3318.66) | 0.16 (0.10, 0.22) | <0.001 | 1457.01 (1435.31, 1478.96) | 1075.83 (1064.65, 1087.11) | -1.01 (-1.13, -0.90) | <0.001 | 21.81 (19.22, 24.66) | 15.04 (13.73, 16.46) | -1.26 (-1.42, -1.09) | <0.001 |
| South Africa | 188.58 (185.58, 191.62) | 169.08 (166.72, 171.46) | -0.37 (-0.40, -0.35) | <0.001 | 2911.83 (2899.94, 2923.75) | 2825.24 (2815.65, 2834.86) | -0.09 (-0.17, -0.02) | 0.016 | 1599.48 (1590.38, 1608.62) | 617.65 (613.24, 622.08) | -3.10 (-4.74, -1.43) | <0.001 | 24.41 (23.29, 25.58) | 7.51 (7.04, 8.01) | -3.88 (-5.82, -1.89) | <0.001 |
| South Sudan | 154.98 (147.45, 162.83) | 155.64 (149.83, 161.64) | 0.02 (-0.00, 0.03) | 0.081 | 3055.80 (3021.69, 3090.25) | 3212.05 (3184.94, 3239.34) | 0.19 (0.12, 0.26) | <0.001 | 915.50 (896.05, 935.30) | 665.57 (653.07, 678.26) | -1.10 (-1.44, -0.77) | <0.001 | 12.37 (10.16, 14.97) | 7.88 (6.56, 9.41) | -1.55 (-1.97, -1.14) | <0.001 |

| | | | | | | | | | | | | | | | | |
|---|---|---|---|---|---|---|---|---|---|---|---|---|---|---|---|---|
| Spain | 61.90 (60.10, 63.74) | 58.37 (56.53, 60.26) | -0.21 (-0.27, -0.14) | <0.001 | 797.46 (790.97, 803.99) | 796.03 (789.23, 802.88) | 0.02 (-0.13, 0.17) | 0.776 | 415.44 (410.76, 420.17) | 180.42 (177.20, 183.69) | -2.87 (-3.11, -2.64) | <0.001 | 5.87 (5.33, 6.46) | 1.96 (1.65, 2.33) | -3.78 (-4.08, -3.48) | <0.001 |
| Sri Lanka | 64.57 (61.96, 67.25) | 63.84 (61.45, 66.31) | -0.03 (-0.06, 0.01) | 0.099 | 760.77 (751.77, 769.84) | 759.99 (751.67, 768.38) | 0.03 (-0.15, 0.21) | 0.766 | 952.45 (942.41, 962.57) | 494.98 (488.26, 501.77) | -2.09 (-3.15, -1.03) | <0.001 | 14.35 (13.14, 15.65) | 6.61 (5.85, 7.43) | -2.48 (-3.72, -1.23) | <0.001 |
| Sudan | 156.56 (152.49, 160.71) | 155.53 (152.87, 158.22) | -0.03 (-0.03, -0.02) | <0.001 | 1863.00 (1848.91, 1877.17) | 1959.76 (1950.30, 1969.24) | 0.19 (0.09, 0.28) | <0.001 | 2360.47 (2344.34, 2376.69) | 1342.12 (1334.22, 1350.05) | -1.91 (-2.00, -1.81) | <0.001 | 38.18 (36.13, 40.32) | 20.50 (19.53, 21.51) | -2.10 (-2.19, -2.01) | <0.001 |
| Suriname | 186.57 (156.72, 220.89) | 191.13 (166.00, 219.04) | 0.09 (0.06, 0.13) | <0.001 | 2291.43 (2182.81, 2404.43) | 2353.58 (2263.24, 2446.64) | 0.12 (0.04, 0.20) | 0.004 | 1190.62 (1110.94, 1274.81) | 903.68 (848.15, 961.93) | -0.71 (-2.66, 1.28) | 0.483 | 18.38 (9.64, 32.19) | 13.38 (7.43, 22.27) | -0.86 (-3.20, 1.54) | 0.479 |
| Sweden | 69.01 (64.83, 73.41) | 76.31 (72.07, 80.74) | 0.35 (0.30, 0.41) | <0.001 | 1231.50 (1213.51, 1249.69) | 1108.78 (1092.46, 1125.30) | -0.30 (-0.48, -0.13) | 0.001 | 336.46 (327.16, 345.98) | 190.90 (184.15, 197.85) | -1.88 (-2.68, -1.08) | <0.001 | 4.00 (3.05, 5.17) | 1.45 (0.93, 2.19) | -3.44 (-4.65, -2.22) | <0.001 |
| Switzerland | 54.12 (50.23, 58.27) | 52.32 (48.61, 56.27) | -0.11 (-0.19, -0.02) | 0.012 | 790.02 (774.99, 805.31) | 748.53 (734.42, 762.88) | -0.15 (-0.35, 0.04) | 0.121 | 282.01 (273.01, 291.27) | 128.17 (122.35, 134.23) | -2.66 (-3.09, -2.23) | <0.001 | 3.71 (2.74, 4.94) | 1.17 (0.69, 1.93) | -3.92 (-4.44, -3.39) | <0.001 |
| Syrian Arab Republic | 147.55 (142.35, 152.90) | 133.50 (129.14, 137.99) | -0.35 (-0.39, -0.31) | <0.001 | 2071.96 (2052.24, 2091.84) | 2079.47 (2062.07, 2097.00) | 0.03 (-0.03, 0.08) | 0.380 | 3128.23 (3103.88, 3152.75) | 1558.76 (1543.65, 1574.00) | -2.32 (-2.64, -2.00) | <0.001 | 50.32 (47.24, 53.57) | 23.75 (21.91, 25.72) | -2.50 (-2.86, -2.13) | <0.001 |
| Taiwan (Province of China) | 62.72 (60.45, 65.06) | 70.05 (67.50, 72.69) | 0.39 (0.36, 0.41) | <0.001 | 919.70 (910.93, 928.54) | 1000.76 (991.10, 1010.51) | 0.30 (0.25, 0.35) | <0.001 | 497.05 (490.60, 503.57) | 280.01 (274.91, 285.19) | -1.92 (-2.19, -1.65) | <0.001 | 6.53 (5.81, 7.32) | 2.80 (2.33, 3.37) | -2.83 (-3.54, -2.11) | <0.001 |
| Tajikistan | 145.74 (138.34, 153.48) | 144.37 (139.11, 149.79) | -0.03 (-0.05, -0.01) | 0.006 | 1743.83 (1717.68, 1770.32) | 1753.87 (1735.38, 1772.51) | 0.03 (-0.00, 0.06) | 0.069 | 1341.03 (1317.27, 1365.12) | 850.76 (837.73, 863.95) | -1.50 (-1.75, -1.25) | <0.001 | 21.25 (18.32, 24.55) | 12.78 (11.22, 14.51) | -1.66 (-1.94, -1.38) | <0.001 |
| Thailand | 105.81 | 102.79 | -0.11 | <0.001 | 1569.85 | 1549.62 | -0.04 | 0.150 | 666.12 | 467.19 | -1.27 | 0.018 | 9.11 | 5.61 | -1.68 | 0.013 |

| | | | | | | | | | | | | | | | | |
|---|---|---|---|---|---|---|---|---|---|---|---|---|---|---|---|---|
| | (104.03, 107.61) | (101.00, 104.61) | (-0.12, -0.09) | | (1562.99, 1576.74) | (1542.67, 1556.59) | (-0.09, 0.01) | | (661.60, 670.67) | (463.42, 470.99) | (-2.31, -0.22) | | (8.59, 9.66) | (5.21, 6.04) | (-2.99, -0.35) | |
| Timor-Leste | 132.53 (114.66, 152.56) | 123.54 (109.96, 138.51) | -0.24 (-0.26, -0.23) | <0.001 | 1550.98 (1488.10, 1615.98) | 1550.91 (1501.22, 1601.97) | 0.02 (-0.11, 0.14) | 0.808 | 879.84 (831.83, 930.03) | 926.19 (886.76, 967.06) | 0.32 (-0.28, 0.93) | 0.301 | 12.59 (7.42, 20.16) | 13.39 (8.96, 19.38) | 0.37 (-0.35, 1.10) | 0.310 |
| Togo | 151.50 (142.40, 161.07) | 151.23 (145.35, 157.30) | -0.01 (-0.02, 0.01) | 0.395 | 2671.88 (2633.31, 2710.91) | 2783.43 (2757.99, 2809.05) | 0.15 (0.10, 0.20) | <0.001 | 1140.54 (1114.59, 1166.99) | 881.86 (867.42, 896.49) | -0.89 (-1.09, -0.70) | <0.001 | 16.52 (13.49, 20.07) | 12.06 (10.41, 13.89) | -1.03 (-1.29, -0.77) | <0.001 |
| Tokelau | 118.22 (0.00, 1911.84) | 112.62 (0.00, 2501.25) | -0.17 (-0.17, -0.16) | <0.001 | 1042.81 (201.30, 3355.81) | 1049.75 (142.16, 3987.06) | 0.03 (-0.01, 0.07) | 0.118 | 2325.44 (868.73, 5174.62) | 2050.04 (586.51, 5425.14) | -0.43 (-0.45, -0.41) | <0.001 | 38.09 (0.00, 1772.65) | 33.23 (0.00, 2366.33) | -0.47 (-0.49, -0.45) | <0.001 |
| Tonga | 143.97 (91.67, 217.65) | 138.78 (90.96, 203.48) | -0.11 (-0.26, 0.05) | 0.188 | 2389.24 (2159.40, 2638.78) | 2401.68 (2186.84, 2632.46) | 0.04 (-0.08, 0.16) | 0.532 | 892.35 (748.39, 1057.31) | 780.94 (658.78, 919.57) | -0.46 (-0.58, -0.34) | <0.001 | 11.97 (1.16, 48.98) | 9.97 (1.03, 38.66) | -0.59 (-0.80, -0.39) | <0.001 |
| Trinidad and Tobago | 200.76 (183.45, 219.32) | 192.12 (175.36, 210.15) | -0.15 (-0.16, -0.14) | <0.001 | 2689.39 (2624.87, 2755.16) | 2694.11 (2630.87, 2758.59) | 0.02 (-0.04, 0.07) | 0.571 | 1020.54 (980.63, 1061.69) | 667.28 (636.42, 699.38) | -1.57 (-2.00, -1.14) | <0.001 | 15.07 (10.55, 20.91) | 8.94 (5.74, 13.47) | -1.93 (-2.43, -1.42) | <0.001 |
| Tunisia | 76.21 (71.89, 80.72) | 78.35 (74.83, 82.00) | 0.10 (0.07, 0.13) | <0.001 | 1008.48 (992.81, 1024.35) | 1133.47 (1119.82, 1147.26) | 0.38 (0.28, 0.48) | <0.001 | 904.68 (889.65, 919.91) | 581.34 (571.70, 591.13) | -1.51 (-1.74, -1.28) | <0.001 | 14.21 (12.37, 16.27) | 8.37 (7.26, 9.62) | -1.79 (-2.05, -1.54) | <0.001 |
| Turkey | 86.75 (85.02, 88.50) | 69.62 (68.37, 70.89) | -0.75 (-0.80, -0.70) | <0.001 | 1426.87 (1419.88, 1433.88) | 1250.32 (1244.98, 1255.67) | -0.44 (-0.52, -0.37) | <0.001 | 750.63 (745.54, 755.75) | 367.10 (364.21, 370.00) | -2.40 (-2.94, -1.85) | <0.001 | 10.72 (10.11, 11.35) | 4.33 (4.02, 4.65) | -3.03 (-3.72, -2.33) | <0.001 |
| Turkmenistan | 144.34 (135.85, | 150.82 (143.11, | 0.16 (0.12, | <0.001 | 1857.12 (1826.11 | 1925.68 (1897.89 | 0.14 (0.06, | 0.001 | 1676.98 (1647.06 | 1594.05 (1568.81 | -0.12 (-0.99, | 0.780 | 26.40 (22.74, | 24.48 (21.45, | -0.20 (-1.13, | 0.675 |

| | | | | | | | | | | | | | | | | |
|---|---|---|---|---|---|---|---|---|---|---|---|---|---|---|---|---|
| | 153.27) | 158.86) | 0.20) | | , 1888.56) | , 1953.79) | 0.23) | | , 1707.33) | , 1619.60) | 0.75) | | 30.53) | 27.84) | 0.74) | |
| Tuvalu | 139.79 (25.39, 438.79) | 114.42 (17.96, 387.38) | -0.69 (-0.72, -0.66) | <0.001 | 1062.27 (651.86, 1639.19) | 985.86 (607.35, 1520.47) | -0.24 (-0.34, -0.13) | <0.001 | 2941.63 (2225.23, 3819.34) | 2265.74 (1665.30, 3018.68) | -0.87 (-0.95, -0.79) | <0.001 | 48.56 (0.98, 292.94) | 37.04 (0.35, 263.69) | -0.90 (-0.99, -0.82) | <0.001 |
| Uganda | 185.12 (180.40, 189.95) | 176.12 (173.26, 179.01) | -0.17 (-0.18, -0.17) | <0.001 | 3262.84 (3242.56, 3283.23) | 3519.28 (3506.20, 3532.40) | 0.30 (0.12, 0.47) | 0.001 | 702.46 (692.59, 712.45) | 671.03 (665.10, 677.00) | -0.19 (-0.46, 0.08) | 0.169 | 8.59 (7.50, 9.81) | 7.78 (7.14, 8.47) | -0.44 (-0.83, -0.04) | 0.032 |
| Ukraine | 96.79 (94.87, 98.75) | 104.65 (102.35, 106.99) | 0.28 (0.24, 0.32) | <0.001 | 972.55 (966.43, 978.69) | 1088.54 (1081.09, 1096.05) | 0.40 (0.24, 0.56) | <0.001 | 571.27 (566.61, 575.97) | 903.34 (896.73, 910.00) | 1.63 (-0.47, 3.77) | 0.129 | 7.93 (7.39, 8.50) | 13.49 (12.71, 14.31) | 1.88 (-0.59, 4.41) | 0.137 |
| United Arab Emirates | 137.50 (124.42, 151.66) | 141.17 (134.12, 148.55) | 0.09 (0.06, 0.11) | <0.001 | 1743.08 (1695.78, 1791.43) | 1751.17 (1726.18, 1776.50) | 0.04 (-0.07, 0.15) | 0.514 | 1017.79 (981.52, 1055.13) | 713.85 (698.37, 729.65) | -1.22 (-1.56, -0.88) | <0.001 | 14.77 (10.69, 19.98) | 9.65 (7.99, 11.65) | -1.49 (-1.90, -1.07) | <0.001 |
| United Kingdom | 72.77 (71.16, 74.41) | 70.34 (68.81, 71.91) | -0.11 (-0.17, -0.06) | <0.001 | 1100.05 (1093.75, 1106.39) | 1125.43 (1119.21, 1131.67) | 0.09 (0.01, 0.18) | 0.037 | 377.22 (373.53, 380.94) | 261.20 (258.24, 264.19) | -1.21 (-1.35, -1.07) | <0.001 | 5.12 (4.70, 5.57) | 3.17 (2.86, 3.52) | -1.62 (-2.10, -1.14) | <0.001 |
| United Republic of Tanzania | 170.20 (166.58, 173.88) | 174.65 (172.24, 177.08) | 0.09 (0.08, 0.10) | <0.001 | 3076.23 (3060.54, 3091.98) | 3338.71 (3328.02, 3349.44) | 0.29 (0.22, 0.37) | <0.001 | 903.59 (894.75, 912.50) | 712.85 (707.78, 717.96) | -0.75 (-0.87, -0.63) | <0.001 | 12.18 (11.15, 13.28) | 8.62 (8.06, 9.21) | -1.13 (-1.29, -0.96) | <0.001 |
| United States of America | 145.65 (144.63, 146.68) | 149.19 (148.18, 150.20) | 0.08 (0.03, 0.13) | 0.001 | 2025.46 (2021.64, 2029.30) | 1959.78 (1956.13, 1963.43) | -0.09 (-0.29, 0.10) | 0.338 | 507.97 (506.05, 509.89) | 460.58 (458.82, 462.35) | -0.34 (-0.65, -0.02) | 0.035 | 6.62 (6.40, 6.84) | 5.81 (5.61, 6.01) | -0.43 (-0.74, -0.11) | 0.008 |
| United States Virgin Islands | 105.46 (66.07, 160.40) | 112.64 (67.15, 178.65) | 0.24 (0.21, 0.27) | <0.001 | 1122.46 (983.60, 1276.00) | 1150.69 (991.94, 1328.70) | 0.10 (0.06, 0.13) | <0.001 | 678.75 (572.14, 800.10) | 370.58 (283.63, 477.08) | -2.04 (-2.30, -1.78) | <0.001 | 10.53 (1.51, 37.13) | 5.17 (0.08, 35.05) | -2.39 (-2.72, -2.07) | <0.001 |

| | | | | | | | | | | | | | | | | |
|---|---|---|---|---|---|---|---|---|---|---|---|---|---|---|---|---|
| Uruguay | 64.36 (57.95, 71.29) | 60.71 (54.74, 67.19) | -0.18 (-0.22, -0.14) | <0.001 | 844.00 (820.34, 868.17) | 873.75 (850.57, 897.43) | 0.15 (-0.02, 0.31) | 0.086 | 717.78 (695.97, 740.10) | 354.00 (339.39, 369.10) | -2.39 (-2.74, -2.04) | <0.001 | 10.97 (8.42, 14.05) | 4.69 (3.15, 6.74) | -2.87 (-3.32, -2.40) | <0.001 |
| Uzbekistan | 147.16 (143.49, 150.91) | 144.42 (141.62, 147.25) | -0.05 (-0.11, 0.01) | 0.098 | 1771.48 (1758.58, 1784.45) | 1872.72 (1862.65, 1882.83) | 0.22 (0.11, 0.32) | <0.001 | 1242.67 (1231.72, 1253.70) | 1229.82 (1221.69, 1237.99) | -0.04 (-0.44, 0.35) | 0.834 | 18.99 (17.65, 20.41) | 18.74 (17.75, 19.78) | -0.06 (-0.50, 0.38) | 0.785 |
| Vanuatu | 198.97 (149.71, 260.12) | 179.85 (146.82, 218.43) | -0.35 (-0.36, -0.34) | <0.001 | 2102.15 (1934.75, 2280.92) | 2011.29 (1897.15, 2130.81) | -0.14 (-0.21, -0.08) | <0.001 | 2078.31 (1909.45, 2258.73) | 2645.97 (2513.32, 2784.07) | 0.81 (0.56, 1.05) | <0.001 | 32.00 (14.21, 62.85) | 42.16 (26.86, 63.35) | 0.92 (0.65, 1.19) | <0.001 |
| Venezuela (Bolivarian Republic of) | 79.09 (76.30, 81.96) | 70.17 (67.99, 72.40) | -0.41 (-0.43, -0.39) | <0.001 | 943.73 (934.06, 953.48) | 903.79 (895.92, 911.70) | -0.10 (-0.33, 0.12) | 0.370 | 909.54 (899.96, 919.20) | 659.91 (653.19, 666.68) | -1.27 (-2.38, -0.15) | 0.026 | 14.58 (13.38, 15.86) | 10.09 (9.28, 10.96) | -1.47 (-2.74, -0.19) | 0.025 |
| Viet Nam | 64.09 (62.74, 65.45) | 63.68 (62.59, 64.79) | 0.03 (-0.06, 0.12) | 0.542 | 739.76 (735.20, 744.35) | 784.16 (780.30, 788.05) | 0.23 (-0.01, 0.47) | 0.059 | 665.40 (661.00, 669.83) | 421.69 (418.89, 424.52) | -1.54 (-1.68, -1.41) | <0.001 | 9.96 (9.42, 10.53) | 5.45 (5.14, 5.78) | -2.03 (-2.12, -1.95) | <0.001 |
| Yemen | 160.50 (155.26, 165.88) | 156.79 (153.72, 159.92) | -0.08 (-0.11, -0.06) | <0.001 | 1901.49 (1883.32, 1919.79) | 1976.78 (1965.81, 1987.80) | 0.17 (-0.06, 0.40) | 0.150 | 1620.81 (1603.61, 1638.15) | 1249.72 (1240.90, 1258.60) | -0.85 (-1.09, -0.61) | <0.001 | 26.13 (23.97, 28.43) | 19.35 (18.26, 20.49) | -1.02 (-1.35, -0.68) | <0.001 |
| Zambia | 190.55 (183.48, 197.87) | 177.91 (173.72, 182.18) | -0.24 (-0.27, -0.21) | <0.001 | 3146.96 (3117.72, 3176.43) | 3178.55 (3160.49, 3196.69) | 0.03 (-0.00, 0.07) | 0.068 | 1341.48 (1321.19, 1362.02) | 886.28 (876.39, 896.26) | -1.44 (-1.66, -1.21) | <0.001 | 19.69 (17.25, 22.41) | 11.85 (10.71, 13.09) | -1.75 (-1.99, -1.51) | <0.001 |
| Zimbabwe | 157.66 (152.17, 163.33) | 174.75 (170.22, 179.39) | 0.36 (0.30, 0.42) | <0.001 | 2787.87 (2764.45, 2811.46) | 2880.10 (2861.56, 2898.74) | 0.12 (0.05, 0.19) | 0.001 | 720.32 (708.16, 732.66) | 919.11 (908.52, 929.81) | 0.82 (0.04, 1.60) | 0.041 | 9.12 (7.77, 10.65) | 12.41 (11.19, 13.73) | 1.05 (-0.39, 2.51) | 0.152 |
| **Men** | | | | | | | | | | | | | | | | |
| Afghanistan | 159.43 (152.59, 166.55) | 166.50 (163.37, 169.69) | 0.15 (0.12, 0.19) | <0.001 | 1675.83 (1653.64, | 1766.16 (1755.97, | 0.20 (0.08, 0.32) | 0.001 | 2576.92 (2547.68, | 1971.14 (1959.71, | -0.91 (-1.12, -0.71) | <0.001 | 43.74 (39.96, 47.82) | 32.95 (31.47, 34.49) | -0.97 (-1.14, -0.80) | <0.001 |

| | | | | | | | | | | | | | | | | |
|---|---|---|---|---|---|---|---|---|---|---|---|---|---|---|---|---|
| | | | | | 1698.27) | 1776.39) | | | 2606.45) | 1982.62) | | | | | | |
| Albania | 145.48 (136.69, 154.71) | 149.26 (138.69, 160.45) | 0.10 (0.06, 0.13) | <0.001 | 1957.61 (1925.05, 1990.62) | 1996.02 (1956.85, 2035.81) | 0.07 (0.04, 0.10) | <0.001 | 1127.23 (1102.38, 1152.51) | 1484.84 (1451.20, 1519.08) | 0.80 (-0.19, 1.80) | 0.114 | 16.95 (14.01, 20.35) | 23.09 (19.05, 27.75) | 0.89 (-0.20, 1.99) | 0.108 |
| Algeria | 134.22 (130.87, 137.64) | 125.67 (123.35, 128.03) | -0.22 (-0.25, -0.20) | <0.001 | 1507.99 (1496.74, 1519.31) | 1538.27 (1530.09, 1546.49) | 0.09 (-0.01, 0.18) | 0.063 | 2125.75 (2112.06, 2139.51) | 1079.26 (1072.50, 1086.05) | -2.22 (-2.52, -1.92) | <0.001 | 35.17 (33.41, 37.01) | 16.90 (16.07, 17.77) | -2.48 (-2.68, -2.28) | <0.001 |
| American Samoa | 152.77 (84.35, 257.22) | 141.91 (76.03, 245.23) | -0.26 (-0.27, -0.24) | <0.001 | 1776.10 (1518.89, 2066.52) | 1744.41 (1489.45, 2033.38) | -0.06 (-0.09, -0.03) | 0.001 | 2409.16 (2101.04, 2751.26) | 2267.09 (1965.12, 2604.31) | -0.23 (-0.38, -0.08) | 0.003 | 39.51 (9.66, 109.22) | 37.24 (8.39, 107.95) | -0.23 (-0.39, -0.07) | 0.005 |
| Andorra | 79.58 (40.05, 147.90) | 75.07 (36.52, 141.93) | -0.20 (-0.23, -0.17) | <0.001 | 485.94 (377.88, 619.94) | 467.28 (361.23, 598.75) | -0.11 (-0.24, 0.01) | 0.080 | 419.88 (320.18, 545.60) | 288.05 (207.41, 394.24) | -1.29 (-1.32, -1.25) | <0.001 | 6.62 (0.14, 49.22) | 4.38 (0.02, 44.24) | -1.42 (-1.47, -1.36) | <0.001 |
| Angola | 187.01 (180.92, 193.26) | 174.62 (171.01, 178.30) | -0.23 (-0.26, -0.21) | <0.001 | 2631.79 (2608.85, 2654.89) | 2666.75 (2652.56, 2681.01) | 0.06 (0.00, 0.11) | 0.047 | 1443.12 (1425.72, 1460.69) | 1147.73 (1138.10, 1157.43) | -0.76 (-1.46, -0.07) | 0.032 | 22.19 (20.06, 24.50) | 17.17 (15.99, 18.41) | -0.85 (-1.62, -0.08) | 0.030 |
| Antigua and Barbuda | 175.47 (108.98, 269.44) | 174.05 (117.32, 249.28) | -0.03 (-0.04, -0.02) | <0.001 | 2290.37 (2029.74, 2576.68) | 2260.98 (2041.38, 2498.25) | -0.05 (-0.07, -0.02) | 0.002 | 1138.63 (953.95, 1349.85) | 774.52 (648.93, 917.91) | -1.33 (-3.42, 0.81) | 0.222 | 17.88 (2.29, 65.52) | 11.42 (1.36, 42.71) | -1.56 (-3.95, 0.89) | 0.211 |
| Argentina | 125.46 (122.64, 128.32) | 119.65 (117.36, 121.97) | -0.17 (-0.18, -0.16) | <0.001 | 1419.63 (1410.12, 1429.19) | 1485.46 (1477.36, 1493.59) | 0.16 (0.11, 0.20) | <0.001 | 1438.93 (1429.31, 1448.60) | 720.55 (714.93, 726.20) | -2.42 (-2.60, -2.25) | <0.001 | 23.47 (22.25, 24.73) | 10.87 (10.19, 11.58) | -2.68 (-2.88, -2.48) | <0.001 |
| Armenia | 174.57 (164.94, 184.63) | 160.62 (150.28, 171.56) | -0.28 (-0.32, -0.24) | <0.001 | 1796.50 (1765.32, 1828.12) | 1698.31 (1664.46, 1732.74) | -0.20 (-0.24, -0.15) | <0.001 | 1366.02 (1338.68, 1393.78) | 905.41 (881.59, 929.80) | -1.44 (-2.40, -0.48) | 0.003 | 22.39 (19.00, 26.22) | 14.21 (11.40, 17.62) | -1.55 (-2.59, -0.49) | 0.004 |
| Australia | 137.46 | 136.97 | -0.03 | 0.422 | 702.09 | 743.38 | 0.19 | <0.001 | 613.91 | 340.47 | -2.01 | <0.001 | 9.96 | 5.30 | -2.15 | <0.001 |

| | | | | | | | | | | | | | | | | |
|---|---|---|---|---|---|---|---|---|---|---|---|---|---|---|---|---|
| | (133.60, 141.41) | (133.54, 140.48) | (-0.09, 0.04) | | (693.34, 710.93) | (735.42, 751.42) | (0.11, 0.28) | | (605.72, 622.19) | (335.08, 345.94) | (-2.44, -1.59) | | (8.95, 11.07) | (4.65, 6.02) | (-2.63, -1.68) | |
| Austria | 98.78 (93.87, 103.90) | 91.60 (86.85, 96.57) | -0.26 (-0.29, -0.23) | <0.001 | 611.43 (599.10, 623.97) | 576.05 (564.09, 588.22) | -0.21 (-0.35, -0.07) | 0.003 | 679.17 (666.12, 692.42) | 271.32 (263.15, 279.70) | -3.12 (-3.69, -2.55) | <0.001 | 11.03 (9.42, 12.86) | 3.98 (3.05, 5.13) | -3.46 (-4.11, -2.81) | <0.001 |
| Azerbaijan | 174.08 (167.39, 180.97) | 165.01 (159.66, 170.51) | -0.17 (-0.21, -0.14) | <0.001 | 1702.75 (1681.69, 1724.02) | 1659.33 (1642.34, 1676.48) | -0.09 (-0.14, -0.04) | <0.001 | 2089.44 (2065.44, 2113.65) | 1565.76 (1549.48, 1582.19) | -1.16 (-2.09, -0.21) | 0.017 | 35.07 (31.99, 38.37) | 25.51 (23.48, 27.69) | -1.32 (-2.30, -0.32) | 0.010 |
| Bahamas | 181.69 (147.94, 221.32) | 188.90 (158.81, 223.13) | 0.13 (0.11, 0.16) | <0.001 | 2026.94 (1910.40, 2149.16) | 2008.97 (1907.84, 2114.17) | -0.03 (-0.05, -0.01) | 0.005 | 1605.80 (1499.15, 1718.35) | 1587.85 (1498.49, 1681.26) | -0.08 (-0.79, 0.64) | 0.835 | 26.53 (14.38, 45.21) | 26.39 (16.03, 41.06) | -0.07 (-0.84, 0.70) | 0.855 |
| Bahrain | 98.20 (83.59, 115.36) | 94.06 (84.34, 104.84) | -0.15 (-0.22, -0.07) | <0.001 | 964.04 (916.98, 1013.51) | 911.10 (880.52, 942.71) | -0.18 (-0.23, -0.13) | <0.001 | 1558.33 (1497.84, 1621.24) | 631.74 (605.37, 659.17) | -3.03 (-3.52, -2.53) | <0.001 | 25.57 (18.44, 35.40) | 9.52 (6.61, 13.60) | -3.32 (-3.80, -2.83) | <0.001 |
| Bangladesh | 119.05 (117.55, 120.57) | 126.48 (125.26, 127.72) | 0.21 (0.20, 0.22) | <0.001 | 1222.51 (1217.65, 1227.39) | 1316.59 (1312.63, 1320.57) | 0.27 (0.20, 0.33) | <0.001 | 1605.78 (1600.13, 1611.45) | 1238.95 (1235.09, 1242.82) | -0.87 (-1.53, -0.20) | 0.011 | 26.33 (25.60, 27.07) | 20.08 (19.59, 20.58) | -0.93 (-1.60, -0.25) | 0.007 |
| Barbados | 182.13 (147.83, 222.11) | 184.74 (148.80, 226.92) | 0.05 (0.04, 0.06) | <0.001 | 2136.36 (2014.52, 2263.74) | 2145.07 (2017.76, 2278.45) | 0.01 (-0.01, 0.03) | 0.557 | 1132.59 (1044.10, 1226.67) | 745.80 (672.14, 825.52) | -1.57 (-2.48, -0.65) | 0.001 | 17.93 (8.42, 33.57) | 11.09 (3.90, 25.12) | -1.64 (-2.70, -0.56) | 0.003 |
| Belarus | 130.46 (125.58, 135.49) | 130.63 (125.25, 136.22) | 0.00 (-0.05, 0.06) | 0.887 | 680.41 (669.26, 691.71) | 709.58 (697.21, 722.15) | 0.14 (0.06, 0.22) | 0.001 | 1930.88 (1912.16, 1949.75) | 1710.54 (1691.71, 1729.57) | -0.39 (-1.39, 0.62) | 0.446 | 33.65 (31.23, 36.23) | 29.76 (27.34, 32.38) | -0.39 (-1.43, 0.66) | 0.461 |
| Belgium | 79.17 (75.29, 83.20) | 74.72 (70.84, 78.78) | -0.20 (-0.22, -0.18) | <0.001 | 558.04 (547.63, 568.60) | 568.40 (557.58, 579.40) | 0.08 (-0.01, 0.16) | 0.069 | 684.10 (672.60, 695.77) | 249.00 (241.90, 256.26) | -3.42 (-4.07, -2.77) | <0.001 | 11.11 (9.69, 12.69) | 3.56 (2.76, 4.53) | -3.86 (-4.53, -3.18) | <0.001 |
| Belize | 178.72 | 189.14 | 0.20 | <0.001 | 1973.90 | 2060.27 | 0.15 | <0.001 | 901.95 | 1016.47 | 0.41 | 0.293 | 13.69 | 15.88 | 0.52 | 0.258 |

| | | | | | | | | | | | | | | | | |
|---|---|---|---|---|---|---|---|---|---|---|---|---|---|---|---|---|
| | (136.42, 231.16) | (161.00, 220.97) | (0.18, 0.21) | | (1827.29, 2130.15) | (1965.07, 2159.05) | (0.14, 0.17) | | (801.62, 1012.27) | (948.71, 1087.91) | (-0.35, 1.18) | | (3.98, 35.38) | (8.42, 27.38) | (-0.38, 1.42) | |
| Benin | 162.16 (153.01, 171.74) | 159.08 (153.83, 164.48) | -0.07 (-0.07, -0.06) | <0.001 | 2486.49 (2450.60, 2522.79) | 2573.32 (2552.29, 2594.49) | 0.13 (0.05, 0.21) | 0.002 | 1229.82 (1203.78, 1256.32) | 1077.50 (1063.32, 1091.84) | -0.42 (-0.60, -0.25) | <0.001 | 18.58 (15.47, 22.17) | 15.93 (14.22, 17.80) | -0.50 (-0.69, -0.30) | <0.001 |
| Bermuda | 111.67 (62.64, 189.58) | 111.43 (54.82, 207.82) | -0.01 (-0.05, 0.03) | 0.705 | 1099.30 (926.11, 1299.18) | 1070.68 (869.27, 1308.76) | -0.10 (-0.15, -0.05) | <0.001 | 1013.07 (849.74, 1202.68) | 475.08 (346.44, 640.71) | -2.75 (-3.12, -2.37) | <0.001 | 16.50 (2.37, 64.80) | 7.21 (0.05, 63.84) | -3.02 (-3.49, -2.54) | <0.001 |
| Bhutan | 131.44 (112.18, 153.41) | 124.31 (108.76, 141.54) | -0.19 (-0.21, -0.17) | <0.001 | 1302.29 (1238.88, 1368.38) | 1280.84 (1229.82, 1333.52) | -0.05 (-0.17, 0.06) | 0.377 | 1350.69 (1284.53, 1419.61) | 991.48 (946.66, 1037.96) | -1.05 (-1.19, -0.91) | <0.001 | 21.93 (14.15, 32.74) | 15.57 (10.41, 22.49) | -1.17 (-1.32, -1.02) | <0.001 |
| Bolivia (Plurinational State of) | 140.42 (133.70, 147.42) | 130.70 (126.18, 135.35) | -0.25 (-0.27, -0.23) | <0.001 | 1825.92 (1801.33, 1850.78) | 1815.59 (1798.62, 1832.69) | -0.02 (-0.04, 0.01) | 0.289 | 1298.31 (1277.28, 1319.61) | 753.50 (742.52, 764.61) | -1.86 (-2.01, -1.70) | <0.001 | 20.34 (17.76, 23.20) | 10.98 (9.69, 12.41) | -2.10 (-2.27, -1.93) | <0.001 |
| Bosnia and Herzegovina | 106.14 (99.88, 112.69) | 112.34 (103.67, 121.59) | 0.20 (0.13, 0.27) | <0.001 | 810.50 (793.02, 828.29) | 877.25 (852.70, 902.38) | 0.27 (0.23, 0.31) | <0.001 | 1170.81 (1149.82, 1192.10) | 726.50 (704.30, 749.27) | -1.84 (-2.32, -1.36) | <0.001 | 19.50 (16.88, 22.43) | 11.37 (8.76, 14.57) | -2.09 (-2.59, -1.57) | <0.001 |
| Botswana | 196.42 (178.23, 216.12) | 175.56 (164.40, 187.30) | -0.39 (-0.42, -0.36) | <0.001 | 2359.55 (2295.50, 2425.07) | 2332.93 (2291.76, 2374.67) | -0.04 (-0.07, -0.02) | <0.001 | 1348.04 (1297.94, 1399.69) | 1041.50 (1014.32, 1069.25) | -0.82 (-1.11, -0.53) | <0.001 | 20.80 (14.91, 28.36) | 15.70 (12.53, 19.44) | -0.90 (-1.22, -0.58) | <0.001 |
| Brazil | 163.49 (162.04, 164.95) | 140.75 (139.63, 141.88) | -0.52 (-0.54, -0.50) | <0.001 | 1990.81 (1985.75, 1995.88) | 1901.74 (1897.62, 1905.86) | -0.15 (-0.17, -0.12) | <0.001 | 1739.95 (1735.09, 1744.83) | 924.64 (921.81, 927.48) | -2.17 (-2.36, -1.98) | <0.001 | 28.87 (28.24, 29.50) | 14.29 (13.94, 14.65) | -2.42 (-2.66, -2.18) | <0.001 |
| Brunei Darussalam | 134.24 (107.85, 165.42) | 122.40 (102.94, 144.78) | -0.32 (-0.34, -0.30) | <0.001 | 779.90 (714.28, 850.20) | 749.04 (700.28, 800.60) | -0.10 (-0.24, 0.05) | 0.178 | 1951.74 (1846.83 , | 1007.45 (950.43, 1067.30) | -2.33 (-2.86, -1.79) | <0.001 | 32.24 (20.06, 49.44) | 16.80 (10.20, 26.48) | -2.30 (-2.84, -1.76) | <0.001 |

| | | | | | | | | | | | | | | | | |
|---|---|---|---|---|---|---|---|---|---|---|---|---|---|---|---|---|
| | | | | | | | | | 2061.31) | | | | | | | |
| Bulgaria | 114.88 (109.60, 120.35) | 112.78 (106.61, 119.26) | -0.07 (-0.08, -0.05) | <0.001 | 1276.20 (1258.30, 1294.30) | 1324.19 (1301.81, 1346.90) | 0.13 (0.06, 0.20) | <0.001 | 2213.36 (2190.11, 2236.80) | 1617.81 (1594.59, 1641.33) | -1.00 (-1.26, -0.73) | <0.001 | 37.40 (34.45, 40.55) | 26.95 (24.07, 30.14) | -1.03 (-1.31, -0.75) | <0.001 |
| Burkina Faso | 150.18 (143.59, 157.03) | 151.67 (147.77, 155.66) | 0.03 (0.02, 0.05) | <0.001 | 2104.19 (2079.42, 2129.19) | 2149.91 (2135.20, 2164.71) | 0.08 (0.06, 0.11) | <0.001 | 969.24 (951.64, 987.10) | 1054.37 (1043.67, 1065.16) | 0.38 (0.12, 0.64) | 0.004 | 14.62 (12.50, 17.02) | 16.06 (14.74, 17.46) | 0.44 (0.13, 0.75) | 0.006 |
| Burundi | 214.84 (205.67, 224.34) | 192.79 (187.04, 198.69) | -0.37 (-0.40, -0.35) | <0.001 | 2673.08 (2640.42, 2706.07) | 2683.92 (2662.18, 2705.80) | 0.02 (-0.05, 0.08) | 0.568 | 1983.47 (1954.79, 2012.50) | 1297.61 (1282.25, 1313.12) | -1.47 (-1.76, -1.18) | <0.001 | 31.06 (27.52, 34.94) | 19.55 (17.69, 21.56) | -1.64 (-2.00, -1.28) | <0.001 |
| Cabo Verde | 164.40 (130.70, 205.81) | 153.79 (133.13, 176.85) | -0.23 (-0.25, -0.21) | <0.001 | 2983.78 (2839.08, 3135.47) | 3050.75 (2955.89, 3147.97) | 0.08 (0.07, 0.09) | <0.001 | 1581.77 (1465.58, 1705.82) | 874.35 (824.19, 926.87) | -2.23 (-2.53, -1.93) | <0.001 | 24.56 (11.82, 46.60) | 11.76 (6.63, 19.46) | -2.78 (-3.12, -2.44) | <0.001 |
| Cambodia | 129.64 (124.02, 135.46) | 118.78 (115.21, 122.43) | -0.30 (-0.31, -0.29) | <0.001 | 1151.37 (1134.57, 1168.37) | 1128.28 (1117.23, 1139.41) | -0.06 (-0.11, -0.00) | 0.035 | 1482.20 (1462.52, 1502.09) | 1003.04 (992.56, 1013.60) | -1.30 (-1.35, -1.24) | <0.001 | 24.23 (21.74, 26.94) | 15.81 (14.52, 17.19) | -1.45 (-1.58, -1.33) | <0.001 |
| Cameroon | 150.80 (144.97, 156.81) | 160.47 (157.17, 163.82) | 0.21 (0.19, 0.24) | <0.001 | 2247.28 (2224.79, 2269.95) | 2464.24 (2451.38, 2477.17) | 0.32 (0.30, 0.35) | <0.001 | 1090.22 (1074.04, 1106.60) | 1205.51 (1196.22, 1214.85) | 0.35 (0.20, 0.50) | <0.001 | 16.38 (14.42, 18.54) | 18.23 (17.09, 19.43) | 0.38 (0.21, 0.54) | <0.001 |
| Canada | 110.91 (108.23, 113.64) | 98.91 (96.42, 101.45) | -0.39 (-0.41, -0.37) | <0.001 | 1070.26 (1061.90, 1078.67) | 939.73 (932.04, 947.48) | -0.40 (-0.75, -0.05) | 0.024 | 575.19 (569.09, 581.34) | 353.67 (348.98, 358.41) | -1.64 (-1.92, -1.36) | <0.001 | 8.58 (7.86, 9.36) | 4.89 (4.35, 5.47) | -1.89 (-2.25, -1.54) | <0.001 |
| Central African Republic | 208.70 (195.93, 222.16) | 208.21 (199.13, 217.63) | -0.01 (-0.02, 0.00) | 0.136 | 2620.85 (2575.36, 2667.02) | 2627.82 (2595.39, 2660.56) | 0.01 (-0.01, 0.03) | 0.196 | 1736.27 (1698.00, 1775.24) | 1587.27 (1561.27, 1613.60) | -0.35 (-0.65, -0.04) | 0.026 | 27.13 (22.46, 32.53) | 24.78 (21.59, 28.33) | -0.36 (-0.68, -0.02) | 0.035 |

| | | | | | | | | | | | | | | | | |
|---|---|---|---|---|---|---|---|---|---|---|---|---|---|---|---|---|
| Chad | 158.44 (150.35, 166.88) | 161.97 (156.96, 167.12) | 0.07 (0.07, 0.08) | <0.001 | 2210.42 (2180.04, 2241.15) | 2291.89 (2273.02, 2310.90) | 0.12 (0.09, 0.16) | <0.001 | 1135.01 (1112.63, 1157.74) | 1193.91 (1179.74, 1208.22) | 0.26 (-0.11, 0.63) | 0.172 | 17.11 (14.43, 20.17) | 18.11 (16.37, 19.99) | 0.29 (-0.12, 0.70) | 0.167 |
| Chile | 72.46 (69.28, 75.76) | 75.98 (73.12, 78.92) | 0.16 (0.14, 0.18) | <0.001 | 479.01 (470.78, 487.36) | 527.17 (519.59, 534.83) | 0.35 (0.25, 0.45) | <0.001 | 657.47 (647.80, 667.26) | 471.47 (464.32, 478.71) | -1.11 (-1.46, -0.76) | <0.001 | 10.62 (9.42, 11.94) | 7.42 (6.55, 8.39) | -1.20 (-1.56, -0.83) | <0.001 |
| China | 116.09 (115.69, 116.50) | 104.59 (104.20, 104.99) | -0.36 (-0.39, -0.34) | <0.001 | 1177.55 (1176.27, 1178.84) | 1066.85 (1065.60, 1068.11) | -0.32 (-0.38, -0.27) | <0.001 | 1284.47 (1283.12, 1285.82) | 1020.97 (1019.77, 1022.17) | -0.77 (-1.39, -0.13) | 0.018 | 20.52 (20.34, 20.69) | 16.27 (16.12, 16.42) | -0.77 (-1.47, -0.07) | 0.031 |
| Colombia | 74.33 (72.22, 76.49) | 67.85 (66.20, 69.53) | -0.32 (-0.35, -0.28) | <0.001 | 652.29 (646.07, 658.56) | 649.86 (644.74, 655.01) | 0.00 (-0.06, 0.07) | 0.899 | 996.10 (988.33, 1003.93) | 554.23 (549.50, 558.99) | -2.07 (-2.35, -1.80) | <0.001 | 16.47 (15.48, 17.52) | 8.85 (8.26, 9.47) | -2.19 (-2.48, -1.90) | <0.001 |
| Comoros | 199.32 (168.76, 234.41) | 180.65 (159.64, 203.76) | -0.34 (-0.36, -0.32) | <0.001 | 2733.79 (2616.62, 2855.35) | 2744.75 (2660.62, 2830.94) | 0.01 (-0.01, 0.04) | 0.277 | 1250.60 (1168.51, 1337.32) | 972.58 (922.00, 1025.28) | -0.88 (-2.88, 1.16) | 0.396 | 18.75 (9.83, 32.93) | 14.04 (8.54, 21.84) | -1.01 (-3.31, 1.34) | 0.396 |
| Congo | 199.61 (186.39, 213.60) | 176.72 (168.65, 185.09) | -0.41 (-0.45, -0.37) | <0.001 | 2758.38 (2709.00, 2808.52) | 2690.55 (2658.72, 2722.67) | -0.10 (-0.15, -0.04) | 0.001 | 1920.80 (1877.54, 1964.86) | 1017.43 (997.73, 1037.44) | -2.24 (-2.81, -1.66) | <0.001 | 30.53 (25.22, 36.70) | 14.97 (12.66, 17.59) | -2.50 (-3.11, -1.89) | <0.001 |
| Cook Islands | 112.83 (31.58, 296.67) | 113.36 (23.69, 333.03) | 0.01 (-0.02, 0.04) | 0.368 | 1074.19 (767.52, 1470.79) | 1093.20 (735.37, 1567.29) | 0.07 (0.02, 0.11) | 0.004 | 2315.47 (1846.07, 2874.30) | 2047.38 (1540.14, 2670.07) | -0.42 (-0.57, -0.26) | <0.001 | 38.60 (2.33, 187.00) | 34.19 (0.63, 207.60) | -0.41 (-0.57, -0.25) | <0.001 |
| Costa Rica | 133.09 (124.17, 142.51) | 134.57 (127.22, 142.25) | 0.04 (0.03, 0.05) | <0.001 | 1794.79 (1761.59, 1828.49) | 1823.59 (1796.32, 1851.19) | 0.05 (0.02, 0.08) | 0.004 | 816.46 (793.65, 839.79) | 685.15 (668.58, 702.05) | -0.36 (-0.93, 0.20) | 0.207 | 12.54 (9.84, 15.79) | 10.17 (8.25, 12.43) | -0.44 (-1.09, 0.21) | 0.185 |
| Cote d'Ivoire | 176.87 (171.42, 182.46) | 168.83 (165.36, 172.36) | -0.16 (-0.18, -0.15) | <0.001 | 2549.72 (2529.08 , | 2547.04 (2533.57 , | 0.01 (-0.04, 0.05) | 0.744 | 1441.24 (1425.33 , | 1172.66 (1163.39 , | -0.71 (-0.94, -0.48) | <0.001 | 21.99 (20.04, 24.09) | 17.51 (16.39, 18.70) | -0.79 (-1.05, -0.52) | <0.001 |

| | | | | | | | | | | | | | | | | |
|---|---|---|---|---|---|---|---|---|---|---|---|---|---|---|---|---|
| | | | | | 2570.49) | 2560.57) | | | 1457.29) | 1182.00) | | | | | | |
| Croatia | 97.18 (91.09, 103.60) | 99.28 (92.06, 106.99) | 0.08 (0.01, 0.14) | 0.021 | 718.04 (701.49, 734.92) | 740.39 (720.75, 760.50) | 0.14 (-0.01, 0.29) | 0.071 | 1115.96 (1095.39 , 1136.85) | 445.63 (430.48, 461.23) | -3.04 (-3.93, -2.15) | <0.001 | 18.84 (16.27, 21.74) | 6.98 (5.22, 9.24) | -3.28 (-4.30, -2.26) | <0.001 |
| Cuba | 171.77 (166.46, 177.22) | 168.20 (162.38, 174.18) | -0.07 (-0.09, -0.06) | <0.001 | 1964.23 (1946.17 , 1982.42) | 1910.45 (1890.73 , 1930.33) | -0.10 (-0.11, -0.08) | <0.001 | 1120.29 (1106.26 , 1134.46) | 642.78 (631.48, 654.24) | -1.87 (-2.27, -1.47) | <0.001 | 17.95 (16.19, 19.85) | 9.61 (8.28, 11.11) | -2.11 (-2.56, -1.66) | <0.001 |
| Cyprus | 66.70 (54.63, 80.73) | 65.46 (56.12, 76.24) | -0.06 (-0.07, -0.06) | <0.001 | 411.00 (380.09, 443.82) | 439.09 (414.46, 465.10) | 0.31 (-0.00, 0.61) | 0.051 | 694.40 (654.01, 736.69) | 489.90 (464.34, 516.81) | -1.29 (-1.76, -0.81) | <0.001 | 11.50 (6.85, 18.22) | 7.92 (5.01, 12.39) | -1.34 (-1.82, -0.86) | <0.001 |
| Czechia | 123.31 (118.41, 128.36) | 122.86 (117.62, 128.30) | -0.00 (-0.02, 0.02) | 0.814 | 1012.03 (997.93, 1026.30) | 1016.26 (1001.13 , 1031.59) | 0.04 (-0.00, 0.08) | 0.069 | 1140.30 (1125.54 , 1155.21) | 422.13 (412.58, 431.88) | -3.40 (-3.84, -2.96) | <0.001 | 19.02 (17.17, 21.03) | 6.21 (5.12, 7.50) | -3.84 (-4.37, -3.31) | <0.001 |
| Democratic People's Republic of Korea | 130.57 (126.97, 134.25) | 130.88 (127.85, 133.98) | 0.01 (-0.00, 0.02) | 0.238 | 1180.23 (1169.34 , 1191.20) | 1181.64 (1172.51 , 1190.84) | 0.01 (-0.02, 0.03) | 0.652 | 1673.20 (1660.18 , 1686.29) | 1669.48 (1658.69 , 1680.34) | -0.01 (-0.13, 0.12) | 0.924 | 27.12 (25.48, 28.84) | 26.95 (25.60, 28.36) | -0.03 (-0.10, 0.05) | 0.522 |
| Democratic Republic of the Congo | 181.99 (178.76, 185.25) | 179.15 (177.14, 181.18) | -0.05 (-0.08, -0.03) | <0.001 | 2582.71 (2570.52 , 2594.95) | 2566.53 (2558.89 , 2574.18) | -0.02 (-0.05, 0.01) | 0.112 | 1198.29 (1189.65 , 1206.98) | 1020.91 (1015.91 , 1025.93) | -0.57 (-0.72, -0.42) | <0.001 | 18.20 (17.13, 19.32) | 15.21 (14.59, 15.84) | -0.64 (-0.81, -0.47) | <0.001 |
| Denmark | 93.20 (87.32, 99.38) | 78.31 (72.73, 84.22) | -0.60 (-0.62, -0.59) | <0.001 | 612.76 (597.54, 628.29) | 528.94 (514.27, 543.94) | -0.50 (-0.59, -0.40) | <0.001 | 575.75 (561.07, 590.74) | 208.50 (199.35, 217.99) | -3.52 (-4.41, -2.61) | <0.001 | 9.43 (7.64, 11.54) | 2.95 (1.96, 4.30) | -4.02 (-5.12, -2.90) | <0.001 |
| Djibouti | 185.61 (160.11, 214.42) | 180.27 (164.38, 197.29) | -0.10 (-0.11, -0.09) | <0.001 | 2634.68 (2535.51 , 2737.08) | 2753.48 (2690.29 , 2817.80) | 0.15 (0.12, 0.19) | <0.001 | 1297.58 (1225.62 , 1372.94) | 1147.51 (1106.74 , 1189.42) | -0.39 (-0.94, 0.15) | 0.159 | 19.63 (11.59, 31.45) | 16.96 (12.33, 22.78) | -0.46 (-1.11, 0.19) | 0.164 |
| Dominica | 181.77 (118.50, | 183.83 (117.98, | 0.04 (0.02, | <0.001 | 1985.21 (1759.99 | 1990.39 (1756.45 | 0.01 (0.00, | 0.038 | 1300.30 (1114.65 | 1045.88 (877.89, | -0.77 (-1.05, | <0.001 | 20.95 (3.93, | 16.55 (2.25, | -0.81 (-1.23, | <0.001 |

| | | | | | | | | | | | | | | | | |
|---|---|---|---|---|---|---|---|---|---|---|---|---|---|---|---|---|
| | 268.89) | 273.44) | 0.05) | | , 2232.91) | , 2247.03) | 0.02) | | , 1509.30) | 1236.83) | -0.49) | | 65.93) | 57.63) | -0.39) | |
| Dominican Republic | 176.04 (169.05, 183.27) | 185.25 (179.67, 190.95) | 0.18 (0.17, 0.18) | <0.001 | 1986.65 (1963.12 , 2010.42) | 2062.03 (2043.35 , 2080.83) | 0.13 (0.11, 0.15) | <0.001 | 1481.86 (1460.95 , 1503.00) | 1549.52 (1533.26 , 1565.91) | 0.22 (-0.31, 0.76) | 0.414 | 23.87 (21.25, 26.74) | 25.31 (23.27, 27.49) | 0.27 (-0.30, 0.85) | 0.356 |
| Ecuador | 134.96 (129.85, 140.23) | 130.06 (126.34, 133.85) | -0.13 (-0.14, -0.12) | <0.001 | 1748.36 (1729.72 , 1767.16) | 1751.83 (1738.09 , 1765.65) | 0.01 (-0.00, 0.03) | 0.102 | 1428.51 (1411.40 , 1445.79) | 1114.55 (1103.53 , 1125.65) | -1.00 (-1.51, -0.48) | <0.001 | 22.62 (20.49, 24.92) | 17.26 (15.90, 18.70) | -1.08 (-1.63, -0.53) | <0.001 |
| Egypt | 144.25 (141.96, 146.57) | 148.98 (147.32, 150.64) | 0.11 (0.08, 0.15) | <0.001 | 2189.92 (2181.08 , 2198.79) | 2349.89 (2343.32 , 2356.46) | 0.25 (0.21, 0.30) | <0.001 | 3411.50 (3400.33 , 3422.70) | 2498.87 (2492.10 , 2505.67) | -1.06 (-1.54, -0.59) | <0.001 | 55.49 (54.06, 56.95) | 39.43 (38.58, 40.29) | -1.17 (-1.66, -0.68) | <0.001 |
| El Salvador | 141.19 (133.64, 149.09) | 137.39 (130.78, 144.27) | -0.09 (-0.11, -0.08) | <0.001 | 1657.44 (1631.28 , 1683.95) | 1721.35 (1697.72 , 1745.24) | 0.13 (0.11, 0.16) | <0.001 | 2009.61 (1980.10 , 2039.48) | 928.61 (910.75, 946.74) | -2.55 (-3.79, -1.29) | <0.001 | 32.47 (28.77, 36.54) | 14.48 (12.30, 16.94) | -2.66 (-4.01, -1.30) | <0.001 |
| Equatorial Guinea | 203.06 (169.45, 241.90) | 156.45 (143.27, 170.70) | -0.90 (-0.94, -0.87) | <0.001 | 2735.67 (2608.55 , 2867.84) | 2585.65 (2531.16 , 2641.18) | -0.19 (-0.23, -0.16) | <0.001 | 1710.01 (1607.13 , 1818.10) | 722.14 (691.99, 753.38) | -2.93 (-3.25, -2.62) | <0.001 | 26.67 (15.15, 44.00) | 9.86 (6.54, 14.40) | -3.38 (-3.74, -3.01) | <0.001 |
| Eritrea | 230.30 (217.70, 243.51) | 202.69 (195.40, 210.21) | -0.45 (-0.50, -0.39) | <0.001 | 2943.64 (2897.98 , 2989.90) | 2904.82 (2876.78 , 2933.07) | -0.05 (-0.10, 0.01) | 0.122 | 1751.25 (1714.71 , 1788.42) | 1513.16 (1492.29 , 1534.26) | -0.54 (-0.90, -0.17) | 0.004 | 27.17 (22.72, 32.29) | 23.32 (20.76, 26.11) | -0.57 (-0.96, -0.18) | 0.004 |
| Estonia | 152.53 (138.84, 167.25) | 147.79 (132.39, 164.79) | -0.09 (-0.14, -0.05) | <0.001 | 789.69 (758.19, 822.21) | 831.98 (795.04, 870.47) | 0.17 (0.12, 0.22) | <0.001 | 1720.38 (1674.07 , 1767.71) | 586.67 (556.41, 618.45) | -3.57 (-4.48, -2.65) | <0.001 | 29.90 (24.09, 36.77) | 9.49 (6.04, 14.65) | -4.40 (-4.95, -3.85) | <0.001 |
| Eswatini | 181.74 (158.54, 207.68) | 189.18 (172.07, 207.60) | 0.14 (0.11, 0.18) | <0.001 | 2251.36 (2167.40 , | 2307.73 (2246.99 , | 0.09 (0.06, 0.11) | <0.001 | 976.60 (919.43, 1036.62) | 1281.72 (1235.44 , | 0.91 (0.63, 1.18) | <0.001 | 14.53 (8.23, 24.01) | 19.95 (14.51, 26.82) | 1.06 (0.74, 1.38) | <0.001 |

| | | | | | | | | | | | | | | | | |
|---|---|---|---|---|---|---|---|---|---|---|---|---|---|---|---|---|
| | | | | | 2338.01) | 2369.78) | | | | 1329.34) | | | | | | |
| Ethiopia | 172.18 (169.38, 175.02) | 167.65 (165.90, 169.42) | -0.08 (-0.11, -0.05) | <0.001 | 2171.05 (2161.00, 2181.15) | 2430.88 (2424.12, 2437.65) | 0.40 (0.35, 0.46) | <0.001 | 1538.34 (1529.67, 1547.06) | 855.59 (851.44, 859.75) | -2.00 (-2.11, -1.88) | <0.001 | 24.10 (23.01, 25.23) | 12.22 (11.72, 12.73) | -2.32 (-2.44, -2.20) | <0.001 |
| Fiji | 130.89 (113.51, 150.29) | 134.05 (117.87, 151.85) | 0.08 (0.07, 0.10) | <0.001 | 1459.47 (1400.17, 1520.73) | 1660.34 (1602.03, 1720.25) | 0.46 (0.32, 0.60) | <0.001 | 3755.35 (3658.34, 3854.35) | 2836.07 (2760.06, 2913.66) | -1.01 (-1.53, -0.48) | <0.001 | 63.48 (51.36, 77.69) | 47.09 (37.76, 58.05) | -1.07 (-1.64, -0.50) | <0.001 |
| Finland | 103.86 (97.61, 110.45) | 90.55 (84.47, 97.00) | -0.47 (-0.50, -0.45) | <0.001 | 615.87 (600.49, 631.58) | 557.86 (542.62, 573.46) | -0.34 (-0.46, -0.21) | <0.001 | 998.82 (979.63, 1018.33) | 364.48 (352.33, 376.98) | -3.48 (-4.12, -2.84) | <0.001 | 17.01 (14.61, 19.76) | 5.67 (4.26, 7.46) | -3.79 (-4.64, -2.93) | <0.001 |
| France | 79.85 (78.21, 81.50) | 73.30 (71.65, 74.98) | -0.29 (-0.32, -0.27) | <0.001 | 633.42 (628.79, 638.08) | 571.14 (566.50, 575.81) | -0.35 (-0.47, -0.24) | <0.001 | 606.09 (601.60, 610.61) | 298.33 (295.01, 301.69) | -2.48 (-2.98, -1.98) | <0.001 | 9.80 (9.24, 10.39) | 4.44 (4.04, 4.86) | -2.78 (-3.37, -2.19) | <0.001 |
| Gabon | 171.97 (153.73, 191.87) | 163.69 (150.50, 177.77) | -0.17 (-0.18, -0.16) | <0.001 | 2609.15 (2536.87, 2683.06) | 2609.91 (2556.50, 2664.19) | 0.00 (-0.02, 0.03) | 0.699 | 1455.55 (1400.42, 1512.36) | 1038.90 (1004.71, 1074.00) | -1.17 (-1.68, -0.66) | <0.001 | 22.50 (16.07, 30.73) | 15.33 (11.40, 20.21) | -1.33 (-1.89, -0.76) | <0.001 |
| Gambia | 167.93 (148.89, 188.94) | 174.43 (162.04, 187.58) | 0.13 (0.12, 0.14) | <0.001 | 2534.42 (2459.40, 2611.35) | 2655.20 (2606.57, 2704.58) | 0.17 (0.13, 0.21) | <0.001 | 1157.50 (1105.30, 1211.69) | 1215.58 (1181.74, 1250.19) | 0.24 (-0.85, 1.34) | 0.671 | 17.20 (11.30, 25.29) | 18.06 (14.10, 22.85) | 0.24 (-0.99, 1.49) | 0.698 |
| Georgia | 190.17 (181.91, 198.71) | 187.47 (176.84, 198.63) | -0.04 (-0.08, -0.01) | 0.027 | 1966.83 (1940.05, 1993.90) | 2029.98 (1994.42, 2066.06) | 0.10 (0.07, 0.14) | <0.001 | 2873.73 (2841.37, 2906.37) | 2002.44 (1968.28, 2037.11) | -1.27 (-2.83, 0.31) | 0.114 | 48.66 (44.52, 53.08) | 33.26 (29.02, 38.02) | -1.34 (-2.92, 0.26) | 0.100 |
| Germany | 96.51 (94.99, 98.06) | 84.19 (82.68, 85.72) | -0.47 (-0.51, -0.43) | <0.001 | 650.44 (646.45, 654.45) | 571.69 (567.74, 575.66) | -0.43 (-0.52, -0.34) | <0.001 | 942.79 (938.01, 947.60) | 341.88 (338.86, 344.93) | -3.47 (-3.70, -3.23) | <0.001 | 15.65 (15.05, 16.28) | 5.21 (4.84, 5.59) | -3.75 (-4.02, -3.49) | <0.001 |
| Ghana | 158.57 (153.79, 163.47) | 166.41 (163.24, 169.64) | 0.17 (0.16, 0.18) | <0.001 | 2492.52 (2473.56, | 2562.09 (2549.66, | 0.10 (0.07, 0.12) | <0.001 | 1066.11 (1053.35, | 1097.92 (1089.61, | 0.10 (-0.07, 0.27) | 0.242 | 15.70 (14.17, 17.36) | 16.19 (15.18, 17.24) | 0.11 (-0.10, 0.31) | 0.302 |

| | | | | | | | | | | | | | | | | |
|---|---|---|---|---|---|---|---|---|---|---|---|---|---|---|---|---|
| | | | | | 2511.60) | 2574.55) | | | 1078.99) | 1106.28) | | | | | | |
| Greece | 82.02 (78.01, 86.20) | 74.76 (70.51, 79.22) | -0.32 (-0.34, -0.30) | <0.001 | 478.48 (468.71, 488.40) | 450.48 (440.05, 461.11) | -0.19 (-0.39, 0.02) | 0.074 | 936.01 (922.34, 949.83) | 680.64 (668.03, 693.46) | -1.22 (-1.57, -0.87) | <0.001 | 15.57 (13.85, 17.44) | 11.37 (9.81, 13.13) | -1.21 (-1.59, -0.84) | <0.001 |
| Greenland | 125.82 (75.07, 205.25) | 105.27 (53.21, 191.91) | -0.62 (-0.64, -0.60) | <0.001 | 1092.99 (930.13, 1281.58) | 937.97 (764.25, 1143.10) | -0.49 (-0.71, -0.26) | <0.001 | 1054.67 (895.09, 1239.98) | 536.55 (407.78, 697.13) | -2.28 (-2.47, -2.09) | <0.001 | 17.31 (2.95, 66.52) | 8.22 (0.15, 58.69) | -2.53 (-2.72, -2.33) | <0.001 |
| Grenada | 194.11 (131.59, 277.84) | 193.70 (138.11, 265.26) | -0.01 (-0.02, 0.00) | 0.145 | 2180.19 (1956.63, 2423.77) | 2184.93 (1987.34, 2397.74) | 0.01 (-0.01, 0.03) | 0.316 | 1827.34 (1616.20, 2059.39) | 933.32 (803.49, 1078.87) | -2.36 (-2.60, -2.12) | <0.001 | 30.56 (9.10, 76.27) | 14.44 (2.77, 44.83) | -2.65 (-2.92, -2.37) | <0.001 |
| Guam | 119.75 (85.81, 163.16) | 139.45 (100.89, 188.29) | 0.53 (0.50, 0.56) | <0.001 | 1467.87 (1341.80, 1603.01) | 1621.51 (1482.65, 1770.24) | 0.35 (0.29, 0.41) | <0.001 | 1506.53 (1378.08, 1644.10) | 2389.77 (2217.78, 2571.77) | 1.59 (1.04, 2.14) | <0.001 | 23.82 (10.25, 47.60) | 40.02 (20.59, 70.40) | 1.79 (1.16, 2.42) | <0.001 |
| Guatemala | 150.14 (143.63, 156.88) | 141.10 (137.31, 144.98) | -0.22 (-0.23, -0.21) | <0.001 | 1724.49 (1702.29, 1746.93) | 1860.95 (1847.09, 1874.89) | 0.26 (0.23, 0.29) | <0.001 | 1745.09 (1722.28, 1768.15) | 1047.09 (1036.39, 1057.89) | -1.80 (-2.31, -1.29) | <0.001 | 28.04 (25.19, 31.14) | 16.36 (15.03, 17.78) | -1.89 (-2.43, -1.34) | <0.001 |
| Guinea | 136.85 (129.44, 144.59) | 151.96 (146.62, 157.45) | 0.36 (0.32, 0.40) | <0.001 | 1966.53 (1938.46, 1994.92) | 2261.18 (2240.76, 2281.75) | 0.47 (0.42, 0.53) | <0.001 | 1044.46 (1023.55, 1065.71) | 1223.24 (1207.67, 1238.97) | 0.58 (0.40, 0.77) | <0.001 | 15.73 (13.23, 18.58) | 18.63 (16.73, 20.71) | 0.63 (0.43, 0.84) | <0.001 |
| Guinea-Bissau | 184.71 (164.06, 207.44) | 185.59 (171.73, 200.36) | 0.01 (0.00, 0.03) | 0.017 | 2056.57 (1986.36, 2128.83) | 2140.04 (2092.39, 2188.58) | 0.14 (0.12, 0.17) | <0.001 | 1778.95 (1712.18, 1847.82) | 1627.94 (1585.28, 1671.51) | -0.29 (-0.37, -0.22) | <0.001 | 28.23 (20.29, 38.43) | 25.78 (20.61, 31.93) | -0.30 (-0.38, -0.22) | <0.001 |
| Guyana | 223.33 (200.48, 248.23) | 211.18 (188.64, 235.79) | -0.19 (-0.22, -0.16) | <0.001 | 2137.86 (2066.12, 2211.60) | 2062.13 (1990.53, 2135.75) | -0.12 (-0.14, -0.09) | <0.001 | 2838.32 (2753.32, 2925.38) | 2050.78 (1977.37, 2126.28) | -0.92 (-1.76, -0.07) | 0.034 | 47.68 (37.15, 60.38) | 34.27 (25.31, 45.45) | -0.93 (-1.79, -0.06) | 0.036 |
| Haiti | 206.42 (197.99, | 204.00 (198.42, | -0.05 (-0.07, | 0.002 | 1981.39 (1955.10 | 2006.19 (1988.61 | 0.05 (0.02, | 0.002 | 1887.63 (1861.46 | 1387.95 (1373.10 | -1.02 (-1.25, | <0.001 | 31.36 (28.05, | 22.69 (20.81, | -1.08 (-1.33, | <0.001 |

| | | | | | | | | | | | | | | | | |
|---|---|---|---|---|---|---|---|---|---|---|---|---|---|---|---|---|
| | 215.14) | 209.71) | -0.02) | | , 2007.96) | , 2023.90) | 0.08) | | , 1914.08) | , 1402.92) | -0.78) | | 34.97) | 24.68) | -0.83) | |
| Honduras | 139.12 (131.00, 147.65) | 138.29 (133.09, 143.65) | -0.02 (-0.04, 0.00) | 0.110 | 1666.79 (1638.25 , 1695.73) | 1725.80 (1707.23 , 1744.53) | 0.13 (0.09, 0.17) | <0.001 | 1257.05 (1231.38 , 1283.13) | 581.25 (570.16, 592.52) | -2.66 (-2.88, -2.44) | <0.001 | 20.31 (17.14, 23.93) | 8.51 (7.20, 10.01) | -3.00 (-3.24, -2.75) | <0.001 |
| Hungary | 129.21 (124.23, 134.34) | 115.41 (110.12, 120.91) | -0.40 (-0.42, -0.38) | <0.001 | 923.99 (910.65, 937.50) | 875.11 (860.52, 889.91) | -0.19 (-0.25, -0.12) | <0.001 | 2228.47 (2208.13 , 2248.96) | 698.38 (685.56, 711.42) | -3.94 (-4.46, -3.42) | <0.001 | 38.87 (36.24, 41.65) | 11.33 (9.76, 13.10) | -4.19 (-4.75, -3.62) | <0.001 |
| Iceland | 90.43 (66.82, 119.89) | 84.92 (63.95, 111.20) | -0.21 (-0.23, -0.20) | <0.001 | 494.89 (437.21, 558.25) | 470.65 (419.33, 527.07) | -0.16 (-0.27, -0.05) | 0.004 | 637.15 (571.34, 708.64) | 276.15 (237.33, 320.10) | -2.89 (-3.29, -2.49) | <0.001 | 10.21 (3.53, 23.31) | 4.17 (0.78, 13.93) | -3.12 (-3.68, -2.56) | <0.001 |
| India | 135.16 (134.62, 135.72) | 136.93 (136.52, 137.35) | 0.05 (-0.02, 0.12) | 0.135 | 1360.61 (1358.85 , 1362.36) | 1382.67 (1381.35 , 1384.00) | 0.10 (-0.06, 0.26) | 0.211 | 1852.16 (1850.08 , 1854.24) | 1537.53 (1536.12 , 1538.93) | -0.59 (-1.23, 0.05) | 0.072 | 30.71 (30.44, 30.98) | 25.54 (25.36, 25.73) | -0.57 (-1.28, 0.15) | 0.119 |
| Indonesia | 102.28 (101.23, 103.33) | 108.57 (107.69, 109.45) | 0.21 (0.20, 0.22) | <0.001 | 944.37 (941.20, 947.55) | 1008.62 (1005.95 , 1011.29) | 0.25 (0.16, 0.33) | <0.001 | 1648.54 (1644.30 , 1652.78) | 1436.37 (1433.19 , 1439.55) | -0.48 (-0.69, -0.27) | <0.001 | 26.83 (26.29, 27.38) | 23.01 (22.61, 23.42) | -0.55 (-0.69, -0.40) | <0.001 |
| Iran (Islamic Republic of) | 166.92 (164.41, 169.46) | 165.61 (163.76, 167.47) | -0.03 (-0.08, 0.02) | 0.291 | 1869.58 (1861.23 , 1877.96) | 1934.80 (1928.41 , 1941.21) | 0.12 (0.10, 0.15) | <0.001 | 1471.62 (1464.01 , 1479.25) | 1043.25 (1038.62 , 1047.90) | -1.18 (-1.29, -1.07) | <0.001 | 23.57 (22.60, 24.57) | 15.80 (15.24, 16.37) | -1.38 (-1.50, -1.26) | <0.001 |
| Iraq | 164.80 (160.25, 169.45) | 165.10 (162.46, 167.78) | 0.00 (-0.01, 0.02) | 0.769 | 1806.67 (1791.51 , 1821.93) | 1834.89 (1826.04 , 1843.78) | 0.06 (-0.02, 0.15) | 0.139 | 1838.72 (1823.11 , 1854.42) | 1193.82 (1186.60 , 1201.08) | -1.46 (-1.58, -1.34) | <0.001 | 29.87 (27.89, 31.96) | 18.36 (17.47, 19.30) | -1.64 (-1.77, -1.51) | <0.001 |
| Ireland | 88.71 (81.71, 96.15) | 78.43 (72.51, 84.72) | -0.43 (-0.45, -0.41) | <0.001 | 568.32 (550.43, 586.66) | 522.20 (506.79, 537.99) | -0.28 (-0.36, -0.19) | <0.001 | 673.56 (654.03, 693.52) | 270.92 (259.96, 282.25) | -3.15 (-3.80, -2.50) | <0.001 | 11.11 (8.73, 13.96) | 4.02 (2.80, 5.63) | -3.51 (-4.24, -2.77) | <0.001 |
| Israel | 76.79 | 71.69 | -0.24 | <0.001 | 480.13 | 471.56 | -0.03 | 0.669 | 683.89 | 233.47 | -3.92 | <0.001 | 11.16 | 3.36 | -4.13 | <0.001 |

| | | | | | | | | | | | | | | | | |
|---|---|---|---|---|---|---|---|---|---|---|---|---|---|---|---|---|
| | (71.25, 82.66) | (67.66, 75.90) | (-0.26, -0.21) | | (466.15, 494.44) | (461.14, 482.15) | (-0.16, 0.11) | | (667.14, 700.97) | (226.16, 240.95) | (-4.35, -3.50) | | (9.11, 13.55) | (2.54, 4.37) | (-4.82, -3.43) | |
| Italy | 127.57 (125.45, 129.73) | 119.78 (117.48, 122.11) | -0.22 (-0.25, -0.19) | <0.001 | 839.05 (833.58, 844.56) | 848.56 (842.47, 854.68) | 0.04 (-0.04, 0.12) | 0.328 | 683.24 (678.30, 688.21) | 326.86 (323.09, 330.67) | -2.57 (-2.88, -2.25) | <0.001 | 11.08 (10.46, 11.73) | 4.93 (4.48, 5.42) | -2.80 (-3.36, -2.25) | <0.001 |
| Jamaica | 164.78 (153.00, 177.28) | 182.49 (171.66, 193.85) | 0.36 (0.33, 0.38) | <0.001 | 1957.79 (1916.61, 1999.69) | 2116.19 (2078.91, 2154.00) | 0.28 (0.26, 0.30) | <0.001 | 700.00 (675.22, 725.52) | 655.56 (634.66, 676.99) | -0.30 (-1.84, 1.27) | 0.710 | 10.22 (7.39, 13.84) | 9.60 (7.20, 12.55) | -0.29 (-2.22, 1.67) | 0.768 |
| Japan | 135.36 (133.85, 136.88) | 143.50 (141.75, 145.27) | 0.21 (0.18, 0.23) | <0.001 | 957.31 (953.32, 961.31) | 1060.36 (1055.63, 1065.10) | 0.36 (0.29, 0.43) | <0.001 | 574.69 (571.60, 577.79) | 381.12 (378.28, 383.97) | -1.40 (-1.65, -1.15) | <0.001 | 9.02 (8.64, 9.42) | 5.55 (5.21, 5.90) | -1.67 (-1.96, -1.38) | <0.001 |
| Jordan | 107.84 (99.82, 116.38) | 100.57 (96.79, 104.47) | -0.24 (-0.28, -0.20) | <0.001 | 1145.54 (1119.33, 1172.27) | 1111.43 (1098.80, 1124.17) | -0.08 (-0.24, 0.07) | 0.303 | 1013.92 (988.38, 1039.99) | 586.69 (577.47, 596.03) | -1.81 (-2.19, -1.43) | <0.001 | 16.26 (13.12, 19.97) | 8.72 (7.62, 9.93) | -2.06 (-2.50, -1.62) | <0.001 |
| Kazakhstan | 135.48 (131.58, 139.48) | 117.83 (114.33, 121.42) | -0.48 (-0.51, -0.45) | <0.001 | 865.99 (856.06, 876.01) | 813.26 (803.95, 822.65) | -0.22 (-0.27, -0.17) | <0.001 | 2211.15 (2195.24, 2227.15) | 1928.43 (1914.33, 1942.62) | -0.48 (-1.51, 0.57) | 0.374 | 38.01 (35.94, 40.16) | 33.30 (31.47, 35.21) | -0.45 (-1.53, 0.63) | 0.412 |
| Kenya | 179.15 (175.00, 183.39) | 177.92 (175.36, 180.52) | -0.01 (-0.04, 0.01) | 0.308 | 2612.00 (2595.93, 2628.16) | 2745.08 (2734.93, 2755.26) | 0.18 (0.16, 0.20) | <0.001 | 866.23 (856.66, 875.89) | 959.22 (953.08, 965.39) | 0.34 (0.16, 0.51) | <0.001 | 12.13 (11.00, 13.35) | 13.59 (12.86, 14.35) | 0.36 (0.17, 0.55) | <0.001 |
| Kiribati | 300.48 (214.77, 410.63) | 247.48 (187.28, 321.39) | -0.67 (-0.72, -0.62) | <0.001 | 2096.22 (1860.29, 2355.33) | 1998.61 (1820.22, 2190.23) | -0.17 (-0.21, -0.14) | <0.001 | 8803.13 (8304.10, 9325.50) | 7889.23 (7527.28, 8264.47) | -0.38 (-0.46, -0.30) | <0.001 | 150.93 (91.70, 235.58) | 134.93 (91.21, 192.76) | -0.39 (-0.47, -0.31) | <0.001 |
| Kuwait | 96.40 (88.26, 105.25) | 101.96 (96.20, 108.08) | 0.21 (0.13, 0.29) | <0.001 | 914.39 (888.79, 940.69) | 950.96 (933.06, 969.21) | 0.15 (0.09, 0.21) | <0.001 | 1145.08 (1116.19, 1174.67) | 990.69 (972.68, 1009.04) | -0.51 (-2.15, 1.16) | 0.546 | 18.34 (14.89, 22.55) | 15.89 (13.73, 18.41) | -0.50 (-2.28, 1.32) | 0.590 |

| | | | | | | | | | | | | | | | | |
|---|---|---|---|---|---|---|---|---|---|---|---|---|---|---|---|---|
| Kyrgyzstan | 182.86 (173.93, 192.15) | 164.02 (157.16, 171.10) | -0.37 (-0.39, -0.35) | <0.001 | 1741.49 (1713.76, 1769.57) | 1646.66 (1624.82, 1668.73) | -0.19 (-0.23, -0.15) | <0.001 | 2216.26 (2184.21, 2248.67) | 1433.86 (1413.23, 1454.74) | -1.44 (-2.58, -0.29) | 0.014 | 37.20 (33.12, 41.66) | 23.85 (21.24, 26.69) | -1.48 (-2.71, -0.23) | 0.020 |
| Lao People's Democratic Republic | 174.82 (165.01, 185.09) | 148.11 (142.11, 154.31) | -0.58 (-0.62, -0.54) | <0.001 | 1509.43 (1480.40, 1538.91) | 1408.50 (1389.89, 1427.31) | -0.21 (-0.34, -0.08) | 0.002 | 1800.38 (1768.07, 1833.16) | 1658.30 (1637.93, 1678.87) | -0.24 (-0.44, -0.05) | 0.013 | 29.06 (25.05, 33.55) | 26.70 (24.16, 29.44) | -0.25 (-0.46, -0.05) | 0.014 |
| Latvia | 121.71 (112.17, 131.87) | 126.60 (114.40, 139.93) | 0.15 (0.12, 0.18) | <0.001 | 631.29 (609.40, 653.80) | 700.62 (672.27, 730.05) | 0.41 (0.21, 0.60) | <0.001 | 2229.45 (2188.16, 2271.36) | 1546.55 (1504.66, 1589.52) | -1.23 (-2.80, 0.37) | 0.132 | 39.06 (33.76, 44.98) | 26.84 (21.61, 33.20) | -1.25 (-2.89, 0.43) | 0.144 |
| Lebanon | 94.83 (86.61, 103.64) | 96.86 (91.06, 102.96) | 0.08 (0.06, 0.10) | <0.001 | 1263.35 (1233.51, 1293.76) | 1368.79 (1346.44, 1391.46) | 0.28 (0.23, 0.33) | <0.001 | 2321.25 (2279.74, 2363.35) | 1613.15 (1589.10, 1637.50) | -1.22 (-1.39, -1.05) | <0.001 | 38.79 (33.54, 44.65) | 26.07 (23.11, 29.34) | -1.34 (-1.52, -1.16) | <0.001 |
| Lesotho | 179.89 (165.37, 195.44) | 202.01 (189.47, 215.18) | 0.40 (0.39, 0.42) | <0.001 | 2174.69 (2123.25, 2227.15) | 2283.40 (2240.84, 2326.59) | 0.17 (0.14, 0.20) | <0.001 | 871.53 (838.19, 905.92) | 1316.25 (1283.64, 1349.49) | 1.43 (1.11, 1.75) | <0.001 | 12.74 (8.95, 17.67) | 20.55 (16.64, 25.12) | 1.66 (1.37, 1.95) | <0.001 |
| Liberia | 161.60 (147.31, 176.97) | 166.96 (158.76, 175.50) | 0.11 (0.10, 0.12) | <0.001 | 2270.32 (2215.81, 2325.90) | 2444.67 (2413.03, 2476.62) | 0.26 (0.24, 0.29) | <0.001 | 1143.41 (1104.67, 1183.22) | 984.30 (963.81, 1005.13) | -0.59 (-0.95, -0.22) | 0.002 | 17.25 (12.78, 22.84) | 14.44 (12.03, 17.20) | -0.69 (-1.10, -0.28) | 0.001 |
| Libya | 125.32 (117.75, 133.27) | 138.84 (133.12, 144.75) | 0.35 (0.31, 0.39) | <0.001 | 1609.51 (1582.12, 1637.29) | 1829.36 (1808.41, 1850.51) | 0.45 (0.36, 0.53) | <0.001 | 1745.15 (1716.37, 1774.31) | 1501.39 (1482.61, 1520.37) | -0.53 (-1.29, 0.23) | 0.171 | 27.88 (24.31, 31.84) | 23.68 (21.38, 26.16) | -0.57 (-1.38, 0.24) | 0.166 |
| Lithuania | 129.38 (121.10, 138.08) | 129.74 (119.44, 140.79) | 0.01 (-0.01, 0.02) | 0.364 | 758.90 (738.62, 779.62) | 788.90 (763.17, 815.37) | 0.15 (0.08, 0.22) | <0.001 | 1767.39 (1736.35, 1798.86) | 1039.31 (1010.11, 1069.25) | -1.96 (-3.49, -0.40) | 0.014 | 30.83 (26.85, 35.26) | 17.75 (14.13, 22.14) | -2.03 (-3.63, -0.39) | 0.015 |
| Luxembourg | 77.95 | 71.24 | -0.31 | <0.001 | 626.94 | 620.64 | -0.01 | 0.883 | 849.08 | 307.05 | -3.41 | <0.001 | 13.80 | 4.48 | -3.77 | <0.001 |

| | | | | | | | | | | | | | | | | |
|---|---|---|---|---|---|---|---|---|---|---|---|---|---|---|---|---|
| | (59.69, 100.89) | (56.64, 88.98) | (-0.35, -0.26) | | (572.31, 686.07) | (575.35, 668.97) | (-0.15, 0.13) | | (785.62, 916.99) | (276.06, 341.05) | (-4.21, -2.61) | | (6.88, 25.91) | (1.53, 11.10) | (-4.65, -2.89) | |
| Madagascar | 228.60 (222.15, 235.21) | 212.72 (208.76, 216.74) | -0.25 (-0.27, -0.24) | <0.001 | 2975.62 (2952.29, 2999.10) | 3068.07 (3052.99, 3083.21) | 0.11 (0.09, 0.14) | <0.001 | 3487.80 (3461.81, 3513.93) | 2344.25 (2330.68, 2357.88) | -1.30 (-1.68, -0.93) | <0.001 | 56.55 (53.25, 60.01) | 37.13 (35.42, 38.91) | -1.38 (-1.78, -0.97) | <0.001 |
| Malawi | 194.20 (187.58, 201.02) | 199.09 (194.42, 203.85) | 0.09 (0.08, 0.10) | <0.001 | 2708.79 (2683.66, 2734.12) | 2818.79 (2800.93, 2836.75) | 0.15 (0.10, 0.20) | <0.001 | 1240.83 (1223.32, 1258.54) | 1268.91 (1256.56, 1281.35) | 0.12 (-0.12, 0.35) | 0.326 | 18.53 (16.41, 20.86) | 18.94 (17.44, 20.54) | 0.11 (-0.13, 0.35) | 0.356 |
| Malaysia | 146.69 (142.76, 150.72) | 133.49 (130.85, 136.17) | -0.32 (-0.34, -0.31) | <0.001 | 1707.91 (1694.43, 1721.48) | 1664.38 (1655.02, 1673.79) | -0.08 (-0.14, -0.03) | 0.003 | 1551.01 (1538.05, 1564.05) | 1366.11 (1357.68, 1374.59) | -0.43 (-1.25, 0.39) | 0.303 | 24.15 (22.55, 25.84) | 21.63 (20.58, 22.72) | -0.38 (-1.28, 0.54) | 0.416 |
| Maldives | 136.06 (99.67, 182.79) | 111.04 (94.82, 130.09) | -0.70 (-0.73, -0.67) | <0.001 | 1640.57 (1508.74, 1782.04) | 1554.37 (1491.48, 1619.94) | -0.19 (-0.22, -0.16) | <0.001 | 2154.23 (1994.80, 2323.86) | 820.67 (777.43, 866.50) | -3.29 (-3.98, -2.60) | <0.001 | 35.60 (17.67, 65.12) | 12.02 (7.43, 19.77) | -3.70 (-4.42, -2.97) | <0.001 |
| Mali | 165.23 (158.36, 172.33) | 161.96 (157.79, 166.22) | -0.07 (-0.08, -0.06) | <0.001 | 2461.07 (2434.40, 2487.97) | 2514.53 (2498.06, 2531.09) | 0.08 (0.04, 0.12) | <0.001 | 979.53 (962.36, 996.94) | 902.07 (891.83, 912.41) | -0.26 (-0.56, 0.04) | 0.084 | 14.13 (12.11, 16.40) | 12.83 (11.61, 14.15) | -0.31 (-0.65, 0.04) | 0.079 |
| Malta | 79.26 (60.18, 102.88) | 72.25 (54.35, 95.23) | -0.32 (-0.33, -0.31) | <0.001 | 502.10 (452.16, 556.40) | 460.21 (413.23, 511.96) | -0.29 (-0.40, -0.18) | <0.001 | 649.44 (592.19, 711.06) | 413.97 (369.74, 462.95) | -1.59 (-2.30, -0.86) | <0.001 | 10.39 (4.38, 21.37) | 6.49 (2.15, 16.85) | -1.65 (-2.40, -0.89) | <0.001 |
| Marshall Islands | 179.98 (99.07, 303.11) | 176.43 (110.07, 268.46) | -0.07 (-0.08, -0.06) | <0.001 | 1626.37 (1360.49, 1931.29) | 1635.29 (1416.72, 1878.35) | 0.02 (-0.00, 0.04) | 0.088 | 4529.22 (4071.51, 5026.04) | 5006.08 (4617.96, 5418.43) | 0.36 (0.30, 0.42) | <0.001 | 75.11 (27.14, 167.69) | 85.90 (42.15, 156.03) | 0.51 (0.45, 0.57) | <0.001 |
| Mauritania | 167.18 (153.88, 181.38) | 152.04 (143.18, 161.34) | -0.33 (-0.34, -0.32) | <0.001 | 2544.60 (2492.25, 2597.84) | 2527.18 (2490.80, 2564.00) | -0.03 (-0.05, -0.02) | <0.001 | 1252.77 (1215.14, 1291.31) | 707.51 (687.73, 727.73) | -1.90 (-2.02, -1.78) | <0.001 | 18.79 (14.39, 24.17) | 9.53 (7.33, 12.22) | -2.24 (-2.38, -2.09) | <0.001 |

| | | | | | | | | | | | | | | | | |
|---|---|---|---|---|---|---|---|---|---|---|---|---|---|---|---|---|
| Mauritius | 136.26 (122.21, 151.51) | 125.26 (111.52, 140.29) | -0.29 (-0.30, -0.28) | <0.001 | 1543.60 (1495.51, 1592.87) | 1456.01 (1408.14, 1505.13) | -0.19 (-0.24, -0.14) | <0.001 | 2224.11 (2165.94, 2283.48) | 1379.45 (1333.52, 1426.63) | -1.56 (-2.10, -1.01) | <0.001 | 37.53 (30.30, 46.00) | 22.26 (16.80, 29.03) | -1.69 (-2.29, -1.10) | <0.001 |
| Mexico | 102.03 (100.45, 103.62) | 99.18 (97.93, 100.43) | -0.10 (-0.13, -0.07) | <0.001 | 1150.61 (1145.35, 1155.89) | 1146.74 (1142.50, 1150.98) | -0.00 (-0.04, 0.04) | 0.971 | 746.07 (741.70, 750.46) | 814.02 (810.44, 817.61) | 0.32 (-0.19, 0.84) | 0.218 | 11.82 (11.27, 12.39) | 13.00 (12.55, 13.46) | 0.37 (-0.16, 0.90) | 0.173 |
| Micronesia (Federated States of) | 191.54 (133.82, 266.51) | 170.48 (118.26, 238.77) | -0.40 (-0.44, -0.37) | <0.001 | 1652.04 (1474.12, 1846.29) | 1591.20 (1423.27, 1774.31) | -0.13 (-0.16, -0.10) | <0.001 | 5684.63 (5342.43, 6043.45) | 5105.10 (4793.51, 5432.16) | -0.37 (-0.45, -0.30) | <0.001 | 97.89 (57.57, 156.18) | 87.03 (50.57, 140.29) | -0.41 (-0.48, -0.34) | <0.001 |
| Monaco | 83.61 (23.07, 239.89) | 81.22 (21.44, 216.22) | -0.11 (-0.16, -0.05) | <0.001 | 445.52 (279.59, 693.36) | 425.89 (261.81, 658.39) | -0.15 (-0.33, 0.02) | 0.084 | 710.91 (495.45, 1006.09) | 465.01 (292.59, 705.61) | -1.44 (-1.49, -1.40) | <0.001 | 11.78 (0.03, 137.21) | 7.53 (0.00, 100.21) | -1.52 (-1.57, -1.47) | <0.001 |
| Mongolia | 193.84 (180.30, 208.22) | 192.10 (181.88, 202.80) | -0.03 (-0.05, -0.01) | 0.009 | 1814.29 (1772.53, 1856.87) | 1846.47 (1814.41, 1879.01) | 0.07 (0.04, 0.10) | <0.001 | 1902.36 (1857.50, 1948.09) | 2351.25 (2316.34, 2386.63) | 0.76 (-0.05, 1.58) | 0.065 | 31.46 (25.85, 37.99) | 39.85 (35.44, 44.72) | 0.84 (-0.02, 1.71) | 0.054 |
| Montenegro | 118.41 (100.51, 138.63) | 109.71 (91.21, 131.03) | -0.26 (-0.30, -0.22) | <0.001 | 791.48 (744.05, 841.20) | 778.56 (728.23, 831.63) | -0.05 (-0.11, 0.02) | 0.142 | 1695.21 (1625.54, 1767.16) | 846.24 (793.94, 901.27) | -3.04 (-5.04, -0.99) | 0.004 | 28.80 (20.37, 39.62) | 13.93 (7.99, 22.83) | -3.16 (-5.16, -1.11) | 0.003 |
| Morocco | 150.85 (147.39, 154.38) | 147.62 (144.86, 150.43) | -0.07 (-0.09, -0.05) | <0.001 | 1840.62 (1828.50, 1852.81) | 1871.28 (1861.40, 1881.20) | 0.08 (0.01, 0.16) | 0.036 | 1771.28 (1759.08, 1783.55) | 1027.18 (1019.87, 1034.53) | -1.82 (-2.10, -1.53) | <0.001 | 28.96 (27.41, 30.59) | 15.63 (14.74, 16.56) | -2.05 (-2.55, -1.55) | <0.001 |
| Mozambique | 209.19 (202.86, 215.68) | 218.49 (214.36, 222.68) | 0.16 (0.13, 0.18) | <0.001 | 2767.50 (2744.12, 2791.04) | 2913.30 (2898.09, 2928.57) | 0.19 (0.08, 0.30) | <0.001 | 1010.14 (995.75, 1024.70) | 1472.52 (1461.28, 1483.82) | 1.34 (1.21, 1.47) | <0.001 | 14.65 (12.95, 16.53) | 22.54 (21.14, 24.00) | 1.54 (1.38, 1.69) | <0.001 |
| Myanmar | 156.28 (153.52, | 137.04 (134.83, | -0.45 (-0.48, | <0.001 | 1391.87 (1383.66 | 1347.31 (1340.35 | -0.09 (-0.16, | 0.007 | 2752.79 (2741.09 | 1852.18 (1843.99 | -1.37 (-1.54, | <0.001 | 45.47 (43.96, | 29.62 (28.59, | -1.48 (-1.64, | <0.001 |

| | | | | | | | | | | | | | | | | |
|---|---|---|---|---|---|---|---|---|---|---|---|---|---|---|---|---|
| | 159.08) | 139.29) | -0.43) | | , 1400.13) | , 1354.31) | -0.03) | | , 2764.52) | , 1860.40) | -1.20) | | 47.01) | 30.67) | -1.32) | |
| Namibia | 177.40 (161.22, 194.92) | 165.99 (154.77, 177.86) | -0.23 (-0.25, -0.21) | <0.001 | 2283.49 (2224.24, 2344.06) | 2277.35 (2235.17, 2320.17) | -0.01 (-0.04, 0.02) | 0.568 | 887.07 (848.99, 926.53) | 847.96 (821.74, 874.84) | -0.11 (-0.39, 0.18) | 0.463 | 12.92 (8.63, 18.73) | 12.35 (9.34, 16.06) | -0.09 (-0.42, 0.25) | 0.610 |
| Nauru | 158.18 (33.21, 468.39) | 152.42 (34.67, 435.52) | -0.12 (-0.21, -0.04) | 0.004 | 1126.44 (704.31, 1715.30) | 1094.17 (699.53, 1637.55) | -0.11 (-0.16, -0.06) | <0.001 | 6466.20 (5375.32, 7717.97) | 6554.01 (5512.65, 7739.28) | 0.05 (-0.10, 0.20) | 0.505 | 111.90 (14.80, 399.80) | 113.42 (17.87, 378.96) | 0.05 (-0.10, 0.20) | 0.517 |
| Nepal | 133.57 (129.69, 137.53) | 124.45 (121.54, 127.40) | -0.24 (-0.26, -0.21) | <0.001 | 1208.27 (1196.41, 1220.21) | 1186.50 (1177.37, 1195.69) | -0.06 (-0.14, 0.02) | 0.128 | 1428.89 (1415.85, 1442.03) | 1078.04 (1069.26, 1086.89) | -0.95 (-1.21, -0.70) | <0.001 | 23.42 (21.76, 25.17) | 17.17 (16.07, 18.33) | -1.03 (-1.22, -0.85) | <0.001 |
| Netherlands | 108.54 (104.94, 112.23) | 98.84 (95.15, 102.64) | -0.32 (-0.34, -0.31) | <0.001 | 576.57 (568.26, 584.98) | 574.61 (565.67, 583.65) | -0.02 (-0.07, 0.02) | 0.274 | 578.41 (570.09, 586.83) | 231.66 (226.01, 237.43) | -3.16 (-3.41, -2.91) | <0.001 | 9.49 (8.45, 10.62) | 3.32 (2.68, 4.08) | -3.63 (-3.89, -3.37) | <0.001 |
| New Zealand | 122.49 (114.36, 131.05) | 114.47 (106.66, 122.70) | -0.21 (-0.29, -0.13) | <0.001 | 573.17 (555.43, 591.33) | 582.86 (565.09, 601.05) | 0.08 (-0.13, 0.29) | 0.452 | 843.33 (821.79, 865.30) | 412.12 (397.19, 427.47) | -2.40 (-2.84, -1.96) | <0.001 | 14.10 (11.44, 17.20) | 6.60 (4.84, 8.81) | -2.58 (-3.11, -2.04) | <0.001 |
| Nicaragua | 139.63 (130.69, 149.07) | 138.95 (132.81, 145.30) | -0.01 (-0.03, 0.00) | 0.056 | 1759.28 (1726.97, 1792.10) | 1827.64 (1805.18, 1850.32) | 0.13 (0.07, 0.19) | <0.001 | 660.70 (640.18, 681.75) | 494.06 (482.37, 505.97) | -1.24 (-1.92, -0.55) | <0.001 | 9.86 (7.46, 12.82) | 6.65 (5.35, 8.18) | -1.55 (-2.19, -0.90) | <0.001 |
| Niger | 157.22 (150.30, 164.40) | 158.26 (154.14, 162.47) | 0.02 (0.02, 0.03) | <0.001 | 2360.82 (2334.00, 2387.89) | 2478.86 (2462.67, 2495.13) | 0.18 (0.11, 0.25) | <0.001 | 1156.79 (1137.46, 1176.39) | 1028.09 (1017.15, 1039.13) | -0.38 (-0.66, -0.11) | 0.007 | 17.34 (15.01, 19.94) | 15.15 (13.82, 16.57) | -0.42 (-0.76, -0.08) | 0.015 |
| Nigeria | 147.56 (145.70, 149.44) | 151.88 (150.63, 153.15) | 0.10 (0.09, 0.12) | <0.001 | 2604.12 (2596.33, 2611.93) | 2837.26 (2831.87, 2842.66) | 0.30 (0.28, 0.32) | <0.001 | 997.38 (992.43, 1002.35) | 841.07 (838.01, 844.14) | -0.60 (-0.83, -0.37) | <0.001 | 14.54 (13.94, 15.16) | 11.66 (11.29, 12.03) | -0.78 (-1.05, -0.52) | <0.001 |
| Niue | 123.99 | 112.89 | -0.33 | <0.001 | 1014.38 | 1002.84 | -0.04 | 0.152 | 3735.84 | 2570.04 | -1.30 | <0.001 | 63.75 | 42.65 | -1.39 | <0.001 |

| | | | | | | | | | | | | | | | | |
|---|---|---|---|---|---|---|---|---|---|---|---|---|---|---|---|---|
| | (0.09, 1266.09) | (0.00, 1680.82) | (-0.34, -0.31) | | (273.04, 2660.11) | (191.94, 3120.35) | (-0.09, 0.01) | | (2049.05, 6277.39) | (1043.32, 5331.45) | (-1.58, -1.02) | | (0.00, 1156.89) | (0.00, 1549.40) | (-1.68, -1.11) | |
| North Macedonia | 103.96 (94.43, 114.19) | 103.08 (93.70, 113.26) | -0.03 (-0.04, -0.02) | <0.001 | 748.91 (722.97, 775.55) | 766.91 (741.04, 793.57) | 0.09 (-0.03, 0.21) | 0.122 | 1362.01 (1326.95, 1397.76) | 925.76 (897.37, 954.94) | -1.20 (-2.36, -0.03) | 0.045 | 22.74 (18.41, 27.79) | 15.08 (11.68, 19.31) | -1.26 (-2.52, 0.02) | 0.054 |
| Northern Mariana Islands | 132.54 (75.78, 220.80) | 133.06 (58.21, 270.08) | 0.02 (0.00, 0.03) | 0.039 | 1659.59 (1434.53, 1913.67) | 1731.87 (1424.63, 2093.13) | 0.15 (0.12, 0.18) | <0.001 | 2005.19 (1766.11, 2272.01) | 1683.99 (1367.50, 2057.29) | -0.62 (-0.83, -0.41) | <0.001 | 32.87 (9.38, 90.97) | 27.05 (2.06, 124.66) | -0.68 (-0.85, -0.52) | <0.001 |
| Norway | 122.45 (115.08, 130.18) | 112.81 (106.14, 119.82) | -0.28 (-0.32, -0.24) | <0.001 | 844.89 (825.39, 864.75) | 809.35 (791.41, 827.63) | -0.13 (-0.17, -0.09) | <0.001 | 645.89 (628.89, 663.25) | 224.02 (214.65, 233.73) | -3.58 (-4.03, -3.12) | <0.001 | 10.45 (8.39, 12.88) | 3.00 (2.01, 4.34) | -4.16 (-4.71, -3.61) | <0.001 |
| Oman | 92.84 (84.85, 101.45) | 95.11 (90.55, 99.95) | 0.09 (0.02, 0.16) | 0.019 | 1069.68 (1041.70, 1098.27) | 1208.27 (1190.31, 1226.53) | 0.44 (0.33, 0.54) | <0.001 | 1462.34 (1429.79, 1495.50) | 795.33 (780.71, 810.25) | -2.09 (-2.34, -1.83) | <0.001 | 23.78 (19.81, 28.39) | 11.85 (10.18, 13.85) | -2.36 (-2.71, -2.01) | <0.001 |
| Pakistan | 159.05 (157.30, 160.82) | 165.30 (164.10, 166.51) | 0.14 (0.13, 0.15) | <0.001 | 1607.42 (1601.76, 1613.09) | 1678.22 (1674.35, 1682.10) | 0.16 (0.10, 0.23) | <0.001 | 1741.13 (1735.08, 1747.20) | 2509.48 (2504.65, 2514.31) | 1.31 (1.11, 1.51) | <0.001 | 28.45 (27.68, 29.25) | 41.62 (41.00, 42.25) | 1.36 (1.16, 1.56) | <0.001 |
| Palau | 133.03 (40.96, 324.72) | 128.28 (41.18, 320.92) | -0.13 (-0.14, -0.12) | <0.001 | 1340.71 (987.24, 1783.51) | 1353.96 (1002.19, 1799.75) | 0.04 (0.00, 0.08) | 0.032 | 3867.26 (3240.11, 4583.08) | 3559.20 (2993.80, 4212.37) | -0.29 (-0.39, -0.19) | <0.001 | 65.67 (9.52, 226.86) | 60.31 (9.59, 222.82) | -0.24 (-0.65, 0.17) | 0.247 |
| Palestine | 136.14 (123.82, 149.51) | 132.89 (125.76, 140.34) | -0.09 (-0.14, -0.03) | 0.002 | 1578.04 (1535.25, 1621.85) | 1603.37 (1578.35, 1628.72) | 0.07 (-0.01, 0.15) | 0.082 | 1243.13 (1203.46, 1283.87) | 780.52 (762.61, 798.76) | -1.65 (-2.13, -1.17) | <0.001 | 19.93 (15.11, 25.90) | 11.82 (9.68, 14.31) | -1.85 (-2.43, -1.27) | <0.001 |
| Panama | 130.19 (120.28, 140.75) | 126.83 (119.17, 134.85) | -0.09 (-0.10, -0.08) | <0.001 | 1629.79 (1594.08, | 1644.14 (1616.24, | 0.03 (0.01, 0.06) | 0.015 | 822.00 (796.20, 848.46) | 592.76 (575.98, 609.90) | -1.05 (-1.65, -0.45) | 0.001 | 12.64 (9.59, 16.38) | 8.71 (6.78, 11.02) | -1.19 (-1.87, -0.51) | 0.001 |

| | | | | | | | | | | | | | | | | |
|---|---|---|---|---|---|---|---|---|---|---|---|---|---|---|---|---|
| | | | | | 1666.15) | 1672.40) | | | | | | | | | | |
| Papua New Guinea | 167.39 (158.37, 176.82) | 168.60 (162.92, 174.44) | 0.03 (0.02, 0.03) | <0.001 | 1496.38 (1469.37, 1523.79) | 1546.01 (1528.78, 1563.38) | 0.11 (0.09, 0.13) | <0.001 | 2763.71 (2726.13, 2801.70) | 2910.33 (2886.36, 2934.45) | 0.11 (-0.06, 0.28) | 0.213 | 45.94 (41.17, 51.14) | 48.38 (45.31, 51.60) | 0.10 (-0.07, 0.28) | 0.230 |
| Paraguay | 136.21 (128.07, 144.76) | 134.77 (128.97, 140.77) | -0.03 (-0.05, -0.01) | 0.007 | 1901.88 (1871.00, 1933.17) | 1930.29 (1908.16, 1952.63) | 0.05 (0.04, 0.07) | <0.001 | 858.67 (837.49, 880.27) | 731.19 (717.52, 745.06) | -0.41 (-1.21, 0.39) | 0.316 | 13.25 (10.72, 16.22) | 10.88 (9.26, 12.70) | -0.47 (-1.21, 0.28) | 0.219 |
| Peru | 128.27 (124.86, 131.75) | 121.41 (118.82, 124.04) | -0.20 (-0.22, -0.17) | <0.001 | 1883.71 (1870.53, 1896.96) | 1939.90 (1929.50, 1950.35) | 0.10 (0.08, 0.12) | <0.001 | 1318.55 (1307.38, 1329.80) | 771.31 (764.75, 777.92) | -1.66 (-2.33, -0.98) | <0.001 | 20.38 (19.00, 21.85) | 11.17 (10.39, 11.99) | -1.89 (-2.64, -1.13) | <0.001 |
| Philippines | 157.50 (155.25, 159.77) | 146.03 (144.47, 147.61) | -0.26 (-0.27, -0.25) | <0.001 | 1250.92 (1244.57, 1257.29) | 1237.81 (1233.26, 1242.38) | -0.03 (-0.08, 0.02) | 0.225 | 846.05 (840.75, 851.38) | 2178.96 (2172.87, 2185.07) | 3.41 (2.69, 4.14) | <0.001 | 12.79 (12.14, 13.46) | 36.25 (35.46, 37.04) | 3.76 (2.90, 4.63) | <0.001 |
| Poland | 99.53 (97.32, 101.77) | 88.08 (85.87, 90.34) | -0.41 (-0.47, -0.34) | <0.001 | 840.11 (833.63, 846.64) | 792.29 (785.52, 799.11) | -0.19 (-0.28, -0.09) | <0.001 | 1884.85 (1875.46, 1894.29) | 577.76 (572.30, 583.27) | -4.16 (-4.97, -3.34) | <0.001 | 32.67 (31.45, 33.93) | 9.19 (8.53, 9.91) | -4.41 (-5.27, -3.54) | <0.001 |
| Portugal | 73.66 (69.81, 77.68) | 60.72 (56.93, 64.71) | -0.67 (-0.71, -0.63) | <0.001 | 589.08 (578.10, 600.21) | 545.01 (533.48, 556.72) | -0.18 (-0.57, 0.23) | 0.392 | 1017.98 (1003.50, 1032.62) | 339.35 (330.47, 348.42) | -3.86 (-4.51, -3.20) | <0.001 | 16.80 (14.98, 18.78) | 5.19 (4.16, 6.42) | -4.01 (-4.87, -3.15) | <0.001 |
| Puerto Rico | 113.80 (105.77, 122.27) | 126.07 (116.91, 135.76) | 0.36 (0.33, 0.40) | <0.001 | 859.49 (837.29, 882.15) | 930.68 (905.51, 956.37) | 0.28 (0.20, 0.37) | <0.001 | 922.54 (899.33, 946.19) | 529.19 (510.30, 548.61) | -1.83 (-2.72, -0.94) | <0.001 | 15.54 (12.65, 18.90) | 8.50 (6.27, 11.29) | -1.99 (-2.97, -1.01) | <0.001 |
| Qatar | 103.14 (88.68, 120.33) | 94.38 (88.90, 100.41) | -0.29 (-0.34, -0.24) | <0.001 | 1121.09 (1071.20, 1173.57) | 1039.37 (1020.03, 1059.23) | -0.25 (-0.29, -0.22) | <0.001 | 836.41 (791.89, 883.62) | 400.94 (388.84, 413.58) | -2.40 (-2.98, -1.82) | <0.001 | 12.69 (7.86, 20.78) | 5.44 (4.16, 7.39) | -2.79 (-3.58, -1.99) | <0.001 |
| Republic of Korea | 115.67 | 106.07 | -0.30 | <0.001 | 741.82 | 744.94 | 0.05 | 0.197 | 1480.95 | 287.89 | -5.51 | <0.001 | 24.30 | 4.17 | -5.92 | <0.001 |

| | | | | | | | | | | | | | | | | |
|---|---|---|---|---|---|---|---|---|---|---|---|---|---|---|---|---|
| | (113.61, 117.75) | (104.04, 108.14) | (-0.34, -0.25) | | (736.58, 747.09) | (739.62, 750.29) | (-0.03, 0.14) | | (1473.57, 1488.36) | (284.58, 291.23) | (-6.03, -4.98) | | (23.36, 25.27) | (3.79, 4.59) | (-6.37, -5.46) | |
| Republic of Moldova | 127.91 (120.62, 135.56) | 125.93 (117.92, 134.42) | -0.06 (-0.09, -0.02) | 0.001 | 690.17 (673.20, 707.49) | 697.98 (679.16, 717.27) | 0.04 (-0.01, 0.08) | 0.109 | 1382.99 (1358.85, 1407.48) | 1052.09 (1029.29, 1075.36) | -1.00 (-2.05, 0.06) | 0.063 | 23.37 (20.34, 26.76) | 17.76 (14.93, 21.07) | -1.01 (-2.12, 0.11) | 0.077 |
| Romania | 100.00 (97.07, 103.00) | 107.87 (104.22, 111.62) | 0.26 (0.24, 0.28) | <0.001 | 719.35 (711.47, 727.30) | 822.24 (812.22, 832.35) | 0.49 (0.40, 0.58) | <0.001 | 1590.55 (1578.89, 1602.27) | 941.87 (931.23, 952.62) | -1.79 (-2.75, -0.82) | <0.001 | 27.33 (25.83, 28.91) | 15.64 (14.31, 17.08) | -1.91 (-2.95, -0.85) | <0.001 |
| Russian Federation | 135.36 (134.07, 136.65) | 145.94 (144.49, 147.40) | 0.26 (0.23, 0.30) | <0.001 | 717.41 (714.45, 720.38) | 785.00 (781.67, 788.35) | 0.31 (0.30, 0.32) | <0.001 | 2179.65 (2174.56, 2184.76) | 2659.26 (2653.26, 2665.26) | 0.90 (-0.16, 1.96) | 0.097 | 37.96 (37.30, 38.64) | 46.60 (45.82, 47.40) | 0.62 (-0.71, 1.97) | 0.365 |
| Rwanda | 212.11 (204.22, 220.25) | 172.69 (167.71, 177.78) | -0.70 (-0.75, -0.65) | <0.001 | 2615.50 (2587.56, 2643.69) | 2514.14 (2494.89, 2533.51) | -0.12 (-0.21, -0.03) | 0.007 | 1833.43 (1809.58, 1857.53) | 979.51 (967.29, 991.86) | -2.15 (-2.30, -2.01) | <0.001 | 28.63 (25.69, 31.83) | 14.33 (12.87, 15.91) | -2.37 (-2.52, -2.23) | <0.001 |
| Saint Kitts and Nevis | 130.37 (63.50, 239.98) | 121.27 (66.77, 204.72) | -0.25 (-0.28, -0.23) | <0.001 | 995.71 (789.83, 1241.06) | 980.65 (809.25, 1179.23) | -0.04 (-0.13, 0.05) | 0.389 | 1882.62 (1588.64, 2216.64) | 715.82 (570.71, 888.26) | -2.95 (-4.30, -1.57) | <0.001 | 32.96 (5.51, 107.78) | 11.42 (0.65, 56.08) | -3.27 (-5.02, -1.47) | <0.001 |
| Saint Lucia | 195.89 (144.53, 261.17) | 196.47 (152.30, 249.79) | 0.01 (-0.01, 0.03) | 0.441 | 2253.94 (2071.31, 2449.72) | 2211.87 (2056.88, 2375.72) | -0.06 (-0.09, -0.03) | <0.001 | 1434.62 (1282.43, 1600.81) | 960.35 (859.78, 1069.78) | -1.43 (-2.31, -0.54) | 0.002 | 23.37 (7.72, 55.42) | 14.79 (4.90, 34.77) | -1.64 (-2.68, -0.60) | 0.002 |
| Saint Vincent and the Grenadines | 182.30 (129.39, 251.86) | 186.97 (133.35, 255.32) | 0.09 (0.07, 0.11) | <0.001 | 2294.85 (2096.63, 2508.63) | 2333.96 (2132.96, 2549.12) | 0.06 (0.03, 0.09) | <0.001 | 1580.07 (1407.48, 1769.28) | 1029.68 (897.41, 1176.26) | -1.32 (-2.42, -0.21) | 0.020 | 25.87 (8.21, 63.18) | 15.93 (3.70, 44.65) | -1.49 (-2.73, -0.22) | 0.021 |
| Samoa | 161.23 (118.25, | 148.17 (112.29, | -0.29 (-0.30, | <0.001 | 1703.82 (1558.80 | 1679.11 (1553.52 | -0.04 (-0.09, | 0.109 | 2659.99 (2469.46 | 2741.45 (2575.49 | 0.12 (0.04, | 0.004 | 44.07 (22.49, | 45.58 (26.38, | 0.13 (0.04, | 0.003 |

| | | | | | | | | | | | | | | | | |
|---|---|---|---|---|---|---|---|---|---|---|---|---|---|---|---|---|
| | 216.18) | 192.43) | -0.28) | | , 1860.01) | , 1812.67) | 0.01) | | , 2862.23) | , 2915.63) | 0.20) | | 78.92) | 73.76) | 0.22) | |
| San Marino | 81.01 (20.49, 217.86) | 75.78 (19.77, 201.10) | -0.23 (-0.26, -0.19) | <0.001 | 759.71 (527.76, 1060.62) | 729.94 (512.48, 1010.16) | -0.13 (-0.17, -0.09) | <0.001 | 397.97 (235.99, 630.49) | 377.27 (227.34, 590.92) | -0.18 (-0.28, -0.08) | <0.001 | 5.94 (0.00, 97.42) | 5.69 (0.00, 89.28) | -0.14 (-0.26, -0.03) | 0.016 |
| Sao Tome and Principe | 169.01 (114.08, 244.83) | 180.20 (142.02, 225.83) | 0.21 (0.16, 0.26) | <0.001 | 2926.97 (2686.59, 3186.13) | 3243.35 (3075.08, 3418.77) | 0.35 (0.33, 0.38) | <0.001 | 1118.35 (958.40, 1299.32) | 1178.40 (1076.60, 1287.50) | 0.31 (-0.07, 0.69) | 0.109 | 16.57 (2.68, 56.84) | 16.46 (6.50, 34.67) | 0.11 (-0.31, 0.54) | 0.607 |
| Saudi Arabia | 87.41 (84.43, 90.48) | 98.14 (96.35, 99.96) | 0.41 (0.37, 0.45) | <0.001 | 984.33 (974.29, 994.46) | 1073.24 (1067.20, 1079.31) | 0.43 (-0.27, 1.14) | 0.226 | 938.83 (928.92, 948.83) | 938.90 (933.46, 944.37) | -0.00 (-0.27, 0.26) | 0.987 | 14.99 (13.75, 16.31) | 14.94 (14.27, 15.64) | -0.03 (-0.41, 0.35) | 0.874 |
| Senegal | 165.14 (157.91, 172.64) | 160.35 (155.72, 165.09) | -0.11 (-0.12, -0.09) | <0.001 | 2437.32 (2409.49, 2465.42) | 2460.03 (2441.87, 2478.31) | 0.04 (-0.03, 0.11) | 0.286 | 1162.07 (1142.23, 1182.19) | 1029.81 (1017.69, 1042.05) | -0.40 (-0.93, 0.13) | 0.136 | 17.36 (14.98, 20.04) | 15.11 (13.65, 16.69) | -0.44 (-1.05, 0.17) | 0.153 |
| Serbia | 113.49 (108.65, 118.50) | 111.73 (106.49, 117.18) | -0.05 (-0.08, -0.02) | 0.005 | 856.62 (843.22, 870.19) | 885.77 (870.89, 900.85) | 0.12 (-0.01, 0.25) | 0.065 | 1358.31 (1341.55, 1375.24) | 634.95 (622.52, 647.58) | -2.54 (-2.97, -2.11) | <0.001 | 22.90 (20.77, 25.19) | 10.10 (8.59, 11.81) | -2.75 (-3.23, -2.26) | <0.001 |
| Seychelles | 136.20 (83.64, 211.58) | 127.06 (84.27, 186.23) | -0.23 (-0.27, -0.20) | <0.001 | 1505.28 (1315.95, 1715.85) | 1440.84 (1285.73, 1611.28) | -0.14 (-0.19, -0.08) | <0.001 | 2108.01 (1876.36, 2361.53) | 1419.10 (1269.21, 1583.86) | -1.37 (-2.28, -0.44) | 0.004 | 35.18 (11.31, 84.39) | 22.61 (7.64, 55.55) | -1.54 (-2.55, -0.51) | 0.003 |
| Sierra Leone | 178.10 (167.68, 189.04) | 177.46 (170.97, 184.16) | -0.01 (-0.03, 0.00) | 0.076 | 2626.67 (2586.45, 2667.40) | 2740.39 (2714.93, 2766.06) | 0.15 (0.13, 0.18) | <0.001 | 1198.70 (1171.00, 1226.91) | 1166.65 (1149.51, 1184.00) | -0.09 (-0.36, 0.19) | 0.536 | 17.79 (14.53, 21.59) | 17.22 (15.17, 19.49) | -0.10 (-0.41, 0.21) | 0.516 |
| Singapore | 125.46 (117.71, 133.61) | 123.95 (117.59, 130.64) | -0.04 (-0.06, -0.02) | <0.001 | 758.35 (739.11, 777.99) | 754.15 (739.35, 769.25) | -0.03 (-0.10, 0.05) | 0.506 | 947.56 (926.06, 969.46) | 378.44 (367.37, 389.82) | -3.10 (-3.79, -2.41) | <0.001 | 15.71 (13.05, 18.78) | 5.77 (4.51, 7.38) | -3.39 (-4.24, -2.54) | <0.001 |
| Slovakia | 105.72 | 102.24 | -0.12 | <0.001 | 935.76 | 927.14 | -0.01 | 0.726 | 1330.15 | 581.17 | -2.95 | <0.001 | 22.53 | 9.09 | -3.23 | <0.001 |

| | | | | | | | | | | | | | | | | |
|---|---|---|---|---|---|---|---|---|---|---|---|---|---|---|---|---|
| | (99.68, 112.05) | (96.03, 108.81) | (-0.14, -0.10) | | (917.53, 954.28) | (907.96, 946.68) | (-0.08, 0.05) | | (1308.84, 1351.75) | (566.59, 596.10) | (-3.51, -2.38) | | (19.84, 25.50) | (7.38, 11.17) | (-3.86, -2.60) | |
| Slovenia | 100.30 (90.85, 110.52) | 103.51 (92.88, 115.21) | 0.11 (0.07, 0.15) | <0.001 | 710.86 (685.53, 736.96) | 731.24 (703.10, 760.40) | 0.08 (0.02, 0.14) | 0.005 | 789.81 (763.36, 817.01) | 253.67 (237.32, 271.04) | -3.88 (-4.31, -3.45) | <0.001 | 13.13 (9.94, 17.11) | 3.60 (1.93, 6.45) | -4.48 (-5.03, -3.93) | <0.001 |
| Solomon Islands | 169.68 (136.63, 208.77) | 157.33 (135.81, 181.38) | -0.26 (-0.29, -0.24) | <0.001 | 1440.45 (1342.97, 1543.63) | 1424.40 (1358.80, 1492.45) | -0.01 (-0.15, 0.14) | 0.930 | 6188.56 (5973.96, 6409.18) | 6401.90 (6257.85, 6548.46) | 0.12 (-0.01, 0.24) | 0.067 | 108.23 (81.30, 141.53) | 112.22 (93.80, 133.24) | 0.13 (-0.00, 0.25) | 0.055 |
| Somalia | 210.05 (201.98, 218.39) | 208.04 (203.49, 212.67) | -0.04 (-0.05, -0.02) | <0.001 | 2604.14 (2575.23, 2633.31) | 2708.08 (2691.47, 2724.76) | 0.14 (0.09, 0.19) | <0.001 | 1632.25 (1609.14, 1655.62) | 1492.14 (1479.44, 1504.92) | -0.28 (-0.44, -0.12) | <0.001 | 25.52 (22.69, 28.62) | 22.94 (21.37, 24.60) | -0.35 (-0.53, -0.17) | <0.001 |
| South Africa | 197.35 (194.16, 200.58) | 186.48 (184.05, 188.93) | -0.19 (-0.22, -0.17) | <0.001 | 2490.22 (2478.88, 2501.60) | 2501.81 (2492.91, 2510.75) | 0.02 (-0.01, 0.05) | 0.160 | 1554.08 (1544.72, 1563.47) | 924.24 (918.92, 929.58) | -1.92 (-2.74, -1.09) | <0.001 | 25.10 (23.92, 26.33) | 13.73 (13.09, 14.39) | -2.24 (-3.22, -1.25) | <0.001 |
| South Sudan | 167.81 (160.35, 175.57) | 167.93 (161.32, 174.76) | 0.00 (-0.00, 0.01) | 0.236 | 2486.79 (2457.65, 2516.24) | 2570.47 (2544.05, 2597.11) | 0.13 (0.09, 0.18) | <0.001 | 1016.35 (996.92, 1036.09) | 858.19 (842.50, 874.11) | -0.50 (-0.88, -0.12) | 0.010 | 15.03 (12.70, 17.68) | 12.17 (10.34, 14.25) | -0.64 (-1.08, -0.19) | 0.005 |
| Spain | 89.26 (87.10, 91.45) | 77.55 (75.49, 79.66) | -0.50 (-0.56, -0.44) | <0.001 | 557.39 (551.99, 562.83) | 524.50 (519.10, 529.96) | -0.19 (-0.30, -0.07) | 0.001 | 953.75 (946.68, 960.86) | 344.72 (340.41, 349.08) | -3.46 (-3.81, -3.12) | <0.001 | 15.62 (14.72, 16.56) | 5.27 (4.75, 5.82) | -3.69 (-4.10, -3.28) | <0.001 |
| Sri Lanka | 83.14 (80.18, 86.18) | 80.27 (77.51, 83.11) | -0.12 (-0.13, -0.11) | <0.001 | 699.86 (691.22, 708.59) | 694.21 (686.06, 702.44) | 0.01 (-0.20, 0.22) | 0.934 | 1741.82 (1728.14, 1755.58) | 1052.97 (1042.93, 1063.09) | -1.60 (-3.47, 0.30) | 0.099 | 28.99 (27.25, 30.82) | 16.93 (15.68, 18.25) | -1.71 (-3.66, 0.27) | 0.090 |
| Sudan | 158.75 (154.48, 163.12) | 162.11 (159.27, 164.98) | 0.07 (0.06, 0.09) | <0.001 | 1710.02 (1695.99, 1724.13) | 1836.83 (1827.30, 1846.39) | 0.27 (0.09, 0.45) | 0.003 | 2583.51 (2565.65, 2601.46) | 1782.75 (1773.13, 1792.41) | -1.29 (-1.39, -1.18) | <0.001 | 43.49 (41.18, 45.90) | 28.88 (27.65, 30.15) | -1.42 (-1.54, -1.31) | <0.001 |

| | | | | | | | | | | | | | | | | |
|---|---|---|---|---|---|---|---|---|---|---|---|---|---|---|---|---|
| Suriname | 189.52 (159.63, 223.94) | 195.65 (169.95, 224.19) | 0.11 (0.07, 0.14) | <0.001 | 1974.07 (1874.96, 2077.55) | 2037.95 (1953.03, 2125.68) | 0.11 (0.09, 0.13) | <0.001 | 1734.64 (1637.19, 1836.73) | 1229.83 (1164.05, 1298.42) | -1.22 (-2.66, 0.23) | 0.099 | 28.87 (17.42, 45.44) | 19.88 (12.33, 30.43) | -1.65 (-3.64, 0.38) | 0.111 |
| Sweden | 89.19 (84.55, 94.02) | 97.42 (92.81, 102.22) | 0.31 (0.29, 0.34) | <0.001 | 789.21 (775.21, 803.41) | 822.75 (809.19, 836.49) | 0.18 (-0.25, 0.62) | 0.414 | 558.34 (546.65, 570.22) | 266.32 (258.64, 274.19) | -2.48 (-2.67, -2.29) | <0.001 | 8.53 (7.14, 10.12) | 3.29 (2.50, 4.28) | -3.26 (-3.97, -2.54) | <0.001 |
| Switzerland | 75.90 (71.44, 80.61) | 69.45 (65.31, 73.81) | -0.31 (-0.34, -0.29) | <0.001 | 471.02 (459.84, 482.44) | 438.88 (428.48, 449.51) | -0.24 (-0.38, -0.10) | 0.001 | 571.11 (558.79, 583.67) | 189.40 (182.62, 196.41) | -3.81 (-4.69, -2.92) | <0.001 | 9.26 (7.76, 11.01) | 2.68 (1.93, 3.66) | -4.28 (-5.31, -3.24) | <0.001 |
| Syrian Arab Republic | 173.16 (167.60, 178.87) | 155.40 (149.93, 161.04) | -0.37 (-0.41, -0.33) | <0.001 | 1877.84 (1859.33, 1896.50) | 1868.73 (1849.60, 1888.04) | -0.02 (-0.06, 0.02) | 0.371 | 3944.55 (3917.62, 3971.64) | 2141.92 (2121.42, 2162.61) | -2.11 (-2.64, -1.58) | <0.001 | 64.71 (61.25, 68.32) | 34.22 (31.66, 36.96) | -2.19 (-2.75, -1.63) | <0.001 |
| Taiwan (Province of China) | 81.58 (79.05, 84.19) | 81.32 (78.64, 84.08) | -0.02 (-0.05, 0.02) | 0.296 | 742.97 (735.27, 750.74) | 804.92 (796.57, 813.35) | 0.27 (0.15, 0.39) | <0.001 | 775.25 (767.39, 783.18) | 619.05 (611.83, 626.33) | -0.85 (-1.42, -0.28) | 0.004 | 12.16 (11.19, 13.19) | 9.64 (8.77, 10.59) | -0.88 (-1.52, -0.24) | 0.007 |
| Tajikistan | 166.54 (158.44, 174.98) | 164.26 (158.69, 169.98) | -0.04 (-0.07, -0.02) | 0.003 | 1591.07 (1565.73, 1616.75) | 1588.20 (1570.79, 1605.77) | -0.01 (-0.06, 0.04) | 0.645 | 1266.92 (1243.18, 1291.01) | 1107.45 (1092.62, 1122.43) | -0.39 (-0.92, 0.14) | 0.146 | 20.84 (17.85, 24.20) | 18.05 (16.19, 20.08) | -0.42 (-0.99, 0.15) | 0.148 |
| Thailand | 132.21 (130.19, 134.26) | 127.66 (125.69, 129.67) | -0.12 (-0.15, -0.10) | <0.001 | 1314.60 (1308.22, 1321.01) | 1278.83 (1272.53, 1285.16) | -0.09 (-0.15, -0.02) | 0.008 | 1353.83 (1347.24, 1360.45) | 1393.60 (1387.14, 1400.08) | 0.25 (-0.57, 1.08) | 0.554 | 21.74 (20.91, 22.60) | 22.42 (21.62, 23.25) | 0.26 (-0.59, 1.11) | 0.552 |
| Timor-Leste | 141.54 (123.34, 161.82) | 143.92 (128.62, 160.70) | 0.06 (0.05, 0.07) | <0.001 | 1356.46 (1298.84, 1416.12) | 1425.31 (1376.27, 1475.80) | 0.17 (0.14, 0.21) | <0.001 | 1007.76 (957.27, 1060.32) | 1352.72 (1303.98, 1402.95) | 1.06 (0.46, 1.66) | 0.001 | 15.52 (9.79, 23.51) | 21.10 (15.33, 28.47) | 1.10 (0.45, 1.75) | 0.001 |
| Togo | 170.98 (160.51, 182.02) | 172.52 (165.82, 179.44) | 0.03 (0.02, 0.04) | <0.001 | 2552.91 (2512.52, 2593.85) | 2655.49 (2629.24, 2681.96) | 0.14 (0.11, 0.17) | <0.001 | 1280.42 (1250.54, 1310.90) | 1278.20 (1259.39, 1297.22) | 0.04 (-0.24, 0.31) | 0.803 | 19.32 (15.75, 23.51) | 19.30 (17.03, 21.81) | 0.05 (-0.22, 0.33) | 0.697 |

| | | | | | | | | | | | | | | | | |
|---|---|---|---|---|---|---|---|---|---|---|---|---|---|---|---|---|
| Tokelau | 119.56 (0.00, 1806.16) | 106.79 (0.00, 1994.15) | -0.39 (-0.41, -0.37) | <0.001 | 999.86 (179.82, 3225.38) | 970.34 (150.87, 3383.49) | -0.10 (-0.14, -0.05) | <0.001 | 2403.91 (903.32, 5242.61) | 2134.67 (705.01, 5077.49) | -0.41 (-0.44, -0.38) | <0.001 | 40.15 (0.00, 1661.49) | 35.54 (0.00, 1865.95) | -0.42 (-0.45, -0.39) | <0.001 |
| Tonga | 141.57 (87.78, 220.09) | 142.84 (92.11, 212.68) | 0.04 (-0.04, 0.13) | 0.322 | 2000.77 (1784.62, 2239.11) | 2055.67 (1849.58, 2279.53) | 0.10 (0.01, 0.18) | 0.023 | 1370.53 (1178.71, 1586.68) | 1548.42 (1364.31, 1751.10) | 0.44 (0.28, 0.61) | <0.001 | 21.64 (4.07, 69.15) | 24.41 (6.58, 63.79) | 0.42 (0.24, 0.60) | <0.001 |
| Trinidad and Tobago | 203.56 (186.15, 222.21) | 197.85 (181.22, 215.72) | -0.10 (-0.11, -0.08) | <0.001 | 2183.68 (2125.77, 2242.82) | 2158.31 (2102.47, 2215.36) | -0.03 (-0.06, -0.00) | 0.032 | 1628.26 (1577.75, 1680.02) | 1084.82 (1046.69, 1124.11) | -1.26 (-2.27, -0.25) | 0.014 | 26.76 (20.61, 34.21) | 17.19 (12.75, 22.86) | -1.49 (-2.10, -0.88) | <0.001 |
| Tunisia | 85.60 (81.03, 90.38) | 89.39 (85.56, 93.34) | 0.16 (0.12, 0.19) | <0.001 | 873.05 (858.45, 887.85) | 982.24 (969.36, 995.27) | 0.39 (0.34, 0.45) | <0.001 | 1109.93 (1093.16, 1126.91) | 940.62 (928.19, 953.19) | -0.57 (-0.67, -0.48) | <0.001 | 18.27 (16.15, 20.60) | 15.11 (13.58, 16.77) | -0.66 (-0.77, -0.55) | <0.001 |
| Turkey | 80.90 (79.27, 82.56) | 73.53 (72.29, 74.79) | -0.32 (-0.39, -0.25) | <0.001 | 1093.86 (1087.87, 1099.87) | 1004.25 (999.62, 1008.89) | -0.29 (-0.35, -0.23) | <0.001 | 1410.48 (1403.54, 1417.44) | 582.44 (578.94, 585.95) | -3.04 (-3.41, -2.66) | <0.001 | 23.53 (22.64, 24.46) | 8.75 (8.33, 9.19) | -3.37 (-3.74, -2.99) | <0.001 |
| Turkmenistan | 176.87 (167.18, 187.01) | 200.01 (191.71, 208.59) | 0.43 (0.40, 0.47) | <0.001 | 1638.05 (1608.29, 1668.26) | 1776.35 (1751.45, 1801.53) | 0.27 (0.26, 0.28) | <0.001 | 2481.90 (2444.06, 2520.22) | 2644.61 (2614.32, 2675.16) | 0.38 (-0.32, 1.07) | 0.291 | 41.89 (37.05, 47.21) | 44.09 (40.25, 48.20) | 0.33 (-0.39, 1.06) | 0.370 |
| Tuvalu | 138.49 (19.81, 482.15) | 123.58 (25.52, 369.18) | -0.40 (-0.41, -0.38) | <0.001 | 986.38 (563.35, 1610.55) | 957.56 (609.40, 1437.96) | -0.10 (-0.15, -0.04) | <0.001 | 4094.15 (3163.18, 5218.88) | 4145.75 (3371.19, 5048.80) | 0.03 (-0.06, 0.13) | 0.505 | 69.95 (2.42, 373.51) | 71.31 (6.54, 290.23) | 0.06 (-0.04, 0.15) | 0.269 |
| Uganda | 196.03 (190.96, 201.22) | 185.31 (182.19, 188.47) | -0.19 (-0.22, -0.16) | <0.001 | 2742.83 (2723.57, 2762.20) | 2936.50 (2923.93, 2949.13) | 0.25 (0.22, 0.28) | <0.001 | 1072.53 (1059.95, 1085.22) | 1169.43 (1161.11, 1177.80) | 0.26 (-0.01, 0.54) | 0.056 | 15.71 (14.19, 17.36) | 17.15 (16.14, 18.22) | 0.23 (-0.12, 0.58) | 0.195 |
| Ukraine | 126.05 | 149.22 | 0.59 | <0.001 | 708.10 | 793.40 | 0.41 | <0.001 | 1731.53 | 3528.09 | 2.36 | 0.055 | 29.82 | 61.67 | 2.55 | <0.001 |

| | | | | | | | | | | | | | | | | |
|---|---|---|---|---|---|---|---|---|---|---|---|---|---|---|---|---|
| | (123.85, 128.28) | (146.57, 151.92) | (0.44, 0.74) | | (702.89, 713.33) | (787.33, 799.52) | (0.30, 0.53) | | (1723.42, 1739.66) | (3515.46, 3540.76) | (-0.05, 4.82) | | (28.77, 30.90) | (60.02, 63.35) | (1.12, 4.01) | |
| United Arab Emirates | 136.44 (127.42, 146.12) | 142.50 (137.00, 148.23) | 0.15 (0.10, 0.20) | <0.001 | 1520.37 (1490.37, 1551.01) | 1550.90 (1533.01, 1569.01) | 0.10 (-0.01, 0.22) | 0.069 | 1135.24 (1109.49, 1161.63) | 934.10 (921.48, 946.94) | -0.70 (-0.96, -0.43) | <0.001 | 17.72 (14.71, 21.42) | 14.41 (12.97, 16.06) | -0.76 (-1.09, -0.42) | <0.001 |
| United Kingdom | 93.12 (91.29, 94.98) | 86.27 (84.59, 87.97) | -0.29 (-0.33, -0.25) | <0.001 | 634.27 (629.48, 639.10) | 622.23 (617.71, 626.78) | -0.06 (-0.11, -0.01) | 0.025 | 702.69 (697.65, 707.77) | 459.60 (455.76, 463.46) | -1.46 (-1.81, -1.10) | <0.001 | 11.59 (10.95, 12.25) | 7.32 (6.84, 7.82) | -1.57 (-1.93, -1.21) | <0.001 |
| United Republic of Tanzania | 179.34 (175.35, 183.41) | 182.73 (180.10, 185.39) | 0.07 (0.03, 0.11) | <0.001 | 2528.82 (2513.65, 2544.07) | 2711.47 (2701.23, 2721.74) | 0.25 (0.18, 0.31) | <0.001 | 1289.85 (1278.67, 1301.10) | 1044.26 (1037.76, 1050.79) | -0.72 (-0.84, -0.61) | <0.001 | 19.54 (18.16, 20.99) | 15.10 (14.32, 15.91) | -0.89 (-1.02, -0.75) | <0.001 |
| United States of America | 138.56 (137.57, 139.56) | 135.12 (134.17, 136.08) | -0.10 (-0.12, -0.07) | <0.001 | 1282.45 (1279.43, 1285.48) | 1313.66 (1310.69, 1316.62) | 0.11 (-0.25, 0.48) | 0.547 | 836.66 (834.23, 839.10) | 668.44 (666.33, 670.55) | -0.78 (-1.01, -0.54) | <0.001 | 13.40 (13.10, 13.71) | 10.23 (9.97, 10.49) | -0.93 (-1.33, -0.54) | <0.001 |
| United States Virgin Islands | 111.17 (68.15, 171.80) | 122.88 (72.95, 195.04) | 0.35 (0.33, 0.38) | <0.001 | 809.24 (684.76, 950.37) | 871.90 (728.04, 1036.77) | 0.25 (0.20, 0.29) | <0.001 | 1571.56 (1395.38, 1764.31) | 814.34 (676.47, 973.06) | -2.18 (-2.79, -1.57) | <0.001 | 26.72 (8.55, 63.72) | 13.62 (1.73, 50.59) | -2.24 (-2.96, -1.52) | <0.001 |
| Uruguay | 74.01 (67.02, 81.54) | 74.60 (67.86, 81.84) | 0.03 (0.01, 0.04) | <0.001 | 534.19 (515.18, 553.71) | 572.11 (553.13, 591.58) | 0.26 (0.14, 0.38) | <0.001 | 1006.99 (980.73, 1033.79) | 500.26 (482.58, 518.44) | -2.14 (-2.59, -1.69) | <0.001 | 16.82 (13.57, 20.62) | 7.83 (5.76, 10.41) | -2.34 (-2.82, -1.85) | <0.001 |
| Uzbekistan | 165.41 (161.46, 169.43) | 170.46 (167.45, 173.52) | 0.11 (0.09, 0.13) | <0.001 | 1594.77 (1582.46, 1607.15) | 1738.31 (1728.65, 1748.01) | 0.31 (0.27, 0.34) | <0.001 | 1628.73 (1615.91, 1641.63) | 1964.59 (1954.32, 1974.91) | 0.72 (0.20, 1.24) | 0.006 | 27.00 (25.36, 28.73) | 32.58 (31.27, 33.93) | 0.73 (0.17, 1.29) | 0.011 |
| Vanuatu | 199.89 (149.68, 262.10) | 188.88 (154.10, 229.43) | -0.20 (-0.21, -0.18) | <0.001 | 1826.89 (1669.25, 1995.97) | 1784.71 (1674.89, 1900.09) | -0.07 (-0.12, -0.03) | 0.003 | 4666.73 (4406.07, 4939.13) | 5579.75 (5380.48, 5784.68) | 0.58 (0.42, 0.75) | <0.001 | 79.51 (48.68, 123.00) | 95.52 (70.93, 126.07) | 0.60 (0.44, 0.76) | <0.001 |

| | | | | | | | | | | | | | | | | |
|---|---|---|---|---|---|---|---|---|---|---|---|---|---|---|---|---|
| Venezuela (Bolivarian Republic of) | 79.39 (76.54, 82.32) | 73.08 (70.79, 75.43) | -0.28 (-0.30, -0.26) | <0.001 | 643.77 (635.65, 651.97) | 612.47 (605.81, 619.19) | -0.17 (-0.26, -0.07) | 0.001 | 1199.50 (1188.29, 1210.79) | 755.98 (748.63, 763.38) | -1.52 (-2.07, -0.96) | <0.001 | 20.28 (18.83, 21.81) | 12.62 (11.69, 13.60) | -1.58 (-2.15, -1.01) | <0.001 |
| Viet Nam | 96.92 (95.17, 98.69) | 89.94 (88.66, 91.24) | -0.25 (-0.30, -0.19) | <0.001 | 766.50 (761.62, 771.40) | 809.31 (805.42, 813.21) | 0.24 (-0.06, 0.54) | 0.123 | 1305.70 (1299.22, 1312.21) | 965.28 (961.09, 969.49) | -1.04 (-1.15, -0.93) | <0.001 | 21.55 (20.71, 22.41) | 15.32 (14.80, 15.85) | -1.18 (-1.29, -1.07) | <0.001 |
| Yemen | 169.37 (163.81, 175.08) | 169.13 (165.93, 172.38) | -0.01 (-0.03, 0.01) | 0.321 | 1832.73 (1814.35, 1851.26) | 1926.00 (1915.16, 1936.89) | 0.19 (0.14, 0.23) | <0.001 | 2040.23 (2020.04, 2060.57) | 1671.51 (1661.23, 1681.85) | -0.64 (-0.90, -0.38) | <0.001 | 34.40 (31.80, 37.15) | 27.36 (26.05, 28.71) | -0.74 (-1.00, -0.48) | <0.001 |
| Zambia | 177.63 (170.54, 184.98) | 180.21 (175.84, 184.67) | 0.04 (0.03, 0.06) | <0.001 | 2478.52 (2451.57, 2505.73) | 2534.40 (2517.80, 2551.08) | 0.07 (0.03, 0.10) | <0.001 | 1183.92 (1164.46, 1203.65) | 1258.51 (1246.46, 1270.66) | 0.20 (0.02, 0.38) | 0.027 | 17.82 (15.45, 20.47) | 19.18 (17.69, 20.75) | 0.25 (0.05, 0.44) | 0.016 |
| Zimbabwe | 166.46 (160.44, 172.67) | 181.24 (176.32, 186.27) | 0.30 (0.28, 0.32) | <0.001 | 2419.70 (2396.53, 2443.05) | 2544.27 (2525.71, 2562.94) | 0.17 (0.15, 0.20) | <0.001 | 942.66 (928.11, 957.41) | 1342.09 (1328.55, 1355.73) | 1.28 (0.83, 1.73) | <0.001 | 13.32 (11.61, 15.23) | 19.94 (18.30, 21.69) | 1.48 (0.98, 1.98) | <0.001 |
| **Rheumatic heart disease** | | | | | | | | | | | | | | | | |
| **Overall** | | | | | | | | | | | | | | | | |
| Afghanistan | 56.67 (54.18, 59.27) | 57.57 (56.35, 58.81) | 0.06 (0.04, 0.08) | <0.001 | 741.71 (732.14, 751.40) | 755.99 (751.35, 760.65) | 0.07 (0.05, 0.09) | <0.001 | 329.89 (323.35, 336.53) | 154.80 (152.64, 156.99) | -2.49 (-2.70, -2.27) | <0.001 | 4.98 (4.19, 5.89) | 2.03 (1.78, 2.30) | -2.95 (-3.18, -2.72) | <0.001 |
| Albania | 56.05 (52.25, 60.07) | 53.16 (48.65, 57.99) | -0.18 (-0.20, -0.16) | <0.001 | 1080.11 (1062.95, 1097.49) | 1065.60 (1045.12, 1086.38) | -0.04 (-0.05, -0.04) | <0.001 | 112.94 (107.42, 118.69) | 67.35 (62.28, 72.74) | -1.81 (-2.02, -1.60) | <0.001 | 1.03 (0.56, 1.75) | 0.27 (0.05, 0.87) | -4.51 (-5.35, -3.67) | <0.001 |
| Algeria | 34.50 (33.36, 35.67) | 35.00 (34.10, 35.92) | 0.04 (0.03, 0.06) | <0.001 | 501.71 (497.17, 506.28) | 566.86 (563.31, 570.44) | 0.44 (0.40, 0.48) | <0.001 | 111.20 (109.06, 113.37) | 45.91 (44.90, 46.93) | -3.02 (-3.18, -2.85) | <0.001 | 1.45 (1.22, 1.73) | 0.32 (0.24, 0.41) | -5.06 (-5.22, -4.90) | <0.001 |

| | | | | | | | | | | | | | | | | |
|---|---|---|---|---|---|---|---|---|---|---|---|---|---|---|---|---|
| American Samoa | 36.92 (15.27, 77.18) | 38.51 (16.46, 79.00) | 0.14 (0.13, 0.16) | <0.001 | 865.33 (740.66, 1006.47) | 889.17 (763.64, 1031.04) | 0.09 (0.09, 0.10) | <0.001 | 295.33 (223.79, 383.97) | 240.83 (176.65, 322.24) | -0.76 (-0.87, -0.64) | <0.001 | 4.23 (0.05, 30.62) | 3.31 (0.01, 29.19) | -0.92 (-1.07, -0.77) | <0.001 |
| Andorra | 0.39 (0.00, 21.07) | 0.35 (0.00, 19.83) | -0.32 (-0.46, -0.18) | <0.001 | 7.09 (0.69, 31.58) | 6.75 (0.62, 30.09) | -0.17 (-0.19, -0.14) | <0.001 | 6.68 (0.63, 30.76) | 3.65 (0.09, 25.07) | -2.05 (-2.20, -1.90) | <0.001 | 0.11 (0.00, 20.61) | 0.06 (0.00, 19.36) | -2.01 (-2.44, -1.58) | <0.001 |
| Angola | 98.37 (95.34, 101.48) | 97.41 (95.63, 99.23) | -0.03 (-0.05, -0.01) | 0.001 | 1791.02 (1777.67, 1804.45) | 1830.05 (1822.02, 1838.11) | 0.08 (0.06, 0.09) | <0.001 | 204.92 (200.41, 209.51) | 127.66 (125.55, 129.81) | -1.58 (-1.89, -1.26) | <0.001 | 1.97 (1.55, 2.49) | 0.67 (0.52, 0.85) | -3.57 (-4.36, -2.78) | <0.001 |
| Antigua and Barbuda | 62.28 (35.77, 101.80) | 60.71 (37.36, 93.60) | -0.09 (-0.10, -0.07) | <0.001 | 1257.02 (1123.39, 1402.82) | 1246.98 (1132.19, 1370.61) | -0.03 (-0.03, -0.02) | <0.001 | 140.97 (98.63, 196.09) | 87.31 (59.03, 124.86) | -1.63 (-2.09, -1.17) | <0.001 | 1.36 (0.00, 19.09) | 0.46 (0.00, 12.68) | -3.65 (-4.53, -2.77) | <0.001 |
| Argentina | 50.87 (49.62, 52.15) | 51.04 (49.98, 52.12) | 0.01 (0.00, 0.02) | 0.055 | 973.79 (968.25, 979.35) | 997.21 (992.53, 1001.91) | 0.08 (0.07, 0.10) | <0.001 | 88.47 (86.81, 90.16) | 58.38 (57.25, 59.52) | -1.44 (-1.56, -1.32) | <0.001 | 0.72 (0.57, 0.89) | 0.18 (0.13, 0.26) | -4.58 (-5.20, -3.96) | <0.001 |
| Armenia | 60.03 (56.05, 64.23) | 56.01 (51.42, 60.94) | -0.24 (-0.26, -0.22) | <0.001 | 1210.78 (1192.82, 1228.95) | 1152.55 (1132.37, 1173.05) | -0.17 (-0.20, -0.14) | <0.001 | 182.71 (175.77, 189.85) | 89.08 (83.59, 94.88) | -2.53 (-2.86, -2.21) | <0.001 | 2.16 (1.46, 3.07) | 0.58 (0.23, 1.30) | -4.45 (-5.82, -3.05) | <0.001 |
| Australia | 1.05 (0.82, 1.32) | 0.94 (0.75, 1.18) | -0.33 (-0.50, -0.17) | <0.001 | 23.40 (22.26, 24.58) | 23.05 (22.03, 24.11) | -0.04 (-0.11, 0.04) | 0.352 | 19.50 (18.47, 20.58) | 9.58 (8.94, 10.26) | -2.40 (-2.97, -1.84) | <0.001 | 0.31 (0.19, 0.47) | 0.14 (0.07, 0.25) | -2.61 (-3.39, -1.81) | <0.001 |
| Austria | 1.11 (0.77, 1.57) | 0.45 (0.24, 0.78) | -3.04 (-3.32, -2.76) | <0.001 | 15.39 (14.01, 16.88) | 7.31 (6.35, 8.40) | -2.59 (-2.74, -2.44) | <0.001 | 9.11 (8.06, 10.27) | 3.84 (3.16, 4.64) | -3.01 (-3.59, -2.43) | <0.001 | 0.14 (0.04, 0.37) | 0.06 (0.01, 0.27) | -3.14 (-3.53, -2.74) | <0.001 |
| Azerbaijan | 58.44 (55.82, 61.16) | 56.49 (54.15, 58.91) | -0.12 (-0.14, -0.10) | <0.001 | 1088.74 (1077.18, 1100.39) | 1067.26 (1057.39, 1077.21) | -0.07 (-0.08, -0.06) | <0.001 | 192.44 (187.57, 197.42) | 101.99 (98.95, 105.12) | -2.34 (-2.91, -1.77) | <0.001 | 2.36 (1.85, 2.99) | 0.84 (0.59, 1.18) | -3.71 (-4.54, -2.86) | <0.001 |
| Bahamas | 65.98 (52.25, | 66.07 (53.61, | 0.01 (-0.04, | 0.799 | 1239.51 (1176.37 | 1242.09 (1186.29 | 0.01 (-0.00, | 0.123 | 133.18 (112.87, | 99.80 (84.48, | -0.96 (-1.25, | <0.001 | 1.26 (0.08, | 0.69 (0.02, | -2.01 (-2.63, | <0.001 |

| | | | | | | | | | | | | | | | | |
|---|---|---|---|---|---|---|---|---|---|---|---|---|---|---|---|---|
| | 82.49) | 80.60) | 0.05) | | , 1305.37) | , 1299.88) | 0.02) | | 156.31) | 117.16) | -0.68) | | 6.05) | 3.89) | -1.38) | |
| Bahrain | 0.94 (0.14, 3.59) | 0.82 (0.26, 2.14) | -0.48 (-0.61, -0.35) | <0.001 | 16.96 (12.23, 23.18) | 14.78 (11.74, 18.47) | -0.47 (-0.58, -0.35) | <0.001 | 30.87 (24.04, 39.23) | 12.17 (9.29, 15.75) | -3.20 (-3.78, -2.62) | <0.001 | 0.47 (0.02, 2.94) | 0.18 (0.00, 1.26) | -3.27 (-3.95, -2.59) | <0.001 |
| Bangladesh | 69.28 (68.51, 70.07) | 64.58 (63.98, 65.19) | -0.25 (-0.26, -0.23) | <0.001 | 845.63 (842.77, 848.50) | 864.67 (862.45, 866.90) | 0.07 (0.06, 0.09) | <0.001 | 225.70 (224.25, 227.15) | 118.15 (117.33, 118.97) | -2.17 (-2.70, -1.64) | <0.001 | 3.00 (2.83, 3.17) | 1.25 (1.16, 1.34) | -2.84 (-3.55, -2.12) | <0.001 |
| Barbados | 63.75 (49.61, 80.73) | 63.44 (48.51, 81.58) | -0.02 (-0.04, 0.01) | 0.150 | 1248.99 (1183.61 , 1317.10) | 1259.76 (1190.71 , 1331.86) | 0.03 (0.02, 0.04) | <0.001 | 131.33 (110.71, 154.74) | 91.87 (73.98, 112.89) | -1.23 (-1.51, -0.94) | <0.001 | 1.20 (0.06, 5.73) | 0.53 (0.00, 5.04) | -2.79 (-3.49, -2.09) | <0.001 |
| Belarus | 6.15 (5.42, 6.95) | 2.93 (2.40, 3.58) | -2.54 (-2.66, -2.43) | <0.001 | 37.54 (35.69, 39.46) | 25.11 (23.41, 26.93) | -1.37 (-1.46, -1.28) | <0.001 | 110.73 (107.55, 113.99) | 20.08 (18.60, 21.66) | -5.71 (-6.79, -4.62) | <0.001 | 1.92 (1.52, 2.39) | 0.33 (0.17, 0.62) | -5.86 (-7.06, -4.65) | <0.001 |
| Belgium | 0.44 (0.26, 0.72) | 0.43 (0.25, 0.71) | -0.16 (-0.50, 0.18) | 0.366 | 8.01 (7.12, 8.97) | 8.16 (7.25, 9.17) | 0.02 (-0.07, 0.11) | 0.663 | 3.84 (3.24, 4.52) | 2.61 (2.11, 3.20) | -1.33 (-1.69, -0.98) | <0.001 | 0.06 (0.01, 0.21) | 0.04 (0.00, 0.19) | -1.23 (-1.74, -0.72) | <0.001 |
| Belize | 71.03 (53.26, 93.72) | 69.18 (57.67, 82.44) | -0.08 (-0.11, -0.06) | <0.001 | 1230.60 (1149.13 , 1316.90) | 1241.17 (1189.69 , 1294.39) | 0.03 (0.02, 0.04) | <0.001 | 123.11 (98.26, 153.02) | 104.22 (89.64, 120.58) | -0.57 (-0.83, -0.32) | <0.001 | 1.06 (0.01, 9.12) | 0.75 (0.04, 3.70) | -1.27 (-1.77, -0.78) | <0.001 |
| Benin | 73.86 (69.87, 78.03) | 75.62 (73.23, 78.09) | 0.08 (0.07, 0.09) | <0.001 | 1183.46 (1166.91 , 1200.20) | 1232.91 (1222.86 , 1243.03) | 0.14 (0.13, 0.15) | <0.001 | 140.65 (135.01, 146.49) | 95.64 (92.86, 98.48) | -1.32 (-1.38, -1.26) | <0.001 | 1.37 (0.86, 2.09) | 0.60 (0.40, 0.88) | -2.80 (-2.92, -2.67) | <0.001 |
| Bermuda | 1.46 (0.00, 22.57) | 0.82 (0.00, 27.60) | -1.98 (-2.14, -1.82) | <0.001 | 25.88 (10.14, 57.35) | 17.51 (3.83, 53.46) | -1.36 (-1.43, -1.29) | <0.001 | 49.53 (26.05, 88.09) | 12.76 (1.87, 46.65) | -4.91 (-5.28, -4.54) | <0.001 | 0.80 (0.00, 21.52) | 0.19 (0.00, 26.54) | -5.16 (-5.64, -4.67) | <0.001 |
| Bhutan | 67.65 (58.09, 78.55) | 64.04 (55.94, 73.01) | -0.18 (-0.22, -0.14) | <0.001 | 892.11 (854.38, 931.24) | 881.77 (851.03, 913.36) | -0.04 (-0.06, -0.02) | <0.001 | 509.42 (480.59, 539.67) | 214.44 (199.38, 230.37) | -2.95 (-3.17, -2.72) | <0.001 | 7.90 (4.65, 12.74) | 2.89 (1.39, 5.34) | -3.40 (-3.63, -3.17) | <0.001 |
| Bolivia | 73.21 | 72.59 | -0.03 | 0.067 | 1196.59 | 1214.59 | 0.05 | <0.001 | 170.42 | 85.74 | -2.34 | <0.001 | 1.92 | 0.47 | -4.75 | <0.001 |

| | | | | | | | | | | | | | | | | |
|---|---|---|---|---|---|---|---|---|---|---|---|---|---|---|---|---|
| (Plurinational State of) | (69.91, 76.63) | (70.22, 75.02) | (-0.06, 0.00) | | (1182.76, 1210.56) | (1204.77, 1224.48) | (0.04, 0.06) | | (165.18, 175.80) | (83.14, 88.41) | (-2.43, -2.26) | | (1.39, 2.58) | (0.30, 0.71) | (-4.85, -4.65) | |
| Bosnia and Herzegovina | 1.03 (0.63, 1.59) | 1.20 (0.65, 2.07) | 0.61 (0.43, 0.80) | <0.001 | 8.13 (6.92, 9.51) | 9.70 (7.94, 11.77) | 0.64 (0.34, 0.94) | <0.001 | 15.49 (13.79, 17.34) | 4.35 (3.19, 5.83) | -4.33 (-4.86, -3.79) | <0.001 | 0.26 (0.08, 0.61) | 0.06 (0.00, 0.54) | -4.67 (-5.67, -3.66) | <0.001 |
| Botswana | 96.77 (88.55, 105.63) | 93.40 (87.59, 99.50) | -0.12 (-0.13, -0.11) | <0.001 | 1609.13 (1573.76, 1645.17) | 1602.68 (1578.57, 1627.08) | -0.01 (-0.02, -0.00) | 0.026 | 215.60 (202.63, 229.26) | 125.50 (118.82, 132.47) | -1.86 (-2.08, -1.65) | <0.001 | 2.32 (1.14, 4.29) | 0.82 (0.37, 1.58) | -3.53 (-3.95, -3.11) | <0.001 |
| Brazil | 74.56 (73.89, 75.23) | 74.41 (73.82, 75.00) | -0.00 (-0.01, 0.01) | 0.620 | 1358.25 (1355.35, 1361.16) | 1377.48 (1374.99, 1379.96) | 0.05 (0.04, 0.05) | <0.001 | 152.86 (151.89, 153.84) | 90.44 (89.80, 91.07) | -1.79 (-1.93, -1.66) | <0.001 | 1.47 (1.37, 1.57) | 0.42 (0.38, 0.47) | -4.23 (-4.50, -3.96) | <0.001 |
| Brunei Darussalam | 1.43 (0.14, 5.79) | 0.82 (0.08, 3.56) | -1.96 (-2.17, -1.74) | <0.001 | 21.24 (13.87, 31.24) | 14.22 (9.52, 20.58) | -1.41 (-1.57, -1.24) | <0.001 | 50.21 (38.49, 64.49) | 19.83 (14.24, 27.04) | -3.16 (-3.56, -2.76) | <0.001 | 0.83 (0.02, 4.79) | 0.33 (0.00, 2.78) | -3.15 (-3.54, -2.76) | <0.001 |
| Bulgaria | 2.94 (2.37, 3.61) | 1.37 (0.93, 1.98) | -2.61 (-2.69, -2.54) | <0.001 | 17.98 (16.52, 19.55) | 10.95 (9.58, 12.48) | -1.72 (-1.86, -1.59) | <0.001 | 111.46 (107.76, 115.25) | 28.41 (26.18, 30.80) | -4.55 (-4.94, -4.15) | <0.001 | 1.92 (1.47, 2.48) | 0.48 (0.24, 0.92) | -4.60 (-5.00, -4.19) | <0.001 |
| Burkina Faso | 77.36 (74.35, 80.47) | 77.32 (75.48, 79.20) | -0.01 (-0.02, 0.01) | 0.419 | 1178.38 (1166.16, 1190.71) | 1195.46 (1188.00, 1202.96) | 0.05 (0.05, 0.05) | <0.001 | 108.56 (104.90, 112.32) | 91.71 (89.67, 93.80) | -0.56 (-0.65, -0.48) | <0.001 | 0.85 (0.56, 1.26) | 0.56 (0.41, 0.75) | -1.38 (-1.63, -1.13) | <0.001 |
| Burundi | 113.89 (109.41, 118.52) | 119.65 (116.58, 122.79) | 0.17 (0.15, 0.19) | <0.001 | 1831.74 (1813.17, 1850.48) | 1919.27 (1906.51, 1932.10) | 0.16 (0.14, 0.18) | <0.001 | 249.85 (243.10, 256.77) | 154.53 (150.96, 158.18) | -1.65 (-1.73, -1.57) | <0.001 | 2.59 (1.94, 3.41) | 1.01 (0.74, 1.35) | -3.21 (-3.34, -3.08) | <0.001 |
| Cabo Verde | 67.25 (53.94, 83.61) | 65.19 (55.48, 76.15) | -0.11 (-0.12, -0.10) | <0.001 | 1296.24 (1232.25, 1363.21) | 1301.50 (1257.03, 1347.17) | 0.01 (0.01, 0.02) | <0.001 | 200.41 (175.66, 228.24) | 75.74 (65.30, 87.42) | -3.33 (-3.48, -3.19) | <0.001 | 2.28 (0.40, 8.32) | 0.22 (0.00, 2.02) | -7.86 (-8.48, -7.24) | <0.001 |
| Cambodia | 45.03 | 42.89 | -0.17 | <0.001 | 639.25 | 630.02 | -0.08 | 0.014 | 347.96 | 131.40 | -3.28 | <0.001 | 5.32 | 1.69 | -3.85 | <0.001 |

| | | | | | | | | | | | | | | | | |
|---|---|---|---|---|---|---|---|---|---|---|---|---|---|---|---|---|
| | (42.94, 47.21) | (41.38, 44.45) | (-0.19, -0.15) | | (631.14, 647.46) | (624.19, 635.88) | (-0.14, -0.02) | | (341.90, 354.11) | (128.74, 134.10) | (-3.42, -3.13) | | (4.59, 6.15) | (1.40, 2.02) | (-4.00, -3.70) | |
| Cameroon | 72.32 (69.66, 75.06) | 77.43 (75.89, 79.01) | 0.23 (0.21, 0.26) | <0.001 | 1143.72 (1132.74 , 1154.78) | 1231.00 (1224.66 , 1237.36) | 0.26 (0.24, 0.27) | <0.001 | 122.70 (119.13, 126.36) | 88.20 (86.52, 89.92) | -1.14 (-1.19, -1.10) | <0.001 | 1.12 (0.80, 1.54) | 0.48 (0.36, 0.63) | -2.87 (-3.06, -2.68) | <0.001 |
| Canada | 1.72 (1.49, 1.98) | 1.50 (1.29, 1.74) | -0.46 (-0.53, -0.39) | <0.001 | 28.52 (27.55, 29.52) | 27.13 (26.20, 28.09) | -0.17 (-0.21, -0.14) | <0.001 | 9.03 (8.49, 9.60) | 5.44 (5.04, 5.88) | -1.70 (-1.94, -1.45) | <0.001 | 0.12 (0.07, 0.21) | 0.07 (0.03, 0.14) | -1.91 (-2.86, -0.96) | <0.001 |
| Central African Republic | 112.67 (106.42, 119.24) | 117.45 (112.94, 122.12) | 0.14 (0.13, 0.16) | <0.001 | 1866.14 (1839.71 , 1892.90) | 1932.35 (1913.34 , 1951.52) | 0.12 (0.10, 0.14) | <0.001 | 247.74 (238.10, 257.70) | 191.47 (185.50, 197.60) | -0.88 (-1.00, -0.75) | <0.001 | 2.62 (1.70, 3.90) | 1.65 (1.13, 2.33) | -1.55 (-1.76, -1.35) | <0.001 |
| Chad | 76.88 (73.19, 80.71) | 76.55 (74.32, 78.84) | -0.01 (-0.03, 0.00) | 0.122 | 1174.85 (1159.88 , 1189.98) | 1194.42 (1185.18 , 1203.71) | 0.06 (0.05, 0.06) | <0.001 | 149.06 (143.79, 154.48) | 107.73 (104.99, 110.53) | -1.08 (-1.20, -0.96) | <0.001 | 1.51 (1.02, 2.17) | 0.82 (0.60, 1.12) | -2.04 (-2.24, -1.83) | <0.001 |
| Chile | 2.89 (2.45, 3.38) | 1.65 (1.36, 1.99) | -1.93 (-2.08, -1.77) | <0.001 | 31.33 (29.87, 32.84) | 24.38 (23.23, 25.58) | -0.88 (-1.00, -0.77) | <0.001 | 70.63 (68.43, 72.89) | 11.96 (11.15, 12.80) | -5.89 (-6.18, -5.59) | <0.001 | 1.19 (0.92, 1.52) | 0.18 (0.09, 0.32) | -6.26 (-6.59, -5.93) | <0.001 |
| China | 34.20 (34.05, 34.36) | 28.71 (28.55, 28.87) | -0.62 (-0.66, -0.57) | <0.001 | 625.60 (624.93, 626.26) | 513.66 (513.01, 514.30) | -0.68 (-0.71, -0.65) | <0.001 | 170.31 (169.96, 170.67) | 42.06 (41.87, 42.24) | -4.72 (-5.12, -4.33) | <0.001 | 2.42 (2.38, 2.46) | 0.29 (0.28, 0.31) | -7.13 (-7.59, -6.67) | <0.001 |
| Colombia | 1.56 (1.35, 1.79) | 1.31 (1.15, 1.48) | -0.59 (-0.87, -0.32) | <0.001 | 22.66 (21.86, 23.48) | 20.74 (20.10, 21.39) | -0.31 (-0.37, -0.24) | <0.001 | 46.90 (45.75, 48.08) | 7.16 (6.79, 7.55) | -6.22 (-7.03, -5.42) | <0.001 | 0.77 (0.63, 0.94) | 0.10 (0.06, 0.16) | -6.62 (-7.54, -5.69) | <0.001 |
| Comoros | 101.32 (87.10, 117.54) | 105.81 (94.57, 118.08) | 0.15 (0.14, 0.16) | <0.001 | 1835.41 (1769.87 , 1902.99) | 1902.86 (1853.30 , 1953.47) | 0.13 (0.10, 0.16) | <0.001 | 175.18 (155.53, 196.92) | 124.72 (112.30, 138.19) | -1.18 (-2.25, -0.10) | 0.032 | 1.42 (0.21, 5.35) | 0.55 (0.05, 2.37) | -3.25 (-6.64, 0.27) | 0.070 |
| Congo | 101.73 (95.50, 108.32) | 103.22 (98.96, 107.62) | 0.06 (0.04, 0.08) | <0.001 | 1864.54 (1836.52 , | 1892.72 (1874.15 , | 0.03 (-0.06, 0.12) | 0.484 | 187.67 (178.78, 196.93) | 121.71 (117.04, 126.53) | -1.42 (-1.66, -1.18) | <0.001 | 1.65 (0.90, 2.82) | 0.53 (0.27, 0.95) | -3.92 (-4.73, -3.11) | <0.001 |

| | | | | | | | | | | | | | | | | |
|---|---|---|---|---|---|---|---|---|---|---|---|---|---|---|---|---|
| | | | | | 1892.91) | 1911.42) | | | | | | | | | | |
| Cook Islands | 5.01 (0.00, 69.63) | 4.54 (0.00, 75.50) | -0.32 (-0.48, -0.16) | <0.001 | 75.06 (26.27, 173.78) | 79.30 (24.33, 192.71) | 0.21 (0.11, 0.30) | <0.001 | 168.82 (88.23, 297.11) | 82.25 (25.90, 196.96) | -2.48 (-2.75, -2.21) | <0.001 | 2.73 (0.00, 65.50) | 1.30 (0.00, 69.17) | -2.56 (-2.86, -2.25) | <0.001 |
| Costa Rica | 66.13 (61.80, 70.71) | 63.53 (59.96, 67.27) | -0.14 (-0.15, -0.12) | <0.001 | 1334.70 (1314.65 , 1355.00) | 1317.37 (1301.18 , 1333.73) | -0.05 (-0.07, -0.03) | <0.001 | 124.06 (117.97, 130.41) | 89.45 (85.28, 93.78) | -1.05 (-1.36, -0.73) | <0.001 | 1.03 (0.54, 1.79) | 0.45 (0.20, 0.88) | -2.64 (-3.28, -2.00) | <0.001 |
| Cote d'Ivoire | 77.27 (74.80, 79.80) | 79.04 (77.38, 80.72) | 0.08 (0.06, 0.09) | <0.001 | 1215.98 (1205.88 , 1226.16) | 1245.76 (1239.07 , 1252.48) | 0.09 (0.08, 0.10) | <0.001 | 130.94 (127.67, 134.29) | 91.10 (89.30, 92.93) | -1.22 (-1.31, -1.13) | <0.001 | 1.18 (0.89, 1.55) | 0.51 (0.39, 0.67) | -2.80 (-3.02, -2.58) | <0.001 |
| Croatia | 0.90 (0.53, 1.45) | 0.72 (0.35, 1.37) | -0.77 (-1.32, -0.21) | 0.007 | 6.89 (5.76, 8.19) | 5.96 (4.74, 7.44) | -0.54 (-0.63, -0.44) | <0.001 | 17.92 (16.06, 19.95) | 3.84 (2.87, 5.07) | -5.25 (-6.13, -4.37) | <0.001 | 0.30 (0.11, 0.69) | 0.06 (0.00, 0.48) | -5.48 (-7.17, -3.76) | <0.001 |
| Cuba | 63.02 (60.83, 65.28) | 63.05 (60.44, 65.74) | 0.00 (-0.02, 0.02) | 0.871 | 1253.73 (1243.65 , 1263.88) | 1262.87 (1251.32 , 1274.51) | 0.03 (0.02, 0.03) | <0.001 | 174.04 (170.25, 177.90) | 92.55 (89.45, 95.73) | -2.15 (-2.47, -1.83) | <0.001 | 1.94 (1.55, 2.39) | 0.55 (0.34, 0.85) | -4.26 (-4.86, -3.65) | <0.001 |
| Cyprus | 0.40 (0.02, 2.02) | 0.32 (0.03, 1.68) | -0.79 (-1.07, -0.51) | <0.001 | 5.58 (3.26, 8.97) | 5.20 (3.37, 7.88) | -0.25 (-0.30, -0.20) | <0.001 | 6.25 (3.79, 9.78) | 2.41 (1.25, 4.46) | -3.27 (-3.80, -2.73) | <0.001 | 0.10 (0.00, 1.52) | 0.04 (0.00, 1.29) | -3.42 (-4.25, -2.58) | <0.001 |
| Czechia | 1.23 (0.91, 1.64) | 1.04 (0.73, 1.46) | -0.63 (-0.85, -0.41) | <0.001 | 8.92 (8.00, 9.91) | 8.39 (7.43, 9.47) | -0.25 (-0.37, -0.14) | <0.001 | 27.46 (25.83, 29.18) | 5.71 (4.93, 6.59) | -5.20 (-5.54, -4.87) | <0.001 | 0.47 (0.28, 0.75) | 0.09 (0.02, 0.30) | -5.36 (-5.91, -4.81) | <0.001 |
| Democratic People's Republic of Korea | 36.70 (35.41, 38.04) | 38.16 (36.94, 39.40) | 0.13 (0.11, 0.15) | <0.001 | 591.25 (585.97, 596.56) | 595.38 (590.61, 600.18) | 0.03 (0.02, 0.03) | <0.001 | 146.12 (143.47, 148.81) | 98.67 (96.75, 100.63) | -1.33 (-1.39, -1.26) | <0.001 | 2.00 (1.70, 2.35) | 1.19 (0.99, 1.42) | -1.76 (-1.84, -1.68) | <0.001 |
| Democratic Republic of the Congo | 103.92 (102.28, 105.58) | 107.36 (106.30, 108.43) | 0.11 (0.09, 0.14) | <0.001 | 1859.53 (1852.34 , 1866.74) | 1885.65 (1881.04 , 1890.28) | 0.05 (0.03, 0.06) | <0.001 | 187.18 (184.89, 189.49) | 136.44 (135.20, 137.69) | -1.08 (-1.14, -1.02) | <0.001 | 1.64 (1.43, 1.88) | 0.78 (0.68, 0.88) | -2.50 (-2.68, -2.31) | <0.001 |
| Denmark | 0.33 | 0.26 | -0.74 | <0.001 | 4.98 | 4.59 | -0.29 | <0.001 | 6.40 | 1.46 | -4.97 | <0.001 | 0.11 | 0.02 | -5.73 | <0.001 |

| | | | | | | | | | | | | | | | | |
|---|---|---|---|---|---|---|---|---|---|---|---|---|---|---|---|---|
| | (0.12, 0.71) | (0.08, 0.64) | (-1.13, -0.36) | | (4.03, 6.09) | (3.66, 5.70) | (-0.33, -0.26) | | (5.33, 7.64) | (0.96, 2.14) | (-6.00, -3.94) | | (0.01, 0.40) | (0.00, 0.27) | (-8.45, -2.92) | |
| Djibouti | 97.41 (84.42, 112.09) | 101.85 (93.15, 111.15) | 0.15 (0.14, 0.17) | <0.001 | 1769.17 (1709.83, 1830.23) | 1878.55 (1840.90, 1916.79) | 0.21 (0.20, 0.21) | <0.001 | 152.63 (135.70, 171.31) | 116.33 (107.09, 126.16) | -0.87 (-1.11, -0.63) | <0.001 | 1.10 (0.14, 4.33) | 0.43 (0.06, 1.52) | -3.10 (-3.59, -2.60) | <0.001 |
| Dominica | 71.75 (44.70, 110.88) | 70.42 (41.87, 111.17) | -0.06 (-0.09, -0.03) | <0.001 | 1267.76 (1139.29, 1407.77) | 1272.17 (1138.14, 1417.73) | 0.01 (-0.01, 0.03) | 0.548 | 188.02 (140.23, 247.95) | 123.46 (84.34, 174.61) | -1.46 (-1.73, -1.19) | <0.001 | 2.14 (0.00, 20.31) | 1.05 (0.00, 16.75) | -2.40 (-2.85, -1.94) | <0.001 |
| Dominican Republic | 68.81 (65.95, 71.78) | 65.34 (63.01, 67.75) | -0.18 (-0.19, -0.17) | <0.001 | 1215.65 (1203.08, 1228.33) | 1205.15 (1195.02, 1215.35) | -0.03 (-0.04, -0.02) | <0.001 | 154.14 (149.70, 158.70) | 106.58 (103.58, 109.64) | -1.25 (-1.46, -1.04) | <0.001 | 1.57 (1.15, 2.11) | 0.81 (0.57, 1.12) | -2.24 (-2.61, -1.87) | <0.001 |
| Ecuador | 66.64 (64.21, 69.16) | 65.67 (63.83, 67.56) | -0.05 (-0.06, -0.04) | <0.001 | 1177.78 (1167.15, 1188.49) | 1178.76 (1170.83, 1186.74) | 0.01 (-0.01, 0.02) | 0.568 | 125.12 (121.65, 128.66) | 76.09 (74.08, 78.14) | -1.74 (-2.00, -1.49) | <0.001 | 1.15 (0.83, 1.55) | 0.33 (0.21, 0.49) | -4.29 (-4.79, -3.79) | <0.001 |
| Egypt | 47.17 (46.28, 48.09) | 48.80 (48.13, 49.48) | 0.12 (0.06, 0.18) | <0.001 | 810.21 (806.38, 814.05) | 812.67 (809.91, 815.44) | 0.01 (-0.02, 0.04) | 0.433 | 182.33 (180.53, 184.14) | 73.81 (72.98, 74.65) | -3.09 (-3.37, -2.82) | <0.001 | 2.33 (2.13, 2.54) | 0.57 (0.50, 0.65) | -4.76 (-5.15, -4.37) | <0.001 |
| El Salvador | 74.49 (70.88, 78.26) | 72.69 (69.47, 76.04) | -0.08 (-0.09, -0.08) | <0.001 | 1250.47 (1234.88, 1266.23) | 1261.51 (1247.72, 1275.43) | 0.04 (0.02, 0.05) | <0.001 | 90.84 (86.64, 95.20) | 69.74 (66.52, 73.09) | -0.92 (-1.04, -0.79) | <0.001 | 0.53 (0.25, 1.01) | 0.16 (0.04, 0.43) | -3.95 (-4.52, -3.39) | <0.001 |
| Equatorial Guinea | 105.97 (90.36, 123.75) | 86.91 (80.00, 94.35) | -0.69 (-0.75, -0.64) | <0.001 | 1890.12 (1820.06, 1962.36) | 1748.58 (1715.84, 1781.86) | -0.28 (-0.30, -0.25) | <0.001 | 216.54 (193.28, 242.02) | 97.56 (89.94, 105.73) | -2.74 (-2.86, -2.62) | <0.001 | 2.10 (0.43, 6.48) | 0.24 (0.01, 1.23) | -7.24 (-7.62, -6.85) | <0.001 |
| Eritrea | 130.43 (124.06, 137.07) | 129.69 (125.64, 133.85) | -0.01 (-0.05, 0.02) | 0.450 | 2187.11 (2159.72, 2214.79) | 2199.84 (2182.52, 2217.26) | 0.00 (-0.06, 0.06) | 0.921 | 236.74 (227.86, 245.91) | 162.54 (157.88, 167.31) | -1.28 (-1.51, -1.05) | <0.001 | 2.15 (1.37, 3.24) | 0.93 (0.61, 1.38) | -2.87 (-3.09, -2.65) | <0.001 |

| | | | | | | | | | | | | | | | | |
|---|---|---|---|---|---|---|---|---|---|---|---|---|---|---|---|---|
| Estonia | 4.61 (3.07, 6.70) | 3.06 (1.68, 5.37) | -1.39 (-1.46, -1.32) | <0.001 | 32.12 (27.73, 37.04) | 26.61 (21.94, 32.13) | -0.63 (-0.71, -0.55) | <0.001 | 53.71 (47.98, 59.97) | 8.87 (6.33, 12.27) | -6.00 (-6.73, -5.26) | <0.001 | 0.91 (0.31, 2.12) | 0.13 (0.00, 1.51) | -7.45 (-8.03, -6.86) | <0.001 |
| Eswatini | 98.25 (87.47, 110.18) | 97.28 (88.95, 106.22) | -0.04 (-0.05, -0.02) | <0.001 | 1585.68 (1539.50 , 1633.04) | 1592.00 (1557.23 , 1627.39) | 0.01 (0.01, 0.02) | <0.001 | 202.60 (186.15, 220.25) | 146.74 (136.30, 157.82) | -1.10 (-1.32, -0.88) | <0.001 | 2.11 (0.74, 4.95) | 1.17 (0.42, 2.66) | -2.00 (-2.39, -1.61) | <0.001 |
| Ethiopia | 100.92 (99.50, 102.35) | 104.49 (103.56, 105.43) | 0.12 (0.09, 0.15) | <0.001 | 1555.95 (1550.16 , 1561.76) | 1749.08 (1745.10 , 1753.06) | 0.40 (0.33, 0.47) | <0.001 | 230.60 (228.40, 232.81) | 120.39 (119.35, 121.43) | -2.21 (-2.31, -2.10) | <0.001 | 2.52 (2.30, 2.77) | 0.59 (0.52, 0.67) | -4.85 (-5.02, -4.69) | <0.001 |
| Fiji | 27.03 (21.62, 33.47) | 31.95 (26.36, 38.38) | 0.58 (0.54, 0.62) | <0.001 | 406.59 (384.65, 429.51) | 626.97 (601.31, 653.45) | 1.46 (1.40, 1.52) | <0.001 | 552.59 (527.06, 579.10) | 340.63 (321.79, 360.30) | -1.64 (-1.72, -1.56) | <0.001 | 8.75 (5.79, 12.76) | 5.09 (3.03, 8.03) | -1.80 (-1.91, -1.70) | <0.001 |
| Finland | 0.32 (0.12, 0.73) | 0.25 (0.07, 0.65) | -0.92 (-1.49, -0.34) | 0.002 | 4.96 (3.99, 6.12) | 4.52 (3.56, 5.67) | -0.36 (-0.46, -0.27) | <0.001 | 5.12 (4.16, 6.27) | 1.30 (0.82, 1.99) | -4.64 (-5.04, -4.25) | <0.001 | 0.08 (0.01, 0.40) | 0.02 (0.00, 0.30) | -5.34 (-6.05, -4.62) | <0.001 |
| France | 0.45 (0.36, 0.54) | 0.37 (0.29, 0.47) | -0.70 (-1.14, -0.26) | 0.002 | 7.05 (6.71, 7.41) | 6.51 (6.16, 6.87) | -0.27 (-0.33, -0.21) | <0.001 | 11.51 (11.07, 11.97) | 5.14 (4.83, 5.46) | -2.71 (-3.08, -2.34) | <0.001 | 0.19 (0.13, 0.25) | 0.08 (0.05, 0.13) | -2.98 (-3.97, -1.97) | <0.001 |
| Gabon | 86.40 (77.45, 96.19) | 88.38 (81.82, 95.34) | 0.08 (0.07, 0.09) | <0.001 | 1758.42 (1716.10 , 1801.61) | 1806.28 (1775.77 , 1837.20) | 0.10 (0.09, 0.11) | <0.001 | 144.19 (132.23, 157.02) | 104.25 (97.02, 111.91) | -1.11 (-1.20, -1.01) | <0.001 | 1.00 (0.25, 2.81) | 0.30 (0.04, 1.09) | -4.14 (-4.33, -3.95) | <0.001 |
| Gambia | 75.44 (67.00, 84.80) | 79.38 (73.85, 85.26) | 0.17 (0.16, 0.19) | <0.001 | 1243.72 (1207.58 , 1280.79) | 1312.72 (1289.21 , 1336.58) | 0.19 (0.17, 0.21) | <0.001 | 120.93 (109.91, 132.89) | 93.01 (86.86, 99.53) | -0.85 (-1.35, -0.35) | 0.001 | 1.00 (0.25, 2.93) | 0.49 (0.15, 1.28) | -2.31 (-3.51, -1.09) | <0.001 |
| Georgia | 57.03 (53.84, 60.37) | 58.41 (53.95, 63.16) | 0.08 (0.07, 0.10) | <0.001 | 1101.89 (1087.83 , 1116.09) | 1123.77 (1104.58 , 1143.24) | 0.07 (0.06, 0.08) | <0.001 | 177.17 (171.55, 182.94) | 132.73 (126.14, 139.59) | -1.04 (-2.28, 0.22) | 0.104 | 2.02 (1.47, 2.73) | 1.29 (0.72, 2.16) | -1.52 (-2.87, -0.14) | 0.031 |
| Germany | 0.50 | 0.39 | -0.91 | <0.001 | 7.62 | 6.80 | -0.44 | <0.001 | 10.96 | 4.44 | -3.14 | <0.001 | 0.18 | 0.07 | -3.27 | <0.001 |

| | | | | | | | | | | | | | | | | |
|---|---|---|---|---|---|---|---|---|---|---|---|---|---|---|---|---|
| | (0.43, 0.59) | (0.32, 0.47) | (-1.19, -0.64) | | (7.31, 7.95) | (6.49, 7.13) | (-0.67, -0.20) | | (10.59, 11.34) | (4.19, 4.70) | (-3.39, -2.89) | | (0.14, 0.24) | (0.04, 0.11) | (-3.78, -2.75) | |
| Ghana | 76.30 (74.08, 78.58) | 74.08 (72.65, 75.52) | -0.10 (-0.13, -0.07) | <0.001 | 1224.90 (1215.71, 1234.14) | 1229.26 (1223.34, 1235.21) | 0.01 (0.01, 0.02) | <0.001 | 119.39 (116.54, 122.29) | 83.65 (82.11, 85.21) | -1.23 (-1.37, -1.08) | <0.001 | 0.99 (0.75, 1.30) | 0.41 (0.31, 0.53) | -2.99 (-3.21, -2.77) | <0.001 |
| Greece | 0.40 (0.23, 0.66) | 0.34 (0.17, 0.64) | -0.49 (-0.88, -0.10) | 0.014 | 6.52 (5.73, 7.39) | 6.13 (5.26, 7.12) | -0.17 (-0.23, -0.10) | <0.001 | 6.53 (5.75, 7.40) | 2.47 (1.94, 3.11) | -3.17 (-3.99, -2.34) | <0.001 | 0.11 (0.03, 0.27) | 0.04 (0.00, 0.22) | -3.18 (-4.03, -2.31) | <0.001 |
| Greenland | 2.31 (0.01, 25.59) | 1.67 (0.00, 25.43) | -1.17 (-1.27, -1.06) | <0.001 | 33.14 (14.81, 67.92) | 27.65 (9.84, 63.81) | -0.63 (-0.69, -0.57) | <0.001 | 22.61 (8.34, 53.76) | 7.13 (0.51, 34.15) | -3.88 (-4.37, -3.39) | <0.001 | 0.36 (0.00, 22.61) | 0.10 (0.00, 22.71) | -4.32 (-4.86, -3.78) | <0.001 |
| Grenada | 70.64 (45.36, 106.26) | 67.26 (44.56, 98.14) | -0.17 (-0.18, -0.16) | <0.001 | 1289.41 (1168.71, 1420.00) | 1277.81 (1169.85, 1393.52) | -0.03 (-0.04, -0.03) | <0.001 | 295.51 (238.41, 362.92) | 136.09 (102.23, 178.10) | -2.61 (-2.91, -2.30) | <0.001 | 3.98 (0.18, 20.91) | 1.27 (0.00, 13.09) | -3.80 (-4.20, -3.41) | <0.001 |
| Guam | 31.50 (19.18, 49.03) | 32.57 (19.80, 50.72) | 0.12 (0.10, 0.14) | <0.001 | 786.27 (718.47, 858.93) | 828.31 (757.16, 904.51) | 0.18 (0.17, 0.19) | <0.001 | 171.92 (141.00, 207.80) | 145.16 (116.14, 179.38) | -0.62 (-0.99, -0.25) | 0.001 | 2.18 (0.13, 10.33) | 1.75 (0.05, 9.86) | -0.83 (-1.36, -0.29) | 0.002 |
| Guatemala | 77.41 (74.28, 80.65) | 78.85 (76.92, 80.81) | 0.06 (0.05, 0.08) | <0.001 | 1212.70 (1199.79, 1225.73) | 1265.28 (1257.37, 1273.22) | 0.15 (0.13, 0.16) | <0.001 | 88.42 (84.95, 92.01) | 69.01 (67.18, 70.89) | -0.86 (-1.18, -0.54) | <0.001 | 0.52 (0.28, 0.89) | 0.15 (0.07, 0.27) | -4.24 (-5.93, -2.52) | <0.001 |
| Guinea | 59.80 (56.59, 63.14) | 66.08 (63.82, 68.40) | 0.33 (0.30, 0.37) | <0.001 | 850.89 (838.44, 863.49) | 1004.99 (995.88, 1014.16) | 0.57 (0.52, 0.62) | <0.001 | 143.15 (138.10, 148.35) | 99.06 (96.23, 101.97) | -1.27 (-1.37, -1.17) | <0.001 | 1.67 (1.16, 2.34) | 0.83 (0.59, 1.15) | -2.37 (-2.51, -2.23) | <0.001 |
| Guinea-Bissau | 79.80 (71.07, 89.43) | 84.08 (77.92, 90.65) | 0.18 (0.17, 0.19) | <0.001 | 1199.95 (1164.32, 1236.47) | 1273.58 (1248.70, 1298.86) | 0.20 (0.20, 0.21) | <0.001 | 222.62 (207.54, 238.60) | 127.04 (119.28, 135.22) | -1.92 (-2.02, -1.82) | <0.001 | 2.69 (1.26, 5.15) | 1.08 (0.48, 2.16) | -3.11 (-3.28, -2.93) | <0.001 |
| Guyana | 81.95 (72.73, 92.14) | 78.82 (69.50, 89.10) | -0.13 (-0.17, -0.09) | <0.001 | 1287.44 (1248.97, | 1279.10 (1239.97, | -0.02 (-0.04, -0.01) | 0.008 | 237.39 (220.90, 254.86) | 151.73 (138.33, 166.12) | -1.49 (-2.09, -0.89) | <0.001 | 2.94 (1.37, 5.65) | 1.54 (0.47, 3.78) | -2.14 (-2.99, -1.29) | <0.001 |

| | | | | | | | | | | | | | | | | |
|---|---|---|---|---|---|---|---|---|---|---|---|---|---|---|---|---|
| | | | | | 1326.88) | 1319.21) | | | | | | | | | | |
| Haiti | 87.75 (84.11, 91.52) | 89.67 (87.16, 92.24) | 0.07 (0.05, 0.10) | <0.001 | 1263.02 (1248.76, 1277.41) | 1298.20 (1288.52, 1307.94) | 0.09 (0.08, 0.10) | <0.001 | 702.56 (691.80, 713.46) | 322.35 (317.52, 327.25) | -2.59 (-2.79, -2.38) | <0.001 | 10.86 (9.55, 12.31) | 4.40 (3.85, 5.01) | -3.00 (-3.22, -2.77) | <0.001 |
| Honduras | 79.61 (75.50, 83.90) | 77.99 (75.39, 80.67) | -0.07 (-0.07, -0.06) | <0.001 | 1278.69 (1261.41, 1296.16) | 1279.06 (1268.14, 1290.05) | 0.00 (-0.01, 0.01) | 0.511 | 72.76 (68.65, 77.06) | 64.41 (61.97, 66.92) | -0.39 (-0.47, -0.32) | <0.001 | 0.21 (0.04, 0.62) | 0.06 (0.01, 0.21) | -4.34 (-4.95, -3.72) | <0.001 |
| Hungary | 1.65 (1.28, 2.10) | 1.20 (0.85, 1.66) | -1.09 (-1.22, -0.95) | <0.001 | 11.80 (10.75, 12.94) | 10.04 (8.94, 11.25) | -0.56 (-0.65, -0.47) | <0.001 | 48.94 (46.78, 51.18) | 7.13 (6.22, 8.16) | -6.50 (-7.12, -5.87) | <0.001 | 0.85 (0.59, 1.20) | 0.12 (0.03, 0.34) | -6.69 (-7.37, -6.01) | <0.001 |
| Iceland | 0.42 (0.00, 4.62) | 0.43 (0.00, 4.50) | 0.13 (-0.11, 0.38) | 0.294 | 7.40 (3.13, 14.91) | 7.65 (3.54, 14.77) | 0.12 (0.06, 0.19) | <0.001 | 4.78 (1.55, 11.33) | 2.31 (0.45, 7.43) | -2.50 (-2.83, -2.18) | <0.001 | 0.07 (0.00, 3.98) | 0.03 (0.00, 3.82) | -3.08 (-4.42, -1.71) | <0.001 |
| India | 76.45 (76.16, 76.75) | 73.82 (73.61, 74.04) | -0.13 (-0.30, 0.04) | 0.129 | 991.14 (990.07, 992.21) | 1001.02 (1000.22, 1001.83) | 0.06 (0.04, 0.08) | <0.001 | 493.94 (493.18, 494.70) | 265.04 (264.63, 265.46) | -2.18 (-2.56, -1.80) | <0.001 | 7.46 (7.37, 7.56) | 3.67 (3.62, 3.72) | -2.44 (-2.86, -2.03) | <0.001 |
| Indonesia | 10.37 (10.14, 10.60) | 11.48 (11.27, 11.68) | 0.35 (0.33, 0.37) | <0.001 | 152.90 (152.02, 153.78) | 179.06 (178.26, 179.87) | 0.55 (0.53, 0.57) | <0.001 | 70.71 (70.10, 71.31) | 25.40 (25.10, 25.71) | -3.46 (-3.58, -3.34) | <0.001 | 1.06 (0.99, 1.14) | 0.28 (0.24, 0.31) | -4.51 (-4.69, -4.32) | <0.001 |
| Iran (Islamic Republic of) | 43.33 (42.48, 44.19) | 42.40 (41.68, 43.13) | -0.07 (-0.08, -0.06) | <0.001 | 722.58 (718.97, 726.21) | 725.23 (722.39, 728.09) | 0.02 (0.01, 0.03) | 0.004 | 91.43 (90.14, 92.72) | 53.27 (52.50, 54.05) | -1.84 (-2.04, -1.63) | <0.001 | 0.94 (0.81, 1.08) | 0.30 (0.25, 0.37) | -3.77 (-4.31, -3.22) | <0.001 |
| Iraq | 46.91 (45.27, 48.60) | 47.04 (46.07, 48.03) | 0.01 (-0.01, 0.02) | 0.402 | 743.47 (736.66, 750.33) | 753.49 (749.50, 757.50) | 0.04 (0.04, 0.05) | <0.001 | 126.94 (124.08, 129.84) | 53.33 (52.26, 54.41) | -2.96 (-3.16, -2.76) | <0.001 | 1.56 (1.25, 1.92) | 0.30 (0.22, 0.39) | -5.52 (-5.93, -5.11) | <0.001 |
| Ireland | 0.47 (0.18, 1.02) | 0.39 (0.15, 0.84) | -0.66 (-0.97, -0.35) | <0.001 | 7.09 (5.75, 8.66) | 6.89 (5.67, 8.31) | -0.08 (-0.13, -0.03) | 0.002 | 7.02 (5.68, 8.59) | 2.43 (1.74, 3.32) | -3.61 (-4.36, -2.86) | <0.001 | 0.11 (0.01, 0.49) | 0.04 (0.00, 0.33) | -3.70 (-5.56, -1.80) | <0.001 |
| Israel | 0.63 (0.33, | 0.46 (0.26, | -1.09 (-1.31, | <0.001 | 8.43 (7.17, | 7.26 (6.36, | -0.52 (-0.59, | <0.001 | 22.04 (19.96, | 9.48 (8.45, | -2.91 (-3.82, | <0.001 | 0.37 (0.15, | 0.15 (0.05, | -3.04 (-4.00, | <0.001 |

| | | | | | | | | | | | | | | | | |
|---|---|---|---|---|---|---|---|---|---|---|---|---|---|---|---|---|
| | 1.11) | 0.76) | -0.86) | | 9.85) | 8.25) | -0.45) | | 24.27) | 10.60) | -2.01) | | 0.77) | 0.36) | -2.08) | |
| Italy | 1.28 (1.13, 1.44) | 0.45 (0.36, 0.57) | -3.50 (-3.85, -3.16) | <0.001 | 20.41 (19.81, 21.03) | 11.06 (10.55, 11.59) | -2.11 (-2.22, -2.00) | <0.001 | 24.31 (23.65, 24.98) | 7.95 (7.53, 8.40) | -3.86 (-4.48, -3.23) | <0.001 | 0.39 (0.31, 0.49) | 0.12 (0.07, 0.19) | -4.18 (-4.61, -3.74) | <0.001 |
| Jamaica | 63.12 (58.32, 68.27) | 64.84 (60.35, 69.59) | 0.09 (0.08, 0.11) | <0.001 | 1230.46 (1208.14, 1253.12) | 1288.85 (1268.53, 1309.43) | 0.16 (0.15, 0.17) | <0.001 | 191.78 (183.14, 200.77) | 121.75 (115.57, 128.19) | -1.52 (-2.04, -0.99) | <0.001 | 2.14 (1.31, 3.34) | 0.99 (0.51, 1.75) | -2.58 (-3.54, -1.62) | <0.001 |
| Japan | 0.63 (0.56, 0.70) | 0.27 (0.21, 0.33) | -2.97 (-3.22, -2.73) | <0.001 | 14.33 (13.98, 14.68) | 9.61 (9.28, 9.96) | -1.37 (-1.41, -1.34) | <0.001 | 8.90 (8.63, 9.18) | 3.28 (3.09, 3.48) | -3.40 (-3.74, -3.06) | <0.001 | 0.14 (0.11, 0.18) | 0.05 (0.03, 0.08) | -3.67 (-4.02, -3.32) | <0.001 |
| Jordan | 0.96 (0.49, 1.74) | 0.77 (0.54, 1.06) | -0.74 (-0.93, -0.54) | <0.001 | 14.34 (12.36, 16.60) | 12.55 (11.58, 13.58) | -0.45 (-0.56, -0.34) | <0.001 | 24.99 (22.33, 27.90) | 6.51 (5.82, 7.27) | -4.43 (-5.00, -3.87) | <0.001 | 0.41 (0.13, 1.00) | 0.10 (0.03, 0.24) | -4.65 (-5.45, -3.84) | <0.001 |
| Kazakhstan | 8.30 (7.63, 9.02) | 3.56 (3.14, 4.03) | -2.88 (-2.96, -2.79) | <0.001 | 61.17 (59.32, 63.06) | 36.41 (35.02, 37.85) | -1.75 (-1.83, -1.67) | <0.001 | 222.60 (219.06, 226.18) | 34.46 (33.12, 35.85) | -6.30 (-6.82, -5.78) | <0.001 | 3.77 (3.32, 4.26) | 0.57 (0.41, 0.77) | -6.36 (-6.90, -5.81) | <0.001 |
| Kenya | 99.78 (97.73, 101.88) | 101.13 (99.82, 102.45) | 0.05 (0.04, 0.05) | <0.001 | 1589.43 (1580.75, 1598.16) | 1741.29 (1735.66, 1746.92) | 0.34 (0.29, 0.38) | <0.001 | 120.69 (118.33, 123.09) | 111.63 (110.22, 113.06) | -0.27 (-0.33, -0.21) | <0.001 | 0.72 (0.55, 0.94) | 0.46 (0.37, 0.56) | -1.53 (-1.73, -1.33) | <0.001 |
| Kiribati | 46.72 (25.51, 79.81) | 48.03 (30.54, 72.20) | 0.10 (0.09, 0.10) | <0.001 | 771.50 (675.22, 878.60) | 790.51 (713.42, 873.91) | 0.08 (0.07, 0.10) | <0.001 | 1231.05 (1106.69, 1366.36) | 835.85 (755.92, 922.15) | -1.33 (-1.39, -1.28) | <0.001 | 20.07 (7.16, 45.72) | 13.40 (5.10, 28.88) | -1.39 (-1.45, -1.34) | <0.001 |
| Kuwait | 0.96 (0.42, 1.96) | 0.75 (0.42, 1.32) | -0.83 (-1.03, -0.63) | <0.001 | 17.69 (14.99, 20.79) | 14.40 (12.76, 16.25) | -0.70 (-0.74, -0.66) | <0.001 | 35.62 (31.63, 40.01) | 7.31 (6.09, 8.75) | -5.25 (-6.49, -4.00) | <0.001 | 0.56 (0.17, 1.44) | 0.11 (0.01, 0.50) | -5.47 (-7.29, -3.61) | <0.001 |
| Kyrgyzstan | 61.56 (58.02, 65.27) | 62.16 (59.18, 65.25) | 0.03 (0.02, 0.05) | <0.001 | 1076.60 (1061.40, 1091.97) | 1081.93 (1069.45, 1094.53) | 0.02 (0.01, 0.02) | <0.001 | 447.14 (437.23, 457.23) | 137.76 (133.31, 142.33) | -3.96 (-4.33, -3.60) | <0.001 | 6.75 (5.58, 8.12) | 1.48 (1.05, 2.04) | -5.03 (-5.58, -4.47) | <0.001 |
| Lao People's | 61.81 | 58.27 | -0.21 | <0.001 | 894.10 | 883.36 | -0.05 | 0.009 | 332.53 | 200.53 | -1.71 | <0.001 | 4.83 | 2.61 | -2.09 | <0.001 |

| | | | | | | | | | | | | | | | | |
|---|---|---|---|---|---|---|---|---|---|---|---|---|---|---|---|---|
| Democratic Republic | (57.96, 65.86) | (55.64, 60.99) | (-0.24, -0.18) | | (878.94, 909.47) | (872.97, 893.84) | (-0.09, -0.01) | | (323.24, 342.03) | (195.59, 205.57) | (-1.92, -1.50) | | (3.76, 6.13) | (2.08, 3.25) | (-2.33, -1.84) | |
| Latvia | 6.87 (5.35, 8.72) | 3.17 (1.95, 5.03) | -2.63 (-2.84, -2.43) | <0.001 | 45.44 (41.33, 49.86) | 28.21 (24.09, 32.92) | -1.64 (-1.75, -1.54) | <0.001 | 108.57 (102.17, 115.29) | 16.45 (13.42, 20.07) | -6.26 (-8.30, -4.18) | <0.001 | 1.86 (1.11, 2.95) | 0.27 (0.02, 1.31) | -6.38 (-8.65, -4.05) | <0.001 |
| Lebanon | 2.00 (1.26, 3.04) | 1.05 (0.66, 1.62) | -2.24 (-2.38, -2.10) | <0.001 | 26.82 (23.90, 30.02) | 16.30 (14.60, 18.16) | -1.73 (-1.81, -1.65) | <0.001 | 62.51 (58.03, 67.25) | 16.98 (15.22, 18.90) | -4.41 (-4.53, -4.28) | <0.001 | 1.01 (0.51, 1.81) | 0.26 (0.09, 0.63) | -4.52 (-4.70, -4.34) | <0.001 |
| Lesotho | 104.19 (96.80, 112.07) | 102.33 (96.04, 108.93) | -0.05 (-0.09, -0.02) | 0.001 | 1608.62 (1578.15, 1639.57) | 1613.36 (1587.88, 1639.16) | 0.01 (0.01, 0.02) | <0.001 | 200.20 (189.49, 211.41) | 169.61 (161.40, 178.14) | -0.57 (-0.67, -0.47) | <0.001 | 2.04 (1.09, 3.57) | 1.54 (0.85, 2.58) | -0.98 (-1.16, -0.79) | <0.001 |
| Liberia | 76.31 (69.83, 83.26) | 82.54 (78.63, 86.61) | 0.27 (0.22, 0.31) | <0.001 | 1198.32 (1171.71, 1225.41) | 1295.96 (1279.96, 1312.12) | 0.27 (0.25, 0.28) | <0.001 | 138.42 (129.50, 147.81) | 94.45 (90.18, 98.88) | -1.31 (-1.48, -1.13) | <0.001 | 1.32 (0.59, 2.58) | 0.53 (0.26, 0.99) | -3.05 (-3.41, -2.69) | <0.001 |
| Libya | 37.92 (34.99, 41.05) | 43.03 (40.69, 45.48) | 0.44 (0.41, 0.47) | <0.001 | 669.21 (656.26, 682.37) | 751.82 (742.09, 761.65) | 0.40 (0.39, 0.41) | <0.001 | 92.80 (88.06, 97.74) | 56.25 (53.60, 58.99) | -1.68 (-1.93, -1.43) | <0.001 | 0.99 (0.56, 1.67) | 0.33 (0.16, 0.62) | -3.63 (-4.27, -2.99) | <0.001 |
| Lithuania | 5.97 (4.76, 7.40) | 2.98 (1.96, 4.41) | -2.43 (-2.77, -2.08) | <0.001 | 37.29 (34.16, 40.64) | 25.06 (21.84, 28.67) | -1.42 (-1.67, -1.17) | <0.001 | 91.17 (86.26, 96.29) | 15.62 (13.15, 18.48) | -6.01 (-7.67, -4.33) | <0.001 | 1.58 (0.99, 2.40) | 0.26 (0.04, 0.98) | -6.28 (-7.85, -4.69) | <0.001 |
| Luxembourg | 0.62 (0.01, 4.60) | 0.53 (0.02, 3.22) | -0.60 (-0.79, -0.41) | <0.001 | 10.40 (5.85, 17.60) | 10.16 (6.34, 15.73) | -0.09 (-0.14, -0.04) | 0.001 | 9.90 (5.50, 16.93) | 3.97 (1.80, 7.97) | -3.06 (-3.51, -2.61) | <0.001 | 0.16 (0.00, 3.89) | 0.06 (0.00, 2.50) | -3.28 (-4.04, -2.50) | <0.001 |
| Madagascar | 102.94 (100.06, 105.90) | 110.21 (108.29, 112.16) | 0.23 (0.22, 0.25) | <0.001 | 1761.82 (1749.36, 1774.35) | 1881.20 (1872.95, 1889.47) | 0.23 (0.21, 0.25) | <0.001 | 335.20 (329.87, 340.60) | 206.92 (204.22, 209.65) | -1.62 (-1.98, -1.26) | <0.001 | 4.01 (3.44, 4.65) | 1.87 (1.62, 2.15) | -2.56 (-3.07, -2.04) | <0.001 |
| Malawi | 121.47 (117.96, 125.07) | 125.69 (123.23, 128.19) | 0.12 (0.11, 0.12) | <0.001 | 1940.09 (1925.46, 1954.82) | 2024.27 (2013.94, 2034.66) | 0.15 (0.13, 0.16) | <0.001 | 186.75 (182.28, 191.31) | 136.16 (133.51, 138.84) | -1.07 (-1.14, -0.99) | <0.001 | 1.51 (1.13, 1.99) | 0.63 (0.47, 0.84) | -2.94 (-3.28, -2.60) | <0.001 |

| | | | | | | | | | | | | | | | | |
|---|---|---|---|---|---|---|---|---|---|---|---|---|---|---|---|---|
| Malaysia | 39.91 (38.50, 41.37) | 38.67 (37.63, 39.73) | -0.11 (-0.12, -0.09) | <0.001 | 756.20 (749.91, 762.53) | 763.21 (758.60, 767.83) | 0.03 (0.03, 0.04) | <0.001 | 154.69 (151.85, 157.57) | 58.76 (57.49, 60.06) | -3.27 (-3.46, -3.08) | <0.001 | 1.96 (1.65, 2.31) | 0.37 (0.27, 0.49) | -5.65 (-5.91, -5.40) | <0.001 |
| Maldives | 40.99 (28.20, 58.77) | 37.58 (29.65, 47.28) | -0.30 (-0.31, -0.29) | <0.001 | 738.91 (678.12, 804.44) | 703.69 (669.79, 739.18) | -0.17 (-0.19, -0.15) | <0.001 | 201.15 (169.76, 237.45) | 53.80 (44.68, 64.60) | -4.32 (-4.48, -4.17) | <0.001 | 2.76 (0.31, 11.78) | 0.33 (0.00, 3.16) | -7.01 (-7.36, -6.65) | <0.001 |
| Mali | 87.87 (84.57, 91.28) | 87.81 (85.80, 89.85) | 0.00 (-0.02, 0.03) | 0.763 | 1388.21 (1374.62, 1401.91) | 1413.43 (1404.99, 1421.92) | 0.07 (0.04, 0.09) | <0.001 | 224.20 (218.78, 229.73) | 124.03 (121.54, 126.56) | -1.98 (-2.24, -1.72) | <0.001 | 2.58 (2.02, 3.25) | 0.92 (0.72, 1.17) | -3.49 (-3.77, -3.22) | <0.001 |
| Malta | 0.33 (0.00, 3.74) | 0.29 (0.00, 4.38) | -0.54 (-0.67, -0.40) | <0.001 | 4.99 (1.99, 10.58) | 4.69 (1.79, 10.66) | -0.23 (-0.27, -0.18) | <0.001 | 8.20 (4.16, 14.75) | 5.07 (2.06, 11.08) | -1.68 (-2.15, -1.22) | <0.001 | 0.13 (0.00, 3.41) | 0.08 (0.00, 4.06) | -1.53 (-2.57, -0.47) | 0.005 |
| Marshall Islands | 44.19 (18.29, 92.06) | 44.67 (21.98, 81.00) | 0.04 (0.03, 0.05) | <0.001 | 786.10 (657.38, 934.34) | 801.37 (691.59, 923.82) | 0.06 (0.04, 0.09) | <0.001 | 815.85 (682.94, 968.57) | 659.67 (560.35, 771.70) | -0.74 (-0.83, -0.65) | <0.001 | 12.90 (1.64, 48.65) | 10.37 (1.69, 33.87) | -0.76 (-0.84, -0.67) | <0.001 |
| Mauritania | 71.42 (65.63, 77.64) | 71.75 (67.71, 75.99) | 0.01 (-0.00, 0.03) | 0.120 | 1231.08 (1205.93, 1256.66) | 1245.83 (1228.25, 1263.61) | 0.04 (0.02, 0.06) | <0.001 | 130.56 (122.52, 139.03) | 76.51 (72.21, 81.02) | -1.84 (-1.93, -1.74) | <0.001 | 1.16 (0.52, 2.29) | 0.28 (0.08, 0.72) | -4.79 (-4.97, -4.60) | <0.001 |
| Mauritius | 40.17 (34.79, 46.17) | 38.77 (33.28, 44.92) | -0.12 (-0.14, -0.11) | <0.001 | 756.50 (732.56, 781.04) | 755.06 (730.37, 780.40) | -0.01 (-0.01, -0.00) | 0.005 | 164.38 (153.29, 176.08) | 67.75 (60.50, 75.65) | -2.94 (-3.63, -2.25) | <0.001 | 2.12 (1.04, 3.87) | 0.52 (0.09, 1.74) | -4.64 (-5.98, -3.27) | <0.001 |
| Mexico | 31.23 (30.66, 31.81) | 26.99 (26.54, 27.45) | -0.49 (-0.52, -0.47) | <0.001 | 566.23 (563.69, 568.77) | 491.29 (489.35, 493.23) | -0.49 (-0.51, -0.46) | <0.001 | 96.95 (95.88, 98.04) | 33.13 (32.63, 33.63) | -3.59 (-3.72, -3.46) | <0.001 | 1.21 (1.09, 1.34) | 0.16 (0.13, 0.20) | -6.65 (-7.13, -6.16) | <0.001 |
| Micronesia (Federated States of) | 44.28 (25.89, 71.60) | 44.41 (26.50, 70.54) | 0.02 (0.00, 0.03) | 0.028 | 782.27 (696.78, 875.99) | 793.30 (709.92, 884.31) | 0.05 (0.04, 0.06) | <0.001 | 851.73 (760.76, 951.11) | 587.90 (515.51, 668.06) | -1.28 (-1.34, -1.22) | <0.001 | 13.71 (4.48, 32.72) | 9.16 (2.30, 25.22) | -1.39 (-1.47, -1.32) | <0.001 |
| Monaco | 0.38 (0.00, 61.97) | 0.36 (0.00, 44.98) | -0.16 (-0.24, -0.08) | <0.001 | 7.08 (0.03, 72.31) | 6.87 (0.03, 56.78) | -0.10 (-0.13, -0.08) | <0.001 | 4.09 (0.00, 67.61) | 3.10 (0.00, 50.06) | -0.95 (-1.01, -0.89) | <0.001 | 0.06 (0.00, 61.49) | 0.05 (0.00, 44.40) | -0.61 (-0.61, -0.61) | <0.001 |
| Mongolia | 70.09 | 66.59 | -0.18 | <0.001 | 1139.88 | 1126.30 | -0.04 | <0.001 | 204.50 | 119.31 | -1.80 | <0.001 | 2.60 | 1.14 | -2.76 | <0.001 |

| | | | | | | | | | | | | | | | | |
|---|---|---|---|---|---|---|---|---|---|---|---|---|---|---|---|---|
| | (64.73, 75.85) | (62.13, 71.32) | (-0.19, -0.16) | | (1117.09, 1163.08) | (1108.31, 1144.52) | (-0.05, -0.03) | | (194.54, 214.90) | (113.63, 125.24) | (-2.02, -1.58) | | (1.57, 4.12) | (0.66, 1.88) | (-3.16, -2.37) | |
| Montenegro | 1.28 (0.29, 3.65) | 1.16 (0.21, 3.85) | -0.30 (-0.52, -0.07) | 0.010 | 10.04 (6.52, 14.81) | 9.76 (6.08, 14.98) | -0.08 (-0.14, -0.02) | 0.009 | 28.88 (22.65, 36.33) | 13.01 (8.71, 18.82) | -2.91 (-3.90, -1.92) | <0.001 | 0.49 (0.02, 2.42) | 0.22 (0.00, 2.37) | -2.99 (-4.24, -1.71) | <0.001 |
| Morocco | 48.98 (47.65, 50.34) | 48.37 (47.24, 49.51) | -0.04 (-0.05, -0.02) | <0.001 | 753.95 (748.57, 759.35) | 767.44 (762.96, 771.94) | 0.07 (0.04, 0.09) | <0.001 | 136.75 (134.46, 139.07) | 64.77 (63.47, 66.09) | -2.56 (-2.71, -2.42) | <0.001 | 1.68 (1.43, 1.96) | 0.47 (0.37, 0.60) | -4.26 (-4.45, -4.08) | <0.001 |
| Mozambique | 123.87 (120.72, 127.09) | 123.89 (121.88, 125.93) | -0.01 (-0.06, 0.04) | 0.815 | 1965.01 (1951.97, 1978.12) | 2022.86 (2014.34, 2031.41) | 0.10 (0.07, 0.14) | <0.001 | 156.60 (152.96, 160.30) | 137.17 (134.97, 139.39) | -0.46 (-0.50, -0.42) | <0.001 | 1.02 (0.75, 1.37) | 0.66 (0.52, 0.83) | -1.49 (-1.67, -1.31) | <0.001 |
| Myanmar | 65.06 (63.86, 66.28) | 56.27 (55.29, 57.27) | -0.51 (-0.54, -0.47) | <0.001 | 920.62 (915.99, 925.26) | 856.77 (852.91, 860.64) | -0.25 (-0.31, -0.19) | <0.001 | 240.65 (238.27, 243.05) | 108.13 (106.76, 109.51) | -2.73 (-2.89, -2.57) | <0.001 | 3.30 (3.03, 3.60) | 1.13 (0.99, 1.27) | -3.62 (-3.77, -3.48) | <0.001 |
| Namibia | 91.43 (83.76, 99.70) | 89.95 (84.28, 95.92) | -0.06 (-0.07, -0.05) | <0.001 | 1582.83 (1549.08, 1617.21) | 1582.86 (1558.45, 1607.58) | -0.00 (-0.02, 0.01) | 0.638 | 186.27 (174.71, 198.47) | 114.27 (107.77, 121.08) | -1.67 (-1.80, -1.55) | <0.001 | 1.85 (0.85, 3.59) | 0.64 (0.24, 1.40) | -3.56 (-3.85, -3.26) | <0.001 |
| Nauru | 14.28 (0.03, 130.44) | 11.43 (0.01, 114.54) | -0.79 (-0.94, -0.65) | <0.001 | 129.05 (42.58, 304.18) | 120.39 (41.22, 278.51) | -0.27 (-0.38, -0.16) | <0.001 | 595.22 (379.70, 892.89) | 491.28 (307.72, 748.36) | -0.66 (-0.84, -0.48) | <0.001 | 9.85 (0.00, 122.18) | 8.06 (0.00, 108.20) | -0.69 (-0.89, -0.50) | <0.001 |
| Nepal | 68.89 (67.02, 70.81) | 65.44 (64.07, 66.83) | -0.17 (-0.21, -0.12) | <0.001 | 810.35 (803.66, 817.09) | 833.46 (828.42, 838.53) | 0.11 (0.06, 0.16) | <0.001 | 514.55 (509.18, 519.96) | 219.01 (216.42, 221.63) | -2.90 (-3.12, -2.68) | <0.001 | 8.07 (7.40, 8.78) | 3.02 (2.72, 3.34) | -3.33 (-3.52, -3.14) | <0.001 |
| Netherlands | 0.37 (0.23, 0.56) | 0.36 (0.21, 0.56) | -0.09 (-0.31, 0.12) | 0.403 | 6.57 (5.94, 7.26) | 6.74 (6.06, 7.47) | 0.10 (0.06, 0.13) | <0.001 | 4.18 (3.68, 4.72) | 2.30 (1.92, 2.75) | -2.06 (-2.35, -1.77) | <0.001 | 0.07 (0.02, 0.17) | 0.03 (0.00, 0.13) | -2.17 (-3.23, -1.10) | <0.001 |
| New Zealand | 1.91 (1.25, 2.79) | 0.93 (0.50, 1.59) | -2.50 (-2.67, -2.33) | <0.001 | 30.41 (27.57, 33.46) | 24.03 (21.54, 26.73) | -0.81 (-0.93, -0.69) | <0.001 | 74.13 (69.67, 78.81) | 30.22 (27.44, 33.21) | -2.97 (-3.52, -2.42) | <0.001 | 1.22 (0.71, 1.96) | 0.50 (0.20, 1.03) | -2.98 (-3.53, -2.42) | <0.001 |
| Nicaragua | 71.97 | 72.34 | 0.03 | 0.506 | 1316.49 | 1340.01 | 0.06 | <0.001 | 120.54 | 75.64 | -1.62 | <0.001 | 0.97 | 0.19 | -5.58 | <0.001 |

| | | | | | | | | | | | | | | | | |
|---|---|---|---|---|---|---|---|---|---|---|---|---|---|---|---|---|
| | (67.76, 76.40) | (69.25, 75.54) | (-0.06, 0.12) | | (1297.40, 1335.83) | (1326.47, 1353.66) | (0.05, 0.07) | | (114.78, 126.54) | (72.45, 78.94) | (-1.88, -1.37) | | (0.51, 1.70) | (0.06, 0.44) | (-6.43, -4.72) | |
| Niger | 74.76 (71.60, 78.04) | 78.70 (76.78, 80.66) | 0.18 (0.17, 0.20) | <0.001 | 1155.78 (1142.84, 1168.83) | 1221.93 (1213.99, 1229.92) | 0.20 (0.18, 0.21) | <0.001 | 166.51 (161.67, 171.48) | 110.21 (107.85, 112.62) | -1.40 (-1.56, -1.24) | <0.001 | 1.80 (1.33, 2.41) | 0.84 (0.65, 1.09) | -2.54 (-2.84, -2.25) | <0.001 |
| Nigeria | 74.44 (73.54, 75.35) | 78.00 (77.41, 78.60) | 0.16 (0.15, 0.17) | <0.001 | 1248.75 (1244.92, 1252.59) | 1338.22 (1335.69, 1340.77) | 0.24 (0.23, 0.25) | <0.001 | 137.99 (136.73, 139.27) | 86.80 (86.16, 87.45) | -1.61 (-1.75, -1.48) | <0.001 | 1.28 (1.16, 1.41) | 0.38 (0.34, 0.42) | -4.11 (-4.30, -3.92) | <0.001 |
| Niue | 8.13 (0.00, 534.04) | 5.96 (0.00, 743.82) | -1.07 (-1.09, -1.04) | <0.001 | 92.18 (0.67, 687.19) | 91.32 (0.10, 902.03) | -0.04 (-0.07, -0.00) | 0.036 | 335.79 (58.97, 1078.69) | 195.86 (6.35, 1082.31) | -1.85 (-2.07, -1.63) | <0.001 | 5.50 (0.00, 528.62) | 3.11 (0.00, 738.22) | -1.95 (-2.17, -1.74) | <0.001 |
| North Macedonia | 1.65 (0.89, 2.79) | 1.13 (0.53, 2.21) | -1.23 (-1.43, -1.04) | <0.001 | 13.07 (10.71, 15.80) | 9.63 (7.62, 12.09) | -1.03 (-1.16, -0.91) | <0.001 | 56.86 (51.81, 62.27) | 13.29 (10.89, 16.14) | -4.91 (-5.22, -4.59) | <0.001 | 0.97 (0.42, 1.92) | 0.22 (0.02, 1.00) | -5.05 (-5.38, -4.73) | <0.001 |
| Northern Mariana Islands | 33.86 (14.38, 69.34) | 35.85 (10.89, 95.43) | 0.20 (0.15, 0.24) | <0.001 | 825.65 (712.57, 952.81) | 859.25 (704.68, 1042.40) | 0.14 (0.09, 0.18) | <0.001 | 187.38 (136.34, 252.85) | 125.48 (69.75, 213.39) | -1.38 (-1.53, -1.22) | <0.001 | 2.48 (0.01, 24.11) | 1.41 (0.00, 45.92) | -1.94 (-2.13, -1.74) | <0.001 |
| Norway | 0.27 (0.08, 0.68) | 0.25 (0.08, 0.64) | -0.02 (-0.45, 0.41) | 0.923 | 4.55 (3.57, 5.73) | 4.78 (3.82, 5.93) | 0.18 (0.16, 0.20) | <0.001 | 4.46 (3.49, 5.62) | 1.28 (0.81, 1.93) | -4.22 (-4.54, -3.90) | <0.001 | 0.07 (0.00, 0.38) | 0.02 (0.00, 0.28) | -4.24 (-5.25, -3.21) | <0.001 |
| Oman | 0.77 (0.29, 1.68) | 0.82 (0.50, 1.35) | 0.26 (0.09, 0.44) | 0.003 | 12.54 (10.20, 15.25) | 13.79 (12.31, 15.45) | 0.37 (0.26, 0.48) | <0.001 | 14.30 (11.81, 17.17) | 3.35 (2.62, 4.26) | -4.86 (-5.22, -4.50) | <0.001 | 0.23 (0.02, 0.89) | 0.04 (0.00, 0.37) | -5.59 (-6.03, -5.14) | <0.001 |
| Pakistan | 87.07 (86.18, 87.97) | 91.93 (91.32, 92.55) | 0.19 (0.07, 0.32) | 0.003 | 1147.27 (1143.86, 1150.70) | 1224.62 (1222.29, 1226.95) | 0.26 (0.17, 0.34) | <0.001 | 569.20 (566.76, 571.64) | 474.21 (472.76, 475.66) | -0.61 (-0.76, -0.47) | <0.001 | 8.68 (8.38, 8.99) | 6.95 (6.77, 7.13) | -0.75 (-0.90, -0.60) | <0.001 |
| Palau | 6.13 (0.00, 70.53) | 4.77 (0.00, 77.45) | -0.85 (-0.90, -0.81) | <0.001 | 72.73 (23.66, 172.45) | 69.40 (19.86, 178.08) | -0.16 (-0.18, -0.15) | <0.001 | 232.47 (133.15, 379.32) | 155.21 (72.51, 294.32) | -1.37 (-1.46, -1.28) | <0.001 | 3.79 (0.00, 66.12) | 2.51 (0.00, 73.53) | -1.41 (-1.51, -1.30) | <0.001 |

| | | | | | | | | | | | | | | | | |
|---|---|---|---|---|---|---|---|---|---|---|---|---|---|---|---|---|
| Palestine | 42.62 (38.10, 47.63) | 42.02 (39.30, 44.89) | -0.05 (-0.07, -0.03) | <0.001 | 728.91 (708.82, 749.50) | 741.53 (729.63, 753.60) | 0.06 (0.05, 0.08) | <0.001 | 61.87 (56.06, 68.18) | 42.63 (39.80, 45.62) | -1.28 (-1.34, -1.22) | <0.001 | 0.46 (0.09, 1.49) | 0.13 (0.02, 0.44) | -4.32 (-4.71, -3.94) | <0.001 |
| Panama | 65.36 (60.54, 70.50) | 61.16 (57.40, 65.11) | -0.23 (-0.25, -0.20) | <0.001 | 1230.13 (1208.30 , 1252.27) | 1202.60 (1185.64 , 1219.75) | -0.08 (-0.09, -0.07) | <0.001 | 150.79 (143.19, 158.71) | 79.76 (75.43, 84.27) | -2.16 (-2.67, -1.64) | <0.001 | 1.53 (0.85, 2.57) | 0.37 (0.14, 0.82) | -4.76 (-5.85, -3.66) | <0.001 |
| Papua New Guinea | 43.76 (40.59, 47.13) | 44.72 (42.69, 46.83) | 0.08 (0.07, 0.09) | <0.001 | 743.41 (730.15, 756.87) | 774.80 (766.27, 783.42) | 0.14 (0.13, 0.16) | <0.001 | 707.24 (694.03, 720.66) | 635.51 (627.72, 643.37) | -0.41 (-0.63, -0.20) | <0.001 | 11.30 (9.67, 13.14) | 9.98 (9.02, 11.01) | -0.48 (-0.70, -0.26) | <0.001 |
| Paraguay | 68.16 (64.19, 72.33) | 65.84 (62.97, 68.82) | -0.12 (-0.14, -0.09) | <0.001 | 1288.30 (1270.38 , 1306.42) | 1279.70 (1266.86 , 1292.64) | -0.02 (-0.03, -0.02) | <0.001 | 105.60 (100.50, 110.91) | 81.20 (77.99, 84.52) | -0.86 (-1.13, -0.59) | <0.001 | 0.74 (0.37, 1.34) | 0.34 (0.16, 0.63) | -2.53 (-3.51, -1.54) | <0.001 |
| Peru | 64.07 (62.43, 65.74) | 63.59 (62.25, 64.94) | -0.03 (-0.06, 0.00) | 0.078 | 1159.60 (1152.39 , 1166.85) | 1183.14 (1177.37 , 1188.92) | 0.06 (0.03, 0.08) | <0.001 | 98.38 (96.27, 100.52) | 68.71 (67.33, 70.11) | -1.25 (-1.51, -0.99) | <0.001 | 0.72 (0.55, 0.94) | 0.20 (0.14, 0.30) | -4.24 (-5.59, -2.87) | <0.001 |
| Philippines | 36.82 (36.09, 37.56) | 35.80 (35.26, 36.34) | -0.10 (-0.11, -0.09) | <0.001 | 540.36 (537.47, 543.26) | 589.42 (587.20, 591.65) | 0.30 (0.23, 0.38) | <0.001 | 102.88 (101.62, 104.15) | 173.97 (172.76, 175.18) | 1.92 (1.37, 2.47) | <0.001 | 1.27 (1.14, 1.43) | 2.39 (2.25, 2.54) | 2.31 (1.62, 3.00) | <0.001 |
| Poland | 2.88 (2.62, 3.16) | 1.35 (1.17, 1.56) | -2.55 (-2.74, -2.35) | <0.001 | 19.83 (19.13, 20.55) | 13.28 (12.66, 13.92) | -1.38 (-1.50, -1.26) | <0.001 | 87.49 (86.02, 88.97) | 9.14 (8.64, 9.67) | -7.81 (-8.32, -7.30) | <0.001 | 1.52 (1.33, 1.73) | 0.14 (0.09, 0.23) | -8.13 (-8.64, -7.63) | <0.001 |
| Portugal | 1.10 (0.79, 1.49) | 0.50 (0.29, 0.82) | -2.76 (-3.09, -2.42) | <0.001 | 12.13 (11.04, 13.29) | 7.83 (6.87, 8.89) | -1.51 (-1.58, -1.44) | <0.001 | 44.17 (42.07, 46.35) | 8.35 (7.38, 9.42) | -5.64 (-6.60, -4.67) | <0.001 | 0.74 (0.49, 1.07) | 0.14 (0.04, 0.35) | -5.62 (-6.78, -4.43) | <0.001 |
| Puerto Rico | 0.98 (0.53, 1.65) | 0.82 (0.38, 1.55) | -0.61 (-0.74, -0.49) | <0.001 | 18.21 (16.05, 20.60) | 16.95 (14.63, 19.53) | -0.25 (-0.31, -0.18) | <0.001 | 25.16 (22.59, 27.94) | 9.83 (8.09, 11.83) | -3.29 (-3.91, -2.67) | <0.001 | 0.41 (0.15, 0.91) | 0.15 (0.01, 0.61) | -3.44 (-4.13, -2.74) | <0.001 |
| Qatar | 1.16 (0.22, 4.45) | 0.71 (0.34, 1.61) | -1.68 (-1.78, -1.57) | <0.001 | 19.36 (14.03, 26.52) | 13.16 (11.27, 15.43) | -1.35 (-1.48, -1.21) | <0.001 | 36.76 (28.87, 46.51) | 7.53 (6.06, 9.43) | -5.38 (-5.71, -5.05) | <0.001 | 0.58 (0.03, 3.72) | 0.11 (0.01, 0.89) | -5.57 (-5.95, -5.18) | <0.001 |

| | | | | | | | | | | | | | | | | |
|---|---|---|---|---|---|---|---|---|---|---|---|---|---|---|---|---|
| Republic of Korea | 0.45 (0.36, 0.55) | 0.33 (0.25, 0.43) | -1.07 (-1.22, -0.92) | <0.001 | 10.23 (9.80, 10.68) | 9.32 (8.87, 9.80) | -0.32 (-0.36, -0.28) | <0.001 | 13.40 (12.91, 13.91) | 2.69 (2.45, 2.95) | -5.36 (-5.79, -4.93) | <0.001 | 0.21 (0.15, 0.29) | 0.04 (0.01, 0.08) | -5.54 (-6.76, -4.30) | <0.001 |
| Republic of Moldova | 9.08 (7.77, 10.56) | 2.75 (1.98, 3.79) | -4.05 (-4.24, -3.86) | <0.001 | 58.84 (55.37, 62.48) | 23.94 (21.42, 26.72) | -3.06 (-3.15, -2.97) | <0.001 | 198.82 (192.36, 205.46) | 30.42 (27.57, 33.53) | -6.42 (-6.86, -5.97) | <0.001 | 3.38 (2.58, 4.35) | 0.50 (0.20, 1.11) | -6.49 (-6.95, -6.02) | <0.001 |
| Romania | 3.65 (3.26, 4.08) | 1.46 (1.17, 1.80) | -3.12 (-3.32, -2.92) | <0.001 | 23.79 (22.78, 24.83) | 13.29 (12.38, 14.26) | -2.05 (-2.27, -1.83) | <0.001 | 118.09 (115.82, 120.39) | 14.61 (13.64, 15.64) | -6.93 (-7.51, -6.35) | <0.001 | 2.05 (1.77, 2.38) | 0.24 (0.13, 0.40) | -7.13 (-7.74, -6.52) | <0.001 |
| Russian Federation | 6.56 (6.36, 6.76) | 3.40 (3.24, 3.57) | -2.23 (-2.43, -2.04) | <0.001 | 52.77 (52.19, 53.36) | 38.24 (37.69, 38.80) | -1.08 (-1.17, -0.99) | <0.001 | 86.88 (86.15, 87.62) | 12.84 (12.53, 13.16) | -6.50 (-8.08, -4.90) | <0.001 | 1.47 (1.38, 1.57) | 0.19 (0.16, 0.23) | -6.98 (-8.74, -5.18) | <0.001 |
| Rwanda | 110.71 (106.87, 114.67) | 110.30 (107.56, 113.09) | -0.02 (-0.03, -0.00) | 0.010 | 1821.65 (1805.47, 1837.94) | 1846.81 (1835.29, 1858.39) | 0.05 (0.04, 0.06) | <0.001 | 265.56 (259.45, 271.78) | 127.11 (124.11, 130.16) | -2.51 (-2.73, -2.30) | <0.001 | 2.88 (2.27, 3.61) | 0.63 (0.44, 0.89) | -5.11 (-5.37, -4.84) | <0.001 |
| Saint Kitts and Nevis | 3.63 (0.01, 32.24) | 1.07 (0.00, 20.22) | -4.12 (-4.35, -3.90) | <0.001 | 40.96 (16.20, 87.01) | 18.97 (5.53, 48.08) | -2.65 (-2.75, -2.55) | <0.001 | 148.27 (95.36, 221.24) | 24.47 (8.50, 55.95) | -5.92 (-6.82, -5.01) | <0.001 | 2.52 (0.00, 30.34) | 0.38 (0.00, 18.95) | -6.09 (-7.14, -5.03) | <0.001 |
| Saint Lucia | 70.21 (50.29, 96.54) | 68.49 (50.01, 91.72) | -0.08 (-0.09, -0.07) | <0.001 | 1328.08 (1231.99, 1430.40) | 1320.82 (1235.55, 1410.58) | -0.02 (-0.02, -0.01) | <0.001 | 216.97 (178.32, 262.20) | 114.80 (90.74, 143.48) | -2.14 (-2.48, -1.79) | <0.001 | 2.62 (0.13, 13.77) | 0.88 (0.00, 7.62) | -3.64 (-4.20, -3.07) | <0.001 |
| Saint Vincent and the Grenadines | 72.64 (50.38, 102.94) | 70.17 (47.15, 100.67) | -0.12 (-0.13, -0.11) | <0.001 | 1288.33 (1183.86, 1400.54) | 1275.95 (1170.09, 1388.93) | -0.03 (-0.04, -0.02) | <0.001 | 210.58 (169.06, 260.25) | 128.14 (96.18, 167.46) | -1.64 (-2.38, -0.89) | <0.001 | 2.51 (0.07, 15.95) | 1.13 (0.00, 11.36) | -2.62 (-3.83, -1.39) | <0.001 |
| Samoa | 40.91 (26.62, 61.18) | 41.28 (28.50, 58.25) | 0.03 (0.03, 0.04) | <0.001 | 815.05 (745.47, 890.13) | 839.31 (777.23, 905.36) | 0.10 (0.10, 0.11) | <0.001 | 414.95 (364.36, 471.29) | 339.29 (299.66, 383.00) | -0.67 (-0.74, -0.60) | <0.001 | 6.30 (1.55, 18.17) | 5.00 (1.31, 13.56) | -0.77 (-0.86, -0.68) | <0.001 |
| San Marino | 0.45 (0.00, 44.70) | 0.42 (0.00, 40.12) | -0.22 (-0.39, -0.05) | 0.011 | 7.32 (0.03, 57.53) | 7.08 (0.06, 52.21) | -0.11 (-0.14, -0.09) | <0.001 | 8.03 (0.06, 58.84) | 6.50 (0.04, 51.10) | -0.74 (-0.95, -0.53) | <0.001 | 0.13 (0.00, 44.05) | 0.10 (0.00, 39.52) | -0.77 (-1.87, 0.35) | 0.178 |

| | | | | | | | | | | | | | | | | |
|---|---|---|---|---|---|---|---|---|---|---|---|---|---|---|---|---|
| Sao Tome and Principe | 68.66 (46.16, 100.38) | 73.14 (56.45, 93.50) | 0.22 (0.20, 0.23) | <0.001 | 1230.36 (1122.77 , 1346.76) | 1333.91 (1257.43 , 1414.00) | 0.28 (0.27, 0.29) | <0.001 | 135.64 (101.18, 179.53) | 113.43 (92.00, 138.58) | -0.70 (-1.17, -0.23) | 0.004 | 1.28 (0.00, 15.84) | 0.81 (0.00, 6.40) | -1.79 (-2.55, -1.02) | <0.001 |
| Saudi Arabia | 1.68 (1.37, 2.04) | 1.21 (1.06, 1.38) | -1.13 (-1.22, -1.03) | <0.001 | 22.21 (21.07, 23.40) | 16.82 (16.23, 17.43) | -0.96 (-1.00, -0.93) | <0.001 | 76.70 (74.56, 78.89) | 20.51 (19.87, 21.16) | -4.46 (-4.88, -4.04) | <0.001 | 1.28 (1.01, 1.59) | 0.34 (0.26, 0.43) | -4.50 (-5.00, -3.99) | <0.001 |
| Senegal | 73.86 (70.71, 77.13) | 75.61 (73.48, 77.81) | 0.08 (0.06, 0.10) | <0.001 | 1233.62 (1220.16 , 1247.21) | 1277.55 (1268.44 , 1286.71) | 0.11 (0.05, 0.17) | <0.001 | 125.85 (121.62, 130.21) | 90.34 (87.94, 92.80) | -1.17 (-1.37, -0.97) | <0.001 | 1.08 (0.72, 1.58) | 0.47 (0.31, 0.69) | -2.80 (-3.24, -2.36) | <0.001 |
| Serbia | 0.89 (0.61, 1.26) | 1.08 (0.75, 1.53) | 0.66 (0.45, 0.87) | <0.001 | 7.15 (6.30, 8.09) | 8.42 (7.41, 9.54) | 0.52 (0.43, 0.61) | <0.001 | 12.70 (11.56, 13.94) | 3.29 (2.67, 4.02) | -4.58 (-5.10, -4.05) | <0.001 | 0.21 (0.09, 0.43) | 0.05 (0.00, 0.24) | -4.80 (-5.43, -4.17) | <0.001 |
| Seychelles | 41.04 (21.79, 72.37) | 38.62 (21.24, 65.24) | -0.21 (-0.22, -0.20) | <0.001 | 773.55 (677.78, 880.31) | 740.03 (656.38, 831.99) | -0.15 (-0.17, -0.14) | <0.001 | 166.79 (123.51, 221.66) | 58.46 (36.84, 88.94) | -3.52 (-3.77, -3.28) | <0.001 | 2.19 (0.01, 19.91) | 0.38 (0.00, 12.31) | -5.77 (-6.11, -5.42) | <0.001 |
| Sierra Leone | 78.21 (73.63, 83.02) | 82.72 (79.76, 85.77) | 0.19 (0.18, 0.20) | <0.001 | 1216.94 (1198.29 , 1235.81) | 1290.41 (1278.31 , 1302.61) | 0.20 (0.19, 0.21) | <0.001 | 137.53 (131.34, 143.94) | 104.97 (101.55, 108.49) | -0.91 (-0.99, -0.83) | <0.001 | 1.29 (0.75, 2.08) | 0.71 (0.45, 1.07) | -2.06 (-2.35, -1.78) | <0.001 |
| Singapore | 1.21 (0.72, 1.93) | 0.38 (0.17, 0.83) | -4.02 (-4.34, -3.70) | <0.001 | 18.11 (16.03, 20.41) | 10.19 (8.82, 11.76) | -2.03 (-2.21, -1.85) | <0.001 | 41.87 (38.69, 45.25) | 4.99 (4.07, 6.11) | -7.12 (-7.87, -6.36) | <0.001 | 0.70 (0.35, 1.29) | 0.07 (0.01, 0.42) | -7.60 (-8.35, -6.83) | <0.001 |
| Slovakia | 1.38 (0.93, 1.98) | 1.54 (1.05, 2.23) | 0.39 (0.28, 0.49) | <0.001 | 12.68 (11.20, 14.30) | 14.15 (12.51, 15.98) | 0.35 (0.29, 0.41) | <0.001 | 17.61 (15.87, 19.49) | 6.87 (5.75, 8.18) | -3.25 (-3.67, -2.82) | <0.001 | 0.30 (0.11, 0.65) | 0.11 (0.01, 0.46) | -3.47 (-4.15, -2.78) | <0.001 |
| Slovenia | 0.92 (0.39, 1.90) | 0.91 (0.34, 2.13) | -0.09 (-0.37, 0.18) | 0.506 | 6.63 (4.97, 8.71) | 7.01 (5.11, 9.51) | 0.16 (0.08, 0.24) | <0.001 | 16.49 (13.83, 19.56) | 5.07 (3.49, 7.25) | -4.02 (-5.07, -2.97) | <0.001 | 0.28 (0.04, 1.02) | 0.08 (0.00, 1.00) | -4.49 (-4.84, -4.15) | <0.001 |
| Solomon Islands | 47.79 (36.19, 62.36) | 48.14 (40.04, 57.46) | 0.03 (0.02, 0.05) | <0.001 | 771.72 (722.83, 823.40) | 785.44 (751.64, 820.43) | 0.06 (0.05, 0.07) | <0.001 | 1136.09 (1073.64 , | 864.51 (828.30, 901.93) | -0.95 (-1.09, -0.80) | <0.001 | 18.77 (11.45, 29.35) | 14.07 (9.77, 19.66) | -1.00 (-1.14, -0.86) | <0.001 |

| | | | | | | | | | | | | | | | | |
|---|---|---|---|---|---|---|---|---|---|---|---|---|---|---|---|---|
| | | | | | | | | | 1201.48) | | | | | | | |
| Somalia | 119.46 (115.27, 123.78) | 127.31 (124.86, 129.79) | 0.21 (0.19, 0.24) | <0.001 | 1874.55 (1857.28 , 1891.95) | 1977.47 (1967.38 , 1987.61) | 0.18 (0.17, 0.19) | <0.001 | 251.24 (245.02, 257.59) | 188.08 (185.02, 191.18) | -0.99 (-1.10, -0.88) | <0.001 | 2.61 (2.01, 3.35) | 1.51 (1.24, 1.81) | -1.87 (-2.03, -1.72) | <0.001 |
| South Africa | 99.23 (97.71, 100.77) | 99.91 (98.62, 101.21) | 0.02 (0.02, 0.03) | <0.001 | 1728.18 (1721.67 , 1734.72) | 1755.31 (1749.99 , 1760.65) | 0.05 (0.03, 0.07) | <0.001 | 236.02 (233.58, 238.48) | 119.46 (118.08, 120.85) | -2.38 (-3.03, -1.73) | <0.001 | 2.61 (2.35, 2.88) | 0.61 (0.52, 0.72) | -4.92 (-6.15, -3.68) | <0.001 |
| South Sudan | 88.15 (84.44, 92.01) | 91.82 (88.67, 95.07) | 0.14 (0.13, 0.15) | <0.001 | 1674.24 (1657.11 , 1691.52) | 1755.51 (1740.86 , 1770.27) | 0.16 (0.15, 0.18) | <0.001 | 155.83 (150.67, 161.13) | 123.76 (119.93, 127.70) | -0.74 (-0.91, -0.57) | <0.001 | 1.24 (0.81, 1.83) | 0.65 (0.41, 1.01) | -2.08 (-2.47, -1.68) | <0.001 |
| Spain | 0.77 (0.63, 0.93) | 0.40 (0.30, 0.52) | -2.27 (-2.50, -2.04) | <0.001 | 8.68 (8.21, 9.17) | 6.73 (6.29, 7.20) | -0.88 (-0.95, -0.82) | <0.001 | 25.50 (24.69, 26.34) | 5.66 (5.26, 6.07) | -5.14 (-5.56, -4.72) | <0.001 | 0.43 (0.33, 0.55) | 0.09 (0.05, 0.16) | -5.26 (-5.95, -4.56) | <0.001 |
| Sri Lanka | 2.70 (2.33, 3.11) | 1.71 (1.44, 2.02) | -1.55 (-1.71, -1.39) | <0.001 | 27.92 (26.72, 29.17) | 24.16 (23.10, 25.25) | -0.48 (-0.57, -0.40) | <0.001 | 81.95 (79.89, 84.06) | 18.44 (17.51, 19.40) | -4.95 (-5.88, -4.02) | <0.001 | 1.34 (1.09, 1.64) | 0.28 (0.18, 0.43) | -5.18 (-6.10, -4.24) | <0.001 |
| Sudan | 53.81 (52.18, 55.49) | 54.60 (53.51, 55.71) | 0.05 (0.03, 0.07) | <0.001 | 767.25 (760.84, 773.70) | 806.58 (802.27, 810.91) | 0.19 (0.13, 0.24) | <0.001 | 183.35 (180.21, 186.53) | 82.06 (80.68, 83.45) | -2.72 (-2.76, -2.68) | <0.001 | 2.44 (2.09, 2.85) | 0.73 (0.60, 0.87) | -4.07 (-4.24, -3.90) | <0.001 |
| Suriname | 69.05 (56.96, 83.29) | 67.72 (57.15, 79.69) | -0.07 (-0.08, -0.05) | <0.001 | 1202.76 (1148.69 , 1258.96) | 1210.74 (1164.63 , 1258.22) | 0.02 (0.01, 0.03) | 0.011 | 151.64 (132.65, 172.85) | 102.05 (88.99, 116.52) | -1.29 (-2.06, -0.51) | 0.001 | 1.58 (0.23, 5.76) | 0.74 (0.06, 3.11) | -2.45 (-3.90, -0.98) | 0.001 |
| Sweden | 0.29 (0.13, 0.56) | 0.26 (0.11, 0.51) | -0.36 (-0.71, -0.01) | 0.045 | 5.21 (4.42, 6.11) | 4.86 (4.13, 5.70) | -0.27 (-0.38, -0.16) | <0.001 | 5.02 (4.25, 5.90) | 1.72 (1.30, 2.24) | -3.63 (-4.12, -3.14) | <0.001 | 0.08 (0.01, 0.27) | 0.02 (0.00, 0.18) | -3.97 (-4.66, -3.28) | <0.001 |
| Switzerland | 0.56 (0.32, 0.95) | 0.46 (0.25, 0.81) | -0.69 (-0.94, -0.44) | <0.001 | 9.64 (8.49, 10.92) | 9.01 (7.93, 10.22) | -0.27 (-0.36, -0.18) | <0.001 | 8.05 (7.01, 9.21) | 1.67 (1.23, 2.24) | -5.45 (-5.92, -4.97) | <0.001 | 0.13 (0.03, 0.39) | 0.02 (0.00, 0.22) | -6.28 (-8.20, -4.33) | <0.001 |
| Syrian Arab | 44.10 | 45.27 | 0.09 | <0.001 | 719.88 | 749.82 | 0.14 | <0.001 | 278.15 | 63.36 | -5.00 | <0.001 | 4.09 | 0.45 | -7.33 | <0.001 |

| | | | | | | | | | | | | | | | | |
|---|---|---|---|---|---|---|---|---|---|---|---|---|---|---|---|---|
| Republic | (42.23, 46.03) | (43.41, 47.19) | (0.08, 0.11) | | (711.94, 727.91) | (741.93, 757.79) | (0.13, 0.15) | | (273.14, 283.22) | (61.09, 65.71) | (-5.45, -4.55) | | (3.50, 4.77) | (0.28, 0.71) | (-7.92, -6.74) | |
| Taiwan (Province of China) | 2.48 (2.17, 2.82) | 2.32 (2.01, 2.67) | -0.23 (-0.32, -0.13) | <0.001 | 24.82 (23.82, 25.86) | 35.54 (34.25, 36.86) | 1.24 (1.10, 1.38) | <0.001 | 36.88 (35.66, 38.14) | 6.37 (5.84, 6.95) | -5.84 (-6.50, -5.18) | <0.001 | 0.60 (0.46, 0.79) | 0.08 (0.03, 0.18) | -6.82 (-7.42, -6.22) | <0.001 |
| Tajikistan | 62.23 (58.95, 65.67) | 64.73 (62.29, 67.26) | 0.14 (0.10, 0.17) | <0.001 | 1088.61 (1074.25, 1103.13) | 1108.45 (1098.19, 1118.79) | 0.06 (0.04, 0.09) | <0.001 | 402.49 (393.52, 411.62) | 154.44 (150.60, 158.36) | -3.20 (-3.56, -2.83) | <0.001 | 5.98 (4.92, 7.23) | 1.74 (1.35, 2.21) | -4.05 (-4.53, -3.58) | <0.001 |
| Thailand | 41.90 (41.12, 42.70) | 39.12 (38.31, 39.93) | -0.24 (-0.29, -0.20) | <0.001 | 699.27 (696.04, 702.52) | 682.13 (678.83, 685.44) | -0.09 (-0.11, -0.06) | <0.001 | 163.89 (162.31, 165.48) | 44.02 (43.18, 44.86) | -4.46 (-4.89, -4.04) | <0.001 | 2.23 (2.04, 2.42) | 0.19 (0.14, 0.25) | -8.25 (-8.98, -7.53) | <0.001 |
| Timor-Leste | 48.07 (40.78, 56.39) | 47.90 (42.19, 54.27) | -0.01 (-0.03, -0.00) | 0.034 | 735.02 (705.21, 765.85) | 762.23 (737.97, 787.17) | 0.13 (0.12, 0.13) | <0.001 | 191.28 (176.06, 207.54) | 142.22 (131.88, 153.26) | -0.89 (-1.66, -0.12) | 0.024 | 2.64 (1.13, 5.34) | 1.74 (0.76, 3.53) | -1.27 (-2.27, -0.25) | 0.015 |
| Togo | 77.17 (72.62, 81.97) | 80.79 (77.73, 83.95) | 0.16 (0.14, 0.17) | <0.001 | 1212.21 (1193.32, 1231.34) | 1277.49 (1265.01, 1290.06) | 0.18 (0.17, 0.19) | <0.001 | 138.46 (132.18, 145.00) | 96.37 (92.97, 99.87) | -1.25 (-1.37, -1.13) | <0.001 | 1.31 (0.76, 2.14) | 0.58 (0.34, 0.92) | -2.78 (-3.09, -2.48) | <0.001 |
| Tokelau | 11.41 (0.00, 841.94) | 7.80 (0.00, 902.29) | -1.31 (-1.41, -1.20) | <0.001 | 101.24 (0.21, 996.62) | 94.65 (0.04, 1060.58) | -0.25 (-0.30, -0.19) | <0.001 | 488.09 (85.89, 1611.91) | 272.05 (14.43, 1356.55) | -2.00 (-2.07, -1.92) | <0.001 | 8.15 (0.00, 835.98) | 4.46 (0.00, 895.69) | -2.06 (-2.14, -1.98) | <0.001 |
| Tonga | 50.82 (29.90, 82.91) | 50.70 (30.67, 79.58) | 0.01 (-0.06, 0.08) | 0.767 | 1188.96 (1075.59, 1312.45) | 1211.34 (1102.63, 1328.32) | 0.10 (0.03, 0.17) | 0.005 | 199.33 (153.25, 256.30) | 149.48 (112.74, 194.87) | -0.97 (-1.12, -0.82) | <0.001 | 2.41 (0.02, 19.64) | 1.52 (0.00, 14.14) | -1.52 (-1.60, -1.45) | <0.001 |
| Trinidad and Tobago | 68.87 (61.83, 76.53) | 65.73 (58.58, 73.54) | -0.17 (-0.19, -0.14) | <0.001 | 1272.43 (1241.31, 1304.16) | 1270.08 (1238.99, 1301.79) | -0.01 (-0.02, 0.01) | 0.532 | 185.69 (173.91, 198.09) | 100.52 (91.96, 109.72) | -2.09 (-2.61, -1.57) | <0.001 | 2.09 (1.01, 3.84) | 0.67 (0.16, 1.94) | -3.87 (-4.76, -2.97) | <0.001 |
| Tunisia | 1.16 (0.81, 1.62) | 0.87 (0.62, 1.19) | -0.98 (-1.07, -0.88) | <0.001 | 17.40 (15.99, 18.91) | 14.09 (13.01, 15.24) | -0.73 (-0.77, -0.68) | <0.001 | 44.80 (42.52, 47.18) | 14.21 (13.13, 15.35) | -3.90 (-4.05, -3.75) | <0.001 | 0.73 (0.47, 1.11) | 0.23 (0.11, 0.42) | -3.96 (-4.18, -3.74) | <0.001 |

| | | | | | | | | | | | | | | | | |
|---|---|---|---|---|---|---|---|---|---|---|---|---|---|---|---|---|
| Turkey | 1.00 (0.88, 1.14) | 0.79 (0.69, 0.88) | -0.80 (-0.93, -0.66) | <0.001 | 14.63 (14.14, 15.13) | 13.70 (13.31, 14.09) | -0.22 (-0.29, -0.14) | <0.001 | 17.50 (16.96, 18.05) | 4.94 (4.71, 5.18) | -4.25 (-4.72, -3.78) | <0.001 | 0.28 (0.22, 0.36) | 0.07 (0.05, 0.11) | -4.56 (-5.51, -3.61) | <0.001 |
| Turkmenistan | 61.38 (57.56, 65.40) | 59.49 (56.15, 62.98) | -0.11 (-0.12, -0.10) | <0.001 | 1060.06 (1043.62, 1076.71) | 1054.88 (1040.81, 1069.10) | -0.02 (-0.04, 0.01) | 0.169 | 318.53 (309.46, 327.81) | 166.24 (160.69, 171.95) | -2.22 (-3.16, -1.27) | <0.001 | 4.47 (3.44, 5.73) | 1.92 (1.37, 2.63) | -2.86 (-3.97, -1.73) | <0.001 |
| Tuvalu | 16.73 (0.05, 140.49) | 10.41 (0.01, 109.08) | -1.63 (-1.69, -1.56) | <0.001 | 109.87 (29.42, 286.71) | 94.75 (28.01, 238.44) | -0.52 (-0.57, -0.47) | <0.001 | 790.14 (525.97, 1142.36) | 431.43 (263.84, 669.94) | -2.06 (-2.17, -1.95) | <0.001 | 13.26 (0.01, 134.07) | 7.14 (0.00, 103.00) | -2.11 (-2.22, -2.00) | <0.001 |
| Uganda | 122.01 (119.37, 124.71) | 118.76 (117.12, 120.42) | -0.09 (-0.11, -0.08) | <0.001 | 1974.98 (1963.76, 1986.27) | 2188.29 (2180.89, 2195.71) | 0.38 (0.33, 0.42) | <0.001 | 156.70 (153.58, 159.88) | 140.13 (138.28, 142.01) | -0.40 (-0.64, -0.16) | 0.001 | 1.02 (0.78, 1.32) | 0.57 (0.46, 0.71) | -2.04 (-2.41, -1.66) | <0.001 |
| Ukraine | 2.93 (2.70, 3.17) | 2.57 (2.33, 2.83) | -0.41 (-0.68, -0.14) | 0.003 | 24.41 (23.72, 25.11) | 23.77 (22.99, 24.57) | -0.01 (-0.27, 0.25) | 0.940 | 23.08 (22.42, 23.77) | 27.37 (26.54, 28.23) | 0.54 (-1.84, 2.98) | 0.658 | 0.38 (0.30, 0.47) | 0.45 (0.35, 0.57) | 0.65 (-1.30, 2.63) | 0.516 |
| United Arab Emirates | 37.31 (33.21, 41.85) | 38.64 (36.05, 41.39) | 0.12 (0.11, 0.13) | <0.001 | 712.16 (694.83, 729.89) | 718.88 (708.50, 729.39) | 0.03 (0.01, 0.06) | 0.006 | 77.79 (72.25, 83.72) | 54.73 (52.01, 57.58) | -1.20 (-1.39, -1.02) | <0.001 | 0.74 (0.31, 1.68) | 0.35 (0.18, 0.68) | -2.55 (-2.91, -2.19) | <0.001 |
| United Kingdom | 0.51 (0.42, 0.61) | 0.38 (0.30, 0.47) | -0.96 (-1.12, -0.79) | <0.001 | 8.01 (7.63, 8.41) | 7.02 (6.67, 7.38) | -0.44 (-0.50, -0.38) | <0.001 | 8.37 (7.99, 8.77) | 3.57 (3.33, 3.82) | -2.92 (-3.26, -2.57) | <0.001 | 0.14 (0.09, 0.20) | 0.06 (0.03, 0.10) | -2.88 (-3.35, -2.40) | <0.001 |
| United Republic of Tanzania | 108.19 (106.16, 110.25) | 111.36 (110.00, 112.73) | 0.11 (0.07, 0.14) | <0.001 | 1810.44 (1801.74, 1819.17) | 1913.06 (1907.20, 1918.93) | 0.19 (0.16, 0.21) | <0.001 | 154.93 (152.43, 157.47) | 122.75 (121.28, 124.24) | -0.80 (-0.87, -0.73) | <0.001 | 1.10 (0.90, 1.34) | 0.50 (0.42, 0.61) | -2.68 (-2.87, -2.49) | <0.001 |
| United States of America | 1.94 (1.85, 2.02) | 1.30 (1.23, 1.36) | -1.37 (-1.45, -1.29) | <0.001 | 29.36 (29.03, 29.69) | 21.17 (20.91, 21.44) | -1.11 (-1.15, -1.06) | <0.001 | 17.14 (16.89, 17.39) | 6.56 (6.41, 6.71) | -3.23 (-3.67, -2.79) | <0.001 | 0.27 (0.24, 0.30) | 0.09 (0.07, 0.11) | -3.61 (-4.87, -2.34) | <0.001 |
| United States Virgin Islands | 1.53 (0.00, 13.08) | 1.17 (0.00, 15.40) | -0.95 (-1.07, -0.83) | <0.001 | 25.34 (12.13, 47.05) | 20.91 (7.98, 45.06) | -0.67 (-0.78, -0.55) | <0.001 | 59.20 (37.71, 88.88) | 20.37 (7.70, 44.23) | -3.60 (-4.55, -2.65) | <0.001 | 0.96 (0.00, 12.07) | 0.33 (0.00, 13.83) | -3.77 (-4.36, -3.17) | <0.001 |

| | | | | | | | | | | | | | | | | |
|---|---|---|---|---|---|---|---|---|---|---|---|---|---|---|---|---|
| Uruguay | 1.65 (0.98, 2.59) | 1.66 (1.02, 2.56) | 0.05 (-0.07, 0.16) | 0.428 | 22.14 (19.48, 25.06) | 23.89 (21.23, 26.81) | 0.28 (0.23, 0.34) | <0.001 | 29.10 (26.03, 32.42) | 14.57 (12.52, 16.88) | -2.35 (-2.80, -1.89) | <0.001 | 0.48 (0.17, 1.09) | 0.23 (0.04, 0.71) | -2.48 (-2.85, -2.11) | <0.001 |
| Uzbekistan | 61.69 (60.06, 63.36) | 63.45 (62.14, 64.78) | 0.10 (0.09, 0.11) | <0.001 | 1040.83 (1033.93, 1047.76) | 1109.19 (1103.73, 1114.67) | 0.22 (0.20, 0.24) | <0.001 | 453.15 (448.54, 457.80) | 206.30 (203.95, 208.67) | -2.61 (-3.45, -1.76) | <0.001 | 6.82 (6.26, 7.42) | 2.60 (2.34, 2.87) | -3.19 (-4.02, -2.36) | <0.001 |
| Vanuatu | 47.84 (31.66, 69.94) | 48.26 (36.49, 62.83) | 0.03 (0.02, 0.05) | <0.001 | 868.67 (794.09, 948.79) | 877.58 (824.66, 933.18) | 0.04 (0.02, 0.06) | <0.001 | 706.68 (638.15, 780.91) | 702.61 (654.83, 753.11) | -0.05 (-0.27, 0.18) | 0.696 | 11.23 (4.15, 24.87) | 11.06 (5.80, 19.34) | -0.08 (-0.31, 0.15) | 0.498 |
| Venezuela (Bolivarian Republic of) | 1.87 (1.57, 2.20) | 1.28 (1.07, 1.51) | -1.30 (-1.39, -1.20) | <0.001 | 27.16 (26.01, 28.35) | 20.45 (19.60, 21.32) | -0.97 (-1.07, -0.87) | <0.001 | 64.57 (62.79, 66.39) | 18.11 (17.31, 18.93) | -4.28 (-5.39, -3.16) | <0.001 | 1.06 (0.85, 1.33) | 0.29 (0.19, 0.41) | -4.39 (-5.50, -3.27) | <0.001 |
| Viet Nam | 3.37 (3.14, 3.60) | 2.46 (2.31, 2.62) | -1.08 (-1.20, -0.96) | <0.001 | 32.63 (31.94, 33.33) | 31.00 (30.45, 31.55) | -0.16 (-0.26, -0.06) | 0.001 | 80.99 (79.90, 82.09) | 26.60 (26.10, 27.11) | -3.76 (-3.87, -3.65) | <0.001 | 1.36 (1.22, 1.52) | 0.43 (0.37, 0.50) | -3.88 (-4.09, -3.67) | <0.001 |
| Yemen | 63.72 (61.44, 66.07) | 66.03 (64.65, 67.44) | 0.12 (0.03, 0.20) | 0.008 | 880.91 (872.13, 889.76) | 946.10 (940.77, 951.46) | 0.24 (0.18, 0.30) | <0.001 | 162.06 (158.25, 165.94) | 101.46 (99.70, 103.23) | -1.59 (-1.79, -1.38) | <0.001 | 2.06 (1.64, 2.55) | 0.96 (0.80, 1.15) | -2.57 (-2.87, -2.27) | <0.001 |
| Zambia | 110.90 (107.23, 114.68) | 114.13 (111.79, 116.52) | 0.09 (0.08, 0.11) | <0.001 | 1881.94 (1865.89, 1898.11) | 1878.97 (1869.08, 1888.90) | -0.01 (-0.07, 0.05) | 0.731 | 188.91 (183.93, 194.01) | 134.81 (132.19, 137.48) | -1.16 (-1.28, -1.05) | <0.001 | 1.59 (1.16, 2.15) | 0.73 (0.55, 0.95) | -2.65 (-2.90, -2.40) | <0.001 |
| Zimbabwe | 95.83 (92.83, 98.91) | 99.99 (97.55, 102.48) | 0.15 (0.13, 0.18) | <0.001 | 1535.52 (1522.92, 1548.21) | 1587.62 (1577.61, 1597.68) | 0.12 (0.11, 0.13) | <0.001 | 280.87 (275.64, 286.18) | 311.68 (307.32, 316.08) | 0.35 (-0.24, 0.95) | 0.244 | 3.29 (2.75, 3.93) | 3.75 (3.28, 4.26) | 0.45 (-0.33, 1.23) | 0.259 |
| **Women** | | | | | | | | | | | | | | | | |
| Afghanistan | 59.24 (55.82, 62.86) | 60.46 (58.66, 62.30) | 0.07 (0.05, 0.09) | <0.001 | 768.75 (755.67, 782.03) | 787.29 (780.49, 794.13) | 0.08 (0.07, 0.10) | <0.001 | 454.88 (444.61, 465.35) | 211.97 (208.34, 215.67) | -2.59 (-2.76, -2.42) | <0.001 | 7.05 (5.81, 8.51) | 2.99 (2.56, 3.47) | -2.91 (-3.10, -2.71) | <0.001 |
| Albania | 55.15 (49.82, | 52.43 (46.03, | -0.17 (-0.18, | <0.001 | 1142.65 (1117.50 | 1141.96 (1111.49 | 0.00 (-0.00, | 0.792 | 127.14 (118.79, | 66.68 (59.47, | -2.26 (-2.52, | <0.001 | 1.24 (0.54, | 0.21 (0.00, | -6.13 (-6.78, | <0.001 |

| | | | | | | | | | | | | | | | | |
|---|---|---|---|---|---|---|---|---|---|---|---|---|---|---|---|---|
| | 60.94) | 59.50) | -0.16) | | , 1168.26) | , 1173.09) | 0.01) | | 135.95) | 74.54) | -1.99) | | 2.47) | 1.25) | -5.49) | |
| Algeria | 34.85 (33.23, 36.54) | 35.21 (33.92, 36.53) | 0.03 (-0.01, 0.07) | 0.145 | 521.16 (514.55, 527.84) | 591.70 (586.54, 596.90) | 0.44 (0.35, 0.52) | <0.001 | 140.61 (137.17, 144.12) | 49.91 (48.42, 51.43) | -3.52 (-3.70, -3.34) | <0.001 | 1.94 (1.55, 2.41) | 0.37 (0.25, 0.52) | -5.58 (-5.71, -5.44) | <0.001 |
| American Samoa | 37.88 (9.82, 105.88) | 39.77 (11.07, 106.54) | 0.17 (0.16, 0.18) | <0.001 | 942.33 (760.49, 1158.03) | 968.53 (786.21, 1183.50) | 0.10 (0.09, 0.11) | <0.001 | 281.82 (185.53, 413.93) | 264.25 (171.66, 391.79) | -0.16 (-0.35, 0.03) | 0.106 | 3.97 (0.00, 54.81) | 3.67 (0.00, 52.37) | -0.21 (-0.44, 0.02) | 0.072 |
| Andorra | 0.42 (0.00, 43.48) | 0.36 (0.00, 40.17) | -0.55 (-0.68, -0.41) | <0.001 | 7.77 (0.13, 55.51) | 7.26 (0.12, 51.57) | -0.24 (-0.25, -0.22) | <0.001 | 9.67 (0.37, 58.18) | 4.75 (0.02, 47.25) | -2.42 (-2.60, -2.24) | <0.001 | 0.16 (0.00, 43.03) | 0.07 (0.00, 39.71) | -2.59 (-2.91, -2.27) | <0.001 |
| Angola | 100.60 (96.27, 105.09) | 100.07 (97.58, 102.62) | -0.02 (-0.03, -0.00) | 0.015 | 1812.43 (1793.34, 1831.69) | 1873.19 (1861.99, 1884.45) | 0.12 (0.10, 0.14) | <0.001 | 248.46 (241.30, 255.78) | 135.41 (132.38, 138.50) | -2.02 (-2.47, -1.57) | <0.001 | 2.76 (2.04, 3.66) | 0.80 (0.58, 1.08) | -4.19 (-4.85, -3.53) | <0.001 |
| Antigua and Barbuda | 64.14 (28.66, 125.38) | 62.24 (30.48, 113.85) | -0.10 (-0.11, -0.09) | <0.001 | 1384.55 (1191.23, 1601.37) | 1380.80 (1212.33, 1567.07) | -0.01 (-0.01, 0.00) | 0.261 | 171.47 (108.12, 259.64) | 97.39 (56.83, 157.02) | -1.97 (-2.18, -1.76) | <0.001 | 1.79 (0.00, 34.67) | 0.53 (0.00, 25.09) | -4.18 (-4.74, -3.62) | <0.001 |
| Argentina | 49.67 (47.94, 51.45) | 49.83 (48.35, 51.35) | 0.01 (0.00, 0.02) | 0.057 | 1033.13 (1025.10, 1041.20) | 1061.78 (1054.98, 1068.61) | 0.09 (0.08, 0.10) | <0.001 | 94.88 (92.46, 97.36) | 61.63 (60.00, 63.29) | -1.50 (-1.62, -1.37) | <0.001 | 0.78 (0.58, 1.04) | 0.19 (0.11, 0.31) | -4.85 (-5.56, -4.13) | <0.001 |
| Armenia | 58.70 (53.17, 64.68) | 55.10 (48.61, 62.31) | -0.22 (-0.25, -0.20) | <0.001 | 1303.91 (1277.72, 1330.52) | 1245.99 (1216.19, 1276.44) | -0.16 (-0.17, -0.14) | <0.001 | 201.37 (191.16, 212.00) | 95.72 (87.71, 104.39) | -2.53 (-3.23, -1.82) | <0.001 | 2.40 (1.40, 3.85) | 0.62 (0.16, 1.92) | -4.56 (-5.94, -3.16) | <0.001 |
| Australia | 1.44 (1.07, 1.90) | 1.28 (0.97, 1.68) | -0.37 (-0.53, -0.21) | <0.001 | 29.40 (27.60, 31.28) | 28.75 (27.15, 30.44) | -0.05 (-0.15, 0.05) | 0.302 | 19.63 (18.17, 21.17) | 9.96 (9.05, 10.95) | -2.31 (-3.01, -1.61) | <0.001 | 0.30 (0.15, 0.56) | 0.15 (0.06, 0.33) | -2.41 (-3.31, -1.50) | <0.001 |
| Austria | 1.06 (0.60, 1.77) | 0.43 (0.16, 0.99) | -2.96 (-3.32, -2.60) | <0.001 | 15.36 (13.41, 17.53) | 7.50 (6.13, 9.13) | -2.45 (-2.74, -2.17) | <0.001 | 8.35 (6.94, 9.98) | 3.47 (2.56, 4.63) | -2.98 (-3.90, -2.06) | <0.001 | 0.13 (0.01, 0.52) | 0.05 (0.00, 0.45) | -3.26 (-5.37, -1.10) | 0.003 |

| | | | | | | | | | | | | | | | | |
|---|---|---|---|---|---|---|---|---|---|---|---|---|---|---|---|---|
| Azerbaijan | 58.14 (54.48, 62.00) | 55.84 (52.51, 59.36) | -0.15 (-0.19, -0.11) | <0.001 | 1164.83 (1148.11, 1181.76) | 1145.26 (1130.64, 1160.05) | -0.06 (-0.08, -0.05) | <0.001 | 213.84 (206.68, 221.21) | 105.78 (101.37, 110.35) | -2.41 (-2.91, -1.91) | <0.001 | 2.67 (1.92, 3.64) | 0.85 (0.50, 1.38) | -3.96 (-4.76, -3.15) | <0.001 |
| Bahamas | 67.34 (48.33, 91.88) | 67.82 (50.46, 89.34) | 0.03 (-0.01, 0.06) | 0.095 | 1346.97 (1255.15, 1444.12) | 1364.42 (1282.85, 1449.93) | 0.05 (0.03, 0.06) | <0.001 | 153.20 (123.06, 188.89) | 108.06 (86.07, 134.09) | -1.34 (-1.53, -1.16) | <0.001 | 1.53 (0.02, 10.24) | 0.74 (0.00, 6.62) | -2.69 (-3.18, -2.19) | <0.001 |
| Bahrain | 1.24 (0.05, 6.78) | 1.07 (0.19, 3.67) | -0.49 (-0.59, -0.40) | <0.001 | 20.79 (12.72, 32.42) | 18.42 (13.36, 24.93) | -0.43 (-0.47, -0.39) | <0.001 | 35.06 (24.23, 49.38) | 13.80 (9.32, 19.82) | -3.23 (-4.52, -1.92) | <0.001 | 0.53 (0.00, 5.59) | 0.20 (0.00, 2.38) | -3.41 (-4.69, -2.11) | <0.001 |
| Bangladesh | 87.82 (86.59, 89.08) | 77.18 (76.28, 78.10) | -0.46 (-0.52, -0.39) | <0.001 | 1075.94 (1071.29, 1080.59) | 1042.31 (1038.93, 1045.70) | -0.13 (-0.18, -0.07) | <0.001 | 216.29 (214.26, 218.34) | 127.82 (126.64, 129.01) | -1.77 (-2.48, -1.05) | <0.001 | 2.68 (2.46, 2.92) | 1.28 (1.16, 1.40) | -2.46 (-3.40, -1.51) | <0.001 |
| Barbados | 66.12 (46.30, 91.72) | 65.84 (44.91, 93.38) | -0.01 (-0.03, -0.00) | 0.029 | 1351.29 (1256.19, 1451.86) | 1374.47 (1273.52, 1481.56) | 0.06 (0.05, 0.06) | <0.001 | 152.20 (121.42, 188.59) | 105.05 (78.64, 137.82) | -1.24 (-1.61, -0.87) | <0.001 | 1.46 (0.02, 9.94) | 0.67 (0.00, 9.50) | -2.64 (-3.49, -1.77) | <0.001 |
| Belarus | 4.92 (4.02, 5.98) | 2.68 (1.98, 3.63) | -2.06 (-2.20, -1.91) | <0.001 | 34.35 (31.86, 36.99) | 24.71 (22.32, 27.34) | -1.12 (-1.31, -0.94) | <0.001 | 78.78 (74.99, 82.73) | 14.48 (12.69, 16.49) | -6.06 (-7.49, -4.61) | <0.001 | 1.35 (0.89, 1.97) | 0.23 (0.06, 0.70) | -6.29 (-7.74, -4.81) | <0.001 |
| Belgium | 0.46 (0.21, 0.91) | 0.45 (0.20, 0.92) | -0.02 (-0.37, 0.32) | 0.890 | 9.22 (7.88, 10.74) | 9.48 (8.09, 11.05) | 0.05 (-0.09, 0.18) | 0.494 | 3.95 (3.10, 4.97) | 2.83 (2.11, 3.75) | -1.21 (-1.58, -0.85) | <0.001 | 0.06 (0.00, 0.35) | 0.04 (0.00, 0.33) | -1.34 (-2.11, -0.55) | 0.001 |
| Belize | 73.24 (48.47, 108.39) | 71.50 (55.39, 91.06) | -0.08 (-0.09, -0.06) | <0.001 | 1355.95 (1234.81, 1487.06) | 1375.30 (1299.59, 1454.42) | 0.05 (0.04, 0.06) | <0.001 | 137.68 (100.74, 185.27) | 112.62 (91.64, 137.11) | -0.70 (-1.04, -0.37) | <0.001 | 1.23 (0.00, 17.28) | 0.79 (0.00, 6.08) | -1.52 (-2.23, -0.81) | <0.001 |
| Benin | 75.06 (69.65, 80.82) | 76.96 (73.62, 80.43) | 0.09 (0.08, 0.10) | <0.001 | 1216.50 (1193.90, 1239.46) | 1272.52 (1258.37, 1286.80) | 0.16 (0.14, 0.17) | <0.001 | 147.07 (139.33, 155.17) | 98.04 (94.16, 102.05) | -1.37 (-1.44, -1.31) | <0.001 | 1.44 (0.77, 2.52) | 0.61 (0.34, 1.03) | -2.93 (-3.10, -2.77) | <0.001 |

| | | | | | | | | | | | | | | | | |
|---|---|---|---|---|---|---|---|---|---|---|---|---|---|---|---|---|
| Bermuda | 1.68 (0.00, 43.73) | 0.94 (0.00, 54.20) | -1.97 (-2.10, -1.85) | <0.001 | 28.88 (7.50, 83.73) | 19.85 (2.09, 84.30) | -1.31 (-1.36, -1.25) | <0.001 | 46.07 (16.85, 107.43) | 10.24 (0.21, 69.48) | -5.59 (-6.10, -5.07) | <0.001 | 0.74 (0.00, 42.25) | 0.15 (0.00, 52.87) | -6.06 (-6.77, -5.35) | <0.001 |
| Bhutan | 78.12 (63.22, 95.94) | 73.35 (60.90, 87.64) | -0.21 (-0.23, -0.18) | <0.001 | 1027.93 (968.53, 1090.33) | 1015.86 (967.89, 1065.63) | -0.04 (-0.05, -0.03) | <0.001 | 524.16 (481.23, 570.16) | 191.67 (171.09, 214.09) | -3.42 (-3.69, -3.15) | <0.001 | 8.10 (3.55, 16.19) | 2.47 (0.68, 6.35) | -4.03 (-4.33, -3.72) | <0.001 |
| Bolivia (Plurinational State of) | 78.33 (73.59, 83.33) | 78.03 (74.55, 81.62) | -0.01 (-0.03, 0.01) | 0.259 | 1296.52 (1276.43, 1316.87) | 1336.01 (1321.42, 1350.72) | 0.10 (0.09, 0.12) | <0.001 | 215.92 (207.66, 224.43) | 98.46 (94.52, 102.53) | -2.69 (-2.79, -2.58) | <0.001 | 2.62 (1.78, 3.75) | 0.60 (0.33, 1.01) | -4.95 (-5.06, -4.85) | <0.001 |
| Bosnia and Herzegovina | 0.85 (0.37, 1.70) | 0.99 (0.35, 2.34) | 0.60 (0.33, 0.87) | <0.001 | 7.05 (5.45, 8.98) | 8.32 (6.06, 11.22) | 0.58 (0.43, 0.73) | <0.001 | 13.97 (11.69, 16.58) | 3.53 (2.12, 5.59) | -4.67 (-5.31, -4.02) | <0.001 | 0.23 (0.03, 0.82) | 0.05 (0.00, 0.98) | -4.91 (-5.84, -3.97) | <0.001 |
| Botswana | 100.46 (89.14, 113.00) | 97.68 (89.36, 106.59) | -0.10 (-0.11, -0.09) | <0.001 | 1686.45 (1637.25, 1736.89) | 1683.39 (1648.64, 1718.71) | -0.01 (-0.05, 0.03) | 0.557 | 216.65 (198.94, 235.65) | 131.18 (121.62, 141.31) | -1.71 (-1.89, -1.53) | <0.001 | 2.30 (0.81, 5.35) | 0.86 (0.26, 2.11) | -3.31 (-3.70, -2.92) | <0.001 |
| Brazil | 77.15 (76.20, 78.10) | 77.92 (77.07, 78.78) | 0.04 (-0.02, 0.10) | 0.206 | 1508.33 (1504.04, 1512.63) | 1554.50 (1550.78, 1558.22) | 0.11 (0.10, 0.11) | <0.001 | 169.00 (167.56, 170.45) | 100.78 (99.84, 101.73) | -1.76 (-1.89, -1.63) | <0.001 | 1.64 (1.50, 1.79) | 0.47 (0.41, 0.53) | -4.22 (-4.57, -3.89) | <0.001 |
| Brunei Darussalam | 2.01 (0.07, 10.97) | 0.99 (0.02, 6.42) | -2.47 (-2.63, -2.31) | <0.001 | 24.85 (13.56, 42.09) | 15.41 (8.55, 25.96) | -1.72 (-1.95, -1.48) | <0.001 | 58.09 (39.91, 82.01) | 23.80 (15.04, 36.15) | -3.07 (-3.24, -2.89) | <0.001 | 0.98 (0.00, 9.18) | 0.39 (0.00, 5.45) | -3.11 (-3.28, -2.94) | <0.001 |
| Bulgaria | 2.75 (1.99, 3.71) | 1.23 (0.66, 2.17) | -2.75 (-2.88, -2.61) | <0.001 | 16.85 (14.86, 19.04) | 9.96 (8.11, 12.17) | -1.81 (-1.94, -1.68) | <0.001 | 115.65 (110.35, 121.14) | 29.01 (25.83, 32.53) | -4.66 (-5.12, -4.20) | <0.001 | 2.01 (1.37, 2.86) | 0.50 (0.17, 1.24) | -4.70 (-5.18, -4.23) | <0.001 |
| Burkina Faso | 79.58 (75.50, 83.84) | 79.47 (76.92, 82.09) | -0.01 (-0.04, 0.02) | 0.437 | 1213.59 (1197.03, 1230.34) | 1234.24 (1223.85, 1244.69) | 0.06 (0.05, 0.07) | <0.001 | 122.38 (117.17, 127.77) | 100.93 (97.99, 103.95) | -0.65 (-0.74, -0.55) | <0.001 | 1.06 (0.63, 1.71) | 0.69 (0.46, 0.99) | -1.44 (-1.69, -1.19) | <0.001 |
| Burundi | 119.92 (113.56, | 127.34 (122.93, | 0.21 (0.19, | <0.001 | 1936.93 (1910.40 | 2039.99 (2021.60 | 0.18 (0.17, | <0.001 | 279.40 (269.41, | 163.62 (158.47, | -1.85 (-1.96, | <0.001 | 3.05 (2.08, | 1.09 (0.70, | -3.53 (-3.73, | <0.001 |

| | | | | | | | | | | | | | | | | |
|---|---|---|---|---|---|---|---|---|---|---|---|---|---|---|---|---|
| | 126.58) | 131.87) | 0.22) | | , 1963.77) | , 2058.52) | 0.19) | | 289.70) | 168.92) | -1.73) | | 4.36) | 1.62) | -3.32) | |
| Cabo Verde | 65.86 (48.27, 88.93) | 63.20 (49.72, 79.29) | -0.14 (-0.16, -0.12) | <0.001 | 1326.74 (1239.48, 1419.29) | 1337.68 (1272.90, 1404.98) | 0.03 (0.02, 0.04) | <0.001 | 230.58 (195.20, 271.39) | 72.97 (58.46, 90.07) | -3.90 (-4.24, -3.57) | <0.001 | 2.74 (0.26, 12.52) | 0.15 (0.00, 3.62) | -9.73 (-10.19, -9.27) | <0.001 |
| Cambodia | 49.04 (46.09, 52.15) | 46.88 (44.64, 49.20) | -0.16 (-0.18, -0.14) | <0.001 | 703.12 (691.61, 714.78) | 704.15 (695.42, 712.95) | -0.02 (-0.07, 0.04) | 0.583 | 431.40 (422.29, 440.68) | 155.90 (151.80, 160.08) | -3.44 (-3.57, -3.30) | <0.001 | 6.70 (5.60, 7.97) | 2.07 (1.62, 2.61) | -3.94 (-4.09, -3.79) | <0.001 |
| Cameroon | 74.58 (70.88, 78.45) | 80.49 (78.29, 82.74) | 0.25 (0.18, 0.33) | <0.001 | 1177.24 (1161.92, 1192.72) | 1273.27 (1264.27, 1282.33) | 0.27 (0.22, 0.32) | <0.001 | 138.96 (133.74, 144.35) | 89.31 (86.95, 91.74) | -1.51 (-1.56, -1.45) | <0.001 | 1.36 (0.89, 2.03) | 0.47 (0.31, 0.69) | -3.60 (-3.73, -3.47) | <0.001 |
| Canada | 2.07 (1.71, 2.48) | 1.76 (1.44, 2.14) | -0.54 (-0.59, -0.49) | <0.001 | 32.84 (31.38, 34.37) | 30.59 (29.20, 32.04) | -0.23 (-0.31, -0.16) | <0.001 | 9.10 (8.35, 9.91) | 5.42 (4.85, 6.04) | -1.76 (-2.08, -1.43) | <0.001 | 0.12 (0.05, 0.26) | 0.06 (0.02, 0.18) | -2.05 (-2.48, -1.62) | <0.001 |
| Central African Republic | 116.31 (107.51, 125.73) | 122.83 (116.48, 129.47) | 0.18 (0.16, 0.21) | <0.001 | 1889.22 (1852.16, 1926.90) | 1987.53 (1960.91, 2014.45) | 0.17 (0.14, 0.21) | <0.001 | 278.97 (264.44, 294.15) | 207.04 (198.33, 216.06) | -1.00 (-1.13, -0.86) | <0.001 | 3.25 (1.83, 5.42) | 1.94 (1.17, 3.06) | -1.75 (-1.93, -1.56) | <0.001 |
| Chad | 79.26 (74.17, 84.64) | 78.69 (75.59, 81.90) | -0.03 (-0.03, -0.02) | <0.001 | 1210.37 (1189.65, 1231.38) | 1233.36 (1220.48, 1246.35) | 0.06 (0.05, 0.08) | <0.001 | 164.42 (156.87, 172.25) | 110.66 (106.85, 114.58) | -1.34 (-1.48, -1.20) | <0.001 | 1.74 (1.04, 2.76) | 0.85 (0.54, 1.28) | -2.44 (-2.69, -2.20) | <0.001 |
| Chile | 3.39 (2.74, 4.15) | 1.90 (1.47, 2.43) | -1.99 (-2.14, -1.83) | <0.001 | 37.20 (34.97, 39.53) | 28.10 (26.36, 29.94) | -0.94 (-1.05, -0.84) | <0.001 | 70.34 (67.26, 73.52) | 12.13 (11.00, 13.36) | -5.90 (-6.24, -5.55) | <0.001 | 1.19 (0.82, 1.67) | 0.18 (0.07, 0.40) | -6.19 (-6.55, -5.82) | <0.001 |
| China | 34.47 (34.25, 34.69) | 29.33 (29.10, 29.56) | -0.57 (-0.62, -0.52) | <0.001 | 655.40 (654.42, 656.38) | 550.43 (549.47, 551.40) | -0.60 (-0.64, -0.56) | <0.001 | 214.49 (213.92, 215.06) | 42.87 (42.60, 43.13) | -5.41 (-6.10, -4.72) | <0.001 | 3.18 (3.11, 3.25) | 0.28 (0.26, 0.30) | -8.41 (-9.04, -7.79) | <0.001 |
| Colombia | 2.02 (1.69, 2.39) | 1.71 (1.46, 1.99) | -0.57 (-0.83, -0.32) | <0.001 | 29.06 (27.80, 30.36) | 26.39 (25.38, 27.44) | -0.34 (-0.40, -0.27) | <0.001 | 52.87 (51.16, 54.62) | 8.66 (8.08, 9.27) | -5.95 (-6.82, -5.07) | <0.001 | 0.87 (0.66, 1.13) | 0.12 (0.06, 0.22) | -6.45 (-7.83, -5.06) | <0.001 |

| | | | | | | | | | | | | | | | | |
|---|---|---|---|---|---|---|---|---|---|---|---|---|---|---|---|---|
| Comoros | 106.43 (86.45, 130.33) | 112.25 (96.00, 130.60) | 0.18 (0.15, 0.21) | <0.001 | 1952.02 (1858.45 , 2049.52) | 2034.70 (1962.06 , 2109.46) | 0.14 (0.14, 0.15) | <0.001 | 210.63 (180.79, 244.52) | 139.83 (121.31, 160.51) | -1.41 (-2.63, -0.17) | 0.026 | 1.92 (0.16, 9.06) | 0.70 (0.02, 4.15) | -3.43 (-6.90, 0.17) | 0.061 |
| Congo | 105.86 (97.03, 115.39) | 108.18 (102.13, 114.51) | 0.08 (0.06, 0.09) | <0.001 | 1887.60 (1848.20 , 1927.70) | 1941.72 (1915.55 , 1968.16) | 0.08 (-0.05, 0.22) | 0.234 | 216.19 (202.63, 230.47) | 131.30 (124.54, 138.33) | -1.67 (-1.94, -1.40) | <0.001 | 2.18 (1.00, 4.22) | 0.68 (0.28, 1.39) | -3.99 (-4.77, -3.20) | <0.001 |
| Cook Islands | 5.51 (0.00, 131.27) | 5.07 (0.00, 133.98) | -0.27 (-0.42, -0.13) | <0.001 | 85.27 (17.91, 258.11) | 90.36 (17.34, 275.79) | 0.22 (0.13, 0.31) | <0.001 | 135.52 (42.41, 331.39) | 58.73 (6.09, 227.10) | -2.83 (-2.98, -2.68) | <0.001 | 2.20 (0.00, 125.08) | 0.91 (0.00, 125.68) | -2.99 (-3.20, -2.77) | <0.001 |
| Costa Rica | 69.35 (63.15, 76.04) | 66.39 (61.28, 71.82) | -0.14 (-0.16, -0.13) | <0.001 | 1500.64 (1470.75 , 1531.01) | 1475.88 (1451.99 , 1500.08) | -0.05 (-0.06, -0.04) | <0.001 | 142.08 (132.92, 151.73) | 96.53 (90.51, 102.85) | -1.28 (-1.62, -0.94) | <0.001 | 1.22 (0.51, 2.50) | 0.45 (0.14, 1.14) | -3.24 (-4.14, -2.33) | <0.001 |
| Cote d'Ivoire | 79.62 (76.07, 83.31) | 82.06 (79.67, 84.52) | 0.11 (0.09, 0.12) | <0.001 | 1248.66 (1234.04 , 1263.44) | 1290.53 (1280.85 , 1300.27) | 0.12 (0.11, 0.12) | <0.001 | 143.67 (138.78, 148.71) | 95.27 (92.66, 97.94) | -1.39 (-1.52, -1.25) | <0.001 | 1.37 (0.93, 1.98) | 0.55 (0.37, 0.80) | -3.05 (-3.39, -2.72) | <0.001 |
| Croatia | 0.73 (0.30, 1.56) | 0.64 (0.19, 1.70) | -0.48 (-0.85, -0.11) | 0.012 | 5.96 (4.49, 7.78) | 5.53 (3.89, 7.72) | -0.31 (-0.48, -0.13) | 0.001 | 14.46 (12.10, 17.17) | 3.08 (1.89, 4.83) | -5.40 (-6.36, -4.43) | <0.001 | 0.24 (0.03, 0.88) | 0.05 (0.00, 0.86) | -5.39 (-7.00, -3.75) | <0.001 |
| Cuba | 65.58 (62.42, 68.87) | 65.64 (61.83, 69.63) | 0.01 (-0.03, 0.04) | 0.624 | 1364.81 (1349.90 , 1379.85) | 1391.55 (1374.16 , 1409.11) | 0.07 (0.06, 0.08) | <0.001 | 210.01 (204.09, 216.06) | 103.81 (99.12, 108.68) | -2.41 (-2.72, -2.09) | <0.001 | 2.48 (1.87, 3.24) | 0.65 (0.33, 1.15) | -4.55 (-5.13, -3.96) | <0.001 |
| Cyprus | 0.39 (0.00, 3.47) | 0.31 (0.00, 2.98) | -0.83 (-1.11, -0.56) | <0.001 | 5.97 (2.72, 11.49) | 5.56 (2.96, 9.97) | -0.26 (-0.30, -0.21) | <0.001 | 5.42 (2.37, 10.77) | 1.46 (0.35, 4.62) | -4.75 (-5.87, -3.61) | <0.001 | 0.09 (0.00, 2.92) | 0.02 (0.00, 2.58) | -5.80 (-6.36, -5.23) | <0.001 |
| Czechia | 0.83 (0.48, 1.36) | 0.88 (0.49, 1.50) | 0.17 (-0.05, 0.40) | 0.131 | 6.81 (5.68, 8.11) | 7.38 (6.09, 8.90) | 0.27 (0.00, 0.53) | 0.049 | 17.68 (15.85, 19.68) | 4.24 (3.29, 5.41) | -4.64 (-5.25, -4.01) | <0.001 | 0.30 (0.11, 0.69) | 0.07 (0.00, 0.45) | -4.78 (-5.45, -4.10) | <0.001 |
| Democratic | 36.21 | 38.17 | 0.18 | <0.001 | 622.48 | 635.52 | 0.08 | 0.002 | 179.65 | 111.16 | -1.62 | <0.001 | 2.57 | 1.39 | -2.07 | <0.001 |

| | | | | | | | | | | | | | | | | |
|---|---|---|---|---|---|---|---|---|---|---|---|---|---|---|---|---|
| People's Republic of Korea | (34.44, 38.05) | (36.41, 39.98) | (0.15, 0.21) | | (614.99, 630.05) | (628.38, 642.73) | (0.03, 0.13) | | (175.55, 183.82) | (108.20, 114.18) | (-1.69, -1.54) | | (2.10, 3.12) | (1.08, 1.77) | (-2.17, -1.98) | |
| Democratic Republic of the Congo | 106.97 (104.64, 109.35) | 111.51 (109.97, 113.06) | 0.15 (0.08, 0.22) | <0.001 | 1891.49 (1881.30, 1901.72) | 1939.88 (1933.23, 1946.55) | 0.09 (0.07, 0.12) | <0.001 | 221.70 (218.16, 225.28) | 142.47 (140.65, 144.32) | -1.54 (-1.61, -1.47) | <0.001 | 2.25 (1.90, 2.65) | 0.87 (0.73, 1.04) | -3.16 (-3.29, -3.03) | <0.001 |
| Denmark | 0.31 (0.06, 0.95) | 0.25 (0.04, 0.90) | -0.74 (-1.22, -0.26) | 0.003 | 5.34 (3.96, 7.06) | 4.96 (3.60, 6.68) | -0.25 (-0.32, -0.18) | <0.001 | 4.42 (3.18, 6.00) | 1.12 (0.54, 2.09) | -4.62 (-5.08, -4.15) | <0.001 | 0.07 (0.00, 0.58) | 0.01 (0.00, 0.51) | -5.28 (-6.66, -3.88) | <0.001 |
| Djibouti | 101.42 (81.99, 124.63) | 107.76 (94.73, 122.12) | 0.20 (0.18, 0.21) | <0.001 | 1887.25 (1796.33, 1981.95) | 2009.87 (1953.54, 2067.49) | 0.22 (0.21, 0.22) | <0.001 | 167.58 (141.30, 197.76) | 122.72 (109.11, 137.64) | -1.02 (-1.28, -0.76) | <0.001 | 1.28 (0.04, 7.74) | 0.45 (0.01, 2.55) | -3.43 (-3.95, -2.91) | <0.001 |
| Dominica | 74.81 (37.11, 138.91) | 73.45 (34.00, 138.63) | -0.05 (-0.10, 0.00) | 0.050 | 1387.19 (1194.72, 1604.13) | 1396.90 (1198.01, 1619.59) | 0.02 (0.02, 0.03) | <0.001 | 239.02 (162.50, 341.60) | 149.34 (89.65, 234.03) | -1.64 (-2.10, -1.18) | <0.001 | 2.92 (0.00, 40.08) | 1.39 (0.00, 32.80) | -2.53 (-3.11, -1.95) | <0.001 |
| Dominican Republic | 70.61 (66.62, 74.81) | 66.83 (63.49, 70.31) | -0.18 (-0.22, -0.15) | <0.001 | 1326.43 (1308.24, 1344.83) | 1327.94 (1312.82, 1343.19) | 0.01 (0.00, 0.01) | 0.059 | 182.86 (176.15, 189.79) | 125.55 (120.93, 130.31) | -1.29 (-1.62, -0.96) | <0.001 | 1.97 (1.32, 2.85) | 1.04 (0.66, 1.56) | -2.21 (-2.80, -1.60) | <0.001 |
| Ecuador | 68.21 (64.78, 71.79) | 67.74 (65.10, 70.47) | -0.03 (-0.04, -0.02) | <0.001 | 1282.39 (1266.86, 1298.08) | 1300.46 (1288.69, 1312.31) | 0.05 (0.03, 0.07) | <0.001 | 140.59 (135.43, 145.90) | 82.28 (79.34, 85.31) | -1.85 (-2.27, -1.44) | <0.001 | 1.34 (0.88, 1.98) | 0.35 (0.18, 0.60) | -4.85 (-5.89, -3.80) | <0.001 |
| Egypt | 47.86 (46.56, 49.18) | 48.95 (47.99, 49.93) | 0.08 (0.06, 0.10) | <0.001 | 856.34 (850.72, 862.00) | 855.27 (851.21, 859.34) | -0.00 (-0.04, 0.03) | 0.824 | 234.21 (231.30, 237.16) | 80.08 (78.84, 81.33) | -3.66 (-4.04, -3.28) | <0.001 | 3.14 (2.81, 3.51) | 0.65 (0.54, 0.77) | -5.30 (-5.80, -4.80) | <0.001 |
| El Salvador | 76.15 (71.15, 81.45) | 74.45 (69.95, 79.17) | -0.08 (-0.09, -0.07) | <0.001 | 1395.87 (1373.16, 1418.89) | 1406.49 (1386.50, 1426.70) | 0.03 (0.02, 0.04) | <0.001 | 101.74 (95.64, 108.17) | 74.32 (69.78, 79.09) | -1.06 (-1.22, -0.89) | <0.001 | 0.60 (0.22, 1.37) | 0.12 (0.01, 0.52) | -5.46 (-6.05, -4.87) | <0.001 |
| Equatorial Guinea | 108.66 | 90.68 | -0.64 | <0.001 | 1912.47 | 1788.79 | -0.23 | <0.001 | 236.97 | 101.62 | -2.90 | <0.001 | 2.51 | 0.30 | -7.21 | <0.001 |

| | | | | | | | | | | | | | | | | |
|---|---|---|---|---|---|---|---|---|---|---|---|---|---|---|---|---|
| | (87.57, 133.66) | (80.15, 102.35) | (-0.71, -0.57) | | (1817.97, 2010.85) | (1739.44, 1839.28) | (-0.27, -0.19) | | (204.01, 273.95) | (90.06, 114.37) | (-3.11, -2.69) | | (0.28, 9.72) | (0.00, 2.22) | (-7.49, -6.93) | |
| Eritrea | 134.29 (125.19, 143.94) | 135.00 (129.13, 141.10) | 0.03 (-0.02, 0.07) | 0.206 | 2294.89 (2255.36, 2334.98) | 2327.89 (2302.45, 2353.56) | 0.04 (-0.06, 0.13) | 0.459 | 249.16 (236.18, 262.71) | 166.62 (159.85, 173.62) | -1.41 (-1.56, -1.26) | <0.001 | 2.33 (1.23, 4.10) | 0.93 (0.49, 1.64) | -3.16 (-3.32, -2.99) | <0.001 |
| Estonia | 3.54 (1.77, 6.50) | 2.82 (1.07, 6.62) | -0.77 (-0.83, -0.72) | <0.001 | 27.93 (22.24, 34.72) | 25.74 (19.28, 34.00) | -0.27 (-0.32, -0.23) | <0.001 | 30.27 (24.42, 37.20) | 8.72 (5.29, 14.00) | -3.75 (-4.86, -2.62) | <0.001 | 0.52 (0.04, 2.36) | 0.13 (0.00, 2.90) | -4.37 (-5.88, -2.83) | <0.001 |
| Eswatini | 102.24 (87.62, 118.96) | 101.31 (89.56, 114.27) | -0.03 (-0.06, -0.01) | 0.003 | 1660.86 (1597.72, 1726.13) | 1665.99 (1616.66, 1716.52) | 0.01 (0.00, 0.03) | 0.022 | 219.92 (197.00, 245.05) | 141.24 (127.10, 156.60) | -1.55 (-1.83, -1.26) | <0.001 | 2.36 (0.56, 6.93) | 1.04 (0.18, 3.40) | -2.86 (-3.36, -2.36) | <0.001 |
| Ethiopia | 108.39 (106.36, 110.45) | 113.20 (111.84, 114.57) | 0.15 (0.14, 0.16) | <0.001 | 1696.24 (1687.92, 1704.59) | 1904.85 (1899.03, 1910.68) | 0.40 (0.36, 0.44) | <0.001 | 263.82 (260.56, 267.13) | 127.51 (126.01, 129.02) | -2.47 (-2.59, -2.36) | <0.001 | 3.01 (2.67, 3.39) | 0.60 (0.51, 0.72) | -5.39 (-5.54, -5.25) | <0.001 |
| Fiji | 28.20 (20.51, 37.96) | 33.46 (25.44, 43.22) | 0.60 (0.58, 0.62) | <0.001 | 438.04 (405.83, 472.25) | 683.17 (645.01, 723.03) | 1.50 (1.42, 1.58) | <0.001 | 584.63 (547.34, 623.91) | 360.48 (332.91, 389.74) | -1.62 (-1.95, -1.29) | <0.001 | 9.33 (5.16, 15.70) | 5.44 (2.56, 10.17) | -1.85 (-2.39, -1.31) | <0.001 |
| Finland | 0.29 (0.05, 0.99) | 0.24 (0.03, 0.94) | -0.76 (-1.56, 0.05) | 0.064 | 5.23 (3.83, 7.01) | 4.75 (3.38, 6.55) | -0.37 (-0.44, -0.30) | <0.001 | 3.98 (2.80, 5.55) | 1.16 (0.54, 2.21) | -4.18 (-4.95, -3.40) | <0.001 | 0.06 (0.00, 0.65) | 0.02 (0.00, 0.59) | -3.72 (-4.78, -2.63) | <0.001 |
| France | 0.43 (0.32, 0.57) | 0.36 (0.26, 0.50) | -0.61 (-1.02, -0.20) | 0.004 | 7.59 (7.08, 8.12) | 6.93 (6.42, 7.46) | -0.32 (-0.45, -0.18) | <0.001 | 11.85 (11.21, 12.50) | 5.19 (4.76, 5.66) | -2.83 (-3.15, -2.50) | <0.001 | 0.19 (0.12, 0.29) | 0.08 (0.03, 0.16) | -3.02 (-3.77, -2.26) | <0.001 |
| Gabon | 87.90 (75.22, 102.39) | 90.87 (81.77, 100.76) | 0.11 (0.10, 0.13) | <0.001 | 1794.40 (1733.06, 1857.56) | 1860.18 (1817.52, 1903.63) | 0.12 (0.11, 0.13) | <0.001 | 158.50 (140.30, 178.60) | 105.72 (95.70, 116.55) | -1.38 (-1.47, -1.29) | <0.001 | 1.27 (0.16, 4.83) | 0.31 (0.01, 1.67) | -4.70 (-5.15, -4.24) | <0.001 |
| Gambia | 76.82 (65.16, | 81.08 (73.39, | 0.19 (0.17, | <0.001 | 1284.46 (1233.65 | 1360.98 (1327.90 | 0.20 (0.19, | <0.001 | 128.62 (112.99, | 93.49 (84.99, | -1.03 (-1.48, | <0.001 | 1.10 (0.14, | 0.47 (0.07, | -2.75 (-3.82, | <0.001 |

| | | | | | | | | | | | | | | | | |
|---|---|---|---|---|---|---|---|---|---|---|---|---|---|---|---|---|
| | 90.28) | 89.43) | 0.20) | | , 1337.07) | , 1394.74) | 0.22) | | 146.09) | 102.68) | -0.58) | | 4.51) | 1.77) | -1.67) | |
| Georgia | 54.64 (50.25, 59.32) | 56.32 (50.04, 63.23) | 0.12 (0.10, 0.13) | <0.001 | 1175.79 (1155.44 , 1196.43) | 1205.91 (1177.41 , 1234.99) | 0.08 (0.04, 0.13) | <0.001 | 167.78 (160.11, 175.74) | 117.24 (108.37, 126.70) | -1.33 (-2.88, 0.25) | 0.099 | 1.81 (1.10, 2.83) | 0.97 (0.33, 2.32) | -2.30 (-4.58, 0.04) | 0.054 |
| Germany | 0.45 (0.35, 0.58) | 0.35 (0.25, 0.47) | -0.90 (-1.13, -0.67) | <0.001 | 7.61 (7.16, 8.08) | 6.78 (6.32, 7.26) | -0.45 (-0.59, -0.32) | <0.001 | 8.45 (7.98, 8.93) | 3.74 (3.41, 4.10) | -2.92 (-3.14, -2.70) | <0.001 | 0.14 (0.08, 0.22) | 0.06 (0.02, 0.12) | -2.86 (-3.35, -2.36) | <0.001 |
| Ghana | 78.00 (74.89, 81.21) | 76.06 (74.06, 78.10) | -0.08 (-0.12, -0.04) | <0.001 | 1260.89 (1247.99 , 1273.91) | 1269.95 (1261.64 , 1278.31) | 0.03 (0.01, 0.05) | 0.004 | 146.61 (142.25, 151.09) | 91.07 (88.86, 93.33) | -1.64 (-1.82, -1.45) | <0.001 | 1.42 (1.02, 1.94) | 0.50 (0.35, 0.70) | -3.49 (-3.79, -3.20) | <0.001 |
| Greece | 0.40 (0.17, 0.81) | 0.33 (0.11, 0.82) | -0.66 (-1.09, -0.23) | 0.003 | 7.06 (5.91, 8.38) | 6.55 (5.29, 8.04) | -0.25 (-0.31, -0.18) | <0.001 | 6.62 (5.52, 7.90) | 2.58 (1.83, 3.55) | -3.06 (-3.88, -2.23) | <0.001 | 0.11 (0.01, 0.39) | 0.04 (0.00, 0.39) | -3.20 (-4.48, -1.91) | <0.001 |
| Greenland | 3.13 (0.00, 51.81) | 2.02 (0.00, 48.48) | -1.51 (-1.62, -1.40) | <0.001 | 42.58 (13.69, 108.63) | 32.17 (7.22, 95.63) | -0.97 (-1.04, -0.90) | <0.001 | 35.52 (10.09, 98.55) | 9.84 (0.26, 61.48) | -4.28 (-4.81, -3.75) | <0.001 | 0.58 (0.00, 47.75) | 0.14 (0.00, 45.13) | -4.81 (-5.68, -3.94) | <0.001 |
| Grenada | 74.01 (38.95, 130.54) | 69.91 (37.97, 119.33) | -0.19 (-0.26, -0.12) | <0.001 | 1400.36 (1223.21 , 1597.55) | 1392.06 (1230.87 , 1569.45) | -0.02 (-0.04, 0.00) | 0.047 | 357.78 (270.07, 466.46) | 147.99 (98.46, 214.72) | -3.04 (-3.47, -2.61) | <0.001 | 4.94 (0.04, 36.85) | 1.39 (0.00, 24.74) | -4.28 (-4.75, -3.80) | <0.001 |
| Guam | 31.88 (14.63, 60.82) | 33.25 (15.60, 62.64) | 0.14 (0.14, 0.15) | <0.001 | 866.80 (762.05, 982.22) | 912.65 (805.08, 1030.97) | 0.18 (0.14, 0.22) | <0.001 | 200.86 (152.22, 260.43) | 159.24 (116.06, 213.59) | -0.71 (-1.17, -0.24) | 0.003 | 2.62 (0.02, 18.77) | 1.91 (0.00, 17.76) | -1.04 (-1.66, -0.43) | 0.001 |
| Guatemala | 82.32 (77.87, 86.99) | 83.97 (81.20, 86.82) | 0.08 (0.05, 0.10) | <0.001 | 1353.95 (1335.00 , 1373.11) | 1413.37 (1401.73 , 1425.07) | 0.15 (0.13, 0.17) | <0.001 | 105.61 (100.34, 111.10) | 76.28 (73.59, 79.04) | -1.14 (-1.47, -0.80) | <0.001 | 0.70 (0.33, 1.33) | 0.15 (0.06, 0.34) | -5.32 (-6.74, -3.89) | <0.001 |
| Guinea | 62.01 (57.59, 66.69) | 68.88 (65.77, 72.12) | 0.36 (0.34, 0.38) | <0.001 | 875.19 (858.11, 892.55) | 1040.36 (1027.86 , | 0.59 (0.55, 0.63) | <0.001 | 172.29 (164.79, 180.05) | 107.05 (103.08, 111.14) | -1.63 (-1.83, -1.43) | <0.001 | 2.13 (1.37, 3.19) | 0.94 (0.60, 1.42) | -2.81 (-2.99, -2.63) | <0.001 |

| | | | | | | | | | | | | | | | | |
|---|---|---|---|---|---|---|---|---|---|---|---|---|---|---|---|---|
| | | | | | | 1052.98) | | | | | | | | | | |
| Guinea-Bissau | 81.55 (69.64, 95.11) | 86.54 (77.97, 95.87) | 0.20 (0.20, 0.21) | <0.001 | 1235.63 (1186.64, 1286.27) | 1319.29 (1284.51, 1354.84) | 0.22 (0.21, 0.24) | <0.001 | 245.64 (224.11, 268.84) | 131.93 (121.09, 143.54) | -2.13 (-2.27, -1.99) | <0.001 | 3.06 (1.11, 6.99) | 1.14 (0.35, 2.86) | -3.37 (-3.56, -3.18) | <0.001 |
| Guyana | 85.93 (72.86, 100.91) | 82.02 (68.79, 97.18) | -0.16 (-0.18, -0.14) | <0.001 | 1406.75 (1350.40, 1465.02) | 1403.30 (1345.83, 1462.69) | -0.01 (-0.03, 0.01) | 0.416 | 247.28 (223.74, 272.79) | 164.26 (144.85, 185.64) | -1.40 (-2.14, -0.65) | <0.001 | 3.04 (0.96, 7.46) | 1.66 (0.28, 5.41) | -2.03 (-3.09, -0.95) | <0.001 |
| Haiti | 95.83 (90.61, 101.29) | 98.47 (94.82, 102.23) | 0.09 (0.04, 0.14) | <0.001 | 1370.97 (1350.57, 1391.63) | 1423.80 (1409.80, 1437.91) | 0.13 (0.12, 0.14) | <0.001 | 1022.55 (1004.69, 1040.66) | 434.51 (426.77, 442.35) | -2.87 (-3.02, -2.72) | <0.001 | 16.21 (14.01, 18.66) | 6.19 (5.30, 7.20) | -3.24 (-3.40, -3.08) | <0.001 |
| Honduras | 87.46 (81.53, 93.76) | 85.29 (81.53, 89.19) | -0.07 (-0.10, -0.04) | <0.001 | 1430.76 (1405.36, 1456.54) | 1429.07 (1413.18, 1445.10) | 0.00 (-0.03, 0.03) | 0.941 | 84.07 (77.94, 90.59) | 72.64 (69.08, 76.34) | -0.45 (-0.54, -0.37) | <0.001 | 0.28 (0.04, 1.03) | 0.09 (0.01, 0.35) | -3.91 (-4.71, -3.11) | <0.001 |
| Hungary | 1.32 (0.86, 1.94) | 0.99 (0.56, 1.66) | -0.94 (-1.11, -0.77) | <0.001 | 10.05 (8.67, 11.59) | 8.77 (7.32, 10.46) | -0.47 (-0.57, -0.37) | <0.001 | 43.35 (40.50, 46.37) | 7.15 (5.87, 8.66) | -5.94 (-6.65, -5.22) | <0.001 | 0.76 (0.43, 1.28) | 0.12 (0.01, 0.52) | -6.27 (-6.88, -5.66) | <0.001 |
| Iceland | 0.43 (0.00, 8.70) | 0.45 (0.00, 8.38) | 0.19 (0.06, 0.32) | 0.005 | 8.39 (2.41, 21.24) | 8.34 (2.64, 20.39) | 0.01 (-0.09, 0.11) | 0.885 | 4.64 (0.72, 15.69) | 2.33 (0.14, 11.49) | -2.39 (-2.65, -2.14) | <0.001 | 0.07 (0.00, 8.01) | 0.03 (0.00, 7.63) | -3.12 (-4.62, -1.60) | <0.001 |
| India | 91.15 (90.69, 91.61) | 86.59 (86.26, 86.93) | -0.17 (-0.25, -0.10) | <0.001 | 1175.11 (1173.43, 1176.79) | 1174.14 (1172.88, 1175.39) | -0.01 (-0.09, 0.08) | 0.884 | 510.69 (509.58, 511.80) | 269.46 (268.86, 270.06) | -2.27 (-2.84, -1.69) | <0.001 | 7.59 (7.46, 7.73) | 3.62 (3.55, 3.69) | -2.63 (-3.28, -1.97) | <0.001 |
| Indonesia | 11.90 (11.56, 12.25) | 13.58 (13.27, 13.90) | 0.46 (0.43, 0.48) | <0.001 | 178.86 (177.53, 180.20) | 213.21 (211.96, 214.46) | 0.61 (0.60, 0.62) | <0.001 | 76.83 (75.95, 77.71) | 29.75 (29.28, 30.22) | -3.20 (-3.28, -3.12) | <0.001 | 1.13 (1.03, 1.25) | 0.32 (0.27, 0.37) | -4.24 (-4.37, -4.11) | <0.001 |
| Iran (Islamic Republic of) | 42.67 (41.47, 43.89) | 41.78 (40.76, 42.82) | -0.07 (-0.08, -0.06) | <0.001 | 749.50 (744.26, 754.77) | 755.80 (751.66, 759.96) | 0.03 (0.02, 0.04) | <0.001 | 103.71 (101.76, 105.68) | 53.59 (52.49, 54.71) | -2.24 (-2.50, -1.99) | <0.001 | 1.12 (0.93, 1.35) | 0.29 (0.21, 0.38) | -4.57 (-4.92, -4.21) | <0.001 |

| | | | | | | | | | | | | | | | | |
|---|---|---|---|---|---|---|---|---|---|---|---|---|---|---|---|---|
| Iraq | 46.74 (44.41, 49.17) | 46.66 (45.27, 48.08) | -0.01 (-0.03, 0.02) | 0.728 | 775.95 (766.00, 786.01) | 788.41 (782.55, 794.31) | 0.06 (0.04, 0.07) | <0.001 | 150.78 (146.33, 155.35) | 58.32 (56.72, 59.95) | -3.22 (-3.56, -2.88) | <0.001 | 1.94 (1.46, 2.55) | 0.36 (0.24, 0.52) | -5.59 (-6.08, -5.09) | <0.001 |
| Ireland | 0.51 (0.12, 1.40) | 0.39 (0.09, 1.14) | -0.90 (-1.05, -0.74) | <0.001 | 7.75 (5.80, 10.17) | 7.32 (5.58, 9.46) | -0.19 (-0.23, -0.14) | <0.001 | 8.01 (6.01, 10.46) | 2.45 (1.50, 3.81) | -4.05 (-5.34, -2.74) | <0.001 | 0.13 (0.00, 0.81) | 0.03 (0.00, 0.60) | -4.31 (-6.07, -2.52) | <0.001 |
| Israel | 0.70 (0.28, 1.48) | 0.43 (0.18, 0.90) | -1.68 (-1.92, -1.44) | <0.001 | 9.33 (7.49, 11.49) | 7.55 (6.27, 9.02) | -0.74 (-0.81, -0.66) | <0.001 | 23.05 (20.10, 26.32) | 7.93 (6.62, 9.43) | -3.63 (-4.26, -2.99) | <0.001 | 0.39 (0.10, 1.04) | 0.13 (0.02, 0.46) | -3.76 (-4.39, -3.14) | <0.001 |
| Italy | 1.29 (1.08, 1.53) | 0.37 (0.26, 0.53) | -4.19 (-4.67, -3.71) | <0.001 | 21.60 (20.72, 22.50) | 11.82 (11.06, 12.61) | -2.08 (-2.19, -1.97) | <0.001 | 22.65 (21.75, 23.57) | 6.80 (6.24, 7.40) | -4.06 (-4.43, -3.70) | <0.001 | 0.37 (0.26, 0.50) | 0.10 (0.04, 0.20) | -4.54 (-4.86, -4.21) | <0.001 |
| Jamaica | 66.32 (59.51, 73.79) | 68.69 (62.19, 75.71) | 0.12 (0.08, 0.16) | <0.001 | 1345.71 (1313.22, 1378.86) | 1426.64 (1396.58, 1457.20) | 0.20 (0.20, 0.21) | <0.001 | 230.60 (217.39, 244.48) | 143.45 (134.04, 153.38) | -1.53 (-2.48, -0.57) | 0.002 | 2.69 (1.44, 4.71) | 1.24 (0.52, 2.54) | -2.51 (-4.35, -0.64) | 0.009 |
| Japan | 0.66 (0.55, 0.77) | 0.27 (0.20, 0.36) | -3.01 (-3.30, -2.72) | <0.001 | 13.93 (13.44, 14.43) | 9.23 (8.76, 9.71) | -1.42 (-1.45, -1.38) | <0.001 | 8.05 (7.68, 8.43) | 2.94 (2.68, 3.21) | -3.42 (-3.74, -3.09) | <0.001 | 0.12 (0.08, 0.18) | 0.04 (0.02, 0.09) | -3.93 (-4.40, -3.46) | <0.001 |
| Jordan | 1.25 (0.51, 2.64) | 0.91 (0.55, 1.41) | -1.11 (-1.40, -0.83) | <0.001 | 17.86 (14.66, 21.63) | 14.67 (13.12, 16.36) | -0.68 (-0.78, -0.57) | <0.001 | 34.77 (30.25, 39.85) | 7.42 (6.33, 8.65) | -5.05 (-5.83, -4.26) | <0.001 | 0.57 (0.13, 1.69) | 0.11 (0.02, 0.37) | -5.38 (-6.36, -4.39) | <0.001 |
| Kazakhstan | 8.61 (7.65, 9.67) | 3.53 (2.94, 4.21) | -3.04 (-3.15, -2.93) | <0.001 | 66.96 (64.23, 69.78) | 38.13 (36.12, 40.23) | -1.92 (-2.07, -1.76) | <0.001 | 220.04 (215.07, 225.10) | 34.84 (32.94, 36.83) | -6.14 (-7.02, -5.26) | <0.001 | 3.71 (3.09, 4.42) | 0.57 (0.35, 0.89) | -6.22 (-7.15, -5.29) | <0.001 |
| Kenya | 103.62 (100.70, 106.62) | 107.08 (105.19, 109.00) | 0.11 (0.10, 0.12) | <0.001 | 1654.30 (1641.88, 1666.80) | 1839.42 (1831.32, 1847.55) | 0.37 (0.33, 0.41) | <0.001 | 131.52 (128.05, 135.07) | 116.57 (114.54, 118.63) | -0.41 (-0.48, -0.35) | <0.001 | 0.87 (0.60, 1.22) | 0.48 (0.36, 0.63) | -2.01 (-2.19, -1.82) | <0.001 |
| Kiribati | 49.11 (20.55, 101.46) | 51.07 (26.96, 88.42) | 0.13 (0.11, 0.15) | <0.001 | 832.92 (694.81, 992.22) | 855.39 (744.30, 978.78) | 0.09 (0.08, 0.10) | <0.001 | 1250.65 (1076.97, 1445.81) | 788.30 (681.06, 907.98) | -1.58 (-1.64, -1.52) | <0.001 | 20.63 (4.28, 62.76) | 12.67 (2.70, 37.10) | -1.67 (-1.73, -1.60) | <0.001 |

| | | | | | | | | | | | | | | | | |
|---|---|---|---|---|---|---|---|---|---|---|---|---|---|---|---|---|
| Kuwait | 1.30 (0.39, 3.23) | 0.90 (0.40, 1.90) | -1.23 (-1.53, -0.94) | <0.001 | 21.58 (17.02, 27.04) | 16.78 (14.27, 19.70) | -0.86 (-0.95, -0.77) | <0.001 | 37.95 (31.83, 44.95) | 5.27 (3.85, 7.14) | -6.43 (-8.42, -4.41) | <0.001 | 0.60 (0.08, 2.20) | 0.07 (0.00, 0.81) | -6.97 (-9.52, -4.36) | <0.001 |
| Kyrgyzstan | 60.65 (55.71, 65.93) | 61.46 (57.28, 65.88) | 0.05 (0.03, 0.06) | <0.001 | 1144.73 (1122.61, 1167.20) | 1156.90 (1138.61, 1175.43) | 0.03 (0.02, 0.04) | <0.001 | 485.43 (470.87, 500.35) | 135.91 (129.66, 142.40) | -4.35 (-4.86, -3.83) | <0.001 | 7.37 (5.67, 9.46) | 1.40 (0.83, 2.22) | -5.56 (-6.21, -4.91) | <0.001 |
| Lao People's Democratic Republic | 70.12 (64.49, 76.15) | 66.25 (62.29, 70.40) | -0.20 (-0.30, -0.10) | <0.001 | 1028.91 (1006.39, 1051.85) | 1022.51 (1006.66, 1038.56) | -0.03 (-0.08, 0.03) | 0.391 | 413.75 (399.29, 428.64) | 248.96 (241.14, 256.98) | -1.72 (-1.94, -1.51) | <0.001 | 6.15 (4.48, 8.27) | 3.35 (2.49, 4.41) | -2.06 (-2.27, -1.84) | <0.001 |
| Latvia | 5.65 (3.77, 8.20) | 2.97 (1.38, 5.93) | -2.24 (-2.43, -2.04) | <0.001 | 40.36 (34.96, 46.41) | 27.96 (22.19, 34.98) | -1.30 (-1.68, -0.93) | <0.001 | 74.27 (66.90, 82.30) | 14.20 (10.29, 19.35) | -5.38 (-7.42, -3.28) | <0.001 | 1.27 (0.48, 2.80) | 0.23 (0.00, 2.22) | -6.02 (-7.37, -4.64) | <0.001 |
| Lebanon | 1.60 (0.74, 3.04) | 0.98 (0.47, 1.87) | -1.64 (-1.80, -1.47) | <0.001 | 22.95 (19.23, 27.21) | 16.54 (14.12, 19.30) | -1.12 (-1.22, -1.03) | <0.001 | 62.67 (56.42, 69.44) | 13.14 (10.99, 15.63) | -5.25 (-5.37, -5.13) | <0.001 | 1.03 (0.38, 2.28) | 0.20 (0.03, 0.82) | -5.51 (-5.67, -5.35) | <0.001 |
| Lesotho | 110.00 (99.52, 121.43) | 108.13 (98.98, 117.94) | -0.06 (-0.07, -0.05) | <0.001 | 1701.86 (1658.33, 1746.34) | 1709.86 (1672.42, 1747.96) | 0.02 (-0.00, 0.04) | 0.098 | 200.58 (185.69, 216.45) | 167.58 (155.96, 179.88) | -0.68 (-0.89, -0.47) | <0.001 | 1.99 (0.76, 4.40) | 1.45 (0.56, 3.11) | -1.10 (-1.67, -0.54) | <0.001 |
| Liberia | 78.99 (70.17, 88.67) | 86.17 (80.61, 92.03) | 0.29 (0.26, 0.32) | <0.001 | 1229.92 (1193.57, 1267.12) | 1335.82 (1313.16, 1358.77) | 0.28 (0.27, 0.29) | <0.001 | 161.52 (148.55, 175.36) | 98.49 (92.43, 104.85) | -1.68 (-1.84, -1.51) | <0.001 | 1.68 (0.61, 3.73) | 0.57 (0.20, 1.30) | -3.63 (-3.95, -3.31) | <0.001 |
| Libya | 37.75 (33.47, 42.51) | 42.94 (39.60, 46.49) | 0.45 (0.44, 0.46) | <0.001 | 711.62 (691.61, 732.12) | 799.31 (784.90, 813.92) | 0.41 (0.39, 0.42) | <0.001 | 116.96 (108.94, 125.47) | 62.14 (58.17, 66.32) | -2.12 (-2.52, -1.72) | <0.001 | 1.38 (0.63, 2.71) | 0.40 (0.15, 0.89) | -4.07 (-4.74, -3.39) | <0.001 |
| Lithuania | 5.15 (3.62, 7.14) | 2.73 (1.42, 4.93) | -2.22 (-2.62, -1.83) | <0.001 | 34.90 (30.66, 39.57) | 24.45 (19.98, 29.74) | -1.29 (-1.51, -1.07) | <0.001 | 65.53 (59.70, 71.79) | 13.63 (10.41, 17.66) | -5.49 (-7.17, -3.77) | <0.001 | 1.13 (0.49, 2.26) | 0.22 (0.01, 1.55) | -5.60 (-7.45, -3.70) | <0.001 |
| Luxembourg | 0.60 (0.00, | 0.52 (0.00, | -0.51 (-0.72, | <0.001 | 11.03 (4.74, | 10.90 (5.52, | -0.06 (-0.12, | 0.043 | 7.70 (2.75, | 3.46 (0.89, | -2.65 (-3.04, | <0.001 | 0.12 (0.00, | 0.05 (0.00, | -3.05 (-3.93, | <0.001 |

| | | | | | | | | | | | | | | | | |
|---|---|---|---|---|---|---|---|---|---|---|---|---|---|---|---|---|
| | 8.44) | 5.76) | -0.29) | | 23.05) | 20.00) | -0.00) | | 18.47) | 10.08) | -2.27) | | 7.70) | 5.02) | -2.17) | |
| Madagascar | 107.76 (103.63, 112.02) | 117.62 (114.83, 120.47) | 0.30 (0.27, 0.33) | <0.001 | 1868.48 (1850.45, 1886.65) | 2012.26 (2000.25, 2024.33) | 0.26 (0.25, 0.27) | <0.001 | 383.78 (375.71, 392.00) | 221.04 (217.08, 225.06) | -1.87 (-2.12, -1.61) | <0.001 | 4.77 (3.90, 5.80) | 2.04 (1.68, 2.47) | -2.84 (-3.22, -2.47) | <0.001 |
| Malawi | 128.05 (123.06, 133.22) | 133.97 (130.47, 137.54) | 0.15 (0.13, 0.17) | <0.001 | 2042.47 (2021.58, 2063.55) | 2150.65 (2135.93, 2165.45) | 0.18 (0.17, 0.19) | <0.001 | 216.59 (209.85, 223.51) | 141.27 (137.53, 145.09) | -1.44 (-1.53, -1.35) | <0.001 | 1.95 (1.36, 2.75) | 0.64 (0.41, 0.96) | -3.73 (-4.05, -3.41) | <0.001 |
| Malaysia | 43.42 (41.33, 45.58) | 42.17 (40.61, 43.79) | -0.10 (-0.11, -0.09) | <0.001 | 883.61 (874.00, 893.31) | 903.66 (896.39, 910.97) | 0.08 (0.07, 0.09) | <0.001 | 175.68 (171.40, 180.05) | 65.66 (63.71, 67.66) | -3.34 (-3.56, -3.13) | <0.001 | 2.23 (1.76, 2.77) | 0.38 (0.24, 0.56) | -5.92 (-6.22, -5.63) | <0.001 |
| Maldives | 47.09 (28.39, 76.31) | 44.75 (31.38, 62.37) | -0.18 (-0.21, -0.16) | <0.001 | 854.44 (762.45, 956.16) | 852.99 (792.74, 917.06) | -0.01 (-0.03, 0.01) | 0.272 | 302.59 (248.14, 367.16) | 66.68 (50.68, 86.70) | -5.05 (-5.19, -4.90) | <0.001 | 4.37 (0.31, 22.26) | 0.43 (0.00, 6.49) | -7.66 (-8.01, -7.29) | <0.001 |
| Mali | 90.87 (86.30, 95.65) | 91.31 (88.50, 94.21) | 0.02 (-0.00, 0.05) | 0.059 | 1414.64 (1395.95, 1433.55) | 1439.27 (1427.55, 1451.06) | 0.07 (0.01, 0.12) | 0.018 | 292.85 (284.40, 301.49) | 139.49 (135.87, 143.18) | -2.51 (-2.72, -2.31) | <0.001 | 3.69 (2.79, 4.80) | 1.16 (0.85, 1.56) | -3.90 (-4.17, -3.63) | <0.001 |
| Malta | 0.32 (0.00, 7.06) | 0.28 (0.00, 8.42) | -0.55 (-0.70, -0.39) | <0.001 | 5.25 (1.29, 14.78) | 4.97 (1.12, 15.43) | -0.20 (-0.25, -0.16) | <0.001 | 8.24 (2.94, 18.87) | 4.87 (1.11, 15.21) | -1.70 (-2.03, -1.36) | <0.001 | 0.13 (0.00, 6.74) | 0.08 (0.00, 8.10) | -1.71 (-2.35, -1.07) | <0.001 |
| Marshall Islands | 46.75 (12.34, 128.81) | 47.55 (16.47, 107.80) | 0.06 (0.05, 0.07) | <0.001 | 859.84 (670.99, 1089.46) | 879.37 (716.52, 1068.64) | 0.08 (0.04, 0.11) | <0.001 | 727.49 (550.17, 947.05) | 710.05 (564.21, 882.50) | -0.07 (-0.14, -0.01) | 0.024 | 11.64 (0.20, 77.24) | 11.24 (0.57, 53.85) | -0.11 (-0.19, -0.04) | 0.003 |
| Mauritania | 74.22 (66.07, 83.19) | 75.11 (69.42, 81.20) | 0.04 (0.03, 0.05) | <0.001 | 1267.22 (1231.80, 1303.47) | 1293.61 (1268.85, 1318.77) | 0.06 (0.03, 0.08) | <0.001 | 150.72 (138.76, 163.51) | 79.85 (73.81, 86.29) | -2.17 (-2.27, -2.08) | <0.001 | 1.46 (0.51, 3.38) | 0.30 (0.05, 1.05) | -5.31 (-5.48, -5.15) | <0.001 |
| Mauritius | 43.69 (35.80, 52.85) | 42.15 (34.13, 51.53) | -0.12 (-0.15, -0.10) | <0.001 | 869.62 (833.11, 907.34) | 872.35 (834.76, 911.25) | 0.01 (-0.00, 0.03) | 0.146 | 208.37 (190.65, 227.32) | 75.90 (65.11, 88.02) | -3.45 (-4.28, -2.61) | <0.001 | 2.76 (1.08, 5.82) | 0.57 (0.03, 2.75) | -5.32 (-6.67, -3.95) | <0.001 |
| Mexico | 33.45 | 28.88 | -0.50 | <0.001 | 640.17 | 558.58 | -0.47 | <0.001 | 119.42 | 36.47 | -3.95 | <0.001 | 1.54 | 0.17 | -7.18 | <0.001 |

| | | | | | | | | | | | | | | | | |
|---|---|---|---|---|---|---|---|---|---|---|---|---|---|---|---|---|
| | (32.63, 34.29) | (28.22, 29.55) | (-0.56, -0.44) | | (636.42, 643.94) | (555.68, 561.48) | (-0.49, -0.44) | | (117.75, 121.11) | (35.73, 37.21) | (-4.11, -3.79) | | (1.36, 1.75) | (0.12, 0.23) | (-7.69, -6.68) | |
| Micronesia (Federated States of) | 46.48 (21.16, 91.06) | 46.92 (21.97, 89.35) | 0.05 (-0.01, 0.10) | 0.129 | 852.67 (726.71, 995.84) | 867.36 (743.46, 1007.10) | 0.06 (0.05, 0.08) | <0.001 | 851.24 (722.11, 998.12) | 600.77 (496.63, 721.20) | -1.20 (-1.25, -1.15) | <0.001 | 13.77 (2.23, 47.01) | 9.40 (0.95, 37.82) | -1.31 (-1.37, -1.25) | <0.001 |
| Monaco | 0.37 (0.00, 126.47) | 0.35 (0.00, 91.77) | -0.14 (-0.35, 0.08) | 0.220 | 7.42 (0.00, 137.30) | 7.30 (0.00, 104.46) | -0.05 (-0.08, -0.01) | 0.005 | 3.74 (0.00, 131.55) | 2.75 (0.00, 96.22) | -1.04 (-1.14, -0.95) | <0.001 | 0.06 (0.00, 126.00) | 0.04 (0.00, 91.20) | -1.44 (-2.03, -0.86) | <0.001 |
| Mongolia | 70.84 (63.29, 79.20) | 66.88 (60.55, 73.74) | -0.20 (-0.20, -0.19) | <0.001 | 1227.00 (1193.58, 1261.23) | 1208.18 (1181.81, 1235.04) | -0.05 (-0.06, -0.05) | <0.001 | 251.23 (235.51, 267.82) | 120.83 (112.74, 129.41) | -2.51 (-2.92, -2.11) | <0.001 | 3.37 (1.75, 5.99) | 1.10 (0.47, 2.30) | -3.85 (-4.45, -3.25) | <0.001 |
| Montenegro | 1.06 (0.06, 5.05) | 0.97 (0.03, 5.76) | -0.27 (-0.37, -0.16) | <0.001 | 8.46 (4.12, 15.47) | 8.56 (3.95, 16.49) | 0.07 (0.03, 0.10) | 0.001 | 22.40 (14.82, 32.54) | 12.31 (6.64, 21.20) | -1.83 (-2.83, -0.83) | <0.001 | 0.38 (0.00, 3.88) | 0.21 (0.00, 4.50) | -1.89 (-2.92, -0.85) | <0.001 |
| Morocco | 50.19 (48.30, 52.13) | 49.46 (47.85, 51.11) | -0.05 (-0.07, -0.04) | <0.001 | 784.96 (777.25, 792.73) | 800.13 (793.66, 806.64) | 0.07 (0.03, 0.12) | 0.001 | 161.91 (158.40, 165.47) | 74.17 (72.21, 76.17) | -2.65 (-2.76, -2.53) | <0.001 | 2.08 (1.69, 2.52) | 0.61 (0.45, 0.82) | -4.13 (-4.35, -3.92) | <0.001 |
| Mozambique | 128.48 (124.17, 132.90) | 130.28 (127.47, 133.15) | 0.04 (-0.01, 0.09) | 0.115 | 2051.38 (2033.50, 2069.39) | 2135.57 (2123.58, 2147.62) | 0.14 (0.13, 0.16) | <0.001 | 164.13 (159.10, 169.29) | 135.26 (132.26, 138.32) | -0.68 (-0.73, -0.62) | <0.001 | 1.11 (0.73, 1.62) | 0.57 (0.39, 0.81) | -2.30 (-2.54, -2.06) | <0.001 |
| Myanmar | 82.27 (80.38, 84.20) | 66.62 (65.13, 68.14) | -0.75 (-0.83, -0.68) | <0.001 | 1177.06 (1169.70, 1184.45) | 1042.61 (1036.68, 1048.56) | -0.43 (-0.50, -0.36) | <0.001 | 214.77 (211.62, 217.95) | 89.25 (87.52, 91.00) | -3.01 (-3.15, -2.87) | <0.001 | 2.64 (2.30, 3.02) | 0.66 (0.52, 0.83) | -4.72 (-5.01, -4.43) | <0.001 |
| Namibia | 94.52 (83.79, 106.43) | 93.39 (85.35, 102.01) | -0.05 (-0.06, -0.03) | <0.001 | 1672.11 (1624.01, 1721.41) | 1670.69 (1635.69, 1706.29) | -0.01 (-0.01, 0.00) | 0.077 | 206.72 (189.79, 224.89) | 112.03 (103.09, 121.57) | -2.12 (-2.37, -1.87) | <0.001 | 2.14 (0.74, 5.05) | 0.55 (0.10, 1.72) | -4.60 (-5.17, -4.02) | <0.001 |
| Nauru | 18.89 (0.00, 243.35) | 14.55 (0.00, 213.71) | -0.92 (-1.03, -0.81) | <0.001 | 164.39 (36.57, 475.57) | 149.36 (34.03, 428.38) | -0.37 (-0.47, -0.26) | <0.001 | 616.30 (319.20, 1083.17) | 510.63 (257.30, 916.14) | -0.66 (-0.90, -0.42) | <0.001 | 10.26 (0.00, 226.93) | 8.42 (0.00, 202.00) | -0.69 (-0.95, -0.44) | <0.001 |

| | | | | | | | | | | | | | | | | |
|---|---|---|---|---|---|---|---|---|---|---|---|---|---|---|---|---|
| Nepal | 73.70 (71.01, 76.46) | 70.68 (68.76, 72.64) | -0.14 (-0.16, -0.12) | <0.001 | 874.75 (865.07, 884.51) | 902.65 (895.60, 909.75) | 0.11 (0.09, 0.13) | <0.001 | 524.25 (516.66, 531.92) | 196.03 (192.70, 199.39) | -3.34 (-3.53, -3.15) | <0.001 | 8.27 (7.33, 9.30) | 2.65 (2.28, 3.08) | -3.85 (-4.15, -3.56) | <0.001 |
| Netherlands | 0.36 (0.18, 0.66) | 0.35 (0.17, 0.67) | -0.08 (-0.20, 0.04) | 0.193 | 6.97 (6.05, 7.99) | 7.18 (6.19, 8.29) | 0.11 (0.08, 0.14) | <0.001 | 4.26 (3.56, 5.07) | 2.34 (1.80, 3.01) | -2.04 (-2.48, -1.59) | <0.001 | 0.07 (0.01, 0.26) | 0.03 (0.00, 0.22) | -2.97 (-5.18, -0.71) | 0.010 |
| New Zealand | 2.38 (1.37, 3.84) | 1.15 (0.51, 2.26) | -2.52 (-2.62, -2.41) | <0.001 | 36.65 (32.29, 41.45) | 29.93 (26.06, 34.24) | -0.70 (-0.84, -0.57) | <0.001 | 68.14 (62.16, 74.55) | 27.23 (23.59, 31.30) | -2.95 (-3.99, -1.89) | <0.001 | 1.12 (0.48, 2.24) | 0.44 (0.10, 1.28) | -3.01 (-3.97, -2.05) | <0.001 |
| Nicaragua | 75.68 (69.77, 82.03) | 75.73 (71.26, 80.41) | -0.01 (-0.08, 0.07) | 0.864 | 1465.42 (1437.60, 1493.69) | 1495.90 (1475.69, 1516.31) | 0.08 (0.06, 0.10) | <0.001 | 151.62 (142.71, 161.00) | 85.21 (80.44, 90.19) | -1.99 (-2.38, -1.60) | <0.001 | 1.37 (0.64, 2.64) | 0.23 (0.05, 0.67) | -6.16 (-7.14, -5.16) | <0.001 |
| Niger | 77.22 (72.81, 81.86) | 81.55 (78.83, 84.36) | 0.19 (0.17, 0.21) | <0.001 | 1186.05 (1167.92, 1204.43) | 1260.28 (1248.97, 1271.68) | 0.21 (0.20, 0.23) | <0.001 | 189.16 (182.03, 196.53) | 114.89 (111.53, 118.34) | -1.70 (-1.86, -1.54) | <0.001 | 2.16 (1.45, 3.13) | 0.89 (0.62, 1.26) | -2.96 (-3.39, -2.54) | <0.001 |
| Nigeria | 75.78 (74.50, 77.08) | 80.61 (79.79, 81.44) | 0.22 (0.20, 0.23) | <0.001 | 1270.75 (1265.26, 1276.26) | 1383.96 (1380.42, 1387.51) | 0.30 (0.28, 0.31) | <0.001 | 154.69 (152.79, 156.61) | 89.03 (88.14, 89.93) | -1.89 (-1.99, -1.79) | <0.001 | 1.54 (1.35, 1.74) | 0.38 (0.33, 0.45) | -4.70 (-5.00, -4.41) | <0.001 |
| Niue | 9.79 (0.00, 1059.76) | 7.37 (0.00, 1476.12) | -0.98 (-1.01, -0.95) | <0.001 | 109.53 (0.02, 1251.11) | 109.13 (0.00, 1671.08) | -0.02 (-0.05, 0.01) | 0.204 | 299.20 (10.48, 1585.56) | 179.47 (0.14, 1801.76) | -1.73 (-1.98, -1.48) | <0.001 | 4.94 (0.00, 1049.80) | 2.88 (0.00, 1467.23) | -1.83 (-2.09, -1.57) | <0.001 |
| North Macedonia | 1.80 (0.74, 3.70) | 0.94 (0.25, 2.74) | -2.17 (-2.42, -1.91) | <0.001 | 12.98 (9.69, 17.04) | 8.21 (5.61, 11.77) | -1.57 (-1.71, -1.43) | <0.001 | 67.85 (60.03, 76.43) | 12.19 (9.03, 16.27) | -5.75 (-6.28, -5.22) | <0.001 | 1.18 (0.37, 2.84) | 0.21 (0.00, 1.69) | -5.86 (-6.50, -5.22) | <0.001 |
| Northern Mariana Islands | 35.12 (9.69, 92.29) | 37.48 (5.28, 153.72) | 0.23 (0.20, 0.25) | <0.001 | 891.85 (728.39, 1082.79) | 936.53 (702.55, 1236.01) | 0.17 (0.15, 0.19) | <0.001 | 264.62 (179.73, 377.80) | 159.95 (70.16, 325.81) | -1.71 (-1.96, -1.45) | <0.001 | 3.76 (0.00, 43.78) | 1.94 (0.00, 102.04) | -2.14 (-2.44, -1.85) | <0.001 |
| Norway | 0.23 (0.02, | 0.21 (0.02, | -0.14 (-0.51, | 0.477 | 4.23 (2.91, | 4.36 (3.08, | 0.11 (0.08, | <0.001 | 4.51 (3.15, | 1.11 (0.53, | -4.76 (-5.17, | <0.001 | 0.07 (0.00, | 0.01 (0.00, | -7.15 (-10.41, | <0.001 |

| | | | | | | | | | | | | | | | | |
|---|---|---|---|---|---|---|---|---|---|---|---|---|---|---|---|---|
| | 0.91) | 0.87) | 0.24) | | 5.95) | 6.04) | 0.13) | | 6.27) | 2.11) | -4.35) | | 0.66) | 0.55) | -3.78) | |
| Oman | 1.01 (0.19, 3.23) | 1.04 (0.46, 2.12) | 0.19 (0.04, 0.34) | 0.011 | 16.30 (11.89, 21.90) | 17.43 (14.62, 20.67) | 0.26 (0.16, 0.36) | <0.001 | 23.79 (18.39, 30.36) | 5.46 (3.95, 7.42) | -4.94 (-5.43, -4.45) | <0.001 | 0.39 (0.01, 2.25) | 0.08 (0.00, 0.76) | -5.22 (-5.56, -4.87) | <0.001 |
| Pakistan | 100.41 (99.02, 101.82) | 105.96 (105.02, 106.90) | 0.21 (0.02, 0.40) | 0.029 | 1314.41 (1309.10 , 1319.74) | 1402.41 (1398.87 , 1405.96) | 0.25 (0.12, 0.39) | <0.001 | 610.89 (607.21, 614.59) | 484.71 (482.61, 486.82) | -0.79 (-0.92, -0.66) | <0.001 | 9.35 (8.89, 9.82) | 7.09 (6.83, 7.35) | -0.94 (-1.10, -0.79) | <0.001 |
| Palau | 7.38 (0.00, 135.20) | 5.94 (0.00, 181.09) | -0.73 (-0.75, -0.70) | <0.001 | 86.90 (17.01, 265.23) | 85.47 (11.67, 311.87) | -0.05 (-0.08, -0.02) | 0.005 | 213.23 (86.06, 442.87) | 130.20 (29.52, 378.89) | -1.68 (-1.82, -1.54) | <0.001 | 3.51 (0.00, 127.69) | 2.11 (0.00, 174.13) | -1.74 (-1.88, -1.59) | <0.001 |
| Palestine | 42.11 (35.79, 49.42) | 41.22 (37.41, 45.35) | -0.07 (-0.08, -0.07) | <0.001 | 779.88 (750.32, 810.44) | 793.18 (775.64, 811.04) | 0.06 (0.06, 0.06) | <0.001 | 70.80 (62.01, 80.62) | 45.85 (41.70, 50.33) | -1.50 (-1.66, -1.33) | <0.001 | 0.57 (0.06, 2.45) | 0.14 (0.01, 0.71) | -4.69 (-5.20, -4.18) | <0.001 |
| Panama | 69.13 (62.16, 76.72) | 64.20 (58.73, 70.04) | -0.25 (-0.28, -0.21) | <0.001 | 1382.29 (1349.54 , 1415.68) | 1349.77 (1324.22 , 1375.69) | -0.08 (-0.09, -0.07) | <0.001 | 188.20 (176.17, 200.88) | 92.26 (85.66, 99.23) | -2.39 (-3.00, -1.78) | <0.001 | 2.06 (0.98, 3.88) | 0.47 (0.12, 1.26) | -4.85 (-6.53, -3.14) | <0.001 |
| Papua New Guinea | 46.59 (41.94, 51.66) | 47.76 (44.78, 50.90) | 0.10 (0.07, 0.12) | <0.001 | 810.80 (790.96, 831.05) | 844.99 (832.28, 857.85) | 0.15 (0.13, 0.16) | <0.001 | 680.11 (661.53, 699.12) | 635.56 (624.47, 646.81) | -0.28 (-0.55, -0.01) | 0.046 | 10.83 (8.58, 13.53) | 10.01 (8.65, 11.52) | -0.32 (-0.60, -0.03) | 0.028 |
| Paraguay | 71.21 (65.50, 77.32) | 68.93 (64.75, 73.32) | -0.11 (-0.12, -0.09) | <0.001 | 1416.56 (1389.93 , 1443.60) | 1416.99 (1397.71 , 1436.47) | 0.01 (-0.00, 0.01) | 0.202 | 119.22 (111.54, 127.31) | 87.98 (83.22, 92.94) | -1.01 (-1.31, -0.72) | <0.001 | 0.88 (0.34, 1.91) | 0.36 (0.12, 0.83) | -2.96 (-3.65, -2.27) | <0.001 |
| Peru | 67.29 (64.95, 69.69) | 67.16 (65.20, 69.16) | -0.01 (-0.04, 0.02) | 0.455 | 1257.68 (1247.16 , 1268.27) | 1299.59 (1291.01 , 1308.21) | 0.11 (0.09, 0.13) | <0.001 | 110.96 (107.83, 114.17) | 74.13 (72.09, 76.21) | -1.38 (-1.77, -0.99) | <0.001 | 0.87 (0.61, 1.21) | 0.21 (0.11, 0.35) | -4.71 (-6.34, -3.06) | <0.001 |
| Philippines | 40.34 (39.26, 41.44) | 39.54 (38.73, 40.37) | -0.08 (-0.09, -0.06) | <0.001 | 619.92 (615.54, 624.32) | 683.36 (679.93, 686.79) | 0.33 (0.19, 0.47) | <0.001 | 112.25 (110.38, 114.14) | 200.32 (198.47, 202.19) | 2.13 (1.49, 2.78) | <0.001 | 1.38 (1.18, 1.61) | 2.77 (2.56, 3.00) | 2.59 (1.72, 3.47) | <0.001 |
| Poland | 2.13 | 1.23 | -1.84 | <0.001 | 15.91 | 12.64 | -0.80 | <0.001 | 61.79 | 6.60 | -7.62 | <0.001 | 1.08 | 0.10 | -8.04 | <0.001 |

| | | | | | | | | | | | | | | | | |
|---|---|---|---|---|---|---|---|---|---|---|---|---|---|---|---|---|
| | (1.82, 2.48) | (0.99, 1.53) | (-2.09, -1.59) | | (15.02, 16.85) | (11.78, 13.55) | (-0.91, -0.68) | | (60.06, 63.57) | (5.98, 7.26) | (-8.11, -7.13) | | (0.86, 1.34) | (0.04, 0.23) | (-8.61, -7.47) | |
| Portugal | 1.19 (0.75, 1.79) | 0.42 (0.17, 0.91) | -3.60 (-3.85, -3.35) | <0.001 | 12.82 (11.26, 14.53) | 7.44 (6.14, 8.95) | -1.88 (-1.97, -1.79) | <0.001 | 46.74 (43.71, 49.92) | 8.17 (6.83, 9.72) | -5.74 (-6.97, -4.50) | <0.001 | 0.79 (0.44, 1.30) | 0.13 (0.02, 0.50) | -6.57 (-6.77, -6.36) | <0.001 |
| Puerto Rico | 1.19 (0.54, 2.30) | 0.95 (0.33, 2.17) | -0.75 (-0.88, -0.61) | <0.001 | 22.06 (18.78, 25.75) | 19.81 (16.35, 23.81) | -0.37 (-0.41, -0.32) | <0.001 | 27.97 (24.26, 32.09) | 7.96 (5.84, 10.62) | -4.73 (-5.31, -4.15) | <0.001 | 0.45 (0.10, 1.27) | 0.12 (0.00, 0.92) | -4.99 (-5.76, -4.21) | <0.001 |
| Qatar | 1.53 (0.05, 9.56) | 0.94 (0.21, 3.21) | -1.68 (-1.91, -1.45) | <0.001 | 21.62 (11.95, 36.66) | 16.86 (12.73, 22.19) | -0.85 (-0.93, -0.76) | <0.001 | 30.81 (19.04, 47.81) | 5.93 (3.66, 9.46) | -5.36 (-7.37, -3.30) | <0.001 | 0.50 (0.00, 7.89) | 0.08 (0.00, 2.02) | -6.00 (-7.30, -4.68) | <0.001 |
| Republic of Korea | 0.51 (0.38, 0.67) | 0.36 (0.24, 0.52) | -1.25 (-1.44, -1.05) | <0.001 | 10.79 (10.16, 11.45) | 9.41 (8.76, 10.11) | -0.49 (-0.54, -0.44) | <0.001 | 17.06 (16.26, 17.88) | 3.13 (2.75, 3.54) | -5.76 (-6.43, -5.08) | <0.001 | 0.27 (0.18, 0.40) | 0.04 (0.01, 0.13) | -6.38 (-6.73, -6.02) | <0.001 |
| Republic of Moldova | 8.71 (6.96, 10.82) | 2.62 (1.59, 4.22) | -4.07 (-4.14, -3.99) | <0.001 | 57.54 (52.78, 62.63) | 23.98 (20.42, 28.09) | -2.98 (-3.02, -2.93) | <0.001 | 164.51 (156.35, 173.02) | 25.16 (21.52, 29.33) | -6.63 (-7.81, -5.44) | <0.001 | 2.82 (1.85, 4.16) | 0.41 (0.08, 1.45) | -6.82 (-8.01, -5.62) | <0.001 |
| Romania | 3.26 (2.75, 3.85) | 1.24 (0.87, 1.71) | -3.26 (-3.45, -3.07) | <0.001 | 21.45 (20.09, 22.88) | 11.98 (10.74, 13.33) | -2.06 (-2.31, -1.82) | <0.001 | 111.20 (108.08, 114.39) | 12.57 (11.29, 13.96) | -7.15 (-7.73, -6.57) | <0.001 | 1.94 (1.55, 2.41) | 0.20 (0.07, 0.47) | -7.55 (-8.24, -6.85) | <0.001 |
| Russian Federation | 5.70 (5.44, 5.98) | 3.35 (3.12, 3.58) | -1.80 (-1.90, -1.70) | <0.001 | 50.75 (49.94, 51.56) | 39.69 (38.89, 40.50) | -0.82 (-0.90, -0.75) | <0.001 | 65.37 (64.46, 66.28) | 9.85 (9.46, 10.25) | -6.74 (-8.14, -5.31) | <0.001 | 1.10 (0.98, 1.22) | 0.14 (0.10, 0.19) | -7.31 (-8.66, -5.95) | <0.001 |
| Rwanda | 115.71 (110.24, 121.42) | 117.37 (113.42, 121.43) | 0.04 (0.02, 0.07) | 0.002 | 1923.79 (1900.50, 1947.32) | 1973.79 (1957.08, 1990.61) | 0.09 (0.07, 0.10) | <0.001 | 308.26 (298.97, 317.80) | 134.61 (130.28, 139.06) | -2.86 (-3.07, -2.65) | <0.001 | 3.56 (2.62, 4.76) | 0.67 (0.40, 1.07) | -5.62 (-5.98, -5.27) | <0.001 |
| Saint Kitts and Nevis | 5.31 (0.00, 60.22) | 1.30 (0.00, 38.80) | -4.86 (-5.11, -4.60) | <0.001 | 57.79 (18.32, 140.19) | 22.63 (3.95, 73.71) | -3.21 (-3.31, -3.11) | <0.001 | 220.51 (131.50, 349.48) | 25.92 (5.21, 78.89) | -7.10 (-7.52, -6.68) | <0.001 | 3.74 (0.00, 57.50) | 0.40 (0.00, 37.12) | -7.39 (-8.03, -6.74) | <0.001 |
| Saint Lucia | 71.60 (44.50, 111.54) | 70.25 (44.51, 105.83) | -0.06 (-0.09, -0.03) | 0.001 | 1461.65 (1321.82 , | 1469.60 (1342.84 , | 0.02 (0.00, 0.04) | 0.049 | 270.63 (211.13, 343.06) | 130.41 (94.82, 175.45) | -2.50 (-2.82, -2.18) | <0.001 | 3.44 (0.05, 24.02) | 1.04 (0.00, 14.15) | -4.09 (-4.61, -3.57) | <0.001 |

| | | | | | | | | | | | | | | | | |
|---|---|---|---|---|---|---|---|---|---|---|---|---|---|---|---|---|
| | | | | | 1613.65) | 1605.48) | | | | | | | | | | |
| Saint Vincent and the Grenadines | 76.15 (44.98, 124.21) | 73.92 (41.53, 121.73) | -0.10 (-0.11, -0.09) | <0.001 | 1399.90 (1245.79, 1569.84) | 1398.56 (1241.95, 1569.61) | -0.01 (-0.04, 0.03) | 0.781 | 210.77 (153.15, 285.23) | 128.60 (84.46, 187.80) | -1.64 (-2.27, -1.00) | <0.001 | 2.42 (0.00, 27.92) | 1.05 (0.00, 20.43) | -2.75 (-3.65, -1.83) | <0.001 |
| Samoa | 42.81 (22.61, 75.79) | 43.27 (25.32, 70.04) | 0.04 (0.02, 0.07) | <0.001 | 894.96 (789.83, 1011.59) | 922.83 (830.30, 1023.51) | 0.11 (0.09, 0.12) | <0.001 | 438.54 (363.23, 526.01) | 373.32 (314.17, 440.94) | -0.52 (-0.64, -0.40) | <0.001 | 6.73 (0.66, 28.24) | 5.57 (0.70, 20.88) | -0.62 (-0.73, -0.50) | <0.001 |
| San Marino | 0.45 (0.00, 94.19) | 0.42 (0.00, 79.74) | -0.21 (-0.37, -0.06) | 0.008 | 7.78 (0.00, 107.90) | 7.54 (0.00, 92.67) | -0.11 (-0.13, -0.09) | <0.001 | 8.06 (0.00, 108.54) | 6.42 (0.00, 90.52) | -0.77 (-0.89, -0.66) | <0.001 | 0.13 (0.00, 93.55) | 0.10 (0.00, 79.15) | -1.01 (-1.74, -0.27) | 0.008 |
| Sao Tome and Principe | 70.20 (39.78, 118.67) | 75.15 (51.84, 106.01) | 0.24 (0.23, 0.25) | <0.001 | 1273.90 (1123.21, 1441.40) | 1391.78 (1281.56, 1509.34) | 0.31 (0.30, 0.32) | <0.001 | 156.85 (106.82, 224.89) | 122.00 (91.00, 160.63) | -0.71 (-1.43, 0.03) | 0.060 | 1.60 (0.00, 28.01) | 0.92 (0.00, 11.75) | -2.11 (-2.99, -1.23) | <0.001 |
| Saudi Arabia | 2.49 (1.91, 3.21) | 1.81 (1.52, 2.13) | -1.08 (-1.20, -0.96) | <0.001 | 31.74 (29.59, 34.02) | 23.02 (21.97, 24.13) | -1.12 (-1.19, -1.05) | <0.001 | 128.70 (124.31, 133.21) | 29.78 (28.59, 31.03) | -4.94 (-5.48, -4.39) | <0.001 | 2.15 (1.61, 2.83) | 0.49 (0.35, 0.68) | -5.01 (-5.48, -4.55) | <0.001 |
| Senegal | 75.42 (71.07, 80.01) | 77.48 (74.44, 80.62) | 0.10 (0.08, 0.11) | <0.001 | 1273.07 (1254.27, 1292.10) | 1324.10 (1311.09, 1337.20) | 0.15 (0.12, 0.17) | <0.001 | 136.46 (130.42, 142.74) | 90.73 (87.37, 94.21) | -1.37 (-1.63, -1.11) | <0.001 | 1.23 (0.71, 2.00) | 0.45 (0.24, 0.77) | -3.46 (-3.86, -3.05) | <0.001 |
| Serbia | 0.71 (0.37, 1.23) | 0.87 (0.47, 1.52) | 0.69 (0.43, 0.95) | <0.001 | 5.86 (4.78, 7.13) | 6.94 (5.65, 8.46) | 0.55 (0.46, 0.64) | <0.001 | 11.44 (9.92, 13.14) | 2.59 (1.84, 3.58) | -5.00 (-5.46, -4.55) | <0.001 | 0.19 (0.05, 0.55) | 0.04 (0.00, 0.39) | -5.15 (-5.97, -4.32) | <0.001 |
| Seychelles | 44.00 (17.43, 97.80) | 41.57 (16.70, 86.66) | -0.20 (-0.21, -0.19) | <0.001 | 894.17 (747.81, 1064.22) | 869.49 (736.02, 1020.97) | -0.10 (-0.10, -0.09) | <0.001 | 147.62 (91.70, 229.18) | 57.90 (27.79, 107.60) | -3.19 (-3.35, -3.03) | <0.001 | 1.76 (0.00, 38.41) | 0.27 (0.00, 24.37) | -6.32 (-7.10, -5.53) | <0.001 |
| Sierra Leone | 80.54 (74.21, 87.31) | 85.76 (81.59, 90.12) | 0.22 (0.20, 0.23) | <0.001 | 1255.29 (1229.37, 1281.65) | 1335.41 (1318.28, 1352.72) | 0.22 (0.19, 0.24) | <0.001 | 151.02 (142.17, 160.32) | 113.05 (108.13, 118.16) | -0.98 (-1.06, -0.90) | <0.001 | 1.48 (0.73, 2.74) | 0.81 (0.44, 1.38) | -2.03 (-2.36, -1.69) | <0.001 |
| Singapore | 1.51 | 0.43 | -4.34 | <0.001 | 20.31 | 10.33 | -2.37 | <0.001 | 48.81 | 5.11 | -7.77 | <0.001 | 0.83 | 0.08 | -8.21 | <0.001 |

| | | | | | | | | | | | | | | | | |
|---|---|---|---|---|---|---|---|---|---|---|---|---|---|---|---|---|
| | (0.76, 2.74) | (0.13, 1.27) | (-4.65, -4.04) | | (17.19, 23.87) | (8.39, 12.69) | (-2.56, -2.17) | | (43.96, 54.10) | (3.83, 6.82) | (-8.72, -6.82) | | (0.31, 1.85) | (0.00, 0.79) | (-9.26, -7.15) | |
| Slovakia | 1.10 (0.56, 1.96) | 1.23 (0.64, 2.26) | 0.38 (0.26, 0.49) | <0.001 | 10.79 (8.89, 13.01) | 11.94 (9.81, 14.48) | 0.32 (0.20, 0.45) | <0.001 | 15.23 (12.96, 17.80) | 6.30 (4.79, 8.22) | -2.92 (-3.62, -2.22) | <0.001 | 0.26 (0.05, 0.83) | 0.10 (0.00, 0.77) | -3.30 (-3.68, -2.91) | <0.001 |
| Slovenia | 0.70 (0.14, 2.27) | 0.76 (0.13, 2.90) | 0.20 (-0.16, 0.56) | 0.279 | 5.72 (3.60, 8.73) | 6.28 (3.78, 10.06) | 0.28 (0.23, 0.34) | <0.001 | 13.04 (9.73, 17.21) | 4.23 (2.22, 7.57) | -4.03 (-4.81, -3.24) | <0.001 | 0.22 (0.00, 1.53) | 0.07 (0.00, 1.92) | -4.19 (-5.06, -3.31) | <0.001 |
| Solomon Islands | 51.00 (34.44, 73.78) | 51.79 (40.15, 65.87) | 0.06 (-0.02, 0.14) | 0.133 | 840.33 (768.16, 918.25) | 855.54 (805.95, 907.49) | 0.06 (0.06, 0.07) | <0.001 | 1333.83 (1236.89, 1436.89) | 1004.92 (950.04, 1062.21) | -0.98 (-1.13, -0.84) | <0.001 | 22.28 (11.29, 40.21) | 16.53 (10.15, 25.50) | -1.03 (-1.17, -0.89) | <0.001 |
| Somalia | 125.67 (119.59, 132.00) | 136.26 (132.63, 139.97) | 0.28 (0.26, 0.30) | <0.001 | 1992.94 (1967.79, 2018.34) | 2121.03 (2105.99, 2136.16) | 0.22 (0.20, 0.24) | <0.001 | 282.59 (273.14, 292.30) | 197.12 (192.57, 201.77) | -1.22 (-1.30, -1.14) | <0.001 | 3.09 (2.17, 4.28) | 1.59 (1.20, 2.07) | -2.27 (-2.40, -2.13) | <0.001 |
| South Africa | 104.87 (102.70, 107.07) | 105.84 (103.96, 107.75) | 0.04 (0.02, 0.06) | <0.001 | 1845.62 (1836.23, 1855.04) | 1877.75 (1869.93, 1885.60) | 0.06 (0.04, 0.08) | <0.001 | 302.83 (298.99, 306.72) | 124.84 (122.84, 126.87) | -3.02 (-3.96, -2.07) | <0.001 | 3.64 (3.22, 4.10) | 0.61 (0.48, 0.77) | -5.86 (-7.58, -4.10) | <0.001 |
| South Sudan | 90.70 (85.26, 96.46) | 95.49 (91.08, 100.07) | 0.18 (0.17, 0.19) | <0.001 | 1777.63 (1751.93, 1803.65) | 1862.85 (1842.30, 1883.60) | 0.16 (0.15, 0.18) | <0.001 | 182.20 (174.08, 190.65) | 135.77 (130.28, 141.46) | -0.98 (-1.14, -0.82) | <0.001 | 1.60 (0.92, 2.67) | 0.78 (0.42, 1.36) | -2.42 (-2.88, -1.96) | <0.001 |
| Spain | 0.79 (0.60, 1.03) | 0.38 (0.25, 0.56) | -2.48 (-2.66, -2.30) | <0.001 | 9.26 (8.57, 9.99) | 7.16 (6.52, 7.86) | -0.90 (-0.97, -0.83) | <0.001 | 23.12 (22.02, 24.27) | 5.21 (4.67, 5.79) | -5.05 (-5.52, -4.58) | <0.001 | 0.39 (0.26, 0.57) | 0.08 (0.03, 0.19) | -5.39 (-5.93, -4.84) | <0.001 |
| Sri Lanka | 3.26 (2.69, 3.91) | 1.92 (1.52, 2.38) | -1.81 (-1.96, -1.67) | <0.001 | 34.21 (32.33, 36.18) | 27.67 (26.10, 29.32) | -0.71 (-0.84, -0.59) | <0.001 | 90.02 (86.97, 93.16) | 18.46 (17.17, 19.82) | -5.20 (-5.90, -4.50) | <0.001 | 1.47 (1.10, 1.92) | 0.28 (0.14, 0.50) | -5.44 (-6.15, -4.71) | <0.001 |
| Sudan | 56.44 (54.13, 58.84) | 57.03 (55.47, 58.62) | 0.04 (0.01, 0.08) | 0.010 | 823.52 (814.31, 832.82) | 864.39 (858.17, 870.64) | 0.17 (0.16, 0.18) | <0.001 | 226.40 (221.57, 231.32) | 90.20 (88.19, 92.25) | -3.09 (-3.21, -2.97) | <0.001 | 3.12 (2.57, 3.76) | 0.82 (0.64, 1.05) | -4.46 (-4.60, -4.33) | <0.001 |

| | | | | | | | | | | | | | | | | |
|---|---|---|---|---|---|---|---|---|---|---|---|---|---|---|---|---|
| Suriname | 71.52 (54.24, 93.20) | 70.15 (55.19, 87.94) | -0.07 (-0.11, -0.03) | 0.001 | 1302.73 (1222.52, 1387.25) | 1314.39 (1246.97, 1384.54) | 0.04 (0.01, 0.06) | 0.003 | 172.75 (144.08, 205.90) | 113.86 (94.66, 135.86) | -1.31 (-2.30, -0.31) | 0.010 | 1.86 (0.10, 9.47) | 0.87 (0.02, 5.15) | -2.42 (-4.52, -0.28) | 0.027 |
| Sweden | 0.29 (0.08, 0.75) | 0.26 (0.07, 0.70) | -0.45 (-1.12, 0.24) | 0.199 | 5.67 (4.51, 7.05) | 5.31 (4.22, 6.61) | -0.25 (-0.35, -0.15) | <0.001 | 5.31 (4.19, 6.65) | 1.62 (1.05, 2.41) | -3.91 (-4.89, -2.93) | <0.001 | 0.08 (0.00, 0.44) | 0.02 (0.00, 0.34) | -4.43 (-5.09, -3.77) | <0.001 |
| Switzerland | 0.55 (0.22, 1.18) | 0.45 (0.18, 1.03) | -0.72 (-1.01, -0.42) | <0.001 | 10.25 (8.56, 12.20) | 9.61 (8.02, 11.46) | -0.25 (-0.32, -0.19) | <0.001 | 6.26 (4.98, 7.81) | 1.49 (0.92, 2.34) | -4.84 (-5.34, -4.34) | <0.001 | 0.10 (0.01, 0.55) | 0.02 (0.00, 0.42) | -5.45 (-7.46, -3.39) | <0.001 |
| Syrian Arab Republic | 44.39 (41.74, 47.18) | 45.35 (42.89, 47.93) | 0.08 (0.06, 0.10) | <0.001 | 750.81 (739.24, 762.53) | 775.48 (764.88, 786.21) | 0.12 (0.10, 0.13) | <0.001 | 320.16 (312.51, 327.97) | 67.35 (64.25, 70.58) | -5.27 (-5.80, -4.75) | <0.001 | 4.78 (3.87, 5.86) | 0.50 (0.27, 0.89) | -7.53 (-8.23, -6.83) | <0.001 |
| Taiwan (Province of China) | 3.37 (2.86, 3.95) | 2.91 (2.42, 3.48) | -0.51 (-0.60, -0.43) | <0.001 | 32.56 (30.93, 34.27) | 42.50 (40.50, 44.58) | 0.90 (0.69, 1.11) | <0.001 | 42.14 (40.27, 44.07) | 6.95 (6.16, 7.82) | -5.96 (-6.56, -5.36) | <0.001 | 0.69 (0.47, 0.98) | 0.08 (0.02, 0.26) | -7.17 (-7.77, -6.56) | <0.001 |
| Tajikistan | 63.72 (59.07, 68.68) | 66.32 (62.80, 70.00) | 0.14 (0.13, 0.15) | <0.001 | 1165.60 (1144.67, 1186.85) | 1190.70 (1175.56, 1205.99) | 0.07 (0.07, 0.08) | <0.001 | 519.45 (505.10, 534.14) | 173.63 (167.84, 179.58) | -3.65 (-4.06, -3.23) | <0.001 | 7.94 (6.23, 10.01) | 2.02 (1.44, 2.77) | -4.52 (-5.05, -3.99) | <0.001 |
| Thailand | 44.97 (43.84, 46.13) | 41.91 (40.74, 43.12) | -0.24 (-0.28, -0.21) | <0.001 | 808.39 (803.50, 813.30) | 792.55 (787.53, 797.60) | -0.07 (-0.09, -0.05) | <0.001 | 126.86 (124.91, 128.83) | 47.41 (46.19, 48.66) | -3.40 (-3.87, -2.93) | <0.001 | 1.49 (1.29, 1.73) | 0.16 (0.10, 0.25) | -7.44 (-8.22, -6.66) | <0.001 |
| Timor-Leste | 55.52 (44.44, 68.74) | 55.12 (46.65, 64.93) | -0.03 (-0.04, -0.01) | <0.001 | 852.29 (806.43, 900.25) | 881.99 (845.47, 919.85) | 0.12 (0.11, 0.13) | <0.001 | 245.48 (220.85, 272.27) | 170.73 (154.72, 188.12) | -1.13 (-1.84, -0.42) | 0.002 | 3.48 (1.14, 8.36) | 2.14 (0.70, 5.28) | -1.52 (-2.44, -0.60) | 0.001 |
| Togo | 79.02 (72.72, 85.76) | 83.08 (78.78, 87.56) | 0.17 (0.16, 0.19) | <0.001 | 1249.42 (1223.24, 1276.07) | 1319.89 (1302.41, 1337.55) | 0.19 (0.18, 0.20) | <0.001 | 159.10 (149.92, 168.75) | 97.58 (92.87, 102.47) | -1.68 (-1.80, -1.55) | <0.001 | 1.62 (0.81, 2.96) | 0.57 (0.26, 1.08) | -3.54 (-3.85, -3.22) | <0.001 |
| Tokelau | 15.41 (0.00, 1731.77 | 11.24 (0.00, 2327.55 | -1.10 (-1.15, -1.04) | <0.001 | 127.28 (0.01, 1924.39) | 125.02 (0.00, 2518.07) | -0.08 (-0.10, -0.05) | <0.001 | 624.06 (53.57, 2742.90) | 341.48 (3.27, 2854.60) | -2.06 (-2.09, -2.03) | <0.001 | 10.52 (0.00, 1723.31 | 5.65 (0.00, 2317.46 | -2.12 (-2.16, -2.08) | <0.001 |

| | | | | | | | | | | | | | | | | |
|---|---|---|---|---|---|---|---|---|---|---|---|---|---|---|---|---|
| | ) | ) | | | | | | | | | | | ) | ) | | |
| Tonga | 52.80 (24.61, 102.83) | 52.72 (25.70, 97.01) | 0.02 (-0.08, 0.12) | 0.708 | 1307.74 (1142.75, 1492.12) | 1327.82 (1170.70, 1500.80) | 0.10 (0.00, 0.19) | 0.042 | 234.05 (165.13, 324.13) | 159.93 (108.19, 228.47) | -1.27 (-1.35, -1.20) | <0.001 | 2.94 (0.00, 33.87) | 1.63 (0.00, 24.31) | -1.99 (-2.16, -1.83) | <0.001 |
| Trinidad and Tobago | 70.92 (60.90, 82.19) | 67.82 (57.58, 79.44) | -0.15 (-0.19, -0.11) | <0.001 | 1396.42 (1350.30, 1443.77) | 1405.85 (1359.44, 1453.53) | 0.02 (0.01, 0.04) | 0.004 | 196.70 (179.61, 215.05) | 103.59 (91.31, 117.16) | -2.22 (-2.79, -1.65) | <0.001 | 2.18 (0.74, 5.06) | 0.61 (0.05, 2.84) | -4.49 (-5.28, -3.68) | <0.001 |
| Tunisia | 1.48 (0.93, 2.24) | 1.02 (0.65, 1.54) | -1.27 (-1.40, -1.13) | <0.001 | 21.87 (19.64, 24.29) | 16.48 (14.85, 18.25) | -0.97 (-1.01, -0.94) | <0.001 | 56.92 (53.28, 60.77) | 15.13 (13.60, 16.81) | -4.45 (-4.56, -4.34) | <0.001 | 0.94 (0.52, 1.58) | 0.24 (0.09, 0.56) | -4.56 (-4.92, -4.20) | <0.001 |
| Turkey | 1.22 (1.02, 1.44) | 0.94 (0.80, 1.10) | -0.86 (-1.05, -0.68) | <0.001 | 17.45 (16.69, 18.23) | 16.24 (15.63, 16.86) | -0.24 (-0.33, -0.16) | <0.001 | 23.69 (22.81, 24.60) | 5.66 (5.31, 6.03) | -4.84 (-5.59, -4.10) | <0.001 | 0.38 (0.28, 0.52) | 0.08 (0.04, 0.14) | -5.22 (-6.92, -3.49) | <0.001 |
| Turkmenistan | 61.77 (56.41, 67.55) | 59.55 (54.70, 64.73) | -0.13 (-0.14, -0.11) | <0.001 | 1128.44 (1104.61, 1152.70) | 1126.20 (1104.93, 1147.77) | -0.01 (-0.02, 0.00) | 0.127 | 380.81 (366.90, 395.14) | 193.33 (184.58, 202.39) | -2.15 (-3.20, -1.09) | <0.001 | 5.47 (3.91, 7.50) | 2.31 (1.45, 3.52) | -2.71 (-3.97, -1.44) | <0.001 |
| Tuvalu | 21.93 (0.01, 245.76) | 13.98 (0.00, 222.20) | -1.55 (-1.63, -1.47) | <0.001 | 137.41 (24.30, 435.69) | 119.98 (20.63, 393.85) | -0.48 (-0.53, -0.44) | <0.001 | 923.42 (544.97, 1468.90) | 454.26 (214.97, 853.69) | -2.41 (-2.55, -2.26) | <0.001 | 15.64 (0.00, 234.03) | 7.57 (0.00, 210.14) | -2.45 (-2.57, -2.33) | <0.001 |
| Uganda | 126.71 (122.97, 130.54) | 125.40 (123.07, 127.77) | -0.04 (-0.09, 0.01) | 0.130 | 2065.75 (2049.79, 2081.83) | 2303.77 (2293.29, 2314.30) | 0.40 (0.27, 0.53) | <0.001 | 158.28 (153.88, 162.79) | 142.04 (139.45, 144.68) | -0.34 (-0.67, -0.02) | 0.041 | 1.01 (0.68, 1.46) | 0.54 (0.39, 0.73) | -2.15 (-2.73, -1.56) | <0.001 |
| Ukraine | 2.67 (2.36, 3.01) | 2.43 (2.10, 2.80) | -0.27 (-0.59, 0.05) | 0.096 | 24.29 (23.32, 25.28) | 24.32 (23.21, 25.48) | 0.08 (-0.16, 0.31) | 0.516 | 16.13 (15.34, 16.94) | 17.71 (16.75, 18.72) | 0.35 (-2.06, 2.82) | 0.777 | 0.26 (0.17, 0.38) | 0.28 (0.17, 0.44) | 0.34 (-2.39, 3.15) | 0.807 |
| United Arab Emirates | 37.03 (30.32, 44.85) | 38.03 (34.00, 42.44) | 0.09 (0.09, 0.10) | <0.001 | 769.81 (738.47, 802.22) | 776.34 (758.78, 794.26) | 0.03 (0.02, 0.04) | <0.001 | 50.76 (42.96, 59.65) | 41.10 (37.12, 45.46) | -0.71 (-0.78, -0.64) | <0.001 | 0.23 (0.00, 1.92) | 0.07 (0.00, 0.68) | -4.07 (-5.25, -2.88) | <0.001 |
| United Kingdom | 0.53 | 0.38 | -1.06 | <0.001 | 8.64 | 7.57 | -0.43 | <0.001 | 8.51 | 3.32 | -3.18 | <0.001 | 0.14 | 0.05 | -3.42 | <0.001 |

| | | | | | | | | | | | | | | | | |
|---|---|---|---|---|---|---|---|---|---|---|---|---|---|---|---|---|
| | (0.40, 0.69) | (0.28, 0.52) | (-1.27, -0.85) | | (8.08, 9.22) | (7.06, 8.11) | (-0.50, -0.37) | | (7.97, 9.09) | (2.99, 3.67) | (-3.55, -2.81) | | (0.08, 0.23) | (0.02, 0.12) | (-4.41, -2.42) | |
| United Republic of Tanzania | 113.61 (110.77, 116.51) | 117.97 (116.05, 119.92) | 0.13 (0.10, 0.17) | <0.001 | 1911.41 (1899.15, 1923.74) | 2027.69 (2019.40, 2036.00) | 0.20 (0.19, 0.21) | <0.001 | 161.85 (158.31, 165.45) | 129.90 (127.82, 132.02) | -0.74 (-0.80, -0.68) | <0.001 | 1.17 (0.88, 1.53) | 0.55 (0.42, 0.71) | -2.53 (-2.70, -2.35) | <0.001 |
| United States of America | 2.31 (2.19, 2.45) | 1.58 (1.47, 1.68) | -1.30 (-1.36, -1.24) | <0.001 | 33.47 (32.98, 33.97) | 24.80 (24.39, 25.22) | -1.02 (-1.06, -0.98) | <0.001 | 18.80 (18.44, 19.17) | 7.15 (6.93, 7.37) | -3.25 (-3.73, -2.77) | <0.001 | 0.29 (0.25, 0.34) | 0.10 (0.07, 0.13) | -3.65 (-5.00, -2.28) | <0.001 |
| United States Virgin Islands | 1.87 (0.00, 22.95) | 1.33 (0.00, 28.52) | -1.19 (-1.33, -1.04) | <0.001 | 29.46 (10.96, 64.50) | 23.56 (6.10, 63.63) | -0.78 (-0.91, -0.66) | <0.001 | 55.72 (28.49, 98.90) | 19.11 (4.09, 56.96) | -3.58 (-3.98, -3.17) | <0.001 | 0.91 (0.00, 21.19) | 0.30 (0.00, 26.61) | -3.78 (-4.05, -3.50) | <0.001 |
| Uruguay | 1.93 (0.96, 3.45) | 1.88 (0.96, 3.34) | -0.06 (-0.20, 0.08) | 0.383 | 26.12 (22.10, 30.67) | 27.20 (23.24, 31.66) | 0.15 (0.13, 0.16) | <0.001 | 33.83 (29.23, 38.96) | 15.32 (12.40, 18.75) | -2.71 (-3.08, -2.35) | <0.001 | 0.56 (0.12, 1.59) | 0.24 (0.02, 1.07) | -2.88 (-3.32, -2.45) | <0.001 |
| Uzbekistan | 62.99 (60.67, 65.39) | 64.01 (62.15, 65.92) | 0.06 (0.05, 0.07) | <0.001 | 1114.44 (1104.37, 1124.59) | 1184.36 (1176.36, 1192.41) | 0.21 (0.20, 0.22) | <0.001 | 523.88 (516.88, 530.95) | 226.01 (222.53, 229.54) | -2.77 (-3.64, -1.88) | <0.001 | 7.96 (7.11, 8.89) | 2.88 (2.50, 3.31) | -3.34 (-4.29, -2.38) | <0.001 |
| Vanuatu | 49.64 (27.47, 84.00) | 50.62 (34.22, 72.65) | 0.06 (0.02, 0.10) | 0.001 | 945.23 (836.74, 1064.87) | 960.91 (883.81, 1043.32) | 0.06 (0.03, 0.08) | <0.001 | 662.38 (569.98, 766.31) | 699.65 (633.20, 771.51) | 0.18 (-0.09, 0.44) | 0.203 | 10.50 (2.05, 32.88) | 11.04 (4.18, 24.12) | 0.16 (-0.12, 0.44) | 0.276 |
| Venezuela (Bolivarian Republic of) | 2.56 (2.08, 3.12) | 1.63 (1.31, 2.00) | -1.55 (-1.69, -1.40) | <0.001 | 36.00 (34.14, 37.94) | 25.60 (24.29, 26.97) | -1.17 (-1.30, -1.04) | <0.001 | 82.62 (79.78, 85.54) | 23.07 (21.82, 24.38) | -4.37 (-5.66, -3.05) | <0.001 | 1.37 (1.03, 1.80) | 0.37 (0.22, 0.56) | -4.46 (-5.84, -3.05) | <0.001 |
| Viet Nam | 4.43 (4.07, 4.81) | 2.84 (2.61, 3.08) | -1.52 (-1.60, -1.44) | <0.001 | 42.28 (41.20, 43.39) | 35.64 (34.82, 36.49) | -0.58 (-0.69, -0.46) | <0.001 | 105.77 (104.04, 107.53) | 34.22 (33.42, 35.05) | -3.80 (-3.97, -3.64) | <0.001 | 1.79 (1.57, 2.03) | 0.56 (0.46, 0.67) | -3.95 (-4.13, -3.77) | <0.001 |
| Yemen | 64.46 (61.26, 67.79) | 66.62 (64.66, 68.63) | 0.11 (-0.03, 0.24) | 0.119 | 909.31 (896.88, 921.88) | 979.56 (971.89, 987.29) | 0.26 (0.15, 0.37) | <0.001 | 184.38 (178.74, 190.17) | 111.26 (108.67, 113.90) | -1.69 (-1.93, -1.46) | <0.001 | 2.42 (1.80, 3.18) | 1.11 (0.86, 1.40) | -2.62 (-2.93, -2.30) | <0.001 |
| Zambia | 117.85 (112.64, | 121.94 (118.57, | 0.12 (0.10, | <0.001 | 2015.76 (1992.73 | 2005.44 (1991.19 | -0.03 (-0.06, | 0.079 | 226.74 (219.11, | 142.65 (138.88, | -1.59 (-1.71, | <0.001 | 2.13 (1.44, | 0.78 (0.52, | -3.41 (-3.59, | <0.001 |

| | | | | | | | | | | | | | | | | |
|---|---|---|---|---|---|---|---|---|---|---|---|---|---|---|---|---|
| | 123.29) | 125.39) | 0.14) | | , 2039.04) | , 2019.77) | 0.00) | | 234.61) | 146.51) | -1.47) | | 3.08) | 1.13) | -3.24) | |
| Zimbabwe | 100.45 (96.22, 104.83) | 105.38 (101.93, 108.92) | 0.17 (0.14, 0.20) | <0.001 | 1614.03 (1596.25 , 1631.98) | 1665.75 (1651.67 , 1679.92) | 0.11 (0.10, 0.13) | <0.001 | 270.55 (263.38, 277.89) | 284.63 (278.86, 290.49) | 0.16 (-0.48, 0.81) | 0.622 | 3.13 (2.39, 4.04) | 3.31 (2.71, 4.01) | 0.17 (-0.89, 1.25) | 0.751 |
| **Men** | | | | | | | | | | | | | | | | |
| Afghanistan | 53.75 (50.16, 57.60) | 54.84 (53.18, 56.55) | 0.08 (0.05, 0.12) | <0.001 | 709.22 (695.18, 723.52) | 726.50 (720.17, 732.88) | 0.09 (0.06, 0.11) | <0.001 | 176.69 (169.64, 184.00) | 100.99 (98.56, 103.46) | -1.82 (-1.98, -1.66) | <0.001 | 2.41 (1.64, 3.49) | 1.13 (0.88, 1.44) | -2.46 (-2.65, -2.27) | <0.001 |
| Albania | 56.91 (51.57, 62.69) | 53.84 (47.60, 60.71) | -0.19 (-0.21, -0.17) | <0.001 | 1019.52 (996.18, 1043.28) | 994.36 (966.92, 1022.41) | -0.09 (-0.09, -0.08) | <0.001 | 99.14 (91.96, 106.76) | 67.93 (60.91, 75.58) | -1.36 (-1.62, -1.11) | <0.001 | 0.82 (0.30, 1.86) | 0.33 (0.03, 1.40) | -3.18 (-3.80, -2.56) | <0.001 |
| Algeria | 34.15 (32.57, 35.80) | 34.81 (33.55, 36.10) | 0.07 (0.04, 0.11) | <0.001 | 482.99 (476.76, 489.28) | 542.38 (537.48, 547.31) | 0.42 (0.33, 0.52) | <0.001 | 82.85 (80.29, 85.48) | 41.96 (40.60, 43.34) | -2.31 (-2.44, -2.17) | <0.001 | 0.99 (0.72, 1.32) | 0.27 (0.17, 0.40) | -4.43 (-4.71, -4.16) | <0.001 |
| American Samoa | 35.97 (8.89, 101.66) | 37.28 (9.59, 104.87) | 0.12 (0.10, 0.14) | <0.001 | 788.50 (623.68, 986.35) | 808.92 (642.42, 1009.11) | 0.09 (0.08, 0.09) | <0.001 | 309.30 (209.12, 443.75) | 216.47 (134.27, 334.49) | -1.23 (-1.50, -0.96) | <0.001 | 4.49 (0.00, 53.43) | 2.93 (0.00, 52.94) | -1.63 (-1.92, -1.35) | <0.001 |
| Andorra | 0.36 (0.00, 39.72) | 0.34 (0.00, 38.07) | -0.12 (-0.26, 0.02) | 0.091 | 6.50 (0.10, 49.50) | 6.26 (0.08, 47.89) | -0.09 (-0.16, -0.03) | 0.005 | 4.15 (0.01, 45.65) | 2.59 (0.00, 41.73) | -1.60 (-1.69, -1.51) | <0.001 | 0.07 (0.00, 39.26) | 0.04 (0.00, 37.59) | -1.83 (-1.83, -1.83) | <0.001 |
| Angola | 96.17 (91.96, 100.53) | 94.61 (92.06, 97.23) | -0.05 (-0.07, -0.04) | <0.001 | 1770.11 (1751.46 , 1788.91) | 1781.88 (1770.39 , 1793.43) | 0.02 (0.02, 0.03) | <0.001 | 162.50 (156.99, 168.17) | 118.52 (115.61, 121.50) | -1.04 (-1.29, -0.79) | <0.001 | 1.21 (0.78, 1.81) | 0.52 (0.35, 0.76) | -2.84 (-3.66, -2.02) | <0.001 |
| Antigua and Barbuda | 60.42 (25.52, 123.75) | 59.27 (28.45, 109.52) | -0.06 (-0.11, -0.00) | 0.034 | 1120.20 (941.45, 1324.72) | 1108.93 (956.73, 1278.95) | -0.03 (-0.05, -0.02) | <0.001 | 107.96 (57.93, 185.88) | 76.83 (41.09, 131.66) | -1.01 (-1.35, -0.66) | <0.001 | 0.90 (0.00, 36.83) | 0.39 (0.00, 23.90) | -2.69 (-3.96, -1.41) | <0.001 |
| Argentina | 52.10 (50.31, 53.94) | 52.27 (50.75, 53.83) | 0.01 (-0.00, 0.02) | 0.062 | 913.25 (905.64, 920.92) | 931.48 (925.07, 937.93) | 0.07 (0.06, 0.08) | <0.001 | 81.91 (79.64, 84.24) | 55.06 (53.51, 56.65) | -1.37 (-1.50, -1.24) | <0.001 | 0.65 (0.46, 0.89) | 0.18 (0.10, 0.29) | -4.34 (-4.81, -3.87) | <0.001 |
| Armenia | 61.45 | 56.99 | -0.26 | <0.001 | 1114.97 | 1058.78 | -0.18 | <0.001 | 163.51 | 82.31 | -2.42 | <0.001 | 1.91 | 0.54 | -4.49 | <0.001 |

| | | | | | | | | | | | | | | | | |
|---|---|---|---|---|---|---|---|---|---|---|---|---|---|---|---|---|
| | (55.79, 67.54) | (50.59, 64.04) | (-0.30, -0.22) | | (1090.50, 1139.87) | (1031.57, 1086.59) | (-0.21, -0.14) | | (154.21, 173.23) | (74.92, 90.32) | (-2.82, -2.02) | | (1.03, 3.26) | (0.11, 1.72) | (-5.12, -3.85) | |
| Australia | 0.66 (0.42, 1.00) | 0.60 (0.39, 0.89) | -0.32 (-0.53, -0.10) | 0.004 | 17.40 (16.02, 18.86) | 17.30 (16.05, 18.63) | -0.01 (-0.09, 0.08) | 0.907 | 19.33 (17.89, 20.87) | 9.18 (8.29, 10.14) | -2.52 (-3.08, -1.97) | <0.001 | 0.30 (0.15, 0.56) | 0.14 (0.05, 0.32) | -2.61 (-3.37, -1.85) | <0.001 |
| Austria | 1.16 (0.68, 1.87) | 0.46 (0.19, 1.00) | -3.13 (-3.34, -2.91) | <0.001 | 15.42 (13.51, 17.55) | 7.13 (5.83, 8.68) | -2.68 (-2.83, -2.53) | <0.001 | 9.85 (8.34, 11.58) | 4.20 (3.22, 5.41) | -3.00 (-3.59, -2.41) | <0.001 | 0.15 (0.02, 0.55) | 0.06 (0.00, 0.44) | -3.23 (-3.71, -2.74) | <0.001 |
| Azerbaijan | 58.84 (55.12, 62.77) | 57.16 (53.90, 60.58) | -0.11 (-0.12, -0.09) | <0.001 | 1008.90 (993.01, 1024.99) | 991.93 (978.62, 1005.39) | -0.06 (-0.08, -0.04) | <0.001 | 169.59 (163.06, 176.33) | 98.20 (94.03, 102.53) | -1.93 (-2.62, -1.22) | <0.001 | 2.03 (1.37, 2.93) | 0.83 (0.49, 1.34) | -3.15 (-4.14, -2.14) | <0.001 |
| Bahamas | 64.64 (45.78, 89.37) | 64.32 (47.22, 85.67) | -0.02 (-0.03, -0.01) | 0.001 | 1127.66 (1042.26, 1218.68) | 1114.35 (1039.27, 1193.51) | -0.04 (-0.05, -0.03) | <0.001 | 112.10 (86.18, 143.94) | 91.13 (70.63, 115.83) | -0.74 (-1.24, -0.23) | 0.005 | 0.97 (0.00, 9.84) | 0.64 (0.00, 6.54) | -1.44 (-2.42, -0.45) | 0.005 |
| Bahrain | 0.77 (0.03, 5.46) | 0.66 (0.09, 2.78) | -0.53 (-0.72, -0.35) | <0.001 | 14.67 (9.12, 23.17) | 12.43 (8.80, 17.30) | -0.57 (-0.68, -0.46) | <0.001 | 27.22 (18.95, 38.46) | 10.87 (7.31, 15.75) | -3.14 (-4.03, -2.25) | <0.001 | 0.42 (0.00, 5.02) | 0.16 (0.00, 2.10) | -3.29 (-4.50, -2.08) | <0.001 |
| Bangladesh | 50.49 (49.55, 51.44) | 50.78 (50.01, 51.55) | 0.02 (0.01, 0.03) | <0.001 | 624.27 (620.84, 627.72) | 672.30 (669.47, 675.13) | 0.26 (0.24, 0.27) | <0.001 | 235.87 (233.81, 237.95) | 107.87 (106.74, 109.00) | -2.53 (-3.07, -1.98) | <0.001 | 3.32 (3.08, 3.58) | 1.22 (1.10, 1.34) | -3.24 (-4.26, -2.19) | <0.001 |
| Barbados | 61.43 (42.31, 86.36) | 61.09 (41.00, 87.71) | -0.03 (-0.08, 0.02) | 0.230 | 1143.29 (1054.81, 1237.31) | 1141.69 (1048.83, 1240.70) | -0.01 (-0.03, 0.01) | 0.413 | 109.85 (83.65, 141.78) | 78.28 (55.47, 107.49) | -1.13 (-1.62, -0.63) | <0.001 | 0.92 (0.00, 8.97) | 0.39 (0.00, 8.78) | -2.80 (-3.74, -1.85) | <0.001 |
| Belarus | 7.38 (6.26, 8.64) | 3.18 (2.41, 4.17) | -2.89 (-3.01, -2.78) | <0.001 | 40.75 (38.03, 43.61) | 25.50 (23.11, 28.11) | -1.61 (-1.69, -1.54) | <0.001 | 142.77 (137.68, 148.01) | 25.60 (23.28, 28.13) | -5.76 (-6.80, -4.72) | <0.001 | 2.49 (1.86, 3.28) | 0.43 (0.18, 0.94) | -5.87 (-7.10, -4.63) | <0.001 |
| Belgium | 0.43 (0.19, 0.86) | 0.41 (0.17, 0.84) | -0.21 (-0.53, 0.11) | 0.199 | 6.84 (5.71, 8.13) | 6.86 (5.70, 8.21) | -0.02 (-0.17, 0.13) | 0.827 | 3.73 (2.91, 4.71) | 2.38 (1.72, 3.22) | -1.58 (-1.93, -1.22) | <0.001 | 0.06 (0.00, 0.33) | 0.03 (0.00, 0.31) | -2.53 (-3.31, -1.75) | <0.001 |
| Belize | 68.79 | 66.83 | -0.10 | <0.001 | 1107.97 | 1101.98 | -0.02 | 0.037 | 109.04 | 95.49 | -0.41 | 0.035 | 0.91 | 0.71 | -0.73 | 0.059 |

| | | | | | | | | | | | | | | | | |
|---|---|---|---|---|---|---|---|---|---|---|---|---|---|---|---|---|
| | (44.87, 102.72) | (51.01, 86.29) | (-0.12, -0.08) | | (1000.34 , 1225.14) | (1033.16 , 1174.37) | (-0.04, -0.00) | | (77.35, 150.81) | (75.97, 118.69) | (-0.80, -0.03) | | (0.00, 15.78) | (0.00, 6.19) | (-1.48, 0.03) | |
| Benin | 72.43 (66.58, 78.70) | 74.20 (70.78, 77.76) | 0.08 (0.08, 0.09) | <0.001 | 1142.52 (1118.28 , 1167.18) | 1189.73 (1175.49 , 1204.12) | 0.14 (0.12, 0.15) | <0.001 | 132.52 (124.39, 141.09) | 93.00 (89.05, 97.10) | -1.22 (-1.29, -1.15) | <0.001 | 1.27 (0.59, 2.44) | 0.59 (0.31, 1.03) | -2.65 (-2.84, -2.46) | <0.001 |
| Bermuda | 1.23 (0.00, 41.90) | 0.70 (0.00, 53.41) | -1.97 (-2.17, -1.77) | <0.001 | 22.79 (4.54, 74.71) | 15.09 (0.84, 77.25) | -1.44 (-1.53, -1.34) | <0.001 | 52.95 (20.60, 116.79) | 15.35 (0.85, 77.97) | -4.38 (-4.73, -4.02) | <0.001 | 0.85 (0.00, 41.27) | 0.24 (0.00, 52.62) | -4.47 (-4.95, -3.99) | <0.001 |
| Bhutan | 58.88 (46.92, 73.44) | 55.69 (45.45, 67.65) | -0.18 (-0.20, -0.17) | <0.001 | 775.88 (728.21, 826.17) | 763.61 (724.45, 804.41) | -0.05 (-0.06, -0.04) | <0.001 | 495.66 (457.29, 536.69) | 235.05 (213.50, 258.28) | -2.53 (-2.68, -2.38) | <0.001 | 7.70 (3.58, 14.94) | 3.26 (1.20, 7.23) | -2.92 (-3.06, -2.77) | <0.001 |
| Bolivia (Plurinational State of) | 67.87 (63.35, 72.66) | 67.24 (64.04, 70.57) | -0.04 (-0.05, -0.02) | <0.001 | 1091.48 (1072.59 , 1110.65) | 1094.09 (1080.94 , 1107.36) | 0.00 (-0.03, 0.03) | 0.996 | 122.39 (116.09, 128.95) | 73.06 (69.69, 76.55) | -1.78 (-1.82, -1.74) | <0.001 | 1.17 (0.63, 2.02) | 0.34 (0.15, 0.68) | -4.17 (-4.29, -4.05) | <0.001 |
| Bosnia and Herzegovina | 1.19 (0.62, 2.09) | 1.40 (0.60, 2.86) | 0.59 (0.35, 0.82) | <0.001 | 9.15 (7.38, 11.23) | 11.06 (8.47, 14.27) | 0.73 (0.53, 0.93) | <0.001 | 16.89 (14.45, 19.65) | 5.14 (3.40, 7.53) | -4.28 (-4.97, -3.59) | <0.001 | 0.28 (0.05, 0.86) | 0.08 (0.00, 0.98) | -4.66 (-5.38, -3.94) | <0.001 |
| Botswana | 92.59 (80.88, 105.75) | 89.07 (81.09, 97.63) | -0.13 (-0.15, -0.12) | <0.001 | 1518.92 (1468.40 , 1570.89) | 1520.52 (1487.22 , 1554.39) | 0.00 (-0.00, 0.01) | 0.346 | 213.73 (194.98, 233.96) | 119.66 (110.46, 129.45) | -1.96 (-2.12, -1.81) | <0.001 | 2.32 (0.76, 5.70) | 0.77 (0.21, 2.00) | -3.71 (-3.98, -3.44) | <0.001 |
| Brazil | 71.93 (71.00, 72.87) | 70.97 (70.17, 71.79) | -0.04 (-0.06, -0.02) | <0.001 | 1202.70 (1198.80 , 1206.60) | 1197.69 (1194.41 , 1200.98) | -0.02 (-0.03, -0.01) | 0.002 | 136.02 (134.72, 137.34) | 79.84 (79.00, 80.69) | -1.84 (-2.02, -1.65) | <0.001 | 1.29 (1.17, 1.43) | 0.37 (0.32, 0.44) | -4.23 (-4.54, -3.92) | <0.001 |
| Brunei Darussalam | 0.96 (0.00, 8.15) | 0.68 (0.01, 5.33) | -1.21 (-1.40, -1.03) | <0.001 | 18.27 (9.44, 32.28) | 13.20 (7.29, 22.34) | -1.13 (-1.18, -1.07) | <0.001 | 44.00 (29.49, 63.48) | 16.41 (9.81, 26.17) | -3.43 (-4.33, -2.53) | <0.001 | 0.71 (0.00, 7.72) | 0.27 (0.00, 4.65) | -3.41 (-4.24, -2.59) | <0.001 |
| Bulgaria | 3.14 (2.33, | 1.50 (0.88, | -2.50 (-2.64, | <0.001 | 19.11 (17.00, | 11.87 (9.91, | -1.64 (-1.77, | <0.001 | 107.13 (102.03, | 27.86 (24.79, | -4.35 (-5.04, | <0.001 | 1.83 (1.22, | 0.47 (0.16, | -4.49 (-4.94, | <0.001 |

| | | | | | | | | | | | | | | | | |
|---|---|---|---|---|---|---|---|---|---|---|---|---|---|---|---|---|
| | 4.15) | 2.47) | -2.37) | | 21.42) | 14.15) | -1.52) | | 112.44) | 31.25) | -3.65) | | 2.65) | 1.17) | -4.05) | |
| Burkina Faso | 74.76 (70.34, 79.42) | 75.00 (72.36, 77.71) | 0.01 (0.00, 0.02) | 0.022 | 1133.48 (1115.41 , 1151.79) | 1151.06 (1140.34 , 1161.85) | 0.05 (0.03, 0.08) | <0.001 | 90.29 (85.35, 95.48) | 80.96 (78.17, 83.83) | -0.36 (-0.43, -0.29) | <0.001 | 0.57 (0.25, 1.18) | 0.41 (0.23, 0.67) | -1.09 (-1.38, -0.81) | <0.001 |
| Burundi | 107.46 (101.23, 114.02) | 111.32 (107.07, 115.71) | 0.12 (0.06, 0.17) | <0.001 | 1718.85 (1692.98 , 1745.04) | 1791.88 (1774.23 , 1809.66) | 0.15 (0.10, 0.20) | <0.001 | 217.40 (208.47, 226.65) | 145.06 (140.14, 150.11) | -1.38 (-1.48, -1.27) | <0.001 | 2.08 (1.30, 3.22) | 0.93 (0.58, 1.43) | -2.73 (-2.94, -2.51) | <0.001 |
| Cabo Verde | 69.00 (49.54, 95.98) | 67.02 (53.52, 82.99) | -0.10 (-0.12, -0.08) | <0.001 | 1257.87 (1164.63 , 1358.18) | 1268.81 (1208.05 , 1331.93) | 0.03 (0.01, 0.04) | <0.001 | 166.30 (133.04, 207.14) | 78.40 (63.85, 95.40) | -2.57 (-2.81, -2.33) | <0.001 | 1.76 (0.03, 14.79) | 0.28 (0.00, 3.73) | -6.18 (-6.89, -5.46) | <0.001 |
| Cambodia | 40.60 (37.67, 43.73) | 39.02 (37.00, 41.13) | -0.14 (-0.17, -0.11) | <0.001 | 563.16 (551.86, 574.67) | 556.64 (548.93, 564.44) | -0.08 (-0.18, 0.02) | 0.130 | 244.59 (237.14, 252.24) | 106.69 (103.32, 110.13) | -2.82 (-2.91, -2.72) | <0.001 | 3.59 (2.73, 4.67) | 1.30 (0.95, 1.74) | -3.40 (-3.48, -3.32) | <0.001 |
| Cameroon | 69.77 (65.98, 73.75) | 74.26 (72.10, 76.47) | 0.21 (0.19, 0.24) | <0.001 | 1105.92 (1090.22 , 1121.80) | 1186.51 (1177.61 , 1195.46) | 0.24 (0.23, 0.25) | <0.001 | 104.20 (99.43, 109.15) | 86.99 (84.60, 89.44) | -0.61 (-0.66, -0.56) | <0.001 | 0.84 (0.46, 1.43) | 0.49 (0.33, 0.72) | -1.79 (-2.03, -1.55) | <0.001 |
| Canada | 1.37 (1.09, 1.71) | 1.25 (0.98, 1.57) | -0.30 (-0.36, -0.23) | <0.001 | 24.18 (22.92, 25.50) | 23.66 (22.44, 24.94) | -0.07 (-0.12, -0.02) | 0.012 | 8.95 (8.19, 9.76) | 5.47 (4.89, 6.10) | -1.63 (-1.94, -1.33) | <0.001 | 0.13 (0.05, 0.27) | 0.07 (0.02, 0.19) | -2.05 (-3.15, -0.94) | <0.001 |
| Central African Republic | 108.85 (100.08, 118.28) | 111.69 (105.34, 118.36) | 0.09 (0.08, 0.11) | <0.001 | 1841.38 (1803.77 , 1879.65) | 1871.11 (1844.04 , 1898.51) | 0.06 (0.05, 0.06) | <0.001 | 213.77 (201.42, 226.79) | 173.07 (165.10, 181.35) | -0.72 (-0.86, -0.57) | <0.001 | 1.94 (0.94, 3.70) | 1.30 (0.71, 2.25) | -1.34 (-1.60, -1.08) | <0.001 |
| Chad | 74.23 (68.95, 79.84) | 74.24 (71.04, 77.56) | -0.00 (-0.02, 0.02) | 0.831 | 1133.66 (1112.09 , 1155.58) | 1150.50 (1137.28 , 1163.85) | 0.05 (0.04, 0.06) | <0.001 | 131.21 (124.01, 138.76) | 104.40 (100.47, 108.46) | -0.72 (-0.84, -0.60) | <0.001 | 1.25 (0.64, 2.25) | 0.80 (0.49, 1.26) | -1.42 (-1.63, -1.20) | <0.001 |
| Chile | 2.36 (1.81, | 1.40 (1.04, | -1.77 (-2.02, | <0.001 | 25.21 (23.36, | 20.69 (19.20, | -0.69 (-0.77, | <0.001 | 70.87 (67.74, | 11.76 (10.65, | -5.95 (-6.38, | <0.001 | 1.19 (0.82, | 0.18 (0.07, | -6.37 (-7.21, | <0.001 |

| | | | | | | | | | | | | | | | | |
|---|---|---|---|---|---|---|---|---|---|---|---|---|---|---|---|---|
| | 3.03) | 1.86) | -1.52) | | 27.17) | 22.27) | -0.61) | | 74.11) | 12.97) | -5.51) | | 1.69) | 0.40) | -5.52) | |
| China | 33.94 (33.73, 34.16) | 28.15 (27.93, 28.37) | -0.65 (-0.71, -0.58) | <0.001 | 597.59 (596.68, 598.49) | 478.94 (478.07, 479.81) | -0.76 (-0.79, -0.74) | <0.001 | 129.03 (128.60, 129.45) | 41.27 (41.02, 41.52) | -3.89 (-4.14, -3.64) | <0.001 | 1.71 (1.66, 1.76) | 0.31 (0.29, 0.33) | -5.82 (-6.18, -5.46) | <0.001 |
| Colombia | 1.07 (0.83, 1.36) | 0.91 (0.73, 1.13) | -0.55 (-0.80, -0.30) | <0.001 | 15.84 (14.89, 16.83) | 14.98 (14.21, 15.78) | -0.19 (-0.26, -0.13) | <0.001 | 40.55 (39.03, 42.12) | 5.63 (5.16, 6.13) | -6.75 (-7.77, -5.71) | <0.001 | 0.66 (0.48, 0.89) | 0.08 (0.03, 0.16) | -7.14 (-8.13, -6.13) | <0.001 |
| Comoros | 96.06 (76.49, 119.94) | 99.45 (84.28, 116.69) | 0.12 (0.11, 0.13) | <0.001 | 1710.33 (1619.58, 1805.40) | 1773.23 (1706.07, 1842.44) | 0.12 (0.11, 0.14) | <0.001 | 136.82 (112.52, 165.53) | 109.84 (93.67, 128.10) | -0.75 (-1.68, 0.19) | 0.116 | 0.87 (0.01, 8.03) | 0.39 (0.00, 3.53) | -2.73 (-6.06, 0.72) | 0.120 |
| Congo | 97.49 (88.82, 106.89) | 98.03 (92.10, 104.25) | 0.03 (-0.05, 0.12) | 0.413 | 1840.42 (1800.70, 1880.88) | 1840.39 (1814.12, 1866.95) | -0.02 (-0.10, 0.07) | 0.732 | 157.13 (145.95, 169.05) | 111.30 (104.93, 117.96) | -1.14 (-1.32, -0.95) | <0.001 | 1.08 (0.36, 2.71) | 0.37 (0.10, 0.98) | -3.72 (-4.49, -2.95) | <0.001 |
| Cook Islands | 4.54 (0.00, 129.37) | 3.93 (0.00, 151.72) | -0.49 (-0.58, -0.40) | <0.001 | 65.45 (11.00, 225.31) | 66.59 (6.89, 259.90) | 0.08 (0.01, 0.16) | 0.035 | 200.30 (84.21, 413.45) | 109.17 (22.28, 325.52) | -2.15 (-2.63, -1.66) | <0.001 | 3.23 (0.00, 126.95) | 1.75 (0.00, 147.37) | -2.17 (-2.66, -1.68) | <0.001 |
| Costa Rica | 62.90 (56.93, 69.36) | 60.66 (55.71, 65.94) | -0.13 (-0.14, -0.11) | <0.001 | 1164.97 (1138.41, 1192.03) | 1148.91 (1127.25, 1170.90) | -0.05 (-0.07, -0.03) | <0.001 | 105.55 (97.64, 113.97) | 81.82 (76.12, 87.84) | -0.83 (-1.00, -0.66) | <0.001 | 0.83 (0.27, 1.99) | 0.45 (0.13, 1.15) | -1.98 (-2.50, -1.46) | <0.001 |
| Cote d'Ivoire | 74.88 (71.47, 78.43) | 76.08 (73.80, 78.42) | 0.05 (0.05, 0.06) | <0.001 | 1184.41 (1170.44, 1198.51) | 1201.65 (1192.41, 1210.95) | 0.05 (0.04, 0.06) | <0.001 | 118.45 (114.09, 122.95) | 86.92 (84.45, 89.45) | -1.05 (-1.14, -0.96) | <0.001 | 1.00 (0.63, 1.51) | 0.47 (0.31, 0.70) | -2.55 (-2.82, -2.28) | <0.001 |
| Croatia | 1.06 (0.52, 1.97) | 0.80 (0.29, 1.88) | -0.97 (-1.42, -0.51) | <0.001 | 7.79 (6.13, 9.78) | 6.37 (4.64, 8.60) | -0.70 (-1.01, -0.39) | <0.001 | 21.26 (18.44, 24.41) | 4.56 (3.12, 6.53) | -5.08 (-6.10, -4.04) | <0.001 | 0.36 (0.09, 1.03) | 0.07 (0.00, 0.86) | -5.52 (-6.44, -4.58) | <0.001 |
| Cuba | 60.57 (57.55, 63.71) | 60.63 (57.09, 64.34) | -0.00 (-0.02, 0.01) | 0.810 | 1143.83 (1130.27, 1157.52) | 1141.50 (1126.21, 1156.95) | -0.01 (-0.02, 0.00) | 0.053 | 137.98 (133.26, 142.82) | 81.87 (77.82, 86.08) | -1.78 (-2.04, -1.52) | <0.001 | 1.38 (0.94, 1.97) | 0.46 (0.21, 0.90) | -3.73 (-4.39, -3.06) | <0.001 |

| | | | | | | | | | | | | | | | | |
|---|---|---|---|---|---|---|---|---|---|---|---|---|---|---|---|---|
| Cyprus | 0.40 (0.00, 3.30) | 0.33 (0.01, 2.87) | -0.71 (-0.97, -0.45) | <0.001 | 5.21 (2.28, 10.28) | 4.86 (2.50, 8.95) | -0.24 (-0.30, -0.19) | <0.001 | 7.02 (3.53, 12.62) | 3.34 (1.49, 6.94) | -2.46 (-3.10, -1.81) | <0.001 | 0.11 (0.00, 2.78) | 0.05 (0.00, 2.47) | -2.69 (-3.83, -1.53) | <0.001 |
| Czechia | 1.63 (1.12, 2.31) | 1.20 (0.75, 1.88) | -1.11 (-1.42, -0.80) | <0.001 | 10.97 (9.56, 12.55) | 9.35 (7.95, 10.96) | -0.60 (-0.83, -0.38) | <0.001 | 36.98 (34.32, 39.80) | 7.10 (5.91, 8.51) | -5.48 (-5.89, -5.06) | <0.001 | 0.64 (0.34, 1.12) | 0.12 (0.01, 0.50) | -5.72 (-6.26, -5.17) | <0.001 |
| Democratic People's Republic of Korea | 37.14 (35.25, 39.11) | 38.08 (36.41, 39.81) | 0.09 (0.08, 0.09) | <0.001 | 557.26 (549.86, 564.75) | 559.39 (553.01, 565.83) | 0.00 (-0.05, 0.05) | 0.896 | 110.96 (107.66, 114.35) | 88.01 (85.50, 90.58) | -0.78 (-0.85, -0.72) | <0.001 | 1.42 (1.06, 1.85) | 1.02 (0.77, 1.34) | -1.11 (-1.21, -1.01) | <0.001 |
| Democratic Republic of the Congo | 100.88 (98.59, 103.22) | 103.27 (101.80, 104.76) | 0.08 (0.07, 0.09) | <0.001 | 1826.28 (1816.15, 1836.47) | 1832.41 (1826.01, 1838.83) | 0.01 (-0.01, 0.03) | 0.267 | 150.77 (147.92, 153.66) | 130.57 (128.89, 132.28) | -0.49 (-0.56, -0.43) | <0.001 | 1.00 (0.78, 1.27) | 0.68 (0.57, 0.82) | -1.35 (-1.62, -1.09) | <0.001 |
| Denmark | 0.34 (0.08, 0.98) | 0.27 (0.05, 0.91) | -0.83 (-1.38, -0.28) | 0.003 | 4.64 (3.39, 6.21) | 4.24 (3.02, 5.82) | -0.31 (-0.46, -0.15) | <0.001 | 8.29 (6.61, 10.29) | 1.79 (1.04, 2.90) | -5.19 (-6.37, -4.00) | <0.001 | 0.14 (0.01, 0.67) | 0.03 (0.00, 0.51) | -5.50 (-6.65, -4.33) | <0.001 |
| Djibouti | 94.21 (77.21, 114.39) | 96.91 (85.40, 109.56) | 0.10 (0.08, 0.12) | <0.001 | 1672.77 (1595.15, 1753.57) | 1756.10 (1705.76, 1807.56) | 0.17 (0.16, 0.18) | <0.001 | 140.23 (118.75, 164.94) | 109.60 (97.31, 123.03) | -0.84 (-1.04, -0.63) | <0.001 | 0.95 (0.03, 6.37) | 0.40 (0.01, 2.18) | -2.87 (-3.36, -2.38) | <0.001 |
| Dominica | 68.88 (33.97, 127.90) | 67.57 (30.82, 128.90) | -0.07 (-0.09, -0.06) | <0.001 | 1158.10 (990.23, 1348.24) | 1152.91 (976.86, 1351.77) | -0.03 (-0.07, 0.02) | 0.274 | 141.40 (86.85, 219.86) | 98.67 (52.52, 169.09) | -1.26 (-1.44, -1.08) | <0.001 | 1.43 (0.00, 34.01) | 0.73 (0.00, 30.25) | -2.32 (-2.53, -2.10) | <0.001 |
| Dominican Republic | 66.87 (62.80, 71.16) | 63.86 (60.62, 67.23) | -0.16 (-0.17, -0.15) | <0.001 | 1095.17 (1077.97, 1112.60) | 1085.25 (1071.74, 1098.88) | -0.03 (-0.04, -0.02) | <0.001 | 122.90 (117.22, 128.82) | 88.08 (84.26, 92.03) | -1.11 (-1.27, -0.94) | <0.001 | 1.14 (0.65, 1.89) | 0.59 (0.32, 1.01) | -2.18 (-2.50, -1.86) | <0.001 |
| Ecuador | 65.02 (61.59, 68.61) | 63.67 (61.11, 66.31) | -0.08 (-0.09, -0.06) | <0.001 | 1068.70 (1054.25, 1083.30) | 1056.27 (1045.65, 1066.98) | -0.04 (-0.05, -0.03) | <0.001 | 108.98 (104.41, 113.72) | 69.78 (67.07, 72.57) | -1.56 (-1.75, -1.37) | <0.001 | 0.95 (0.56, 1.51) | 0.31 (0.16, 0.56) | -3.86 (-4.32, -3.41) | <0.001 |
| Egypt | 46.55 (45.30, | 48.67 (47.73, | 0.16 (0.13, | <0.001 | 766.00 (760.80, | 772.10 (768.34, | 0.02 (-0.01, | 0.205 | 132.80 (130.66, | 67.81 (66.70, | -2.33 (-2.64, | <0.001 | 1.55 (1.33, | 0.49 (0.40, | -3.91 (-4.44, | <0.001 |

| | | | | | | | | | | | | | | | | |
|---|---|---|---|---|---|---|---|---|---|---|---|---|---|---|---|---|
| | 47.82) | 49.62) | 0.18) | | 771.23) | 775.88) | 0.06) | | 134.96) | 68.93) | -2.02) | | 1.80) | 0.60) | -3.38) | |
| El Salvador | 72.79 (67.61, 78.31) | 71.10 (66.49, 75.98) | -0.08 (-0.10, -0.06) | <0.001 | 1088.74 (1067.67, 1110.17) | 1095.98 (1077.23, 1114.98) | 0.02 (0.01, 0.04) | 0.001 | 78.70 (73.07, 84.68) | 64.55 (60.04, 69.33) | -0.66 (-0.95, -0.37) | <0.001 | 0.45 (0.12, 1.22) | 0.21 (0.03, 0.73) | -2.67 (-4.04, -1.27) | <0.001 |
| Equatorial Guinea | 102.97 (80.50, 130.50) | 84.21 (75.14, 94.29) | -0.70 (-0.74, -0.66) | <0.001 | 1861.30 (1757.65, 1969.95) | 1715.15 (1671.43, 1759.89) | -0.29 (-0.31, -0.27) | <0.001 | 185.81 (155.25, 221.32) | 93.56 (83.64, 104.53) | -2.35 (-2.48, -2.23) | <0.001 | 1.49 (0.05, 9.69) | 0.18 (0.00, 1.95) | -6.99 (-7.77, -6.20) | <0.001 |
| Eritrea | 126.79 (117.95, 136.20) | 124.58 (119.03, 130.34) | -0.06 (-0.10, -0.02) | 0.002 | 2078.74 (2040.91, 2117.16) | 2076.91 (2053.39, 2100.65) | -0.02 (-0.07, 0.02) | 0.240 | 222.38 (210.44, 234.89) | 158.65 (152.26, 165.26) | -1.13 (-1.38, -0.88) | <0.001 | 1.93 (0.98, 3.56) | 0.93 (0.51, 1.61) | -2.41 (-2.76, -2.06) | <0.001 |
| Estonia | 5.70 (3.34, 9.16) | 3.29 (1.40, 7.08) | -1.86 (-1.98, -1.74) | <0.001 | 36.37 (29.85, 43.95) | 27.42 (20.95, 35.59) | -0.96 (-1.03, -0.89) | <0.001 | 76.92 (67.25, 87.65) | 9.01 (5.54, 14.26) | -6.94 (-8.32, -5.54) | <0.001 | 1.30 (0.34, 3.48) | 0.13 (0.00, 2.77) | -8.77 (-9.32, -8.21) | <0.001 |
| Eswatini | 93.52 (77.97, 111.72) | 93.09 (81.51, 105.96) | -0.02 (-0.02, -0.01) | <0.001 | 1491.59 (1424.40, 1561.43) | 1512.40 (1463.68, 1562.40) | 0.04 (0.00, 0.08) | 0.034 | 179.60 (156.82, 205.10) | 152.37 (137.17, 168.88) | -0.57 (-0.73, -0.41) | <0.001 | 1.78 (0.24, 6.86) | 1.32 (0.28, 3.94) | -1.07 (-1.32, -0.82) | <0.001 |
| Ethiopia | 92.95 (90.98, 94.96) | 95.76 (94.49, 97.05) | 0.11 (0.08, 0.14) | <0.001 | 1400.27 (1392.28, 1408.31) | 1588.39 (1582.99, 1593.81) | 0.43 (0.38, 0.49) | <0.001 | 190.68 (187.84, 193.56) | 112.73 (111.31, 114.16) | -1.77 (-1.92, -1.62) | <0.001 | 1.93 (1.66, 2.25) | 0.58 (0.48, 0.69) | -4.02 (-4.22, -3.82) | <0.001 |
| Fiji | 25.90 (18.62, 35.23) | 30.50 (23.03, 39.65) | 0.57 (0.52, 0.63) | <0.001 | 375.81 (346.34, 407.24) | 573.22 (539.06, 608.99) | 1.47 (1.39, 1.56) | <0.001 | 521.24 (486.67, 557.76) | 321.83 (296.36, 348.92) | -1.57 (-1.76, -1.38) | <0.001 | 8.18 (4.37, 14.15) | 4.76 (2.14, 9.14) | -1.85 (-2.38, -1.32) | <0.001 |
| Finland | 0.35 (0.08, 1.04) | 0.26 (0.04, 0.95) | -1.03 (-1.43, -0.64) | <0.001 | 4.71 (3.42, 6.37) | 4.29 (3.02, 5.95) | -0.36 (-0.44, -0.28) | <0.001 | 6.21 (4.76, 8.03) | 1.44 (0.76, 2.52) | -5.01 (-5.64, -4.38) | <0.001 | 0.10 (0.00, 0.68) | 0.02 (0.00, 0.57) | -5.96 (-6.64, -5.27) | <0.001 |
| France | 0.46 (0.34, 0.60) | 0.38 (0.27, 0.52) | -0.64 (-0.82, -0.46) | <0.001 | 6.52 (6.06, 7.02) | 6.09 (5.62, 6.59) | -0.23 (-0.29, -0.17) | <0.001 | 11.19 (10.58, 11.82) | 5.11 (4.68, 5.57) | -2.67 (-3.42, -1.93) | <0.001 | 0.18 (0.11, 0.28) | 0.08 (0.04, 0.16) | -2.74 (-3.56, -1.91) | <0.001 |

| | | | | | | | | | | | | | | | | |
|---|---|---|---|---|---|---|---|---|---|---|---|---|---|---|---|---|
| Gabon | 84.76 (72.43, 98.72) | 85.62 (76.33, 95.79) | 0.04 (0.03, 0.04) | <0.001 | 1725.73 (1667.38, 1785.70) | 1746.45 (1703.00, 1790.77) | 0.05 (0.03, 0.06) | <0.001 | 132.88 (117.20, 150.19) | 102.62 (92.32, 113.79) | -0.92 (-1.14, -0.70) | <0.001 | 0.78 (0.06, 3.59) | 0.30 (0.01, 1.73) | -3.29 (-3.89, -2.69) | <0.001 |
| Gambia | 73.93 (62.00, 87.77) | 77.56 (69.71, 86.14) | 0.16 (0.16, 0.17) | <0.001 | 1199.20 (1148.13, 1252.16) | 1259.84 (1226.61, 1293.82) | 0.17 (0.16, 0.18) | <0.001 | 112.42 (97.26, 129.52) | 92.43 (83.64, 101.98) | -0.65 (-1.19, -0.10) | 0.020 | 0.88 (0.07, 4.29) | 0.51 (0.07, 1.92) | -1.78 (-3.12, -0.42) | 0.010 |
| Georgia | 59.56 (54.95, 64.44) | 60.42 (54.19, 67.21) | 0.05 (0.05, 0.06) | <0.001 | 1024.97 (1005.67, 1044.56) | 1045.00 (1019.23, 1071.30) | 0.07 (0.06, 0.08) | <0.001 | 187.01 (178.81, 195.49) | 147.53 (137.92, 157.68) | -0.94 (-2.40, 0.55) | 0.214 | 2.25 (1.43, 3.37) | 1.59 (0.75, 3.04) | -1.28 (-2.66, 0.13) | 0.076 |
| Germany | 0.55 (0.44, 0.69) | 0.42 (0.32, 0.55) | -0.98 (-1.17, -0.79) | <0.001 | 7.64 (7.20, 8.10) | 6.83 (6.39, 7.29) | -0.44 (-0.67, -0.22) | <0.001 | 13.35 (12.78, 13.94) | 5.08 (4.72, 5.47) | -3.37 (-3.64, -3.10) | <0.001 | 0.22 (0.15, 0.31) | 0.08 (0.04, 0.15) | -3.59 (-3.82, -3.35) | <0.001 |
| Ghana | 74.52 (71.36, 77.80) | 72.00 (69.97, 74.07) | -0.12 (-0.15, -0.08) | <0.001 | 1185.47 (1172.41, 1198.64) | 1184.09 (1175.67, 1192.55) | -0.00 (-0.03, 0.02) | 0.801 | 89.42 (85.88, 93.08) | 75.43 (73.32, 77.59) | -0.59 (-0.65, -0.53) | <0.001 | 0.53 (0.29, 0.90) | 0.30 (0.18, 0.48) | -1.87 (-2.18, -1.56) | <0.001 |
| Greece | 0.40 (0.17, 0.80) | 0.35 (0.12, 0.83) | -0.44 (-0.61, -0.28) | <0.001 | 5.99 (4.94, 7.20) | 5.73 (4.56, 7.11) | -0.12 (-0.17, -0.08) | <0.001 | 6.43 (5.34, 7.68) | 2.36 (1.65, 3.29) | -3.40 (-4.10, -2.70) | <0.001 | 0.10 (0.01, 0.38) | 0.04 (0.00, 0.37) | -3.77 (-4.66, -2.86) | <0.001 |
| Greenland | 1.64 (0.00, 44.16) | 1.34 (0.00, 47.57) | -0.70 (-0.78, -0.62) | <0.001 | 25.60 (6.37, 78.50) | 23.39 (3.82, 82.22) | -0.32 (-0.33, -0.30) | <0.001 | 12.23 (1.28, 59.24) | 4.57 (0.00, 52.90) | -3.32 (-3.52, -3.12) | <0.001 | 0.19 (0.00, 42.00) | 0.06 (0.00, 45.37) | -3.91 (-4.87, -2.94) | <0.001 |
| Grenada | 67.37 (34.37, 121.86) | 64.87 (35.36, 110.73) | -0.14 (-0.20, -0.09) | <0.001 | 1180.53 (1019.89, 1361.04) | 1172.20 (1030.19, 1329.35) | -0.03 (-0.06, -0.01) | 0.020 | 234.38 (164.97, 324.90) | 124.99 (81.52, 184.59) | -2.09 (-2.60, -1.58) | <0.001 | 3.04 (0.00, 33.57) | 1.16 (0.00, 23.12) | -3.11 (-3.70, -2.52) | <0.001 |
| Guam | 31.19 (15.38, 56.95) | 31.97 (15.41, 59.26) | 0.09 (0.07, 0.11) | <0.001 | 719.61 (632.63, 815.65) | 752.26 (659.72, 854.60) | 0.16 (0.15, 0.16) | <0.001 | 148.10 (110.07, 195.51) | 132.37 (95.14, 179.80) | -0.43 (-0.62, -0.24) | <0.001 | 1.81 (0.01, 15.52) | 1.59 (0.00, 16.08) | -0.44 (-0.69, -0.20) | <0.001 |
| Guatemala | 72.18 | 73.84 | 0.08 | <0.001 | 1060.75 | 1107.44 | 0.16 | <0.001 | 69.94 | 61.29 | -0.46 | 0.001 | 0.33 | 0.14 | -2.93 | <0.001 |

| | | | | | | | | | | | | | | | | |
|---|---|---|---|---|---|---|---|---|---|---|---|---|---|---|---|---|
| | (67.83, 76.76) | (71.19, 76.59) | (0.07, 0.10) | | (1043.39 , 1078.34) | (1096.81 , 1118.15) | (0.14, 0.16) | | (65.53, 74.58) | (58.81, 63.86) | (-0.74, -0.18) | | (0.09, 0.84) | (0.05, 0.34) | (-3.80, -2.05) | |
| Guinea | 57.23 (52.62, 62.15) | 62.81 (59.56, 66.22) | 0.31 (0.26, 0.37) | <0.001 | 821.39 (803.26, 839.85) | 961.82 (948.57, 975.23) | 0.54 (0.50, 0.59) | <0.001 | 107.95 (101.50, 114.72) | 89.27 (85.28, 93.42) | -0.64 (-0.76, -0.52) | <0.001 | 1.11 (0.55, 2.04) | 0.70 (0.39, 1.20) | -1.57 (-1.76, -1.38) | <0.001 |
| Guinea-Bissau | 77.89 (65.36, 92.42) | 81.47 (72.73, 91.08) | 0.15 (0.14, 0.16) | <0.001 | 1157.87 (1106.32 , 1211.42) | 1222.38 (1186.96 , 1258.67) | 0.19 (0.17, 0.20) | <0.001 | 194.26 (173.87, 216.64) | 121.32 (110.38, 133.13) | -1.61 (-1.67, -1.55) | <0.001 | 2.22 (0.57, 6.37) | 1.02 (0.26, 2.87) | -2.65 (-2.78, -2.52) | <0.001 |
| Guyana | 77.83 (65.16, 92.48) | 75.70 (62.91, 90.49) | -0.10 (-0.12, -0.08) | <0.001 | 1164.47 (1112.57 , 1218.33) | 1152.02 (1099.39 , 1206.64) | -0.04 (-0.06, -0.03) | <0.001 | 227.26 (204.54, 251.99) | 138.83 (120.78, 158.95) | -1.59 (-2.10, -1.07) | <0.001 | 2.85 (0.85, 7.25) | 1.42 (0.19, 5.20) | -2.17 (-2.87, -1.48) | <0.001 |
| Haiti | 78.79 (73.78, 84.06) | 80.33 (76.91, 83.88) | 0.06 (0.03, 0.08) | <0.001 | 1141.72 (1122.00 , 1161.72) | 1160.04 (1146.77 , 1173.43) | 0.04 (-0.02, 0.11) | 0.191 | 342.52 (331.67, 353.65) | 197.48 (191.99, 203.08) | -1.80 (-2.00, -1.60) | <0.001 | 4.84 (3.62, 6.36) | 2.39 (1.82, 3.10) | -2.31 (-2.56, -2.06) | <0.001 |
| Honduras | 71.22 (65.65, 77.18) | 70.28 (66.71, 74.02) | -0.04 (-0.07, -0.02) | <0.001 | 1115.55 (1092.39 , 1139.11) | 1112.07 (1097.28 , 1127.01) | -0.02 (-0.05, 0.02) | 0.326 | 60.65 (55.32, 66.39) | 55.20 (51.94, 58.62) | -0.33 (-0.37, -0.29) | <0.001 | 0.13 (0.00, 0.81) | 0.04 (0.00, 0.30) | -3.89 (-4.68, -3.10) | <0.001 |
| Hungary | 1.97 (1.41, 2.71) | 1.41 (0.89, 2.16) | -1.16 (-1.37, -0.96) | <0.001 | 13.55 (11.97, 15.29) | 11.28 (9.67, 13.11) | -0.64 (-0.73, -0.55) | <0.001 | 54.41 (51.20, 57.78) | 7.11 (5.84, 8.60) | -6.91 (-7.61, -6.20) | <0.001 | 0.94 (0.57, 1.50) | 0.11 (0.01, 0.50) | -7.26 (-7.95, -6.57) | <0.001 |
| Iceland | 0.40 (0.00, 8.26) | 0.41 (0.00, 8.18) | 0.12 (-0.19, 0.43) | 0.458 | 6.45 (1.53, 18.02) | 7.01 (2.05, 18.20) | 0.29 (0.24, 0.34) | <0.001 | 4.92 (0.86, 15.74) | 2.30 (0.16, 11.23) | -2.57 (-3.00, -2.14) | <0.001 | 0.08 (0.00, 7.64) | 0.03 (0.00, 7.54) | -3.49 (-4.34, -2.63) | <0.001 |
| India | 62.76 (62.39, 63.12) | 61.89 (61.61, 62.17) | 0.02 (-0.36, 0.40) | 0.927 | 819.08 (817.73, 820.44) | 837.77 (836.75, 838.80) | 0.14 (0.07, 0.21) | <0.001 | 478.38 (477.35, 479.42) | 260.88 (260.31, 261.46) | -2.04 (-2.43, -1.66) | <0.001 | 7.34 (7.21, 7.47) | 3.72 (3.65, 3.79) | -2.30 (-2.71, -1.88) | <0.001 |
| Indonesia | 8.78 (8.48, | 9.42 (9.16, | 0.24 (0.20, | <0.001 | 126.17 (125.04, | 145.71 (144.69, | 0.49 (0.46, | <0.001 | 64.45 (63.63, | 21.17 (20.78, | -3.78 (-3.85, | <0.001 | 0.99 (0.89, | 0.23 (0.19, | -4.88 (-5.12, | <0.001 |

| | | | | | | | | | | | | | | | | |
|---|---|---|---|---|---|---|---|---|---|---|---|---|---|---|---|---|
| | 9.08) | 9.69) | 0.29) | | 127.31) | 146.73) | 0.52) | | 65.27) | 21.56) | -3.70) | | 1.10) | 0.28) | -4.64) | |
| Iran (Islamic Republic of) | 43.98 (42.78, 45.21) | 43.00 (41.99, 44.03) | -0.06 (-0.08, -0.03) | <0.001 | 696.31 (691.33, 701.32) | 695.65 (691.74, 699.58) | 0.00 (-0.01, 0.01) | 0.883 | 79.45 (77.77, 81.15) | 52.97 (51.89, 54.06) | -1.38 (-1.52, -1.25) | <0.001 | 0.76 (0.60, 0.95) | 0.32 (0.24, 0.42) | -2.92 (-3.45, -2.40) | <0.001 |
| Iraq | 47.07 (44.77, 49.46) | 47.40 (46.04, 48.79) | 0.02 (0.00, 0.05) | 0.053 | 712.48 (703.18, 721.88) | 720.56 (715.12, 726.03) | 0.03 (0.01, 0.05) | <0.001 | 104.16 (100.57, 107.84) | 48.62 (47.21, 50.07) | -2.61 (-2.75, -2.47) | <0.001 | 1.19 (0.83, 1.66) | 0.24 (0.15, 0.37) | -5.31 (-5.65, -4.96) | <0.001 |
| Ireland | 0.44 (0.09, 1.32) | 0.39 (0.09, 1.14) | -0.43 (-0.58, -0.28) | <0.001 | 6.43 (4.67, 8.66) | 6.45 (4.82, 8.49) | 0.03 (-0.04, 0.09) | 0.426 | 6.05 (4.33, 8.24) | 2.41 (1.47, 3.76) | -3.26 (-4.08, -2.43) | <0.001 | 0.10 (0.00, 0.77) | 0.04 (0.00, 0.59) | -3.07 (-3.30, -2.85) | <0.001 |
| Israel | 0.56 (0.19, 1.30) | 0.48 (0.21, 0.96) | -0.52 (-0.73, -0.31) | <0.001 | 7.51 (5.86, 9.49) | 6.98 (5.76, 8.38) | -0.26 (-0.32, -0.20) | <0.001 | 20.95 (18.10, 24.12) | 11.00 (9.46, 12.74) | -2.12 (-3.17, -1.07) | <0.001 | 0.34 (0.08, 0.99) | 0.18 (0.04, 0.54) | -2.20 (-3.27, -1.11) | <0.001 |
| Italy | 1.27 (1.06, 1.50) | 0.53 (0.39, 0.71) | -2.99 (-3.11, -2.87) | <0.001 | 19.24 (18.42, 20.09) | 10.35 (9.66, 11.07) | -2.13 (-2.32, -1.94) | <0.001 | 25.91 (24.96, 26.89) | 9.06 (8.43, 9.73) | -3.84 (-4.61, -3.06) | <0.001 | 0.42 (0.31, 0.56) | 0.14 (0.07, 0.25) | -3.97 (-4.84, -3.09) | <0.001 |
| Jamaica | 59.79 (53.13, 67.16) | 61.09 (54.97, 67.73) | 0.07 (0.05, 0.09) | <0.001 | 1107.97 (1077.65, 1139.00) | 1148.29 (1121.08, 1176.02) | 0.12 (0.12, 0.13) | <0.001 | 150.66 (139.80, 162.23) | 99.66 (91.76, 108.08) | -1.43 (-2.56, -0.28) | 0.015 | 1.55 (0.64, 3.29) | 0.73 (0.21, 1.87) | -2.60 (-4.99, -0.15) | 0.038 |
| Japan | 0.60 (0.50, 0.71) | 0.26 (0.19, 0.35) | -2.93 (-3.36, -2.51) | <0.001 | 14.71 (14.22, 15.22) | 9.98 (9.50, 10.47) | -1.35 (-1.48, -1.22) | <0.001 | 9.72 (9.32, 10.14) | 3.62 (3.34, 3.91) | -3.35 (-3.68, -3.02) | <0.001 | 0.15 (0.10, 0.21) | 0.05 (0.02, 0.10) | -3.81 (-4.17, -3.44) | <0.001 |
| Jordan | 0.71 (0.22, 1.84) | 0.66 (0.39, 1.05) | -0.22 (-0.32, -0.11) | <0.001 | 11.24 (8.89, 14.11) | 10.80 (9.59, 12.12) | -0.12 (-0.17, -0.07) | <0.001 | 16.38 (13.48, 19.79) | 5.75 (4.88, 6.74) | -3.53 (-3.77, -3.29) | <0.001 | 0.26 (0.03, 1.19) | 0.09 (0.01, 0.29) | -3.63 (-3.93, -3.33) | <0.001 |
| Kazakhstan | 8.00 (7.07, 9.01) | 3.60 (3.00, 4.29) | -2.75 (-2.96, -2.55) | <0.001 | 55.38 (52.89, 57.95) | 34.68 (32.76, 36.69) | -1.60 (-1.65, -1.55) | <0.001 | 225.32 (220.29, 230.44) | 34.09 (32.20, 36.07) | -6.38 (-7.26, -5.49) | <0.001 | 3.83 (3.19, 4.55) | 0.56 (0.34, 0.88) | -6.49 (-7.41, -5.57) | <0.001 |
| Kenya | 95.74 (92.86, 98.69) | 95.07 (93.27, 96.91) | -0.02 (-0.03, -0.01) | <0.001 | 1521.64 (1509.52, 1533.84) | 1639.79 (1632.01, 1647.59) | 0.26 (0.23, 0.28) | <0.001 | 109.31 (106.14, 112.57) | 106.26 (104.31, 108.24) | -0.10 (-0.17, -0.03) | 0.008 | 0.57 (0.37, 0.87) | 0.43 (0.32, 0.58) | -1.01 (-1.16, -0.86) | <0.001 |

| | | | | | | | | | | | | | | | | |
|---|---|---|---|---|---|---|---|---|---|---|---|---|---|---|---|---|
| Kiribati | 44.27 (16.91, 97.56) | 44.89 (22.06, 82.08) | 0.05 (0.03, 0.07) | <0.001 | 707.43 (577.53, 860.09) | 722.62 (618.45, 839.96) | 0.07 (0.06, 0.09) | <0.001 | 1201.97 (1029.02, 1397.67) | 884.15 (767.66, 1013.88) | -1.06 (-1.16, -0.96) | <0.001 | 19.35 (3.56, 63.09) | 14.14 (3.20, 40.64) | -1.10 (-1.20, -1.00) | <0.001 |
| Kuwait | 0.74 (0.19, 2.28) | 0.62 (0.24, 1.50) | -0.58 (-0.73, -0.44) | <0.001 | 15.18 (11.92, 19.22) | 12.20 (10.12, 14.68) | -0.77 (-0.86, -0.68) | <0.001 | 35.13 (29.72, 41.37) | 9.23 (7.32, 11.57) | -4.61 (-5.79, -3.42) | <0.001 | 0.54 (0.08, 2.05) | 0.14 (0.01, 0.87) | -4.53 (-5.80, -3.24) | <0.001 |
| Kyrgyzstan | 62.49 (57.47, 67.86) | 62.86 (58.67, 67.29) | 0.02 (0.00, 0.03) | 0.049 | 1008.15 (987.38, 1029.28) | 1007.73 (990.77, 1024.92) | -0.00 (-0.01, 0.00) | 0.325 | 408.46 (395.09, 422.19) | 139.61 (133.31, 146.14) | -3.55 (-4.18, -2.91) | <0.001 | 6.13 (4.58, 8.06) | 1.57 (0.97, 2.43) | -4.52 (-5.42, -3.60) | <0.001 |
| Lao People's Democratic Republic | 52.70 (47.59, 58.25) | 50.39 (46.97, 54.01) | -0.14 (-0.18, -0.11) | <0.001 | 747.42 (727.46, 767.81) | 746.46 (733.04, 760.08) | 0.00 (-0.04, 0.04) | 0.896 | 244.90 (233.61, 256.64) | 152.99 (146.96, 159.22) | -1.59 (-1.77, -1.41) | <0.001 | 3.41 (2.19, 5.11) | 1.90 (1.28, 2.72) | -1.97 (-2.16, -1.78) | <0.001 |
| Latvia | 8.12 (5.81, 11.08) | 3.37 (1.68, 6.36) | -3.00 (-3.12, -2.87) | <0.001 | 50.59 (44.50, 57.32) | 28.45 (22.76, 35.32) | -2.03 (-2.32, -1.74) | <0.001 | 143.05 (132.68, 154.05) | 18.61 (14.17, 24.23) | -6.83 (-9.87, -3.68) | <0.001 | 2.45 (1.27, 4.32) | 0.30 (0.01, 2.25) | -7.55 (-8.90, -6.17) | <0.001 |
| Lebanon | 2.42 (1.29, 4.19) | 1.13 (0.58, 2.02) | -2.61 (-2.82, -2.39) | <0.001 | 30.78 (26.33, 35.82) | 16.06 (13.71, 18.72) | -2.25 (-2.37, -2.13) | <0.001 | 61.54 (55.29, 68.37) | 20.63 (17.92, 23.65) | -3.70 (-3.88, -3.52) | <0.001 | 0.98 (0.34, 2.29) | 0.32 (0.07, 0.95) | -3.77 (-4.03, -3.52) | <0.001 |
| Lesotho | 97.99 (87.74, 109.23) | 96.64 (88.12, 105.78) | -0.05 (-0.08, -0.01) | 0.010 | 1508.51 (1466.15, 1551.88) | 1520.67 (1486.10, 1555.86) | 0.03 (0.00, 0.07) | 0.027 | 199.45 (184.23, 215.68) | 171.70 (160.20, 183.82) | -0.52 (-0.65, -0.38) | <0.001 | 2.09 (0.80, 4.58) | 1.63 (0.69, 3.26) | -0.86 (-1.05, -0.67) | <0.001 |
| Liberia | 72.99 (63.61, 83.45) | 78.76 (73.31, 84.52) | 0.25 (0.22, 0.28) | <0.001 | 1159.85 (1120.86, 1199.92) | 1253.77 (1231.24, 1276.62) | 0.26 (0.25, 0.28) | <0.001 | 109.49 (97.87, 122.20) | 90.19 (84.23, 96.47) | -0.66 (-0.90, -0.43) | <0.001 | 0.87 (0.15, 2.91) | 0.49 (0.15, 1.23) | -2.00 (-2.59, -1.40) | <0.001 |
| Libya | 38.02 (34.07, 42.33) | 43.10 (39.86, 46.55) | 0.44 (0.42, 0.45) | <0.001 | 635.08 (618.16, 652.38) | 707.83 (694.70, 721.15) | 0.37 (0.37, 0.38) | <0.001 | 73.58 (67.99, 79.55) | 50.77 (47.29, 54.44) | -1.25 (-1.52, -0.98) | <0.001 | 0.69 (0.25, 1.56) | 0.27 (0.08, 0.70) | -3.12 (-3.76, -2.47) | <0.001 |
| Lithuania | 6.79 (5.00, 9.04) | 3.21 (1.79, 5.48) | -2.59 (-2.78, -2.40) | <0.001 | 39.74 (35.19, 44.72) | 25.65 (21.15, 30.92) | -1.54 (-1.84, -1.24) | <0.001 | 117.06 (109.20, 125.34) | 17.53 (13.92, 21.92) | -6.50 (-8.15, -4.82) | <0.001 | 2.04 (1.12, 3.42) | 0.29 (0.02, 1.59) | -6.55 (-8.20, -4.88) | <0.001 |

| | | | | | | | | | | | | | | | | |
|---|---|---|---|---|---|---|---|---|---|---|---|---|---|---|---|---|
| Luxembourg | 0.64 (0.00, 8.08) | 0.53 (0.00, 5.54) | -0.72 (-1.12, -0.32) | <0.001 | 9.79 (4.03, 21.00) | 9.45 (4.58, 17.89) | -0.14 (-0.21, -0.06) | <0.001 | 12.01 (5.48, 23.91) | 4.47 (1.47, 11.21) | -3.30 (-4.09, -2.50) | <0.001 | 0.19 (0.00, 7.39) | 0.07 (0.00, 4.82) | -3.28 (-4.61, -1.94) | <0.001 |
| Madagascar | 98.00 (94.00, 102.14) | 102.77 (100.13, 105.46) | 0.16 (0.16, 0.17) | <0.001 | 1652.18 (1635.02, 1669.49) | 1747.88 (1736.60, 1759.21) | 0.20 (0.18, 0.21) | <0.001 | 285.02 (278.12, 292.06) | 192.18 (188.52, 195.89) | -1.32 (-1.63, -1.00) | <0.001 | 3.22 (2.52, 4.07) | 1.70 (1.37, 2.09) | -2.12 (-2.59, -1.64) | <0.001 |
| Malawi | 114.41 (109.53, 119.49) | 116.79 (113.36, 120.30) | 0.07 (0.06, 0.08) | <0.001 | 1830.66 (1810.26, 1851.26) | 1885.38 (1870.93, 1899.93) | 0.10 (0.09, 0.11) | <0.001 | 154.73 (148.97, 160.69) | 130.18 (126.47, 133.97) | -0.59 (-0.67, -0.51) | <0.001 | 1.04 (0.63, 1.67) | 0.62 (0.40, 0.94) | -1.74 (-1.94, -1.54) | <0.001 |
| Malaysia | 36.42 (34.51, 38.41) | 35.41 (34.05, 36.83) | -0.09 (-0.11, -0.08) | <0.001 | 628.88 (620.79, 637.06) | 636.97 (631.16, 642.81) | 0.05 (0.04, 0.05) | <0.001 | 133.74 (130.03, 137.54) | 52.62 (50.96, 54.32) | -3.18 (-3.37, -2.98) | <0.001 | 1.69 (1.30, 2.17) | 0.36 (0.24, 0.53) | -5.16 (-5.43, -4.89) | <0.001 |
| Maldives | 34.78 (18.90, 61.11) | 32.92 (23.58, 45.52) | -0.20 (-0.29, -0.11) | <0.001 | 622.80 (544.86, 710.22) | 619.08 (578.56, 662.39) | -0.03 (-0.04, -0.01) | 0.002 | 99.60 (70.03, 139.25) | 46.91 (36.07, 60.81) | -2.45 (-2.91, -1.98) | <0.001 | 1.15 (0.00, 16.49) | 0.27 (0.00, 5.38) | -4.62 (-5.36, -3.88) | <0.001 |
| Mali | 84.51 (79.78, 89.47) | 84.04 (81.20, 86.98) | -0.02 (-0.04, -0.01) | 0.010 | 1357.34 (1337.59, 1377.32) | 1384.14 (1371.97, 1396.41) | 0.07 (0.05, 0.08) | <0.001 | 144.01 (137.67, 150.58) | 106.86 (103.50, 110.31) | -1.02 (-1.20, -0.85) | <0.001 | 1.28 (0.75, 2.07) | 0.66 (0.42, 1.00) | -2.25 (-2.72, -1.78) | <0.001 |
| Malta | 0.34 (0.00, 6.80) | 0.30 (0.00, 8.22) | -0.43 (-0.76, -0.10) | 0.011 | 4.73 (1.09, 13.73) | 4.43 (0.95, 14.37) | -0.24 (-0.28, -0.19) | <0.001 | 8.14 (2.90, 18.56) | 5.25 (1.38, 15.39) | -1.47 (-2.15, -0.78) | <0.001 | 0.13 (0.00, 6.44) | 0.08 (0.00, 7.90) | -1.54 (-2.50, -0.57) | 0.002 |
| Marshall Islands | 41.67 (10.12, 118.25) | 41.93 (13.87, 97.37) | 0.02 (0.02, 0.03) | <0.001 | 714.57 (546.14, 921.73) | 727.89 (584.60, 896.01) | 0.06 (0.06, 0.07) | <0.001 | 906.70 (714.73, 1137.34) | 613.48 (482.57, 769.28) | -1.35 (-1.56, -1.14) | <0.001 | 14.21 (0.58, 76.94) | 9.58 (0.39, 48.68) | -1.37 (-1.52, -1.23) | <0.001 |
| Mauritania | 68.45 (60.37, 77.40) | 68.11 (62.45, 74.19) | -0.03 (-0.05, 0.00) | 0.055 | 1192.24 (1156.71, 1228.65) | 1193.48 (1168.65, 1218.74) | -0.00 (-0.02, 0.01) | 0.767 | 108.93 (98.45, 120.29) | 72.86 (66.83, 79.33) | -1.36 (-1.46, -1.27) | <0.001 | 0.83 (0.17, 2.59) | 0.25 (0.03, 1.05) | -3.93 (-4.23, -3.63) | <0.001 |
| Mauritius | 36.76 (29.66, | 35.44 (28.20, | -0.12 (-0.14, | <0.001 | 647.09 (616.15, | 640.12 (608.24, | -0.04 (-0.06, | <0.001 | 121.85 (108.58, | 59.76 (50.30, | -2.29 (-2.82, | <0.001 | 1.50 (0.39, | 0.48 (0.02, | -3.51 (-4.28, | <0.001 |

| | | | | | | | | | | | | | | | | |
|---|---|---|---|---|---|---|---|---|---|---|---|---|---|---|---|---|
| | 45.09) | 44.01) | -0.11) | | 679.21) | 673.27) | -0.02) | | 136.32) | 70.53) | -1.76) | | 4.00) | 2.57) | -2.73) | |
| Mexico | 28.88 (28.09, 29.68) | 25.09 (24.47, 25.72) | -0.48 (-0.52, -0.44) | <0.001 | 487.05 (483.68, 490.45) | 421.16 (418.60, 423.73) | -0.50 (-0.51, -0.49) | <0.001 | 72.83 (71.50, 74.18) | 29.62 (28.94, 30.31) | -3.01 (-3.14, -2.88) | <0.001 | 0.84 (0.70, 1.00) | 0.16 (0.11, 0.22) | -5.47 (-5.88, -5.05) | <0.001 |
| Micronesia (Federated States of) | 42.10 (18.49, 83.62) | 42.05 (19.16, 81.59) | -0.00 (-0.02, 0.01) | 0.946 | 713.82 (600.80, 842.98) | 723.56 (614.03, 848.08) | 0.05 (0.04, 0.06) | <0.001 | 855.14 (729.43, 997.18) | 575.85 (477.87, 689.03) | -1.36 (-1.43, -1.29) | <0.001 | 13.70 (2.39, 44.61) | 8.93 (0.95, 35.70) | -1.47 (-1.54, -1.41) | <0.001 |
| Monaco | 0.39 (0.00, 120.41) | 0.36 (0.00, 86.95) | -0.28 (-0.37, -0.19) | <0.001 | 6.74 (0.00, 130.42) | 6.44 (0.00, 98.54) | -0.15 (-0.18, -0.12) | <0.001 | 4.45 (0.00, 126.68) | 3.48 (0.00, 92.92) | -0.84 (-0.89, -0.79) | <0.001 | 0.07 (0.00, 119.92) | 0.05 (0.00, 86.36) | -1.00 (-1.00, -1.00) | <0.001 |
| Mongolia | 69.36 (61.86, 77.66) | 66.34 (60.12, 73.07) | -0.16 (-0.18, -0.14) | <0.001 | 1052.85 (1021.97, 1084.53) | 1044.88 (1020.49, 1069.76) | -0.03 (-0.04, -0.02) | <0.001 | 157.88 (145.76, 170.85) | 117.86 (109.95, 126.25) | -1.03 (-1.45, -0.62) | <0.001 | 1.83 (0.73, 3.94) | 1.18 (0.53, 2.37) | -1.48 (-2.23, -0.73) | <0.001 |
| Montenegro | 1.49 (0.17, 5.58) | 1.35 (0.10, 6.10) | -0.34 (-0.52, -0.17) | <0.001 | 11.52 (6.46, 19.06) | 10.93 (5.70, 19.21) | -0.16 (-0.26, -0.05) | 0.003 | 34.92 (25.55, 46.68) | 13.67 (7.72, 22.64) | -3.33 (-4.02, -2.62) | <0.001 | 0.59 (0.01, 4.10) | 0.23 (0.00, 4.25) | -3.99 (-5.85, -2.10) | <0.001 |
| Morocco | 47.75 (45.89, 49.67) | 47.29 (45.73, 48.90) | -0.02 (-0.04, 0.00) | 0.076 | 722.27 (714.80, 729.80) | 734.76 (728.57, 740.99) | 0.07 (0.05, 0.08) | <0.001 | 111.05 (108.12, 114.04) | 55.32 (53.63, 57.05) | -2.34 (-2.63, -2.05) | <0.001 | 1.27 (0.97, 1.64) | 0.33 (0.21, 0.49) | -4.47 (-4.96, -3.98) | <0.001 |
| Mozambique | 118.35 (113.76, 123.09) | 116.62 (113.77, 119.54) | -0.05 (-0.08, -0.01) | 0.011 | 1857.74 (1838.75, 1876.88) | 1893.55 (1881.48, 1905.68) | 0.07 (0.04, 0.09) | <0.001 | 146.06 (140.89, 151.39) | 138.99 (135.79, 142.25) | -0.16 (-0.24, -0.09) | <0.001 | 0.90 (0.55, 1.42) | 0.76 (0.55, 1.05) | -0.54 (-0.70, -0.38) | <0.001 |
| Myanmar | 47.55 (46.08, 49.05) | 45.49 (44.22, 46.78) | -0.15 (-0.17, -0.14) | <0.001 | 658.54 (652.99, 664.13) | 658.99 (654.13, 663.87) | -0.00 (-0.01, 0.01) | 0.831 | 267.26 (263.68, 270.87) | 128.51 (126.36, 130.68) | -2.48 (-2.61, -2.34) | <0.001 | 3.98 (3.55, 4.45) | 1.62 (1.39, 1.89) | -3.06 (-3.25, -2.87) | <0.001 |
| Namibia | 88.18 (77.43, 100.20) | 86.40 (78.51, 94.92) | -0.07 (-0.08, -0.06) | <0.001 | 1486.86 (1439.84, 1535.17) | 1490.61 (1456.76, 1525.09) | 0.01 (-0.01, 0.03) | 0.347 | 163.96 (148.60, 180.63) | 116.58 (107.24, 126.56) | -1.17 (-1.31, -1.02) | <0.001 | 1.52 (0.39, 4.31) | 0.74 (0.18, 2.07) | -2.42 (-2.73, -2.11) | <0.001 |
| Nauru | 9.70 (0.00, | 8.33 (0.00, | -0.54 (-0.61, | <0.001 | 94.02 (10.41, | 91.79 (11.95, | -0.11 (-0.26, | 0.128 | 574.42 (291.99, | 471.48 (232.67, | -0.66 (-0.84, | <0.001 | 9.45 (0.00, | 7.69 (0.00, | -0.69 (-0.87, | <0.001 |

| | | | | | | | | | | | | | | | | |
|---|---|---|---|---|---|---|---|---|---|---|---|---|---|---|---|---|
| | 226.27) | 202.67) | -0.46) | | 366.93) | 339.82) | 0.03) | | 1025.52) | 861.40) | -0.49) | | 225.49) | 201.17) | -0.51) | |
| Nepal | 63.68 (61.09, 66.37) | 59.42 (57.49, 61.40) | -0.23 (-0.26, -0.20) | <0.001 | 741.13 (731.93, 750.41) | 746.77 (739.62, 753.96) | 0.04 (-0.02, 0.09) | 0.199 | 505.01 (497.42, 512.68) | 243.15 (239.12, 247.23) | -2.47 (-2.65, -2.28) | <0.001 | 7.87 (6.94, 8.89) | 3.40 (2.94, 3.93) | -2.84 (-2.97, -2.71) | <0.001 |
| Netherlands | 0.38 (0.20, 0.68) | 0.36 (0.17, 0.67) | -0.19 (-0.56, 0.18) | 0.315 | 6.20 (5.35, 7.15) | 6.31 (5.39, 7.33) | 0.08 (0.02, 0.14) | 0.013 | 4.10 (3.42, 4.88) | 2.27 (1.74, 2.91) | -2.03 (-2.33, -1.73) | <0.001 | 0.06 (0.01, 0.25) | 0.03 (0.00, 0.21) | -2.86 (-4.93, -0.74) | 0.008 |
| New Zealand | 1.43 (0.68, 2.65) | 0.71 (0.23, 1.67) | -2.42 (-2.94, -1.91) | <0.001 | 24.05 (20.53, 28.01) | 17.83 (14.83, 21.27) | -1.04 (-1.10, -0.98) | <0.001 | 80.16 (73.61, 87.14) | 33.37 (29.22, 37.95) | -2.99 (-3.68, -2.30) | <0.001 | 1.32 (0.61, 2.51) | 0.56 (0.15, 1.45) | -2.96 (-3.68, -2.22) | <0.001 |
| Nicaragua | 68.01 (62.11, 74.40) | 69.07 (64.82, 73.54) | 0.05 (0.04, 0.06) | <0.001 | 1152.51 (1126.69, 1178.82) | 1183.09 (1165.10, 1201.29) | 0.08 (0.05, 0.11) | <0.001 | 86.11 (79.16, 93.56) | 65.95 (61.76, 70.36) | -0.95 (-1.15, -0.75) | <0.001 | 0.52 (0.12, 1.55) | 0.15 (0.02, 0.54) | -4.23 (-4.88, -3.58) | <0.001 |
| Niger | 72.00 (67.53, 76.72) | 75.70 (73.01, 78.47) | 0.17 (0.17, 0.18) | <0.001 | 1122.62 (1104.21, 1141.28) | 1182.02 (1170.88, 1193.25) | 0.18 (0.17, 0.19) | <0.001 | 141.26 (134.84, 147.94) | 105.25 (101.95, 108.64) | -0.98 (-1.08, -0.87) | <0.001 | 1.41 (0.84, 2.28) | 0.79 (0.53, 1.16) | -1.94 (-2.16, -1.72) | <0.001 |
| Nigeria | 72.98 (71.70, 74.27) | 75.05 (74.21, 75.91) | 0.10 (0.08, 0.11) | <0.001 | 1227.58 (1222.22, 1232.95) | 1286.14 (1282.51, 1289.78) | 0.16 (0.15, 0.18) | <0.001 | 121.00 (119.32, 122.69) | 84.22 (83.29, 85.16) | -1.24 (-1.36, -1.12) | <0.001 | 1.02 (0.87, 1.18) | 0.37 (0.31, 0.44) | -3.45 (-3.71, -3.20) | <0.001 |
| Niue | 6.54 (0.00, 1044.21) | 4.56 (0.00, 1475.86) | -1.25 (-1.28, -1.22) | <0.001 | 75.45 (0.00, 1170.70) | 73.75 (0.00, 1605.77) | -0.08 (-0.10, -0.06) | <0.001 | 367.57 (26.10, 1665.37) | 210.43 (0.61, 1850.05) | -1.95 (-2.20, -1.70) | <0.001 | 6.00 (0.00, 1042.86) | 3.32 (0.00, 1473.39) | -2.03 (-2.26, -1.80) | <0.001 |
| North Macedonia | 1.49 (0.56, 3.22) | 1.30 (0.47, 3.13) | -0.46 (-0.61, -0.31) | <0.001 | 13.16 (9.92, 17.13) | 10.95 (8.03, 14.73) | -0.61 (-0.71, -0.51) | <0.001 | 46.35 (40.05, 53.37) | 14.33 (10.87, 18.67) | -3.90 (-4.48, -3.32) | <0.001 | 0.77 (0.17, 2.19) | 0.23 (0.00, 1.65) | -4.05 (-4.70, -3.39) | <0.001 |
| Northern Mariana Islands | 32.45 (8.14, 92.85) | 34.31 (5.21, 131.56) | 0.19 (0.17, 0.22) | <0.001 | 758.57 (606.71, 940.52) | 791.46 (593.58, 1043.37) | 0.14 (0.14, 0.16) | <0.001 | 114.17 (61.29, 199.36) | 96.53 (36.43, 219.20) | -0.59 (-0.68, -0.50) | <0.001 | 1.29 (0.00, 45.30) | 0.97 (0.00, 81.43) | -1.16 (-1.39, -0.92) | <0.001 |
| Norway | 0.30 | 0.29 | 0.17 | 0.242 | 4.87 | 5.17 | 0.23 | <0.001 | 4.42 | 1.43 | -3.78 | <0.001 | 0.07 | 0.02 | -4.13 | 0.007 |

| | | | | | | | | | | | | | | | | |
|---|---|---|---|---|---|---|---|---|---|---|---|---|---|---|---|---|
| | (0.05, 1.00) | (0.05, 0.95) | (-0.11, 0.45) | | (3.48, 6.64) | (3.80, 6.91) | (0.14, 0.32) | | (3.11, 6.12) | (0.77, 2.47) | (-4.43, -3.12) | | (0.00, 0.63) | (0.00, 0.52) | (-7.04, -1.13) | |
| Oman | 0.64 (0.15, 1.92) | 0.72 (0.36, 1.50) | 0.50 (0.18, 0.82) | 0.002 | 10.58 (7.95, 13.87) | 12.18 (10.44, 14.25) | 0.54 (0.44, 0.65) | <0.001 | 9.51 (7.02, 12.66) | 2.39 (1.64, 3.53) | -4.59 (-4.91, -4.26) | <0.001 | 0.15 (0.00, 1.17) | 0.03 (0.00, 0.65) | -5.86 (-7.29, -4.40) | <0.001 |
| Pakistan | 75.15 (74.01, 76.31) | 78.40 (77.60, 79.20) | 0.15 (0.14, 0.17) | <0.001 | 997.42 (993.04, 1001.81) | 1050.68 (1047.65, 1053.71) | 0.21 (0.15, 0.27) | <0.001 | 530.41 (527.20, 533.64) | 463.20 (461.20, 465.20) | -0.45 (-0.62, -0.28) | <0.001 | 8.06 (7.67, 8.47) | 6.80 (6.56, 7.05) | -0.57 (-0.70, -0.45) | <0.001 |
| Palau | 4.96 (0.00, 124.48) | 4.00 (0.00, 137.39) | -0.74 (-0.78, -0.69) | <0.001 | 59.58 (8.05, 215.14) | 58.59 (7.44, 225.67) | -0.06 (-0.07, -0.04) | <0.001 | 249.61 (114.43, 479.27) | 173.91 (64.44, 389.83) | -1.25 (-1.32, -1.18) | <0.001 | 4.04 (0.00, 122.59) | 2.80 (0.00, 135.57) | -1.26 (-1.34, -1.19) | <0.001 |
| Palestine | 43.13 (36.81, 50.43) | 42.79 (38.97, 46.93) | -0.03 (-0.05, 0.00) | 0.048 | 679.63 (652.50, 707.74) | 691.46 (675.37, 707.87) | 0.06 (0.05, 0.07) | <0.001 | 53.28 (45.81, 61.77) | 39.51 (35.72, 43.62) | -1.05 (-1.19, -0.91) | <0.001 | 0.35 (0.01, 2.14) | 0.11 (0.00, 0.66) | -3.96 (-4.62, -3.29) | <0.001 |
| Panama | 61.64 (55.07, 68.84) | 58.25 (53.13, 63.74) | -0.19 (-0.23, -0.16) | <0.001 | 1078.99 (1050.22, 1108.39) | 1058.95 (1036.63, 1081.63) | -0.07 (-0.07, -0.06) | <0.001 | 113.55 (104.38, 123.36) | 67.52 (61.98, 73.44) | -1.76 (-2.21, -1.31) | <0.001 | 1.01 (0.32, 2.45) | 0.28 (0.04, 0.95) | -4.26 (-5.32, -3.19) | <0.001 |
| Papua New Guinea | 41.09 (36.83, 45.74) | 41.86 (39.11, 44.76) | 0.07 (0.06, 0.08) | <0.001 | 679.80 (662.14, 697.84) | 707.20 (695.77, 718.77) | 0.14 (0.13, 0.14) | <0.001 | 732.57 (713.89, 751.64) | 632.99 (622.11, 644.01) | -0.55 (-0.74, -0.37) | <0.001 | 11.73 (9.45, 14.43) | 9.91 (8.58, 11.39) | -0.63 (-0.82, -0.44) | <0.001 |
| Paraguay | 65.09 (59.63, 70.94) | 62.87 (58.95, 66.99) | -0.12 (-0.13, -0.11) | <0.001 | 1161.37 (1137.42, 1185.71) | 1147.52 (1130.51, 1164.73) | -0.05 (-0.05, -0.04) | <0.001 | 92.18 (85.51, 99.25) | 74.69 (70.39, 79.18) | -0.69 (-1.11, -0.26) | 0.001 | 0.61 (0.18, 1.51) | 0.33 (0.10, 0.78) | -2.01 (-2.70, -1.32) | <0.001 |
| Peru | 60.75 (58.48, 63.09) | 60.27 (58.45, 62.13) | -0.04 (-0.07, -0.01) | 0.010 | 1057.53 (1047.70, 1067.43) | 1068.08 (1060.37, 1075.83) | 0.02 (-0.02, 0.06) | 0.328 | 85.26 (82.48, 88.11) | 63.32 (61.46, 65.23) | -0.95 (-1.25, -0.65) | <0.001 | 0.57 (0.36, 0.87) | 0.20 (0.11, 0.34) | -3.42 (-4.44, -2.39) | <0.001 |
| Philippines | 33.30 (32.31, 34.30) | 32.20 (31.49, 32.93) | -0.12 (-0.13, -0.11) | <0.001 | 461.11 (457.35, 464.90) | 499.62 (496.76, 502.49) | 0.28 (0.18, 0.38) | <0.001 | 93.61 (91.92, 95.32) | 148.82 (147.27, 150.39) | 1.67 (1.19, 2.16) | <0.001 | 1.17 (0.99, 1.38) | 2.02 (1.85, 2.22) | 1.98 (1.35, 2.60) | <0.001 |

| | | | | | | | | | | | | | | | | |
|---|---|---|---|---|---|---|---|---|---|---|---|---|---|---|---|---|
| Poland | 3.62 (3.21, 4.06) | 1.47 (1.20, 1.79) | -3.02 (-3.24, -2.80) | <0.001 | 23.67 (22.60, 24.78) | 13.90 (13.03, 14.83) | -1.81 (-2.00, -1.61) | <0.001 | 112.57 (110.23, 114.95) | 11.60 (10.82, 12.44) | -7.92 (-8.50, -7.34) | <0.001 | 1.95 (1.65, 2.29) | 0.19 (0.10, 0.33) | -8.17 (-8.81, -7.53) | <0.001 |
| Portugal | 1.00 (0.60, 1.57) | 0.57 (0.26, 1.10) | -1.95 (-2.10, -1.80) | <0.001 | 11.42 (9.94, 13.06) | 8.23 (6.86, 9.81) | -1.12 (-1.21, -1.03) | <0.001 | 41.50 (38.63, 44.53) | 8.54 (7.16, 10.13) | -5.41 (-6.59, -4.22) | <0.001 | 0.69 (0.37, 1.19) | 0.14 (0.02, 0.50) | -5.47 (-7.12, -3.79) | <0.001 |
| Puerto Rico | 0.74 (0.24, 1.75) | 0.68 (0.17, 1.79) | -0.28 (-0.36, -0.20) | <0.001 | 14.01 (11.33, 17.15) | 13.92 (10.98, 17.41) | -0.02 (-0.09, 0.06) | 0.667 | 22.22 (18.77, 26.13) | 11.80 (9.11, 15.04) | -2.16 (-2.83, -1.49) | <0.001 | 0.37 (0.06, 1.21) | 0.19 (0.01, 1.04) | -2.33 (-2.98, -1.67) | <0.001 |
| Qatar | 1.02 (0.09, 6.42) | 0.65 (0.25, 1.98) | -1.56 (-1.77, -1.36) | <0.001 | 18.72 (12.40, 28.27) | 12.11 (9.98, 14.90) | -1.52 (-1.63, -1.40) | <0.001 | 41.74 (30.95, 55.84) | 8.25 (6.31, 10.91) | -5.62 (-6.07, -5.17) | <0.001 | 0.65 (0.01, 6.07) | 0.12 (0.00, 1.39) | -5.78 (-6.34, -5.21) | <0.001 |
| Republic of Korea | 0.39 (0.28, 0.53) | 0.31 (0.21, 0.45) | -0.79 (-0.96, -0.61) | <0.001 | 9.70 (9.12, 10.31) | 9.24 (8.63, 9.90) | -0.15 (-0.21, -0.09) | <0.001 | 9.90 (9.31, 10.52) | 2.31 (2.01, 2.64) | -4.87 (-5.17, -4.56) | <0.001 | 0.15 (0.09, 0.25) | 0.03 (0.01, 0.10) | -5.35 (-6.72, -3.95) | <0.001 |
| Republic of Moldova | 9.45 (7.57, 11.70) | 2.88 (1.81, 4.51) | -4.03 (-4.17, -3.88) | <0.001 | 60.16 (55.17, 65.50) | 23.90 (20.41, 27.90) | -3.15 (-3.29, -3.02) | <0.001 | 234.44 (224.41, 244.84) | 35.48 (31.18, 40.28) | -6.53 (-7.02, -6.03) | <0.001 | 3.96 (2.75, 5.54) | 0.58 (0.16, 1.65) | -6.55 (-7.06, -6.04) | <0.001 |
| Romania | 4.03 (3.46, 4.67) | 1.67 (1.25, 2.19) | -3.02 (-3.20, -2.84) | <0.001 | 26.09 (24.61, 27.64) | 14.55 (13.22, 15.98) | -2.03 (-2.33, -1.74) | <0.001 | 124.82 (121.55, 128.17) | 16.56 (15.13, 18.10) | -6.75 (-7.58, -5.92) | <0.001 | 2.17 (1.76, 2.65) | 0.27 (0.12, 0.54) | -6.98 (-7.84, -6.12) | <0.001 |
| Russian Federation | 7.41 (7.11, 7.71) | 3.46 (3.23, 3.70) | -2.59 (-2.71, -2.47) | <0.001 | 54.79 (53.96, 55.62) | 36.82 (36.06, 37.60) | -1.35 (-1.49, -1.21) | <0.001 | 108.16 (107.01, 109.32) | 15.84 (15.36, 16.33) | -6.57 (-8.23, -4.87) | <0.001 | 1.85 (1.70, 2.00) | 0.25 (0.19, 0.32) | -6.87 (-8.64, -5.07) | <0.001 |
| Rwanda | 105.49 (100.15, 111.07) | 103.04 (99.28, 106.91) | -0.08 (-0.10, -0.06) | <0.001 | 1715.72 (1693.34, 1738.34) | 1715.87 (1700.06, 1731.79) | 0.00 (-0.01, 0.01) | 0.961 | 221.31 (213.51, 229.36) | 119.33 (115.22, 123.56) | -2.12 (-2.23, -2.00) | <0.001 | 2.17 (1.46, 3.13) | 0.59 (0.34, 0.98) | -4.34 (-4.56, -4.13) | <0.001 |
| Saint Kitts and Nevis | 1.91 (0.00, 55.90) | 0.84 (0.00, 38.12) | -2.82 (-2.98, -2.67) | <0.001 | 23.77 (2.81, 91.92) | 15.30 (1.47, 62.68) | -1.53 (-1.64, -1.43) | <0.001 | 74.27 (27.26, 165.11) | 23.04 (3.99, 74.85) | -3.37 (-4.65, -2.07) | <0.001 | 1.26 (0.00, 54.71) | 0.36 (0.00, 37.22) | -2.76 (-5.00, -0.47) | 0.018 |
| Saint Lucia | 68.81 (41.58, | 66.81 (42.02, | -0.10 (-0.12, | <0.001 | 1186.57 (1057.72 | 1173.10 (1060.64 | -0.04 (-0.06, | <0.001 | 159.84 (114.01, | 99.21 (68.60, | -1.68 (-1.98, | <0.001 | 1.75 (0.00, | 0.72 (0.00, | -3.09 (-4.02, | <0.001 |

| | | | | | | | | | | | | | | | | |
|---|---|---|---|---|---|---|---|---|---|---|---|---|---|---|---|---|
| | 109.82) | 101.15) | -0.09) | | , 1328.39) | , 1294.50) | -0.03) | | 219.64) | 139.15) | -1.38) | | 22.45) | 13.17) | -2.16) | |
| Saint Vincent and the Grenadines | 69.24 (39.94, 115.22) | 66.65 (36.65, 111.83) | -0.13 (-0.15, -0.12) | <0.001 | 1180.17 (1041.35, 1334.45) | 1156.36 (1016.27, 1310.68) | -0.08 (-0.12, -0.04) | <0.001 | 210.42 (153.34, 284.03) | 127.64 (84.17, 186.00) | -1.58 (-2.33, -0.82) | <0.001 | 2.59 (0.00, 27.78) | 1.21 (0.00, 20.77) | -2.42 (-3.67, -1.16) | <0.001 |
| Samoa | 39.29 (20.82, 69.97) | 39.41 (22.65, 64.68) | 0.01 (-0.03, 0.04) | 0.740 | 744.37 (653.69, 845.84) | 759.69 (677.89, 849.32) | 0.07 (0.07, 0.08) | <0.001 | 391.22 (325.29, 468.25) | 306.07 (254.63, 365.51) | -0.84 (-0.96, -0.73) | <0.001 | 5.87 (0.59, 25.94) | 4.44 (0.44, 18.52) | -0.94 (-1.08, -0.81) | <0.001 |
| San Marino | 0.46 (0.00, 86.69) | 0.41 (0.00, 79.15) | -0.34 (-0.61, -0.07) | 0.013 | 6.87 (0.00, 99.14) | 6.58 (0.00, 91.09) | -0.14 (-0.16, -0.14) | <0.001 | 8.01 (0.00, 101.27) | 6.58 (0.00, 91.04) | -0.66 (-0.77, -0.54) | <0.001 | 0.13 (0.00, 86.04) | 0.11 (0.00, 78.55) | -0.51 (-0.75, -0.26) | <0.001 |
| Sao Tome and Principe | 67.12 (36.62, 118.54) | 71.13 (48.53, 101.16) | 0.20 (0.17, 0.22) | <0.001 | 1183.19 (1032.64, 1352.77) | 1276.77 (1171.98, 1388.74) | 0.26 (0.23, 0.28) | <0.001 | 113.24 (69.50, 178.26) | 105.04 (76.75, 140.77) | -0.24 (-0.56, 0.07) | 0.125 | 0.95 (0.00, 31.48) | 0.70 (0.00, 11.01) | -1.09 (-1.65, -0.52) | <0.001 |
| Saudi Arabia | 1.13 (0.81, 1.53) | 0.81 (0.65, 1.00) | -1.15 (-1.26, -1.04) | <0.001 | 15.78 (14.54, 17.10) | 12.58 (11.92, 13.29) | -0.79 (-0.87, -0.70) | <0.001 | 41.49 (39.45, 43.60) | 14.14 (13.46, 14.86) | -3.65 (-3.93, -3.37) | <0.001 | 0.69 (0.45, 1.02) | 0.23 (0.15, 0.35) | -3.70 (-4.30, -3.10) | <0.001 |
| Senegal | 72.13 (67.61, 76.91) | 73.82 (70.82, 76.92) | 0.07 (0.04, 0.10) | <0.001 | 1189.13 (1169.90, 1208.63) | 1229.12 (1216.38, 1241.97) | 0.13 (0.06, 0.19) | <0.001 | 113.86 (108.02, 119.97) | 89.95 (86.53, 93.48) | -0.86 (-1.08, -0.63) | <0.001 | 0.91 (0.46, 1.67) | 0.50 (0.28, 0.85) | -1.92 (-2.48, -1.37) | <0.001 |
| Serbia | 1.07 (0.66, 1.68) | 1.29 (0.79, 2.01) | 0.62 (0.37, 0.88) | <0.001 | 8.40 (7.12, 9.85) | 9.84 (8.33, 11.56) | 0.50 (0.38, 0.62) | <0.001 | 13.93 (12.25, 15.78) | 3.96 (3.02, 5.12) | -4.22 (-4.80, -3.64) | <0.001 | 0.23 (0.06, 0.60) | 0.06 (0.00, 0.40) | -4.46 (-5.04, -3.88) | <0.001 |
| Seychelles | 38.07 (14.06, 86.06) | 36.02 (14.74, 75.61) | -0.19 (-0.21, -0.17) | <0.001 | 658.79 (537.58, 801.18) | 635.94 (532.49, 755.27) | -0.12 (-0.13, -0.12) | <0.001 | 184.49 (122.63, 268.71) | 58.93 (30.84, 104.39) | -3.75 (-4.07, -3.42) | <0.001 | 2.58 (0.00, 34.33) | 0.47 (0.00, 23.50) | -5.58 (-6.25, -4.90) | <0.001 |
| Sierra Leone | 75.56 (69.01, 82.59) | 79.48 (75.33, 83.83) | 0.18 (0.17, 0.19) | <0.001 | 1173.10 (1146.37, 1200.32) | 1242.28 (1225.24, 1259.52) | 0.21 (0.19, 0.22) | <0.001 | 121.88 (113.43, 130.83) | 96.28 (91.59, 101.17) | -0.79 (-0.97, -0.62) | <0.001 | 1.06 (0.41, 2.28) | 0.60 (0.28, 1.14) | -1.95 (-2.18, -1.73) | <0.001 |

| | | | | | | | | | | | | | | | | |
|---|---|---|---|---|---|---|---|---|---|---|---|---|---|---|---|---|
| Singapore | 0.92 (0.37, 1.93) | 0.34 (0.08, 1.06) | -3.38 (-3.77, -2.99) | <0.001 | 15.96 (13.26, 19.07) | 10.08 (8.18, 12.36) | -1.60 (-1.77, -1.44) | <0.001 | 35.21 (31.15, 39.68) | 4.89 (3.60, 6.57) | -6.44 (-6.99, -5.89) | <0.001 | 0.59 (0.18, 1.47) | 0.07 (0.00, 0.69) | -7.08 (-9.48, -4.62) | <0.001 |
| Slovakia | 1.66 (0.99, 2.64) | 1.84 (1.11, 2.97) | 0.38 (0.24, 0.52) | <0.001 | 14.51 (12.32, 16.99) | 16.25 (13.84, 19.04) | 0.38 (0.34, 0.42) | <0.001 | 19.92 (17.34, 22.78) | 7.41 (5.82, 9.39) | -3.49 (-3.98, -2.99) | <0.001 | 0.33 (0.08, 0.93) | 0.11 (0.00, 0.76) | -3.72 (-4.66, -2.78) | <0.001 |
| Slovenia | 1.13 (0.36, 2.81) | 1.04 (0.27, 3.17) | -0.33 (-0.54, -0.11) | 0.003 | 7.49 (5.09, 10.72) | 7.69 (5.01, 11.53) | 0.05 (-0.05, 0.15) | 0.314 | 19.78 (15.76, 24.58) | 5.84 (3.59, 9.26) | -4.12 (-5.15, -3.09) | <0.001 | 0.34 (0.02, 1.67) | 0.10 (0.00, 1.84) | -4.20 (-6.11, -2.25) | <0.001 |
| Solomon Islands | 44.66 (29.38, 65.92) | 44.52 (33.70, 57.84) | -0.00 (-0.02, 0.02) | 0.953 | 704.79 (639.61, 775.48) | 715.08 (669.59, 762.96) | 0.05 (0.05, 0.06) | <0.001 | 944.65 (866.31, 1028.70) | 718.25 (671.85, 767.10) | -0.94 (-1.05, -0.83) | <0.001 | 15.38 (6.84, 30.52) | 11.52 (6.31, 19.41) | -0.99 (-1.11, -0.88) | <0.001 |
| Somalia | 113.70 (107.96, 119.71) | 119.00 (115.72, 122.37) | 0.14 (0.08, 0.21) | <0.001 | 1756.89 (1733.23, 1780.80) | 1843.41 (1829.87, 1857.03) | 0.17 (0.15, 0.19) | <0.001 | 217.12 (209.15, 225.35) | 179.46 (175.37, 183.63) | -0.63 (-0.76, -0.50) | <0.001 | 2.09 (1.38, 3.06) | 1.43 (1.09, 1.85) | -1.26 (-1.48, -1.05) | <0.001 |
| South Africa | 93.19 (91.07, 95.35) | 94.15 (92.39, 95.92) | 0.03 (-0.01, 0.07) | 0.097 | 1603.20 (1594.19, 1612.25) | 1635.77 (1628.55, 1643.01) | 0.07 (0.06, 0.07) | <0.001 | 164.93 (162.00, 167.90) | 114.20 (112.31, 116.12) | -1.29 (-1.65, -0.93) | <0.001 | 1.51 (1.24, 1.83) | 0.61 (0.48, 0.77) | -3.13 (-3.93, -2.33) | <0.001 |
| South Sudan | 85.88 (80.86, 91.19) | 88.13 (83.62, 92.84) | 0.09 (0.07, 0.11) | <0.001 | 1581.86 (1559.00, 1605.00) | 1630.41 (1609.60, 1651.44) | 0.11 (0.10, 0.12) | <0.001 | 132.19 (125.73, 138.95) | 108.87 (103.65, 114.29) | -0.61 (-0.83, -0.39) | <0.001 | 0.91 (0.45, 1.71) | 0.49 (0.21, 1.02) | -2.03 (-2.91, -1.14) | <0.001 |
| Spain | 0.74 (0.56, 0.97) | 0.41 (0.28, 0.60) | -2.05 (-2.19, -1.91) | <0.001 | 8.10 (7.47, 8.78) | 6.31 (5.72, 6.95) | -0.85 (-0.94, -0.77) | <0.001 | 27.81 (26.62, 29.04) | 6.10 (5.53, 6.72) | -5.11 (-5.65, -4.56) | <0.001 | 0.47 (0.32, 0.65) | 0.10 (0.04, 0.21) | -5.46 (-6.00, -4.92) | <0.001 |
| Sri Lanka | 2.15 (1.69, 2.69) | 1.49 (1.14, 1.92) | -1.22 (-1.40, -1.05) | <0.001 | 21.64 (20.15, 23.21) | 20.42 (19.04, 21.88) | -0.19 (-0.27, -0.11) | <0.001 | 73.89 (71.13, 76.74) | 18.44 (17.12, 19.83) | -4.69 (-6.49, -2.86) | <0.001 | 1.21 (0.88, 1.63) | 0.29 (0.15, 0.51) | -4.72 (-6.49, -2.91) | <0.001 |
| Sudan | 51.05 (48.76, 53.42) | 52.22 (50.70, 53.78) | 0.07 (0.01, 0.14) | 0.026 | 706.66 (697.81, 715.59) | 745.29 (739.34, 751.27) | 0.18 (0.15, 0.22) | <0.001 | 136.81 (132.91, 140.80) | 73.26 (71.39, 75.16) | -2.14 (-2.25, -2.03) | <0.001 | 1.72 (1.30, 2.23) | 0.62 (0.46, 0.83) | -3.43 (-3.60, -3.26) | <0.001 |

| | | | | | | | | | | | | | | | | |
|---|---|---|---|---|---|---|---|---|---|---|---|---|---|---|---|---|
| Suriname | 66.82 (50.54, 87.51) | 65.36 (50.94, 82.67) | -0.09 (-0.11, -0.06) | <0.001 | 1107.76 (1035.63, 1184.17) | 1104.87 (1042.58, 1169.97) | -0.02 (-0.04, 0.00) | 0.119 | 131.35 (107.09, 160.09) | 89.95 (72.83, 109.95) | -1.28 (-2.13, -0.43) | 0.003 | 1.30 (0.03, 8.74) | 0.62 (0.00, 4.84) | -2.49 (-4.33, -0.61) | 0.010 |
| Sweden | 0.28 (0.08, 0.72) | 0.25 (0.07, 0.65) | -0.44 (-0.97, 0.10) | 0.111 | 4.78 (3.74, 6.03) | 4.45 (3.49, 5.61) | -0.28 (-0.46, -0.10) | 0.002 | 4.75 (3.72, 5.99) | 1.81 (1.22, 2.60) | -3.33 (-4.10, -2.55) | <0.001 | 0.07 (0.00, 0.41) | 0.03 (0.00, 0.32) | -2.97 (-4.77, -1.13) | 0.002 |
| Switzerland | 0.57 (0.25, 1.18) | 0.46 (0.19, 1.02) | -0.76 (-1.07, -0.45) | <0.001 | 9.07 (7.54, 10.85) | 8.45 (7.01, 10.14) | -0.28 (-0.39, -0.18) | <0.001 | 9.72 (8.15, 11.53) | 1.84 (1.22, 2.72) | -5.78 (-6.47, -5.10) | <0.001 | 0.16 (0.02, 0.61) | 0.02 (0.00, 0.40) | -7.22 (-8.45, -5.97) | <0.001 |
| Syrian Arab Republic | 43.82 (41.22, 46.56) | 45.21 (42.40, 48.18) | 0.10 (0.09, 0.11) | <0.001 | 689.96 (679.07, 701.01) | 716.42 (704.61, 728.40) | 0.13 (0.12, 0.14) | <0.001 | 237.39 (230.93, 243.99) | 57.99 (54.70, 61.45) | -4.72 (-5.11, -4.32) | <0.001 | 3.43 (2.68, 4.34) | 0.39 (0.17, 0.83) | -7.15 (-7.69, -6.62) | <0.001 |
| Taiwan (Province of China) | 1.62 (1.28, 2.03) | 1.76 (1.39, 2.20) | 0.28 (0.23, 0.33) | <0.001 | 17.41 (16.25, 18.65) | 28.81 (27.19, 30.50) | 1.75 (1.64, 1.86) | <0.001 | 31.86 (30.28, 33.51) | 5.82 (5.11, 6.60) | -5.79 (-6.24, -5.34) | <0.001 | 0.53 (0.34, 0.78) | 0.07 (0.02, 0.24) | -6.88 (-7.49, -6.25) | <0.001 |
| Tajikistan | 60.73 (56.15, 65.64) | 63.22 (59.84, 66.75) | 0.14 (0.11, 0.16) | <0.001 | 1010.55 (990.96, 1030.47) | 1028.45 (1014.59, 1042.47) | 0.06 (0.05, 0.08) | <0.001 | 283.59 (272.96, 294.55) | 135.60 (130.57, 140.79) | -2.41 (-2.90, -1.92) | <0.001 | 4.00 (2.81, 5.56) | 1.46 (0.98, 2.11) | -3.31 (-3.93, -2.68) | <0.001 |
| Thailand | 38.83 (37.76, 39.93) | 36.36 (35.27, 37.47) | -0.22 (-0.24, -0.21) | <0.001 | 587.65 (583.43, 591.88) | 571.04 (566.77, 575.34) | -0.10 (-0.14, -0.07) | <0.001 | 202.42 (199.91, 204.96) | 40.60 (39.47, 41.76) | -5.41 (-5.91, -4.90) | <0.001 | 2.99 (2.69, 3.32) | 0.21 (0.14, 0.32) | -8.72 (-9.77, -7.66) | <0.001 |
| Timor-Leste | 41.07 (31.86, 52.34) | 40.60 (33.21, 49.40) | -0.04 (-0.06, -0.03) | <0.001 | 623.46 (585.34, 663.57) | 638.69 (607.20, 671.58) | 0.08 (0.06, 0.10) | <0.001 | 139.50 (121.68, 159.38) | 112.42 (99.74, 126.49) | -0.73 (-1.67, 0.22) | 0.134 | 1.83 (0.35, 5.81) | 1.31 (0.29, 4.12) | -1.14 (-2.04, -0.24) | 0.013 |
| Togo | 75.21 (68.69, 82.28) | 78.48 (74.14, 83.03) | 0.15 (0.13, 0.17) | <0.001 | 1169.54 (1142.39, 1197.25) | 1229.85 (1212.04, 1247.87) | 0.17 (0.16, 0.18) | <0.001 | 115.04 (106.71, 123.93) | 95.04 (90.14, 100.15) | -0.65 (-0.76, -0.53) | <0.001 | 0.95 (0.34, 2.24) | 0.59 (0.26, 1.16) | -1.62 (-1.82, -1.42) | <0.001 |
| Tokelau | 7.26 (0.00, 1597.61 | 4.67 (0.00, 1808.48 | -1.51 (-1.55, -1.47) | <0.001 | 74.68 (0.00, 1717.83) | 67.51 (0.00, 1921.27) | -0.36 (-0.47, -0.24) | <0.001 | 349.51 (7.93, 2189.68) | 205.11 (0.20, 2165.30) | -1.82 (-1.87, -1.77) | <0.001 | 5.71 (0.00, 1594.50 | 3.31 (0.00, 1805.99 | -1.87 (-1.92, -1.82) | <0.001 |

| | | | | | | | | | | | | | | | | |
|---|---|---|---|---|---|---|---|---|---|---|---|---|---|---|---|---|
| | ) | ) | | | | | | | | | | | ) | ) | | |
| Tonga | 49.01 (21.38, 102.23) | 48.74 (22.18, 94.91) | -0.01 (-0.06, 0.04) | 0.741 | 1067.68 (915.93, 1241.21) | 1087.48 (941.04, 1251.55) | 0.09 (0.04, 0.15) | 0.002 | 161.00 (105.10, 240.64) | 136.65 (88.43, 203.44) | -0.57 (-0.78, -0.36) | <0.001 | 1.81 (0.00, 37.07) | 1.36 (0.00, 26.96) | -0.93 (-1.19, -0.66) | <0.001 |
| Trinidad and Tobago | 66.86 (57.19, 77.77) | 63.70 (53.96, 74.76) | -0.17 (-0.20, -0.15) | <0.001 | 1149.45 (1107.85 , 1192.26) | 1137.67 (1096.41 , 1180.17) | -0.04 (-0.05, -0.02) | <0.001 | 174.81 (158.82, 192.04) | 97.55 (85.81, 110.55) | -2.01 (-2.83, -1.18) | <0.001 | 1.99 (0.64, 4.76) | 0.72 (0.08, 2.94) | -3.56 (-5.14, -1.96) | <0.001 |
| Tunisia | 0.85 (0.45, 1.47) | 0.72 (0.41, 1.18) | -0.55 (-0.67, -0.43) | <0.001 | 12.95 (11.27, 14.84) | 11.61 (10.23, 13.14) | -0.37 (-0.43, -0.32) | <0.001 | 32.69 (29.98, 35.61) | 13.21 (11.74, 14.83) | -3.09 (-3.17, -3.00) | <0.001 | 0.53 (0.24, 1.06) | 0.21 (0.07, 0.52) | -3.19 (-3.40, -2.99) | <0.001 |
| Turkey | 0.79 (0.64, 0.98) | 0.64 (0.53, 0.77) | -0.71 (-0.85, -0.57) | <0.001 | 11.93 (11.32, 12.56) | 11.32 (10.83, 11.82) | -0.16 (-0.24, -0.08) | <0.001 | 11.55 (10.95, 12.18) | 4.27 (3.98, 4.59) | -3.26 (-3.70, -2.81) | <0.001 | 0.19 (0.12, 0.28) | 0.06 (0.03, 0.11) | -3.87 (-5.29, -2.42) | <0.001 |
| Turkmenistan | 61.04 (55.67, 66.84) | 59.41 (54.87, 64.25) | -0.10 (-0.12, -0.08) | <0.001 | 990.26 (967.72, 1013.25) | 992.73 (974.08, 1011.67) | 0.01 (-0.00, 0.02) | 0.080 | 254.32 (242.86, 266.22) | 142.66 (135.64, 149.96) | -2.05 (-3.24, -0.85) | 0.001 | 3.43 (2.21, 5.15) | 1.58 (0.93, 2.54) | -2.72 (-4.08, -1.34) | <0.001 |
| Tuvalu | 10.53 (0.00, 266.99) | 7.34 (0.00, 179.50) | -1.24 (-1.29, -1.19) | <0.001 | 77.06 (3.94, 381.66) | 73.11 (7.77, 289.54) | -0.19 (-0.25, -0.13) | <0.001 | 626.28 (305.83, 1149.13) | 412.49 (201.00, 759.04) | -1.43 (-1.53, -1.34) | <0.001 | 10.34 (0.00, 266.32) | 6.79 (0.00, 178.26) | -1.44 (-1.54, -1.34) | <0.001 |
| Uganda | 116.93 (113.20, 120.78) | 111.79 (109.50, 114.13) | -0.15 (-0.16, -0.14) | <0.001 | 1877.27 (1861.53 , 1893.12) | 2061.95 (2051.55 , 2072.39) | 0.32 (0.26, 0.39) | <0.001 | 154.93 (150.52, 159.45) | 137.49 (134.86, 140.17) | -0.44 (-0.64, -0.23) | <0.001 | 1.03 (0.71, 1.49) | 0.61 (0.45, 0.81) | -1.91 (-2.41, -1.40) | <0.001 |
| Ukraine | 3.19 (2.85, 3.56) | 2.72 (2.37, 3.10) | -0.51 (-0.76, -0.26) | <0.001 | 24.54 (23.57, 25.54) | 23.22 (22.15, 24.34) | -0.15 (-0.32, 0.02) | 0.081 | 30.18 (29.10, 31.29) | 36.94 (35.59, 38.34) | 0.75 (-1.01, 2.55) | 0.405 | 0.50 (0.37, 0.67) | 0.62 (0.46, 0.83) | 0.76 (-1.20, 2.76) | 0.449 |
| United Arab Emirates | 37.36 (32.17, 43.31) | 38.92 (35.47, 42.66) | 0.14 (0.12, 0.17) | <0.001 | 686.49 (665.30, 708.36) | 691.88 (678.57, 705.45) | 0.03 (0.00, 0.06) | 0.037 | 89.06 (81.79, 97.01) | 60.56 (56.85, 64.51) | -1.32 (-1.51, -1.13) | <0.001 | 0.95 (0.36, 2.45) | 0.47 (0.22, 1.03) | -2.41 (-2.70, -2.12) | <0.001 |
| United Kingdom | 0.49 (0.36, 0.64) | 0.37 (0.27, 0.51) | -0.92 (-1.11, -0.72) | <0.001 | 7.39 (6.87, 7.93) | 6.48 (6.01, 6.97) | -0.44 (-0.50, -0.38) | <0.001 | 8.23 (7.69, 8.80) | 3.82 (3.47, 4.19) | -2.65 (-2.93, -2.37) | <0.001 | 0.13 (0.07, 0.23) | 0.06 (0.02, 0.13) | -2.64 (-3.58, -1.69) | <0.001 |

| | | | | | | | | | | | | | | | | |
|---|---|---|---|---|---|---|---|---|---|---|---|---|---|---|---|---|
| United Republic of Tanzania | 102.09 (99.21, 105.04) | 104.02 (102.11, 105.97) | 0.08 (0.01, 0.15) | 0.020 | 1695.35 (1683.05, 1707.72) | 1783.78 (1775.54, 1792.05) | 0.18 (0.16, 0.20) | <0.001 | 146.42 (142.92, 150.00) | 114.37 (112.32, 116.45) | -0.85 (-0.93, -0.76) | <0.001 | 1.02 (0.75, 1.37) | 0.45 (0.33, 0.60) | -2.76 (-2.93, -2.58) | <0.001 |
| United States of America | 1.56 (1.46, 1.67) | 1.02 (0.94, 1.11) | -1.43 (-1.53, -1.33) | <0.001 | 25.25 (24.82, 25.69) | 17.58 (17.24, 17.93) | -1.23 (-1.26, -1.19) | <0.001 | 15.48 (15.15, 15.82) | 5.97 (5.77, 6.18) | -3.21 (-3.83, -2.58) | <0.001 | 0.24 (0.20, 0.29) | 0.08 (0.06, 0.11) | -3.74 (-4.55, -2.92) | <0.001 |
| United States Virgin Islands | 1.13 (0.00, 23.87) | 0.98 (0.00, 29.59) | -0.48 (-0.63, -0.33) | <0.001 | 20.56 (5.38, 54.79) | 17.93 (3.14, 57.65) | -0.51 (-0.67, -0.35) | <0.001 | 62.80 (32.15, 111.27) | 21.63 (4.74, 63.05) | -3.54 (-4.86, -2.20) | <0.001 | 1.02 (0.00, 23.67) | 0.35 (0.00, 28.38) | -3.59 (-5.26, -1.88) | <0.001 |
| Uruguay | 1.36 (0.57, 2.74) | 1.43 (0.64, 2.76) | 0.22 (0.10, 0.34) | <0.001 | 18.04 (14.69, 21.93) | 20.46 (17.00, 24.42) | 0.45 (0.42, 0.48) | <0.001 | 24.27 (20.35, 28.74) | 13.79 (10.99, 17.10) | -1.77 (-2.78, -0.74) | 0.001 | 0.41 (0.06, 1.39) | 0.22 (0.01, 1.03) | -1.91 (-3.06, -0.75) | 0.001 |
| Uzbekistan | 60.40 (58.11, 62.76) | 62.93 (61.10, 64.80) | 0.14 (0.10, 0.17) | <0.001 | 966.83 (957.43, 976.31) | 1035.13 (1027.71, 1042.60) | 0.24 (0.22, 0.25) | <0.001 | 382.00 (376.01, 388.07) | 186.70 (183.55, 189.89) | -2.34 (-3.42, -1.24) | <0.001 | 5.67 (4.96, 6.47) | 2.31 (1.97, 2.69) | -2.94 (-4.19, -1.67) | <0.001 |
| Vanuatu | 46.00 (24.48, 79.81) | 45.81 (29.94, 67.53) | -0.02 (-0.02, -0.01) | <0.001 | 790.14 (690.01, 901.51) | 790.44 (719.19, 867.16) | 0.00 (-0.00, 0.01) | 0.509 | 751.70 (652.30, 862.61) | 705.67 (637.93, 778.95) | -0.21 (-0.39, -0.03) | 0.021 | 11.97 (2.65, 34.96) | 11.07 (4.13, 24.37) | -0.26 (-0.45, -0.07) | 0.008 |
| Venezuela (Bolivarian Republic of) | 1.16 (0.84, 1.56) | 0.91 (0.67, 1.21) | -0.79 (-0.98, -0.60) | <0.001 | 18.07 (16.75, 19.47) | 14.94 (13.90, 16.03) | -0.66 (-0.73, -0.60) | <0.001 | 45.96 (43.85, 48.15) | 12.74 (11.79, 13.75) | -4.30 (-4.99, -3.61) | <0.001 | 0.74 (0.50, 1.08) | 0.20 (0.10, 0.37) | -4.44 (-5.15, -3.73) | <0.001 |
| Viet Nam | 2.21 (1.95, 2.49) | 2.09 (1.90, 2.30) | -0.17 (-0.35, 0.00) | 0.054 | 22.10 (21.29, 22.93) | 26.44 (25.73, 27.16) | 0.62 (0.58, 0.67) | <0.001 | 54.03 (52.76, 55.33) | 19.13 (18.53, 19.73) | -3.53 (-3.72, -3.33) | <0.001 | 0.90 (0.74, 1.09) | 0.30 (0.23, 0.39) | -3.69 (-3.85, -3.52) | <0.001 |
| Yemen | 63.00 (59.77, 66.37) | 65.47 (63.53, 67.46) | 0.12 (0.04, 0.20) | 0.002 | 850.71 (838.34, 863.22) | 912.53 (905.12, 919.99) | 0.26 (0.21, 0.32) | <0.001 | 137.92 (132.88, 143.11) | 91.54 (89.19, 93.94) | -1.40 (-1.66, -1.14) | <0.001 | 1.67 (1.15, 2.35) | 0.81 (0.60, 1.07) | -2.44 (-2.68, -2.19) | <0.001 |
| Zambia | 103.34 (98.23, 108.70) | 105.82 (102.58, 109.14) | 0.08 (0.06, 0.09) | <0.001 | 1736.56 (1714.33, 1759.03) | 1744.80 (1731.15, 1758.54) | 0.01 (-0.00, 0.03) | 0.140 | 147.47 (141.27, 153.91) | 126.38 (122.77, 130.07) | -0.54 (-0.61, -0.46) | <0.001 | 1.00 (0.56, 1.71) | 0.68 (0.44, 1.01) | -1.33 (-1.49, -1.18) | <0.001 |

| | | | | | | | | | | | | | | | | |
|---|---|---|---|---|---|---|---|---|---|---|---|---|---|---|---|---|
| Zimbabwe | 90.82 (86.59, 95.23) | 94.09 (90.65, 97.63) | 0.13 (0.07, 0.20) | <0.001 | 1448.55 (1430.77, 1466.52) | 1499.66 (1485.49, 1513.94) | 0.12 (0.11, 0.13) | <0.001 | 290.16 (282.60, 297.91) | 339.31 (332.77, 345.96) | 0.53 (0.34, 0.73) | <0.001 | 3.44 (2.66, 4.42) | 4.19 (3.49, 5.00) | 0.66 (0.43, 0.90) | <0.001 |
| **Ischemic heart disease** | | | | | | | | | | | | | | | | |
| **Overall** | | | | | | | | | | | | | | | | |
| Afghanistan | 40.07 (37.69, 42.58) | 42.39 (41.20, 43.60) | 0.19 (0.15, 0.23) | <0.001 | 236.03 (230.06, 242.12) | 259.26 (256.21, 262.35) | 0.36 (0.25, 0.47) | <0.001 | 1582.86 (1567.54, 1598.30) | 1052.60 (1046.51, 1058.71) | -1.31 (-1.49, -1.13) | <0.001 | 28.21 (26.19, 30.36) | 18.81 (18.00, 19.65) | -1.30 (-1.48, -1.13) | <0.001 |
| Albania | 21.19 (18.79, 23.83) | 19.12 (16.46, 22.11) | -0.35 (-0.37, -0.33) | <0.001 | 151.55 (144.88, 158.47) | 140.25 (132.79, 148.04) | -0.27 (-0.28, -0.26) | <0.001 | 343.27 (333.43, 353.35) | 486.75 (472.93, 500.87) | 0.99 (0.43, 1.56) | 0.001 | 5.82 (4.60, 7.29) | 8.28 (6.56, 10.32) | 1.00 (0.43, 1.57) | 0.001 |
| Algeria | 34.55 (33.30, 35.83) | 34.07 (33.23, 34.92) | -0.05 (-0.08, -0.03) | <0.001 | 222.41 (219.14, 225.72) | 224.09 (221.96, 226.23) | 0.04 (-0.04, 0.13) | 0.339 | 1092.75 (1085.65, 1099.88) | 489.61 (486.41, 492.83) | -2.64 (-2.91, -2.37) | <0.001 | 19.18 (18.24, 20.16) | 8.52 (8.11, 8.96) | -2.67 (-2.94, -2.41) | <0.001 |
| American Samoa | 25.23 (7.66, 62.66) | 26.27 (8.16, 64.32) | 0.14 (0.13, 0.15) | <0.001 | 217.53 (153.68, 299.71) | 232.96 (166.64, 317.62) | 0.23 (0.22, 0.24) | <0.001 | 499.21 (399.26, 617.20) | 575.23 (467.74, 700.69) | 0.50 (0.25, 0.74) | <0.001 | 8.96 (0.67, 39.04) | 10.26 (1.01, 41.12) | 0.44 (0.16, 0.73) | 0.002 |
| Andorra | 28.86 (12.26, 60.98) | 26.36 (10.67, 57.03) | -0.31 (-0.36, -0.26) | <0.001 | 159.66 (115.90, 217.16) | 165.13 (122.20, 220.81) | 0.11 (0.08, 0.14) | <0.001 | 116.75 (79.57, 167.98) | 66.45 (40.64, 105.61) | -1.92 (-2.05, -1.79) | <0.001 | 2.05 (0.00, 23.63) | 1.16 (0.00, 21.00) | -1.94 (-2.12, -1.76) | <0.001 |
| Angola | 29.74 (27.96, 31.61) | 28.07 (27.03, 29.14) | -0.20 (-0.22, -0.19) | <0.001 | 136.53 (132.59, 140.56) | 131.17 (128.84, 133.53) | -0.14 (-0.15, -0.13) | <0.001 | 297.79 (292.05, 303.62) | 229.68 (226.66, 232.73) | -0.82 (-1.55, -0.08) | 0.029 | 5.23 (4.49, 6.06) | 4.02 (3.62, 4.44) | -0.83 (-1.56, -0.10) | 0.027 |
| Antigua and Barbuda | 59.94 (33.52, 99.68) | 58.31 (35.97, 89.94) | -0.10 (-0.11, -0.08) | <0.001 | 317.98 (250.68, 398.18) | 304.49 (250.27, 367.50) | -0.17 (-0.26, -0.08) | <0.001 | 252.88 (193.49, 325.20) | 111.38 (79.58, 152.22) | -2.78 (-4.05, -1.50) | <0.001 | 4.44 (0.14, 24.58) | 1.91 (0.01, 15.21) | -2.87 (-4.12, -1.60) | <0.001 |
| Argentina | 15.93 (15.22, 16.66) | 14.96 (14.40, 15.54) | -0.22 (-0.23, -0.20) | <0.001 | 83.78 (82.15, 85.44) | 82.31 (80.98, 83.65) | -0.06 (-0.08, -0.05) | <0.001 | 371.23 (367.79, 374.70) | 156.51 (154.67, 158.36) | -2.98 (-3.17, -2.79) | <0.001 | 6.53 (6.08, 7.00) | 2.73 (2.49, 2.99) | -3.04 (-3.24, -2.84) | <0.001 |

| | | | | | | | | | | | | | | | | |
|---|---|---|---|---|---|---|---|---|---|---|---|---|---|---|---|---|
| Armenia | 37.02 (33.95, 40.29) | 35.54 (32.28, 39.08) | -0.14 (-0.15, -0.12) | <0.001 | 229.83 (222.03, 237.83) | 222.67 (214.59, 231.03) | -0.11 (-0.13, -0.10) | <0.001 | 482.49 (471.15, 494.05) | 304.71 (295.15, 314.56) | -1.55 (-2.66, -0.43) | 0.007 | 8.66 (7.20, 10.34) | 5.43 (4.22, 6.94) | -1.58 (-2.67, -0.48) | 0.005 |
| Australia | 55.22 (53.49, 56.99) | 48.97 (47.53, 50.44) | -0.43 (-0.50, -0.37) | <0.001 | 250.09 (246.42, 253.80) | 235.70 (232.58, 238.86) | -0.25 (-0.48, -0.02) | 0.036 | 173.92 (170.86, 177.02) | 84.37 (82.52, 86.27) | -2.45 (-2.92, -1.98) | <0.001 | 3.10 (2.71, 3.54) | 1.51 (1.27, 1.78) | -2.46 (-3.12, -1.80) | <0.001 |
| Austria | 34.78 (32.73, 36.93) | 31.42 (29.45, 33.51) | -0.35 (-0.37, -0.33) | <0.001 | 178.89 (174.15, 183.73) | 176.73 (172.11, 181.46) | -0.04 (-0.07, -0.02) | 0.001 | 180.62 (175.84, 185.51) | 68.41 (65.55, 71.38) | -3.31 (-4.03, -2.58) | <0.001 | 3.22 (2.61, 3.94) | 1.20 (0.85, 1.67) | -3.59 (-4.13, -3.05) | <0.001 |
| Azerbaijan | 38.17 (35.97, 40.47) | 35.75 (34.05, 37.53) | -0.22 (-0.24, -0.21) | <0.001 | 229.39 (223.80, 235.09) | 215.33 (211.15, 219.59) | -0.22 (-0.23, -0.21) | <0.001 | 740.76 (730.75, 750.87) | 532.21 (525.48, 539.00) | -1.11 (-2.09, -0.12) | 0.027 | 13.18 (11.87, 14.61) | 9.31 (8.45, 10.26) | -1.17 (-2.14, -0.20) | 0.019 |
| Bahamas | 61.45 (47.76, 78.04) | 62.41 (50.50, 76.35) | 0.06 (0.04, 0.08) | <0.001 | 298.07 (266.01, 333.06) | 299.02 (272.31, 327.72) | 0.02 (0.00, 0.03) | 0.016 | 362.82 (327.36, 401.19) | 266.43 (241.25, 293.60) | -1.13 (-2.20, -0.05) | 0.040 | 6.45 (2.54, 13.67) | 4.73 (1.94, 9.74) | -1.20 (-2.40, 0.01) | 0.052 |
| Bahrain | 42.47 (35.11, 51.20) | 38.25 (33.77, 43.31) | -0.35 (-0.39, -0.32) | <0.001 | 307.26 (286.92, 328.90) | 276.99 (265.48, 289.01) | -0.35 (-0.39, -0.31) | <0.001 | 867.81 (832.55, 904.39) | 245.19 (233.45, 257.47) | -4.28 (-4.82, -3.74) | <0.001 | 15.00 (10.71, 20.75) | 4.17 (2.80, 6.17) | -4.34 (-4.93, -3.75) | <0.001 |
| Bangladesh | 23.53 (23.04, 24.04) | 23.61 (23.24, 23.98) | 0.01 (-0.01, 0.02) | 0.322 | 156.56 (155.24, 157.89) | 162.61 (161.63, 163.60) | 0.12 (0.10, 0.15) | <0.001 | 459.69 (457.48, 461.91) | 314.53 (313.17, 315.89) | -1.24 (-1.61, -0.87) | <0.001 | 8.02 (7.73, 8.32) | 5.51 (5.33, 5.70) | -1.23 (-1.59, -0.86) | <0.001 |
| Barbados | 61.19 (47.47, 77.73) | 62.65 (48.26, 80.14) | 0.09 (0.04, 0.15) | 0.002 | 321.94 (289.21, 357.43) | 328.22 (294.61, 364.80) | 0.06 (0.05, 0.08) | <0.001 | 219.65 (192.74, 249.33) | 109.95 (90.79, 132.13) | -2.64 (-3.66, -1.60) | <0.001 | 3.85 (1.09, 9.76) | 1.88 (0.22, 7.21) | -2.69 (-3.70, -1.67) | <0.001 |
| Belarus | 34.88 (33.12, 36.73) | 34.08 (32.15, 36.12) | -0.09 (-0.11, -0.07) | <0.001 | 193.82 (189.66, 198.06) | 190.72 (186.29, 195.25) | -0.06 (-0.08, -0.04) | <0.001 | 537.31 (530.37, 544.32) | 411.60 (405.16, 418.14) | -0.85 (-2.36, 0.68) | 0.273 | 9.76 (8.85, 10.75) | 7.47 (6.63, 8.42) | -0.85 (-2.38, 0.70) | 0.280 |
| Belgium | 31.58 (29.85, 33.40) | 29.76 (28.03, 31.58) | -0.20 (-0.23, -0.16) | <0.001 | 171.57 (167.53, 175.70) | 183.15 (178.91, 187.47) | 0.23 (0.18, 0.28) | <0.001 | 228.14 (223.44, 232.93) | 63.58 (61.08, 66.16) | -4.28 (-5.08, -3.48) | <0.001 | 4.01 (3.41, 4.69) | 1.10 (0.79, 1.49) | -4.35 (-5.18, -3.51) | <0.001 |
| Belize | 62.66 (44.53, 86.23) | 66.77 (54.99, 80.40) | 0.22 (0.21, 0.23) | <0.001 | 304.38 (260.79, 353.46) | 327.38 (300.13, 356.46) | 0.26 (0.24, 0.28) | <0.001 | 229.67 (193.02, 271.64) | 201.13 (180.04, 224.05) | -0.50 (-1.31, 0.31) | 0.226 | 3.95 (0.60, 13.84) | 3.49 (1.24, 7.80) | -0.61 (-1.42, 0.22) | 0.147 |

| | | | | | | | | | | | | | | | | |
|---|---|---|---|---|---|---|---|---|---|---|---|---|---|---|---|---|
| Benin | 25.00 (22.54, 27.68) | 26.58 (25.04, 28.19) | 0.21 (0.19, 0.24) | <0.001 | 137.41 (131.38, 143.66) | 145.81 (142.05, 149.64) | 0.22 (0.15, 0.28) | <0.001 | 196.50 (189.36, 203.85) | 200.64 (196.31, 205.05) | 0.06 (-0.23, 0.35) | 0.680 | 3.45 (2.56, 4.57) | 3.51 (2.95, 4.14) | 0.05 (-0.24, 0.34) | 0.743 |
| Bermuda | 56.80 (32.32, 95.69) | 53.50 (26.09, 100.75) | -0.21 (-0.24, -0.18) | <0.001 | 311.66 (250.62, 385.66) | 288.10 (220.73, 372.81) | -0.30 (-0.45, -0.16) | <0.001 | 291.56 (231.72, 364.58) | 87.53 (52.04, 141.70) | -4.47 (-5.00, -3.93) | <0.001 | 5.07 (0.33, 27.83) | 1.49 (0.00, 28.51) | -4.53 (-5.25, -3.81) | <0.001 |
| Bhutan | 26.00 (19.61, 33.95) | 25.02 (20.06, 30.87) | -0.13 (-0.14, -0.11) | <0.001 | 168.14 (150.53, 187.31) | 165.47 (152.19, 179.63) | -0.06 (-0.07, -0.04) | <0.001 | 469.36 (440.25, 499.97) | 395.74 (375.13, 417.21) | -0.58 (-0.74, -0.42) | <0.001 | 8.27 (4.80, 13.40) | 6.89 (4.41, 10.29) | -0.63 (-0.79, -0.47) | <0.001 |
| Bolivia (Plurinational State of) | 11.14 (9.81, 12.62) | 10.98 (10.05, 11.97) | -0.05 (-0.07, -0.03) | <0.001 | 89.37 (85.40, 93.47) | 91.11 (88.37, 93.92) | 0.07 (0.05, 0.09) | <0.001 | 362.00 (354.18, 369.94) | 192.84 (188.89, 196.85) | -2.12 (-2.33, -1.91) | <0.001 | 6.21 (5.21, 7.34) | 3.27 (2.77, 3.83) | -2.15 (-2.36, -1.95) | <0.001 |
| Bosnia and Herzegovina | 25.48 (23.29, 27.81) | 23.44 (20.67, 26.51) | -0.28 (-0.30, -0.27) | <0.001 | 202.66 (196.41, 209.06) | 194.07 (186.08, 202.36) | -0.14 (-0.18, -0.11) | <0.001 | 385.99 (377.34, 394.80) | 199.30 (191.15, 207.74) | -2.50 (-3.02, -1.98) | <0.001 | 6.84 (5.73, 8.11) | 3.47 (2.47, 4.77) | -2.57 (-3.09, -2.05) | <0.001 |
| Botswana | 30.47 (25.47, 36.22) | 31.95 (28.67, 35.50) | 0.16 (0.15, 0.17) | <0.001 | 132.83 (121.78, 144.64) | 146.58 (139.52, 153.92) | 0.34 (0.32, 0.35) | <0.001 | 198.90 (185.39, 213.17) | 229.13 (220.25, 238.30) | 0.56 (0.33, 0.78) | <0.001 | 3.54 (1.94, 5.97) | 4.06 (2.96, 5.46) | 0.53 (0.30, 0.75) | <0.001 |
| Brazil | 18.17 (17.83, 18.52) | 16.12 (15.86, 16.39) | -0.42 (-0.52, -0.32) | <0.001 | 133.71 (132.76, 134.67) | 132.04 (131.30, 132.78) | -0.05 (-0.07, -0.03) | <0.001 | 427.87 (426.16, 429.57) | 228.71 (227.72, 229.69) | -2.12 (-2.40, -1.84) | <0.001 | 7.64 (7.42, 7.88) | 3.98 (3.85, 4.11) | -2.22 (-2.49, -1.94) | <0.001 |
| Brunei Darussalam | 26.58 (18.30, 37.46) | 25.38 (19.08, 33.29) | -0.16 (-0.18, -0.14) | <0.001 | 148.70 (127.83, 172.10) | 151.50 (135.63, 168.88) | 0.10 (-0.15, 0.35) | 0.434 | 681.42 (635.95, 729.36) | 349.58 (325.01, 375.67) | -2.29 (-2.80, -1.78) | <0.001 | 11.70 (6.44, 19.65) | 6.14 (3.30, 10.68) | -2.28 (-2.72, -1.83) | <0.001 |
| Bulgaria | 22.50 (20.85, 24.24) | 22.44 (20.49, 24.54) | -0.01 (-0.02, 0.00) | 0.127 | 169.66 (165.16, 174.26) | 175.99 (170.68, 181.46) | 0.14 (0.09, 0.19) | <0.001 | 671.37 (662.35, 680.50) | 455.09 (446.43, 463.90) | -1.25 (-1.75, -0.74) | <0.001 | 11.94 (10.77, 13.21) | 8.11 (7.00, 9.38) | -1.23 (-1.74, -0.72) | <0.001 |
| Burkina Faso | 23.70 (21.89, 25.62) | 24.63 (23.52, 25.79) | 0.13 (0.12, 0.14) | <0.001 | 130.22 (125.81, 134.75) | 135.72 (133.02, 138.46) | 0.16 (0.12, 0.19) | <0.001 | 219.94 (214.30, 225.70) | 242.12 (238.58, 245.70) | 0.36 (-0.08, 0.79) | 0.109 | 3.86 (3.14, 4.70) | 4.23 (3.77, 4.74) | 0.36 (-0.09, 0.80) | 0.115 |
| Burundi | 27.76 (25.41, 30.28) | 28.77 (27.16, 30.46) | 0.12 (0.11, 0.13) | <0.001 | 147.76 (142.11, 153.59) | 152.95 (149.11, 156.88) | 0.13 (0.09, 0.17) | <0.001 | 328.91 (320.59, 337.41) | 287.86 (282.69, 293.11) | -0.49 (-0.84, -0.15) | 0.005 | 5.75 (4.69, 6.99) | 4.99 (4.33, 5.73) | -0.52 (-0.87, -0.17) | 0.003 |

| | | | | | | | | | | | | | | | | |
|---|---|---|---|---|---|---|---|---|---|---|---|---|---|---|---|---|
| | 24.91 | 27.59 | 0.35 | | 149.10 | 168.86 | 0.43 | | 228.68 | 163.76 | -1.16 | | 4.07 | 2.83 | -1.36 | |
| Cabo Verde | (16.16, | (21.49, | (0.33, | <0.001 | (125.09, | (153.12, | (0.41, | <0.001 | (198.74, | (148.20, | (-1.55, | <0.001 | (1.02, | (1.14, | (-1.79, | <0.001 |
| | 37.22) | 34.94) | 0.36) | | 176.66) | 185.83) | 0.45) | | 262.13) | 180.54) | -0.77) | | 11.34) | 5.90) | -0.93) | |
| | 23.70 | 22.63 | -0.16 | | 147.14 | 140.92 | -0.13 | | 412.81 | 300.98 | -1.09 | | 7.27 | 5.25 | -1.11 | |
| Cambodia | (22.07, | (21.53, | (-0.19, | <0.001 | (142.94, | (138.14, | (-0.19, | <0.001 | (405.87, | (296.93, | (-1.22, | <0.001 | (6.37, | (4.73, | (-1.29, | <0.001 |
| | 25.41) | 23.77) | -0.13) | | 151.44) | 143.75) | -0.07) | | 419.85) | 305.07) | -0.95) | | 8.27) | 5.82) | -0.94) | |
| | 22.37 | 25.24 | 0.42 | | 113.79 | 129.36 | 0.45 | | 153.16 | 211.47 | 1.12 | | 2.67 | 3.69 | 1.12 | |
| Cameroon | (20.77, | (24.30, | (0.37, | <0.001 | (110.03, | (127.16, | (0.40, | <0.001 | (148.88, | (208.71, | (0.94, | <0.001 | (2.13, | (3.33, | (0.94, | <0.001 |
| | 24.07) | 26.21) | 0.47) | | 117.65) | 131.58) | 0.50) | | 157.53) | 214.26) | 1.29) | | 3.31) | 4.08) | 1.31) | |
| | 40.85 | 31.90 | -0.84 | | 186.17 | 162.78 | -0.47 | | 165.78 | 84.01 | -2.32 | | 2.89 | 1.46 | -2.34 | |
| Canada | (39.71, | (30.91, | (-0.86, | <0.001 | (183.77, | (160.57, | (-0.51, | <0.001 | (163.50, | (82.42, | (-2.69, | <0.001 | (2.60, | (1.25, | (-2.74, | <0.001 |
| | 42.01) | 32.92) | -0.83) | | 188.60) | 165.02) | -0.44) | | 168.08) | 85.62) | -1.95) | | 3.21) | 1.68) | -1.94) | |
| | 30.97 | 31.23 | 0.03 | | 126.41 | 128.63 | 0.08 | | 349.00 | 357.79 | 0.12 | | 6.16 | 6.33 | 0.13 | |
| Central African Republic | (27.44, | (28.71, | (0.02, | <0.001 | (118.92, | (123.34, | (-0.02, | 0.103 | (336.68, | (349.07, | (-0.16, | 0.403 | (4.60, | (5.21, | (-0.14, | 0.352 |
| | 34.85) | 33.91) | 0.04) | | 134.27) | 134.09) | 0.17) | | 361.68) | 366.68) | 0.40) | | 8.09) | 7.62) | 0.40) | |
| | 23.94 | 26.37 | 0.33 | | 132.09 | 147.06 | 0.39 | | 171.81 | 209.86 | 0.82 | | 3.00 | 3.66 | 0.81 | |
| Chad | (21.74, | (24.92, | (0.32, | <0.001 | (126.70, | (143.48, | (0.34, | <0.001 | (165.77, | (205.69, | (0.67, | <0.001 | (2.24, | (3.12, | (0.67, | <0.001 |
| | 26.31) | 27.88) | 0.34) | | 137.64) | 150.72) | 0.43) | | 178.01) | 214.10) | 0.97) | | 3.93) | 4.26) | 0.96) | |
| | 20.13 | 19.09 | -0.18 | | 117.31 | 116.57 | -0.02 | | 114.73 | 87.90 | -0.92 | | 2.02 | 1.55 | -0.92 | |
| Chile | (18.96, | (18.09, | (-0.21, | <0.001 | (114.42, | (114.08, | (-0.03, | 0.023 | (111.88, | (85.74, | (-1.28, | <0.001 | (1.66, | (1.27, | (-1.26, | <0.001 |
| | 21.35) | 20.14) | -0.16) | | 120.25) | 119.11) | -0.00) | | 117.64) | 90.10) | -0.56) | | 2.45) | 1.87) | -0.57) | |
| | 20.05 | 19.86 | -0.04 | | 212.48 | 223.40 | 0.17 | | 356.38 | 298.66 | -0.48 | | 6.09 | 5.09 | -0.49 | |
| China | (19.93, | (19.74, | (-0.08, | 0.037 | (212.07, | (223.01, | (0.12, | <0.001 | (355.87, | (298.20, | (-0.80, | 0.003 | (6.02, | (5.03, | (-0.81, | 0.002 |
| | 20.18) | 19.98) | -0.00) | | 212.88) | 223.79) | 0.21) | | 356.90) | 299.12) | -0.17) | | 6.15) | 5.15) | -0.18) | |
| | 24.51 | 22.11 | -0.34 | | 138.42 | 130.73 | -0.20 | | 337.22 | 169.17 | -2.34 | | 5.87 | 2.91 | -2.51 | |
| Colombia | (23.66, | (21.45, | (-0.38, | <0.001 | (136.35, | (129.11, | (-0.21, | <0.001 | (334.03, | (167.33, | (-3.08, | <0.001 | (5.46, | (2.67, | (-2.78, | <0.001 |
| | 25.37) | 22.78) | -0.31) | | 140.51) | 132.36) | -0.18) | | 340.42) | 171.02) | -1.59) | | 6.31) | 3.16) | -2.24) | |
| | 27.66 | 27.94 | 0.03 | | 168.27 | 170.40 | 0.05 | | 215.68 | 243.32 | 0.45 | | 3.75 | 4.22 | 0.44 | |
| Comoros | (19.65, | (22.08, | (0.02, | <0.001 | (146.69, | (155.16, | (0.04, | <0.001 | (191.68, | (225.25, | (-1.99, | 0.722 | (1.22, | (2.14, | (-1.94, | 0.720 |
| | 38.04) | 34.93) | 0.04) | | 192.22) | 186.76) | 0.06) | | 241.98) | 262.48) | 2.95) | | 8.97) | 7.49) | 2.87) | |
| | 31.34 | 30.52 | -0.10 | | 141.98 | 140.03 | -0.04 | | 429.36 | 254.13 | -1.86 | | 7.62 | 4.49 | -1.87 | |
| Congo | (27.55, | (28.17, | (-0.11, | <0.001 | (133.51, | (134.92, | (-0.10, | 0.178 | (414.73, | (247.26, | (-2.70, | <0.001 | (5.77, | (3.61, | (-2.71, | <0.001 |
| | 35.52) | 33.02) | -0.08) | | 150.87) | 145.28) | 0.02) | | 444.39) | 261.14) | -1.01) | | 9.90) | 5.51) | -1.03) | |

| | | | | | | | | | | | | | | | | |
|---|---|---|---|---|---|---|---|---|---|---|---|---|---|---|---|---|
| Cook Islands | 24.68 (2.32, 102.49) | 24.67 (1.56, 111.02) | 0.00 (-0.02, 0.02) | 0.901 | 214.98 (119.25, 359.59) | 222.40 (117.29, 383.67) | 0.11 (0.09, 0.14) | <0.001 | 541.39 (380.52, 749.56) | 452.94 (295.32, 665.32) | -0.60 (-0.81, -0.39) | <0.001 | 9.68 (0.04, 78.28) | 8.07 (0.00, 82.26) | -0.61 (-0.82, -0.41) | <0.001 |
| Costa Rica | 24.37 (21.67, 27.33) | 22.91 (20.85, 25.13) | -0.21 (-0.22, -0.19) | <0.001 | 150.26 (143.28, 157.51) | 150.93 (145.59, 156.43) | 0.01 (-0.01, 0.03) | 0.212 | 232.86 (224.19, 241.79) | 144.52 (139.29, 149.92) | -1.58 (-2.54, -0.61) | 0.001 | 4.11 (3.02, 5.47) | 2.52 (1.87, 3.33) | -1.62 (-2.61, -0.62) | 0.002 |
| Cote d'Ivoire | 28.07 (26.47, 29.75) | 29.84 (28.79, 30.93) | 0.21 (0.19, 0.22) | <0.001 | 148.77 (144.92, 152.70) | 156.32 (153.86, 158.81) | 0.19 (0.14, 0.24) | <0.001 | 258.46 (253.43, 263.56) | 230.96 (227.99, 233.96) | -0.38 (-0.63, -0.12) | 0.004 | 4.56 (3.91, 5.29) | 4.05 (3.66, 4.47) | -0.40 (-0.65, -0.14) | 0.002 |
| Croatia | 22.21 (20.16, 24.43) | 18.76 (16.57, 21.21) | -0.58 (-0.60, -0.56) | <0.001 | 209.81 (203.51, 216.27) | 182.00 (175.18, 189.06) | -0.48 (-0.53, -0.43) | <0.001 | 375.23 (366.77, 383.86) | 121.41 (115.88, 127.18) | -3.74 (-4.44, -3.04) | <0.001 | 6.65 (5.57, 7.90) | 2.12 (1.45, 3.04) | -3.78 (-4.54, -3.02) | <0.001 |
| Cuba | 57.36 (55.16, 59.63) | 54.43 (52.10, 56.84) | -0.16 (-0.23, -0.10) | <0.001 | 305.32 (300.05, 310.65) | 281.75 (276.44, 287.13) | -0.29 (-0.36, -0.21) | <0.001 | 373.79 (367.98, 379.68) | 152.52 (148.62, 156.51) | -3.06 (-3.56, -2.57) | <0.001 | 6.64 (5.89, 7.47) | 2.68 (2.18, 3.26) | -3.11 (-3.59, -2.62) | <0.001 |
| Cyprus | 18.91 (14.41, 24.42) | 18.13 (14.75, 22.26) | -0.14 (-0.17, -0.12) | <0.001 | 130.75 (118.41, 144.08) | 160.72 (150.69, 171.41) | 0.85 (0.37, 1.34) | 0.001 | 286.02 (267.56, 305.46) | 163.69 (153.53, 174.53) | -1.84 (-2.42, -1.26) | <0.001 | 5.10 (2.91, 8.36) | 2.89 (1.69, 4.90) | -1.88 (-2.56, -1.20) | <0.001 |
| Czechia | 37.03 (35.11, 39.02) | 33.11 (31.19, 35.13) | -0.38 (-0.39, -0.37) | <0.001 | 250.24 (245.35, 255.22) | 220.37 (215.57, 225.26) | -0.45 (-0.51, -0.39) | <0.001 | 346.48 (340.77, 352.27) | 87.37 (84.38, 90.46) | -4.65 (-5.13, -4.16) | <0.001 | 6.23 (5.49, 7.05) | 1.50 (1.14, 1.98) | -4.80 (-5.28, -4.32) | <0.001 |
| Democratic People's Republic of Korea | 18.00 (17.06, 18.97) | 19.10 (18.27, 19.95) | 0.21 (0.19, 0.22) | <0.001 | 180.11 (177.08, 183.18) | 192.34 (189.71, 194.99) | 0.23 (0.21, 0.25) | <0.001 | 502.15 (497.18, 507.17) | 549.70 (545.20, 554.22) | 0.30 (0.21, 0.39) | <0.001 | 8.63 (7.98, 9.31) | 9.40 (8.82, 10.01) | 0.30 (0.21, 0.40) | <0.001 |
| Democratic Republic of the Congo | 29.17 (28.22, 30.14) | 28.57 (27.97, 29.18) | -0.07 (-0.08, -0.06) | <0.001 | 132.56 (130.46, 134.68) | 130.50 (129.18, 131.83) | -0.05 (-0.07, -0.04) | <0.001 | 261.01 (258.11, 263.93) | 223.13 (221.43, 224.84) | -0.54 (-0.64, -0.44) | <0.001 | 4.60 (4.22, 5.00) | 3.93 (3.71, 4.17) | -0.54 (-0.64, -0.44) | <0.001 |
| Denmark | 41.37 (38.57, 44.32) | 32.20 (29.67, 34.91) | -0.85 (-0.88, -0.83) | <0.001 | 219.16 (212.70, 225.77) | 185.97 (179.80, 192.32) | -0.57 (-0.58, -0.55) | <0.001 | 160.15 (154.66, 165.80) | 33.20 (30.63, 35.95) | -5.41 (-6.05, -4.77) | <0.001 | 2.87 (2.18, 3.73) | 0.57 (0.28, 1.06) | -5.45 (-6.20, -4.68) | <0.001 |
| Djibouti | 26.70 (19.44, 35.93) | 28.14 (23.75, 33.12) | 0.18 (0.16, 0.20) | <0.001 | 158.21 (139.02, 179.38) | 166.96 (156.05, 178.45) | 0.19 (0.17, 0.21) | <0.001 | 204.24 (182.79, 227.62) | 248.11 (234.69, 262.10) | 0.60 (0.36, 0.84) | <0.001 | 3.55 (1.25, 8.07) | 4.28 (2.69, 6.50) | 0.57 (0.34, 0.81) | <0.001 |

| | | | | | | | | | | | | | | | | |
|---|---|---|---|---|---|---|---|---|---|---|---|---|---|---|---|---|
| Dominica | 60.31 (34.16, 99.56) | 61.32 (34.87, 100.08) | 0.06 (0.03, 0.08) | <0.001 | 295.27 (230.84, 372.54) | 299.08 (235.86, 374.11) | 0.04 (0.03, 0.05) | <0.001 | 237.07 (180.17, 306.77) | 136.96 (95.43, 190.52) | -1.92 (-2.43, -1.41) | <0.001 | 4.17 (0.11, 24.05) | 2.38 (0.01, 19.25) | -1.94 (-2.42, -1.45) | <0.001 |
| Dominican Republic | 55.21 (52.44, 58.10) | 60.26 (58.00, 62.59) | 0.30 (0.29, 0.32) | <0.001 | 260.86 (254.57, 267.26) | 292.20 (287.15, 297.32) | 0.39 (0.36, 0.41) | <0.001 | 584.48 (575.31, 593.78) | 672.67 (665.03, 680.37) | 0.56 (0.24, 0.88) | 0.001 | 10.16 (8.97, 11.46) | 11.78 (10.79, 12.85) | 0.58 (0.25, 0.91) | <0.001 |
| Ecuador | 11.02 (9.98, 12.14) | 11.28 (10.51, 12.10) | 0.09 (0.07, 0.11) | <0.001 | 90.43 (87.30, 93.64) | 98.47 (96.13, 100.85) | 0.30 (0.27, 0.34) | <0.001 | 405.48 (399.03, 412.01) | 316.06 (311.91, 320.25) | -0.92 (-1.45, -0.40) | 0.001 | 6.96 (6.13, 7.87) | 5.39 (4.86, 5.96) | -0.94 (-1.46, -0.42) | <0.001 |
| Egypt | 38.75 (37.88, 39.63) | 38.45 (37.84, 39.06) | -0.03 (-0.04, -0.01) | <0.001 | 260.05 (257.76, 262.35) | 267.72 (266.11, 269.33) | 0.12 (-0.01, 0.25) | 0.071 | 1498.01 (1492.66, 1503.38) | 980.67 (977.62, 983.72) | -1.43 (-1.91, -0.95) | <0.001 | 25.89 (25.18, 26.60) | 16.81 (16.42, 17.22) | -1.46 (-1.93, -0.99) | <0.001 |
| El Salvador | 21.27 (19.18, 23.54) | 21.98 (20.15, 23.94) | 0.12 (0.09, 0.15) | <0.001 | 108.22 (103.26, 113.35) | 124.03 (119.53, 128.66) | 0.47 (0.45, 0.49) | <0.001 | 678.33 (666.42, 690.41) | 319.57 (312.44, 326.82) | -2.51 (-3.35, -1.65) | <0.001 | 11.58 (10.05, 13.28) | 5.52 (4.62, 6.56) | -2.46 (-3.31, -1.61) | <0.001 |
| Equatorial Guinea | 29.80 (21.20, 40.84) | 27.29 (23.06, 32.12) | -0.31 (-0.33, -0.29) | <0.001 | 132.06 (112.78, 153.75) | 127.58 (117.87, 137.92) | -0.12 (-0.13, -0.10) | <0.001 | 332.93 (302.36, 365.83) | 148.89 (138.68, 159.69) | -2.68 (-3.08, -2.27) | <0.001 | 5.87 (2.46, 11.90) | 2.57 (1.38, 4.43) | -2.74 (-3.15, -2.33) | <0.001 |
| Eritrea | 25.47 (22.42, 28.84) | 25.37 (23.45, 27.41) | -0.02 (-0.04, 0.01) | 0.263 | 132.82 (125.49, 140.48) | 132.60 (128.04, 137.29) | -0.00 (-0.01, 0.00) | 0.221 | 250.80 (240.86, 261.06) | 311.10 (304.22, 318.10) | 0.69 (0.15, 1.23) | 0.012 | 4.40 (3.16, 5.98) | 5.45 (4.57, 6.46) | 0.69 (0.16, 1.22) | 0.011 |
| Estonia | 50.44 (44.91, 56.49) | 47.79 (41.59, 54.81) | -0.19 (-0.24, -0.15) | <0.001 | 254.51 (242.07, 267.47) | 241.99 (228.21, 256.55) | -0.21 (-0.37, -0.05) | 0.009 | 471.91 (454.97, 489.37) | 104.53 (95.57, 114.27) | -5.42 (-6.47, -4.37) | <0.001 | 8.57 (6.43, 11.25) | 1.83 (0.83, 3.79) | -5.58 (-6.60, -4.55) | <0.001 |
| Eswatini | 30.92 (24.32, 38.86) | 32.84 (27.87, 38.48) | 0.21 (0.20, 0.23) | <0.001 | 137.61 (122.70, 153.88) | 151.44 (140.29, 163.26) | 0.34 (0.31, 0.36) | <0.001 | 150.21 (134.79, 166.97) | 237.92 (223.94, 252.56) | 1.53 (0.99, 2.08) | <0.001 | 2.64 (0.97, 5.85) | 4.22 (2.54, 6.61) | 1.56 (0.98, 2.14) | <0.001 |
| Ethiopia | 22.07 (21.35, 22.81) | 24.03 (23.54, 24.53) | 0.29 (0.27, 0.31) | <0.001 | 111.76 (110.08, 113.46) | 125.23 (124.07, 126.41) | 0.39 (0.38, 0.41) | <0.001 | 358.30 (355.38, 361.24) | 182.42 (181.06, 183.79) | -2.31 (-2.49, -2.13) | <0.001 | 6.22 (5.84, 6.62) | 3.13 (2.95, 3.32) | -2.35 (-2.53, -2.17) | <0.001 |
| Fiji | 27.10 (21.53, | 27.27 (22.17, | 0.03 (0.02, | <0.001 | 229.70 (212.61, | 236.74 (221.25, | 0.10 (0.10, | <0.001 | 1149.71 (1111.07 | 915.92 (885.18, | -0.70 (-1.42, | 0.060 | 20.70 (15.78, | 16.36 (12.49, | -0.73 (-1.46, | 0.053 |

| | | | | | | | | | | | | | | | | |
|---|---|---|---|---|---|---|---|---|---|---|---|---|---|---|---|---|
| | 33.71) | 33.21) | 0.03) | | 247.84) | 253.04) | 0.11) | | , 1189.38) | 947.46) | 0.03) | | 26.68) | 21.07) | 0.01) | |
| Finland | 44.02 (41.12, 47.09) | 35.75 (33.01, 38.67) | -0.71 (-0.73, -0.69) | <0.001 | 229.28 (222.77, 235.95) | 200.47 (194.08, 207.05) | -0.47 (-0.48, -0.45) | <0.001 | 226.50 (220.11, 233.06) | 59.51 (56.08, 63.12) | -4.57 (-5.24, -3.91) | <0.001 | 4.10 (3.28, 5.09) | 1.05 (0.64, 1.65) | -4.66 (-5.36, -3.95) | <0.001 |
| France | 28.59 (27.90, 29.30) | 25.86 (25.17, 26.57) | -0.33 (-0.36, -0.30) | <0.001 | 161.96 (160.33, 163.61) | 164.98 (163.25, 166.71) | 0.08 (0.01, 0.14) | 0.017 | 137.58 (136.09, 139.09) | 62.70 (61.64, 63.77) | -2.69 (-3.11, -2.27) | <0.001 | 2.45 (2.25, 2.66) | 1.10 (0.96, 1.25) | -2.74 (-3.17, -2.30) | <0.001 |
| Gabon | 28.28 (22.89, 34.61) | 27.91 (24.12, 32.13) | -0.05 (-0.06, -0.03) | <0.001 | 134.04 (121.64, 147.38) | 134.46 (125.84, 143.52) | 0.01 (-0.03, 0.04) | 0.765 | 303.71 (285.14, 323.21) | 204.89 (194.32, 215.89) | -1.48 (-1.67, -1.29) | <0.001 | 5.36 (3.14, 8.59) | 3.58 (2.31, 5.33) | -1.47 (-1.66, -1.29) | <0.001 |
| Gambia | 28.71 (23.16, 35.30) | 31.34 (27.62, 35.45) | 0.30 (0.28, 0.31) | <0.001 | 167.71 (153.31, 183.15) | 181.55 (172.15, 191.35) | 0.29 (0.21, 0.37) | <0.001 | 188.46 (173.35, 204.60) | 226.28 (215.92, 237.02) | 0.69 (-0.40, 1.78) | 0.215 | 3.31 (1.58, 6.19) | 3.96 (2.69, 5.65) | 0.67 (-0.41, 1.77) | 0.224 |
| Georgia | 36.89 (34.38, 39.53) | 38.49 (35.17, 42.06) | 0.16 (0.12, 0.19) | <0.001 | 219.50 (213.32, 225.83) | 235.11 (226.98, 243.50) | 0.24 (0.22, 0.26) | <0.001 | 1104.65 (1090.69 , 1118.75) | 512.08 (499.96, 524.46) | -2.71 (-4.07, -1.33) | <0.001 | 19.73 (17.91, 21.70) | 9.14 (7.59, 10.96) | -2.71 (-4.03, -1.37) | <0.001 |
| Germany | 41.67 (40.96, 42.39) | 34.31 (33.62, 35.02) | -0.67 (-0.71, -0.63) | <0.001 | 217.32 (215.70, 218.95) | 202.58 (200.94, 204.24) | -0.24 (-0.31, -0.18) | <0.001 | 290.04 (288.16, 291.94) | 79.66 (78.63, 80.71) | -4.39 (-4.67, -4.11) | <0.001 | 5.13 (4.88, 5.39) | 1.39 (1.26, 1.54) | -4.44 (-4.71, -4.16) | <0.001 |
| Ghana | 26.84 (25.43, 28.31) | 28.29 (27.38, 29.24) | 0.18 (0.10, 0.26) | <0.001 | 144.81 (141.42, 148.26) | 152.65 (150.46, 154.86) | 0.20 (0.14, 0.26) | <0.001 | 252.65 (248.24, 257.12) | 216.62 (214.05, 219.23) | -0.52 (-0.84, -0.20) | 0.001 | 4.44 (3.87, 5.07) | 3.78 (3.45, 4.14) | -0.54 (-0.86, -0.22) | 0.001 |
| Greece | 26.98 (25.36, 28.69) | 26.29 (24.51, 28.19) | -0.08 (-0.10, -0.07) | <0.001 | 145.36 (141.57, 149.23) | 161.96 (157.68, 166.34) | 0.37 (0.34, 0.41) | <0.001 | 330.13 (324.40, 335.94) | 249.96 (244.65, 255.38) | -0.98 (-1.84, -0.12) | 0.027 | 5.83 (5.09, 6.64) | 4.46 (3.78, 5.24) | -0.96 (-1.55, -0.36) | 0.002 |
| Greenland | 42.61 (21.66, 79.43) | 34.12 (14.01, 72.09) | -0.76 (-0.79, -0.72) | <0.001 | 161.46 (116.64, 220.91) | 147.43 (100.52, 210.72) | -0.31 (-0.34, -0.29) | <0.001 | 243.83 (187.39, 314.74) | 89.03 (53.57, 141.31) | -3.44 (-3.66, -3.21) | <0.001 | 4.42 (0.16, 28.75) | 1.60 (0.00, 25.27) | -3.47 (-3.71, -3.22) | <0.001 |
| Grenada | 62.55 (37.65, 98.47) | 64.92 (42.19, 96.06) | 0.13 (0.12, 0.14) | <0.001 | 318.36 (256.57, 390.96) | 334.02 (277.94, 398.37) | 0.17 (0.15, 0.18) | <0.001 | 423.44 (351.71, 505.91) | 158.56 (120.69, 204.84) | -3.32 (-4.04, -2.59) | <0.001 | 7.57 (1.03, 26.91) | 2.77 (0.08, 15.79) | -3.38 (-4.10, -2.66) | <0.001 |

| | | | | | | | | | | | | | | | | |
|---|---|---|---|---|---|---|---|---|---|---|---|---|---|---|---|---|
| Guam | 22.03 (11.97, 37.40) | 24.89 (13.76, 41.56) | 0.42 (0.40, 0.44) | <0.001 | 188.13 (155.30, 226.00) | 226.67 (189.24, 269.41) | 0.63 (0.59, 0.67) | <0.001 | 403.92 (355.19, 457.63) | 829.01 (755.90, 907.34) | 2.47 (2.02, 2.93) | <0.001 | 7.12 (2.08, 17.84) | 14.86 (6.58, 28.87) | 2.53 (2.05, 3.01) | <0.001 |
| Guatemala | 19.70 (18.02, 21.50) | 21.32 (20.27, 22.41) | 0.28 (0.26, 0.31) | <0.001 | 92.12 (88.35, 96.02) | 110.45 (107.99, 112.95) | 0.63 (0.60, 0.66) | <0.001 | 872.34 (861.09, 883.70) | 379.51 (375.06, 384.01) | -2.94 (-4.34, -1.53) | <0.001 | 14.88 (13.43, 16.45) | 6.53 (5.96, 7.15) | -2.92 (-4.34, -1.48) | <0.001 |
| Guinea | 22.29 (20.24, 24.49) | 25.59 (24.09, 27.18) | 0.47 (0.44, 0.50) | <0.001 | 114.77 (109.96, 119.75) | 133.09 (129.52, 136.74) | 0.53 (0.48, 0.58) | <0.001 | 172.85 (167.03, 178.82) | 241.05 (236.32, 245.85) | 1.21 (1.04, 1.38) | <0.001 | 3.02 (2.29, 3.91) | 4.22 (3.61, 4.90) | 1.23 (1.06, 1.39) | <0.001 |
| Guinea-Bissau | 27.78 (22.31, 34.24) | 30.04 (26.17, 34.35) | 0.27 (0.26, 0.28) | <0.001 | 145.28 (132.01, 159.57) | 156.89 (147.60, 166.63) | 0.29 (0.18, 0.40) | <0.001 | 333.58 (313.56, 354.58) | 363.72 (349.63, 378.24) | 0.31 (0.18, 0.43) | <0.001 | 5.91 (3.51, 9.39) | 6.44 (4.68, 8.68) | 0.30 (0.17, 0.43) | <0.001 |
| Guyana | 68.12 (59.17, 78.10) | 67.46 (58.48, 77.48) | -0.03 (-0.06, 0.00) | 0.062 | 308.31 (288.30, 329.38) | 305.18 (285.21, 326.20) | -0.02 (-0.06, 0.02) | 0.284 | 681.79 (652.23, 712.38) | 426.91 (403.38, 451.48) | -1.68 (-2.41, -0.95) | <0.001 | 12.04 (8.38, 16.81) | 7.57 (4.72, 11.53) | -1.67 (-2.39, -0.93) | <0.001 |
| Haiti | 64.74 (61.45, 68.16) | 65.42 (63.24, 67.66) | 0.04 (0.00, 0.07) | 0.053 | 273.19 (266.26, 280.26) | 273.82 (269.29, 278.40) | 0.02 (-0.05, 0.10) | 0.567 | 580.52 (570.46, 590.72) | 405.91 (400.41, 411.47) | -1.23 (-1.48, -0.98) | <0.001 | 10.31 (9.00, 11.76) | 7.20 (6.48, 7.97) | -1.23 (-1.48, -0.99) | <0.001 |
| Honduras | 24.72 (22.26, 27.39) | 24.82 (23.27, 26.45) | 0.01 (-0.00, 0.03) | 0.087 | 132.86 (126.91, 139.03) | 145.44 (141.55, 149.42) | 0.31 (0.28, 0.33) | <0.001 | 465.47 (454.51, 476.63) | 181.77 (177.47, 186.15) | -3.23 (-3.74, -2.71) | <0.001 | 8.12 (6.73, 9.74) | 3.17 (2.62, 3.80) | -3.19 (-3.63, -2.75) | <0.001 |
| Hungary | 31.35 (29.61, 33.18) | 25.64 (23.88, 27.50) | -0.69 (-0.72, -0.67) | <0.001 | 259.58 (254.66, 264.58) | 210.50 (205.54, 215.57) | -0.71 (-0.75, -0.68) | <0.001 | 743.72 (735.48, 752.04) | 178.05 (173.53, 182.68) | -4.86 (-5.54, -4.18) | <0.001 | 13.51 (12.42, 14.67) | 3.16 (2.59, 3.84) | -4.95 (-5.64, -4.25) | <0.001 |
| Iceland | 37.01 (26.34, 50.69) | 32.82 (23.58, 44.79) | -0.42 (-0.51, -0.33) | <0.001 | 182.44 (157.60, 210.19) | 181.22 (158.69, 206.34) | -0.04 (-0.10, 0.02) | 0.231 | 235.12 (206.72, 266.43) | 89.80 (74.13, 108.09) | -3.32 (-3.70, -2.95) | <0.001 | 4.07 (1.17, 10.31) | 1.56 (0.19, 6.25) | -3.30 (-3.81, -2.79) | <0.001 |
| India | 25.67 (25.50, 25.85) | 26.34 (26.21, 26.48) | 0.09 (0.08, 0.10) | <0.001 | 149.16 (148.73, 149.60) | 158.35 (158.03, 158.68) | 0.21 (0.19, 0.22) | <0.001 | 774.50 (773.53, 775.47) | 679.43 (678.75, 680.10) | -0.43 (-1.03, 0.17) | 0.161 | 13.53 (13.40, 13.66) | 11.99 (11.91, 12.08) | -0.39 (-1.03, 0.25) | 0.235 |
| Indonesia | 12.12 (11.86, 12.38) | 13.51 (13.29, 13.73) | 0.38 (0.28, 0.47) | <0.001 | 116.42 (115.61, 117.24) | 133.13 (132.45, 133.82) | 0.47 (0.41, 0.54) | <0.001 | 608.64 (606.82, 610.46) | 534.26 (532.88, 535.64) | -0.44 (-0.61, -0.27) | <0.001 | 10.62 (10.38, 10.87) | 9.31 (9.13, 9.49) | -0.45 (-0.62, -0.28) | <0.001 |

| | | | | | | | | | | | | | | | | |
|---|---|---|---|---|---|---|---|---|---|---|---|---|---|---|---|---|
| Iran (Islamic Republic of) | 60.45 (59.34, 61.58) | 58.97 (58.22, 59.73) | -0.09 (-0.10, -0.07) | <0.001 | 328.20 (325.54, 330.87) | 332.69 (330.96, 334.43) | 0.06 (0.02, 0.10) | 0.002 | 712.29 (708.50, 716.10) | 395.41 (393.41, 397.43) | -2.02 (-2.15, -1.89) | <0.001 | 12.38 (11.88, 12.90) | 6.73 (6.47, 6.99) | -2.09 (-2.23, -1.96) | <0.001 |
| Iraq | 42.60 (40.89, 44.36) | 41.03 (40.06, 42.01) | -0.14 (-0.16, -0.11) | <0.001 | 288.93 (284.37, 293.54) | 283.38 (280.78, 286.00) | -0.04 (-0.20, 0.12) | 0.658 | 735.99 (728.83, 743.19) | 363.27 (360.35, 366.20) | -2.40 (-2.73, -2.08) | <0.001 | 12.93 (11.99, 13.92) | 6.34 (5.96, 6.74) | -2.43 (-2.76, -2.09) | <0.001 |
| Ireland | 34.41 (31.33, 37.73) | 28.81 (26.28, 31.53) | -0.61 (-0.62, -0.60) | <0.001 | 200.73 (193.18, 208.50) | 186.65 (180.37, 193.10) | -0.23 (-0.30, -0.16) | <0.001 | 199.01 (191.51, 206.73) | 57.80 (54.40, 61.38) | -4.17 (-4.90, -3.45) | <0.001 | 3.58 (2.64, 4.76) | 1.02 (0.61, 1.61) | -4.24 (-5.01, -3.46) | <0.001 |
| Israel | 26.58 (24.28, 29.04) | 24.20 (22.54, 25.96) | -0.32 (-0.36, -0.28) | <0.001 | 159.63 (153.95, 165.47) | 163.30 (158.96, 167.73) | 0.08 (0.03, 0.12) | 0.001 | 236.63 (229.70, 243.71) | 52.21 (49.78, 54.73) | -5.10 (-5.77, -4.44) | <0.001 | 4.15 (3.28, 5.19) | 0.90 (0.61, 1.29) | -5.16 (-5.84, -4.48) | <0.001 |
| Italy | 34.93 (34.14, 35.73) | 31.26 (30.43, 32.12) | -0.38 (-0.42, -0.34) | <0.001 | 197.32 (195.44, 199.22) | 188.36 (186.37, 190.37) | -0.15 (-0.19, -0.11) | <0.001 | 182.50 (180.69, 184.33) | 80.56 (79.25, 81.88) | -2.84 (-3.50, -2.18) | <0.001 | 3.20 (2.97, 3.45) | 1.40 (1.23, 1.58) | -2.86 (-3.45, -2.26) | <0.001 |
| Jamaica | 57.85 (52.89, 63.17) | 64.84 (60.32, 69.61) | 0.40 (0.38, 0.43) | <0.001 | 303.99 (292.06, 316.30) | 349.13 (338.40, 360.11) | 0.47 (0.43, 0.52) | <0.001 | 121.36 (113.89, 129.22) | 127.36 (120.91, 134.07) | 0.48 (-0.57, 1.54) | 0.373 | 2.11 (1.22, 3.41) | 2.21 (1.43, 3.26) | 0.50 (-0.60, 1.61) | 0.376 |
| Japan | 22.63 (22.19, 23.08) | 20.77 (20.29, 21.25) | -0.29 (-0.31, -0.28) | <0.001 | 130.48 (129.43, 131.52) | 122.18 (121.05, 123.31) | -0.23 (-0.25, -0.21) | <0.001 | 114.49 (113.52, 115.48) | 79.80 (78.88, 80.72) | -1.21 (-1.53, -0.89) | <0.001 | 1.97 (1.85, 2.11) | 1.38 (1.26, 1.50) | -1.21 (-1.54, -0.87) | <0.001 |
| Jordan | 40.37 (36.76, 44.26) | 38.50 (36.75, 40.30) | -0.17 (-0.23, -0.10) | <0.001 | 284.41 (274.37, 294.75) | 276.25 (271.52, 281.05) | -0.09 (-0.19, 0.01) | 0.081 | 441.72 (429.32, 454.41) | 228.60 (224.31, 232.96) | -2.19 (-2.67, -1.71) | <0.001 | 7.80 (6.22, 9.69) | 3.97 (3.42, 4.58) | -2.25 (-2.73, -1.76) | <0.001 |
| Kazakhstan | 38.61 (37.14, 40.11) | 34.81 (33.48, 36.18) | -0.36 (-0.42, -0.30) | <0.001 | 215.85 (212.36, 219.39) | 195.31 (192.17, 198.48) | -0.34 (-0.36, -0.33) | <0.001 | 698.97 (692.66, 705.32) | 320.12 (316.10, 324.19) | -2.71 (-3.83, -1.58) | <0.001 | 12.61 (11.77, 13.49) | 5.75 (5.23, 6.33) | -2.72 (-3.86, -1.57) | <0.001 |
| Kenya | 26.41 (25.21, 27.65) | 26.87 (26.14, 27.61) | 0.06 (0.04, 0.07) | <0.001 | 155.48 (152.43, 158.58) | 158.17 (156.35, 160.01) | 0.06 (0.04, 0.08) | <0.001 | 104.22 (101.81, 106.68) | 172.19 (170.34, 174.06) | 1.75 (1.62, 1.87) | <0.001 | 1.75 (1.45, 2.10) | 2.94 (2.70, 3.19) | 1.80 (1.67, 1.94) | <0.001 |
| Kiribati | 27.42 (11.49, 56.06) | 28.50 (15.31, 48.65) | 0.13 (0.12, 0.14) | <0.001 | 188.66 (139.79, 249.56) | 200.48 (161.54, 246.08) | 0.22 (0.19, 0.25) | <0.001 | 1919.32 (1755.21 , | 1783.67 (1663.53 , | -0.26 (-0.38, -0.13) | <0.001 | 34.82 (15.96, 66.54) | 32.13 (17.85, 53.42) | -0.28 (-0.41, -0.16) | <0.001 |

| | | | | | | | | | | | | | | | | |
|---|---|---|---|---|---|---|---|---|---|---|---|---|---|---|---|---|
| | | | | | | | | | 2095.04) | 1910.31) | | | | | | |
| Kuwait | 39.53 (35.59, 43.86) | 38.71 (36.26, 41.33) | -0.08 (-0.13, -0.04) | 0.001 | 288.73 (277.91, 299.92) | 289.98 (283.52, 296.61) | 0.03 (-0.06, 0.11) | 0.525 | 566.72 (551.32, 582.50) | 393.87 (385.96, 401.95) | -1.06 (-2.58, 0.49) | 0.178 | 9.79 (7.86, 12.12) | 6.83 (5.84, 8.01) | -1.03 (-2.59, 0.55) | 0.201 |
| Kyrgyzstan | 35.47 (32.68, 38.45) | 32.93 (30.77, 35.20) | -0.26 (-0.27, -0.24) | <0.001 | 196.95 (190.18, 203.92) | 180.86 (175.69, 186.14) | -0.30 (-0.32, -0.27) | <0.001 | 590.43 (578.68, 602.36) | 334.51 (327.46, 341.68) | -1.87 (-3.21, -0.52) | 0.007 | 10.59 (9.06, 12.31) | 6.01 (5.10, 7.05) | -1.87 (-3.22, -0.49) | 0.008 |
| Lao People's Democratic Republic | 24.89 (22.29, 27.72) | 22.68 (21.01, 24.45) | -0.32 (-0.33, -0.31) | <0.001 | 146.69 (140.16, 153.45) | 132.50 (128.38, 136.73) | -0.34 (-0.46, -0.21) | <0.001 | 503.03 (491.01, 515.29) | 520.69 (512.56, 528.91) | 0.15 (-0.06, 0.35) | 0.157 | 8.91 (7.36, 10.69) | 9.11 (8.06, 10.26) | 0.10 (-0.10, 0.30) | 0.319 |
| Latvia | 31.24 (27.87, 34.92) | 32.02 (27.73, 36.89) | 0.08 (0.04, 0.12) | <0.001 | 194.53 (186.03, 203.35) | 201.75 (191.06, 212.99) | 0.13 (0.09, 0.17) | <0.001 | 580.91 (566.18, 595.95) | 174.33 (164.50, 184.71) | -4.05 (-6.12, -1.94) | <0.001 | 10.61 (8.70, 12.83) | 3.13 (1.94, 4.93) | -4.11 (-6.22, -1.94) | <0.001 |
| Lebanon | 35.37 (31.88, 39.14) | 36.85 (34.37, 39.48) | 0.14 (0.12, 0.16) | <0.001 | 236.25 (226.96, 245.84) | 257.03 (250.48, 263.74) | 0.30 (0.24, 0.36) | <0.001 | 1101.93 (1082.12, 1122.03) | 726.04 (714.68, 737.55) | -1.41 (-1.56, -1.26) | <0.001 | 19.26 (16.71, 22.11) | 12.51 (11.06, 14.11) | -1.46 (-1.61, -1.31) | <0.001 |
| Lesotho | 28.27 (24.12, 32.97) | 31.42 (27.88, 35.29) | 0.36 (0.34, 0.38) | <0.001 | 113.58 (104.84, 122.88) | 133.35 (125.85, 141.17) | 0.55 (0.51, 0.58) | <0.001 | 101.40 (93.22, 110.13) | 248.70 (238.43, 259.29) | 3.24 (2.97, 3.51) | <0.001 | 1.78 (0.84, 3.32) | 4.44 (3.16, 6.07) | 3.32 (3.03, 3.61) | <0.001 |
| Liberia | 27.81 (23.81, 32.31) | 30.42 (27.92, 33.09) | 0.30 (0.28, 0.33) | <0.001 | 157.60 (147.87, 167.83) | 171.26 (165.15, 177.54) | 0.30 (0.17, 0.44) | <0.001 | 213.59 (202.33, 225.32) | 203.27 (196.69, 210.01) | -0.21 (-0.45, 0.03) | 0.088 | 3.75 (2.39, 5.62) | 3.56 (2.73, 4.56) | -0.22 (-0.47, 0.02) | 0.074 |
| Libya | 34.67 (31.59, 37.97) | 37.11 (35.03, 39.28) | 0.22 (0.15, 0.29) | <0.001 | 225.48 (217.35, 233.84) | 251.76 (246.36, 257.24) | 0.39 (0.31, 0.47) | <0.001 | 871.09 (855.68, 886.72) | 691.13 (682.00, 700.36) | -0.90 (-1.57, -0.22) | 0.010 | 14.99 (13.01, 17.20) | 11.89 (10.72, 13.16) | -0.90 (-1.60, -0.20) | 0.012 |
| Lithuania | 32.65 (29.74, 35.77) | 30.30 (26.81, 34.17) | -0.27 (-0.31, -0.23) | <0.001 | 198.79 (191.51, 206.29) | 181.81 (173.16, 190.83) | -0.30 (-0.33, -0.27) | <0.001 | 566.41 (554.05, 578.98) | 240.55 (230.62, 250.84) | -3.03 (-4.31, -1.73) | <0.001 | 10.30 (8.69, 12.13) | 4.35 (3.11, 5.99) | -3.04 (-4.34, -1.73) | <0.001 |
| Luxembourg | 23.36 (16.47, 32.68) | 21.50 (15.92, 28.69) | -0.28 (-0.30, -0.25) | <0.001 | 145.29 (127.43, 165.39) | 151.77 (136.73, 168.30) | 0.18 (0.03, 0.33) | 0.017 | 231.01 (208.00, 256.27) | 66.61 (56.72, 78.01) | -4.25 (-4.92, -3.59) | <0.001 | 4.07 (1.57, 9.33) | 1.15 (0.22, 4.08) | -4.33 (-5.01, -3.65) | <0.001 |
| Madagascar | 24.96 | 27.19 | 0.29 | <0.001 | 140.32 | 154.67 | 0.34 | <0.001 | 472.29 | 383.36 | -0.66 | 0.006 | 8.30 | 6.73 | -0.67 | 0.006 |

| | | | | | | | | | | | | | | | | |
|---|---|---|---|---|---|---|---|---|---|---|---|---|---|---|---|---|
| | (23.41, 26.60) | (26.16, 28.26) | (0.27, 0.31) | | (136.48, 144.25) | (152.12, 157.25) | (0.31, 0.37) | | (465.38, 479.28) | (379.42, 387.34) | (-1.13, -0.19) | | (7.40, 9.29) | (6.21, 7.28) | (-1.14, -0.19) | |
| Malawi | 29.30 (27.41, 31.29) | 29.68 (28.37, 31.05) | 0.04 (0.03, 0.05) | <0.001 | 160.99 (156.34, 165.73) | 163.33 (160.13, 166.59) | 0.07 (-0.03, 0.16) | 0.160 | 294.35 (288.27, 300.53) | 256.52 (252.63, 260.46) | -0.42 (-0.63, -0.20) | <0.001 | 5.06 (4.29, 5.95) | 4.41 (3.91, 4.96) | -0.42 (-0.63, -0.21) | <0.001 |
| Malaysia | 25.92 (24.74, 27.15) | 26.26 (25.42, 27.13) | 0.05 (0.02, 0.08) | 0.002 | 203.21 (199.84, 206.63) | 208.80 (206.40, 211.22) | 0.11 (0.03, 0.19) | 0.008 | 285.51 (281.52, 289.54) | 390.16 (386.88, 393.46) | 1.12 (0.30, 1.95) | 0.007 | 5.01 (4.49, 5.57) | 6.92 (6.49, 7.38) | 1.17 (0.34, 2.01) | 0.006 |
| Maldives | 24.13 (13.53, 40.45) | 23.79 (18.46, 30.70) | -0.06 (-0.08, -0.04) | <0.001 | 179.67 (146.95, 217.90) | 176.28 (161.59, 192.40) | -0.06 (-0.08, -0.05) | <0.001 | 723.77 (657.14, 795.72) | 257.79 (239.50, 277.52) | -3.46 (-4.08, -2.84) | <0.001 | 12.87 (5.39, 26.48) | 4.47 (2.38, 8.37) | -3.55 (-4.16, -2.93) | <0.001 |
| Mali | 24.29 (22.44, 26.26) | 25.46 (24.26, 26.71) | 0.16 (0.15, 0.18) | <0.001 | 124.26 (119.92, 128.71) | 129.05 (126.23, 131.92) | 0.14 (0.09, 0.20) | <0.001 | 222.94 (217.23, 228.77) | 195.06 (191.69, 198.48) | -0.42 (-0.70, -0.14) | 0.004 | 3.90 (3.17, 4.75) | 3.39 (2.96, 3.88) | -0.44 (-0.72, -0.15) | 0.003 |
| Malta | 24.56 (17.16, 34.30) | 21.58 (14.80, 31.00) | -0.44 (-0.48, -0.41) | <0.001 | 157.96 (138.56, 179.54) | 152.92 (134.32, 173.89) | -0.09 (-0.24, 0.06) | 0.229 | 238.31 (214.01, 264.83) | 131.03 (113.66, 150.80) | -2.09 (-2.72, -1.45) | <0.001 | 4.14 (1.54, 9.30) | 2.29 (0.57, 7.17) | -2.06 (-2.65, -1.47) | <0.001 |
| Marshall Islands | 24.70 (6.29, 66.98) | 25.44 (9.35, 55.59) | 0.10 (0.09, 0.12) | <0.001 | 176.87 (115.06, 260.69) | 187.14 (136.20, 251.09) | 0.19 (0.19, 0.20) | <0.001 | 1018.50 (861.59, 1196.38) | 1499.96 (1348.14 , 1664.37) | 1.40 (1.23, 1.57) | <0.001 | 18.15 (3.23, 57.74) | 27.11 (10.39, 57.82) | 1.46 (1.27, 1.66) | <0.001 |
| Mauritania | 27.67 (23.85, 31.96) | 28.87 (26.12, 31.85) | 0.14 (0.13, 0.15) | <0.001 | 161.70 (152.01, 171.85) | 167.55 (160.64, 174.70) | 0.13 (0.08, 0.18) | <0.001 | 237.66 (226.04, 249.74) | 135.89 (129.77, 142.23) | -1.84 (-1.96, -1.72) | <0.001 | 4.18 (2.76, 6.09) | 2.35 (1.61, 3.34) | -1.90 (-2.02, -1.77) | <0.001 |
| Mauritius | 25.18 (20.96, 30.02) | 22.92 (18.86, 27.63) | -0.32 (-0.34, -0.31) | <0.001 | 196.45 (184.25, 209.27) | 176.51 (164.99, 188.66) | -0.36 (-0.39, -0.33) | <0.001 | 779.84 (755.28, 805.00) | 353.83 (337.41, 370.87) | -2.62 (-3.11, -2.13) | <0.001 | 14.04 (10.91, 17.79) | 6.26 (4.25, 8.93) | -2.68 (-3.18, -2.17) | <0.001 |
| Mexico | 26.93 (26.35, 27.52) | 24.31 (23.88, 24.74) | -0.35 (-0.36, -0.33) | <0.001 | 162.70 (161.23, 164.17) | 150.30 (149.23, 151.38) | -0.27 (-0.30, -0.25) | <0.001 | 252.46 (250.67, 254.27) | 299.62 (298.11, 301.14) | 0.64 (0.08, 1.20) | 0.025 | 4.39 (4.16, 4.64) | 5.19 (4.99, 5.39) | 0.61 (0.04, 1.19) | 0.035 |
| Micronesia (Federated States of) | 25.98 (11.96, 49.60) | 24.63 (11.45, 46.65) | -0.19 (-0.20, -0.17) | <0.001 | 184.38 (141.62, 236.25) | 175.61 (135.35, 224.28) | -0.18 (-0.20, -0.15) | <0.001 | 1422.99 (1298.87 , | 1488.85 (1367.05 , | 0.16 (0.08, 0.25) | <0.001 | 25.88 (11.67, 49.90) | 26.67 (12.67, 49.60) | 0.11 (0.02, 0.20) | 0.016 |

| | | | | | | | | | | | | | | | | |
|---|---|---|---|---|---|---|---|---|---|---|---|---|---|---|---|---|
| | | | | | | | | | 1555.99) | 1618.78) | | | | | | |
| Monaco | 30.14 (6.22, 104.42) | 26.39 (4.46, 87.70) | -0.46 (-0.47, -0.44) | <0.001 | 169.36 (99.75, 280.78) | 165.64 (96.07, 269.43) | -0.07 (-0.11, -0.04) | <0.001 | 192.93 (117.56, 309.86) | 107.83 (53.69, 196.95) | -1.99 (-2.08, -1.90) | <0.001 | 3.41 (0.00, 66.28) | 1.90 (0.00, 47.70) | -2.01 (-2.12, -1.89) | <0.001 |
| Mongolia | 39.21 (34.82, 44.04) | 38.81 (35.69, 42.16) | -0.02 (-0.06, 0.01) | 0.139 | 213.09 (202.27, 224.36) | 217.14 (209.76, 224.74) | 0.08 (-0.00, 0.17) | 0.054 | 847.31 (825.96, 869.11) | 747.46 (733.62, 761.52) | -0.39 (-0.93, 0.15) | 0.156 | 15.04 (12.29, 18.26) | 13.33 (11.54, 15.36) | -0.38 (-0.92, 0.18) | 0.182 |
| Montenegro | 26.90 (20.91, 34.11) | 24.37 (18.31, 31.90) | -0.34 (-0.35, -0.33) | <0.001 | 220.19 (202.35, 239.20) | 201.92 (183.98, 221.25) | -0.30 (-0.32, -0.28) | <0.001 | 419.11 (394.36, 445.04) | 196.91 (179.23, 215.97) | -2.89 (-4.83, -0.91) | 0.004 | 7.44 (4.48, 11.66) | 3.47 (1.51, 7.02) | -2.91 (-4.83, -0.94) | 0.004 |
| Morocco | 38.75 (37.49, 40.05) | 37.30 (36.32, 38.30) | -0.13 (-0.17, -0.10) | <0.001 | 268.57 (265.16, 272.01) | 265.40 (262.79, 268.04) | -0.03 (-0.10, 0.05) | 0.473 | 801.55 (795.74, 807.39) | 483.03 (479.50, 486.59) | -1.69 (-1.96, -1.42) | <0.001 | 14.09 (13.32, 14.89) | 8.39 (7.93, 8.87) | -1.73 (-2.01, -1.45) | <0.001 |
| Mozambique | 29.27 (27.63, 30.99) | 30.58 (29.47, 31.73) | 0.15 (0.13, 0.17) | <0.001 | 152.64 (148.79, 156.56) | 162.72 (160.05, 165.43) | 0.24 (0.12, 0.37) | <0.001 | 158.14 (154.31, 162.04) | 240.18 (237.03, 243.35) | 1.48 (1.31, 1.64) | <0.001 | 2.71 (2.23, 3.27) | 4.15 (3.74, 4.59) | 1.51 (1.34, 1.67) | <0.001 |
| Myanmar | 26.27 (25.47, 27.10) | 23.40 (22.77, 24.05) | -0.40 (-0.41, -0.39) | <0.001 | 153.06 (151.07, 155.07) | 135.31 (133.77, 136.86) | -0.41 (-0.53, -0.30) | <0.001 | 728.93 (724.67, 733.21) | 367.21 (364.68, 369.76) | -2.37 (-2.52, -2.22) | <0.001 | 12.69 (12.13, 13.27) | 6.23 (5.90, 6.57) | -2.46 (-2.61, -2.32) | <0.001 |
| Namibia | 31.24 (26.35, 36.82) | 29.65 (26.31, 33.33) | -0.18 (-0.19, -0.17) | <0.001 | 150.01 (138.64, 162.09) | 144.54 (136.87, 152.53) | -0.15 (-0.26, -0.04) | 0.009 | 138.64 (127.78, 150.20) | 143.41 (135.79, 151.36) | 0.20 (-0.23, 0.62) | 0.368 | 2.44 (1.19, 4.47) | 2.52 (1.60, 3.79) | 0.20 (-0.24, 0.63) | 0.381 |
| Nauru | 25.89 (0.63, 150.88) | 26.53 (0.97, 140.93) | 0.08 (0.08, 0.09) | <0.001 | 189.66 (76.68, 391.76) | 195.96 (85.40, 385.94) | 0.11 (0.10, 0.13) | <0.001 | 1834.85 (1426.26 , 2325.72) | 2153.23 (1731.72 , 2647.98) | 0.56 (0.44, 0.68) | <0.001 | 33.20 (1.53, 163.91) | 38.71 (3.22, 161.65) | 0.53 (0.41, 0.66) | <0.001 |
| Nepal | 24.73 (23.54, 25.97) | 22.42 (21.58, 23.29) | -0.34 (-0.35, -0.32) | <0.001 | 153.63 (150.58, 156.74) | 139.20 (137.03, 141.38) | -0.34 (-0.37, -0.31) | <0.001 | 466.08 (460.82, 471.39) | 356.09 (352.70, 359.50) | -0.92 (-1.07, -0.77) | <0.001 | 8.21 (7.52, 8.95) | 6.18 (5.74, 6.65) | -0.97 (-1.13, -0.82) | <0.001 |
| Netherlands | 51.39 (49.63, 53.20) | 44.47 (42.71, 46.28) | -0.49 (-0.52, -0.46) | <0.001 | 239.15 (235.35, 242.99) | 240.67 (236.60, 244.80) | -0.01 (-0.11, 0.10) | 0.906 | 192.94 (189.53, 196.40) | 55.43 (53.49, 57.43) | -4.27 (-4.55, -4.00) | <0.001 | 3.46 (3.02, 3.95) | 0.96 (0.72, 1.26) | -4.40 (-4.69, -4.11) | <0.001 |
| New Zealand | 45.67 | 37.15 | -0.69 | <0.001 | 212.02 | 193.06 | -0.32 | <0.001 | 226.66 | 89.32 | -3.12 | <0.001 | 4.06 | 1.60 | -3.12 | <0.001 |

| | | | | | | | | | | | | | | | | |
|---|---|---|---|---|---|---|---|---|---|---|---|---|---|---|---|---|
| | (42.19, 49.36) | (34.07, 40.45) | (-0.77, -0.62) | | (204.44, 219.81) | (185.97, 200.34) | (-0.38, -0.27) | | (218.82, 234.72) | (84.54, 94.32) | (-3.75, -2.47) | | (3.07, 5.27) | (1.02, 2.40) | (-3.76, -2.47) | |
| Nicaragua | 24.19 (21.53, 27.11) | 24.09 (22.29, 26.01) | -0.01 (-0.05, 0.03) | 0.664 | 143.69 (136.86, 150.80) | 154.10 (149.42, 158.90) | 0.24 (0.22, 0.26) | <0.001 | 241.17 (232.53, 250.06) | 190.63 (185.48, 195.88) | -1.10 (-1.98, -0.22) | 0.015 | 4.18 (3.10, 5.53) | 3.22 (2.58, 3.97) | -1.21 (-2.10, -0.30) | 0.009 |
| Niger | 24.55 (22.59, 26.64) | 26.18 (24.95, 27.47) | 0.21 (0.18, 0.25) | <0.001 | 133.18 (128.40, 138.10) | 139.72 (136.72, 142.77) | 0.18 (0.11, 0.25) | <0.001 | 200.06 (194.31, 205.95) | 192.14 (188.72, 195.62) | -0.08 (-0.40, 0.23) | 0.603 | 3.50 (2.77, 4.37) | 3.36 (2.91, 3.85) | -0.09 (-0.41, 0.24) | 0.599 |
| Nigeria | 23.50 (22.95, 24.06) | 25.50 (25.13, 25.87) | 0.28 (0.24, 0.32) | <0.001 | 126.31 (124.99, 127.65) | 138.37 (137.48, 139.26) | 0.32 (0.28, 0.35) | <0.001 | 192.09 (190.48, 193.70) | 163.14 (162.19, 164.10) | -0.60 (-0.97, -0.22) | 0.002 | 3.38 (3.17, 3.60) | 2.85 (2.73, 2.98) | -0.62 (-0.99, -0.24) | 0.001 |
| Niue | 25.06 (0.00, 567.73) | 25.38 (0.00, 781.63) | 0.04 (0.03, 0.06) | <0.001 | 205.75 (14.71, 888.85) | 214.84 (8.73, 1116.21) | 0.14 (0.12, 0.17) | <0.001 | 976.71 (395.83, 2007.59) | 756.41 (211.86, 1941.64) | -0.89 (-1.16, -0.62) | <0.001 | 17.58 (0.00, 553.58) | 13.40 (0.00, 758.38) | -0.95 (-1.21, -0.68) | <0.001 |
| North Macedonia | 23.53 (20.33, 27.10) | 21.63 (18.61, 25.08) | -0.30 (-0.32, -0.27) | <0.001 | 183.84 (174.70, 193.33) | 172.13 (163.57, 181.08) | -0.23 (-0.25, -0.21) | <0.001 | 503.82 (488.61, 519.40) | 293.95 (282.58, 305.73) | -1.71 (-2.60, -0.81) | <0.001 | 8.88 (6.97, 11.17) | 5.16 (3.76, 7.00) | -1.71 (-2.64, -0.76) | <0.001 |
| Northern Mariana Islands | 22.82 (8.02, 53.64) | 24.93 (4.32, 83.57) | 0.38 (0.12, 0.65) | 0.004 | 192.31 (141.58, 257.14) | 217.54 (134.95, 334.24) | 0.45 (0.26, 0.64) | <0.001 | 449.60 (370.06, 542.86) | 472.81 (345.63, 633.36) | 0.38 (-0.17, 0.94) | 0.173 | 8.07 (0.98, 32.67) | 8.45 (0.10, 58.71) | 0.40 (-0.21, 1.00) | 0.200 |
| Norway | 32.38 (29.69, 35.26) | 27.72 (25.37, 30.24) | -0.53 (-0.56, -0.51) | <0.001 | 181.67 (175.25, 188.28) | 168.62 (162.84, 174.58) | -0.25 (-0.28, -0.23) | <0.001 | 208.47 (201.59, 215.53) | 45.34 (42.38, 48.48) | -5.08 (-5.74, -4.41) | <0.001 | 3.72 (2.86, 4.78) | 0.78 (0.44, 1.31) | -5.22 (-5.91, -4.53) | <0.001 |
| Oman | 38.63 (34.49, 43.16) | 42.99 (40.62, 45.51) | 0.37 (0.18, 0.57) | <0.001 | 256.96 (246.05, 268.23) | 304.11 (297.89, 310.49) | 0.61 (0.45, 0.77) | <0.001 | 710.35 (692.09, 728.99) | 353.67 (346.37, 361.13) | -2.35 (-2.89, -1.80) | <0.001 | 12.47 (10.15, 15.17) | 6.00 (5.09, 7.07) | -2.51 (-2.84, -2.18) | <0.001 |
| Pakistan | 28.75 (28.18, 29.32) | 29.85 (29.48, 30.23) | 0.13 (0.11, 0.14) | <0.001 | 182.68 (181.21, 184.16) | 189.58 (188.61, 190.55) | 0.13 (0.12, 0.15) | <0.001 | 633.52 (630.82, 636.23) | 1049.63 (1047.39 , 1051.88) | 1.79 (1.60, 1.98) | <0.001 | 11.24 (10.88, 11.60) | 18.48 (18.18, 18.78) | 1.77 (1.58, 1.95) | <0.001 |
| Palau | 25.36 (2.34, 103.22) | 26.83 (2.61, 113.23) | 0.19 (0.17, 0.22) | <0.001 | 198.16 (105.60, 339.86) | 213.27 (118.46, 360.44) | 0.26 (0.24, 0.27) | <0.001 | 1123.00 (881.76, 1410.85) | 1231.94 (982.69, 1530.19) | 0.21 (-0.21, 0.64) | 0.318 | 20.22 (1.11, 95.33) | 22.13 (1.65, 104.90) | 0.20 (-0.24, 0.64) | 0.371 |

| | | | | | | | | | | | | | | | | |
|---|---|---|---|---|---|---|---|---|---|---|---|---|---|---|---|---|
| Palestine | 38.04 (33.22, 43.41) | 38.00 (35.21, 40.95) | -0.00 (-0.02, 0.01) | 0.635 | 260.71 (247.42, 274.56) | 268.40 (260.74, 276.22) | 0.11 (0.05, 0.18) | 0.001 | 528.39 (509.75, 547.58) | 320.23 (311.98, 328.65) | -1.75 (-2.17, -1.33) | <0.001 | 9.30 (6.95, 12.23) | 5.58 (4.53, 6.81) | -1.78 (-2.18, -1.38) | <0.001 |
| Panama | 22.57 (19.61, 25.88) | 22.59 (20.31, 25.07) | 0.01 (-0.02, 0.04) | 0.496 | 128.87 (121.45, 136.63) | 140.71 (134.89, 146.71) | 0.30 (0.25, 0.35) | <0.001 | 209.02 (199.69, 218.70) | 117.49 (112.19, 122.98) | -1.94 (-2.63, -1.24) | <0.001 | 3.64 (2.50, 5.15) | 2.03 (1.39, 2.87) | -1.96 (-2.67, -1.24) | <0.001 |
| Papua New Guinea | 25.82 (23.27, 28.59) | 26.31 (24.72, 27.98) | 0.06 (0.04, 0.08) | <0.001 | 189.01 (181.81, 196.43) | 197.48 (193.00, 202.03) | 0.15 (0.14, 0.16) | <0.001 | 530.18 (518.04, 542.53) | 658.64 (650.45, 666.91) | 0.70 (0.54, 0.86) | <0.001 | 9.55 (7.97, 11.35) | 11.81 (10.74, 12.97) | 0.68 (0.52, 0.85) | <0.001 |
| Paraguay | 16.50 (14.48, 18.73) | 16.64 (15.20, 18.18) | 0.04 (0.01, 0.06) | 0.002 | 146.79 (140.47, 153.34) | 158.55 (153.98, 163.22) | 0.27 (0.24, 0.29) | <0.001 | 249.33 (241.13, 257.75) | 197.17 (192.09, 202.35) | -0.62 (-1.34, 0.11) | 0.096 | 4.38 (3.35, 5.63) | 3.40 (2.77, 4.15) | -0.67 (-1.40, 0.06) | 0.071 |
| Peru | 9.74 (9.07, 10.45) | 9.59 (9.08, 10.12) | -0.04 (-0.14, 0.06) | 0.428 | 78.95 (76.94, 81.00) | 80.13 (78.64, 81.64) | 0.05 (0.03, 0.07) | <0.001 | 373.44 (369.21, 377.70) | 184.50 (182.23, 186.79) | -2.24 (-3.46, -1.01) | <0.001 | 6.38 (5.84, 6.97) | 3.11 (2.82, 3.42) | -2.29 (-3.51, -1.05) | <0.001 |
| Philippines | 41.23 (40.40, 42.07) | 41.92 (41.32, 42.53) | 0.06 (0.04, 0.07) | <0.001 | 210.30 (208.39, 212.23) | 228.73 (227.31, 230.17) | 0.29 (0.25, 0.34) | <0.001 | 261.64 (259.53, 263.76) | 800.45 (797.81, 803.11) | 4.01 (3.43, 4.59) | <0.001 | 4.56 (4.28, 4.85) | 14.00 (13.65, 14.36) | 4.03 (3.41, 4.65) | <0.001 |
| Poland | 14.78 (14.18, 15.39) | 13.51 (12.90, 14.14) | -0.31 (-0.34, -0.28) | <0.001 | 149.02 (147.16, 150.91) | 144.06 (142.16, 145.99) | -0.10 (-0.22, 0.02) | 0.094 | 624.06 (620.28, 627.86) | 98.68 (97.13, 100.25) | -6.32 (-7.34, -5.28) | <0.001 | 11.34 (10.84, 11.86) | 1.73 (1.53, 1.95) | -6.44 (-7.50, -5.36) | <0.001 |
| Portugal | 14.05 (12.87, 15.30) | 12.42 (11.22, 13.71) | -0.42 (-0.48, -0.37) | <0.001 | 116.59 (113.15, 120.10) | 138.21 (134.27, 142.24) | 0.69 (0.16, 1.22) | 0.011 | 253.00 (247.93, 258.15) | 70.99 (68.21, 73.85) | -4.34 (-5.21, -3.46) | <0.001 | 4.46 (3.81, 5.19) | 1.26 (0.91, 1.70) | -4.33 (-5.24, -3.41) | <0.001 |
| Puerto Rico | 61.00 (56.95, 65.27) | 62.68 (58.19, 67.45) | 0.09 (0.07, 0.12) | <0.001 | 323.19 (313.72, 332.88) | 331.35 (321.00, 341.96) | 0.08 (0.06, 0.11) | <0.001 | 265.84 (257.27, 274.62) | 120.23 (114.03, 126.69) | -2.88 (-4.12, -1.63) | <0.001 | 4.68 (3.60, 5.98) | 2.07 (1.33, 3.09) | -2.94 (-4.25, -1.60) | <0.001 |
| Qatar | 36.89 (29.99, 45.43) | 34.92 (32.32, 37.86) | -0.20 (-0.25, -0.15) | <0.001 | 247.54 (229.62, 266.98) | 236.82 (230.17, 243.78) | -0.16 (-0.19, -0.13) | <0.001 | 355.00 (332.24, 379.35) | 141.89 (136.24, 147.87) | -3.01 (-4.04, -1.96) | <0.001 | 6.10 (3.48, 10.62) | 2.39 (1.72, 3.48) | -3.06 (-4.12, -2.00) | <0.001 |
| Republic of Korea | 17.60 (17.03, 18.19) | 16.28 (15.71, 16.87) | -0.27 (-0.29, -0.25) | <0.001 | 102.53 (101.13, 103.94) | 105.05 (103.62, 106.49) | 0.11 (0.02, 0.20) | 0.013 | 538.53 (535.36, 541.71) | 57.57 (56.51, 58.65) | -7.44 (-8.10, -6.77) | <0.001 | 9.11 (8.70, 9.54) | 1.00 (0.86, 1.15) | -7.36 (-8.05, -6.67) | <0.001 |

| | | | | | | | | | | | | | | | | |
|---|---|---|---|---|---|---|---|---|---|---|---|---|---|---|---|---|
| Republic of Moldova | 33.62 (31.03, 36.39) | 35.02 (32.05, 38.23) | 0.13 (0.08, 0.19) | <0.001 | 185.26 (179.21, 191.48) | 193.33 (186.52, 200.38) | 0.14 (0.11, 0.18) | <0.001 | 377.04 (368.31, 385.94) | 311.35 (302.65, 320.29) | -0.61 (-1.74, 0.54) | 0.297 | 6.71 (5.59, 8.00) | 5.57 (4.47, 6.93) | -0.62 (-1.94, 0.72) | 0.364 |
| Romania | 20.54 (19.60, 21.52) | 20.59 (19.46, 21.77) | 0.01 (-0.03, 0.04) | 0.805 | 177.53 (174.77, 180.32) | 174.99 (171.76, 178.26) | -0.03 (-0.14, 0.08) | 0.592 | 485.44 (480.88, 490.04) | 258.36 (254.43, 262.35) | -2.22 (-3.06, -1.37) | <0.001 | 8.70 (8.10, 9.34) | 4.57 (4.07, 5.13) | -2.20 (-3.17, -1.22) | <0.001 |
| Russian Federation | 36.39 (35.92, 36.86) | 36.40 (35.89, 36.92) | 0.00 (-0.04, 0.05) | 0.867 | 178.43 (177.40, 179.46) | 188.74 (187.62, 189.85) | 0.19 (0.14, 0.24) | <0.001 | 620.74 (618.83, 622.65) | 479.37 (477.59, 481.16) | -0.94 (-2.35, 0.50) | 0.199 | 11.28 (11.03, 11.54) | 8.62 (8.38, 8.86) | -0.97 (-2.39, 0.46) | 0.182 |
| Rwanda | 23.24 (21.35, 25.25) | 22.83 (21.52, 24.20) | -0.06 (-0.09, -0.04) | <0.001 | 120.16 (115.72, 124.74) | 117.59 (114.55, 120.70) | -0.08 (-0.11, -0.04) | <0.001 | 251.39 (245.09, 257.82) | 158.39 (154.93, 161.92) | -1.62 (-1.81, -1.43) | <0.001 | 4.36 (3.56, 5.30) | 2.72 (2.28, 3.22) | -1.65 (-1.85, -1.46) | <0.001 |
| Saint Kitts and Nevis | 60.91 (29.13, 113.66) | 62.10 (34.47, 104.15) | 0.07 (0.03, 0.11) | 0.001 | 300.74 (221.01, 400.62) | 308.31 (242.44, 387.59) | 0.09 (0.08, 0.09) | <0.001 | 522.74 (415.26, 650.19) | 107.34 (69.88, 158.79) | -5.12 (-6.50, -3.72) | <0.001 | 9.43 (0.63, 42.38) | 1.82 (0.00, 21.48) | -5.32 (-6.91, -3.71) | <0.001 |
| Saint Lucia | 63.90 (43.41, 91.38) | 64.55 (47.02, 86.71) | 0.04 (-0.01, 0.09) | 0.082 | 329.04 (278.04, 387.05) | 329.10 (288.05, 374.59) | -0.00 (-0.01, 0.01) | 0.590 | 251.25 (206.84, 302.74) | 106.42 (83.65, 133.74) | -2.90 (-3.44, -2.36) | <0.001 | 4.47 (0.53, 16.97) | 1.83 (0.09, 9.18) | -3.02 (-3.63, -2.41) | <0.001 |
| Saint Vincent and the Grenadines | 61.99 (39.90, 92.89) | 62.24 (40.68, 91.30) | 0.02 (-0.00, 0.04) | 0.119 | 304.52 (250.36, 367.46) | 304.81 (254.38, 362.45) | 0.01 (-0.01, 0.02) | 0.590 | 443.22 (377.78, 517.30) | 221.04 (178.44, 270.89) | -2.29 (-3.51, -1.06) | <0.001 | 7.87 (1.53, 24.59) | 3.87 (0.33, 16.03) | -2.33 (-3.62, -1.03) | <0.001 |
| Samoa | 26.94 (14.78, 45.70) | 26.49 (15.99, 41.52) | -0.06 (-0.07, -0.05) | <0.001 | 221.37 (181.70, 267.43) | 221.37 (187.81, 259.32) | 0.00 (-0.01, 0.02) | 0.650 | 653.64 (583.93, 729.65) | 822.20 (756.51, 892.20) | 0.80 (0.73, 0.87) | <0.001 | 11.75 (4.16, 26.54) | 14.69 (7.12, 27.00) | 0.78 (0.71, 0.85) | <0.001 |
| San Marino | 30.71 (5.71, 95.35) | 26.47 (5.04, 82.84) | -0.51 (-0.54, -0.47) | <0.001 | 174.20 (97.89, 287.08) | 169.36 (100.97, 268.67) | -0.09 (-0.15, -0.04) | 0.001 | 75.11 (29.01, 159.25) | 74.33 (32.33, 148.48) | -0.01 (-0.19, 0.16) | 0.888 | 1.30 (0.00, 46.43) | 1.31 (0.00, 41.74) | 0.05 (-0.14, 0.25) | 0.609 |
| Sao Tome and Principe | 25.74 (11.56, 50.79) | 28.23 (17.81, 42.64) | 0.32 (0.30, 0.34) | <0.001 | 154.69 (113.96, 205.88) | 171.48 (143.60, 203.25) | 0.37 (0.31, 0.42) | <0.001 | 176.53 (132.55, 231.00) | 197.86 (168.21, 231.34) | 0.53 (0.01, 1.05) | 0.046 | 3.16 (0.07, 19.37) | 3.44 (0.63, 10.71) | 0.57 (-0.08, 1.22) | 0.084 |
| Saudi Arabia | 35.63 (34.13, 37.17) | 38.40 (37.57, 39.26) | 0.25 (0.21, 0.29) | <0.001 | 228.69 (224.82, 232.61) | 261.22 (259.08, 263.39) | 0.47 (0.35, 0.59) | <0.001 | 445.83 (440.47, 451.23) | 452.81 (449.94, 455.69) | 0.04 (-0.36, 0.44) | 0.851 | 7.86 (7.16, 8.62) | 8.01 (7.64, 8.41) | 0.05 (-0.36, 0.45) | 0.816 |

| | | | | | | | | | | | | | | | | |
|---|---|---|---|---|---|---|---|---|---|---|---|---|---|---|---|---|
| Senegal | 28.32 (26.19, 30.59) | 29.12 (27.69, 30.60) | 0.09 (0.06, 0.12) | <0.001 | 169.83 (164.37, 175.44) | 171.44 (167.84, 175.10) | 0.05 (0.00, 0.09) | 0.030 | 204.41 (198.51, 210.46) | 188.04 (184.34, 191.80) | -0.28 (-0.47, -0.09) | 0.006 | 3.58 (2.83, 4.47) | 3.26 (2.79, 3.80) | -0.31 (-0.51, -0.12) | 0.003 |
| Serbia | 32.30 (30.46, 34.22) | 33.88 (31.82, 36.03) | 0.15 (0.10, 0.21) | <0.001 | 222.88 (218.08, 227.75) | 234.86 (229.51, 240.30) | 0.17 (0.11, 0.23) | <0.001 | 419.58 (412.99, 426.26) | 162.32 (157.90, 166.85) | -3.18 (-3.71, -2.64) | <0.001 | 7.50 (6.64, 8.44) | 2.86 (2.30, 3.52) | -3.23 (-3.79, -2.66) | <0.001 |
| Seychelles | 24.58 (9.73, 52.61) | 23.72 (11.30, 45.13) | -0.12 (-0.14, -0.11) | <0.001 | 179.04 (131.75, 238.58) | 170.85 (133.90, 215.87) | -0.14 (-0.21, -0.07) | <0.001 | 499.34 (417.72, 592.96) | 338.84 (285.46, 400.23) | -1.20 (-2.19, -0.21) | 0.018 | 8.94 (1.35, 31.03) | 5.95 (1.00, 20.92) | -1.26 (-2.27, -0.25) | 0.015 |
| Sierra Leone | 30.02 (27.05, 33.24) | 31.06 (29.11, 33.10) | 0.12 (0.10, 0.14) | <0.001 | 172.27 (164.86, 179.92) | 175.35 (170.53, 180.28) | 0.08 (-0.04, 0.20) | 0.187 | 214.85 (206.65, 223.30) | 257.47 (251.72, 263.32) | 0.65 (0.39, 0.90) | <0.001 | 3.77 (2.75, 5.06) | 4.50 (3.77, 5.35) | 0.64 (0.38, 0.89) | <0.001 |
| Singapore | 31.73 (28.99, 34.67) | 25.14 (23.12, 27.32) | -0.79 (-0.86, -0.72) | <0.001 | 178.22 (171.63, 185.02) | 140.23 (135.75, 144.88) | -0.83 (-0.84, -0.81) | <0.001 | 187.36 (180.59, 194.32) | 76.67 (73.42, 80.08) | -2.99 (-4.02, -1.94) | <0.001 | 3.36 (2.50, 4.42) | 1.36 (0.96, 1.95) | -3.03 (-4.09, -1.95) | <0.001 |
| Slovakia | 26.42 (24.29, 28.71) | 20.51 (18.57, 22.64) | -0.87 (-0.91, -0.84) | <0.001 | 212.52 (206.49, 218.69) | 166.55 (161.11, 172.16) | -0.84 (-0.86, -0.82) | <0.001 | 437.10 (428.50, 445.85) | 140.40 (135.44, 145.53) | -3.96 (-4.67, -3.25) | <0.001 | 7.90 (6.78, 9.16) | 2.48 (1.87, 3.29) | -4.06 (-5.07, -3.04) | <0.001 |
| Slovenia | 26.50 (23.08, 30.32) | 22.36 (18.89, 26.37) | -0.58 (-0.62, -0.54) | <0.001 | 216.57 (206.73, 226.80) | 179.36 (169.67, 189.57) | -0.66 (-0.70, -0.62) | <0.001 | 181.61 (172.68, 190.92) | 50.53 (45.55, 56.03) | -4.50 (-5.01, -4.00) | <0.001 | 3.23 (2.14, 4.73) | 0.85 (0.32, 2.02) | -4.69 (-5.28, -4.10) | <0.001 |
| Solomon Islands | 26.74 (17.66, 39.10) | 26.42 (20.32, 33.80) | -0.04 (-0.06, -0.03) | <0.001 | 177.34 (151.67, 206.25) | 177.99 (161.33, 195.92) | 0.02 (-0.02, 0.05) | 0.425 | 2525.73 (2426.80, 2627.79) | 2733.27 (2667.05, 2800.74) | 0.28 (0.14, 0.42) | <0.001 | 45.46 (32.99, 61.26) | 49.03 (40.49, 58.85) | 0.27 (0.14, 0.40) | <0.001 |
| Somalia | 26.98 (24.88, 29.21) | 29.29 (27.99, 30.63) | 0.28 (0.27, 0.29) | <0.001 | 141.80 (136.95, 146.78) | 155.22 (152.14, 158.36) | 0.33 (0.28, 0.38) | <0.001 | 262.62 (256.07, 269.30) | 339.40 (334.95, 343.91) | 0.94 (0.77, 1.11) | <0.001 | 4.62 (3.78, 5.58) | 5.92 (5.34, 6.55) | 0.91 (0.68, 1.15) | <0.001 |
| South Africa | 37.28 (36.29, 38.30) | 34.67 (33.94, 35.41) | -0.25 (-0.27, -0.22) | <0.001 | 165.22 (163.07, 167.40) | 155.86 (154.32, 157.42) | -0.19 (-0.22, -0.16) | <0.001 | 358.00 (354.82, 361.20) | 179.73 (178.08, 181.40) | -2.28 (-3.81, -0.73) | 0.004 | 6.44 (6.02, 6.88) | 3.18 (2.97, 3.41) | -2.35 (-3.88, -0.79) | 0.003 |
| South Sudan | 24.45 (22.27, | 26.25 (24.38, | 0.24 (0.22, | <0.001 | 144.42 (138.81, | 155.49 (150.84, | 0.25 (0.21, | <0.001 | 147.79 (142.25, | 165.89 (161.21, | 0.41 (-0.04, | 0.074 | 2.55 (1.87, | 2.84 (2.25, | 0.37 (-0.08, | 0.105 |

| | | | | | | | | | | | | | | | | |
|---|---|---|---|---|---|---|---|---|---|---|---|---|---|---|---|---|
| | 26.80) | 28.22) | 0.27) | | 150.20) | 160.26) | 0.28) | | 153.49) | 170.67) | 0.85) | | 3.42) | 3.54) | 0.82) | |
| Spain | 33.18 (32.25, 34.13) | 30.36 (29.44, 31.30) | -0.30 (-0.32, -0.28) | <0.001 | 168.23 (166.11, 170.38) | 176.28 (174.17, 178.42) | 0.17 (0.08, 0.26) | <0.001 | 251.37 (248.78, 253.97) | 83.90 (82.43, 85.38) | -3.77 (-4.40, -3.13) | <0.001 | 4.39 (4.05, 4.75) | 1.46 (1.28, 1.67) | -3.78 (-4.41, -3.13) | <0.001 |
| Sri Lanka | 24.31 (23.18, 25.49) | 22.89 (21.87, 23.95) | -0.21 (-0.23, -0.19) | <0.001 | 175.00 (171.91, 178.13) | 165.75 (163.00, 168.52) | -0.18 (-0.21, -0.16) | <0.001 | 580.06 (574.46, 585.70) | 291.61 (287.94, 295.31) | -2.25 (-3.99, -0.47) | 0.013 | 10.15 (9.42, 10.92) | 5.05 (4.58, 5.56) | -2.28 (-4.01, -0.52) | 0.011 |
| Sudan | 36.32 (34.87, 37.83) | 38.36 (37.39, 39.36) | 0.19 (0.17, 0.21) | <0.001 | 220.91 (217.21, 224.65) | 246.56 (244.04, 249.11) | 0.40 (0.26, 0.55) | <0.001 | 1249.59 (1240.94, 1258.28) | 767.15 (762.78, 771.54) | -1.67 (-1.77, -1.57) | <0.001 | 22.07 (20.93, 23.27) | 13.39 (12.81, 13.98) | -1.71 (-1.81, -1.62) | <0.001 |
| Suriname | 61.78 (49.54, 76.35) | 68.66 (58.03, 80.70) | 0.38 (0.34, 0.41) | <0.001 | 300.29 (271.27, 331.68) | 340.59 (316.44, 366.12) | 0.44 (0.38, 0.49) | <0.001 | 504.98 (467.24, 545.09) | 294.03 (271.62, 317.82) | -1.81 (-3.09, -0.50) | 0.007 | 9.01 (4.60, 16.05) | 5.20 (2.62, 9.28) | -1.83 (-3.57, -0.07) | 0.042 |
| Sweden | 32.23 (30.25, 34.32) | 37.21 (35.18, 39.33) | 0.50 (0.43, 0.56) | <0.001 | 189.60 (184.79, 194.51) | 209.74 (204.97, 214.60) | 0.35 (0.31, 0.39) | <0.001 | 134.29 (130.25, 138.44) | 40.72 (38.63, 42.91) | -4.01 (-4.33, -3.69) | <0.001 | 2.36 (1.85, 2.97) | 0.68 (0.43, 1.03) | -4.10 (-4.41, -3.79) | <0.001 |
| Switzerland | 28.69 (26.75, 30.76) | 25.09 (23.33, 26.97) | -0.45 (-0.47, -0.44) | <0.001 | 155.09 (150.55, 159.75) | 151.07 (146.84, 155.41) | -0.09 (-0.13, -0.05) | <0.001 | 181.38 (176.44, 186.44) | 43.50 (41.24, 45.87) | -4.88 (-5.88, -3.87) | <0.001 | 3.19 (2.56, 3.94) | 0.75 (0.48, 1.14) | -4.95 (-6.00, -3.89) | <0.001 |
| Syrian Arab Republic | 34.77 (32.91, 36.70) | 35.27 (33.53, 37.08) | 0.05 (0.03, 0.07) | <0.001 | 228.16 (223.24, 233.16) | 242.33 (237.78, 246.95) | 0.23 (0.13, 0.33) | <0.001 | 1929.75 (1916.16, 1943.41) | 1080.27 (1070.75, 1089.86) | -2.04 (-2.32, -1.76) | <0.001 | 33.09 (31.31, 34.95) | 18.46 (17.23, 19.77) | -2.06 (-2.34, -1.78) | <0.001 |
| Taiwan (Province of China) | 19.72 (18.83, 20.63) | 18.87 (17.97, 19.81) | -0.15 (-0.18, -0.13) | <0.001 | 232.16 (229.11, 235.25) | 243.74 (240.53, 246.98) | 0.17 (0.04, 0.31) | 0.013 | 149.68 (147.22, 152.18) | 118.42 (116.20, 120.67) | -0.90 (-1.54, -0.27) | 0.006 | 2.47 (2.17, 2.82) | 1.96 (1.68, 2.27) | -0.83 (-1.49, -0.18) | 0.013 |
| Tajikistan | 36.60 (33.86, 39.52) | 35.72 (33.87, 37.65) | -0.09 (-0.13, -0.05) | <0.001 | 208.41 (201.57, 215.44) | 202.87 (198.35, 207.48) | -0.10 (-0.13, -0.06) | <0.001 | 478.93 (468.50, 489.54) | 475.33 (468.40, 482.34) | 0.06 (-0.52, 0.64) | 0.841 | 8.62 (7.26, 10.17) | 8.55 (7.64, 9.54) | 0.08 (-0.48, 0.63) | 0.791 |
| Thailand | 21.02 (20.44, 21.60) | 20.45 (19.91, 21.01) | -0.09 (-0.13, -0.06) | <0.001 | 135.19 (133.71, 136.68) | 133.40 (132.02, 134.78) | -0.03 (-0.13, 0.07) | 0.536 | 282.81 (280.69, 284.95) | 269.81 (267.83, 271.81) | -0.04 (-0.92, 0.84) | 0.927 | 4.96 (4.68, 5.25) | 4.70 (4.44, 4.97) | -0.07 (-0.93, 0.80) | 0.876 |

| | | | | | | | | | | | | | | | | |
|---|---|---|---|---|---|---|---|---|---|---|---|---|---|---|---|---|
| Timor-Leste | 22.92 (17.74, 29.22) | 22.60 (18.28, 27.69) | -0.05 (-0.06, -0.04) | <0.001 | 149.90 (135.85, 165.07) | 148.96 (137.16, 161.53) | -0.00 (-0.12, 0.12) | 0.991 | 250.17 (232.12, 269.30) | 373.00 (354.90, 391.84) | 1.41 (0.82, 2.00) | <0.001 | 4.38 (2.28, 7.67) | 6.45 (4.24, 9.46) | 1.36 (0.79, 1.94) | <0.001 |
| Togo | 27.25 (24.32, 30.46) | 29.09 (27.17, 31.10) | 0.22 (0.19, 0.26) | <0.001 | 148.85 (141.64, 156.35) | 158.10 (153.52, 162.79) | 0.23 (0.18, 0.29) | <0.001 | 238.40 (229.39, 247.69) | 254.77 (249.00, 260.64) | 0.27 (-0.08, 0.61) | 0.127 | 4.19 (3.06, 5.61) | 4.47 (3.73, 5.32) | 0.26 (-0.08, 0.60) | 0.135 |
| Tokelau | 25.31 (0.00, 867.77) | 26.04 (0.00, 937.88) | 0.10 (0.07, 0.13) | <0.001 | 203.43 (6.00, 1177.63) | 218.94 (5.06, 1287.37) | 0.26 (0.21, 0.31) | <0.001 | 657.01 (152.74, 1877.96) | 759.49 (178.51, 2128.13) | 0.52 (0.42, 0.61) | <0.001 | 11.78 (0.00, 843.23) | 13.57 (0.00, 914.13) | 0.50 (0.39, 0.62) | <0.001 |
| Tonga | 26.31 (11.01, 54.22) | 26.46 (12.19, 50.30) | 0.02 (-0.01, 0.04) | 0.243 | 231.28 (177.24, 297.22) | 238.43 (189.10, 296.82) | 0.10 (0.06, 0.14) | <0.001 | 389.27 (317.58, 472.82) | 445.26 (376.93, 522.54) | 0.45 (0.30, 0.61) | <0.001 | 6.96 (0.72, 27.55) | 7.88 (1.41, 24.74) | 0.43 (0.30, 0.56) | <0.001 |
| Trinidad and Tobago | 73.75 (66.34, 81.80) | 72.62 (65.64, 80.20) | -0.05 (-0.06, -0.04) | <0.001 | 390.05 (372.46, 408.27) | 382.68 (367.03, 398.90) | -0.05 (-0.11, 0.00) | 0.066 | 436.35 (417.70, 455.63) | 247.87 (235.31, 261.02) | -1.95 (-2.75, -1.13) | <0.001 | 7.80 (5.48, 10.79) | 4.38 (2.86, 6.53) | -1.99 (-2.82, -1.14) | <0.001 |
| Tunisia | 33.06 (31.03, 35.19) | 33.22 (31.61, 34.89) | 0.02 (0.00, 0.03) | 0.038 | 213.66 (208.33, 219.09) | 223.91 (219.78, 228.11) | 0.17 (0.13, 0.21) | <0.001 | 510.83 (502.72, 519.04) | 377.28 (371.81, 382.81) | -1.03 (-1.16, -0.91) | <0.001 | 8.95 (7.90, 10.11) | 6.57 (5.87, 7.34) | -1.05 (-1.19, -0.92) | <0.001 |
| Turkey | 24.36 (23.72, 25.02) | 19.88 (19.42, 20.35) | -0.71 (-0.74, -0.68) | <0.001 | 223.62 (221.63, 225.63) | 180.06 (178.68, 181.44) | -0.71 (-0.79, -0.63) | <0.001 | 538.19 (535.10, 541.30) | 179.27 (177.88, 180.66) | -3.69 (-4.05, -3.32) | <0.001 | 9.62 (9.21, 10.05) | 3.11 (2.93, 3.30) | -3.76 (-4.17, -3.36) | <0.001 |
| Turkmenistan | 36.21 (33.11, 39.55) | 36.96 (34.40, 39.68) | 0.08 (0.06, 0.10) | <0.001 | 201.24 (193.62, 209.10) | 210.61 (204.40, 216.96) | 0.16 (0.14, 0.18) | <0.001 | 759.25 (744.38, 774.37) | 697.31 (685.96, 708.82) | -0.15 (-1.26, 0.97) | 0.791 | 13.66 (11.71, 15.84) | 12.39 (10.92, 14.02) | -0.20 (-1.30, 0.91) | 0.722 |
| Tuvalu | 25.52 (0.46, 156.07) | 26.81 (1.14, 137.54) | 0.17 (0.16, 0.18) | <0.001 | 189.12 (73.49, 399.23) | 205.45 (93.57, 393.24) | 0.29 (0.27, 0.31) | <0.001 | 987.59 (685.81, 1378.01) | 1302.18 (985.67, 1689.75) | 0.95 (0.86, 1.04) | <0.001 | 17.81 (0.06, 142.76) | 23.44 (0.62, 132.63) | 0.94 (0.82, 1.06) | <0.001 |
| Uganda | 23.79 (22.47, 25.17) | 23.80 (22.97, 24.66) | -0.00 (-0.01, 0.01) | 0.788 | 117.59 (114.50, 120.74) | 118.21 (116.27, 120.17) | 0.02 (0.01, 0.03) | <0.001 | 131.78 (128.61, 135.02) | 202.42 (199.99, 204.88) | 1.46 (1.12, 1.81) | <0.001 | 2.27 (1.87, 2.74) | 3.48 (3.16, 3.82) | 1.44 (1.10, 1.78) | <0.001 |
| Ukraine | 35.89 (35.07, 36.73) | 36.55 (35.63, 37.49) | 0.06 (0.02, 0.09) | 0.001 | 195.50 (193.60, 197.42) | 195.67 (193.61, 197.76) | 0.01 (-0.03, 0.05) | 0.704 | 445.13 (442.25, 448.02) | 892.33 (887.84, 896.84) | 2.43 (0.74, 4.15) | 0.005 | 7.99 (7.61, 8.38) | 15.89 (15.30, 16.50) | 2.40 (0.67, 4.16) | 0.007 |

| | | | | | | | | | | | | | | | | |
|---|---|---|---|---|---|---|---|---|---|---|---|---|---|---|---|---|
| United Arab Emirates | 37.99 (34.41, 41.95) | 39.01 (37.10, 41.04) | 0.09 (0.05, 0.13) | <0.001 | 255.00 (245.79, 264.57) | 269.49 (265.03, 274.05) | 0.20 (0.15, 0.24) | <0.001 | 437.45 (424.57, 450.71) | 332.87 (327.20, 338.65) | -1.00 (-1.31, -0.69) | <0.001 | 7.54 (5.95, 9.53) | 5.76 (5.06, 6.58) | -0.97 (-1.29, -0.65) | <0.001 |
| United Kingdom | 31.69 (30.95, 32.46) | 27.58 (26.91, 28.26) | -0.48 (-0.50, -0.45) | <0.001 | 182.78 (180.97, 184.60) | 166.35 (164.73, 167.98) | -0.32 (-0.34, -0.31) | <0.001 | 207.26 (205.33, 209.20) | 110.72 (109.41, 112.04) | -2.14 (-2.53, -1.75) | <0.001 | 3.74 (3.48, 4.01) | 1.98 (1.80, 2.16) | -2.17 (-2.57, -1.76) | <0.001 |
| United Republic of Tanzania | 24.62 (23.54, 25.73) | 28.05 (27.30, 28.81) | 0.45 (0.43, 0.47) | <0.001 | 123.22 (120.70, 125.77) | 143.72 (141.99, 145.47) | 0.54 (0.46, 0.62) | <0.001 | 182.60 (179.67, 185.56) | 191.51 (189.57, 193.46) | 0.24 (-0.04, 0.52) | 0.098 | 3.11 (2.74, 3.53) | 3.27 (3.02, 3.53) | 0.20 (0.00, 0.39) | 0.048 |
| United States of America | 37.35 (36.99, 37.71) | 35.86 (35.52, 36.21) | -0.14 (-0.19, -0.09) | <0.001 | 171.82 (171.05, 172.59) | 188.07 (187.29, 188.86) | 0.31 (0.18, 0.45) | <0.001 | 222.85 (221.97, 223.73) | 167.36 (166.62, 168.11) | -0.94 (-1.32, -0.56) | <0.001 | 3.98 (3.86, 4.10) | 2.95 (2.85, 3.05) | -0.99 (-1.36, -0.62) | <0.001 |
| United States Virgin Islands | 57.14 (35.90, 86.66) | 58.29 (34.60, 92.57) | 0.08 (0.04, 0.11) | <0.001 | 285.95 (235.76, 343.98) | 292.57 (236.65, 358.34) | 0.07 (0.06, 0.09) | <0.001 | 415.03 (354.02, 483.83) | 191.42 (146.78, 245.99) | -2.56 (-3.29, -1.83) | <0.001 | 7.32 (1.46, 22.32) | 3.38 (0.11, 19.06) | -2.56 (-3.44, -1.68) | <0.001 |
| Uruguay | 20.49 (17.93, 23.32) | 18.71 (16.37, 21.30) | -0.31 (-0.33, -0.30) | <0.001 | 119.28 (112.96, 125.85) | 113.39 (107.54, 119.49) | -0.16 (-0.24, -0.09) | <0.001 | 219.25 (210.66, 228.10) | 83.60 (78.59, 88.85) | -2.99 (-3.50, -2.48) | <0.001 | 3.93 (2.85, 5.27) | 1.47 (0.87, 2.32) | -3.06 (-3.57, -2.53) | <0.001 |
| Uzbekistan | 32.64 (31.38, 33.94) | 33.63 (32.68, 34.59) | 0.11 (0.09, 0.12) | <0.001 | 181.96 (178.90, 185.06) | 193.92 (191.63, 196.23) | 0.22 (0.20, 0.24) | <0.001 | 540.51 (535.24, 545.82) | 903.03 (898.11, 907.97) | 1.88 (1.34, 2.43) | <0.001 | 9.70 (9.00, 10.43) | 15.89 (15.25, 16.56) | 1.82 (1.27, 2.37) | <0.001 |
| Vanuatu | 29.51 (16.67, 48.69) | 29.11 (19.88, 41.31) | -0.05 (-0.06, -0.03) | <0.001 | 228.76 (188.85, 274.79) | 227.46 (199.53, 258.28) | -0.01 (-0.03, 0.00) | 0.130 | 1068.90 (980.18, 1163.64) | 1496.87 (1423.94 , 1572.64) | 1.13 (0.95, 1.32) | <0.001 | 19.34 (9.11, 36.20) | 26.97 (17.95, 39.00) | 1.12 (0.93, 1.30) | <0.001 |
| Venezuela (Bolivarian Republic of) | 27.61 (26.43, 28.83) | 24.59 (23.67, 25.54) | -0.40 (-0.41, -0.38) | <0.001 | 163.80 (160.85, 166.79) | 151.33 (149.06, 153.62) | -0.27 (-0.30, -0.24) | <0.001 | 464.90 (459.96, 469.87) | 318.69 (315.37, 322.03) | -1.11 (-2.27, 0.06) | 0.063 | 8.20 (7.55, 8.89) | 5.58 (5.15, 6.03) | -1.16 (-2.22, -0.09) | 0.034 |
| Viet Nam | 22.10 (21.52, 22.69) | 21.56 (21.12, 22.00) | -0.09 (-0.10, -0.07) | <0.001 | 156.62 (155.04, 158.20) | 154.98 (153.81, 156.16) | -0.03 (-0.05, -0.01) | 0.012 | 248.83 (246.86, 250.81) | 163.15 (161.93, 164.37) | -1.46 (-1.60, -1.31) | <0.001 | 4.38 (4.12, 4.66) | 2.82 (2.66, 2.99) | -1.52 (-1.67, -1.36) | <0.001 |
| Yemen | 36.61 (34.76, | 38.04 (36.95, | 0.13 (0.10, | <0.001 | 221.43 (216.79, | 237.94 (235.17, | 0.28 (0.08, | 0.006 | 909.47 (900.07, | 719.53 (714.74, | -0.78 (-1.06, | <0.001 | 16.34 (15.10, | 12.78 (12.14, | -0.81 (-1.02, | <0.001 |

| | | | | | | | | | | | | | | | | |
|---|---|---|---|---|---|---|---|---|---|---|---|---|---|---|---|---|
| | 38.53) | 39.15) | 0.15) | | 226.15) | 240.72) | 0.48) | | 918.95) | 724.33) | -0.51) | | 17.67) | 13.43) | -0.59) | |
| Zambia | 26.04 (24.00, 28.21) | 26.47 (25.25, 27.75) | 0.05 (0.04, 0.07) | <0.001 | 122.15 (117.49, 126.96) | 125.71 (122.94, 128.54) | 0.09 (0.03, 0.15) | 0.002 | 317.35 (310.10, 324.73) | 268.19 (264.24, 272.18) | -0.58 (-0.73, -0.43) | <0.001 | 5.51 (4.59, 6.59) | 4.64 (4.13, 5.20) | -0.59 (-0.74, -0.44) | <0.001 |
| Zimbabwe | 30.50 (28.62, 32.48) | 33.47 (31.97, 35.02) | 0.32 (0.30, 0.35) | <0.001 | 136.71 (132.55, 140.97) | 154.16 (150.88, 157.50) | 0.42 (0.40, 0.44) | <0.001 | 103.75 (100.22, 107.38) | 177.82 (174.34, 181.35) | 2.00 (1.44, 2.56) | <0.001 | 1.78 (1.34, 2.32) | 3.09 (2.64, 3.59) | 2.06 (1.49, 2.63) | <0.001 |
| **Women** | | | | | | | | | | | | | | | | |
| Afghanistan | 34.34 (31.43, 37.48) | 34.76 (33.22, 36.35) | 0.04 (0.01, 0.07) | 0.011 | 194.24 (187.11, 201.61) | 203.66 (199.79, 207.60) | 0.20 (0.01, 0.39) | 0.043 | 1686.25 (1665.35 , 1707.37) | 1053.52 (1044.83 , 1062.27) | -1.57 (-1.73, -1.41) | <0.001 | 29.89 (27.15, 32.85) | 18.77 (17.61, 19.98) | -1.55 (-1.71, -1.39) | <0.001 |
| Albania | 15.55 (12.70, 18.88) | 14.31 (11.10, 18.20) | -0.29 (-0.31, -0.27) | <0.001 | 123.82 (115.27, 132.86) | 118.03 (108.33, 128.38) | -0.16 (-0.18, -0.13) | <0.001 | 232.32 (220.83, 244.30) | 192.81 (180.41, 205.86) | -0.86 (-1.21, -0.51) | <0.001 | 3.92 (2.55, 5.81) | 3.25 (1.83, 5.39) | -0.88 (-1.23, -0.52) | <0.001 |
| Algeria | 28.61 (26.99, 30.30) | 27.96 (26.89, 29.07) | -0.08 (-0.09, -0.07) | <0.001 | 182.05 (177.83, 186.35) | 181.97 (179.26, 184.73) | 0.02 (-0.07, 0.10) | 0.732 | 901.06 (891.92, 910.29) | 381.78 (377.75, 385.84) | -2.90 (-3.14, -2.66) | <0.001 | 15.72 (14.52, 16.99) | 6.61 (6.10, 7.17) | -2.92 (-3.16, -2.68) | <0.001 |
| American Samoa | 20.02 (2.06, 81.32) | 20.88 (2.34, 81.04) | 0.15 (0.12, 0.17) | <0.001 | 186.97 (107.03, 305.34) | 199.39 (117.39, 318.31) | 0.22 (0.21, 0.23) | <0.001 | 138.65 (71.73, 244.34) | 241.26 (150.12, 368.95) | 1.99 (1.75, 2.24) | <0.001 | 2.37 (0.00, 52.26) | 4.19 (0.00, 53.59) | 2.04 (1.76, 2.33) | <0.001 |
| Andorra | 23.10 (4.46, 77.77) | 23.18 (4.57, 75.49) | 0.03 (0.01, 0.05) | 0.002 | 148.16 (88.50, 238.49) | 166.30 (106.46, 253.46) | 0.40 (0.36, 0.44) | <0.001 | 52.52 (20.05, 118.43) | 29.96 (8.57, 83.03) | -1.87 (-2.15, -1.59) | <0.001 | 0.89 (0.00, 44.22) | 0.50 (0.00, 40.36) | -1.91 (-2.26, -1.57) | <0.001 |
| Angola | 23.78 (21.54, 26.20) | 22.61 (21.34, 23.95) | -0.18 (-0.20, -0.16) | <0.001 | 105.77 (100.86, 110.87) | 102.46 (99.65, 105.34) | -0.11 (-0.12, -0.10) | <0.001 | 156.92 (151.02, 162.99) | 113.42 (110.52, 116.38) | -1.06 (-1.46, -0.66) | <0.001 | 2.74 (2.01, 3.67) | 1.96 (1.60, 2.39) | -1.10 (-1.49, -0.70) | <0.001 |
| Antigua and Barbuda | 55.52 (22.78, 114.50) | 53.43 (25.29, 100.92) | -0.13 (-0.15, -0.12) | <0.001 | 300.81 (212.90, 413.60) | 284.33 (213.07, 373.33) | -0.22 (-0.32, -0.11) | <0.001 | 157.28 (96.05, 243.85) | 56.81 (27.78, 104.99) | -3.52 (-4.41, -2.62) | <0.001 | 2.73 (0.00, 36.52) | 0.95 (0.00, 25.81) | -3.64 (-4.65, -2.62) | <0.001 |
| Argentina | 12.44 (11.57, 13.37) | 11.55 (10.86, 12.28) | -0.26 (-0.27, -0.25) | <0.001 | 74.88 (72.71, 77.09) | 74.03 (72.27, 75.83) | -0.04 (-0.06, -0.01) | 0.002 | 213.57 (209.92, 217.28) | 80.29 (78.44, 82.17) | -3.30 (-3.73, -2.86) | <0.001 | 3.68 (3.21, 4.19) | 1.38 (1.15, 1.64) | -3.38 (-3.87, -2.89) | <0.001 |

| | | | | | | | | | | | | | | | | |
|---|---|---|---|---|---|---|---|---|---|---|---|---|---|---|---|---|
| | 30.54 | 29.56 | -0.11 | | 216.09 | 207.08 | -0.15 | | 172.65 | 75.67 | -2.80 | | 2.95 | 1.29 | -2.80 | |
| Armenia | (26.70, | (25.40, | (-0.12, | <0.001 | (205.60, | (196.17, | (-0.17, | <0.001 | (163.27, | (68.98, | (-3.63, | <0.001 | (1.84, | (0.56, | (-3.65, | <0.001 |
| | 34.80) | 34.35) | -0.10) | | 227.00) | 218.58) | -0.13) | | 182.46) | 82.96) | -1.97) | | 4.52) | 2.77) | -1.94) | |
| | 22.96 | 18.71 | -0.72 | | 141.22 | 121.68 | -0.49 | | 66.40 | 30.70 | -2.59 | | 1.15 | 0.54 | -2.54 | |
| Australia | (21.39, | (17.46, | (-0.77, | <0.001 | (137.34, | (118.51, | (-0.59, | <0.001 | (63.74, | (29.13, | (-3.10, | <0.001 | (0.82, | (0.35, | (-3.04, | <0.001 |
| | 24.61) | 20.04) | -0.68) | | 145.20) | 124.93) | -0.39) | | 69.15) | 32.34) | -2.07) | | 1.57) | 0.81) | -2.03) | |
| | 31.02 | 30.32 | -0.07 | | 174.60 | 186.99 | 0.24 | | 71.46 | 28.73 | -3.15 | | 1.24 | 0.48 | -3.25 | |
| Austria | (28.29, | (27.54, | (-0.09, | <0.001 | (167.96, | (180.20, | (0.22, | <0.001 | (67.21, | (26.09, | (-4.57, | <0.001 | (0.74, | (0.20, | (-4.63, | <0.001 |
| | 33.96) | 33.34) | -0.04) | | 181.45) | 194.00) | 0.25) | | 75.93) | 31.60) | -1.71) | | 1.98) | 1.05) | -1.84) | |
| | 31.39 | 29.36 | -0.23 | | 212.88 | 196.25 | -0.28 | | 428.85 | 291.46 | -1.35 | | 7.37 | 4.91 | -1.43 | |
| Azerbaijan | (28.67, | (27.17, | (-0.25, | <0.001 | (205.43, | (190.58, | (-0.29, | <0.001 | (418.51, | (284.28, | (-1.94, | <0.001 | (6.06, | (4.02, | (-2.00, | <0.001 |
| | 34.33) | 31.71) | -0.21) | | 220.54) | 202.07) | -0.26) | | 439.41) | 298.80) | -0.76) | | 8.89) | 5.97) | -0.86) | |
| | 56.38 | 56.54 | 0.02 | | 277.70 | 275.92 | -0.02 | | 209.55 | 126.94 | -1.66 | | 3.69 | 2.22 | -1.69 | |
| Bahamas | (38.67, | (41.11, | (-0.00, | 0.120 | (235.23, | (240.56, | (-0.03, | 0.025 | (172.97, | (103.32, | (-2.43, | <0.001 | (0.45, | (0.22, | (-2.49, | <0.001 |
| | 79.84) | 76.05) | 0.04) | | 325.88) | 315.21) | -0.00) | | 251.85) | 154.54) | -0.89) | | 13.83) | 9.04) | -0.88) | |
| | 33.54 | 30.09 | -0.37 | | 237.99 | 212.98 | -0.38 | | 379.46 | 95.73 | -4.59 | | 6.19 | 1.55 | -4.63 | |
| Bahrain | (22.97, | (23.82, | (-0.41, | <0.001 | (207.62, | (196.31, | (-0.40, | <0.001 | (341.10, | (83.86, | (-5.66, | <0.001 | (2.22, | (0.41, | (-5.61, | <0.001 |
| | 47.61) | 37.71) | -0.34) | | 271.76) | 230.91) | -0.36) | | 421.17) | 108.97) | -3.50) | | 14.03) | 4.36) | -3.65) | |
| | 19.24 | 19.47 | 0.04 | | 133.72 | 139.09 | 0.13 | | 504.01 | 265.15 | -2.06 | | 8.71 | 4.58 | -2.07 | |
| Bangladesh | (18.60, | (19.00, | (0.03, | <0.001 | (131.95, | (137.82, | (0.07, | <0.001 | (500.71, | (263.43, | (-3.08, | <0.001 | (8.28, | (4.35, | (-3.14, | <0.001 |
| | 19.90) | 19.94) | 0.05) | | 135.51) | 140.36) | 0.18) | | 507.34) | 266.89) | -1.03) | | 9.16) | 4.81) | -0.98) | |
| | 56.42 | 57.19 | 0.05 | | 298.09 | 301.12 | 0.04 | | 111.09 | 61.37 | -2.07 | | 1.90 | 1.03 | -2.13 | |
| Barbados | (38.53, | (38.50, | (0.04, | <0.001 | (254.72, | (256.68, | (0.03, | <0.001 | (85.19, | (42.20, | (-2.69, | <0.001 | (0.06, | (0.00, | (-2.79, | <0.001 |
| | 80.01) | 82.24) | 0.06) | | 346.96) | 351.51) | 0.04) | | 142.62) | 86.78) | -1.45) | | 10.66) | 10.09) | -1.48) | |
| | 25.59 | 25.91 | 0.04 | | 180.59 | 184.10 | 0.08 | | 139.79 | 113.70 | -0.66 | | 2.46 | 2.02 | -0.62 | |
| Belarus | (23.46, | (23.49, | (-0.00, | 0.055 | (174.92, | (177.89, | (0.05, | <0.001 | (134.78, | (108.89, | (-2.07, | 0.364 | (1.84, | (1.43, | (-2.09, | 0.410 |
| | 27.88) | 28.56) | 0.08) | | 186.41) | 190.53) | 0.10) | | 144.95) | 118.72) | 0.77) | | 3.24) | 2.85) | 0.87) | |
| | 25.47 | 26.08 | 0.09 | | 160.91 | 184.92 | 0.49 | | 115.01 | 31.77 | -4.36 | | 1.98 | 0.53 | -4.44 | |
| Belgium | (23.25, | (23.78, | (0.05, | <0.001 | (155.33, | (178.88, | (0.44, | <0.001 | (110.24, | (29.27, | (-5.18, | <0.001 | (1.40, | (0.25, | (-5.13, | <0.001 |
| | 27.85) | 28.56) | 0.13) | | 166.66) | 191.13) | 0.54) | | 119.95) | 34.44) | -3.53) | | 2.74) | 1.00) | -3.74) | |
| | 57.25 | 60.42 | 0.20 | | 286.45 | 305.61 | 0.23 | | 136.46 | 104.04 | -1.29 | | 2.36 | 1.77 | -1.38 | |
| Belize | (33.74, | (45.13, | (0.18, | <0.001 | (227.26, | (269.22, | (0.20, | <0.001 | (97.39, | (83.46, | (-2.10, | 0.002 | (0.01, | (0.13, | (-2.25, | 0.002 |
| | 92.25) | 79.35) | 0.22) | | 357.03) | 345.59) | 0.26) | | 186.93) | 128.24) | -0.47) | | 19.38) | 7.76) | -0.52) | |

| | | | | | | | | | | | | | | | | |
|---|---|---|---|---|---|---|---|---|---|---|---|---|---|---|---|---|
| Benin | 19.93 (17.01, 23.23) | 20.78 (18.92, 22.79) | 0.14 (0.12, 0.17) | <0.001 | 103.23 (96.24, 110.62) | 106.65 (102.24, 111.21) | 0.13 (0.07, 0.19) | <0.001 | 107.24 (100.23, 114.64) | 120.75 (116.17, 125.46) | 0.42 (0.10, 0.74) | 0.010 | 1.85 (1.03, 3.10) | 2.08 (1.51, 2.80) | 0.41 (0.04, 0.78) | 0.031 |
| Bermuda | 52.93 (21.95, 114.89) | 49.38 (15.64, 125.41) | -0.24 (-0.27, -0.22) | <0.001 | 295.20 (213.84, 403.42) | 269.48 (180.73, 394.09) | -0.35 (-0.49, -0.20) | <0.001 | 131.90 (78.67, 213.92) | 32.19 (7.48, 100.13) | -5.17 (-5.84, -4.50) | <0.001 | 2.26 (0.00, 44.50) | 0.51 (0.00, 53.41) | -5.42 (-6.55, -4.28) | <0.001 |
| Bhutan | 21.05 (13.02, 32.49) | 20.29 (13.99, 28.48) | -0.12 (-0.17, -0.07) | <0.001 | 144.08 (120.70, 170.83) | 141.10 (123.39, 160.65) | -0.07 (-0.09, -0.05) | <0.001 | 422.99 (383.08, 466.12) | 273.60 (248.79, 300.24) | -1.52 (-1.82, -1.22) | <0.001 | 7.42 (3.01, 15.51) | 4.78 (2.03, 9.56) | -1.54 (-1.83, -1.24) | <0.001 |
| Bolivia (Plurinational State of) | 8.28 (6.72, 10.12) | 8.02 (6.92, 9.24) | -0.11 (-0.12, -0.10) | <0.001 | 69.88 (65.03, 74.99) | 69.26 (65.90, 72.74) | -0.02 (-0.09, 0.06) | 0.644 | 322.59 (312.38, 333.06) | 144.34 (139.52, 149.28) | -2.74 (-2.89, -2.59) | <0.001 | 5.49 (4.23, 7.04) | 2.45 (1.86, 3.18) | -2.74 (-2.90, -2.59) | <0.001 |
| Bosnia and Herzegovina | 18.80 (16.13, 21.78) | 17.82 (14.43, 21.83) | -0.19 (-0.21, -0.17) | <0.001 | 162.97 (154.94, 171.32) | 161.23 (150.90, 172.15) | -0.02 (-0.09, 0.04) | 0.486 | 155.24 (147.39, 163.41) | 99.45 (91.30, 108.20) | -1.42 (-2.46, -0.37) | 0.008 | 2.65 (1.72, 3.93) | 1.69 (0.79, 3.27) | -1.42 (-2.53, -0.29) | 0.014 |
| Botswana | 24.63 (18.68, 32.00) | 24.85 (20.85, 29.42) | 0.03 (0.01, 0.04) | 0.006 | 95.60 (83.07, 109.56) | 99.97 (91.82, 108.69) | 0.14 (0.08, 0.21) | <0.001 | 106.79 (93.65, 121.33) | 157.41 (147.08, 168.31) | 1.37 (0.62, 2.12) | <0.001 | 1.88 (0.52, 4.87) | 2.76 (1.55, 4.59) | 1.26 (0.60, 1.93) | <0.001 |
| Brazil | 13.16 (12.75, 13.57) | 11.87 (11.55, 12.19) | -0.36 (-0.45, -0.26) | <0.001 | 92.23 (91.13, 93.34) | 90.10 (89.24, 90.96) | -0.08 (-0.10, -0.06) | <0.001 | 252.44 (250.62, 254.27) | 120.92 (119.92, 121.92) | -2.46 (-2.71, -2.21) | <0.001 | 4.49 (4.25, 4.74) | 2.11 (1.98, 2.25) | -2.53 (-2.78, -2.27) | <0.001 |
| Brunei Darussalam | 20.75 (10.75, 36.67) | 20.83 (12.76, 32.51) | 0.01 (-0.02, 0.05) | 0.406 | 140.08 (110.65, 175.19) | 152.90 (129.64, 179.47) | 0.35 (-0.01, 0.71) | 0.053 | 325.96 (280.29, 377.18) | 145.47 (122.29, 172.05) | -2.80 (-2.98, -2.62) | <0.001 | 5.60 (1.20, 16.54) | 2.46 (0.38, 8.72) | -2.85 (-3.19, -2.50) | <0.001 |
| Bulgaria | 16.58 (14.59, 18.77) | 16.75 (14.33, 19.52) | 0.03 (0.01, 0.05) | 0.003 | 140.23 (134.44, 146.21) | 148.81 (141.75, 156.20) | 0.24 (0.16, 0.31) | <0.001 | 261.34 (253.34, 269.53) | 172.04 (164.32, 180.09) | -1.54 (-2.34, -0.73) | <0.001 | 4.55 (3.55, 5.74) | 3.00 (2.07, 4.29) | -1.55 (-2.37, -0.71) | <0.001 |
| Burkina Faso | 19.07 (16.94, 21.40) | 19.51 (18.17, 20.93) | 0.08 (0.07, 0.08) | <0.001 | 96.99 (91.99, 102.21) | 97.95 (94.85, 101.14) | 0.04 (0.01, 0.07) | 0.008 | 171.27 (164.76, 177.99) | 164.71 (160.79, 168.70) | -0.10 (-0.62, 0.42) | 0.703 | 2.97 (2.16, 3.99) | 2.82 (2.33, 3.40) | -0.14 (-0.66, 0.39) | 0.606 |
| Burundi | 21.69 (18.84, 24.88) | 22.25 (20.27, 24.39) | 0.08 (0.05, 0.11) | <0.001 | 104.11 (97.56, 111.01) | 104.97 (100.48, 109.61) | 0.03 (0.02, 0.04) | <0.001 | 200.37 (191.43, 209.63) | 173.92 (168.33, 179.66) | -0.49 (-0.81, -0.18) | 0.002 | 3.47 (2.38, 4.92) | 2.97 (2.27, 3.82) | -0.55 (-0.87, -0.22) | 0.001 |

| | | | | | | | | | | | | | | | | |
|---|---|---|---|---|---|---|---|---|---|---|---|---|---|---|---|---|
| Cabo Verde | 19.82 (10.02, 36.03) | 21.04 (13.63, 31.12) | 0.20 (0.19, 0.21) | <0.001 | 117.23 (89.81, 150.83) | 124.45 (105.11, 146.37) | 0.20 (0.18, 0.22) | <0.001 | 100.58 (75.75, 131.48) | 80.61 (65.20, 98.63) | -0.66 (-1.48, 0.17) | 0.120 | 1.75 (0.04, 11.30) | 1.38 (0.12, 5.77) | -0.72 (-1.57, 0.14) | 0.099 |
| Cambodia | 18.13 (16.25, 20.17) | 17.13 (15.79, 18.55) | -0.19 (-0.22, -0.16) | <0.001 | 117.12 (112.18, 122.23) | 110.44 (106.99, 113.99) | -0.18 (-0.26, -0.09) | <0.001 | 310.34 (302.36, 318.48) | 182.85 (178.41, 187.39) | -1.78 (-1.97, -1.59) | <0.001 | 5.44 (4.43, 6.63) | 3.18 (2.61, 3.83) | -1.81 (-2.00, -1.62) | <0.001 |
| Cameroon | 17.73 (15.79, 19.85) | 19.79 (18.64, 21.01) | 0.38 (0.34, 0.43) | <0.001 | 82.53 (78.14, 87.12) | 92.03 (89.45, 94.66) | 0.38 (0.35, 0.41) | <0.001 | 109.87 (104.93, 114.99) | 126.95 (124.00, 129.96) | 0.52 (0.29, 0.75) | <0.001 | 1.89 (1.29, 2.70) | 2.18 (1.80, 2.61) | 0.52 (0.31, 0.74) | <0.001 |
| Canada | 33.90 (32.43, 35.42) | 28.31 (26.98, 29.69) | -0.60 (-0.64, -0.56) | <0.001 | 170.03 (166.79, 173.33) | 160.49 (157.39, 163.64) | -0.22 (-0.26, -0.17) | <0.001 | 67.61 (65.55, 69.73) | 36.13 (34.67, 37.65) | -2.10 (-2.92, -1.28) | <0.001 | 1.11 (0.86, 1.42) | 0.60 (0.42, 0.83) | -2.06 (-2.99, -1.11) | <0.001 |
| Central African Republic | 25.16 (20.80, 30.21) | 25.13 (22.06, 28.53) | -0.00 (-0.03, 0.02) | 0.719 | 98.23 (89.12, 108.06) | 99.58 (93.21, 106.29) | 0.07 (-0.04, 0.17) | 0.194 | 175.00 (162.94, 187.76) | 197.75 (188.84, 206.98) | 0.43 (-0.20, 1.07) | 0.180 | 3.08 (1.67, 5.28) | 3.49 (2.39, 4.93) | 0.42 (-0.05, 0.89) | 0.080 |
| Chad | 19.01 (16.38, 21.95) | 20.78 (19.04, 22.65) | 0.30 (0.27, 0.33) | <0.001 | 97.87 (91.64, 104.43) | 107.21 (103.06, 111.49) | 0.33 (0.28, 0.37) | <0.001 | 115.23 (108.60, 122.18) | 131.23 (126.77, 135.82) | 0.54 (0.28, 0.81) | <0.001 | 2.00 (1.21, 3.12) | 2.27 (1.71, 2.96) | 0.54 (0.22, 0.86) | 0.001 |
| Chile | 15.84 (14.42, 17.38) | 14.78 (13.54, 16.12) | -0.24 (-0.27, -0.21) | <0.001 | 108.22 (104.36, 112.18) | 108.14 (104.76, 111.62) | 0.02 (-0.08, 0.11) | 0.733 | 49.10 (46.51, 51.80) | 22.52 (20.98, 24.14) | -2.52 (-3.01, -2.04) | <0.001 | 0.85 (0.54, 1.28) | 0.38 (0.21, 0.66) | -2.67 (-3.51, -1.82) | <0.001 |
| China | 16.20 (16.04, 16.36) | 17.32 (17.16, 17.48) | 0.23 (0.22, 0.25) | <0.001 | 191.08 (190.53, 191.63) | 216.51 (215.96, 217.05) | 0.43 (0.41, 0.44) | <0.001 | 260.48 (259.84, 261.11) | 137.56 (137.10, 138.01) | -2.21 (-3.00, -1.41) | <0.001 | 4.42 (4.33, 4.50) | 2.24 (2.18, 2.30) | -2.36 (-3.14, -1.57) | <0.001 |
| Colombia | 19.91 (18.86, 21.00) | 17.24 (16.43, 18.09) | -0.48 (-0.56, -0.41) | <0.001 | 131.65 (128.85, 134.50) | 118.96 (116.80, 121.15) | -0.35 (-0.37, -0.33) | <0.001 | 235.69 (231.99, 239.44) | 94.89 (92.96, 96.85) | -3.07 (-3.51, -2.63) | <0.001 | 4.09 (3.61, 4.61) | 1.60 (1.36, 1.88) | -3.16 (-3.61, -2.70) | <0.001 |
| Comoros | 21.67 (12.37, 35.64) | 21.39 (14.37, 30.73) | -0.05 (-0.08, -0.02) | <0.001 | 119.27 (94.84, 148.28) | 115.83 (98.27, 135.68) | -0.11 (-0.16, -0.07) | <0.001 | 134.19 (108.94, 163.85) | 174.44 (153.08, 198.01) | 0.69 (-2.07, 3.53) | 0.628 | 2.31 (0.20, 9.89) | 2.99 (0.83, 7.71) | 0.68 (-1.95, 3.38) | 0.615 |
| Congo | 25.51 (20.83, 30.97) | 24.51 (21.61, 27.69) | -0.14 (-0.17, -0.11) | <0.001 | 109.54 (99.30, 120.58) | 106.57 (100.41, 113.01) | -0.07 (-0.15, 0.01) | 0.095 | 212.28 (198.07, 227.27) | 181.09 (173.06, 189.41) | -0.60 (-1.39, 0.19) | 0.135 | 3.76 (2.08, 6.30) | 3.21 (2.22, 4.49) | -0.60 (-1.40, 0.20) | 0.141 |

| | | | | | | | | | | | | | | | | |
|---|---|---|---|---|---|---|---|---|---|---|---|---|---|---|---|---|
| Cook Islands | 19.47 (0.09, 156.54) | 19.88 (0.06, 161.87) | 0.07 (0.03, 0.12) | 0.002 | 184.70 (68.27, 404.75) | 192.88 (70.00, 424.15) | 0.15 (0.14, 0.16) | <0.001 | 200.06 (77.77, 425.33) | 124.43 (32.45, 327.45) | -1.61 (-1.77, -1.44) | <0.001 | 3.46 (0.00, 127.70) | 2.10 (0.00, 128.12) | -1.69 (-1.88, -1.49) | <0.001 |
| Costa Rica | 19.92 (16.56, 23.79) | 18.25 (15.73, 21.09) | -0.30 (-0.31, -0.29) | <0.001 | 139.59 (130.21, 149.50) | 134.79 (127.82, 142.06) | -0.12 (-0.14, -0.10) | <0.001 | 135.06 (125.86, 144.77) | 67.84 (62.90, 73.09) | -2.15 (-2.58, -1.71) | <0.001 | 2.36 (1.28, 4.00) | 1.16 (0.60, 2.07) | -2.17 (-2.64, -1.69) | <0.001 |
| Cote d'Ivoire | 21.80 (19.79, 23.98) | 23.20 (21.88, 24.57) | 0.20 (0.18, 0.23) | <0.001 | 105.09 (100.42, 109.92) | 109.95 (107.02, 112.95) | 0.18 (0.13, 0.23) | <0.001 | 146.43 (141.02, 152.00) | 141.06 (137.77, 144.40) | -0.13 (-0.52, 0.27) | 0.530 | 2.56 (1.88, 3.41) | 2.45 (2.03, 2.93) | -0.15 (-0.48, 0.18) | 0.376 |
| Croatia | 15.63 (13.20, 18.42) | 13.11 (10.51, 16.25) | -0.60 (-0.65, -0.56) | <0.001 | 170.03 (161.93, 178.46) | 151.35 (142.44, 160.75) | -0.37 (-0.53, -0.22) | <0.001 | 137.63 (130.32, 145.27) | 41.21 (36.63, 46.28) | -4.30 (-5.30, -3.30) | <0.001 | 2.37 (1.51, 3.60) | 0.67 (0.21, 1.75) | -4.49 (-5.51, -3.46) | <0.001 |
| Cuba | 52.94 (49.97, 56.04) | 49.60 (46.43, 52.94) | -0.23 (-0.24, -0.21) | <0.001 | 280.38 (273.29, 287.61) | 255.11 (247.90, 262.49) | -0.33 (-0.39, -0.27) | <0.001 | 223.30 (216.97, 229.77) | 66.35 (62.68, 70.19) | -3.99 (-4.44, -3.53) | <0.001 | 3.96 (3.15, 4.91) | 1.14 (0.70, 1.76) | -4.12 (-4.62, -3.62) | <0.001 |
| Cyprus | 13.99 (8.71, 21.44) | 14.65 (10.35, 20.59) | 0.18 (0.14, 0.21) | <0.001 | 119.99 (103.30, 138.72) | 167.70 (153.19, 183.62) | 1.36 (0.70, 2.02) | <0.001 | 137.66 (119.70, 157.66) | 40.07 (33.21, 48.38) | -4.16 (-5.35, -2.97) | <0.001 | 2.48 (0.64, 6.70) | 0.69 (0.09, 3.44) | -4.31 (-5.67, -2.92) | <0.001 |
| Czechia | 27.61 (25.25, 30.13) | 25.35 (22.93, 28.00) | -0.29 (-0.31, -0.27) | <0.001 | 212.91 (206.48, 219.51) | 191.11 (184.66, 197.76) | -0.38 (-0.42, -0.35) | <0.001 | 98.30 (93.96, 102.79) | 31.79 (29.19, 34.60) | -3.88 (-4.71, -3.03) | <0.001 | 1.69 (1.17, 2.38) | 0.51 (0.23, 1.04) | -4.11 (-5.02, -3.19) | <0.001 |
| Democratic People's Republic of Korea | 14.15 (13.00, 15.38) | 15.41 (14.33, 16.55) | 0.30 (0.27, 0.32) | <0.001 | 158.58 (154.60, 162.64) | 171.55 (167.91, 175.26) | 0.28 (0.26, 0.29) | <0.001 | 296.91 (291.59, 302.31) | 303.51 (298.63, 308.45) | 0.09 (-0.07, 0.24) | 0.271 | 5.03 (4.36, 5.79) | 5.14 (4.52, 5.82) | 0.08 (-0.06, 0.22) | 0.261 |
| Democratic Republic of the Congo | 23.56 (22.37, 24.80) | 22.75 (21.99, 23.53) | -0.12 (-0.15, -0.10) | <0.001 | 103.86 (101.28, 106.49) | 99.91 (98.28, 101.57) | -0.13 (-0.14, -0.12) | <0.001 | 160.23 (157.07, 163.43) | 130.00 (128.17, 131.86) | -0.83 (-1.11, -0.54) | <0.001 | 2.80 (2.40, 3.26) | 2.27 (2.04, 2.53) | -0.83 (-1.12, -0.54) | <0.001 |
| Denmark | 35.72 (32.02, 39.75) | 30.27 (26.79, 34.10) | -0.55 (-0.59, -0.52) | <0.001 | 212.69 (203.60, 222.10) | 194.94 (185.94, 204.28) | -0.30 (-0.32, -0.28) | <0.001 | 64.71 (59.76, 69.99) | 11.84 (9.70, 14.33) | -5.65 (-6.97, -4.31) | <0.001 | 1.12 (0.56, 2.05) | 0.19 (0.02, 0.80) | -5.91 (-7.94, -3.82) | <0.001 |
| Djibouti | 20.26 (11.44, 33.52) | 21.67 (16.34, 28.30) | 0.23 (0.21, 0.24) | <0.001 | 109.16 (86.28, 136.39) | 116.76 (104.05, 130.70) | 0.23 (0.21, 0.25) | <0.001 | 89.20 (69.15, 113.49) | 119.90 (106.77, 134.30) | 1.17 (0.37, 1.98) | 0.004 | 1.52 (0.06, 8.30) | 2.05 (0.68, 4.88) | 1.18 (0.44, 1.92) | 0.002 |

| | | | | | | | | | | | | | | | | |
|---|---|---|---|---|---|---|---|---|---|---|---|---|---|---|---|---|
| Dominica | 55.09 (21.82, 117.11) | 55.60 (22.37, 114.88) | 0.04 (0.02, 0.05) | <0.001 | 268.91 (182.80, 382.65) | 270.66 (187.53, 378.32) | 0.02 (0.01, 0.03) | <0.001 | 169.53 (103.67, 263.10) | 90.88 (46.10, 161.14) | -2.15 (-2.63, -1.68) | <0.001 | 2.96 (0.00, 40.42) | 1.55 (0.00, 33.12) | -2.24 (-2.77, -1.71) | <0.001 |
| Dominican Republic | 50.84 (47.20, 54.70) | 54.55 (51.51, 57.74) | 0.24 (0.23, 0.25) | <0.001 | 242.94 (234.58, 251.52) | 267.34 (260.46, 274.35) | 0.33 (0.32, 0.34) | <0.001 | 463.18 (451.91, 474.66) | 509.14 (499.70, 518.71) | 0.32 (-0.23, 0.88) | 0.257 | 8.03 (6.60, 9.70) | 8.83 (7.62, 10.18) | 0.32 (-0.23, 0.87) | 0.260 |
| Ecuador | 8.10 (6.89, 9.49) | 8.18 (7.26, 9.18) | 0.04 (0.00, 0.07) | 0.038 | 73.92 (69.99, 78.01) | 79.25 (76.31, 82.27) | 0.23 (0.17, 0.29) | <0.001 | 297.35 (289.69, 305.17) | 151.73 (147.69, 155.85) | -2.31 (-3.15, -1.47) | <0.001 | 5.06 (4.09, 6.19) | 2.57 (2.07, 3.16) | -2.37 (-3.25, -1.47) | <0.001 |
| Egypt | 32.11 (30.99, 33.26) | 31.51 (30.73, 32.30) | -0.07 (-0.08, -0.05) | <0.001 | 216.20 (213.23, 219.19) | 218.36 (216.29, 220.45) | 0.05 (-0.07, 0.17) | 0.396 | 1312.64 (1305.55 , 1319.76) | 711.60 (707.88, 715.33) | -1.92 (-2.65, -1.19) | <0.001 | 22.34 (21.42, 23.30) | 12.16 (11.68, 12.66) | -1.91 (-2.64, -1.17) | <0.001 |
| El Salvador | 17.24 (14.71, 20.12) | 17.33 (15.15, 19.74) | 0.03 (-0.02, 0.07) | 0.229 | 103.22 (96.61, 110.17) | 114.56 (108.75, 120.60) | 0.36 (0.33, 0.38) | <0.001 | 440.53 (427.38, 454.00) | 164.62 (157.73, 171.74) | -3.43 (-4.21, -2.65) | <0.001 | 7.50 (5.85, 9.48) | 2.80 (1.97, 3.88) | -3.43 (-4.22, -2.64) | <0.001 |
| Equatorial Guinea | 24.59 (14.62, 38.95) | 21.51 (16.17, 28.13) | -0.47 (-0.49, -0.44) | <0.001 | 104.64 (82.31, 131.27) | 95.53 (83.55, 108.80) | -0.31 (-0.34, -0.28) | <0.001 | 175.57 (146.48, 208.87) | 82.35 (71.47, 94.48) | -2.48 (-3.29, -1.66) | <0.001 | 3.10 (0.44, 10.72) | 1.42 (0.34, 3.98) | -2.55 (-3.38, -1.72) | <0.001 |
| Eritrea | 19.73 (16.04, 24.05) | 19.35 (16.99, 21.97) | -0.07 (-0.11, -0.04) | <0.001 | 96.17 (87.57, 105.41) | 93.21 (87.78, 98.89) | -0.11 (-0.12, -0.10) | <0.001 | 101.44 (92.76, 110.74) | 128.48 (122.25, 134.97) | 0.85 (0.65, 1.04) | <0.001 | 1.75 (0.78, 3.43) | 2.22 (1.46, 3.24) | 0.85 (0.65, 1.05) | <0.001 |
| Estonia | 39.04 (32.24, 46.94) | 38.64 (30.68, 48.37) | -0.04 (-0.07, -0.01) | 0.023 | 241.57 (224.54, 259.65) | 238.64 (218.96, 259.94) | -0.08 (-0.23, 0.07) | 0.286 | 108.29 (96.88, 120.76) | 34.49 (27.17, 43.54) | -3.72 (-5.94, -1.43) | 0.002 | 1.87 (0.67, 4.31) | 0.56 (0.03, 3.52) | -3.97 (-6.08, -1.81) | <0.001 |
| Eswatini | 25.20 (17.50, 35.36) | 25.72 (19.78, 32.95) | 0.07 (0.04, 0.10) | <0.001 | 100.51 (83.81, 119.68) | 104.71 (92.07, 118.66) | 0.14 (0.12, 0.17) | <0.001 | 107.05 (90.14, 126.37) | 117.35 (104.09, 131.89) | 0.25 (-0.37, 0.87) | 0.425 | 1.86 (0.30, 6.36) | 2.03 (0.65, 4.88) | 0.24 (-0.40, 0.88) | 0.469 |
| Ethiopia | 17.36 (16.49, 18.27) | 17.80 (17.21, 18.41) | 0.08 (0.06, 0.10) | <0.001 | 79.71 (77.77, 81.68) | 80.96 (79.66, 82.28) | 0.05 (0.03, 0.07) | <0.001 | 284.28 (280.74, 287.86) | 112.96 (111.49, 114.45) | -3.13 (-3.34, -2.92) | <0.001 | 4.90 (4.44, 5.39) | 1.91 (1.72, 2.12) | -3.17 (-3.42, -2.92) | <0.001 |
| Fiji | 21.58 (14.78, | 21.65 (15.35, | 0.02 (-0.03, | 0.356 | 192.64 (170.66, | 196.29 (176.29, | 0.08 (0.03, | 0.004 | 378.75 (347.98, | 343.67 (316.95, | -0.35 (-0.67, | 0.032 | 6.60 (3.11, | 5.96 (2.93, | -0.37 (-0.72, | 0.041 |

| | | | | | | | | | | | | | | | | |
|---|---|---|---|---|---|---|---|---|---|---|---|---|---|---|---|---|
| | 30.53) | 29.70) | 0.07) | | 216.73) | 217.97) | 0.13) | | 411.59) | 372.07) | -0.03) | | 12.37) | 10.82) | -0.02) | |
| Finland | 38.65 (34.75, 42.92) | 34.02 (30.19, 38.24) | -0.42 (-0.47, -0.38) | <0.001 | 225.93 (216.68, 235.52) | 214.47 (204.97, 224.34) | -0.19 (-0.23, -0.15) | <0.001 | 58.25 (53.63, 63.22) | 18.58 (15.86, 21.67) | -4.25 (-4.86, -3.63) | <0.001 | 1.00 (0.48, 1.92) | 0.30 (0.06, 1.02) | -4.47 (-5.11, -3.81) | <0.001 |
| France | 23.21 (22.33, 24.13) | 22.63 (21.71, 23.58) | -0.07 (-0.12, -0.01) | 0.034 | 153.08 (150.84, 155.36) | 167.65 (165.20, 170.12) | 0.33 (0.25, 0.41) | <0.001 | 42.84 (41.65, 44.05) | 23.25 (22.35, 24.19) | -2.09 (-2.44, -1.73) | <0.001 | 0.72 (0.57, 0.89) | 0.39 (0.28, 0.53) | -2.08 (-2.47, -1.69) | <0.001 |
| Gabon | 22.47 (15.72, 31.29) | 22.48 (17.89, 27.93) | 0.00 (-0.00, 0.01) | 0.465 | 103.26 (87.60, 121.00) | 104.73 (94.33, 115.98) | 0.04 (0.00, 0.08) | 0.039 | 113.80 (97.58, 132.05) | 84.63 (75.39, 94.71) | -1.06 (-1.48, -0.65) | <0.001 | 1.99 (0.41, 5.99) | 1.47 (0.49, 3.40) | -1.09 (-1.50, -0.67) | <0.001 |
| Gambia | 22.55 (15.93, 31.23) | 24.59 (20.12, 29.81) | 0.30 (0.29, 0.30) | <0.001 | 123.72 (106.78, 142.73) | 132.66 (121.67, 144.41) | 0.26 (0.18, 0.34) | <0.001 | 110.84 (95.07, 128.63) | 140.03 (128.84, 151.98) | 0.89 (-0.08, 1.86) | 0.071 | 1.93 (0.40, 5.91) | 2.46 (1.18, 4.57) | 0.93 (-0.06, 1.92) | 0.065 |
| Georgia | 30.37 (27.23, 33.80) | 31.25 (27.01, 36.05) | 0.10 (0.09, 0.12) | <0.001 | 206.72 (198.39, 215.33) | 219.17 (208.03, 230.84) | 0.21 (0.17, 0.24) | <0.001 | 565.42 (551.51, 579.61) | 146.57 (137.16, 156.53) | -4.46 (-5.53, -3.38) | <0.001 | 9.79 (8.04, 11.83) | 2.48 (1.41, 4.19) | -4.54 (-5.68, -3.38) | <0.001 |
| Germany | 34.83 (33.90, 35.78) | 30.52 (29.56, 31.50) | -0.44 (-0.48, -0.41) | <0.001 | 208.10 (205.84, 210.39) | 206.77 (204.37, 209.20) | -0.02 (-0.07, 0.02) | 0.261 | 112.33 (110.64, 114.04) | 35.64 (34.64, 36.68) | -3.95 (-4.28, -3.61) | <0.001 | 1.93 (1.72, 2.17) | 0.60 (0.48, 0.75) | -4.05 (-4.41, -3.68) | <0.001 |
| Ghana | 21.07 (19.36, 22.90) | 22.30 (21.18, 23.46) | 0.20 (0.14, 0.26) | <0.001 | 110.60 (106.51, 114.81) | 116.22 (113.61, 118.88) | 0.19 (0.14, 0.24) | <0.001 | 259.05 (252.87, 265.34) | 176.69 (173.51, 179.92) | -1.35 (-1.72, -0.98) | <0.001 | 4.53 (3.74, 5.45) | 3.06 (2.65, 3.51) | -1.39 (-1.77, -1.00) | <0.001 |
| Greece | 21.97 (19.90, 24.19) | 23.16 (20.75, 25.79) | 0.20 (0.15, 0.26) | <0.001 | 138.24 (133.02, 143.62) | 166.03 (159.87, 172.40) | 0.64 (0.59, 0.69) | <0.001 | 124.66 (119.70, 129.78) | 89.51 (84.98, 94.24) | -0.95 (-1.68, -0.22) | 0.011 | 2.14 (1.54, 2.91) | 1.56 (1.02, 2.33) | -0.92 (-1.65, -0.19) | 0.014 |
| Greenland | 34.21 (9.63, 96.27) | 29.98 (6.40, 92.00) | -0.43 (-0.47, -0.39) | <0.001 | 142.67 (82.31, 236.73) | 142.42 (79.30, 239.87) | -0.01 (-0.03, 0.01) | 0.330 | 146.87 (84.92, 242.76) | 58.75 (21.75, 132.16) | -3.13 (-3.39, -2.86) | <0.001 | 2.64 (0.00, 51.11) | 1.05 (0.00, 46.72) | -3.17 (-3.45, -2.89) | <0.001 |
| Grenada | 57.74 (26.07, 112.35) | 58.94 (29.67, 106.09) | 0.08 (0.06, 0.09) | <0.001 | 288.59 (207.58, 391.62) | 297.97 (223.89, 389.34) | 0.12 (0.10, 0.14) | <0.001 | 227.53 (157.03, 319.98) | 99.20 (58.70, 157.65) | -2.69 (-3.27, -2.12) | <0.001 | 3.97 (0.01, 35.42) | 1.72 (0.00, 25.42) | -2.70 (-3.25, -2.14) | <0.001 |
| Guam | 17.26 (5.52, | 19.61 (6.75, | 0.46 (0.40, | <0.001 | 162.94 (119.10, | 196.12 (147.17, | 0.64 (0.57, | <0.001 | 180.70 (134.51, | 348.44 (282.10, | 2.19 (1.32, | <0.001 | 3.08 (0.05, | 6.07 (0.53, | 2.25 (1.15, | <0.001 |

| | | | | | | | | | | | | | | | | |
|---|---|---|---|---|---|---|---|---|---|---|---|---|---|---|---|---|
| | 41.09) | 44.72) | 0.52) | | 217.89) | 256.37) | 0.70) | | 237.92) | 425.92) | 3.06) | | 19.60) | 24.88) | 3.36) | |
| | 15.96 | 16.74 | 0.17 | | 84.74 | 98.11 | 0.51 | | 768.38 | 254.52 | -4.02 | | 13.07 | 4.32 | -4.04 | |
| Guatemala | (13.89, | (15.48, | (0.15, | <0.001 | (79.75, | (94.94, | (0.49, | <0.001 | (753.77, | (249.51, | (-5.92, | <0.001 | (11.21, | (3.68, | (-5.97, | <0.001 |
| | 18.26) | 18.09) | 0.19) | | 89.98) | 101.35) | 0.53) | | 783.22) | 259.60) | -2.08) | | 15.16) | 5.03) | -2.07) | |
| | 17.79 | 20.41 | 0.46 | | 84.67 | 97.07 | 0.49 | | 142.16 | 179.26 | 0.84 | | 2.47 | 3.12 | 0.85 | |
| Guinea | (15.35, | (18.62, | (0.40, | <0.001 | (79.14, | (93.00, | (0.41, | <0.001 | (135.09, | (173.84, | (0.61, | <0.001 | (1.61, | (2.43, | (0.63, | <0.001 |
| | 20.51) | 22.34) | 0.52) | | 90.49) | 101.27) | 0.56) | | 149.52) | 184.82) | 1.07) | | 3.63) | 3.94) | 1.06) | |
| | 22.05 | 23.66 | 0.24 | | 108.94 | 115.18 | 0.22 | | 246.71 | 261.79 | 0.23 | | 4.36 | 4.64 | 0.23 | |
| Guinea-Bissau | (15.68, | (19.05, | (0.23, | <0.001 | (93.71, | (104.44, | (0.11, | <0.001 | (223.76, | (245.58, | (0.12, | <0.001 | (1.81, | (2.70, | (0.11, | <0.001 |
| | 30.27) | 29.10) | 0.25) | | 126.00) | 126.75) | 0.33) | | 271.44) | 278.83) | 0.33) | | 8.94) | 7.48) | 0.35) | |
| | 62.11 | 61.01 | -0.06 | | 284.78 | 279.41 | -0.05 | | 371.16 | 256.47 | -1.25 | | 6.49 | 4.50 | -1.25 | |
| Guyana | (50.36, | (49.29, | (-0.09, | <0.001 | (258.01, | (252.98, | (-0.09, | 0.070 | (340.98, | (231.40, | (-2.34, | 0.025 | (3.06, | (1.75, | (-2.33, | 0.027 |
| | 75.93) | 74.75) | -0.04) | | 313.66) | 307.89) | 0.00) | | 403.41) | 283.58) | -0.16) | | 12.23) | 9.51) | -0.14) | |
| | 60.26 | 60.39 | 0.01 | | 240.44 | 239.31 | 0.01 | | 578.29 | 385.45 | -1.39 | | 10.20 | 6.78 | -1.35 | |
| Haiti | (55.93, | (57.53, | (-0.01, | 0.348 | (231.54, | (233.55, | (-0.14, | 0.888 | (564.55, | (378.13, | (-1.77, | <0.001 | (8.44, | (5.84, | (-1.72, | <0.001 |
| | 64.84) | 63.36) | 0.03) | | 249.61) | 245.19) | 0.16) | | 592.28) | 392.88) | -1.00) | | 12.22) | 7.83) | -0.97) | |
| | 20.31 | 19.88 | -0.07 | | 118.83 | 125.69 | 0.21 | | 436.14 | 168.21 | -3.20 | | 7.57 | 2.93 | -3.20 | |
| Honduras | (17.27, | (18.00, | (-0.09, | <0.001 | (111.04, | (120.78, | (0.06, | 0.008 | (421.49, | (162.58, | (-4.05, | <0.001 | (5.74, | (2.23, | (-4.05, | <0.001 |
| | 23.75) | 21.91) | -0.06) | | 127.03) | 130.76) | 0.37) | | 451.20) | 173.99) | -2.34) | | 9.83) | 3.79) | -2.34) | |
| | 23.35 | 19.33 | -0.65 | | 214.79 | 179.95 | -0.59 | | 285.82 | 78.99 | -4.29 | | 5.16 | 1.38 | -4.40 | |
| Hungary | (21.20, | (17.16, | (-0.68, | <0.001 | (208.42, | (173.39, | (-0.64, | <0.001 | (278.62, | (74.73, | (-5.19, | <0.001 | (4.23, | (0.87, | (-5.31, | <0.001 |
| | 25.66) | 21.73) | -0.63) | | 221.32) | 186.73) | -0.54) | | 293.18) | 83.47) | -3.39) | | 6.24) | 2.12) | -3.48) | |
| | 27.93 | 26.14 | -0.21 | | 166.26 | 174.36 | 0.15 | | 97.75 | 32.68 | -3.73 | | 1.69 | 0.54 | -3.89 | |
| Iceland | (15.46, | (14.82, | (-0.25, | <0.001 | (132.93, | (142.86, | (0.12, | <0.001 | (72.60, | (19.91, | (-3.96, | <0.001 | (0.03, | (0.00, | (-4.20, | <0.001 |
| | 46.78) | 43.30) | -0.18) | | 205.64) | 211.25) | 0.19) | | 129.04) | 51.19) | -3.50) | | 10.94) | 8.52) | -3.57) | |
| | 21.48 | 21.89 | 0.06 | | 134.69 | 140.93 | 0.16 | | 579.65 | 425.43 | -1.11 | | 9.87 | 7.33 | -1.08 | |
| India | (21.25, | (21.72, | (0.05, | <0.001 | (134.10, | (140.49, | (0.12, | <0.001 | (578.45, | (424.68, | (-1.75, | 0.001 | (9.72, | (7.23, | (-1.73, | 0.001 |
| | 21.71) | 22.07) | 0.08) | | 135.28) | 141.37) | 0.19) | | 580.84) | 426.19) | -0.47) | | 10.03) | 7.43) | -0.41) | |
| | 7.45 | 8.18 | 0.36 | | 83.09 | 95.92 | 0.53 | | 514.25 | 367.70 | -1.10 | | 8.92 | 6.38 | -1.10 | |
| Indonesia | (7.17, | (7.94, | (0.25, | <0.001 | (82.12, | (95.09, | (0.41, | <0.001 | (511.90, | (366.08, | (-1.40, | <0.001 | (8.61, | (6.17, | (-1.39, | <0.001 |
| | 7.74) | 8.43) | 0.46) | | 84.06) | 96.75) | 0.64) | | 516.61) | 369.33) | -0.81) | | 9.24) | 6.60) | -0.81) | |
| Iran (Islamic | 46.49 | 43.82 | -0.21 | <0.001 | 265.01 | 252.11 | -0.17 | <0.001 | 541.40 | 235.92 | -2.82 | <0.001 | 9.30 | 3.94 | -2.92 | <0.001 |
| Republic of) | (45.12, | (42.90, | (-0.23, | | (261.63, | (249.96, | (-0.19, | | (536.75, | (233.68, | (-3.05, | | (8.69, | (3.66, | (-3.13, | |

| | | | | | | | | | | | | | | | | |
|---|---|---|---|---|---|---|---|---|---|---|---|---|---|---|---|---|
| | 47.90) | 44.75) | -0.19) | | 268.43) | 254.27) | -0.15) | | 546.08) | 238.18) | -2.59) | | 9.94) | 4.24) | -2.70) | |
| | 35.24 | 33.60 | -0.16 | | 236.50 | 229.16 | -0.09 | | 610.86 | 307.22 | -2.40 | | 10.61 | 5.34 | -2.40 | |
| Iraq | (33.03, | (32.36, | (-0.18, | <0.001 | (230.61, | (225.81, | (-0.19, | 0.123 | (601.62, | (303.41, | (-2.85, | <0.001 | (9.42, | (4.84, | (-2.86, | <0.001 |
| | 37.56) | 34.89) | -0.15) | | 242.50) | 232.54) | 0.02) | | 620.21) | 311.07) | -1.94) | | 11.92) | 5.87) | -1.93) | |
| | 28.05 | 25.48 | -0.31 | | 191.29 | 190.68 | 0.01 | | 80.48 | 22.46 | -4.30 | | 1.40 | 0.37 | -4.44 | |
| Ireland | (24.16, | (22.11, | (-0.39, | <0.001 | (180.92, | (181.78, | (-0.06, | 0.736 | (73.82, | (19.52, | (-5.64, | <0.001 | (0.65, | (0.09, | (-5.79, | <0.001 |
| | 32.39) | 29.25) | -0.22) | | 202.10) | 199.93) | 0.08) | | 87.59) | 25.76) | -2.94) | | 2.64) | 1.10) | -3.08) | |
| | 21.63 | 21.11 | -0.04 | | 147.33 | 161.79 | 0.32 | | 100.28 | 17.07 | -5.88 | | 1.71 | 0.27 | -6.12 | |
| Israel | (18.75, | (18.91, | (-0.13, | 0.371 | (139.71, | (155.67, | (0.26, | <0.001 | (94.02, | (15.12, | (-6.57, | <0.001 | (0.98, | (0.08, | (-6.85, | <0.001 |
| | 24.83) | 23.50) | 0.05) | | 155.26) | 168.09) | 0.38) | | 106.86) | 19.21) | -5.18) | | 2.77) | 0.67) | -5.38) | |
| | 28.54 | 28.51 | 0.00 | | 202.13 | 202.57 | 0.02 | | 63.92 | 25.24 | -3.22 | | 1.08 | 0.41 | -3.40 | |
| Italy | (27.54, | (27.35, | (-0.07, | 0.970 | (199.44, | (199.61, | (-0.05, | 0.587 | (62.41, | (24.21, | (-3.47, | <0.001 | (0.89, | (0.29, | (-3.61, | <0.001 |
| | 29.57) | 29.70) | 0.07) | | 204.86) | 205.56) | 0.09) | | 65.46) | 26.32) | -2.96) | | 1.30) | 0.58) | -3.19) | |
| | 53.55 | 59.36 | 0.37 | | 276.37 | 313.04 | 0.43 | | 114.19 | 118.18 | 0.60 | | 2.02 | 2.08 | 0.60 | |
| Jamaica | (47.02, | (53.38, | (0.34, | <0.001 | (260.65, | (298.97, | (0.40, | <0.001 | (104.08, | (109.59, | (-0.57, | 0.320 | (0.88, | (1.08, | (-0.59, | 0.323 |
| | 60.80) | 65.85) | 0.40) | | 292.83) | 327.63) | 0.46) | | 125.05) | 127.29) | 1.78) | | 4.00) | 3.63) | 1.81) | |
| | 18.51 | 17.94 | -0.10 | | 126.97 | 127.89 | 0.03 | | 48.29 | 28.52 | -1.79 | | 0.80 | 0.48 | -1.75 | |
| Japan | (17.94, | (17.30, | (-0.12, | <0.001 | (125.51, | (126.24, | (-0.00, | 0.079 | (47.39, | (27.74, | (-2.09, | <0.001 | (0.69, | (0.38, | (-2.11, | <0.001 |
| | 19.09) | 18.60) | -0.08) | | 128.45) | 129.57) | 0.07) | | 49.21) | 29.33) | -1.49) | | 0.93) | 0.59) | -1.40) | |
| | 33.15 | 31.03 | -0.23 | | 227.64 | 215.32 | -0.18 | | 230.97 | 83.76 | -3.36 | | 4.05 | 1.42 | -3.48 | |
| Jordan | (28.47, | (28.71, | (-0.25, | <0.001 | (214.72, | (209.09, | (-0.21, | <0.001 | (218.14, | (79.91, | (-4.10, | <0.001 | (2.50, | (0.95, | (-4.22, | <0.001 |
| | 38.41) | 33.48) | -0.22) | | 241.16) | 221.68) | -0.15) | | 244.39) | 87.75) | -2.62) | | 6.24) | 2.03) | -2.73) | |
| | 31.43 | 28.54 | -0.34 | | 202.84 | 180.51 | -0.40 | | 244.45 | 131.30 | -2.06 | | 4.26 | 2.30 | -2.04 | |
| Kazakhstan | (29.58, | (26.85, | (-0.36, | <0.001 | (198.08, | (176.28, | (-0.43, | <0.001 | (239.21, | (127.67, | (-2.69, | <0.001 | (3.59, | (1.85, | (-2.69, | <0.001 |
| | 33.36) | 30.32) | -0.31) | | 207.69) | 184.84) | -0.38) | | 249.78) | 135.02) | -1.43) | | 5.02) | 2.85) | -1.39) | |
| | 20.02 | 20.03 | -0.00 | | 102.05 | 101.49 | -0.03 | | 56.24 | 96.80 | 1.90 | | 0.92 | 1.63 | 2.01 | |
| Kenya | (18.57, | (19.16, | (-0.04, | 0.836 | (98.58, | (99.46, | (-0.11, | 0.460 | (53.80, | (94.87, | (1.73, | <0.001 | (0.63, | (1.39, | (1.83, | <0.001 |
| | 21.57) | 20.94) | 0.03) | | 105.62) | 103.55) | 0.05) | | 58.76) | 98.76) | 2.08) | | 1.31) | 1.91) | 2.18) | |
| | 21.91 | 22.74 | 0.13 | | 159.43 | 168.20 | 0.20 | | 773.73 | 715.63 | -0.29 | | 13.77 | 12.69 | -0.30 | |
| Kiribati | (4.74, | (7.89, | (0.12, | <0.001 | (99.67, | (120.11, | (0.11, | <0.001 | (634.53, | (612.43, | (-0.44, | <0.001 | (1.53, | (2.64, | (-0.45, | <0.001 |
| | 64.94) | 51.58) | 0.14) | | 242.85) | 229.32) | 0.29) | | 935.37) | 831.47) | -0.13) | | 53.07) | 37.32) | -0.14) | |
| | 31.72 | 31.65 | -0.00 | | 229.92 | 235.59 | 0.09 | | 263.86 | 82.50 | -3.78 | | 4.48 | 1.35 | -3.89 | |
| Kuwait | (26.13, | (28.48, | (-0.03, | 0.814 | (214.23, | (227.14, | (0.04, | <0.001 | (247.16, | (77.13, | (-4.99, | <0.001 | (2.55, | (0.75, | (-5.24, | <0.001 |

| | | | | | | | | | | | | | | | | |
|---|---|---|---|---|---|---|---|---|---|---|---|---|---|---|---|---|
| | 38.20) | 35.20) | 0.02) | | 246.49) | 244.40) | 0.14) | | 281.44) | 88.25) | -2.55) | | 7.37) | 2.42) | -2.53) | |
| | 29.03 | 27.02 | -0.25 | | 182.33 | 165.52 | -0.33 | | 244.51 | 134.33 | -2.08 | | 4.26 | 2.36 | -2.05 | |
| Kyrgyzstan | (25.54, | (24.30, | (-0.26, | <0.001 | (173.21, | (158.57, | (-0.35, | <0.001 | (234.03, | (128.05, | (-2.81, | <0.001 | (2.98, | (1.59, | (-2.78, | <0.001 |
| | 32.88) | 29.97) | -0.23) | | 191.81) | 172.70) | -0.31) | | 255.36) | 140.84) | -1.35) | | 5.94) | 3.37) | -1.32) | |
| Lao People's | 18.58 | 17.19 | -0.27 | | 115.87 | 106.03 | -0.27 | | 352.04 | 335.54 | -0.13 | | 6.24 | 5.84 | -0.20 | |
| Democratic | (15.52, | (15.16, | (-0.28, | <0.001 | (107.87, | (100.81, | (-0.46, | 0.005 | (338.09, | (326.30, | (-0.40, | 0.314 | (4.50, | (4.67, | (-0.44, | 0.112 |
| Republic | 22.09) | 19.43) | -0.25) | | 124.33) | 111.45) | -0.09) | | 366.44) | 344.98) | 0.13) | | 8.46) | 7.22) | 0.05) | |
| | 21.96 | 23.01 | 0.15 | | 176.82 | 190.22 | 0.26 | | 147.06 | 48.07 | -3.65 | | 2.64 | 0.82 | -3.84 | |
| Latvia | (18.03, | (17.87, | (0.09, | <0.001 | (165.45, | (175.39, | (0.18, | <0.001 | (136.71, | (40.80, | (-5.51, | <0.001 | (1.42, | (0.15, | (-5.89, | <0.001 |
| | 26.54) | 29.39) | 0.21) | | 188.82) | 206.19) | 0.35) | | 158.04) | 56.52) | -1.76) | | 4.55) | 3.04) | -1.74) | |
| | 29.68 | 30.48 | 0.09 | | 197.44 | 210.02 | 0.22 | | 536.01 | 316.29 | -1.79 | | 9.33 | 5.41 | -1.85 | |
| Lebanon | (25.33, | (27.32, | (0.08, | <0.001 | (185.85, | (201.73, | (0.16, | <0.001 | (517.04, | (305.71, | (-1.93, | <0.001 | (6.97, | (4.12, | (-2.02, | <0.001 |
| | 34.56) | 33.95) | 0.11) | | 209.57) | 218.61) | 0.28) | | 555.52) | 327.20) | -1.64) | | 12.25) | 7.05) | -1.68) | |
| | 22.58 | 24.25 | 0.25 | | 80.26 | 89.88 | 0.39 | | 60.48 | 145.29 | 2.92 | | 1.04 | 2.55 | 2.99 | |
| Lesotho | (17.53, | (19.87, | (0.22, | <0.001 | (70.15, | (81.11, | (0.36, | <0.001 | (51.93, | (134.16, | (2.34, | <0.001 | (0.22, | (1.27, | (2.37, | <0.001 |
| | 28.71) | 29.33) | 0.27) | | 91.47) | 99.34) | 0.41) | | 70.10) | 157.11) | 3.51) | | 3.15) | 4.59) | 3.62) | |
| | 22.13 | 23.92 | 0.26 | | 114.33 | 121.38 | 0.23 | | 169.68 | 159.66 | -0.23 | | 2.96 | 2.79 | -0.22 | |
| Liberia | (17.38, | (20.87, | (0.22, | <0.001 | (103.05, | (114.26, | (0.09, | 0.002 | (156.04, | (151.59, | (-0.35, | 0.001 | (1.41, | (1.81, | (-0.36, | 0.002 |
| | 27.81) | 27.30) | 0.30) | | 126.51) | 128.84) | 0.37) | | 184.21) | 168.06) | -0.09) | | 5.49) | 4.12) | -0.08) | |
| | 28.06 | 30.19 | 0.25 | | 181.60 | 203.79 | 0.40 | | 678.33 | 507.71 | -0.96 | | 11.63 | 8.69 | -0.93 | |
| Libya | (23.93, | (27.50, | (0.21, | <0.001 | (170.56, | (196.77, | (0.32, | <0.001 | (657.89, | (496.41, | (-1.61, | 0.004 | (9.06, | (7.27, | (-1.67, | 0.015 |
| | 32.73) | 33.08) | 0.29) | | 193.19) | 211.00) | 0.48) | | 699.28) | 519.22) | -0.31) | | 14.75) | 10.31) | -0.18) | |
| | 24.13 | 22.66 | -0.24 | | 189.07 | 178.30 | -0.19 | | 142.87 | 71.19 | -2.27 | | 2.51 | 1.24 | -2.26 | |
| Lithuania | (20.66, | (18.42, | (-0.30, | <0.001 | (179.11, | (166.13, | (-0.28, | <0.001 | (134.19, | (63.60, | (-4.26, | 0.029 | (1.48, | (0.44, | (-4.36, | 0.039 |
| | 28.04) | 27.71) | -0.17) | | 199.46) | 191.23) | -0.09) | | 151.98) | 79.57) | -0.23) | | 4.01) | 3.00) | -0.12) | |
| | 19.92 | 19.89 | 0.01 | | 142.03 | 160.77 | 0.45 | | 85.51 | 25.52 | -4.07 | | 1.47 | 0.42 | -4.24 | |
| Luxembourg | (11.25, | (12.44, | (-0.03, | 0.690 | (117.14, | (138.87, | (0.29, | <0.001 | (66.25, | (17.16, | (-4.65, | <0.001 | (0.06, | (0.00, | (-4.85, | <0.001 |
| | 33.89) | 30.83) | 0.04) | | 171.63) | 185.74) | 0.62) | | 109.63) | 37.25) | -3.50) | | 9.70) | 5.57) | -3.63) | |
| | 19.44 | 20.96 | 0.25 | | 99.19 | 107.50 | 0.28 | | 187.52 | 161.42 | -0.48 | | 3.25 | 2.81 | -0.46 | |
| Madagascar | (17.53, | (19.69, | (0.23, | <0.001 | (94.68, | (104.53, | (0.25, | <0.001 | (181.47, | (157.84, | (-1.04, | 0.088 | (2.49, | (2.35, | (-1.02, | 0.105 |
| | 21.51) | 22.29) | 0.28) | | 103.87) | 110.53) | 0.31) | | 193.73) | 165.06) | 0.07) | | 4.18) | 3.34) | 0.10) | |
| | 22.96 | 23.22 | 0.04 | | 112.02 | 112.92 | 0.04 | | 226.97 | 121.63 | -2.08 | | 3.86 | 2.05 | -2.12 | |
| Malawi | (20.65, | (21.62, | (0.01, | 0.003 | (106.65, | (109.26, | (-0.03, | 0.266 | (219.64, | (117.99, | (-2.28, | <0.001 | (2.95, | (1.59, | (-2.32, | <0.001 |

| | | | | | | | | | | | | | | | | |
|---|---|---|---|---|---|---|---|---|---|---|---|---|---|---|---|---|
| | 25.47) | 24.90) | 0.06) | | 117.60) | 116.67) | 0.12) | | 234.50) | 125.35) | -1.88) | | 4.99) | 2.59) | -1.91) | |
| | 19.22 | 19.64 | 0.08 | | 168.81 | 174.56 | 0.13 | | 130.12 | 118.10 | -0.37 | | 2.23 | 2.01 | -0.42 | |
| Malaysia | (17.80, | (18.58, | (0.03, | 0.002 | (164.48, | (171.36, | (0.04, | 0.005 | (126.34, | (115.47, | (-0.77, | 0.068 | (1.76, | (1.68, | (-0.81, | 0.037 |
| | 20.74) | 20.74) | 0.14) | | 173.23) | 177.80) | 0.22) | | 133.99) | 120.77) | 0.03) | | 2.79) | 2.39) | -0.03) | |
| | 17.89 | 16.76 | -0.22 | | 134.62 | 125.06 | -0.24 | | 426.99 | 71.09 | -6.02 | | 7.49 | 1.20 | -6.10 | |
| Maldives | (6.29, | (9.61, | (-0.24, | <0.001 | (95.83, | (104.07, | (-0.30, | <0.001 | (356.78, | (55.21, | (-6.29, | <0.001 | (1.06, | (0.05, | (-6.32, | <0.001 |
| | 41.95) | 28.04) | -0.21) | | 184.91) | 149.75) | -0.19) | | 508.08) | 90.81) | -5.75) | | 27.55) | 7.62) | -5.87) | |
| | 19.40 | 20.14 | 0.13 | | 89.99 | 92.02 | 0.09 | | 262.17 | 201.25 | -0.91 | | 4.60 | 3.51 | -0.93 | |
| Mali | (17.17, | (18.69, | (0.12, | <0.001 | (84.99, | (88.78, | (0.03, | 0.003 | (253.74, | (196.58, | (-1.19, | <0.001 | (3.54, | (2.91, | (-1.22, | <0.001 |
| | 21.84) | 21.68) | 0.14) | | 95.21) | 95.35) | 0.14) | | 270.83) | 206.01) | -0.63) | | 5.90) | 4.20) | -0.64) | |
| | 17.52 | 16.66 | -0.17 | | 140.77 | 147.38 | 0.20 | | 124.71 | 57.58 | -2.59 | | 2.14 | 0.97 | -2.65 | |
| Malta | (9.09, | (8.52, | (-0.23, | <0.001 | (115.06, | (121.37, | (-0.05, | 0.123 | (100.28, | (41.46, | (-2.96, | <0.001 | (0.17, | (0.01, | (-3.25, | <0.001 |
| | 31.04) | 30.62) | -0.12) | | 171.01) | 178.38) | 0.45) | | 153.75) | 79.00) | -2.22) | | 9.98) | 9.43) | -2.04) | |
| | 19.45 | 19.95 | 0.09 | | 148.39 | 155.58 | 0.17 | | 389.89 | 656.32 | 1.78 | | 6.95 | 11.71 | 1.78 | |
| Marshall Islands | (1.27, | (2.92, | (0.07, | <0.001 | (72.50, | (91.87, | (0.13, | <0.001 | (258.18, | (516.11, | (1.55, | <0.001 | (0.01, | (0.65, | (1.53, | <0.001 |
| | 90.50) | 67.87) | 0.10) | | 271.49) | 247.04) | 0.20) | | 566.86) | 823.26) | 2.01) | | 69.94) | 54.65) | 2.03) | |
| | 21.76 | 22.74 | 0.14 | | 116.44 | 118.84 | 0.08 | | 178.73 | 109.61 | -1.65 | | 3.13 | 1.89 | -1.73 | |
| Mauritania | (17.15, | (19.42, | (0.13, | <0.001 | (105.16, | (110.86, | (0.03, | 0.002 | (164.90, | (102.11, | (-1.80, | <0.001 | (1.54, | (1.02, | (-1.93, | <0.001 |
| | 27.27) | 26.50) | 0.16) | | 128.63) | 127.25) | 0.13) | | 193.45) | 117.53) | -1.50) | | 5.70) | 3.23) | -1.52) | |
| | 18.50 | 17.27 | -0.21 | | 145.05 | 132.64 | -0.30 | | 321.05 | 122.20 | -3.29 | | 5.65 | 2.11 | -3.40 | |
| Mauritius | (13.50, | (12.41, | (-0.30, | <0.001 | (130.27, | (118.56, | (-0.34, | <0.001 | (298.82, | (108.65, | (-3.93, | <0.001 | (3.06, | (0.70, | (-4.11, | <0.001 |
| | 24.77) | 23.47) | -0.13) | | 161.09) | 148.00) | -0.25) | | 344.52) | 137.05) | -2.65) | | 9.59) | 5.00) | -2.68) | |
| | 23.45 | 19.70 | -0.60 | | 165.38 | 139.83 | -0.58 | | 162.07 | 130.35 | -0.66 | | 2.78 | 2.22 | -0.68 | |
| Mexico | (22.71, | (19.16, | (-0.61, | <0.001 | (163.33, | (138.38, | (-0.63, | <0.001 | (160.09, | (128.96, | (-1.39, | 0.079 | (2.52, | (2.04, | (-1.38, | 0.056 |
| | 24.22) | 20.25) | -0.58) | | 167.45) | 141.28) | -0.52) | | 164.07) | 131.76) | 0.08) | | 3.05) | 2.41) | 0.02) | |
| Micronesia | 20.36 | 19.21 | -0.20 | | 155.87 | 146.89 | -0.20 | | 486.19 | 603.49 | 0.73 | | 8.68 | 10.65 | 0.70 | |
| (Federated States | (4.90, | (4.69, | (-0.23, | <0.001 | (101.50, | (96.15, | (-0.25, | <0.001 | (385.97, | (495.78, | (0.63, | <0.001 | (0.56, | (1.21, | (0.61, | <0.001 |
| of) | 57.15) | 52.95) | -0.17) | | 229.76) | 215.32) | -0.16) | | 605.24) | 728.13) | 0.84) | | 39.73) | 40.26) | 0.79) | |
| | 24.55 | 23.42 | -0.13 | | 158.74 | 167.41 | 0.19 | | 72.38 | 40.13 | -2.02 | | 1.23 | 0.68 | -2.02 | |
| Monaco | (1.04, | (0.78, | (-0.18, | <0.001 | (69.60, | (74.98, | (0.15, | <0.001 | (18.00, | (4.99, | (-2.12, | <0.001 | (0.00, | (0.00, | (-2.25, | <0.001 |
| | 161.58) | 132.05) | -0.09) | | 339.50) | 331.73) | 0.23) | | 227.67) | 156.15) | -1.91) | | 127.68) | 92.32) | -1.79) | |
| Mongolia | 32.00 | 31.93 | -0.01 | 0.627 | 195.98 | 197.44 | 0.05 | 0.344 | 812.72 | 401.65 | -2.48 | <0.001 | 14.22 | 7.02 | -2.49 | <0.001 |
| | (26.58, | (27.95, | (-0.04, | | (181.48, | (187.53, | (-0.06, | | (783.73, | (387.19, | (-3.19, | | (10.58, | (5.23, | (-3.21, | |

| | | | | | | | | | | | | | | | | |
|---|---|---|---|---|---|---|---|---|---|---|---|---|---|---|---|---|
| | 38.32) | 36.39) | 0.02) | | 211.40) | 207.82) | 0.16) | | 842.58) | 416.58) | -1.76) | | 18.80) | 9.31) | -1.77) | |
| Montenegro | 19.78 (12.70, 29.43) | 18.38 (11.17, 28.77) | -0.26 (-0.27, -0.24) | <0.001 | 180.52 (157.58, 205.90) | 170.96 (147.64, 197.18) | -0.17 (-0.21, -0.13) | <0.001 | 100.87 (83.91, 120.31) | 77.15 (61.78, 95.47) | -0.81 (-1.86, 0.25) | 0.136 | 1.72 (0.23, 6.12) | 1.32 (0.09, 6.29) | -0.80 (-1.87, 0.28) | 0.146 |
| Morocco | 32.25 (30.64, 33.93) | 30.91 (29.66, 32.20) | -0.15 (-0.16, -0.14) | <0.001 | 213.57 (209.31, 217.90) | 207.98 (204.72, 211.28) | -0.08 (-0.14, -0.01) | 0.024 | 601.99 (594.98, 609.07) | 411.19 (406.58, 415.84) | -1.26 (-1.49, -1.03) | <0.001 | 10.45 (9.54, 11.43) | 7.14 (6.54, 7.77) | -1.26 (-1.49, -1.03) | <0.001 |
| Mozambique | 23.29 (21.34, 25.38) | 23.90 (22.56, 25.30) | 0.08 (0.06, 0.10) | <0.001 | 111.29 (106.91, 115.81) | 115.06 (112.00, 118.19) | 0.14 (0.04, 0.23) | 0.005 | 117.01 (112.61, 121.54) | 114.18 (111.24, 117.18) | -0.14 (-0.37, 0.09) | 0.236 | 2.00 (1.46, 2.68) | 1.94 (1.57, 2.38) | -0.09 (-0.33, 0.16) | 0.490 |
| Myanmar | 19.53 (18.56, 20.53) | 17.90 (17.13, 18.69) | -0.30 (-0.32, -0.28) | <0.001 | 120.95 (118.48, 123.46) | 109.34 (107.42, 111.27) | -0.31 (-0.50, -0.13) | 0.001 | 891.38 (884.77, 898.02) | 246.16 (243.28, 249.06) | -4.43 (-4.77, -4.08) | <0.001 | 15.50 (14.63, 16.40) | 4.16 (3.79, 4.55) | -4.52 (-4.88, -4.16) | <0.001 |
| Namibia | 25.02 (19.11, 32.30) | 23.16 (19.11, 27.84) | -0.27 (-0.31, -0.24) | <0.001 | 107.01 (93.91, 121.49) | 99.98 (91.20, 109.39) | -0.26 (-0.39, -0.12) | <0.001 | 94.22 (82.09, 107.72) | 68.16 (61.00, 75.94) | -1.02 (-1.55, -0.49) | <0.001 | 1.65 (0.42, 4.48) | 1.17 (0.41, 2.65) | -1.08 (-1.66, -0.49) | <0.001 |
| Nauru | 20.38 (0.00, 246.24) | 20.89 (0.01, 225.57) | 0.09 (0.06, 0.11) | <0.001 | 160.98 (33.16, 476.11) | 164.96 (39.09, 457.27) | 0.10 (0.05, 0.14) | <0.001 | 663.63 (347.63, 1152.64) | 950.69 (581.17, 1471.80) | 1.23 (0.92, 1.55) | <0.001 | 11.83 (0.00, 230.52) | 16.83 (0.00, 218.43) | 1.21 (0.89, 1.53) | <0.001 |
| Nepal | 20.42 (18.92, 22.00) | 18.79 (17.76, 19.86) | -0.28 (-0.31, -0.26) | <0.001 | 133.83 (129.85, 137.90) | 122.20 (119.52, 124.93) | -0.31 (-0.34, -0.28) | <0.001 | 389.58 (382.90, 396.36) | 200.85 (197.45, 204.30) | -2.25 (-2.50, -1.99) | <0.001 | 6.83 (5.97, 7.79) | 3.50 (3.06, 3.98) | -2.27 (-2.53, -2.00) | <0.001 |
| Netherlands | 39.44 (37.25, 41.74) | 35.18 (32.96, 37.51) | -0.38 (-0.39, -0.36) | <0.001 | 217.31 (212.14, 222.58) | 227.91 (222.29, 233.65) | 0.15 (0.09, 0.21) | <0.001 | 83.21 (80.02, 86.51) | 26.29 (24.40, 28.29) | -3.80 (-4.80, -2.79) | <0.001 | 1.44 (1.05, 1.94) | 0.44 (0.23, 0.78) | -3.93 (-4.96, -2.89) | <0.001 |
| New Zealand | 27.09 (23.38, 31.24) | 20.58 (17.43, 24.16) | -0.93 (-0.98, -0.88) | <0.001 | 154.52 (145.47, 164.00) | 134.63 (126.45, 143.23) | -0.47 (-0.54, -0.39) | <0.001 | 83.98 (77.34, 91.04) | 32.34 (28.40, 36.68) | -3.18 (-3.79, -2.57) | <0.001 | 1.47 (0.71, 2.69) | 0.56 (0.16, 1.45) | -3.17 (-4.38, -1.93) | <0.001 |
| Nicaragua | 19.93 (16.68, 23.68) | 19.05 (16.81, 21.50) | -0.15 (-0.17, -0.13) | <0.001 | 131.80 (122.84, 141.25) | 135.60 (129.47, 141.96) | 0.09 (0.07, 0.11) | <0.001 | 208.03 (197.02, 219.51) | 141.60 (135.37, 148.06) | -1.40 (-2.58, -0.20) | 0.023 | 3.60 (2.27, 5.45) | 2.42 (1.67, 3.40) | -1.44 (-2.64, -0.23) | 0.019 |
| Niger | 19.26 (16.88, | 20.34 (18.82, | 0.18 (0.14, | <0.001 | 96.52 (90.88, | 99.92 (96.39, | 0.13 (0.08, | <0.001 | 145.65 (138.91, | 127.61 (123.74, | -0.43 (-0.73, | 0.005 | 2.53 (1.70, | 2.21 (1.72, | -0.45 (-0.72, | 0.001 |

| | | | | | | | | | | | | | | | | |
|---|---|---|---|---|---|---|---|---|---|---|---|---|---|---|---|---|
| | 21.91) | 21.96) | 0.22) | | 102.43) | 103.56) | 0.18) | | 152.66) | 131.56) | -0.13) | | 3.64) | 2.80) | -0.19) | |
| Nigeria | 17.68 (16.99, 18.39) | 19.58 (19.14, 20.04) | 0.34 (0.26, 0.42) | <0.001 | 86.10 (84.52, 87.71) | 96.66 (95.64, 97.68) | 0.41 (0.39, 0.43) | <0.001 | 126.98 (125.11, 128.87) | 92.43 (91.46, 93.41) | -1.05 (-1.51, -0.60) | <0.001 | 2.21 (1.97, 2.47) | 1.60 (1.47, 1.73) | -1.07 (-1.54, -0.61) | <0.001 |
| Niue | 19.83 (0.00, 1080.30) | 20.11 (0.00, 1501.26) | 0.05 (0.04, 0.06) | <0.001 | 177.81 (0.74, 1382.69) | 184.00 (0.19, 1807.72) | 0.12 (0.11, 0.12) | <0.001 | 291.94 (8.68, 1582.74) | 322.71 (5.19, 2053.46) | 0.35 (0.05, 0.64) | 0.021 | 5.12 (0.00, 1050.39) | 5.61 (0.00, 1472.62) | 0.31 (0.03, 0.60) | 0.032 |
| North Macedonia | 17.35 (13.51, 21.95) | 16.17 (12.46, 20.81) | -0.25 (-0.27, -0.23) | <0.001 | 149.51 (137.81, 161.96) | 143.06 (131.82, 155.15) | -0.15 (-0.17, -0.12) | <0.001 | 307.98 (291.04, 325.67) | 153.00 (141.11, 165.75) | -2.34 (-3.36, -1.31) | <0.001 | 5.30 (3.29, 8.10) | 2.62 (1.29, 4.96) | -2.35 (-3.34, -1.34) | <0.001 |
| Northern Mariana Islands | 17.86 (2.31, 66.65) | 19.28 (0.33, 131.50) | 0.27 (0.23, 0.32) | <0.001 | 160.73 (95.91, 254.71) | 179.08 (75.20, 364.80) | 0.37 (0.36, 0.38) | <0.001 | 186.80 (116.53, 285.90) | 181.58 (77.20, 367.38) | -0.03 (-0.20, 0.14) | 0.757 | 3.25 (0.00, 42.95) | 3.15 (0.00, 104.64) | -0.04 (-0.21, 0.13) | 0.640 |
| Norway | 26.94 (23.45, 30.81) | 24.60 (21.45, 28.12) | -0.30 (-0.35, -0.25) | <0.001 | 177.27 (168.21, 186.72) | 174.95 (166.50, 183.75) | -0.04 (-0.07, -0.00) | 0.038 | 74.85 (69.00, 81.08) | 16.53 (14.00, 19.41) | -5.16 (-5.70, -4.62) | <0.001 | 1.30 (0.64, 2.38) | 0.26 (0.04, 0.93) | -5.49 (-6.35, -4.63) | <0.001 |
| Oman | 30.19 (23.92, 37.67) | 33.00 (29.23, 37.20) | 0.30 (0.25, 0.36) | <0.001 | 201.63 (184.61, 219.83) | 231.28 (221.16, 241.80) | 0.49 (0.44, 0.53) | <0.001 | 478.54 (452.58, 505.65) | 256.77 (245.74, 268.22) | -1.99 (-2.26, -1.73) | <0.001 | 8.30 (5.19, 12.66) | 4.32 (3.01, 6.09) | -2.12 (-2.39, -1.86) | <0.001 |
| Pakistan | 24.63 (23.88, 25.41) | 25.51 (25.01, 26.01) | 0.12 (0.11, 0.13) | <0.001 | 165.15 (163.13, 167.18) | 172.09 (170.78, 173.41) | 0.15 (0.12, 0.18) | <0.001 | 535.69 (532.10, 539.31) | 689.01 (686.42, 691.60) | 0.89 (0.74, 1.03) | <0.001 | 9.52 (9.04, 10.02) | 12.19 (11.84, 12.54) | 0.87 (0.73, 1.02) | <0.001 |
| Palau | 19.85 (0.08, 158.16) | 20.13 (0.03, 205.85) | 0.05 (0.04, 0.07) | <0.001 | 170.50 (58.64, 387.87) | 177.02 (55.85, 438.96) | 0.13 (0.11, 0.15) | <0.001 | 382.07 (199.08, 666.08) | 376.99 (180.73, 708.15) | -0.05 (-0.12, 0.02) | 0.195 | 6.73 (0.00, 134.11) | 6.60 (0.00, 181.94) | -0.07 (-0.14, -0.00) | 0.040 |
| Palestine | 31.49 (25.40, 38.68) | 31.28 (27.74, 35.18) | -0.03 (-0.04, -0.01) | 0.001 | 216.81 (199.84, 234.90) | 219.55 (209.78, 229.66) | 0.05 (0.03, 0.07) | <0.001 | 322.17 (302.04, 343.38) | 191.98 (183.09, 201.21) | -1.76 (-1.89, -1.63) | <0.001 | 5.58 (3.20, 9.14) | 3.27 (2.19, 4.71) | -1.81 (-1.94, -1.69) | <0.001 |
| Panama | 18.34 (14.65, 22.72) | 17.65 (14.83, 20.85) | -0.12 (-0.16, -0.08) | <0.001 | 118.46 (108.47, 129.14) | 123.94 (116.24, 132.03) | 0.15 (0.13, 0.17) | <0.001 | 132.62 (122.22, 143.69) | 67.16 (61.53, 73.17) | -2.34 (-3.62, -1.03) | <0.001 | 2.28 (1.10, 4.22) | 1.13 (0.51, 2.17) | -2.39 (-3.79, -0.96) | 0.001 |
| Papua New Guinea | 20.51 | 21.00 | 0.09 | <0.001 | 155.75 | 162.68 | 0.17 | <0.001 | 214.36 | 291.33 | 1.04 | <0.001 | 3.76 | 5.11 | 1.04 | <0.001 |

| | | | | | | | | | | | | | | | | |
|---|---|---|---|---|---|---|---|---|---|---|---|---|---|---|---|---|
| | (17.30, 24.16) | (19.00, 23.15) | (0.07, 0.10) | | (146.43, 165.52) | (156.96, 168.56) | (0.09, 0.24) | | (203.49, 225.68) | (283.69, 299.11) | (0.78, 1.30) | | (2.44, 5.56) | (4.14, 6.24) | (0.78, 1.31) | |
| Paraguay | 12.24 (9.83, 15.08) | 12.41 (10.66, 14.36) | 0.06 (0.02, 0.10) | 0.003 | 89.26 (82.33, 96.63) | 97.68 (92.60, 102.97) | 0.31 (0.29, 0.34) | <0.001 | 191.41 (181.26, 202.01) | 103.92 (98.68, 109.37) | -1.95 (-2.67, -1.21) | <0.001 | 3.37 (2.14, 5.07) | 1.81 (1.18, 2.66) | -2.02 (-3.06, -0.96) | <0.001 |
| Peru | 7.19 (6.40, 8.07) | 6.98 (6.36, 7.63) | -0.11 (-0.14, -0.07) | <0.001 | 62.49 (60.01, 65.05) | 62.20 (60.36, 64.09) | -0.02 (-0.04, 0.00) | 0.084 | 266.32 (261.34, 271.38) | 96.25 (93.94, 98.61) | -3.34 (-4.80, -1.86) | <0.001 | 4.55 (3.91, 5.26) | 1.59 (1.31, 1.92) | -3.45 (-4.98, -1.90) | <0.001 |
| Philippines | 33.80 (32.74, 34.88) | 36.69 (35.88, 37.50) | 0.28 (0.25, 0.32) | <0.001 | 176.69 (174.21, 179.21) | 206.90 (204.96, 208.86) | 0.55 (0.51, 0.57) | <0.001 | 123.46 (121.42, 125.53) | 417.92 (415.19, 420.66) | 4.42 (3.85, 4.99) | <0.001 | 2.12 (1.86, 2.41) | 7.23 (6.88, 7.61) | 4.45 (3.88, 5.03) | <0.001 |
| Poland | 10.31 (9.60, 11.07) | 9.70 (8.96, 10.50) | -0.21 (-0.24, -0.18) | <0.001 | 126.90 (124.45, 129.40) | 133.42 (130.78, 136.11) | 0.20 (0.08, 0.32) | 0.001 | 164.24 (161.47, 167.05) | 31.73 (30.46, 33.06) | -5.67 (-6.65, -4.68) | <0.001 | 2.91 (2.56, 3.31) | 0.51 (0.36, 0.71) | -6.02 (-7.38, -4.63) | <0.001 |
| Portugal | 10.94 (9.50, 12.54) | 10.22 (8.69, 11.97) | -0.23 (-0.30, -0.15) | <0.001 | 112.33 (107.61, 117.20) | 143.60 (137.96, 149.43) | 0.91 (0.68, 1.15) | <0.001 | 102.08 (97.58, 106.73) | 23.33 (21.12, 25.72) | -4.95 (-5.64, -4.26) | <0.001 | 1.77 (1.23, 2.48) | 0.39 (0.16, 0.85) | -5.05 (-5.79, -4.30) | <0.001 |
| Puerto Rico | 56.38 (51.04, 62.13) | 57.16 (51.22, 63.63) | 0.05 (0.02, 0.08) | <0.001 | 315.42 (302.59, 328.66) | 319.49 (305.42, 334.08) | 0.04 (0.02, 0.06) | <0.001 | 141.09 (132.56, 150.02) | 53.97 (48.28, 60.18) | -4.01 (-4.30, -3.71) | <0.001 | 2.41 (1.42, 3.84) | 0.90 (0.31, 2.08) | -4.15 (-4.46, -3.84) | <0.001 |
| Qatar | 28.29 (17.24, 44.51) | 26.01 (21.25, 31.86) | -0.29 (-0.31, -0.28) | <0.001 | 187.35 (157.10, 222.35) | 173.41 (161.28, 186.54) | -0.27 (-0.31, -0.24) | <0.001 | 294.49 (255.50, 338.29) | 83.17 (73.88, 93.58) | -4.07 (-5.58, -2.53) | <0.001 | 5.06 (1.24, 14.75) | 1.36 (0.44, 3.74) | -4.22 (-5.74, -2.68) | <0.001 |
| Republic of Korea | 13.65 (12.95, 14.38) | 12.90 (12.14, 13.69) | -0.20 (-0.23, -0.17) | <0.001 | 97.16 (95.23, 99.13) | 105.24 (103.14, 107.38) | 0.31 (0.03, 0.58) | 0.028 | 340.40 (336.82, 344.02) | 19.32 (18.41, 20.27) | -9.50 (-10.10, -8.91) | <0.001 | 5.66 (5.20, 6.14) | 0.31 (0.21, 0.46) | -9.61 (-10.29, -8.93) | <0.001 |
| Republic of Moldova | 24.88 (21.78, 28.33) | 26.53 (22.84, 30.75) | 0.21 (0.17, 0.24) | <0.001 | 171.42 (163.29, 179.88) | 185.40 (175.84, 195.44) | 0.27 (0.25, 0.30) | <0.001 | 169.53 (161.33, 178.07) | 93.95 (87.11, 101.29) | -1.79 (-3.09, -0.48) | 0.008 | 2.96 (1.97, 4.32) | 1.64 (0.86, 3.02) | -1.76 (-3.06, -0.44) | 0.009 |
| Romania | 16.06 (14.87, 17.32) | 16.73 (15.27, 18.31) | 0.13 (0.10, 0.16) | <0.001 | 149.51 (145.92, 153.18) | 152.97 (148.64, 157.40) | 0.10 (-0.04, 0.24) | 0.150 | 181.98 (178.01, 186.03) | 103.36 (99.78, 107.05) | -1.77 (-2.46, -1.07) | <0.001 | 3.19 (2.68, 3.77) | 1.79 (1.35, 2.34) | -1.82 (-2.50, -1.14) | <0.001 |
| Russian Federation | 27.89 | 29.99 | 0.23 | <0.001 | 180.45 | 201.98 | 0.43 | <0.001 | 161.88 | 161.95 | 0.13 | 0.845 | 2.86 | 2.86 | 0.14 | 0.837 |

| | | | | | | | | | | | | | | | | |
|---|---|---|---|---|---|---|---|---|---|---|---|---|---|---|---|---|
| | (27.30, 28.49) | (29.32, 30.67) | (0.19, 0.28) | | (178.99, 181.92) | (200.33, 203.63) | (0.29, 0.57) | | (160.49, 163.29) | (160.48, 163.44) | (-1.15, 1.42) | | (2.68, 3.06) | (2.67, 3.07) | (-1.16, 1.45) | |
| Rwanda | 18.02 (15.72, 20.58) | 17.63 (16.03, 19.35) | -0.08 (-0.11, -0.05) | <0.001 | 84.54 (79.34, 90.01) | 81.43 (77.90, 85.08) | -0.14 (-0.21, -0.08) | <0.001 | 151.33 (144.56, 158.34) | 83.30 (79.84, 86.88) | -2.09 (-2.34, -1.84) | <0.001 | 2.58 (1.76, 3.67) | 1.39 (0.97, 1.93) | -2.17 (-2.41, -1.92) | <0.001 |
| Saint Kitts and Nevis | 55.79 (17.12, 137.65) | 56.18 (21.85, 120.66) | 0.03 (0.00, 0.06) | 0.024 | 280.64 (176.66, 425.12) | 283.58 (196.64, 398.03) | 0.04 (0.03, 0.05) | <0.001 | 372.13 (251.03, 533.12) | 45.58 (15.56, 106.14) | -7.04 (-7.44, -6.63) | <0.001 | 6.59 (0.01, 62.82) | 0.74 (0.00, 37.72) | -7.32 (-7.90, -6.73) | <0.001 |
| Saint Lucia | 58.94 (32.94, 98.88) | 58.54 (35.84, 90.88) | -0.02 (-0.04, 0.00) | 0.067 | 312.06 (244.48, 393.32) | 308.64 (253.34, 373.01) | -0.04 (-0.05, -0.03) | <0.001 | 136.62 (93.50, 193.70) | 51.36 (30.46, 81.87) | -3.34 (-3.78, -2.89) | <0.001 | 2.39 (0.00, 22.48) | 0.86 (0.00, 13.81) | -3.50 (-3.96, -3.04) | <0.001 |
| Saint Vincent and the Grenadines | 56.98 (28.66, 103.79) | 56.57 (28.99, 99.64) | -0.02 (-0.04, 0.01) | 0.122 | 277.44 (205.87, 367.04) | 275.62 (209.32, 356.54) | -0.02 (-0.04, 0.02) | 0.322 | 297.07 (224.17, 387.57) | 158.40 (109.21, 222.46) | -2.14 (-2.80, -1.48) | <0.001 | 5.18 (0.11, 32.82) | 2.75 (0.01, 23.57) | -2.16 (-2.83, -1.50) | <0.001 |
| Samoa | 21.27 (7.30, 49.54) | 20.99 (8.75, 42.88) | -0.04 (-0.05, -0.03) | <0.001 | 186.66 (136.01, 250.61) | 186.17 (143.34, 238.06) | -0.00 (-0.02, 0.01) | 0.662 | 296.11 (231.45, 373.85) | 448.41 (380.87, 524.78) | 1.44 (1.38, 1.51) | <0.001 | 5.23 (0.25, 26.35) | 7.91 (1.47, 24.76) | 1.44 (1.37, 1.51) | <0.001 |
| San Marino | 24.74 (0.84, 136.99) | 23.57 (1.19, 119.53) | -0.15 (-0.17, -0.12) | <0.001 | 162.01 (64.28, 339.45) | 170.74 (81.03, 323.08) | 0.19 (0.13, 0.25) | <0.001 | 27.14 (1.03, 142.30) | 24.32 (1.48, 119.73) | -0.37 (-0.51, -0.24) | <0.001 | 0.44 (0.00, 94.18) | 0.40 (0.00, 79.68) | -0.28 (-0.66, 0.11) | 0.159 |
| Sao Tome and Principe | 20.20 (5.00, 56.97) | 21.87 (9.83, 42.35) | 0.27 (0.26, 0.28) | <0.001 | 115.46 (69.61, 181.36) | 123.54 (90.95, 164.19) | 0.24 (0.19, 0.28) | <0.001 | 111.47 (66.67, 176.27) | 133.04 (99.39, 174.64) | 0.77 (-0.04, 1.59) | 0.061 | 1.97 (0.00, 29.00) | 2.34 (0.04, 14.37) | 0.74 (-0.06, 1.55) | 0.068 |
| Saudi Arabia | 28.45 (26.34, 30.69) | 30.63 (29.45, 31.85) | 0.26 (0.24, 0.27) | <0.001 | 179.74 (174.26, 185.36) | 203.25 (200.24, 206.30) | 0.43 (0.40, 0.47) | <0.001 | 430.35 (422.07, 438.77) | 382.52 (378.33, 386.75) | -0.43 (-0.77, -0.09) | 0.014 | 7.48 (6.42, 8.69) | 6.74 (6.19, 7.32) | -0.38 (-0.72, -0.05) | 0.025 |
| Senegal | 22.38 (19.81, 25.21) | 22.72 (20.98, 24.58) | 0.05 (0.04, 0.06) | <0.001 | 124.29 (117.92, 130.92) | 122.63 (118.42, 126.95) | -0.04 (-0.06, -0.02) | 0.001 | 137.18 (130.61, 144.00) | 114.77 (110.77, 118.88) | -0.66 (-0.97, -0.35) | <0.001 | 2.38 (1.58, 3.47) | 1.98 (1.48, 2.59) | -0.53 (-0.99, -0.07) | 0.024 |
| Serbia | 25.79 (23.46, 28.30) | 28.37 (25.69, 31.27) | 0.32 (0.27, 0.36) | <0.001 | 190.40 (184.09, 196.88) | 207.57 (200.38, 214.97) | 0.28 (0.22, 0.35) | <0.001 | 192.34 (185.96, 198.89) | 70.30 (66.15, 74.67) | -3.38 (-3.75, -3.01) | <0.001 | 3.35 (2.56, 4.33) | 1.20 (0.71, 1.92) | -3.44 (-3.85, -3.04) | <0.001 |
| Seychelles | 17.92 | 17.32 | -0.11 | <0.001 | 141.28 | 135.31 | -0.13 | 0.001 | 253.52 | 141.17 | -2.05 | <0.001 | 4.41 | 2.42 | -2.10 | <0.001 |

| | | | | | | | | | | | | | | | | |
|---|---|---|---|---|---|---|---|---|---|---|---|---|---|---|---|---|
| | (2.80, 63.98) | (3.73, 51.71) | (-0.15, -0.07) | | (83.43, 226.49) | (87.47, 201.42) | (-0.21, -0.05) | | (174.54, 358.72) | (91.80, 208.94) | (-2.74, -1.35) | | (0.01, 43.13) | (0.00, 28.21) | (-2.80, -1.39) | |
| Sierra Leone | 23.75 (20.18, 27.79) | 24.34 (21.96, 26.91) | 0.08 (0.07, 0.10) | <0.001 | 126.45 (117.80, 135.57) | 126.17 (120.51, 132.04) | 0.02 (-0.08, 0.11) | 0.754 | 156.48 (146.95, 166.48) | 207.86 (200.73, 215.19) | 1.01 (0.78, 1.24) | <0.001 | 2.73 (1.60, 4.39) | 3.62 (2.72, 4.72) | 1.01 (0.75, 1.27) | <0.001 |
| Singapore | 26.45 (22.93, 30.40) | 21.32 (18.61, 24.42) | -0.74 (-0.77, -0.70) | <0.001 | 172.54 (163.37, 182.15) | 142.42 (135.85, 149.35) | -0.66 (-0.68, -0.64) | <0.001 | 55.97 (50.79, 61.58) | 11.39 (9.60, 13.56) | -5.46 (-6.65, -4.25) | <0.001 | 0.98 (0.41, 2.05) | 0.19 (0.03, 0.92) | -5.68 (-7.13, -4.21) | <0.001 |
| Slovakia | 19.89 (17.26, 22.83) | 15.32 (12.91, 18.12) | -0.90 (-0.92, -0.88) | <0.001 | 179.24 (171.37, 187.41) | 142.96 (135.71, 150.58) | -0.78 (-0.81, -0.75) | <0.001 | 153.89 (146.61, 161.45) | 57.61 (53.01, 62.59) | -3.22 (-4.39, -2.03) | <0.001 | 2.70 (1.82, 3.89) | 0.97 (0.47, 1.93) | -3.35 (-4.53, -2.16) | <0.001 |
| Slovenia | 18.36 (14.37, 23.19) | 15.59 (11.48, 20.94) | -0.57 (-0.63, -0.51) | <0.001 | 183.50 (170.56, 197.23) | 155.19 (142.13, 169.33) | -0.58 (-0.61, -0.56) | <0.001 | 58.76 (51.56, 66.75) | 15.18 (11.30, 20.23) | -4.76 (-5.62, -3.89) | <0.001 | 0.98 (0.27, 2.65) | 0.21 (0.00, 2.12) | -5.40 (-6.46, -4.33) | <0.001 |
| Solomon Islands | 21.16 (10.42, 38.92) | 21.05 (13.72, 30.98) | -0.02 (-0.05, 0.02) | 0.421 | 146.64 (114.08, 185.94) | 146.79 (125.88, 170.23) | 0.02 (-0.07, 0.10) | 0.707 | 1721.53 (1606.89, 1842.55) | 1849.19 (1773.44, 1927.40) | 0.24 (0.12, 0.36) | <0.001 | 30.61 (17.01, 51.27) | 32.74 (23.34, 44.73) | 0.23 (0.11, 0.35) | <0.001 |
| Somalia | 20.95 (18.40, 23.77) | 22.29 (20.68, 23.99) | 0.21 (0.18, 0.23) | <0.001 | 101.20 (95.51, 107.16) | 107.28 (103.61, 111.05) | 0.20 (0.18, 0.22) | <0.001 | 154.83 (147.81, 162.10) | 200.59 (195.72, 205.55) | 0.92 (0.79, 1.05) | <0.001 | 2.69 (1.83, 3.81) | 3.47 (2.85, 4.18) | 0.90 (0.77, 1.03) | <0.001 |
| South Africa | 30.36 (29.11, 31.65) | 26.27 (25.37, 27.19) | -0.50 (-0.55, -0.46) | <0.001 | 125.81 (123.18, 128.47) | 106.97 (105.16, 108.82) | -0.55 (-0.60, -0.51) | <0.001 | 276.99 (273.14, 280.88) | 99.40 (97.65, 101.17) | -3.36 (-5.54, -1.13) | 0.003 | 4.89 (4.39, 5.44) | 1.73 (1.51, 1.98) | -3.42 (-5.63, -1.15) | 0.003 |
| South Sudan | 18.69 (15.96, 21.79) | 20.64 (18.44, 23.05) | 0.34 (0.31, 0.36) | <0.001 | 101.22 (94.44, 108.37) | 113.02 (107.72, 118.52) | 0.37 (0.31, 0.43) | <0.001 | 80.78 (74.98, 86.94) | 100.13 (95.32, 105.15) | 0.61 (0.15, 1.06) | 0.009 | 1.36 (0.70, 2.43) | 1.68 (1.11, 2.47) | 0.60 (0.16, 1.04) | 0.007 |
| Spain | 27.75 (26.56, 28.99) | 26.70 (25.45, 27.99) | -0.13 (-0.19, -0.06) | <0.001 | 163.68 (160.72, 166.69) | 182.32 (179.25, 185.43) | 0.38 (0.32, 0.43) | <0.001 | 82.22 (80.14, 84.35) | 27.69 (26.49, 28.94) | -3.73 (-4.04, -3.42) | <0.001 | 1.38 (1.12, 1.68) | 0.46 (0.32, 0.65) | -3.77 (-4.11, -3.42) | <0.001 |
| Sri Lanka | 17.93 (16.57, 19.38) | 17.37 (16.13, 18.67) | -0.11 (-0.12, -0.10) | <0.001 | 137.19 (133.34, 141.13) | 132.68 (129.27, 136.16) | -0.11 (-0.13, -0.08) | <0.001 | 313.96 (308.19, 319.82) | 137.63 (134.12, 141.22) | -2.67 (-4.13, -1.19) | <0.001 | 5.34 (4.61, 6.16) | 2.32 (1.88, 2.82) | -2.70 (-4.19, -1.19) | <0.001 |

| | | | | | | | | | | | | | | | | |
|---|---|---|---|---|---|---|---|---|---|---|---|---|---|---|---|---|
| Sudan | 30.29 (28.46, 32.21) | 31.95 (30.73, 33.21) | 0.18 (0.18, 0.19) | <0.001 | 181.02 (176.40, 185.73) | 199.86 (196.75, 203.01) | 0.37 (0.23, 0.50) | <0.001 | 982.80 (972.29, 993.39) | 558.78 (553.66, 563.94) | -1.91 (-2.01, -1.81) | <0.001 | 17.11 (15.73, 18.57) | 9.68 (9.02, 10.39) | -1.92 (-2.01, -1.84) | <0.001 |
| Suriname | 56.49 (40.34, 77.37) | 62.35 (48.43, 79.08) | 0.35 (0.32, 0.38) | <0.001 | 274.69 (235.98, 318.16) | 309.24 (277.27, 343.95) | 0.42 (0.33, 0.52) | <0.001 | 271.95 (233.76, 314.86) | 169.12 (145.67, 195.32) | -1.33 (-3.96, 1.37) | 0.331 | 4.78 (1.03, 14.13) | 2.94 (0.66, 8.41) | -1.35 (-4.17, 1.56) | 0.361 |
| Sweden | 27.46 (24.85, 30.29) | 33.01 (30.27, 35.95) | 0.64 (0.61, 0.67) | <0.001 | 187.08 (180.24, 194.12) | 211.60 (204.74, 218.67) | 0.42 (0.39, 0.46) | <0.001 | 62.20 (58.29, 66.32) | 18.58 (16.57, 20.78) | -4.07 (-4.95, -3.19) | <0.001 | 1.06 (0.60, 1.74) | 0.28 (0.09, 0.72) | -4.51 (-5.90, -3.09) | <0.001 |
| Switzerland | 23.15 (20.65, 25.91) | 22.04 (19.66, 24.67) | -0.15 (-0.19, -0.10) | <0.001 | 144.63 (138.34, 151.16) | 152.41 (146.32, 158.72) | 0.19 (0.14, 0.24) | <0.001 | 52.63 (48.81, 56.70) | 14.71 (12.85, 16.81) | -4.29 (-4.74, -3.83) | <0.001 | 0.88 (0.46, 1.61) | 0.23 (0.06, 0.72) | -4.42 (-5.05, -3.79) | <0.001 |
| Syrian Arab Republic | 28.85 (26.46, 31.40) | 29.90 (27.80, 32.13) | 0.12 (0.10, 0.14) | <0.001 | 186.00 (179.70, 192.48) | 201.43 (195.98, 207.02) | 0.30 (0.19, 0.41) | <0.001 | 1586.85 (1569.36, 1604.50) | 893.57 (882.10, 905.17) | -1.91 (-2.49, -1.33) | <0.001 | 27.12 (24.85, 29.55) | 15.23 (13.76, 16.83) | -1.93 (-2.51, -1.34) | <0.001 |
| Taiwan (Province of China) | 15.38 (14.27, 16.55) | 15.42 (14.26, 16.66) | 0.02 (-0.01, 0.04) | 0.255 | 207.38 (203.26, 211.57) | 214.90 (210.59, 219.30) | 0.13 (0.03, 0.24) | 0.012 | 85.93 (83.28, 88.66) | 43.06 (41.13, 45.07) | -2.28 (-2.96, -1.59) | <0.001 | 1.36 (1.04, 1.74) | 0.61 (0.40, 0.91) | -2.69 (-3.66, -1.72) | <0.001 |
| Tajikistan | 30.08 (26.67, 33.84) | 29.38 (27.03, 31.89) | -0.08 (-0.11, -0.05) | <0.001 | 188.79 (179.69, 198.24) | 181.02 (174.98, 187.22) | -0.13 (-0.18, -0.09) | <0.001 | 370.46 (357.76, 383.51) | 333.35 (325.17, 341.68) | -0.29 (-0.91, 0.33) | 0.361 | 6.57 (4.97, 8.56) | 5.94 (4.89, 7.16) | -0.27 (-0.87, 0.34) | 0.387 |
| Thailand | 15.47 (14.78, 16.17) | 15.36 (14.70, 16.05) | -0.02 (-0.04, 0.01) | 0.232 | 109.84 (107.98, 111.72) | 109.64 (107.88, 111.42) | 0.00 (-0.04, 0.05) | 0.962 | 161.42 (159.18, 163.69) | 86.41 (84.81, 88.03) | -2.14 (-3.61, -0.64) | 0.005 | 2.81 (2.52, 3.12) | 1.45 (1.25, 1.68) | -2.26 (-3.77, -0.73) | 0.004 |
| Timor-Leste | 16.97 (10.89, 25.40) | 17.30 (12.22, 23.93) | 0.07 (0.04, 0.09) | <0.001 | 112.55 (95.45, 131.93) | 114.90 (100.66, 130.68) | 0.10 (-0.09, 0.29) | 0.283 | 162.62 (142.18, 185.30) | 224.88 (205.47, 245.76) | 1.30 (0.58, 2.02) | <0.001 | 2.82 (0.75, 7.52) | 3.87 (1.70, 7.72) | 1.23 (0.54, 1.93) | <0.001 |
| Togo | 21.64 (18.14, 25.64) | 23.04 (20.74, 25.52) | 0.22 (0.20, 0.23) | <0.001 | 110.61 (102.27, 119.47) | 116.11 (110.83, 121.58) | 0.19 (0.10, 0.28) | <0.001 | 167.01 (156.93, 177.59) | 152.98 (146.96, 159.19) | -0.30 (-0.68, 0.08) | 0.120 | 2.91 (1.71, 4.66) | 2.66 (1.91, 3.59) | -0.30 (-0.66, 0.06) | 0.106 |
| Tokelau | 20.34 (0.00, | 20.52 (0.00, | 0.03 (0.00, | 0.035 | 167.71 (0.07, | 174.69 (0.01, | 0.15 (0.12, | <0.001 | 434.52 (15.92, | 531.40 (16.96, | 0.70 (0.66, | <0.001 | 7.73 (0.00, | 9.40 (0.00, | 0.68 (0.64, | <0.001 |

| | | | | | | | | | | | | | | | | |
|---|---|---|---|---|---|---|---|---|---|---|---|---|---|---|---|---|
| | 1740.70 ) | 2344.12 ) | 0.06) | | 1998.35) | 2618.99) | 0.17) | | 2445.71) | 3193.63) | 0.75) | | 1718.23 ) | 2324.81 ) | 0.73) | |
| Tonga | 21.25 (4.70, 62.69) | 21.39 (5.71, 55.87) | 0.03 (-0.03, 0.10) | 0.349 | 199.07 (132.62, 288.16) | 203.22 (142.63, 281.05) | 0.07 (0.05, 0.10) | <0.001 | 144.55 (88.83, 223.22) | 139.35 (90.21, 205.89) | -0.10 (-0.35, 0.14) | 0.401 | 2.50 (0.00, 33.35) | 2.37 (0.00, 25.80) | -0.16 (-0.42, 0.11) | 0.254 |
| Trinidad and Tobago | 67.58 (57.67, 78.75) | 65.81 (56.44, 76.43) | -0.09 (-0.11, -0.08) | <0.001 | 364.33 (340.41, 389.52) | 353.04 (331.70, 375.56) | -0.09 (-0.17, -0.01) | 0.028 | 194.08 (176.72, 212.73) | 109.49 (97.59, 122.61) | -2.15 (-2.70, -1.60) | <0.001 | 3.39 (1.46, 6.75) | 1.88 (0.64, 4.57) | -2.13 (-2.75, -1.50) | <0.001 |
| Tunisia | 27.46 (24.87, 30.26) | 27.44 (25.41, 29.61) | -0.00 (-0.02, 0.01) | 0.816 | 176.24 (169.44, 183.26) | 182.50 (177.29, 187.84) | 0.12 (0.10, 0.14) | <0.001 | 373.14 (363.45, 383.04) | 223.68 (217.76, 229.75) | -1.69 (-1.91, -1.47) | <0.001 | 6.47 (5.24, 7.92) | 3.84 (3.10, 4.72) | -1.73 (-1.94, -1.51) | <0.001 |
| Turkey | 21.53 (20.66, 22.42) | 17.59 (16.97, 18.23) | -0.70 (-0.72, -0.67) | <0.001 | 188.70 (186.09, 191.34) | 148.35 (146.56, 150.17) | -0.81 (-0.87, -0.75) | <0.001 | 223.42 (220.60, 226.26) | 85.29 (83.91, 86.69) | -3.29 (-4.02, -2.55) | <0.001 | 3.87 (3.51, 4.27) | 1.42 (1.25, 1.62) | -3.42 (-4.12, -2.71) | <0.001 |
| Turkmenistan | 29.71 (25.86, 34.01) | 30.13 (26.77, 33.81) | 0.05 (0.02, 0.07) | <0.001 | 183.50 (173.40, 194.06) | 188.12 (179.55, 197.00) | 0.09 (0.03, 0.16) | 0.004 | 437.52 (422.00, 453.50) | 411.71 (398.94, 424.79) | -0.14 (-1.36, 1.11) | 0.831 | 7.71 (5.76, 10.14) | 7.15 (5.56, 9.08) | -0.17 (-1.39, 1.06) | 0.784 |
| Tuvalu | 20.60 (0.00, 243.27) | 20.88 (0.01, 234.98) | 0.05 (0.03, 0.07) | <0.001 | 161.39 (34.47, 471.22) | 168.61 (39.30, 472.67) | 0.17 (0.08, 0.26) | <0.001 | 517.46 (247.66, 958.36) | 552.57 (277.87, 989.89) | 0.24 (0.16, 0.32) | <0.001 | 9.20 (0.00, 221.79) | 9.79 (0.00, 214.89) | 0.23 (0.15, 0.31) | <0.001 |
| Uganda | 18.66 (17.04, 20.40) | 18.58 (17.58, 19.63) | -0.02 (-0.04, 0.01) | 0.236 | 82.35 (78.76, 86.08) | 82.32 (80.11, 84.58) | -0.01 (-0.04, 0.02) | 0.653 | 54.21 (51.45, 57.10) | 93.29 (91.06, 95.56) | 1.95 (1.57, 2.33) | <0.001 | 0.90 (0.58, 1.37) | 1.56 (1.28, 1.89) | 1.96 (1.54, 2.37) | <0.001 |
| Ukraine | 27.90 (26.88, 28.96) | 29.84 (28.64, 31.09) | 0.23 (0.21, 0.25) | <0.001 | 195.38 (192.71, 198.09) | 209.43 (206.39, 212.52) | 0.26 (0.18, 0.35) | <0.001 | 136.26 (134.01, 138.55) | 254.35 (250.90, 257.84) | 2.21 (-0.70, 5.21) | 0.139 | 2.36 (2.08, 2.68) | 4.45 (4.01, 4.94) | 2.24 (-0.72, 5.29) | 0.140 |
| United Arab Emirates | 29.31 (23.51, 36.20) | 29.75 (26.76, 33.07) | 0.05 (0.03, 0.08) | <0.001 | 192.74 (177.26, 209.29) | 199.85 (192.58, 207.40) | 0.13 (0.09, 0.17) | <0.001 | 240.09 (222.78, 258.46) | 165.99 (158.06, 174.27) | -1.30 (-1.68, -0.92) | <0.001 | 3.92 (2.01, 7.02) | 2.73 (1.81, 4.03) | -1.28 (-1.70, -0.85) | <0.001 |
| United Kingdom | 26.33 (25.37, 27.32) | 24.99 (24.07, 25.93) | -0.17 (-0.21, -0.14) | <0.001 | 168.27 (165.83, 170.74) | 173.24 (170.89, 175.61) | 0.12 (-0.00, 0.23) | 0.051 | 75.56 (73.92, 77.22) | 38.74 (37.64, 39.86) | -2.20 (-2.36, -2.04) | <0.001 | 1.32 (1.11, 1.55) | 0.67 (0.54, 0.84) | -2.19 (-2.39, -1.99) | <0.001 |
| United Republic of | 19.41 | 21.99 | 0.43 | <0.001 | 88.99 | 102.01 | 0.48 | <0.001 | 109.09 | 112.60 | 0.14 | 0.302 | 1.81 | 1.89 | 0.19 | 0.224 |

| | | | | | | | | | | | | | | | | |
|---|---|---|---|---|---|---|---|---|---|---|---|---|---|---|---|---|
| Tanzania | (18.10, 20.79) | (21.09, 22.92) | (0.41, 0.44) | | (86.06, 92.00) | (100.01, 104.05) | (0.44, 0.51) | | (106.07, 112.19) | (110.58, 114.65) | (-0.13, 0.42) | | (1.43, 2.26) | (1.64, 2.18) | (-0.12, 0.50) | |
| United States of America | 29.95 (29.49, 30.41) | 33.01 (32.53, 33.49) | 0.36 (0.26, 0.46) | <0.001 | 151.89 (150.87, 152.92) | 189.73 (188.61, 190.86) | 0.77 (0.68, 0.87) | <0.001 | 101.56 (100.72, 102.41) | 95.64 (94.85, 96.44) | -0.18 (-0.49, 0.14) | 0.270 | 1.76 (1.65, 1.87) | 1.67 (1.56, 1.78) | -0.16 (-0.50, 0.17) | 0.334 |
| United States Virgin Islands | 52.73 (26.23, 95.30) | 53.37 (24.01, 104.02) | 0.04 (0.03, 0.06) | <0.001 | 279.90 (213.49, 361.20) | 282.70 (208.87, 375.80) | 0.03 (0.00, 0.05) | 0.032 | 190.96 (136.90, 260.07) | 83.33 (45.85, 141.14) | -2.78 (-3.16, -2.41) | <0.001 | 3.34 (0.02, 25.48) | 1.43 (0.00, 28.60) | -2.84 (-3.27, -2.41) | <0.001 |
| Uruguay | 16.17 (13.04, 19.83) | 14.59 (11.74, 17.95) | -0.35 (-0.37, -0.34) | <0.001 | 108.73 (100.34, 117.63) | 103.74 (95.94, 112.03) | -0.14 (-0.26, -0.02) | 0.022 | 99.54 (91.52, 108.07) | 34.68 (30.24, 39.60) | -3.66 (-4.05, -3.26) | <0.001 | 1.73 (0.82, 3.20) | 0.59 (0.15, 1.61) | -3.70 (-4.11, -3.29) | <0.001 |
| Uzbekistan | 26.66 (25.08, 28.32) | 27.70 (26.50, 28.95) | 0.13 (0.12, 0.14) | <0.001 | 165.39 (161.29, 169.57) | 174.41 (171.35, 177.51) | 0.19 (0.13, 0.26) | <0.001 | 311.10 (305.55, 316.73) | 603.80 (598.12, 609.52) | 2.43 (1.76, 3.10) | <0.001 | 5.45 (4.73, 6.25) | 10.48 (9.74, 11.26) | 2.40 (1.73, 3.07) | <0.001 |
| Vanuatu | 23.42 (8.79, 51.33) | 22.99 (12.19, 39.83) | -0.06 (-0.09, -0.04) | <0.001 | 196.28 (145.62, 259.34) | 193.82 (158.38, 235.03) | -0.03 (-0.07, 0.01) | 0.100 | 323.40 (257.67, 401.31) | 539.10 (479.26, 604.57) | 1.73 (1.43, 2.03) | <0.001 | 5.69 (0.38, 25.86) | 9.52 (3.16, 22.32) | 1.74 (1.43, 2.05) | <0.001 |
| Venezuela (Bolivarian Republic of) | 22.47 (20.99, 24.03) | 19.38 (18.25, 20.57) | -0.51 (-0.56, -0.46) | <0.001 | 153.16 (149.18, 157.23) | 136.06 (133.08, 139.10) | -0.40 (-0.44, -0.37) | <0.001 | 291.05 (285.59, 296.60) | 229.56 (225.62, 233.54) | -1.07 (-2.47, 0.35) | 0.139 | 5.11 (4.40, 5.89) | 3.93 (3.43, 4.48) | -1.16 (-2.60, 0.30) | 0.119 |
| Viet Nam | 16.53 (15.85, 17.24) | 16.37 (15.83, 16.93) | -0.02 (-0.05, 0.00) | 0.047 | 130.06 (128.09, 132.06) | 129.52 (128.00, 131.05) | -0.01 (-0.04, 0.02) | 0.605 | 144.14 (142.07, 146.23) | 74.51 (73.34, 75.70) | -2.24 (-2.38, -2.11) | <0.001 | 2.52 (2.25, 2.81) | 1.26 (1.11, 1.42) | -2.37 (-2.52, -2.22) | <0.001 |
| Yemen | 30.67 (28.35, 33.14) | 31.36 (29.97, 32.80) | 0.08 (0.06, 0.09) | <0.001 | 181.89 (176.07, 187.85) | 191.15 (187.67, 194.68) | 0.20 (0.02, 0.38) | 0.027 | 652.16 (641.20, 663.28) | 499.84 (494.24, 505.49) | -0.87 (-1.39, -0.34) | 0.001 | 11.60 (10.17, 13.17) | 8.80 (8.07, 9.58) | -0.90 (-1.09, -0.72) | <0.001 |
| Zambia | 20.46 (17.98, 23.21) | 20.50 (19.00, 22.10) | 0.01 (-0.02, 0.03) | 0.596 | 86.52 (81.09, 92.23) | 87.49 (84.27, 90.82) | 0.04 (0.02, 0.06) | <0.001 | 271.17 (261.95, 280.66) | 176.32 (171.88, 180.85) | -1.48 (-1.83, -1.13) | <0.001 | 4.69 (3.53, 6.13) | 3.03 (2.47, 3.69) | -1.50 (-1.85, -1.15) | <0.001 |
| Zimbabwe | 24.65 (22.35, 27.14) | 26.43 (24.62, 28.34) | 0.24 (0.21, 0.28) | <0.001 | 94.86 (90.13, 99.77) | 103.72 (100.05, 107.49) | 0.31 (0.29, 0.32) | <0.001 | 61.62 (57.95, 65.48) | 105.63 (101.99, 109.37) | 1.86 (0.07, 3.69) | 0.042 | 1.05 (0.61, 1.68) | 1.82 (1.37, 2.38) | 1.95 (0.70, 3.22) | 0.002 |
| **Men** | | | | | | | | | | | | | | | | |

| | | | | | | | | | | | | | | | | |
|---|---|---|---|---|---|---|---|---|---|---|---|---|---|---|---|---|
| Afghanistan | 47.50 (43.56, 51.72) | 49.56 (47.77, 51.40) | 0.14 (0.12, 0.16) | <0.001 | 291.57 (281.43, 302.00) | 311.44 (306.77, 316.16) | 0.25 (0.11, 0.40) | 0.001 | 1459.36 (1436.89, 1482.13) | 1051.58 (1043.06, 1060.15) | -1.12 (-1.37, -0.88) | <0.001 | 26.19 (23.24, 29.45) | 18.85 (17.71, 20.04) | -1.13 (-1.37, -0.88) | <0.001 |
| Albania | 26.56 (22.81, 30.77) | 23.78 (19.65, 28.54) | -0.38 (-0.40, -0.35) | <0.001 | 177.72 (167.64, 188.27) | 162.11 (150.88, 173.98) | -0.32 (-0.34, -0.30) | <0.001 | 449.97 (434.21, 466.18) | 762.43 (738.35, 787.11) | 1.67 (0.61, 2.75) | 0.002 | 7.64 (5.70, 10.05) | 13.01 (10.02, 16.63) | 1.69 (0.63, 2.75) | 0.002 |
| Algeria | 40.23 (38.35, 42.19) | 40.14 (38.86, 41.45) | -0.01 (-0.05, 0.02) | 0.386 | 260.87 (255.92, 265.90) | 266.06 (262.79, 269.37) | 0.08 (0.00, 0.17) | 0.047 | 1275.58 (1264.82, 1286.41) | 596.86 (591.88, 601.87) | -2.59 (-2.80, -2.38) | <0.001 | 22.48 (21.06, 23.98) | 10.43 (9.78, 11.11) | -2.62 (-2.83, -2.40) | <0.001 |
| American Samoa | 30.39 (5.70, 95.49) | 31.83 (6.07, 99.89) | 0.15 (0.11, 0.18) | <0.001 | 247.36 (154.26, 377.17) | 267.86 (169.17, 404.76) | 0.27 (0.24, 0.29) | <0.001 | 852.14 (669.22, 1070.62) | 921.76 (728.54, 1151.89) | 0.25 (-0.04, 0.55) | 0.090 | 15.39 (0.85, 72.93) | 16.57 (1.02, 77.00) | 0.24 (-0.06, 0.53) | 0.119 |
| Andorra | 33.60 (10.78, 87.16) | 29.63 (8.45, 81.11) | -0.43 (-0.48, -0.39) | <0.001 | 169.11 (109.70, 255.19) | 164.41 (106.57, 247.99) | -0.10 (-0.14, -0.05) | <0.001 | 170.30 (110.23, 257.22) | 102.47 (58.49, 172.86) | -1.74 (-1.80, -1.69) | <0.001 | 3.02 (0.00, 43.74) | 1.82 (0.00, 40.25) | -1.75 (-1.80, -1.69) | <0.001 |
| Angola | 35.55 (32.83, 38.45) | 34.21 (32.54, 35.95) | -0.13 (-0.14, -0.13) | <0.001 | 166.41 (160.31, 172.68) | 163.58 (159.79, 167.44) | -0.06 (-0.07, -0.05) | <0.001 | 434.80 (425.07, 444.71) | 360.02 (354.50, 365.61) | -0.59 (-1.28, 0.10) | 0.093 | 7.65 (6.40, 9.08) | 6.33 (5.61, 7.11) | -0.60 (-1.29, 0.09) | 0.090 |
| Antigua and Barbuda | 64.85 (27.44, 131.42) | 63.61 (31.78, 114.57) | -0.07 (-0.09, -0.05) | <0.001 | 337.26 (239.04, 463.27) | 326.72 (247.36, 424.11) | -0.13 (-0.20, -0.05) | 0.001 | 358.45 (257.32, 487.20) | 169.82 (114.20, 243.80) | -2.44 (-4.16, -0.68) | 0.007 | 6.33 (0.05, 46.84) | 2.93 (0.00, 28.61) | -2.52 (-4.17, -0.84) | 0.003 |
| Argentina | 19.50 (18.39, 20.66) | 18.51 (17.62, 19.43) | -0.18 (-0.20, -0.16) | <0.001 | 92.91 (90.46, 95.40) | 90.95 (88.97, 92.96) | -0.08 (-0.15, -0.02) | 0.012 | 532.53 (526.67, 538.43) | 235.01 (231.81, 238.24) | -2.79 (-3.09, -2.48) | <0.001 | 9.44 (8.68, 10.26) | 4.13 (3.71, 4.58) | -2.83 (-3.15, -2.52) | <0.001 |
| Armenia | 43.90 (39.14, 49.09) | 41.87 (36.91, 47.42) | -0.16 (-0.17, -0.14) | <0.001 | 244.61 (233.11, 256.56) | 239.43 (227.53, 251.89) | -0.08 (-0.11, -0.06) | <0.001 | 810.42 (789.33, 831.95) | 544.12 (525.95, 562.85) | -1.36 (-2.45, -0.26) | 0.015 | 14.70 (11.98, 17.88) | 9.76 (7.47, 12.65) | -1.41 (-2.48, -0.32) | 0.011 |
| Australia | 87.65 (84.57, 90.81) | 79.67 (77.08, 82.34) | -0.33 (-0.37, -0.29) | <0.001 | 359.84 (353.62, 366.15) | 351.97 (346.57, 357.45) | -0.14 (-0.48, 0.20) | 0.420 | 282.22 (276.72, 287.82) | 139.08 (135.70, 142.53) | -2.40 (-3.03, -1.78) | <0.001 | 5.07 (4.36, 5.87) | 2.50 (2.06, 3.01) | -2.41 (-2.95, -1.86) | <0.001 |
| Austria | 38.48 | 32.61 | -0.59 | <0.001 | 183.19 | 167.06 | -0.31 | <0.001 | 286.55 | 106.83 | -3.38 | <0.001 | 5.14 | 1.90 | -3.42 | <0.001 |

| | | | | | | | | | | | | | | | | |
|---|---|---|---|---|---|---|---|---|---|---|---|---|---|---|---|---|
| | (35.46, 41.71) | (29.84, 35.60) | (-0.63, -0.54) | | (176.47, 190.12) | (160.80, 173.53) | (-0.38, -0.25) | | (278.09, 295.22) | (101.84, 112.03) | (-4.31, -2.45) | | (4.06, 6.44) | (1.29, 2.74) | (-4.34, -2.48) | |
| Azerbaijan | 45.36 (41.91, 49.04) | 42.04 (39.45, 44.78) | -0.26 (-0.28, -0.23) | <0.001 | 246.95 (238.61, 255.52) | 234.15 (228.02, 240.42) | -0.19 (-0.20, -0.17) | <0.001 | 1072.89 (1055.45, 1090.56) | 768.50 (757.22, 779.93) | -1.37 (-2.47, -0.26) | 0.016 | 19.37 (17.08, 21.90) | 13.64 (12.17, 15.25) | -1.14 (-2.44, 0.18) | 0.090 |
| Bahamas | 66.85 (46.82, 93.02) | 68.68 (51.13, 90.43) | 0.10 (0.06, 0.13) | <0.001 | 319.91 (272.61, 373.32) | 323.88 (284.28, 367.56) | 0.05 (0.03, 0.06) | <0.001 | 525.53 (464.41, 592.73) | 415.31 (370.31, 464.39) | -0.93 (-2.21, 0.36) | 0.157 | 9.39 (2.92, 22.85) | 7.41 (2.58, 16.83) | -0.95 (-2.26, 0.38) | 0.161 |
| Bahrain | 47.25 (37.61, 59.44) | 42.90 (36.74, 50.11) | -0.34 (-0.35, -0.32) | <0.001 | 341.75 (315.36, 370.47) | 310.17 (294.61, 326.65) | -0.33 (-0.39, -0.26) | <0.001 | 1114.91 (1065.20, 1166.98) | 331.60 (313.61, 350.61) | -4.03 (-4.80, -3.25) | <0.001 | 19.42 (13.41, 28.15) | 5.65 (3.57, 8.91) | -4.18 (-5.02, -3.34) | <0.001 |
| Bangladesh | 27.52 (26.78, 28.28) | 28.04 (27.46, 28.63) | 0.06 (0.05, 0.07) | <0.001 | 177.22 (175.28, 179.18) | 187.62 (186.11, 189.15) | 0.19 (0.16, 0.22) | <0.001 | 414.32 (411.39, 417.27) | 366.41 (364.30, 368.53) | -0.41 (-0.94, 0.13) | 0.140 | 7.32 (6.93, 7.72) | 6.49 (6.22, 6.78) | -0.41 (-0.92, 0.11) | 0.119 |
| Barbados | 66.28 (46.34, 92.03) | 68.45 (47.51, 95.72) | 0.12 (0.09, 0.14) | <0.001 | 347.50 (299.17, 401.51) | 357.19 (307.47, 412.94) | 0.09 (0.07, 0.11) | <0.001 | 334.91 (287.49, 388.00) | 161.31 (128.37, 200.38) | -2.44 (-3.67, -1.19) | <0.001 | 5.92 (1.28, 17.08) | 2.78 (0.18, 12.86) | -2.56 (-4.00, -1.10) | 0.001 |
| Belarus | 44.22 (41.42, 47.18) | 42.19 (39.22, 45.37) | -0.17 (-0.20, -0.14) | <0.001 | 207.20 (201.13, 213.43) | 197.62 (191.33, 204.11) | -0.17 (-0.21, -0.12) | <0.001 | 936.99 (924.03, 950.11) | 706.34 (694.48, 718.40) | -0.92 (-2.04, 0.21) | 0.112 | 17.11 (15.40, 18.97) | 12.88 (11.33, 14.64) | -0.92 (-2.06, 0.23) | 0.116 |
| Belgium | 37.47 (34.84, 40.26) | 33.44 (30.88, 36.17) | -0.39 (-0.42, -0.37) | <0.001 | 181.84 (176.03, 187.81) | 181.42 (175.49, 187.52) | -0.01 (-0.04, 0.03) | 0.738 | 337.03 (329.04, 345.17) | 95.27 (90.98, 99.73) | -4.26 (-5.05, -3.47) | <0.001 | 5.97 (4.95, 7.15) | 1.66 (1.14, 2.37) | -4.32 (-4.76, -3.89) | <0.001 |
| Belize | 67.82 (42.10, 104.46) | 73.45 (56.06, 94.67) | 0.28 (0.26, 0.29) | <0.001 | 321.29 (259.41, 394.00) | 350.40 (310.32, 394.28) | 0.30 (0.29, 0.32) | <0.001 | 319.97 (260.07, 390.31) | 302.95 (266.02, 343.65) | -0.20 (-1.17, 0.79) | 0.695 | 5.50 (0.49, 23.52) | 5.30 (1.51, 13.41) | -0.13 (-1.16, 0.92) | 0.808 |
| Benin | 31.32 (27.23, 35.88) | 32.93 (30.46, 35.55) | 0.17 (0.16, 0.19) | <0.001 | 179.27 (169.02, 189.99) | 189.00 (182.80, 195.36) | 0.19 (0.13, 0.26) | <0.001 | 305.80 (292.52, 319.54) | 288.75 (281.19, 296.47) | -0.15 (-0.44, 0.14) | 0.316 | 5.41 (3.76, 7.55) | 5.08 (4.12, 6.22) | -0.17 (-0.46, 0.12) | 0.257 |
| Bermuda | 60.82 (26.86, | 57.76 (20.20, | -0.18 (-0.26, | <0.001 | 328.80 (241.39, | 307.39 (210.93, | -0.27 (-0.41, | <0.001 | 456.50 (350.98, | 144.96 (80.81, | -4.21 (-4.80, | <0.001 | 7.98 (0.28, | 2.49 (0.00, | -4.68 (-5.08, | <0.001 |

| | | | | | | | | | | | | | | | | |
|---|---|---|---|---|---|---|---|---|---|---|---|---|---|---|---|---|
| | 124.85) | 137.02) | -0.10) | | 442.77) | 439.66) | -0.12) | | 588.54) | 247.09) | -3.61) | | 52.05) | 56.13) | -4.27) | |
| Bhutan | 30.31 (21.03, 42.58) | 29.18 (21.95, 38.13) | -0.13 (-0.14, -0.12) | <0.001 | 189.36 (163.90, 217.81) | 186.92 (167.68, 207.85) | -0.05 (-0.10, -0.00) | 0.043 | 510.17 (468.66, 554.56) | 503.19 (471.43, 536.60) | -0.07 (-0.20, 0.07) | 0.312 | 9.01 (4.26, 17.01) | 8.74 (5.02, 14.28) | -0.13 (-0.26, 0.01) | 0.063 |
| Bolivia (Plurinational State of) | 14.18 (12.03, 16.61) | 13.94 (12.48, 15.54) | -0.05 (-0.07, -0.03) | <0.001 | 110.07 (103.76, 116.67) | 113.07 (108.75, 117.51) | 0.09 (0.08, 0.11) | <0.001 | 403.85 (392.00, 415.99) | 241.20 (234.97, 247.56) | -1.76 (-2.00, -1.51) | <0.001 | 6.96 (5.47, 8.74) | 4.09 (3.31, 5.00) | -1.82 (-2.06, -1.57) | <0.001 |
| Bosnia and Herzegovina | 31.72 (28.35, 35.39) | 29.03 (24.74, 33.89) | -0.30 (-0.31, -0.29) | <0.001 | 239.86 (230.42, 249.60) | 226.78 (214.67, 239.45) | -0.19 (-0.22, -0.17) | <0.001 | 601.87 (586.85, 617.19) | 297.95 (283.96, 312.50) | -2.69 (-3.19, -2.18) | <0.001 | 10.77 (8.84, 13.00) | 5.22 (3.53, 7.53) | -2.77 (-3.27, -2.26) | <0.001 |
| Botswana | 37.41 (29.33, 47.13) | 39.17 (34.06, 44.85) | 0.16 (0.13, 0.19) | <0.001 | 177.00 (158.23, 197.45) | 193.68 (182.20, 205.71) | 0.31 (0.30, 0.32) | <0.001 | 307.58 (282.75, 334.06) | 301.10 (286.72, 316.04) | 0.01 (-0.36, 0.38) | 0.959 | 5.50 (2.64, 10.20) | 5.37 (3.61, 7.72) | 0.00 (-0.37, 0.38) | 0.989 |
| Brazil | 23.41 (22.85, 23.97) | 20.51 (20.09, 20.93) | -0.45 (-0.50, -0.40) | <0.001 | 177.12 (175.55, 178.71) | 175.63 (174.42, 176.85) | -0.03 (-0.06, -0.00) | 0.024 | 611.23 (608.31, 614.15) | 339.78 (338.07, 341.49) | -2.00 (-2.24, -1.77) | <0.001 | 10.94 (10.55, 11.34) | 5.91 (5.69, 6.14) | -2.11 (-2.36, -1.86) | <0.001 |
| Brunei Darussalam | 31.19 (19.29, 48.09) | 29.17 (20.19, 41.17) | -0.23 (-0.25, -0.21) | <0.001 | 155.16 (126.70, 188.39) | 150.08 (128.87, 174.13) | -0.08 (-0.23, 0.08) | 0.332 | 982.64 (908.66, 1061.30) | 522.84 (482.20, 566.31) | -2.25 (-2.88, -1.62) | <0.001 | 16.83 (8.46, 30.38) | 9.26 (4.61, 17.12) | -2.14 (-2.77, -1.51) | <0.001 |
| Bulgaria | 28.42 (25.84, 31.21) | 27.74 (24.77, 31.01) | -0.08 (-0.12, -0.05) | <0.001 | 199.25 (192.38, 206.30) | 201.30 (193.47, 209.43) | 0.04 (0.02, 0.07) | 0.003 | 1081.30 (1065.15, 1097.65) | 719.27 (704.20, 734.64) | -1.31 (-1.60, -1.02) | <0.001 | 19.35 (17.24, 21.64) | 12.87 (10.94, 15.11) | -1.30 (-1.60, -1.00) | <0.001 |
| Burkina Faso | 29.80 (26.73, 33.14) | 30.61 (28.79, 32.52) | 0.09 (0.09, 0.10) | <0.001 | 174.45 (166.67, 182.51) | 180.08 (175.50, 184.75) | 0.13 (0.07, 0.19) | <0.001 | 285.42 (275.56, 295.56) | 333.37 (327.21, 339.63) | 0.64 (0.30, 0.98) | <0.001 | 5.06 (3.82, 6.60) | 5.90 (5.10, 6.79) | 0.63 (0.28, 0.98) | <0.001 |
| Burundi | 34.27 (30.54, 38.35) | 35.45 (32.91, 38.13) | 0.11 (0.10, 0.13) | <0.001 | 194.24 (184.94, 203.90) | 200.51 (194.30, 206.87) | 0.12 (0.05, 0.19) | 0.001 | 465.70 (451.44, 480.30) | 402.07 (393.40, 410.90) | -0.55 (-0.87, -0.23) | 0.001 | 8.18 (6.37, 10.35) | 7.01 (5.90, 8.27) | -0.57 (-0.89, -0.26) | <0.001 |
| Cabo Verde | 31.35 (16.98, 54.65) | 33.51 (24.37, 45.08) | 0.23 (0.21, 0.24) | <0.001 | 191.93 (150.47, 242.16) | 208.41 (184.49, 234.69) | 0.29 (0.28, 0.31) | <0.001 | 402.31 (340.60, 472.71) | 238.73 (212.91, 266.93) | -1.96 (-2.33, -1.59) | <0.001 | 7.25 (1.30, 23.87) | 4.13 (1.41, 9.57) | -2.09 (-2.51, -1.65) | <0.001 |
| Cambodia | 30.84 | 28.19 | -0.31 | <0.001 | 186.89 | 171.93 | -0.28 | <0.001 | 545.87 | 420.08 | -0.90 | <0.001 | 9.66 | 7.35 | -0.94 | <0.001 |

| | | | | | | | | | | | | | | | | |
|---|---|---|---|---|---|---|---|---|---|---|---|---|---|---|---|---|
| | (28.04, 33.87) | (26.46, 30.01) | (-0.35, -0.27) | | (179.66, 194.34) | (167.58, 176.38) | (-0.32, -0.24) | | (533.71, 558.26) | (413.30, 426.94) | (-1.03, -0.76) | | (8.09, 11.46) | (6.48, 8.32) | (-1.07, -0.80) | |
| Cameroon | 27.57 (25.01, 30.34) | 31.00 (29.51, 32.55) | 0.41 (0.39, 0.44) | <0.001 | 148.20 (141.99, 154.61) | 168.62 (165.03, 172.27) | 0.46 (0.41, 0.50) | <0.001 | 200.92 (193.80, 208.24) | 300.25 (295.52, 305.04) | 1.40 (1.25, 1.55) | <0.001 | 3.52 (2.63, 4.63) | 5.28 (4.66, 5.95) | 1.41 (1.26, 1.57) | <0.001 |
| Canada | 47.90 (46.17, 49.68) | 35.65 (34.18, 37.18) | -1.02 (-1.04, -0.99) | <0.001 | 202.53 (198.99, 206.13) | 165.36 (162.21, 168.57) | -0.71 (-0.73, -0.68) | <0.001 | 264.91 (260.84, 269.03) | 132.71 (129.89, 135.59) | -2.34 (-2.79, -1.90) | <0.001 | 4.69 (4.16, 5.27) | 2.33 (1.97, 2.74) | -2.37 (-2.86, -1.88) | <0.001 |
| Central African Republic | 37.15 (31.65, 43.38) | 38.01 (34.00, 42.37) | 0.07 (0.05, 0.09) | <0.001 | 156.26 (144.35, 168.94) | 160.75 (152.19, 169.67) | 0.12 (0.02, 0.21) | 0.015 | 532.76 (510.94, 555.31) | 533.37 (517.95, 549.14) | -0.01 (-0.31, 0.30) | 0.974 | 9.40 (6.68, 12.91) | 9.44 (7.48, 11.77) | 0.01 (-0.29, 0.31) | 0.970 |
| Chad | 29.78 (26.18, 33.77) | 32.78 (30.42, 35.29) | 0.33 (0.31, 0.36) | <0.001 | 173.10 (163.97, 182.61) | 193.33 (187.29, 199.51) | 0.40 (0.34, 0.45) | <0.001 | 239.19 (228.64, 250.12) | 300.49 (293.13, 307.99) | 0.99 (0.83, 1.16) | <0.001 | 4.19 (2.89, 5.89) | 5.26 (4.32, 6.35) | 0.98 (0.82, 1.15) | <0.001 |
| Chile | 24.65 (22.79, 26.62) | 23.44 (21.88, 25.09) | -0.17 (-0.20, -0.15) | <0.001 | 126.95 (122.65, 131.37) | 125.21 (121.57, 128.93) | -0.05 (-0.06, -0.03) | <0.001 | 183.41 (178.25, 188.69) | 153.38 (149.34, 157.50) | -0.63 (-1.03, -0.24) | 0.002 | 3.25 (2.59, 4.03) | 2.72 (2.21, 3.32) | -0.63 (-0.99, -0.27) | 0.001 |
| China | 23.65 (23.47, 23.84) | 22.30 (22.13, 22.48) | -0.19 (-0.27, -0.12) | <0.001 | 232.39 (231.80, 232.97) | 230.16 (229.61, 230.71) | -0.03 (-0.09, 0.02) | 0.260 | 446.10 (445.30, 446.90) | 453.03 (452.24, 453.83) | 0.13 (-0.63, 0.89) | 0.744 | 7.65 (7.54, 7.75) | 7.83 (7.73, 7.94) | 0.15 (-0.62, 0.94) | 0.701 |
| Colombia | 29.39 (28.06, 30.77) | 27.12 (26.08, 28.19) | -0.27 (-0.29, -0.26) | <0.001 | 145.52 (142.48, 148.62) | 143.05 (140.64, 145.49) | -0.06 (-0.08, -0.04) | <0.001 | 445.17 (439.92, 450.47) | 245.54 (242.39, 248.72) | -2.18 (-2.58, -1.78) | <0.001 | 7.77 (7.09, 8.51) | 4.25 (3.85, 4.69) | -2.20 (-2.61, -1.79) | <0.001 |
| Comoros | 34.23 (21.65, 51.91) | 34.36 (25.36, 45.59) | 0.01 (0.01, 0.02) | <0.001 | 222.67 (186.88, 263.53) | 223.60 (199.18, 250.22) | 0.02 (-0.00, 0.04) | 0.078 | 305.87 (264.40, 352.28) | 310.48 (281.83, 341.31) | -0.47 (-2.67, 1.79) | 0.682 | 5.36 (1.24, 15.33) | 5.41 (2.25, 10.99) | -0.48 (-2.59, 1.66) | 0.656 |
| Congo | 37.49 (31.61, 44.22) | 36.98 (33.27, 40.99) | -0.05 (-0.07, -0.04) | <0.001 | 176.63 (163.07, 191.05) | 175.90 (167.68, 184.42) | -0.01 (-0.06, 0.05) | 0.849 | 660.26 (634.20, 687.17) | 332.17 (320.89, 343.74) | -2.35 (-3.03, -1.66) | <0.001 | 11.74 (8.47, 15.90) | 5.85 (4.44, 7.58) | -2.38 (-3.05, -1.70) | <0.001 |
| Cook Islands | 29.55 (0.89, 172.38) | 30.22 (0.35, 200.66) | 0.07 (0.05, 0.09) | <0.001 | 243.05 (108.27, 477.04) | 256.57 (101.24, 536.75) | 0.18 (0.13, 0.22) | <0.001 | 859.02 (579.54, 1232.29) | 833.18 (522.08, 1263.18) | -0.10 (-0.34, 0.13) | 0.387 | 15.45 (0.02, 149.22) | 14.97 (0.00, 173.18) | -0.11 (-0.35, 0.13) | 0.373 |
| Costa Rica | 28.95 | 27.96 | -0.11 | <0.001 | 161.23 | 168.64 | 0.15 | <0.001 | 333.33 | 227.65 | -1.21 | 0.033 | 5.91 | 3.99 | -1.25 | 0.032 |

| | | | | | | | | | | | | | | | | |
|---|---|---|---|---|---|---|---|---|---|---|---|---|---|---|---|---|
| | (24.79, 33.64) | (24.70, 31.54) | (-0.13, -0.09) | | (150.96, 172.04) | (160.51, 177.09) | (0.12, 0.18) | | (318.58, 348.60) | (218.18, 237.44) | (-2.31, -0.10) | | (4.08, 8.29) | (2.83, 5.50) | (-2.39, -0.11) | |
| Cote d'Ivoire | 33.95 (31.52, 36.54) | 36.37 (34.73, 38.06) | 0.23 (0.22, 0.25) | <0.001 | 188.39 (182.42, 194.52) | 201.05 (197.14, 205.02) | 0.24 (0.20, 0.29) | <0.001 | 361.19 (352.98, 369.55) | 317.47 (312.59, 322.40) | -0.45 (-0.71, -0.20) | 0.001 | 6.38 (5.32, 7.60) | 5.59 (4.96, 6.28) | -0.46 (-0.73, -0.18) | 0.001 |
| Croatia | 28.52 (25.30, 32.08) | 24.18 (20.74, 28.11) | -0.56 (-0.58, -0.53) | <0.001 | 247.88 (238.34, 257.74) | 211.39 (201.18, 222.06) | -0.54 (-0.60, -0.47) | <0.001 | 603.28 (588.29, 618.60) | 198.30 (188.44, 208.61) | -3.66 (-4.43, -2.89) | <0.001 | 10.76 (8.84, 13.00) | 3.50 (2.31, 5.20) | -3.75 (-4.83, -2.66) | <0.001 |
| Cuba | 61.84 (58.60, 65.21) | 59.04 (55.67, 62.57) | -0.15 (-0.19, -0.11) | <0.001 | 330.63 (322.86, 338.54) | 307.24 (299.51, 315.13) | -0.27 (-0.35, -0.18) | <0.001 | 525.41 (515.65, 535.31) | 234.65 (227.90, 241.57) | -2.72 (-3.17, -2.27) | <0.001 | 9.35 (8.08, 10.77) | 4.14 (3.29, 5.17) | -2.81 (-3.28, -2.34) | <0.001 |
| Cyprus | 23.66 (16.74, 32.58) | 21.76 (16.69, 28.29) | -0.29 (-0.30, -0.28) | <0.001 | 141.28 (123.45, 161.03) | 154.49 (140.76, 169.55) | 0.40 (0.09, 0.72) | 0.012 | 428.17 (396.63, 461.62) | 287.48 (268.49, 307.81) | -1.44 (-2.00, -0.88) | <0.001 | 7.62 (3.96, 13.39) | 5.10 (2.88, 8.94) | -1.42 (-1.97, -0.85) | <0.001 |
| Czechia | 46.23 (43.26, 49.37) | 40.41 (37.49, 43.53) | -0.46 (-0.48, -0.44) | <0.001 | 286.84 (279.51, 294.33) | 247.86 (240.83, 255.09) | -0.51 (-0.59, -0.44) | <0.001 | 589.32 (578.87, 599.92) | 139.73 (134.48, 145.17) | -4.80 (-5.43, -4.17) | <0.001 | 10.68 (9.32, 12.20) | 2.44 (1.79, 3.29) | -4.93 (-5.58, -4.27) | <0.001 |
| Democratic People's Republic of Korea | 21.99 (20.52, 23.54) | 22.29 (21.07, 23.56) | 0.04 (0.03, 0.06) | <0.001 | 201.85 (197.28, 206.50) | 209.83 (206.10, 213.62) | 0.13 (0.09, 0.17) | <0.001 | 717.74 (709.22, 726.35) | 765.89 (758.61, 773.23) | 0.23 (0.12, 0.34) | <0.001 | 12.37 (11.27, 13.56) | 13.13 (12.19, 14.12) | 0.21 (0.10, 0.32) | <0.001 |
| Democratic Republic of the Congo | 35.02 (33.54, 36.56) | 34.28 (33.36, 35.22) | -0.07 (-0.08, -0.06) | <0.001 | 162.74 (159.41, 166.12) | 160.45 (158.39, 162.53) | -0.05 (-0.08, -0.01) | 0.012 | 366.38 (361.46, 371.35) | 314.37 (311.52, 317.23) | -0.50 (-0.60, -0.40) | <0.001 | 6.47 (5.83, 7.17) | 5.55 (5.18, 5.95) | -0.50 (-0.61, -0.40) | <0.001 |
| Denmark | 46.82 (42.70, 51.25) | 34.13 (30.50, 38.09) | -1.09 (-1.11, -1.06) | <0.001 | 225.50 (216.37, 234.92) | 177.51 (169.08, 186.27) | -0.82 (-0.83, -0.81) | <0.001 | 251.62 (242.00, 261.55) | 53.93 (49.33, 58.87) | -5.21 (-6.21, -4.19) | <0.001 | 4.54 (3.34, 6.08) | 0.95 (0.43, 1.84) | -5.27 (-6.33, -4.20) | <0.001 |
| Djibouti | 32.07 (21.48, 46.37) | 34.55 (27.77, 42.49) | 0.25 (0.23, 0.28) | <0.001 | 199.83 (170.65, 232.71) | 218.44 (200.77, 237.25) | 0.31 (0.27, 0.35) | <0.001 | 301.17 (265.77, 340.17) | 376.14 (352.90, 400.52) | 0.74 (0.51, 0.97) | <0.001 | 5.27 (1.58, 13.15) | 6.53 (3.80, 10.48) | 0.72 (0.49, 0.94) | <0.001 |
| Dominica | 65.03 (29.31, 126.50) | 66.86 (30.15, 128.34) | 0.10 (0.02, 0.18) | 0.014 | 318.99 (228.40, 434.43) | 326.61 (235.87, 440.88) | 0.08 (0.06, 0.09) | <0.001 | 298.17 (211.29, 409.77) | 181.53 (115.84, 271.14) | -1.75 (-2.31, -1.19) | <0.001 | 5.28 (0.03, 41.20) | 3.18 (0.00, 35.05) | -1.76 (-2.42, -1.11) | <0.001 |
| Dominican | 59.99 | 65.75 | 0.32 | <0.001 | 280.42 | 316.05 | 0.41 | <0.001 | 716.74 | 830.21 | 0.60 | 0.058 | 12.47 | 14.63 | 0.64 | 0.043 |

| | | | | | | | | | | | | | | | | |
|---|---|---|---|---|---|---|---|---|---|---|---|---|---|---|---|---|
| Republic | (55.81, 64.40) | (62.45, 69.19) | (0.30, 0.34) | | (270.99, 290.10) | (308.70, 323.54) | (0.37, 0.44) | | (702.04, 731.68) | (818.30, 842.25) | (-0.02, 1.22) | | (10.59, 14.61) | (13.08, 16.31) | (0.02, 1.27) | |
| Ecuador | 14.06 (12.39, 15.90) | 14.45 (13.21, 15.77) | 0.10 (0.08, 0.13) | <0.001 | 107.58 (102.71, 112.62) | 118.28 (114.64, 122.01) | 0.33 (0.31, 0.35) | <0.001 | 518.14 (507.69, 528.74) | 482.77 (475.49, 490.13) | -0.33 (-0.85, 0.19) | 0.209 | 8.94 (7.61, 10.45) | 8.25 (7.32, 9.27) | -0.36 (-0.88, 0.16) | 0.177 |
| Egypt | 45.17 (43.86, 46.51) | 45.03 (44.12, 45.96) | -0.01 (-0.07, 0.06) | 0.841 | 302.69 (299.23, 306.19) | 314.37 (311.95, 316.80) | 0.15 (0.02, 0.29) | 0.029 | 1679.95 (1671.95, 1687.99) | 1236.04 (1231.26, 1240.83) | -1.05 (-1.54, -0.56) | <0.001 | 29.36 (28.30, 30.44) | 21.23 (20.60, 21.87) | -1.11 (-1.60, -0.62) | <0.001 |
| El Salvador | 25.80 (22.46, 29.52) | 27.56 (24.54, 30.86) | 0.23 (0.21, 0.25) | <0.001 | 113.82 (106.43, 121.59) | 135.86 (128.86, 143.16) | 0.61 (0.59, 0.63) | <0.001 | 944.54 (924.07, 965.37) | 503.62 (490.35, 517.16) | -2.04 (-3.68, -0.37) | 0.017 | 16.15 (13.55, 19.13) | 8.77 (7.09, 10.75) | -1.98 (-3.62, -0.31) | 0.020 |
| Equatorial Guinea | 36.53 (22.56, 56.31) | 32.31 (26.04, 39.75) | -0.43 (-0.44, -0.41) | <0.001 | 167.89 (135.30, 206.18) | 156.86 (141.96, 172.98) | -0.23 (-0.24, -0.21) | <0.001 | 532.96 (474.72, 596.66) | 207.46 (190.82, 225.28) | -3.20 (-3.90, -2.48) | <0.001 | 9.42 (3.18, 21.92) | 3.60 (1.71, 6.84) | -3.26 (-3.98, -2.52) | <0.001 |
| Eritrea | 31.44 (26.62, 36.92) | 31.14 (28.18, 34.34) | -0.04 (-0.05, -0.02) | <0.001 | 171.75 (159.80, 184.40) | 170.32 (163.10, 177.79) | -0.02 (-0.08, 0.04) | 0.550 | 407.63 (389.42, 426.50) | 486.04 (473.99, 498.33) | 0.57 (0.14, 1.01) | 0.009 | 7.19 (4.94, 10.16) | 8.55 (7.01, 10.34) | 0.57 (0.14, 1.00) | 0.010 |
| Estonia | 62.13 (53.54, 71.76) | 56.35 (47.16, 67.14) | -0.35 (-0.40, -0.30) | <0.001 | 268.27 (250.25, 287.32) | 245.24 (226.16, 265.84) | -0.34 (-0.51, -0.18) | <0.001 | 845.00 (812.83, 878.17) | 169.86 (154.08, 187.15) | -5.72 (-6.56, -4.87) | <0.001 | 15.46 (11.38, 20.59) | 3.02 (1.27, 6.66) | -5.84 (-6.68, -4.99) | <0.001 |
| Eswatini | 38.30 (27.43, 52.26) | 40.68 (32.75, 50.00) | 0.21 (0.19, 0.23) | <0.001 | 185.06 (159.17, 214.05) | 202.71 (184.11, 222.71) | 0.32 (0.30, 0.33) | <0.001 | 204.37 (177.31, 234.50) | 369.28 (344.07, 395.87) | 1.97 (1.49, 2.45) | <0.001 | 3.62 (0.89, 9.92) | 6.60 (3.62, 11.09) | 2.01 (1.49, 2.53) | <0.001 |
| Ethiopia | 27.46 (26.28, 28.68) | 30.62 (29.82, 31.44) | 0.38 (0.36, 0.39) | <0.001 | 148.52 (145.68, 151.40) | 172.55 (170.58, 174.54) | 0.53 (0.50, 0.55) | <0.001 | 442.29 (437.52, 447.10) | 256.28 (253.94, 258.63) | -1.87 (-2.00, -1.73) | <0.001 | 7.73 (7.10, 8.39) | 4.43 (4.13, 4.75) | -1.91 (-2.04, -1.77) | <0.001 |
| Fiji | 32.51 (24.07, 43.06) | 32.64 (24.95, 41.98) | 0.01 (0.00, 0.02) | 0.027 | 266.08 (240.31, 293.92) | 275.27 (252.05, 300.09) | 0.12 (0.09, 0.14) | <0.001 | 1906.58 (1836.43, 1978.76) | 1461.29 (1407.11, 1517.05) | -0.97 (-1.62, -0.31) | 0.004 | 34.54 (25.65, 45.58) | 26.26 (19.46, 34.72) | -0.99 (-1.66, -0.32) | 0.004 |
| Finland | 49.15 (44.93, | 37.34 (33.51, | -0.95 (-0.99, | <0.001 | 232.48 (223.38, | 187.24 (178.69, | -0.74 (-0.77, | <0.001 | 387.44 (375.76, | 97.97 (91.86, | -4.68 (-5.38, | <0.001 | 7.06 (5.57, | 1.75 (1.02, | -4.74 (-5.47, | <0.001 |

| | | | | | | | | | | | | | | | | |
|---|---|---|---|---|---|---|---|---|---|---|---|---|---|---|---|---|
| | 53.71) | 41.53) | -0.92) | | 241.91) | 196.14) | -0.70) | | 399.43) | 104.43) | -3.97) | | 8.89) | 2.86) | -4.01) | |
| France | 33.96 (32.91, 35.05) | 29.26 (28.22, 30.33) | -0.51 (-0.53, -0.50) | <0.001 | 170.83 (168.47, 173.22) | 162.39 (159.97, 164.85) | -0.16 (-0.23, -0.10) | <0.001 | 232.14 (229.39, 234.91) | 103.39 (101.46, 105.35) | -2.84 (-3.55, -2.13) | <0.001 | 4.18 (3.82, 4.57) | 1.83 (1.58, 2.11) | -2.91 (-3.64, -2.17) | <0.001 |
| Gabon | 33.53 (25.56, 43.27) | 33.91 (27.93, 40.83) | 0.04 (0.02, 0.06) | <0.001 | 160.81 (142.34, 181.04) | 167.19 (153.32, 181.99) | 0.13 (0.11, 0.16) | <0.001 | 473.20 (441.47, 506.65) | 337.41 (317.79, 357.95) | -1.14 (-1.76, -0.53) | <0.001 | 8.35 (4.61, 13.97) | 5.92 (3.57, 9.25) | -1.16 (-1.77, -0.55) | <0.001 |
| Gambia | 35.41 (26.66, 46.29) | 38.78 (32.83, 45.55) | 0.31 (0.30, 0.33) | <0.001 | 214.61 (191.34, 240.02) | 235.43 (219.95, 251.75) | 0.34 (0.26, 0.42) | <0.001 | 271.27 (245.30, 299.35) | 321.00 (303.19, 339.62) | 0.65 (-0.50, 1.81) | 0.271 | 4.78 (1.91, 10.05) | 5.61 (3.47, 8.63) | 0.62 (-0.53, 1.79) | 0.292 |
| Georgia | 43.88 (39.97, 48.08) | 45.78 (40.76, 51.31) | 0.16 (0.11, 0.21) | <0.001 | 233.38 (224.23, 242.82) | 251.46 (239.65, 263.77) | 0.26 (0.24, 0.28) | <0.001 | 1682.76 (1658.00, 1707.80) | 876.54 (854.29, 899.28) | -2.10 (-3.51, -0.68) | 0.004 | 30.40 (27.15, 33.94) | 15.80 (12.94, 19.17) | -2.25 (-3.76, -0.73) | 0.004 |
| Germany | 48.24 (47.18, 49.32) | 38.00 (37.00, 39.02) | -0.83 (-0.86, -0.80) | <0.001 | 226.28 (223.98, 228.61) | 199.16 (196.91, 201.43) | -0.42 (-0.54, -0.31) | <0.001 | 459.48 (456.16, 462.81) | 120.65 (118.89, 122.44) | -4.59 (-4.91, -4.27) | <0.001 | 8.18 (7.74, 8.64) | 2.13 (1.90, 2.38) | -4.61 (-4.94, -4.29) | <0.001 |
| Ghana | 33.17 (30.92, 35.56) | 35.05 (33.56, 36.58) | 0.18 (0.11, 0.26) | <0.001 | 181.72 (176.25, 187.31) | 193.95 (190.35, 197.60) | 0.25 (0.19, 0.31) | <0.001 | 245.94 (239.66, 252.35) | 262.24 (258.10, 266.44) | 0.22 (-0.03, 0.47) | 0.078 | 4.33 (3.53, 5.27) | 4.61 (4.07, 5.20) | 0.21 (-0.11, 0.52) | 0.192 |
| Greece | 32.00 (29.51, 34.64) | 29.51 (26.89, 32.33) | -0.28 (-0.29, -0.27) | <0.001 | 152.46 (146.98, 158.08) | 158.04 (152.12, 164.15) | 0.14 (0.04, 0.25) | 0.009 | 535.46 (525.15, 545.92) | 411.18 (401.57, 420.99) | -1.00 (-1.56, -0.43) | 0.001 | 9.51 (8.18, 11.00) | 7.37 (6.15, 8.81) | -0.96 (-1.52, -0.39) | 0.001 |
| Greenland | 48.84 (20.16, 108.47) | 38.03 (10.74, 102.50) | -0.86 (-0.88, -0.84) | <0.001 | 175.33 (114.58, 263.91) | 152.21 (88.25, 249.68) | -0.49 (-0.51, -0.46) | <0.001 | 317.41 (232.77, 429.08) | 117.63 (62.40, 206.98) | -3.37 (-3.67, -3.06) | <0.001 | 5.77 (0.08, 50.16) | 2.13 (0.00, 48.85) | -3.38 (-3.70, -3.06) | <0.001 |
| Grenada | 67.32 (32.60, 124.76) | 70.57 (38.76, 119.13) | 0.16 (0.14, 0.18) | <0.001 | 347.93 (258.42, 459.33) | 368.09 (287.20, 465.27) | 0.20 (0.19, 0.20) | <0.001 | 617.73 (495.96, 761.16) | 214.46 (153.91, 291.52) | -3.72 (-4.75, -2.69) | <0.001 | 11.13 (0.90, 47.55) | 3.77 (0.03, 27.95) | -3.82 (-4.90, -2.73) | <0.001 |
| Guam | 26.04 (11.79, 50.37) | 29.73 (13.60, 56.85) | 0.45 (0.37, 0.53) | <0.001 | 209.46 (163.05, 265.33) | 255.09 (200.73, 319.85) | 0.67 (0.63, 0.70) | <0.001 | 590.95 (511.21, 679.96) | 1274.75 (1149.12, 1410.55) | 2.66 (1.98, 3.36) | <0.001 | 10.50 (2.52, 29.27) | 23.02 (9.07, 48.29) | 2.72 (1.95, 3.50) | <0.001 |

| | | | | | | | | | | | | | | | | |
|---|---|---|---|---|---|---|---|---|---|---|---|---|---|---|---|---|
| Guatemala | 23.73 (21.08, 26.63) | 26.45 (24.76, 28.24) | 0.38 (0.36, 0.41) | <0.001 | 100.04 (94.38, 105.94) | 124.73 (120.91, 128.65) | 0.77 (0.72, 0.82) | <0.001 | 984.52 (967.29, 1001.98) | 519.56 (511.95, 527.26) | -2.18 (-2.64, -1.71) | <0.001 | 16.83 (14.63, 19.29) | 9.03 (8.05, 10.11) | -2.12 (-2.60, -1.64) | <0.001 |
| Guinea | 27.84 (24.45, 31.58) | 32.07 (29.55, 34.76) | 0.49 (0.47, 0.52) | <0.001 | 151.69 (143.46, 160.27) | 178.56 (172.34, 184.96) | 0.58 (0.53, 0.64) | <0.001 | 210.46 (200.90, 220.38) | 318.77 (310.59, 327.12) | 1.49 (1.29, 1.68) | <0.001 | 3.69 (2.51, 5.23) | 5.60 (4.56, 6.83) | 1.50 (1.31, 1.69) | <0.001 |
| Guinea-Bissau | 34.75 (25.80, 45.97) | 37.36 (31.09, 44.60) | 0.25 (0.23, 0.27) | <0.001 | 190.25 (167.66, 215.12) | 205.49 (189.89, 222.07) | 0.29 (0.18, 0.41) | <0.001 | 439.55 (405.33, 475.99) | 481.09 (457.33, 505.80) | 0.32 (0.20, 0.44) | <0.001 | 7.81 (3.84, 14.24) | 8.53 (5.61, 12.47) | 0.31 (0.18, 0.43) | <0.001 |
| Guyana | 74.26 (61.09, 89.56) | 74.27 (60.93, 89.76) | 0.01 (-0.02, 0.03) | 0.567 | 332.31 (302.88, 363.89) | 332.56 (302.81, 364.49) | 0.01 (-0.02, 0.04) | 0.484 | 1000.34 (949.40, 1053.38) | 606.78 (566.58, 649.13) | -1.58 (-2.55, -0.60) | 0.002 | 17.74 (11.51, 26.23) | 10.82 (6.05, 17.92) | -1.56 (-2.51, -0.61) | 0.001 |
| Haiti | 69.76 (64.79, 75.01) | 71.26 (67.94, 74.70) | 0.07 (0.03, 0.12) | 0.001 | 309.85 (299.11, 320.89) | 314.06 (306.94, 321.30) | 0.05 (0.04, 0.06) | <0.001 | 582.52 (567.80, 597.53) | 431.67 (423.33, 440.13) | -1.03 (-1.28, -0.79) | <0.001 | 10.43 (8.54, 12.62) | 7.71 (6.63, 8.92) | -1.02 (-1.25, -0.78) | <0.001 |
| Honduras | 29.43 (25.59, 33.71) | 30.51 (27.99, 33.20) | 0.12 (0.09, 0.16) | <0.001 | 147.72 (138.73, 157.16) | 168.48 (162.32, 174.82) | 0.44 (0.41, 0.48) | <0.001 | 496.25 (479.96, 512.97) | 197.28 (190.72, 204.02) | -3.18 (-3.43, -2.93) | <0.001 | 8.70 (6.66, 11.20) | 3.43 (2.61, 4.44) | -3.21 (-3.47, -2.95) | <0.001 |
| Hungary | 39.31 (36.59, 42.19) | 31.81 (29.10, 34.73) | -0.73 (-0.74, -0.72) | <0.001 | 304.31 (296.84, 311.94) | 240.53 (233.13, 248.14) | -0.81 (-0.83, -0.78) | <0.001 | 1199.14 (1184.37 , 1214.06) | 274.84 (266.95, 282.93) | -5.00 (-5.67, -4.32) | <0.001 | 21.82 (19.87, 23.92) | 4.90 (3.90, 6.12) | -5.07 (-5.77, -4.37) | <0.001 |
| Iceland | 45.55 (29.38, 67.68) | 38.95 (25.37, 57.97) | -0.54 (-0.55, -0.52) | <0.001 | 197.49 (161.88, 238.83) | 187.48 (156.18, 223.87) | -0.20 (-0.29, -0.11) | <0.001 | 365.88 (316.57, 420.90) | 142.33 (115.15, 174.66) | -3.24 (-3.80, -2.69) | <0.001 | 6.33 (1.49, 17.79) | 2.50 (0.22, 11.44) | -3.19 (-3.76, -2.61) | <0.001 |
| India | 29.60 (29.33, 29.86) | 30.56 (30.37, 30.76) | 0.11 (0.09, 0.13) | <0.001 | 162.68 (162.05, 163.31) | 174.91 (174.43, 175.38) | 0.25 (0.22, 0.28) | <0.001 | 956.57 (955.07, 958.08) | 920.95 (919.86, 922.05) | -0.06 (-0.84, 0.73) | 0.882 | 16.95 (16.75, 17.15) | 16.43 (16.29, 16.58) | -0.03 (-0.83, 0.78) | 0.948 |
| Indonesia | 16.80 (16.37, 17.24) | 18.72 (18.35, 19.08) | 0.36 (0.27, 0.45) | <0.001 | 149.49 (148.19, 150.79) | 169.43 (168.35, 170.52) | 0.44 (0.41, 0.47) | <0.001 | 703.42 (700.64, 706.20) | 697.00 (694.78, 699.21) | -0.06 (-0.26, 0.15) | 0.594 | 12.32 (11.95, 12.70) | 12.17 (11.88, 12.46) | -0.07 (-0.28, 0.14) | 0.511 |
| Iran (Islamic Republic of) | 74.13 (72.40, | 73.68 (72.51, | -0.02 (-0.06, | 0.216 | 390.19 (386.11, | 411.01 (408.32, | 0.19 (0.15, | <0.001 | 879.75 (873.78, | 550.07 (546.78, | -1.62 (-1.85, | <0.001 | 15.40 (14.61, | 9.43 (9.01, | -1.69 (-1.93, | <0.001 |

| | | | | | | | | | | | | | | | | |
|---|---|---|---|---|---|---|---|---|---|---|---|---|---|---|---|---|
| | 75.89) | 74.86) | 0.01) | | 394.31) | 413.72) | 0.23) | | 885.75) | 553.38) | -1.38) | | 16.22) | 9.87) | -1.45) | |
| Iraq | 49.59 (47.03, 52.26) | 48.03 (46.58, 49.52) | -0.11 (-0.15, -0.08) | <0.001 | 338.58 (331.70, 345.57) | 334.57 (330.63, 338.55) | -0.01 (-0.21, 0.19) | 0.915 | 854.03 (843.22, 864.95) | 416.11 (411.74, 420.51) | -2.41 (-2.67, -2.16) | <0.001 | 15.11 (13.69, 16.64) | 7.29 (6.72, 7.90) | -2.44 (-2.70, -2.19) | <0.001 |
| Ireland | 40.77 (36.05, 45.94) | 32.48 (28.73, 36.60) | -0.79 (-0.82, -0.77) | <0.001 | 210.08 (199.20, 221.41) | 182.77 (173.97, 191.92) | -0.46 (-0.55, -0.37) | <0.001 | 317.38 (304.00, 331.20) | 95.39 (89.15, 101.99) | -4.13 (-4.94, -3.31) | <0.001 | 5.76 (4.09, 7.90) | 1.70 (0.96, 2.83) | -4.18 (-5.04, -3.30) | <0.001 |
| Israel | 31.79 (28.24, 35.67) | 27.29 (24.82, 29.94) | -0.53 (-0.55, -0.50) | <0.001 | 172.60 (164.20, 181.33) | 164.89 (158.77, 171.19) | -0.15 (-0.19, -0.12) | <0.001 | 378.59 (366.10, 391.41) | 86.97 (82.56, 91.57) | -4.99 (-5.79, -4.19) | <0.001 | 6.70 (5.13, 8.61) | 1.52 (0.99, 2.25) | -5.04 (-5.85, -4.22) | <0.001 |
| Italy | 41.33 (40.13, 42.57) | 34.07 (32.86, 35.32) | -0.68 (-0.74, -0.62) | <0.001 | 192.74 (190.11, 195.40) | 174.75 (172.06, 177.47) | -0.33 (-0.38, -0.29) | <0.001 | 300.46 (297.18, 303.77) | 134.61 (132.24, 137.02) | -2.79 (-3.49, -2.09) | <0.001 | 5.31 (4.89, 5.77) | 2.36 (2.06, 2.70) | -2.83 (-3.21, -2.45) | <0.001 |
| Jamaica | 62.43 (55.05, 70.57) | 70.72 (64.01, 77.97) | 0.45 (0.41, 0.48) | <0.001 | 333.45 (315.50, 352.19) | 388.11 (371.88, 404.89) | 0.51 (0.46, 0.56) | <0.001 | 128.95 (118.02, 140.66) | 136.88 (127.32, 146.98) | 0.22 (-1.75, 2.22) | 0.831 | 2.20 (0.98, 4.29) | 2.35 (1.25, 4.04) | 0.23 (-1.88, 2.39) | 0.829 |
| Japan | 26.70 (26.03, 27.38) | 23.54 (22.84, 24.25) | -0.43 (-0.44, -0.42) | <0.001 | 133.99 (132.51, 135.48) | 116.74 (115.21, 118.29) | -0.48 (-0.50, -0.45) | <0.001 | 179.34 (177.61, 181.07) | 129.33 (127.70, 130.98) | -1.10 (-1.44, -0.75) | <0.001 | 3.12 (2.90, 3.35) | 2.24 (2.03, 2.47) | -1.11 (-1.46, -0.76) | <0.001 |
| Jordan | 46.83 (41.49, 52.71) | 44.55 (42.04, 47.18) | -0.18 (-0.29, -0.06) | 0.002 | 335.93 (320.84, 351.58) | 325.60 (318.69, 332.62) | -0.09 (-0.22, 0.04) | 0.184 | 632.35 (611.79, 653.45) | 346.38 (339.27, 353.61) | -1.99 (-2.44, -1.54) | <0.001 | 11.21 (8.60, 14.41) | 6.05 (5.14, 7.07) | -2.04 (-2.49, -1.59) | <0.001 |
| Kazakhstan | 45.94 (43.68, 48.29) | 41.22 (39.18, 43.35) | -0.37 (-0.39, -0.35) | <0.001 | 229.34 (224.23, 234.55) | 210.52 (205.91, 215.22) | -0.30 (-0.33, -0.27) | <0.001 | 1162.44 (1150.87 , 1174.10) | 513.15 (505.93, 520.47) | -2.87 (-4.11, -1.62) | <0.001 | 21.13 (19.59, 22.76) | 9.28 (8.33, 10.32) | -2.88 (-4.13, -1.62) | <0.001 |
| Kenya | 32.96 (31.06, 34.95) | 34.01 (32.84, 35.22) | 0.11 (0.10, 0.12) | <0.001 | 209.46 (204.45, 214.57) | 217.50 (214.45, 220.59) | 0.14 (0.12, 0.16) | <0.001 | 152.95 (148.78, 157.21) | 250.82 (247.62, 254.06) | 1.71 (1.58, 1.84) | <0.001 | 2.59 (2.07, 3.21) | 4.29 (3.88, 4.74) | 1.75 (1.62, 1.88) | <0.001 |
| Kiribati | 33.37 (9.85, 84.32) | 34.67 (14.80, 69.43) | 0.13 (0.10, 0.15) | <0.001 | 220.38 (146.04, 320.24) | 235.31 (175.59, 309.10) | 0.23 (0.21, 0.24) | <0.001 | 3160.96 (2856.81 , 3489.43) | 2933.05 (2711.27 , 3168.38) | -0.26 (-0.35, -0.17) | <0.001 | 57.65 (23.43, 119.10) | 53.08 (27.11, 93.84) | -0.29 (-0.36, -0.21) | <0.001 |

| | | | | | | | | | | | | | | | | |
|---|---|---|---|---|---|---|---|---|---|---|---|---|---|---|---|---|
| Kuwait | 44.31 (39.00, 50.34) | 45.17 (41.53, 49.16) | 0.07 (-0.02, 0.15) | 0.117 | 322.94 (308.49, 338.07) | 339.09 (329.47, 349.04) | 0.19 (0.09, 0.29) | <0.001 | 757.45 (734.48, 781.12) | 680.29 (665.91, 695.01) | -0.35 (-2.31, 1.64) | 0.725 | 13.08 (10.24, 16.67) | 11.84 (10.04, 14.01) | -0.32 (-2.31, 1.72) | 0.757 |
| Kyrgyzstan | 42.02 (37.72, 46.70) | 38.87 (35.56, 42.41) | -0.27 (-0.29, -0.25) | <0.001 | 211.88 (201.91, 222.24) | 196.35 (188.73, 204.21) | -0.28 (-0.31, -0.24) | <0.001 | 941.75 (920.64, 963.24) | 535.35 (522.73, 548.21) | -1.88 (-3.35, -0.39) | 0.014 | 17.01 (14.27, 20.15) | 9.69 (8.05, 11.57) | -1.88 (-3.35, -0.37) | 0.015 |
| Lao People's Democratic Republic | 31.65 (27.46, 36.33) | 28.06 (25.46, 30.86) | -0.41 (-0.43, -0.39) | <0.001 | 179.47 (169.11, 190.32) | 158.40 (152.06, 164.95) | -0.42 (-0.49, -0.36) | <0.001 | 664.95 (645.11, 685.27) | 702.21 (688.93, 715.68) | 0.22 (0.03, 0.42) | 0.027 | 11.77 (9.25, 14.79) | 12.31 (10.60, 14.22) | 0.19 (-0.01, 0.38) | 0.056 |
| Latvia | 40.74 (35.32, 46.78) | 40.72 (34.06, 48.52) | 0.00 (-0.04, 0.04) | 0.995 | 213.01 (200.42, 226.23) | 213.08 (197.83, 229.39) | -0.01 (-0.05, 0.02) | 0.459 | 1026.47 (998.62, 1054.93) | 296.24 (278.33, 315.21) | -4.25 (-6.39, -2.05) | <0.001 | 18.80 (15.20, 23.04) | 5.36 (3.21, 8.74) | -4.27 (-6.45, -2.05) | <0.001 |
| Lebanon | 41.72 (36.25, 47.82) | 43.41 (39.61, 47.52) | 0.14 (0.11, 0.17) | <0.001 | 280.57 (265.78, 295.99) | 306.64 (296.43, 317.15) | 0.32 (0.25, 0.39) | <0.001 | 1726.90 (1690.75, 1763.66) | 1140.70 (1120.59, 1161.12) | -1.40 (-1.61, -1.18) | <0.001 | 30.32 (25.66, 35.60) | 19.75 (17.19, 22.62) | -1.45 (-1.66, -1.24) | <0.001 |
| Lesotho | 34.36 (27.86, 41.98) | 38.16 (32.78, 44.19) | 0.36 (0.32, 0.41) | <0.001 | 148.65 (134.38, 164.06) | 173.57 (161.74, 186.04) | 0.53 (0.52, 0.55) | <0.001 | 144.00 (130.04, 159.10) | 343.75 (327.00, 361.13) | 3.04 (2.54, 3.55) | <0.001 | 2.54 (1.00, 5.34) | 6.18 (4.12, 8.92) | 3.11 (2.59, 3.64) | <0.001 |
| Liberia | 34.52 (27.99, 42.18) | 37.32 (33.37, 41.62) | 0.27 (0.26, 0.28) | <0.001 | 206.20 (189.98, 223.51) | 224.03 (214.02, 234.39) | 0.30 (0.19, 0.42) | <0.001 | 262.75 (244.51, 282.05) | 249.33 (238.88, 260.12) | -0.26 (-0.62, 0.09) | 0.149 | 4.62 (2.50, 7.92) | 4.37 (3.08, 6.02) | -0.28 (-0.64, 0.08) | 0.128 |
| Libya | 39.75 (35.40, 44.51) | 43.44 (40.34, 46.73) | 0.28 (0.13, 0.42) | <0.001 | 258.37 (246.87, 270.28) | 295.25 (287.19, 303.49) | 0.48 (0.39, 0.57) | <0.001 | 1022.33 (1000.07, 1044.99) | 859.13 (845.04, 873.40) | -0.64 (-1.46, 0.20) | 0.134 | 17.60 (14.76, 20.84) | 14.82 (13.03, 16.80) | -0.62 (-1.45, 0.21) | 0.140 |
| Lithuania | 41.36 (36.75, 46.40) | 37.75 (32.35, 43.91) | -0.32 (-0.37, -0.28) | <0.001 | 209.09 (198.51, 220.10) | 185.50 (173.32, 198.42) | -0.42 (-0.46, -0.38) | <0.001 | 1000.03 (976.68, 1023.81) | 405.42 (387.35, 424.21) | -3.16 (-4.51, -1.78) | <0.001 | 18.29 (15.25, 21.78) | 7.37 (5.12, 10.42) | -3.17 (-4.53, -1.79) | <0.001 |
| Luxembourg | 26.61 (16.65, 41.44) | 23.10 (15.30, 34.12) | -0.49 (-0.52, -0.47) | <0.001 | 148.24 (123.36, 177.57) | 143.06 (122.85, 166.21) | -0.10 (-0.23, 0.03) | 0.130 | 370.40 (329.74, 415.48) | 106.72 (89.29, 127.15) | -4.22 (-5.03, -3.40) | <0.001 | 6.55 (2.24, 16.35) | 1.87 (0.28, 7.44) | -4.27 (-5.04, -3.48) | <0.001 |
| Madagascar | 30.62 | 33.59 | 0.32 | <0.001 | 182.19 | 203.21 | 0.39 | <0.001 | 762.96 | 611.09 | -0.64 | 0.095 | 13.46 | 10.75 | -0.72 | 0.003 |

| | | | | | | | | | | | | | | | | |
|---|---|---|---|---|---|---|---|---|---|---|---|---|---|---|---|---|
| | (28.19, 33.21) | (31.96, 35.28) | (0.31, 0.33) | | (175.98, 188.57) | (199.05, 207.43) | (0.32, 0.45) | | (750.45, 775.63) | (603.99, 618.25) | (-1.39, 0.11) | | (11.83, 15.26) | (9.82, 11.74) | (-1.20, -0.24) | |
| Malawi | 36.02 (33.03, 39.23) | 36.87 (34.74, 39.10) | 0.08 (0.06, 0.10) | <0.001 | 212.54 (204.91, 220.40) | 219.42 (214.02, 224.92) | 0.14 (0.01, 0.27) | 0.035 | 365.24 (355.48, 375.22) | 405.97 (398.83, 413.22) | 0.41 (0.20, 0.63) | <0.001 | 6.32 (5.09, 7.78) | 7.03 (6.11, 8.06) | 0.42 (0.20, 0.63) | <0.001 |
| Malaysia | 32.60 (30.73, 34.55) | 32.09 (30.81, 33.41) | -0.05 (-0.10, 0.00) | 0.049 | 237.39 (232.24, 242.62) | 238.69 (235.18, 242.24) | 0.04 (-0.03, 0.11) | 0.313 | 440.24 (433.24, 447.32) | 628.96 (623.25, 634.70) | 1.29 (0.35, 2.24) | 0.007 | 7.77 (6.87, 8.77) | 11.23 (10.48, 12.02) | 1.34 (0.39, 2.31) | 0.006 |
| Maldives | 30.25 (14.18, 57.87) | 27.30 (20.30, 37.16) | -0.36 (-0.38, -0.34) | <0.001 | 223.40 (172.75, 285.00) | 201.20 (182.01, 222.88) | -0.36 (-0.38, -0.33) | <0.001 | 1012.22 (901.51, 1133.48) | 352.67 (326.02, 381.84) | -3.59 (-4.29, -2.87) | <0.001 | 18.10 (6.15, 42.32) | 6.11 (3.11, 12.49) | -3.69 (-4.40, -2.98) | <0.001 |
| Mali | 30.07 (27.05, 33.34) | 31.56 (29.60, 33.61) | 0.17 (0.16, 0.18) | <0.001 | 164.23 (156.91, 171.81) | 171.82 (167.04, 176.71) | 0.16 (0.15, 0.17) | <0.001 | 177.66 (170.19, 185.39) | 188.33 (183.47, 193.29) | 0.24 (-0.13, 0.61) | 0.207 | 3.08 (2.17, 4.27) | 3.26 (2.64, 3.99) | 0.23 (-0.15, 0.61) | 0.234 |
| Malta | 31.48 (20.05, 47.57) | 26.17 (16.15, 41.48) | -0.64 (-0.66, -0.62) | <0.001 | 174.94 (146.63, 207.58) | 158.10 (132.24, 188.66) | -0.34 (-0.39, -0.28) | <0.001 | 349.24 (308.11, 394.72) | 199.67 (170.11, 233.95) | -1.95 (-2.79, -1.10) | <0.001 | 6.10 (1.86, 15.47) | 3.52 (0.70, 12.71) | -1.91 (-2.73, -1.09) | <0.001 |
| Marshall Islands | 29.70 (4.36, 102.79) | 30.54 (7.84, 81.13) | 0.09 (0.06, 0.11) | <0.001 | 203.67 (114.27, 336.51) | 216.41 (142.18, 316.10) | 0.20 (0.19, 0.22) | <0.001 | 1617.05 (1342.60, 1932.23) | 2282.68 (2023.55, 2566.16) | 1.28 (1.07, 1.49) | <0.001 | 28.78 (3.90, 101.90) | 41.40 (13.64, 96.49) | 1.36 (1.12, 1.61) | <0.001 |
| Mauritania | 34.05 (28.00, 41.07) | 35.61 (31.21, 40.48) | 0.15 (0.13, 0.17) | <0.001 | 210.44 (194.57, 227.29) | 221.22 (209.72, 233.20) | 0.19 (0.15, 0.22) | <0.001 | 301.08 (282.25, 320.87) | 164.91 (155.13, 175.16) | -2.02 (-2.16, -1.89) | <0.001 | 5.31 (3.07, 8.59) | 2.86 (1.70, 4.53) | -2.06 (-2.19, -1.93) | <0.001 |
| Mauritius | 31.64 (25.08, 39.43) | 28.43 (22.17, 35.99) | -0.36 (-0.39, -0.34) | <0.001 | 246.13 (227.04, 266.43) | 219.20 (201.27, 238.38) | -0.39 (-0.42, -0.37) | <0.001 | 1223.37 (1180.27, 1267.67) | 579.60 (550.13, 610.32) | -2.49 (-3.06, -1.91) | <0.001 | 22.14 (16.67, 28.86) | 10.30 (6.72, 15.21) | -2.55 (-3.13, -1.97) | <0.001 |
| Mexico | 30.66 (29.77, 31.57) | 29.20 (28.52, 29.89) | -0.17 (-0.19, -0.15) | <0.001 | 159.80 (157.70, 161.91) | 161.58 (159.97, 163.19) | 0.04 (-0.02, 0.09) | 0.173 | 349.53 (346.49, 352.60) | 478.53 (475.78, 481.29) | 1.09 (0.50, 1.68) | <0.001 | 6.13 (5.73, 6.55) | 8.33 (7.97, 8.70) | 1.06 (0.45, 1.66) | 0.001 |
| Micronesia (Federated States | 31.27 (11.03, | 29.75 (10.67, | -0.18 (-0.21, | <0.001 | 210.75 (148.50, | 202.73 (143.57, | -0.14 (-0.17, | <0.001 | 2292.04 (2073.61 | 2323.73 (2111.84 | 0.05 (-0.02, | 0.170 | 41.79 (17.21, | 41.78 (17.90, | -0.01 (-0.09, | 0.877 |

| | | | | | | | | | | | | | | | | |
|---|---|---|---|---|---|---|---|---|---|---|---|---|---|---|---|---|
| of) | 70.43) | 66.51) | -0.15) | | 290.70) | 278.49) | -0.10) | | , 2527.51) | , 2551.49) | 0.12) | | 85.27) | 83.27) | 0.08) | |
| Monaco | 35.82 (3.67, 171.87) | 30.10 (1.99, 138.56) | -0.60 (-0.65, -0.55) | <0.001 | 180.08 (83.91, 363.08) | 165.37 (71.78, 330.01) | -0.30 (-0.35, -0.24) | <0.001 | 314.89 (180.79, 531.88) | 182.93 (83.43, 353.03) | -1.85 (-1.92, -1.78) | <0.001 | 5.63 (0.00, 127.98) | 3.27 (0.00, 92.36) | -1.85 (-1.91, -1.79) | <0.001 |
| Mongolia | 46.37 (39.61, 54.02) | 45.77 (41.02, 50.99) | -0.01 (-0.14, 0.12) | 0.871 | 229.99 (214.12, 246.78) | 237.15 (226.27, 248.48) | 0.11 (0.10, 0.12) | <0.001 | 879.89 (848.75, 911.93) | 1096.91 (1073.30, 1120.98) | 0.79 (0.04, 1.54) | 0.039 | 15.81 (11.85, 20.74) | 19.71 (16.67, 23.23) | 0.78 (0.00, 1.57) | 0.049 |
| Montenegro | 33.59 (24.42, 45.14) | 30.24 (21.01, 42.37) | -0.36 (-0.37, -0.35) | <0.001 | 257.50 (230.82, 286.48) | 232.52 (205.75, 262.03) | -0.35 (-0.37, -0.33) | <0.001 | 717.39 (672.37, 764.70) | 314.13 (282.89, 348.09) | -3.54 (-5.72, -1.30) | 0.002 | 12.81 (7.44, 20.62) | 5.59 (2.17, 12.12) | -3.54 (-5.67, -1.36) | 0.002 |
| Morocco | 45.40 (43.46, 47.41) | 43.74 (42.24, 45.28) | -0.13 (-0.15, -0.11) | <0.001 | 324.76 (319.43, 330.16) | 323.43 (319.34, 327.56) | 0.00 (-0.08, 0.08) | 0.984 | 1005.55 (996.26, 1014.91) | 555.50 (550.14, 560.91) | -1.98 (-2.29, -1.66) | <0.001 | 17.80 (16.58, 19.10) | 9.65 (8.96, 10.39) | -2.04 (-2.35, -1.73) | <0.001 |
| Mozambique | 36.80 (34.04, 39.72) | 38.24 (36.43, 40.13) | 0.13 (0.12, 0.14) | <0.001 | 204.59 (197.91, 211.45) | 217.05 (212.54, 221.63) | 0.24 (0.09, 0.38) | 0.002 | 209.22 (202.62, 215.98) | 383.74 (377.92, 389.63) | 2.15 (1.96, 2.34) | <0.001 | 3.59 (2.77, 4.59) | 6.66 (5.91, 7.49) | 2.17 (1.99, 2.36) | <0.001 |
| Myanmar | 33.19 (31.90, 34.52) | 29.39 (28.36, 30.45) | -0.42 (-0.46, -0.39) | <0.001 | 186.01 (182.89, 189.18) | 163.70 (161.26, 166.17) | -0.43 (-0.50, -0.36) | <0.001 | 562.66 (557.33, 568.04) | 498.02 (493.77, 502.30) | -0.36 (-0.59, -0.12) | 0.003 | 9.82 (9.12, 10.56) | 8.47 (7.93, 9.05) | -0.45 (-0.67, -0.23) | <0.001 |
| Namibia | 37.98 (30.30, 47.14) | 36.55 (31.26, 42.52) | -0.13 (-0.15, -0.12) | <0.001 | 196.72 (177.98, 216.95) | 192.17 (179.49, 205.54) | -0.10 (-0.17, -0.02) | 0.010 | 186.65 (168.48, 206.31) | 223.76 (210.06, 238.14) | 0.72 (0.31, 1.12) | <0.001 | 3.29 (1.29, 6.98) | 3.96 (2.33, 6.33) | 0.74 (0.31, 1.17) | 0.001 |
| Nauru | 31.35 (0.09, 266.30) | 32.15 (0.19, 246.33) | 0.07 (0.04, 0.11) | <0.001 | 218.17 (60.37, 559.82) | 226.97 (70.59, 547.23) | 0.14 (0.13, 0.14) | <0.001 | 2998.34 (2265.23, 3896.69) | 3353.47 (2614.36, 4240.09) | 0.39 (0.26, 0.52) | <0.001 | 54.45 (1.44, 307.70) | 60.57 (2.92, 296.12) | 0.37 (0.24, 0.50) | <0.001 |
| Nepal | 29.31 (27.46, 31.26) | 27.11 (25.71, 28.57) | -0.27 (-0.28, -0.26) | <0.001 | 174.40 (169.75, 179.16) | 161.52 (157.98, 165.12) | -0.27 (-0.31, -0.23) | <0.001 | 546.81 (538.63, 555.09) | 553.42 (547.04, 559.86) | 0.06 (-0.19, 0.31) | 0.651 | 9.67 (8.60, 10.83) | 9.62 (8.79, 10.51) | -0.00 (-0.25, 0.24) | 0.978 |
| Netherlands | 62.84 (60.13, | 53.68 (50.98, | -0.54 (-0.56, | <0.001 | 260.08 (254.55, | 253.48 (247.60, | -0.13 (-0.28, | 0.104 | 298.16 (292.24, | 84.25 (80.88, | -4.21 (-4.63, | <0.001 | 5.39 (4.62, | 1.48 (1.06, | -4.39 (-4.75, | <0.001 |

| | | | | | | | | | | | | | | | | |
|---|---|---|---|---|---|---|---|---|---|---|---|---|---|---|---|---|
| | 65.66) | 56.49) | -0.52) | | 265.71) | 259.47) | 0.03) | | 304.19) | 87.73) | -3.78) | | 6.26) | 2.01) | -4.02) | |
| New Zealand | 64.63 (58.77, 70.93) | 54.88 (49.52, 60.67) | -0.55 (-0.60, -0.51) | <0.001 | 270.75 (258.61, 283.32) | 256.68 (244.93, 268.84) | -0.18 (-0.22, -0.15) | <0.001 | 372.16 (357.89, 386.85) | 151.30 (142.32, 160.70) | -3.00 (-3.67, -2.32) | <0.001 | 6.71 (4.92, 8.95) | 2.73 (1.65, 4.27) | -3.05 (-3.82, -2.26) | <0.001 |
| Nicaragua | 28.92 (24.72, 33.67) | 29.27 (26.45, 32.31) | 0.05 (0.03, 0.06) | <0.001 | 156.91 (146.55, 167.83) | 173.26 (166.20, 180.55) | 0.33 (0.29, 0.37) | <0.001 | 277.93 (264.49, 291.91) | 239.82 (231.66, 248.20) | -0.79 (-1.69, 0.12) | 0.090 | 4.83 (3.19, 7.04) | 4.02 (3.02, 5.25) | -0.92 (-1.84, 0.01) | 0.053 |
| Niger | 30.27 (27.15, 33.66) | 32.24 (30.28, 34.29) | 0.22 (0.19, 0.24) | <0.001 | 171.93 (164.16, 179.99) | 180.86 (176.00, 185.82) | 0.19 (0.11, 0.27) | <0.001 | 257.63 (248.23, 267.30) | 259.02 (253.31, 264.82) | 0.07 (-0.31, 0.45) | 0.720 | 4.53 (3.35, 6.00) | 4.55 (3.81, 5.38) | 0.07 (-0.31, 0.44) | 0.728 |
| Nigeria | 28.97 (28.12, 29.84) | 32.23 (31.62, 32.85) | 0.37 (0.34, 0.40) | <0.001 | 162.36 (160.30, 164.44) | 185.62 (184.11, 187.13) | 0.47 (0.40, 0.55) | <0.001 | 250.64 (248.11, 253.20) | 243.26 (241.57, 244.97) | -0.14 (-0.53, 0.25) | 0.492 | 4.43 (4.09, 4.78) | 4.27 (4.05, 4.51) | -0.16 (-0.55, 0.23) | 0.428 |
| Niue | 30.12 (0.00, 1091.41 ) | 30.63 (0.00, 1526.93 ) | 0.05 (0.04, 0.07) | <0.001 | 233.28 (3.46, 1470.07) | 245.61 (1.32, 1920.37) | 0.17 (0.13, 0.21) | <0.001 | 1647.02 (606.83, 3589.25) | 1187.96 (266.98, 3410.69) | -1.19 (-1.58, -0.79) | <0.001 | 29.81 (0.00, 1092.01 ) | 21.15 (0.00, 1508.38 ) | -1.19 (-1.51, -0.87) | <0.001 |
| North Macedonia | 29.44 (24.48, 35.11) | 26.66 (22.08, 32.07) | -0.34 (-0.36, -0.33) | <0.001 | 216.67 (202.86, 231.18) | 198.97 (186.31, 212.39) | -0.31 (-0.36, -0.26) | <0.001 | 691.11 (666.24, 716.68) | 424.11 (405.28, 443.73) | -1.50 (-2.83, -0.15) | 0.029 | 12.31 (9.20, 16.17) | 7.50 (5.20, 10.67) | -1.51 (-2.88, -0.11) | 0.035 |
| Northern Mariana Islands | 27.26 (6.55, 83.31) | 29.47 (2.69, 128.23) | 0.27 (0.26, 0.28) | <0.001 | 219.08 (146.65, 320.95) | 246.89 (133.39, 423.34) | 0.40 (0.36, 0.44) | <0.001 | 673.07 (540.95, 833.06) | 696.69 (493.05, 960.73) | 0.07 (-0.12, 0.25) | 0.478 | 12.14 (1.02, 61.24) | 12.50 (0.07, 101.95) | 0.05 (-0.15, 0.24) | 0.648 |
| Norway | 37.55 (33.54, 41.93) | 30.63 (27.23, 34.36) | -0.72 (-0.75, -0.68) | <0.001 | 185.83 (176.81, 195.22) | 162.56 (154.69, 170.76) | -0.47 (-0.49, -0.44) | <0.001 | 335.30 (323.14, 347.81) | 72.43 (67.22, 77.96) | -5.09 (-5.91, -4.26) | <0.001 | 6.03 (4.50, 7.94) | 1.27 (0.67, 2.24) | -5.17 (-6.04, -4.30) | <0.001 |
| Oman | 42.62 (37.34, 48.53) | 46.96 (43.99, 50.21) | 0.33 (0.29, 0.38) | <0.001 | 281.96 (268.16, 296.35) | 332.14 (324.41, 340.14) | 0.57 (0.52, 0.63) | <0.001 | 819.11 (795.15, 843.68) | 394.13 (384.59, 403.96) | -2.46 (-3.05, -1.87) | <0.001 | 14.40 (11.38, 18.05) | 6.69 (5.52, 8.18) | -2.58 (-3.19, -1.96) | <0.001 |
| Pakistan | 32.48 (31.65, 33.33) | 34.14 (33.58, 34.72) | 0.17 (0.12, 0.21) | <0.001 | 198.79 (196.66, 200.93) | 206.90 (205.47, 208.33) | 0.14 (0.13, 0.16) | <0.001 | 721.02 (717.04, 725.02) | 1404.81 (1401.16 , 1408.47) | 2.38 (2.14, 2.63) | <0.001 | 12.77 (12.24, 13.32) | 24.69 (24.20, 25.18) | 2.36 (2.11, 2.60) | <0.001 |

| | | | | | | | | | | | | | | | |
|---|---|---|---|---|---|---|---|---|---|---|---|---|---|---|---|
| Palau | 30.48 (0.92, 169.92) | 31.26 (1.42, 180.19) | 0.08 (0.06, 0.10) | <0.001 | 224.16 (94.23, 452.18) | 237.04 (112.92, 458.65) | 0.19 (0.18, 0.20) | <0.001 | 1817.20 (1392.12, 2333.32) | 1794.66 (1409.28, 2267.38) | 0.01 (-0.44, 0.46) | 0.972 | 32.88 (1.14, 174.87) | 32.36 (1.85, 180.32) | 0.00 (-0.44, 0.44) | 0.999 |
| Palestine | 44.50 (37.21, 52.91) | 44.57 (40.35, 49.14) | 0.00 (-0.02, 0.02) | 0.786 | 304.57 (284.27, 326.00) | 316.51 (304.81, 328.56) | 0.15 (0.05, 0.26) | 0.003 | 734.55 (703.25, 766.97) | 446.56 (432.76, 460.70) | -1.75 (-2.34, -1.16) | <0.001 | 13.03 (9.12, 18.13) | 7.86 (6.12, 9.97) | -1.78 (-2.40, -1.15) | <0.001 |
| Panama | 26.78 (22.26, 31.98) | 27.46 (23.94, 31.36) | 0.09 (0.08, 0.10) | <0.001 | 139.19 (128.34, 150.73) | 157.32 (148.68, 166.33) | 0.41 (0.33, 0.49) | <0.001 | 284.92 (269.54, 300.98) | 167.12 (158.23, 176.38) | -1.64 (-2.64, -0.63) | 0.001 | 5.00 (3.14, 7.58) | 2.92 (1.85, 4.38) | -1.66 (-2.69, -0.61) | 0.002 |
| Papua New Guinea | 30.84 (26.98, 35.14) | 31.62 (29.15, 34.24) | 0.08 (0.07, 0.10) | <0.001 | 220.35 (209.53, 231.60) | 232.54 (225.66, 239.57) | 0.18 (0.17, 0.20) | <0.001 | 827.59 (806.42, 849.18) | 1027.75 (1013.25, 1042.41) | 0.68 (0.52, 0.84) | <0.001 | 15.00 (12.26, 18.19) | 18.56 (16.65, 20.63) | 0.67 (0.51, 0.82) | <0.001 |
| Paraguay | 20.67 (17.50, 24.25) | 20.71 (18.47, 23.15) | 0.01 (-0.01, 0.04) | 0.300 | 202.57 (192.15, 213.42) | 217.05 (209.58, 224.73) | 0.25 (0.23, 0.27) | <0.001 | 305.56 (292.81, 318.72) | 286.87 (278.30, 295.64) | -0.02 (-0.96, 0.93) | 0.969 | 5.35 (3.78, 7.37) | 4.94 (3.87, 6.21) | -0.07 (-1.01, 0.88) | 0.886 |
| Peru | 12.40 (11.32, 13.57) | 12.26 (11.44, 13.12) | -0.03 (-0.10, 0.04) | 0.438 | 96.17 (93.00, 99.42) | 98.75 (96.39, 101.15) | 0.09 (0.07, 0.11) | <0.001 | 485.12 (478.23, 492.09) | 273.86 (269.94, 277.82) | -1.74 (-2.57, -0.90) | <0.001 | 8.30 (7.41, 9.27) | 4.65 (4.15, 5.19) | -1.78 (-2.62, -0.94) | <0.001 |
| Philippines | 48.57 (47.30, 49.86) | 46.89 (46.00, 47.79) | -0.12 (-0.14, -0.09) | <0.001 | 243.41 (240.50, 246.34) | 249.44 (247.36, 251.54) | 0.09 (0.04, 0.13) | <0.001 | 398.44 (394.77, 402.14) | 1164.90 (1160.43, 1169.37) | 3.85 (3.20, 4.51) | <0.001 | 6.97 (6.49, 7.48) | 20.44 (19.85, 21.04) | 3.87 (3.16, 4.57) | <0.001 |
| Poland | 19.16 (18.22, 20.15) | 17.20 (16.25, 18.20) | -0.37 (-0.40, -0.34) | <0.001 | 170.87 (168.07, 173.70) | 154.46 (151.72, 157.25) | -0.35 (-0.38, -0.31) | <0.001 | 1076.51 (1069.52, 1083.54) | 163.71 (160.93, 166.54) | -6.45 (-7.51, -5.37) | <0.001 | 19.63 (18.70, 20.60) | 2.91 (2.55, 3.32) | -6.53 (-7.63, -5.42) | <0.001 |
| Portugal | 17.28 (15.44, 19.29) | 14.75 (12.94, 16.77) | -0.55 (-0.59, -0.51) | <0.001 | 121.14 (116.16, 126.28) | 132.91 (127.45, 138.55) | 0.58 (-0.15, 1.31) | 0.119 | 408.51 (399.33, 417.85) | 120.95 (115.79, 126.30) | -4.20 (-5.20, -3.19) | <0.001 | 7.24 (6.06, 8.58) | 2.16 (1.53, 3.00) | -4.19 (-5.24, -3.13) | <0.001 |
| Puerto Rico | 66.24 (60.14, | 68.69 (61.98, | 0.13 (0.08, | <0.001 | 332.29 (318.34, | 344.59 (329.43, | 0.12 (0.10, | <0.001 | 406.22 (390.80, | 191.82 (180.55, | -2.50 (-3.43, | <0.001 | 7.23 (5.31, | 3.35 (2.01, | -2.48 (-3.97, | 0.001 |

| | | | | | | | | | | | | | | | | |
|---|---|---|---|---|---|---|---|---|---|---|---|---|---|---|---|---|
| | 72.79) | 75.94) | 0.17) | | 346.69) | 360.28) | 0.15) | | 422.09) | 203.62) | -1.57) | | 9.64) | 5.25) | -0.97) | |
| Qatar | 39.84 (31.50, 51.00) | 37.34 (34.26, 40.96) | -0.23 (-0.28, -0.17) | <0.001 | 267.51 (245.90, 291.59) | 254.61 (246.74, 262.97) | -0.17 (-0.20, -0.14) | <0.001 | 377.07 (349.09, 407.65) | 159.06 (152.12, 166.52) | -2.86 (-3.56, -2.15) | <0.001 | 6.47 (3.34, 13.05) | 2.69 (1.87, 4.24) | -2.90 (-3.66, -2.14) | <0.001 |
| Republic of Korea | 21.33 (20.46, 22.24) | 19.31 (18.47, 20.19) | -0.35 (-0.37, -0.32) | <0.001 | 107.53 (105.53, 109.56) | 105.00 (103.05, 106.98) | -0.09 (-0.12, -0.06) | <0.001 | 727.76 (722.60, 732.96) | 91.57 (89.74, 93.43) | -6.93 (-7.78, -6.08) | <0.001 | 12.41 (11.75, 13.11) | 1.60 (1.37, 1.87) | -6.85 (-7.74, -5.96) | <0.001 |
| Republic of Moldova | 42.83 (38.69, 47.34) | 43.20 (38.66, 48.23) | 0.02 (-0.04, 0.09) | 0.524 | 199.95 (190.99, 209.26) | 201.02 (191.37, 211.12) | 0.00 (-0.08, 0.08) | 0.951 | 595.76 (580.09, 611.78) | 520.87 (505.14, 537.05) | -0.45 (-1.67, 0.79) | 0.477 | 10.66 (8.66, 13.02) | 9.36 (7.37, 11.85) | -0.47 (-1.89, 0.97) | 0.522 |
| Romania | 24.96 (23.50, 26.48) | 24.28 (22.59, 26.07) | -0.09 (-0.12, -0.07) | <0.001 | 205.11 (200.95, 209.34) | 196.12 (191.38, 200.96) | -0.15 (-0.18, -0.12) | <0.001 | 783.78 (775.63, 792.00) | 406.61 (399.72, 413.59) | -2.29 (-3.36, -1.21) | <0.001 | 14.13 (13.06, 15.27) | 7.24 (6.35, 8.23) | -2.34 (-3.44, -1.23) | <0.001 |
| Russian Federation | 44.86 (44.13, 45.60) | 42.91 (42.15, 43.69) | -0.15 (-0.20, -0.10) | <0.001 | 176.59 (175.15, 178.03) | 175.79 (174.29, 177.31) | -0.03 (-0.10, 0.04) | 0.411 | 1077.89 (1074.35, 1081.44) | 798.03 (794.80, 801.28) | -1.10 (-2.56, 0.38) | 0.143 | 19.67 (19.20, 20.16) | 14.41 (13.98, 14.85) | -1.13 (-2.58, 0.34) | 0.130 |
| Rwanda | 28.61 (25.65, 31.83) | 28.20 (26.14, 30.40) | -0.05 (-0.07, -0.02) | <0.001 | 156.61 (149.39, 164.08) | 155.16 (150.17, 160.27) | -0.03 (-0.04, -0.02) | <0.001 | 353.92 (343.24, 364.86) | 236.27 (230.20, 242.46) | -1.41 (-1.63, -1.18) | <0.001 | 6.19 (4.84, 7.81) | 4.09 (3.33, 4.99) | -1.44 (-1.66, -1.22) | <0.001 |
| Saint Kitts and Nevis | 66.21 (22.42, 154.31) | 67.95 (29.39, 136.34) | 0.10 (0.08, 0.11) | <0.001 | 321.61 (207.45, 477.65) | 332.60 (238.27, 454.19) | 0.11 (0.09, 0.13) | <0.001 | 678.46 (505.54, 892.70) | 168.71 (103.39, 262.27) | -4.39 (-6.08, -2.68) | <0.001 | 12.36 (0.24, 75.02) | 2.90 (0.00, 41.72) | -4.55 (-6.16, -2.90) | <0.001 |
| Saint Lucia | 69.23 (39.62, 113.82) | 70.63 (45.47, 105.14) | 0.07 (0.05, 0.09) | <0.001 | 347.31 (272.96, 436.41) | 349.95 (290.76, 418.05) | 0.02 (0.02, 0.03) | <0.001 | 374.11 (296.49, 466.52) | 161.91 (122.49, 210.41) | -2.79 (-3.78, -1.78) | <0.001 | 6.70 (0.42, 31.13) | 2.80 (0.07, 16.79) | -2.90 (-3.99, -1.80) | <0.001 |
| Saint Vincent and the Grenadines | 66.87 (35.85, 115.93) | 67.96 (37.44, 113.77) | 0.06 (0.01, 0.11) | 0.017 | 330.94 (252.78, 426.74) | 334.37 (260.84, 422.50) | 0.04 (0.02, 0.05) | <0.001 | 585.57 (480.04, 708.47) | 284.14 (216.77, 366.14) | -2.22 (-3.86, -0.55) | 0.009 | 10.49 (1.17, 41.08) | 5.01 (0.15, 27.57) | -2.26 (-4.01, -0.47) | 0.013 |
| Samoa | 32.17 (14.48, 63.23) | 31.80 (16.27, 56.43) | -0.04 (-0.06, -0.03) | <0.001 | 254.25 (195.70, 325.54) | 255.36 (205.42, 314.00) | 0.01 (0.01, 0.02) | 0.001 | 991.27 (871.42, 1123.58) | 1182.68 (1072.42, 1301.43) | 0.63 (0.57, 0.69) | <0.001 | 17.93 (5.29, 45.03) | 21.22 (8.86, 43.05) | 0.60 (0.54, 0.67) | <0.001 |

| | | | | | | | | | | | | | | | | |
|---|---|---|---|---|---|---|---|---|---|---|---|---|---|---|---|---|
| San Marino | 36.02 (3.08, 149.31) | 29.96 (2.19, 131.25) | -0.63 (-0.68, -0.58) | <0.001 | 184.79 (81.43, 361.25) | 168.36 (75.30, 327.13) | -0.32 (-0.37, -0.28) | <0.001 | 119.46 (40.86, 272.86) | 131.59 (51.77, 277.91) | 0.33 (0.18, 0.48) | <0.001 | 2.10 (0.00, 89.98) | 2.35 (0.00, 82.91) | 0.38 (0.24, 0.52) | <0.001 |
| Sao Tome and Principe | 31.88 (10.41, 77.84) | 34.48 (18.81, 58.19) | 0.26 (0.24, 0.28) | <0.001 | 199.42 (133.08, 288.81) | 218.31 (174.55, 269.80) | 0.33 (0.27, 0.38) | <0.001 | 251.51 (175.30, 350.90) | 261.65 (214.20, 316.67) | 0.06 (-0.78, 0.91) | 0.889 | 4.53 (0.02, 38.19) | 4.51 (0.46, 17.61) | -0.14 (-1.44, 1.18) | 0.838 |
| Saudi Arabia | 40.23 (38.21, 42.35) | 43.55 (42.40, 44.74) | 0.27 (0.24, 0.30) | <0.001 | 259.48 (254.22, 264.83) | 298.58 (295.63, 301.56) | 0.50 (0.39, 0.62) | <0.001 | 451.16 (444.22, 458.19) | 496.90 (493.04, 500.78) | 0.31 (-0.28, 0.91) | 0.307 | 8.04 (7.13, 9.03) | 8.81 (8.31, 9.34) | 0.29 (-0.30, 0.89) | 0.335 |
| Senegal | 35.05 (31.61, 38.78) | 35.92 (33.65, 38.32) | 0.08 (0.06, 0.10) | <0.001 | 221.33 (212.23, 230.72) | 223.95 (218.02, 230.00) | 0.06 (0.00, 0.11) | 0.034 | 280.31 (270.20, 290.71) | 266.19 (259.84, 272.65) | -0.04 (-0.24, 0.16) | 0.659 | 4.93 (3.66, 6.51) | 4.63 (3.82, 5.57) | -0.08 (-0.28, 0.12) | 0.428 |
| Serbia | 38.62 (35.83, 41.58) | 39.22 (36.16, 42.48) | 0.05 (0.03, 0.07) | <0.001 | 254.43 (247.27, 261.75) | 261.36 (253.50, 269.42) | 0.08 (0.02, 0.14) | 0.010 | 640.11 (628.72, 651.68) | 251.25 (243.56, 259.15) | -3.09 (-3.78, -2.41) | <0.001 | 11.53 (10.05, 13.18) | 4.46 (3.49, 5.64) | -3.14 (-3.85, -2.43) | <0.001 |
| Seychelles | 30.62 (9.08, 77.42) | 28.60 (11.23, 63.56) | -0.23 (-0.25, -0.21) | <0.001 | 212.40 (142.98, 305.04) | 197.55 (145.66, 264.73) | -0.24 (-0.29, -0.20) | <0.001 | 716.12 (582.23, 872.61) | 489.10 (404.31, 588.84) | -1.14 (-2.34, 0.07) | 0.064 | 12.91 (1.28, 51.93) | 8.63 (1.11, 35.76) | -1.21 (-2.41, 0.01) | 0.051 |
| Sierra Leone | 37.18 (32.38, 42.51) | 38.24 (35.15, 41.54) | 0.10 (0.09, 0.11) | <0.001 | 223.98 (211.70, 236.81) | 227.68 (219.80, 235.78) | 0.08 (-0.06, 0.22) | 0.275 | 280.65 (267.00, 294.84) | 310.26 (301.17, 319.56) | 0.34 (0.04, 0.64) | 0.025 | 4.94 (3.27, 7.18) | 5.44 (4.29, 6.82) | 0.33 (0.02, 0.64) | 0.038 |
| Singapore | 37.10 (32.96, 41.64) | 27.96 (25.09, 31.15) | -0.95 (-1.04, -0.87) | <0.001 | 184.39 (174.96, 194.21) | 136.57 (130.53, 142.91) | -1.04 (-1.11, -0.97) | <0.001 | 316.92 (304.52, 329.71) | 135.70 (129.64, 142.06) | -2.85 (-4.01, -1.68) | <0.001 | 5.71 (4.15, 7.68) | 2.41 (1.68, 3.50) | -2.91 (-4.14, -1.66) | <0.001 |
| Slovakia | 32.81 (29.50, 36.41) | 25.44 (22.46, 28.77) | -0.87 (-0.91, -0.84) | <0.001 | 245.07 (236.02, 254.41) | 188.90 (180.89, 197.25) | -0.90 (-0.92, -0.88) | <0.001 | 713.87 (698.44, 729.57) | 218.92 (210.34, 227.84) | -4.18 (-4.92, -3.44) | <0.001 | 12.98 (10.98, 15.27) | 3.91 (2.85, 5.35) | -4.25 (-5.01, -3.48) | <0.001 |
| Slovenia | 34.21 (28.83, 40.36) | 28.53 (23.21, 34.92) | -0.63 (-0.65, -0.60) | <0.001 | 247.61 (233.00, 262.97) | 201.29 (187.23, 216.33) | -0.73 (-0.84, -0.63) | <0.001 | 297.26 (281.36, 313.89) | 82.72 (73.95, 92.47) | -4.50 (-5.09, -3.92) | <0.001 | 5.35 (3.41, 8.09) | 1.42 (0.50, 3.63) | -4.65 (-5.32, -3.98) | <0.001 |
| Solomon Islands | 32.13 (18.59, 52.20) | 31.95 (22.58, 43.97) | -0.03 (-0.08, 0.02) | 0.244 | 206.65 (168.30, 251.36) | 210.32 (184.66, 238.57) | 0.06 (0.05, 0.07) | <0.001 | 3292.17 (3133.81 , | 3648.83 (3539.58 , | 0.37 (0.12, 0.61) | 0.003 | 59.59 (39.91, 85.84) | 65.89 (51.91, 82.52) | 0.36 (0.12, 0.60) | 0.004 |

| | | | | | | | | | | | | | | | | |
|---|---|---|---|---|---|---|---|---|---|---|---|---|---|---|---|---|
| | | | | | | | | | 3456.68) | 3760.62) | | | | | | |
| Somalia | 33.23 (29.92, 36.81) | 35.86 (33.87, 37.94) | 0.26 (0.24, 0.28) | <0.001 | 184.34 (176.45, 192.51) | 200.28 (195.40, 205.25) | 0.30 (0.24, 0.37) | <0.001 | 374.47 (363.31, 385.91) | 469.68 (462.36, 477.09) | 0.80 (0.61, 0.99) | <0.001 | 6.62 (5.21, 8.31) | 8.23 (7.28, 9.27) | 0.80 (0.64, 0.95) | <0.001 |
| South Africa | 44.57 (43.02, 46.17) | 42.89 (41.76, 44.05) | -0.13 (-0.15, -0.11) | <0.001 | 206.38 (202.93, 209.86) | 203.64 (201.17, 206.14) | -0.04 (-0.08, 0.01) | 0.123 | 441.37 (436.29, 446.50) | 258.19 (255.41, 261.01) | -2.03 (-3.51, -0.53) | 0.008 | 8.03 (7.36, 8.75) | 4.60 (4.24, 5.00) | -2.11 (-3.68, -0.53) | 0.009 |
| South Sudan | 29.60 (26.32, 33.20) | 33.12 (30.00, 36.49) | 0.39 (0.38, 0.40) | <0.001 | 182.84 (174.17, 191.83) | 208.73 (200.64, 217.07) | 0.45 (0.43, 0.48) | <0.001 | 207.40 (198.34, 216.78) | 247.60 (238.98, 256.46) | 0.71 (0.28, 1.13) | 0.001 | 3.61 (2.50, 5.08) | 4.28 (3.21, 5.60) | 0.68 (0.25, 1.10) | 0.002 |
| Spain | 38.61 (37.19, 40.07) | 34.02 (32.68, 35.41) | -0.44 (-0.48, -0.41) | <0.001 | 172.87 (169.83, 175.95) | 170.31 (167.41, 173.26) | -0.04 (-0.11, 0.03) | 0.227 | 418.73 (414.02, 423.48) | 139.66 (137.00, 142.36) | -3.79 (-4.48, -3.09) | <0.001 | 7.37 (6.75, 8.03) | 2.46 (2.12, 2.84) | -3.78 (-4.48, -3.07) | <0.001 |
| Sri Lanka | 30.69 (28.90, 32.58) | 28.81 (27.17, 30.52) | -0.22 (-0.27, -0.17) | <0.001 | 212.78 (207.97, 217.68) | 201.28 (196.94, 205.69) | -0.19 (-0.22, -0.17) | <0.001 | 846.08 (836.51, 855.74) | 456.36 (449.77, 463.02) | -1.99 (-4.21, 0.29) | 0.086 | 14.95 (13.69, 16.28) | 7.98 (7.13, 8.90) | -2.02 (-4.20, 0.21) | 0.076 |
| Sudan | 42.88 (40.60, 45.26) | 45.52 (43.98, 47.11) | 0.21 (0.19, 0.23) | <0.001 | 264.23 (258.39, 270.16) | 299.90 (295.82, 304.02) | 0.46 (0.31, 0.62) | <0.001 | 1540.28 (1526.31 , 1554.35) | 1001.57 (994.26, 1008.91) | -1.50 (-1.58, -1.41) | <0.001 | 27.48 (25.63, 29.43) | 17.58 (16.61, 18.58) | -1.56 (-1.64, -1.48) | <0.001 |
| Suriname | 67.05 (49.39, 89.50) | 75.22 (59.59, 93.75) | 0.40 (0.35, 0.44) | <0.001 | 326.12 (283.64, 373.46) | 373.17 (337.31, 411.87) | 0.47 (0.44, 0.50) | <0.001 | 736.96 (672.36, 806.36) | 423.78 (385.54, 464.85) | -1.94 (-3.57, -0.28) | 0.022 | 13.23 (5.87, 26.03) | 7.54 (3.26, 14.95) | -1.98 (-3.67, -0.25) | 0.025 |
| Sweden | 36.82 (33.88, 39.95) | 41.22 (38.27, 44.35) | 0.39 (0.36, 0.41) | <0.001 | 192.09 (185.34, 199.03) | 208.06 (201.46, 214.85) | 0.27 (0.23, 0.32) | <0.001 | 203.28 (196.34, 210.42) | 61.68 (58.10, 65.45) | -3.89 (-4.19, -3.58) | <0.001 | 3.61 (2.74, 4.69) | 1.05 (0.63, 1.67) | -4.06 (-4.38, -3.74) | <0.001 |
| Switzerland | 33.91 (30.99, 37.07) | 28.05 (25.49, 30.83) | -0.65 (-0.67, -0.64) | <0.001 | 164.99 (158.50, 171.71) | 149.83 (143.97, 155.90) | -0.33 (-0.35, -0.30) | <0.001 | 302.05 (293.19, 311.15) | 71.18 (67.16, 75.43) | -4.97 (-6.12, -3.79) | <0.001 | 5.35 (4.23, 6.71) | 1.24 (0.77, 1.97) | -5.01 (-6.23, -3.78) | <0.001 |
| Syrian Arab Republic | 40.49 (37.70, 43.45) | 42.49 (39.55, 45.61) | 0.17 (0.14, 0.19) | <0.001 | 268.86 (261.38, 276.52) | 297.36 (289.59, 305.30) | 0.38 (0.26, 0.50) | <0.001 | 2260.62 (2239.96 , 2281.45) | 1321.37 (1305.24 , 1337.69) | -1.81 (-2.46, -1.15) | <0.001 | 38.84 (36.14, 41.71) | 22.66 (20.59, 24.92) | -1.87 (-2.46, -1.28) | <0.001 |

| | | | | | | | | | | | | | | | | |
|---|---|---|---|---|---|---|---|---|---|---|---|---|---|---|---|---|
| Taiwan (Province of China) | 23.87 (22.52, 25.29) | 22.24 (20.88, 23.68) | -0.24 (-0.29, -0.19) | <0.001 | 255.92 (251.44, 260.47) | 272.50 (267.76, 277.31) | 0.20 (-0.02, 0.42) | 0.077 | 210.71 (206.63, 214.85) | 192.77 (188.81, 196.80) | -0.33 (-1.02, 0.36) | 0.345 | 3.54 (3.03, 4.12) | 3.29 (2.79, 3.86) | -0.38 (-1.17, 0.42) | 0.350 |
| Tajikistan | 43.21 (38.97, 47.81) | 41.94 (39.12, 44.92) | -0.10 (-0.13, -0.08) | <0.001 | 228.31 (218.13, 238.86) | 224.31 (217.61, 231.16) | -0.06 (-0.09, -0.03) | <0.001 | 588.73 (572.19, 605.64) | 614.48 (603.37, 625.75) | 0.22 (-0.39, 0.84) | 0.477 | 10.69 (8.55, 13.22) | 11.10 (9.65, 12.72) | 0.21 (-0.40, 0.82) | 0.508 |
| Thailand | 26.78 (25.86, 27.73) | 25.62 (24.76, 26.50) | -0.16 (-0.18, -0.13) | <0.001 | 161.68 (159.37, 164.02) | 157.53 (155.42, 159.67) | -0.08 (-0.16, -0.00) | 0.041 | 409.15 (405.50, 412.82) | 456.12 (452.46, 459.80) | 0.52 (-0.30, 1.34) | 0.212 | 7.20 (6.72, 7.71) | 7.99 (7.52, 8.49) | 0.50 (-0.29, 1.29) | 0.218 |
| Timor-Leste | 28.62 (20.64, 38.81) | 28.14 (21.33, 36.56) | -0.06 (-0.07, -0.05) | <0.001 | 185.60 (163.83, 209.53) | 184.60 (165.88, 204.94) | -0.00 (-0.08, 0.08) | 0.958 | 334.07 (304.96, 365.30) | 527.36 (496.61, 559.63) | 1.63 (0.98, 2.28) | <0.001 | 5.86 (2.60, 11.52) | 9.13 (5.46, 14.48) | 1.57 (0.94, 2.22) | <0.001 |
| Togo | 33.89 (29.09, 39.31) | 36.23 (33.08, 39.60) | 0.22 (0.15, 0.29) | <0.001 | 194.86 (182.62, 207.73) | 209.21 (201.37, 217.28) | 0.27 (0.22, 0.33) | <0.001 | 324.03 (308.40, 340.27) | 376.72 (366.29, 387.37) | 0.59 (0.28, 0.90) | <0.001 | 5.74 (3.81, 8.33) | 6.66 (5.33, 8.23) | 0.57 (0.21, 0.94) | 0.002 |
| Tokelau | 30.51 (0.00, 1642.92 ) | 30.85 (0.00, 1857.45 ) | 0.03 (0.00, 0.06) | 0.028 | 241.89 (0.74, 2037.58) | 255.42 (0.82, 2256.95) | 0.17 (0.12, 0.23) | <0.001 | 897.22 (127.91, 3116.82) | 947.67 (136.24, 3372.54) | 0.19 (0.13, 0.24) | <0.001 | 16.17 (0.00, 1616.12 ) | 16.96 (0.00, 1831.72 ) | 0.16 (0.11, 0.22) | <0.001 |
| Tonga | 31.86 (9.21, 83.02) | 32.15 (10.76, 74.71) | 0.02 (-0.01, 0.06) | 0.149 | 267.84 (184.75, 377.21) | 278.74 (202.29, 374.91) | 0.13 (0.10, 0.15) | <0.001 | 666.32 (529.37, 829.19) | 791.62 (658.87, 943.62) | 0.59 (0.42, 0.76) | <0.001 | 12.04 (0.81, 55.12) | 14.14 (2.00, 48.88) | 0.55 (0.38, 0.71) | <0.001 |
| Trinidad and Tobago | 79.89 (69.08, 91.97) | 79.26 (69.14, 90.57) | -0.03 (-0.04, -0.02) | <0.001 | 415.59 (390.03, 442.42) | 411.56 (388.88, 435.37) | -0.02 (-0.05, 0.01) | 0.214 | 677.30 (644.52, 711.34) | 382.94 (361.13, 405.89) | -1.92 (-2.59, -1.24) | <0.001 | 12.19 (8.14, 17.58) | 6.83 (4.21, 10.70) | -1.95 (-2.64, -1.25) | <0.001 |
| Tunisia | 38.66 (35.57, 41.97) | 39.27 (36.78, 41.90) | 0.05 (0.03, 0.08) | <0.001 | 251.18 (243.01, 259.57) | 267.48 (261.03, 274.06) | 0.23 (0.18, 0.28) | <0.001 | 648.79 (635.81, 661.98) | 538.47 (529.17, 547.91) | -0.63 (-0.72, -0.54) | <0.001 | 11.44 (9.76, 13.34) | 9.43 (8.24, 10.76) | -0.66 (-0.76, -0.56) | <0.001 |
| Turkey | 27.07 (26.12, 28.05) | 22.04 (21.37, 22.73) | -0.71 (-0.74, -0.69) | <0.001 | 256.90 (253.91, 259.91) | 209.88 (207.81, 211.97) | -0.65 (-0.76, -0.53) | <0.001 | 838.13 (832.72, 843.57) | 267.69 (265.34, 270.05) | -3.88 (-4.29, -3.46) | <0.001 | 15.09 (14.37, 15.84) | 4.70 (4.39, 5.02) | -3.93 (-4.40, -3.47) | <0.001 |
| Turkmenistan | 43.04 (38.19, | 42.88 (39.12, | -0.00 (-0.03, | 0.721 | 219.97 (208.56, | 230.11 (221.26, | 0.16 (0.14, | <0.001 | 1096.24 (1070.53 | 944.56 (926.52, | -0.49 (-1.60, | 0.388 | 19.89 (16.54, | 16.93 (14.59, | -0.54 (-1.64, | 0.338 |

| | | | | | | | | | | | | | | | | |
|---|---|---|---|---|---|---|---|---|---|---|---|---|---|---|---|---|
| | 48.37) | 46.92) | 0.02) | | 231.87) | 239.23) | 0.17) | | , 1122.43) | 962.87) | 0.63) | | 23.74) | 19.56) | 0.57) | |
| Tuvalu | 31.46 (0.03, 306.11) | 31.88 (0.31, 224.18) | 0.04 (0.03, 0.06) | <0.001 | 223.49 (53.79, 614.11) | 236.73 (82.60, 534.69) | 0.19 (0.18, 0.21) | <0.001 | 1568.75 (1009.85 , 2329.38) | 1939.78 (1418.04 , 2593.68) | 0.71 (0.60, 0.82) | <0.001 | 28.48 (0.01, 301.73) | 35.05 (0.44, 230.72) | 0.69 (0.58, 0.80) | <0.001 |
| Uganda | 29.22 (27.13, 31.44) | 29.66 (28.31, 31.07) | 0.05 (0.02, 0.09) | 0.001 | 154.44 (149.39, 159.64) | 158.72 (155.44, 162.06) | 0.10 (0.08, 0.12) | <0.001 | 213.31 (207.51, 219.24) | 324.66 (320.14, 329.24) | 1.41 (1.12, 1.69) | <0.001 | 3.71 (2.97, 4.58) | 5.63 (5.04, 6.27) | 1.39 (1.11, 1.68) | <0.001 |
| Ukraine | 44.15 (42.86, 45.47) | 43.38 (41.98, 44.81) | -0.07 (-0.10, -0.04) | <0.001 | 195.93 (193.23, 198.66) | 182.28 (179.49, 185.11) | -0.25 (-0.26, -0.23) | <0.001 | 763.16 (757.81, 768.53) | 1531.03 (1522.75 , 1539.35) | 2.41 (0.67, 4.18) | 0.006 | 13.78 (13.08, 14.52) | 27.36 (26.27, 28.49) | 2.37 (0.59, 4.18) | 0.009 |
| United Arab Emirates | 41.24 (36.81, 46.31) | 42.58 (40.06, 45.30) | 0.11 (0.08, 0.14) | <0.001 | 276.50 (265.27, 288.33) | 294.12 (288.41, 300.01) | 0.22 (0.16, 0.29) | <0.001 | 506.36 (489.60, 523.75) | 392.65 (385.04, 400.47) | -0.93 (-1.30, -0.56) | <0.001 | 8.77 (6.73, 11.52) | 6.84 (5.91, 7.99) | -0.90 (-1.30, -0.50) | <0.001 |
| United Kingdom | 37.10 (35.95, 38.28) | 30.17 (29.20, 31.18) | -0.71 (-0.76, -0.67) | <0.001 | 197.40 (194.74, 200.08) | 159.61 (157.39, 161.86) | -0.73 (-0.78, -0.68) | <0.001 | 340.02 (336.52, 343.54) | 181.93 (179.57, 184.32) | -2.13 (-2.56, -1.70) | <0.001 | 6.18 (5.71, 6.67) | 3.26 (2.95, 3.60) | -2.18 (-2.63, -1.73) | <0.001 |
| United Republic of Tanzania | 30.50 (28.76, 32.33) | 34.89 (33.69, 36.14) | 0.47 (0.46, 0.48) | <0.001 | 161.19 (157.02, 165.44) | 190.46 (187.55, 193.40) | 0.60 (0.46, 0.73) | <0.001 | 264.28 (259.11, 269.54) | 280.06 (276.63, 283.52) | 0.20 (-0.07, 0.46) | 0.143 | 4.56 (3.90, 5.31) | 4.80 (4.36, 5.28) | 0.17 (-0.11, 0.44) | 0.228 |
| United States of America | 44.83 (44.27, 45.39) | 38.77 (38.27, 39.29) | -0.50 (-0.55, -0.45) | <0.001 | 191.99 (190.84, 193.14) | 186.62 (185.51, 187.73) | -0.07 (-0.26, 0.11) | 0.452 | 345.00 (343.46, 346.55) | 238.92 (237.66, 240.18) | -1.22 (-1.67, -0.78) | <0.001 | 6.21 (6.01, 6.42) | 4.23 (4.06, 4.40) | -1.27 (-1.75, -0.79) | <0.001 |
| United States Virgin Islands | 62.39 (31.46, 111.45) | 63.95 (29.81, 121.06) | 0.10 (0.04, 0.15) | 0.001 | 293.48 (220.22, 383.82) | 304.14 (222.77, 406.75) | 0.12 (0.11, 0.13) | <0.001 | 677.51 (563.36, 808.47) | 314.62 (231.84, 418.57) | -2.52 (-3.31, -1.73) | <0.001 | 12.00 (1.67, 42.20) | 5.60 (0.08, 37.63) | -2.52 (-3.53, -1.50) | <0.001 |
| Uruguay | 25.02 (21.02, 29.56) | 23.07 (19.39, 27.24) | -0.28 (-0.29, -0.27) | <0.001 | 130.40 (121.02, 140.32) | 123.72 (115.02, 132.91) | -0.18 (-0.20, -0.15) | <0.001 | 344.03 (328.68, 359.92) | 134.95 (125.86, 144.52) | -2.94 (-3.34, -2.54) | <0.001 | 6.22 (4.31, 8.69) | 2.39 (1.32, 3.98) | -3.02 (-3.43, -2.61) | <0.001 |
| Uzbekistan | 38.67 (36.72, | 39.60 (38.15, | 0.09 (0.07, | <0.001 | 198.69 (194.15, | 213.66 (210.27, | 0.25 (0.23, | <0.001 | 771.73 (762.76, | 1203.01 (1194.97 | 1.67 (1.07, | <0.001 | 13.98 (12.79, | 21.33 (20.27, | 1.60 (0.99, | <0.001 |

| | | | | | | | | | | | | | | | | |
|---|---|---|---|---|---|---|---|---|---|---|---|---|---|---|---|---|
| | 40.70) | 41.08) | 0.10) | | 203.31) | 217.10) | 0.28) | | 780.79) | , 1211.09) | 2.27) | | 15.25) | 22.43) | 2.20) | |
| Vanuatu | 35.77 (16.53, 68.03) | 35.53 (21.35, 55.83) | -0.03 (-0.06, -0.01) | 0.007 | 261.77 (202.04, 333.89) | 262.39 (219.98, 310.71) | 0.01 (-0.00, 0.01) | 0.107 | 1828.69 (1663.94 , 2005.54) | 2495.48 (2360.99 , 2635.76) | 1.04 (0.89, 1.19) | <0.001 | 33.22 (14.49, 65.30) | 45.12 (28.64, 67.77) | 1.03 (0.88, 1.17) | <0.001 |
| Venezuela (Bolivarian Republic of) | 32.92 (31.08, 34.83) | 30.19 (28.72, 31.71) | -0.30 (-0.32, -0.27) | <0.001 | 174.82 (170.48, 179.25) | 167.56 (164.14, 171.03) | -0.14 (-0.23, -0.06) | 0.001 | 644.32 (636.04, 652.68) | 413.74 (408.33, 419.20) | -1.46 (-2.24, -0.68) | <0.001 | 11.39 (10.31, 12.56) | 7.33 (6.63, 8.09) | -1.48 (-2.26, -0.68) | <0.001 |
| Viet Nam | 28.20 (27.26, 29.17) | 26.66 (25.98, 27.37) | -0.19 (-0.22, -0.17) | <0.001 | 185.88 (183.39, 188.39) | 180.22 (178.45, 182.01) | -0.10 (-0.13, -0.08) | <0.001 | 363.66 (360.21, 367.13) | 250.42 (248.31, 252.55) | -1.28 (-1.43, -1.13) | <0.001 | 6.43 (5.97, 6.91) | 4.36 (4.09, 4.65) | -1.33 (-1.50, -1.16) | <0.001 |
| Yemen | 43.02 (40.14, 46.05) | 44.78 (43.11, 46.51) | 0.13 (0.10, 0.16) | <0.001 | 264.21 (256.90, 271.68) | 285.26 (280.97, 289.59) | 0.30 (0.11, 0.49) | 0.002 | 1187.50 (1171.96 , 1203.19) | 941.57 (933.81, 949.39) | -0.75 (-0.97, -0.53) | <0.001 | 21.47 (19.42, 23.69) | 16.79 (15.77, 17.87) | -0.80 (-1.02, -0.58) | <0.001 |
| Zambia | 32.03 (28.81, 35.54) | 32.75 (30.80, 34.80) | 0.07 (0.06, 0.09) | <0.001 | 159.87 (152.24, 167.80) | 165.39 (160.86, 170.03) | 0.11 (0.04, 0.19) | 0.003 | 366.10 (354.88, 377.60) | 364.06 (357.48, 370.74) | -0.02 (-0.14, 0.11) | 0.778 | 6.39 (4.97, 8.11) | 6.33 (5.48, 7.27) | -0.03 (-0.15, 0.09) | 0.632 |
| Zimbabwe | 37.09 (34.08, 40.31) | 41.54 (39.11, 44.09) | 0.40 (0.38, 0.41) | <0.001 | 183.80 (176.77, 191.04) | 212.22 (206.57, 217.99) | 0.51 (0.46, 0.56) | <0.001 | 150.86 (144.64, 157.29) | 260.23 (254.06, 266.51) | 1.91 (1.02, 2.82) | <0.001 | 2.60 (1.83, 3.59) | 4.54 (3.75, 5.44) | 1.95 (1.05, 2.86) | <0.001 |
| **Stroke** | | | | | | | | | | | | | | | | |
| **Overall** | | | | | | | | | | | | | | | | |
| Afghanistan | 57.63 (54.78, 60.60) | 56.66 (55.29, 58.06) | -0.37 (-0.58, -0.15) | 0.002 | 530.10 (521.57, 538.74) | 526.19 (522.09, 530.32) | -0.00 (-0.08, 0.08) | 0.979 | 1101.29 (1088.83 , 1113.86) | 729.49 (724.56, 734.45) | -1.77 (-2.06, -1.49) | <0.001 | 17.98 (16.40, 19.68) | 11.50 (10.88, 12.15) | -1.93 (-2.25, -1.62) | <0.001 |
| Albania | 27.03 (24.33, 29.97) | 26.57 (23.41, 30.05) | -0.04 (-0.11, 0.04) | 0.301 | 307.82 (298.47, 317.40) | 302.35 (291.42, 313.60) | -0.03 (-0.10, 0.04) | 0.362 | 381.23 (370.94, 391.75) | 348.86 (337.19, 360.85) | -0.23 (-0.46, -0.01) | 0.043 | 5.37 (4.21, 6.77) | 4.84 (3.56, 6.46) | -0.26 (-0.54, 0.02) | 0.065 |
| Algeria | 44.65 (43.25, | 32.74 (31.90, | -1.27 (-1.33, | <0.001 | 490.03 (485.39, | 430.72 (427.68, | -0.47 (-0.55, | <0.001 | 580.95 (575.89, | 278.72 (276.28, | -2.73 (-2.88, | <0.001 | 8.78 (8.16, | 3.69 (3.42, | -3.22 (-3.39, | <0.001 |

| | | | | | | | | | | | | | | | | |
|---|---|---|---|---|---|---|---|---|---|---|---|---|---|---|---|---|
| | 46.10) | 33.59) | -1.20) | | 494.72) | 433.78) | -0.38) | | 586.05) | 281.19) | -2.58) | | 9.44) | 3.98) | -3.05) | |
| American Samoa | 56.00 (27.27, 103.48) | 51.85 (24.36, 98.23) | -0.32 (-0.38, -0.25) | <0.001 | 607.11 (499.75, 731.75) | 585.34 (479.89, 708.13) | -0.13 (-0.19, -0.08) | <0.001 | 670.09 (556.88, 800.58) | 600.71 (493.87, 724.89) | -0.68 (-0.92, -0.45) | <0.001 | 9.63 (0.92, 39.66) | 8.45 (0.63, 37.83) | -0.85 (-1.14, -0.56) | <0.001 |
| Andorra | 16.54 (4.75, 44.87) | 14.69 (3.93, 41.25) | -0.60 (-0.70, -0.50) | <0.001 | 227.99 (173.56, 296.31) | 222.63 (169.74, 288.76) | -0.20 (-0.31, -0.08) | 0.002 | 116.06 (78.34, 168.09) | 77.47 (47.92, 120.87) | -1.65 (-1.77, -1.52) | <0.001 | 1.40 (0.00, 22.66) | 0.74 (0.00, 20.44) | -2.55 (-2.70, -2.39) | <0.001 |
| Angola | 39.15 (37.10, 41.29) | 26.00 (25.00, 27.02) | -1.71 (-1.83, -1.60) | <0.001 | 459.43 (452.45, 466.50) | 418.50 (414.53, 422.50) | -0.35 (-0.42, -0.28) | <0.001 | 493.63 (486.37, 500.97) | 321.11 (317.61, 324.64) | -1.58 (-1.70, -1.46) | <0.001 | 7.46 (6.58, 8.42) | 4.57 (4.15, 5.01) | -1.79 (-1.94, -1.65) | <0.001 |
| Antigua and Barbuda | 33.75 (14.82, 66.52) | 29.96 (14.74, 54.79) | -0.52 (-0.65, -0.39) | <0.001 | 438.30 (359.88, 529.30) | 426.26 (360.85, 500.52) | -0.08 (-0.14, -0.02) | 0.013 | 314.15 (248.07, 392.95) | 183.28 (141.45, 234.09) | -2.09 (-2.36, -1.81) | <0.001 | 4.78 (0.19, 25.08) | 2.50 (0.04, 16.24) | -2.49 (-2.81, -2.17) | <0.001 |
| Argentina | 32.08 (31.08, 33.11) | 21.01 (20.34, 21.70) | -1.78 (-1.94, -1.62) | <0.001 | 298.34 (295.25, 301.44) | 243.11 (240.82, 245.41) | -0.83 (-0.91, -0.75) | <0.001 | 542.52 (538.36, 546.69) | 217.95 (215.78, 220.14) | -3.59 (-3.98, -3.20) | <0.001 | 8.57 (8.06, 9.11) | 3.04 (2.79, 3.31) | -4.12 (-4.54, -3.70) | <0.001 |
| Armenia | 35.16 (32.16, 38.37) | 27.38 (24.46, 30.60) | -0.90 (-0.97, -0.82) | <0.001 | 376.47 (366.50, 386.64) | 326.61 (316.43, 337.08) | -0.48 (-0.54, -0.43) | <0.001 | 228.20 (220.43, 236.18) | 139.06 (132.41, 146.00) | -2.40 (-2.81, -2.00) | <0.001 | 2.70 (1.92, 3.71) | 1.28 (0.73, 2.18) | -3.42 (-4.07, -2.76) | <0.001 |
| Australia | 18.47 (17.47, 19.51) | 16.07 (15.24, 16.95) | -0.60 (-0.67, -0.52) | <0.001 | 257.48 (253.72, 261.29) | 235.71 (232.48, 238.97) | -0.19 (-0.30, -0.08) | 0.002 | 138.00 (135.25, 140.80) | 77.03 (75.20, 78.90) | -2.12 (-2.26, -1.98) | <0.001 | 1.66 (1.37, 1.99) | 0.66 (0.50, 0.86) | -3.34 (-3.59, -3.10) | <0.001 |
| Austria | 22.44 (20.78, 24.21) | 17.34 (15.86, 18.93) | -1.13 (-1.23, -1.03) | <0.001 | 307.19 (300.95, 313.53) | 268.04 (262.15, 274.05) | -0.56 (-0.66, -0.46) | <0.001 | 184.70 (179.87, 189.64) | 72.39 (69.35, 75.55) | -3.84 (-4.07, -3.61) | <0.001 | 2.44 (1.91, 3.08) | 0.57 (0.33, 0.93) | -5.83 (-6.15, -5.51) | <0.001 |
| Azerbaijan | 37.11 (34.93, 39.39) | 29.64 (28.06, 31.30) | -0.68 (-0.77, -0.60) | <0.001 | 375.20 (368.22, 382.27) | 341.44 (336.05, 346.91) | -0.24 (-0.32, -0.17) | <0.001 | 329.05 (322.48, 335.72) | 219.15 (214.81, 223.56) | -1.85 (-2.33, -1.38) | <0.001 | 4.49 (3.75, 5.35) | 2.66 (2.20, 3.19) | -2.29 (-2.89, -1.69) | <0.001 |
| Bahamas | 34.12 (24.03, 47.21) | 31.08 (22.85, 41.37) | -0.25 (-0.35, -0.14) | <0.001 | 453.15 (414.43, 494.69) | 424.44 (392.27, 458.60) | -0.14 (-0.24, -0.04) | 0.008 | 366.19 (331.07, 404.19) | 300.23 (273.38, 329.09) | -0.97 (-1.21, -0.72) | <0.001 | 5.72 (2.12, 12.60) | 4.62 (1.87, 9.60) | -1.10 (-1.38, -0.81) | <0.001 |
| Bahrain | 30.68 (24.29, | 28.60 (24.48, | -0.49 (-0.66, | <0.001 | 451.31 (425.77, | 418.45 (402.09, | -0.33 (-0.42, | <0.001 | 307.57 (285.86, | 170.37 (159.90, | -2.09 (-2.46, | <0.001 | 3.98 (1.88, | 1.84 (0.92, | -2.83 (-3.34, | <0.001 |

| | | | | | | | | | | | | | | | | |
|---|---|---|---|---|---|---|---|---|---|---|---|---|---|---|---|---|
| | 38.51) | 33.33) | -0.32) | | 478.20) | 435.40) | -0.25) | | 330.68) | 181.44) | -1.72) | | 7.72) | 3.45) | -2.31) | |
| Bangladesh | 24.85 (24.34, 25.37) | 28.76 (28.36, 29.18) | 0.46 (0.37, 0.56) | <0.001 | 369.91 (367.97, 371.85) | 391.35 (389.85, 392.86) | 0.48 (0.21, 0.74) | 0.001 | 729.00 (726.25, 731.75) | 466.40 (464.75, 468.05) | -1.54 (-1.73, -1.35) | <0.001 | 11.69 (11.34, 12.04) | 7.09 (6.89, 7.30) | -1.77 (-1.98, -1.55) | <0.001 |
| Barbados | 33.69 (23.69, 46.55) | 30.93 (21.12, 43.91) | -0.35 (-0.50, -0.20) | <0.001 | 466.36 (426.76, 508.70) | 452.84 (412.40, 496.32) | -0.11 (-0.18, -0.03) | 0.010 | 291.49 (260.35, 325.39) | 193.30 (167.32, 222.31) | -2.06 (-2.34, -1.79) | <0.001 | 4.34 (1.36, 10.47) | 2.65 (0.49, 8.40) | -2.43 (-2.75, -2.11) | <0.001 |
| Belarus | 35.85 (34.04, 37.73) | 32.22 (30.32, 34.24) | -0.04 (-0.25, 0.16) | 0.660 | 372.51 (366.64, 378.46) | 363.25 (356.75, 369.85) | 0.06 (-0.02, 0.13) | 0.122 | 285.15 (280.03, 290.36) | 236.61 (231.51, 241.80) | -0.79 (-1.44, -0.13) | 0.021 | 3.76 (3.20, 4.40) | 2.96 (2.43, 3.60) | -0.96 (-1.83, -0.09) | 0.032 |
| Belgium | 18.50 (17.16, 19.92) | 14.10 (12.91, 15.38) | -1.22 (-1.34, -1.10) | <0.001 | 243.60 (238.69, 248.58) | 240.25 (235.24, 245.35) | 0.04 (-0.10, 0.18) | 0.520 | 231.38 (226.59, 236.26) | 87.97 (84.96, 91.07) | -3.44 (-3.68, -3.19) | <0.001 | 3.33 (2.78, 3.97) | 0.89 (0.61, 1.26) | -4.74 (-4.98, -4.50) | <0.001 |
| Belize | 28.41 (16.72, 45.73) | 30.61 (22.83, 40.25) | 0.28 (0.17, 0.38) | <0.001 | 405.29 (357.24, 458.48) | 429.54 (398.96, 461.90) | 0.25 (0.21, 0.30) | <0.001 | 223.96 (188.66, 264.45) | 235.59 (212.98, 260.00) | -1.04 (-1.47, -0.62) | <0.001 | 3.20 (0.38, 12.58) | 3.40 (1.20, 7.64) | -1.32 (-1.82, -0.82) | <0.001 |
| Benin | 35.92 (32.97, 39.09) | 31.34 (29.67, 33.09) | -0.55 (-0.59, -0.50) | <0.001 | 465.62 (454.92, 476.52) | 441.39 (435.16, 447.69) | -0.11 (-0.21, -0.01) | 0.031 | 369.22 (359.58, 379.06) | 323.96 (318.57, 329.43) | -0.57 (-0.67, -0.47) | <0.001 | 5.38 (4.26, 6.71) | 4.61 (3.98, 5.31) | -0.69 (-0.81, -0.57) | <0.001 |
| Bermuda | 30.91 (13.63, 63.46) | 29.80 (10.68, 69.71) | -0.18 (-0.33, -0.04) | 0.013 | 434.36 (358.76, 523.31) | 406.68 (320.95, 510.60) | -0.25 (-0.30, -0.20) | <0.001 | 175.24 (128.72, 235.49) | 85.69 (49.37, 141.37) | -2.82 (-3.07, -2.57) | <0.001 | 2.33 (0.01, 23.84) | 0.83 (0.00, 27.55) | -4.23 (-4.54, -3.92) | <0.001 |
| Bhutan | 27.81 (21.19, 35.97) | 20.59 (16.10, 25.98) | -1.28 (-1.42, -1.15) | <0.001 | 329.50 (305.95, 354.50) | 301.89 (283.94, 320.70) | -0.27 (-0.41, -0.13) | 0.001 | 268.36 (246.81, 291.40) | 160.65 (147.62, 174.56) | -2.10 (-2.23, -1.97) | <0.001 | 3.83 (1.64, 7.73) | 1.96 (0.78, 4.13) | -2.69 (-2.84, -2.53) | <0.001 |
| Bolivia (Plurinational State of) | 51.98 (49.05, 55.03) | 33.39 (31.77, 35.09) | -1.78 (-1.95, -1.61) | <0.001 | 521.43 (512.03, 530.97) | 433.14 (427.22, 439.12) | -0.66 (-0.76, -0.56) | <0.001 | 677.87 (667.27, 688.61) | 319.82 (314.75, 324.95) | -2.77 (-2.87, -2.67) | <0.001 | 10.71 (9.40, 12.15) | 4.74 (4.14, 5.40) | -2.99 (-3.10, -2.89) | <0.001 |
| Bosnia and Herzegovina | 33.55 (31.03, 36.22) | 35.32 (31.88, 39.06) | 0.41 (0.32, 0.50) | <0.001 | 371.23 (362.74, 379.88) | 394.35 (382.69, 406.30) | 0.29 (0.24, 0.33) | <0.001 | 307.10 (299.38, 314.98) | 234.52 (225.49, 243.84) | -1.51 (-1.78, -1.25) | <0.001 | 4.07 (3.23, 5.07) | 2.69 (1.81, 3.88) | -2.24 (-2.58, -1.89) | <0.001 |
| Botswana | 33.53 (28.29, | 25.05 (22.16, | -1.60 (-1.89, | <0.001 | 490.67 (470.43, | 460.12 (447.35, | -0.28 (-0.36, | <0.001 | 409.56 (390.83, | 303.87 (293.54, | -2.00 (-2.50, | <0.001 | 5.99 (3.89, | 4.21 (3.08, | -2.34 (-2.94, | <0.001 |

| | | | | | | | | | | | | | | | | |
|---|---|---|---|---|---|---|---|---|---|---|---|---|---|---|---|---|
| | 39.51) | 28.24) | -1.30) | | 511.61) | 473.17) | -0.19) | | 429.01) | 314.49) | -1.49) | | 8.86) | 5.64) | -1.74) | |
| Brazil | 52.45 (51.86, 53.04) | 29.42 (29.06, 29.78) | -2.45 (-2.75, -2.15) | <0.001 | 574.77 (572.84, 576.71) | 451.25 (449.85, 452.65) | -1.03 (-1.25, -0.80) | <0.001 | 627.59 (625.55, 629.64) | 236.04 (235.03, 237.05) | -3.56 (-3.81, -3.32) | <0.001 | 10.23 (9.97, 10.50) | 3.40 (3.28, 3.53) | -4.01 (-4.25, -3.77) | <0.001 |
| Brunei Darussalam | 35.07 (25.33, 47.42) | 21.21 (15.49, 28.54) | -2.11 (-2.34, -1.88) | <0.001 | 344.63 (312.49, 379.28) | 297.44 (274.57, 321.84) | -0.56 (-0.74, -0.37) | <0.001 | 409.47 (374.32, 447.12) | 162.25 (145.60, 180.44) | -3.91 (-4.28, -3.53) | <0.001 | 6.10 (2.53, 12.46) | 1.93 (0.55, 5.16) | -4.92 (-5.34, -4.50) | <0.001 |
| Bulgaria | 39.25 (37.07, 41.52) | 32.10 (29.75, 34.60) | -0.78 (-0.82, -0.73) | <0.001 | 431.37 (424.06, 438.78) | 397.01 (388.58, 405.61) | -0.28 (-0.39, -0.16) | <0.001 | 540.51 (532.35, 548.77) | 361.78 (353.80, 369.93) | -2.07 (-2.32, -1.82) | <0.001 | 8.00 (7.04, 9.06) | 5.00 (4.11, 6.04) | -2.45 (-2.75, -2.14) | <0.001 |
| Burkina Faso | 26.75 (24.85, 28.77) | 25.21 (24.09, 26.36) | -0.20 (-0.31, -0.09) | 0.001 | 390.72 (383.45, 398.10) | 375.09 (370.80, 379.43) | -0.08 (-0.15, -0.00) | 0.042 | 326.22 (319.45, 333.11) | 310.52 (306.56, 314.51) | -0.10 (-0.24, 0.03) | 0.122 | 4.80 (4.00, 5.72) | 4.52 (4.05, 5.03) | -0.14 (-0.29, 0.01) | 0.074 |
| Burundi | 53.75 (50.44, 57.23) | 26.29 (24.76, 27.90) | -2.99 (-3.25, -2.73) | <0.001 | 601.12 (590.14, 612.25) | 492.16 (485.56, 498.83) | -0.77 (-0.90, -0.64) | <0.001 | 810.81 (797.97, 823.82) | 423.40 (417.22, 429.65) | -2.66 (-2.90, -2.41) | <0.001 | 12.64 (11.07, 14.39) | 6.16 (5.43, 6.97) | -2.94 (-3.20, -2.68) | <0.001 |
| Cabo Verde | 38.49 (27.37, 53.11) | 30.37 (23.91, 38.07) | -0.93 (-0.98, -0.87) | <0.001 | 493.24 (451.75, 537.94) | 460.28 (434.03, 487.75) | -0.19 (-0.22, -0.15) | <0.001 | 498.86 (456.27, 544.73) | 250.73 (231.46, 271.23) | -2.18 (-2.36, -2.00) | <0.001 | 7.58 (3.14, 15.91) | 3.25 (1.41, 6.44) | -2.59 (-2.83, -2.34) | <0.001 |
| Cambodia | 38.77 (36.68, 40.96) | 28.83 (27.59, 30.12) | -1.25 (-1.33, -1.16) | <0.001 | 379.11 (372.58, 385.72) | 354.60 (350.22, 359.03) | -0.23 (-0.37, -0.10) | 0.001 | 576.14 (567.97, 584.41) | 338.41 (334.10, 342.75) | -2.05 (-2.17, -1.93) | <0.001 | 8.84 (7.84, 9.93) | 4.70 (4.20, 5.24) | -2.40 (-2.55, -2.27) | <0.001 |
| Cameroon | 32.63 (30.71, 34.64) | 32.23 (31.17, 33.32) | -0.28 (-0.56, 0.01) | 0.057 | 428.37 (421.40, 435.43) | 432.09 (428.22, 435.98) | 0.00 (-0.10, 0.10) | 0.964 | 442.69 (435.51, 449.96) | 408.73 (404.93, 412.54) | -0.52 (-0.79, -0.25) | 0.001 | 6.72 (5.85, 7.69) | 6.10 (5.64, 6.58) | -0.61 (-0.91, -0.31) | <0.001 |
| Canada | 15.76 (15.04, 16.50) | 13.55 (12.89, 14.23) | -0.53 (-0.68, -0.38) | <0.001 | 357.75 (354.29, 361.23) | 312.48 (309.31, 315.68) | -0.33 (-0.61, -0.06) | 0.020 | 156.73 (154.46, 159.02) | 91.64 (89.93, 93.37) | -1.87 (-2.09, -1.66) | <0.001 | 1.78 (1.55, 2.04) | 0.75 (0.61, 0.93) | -3.10 (-3.36, -2.83) | <0.001 |
| Central African Republic | 40.95 (36.84, 45.42) | 39.76 (36.90, 42.79) | -0.21 (-0.26, -0.15) | <0.001 | 450.64 (437.10, 464.54) | 437.91 (428.52, 447.46) | -0.08 (-0.14, -0.01) | 0.029 | 570.15 (554.79, 585.85) | 506.24 (496.08, 516.57) | -0.46 (-0.56, -0.36) | <0.001 | 8.82 (6.98, 11.02) | 7.76 (6.53, 9.15) | -0.50 (-0.61, -0.40) | <0.001 |
| Chad | 34.11 (31.48, | 33.99 (32.35, | -0.09 (-0.20, | 0.133 | 437.10 (427.68, | 445.41 (439.52, | 0.15 (0.09, | <0.001 | 447.99 (438.36, | 422.36 (416.57, | -0.22 (-0.39, | 0.011 | 6.77 (5.63, | 6.29 (5.59, | -0.29 (-0.48, | 0.004 |

| | | | | | | | | | | | | | | | | |
|---|---|---|---|---|---|---|---|---|---|---|---|---|---|---|---|---|
| | 36.90) | 35.70) | 0.03) | | 446.68) | 451.36) | 0.21) | | 457.78) | 428.22) | -0.06) | | 8.10) | 7.06) | -0.10) | |
| Chile | 24.44 (23.15, 25.79) | 21.09 (20.02, 22.20) | -0.28 (-0.53, -0.03) | 0.030 | 300.89 (296.32, 305.51) | 273.23 (269.37, 277.14) | -0.12 (-0.30, 0.05) | 0.164 | 250.44 (246.28, 254.66) | 147.80 (144.96, 150.69) | -1.52 (-1.65, -1.39) | <0.001 | 3.49 (3.01, 4.03) | 1.80 (1.50, 2.14) | -1.92 (-2.07, -1.78) | <0.001 |
| China | 28.76 (28.62, 28.91) | 23.59 (23.45, 23.72) | -1.23 (-1.44, -1.02) | <0.001 | 352.47 (351.96, 352.98) | 315.25 (314.77, 315.74) | -0.59 (-0.85, -0.33) | <0.001 | 491.25 (490.65, 491.85) | 318.68 (318.20, 319.16) | -1.76 (-1.97, -1.55) | <0.001 | 7.27 (7.20, 7.35) | 4.38 (4.32, 4.43) | -2.01 (-2.24, -1.79) | <0.001 |
| Colombia | 34.34 (33.34, 35.37) | 23.81 (23.13, 24.52) | -1.54 (-1.77, -1.30) | <0.001 | 454.15 (450.51, 457.81) | 386.63 (383.85, 389.42) | -0.53 (-0.66, -0.41) | <0.001 | 348.16 (344.96, 351.37) | 166.20 (164.37, 168.03) | -2.64 (-2.96, -2.32) | <0.001 | 5.28 (4.89, 5.69) | 2.23 (2.02, 2.45) | -3.08 (-3.44, -2.73) | <0.001 |
| Comoros | 51.55 (40.30, 65.13) | 28.77 (22.82, 35.83) | -2.34 (-2.54, -2.14) | <0.001 | 642.77 (602.49, 685.23) | 505.67 (479.91, 532.51) | -0.98 (-1.11, -0.85) | <0.001 | 514.07 (477.64, 552.71) | 327.85 (307.04, 349.73) | -2.08 (-2.69, -1.47) | <0.001 | 7.45 (3.63, 13.79) | 4.50 (2.36, 7.82) | -2.30 (-3.00, -1.60) | <0.001 |
| Congo | 50.35 (45.48, 55.62) | 27.65 (25.42, 30.02) | -2.49 (-2.63, -2.34) | <0.001 | 546.07 (530.14, 562.38) | 446.01 (436.95, 455.21) | -0.75 (-0.85, -0.66) | <0.001 | 623.79 (606.61, 641.35) | 326.66 (318.90, 334.57) | -2.52 (-2.75, -2.28) | <0.001 | 9.60 (7.55, 12.06) | 4.65 (3.76, 5.68) | -2.81 (-3.07, -2.54) | <0.001 |
| Cook Islands | 51.55 (13.01, 142.24) | 50.84 (10.22, 151.83) | -0.18 (-0.37, 0.00) | 0.052 | 571.28 (409.01, 779.63) | 567.50 (389.92, 799.12) | -0.05 (-0.13, 0.04) | 0.274 | 668.64 (492.56, 890.38) | 456.62 (299.09, 668.56) | -1.32 (-1.64, -0.99) | <0.001 | 9.63 (0.05, 77.70) | 5.99 (0.00, 78.30) | -1.70 (-2.10, -1.29) | <0.001 |
| Costa Rica | 28.01 (25.11, 31.17) | 25.50 (23.31, 27.86) | -0.35 (-0.52, -0.19) | <0.001 | 424.64 (413.17, 436.36) | 409.37 (400.43, 418.46) | -0.09 (-0.15, -0.03) | 0.004 | 205.91 (197.88, 214.19) | 138.19 (133.03, 143.52) | -1.53 (-1.94, -1.12) | <0.001 | 2.89 (2.00, 4.05) | 1.72 (1.19, 2.42) | -1.99 (-2.52, -1.46) | <0.001 |
| Cote d'Ivoire | 42.78 (40.80, 44.84) | 35.11 (33.96, 36.28) | -0.94 (-1.10, -0.78) | <0.001 | 519.52 (512.65, 526.47) | 478.00 (473.79, 482.23) | -0.33 (-0.40, -0.27) | <0.001 | 514.70 (507.83, 521.63) | 389.06 (385.25, 392.89) | -1.16 (-1.37, -0.94) | <0.001 | 7.72 (6.89, 8.63) | 5.62 (5.17, 6.10) | -1.29 (-1.53, -1.05) | <0.001 |
| Croatia | 27.15 (24.87, 29.60) | 20.55 (18.22, 23.13) | -1.27 (-1.46, -1.07) | <0.001 | 331.72 (323.60, 340.00) | 279.69 (270.93, 288.69) | -0.72 (-0.87, -0.58) | <0.001 | 308.89 (301.10, 316.84) | 129.89 (123.96, 136.07) | -3.37 (-3.54, -3.20) | <0.001 | 4.29 (3.42, 5.32) | 1.25 (0.74, 2.03) | -4.67 (-4.91, -4.43) | <0.001 |
| Cuba | 34.67 (32.95, 36.47) | 28.71 (27.01, 30.49) | -0.78 (-0.92, -0.64) | <0.001 | 452.36 (446.16, 458.64) | 401.68 (395.24, 408.20) | -0.41 (-0.52, -0.30) | <0.001 | 296.52 (291.44, 301.68) | 149.99 (146.08, 153.98) | -2.72 (-3.00, -2.44) | <0.001 | 4.48 (3.87, 5.17) | 1.98 (1.56, 2.50) | -3.28 (-3.60, -2.96) | <0.001 |
| Cyprus | 16.87 (12.61, | 14.97 (11.83, | -0.74 (-0.86, | <0.001 | 238.97 (222.11, | 257.02 (243.41, | 0.29 (0.16, | <0.001 | 136.60 (123.88, | 79.57 (72.05, | -2.71 (-3.08, | <0.001 | 1.72 (0.59, | 0.71 (0.18, | -4.08 (-4.70, | <0.001 |

| | | | | | | | | | | | | | | | | |
|---|---|---|---|---|---|---|---|---|---|---|---|---|---|---|---|---|
| | 22.15) | 18.88) | -0.61) | | 256.81) | 271.34) | 0.42) | | 150.31) | 87.82) | -2.33) | | 4.00) | 2.21) | -3.46) | |
| Czechia | 30.63 (28.91, 32.44) | 24.82 (23.13, 26.61) | -0.95 (-1.16, -0.73) | <0.001 | 364.62 (358.59, 370.73) | 317.75 (311.68, 323.92) | -0.54 (-0.65, -0.42) | <0.001 | 271.99 (266.80, 277.26) | 113.58 (109.96, 117.32) | -3.43 (-3.63, -3.23) | <0.001 | 3.50 (2.94, 4.14) | 0.85 (0.58, 1.25) | -5.38 (-5.58, -5.18) | <0.001 |
| Democratic People's Republic of Korea | 41.41 (39.99, 42.87) | 41.18 (39.95, 42.43) | -0.04 (-0.16, 0.08) | 0.504 | 385.00 (380.65, 389.38) | 388.22 (384.44, 392.04) | 0.08 (0.02, 0.14) | 0.010 | 602.41 (596.95, 607.91) | 567.94 (563.37, 572.55) | -0.14 (-0.26, -0.03) | 0.019 | 9.12 (8.46, 9.83) | 8.49 (7.94, 9.07) | -0.20 (-0.34, -0.06) | 0.006 |
| Democratic Republic of the Congo | 29.84 (28.88, 30.82) | 23.68 (23.14, 24.23) | -0.96 (-1.04, -0.88) | <0.001 | 393.56 (390.12, 397.03) | 354.20 (352.13, 356.28) | -0.36 (-0.46, -0.27) | <0.001 | 406.83 (403.31, 410.39) | 297.56 (295.64, 299.48) | -1.12 (-1.17, -1.07) | <0.001 | 6.12 (5.69, 6.58) | 4.32 (4.09, 4.56) | -1.25 (-1.31, -1.18) | <0.001 |
| Denmark | 20.89 (18.92, 23.03) | 14.12 (12.44, 15.97) | -1.53 (-1.65, -1.40) | <0.001 | 296.03 (288.43, 303.78) | 253.73 (246.47, 261.16) | -0.57 (-0.66, -0.48) | <0.001 | 191.14 (185.08, 197.35) | 76.14 (72.19, 80.25) | -3.73 (-3.95, -3.51) | <0.001 | 2.63 (1.96, 3.46) | 0.68 (0.35, 1.19) | -5.38 (-5.74, -5.01) | <0.001 |
| Djibouti | 35.87 (27.36, 46.33) | 26.42 (22.16, 31.28) | -1.21 (-1.35, -1.08) | <0.001 | 516.00 (482.88, 550.93) | 505.75 (486.42, 525.67) | 0.01 (-0.10, 0.11) | 0.914 | 449.83 (418.64, 482.87) | 323.03 (307.63, 339.01) | -1.29 (-1.44, -1.15) | <0.001 | 6.60 (3.29, 11.99) | 4.40 (2.78, 6.65) | -1.58 (-1.74, -1.42) | <0.001 |
| Dominica | 32.38 (14.21, 64.15) | 29.34 (12.28, 59.11) | -0.44 (-0.52, -0.35) | <0.001 | 437.86 (361.49, 526.40) | 430.86 (354.34, 519.11) | -0.01 (-0.09, 0.07) | 0.812 | 272.28 (212.18, 344.85) | 197.23 (146.67, 259.69) | -1.85 (-2.14, -1.56) | <0.001 | 4.06 (0.11, 23.74) | 2.76 (0.02, 19.93) | -2.28 (-2.66, -1.90) | <0.001 |
| Dominican Republic | 35.59 (33.35, 37.96) | 37.71 (35.91, 39.57) | 0.18 (0.12, 0.24) | <0.001 | 414.98 (407.35, 422.73) | 429.18 (423.10, 435.33) | 0.18 (0.12, 0.24) | <0.001 | 413.11 (405.45, 420.88) | 391.61 (385.79, 397.50) | -0.03 (-0.34, 0.29) | 0.865 | 6.52 (5.58, 7.58) | 6.18 (5.47, 6.96) | -0.01 (-0.35, 0.34) | 0.969 |
| Ecuador | 43.47 (41.38, 45.63) | 32.81 (31.48, 34.17) | -1.10 (-1.21, -0.99) | <0.001 | 499.30 (492.15, 506.53) | 454.28 (449.30, 459.29) | -0.35 (-0.38, -0.31) | <0.001 | 544.20 (536.81, 551.66) | 320.79 (316.63, 324.99) | -2.13 (-2.38, -1.88) | <0.001 | 8.46 (7.56, 9.45) | 4.71 (4.22, 5.25) | -2.35 (-2.62, -2.08) | <0.001 |
| Egypt | 37.15 (36.31, 38.00) | 36.20 (35.61, 36.79) | 0.08 (-0.06, 0.22) | 0.234 | 450.28 (447.36, 453.22) | 468.55 (466.44, 470.66) | 0.37 (0.25, 0.49) | <0.001 | 1022.02 (1017.73, 1026.32) | 651.05 (648.58, 653.53) | -1.08 (-1.42, -0.73) | <0.001 | 15.66 (15.13, 16.20) | 9.52 (9.22, 9.82) | -1.24 (-1.61, -0.87) | <0.001 |
| El Salvador | 31.37 (28.85, 34.07) | 24.05 (22.14, 26.09) | -1.14 (-1.34, -0.93) | <0.001 | 398.19 (389.07, 407.47) | 376.90 (369.25, 384.68) | -0.17 (-0.27, -0.08) | 0.001 | 661.87 (650.34, 673.57) | 211.86 (206.13, 217.71) | -4.39 (-5.13, -3.64) | <0.001 | 10.45 (9.03, 12.04) | 3.00 (2.35, 3.78) | -4.90 (-5.69, -4.10) | <0.001 |
| Equatorial Guinea | 44.23 | 21.32 | -2.83 | <0.001 | 489.96 | 401.93 | -0.72 | <0.001 | 554.23 | 209.79 | -3.72 | <0.001 | 8.46 | 2.69 | -4.40 | <0.001 |

| | | | | | | | | | | | | | | | | |
|---|---|---|---|---|---|---|---|---|---|---|---|---|---|---|---|---|
| | (33.61, | (17.64, | (-3.13, | | (453.39, | (385.62, | (-0.84, | | (515.28, | (197.93, | (-4.06, | | (4.26, | (1.48, | (-4.77, | |
| | 57.27) | 25.59) | -2.53) | | 528.81) | 418.82) | -0.60) | | 595.48) | 222.22) | -3.38) | | 15.19) | 4.54) | -4.04) | |
| | 45.50 | 26.67 | -2.10 | | 498.99 | 437.25 | -0.48 | | 622.75 | 430.27 | -1.38 | | 9.67 | 6.45 | -1.50 | |
| Eritrea | (41.35, | (24.71, | (-2.24, | <0.001 | (485.40, | (429.34, | (-0.58, | <0.001 | (607.37, | (422.32, | (-1.47, | <0.001 | (7.82, | (5.50, | (-1.59, | <0.001 |
| | 49.96) | 28.76) | -1.96) | | 512.90) | 445.28) | -0.39) | | 638.43) | 438.35) | -1.29) | | 11.85) | 7.53) | -1.40) | |
| | 35.43 | 28.57 | -1.11 | | 337.63 | 308.74 | -0.45 | | 315.60 | 114.18 | -4.70 | | 4.35 | 0.90 | -6.32 | |
| Estonia | (30.82, | (23.75, | (-1.33, | <0.001 | (323.04, | (292.41, | (-0.55, | <0.001 | (301.48, | (104.32, | (-5.31, | <0.001 | (2.85, | (0.25, | (-7.37, | <0.001 |
| | 40.57) | 34.24) | -0.89) | | 352.74) | 325.87) | -0.35) | | 330.23) | 124.86) | -4.08) | | 6.41) | 2.59) | -5.27) | |
| | 26.72 | 28.36 | 0.19 | | 450.70 | 440.86 | -0.04 | | 330.41 | 329.67 | 0.16 | | 4.67 | 4.69 | 0.15 | |
| Eswatini | (20.68, | (23.75, | (-0.42, | 0.532 | (425.19, | (422.29, | (-0.15, | 0.526 | (308.30, | (313.53, | (-0.57, | 0.662 | (2.36, | (2.94, | (-0.71, | 0.723 |
| | 34.08) | 33.64) | 0.80) | | 477.45) | 460.06) | 0.08) | | 353.80) | 346.45) | 0.90) | | 8.43) | 7.15) | 1.02) | |
| | 36.10 | 19.80 | -2.52 | | 468.35 | 413.68 | -0.42 | | 594.17 | 252.81 | -3.45 | | 9.23 | 3.39 | -4.02 | |
| Ethiopia | (35.18, | (19.36, | (-2.76, | <0.001 | (465.07, | (411.69, | (-0.56, | <0.001 | (590.43, | (251.23, | (-3.65, | <0.001 | (8.76, | (3.21, | (-4.24, | <0.001 |
| | 37.05) | 20.25) | -2.27) | | 471.65) | 415.68) | -0.27) | | 597.92) | 254.39) | -3.25) | | 9.71) | 3.58) | -3.80) | |
| | 51.18 | 45.98 | -0.52 | | 579.33 | 552.79 | -0.18 | | 818.66 | 618.46 | -1.27 | | 12.10 | 8.75 | -1.47 | |
| Fiji | (43.48, | (39.26, | (-0.58, | <0.001 | (552.58, | (528.84, | (-0.25, | <0.001 | (786.95, | (593.10, | (-1.45, | <0.001 | (8.50, | (5.98, | (-1.67, | <0.001 |
| | 59.91) | 53.54) | -0.46) | | 607.08) | 577.55) | -0.11) | | 851.37) | 644.64) | -1.09) | | 16.75) | 12.39) | -1.26) | |
| | 22.43 | 19.16 | -0.60 | | 336.92 | 310.52 | -0.08 | | 291.30 | 106.69 | -3.51 | | 4.31 | 1.08 | -4.86 | |
| Finland | (20.36, | (17.17, | (-0.75, | <0.001 | (328.70, | (302.29, | (-0.33, | 0.538 | (283.81, | (101.93, | (-3.80, | <0.001 | (3.45, | (0.66, | (-5.13, | <0.001 |
| | 24.67) | 21.34) | -0.44) | | 345.32) | 318.94) | 0.18) | | 298.95) | 111.63) | -3.22) | | 5.35) | 1.70) | -4.60) | |
| | 16.36 | 14.04 | -0.26 | | 245.27 | 211.37 | -0.39 | | 183.84 | 90.31 | -2.69 | | 2.55 | 0.99 | -3.59 | |
| France | (15.84, | (13.53, | (-0.38, | <0.001 | (243.23, | (209.38, | (-0.53, | <0.001 | (182.08, | (89.02, | (-2.80, | <0.001 | (2.35, | (0.86, | (-3.71, | <0.001 |
| | 16.90) | 14.56) | -0.14) | | 247.32) | 213.37) | -0.25) | | 185.61) | 91.62) | -2.58) | | 2.77) | 1.13) | -3.47) | |
| | 31.35 | 23.38 | -1.20 | | 447.35 | 416.71 | -0.25 | | 404.25 | 239.34 | -1.95 | | 5.97 | 3.15 | -2.35 | |
| Gabon | (25.66, | (19.94, | (-1.28, | <0.001 | (425.44, | (401.87, | (-0.31, | <0.001 | (383.28, | (228.08, | (-2.07, | <0.001 | (3.65, | (1.98, | (-2.51, | <0.001 |
| | 37.97) | 27.26) | -1.12) | | 470.15) | 431.99) | -0.18) | | 426.12) | 251.03) | -1.83) | | 9.27) | 4.80) | -2.18) | |
| | 36.28 | 35.10 | -0.01 | | 514.54 | 507.45 | 0.08 | | 406.22 | 385.32 | -0.19 | | 5.87 | 5.50 | -0.26 | |
| Gambia | (30.00, | (31.16, | (-0.07, | 0.720 | (490.47, | (492.39, | (0.01, | 0.026 | (384.65, | (372.11, | (-0.42, | 0.097 | (3.51, | (4.01, | (-0.53, | 0.062 |
| | 43.57) | 39.42) | 0.05) | | 539.58) | 522.88) | 0.14) | | 428.78) | 398.90) | 0.04) | | 9.31) | 7.39) | 0.01) | |
| | 46.65 | 34.30 | -0.76 | | 444.92 | 393.29 | -0.44 | | 654.69 | 458.29 | -1.39 | | 9.81 | 6.74 | -1.35 | |
| Georgia | (43.81, | (31.14, | (-1.09, | <0.001 | (436.08, | (382.42, | (-0.52, | <0.001 | (643.94, | (446.62, | (-2.38, | 0.009 | (8.53, | (5.41, | (-2.48, | 0.023 |
| | 49.62) | 37.72) | -0.42) | | 453.91) | 404.42) | -0.37) | | 665.59) | 470.22) | -0.38) | | 11.23) | 8.36) | -0.20) | |
| Germany | 19.55 | 15.65 | -0.87 | <0.001 | 284.42 | 248.20 | -0.44 | <0.001 | 242.02 | 91.89 | -3.78 | <0.001 | 3.46 | 0.94 | -5.22 | <0.001 |

| | | | | | | | | | | | | | | | | |
|---|---|---|---|---|---|---|---|---|---|---|---|---|---|---|---|---|
| | (19.06, | (15.18, | (-0.97, | | (282.52, | (246.31, | (-0.57, | | (240.28, | (90.75, | (-4.20, | | (3.25, | (0.83, | (-5.70, | |
| | 20.06) | 16.14) | -0.77) | | 286.33) | 250.10) | -0.30) | | 243.77) | 93.05) | -3.35) | | 3.67) | 1.06) | -4.73) | |
| | 39.20 | 37.23 | -0.27 | | 516.52 | 501.43 | -0.01 | | 540.99 | 414.42 | -0.84 | | 8.29 | 6.08 | -0.98 | |
| Ghana | (37.50, | (36.18, | (-0.45, | 0.005 | (510.37, | (497.57, | (-0.08, | 0.731 | (534.60, | (410.89, | (-1.09, | <0.001 | (7.51, | (5.65, | (-1.26, | <0.001 |
| | 40.97) | 38.31) | -0.09) | | 522.73) | 505.31) | 0.05) | | 547.44) | 417.98) | -0.59) | | 9.13) | 6.53) | -0.70) | |
| | 20.07 | 15.33 | -1.03 | | 289.42 | 271.00 | -0.22 | | 249.53 | 135.37 | -2.44 | | 3.52 | 1.66 | -3.00 | |
| Greece | (18.67, | (13.98, | (-1.06, | <0.001 | (284.03, | (265.18, | (-0.35, | 0.002 | (244.52, | (131.29, | (-2.60, | <0.001 | (2.95, | (1.25, | (-3.21, | <0.001 |
| | 21.55) | 16.80) | -1.00) | | 294.88) | 276.94) | -0.08) | | 254.61) | 139.54) | -2.28) | | 4.17) | 2.19) | -2.80) | |
| | 28.34 | 17.98 | -1.86 | | 506.85 | 425.15 | -0.53 | | 481.03 | 217.52 | -3.18 | | 7.31 | 2.79 | -3.75 | |
| Greenland | (11.67, | (4.62, | (-1.98, | <0.001 | (423.85, | (341.03, | (-0.78, | <0.001 | (400.63, | (159.13, | (-3.45, | <0.001 | (0.82, | (0.01, | (-4.09, | <0.001 |
| | 61.70) | 50.30) | -1.74) | | 603.95) | 525.34) | -0.28) | | 575.49) | 292.16) | -2.91) | | 32.89) | 27.22) | -3.41) | |
| | 40.76 | 33.33 | -0.85 | | 504.21 | 475.52 | -0.14 | | 509.08 | 255.05 | -2.76 | | 8.18 | 3.74 | -3.21 | |
| Grenada | (21.15, | (17.62, | (-1.04, | <0.001 | (427.76, | (409.53, | (-0.23, | 0.008 | (431.28, | (206.88, | (-3.08, | <0.001 | (1.29, | (0.23, | (-3.56, | <0.001 |
| | 71.74) | 57.78) | -0.66) | | 591.01) | 549.53) | -0.04) | | 597.40) | 311.43) | -2.45) | | 27.71) | 17.34) | -2.86) | |
| | 42.12 | 52.27 | 0.78 | | 506.81 | 571.45 | 0.55 | | 403.64 | 520.87 | 0.65 | | 5.16 | 7.13 | 0.76 | |
| Guam | (27.56, | (35.41, | (0.74, | <0.001 | (452.43, | (511.77, | (0.50, | <0.001 | (355.35, | (463.97, | (0.42, | <0.001 | (1.14, | (2.00, | (0.45, | <0.001 |
| | 61.85) | 74.52) | 0.82) | | 566.10) | 636.29) | 0.59) | | 456.85) | 582.93) | 0.88) | | 14.94) | 18.17) | 1.06) | |
| | 47.87 | 26.32 | -2.53 | | 486.42 | 395.97 | -0.86 | | 576.80 | 318.54 | -3.39 | | 9.02 | 4.79 | -3.62 | |
| Guatemala | (45.26, | (25.16, | (-2.71, | <0.001 | (477.99, | (391.46, | (-0.98, | <0.001 | (567.71, | (314.50, | (-3.87, | <0.001 | (7.91, | (4.31, | (-4.14, | <0.001 |
| | 50.60) | 27.52) | -2.35) | | 494.97) | 400.53) | -0.73) | | 586.01) | 322.63) | -2.90) | | 10.26) | 5.32) | -3.10) | |
| | 33.44 | 36.08 | 0.45 | | 394.58 | 416.66 | 0.38 | | 444.58 | 436.01 | 0.31 | | 6.78 | 6.56 | 0.28 | |
| Guinea | (30.93, | (34.28, | (0.36, | <0.001 | (385.90, | (410.58, | (0.30, | <0.001 | (435.33, | (429.77, | (0.18, | <0.001 | (5.67, | (5.81, | (0.15, | <0.001 |
| | 36.11) | 37.94) | 0.54) | | 403.41) | 422.80) | 0.47) | | 453.99) | 442.32) | 0.43) | | 8.04) | 7.39) | 0.42) | |
| | 52.56 | 48.40 | -0.28 | | 548.15 | 524.82 | -0.06 | | 725.99 | 609.40 | -0.37 | | 11.36 | 9.39 | -0.41 | |
| Guinea-Bissau | (44.91, | (43.41, | (-0.33, | <0.001 | (523.15, | (508.36, | (-0.11, | 0.048 | (696.99, | (591.51, | (-0.51, | <0.001 | (7.97, | (7.27, | (-0.56, | <0.001 |
| | 61.21) | 53.83) | -0.23) | | 574.09) | 541.72) | 0.00) | | 755.93) | 627.71) | -0.24) | | 15.78) | 11.96) | -0.26) | |
| | 50.86 | 37.94 | -1.23 | | 594.41 | 519.52 | -0.41 | | 806.33 | 532.88 | -1.76 | | 13.10 | 8.45 | -1.88 | |
| Guyana | (43.09, | (31.23, | (-1.32, | <0.001 | (567.45, | (494.09, | (-0.53, | <0.001 | (774.71, | (507.01, | (-2.07, | <0.001 | (9.32, | (5.46, | (-2.21, | <0.001 |
| | 59.69) | 45.71) | -1.15) | | 622.37) | 545.96) | -0.29) | | 838.96) | 559.75) | -1.45) | | 17.96) | 12.53) | -1.54) | |
| | 61.83 | 47.12 | -0.98 | | 545.09 | 491.81 | -0.26 | | 1005.86 | 666.63 | -1.08 | | 16.66 | 10.84 | -1.13 | |
| Haiti | (58.59, | (45.26, | (-1.07, | <0.001 | (535.48, | (485.80, | (-0.39, | <0.001 | (992.77, | (659.61, | (-1.28, | <0.001 | (15.01, | (9.96, | (-1.34, | <0.001 |
| | 65.20) | 49.04) | -0.89) | | 554.83) | 497.88) | -0.14) | | 1019.08) | 673.71) | -0.89) | | 18.46) | 11.78) | -0.93) | |
| Honduras | 29.53 | 23.40 | -0.96 | <0.001 | 389.85 | 391.43 | 0.09 | 0.110 | 692.30 | 327.05 | -2.95 | <0.001 | 11.22 | 5.03 | -3.15 | <0.001 |

| | | | | | | | | | | | | | | | | |
|---|---|---|---|---|---|---|---|---|---|---|---|---|---|---|---|---|
| | (26.85, 32.42) | (21.90, 24.97) | (-1.15, -0.77) | | (380.03, 399.86) | (385.27, 397.68) | (-0.02, 0.20) | | (679.19, 705.61) | (321.36, 332.81) | (-3.36, -2.55) | | (9.59, 13.06) | (4.34, 5.80) | (-3.61, -2.69) | |
| Hungary | 43.68 (41.65, 45.80) | 26.75 (24.93, 28.68) | -2.06 (-2.20, -1.91) | <0.001 | 463.14 (456.38, 469.98) | 368.93 (362.11, 375.86) | -0.77 (-0.91, -0.63) | <0.001 | 467.33 (460.66, 474.08) | 161.83 (157.36, 166.40) | -4.50 (-4.81, -4.20) | <0.001 | 6.79 (6.02, 7.64) | 1.57 (1.17, 2.09) | -6.19 (-6.56, -5.82) | <0.001 |
| Iceland | 16.64 (9.77, 26.61) | 13.36 (7.71, 21.87) | -1.11 (-1.35, -0.87) | <0.001 | 227.84 (199.88, 258.72) | 208.94 (184.27, 236.24) | -0.39 (-0.55, -0.23) | <0.001 | 165.35 (141.63, 192.00) | 72.48 (58.25, 89.41) | -3.21 (-3.46, -2.96) | <0.001 | 2.22 (0.33, 7.58) | 0.67 (0.01, 4.89) | -4.71 (-5.00, -4.43) | <0.001 |
| India | 25.43 (25.25, 25.60) | 24.26 (24.13, 24.39) | -0.24 (-0.40, -0.08) | 0.005 | 337.69 (337.05, 338.32) | 343.63 (343.16, 344.11) | 0.16 (0.03, 0.29) | 0.021 | 310.20 (309.59, 310.81) | 235.61 (235.21, 236.00) | -1.11 (-1.37, -0.85) | <0.001 | 4.49 (4.42, 4.57) | 3.22 (3.17, 3.26) | -1.31 (-1.63, -0.99) | <0.001 |
| Indonesia | 55.24 (54.70, 55.79) | 52.36 (51.93, 52.80) | -0.21 (-0.23, -0.19) | <0.001 | 602.16 (600.36, 603.95) | 576.50 (575.07, 577.94) | -0.11 (-0.17, -0.04) | 0.002 | 773.32 (771.28, 775.36) | 523.52 (522.16, 524.89) | -1.22 (-1.35, -1.10) | <0.001 | 11.46 (11.21, 11.71) | 7.19 (7.03, 7.35) | -1.44 (-1.60, -1.27) | <0.001 |
| Iran (Islamic Republic of) | 31.05 (30.27, 31.85) | 25.73 (25.22, 26.25) | -0.75 (-0.89, -0.61) | <0.001 | 399.52 (396.73, 402.32) | 382.93 (380.93, 384.93) | -0.12 (-0.28, 0.03) | 0.114 | 334.61 (332.06, 337.17) | 222.38 (220.84, 223.93) | -1.33 (-1.45, -1.21) | <0.001 | 4.69 (4.39, 5.00) | 2.76 (2.59, 2.94) | -1.73 (-1.86, -1.61) | <0.001 |
| Iraq | 52.26 (50.40, 54.17) | 46.92 (45.90, 47.96) | -0.52 (-0.58, -0.45) | <0.001 | 612.45 (606.08, 618.88) | 575.51 (571.94, 579.10) | -0.21 (-0.27, -0.15) | <0.001 | 642.92 (636.40, 649.50) | 503.54 (500.21, 506.89) | -1.04 (-1.20, -0.88) | <0.001 | 9.53 (8.74, 10.37) | 7.17 (6.77, 7.58) | -1.22 (-1.41, -1.03) | <0.001 |
| Ireland | 19.61 (17.31, 22.13) | 14.64 (12.86, 16.61) | -1.41 (-1.58, -1.25) | <0.001 | 262.05 (253.46, 270.86) | 242.47 (235.05, 250.07) | -0.42 (-0.55, -0.30) | <0.001 | 186.56 (179.31, 194.03) | 78.45 (74.27, 82.82) | -3.26 (-3.49, -3.03) | <0.001 | 2.57 (1.78, 3.59) | 0.72 (0.38, 1.27) | -4.53 (-4.89, -4.17) | <0.001 |
| Israel | 16.52 (14.73, 18.46) | 14.49 (13.21, 15.85) | -0.71 (-1.03, -0.39) | <0.001 | 233.68 (226.82, 240.70) | 231.15 (225.96, 236.43) | -0.08 (-0.25, 0.09) | 0.327 | 146.78 (141.36, 152.35) | 66.69 (63.92, 69.56) | -3.22 (-3.50, -2.94) | <0.001 | 1.87 (1.30, 2.60) | 0.53 (0.31, 0.85) | -5.12 (-5.41, -4.82) | <0.001 |
| Italy | 17.50 (16.94, 18.07) | 13.61 (13.06, 14.19) | -1.45 (-1.67, -1.22) | <0.001 | 232.23 (230.19, 234.29) | 235.83 (233.51, 238.17) | 0.02 (-0.05, 0.10) | 0.519 | 195.03 (193.16, 196.92) | 92.20 (90.75, 93.66) | -2.96 (-3.13, -2.79) | <0.001 | 2.73 (2.52, 2.96) | 0.97 (0.83, 1.13) | -4.10 (-4.30, -3.91) | <0.001 |
| Jamaica | 28.78 (25.30, 32.63) | 32.43 (29.24, 35.88) | 0.43 (0.31, 0.56) | <0.001 | 417.36 (403.95, 431.13) | 463.32 (451.08, 475.82) | 0.46 (0.38, 0.53) | <0.001 | 296.19 (284.87, 307.88) | 257.14 (248.01, 266.52) | -1.14 (-1.71, -0.57) | <0.001 | 4.44 (3.14, 6.13) | 3.76 (2.73, 5.06) | -1.33 (-1.98, -0.67) | <0.001 |
| Japan | 21.51 | 24.56 | 0.46 | <0.001 | 242.15 | 292.97 | 0.70 | <0.001 | 196.77 | 127.21 | -1.58 | <0.001 | 2.67 | 1.32 | -2.40 | <0.001 |

| | | | | | | | | | | | | | | | | |
|---|---|---|---|---|---|---|---|---|---|---|---|---|---|---|---|---|
| | (21.09, 21.94) | (24.05, 25.08) | (0.28, 0.63) | | (240.72, 243.59) | (291.18, 294.77) | (0.47, 0.92) | | (195.49, 198.06) | (126.04, 128.39) | (-1.70, -1.47) | | (2.52, 2.82) | (1.20, 1.44) | (-2.63, -2.17) | |
| Jordan | 43.83 (40.14, 47.80) | 34.93 (33.29, 36.64) | -0.95 (-1.10, -0.80) | <0.001 | 592.87 (579.27, 606.73) | 536.38 (529.88, 542.94) | -0.33 (-0.40, -0.25) | <0.001 | 278.93 (269.49, 288.63) | 148.92 (145.50, 152.40) | -2.71 (-3.03, -2.39) | <0.001 | 3.37 (2.38, 4.65) | 1.23 (0.94, 1.59) | -4.37 (-4.91, -3.84) | <0.001 |
| Kazakhstan | 45.62 (44.03, 47.26) | 32.81 (31.51, 34.16) | -1.15 (-1.63, -0.68) | <0.001 | 451.39 (446.34, 456.48) | 389.35 (384.82, 393.92) | -0.54 (-0.72, -0.36) | <0.001 | 430.76 (425.82, 435.74) | 358.88 (354.56, 363.25) | -1.34 (-2.21, -0.45) | 0.004 | 6.00 (5.43, 6.62) | 5.04 (4.55, 5.59) | -1.41 (-2.42, -0.38) | 0.009 |
| Kenya | 29.24 (28.00, 30.53) | 25.61 (24.91, 26.33) | -0.65 (-0.81, -0.50) | <0.001 | 536.99 (531.75, 542.26) | 530.97 (527.79, 534.16) | -0.03 (-0.09, 0.04) | 0.406 | 336.21 (331.99, 340.48) | 315.27 (312.80, 317.75) | -0.27 (-0.57, 0.03) | 0.071 | 4.59 (4.10, 5.13) | 4.22 (3.94, 4.53) | -0.36 (-0.74, 0.02) | 0.064 |
| Kiribati | 167.41 (122.63, 223.91) | 126.55 (96.47, 163.25) | -1.19 (-1.27, -1.12) | <0.001 | 974.20 (860.54, 1099.27) | 840.57 (759.50, 928.10) | -0.57 (-0.61, -0.53) | <0.001 | 2674.26 (2485.66, 2874.01) | 2350.97 (2214.68, 2493.64) | -0.70 (-0.81, -0.59) | <0.001 | 43.37 (22.36, 76.76) | 38.04 (22.49, 60.46) | -0.73 (-0.84, -0.61) | <0.001 |
| Kuwait | 30.15 (26.64, 34.05) | 31.80 (29.45, 34.35) | 0.04 (-0.18, 0.26) | 0.733 | 420.68 (407.31, 434.44) | 440.98 (432.06, 450.09) | 0.17 (0.10, 0.24) | <0.001 | 212.91 (203.32, 222.88) | 169.40 (163.81, 175.17) | -1.10 (-1.59, -0.60) | <0.001 | 2.51 (1.58, 3.88) | 1.76 (1.25, 2.49) | -1.63 (-2.38, -0.88) | <0.001 |
| Kyrgyzstan | 45.03 (41.87, 48.37) | 28.56 (26.54, 30.69) | -1.90 (-2.10, -1.70) | <0.001 | 400.75 (391.23, 410.46) | 315.56 (308.78, 322.45) | -1.05 (-1.16, -0.94) | <0.001 | 527.37 (516.41, 538.51) | 311.78 (304.99, 318.68) | -2.93 (-3.48, -2.38) | <0.001 | 7.92 (6.62, 9.41) | 4.44 (3.66, 5.34) | -3.21 (-3.85, -2.57) | <0.001 |
| Lao People's Democratic Republic | 68.56 (64.22, 73.13) | 44.53 (42.17, 46.98) | -1.79 (-1.91, -1.67) | <0.001 | 552.06 (539.68, 564.67) | 437.89 (430.47, 445.40) | -0.88 (-0.96, -0.80) | <0.001 | 667.80 (654.08, 681.75) | 566.20 (557.73, 574.77) | -0.55 (-0.81, -0.29) | <0.001 | 9.87 (8.25, 11.73) | 8.38 (7.37, 9.49) | -0.55 (-0.86, -0.24) | 0.001 |
| Latvia | 31.04 (27.67, 34.73) | 27.90 (23.84, 32.55) | -0.44 (-0.52, -0.36) | <0.001 | 333.67 (322.38, 345.27) | 338.41 (323.87, 353.53) | 0.12 (0.03, 0.21) | 0.011 | 263.56 (253.50, 273.93) | 158.65 (148.80, 169.08) | -2.63 (-3.24, -2.02) | <0.001 | 3.44 (2.39, 4.84) | 1.61 (0.78, 3.10) | -3.52 (-4.42, -2.60) | <0.001 |
| Lebanon | 32.13 (28.86, 35.69) | 30.65 (28.34, 33.12) | -0.02 (-0.24, 0.20) | 0.831 | 440.96 (428.64, 453.56) | 448.19 (439.21, 457.33) | 0.24 (0.13, 0.36) | <0.001 | 222.92 (214.18, 231.93) | 152.36 (147.13, 157.75) | -1.16 (-1.48, -0.84) | <0.001 | 2.70 (1.81, 3.89) | 1.45 (0.98, 2.08) | -2.11 (-2.54, -1.68) | <0.001 |
| Lesotho | 24.42 (20.61, 28.76) | 38.48 (34.54, 42.75) | 1.93 (1.59, 2.26) | <0.001 | 395.77 (380.21, 411.85) | 445.61 (432.08, 459.47) | 0.57 (0.50, 0.65) | <0.001 | 271.29 (258.29, 284.82) | 404.83 (391.87, 418.12) | 2.35 (1.84, 2.86) | <0.001 | 3.76 (2.36, 5.74) | 6.04 (4.54, 7.88) | 2.70 (2.09, 3.32) | <0.001 |

| | | | | | | | | | | | | | | | | |
|---|---|---|---|---|---|---|---|---|---|---|---|---|---|---|---|---|
| Liberia | 36.34 (31.78, 41.39) | 30.86 (28.36, 33.53) | -0.54 (-0.65, -0.44) | <0.001 | 472.84 (456.03, 490.12) | 447.17 (437.59, 456.91) | -0.03 (-0.14, 0.09) | 0.608 | 466.62 (449.86, 483.86) | 356.82 (348.22, 365.59) | -0.90 (-1.09, -0.72) | <0.001 | 7.00 (5.08, 9.42) | 5.14 (4.15, 6.31) | -1.06 (-1.26, -0.86) | <0.001 |
| Libya | 34.66 (31.66, 37.89) | 38.54 (36.40, 40.78) | 0.50 (0.41, 0.59) | <0.001 | 432.60 (421.83, 443.60) | 492.91 (485.16, 500.75) | 0.58 (0.51, 0.65) | <0.001 | 461.31 (450.31, 472.53) | 385.52 (378.65, 392.49) | -0.55 (-0.86, -0.24) | 0.001 | 6.72 (5.44, 8.23) | 5.32 (4.54, 6.19) | -0.78 (-1.13, -0.43) | <0.001 |
| Lithuania | 34.44 (31.44, 37.65) | 30.87 (27.30, 34.82) | -0.26 (-0.38, -0.15) | <0.001 | 367.06 (357.12, 377.22) | 349.62 (337.36, 362.27) | -0.09 (-0.16, -0.02) | 0.010 | 251.14 (242.91, 259.58) | 185.38 (176.53, 194.61) | -0.98 (-1.43, -0.53) | <0.001 | 3.15 (2.29, 4.23) | 2.07 (1.24, 3.31) | -1.24 (-1.89, -0.58) | 0.001 |
| Luxembourg | 18.72 (12.52, 27.41) | 13.52 (9.14, 19.59) | -1.44 (-1.63, -1.25) | <0.001 | 255.45 (230.86, 282.32) | 239.09 (219.25, 260.47) | -0.20 (-0.33, -0.07) | 0.005 | 244.86 (220.57, 271.47) | 90.52 (78.48, 104.12) | -4.04 (-4.35, -3.73) | <0.001 | 3.55 (1.22, 8.71) | 0.93 (0.13, 3.80) | -5.49 (-5.85, -5.13) | <0.001 |
| Madagascar | 72.93 (70.25, 75.69) | 52.17 (50.73, 53.65) | -1.36 (-1.44, -1.29) | <0.001 | 669.35 (661.31, 677.46) | 588.96 (584.19, 593.76) | -0.47 (-0.53, -0.41) | <0.001 | 1363.79 (1352.33 , 1375.33) | 844.20 (838.45, 849.97) | -1.46 (-1.72, -1.20) | <0.001 | 21.95 (20.50, 23.48) | 13.25 (12.53, 14.00) | -1.53 (-1.81, -1.26) | <0.001 |
| Malawi | 28.16 (26.32, 30.11) | 23.48 (22.33, 24.68) | -0.91 (-1.05, -0.77) | <0.001 | 481.92 (474.38, 489.55) | 478.80 (473.63, 484.01) | -0.04 (-0.14, 0.06) | 0.447 | 444.42 (437.08, 451.87) | 341.35 (336.95, 345.80) | -1.22 (-1.43, -1.01) | <0.001 | 6.55 (5.68, 7.53) | 4.75 (4.23, 5.31) | -1.46 (-1.70, -1.22) | <0.001 |
| Malaysia | 45.44 (43.89, 47.04) | 35.26 (34.27, 36.26) | -0.94 (-1.17, -0.71) | <0.001 | 556.29 (550.81, 561.81) | 478.95 (475.32, 482.61) | -0.52 (-0.66, -0.38) | <0.001 | 582.82 (577.22, 588.47) | 356.87 (353.73, 360.03) | -1.61 (-1.99, -1.23) | <0.001 | 8.15 (7.49, 8.84) | 4.56 (4.21, 4.93) | -1.92 (-2.38, -1.45) | <0.001 |
| Maldives | 45.06 (30.12, 65.51) | 26.39 (20.48, 33.94) | -2.15 (-2.51, -1.78) | <0.001 | 474.11 (422.63, 530.71) | 366.67 (343.81, 391.02) | -0.87 (-1.05, -0.69) | <0.001 | 718.61 (654.23, 788.12) | 229.12 (211.03, 248.73) | -4.03 (-4.39, -3.67) | <0.001 | 10.94 (4.30, 23.63) | 2.70 (1.11, 6.24) | -5.07 (-5.47, -4.67) | <0.001 |
| Mali | 44.98 (42.46, 47.62) | 34.22 (32.83, 35.66) | -1.09 (-1.20, -0.98) | <0.001 | 477.35 (469.12, 485.70) | 419.87 (415.05, 424.74) | -0.42 (-0.51, -0.33) | <0.001 | 626.23 (616.73, 635.84) | 423.34 (418.45, 428.27) | -1.48 (-1.69, -1.27) | <0.001 | 9.79 (8.63, 11.08) | 6.35 (5.76, 6.99) | -1.65 (-1.87, -1.42) | <0.001 |
| Malta | 19.65 (13.07, 28.60) | 15.77 (9.97, 24.28) | -1.03 (-1.15, -0.90) | <0.001 | 258.56 (232.93, 286.43) | 240.42 (215.91, 267.38) | -0.25 (-0.35, -0.15) | <0.001 | 181.06 (159.55, 204.83) | 103.72 (87.73, 122.22) | -2.16 (-2.30, -2.02) | <0.001 | 2.41 (0.58, 6.96) | 1.14 (0.10, 5.63) | -2.89 (-3.07, -2.70) | <0.001 |
| Marshall Islands | 86.55 (46.24, | 85.07 (52.07, | -0.25 (-0.49, | 0.038 | 659.58 (536.67, | 655.22 (556.21, | -0.12 (-0.24, | 0.046 | 1205.84 (1038.59 | 1340.43 (1196.89 | 0.22 (-0.00, | 0.053 | 18.56 (3.58, | 21.26 (6.93, | 0.25 (0.00, | 0.049 |

| | | | | | | | | | | | | | | | | |
|---|---|---|---|---|---|---|---|---|---|---|---|---|---|---|---|---|
| | 148.96) | 131.39) | -0.02) | | 803.18) | 766.96) | -0.00) | | , 1393.40) | , 1496.60) | 0.44) | | 57.77) | 49.78) | 0.49) | |
| Mauritania | 43.55 (38.73, 48.84) | 28.66 (25.94, 31.59) | -1.64 (-1.81, -1.46) | <0.001 | 546.15 (528.91, 563.84) | 473.12 (462.00, 484.46) | -0.49 (-0.59, -0.40) | <0.001 | 510.95 (494.18, 528.16) | 255.91 (247.66, 264.37) | -2.14 (-2.27, -2.01) | <0.001 | 7.60 (5.66, 10.01) | 3.33 (2.44, 4.46) | -2.53 (-2.67, -2.39) | <0.001 |
| Mauritius | 43.53 (37.91, 49.77) | 33.43 (28.46, 39.05) | -1.23 (-1.34, -1.11) | <0.001 | 515.39 (495.60, 535.79) | 441.27 (422.67, 460.50) | -0.74 (-0.85, -0.64) | <0.001 | 530.71 (510.54, 551.49) | 337.05 (320.91, 353.81) | -1.91 (-2.10, -1.71) | <0.001 | 7.62 (5.37, 10.50) | 4.41 (2.75, 6.75) | -2.23 (-2.45, -2.00) | <0.001 |
| Mexico | 33.13 (32.49, 33.77) | 26.41 (25.96, 26.87) | -0.94 (-1.22, -0.66) | <0.001 | 506.39 (503.92, 508.87) | 470.75 (468.86, 472.66) | -0.22 (-0.36, -0.07) | 0.005 | 259.89 (258.12, 261.68) | 180.37 (179.20, 181.55) | -1.10 (-1.38, -0.81) | <0.001 | 3.67 (3.46, 3.89) | 2.36 (2.22, 2.50) | -1.36 (-1.69, -1.02) | <0.001 |
| Micronesia (Federated States of) | 102.90 (72.24, 142.62) | 74.91 (50.07, 108.13) | -1.26 (-1.35, -1.18) | <0.001 | 715.13 (629.82, 809.15) | 604.89 (529.49, 688.31) | -0.63 (-0.69, -0.57) | <0.001 | 1550.55 (1423.44 , 1686.32) | 1334.00 (1221.26 , 1454.66) | -0.65 (-0.70, -0.60) | <0.001 | 24.80 (11.14, 48.13) | 21.07 (9.13, 41.90) | -0.70 (-0.76, -0.64) | <0.001 |
| Monaco | 16.05 (1.20, 85.16) | 14.88 (0.97, 69.82) | -0.30 (-0.36, -0.25) | <0.001 | 243.03 (154.37, 373.74) | 239.95 (152.33, 362.04) | -0.01 (-0.13, 0.11) | 0.822 | 187.97 (111.58, 307.01) | 111.20 (55.00, 203.09) | -1.75 (-1.99, -1.51) | <0.001 | 2.61 (0.00, 65.26) | 1.29 (0.00, 46.69) | -2.36 (-2.64, -2.08) | <0.001 |
| Mongolia | 51.34 (46.25, 56.87) | 41.48 (38.22, 44.97) | -1.02 (-1.22, -0.82) | <0.001 | 417.61 (402.97, 432.69) | 394.48 (384.26, 404.93) | -0.27 (-0.36, -0.17) | <0.001 | 668.26 (649.55, 687.40) | 671.43 (658.18, 684.91) | -0.62 (-0.98, -0.27) | 0.001 | 10.33 (8.10, 13.04) | 10.60 (9.00, 12.44) | -0.62 (-1.03, -0.21) | 0.005 |
| Montenegro | 40.27 (32.84, 48.91) | 29.69 (22.94, 37.92) | -1.39 (-1.56, -1.22) | <0.001 | 410.15 (385.59, 435.89) | 370.68 (345.71, 397.08) | -0.47 (-0.56, -0.39) | <0.001 | 468.69 (442.43, 496.11) | 266.18 (245.19, 288.58) | -2.37 (-2.88, -1.85) | <0.001 | 6.86 (4.03, 10.95) | 3.40 (1.44, 6.96) | -2.83 (-3.47, -2.18) | <0.001 |
| Morocco | 43.35 (42.02, 44.71) | 41.79 (40.75, 42.84) | -0.17 (-0.24, -0.11) | <0.001 | 501.18 (496.69, 505.72) | 493.26 (489.68, 496.86) | -0.03 (-0.10, 0.04) | 0.402 | 476.39 (471.99, 480.83) | 300.27 (297.48, 303.08) | -1.67 (-1.90, -1.44) | <0.001 | 6.99 (6.46, 7.55) | 3.91 (3.60, 4.24) | -2.03 (-2.32, -1.74) | <0.001 |
| Mozambique | 33.73 (31.97, 35.56) | 35.25 (34.05, 36.48) | 0.35 (0.24, 0.46) | <0.001 | 539.60 (532.62, 546.65) | 568.20 (563.52, 572.92) | 0.36 (0.24, 0.47) | <0.001 | 357.93 (352.18, 363.75) | 436.97 (432.78, 441.20) | 1.22 (1.00, 1.44) | <0.001 | 5.01 (4.35, 5.75) | 6.32 (5.82, 6.86) | 1.41 (1.15, 1.67) | <0.001 |
| Myanmar | 39.45 (38.46, 40.45) | 30.66 (29.93, 31.40) | -1.01 (-1.08, -0.94) | <0.001 | 389.34 (386.26, 392.45) | 362.53 (360.02, 365.06) | -0.16 (-0.29, -0.04) | 0.011 | 1546.91 (1540.75 , | 806.97 (803.22, 810.74) | -2.44 (-2.64, -2.25) | <0.001 | 25.39 (24.60, 26.20) | 12.60 (12.13, 13.08) | -2.61 (-2.83, -2.38) | <0.001 |

| | | | | | | | | | | | | | | | | |
|---|---|---|---|---|---|---|---|---|---|---|---|---|---|---|---|---|
| | | | | | | | | | 1553.09) | | | | | | | |
| Namibia | 29.95 (25.20, 35.40) | 21.55 (18.72, 24.70) | -1.60 (-1.83, -1.37) | <0.001 | 468.47 (449.45, 488.14) | 437.71 (424.71, 451.02) | -0.28 (-0.35, -0.20) | <0.001 | 307.71 (292.11, 323.99) | 222.38 (213.08, 232.01) | -1.66 (-2.24, -1.08) | <0.001 | 4.26 (2.58, 6.68) | 2.83 (1.86, 4.13) | -2.09 (-2.83, -1.35) | <0.001 |
| Nauru | 90.36 (21.59, 251.10) | 83.67 (20.70, 228.91) | -0.31 (-0.76, 0.16) | 0.187 | 724.41 (480.38, 1051.53) | 683.63 (459.03, 982.48) | -0.24 (-0.41, -0.08) | 0.006 | 1846.47 (1441.53 , 2332.43) | 1837.52 (1455.20 , 2292.04) | -0.04 (-0.51, 0.42) | 0.846 | 29.76 (1.09, 157.44) | 29.62 (1.45, 145.99) | -0.05 (-0.54, 0.44) | 0.843 |
| Nepal | 21.83 (20.72, 22.99) | 18.48 (17.72, 19.26) | -0.63 (-0.81, -0.44) | <0.001 | 267.16 (263.26, 271.10) | 248.64 (245.84, 251.46) | -0.14 (-0.25, -0.02) | 0.019 | 291.70 (287.57, 295.88) | 169.10 (166.78, 171.44) | -1.91 (-2.15, -1.66) | <0.001 | 4.39 (3.90, 4.94) | 2.22 (1.96, 2.51) | -2.40 (-2.68, -2.13) | <0.001 |
| Netherlands | 18.93 (17.86, 20.05) | 15.43 (14.39, 16.52) | -0.97 (-1.08, -0.86) | <0.001 | 253.11 (249.15, 257.11) | 255.48 (251.23, 259.79) | -0.00 (-0.08, 0.08) | 0.977 | 170.08 (166.85, 173.36) | 84.56 (82.12, 87.05) | -2.94 (-3.22, -2.65) | <0.001 | 2.29 (1.93, 2.70) | 0.81 (0.59, 1.09) | -4.23 (-4.67, -3.79) | <0.001 |
| New Zealand | 18.21 (16.04, 20.60) | 16.88 (14.81, 19.16) | -0.31 (-0.56, -0.06) | 0.018 | 227.21 (219.35, 235.29) | 274.97 (266.44, 283.71) | 0.74 (0.50, 0.98) | <0.001 | 165.77 (159.07, 172.69) | 95.43 (90.44, 100.63) | -2.23 (-2.41, -2.05) | <0.001 | 2.22 (1.50, 3.15) | 0.89 (0.47, 1.54) | -3.55 (-3.71, -3.39) | <0.001 |
| Nicaragua | 32.00 (28.94, 35.30) | 25.75 (23.88, 27.72) | -0.82 (-0.97, -0.67) | <0.001 | 456.88 (445.25, 468.76) | 426.55 (418.86, 434.35) | -0.17 (-0.26, -0.07) | 0.002 | 244.85 (236.19, 253.76) | 122.49 (118.38, 126.71) | -2.31 (-2.71, -1.91) | <0.001 | 3.56 (2.57, 4.82) | 1.42 (1.01, 1.95) | -3.11 (-3.59, -2.63) | <0.001 |
| Niger | 37.93 (35.49, 40.50) | 30.69 (29.35, 32.07) | -0.83 (-0.96, -0.70) | <0.001 | 446.58 (438.22, 455.08) | 408.79 (403.98, 413.65) | -0.24 (-0.35, -0.13) | <0.001 | 488.86 (480.03, 497.82) | 375.54 (370.86, 380.27) | -1.00 (-1.19, -0.80) | <0.001 | 7.45 (6.38, 8.66) | 5.57 (5.00, 6.18) | -1.11 (-1.33, -0.90) | <0.001 |
| Nigeria | 26.63 (26.05, 27.22) | 23.57 (23.22, 23.93) | -0.57 (-0.72, -0.43) | <0.001 | 413.08 (410.81, 415.36) | 436.27 (434.78, 437.77) | 0.24 (0.13, 0.36) | <0.001 | 303.43 (301.45, 305.42) | 231.03 (229.92, 232.14) | -1.26 (-1.45, -1.06) | <0.001 | 4.33 (4.10, 4.58) | 3.00 (2.88, 3.14) | -1.65 (-1.89, -1.41) | <0.001 |
| Niue | 57.32 (0.02, 627.95) | 50.40 (0.00, 828.54) | -0.52 (-0.57, -0.47) | <0.001 | 574.66 (166.62, 1439.39) | 554.47 (116.00, 1645.86) | -0.14 (-0.18, -0.09) | <0.001 | 973.30 (400.56, 1989.36) | 699.52 (184.49, 1856.32) | -1.48 (-1.66, -1.31) | <0.001 | 14.97 (0.00, 547.76) | 10.13 (0.00, 751.92) | -1.67 (-1.90, -1.44) | <0.001 |
| North Macedonia | 39.11 (34.95, 43.64) | 29.98 (26.36, 34.03) | -1.10 (-1.16, -1.05) | <0.001 | 433.96 (419.83, 448.46) | 399.41 (385.89, 413.34) | -0.34 (-0.44, -0.24) | <0.001 | 504.97 (489.69, 520.60) | 324.40 (312.13, 337.08) | -1.90 (-2.08, -1.71) | <0.001 | 7.25 (5.53, 9.35) | 4.26 (2.97, 6.00) | -2.26 (-2.48, -2.04) | <0.001 |
| Northern Mariana | 56.46 | 45.88 | -0.93 | <0.001 | 568.94 | 536.58 | -0.28 | <0.001 | 850.29 | 527.75 | -2.10 | <0.001 | 12.95 | 7.38 | -2.43 | <0.001 |

| | | | | | | | | | | | | | | | | |
|---|---|---|---|---|---|---|---|---|---|---|---|---|---|---|---|---|
| Islands | (30.62, | (14.85, | (-1.00, | | (477.90, | (407.53, | (-0.35, | | (738.39, | (399.74, | (-2.35, | | (2.83, | (0.07, | (-2.73, | |
| | 97.41) | 112.56) | -0.85) | | 673.81) | 696.88) | -0.20) | | 975.85) | 687.04) | -1.86) | | 39.91) | 56.13) | -2.14) | |
| | 23.21 | 22.68 | -0.66 | | 334.17 | 321.46 | -0.30 | | 169.07 | 73.50 | -3.06 | | 2.13 | 0.48 | -5.21 | |
| Norway | (20.94, | (20.54, | (-0.88, | <0.001 | (325.36, | (313.26, | (-0.45, | 0.001 | (162.83, | (69.62, | (-3.23, | <0.001 | (1.48, | (0.22, | (-5.42, | <0.001 |
| | 25.68) | 25.01) | -0.43) | | 343.17) | 329.82) | -0.14) | | 175.49) | 77.56) | -2.90) | | 2.97) | 0.94) | -5.00) | |
| | 33.85 | 27.84 | -0.79 | | 441.38 | 444.54 | 0.11 | | 436.59 | 246.31 | -1.67 | | 6.25 | 2.97 | -2.19 | |
| Oman | (29.95, | (25.77, | (-0.86, | <0.001 | (427.02, | (436.26, | (0.01, | 0.029 | (422.21, | (239.71, | (-1.97, | <0.001 | (4.64, | (2.29, | (-2.59, | <0.001 |
| | 38.13) | 30.08) | -0.72) | | 456.13) | 452.99) | 0.21) | | 451.35) | 253.08) | -1.37) | | 8.26) | 3.84) | -1.79) | |
| | 38.85 | 36.93 | -0.38 | | 436.60 | 447.21 | 0.09 | | 402.42 | 468.91 | 0.10 | | 5.92 | 6.97 | 0.05 | |
| Pakistan | (38.19, | (36.51, | (-0.50, | <0.001 | (434.43, | (445.77, | (0.02, | 0.009 | (400.31, | (467.43, | (-0.14, | 0.409 | (5.66, | (6.79, | (-0.23, | 0.696 |
| | 39.51) | 37.35) | -0.25) | | 438.78) | 448.65) | 0.15) | | 404.54) | 470.39) | 0.35) | | 6.18) | 7.16) | 0.34) | |
| | 60.90 | 60.66 | -0.05 | | 598.12 | 590.65 | -0.02 | | 1063.67 | 1004.88 | -0.03 | | 16.44 | 15.46 | -0.02 | |
| Palau | (16.99, | (16.25, | (-0.10, | 0.034 | (428.04, | (418.14, | (-0.08, | 0.368 | (832.13, | (774.49, | (-0.12, | 0.561 | (0.56, | (0.40, | (-0.14, | 0.754 |
| | 156.45) | 163.55) | -0.00) | | 814.84) | 814.77) | 0.03) | | 1341.18) | 1286.23) | 0.07) | | 88.60) | 95.29) | 0.10) | |
| | 33.04 | 28.31 | -0.60 | | 438.84 | 429.61 | 0.06 | | 281.26 | 174.37 | -1.86 | | 3.70 | 1.88 | -2.70 | |
| Palestine | (28.67, | (25.96, | (-0.76, | <0.001 | (422.56, | (420.32, | (-0.04, | 0.241 | (268.29, | (168.48, | (-2.11, | <0.001 | (2.33, | (1.31, | (-3.05, | <0.001 |
| | 37.94) | 30.83) | -0.44) | | 455.63) | 439.06) | 0.15) | | 294.75) | 180.43) | -1.61) | | 5.64) | 2.63) | -2.35) | |
| | 28.43 | 24.53 | -0.53 | | 420.08 | 407.78 | -0.03 | | 260.56 | 158.61 | -1.55 | | 3.81 | 2.07 | -1.92 | |
| Panama | (25.10, | (22.15, | (-0.68, | <0.001 | (407.06, | (397.89, | (-0.14, | 0.651 | (250.31, | (152.47, | (-1.87, | <0.001 | (2.65, | (1.43, | (-2.31, | <0.001 |
| | 32.10) | 27.10) | -0.39) | | 433.42) | 417.85) | 0.09) | | 271.15) | 164.95) | -1.22) | | 5.32) | 2.92) | -1.54) | |
| | 74.67 | 76.03 | 0.09 | | 579.38 | 586.90 | 0.08 | | 764.30 | 799.73 | 0.22 | | 11.37 | 11.90 | 0.22 | |
| Papua New Guinea | (70.30, | (73.31, | (0.01, | 0.035 | (567.10, | (579.29, | (0.05, | <0.001 | (750.21, | (790.87, | (0.08, | 0.004 | (9.69, | (10.84, | (0.06, | 0.009 |
| | 79.25) | 78.84) | 0.18) | | 591.88) | 594.60) | 0.12) | | 778.60) | 808.68) | 0.36) | | 13.26) | 13.04) | 0.39) | |
| | 35.28 | 31.17 | -0.54 | | 456.22 | 452.80 | -0.07 | | 356.52 | 209.99 | -2.15 | | 5.51 | 2.95 | -2.53 | |
| Paraguay | (32.28, | (29.17, | (-0.64, | <0.001 | (445.34, | (445.13, | (-0.12, | 0.032 | (346.83, | (204.77, | (-2.38, | <0.001 | (4.35, | (2.36, | (-2.80, | <0.001 |
| | 38.50) | 33.26) | -0.44) | | 467.31) | 460.58) | -0.01) | | 366.41) | 215.32) | -1.92) | | 6.88) | 3.65) | -2.26) | |
| | 41.46 | 30.01 | -1.19 | | 473.09 | 406.15 | -0.47 | | 529.03 | 264.03 | -2.27 | | 8.13 | 3.79 | -2.50 | |
| Peru | (40.07, | (29.10, | (-1.31, | <0.001 | (468.33, | (402.78, | (-0.55, | <0.001 | (524.10, | (261.31, | (-2.56, | <0.001 | (7.52, | (3.47, | (-2.83, | <0.001 |
| | 42.88) | 30.95) | -1.08) | | 477.89) | 409.54) | -0.39) | | 534.01) | 266.77) | -1.97) | | 8.78) | 4.13) | -2.17) | |
| | 45.38 | 39.59 | -0.40 | | 471.98 | 453.39 | -0.03 | | 238.06 | 499.94 | 3.46 | | 2.57 | 7.23 | 4.33 | |
| Philippines | (44.51, | (39.00, | (-0.48, | <0.001 | (469.20, | (451.41, | (-0.13, | 0.566 | (236.07, | (497.85, | (2.64, | <0.001 | (2.37, | (6.98, | (3.14, | <0.001 |
| | 46.25) | 40.18) | -0.32) | | 474.78) | 455.37) | 0.07) | | 240.06) | 502.04) | 4.29) | | 2.79) | 7.48) | 5.53) | |
| Poland | 31.86 | 21.34 | -1.73 | <0.001 | 344.65 | 309.88 | -0.39 | 0.002 | 379.24 | 176.85 | -2.92 | <0.001 | 5.49 | 2.00 | -3.80 | <0.001 |

| | | | | | | | | | | | | | | | | |
|---|---|---|---|---|---|---|---|---|---|---|---|---|---|---|---|---|
| | (30.97, | (20.57, | (-1.96, | | (341.72, | (306.91, | (-0.63, | | (376.19, | (174.65, | (-3.07, | | (5.13, | (1.78, | (-3.94, | |
| | 32.76) | 22.15) | -1.49) | | 347.61) | 312.88) | -0.16) | | 382.30) | 179.08) | -2.77) | | 5.87) | 2.24) | -3.65) | |
| | 24.04 | 12.81 | -2.58 | | 329.36 | 284.12 | -0.52 | | 337.80 | 97.34 | -5.33 | | 5.04 | 0.99 | -6.65 | |
| Portugal | (22.50, | (11.60, | (-2.75, | <0.001 | (323.59, | (278.27, | (-0.65, | <0.001 | (331.95, | (93.99, | (-5.76, | <0.001 | (4.35, | (0.69, | (-7.29, | <0.001 |
| | 25.66) | 14.13) | -2.41) | | 335.22) | 290.07) | -0.38) | | 343.73) | 100.80) | -4.89) | | 5.81) | 1.40) | -6.01) | |
| | 29.81 | 29.26 | -0.16 | | 413.15 | 411.07 | 0.27 | | 197.41 | 111.88 | -2.46 | | 2.77 | 1.29 | -3.41 | |
| Puerto Rico | (27.00, | (26.21, | (-0.30, | 0.031 | (402.52, | (399.39, | (0.14, | <0.001 | (190.07, | (105.85, | (-2.67, | <0.001 | (1.96, | (0.72, | (-3.70, | <0.001 |
| | 32.83) | 32.57) | -0.02) | | 423.98) | 423.00) | 0.40) | | 204.96) | 118.17) | -2.25) | | 3.80) | 2.14) | -3.12) | |
| | 39.55 | 30.68 | -1.10 | | 499.18 | 429.90 | -0.59 | | 321.21 | 155.89 | -2.84 | | 4.24 | 1.60 | -3.81 | |
| Qatar | (32.01, | (27.92, | (-1.27, | <0.001 | (471.31, | (419.41, | (-0.63, | <0.001 | (298.26, | (149.38, | (-3.05, | <0.001 | (2.02, | (1.02, | (-4.11, | <0.001 |
| | 48.81) | 33.81) | -0.94) | | 528.65) | 440.74) | -0.55) | | 345.83) | 162.77) | -2.64) | | 8.50) | 2.65) | -3.52) | |
| | 34.50 | 18.13 | -2.85 | | 330.43 | 241.72 | -1.44 | | 498.53 | 116.85 | -5.50 | | 7.62 | 1.27 | -6.74 | |
| Republic of Korea | (33.69, | (17.53, | (-3.08, | <0.001 | (327.93, | (239.48, | (-1.61, | <0.001 | (495.46, | (115.31, | (-5.82, | <0.001 | (7.25, | (1.12, | (-7.04, | <0.001 |
| | 35.32) | 18.76) | -2.61) | | 332.93) | 243.98) | -1.26) | | 501.61) | 118.41) | -5.17) | | 8.01) | 1.44) | -6.45) | |
| | 33.68 | 29.96 | -0.35 | | 335.35 | 324.40 | -0.12 | | 306.64 | 197.97 | -1.84 | | 4.22 | 2.35 | -2.30 | |
| Republic of Moldova | (31.08, | (27.18, | (-0.41, | <0.001 | (327.02, | (315.09, | (-0.15, | <0.001 | (298.68, | (190.78, | (-2.23, | <0.001 | (3.34, | (1.64, | (-2.83, | <0.001 |
| | 36.46) | 33.00) | -0.30) | | 343.86) | 333.97) | -0.10) | | 314.79) | 205.42) | -1.45) | | 5.29) | 3.33) | -1.77) | |
| | 33.23 | 24.74 | -1.23 | | 378.49 | 356.89 | -0.11 | | 343.14 | 235.00 | -1.66 | | 4.77 | 2.88 | -2.19 | |
| Romania | (32.04, | (23.49, | (-1.30, | <0.001 | (374.41, | (352.11, | (-0.23, | 0.056 | (339.28, | (231.15, | (-1.92, | <0.001 | (4.33, | (2.48, | (-2.55, | <0.001 |
| | 34.47) | 26.04) | -1.15) | | 382.60) | 361.73) | 0.00) | | 347.04) | 238.91) | -1.39) | | 5.25) | 3.34) | -1.83) | |
| | 35.90 | 31.99 | -0.38 | | 367.75 | 381.04 | 0.15 | | 322.74 | 358.11 | -0.11 | | 4.38 | 5.04 | -0.14 | |
| Russian Federation | (35.43, | (31.51, | (-0.67, | 0.013 | (366.24, | (379.35, | (0.05, | 0.005 | (321.32, | (356.52, | (-0.84, | 0.770 | (4.22, | (4.85, | (-1.01, | 0.757 |
| | 36.37) | 32.48) | -0.09) | | 369.26) | 382.73) | 0.26) | | 324.16) | 359.70) | 0.64) | | 4.55) | 5.23) | 0.75) | |
| | 60.22 | 20.77 | -4.53 | | 568.84 | 408.19 | -1.39 | | 825.03 | 303.98 | -4.47 | | 13.02 | 4.30 | -4.94 | |
| Rwanda | (57.15, | (19.53, | (-4.92, | <0.001 | (559.49, | (402.70, | (-1.56, | <0.001 | (813.69, | (299.20, | (-4.93, | <0.001 | (11.62, | (3.74, | (-5.44, | <0.001 |
| | 63.41) | 22.06) | -4.14) | | 578.32) | 413.75) | -1.22) | | 836.50) | 308.81) | -4.01) | | 14.56) | 4.91) | -4.44) | |
| | 52.14 | 32.73 | -2.01 | | 574.41 | 496.45 | -0.54 | | 836.88 | 231.55 | -4.17 | | 13.98 | 3.21 | -4.92 | |
| Saint Kitts and Nevis | (22.89, | (13.87, | (-2.22, | <0.001 | (463.95, | (410.13, | (-0.66, | <0.001 | (700.76, | (173.89, | (-4.71, | <0.001 | (1.95, | (0.03, | (-5.47, | <0.001 |
| | 102.81) | 66.52) | -1.79) | | 704.26) | 596.34) | -0.43) | | 992.52) | 303.05) | -3.63) | | 49.39) | 23.91) | -4.36) | |
| | 40.84 | 32.76 | -0.93 | | 547.42 | 489.18 | -0.36 | | 411.08 | 230.31 | -2.22 | | 6.41 | 3.27 | -2.60 | |
| Saint Lucia | (24.66, | (20.68, | (-1.10, | <0.001 | (483.86, | (438.27, | (-0.47, | <0.001 | (355.01, | (195.95, | (-2.47, | <0.001 | (1.28, | (0.48, | (-2.89, | <0.001 |
| | 64.27) | 49.58) | -0.75) | | 617.57) | 544.56) | -0.24) | | 473.95) | 269.17) | -1.96) | | 19.77) | 11.45) | -2.31) | |
| Saint Vincent and | 33.00 | 33.19 | -0.16 | 0.001 | 436.21 | 458.54 | 0.20 | <0.001 | 418.90 | 278.35 | -2.05 | <0.001 | 6.63 | 4.13 | -2.35 | <0.001 |

| | | | | | | | | | | | | | | | | |
|---|---|---|---|---|---|---|---|---|---|---|---|---|---|---|---|---|
| the Grenadines | (17.44, 57.75) | (18.13, 55.90) | (-0.24, -0.07) | | (373.83, 506.83) | (396.10, 528.17) | (0.14, 0.25) | | (356.94, 489.25) | (230.22, 333.71) | (-2.35, -1.75) | | (1.11, 22.52) | (0.40, 16.45) | (-2.68, -2.01) | |
| Samoa | 66.50 (46.47, 92.82) | 59.31 (43.05, 79.93) | -0.45 (-0.50, -0.40) | <0.001 | 624.33 (559.28, 695.35) | 599.50 (544.95, 658.24) | -0.13 (-0.18, -0.07) | <0.001 | 840.21 (764.29, 922.11) | 853.51 (788.03, 923.17) | -0.01 (-0.10, 0.09) | 0.904 | 12.56 (4.86, 27.21) | 12.83 (5.96, 24.39) | -0.01 (-0.13, 0.11) | 0.863 |
| San Marino | 16.14 (1.05, 72.60) | 14.91 (1.18, 65.04) | -0.31 (-0.37, -0.26) | <0.001 | 235.78 (145.88, 361.45) | 230.51 (148.20, 343.78) | -0.01 (-0.11, 0.10) | 0.906 | 110.26 (52.35, 205.13) | 89.58 (41.97, 169.44) | -0.67 (-0.80, -0.54) | <0.001 | 1.29 (0.00, 46.36) | 0.93 (0.00, 41.08) | -1.11 (-1.30, -0.92) | <0.001 |
| Sao Tome and Principe | 58.24 (35.41, 91.42) | 53.41 (38.69, 72.03) | -0.50 (-0.61, -0.40) | <0.001 | 640.49 (558.93, 731.56) | 650.66 (596.49, 708.55) | -0.01 (-0.06, 0.05) | 0.799 | 479.97 (408.00, 561.77) | 480.91 (434.48, 531.08) | -0.62 (-1.13, -0.11) | 0.020 | 7.01 (1.00, 25.25) | 6.81 (2.36, 15.52) | -0.88 (-1.45, -0.30) | 0.004 |
| Saudi Arabia | 28.08 (26.77, 29.44) | 34.99 (34.17, 35.83) | 0.92 (0.67, 1.16) | <0.001 | 637.13 (630.92, 643.38) | 683.81 (680.07, 687.58) | 0.44 (-0.02, 0.89) | 0.060 | 421.80 (416.68, 426.97) | 347.28 (344.69, 349.89) | -0.41 (-0.63, -0.20) | <0.001 | 5.79 (5.20, 6.43) | 4.42 (4.14, 4.72) | -0.61 (-0.86, -0.35) | <0.001 |
| Senegal | 37.13 (34.70, 39.70) | 30.52 (29.06, 32.03) | -0.80 (-0.90, -0.70) | <0.001 | 505.72 (496.77, 514.81) | 461.07 (455.43, 466.76) | -0.27 (-0.36, -0.19) | <0.001 | 435.41 (427.03, 443.92) | 325.58 (320.82, 330.40) | -0.86 (-1.03, -0.69) | <0.001 | 6.38 (5.39, 7.51) | 4.55 (4.00, 5.16) | -1.00 (-1.20, -0.80) | <0.001 |
| Serbia | 36.53 (34.58, 38.56) | 25.77 (23.98, 27.67) | -1.51 (-1.63, -1.38) | <0.001 | 397.16 (390.66, 403.75) | 390.19 (383.11, 397.38) | -0.05 (-0.17, 0.07) | 0.433 | 467.28 (460.23, 474.41) | 206.85 (201.74, 212.07) | -3.30 (-3.54, -3.05) | <0.001 | 6.84 (6.02, 7.76) | 2.30 (1.80, 2.92) | -4.33 (-4.67, -3.98) | <0.001 |
| Seychelles | 37.25 (18.17, 69.12) | 33.79 (18.31, 58.17) | -0.46 (-0.57, -0.34) | <0.001 | 462.55 (386.94, 549.67) | 445.40 (382.59, 516.36) | -0.15 (-0.24, -0.07) | 0.001 | 570.25 (484.33, 667.88) | 367.25 (310.72, 431.91) | -1.57 (-1.78, -1.37) | <0.001 | 8.47 (1.23, 30.15) | 4.86 (0.60, 19.39) | -2.01 (-2.25, -1.77) | <0.001 |
| Sierra Leone | 42.90 (39.33, 46.72) | 42.24 (39.96, 44.63) | -0.02 (-0.14, 0.10) | 0.763 | 539.68 (526.95, 552.64) | 525.39 (517.40, 533.48) | 0.01 (-0.05, 0.07) | 0.760 | 485.68 (473.55, 498.05) | 476.49 (468.84, 484.25) | 0.14 (-0.05, 0.34) | 0.145 | 7.19 (5.77, 8.87) | 7.05 (6.13, 8.06) | 0.16 (-0.07, 0.38) | 0.165 |
| Singapore | 24.70 (22.27, 27.34) | 18.06 (16.39, 19.91) | -1.30 (-1.48, -1.13) | <0.001 | 265.86 (257.75, 274.17) | 221.58 (215.48, 227.84) | -0.66 (-0.77, -0.54) | <0.001 | 193.02 (186.11, 200.13) | 73.92 (70.36, 77.66) | -3.43 (-3.66, -3.20) | <0.001 | 2.52 (1.79, 3.47) | 0.53 (0.28, 0.99) | -5.57 (-5.80, -5.34) | <0.001 |
| Slovakia | 32.11 (29.75, 34.61) | 24.93 (22.75, 27.30) | -1.03 (-1.15, -0.91) | <0.001 | 365.45 (357.39, 373.66) | 311.68 (303.87, 319.67) | -0.62 (-0.72, -0.51) | <0.001 | 309.77 (302.38, 317.30) | 148.52 (143.13, 154.09) | -2.58 (-2.73, -2.43) | <0.001 | 4.19 (3.37, 5.15) | 1.48 (1.00, 2.16) | -3.63 (-3.81, -3.45) | <0.001 |
| Slovenia | 24.40 | 19.29 | -1.20 | <0.001 | 280.08 | 233.62 | -0.73 | <0.001 | 233.23 | 89.19 | -3.85 | <0.001 | 3.09 | 0.66 | -5.95 | <0.001 |

| | | | | | | | | | | | | | | | | |
|---|---|---|---|---|---|---|---|---|---|---|---|---|---|---|---|---|
| | (21.11, 28.09) | (16.03, 23.11) | (-1.43, -0.96) | | (268.66, 291.89) | (222.04, 245.73) | (-0.86, -0.60) | | (222.86, 244.00) | (81.97, 96.95) | (-4.05, -3.66) | | (2.01, 4.59) | (0.19, 1.82) | (-6.23, -5.67) | |
| Solomon Islands | 91.55 (73.78, 112.51) | 77.14 (66.41, 89.13) | -0.59 (-0.63, -0.56) | <0.001 | 607.69 (560.97, 657.46) | 557.83 (528.49, 588.41) | -0.25 (-0.32, -0.18) | <0.001 | 1470.71 (1396.22, 1548.31) | 1511.25 (1462.34, 1561.40) | 0.27 (0.16, 0.38) | <0.001 | 24.19 (15.43, 36.33) | 25.02 (19.06, 32.28) | 0.30 (0.18, 0.42) | <0.001 |
| Somalia | 47.02 (44.26, 49.90) | 34.44 (33.03, 35.88) | -1.37 (-1.52, -1.23) | <0.001 | 508.28 (499.20, 517.49) | 473.44 (468.34, 478.60) | -0.24 (-0.34, -0.15) | <0.001 | 652.30 (641.97, 662.77) | 495.13 (489.85, 500.45) | -0.99 (-1.13, -0.85) | <0.001 | 10.19 (8.93, 11.58) | 7.47 (6.83, 8.16) | -1.11 (-1.26, -0.95) | <0.001 |
| South Africa | 35.81 (34.84, 36.81) | 21.46 (20.88, 22.05) | -2.18 (-2.63, -1.74) | <0.001 | 588.02 (584.13, 591.94) | 496.78 (493.97, 499.60) | -0.73 (-0.83, -0.63) | <0.001 | 574.78 (570.83, 578.74) | 252.15 (250.18, 254.14) | -3.31 (-4.41, -2.19) | <0.001 | 8.82 (8.33, 9.33) | 3.30 (3.08, 3.53) | -3.82 (-5.16, -2.46) | <0.001 |
| South Sudan | 33.05 (30.50, 35.77) | 27.17 (25.30, 29.14) | -0.82 (-0.94, -0.70) | <0.001 | 485.47 (475.83, 495.26) | 493.09 (485.13, 501.14) | 0.07 (-0.02, 0.16) | 0.118 | 399.47 (390.57, 408.53) | 281.26 (275.23, 287.40) | -1.40 (-1.67, -1.13) | <0.001 | 5.83 (4.79, 7.06) | 3.73 (3.05, 4.51) | -1.78 (-2.10, -1.46) | <0.001 |
| Spain | 19.43 (18.72, 20.16) | 13.45 (12.84, 14.08) | -1.91 (-2.14, -1.67) | <0.001 | 267.70 (265.04, 270.37) | 240.60 (237.99, 243.23) | -0.44 (-0.57, -0.31) | <0.001 | 266.92 (264.28, 269.59) | 86.05 (84.50, 87.63) | -4.36 (-4.66, -4.06) | <0.001 | 3.85 (3.54, 4.18) | 0.86 (0.72, 1.03) | -5.85 (-6.13, -5.57) | <0.001 |
| Sri Lanka | 26.15 (24.98, 27.36) | 24.58 (23.51, 25.68) | -0.30 (-0.38, -0.21) | <0.001 | 382.72 (378.21, 387.27) | 381.75 (377.53, 386.02) | 0.03 (-0.07, 0.14) | 0.521 | 315.02 (310.94, 319.15) | 236.98 (233.65, 240.34) | -0.92 (-1.21, -0.64) | <0.001 | 4.06 (3.60, 4.55) | 2.74 (2.39, 3.12) | -1.29 (-1.69, -0.89) | <0.001 |
| Sudan | 51.01 (49.30, 52.78) | 47.67 (46.59, 48.77) | -0.29 (-0.39, -0.19) | <0.001 | 502.62 (497.25, 508.04) | 515.24 (511.70, 518.80) | 0.13 (0.04, 0.22) | 0.005 | 756.89 (750.30, 763.53) | 478.91 (475.50, 482.33) | -1.52 (-1.62, -1.42) | <0.001 | 11.80 (10.99, 12.67) | 6.93 (6.52, 7.36) | -1.77 (-1.88, -1.65) | <0.001 |
| Suriname | 40.42 (30.49, 52.74) | 36.42 (28.82, 45.44) | -0.50 (-0.57, -0.43) | <0.001 | 480.05 (444.66, 517.70) | 485.50 (456.52, 515.87) | 0.06 (-0.03, 0.15) | 0.197 | 539.91 (502.01, 580.08) | 403.67 (377.30, 431.42) | -1.48 (-1.89, -1.06) | <0.001 | 8.64 (4.43, 15.42) | 6.29 (3.40, 10.67) | -1.60 (-2.05, -1.15) | <0.001 |
| Sweden | 19.39 (17.85, 21.04) | 19.05 (17.58, 20.63) | 0.03 (-0.33, 0.38) | 0.879 | 397.26 (390.15, 404.48) | 534.76 (526.82, 542.80) | 1.37 (1.12, 1.63) | <0.001 | 157.15 (152.71, 161.69) | 103.51 (100.05, 107.07) | -1.36 (-1.62, -1.10) | <0.001 | 1.78 (1.34, 2.33) | 0.54 (0.33, 0.87) | -4.14 (-4.32, -3.97) | <0.001 |
| Switzerland | 15.91 (14.44, 17.50) | 13.04 (11.76, 14.44) | -0.82 (-0.96, -0.69) | <0.001 | 218.17 (212.67, 223.78) | 196.99 (191.95, 202.14) | -0.36 (-0.50, -0.21) | <0.001 | 131.85 (127.58, 136.24) | 53.39 (50.77, 56.12) | -3.70 (-4.01, -3.40) | <0.001 | 1.68 (1.24, 2.26) | 0.38 (0.19, 0.70) | -6.12 (-6.49, -5.74) | <0.001 |

| | | | | | | | | | | | | | | | | |
|---|---|---|---|---|---|---|---|---|---|---|---|---|---|---|---|---|
| Syrian Arab Republic | 63.73 (61.30, 66.24) | 43.32 (41.42, 45.29) | -1.66 (-1.87, -1.45) | <0.001 | 632.82 (625.02, 640.70) | 544.42 (537.62, 551.29) | -0.65 (-0.72, -0.58) | <0.001 | 1012.58 (1002.98, 1022.24) | 500.36 (493.92, 506.88) | -2.81 (-3.18, -2.43) | <0.001 | 15.46 (14.28, 16.72) | 7.02 (6.28, 7.85) | -3.16 (-3.58, -2.74) | <0.001 |
| Taiwan (Province of China) | 27.44 (26.39, 28.53) | 24.27 (23.22, 25.36) | -0.51 (-0.58, -0.44) | <0.001 | 341.95 (338.21, 345.72) | 341.96 (338.00, 345.97) | -0.03 (-0.12, 0.06) | 0.504 | 343.42 (339.67, 347.19) | 204.72 (201.74, 207.75) | -1.98 (-2.12, -1.85) | <0.001 | 4.73 (4.30, 5.19) | 2.36 (2.06, 2.71) | -2.65 (-2.83, -2.47) | <0.001 |
| Tajikistan | 30.79 (28.29, 33.48) | 27.15 (25.54, 28.84) | -0.35 (-0.62, -0.07) | 0.015 | 278.26 (270.62, 286.07) | 264.00 (258.92, 269.16) | -0.11 (-0.26, 0.05) | 0.168 | 309.02 (300.82, 317.40) | 236.65 (231.81, 241.56) | -1.95 (-2.34, -1.56) | <0.001 | 4.50 (3.55, 5.65) | 3.22 (2.68, 3.85) | -2.37 (-2.82, -1.92) | <0.001 |
| Thailand | 33.26 (32.55, 33.99) | 31.35 (30.66, 32.04) | -0.60 (-0.78, -0.42) | <0.001 | 384.39 (381.94, 386.84) | 360.61 (358.29, 362.95) | -0.36 (-0.48, -0.25) | <0.001 | 406.18 (403.65, 408.72) | 458.81 (456.19, 461.43) | -0.72 (-1.39, -0.05) | 0.037 | 5.80 (5.49, 6.11) | 6.73 (6.42, 7.05) | -0.83 (-1.59, -0.05) | 0.037 |
| Timor-Leste | 48.64 (40.97, 57.40) | 43.63 (37.63, 50.39) | -0.40 (-0.48, -0.31) | <0.001 | 482.08 (457.27, 507.95) | 471.59 (451.43, 492.48) | 0.01 (-0.08, 0.09) | 0.893 | 403.98 (381.11, 427.93) | 485.96 (465.40, 507.25) | 0.89 (0.60, 1.18) | <0.001 | 5.47 (3.09, 9.03) | 6.84 (4.57, 9.91) | 1.08 (0.74, 1.43) | <0.001 |
| Togo | 40.22 (36.65, 44.06) | 34.51 (32.43, 36.69) | -0.57 (-0.60, -0.54) | <0.001 | 517.03 (504.21, 530.12) | 484.32 (476.50, 492.24) | -0.16 (-0.23, -0.10) | <0.001 | 506.69 (493.88, 519.77) | 414.02 (406.75, 421.38) | -0.64 (-0.73, -0.55) | <0.001 | 7.60 (6.08, 9.39) | 6.05 (5.19, 7.01) | -0.72 (-0.82, -0.62) | <0.001 |
| Tokelau | 60.43 (0.00, 929.92) | 51.12 (0.00, 984.43) | -0.66 (-0.70, -0.63) | <0.001 | 578.42 (122.67, 1747.97) | 541.03 (89.58, 1790.45) | -0.24 (-0.28, -0.21) | <0.001 | 906.68 (286.88, 2216.82) | 765.12 (184.79, 2126.41) | -0.58 (-0.69, -0.48) | <0.001 | 13.89 (0.00, 846.61) | 11.49 (0.00, 909.74) | -0.64 (-0.76, -0.52) | <0.001 |
| Tonga | 44.20 (23.60, 76.83) | 40.45 (22.31, 67.83) | -0.35 (-0.50, -0.20) | <0.001 | 517.60 (438.75, 607.52) | 509.55 (437.73, 590.06) | -0.01 (-0.10, 0.09) | 0.894 | 386.22 (317.97, 465.70) | 386.58 (324.53, 457.35) | 0.04 (-0.01, 0.09) | 0.084 | 4.98 (0.31, 24.04) | 4.93 (0.46, 19.94) | -0.03 (-0.08, 0.02) | 0.214 |
| Trinidad and Tobago | 42.06 (36.46, 48.30) | 34.65 (29.89, 40.01) | -0.83 (-0.91, -0.74) | <0.001 | 539.47 (519.03, 560.54) | 507.65 (488.70, 527.20) | -0.26 (-0.32, -0.21) | <0.001 | 398.94 (381.37, 417.14) | 237.41 (224.54, 250.87) | -2.27 (-2.56, -1.97) | <0.001 | 6.05 (4.06, 8.71) | 3.31 (1.96, 5.31) | -2.63 (-2.98, -2.28) | <0.001 |
| Tunisia | 29.28 (27.40, 31.27) | 30.03 (28.48, 31.66) | 0.10 (0.04, 0.16) | 0.003 | 277.28 (271.43, 283.22) | 334.15 (328.92, 339.45) | 0.27 (0.13, 0.41) | <0.001 | 282.27 (276.34, 288.30) | 213.16 (209.00, 217.39) | -1.10 (-1.18, -1.02) | <0.001 | 4.13 (3.43, 4.93) | 2.79 (2.33, 3.31) | -1.46 (-1.54, -1.37) | <0.001 |
| Turkey | 37.81 (37.02, | 29.71 (29.14, | -1.05 (-1.16, | <0.001 | 467.80 (465.00, | 409.01 (406.90, | -0.47 (-0.52, | <0.001 | 293.63 (291.40, | 171.49 (170.12, | -1.97 (-2.14, | <0.001 | 3.90 (3.64, | 1.90 (1.76, | -2.66 (-2.90, | <0.001 |

| | | | | | | | | | | | | | | | | |
|---|---|---|---|---|---|---|---|---|---|---|---|---|---|---|---|---|
| | 38.62) | 30.28) | -0.93) | | 470.62) | 411.13) | -0.42) | | 295.87) | 172.86) | -1.81) | | 4.17) | 2.05) | -2.43) | |
| Turkmenistan | 35.61 (32.52, 38.93) | 50.04 (47.04, 53.20) | 1.24 (1.04, 1.44) | <0.001 | 351.25 (341.41, 361.31) | 421.70 (412.89, 430.67) | 0.68 (0.60, 0.76) | <0.001 | 395.94 (385.41, 406.69) | 611.64 (600.99, 622.43) | 1.56 (1.08, 2.05) | <0.001 | 5.77 (4.55, 7.23) | 9.27 (8.00, 10.69) | 1.70 (1.15, 2.26) | <0.001 |
| Tuvalu | 75.57 (13.67, 236.45) | 58.47 (10.37, 187.15) | -1.01 (-1.08, -0.93) | <0.001 | 616.76 (385.58, 937.22) | 543.55 (350.13, 808.43) | -0.47 (-0.53, -0.41) | <0.001 | 1305.95 (956.75, 1742.48) | 1150.00 (858.29, 1511.68) | -0.25 (-0.32, -0.18) | <0.001 | 20.67 (0.16, 147.63) | 18.25 (0.22, 123.10) | -0.22 (-0.30, -0.14) | <0.001 |
| Uganda | 29.65 (28.18, 31.19) | 21.93 (21.14, 22.74) | -1.52 (-1.73, -1.31) | <0.001 | 461.08 (455.41, 466.82) | 434.05 (430.60, 437.52) | -0.29 (-0.39, -0.19) | <0.001 | 366.08 (360.92, 371.30) | 329.94 (326.89, 333.01) | -1.38 (-1.81, -0.96) | <0.001 | 5.29 (4.67, 5.96) | 4.66 (4.30, 5.05) | -1.62 (-2.11, -1.13) | <0.001 |
| Ukraine | 33.54 (32.74, 34.36) | 39.90 (38.92, 40.90) | 0.73 (0.58, 0.88) | <0.001 | 426.13 (423.25, 429.02) | 495.95 (492.41, 499.51) | 0.59 (0.47, 0.70) | <0.001 | 271.37 (269.08, 273.67) | 453.96 (450.68, 457.26) | 1.51 (0.98, 2.04) | <0.001 | 3.32 (3.07, 3.58) | 6.38 (6.01, 6.78) | 1.85 (1.18, 2.53) | <0.001 |
| United Arab Emirates | 40.66 (36.80, 44.92) | 40.95 (38.87, 43.16) | 0.02 (-0.12, 0.16) | 0.750 | 553.22 (538.58, 568.25) | 548.36 (540.18, 556.66) | -0.04 (-0.17, 0.09) | 0.531 | 414.23 (401.48, 427.38) | 304.20 (298.53, 309.98) | -0.97 (-1.38, -0.56) | <0.001 | 5.82 (4.42, 7.65) | 3.98 (3.40, 4.68) | -1.15 (-1.66, -0.64) | <0.001 |
| United Kingdom | 17.95 (17.38, 18.53) | 13.52 (13.05, 14.01) | -1.24 (-1.40, -1.09) | <0.001 | 262.07 (259.89, 264.26) | 238.39 (236.38, 240.41) | -0.33 (-0.50, -0.16) | <0.001 | 181.25 (179.44, 183.08) | 106.96 (105.63, 108.30) | -2.11 (-2.26, -1.95) | <0.001 | 2.47 (2.26, 2.69) | 1.24 (1.10, 1.39) | -2.73 (-2.90, -2.56) | <0.001 |
| United Republic of Tanzania | 26.19 (25.09, 27.33) | 23.14 (22.47, 23.82) | -0.57 (-0.75, -0.40) | <0.001 | 449.30 (444.81, 453.83) | 466.15 (463.18, 469.12) | 0.21 (0.08, 0.35) | 0.003 | 450.39 (445.82, 455.01) | 325.71 (323.20, 328.22) | -1.40 (-1.67, -1.13) | <0.001 | 6.75 (6.20, 7.35) | 4.52 (4.23, 4.83) | -1.74 (-2.04, -1.43) | <0.001 |
| United States of America | 18.44 (18.18, 18.70) | 18.08 (17.83, 18.33) | -0.25 (-0.43, -0.06) | 0.012 | 357.49 (356.35, 358.63) | 444.86 (443.62, 446.09) | 1.43 (0.98, 1.88) | <0.001 | 184.50 (183.69, 185.32) | 148.63 (147.92, 149.34) | -0.46 (-0.57, -0.34) | <0.001 | 2.28 (2.19, 2.37) | 1.46 (1.39, 1.53) | -1.48 (-1.65, -1.31) | <0.001 |
| United States Virgin Islands | 29.41 (15.02, 52.22) | 29.42 (13.62, 56.09) | -0.07 (-0.16, 0.03) | 0.147 | 407.87 (347.24, 476.32) | 413.80 (345.34, 492.28) | 0.15 (0.08, 0.21) | <0.001 | 240.65 (194.73, 294.44) | 133.63 (96.40, 181.05) | -2.72 (-3.28, -2.15) | <0.001 | 3.53 (0.22, 16.45) | 1.68 (0.00, 16.24) | -3.15 (-3.86, -2.42) | <0.001 |
| Uruguay | 27.21 (24.25, 30.43) | 21.70 (19.17, 24.48) | -1.12 (-1.27, -0.97) | <0.001 | 309.73 (299.53, 320.18) | 292.49 (282.99, 302.24) | -0.38 (-0.51, -0.26) | <0.001 | 440.04 (427.88, 452.47) | 199.08 (191.27, 207.14) | -2.96 (-3.17, -2.74) | <0.001 | 6.78 (5.35, 8.49) | 2.64 (1.81, 3.72) | -3.52 (-3.76, -3.28) | <0.001 |
| Uzbekistan | 36.25 (34.92, | 32.01 (31.09, | -0.34 (-0.42, | <0.001 | 342.16 (338.06, | 355.39 (352.30, | 0.15 (0.08, | <0.001 | 352.22 (348.03, | 344.73 (341.68, | -0.24 (-0.55, | 0.132 | 5.04 (4.55, | 4.85 (4.50, | -0.34 (-0.73, | 0.093 |

| | | | | | | | | | | | | | | | | |
|---|---|---|---|---|---|---|---|---|---|---|---|---|---|---|---|---|
| | 37.62) | 32.95) | -0.27) | | 346.31) | 358.50) | 0.23) | | 356.45) | 347.79) | 0.08) | | 5.58) | 5.23) | 0.06) | |
| Vanuatu | 101.36 (75.97, 132.80) | 84.73 (68.36, 103.97) | -0.72 (-0.77, -0.67) | <0.001 | 785.27 (711.42, 864.96) | 708.46 (659.52, 760.18) | -0.37 (-0.40, -0.33) | <0.001 | 1294.44 (1199.00 , 1395.70) | 1482.50 (1411.36 , 1556.42) | 0.03 (-0.12, 0.17) | 0.718 | 20.05 (9.78, 36.79) | 23.47 (15.26, 34.67) | 0.04 (-0.11, 0.20) | 0.566 |
| Venezuela (Bolivarian Republic of) | 34.09 (32.78, 35.44) | 28.34 (27.35, 29.36) | -0.76 (-0.94, -0.58) | <0.001 | 496.67 (491.66, 501.71) | 451.59 (447.60, 455.61) | -0.30 (-0.44, -0.16) | <0.001 | 375.31 (370.94, 379.72) | 255.34 (252.34, 258.36) | -1.52 (-2.05, -0.99) | <0.001 | 5.72 (5.19, 6.29) | 3.67 (3.32, 4.05) | -1.77 (-2.35, -1.19) | <0.001 |
| Viet Nam | 35.65 (34.91, 36.39) | 31.88 (31.33, 32.43) | -0.49 (-0.70, -0.27) | <0.001 | 385.40 (383.01, 387.80) | 406.71 (404.77, 408.67) | 0.26 (0.09, 0.44) | 0.005 | 480.24 (477.52, 482.97) | 369.20 (367.35, 371.05) | -0.43 (-0.66, -0.21) | 0.001 | 7.15 (6.82, 7.50) | 5.06 (4.85, 5.28) | -0.60 (-0.89, -0.31) | <0.001 |
| Yemen | 48.13 (46.03, 50.31) | 41.16 (40.03, 42.31) | -0.60 (-0.63, -0.58) | <0.001 | 478.23 (471.60, 484.93) | 454.93 (451.18, 458.71) | -0.11 (-0.19, -0.03) | 0.013 | 542.96 (535.80, 550.21) | 426.16 (422.51, 429.83) | -0.88 (-1.01, -0.74) | <0.001 | 8.39 (7.51, 9.34) | 6.27 (5.84, 6.74) | -1.08 (-1.23, -0.93) | <0.001 |
| Zambia | 32.25 (29.98, 34.65) | 22.64 (21.51, 23.81) | -1.68 (-1.86, -1.50) | <0.001 | 464.93 (456.51, 473.48) | 440.70 (435.78, 445.66) | -0.24 (-0.34, -0.14) | <0.001 | 462.88 (454.31, 471.58) | 398.21 (393.45, 403.01) | -0.90 (-1.09, -0.70) | <0.001 | 6.93 (5.90, 8.11) | 5.88 (5.31, 6.50) | -0.99 (-1.21, -0.77) | <0.001 |
| Zimbabwe | 19.04 (17.59, 20.59) | 27.36 (26.02, 28.75) | 1.22 (0.63, 1.81) | <0.001 | 380.77 (374.28, 387.36) | 422.96 (417.69, 428.27) | 0.37 (0.23, 0.50) | <0.001 | 216.64 (211.75, 221.63) | 316.41 (311.87, 321.00) | 1.81 (1.16, 2.47) | <0.001 | 2.78 (2.25, 3.42) | 4.39 (3.86, 4.97) | 2.15 (1.33, 2.97) | <0.001 |
| **Women** | | | | | | | | | | | | | | | | |
| Afghanistan | 72.22 (67.98, 76.67) | 73.30 (71.06, 75.60) | -0.41 (-0.72, -0.10) | 0.012 | 619.28 (607.00, 631.78) | 632.07 (625.58, 638.60) | -0.01 (-0.13, 0.10) | 0.798 | 1455.91 (1436.89 , 1475.15) | 936.40 (928.36, 944.50) | -1.95 (-2.30, -1.60) | <0.001 | 23.87 (21.47, 26.50) | 14.82 (13.81, 15.89) | -2.10 (-2.48, -1.73) | <0.001 |
| Albania | 30.84 (26.75, 35.41) | 27.52 (22.96, 32.75) | -0.39 (-0.44, -0.34) | <0.001 | 408.09 (392.79, 423.86) | 395.13 (377.27, 413.63) | -0.09 (-0.18, -0.01) | 0.028 | 379.84 (365.20, 394.94) | 271.76 (257.01, 287.16) | -0.92 (-1.14, -0.69) | <0.001 | 5.01 (3.45, 7.07) | 3.22 (1.80, 5.34) | -1.15 (-1.47, -0.84) | <0.001 |
| Algeria | 50.17 (48.04, 52.38) | 36.85 (35.60, 38.13) | -1.26 (-1.34, -1.18) | <0.001 | 556.26 (549.19, 563.41) | 493.32 (488.70, 497.97) | -0.40 (-0.49, -0.31) | <0.001 | 661.94 (654.18, 669.77) | 299.91 (296.32, 303.54) | -2.86 (-3.00, -2.72) | <0.001 | 9.99 (9.04, 11.01) | 3.86 (3.47, 4.29) | -3.45 (-3.60, -3.30) | <0.001 |
| American Samoa | 50.26 (15.51, | 58.69 (20.34, | 0.64 (0.57, | <0.001 | 629.51 (477.68, | 670.29 (514.23, | 0.28 (0.22, | <0.001 | 463.62 (334.57, | 524.88 (387.92, | 0.13 (-0.08, | 0.222 | 5.79 (0.01, | 6.70 (0.02, | 0.03 (-0.26, | 0.817 |

| | | | | | | | | | | | | | | | | |
|---|---|---|---|---|---|---|---|---|---|---|---|---|---|---|---|---|
| | 125.07) | 135.39) | 0.72) | | 816.93) | 860.87) | 0.33) | | 628.67) | 696.61) | 0.34) | | 58.21) | 57.84) | 0.33) | |
| Andorra | 17.94 (2.38, 70.72) | 16.43 (2.19, 65.03) | -0.46 (-0.56, -0.35) | <0.001 | 274.77 (188.75, 391.10) | 272.61 (190.32, 382.74) | -0.12 (-0.23, -0.02) | 0.018 | 114.74 (62.17, 199.12) | 80.38 (39.31, 151.31) | -1.45 (-1.60, -1.31) | <0.001 | 1.24 (0.00, 44.80) | 0.66 (0.00, 40.63) | -2.55 (-2.76, -2.34) | <0.001 |
| Angola | 40.49 (37.55, 43.61) | 24.93 (23.59, 26.32) | -2.02 (-2.18, -1.85) | <0.001 | 541.90 (531.16, 552.81) | 481.84 (476.01, 487.73) | -0.44 (-0.50, -0.37) | <0.001 | 465.87 (455.80, 476.12) | 271.25 (266.80, 275.75) | -2.11 (-2.35, -1.86) | <0.001 | 6.83 (5.64, 8.19) | 3.55 (3.06, 4.11) | -2.51 (-2.83, -2.20) | <0.001 |
| Antigua and Barbuda | 38.48 (12.38, 91.61) | 35.96 (13.86, 77.94) | -0.31 (-0.48, -0.15) | <0.001 | 491.55 (378.18, 629.17) | 486.82 (390.41, 601.07) | 0.01 (-0.07, 0.10) | 0.755 | 293.34 (207.32, 404.02) | 151.94 (100.55, 221.77) | -2.45 (-2.70, -2.19) | <0.001 | 4.27 (0.01, 39.28) | 1.81 (0.00, 27.35) | -3.32 (-3.61, -3.03) | <0.001 |
| Argentina | 34.23 (32.77, 35.73) | 23.02 (22.04, 24.04) | -1.64 (-1.81, -1.46) | <0.001 | 350.84 (346.14, 355.58) | 290.67 (287.15, 294.22) | -0.72 (-0.80, -0.63) | <0.001 | 512.09 (506.42, 517.81) | 205.54 (202.57, 208.54) | -3.44 (-3.86, -3.03) | <0.001 | 7.88 (7.19, 8.62) | 2.67 (2.34, 3.03) | -4.16 (-4.62, -3.69) | <0.001 |
| Armenia | 34.72 (30.58, 39.29) | 29.96 (25.66, 34.90) | -0.58 (-0.76, -0.41) | <0.001 | 448.68 (433.45, 464.33) | 406.67 (390.52, 423.44) | -0.30 (-0.37, -0.22) | <0.001 | 206.90 (196.56, 217.67) | 121.36 (112.58, 130.77) | -2.12 (-2.27, -1.98) | <0.001 | 2.01 (1.11, 3.37) | 0.67 (0.18, 1.97) | -4.02 (-4.43, -3.61) | <0.001 |
| Australia | 20.67 (19.18, 22.25) | 19.03 (17.75, 20.39) | -0.56 (-0.68, -0.43) | <0.001 | 329.60 (323.57, 335.72) | 310.40 (305.16, 315.71) | -0.09 (-0.19, 0.01) | 0.065 | 141.76 (137.82, 145.79) | 87.71 (84.95, 90.54) | -1.76 (-1.88, -1.63) | <0.001 | 1.51 (1.13, 1.99) | 0.63 (0.42, 0.92) | -3.15 (-3.41, -2.90) | <0.001 |
| Austria | 24.79 (22.31, 27.49) | 19.67 (17.44, 22.15) | -1.04 (-1.14, -0.94) | <0.001 | 366.71 (357.00, 376.63) | 329.99 (320.61, 339.61) | -0.42 (-0.53, -0.32) | <0.001 | 185.41 (178.54, 192.50) | 78.80 (74.26, 83.57) | -3.47 (-3.68, -3.25) | <0.001 | 2.30 (1.59, 3.24) | 0.52 (0.22, 1.10) | -5.81 (-6.16, -5.47) | <0.001 |
| Azerbaijan | 37.84 (34.81, 41.08) | 30.34 (28.06, 32.77) | -0.67 (-0.76, -0.58) | <0.001 | 447.54 (436.97, 458.32) | 421.29 (412.71, 430.02) | -0.11 (-0.19, -0.03) | 0.012 | 322.66 (313.66, 331.86) | 209.13 (203.08, 215.33) | -1.67 (-2.00, -1.34) | <0.001 | 4.05 (3.09, 5.22) | 2.15 (1.58, 2.89) | -2.34 (-2.81, -1.86) | <0.001 |
| Bahamas | 39.30 (24.65, 59.86) | 34.82 (22.97, 50.82) | -0.45 (-0.59, -0.31) | <0.001 | 508.07 (451.15, 570.49) | 470.78 (423.79, 521.71) | -0.18 (-0.30, -0.06) | 0.006 | 349.37 (302.09, 402.25) | 248.51 (214.82, 286.16) | -1.71 (-2.02, -1.39) | <0.001 | 5.27 (1.05, 16.17) | 3.56 (0.68, 11.10) | -2.04 (-2.41, -1.67) | <0.001 |
| Bahrain | 35.92 (24.95, 50.39) | 34.08 (27.18, 42.37) | -0.38 (-0.57, -0.20) | <0.001 | 550.66 (504.81, 599.79) | 510.97 (483.06, 540.23) | -0.25 (-0.32, -0.17) | <0.001 | 384.36 (345.86, 426.20) | 178.82 (162.28, 196.72) | -2.47 (-2.95, -1.98) | <0.001 | 4.86 (1.47, 12.19) | 1.66 (0.46, 4.53) | -3.67 (-4.41, -2.92) | <0.001 |
| Bangladesh | 26.46 (25.70, | 28.79 (28.23, | 0.27 (0.21, | <0.001 | 459.63 (456.53, | 469.92 (467.63, | 0.45 (0.07, | 0.020 | 621.72 (618.07, | 300.33 (298.50, | -2.15 (-2.42, | <0.001 | 9.53 (9.07, | 3.91 (3.70, | -2.71 (-2.99, | <0.001 |

| | | | | | | | | | | | | | | | | |
|---|---|---|---|---|---|---|---|---|---|---|---|---|---|---|---|---|
| | 27.23) | 29.37) | 0.34) | | 462.75) | 472.21) | 0.82) | | 625.39) | 302.17) | -1.88) | | 10.00) | 4.12) | -2.42) | |
| | 37.27 | 36.82 | -0.02 | | 511.85 | 507.59 | 0.10 | | 249.79 | 177.34 | -1.79 | | 3.45 | 2.22 | -2.32 | |
| Barbados | (23.01, | (22.21, | (-0.21, | 0.794 | (454.22, | (448.08, | (-0.03, | 0.109 | (209.99, | (143.05, | (-2.05, | <0.001 | (0.39, | (0.09, | (-2.66, | <0.001 |
| | 57.34) | 57.91) | 0.16) | | 574.96) | 573.16) | 0.23) | | 295.14) | 217.79) | -1.52) | | 13.17) | 12.09) | -1.99) | |
| | 34.74 | 33.59 | 0.06 | | 460.26 | 470.22 | 0.17 | | 218.90 | 190.85 | -0.36 | | 2.20 | 1.73 | -0.54 | |
| Belarus | (32.23, | (30.79, | (-0.06, | 0.312 | (451.01, | (459.55, | (0.12, | <0.001 | (212.52, | (184.23, | (-0.80, | 0.114 | (1.61, | (1.18, | (-1.31, | 0.160 |
| | 37.40) | 36.61) | 0.18) | | 469.67) | 481.12) | 0.21) | | 225.44) | 197.68) | 0.09) | | 2.96) | 2.52) | 0.23) | |
| | 21.08 | 16.12 | -1.20 | | 298.15 | 298.80 | 0.10 | | 233.20 | 97.69 | -3.08 | | 3.24 | 0.90 | -4.58 | |
| Belgium | (19.06, | (14.32, | (-1.31, | <0.001 | (290.40, | (290.87, | (-0.05, | 0.175 | (226.35, | (93.20, | (-3.31, | <0.001 | (2.48, | (0.53, | (-4.80, | <0.001 |
| | 23.27) | 18.09) | -1.09) | | 306.06) | 306.90) | 0.24) | | 240.22) | 102.37) | -2.86) | | 4.17) | 1.48) | -4.35) | |
| | 33.55 | 36.04 | 0.33 | | 462.85 | 489.54 | 0.29 | | 205.38 | 192.46 | -1.41 | | 2.76 | 2.49 | -2.03 | |
| Belize | (16.27, | (24.52, | (0.21, | <0.001 | (390.06, | (444.05, | (0.23, | <0.001 | (157.78, | (164.28, | (-1.85, | <0.001 | (0.03, | (0.33, | (-2.58, | <0.001 |
| | 62.75) | 51.23) | 0.45) | | 546.33) | 538.52) | 0.36) | | 263.93) | 224.18) | -0.97) | | 19.96) | 8.89) | -1.48) | |
| | 32.66 | 29.96 | -0.34 | | 473.01 | 458.26 | -0.04 | | 310.36 | 281.56 | -0.40 | | 4.29 | 3.78 | -0.53 | |
| Benin | (28.89, | (27.72, | (-0.40, | <0.001 | (458.57, | (449.53, | (-0.13, | 0.320 | (298.45, | (274.62, | (-0.49, | <0.001 | (2.98, | (3.00, | (-0.63, | <0.001 |
| | 36.82) | 32.35) | -0.29) | | 487.83) | 467.13) | 0.04) | | 322.65) | 288.64) | -0.31) | | 6.01) | 4.70) | -0.42) | |
| | 36.57 | 37.94 | 0.12 | | 484.08 | 456.10 | -0.24 | | 157.47 | 76.23 | -2.98 | | 1.89 | 0.54 | -5.40 | |
| Bermuda | (11.80, | (9.70, | (-0.04, | 0.143 | (373.99, | (331.86, | (-0.29, | <0.001 | (97.85, | (31.67, | (-3.25, | <0.001 | (0.00, | (0.00, | (-5.76, | <0.001 |
| | 93.74) | 109.48) | 0.28) | | 621.38) | 617.04) | -0.18) | | 245.86) | 161.59) | -2.71) | | 43.98) | 53.48) | -5.05) | |
| | 31.49 | 21.12 | -1.73 | | 379.61 | 339.42 | -0.37 | | 279.17 | 147.88 | -2.66 | | 3.85 | 1.62 | -3.58 | |
| Bhutan | (21.43, | (14.67, | (-1.91, | <0.001 | (342.84, | (311.82, | (-0.52, | <0.001 | (247.24, | (129.82, | (-2.87, | <0.001 | (0.98, | (0.29, | (-3.83, | <0.001 |
| | 44.91) | 29.48) | -1.54) | | 419.49) | 368.84) | -0.22) | | 314.31) | 167.79) | -2.45) | | 10.55) | 5.11) | -3.33) | |
| Bolivia | 60.58 | 35.92 | -2.10 | | 586.52 | 466.25 | -0.83 | | 778.84 | 343.37 | -3.08 | | 12.29 | 5.08 | -3.31 | |
| (Plurinational | (56.19, | (33.54, | (-2.28, | <0.001 | (572.61, | (457.57, | (-0.95, | <0.001 | (762.98, | (335.93, | (-3.22, | <0.001 | (10.36, | (4.21, | (-3.46, | <0.001 |
| State of) | 65.24) | 38.42) | -1.92) | | 600.69) | 475.06) | -0.71) | | 794.97) | 350.93) | -2.95) | | 14.50) | 6.08) | -3.17) | |
| | 34.29 | 36.88 | 0.46 | | 474.18 | 500.89 | 0.29 | | 253.53 | 222.14 | -0.91 | | 2.72 | 2.08 | -1.71 | |
| Bosnia and Herzegovina | (30.65, | (31.93, | (0.37, | <0.001 | (460.37, | (482.16, | (0.21, | <0.001 | (243.45, | (209.69, | (-1.10, | <0.001 | (1.77, | (1.05, | (-2.04, | <0.001 |
| | 38.25) | 42.45) | 0.54) | | 488.32) | 520.21) | 0.37) | | 263.92) | 235.19) | -0.73) | | 4.01) | 3.79) | -1.37) | |
| | 23.47 | 24.03 | 0.24 | | 521.32 | 523.54 | 0.15 | | 319.33 | 260.83 | -0.37 | | 4.34 | 3.28 | -0.56 | |
| Botswana | (17.70, | (20.08, | (0.12, | <0.001 | (493.31, | (504.36, | (0.05, | 0.005 | (296.85, | (247.39, | (-0.59, | 0.002 | (2.06, | (1.94, | (-0.85, | <0.001 |
| | 30.63) | 28.55) | 0.37) | | 550.62) | 543.28) | 0.25) | | 343.15) | 274.82) | -0.15) | | 8.15) | 5.23) | -0.27) | |
| Brazil | 57.05 | 33.04 | -2.40 | <0.001 | 654.50 | 522.29 | -0.98 | <0.001 | 619.79 | 238.47 | -3.57 | <0.001 | 9.88 | 3.29 | -4.08 | <0.001 |
| | (56.19, | (32.52, | (-2.72, | | (651.61, | (520.18, | (-1.20, | | (616.96, | (237.05, | (-3.80, | | (9.53, | (3.13, | (-4.29, | |

| | | | | | | | | | | | | | | | | |
|---|---|---|---|---|---|---|---|---|---|---|---|---|---|---|---|---|
| | 57.91) | 33.58) | -2.08) | | 657.40) | 524.41) | -0.76) | | 622.64) | 239.90) | -3.35) | | 10.25) | 3.46) | -3.86) | |
| Brunei Darussalam | 34.34 (20.64, 53.98) | 21.17 (13.04, 32.89) | -2.03 (-2.28, -1.78) | <0.001 | 415.13 (363.41, 472.39) | 368.19 (330.85, 408.88) | -0.36 (-0.57, -0.16) | 0.001 | 377.67 (328.08, 432.86) | 156.15 (132.27, 183.40) | -3.32 (-3.68, -2.96) | <0.001 | 5.37 (1.08, 16.27) | 1.56 (0.12, 7.31) | -4.86 (-5.26, -4.45) | <0.001 |
| Bulgaria | 37.40 (34.39, 40.60) | 32.59 (29.18, 36.33) | -0.51 (-0.54, -0.48) | <0.001 | 540.12 (528.50, 551.94) | 510.56 (496.70, 524.77) | -0.14 (-0.26, -0.03) | 0.016 | 445.20 (434.67, 455.92) | 318.28 (307.38, 329.50) | -1.82 (-2.13, -1.52) | <0.001 | 5.84 (4.70, 7.19) | 3.75 (2.67, 5.19) | -2.38 (-2.82, -1.95) | <0.001 |
| Burkina Faso | 25.92 (23.44, 28.60) | 23.39 (21.93, 24.93) | -0.37 (-0.50, -0.24) | <0.001 | 426.23 (416.18, 436.47) | 406.72 (400.65, 412.87) | -0.11 (-0.18, -0.05) | 0.001 | 315.95 (307.07, 325.04) | 273.31 (268.24, 278.46) | -0.43 (-0.57, -0.29) | <0.001 | 4.53 (3.51, 5.77) | 3.78 (3.20, 4.45) | -0.54 (-0.71, -0.38) | <0.001 |
| Burundi | 53.63 (49.04, 58.54) | 24.84 (22.75, 27.08) | -3.30 (-3.56, -3.04) | <0.001 | 704.87 (688.43, 721.63) | 589.42 (579.34, 599.65) | -0.71 (-0.82, -0.59) | <0.001 | 719.85 (702.82, 737.21) | 362.92 (354.80, 371.19) | -2.89 (-3.12, -2.66) | <0.001 | 11.01 (8.97, 13.40) | 4.95 (4.03, 6.03) | -3.37 (-3.64, -3.11) | <0.001 |
| Cabo Verde | 32.92 (19.76, 52.31) | 27.18 (18.62, 38.42) | -0.77 (-0.83, -0.71) | <0.001 | 508.67 (453.22, 569.69) | 485.31 (446.52, 526.64) | -0.13 (-0.15, -0.10) | <0.001 | 374.95 (326.41, 429.24) | 187.26 (163.41, 213.69) | -2.36 (-2.63, -2.10) | <0.001 | 5.31 (1.07, 16.71) | 2.06 (0.33, 6.84) | -3.06 (-3.44, -2.68) | <0.001 |
| Cambodia | 39.52 (36.72, 42.48) | 27.65 (25.94, 29.45) | -1.57 (-1.69, -1.45) | <0.001 | 418.45 (409.31, 427.75) | 393.90 (387.38, 400.52) | -0.27 (-0.40, -0.14) | <0.001 | 606.69 (595.52, 618.02) | 325.79 (319.84, 331.83) | -2.46 (-2.62, -2.30) | <0.001 | 9.16 (7.83, 10.67) | 4.28 (3.62, 5.02) | -2.96 (-3.15, -2.77) | <0.001 |
| Cameroon | 30.50 (27.95, 33.25) | 28.96 (27.56, 30.42) | -0.42 (-0.70, -0.14) | 0.005 | 459.46 (449.58, 469.51) | 460.34 (454.80, 465.94) | -0.01 (-0.11, 0.10) | 0.904 | 419.19 (409.51, 429.04) | 352.21 (347.29, 357.19) | -0.82 (-1.11, -0.52) | <0.001 | 6.24 (5.09, 7.58) | 5.02 (4.44, 5.66) | -1.00 (-1.34, -0.66) | <0.001 |
| Canada | 18.16 (17.08, 19.30) | 15.68 (14.69, 16.73) | -0.48 (-0.64, -0.32) | <0.001 | 348.33 (343.52, 353.20) | 309.55 (305.09, 314.06) | -0.25 (-0.46, -0.05) | 0.017 | 159.24 (156.01, 162.52) | 94.28 (91.84, 96.78) | -1.90 (-2.14, -1.67) | <0.001 | 1.77 (1.45, 2.15) | 0.73 (0.54, 0.99) | -3.33 (-3.64, -3.02) | <0.001 |
| Central African Republic | 37.02 (31.68, 43.07) | 38.86 (35.01, 43.03) | 0.10 (0.03, 0.16) | 0.005 | 512.93 (492.96, 533.56) | 510.23 (496.32, 524.44) | 0.01 (-0.06, 0.08) | 0.718 | 490.12 (470.07, 510.84) | 443.50 (430.28, 457.03) | -0.27 (-0.36, -0.18) | <0.001 | 7.38 (5.08, 10.43) | 6.56 (5.02, 8.43) | -0.33 (-0.44, -0.23) | <0.001 |
| Chad | 31.97 (28.54, 35.71) | 31.92 (29.75, 34.21) | -0.01 (-0.08, 0.07) | 0.896 | 470.03 (456.83, 483.53) | 477.01 (468.73, 485.41) | 0.18 (0.10, 0.26) | <0.001 | 416.32 (403.68, 429.27) | 368.85 (361.41, 376.40) | -0.34 (-0.50, -0.19) | <0.001 | 6.16 (4.69, 7.95) | 5.28 (4.41, 6.28) | -0.46 (-0.64, -0.28) | <0.001 |
| Chile | 28.13 (26.20, | 24.56 (22.93, | -0.22 (-0.49, | 0.091 | 374.05 (366.93, | 341.28 (335.17, | -0.09 (-0.28, | 0.296 | 249.59 (243.77, | 144.65 (140.67, | -1.62 (-1.76, | <0.001 | 3.27 (2.64, | 1.54 (1.16, | -2.27 (-2.41, | <0.001 |

| | | | | | | | | | | | | | | | | |
|---|---|---|---|---|---|---|---|---|---|---|---|---|---|---|---|---|
| | 30.17) | 26.28) | 0.04) | | 381.28) | 347.49) | 0.09) | | 255.52) | 148.72) | -1.48) | | 4.03) | 2.02) | -2.12) | |
| China | 28.09 (27.89, 28.30) | 20.08 (19.90, 20.26) | -1.87 (-2.13, -1.61) | <0.001 | 408.71 (407.92, 409.50) | 365.55 (364.80, 366.31) | -0.66 (-0.94, -0.38) | <0.001 | 411.92 (411.13, 412.71) | 204.45 (203.89, 205.02) | -3.01 (-3.48, -2.53) | <0.001 | 5.62 (5.53, 5.72) | 2.08 (2.02, 2.13) | -3.96 (-4.59, -3.33) | <0.001 |
| Colombia | 42.11 (40.56, 43.70) | 28.77 (27.71, 29.86) | -1.61 (-1.87, -1.36) | <0.001 | 548.95 (543.38, 554.58) | 461.14 (456.86, 465.44) | -0.59 (-0.72, -0.46) | <0.001 | 368.98 (364.39, 373.62) | 166.00 (163.44, 168.59) | -2.95 (-3.34, -2.56) | <0.001 | 5.47 (4.92, 6.07) | 2.06 (1.79, 2.37) | -3.64 (-4.09, -3.19) | <0.001 |
| Comoros | 52.98 (37.54, 72.97) | 29.90 (21.47, 40.64) | -2.32 (-2.46, -2.17) | <0.001 | 796.17 (734.80, 861.66) | 624.65 (584.19, 667.27) | -1.05 (-1.18, -0.92) | <0.001 | 515.91 (465.35, 570.76) | 333.08 (303.34, 365.01) | -2.01 (-2.59, -1.44) | <0.001 | 7.17 (2.36, 17.04) | 4.33 (1.57, 9.60) | -2.26 (-2.95, -1.57) | <0.001 |
| Congo | 54.67 (47.67, 62.45) | 31.90 (28.59, 35.49) | -2.30 (-2.51, -2.10) | <0.001 | 649.01 (624.93, 673.83) | 541.26 (527.41, 555.40) | -0.70 (-0.77, -0.63) | <0.001 | 550.73 (528.08, 574.15) | 342.87 (331.82, 354.21) | -1.96 (-2.30, -1.61) | <0.001 | 8.16 (5.58, 11.56) | 4.74 (3.52, 6.25) | -2.23 (-2.64, -1.82) | <0.001 |
| Cook Islands | 48.55 (4.38, 203.83) | 49.17 (3.63, 211.81) | -0.04 (-0.23, 0.15) | 0.667 | 603.45 (372.28, 930.34) | 604.76 (362.21, 949.91) | 0.02 (-0.07, 0.10) | 0.681 | 565.65 (343.01, 884.16) | 337.25 (164.55, 614.93) | -1.83 (-2.09, -1.57) | <0.001 | 7.60 (0.00, 135.25) | 3.64 (0.00, 131.18) | -2.71 (-3.02, -2.39) | <0.001 |
| Costa Rica | 33.80 (29.36, 38.75) | 30.38 (27.07, 34.02) | -0.42 (-0.58, -0.27) | <0.001 | 508.07 (490.45, 526.19) | 482.78 (469.30, 496.57) | -0.16 (-0.23, -0.09) | <0.001 | 221.26 (209.59, 233.44) | 131.85 (124.86, 139.16) | -1.99 (-2.37, -1.61) | <0.001 | 2.98 (1.76, 4.75) | 1.46 (0.82, 2.45) | -2.74 (-3.28, -2.21) | <0.001 |
| Cote d'Ivoire | 35.87 (33.26, 38.65) | 33.02 (31.45, 34.66) | -0.49 (-0.67, -0.31) | <0.001 | 530.59 (520.68, 540.65) | 514.60 (508.41, 520.86) | -0.11 (-0.18, -0.04) | 0.005 | 411.82 (402.92, 420.89) | 328.48 (323.50, 333.53) | -0.52 (-0.73, -0.31) | <0.001 | 5.90 (4.86, 7.11) | 4.46 (3.89, 5.09) | -0.64 (-0.89, -0.39) | <0.001 |
| Croatia | 29.19 (25.82, 32.90) | 23.88 (20.31, 27.97) | -0.91 (-1.06, -0.76) | <0.001 | 436.03 (422.70, 449.69) | 379.98 (365.32, 395.15) | -0.55 (-0.67, -0.42) | <0.001 | 274.45 (263.93, 285.31) | 135.37 (126.69, 144.56) | -2.64 (-2.78, -2.50) | <0.001 | 3.25 (2.20, 4.65) | 0.98 (0.39, 2.17) | -4.17 (-4.46, -3.87) | <0.001 |
| Cuba | 39.73 (37.14, 42.46) | 33.80 (31.17, 36.60) | -0.71 (-0.88, -0.53) | <0.001 | 505.73 (496.44, 515.15) | 455.56 (445.75, 465.53) | -0.36 (-0.49, -0.23) | <0.001 | 294.64 (287.47, 301.94) | 138.01 (132.65, 143.55) | -3.12 (-3.46, -2.77) | <0.001 | 4.32 (3.48, 5.30) | 1.63 (1.10, 2.35) | -4.05 (-4.47, -3.63) | <0.001 |
| Cyprus | 19.19 (12.86, 27.67) | 16.43 (11.84, 22.65) | -0.95 (-1.10, -0.79) | <0.001 | 293.27 (266.65, 321.92) | 316.38 (294.79, 339.45) | 0.29 (0.16, 0.43) | <0.001 | 151.87 (132.85, 172.95) | 76.94 (66.48, 88.94) | -3.12 (-3.53, -2.72) | <0.001 | 1.86 (0.36, 5.80) | 0.50 (0.03, 3.24) | -4.90 (-5.74, -4.05) | <0.001 |
| Czechia | 33.20 (30.64, | 29.32 (26.69, | -0.57 (-0.76, | <0.001 | 473.14 (463.33, | 433.14 (422.90, | -0.36 (-0.46, | <0.001 | 259.24 (252.00, | 126.35 (120.82, | -2.92 (-3.11, | <0.001 | 2.85 (2.14, | 0.67 (0.33, | -5.59 (-5.85, | <0.001 |

| | | | | | | | | | | | | | | | | |
|---|---|---|---|---|---|---|---|---|---|---|---|---|---|---|---|---|
| | 35.93) | 32.17) | -0.37) | | 483.12) | 443.61) | -0.26) | | 266.64) | 132.10) | -2.73) | | 3.73) | 1.26) | -5.32) | |
| Democratic | 35.54 | 34.75 | -0.12 | | 408.27 | 424.30 | 0.21 | | 503.58 | 439.90 | -0.48 | | 7.25 | 6.06 | -0.65 | |
| People's Republic | (33.71, | (33.11, | (-0.24, | 0.046 | (402.05, | (418.51, | (0.13, | <0.001 | (496.60, | (434.01, | (-0.66, | <0.001 | (6.43, | (5.39, | (-0.88, | <0.001 |
| of Korea | 37.46) | 36.46) | -0.00) | | 414.57) | 430.15) | 0.30) | | 510.65) | 445.84) | -0.30) | | 8.16) | 6.80) | -0.42) | |
| Democratic | 30.93 | 23.63 | -1.15 | | 468.22 | 422.11 | -0.34 | | 407.71 | 276.80 | -1.43 | | 5.98 | 3.80 | -1.66 | |
| Republic of the | (29.57, | (22.87, | (-1.24, | <0.001 | (462.99, | (418.92, | (-0.44, | <0.001 | (402.75, | (274.16, | (-1.56, | <0.001 | (5.39, | (3.49, | (-1.83, | <0.001 |
| Congo | 32.34) | 24.42) | -1.05) | | 473.49) | 425.33) | -0.25) | | 412.72) | 279.46) | -1.30) | | 6.63) | 4.13) | -1.49) | |
| | 24.22 | 16.12 | -1.64 | | 362.71 | 313.35 | -0.58 | | 195.24 | 80.90 | -3.69 | | 2.52 | 0.60 | -5.80 | |
| Denmark | (21.20, | (13.59, | (-1.76, | <0.001 | (350.68, | (301.83, | (-0.68, | <0.001 | (186.50, | (75.12, | (-3.94, | <0.001 | (1.62, | (0.21, | (-6.25, | <0.001 |
| | 27.58) | 19.00) | -1.51) | | 375.05) | 325.21) | -0.48) | | 204.30) | 87.04) | -3.45) | | 3.76) | 1.40) | -5.35) | |
| | 30.68 | 23.58 | -1.07 | | 609.93 | 611.15 | 0.09 | | 356.82 | 247.45 | -1.41 | | 4.82 | 2.89 | -1.99 | |
| Djibouti | (19.49, | (17.96, | (-1.21, | <0.001 | (557.31, | (580.59, | (-0.01, | 0.082 | (315.60, | (228.39, | (-1.55, | <0.001 | (1.17, | (1.20, | (-2.17, | <0.001 |
| | 46.25) | 30.51) | -0.94) | | 666.49) | 642.98) | 0.20) | | 402.17) | 267.75) | -1.27) | | 13.39) | 6.00) | -1.82) | |
| | 38.43 | 34.91 | -0.45 | | 500.89 | 494.06 | -0.02 | | 281.75 | 196.43 | -2.17 | | 4.05 | 2.57 | -2.80 | |
| Dominica | (11.92, | (10.28, | (-0.55, | <0.001 | (384.25, | (378.93, | (-0.12, | 0.685 | (195.71, | (126.78, | (-2.52, | <0.001 | (0.00, | (0.00, | (-3.27, | <0.001 |
| | 94.54) | 86.25) | -0.34) | | 643.59) | 633.39) | 0.08) | | 394.52) | 290.86) | -1.82) | | 42.29) | 35.08) | -2.32) | |
| | 39.64 | 43.21 | 0.34 | | 458.86 | 483.21 | 0.28 | | 412.50 | 395.75 | 0.07 | | 6.39 | 6.11 | 0.08 | |
| Dominican Republic | (36.39, | (40.48, | (0.28, | <0.001 | (447.74, | (474.03, | (0.21, | <0.001 | (401.90, | (387.42, | (-0.20, | 0.609 | (5.12, | (5.11, | (-0.22, | 0.591 |
| | 43.12) | 46.07) | 0.40) | | 470.21) | 492.53) | 0.34) | | 423.31) | 404.22) | 0.33) | | 7.89) | 7.25) | 0.38) | |
| | 46.63 | 34.94 | -1.17 | | 540.86 | 490.80 | -0.37 | | 502.41 | 271.31 | -2.47 | | 7.69 | 3.78 | -2.81 | |
| Ecuador | (43.62, | (33.02, | (-1.28, | <0.001 | (530.44, | (483.53, | (-0.42, | <0.001 | (492.44, | (265.91, | (-2.74, | <0.001 | (6.49, | (3.17, | (-3.12, | <0.001 |
| | 49.81) | 36.95) | -1.06) | | 551.44) | 498.17) | -0.31) | | 512.54) | 276.78) | -2.19) | | 9.05) | 4.48) | -2.49) | |
| | 42.33 | 40.59 | 0.04 | | 523.31 | 540.94 | 0.35 | | 850.94 | 475.54 | -1.70 | | 12.71 | 6.54 | -1.98 | |
| Egypt | (41.05, | (39.70, | (-0.11, | 0.631 | (518.81, | (537.70, | (0.21, | <0.001 | (845.33, | (472.51, | (-2.07, | <0.001 | (12.02, | (6.18, | (-2.39, | <0.001 |
| | 43.64) | 41.49) | 0.18) | | 527.83) | 544.19) | 0.48) | | 856.59) | 478.58) | -1.32) | | 13.42) | 6.90) | -1.57) | |
| | 36.18 | 28.06 | -1.10 | | 471.39 | 441.88 | -0.18 | | 527.97 | 179.63 | -4.13 | | 8.10 | 2.31 | -4.87 | |
| El Salvador | (32.46, | (25.27, | (-1.28, | <0.001 | (457.73, | (430.59, | (-0.29, | 0.001 | (513.75, | (172.45, | (-4.64, | <0.001 | (6.41, | (1.56, | (-5.43, | <0.001 |
| | 40.24) | 31.09) | -0.92) | | 485.37) | 453.40) | -0.08) | | 542.50) | 187.04) | -3.61) | | 10.13) | 3.31) | -4.31) | |
| | 43.06 | 21.91 | -2.69 | | 555.21 | 487.45 | -0.46 | | 487.47 | 199.81 | -3.38 | | 7.22 | 2.32 | -4.28 | |
| Equatorial Guinea | (29.45, | (16.57, | (-2.93, | <0.001 | (503.62, | (461.20, | (-0.56, | <0.001 | (438.68, | (182.83, | (-3.61, | <0.001 | (2.45, | (0.82, | (-4.53, | <0.001 |
| | 60.96) | 28.51) | -2.45) | | 610.83) | 514.89) | -0.36) | | 540.34) | 218.03) | -3.16) | | 16.68) | 5.25) | -4.02) | |
| Eritrea | 37.87 | 24.67 | -1.61 | <0.001 | 572.16 | 524.18 | -0.28 | <0.001 | 486.37 | 312.86 | -1.23 | <0.001 | 7.28 | 4.29 | -1.48 | <0.001 |
| | (32.64, | (21.99, | (-1.79, | | (551.95, | (511.88, | (-0.37, | | (467.11, | (303.07, | (-1.42, | | (5.07, | (3.19, | (-1.70, | |

| | | | | | | | | | | | | | | | | |
|---|---|---|---|---|---|---|---|---|---|---|---|---|---|---|---|---|
| | 43.72) | 27.61) | -1.42) | | 592.96) | 536.72) | -0.18) | | 506.25) | 322.91) | -1.04) | | 10.16) | 5.64) | -1.26) | |
| Estonia | 33.39 (27.13, 40.74) | 32.03 (24.77, 41.08) | -0.37 (-0.59, -0.16) | 0.001 | 418.08 (395.05, 442.17) | 406.15 (379.03, 434.96) | -0.17 (-0.27, -0.07) | 0.002 | 256.66 (238.62, 275.76) | 116.97 (102.55, 133.13) | -3.69 (-4.18, -3.21) | <0.001 | 2.97 (1.35, 5.81) | 0.58 (0.03, 3.57) | -6.35 (-7.47, -5.23) | <0.001 |
| Eswatini | 23.54 (16.18, 33.35) | 22.64 (17.08, 29.50) | 0.02 (-0.54, 0.57) | 0.956 | 503.88 (468.19, 541.80) | 487.95 (461.05, 516.07) | -0.04 (-0.14, 0.06) | 0.409 | 306.37 (277.96, 337.09) | 234.03 (215.34, 253.97) | -0.36 (-1.03, 0.32) | 0.287 | 4.12 (1.43, 9.54) | 2.88 (1.16, 6.04) | -0.49 (-1.37, 0.40) | 0.271 |
| Ethiopia | 38.72 (37.41, 40.07) | 17.78 (17.20, 18.38) | -3.32 (-3.60, -3.03) | <0.001 | 564.80 (559.87, 569.76) | 478.92 (475.94, 481.91) | -0.57 (-0.71, -0.44) | <0.001 | 581.84 (576.71, 587.01) | 215.74 (213.69, 217.80) | -4.09 (-4.37, -3.82) | <0.001 | 8.92 (8.29, 9.59) | 2.61 (2.38, 2.85) | -5.01 (-5.34, -4.67) | <0.001 |
| Fiji | 54.29 (43.20, 67.46) | 48.69 (38.95, 60.16) | -0.51 (-0.59, -0.42) | <0.001 | 638.57 (598.83, 680.34) | 609.89 (574.04, 647.42) | -0.16 (-0.22, -0.10) | <0.001 | 790.36 (746.23, 836.51) | 637.03 (600.37, 675.37) | -1.18 (-1.39, -0.96) | <0.001 | 11.29 (6.57, 18.22) | 8.76 (4.96, 14.37) | -1.39 (-1.64, -1.14) | <0.001 |
| Finland | 23.15 (20.16, 26.50) | 20.88 (17.92, 24.23) | -0.34 (-0.46, -0.23) | <0.001 | 426.52 (413.26, 440.13) | 403.48 (389.99, 417.35) | 0.12 (-0.19, 0.43) | 0.445 | 232.34 (222.74, 242.31) | 110.03 (103.08, 117.37) | -2.65 (-2.88, -2.42) | <0.001 | 3.00 (2.01, 4.36) | 0.88 (0.37, 1.83) | -4.35 (-4.58, -4.13) | <0.001 |
| France | 17.56 (16.79, 18.35) | 15.77 (15.02, 16.56) | 0.10 (-0.08, 0.28) | 0.277 | 292.52 (289.36, 295.70) | 263.19 (260.06, 266.35) | -0.19 (-0.35, -0.04) | 0.015 | 163.29 (160.94, 165.67) | 87.37 (85.57, 89.19) | -2.39 (-2.49, -2.29) | <0.001 | 2.05 (1.79, 2.33) | 0.80 (0.64, 0.99) | -3.55 (-3.74, -3.36) | <0.001 |
| Gabon | 27.57 (20.06, 37.14) | 22.45 (17.88, 27.87) | -0.93 (-1.04, -0.82) | <0.001 | 512.92 (479.27, 548.47) | 486.41 (464.39, 509.25) | -0.14 (-0.21, -0.07) | <0.001 | 313.64 (286.74, 342.52) | 183.83 (170.23, 198.26) | -2.04 (-2.34, -1.74) | <0.001 | 4.28 (1.66, 9.20) | 2.04 (0.84, 4.18) | -2.75 (-3.20, -2.30) | <0.001 |
| Gambia | 32.76 (24.63, 42.93) | 31.99 (26.86, 37.87) | -0.01 (-0.05, 0.04) | 0.757 | 554.58 (520.23, 590.82) | 548.31 (526.83, 570.49) | 0.07 (-0.00, 0.14) | 0.059 | 334.00 (306.80, 363.14) | 314.83 (298.29, 332.10) | -0.15 (-0.34, 0.05) | 0.131 | 4.51 (1.84, 9.46) | 4.19 (2.46, 6.72) | -0.21 (-0.46, 0.04) | 0.097 |
| Georgia | 41.91 (38.17, 45.93) | 29.90 (25.69, 34.69) | -0.87 (-1.09, -0.65) | <0.001 | 504.89 (491.73, 518.34) | 454.52 (437.73, 471.86) | -0.36 (-0.40, -0.33) | <0.001 | 527.24 (513.73, 541.04) | 262.10 (249.30, 275.47) | -2.35 (-3.22, -1.47) | <0.001 | 7.17 (5.68, 8.96) | 2.91 (1.72, 4.74) | -2.56 (-3.71, -1.40) | <0.001 |
| Germany | 22.28 (21.53, 23.06) | 18.18 (17.44, 18.94) | -0.77 (-0.88, -0.66) | <0.001 | 349.55 (346.53, 352.59) | 316.08 (312.98, 319.20) | -0.30 (-0.45, -0.16) | <0.001 | 230.43 (227.99, 232.88) | 97.39 (95.69, 99.13) | -3.26 (-3.61, -2.90) | <0.001 | 3.10 (2.83, 3.40) | 0.86 (0.71, 1.04) | -4.98 (-5.37, -4.59) | <0.001 |
| Ghana | 45.54 (42.97, | 35.25 (33.83, | -0.99 (-1.11, | <0.001 | 601.35 (592.16, | 548.44 (542.90, | -0.20 (-0.29, | <0.001 | 634.90 (625.23, | 391.03 (386.29, | -1.59 (-1.76, | <0.001 | 9.80 (8.62, | 5.58 (5.02, | -1.84 (-2.04, | <0.001 |

| | | | | | | | | | | | | | | | | |
|---|---|---|---|---|---|---|---|---|---|---|---|---|---|---|---|---|
| | 48.22) | 36.71) | -0.86) | | 610.64) | 554.03) | -0.12) | | 644.70) | 395.82) | -1.42) | | 11.11) | 6.19) | -1.64) | |
| | 19.98 | 16.82 | -0.68 | | 348.14 | 338.09 | -0.01 | | 226.69 | 129.05 | -2.14 | | 2.98 | 1.38 | -2.96 | |
| Greece | (18.01, | (14.82, | (-0.76, | <0.001 | (339.78, | (328.84, | (-0.18, | 0.900 | (219.95, | (123.42, | (-2.29, | <0.001 | (2.26, | (0.86, | (-3.15, | <0.001 |
| | 22.10) | 19.04) | -0.61) | | 356.66) | 347.57) | 0.16) | | 233.58) | 134.89) | -1.99) | | 3.87) | 2.12) | -2.77) | |
| | 31.68 | 20.68 | -1.70 | | 502.40 | 429.22 | -0.45 | | 560.39 | 245.49 | -3.46 | | 8.57 | 3.15 | -4.00 | |
| Greenland | (7.86, | (2.69, | (-1.81, | <0.001 | (381.41, | (311.02, | (-0.73, | 0.003 | (432.10, | (159.04, | (-3.83, | <0.001 | (0.20, | (0.00, | (-4.47, | <0.001 |
| | 93.95) | 78.55) | -1.58) | | 654.97) | 580.59) | -0.16) | | 720.14) | 365.60) | -3.09) | | 60.22) | 50.37) | -3.53) | |
| | 45.05 | 38.07 | -0.79 | | 560.39 | 531.32 | -0.10 | | 484.64 | 264.74 | -2.90 | | 7.50 | 3.75 | -3.47 | |
| Grenada | (17.71, | (15.44, | (-1.03, | <0.001 | (447.52, | (432.26, | (-0.23, | 0.099 | (379.62, | (195.70, | (-3.31, | <0.001 | (0.26, | (0.02, | (-3.97, | <0.001 |
| | 95.82) | 79.19) | -0.55) | | 694.32) | 647.11) | 0.02) | | 610.90) | 350.99) | -2.48) | | 41.33) | 29.04) | -2.98) | |
| | 46.21 | 55.15 | 0.66 | | 570.59 | 631.49 | 0.54 | | 344.39 | 426.38 | 0.39 | | 3.82 | 5.08 | 0.34 | |
| Guam | (24.68, | (31.14, | (0.57, | <0.001 | (486.01, | (541.58, | (0.48, | <0.001 | (279.58, | (353.25, | (0.16, | 0.002 | (0.12, | (0.32, | (0.01, | 0.047 |
| | 79.24) | 90.77) | 0.75) | | 665.93) | 732.33) | 0.59) | | 420.03) | 510.49) | 0.61) | | 20.83) | 23.18) | 0.68) | |
| | 57.20 | 29.97 | -2.71 | | 593.23 | 463.73 | -1.01 | | 635.93 | 306.18 | -3.52 | | 9.88 | 4.41 | -3.88 | |
| Guatemala | (53.25, | (28.27, | (-2.90, | <0.001 | (580.29, | (456.97, | (-1.16, | <0.001 | (622.64, | (300.71, | (-3.92, | <0.001 | (8.27, | (3.77, | (-4.32, | <0.001 |
| | 61.37) | 31.74) | -2.52) | | 606.40) | 470.56) | -0.86) | | 649.44) | 311.73) | -3.11) | | 11.72) | 5.12) | -3.44) | |
| | 33.08 | 34.48 | 0.35 | | 430.29 | 445.83 | 0.32 | | 462.44 | 413.99 | 0.06 | | 7.01 | 6.10 | -0.00 | |
| Guinea | (29.73, | (32.13, | (0.22, | <0.001 | (418.09, | (437.43, | (0.22, | <0.001 | (449.66, | (405.81, | (-0.09, | 0.442 | (5.51, | (5.13, | (-0.17, | 0.967 |
| | 36.72) | 36.96) | 0.47) | | 442.76) | 454.36) | 0.41) | | 475.50) | 422.30) | 0.21) | | 8.82) | 7.20) | 0.16) | |
| | 51.41 | 49.00 | -0.15 | | 590.39 | 572.02 | -0.02 | | 691.93 | 573.56 | -0.39 | | 10.74 | 8.71 | -0.44 | |
| Guinea-Bissau | (41.32, | (42.19, | (-0.25, | 0.002 | (555.60, | (548.59, | (-0.07, | 0.576 | (653.57, | (549.73, | (-0.59, | <0.001 | (6.41, | (5.97, | (-0.66, | <0.001 |
| | 63.31) | 56.65) | -0.06) | | 626.91) | 596.24) | 0.04) | | 732.06) | 598.20) | -0.19) | | 16.98) | 12.31) | -0.22) | |
| | 50.14 | 41.31 | -0.81 | | 646.06 | 574.51 | -0.27 | | 631.16 | 488.41 | -1.40 | | 9.84 | 7.50 | -1.51 | |
| Guyana | (39.55, | (31.70, | (-0.88, | <0.001 | (606.70, | (537.19, | (-0.40, | <0.001 | (592.24, | (453.99, | (-1.82, | <0.001 | (5.51, | (3.79, | (-1.99, | <0.001 |
| | 62.82) | 52.98) | -0.74) | | 687.42) | 613.82) | -0.14) | | 672.09) | 524.81) | -0.97) | | 16.40) | 13.40) | -1.02) | |
| | 81.21 | 61.00 | -1.03 | | 674.12 | 587.53 | -0.39 | | 1363.71 | 887.56 | -1.14 | | 22.57 | 14.40 | -1.19 | |
| Haiti | (76.14, | (58.11, | (-1.09, | <0.001 | (659.41, | (578.50, | (-0.52, | <0.001 | (1342.80 , | (876.46, | (-1.29, | <0.001 | (19.94, | (13.01, | (-1.35, | <0.001 |
| | 86.55) | 64.00) | -0.98) | | 689.08) | 596.67) | -0.25) | | 1384.89) | 898.77) | -0.99) | | 25.47) | 15.89) | -1.03) | |
| | 37.03 | 27.75 | -1.23 | | 475.82 | 465.44 | -0.02 | | 835.17 | 396.83 | -2.95 | | 13.46 | 6.10 | -3.14 | |
| Honduras | (32.86, | (25.52, | (-1.42, | <0.001 | (460.71, | (456.21, | (-0.15, | 0.726 | (815.20, | (388.24, | (-3.32, | <0.001 | (11.01, | (5.07, | (-3.55, | <0.001 |
| | 41.60) | 30.12) | -1.04) | | 491.33) | 474.81) | 0.11) | | 855.53) | 405.56) | -2.59) | | 16.32) | 7.29) | -2.73) | |
| Hungary | 45.95 | 31.56 | -1.57 | <0.001 | 591.27 | 497.92 | -0.55 | <0.001 | 424.57 | 173.63 | -3.78 | <0.001 | 5.49 | 1.29 | -6.07 | <0.001 |

| | | | | | | | | | | | | | | | | |
|---|---|---|---|---|---|---|---|---|---|---|---|---|---|---|---|---|
| | (42.97, 49.09) | (28.74, 34.61) | (-1.71, -1.43) | | (580.37, 602.33) | (486.55, 509.51) | (-0.66, -0.44) | | (415.51, 433.80) | (166.98, 180.50) | (-4.04, -3.52) | | (4.52, 6.63) | (0.79, 2.04) | (-6.43, -5.71) | |
| Iceland | 18.57 (8.71, 34.98) | 14.76 (6.67, 28.92) | -1.19 (-1.47, -0.91) | <0.001 | 273.81 (230.39, 323.26) | 257.78 (218.59, 302.42) | -0.32 (-0.47, -0.16) | <0.001 | 160.21 (127.45, 199.04) | 77.21 (56.50, 103.54) | -2.92 (-3.16, -2.68) | <0.001 | 2.05 (0.06, 11.54) | 0.62 (0.00, 8.65) | -4.80 (-5.09, -4.52) | <0.001 |
| India | 27.40 (27.14, 27.67) | 24.85 (24.66, 25.03) | -0.48 (-0.71, -0.25) | <0.001 | 368.65 (367.70, 369.60) | 381.46 (380.75, 382.18) | 0.18 (0.04, 0.32) | 0.015 | 310.13 (309.25, 311.00) | 213.55 (213.01, 214.09) | -1.62 (-1.85, -1.40) | <0.001 | 4.31 (4.20, 4.41) | 2.64 (2.58, 2.70) | -2.09 (-2.38, -1.80) | <0.001 |
| Indonesia | 56.38 (55.60, 57.16) | 48.76 (48.17, 49.36) | -0.62 (-0.66, -0.59) | <0.001 | 674.68 (672.01, 677.36) | 620.17 (618.06, 622.30) | -0.28 (-0.35, -0.21) | <0.001 | 846.58 (843.57, 849.60) | 491.87 (489.99, 493.76) | -1.80 (-1.97, -1.63) | <0.001 | 12.33 (11.96, 12.70) | 6.35 (6.14, 6.57) | -2.14 (-2.37, -1.92) | <0.001 |
| Iran (Islamic Republic of) | 35.61 (34.43, 36.83) | 27.16 (26.40, 27.93) | -1.11 (-1.28, -0.94) | <0.001 | 467.45 (463.17, 471.77) | 421.29 (418.30, 424.29) | -0.37 (-0.53, -0.21) | <0.001 | 345.64 (341.96, 349.35) | 199.01 (196.94, 201.10) | -1.99 (-2.10, -1.89) | <0.001 | 4.65 (4.23, 5.11) | 2.20 (1.99, 2.43) | -2.67 (-2.79, -2.55) | <0.001 |
| Iraq | 57.60 (54.82, 60.50) | 46.53 (45.07, 48.02) | -1.04 (-1.18, -0.89) | <0.001 | 694.31 (684.60, 704.13) | 620.44 (615.12, 625.81) | -0.46 (-0.53, -0.39) | <0.001 | 627.47 (618.26, 636.79) | 417.33 (412.95, 421.74) | -1.92 (-2.25, -1.59) | <0.001 | 8.95 (7.87, 10.14) | 5.53 (5.03, 6.06) | -2.24 (-2.63, -1.84) | <0.001 |
| Ireland | 22.05 (18.64, 25.92) | 16.36 (13.74, 19.36) | -1.48 (-1.67, -1.29) | <0.001 | 320.77 (307.35, 334.62) | 300.57 (288.95, 312.57) | -0.37 (-0.50, -0.23) | <0.001 | 198.63 (188.09, 209.61) | 83.34 (77.30, 89.75) | -3.17 (-3.43, -2.91) | <0.001 | 2.62 (1.54, 4.18) | 0.66 (0.24, 1.51) | -4.79 (-5.25, -4.34) | <0.001 |
| Israel | 18.02 (15.42, 20.94) | 16.24 (14.33, 18.33) | -0.61 (-1.01, -0.21) | 0.004 | 277.39 (266.90, 288.19) | 282.64 (274.50, 290.97) | -0.01 (-0.20, 0.18) | 0.913 | 126.21 (119.18, 133.55) | 64.15 (60.30, 68.18) | -2.67 (-2.95, -2.39) | <0.001 | 1.41 (0.76, 2.40) | 0.35 (0.12, 0.78) | -5.45 (-5.75, -5.15) | <0.001 |
| Italy | 18.56 (17.75, 19.40) | 14.64 (13.82, 15.51) | -1.34 (-1.52, -1.17) | <0.001 | 288.53 (285.31, 291.78) | 289.76 (286.08, 293.48) | 0.06 (-0.03, 0.16) | 0.201 | 181.73 (179.17, 184.32) | 89.75 (87.71, 91.82) | -2.89 (-3.07, -2.70) | <0.001 | 2.35 (2.07, 2.67) | 0.78 (0.60, 0.99) | -4.55 (-4.81, -4.29) | <0.001 |
| Jamaica | 33.86 (28.67, 39.78) | 39.21 (34.34, 44.59) | 0.63 (0.48, 0.78) | <0.001 | 473.02 (453.13, 493.62) | 527.24 (508.98, 546.00) | 0.50 (0.41, 0.60) | <0.001 | 325.87 (309.29, 343.16) | 292.08 (278.52, 306.14) | -0.51 (-0.97, -0.06) | 0.028 | 4.84 (3.00, 7.47) | 4.21 (2.72, 6.23) | -0.65 (-1.17, -0.11) | 0.019 |
| Japan | 21.27 (20.68, 21.89) | 22.64 (21.94, 23.36) | 0.11 (-0.10, 0.33) | 0.286 | 299.97 (297.70, 302.25) | 344.37 (341.58, 347.18) | 0.51 (0.32, 0.70) | <0.001 | 167.15 (165.47, 168.85) | 113.11 (111.52, 114.72) | -1.39 (-1.47, -1.32) | <0.001 | 1.93 (1.76, 2.12) | 0.87 (0.74, 1.02) | -2.63 (-2.80, -2.46) | <0.001 |
| Jordan | 50.87 | 39.78 | -1.08 | <0.001 | 678.68 | 616.00 | -0.33 | <0.001 | 346.27 | 166.72 | -3.13 | <0.001 | 4.22 | 1.25 | -5.12 | <0.001 |

| | | | | | | | | | | | | | | | | |
|---|---|---|---|---|---|---|---|---|---|---|---|---|---|---|---|---|
| | (45.12, 57.19) | (37.18, 42.53) | (-1.24, -0.92) | | (657.55, 700.37) | (605.64, 626.51) | (-0.40, -0.25) | | (331.04, 362.07) | (161.34, 172.24) | (-3.52, -2.74) | | (2.67, 6.40) | (0.82, 1.83) | (-5.82, -4.42) | |
| Kazakhstan | 44.66 (42.43, 46.97) | 33.06 (31.22, 35.00) | -1.06 (-1.46, -0.67) | <0.001 | 530.28 (522.55, 538.09) | 468.91 (461.89, 476.03) | -0.42 (-0.54, -0.30) | <0.001 | 374.18 (367.68, 380.77) | 284.61 (279.17, 290.14) | -1.60 (-2.31, -0.89) | <0.001 | 4.64 (3.94, 5.43) | 3.38 (2.81, 4.03) | -1.89 (-2.81, -0.96) | <0.001 |
| Kenya | 27.67 (25.97, 29.46) | 22.86 (21.93, 23.82) | -0.96 (-1.13, -0.80) | <0.001 | 629.71 (621.81, 637.70) | 616.26 (611.49, 621.06) | -0.07 (-0.14, 0.01) | 0.079 | 298.22 (292.57, 303.97) | 258.14 (254.98, 261.33) | -0.59 (-0.74, -0.43) | <0.001 | 3.72 (3.10, 4.44) | 3.07 (2.72, 3.44) | -0.80 (-1.04, -0.55) | <0.001 |
| Kiribati | 141.11 (86.84, 218.37) | 113.13 (74.89, 164.36) | -0.82 (-0.85, -0.80) | <0.001 | 931.77 (779.58, 1106.11) | 801.64 (692.63, 923.19) | -0.50 (-0.51, -0.49) | <0.001 | 1699.03 (1493.10, 1926.65) | 1452.48 (1304.95, 1612.41) | -0.62 (-0.69, -0.56) | <0.001 | 26.29 (6.78, 71.33) | 22.42 (7.71, 51.13) | -0.64 (-0.72, -0.57) | <0.001 |
| Kuwait | 35.73 (29.76, 42.59) | 35.27 (31.71, 39.24) | -0.14 (-0.28, -0.00) | 0.045 | 489.99 (467.22, 513.64) | 496.78 (483.21, 510.74) | 0.04 (-0.04, 0.12) | 0.290 | 232.28 (216.64, 248.79) | 149.81 (142.31, 157.71) | -2.17 (-2.59, -1.74) | <0.001 | 2.64 (1.22, 5.03) | 1.22 (0.65, 2.28) | -3.50 (-4.23, -2.75) | <0.001 |
| Kyrgyzstan | 44.52 (40.14, 49.27) | 28.01 (25.20, 31.04) | -2.02 (-2.20, -1.84) | <0.001 | 468.68 (454.23, 483.49) | 380.26 (369.75, 390.99) | -0.92 (-1.02, -0.82) | <0.001 | 470.70 (456.19, 485.59) | 230.28 (222.07, 238.72) | -3.53 (-4.04, -3.02) | <0.001 | 6.58 (4.96, 8.59) | 2.68 (1.86, 3.76) | -4.24 (-4.90, -3.58) | <0.001 |
| Lao People's Democratic Republic | 67.01 (61.10, 73.37) | 41.20 (38.01, 44.59) | -2.01 (-2.20, -1.83) | <0.001 | 590.84 (573.12, 609.00) | 464.70 (453.88, 475.73) | -0.93 (-1.01, -0.84) | <0.001 | 627.16 (608.69, 646.08) | 511.74 (500.31, 523.36) | -0.80 (-1.12, -0.48) | <0.001 | 8.91 (6.81, 11.48) | 7.24 (5.93, 8.75) | -0.85 (-1.23, -0.46) | <0.001 |
| Latvia | 31.93 (27.15, 37.37) | 30.45 (24.42, 37.71) | -0.24 (-0.32, -0.15) | <0.001 | 416.97 (399.12, 435.45) | 442.90 (418.94, 468.06) | 0.28 (0.20, 0.37) | <0.001 | 225.00 (211.88, 238.76) | 147.69 (133.96, 162.62) | -2.27 (-2.75, -1.80) | <0.001 | 2.41 (1.24, 4.30) | 0.99 (0.20, 3.33) | -3.86 (-4.82, -2.89) | <0.001 |
| Lebanon | 37.66 (32.79, 43.07) | 35.88 (32.35, 39.75) | -0.07 (-0.28, 0.15) | 0.538 | 516.34 (497.89, 535.30) | 526.34 (512.55, 540.45) | 0.25 (0.14, 0.36) | <0.001 | 246.83 (234.10, 260.09) | 158.01 (150.46, 165.89) | -1.46 (-1.74, -1.18) | <0.001 | 2.88 (1.65, 4.68) | 1.28 (0.70, 2.24) | -2.91 (-3.28, -2.54) | <0.001 |
| Lesotho | 19.99 (15.32, 25.73) | 29.19 (24.36, 34.73) | 1.83 (1.60, 2.07) | <0.001 | 449.16 (426.28, 473.03) | 485.27 (465.14, 506.06) | 0.45 (0.37, 0.54) | <0.001 | 226.73 (210.22, 244.27) | 300.86 (284.91, 317.49) | 2.38 (1.78, 2.98) | <0.001 | 2.85 (1.26, 5.63) | 4.09 (2.42, 6.51) | 2.97 (2.17, 3.78) | <0.001 |
| Liberia | 36.69 (30.52, 43.77) | 29.91 (26.50, 33.65) | -0.72 (-0.80, -0.64) | <0.001 | 519.56 (495.72, 544.27) | 490.11 (476.18, 504.35) | -0.05 (-0.15, 0.05) | 0.299 | 468.37 (445.59, 492.02) | 354.64 (342.70, 366.91) | -0.96 (-1.11, -0.81) | <0.001 | 6.91 (4.38, 10.37) | 4.97 (3.64, 6.65) | -1.16 (-1.33, -1.00) | <0.001 |

| | | | | | | | | | | | | | | | | |
|---|---|---|---|---|---|---|---|---|---|---|---|---|---|---|---|---|
| Libya | 44.55 (39.41, 50.23) | 47.65 (44.23, 51.28) | 0.44 (0.32, 0.57) | <0.001 | 536.45 (518.29, 555.14) | 604.21 (591.87, 616.76) | 0.62 (0.54, 0.70) | <0.001 | 521.35 (503.58, 539.64) | 409.69 (399.51, 420.08) | -0.62 (-1.06, -0.19) | 0.007 | 7.49 (5.47, 10.06) | 5.40 (4.29, 6.71) | -0.98 (-1.51, -0.44) | 0.001 |
| Lithuania | 36.77 (32.43, 41.56) | 34.50 (29.16, 40.64) | -0.16 (-0.32, 0.01) | 0.060 | 478.84 (462.81, 495.31) | 467.59 (447.28, 488.69) | 0.00 (-0.08, 0.08) | 0.985 | 215.00 (204.28, 226.15) | 170.65 (158.53, 183.55) | -0.81 (-1.11, -0.51) | <0.001 | 2.06 (1.14, 3.46) | 1.34 (0.49, 3.13) | -1.34 (-1.95, -0.72) | <0.001 |
| Luxembourg | 19.25 (10.67, 33.20) | 15.09 (8.72, 25.01) | -1.06 (-1.20, -0.93) | <0.001 | 300.46 (262.66, 343.01) | 289.49 (258.50, 323.66) | -0.11 (-0.27, 0.05) | 0.184 | 211.31 (179.85, 247.56) | 90.81 (73.81, 111.07) | -3.38 (-3.65, -3.12) | <0.001 | 2.88 (0.41, 11.75) | 0.79 (0.01, 6.16) | -5.01 (-5.43, -4.59) | <0.001 |
| Madagascar | 67.93 (64.28, 71.74) | 49.08 (47.11, 51.11) | -1.32 (-1.38, -1.25) | <0.001 | 748.66 (736.79, 760.69) | 677.99 (670.83, 685.20) | -0.32 (-0.39, -0.26) | <0.001 | 1155.93 (1140.89, 1171.12) | 750.30 (742.60, 758.06) | -1.35 (-1.55, -1.15) | <0.001 | 18.44 (16.56, 20.48) | 11.58 (10.63, 12.59) | -1.46 (-1.67, -1.25) | <0.001 |
| Malawi | 29.99 (27.33, 32.85) | 20.44 (18.96, 22.00) | -1.77 (-1.97, -1.57) | <0.001 | 586.50 (574.98, 598.21) | 564.43 (556.74, 572.20) | -0.15 (-0.25, -0.04) | 0.008 | 433.30 (423.07, 443.74) | 245.70 (240.50, 250.99) | -2.40 (-2.63, -2.18) | <0.001 | 6.19 (5.00, 7.59) | 2.94 (2.38, 3.59) | -3.13 (-3.41, -2.85) | <0.001 |
| Malaysia | 41.33 (39.24, 43.51) | 32.78 (31.41, 34.20) | -0.85 (-1.09, -0.61) | <0.001 | 574.54 (566.67, 582.49) | 515.40 (509.91, 520.93) | -0.33 (-0.45, -0.20) | <0.001 | 466.83 (459.73, 474.02) | 265.61 (261.67, 269.60) | -1.56 (-1.96, -1.17) | <0.001 | 5.99 (5.20, 6.86) | 2.71 (2.32, 3.14) | -2.28 (-2.82, -1.73) | <0.001 |
| Maldives | 40.70 (21.72, 71.31) | 24.33 (15.27, 37.53) | -2.21 (-2.60, -1.82) | <0.001 | 485.38 (412.61, 568.62) | 395.59 (356.05, 438.86) | -0.71 (-0.89, -0.52) | <0.001 | 595.97 (514.33, 688.13) | 146.32 (122.65, 173.82) | -5.34 (-5.70, -4.98) | <0.001 | 8.58 (1.53, 28.98) | 1.06 (0.03, 7.43) | -7.95 (-8.29, -7.62) | <0.001 |
| Mali | 55.40 (51.57, 59.44) | 39.78 (37.72, 41.92) | -1.37 (-1.45, -1.29) | <0.001 | 566.63 (554.43, 579.05) | 486.51 (479.43, 493.69) | -0.54 (-0.61, -0.47) | <0.001 | 826.74 (811.80, 841.90) | 505.40 (498.04, 512.84) | -1.90 (-2.06, -1.73) | <0.001 | 13.13 (11.30, 15.20) | 7.64 (6.74, 8.62) | -2.09 (-2.27, -1.92) | <0.001 |
| Malta | 20.93 (11.67, 35.19) | 17.17 (8.88, 31.28) | -0.91 (-1.05, -0.78) | <0.001 | 303.31 (264.01, 347.20) | 292.04 (253.45, 335.72) | -0.10 (-0.22, 0.02) | 0.093 | 186.95 (156.36, 222.19) | 107.52 (84.50, 135.82) | -1.73 (-1.92, -1.54) | <0.001 | 2.41 (0.23, 10.44) | 1.06 (0.01, 9.59) | -2.59 (-2.87, -2.32) | <0.001 |
| Marshall Islands | 86.58 (33.17, 187.41) | 90.18 (43.78, 165.00) | -0.16 (-0.54, 0.22) | 0.400 | 711.06 (532.08, 933.39) | 730.67 (582.42, 905.56) | -0.08 (-0.27, 0.10) | 0.364 | 834.40 (638.60, 1073.60) | 1078.08 (895.90, 1286.78) | 0.46 (0.07, 0.85) | 0.022 | 12.10 (0.19, 78.64) | 16.25 (1.73, 62.05) | 0.43 (0.01, 0.84) | 0.044 |
| Mauritania | 41.56 (35.08, | 28.44 (24.72, | -1.51 (-1.63, | <0.001 | 590.41 (565.68, | 524.80 (508.69, | -0.40 (-0.49, | <0.001 | 503.64 (480.51, | 261.70 (250.20, | -2.04 (-2.12, | <0.001 | 7.36 (4.79, | 3.28 (2.09, | -2.49 (-2.58, | <0.001 |

| | | | | | | | | | | | | | | | | |
|---|---|---|---|---|---|---|---|---|---|---|---|---|---|---|---|---|
| | 48.95) | 32.59) | -1.39) | | 616.00) | 541.32) | -0.32) | | 527.64) | 273.62) | -1.96) | | 10.88) | 4.91) | -2.39) | |
| Mauritius | 41.36 (33.68, 50.31) | 30.99 (24.30, 39.01) | -1.40 (-1.55, -1.25) | <0.001 | 552.37 (523.26, 582.70) | 466.05 (438.96, 494.43) | -0.85 (-0.96, -0.73) | <0.001 | 436.80 (410.89, 463.94) | 259.45 (239.43, 280.76) | -2.37 (-2.62, -2.12) | <0.001 | 5.57 (3.00, 9.48) | 2.80 (1.11, 5.95) | -3.01 (-3.34, -2.69) | <0.001 |
| Mexico | 41.64 (40.65, 42.64) | 30.70 (30.02, 31.38) | -1.28 (-1.56, -1.00) | <0.001 | 645.90 (642.02, 649.80) | 571.12 (568.20, 574.06) | -0.39 (-0.54, -0.25) | <0.001 | 293.43 (290.81, 296.06) | 163.08 (161.52, 164.66) | -1.89 (-2.20, -1.59) | <0.001 | 3.98 (3.68, 4.30) | 1.85 (1.69, 2.02) | -2.50 (-2.86, -2.14) | <0.001 |
| Micronesia (Federated States of) | 109.77 (66.11, 172.70) | 75.30 (41.30, 126.98) | -1.56 (-1.71, -1.41) | <0.001 | 804.76 (676.27, 951.53) | 657.16 (545.82, 785.16) | -0.77 (-0.86, -0.67) | <0.001 | 1060.72 (912.27, 1227.38) | 974.17 (837.97, 1126.86) | -0.57 (-0.69, -0.46) | <0.001 | 15.69 (2.81, 50.39) | 14.48 (2.63, 45.90) | -0.61 (-0.74, -0.48) | <0.001 |
| Monaco | 18.01 (0.27, 152.53) | 16.77 (0.21, 120.26) | -0.29 (-0.34, -0.25) | <0.001 | 299.08 (164.09, 523.02) | 300.85 (166.62, 506.50) | 0.05 (-0.09, 0.19) | 0.473 | 189.74 (87.11, 384.56) | 118.20 (42.18, 269.60) | -1.51 (-1.74, -1.27) | <0.001 | 2.48 (0.00, 129.54) | 1.25 (0.00, 93.37) | -2.21 (-2.51, -1.90) | <0.001 |
| Mongolia | 58.34 (50.76, 66.83) | 39.76 (35.26, 44.74) | -1.70 (-1.85, -1.54) | <0.001 | 516.76 (493.90, 540.50) | 467.31 (451.49, 483.62) | -0.41 (-0.51, -0.31) | <0.001 | 825.05 (795.54, 855.45) | 539.53 (522.70, 556.83) | -1.99 (-2.29, -1.69) | <0.001 | 12.81 (9.32, 17.25) | 7.91 (6.00, 10.31) | -2.20 (-2.55, -1.85) | <0.001 |
| Montenegro | 40.60 (30.10, 53.62) | 34.13 (24.00, 47.37) | -0.89 (-1.07, -0.70) | <0.001 | 531.73 (491.63, 574.27) | 506.10 (464.36, 550.79) | -0.26 (-0.36, -0.17) | <0.001 | 371.65 (338.28, 407.45) | 286.61 (255.65, 320.51) | -1.19 (-1.93, -0.45) | 0.003 | 4.67 (1.66, 10.40) | 3.28 (0.82, 9.23) | -1.49 (-2.54, -0.44) | 0.007 |
| Morocco | 48.89 (46.91, 50.93) | 49.16 (47.57, 50.78) | -0.05 (-0.16, 0.07) | 0.440 | 564.68 (557.97, 571.46) | 573.01 (567.57, 578.50) | 0.07 (-0.02, 0.15) | 0.104 | 515.09 (508.66, 521.58) | 347.03 (342.80, 351.31) | -1.45 (-1.67, -1.23) | <0.001 | 7.39 (6.63, 8.22) | 4.46 (4.00, 4.97) | -1.81 (-2.09, -1.52) | <0.001 |
| Mozambique | 31.22 (28.96, 33.61) | 26.96 (25.54, 28.44) | -0.48 (-0.70, -0.27) | <0.001 | 623.76 (613.75, 633.90) | 638.77 (632.03, 645.56) | 0.22 (0.12, 0.33) | <0.001 | 302.35 (295.25, 309.57) | 262.78 (258.32, 267.30) | -0.10 (-0.33, 0.14) | 0.412 | 3.84 (3.07, 4.75) | 3.10 (2.62, 3.65) | -0.23 (-0.56, 0.09) | 0.151 |
| Myanmar | 24.94 (23.86, 26.06) | 22.67 (21.80, 23.56) | -0.41 (-0.62, -0.20) | <0.001 | 342.90 (338.85, 346.99) | 343.93 (340.53, 347.35) | 0.07 (-0.05, 0.20) | 0.237 | 1446.69 (1438.33 , 1455.10) | 616.40 (611.85, 620.98) | -3.24 (-3.61, -2.87) | <0.001 | 23.68 (22.61, 24.79) | 9.37 (8.82, 9.95) | -3.48 (-3.90, -3.06) | <0.001 |
| Namibia | 28.23 (21.95, 35.87) | 20.55 (16.76, 24.96) | -1.49 (-1.68, -1.31) | <0.001 | 538.83 (510.80, 568.12) | 505.52 (486.12, 525.54) | -0.21 (-0.28, -0.13) | <0.001 | 289.78 (268.74, 312.12) | 167.02 (155.85, 178.80) | -2.12 (-2.39, -1.84) | <0.001 | 3.79 (1.71, 7.36) | 1.68 (0.73, 3.33) | -3.08 (-3.49, -2.67) | <0.001 |
| Nauru | 87.49 | 80.94 | -0.27 | 0.380 | 757.20 | 722.10 | -0.21 | 0.075 | 1351.58 | 1526.74 | 0.29 | 0.358 | 20.91 | 23.94 | 0.26 | 0.451 |

| | | | | | | | | | | | | | | | | |
|---|---|---|---|---|---|---|---|---|---|---|---|---|---|---|---|---|
| | (7.79, 360.25) | (7.70, 326.73) | (-0.90, 0.35) | | (418.51, 1266.35) | (408.13, 1188.16) | (-0.43, 0.02) | | (880.80, 1989.36) | (1049.16, 2152.24) | (-0.34, 0.93) | | (0.00, 247.35) | (0.03, 231.12) | (-0.43, 0.95) | |
| Nepal | 20.61 (19.12, 22.20) | 17.90 (16.91, 18.94) | -0.52 (-0.73, -0.31) | <0.001 | 285.97 (280.36, 291.66) | 267.56 (263.69, 271.49) | -0.10 (-0.21, 0.02) | 0.091 | 287.56 (281.83, 293.38) | 139.14 (136.32, 142.01) | -2.53 (-2.82, -2.23) | <0.001 | 4.25 (3.57, 5.02) | 1.64 (1.35, 1.99) | -3.36 (-3.71, -3.01) | <0.001 |
| Netherlands | 22.02 (20.38, 23.77) | 17.57 (16.01, 19.24) | -1.09 (-1.23, -0.94) | <0.001 | 308.86 (302.63, 315.20) | 318.31 (311.56, 325.18) | 0.04 (-0.07, 0.15) | 0.445 | 189.72 (184.86, 194.69) | 95.03 (91.37, 98.81) | -3.08 (-3.42, -2.73) | <0.001 | 2.49 (1.96, 3.12) | 0.82 (0.52, 1.25) | -4.67 (-5.24, -4.10) | <0.001 |
| New Zealand | 21.13 (17.86, 24.83) | 20.06 (16.94, 23.62) | -0.18 (-0.48, 0.11) | 0.219 | 299.29 (286.60, 312.41) | 374.07 (360.15, 388.39) | 0.86 (0.60, 1.12) | <0.001 | 180.00 (170.20, 190.23) | 109.80 (102.36, 117.66) | -2.00 (-2.20, -1.80) | <0.001 | 2.28 (1.30, 3.72) | 0.87 (0.33, 1.87) | -3.65 (-3.85, -3.46) | <0.001 |
| Nicaragua | 38.03 (33.47, 43.07) | 30.90 (28.04, 33.99) | -0.82 (-0.98, -0.65) | <0.001 | 543.17 (525.67, 561.15) | 505.31 (493.53, 517.32) | -0.17 (-0.29, -0.05) | 0.006 | 277.15 (264.50, 290.28) | 134.45 (128.39, 140.73) | -2.44 (-2.88, -2.00) | <0.001 | 3.93 (2.54, 5.85) | 1.47 (0.90, 2.28) | -3.37 (-3.90, -2.84) | <0.001 |
| Niger | 38.68 (35.26, 42.36) | 29.76 (27.92, 31.69) | -1.09 (-1.22, -0.97) | <0.001 | 493.82 (481.66, 506.23) | 448.51 (441.50, 455.61) | -0.30 (-0.40, -0.19) | <0.001 | 502.20 (489.70, 514.97) | 360.79 (354.34, 367.33) | -1.43 (-1.64, -1.22) | <0.001 | 7.58 (6.09, 9.34) | 5.20 (4.44, 6.06) | -1.64 (-1.88, -1.41) | <0.001 |
| Nigeria | 27.90 (27.04, 28.79) | 23.33 (22.85, 23.82) | -0.88 (-1.04, -0.72) | <0.001 | 482.25 (478.74, 485.78) | 495.99 (493.81, 498.17) | 0.12 (0.04, 0.20) | 0.003 | 290.03 (287.23, 292.87) | 209.13 (207.69, 210.59) | -1.49 (-1.72, -1.26) | <0.001 | 3.93 (3.60, 4.28) | 2.46 (2.30, 2.62) | -2.10 (-2.40, -1.79) | <0.001 |
| Niue | 50.88 (0.00, 1141.41) | 48.44 (0.00, 1556.39) | -0.19 (-0.23, -0.16) | <0.001 | 593.87 (81.81, 2068.44) | 593.91 (49.48, 2502.25) | 0.02 (-0.04, 0.07) | 0.507 | 617.39 (89.54, 2106.38) | 544.73 (38.24, 2422.08) | -0.73 (-0.89, -0.58) | <0.001 | 8.60 (0.00, 1057.42) | 7.27 (0.00, 1475.87) | -0.92 (-1.13, -0.70) | <0.001 |
| North Macedonia | 45.43 (39.06, 52.55) | 33.45 (27.97, 39.83) | -1.28 (-1.35, -1.21) | <0.001 | 569.12 (545.98, 593.00) | 533.02 (510.37, 556.54) | -0.30 (-0.41, -0.20) | <0.001 | 566.57 (543.47, 590.42) | 343.52 (325.31, 362.60) | -2.08 (-2.34, -1.82) | <0.001 | 7.75 (5.26, 11.01) | 4.08 (2.34, 6.81) | -2.62 (-2.97, -2.27) | <0.001 |
| Northern Mariana Islands | 64.10 (26.71, 131.85) | 47.27 (7.09, 173.36) | -1.32 (-1.40, -1.24) | <0.001 | 651.64 (513.12, 817.83) | 590.23 (393.14, 859.94) | -0.43 (-0.51, -0.36) | <0.001 | 750.38 (601.38, 926.90) | 401.42 (241.92, 635.21) | -2.69 (-2.97, -2.40) | <0.001 | 10.76 (0.51, 55.56) | 4.84 (0.00, 107.32) | -3.31 (-3.69, -2.94) | <0.001 |
| Norway | 23.32 (20.09, | 22.79 (19.73, | -0.71 (-0.97, | <0.001 | 363.58 (350.44, | 356.99 (344.61, | -0.14 (-0.27, | 0.042 | 163.59 (154.82, | 75.80 (70.15, | -2.83 (-3.01, | <0.001 | 1.91 (1.07, | 0.39 (0.09, | -5.65 (-5.85, | <0.001 |

| | | | | | | | | | | | | | | | | |
|---|---|---|---|---|---|---|---|---|---|---|---|---|---|---|---|---|
| | 26.95) | 26.21) | -0.46) | | 377.11) | 369.74) | -0.01) | | 172.74) | 81.81) | -2.65) | | 3.16) | 1.12) | -5.45) | |
| | 43.37 | 35.46 | -0.77 | | 552.40 | 553.00 | 0.16 | | 445.70 | 274.25 | -1.24 | | 6.12 | 3.12 | -1.78 | |
| Oman | (35.86, | (31.43, | (-0.86, | <0.001 | (524.69, | (536.83, | (0.08, | <0.001 | (420.95, | (262.68, | (-1.47, | <0.001 | (3.53, | (2.00, | (-2.10, | <0.001 |
| | 52.05) | 39.92) | -0.68) | | 581.25) | 569.57) | 0.23) | | 471.59) | 286.24) | -1.01) | | 9.97) | 4.70) | -1.46) | |
| | 46.59 | 42.40 | -0.63 | | 523.45 | 533.70 | -0.02 | | 433.45 | 466.75 | -0.20 | | 6.15 | 6.64 | -0.29 | |
| Pakistan | (45.56, | (41.78, | (-0.81, | <0.001 | (520.00, | (531.48, | (-0.11, | 0.671 | (430.28, | (464.66, | (-0.42, | 0.065 | (5.78, | (6.39, | (-0.55, | 0.027 |
| | 47.65) | 43.04) | -0.46) | | 526.91) | 535.93) | 0.07) | | 436.63) | 468.85) | 0.01) | | 6.55) | 6.90) | -0.04) | |
| | 46.22 | 48.30 | 0.14 | | 573.92 | 591.12 | 0.13 | | 567.63 | 527.19 | -0.15 | | 7.79 | 6.99 | -0.24 | |
| Palau | (3.37, | (2.24, | (0.04, | 0.005 | (343.88, | (332.83, | (0.04, | 0.007 | (338.80, | (285.03, | (-0.23, | 0.001 | (0.00, | (0.00, | (-0.33, | <0.001 |
| | 202.48) | 252.90) | 0.23) | | 902.60) | 982.55) | 0.22) | | 895.26) | 903.54) | -0.07) | | 136.04) | 182.87) | -0.14) | |
| | 38.17 | 33.51 | -0.44 | | 510.72 | 508.90 | 0.11 | | 275.82 | 177.06 | -1.60 | | 3.37 | 1.67 | -2.69 | |
| Palestine | (31.57, | (29.90, | (-0.70, | 0.002 | (485.83, | (494.53, | (0.01, | 0.033 | (257.61, | (168.63, | (-1.91, | <0.001 | (1.62, | (0.94, | (-3.14, | <0.001 |
| | 45.85) | 37.45) | -0.17) | | 536.64) | 523.60) | 0.22) | | 295.08) | 185.83) | -1.28) | | 6.33) | 2.77) | -2.24) | |
| | 33.17 | 29.47 | -0.46 | | 494.53 | 477.57 | -0.05 | | 257.63 | 150.59 | -1.70 | | 3.59 | 1.77 | -2.27 | |
| Panama | (28.12, | (25.79, | (-0.61, | <0.001 | (474.54, | (462.38, | (-0.18, | 0.419 | (243.26, | (142.11, | (-2.01, | <0.001 | (2.07, | (0.96, | (-2.67, | <0.001 |
| | 38.90) | 33.53) | -0.31) | | 515.19) | 493.13) | 0.08) | | 272.68) | 159.44) | -1.38) | | 5.84) | 2.98) | -1.88) | |
| | 75.25 | 79.35 | 0.23 | | 622.73 | 640.05 | 0.14 | | 555.59 | 626.80 | 0.54 | | 7.41 | 8.57 | 0.64 | |
| Papua New Guinea | (69.03, | (75.43, | (0.12, | <0.001 | (604.49, | (628.80, | (0.10, | <0.001 | (538.44, | (615.69, | (0.33, | <0.001 | (5.53, | (7.31, | (0.37, | <0.001 |
| | 81.91) | 83.43) | 0.35) | | 641.41) | 651.47) | 0.19) | | 573.18) | 638.07) | 0.76) | | 9.74) | 9.98) | 0.91) | |
| | 38.65 | 32.79 | -0.72 | | 511.19 | 511.11 | -0.05 | | 380.65 | 188.25 | -2.78 | | 5.75 | 2.44 | -3.36 | |
| Paraguay | (34.23, | (29.89, | (-0.83, | <0.001 | (494.84, | (499.48, | (-0.13, | 0.214 | (366.49, | (181.20, | (-3.04, | <0.001 | (4.12, | (1.69, | (-3.69, | <0.001 |
| | 43.51) | 35.89) | -0.61) | | 527.96) | 522.93) | 0.03) | | 395.24) | 195.51) | -2.52) | | 7.84) | 3.40) | -3.03) | |
| | 43.61 | 30.77 | -1.37 | | 501.81 | 429.52 | -0.52 | | 505.66 | 220.77 | -3.02 | | 7.69 | 2.97 | -3.45 | |
| Peru | (41.62, | (29.47, | (-1.52, | <0.001 | (494.94, | (424.63, | (-0.61, | <0.001 | (498.88, | (217.25, | (-3.35, | <0.001 | (6.87, | (2.58, | (-3.83, | <0.001 |
| | 45.68) | 32.12) | -1.22) | | 508.76) | 434.46) | -0.43) | | 512.52) | 224.33) | -2.69) | | 8.59) | 3.41) | -3.07) | |
| | 36.87 | 36.79 | 0.05 | | 488.03 | 514.43 | 0.28 | | 206.94 | 394.03 | 2.86 | | 1.84 | 5.02 | 3.88 | |
| Philippines | (35.77, | (35.99, | (-0.00, | 0.059 | (484.04, | (511.42, | (0.21, | <0.001 | (204.32, | (391.39, | (2.12, | <0.001 | (1.59, | (4.73, | (2.64, | <0.001 |
| | 37.99) | 37.61) | 0.11) | | 492.05) | 517.46) | 0.35) | | 209.58) | 396.70) | 3.61) | | 2.11) | 5.33) | 5.13) | |
| | 33.72 | 25.12 | -1.36 | | 456.20 | 456.21 | 0.02 | | 325.98 | 164.21 | -2.66 | | 4.12 | 1.26 | -4.42 | |
| Poland | (32.42, | (23.90, | (-1.65, | <0.001 | (451.37, | (451.01, | (-0.20, | 0.878 | (321.94, | (161.13, | (-2.85, | <0.001 | (3.68, | (1.01, | (-4.56, | <0.001 |
| | 35.06) | 26.39) | -1.07) | | 461.07) | 461.47) | 0.24) | | 330.05) | 167.35) | -2.47) | | 4.60) | 1.56) | -4.28) | |
| | 23.46 | 14.77 | -1.97 | | 385.32 | 347.47 | -0.30 | | 268.81 | 90.66 | -4.43 | | 3.69 | 0.69 | -6.40 | |
| Portugal | (21.33, | (12.93, | (-2.10, | <0.001 | (376.53, | (338.33, | (-0.44, | <0.001 | (261.48, | (86.08, | (-4.76, | <0.001 | (2.88, | (0.36, | (-6.99, | <0.001 |

| | | | | | | | | | | | | | | | | |
|---|---|---|---|---|---|---|---|---|---|---|---|---|---|---|---|---|
| | 25.74) | 16.81) | -1.85) | | 394.25) | 356.81) | -0.17) | | 276.29) | 95.43) | -4.11) | | 4.66) | 1.24) | -5.80) | |
| Puerto Rico | 35.20 (31.01, 39.80) | 37.30 (32.53, 42.60) | 0.15 (0.01, 0.29) | 0.034 | 458.45 (442.99, 474.31) | 456.81 (439.70, 474.43) | 0.19 (0.13, 0.24) | <0.001 | 179.05 (169.44, 189.06) | 104.20 (96.13, 112.79) | -2.35 (-2.56, -2.14) | <0.001 | 2.29 (1.32, 3.69) | 1.03 (0.38, 2.26) | -3.56 (-3.91, -3.20) | <0.001 |
| Qatar | 51.50 (35.87, 72.24) | 39.09 (32.83, 46.49) | -1.18 (-1.35, -1.02) | <0.001 | 641.07 (582.27, 704.67) | 564.21 (539.76, 589.73) | -0.53 (-0.56, -0.50) | <0.001 | 451.90 (402.40, 506.28) | 198.08 (183.34, 213.93) | -3.13 (-3.53, -2.74) | <0.001 | 6.06 (1.69, 16.29) | 1.88 (0.73, 4.46) | -4.50 (-5.06, -3.94) | <0.001 |
| Republic of Korea | 33.02 (31.89, 34.17) | 18.91 (18.01, 19.85) | -2.54 (-2.81, -2.26) | <0.001 | 397.42 (393.53, 401.35) | 297.87 (294.22, 301.55) | -1.37 (-1.56, -1.17) | <0.001 | 440.00 (435.89, 444.14) | 105.36 (103.21, 107.56) | -5.34 (-5.80, -4.88) | <0.001 | 6.37 (5.88, 6.89) | 0.85 (0.67, 1.07) | -7.47 (-7.95, -7.00) | <0.001 |
| Republic of Moldova | 33.63 (30.01, 37.61) | 31.98 (27.86, 36.63) | -0.21 (-0.31, -0.11) | <0.001 | 415.40 (402.37, 428.77) | 425.07 (409.68, 440.98) | 0.10 (0.07, 0.13) | <0.001 | 256.60 (246.36, 267.19) | 165.26 (155.76, 175.28) | -1.83 (-2.22, -1.44) | <0.001 | 3.00 (2.00, 4.37) | 1.39 (0.67, 2.72) | -2.73 (-3.39, -2.06) | <0.001 |
| Romania | 34.99 (33.25, 36.81) | 28.10 (26.19, 30.12) | -0.92 (-1.00, -0.85) | <0.001 | 497.98 (491.32, 504.71) | 488.02 (479.96, 496.20) | 0.06 (-0.06, 0.19) | 0.320 | 313.91 (308.65, 319.24) | 220.05 (214.68, 225.53) | -1.30 (-1.52, -1.09) | <0.001 | 3.79 (3.24, 4.42) | 2.14 (1.65, 2.75) | -2.08 (-2.42, -1.74) | <0.001 |
| Russian Federation | 34.72 (34.07, 35.39) | 32.41 (31.72, 33.12) | -0.26 (-0.42, -0.10) | 0.003 | 460.50 (458.09, 462.92) | 518.39 (515.56, 521.24) | 0.48 (0.40, 0.56) | <0.001 | 262.89 (261.07, 264.73) | 271.99 (269.99, 274.01) | -0.15 (-0.70, 0.41) | 0.591 | 2.92 (2.73, 3.12) | 2.97 (2.77, 3.18) | -0.37 (-1.17, 0.44) | 0.354 |
| Rwanda | 61.57 (57.23, 66.17) | 18.93 (17.28, 20.69) | -5.01 (-5.43, -4.59) | <0.001 | 666.37 (652.23, 680.75) | 485.24 (476.87, 493.73) | -1.31 (-1.47, -1.15) | <0.001 | 819.13 (803.10, 835.41) | 250.12 (244.03, 256.32) | -5.22 (-5.74, -4.71) | <0.001 | 12.87 (10.91, 15.09) | 3.21 (2.54, 3.99) | -6.08 (-6.67, -5.49) | <0.001 |
| Saint Kitts and Nevis | 61.08 (19.69, 145.83) | 36.52 (10.53, 93.59) | -2.23 (-2.46, -2.00) | <0.001 | 672.01 (506.61, 876.16) | 557.61 (430.53, 712.13) | -0.66 (-0.79, -0.54) | <0.001 | 948.09 (748.57, 1186.24) | 187.16 (116.89, 285.95) | -6.25 (-6.74, -5.76) | <0.001 | 15.59 (0.75, 78.04) | 2.27 (0.00, 40.54) | -7.69 (-8.16, -7.22) | <0.001 |
| Saint Lucia | 45.37 (22.79, 82.28) | 38.09 (20.33, 65.67) | -0.77 (-0.98, -0.56) | <0.001 | 623.35 (529.64, 729.98) | 557.69 (481.51, 642.97) | -0.35 (-0.49, -0.22) | <0.001 | 382.84 (309.12, 469.85) | 195.96 (152.13, 249.03) | -2.41 (-2.82, -2.00) | <0.001 | 5.70 (0.33, 27.90) | 2.50 (0.04, 16.64) | -3.04 (-3.53, -2.54) | <0.001 |
| Saint Vincent and the Grenadines | 39.35 (16.51, 81.31) | 37.88 (16.27, 75.22) | -0.35 (-0.46, -0.25) | <0.001 | 497.81 (403.85, 608.74) | 516.67 (423.76, 624.12) | 0.14 (0.07, 0.21) | 0.001 | 429.72 (342.64, 533.86) | 289.39 (221.07, 372.39) | -2.53 (-3.00, -2.05) | <0.001 | 6.59 (0.32, 34.90) | 4.15 (0.07, 26.07) | -2.97 (-3.52, -2.41) | <0.001 |
| Samoa | 66.33 (38.82, | 65.98 (42.23, | 0.04 (-0.03, | 0.275 | 677.91 (581.32, | 677.18 (595.04, | 0.04 (-0.02, | 0.167 | 694.76 (596.21, | 787.10 (697.97, | 0.56 (0.46, | <0.001 | 9.72 (1.55, | 11.30 (3.07, | 0.66 (0.54, | <0.001 |

| | | | | | | | | | | | | | | | | |
|---|---|---|---|---|---|---|---|---|---|---|---|---|---|---|---|---|
| | 107.07) | 98.82) | 0.11) | | 786.87) | 767.92) | 0.09) | | 805.79) | 884.86) | 0.65) | | 33.15) | 29.61) | 0.78) | |
| San Marino | 18.26 (0.20, 126.63) | 16.76 (0.33, 108.16) | -0.33 (-0.40, -0.27) | <0.001 | 288.68 (151.76, 501.92) | 284.70 (161.76, 469.57) | 0.01 (-0.10, 0.12) | 0.848 | 105.97 (32.25, 260.53) | 89.76 (29.00, 216.81) | -0.49 (-0.58, -0.41) | <0.001 | 1.06 (0.00, 95.39) | 0.79 (0.00, 80.39) | -0.93 (-1.08, -0.79) | <0.001 |
| Sao Tome and Principe | 63.99 (32.34, 115.60) | 52.42 (32.41, 80.53) | -1.02 (-1.20, -0.85) | <0.001 | 729.39 (611.19, 865.50) | 707.14 (627.78, 794.05) | -0.24 (-0.33, -0.16) | <0.001 | 480.44 (383.52, 595.86) | 469.48 (404.75, 541.87) | -0.94 (-1.58, -0.31) | 0.005 | 6.70 (0.25, 36.92) | 6.52 (1.12, 21.02) | -1.26 (-1.99, -0.53) | 0.002 |
| Saudi Arabia | 33.06 (30.81, 35.44) | 44.40 (42.95, 45.89) | 1.24 (0.97, 1.51) | <0.001 | 775.44 (764.60, 786.40) | 836.29 (829.86, 842.76) | 0.35 (-0.02, 0.72) | 0.064 | 566.28 (556.83, 575.86) | 421.66 (417.18, 426.18) | -0.80 (-1.01, -0.60) | <0.001 | 7.84 (6.75, 9.07) | 5.19 (4.71, 5.71) | -1.12 (-1.38, -0.86) | <0.001 |
| Senegal | 34.87 (31.64, 38.35) | 29.73 (27.73, 31.84) | -0.71 (-0.78, -0.63) | <0.001 | 549.83 (537.10, 562.81) | 510.51 (502.26, 518.86) | -0.21 (-0.29, -0.14) | <0.001 | 388.28 (377.39, 399.42) | 273.77 (267.66, 279.98) | -1.15 (-1.29, -1.01) | <0.001 | 5.44 (4.20, 6.95) | 3.51 (2.85, 4.30) | -1.44 (-1.62, -1.26) | <0.001 |
| Serbia | 40.35 (37.45, 43.44) | 29.38 (26.64, 32.33) | -1.43 (-1.58, -1.28) | <0.001 | 517.40 (506.80, 528.19) | 531.80 (519.93, 543.89) | 0.09 (-0.03, 0.21) | 0.130 | 466.19 (456.15, 476.41) | 218.50 (210.97, 226.25) | -3.24 (-3.54, -2.93) | <0.001 | 6.36 (5.25, 7.66) | 1.99 (1.34, 2.87) | -4.79 (-5.26, -4.32) | <0.001 |
| Seychelles | 31.30 (9.01, 82.83) | 30.34 (10.36, 70.47) | -0.11 (-0.22, -0.00) | 0.047 | 472.94 (364.96, 605.88) | 472.89 (377.13, 586.60) | 0.01 (-0.06, 0.08) | 0.808 | 417.47 (315.54, 544.73) | 244.67 (177.42, 330.15) | -1.34 (-1.64, -1.04) | <0.001 | 5.48 (0.04, 44.82) | 2.48 (0.00, 28.34) | -2.02 (-2.45, -1.59) | <0.001 |
| Sierra Leone | 39.00 (34.37, 44.11) | 43.59 (40.37, 47.01) | 0.43 (0.23, 0.63) | <0.001 | 573.75 (555.87, 592.09) | 581.16 (569.52, 592.99) | 0.17 (0.09, 0.25) | <0.001 | 460.64 (444.42, 477.33) | 494.70 (483.82, 505.78) | 0.44 (0.14, 0.74) | 0.005 | 6.66 (4.82, 9.00) | 7.20 (5.93, 8.68) | 0.48 (0.13, 0.83) | 0.010 |
| Singapore | 25.41 (21.93, 29.33) | 18.71 (16.25, 21.55) | -1.27 (-1.43, -1.11) | <0.001 | 328.70 (315.86, 341.96) | 274.15 (264.30, 284.38) | -0.62 (-0.74, -0.49) | <0.001 | 181.65 (172.12, 191.61) | 73.65 (68.50, 79.18) | -3.08 (-3.23, -2.92) | <0.001 | 2.09 (1.19, 3.47) | 0.34 (0.09, 1.14) | -5.92 (-6.15, -5.69) | <0.001 |
| Slovakia | 35.19 (31.69, 38.99) | 28.79 (25.44, 32.54) | -0.83 (-0.97, -0.69) | <0.001 | 485.27 (472.00, 498.83) | 425.33 (412.15, 438.89) | -0.51 (-0.61, -0.40) | <0.001 | 283.19 (273.12, 293.56) | 151.77 (143.90, 160.02) | -2.16 (-2.36, -1.96) | <0.001 | 3.26 (2.27, 4.56) | 1.12 (0.55, 2.13) | -3.74 (-4.01, -3.47) | <0.001 |
| Slovenia | 27.38 (22.44, 33.14) | 22.77 (17.72, 29.02) | -0.93 (-1.14, -0.72) | <0.001 | 373.99 (355.10, 393.68) | 321.84 (302.06, 342.74) | -0.62 (-0.74, -0.50) | <0.001 | 213.58 (199.39, 228.58) | 96.24 (85.38, 108.28) | -3.10 (-3.30, -2.89) | <0.001 | 2.38 (1.12, 4.55) | 0.46 (0.03, 2.51) | -6.13 (-6.41, -5.84) | <0.001 |
| Solomon Islands | 112.17 (84.68, | 95.35 (78.80, | -0.58 (-0.68, | <0.001 | 741.51 (668.12, | 673.46 (628.31, | -0.30 (-0.35, | <0.001 | 1354.65 (1253.46 | 1409.83 (1343.87 | 0.30 (0.17, | <0.001 | 21.42 (10.38, | 22.57 (14.90, | 0.35 (0.20, | <0.001 |

| | | | | | | | | | | | | | | | | |
|---|---|---|---|---|---|---|---|---|---|---|---|---|---|---|---|---|
| | 146.25) | 114.40) | -0.49) | | 821.22) | 721.06) | -0.26) | | , 1462.21) | , 1478.25) | 0.44) | | 39.64) | 32.84) | 0.51) | |
| Somalia | 48.58 (44.67, 52.74) | 33.57 (31.58, 35.65) | -1.61 (-1.76, -1.45) | <0.001 | 613.58 (599.58, 627.82) | 570.22 (562.22, 578.31) | -0.24 (-0.32, -0.16) | <0.001 | 614.01 (599.93, 628.34) | 422.95 (415.85, 430.14) | -1.25 (-1.36, -1.14) | <0.001 | 9.41 (7.74, 11.35) | 6.09 (5.25, 7.03) | -1.46 (-1.59, -1.34) | <0.001 |
| South Africa | 34.67 (33.34, 36.04) | 20.08 (19.28, 20.90) | -2.19 (-2.75, -1.63) | <0.001 | 691.68 (685.81, 697.60) | 573.33 (569.04, 577.65) | -0.73 (-0.85, -0.62) | <0.001 | 575.81 (570.33, 581.32) | 210.05 (207.48, 212.64) | -3.55 (-4.97, -2.11) | <0.001 | 8.46 (7.80, 9.16) | 2.30 (2.04, 2.59) | -4.22 (-6.12, -2.27) | <0.001 |
| South Sudan | 32.20 (28.55, 36.22) | 26.21 (23.75, 28.86) | -0.85 (-0.99, -0.71) | <0.001 | 587.45 (572.17, 603.08) | 585.71 (574.05, 597.57) | 0.03 (-0.06, 0.12) | 0.453 | 377.01 (364.31, 390.06) | 256.21 (248.42, 264.19) | -1.37 (-1.63, -1.11) | <0.001 | 5.23 (3.80, 7.05) | 3.09 (2.28, 4.10) | -1.90 (-2.23, -1.57) | <0.001 |
| Spain | 18.97 (17.97, 20.00) | 15.32 (14.40, 16.30) | -1.30 (-1.50, -1.11) | <0.001 | 308.74 (304.70, 312.82) | 295.25 (291.13, 299.42) | -0.14 (-0.28, 0.01) | 0.063 | 216.75 (213.37, 220.17) | 85.26 (83.06, 87.50) | -3.45 (-3.65, -3.25) | <0.001 | 2.88 (2.50, 3.30) | 0.70 (0.52, 0.94) | -5.28 (-5.47, -5.09) | <0.001 |
| Sri Lanka | 26.61 (24.95, 28.35) | 25.47 (23.96, 27.05) | -0.20 (-0.28, -0.12) | <0.001 | 415.57 (408.94, 422.29) | 416.09 (409.94, 422.31) | 0.04 (-0.04, 0.12) | 0.299 | 287.90 (282.40, 293.48) | 194.73 (190.53, 199.01) | -1.27 (-1.48, -1.07) | <0.001 | 3.34 (2.77, 3.99) | 1.79 (1.41, 2.24) | -1.99 (-2.34, -1.63) | <0.001 |
| Sudan | 56.31 (53.82, 58.89) | 51.82 (50.27, 53.41) | -0.36 (-0.48, -0.25) | <0.001 | 569.74 (561.82, 577.75) | 581.57 (576.38, 586.81) | 0.08 (-0.01, 0.17) | 0.082 | 871.29 (861.51, 881.17) | 501.09 (496.27, 505.95) | -1.86 (-1.95, -1.77) | <0.001 | 13.50 (12.29, 14.80) | 7.08 (6.51, 7.68) | -2.16 (-2.27, -2.06) | <0.001 |
| Suriname | 44.26 (29.98, 63.37) | 40.68 (29.61, 54.58) | -0.41 (-0.54, -0.28) | <0.001 | 531.69 (478.96, 588.96) | 535.43 (492.90, 580.70) | 0.05 (-0.06, 0.16) | 0.337 | 496.99 (445.93, 552.62) | 375.62 (340.15, 413.82) | -1.49 (-2.07, -0.90) | <0.001 | 7.71 (2.59, 18.17) | 5.62 (2.10, 12.21) | -1.66 (-2.31, -1.00) | <0.001 |
| Sweden | 22.41 (20.06, 24.98) | 21.20 (18.98, 23.63) | -0.19 (-0.62, 0.24) | 0.366 | 441.95 (431.23, 452.88) | 591.86 (579.88, 604.05) | 1.37 (1.13, 1.62) | <0.001 | 155.10 (148.81, 161.59) | 109.45 (104.35, 114.76) | -1.08 (-1.33, -0.84) | <0.001 | 1.59 (1.01, 2.38) | 0.44 (0.18, 0.93) | -4.48 (-4.68, -4.28) | <0.001 |
| Switzerland | 17.64 (15.44, 20.10) | 14.38 (12.47, 16.53) | -0.83 (-0.97, -0.69) | <0.001 | 264.79 (256.07, 273.76) | 241.91 (233.88, 250.17) | -0.31 (-0.47, -0.15) | <0.001 | 131.58 (125.47, 137.94) | 59.23 (55.30, 63.41) | -3.28 (-3.56, -2.99) | <0.001 | 1.57 (0.97, 2.45) | 0.36 (0.12, 0.89) | -6.05 (-6.42, -5.67) | <0.001 |
| Syrian Arab Republic | 59.38 (56.04, 62.88) | 42.10 (39.65, 44.69) | -1.52 (-1.74, -1.29) | <0.001 | 643.70 (632.51, 655.05) | 564.76 (555.66, 574.00) | -0.56 (-0.65, -0.48) | <0.001 | 857.17 (844.52, 869.98) | 421.20 (413.40, 429.13) | -2.92 (-3.36, -2.47) | <0.001 | 12.78 (11.26, 14.47) | 5.60 (4.73, 6.60) | -3.41 (-3.92, -2.90) | <0.001 |
| Taiwan (Province | 25.49 | 24.37 | -0.25 | <0.001 | 395.38 | 412.66 | 0.11 | 0.022 | 289.19 | 159.04 | -2.15 | <0.001 | 3.45 | 1.17 | -3.92 | <0.001 |

| | | | | | | | | | | | | | | | | |
|---|---|---|---|---|---|---|---|---|---|---|---|---|---|---|---|---|
| of China) | (24.04, 27.00) | (22.85, 25.97) | (-0.35, -0.15) | | (389.63, 401.20) | (406.39, 419.02) | (0.02, 0.20) | | (284.28, 294.18) | (155.21, 162.96) | (-2.33, -1.97) | | (2.93, 4.03) | (0.88, 1.56) | (-4.17, -3.66) | |
| Tajikistan | 31.79 (28.25, 35.68) | 28.21 (25.89, 30.69) | -0.31 (-0.60, -0.01) | 0.041 | 335.30 (323.61, 347.33) | 329.72 (321.67, 337.92) | 0.03 (-0.11, 0.18) | 0.632 | 363.44 (350.95, 376.28) | 261.02 (253.82, 268.38) | -1.95 (-2.35, -1.54) | <0.001 | 5.25 (3.83, 7.06) | 3.42 (2.64, 4.37) | -2.47 (-2.95, -1.99) | <0.001 |
| Thailand | 26.08 (25.19, 26.99) | 25.37 (24.50, 26.27) | -0.50 (-0.69, -0.31) | <0.001 | 387.65 (384.22, 391.11) | 376.14 (372.77, 379.53) | -0.19 (-0.30, -0.09) | 0.001 | 251.39 (248.61, 254.20) | 232.92 (230.27, 235.59) | -0.63 (-1.41, 0.15) | 0.107 | 2.95 (2.65, 3.27) | 2.61 (2.34, 2.91) | -0.92 (-2.01, 0.19) | 0.100 |
| Timor-Leste | 45.79 (35.39, 58.46) | 34.98 (27.65, 43.80) | -1.14 (-1.24, -1.03) | <0.001 | 512.67 (476.33, 551.18) | 468.50 (440.69, 497.73) | -0.28 (-0.39, -0.18) | <0.001 | 386.37 (354.58, 420.37) | 416.00 (389.40, 444.06) | 0.33 (0.01, 0.65) | 0.043 | 4.91 (1.94, 10.43) | 5.53 (2.82, 9.90) | 0.52 (0.10, 0.93) | 0.016 |
| Togo | 37.40 (32.76, 42.55) | 31.12 (28.45, 33.99) | -0.65 (-0.72, -0.57) | <0.001 | 551.08 (533.20, 569.45) | 513.09 (502.12, 524.25) | -0.17 (-0.25, -0.09) | <0.001 | 487.83 (470.72, 505.43) | 358.22 (349.00, 367.61) | -0.86 (-0.99, -0.74) | <0.001 | 7.17 (5.21, 9.67) | 4.99 (3.95, 6.22) | -1.01 (-1.16, -0.87) | <0.001 |
| Tokelau | 61.85 (0.00, 1813.98 ) | 55.33 (0.00, 2403.77 ) | -0.43 (-0.46, -0.41) | <0.001 | 618.21 (54.17, 2723.57) | 601.48 (28.01, 3296.66) | -0.09 (-0.14, -0.04) | 0.001 | 931.05 (154.82, 3197.14) | 854.85 (81.60, 3698.54) | -0.28 (-0.39, -0.18) | <0.001 | 14.10 (0.00, 1729.37 ) | 12.76 (0.00, 2330.84 ) | -0.32 (-0.44, -0.20) | <0.001 |
| Tonga | 50.19 (21.35, 102.23) | 42.91 (18.53, 85.20) | -0.64 (-0.81, -0.47) | <0.001 | 593.64 (478.17, 730.13) | 569.05 (465.63, 689.01) | -0.11 (-0.20, -0.02) | 0.024 | 398.35 (303.94, 514.24) | 362.33 (280.71, 460.67) | -0.38 (-0.53, -0.22) | <0.001 | 4.84 (0.03, 37.34) | 4.23 (0.04, 29.09) | -0.55 (-0.76, -0.34) | <0.001 |
| Trinidad and Tobago | 47.18 (38.90, 56.75) | 39.59 (32.40, 48.05) | -0.82 (-0.93, -0.70) | <0.001 | 594.59 (564.27, 626.16) | 563.33 (535.09, 592.80) | -0.26 (-0.34, -0.19) | <0.001 | 355.46 (332.04, 380.13) | 213.00 (195.70, 231.54) | -2.70 (-3.14, -2.27) | <0.001 | 5.14 (2.68, 8.99) | 2.71 (1.11, 5.76) | -3.28 (-3.82, -2.74) | <0.001 |
| Tunisia | 32.82 (30.01, 35.82) | 33.58 (31.27, 36.02) | 0.08 (-0.00, 0.16) | 0.061 | 312.67 (303.91, 321.63) | 375.52 (367.75, 383.41) | 0.25 (0.10, 0.40) | 0.002 | 304.06 (295.35, 312.98) | 204.33 (198.64, 210.16) | -1.37 (-1.45, -1.29) | <0.001 | 4.39 (3.39, 5.61) | 2.48 (1.90, 3.21) | -1.89 (-2.00, -1.78) | <0.001 |
| Turkey | 47.42 (46.15, 48.72) | 33.31 (32.45, 34.19) | -1.60 (-1.75, -1.44) | <0.001 | 563.84 (559.44, 568.27) | 472.03 (468.77, 475.32) | -0.69 (-0.74, -0.63) | <0.001 | 325.69 (322.35, 329.06) | 167.88 (165.94, 169.85) | -2.48 (-2.60, -2.35) | <0.001 | 4.11 (3.74, 4.51) | 1.61 (1.42, 1.81) | -3.44 (-3.63, -3.26) | <0.001 |
| Turkmenistan | 32.19 (28.15, 36.68) | 38.35 (34.53, 42.50) | 0.76 (0.66, 0.86) | <0.001 | 404.01 (389.35, 419.10) | 453.09 (439.70, 466.80) | 0.56 (0.49, 0.64) | <0.001 | 388.15 (373.66, 403.08) | 475.33 (461.59, 489.39) | 0.95 (0.55, 1.35) | <0.001 | 5.38 (3.78, 7.45) | 6.60 (5.07, 8.45) | 0.97 (0.48, 1.47) | <0.001 |

| | | | | | | | | | | | | | | | | |
|---|---|---|---|---|---|---|---|---|---|---|---|---|---|---|---|---|
| Tuvalu | 76.88 (5.36, 340.22) | 56.10 (2.25, 295.23) | -1.30 (-1.41, -1.19) | <0.001 | 657.69 (346.43, 1138.41) | 580.12 (300.71, 1019.51) | -0.51 (-0.58, -0.43) | <0.001 | 1133.82 (708.76, 1724.68) | 924.00 (558.04, 1446.99) | -0.62 (-0.67, -0.58) | <0.001 | 17.44 (0.00, 237.43) | 14.02 (0.00, 222.52) | -0.65 (-0.70, -0.60) | <0.001 |
| Uganda | 27.26 (25.29, 29.36) | 18.99 (17.99, 20.04) | -1.78 (-2.01, -1.56) | <0.001 | 542.38 (533.88, 551.00) | 506.96 (501.87, 512.09) | -0.30 (-0.39, -0.20) | <0.001 | 291.27 (284.78, 297.89) | 243.73 (240.08, 247.42) | -1.73 (-2.26, -1.20) | <0.001 | 3.83 (3.10, 4.69) | 3.04 (2.63, 3.49) | -2.23 (-2.92, -1.55) | <0.001 |
| Ukraine | 33.03 (31.91, 34.18) | 36.38 (35.02, 37.79) | 0.45 (0.28, 0.62) | <0.001 | 540.24 (535.65, 544.85) | 645.69 (639.85, 651.57) | 0.70 (0.58, 0.81) | <0.001 | 229.35 (226.38, 232.36) | 311.51 (307.56, 315.51) | 0.88 (0.54, 1.22) | <0.001 | 2.12 (1.85, 2.43) | 3.25 (2.87, 3.67) | 1.09 (0.53, 1.66) | <0.001 |
| United Arab Emirates | 54.89 (46.76, 64.10) | 55.86 (51.70, 60.34) | 0.10 (-0.11, 0.30) | 0.346 | 705.22 (675.28, 736.21) | 703.46 (688.02, 719.22) | 0.05 (-0.03, 0.13) | 0.249 | 584.40 (556.90, 612.97) | 393.11 (382.22, 404.31) | -1.34 (-2.01, -0.67) | <0.001 | 8.32 (5.34, 12.47) | 5.04 (3.94, 6.48) | -1.70 (-2.59, -0.80) | 0.001 |
| United Kingdom | 19.79 (18.95, 20.65) | 15.28 (14.56, 16.03) | -1.17 (-1.35, -1.00) | <0.001 | 317.41 (314.03, 320.83) | 303.63 (300.41, 306.89) | -0.11 (-0.28, 0.07) | 0.218 | 182.65 (180.09, 185.25) | 107.07 (105.18, 108.99) | -2.14 (-2.31, -1.97) | <0.001 | 2.35 (2.06, 2.66) | 1.07 (0.90, 1.28) | -3.09 (-3.28, -2.90) | <0.001 |
| United Republic of Tanzania | 24.37 (22.90, 25.91) | 21.36 (20.48, 22.27) | -0.61 (-0.80, -0.43) | <0.001 | 529.55 (522.90, 536.26) | 550.45 (546.05, 554.88) | 0.23 (0.10, 0.35) | 0.001 | 374.82 (369.00, 380.71) | 270.80 (267.63, 274.00) | -1.10 (-1.35, -0.85) | <0.001 | 5.32 (4.64, 6.09) | 3.42 (3.07, 3.81) | -1.52 (-1.81, -1.22) | <0.001 |
| United States of America | 21.20 (20.81, 21.60) | 21.04 (20.66, 21.42) | -0.18 (-0.37, 0.01) | 0.067 | 426.54 (424.77, 428.31) | 526.24 (524.33, 528.15) | 1.37 (0.99, 1.76) | <0.001 | 193.42 (192.24, 194.61) | 160.46 (159.41, 161.51) | -0.45 (-0.56, -0.34) | <0.001 | 2.19 (2.07, 2.32) | 1.39 (1.29, 1.49) | -1.72 (-1.92, -1.53) | <0.001 |
| United States Virgin Islands | 33.73 (13.67, 70.13) | 35.15 (12.58, 79.53) | 0.13 (0.00, 0.26) | 0.050 | 465.19 (377.44, 567.82) | 475.96 (376.06, 595.42) | 0.22 (0.14, 0.30) | <0.001 | 186.90 (133.21, 255.75) | 110.78 (66.04, 175.89) | -1.96 (-2.08, -1.85) | <0.001 | 2.46 (0.00, 23.97) | 1.12 (0.00, 28.07) | -2.98 (-3.16, -2.80) | <0.001 |
| Uruguay | 31.68 (27.23, 36.65) | 25.09 (21.31, 29.37) | -1.11 (-1.25, -0.97) | <0.001 | 386.36 (370.40, 402.83) | 362.76 (347.94, 378.07) | -0.38 (-0.50, -0.26) | <0.001 | 437.45 (420.46, 454.95) | 203.68 (192.63, 215.21) | -2.89 (-3.07, -2.71) | <0.001 | 6.52 (4.60, 8.99) | 2.52 (1.44, 4.13) | -3.61 (-3.82, -3.40) | <0.001 |
| Uzbekistan | 37.92 (36.01, 39.90) | 31.22 (29.93, 32.55) | -0.68 (-0.80, -0.57) | <0.001 | 412.36 (406.04, 418.76) | 417.45 (412.71, 422.23) | 0.09 (-0.00, 0.17) | 0.053 | 342.20 (336.41, 348.06) | 296.03 (292.05, 300.06) | -0.85 (-1.17, -0.53) | <0.001 | 4.54 (3.89, 5.27) | 3.71 (3.27, 4.19) | -1.16 (-1.60, -0.72) | <0.001 |
| Vanuatu | 106.75 (71.28, 154.50) | 85.22 (62.84, 113.33) | -0.90 (-0.98, -0.82) | <0.001 | 883.27 (774.06, 1004.21) | 776.30 (705.10, 853.02) | -0.46 (-0.50, -0.43) | <0.001 | 888.67 (779.20, 1009.84) | 1101.57 (1016.49 , | 0.10 (-0.11, 0.31) | 0.352 | 12.37 (2.78, 35.87) | 16.41 (7.55, 31.44) | 0.15 (-0.09, 0.40) | 0.207 |

| | | | | | | | | | | | | | | | | |
|---|---|---|---|---|---|---|---|---|---|---|---|---|---|---|---|---|
| | | | | | | | | | | 1192.14) | | | | | | |
| Venezuela | 40.54 | 34.11 | -0.69 | | 593.91 | 536.66 | -0.31 | | 396.33 | 290.23 | -0.90 | | 5.88 | 4.05 | -1.13 | |
| (Bolivarian | (38.54, | (32.60, | (-0.90, | <0.001 | (586.22, | (530.61, | (-0.46, | <0.001 | (390.03, | (285.77, | (-1.61, | 0.015 | (5.13, | (3.54, | (-1.94, | 0.009 |
| Republic of) | 42.62) | 35.67) | -0.48) | | 601.67) | 542.76) | -0.17) | | 402.72) | 294.73) | -0.19) | | 6.72) | 4.61) | -0.31) | |
| | 27.81 | 26.89 | -0.29 | | 392.28 | 429.10 | 0.36 | | 300.83 | 215.90 | -0.80 | | 3.86 | 2.18 | -1.43 | |
| Viet Nam | (26.93, | (26.17, | (-0.57, | 0.041 | (388.96, | (426.25, | (0.22, | <0.001 | (297.86, | (213.90, | (-0.96, | <0.001 | (3.52, | (1.99, | (-1.65, | <0.001 |
| | 28.72) | 27.62) | -0.01) | | 395.63) | 431.97) | 0.51) | | 303.82) | 217.92) | -0.65) | | 4.22) | 2.39) | -1.20) | |
| | 51.92 | 44.31 | -0.65 | | 530.39 | 506.89 | -0.09 | | 579.98 | 444.19 | -0.96 | | 8.84 | 6.40 | -1.19 | |
| Yemen | (48.90, | (42.66, | (-0.69, | <0.001 | (520.70, | (501.30, | (-0.18, | 0.042 | (569.70, | (438.94, | (-1.10, | <0.001 | (7.60, | (5.78, | (-1.35, | <0.001 |
| | 55.10) | 46.01) | -0.61) | | 540.23) | 512.53) | -0.00) | | 590.41) | 449.50) | -0.82) | | 10.23) | 7.07) | -1.03) | |
| | 39.28 | 22.30 | -2.60 | | 585.15 | 538.63 | -0.37 | | 494.22 | 327.57 | -1.82 | | 7.29 | 4.47 | -2.14 | |
| Zambia | (35.78, | (20.74, | (-2.85, | <0.001 | (572.06, | (531.08, | (-0.47, | <0.001 | (481.66, | (321.48, | (-2.02, | <0.001 | (5.81, | (3.77, | (-2.38, | <0.001 |
| | 43.07) | 23.95) | -2.35) | | 598.50) | 546.27) | -0.28) | | 507.05) | 333.75) | -1.63) | | 9.07) | 5.26) | -1.91) | |
| | 18.13 | 28.11 | 1.75 | | 459.22 | 491.82 | 0.35 | | 189.42 | 264.90 | 2.72 | | 2.16 | 3.39 | 3.63 | |
| Zimbabwe | (16.20, | (26.25, | (1.13, | <0.001 | (449.47, | (484.06, | (0.23, | <0.001 | (183.07, | (259.17, | (1.96, | <0.001 | (1.52, | (2.76, | (2.50, | <0.001 |
| | 20.24) | 30.07) | 2.38) | | 469.15) | 499.67) | 0.47) | | 195.96) | 270.73) | 3.50) | | 2.99) | 4.11) | 4.78) | |
| **Men** | | | | | | | | | | | | | | | | |
| | 38.90 | 41.03 | 0.04 | | 417.01 | 426.68 | 0.22 | | 650.21 | 534.95 | -1.03 | | 10.40 | 8.38 | -1.16 | |
| Afghanistan | (35.40, | (39.41, | (-0.12, | 0.631 | (405.65, | (421.56, | (0.13, | <0.001 | (635.80, | (529.10, | (-1.34, | <0.001 | (8.62, | (7.65, | (-1.50, | <0.001 |
| | 42.68) | 42.70) | 0.19) | | 428.64) | 431.84) | 0.31) | | 664.89) | 540.85) | -0.72) | | 12.47) | 9.16) | -0.82) | |
| | 23.35 | 25.70 | 0.38 | | 210.81 | 215.92 | 0.12 | | 382.59 | 420.69 | 0.31 | | 5.72 | 6.36 | 0.35 | |
| Albania | (19.87, | (21.42, | (0.28, | <0.001 | (199.97, | (203.03, | (0.06, | <0.001 | (368.18, | (402.88, | (0.05, | 0.023 | (4.08, | (4.34, | (0.05, | 0.025 |
| | 27.28) | 30.61) | 0.48) | | 222.11) | 229.43) | 0.19) | | 397.44) | 439.10) | 0.57) | | 7.84) | 9.04) | 0.65) | |
| | 39.37 | 28.67 | -1.29 | | 426.41 | 368.88 | -0.56 | | 503.41 | 257.57 | -2.59 | | 7.62 | 3.52 | -2.98 | |
| Algeria | (37.53, | (27.58, | (-1.34, | <0.001 | (420.35, | (364.92, | (-0.64, | <0.001 | (496.87, | (254.26, | (-2.77, | <0.001 | (6.83, | (3.14, | (-3.17, | <0.001 |
| | 41.28) | 29.80) | -1.23) | | 432.55) | 372.88) | -0.48) | | 510.03) | 260.92) | -2.42) | | 8.49) | 3.93) | -2.78) | |
| | 61.59 | 44.87 | -1.27 | | 584.69 | 498.23 | -0.63 | | 874.95 | 678.19 | -1.22 | | 13.42 | 10.24 | -1.36 | |
| American Samoa | (21.86, | (12.40, | (-1.33, | <0.001 | (439.11, | (363.67, | (-0.68, | <0.001 | (694.57, | (519.43, | (-1.49, | <0.001 | (0.60, | (0.19, | (-1.67, | <0.001 |
| | 139.58) | 118.13) | -1.21) | | 764.97) | 668.84) | -0.57) | | 1089.67) | 872.67) | -0.96) | | 68.99) | 65.80) | -1.05) | |
| | 15.34 | 13.02 | -0.78 | | 188.25 | 174.76 | -0.39 | | 117.12 | 74.79 | -1.81 | | 1.54 | 0.83 | -2.47 | |
| Andorra | (2.06, | (1.29, | (-0.88, | <0.001 | (123.40, | (112.09, | (-0.52, | <0.001 | (67.48, | (36.55, | (-1.92, | <0.001 | (0.00, | (0.00, | (-2.59, | <0.001 |
| | 62.32) | 57.89) | -0.69) | | 280.32) | 264.24) | -0.26) | | 194.79) | 141.19) | -1.70) | | 41.55) | 38.83) | -2.34) | |
| Angola | 37.84 | 27.22 | -1.39 | <0.001 | 378.66 | 348.57 | -0.31 | <0.001 | 521.00 | 376.54 | -1.07 | <0.001 | 8.08 | 5.70 | -1.15 | <0.001 |

| | | | | | | | | | | | | | | | | |
|---|---|---|---|---|---|---|---|---|---|---|---|---|---|---|---|---|
| | (35.02, 40.83) | (25.73, 28.77) | (-1.49, -1.30) | | (369.72, 387.76) | (343.27, 353.93) | (-0.39, -0.23) | | (510.59, 531.57) | (371.05, 382.09) | (-1.20, -0.94) | | (6.82, 9.52) | (5.03, 6.43) | (-1.29, -1.00) | |
| Antigua and Barbuda | 28.68 (6.60, 81.74) | 23.73 (6.60, 61.12) | -0.78 (-0.88, -0.68) | <0.001 | 379.99 (277.57, 509.37) | 361.76 (277.55, 464.08) | -0.19 (-0.24, -0.14) | <0.001 | 336.94 (240.27, 460.91) | 216.22 (152.60, 298.13) | -1.85 (-2.26, -1.43) | <0.001 | 5.34 (0.02, 44.97) | 3.23 (0.01, 29.16) | -2.05 (-2.51, -1.58) | <0.001 |
| Argentina | 29.90 (28.52, 31.32) | 18.95 (18.05, 19.89) | -1.95 (-2.12, -1.79) | <0.001 | 244.74 (240.77, 248.75) | 194.53 (191.62, 197.47) | -0.99 (-1.09, -0.89) | <0.001 | 573.56 (567.49, 579.67) | 230.68 (227.51, 233.89) | -3.72 (-4.10, -3.33) | <0.001 | 9.28 (8.52, 10.08) | 3.42 (3.04, 3.83) | -4.10 (-4.50, -3.70) | <0.001 |
| Armenia | 35.71 (31.44, 40.42) | 24.84 (20.98, 29.31) | -1.22 (-1.29, -1.14) | <0.001 | 302.64 (289.91, 315.81) | 246.24 (233.87, 259.19) | -0.77 (-0.81, -0.73) | <0.001 | 251.30 (239.70, 263.34) | 157.64 (147.68, 168.17) | -2.62 (-3.24, -2.00) | <0.001 | 3.44 (2.20, 5.14) | 1.92 (0.98, 3.54) | -3.13 (-3.94, -2.32) | <0.001 |
| Australia | 16.28 (14.96, 17.68) | 13.10 (12.04, 14.23) | -0.65 (-0.72, -0.58) | <0.001 | 185.61 (181.11, 190.20) | 160.65 (156.90, 164.48) | -0.36 (-0.51, -0.22) | <0.001 | 134.25 (130.42, 138.17) | 66.32 (63.93, 68.79) | -2.55 (-2.72, -2.38) | <0.001 | 1.80 (1.39, 2.31) | 0.69 (0.47, 0.99) | -3.48 (-3.73, -3.22) | <0.001 |
| Austria | 20.18 (17.99, 22.59) | 15.11 (13.21, 17.24) | -1.26 (-1.37, -1.14) | <0.001 | 249.90 (242.04, 257.97) | 209.23 (202.01, 216.68) | -0.76 (-0.87, -0.65) | <0.001 | 184.00 (177.24, 190.96) | 66.32 (62.27, 70.59) | -4.25 (-4.51, -3.99) | <0.001 | 2.58 (1.84, 3.55) | 0.62 (0.29, 1.20) | -5.84 (-6.17, -5.51) | <0.001 |
| Azerbaijan | 36.36 (33.28, 39.66) | 29.01 (26.83, 31.33) | -0.68 (-0.77, -0.59) | <0.001 | 299.33 (290.36, 308.53) | 264.29 (257.69, 271.04) | -0.35 (-0.44, -0.26) | <0.001 | 335.98 (326.42, 345.77) | 229.10 (222.91, 235.45) | -2.05 (-2.69, -1.41) | <0.001 | 4.97 (3.86, 6.32) | 3.15 (2.47, 3.99) | -2.32 (-3.05, -1.59) | <0.001 |
| Bahamas | 28.72 (16.07, 47.83) | 27.18 (16.63, 42.06) | 0.03 (-0.09, 0.14) | 0.600 | 395.11 (344.04, 452.05) | 375.34 (332.44, 422.33) | -0.10 (-0.17, -0.02) | 0.019 | 383.95 (332.81, 441.08) | 355.32 (313.73, 400.99) | -0.35 (-0.62, -0.07) | 0.015 | 6.19 (1.37, 18.14) | 5.75 (1.66, 14.51) | -0.38 (-0.70, -0.07) | 0.018 |
| Bahrain | 27.37 (19.81, 37.66) | 24.99 (20.03, 31.09) | -0.51 (-0.64, -0.39) | <0.001 | 391.26 (360.96, 424.04) | 359.01 (339.12, 379.98) | -0.32 (-0.37, -0.26) | <0.001 | 248.72 (224.12, 275.89) | 163.93 (150.45, 178.52) | -1.77 (-2.11, -1.42) | <0.001 | 3.28 (1.07, 8.84) | 1.94 (0.77, 4.47) | -2.32 (-2.77, -1.86) | <0.001 |
| Bangladesh | 23.39 (22.71, 24.09) | 28.75 (28.16, 29.35) | 0.64 (0.51, 0.77) | <0.001 | 283.85 (281.48, 286.23) | 306.36 (304.44, 308.28) | 0.41 (0.31, 0.52) | <0.001 | 829.18 (825.10, 833.27) | 644.75 (641.96, 647.55) | -1.07 (-1.34, -0.79) | <0.001 | 13.70 (13.18, 14.24) | 10.51 (10.16, 10.87) | -1.14 (-1.43, -0.85) | <0.001 |
| Barbados | 30.04 (17.16, 48.90) | 24.87 (13.08, 43.22) | -0.81 (-0.92, -0.69) | <0.001 | 418.42 (365.43, 477.03) | 395.19 (341.88, 454.64) | -0.37 (-0.45, -0.29) | <0.001 | 335.91 (288.54, 388.94) | 210.21 (172.04, 254.54) | -2.29 (-2.64, -1.94) | <0.001 | 5.30 (1.02, 16.13) | 3.10 (0.25, 13.41) | -2.51 (-2.90, -2.11) | <0.001 |
| Belarus | 36.98 | 30.98 | -0.12 | 0.450 | 284.90 | 260.12 | -0.08 | 0.219 | 351.80 | 282.02 | -1.04 | 0.013 | 5.33 | 4.18 | -1.12 | 0.021 |

| | | | | | | | | | | | | | | | | |
|---|---|---|---|---|---|---|---|---|---|---|---|---|---|---|---|---|
| | (34.41, 39.70) | (28.41, 33.76) | (-0.46, 0.21) | | (277.69, 292.27) | (252.59, 267.85) | (-0.22, 0.05) | | (343.79, 359.96) | (274.31, 289.94) | (-1.84, -0.24) | | (4.39, 6.43) | (3.30, 5.27) | (-2.04, -0.19) | |
| Belgium | 16.02 (14.29, 17.90) | 12.11 (10.57, 13.83) | -1.26 (-1.41, -1.12) | <0.001 | 191.12 (185.05, 197.35) | 182.37 (176.26, 188.66) | -0.07 (-0.21, 0.07) | 0.320 | 229.63 (222.94, 236.48) | 78.38 (74.39, 82.54) | -3.84 (-4.11, -3.57) | <0.001 | 3.42 (2.65, 4.36) | 0.88 (0.51, 1.44) | -4.91 (-5.19, -4.64) | <0.001 |
| Belize | 23.39 (9.62, 48.76) | 24.99 (15.35, 38.58) | 0.17 (0.06, 0.28) | 0.004 | 349.79 (288.50, 421.22) | 366.87 (326.98, 410.43) | 0.16 (0.12, 0.19) | <0.001 | 242.47 (192.06, 303.08) | 280.73 (245.68, 319.49) | -0.75 (-1.21, -0.28) | 0.003 | 3.62 (0.14, 20.36) | 4.34 (1.06, 11.97) | -0.85 (-1.36, -0.34) | 0.002 |
| Benin | 40.04 (35.42, 45.12) | 32.85 (30.39, 35.46) | -0.79 (-0.83, -0.75) | <0.001 | 457.03 (441.18, 473.34) | 423.29 (414.44, 432.30) | -0.17 (-0.30, -0.05) | 0.007 | 443.54 (427.83, 459.71) | 370.49 (362.15, 378.98) | -0.79 (-0.92, -0.67) | <0.001 | 6.75 (4.92, 9.05) | 5.52 (4.53, 6.67) | -0.90 (-1.04, -0.75) | <0.001 |
| Bermuda | 25.12 (5.86, 77.26) | 21.35 (2.71, 85.59) | -0.69 (-0.82, -0.57) | <0.001 | 382.85 (284.09, 509.09) | 355.25 (244.01, 504.43) | -0.26 (-0.30, -0.21) | <0.001 | 193.43 (125.92, 289.10) | 95.48 (43.60, 187.81) | -2.69 (-2.94, -2.45) | <0.001 | 2.78 (0.00, 44.26) | 1.14 (0.00, 54.08) | -3.54 (-3.85, -3.22) | <0.001 |
| Bhutan | 24.58 (16.35, 35.80) | 20.12 (14.17, 27.82) | -0.84 (-0.92, -0.76) | <0.001 | 286.26 (256.48, 318.81) | 268.59 (245.50, 293.35) | -0.16 (-0.28, -0.03) | 0.018 | 258.90 (230.20, 290.43) | 172.17 (153.76, 192.26) | -1.65 (-1.75, -1.54) | <0.001 | 3.81 (1.08, 9.92) | 2.27 (0.64, 5.86) | -2.05 (-2.18, -1.92) | <0.001 |
| Bolivia (Plurinational State of) | 42.87 (39.10, 46.92) | 30.87 (28.68, 33.20) | -1.33 (-1.48, -1.17) | <0.001 | 452.44 (439.94, 465.21) | 399.99 (391.97, 408.14) | -0.42 (-0.50, -0.34) | <0.001 | 571.02 (557.14, 585.17) | 296.06 (289.20, 303.05) | -2.32 (-2.40, -2.24) | <0.001 | 9.03 (7.34, 10.99) | 4.39 (3.59, 5.33) | -2.53 (-2.60, -2.46) | <0.001 |
| Bosnia and Herzegovina | 32.89 (29.45, 36.63) | 33.80 (29.12, 39.06) | 0.35 (0.25, 0.46) | <0.001 | 275.61 (265.48, 286.03) | 290.07 (276.15, 304.56) | 0.29 (0.25, 0.34) | <0.001 | 357.14 (345.59, 368.99) | 246.59 (233.64, 260.13) | -2.00 (-2.38, -1.62) | <0.001 | 5.33 (4.00, 6.97) | 3.28 (1.95, 5.24) | -2.58 (-3.00, -2.15) | <0.001 |
| Botswana | 45.45 (36.50, 56.03) | 26.11 (21.96, 30.83) | -2.86 (-3.39, -2.32) | <0.001 | 455.80 (426.82, 486.36) | 395.68 (378.96, 412.97) | -0.79 (-0.94, -0.64) | <0.001 | 514.96 (484.20, 547.27) | 347.25 (331.61, 363.46) | -3.08 (-3.87, -2.28) | <0.001 | 7.91 (4.49, 13.06) | 5.15 (3.41, 7.48) | -3.40 (-4.27, -2.52) | <0.001 |
| Brazil | 47.68 (46.87, 48.49) | 25.71 (25.24, 26.19) | -2.51 (-2.79, -2.23) | <0.001 | 491.75 (489.20, 494.32) | 378.45 (376.63, 380.28) | -1.08 (-1.31, -0.85) | <0.001 | 635.81 (632.87, 638.76) | 233.46 (232.04, 234.89) | -3.55 (-3.83, -3.27) | <0.001 | 10.59 (10.21, 10.98) | 3.52 (3.34, 3.69) | -3.92 (-4.20, -3.64) | <0.001 |
| Brunei Darussalam | 35.59 (22.71, 53.48) | 21.21 (13.68, 31.79) | -2.17 (-2.38, -1.96) | <0.001 | 283.65 (244.60, 327.44) | 236.45 (209.12, 266.66) | -0.77 (-0.95, -0.58) | <0.001 | 437.87 (388.93, 491.51) | 167.19 (144.51, 192.74) | -4.34 (-4.78, -3.91) | <0.001 | 6.74 (2.01, 16.94) | 2.25 (0.40, 7.68) | -5.01 (-5.49, -4.52) | <0.001 |
| Bulgaria | 41.15 | 31.62 | -1.04 | <0.001 | 324.26 | 290.61 | -0.42 | <0.001 | 636.03 | 402.28 | -2.30 | <0.001 | 10.16 | 6.16 | -2.54 | <0.001 |

| | | | | | | | | | | | | | | | | |
|---|---|---|---|---|---|---|---|---|---|---|---|---|---|---|---|---|
| | (38.04, | (28.45, | (-1.13, | | (315.38, | (280.78, | (-0.54, | | (623.58, | (390.70, | (-2.57, | | (8.65, | (4.82, | (-2.84, | |
| | 44.47) | 35.10) | -0.96) | | 333.33) | 300.75) | -0.30) | | 648.68) | 414.15) | -2.03) | | 11.87) | 7.81) | -2.23) | |
| | 27.82 | 27.32 | -0.05 | | 347.39 | 339.95 | 0.01 | | 339.03 | 353.53 | 0.19 | | 5.13 | 5.37 | 0.20 | |
| Burkina Faso | (24.91, | (25.62, | (-0.14, | 0.291 | (336.93, | (333.91, | (-0.09, | 0.877 | (328.61, | (347.35, | (0.05, | 0.008 | (3.91, | (4.62, | (0.05, | 0.011 |
| | 31.01) | 29.11) | 0.04) | | 358.10) | 346.07) | 0.10) | | 349.71) | 359.81) | 0.33) | | 6.63) | 6.20) | 0.35) | |
| | 53.91 | 27.83 | -2.66 | | 489.93 | 389.13 | -0.83 | | 909.18 | 489.33 | -2.46 | | 14.40 | 7.47 | -2.60 | |
| Burundi | (49.19, | (25.60, | (-2.95, | <0.001 | (475.63, | (380.73, | (-0.98, | <0.001 | (889.86, | (479.94, | (-2.75, | <0.001 | (12.04, | (6.35, | (-2.91, | <0.001 |
| | 58.98) | 30.20) | -2.37) | | 504.58) | 397.67) | -0.68) | | 928.84) | 498.87) | -2.16) | | 17.12) | 8.75) | -2.30) | |
| | 45.54 | 33.26 | -1.18 | | 480.09 | 436.25 | -0.25 | | 656.78 | 308.94 | -2.28 | | 10.48 | 4.33 | -2.58 | |
| Cabo Verde | (27.75, | (24.11, | (-1.25, | <0.001 | (417.89, | (401.15, | (-0.30, | <0.001 | (582.57, | (279.51, | (-2.46, | <0.001 | (3.07, | (1.53, | (-2.82, | <0.001 |
| | 72.05) | 44.87) | -1.10) | | 550.19) | 473.71) | -0.20) | | 738.95) | 340.74) | -2.10) | | 27.81) | 9.84) | -2.34) | |
| | 38.03 | 30.05 | -0.91 | | 331.70 | 315.85 | -0.10 | | 537.35 | 350.96 | -1.58 | | 8.41 | 5.11 | -1.83 | |
| Cambodia | (34.90, | (28.26, | (-0.96, | <0.001 | (322.48, | (309.99, | (-0.25, | 0.157 | (525.42, | (344.76, | (-1.67, | <0.001 | (6.95, | (4.39, | (-1.95, | <0.001 |
| | 41.39) | 31.93) | -0.85) | | 341.13) | 321.78) | 0.04) | | 549.50) | 357.24) | -1.48) | | 10.09) | 5.93) | -1.71) | |
| | 35.06 | 35.70 | -0.18 | | 393.25 | 402.65 | 0.04 | | 470.92 | 468.59 | -0.29 | | 7.29 | 7.23 | -0.33 | |
| Cameroon | (32.19, | (34.10, | (-0.47, | 0.229 | (383.50, | (397.29, | (-0.05, | 0.429 | (460.23, | (462.79, | (-0.55, | 0.028 | (6.01, | (6.52, | (-0.61, | 0.020 |
| | 38.12) | 37.35) | 0.12) | | 403.20) | 408.07) | 0.12) | | 481.81) | 474.45) | -0.03) | | 8.79) | 8.00) | -0.06) | |
| | 13.34 | 11.41 | -0.60 | | 366.95 | 315.04 | -0.41 | | 154.17 | 89.00 | -1.84 | | 1.80 | 0.77 | -2.86 | |
| Canada | (12.42, | (10.57, | (-0.75, | <0.001 | (362.00, | (310.53, | (-0.76, | 0.023 | (150.99, | (86.63, | (-2.06, | <0.001 | (1.47, | (0.57, | (-3.13, | <0.001 |
| | 14.32) | 12.31) | -0.45) | | 371.96) | 319.60) | -0.06) | | 157.40) | 91.42) | -1.62) | | 2.18) | 1.03) | -2.59) | |
| | 45.11 | 40.75 | -0.49 | | 384.48 | 358.34 | -0.25 | | 655.63 | 575.33 | -0.60 | | 10.35 | 9.08 | -0.61 | |
| Central African Republic | (38.94, | (36.57, | (-0.54, | <0.001 | (366.43, | (345.98, | (-0.30, | <0.001 | (632.27, | (559.76, | (-0.73, | <0.001 | (7.57, | (7.20, | (-0.75, | <0.001 |
| | 52.02) | 45.29) | -0.43) | | 403.25) | 371.05) | -0.19) | | 679.68) | 591.25) | -0.46) | | 13.88) | 11.32) | -0.47) | |
| | 36.60 | 36.36 | -0.18 | | 399.96 | 410.75 | 0.12 | | 484.29 | 483.07 | -0.12 | | 7.48 | 7.44 | -0.16 | |
| Chad | (32.63, | (33.89, | (-0.34, | 0.035 | (386.60, | (402.41, | (0.07, | <0.001 | (469.62, | (474.05, | (-0.33, | 0.216 | (5.74, | (6.34, | (-0.37, | 0.150 |
| | 40.95) | 38.97) | -0.01) | | 413.67) | 419.22) | 0.17) | | 499.33) | 492.24) | 0.08) | | 9.61) | 8.68) | 0.06) | |
| | 20.61 | 17.64 | -0.34 | | 225.00 | 205.82 | -0.14 | | 251.19 | 150.95 | -1.42 | | 3.71 | 2.05 | -1.64 | |
| Chile | (18.93, | (16.28, | (-0.57, | 0.006 | (219.35, | (201.11, | (-0.30, | 0.107 | (245.25, | (146.91, | (-1.57, | <0.001 | (3.02, | (1.60, | (-1.82, | <0.001 |
| | 22.40) | 19.10) | -0.11) | | 230.76) | 210.63) | 0.03) | | 257.24) | 155.09) | -1.27) | | 4.52) | 2.59) | -1.46) | |
| | 29.38 | 26.99 | -0.71 | | 299.64 | 268.31 | -0.51 | | 565.58 | 428.15 | -0.98 | | 8.81 | 6.58 | -1.01 | |
| China | (29.18, | (26.79, | (-0.88, | <0.001 | (298.99, | (267.69, | (-0.77, | <0.001 | (564.68, | (427.37, | (-1.13, | <0.001 | (8.70, | (6.49, | (-1.16, | <0.001 |
| | 29.59) | 27.19) | -0.54) | | 300.30) | 268.93) | -0.26) | | 566.48) | 428.93) | -0.82) | | 8.93) | 6.68) | -0.86) | |
| Colombia | 26.09 | 18.78 | -1.35 | <0.001 | 353.22 | 310.44 | -0.40 | <0.001 | 326.18 | 166.38 | -2.31 | <0.001 | 5.07 | 2.39 | -2.57 | <0.001 |

| | | | | | | | | | | | | | | | | |
|---|---|---|---|---|---|---|---|---|---|---|---|---|---|---|---|---|
| | (24.84, 27.38) | (17.91, 19.67) | (-1.55, -1.15) | | (348.64, 357.86) | (306.90, 314.00) | (-0.53, -0.27) | | (321.77, 330.65) | (163.79, 168.99) | (-2.58, -2.05) | | (4.53, 5.67) | (2.09, 2.73) | (-2.86, -2.29) | |
| Comoros | 49.92 (34.42, 70.44) | 27.67 (19.69, 37.87) | -2.32 (-2.59, -2.06) | <0.001 | 478.75 (428.37, 533.83) | 388.68 (357.03, 422.46) | -0.75 (-0.89, -0.62) | <0.001 | 510.47 (458.91, 566.67) | 323.06 (294.30, 353.94) | -2.13 (-2.79, -1.46) | <0.001 | 7.72 (2.60, 18.35) | 4.68 (1.83, 9.93) | -2.29 (-3.01, -1.56) | <0.001 |
| Congo | 45.79 (39.21, 53.23) | 23.10 (20.21, 26.29) | -2.79 (-3.06, -2.51) | <0.001 | 438.32 (417.82, 459.63) | 344.52 (333.11, 356.23) | -0.88 (-1.03, -0.74) | <0.001 | 699.20 (673.39, 725.80) | 309.34 (298.51, 320.46) | -3.16 (-3.49, -2.82) | <0.001 | 11.09 (8.02, 15.02) | 4.55 (3.32, 6.09) | -3.44 (-3.79, -3.09) | <0.001 |
| Cook Islands | 54.29 (6.49, 210.80) | 52.79 (3.33, 238.66) | -0.26 (-0.44, -0.08) | 0.007 | 540.85 (329.07, 846.07) | 524.73 (287.19, 882.27) | -0.17 (-0.24, -0.09) | <0.001 | 764.59 (508.96, 1111.74) | 594.56 (339.06, 969.60) | -0.83 (-1.22, -0.43) | <0.001 | 11.52 (0.00, 141.64) | 8.71 (0.00, 161.13) | -0.94 (-1.42, -0.47) | <0.001 |
| Costa Rica | 22.09 (18.49, 26.23) | 20.29 (17.52, 23.40) | -0.29 (-0.46, -0.12) | 0.001 | 339.03 (324.54, 354.03) | 330.41 (318.89, 342.25) | -0.03 (-0.08, 0.02) | 0.234 | 190.32 (179.42, 201.75) | 144.96 (137.38, 152.86) | -1.03 (-1.47, -0.59) | <0.001 | 2.80 (1.61, 4.55) | 2.01 (1.21, 3.16) | -1.27 (-1.80, -0.74) | <0.001 |
| Cote d'Ivoire | 49.38 (46.45, 52.47) | 37.21 (35.57, 38.91) | -1.29 (-1.44, -1.14) | <0.001 | 506.12 (496.64, 515.75) | 442.15 (436.45, 447.91) | -0.57 (-0.64, -0.50) | <0.001 | 614.63 (604.26, 625.15) | 449.27 (443.53, 455.07) | -1.56 (-1.82, -1.30) | <0.001 | 9.48 (8.22, 10.89) | 6.78 (6.08, 7.53) | -1.67 (-1.95, -1.39) | <0.001 |
| Croatia | 25.17 (22.14, 28.54) | 17.36 (14.43, 20.78) | -1.70 (-1.95, -1.45) | <0.001 | 231.03 (221.66, 240.71) | 183.62 (173.88, 193.82) | -1.04 (-1.22, -0.86) | <0.001 | 341.69 (330.31, 353.39) | 124.66 (116.61, 133.18) | -4.06 (-4.31, -3.81) | <0.001 | 5.29 (3.96, 6.94) | 1.51 (0.76, 2.81) | -4.99 (-5.29, -4.70) | <0.001 |
| Cuba | 29.65 (27.40, 32.04) | 23.90 (21.76, 26.21) | -0.83 (-0.93, -0.74) | <0.001 | 398.83 (390.62, 407.17) | 350.56 (342.17, 359.11) | -0.45 (-0.54, -0.36) | <0.001 | 298.30 (291.10, 305.64) | 161.39 (155.75, 167.20) | -2.38 (-2.62, -2.14) | <0.001 | 4.65 (3.78, 5.66) | 2.32 (1.69, 3.12) | -2.71 (-2.98, -2.44) | <0.001 |
| Cyprus | 14.66 (9.32, 22.03) | 13.54 (9.48, 19.17) | -0.50 (-0.60, -0.41) | <0.001 | 187.45 (166.82, 210.00) | 199.68 (183.09, 217.67) | 0.26 (0.14, 0.38) | <0.001 | 121.94 (105.34, 140.48) | 82.32 (71.71, 94.36) | -2.23 (-2.62, -1.85) | <0.001 | 1.60 (0.27, 5.23) | 0.91 (0.15, 3.67) | -3.07 (-3.64, -2.50) | <0.001 |
| Czechia | 28.16 (25.87, 30.62) | 20.55 (18.45, 22.87) | -1.40 (-1.63, -1.17) | <0.001 | 259.42 (252.33, 266.66) | 208.55 (201.85, 215.45) | -0.83 (-0.97, -0.68) | <0.001 | 284.61 (277.19, 292.19) | 101.47 (96.76, 106.37) | -3.97 (-4.20, -3.75) | <0.001 | 4.13 (3.29, 5.14) | 1.03 (0.62, 1.67) | -5.26 (-5.49, -5.04) | <0.001 |
| Democratic People's Republic of Korea | 47.57 (45.39, 49.82) | 46.75 (44.97, 48.60) | -0.09 (-0.22, 0.03) | 0.143 | 357.81 (351.80, 363.90) | 354.99 (350.04, 359.99) | -0.01 (-0.04, 0.03) | 0.734 | 707.93 (699.47, 716.48) | 681.32 (674.44, 688.26) | -0.05 (-0.14, 0.04) | 0.265 | 11.11 (10.07, 12.24) | 10.63 (9.79, 11.53) | -0.08 (-0.17, 0.02) | 0.116 |
| Democratic | 28.72 | 23.72 | -0.77 | <0.001 | 316.85 | 287.46 | -0.36 | <0.001 | 405.40 | 317.98 | -0.81 | <0.001 | 6.26 | 4.82 | -0.86 | <0.001 |

| | | | | | | | | | | | | | | | | |
|---|---|---|---|---|---|---|---|---|---|---|---|---|---|---|---|---|
| Republic of the Congo | (27.38, 30.11) | (22.96, 24.51) | (-0.84, -0.70) | | (312.42, 321.33) | (284.82, 290.11) | (-0.45, -0.27) | | (400.40, 410.45) | (315.20, 320.77) | (-0.89, -0.72) | | (5.65, 6.93) | (4.48, 5.18) | (-0.95, -0.77) | |
| Denmark | 17.72 (15.21, 20.55) | 12.20 (10.05, 14.68) | -1.40 (-1.54, -1.27) | <0.001 | 232.54 (223.18, 242.22) | 196.54 (187.63, 205.79) | -0.59 (-0.67, -0.51) | <0.001 | 187.26 (178.90, 195.92) | 71.57 (66.25, 77.23) | -3.77 (-4.02, -3.52) | <0.001 | 2.73 (1.81, 3.98) | 0.75 (0.30, 1.58) | -4.98 (-5.33, -4.62) | <0.001 |
| Djibouti | 40.16 (28.21, 55.77) | 29.19 (22.99, 36.55) | -1.25 (-1.39, -1.10) | <0.001 | 439.92 (398.39, 484.89) | 410.10 (385.88, 435.46) | -0.19 (-0.30, -0.08) | 0.001 | 525.30 (480.21, 573.78) | 392.99 (369.30, 417.82) | -1.14 (-1.29, -0.99) | <0.001 | 8.04 (3.35, 16.68) | 5.82 (3.27, 9.59) | -1.26 (-1.42, -1.10) | <0.001 |
| Dominica | 26.86 (6.59, 74.52) | 24.00 (5.19, 68.96) | -0.48 (-0.54, -0.41) | <0.001 | 380.65 (284.85, 500.00) | 369.84 (273.14, 489.88) | -0.04 (-0.10, 0.02) | 0.237 | 263.63 (183.92, 367.61) | 198.09 (129.25, 290.71) | -1.54 (-1.80, -1.28) | <0.001 | 4.07 (0.01, 38.91) | 2.94 (0.00, 34.58) | -1.79 (-2.12, -1.46) | <0.001 |
| Dominican Republic | 31.19 (28.17, 34.47) | 32.34 (30.02, 34.79) | 0.04 (-0.03, 0.10) | 0.254 | 367.14 (356.82, 377.70) | 376.87 (368.89, 384.99) | 0.12 (0.06, 0.19) | <0.001 | 413.77 (402.73, 425.06) | 387.65 (379.54, 395.89) | -0.11 (-0.54, 0.33) | 0.625 | 6.66 (5.31, 8.26) | 6.25 (5.25, 7.38) | -0.09 (-0.54, 0.37) | 0.699 |
| Ecuador | 40.17 (37.33, 43.17) | 30.63 (28.83, 32.53) | -1.02 (-1.13, -0.90) | <0.001 | 455.98 (446.25, 465.87) | 417.04 (410.29, 423.87) | -0.31 (-0.34, -0.29) | <0.001 | 587.88 (576.98, 598.95) | 370.39 (364.07, 376.80) | -1.87 (-2.12, -1.61) | <0.001 | 9.28 (7.94, 10.78) | 5.65 (4.89, 6.50) | -2.01 (-2.28, -1.74) | <0.001 |
| Egypt | 32.16 (31.07, 33.29) | 32.04 (31.27, 32.82) | 0.15 (0.02, 0.28) | 0.026 | 380.03 (376.27, 383.82) | 399.76 (397.05, 402.49) | 0.41 (0.30, 0.53) | <0.001 | 1184.40 (1177.96, 1190.86) | 818.94 (815.08, 822.82) | -0.71 (-1.06, -0.36) | <0.001 | 18.47 (17.67, 19.30) | 12.37 (11.90, 12.86) | -0.83 (-1.20, -0.47) | <0.001 |
| El Salvador | 26.01 (22.71, 29.67) | 19.41 (16.91, 22.19) | -1.24 (-1.51, -0.97) | <0.001 | 316.32 (304.60, 328.42) | 300.51 (290.51, 310.77) | -0.20 (-0.29, -0.10) | <0.001 | 811.96 (793.46, 830.81) | 249.04 (239.91, 258.45) | -4.59 (-5.53, -3.64) | <0.001 | 13.09 (10.81, 15.74) | 3.80 (2.74, 5.16) | -4.88 (-5.87, -3.88) | <0.001 |
| Equatorial Guinea | 45.83 (29.99, 67.41) | 20.87 (15.96, 26.96) | -2.95 (-3.32, -2.57) | <0.001 | 409.91 (359.74, 465.45) | 332.87 (312.54, 354.30) | -0.73 (-0.91, -0.56) | <0.001 | 634.22 (572.29, 701.40) | 216.26 (199.99, 233.64) | -4.13 (-4.59, -3.67) | <0.001 | 9.96 (3.62, 22.40) | 2.96 (1.33, 5.90) | -4.68 (-5.15, -4.19) | <0.001 |
| Eritrea | 53.43 (47.05, 60.49) | 28.59 (25.77, 31.65) | -2.50 (-2.64, -2.36) | <0.001 | 426.90 (408.79, 445.66) | 353.77 (343.76, 364.01) | -0.75 (-0.86, -0.65) | <0.001 | 758.12 (734.22, 782.65) | 542.99 (530.58, 555.62) | -1.50 (-1.65, -1.34) | <0.001 | 12.06 (9.19, 15.62) | 8.53 (7.03, 10.28) | -1.56 (-1.72, -1.40) | <0.001 |
| Estonia | 37.60 (30.99, 45.28) | 25.34 (19.20, 33.16) | -1.86 (-2.09, -1.63) | <0.001 | 258.65 (240.81, 277.52) | 217.77 (199.22, 237.88) | -0.83 (-0.94, -0.71) | <0.001 | 376.16 (354.52, 398.83) | 111.50 (98.28, 126.31) | -5.45 (-6.20, -4.68) | <0.001 | 5.76 (3.38, 9.26) | 1.20 (0.24, 4.27) | -6.24 (-7.33, -5.13) | <0.001 |

| | | | | | | | | | | | | | | | | |
|---|---|---|---|---|---|---|---|---|---|---|---|---|---|---|---|---|
| Eswatini | 30.77 (21.25, 43.34) | 34.63 (27.35, 43.31) | 0.17 (-0.48, 0.83) | 0.598 | 384.82 (349.23, 423.26) | 390.96 (365.71, 417.55) | 0.02 (-0.13, 0.17) | 0.761 | 359.13 (324.84, 396.28) | 432.94 (406.30, 460.92) | 0.33 (-0.51, 1.17) | 0.431 | 5.35 (1.92, 12.13) | 6.65 (3.71, 11.06) | 0.26 (-0.66, 1.19) | 0.570 |
| Ethiopia | 33.08 (31.80, 34.40) | 21.91 (21.25, 22.59) | -1.67 (-1.86, -1.47) | <0.001 | 361.15 (356.92, 365.42) | 346.98 (344.35, 349.62) | -0.11 (-0.27, 0.05) | 0.162 | 604.07 (598.66, 609.53) | 290.88 (288.48, 293.30) | -2.85 (-2.99, -2.71) | <0.001 | 9.51 (8.83, 10.23) | 4.20 (3.91, 4.50) | -3.17 (-3.31, -3.03) | <0.001 |
| Fiji | 48.14 (37.81, 60.53) | 43.39 (34.43, 54.00) | -0.52 (-0.60, -0.44) | <0.001 | 521.30 (485.80, 558.81) | 498.24 (466.62, 531.46) | -0.20 (-0.29, -0.12) | <0.001 | 846.45 (801.28, 893.60) | 600.84 (566.03, 637.25) | -1.37 (-1.61, -1.13) | <0.001 | 12.89 (7.85, 20.08) | 8.75 (5.02, 14.19) | -1.54 (-1.81, -1.27) | <0.001 |
| Finland | 21.74 (18.93, 24.90) | 17.54 (14.92, 20.53) | -0.85 (-1.06, -0.64) | <0.001 | 251.18 (241.36, 261.35) | 222.77 (213.15, 232.75) | -0.37 (-0.53, -0.20) | <0.001 | 347.68 (336.33, 359.37) | 103.50 (97.04, 110.31) | -4.21 (-4.55, -3.87) | <0.001 | 5.57 (4.23, 7.26) | 1.27 (0.66, 2.28) | -5.20 (-5.56, -4.85) | <0.001 |
| France | 15.18 (14.47, 15.91) | 12.28 (11.61, 12.98) | -0.71 (-0.80, -0.62) | <0.001 | 198.37 (195.78, 200.98) | 159.00 (156.57, 161.47) | -0.70 (-0.84, -0.57) | <0.001 | 204.33 (201.71, 206.97) | 93.45 (91.59, 95.35) | -2.96 (-3.10, -2.82) | <0.001 | 3.05 (2.74, 3.39) | 1.18 (0.98, 1.41) | -3.62 (-3.76, -3.47) | <0.001 |
| Gabon | 34.66 (26.54, 44.55) | 24.40 (19.37, 30.36) | -1.39 (-1.47, -1.31) | <0.001 | 383.64 (355.59, 413.39) | 339.39 (319.99, 359.70) | -0.48 (-0.56, -0.41) | <0.001 | 490.75 (459.09, 524.08) | 300.96 (282.76, 320.06) | -1.83 (-1.93, -1.72) | <0.001 | 7.56 (4.09, 12.88) | 4.39 (2.44, 7.32) | -2.02 (-2.14, -1.91) | <0.001 |
| Gambia | 40.16 (30.84, 51.59) | 38.53 (32.63, 45.23) | -0.01 (-0.08, 0.06) | 0.842 | 470.60 (437.36, 505.85) | 463.19 (442.29, 484.88) | 0.08 (0.02, 0.15) | 0.017 | 486.21 (452.50, 521.93) | 462.79 (441.98, 484.38) | -0.22 (-0.48, 0.04) | 0.098 | 7.37 (3.74, 13.27) | 6.94 (4.59, 10.14) | -0.28 (-0.58, 0.01) | 0.061 |
| Georgia | 51.84 (47.58, 56.38) | 38.84 (34.21, 44.00) | -0.72 (-1.16, -0.27) | 0.003 | 383.23 (371.48, 395.25) | 335.64 (321.71, 350.08) | -0.51 (-0.68, -0.33) | <0.001 | 792.47 (775.55, 809.67) | 653.65 (634.22, 673.58) | -0.98 (-2.08, 0.13) | 0.080 | 12.64 (10.59, 14.99) | 10.56 (8.23, 13.41) | -0.87 (-2.05, 0.31) | 0.142 |
| Germany | 16.97 (16.33, 17.63) | 13.35 (12.75, 13.97) | -0.99 (-1.09, -0.89) | <0.001 | 222.69 (220.35, 225.04) | 186.39 (184.14, 188.67) | -0.64 (-0.78, -0.50) | <0.001 | 252.97 (250.48, 255.49) | 86.92 (85.39, 88.48) | -4.28 (-4.78, -3.78) | <0.001 | 3.79 (3.50, 4.11) | 1.01 (0.85, 1.19) | -5.41 (-5.96, -4.86) | <0.001 |
| Ghana | 32.49 (30.29, 34.81) | 39.41 (37.84, 41.03) | 0.56 (0.30, 0.83) | <0.001 | 425.08 (417.01, 433.26) | 450.55 (445.21, 455.93) | 0.25 (0.19, 0.31) | <0.001 | 441.87 (433.63, 450.23) | 440.10 (434.83, 445.41) | 0.01 (-0.36, 0.37) | 0.977 | 6.70 (5.71, 7.81) | 6.62 (5.99, 7.31) | -0.03 (-0.43, 0.39) | 0.902 |
| Greece | 20.17 (18.20, 22.30) | 13.86 (12.06, 15.87) | -1.39 (-1.47, -1.30) | <0.001 | 231.17 (224.39, 238.10) | 204.54 (197.47, 211.82) | -0.53 (-0.62, -0.43) | <0.001 | 272.16 (264.79, 279.68) | 141.71 (135.85, 147.77) | -2.69 (-2.89, -2.49) | <0.001 | 4.06 (3.20, 5.07) | 1.94 (1.32, 2.79) | -3.04 (-3.28, -2.80) | <0.001 |

| | | | | | | | | | | | | | | | | |
|---|---|---|---|---|---|---|---|---|---|---|---|---|---|---|---|---|
| Greenland | 25.71 (6.53, 78.41) | 15.44 (1.33, 70.14) | -2.12 (-2.25, -1.98) | <0.001 | 511.21 (400.78, 648.35) | 421.32 (307.05, 568.06) | -0.62 (-0.85, -0.39) | <0.001 | 416.30 (318.38, 540.90) | 191.17 (117.87, 297.79) | -2.92 (-3.06, -2.78) | <0.001 | 6.29 (0.12, 50.88) | 2.44 (0.00, 49.36) | -3.47 (-3.67, -3.28) | <0.001 |
| Grenada | 36.57 (12.52, 84.47) | 28.97 (10.22, 65.56) | -0.91 (-1.05, -0.77) | <0.001 | 448.78 (349.41, 569.08) | 423.25 (338.30, 523.96) | -0.17 (-0.24, -0.10) | <0.001 | 533.74 (422.57, 666.26) | 246.02 (181.69, 326.54) | -2.63 (-2.92, -2.34) | <0.001 | 8.87 (0.46, 43.68) | 3.73 (0.03, 27.78) | -3.02 (-3.33, -2.71) | <0.001 |
| Guam | 38.73 (20.66, 66.70) | 49.80 (27.96, 82.37) | 0.90 (0.83, 0.96) | <0.001 | 453.26 (384.46, 531.26) | 517.01 (439.47, 604.60) | 0.51 (0.47, 0.55) | <0.001 | 453.13 (384.33, 531.12) | 608.13 (523.61, 702.73) | 0.86 (0.61, 1.12) | <0.001 | 6.27 (0.83, 22.81) | 9.02 (1.66, 28.22) | 1.05 (0.73, 1.37) | <0.001 |
| Guatemala | 37.84 (34.53, 41.40) | 22.39 (20.85, 24.02) | -2.25 (-2.43, -2.08) | <0.001 | 371.53 (360.98, 382.33) | 321.82 (315.95, 327.78) | -0.62 (-0.72, -0.53) | <0.001 | 513.78 (501.50, 526.29) | 333.06 (327.06, 339.15) | -3.26 (-3.88, -2.63) | <0.001 | 8.11 (6.62, 9.85) | 5.24 (4.50, 6.06) | -3.37 (-4.03, -2.72) | <0.001 |
| Guinea | 33.93 (30.21, 38.01) | 38.04 (35.31, 40.95) | 0.57 (0.50, 0.65) | <0.001 | 352.79 (340.56, 365.36) | 382.44 (373.68, 391.36) | 0.45 (0.38, 0.52) | <0.001 | 423.76 (410.40, 437.45) | 463.31 (453.73, 473.06) | 0.60 (0.46, 0.74) | <0.001 | 6.50 (4.93, 8.43) | 7.13 (5.97, 8.45) | 0.62 (0.46, 0.77) | <0.001 |
| Guinea-Bissau | 53.91 (42.60, 67.46) | 47.63 (40.50, 55.72) | -0.43 (-0.46, -0.39) | <0.001 | 499.60 (464.10, 537.25) | 472.64 (449.76, 496.45) | -0.10 (-0.17, -0.03) | 0.004 | 763.31 (719.70, 809.04) | 648.08 (621.36, 675.72) | -0.35 (-0.44, -0.26) | <0.001 | 12.05 (7.10, 19.33) | 10.13 (7.02, 14.24) | -0.37 (-0.47, -0.27) | <0.001 |
| Guyana | 51.58 (40.57, 64.76) | 34.53 (25.55, 45.71) | -1.72 (-1.91, -1.54) | <0.001 | 541.31 (504.93, 579.75) | 462.09 (428.10, 498.16) | -0.60 (-0.72, -0.48) | <0.001 | 986.15 (936.40, 1037.97) | 579.73 (541.28, 620.27) | -2.06 (-2.47, -1.65) | <0.001 | 16.43 (10.54, 24.56) | 9.46 (5.11, 16.10) | -2.16 (-2.59, -1.73) | <0.001 |
| Haiti | 39.99 (36.22, 44.04) | 31.35 (29.15, 33.68) | -0.88 (-1.06, -0.69) | <0.001 | 399.93 (388.03, 412.11) | 382.34 (374.63, 390.17) | -0.08 (-0.21, 0.05) | 0.221 | 603.50 (588.74, 618.55) | 418.90 (410.76, 427.17) | -0.96 (-1.34, -0.58) | <0.001 | 10.02 (8.19, 12.15) | 6.83 (5.83, 7.97) | -1.02 (-1.41, -0.62) | <0.001 |
| Honduras | 21.54 (18.31, 25.20) | 18.47 (16.56, 20.56) | -0.56 (-0.77, -0.36) | <0.001 | 298.02 (285.78, 310.68) | 307.38 (299.45, 315.47) | 0.20 (0.12, 0.28) | <0.001 | 539.49 (522.85, 556.55) | 247.33 (240.16, 254.68) | -3.03 (-3.53, -2.53) | <0.001 | 8.83 (6.80, 11.30) | 3.80 (2.95, 4.83) | -3.25 (-3.81, -2.70) | <0.001 |
| Hungary | 41.52 (38.76, 44.45) | 22.13 (19.84, 24.63) | -2.63 (-2.78, -2.48) | <0.001 | 337.70 (329.65, 345.90) | 244.52 (236.84, 252.41) | -1.15 (-1.33, -0.97) | <0.001 | 510.27 (500.51, 520.19) | 150.67 (144.70, 156.86) | -5.19 (-5.53, -4.85) | <0.001 | 8.08 (6.91, 9.42) | 1.84 (1.24, 2.66) | -6.29 (-6.68, -5.90) | <0.001 |
| Iceland | 14.80 (6.36, 29.53) | 12.05 (5.11, 24.90) | -1.02 (-1.21, -0.83) | <0.001 | 183.87 (149.41, 224.10) | 163.61 (133.93, 198.51) | -0.46 (-0.62, -0.29) | <0.001 | 170.50 (137.25, 209.55) | 68.09 (49.42, 92.14) | -3.50 (-3.77, -3.23) | <0.001 | 2.38 (0.12, 11.73) | 0.72 (0.00, 8.69) | -4.69 (-5.00, -4.38) | <0.001 |

| | | | | | | | | | | | | | | | | |
|---|---|---|---|---|---|---|---|---|---|---|---|---|---|---|---|---|
| India | 23.58 (23.35, 23.81) | 23.71 (23.54, 23.89) | 0.01 (-0.10, 0.11) | 0.930 | 308.76 (307.92, 309.60) | 308.00 (307.37, 308.62) | 0.14 (0.01, 0.27) | 0.040 | 310.19 (309.34, 311.04) | 256.65 (256.07, 257.22) | -0.67 (-1.00, -0.34) | <0.001 | 4.67 (4.56, 4.78) | 3.77 (3.70, 3.84) | -0.71 (-1.10, -0.32) | 0.001 |
| Indonesia | 53.97 (53.21, 54.75) | 55.88 (55.26, 56.51) | 0.18 (0.14, 0.22) | <0.001 | 528.14 (525.76, 530.53) | 533.90 (531.96, 535.85) | 0.13 (0.07, 0.18) | <0.001 | 698.92 (696.17, 701.68) | 554.44 (552.46, 556.41) | -0.60 (-0.72, -0.49) | <0.001 | 10.58 (10.24, 10.92) | 8.00 (7.77, 8.24) | -0.73 (-0.88, -0.59) | <0.001 |
| Iran (Islamic Republic of) | 26.60 (25.58, 27.64) | 24.36 (23.66, 25.07) | -0.31 (-0.42, -0.21) | <0.001 | 333.15 (329.57, 336.76) | 345.82 (343.17, 348.50) | 0.19 (0.04, 0.34) | 0.014 | 323.73 (320.21, 327.27) | 244.94 (242.67, 247.23) | -0.71 (-0.88, -0.54) | <0.001 | 4.72 (4.30, 5.18) | 3.30 (3.04, 3.57) | -0.95 (-1.14, -0.76) | <0.001 |
| Iraq | 47.17 (44.72, 49.73) | 47.29 (45.87, 48.74) | 0.04 (0.01, 0.07) | 0.012 | 534.48 (526.18, 542.89) | 533.11 (528.32, 537.94) | 0.07 (0.01, 0.13) | 0.036 | 657.75 (648.54, 667.07) | 584.78 (579.80, 589.81) | -0.27 (-0.43, -0.10) | 0.002 | 10.09 (8.97, 11.32) | 8.72 (8.11, 9.36) | -0.37 (-0.55, -0.19) | <0.001 |
| Ireland | 17.17 (14.18, 20.62) | 12.86 (10.53, 15.58) | -1.34 (-1.47, -1.20) | <0.001 | 203.51 (192.86, 214.61) | 182.69 (173.60, 192.14) | -0.55 (-0.66, -0.44) | <0.001 | 174.46 (164.59, 184.77) | 73.44 (67.72, 79.54) | -3.36 (-3.60, -3.12) | <0.001 | 2.51 (1.46, 4.05) | 0.79 (0.31, 1.70) | -4.24 (-4.58, -3.90) | <0.001 |
| Israel | 14.99 (12.60, 17.70) | 12.77 (11.10, 14.62) | -0.83 (-1.05, -0.60) | <0.001 | 189.11 (180.36, 198.18) | 180.74 (174.31, 187.36) | -0.15 (-0.29, -0.02) | 0.029 | 167.50 (159.31, 176.02) | 69.23 (65.27, 73.37) | -3.67 (-3.97, -3.38) | <0.001 | 2.34 (1.46, 3.56) | 0.71 (0.37, 1.26) | -4.97 (-5.29, -4.64) | <0.001 |
| Italy | 16.46 (15.70, 17.24) | 12.64 (11.89, 13.42) | -1.56 (-1.84, -1.27) | <0.001 | 176.84 (174.33, 179.37) | 184.03 (181.17, 186.92) | -0.01 (-0.11, 0.08) | 0.779 | 208.23 (205.50, 210.98) | 94.69 (92.65, 96.76) | -3.03 (-3.22, -2.84) | <0.001 | 3.11 (2.78, 3.46) | 1.16 (0.95, 1.41) | -3.80 (-4.00, -3.59) | <0.001 |
| Jamaica | 23.36 (18.92, 28.59) | 25.43 (21.44, 29.97) | 0.16 (0.04, 0.28) | 0.014 | 357.78 (340.08, 376.21) | 396.20 (380.10, 412.83) | 0.40 (0.35, 0.46) | <0.001 | 264.54 (249.35, 280.47) | 220.72 (208.66, 233.31) | -1.94 (-2.74, -1.14) | <0.001 | 4.01 (2.32, 6.51) | 3.29 (1.97, 5.20) | -2.18 (-3.08, -1.27) | <0.001 |
| Japan | 21.76 (21.17, 22.37) | 26.45 (25.72, 27.20) | 0.76 (0.60, 0.92) | <0.001 | 185.86 (184.11, 187.63) | 243.75 (241.49, 246.03) | 0.99 (0.72, 1.26) | <0.001 | 225.89 (223.96, 227.83) | 140.96 (139.26, 142.69) | -1.73 (-1.90, -1.55) | <0.001 | 3.39 (3.16, 3.63) | 1.75 (1.57, 1.95) | -2.29 (-2.55, -2.02) | <0.001 |
| Jordan | 37.55 (32.90, 42.74) | 30.96 (28.88, 33.15) | -0.74 (-0.92, -0.56) | <0.001 | 516.89 (499.47, 534.82) | 471.11 (462.91, 479.43) | -0.28 (-0.37, -0.20) | <0.001 | 218.87 (207.41, 230.85) | 134.19 (129.82, 138.67) | -2.13 (-2.39, -1.87) | <0.001 | 2.60 (1.47, 4.33) | 1.22 (0.83, 1.72) | -3.38 (-3.79, -2.97) | <0.001 |
| Kazakhstan | 46.69 (44.41, 49.07) | 32.60 (30.77, 34.52) | -1.23 (-1.79, -0.68) | <0.001 | 372.86 (366.36, 379.45) | 309.09 (303.40, 314.87) | -0.71 (-0.98, -0.45) | <0.001 | 488.57 (481.13, 496.10) | 434.85 (428.13, 441.66) | -1.16 (-2.15, -0.15) | 0.025 | 7.39 (6.50, 8.37) | 6.75 (5.94, 7.65) | -1.13 (-2.21, -0.04) | 0.042 |

| | | | | | | | | | | | | | | | | |
|---|---|---|---|---|---|---|---|---|---|---|---|---|---|---|---|---|
| Kenya | 30.89 (29.08, 32.79) | 28.47 (27.42, 29.56) | -0.39 (-0.55, -0.22) | <0.001 | 440.04 (433.23, 446.94) | 443.36 (439.19, 447.56) | 0.05 (-0.00, 0.10) | 0.056 | 376.56 (370.27, 382.94) | 374.17 (370.35, 378.01) | -0.06 (-0.46, 0.35) | 0.769 | 5.51 (4.76, 6.35) | 5.42 (4.97, 5.91) | -0.12 (-0.59, 0.35) | 0.614 |
| Kiribati | 195.84 (127.44, 289.32) | 140.91 (96.38, 199.44) | -1.49 (-1.62, -1.37) | <0.001 | 1019.89 (854.22, 1209.57) | 882.03 (763.86, 1013.72) | -0.64 (-0.72, -0.55) | <0.001 | 3714.70 (3395.72, 4056.94) | 3305.41 (3073.91, 3550.15) | -0.76 (-0.90, -0.61) | <0.001 | 61.65 (26.87, 122.76) | 54.67 (28.61, 95.24) | -0.78 (-0.93, -0.64) | <0.001 |
| Kuwait | 26.55 (22.30, 31.55) | 28.53 (25.47, 31.95) | 0.03 (-0.28, 0.35) | 0.826 | 375.90 (359.46, 393.05) | 389.20 (377.54, 401.23) | 0.11 (0.01, 0.22) | 0.039 | 203.75 (191.35, 216.88) | 187.96 (179.78, 196.51) | -0.36 (-0.94, 0.22) | 0.211 | 2.50 (1.31, 4.55) | 2.27 (1.47, 3.50) | -0.47 (-1.27, 0.34) | 0.242 |
| Kyrgyzstan | 45.58 (41.09, 50.45) | 29.15 (26.28, 32.26) | -1.79 (-2.05, -1.52) | <0.001 | 332.53 (320.21, 345.22) | 251.64 (243.09, 260.42) | -1.22 (-1.35, -1.08) | <0.001 | 585.12 (568.74, 601.87) | 393.68 (382.88, 404.70) | -2.54 (-3.14, -1.94) | <0.001 | 9.28 (7.30, 11.63) | 6.20 (4.91, 7.74) | -2.65 (-3.29, -1.99) | <0.001 |
| Lao People's Democratic Republic | 70.18 (63.88, 76.95) | 47.78 (44.36, 51.39) | -1.57 (-1.65, -1.50) | <0.001 | 510.00 (492.85, 527.61) | 411.45 (401.33, 421.76) | -0.80 (-0.90, -0.71) | <0.001 | 711.37 (691.04, 732.16) | 619.67 (607.22, 632.32) | -0.34 (-0.56, -0.12) | 0.003 | 10.90 (8.49, 13.80) | 9.49 (8.00, 11.19) | -0.33 (-0.59, -0.08) | 0.013 |
| Latvia | 30.22 (25.57, 35.51) | 25.49 (20.21, 31.94) | -0.66 (-0.75, -0.57) | <0.001 | 250.94 (237.19, 265.32) | 238.68 (222.00, 256.48) | -0.10 (-0.21, 0.01) | 0.070 | 303.33 (288.13, 319.16) | 169.49 (155.51, 184.57) | -2.91 (-3.63, -2.18) | <0.001 | 4.50 (2.83, 6.84) | 2.20 (0.90, 4.84) | -3.29 (-4.21, -2.36) | <0.001 |
| Lebanon | 26.10 (21.91, 30.90) | 25.45 (22.50, 28.70) | 0.11 (-0.13, 0.34) | 0.356 | 359.18 (343.19, 375.76) | 369.56 (358.06, 381.35) | 0.28 (0.16, 0.41) | <0.001 | 195.97 (184.25, 208.28) | 146.37 (139.17, 153.87) | -0.75 (-1.11, -0.38) | <0.001 | 2.48 (1.32, 4.30) | 1.61 (0.94, 2.61) | -1.26 (-1.74, -0.77) | <0.001 |
| Lesotho | 29.10 (23.21, 36.11) | 47.08 (41.05, 53.75) | 1.83 (1.41, 2.26) | <0.001 | 338.46 (317.71, 360.26) | 406.40 (388.39, 425.04) | 0.76 (0.67, 0.85) | <0.001 | 318.22 (298.13, 339.37) | 502.51 (482.44, 523.22) | 2.20 (1.70, 2.71) | <0.001 | 4.73 (2.55, 8.08) | 7.87 (5.53, 10.87) | 2.37 (1.80, 2.94) | <0.001 |
| Liberia | 36.10 (29.48, 43.83) | 31.89 (28.28, 35.85) | -0.36 (-0.49, -0.23) | <0.001 | 415.88 (392.64, 440.21) | 402.25 (389.19, 415.64) | 0.07 (-0.06, 0.20) | 0.302 | 469.05 (444.15, 495.04) | 359.62 (347.26, 372.32) | -0.84 (-1.08, -0.60) | <0.001 | 7.18 (4.41, 11.13) | 5.32 (3.91, 7.10) | -0.97 (-1.22, -0.72) | <0.001 |
| Libya | 27.01 (23.54, 30.89) | 30.16 (27.55, 32.96) | 0.52 (0.45, 0.59) | <0.001 | 350.86 (337.95, 364.16) | 390.49 (380.93, 400.24) | 0.48 (0.41, 0.56) | <0.001 | 416.43 (402.55, 430.68) | 363.53 (354.27, 372.98) | -0.51 (-0.76, -0.26) | <0.001 | 6.16 (4.56, 8.16) | 5.25 (4.20, 6.50) | -0.63 (-0.90, -0.36) | <0.001 |
| Lithuania | 32.18 (28.12, | 27.41 (22.80, | -0.37 (-0.47, | <0.001 | 256.05 (244.35, | 236.58 (222.64, | -0.20 (-0.26, | <0.001 | 288.38 (275.92, | 199.95 (187.17, | -1.11 (-1.68, | 0.001 | 4.25 (2.86, | 2.77 (1.46, | -1.20 (-1.90, | 0.002 |

| | | | | | | | | | | | | | | | | |
|---|---|---|---|---|---|---|---|---|---|---|---|---|---|---|---|---|
| | 36.68) | 32.80) | -0.28) | | 268.18) | 251.25) | -0.15) | | 301.27) | 213.47) | -0.53) | | 6.10) | 4.91) | -0.49) | |
| Luxembourg | 18.20 (10.03, 31.48) | 12.00 (6.50, 20.95) | -1.86 (-2.11, -1.61) | <0.001 | 211.90 (181.00, 247.35) | 190.22 (165.79, 217.72) | -0.34 (-0.45, -0.23) | <0.001 | 277.18 (241.02, 317.94) | 90.28 (73.71, 109.96) | -4.67 (-5.16, -4.17) | <0.001 | 4.19 (0.94, 13.28) | 1.06 (0.05, 6.33) | -5.88 (-6.42, -5.33) | <0.001 |
| Madagascar | 78.10 (74.20, 82.17) | 55.31 (53.22, 57.47) | -1.42 (-1.52, -1.31) | <0.001 | 587.79 (577.02, 598.72) | 498.79 (492.50, 505.14) | -0.66 (-0.74, -0.58) | <0.001 | 1579.55 (1562.20, 1597.06) | 938.90 (930.38, 947.49) | -1.57 (-1.89, -1.24) | <0.001 | 25.59 (23.40, 27.94) | 14.94 (13.87, 16.07) | -1.61 (-1.95, -1.27) | <0.001 |
| Malawi | 26.26 (23.74, 28.99) | 26.85 (25.07, 28.73) | -0.10 (-0.28, 0.10) | 0.313 | 370.25 (360.71, 379.99) | 385.28 (378.50, 392.16) | 0.11 (0.01, 0.21) | 0.027 | 457.33 (446.80, 468.06) | 446.77 (439.51, 454.13) | -0.38 (-0.69, -0.06) | 0.020 | 6.95 (5.70, 8.43) | 6.74 (5.87, 7.72) | -0.43 (-0.77, -0.09) | 0.014 |
| Malaysia | 49.54 (47.25, 51.91) | 37.40 (36.01, 38.83) | -1.05 (-1.27, -0.82) | <0.001 | 538.07 (530.45, 545.78) | 446.02 (441.20, 450.88) | -0.70 (-0.86, -0.54) | <0.001 | 698.61 (689.95, 707.36) | 437.71 (432.93, 442.53) | -1.71 (-2.11, -1.31) | <0.001 | 10.30 (9.27, 11.42) | 6.20 (5.65, 6.80) | -1.84 (-2.29, -1.39) | <0.001 |
| Maldives | 49.28 (27.98, 81.75) | 27.28 (19.88, 37.66) | -2.29 (-2.61, -1.97) | <0.001 | 462.28 (391.22, 543.55) | 348.87 (321.23, 379.12) | -0.93 (-1.12, -0.74) | <0.001 | 838.10 (740.60, 945.82) | 273.75 (248.74, 301.43) | -3.78 (-4.08, -3.48) | <0.001 | 13.23 (3.68, 35.11) | 3.57 (1.31, 9.53) | -4.50 (-4.83, -4.18) | <0.001 |
| Mali | 32.83 (29.71, 36.19) | 27.79 (25.99, 29.69) | -0.54 (-0.72, -0.36) | <0.001 | 373.96 (363.25, 384.92) | 345.38 (338.96, 351.89) | -0.19 (-0.31, -0.08) | 0.002 | 393.89 (382.95, 405.08) | 329.70 (323.48, 336.02) | -0.56 (-0.83, -0.29) | <0.001 | 5.92 (4.64, 7.46) | 4.88 (4.14, 5.72) | -0.62 (-0.91, -0.32) | <0.001 |
| Malta | 18.39 (9.89, 31.73) | 14.45 (7.16, 27.39) | -1.14 (-1.27, -1.02) | <0.001 | 215.20 (182.89, 251.94) | 192.05 (162.12, 226.86) | -0.42 (-0.51, -0.34) | <0.001 | 175.07 (145.67, 209.00) | 100.14 (78.70, 126.60) | -2.58 (-2.89, -2.27) | <0.001 | 2.41 (0.23, 10.22) | 1.22 (0.02, 9.58) | -3.21 (-3.65, -2.77) | <0.001 |
| Marshall Islands | 86.31 (33.77, 183.23) | 80.33 (38.29, 148.84) | -0.35 (-0.42, -0.27) | <0.001 | 610.25 (448.91, 812.48) | 584.94 (457.43, 737.35) | -0.15 (-0.19, -0.11) | <0.001 | 1569.18 (1305.74, 1872.13) | 1585.57 (1370.30, 1825.37) | 0.02 (-0.11, 0.15) | 0.732 | 24.86 (2.90, 94.75) | 25.94 (5.69, 74.34) | 0.06 (-0.09, 0.21) | 0.427 |
| Mauritania | 45.71 (38.70, 53.68) | 28.88 (24.99, 33.24) | -1.78 (-2.02, -1.54) | <0.001 | 499.05 (475.27, 523.77) | 416.83 (401.68, 432.43) | -0.62 (-0.75, -0.49) | <0.001 | 519.27 (495.09, 544.37) | 249.71 (237.98, 261.90) | -2.27 (-2.46, -2.08) | <0.001 | 7.86 (5.13, 11.60) | 3.40 (2.15, 5.15) | -2.59 (-2.79, -2.39) | <0.001 |
| Mauritius | 45.63 (37.65, 54.84) | 35.79 (28.69, 44.19) | -1.07 (-1.17, -0.96) | <0.001 | 479.62 (452.94, 507.48) | 416.91 (391.64, 443.44) | -0.63 (-0.73, -0.53) | <0.001 | 621.42 (590.86, 653.18) | 412.55 (387.56, 438.78) | -1.59 (-1.79, -1.38) | <0.001 | 9.59 (6.14, 14.33) | 5.98 (3.34, 9.99) | -1.78 (-2.01, -1.56) | <0.001 |

| | | | | | | | | | | | | | | | | |
|---|---|---|---|---|---|---|---|---|---|---|---|---|---|---|---|---|
| Mexico | 24.01 (23.24, 24.81) | 21.94 (21.36, 22.54) | -0.34 (-0.63, -0.05) | 0.022 | 356.80 (353.82, 359.80) | 365.56 (363.17, 367.96) | 0.13 (-0.02, 0.29) | 0.085 | 223.97 (221.60, 226.36) | 198.65 (196.88, 200.43) | -0.25 (-0.51, 0.01) | 0.062 | 3.33 (3.05, 3.64) | 2.89 (2.68, 3.11) | -0.32 (-0.60, -0.03) | 0.031 |
| Micronesia (Federated States of) | 95.90 (56.26, 153.41) | 74.56 (41.38, 124.55) | -0.95 (-0.97, -0.92) | <0.001 | 630.31 (520.36, 757.19) | 555.68 (456.45, 670.71) | -0.45 (-0.48, -0.42) | <0.001 | 2014.41 (1813.38, 2232.15) | 1673.18 (1498.13, 1863.70) | -0.72 (-0.80, -0.65) | <0.001 | 33.39 (12.28, 73.38) | 27.28 (9.30, 62.96) | -0.78 (-0.86, -0.69) | <0.001 |
| Monaco | 14.07 (0.07, 141.21) | 12.93 (0.04, 110.06) | -0.35 (-0.41, -0.28) | <0.001 | 186.82 (85.44, 376.86) | 177.96 (78.99, 348.61) | -0.18 (-0.27, -0.09) | <0.001 | 186.23 (85.19, 375.84) | 104.47 (33.80, 249.05) | -2.01 (-2.25, -1.77) | <0.001 | 2.74 (0.00, 123.93) | 1.35 (0.00, 88.83) | -2.48 (-2.75, -2.21) | <0.001 |
| Mongolia | 44.33 (37.72, 51.85) | 43.28 (38.63, 48.39) | -0.30 (-0.58, -0.03) | 0.033 | 318.69 (300.54, 337.72) | 322.33 (309.43, 335.69) | -0.03 (-0.12, 0.06) | 0.470 | 511.40 (488.55, 535.12) | 804.66 (784.23, 825.55) | 0.67 (0.16, 1.18) | 0.013 | 7.87 (5.23, 11.45) | 13.32 (10.82, 16.31) | 0.76 (0.17, 1.35) | 0.013 |
| Montenegro | 39.98 (29.90, 52.43) | 25.45 (17.02, 36.82) | -1.96 (-2.13, -1.79) | <0.001 | 296.69 (267.95, 327.72) | 241.21 (213.57, 271.61) | -0.92 (-1.02, -0.82) | <0.001 | 559.59 (519.86, 601.61) | 246.90 (218.92, 277.65) | -3.46 (-3.91, -3.01) | <0.001 | 8.91 (4.56, 15.75) | 3.53 (0.98, 9.33) | -3.86 (-4.37, -3.34) | <0.001 |
| Morocco | 37.69 (35.94, 39.51) | 34.38 (33.06, 35.75) | -0.34 (-0.38, -0.31) | <0.001 | 436.35 (430.39, 442.37) | 413.20 (408.57, 417.87) | -0.15 (-0.23, -0.07) | 0.001 | 436.91 (430.91, 442.98) | 253.21 (249.59, 256.87) | -1.95 (-2.20, -1.70) | <0.001 | 6.57 (5.85, 7.36) | 3.35 (2.94, 3.79) | -2.32 (-2.65, -1.99) | <0.001 |
| Mozambique | 36.89 (34.14, 39.81) | 44.72 (42.74, 46.77) | 0.95 (0.86, 1.05) | <0.001 | 435.69 (426.24, 445.30) | 487.36 (480.93, 493.86) | 0.62 (0.50, 0.74) | <0.001 | 427.20 (417.81, 436.76) | 636.49 (629.09, 643.95) | 1.94 (1.68, 2.19) | <0.001 | 6.47 (5.36, 7.76) | 10.00 (9.09, 10.99) | 2.09 (1.80, 2.37) | <0.001 |
| Myanmar | 54.32 (52.67, 56.02) | 39.38 (38.19, 40.60) | -1.25 (-1.30, -1.20) | <0.001 | 437.02 (432.35, 441.73) | 383.20 (379.47, 386.95) | -0.36 (-0.48, -0.23) | <0.001 | 1649.35 (1640.30, 1658.44) | 1010.77 (1004.73, 1016.85) | -1.76 (-1.83, -1.69) | <0.001 | 27.13 (25.97, 28.33) | 16.06 (15.30, 16.84) | -1.87 (-1.95, -1.79) | <0.001 |
| Namibia | 31.81 (24.87, 40.22) | 22.61 (18.51, 27.39) | -1.71 (-2.04, -1.39) | <0.001 | 393.11 (367.90, 419.70) | 366.70 (349.65, 384.41) | -0.36 (-0.44, -0.27) | <0.001 | 326.53 (303.62, 350.84) | 280.85 (265.88, 296.48) | -1.42 (-2.24, -0.59) | 0.002 | 4.76 (2.35, 8.75) | 4.04 (2.42, 6.37) | -1.69 (-2.65, -0.72) | 0.001 |
| Nauru | 93.23 (9.40, 369.25) | 86.38 (9.27, 335.68) | -0.33 (-0.63, -0.03) | 0.034 | 691.97 (371.47, 1183.17) | 645.69 (352.07, 1092.03) | -0.28 (-0.38, -0.18) | <0.001 | 2337.50 (1705.21, 3133.17) | 2144.57 (1572.54, 2862.62) | -0.29 (-0.64, 0.05) | 0.095 | 38.54 (0.31, 278.92) | 35.24 (0.32, 251.54) | -0.29 (-0.66, 0.07) | 0.109 |

| | | | | | | | | | | | | | | | | |
|---|---|---|---|---|---|---|---|---|---|---|---|---|---|---|---|---|
| Nepal | 23.11 (21.47, 24.84) | 19.28 (18.12, 20.51) | -0.68 (-0.85, -0.51) | <0.001 | 246.81 (241.43, 252.29) | 225.72 (221.69, 229.80) | -0.24 (-0.34, -0.13) | <0.001 | 296.07 (290.13, 302.12) | 205.37 (201.54, 209.25) | -1.29 (-1.51, -1.06) | <0.001 | 4.55 (3.83, 5.36) | 2.93 (2.49, 3.44) | -1.56 (-1.81, -1.31) | <0.001 |
| Netherlands | 15.96 (14.59, 17.42) | 13.33 (11.99, 14.78) | -0.83 (-0.90, -0.75) | <0.001 | 199.64 (194.73, 204.64) | 194.13 (188.93, 199.44) | -0.10 (-0.15, -0.05) | <0.001 | 151.25 (146.99, 155.61) | 74.30 (71.10, 77.61) | -2.78 (-3.01, -2.55) | <0.001 | 2.10 (1.63, 2.68) | 0.79 (0.49, 1.20) | -3.75 (-4.09, -3.42) | <0.001 |
| New Zealand | 15.26 (12.48, 18.47) | 13.58 (10.98, 16.62) | -0.51 (-0.72, -0.30) | <0.001 | 154.19 (145.05, 163.75) | 171.67 (162.08, 181.68) | 0.44 (0.21, 0.67) | <0.001 | 151.30 (142.25, 160.78) | 80.40 (73.88, 87.35) | -2.55 (-2.73, -2.37) | <0.001 | 2.15 (1.20, 3.56) | 0.92 (0.35, 1.96) | -3.42 (-3.60, -3.23) | <0.001 |
| Nicaragua | 25.34 (21.46, 29.76) | 20.53 (18.19, 23.10) | -0.74 (-0.88, -0.61) | <0.001 | 361.36 (346.46, 376.77) | 346.48 (336.69, 356.50) | -0.07 (-0.14, -0.00) | 0.038 | 209.18 (197.60, 221.29) | 110.04 (104.55, 115.75) | -2.11 (-2.47, -1.75) | <0.001 | 3.15 (1.86, 5.03) | 1.36 (0.81, 2.15) | -2.80 (-3.22, -2.38) | <0.001 |
| Niger | 37.20 (33.76, 40.91) | 31.65 (29.73, 33.67) | -0.57 (-0.71, -0.44) | <0.001 | 394.60 (383.24, 406.22) | 367.17 (360.63, 373.82) | -0.16 (-0.29, -0.02) | 0.023 | 476.39 (463.93, 489.12) | 391.02 (384.24, 397.90) | -0.56 (-0.76, -0.36) | <0.001 | 7.35 (5.86, 9.12) | 5.95 (5.13, 6.87) | -0.60 (-0.81, -0.39) | <0.001 |
| Nigeria | 25.50 (24.71, 26.30) | 23.86 (23.34, 24.38) | -0.26 (-0.41, -0.11) | 0.001 | 344.18 (341.27, 347.10) | 368.48 (366.46, 370.50) | 0.34 (0.16, 0.51) | <0.001 | 318.27 (315.47, 321.10) | 256.14 (254.44, 257.84) | -1.03 (-1.19, -0.86) | <0.001 | 4.76 (4.42, 5.12) | 3.63 (3.43, 3.84) | -1.26 (-1.45, -1.07) | <0.001 |
| Niue | 63.60 (0.00, 1154.81) | 52.35 (0.00, 1567.76) | -0.79 (-0.86, -0.72) | <0.001 | 557.26 (74.29, 1987.19) | 515.94 (33.26, 2372.45) | -0.31 (-0.35, -0.26) | <0.001 | 1311.18 (427.28, 3090.54) | 849.66 (131.36, 2892.40) | -1.88 (-2.07, -1.68) | <0.001 | 21.06 (0.00, 1073.21) | 12.92 (0.00, 1492.11) | -2.03 (-2.28, -1.79) | <0.001 |
| North Macedonia | 33.11 (27.84, 39.10) | 26.78 (22.14, 32.26) | -0.86 (-0.90, -0.82) | <0.001 | 305.25 (288.78, 322.42) | 276.15 (260.86, 292.23) | -0.34 (-0.42, -0.25) | <0.001 | 446.53 (426.53, 467.23) | 306.88 (290.46, 324.11) | -1.69 (-1.96, -1.42) | <0.001 | 6.78 (4.53, 9.79) | 4.42 (2.67, 7.08) | -1.92 (-2.22, -1.61) | <0.001 |
| Northern Mariana Islands | 48.99 (18.20, 113.40) | 44.30 (7.86, 149.65) | -0.46 (-0.51, -0.41) | <0.001 | 493.14 (377.45, 637.96) | 491.45 (329.91, 711.48) | -0.05 (-0.12, 0.01) | 0.106 | 943.00 (780.57, 1133.83) | 632.27 (447.65, 874.48) | -1.73 (-1.98, -1.48) | <0.001 | 14.95 (1.66, 65.83) | 9.45 (0.02, 95.86) | -1.92 (-2.22, -1.63) | <0.001 |
| Norway | 23.11 (19.97, 26.62) | 22.58 (19.63, 25.89) | -0.60 (-0.80, -0.40) | <0.001 | 306.22 (294.49, 318.32) | 287.95 (277.20, 299.03) | -0.48 (-0.67, -0.28) | <0.001 | 174.24 (165.44, 183.41) | 71.33 (66.05, 76.94) | -3.28 (-3.45, -3.10) | <0.001 | 2.34 (1.43, 3.65) | 0.57 (0.20, 1.34) | -4.90 (-5.16, -4.65) | <0.001 |
| Oman | 28.94 (24.53, | 24.33 (21.94, | -0.67 (-0.75, | <0.001 | 385.26 (368.73, | 397.72 (387.99, | 0.25 (0.16, | <0.001 | 438.45 (420.42, | 239.18 (230.65, | -1.81 (-2.13, | <0.001 | 6.41 (4.41, | 2.98 (2.10, | -2.28 (-2.71, | <0.001 |

| | | | | | | | | | | | | | | | | |
|---|---|---|---|---|---|---|---|---|---|---|---|---|---|---|---|---|
| | 33.99) | 27.02) | -0.60) | | 402.41) | 407.74) | 0.34) | | 457.12) | 248.03) | -1.49) | | 9.09) | 4.24) | -1.85) | |
| Pakistan | 31.84 (31.02, 32.67) | 31.58 (31.03, 32.13) | -0.11 (-0.17, -0.06) | <0.001 | 358.11 (355.40, 360.84) | 362.55 (360.72, 364.38) | 0.14 (0.05, 0.23) | 0.004 | 374.47 (371.65, 377.31) | 471.19 (469.11, 473.29) | 0.39 (0.11, 0.67) | 0.008 | 5.71 (5.36, 6.07) | 7.31 (7.04, 7.57) | 0.38 (0.06, 0.70) | 0.021 |
| Palau | 74.61 (13.04, 239.79) | 68.89 (12.43, 236.67) | -0.32 (-0.34, -0.30) | <0.001 | 620.94 (387.67, 946.60) | 589.28 (372.39, 900.82) | -0.17 (-0.21, -0.14) | <0.001 | 1522.39 (1141.73 , 1992.72) | 1327.90 (987.31, 1760.34) | -0.25 (-0.40, -0.09) | 0.003 | 24.45 (0.36, 159.61) | 21.16 (0.27, 164.86) | -0.25 (-0.44, -0.06) | 0.011 |
| Palestine | 28.00 (22.46, 34.63) | 23.27 (20.30, 26.57) | -0.83 (-0.98, -0.68) | <0.001 | 368.53 (347.68, 390.42) | 352.42 (340.64, 364.53) | -0.05 (-0.17, 0.07) | 0.421 | 286.23 (267.96, 305.55) | 171.68 (163.50, 180.18) | -2.09 (-2.39, -1.79) | <0.001 | 4.01 (2.10, 7.12) | 2.09 (1.27, 3.27) | -2.68 (-3.08, -2.28) | <0.001 |
| Panama | 23.73 (19.50, 28.64) | 19.69 (16.73, 23.02) | -0.63 (-0.77, -0.49) | <0.001 | 346.09 (329.52, 363.31) | 339.27 (326.64, 352.28) | 0.01 (-0.09, 0.11) | 0.857 | 263.44 (248.94, 278.61) | 166.57 (157.73, 175.77) | -1.41 (-1.80, -1.03) | <0.001 | 4.03 (2.41, 6.37) | 2.38 (1.43, 3.72) | -1.66 (-2.10, -1.21) | <0.001 |
| Papua New Guinea | 74.03 (67.98, 80.50) | 72.76 (69.00, 76.67) | -0.05 (-0.11, 0.01) | 0.099 | 538.41 (521.93, 555.30) | 534.07 (523.83, 544.46) | -0.01 (-0.04, 0.02) | 0.593 | 960.96 (938.93, 983.39) | 969.66 (955.89, 983.58) | 0.04 (-0.07, 0.14) | 0.468 | 15.10 (12.43, 18.19) | 15.20 (13.51, 17.04) | 0.03 (-0.09, 0.14) | 0.653 |
| Paraguay | 31.96 (27.98, 36.37) | 29.61 (26.90, 32.51) | -0.32 (-0.41, -0.23) | <0.001 | 402.27 (387.93, 417.01) | 396.71 (386.67, 406.94) | -0.07 (-0.11, -0.03) | 0.001 | 332.46 (319.32, 346.02) | 230.93 (223.27, 238.78) | -1.56 (-1.82, -1.29) | <0.001 | 5.26 (3.71, 7.24) | 3.44 (2.56, 4.53) | -1.80 (-2.10, -1.50) | <0.001 |
| Peru | 39.20 (37.28, 41.20) | 29.25 (27.98, 30.56) | -0.99 (-1.08, -0.90) | <0.001 | 443.05 (436.48, 449.69) | 382.29 (377.66, 386.96) | -0.41 (-0.49, -0.33) | <0.001 | 552.92 (545.75, 560.17) | 307.07 (302.94, 311.25) | -1.64 (-1.95, -1.33) | <0.001 | 8.58 (7.70, 9.54) | 4.60 (4.11, 5.14) | -1.75 (-2.09, -1.42) | <0.001 |
| Philippines | 53.79 (52.45, 55.14) | 42.23 (41.39, 43.09) | -0.76 (-0.87, -0.64) | <0.001 | 455.76 (451.89, 459.66) | 395.01 (392.42, 397.61) | -0.37 (-0.52, -0.23) | <0.001 | 268.79 (265.80, 271.81) | 600.69 (597.49, 603.91) | 3.86 (2.96, 4.76) | <0.001 | 3.30 (2.97, 3.66) | 9.32 (8.93, 9.73) | 4.55 (3.37, 5.74) | <0.001 |
| Poland | 30.06 (28.87, 31.30) | 17.72 (16.74, 18.74) | -2.15 (-2.32, -1.97) | <0.001 | 236.11 (232.74, 239.51) | 168.75 (165.77, 171.78) | -1.27 (-1.53, -1.02) | <0.001 | 431.63 (427.08, 436.23) | 189.26 (186.13, 192.45) | -3.13 (-3.27, -2.98) | <0.001 | 6.84 (6.28, 7.44) | 2.72 (2.36, 3.12) | -3.48 (-3.64, -3.33) | <0.001 |
| Portugal | 24.66 (22.45, 27.03) | 10.84 (9.28, 12.60) | -3.23 (-3.49, -2.97) | <0.001 | 272.81 (265.34, 280.43) | 219.95 (212.68, 227.41) | -0.85 (-0.99, -0.71) | <0.001 | 408.31 (399.16, 417.61) | 104.56 (99.65, 109.66) | -5.97 (-6.50, -5.45) | <0.001 | 6.42 (5.32, 7.69) | 1.31 (0.82, 2.00) | -6.76 (-7.43, -6.08) | <0.001 |
| Puerto Rico | 23.97 | 20.77 | -0.65 | <0.001 | 362.50 | 361.54 | 0.40 | 0.003 | 218.69 | 120.05 | -2.59 | <0.001 | 3.32 | 1.56 | -3.34 | <0.001 |

| | | | | | | | | | | | | | | | | |
|---|---|---|---|---|---|---|---|---|---|---|---|---|---|---|---|---|
| | (20.36, 28.03) | (17.16, 24.92) | (-0.79, -0.51) | | (348.17, 377.27) | (345.89, 377.72) | (0.15, 0.66) | | (207.50, 230.33) | (111.13, 129.50) | (-2.88, -2.30) | | (2.07, 5.05) | (0.71, 3.01) | (-3.69, -2.99) | |
| Qatar | 34.90 (26.60, 46.16) | 28.26 (25.18, 31.92) | -0.83 (-0.96, -0.69) | <0.001 | 446.19 (414.77, 480.25) | 392.31 (380.55, 404.60) | -0.38 (-0.41, -0.35) | <0.001 | 272.88 (247.20, 301.35) | 143.00 (135.70, 150.86) | -2.48 (-2.59, -2.37) | <0.001 | 3.58 (1.26, 9.75) | 1.51 (0.86, 2.97) | -3.36 (-3.54, -3.17) | <0.001 |
| Republic of Korea | 35.89 (34.74, 37.06) | 17.45 (16.64, 18.29) | -3.12 (-3.33, -2.90) | <0.001 | 266.32 (263.18, 269.48) | 191.79 (189.07, 194.55) | -1.47 (-1.63, -1.31) | <0.001 | 554.38 (549.86, 558.94) | 127.09 (124.91, 129.31) | -5.61 (-5.86, -5.37) | <0.001 | 8.82 (8.26, 9.41) | 1.64 (1.40, 1.91) | -6.37 (-6.59, -6.15) | <0.001 |
| Republic of Moldova | 33.78 (30.10, 37.83) | 28.04 (24.35, 32.23) | -0.50 (-0.58, -0.43) | <0.001 | 252.51 (242.30, 263.08) | 227.75 (217.10, 238.87) | -0.40 (-0.43, -0.38) | <0.001 | 359.92 (347.66, 372.54) | 229.56 (218.86, 240.74) | -1.91 (-2.31, -1.50) | <0.001 | 5.52 (4.10, 7.31) | 3.28 (2.13, 4.96) | -2.13 (-2.61, -1.64) | <0.001 |
| Romania | 31.53 (29.90, 33.23) | 21.55 (19.94, 23.26) | -1.57 (-1.65, -1.48) | <0.001 | 261.59 (256.84, 266.40) | 232.14 (226.84, 237.54) | -0.40 (-0.50, -0.29) | <0.001 | 371.97 (366.33, 377.69) | 249.39 (243.89, 255.00) | -1.95 (-2.28, -1.62) | <0.001 | 5.74 (5.06, 6.49) | 3.59 (2.96, 4.32) | -2.28 (-2.68, -1.88) | <0.001 |
| Russian Federation | 37.12 (36.45, 37.79) | 31.67 (31.01, 32.35) | -0.47 (-0.88, -0.06) | 0.026 | 276.74 (274.92, 278.58) | 246.16 (244.30, 248.02) | -0.42 (-0.60, -0.23) | <0.001 | 382.43 (380.27, 384.60) | 445.00 (442.53, 447.48) | -0.07 (-0.92, 0.79) | 0.870 | 5.83 (5.57, 6.10) | 7.11 (6.81, 7.43) | -0.01 (-0.93, 0.92) | 0.984 |
[truncated: 507,811 more chars]
