# Supplementary material for: Global, regional, and national burden of cardiovascular diseases in youths and young adults aged 15–39 years in 204 countries/territories, 1990–2019: a systematic analysis of Global Burden of Disease Study 2019
Source: BMC Med. 2023 Jun 26;21:222. doi: 10.1186/s12916-023-02925-4 (PMC10294522; doi:10.1186/s12916-023-02925-4)
Supplement: Supplementary file 2 — Additional file 2: Fig S1. Temporal trends in the burden of overall cardiovascular disease by sex and region from 1990 to 2019. Fig S2. Burden of overall cardiovascular disease across 204 countries/territories. Fig S3-13. Temporal trends in burden of overall and type-specific cardiovascular disease by age and sociodemographic index, from 1990 to 2019. Fig S14-24. Number and burden of overall and type-specific cardiovascular disease in 2019 by age and sex. Fig S25-35. Burden of overall and type-specific cardiovascular disease in 2019 by age, sex, and sociodemographic index. Fig S36-45. Difference in burden of type-specific cardiovascular disease by age and sociodemographic index, from 1990 to 2019. Fig S46. Burden of overall and type-specific cardiovascular diseases in 2019 by sex. Fig S47-56. Temporal trends in burden of type-specific cardiovascular disease by sex and regions from 1990 to 2019. Fig S57-79. Average annual percent change in burden of overall and type-specific cardiovascular disease by age sex, and sociodemographic index or region or countries/territories. Fig S80. Proportion of disability-adjusted life years attributed to 20 risk factors in 2019 by age. Fig S81-82. Burden of overall and type-specific cardiovascular disease attributed to systolic blood pressure. Fig S83. Burden of overall cardiovascular disease worldwide and in regions by sociodemographic index, 1990 to 2019. [file 12916_2023_2925_MOESM2_ESM.pdf]

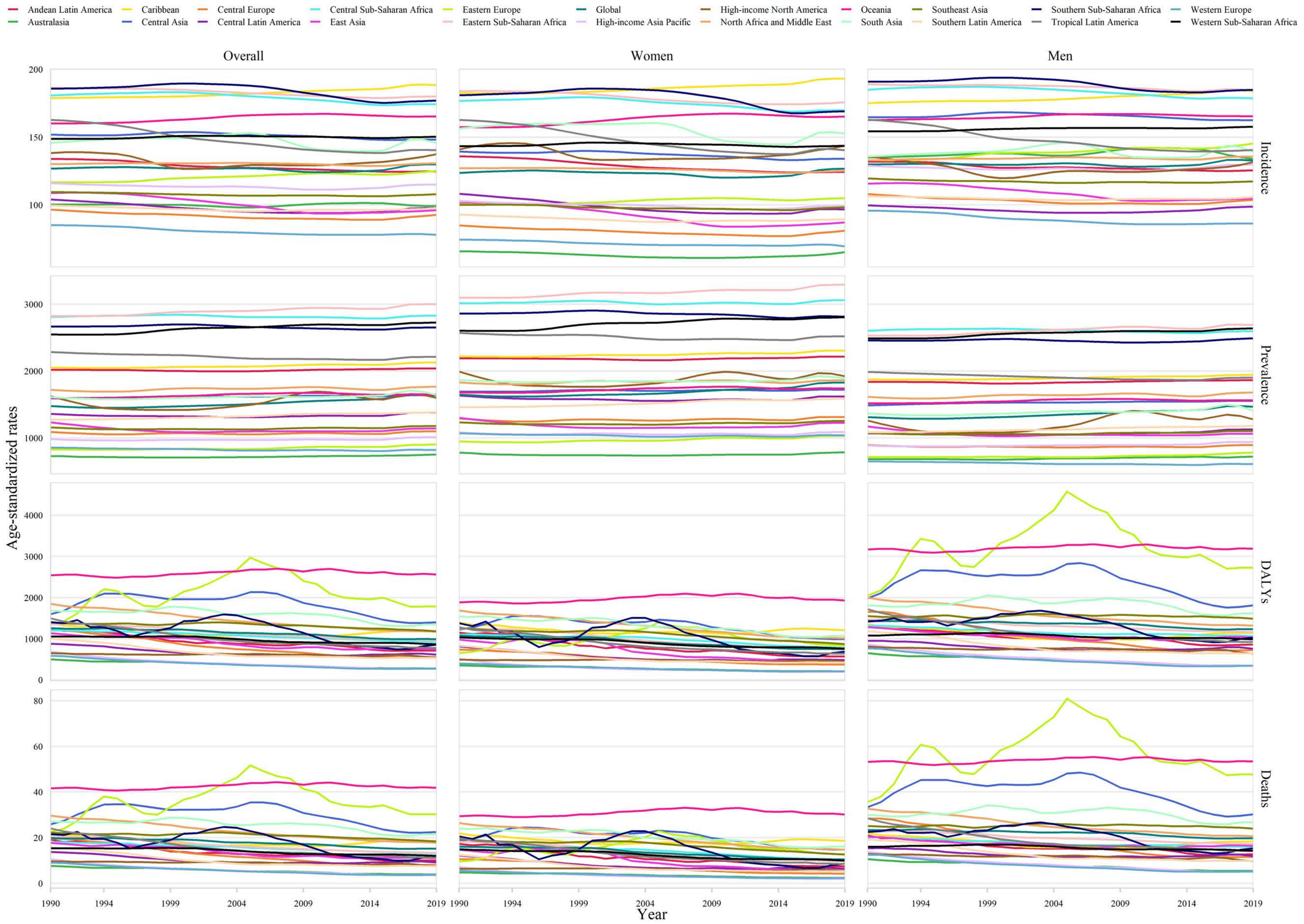

Fig S1 Temporal trends in the burden of overall cardiovascular disease in youths and young adults overall and by sex and 21 GBD region, from 1990 to 2019. DALY, disability-adjusted life years.

A

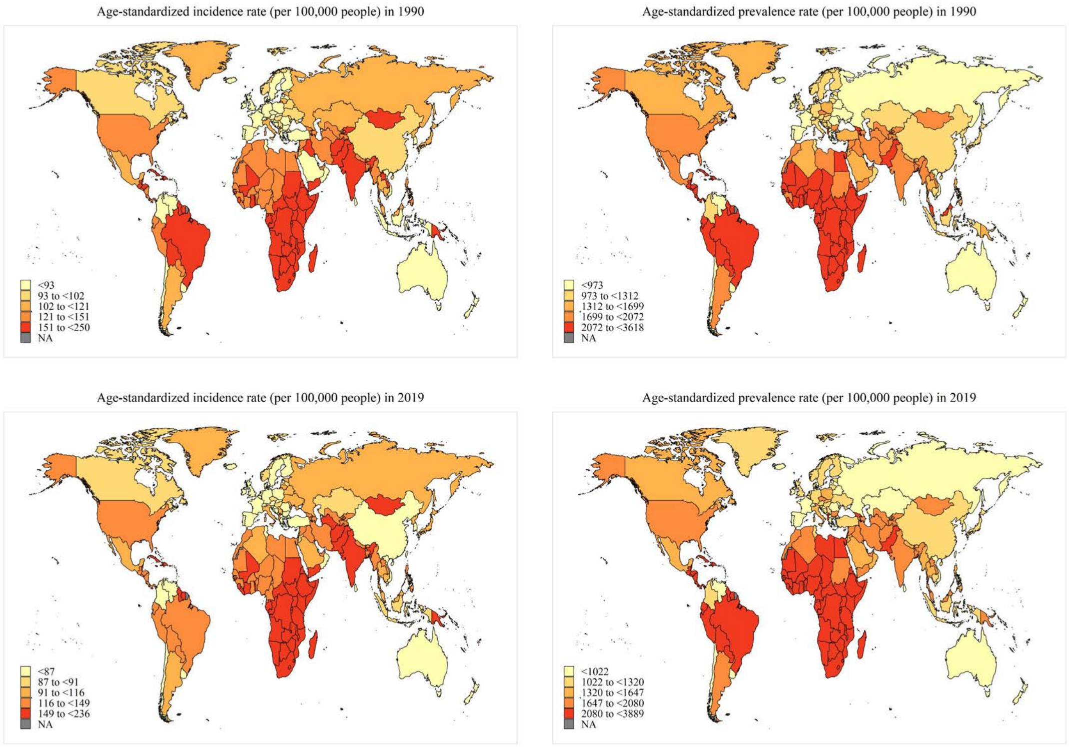

Age-standardized incidence rate (per 100,000 people) in 1990
<93
93 to <102
102 to <121
121 to <151
151 to <250
NA
Age-standardized prevalence rate (per 100,000 people) in 1990
<973
973 to <1312
1312 to <1699
1699 to <2072
2072 to <3618
NA
Age-standardized incidence rate (per 100,000 people) in 2019
<87
87 to <91
91 to <116
116 to <149
149 to <236
NA
Age-standardized prevalence rate (per 100,000 people) in 2019
<1022
1022 to <1320
1320 to <1647
1647 to <2080
2080 to <3889
NA

B

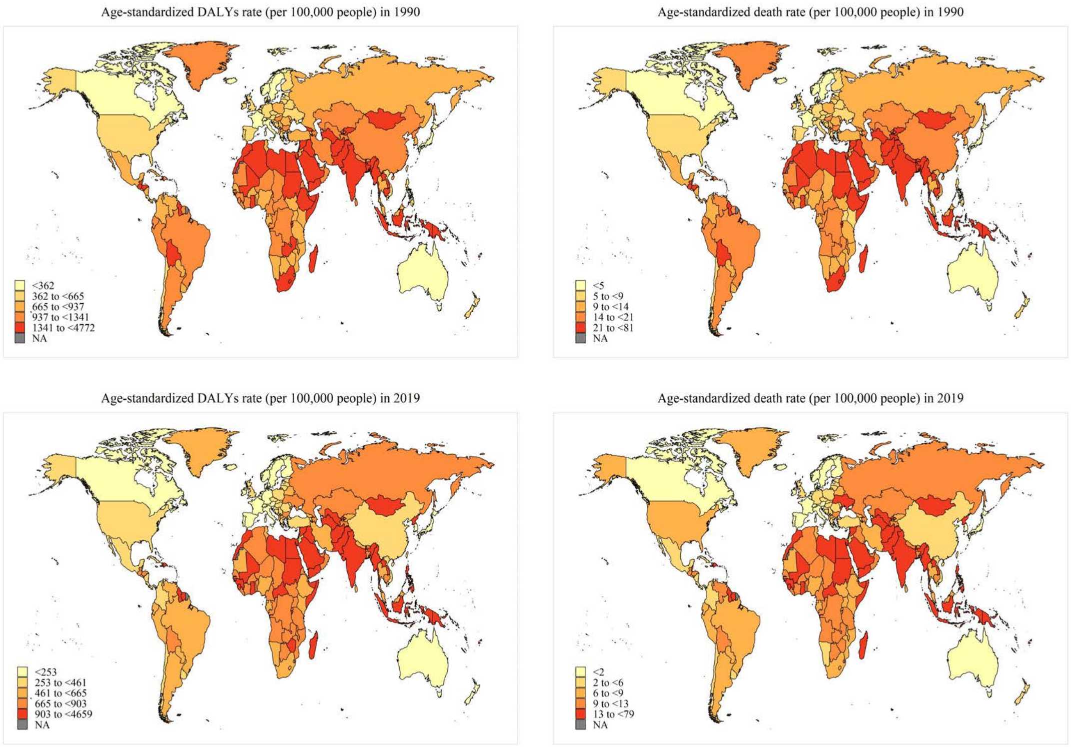

Age-standardized DALYs rate (per 100,000 people) in 1990
<362
362 to <665
665 to <937
937 to <1341
1341 to <4772
NA
Age-standardized death rate (per 100,000 people) in 1990
<5
5 to <9
9 to <14
14 to <21
21 to <81
NA
Age-standardized DALYs rate (per 100,000 people) in 2019
<253
253 to <461
461 to <665
665 to <903
903 to <4659
NA
Age-standardized death rate (per 100,000 people) in 2019
<2
2 to <6
6 to <9
9 to <13
13 to <79
NA

C

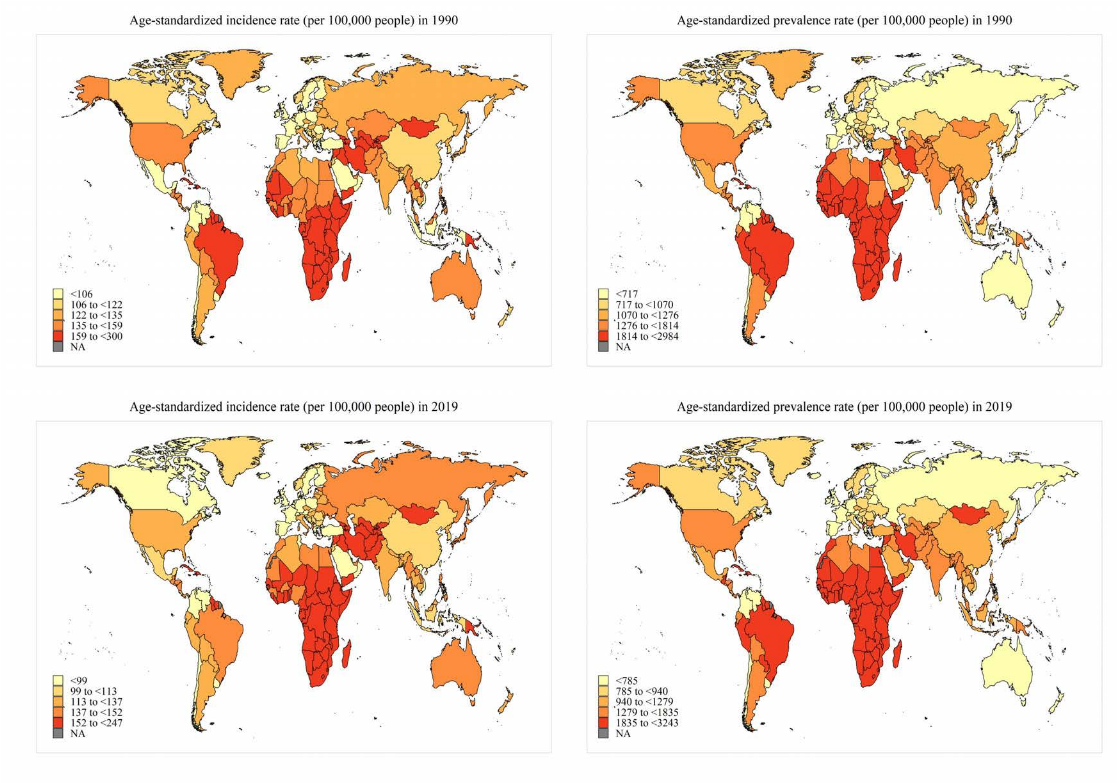

D

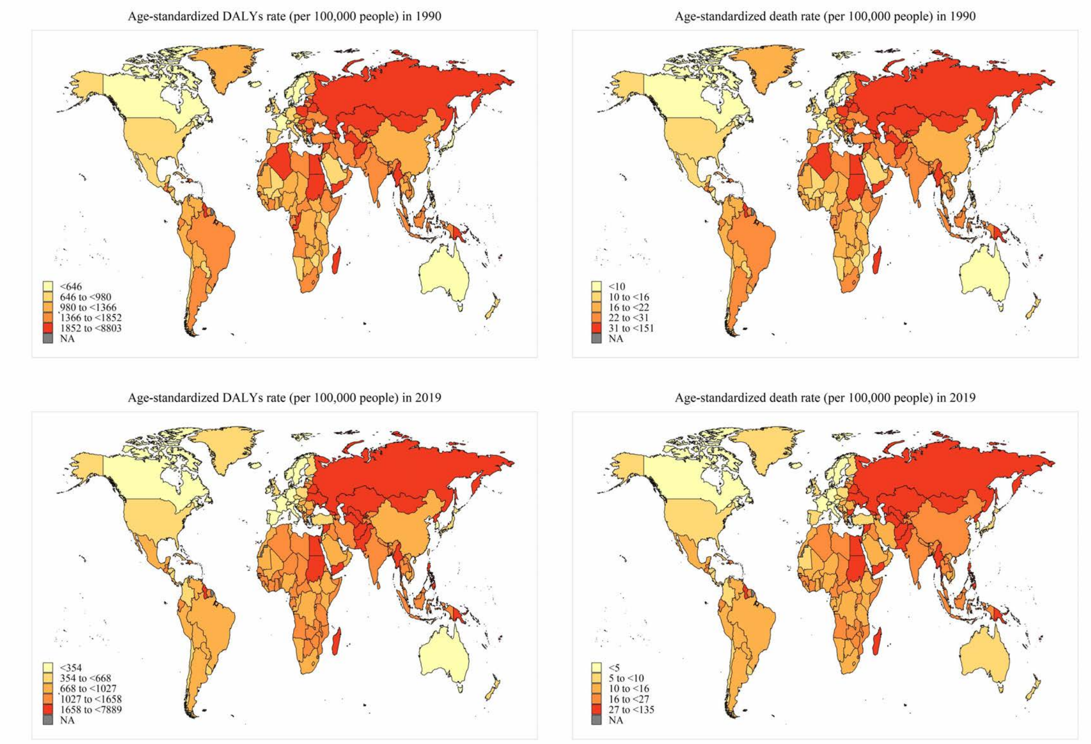

Fig S2 Age-standardized burden of overall cardiovascular disease across 204 countries/territories among youths and young adults by sex, in 1990 and 2019. A, age-standardized incidence and prevalence in women; B, age-standardized DALYs and mortality rate in women; C, age-standardized incidence and prevalence in men; D, age-standardized DALYs and mortality rate in men. DALY, disability-adjusted life years.

A

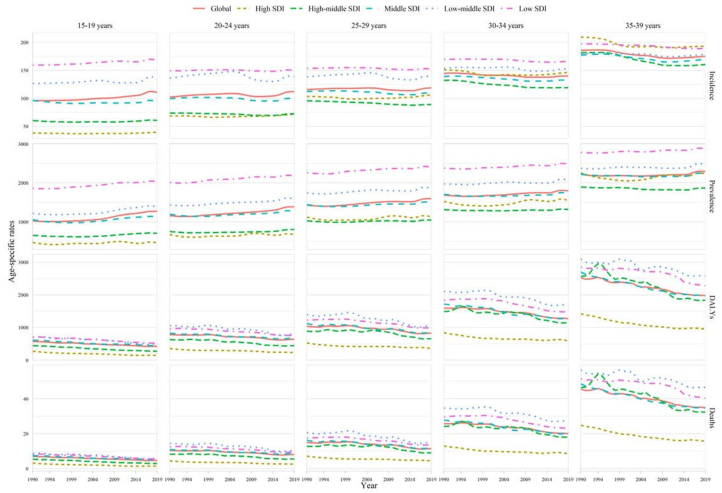

B

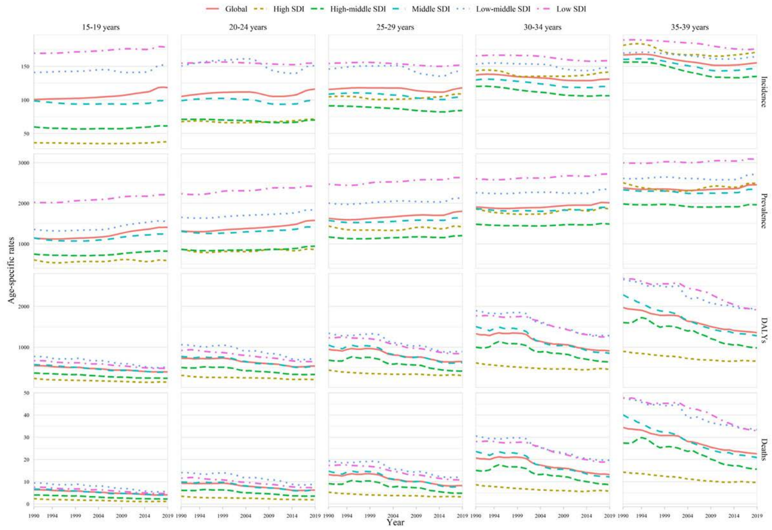

C

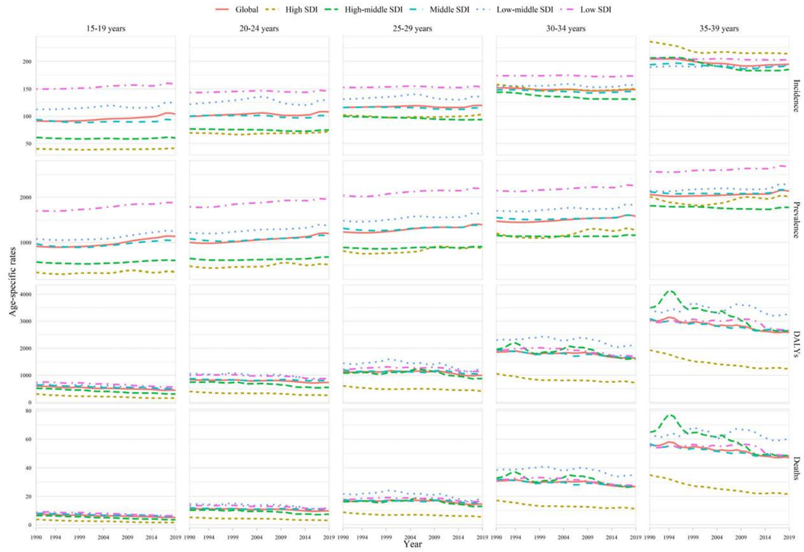

Fig S3 Temporal trends in age-specific incidence, prevalence, disability-adjusted life years (DALYs), and death rate of overall cardiovascular disease in youths and young adults by age and sociodemographic index, from 1990 to 2019. A, overall; B, women; C, men.

A

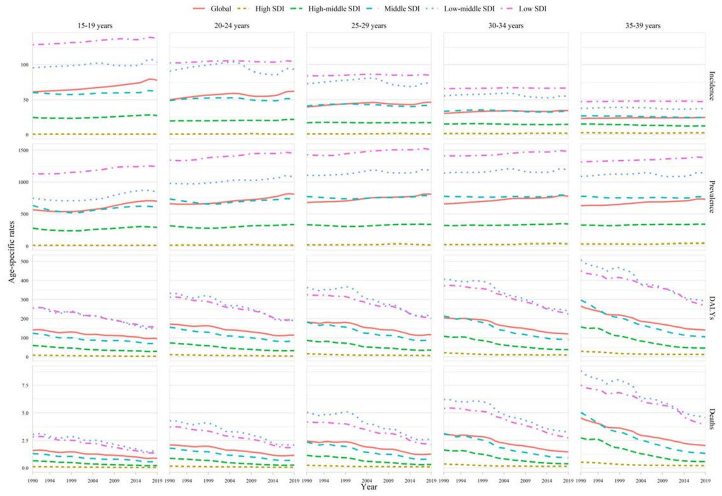

B

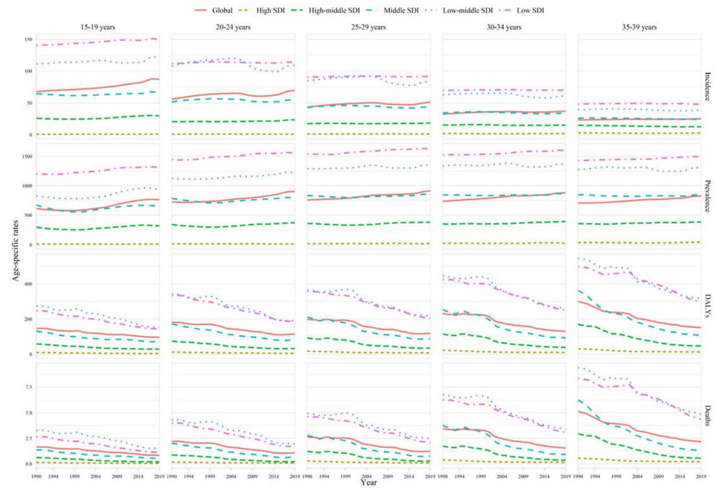

C

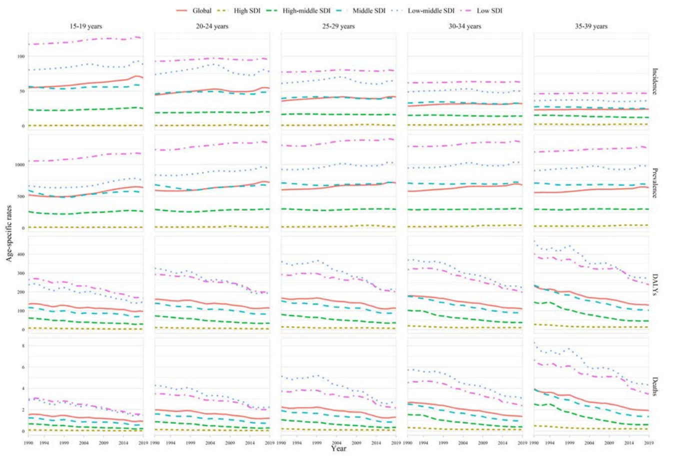

Fig S4 Temporal trends in age-specific incidence, prevalence, disability-adjusted life years (DALYs), and death rate of rheumatic heart disease in youths and young adults by age and sociodemographic index, from 1990 to 2019. A, overall; B, women; C, men.

A

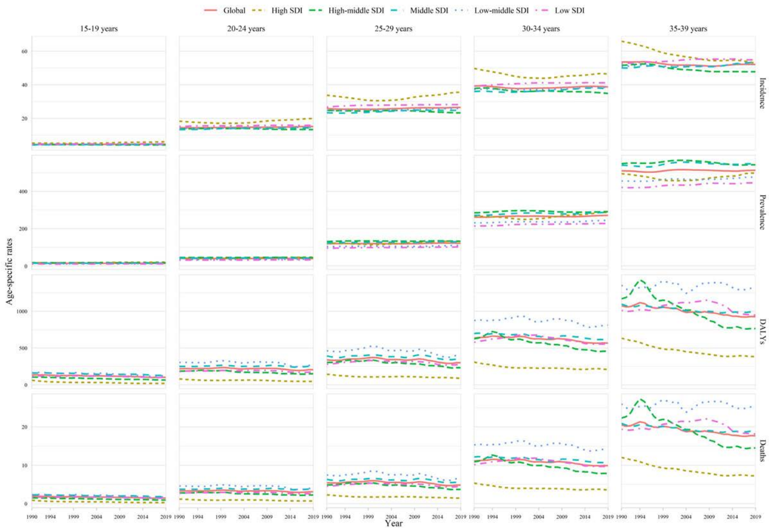

B

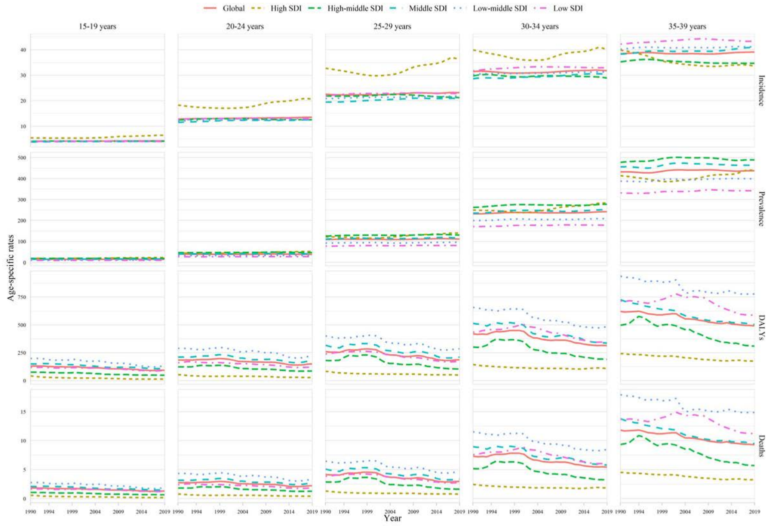

C

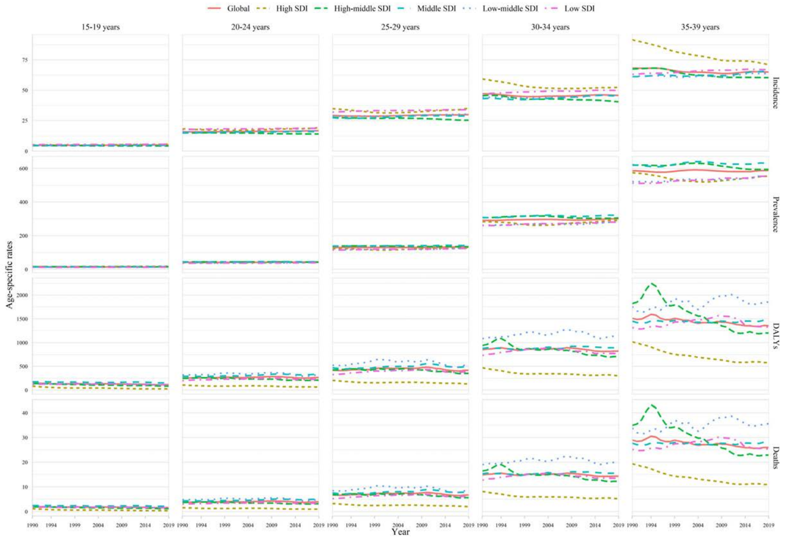

Fig S5 Temporal trends in age-specific incidence, prevalence, disability-adjusted life years (DALYs), and death rate of ischemic heart disease in youths and young adults by age and sociodemographic index, from 1990 to 2019. A, overall; B, women; C, men.

A

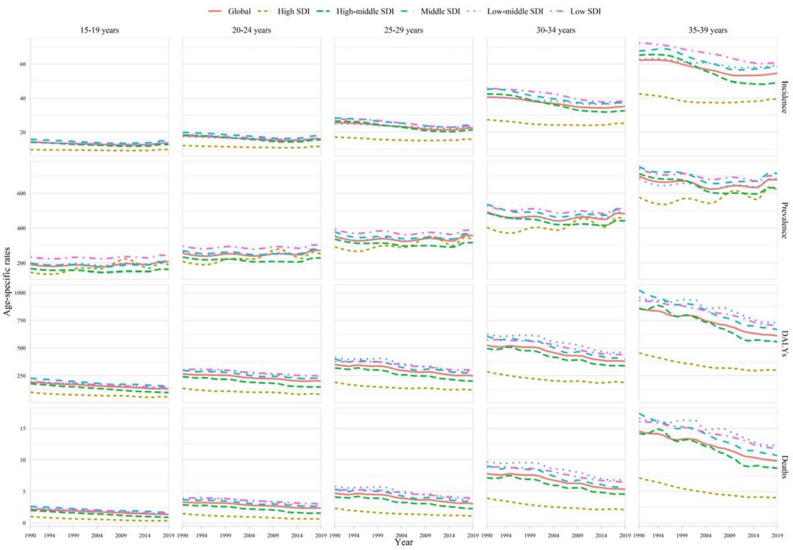

B

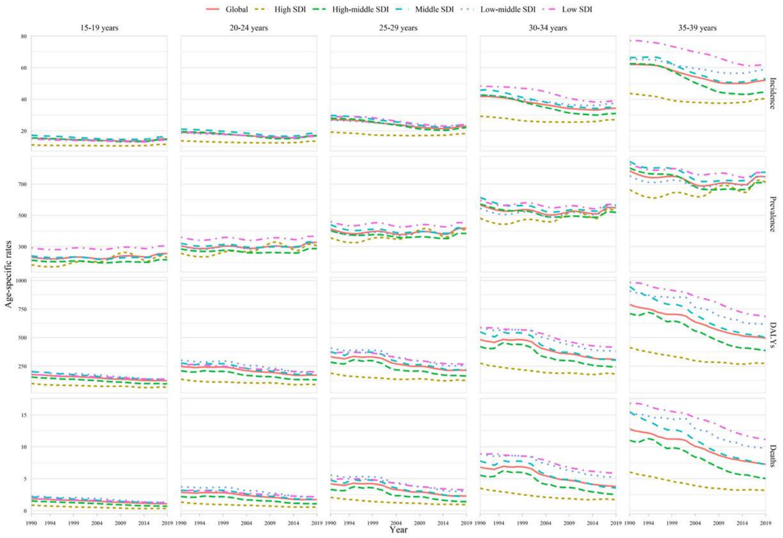

C

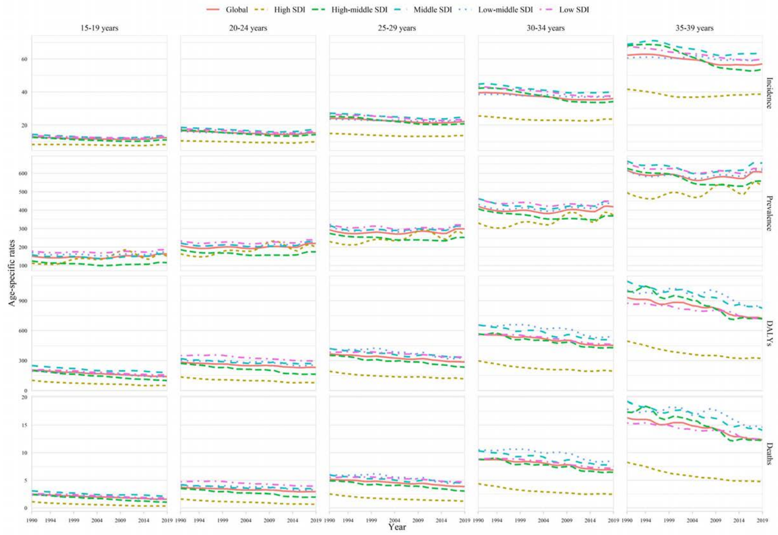

Fig S6 Temporal trends in age-specific incidence, prevalence, disability-adjusted life years (DALYs), and death rate of stroke in youths and young adults by age and sociodemographic index, from 1990 to 2019. A, overall; B, women; C, men.

A

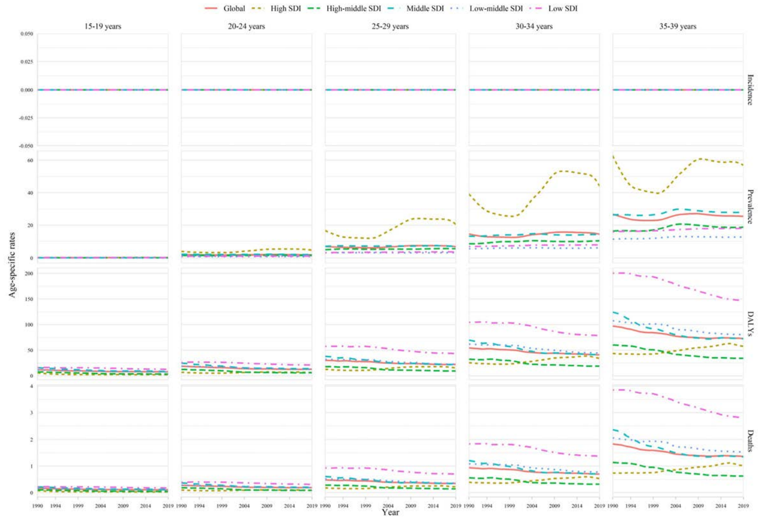

B

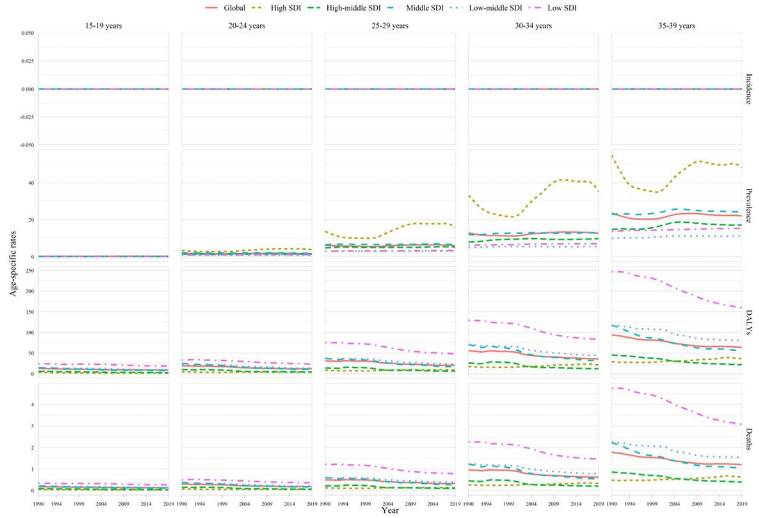

C

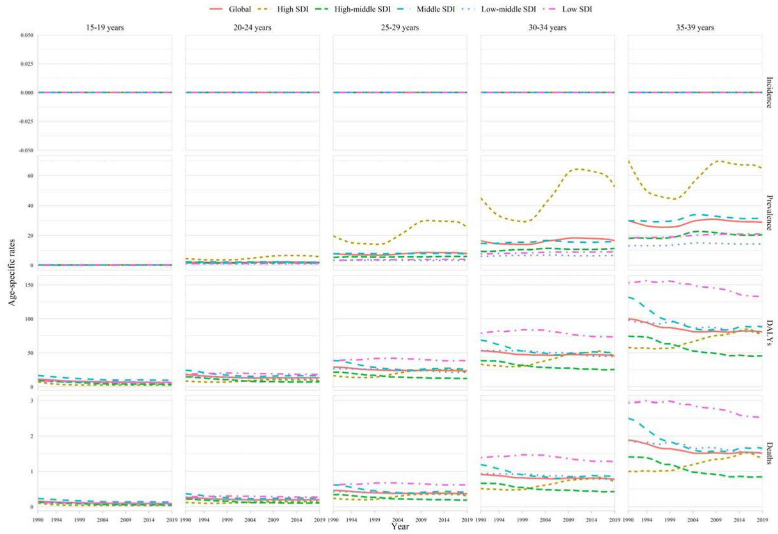

Fig S7 Temporal trends in age-specific prevalence, disability-adjusted life years (DALYs), and death rate of hypertensive heart disease in youths and young adults by age and sociodemographic index, from 1990 to 2019. A, overall; B, women; C, men. Data for incidence are unavailable.

A

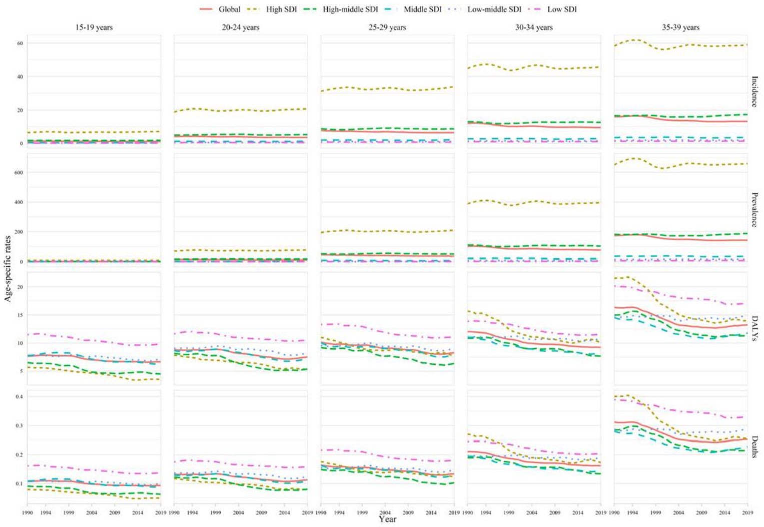

B

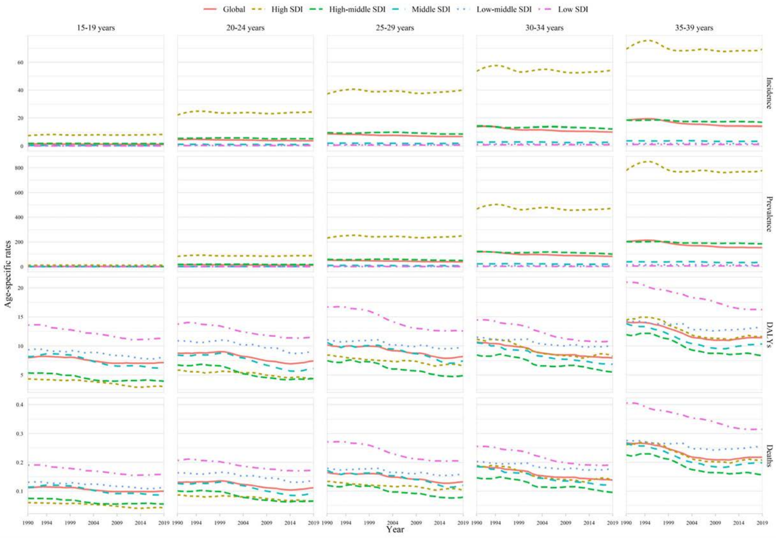

C

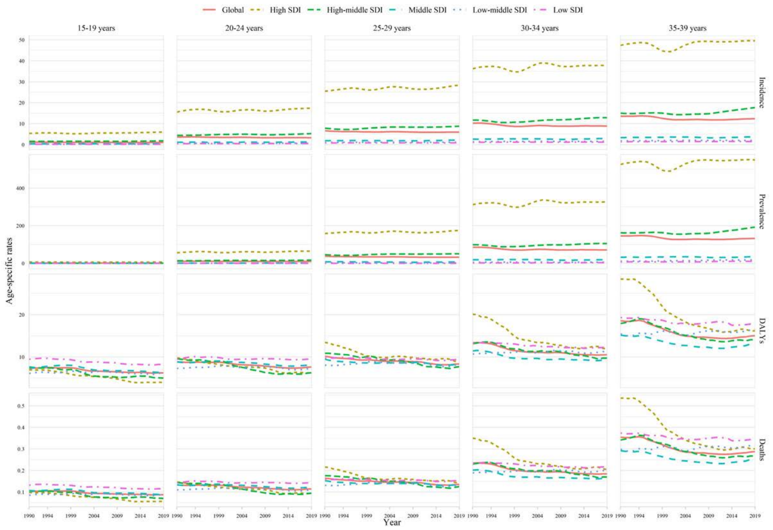

Fig S8 Temporal trends in age-specific incidence, prevalence, disability-adjusted life years (DALYs), and death rate of non-rheumatic valvular heart disease in youths and young adults by age and sociodemographic index, from 1990 to 2019. A, overall; B, women; C, men.

A

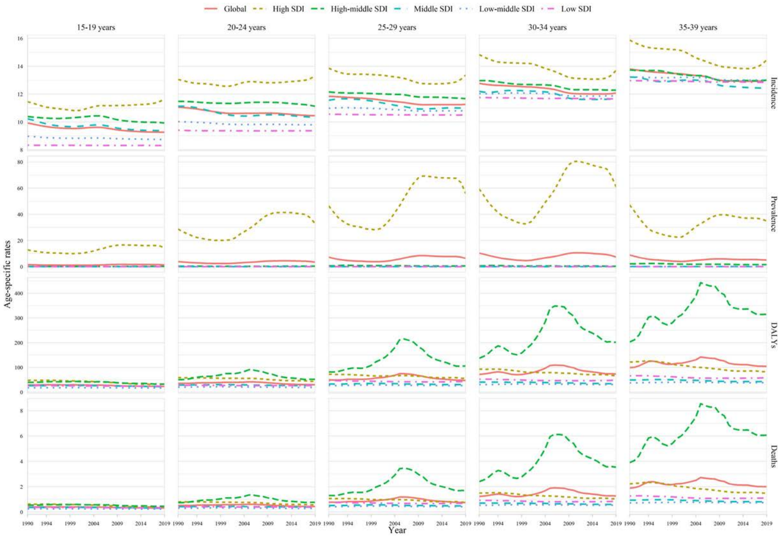

B

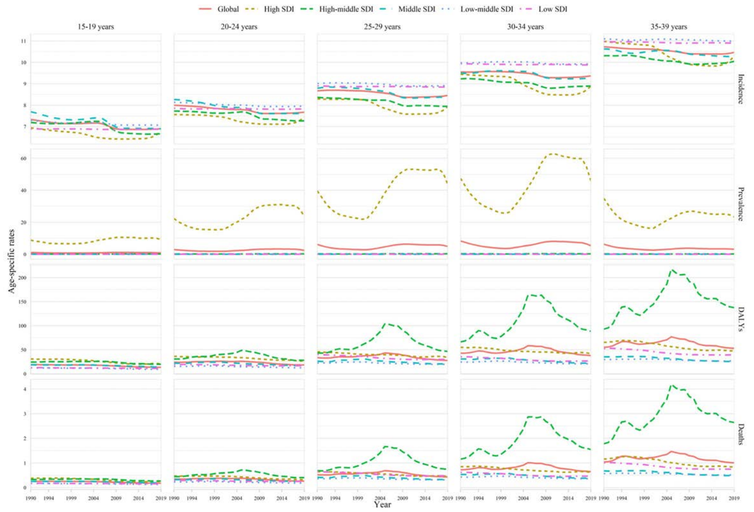

C

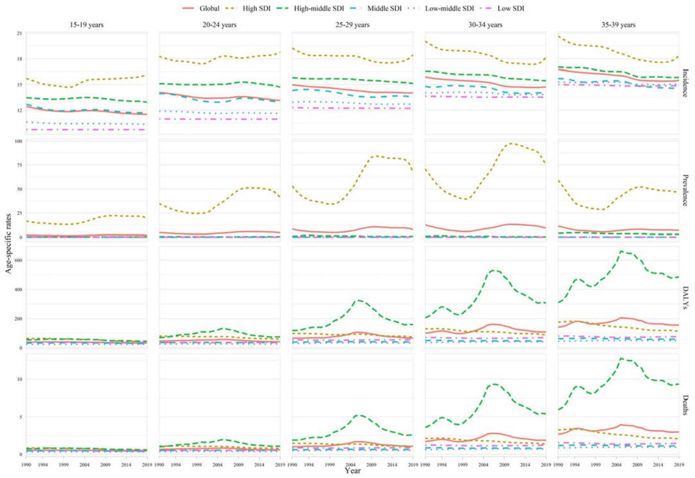

Fig S9 Temporal trends in age-specific incidence, prevalence, disability-adjusted life years (DALYs), and death rate of cardiomyopathy and myocarditis in youths and young adults by age and sociodemographic index, from 1990 to 2019. A, overall; B, women; C, men.

A

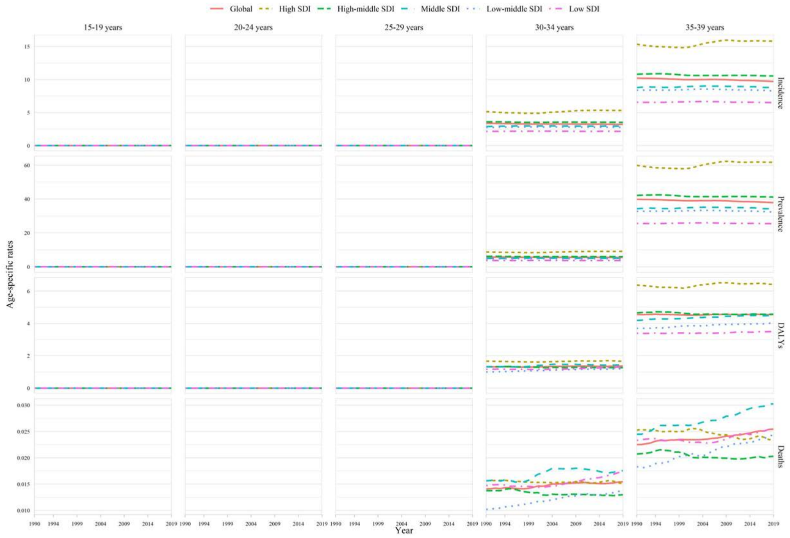

B

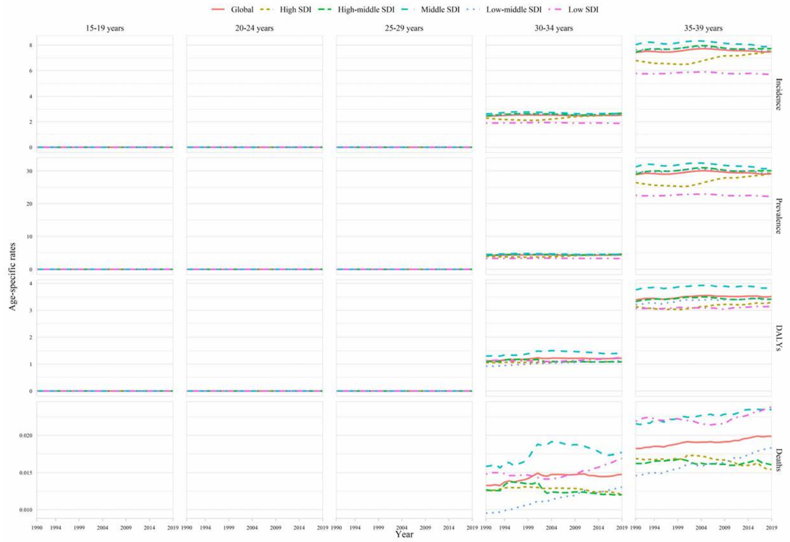

C

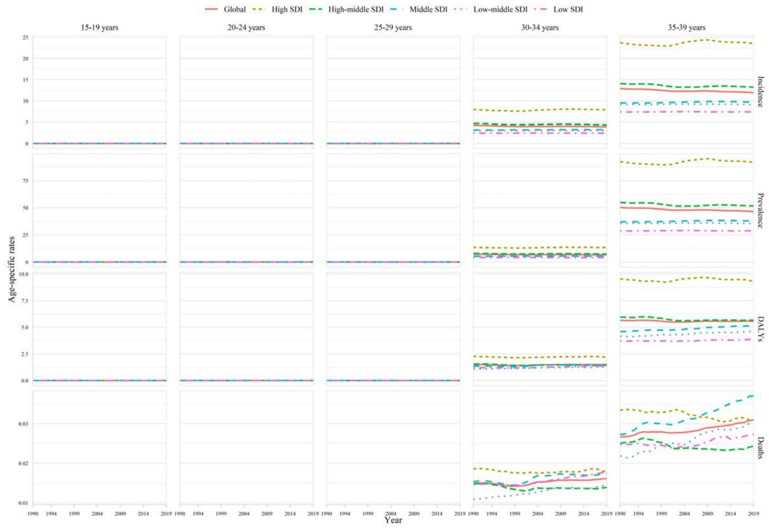

Fig S10 Temporal trends in age-specific incidence, prevalence, disability-adjusted life years (DALYs), and death rate of atrial fibrillation and flutter in youths and young adults by age and sociodemographic index, from 1990 to 2019. A, overall; B, women; C, men. Data for the age of 15-29 years are unavailable.

A

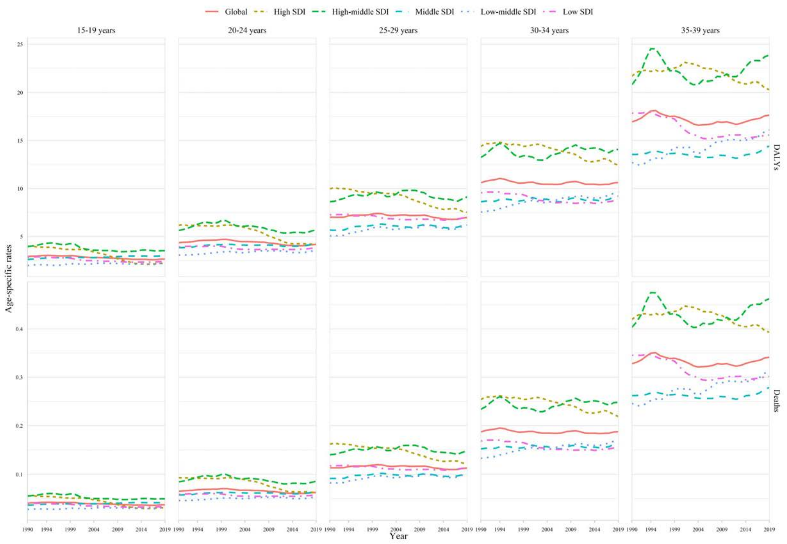

B

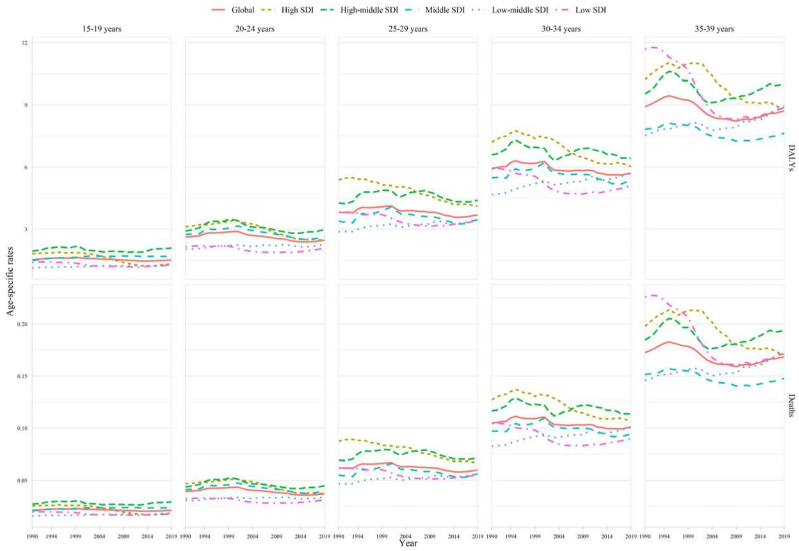

C

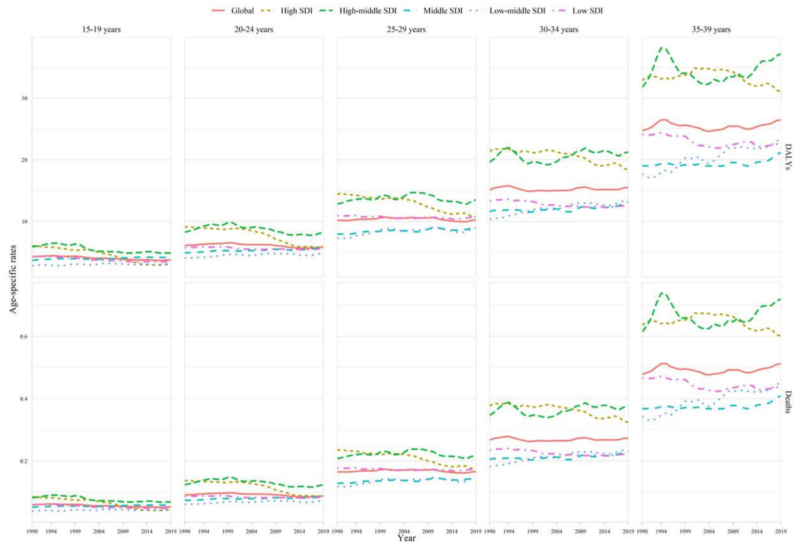

Fig S11 Temporal trends in age-specific disability-adjusted life years (DALYs) and death rate of aortic aneurysm in youths and young adults by age and sociodemographic index, from 1990 to 2019. A, overall; B, women; C, men. Data for incidence and prevalence are unavailable.

A

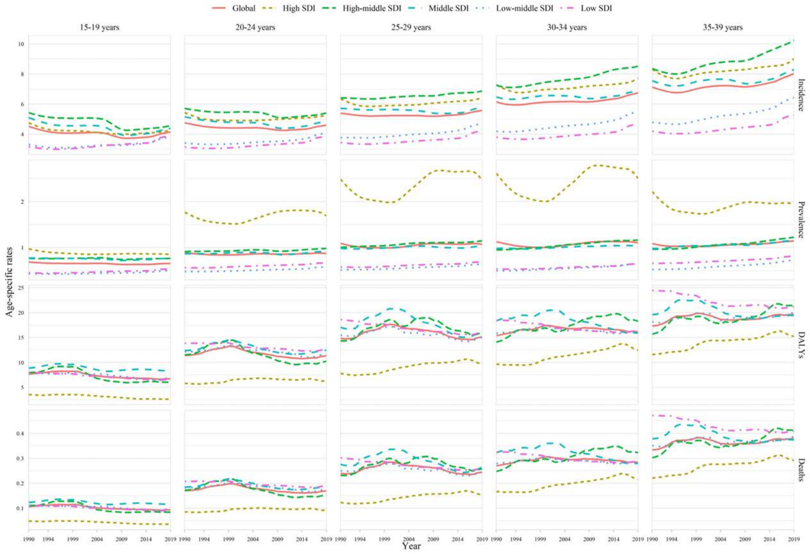

B

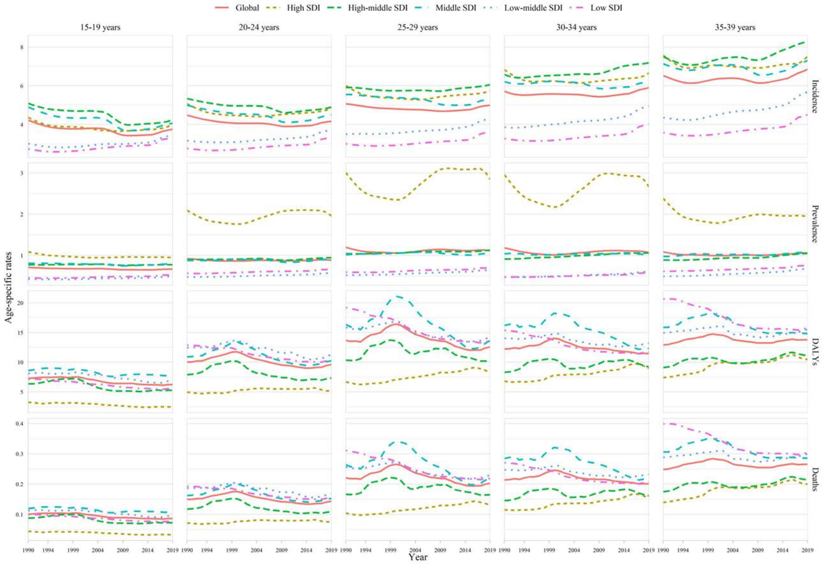

C

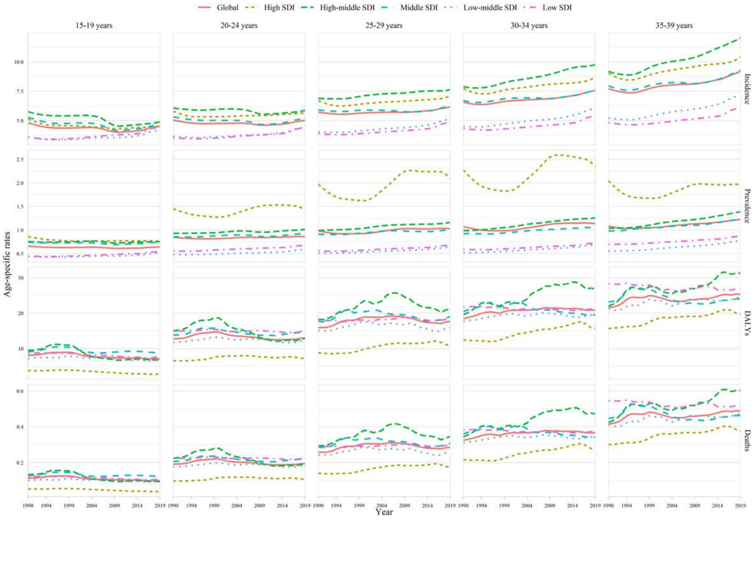

Fig S12 Temporal trends in age-specific incidence, prevalence, disability-adjusted life years (DALYs), and death rate of endocarditis in youths and young adults by age and sociodemographic index, from 1990 to 2019. A, overall; B, women; C, men.

A

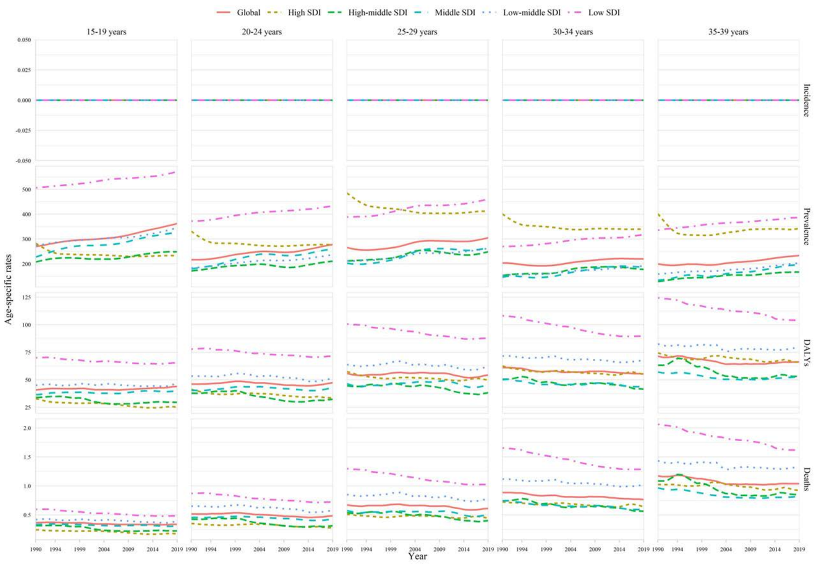

B

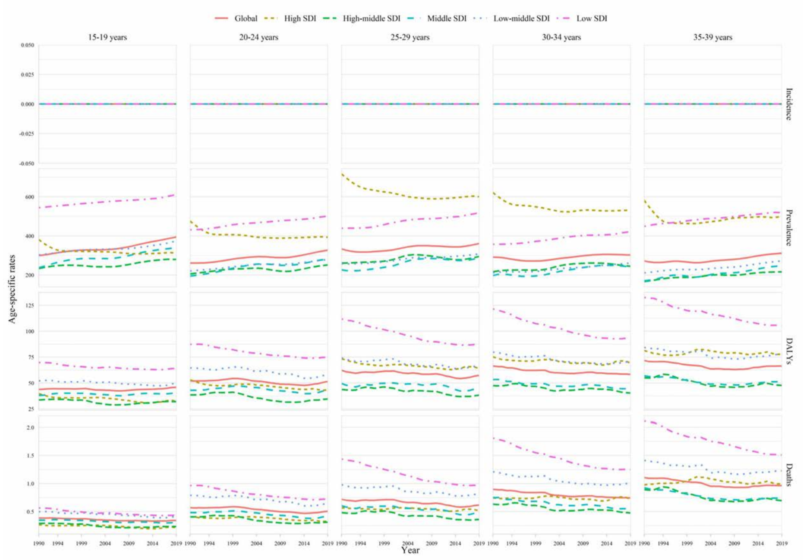

C

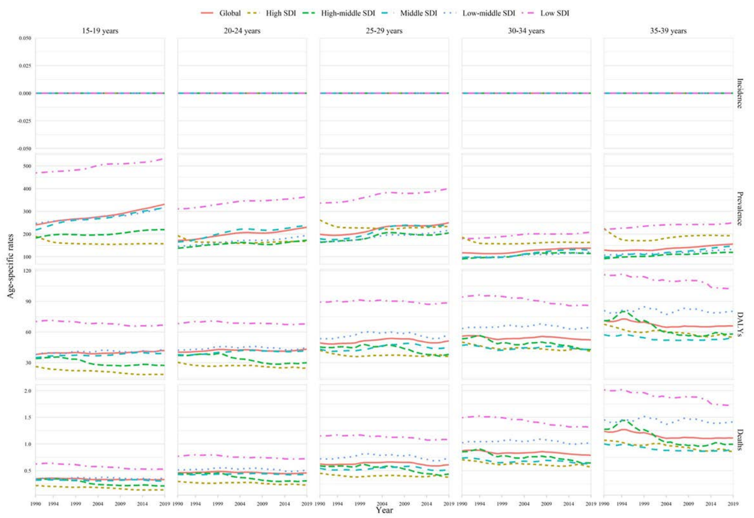

Fig S13 Temporal trends in age-specific prevalence, disability-adjusted life years (DALYs), and death rate of other cardiovascular and circulatory diseases in youths and young adults by age and sociodemographic index, from 1990 to 2019. A, overall; B, women; C, men. Data for incidence are unavailable.

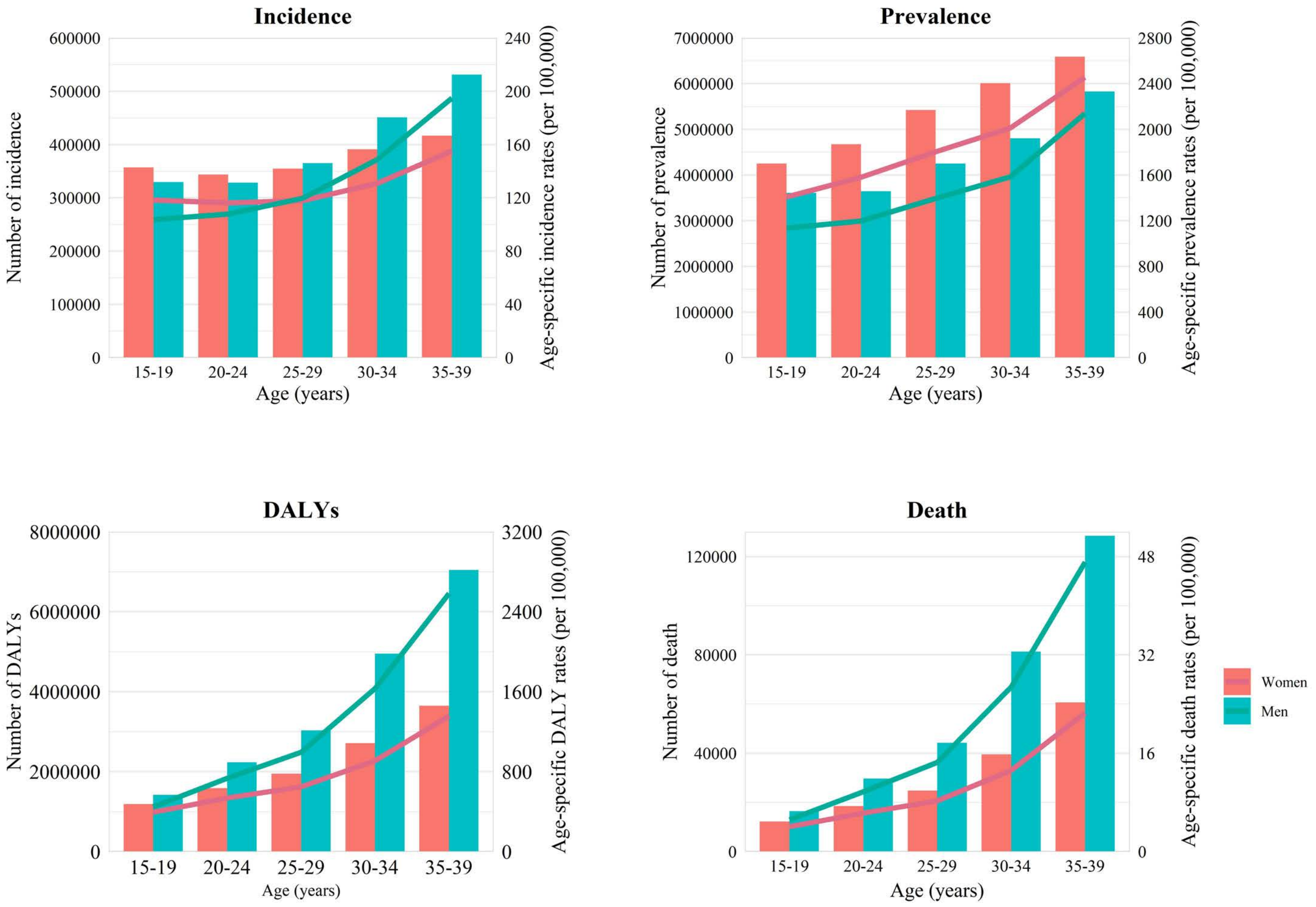

Fig S14 Number and age-specific incidence, prevalence, disability-adjusted life years (DALYs), and death rate of overall cardiovascular disease among youths and young adults in 2019 by age and sex.

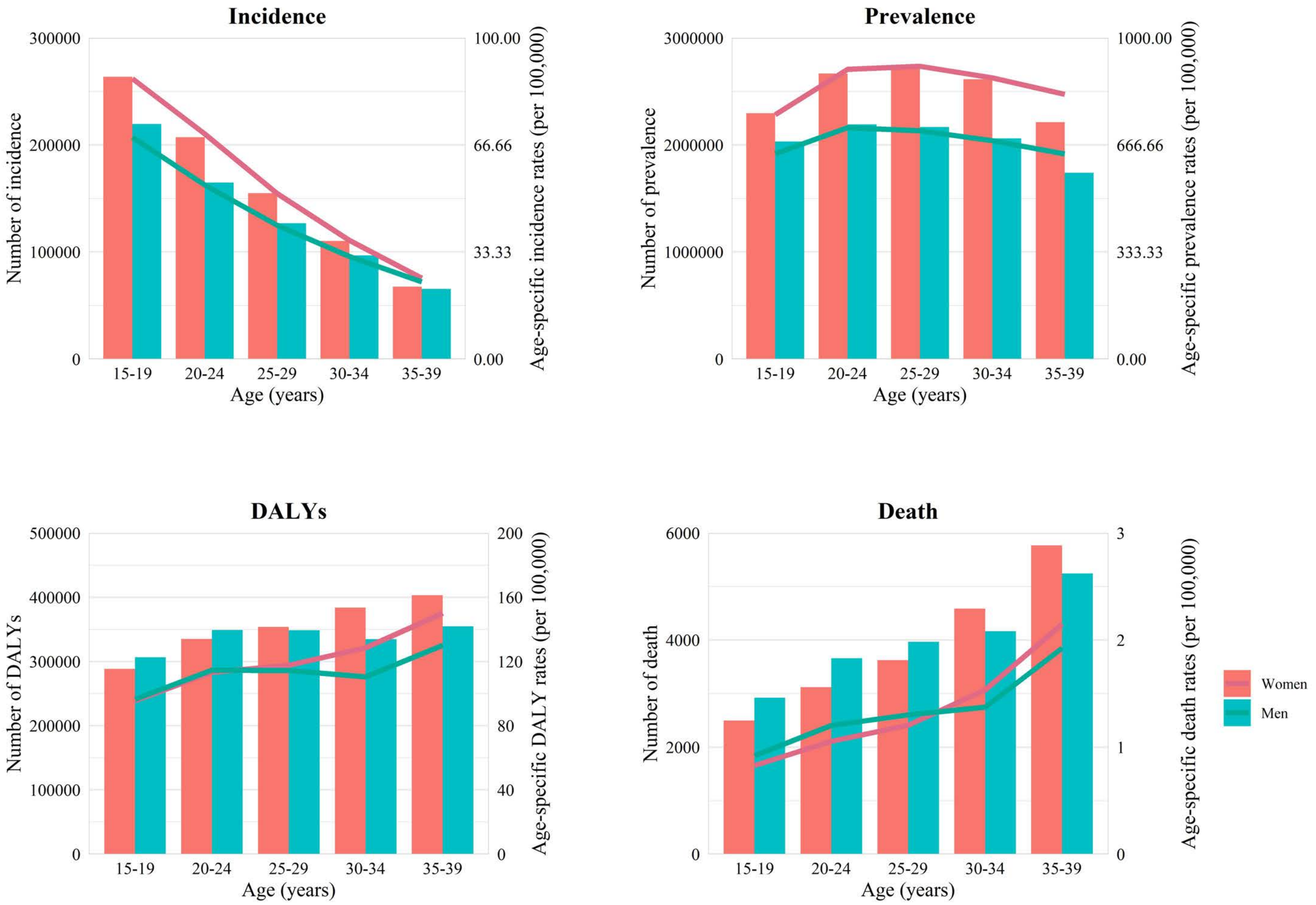

Fig S15 Number and age-specific incidence, prevalence, disability-adjusted life years (DALYs), and death rate of rheumatic heart disease among youths and young adults in 2019 by age and sex.

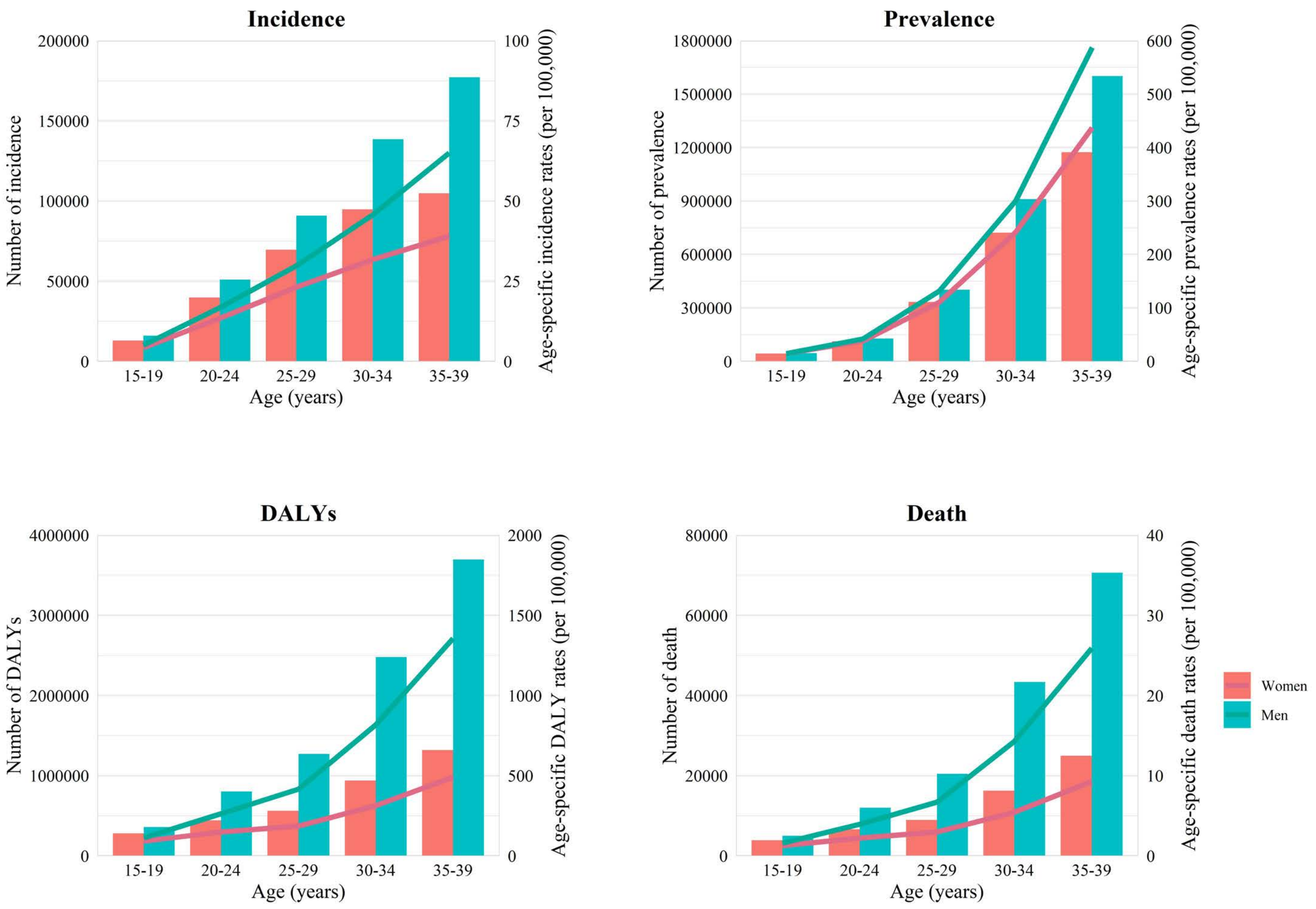

Fig S16 Number and age-specific incidence, prevalence, disability-adjusted life years (DALYs), and death rate of ischemic heart disease among youths and young adults in 2019 by age and sex.

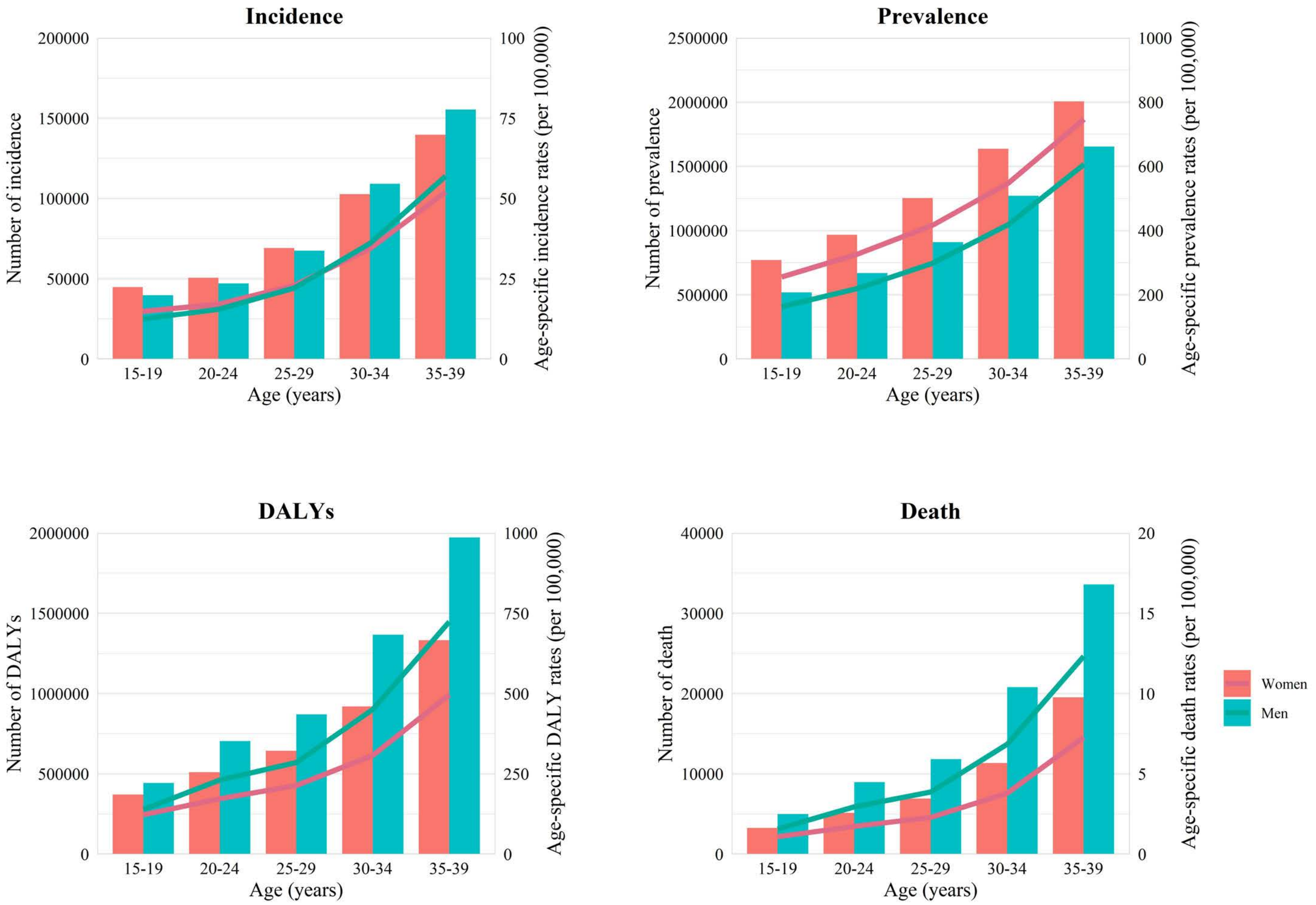

Fig S17 Number and age-specific incidence, prevalence, disability-adjusted life years (DALYs), and death rate of stroke among youths and young adults in 2019 by age and sex.

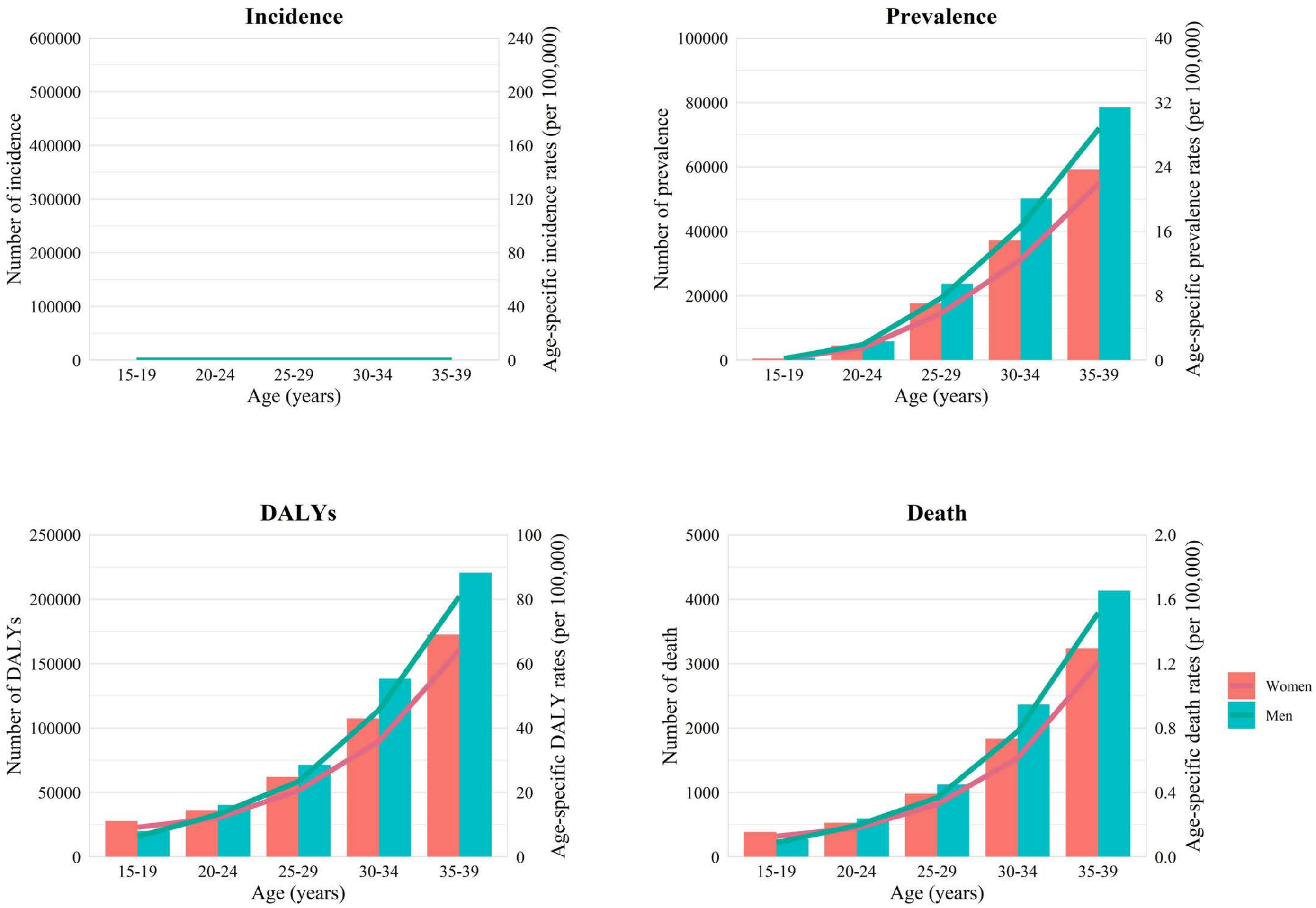

Fig S18 Number and age-specific prevalence, disability-adjusted life years (DALYs), and death rate of hypertensive heart disease among youths and young adults in 2019 by age and sex. Data for incidence are unavailable.

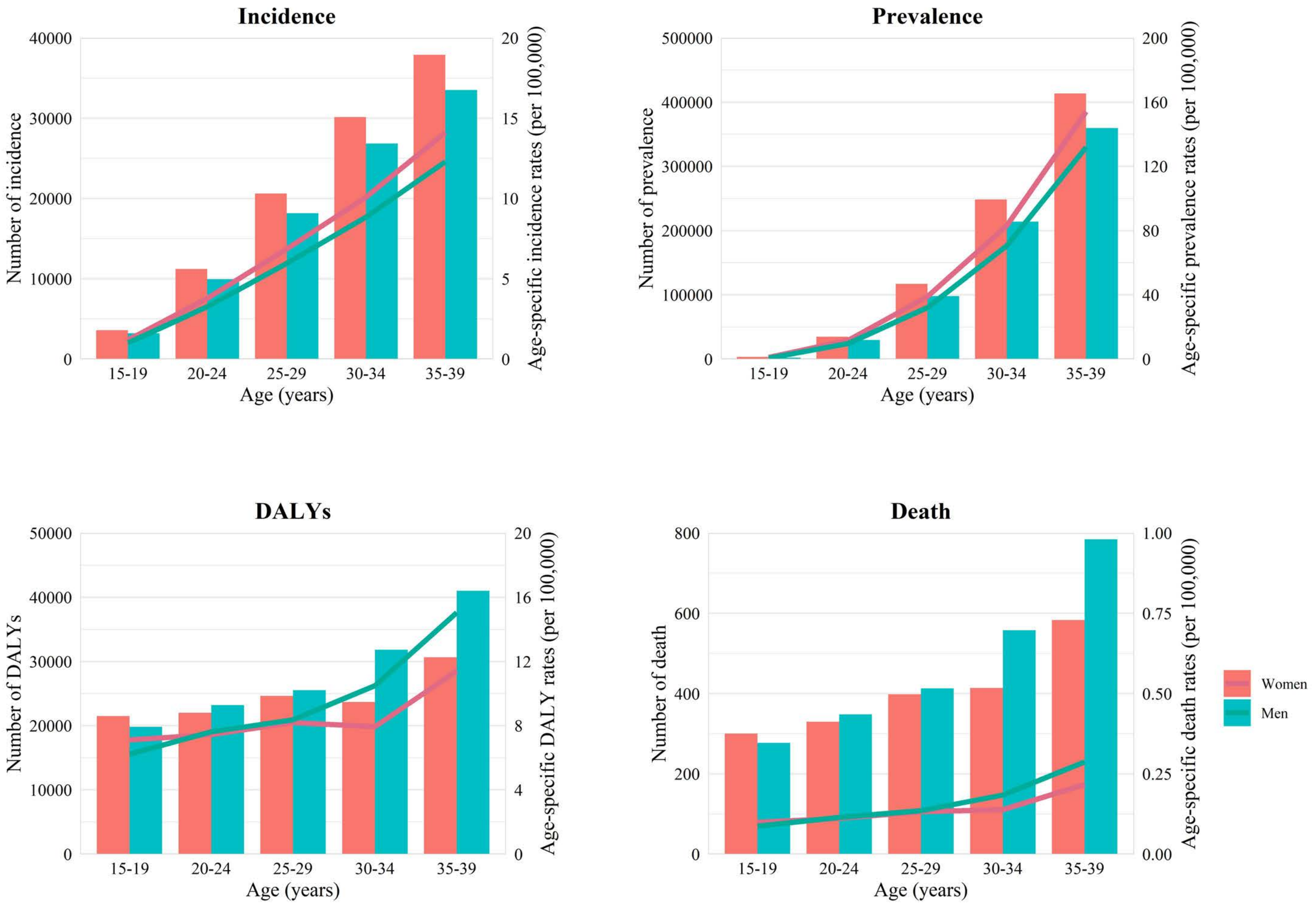

Fig S19 Number and age-specific incidence, prevalence, disability-adjusted life years (DALYs), and death rate of non-rheumatic valvular heart disease among youths and young adults in 2019 by age and sex.

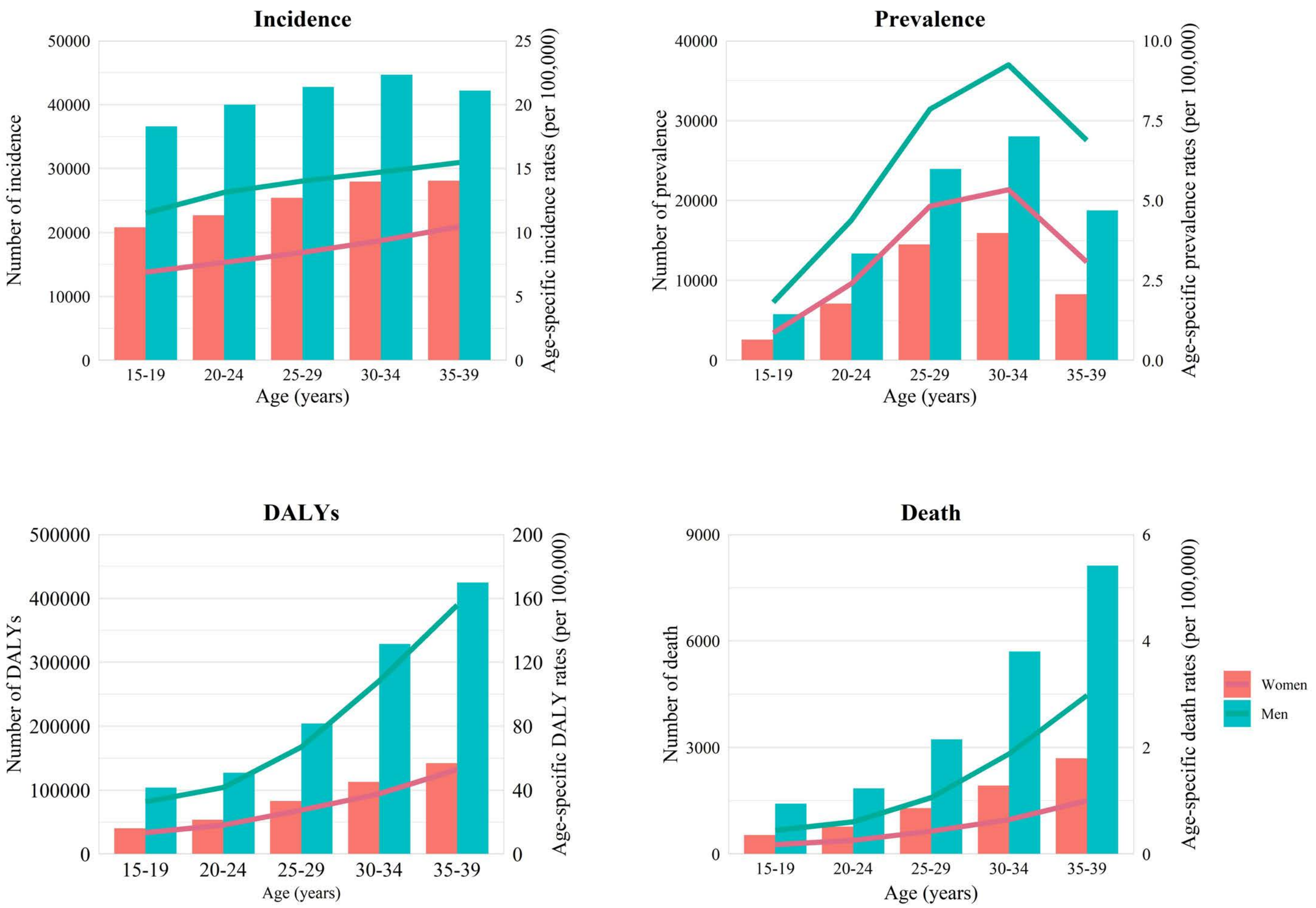

Fig S20 Number and age-specific incidence, prevalence, disability-adjusted life years (DALYs), and death rate of cardiomyopathy and myocarditis among youths and young adults in 2019 by age and sex.

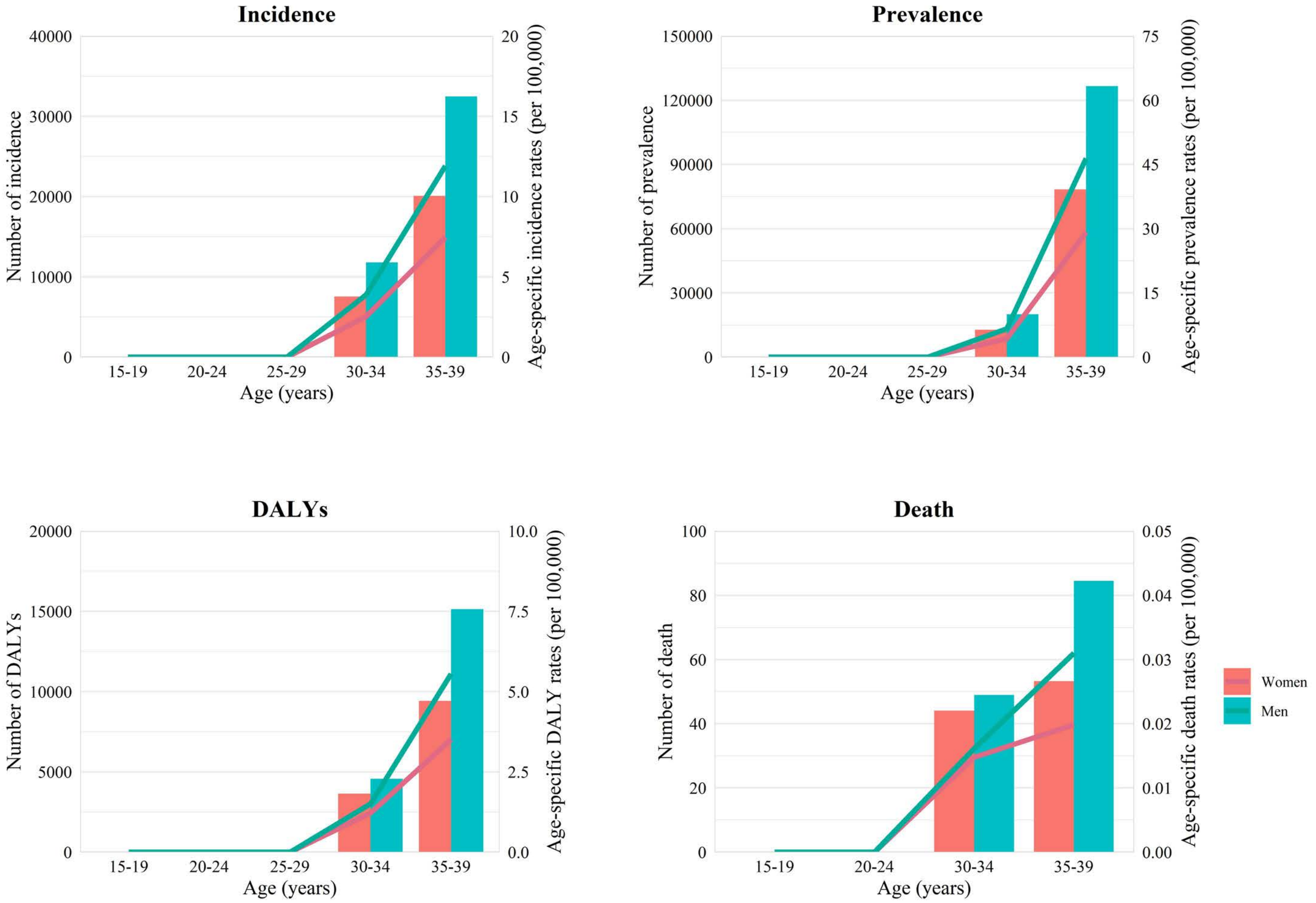

Fig S21 Number and age-specific incidence, prevalence, disability-adjusted life years (DALYs), and death rate of atrial fibrillation and flutter among youths and young adults in 2019 by age and sex. Data for the age of 15-29 are unavailable.

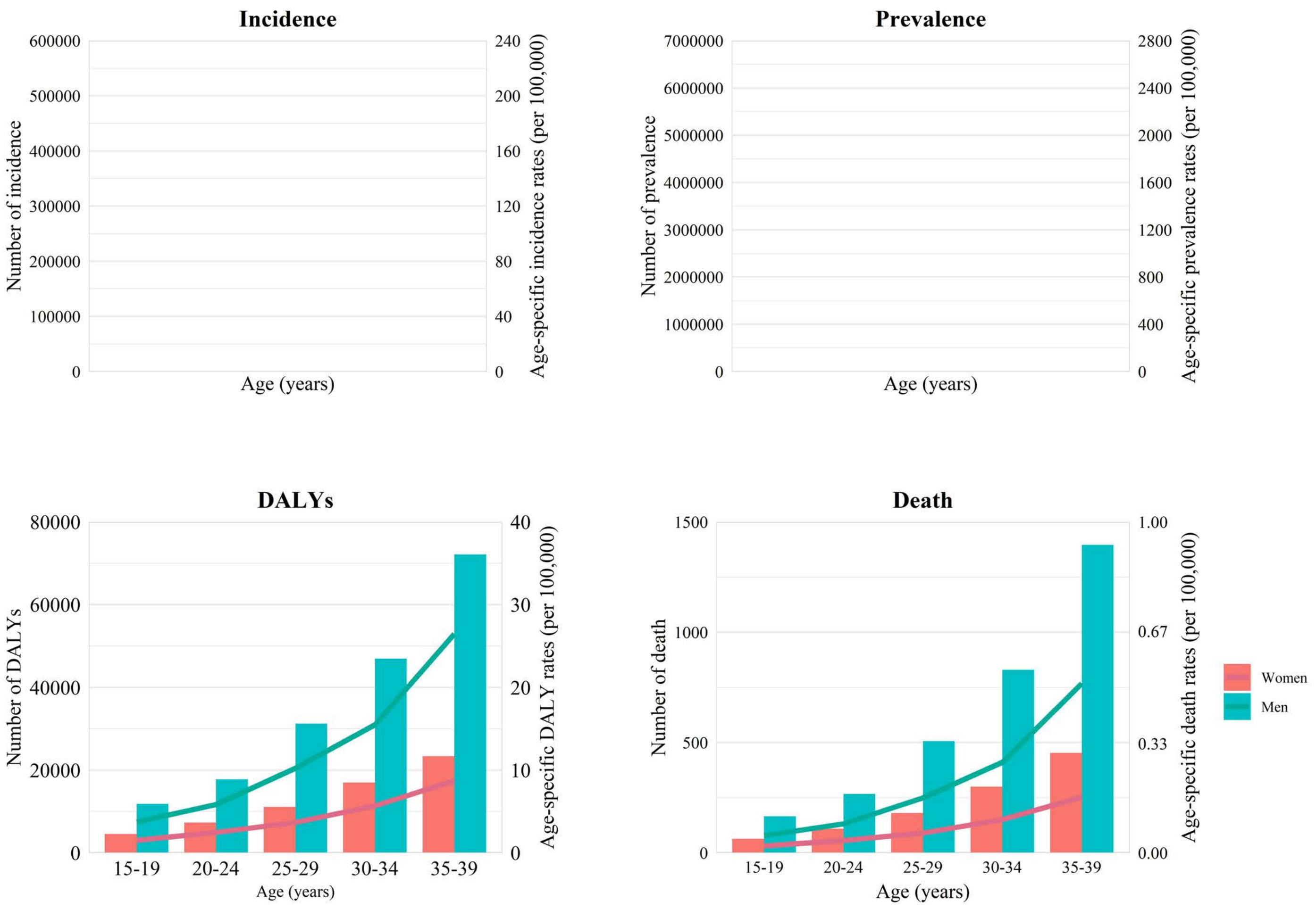

Fig S22 Number and age-specific incidence, prevalence, disability-adjusted life years (DALYs), and death rate of aortic aneurysm among youths and young adults in 2019 by age and sex. Data for incidence and prevalence are unavailable.

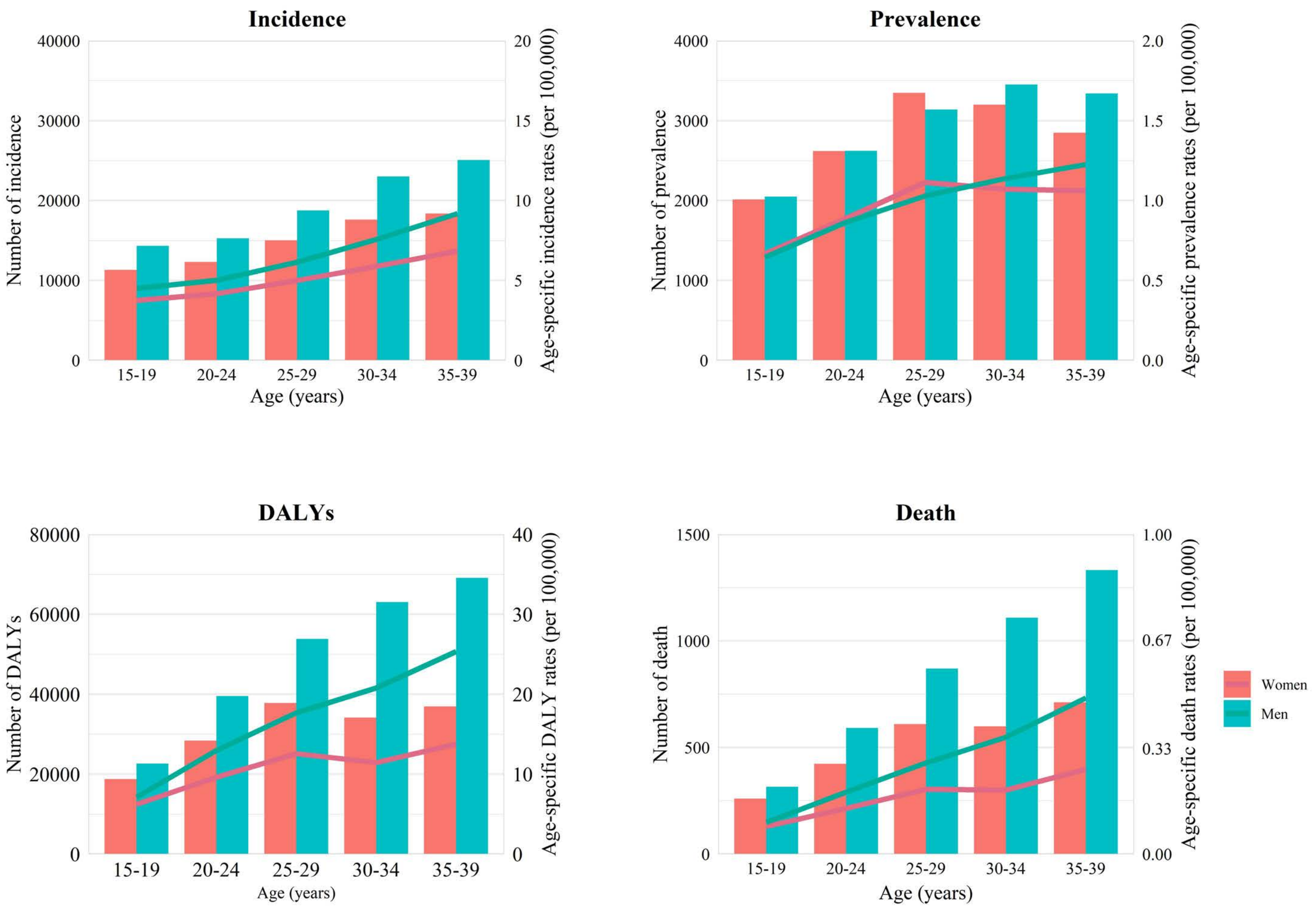

Fig S23 Number and age-specific incidence, prevalence, disability-adjusted life years (DALYs), and death rate of endocarditis among youths and young adults in 2019 by age and sex.

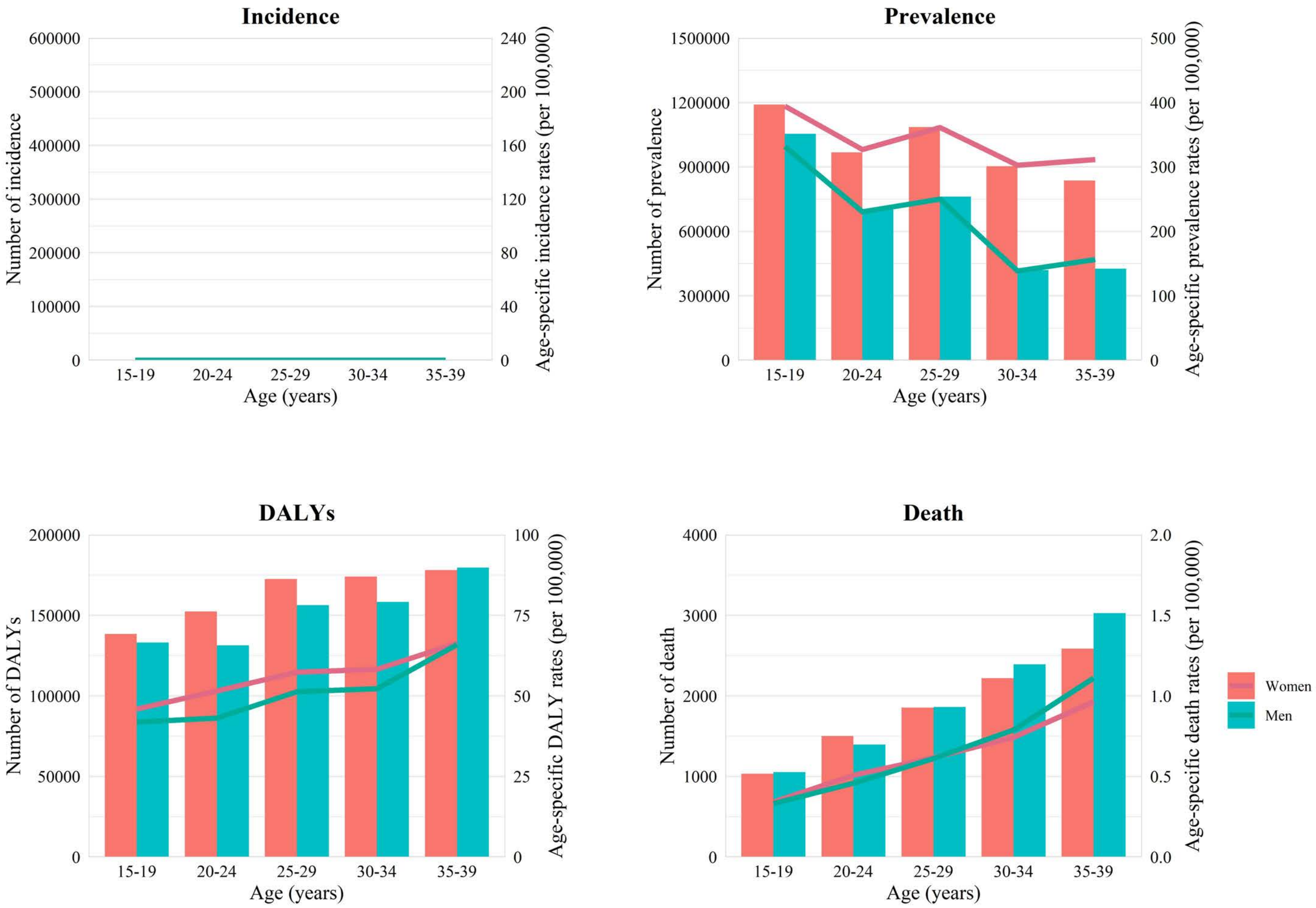

Fig S24 Number and age-specific prevalence, disability-adjusted life years (DALYs), and death rate of other cardiovascular and circulatory diseases among youths and young adults in 2019 by age and sex. Data for incidence are unavailable.

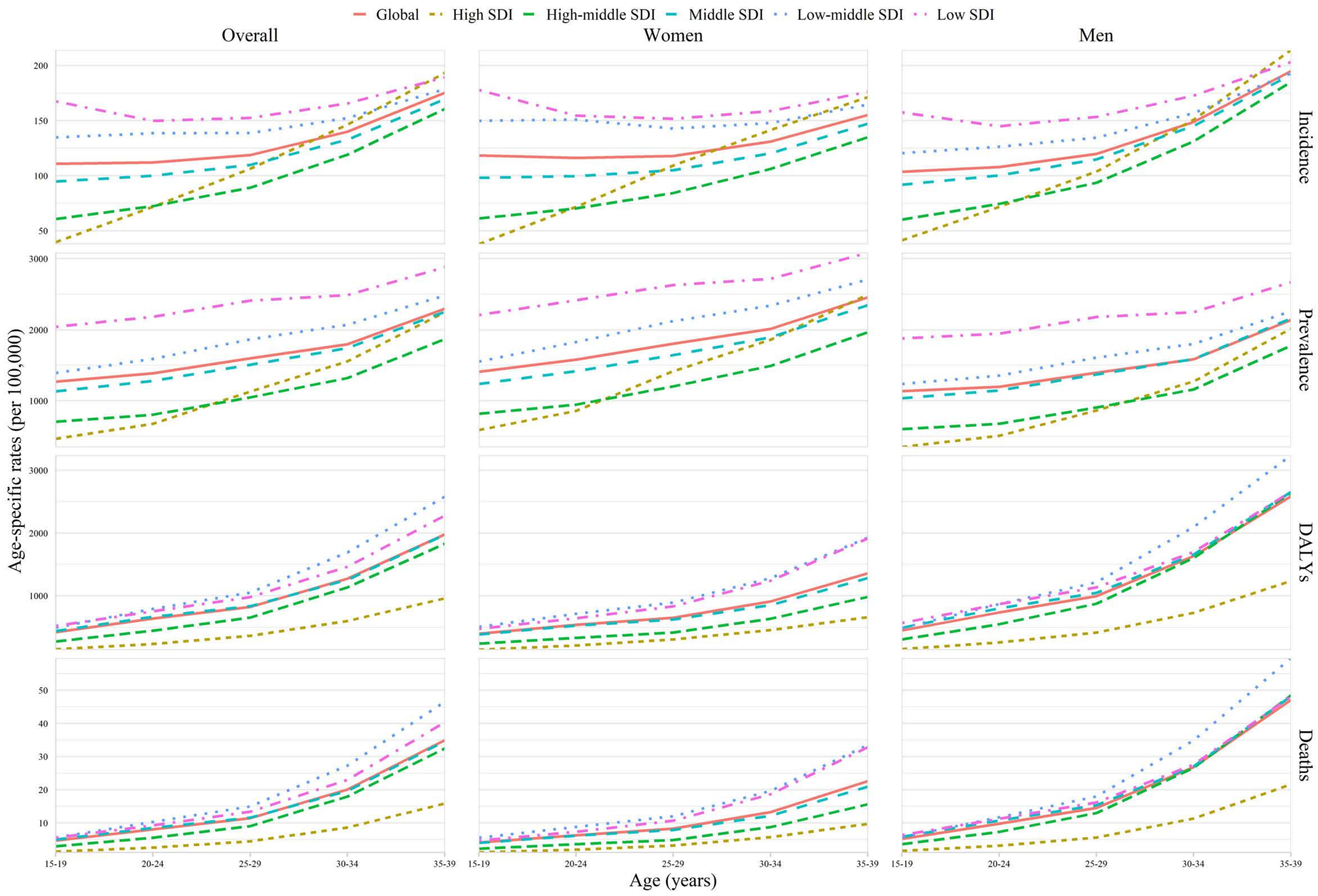

Fig S25 Age-specific incidence, prevalence, disability-adjusted life years (DALYs), and death rate of overall cardiovascular disease among youths and young adults in 2019 by age, sex, and sociodemographic index.

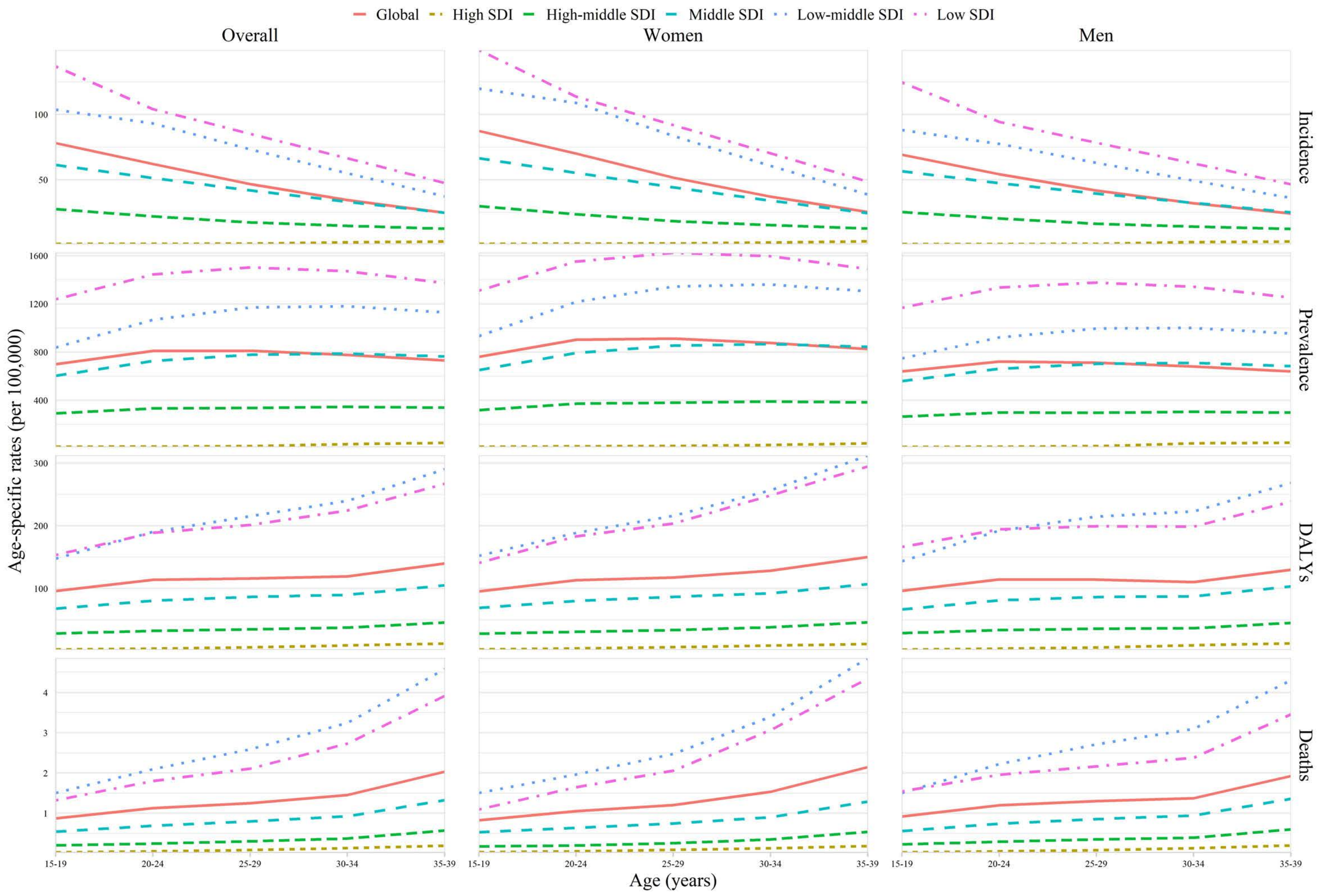

Fig S26 Age-specific incidence, prevalence, disability-adjusted life years (DALYs), and death rate of rheumatic heart disease among youths and young adults in 2019 by age, sex, and sociodemographic index.

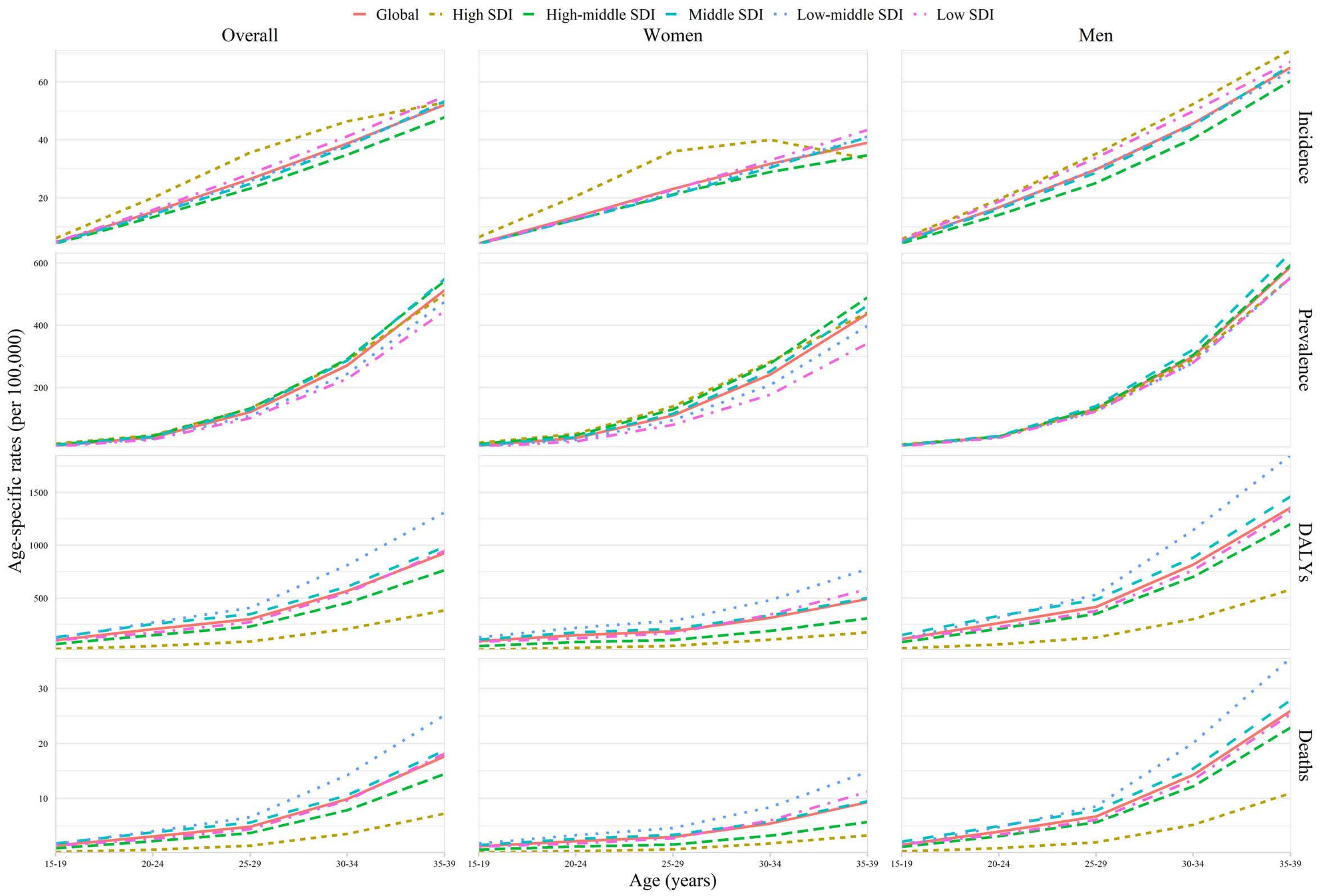

Fig S27 Age-specific incidence, prevalence, disability-adjusted life years (DALYs), and death rate of ischemic heart disease among youths and young adults in 2019 by age, sex, and sociodemographic index.

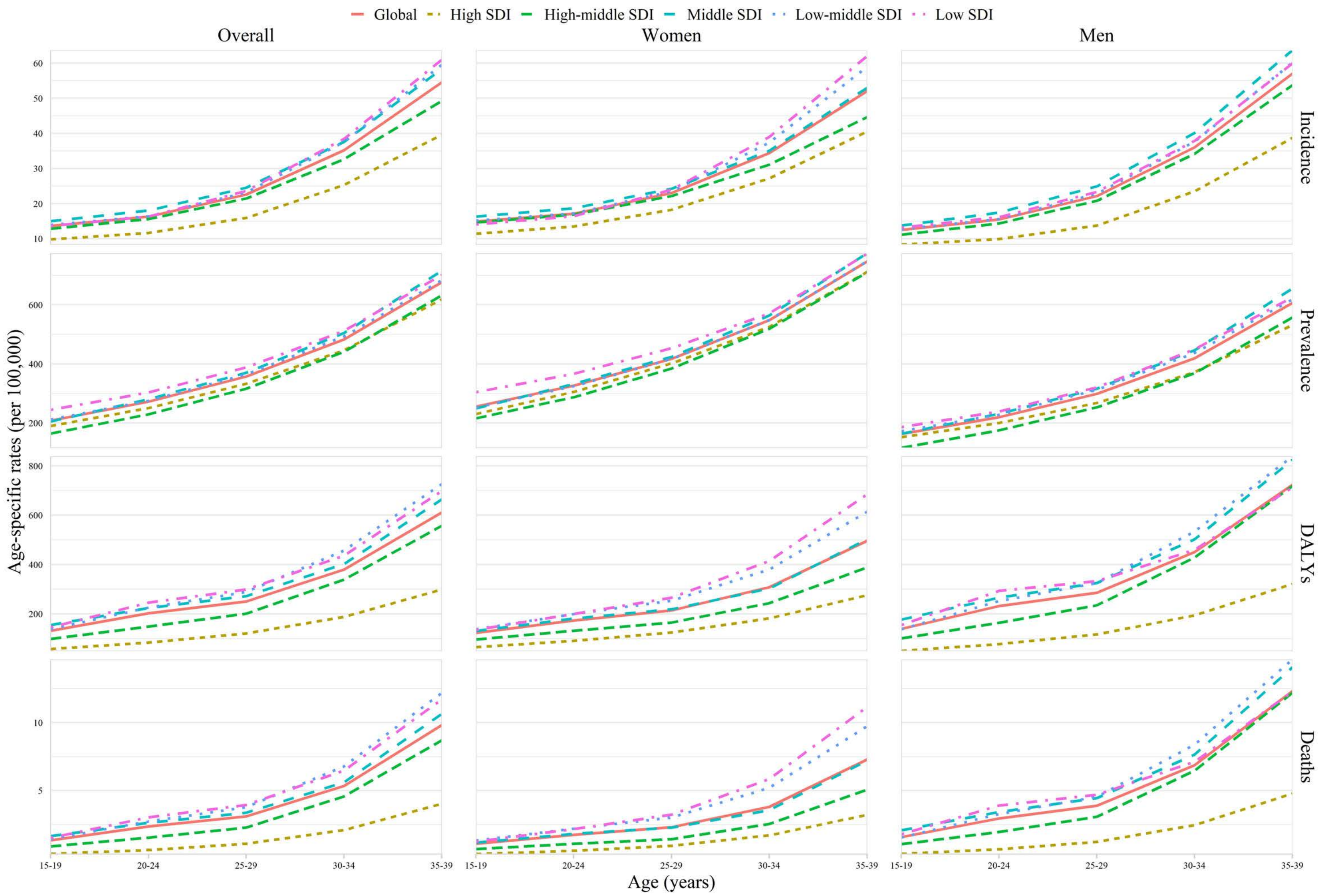

Fig S28 Age-specific incidence, prevalence, disability-adjusted life years (DALYs), and death rate of stroke among youths and young adults in 2019 by age, sex, and sociodemographic index.

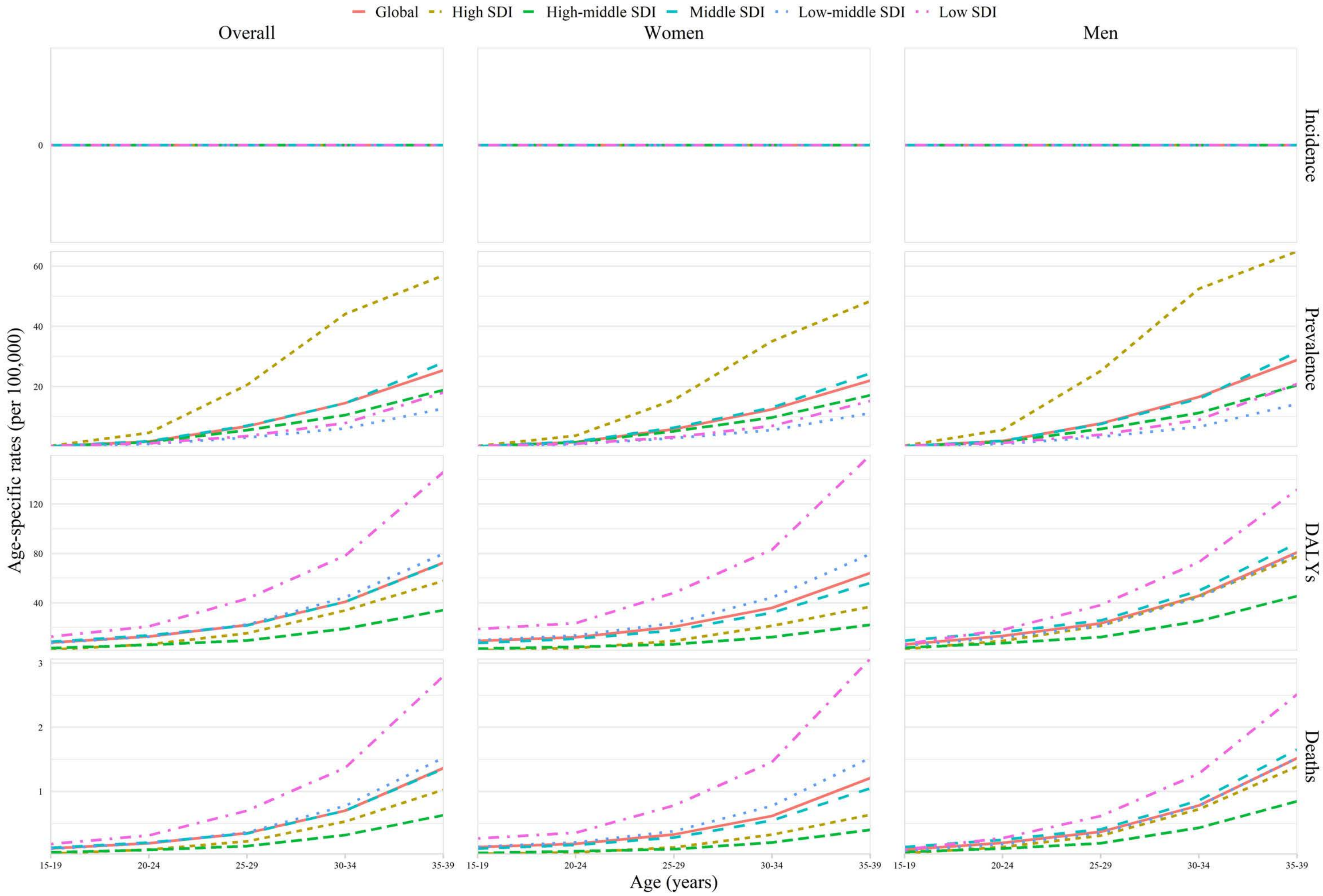

Fig S29 Age-specific prevalence, disability-adjusted life years (DALYs), and death rate of hypertensive heart disease among youths and young adults in 2019 by age, sex, and sociodemographic index. Data for incidence are unavailable.

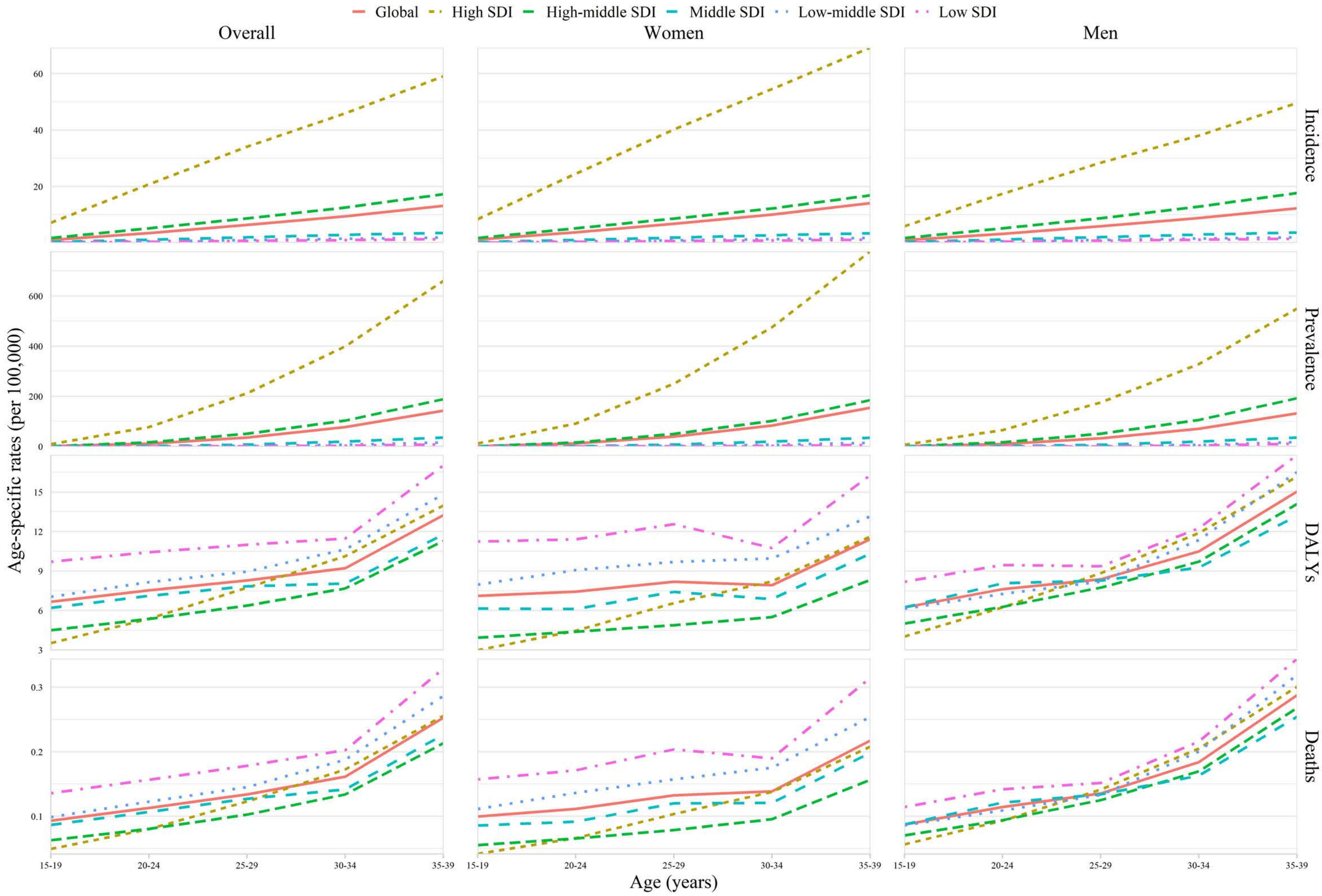

Fig S30 Age-specific incidence, prevalence, disability-adjusted life years (DALYs), and death rate of non-rheumatic valvular heart disease among youths and young adults in 2019 by age, sex, and sociodemographic index.

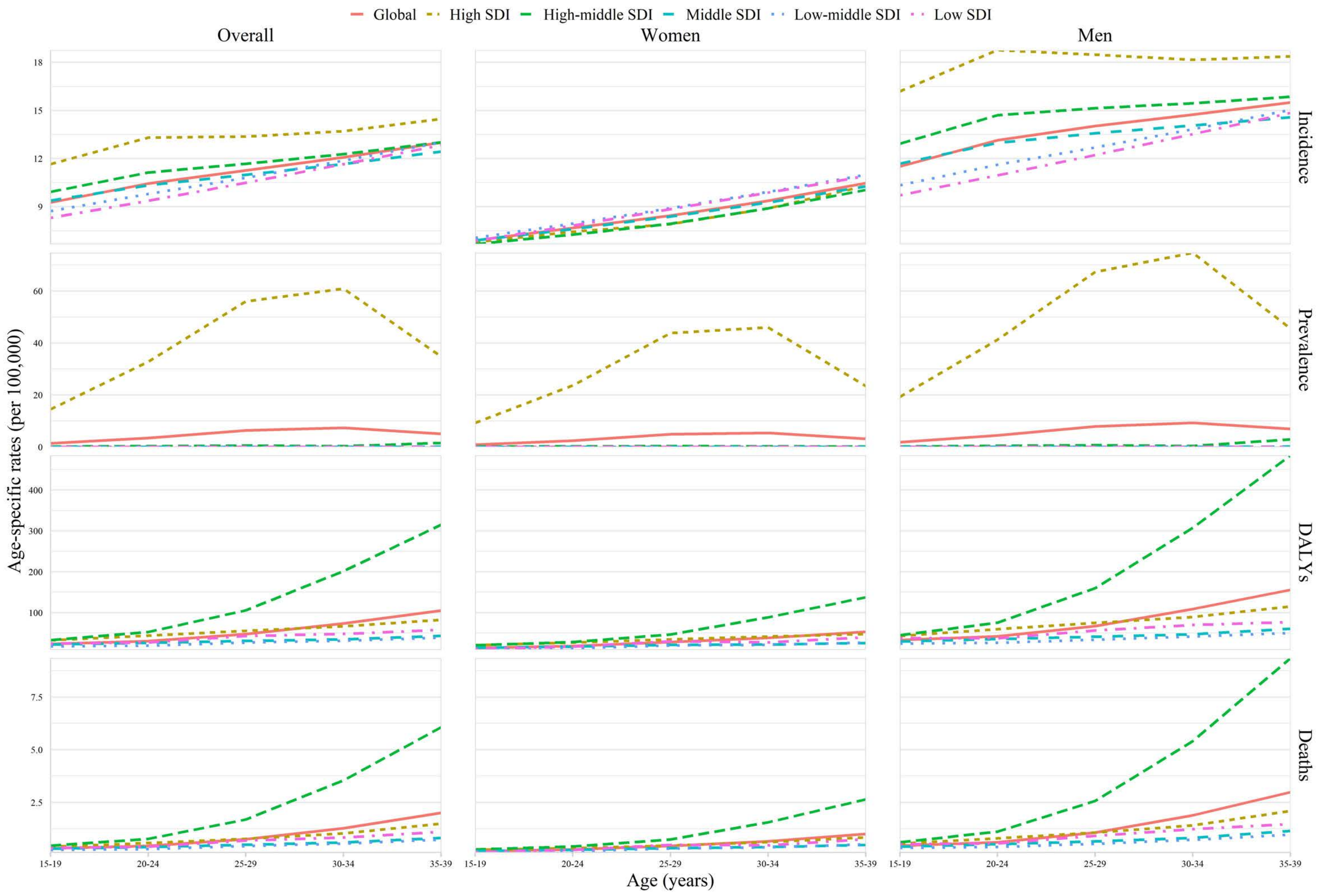

Fig S31 Age-specific incidence, prevalence, disability-adjusted life years (DALYs), and death rate of cardiomyopathy and myocarditis among youths and young adults in 2019 by age, sex, and sociodemographic index.

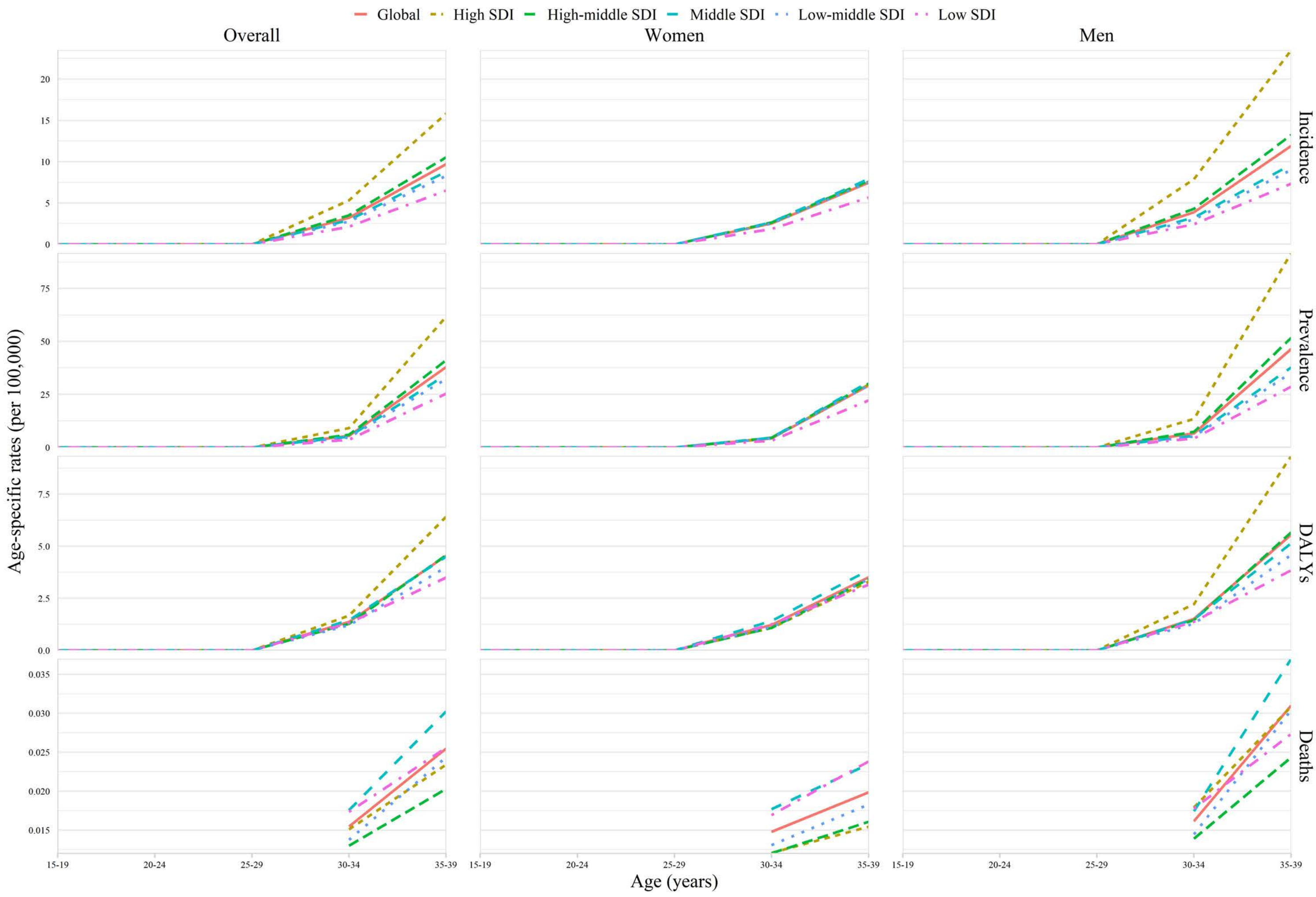

Fig S32 Age-specific incidence, prevalence, disability-adjusted life years (DALYs), and death rate of atrial fibrillation and flutter among youths and young adults in 2019 by age, sex, and sociodemographic index. Data for the age of 15-29 are unavailable.

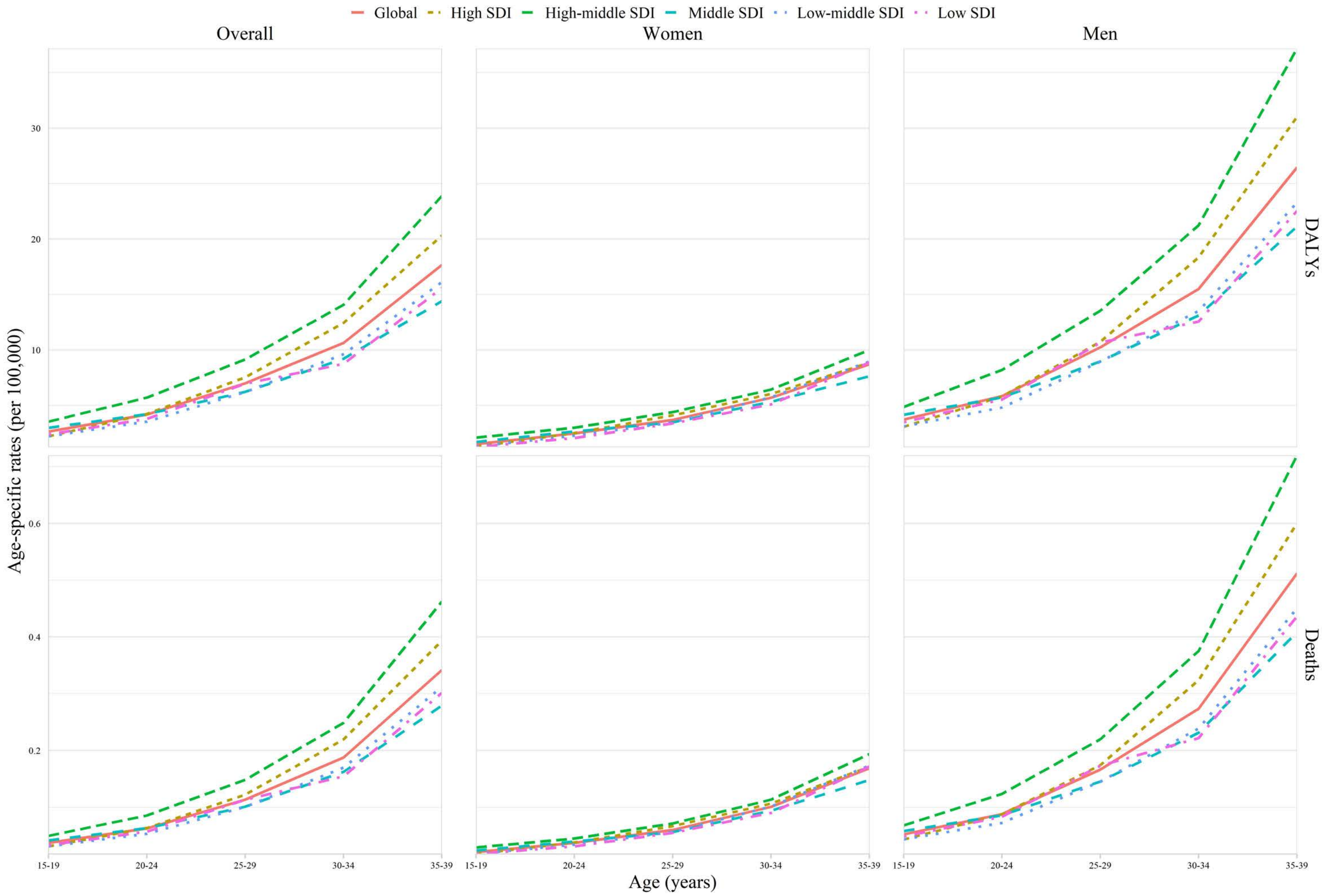

Fig S33 Age-specific disability-adjusted life years (DALYs) and death rate of aortic aneurysm among youths and young adults in 2019 by age, sex, and sociodemographic index. Data for incidence and prevalence are unavailable.

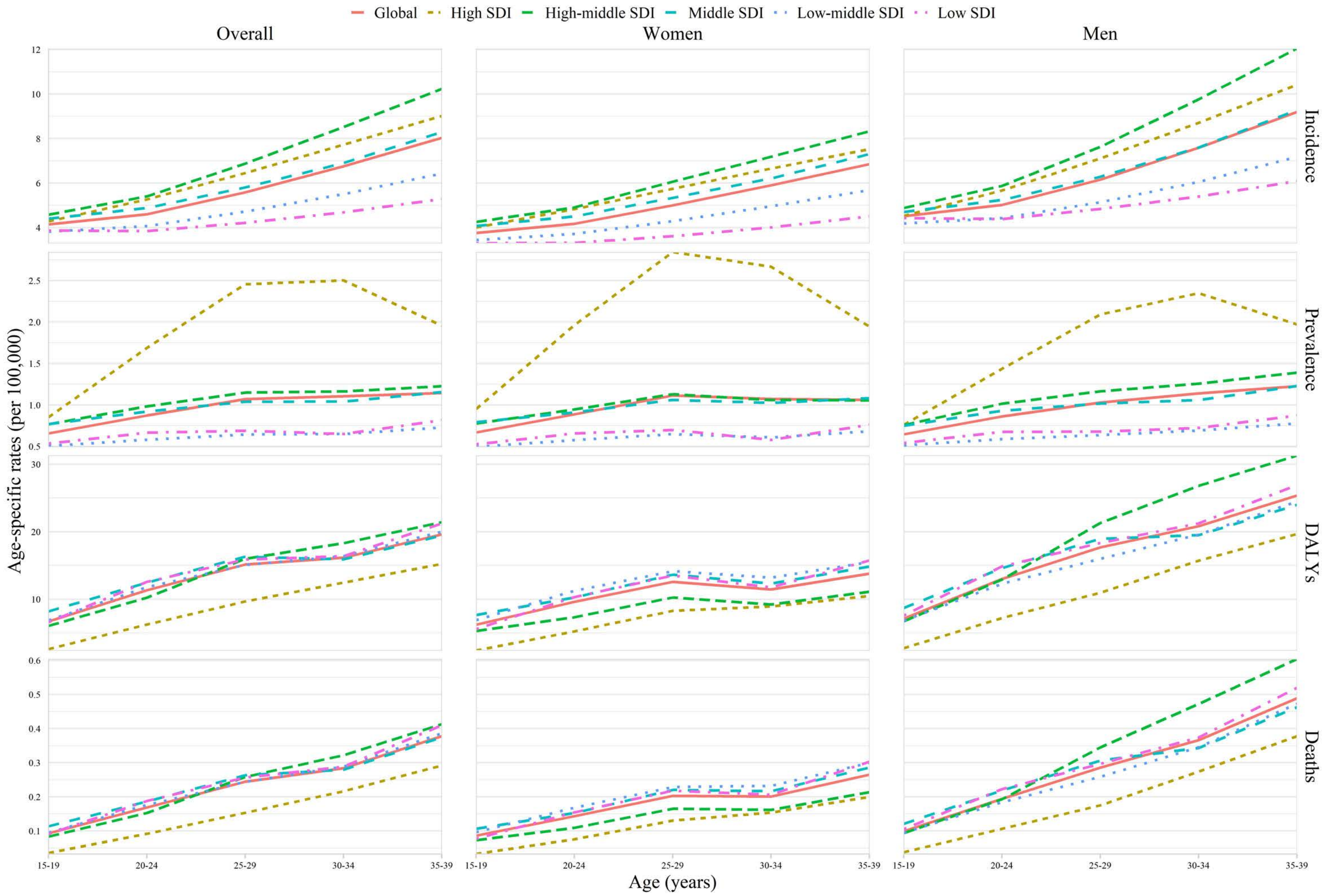

Fig S34 Age-specific incidence, prevalence, disability-adjusted life years (DALYs), and death rate of endocarditis among youths and young adults in 2019 by age, sex, and sociodemographic index.

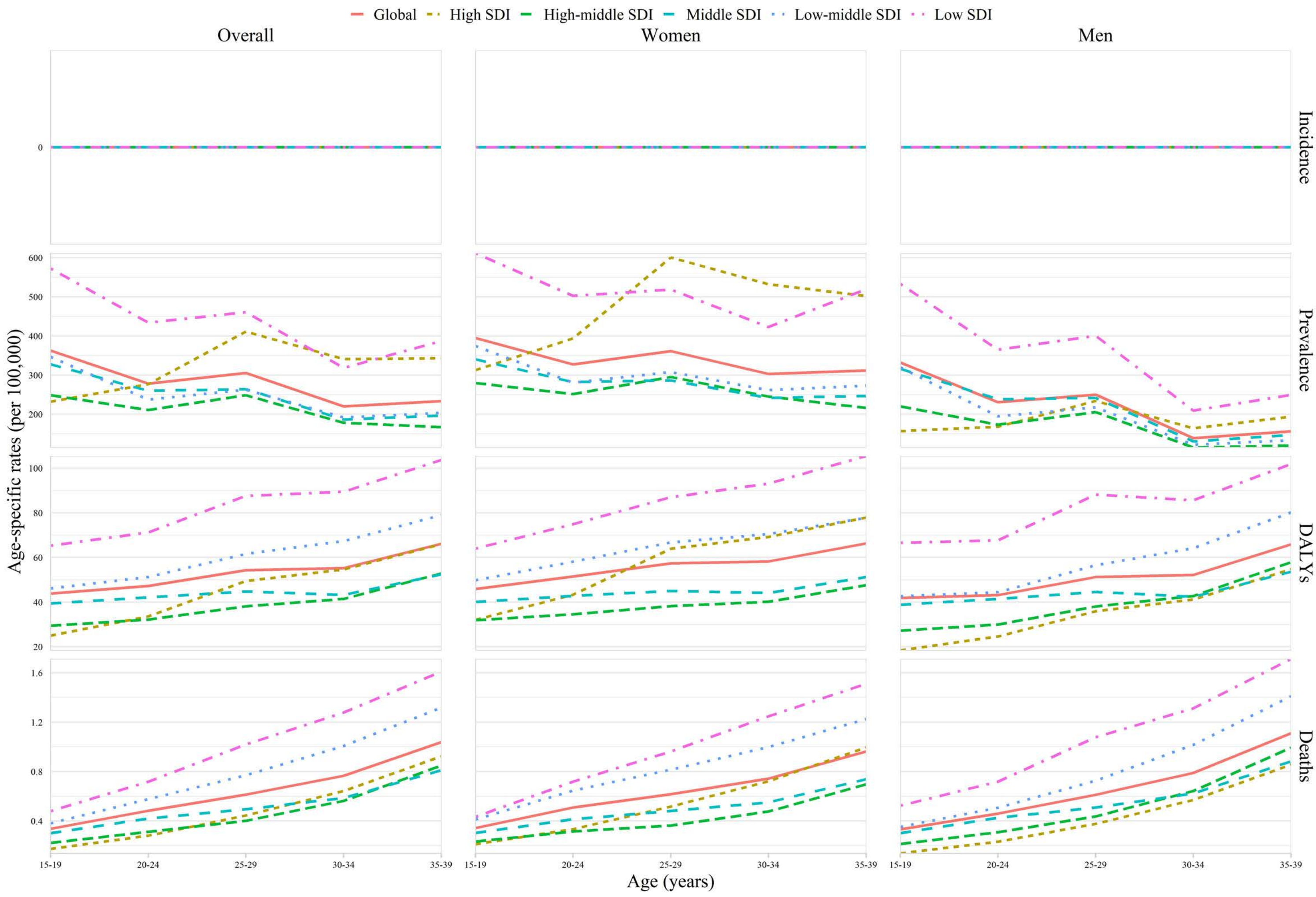

Fig S35 Age-specific prevalence, disability-adjusted life years (DALYs), and death rate of other cardiovascular and circulatory diseases among youths and young adults in 2019 by age, sex, and sociodemographic index. Data for incidence are unavailable.

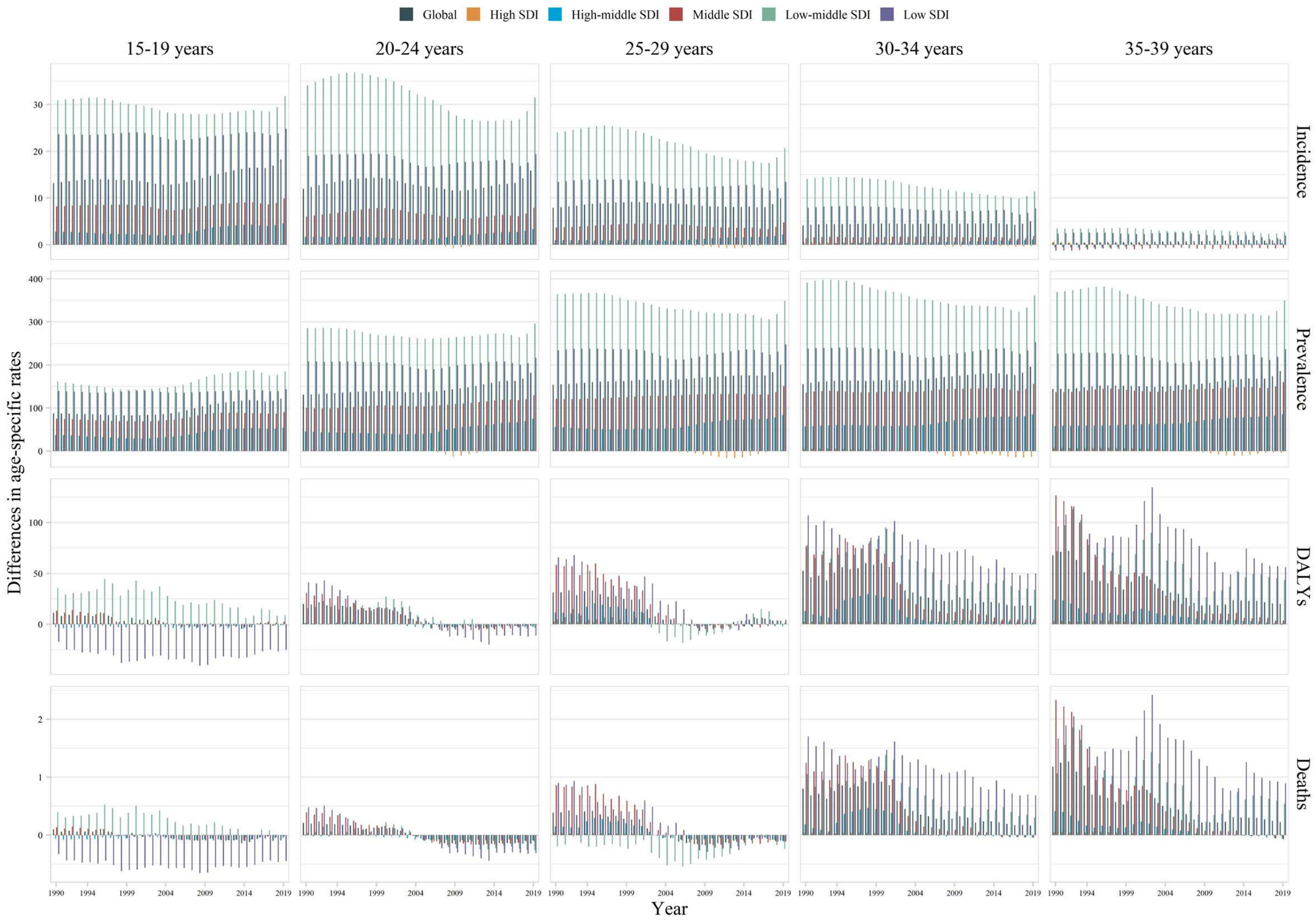

Fig 36 Difference in age-specific incidence, prevalence, disability-adjusted life years (DALYs), and death rate of rheumatic heart disease in youths and young adults between men and women by age and sociodemographic index, from 1990 to 2019. The difference indicates age-standardized rate in women minus that in men. A difference >0 suggests that women have a higher rate than men.

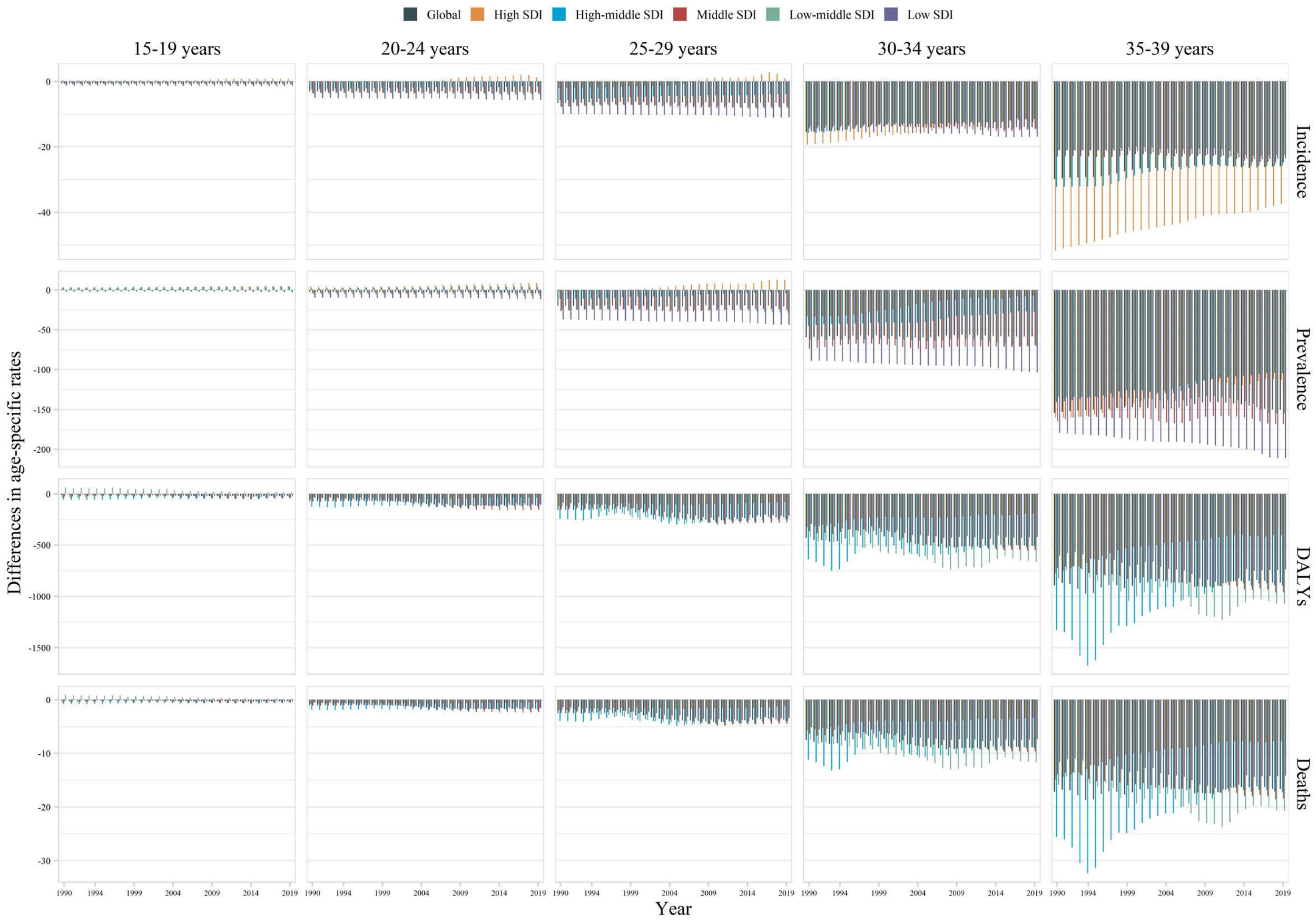

Fig 37 Difference in age-specific incidence, prevalence, disability-adjusted life years (DALYs), and death rate of ischemic heart disease in youths and young adults between men and women by age and sociodemographic index, from 1990 to 2019. The difference indicates age-standardized rate in women minus that in men. A difference >0 suggests that women have higher a rate than men.

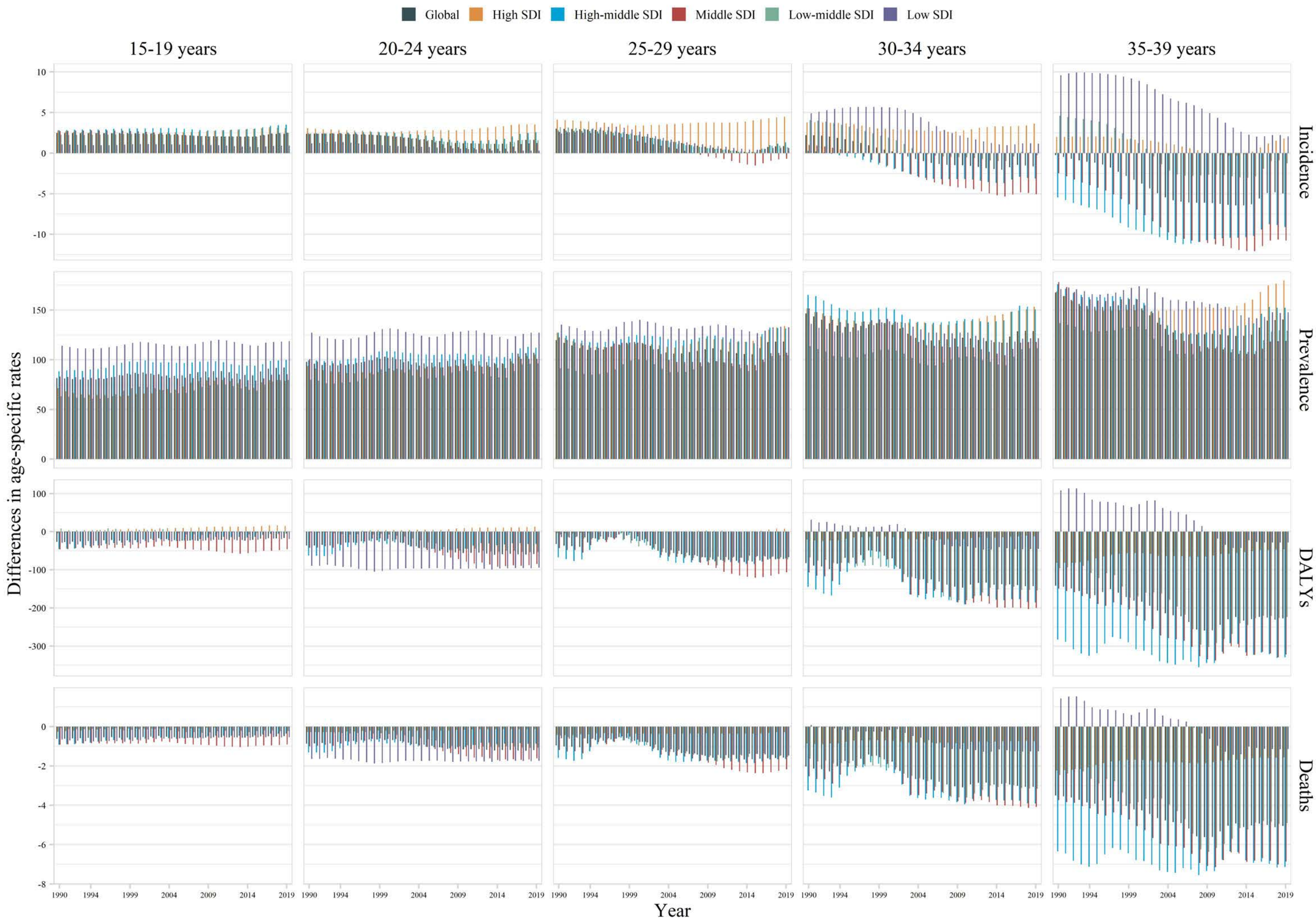

Fig 38 Difference in age-specific incidence, prevalence, disability-adjusted life years (DALYs), and death rate of stroke in youths and young adults between men and women by age and sociodemographic index, from 1990 to 2019. The difference indicates age-standardized rate in women minus that in men. A difference >0 suggests that women have a higher rate than men.

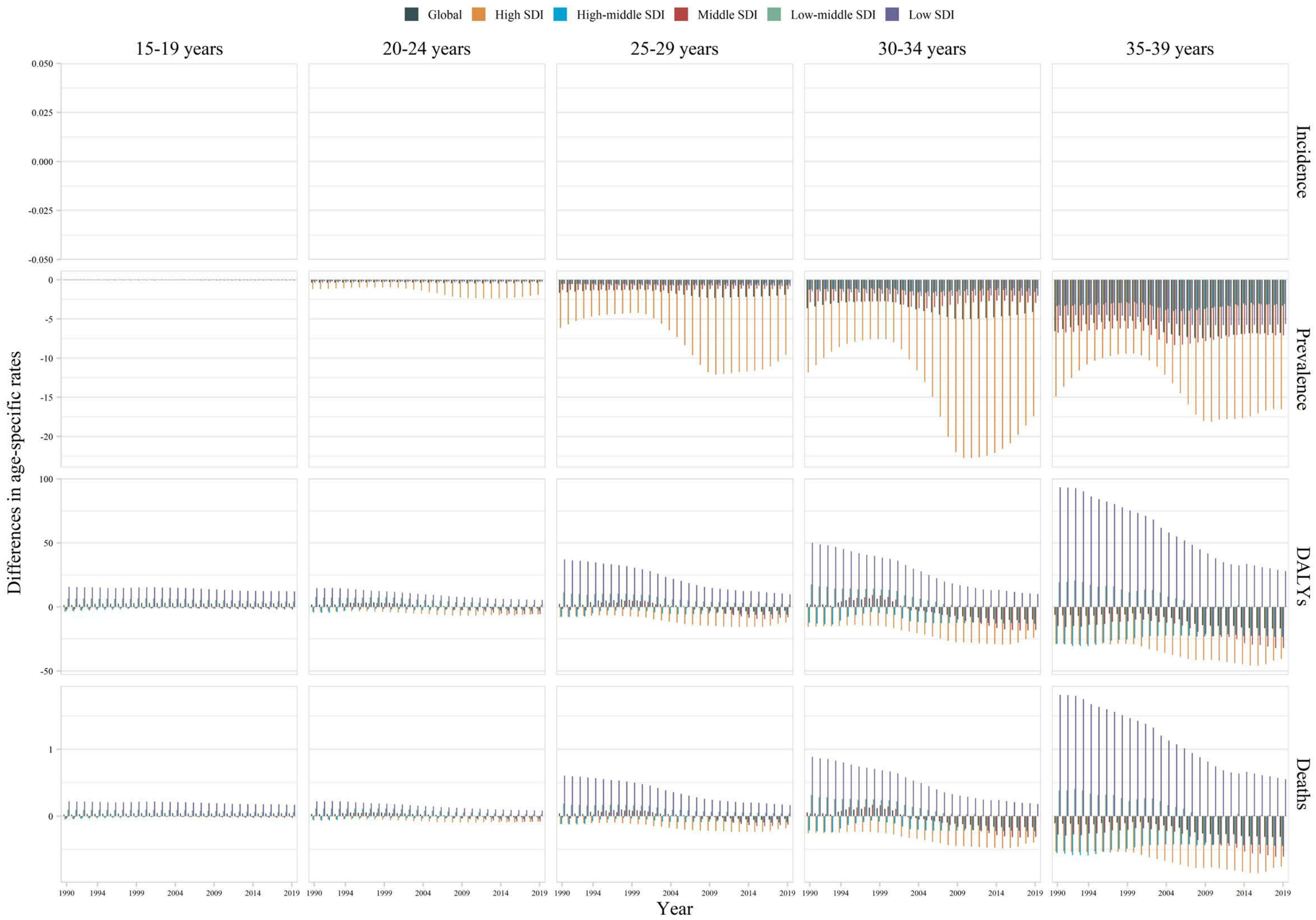

Fig 39 Difference in age-specific prevalence, disability-adjusted life years (DALYs), and death rate of hypertensive heart disease in youths and young adults between men and women by age and sociodemographic index, from 1990 to 2019. Data for incidence are unavailable. The difference indicates age-standardized rate in women minus that in men. A difference >0 suggests that women have a higher rate than men.

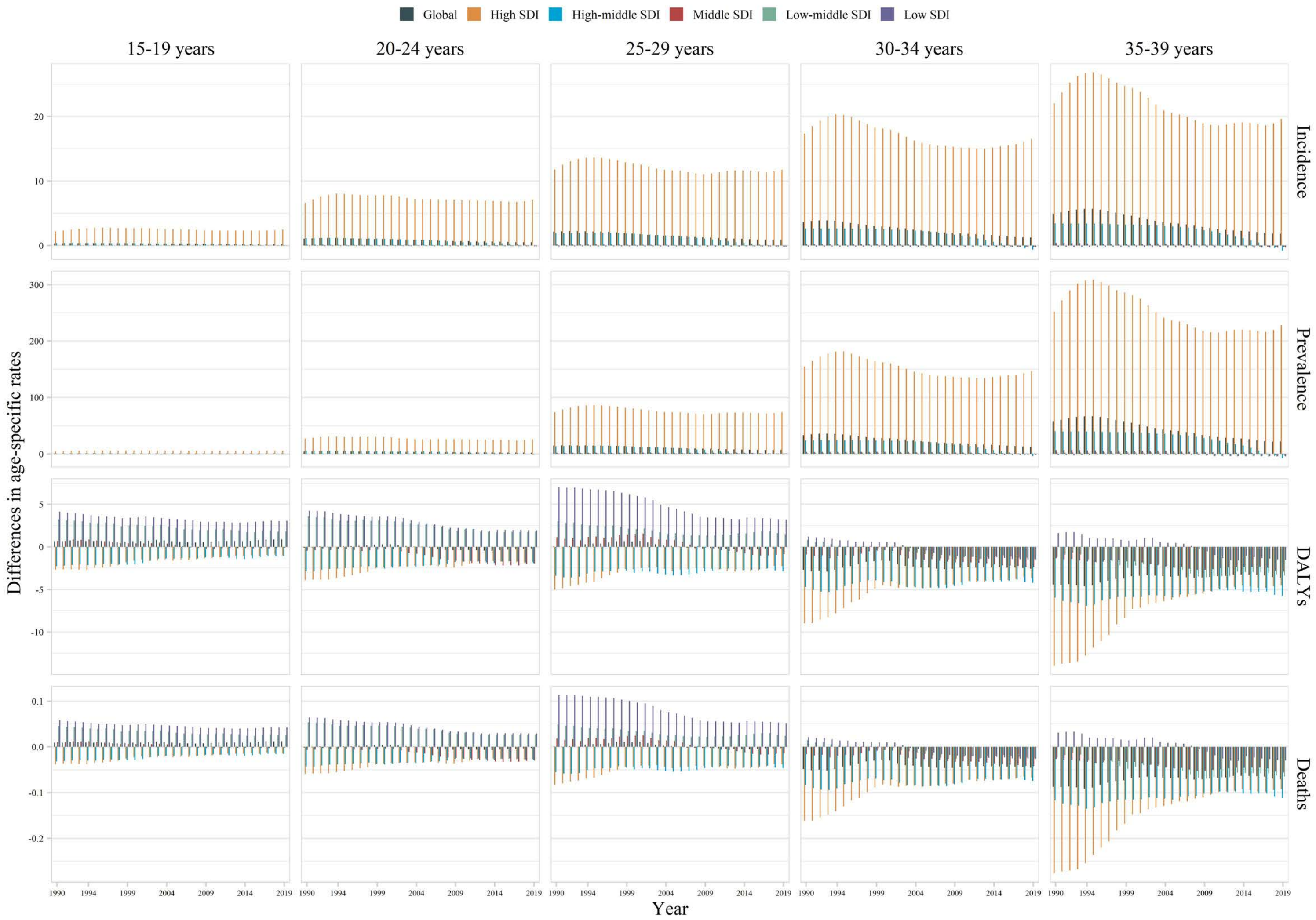

Fig 40 Difference in age-specific incidence, prevalence, disability-adjusted life years (DALYs), and death rate of non-rheumatic valvular heart disease in youths and young adults between men and women by age and sociodemographic index, from 1990 to 2019. The difference indicates age-standardized rate in women minus that in men. A difference >0 suggests that women have a higher rate than men.

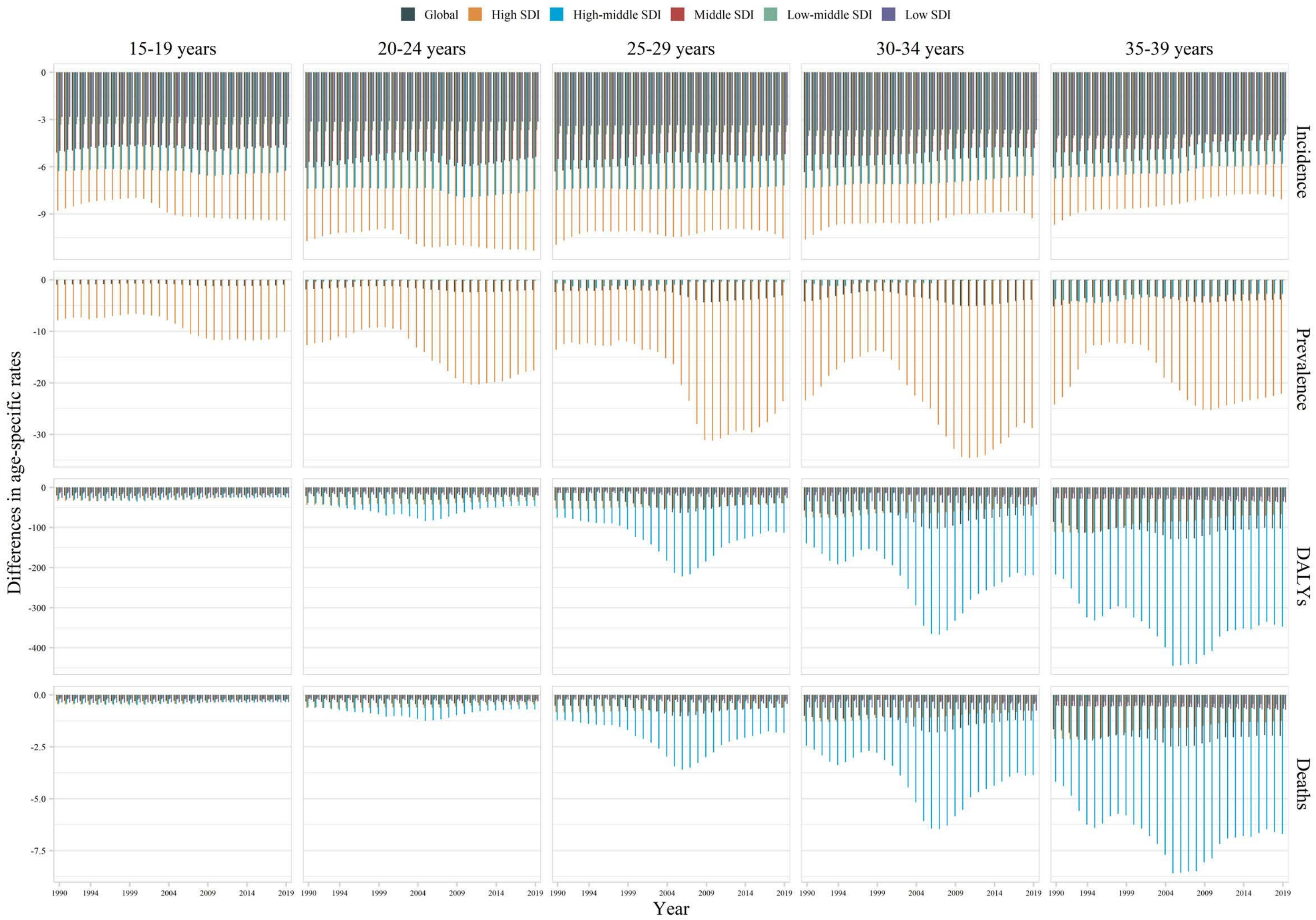

Fig 41 Difference in age-specific incidence, prevalence, disability-adjusted life years (DALYs), and death rate of cardiomyopathy and myocarditis in youths and young adults between men and women by age and sociodemographic index, from 1990 to 2019. The difference indicates age-standardized rate in women minus that in men. A difference >0 suggests that women have a higher rate than men.

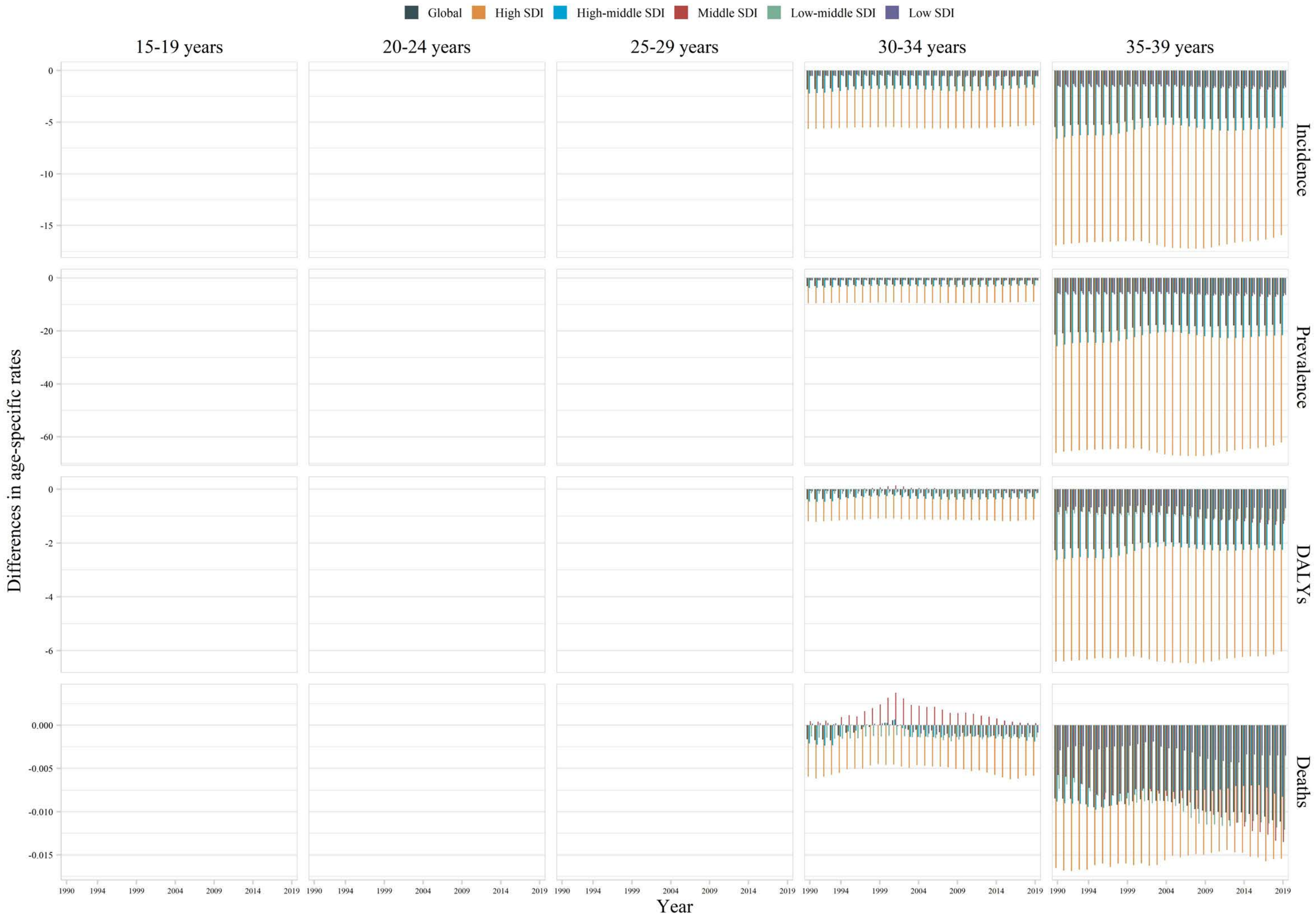

Fig 42 Difference in age-specific incidence, prevalence, disability-adjusted life years (DALYs), and death rate of atrial fibrillation and flutter in youths and young adults between men and women by age and sociodemographic index, from 1990 to 2019. Data for the age of 15-29 are unavailable. The difference indicates age-standardized rate in women minus that in men. A difference >0 suggests that women have a higher rate than men.

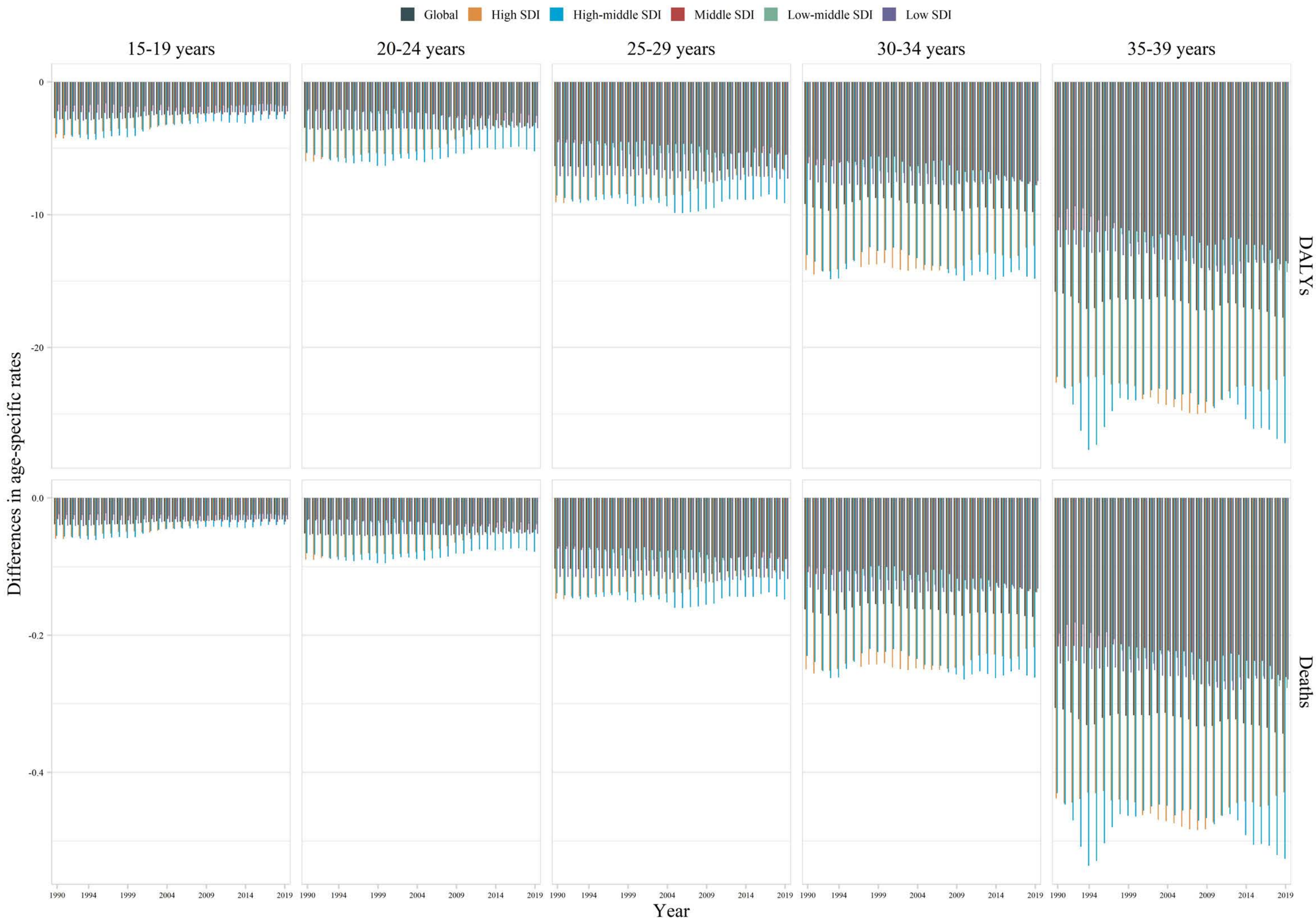

Fig 43 Difference in age-specific disability-adjusted life years (DALYs) and death rate of aortic aneurysm in youths and young adults between men and women by age and sociodemographic index, from 1990 to 2019. Data for incidence and prevalence are unavailable. The difference indicates age-standardized rate in women minus that in men. A difference >0 suggests that women have a higher rate than men.

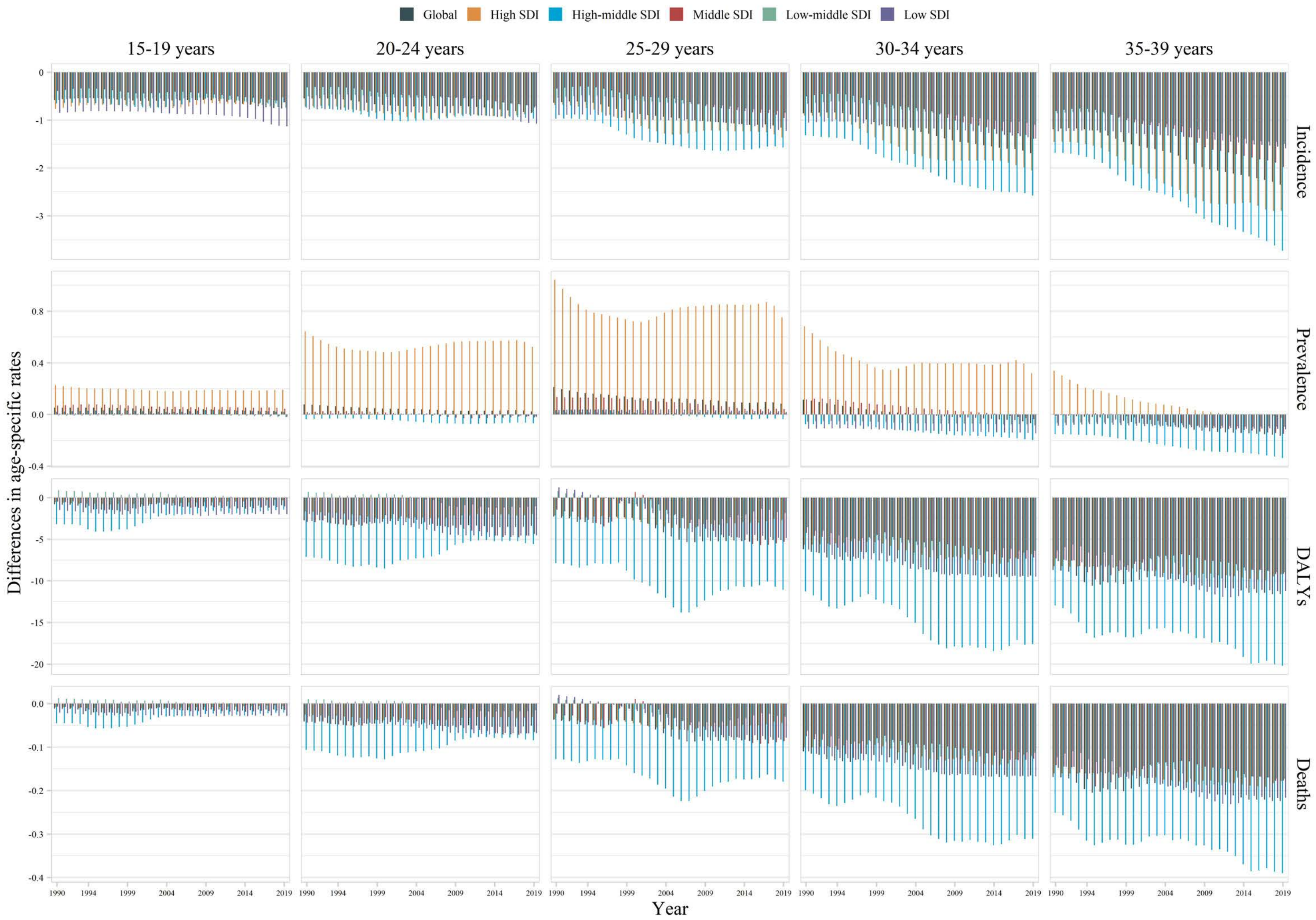

Fig 44 Difference in age-specific incidence, prevalence, disability-adjusted life years (DALYs), and death rate of endocarditis in youths and young adults between men and women by age and sociodemographic index, from 1990 to 2019. The difference indicates age-standardized rate in women minus that in men. A difference >0 suggests that women have a higher rate than men.

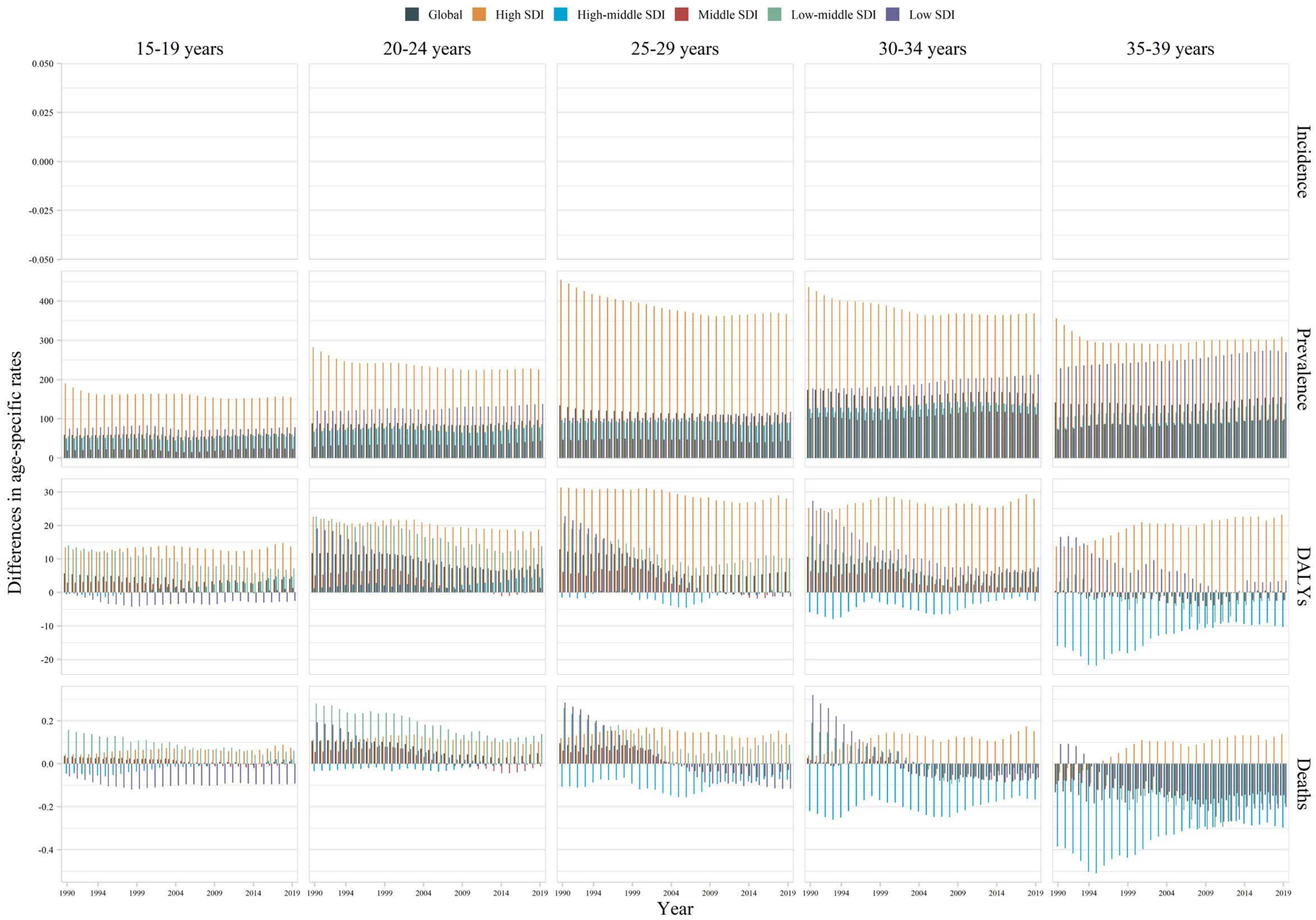

Fig 45 Difference in age-specific prevalence, disability-adjusted life years (DALYs), and death rate of other cardiovascular and circulatory diseases in youths and young adults between men and women by age and sociodemographic index, from 1990 to 2019. Data for incidence are unavailable. The difference indicates age-standardized rate in women minus that in men. A difference >0 suggests that women have a higher rate than men.

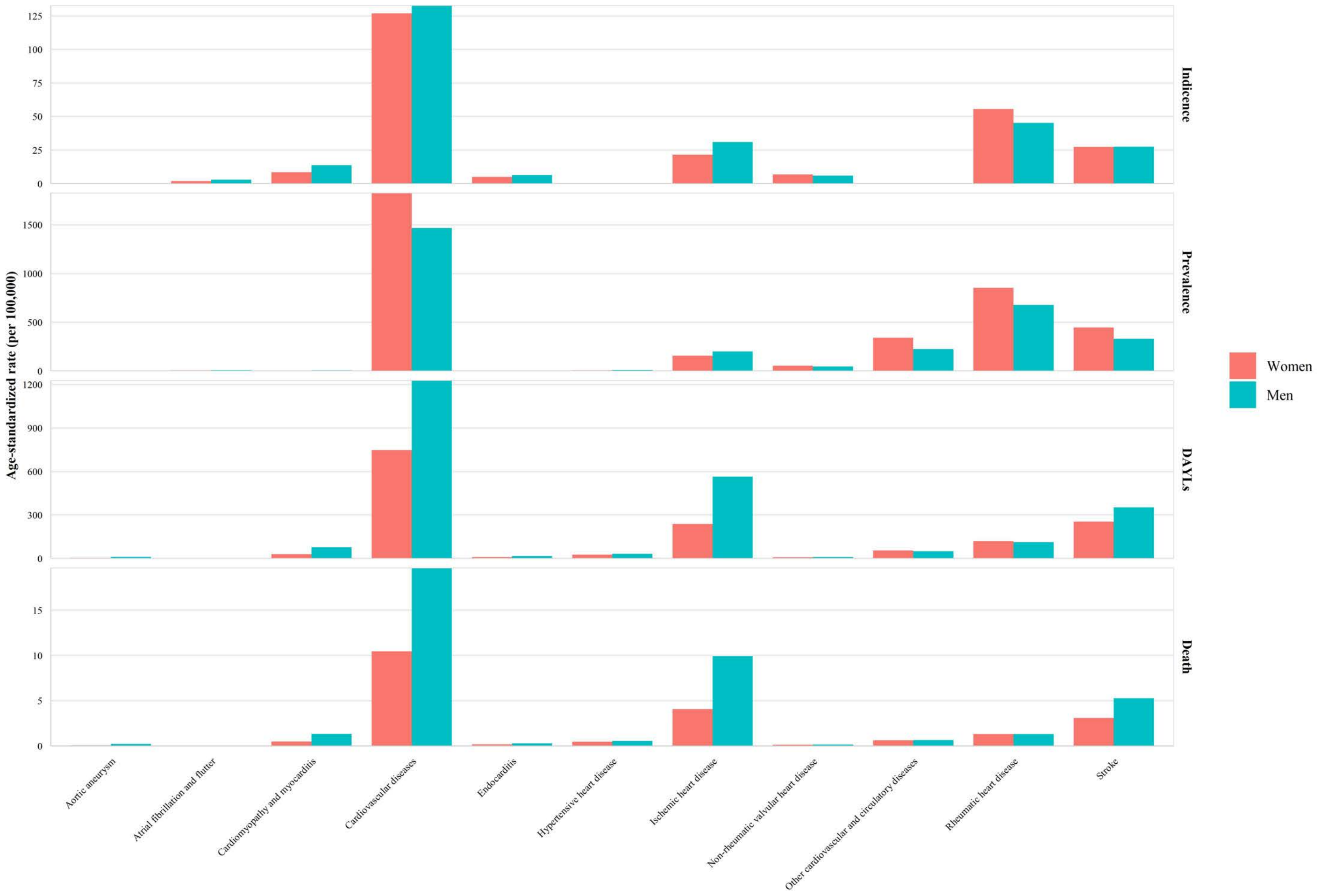

Fig S46 Age-standardized incidence, prevalence, disability-adjusted life years (DALYs), and death rate of overall and type-specific cardiovascular disease among youths and young adults in 2019 by sex. Data for several type-specific cardiovascular diseases are unavailable.

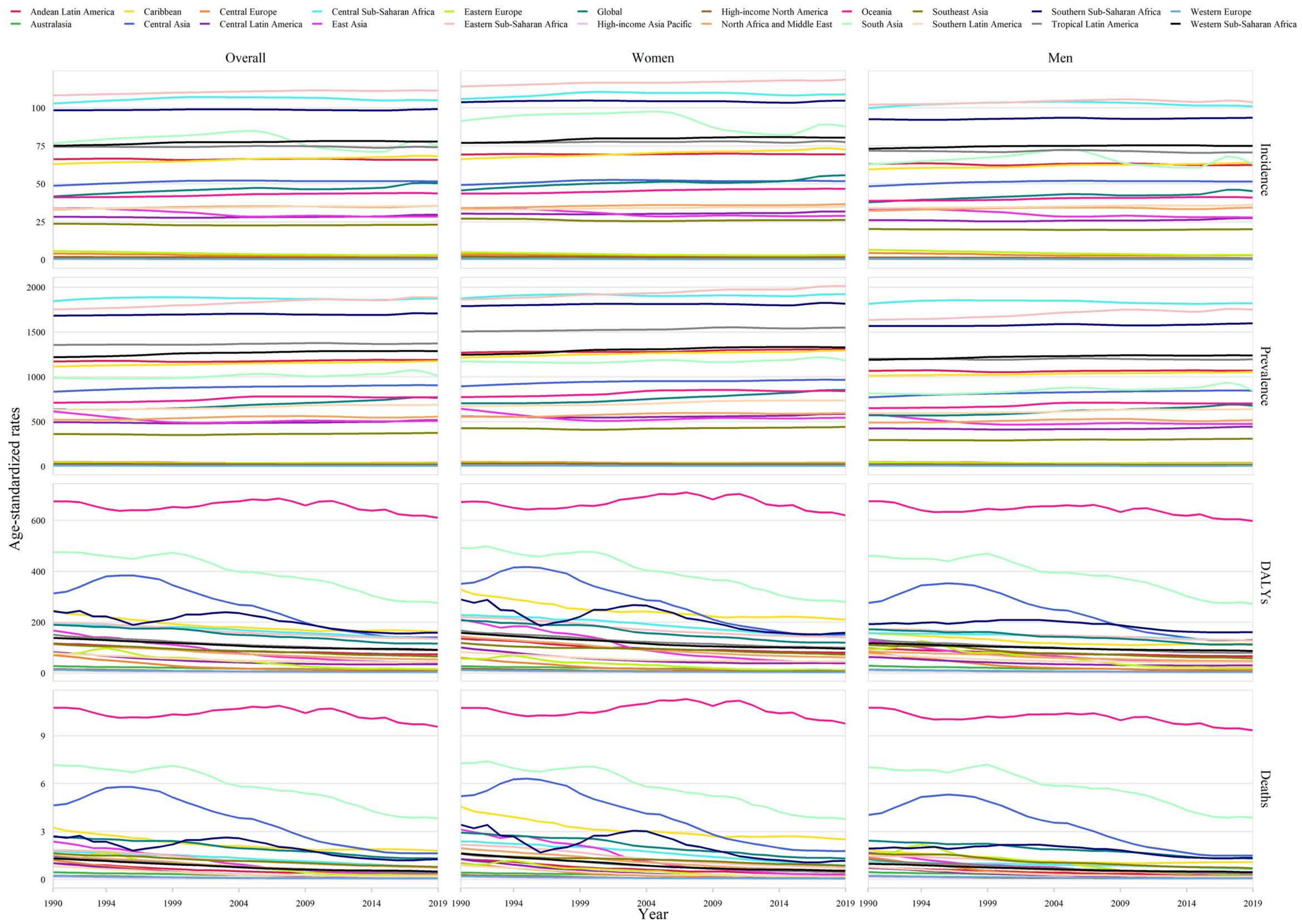

Fig S47 Temporal trends in age-standardized incidence, prevalence, disability-adjusted life years (DALYs), and death rate of rheumatic heart disease in youths and young adults overall and by sex and 21 GBD regions, from 1990 to 2019.

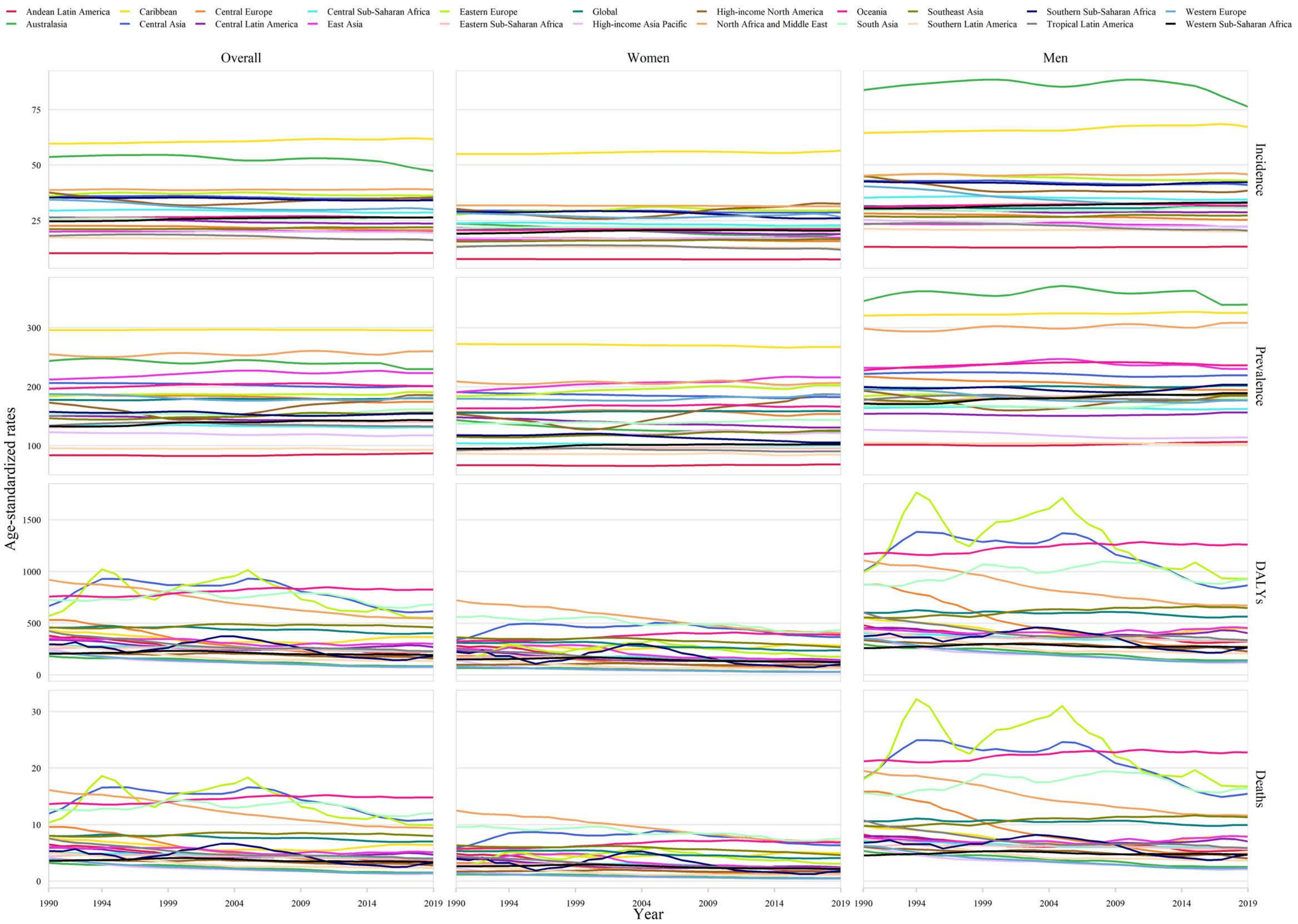

Fig S48 Temporal trends in age-standardized incidence, prevalence, disability-adjusted life years (DALYs), and death rate of ischemic heart disease in youths and young adults overall and by sex and 21 GBD regions, from 1990 to 2019.

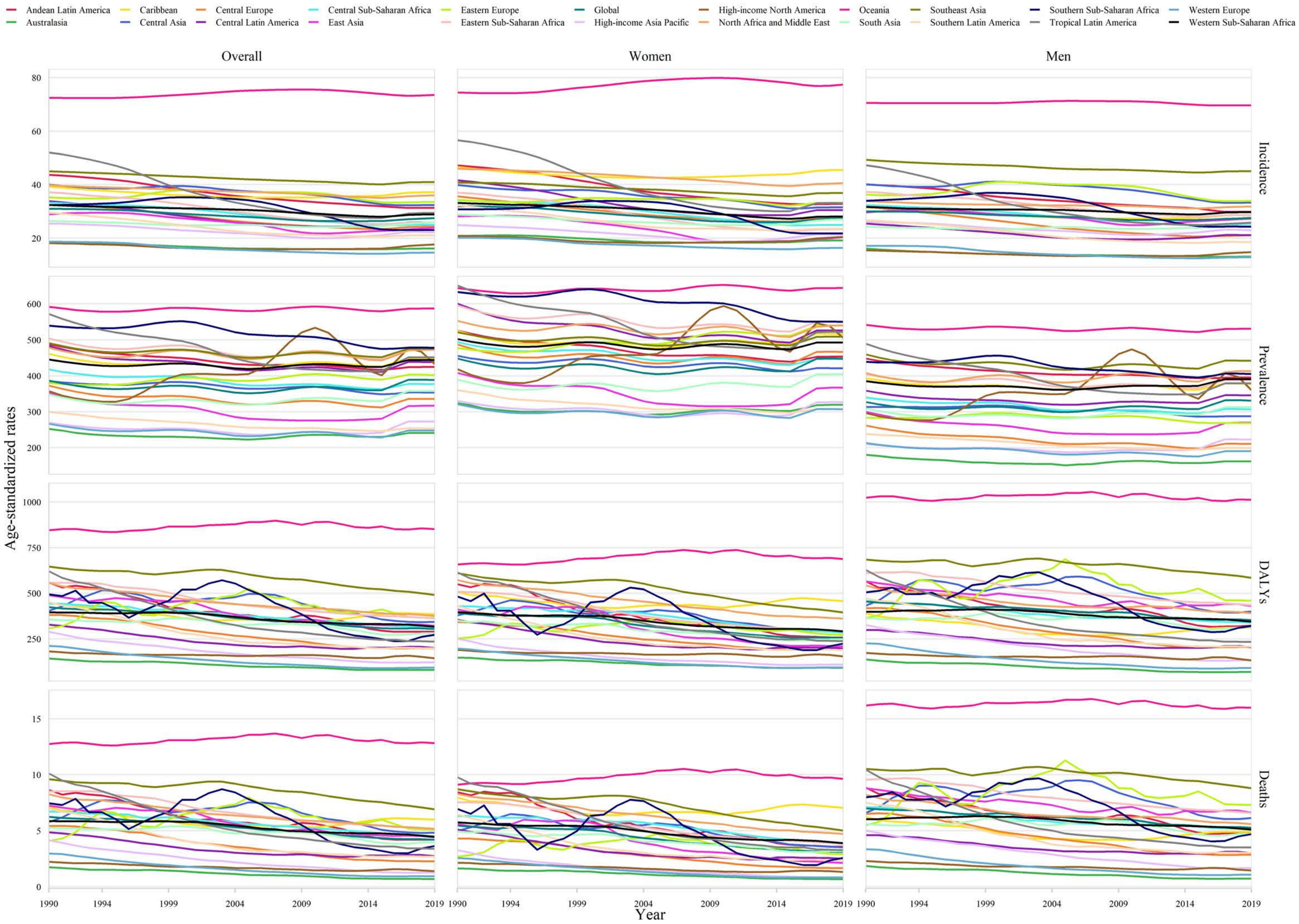

Fig S49 Temporal trends in age-standardized incidence, prevalence, disability-adjusted life years (DALYs), and death rate of stroke in youths and young adults overall and by sex and 21 GBD regions, from 1990 to 2019.

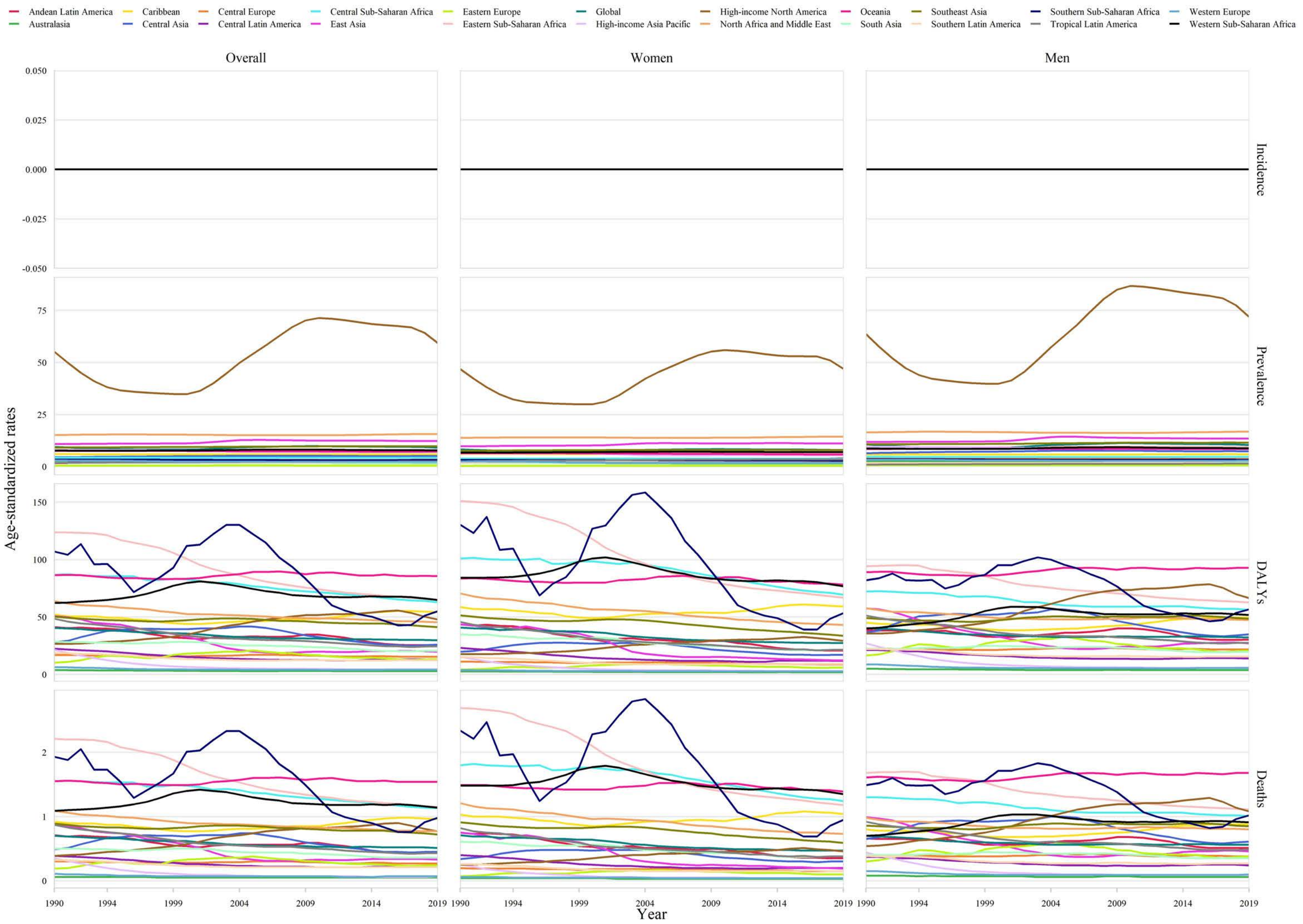

Fig S50 Temporal trends in age-standardized prevalence, disability-adjusted life years (DALYs), and death rate of hypertensive heart disease in youths and young adults overall and by sex and 21 GBD regions, from 1990 to 2019. Data for incidence are unavailable.

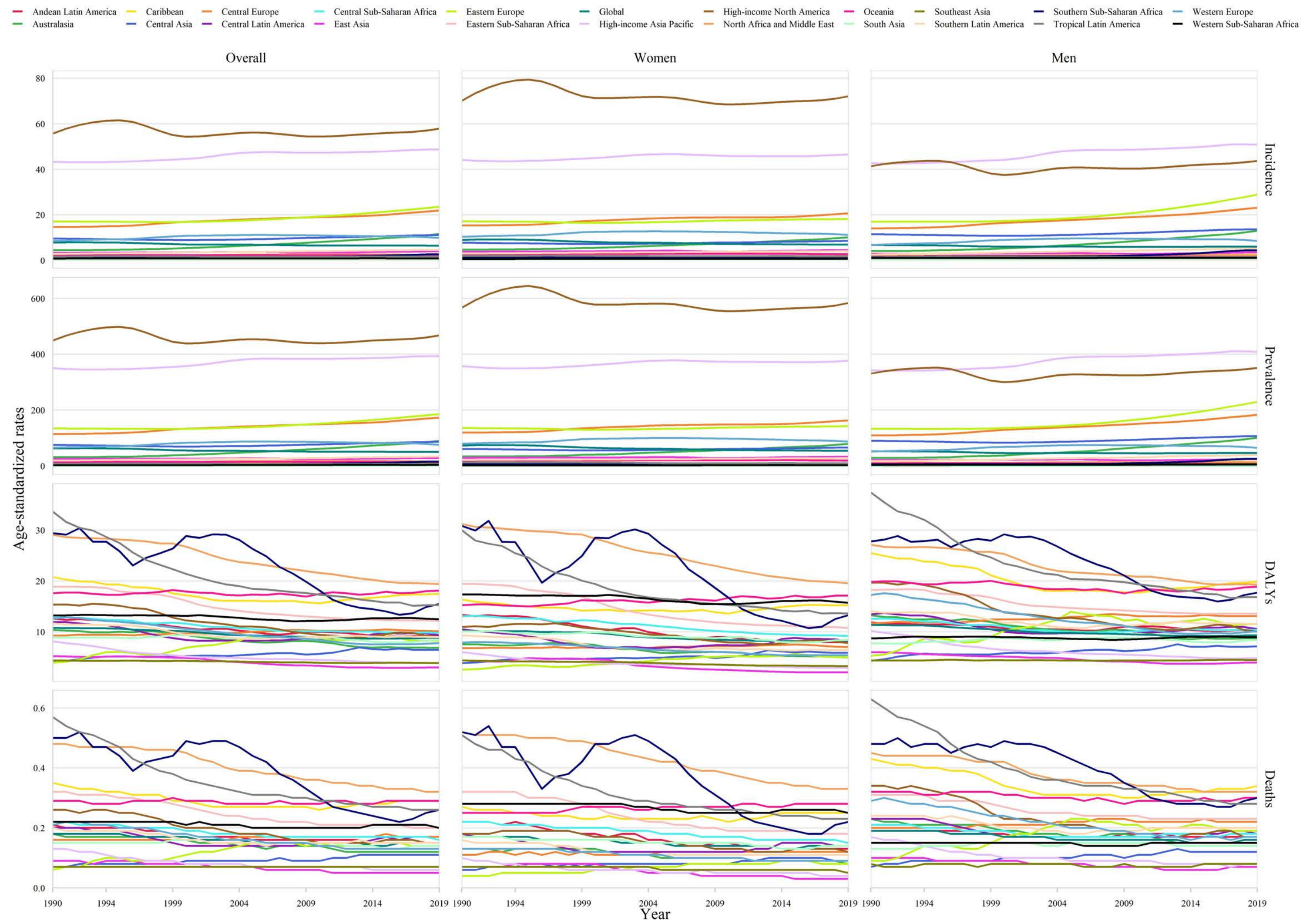

Fig S51 Temporal trends in age-standardized incidence, prevalence, disability-adjusted life years (DALYs), and death rate of non-rheumatic valvular heart disease in youths and young adults overall and by sex and 21 GBD regions, from 1990 to 2019.

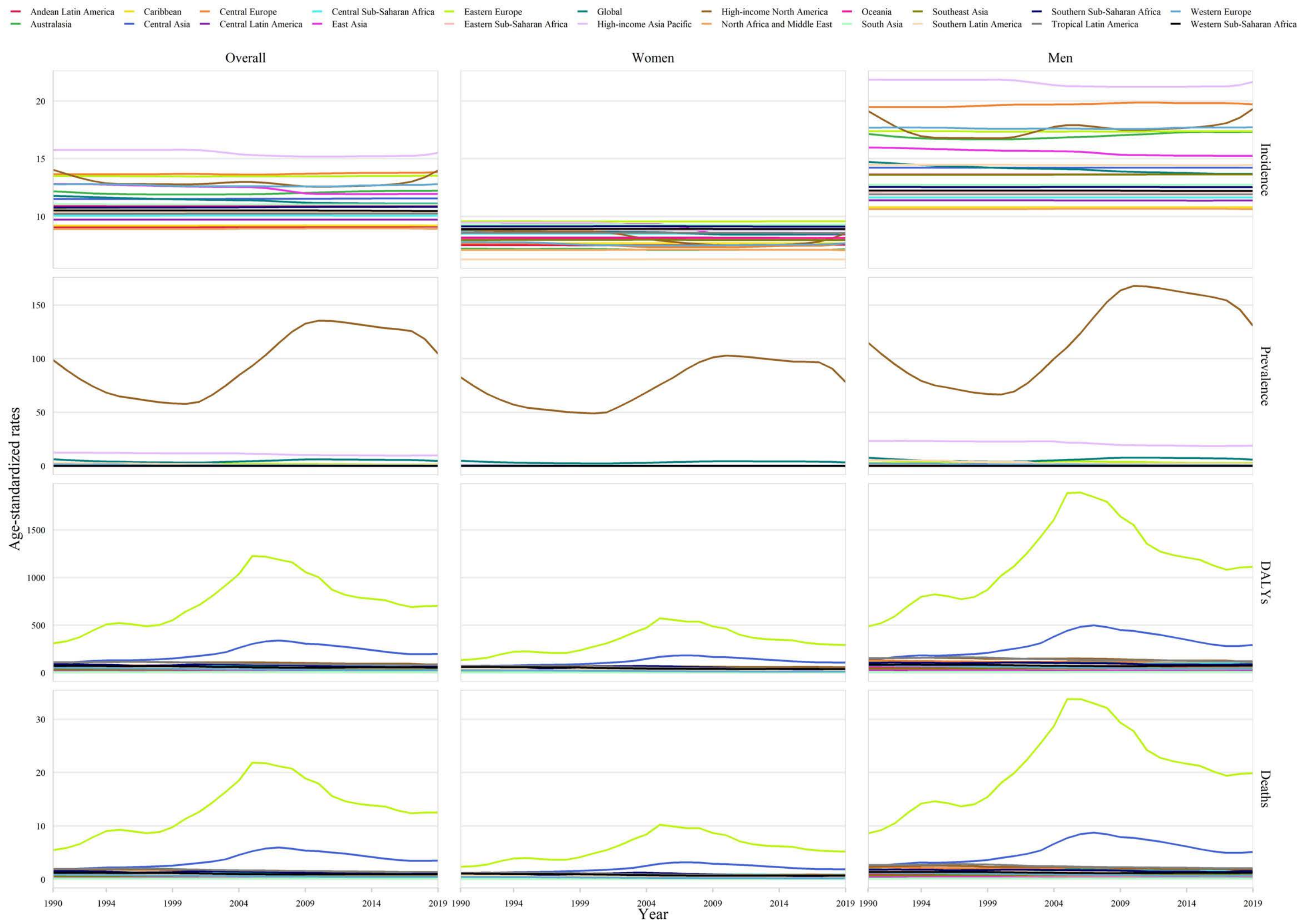

Fig S52 Temporal trends in age-standardized incidence, prevalence, disability-adjusted life years (DALYs), and death rate of cardiomyopathy and myocarditis in youths and young adults overall and by sex and 21 GBD regions, from 1990 to 2019.

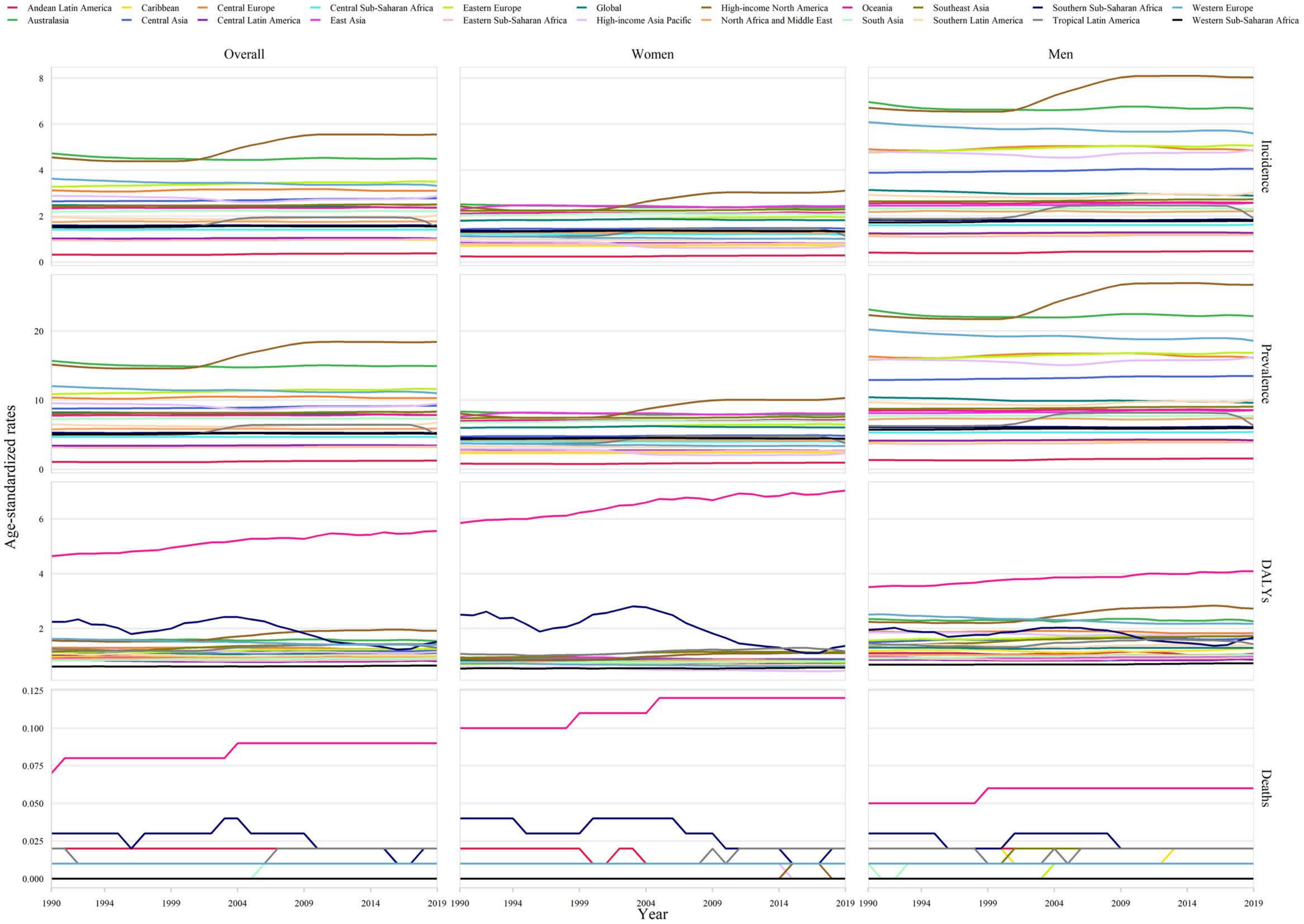

Fig S53 Temporal trends in age-standardized incidence, prevalence, disability-adjusted life years (DALYs), and death rate of atrial fibrillation and flutter in youths and young adults overall and by sex and 21 GBD regions, from 1990 to 2019.

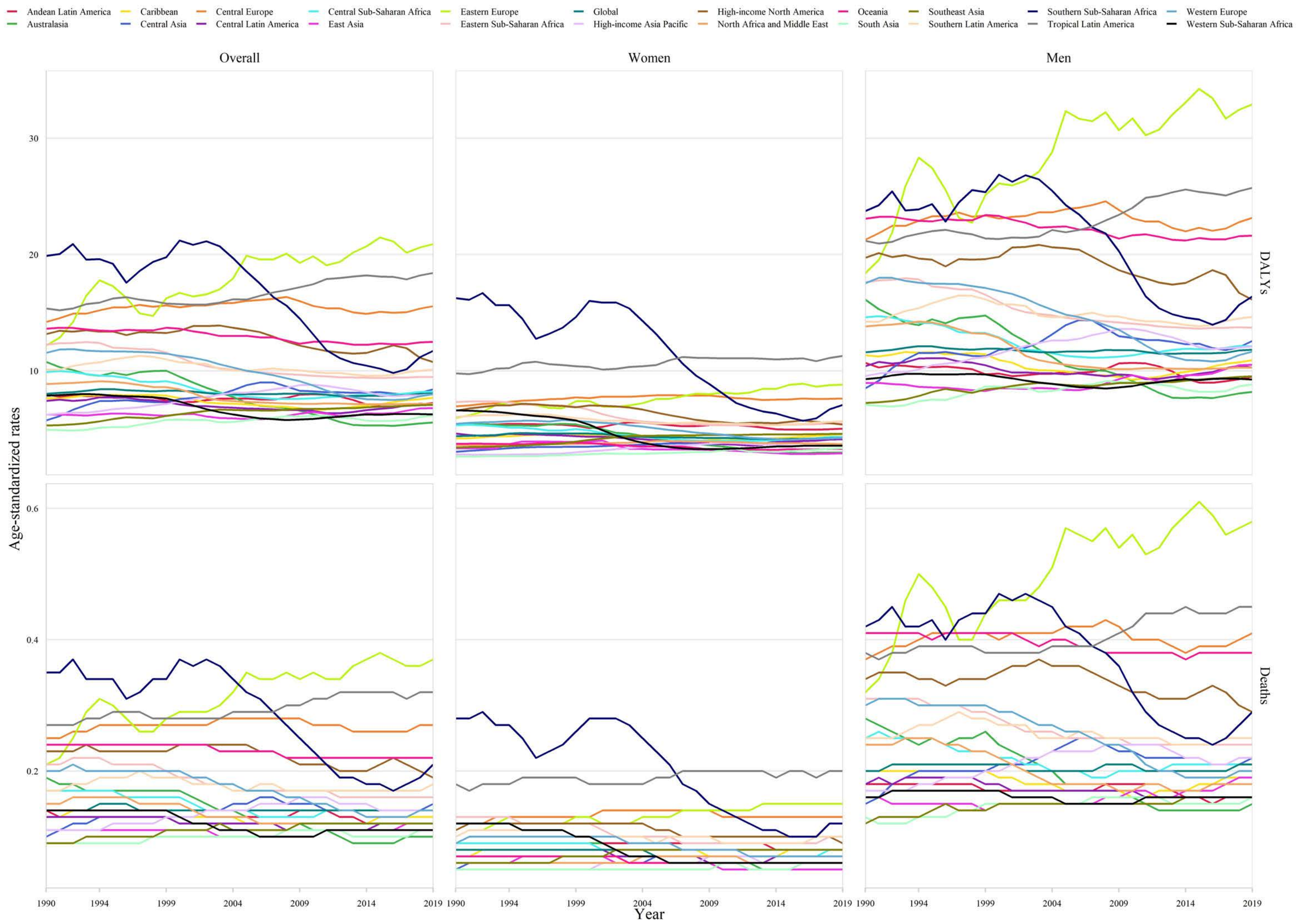

Fig S54 Temporal trends in age-standardized disability-adjusted life years (DALYs) and death rate of aortic aneurysm in youths and young adults overall and by sex and 21 GBD regions, from 1990 to 2019. Data for incidence and prevalence are unavailable.

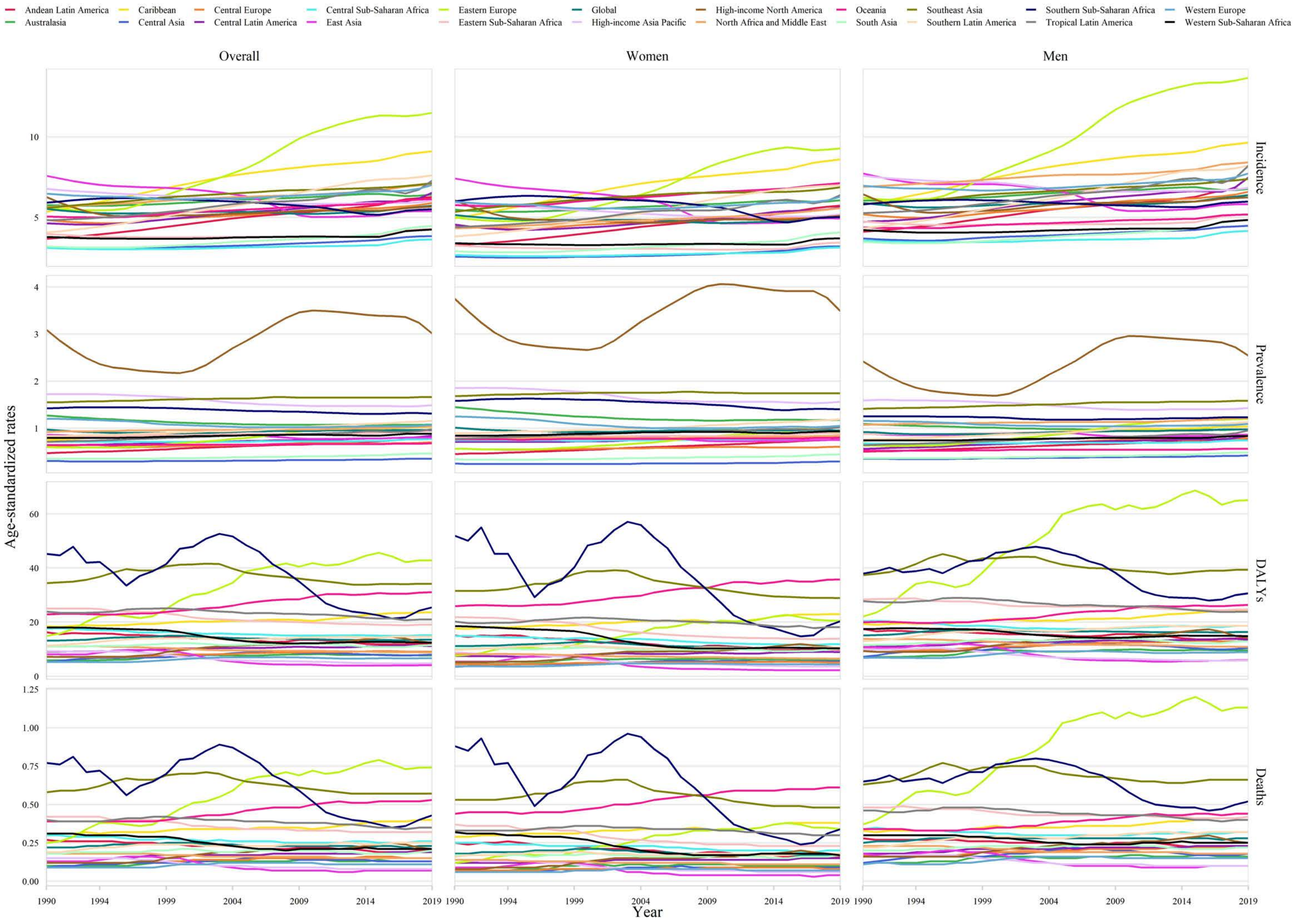

Fig S55 Temporal trends in age-standardized incidence, prevalence, disability-adjusted life years (DALYs), and death rate of endocarditis in youths and young adults overall and by sex and 21 GBD regions, from 1990 to 2019.

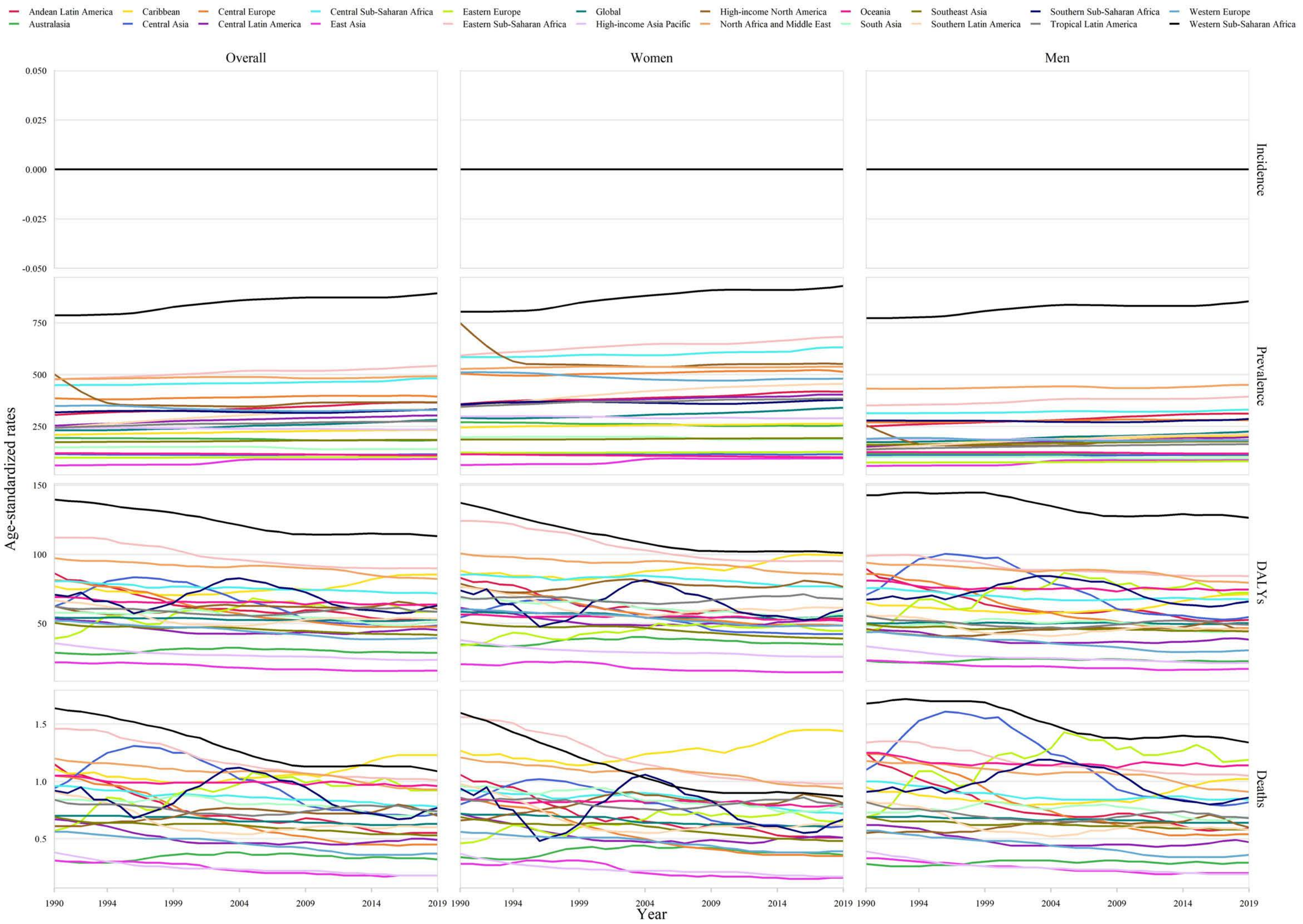

Fig S56 Temporal trends in age-standardized prevalence, disability-adjusted life years (DALYs), and death rate of other cardiovascular and circulatory diseases in youths and young adults overall and by sex and 21 GBD regions, from 1990 to 2019. Data for incidence are unavailable.

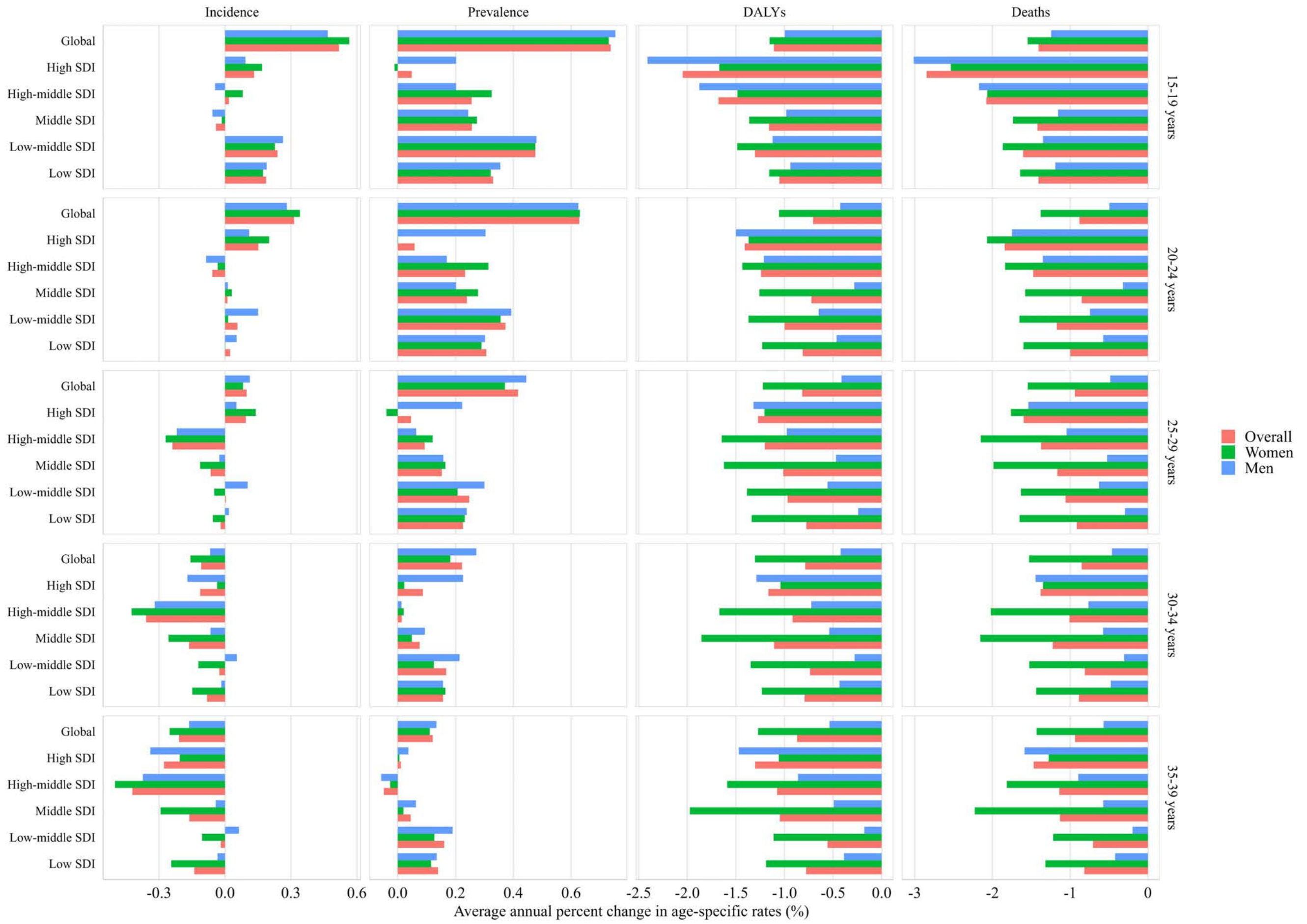

Fig S57 Average annual percent change in age-specific incidence, prevalence, disability-adjusted life years (DALYs), and death rate of overall cardiovascular disease in youths and young adults by age, sex, and sociodemographic index, from 1990 to 2019.

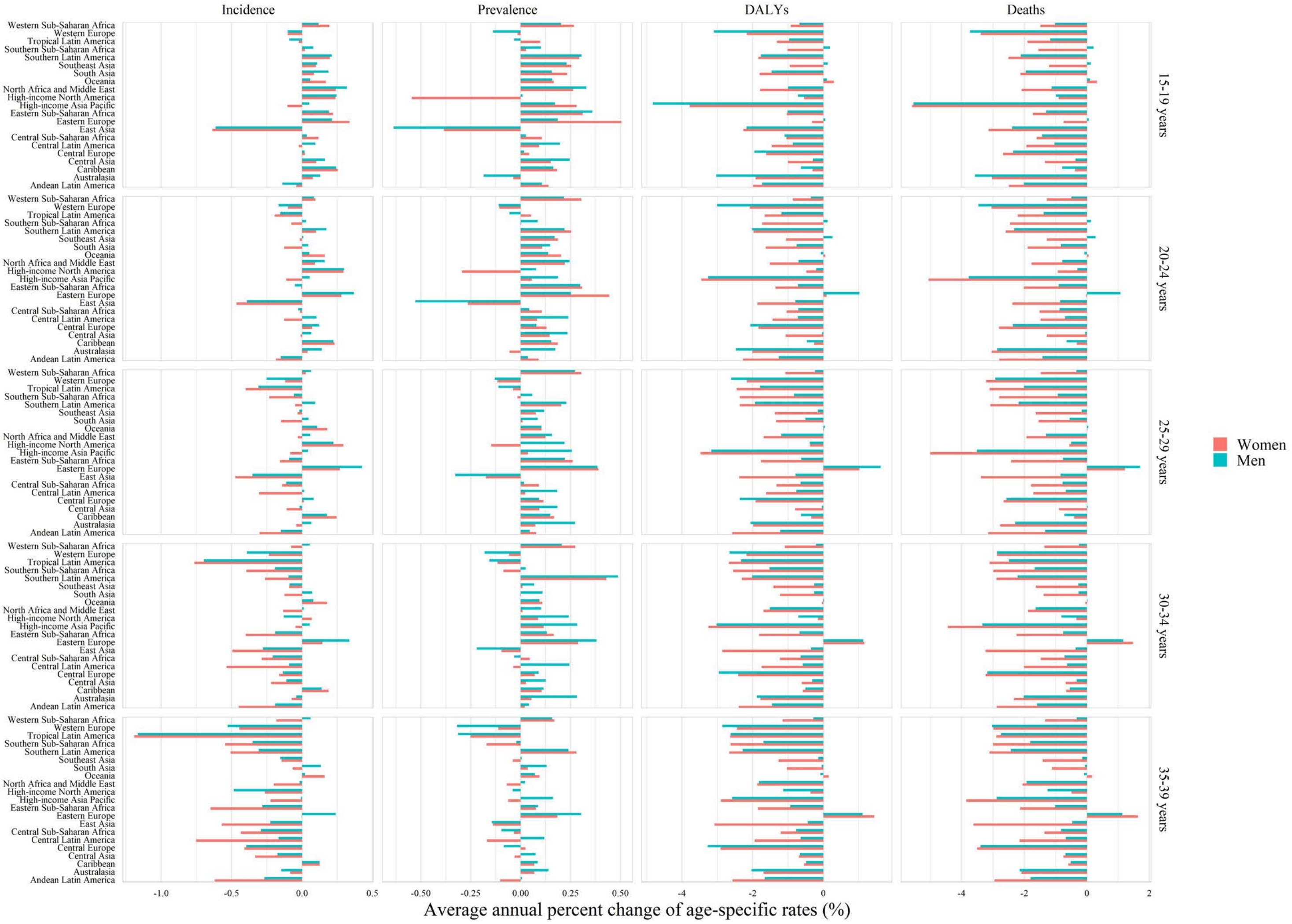

Fig S58 Average annual percent change of age-specific incidence, prevalence, disability-adjusted life years (DALYs), and death rate of overall cardiovascular disease in youths and young adults by age, sex, and region, from 1990 to 2019.

A

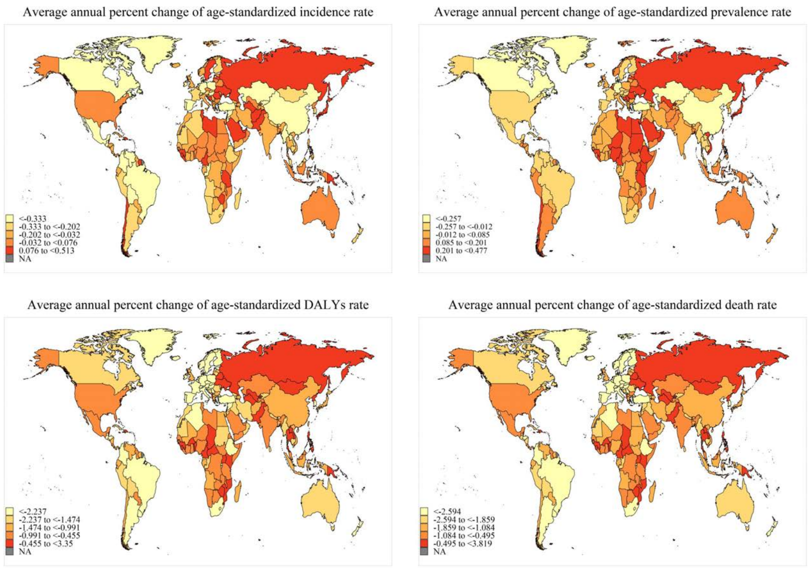

B

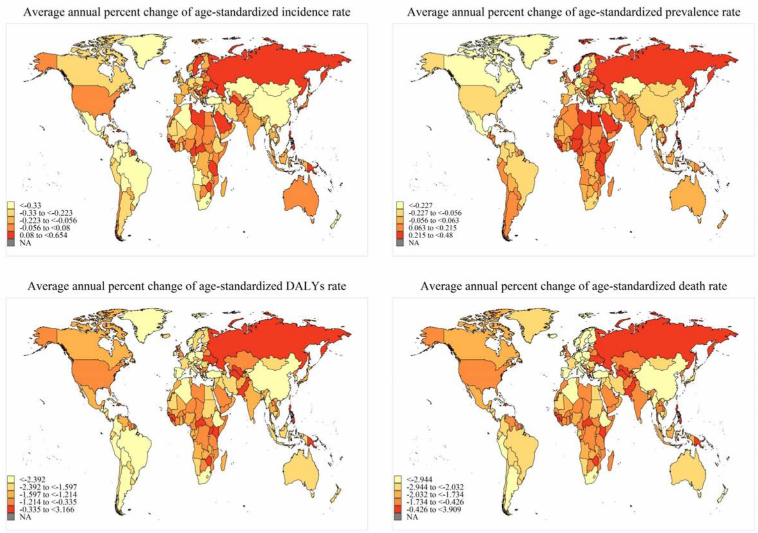

C

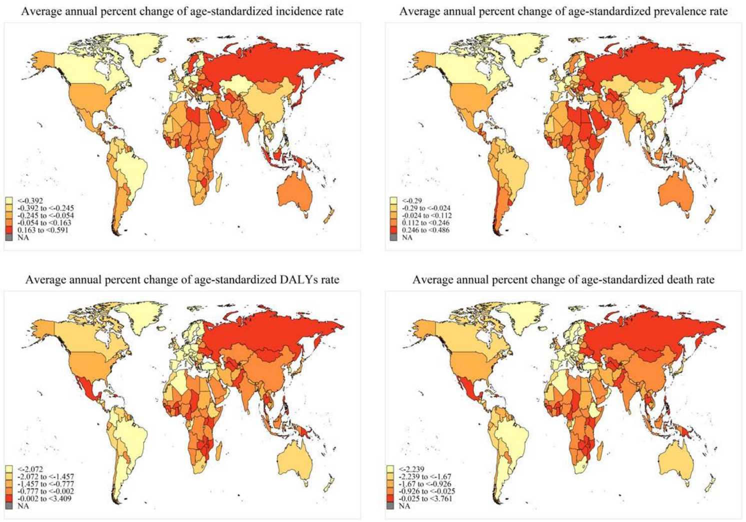

Fig S59 Annual percent change of age-standardized incidence, prevalence, disability-adjusted life years (DALYs), and death rate of overall cardiovascular disease across 204 countries/territories among youths and young adults by sex, from 1990 to 2019. A, overall; B, women; C, men

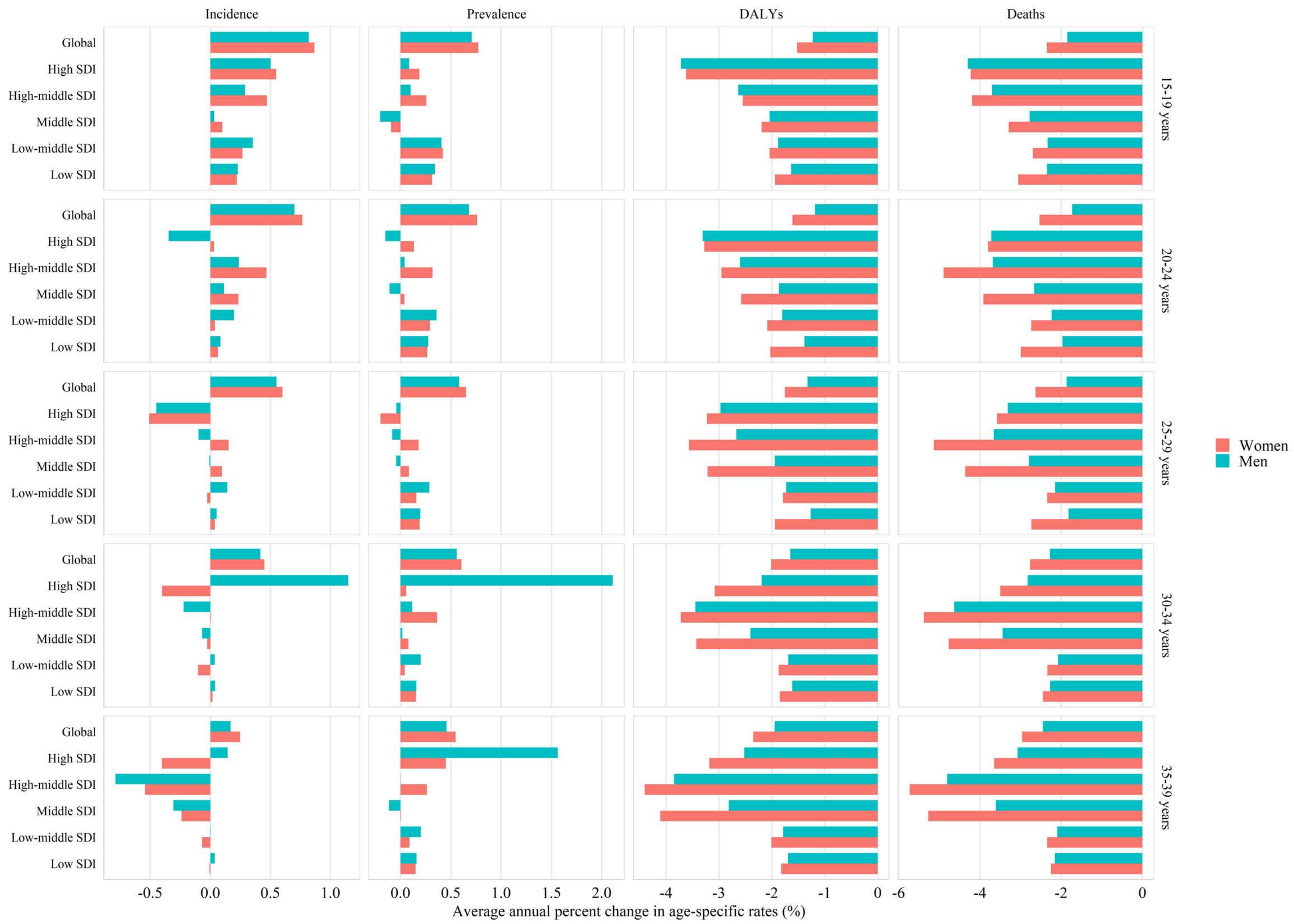

Fig S60 Average annual percent change in age-specific incidence, prevalence, disability-adjusted life years (DALYs), and death rate of rheumatic heart disease in youths and young adults by age, sex, and sociodemographic index, from 1990 to 2019.

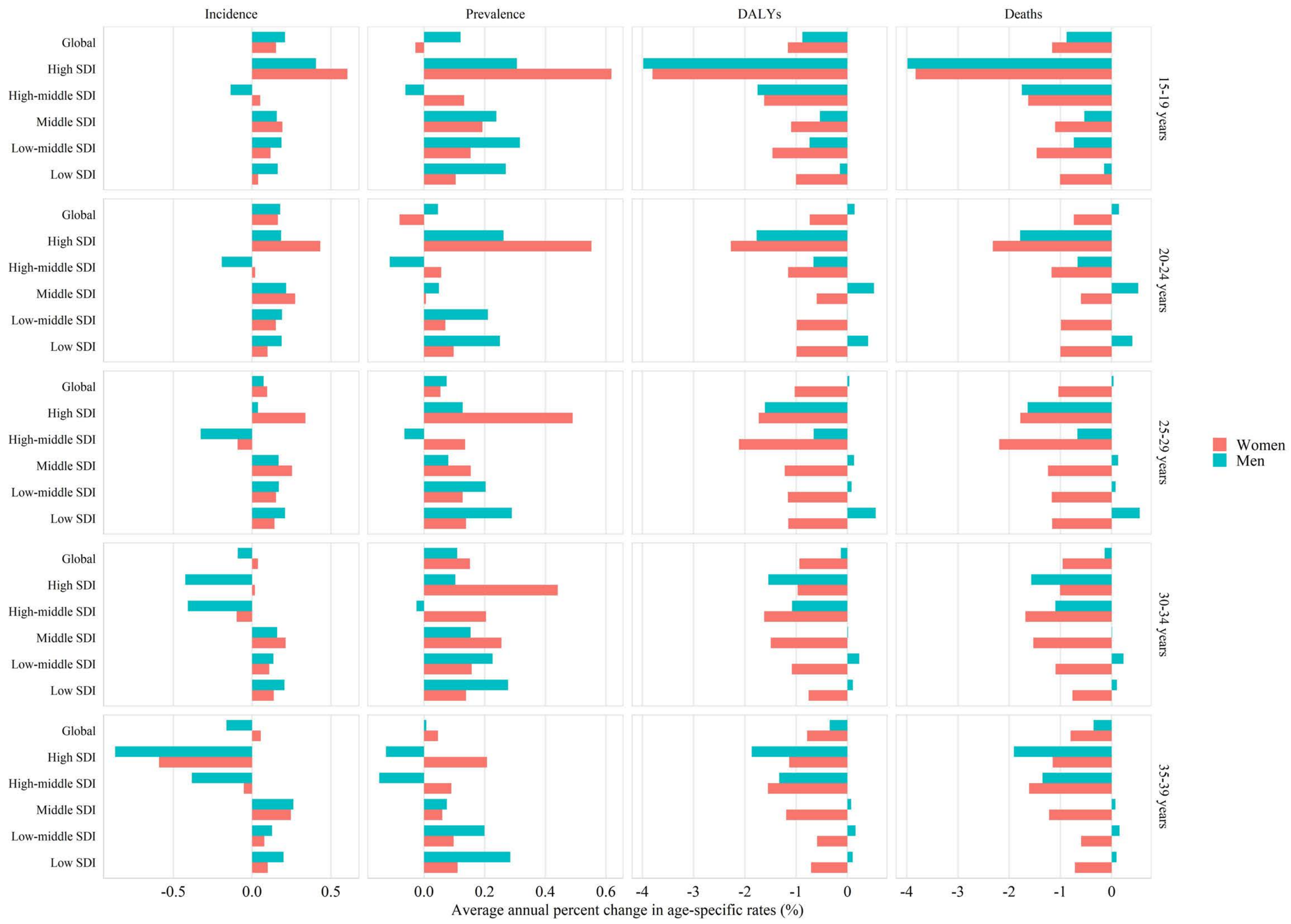

Fig S61 Average annual percent change in age-specific incidence, prevalence, disability-adjusted life years (DALYs), and death rate of overall ischemic heart disease in youths and young adults by age, sex, and sociodemographic index, from 1990 to 2019.

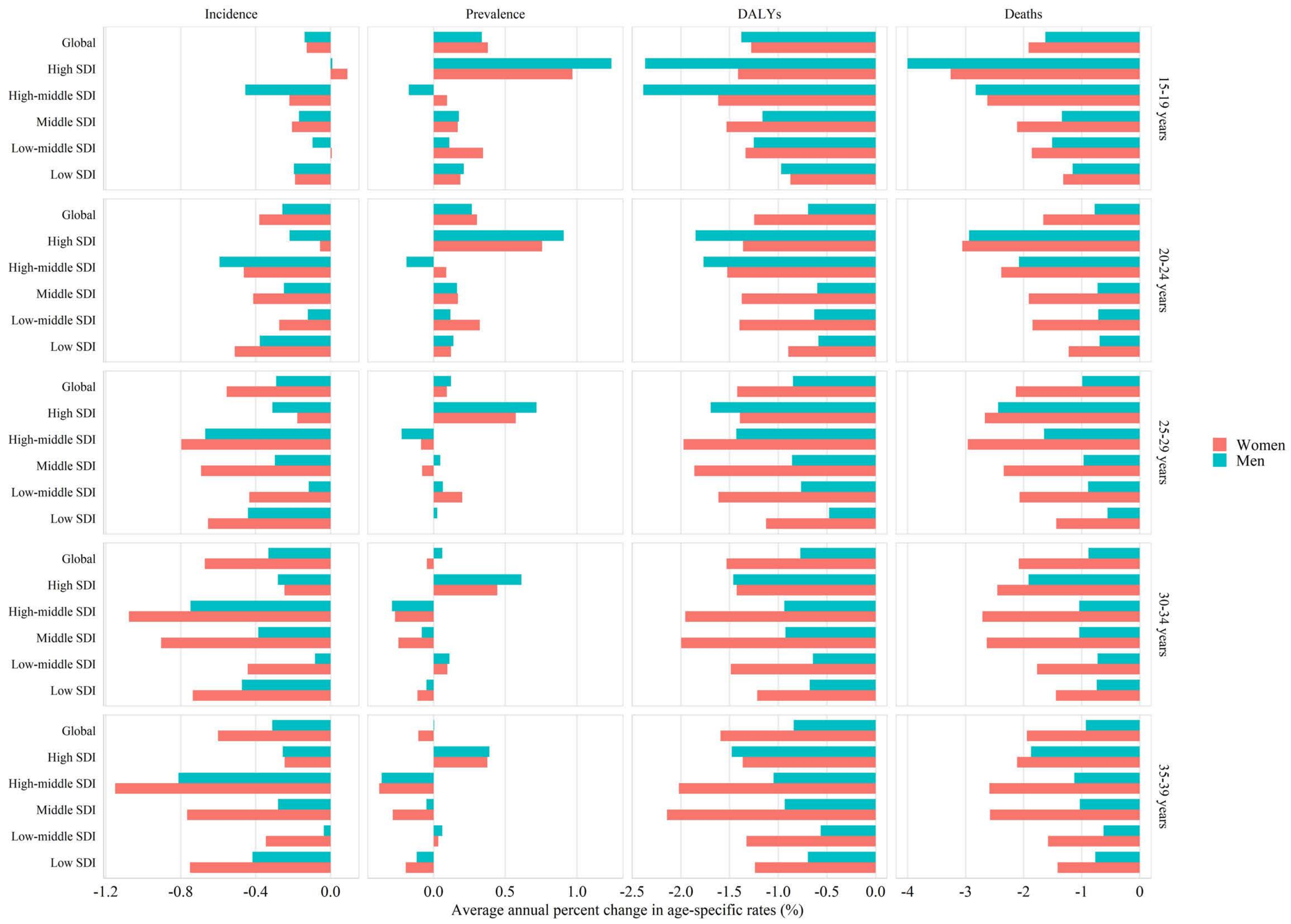

Fig S62 Average annual percent change in age-specific incidence, prevalence, disability-adjusted life years (DALYs), and death rate of stroke in youths and young adults by age, sex, and sociodemographic index, from 1990 to 2019.

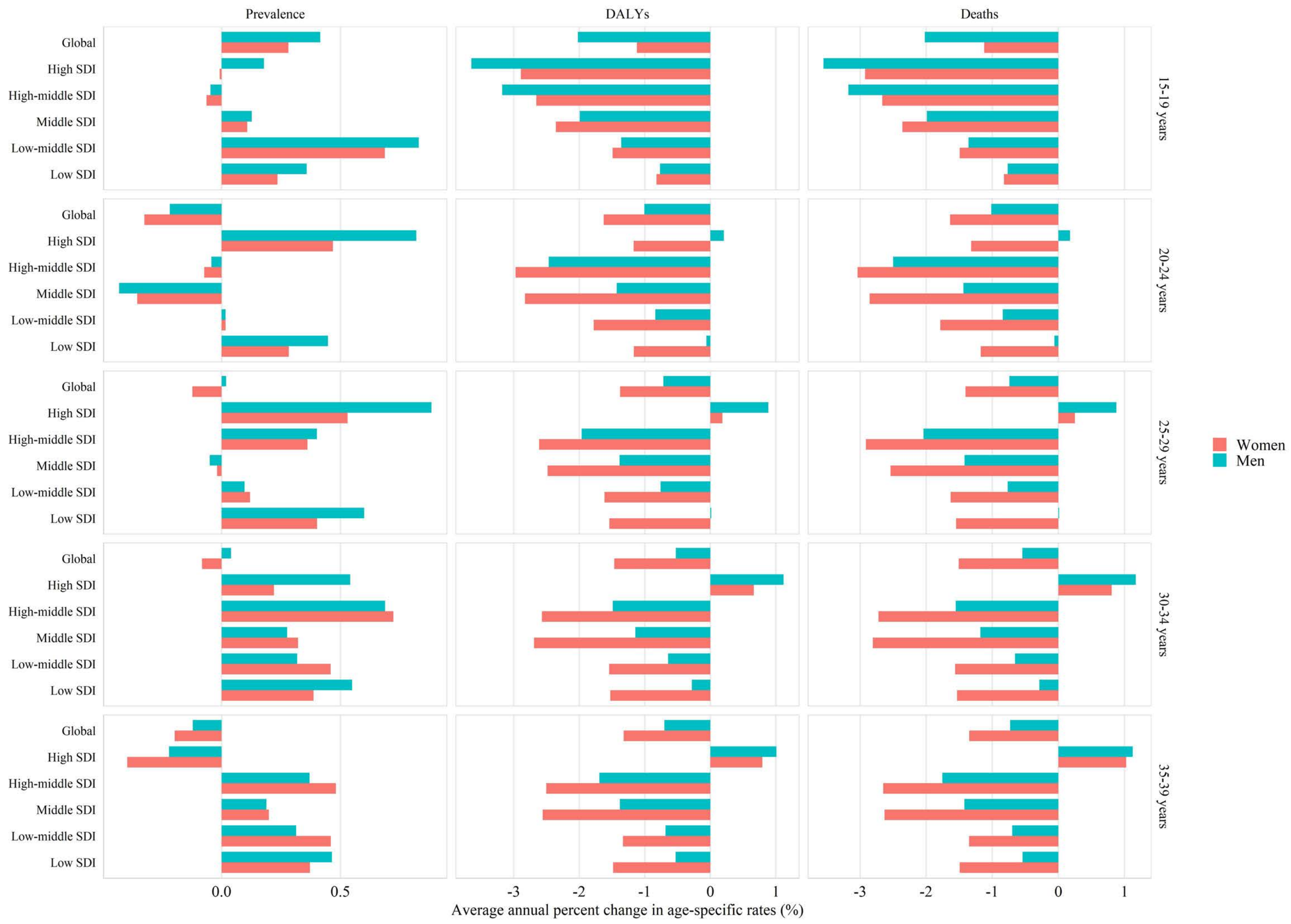

Fig S63 Average annual percent change in age-specific prevalence, disability-adjusted life years (DALYs), and death rate of hypertensive heart disease in youths and young adults by age, sex, and sociodemographic index, from 1990 to 2019. Data for incidence are unavailable.

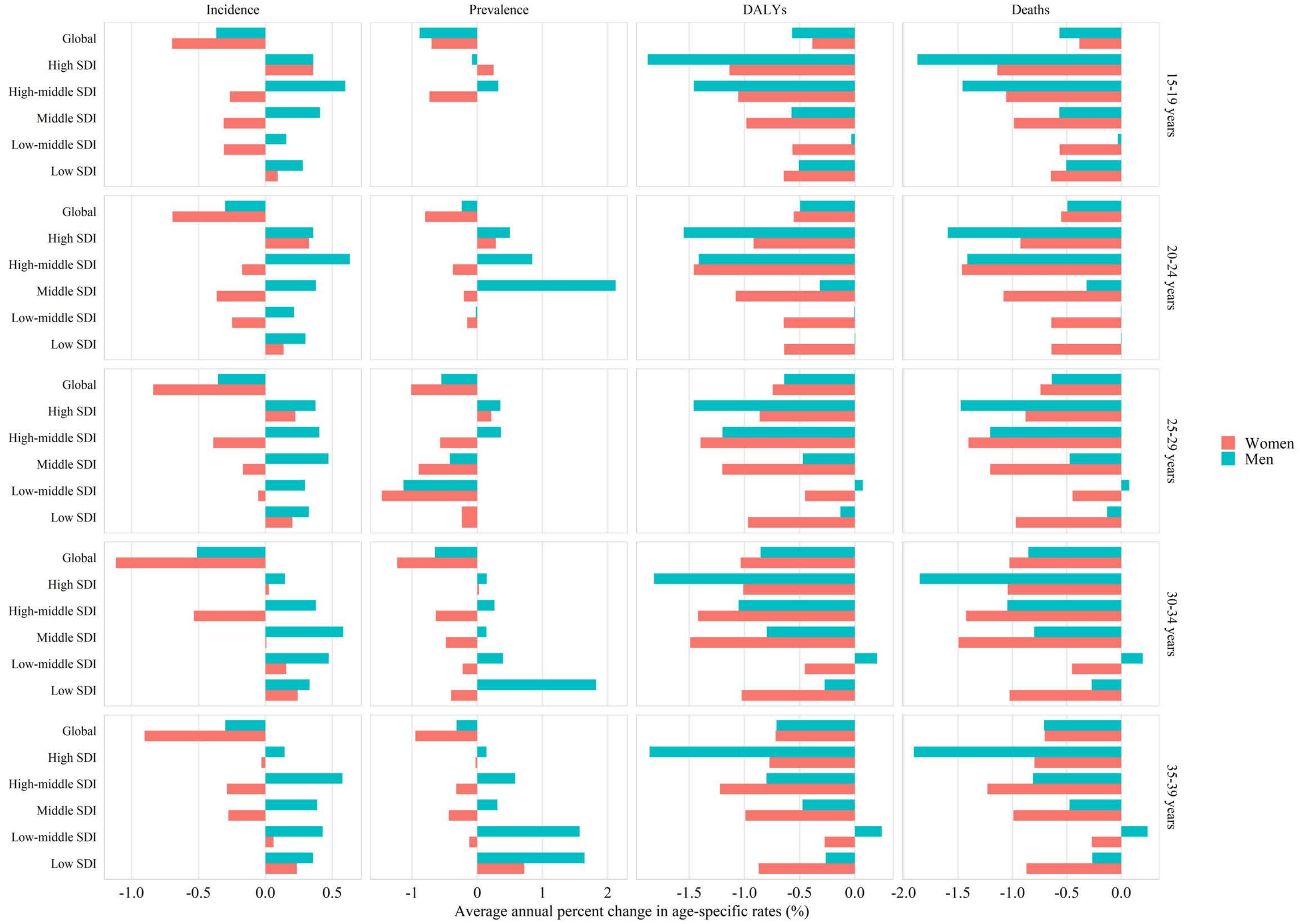

Fig S64 Average annual percent change in age-specific incidence, prevalence, disability-adjusted life years (DALYs), and death rate of non-rheumatic valvular heart disease in youths and young adults by age, sex, and sociodemographic index, from 1990 to 2019. Data for prevalence in several subgroups are unavailable.

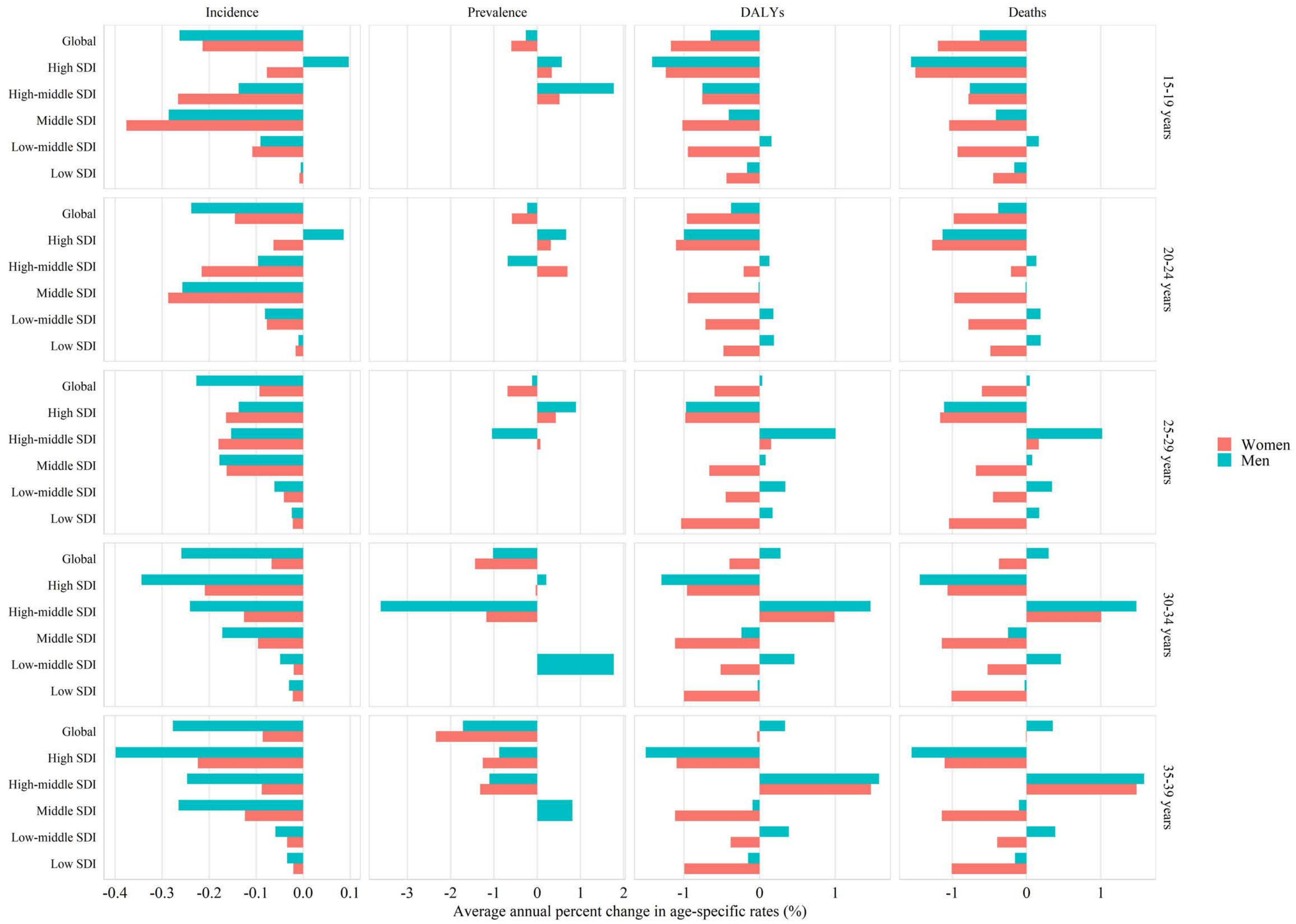

Fig S65 Average annual percent change in age-specific incidence, prevalence, disability-adjusted life years (DALYs), and death rate of cardiomyopathy and myocarditis in youths and young adults by age, sex, and sociodemographic index, from 1990 to 2019. Data for prevalence in several subgroups are unavailable.

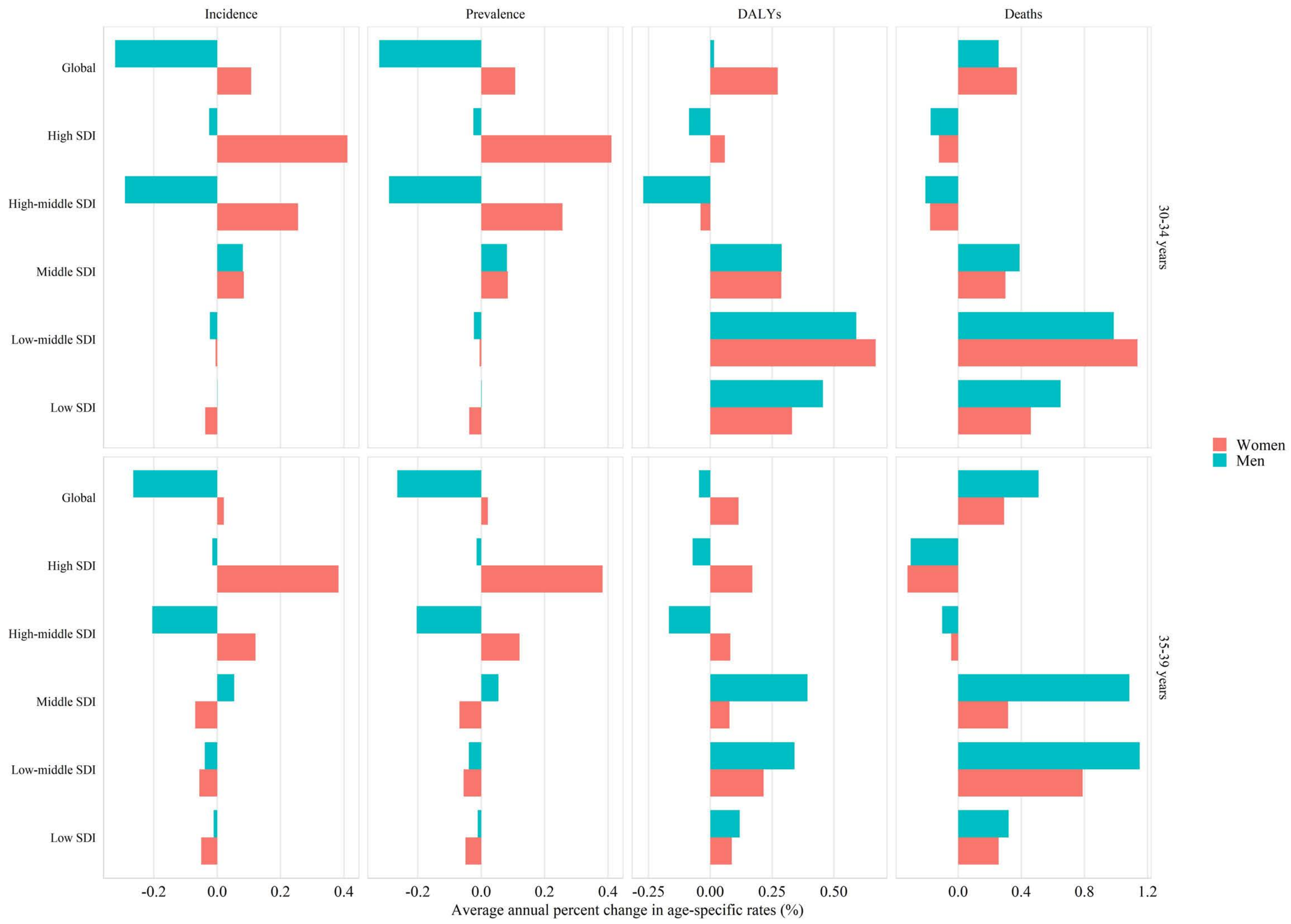

Fig S66 Average annual percent change in age-specific incidence, prevalence, disability-adjusted life years (DALYs), and death rate of atrial fibrillation and flutter in youths and young adults by age, sex, and sociodemographic index, from 1990 to 2019. Data for the age of 15-29 are unavailable.

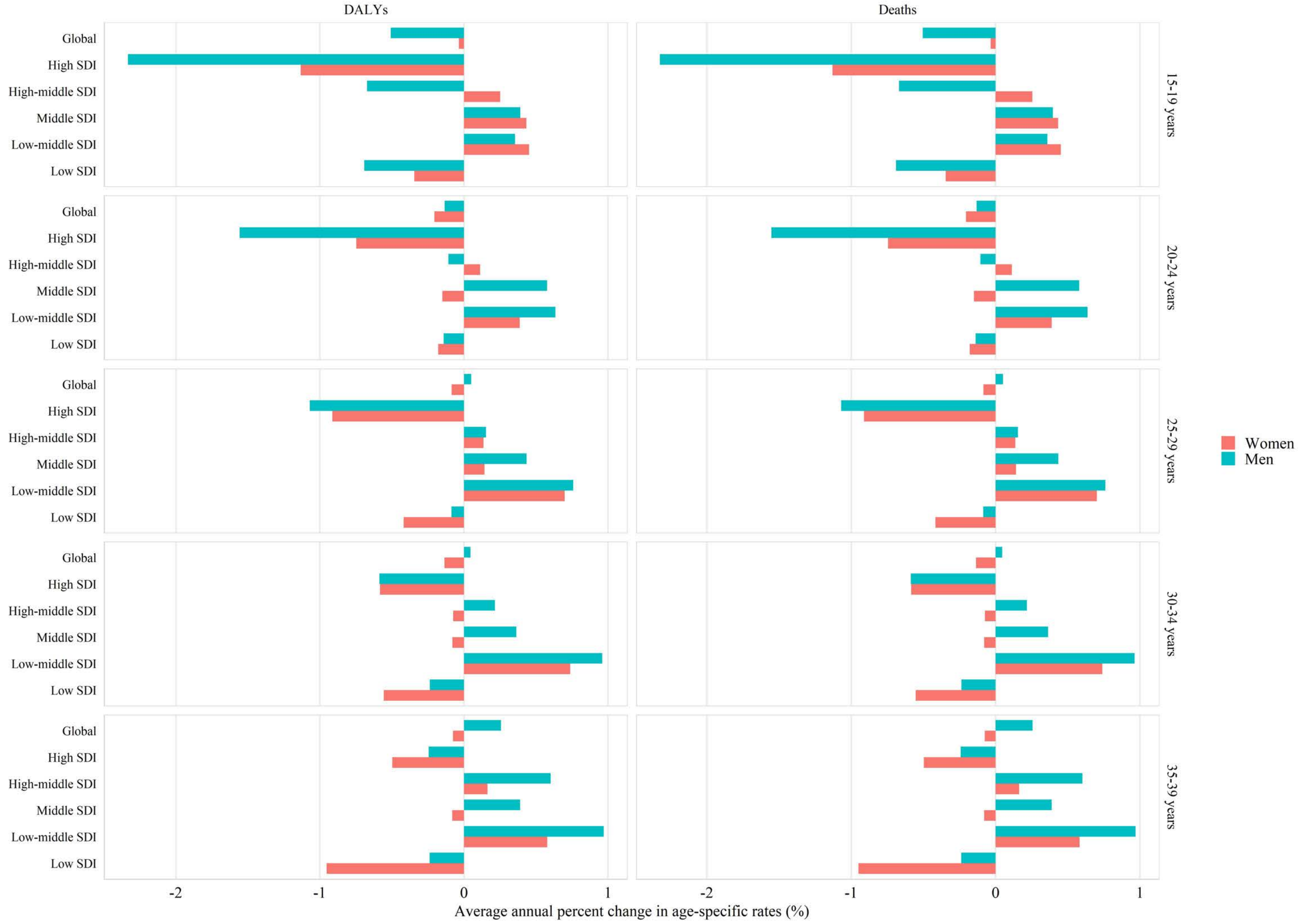

Fig S67 Average annual percent change in age-specific disability-adjusted life years (DALYs) and death rate of aortic aneurysm in youths and young adults by age, sex, and sociodemographic index, from 1990 to 2019. Data for incidence and prevalence are unavailable.

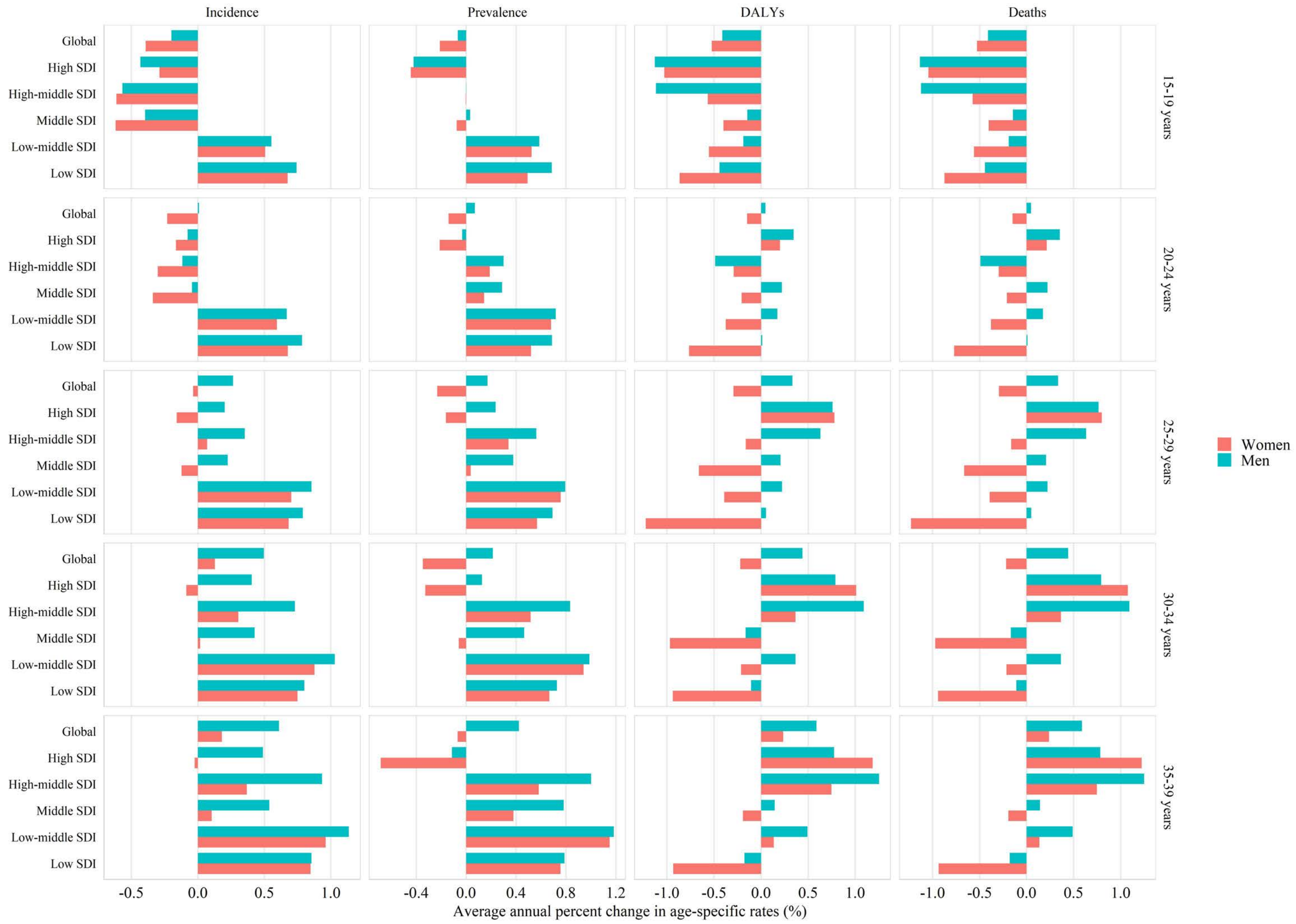

Fig S68 Average annual percent change in age-specific incidence, prevalence, disability-adjusted life years (DALYs), and death rate of endocarditis in youths and young adults by age, sex, and sociodemographic index, from 1990 to 2019.

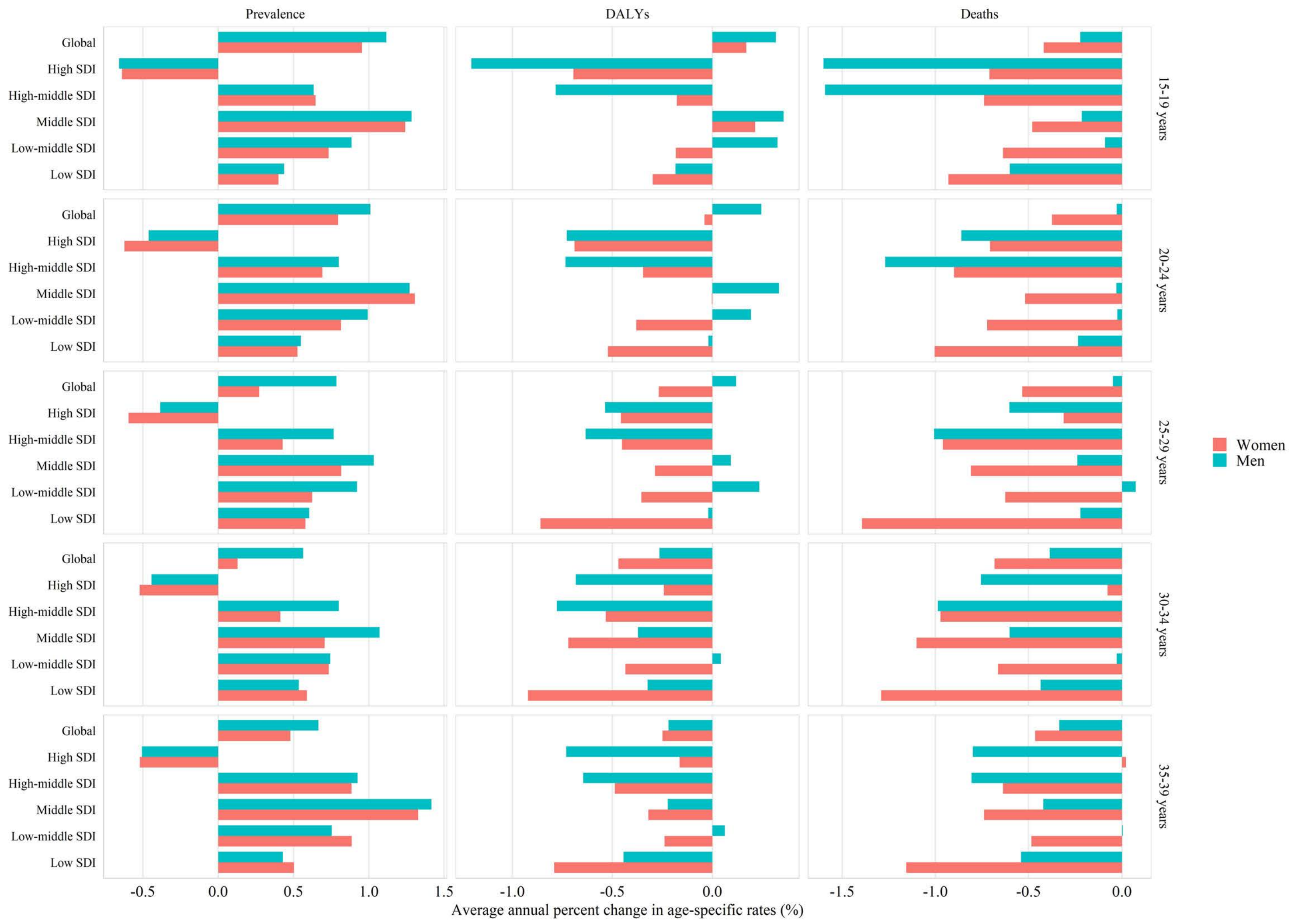

Fig S69 Average annual percent change in age-specific prevalence, disability-adjusted life years (DALYs), and death rate of other cardiovascular and circulatory diseases in youths and young adults by age, sex, and sociodemographic index, from 1990 to 2019. Data for incidence are unavailable.

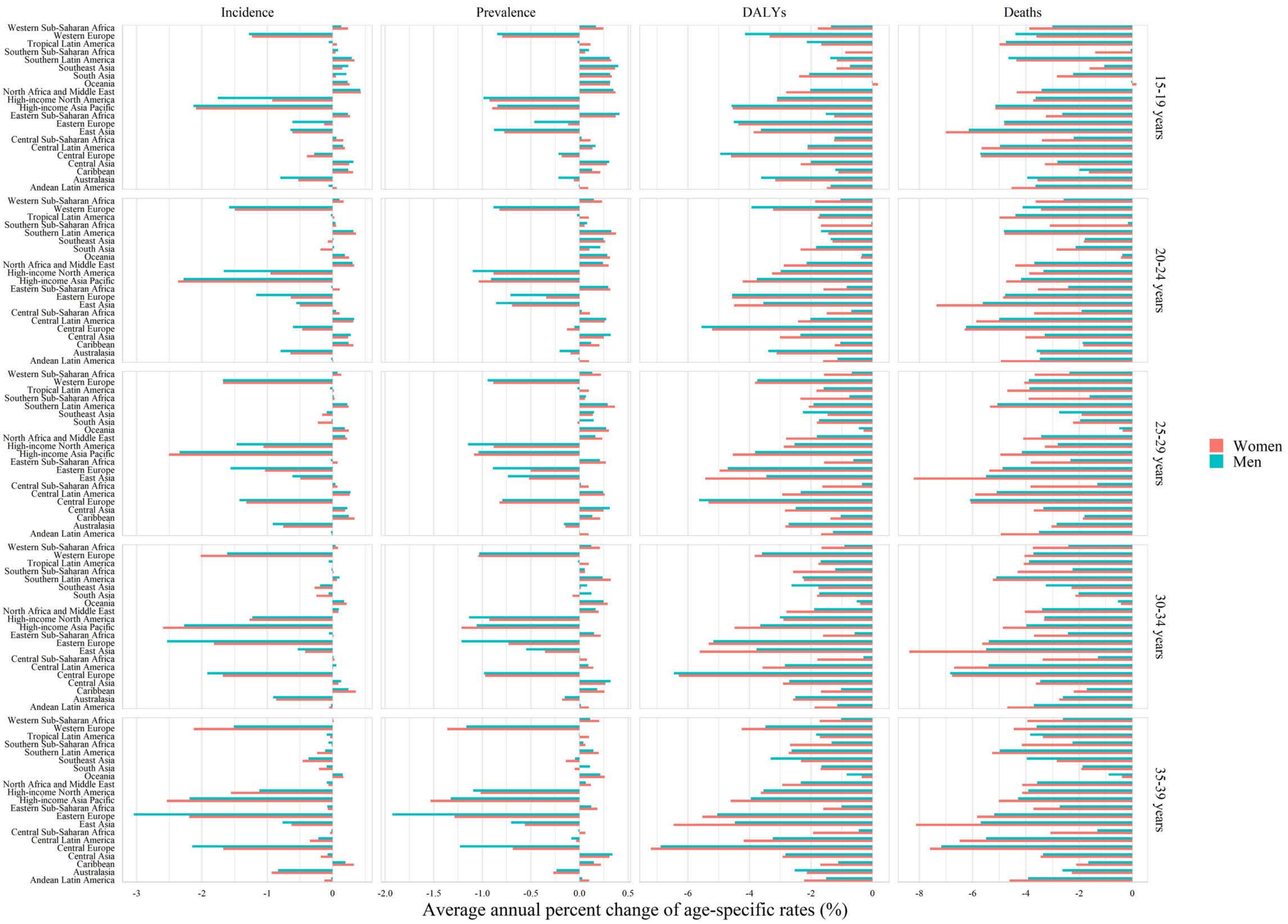

Fig S70 Average annual percent change of age-specific incidence, prevalence, disability-adjusted life years (DALYs), and death rate of rheumatic heart disease in youths and young adults by age, sex, and region, from 1990 to 2019.

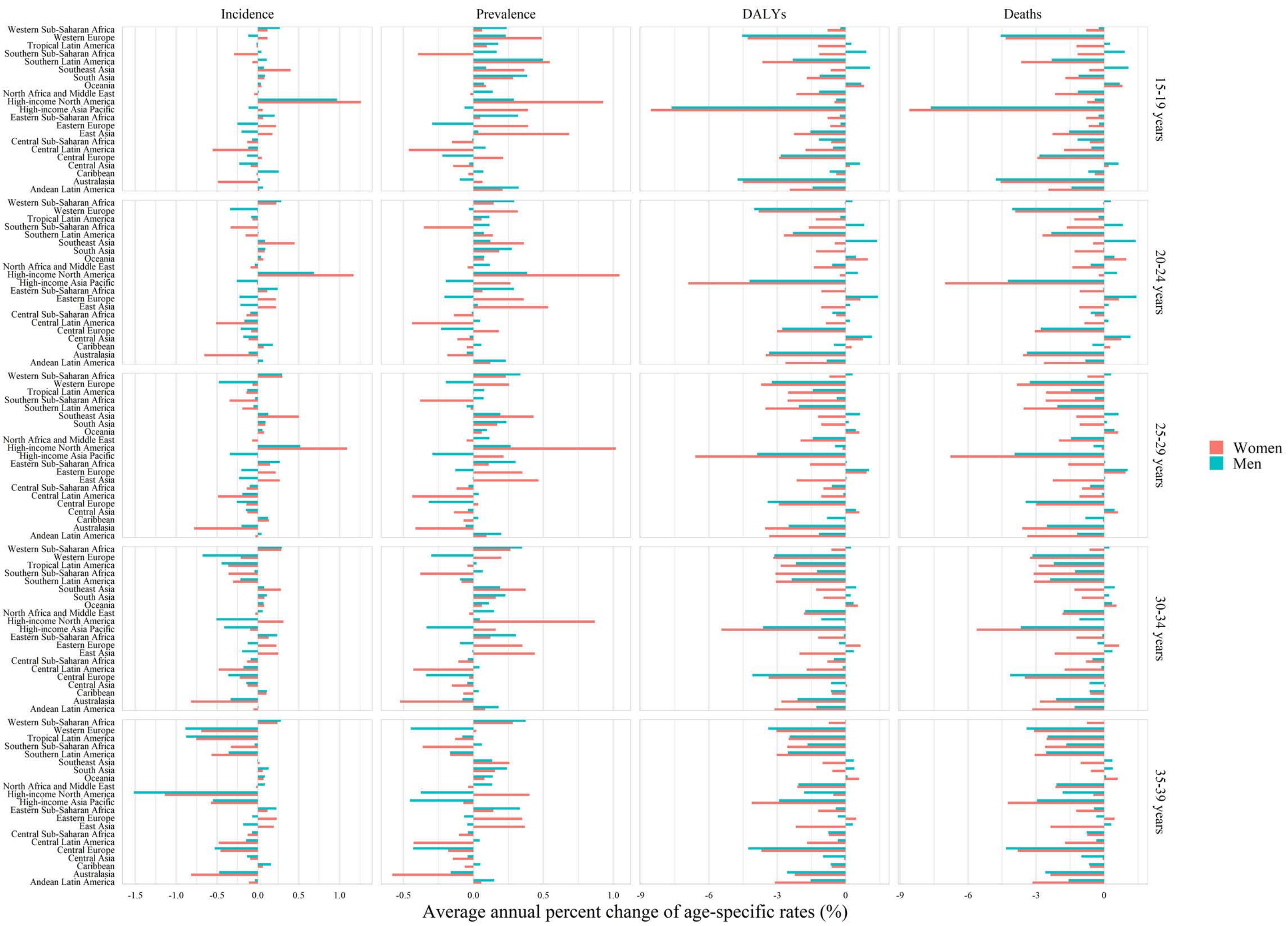

Fig S71 Average annual percent change of age-specific incidence, prevalence, disability-adjusted life years (DALYs), and death rate of ischemic heart disease in youths and young adults by age, sex, and region, from 1990 to 2019.

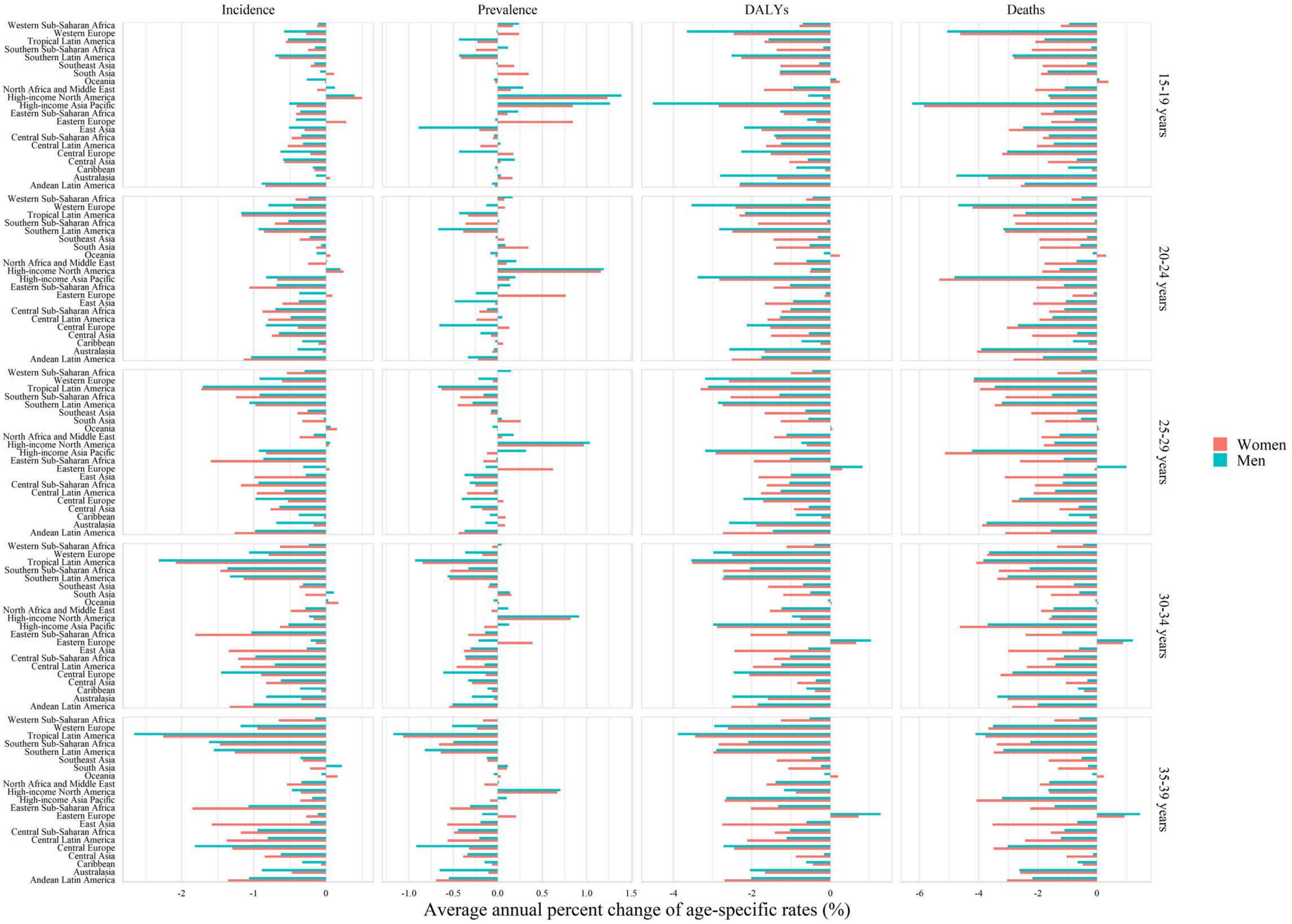

Fig S72 Average annual percent change of age-specific incidence, prevalence, disability-adjusted life years (DALYs), and death rate of stroke in youths and young adults by age, sex, and region, from 1990 to 2019.

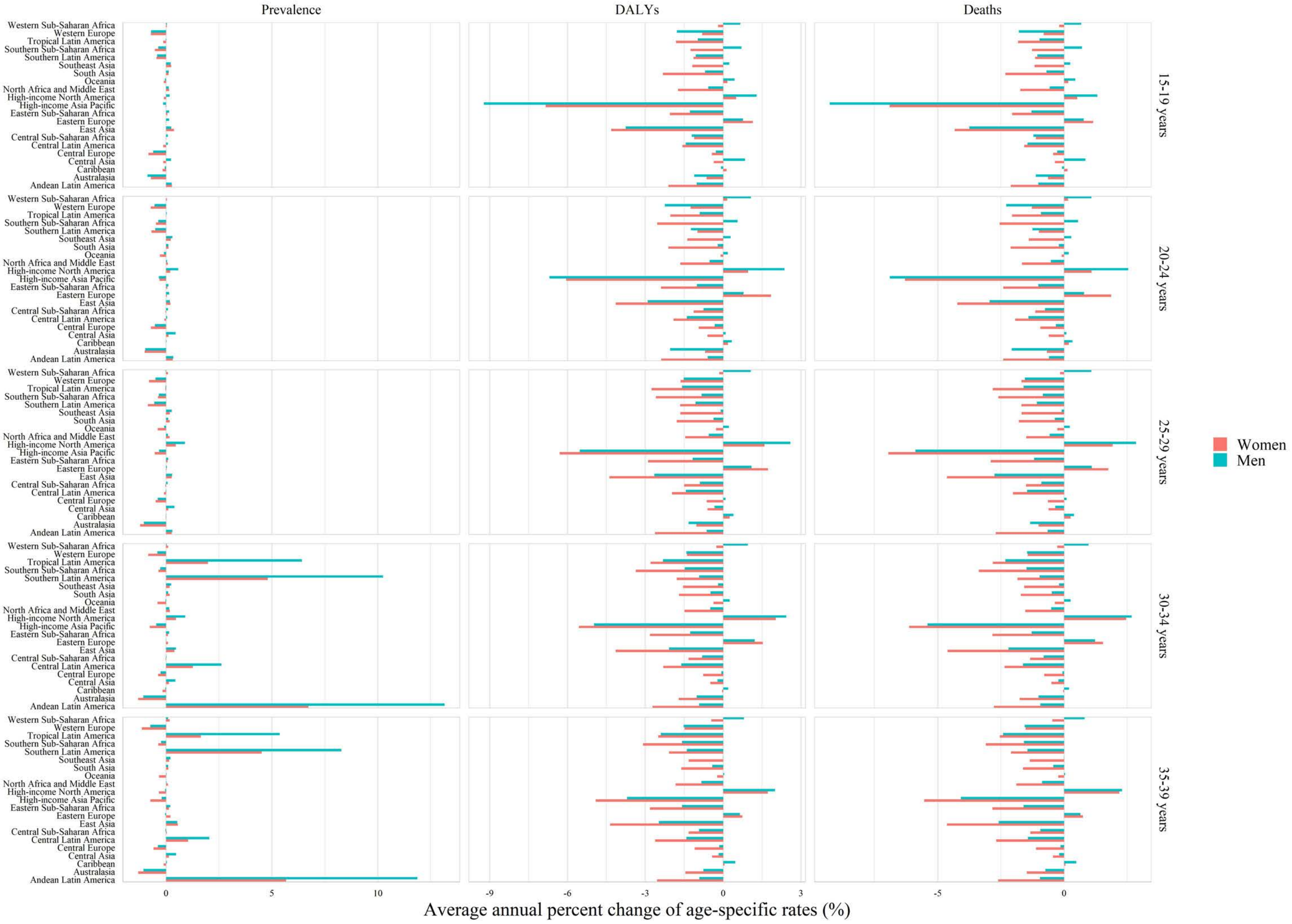

Fig S73 Average annual percent change of age-specific prevalence, disability-adjusted life years (DALYs), and death rate of hypertensive heart disease in youths and young adults by age, sex, and region, from 1990 to 2019. Data for incidence are unavailable.

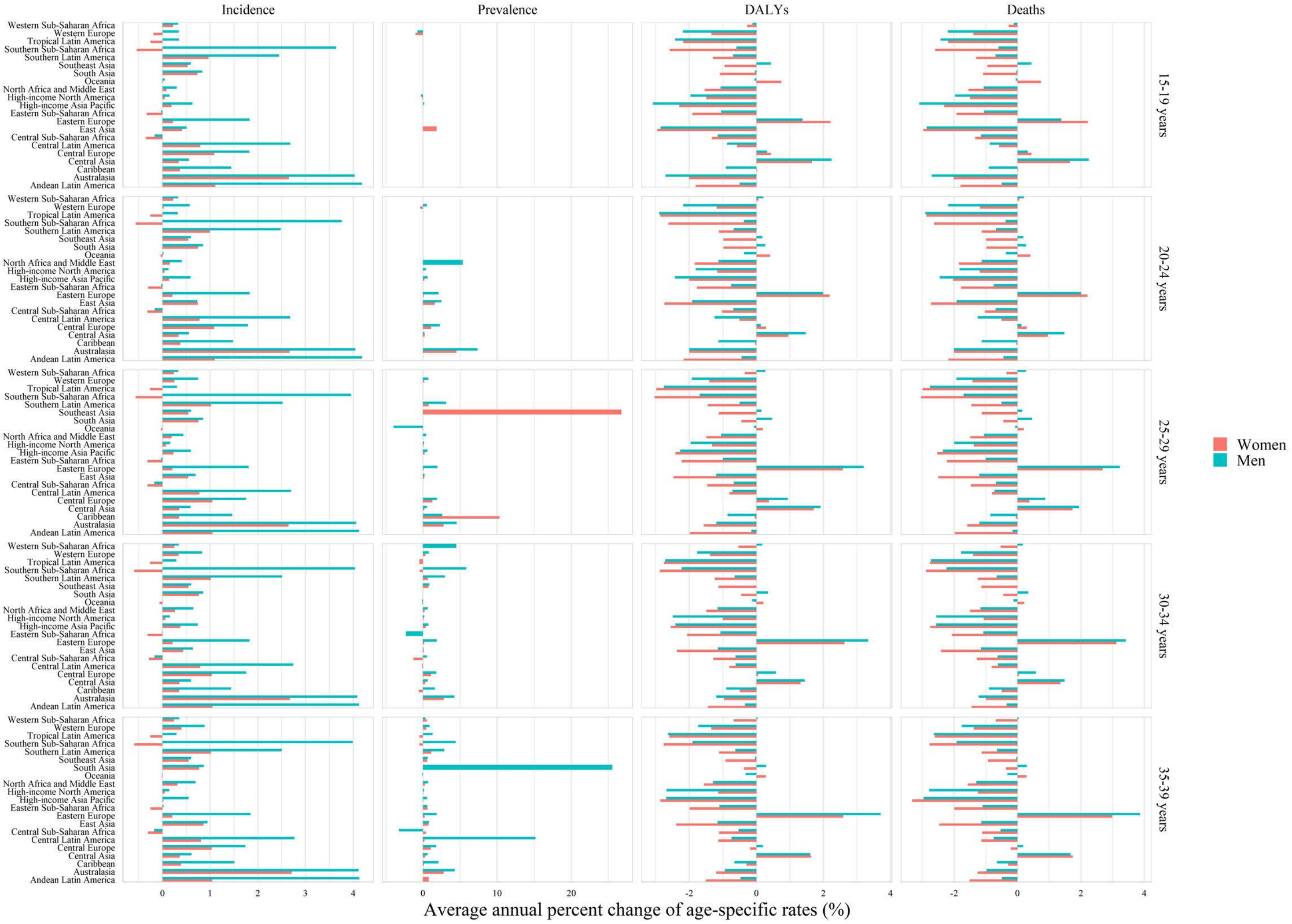

Fig S74 Average annual percent change of age-specific incidence, prevalence, disability-adjusted life years (DALYs), and death rate of non-rheumatic valvular heart disease in youths and young adults by age, sex, and region, from 1990 to 2019. Data for prevalence in several subgroups are unavailable.

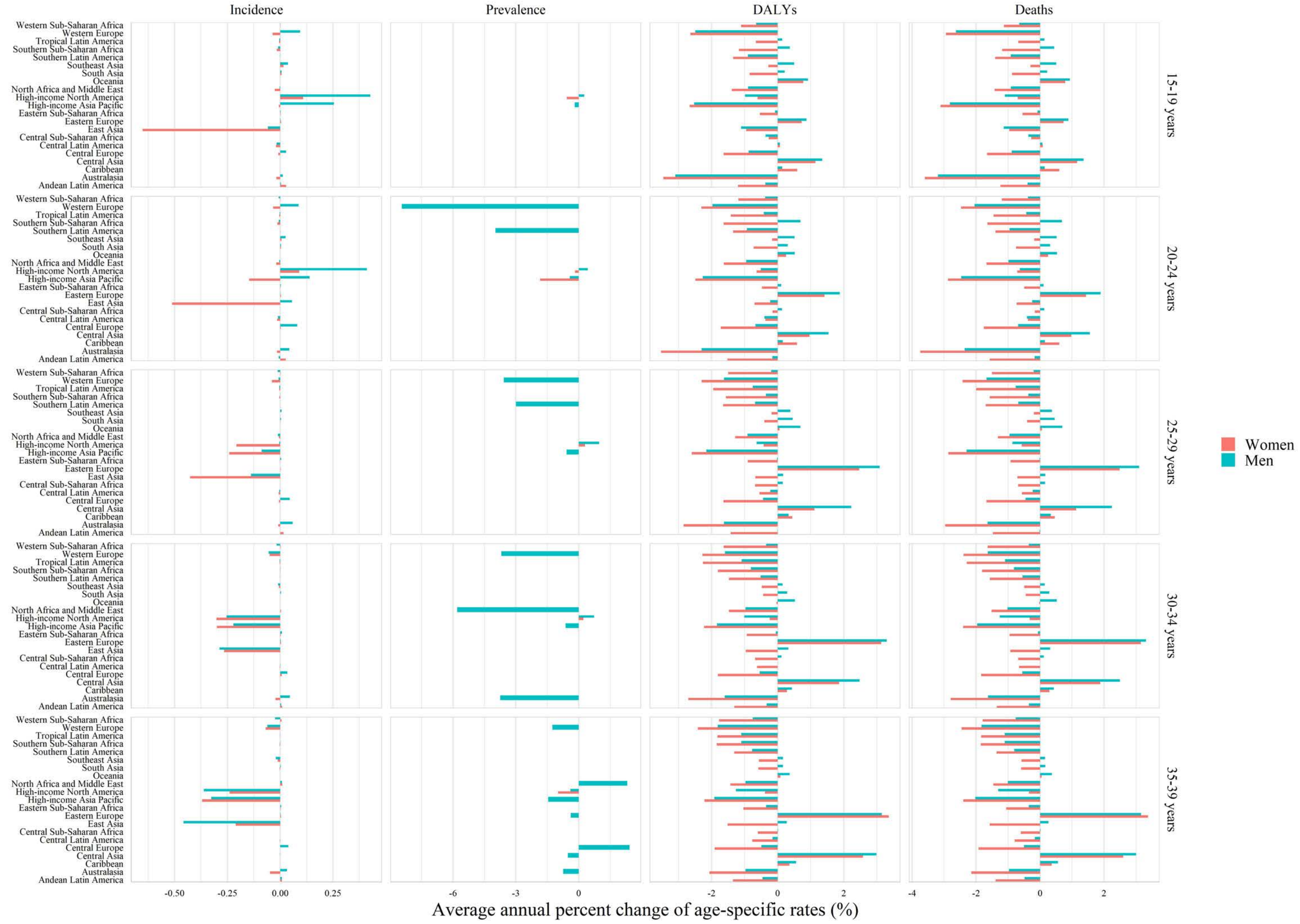

Fig S75 Average annual percent change of age-specific incidence, prevalence, disability-adjusted life years (DALYs), and death rate of cardiomyopathy and myocarditis in youths and young adults by age, sex, and region, from 1990 to 2019. Data for incidence and prevalence in several subgroups are unavailable.

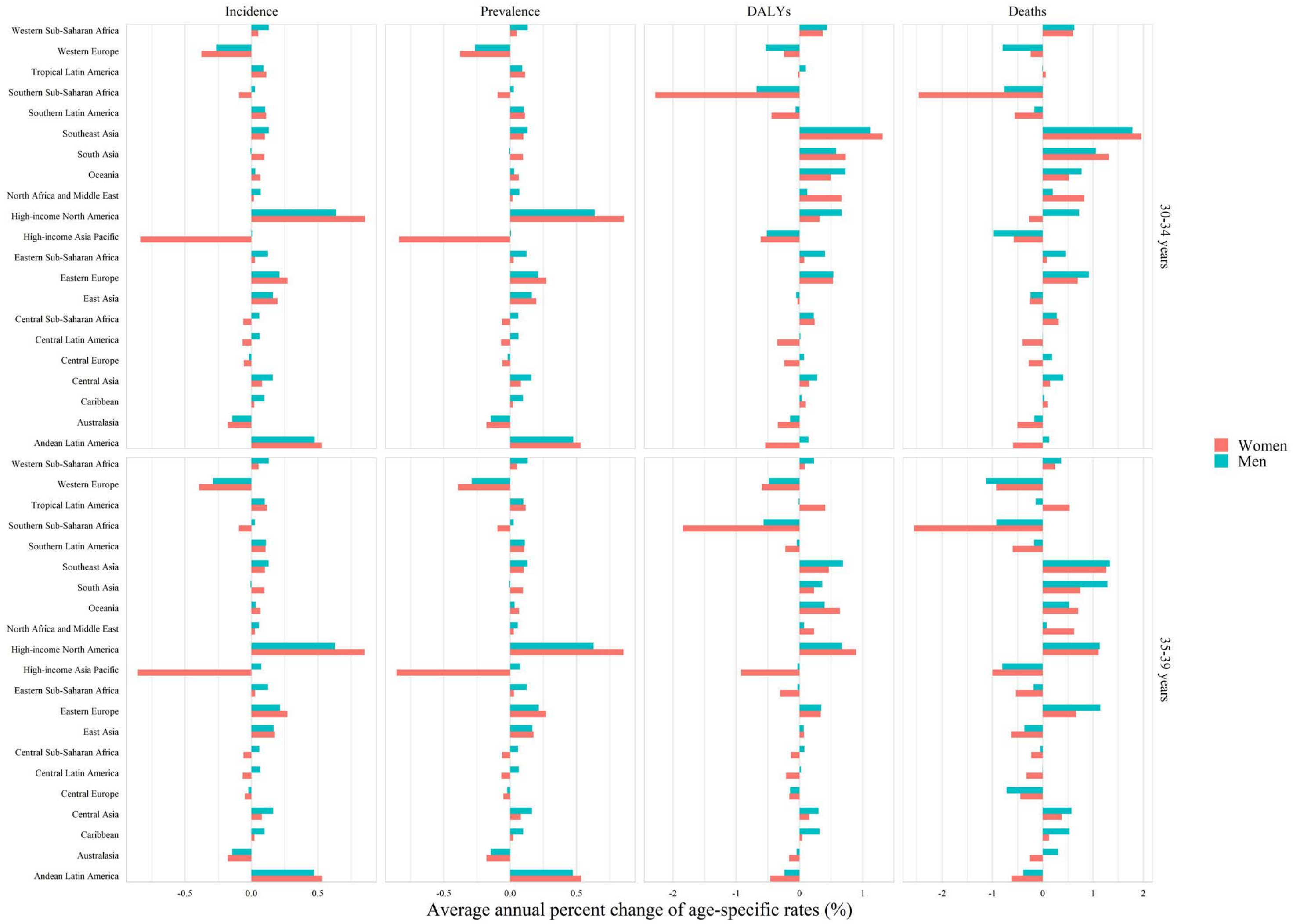

Fig S76 Average annual percent change of age-specific incidence, prevalence, disability-adjusted life years (DALYs), and death rate of atrial fibrillation and flutter in youths and young adults by age, sex, and region, from 1990 to 2019. Data for the age of 15-29 are unavailable.

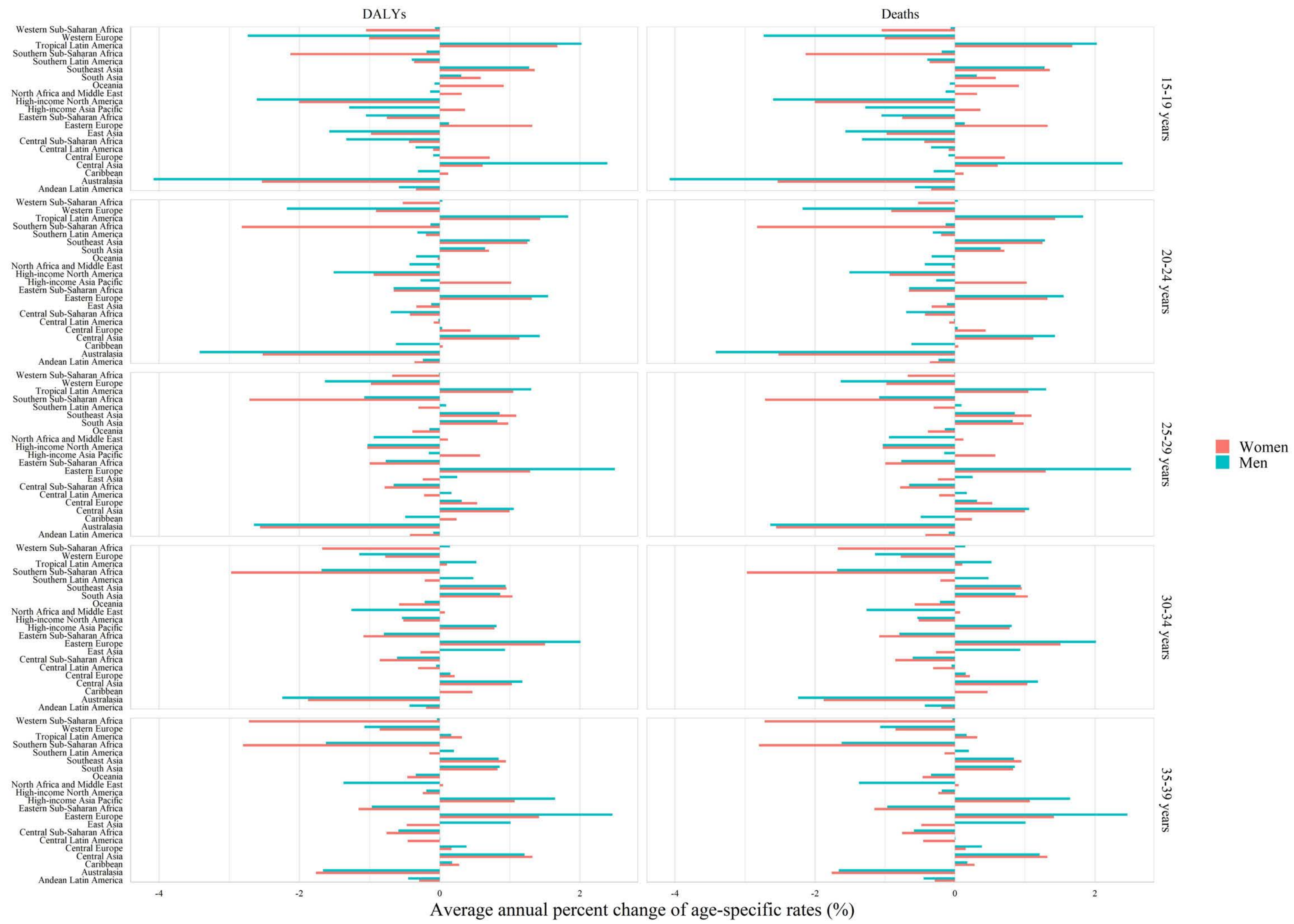

Fig S77 Average annual percent change of age-specific disability-adjusted life years (DALYs) and death rate of aortic aneurysm in youths and young adults by age, sex, and region, from 1990 to 2019. Data for incidence and prevalence are unavailable.

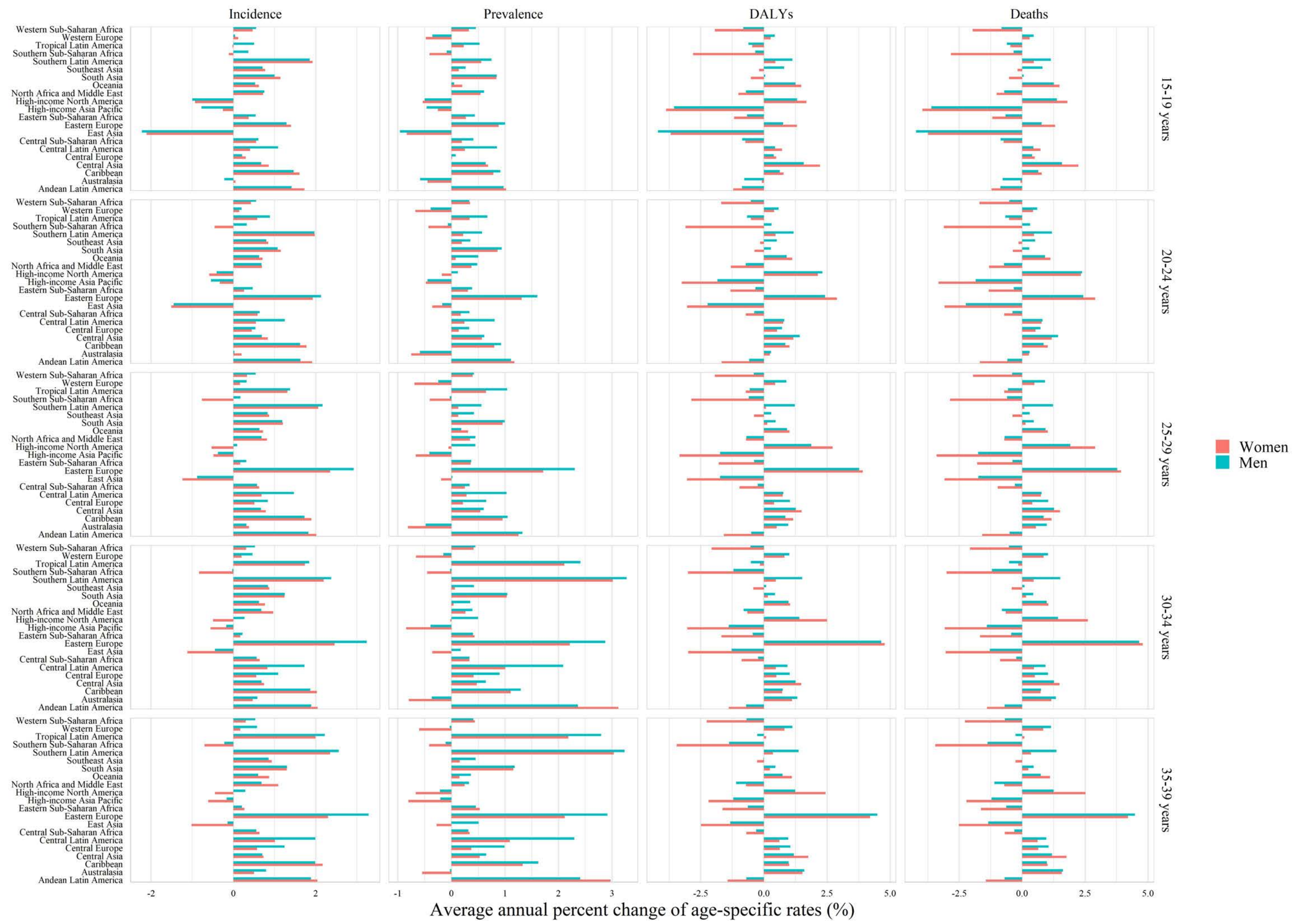

Fig S78 Average annual percent change of age-specific incidence, prevalence, disability-adjusted life years (DALYs), and death rate of endocarditis in youths and young adults by age, sex, and region, from 1990 to 2019.

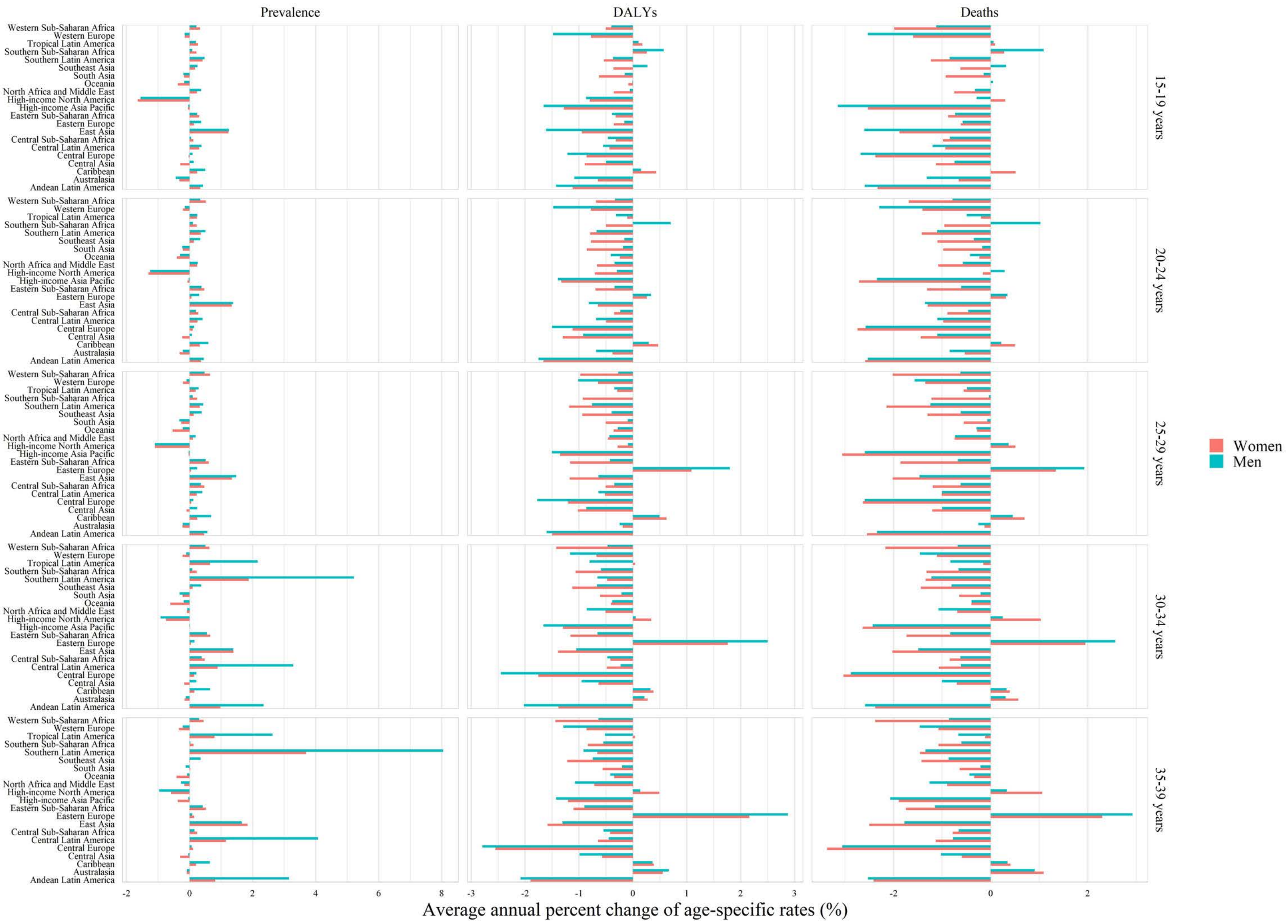

Fig S79 Average annual percent change of age-specific prevalence, disability-adjusted life years (DALYs), and death rate of other cardiovascular and circulatory diseases in youths and young adults by age, sex, and region, from 1990 to 2019. Data for incidence are unavailable.

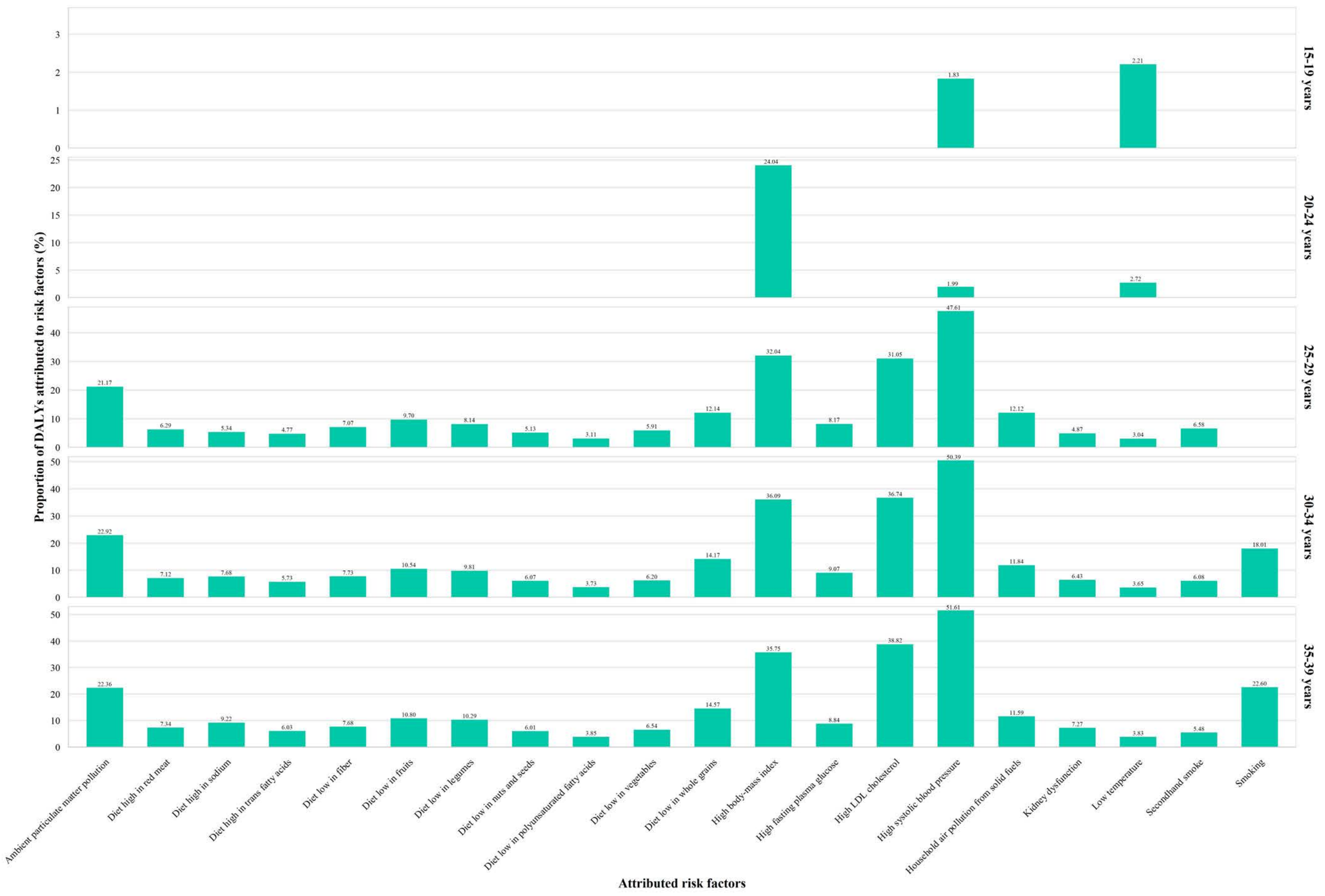

Fig S80 Proportion of disability-adjusted life years of overall cardiovascular disease attributed to 20 risk factors in 2019 by age (15-19, 20-24, 25-29, 30-34, and 35-39 years). Data for several risk factors are unavailable.

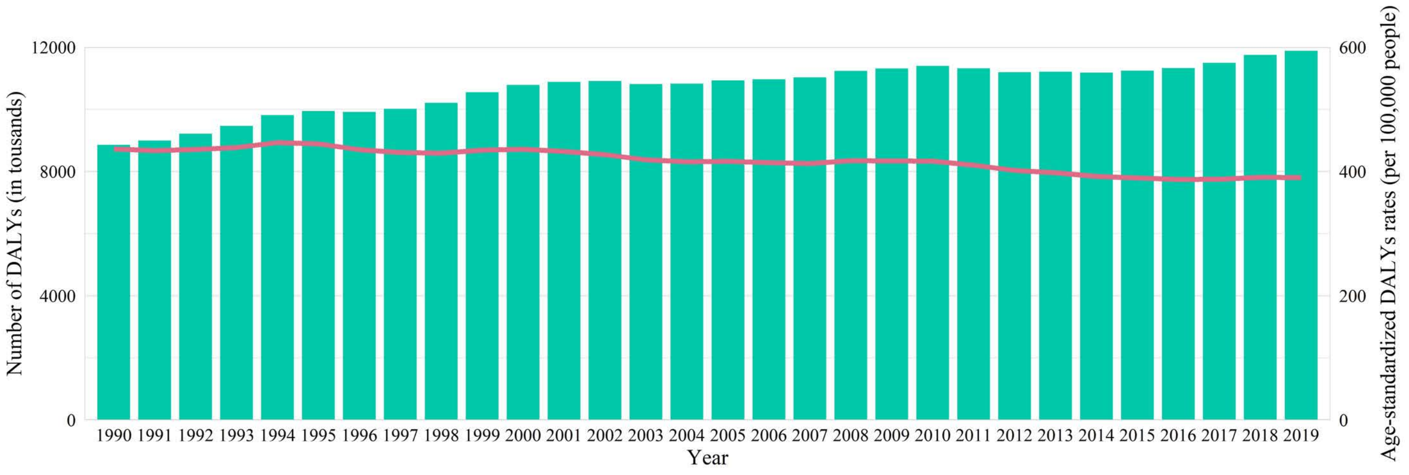

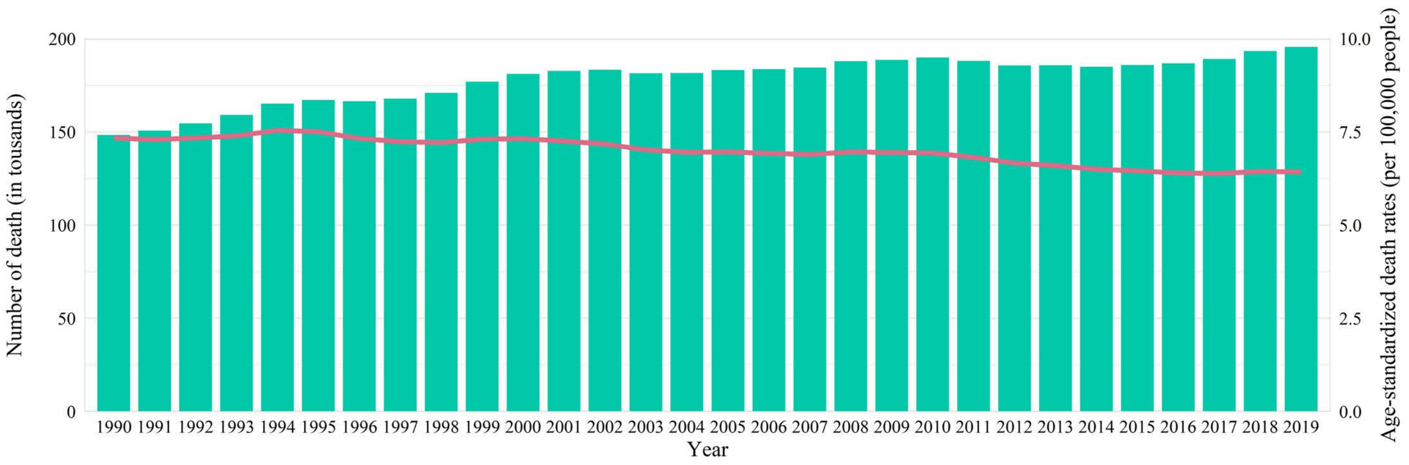

Fig S81 Burden of overall cardiovascular disease attributed to high systolic blood pressure, 1990-2019 A, total number and age-standardized rate of DALYs; B, total number and age-standardized rate of death. DALYs, disability-adjusted life years.

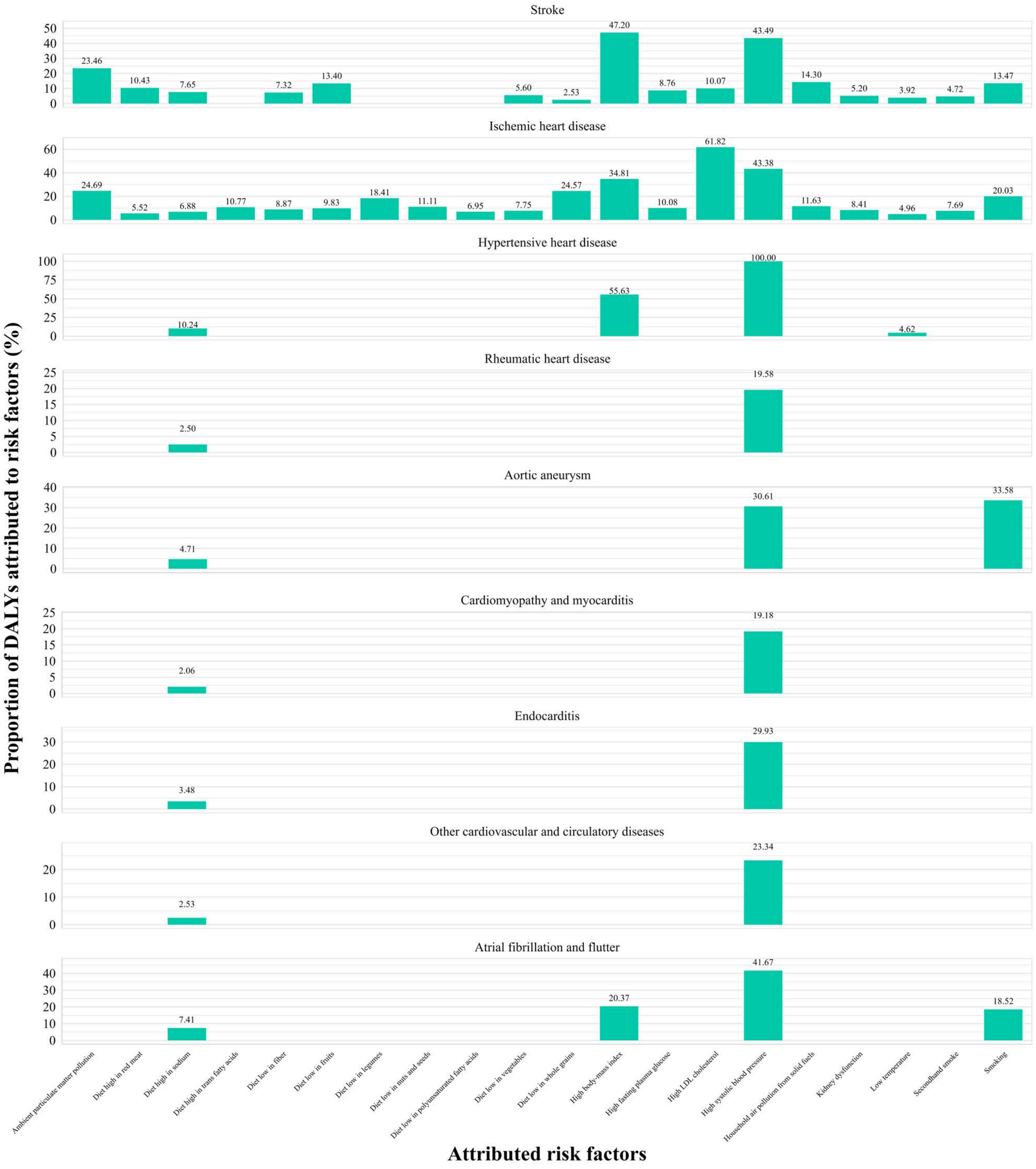

Fig S82 Proportion of DALYs of type-specific cardiovascular disease attributed to high systolic blood pressure in 2019. DALYs, disability-adjusted life years.

A

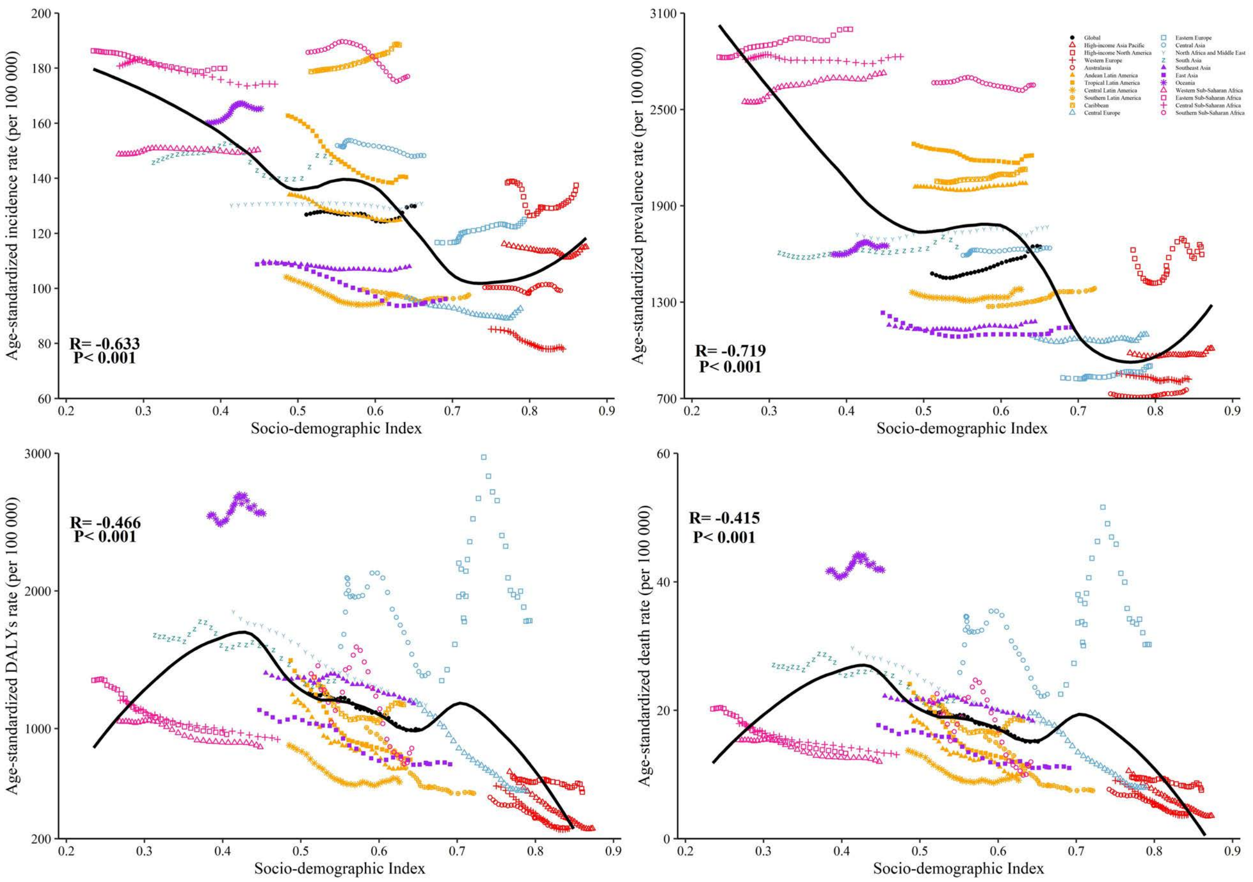

B

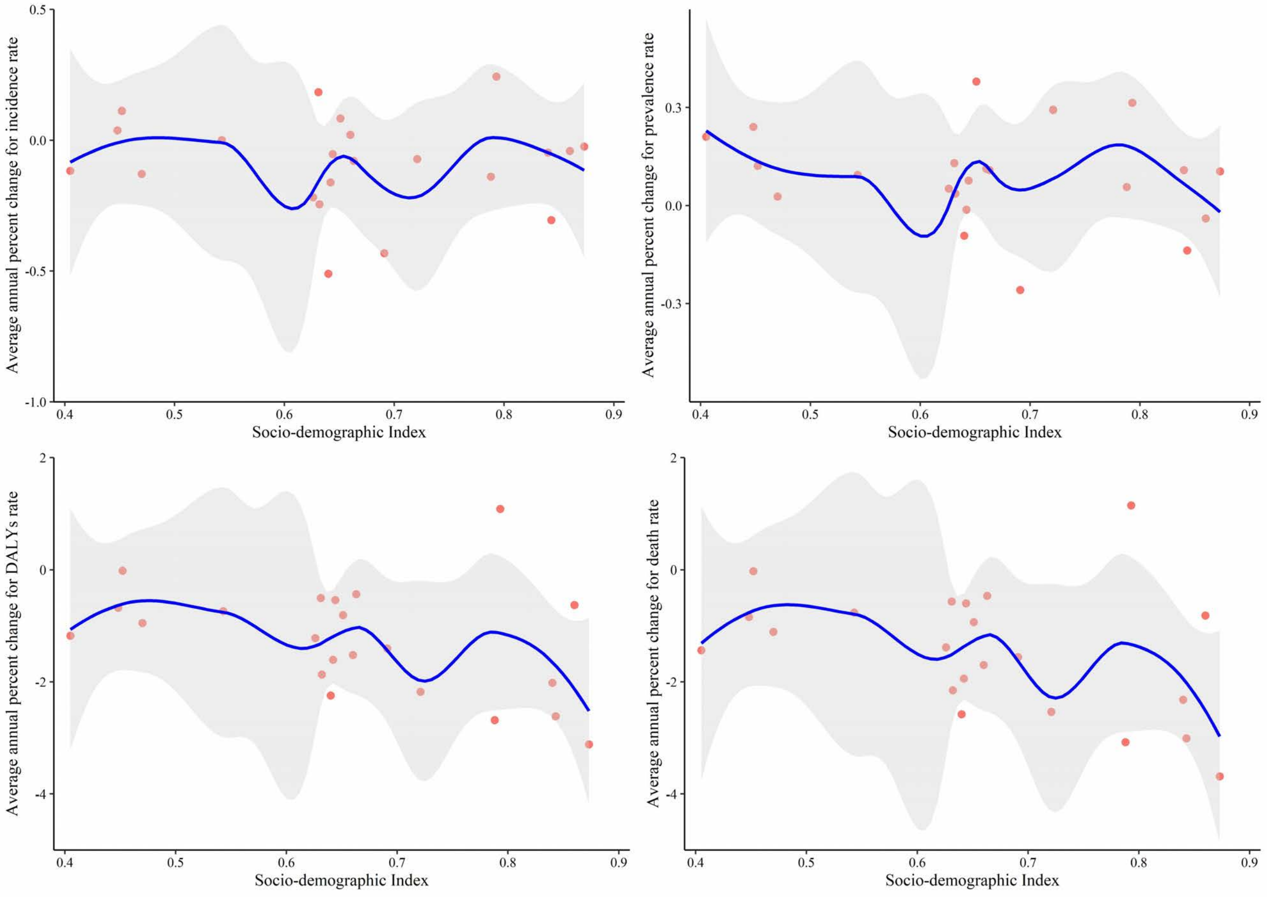

Fig S83 Burden of overall cardiovascular disease among youths and young adults worldwide and in 21 GBD regions by sociodemographic index, 1990 to 2019. A. age-standardized incidence, prevalence, disability-adjusted life years (DALYs), and death rate; B, average annual percent change for these metrics
